# Network Models of BACE-1 Inhibitors: Exploring Structural and Biochemical Relationships

**DOI:** 10.3390/ijms25136890

**Published:** 2024-06-23

**Authors:** Ömer Akgüller, Mehmet Ali Balcı, Gabriela Cioca

**Affiliations:** 1 Department of Mathematics, Faculty of Science, Mugla Sitki Kocman University, 48000 Mugla, Turkey; oakguller@mu.edu.tr; 2 Preclinical Department, Faculty of Medicine, Lucian Blaga University of Sibiu, 550024 Sibiu, Romania; gabriela.cioca@ulbsibiu.ro

**Keywords:** complex networks, molecular descriptors, BACE-1 inhibitors, clustering

## Abstract

This study investigates the clustering patterns of human β-secretase 1 (BACE-1) inhibitors using complex network methodologies based on various distance functions, including Euclidean, Tanimoto, Hamming, and Levenshtein distances. Molecular descriptor vectors such as molecular mass, Merck Molecular Force Field (MMFF) energy, Crippen partition coefficient (ClogP), Crippen molar refractivity (MR), eccentricity, Kappa indices, Synthetic Accessibility Score, Topological Polar Surface Area (TPSA), and 2D/3D autocorrelation entropies are employed to capture the diverse properties of these inhibitors. The Euclidean distance network demonstrates the most reliable clustering results, with strong agreement metrics and minimal information loss, indicating its robustness in capturing essential structural and physicochemical properties. Tanimoto and Hamming distance networks yield valuable clustering outcomes, albeit with moderate performance, while the Levenshtein distance network shows significant discrepancies. The analysis of eigenvector centrality across different networks identifies key inhibitors acting as hubs, which are likely critical in biochemical pathways. Community detection results highlight distinct clustering patterns, with well-defined communities providing insights into the functional and structural groupings of BACE-1 inhibitors. The study also conducts non-parametric tests, revealing significant differences in molecular descriptors, validating the clustering methodology. Despite its limitations, including reliance on specific descriptors and computational complexity, this study offers a comprehensive framework for understanding molecular interactions and guiding therapeutic interventions. Future research could integrate additional descriptors, advanced machine learning techniques, and dynamic network analysis to enhance clustering accuracy and applicability.

## 1. Introduction

Network science is a multidisciplinary field of research that examines complex networks found in different areas of science, engineering, and social sciences. The field involves the utilization of theoretical and analytical methods to comprehend the organization, functioning, and changes in networks consisting of interconnected elements or nodes. These networks can depict several complex systems in the physical world, including but not limited to the social networks [1,2], biological networks [3,4,5], transportation systems [6,7], and the interconnections within financial markets [8,9,10]. The fundamental principle of network science revolves around graph theory, which represents networks as graphs consisting of nodes (representing entities) and edges (indicating interactions or connections between these entities). Researchers can utilize this to analyze different characteristics of networks, including the distribution of connections (degree distribution), the shortest paths between nodes (which determine the network’s navigability), and the clustering or community structures that identify subgroups within the network with more connections among their members than with the rest of the network.

The statistical study of networks and their communities encompasses a wide range of methodologies aimed at quantifying and modeling the inherent complexity in these systems. One commonly utilized approach to identify influential nodes within a network involves employing centrality measures. These measures include degree centrality, which highlights the most connected nodes; betweenness centrality, which focuses on nodes located on the most traveled paths; and eigenvector centrality, which emphasizes nodes connected to other high-degree nodes as being highly central. These centrality metrics are instrumental in determining the importance and influence of nodes within the network structure. For instance, in ref. [11] the application of these centrality measures is discussed in the context of brain networks, and a new centrality metric is proposed known as leverage centrality to identify neighborhood hubs more accurately. Similarly, authors in ref. [12] provide a comparative analysis of various centrality measures, including degree, betweenness, and eigenvector centrality, demonstrating their efficacy in identifying the most influential nodes within social networks.

Statistical analysis in network research also includes the investigation of network architecture and its influence on function and dynamics. Scale-free networks, which have a power-law degree distribution, are recognized for their resilience to random failures but susceptibility to deliberate attacks [13,14]. Small-world networks are characterized by a high clustering coefficient and a short average path length, which allows for efficient information movement within the network [15,16]. Understanding the durability, efficiency, and evolution of complex networks relies heavily on these statistical features and others. Network science, with its combination of theoretical insights and statistical analysis, offers a robust framework for understanding the complex connections that form the basis of natural and man-made systems. It emphasizes the importance of network communities as essential components in the organization and dynamics of complex networks.

Network science methods are commonly employed in molecular studies, much like in other fields. Authors of study [17] applied a modularity optimization algorithm to identify clusters in chemical networks, highlighting its broad generalizability and effectiveness across different scales and systems. This approach enables the identification of subgraphs based on internal and external connectivities, proving valuable in analyzing chemical heterogeneity in complex solutions. In ref. [18], authors introduced a method for clustering structures from atomic trajectories of chemical reactions, even in the presence of hundreds of solvent molecules. This approach simplifies the analysis of large trajectories, paving the way for constructing kinetic network models of activated processes in solution. In ref. [19], size-selective infrared spectroscopy was conducted on gas-phase water clusters to probe the microscopic nature of hydrogen-bonded water networks. The work bridges the gap between simple networks in small-sized clusters and bulk water, providing insights into the structural features of hydrogen bond networks. Authors in ref. [20] characterized hydrogen bonds and their networks in water clusters, utilizing charge-transfer and dispersion terms to evaluate pair-wise interaction energies. This study provides a detailed understanding of hydrogen bond networks and their effects on cluster structures. Authors of ref. [21] demonstrated the combination of Feature-based Molecular Networking (FBMN) and Mass Spec Query Language (MassQL) for studying mass fragmentation pathways of depsipeptides. This integrated approach accelerates the identification of molecules with similar fragmentation patterns, showcasing its utility in the analysis of complex chemical data. In ref. [22], the isomer distribution and folding of the hydrogen bond network were studied in protonated methanol clusters using infrared spectroscopy and density functional theory calculations. This work demonstrates the control over isomer distribution through rare gas tagging, a technique that switches isomer structures to more compactly folded ones composed of closed multiple rings. In ref. [23], authors investigated2 hydrogen bond networks stabilizing small water clusters on glycoaldehyde substrates using rotational spectroscopy. The study identifies the preferred structural motifs and hydrogen bond networks in small hydrates, highlighting the early stages of solvation and the insertion of organic molecules into the hydrogen bond network.

The structural diversity of small molecules is fundamental in enabling a broad spectrum of biological functions within the intricate network of metabolic processes that sustain life. The diverse compounds found in the BACE-1 dataset, particularly those modeled for β-secretase 1 (BACE-1) inhibition, play critical roles in metabolic pathways, signaling cascades, and serve as precursors for the synthesis of complex biomolecules. These molecules, despite their varied structures, exhibit significant differences in their atomic arrangements, resulting in notable variations in their chemical properties, biological activities, and functions within metabolic networks. These differences influence their interactions with enzymes, receptors, and other cellular components, thereby impacting processes such as enzyme inhibition, signal transduction, and molecular recognition. Additionally, the energetic properties of these molecules, including differences in stability and reactivity, highlight their unique functional roles. Understanding these subtle distinctions enhances our knowledge of molecular diversity and its implications for health and disease, providing new opportunities for the development of targeted therapeutic interventions.

Study [24] provided a comprehensive computational modeling approach to predict the binding affinities of human β-secretase 1 (BACE-1) inhibitors. By employing various in silico ligand-based modeling approaches and statistical techniques, the authors developed models that achieve an approximately 70% accuracy in classification tasks and a root mean square error of around one log in quantitative predictions. Notably, the 2D descriptor-based machine learning methods show considerable success, indicating their potential utility in the lead identification and optimization efforts for BACE-1 inhibitors. In ref. [25], authors designed a multiparameter lead generation workflow aimed at identifying optimal core structures for BACE-1 inhibitors as potential treatments for Alzheimer’s disease. This workflow integrated a de novo design of core fragments with predictive in silico models addressing target affinity, permeability, and hERG activity. The study successfully identified core structures that, when decorated with well-characterized substituents from known BACE-1 inhibitors, demonstrated promising activity. This multiparameter approach underscores the importance of combining various predictive models to guide the synthesis of effective inhibitors. In review [26], authors summarize the computational methods used in the discovery and design of BACE-1 inhibitors. BACE-1, a membrane-associated aspartyl protease, plays a central role in the generation of amyloid-β peptide, making it a primary drug target for Alzheimer’s disease. The availability of numerous crystal structures of the catalytic domain of BACE-1 has enabled extensive use of computational methods such as molecular dynamics simulations, quantum mechanical calculations, and ligand docking in almost every hit discovery and optimization campaign targeting BACE-1. Authors of ref. [27] employed ligand-based computational approaches to identify molecular features required for the inhibition of the BACE-1 enzyme. By using a training set of compounds with known experimental activity, they generated pharmacophore hypotheses and validated them through multiple methods. The best hypothesis was then used for database screening and molecular docking studies, leading to the identification of potential virtual leads. These leads, characterized by diverse chemical scaffolds, hold promise for further optimization as BACE-1 inhibitors. In ref. [28], a robust quantitative structure-activity relationship (QSAR) model was developed using a dataset of heterocyclic compounds to predict the inhibitory activity against BACE-1. By focusing on 2D descriptors, the study avoided complications arising from 3D geometry considerations. The model was validated using stringent parameters and revealed key structural features responsible for enhancing BACE-1 enzyme inhibitory activity. Insights from this study provide a foundation for the design of novel BACE-1 inhibitors. Authors of ref. [29] demonstrated the use of free energy perturbation (FEP) calculations in the design of BACE-1 inhibitors. They designed novel spiroaminodihydropyrroles targeting the P3 pocket of BACE-1, with pIC50 potencies ranging from approximately five to seven. The FEP calculations showed good correlation with experimental activity, highlighting the value of FEP as a computational tool for drug discovery. The study emphasizes the importance of computational methods in predicting the activity of potential inhibitors. Authors of ref. [30] explored the effect of pH on the affinities of BACE-1 inhibitors using a surface plasmon resonance biosensor-based assay. They found that the pK(a) values of the titratable residues of BACE-1 depend on the nature of the ligand involved. Their findings suggest that understanding the protonation states of residues at different pH levels is crucial for optimizing inhibitor binding to BACE-1. Authors of ref. [31] applied fragment-based NMR screening, X-ray crystallography, and structure-based design to identify novel inhibitors for BACE-1. They optimized an initial NMR hit through a combination of NMR data and functional assays, leading to the identification of several classes of BACE-1 active site-directed compounds. This approach highlights the effectiveness of integrating multiple techniques for the rapid optimization of potential drug candidates. In ref. [32], a comprehensive 3D-QSAR study was conducted and the binding mode of BACE-1 inhibitors was explored using R-group search and molecular docking. The study built a 3D-QSAR model based on Topomer CoMFA, validated it, and successfully designed new molecules with higher activity than the training inhibitors. The docking studies provided insights into the key interactions between inhibitors and BACE-1, contributing to the development of more potent inhibitors. Ref. [33] presented a new methodology that integrates ligand-based methods with structural information derived from the receptor in the authors’ study on BACE-1 inhibitors. By using a fragment-guided approach, the authors incorporated structural information into a CoMFA study, resulting in predictive models with high external predictive power. The study emphasizes the benefits of adding structural information to enhance the predictive accuracy of 3D-QSAR studies.

These studies collectively demonstrate the significant progress made in the computational and experimental methodologies for the discovery and optimization of BACE-1 inhibitors. They highlight the importance of integrating various computational tools, structural information, and experimental validation in the drug discovery process, particularly for targets like BACE-1 involved in Alzheimer’s disease.

In this study, we employ complex network methodologies to analyze and evaluate molecules from the BACE-1 dataset using various network metrics. By representing each molecule as a node and defining edges based on different distance measures such as Euclidean, Tanimoto, Hamming, and Levenshtein distances, we construct intricate molecular networks that capture the multifaceted relationships among the inhibitors. Graph theory, a fundamental aspect of this approach, provides the mathematical framework necessary to analyze these networks, allowing for us to uncover both global and local structural patterns that might not be apparent through traditional chemical analysis alone.

Utilizing advanced graph theoretical tools, we perform community detection to identify clusters of molecules with similar properties. Techniques such as the Leading Eigenvector method and the Leiden algorithm enable us to discern large-scale structural classifications and finer distinctions within the network. These clustering methods reveal how variations in molecular structure influence their roles and interactions within the biological system, highlighting significant groupings based on shared chemical characteristics and biological functions.

Moreover, we measure the networks using key graph metrics such as node centrality, clustering coefficients, and path lengths. These metrics provide deeper insights into the importance of individual molecules within the network, the degree of interconnectedness among clusters, and the overall topology of the molecular landscape. By integrating these complex network analyses, we gain a comprehensive understanding of the molecular diversity in the BACE-1 dataset, offering new perspectives on how structural variations impact biological activity and potential therapeutic efficacy.

This network-based approach not only enhances our ability to classify and analyze molecules, but also bridges the gap between computational chemistry and systems biology. It allows us the exploration of the intricate web of molecular interactions that drive biological functions and chemical phenomena. By demonstrating the practical applications and biological relevance of our findings, we aim to contribute to the development of more effective BACE-1 inhibitors and advance our understanding of molecular mechanisms in drug discovery. Through this comprehensive network analysis, we provide clear and actionable insights that can guide future therapeutic interventions and the rational design of novel inhibitors.

The selection of a distance metric—be it Euclidean, Tanimoto, Hamming, or Levenshtein—serves as a foundational decision in the construction of these networks, each metric casting the molecular landscape through a distinct analytical prism. Euclidean distance examines the continuum of physicochemical properties, revealing gradations in molecular attributes; Tanimoto distance provides insight into the structural features shared among molecules; Hamming distance quantifies differences in molecular fingerprints, offering a binary perspective on molecular diversity; and Levenshtein distance gauges the similarity between molecules based on the minimal number of transformations required to convert one molecule’s SMILES representation into another’s. Together, these metrics afford a multifaceted examination of molecular relationships, enriching our comprehension through diverse yet complementary lenses.

Choosing the appropriate distance metric is not merely a technical detail but a strategic decision that shapes the outcome of the analysis. Euclidean distance, with its focus on continuous variables, is particularly useful in highlighting subtle changes in properties such as molecular weight, polarity, and solubility. This can elucidate the gradient nature of biological activity and reactivity among molecules. In contrast, Tanimoto distance emphasizes shared structural features, making it an ideal tool for identifying molecules with similar functional groups or ring structures, which are critical in biochemical interactions. Hamming distance, by reducing molecular comparison to binary data, allows for a high-level overview of structural diversity. This can be particularly advantageous in identifying major structural variants that might have significant biological implications, even if the exact nature of the differences is not immediately apparent. Levenshtein distance, on the other hand, offers a more nuanced perspective by focusing on the edit distance between SMILES strings. This method can uncover molecular relationships that are not evident through traditional structural comparison, highlighting the importance of molecular transformations and their potential biological impacts. The integration of these distance metrics within a network framework enables a holistic understanding of molecular diversity. By mapping the multidimensional relationships among molecules, researchers can identify clusters of structurally and functionally similar molecules, trace pathways of molecular evolution, and predict the biological roles of less studied molecules. This approach also facilitates the detection of outliers—unique molecules that may possess novel properties or functions, warranting further investigation.

Incorporating community detection algorithms such as the Leading Eigenvector and Leiden methods into this analytical framework amplifies the depth of the investigation, enabling the identification of molecular clusters or communities within the overarching network. These communities encapsulate molecules that exhibit closer affiliations with one another than with those outside their group, as dictated by the selected similarity measure. A thorough examination of these communities not only delineates the structural and functional contours of molecular diversity, but also unravels the intricate tapestry of potential biological similarities and disparities.

This study employs advanced network and graph theory tools to comprehensively analyze the structural and biochemical properties of BACE-1 inhibitors, which play a critical role in the treatment of Alzheimer’s disease. Utilizing a variety of molecular descriptors, including MMFF energy, cLogP, and TPSA, we construct complex networks that reveal distinct clustering patterns among the inhibitors. By examining these patterns, we are able to elucidate how specific structural features correlate with biochemical activity and efficacy. This approach not only highlights the key molecular characteristics that contribute to effective BACE-1 inhibition, but also identifies potential lead compounds for further development. By linking molecular structure to biochemical function, our study provides deeper insights into the mechanisms of BACE-1 inhibition, offering valuable guidance for future drug design efforts. These insights can enhance the development of targeted therapies, ultimately contributing to more effective treatments for Alzheimer’s disease. Through this network-based analysis, we aim to bridge the gap between molecular structure and biochemical activity, providing a robust framework for understanding and optimizing BACE-1 inhibitors.

The ambition of this study transcends mere classification, aspiring to unravel the complex network of molecular diversity through an integrative strategy that marries advanced distance metrics with cutting-edge community detection techniques. By cataloging molecules into well-defined communities that mirror their intrinsic chemical and physical traits, the research seeks to not only delineate the structural foundations of molecular diversity but also elucidate their biological significance. The exploration aims to expose the underpinnings of biochemical pathways and interaction dynamics, potentially guiding the synthesis of novel therapeutic compounds and enriching our understanding of metabolic processes. Beyond its immediate scope, this endeavor paves the way for the application of network science methodologies to the broader domain of biochemical systems, promising new insights into the molecular diversity that underlies the complexity of life.

The structure of this paper is as follows: Section 2 provides an initial presentation of the dataset employed in this study. Next, we provide fundamental findings on emerging networks and communities that are based on distance. The comprehensive statistical analyses are presented in Section 3 on the outcomes on network and communities. In Section 4, we offer the basic approaches that we applied. Initially, we provide fundamental mathematical concepts related to networks, including network characterization measures and the approach for filtering distance-based networks. In addition, we present specific details regarding the clustering approaches of molecules on networks. We also include metrics to evaluate and contrast new communities. Moreover, Section 5 offers final remarks.

## 2. Results

### 2.1. Dataset

The BACE-1 dataset is a comprehensive collection of molecular data specifically curated for the study of β-secretase 1 (BACE-1) inhibitors in [24]. BACE-1 is an aspartyl protease enzyme that plays a pivotal role in the pathogenesis of Alzheimer’s disease by catalyzing the first step in the production of amyloid-beta peptides. Inhibiting BACE-1 has been identified as a promising therapeutic strategy to reduce amyloid-beta levels and potentially slow or prevent the progression of Alzheimer’s disease. The dataset was meticulously assembled to facilitate the computational modeling and analysis of molecules that inhibit BACE-1. It includes a diverse array of compounds with varied structural characteristics, allowing for a comprehensive examination of the chemical and biological properties that contribute to BACE-1 inhibition.

The existing dataset comprises 1547 artificial human BACE-1 inhibitors that have been recorded in the scientific literature. Among these inhibitors, more than 250 have their protein–ligand X-ray structures accessible in the Protein Data Bank (PDB). IC50 values have been experimentally determined for 1486 of these compounds, and Kd/Ki values are provided for an additional 43 compounds. The structures and documented activities for all compounds were acquired from either ChEMBL or the primary source publication, utilizing citation information from the PDB. When compiling the ligand dataset, only publications that contained a solved X-ray ligand-BACE-1 structure were taken into account by the authors. This is because it provides a reasonable estimation of the potential binding mode (bioactive conformation) of closely related medicinal chemistry analogs mentioned in the same article as part of the structure–activity relationship. The ligand dataset is accessible to the public in an intuitive CSV format in DeepChem datasets (“https://github.com/deepchem/deepchem/tree/master/datasets”, accessed on 2 June 2024).

The molecular values of all inhibitors utilized in this study, together with the corresponding indexes indicating their network communities, can be found in the Supplementary Information file.

**Figure 1:**
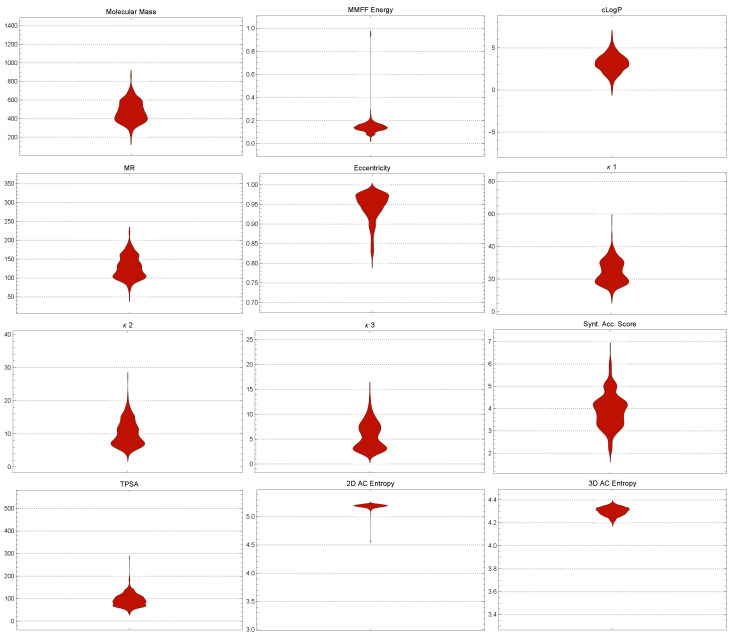
Distributions of descriptor values of BACE-1 inhibitors.

Figure 1 displays the distributions of the descriptor values of the molecules in the BACE-1 dataset, highlighting variety and complexity.

### 2.2. Network Results

The challenge of comprehending the intricate connections and differences between molecules is a major obstacle in the field of computational chemistry’s investigation of molecular structures. In this section, we present complex network-based methods for molecule clustering that makes use of a wide range of distance metrics to chart the complex terrain of molecular similarities and differences. The diverse set of molecules in the BACE-1 dataset presents an ideal case for this analysis due to their shared biological target but varied chemical structures, resulting in significant differences in their chemical properties and biological activities.

Following the formation of a distance-based network for each of the available molecule distance metrics, such as Euclidean, Tanimoto, Hamming, and Levenshtein distances, Triangulated Maximally Filtered Graph (TMFG) filtration is carried out. This filtration method is used to simplify the complex network by retaining the most significant connections while eliminating less relevant links. TMFG creates a sparse yet informative representation of the original network, preserving the essential topological features that highlight the relationships between molecules. By applying TMFG filtration, the study aims to reduce noise and focus on the most pertinent structural similarities and differences among the molecules. This filtration technique ensures that the resulting network maintains a balance between complexity and interpretability, making it easier to identify clusters of molecules that share common structural motifs or functional properties. The TMFG-filtered networks provide a clearer visualization of the molecular landscape, revealing patterns that might be obscured in a more densely connected network.

**Figure 2:**
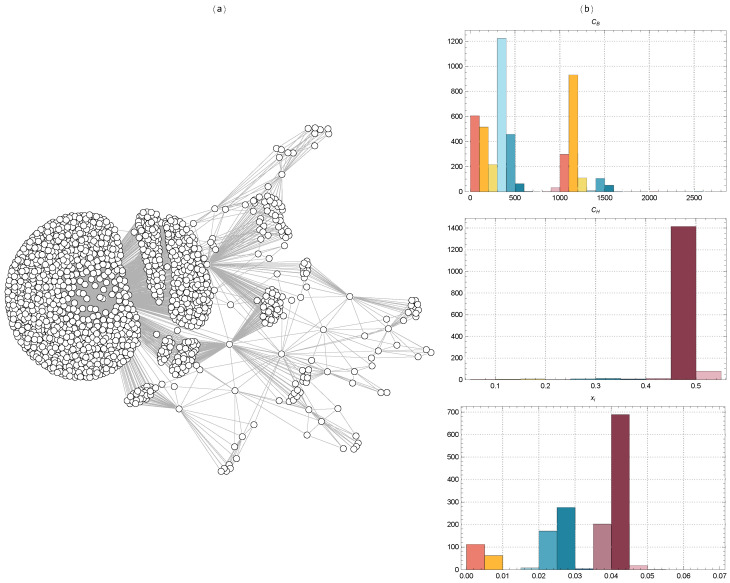
(**a**) Filtered network based on Euclidean distance and (**b**) distributions of network metrics for BACE-1 inhibitors.

**Figure 3:**
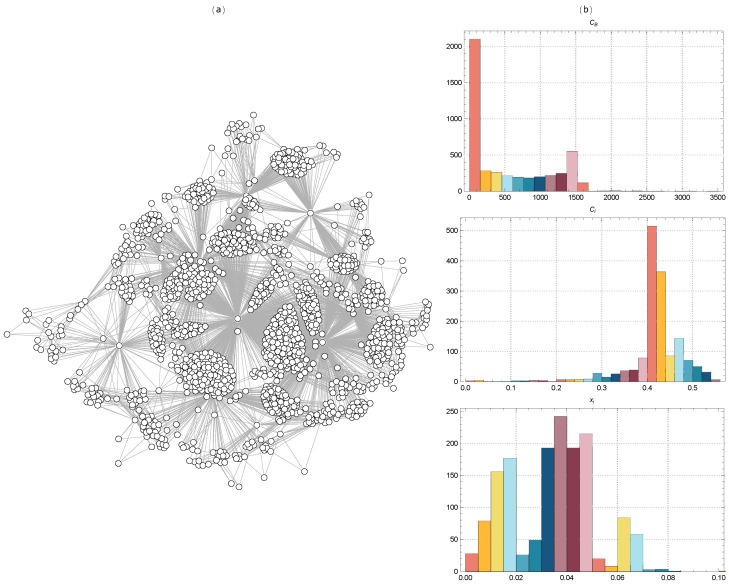
(**a**) Filtered network based on Tanimoto distance and (**b**) distributions of network metrics for BACE-1 inhibitors.

**Figure 4:**
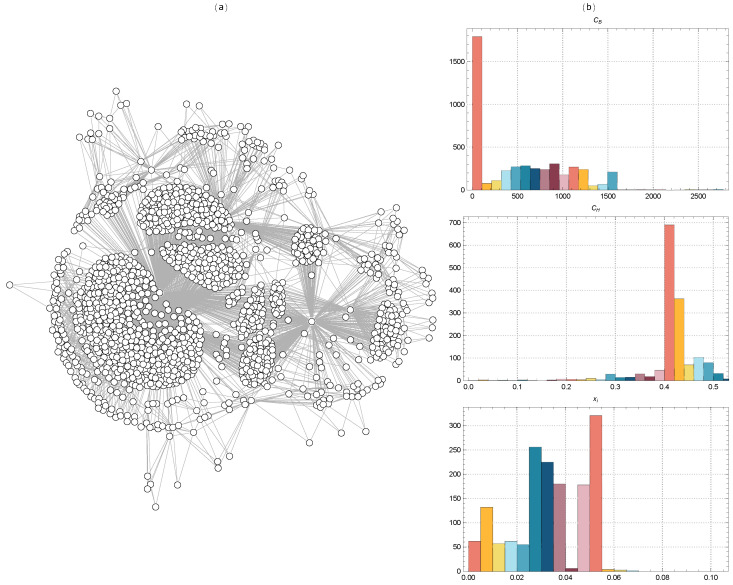
(**a**) Filtered network based on Hamming distance and (**b**) distributions of network metrics for BACE-1 inhibitors.

**Figure 5:**
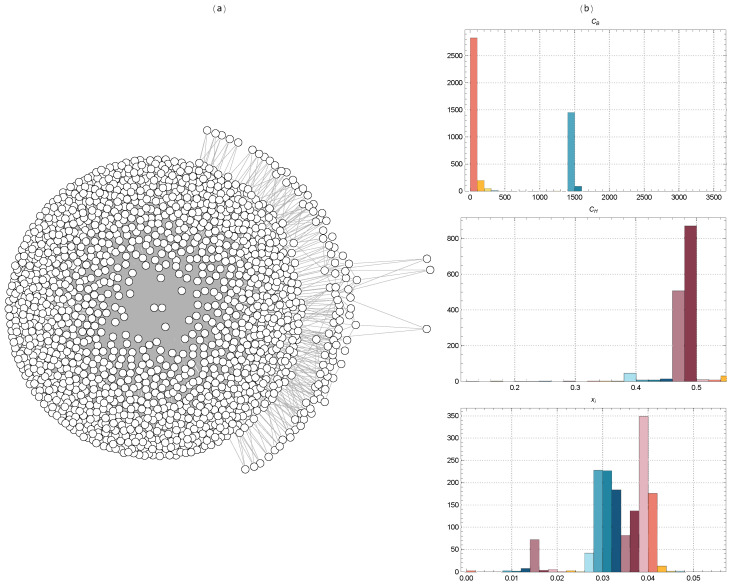
(**a**) Filtered network based on Levenshtein distance and (**b**) distributions of network metrics for BACE-1 inhibitors.

Figure 2 displays the TMFG filtered networks of the BACE-1 dataset molecules, together with the distributions of $C_{B}$, $C_{i}$, and $x_{i}$ values for Euclidean distance. Figure 3 shows the same for Tanimoto distance, Figure 4 for Hamming distance, and Figure 5 for Levenshtein distance.

The TMFG representation in Figure 2 reveals a dense core region with a high concentration of interconnected nodes. This dense core indicates a group of molecules with significant structural similarities, suggesting they share key chemical and physical properties. These core molecules likely play a central role in the network, potentially representing the most active or effective inhibitors within the BACE-1 dataset. Surrounding this dense core are several peripheral clusters. These clusters are less densely connected to the core and more interconnected within themselves, indicating subgroups of molecules that, while still similar to the core, have distinct structural features or properties.

Betweenness centrality measures the extent to which a node lies on the shortest paths between other nodes. Nodes with high betweenness centrality act as crucial connectors or bridges within the network. The distribution of $C_{B}$ on the top right shows a significant variation, indicating the presence of certain molecules that play a key role in connecting different clusters. These molecules might be crucial for maintaining the network’s integrity and could represent potential targets for inhibiting BACE-1 activity. The constraint score quantifies the extent to which a node’s connections are concentrated within a local neighborhood. A high constraint score suggests that a node’s connections are mostly to nodes that are themselves interconnected. The distribution of $C_{H}$ in the middle right panel shows a large concentration around the value of 0.5, indicating that many molecules are part of tightly knit local clusters. This suggests a strong modular structure within the network, where molecules are grouped into distinct communities with dense internal connections. Eigenvector centrality measures a node’s influence based on the influence of its neighbors. Nodes with high eigenvector centrality are connected to other nodes that are themselves well-connected. The distribution of $x_{i}$ in the bottom right panel shows that a significant number of nodes have moderate to high centrality values. This indicates that there are several influential molecules within the network, which play a significant role in the overall connectivity and stability of the network.

The TMFG in Figure 3 reveals a dense core region with a high concentration of interconnected nodes. This dense core indicates a subset of molecules with significant structural similarities, suggesting they share key chemical and physical properties critical for BACE-1 inhibition. Surrounding the dense core, several peripheral clusters are evident, which are less densely connected to the core but more interconnected within themselves. These peripheral clusters likely represent groups of molecules with unique structural features that, while related to the core molecules, exhibit distinct variations. Several nodes act as bridges between the dense core and the peripheral clusters. These key connectors are essential for maintaining the overall network connectivity, indicating that these molecules have unique structural features that allow for them to interact with multiple clusters. The presence of these connector nodes suggests their central role in the network’s integrity and potentially highlights their importance as targets for further study. The network also includes sparsely connected outliers, nodes that are only weakly connected to the main network. These outliers may represent molecules with unique or rare structural features that do not fit neatly into the primary clusters. Their sparse connectivity could indicate novel inhibitor candidates or less relevant molecules with minimal impact on the network’s overall activity.

The distribution of $C_{B}$ (top right panel) shows significant variation, with a few nodes exhibiting very high betweenness centrality. These nodes are critical connectors or bridges within the network, facilitating communication between different clusters. The distribution of $C_{i}$ (middle right panel) shows a large concentration around the value of 0.4, indicating that many nodes are part of tightly knit local clusters. This high constraint score suggests strong modularity in the network, with nodes heavily interconnected within their clusters but having fewer connections outside them. The distribution of $x_{i}$ (bottom right panel) shows a range of centrality values, with a significant number of nodes having moderate to high centrality values.

The TMFG reveals a densely connected core region, indicating a group of molecules with highly similar fingerprints in Figure 4. These molecules likely share key chemical properties and structural motifs that are critical for BACE-1 inhibition. Surrounding the dense core are several peripheral clusters, which are less densely connected to the core but more interconnected within themselves. These peripheral clusters suggest the presence of subgroups of molecules that, while related to the core, exhibit distinct structural variations that may influence their inhibitory properties. Multiple nodes serve as connectors between the compact central region and the outer clusters. These important connectors are crucial for preserving overall network connectivity, suggesting that these molecules possess distinctive structural characteristics that allow them interaction with many clusters. The existence of these connecting nodes emphasizes their significance in maintaining the structural integrity of the network and their potential as crucial places for therapeutic intervention.

The distribution of $C_{B}$ (top right panel) shows significant variation, with a few nodes exhibiting very high betweenness centrality. These nodes are critical connectors within the network, facilitating communication between different clusters. The distribution of $C_{i}$ (middle right panel) shows a large concentration around the value of 0.4, indicating that many nodes are part of tightly knit local clusters. This high constraint score suggests strong modularity in the network, with nodes heavily interconnected within their clusters but having fewer connections outside them. The distribution of $x_{i}$ in the bottom right panel exhibits a wide range of centrality values, with a notable proportion of nodes displaying moderate to high centrality values. They operate as focal hubs that concentrate interactions.

The TMFG filtration on the left side of Figure 5 captures the network structure of BACE-1 inhibitors based on Levenshtein distances between their SMILES codes. The TMFG shows a densely connected core, indicating a large group of molecules with highly similar SMILES codes. This dense core suggests that these molecules share significant structural features. Surrounding this core are several less densely connected nodes, forming a periphery that consists of molecules with more distinct structural features compared to the core group. Several nodes act as bridges between the dense core and the peripheral nodes.

Betweenness centrality measures the extent to which a node lies on the shortest paths between other nodes. The distribution of $C_{B}$ (top right panel) shows significant variation, with a few nodes exhibiting very high betweenness centrality. The constraint score quantifies the extent to which a node’s connections are concentrated within a local neighborhood. The distribution of $C_{i}$ (middle right panel) shows a large concentration around the value of 0.5, indicating that many nodes are part of tightly knit local clusters. This high constraint score suggests strong modularity in the network, with nodes heavily interconnected within their clusters but having fewer connections outside them. The presence of nodes with lower constraint scores indicates some molecules are more globally connected, interacting with multiple clusters. The distribution of $x_{i}$ (bottom right panel) shows a range of centrality values, with a significant number of nodes having moderate to high centrality values.

### 2.3. Community Results

Performing a community analysis on the distance-based (or similarity-based) networks of the BACE-1 molecule using Leading Eigenvector and Leiden methodologies is of great importance for several reasons, as previously indicated in network analyses. These approaches are effective instruments for revealing the fundamental modular structures inside intricate networks, aiding in the identification of groupings of molecules that share closer relationships in terms of their structural and functional qualities. The networks’ architecture, as indicated by various distance measures, display complex patterns of molecule interactions that are crucial for understanding the chemical and biological behavior of these molecules.

Upon analyzing different distance-based networks, it becomes apparent through visual observation that BACE-1 inhibitors organize into discrete communities. These communities consist of groups of molecules that have notable similarities in structure or functional traits, which could be associated with their functions in biological systems or their stability and reactivity. The utilization of Leading Eigenvector and Leiden methods improves the identification of these communities by employing spectrum features and optimizing modularity, respectively.

The Leading Eigenvector technique detects communities by dividing the network using the eigenvectors of the modularity matrix, hence emphasizing divisions where there are more connections within groups compared to between groups. This approach is especially efficient for networks that are both big and sparse, as it offers a precise understanding of the hierarchical organization inside the molecular network. The Leiden algorithm is an enhanced version of the Louvain method that enhances the process of identifying communities by iteratively optimizing modularity and maintaining the stability and clarity of the detected communities. It overcomes the shortcomings of prior technologies by enabling more precise and higher-resolution identification of smaller communities. This is crucial for comprehending the intricate connections between molecules.

The results, including the total number of communities and the number of nodes (inhibitors) in each community as community size, are presented in Table 1.

In Table 1, for the Euclidean distance-based network, both methods identified the same number of communities (6). The sizes of the communities are relatively similar, but the Leiden method resulted in a more balanced distribution, with a larger third community and slightly smaller other communities compared to the Leading Eigenvector method. For the Tanimoto distance-based network, the Leiden method detected more communities (nine compared to seven). This method resulted in smaller and more granular communities, indicating that it might be capturing more subtle structural differences between the molecules compared to the Leading Eigenvector method. For the Hamming distance-based network, the Leiden method identified twice as many communities (12 compared to 6). The community sizes were more evenly distributed, suggesting that the Leiden method is more effective at identifying smaller, distinct groups within the network, whereas the Leading Eigenvector method tends to form larger communities. Different than the others, for the Levenshtein distance-based network, the Leading Eigenvector method identified only one community, suggesting it was unable to differentiate between the molecules based on Levenshtein distance. In contrast, the Leiden method detected a highly granular structure with 23 communities, indicating it can capture a more nuanced differentiation based on SMILES code similarity.

In summary, the Leiden method consistently identified more communities across all distance metrics, suggesting it is more sensitive to subtle structural differences and can provide a more detailed partitioning of the network. The Leading Eigenvector method tends to form fewer, larger communities, indicating a preference for broader structural similarities over finer distinctions. Both methods show the ability to identify meaningful clusters, but the choice of method can significantly impact the granularity and interpretation of the community structure within the molecular networks. The Leiden method, with its higher sensitivity, might be more suitable for detailed exploratory analysis, while the Leading Eigenvector method could be more appropriate for identifying broader structural trends.

The following figures display nodes that are color-coded to represent distinct communities within the resulting networks, providing a clear and intuitive visualization of the complex molecular relationships. Each color corresponds to a specific community, with nodes of the same color indicating a closely connected set of molecules that share significant structural similarities. This color-coding not only highlights the modular structure of the molecule networks, but also facilitates the identification of key clusters at a glance. The visual representations emphasize the inherent modularity of the molecular networks, where distinct hues delineate the boundaries of various communities. This modular structure is crucial for understanding how molecules group together based on their structural characteristics. Each community, represented by a unique color, consists of molecules that are more densely interconnected with each other than with molecules outside the community. This indicates that molecules within the same community are likely to have similar chemical properties, biological activities, or functional roles.

**Figure 6:**
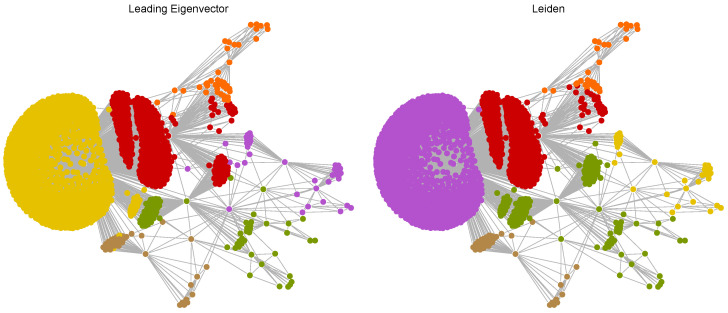
Communities of the filtered network based on Euclidean distance between BACE-1 inhibitors. Nodes of varying colors represent nodes that belong to distinct communities within the resulting network.

Figure 6 showcases the community detection results using the Leading Eigenvector and Leiden methods applied to the Euclidean distance-based network of inhibitors. The yellow community is the largest and most densely packed community, indicating a significant number of molecules with very similar structural characteristics. This community forms the core of the network, signifying a central, highly interconnected group of molecules. Red and orange communities are adjacent to the yellow core. They represent molecules with structural features that are similar to but slightly different from those in the yellow community. The proximity and size of these communities suggest strong intra-community connectivity and moderate inter-community interactions. Green and brown communities are smaller, more peripheral communities. Their position and size suggest molecules with distinct structural features, differentiating them from the larger central communities. The purple community is relatively small and scattered, indicating molecules that are structurally distinct from the core and other larger communities. In the Leiden method, the network is also divided into six communities, but the distribution and composition differ slightly from the Leading Eigenvector method. The purple community, analogous to the yellow community in the Leading Eigenvector method, is large and densely packed. It forms the core of the network, comprising molecules with highly similar structures. Similar to their counterparts in the Leading Eigenvector method, red and orange communities are large and situated near the core. They represent molecules with structural similarities that differentiate them from the core community. Green and brown communities are smaller and more dispersed compared to the Leading Eigenvector results. The Leiden method captures more subtle structural differences, resulting in a more granular distribution of molecules. The yellow community is relatively small and more scattered than in the Leading Eigenvector method, indicating molecules with unique structural features that distinguish them from the rest.

Both methods identify six communities, but the sizes and compositions vary. The Leading Eigenvector method tends to form larger, more cohesive communities, while the Leiden method results in a more granular partitioning, capturing finer structural distinctions. In both methods, a large core community is present (yellow in Leading Eigenvector and purple in Leiden), indicating a central group of structurally similar molecules. The peripheral communities are more varied in size and distribution, with the Leiden method identifying smaller, more distinct peripheral communities. The Leiden method appears to be more sensitive to subtle structural differences, resulting in a more detailed and diverse community structure. This is evidenced by the more dispersed and numerous smaller communities compared to the Leading Eigenvector method.

**Figure 7:**
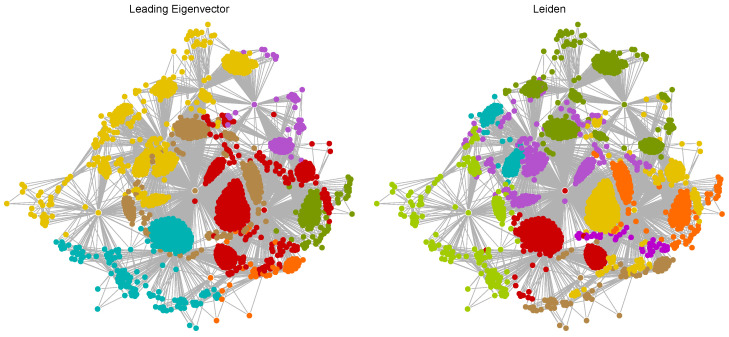
Communities of the filtered network based on Tanimoto distance between BACE-1 inhibitors. Nodes of varying colors represent nodes that belong to distinct communities within the resulting network.

Figure 7 shows the communities detected within the TMFG of the Tanimoto distance-based network using the Leading Eigenvector and Leiden methods. The Leading Eigenvector method identified fewer, larger communities. Notable communities include a large yellow community and substantial red and teal communities. These large communities indicate groups of molecules with similar structural characteristics that are well-connected within each group. The yellow community appears to be centrally located, suggesting it forms the core of the network. The red and teal communities are also prominent, indicating significant intra-community connectivity. Smaller communities like the orange, brown, and purple ones are scattered around the periphery, indicating more specialized or distinct molecular features. The network displays clear modularity with large, well-defined communities. This suggests that the Leading Eigenvector method captures broad structural similarities effectively but may overlook finer distinctions between molecules. The Leiden method revealed a larger number of smaller, more evenly distributed communities compared to the Leading Eigenvector method. Notable communities include several medium-sized ones in orange, green, purple, and red, with a mix of small communities dispersed throughout the network. Unlike the Leading Eigenvector method, the Leiden method does not exhibit a single dominant core community. Instead, it shows a more balanced distribution of communities, indicating a more nuanced detection of structural variations across the network. The Leiden method’s ability to detect smaller communities reflects its sensitivity to subtle structural differences. This granularity provides a more detailed and complex view of the molecular network, capturing a wider range of interactions and similarities.

The Leading Eigenvector method tends to form fewer but larger communities, highlighting broad structural similarities among molecules. In contrast, the Leiden method identifies more communities, which are smaller and more nuanced, indicating its higher sensitivity to minor structural differences. The Leading Eigenvector method’s communities are more centralized, with a clear core–periphery distinction. The Leiden method results in a more dispersed community structure, suggesting a more intricate and interconnected network without a single dominant core. The Leiden method’s higher sensitivity makes it more suitable for identifying subtle structural distinctions, providing a richer and more detailed partitioning of the molecular network. The Leading Eigenvector method, while effective in capturing major structural trends, may miss these finer details. The large, cohesive communities identified by the Leading Eigenvector method suggest that certain groups of molecules share very similar structural features, which are crucial for their biological function. The more numerous and smaller communities detected by the Leiden method indicate a broader diversity in molecular structures, highlighting the method’s ability to capture detailed variations. The detailed community structure provided by the Leiden method can offer deeper insights into the functional roles of different molecular groups. By identifying smaller, more specific communities, researchers can explore how subtle structural differences impact biological activity and efficacy. For drug discovery, the Leiden method’s detailed community detection can help in identifying unique molecular features that might be overlooked by broader classifications. This granularity can aid in the design of more targeted and effective therapeutic agents.

**Figure 8:**
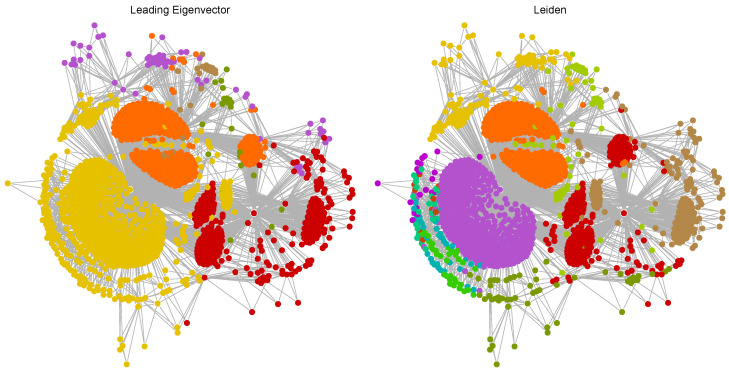
Communities of the filtered network based on Hamming distance between BACE-1 inhibitors. Nodes of varying colors represent nodes that belong to distinct communities within the resulting network.

Figure 8 showcases the community detection results using the Leading Eigenvector and Leiden methods applied to the Hamming distance-based network of BACE-1 inhibitors. The Leading Eigenvector method identified six distinct communities, each represented by a different color. Notable communities include a large yellow community and substantial orange and red communities. These large communities indicate groups of molecules with similar structural characteristics that are well-connected within each group. The yellow and orange communities form the core of the network, suggesting they represent the most structurally similar molecules. The red community is also significant and centrally located. Smaller communities such as the purple and brown ones are found at the periphery, indicating more specialized or distinct molecular features. The network displays clear modularity with large, well-defined communities. This suggests that the Leading Eigenvector method captures broad structural similarities effectively but may overlook finer distinctions between molecules. The Leiden method revealed a larger number of smaller, more evenly distributed communities compared to the Leading Eigenvector method. Notable communities include several medium-sized ones in orange, green, and purple, with a mix of small communities dispersed throughout the network. Unlike the Leading Eigenvector method, the Leiden method does not exhibit a single dominant core community. Instead, it shows a more balanced distribution of communities, indicating a more nuanced detection of structural variations across the network. The Leiden method’s ability to detect smaller communities reflects its sensitivity to subtle structural differences. This granularity provides a more detailed and complex view of the molecular network, capturing a wider range of interactions and similarities.

**Figure 9:**
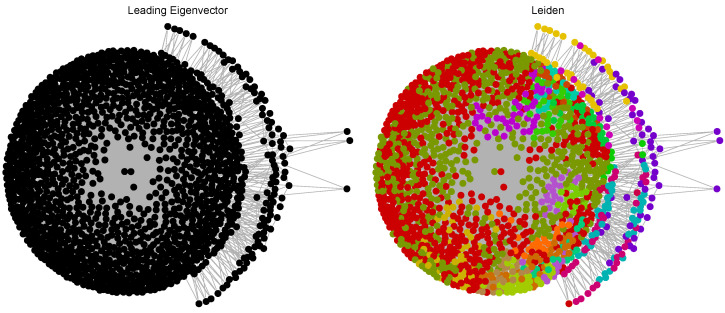
Communities of the filtered network based on Levenshtein distance between BACE-1 inhibitors. Nodes of varying colors represent nodes that belong to distinct communities within the resulting network.

Figure 9 depicts the communities detected within the TMFG of the Levenshtein distance-based network using the Leading Eigenvector and Leiden methods. Each color in the Leiden method graph represents a distinct community, allowing us interpretation of the structural and topological distributions of the communities. The Leading Eigenvector method resulted in a single large community, as indicated by the uniform black color. This suggests that the method was unable to differentiate the molecules into distinct communities based on Levenshtein distances of their SMILES codes. The network appears as a densely packed core with a few peripheral nodes, indicating that the majority of molecules are considered similar enough to belong to a single cluster. There is a lack of modularity in this representation, as all molecules are grouped into a single community. This suggests that the Leading Eigenvector method may not be sensitive enough to detect subtle differences in molecular structures when using Levenshtein distances. The Leiden method identified multiple distinct communities, each represented by a different color. This method revealed a highly modular network with numerous small- and medium-sized communities. The network shows a densely packed core with several smaller communities radiating outward. This indicates a more nuanced detection of structural differences among the molecules. The Leiden method’s granularity captures subtle structural variations, resulting in a more complex and detailed community structure. This method is evidently more sensitive to the differences in SMILES codes, providing a clearer picture of molecular diversity.

The Leading Eigenvector method grouped all molecules into a single large community, while the Leiden method identified numerous communities. This stark contrast highlights the Leiden method’s superior sensitivity to structural differences. The Leading Eigenvector method showed a single, dense core, whereas the Leiden method displayed a more balanced distribution with a clear distinction between the core and peripheral communities. The Leiden method’s ability to detect multiple communities suggests it is better suited for identifying fine-grained structural distinctions in the dataset. The Leading Eigenvector method’s single community indicates it may be too coarse for this level of analysis. The Leiden method’s detailed partitioning suggests a high degree of structural diversity within the BACE-1 dataset. Each color-coded community likely represents molecules with distinct structural motifs or functional groups. The identification of numerous small communities by the Leiden method can provide insights into the specific structural features that influence biological activity and efficacy. This can guide targeted therapeutic interventions. The Leiden method’s ability to identify intricate community structure can assist in identifying distinct molecular characteristics that may be disregarded by more general classifications. This level of detail can aid in the development of more accurate and efficient medicinal drugs.

The network community comparison metrics, namely VOI, NMI, SJD, RI, and ARI, are shown in Table 2.

## 3. Discussions

### 3.1. Discussions on Resulting Distance Based Networks

The synthesis of results from various TMFG network analyses based on Euclidean, Tanimoto, Hamming, and Levenshtein distances reveals a comprehensive view of the BACE-1 inhibitor landscape and their intricate network of relationships.

The Euclidean distance-based network illustrates a complex web of BACE-1 inhibitors, demonstrating how diverse molecular descriptors can group these molecules into distinct, identifiable clusters. This method emphasizes the shared physicochemical properties of the inhibitors, suggesting potential similarities in their reactivity and inhibitory mechanisms. High edge betweenness within the network indicates critical pathways among these clusters, highlighting molecules that could serve as key intermediates in the inhibition process. Notably, inhibitors with high constraint scores appear to be central within the network, potentially acting as crucial control points in BACE-1 inhibition. The distribution of eigenvector centrality further enhances our understanding by identifying inhibitors with high connectivity, which may play significant roles in biochemical interactions or synthetic pathways.

When examined using Tanimoto distance, the network reveals dense clustering, reflecting strong structural similarities among the BACE-1 inhibitors. This metric highlights subtle molecular differences that contribute to the functionality of the inhibitors. The network’s robust overall structure, coupled with the presence of bottleneck inhibitors with high constraint scores, indicates points of potential fragility or regulatory importance. The eigenvector centrality in this context refines our understanding of inhibitor significance, marking certain molecules as influential and potentially critical for the biochemical inhibition of BACE-1.

The Hamming distance-based analysis, which focuses on molecular fingerprints, uncovers how minor variations can lead to significant differences in the structural composition of the network. Edges with high betweenness centrality are particularly noteworthy, as they may represent vital functional transformations within the diverse set of BACE-1 inhibitors. Inhibitors with high constraint scores are highlighted as significant for network stability and function. The centrality distribution once again pinpoints key inhibitors that could be central to biochemical pathways or targets for further synthetic exploration.

The Levenshtein distance analysis provides a unique perspective by focusing on SMILES string edits to discern relationships among BACE-1 inhibitors. This analysis emphasizes critical connections corresponding to significant chemical modifications. The findings reaffirm the presence of central inhibitors, as identified by eigenvector centrality, which likely have important roles in biochemical transformations. The analysis highlights key inhibitors that stand out due to their structural importance and potential biological activity.

Across all metrics, a consistent theme of interconnectedness and the pivotal roles played by certain BACE-1 inhibitors emerges. Both strong and subtle molecular features significantly define the functionality within the network. Each metric offers a distinct viewpoint—whether based on physicochemical properties, structural resemblance, or molecular edits—collectively contributing to a deeper understanding of the BACE-1 inhibitor landscape. These network analyses highlight the chemical diversity among the inhibitors and underscore their potential biological significance, which is crucial for advancements in drug discovery, therapeutic interventions, and the development of new BACE-1 inhibitors. The analyses not only emphasize the structural diversity but also point to their critical roles in biochemical inhibition, providing invaluable insights for biological investigations and the synthesis of novel therapeutic compounds targeting BACE-1.

Eigenvector centrality is a metric that not only accounts for the number of connections a BACE-1 inhibitor has, but also considers the significance of those connections. A BACE-1 inhibitor with high eigenvector centrality is not merely well-connected; it is typically linked to other well-connected inhibitors, amplifying its importance in the network. These central inhibitors may act as hubs within biochemical pathways, suggesting they are involved in multiple inhibitory mechanisms or serve as intermediary compounds bridging critical biochemical transformations.

Their central position in the network implies they could influence the behavior of many other inhibitors in their vicinity, potentially affecting the stability and dynamics of the entire system. In biochemical contexts, such as in the inhibition of BACE-1, these inhibitors could be substrates or products of key enzymatic reactions, playing crucial roles in the overall inhibitory process. Understanding these central inhibitors can provide insights into regulatory mechanisms or reveal potential targets for therapeutic interventions, particularly in conditions related to Alzheimer’s disease.

From a chemical synthesis perspective, inhibitors with high centrality may serve as valuable starting points for the synthesis of complex BACE-1 inhibitory molecules. Their connectivity implies they can be easily transformed into a variety of structurally related compounds, making them prime candidates for the development of new chemical entities or the exploration of novel synthetic pathways. This makes them ideal for further investigation in drug development efforts. In Table 3, Table 4, Table 5 and Table 6, we present nodes (inhibitors) in networks that have higher eigenvector centrality values.

Table 3 highlights the top three BACE-1 inhibitors with the highest eigenvector centrality in the Euclidean distance-based network. The inhibitor with formula C33H47F2N5O4+2 and an eigenvector centrality with 1.0 exhibits the highest eigenvector centrality, indicating its pivotal role within the network. The high centrality suggests that the inhibitor is not only well-connected, but also linked to other well-connected inhibitors, amplifying its importance. The high MMFF energy and significant TPSA suggest substantial interaction potential with the BACE-1 enzyme, likely contributing to its inhibitory efficacy. Its moderate cLogP value indicates balanced lipophilicity, essential for bioavailability. The 3D structure shows a complex spatial configuration, which may enhance its binding affinity through multiple interaction points. The second inhibitor with formula C33H47F2N5O4+2 is identical in formula to the first one, but with slightly different structural configurations. It holds significant network centrality. The slight differences in MMFF energy and eccentricity suggest minor conformational variations that impact its network connectivity. Its structural similarity to the top inhibitor indicates it shares many functional characteristics, making it a critical molecule for understanding BACE-1 inhibition mechanisms. The identical synthetic accessibility and TPSA further reinforce its potential role in biochemical pathways. The inhibitor with formula C41H50F5N4O5+, although lower in centrality compared to the top two, still plays a significant role in the network. Its larger molecular mass and higher cLogP suggest increased lipophilicity, which might affect its bioavailability and binding characteristics. The high MMFF energy and distinct Kappa indices indicate a complex structure, potentially offering multiple interaction sites with the BACE-1 enzyme. Its substantial MR and TPSA values highlight its potential for effective enzyme interaction. The lower centrality may suggest it interacts with a different subset of inhibitors or has a more specialized role within the network.

Table 4 displays the three most potent BACE-1 inhibitors that have the highest eigenvector centrality in the Tanimoto distance-based network. The inhibitor with formula C8H12NO+ exhibits the highest eigenvector centrality, indicating its pivotal role within the network. Its high centrality suggests that it is not only well-connected, but also linked to other well-connected inhibitors, amplifying its importance. The low MMFF energy and moderate TPSA suggest a molecule that is likely highly interactive with the BACE-1 enzyme, possibly contributing to its high inhibitory efficacy. The 3D structure displays a simple yet effective configuration that might enhance its binding affinity through precise interactions. The inhibitor with formula C7H11N3O, while slightly different in structure from the first, also holds significant network centrality. The moderate MMFF energy and TPSA indicate its strong potential for interaction with the BACE-1 enzyme. Its structural simplicity, coupled with a slightly higher cLogP, suggests good solubility and bioavailability. The slightly lower centrality compared to the top inhibitor indicates that while it is crucial, it may not be as influential in the network as the top inhibitor. The inhibitor with formula C66H87N13O18-2 is vastly different in terms of molecular complexity and size. Its large molecular mass and high TPSA suggest significant interaction potential with the BACE-1 enzyme, albeit likely with a different mode of binding compared to smaller inhibitors. The high MMFF energy indicates a more complex structure with numerous potential interaction points. Its negative cLogP suggests challenges in solubility, but its structural complexity might offer unique inhibitory mechanisms.

Table 5 highlights the top three BACE-1 inhibitors with the highest eigenvector centrality in the Hamming distance-based network. The inhibitor with formula C9H8N2 and an eigenvector centrality with 1.0 has the highest eigenvector centrality, highlighting its pivotal role within the network. Its high centrality suggests that it is not only well-connected, but also linked to other well-connected inhibitors, amplifying its importance. The relatively low MMFF energy and moderate TPSA indicate strong interaction potential with the BACE-1 enzyme, likely contributing to its high inhibitory efficacy. The 3D structure displays a compact configuration that might enhance its binding affinity through precise interactions. The inhibitor with formula C9H8N2 with the second highest eigenvector centrality, while slightly different in structure from the first, also holds significant network centrality. The negative MMFF energy suggests a very stable structure. The slight differences in eccentricity and entropy indicate minor conformational variations that impact its network connectivity. Its high centrality indicates it shares many functional characteristics with the top inhibitor, making it a critical molecule for understanding BACE-1 inhibition mechanisms. The inhibitor with formula C7H6ClN3, although with a lower centrality compared to the top two, still plays a significant role in the network. Its negative MMFF energy indicates a stable structure with potential strong interactions. The presence of chlorine adds to its uniqueness in the network. Its relatively high TPSA suggests strong interaction potential with the BACE-1 enzyme. The lower centrality may suggest it interacts with a different subset of inhibitors or has a more specialized role within the network.

Table 6 showcases the three most prominent BACE-1 inhibitors that possess the greatest eigenvector centrality within the Levenshtein distance-based network. The inhibitor with formula C66H87N13O18-2 has the highest eigenvector centrality, indicating its critical role within the network. Its high centrality suggests extensive connectivity with other well-connected inhibitors, amplifying its importance. The substantial molecular mass and high TPSA indicate a large, complex molecule with significant interaction potential with the BACE-1 enzyme. The negative cLogP points to challenges in solubility but could imply a specialized binding mechanism. The high MMFF energy and diverse interaction sites suggest it could play a pivotal role in the inhibition process. The inhibitor with formula C53H91N7O11S also shows high eigenvector centrality, reflecting its significant role in the network. Its molecular descriptors suggest a large, structurally complex molecule. The high MMFF energy and TPSA indicate potential for strong and diverse interactions with the BACE-1 enzyme. The relatively high cLogP suggests good lipophilicity, which may enhance bioavailability. Its significant connectivity within the network highlights its importance in understanding BACE-1 inhibition mechanisms. Despite having a lower centrality compared to the top inhibitors, the inhibitor with formula C7H6ClN3 still plays a noteworthy role within the network. Its negative MMFF energy points to a highly stable structure. The moderate cLogP and TPSA values suggest good solubility and interaction potential with the BACE-1 enzyme. Its relatively high synthetic accessibility score implies ease of synthesis, making it an attractive candidate for further exploration.

### 3.2. Discussions on Clusters

The metrics for network community clustering are useful for an in-depth analysis of clustering behavior by taking into account the topology of the filtered networks that are underlying the network. Table 2 presents various community comparison metrics for the network of BACE-1 ingihitors obtained using several distances, with communities identified through specific methods as previously mentioned. The metrics include Variation of Information (VOI), Normalized Mutual Information (NMI), Split Joint Distance (SJD), Rand Index (RI), and Adjusted Rand Index (ARI). Each metric provides a different perspective on the similarity and quality of the community structures identified within the network.

For the Euclidean distance network, the VOI is 0.266662, which is relatively low, indicating that the clustering information loss is minimal. The NMI is high at 0.881824, suggesting a strong agreement between the clustering results and the reference classification. The SJD is 130, showing the differences in cluster assignments. The RI is very high at 0.964622, implying a strong similarity between the clustering results and the reference classification. The ARI is also high at 0.927738, indicating a strong adjustment for chance, reinforcing the high level of agreement between the clustering results and the reference classification.

In the Tanimoto distance network, the VOI is significantly higher at 1.95333, indicating more information loss compared to the Euclidean network. The NMI is lower at 0.499584, suggesting a moderate agreement between the clustering results and the reference classification. The SJD is 1321, much higher than that of the Euclidean network, indicating greater differences in cluster assignments. The RI is lower at 0.802102, showing a weaker similarity between the clustering results and the reference classification. The ARI is also lower at 0.280018, indicating a weaker adjustment for chance.

For the Hamming distance network, the VOI is 1.38697, indicating moderate information loss. The NMI is 0.571856, showing a moderate agreement between the clustering results and the reference classification. The SJD is 651, which is higher than the Euclidean network but lower than the Tanimoto network, indicating moderate differences in cluster assignments. The RI is 0.807944, which is slightly higher than the Tanimoto network, indicating a better similarity. The ARI is 0.521605, indicating a moderate adjustment for chance.

In the Levenshtein distance network, the VOI is the highest at 2.15364, indicating the greatest information loss among the four networks. The NMI is zero, suggesting no agreement between the clustering results and the reference classification. The SJD is 1009, which is lower than the Tanimoto network but higher than the Euclidean and Hamming networks, indicating substantial differences in cluster assignments. The RI is the lowest at 0.217662, showing poor similarity between the clustering results and the reference classification. The ARI is zero, indicating no adjustment for chance, suggesting a significant discrepancy between the clustering results and the reference classification.

The Euclidean distance network shows the best performance across all metrics, indicating strong agreement with the reference classification and minimal information loss. The Tanimoto and Hamming distance networks show moderate performance, with higher information loss and lower agreement metrics compared to the Euclidean network. The Levenshtein distance network performs the worst, with the highest information loss and lowest agreement metrics, indicating significant discrepancies in clustering results. These metrics collectively highlight the effectiveness of different distance measures in clustering BACE-1 molecules, with Euclidean distance providing the most reliable clustering results.

Non-parametric statistics are employed in the analysis of inhibitor clusters that arise in filtered networks created using various distance functions. The numerical descriptor values of each inhibitor are determined using the same descriptor values calculated for the Euclidean distance. This study employs the Local Equivalence Test with Kruskal–Wallis, Variance Equivalence Test with Conover, and Log Rank Test with Gehan. These methods are advanced and specifically developed to assess different parts of the data. These tests can provide profound insights into the distribution, variance, and survival functions within the communities of BACE-1 inhibitors, allowing for a comprehensive understanding of their features and behaviors.

The Kruskal–Wallis test is a non-parametric statistical technique employed to ascertain whether there are statistically significant distinctions among two or more groups of an independent variable on a continuous or ordinal dependent variable. The test is an extension of the Mann–Whitney U test that may be applied to more than two groups. It does not make any assumptions about the normal distribution of the data. Within the framework of local Equivalence Testing, this methodology enables the evaluation of whether distinct inhibitor communities exhibit comparable distributions for a specific molecular attribute, without making any assumptions about the normality of these distributions. It is especially crucial for inhibitor data that may not satisfy the normalcy condition, as it offers a reliable approach to compare medians among the inhibitor communities.

The Conover test is a non-parametric approach used to compare variances among two or more independent groups. It is an extension of Levene’s test, which checks for homogeneity of variances, but does not require the samples to have a normal distribution. The Conover test can be used to assess the “Variance Equivalence” among distinct inhibitor communities, allowing determination if there is consistent variability in molecular parameters such as cLogP, MR, or TPSA among groups. It is important to determine whether the differences observed between communities are a result of actual differences in inhibitor properties or simply due to variations within the data. This ensures that any differences in central tendencies (such as means or medians) are meaningful and not affected by inconsistent variances.

The Gehan test, which falls under the broader category of Log Rank Tests, is largely utilized in survival analysis to compare the survival distributions of many groups. This analysis could yield valuable information about the duration of specific molecular attributes within and between communities, determining the rate at which features evolve or cease to match predefined criteria within the inhibitor groupings. This approach is particularly relevant when analyzing the stability or reactivity patterns of inhibitors, which are impacted by their molecular descriptors.

The non-parametric test findings for different communities are shown in Table 7, Table 8, Table 9 and Table 10.

Table 7 shows the results of the Kruskal–Wallis, Conover, and Gehan non-parametric tests for various molecular descriptors in the Euclidean distance-based network, considering different clustering methods: Leading Eigenvector and Leiden. The Kruskal–Wallis test evaluates whether there are statistically significant differences between the communities for each molecular descriptor. For both Leading Eigenvector and Leiden methods, molecular mass shows highly significant differences across communities, indicating that molecular mass is a key differentiator in the clustering of BACE-1 molecules. MMFF energy also shows significant differences in both methods, suggesting that energy profiles vary significantly across communities. The cLogP descriptor is significant in both methods, indicating that lipophilicity is an important factor in clustering. MR is highly significant in both methods, highlighting its importance in defining communities. Eccentricity is significant in the Leading Eigenvector method and marginally significant in the Leiden method, suggesting that structural compactness is more pronounced as a differentiator in the former method. All Kappa indices show high significance in both methods, indicating that molecular shape and branching are critical in clustering. The Synthetic Accessibility Score is significant in the Leading Eigenvector method but not in the Leiden method, implying its role is more pronounced in the former. TPSA shows high significance in both methods, indicating that polar surface area is crucial for community differentiation. The 2D AC Entropy is significant in the Leading Eigenvector method but not in the Leiden method, suggesting it is a better differentiator in the former method. The 3D AC Entropy is not significant in either method, indicating that 3D autocorrelation entropy does not play a major role in differentiating the communities.

The Conover test provides pairwise comparisons following the Kruskal–Wallis test, highlighting where significant differences lie between specific groups. For molecular mass, the test shows extreme significance in the Leading Eigenvector method and high significance in the Leiden method. MMFF energy remains very significant in both methods, confirming energy differences between communities. The cLogP descriptor is not significant in the Leading Eigenvector method but is significant in the Leiden method, showing that lipophilicity differences are better captured by the Leiden method. MR is highly significant in both methods, reaffirming its importance. Eccentricity is significant in both methods. All Kappa indices are highly significant in both methods, reinforcing their importance in clustering. The Synthetic Accessibility Score is significant in the Leading Eigenvector method but less so in the Leiden method. TPSA is highly significant in both methods, indicating its role in community differentiation. The 2D AC Entropy is significant in the Leading Eigenvector method but not in the Leiden method. The 3D AC Entropy is not significant in either method.

The Gehan test, like the Conover test, evaluates differences across groups but focuses on survival analysis contexts, offering robustness for non-parametric data. For molecular mass, the test shows high significance in both methods. MMFF energy is very significant in both methods. The cLogP descriptor is significant in both methods, especially in the Leiden method. MR is highly significant in both methods. Eccentricity is significant in both methods. All Kappa indices are highly significant, indicating their consistent importance. The Synthetic Accessibility Score is significant in the Leading Eigenvector method but not in the Leiden method. TPSA is highly significant in both methods. The 2D AC Entropy is significant in the Leading Eigenvector method but not in the Leiden method. The 3D AC Entropy is not significant in either method.

Non-parametric tests reveal consistent patterns of significance for molecular mass, MMFF energy, MR, Kappa indices, and TPSA across different clustering methods. These descriptors are critical for defining the community structure in the Euclidean distance-based network of BACE-1 inhibitors. While some descriptors like cLogP and 2D AC entropy show varying levels of significance between the Leading Eigenvector and Leiden methods, overall, the tests underscore the multidimensional nature of molecular clustering and the importance of using multiple descriptors to capture the complexity of BACE-1 inhibitor interactions.

For several molecular descriptors in the Tanimoto distance-based network, the results of the Kruskal–Wallis, Conover, and Gehan non-parametric tests are presented in Table 8. These tests take into account two distinct clustering methods: Leading Eigenvector and Leiden.

The Kruskal–Wallis test determines whether there are substantial statistical variations among the communities for each molecular description. Both the Leading Eigenvector and Leiden techniques demonstrate notable variations in molecular mass among communities, suggesting that molecular mass plays a crucial role in the clustering of BACE-1 molecules. The MMFF energy exhibits notable disparities in both approaches, indicating substantial variations in energy profiles among different populations. The cLogP descriptor is crucial in both techniques, suggesting that lipophilicity plays a vital role in clustering. MR is extremely important in both methodologies, emphasizing its significance in determining communities. The presence of eccentricity is important in both techniques, indicating that structural compactness plays a crucial role in distinguishing between them. The Kappa indices have strong statistical significance in both techniques, suggesting that molecule structure and branching play a crucial role in clustering. The Synthetic Accessibility Score has a large part in the Leading Eigenvector technique, but it does not have the same impact in the Leiden method. This suggests that its influence is more prominent in the former method. The TPSA exhibits a strong level of relevance in both techniques, suggesting that polar surface area plays a critical role in distinguishing across communities. Both the 2D AC Entropy and 3D AC Entropy do not have a substantial impact in either technique, suggesting that these entropies do not play a prominent role in distinguishing the communities.

The Conover test offers pairwise comparisons subsequent to the Kruskal–Wallis test, identifying significant differences between certain groups. The test demonstrates significant importance in both the Leading Eigenvector and Leiden techniques for determining molecular mass. The MMFF energy remains highly significant in both techniques, indicating the presence of energy disparities within communities. The cLogP descriptor plays a crucial role in both techniques, effectively capturing changes in lipophilicity in both clustering algorithms. The significance of MR is strongly pronounced in both approaches, therefore reinforcing its importance. Eccentricity has a crucial role in both strategies. All Kappa indices exhibit a high level of statistical significance in both techniques, hence reaffirming their crucial role in clustering. The Synthetic Accessibility Score is a relevant factor in the Leading Eigenvector approach, but it does not play a big role in the Leiden method. The TPSA is of great importance in both techniques, clearly illustrating its function in the differentiation of communities. Both the 2D AC Entropy and 3D AC Entropy do not hold much significance in either of the methods.

The Gehan test, similar to the Conover test, assesses disparities between groups, with a specific emphasis on survival analysis scenarios. It provides resilience for non-parametric data. Both approaches yield highly significant results for molecular mass. The MMFF energy plays a crucial role in both procedures. The cLogP descriptor holds particular importance in both techniques, particularly in the Leiden method. Molar refractivity (MR) is of great importance in both approaches. Eccentricity has a crucial role in both strategies. All Kappa indices have a high level of statistical significance, showing their consistent and considerable importance. The Synthetic Accessibility Score is a relevant factor in the Leading Eigenvector approach, but it does not play a big role in the Leiden method. The significance of TPSA is substantial in both techniques. Both the 2D AC Entropy and 3D AC Entropy do not hold much significance in either of the methods.

Non-parametric tests consistently demonstrate significant patterns for molecular mass, MMFF energy, MR, Kappa indices, and TPSA across various clustering approaches. These descriptors play a crucial role in determining the community structure in the network of BACE-1 inhibitors based on the Tanimoto distance. Although the cLogP descriptor may have different levels of significance when comparing the Leading Eigenvector and Leiden methods, the tests overall emphasize the multidimensional nature of molecular clustering. They also highlight the importance of using multiple descriptors to accurately capture the complexity of BACE-1 inhibitor interactions.

Table 9 shows the results of the Kruskal–Wallis, Conover, and Gehan non-parametric tests for various molecular descriptors in the Hamming distance-based network, considering different clustering methods. The Kruskal–Wallis test evaluates whether there are statistically significant differences between the communities for each molecular descriptor. Because there are no statistically significant changes in molecular mass when using the Leading Eigenvector approach (*p* = 1), we can conclude that molecular mass is not an important factor in the clustering of BACE-1 molecules. The MMFF energy may have an effect on clustering since it is marginally significant (*p*-value = 0.086). With a *p*-value of 0.00015, the cLogP descriptor is statistically significant, suggesting that lipophilicity plays a major role in clustering. With a *p*-value of only 0.1273, MR does not indicate significance. The fact that structural compactness is a crucial differentiator is supported by substantial eccentricity (*p*-value = 0.00009). A lack of statistical significance in the Kappa indices suggests that the importance of molecular branching and shape in this grouping is low. With a *p*-value of only 0.0146, the Synthetic Accessibility Score is statistically significant. Polar surface area is critical for community difference, as shown by TPSA’s significance (*p*-value = 0.00016). Both the 2D and 3D AC entropies do not amount to anything.

There is no statistically significant relationship between molecular mass and the Leiden technique. The energy from MMFFs is not statistically significant (*p* = 0.2056). Clustering relies on lipophilicity, since the cLogP descriptor is very significant (*p*-value = 0.000183). An important finding is the MR, with a *p*-value of 0.003318. A person’s eccentricity is a major factor. Clustering relies on the Kappa indices, which are noteworthy because of their significance. No meaningful results were found for the Synthetic Accessibility Score. Significant results are shown by TPSA. Both the 2D and 3D AC entropies do not amount to anything.

When used in conjunction with the Kruskal–Wallis test, the Conover test allows for pairwise comparisons, drawing attention to the areas where certain groups differ significantly. There are no statistically significant changes in molecular mass when using the Leading Eigenvector technique (*p* = 1). At the 0.0002 level, MMFF energy is statistically significant. Of paramount importance is the cLogP descriptor. Statistically, MR is noteworthy (*p*-value = 0.000389). There is a large amount of eccentricity ($7.6\times 10^{-8}$). Only $\kappa_{2}$ is statistically significant (*p*-value = 0.001309) among the Kappa indices. With a *p*-value of 0.00064, the Synthetic Accessibility Score is statistically significant. With a *p*-value of 0.00009, TPSA is statistically significant. Both the 2D and 3D AC entropies do not amount to anything.

Molecular mass does not have a substantial impact on the Leiden technique (*p*-value = 0.601). At the 0.0043 level, MMFF energy is statistically significant. Of paramount importance is the cLogP descriptor. MR holds great importance. A person’s eccentricity is a major factor. Significant Kappa indices were found. With a *p*-value of $2.3\times 10^{-11}$, the Synthetic Accessibility Score is statistically significant. The results show that TPSA is quite significant (*p*-value = $4.1\times 10^{-8}$). Both the 2D and 3D AC entropies do not amount to anything.

While both the Conover and Gehan tests assess group differences, the former is more applicable to survival analysis and the latter provides more robustness for non-parametric data. Differences in molecular mass are statistically significant (*p* = 0.0002) when using the Leading Eigenvector technique. With a *p*-value of 0.0001, the cLogP descriptor is statistically significant, but the MMFF energy is not. A *p*-value of 0.1225 indicates that MR is not statistically significant. There is a great deal of eccentricity (*p*-value = 0.00003). Significant Kappa indices do not exist. An important finding is the Synthetic Accessibility Score (*p*-value = 0.011). The TPSA statistic is quite noteworthy (*p*-value = 0.00088). Both the 2D and 3D AC entropies do not amount to anything.

With a *p*-value of only 0.3277, molecular mass does not prove anything when using the Leiden technique. The energy of MMFF does not have a significant impact (*p*-value = 0.2377). With a *p*-value of 0.00013, the cLogP descriptor is statistically significant. A *p*-value of 0.00282 indicates that MR is statistically significant. A person’s eccentricity is a major factor. There is a range of significance levels shown by the Kappa indices. No meaningful results were found for the Synthetic Accessibility Score. The results show that TPSA is quite significant (*p*-value = $3.2\times 10^{-6}$). Both the 2D and 3D AC entropies do not amount to anything.

MMFF energy, MR, cLogP, and TPSA all show consistent patterns of significance across different clustering approaches, according to the non-parametric tests. The community structure in the BACE-1 inhibitor Hamming distance-based network cannot be defined without these descriptions. The tests highlight the multidimensionality of molecular clustering and the significance of employing various descriptors to capture the complexity of BACE-1 inhibitor interactions. However, there are descriptors that exhibit differing degrees of significance between the Leading Eigenvector and Leiden methods.

Non-parametric tests are not available for the Leading eigenvector centrality for Levenshtein distance-based networks because there is only one community that emerges from them. Table 10 shows the results of the Kruskal–Wallis, Conover, and Gehan non-parametric tests for various molecular descriptors in the Levenshtein distance-based network, considering the Leiden clustering method. The Kruskal–Wallis test evaluates whether there are statistically significant differences between the communities for each molecular descriptor. For the Leiden method, molecular mass shows highly significant differences across communities, indicating that molecular mass is a key differentiator in the clustering of BACE-1 molecules. MMFF energy also exhibits high significance, suggesting that energy profiles vary significantly across communities. The cLogP descriptor is highly significant, indicating that lipophilicity is an important factor in clustering. MR is extremely significant, highlighting the importance of molar refractivity in defining communities. Eccentricity is significant, suggesting that structural compactness is a critical differentiator. All Kappa indices show high significance, with *p*-values of less than $10^{-10}$, indicating that molecular shape and branching are critical in clustering. The Synthetic Accessibility Score is highly significant, implying its important role in clustering. TPSA is also highly significant, indicating that polar surface area is crucial for community differentiation. The 2D AC Entropy and 3D AC Entropy are significant, with *p*-values of 0.0449 and 0.022, respectively, suggesting their roles in differentiating communities are less pronounced but still relevant.

The Conover test provides pairwise comparisons following the Kruskal–Wallis test, highlighting where significant differences lie between specific groups. For molecular mass, the Conover test confirms extreme significance. MMFF energy remains highly significant, confirming energy differences between communities. The cLogP descriptor is significant, indicating lipophilicity differences are well captured. MR is extremely significant, reaffirming its importance. Eccentricity is significant. All Kappa indices show high significance indicating their consistent importance in clustering. The Synthetic Accessibility Score is significant. TPSA remains highly significant. The 2D AC Entropy and 3D AC Entropy are significant, with *p*-values of 0.0042 and 0.08, respectively.

The Gehan test, like the Conover test, evaluates differences across groups but focuses on survival analysis contexts, offering robustness for non-parametric data. For molecular mass, the Gehan test shows high significance. MMFF energy is very significant. The cLogP descriptor is significant. MR is extremely significant (*p*-value = $6.1\times 10^{-80}$), confirming its critical role. Eccentricity is significant (*p*-value = 0.00003). All Kappa indices show high significance indicating their consistent importance. The Synthetic Accessibility Score is highly significant (*p*-value = $1.7\times 10^{-56}$). TPSA is very significant (*p*-value = $1.6\times 10^{-46}$). The 2D AC Entropy and 3D AC Entropy are significant, with *p*-values of 0.0142 and 0.007, respectively.

Non-parametric tests reveal consistent patterns of significance for molecular mass, MMFF energy, MR, cLogP, and TPSA across the Leiden clustering method. These descriptors are critical for defining the community structure in the Levenshtein distance-based network of BACE-1 inhibitors. While some descriptors show varying levels of significance, overall, the tests underscore the multidimensional nature of molecular clustering and the importance of using multiple descriptors to capture the complexity of BACE-1 inhibitor interactions.

Our findings demonstrate that the identified clusters of BACE-1 inhibitors have a significant correlation with their biochemical functions, providing valuable insights into the structural and functional relationships within these molecules. For instance, inhibitors within specific clusters often share common mechanisms of action, such as binding affinity to the BACE-1 enzyme. This commonality suggests that these clusters are not arbitrary but are instead based on meaningful biochemical interactions that dictate their inhibitory efficacy. By understanding these shared mechanisms, we can better predict how new or untested inhibitors might behave, thus enhancing the efficiency of the drug discovery process.

Inhibitors with high eigenvector centrality within these networks are of particular interest. Eigenvector centrality is a measure of a node’s influence within a network, considering both the number and the quality of its connections. In this context, inhibitors with high eigenvector centrality are likely to be critical for effective binding and inhibition of BACE-1. These central inhibitors are well-connected to other significant molecules, suggesting that they play a pivotal role in the overall inhibition process. As such, they are prime candidates for further drug development and optimization. Focusing on these central inhibitors could streamline the identification of lead compounds with the highest potential for therapeutic efficacy.

Moreover, the significance of molecular descriptors, such as Topological Polar Surface Area (TPSA), further underscores their importance in influencing the pharmacokinetics of these inhibitors. TPSA is a key factor that affects a molecule’s ability to interact with polar environments, impacting properties like solubility, permeability, and ultimately bioavailability. Our analysis highlights that inhibitors with optimal TPSA values tend to cluster together, indicating their likely superior pharmacokinetic profiles. This finding is crucial for the rational design of BACE-1 inhibitors, as it provides a clear target for optimizing molecular structures to achieve better drug-like properties.

## 4. Materials and Methods

### 4.1. Network Formation

Networks, often called graphs, are abstract representations of interrelated elements in mathematics and computer science. They are excellent models for understanding and analyzing complex systems. These systems encompass social interactions, information flow in systems [34,35], biological interactions in ecological systems [36,37], and molecular structures in chemistry [38,39,40]. The core elements of a network are nodes (or vertices) and edges (or links). Nodes symbolize the entities in the system, such as individuals, cities, genes, or abstract notions, while edges define the links or relationships between these entities. A network can be described mathematically as G=(V,E), where *V* represents the nodes and *E* represents the edges. Every edge in *E* links two nodes from *V*, representing a type of interaction, reliance, or connection between them. This mathematical framework enables the formalization and examination of intricate systems, offering insights into their structure, behavior, and emergent characteristics.

Moreover, in weighted networks, weights are numerical values assigned to the edges, introducing an additional dimension. The weights indicate a level of importance, ability, or expense related to moving across the edge. A weighted network can be represented mathematically as G=(V,E,ω), where $w:E\to R^{+}$ is a function that assigns weights to edges [41,42].

Examining systems using weighted network topology is a fundamental aspect of contemporary network science, providing deep understanding of the structural and dynamic characteristics of intricate networks. This technique extends beyond the basic binary perspective of connections in traditional network analysis by recognizing that not all interactions or links hold the same level of importance. Weighted networks assign a weight to edges to represent the intensity, capacity, or frequency of interactions between nodes. This multifaceted perspective allows for a more precise and complete comprehension of the network’s fundamental structure and operation.

The weighted degree $d_{i}$ of node *i* in a weighted network is defined as the total of the weights of all edges connected to *i*, represented mathematically as

(1) $$d_{i}=\sum_{j\in N(i)}w_{ij},$$

where N(i) is the set of neighbors of node *i* (or the set of adjacent nodes) and $w_{ij}$ is the weight of the edge between nodes *i* and *j*. This formulation offers a more detailed assessment of node centrality by considering the intensity of connections [43,44].

Understanding the concept of shortest paths in weighted networks is essential for grasping the efficiency of information or resource transfer. The weighted shortest path length between two nodes, *i* and *j*, is the minimum total weight of the edges traveled, represented as $g_{ij}$. If $P_{ij}$ denotes all feasible paths from *i* to *j*, then

(2) $$g_{ij}=\min_{p\in P_{ij}}\sum_{(u,v)\in p}w_{uv}$$

where *p* represents a path in $P_{ij}$ made up of edges (u,v) with weights $w_{uv}$. Dijkstra’s and the Bellman–Ford algorithm are frequently employed to determine the shortest paths in weighted graphs [45].

A clique or a topological triangle in weighted networks can be defined by including edge weights [46,47,48]. A weighted clique with threshold *t* is a subset of nodes *C* from the set *V* of all network nodes, where for every pair of different nodes (i,j) in *C*, there is an edge $e_{ij}$ with weight $w_{ij}\geq t$. This term encapsulates the intuitive notion of closely interconnected groupings with strong internal bonds.

The Minimum Spanning Tree (MST) is a fundamental concept in graph theory, specifically in the examination and improvement of networks where links are assigned weights that represent distances or costs [49,50]. In a graph, a MST is a set of edges that forms a tree and includes all the vertices. The total weight of the edges in the MST is minimized, making it a direct representation of hierarchical clustering in a weighted network. The tree structure is crucial in situations where the objective is to minimize the cost or distance of linking different vertices. The lowest $g_{ij}$ values are crucial in the construction of a MST, especially through algorithms such as Prim’s and Kruskal’s [51]. These algorithms aim to include edges that help achieve the lowest $g_{ij}$ between any two vertices in the tree. This technique guarantees that the tree covers all vertices in the graph while minimizing the total weight, effectively utilizing the concept that shorter distances generally indicate stronger or more desired connections. When building an MST, the rule is to choose edges with smaller weights, as these reflect stronger relationships or more desirable connections [52,53]. The preference for minimizing edge weights guarantees that the resulting tree will cover all vertices while incurring the lowest possible total cost. The MST possesses mathematical elegance by efficiently representing the most optimal route through a network. It showcases a crucial characteristic: out of all the potential spanning trees, the MST ensures the lowest total of distances or expenses, thereby enhancing connection.

Assigning weights to network edges based on distances is a crucial method in network analysis, providing an advanced way to measure the connections or interactions between nodes in a network. The weight of an edge often denotes the distance between nodes, using different distance metrics based on the network’s characteristics and the data it conveys. This method allows for the quantification of the strength, similarity, or dissimilarity of nodes using numerical values, which helps in gaining a more profound insight into the structure and behavior of the network.

The selection of a distance metric significantly impacts the success of this strategy. Euclidean distance is often used in spatial networks, whereas Hamming or cosine distances are better suited for networks that involve text or category data [54,55]. Integrating these weighted edges alters the network analysis, enabling algorithms to take into account not only the existence of a connection but also its qualitative intensity or importance. This sophisticated method of network analysis presents difficulties. Choosing a suitable distance measure and properly scaling distances can greatly influence the results of the investigation. In highly dynamic networks, it is essential to have strong mechanisms in place to update weights in response to changes over time. However, the advantages of edge weighting using distances, such as increased analytical accuracy, greater model realism, and the capacity to provide profound insights into network activity, establish it as a fundamental technique in the realm of network science.

Euclidean distance in descriptor space is particularly useful in molecular networks where nodes represent molecules, and edges signify the similarity or dissimilarity between their chemical properties. For molecules represented by numerical descriptors, the Euclidean distance between two molecules, *A* and *B* with descriptor vectors $\vec{a}$ and $\vec{b}$ can be calculated as

(3) $$d_{Euclidean}={\left(\sum_{i=1}^{n}{\left(a_{i}-b_{i}\right)}^{2}\right)}^{1/2},$$

where *n* is the number of descriptors, and $a_{i}$ and $b_{i}$ are the values of the *i*th descriptor for molecules *A* and *B*, respectively.

This work considers not only the general descriptors of molecules, such as physicochemical properties, but also geometric and topological descriptors, which delve into the spatial arrangement and connectivity of atoms within a molecule.

In the context of clustering BACE-1 inhibitors using Euclidean distance, several molecular descriptor vectors are employed to capture the diverse properties of these inhibitors. The first descriptor considered is the molecular mass, reflecting the overall size and weight of the molecule. This descriptor is fundamental as it influences many physical and chemical properties, including the molecule’s behavior in biological systems.

The Merck Molecular Force Field (MMFF) Energy is a computational estimate of a molecule’s potential energy, reflecting its stability and reactivity. Lower energy indicates a more stable structure, which is crucial for understanding the energetics of BACE-1 inhibitors. The MMFF energy is computed using several components:

(4) $$E_{\mathrm{total}}=E_{\mathrm{bond}}+E_{\mathrm{angle}}+E_{\mathrm{torsion}}+E_{\mathrm{oop}}+E_{\mathrm{vdW}}+E_{\mathrm{electrostatic}}.$$

In equation

(5) $$E_{\mathrm{bond}}=\sum_{\mathrm{bonds}}k_{b}{\left(r-r_{0}\right)}^{2},$$

$k_{b}$ is the bond force constant, *r* is the bond length, $r_{0}$ is the equilibrium bond length. This term accounts for the energy associated with the stretching of bonds between atoms. Angle bending energy is defined by

(6) $$E_{\mathrm{angle}}=\sum_{\mathrm{angles}}k_{\theta }{\left(\theta -\theta_{0}\right)}^{2},$$

where $k_{\theta }$ is the angle force constant, θ is the bond angle, $\theta_{0}$ is the equilibrium bond angle. This term represents the energy required to bend bond angles from their equilibrium positions. Torsional (dihedral) energy is also defined by

(7) $$E_{\mathrm{torsion}}=\sum_{\mathrm{torsions}}V_{n}\left[1+\cos(n\phi -\gamma )\right],$$

where $V_{n}$ is the torsional barrier height, *n* is the periodicity, ϕ is the dihedral angle, γ is the phase angle. This term describes the energy associated with the rotation around bonds. Out-of-plane bending energy is defined by

(8) $$E_{\mathrm{oop}}=\sum_{\mathrm{impropers}}k_{\phi }\phi^{2},$$

where $k_{\phi }$ is the out-of-plane bending force constant, ϕ is the out-of-plane angle. This term accounts for the energy needed to distort atoms out of the plane defined by their neighboring atoms. Van der Waals energy is defined by

(9) $$E_{\mathrm{vdW}}=\sum_{i<j}\left[\frac{A_{ij}}{r_{ij}^{12}}-\frac{B_{ij}}{r_{ij}^{6}}\right],$$

where $A_{ij}$ and $B_{ij}$ are van der Waals parameters for the interaction between atoms *i* and *j*, $r_{ij}$ is the distance between atoms *i* and *j*. This term describes the van der Waals forces, including repulsive and attractive interactions. Finally, electrostatic energy is defined by

(10) $$E_{\mathrm{electrostatic}}=\sum_{i<j}\frac{q_{i}q_{j}}{\epsilon r_{ij}},$$

where $q_{i}$ and $q_{j}$ are the partial charges on atoms *i* and *j*, ϵ is the dielectric constant. This term accounts for the electrostatic interactions between charged atoms.

The MMFF energy descriptor provides a comprehensive estimate of the potential energy of a molecule, encompassing various intra- and intermolecular interactions such as bond stretching, angle bending, torsional strain, van der Waals forces, and electrostatic interactions. These energy components are fundamental to understanding the overall stability and reactivity of the inhibitors. Even though BACE-1 inhibitors are not isomers, the MMFF energy offers insights into how stable and reactive these molecules are under standard conditions which are crucial for their biochemical efficacy. To address the challenge of comparing MMFF energies across non-isomeric compounds, we normalize the energy values. This involves calculating the relative MMFF energy by subtracting the energy of a reference structure within the same class of compounds from the MMFF energy of each inhibitor. This normalization mitigates the differences in the number and types of atoms, allowing for a more meaningful comparison that focuses on the relative stability and reactivity of the molecules.

Crippen ClogP, the calculated logarithm of the partition coefficient, measures the lipophilicity of a compound, influencing its solubility, permeability, and interaction with biological membranes. Different inhibitors can exhibit varying logP values, affecting their bioavailability and distribution within biological systems. Crippen MR, or molar refractivity, measures the volume occupied by a molecule’s electrons, relating to molecular size and polarizability, which impacts interactions with the environment.

Eccentricity is a topological descriptor that measures how much a molecule’s shape deviates from being circular or spherical, affecting how it fits into binding sites and interacts with other molecules. This provides insight into the spatial configuration of inhibitors, which is important for understanding their functional roles. The Kappa indices (κ1, κ2, and κ3) are topological descriptors that measure molecular shape based on the number and arrangement of atoms and bonds. κ1 provides information on the complexity and compactness of a molecule’s structure, while κ2 and κ3 offer additional details on molecular shape and branching patterns, essential for differentiating inhibitors based on their structural intricacies and potential interactions.

The Synthetic Accessibility Score estimates the ease with which a molecule can be synthesized based on its structural complexity, indicating how readily a molecule can be produced for experimental or therapeutic use. Inhibitors with lower synthetic accessibility might be more challenging to work with but could offer unique properties. The Topological Polar Surface Area (TPSA) measures the surface area occupied by polar atoms, affecting a molecule’s ability to interact with polar environments, influencing properties like solubility, permeability, and transport. Inhibitors with different TPSA values have varied interactions with biological systems, impacting their pharmacokinetic and pharmacodynamic profiles.

Two-dimensional autocorrelation refers to the analysis of the spatial distribution of molecular properties (e.g., atomic charges, polarizabilities) on a two-dimensional representation of the molecule, such as its connectivity graph. It involves calculating the correlation of a property at one atom with the same property at another atom, separated by a certain topological distance (number of bonds). This descriptor can include the autocorrelation of atomic charges, masses, electronegativities, or other properties over the molecular graph. Three-dimensional autocorrelation extends this analysis to the three-dimensional structure of the molecule, taking into account the spatial arrangement of atoms in three-dimensional space. It involves calculating the correlation of a property at one atom with the same property at another atom, separated by a certain Euclidean distance in 3D space. This descriptor can include the autocorrelation of properties such as van der Waals volumes, solvent-accessible surface areas, or electrostatic potentials.

When using 2D and 3D autocorrelations as descriptors, their entropy is considered because they are obtained vectorially. Entropy, in the context of autocorrelation, measures the randomness or complexity of the distribution of a particular property across the molecule. High entropy indicates a more random and complex distribution, while low entropy indicates a more ordered distribution. In this study, the Shannon entropy function is used for the entropies of 2D and 3D autocorrelation vectors.

Each of these descriptors is linked to the molecular energies of BACE-1 inhibitors in different ways. For instance, MMFF Energy directly measures the potential energy, while descriptors like Crippen ClogP and TPSA relate to how the structure of the molecule influences its interaction with the environment, indirectly affecting its energy state. Eccentricity and Kappa indices describe shape and branching, which can affect molecular stability and reactivity, thereby influencing the energy profile. The Synthetic Accessibility Score, although not directly related to energy, provides a practical aspect of how molecular properties translate to synthetic feasibility, which is crucial for the experimental validation of theoretical models. Entropy is a fundamental thermodynamic property contributing to the molecule’s free energy. In this context, higher entropy can lower the Gibbs free energy, influencing molecular stability and reaction favorability. Therefore, the entropy of 2D and 3D autocorrelation provides valuable information about molecular complexity and distribution, crucial for understanding molecular stability, reactivity, and overall energy in chemical and biological systems.

We use various molecular descriptors, including MMFF energy, cLogP, and TPSA, to capture the biochemical properties of BACE-1 inhibitors comprehensively. Each of these descriptors provides critical insights into different aspects of the molecular behavior and potential efficacy of the inhibitors. MMFF Energy is crucial for understanding the stability and binding affinity of BACE-1 inhibitors. The MMFF energy calculation provides an estimate of the potential energy of the molecule, taking into account various intra- and intermolecular interactions such as bond stretching, angle bending, torsional strain, van der Waals forces, and electrostatic interactions. A lower MMFF energy value indicates a more stable molecular structure, which is often associated with higher binding affinity to the target enzyme, making it a key predictor of inhibitory activity. cLogP measures the lipophilicity of the inhibitors. Lipophilicity is a critical factor that influences a molecule’s ability to permeate cellular membranes, which is essential for its bioavailability and distribution within biological systems. High cLogP values suggest that a molecule is more hydrophobic, enhancing its potential to traverse lipid-rich environments such as cell membranes. However, excessive lipophilicity can also lead to issues such as poor solubility in aqueous environments and increased risk of off-target effects. Therefore, an optimal cLogP balance is necessary for effective BACE-1 inhibition. TPSA measures the surface area of a molecule occupied by polar atoms, typically oxygen and nitrogen, and their attached hydrogen atoms. TPSA is a significant descriptor as it affects a molecule’s ability to form hydrogen bonds, which in turn influences its solubility, permeability, and overall interaction with biological environments. Molecules with an appropriate TPSA are more likely to exhibit favorable pharmacokinetic properties, such as adequate absorption and permeation, while maintaining sufficient solubility. This balance is particularly important for achieving effective inhibition of BACE-1, as it ensures that the inhibitors can reach and interact with their target site efficiently. By integrating these descriptors into our network analysis, we can map out the structural and functional landscape of BACE-1 inhibitors, highlighting how specific molecular features contribute to their biochemical performance. This approach enables us to identify key structural attributes that enhance inhibitory potency and provides a robust framework for optimizing these molecules for therapeutic applications.

The Tanimoto molecule distance function offers another approach, specifically tailored for comparing molecular structures [56]. It is used to calculate the similarity between two molecules based on their chemical fingerprints, which are binary vectors representing the presence or absence of particular structural elements. The Tanimoto coefficient (also known as the Jaccard index) is widely used for this purpose and is defined for two molecules with fingerprint vectors $\vec{A}$ and $\vec{B}$ as

(11) $$T\left(\vec{A},\vec{B}\right)=\frac{\vec{A}\cdot \vec{B}}{|\vec{A}{|}^{2}+{\left|\vec{B}\right|}^{2}-\vec{A}\cdot \vec{B}},$$

where $\vec{A}\cdot \vec{B}$ is the dot product of the fingerprints and $|\vec{A}{|}^{2}$ and $|\vec{B}{|}^{2}$ are the squares of the norms of the fingerprints (essentially the count of bits set to 1). The Tanimoto coefficient ranges from 0 (completely different) to 1 (identical), and a distance measure can be derived as

(12) $$d_{Tanimoto}=1-T.$$

The Tanimoto coefficient is a ratio that measures the overlap between the fingerprints of two molecules, making it an ideal distance measure for weighting edges in molecular networks, particularly when assessing structural similarity.

Hamming distance for fingerprint differences is relevant for networks where the focus is on the binary representation of molecular features. The Hamming distance counts the number of positions at which the corresponding elements (bits) of two fingerprints differ [57]. For two molecular fingerprints $\vec{A}$ and $\vec{B}$, the Hamming distance is given by

(13) $$d_{Hamming}=\sum_{i=1}^{n}\left|a_{i}-b_{i}\right|,$$

where *n* is the length of the fingerprints and $a_{i}$ and $b_{i}$ are the bits at position *i* in fingerprints $\vec{A}$ and $\vec{B}$, respectively. This measure is particularly useful for quickly assessing the similarity between molecules in large datasets, providing a basis for weighting network edges that reflect minor structural differences or mutations [58,59].

Edit distance for structural comparisons is crucial for analyzing networks of molecules. The edit distance quantifies the minimum number of operations (such as the addition, removal, or substitution of a bond or atom) required to transform one molecular structure into another through string-based representations [60]. In scenarios where molecules are compared based on their structural representations, Simplified Molecular Input Line Entry System (SMILES) strings, the Levenshtein distance [61,62] can quantify how many edits (insertions, deletions, substitutions) are required to convert one molecular representation into another. This measure is more qualitative and depends heavily on the representation scheme. Since it reveals the structural similarities and differences among molecules with the same chemical formula but different spatial configuration, this distance metric is very helpful for building weighted networks of molecules.

The network formed by utilizing the molecular distances between molecules is mathematically represented by a graph that consists of nodes equal in number to the total number of molecules. Among the molecules, the ones that are nearest to each other in terms of distance are connected by edges that have low values, which depend on how the distance functions are defined. These distances are non-zero in practice, except for identical molecules. Therefore, it is anticipated that there will be distinctions established for each node in the graph model that is constructed. The mathematical term for these types of graph structures is *n*-complete graphs. To provide consistent network analysis between molecules, it is imperative to exclude high-weighted edges that signify a weak edge.

The Triangulated Maximally Filtered Graph (TMFG) is a network analysis technique that simplifies intricate networks while retaining crucial information [63]. It is also valuable for examining entire graphs that depict intricate systems, such as molecule networks, where the number of connections increases exponentially with the number of components, resulting in a significant degree of intricacy.

A complete graph G=(V,E,w) represents a network in which each node v∈V represents a molecule, and every pair of nodes is connected by edge e∈E. The dissimilarity of the molecules is quantified by a distance measure (such as Euclidean, Tanimoto, Hamming, Levenshtein), and each edge is weighted accordingly. The TMFG chooses a subset of edges *E* from the complete graph $K_{\left|V\right|}$ to create a minimum spanning tree. This is accomplished by assigning priority to edges based on their weight, which indicates the intensity of the connection (inversely proportional to the distance) between nodes, and choosing those that contribute to a network that is as planar as possible.

A graph is considered planar if it can be represented on a two-dimensional plane without any edges intersecting. The TMFG guarantees planarity by following Euler’s formula for planar graphs, which establishes a relationship between the number of vertices *V*, edges *E*, and faces *F* in the graph: |V|−|E|+|F|=2. The TMFG is a triangulated graph, which means that every face of the graph, except for the outer face, is a 3-clique. This is accomplished by augmenting the initial spanning tree with more edges until the requirement of maximal planarity is achieved without any violations.

The reason for using TMFG filtration for molecule networks is its ability to extract the core of the network, reducing complexity while preserving important information about chemical similarities and differences. The decision to utilize this methodology is justified by its capacity to highlight important molecular connections through a simplified yet informative network structure, improving analytical precision and enabling a more targeted investigation of molecular similarities. Moreover, the TMFG’s ability to effectively process extensive datasets, along with its resilience to interference and slight deviations, establishes it as an indispensable tool in the investigation of molecular networks. It skillfully emphasizes the significant relationships between molecules that are crucial in chemistry and biology, allowing for insightful interpretations that are both computationally possible and scientifically substantial. Therefore, the TMFG filtration not only simplifies but also strategically enhances the analysis, allowing for a more profound comprehension of the intricate connections within molecule networks.

The network representations obtained by filtering the structures of molecules provide a limited set of visual data in this study. To enhance comprehension and offer a more thorough analysis, this study includes quantitative measures that illustrate the interactions between molecules, along with graphical representations. This methodology guarantees a more profound understanding of the intricate dynamics and connections among the molecules, surpassing a simple visual study to encompass a thorough analysis of the interactions between these molecules.

Edge betweenness centrality of a node is a metric employed in network analysis to evaluate the significance of an edge in enabling communication or connectivity within the network. The term refers to the count of the most direct routes connecting pairs of points that traverse a specific link. In essence, it quantifies the frequency with which an edge serves as a bridge on the shortest route between two nodes [64,65]. Mathematically, for a given edge *e* in a network, the edge betweenness centrality can be calculated using the following formula:

(14) $$C_{B}\left(v\right)=\sum_{s\neq v\neq t}\frac{\sigma_{st}\left(v\right)}{\sigma_{st}},$$

where $\sigma_{st}$ is the total number of shortest paths from node *s* to node *t* and $\sigma_{st}$ is the number of those shortest paths that passes through node *v*.

A high betweenness edge in a molecule network signifies a crucial connection or pathway that is traversed by numerous shortest paths. These findings indicate that the two molecules connected by this edge have important functions in connecting other molecules in the network. They may act as central points or connectors that aid in the communication or transitions between different sections of the network. In the context of molecule networks, a high betweenness edge can indicate a pair of molecules that play a crucial role as intermediates in the transformation between two other molecules, or it can emphasize a significant similarity that is shared by many other molecules. Edges with high betweenness play a crucial role in maintaining the integrity and connection of the network from a structural standpoint. Eliminating these edges can greatly disturb the network, potentially causing it to be split into separate and isolated parts. Within the realm of molecules, this implies that the elimination or alteration of specific crucial molecules or transformations could significantly impact the range of potential isomermolecule transformations or resemblances.

In the field of network analysis, particularly when examining networks among molecules, the constraint score is a metric that measures the extent to which a node relies on other nodes in the network for its connections. The evaluation primarily assesses the distribution of a node’s connections among its neighbors, specifically examining whether the node’s network relationships are concentrated on a small number of nodes or spread out among a larger number. A higher constraint score signifies a higher degree of reliance on a smaller number of nodes, implying that the node has fewer but more concentrated connections. Conversely, a lower score implies that a node is more uniformly connected across multiple nodes, indicating a lesser amount of dependency.

The constraint score for node *i* in a network can be calculated using the following formula:

(15) $$C_{i}=\sum_{j}{\left(p_{ij}+\sum_{k}p_{ik}p_{jk}\right)}^{2},$$

where $p_{ij}$ is the proportion of node *i*’s direct ties that is allocated to node *j* and $p_{ik}p_{jk}$ represents the indirect ties from *i* to *j* through a third node, *k* [66].

Within a network of molecules, a high constraint score for a certain molecule signifies its strong reliance on a limited number of other molecules. This reliance can be due to direct transformation pathways, structural similarities, or other types of interactions. This indicates that the molecule holds a specific position or specialized function within the network, potentially serving as a vital mediator in particular reactions or transformations. An analysis of the constraint scores throughout the network can provide insights into the general organization and behavior of the network. A network containing numerous nodes with high constraints tends to exhibit a tightly ordered structure centered around a limited number of crucial pathways or links. On the other hand, a network with lower overall constraint scores tends to be more flexible, featuring multiple pathways and linkages between molecules. Molecules that have high constraint ratings may be promising candidates for chemical synthesis or modification endeavors. Modifying these molecules or their connections, given their crucial location and interdependencies in the network, could have substantial consequences on the network’s behavior. This could potentially lead to the emergence of new routes or changes in the distribution of molecules.

The scores of the constraints can also offer valuable information concerning the network’s resiliency. Networks that consist of numerous nodes with high constraint scores may exhibit greater susceptibility to disruptions. The removal of a crucial molecule or connection could have a substantial influence on the network. On the other hand, networks with nodes that have lower constraint scores are probably more resilient, as molecules rely less on specific connections.

Eigenvector centrality is a metric that quantifies the impact of a node in a network, taking into account both the quantity and quality of its connections [67]. The idea underlying its operation is that connections to nodes that are highly linked or influential have a greater impact on the centrality of a node compared to connections of similar strength to less influential nodes. Eigenvector centrality for node *i*, denoted as $x_{i}$, is defined as

(16) $$x_{i}=\frac{1}{\lambda }\sum_{j=1}^{\left|V\right|}W_{ij}x_{j},$$

where *W* is the weighted adjacency matrix, λ is the largest eigenvalue of *W*.

Within a molecule network, a node with a high eigenvector centrality is linked not only to several other nodes but also specifically to nodes that are themselves highly connected or important. This implies that the molecule has a substantial impact on the network, maybe serving as a crucial intermediary in reaction pathways or as a pivotal hub in a network of structural resemblances. A molecule with high eigenvector centrality suggests that it is a pivotal component in a sequence of reactions, capable of producing numerous other molecules either directly or through densely interconnected intermediary compounds. It can identify molecules that play a crucial role in the synthesis of a diverse range of other chemicals. Eigenvector centrality can be used to find molecules that are not only structurally related to many others but also central in a cluster of very similar molecules, when edges signify structural similarity. This can be beneficial for discerning characteristic structures or comprehending the arrangement of chemical space surrounding fundamental patterns.

### 4.2. Network Communities

Network communities in the context of molecule networks can be mathematically defined using notions of graph theory. In such a network, nodes represent molecules, and edges represent the similarity or dissimilarity between these molecules, often quantified by measures such as Euclidean distance in descriptor space, Tanimoto molecule distance, Hamming distance for fingerprint differences, or edit distance for structural comparisons. Identifying communities involves partitioning the network into subgraphs (communities) where nodes (molecules) are densely connected internally but sparsely connected to nodes outside the community. This clustering based on structural similarities or differences is crucial for understanding the collective behaviors of molecules in chemical reactions or biological systems. Mathematically, if we represent our network as a filtered weighted graph $G_{F}=\left(V,E_{F},w\right)$ where *V* is the set of nodes and $E_{F}$ is the set of filtered edges (distances between molecules), community detection seeks to maximize modularity *Q* of a partition of *V* defined as

(17) $$Q=\frac{1}{2m}\sum_{ij}\left[A_{ij}-\frac{k_{i}k_{j}}{2m}\right]\delta \left(c_{i},c_{j}\right),$$

where $A_{ij}$ represents the edge weight between nodes *i* and *j*, $k_{i}$ and $k_{j}$ are the sum of the weights of the edges attached to nodes *i* and *j*, respectively, *m* is the sum of all of the edge weights in the network, $c_{i}$ and $c_{j}$ are the communities containing nodes *i* and *j*, and δ is the Kronecker delta function, which is 1 if $c_{i}=c_{j}$ and 0 otherwise. We can better understand and control the features and interactions of molecules by classifying them into communities that reflect their significant chemical or structural similarities.

The Leading Eigenvector algorithm [68] utilizes the eigenspectrum of the modularity matrix *B* of a network, where $B_{ij}=A_{ij}-\frac{d_{i}d_{j}}{2m}$. Here, $A_{ij}$ is the adjacency matrix, $d_{i}$ and $d_{j}$ are the weighted degrees of nodes *i* and *j*, and *m* is the total number of edges. The network is partitioned by the sign of the components of the leading eigenvector of *B*, corresponding to its largest eigenvalue, to identify communities.

The Leiden algorithm [69] enhances this by iteratively refining communities through a three-phase process: (1) local moving of nodes to improve modularity, (2) aggregation of nodes into super nodes based on the refined communities, and (3) the creation of a new network of these super nodes. This process, which ensures connectivity within identified communities, repeats until modularity *Q* can no longer be increased, leading to more accurate and finely grained community detection.

Within the scope of molecule networks, the Leading Eigenvector and Leiden algorithms employ mathematical models to group molecules together based on their similarity in structure or properties. The Leading Eigenvector technique includes dividing the network based on the positive and negative components of the principal eigenvector of the modularity matrix. This grouping process forms communities that maximize the density of edge weights inside each community. The Leiden algorithm utilizes iterative refinement to enhance modularity by locally moving nodes and subsequently aggregating communities into super nodes for further partitioning. This approach not only groups molecules based on their similarities, but also guarantees that these groups have significance in terms of chemical behavior or physical properties. This results in more refined classifications that can indicate shared or distinct characteristics among the molecules, such as patterns of reactivity or solubility.

The clustering process is improved when these approaches are applied to distance-based molecule networks because it allows for the utilization of the particular advantages that are associated with each distance measure. For instance, the Euclidean Distance in Descriptor Space makes it possible to cluster based on subtle physicochemical features, which allows for the capture of minor changes in the molecular composition of molecules. By comparing molecular fingerprints, Tanimoto distance makes it possible to categorize molecules according to their more general structural characteristics. In the meantime, the Hamming distance places an emphasis on minute, precise changes in the structure of molecules, and the edit distance offers insight into the degree of structural modification that is required between molecules. An improved comprehension of molecular variety can be achieved by the utilization of this multi-faceted method, which provides a comprehensive and thorough panorama of molecule similarities and variances.

This study examines two distinct network communities within molecule networks and uses a range of network metrics to assess the performance differences between these communities.

The Variation of Information (VI) is a metric derived from information theory that precisely measures the quantity of information that is both lost and gained when comparing two community architectures [70,71]. Given two partitions of the same dataset, $C_{1}$ and $C_{2}$, the Variation of Information is defined as

(18) $$VOI\left(C_{1},C_{2}\right)=H\left(C_{1}\right)+H\left(C_{2}\right)-2I\left(C_{1};C_{2}\right)$$

where $H(C_{1})$ and $H(C_{2})$ are the entropies of communities $C_{1}$ and $C_{2}$, respectively, and $I(C_{1};C_{2})$ is the mutual information between $C_{1}$ and $C_{2}$. The entropy of a partition measures the uncertainty or randomness in the partition, and the mutual information quantifies the amount of shared information. A lower VI indicates a higher similarity between two community structures, with a VI of zero denoting identical community assignments.

Normalized Mutual Information (NMI) is a metric based on information theory that measures the similarity between two sets of community structures [72]. For two communities $C_{1}$ and $C_{2}$, NMI is defined as

(19) $$NMI\left(C_{1},C_{2}\right)=\frac{2I(C_{1};C_{2})}{H\left(C_{1}\right)+C_{2}}.$$

NMI values range from 0 to 1, where 1 indicates perfect agreement between two community structures and 0 denotes no mutual information.

The Split–Join distance measures the similarity between two clusterings by counting the minimum number of split and join operations required to transform one clustering into another. It effectively counts the number of elements that need to be moved to reconcile the differences between two partitions [73]. Given two communities, the Split-Join distance (SJ) is defined as

(20) $$SJD\left(C_{1},C_{2}\right)=S\left(C_{1}|C_{2}\right)+S\left(C_{2}|C_{1}\right),$$

where $S(C_{1}|C_{2})$ is the number of splits required to transform $C_{1}$ into $C_{2}$, and $+S(C_{2}|C_{1})$ is the number of joins required to transform $C_{2}$ into $C_{1}$. A SJ of zero means that the two community structures are identical.

The Rand Index (RI) is a measure of the similarity between two data clusterings. It is calculated by considering all pairs of elements in the dataset and counting the pairs that are either assigned to the same or different clusters in both clusterings [74]. The RI between two communities is defined as

(21) RI(C1,C2)=c1+c2n2,

where $c_{1}$ is the number of pairs of elements that are in the same subset in $C_{1}$ and in the same subset in $C_{2}$, $c_{2}$ is the number of pairs of elements that are in different subsets in $C_{1}$ and different subsets in $C_{2}$, and the denominator is the total number of possible pairs. RI is a measure that ranges from 0 to 1. A value of 1 indicates that the clusterings are completely identical, while a value of 0 indicates that there is no agreement between the clusterings beyond what would be expected by chance.

The Adjusted Rand Index (ARI) is a corrected-for-chance version of the Rand Index that adjusts for the fact that the RI tends to increase simply with the number of clusters, even if the clusterings are randomly generated, and it is defined as

(22) $$ARI\left(C_{1},C_{2}\right)=\frac{RI\left(C_{1},C_{2}\right)-E\left(RI\left(C_{1},C_{2}\right)\right)}{\max RI(C_{1},C_{2})-E\left(RI\left(C_{1},C_{2}\right)\right)},$$

where $E(RI\left(C_{1},C_{2}\right))$ is the expected value of the RI under random classifications and $\max RI(C_{1},C_{2})$ is the maximum possible value of the RI.

## 5. Conclusions

This study demonstrated the efficacy of employing complex network methodologies to analyze and evaluate molecules from the BACE-1 dataset. By representing each molecule as a node and defining edges based on various distance measures such as Euclidean, Tanimoto, Hamming, and Levenshtein distances, we constructed detailed molecular networks that reveal the multifaceted relationships among the inhibitors. Through the application of graph theory, we uncovered both global and local structural patterns that traditional chemical analysis may overlook.

Our advanced graph theoretical tools enabled us to perform community detection, identifying clusters of molecules with similar properties using techniques like the Leading Eigenvector method and the Leiden algorithm. These methods allowed for us to discern large-scale structural classifications and finer distinctions within the network, revealing how variations in molecular structure influence their roles and interactions within the biological system. This highlights significant groupings based on shared chemical characteristics and biological functions.

By employing key graph metrics such as node centrality, clustering coefficients, and path lengths, we gained deeper insights into the importance of individual molecules within the network, the degree of interconnectedness among clusters, and the overall topology of the molecular landscape. These metrics provided a comprehensive understanding of the molecular diversity in the BACE-1 dataset, offering new perspectives on how structural variations impact biological activity and potential therapeutic efficacy.

Our network-based approach not only enhances our ability to classify and analyze molecules, but also bridges the gap between computational chemistry and systems biology. It allows for us to explore the intricate web of molecular interactions that drive biological functions and chemical phenomena. This comprehensive analysis contributes to the development of more effective BACE-1 inhibitors and advances our understanding of molecular mechanisms in drug discovery. By providing clear and actionable insights into the structure-function relationships of these inhibitors, we lay the groundwork for future therapeutic interventions and the rational design of novel inhibitors, thereby addressing significant biochemical challenges in Alzheimer’s disease research.

The Euclidean distance network, in particular, provided the most reliable clustering results, with strong agreement metrics and minimal information loss. This indicates its robustness in capturing the essential structural and physicochemical properties of BACE-1 inhibitors, making it a powerful tool for distinguishing subtle variations among molecules. The high performance of the Euclidean distance metric suggests that it effectively reflects the spatial and geometrical characteristics that are critical for the biological activity of these inhibitors. Tanimoto and Hamming distances also yielded valuable clustering outcomes, albeit with moderate performance. The Tanimoto distance, which is based on molecular fingerprint similarity, highlights the importance of common substructures and functional groups in defining molecular relationships. This metric is particularly useful in identifying compounds with similar pharmacophoric features, which can be crucial for understanding their biological activities. However, its moderate performance indicates that while structural similarity is important, it might not capture all the nuances of molecular interactions. Similarly, the Hamming distance, which measures differences in molecular fingerprints, provides insights into the specific structural variations that differentiate molecules. This distance function is beneficial for pinpointing minor structural changes that can have significant biological implications. Despite its utility, the moderate performance suggests that additional factors beyond simple structural variations need to be considered for a more complete understanding of molecular interactions. In contrast, the Levenshtein distance network shows significant discrepancies, highlighting its limitations in accurately representing the molecular relationships of BACE-1 inhibitors. The Levenshtein distance, based on the edit distance between SMILES strings, is sensitive to small changes in the sequence of atoms and bonds. While this can be useful for identifying exact matches or small modifications, it appears less effective in capturing the broader structural and functional similarities necessary for effective clustering. The higher information loss and lower agreement metrics associated with the Levenshtein distance underscore its inadequacy for this particular application.

The analysis of eigenvector centrality across different networks revealed key inhibitors acting as hubs within the BACE-1 inhibitor networks. Eigenvector centrality, which measures a node’s influence based on its connections and the connections of its neighbors, proved instrumental in identifying these central molecules. In the Euclidean distance-based network, inhibitors with high eigenvector centrality were identified as central players due to their extensive connections with other well-connected molecules. This suggests that these inhibitors occupy significant positions within the network, bridging multiple clusters and facilitating interactions across diverse molecular groups.

These central inhibitors are likely critical in biochemical pathways, serving as pivotal points in metabolic processes or as prime candidates for synthetic transformations. Their high centrality implies that they play essential roles in maintaining the structural and functional integrity of the network, potentially influencing a wide range of biological activities. In biochemical pathways, these inhibitors could act as key substrates or intermediates, participating in various reactions and interacting with multiple enzymes and receptors. This centrality makes them prime targets for drug development, as modifying or inhibiting these molecules could have significant downstream effects on metabolic processes.

The variation in eigenvector centrality across different distance-based networks underscores the importance of selecting appropriate distance metrics to accurately capture the centrality and influence of specific inhibitors within the network. For instance, in the Tanimoto and Hamming distance-based networks, the distribution of eigenvector centrality might highlight different sets of central inhibitors compared to the Euclidean network. This variation reflects the different aspects of molecular similarity that each distance metric emphasizes, such as structural subunits in the case of Tanimoto distance or binary fingerprint similarities for Hamming distance.

Understanding these differences is crucial for accurately mapping the roles of BACE-1 inhibitors within the network. The choice of distance metric can significantly impact which molecules are identified as central, thereby influencing the subsequent interpretation of their roles in biochemical pathways and their potential as therapeutic targets. In the Levenshtein distance-based network, for example, the central molecules identified might be those that share specific sequences of atoms and bonds, highlighting the importance of sequence-specific interactions.

By comparing eigenvector centrality across different networks, researchers can gain a more comprehensive understanding of the multifaceted roles that BACE-1 inhibitors play within biological systems. This comparative approach allows for the identification of robust central inhibitors that are consistently central across multiple network representations, as well as context-specific hubs that may be critical in particular structural or functional contexts. Ultimately, this analysis enhances our ability to identify key molecular targets for therapeutic intervention, offering a nuanced perspective on the intricate web of interactions that underpin the functionality of BACE-1 inhibitors.

The community detection results highlight the distinct clustering patterns formed by BACE-1 inhibitors when different distance metrics are applied, providing a detailed map of molecular interactions and relationships. The Leading Eigenvector and Leiden methods both identified well-defined communities, each method offering unique insights into the structure of the networks. In particular, the Euclidean distance network showed the most coherent and stable community structures, reflecting its effectiveness in capturing the essential physicochemical properties of the molecules. This stability is indicative of the robustness of the Euclidean metric in grouping molecules with similar properties, making it a reliable choice for analyzing complex biochemical datasets.

These communities provide valuable insights into the functional and structural groupings of BACE-1 inhibitors, which are crucial for understanding their roles in biological systems. By identifying clusters of molecules that share similar properties, researchers can infer potential similarities in their biological activities, mechanisms of action, and interactions with other biomolecules. This information is invaluable for guiding targeted therapeutic interventions, as it allows for the identification of specific molecular targets within each community that could be leveraged for drug development.

The detailed community structures revealed by the analysis also highlight key inhibitors within each community. These central molecules often play crucial roles in maintaining the structural integrity and functional dynamics of their respective communities. As such, they serve as focal points for further biochemical and pharmacological studies. Investigating these key inhibitors can provide deeper insights into their specific roles in metabolic pathways, their interactions with enzymes and receptors, and their potential as drug targets.

Moreover, the differences in community structures across the various distance metrics underscore the importance of selecting appropriate metrics based on the specific aspects of molecular similarity that are most relevant to the study at hand. For example, while the Euclidean distance metric may excel in capturing overall physicochemical properties, the Tanimoto and Hamming metrics may provide more detailed insights into structural similarities and differences at the level of specific molecular subunits and fingerprints. This multifaceted approach allows for a more comprehensive understanding of the molecular landscape.

By combining the insights gained from different community detection methods and distance metrics, researchers can construct a multi-dimensional picture of the BACE-1 inhibitor network. This holistic view facilitates the identification of robust, high-priority targets for therapeutic intervention, as well as context-specific targets that may be critical in certain biochemical scenarios. Ultimately, the community detection results from this study not only enhance our understanding of the structural and functional diversity of BACE-1 inhibitors, but also pave the way for more effective and targeted approaches to drug development and therapeutic interventions.

The community comparison metrics, including Variation of Information (VOI), Normalized Mutual Information (NMI), Split Joint Distance (SJD), Rand Index (RI), and Adjusted Rand Index (ARI), collectively highlight the effectiveness of different distance functions in clustering BACE-1 inhibitors. The Euclidean distance network outperformed the others, demonstrating the lowest information loss and highest agreement with the reference classification. The Tanimoto and Hamming distance networks showed moderate performance, while the Levenshtein distance network exhibited the poorest performance, emphasizing the need for careful selection of distance metrics to achieve accurate and meaningful clustering results.

The non-parametric tests (Kruskal–Wallis, Conover, and Gehan) for various molecular descriptors across different networks provided robust statistical validation of the clustering results. Significant differences were observed for key descriptors such as molecular mass, MMFF energy, cLogP, molar refractivity (MR), and TPSA, particularly in the Euclidean distance network. These findings underscore the importance of these descriptors in defining the community structure and validate the clustering methodology used in this study. The results also highlight the multidimensional nature of molecular clustering, necessitating the use of multiple descriptors to capture the complexity of BACE-1 inhibitor interactions.

While this study provides comprehensive insights into the clustering of BACE-1 inhibitors using complex networks, several limitations should be acknowledged to contextualize the findings and suggest areas for improvement. Firstly, the reliance on specific molecular descriptors, such as molecular mass, MMFF energy, cLogP, molar refractivity, and others, may not capture all relevant aspects of molecular similarity and functionality. This choice of descriptors, while useful, inherently limits the scope of the analysis to the selected features, potentially overlooking other critical molecular characteristics that could influence clustering outcomes.

Additionally, the performance of different distance metrics varies significantly, indicating that no single metric is universally optimal for all types of molecular data. For example, while the Euclidean distance metric demonstrated robustness in capturing essential structural and physicochemical properties, the Levenshtein distance metric showed significant discrepancies in accurately representing molecular relationships. This variability suggests that the choice of distance metric should be tailored to the specific context and objectives of the study.

The study also assumed that the clustering results are directly applicable to biochemical and pharmacological contexts, which may not always be the case. The transition from computational models to practical applications in drug development and therapeutic interventions involves numerous additional factors and complexities that are not captured in the network analysis. Therefore, while the clustering provides valuable insights, these results should be interpreted with caution when applied to real-world biochemical scenarios.

Furthermore, the computational complexity of the network analyses may limit scalability to larger datasets. As the size and complexity of the molecular dataset increase, the computational resources and time required for analysis also grow, potentially becoming prohibitive. This limitation highlights the need for more efficient algorithms and computational techniques to handle large-scale network analyses effectively.

Future research could explore the integration of additional molecular descriptors and advanced machine learning techniques to enhance clustering accuracy and robustness. Incorporating a broader range of descriptors, such as quantum mechanical properties, dynamic molecular interactions, and more complex topological features, could provide a more comprehensive understanding of molecular similarities and differences. Advanced machine learning models, particularly those capable of handling high-dimensional and complex data, could further improve clustering outcomes and reveal deeper insights.

The application of dynamic and temporal network analysis could provide deeper insights into the evolution of BACE-1 inhibitor interactions over time. By examining how molecular interactions change under different conditions or over the course of biochemical processes, researchers can gain a more nuanced understanding of the dynamic nature of these interactions and their implications for drug development.

Additionally, extending the study to include other types of molecules and biological targets could validate the generalizability of the findings. This broader application could help determine whether the insights gained from BACE-1 inhibitors are applicable to other molecular systems and therapeutic areas. Such extensions would also provide opportunities to refine the methodologies and address any limitations identified in the current study.

Collaborative efforts with experimental biologists and pharmacologists could also help translate the computational insights into practical therapeutic strategies. By combining computational predictions with experimental validation, researchers can more effectively identify promising drug candidates and optimize therapeutic interventions. This interdisciplinary approach would bridge the gap between computational models and real-world applications, enhancing the overall impact of the research.

A promising direction for future work is the integration of molecular docking studies to model the binding interactions of BACE-1 inhibitors with the BACE-1 enzyme. This approach involves simulating the physical interaction between the inhibitors and the active site of the enzyme, providing detailed insights into how these molecules bind and exert their inhibitory effects. By performing comprehensive docking simulations, we can elucidate the structural basis of inhibition, identifying key binding interactions such as hydrogen bonds, hydrophobic contacts, and electrostatic interactions that are critical for effective inhibition.

Incorporating molecular docking studies can enable us to validate and enhance the significance of the molecular descriptors used in our network analysis, such as MMFF energy, cLogP, and TPSA. The docking simulations generate binding affinity scores, which quantify the strength and stability of the inhibitor–enzyme interactions. By correlating these scores with our existing molecular descriptors, we can confirm which structural features are most influential in determining binding efficacy. This correlation can provide a more robust validation of our network analysis and highlight the biochemical relevance of the descriptors.

Moreover, analyzing the binding poses obtained from docking studies offers a visual and structural perspective on how inhibitors interact with the BACE-1 enzyme. This can reveal specific regions within the enzyme’s active site that are critical for binding, allowing for us to identify hotspots for interaction. Understanding these interactions at a detailed molecular level can guide the optimization of existing inhibitors and the design of new ones with improved efficacy and selectivity.

This study successfully established a link between the molecular structure of BACE-1 inhibitors and their biochemical functions through the application of advanced network analysis techniques. By constructing and analyzing complex molecular networks, we identified key clusters of BACE-1 inhibitors that exhibit similar structural and functional properties. These clusters reveal important patterns and relationships that enhance our understanding of how specific molecular features contribute to the efficacy of BACE-1 inhibition.

In addition to clustering, we pinpointed central inhibitors within these networks using eigenvector centrality and other network metrics. These central inhibitors, due to their significant connectivity and influence within the network, are likely to play crucial roles in the inhibition process of the BACE-1 enzyme. Identifying these key molecules provides valuable insights into the structure–function relationship, which is essential for the rational design of more effective therapeutic agents.

Our findings offered a comprehensive framework that can guide future therapeutic interventions for Alzheimer’s disease. By understanding which molecular features are most important for effective BACE-1 inhibition, researchers can design new inhibitors that are more likely to succeed in clinical applications. This approach not only helps in the optimization of existing compounds, but also facilitates the discovery of novel inhibitors with improved biochemical properties.

Moreover, the methodologies and insights gained from this study are not limited to BACE-1 inhibitors alone. The network analysis approach we employed can be extended to other enzyme inhibitors and different classes of molecules, demonstrating its broader applicability in biochemical research. This versatility underscores the potential of our approach to contribute significantly to various fields of drug discovery and development. By applying these techniques to other targets, researchers can uncover new therapeutic strategies and improve the understanding of the molecular underpinnings of various diseases. 

## Figures and Tables

**Table 1:** Total number of communities and emerging community sizes for various distance-based filtered networks and community detection algorithms.

	Leading Eigenvector		Leiden	
	Count	Community Sizes	Count	Community Sizes
**Euclidean**	6	933, 412, 83, 41, 40, 38	6	910, 383, 149, 41, 34, 30
**Tanimoto**	7	426, 408, 237, 236, 109, 81, 50	9	309, 277, 208, 203, 185, 170, 83, 77, 35
**Hamming**	6	757, 383, 306, 59, 22, 20	12	470, 319, 251, 165, 106, 63, 54, 34, 33, 23, 20, 9
**Levenshtein**	1	1547	23	553, 488, 52, 42, 39, 36, 34, 33, 31, 31, 29, 26, 23, 22, 22, 19, 18, 16, 12, 12, 3, 3, 3

**Table 2:** Network community comparison metric results.

	VOI	NMI	SJD	RI	ARI
**Euclidean**	0.266662	0.881824	130	0.964622	0.927738
**Tanimoto**	1.95333	0.499584	1321	0.802102	0.280018
**Hamming**	1.38697	0.571856	651	0.807944	0.521605
**Levenshtein**	2.15364	0	1009	0.217662	0

**Table 3:** Inhibitors with the three highest eigenvector centralities in the Euclidean distance-based network. Molecular mass values are in atomic mass units, MMFF energy values are in ${\mathrm{kcal}}_{\mathrm{th}}/\mathrm{mol}$ units, and TPSA values are in Å^2^ units.

Formula	2D Plot	3D Plot	Descriptors	Centrality
C33H47F2N5O4+2	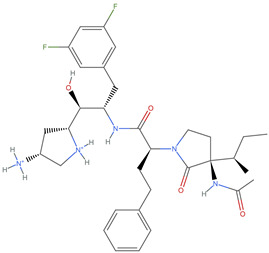	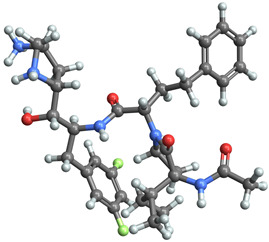	Molecular Mass = 615.766 MMFF Energy = 1697.4 cLogP = 0.454 MR = 160.757 Eccentricity = 0.841 $\kappa_{1}$ = 33.482 $\kappa_{2}$ = 14.58 $\kappa_{3}$ = 7.804 Synt. Acc. Score = 5.27 TPSA = 142.99 2D AC Entropy = 5.221 3D AC Entropy = 4.347	1.0
C33H47F2N5O4+2	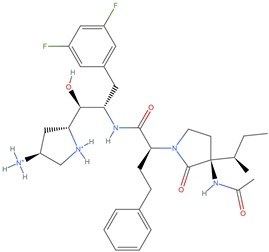	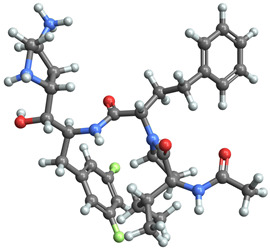	Molecular Mass = 615.766 MMFF Energy = 1692.2 cLogP = 0.454 MR = 160.757 Eccentricity = 0.828 $\kappa_{1}$ = 33.482 $\kappa_{2}$ = 14.58 $\kappa_{3}$ = 7.804 Synt. Acc. Score = 5.27 TPSA = 142.99 2D AC Entropy = 5.221 3D AC Entropy = 4.347	0.89
C41H50F5N4O5+	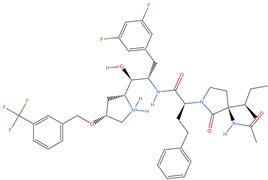	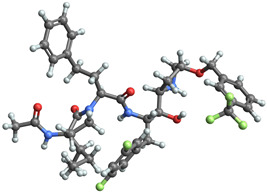	Molecular Mass = 773.865 MMFF Energy = 1663.5 cLogP = 4.4476 MR = 194.102 Eccentricity = 0.931 $\kappa_{1}$ = 41.757 $\kappa_{2}$ = 18.034 $\kappa_{3}$ = 10.471 Synt. Acc. Score = 5.23 TPSA = 124.58 2D AC Entropy = 5.192 3D AC Entropy = 4.313	0.222

**Table 4:** Inhibitors with the three highest eigenvector centralities in the Tanimoto distance-based network. Molecular mass values are in atomic mass units, MMFF energy values are in ${\mathrm{kcal}}_{\mathrm{th}}/\mathrm{mol}$ units and TPSA values are in Å^2^ units.

Formula	2D Plot	3D Plot	Descriptors	Centrality
C8H12NO+	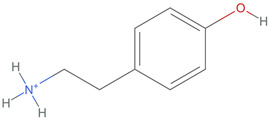	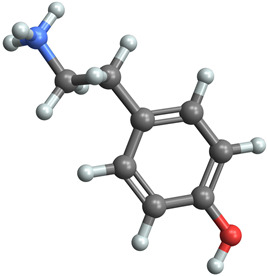	Molecular Mass = 138.189 MMFF Energy = 25.9 cLogP = 0.177 MR = 39.566 Eccentricity = 0.991 $\kappa_{1}$ = 7.091 $\kappa_{2}$ = 3.225 $\kappa_{3}$ = 1.739 Synt. Acc. Score = 2.78 TPSA = 47.87 2D AC Entropy = 4.651 3D AC Entropy = 4.105	1.0
C7H11N3O	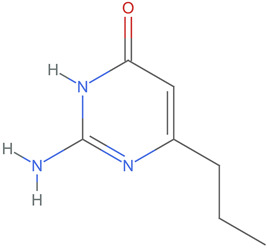	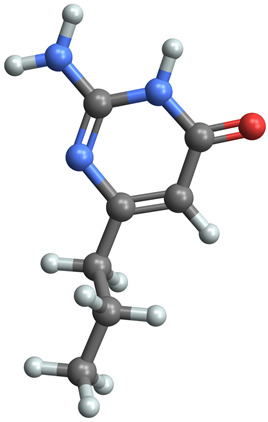	Molecular Mass = 138.189 MMFF Energy = −121.6 cLogP = 0.304 MR = 43.265 Eccentricity = 0.961 $\kappa_{1}$ = 7.783 $\kappa_{2}$ = 3.184 $\kappa_{3}$ = 2.07 Synt. Acc. Score = 2.71 TPSA = 71.77 2D AC Entropy = 4.275 3D AC Entropy = 3.775	0.8931
C66H87N13O18-2	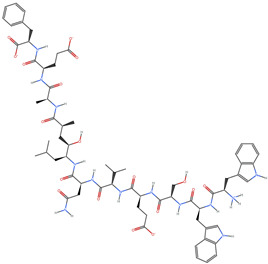	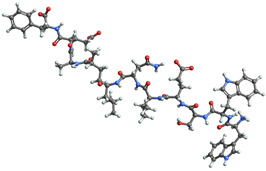	Molecular Mass = 13505 MMFF Energy = 336.6 cLogP = −5.562 MR = 342.345 Eccentricity = 0.995 $\kappa_{1}$ = 77.434 $\kappa_{2}$ = 37.14 $\kappa_{3}$ = 24.16 Synt. Acc. Score = 6.708 TPSA = 525.06 2D AC Entropy = 5.22 2D AC Entropy = 4.24	0.478

**Table 5:** Inhibitors with the three highest eigenvector centralities in the Hamming distance-based network. Molecular mass values are in atomic mass units, MMFF energy values are in ${\mathrm{kcal}}_{\mathrm{th}}/\mathrm{mol}$ units, and TPSA values are in Å^2^ units.

Formula	2D Plot	3D Plot	Descriptors	Centrality
C9H8N2	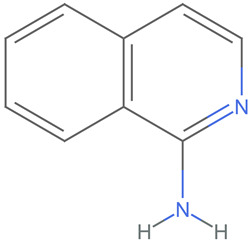	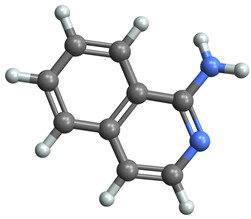	Molecular Mass = 144.177 MMFF Energy = 8.3 cLogP = 1.817 MR = 46.155 Eccentricity = 0.933 $\kappa_{1}$ = 6.16 $\kappa_{2}$ = 2.235 $\kappa_{3}$ = 0.923 Synt. Acc. Score = 1.676 TPSA = 38.91 2D AC Entropy = 3.213 3D AC Entropy = 3.376	1.0
C9H8N2	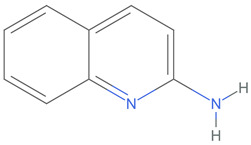	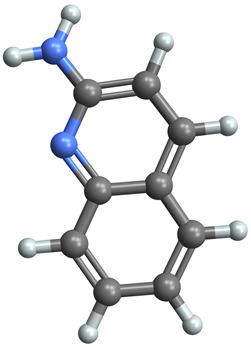	Molecular Mass = 144.177 MMFF Energy = −2.3 cLogP = 1.817 MR = 46.155 Eccentricity = 0.971 $\kappa_{1}$ = 6.16 $\kappa_{2}$ = 2.235 $\kappa_{3}$ = 1.026 Synt. Acc. Score = 1.673 TPSA = 38.91 2D AC Entropy = 3.712 3D AC Entropy = 3.775	0.9062
C7H6ClN3	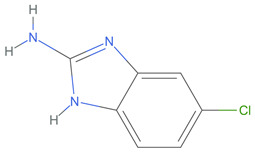	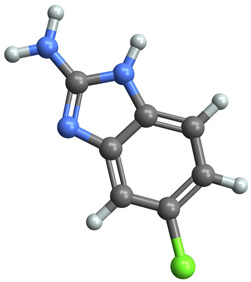	Molecular Mass = 167.596 MMFF Energy = −33.8 cLogP = 1.798 MR = 45.516 Eccentricity = 0.99 $\kappa_{1}$ = 6.487 $\kappa_{2}$ = 2.134 $\kappa_{3}$ = 1.031 Synt. Acc. Score = 2.113 TPSA = 54.7 2D AC Entropy = 4.268 3D AC Entropy = 3.412	0.3781

**Table 6:** Inhibitors with the three highest eigenvector centralities in the Levenshtein distance-based network. Molecular mass values are in atomic mass units, MMFF energy values are in ${\mathrm{kcal}}_{\mathrm{th}}/\mathrm{mol}$ units, and TPSA values are in Å^2^ units.

Formula	2D Plot	3D Plot	Descriptors	Centrality
C66H87N13O18-2	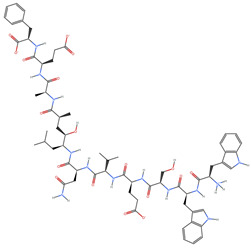	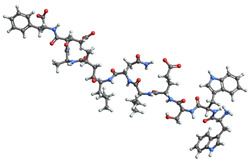	Molecular Mass = 1350.5 MMFF Energy = 336.6 cLogP = −5.562 MR = 342.345 Eccentricity = 0.995 $\kappa_{1}$ = 77.434 $\kappa_{2}$ = 37.14.435 $\kappa_{3}$ = 24.158 Synt. Acc. Score = 6.708 TPSA = 525.06 2D AC Entropy = 5.221 3D AC Entropy = 4.243	1.0
C53H91N7O11S	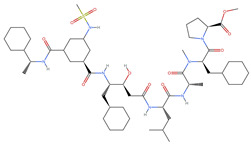	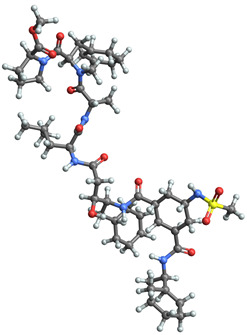	Molecular Mass = 1034.41 MMFF Energy = 327.5 cLogP = 4.609 MR = 274.016 Eccentricity = 0.98 $\kappa_{1}$ = 59.041 $\kappa_{2}$ = 28.167 $\kappa_{3}$ = 17.932 Synt. Acc. Score = 5.596 TPSA = 249.72 2D AC Entropy = 5.228 3D AC Entropy = 4.219	C7H6ClN3
C7H6ClN3	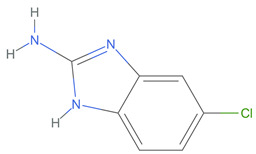	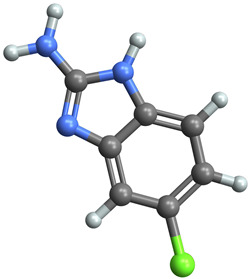	Molecular Mass = 167.6 MMFF Energy = −33.8 cLogP = 1.798 MR = 45.51 Eccentricity = 0.99 $\kappa_{1}$ = 6.487 $\kappa_{2}$ = 2.134 $\kappa_{3}$ = 1.031 Synt. Acc. Score = 2.113 TPSA = 54.7 2D AC Entropy = 4.268 3D AC Entropy = 3.411	0.0474

**Table 7:** Non-parametric test results for the communities in the Euclidean distance-based network.

	**Leading Eigenvector**					
	**Kruskal–Wallis**		**Conover**		**Gehan**	
	**Statistics**	***p*-Value**	**Statistics**	***p*-Value**	**Statistics**	***p*-Value**
**Mass**	72	$2\times 10^{-14}$	80	$0.1\times 10^{-16}$	60	$0.1\times 10^{-12}$
**MMFF Energy**	42.7341	$3.3\times 10^{-8}$	118.651	$6\times 10^{-24}$	42.8741	$3.9\times 10^{-8}$
**cLogP**	24.7661	0.00014	6.76192	0.238957	28.8541	0.00002
**MR**	83.4773	$5.5\times 10^{-17}$	75.6687	$6.7\times 10^{-15}$	93.3778	$1.3\times 10^{-18}$
**Eccentricity**	13.3118	0.0204	19.969	0.00126	14.4728	0.01287
$\kappa_{1}$	71.8606	$1.9\times 10^{-14}$	91.6546	$3\times 10^{-18}$	80.4121	$6.8\times 10^{-16}$
$\kappa_{2}$	65.6224	$4.5\times 10^{-13}$	101.046	$3.1\times 10^{-20}$	73.5804	$1.8\times 10^{-14}$
$\kappa_{3}$	49.2898	$1.4\times 10^{-9}$	92.459	$2\times 10^{-18}$	53.3259	$2.8\times 10^{-10}$
**Synt. Acc. Score**	13.4038	0.01965	23.8343	0.00023	13.2349	0.02127
**TPSA**	55.1682	$7.9\times 10^{-11}$	37.8228	$4.1\times 10^{-7}$	75.7569	$6.4\times 10^{-15}$
**2D AC Entropy**	9.23381	0.09984	14.9943	0.010387	9.56612	0.0885
**3D AC Entropy**	5.37817	0.37180	10.7768	0.05599	5.82368	0.32375
	**Leiden**					
	**Kruskal–Wallis**		**Conover**		**Gehan**	
	**Statistics**	***p*-Value**	**Statistics**	***p*-Value**	**Statistics**	***p*-Value**
**Mass**	65	$5\times 10^{-13}$	70	$0.1\times 10^{-13}$	50	$0.1\times 10^{-9}$
**MMFF Energy**	31.2411	$7.5\times 10^{-6}$	112.89	$1\times 10^{-22}$	30.9764	$9.4\times 10^{-6}$
**cLogP**	21.9618	0.000508	6.47556	0.26265	24.9907	0.00014
**MR**	75.6012	$3\times 10^{-15}$	62.992	$2.9\times 10^{-12}$	82.5172	$2.5\times 10^{-16}$
**Eccentricity**	10.5102	0.0617204	20.8654	0.00086	11.9955	0.03485
$\kappa_{1}$	65.7092	$4.3\times 10^{-13}$	74.6863	$1.1\times 10^{-14}$	71.8463	$4.2\times 10^{-14}$
$\kappa_{2}$	58.846	$1.3\times 10^{-11}$	83.3413	$1.7\times 10^{-16}$	63.8068	$1.9\times 10^{-12}$
$\kappa_{3}$	43.7094	$2.1\times 10^{-8}$	81.9458	$3.3\times 10^{-16}$	45.757	$1\times 10^{-8}$
**Synt. Acc. Score**	8.10002	0.1506	26.8684	0.00006	7.76348	0.16976
**TPSA**	52.8569	$2.4\times 10^{-10}$	28.1617	0.000034	70.9635	$6.4\times 10^{-14}$
**2D AC Entropy**	8.37637	0.1365	15.3493	0.009	8.63544	0.12452
**3D AC Entropy**	3.40426	0.6385	11.6832	0.0394	3.57696	0.61177

**Table 8:** Non-parametric test results for the communities in the Tanimoto distance-based network.

	**Leading Eigenvector**					
	**Kruskal–Wallis**		**Conover**		**Gehan**	
	**Statistics**	***p*-Value**	**Statistics**	***p*-Value**	**Statistics**	***p*-Value**
**Mass**	30	1	20	Indeterminate	22	0.001
**MMFF Energy**	20.8873	0.001853	14.1508	0.028	20.3234	0.0024
**cLogP**	36.139	$2.2\times 10^{-6}$	16.3023	0.01222	37.8253	$1.2\times 10^{-6}$
**MR**	37.872	$1\times 10^{-6}$	32.5278	0.000013	38.1831	$1.1\times 10^{-6}$
**Eccentricity**	29.0763	0.000054	35.1507	$4\times 10^{-6}$	28.4294	0.00008
$\kappa_{1}$	29.7702	0.00004	25.5225	0.0003	32.0708	0.00002
$\kappa_{2}$	37.0264	$1.5\times 10^{-6}$	37.2655	$1.6\times 10^{-6}$	41.9362	$1.9\times 10^{-7}$
$\kappa_{3}$	26.4387	0.00017	27.2663	0.00013	28.5899	0.000073
**Synt. Acc. Score**	6.30621	0.3901	39.987	$4.6\times 10^{-7}$	6.25898	0.3948
**TPSA**	16.2791	0.01212	28.0275	0.00009	19.5257	0.00336
**2D AC Entropy**	8.00562	0.23769	7.24192	0.299	7.80167	0.253
**3D AC Entropy**	7.7852	0.2543	4.19603	0.6502	7.9358	0.2428
	**Leiden**					
	**Kruskal–Wallis**		**Conover**		**Gehan**	
	**Statistics**	***p*-Value**	**Statistics**	***p*-Value**	**Statistics**	***p*-Value**
**Mass**	30	1	20	Indeterminate	401	$0.1\times 10^{-7}$
**MMFF Energy**	25.4477	0.00124	27.5241	0.0005	24.3696	0.002
**cLogP**	32.3444	0.00007	38.5932	$5.8\times 10^{-6}$	30.6288	0.00016
**MR**	53.672	$5.6\times 10^{-9}$	144.707	$2.5\times 10^{-27}$	57.3919	$1.5\times 10^{-9}$
**Eccentricity**	64.9911	$2.8\times 10^{-11}$	75.8778	$3.3\times 10^{-13}$	63.5702	$9.2\times 10^{-11}$
$\kappa_{1}$	41.9088	$1.1\times 10^{-6}$	111.673	$1.7\times 10^{-20}$	44.4378	$4.7\times 10^{-7}$
$\kappa_{2}$	50.5423	$2.4\times 10^{-8}$	132.952	$6.9\times 10^{-25}$	55.7752	$3.1\times 10^{-9}$
$\kappa_{3}$	36.8511	0.00001	81.9263	$2\times 10^{-14}$	38.7391	$5.5\times 10^{-6}$
**Synt. Acc. Score**	12.5741	0.127	31.1684	0.0001	12.7009	0.122
**TPSA**	39.0474	$4.1\times 10^{-6}$	86.8948	$1.9\times 10^{-15}$	39.6856	$3.6\times 10^{-6}$
**2D AC Entropy**	8.44045	0.39206	4.72504	0.7865	8.43121	0.3925
**3D AC Entropy**	12.1642	0.14366	3.43493	0.90418	12.617	0.12572

**Table 9:** Non-parametric test results for the communities in the Hamming distance-based network.

	**Leading Eigenvector**					
	**Kruskal–Wallis**		**Conover**		**Gehan**	
	**Statistics**	***p*-Value**	**Statistics**	***p*-Value**	**Statistics**	***p*-Value**
**Mass**	6	1	10	0.02	25.6	0.0002
**MMFF Energy**	9.62516	0.086	23.7541	0.0002	10.6499	0.059
**cLogP**	24.6365	0.00015	34.7027	$1.7\times 10^{-6}$	25.5948	0.0001
**MR**	8.57345	0.12713	17.3447	0.00389	8.67931	0.1225
**Eccentricity**	25.7991	0.00009	41.4373	$7.6\times 10^{-8}$	27.9279	0.00003
$\kappa_{1}$	6.64146	0.24876	7.05839	0.21633	7.23554	0.2037
$\kappa_{2}$	8.01238	0.15541	19.8928	0.001309	7.29374	0.19969
$\kappa_{3}$	5.52789	0.35518	22.2442	0.00047	5.55959	0.3514
**Synt. Acc. Score**	14.1234	0.0146	21.5493	0.00064	14.8409	0.011
**TPSA**	24.5465	0.00016	13.6856	0.0177	20.8105	0.00088
**2D AC Entropy**	5.04421	0.411	3.92069	0.561	5.0375	0.4113
**3D AC Entropy**	6.22668	0.2849	6.85186	0.2319	6.16288	0.291
	**Leiden**					
	**Kruskal–Wallis**		**Conover**		**Gehan**	
	**Statistics**	***p*-Value**	**Statistics**	***p*-Value**	**Statistics**	***p*-Value**
**Mass**	18	1	10.273	0.601	14.2	0.3277
**MMFF Energy**	14.5109	0.2056	27.1649	0.0043	13.9158	0.2377
**cLogP**	35.5244	0.000183	53.4726	$1.4\times 10^{-7}$	36.6265	0.00013
**MR**	27.7947	0.003318	79.6809	$1.7\times 10^{-12}$	28.3852	0.00282
**Eccentricity**	52.422	$1.7\times 10^{-7}$	62.4345	$3.2\times 10^{-9}$	56.5451	$4\times 10^{-8}$
$\kappa_{1}$	17.5218	0.092688	49.5821	$7.4\times 10^{-7}$	16.3539	0.1285
$\kappa_{2}$	27.0971	0.00425	47.5329	$1.7\times 10^{-6}$	25.3634	0.008
$\kappa_{3}$	21.7383	0.026	29.5024	0.0019	20.09	0.044
**Synt. Acc. Score**	10.345	0.5	73.8085	$2.3\times 10^{-11}$	9.88607	0.5406
**TPSA**	55.947	$3.7\times 10^{-8}$	45.0333	$4.8\times 10^{-6}$	83.4334	$3.2\times 10^{-13}$
**2D AC Entropy**	29.0569	0.0021	9.48592	0.577	28.3405	0.0029
**3D AC Entropy**	19.3205	0.055	10.8827	0.45314	19.5016	0.0527

**Table 10:** Non-parametric test results for the communities in the Levenshtein distance-based network.

	Leiden					
	Kruskal–Wallis		Conover		Gehan	
	Statistics	*p*-Value	Statistics	*p*-Value	Statistics	*p*-Value
**Mass**	455	$4\times 10^{-99}$	40	$1\times 10^{-38}$	40	Indeterminate
**MMFF Energy**	236.364	$1.8\times 10^{-41}$	85.344	$2.1\times 10^{-9}$	214.425	$1.7\times 10^{-33}$
**cLogP**	74.5757	$7.7\times 10^{-8}$	37.2395	0.02226	73.0477	$2.2\times 10^{-7}$
**MR**	476.276	$2.1\times 10^{-105}$	505.476	$5.3\times 10^{-93}$	442.651	$6.1\times 10^{-80}$
**Eccentricity**	46.0728	0.00175	47.2237	0.00137	51.9023	0.0003
$\kappa_{1}$	482.99	$1.9\times 10^{-107}$	526.291	$2.4\times 10^{-97}$	477.338	$3.8\times 10^{-87}$
$\kappa_{2}$	490.049	$1.4\times 10^{-109}$	482.514	$3.2\times 10^{-88}$	472.172	$4.5\times 10^{-86}$
$\kappa_{3}$	467.243	$1.1\times 10^{-102}$	434.316	$3.3\times 10^{-78}$	448.261	$4.2\times 10^{-81}$
**Synt. Acc. Score**	326.354	$1.2\times 10^{-63}$	151.295	$2.7\times 10^{-21}$	329.672	$1.1\times 10^{-56}$
**TPSA**	285.428	$2.7\times 10^{-53}$	198.442	$2.3\times 10^{-30}$	279.618	$1.6\times 10^{-46}$
**2D AC Entropy**	34.2592	0.0449	43.4029	0.0042	38.9614	0.0142
**3D AC Entropy**	37.1441	0.022	31.815	0.08	41.6561	0.007

## Data Availability

The original contributions presented in the study are included in the Supplementary Information file.

## Supplementary Materials

The following supporting information can be downloaded at: https://www.mdpi.com/article/10.3390/ijms25136890/s1.

## References

[1] Ali M., Hassan M., Kifayat K., Kim J.Y., Hakak S., Khan M.K. (2023). Social media content classification and community detection using deep learning and graph analytics. Technol. Forecast. Soc. Chang..

[2] Borgatti S.P., Everett M.G., Johnson J.C., Agneessens F. (2024). Analyzing Social Networks.

[3] Guo M.G., Sosa D.N., Altman R.B. (2022). Challenges and opportunities in network-based solutions for biological questions. Briefings Bioinform..

[4] Liu C., Ma Y., Zhao J., Nussinov R., Zhang Y.C., Cheng F., Zhang Z.K. (2020). Computational network biology: Data, models, and applications. Phys. Rep..

[5] Muzio G., O’Bray L., Borgwardt K. (2021). Biological network analysis with deep learning. Briefings Bioinform..

[6] Sun X., Wandelt S., Zhang A. (2020). How did COVID-19 impact air transportation? A first peek through the lens of complex networks. J. Air Transp. Manag..

[7] Sun X., Wandelt S. (2021). Robustness of air transportation as complex networks: Systematic review of 15 years of research and outlook into the future. Sustainability.

[8] Akgüller Ö., Balcı M.A., Batrancea L.M., Gaban L. (2023). Path-Based Visibility Graph Kernel and Application for the Borsa Istanbul Stock Network. Mathematics.

[9] Magner N.S., Lavin J.F., Valle M.A., Hardy N. (2020). The volatility forecasting power of financial network analysis. Complexity.

[10] Onnela J.P. (2006). Complex Networks in the Study of Financial and Social Systems.

[11] Joyce K.E., Laurienti P.J., Burdette J.H., Hayasaka S. (2010). A new measure of centrality for brain networks. PLoS ONE.

[12] Ghazzali N., Ouellet A. (2017). Comparative Study of Centrality Measures on Social Networks. Information Systems for Crisis Response and Management in Mediterranean Countries, Proceedings of the 4th International Conference, ISCRAM-Med 2017, Xanthi, Greece, 18–20 October 2017.

[13] Albert R. (2005). Scale-free networks in cell biology. J. Cell Sci..

[14] Voitalov I., Van Der Hoorn P., Van Der Hofstad R., Krioukov D. (2019). Scale-free networks well done. Phys. Rev. Res..

[15] Bassett D.S., Bullmore E. (2006). Small-world brain networks. Neuroscientist.

[16] Telesford Q.K., Joyce K.E., Hayasaka S., Burdette J.H., Laurienti P.J. (2011). The ubiquity of small-world networks. Brain Connect..

[17] Servis M.J., Clark A.E. (2021). Cluster identification using modularity optimization to uncover chemical heterogeneity in complex solutions. J. Phys. Chem. A.

[18] Gallet G.A., Pietrucci F. (2013). Structural cluster analysis of chemical reactions in solution. J. Chem. Phys..

[19] Fujii A., Mizuse K. (2013). Infrared spectroscopic studies on hydrogen-bonded water networks in gas phase clusters. Int. Rev. Phys. Chem..

[20] Iwata S. (2014). Analysis of hydrogen bond energies and hydrogen bonded networks in water clusters (H_2_O)_20_ and (H_2_O)_25_ using the charge-transfer and dispersion terms. Phys. Chem. Chem. Phys..

[21] Selegato D.M., Zanatta A.C., Pilon A.C., Veloso J.H., Castro-Gamboa I. (2023). Application of feature-based molecular networking and MassQL for the MS/MS fragmentation study of depsipeptides. Front. Mol. Biosci..

[22] Hamashima T., Li Y.C., Wu M.C., Mizuse K., Kobayashi T., Fujii A., Kuo J.L. (2013). Folding of the hydrogen bond network of h+ (ch3oh) 7 with rare gas tagging. J. Phys. Chem. A.

[23] Steber A.L., Temelso B., Kisiel Z., Schnell M., Pérez C. (2023). Rotational dive into the water clusters on a simple sugar substrate. Proc. Natl. Acad. Sci. USA.

[24] Subramanian G., Ramsundar B., Pande V., Denny R.A. (2016). Computational modeling of *β*-secretase 1 (BACE-1) inhibitors using ligand based approaches. J. Chem. Inf. Model..

[25] Viklund J., Kolmodin K., Nordvall G., Swahn B.M., Svensson M., Gravenfors Y., Rahm F. (2014). Creation of novel cores for *β*-Secretase (BACE-1) inhibitors: A multiparameter lead generation strategy. ACS Med. Chem. Lett..

[26] Kacker P., Bottegoni G., Cavalli A. (2012). Computational methods in the discovery and design of BACE-1 inhibitors. Curr. Med. Chem..

[27] John S., Thangapandian S., Sakkiah S., Lee K.W. (2011). Potent BACE-1 inhibitor design using pharmacophore modeling, in silico screening and molecular docking studies. BMC Bioinform..

[28] Kumar V., Ojha P., Saha A., Roy K. (2020). Exploring 2D-QSAR for prediction of beta-secretase 1 (BACE1) inhibitory activity against Alzheimer’s disease. SAR QSAR Environ. Res..

[29] Ciordia M., Pérez-Benito L., Delgado F., Trabanco A.A., Tresadern G. (2016). Application of free energy perturbation for the design of BACE1 inhibitors. J. Chem. Inf. Model..

[30] Domínguez J.L., Christopeit T., Villaverde M.C., Gossas T., Otero J.M., Nyström S., Baraznenok V., Lindström E., Danielson U.H., Sussman F. (2010). Effect of the protonation state of the titratable residues on the inhibitor affinity to BACE-1. Biochemistry.

[31] Wang Y.S., Strickland C., Voigt J.H., Kennedy M.E., Beyer B.M., Senior M.M., Smith E.M., Nechuta T.L., Madison V.S., Czarniecki M. (2010). Application of fragment-based NMR screening, X-ray crystallography, structure-based design, and focused chemical library design to identify novel *μ*M leads for the development of nM BACE-1 (*β*-site APP cleaving enzyme 1) inhibitors. J. Med. Chem..

[32] Huang D., Liu Y., Shi B., Li Y., Wang G., Liang G. (2013). Comprehensive 3D-QSAR and binding mode of BACE-1 inhibitors using R-group search and molecular docking. J. Mol. Graph. Model..

[33] Salum L.B., Valadares N.F. (2010). Fragment-guided approach to incorporating structural information into a CoMFA study: BACE-1 as an example. J. Comput.-Aided Mol. Des..

[34] Harush U., Barzel B. (2017). Dynamic patterns of information flow in complex networks. Nat. Commun..

[35] Zhu L., Yang F., Guan G., Zhang Z. (2021). Modeling the dynamics of rumor diffusion over complex networks. Inf. Sci..

[36] Guimaraes P.R. (2020). The structure of ecological networks across levels of organization. Annu. Rev. Ecol. Evol. Syst..

[37] Olutola T., Balen J., Lotisa V., Johnima A., Browndi I. (2023). Systems Biology and Cell Signaling: A Comprehensive Review. Asian J. Basic Appl. Sci..

[38] Chen D., Liu J., Wu J., Wei G.W., Pan F., Yau S.T. (2023). Path topology in molecular and materials sciences. J. Phys. Chem. Lett..

[39] Liu J., Chen D., Pan F., Wu J. (2023). Neighborhood Path Complex for the Quantitative Analysis of the Structure and Stability of Carboranes. J. Comput. Biophys. Chem..

[40] Silverman E.K., Schmidt H.H., Anastasiadou E., Altucci L., Angelini M., Badimon L., Balligand J.L., Benincasa G., Capasso G., Conte F. (2020). Molecular networks in Network Medicine: Development and applications. Wiley Interdiscip. Rev. Syst. Biol. Med..

[41] Barthélemy M., Barrat A., Pastor-Satorras R., Vespignani A. (2005). Characterization and modeling of weighted networks. Phys. A Stat. Mech. Its Appl..

[42] Newman M.E. (2004). Analysis of weighted networks. Phys. Rev. E.

[43] Fukunaga T., Nagamochi H. (2010). Network design with weighted degree constraints. Discret. Optim..

[44] Wei D., Li Y., Zhang Y., Deng Y. Degree centrality based on the weighted network. Proceedings of the 2012 24th Chinese Control and Decision Conference (CCDC).

[45] AbuSalim S.W., Ibrahim R., Saringat M.Z., Jamel S., Wahab J.A. (2020). Comparative analysis between dijkstra and bellman-ford algorithms in shortest path optimization. Iop Conf. Ser. Mater. Sci. Eng..

[46] Akgüller Ö. (2019). A threshold method for financial networks and geometric scattering of agents. Commun. Stat. Case Stud. Data Anal. Appl..

[47] Unicomb S., Iñiguez G., Karsai M. (2018). Threshold driven contagion on weighted networks. Sci. Rep..

[48] Yang X.H., Jiang F.L., Chen S.Y., Wang W.L. (2011). Modeling evolution of weighted clique networks. Commun. Theor. Phys..

[49] Stam C., Tewarie P., Van Dellen E., Van Straaten E., Hillebrand A., Van Mieghem P. (2014). The trees and the forest: Characterization of complex brain networks with minimum spanning trees. Int. J. Psychophysiol..

[50] Yu M., Hillebrand A., Tewarie P., Meier J., van Dijk B., Van Mieghem P., Stam C.J. (2015). Hierarchical clustering in minimum spanning trees. Chaos.

[51] Maurya R., Sharma R. (2023). Comparison of Prim and Kruskal’s Algorithm. Glob. J. Comput. Sci. Technol..

[52] Todeschini R., Valsecchi C. (2022). Evaluation of classification performances of minimum spanning trees by 13 different metrics. MATCH.

[53] Wang Y., Yu S., Gu Y., Shun J. Fast parallel algorithms for euclidean minimum spanning tree and hierarchical spatial clustering. Proceedings of the 2021 International Conference on Management of Data.

[54] Sharma K.K., Seal A. (2020). Clustering analysis using an adaptive fused distance. Eng. Appl. Artif. Intell..

[55] Subramanian B., Paul A., Kim J., Chee K.W.A. (2022). Metrics space and norm: Taxonomy to distance metrics. Sci. Program..

[56] Bajusz D., Rácz A., Héberger K. (2015). Why is Tanimoto index an appropriate choice for fingerprint-based similarity calculations?. J. Cheminform..

[57] Martin E., Cao E. (2015). Euclidean chemical spaces from molecular fingerprints: Hamming distance and Hempel’s ravens. J. Comput.-Aided Mol. Des..

[58] Fechner U., Paetz J., Schneider G. (2005). Comparison of three holographic fingerprint descriptors and their binary counterparts. QSAR Comb. Sci..

[59] Laboulais C., Ouali M., Le Bret M., Gabarro-Arpa J. (2002). Hamming distance geometry of a protein conformational space: Application to the clustering of a 4-ns molecular dynamics trajectory of the HIV-1 integrase catalytic core. Proteins Struct. Funct. Bioinform..

[60] Ristad E.S., Yianilos P.N. (1998). Learning string-edit distance. IEEE Trans. Pattern Anal. Mach. Intell..

[61] Berger B., Waterman M.S., Yu Y.W. (2020). Levenshtein distance, sequence comparison and biological database search. IEEE Trans. Inf. Theory.

[62] Öztürk H., Ozkirimli E., Özgür A. (2016). A comparative study of SMILES-based compound similarity functions for drug-target interaction prediction. BMC Bioinform..

[63] Massara G.P., Di Matteo T., Aste T. (2016). Network filtering for big data: Triangulated maximally filtered graph. J. Complex Netw..

[64] Cuzzocrea A., Papadimitriou A., Katsaros D., Manolopoulos Y. (2012). Edge betweenness centrality: A novel algorithm for QoS-based topology control over wireless sensor networks. J. Netw. Comput. Appl..

[65] Hanzelka J., Běloch M., Martinovič J., Slaninová K. (2019). Vertex importance extension of betweenness centrality algorithm. Data Management, Analytics and Innovation: Proceedings of ICDMAI 2018.

[66] Jia Y., Yi J., Yan S. (2020). Constraint Inversion Model of Core Science Complex Network. J. Phys. Conf. Ser..

[67] Martin T., Zhang X., Newman M.E. (2014). Localization and centrality in networks. Phys. Rev. E.

[68] Tudisco F., Mercado P., Hein M. (2018). Community detection in networks via nonlinear modularity eigenvectors. SIAM J. Appl. Math..

[69] Waltman L., Van Eck N.J. (2013). A smart local moving algorithm for large-scale modularity-based community detection. Eur. Phys. J. B.

[70] Meilă M. (2003). Comparing clusterings by the variation of information. Proceedings of the Learning Theory and Kernel Machines: 16th Annual Conference on Learning Theory and 7th Kernel Workshop, COLT/Kernel 2003.

[71] Meilă M. (2007). Comparing clusterings—An information based distance. J. Multivar. Anal..

[72] Amelio A., Pizzuti C. (2017). Correction for closeness: Adjusting normalized mutual information measure for clustering comparison. Comput. Intell..

[73] Shirkhorshidi A.S., Aghabozorgi S., Wah T.Y. (2015). A comparison study on similarity and dissimilarity measures in clustering continuous data. PLoS ONE.

[74] Zhang S., Wong H.S., Shen Y. (2012). Generalized adjusted rand indices for cluster ensembles. Pattern Recognit..

