# Supplementary material for: Network Models of BACE-1 Inhibitors: Exploring Structural and Biochemical Relationships
Source: Int J Mol Sci. 2024 Jun 23;25(13):6890. doi: 10.3390/ijms25136890 (PMC11240958; doi:10.3390/ijms25136890)

2D Plot

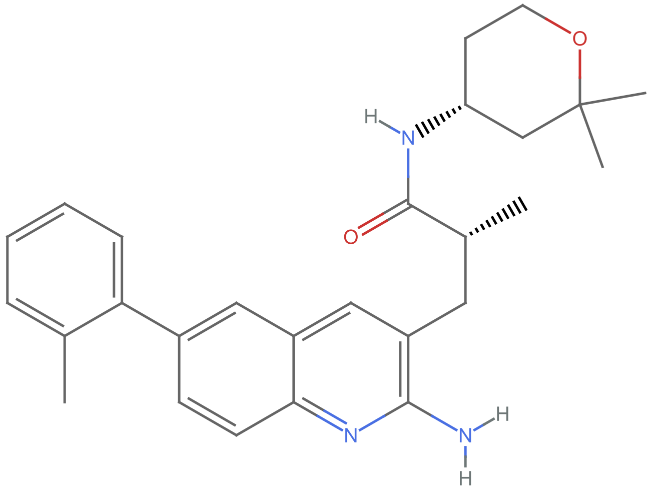

2D Autocorrelation

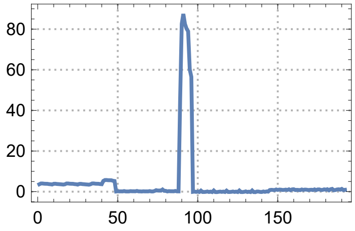

3D Plot

Dataset Index= 1

Molecule Formula= $C_{27}H_{33}N_{3}O_{2}$

3D Autocorrelation

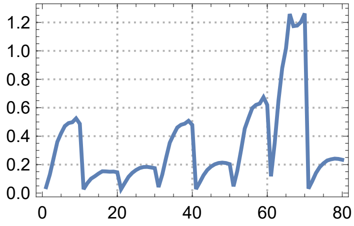

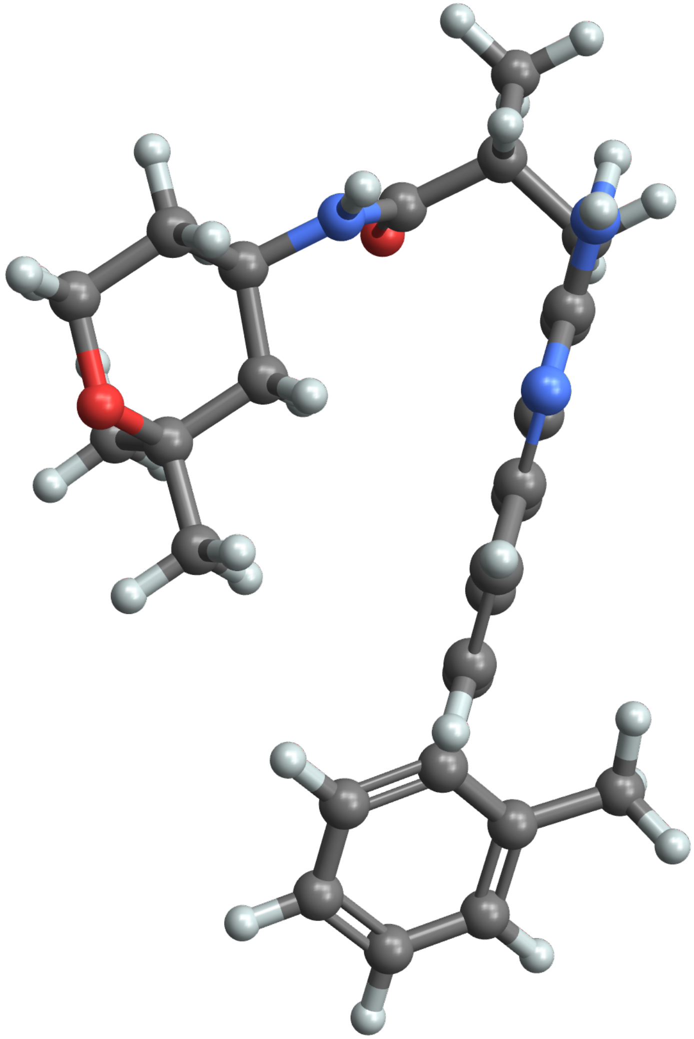

2D Plot

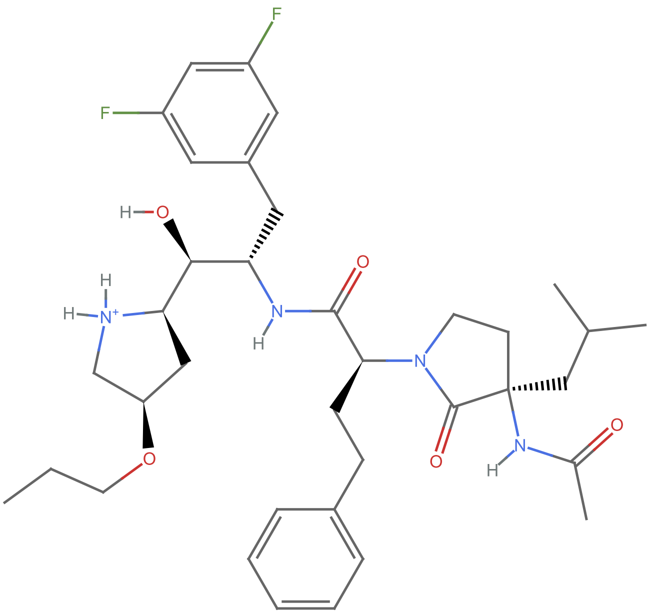

2D Autocorrelation

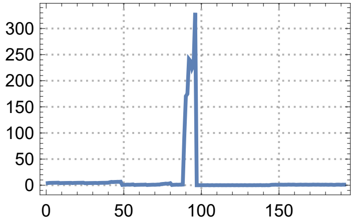

3D Plot

Dataset Index= 2

Molecule Formula= $C_{36}H_{51}F_2N_4O_5^+$

3D Autocorrelation

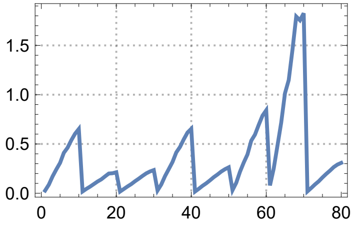

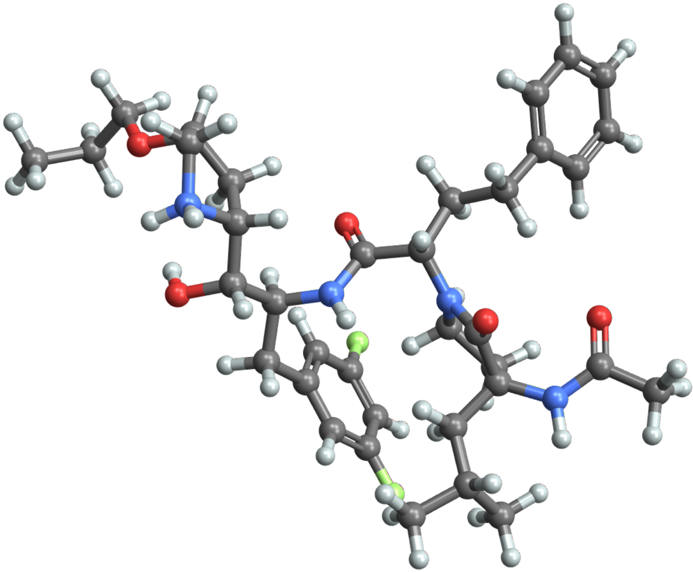

2D Plot

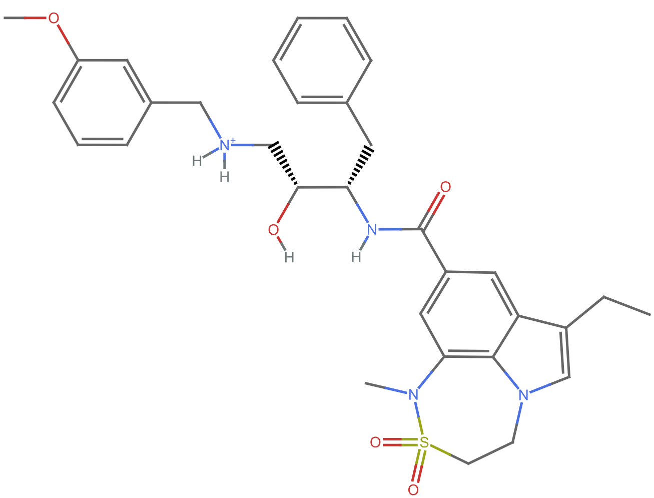

2D Autocorrelation

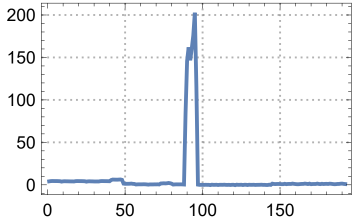

Dataset Index= 3

Molecule Formula= $C_{32}H_{39}N_4O_5S^+$

3D Plot

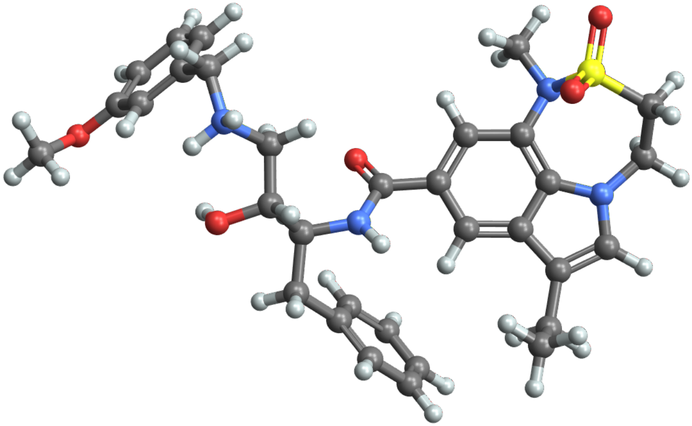

3D Autocorrelation

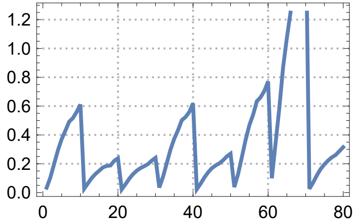

2D Plot

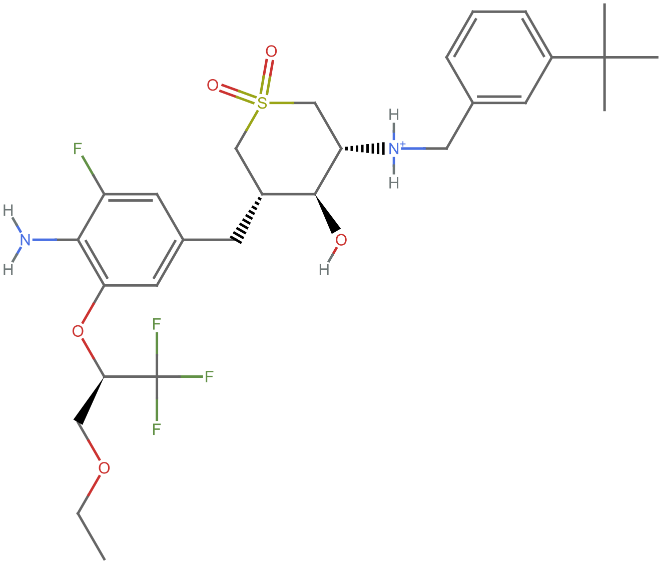

2D Autocorrelation

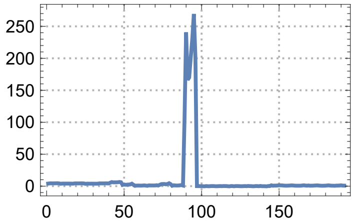

Dataset Index= 4

Molecule Formula= $C_{28}H_{39}F_4N_2O_5S^+$

3D Autocorrelation

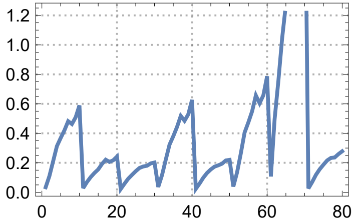

3D Plot

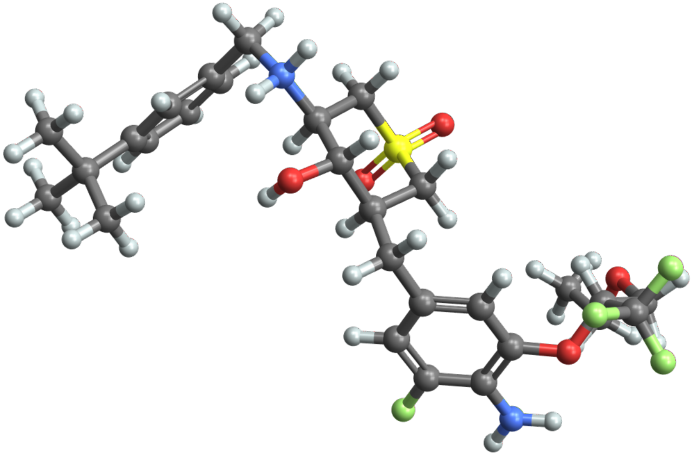

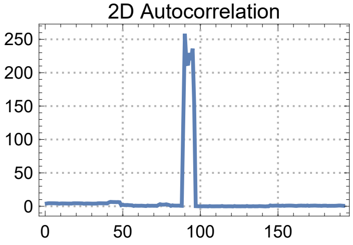

Dataset Index= 5

Molecule Formula= $C_{32}H_{36}F_3N_4O_4S^+$

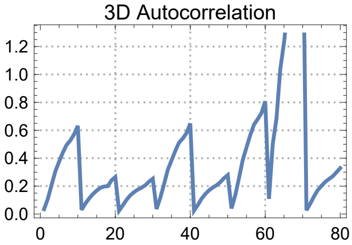

2D Plot

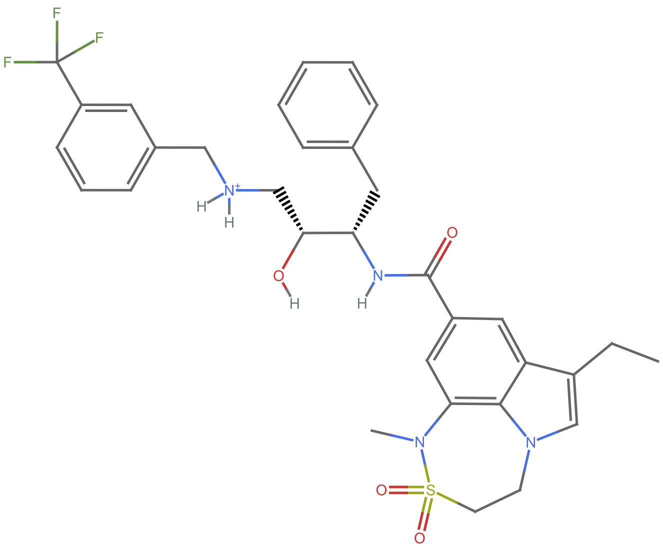

3D Plot

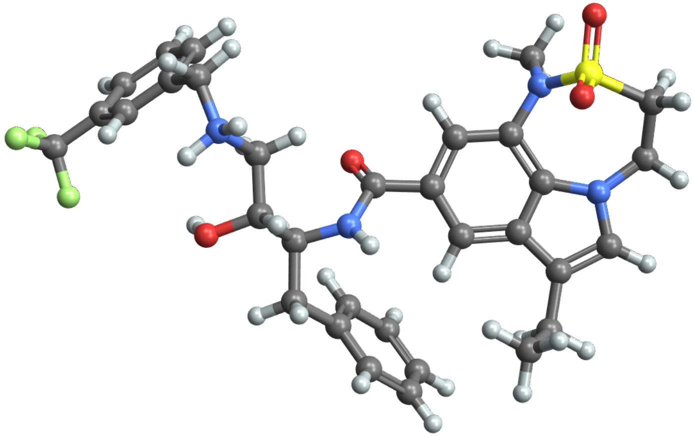

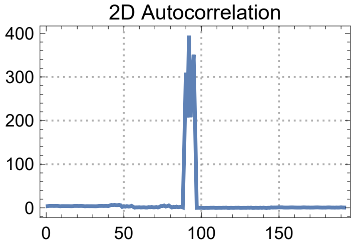

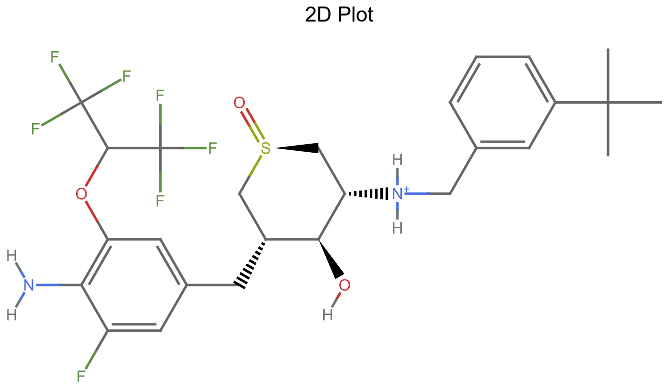

Dataset Index= 6

Molecule Formula= $C_{26}H_{32}F_7N_2O_3S^+$

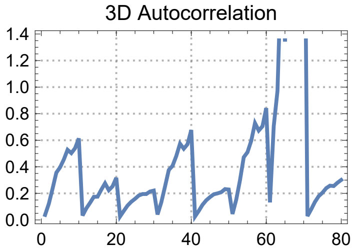

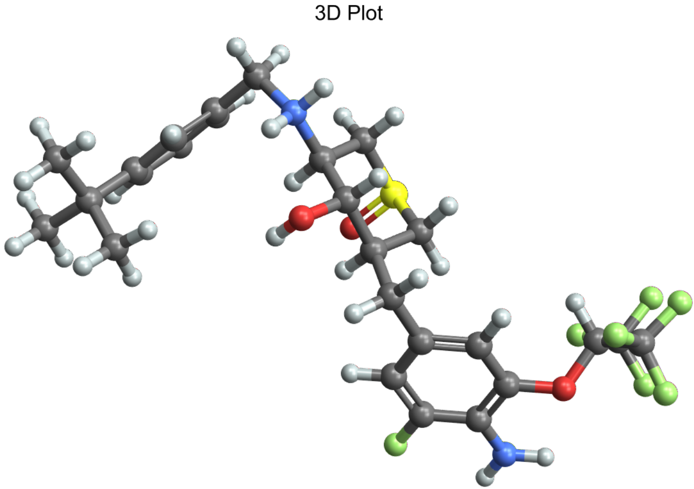

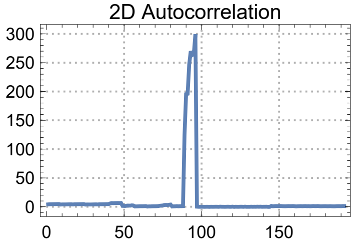

Dataset Index= 7

Molecule Formula= $C_{33}H_{43}F_2N_4O_5S^+$

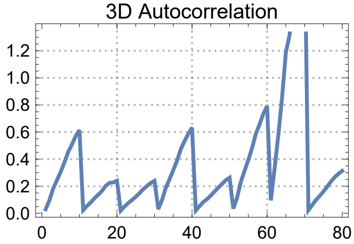

2D Plot

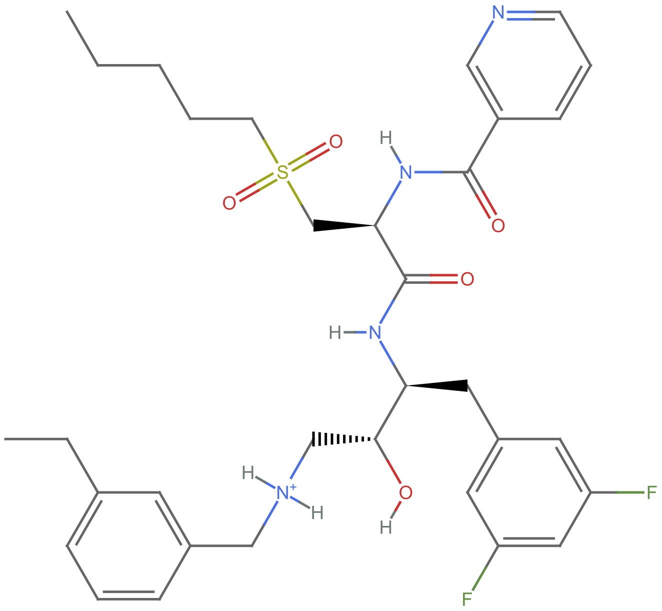

3D Plot

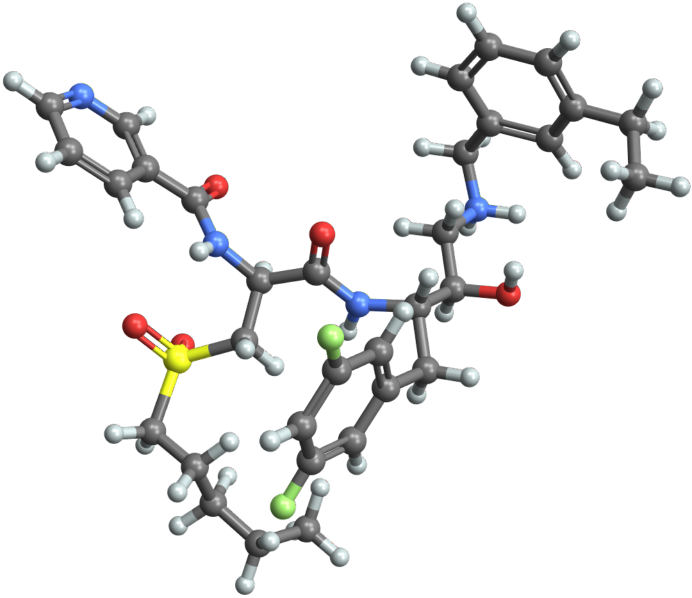

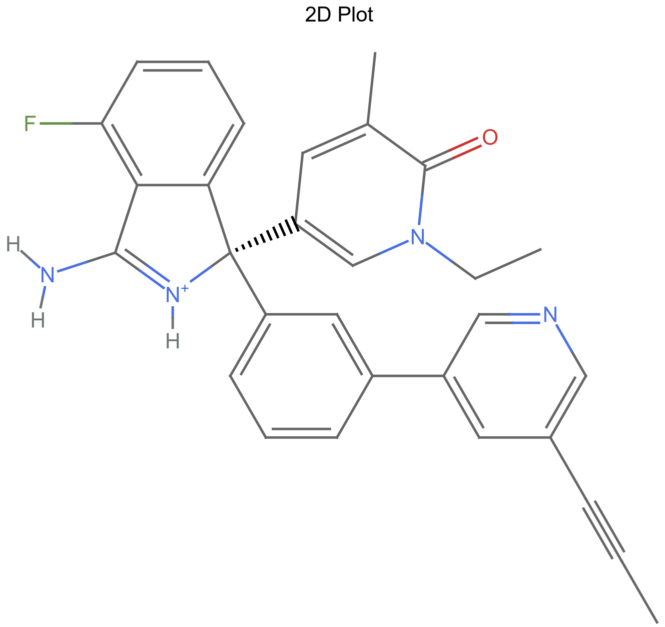

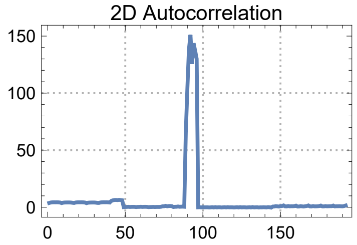

Dataset Index= 8

Molecule Formula= $C_{30}H_{26}FN_4O^+$

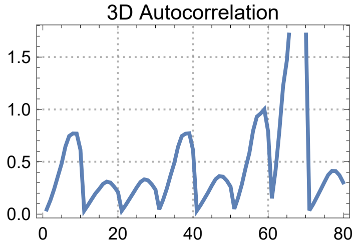

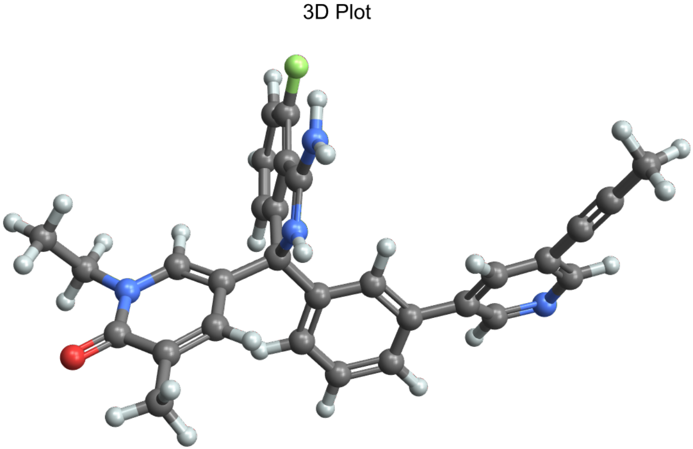

2D Plot

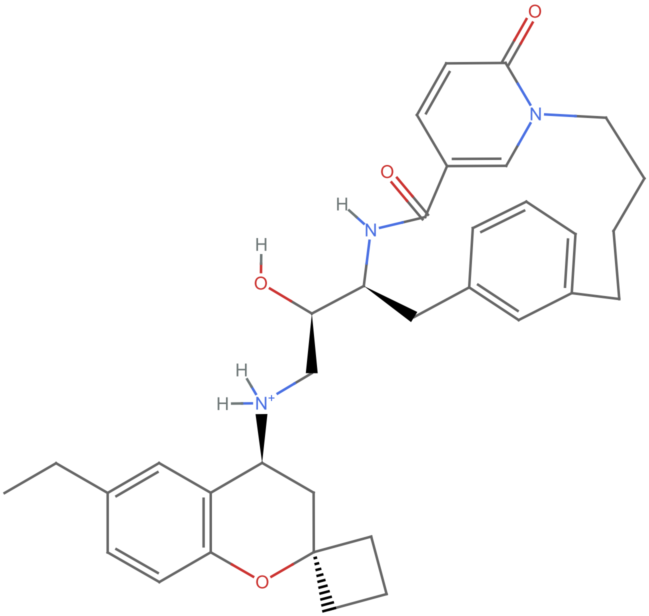

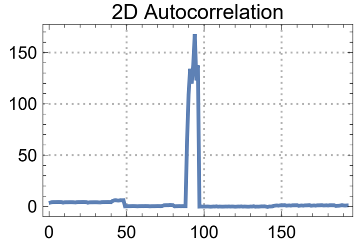

Dataset Index= 9

Molecule Formula= $C_{34}H_{42}N_3O_4^+$

3D Plot

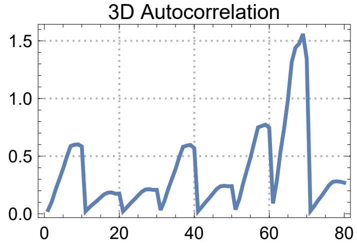

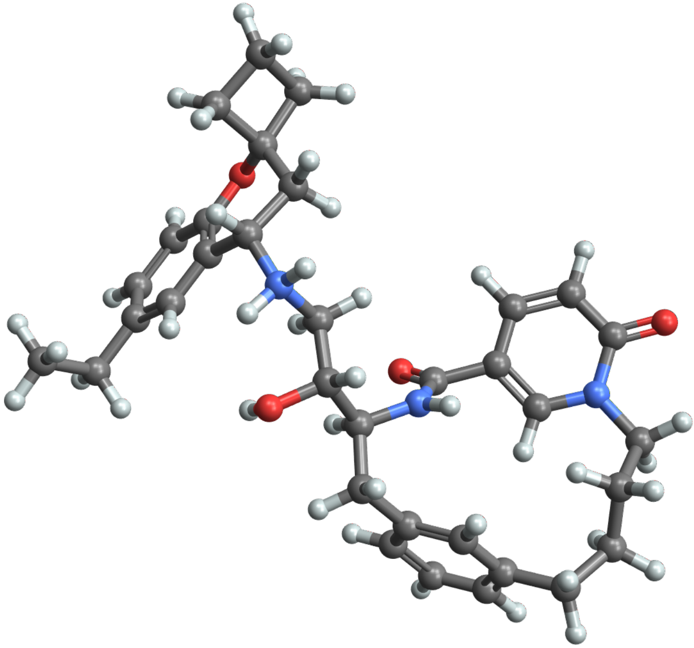

2D Plot

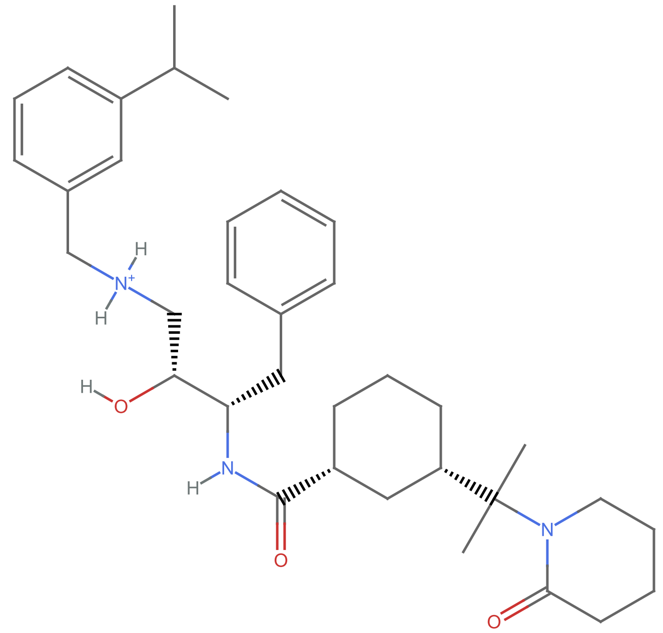

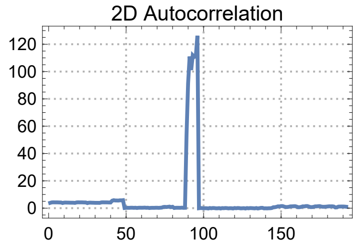

Dataset Index= 10

Molecule Formula= $C_{35}H_{52}N_3O_3^+$

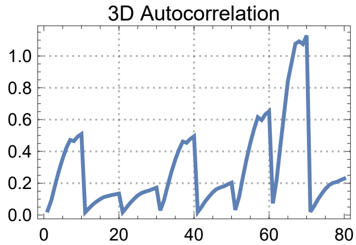

3D Plot

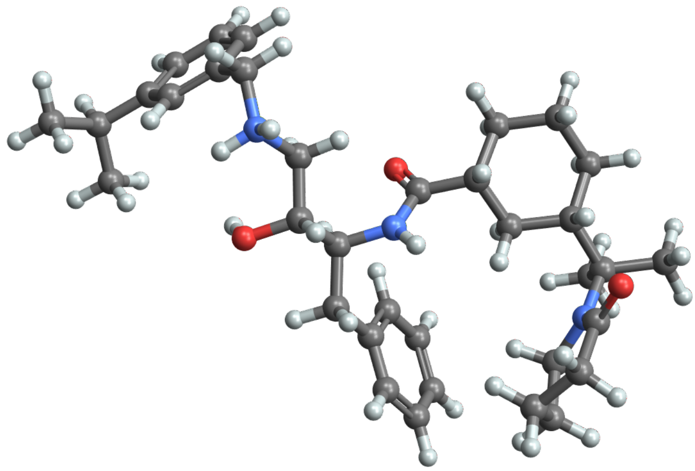

2D Autocorrelation

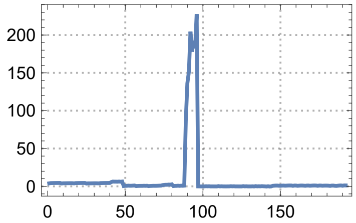

Dataset Index= 11

Molecule Formula= $C_{34}H_{42}F_2N_3O_4^+$

3D Autocorrelation

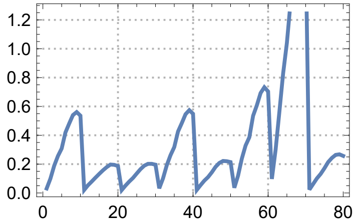

2D Plot

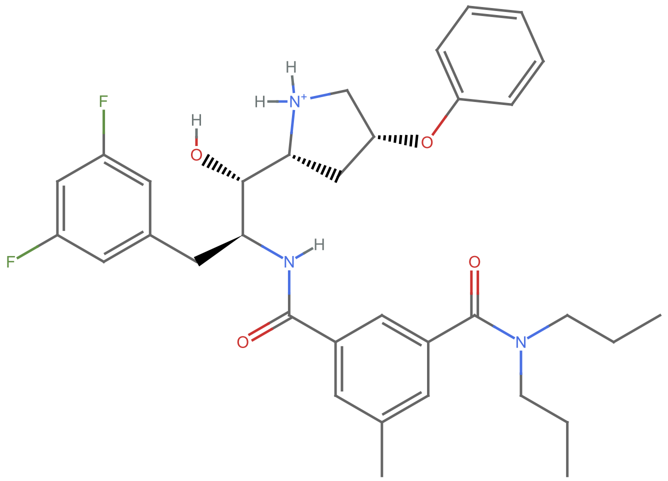

3D Plot

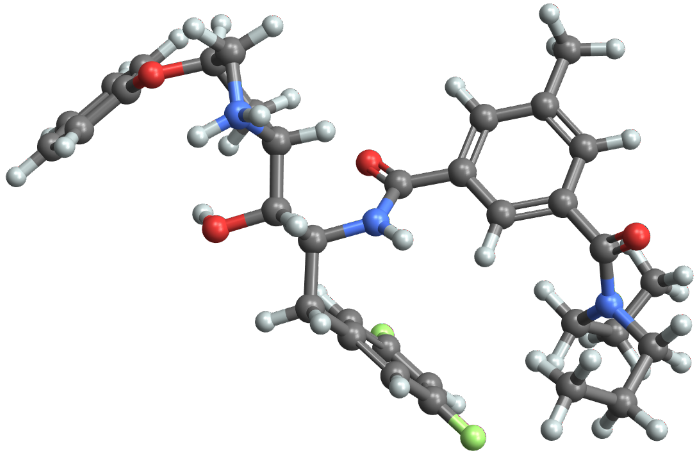

2D Plot

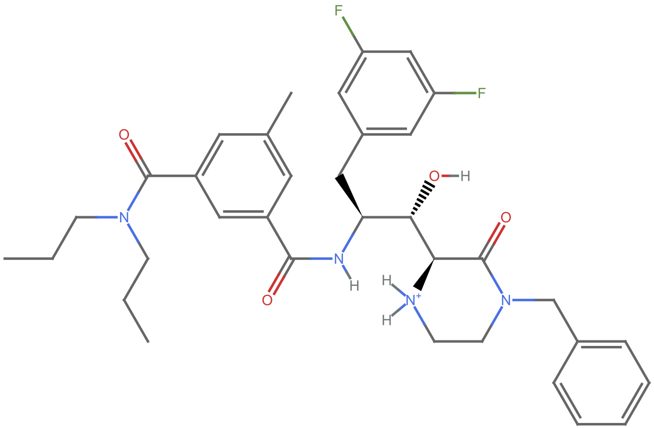

2D Autocorrelation

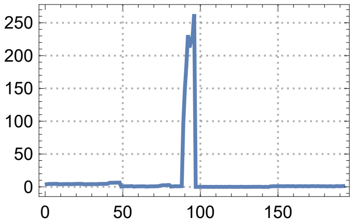

3D Plot

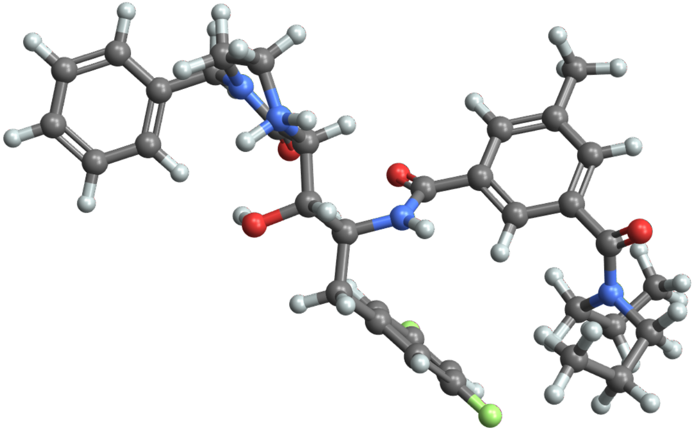

Dataset Index= 12

Molecule Formula= $C_{35}H_{43}F_2N_4O_4^+$

3D Autocorrelation

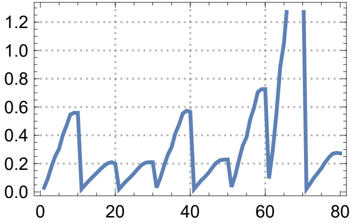

2D Autocorrelation

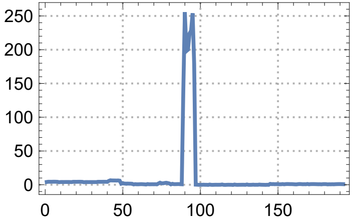

Dataset Index= 13

Molecule Formula= $C_{31}H_{38}F_3N_4O_4S^+$

3D Autocorrelation

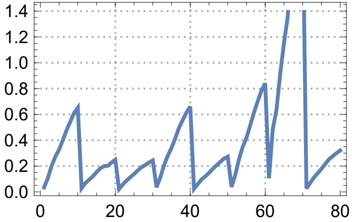

2D Plot

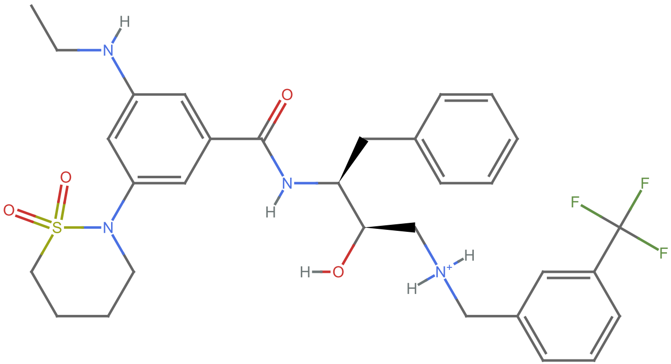

3D Plot

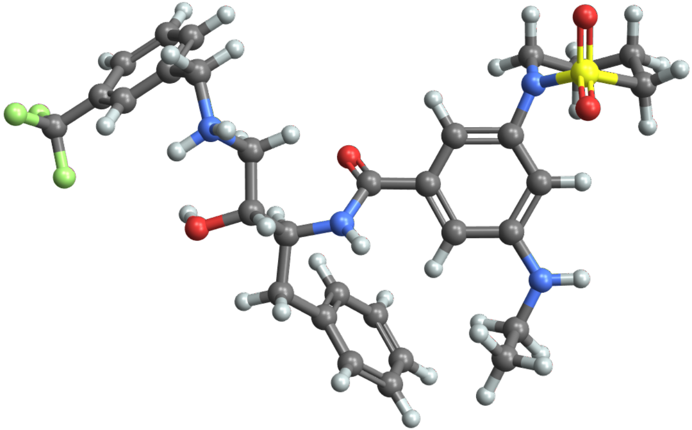

2D Plot

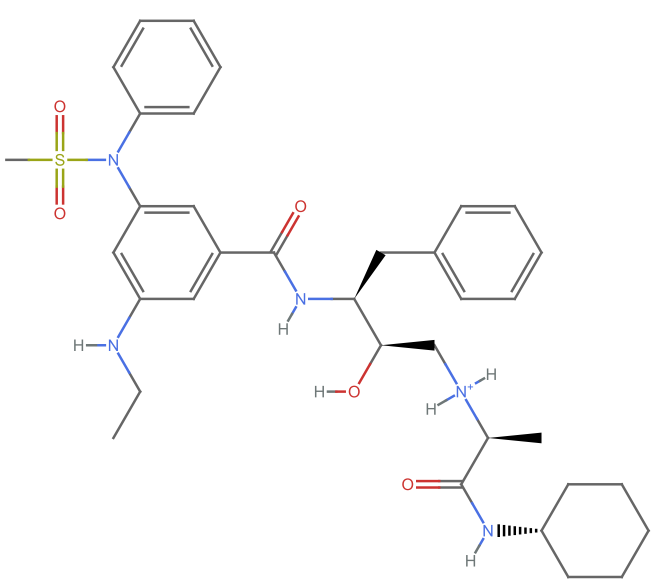

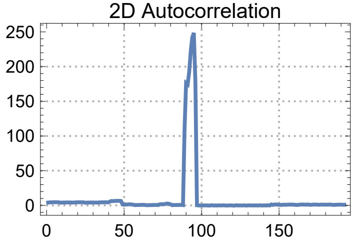

Dataset Index= 14

Molecule Formula= $C_{35}H_{48}N_5O_5S^+$

3D Plot

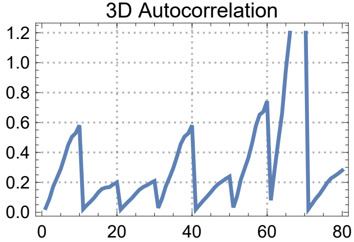

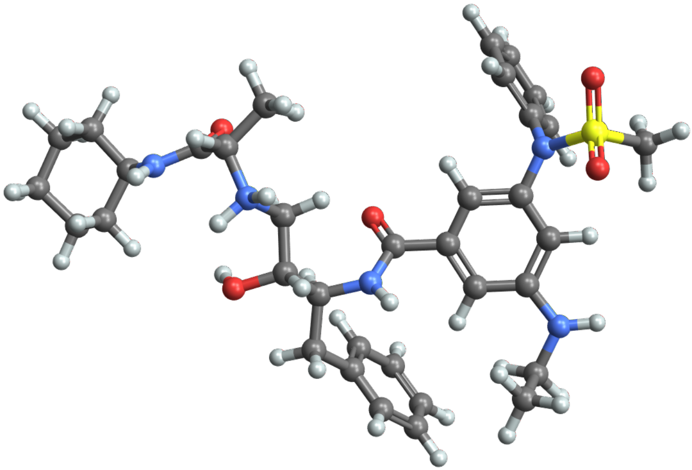

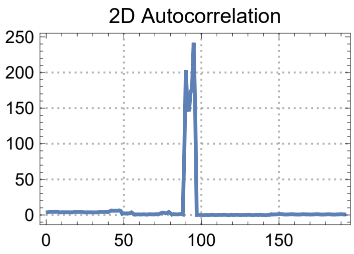

Dataset Index= 15

Molecule Formula= $C_{27}H_{37}F_4N_2O_4S^+$

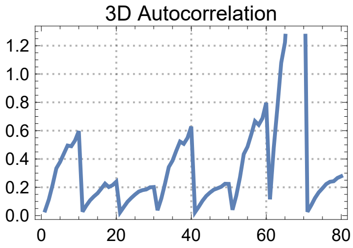

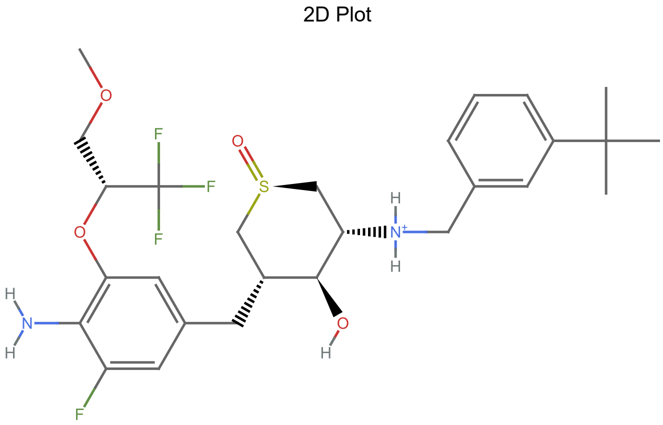

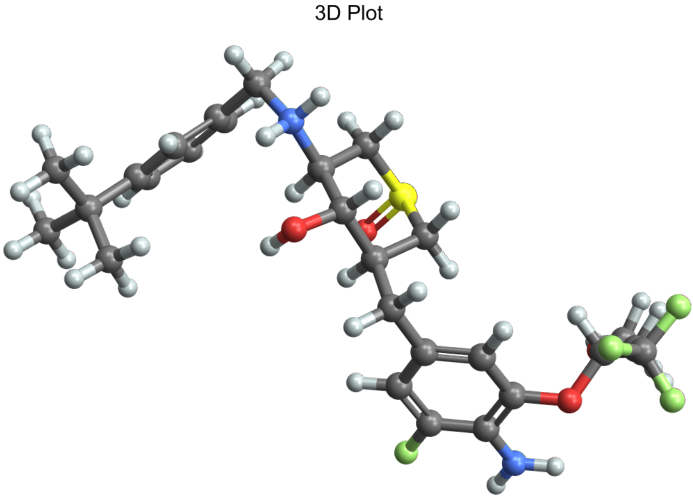

2D Plot

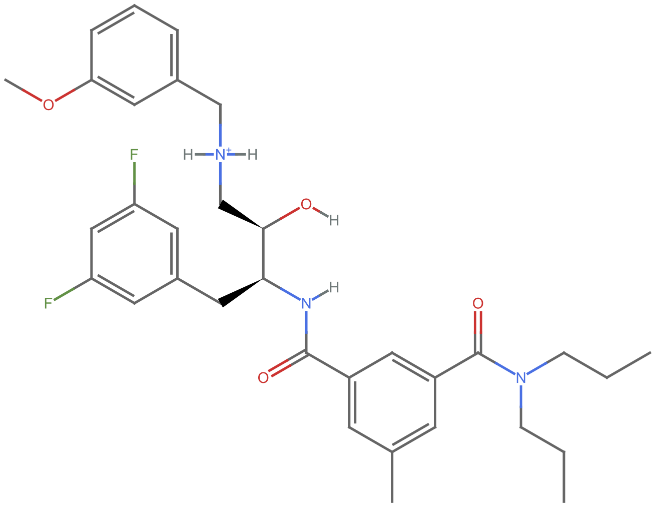

2D Autocorrelation

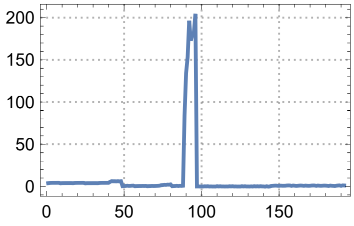

Dataset Index= 16

Molecule Formula= $C_{33}H_{42}F_2N_3O_4^+$

3D Plot

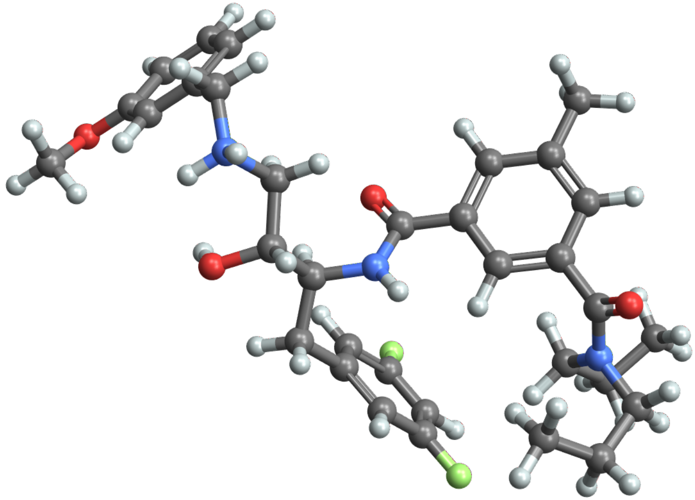

3D Autocorrelation

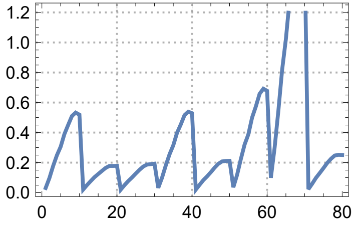

2D Autocorrelation

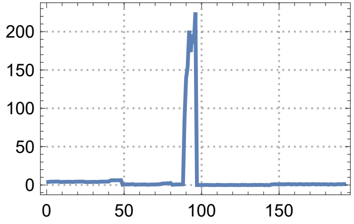

2D Plot

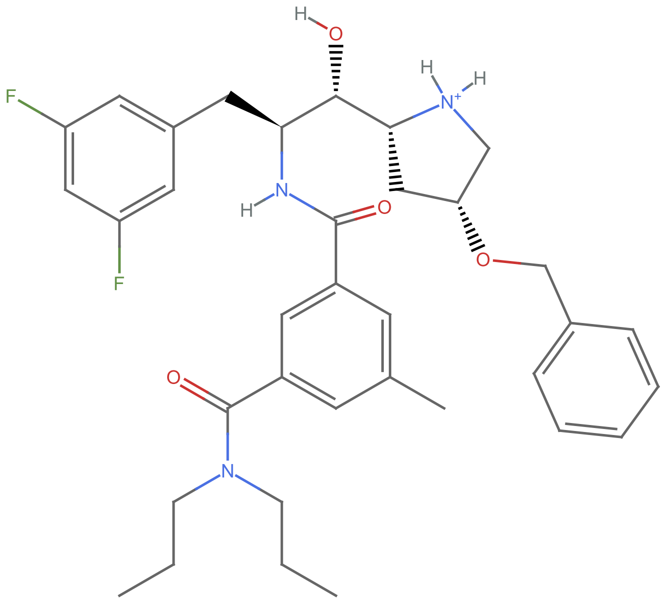

Dataset Index= 17

Molecule Formula= $C_{35}H_{44}F_2N_3O_4^{+}$

3D Autocorrelation

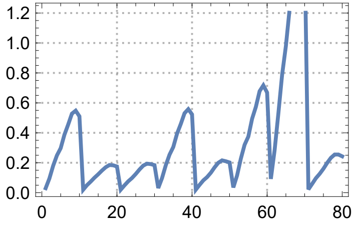

3D Plot

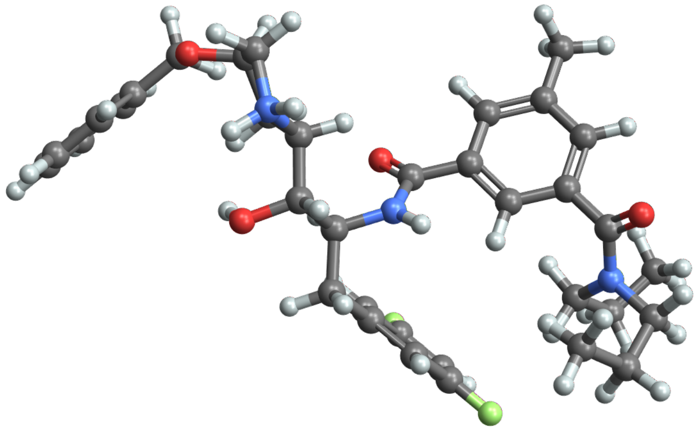

2D Plot

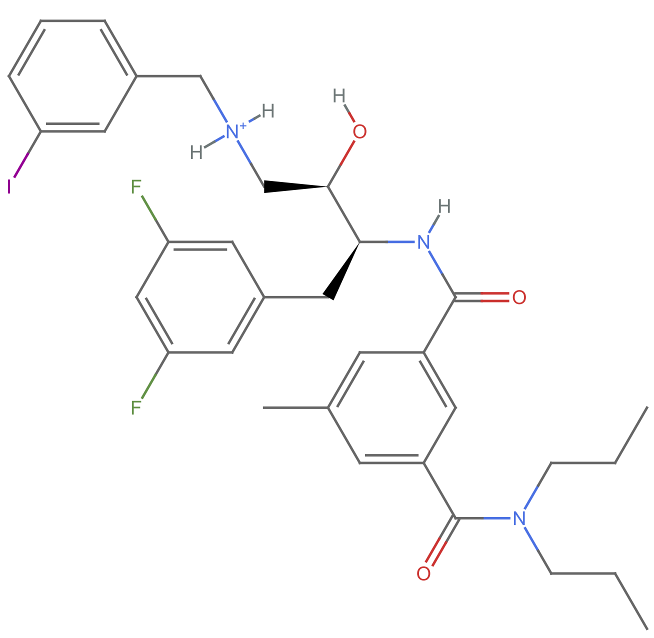

2D Autocorrelation

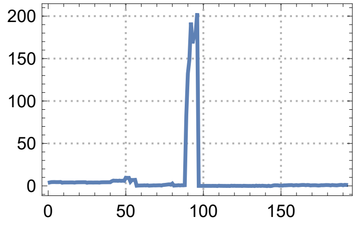

Dataset Index= 18

Molecule Formula= $C_{32}H_{39}F_{2}IN_{3}O_{3}^{+}$

3D Plot

3D Autocorrelation

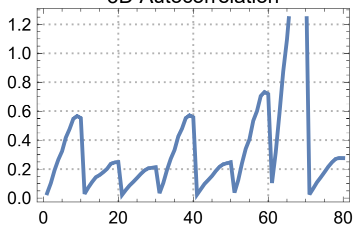

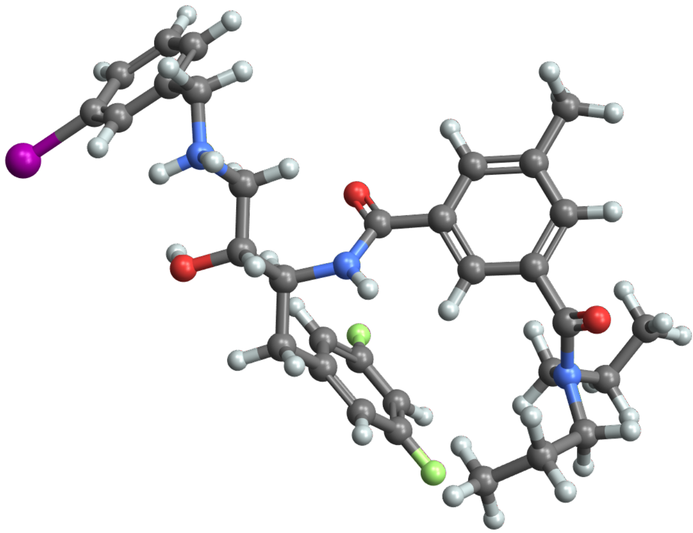

2D Plot

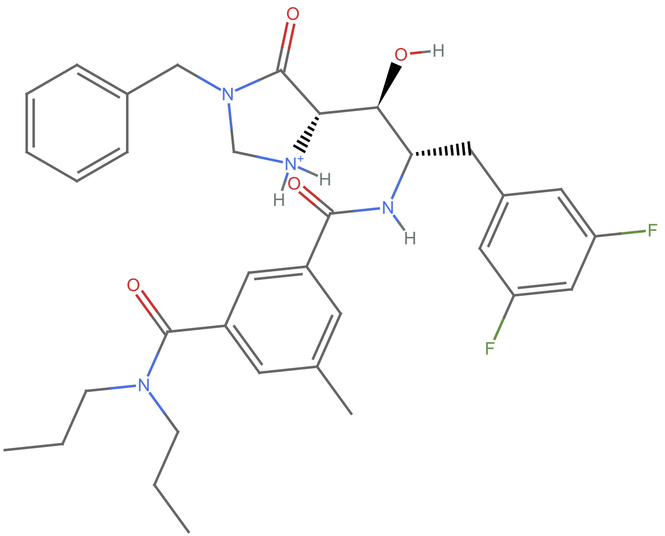

2D Autocorrelation

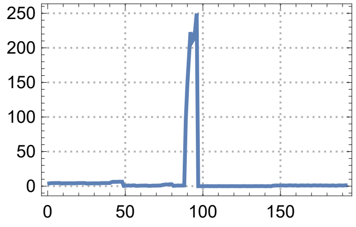

Dataset Index= 19

Molecule Formula= $C_{34}H_{41}F_{2}N_{4}O_{4}^{+}$

3D Plot

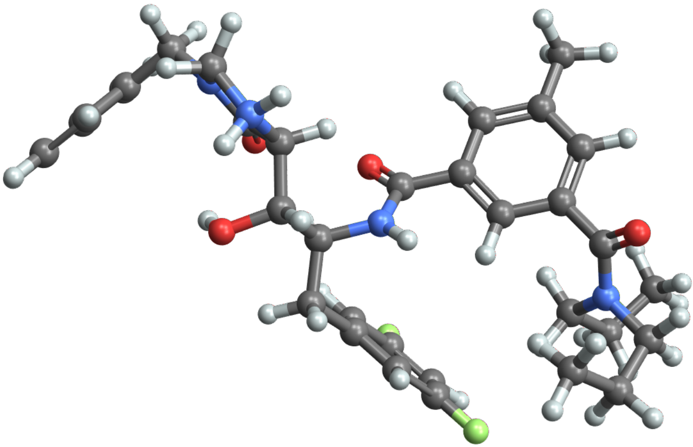

3D Autocorrelation

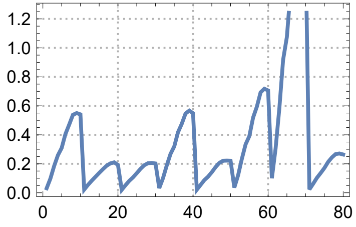

2D Plot

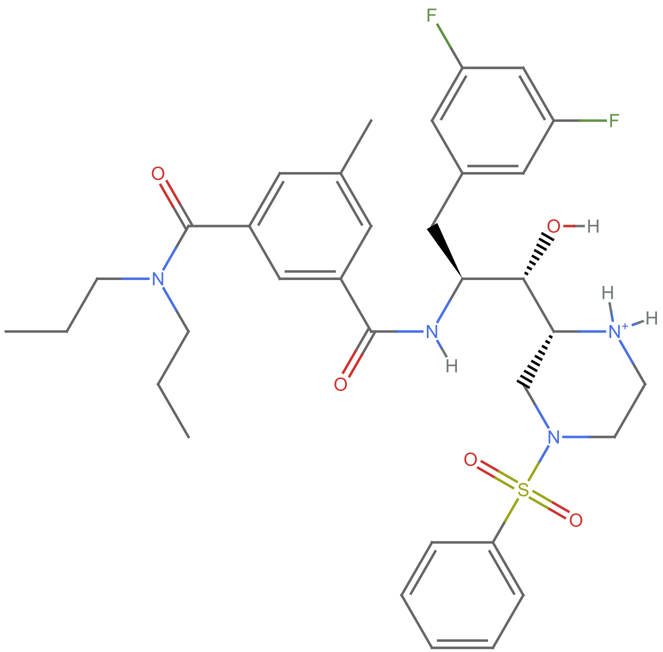

2D Autocorrelation

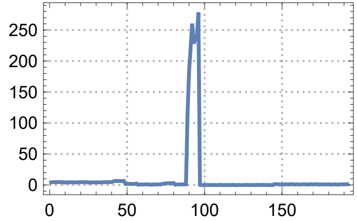

Dataset Index= 20

Molecule Formula= $C_{34}H_{43}F_2N_4O_5S^+$

3D Autocorrelation

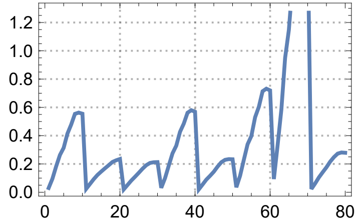

3D Plot

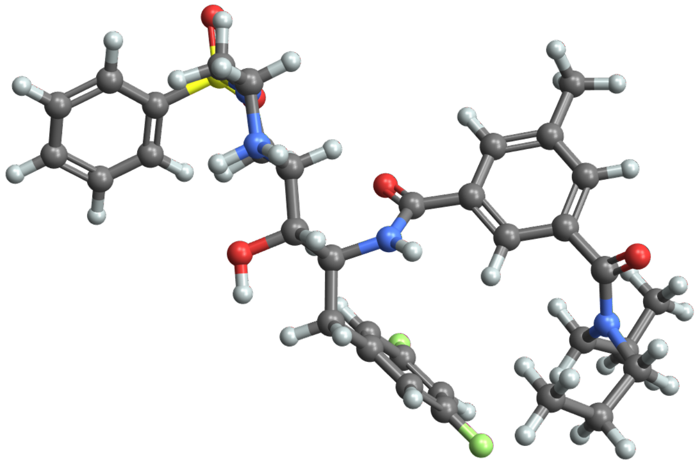

2D Autocorrelation

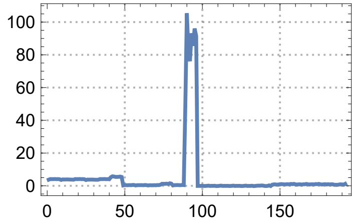

Dataset Index= 21

Molecule Formula= $C_{28}H_{39}FN_3O_3^+$

3D Autocorrelation

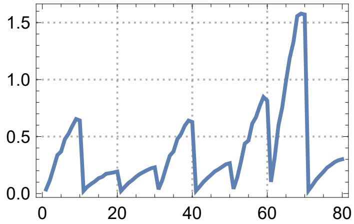

2D Plot

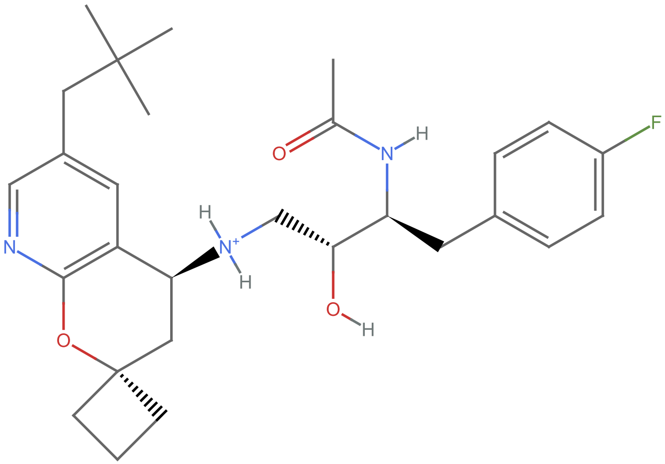

3D Plot

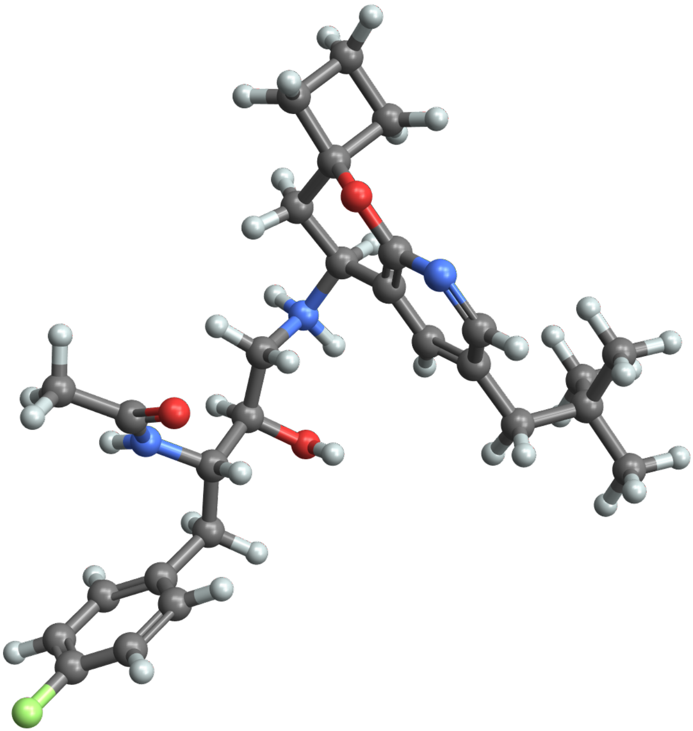

2D Plot

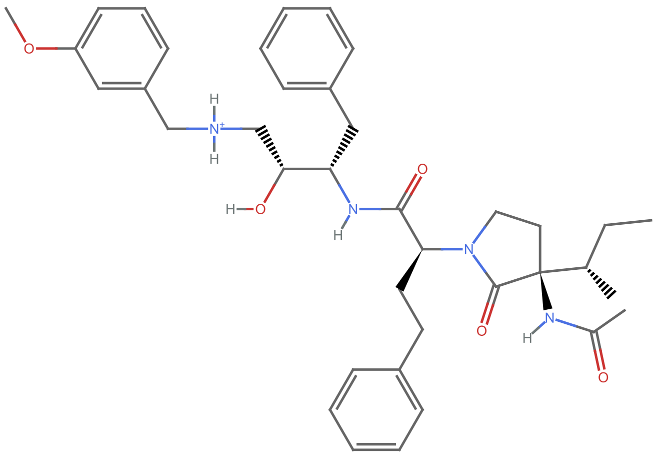

2D Autocorrelation

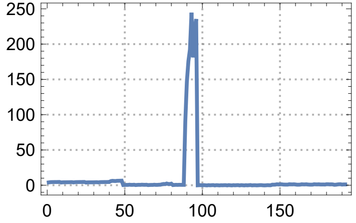

Dataset Index= 22

Molecule Formula= $C_{38}H_{51}N_4O_5^+$

3D Autocorrelation

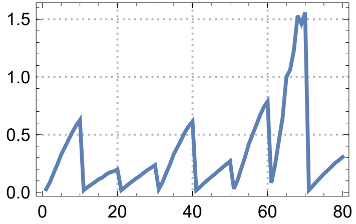

3D Plot

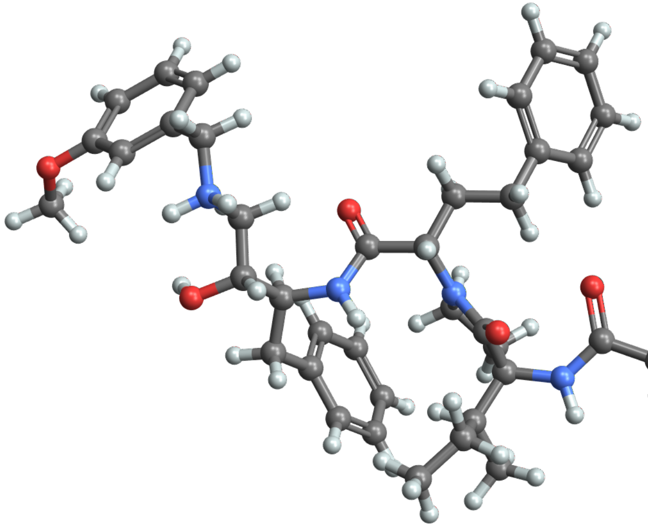

2D Autocorrelation

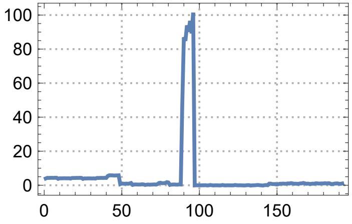

Dataset Index= 23

Molecule Formula= $C_{33}H_{45}N_4O_4S^+$

3D Autocorrelation

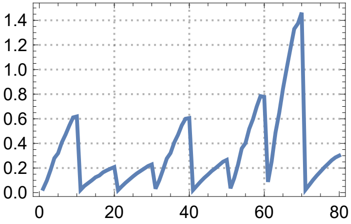

2D Plot

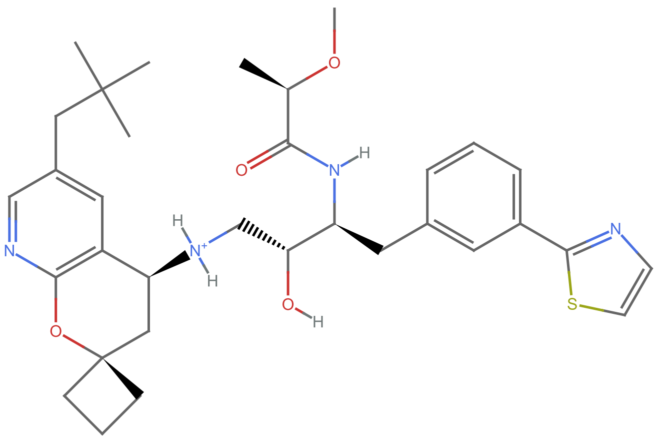

3D Plot

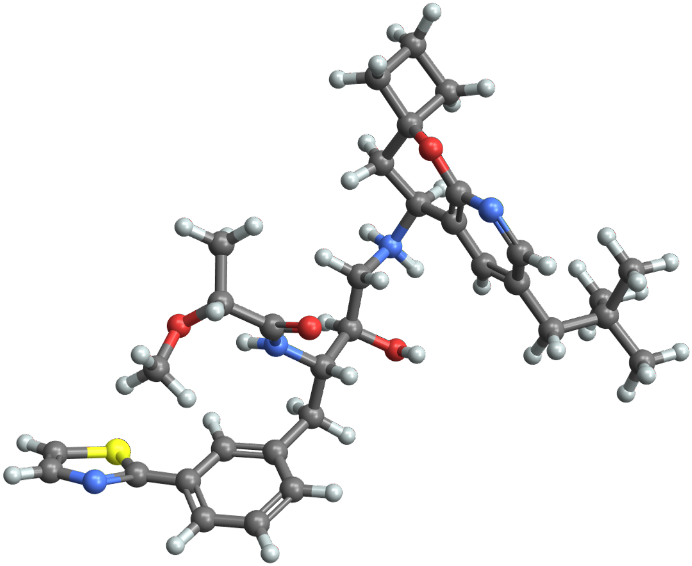

2D Autocorrelation

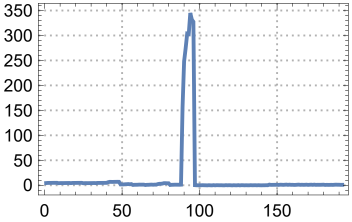

Dataset Index= 24

Molecule Formula= $C_{44}H_{53}F_2N_5O_8S$

3D Autocorrelation

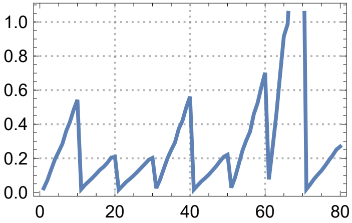

2D Plot

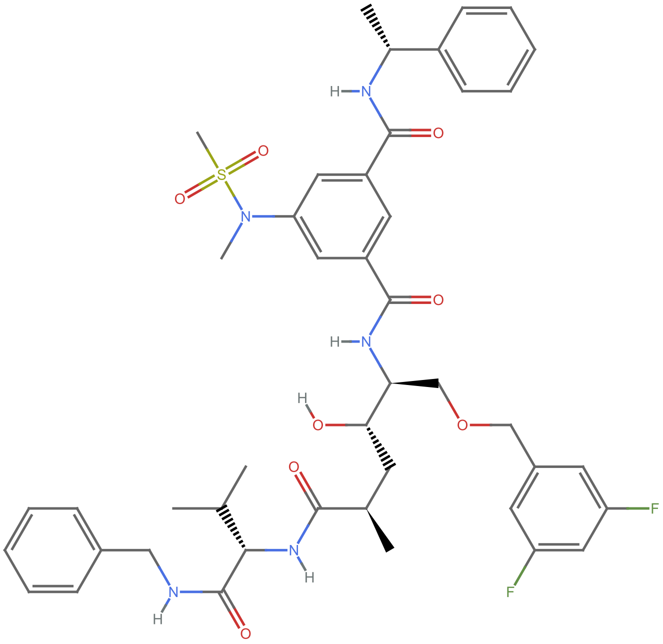

3D Plot

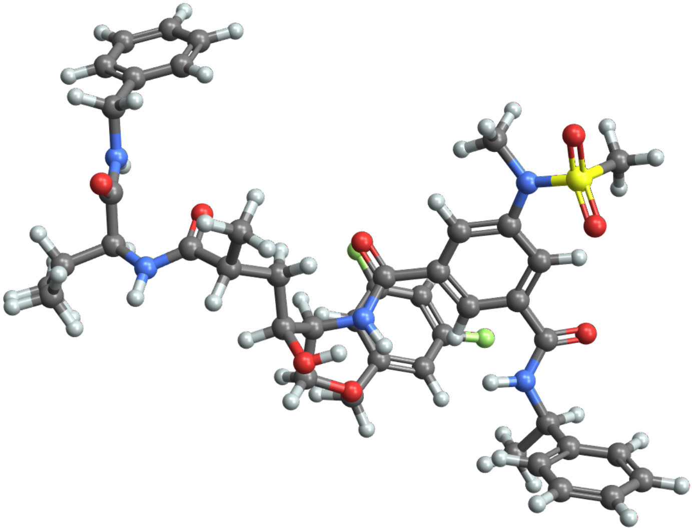

2D Plot

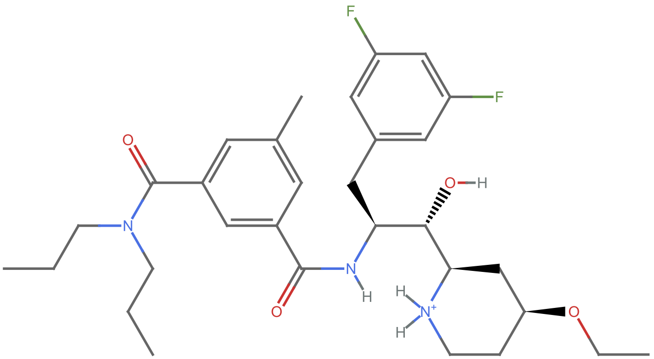

2D Autocorrelation

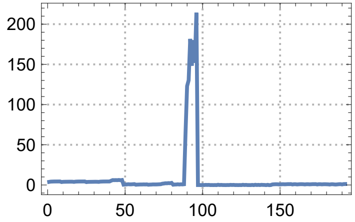

3D Plot

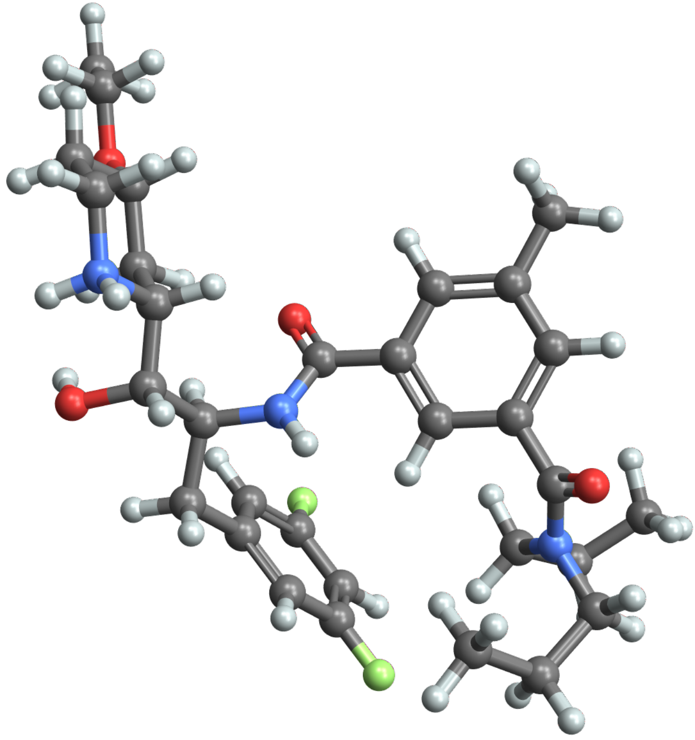

Dataset Index= 25

Molecule Formula= $C_{31}H_{44}F_2N_3O_4^+$

3D Autocorrelation

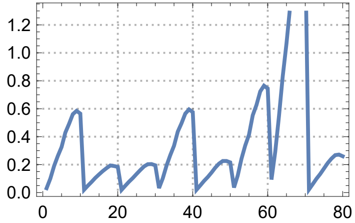

2D Autocorrelation

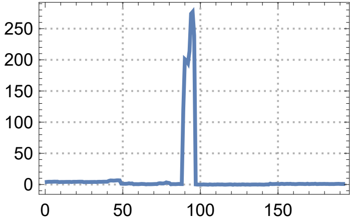

Dataset Index= 26

Molecule Formula= $C_{34}H_{45}FN_5O_5S^+$

3D Autocorrelation

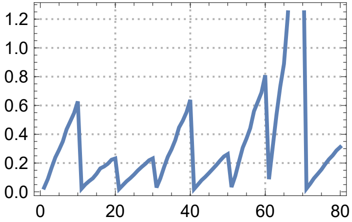

2D Plot

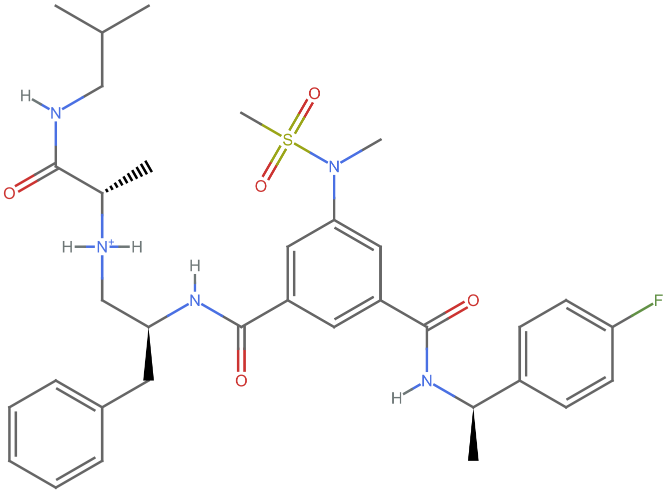

3D Plot

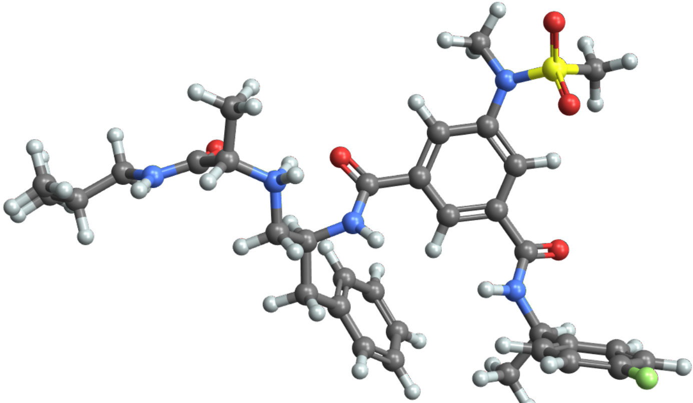

2D Plot

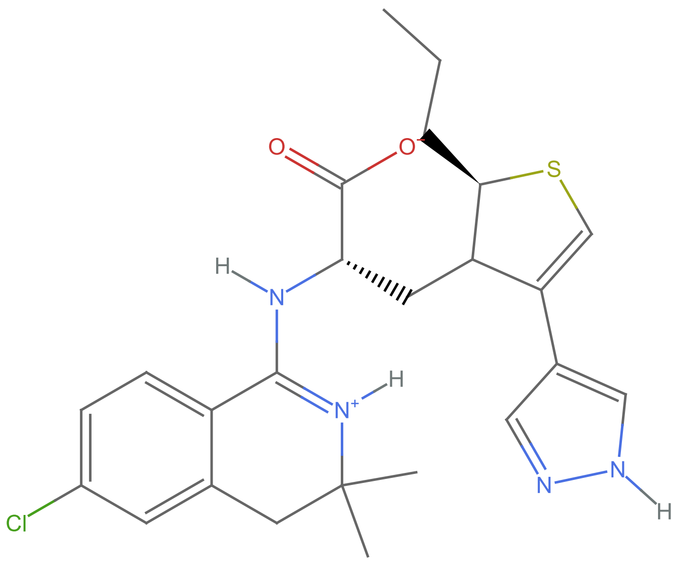

3D Plot

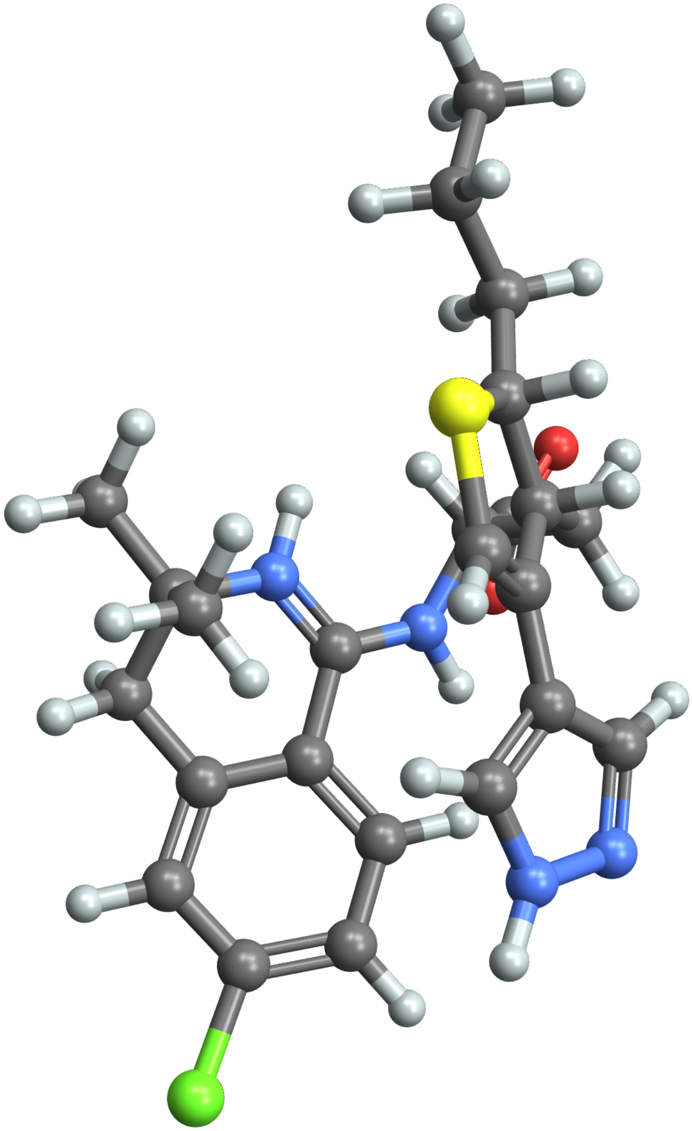

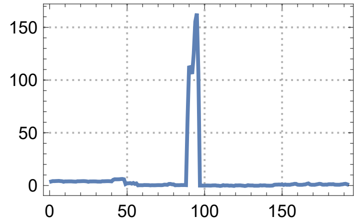

Dataset Index= 27

Molecule Formula= $C_{24}H_{29}ClN_4O_2S$

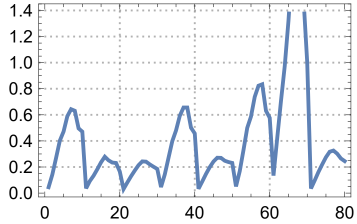

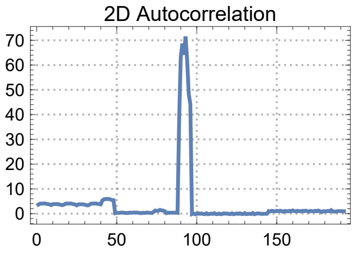

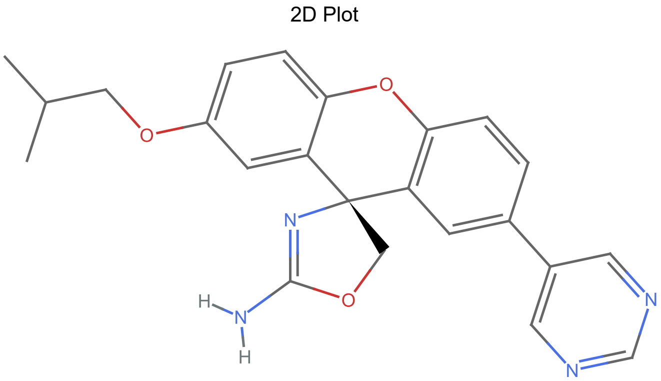

Dataset Index= 28

Molecule Formula= $C_{23}H_{22}N_4O_3$

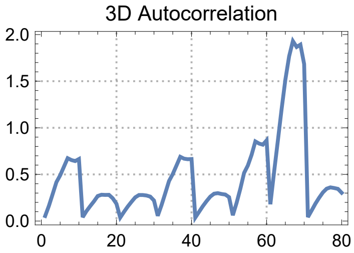

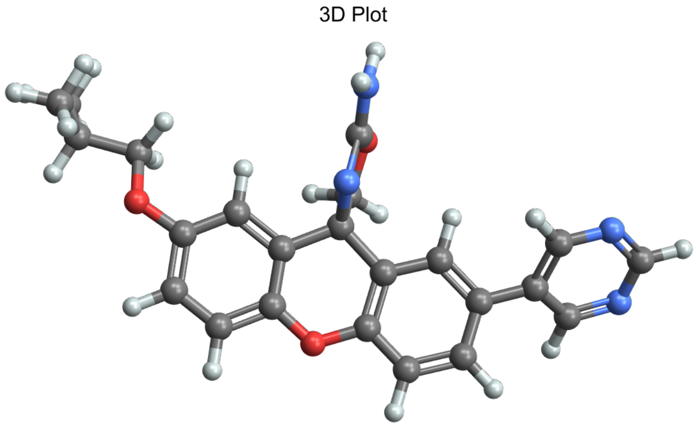

2D Plot

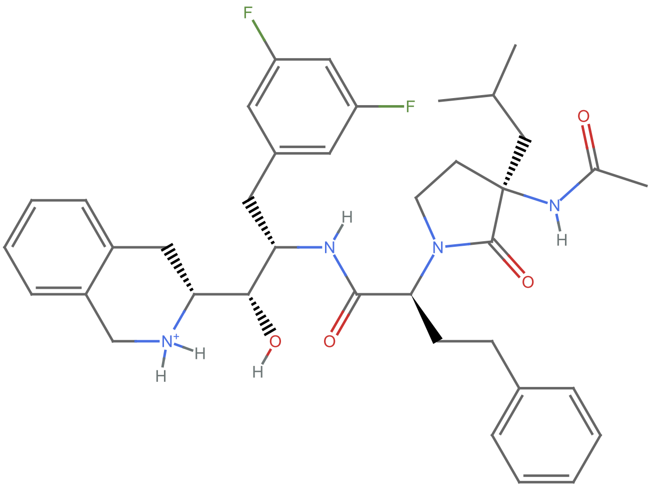

3D Plot

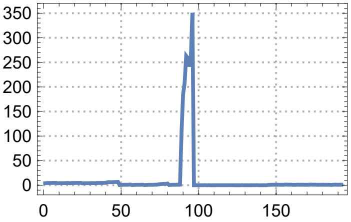

Dataset Index= 29

Molecule Formula= $C_{38}H_{47}F_2N_4O_4^+$

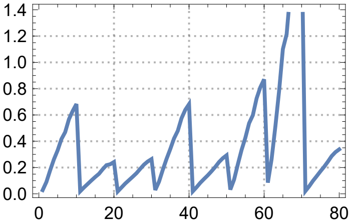

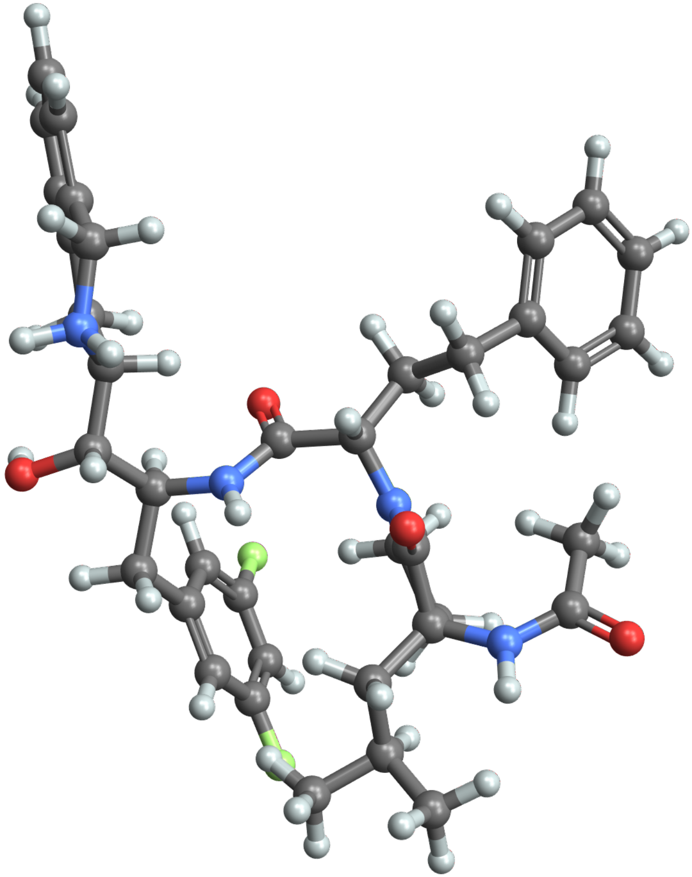

2D Plot

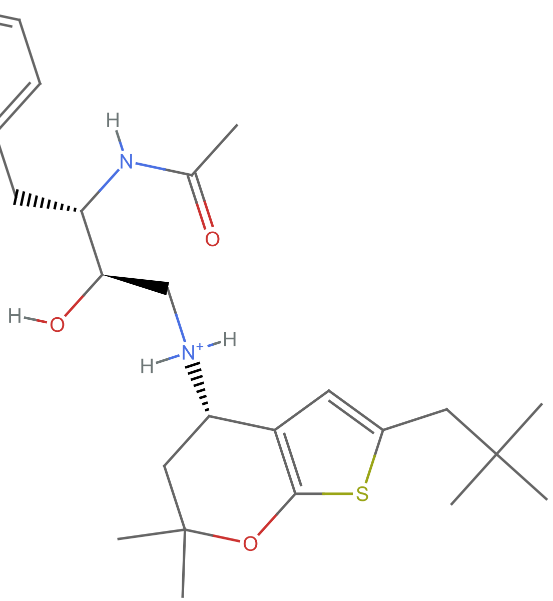

2D Autocorrelation

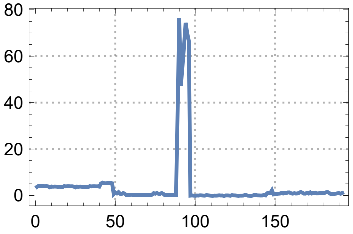

3D Plot

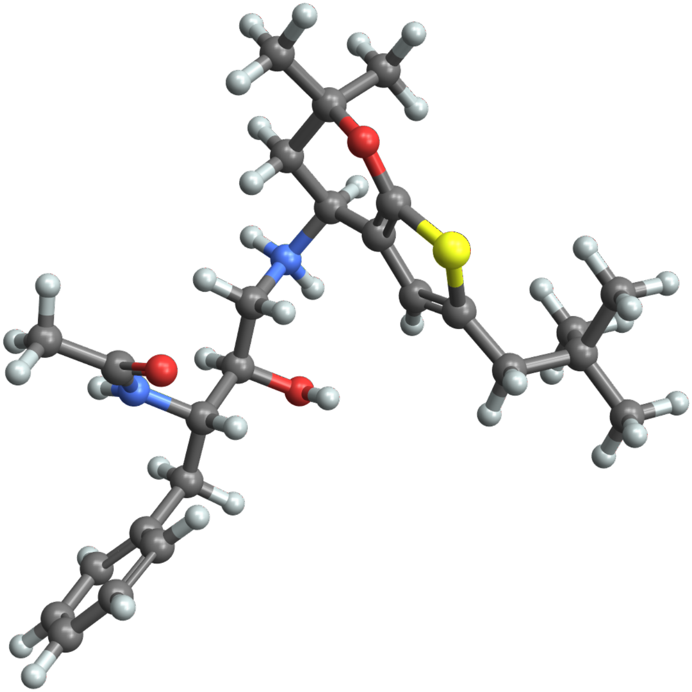

Dataset Index= 30

Molecule Formula= $C_{26}H_{39}N_2O_3S^+$

3D Autocorrelation

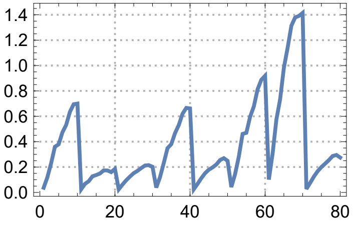

2D Plot

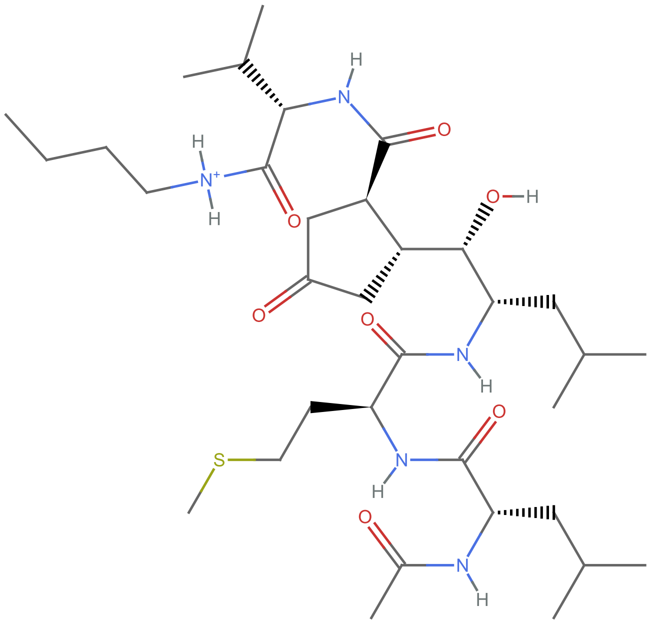

2D Autocorrelation

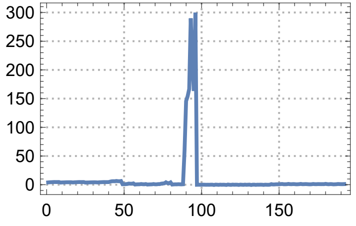

Dataset Index= 31

Molecule Formula= $C_{34}H_{62}N_5O_7S^+$

3D Autocorrelation

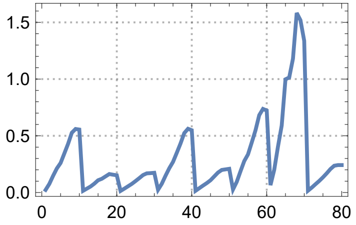

3D Plot

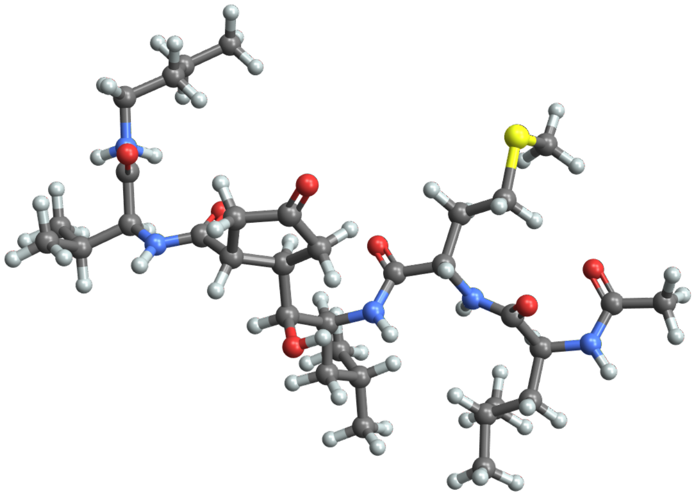

2D Plot

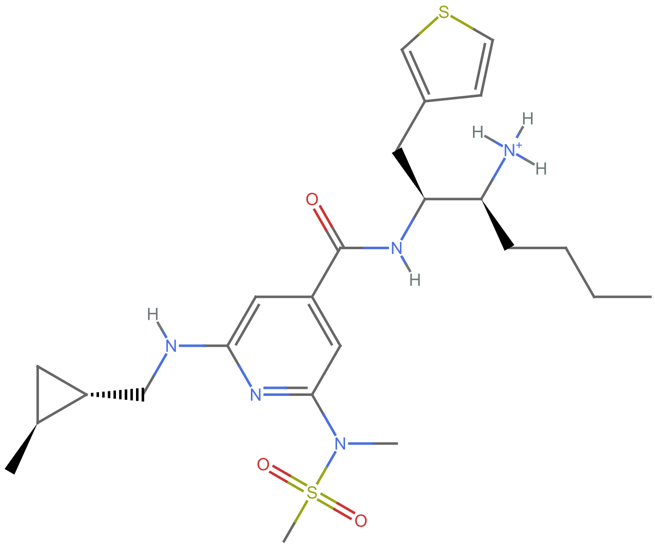

2D Autocorrelation

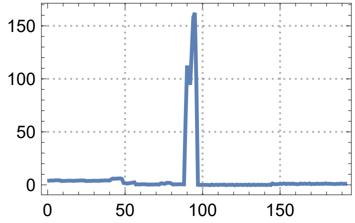

Dataset Index= 32

Molecule Formula= $C_{24}H_{38}N_5O_3S_2^+$

3D Plot

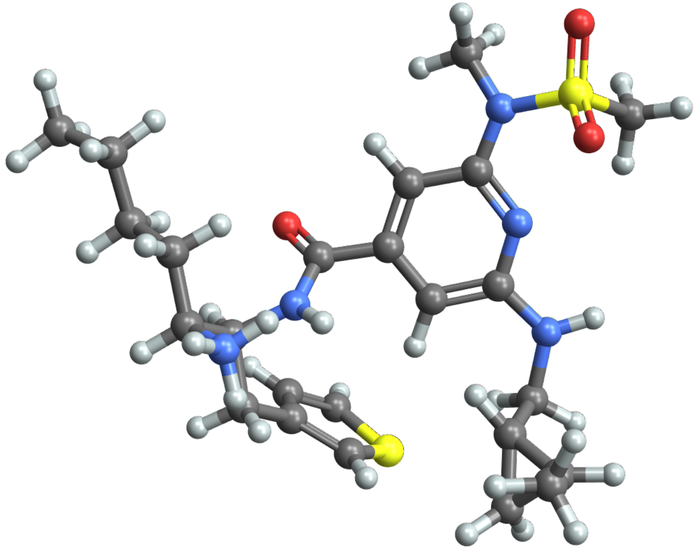

3D Autocorrelation

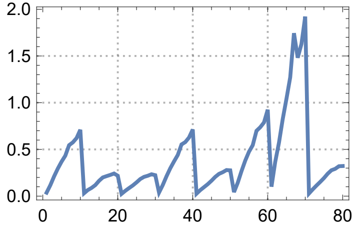

2D Plot

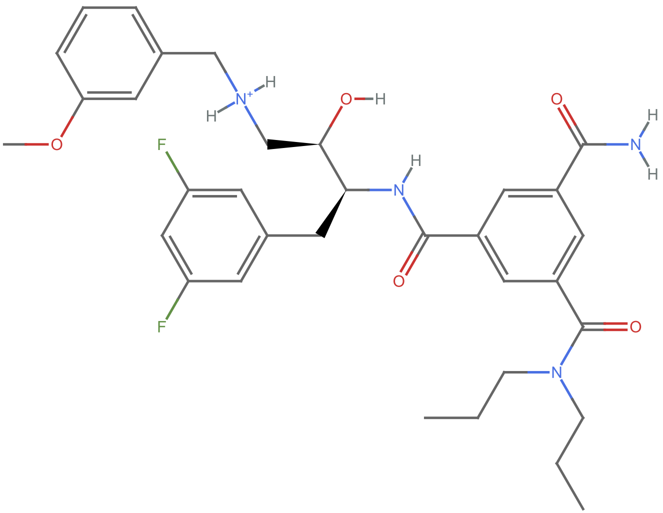

2D Autocorrelation

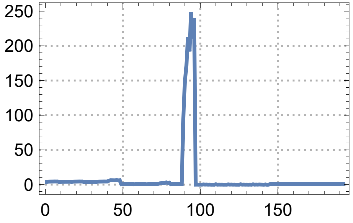

3D Plot

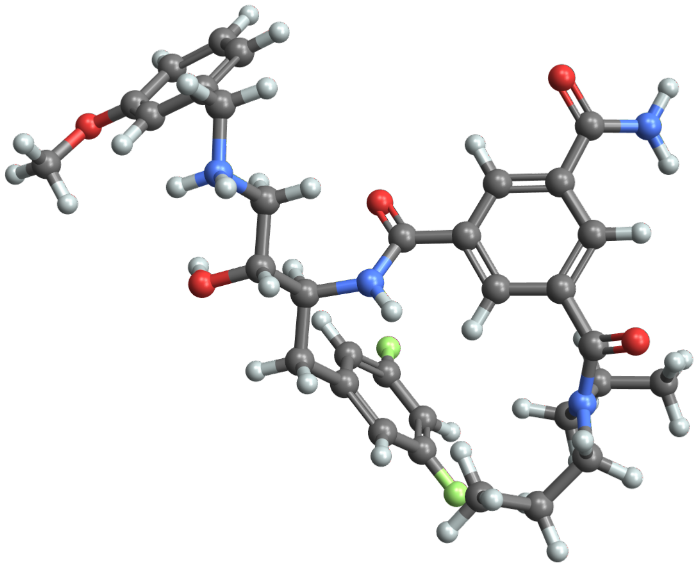

Dataset Index= 33

Molecule Formula= $C_{33}H_{41}F_2N_4O_5^+$

3D Autocorrelation

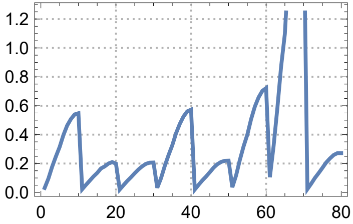

2D Plot

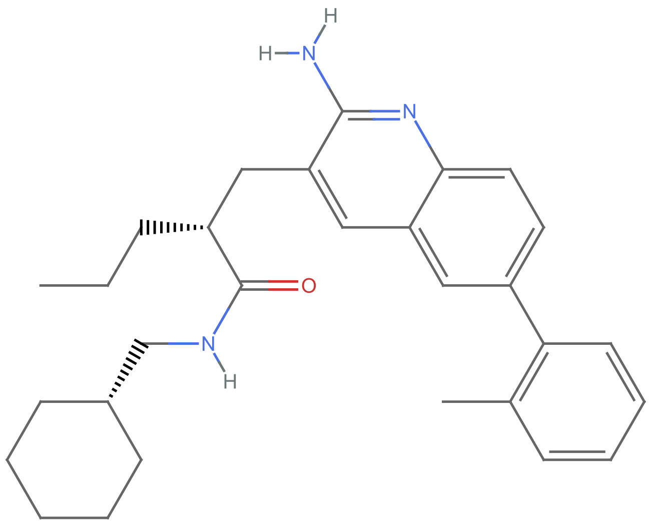

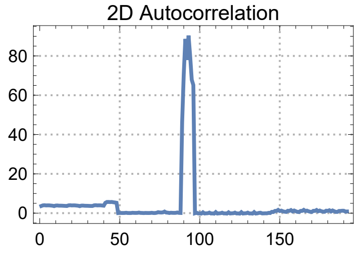

3D Plot

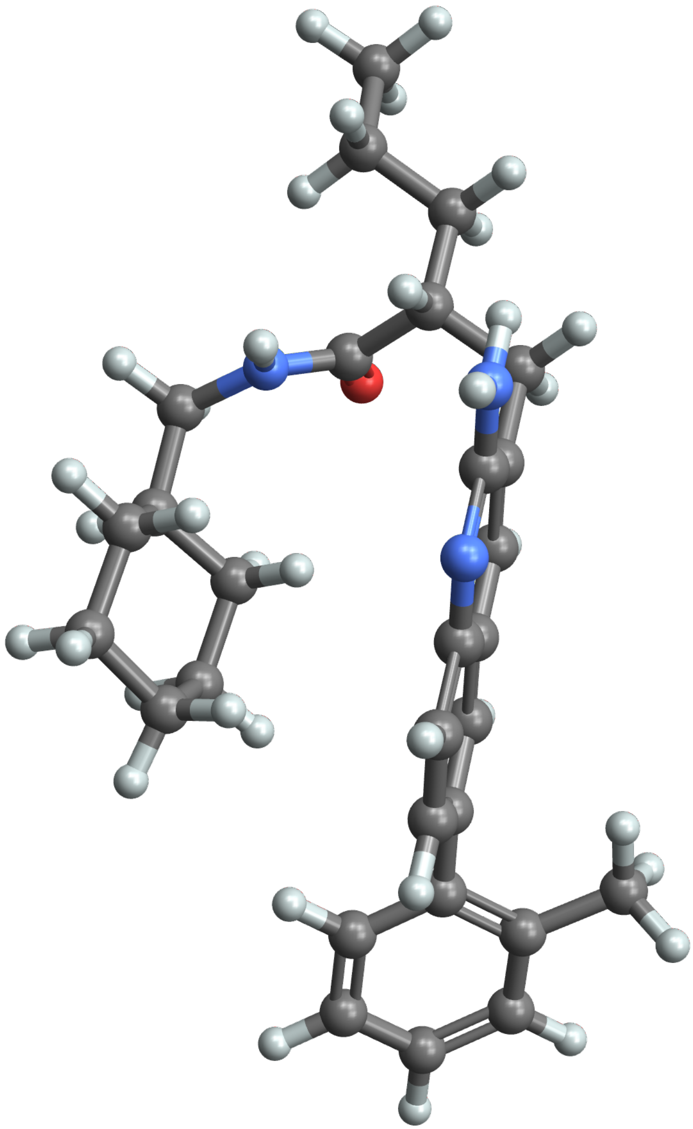

Dataset Index= 34

Molecule Formula= $C_{29}H_{37}N_3O$

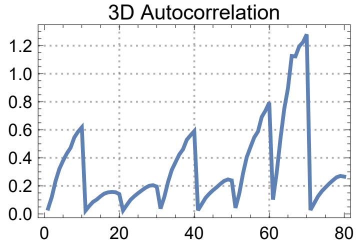

2D Plot

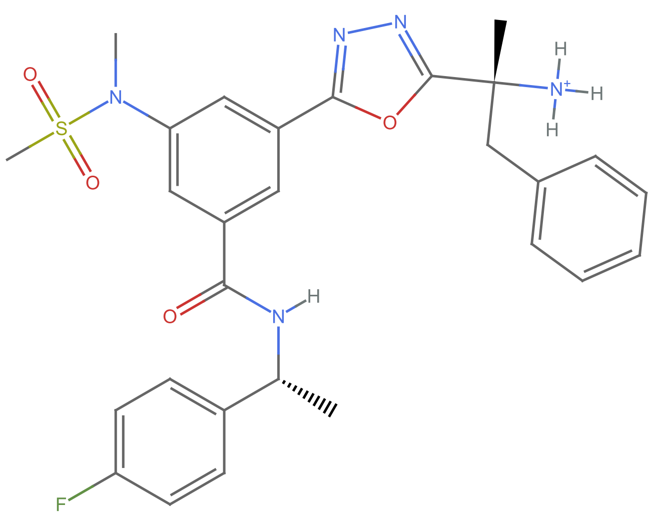

2D Autocorrelation

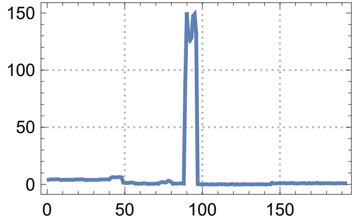

Dataset Index= 35

Molecule Formula= $C_{28}H_{31}FN_5O_4S^+$

3D Autocorrelation

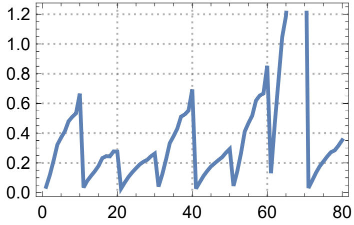

3D Plot

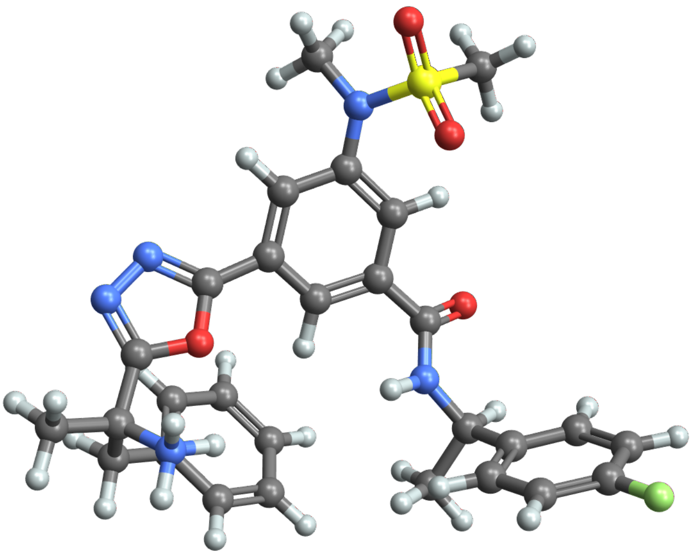

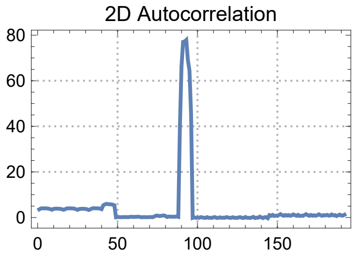

Dataset Index= 36

Molecule Formula= $C_{23}H_{24}N_5O^+$

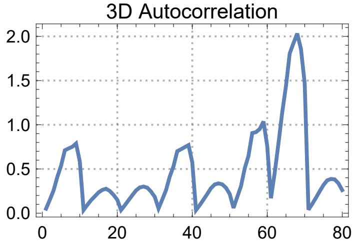

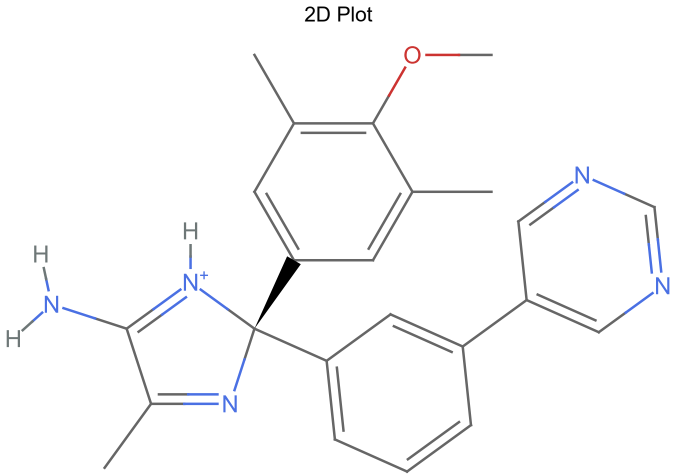

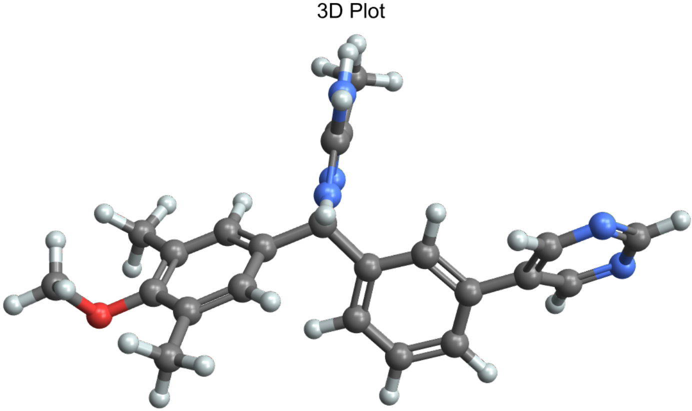

2D Autocorrelation

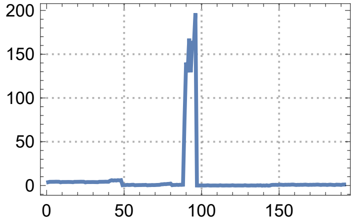

Dataset Index= 37

Molecule Formula= $C_{29}H_{37}F_2N_4O_2^+$

3D Autocorrelation

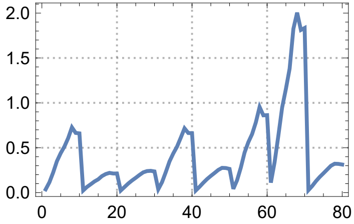

2D Plot

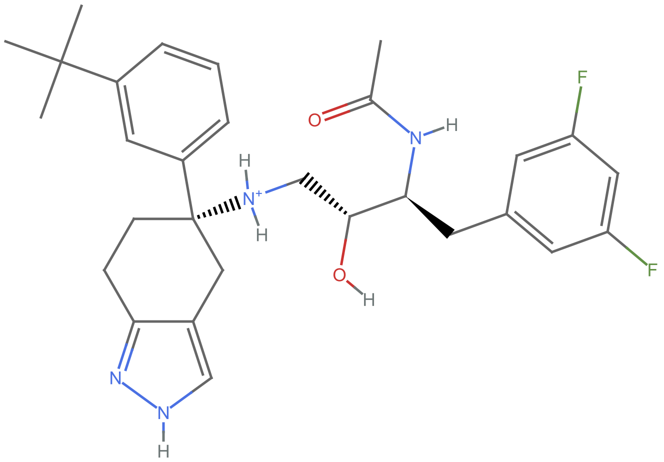

3D Plot

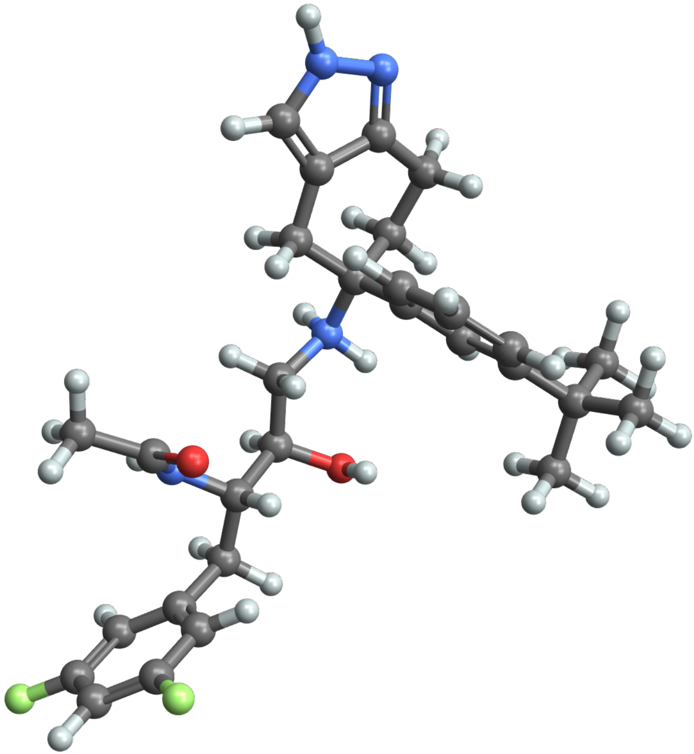

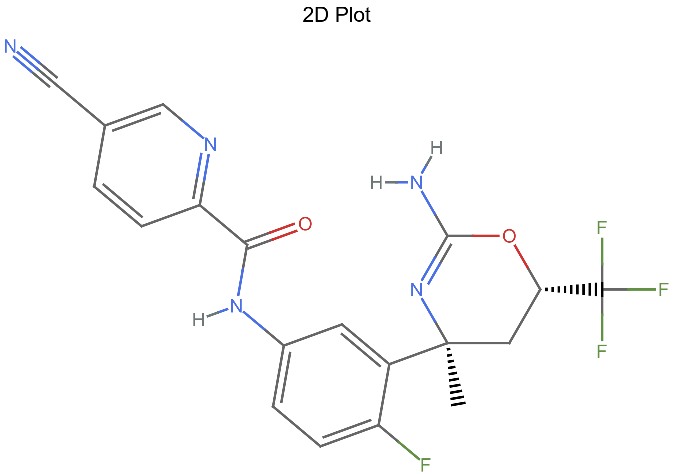

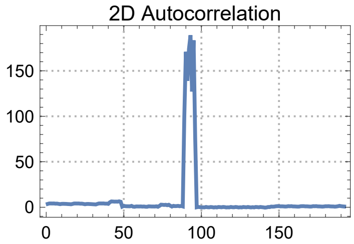

Dataset Index= 38

Molecule Formula= $C_{19}H_{15}F_4N_5O_2$

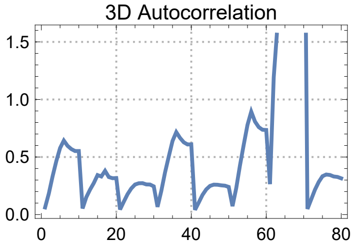

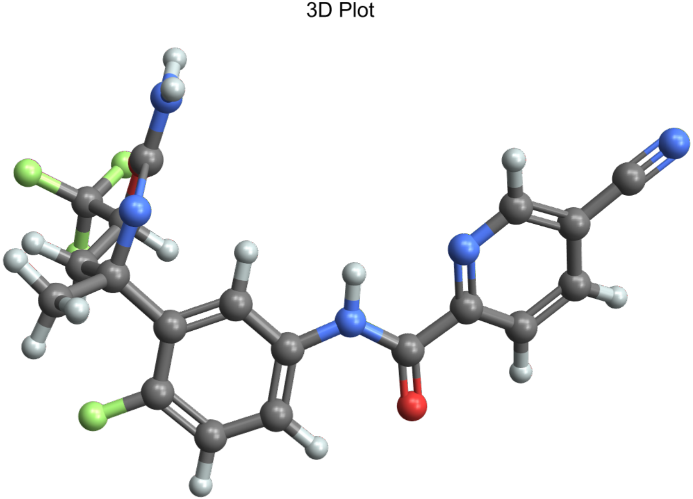

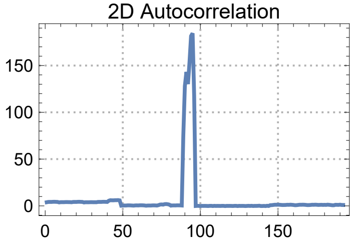

Dataset Index= 39

Molecule Formula= $C_{32}H_{46}N_5O_4^+$

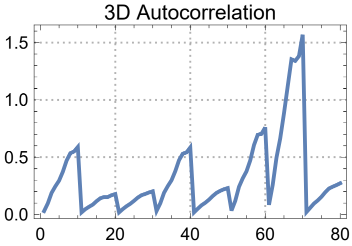

2D Plot

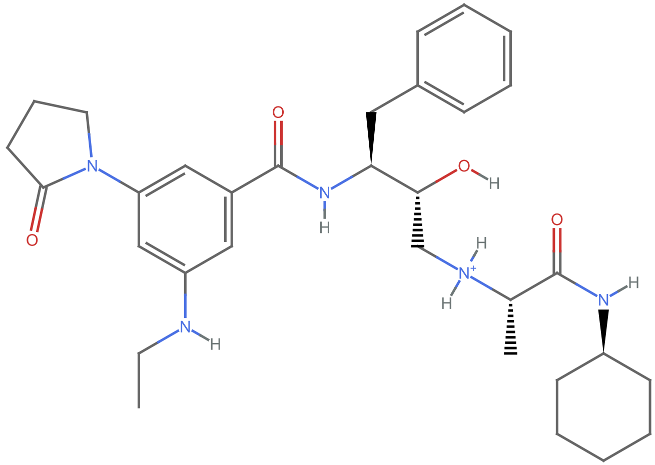

3D Plot

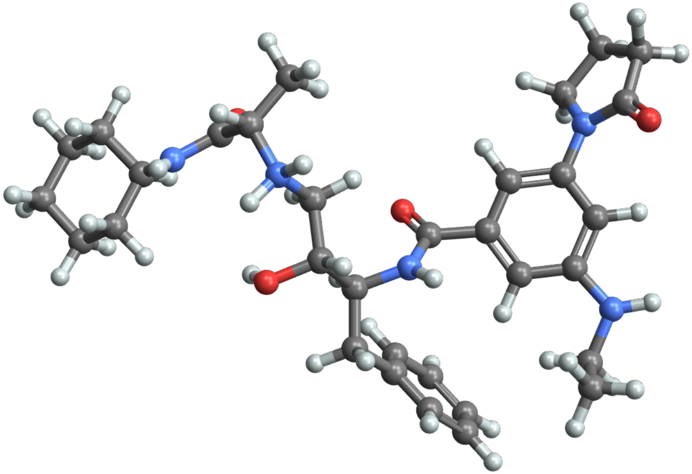

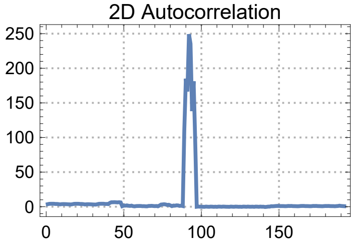

Dataset Index= 40

Molecule Formula= $C_{19}H_{14}F_5N_5O_2$

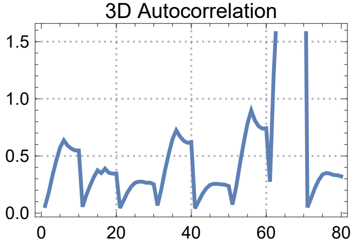

2D Plot

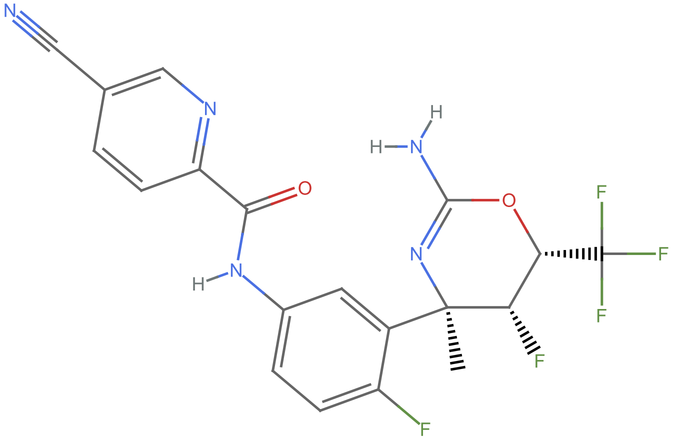

3D Plot

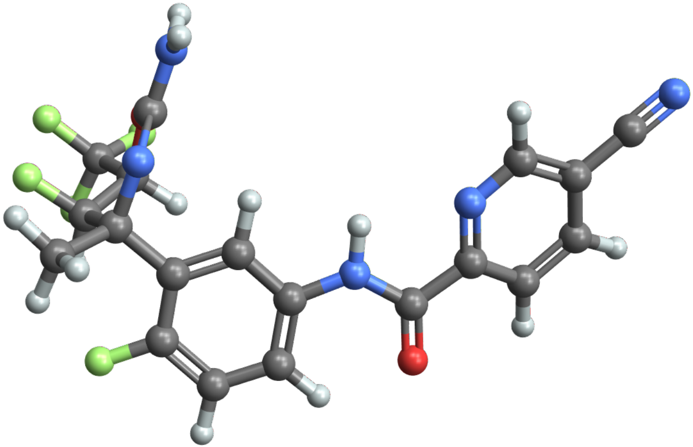

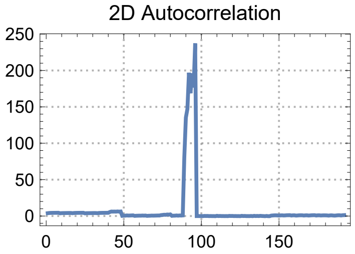

Dataset Index= 41

Molecule Formula= $C_{35}H_{48}F_2N_3O_3^+$

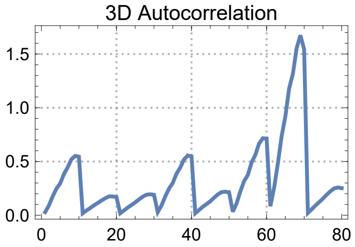

2D Plot

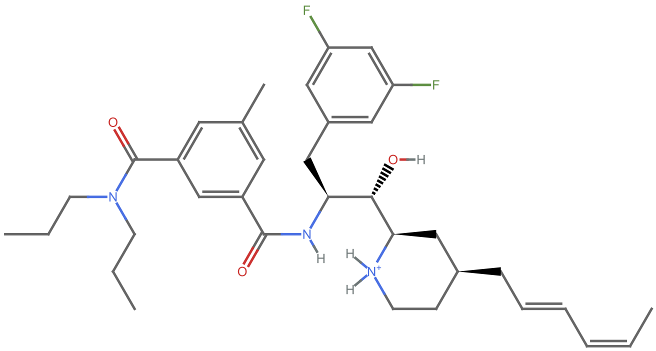

3D Plot

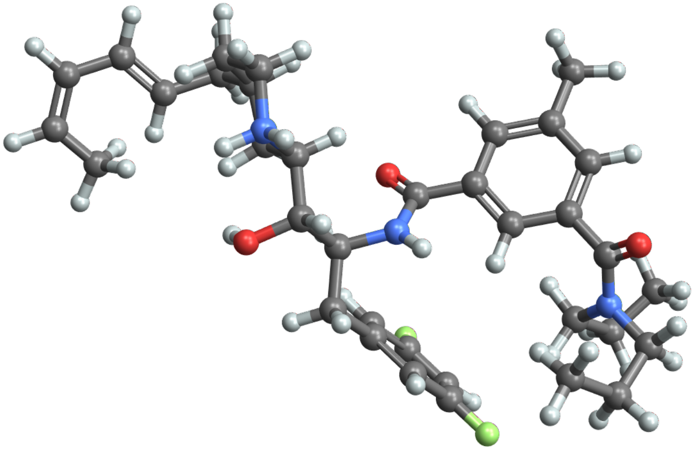

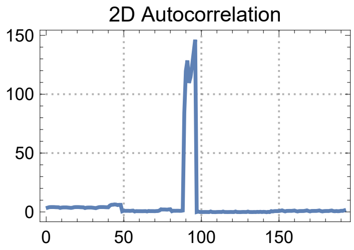

Dataset Index= 42

Molecule Formula= $C_{22}H_{19}F_4N_3O_2$

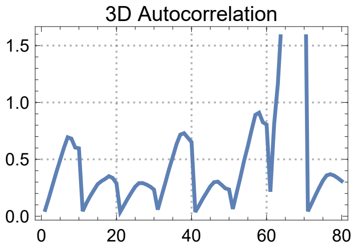

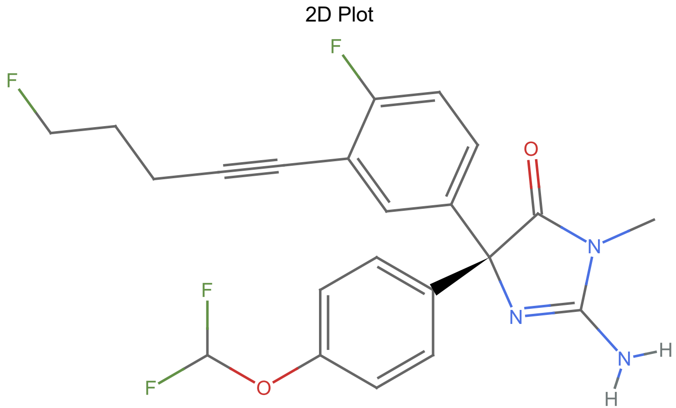

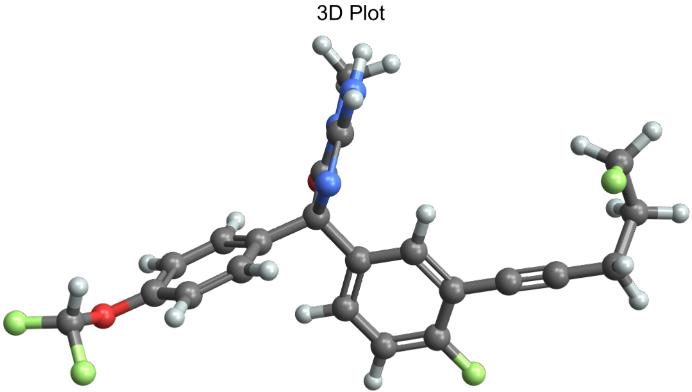

2D Plot

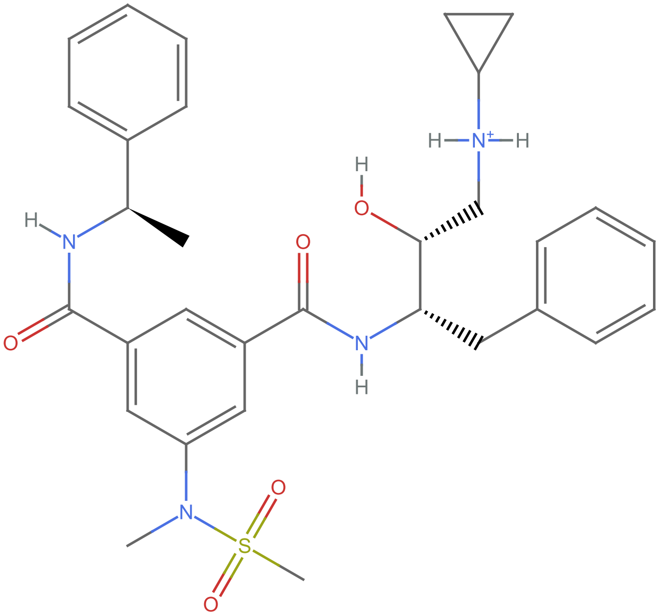

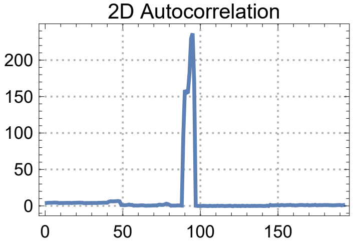

Dataset Index= 43

Molecule Formula= $C_{31}H_{39}N_4O_5S^+$

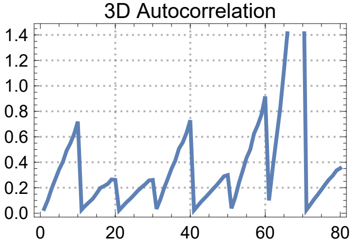

3D Plot

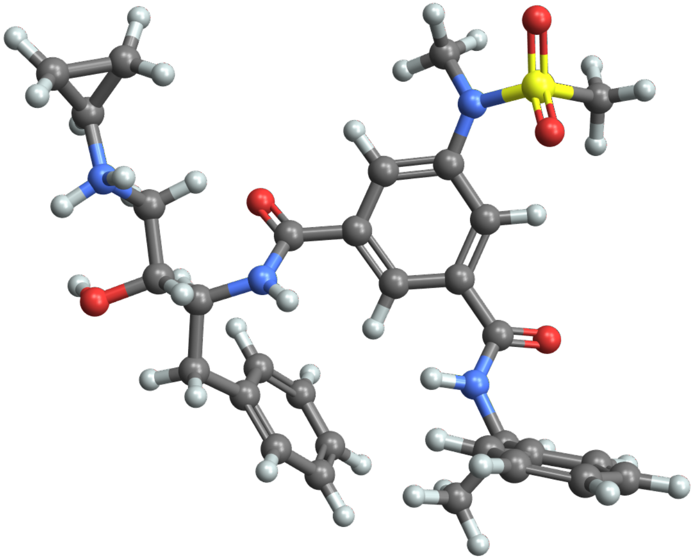

2D Plot

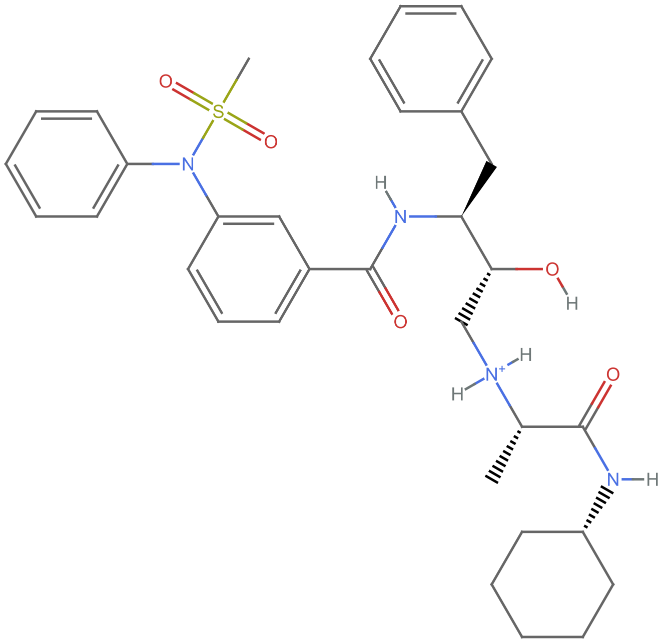

2D Autocorrelation

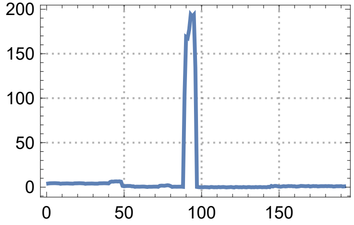

Dataset Index= 44

Molecule Formula= $C_{33}H_{43}N_4O_5S^+$

3D Autocorrelation

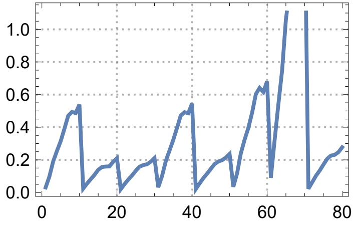

3D Plot

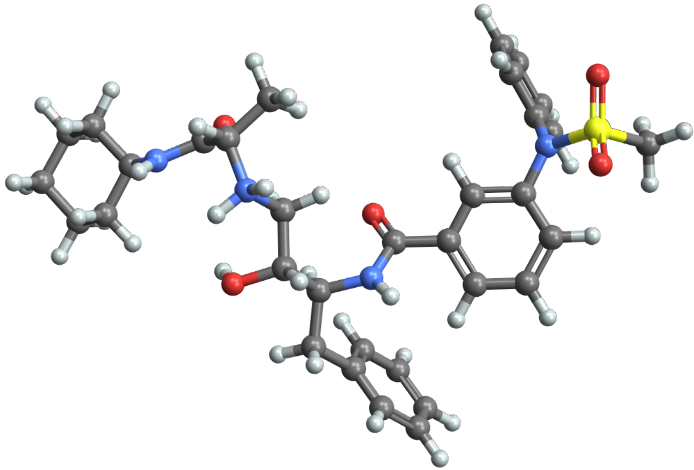

2D Plot

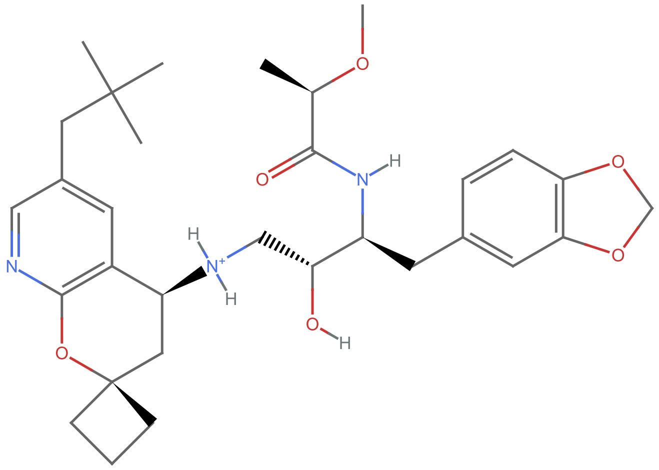

2D Autocorrelation

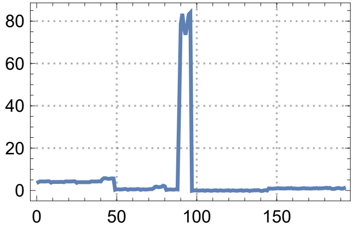

3D Plot

Dataset Index= 45

Molecule Formula= $C_{31}H_{44}N_3O_6^+$

3D Autocorrelation

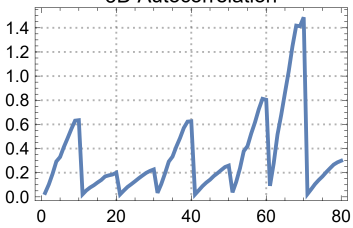

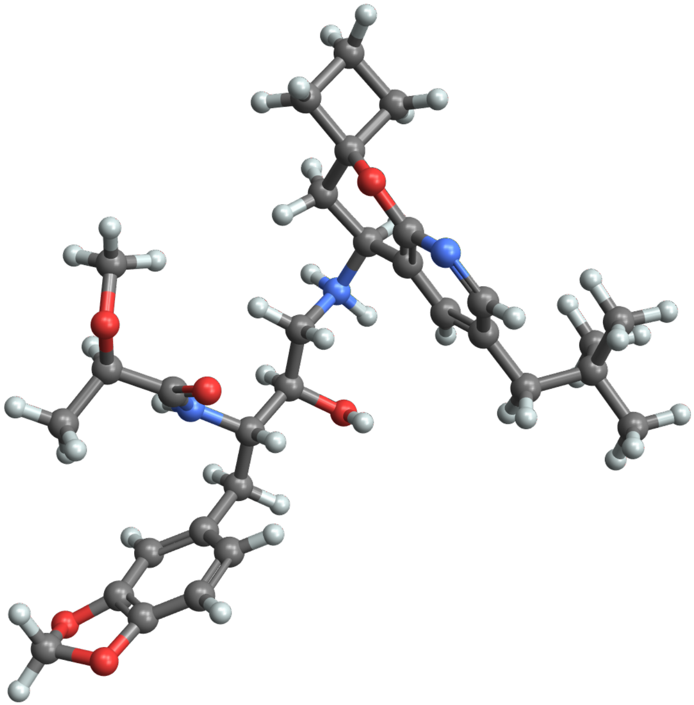

2D Plot

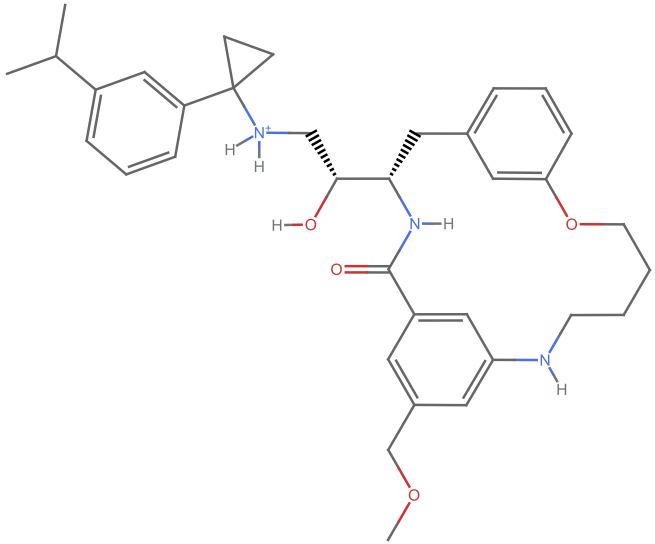

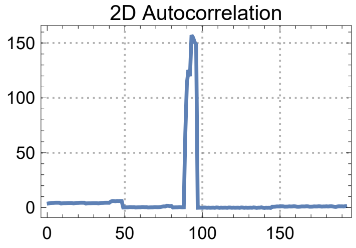

3D Plot

Dataset Index= 46

Molecule Formula= $C_{35}H_{46}N_3O_4^+$

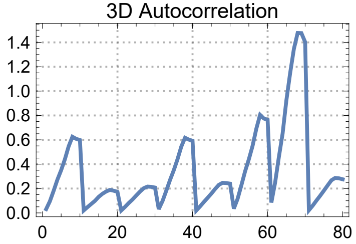

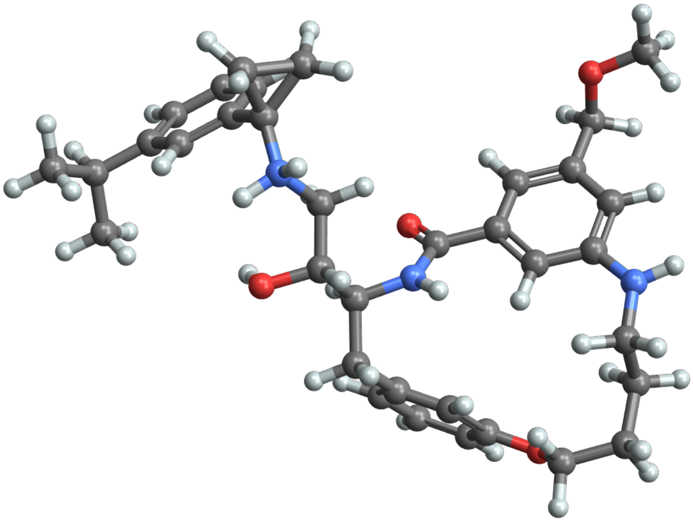

2D Plot

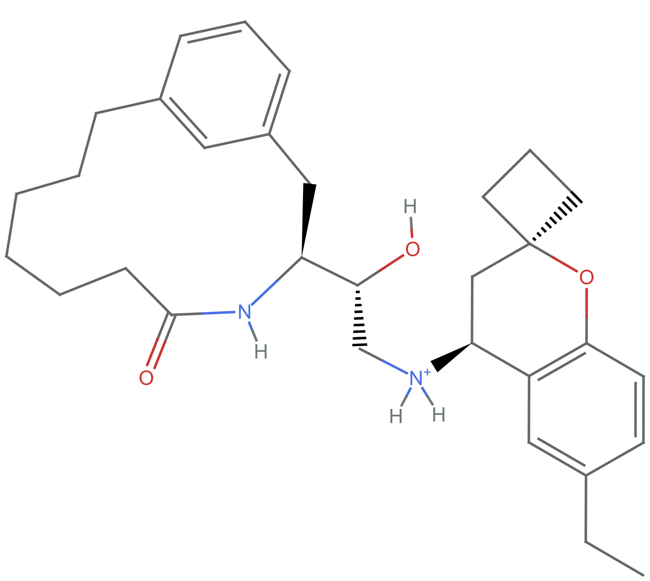

2D Autocorrelation

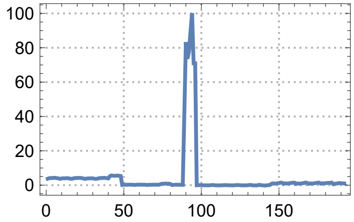

Dataset Index= 47

Molecule Formula= $C_{31}H_{43}N_2O_3^+$

3D Autocorrelation

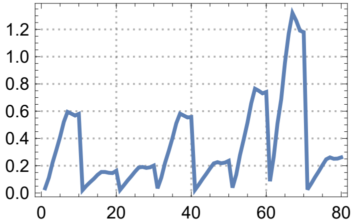

3D Plot

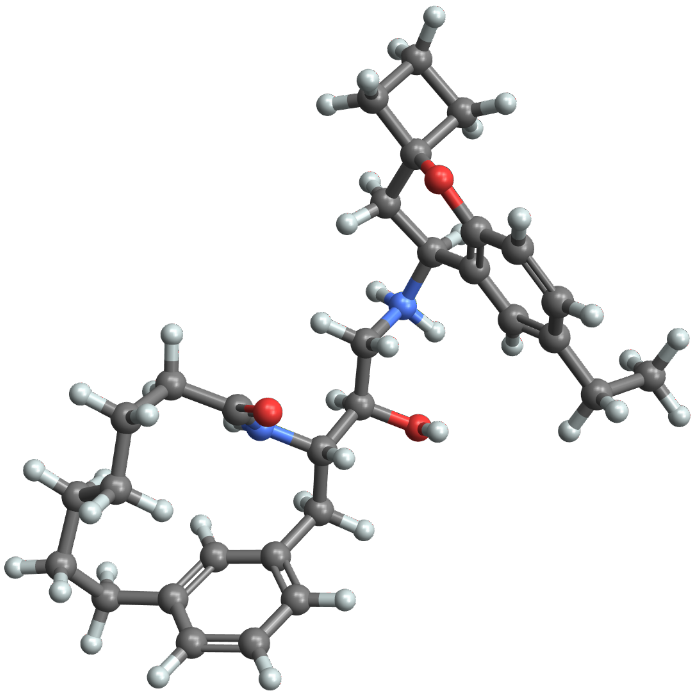

2D Plot

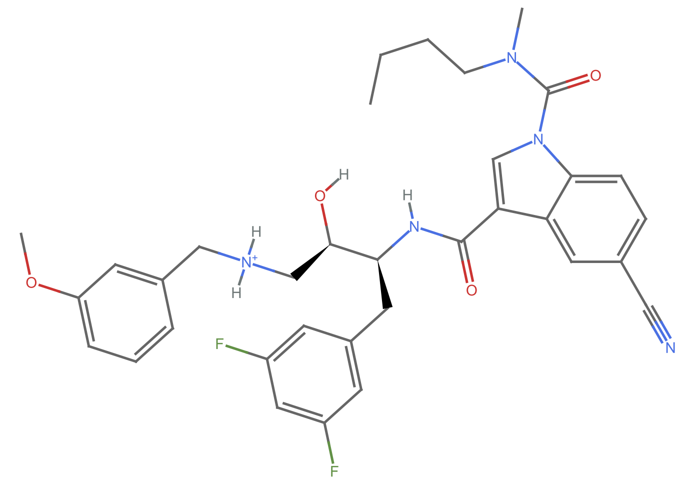

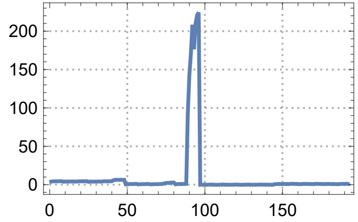

3D Plot

Dataset Index= 48

Molecule Formula= $C_{34}H_{38}F_2N_5O_4^+$

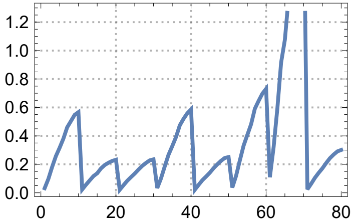

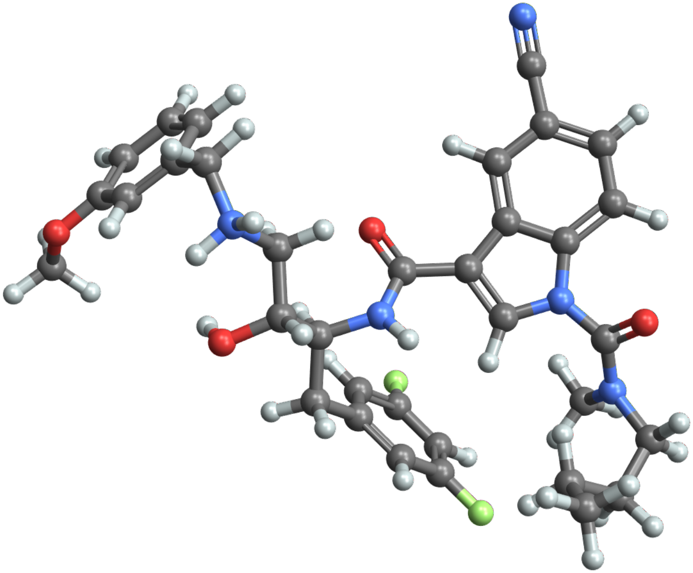

2D Autocorrelation

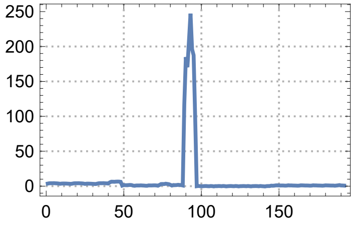

Dataset Index= 49

Molecule Formula= $C_{19}H_{14}F_5N_5O_2$

3D Autocorrelation

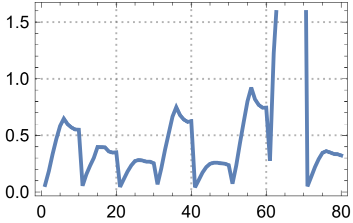

2D Plot

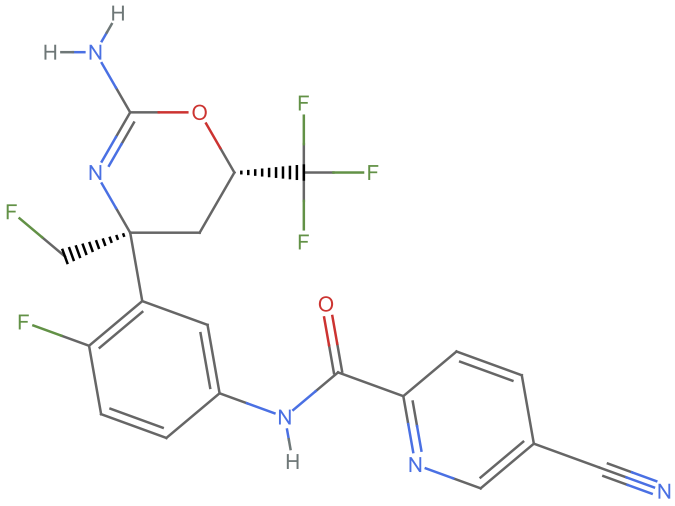

3D Plot

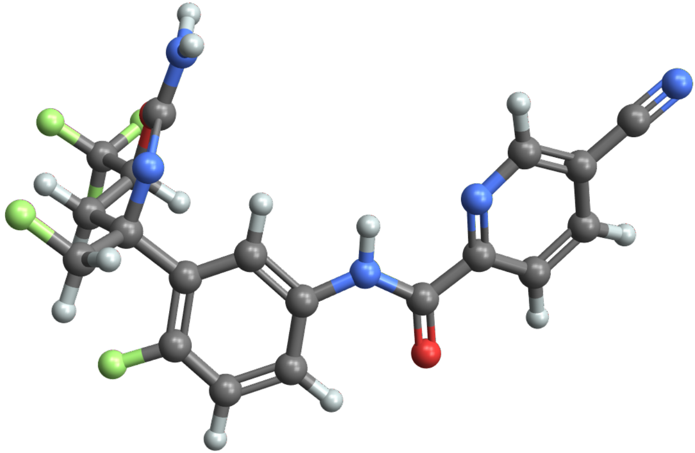

2D Plot

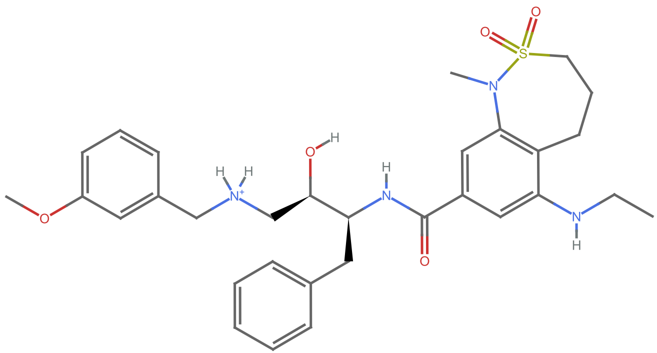

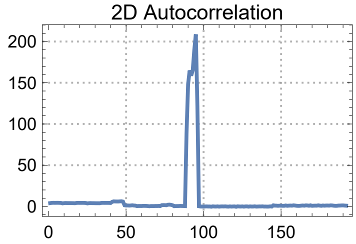

3D Plot

Dataset Index= 50

Molecule Formula= $C_{31}H_{41}N_4O_5S^+$

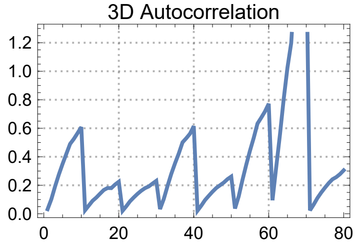

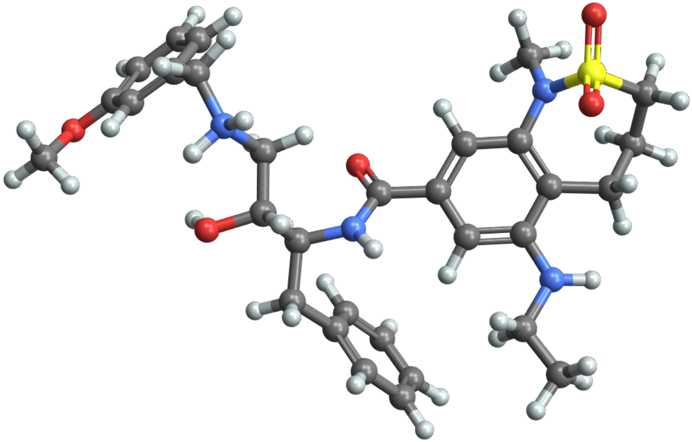

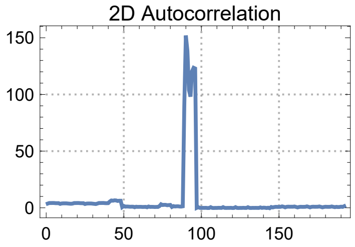

Dataset Index= 51

Molecule Formula= $C_{21}H_{15}F_4N_5O_2$

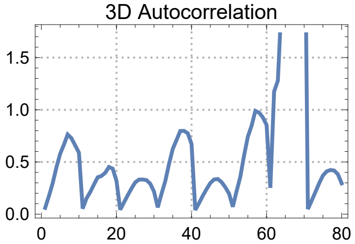

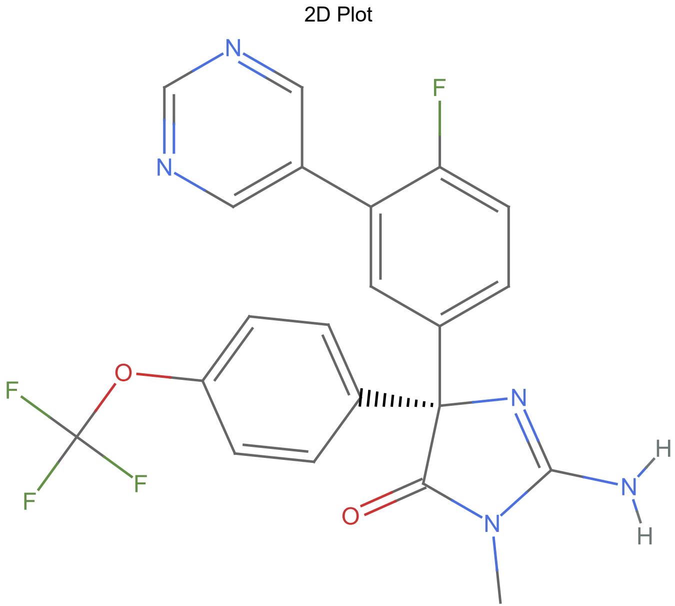

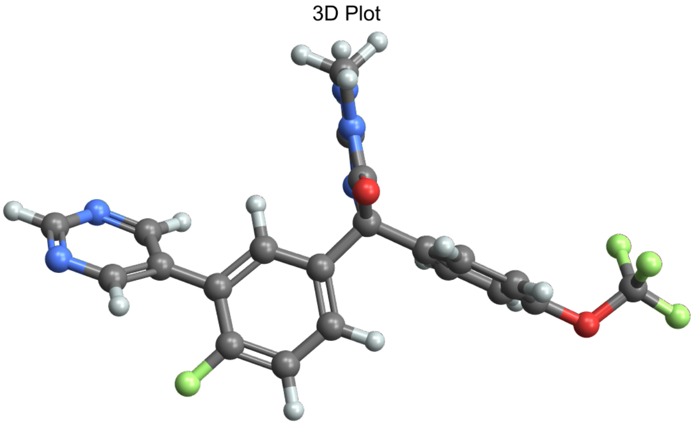

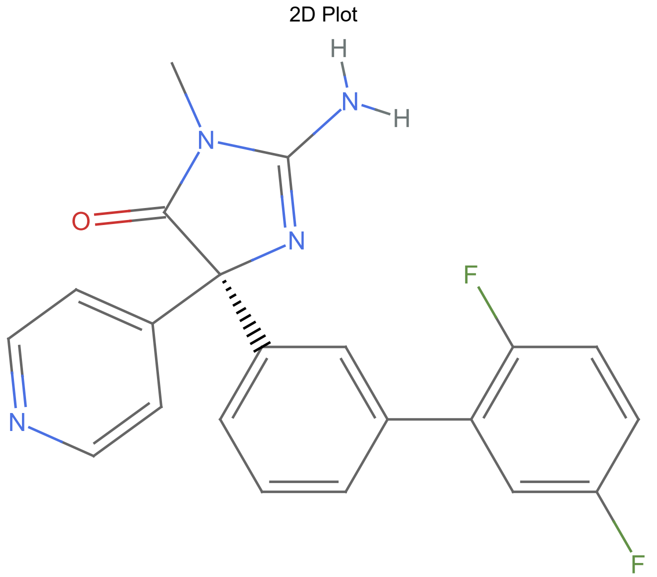

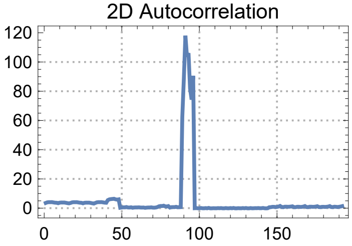

Dataset Index= 52

Molecule Formula= $C_{21}H_{16}F_2N_4O$

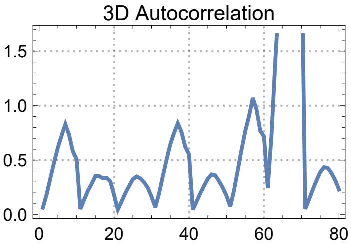

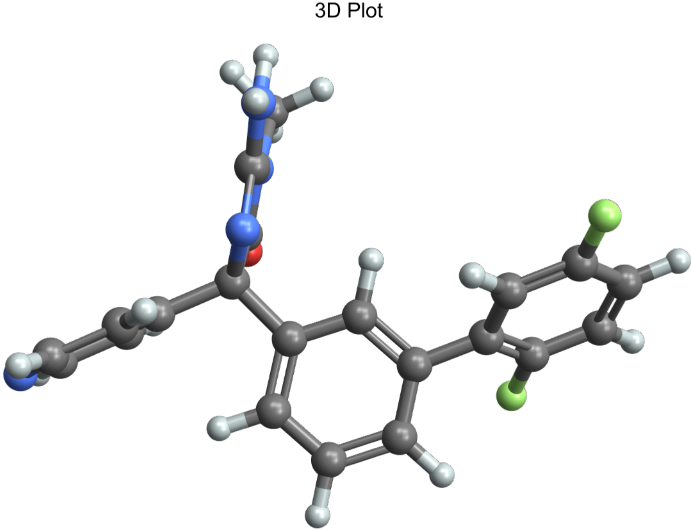

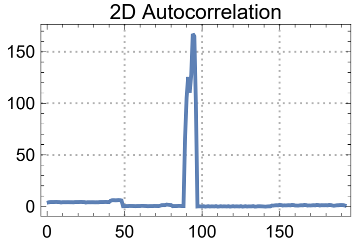

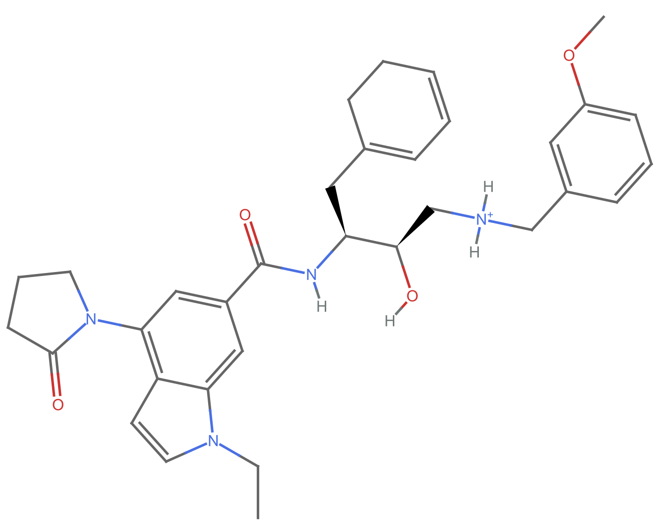

Dataset Index= 53

Molecule Formula= $C_{33}H_{41}N_4O_4^+$

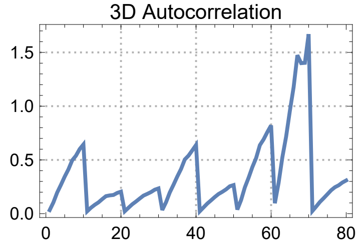

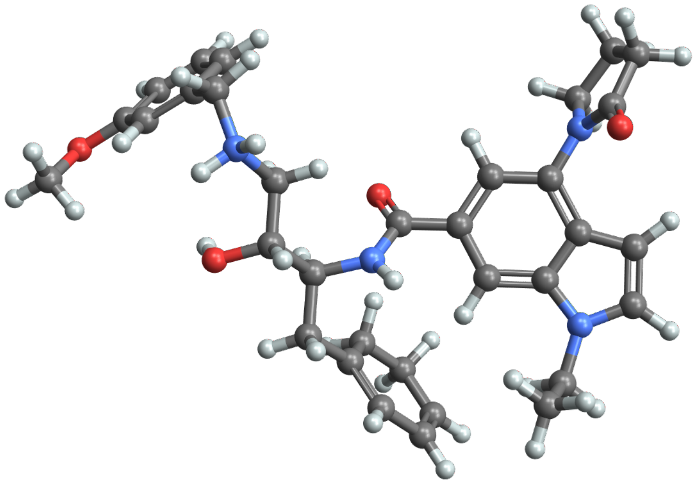

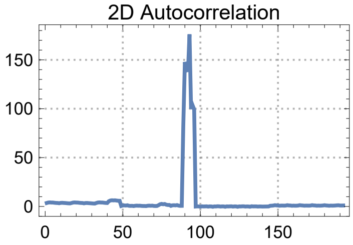

Dataset Index= 54

Molecule Formula= $C_{18}H_{14}F_3N_5O_2$

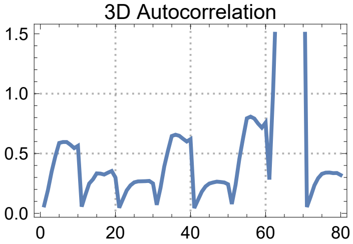

2D Plot

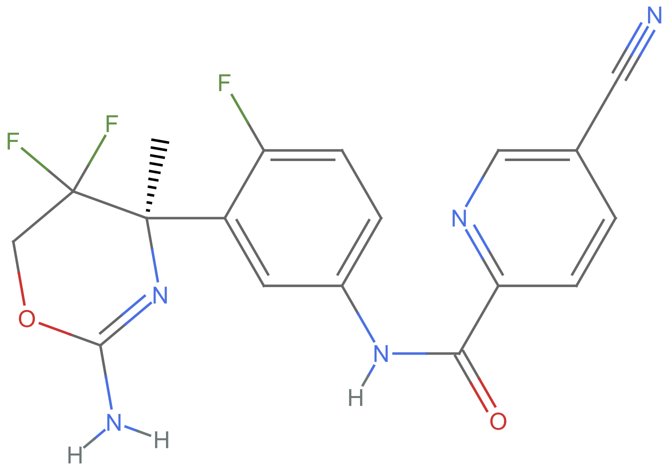

3D Plot

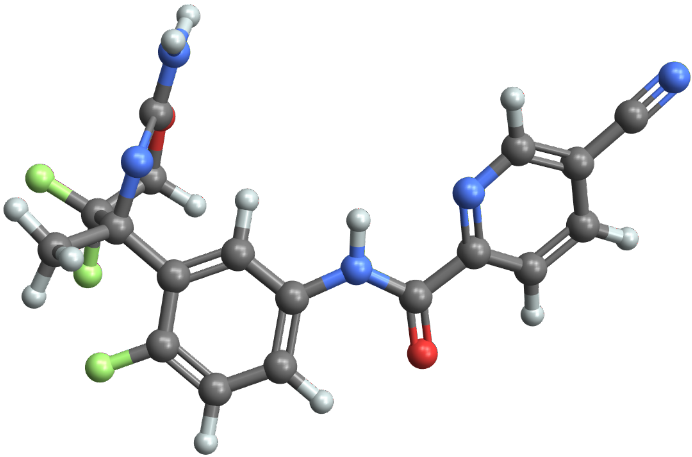

2D Autocorrelation

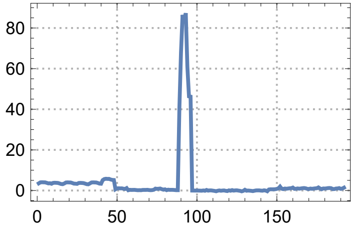

2D Plot

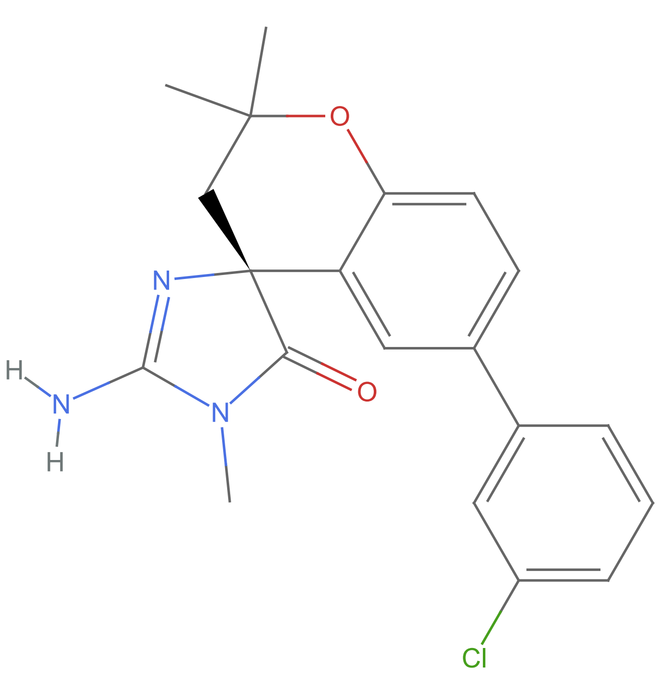

Dataset Index= 55

Molecule Formula= $C_{20}H_{20}ClN_3O_2$

3D Autocorrelation

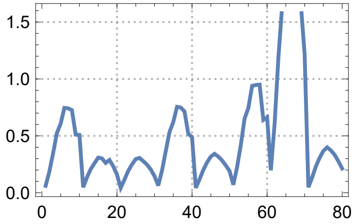

3D Plot

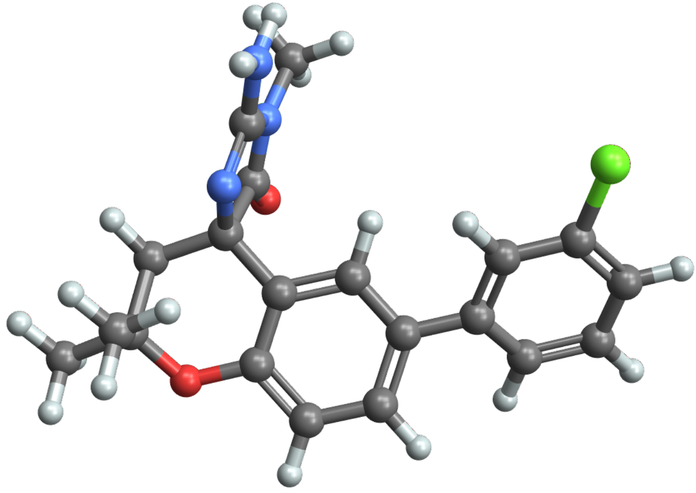

2D Plot

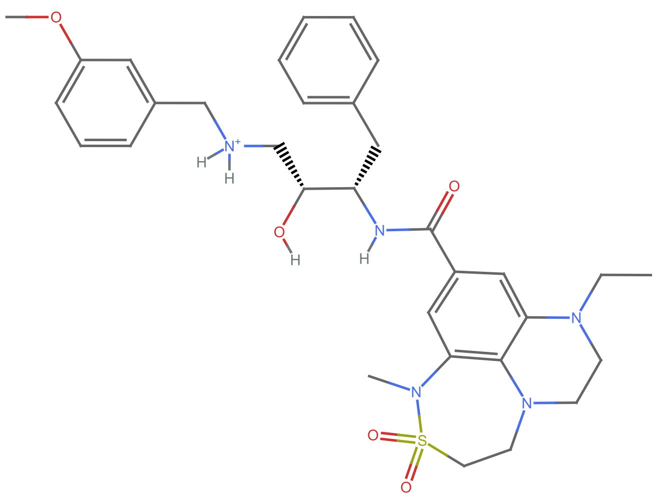

2D Autocorrelation

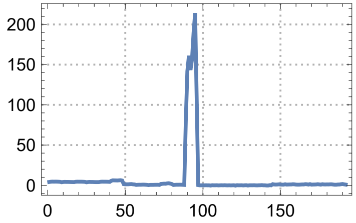

3D Plot

Dataset Index= 56

Molecule Formula= $C_{32}H_{42}N_5O_5S^+$

3D Autocorrelation

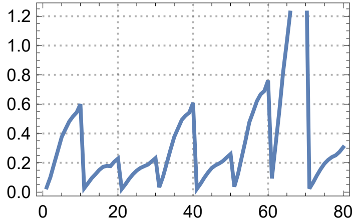

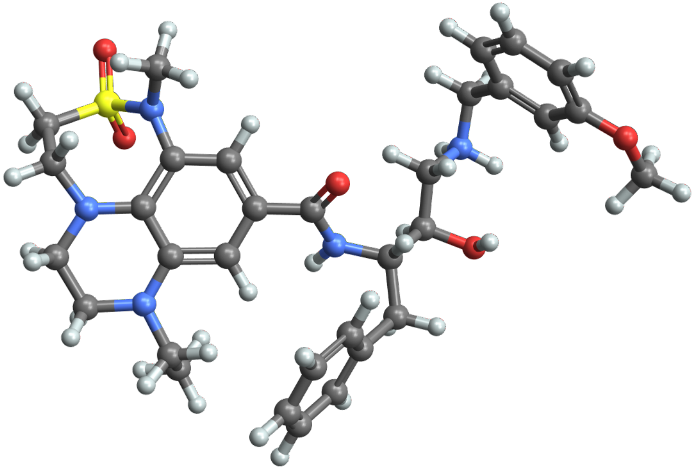

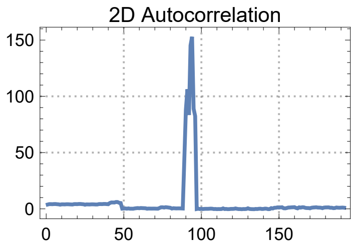

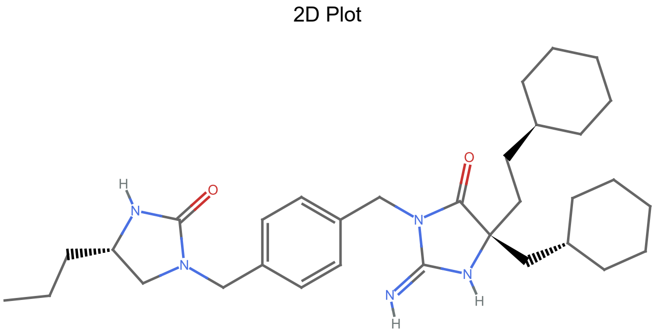

Dataset Index= 57

Molecule Formula= $C_{32}H_{49}N_5O_2$

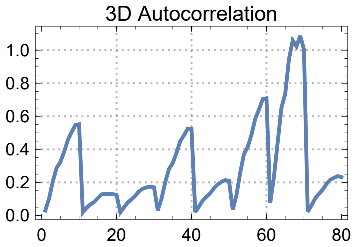

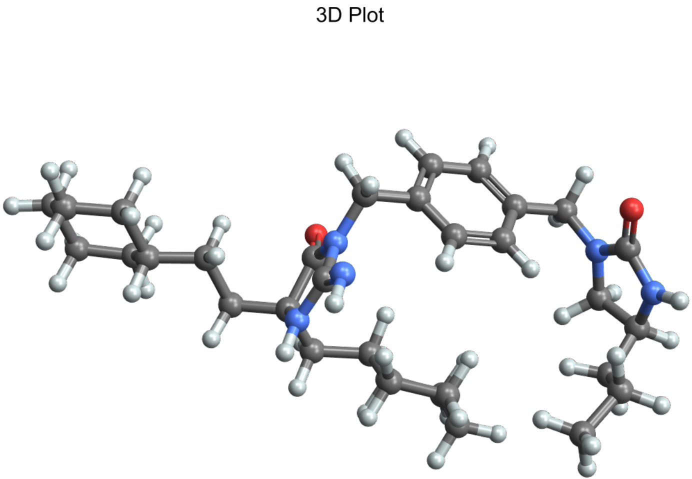

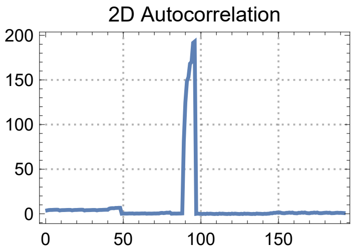

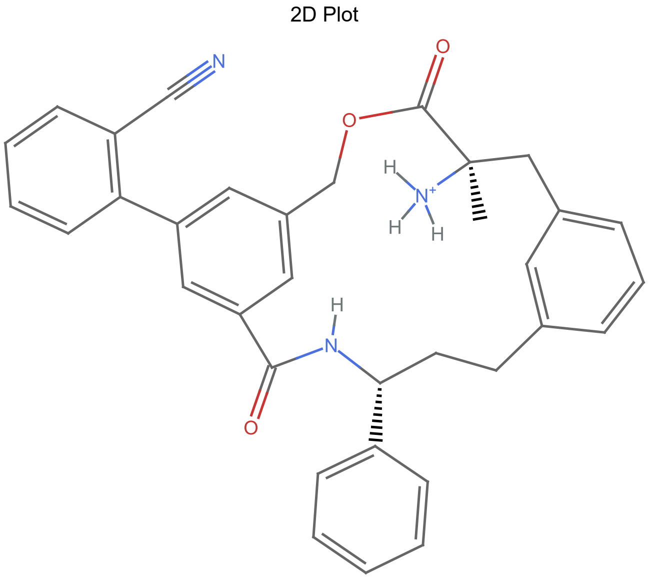

Dataset Index= 58

Molecule Formula= $C_{34}H_{32}N_3O_3^+$

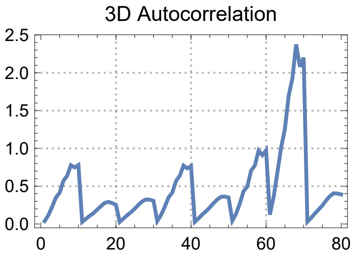

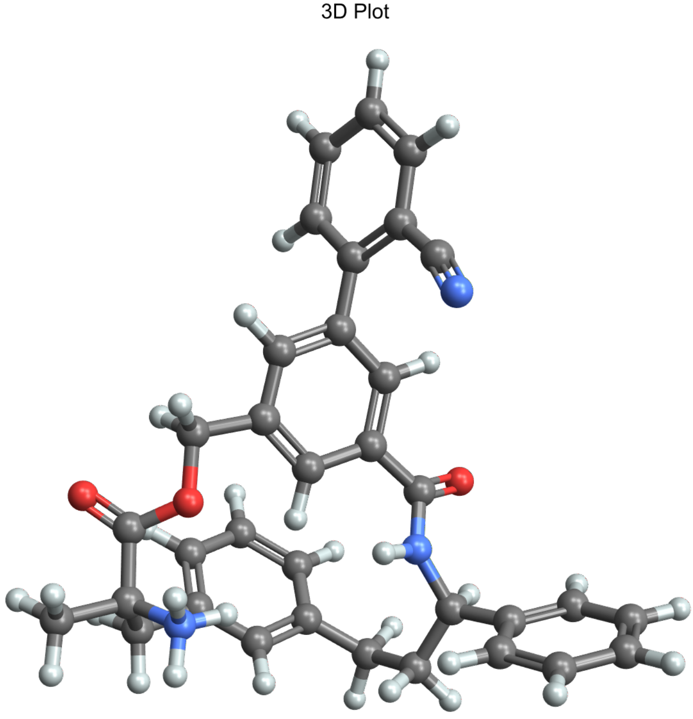

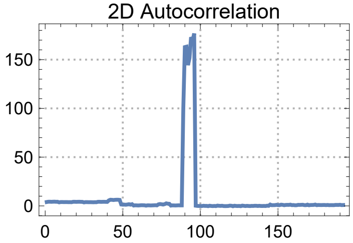

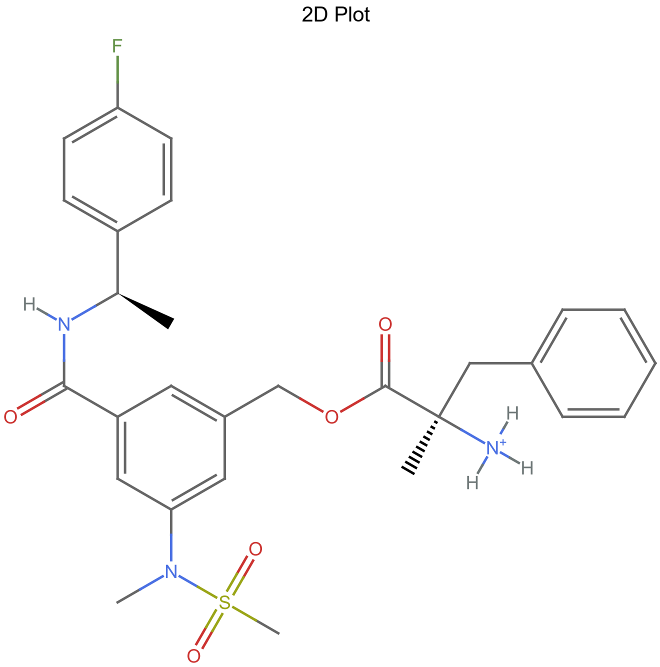

Dataset Index= 59

Molecule Formula= $C_{28}H_{33}FN_3O_5S^+$

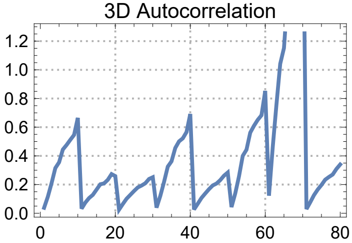

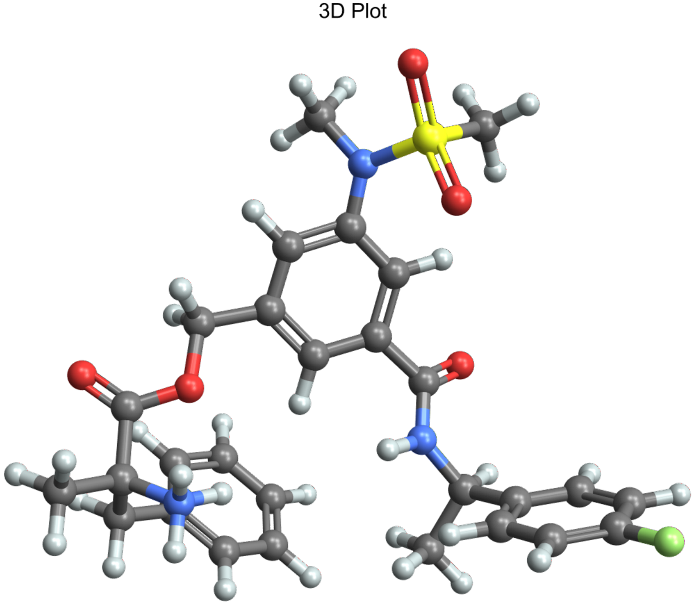

2D Plot

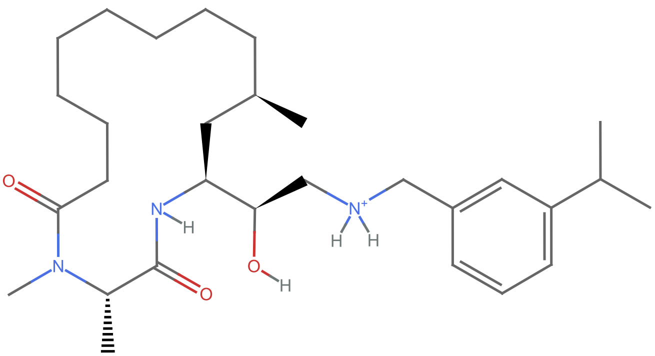

3D Plot

2D Autocorrelation

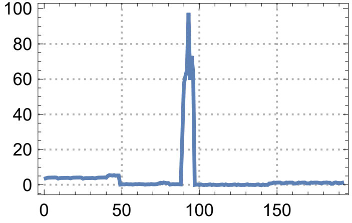

Dataset Index= 60

Molecule Formula= $C_{29}H_{50}N_3O_3^+$

3D Autocorrelation

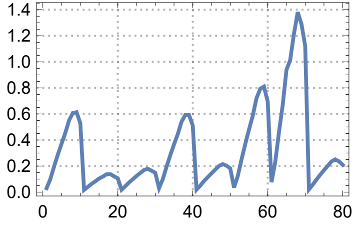

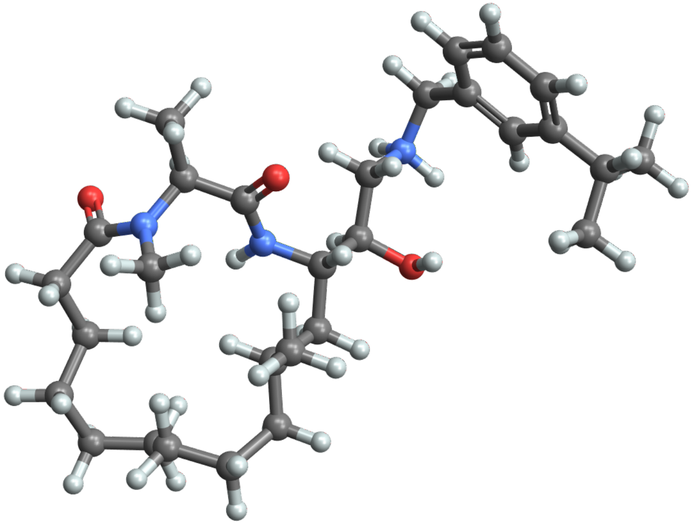

2D Autocorrelation

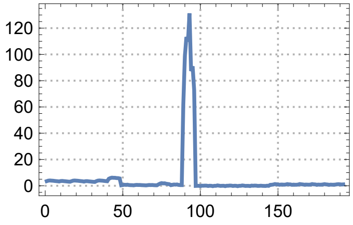

Dataset Index= 61

Molecule Formula= $C_{18}H_{15}F_2N_5O_2$

3D Autocorrelation

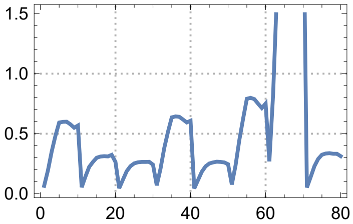

2D Plot

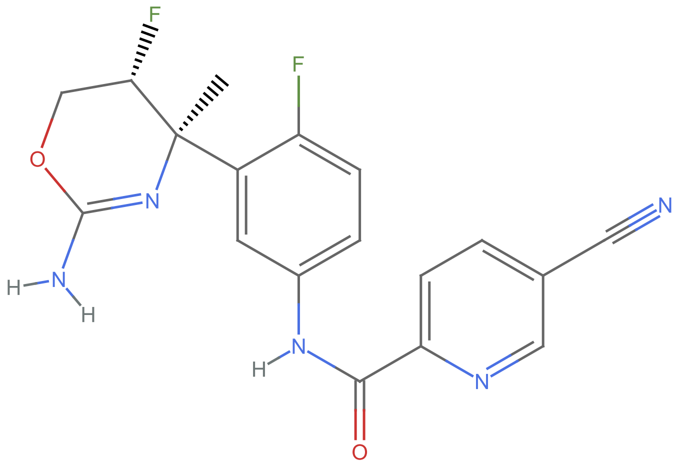

3D Plot

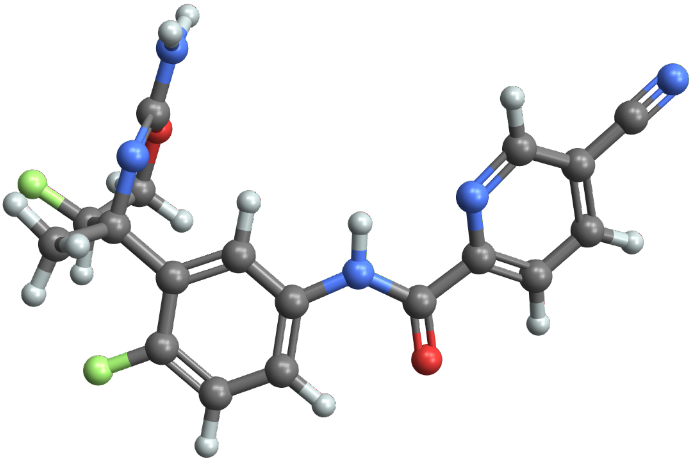

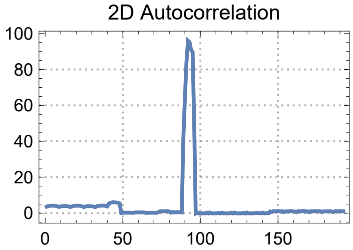

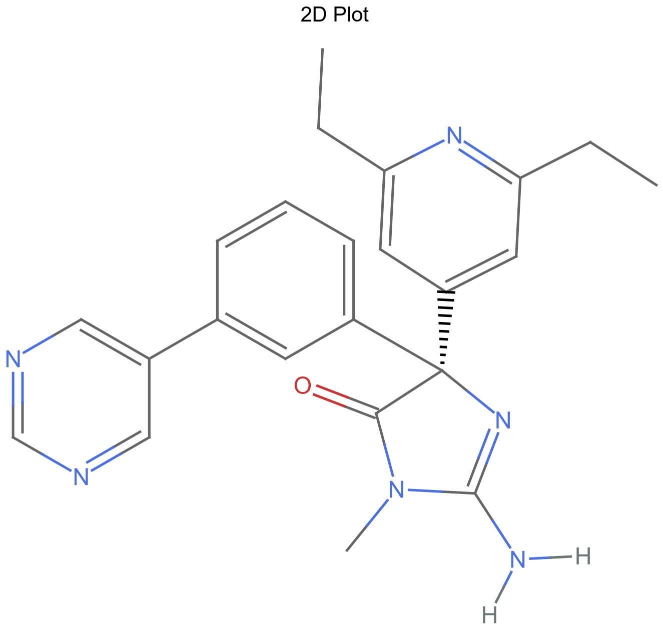

Dataset Index= 62

Molecule Formula= $C_{23}H_{24}N_6O$

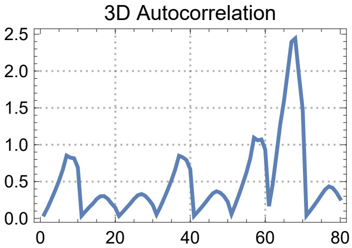

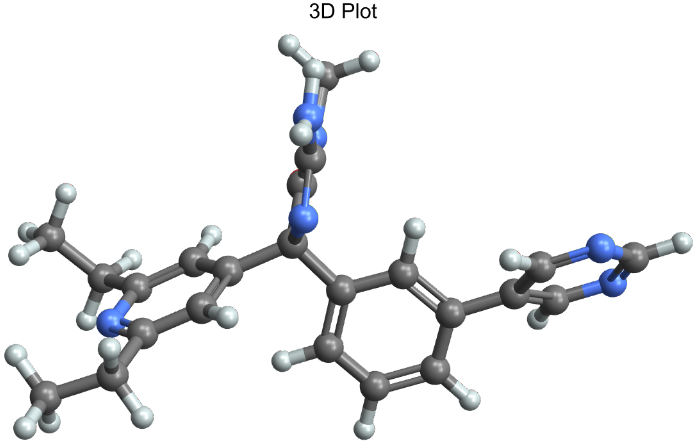

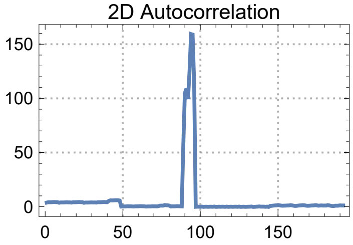

Dataset Index= 63

Molecule Formula= $C_{32}H_{49}N_4O_3^+$

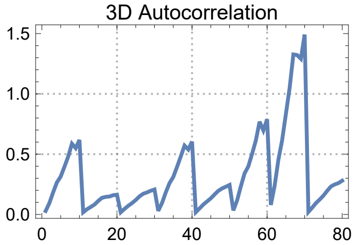

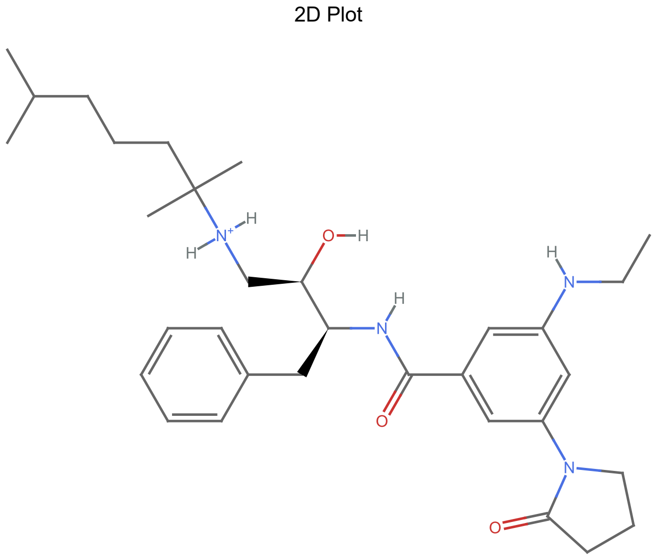

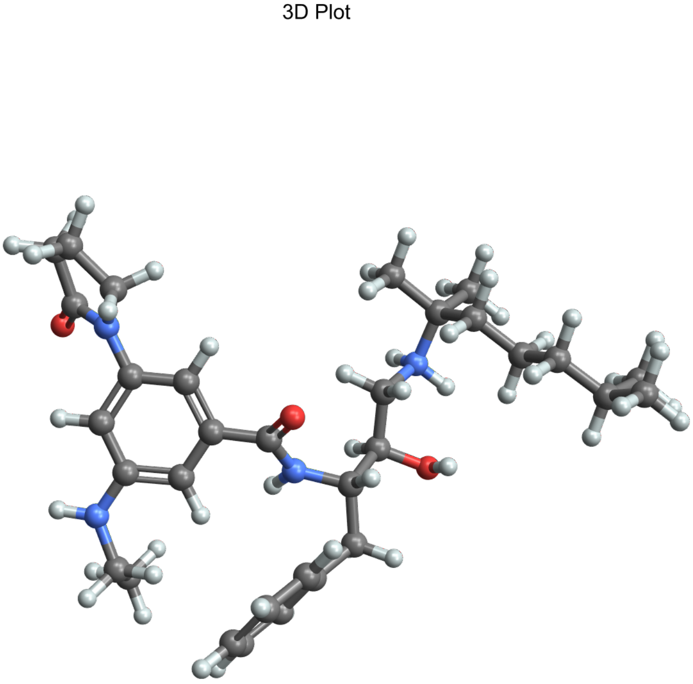

2D Plot

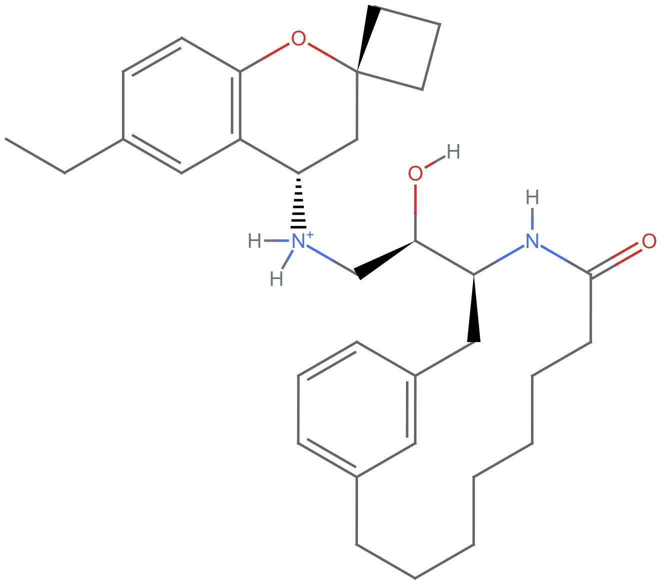

2D Autocorrelation

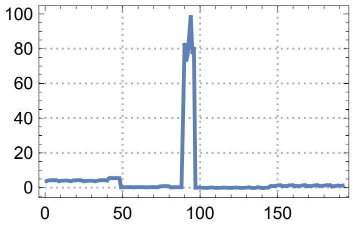

Dataset Index= 64

Molecule Formula= $C_{32}H_{45}N_2O_3^+$

3D Autocorrelation

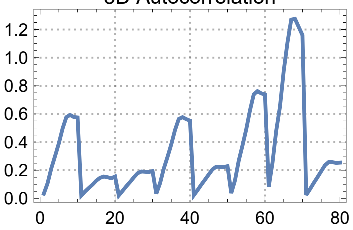

3D Plot

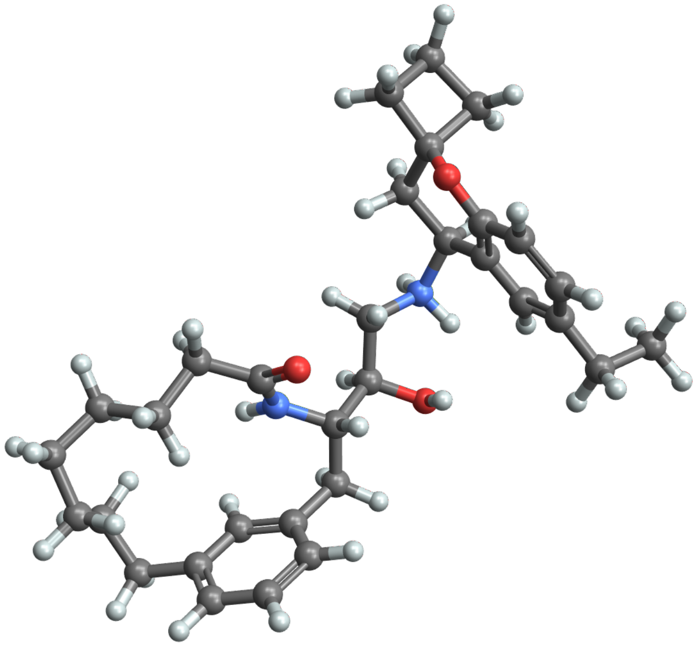

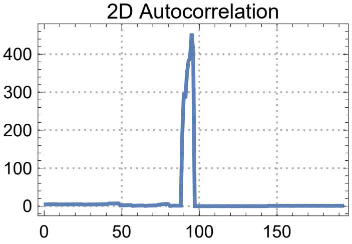

Dataset Index= 65

Molecule Formula= $C_{42}H_{43}F_2N_5O_{12}S^{2-}$

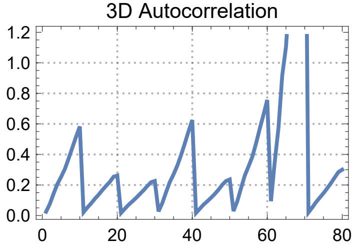

2D Plot

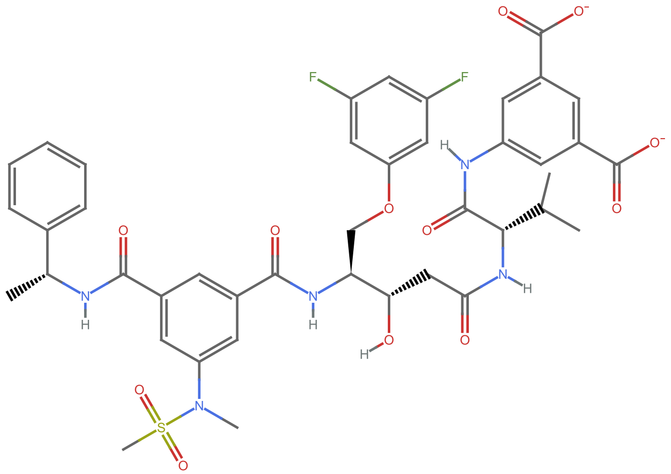

3D Plot

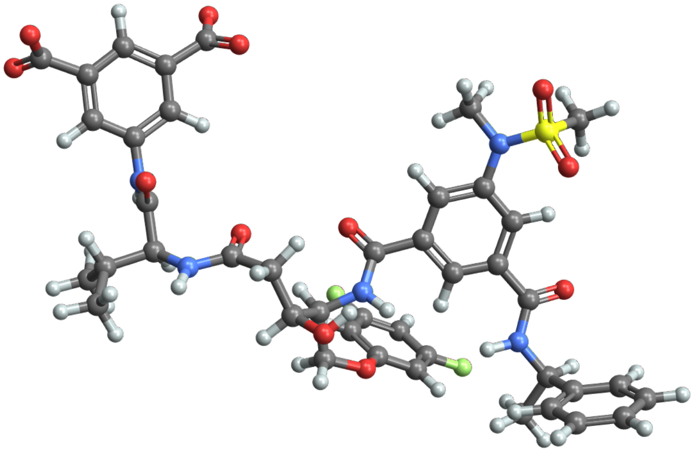

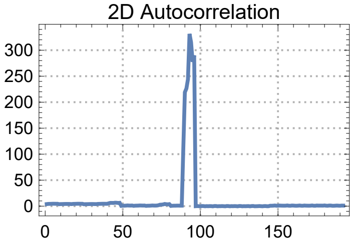

Dataset Index= 66

Molecule Formula= $C_{29}H_{49}N_6O_{10}^-$

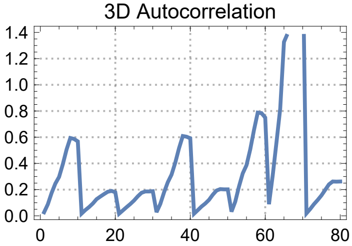

2D Plot

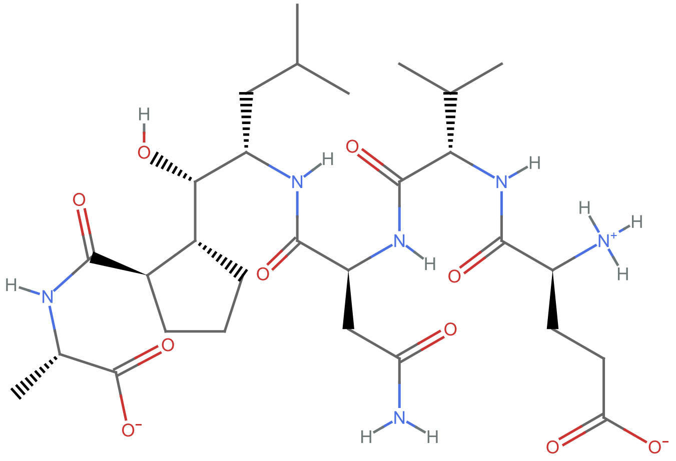

3D Plot

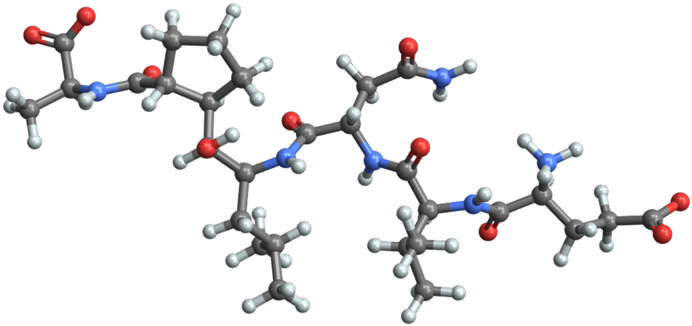

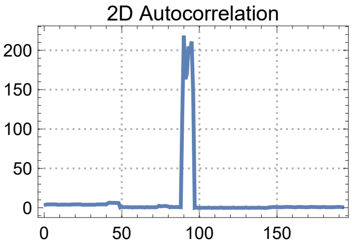

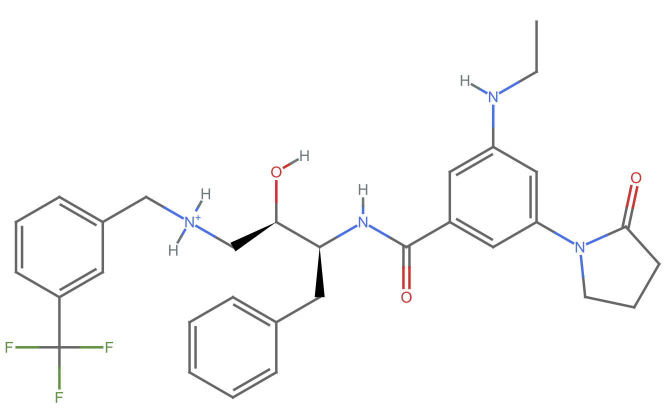

Dataset Index= 67

Molecule Formula= $C_{31}H_{36}F_3N_4O_3^+$

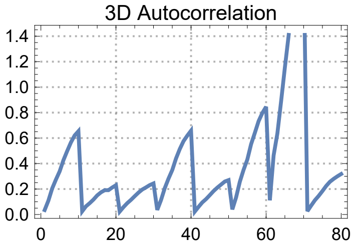

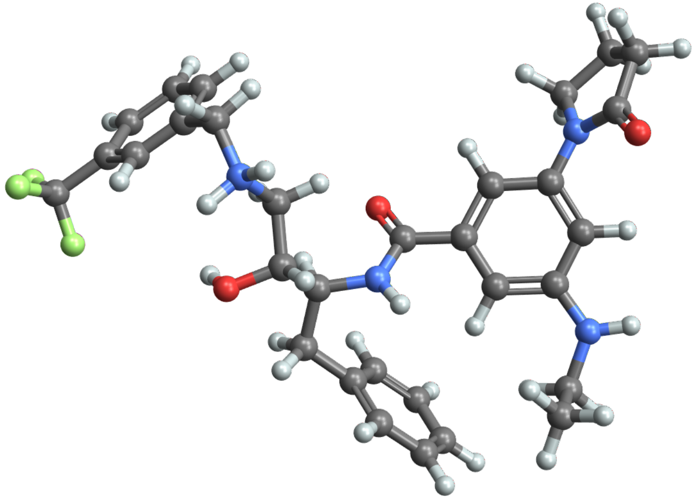

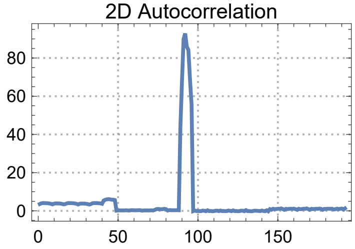

Dataset Index= 68

Molecule Formula= $C_{23}H_{22}N_4O_2$

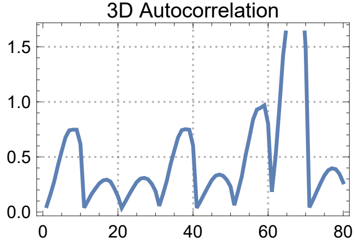

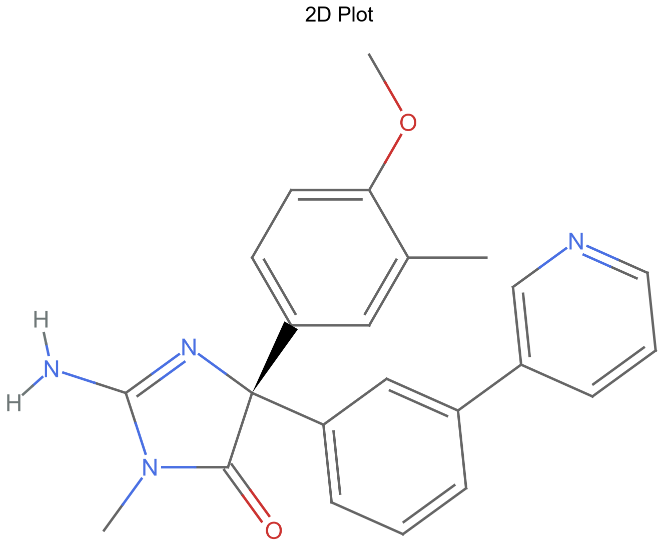

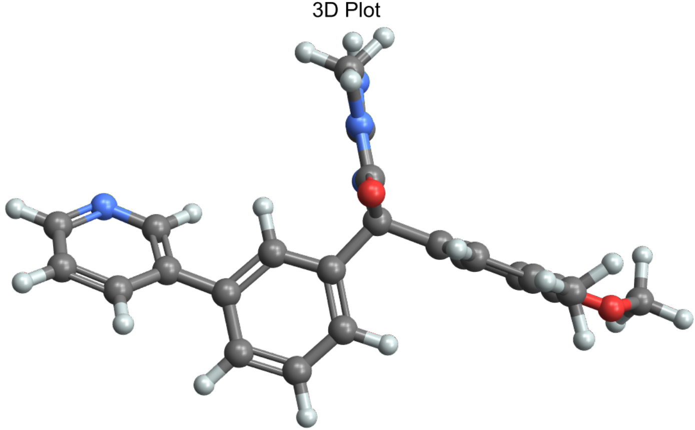

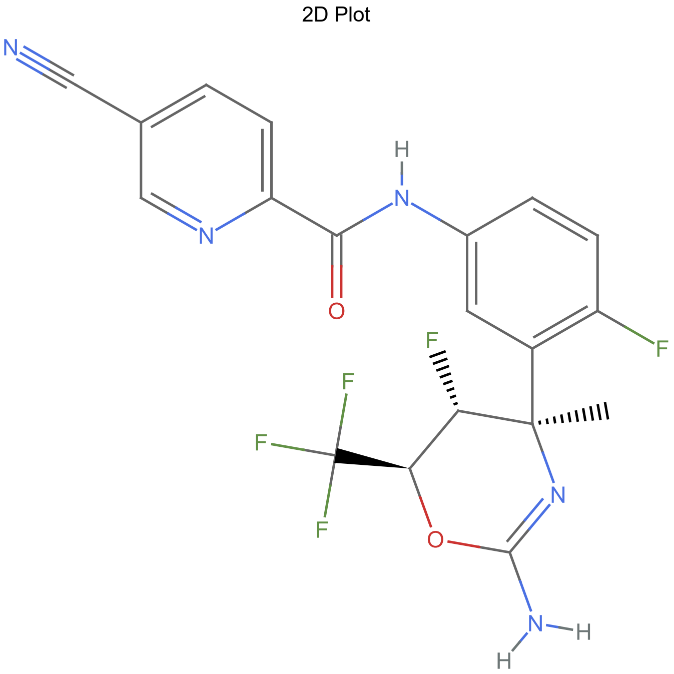

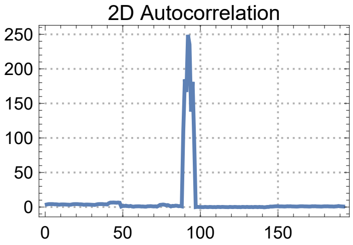

Dataset Index= 69

Molecule Formula= $C_{19}H_{14}F_5N_5O_2$

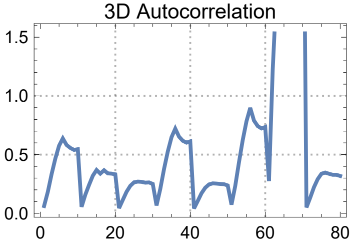

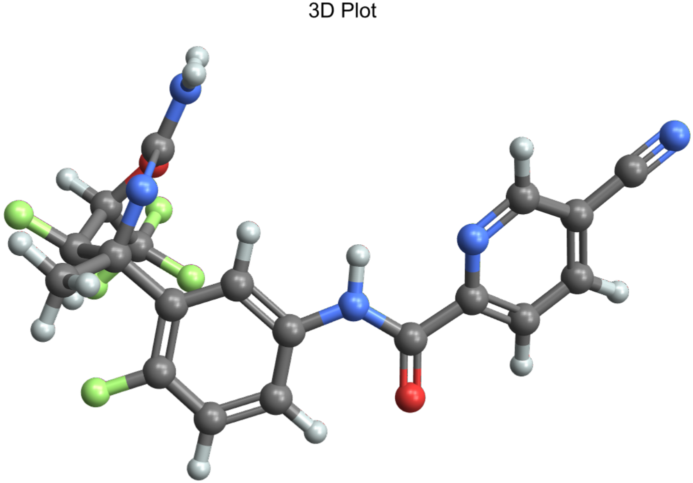

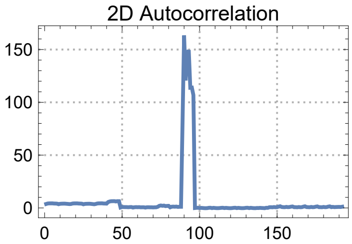

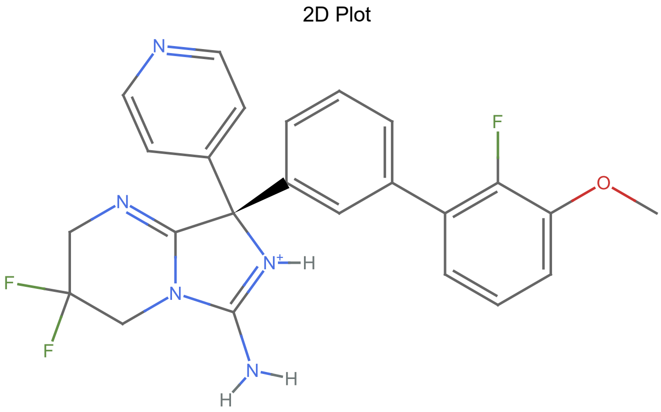

Dataset Index= 70

Molecule Formula= $C_{24}H_{21}F_3N_5O^+$

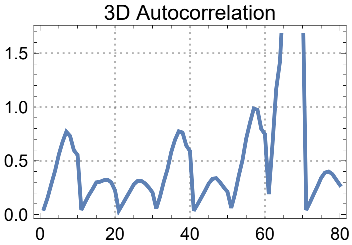

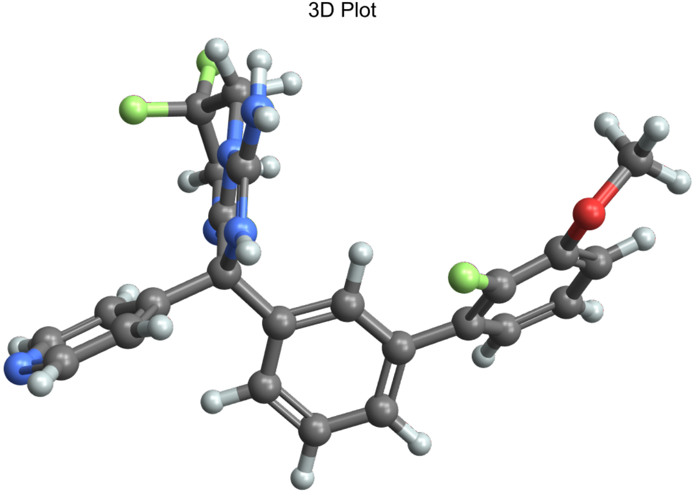

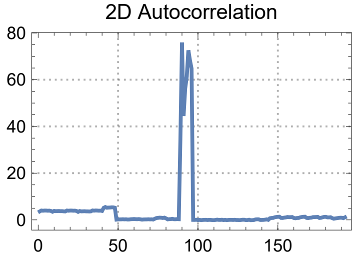

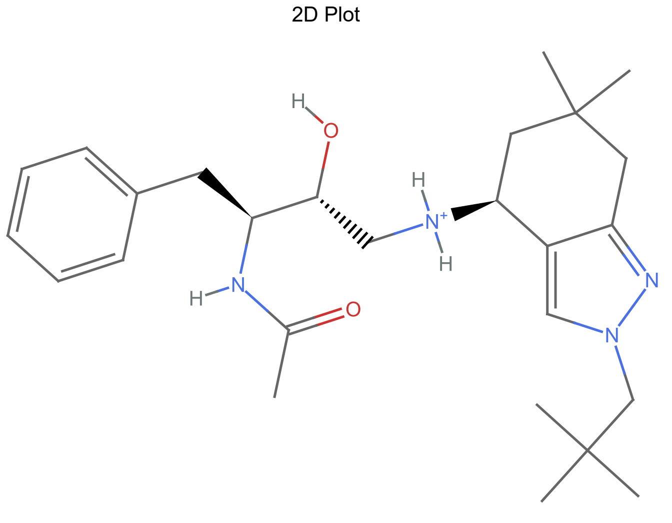

Dataset Index= 71

Molecule Formula= $C_{26}H_{41}N_4O_2^+$

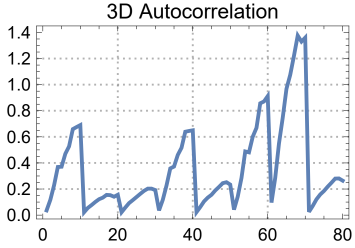

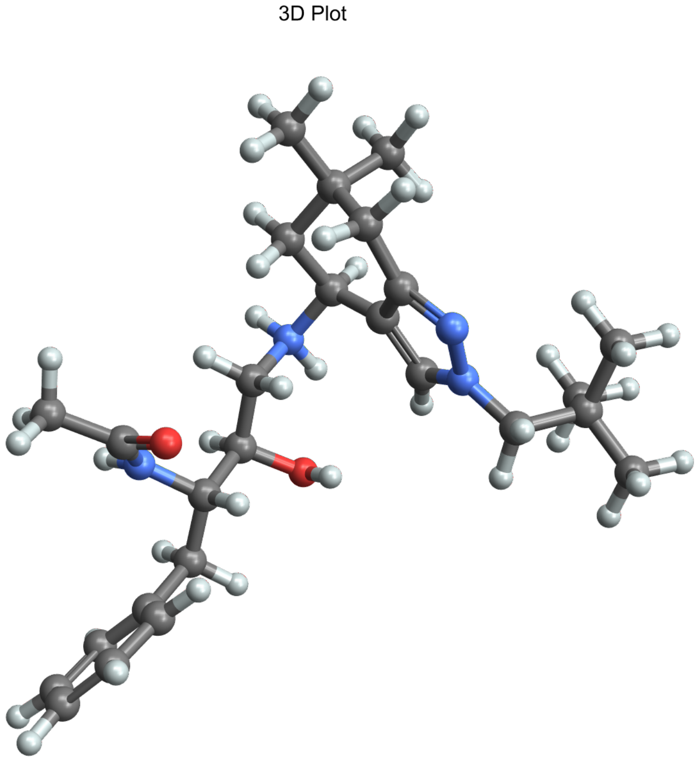

2D Plot

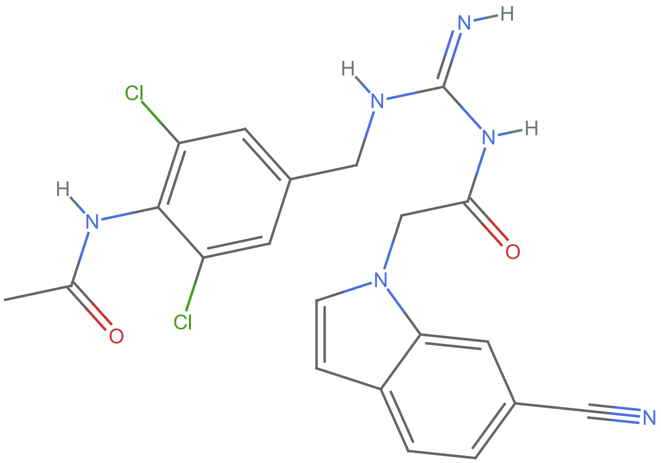

2D Autocorrelation

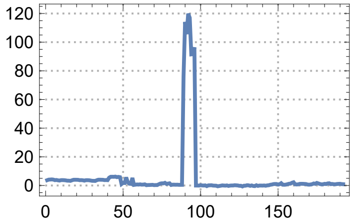

3D Plot

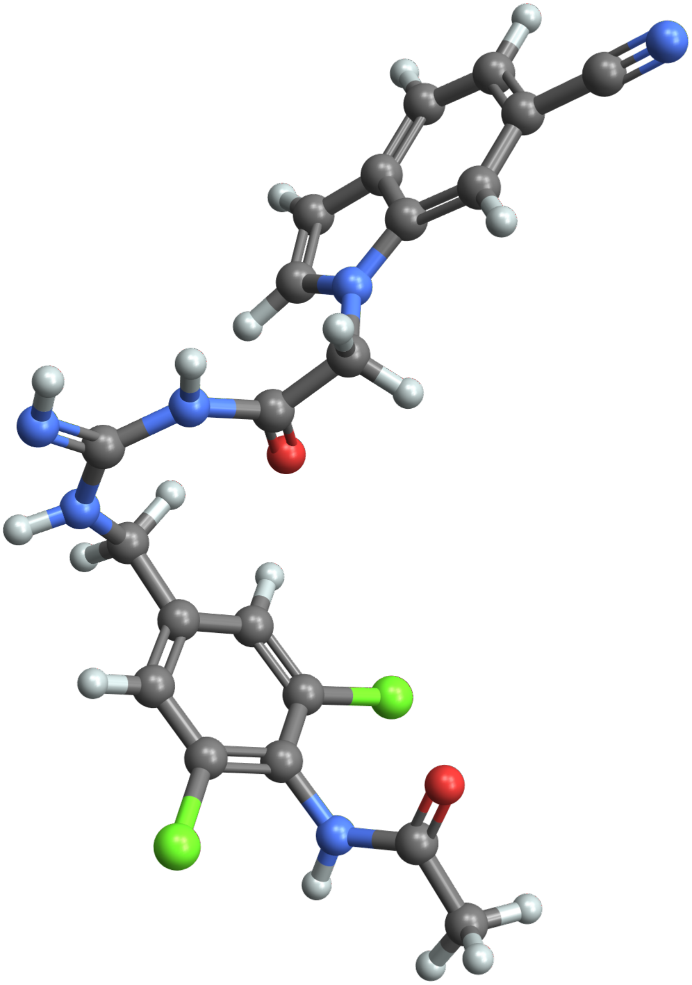

Dataset Index= 72

Molecule Formula= $C_{21}H_{18}Cl_2N_6O_2$

3D Autocorrelation

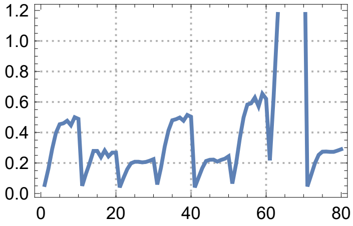

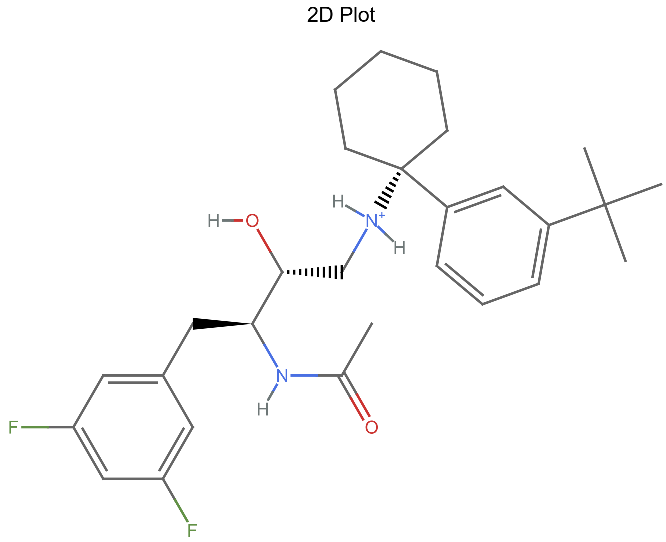

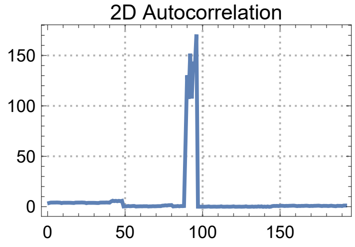

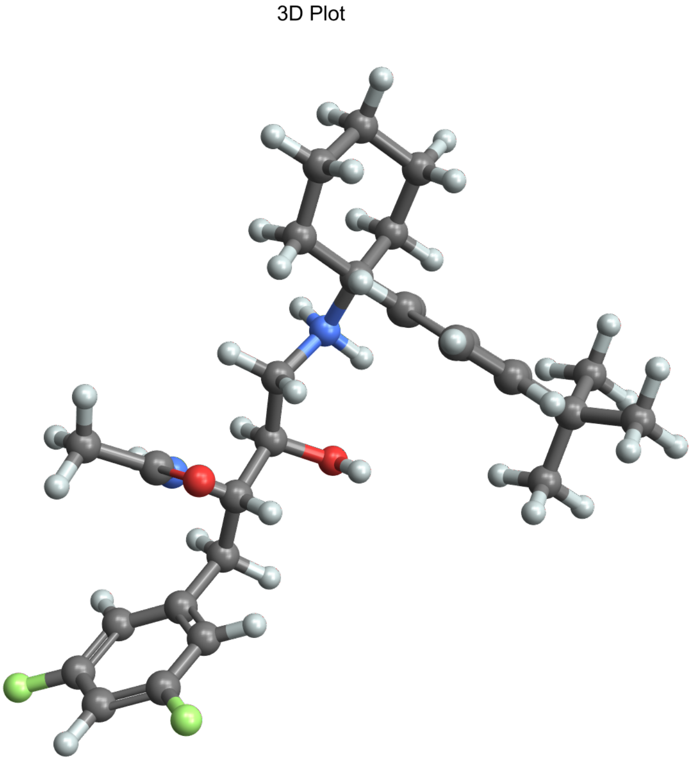

Dataset Index= 73

Molecule Formula= $C_{28}H_{39}F_2N_2O_2^+$

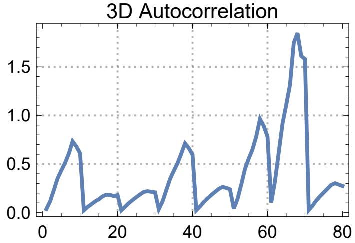

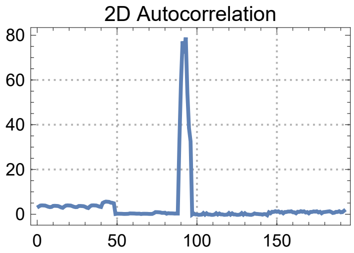

Dataset Index= 74

Molecule Formula= $C_{19}H_{21}N_5O$

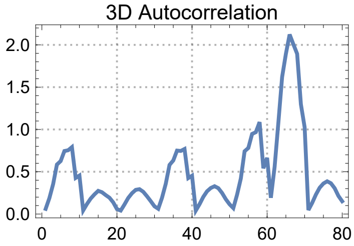

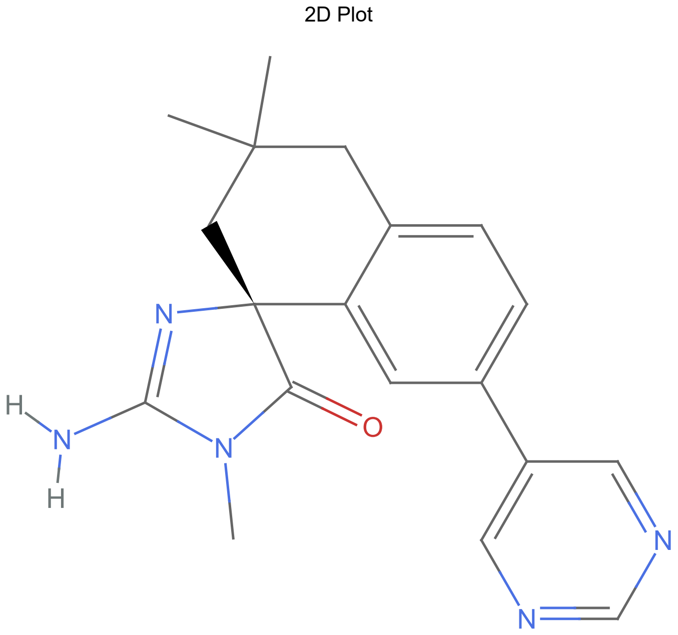

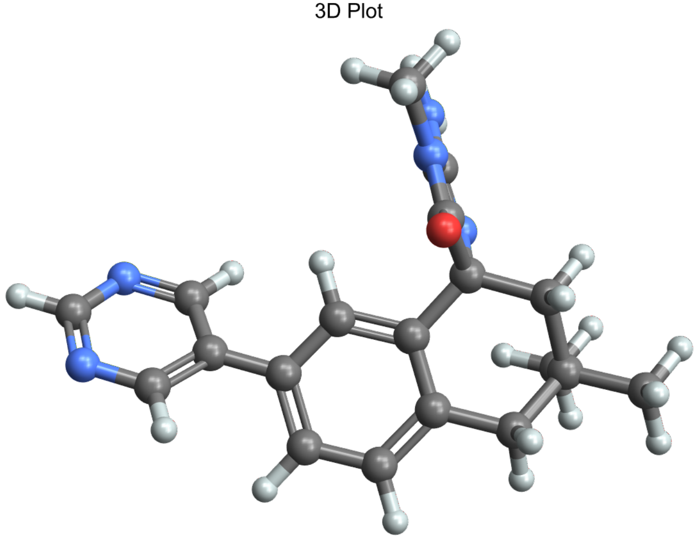

2D Plot

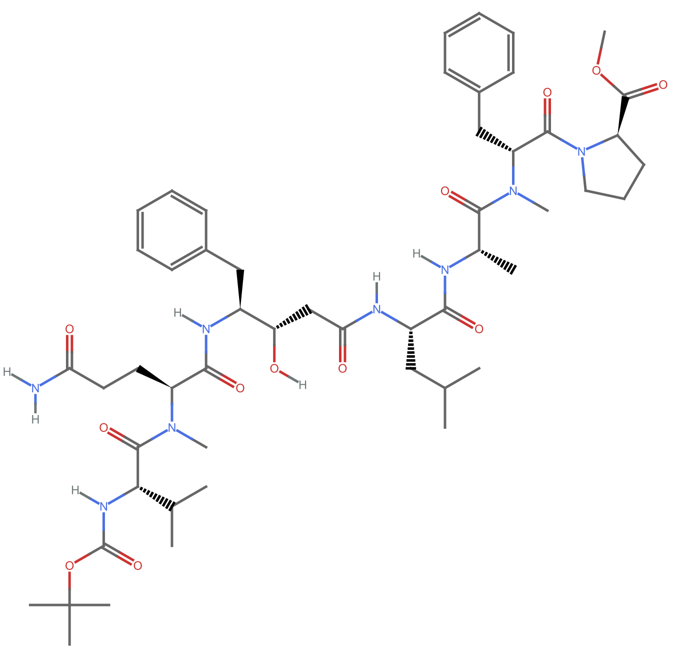

2D Autocorrelation

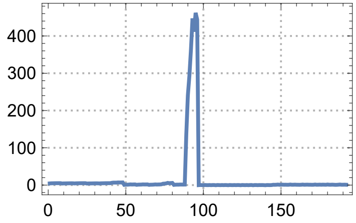

Dataset Index= 75

Molecule Formula= $C_{52}H_{78}N_8O_{12}$

3D Plot

3D Autocorrelation

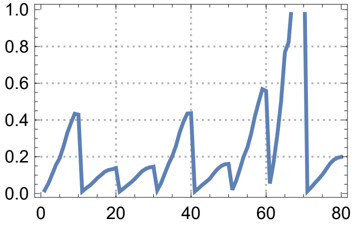

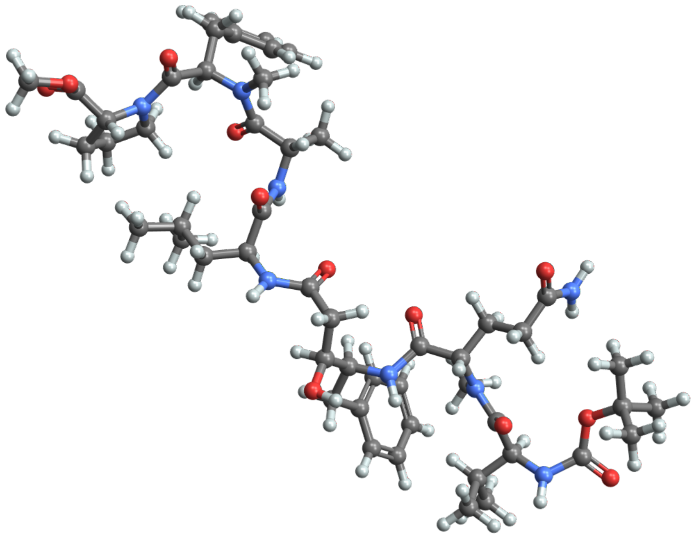

2D Autocorrelation

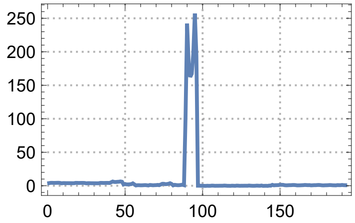

Dataset Index= 76

Molecule Formula= $C_{26}H_{35}F_4N_2O_4S^+$

3D Autocorrelation

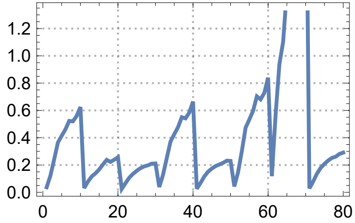

2D Plot

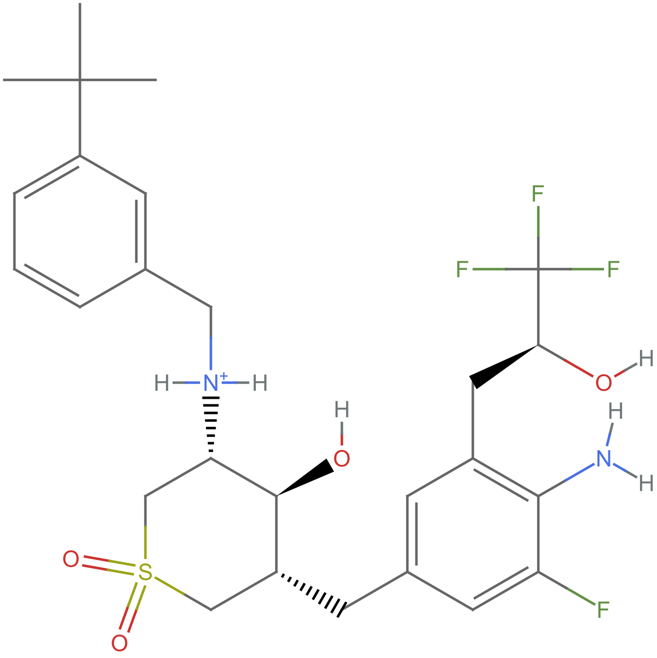

3D Plot

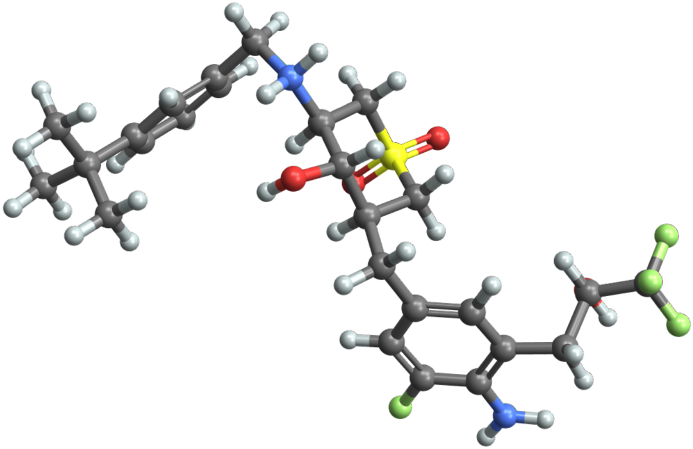

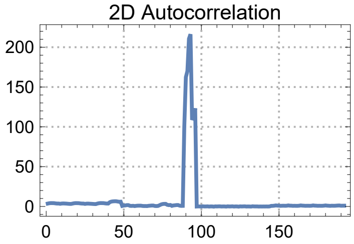

Dataset Index= 77

Molecule Formula= $C_{18}H_{13}F_4N_5O_2$

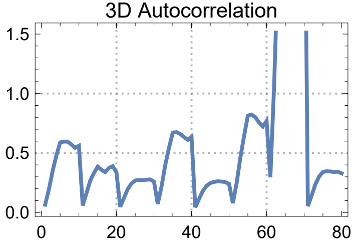

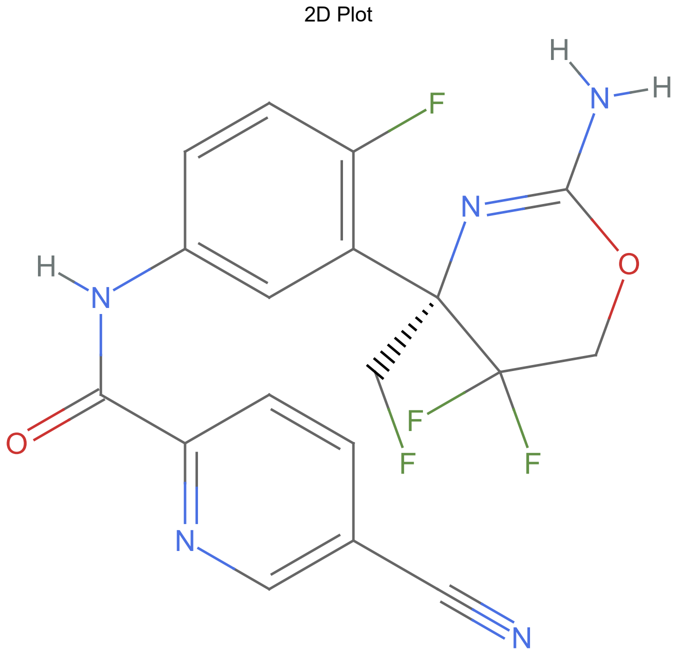

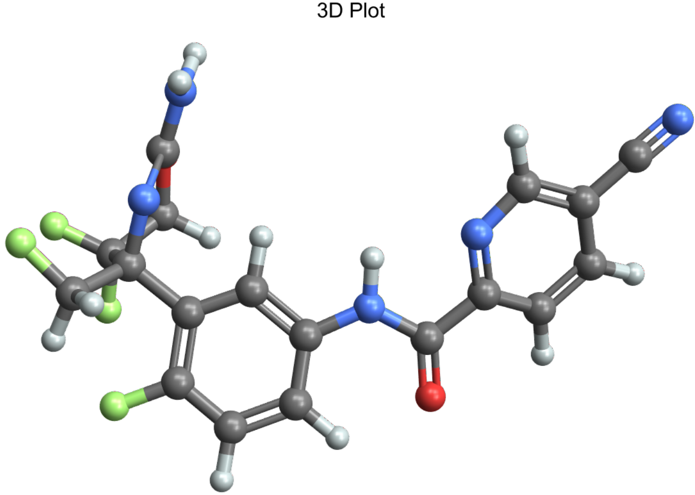

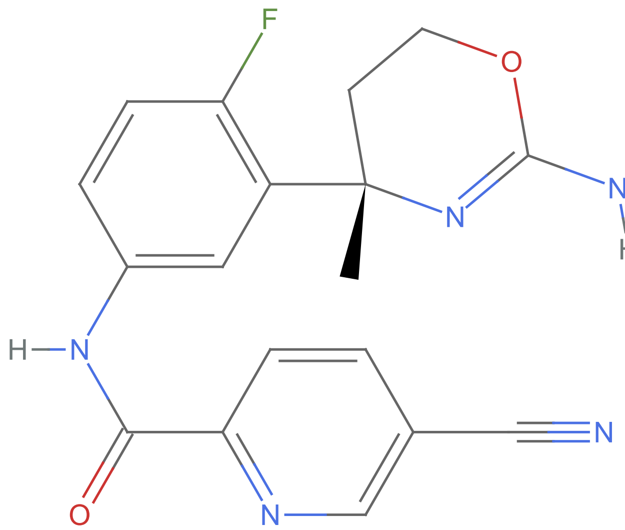

2D Autocorrelation

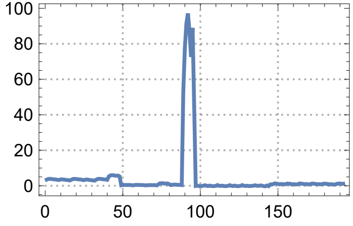

Dataset Index= 78

Molecule Formula= $C_{18}H_{16}FN_5O_2$

3D Plot

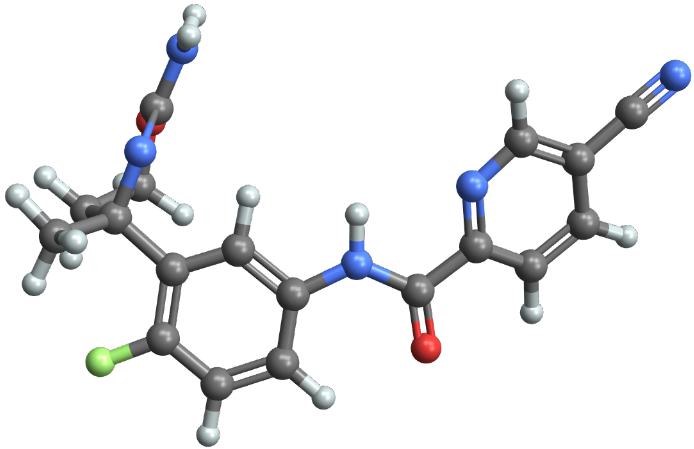

3D Autocorrelation

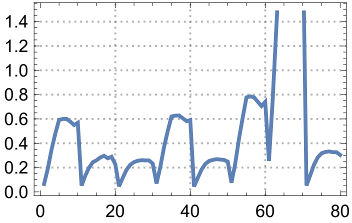

2D Plot

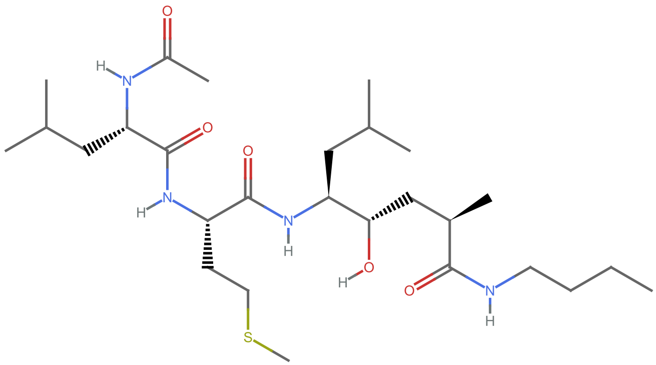

2D Autocorrelation

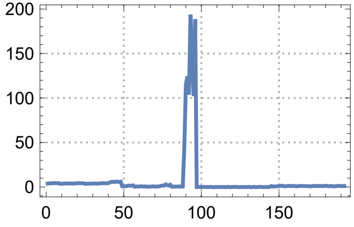

3D Plot

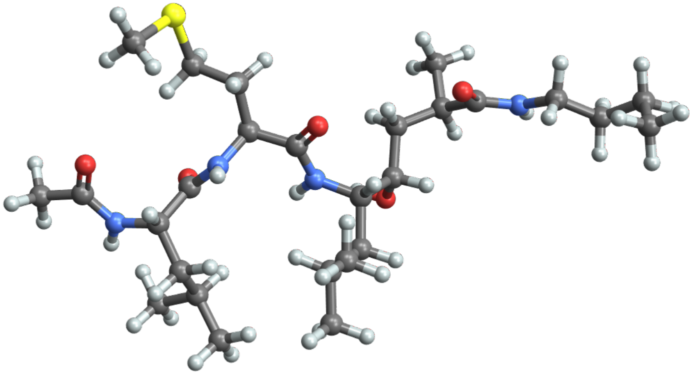

Dataset Index= 79

Molecule Formula= $C_{27}H_{52}N_4O_5S$

3D Autocorrelation

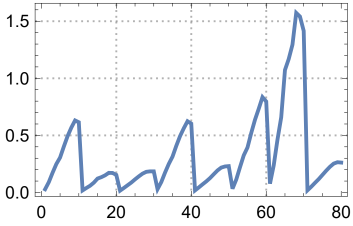

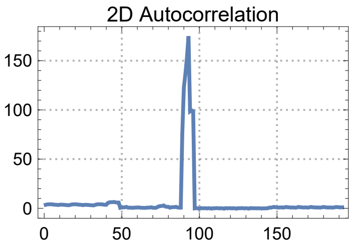

Dataset Index= 80

Molecule Formula= $C_{18}H_{14}F_3N_5O_2$

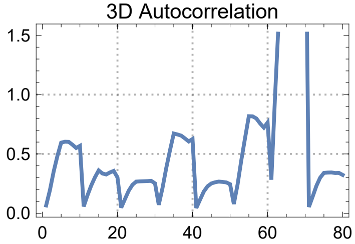

2D Plot

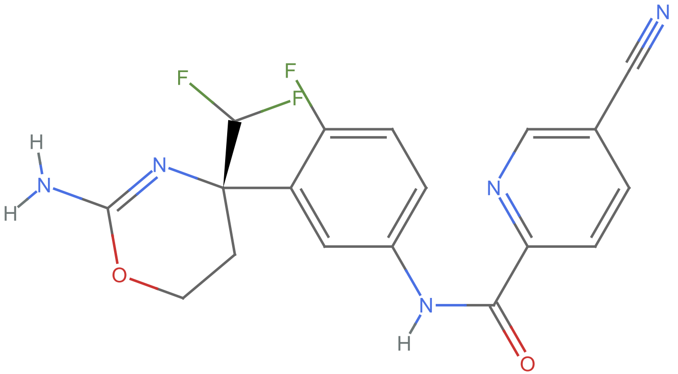

3D Plot

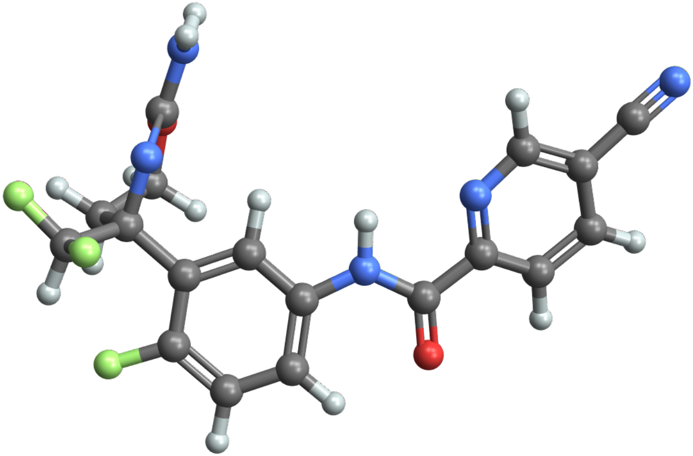

2D Autocorrelation

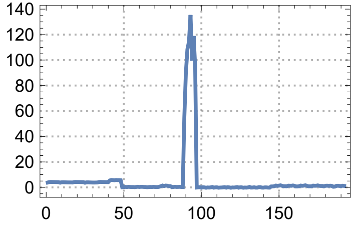

Dataset Index= 81

Molecule Formula= $C_{33}H_{50}N_4O_3^{2+}$

3D Autocorrelation

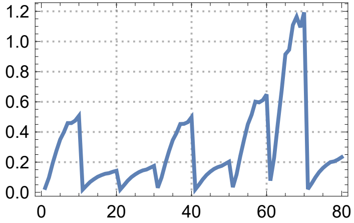

2D Plot

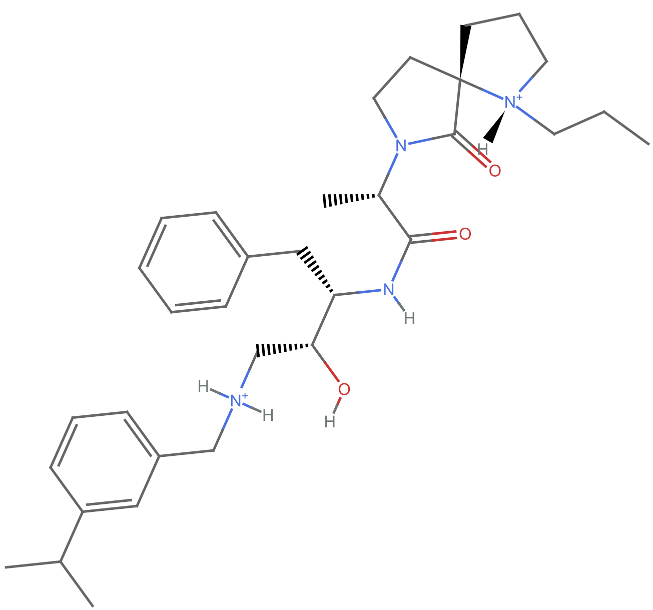

3D Plot

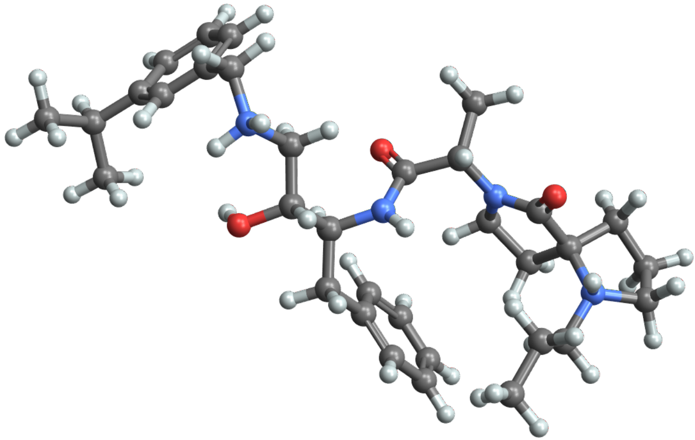

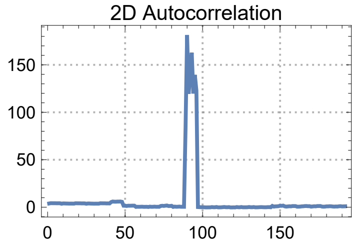

Dataset Index= 82

Molecule Formula= $C_{27}H_{35}F_2N_2O_3S^+$

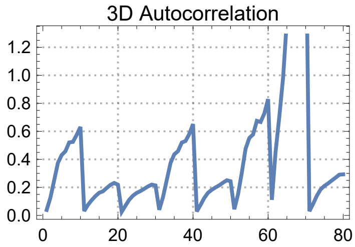

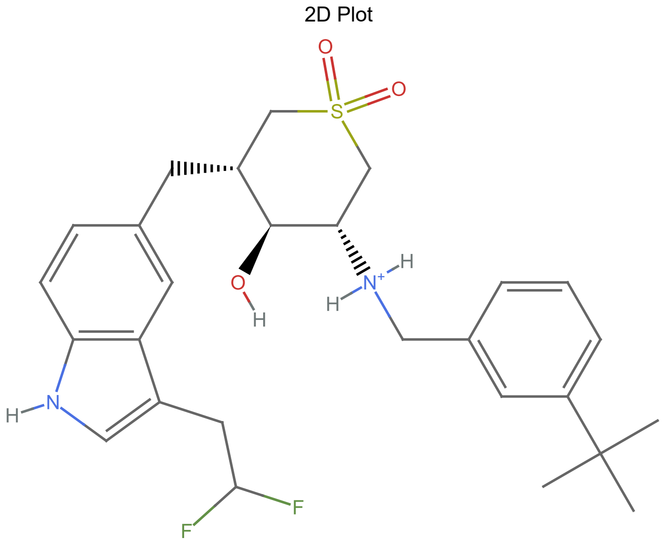

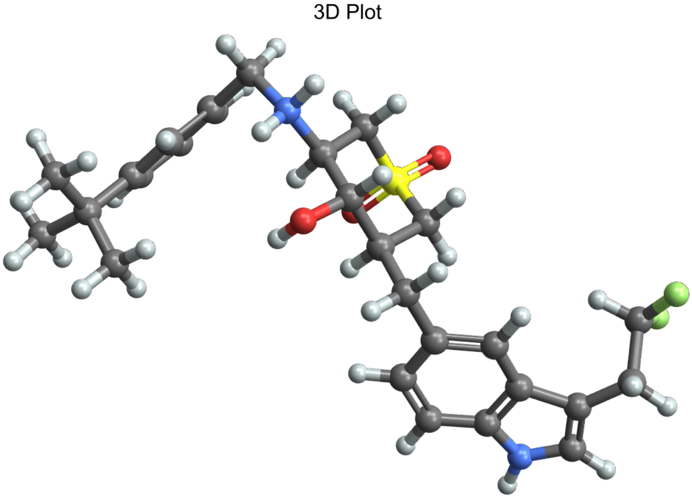

2D Plot

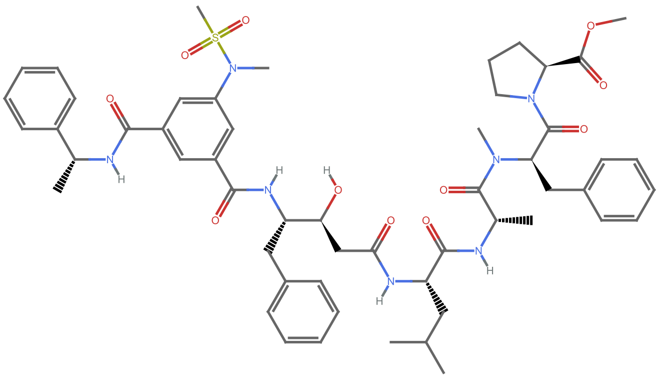

2D Autocorrelation

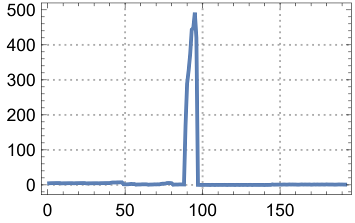

3D Plot

Dataset Index= 83

Molecule Formula= $C_{54}H_{69}N_7O_{11}S$

3D Autocorrelation

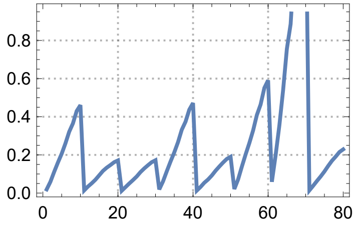

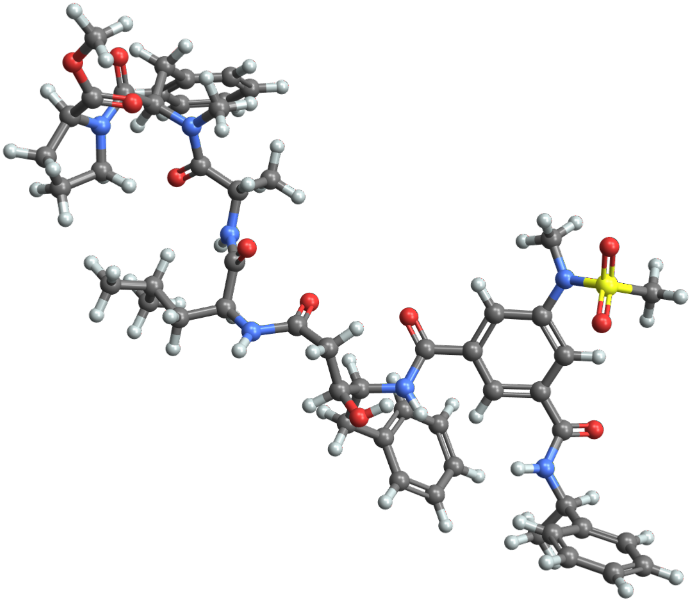

2D Plot

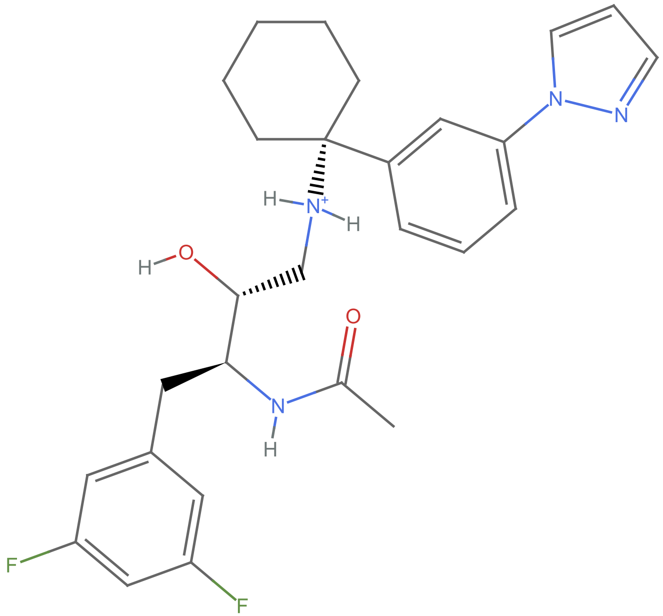

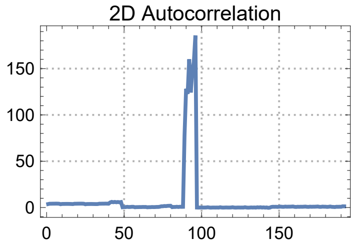

3D Plot

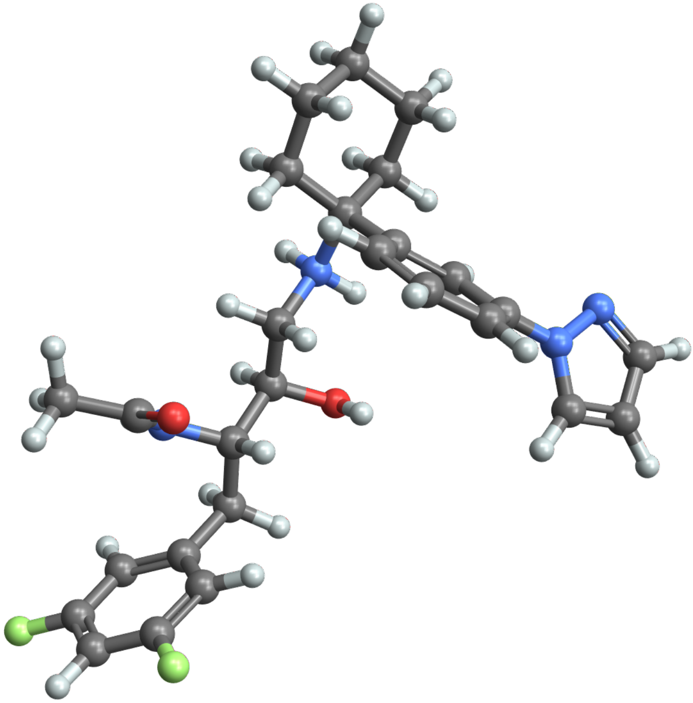

Dataset Index= 84

Molecule Formula= $C_{27}H_{33}F_2N_4O_2^+$

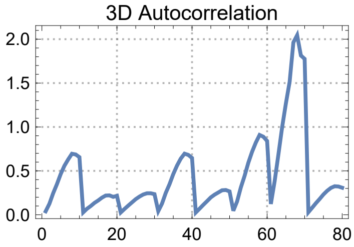

2D Plot

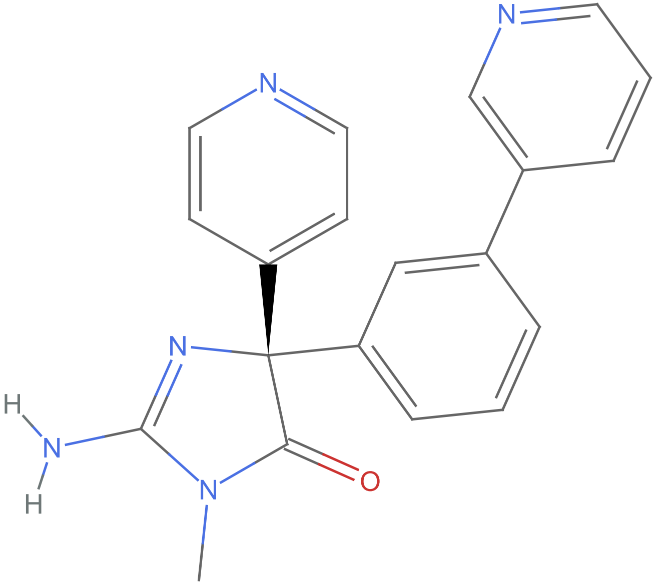

2D Autocorrelation

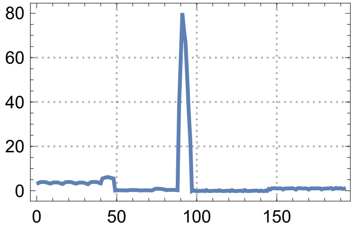

Dataset Index= 85

Molecule Formula= $C_{20}H_{17}N_5O$

3D Autocorrelation

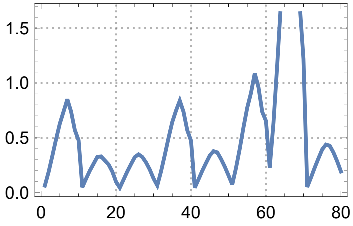

3D Plot

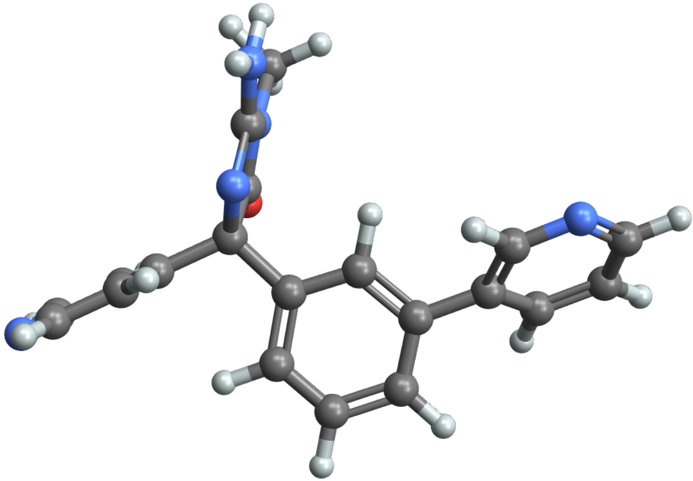

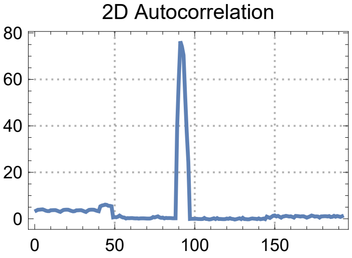

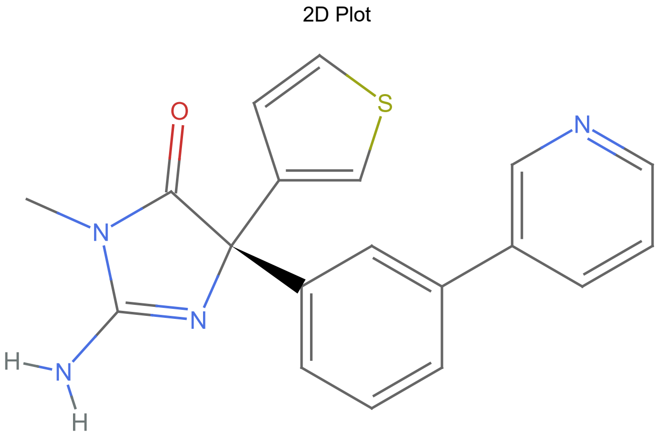

Dataset Index= 86

Molecule Formula= $C_{19}H_{16}N_4OS$

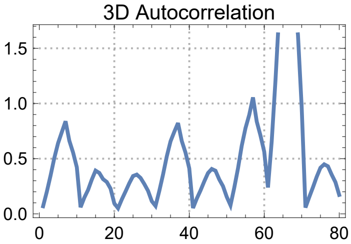

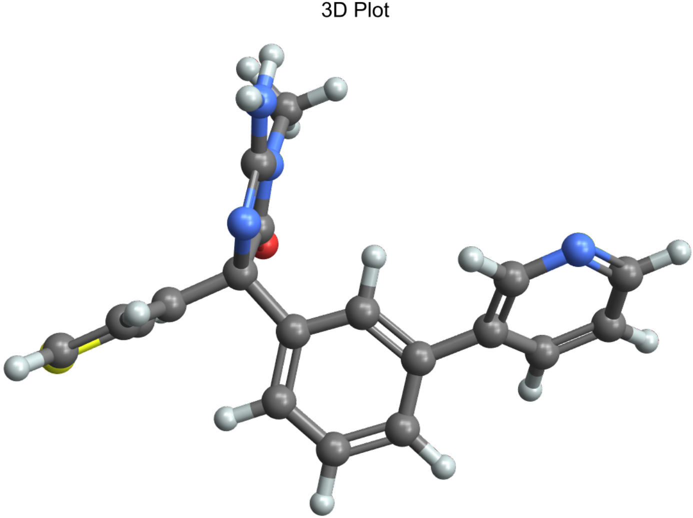

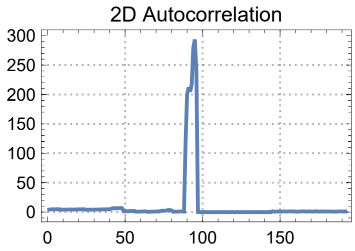

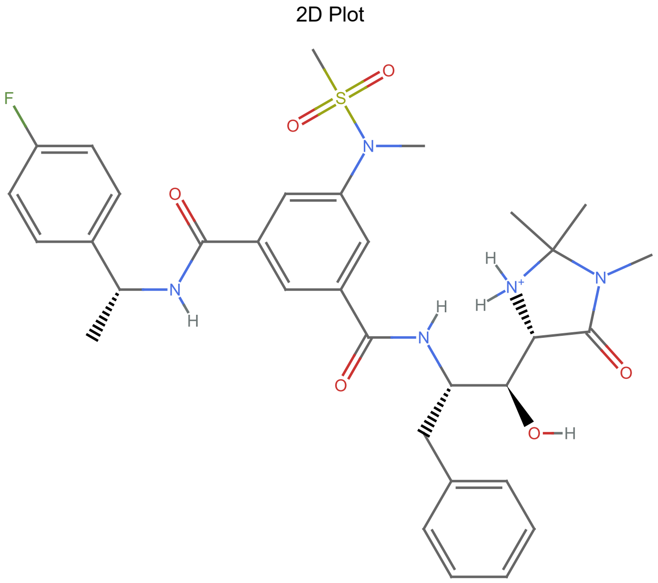

Dataset Index= 87

Molecule Formula= $C_{33}H_{41}FN_5O_6S^+$

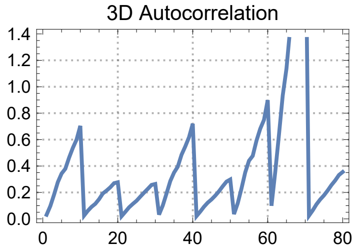

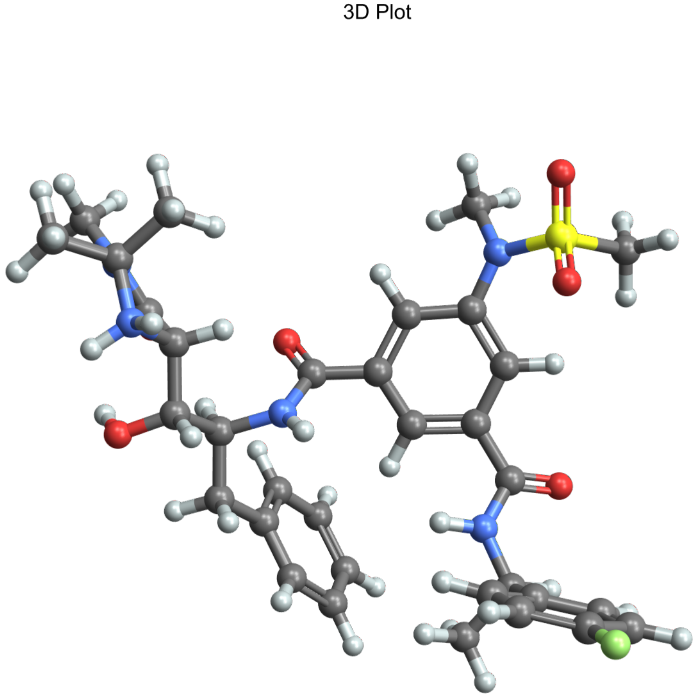

2D Plot

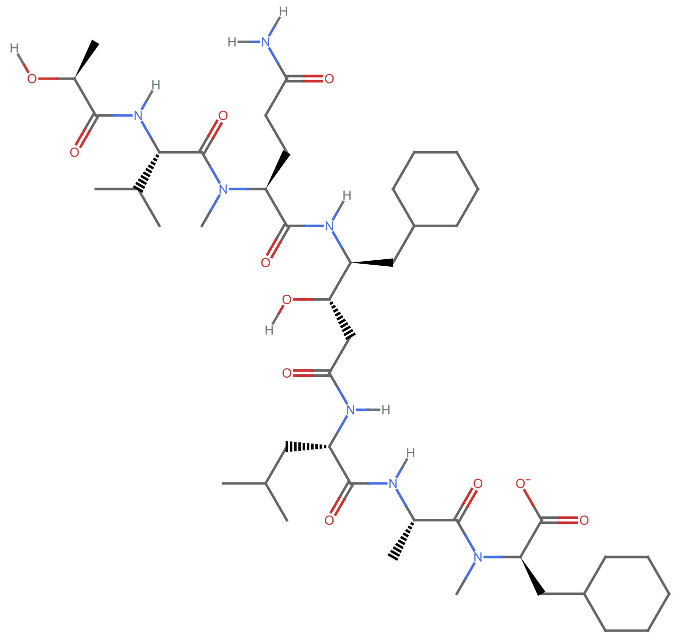

2D Autocorrelation

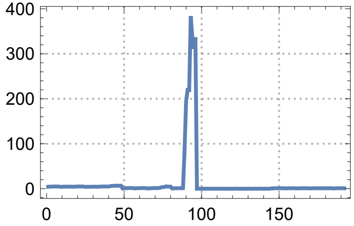

Dataset Index= 88

Molecule Formula= $C_{44}H_{76}N_7O_{11}^-$

3D Autocorrelation

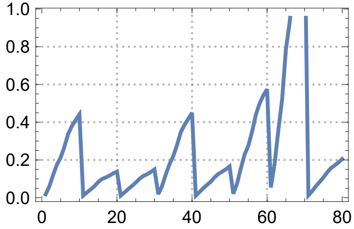

3D Plot

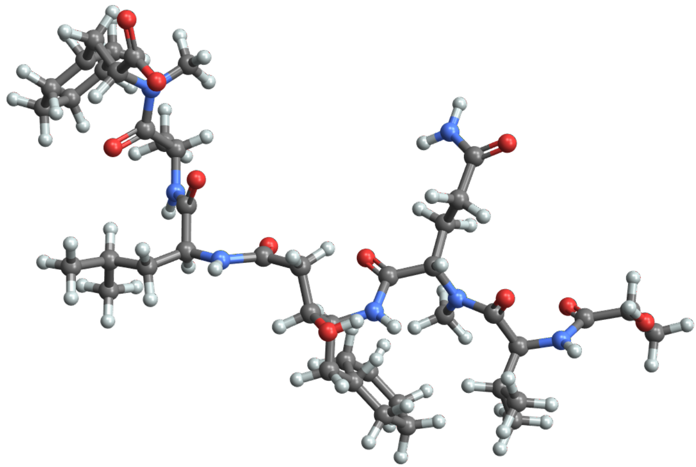

2D Plot

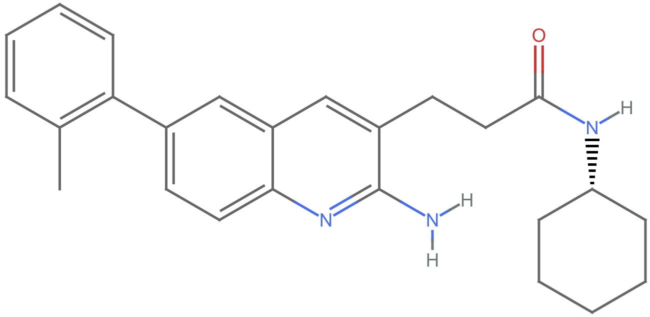

3D Plot

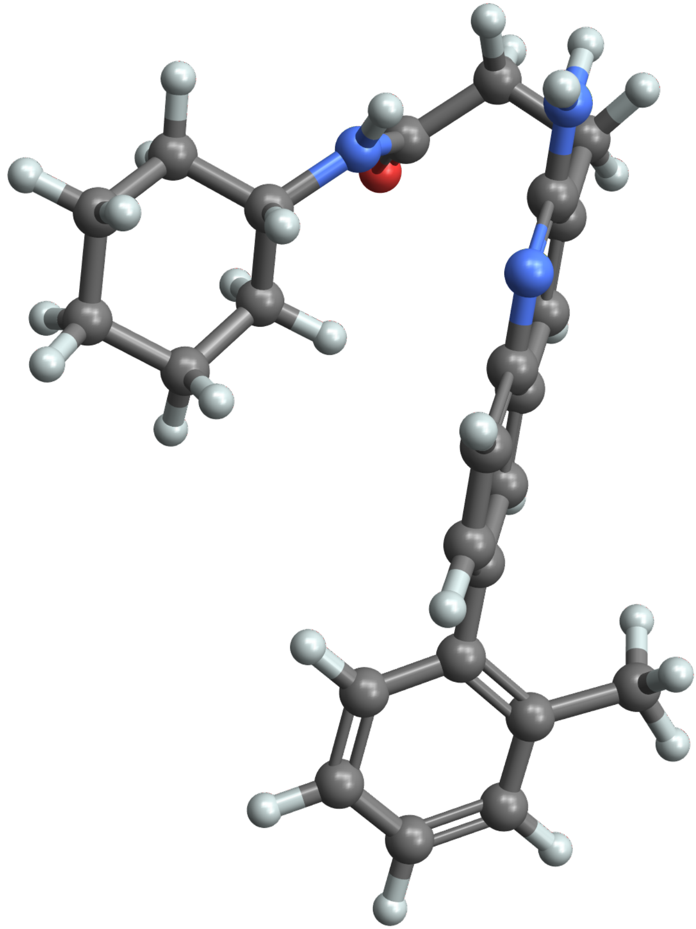

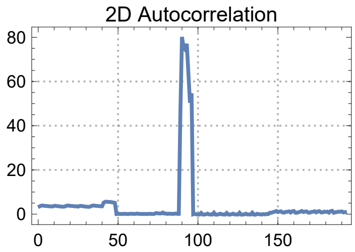

Dataset Index= 89

Molecule Formula= $C_{25}H_{29}N_3O$

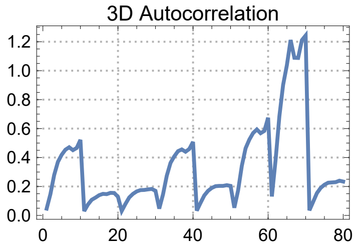

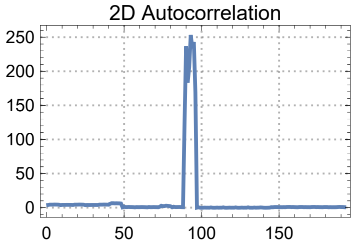

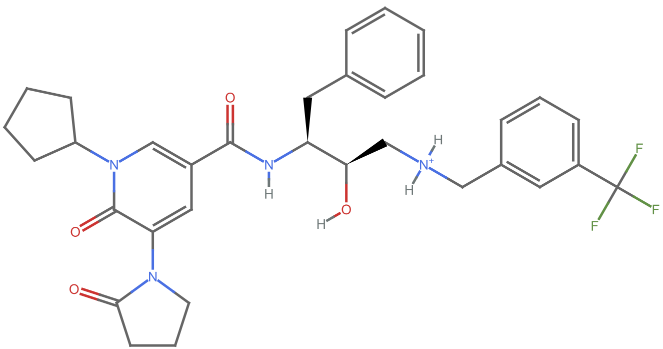

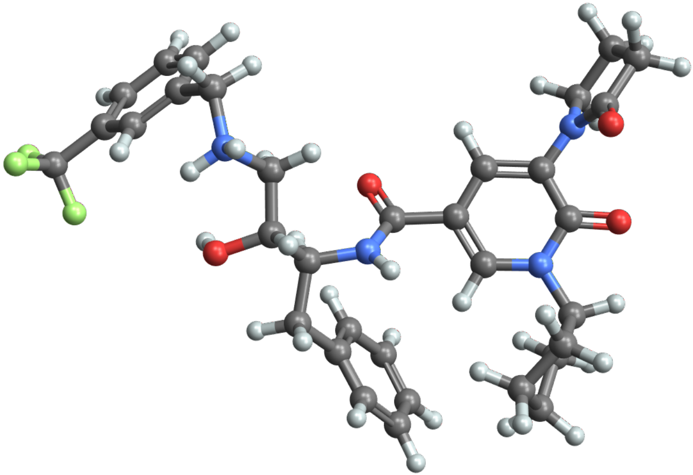

Dataset Index= 90

Molecule Formula= $C_{33}H_{38}F_3N_4O_4^+$

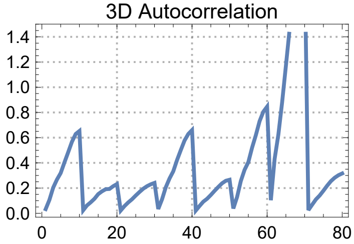

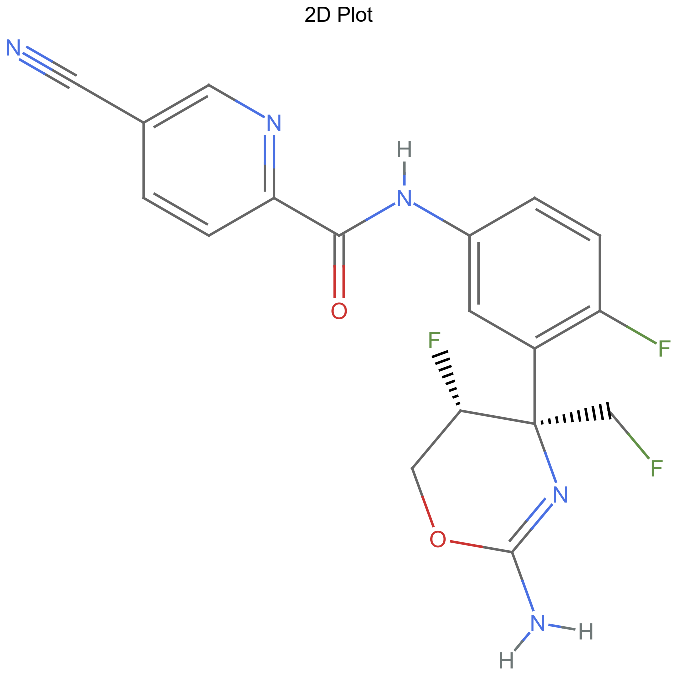

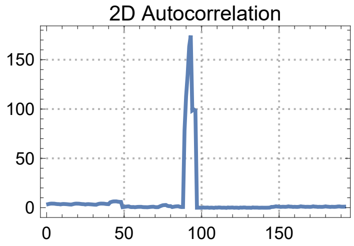

Dataset Index= 91

Molecule Formula= $C_{18}H_{14}F_3N_5O_2$

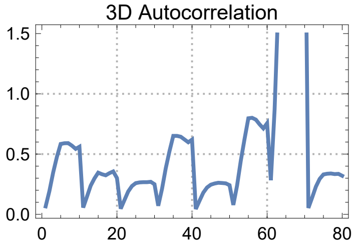

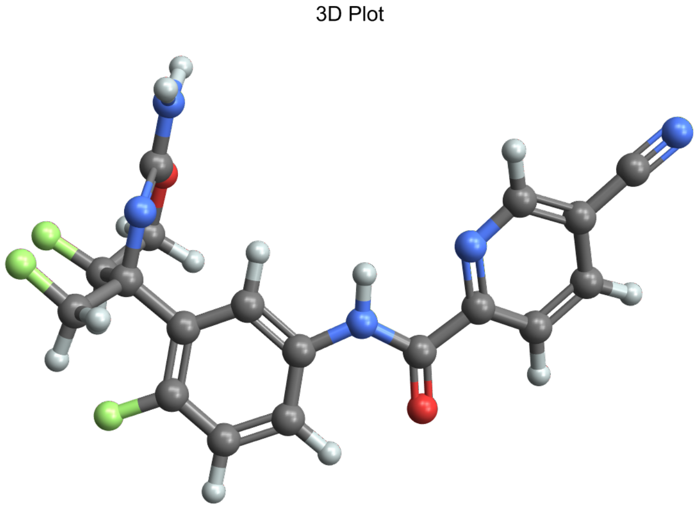

2D Plot

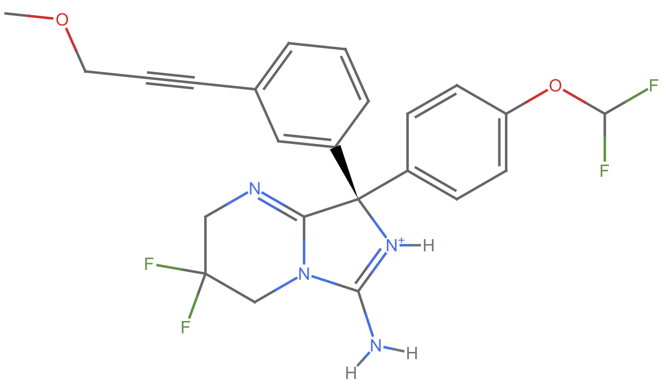

2D Autocorrelation

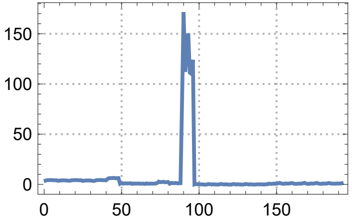

Dataset Index= 92

Molecule Formula= $C_{23}H_{21}F_4N_4O_2^+$

3D Plot

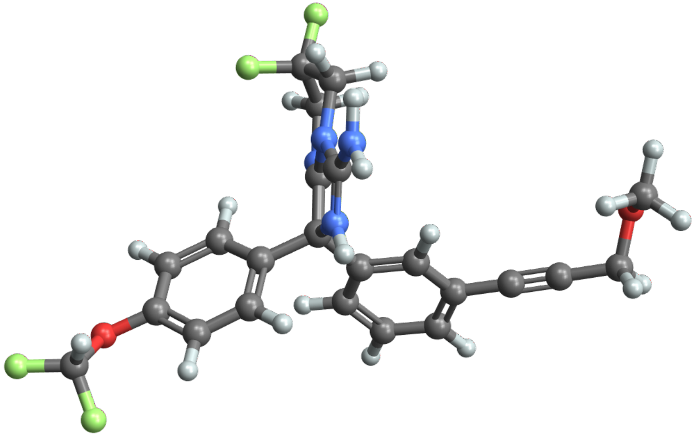

3D Autocorrelation

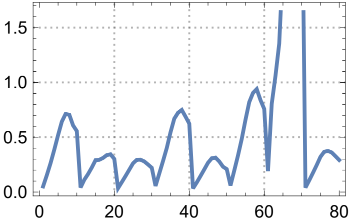

2D Autocorrelation

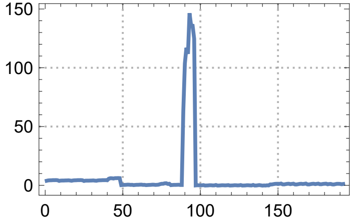

Dataset Index= 93

Molecule Formula= $C_{34}H_{45}N_3O_4{}^{2+}$

3D Autocorrelation

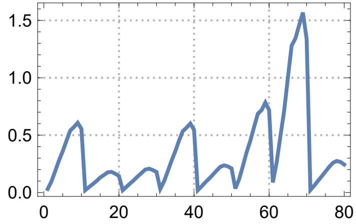

2D Plot

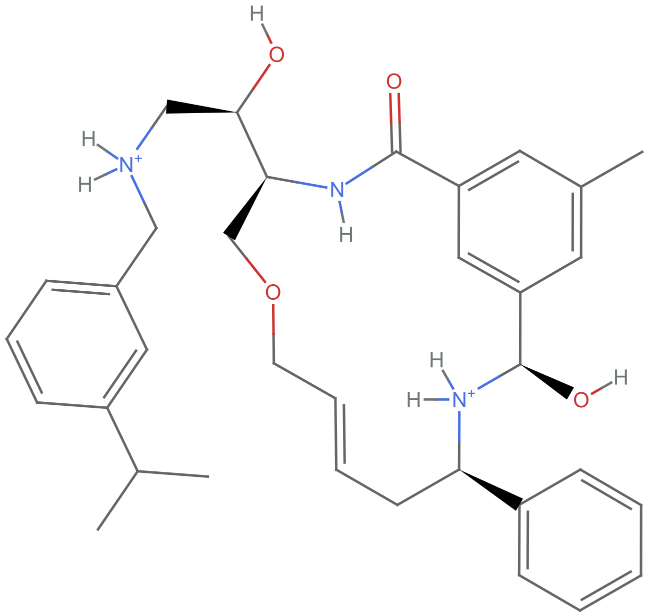

3D Plot

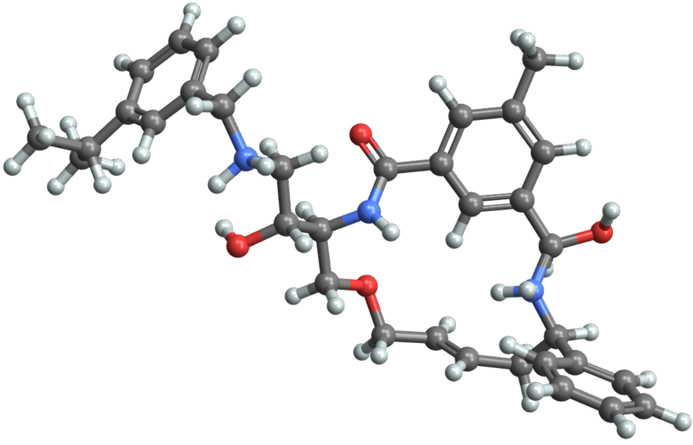

2D Autocorrelation

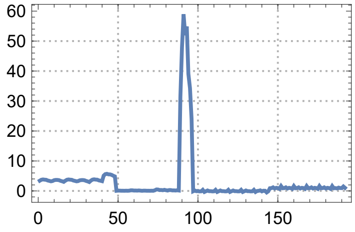

2D Plot

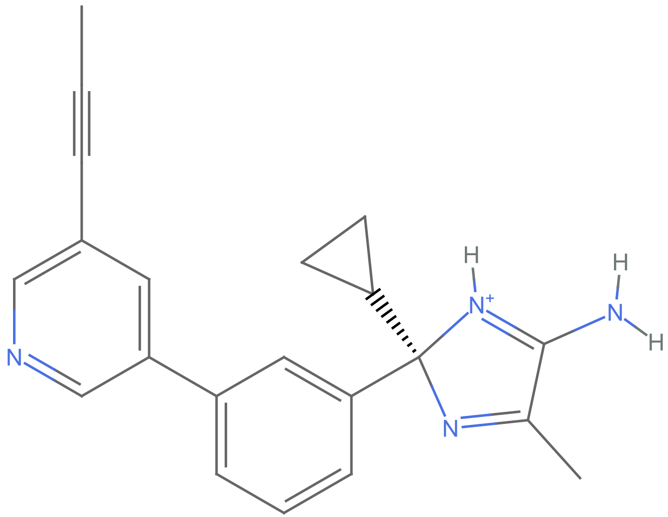

Dataset Index= 94

Molecule Formula= $C_{21}H_{21}N_4^+$

3D Autocorrelation

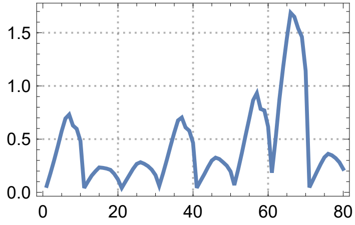

3D Plot

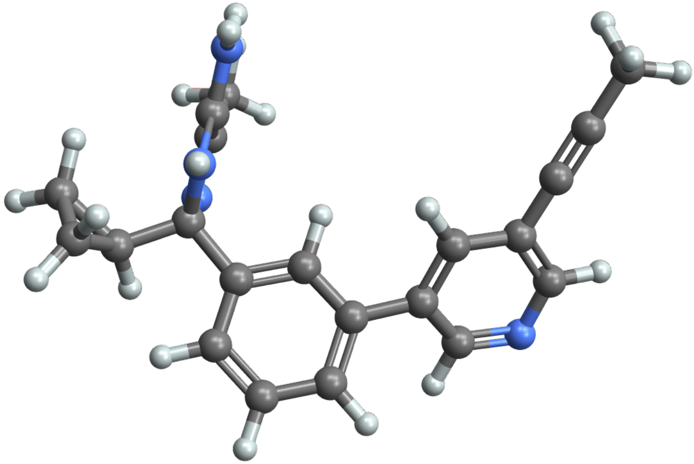

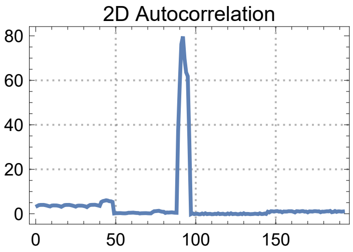

Dataset Index= 95

Molecule Formula= $C_{19}H_{19}N_7O$

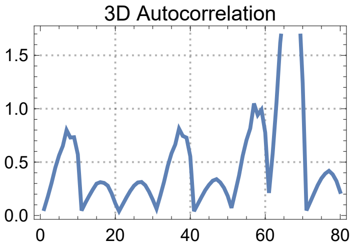

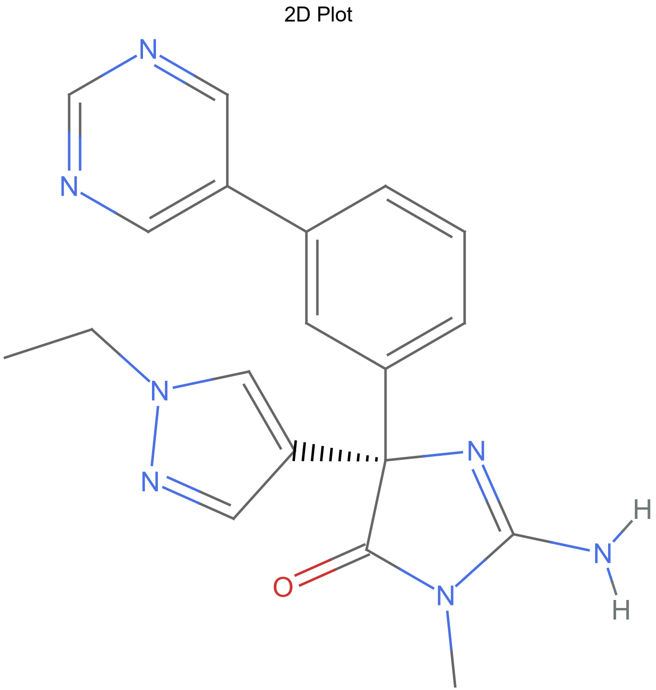

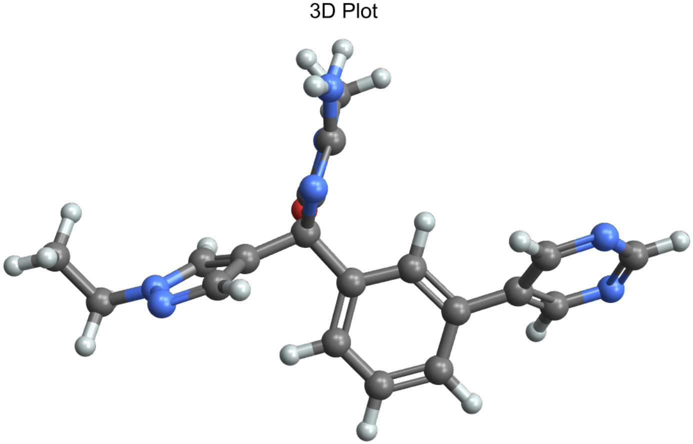

2D Autocorrelation

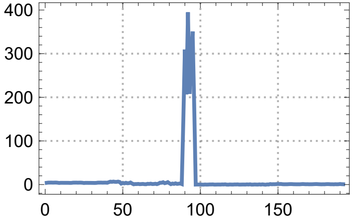

2D Plot

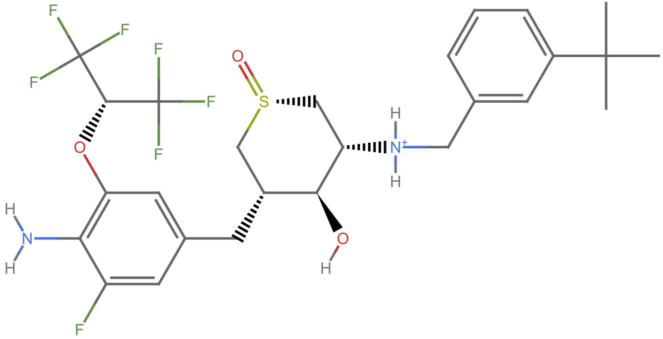

Dataset Index= 96

Molecule Formula= $C_{26}H_{32}F_7N_2O_3S^+$

3D Autocorrelation

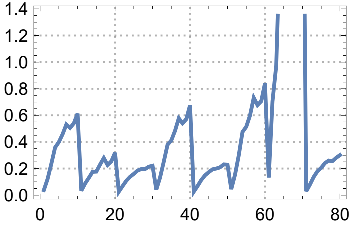

3D Plot

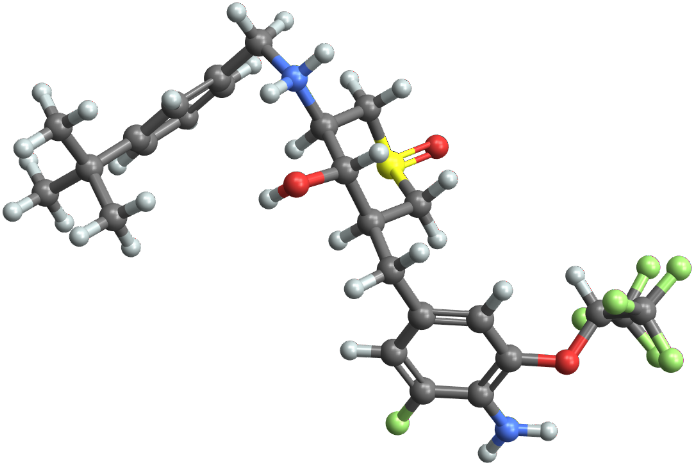

2D Plot

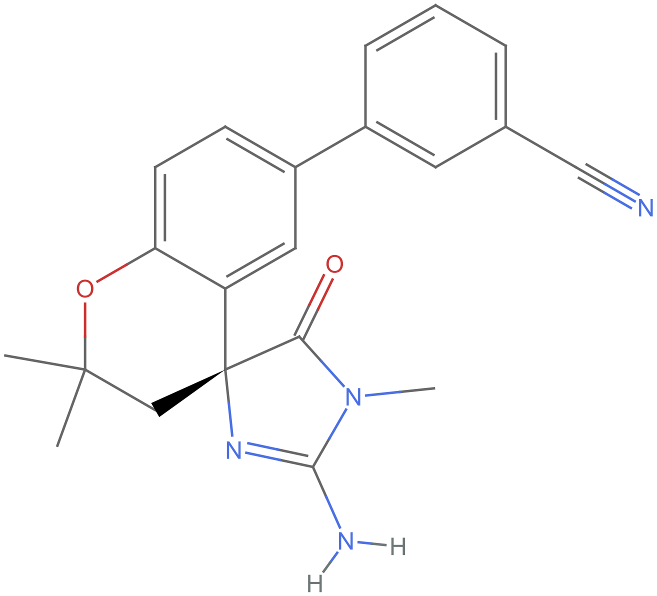

2D Autocorrelation

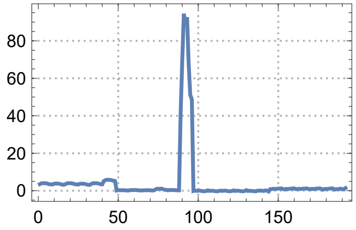

Dataset Index= 97

Molecule Formula= $C_{21}H_{20}N_4O_2$

3D Autocorrelation

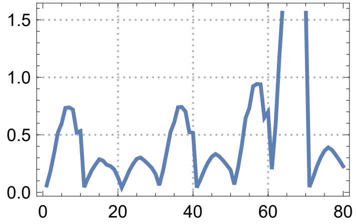

3D Plot

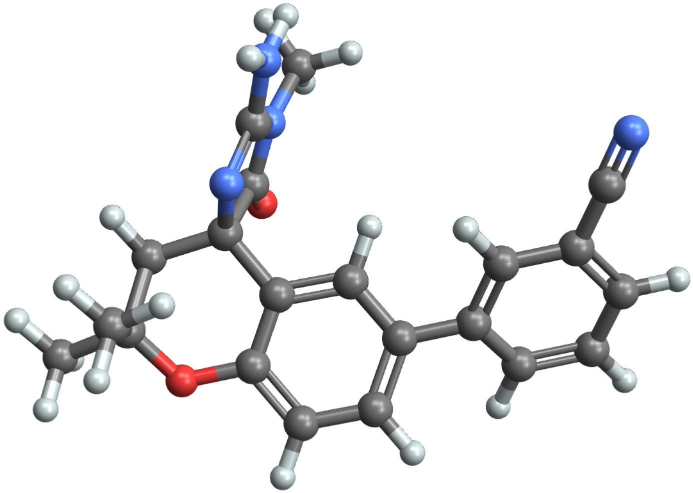

2D Plot

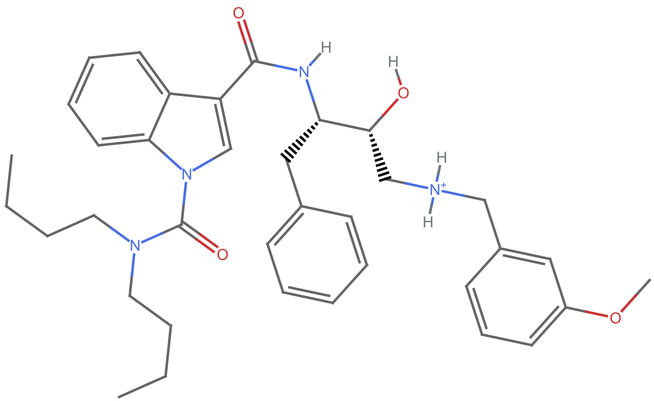

2D Autocorrelation

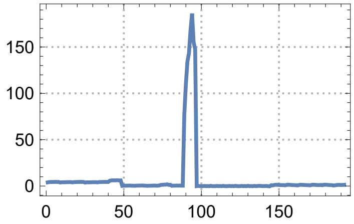

3D Plot

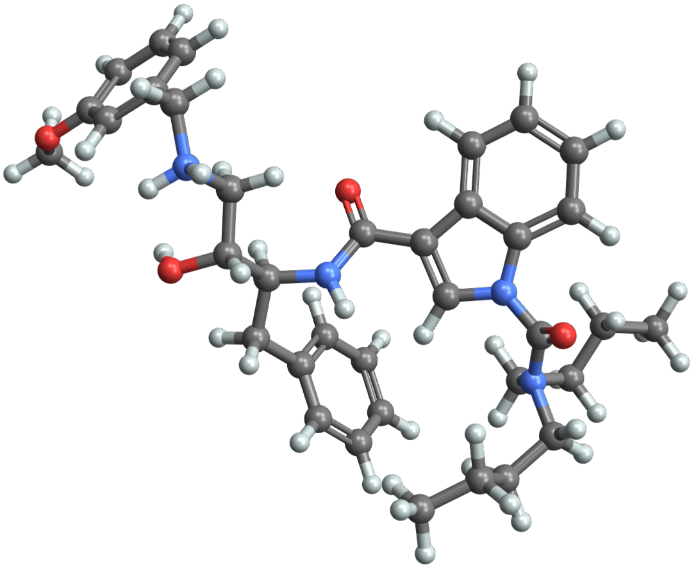

Dataset Index= 98

Molecule Formula= $C_{36}H_{47}N_4O_4^+$

3D Autocorrelation

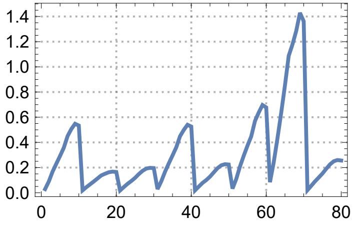

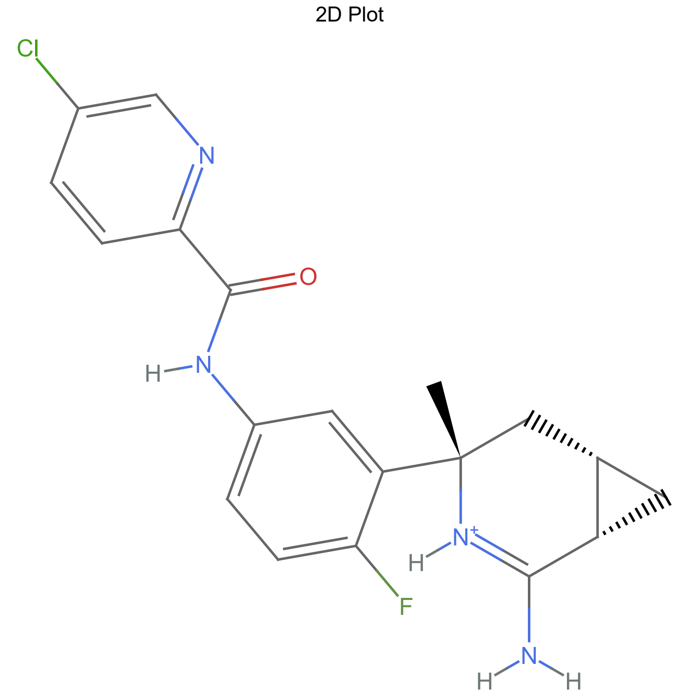

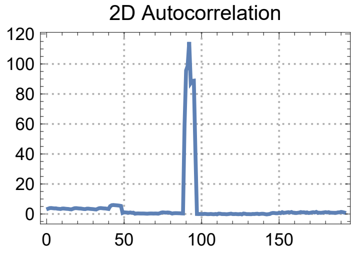

Dataset Index= 99

Molecule Formula= $C_{19}H_{19}ClFN_4O^+$

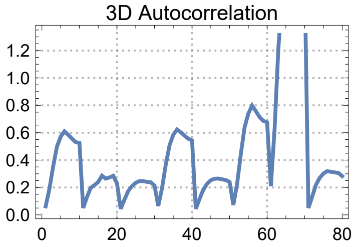

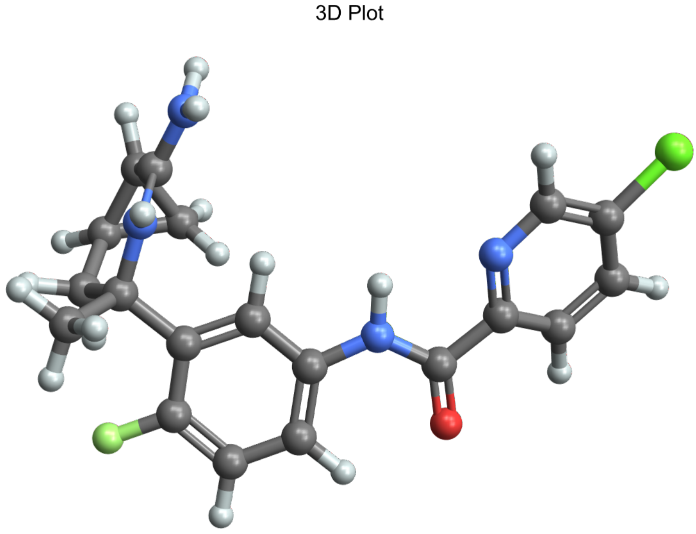

2D Plot

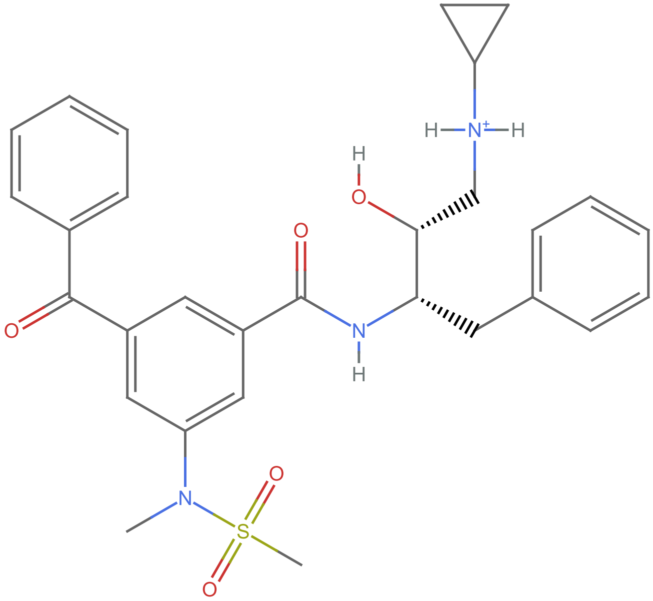

2D Autocorrelation

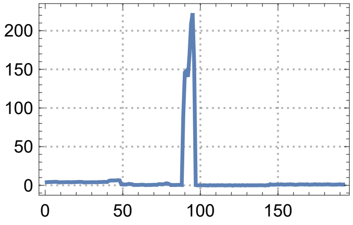

3D Plot

Dataset Index= 100

Molecule Formula= $C_{29}H_{34}N_{3}O_{5}S^{+}$

3D Autocorrelation

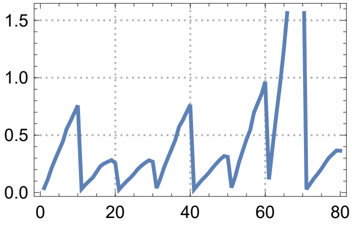

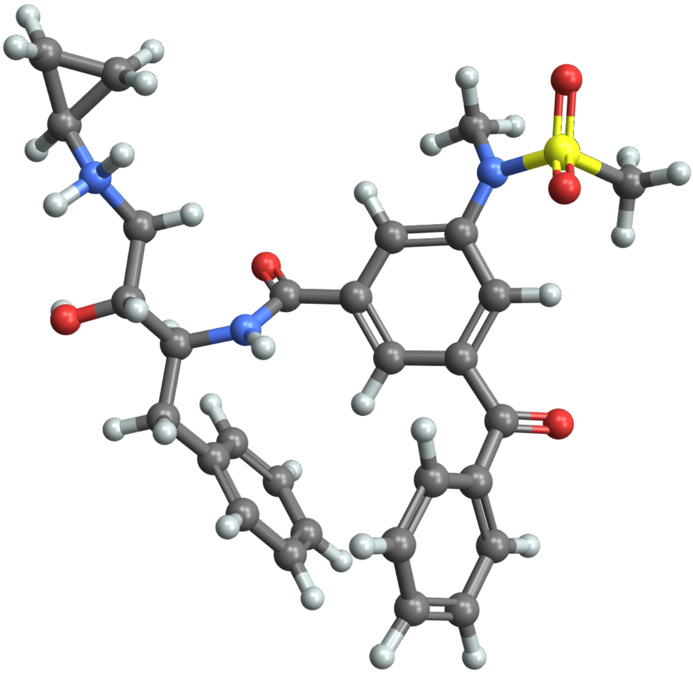

2D Plot

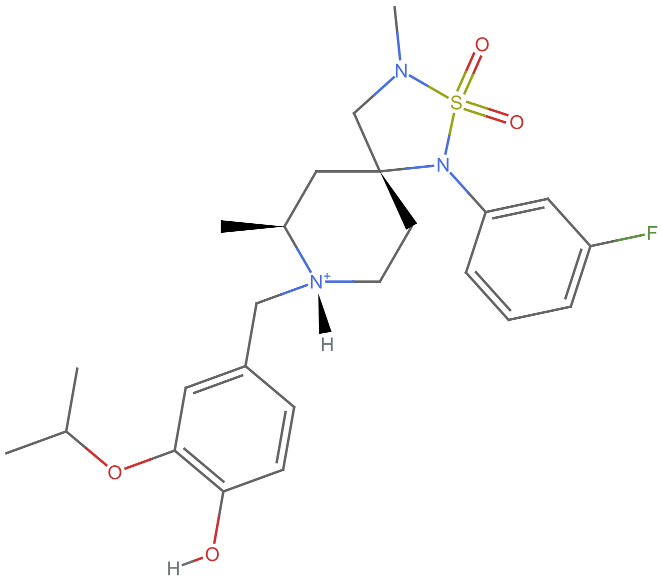

2D Autocorrelation

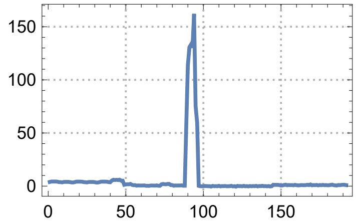

Dataset Index= 101

Molecule Formula= $C_{24}H_{33}FN_3O_4S^+$

3D Autocorrelation

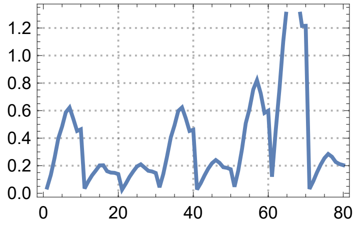

3D Plot

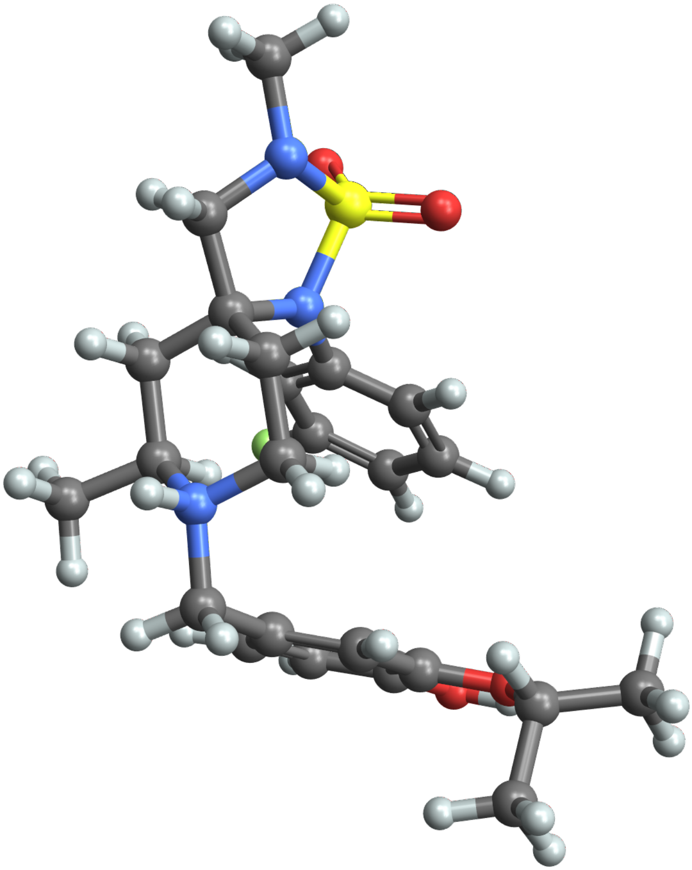

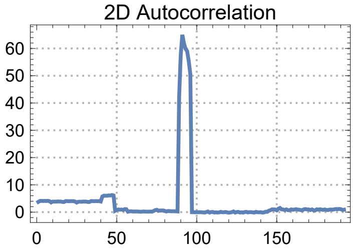

Dataset Index= 102

Molecule Formula= $C_{26}H_{20}ClN_5O$

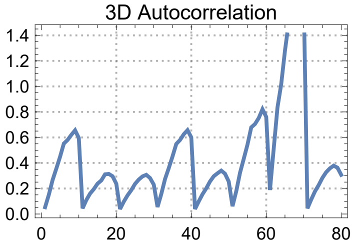

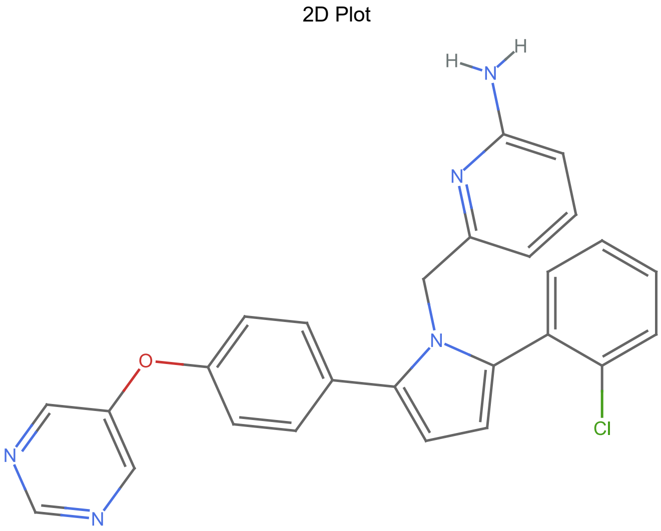

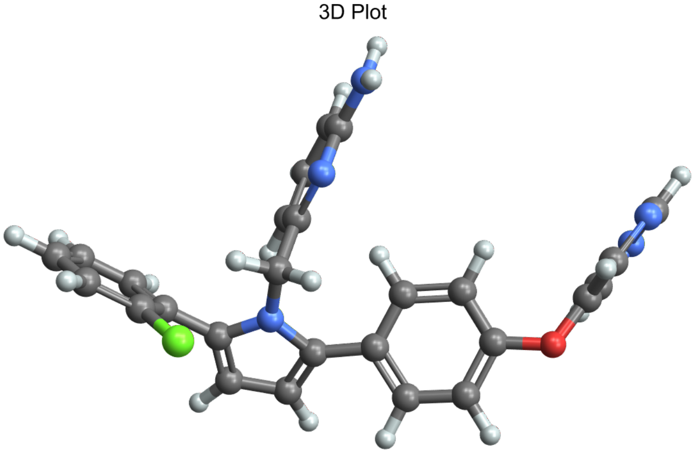

2D Autocorrelation

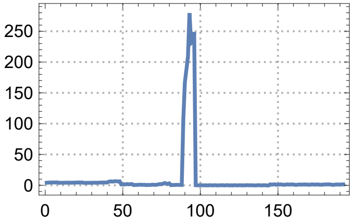

Dataset Index= 103

Molecule Formula= $C_{36}H_{50}N_3O_7S^+$

3D Autocorrelation

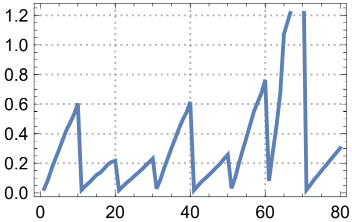

2D Plot

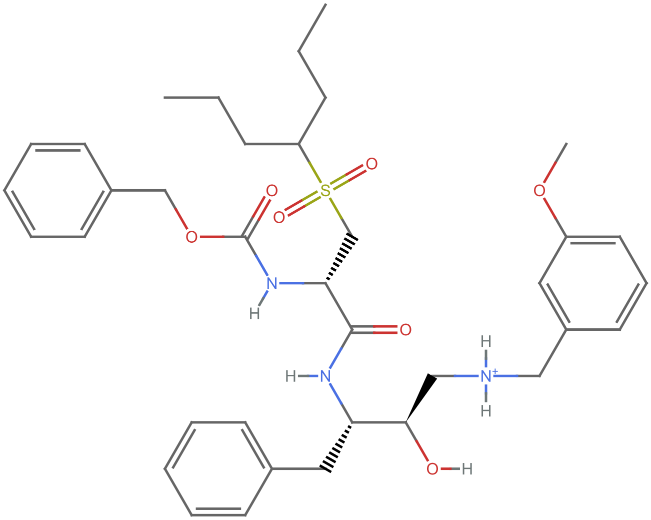

3D Plot

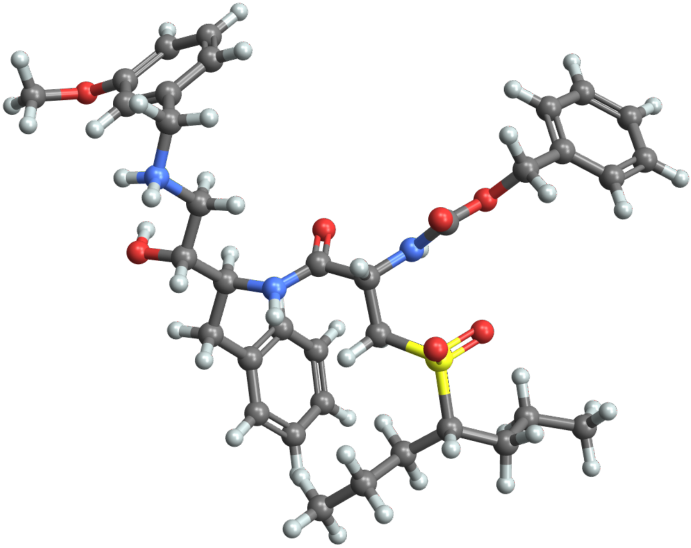

2D Plot

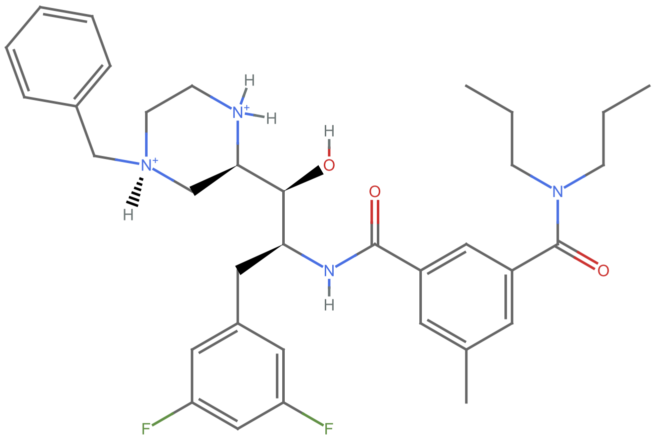

2D Autocorrelation

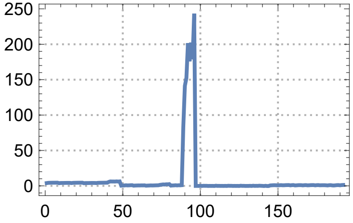

Dataset Index= 104

Molecule Formula= $C_{35}H_{46}F_2N_4O_3{}^{2+}$

3D Autocorrelation

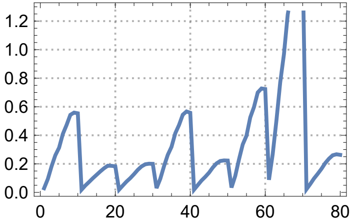

3D Plot

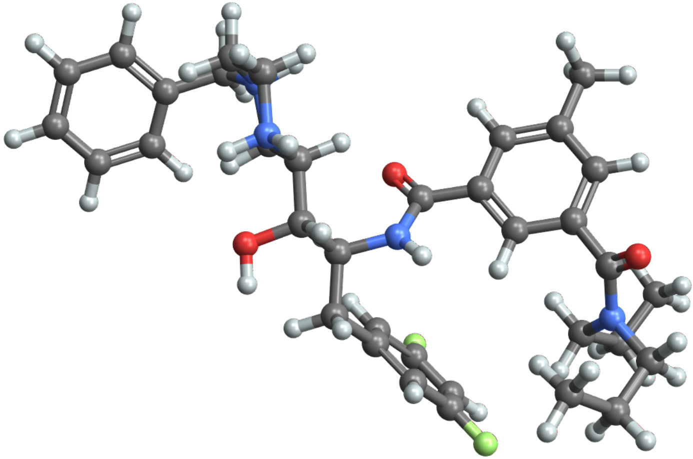

2D Plot

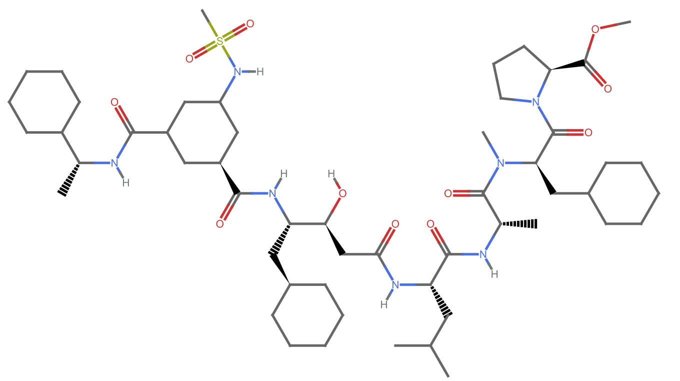

2D Autocorrelation

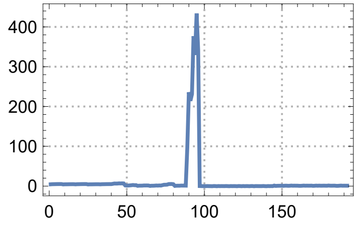

Dataset Index= 105

Molecule Formula= $C_{53}H_{91}N_7O_{11}S$

3D Autocorrelation

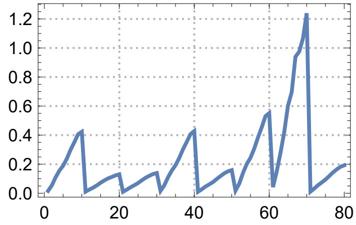

3D Plot

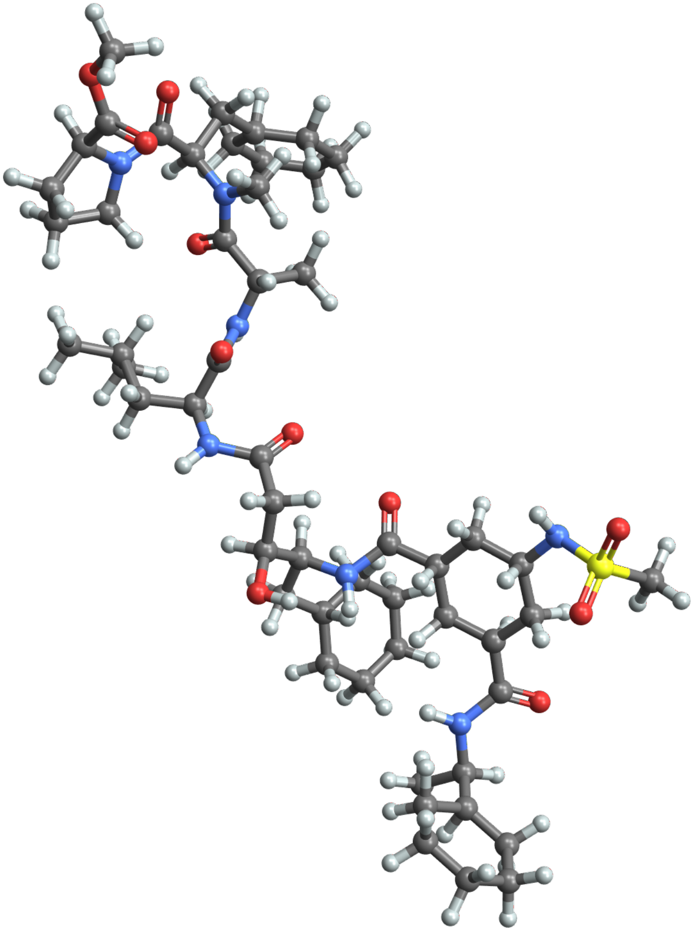

2D Autocorrelation

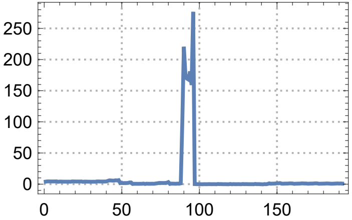

Dataset Index= 106

Molecule Formula= $C_{30}H_{40}F_3N_2O_3S^+$

3D Autocorrelation

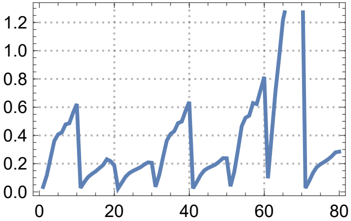

2D Plot

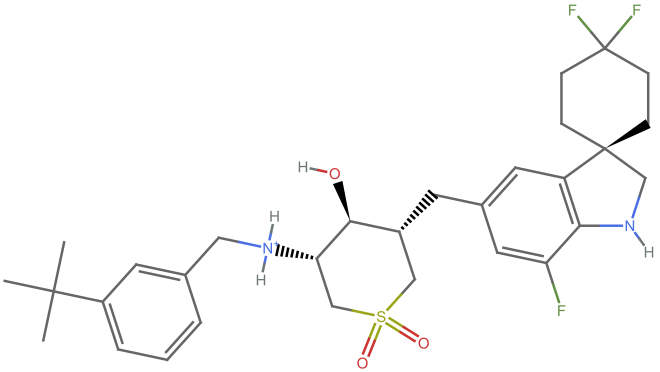

3D Plot

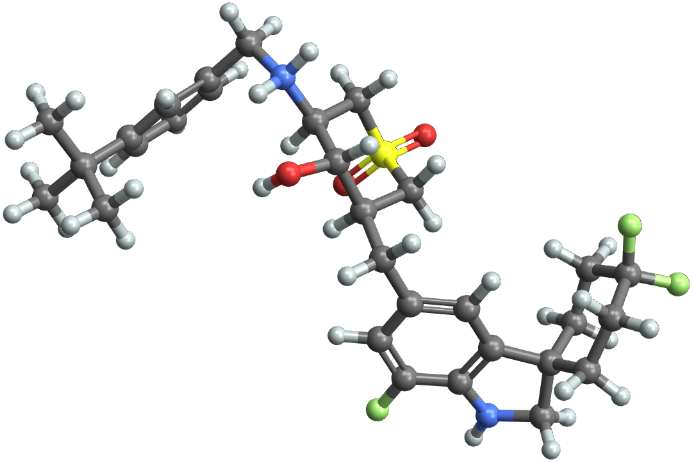

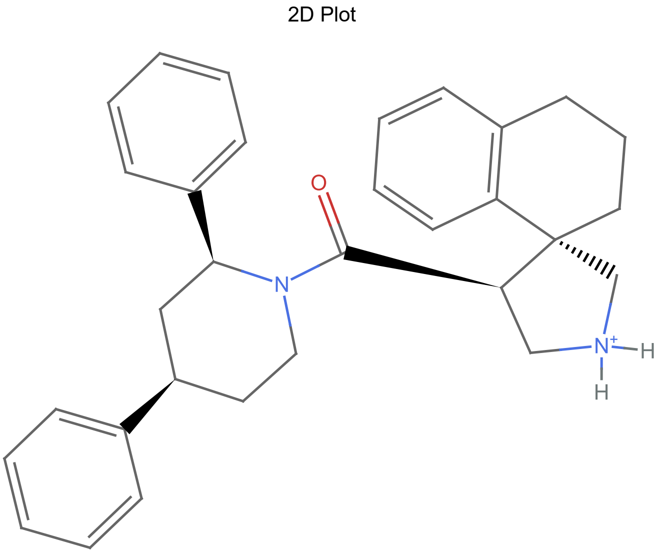

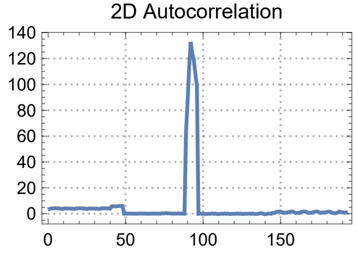

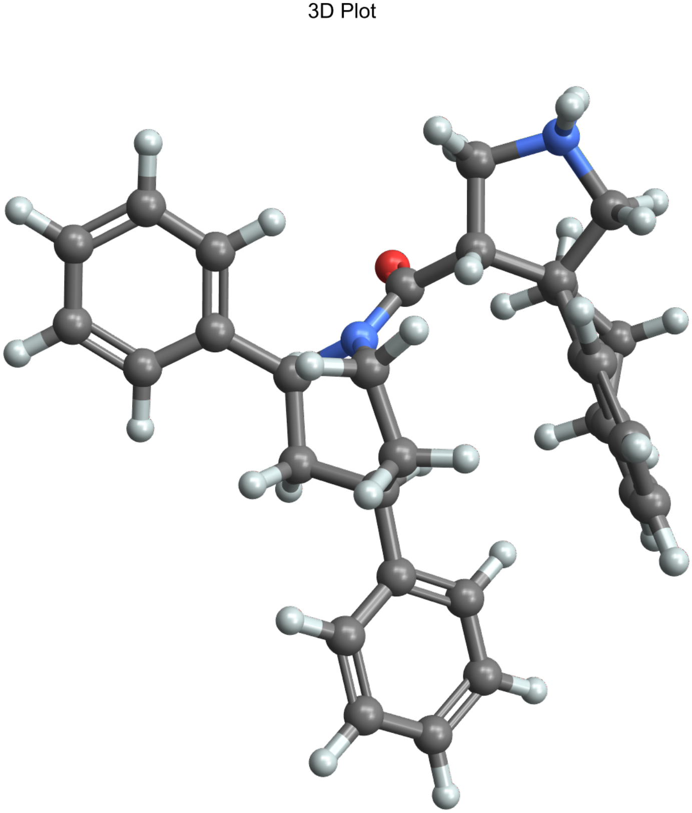

Dataset Index= 107

Molecule Formula= $C_{31}H_{35}N_2O^+$

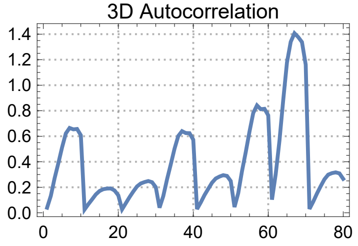

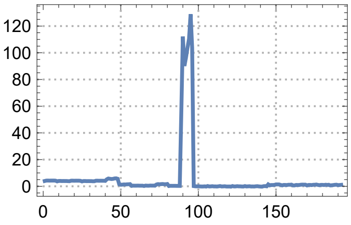

Dataset Index= 108

Molecule Formula= $C_{29}H_{41}N_2O_6S^+$

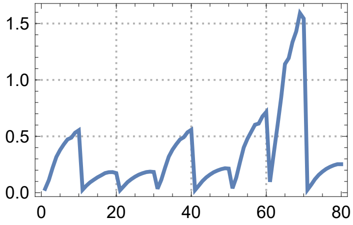

2D Plot

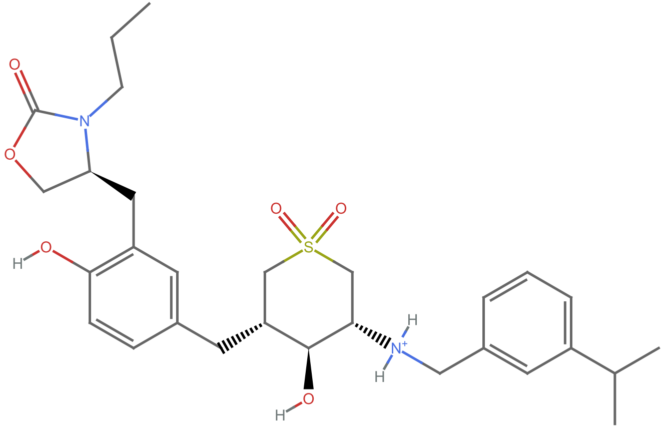

3D Plot

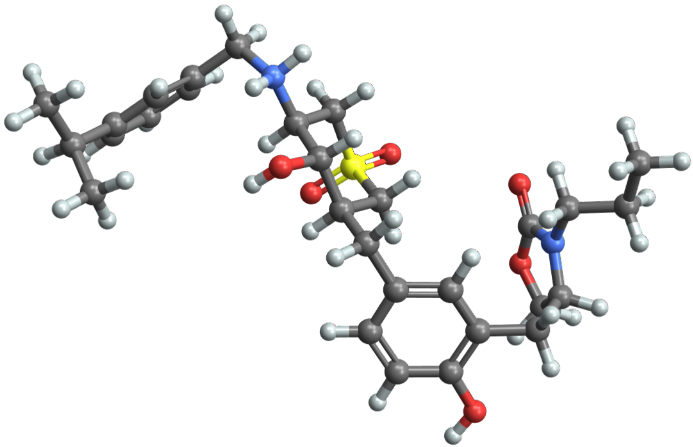

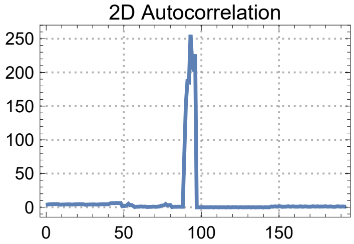

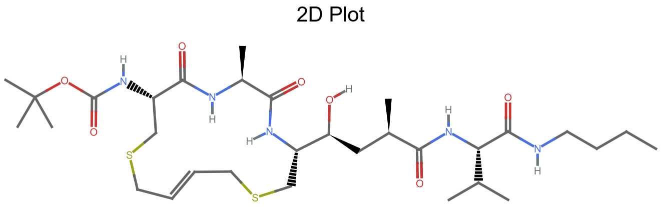

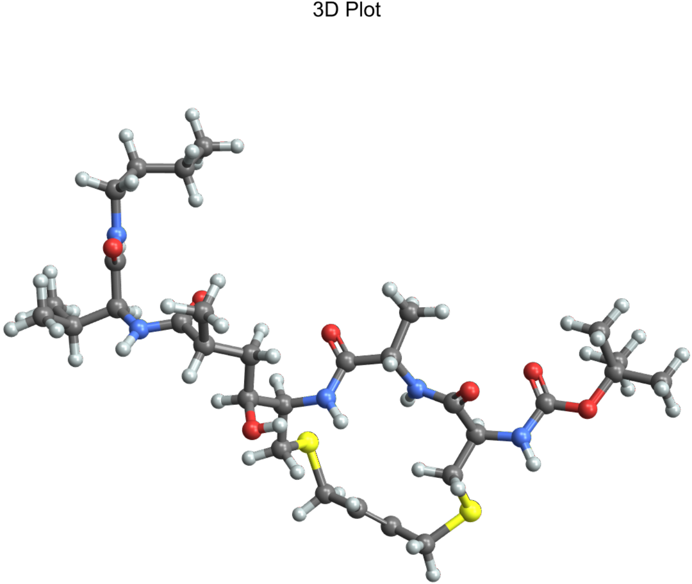

Dataset Index= 109

Molecule Formula= $C_{31}H_{55}N_{5}O_{7}S_{2}$

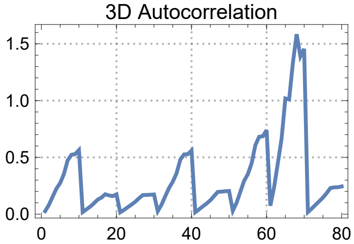

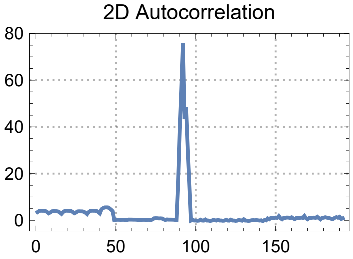

Dataset Index= 110

Molecule Formula= $C_{22}H_{29}N_3O_2$

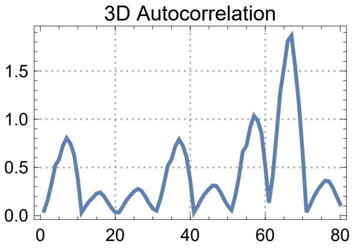

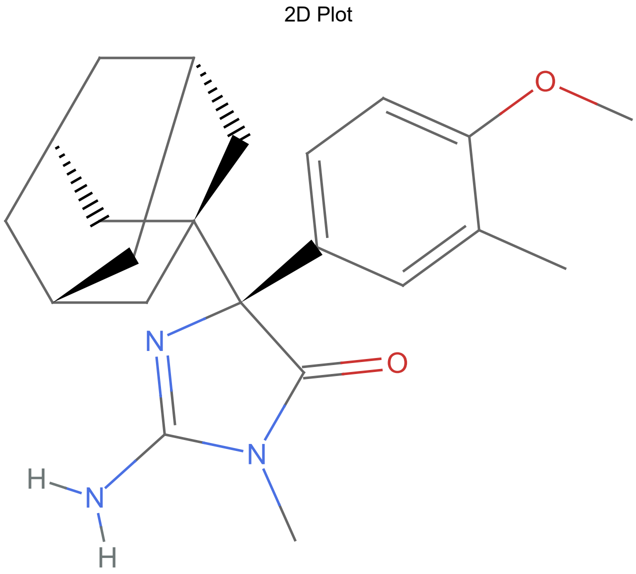

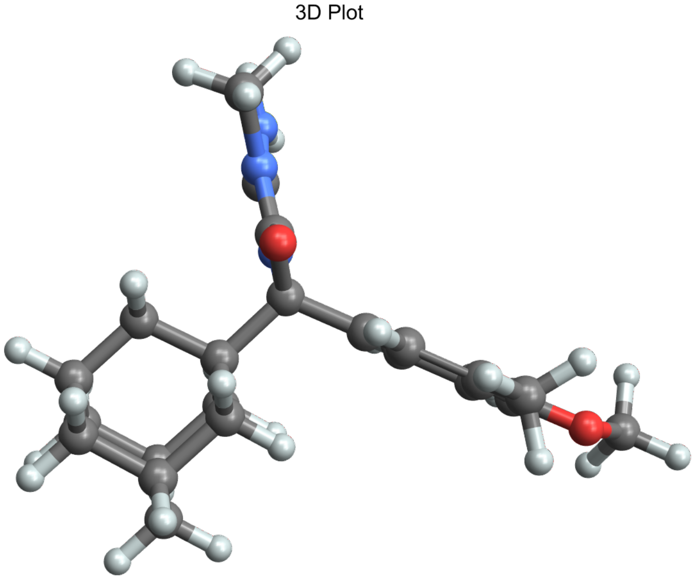

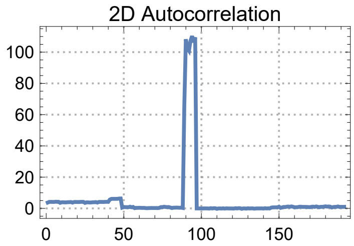

Dataset Index= 111

Molecule Formula= $C_{26}H_{25}ClN_5O^+$

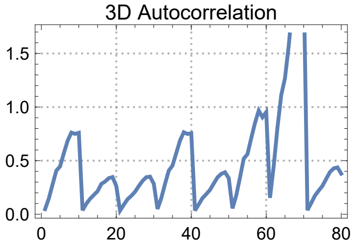

2D Plot

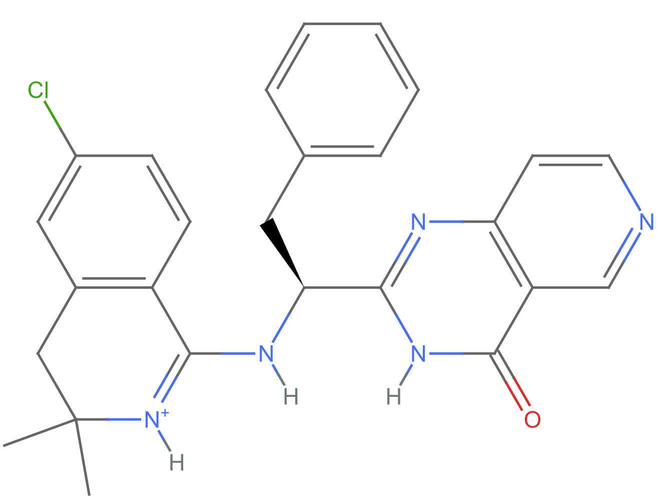

3D Plot

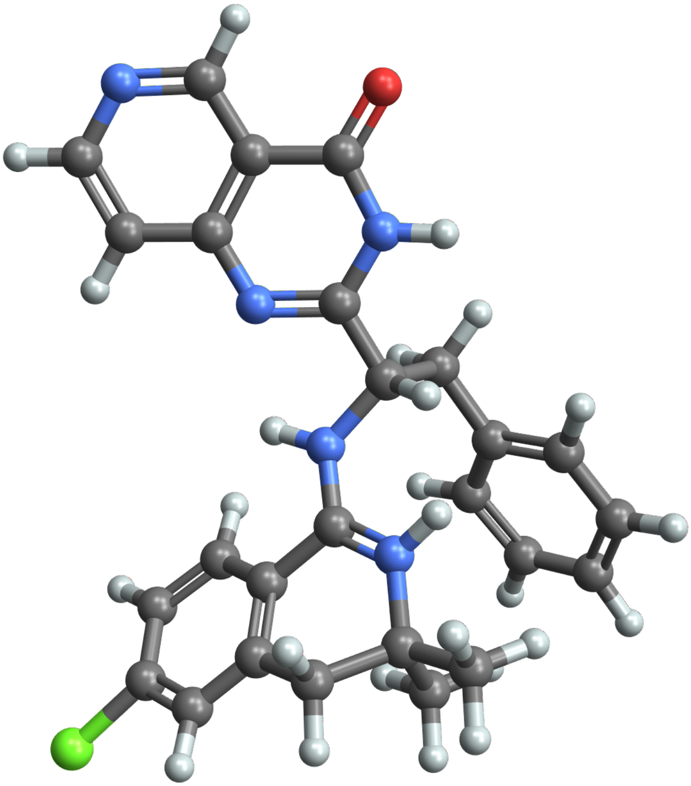

2D Plot

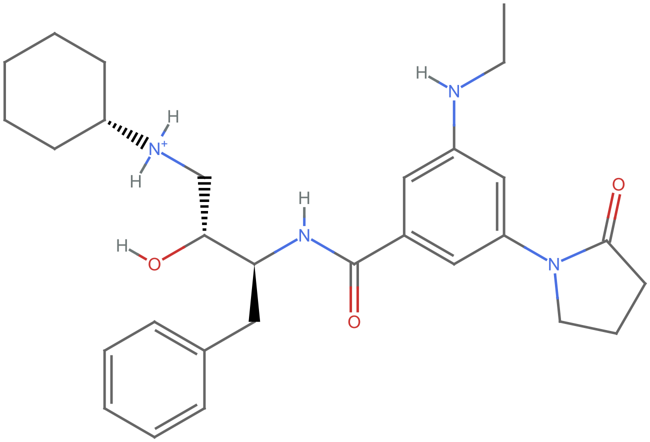

2D Autocorrelation

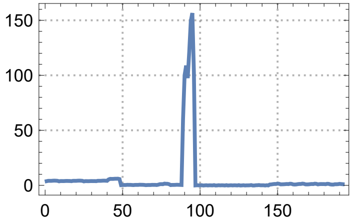

3D Plot

Dataset Index= 112

Molecule Formula= $C_{29}H_{41}N_4O_3^+$

3D Autocorrelation

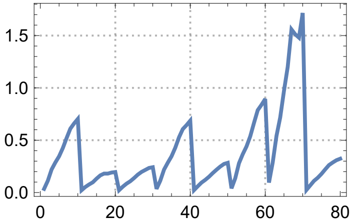

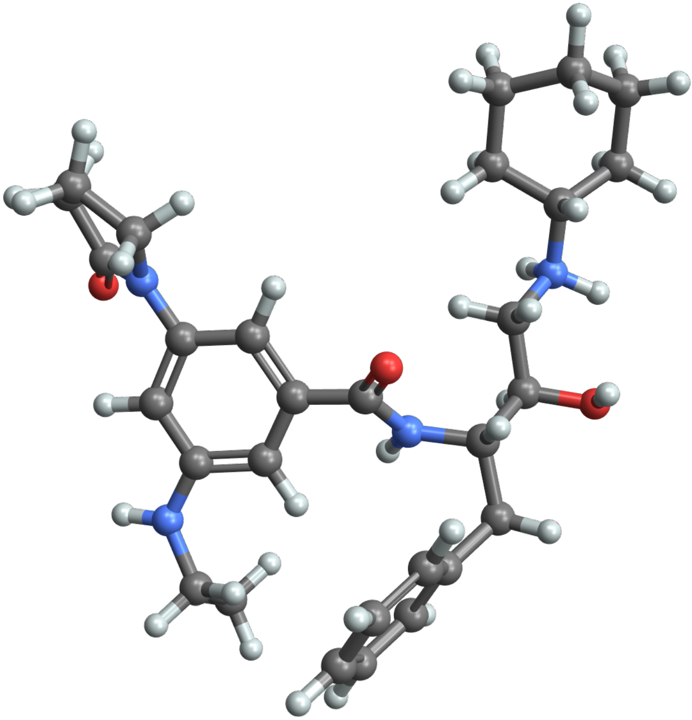

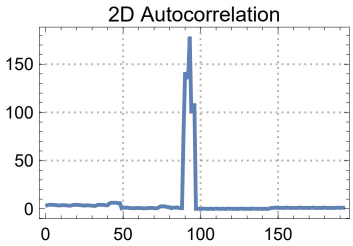

Dataset Index= 113

Molecule Formula= $C_{19}H_{19}F_3N_4O_3$

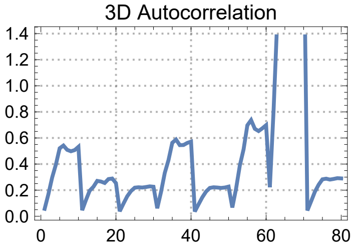

2D Plot

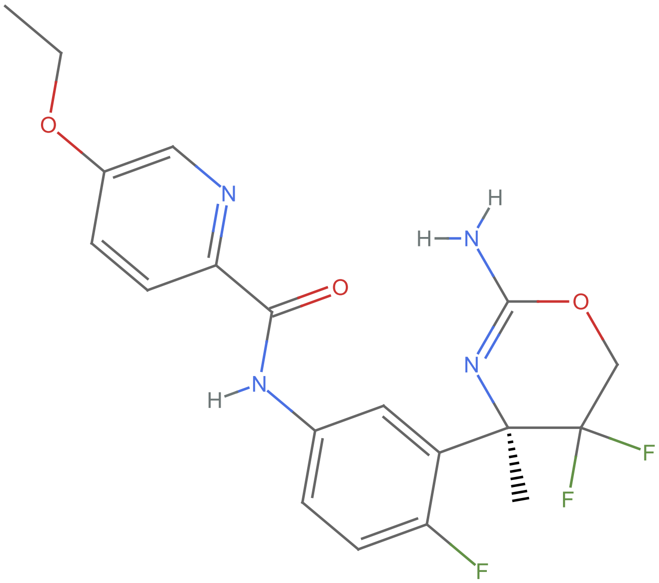

3D Plot

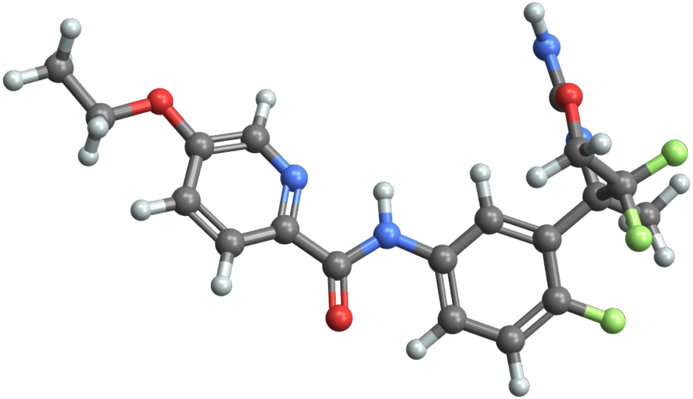

2D Plot

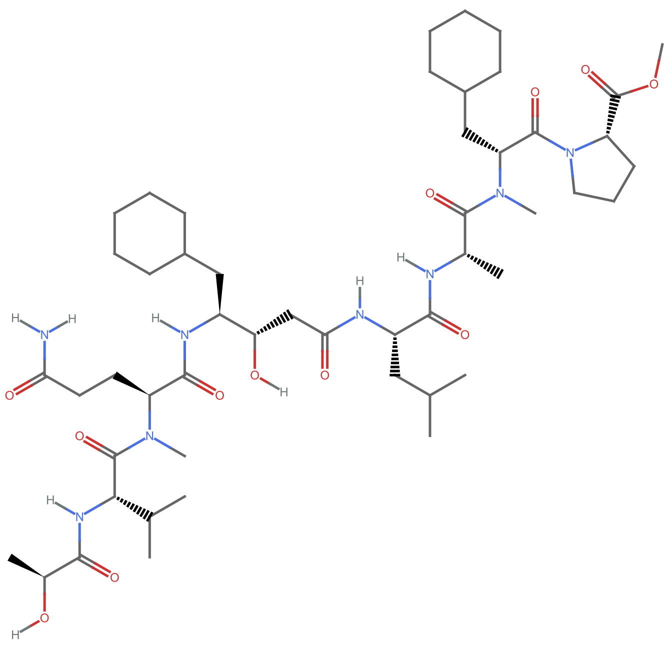

2D Autocorrelation

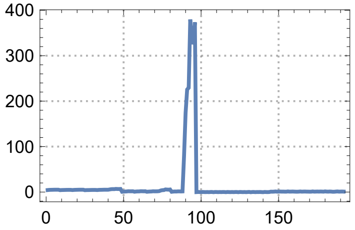

3D Plot

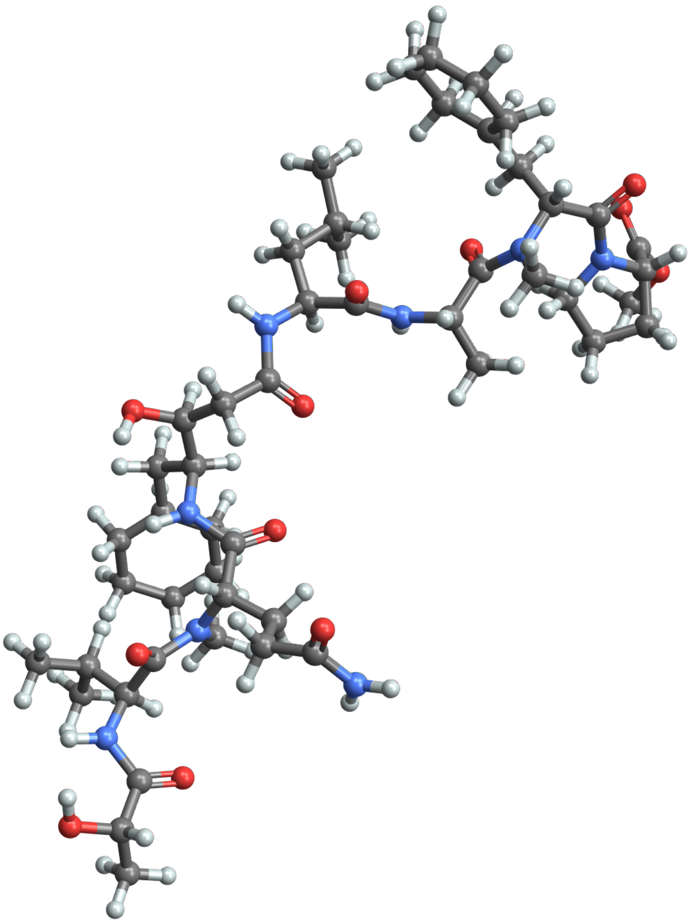

Dataset Index= 114

Molecule Formula= $C_{50}H_{86}N_8O_{12}$

3D Autocorrelation

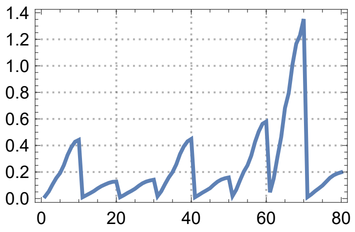

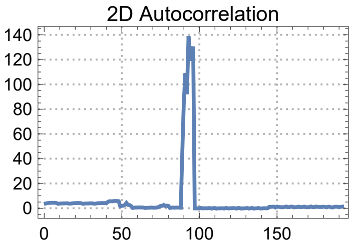

Dataset Index= 115

Molecule Formula= $C_{24}H_{43}N_4O_4S_2^+$

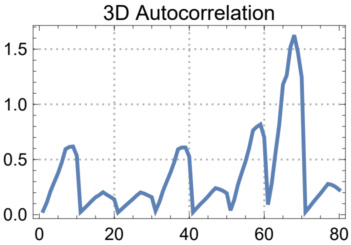

2D Plot

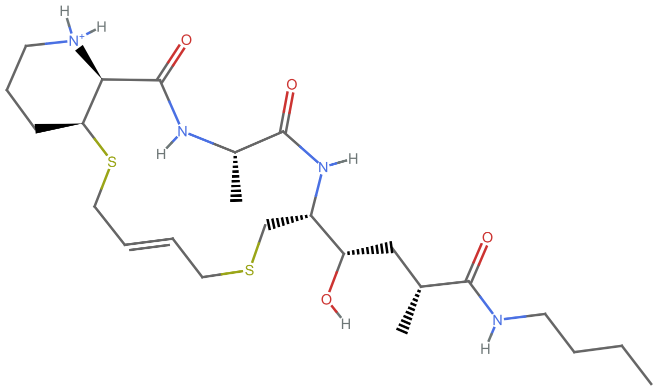

3D Plot

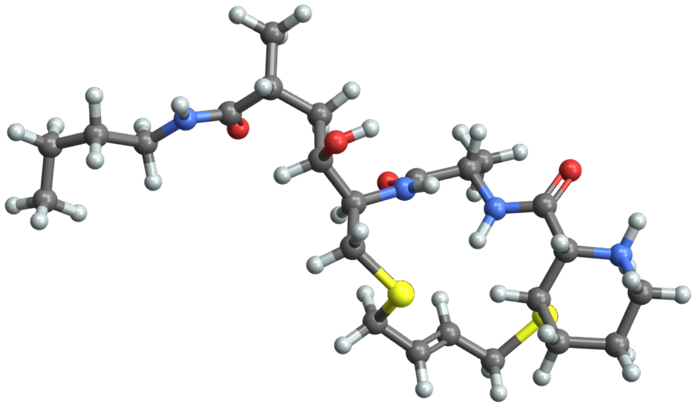

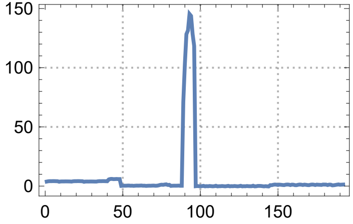

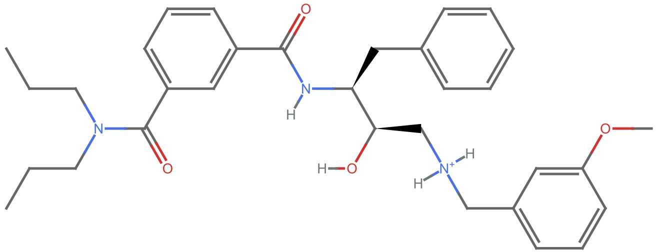

Dataset Index= 116

Molecule Formula= $C_{32}H_{42}N_3O_4^+$

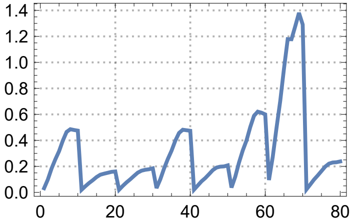

3D Plot

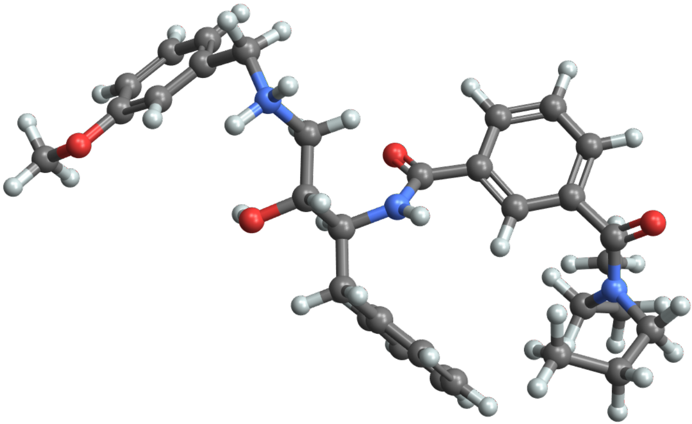

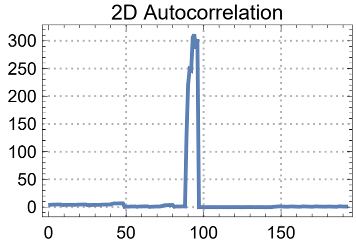

Dataset Index= 117

Molecule Formula= $C_{34}H_{45}N_{10}O_8^-$

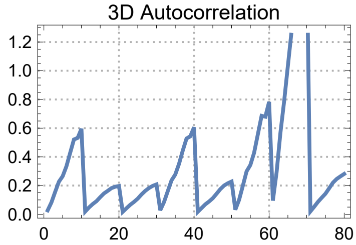

2D Plot

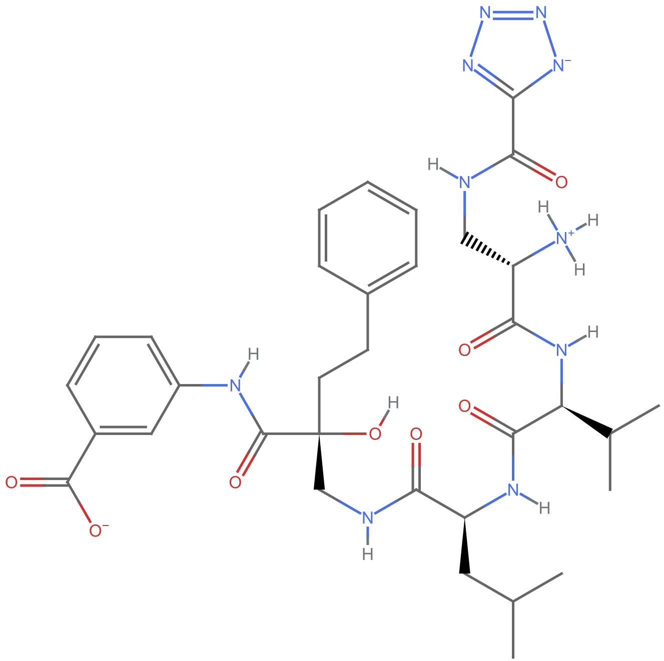

3D Plot

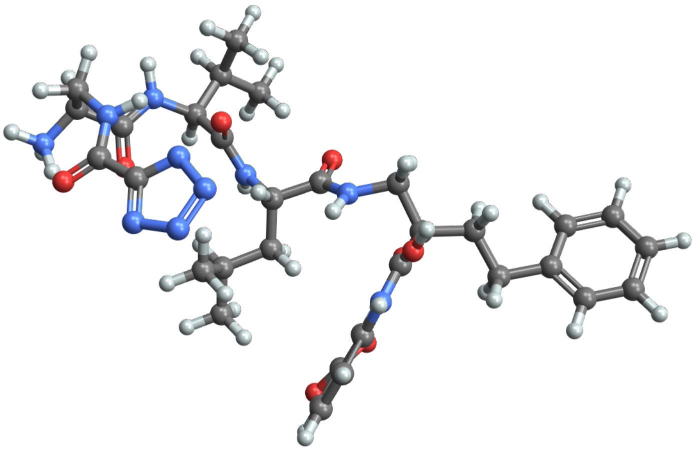

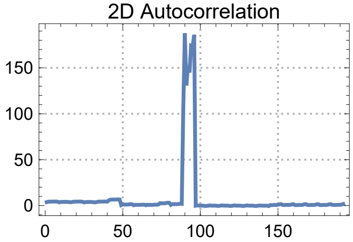

Dataset Index= 118

Molecule Formula= $C_{24}H_{15}F_5N_5^+$

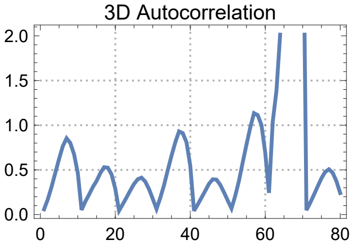

2D Plot

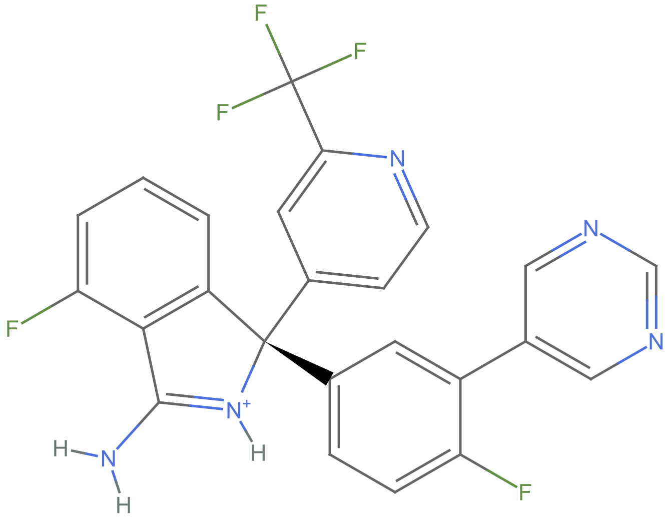

3D Plot

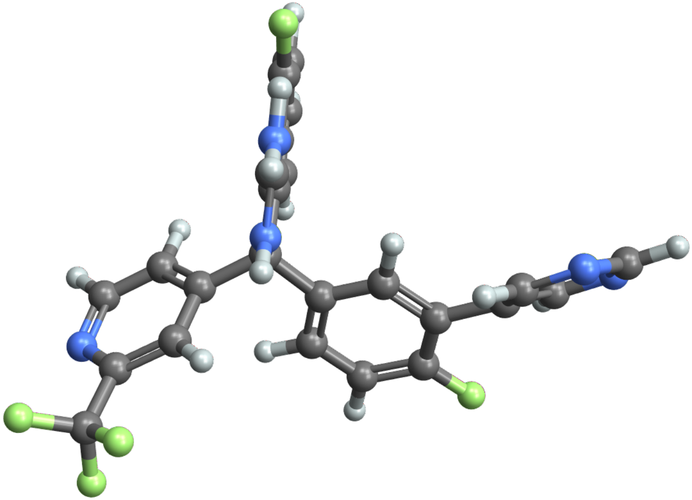

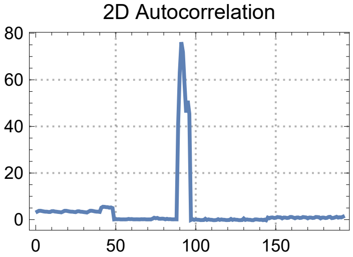

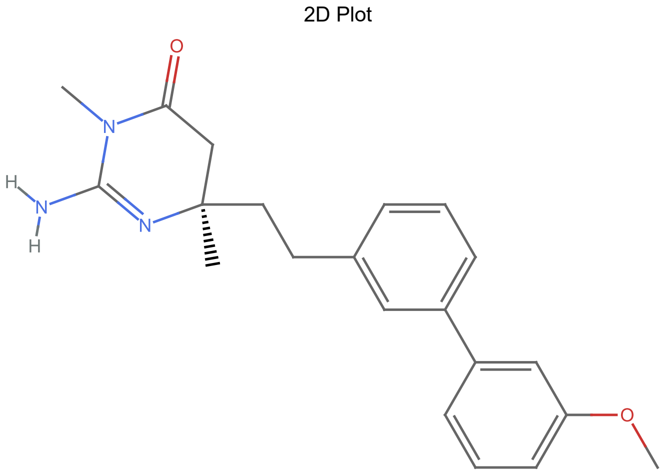

Dataset Index= 119

Molecule Formula= $C_{21}H_{25}N_3O_2$

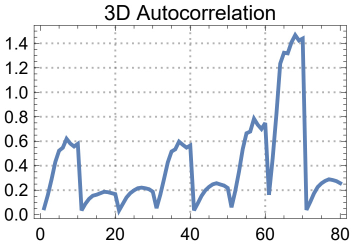

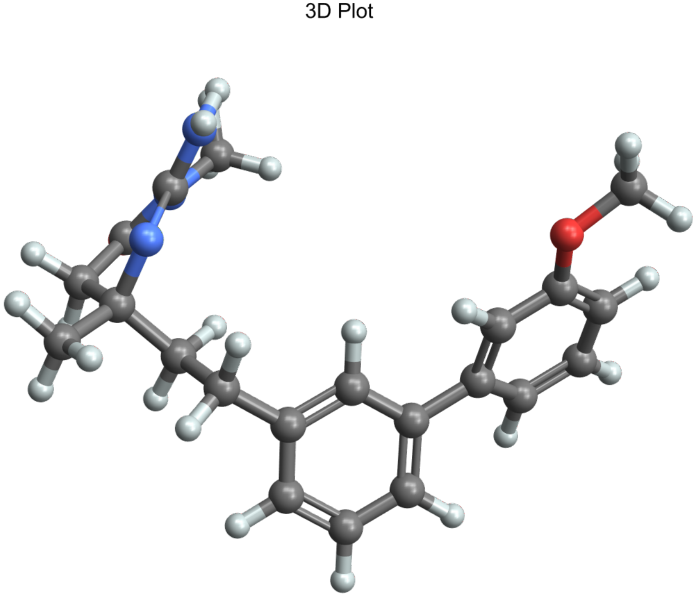

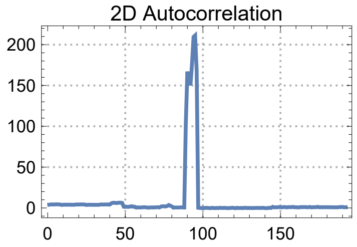

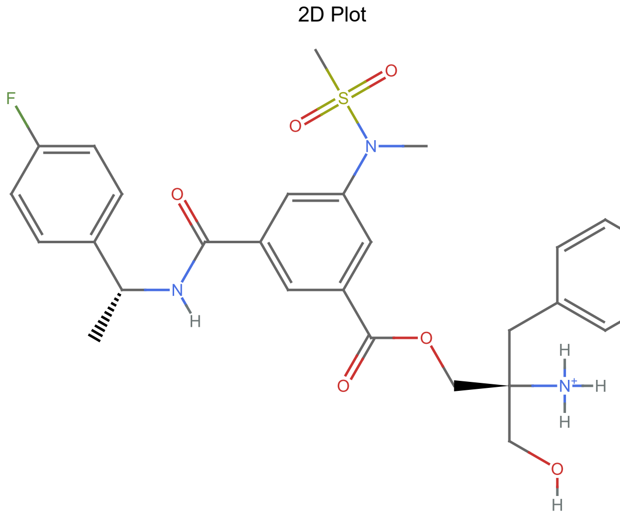

Dataset Index= 120

Molecule Formula= $C_{28}H_{33}FN_3O_6S^+$

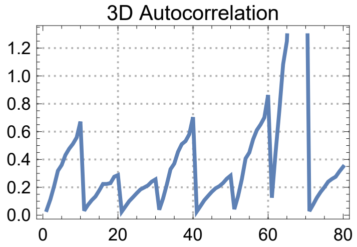

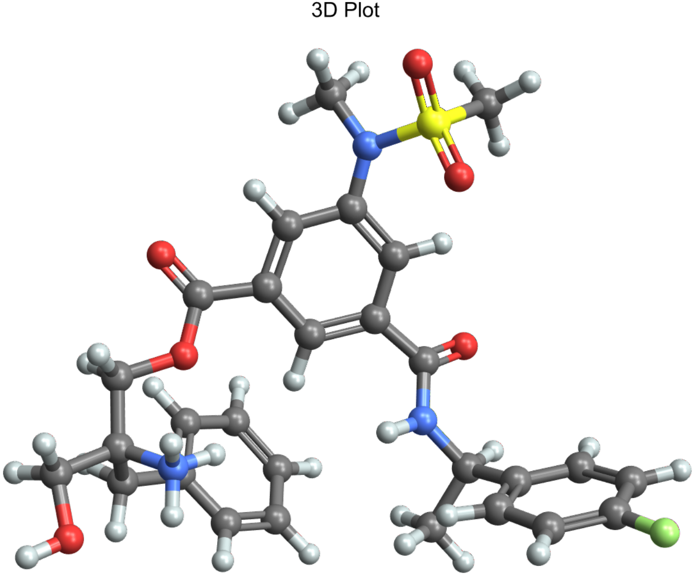

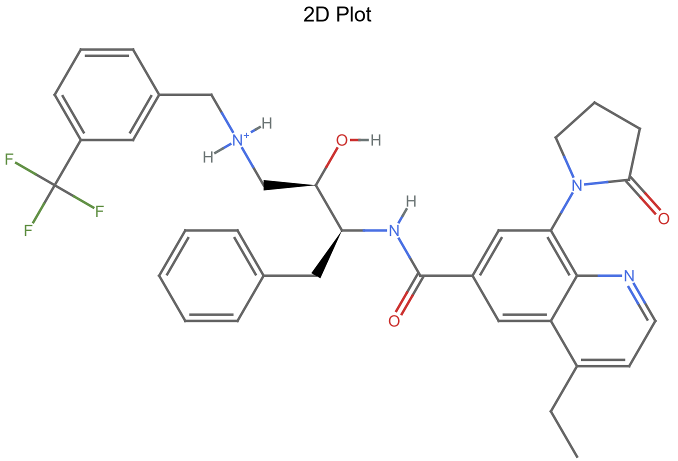

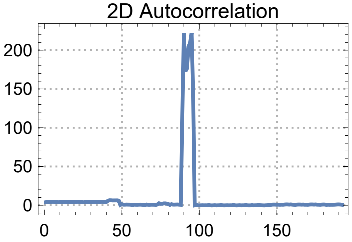

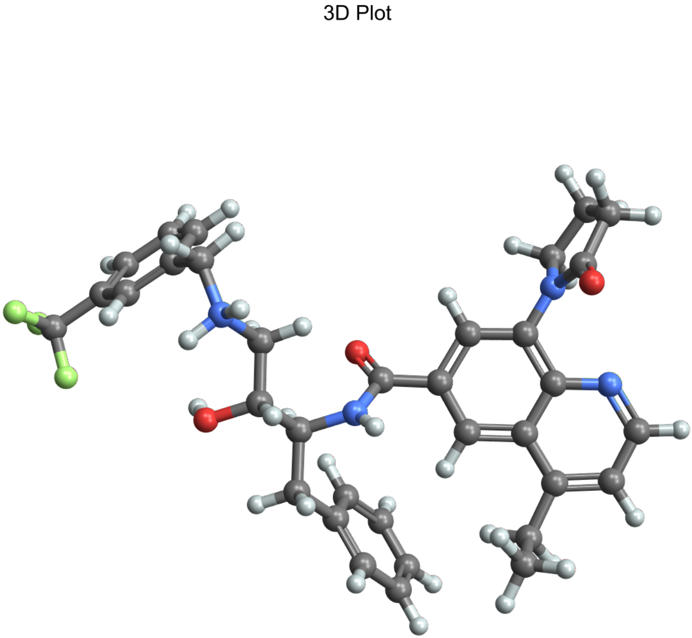

Dataset Index= 121

Molecule Formula= $C_{34}H_{36}F_3N_4O_3^+$

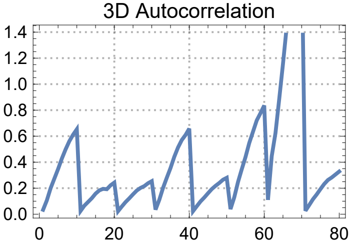

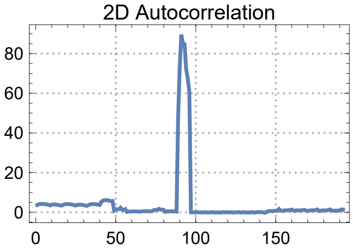

Dataset Index= 122

Molecule Formula= $C_{22}H_{16}ClFN_2O_3$

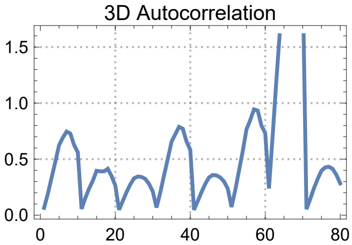

2D Plot

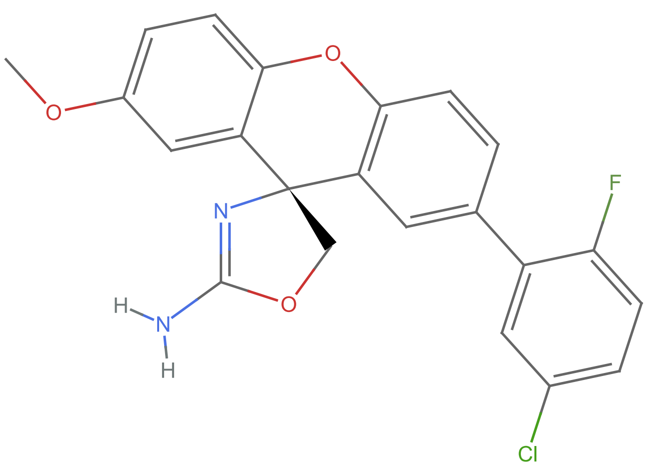

3D Plot

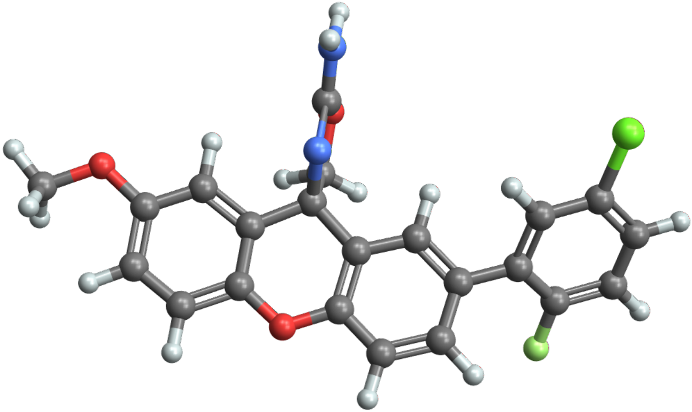

2D Plot

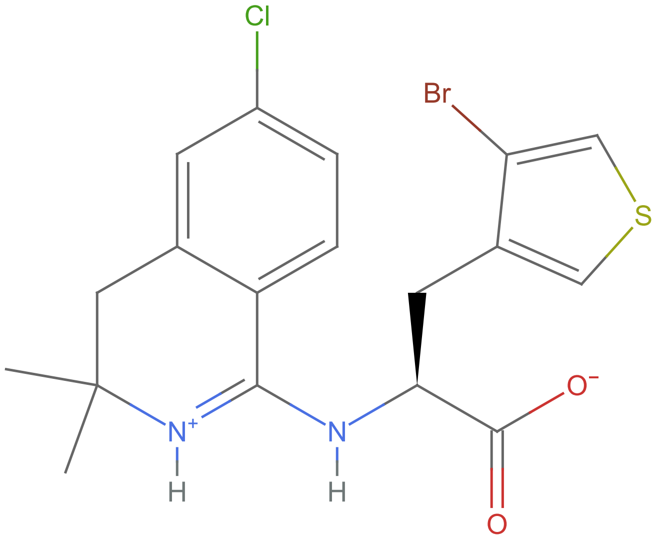

2D Autocorrelation

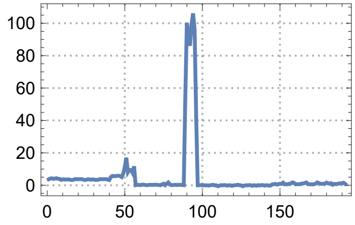

3D Plot

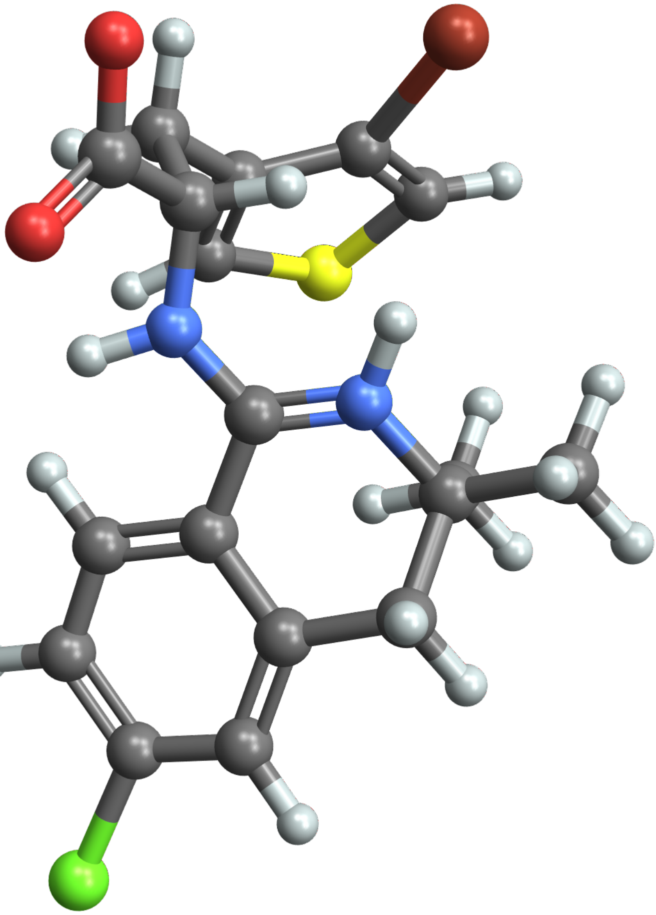

Dataset Index= 123

Molecule Formula= $C_{18}H_{18}BrClN_2O_2S$

3D Autocorrelation

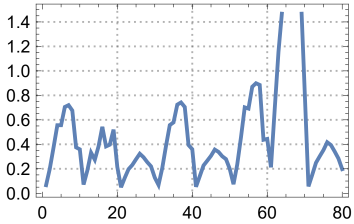

2D Autocorrelation

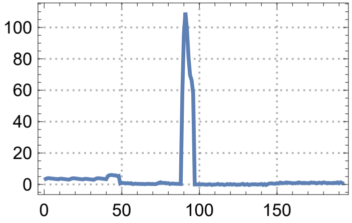

Dataset Index= 124

Molecule Formula= $C_{18}H_{19}ClN_5O_2^+$

3D Autocorrelation

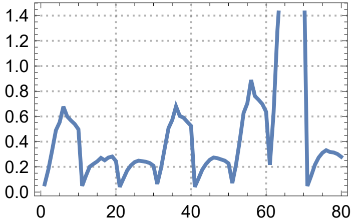

2D Plot

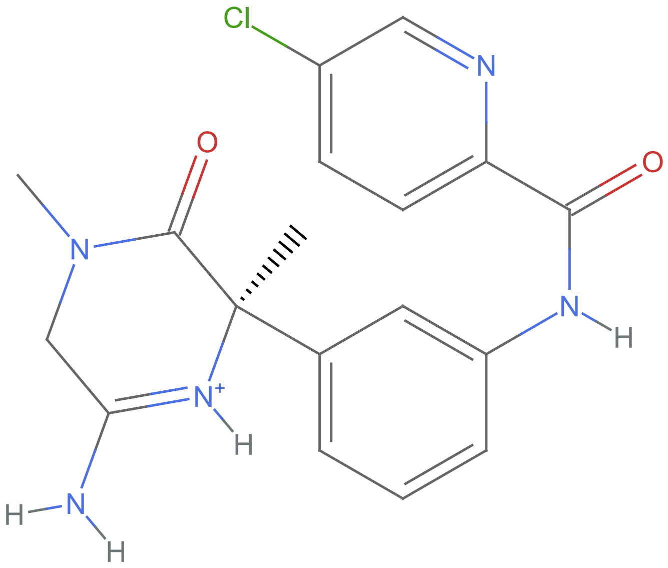

3D Plot

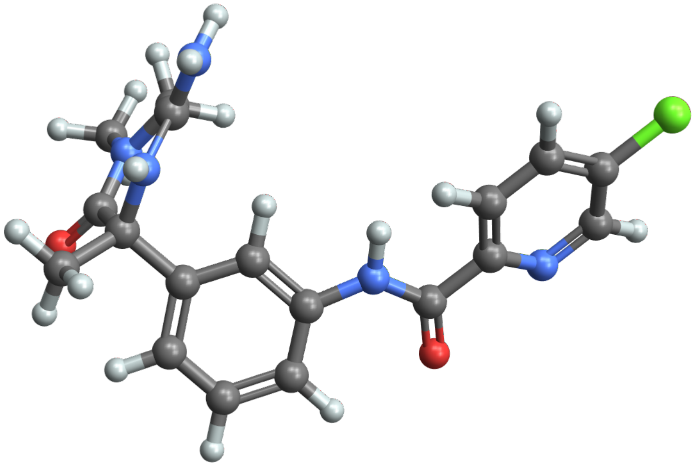

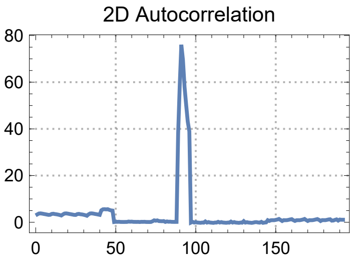

Dataset Index= 125

Molecule Formula= $C_{19}H_{21}N_3O_2$

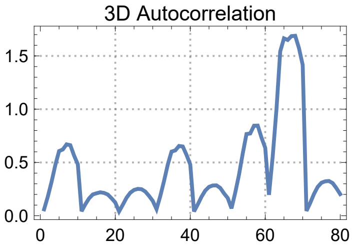

2D Plot

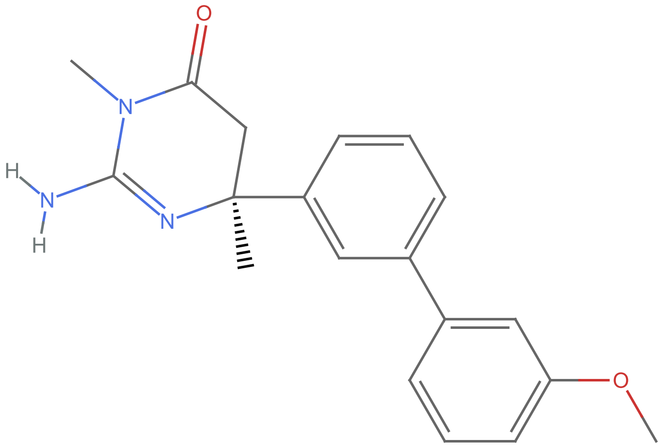

3D Plot

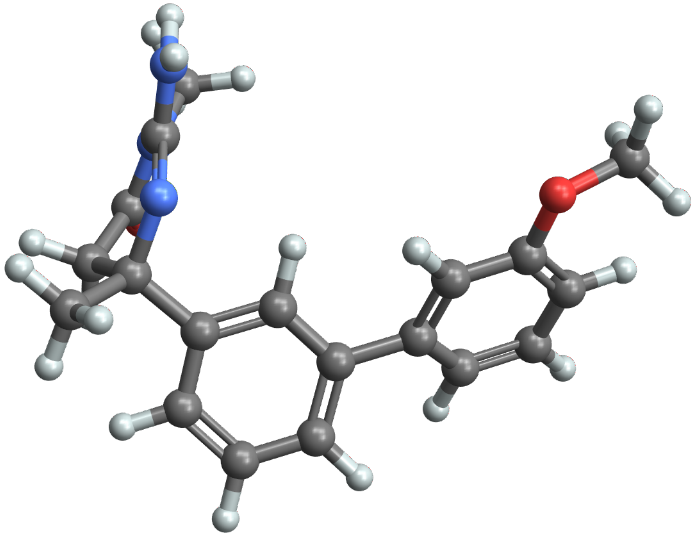

2D Autocorrelation

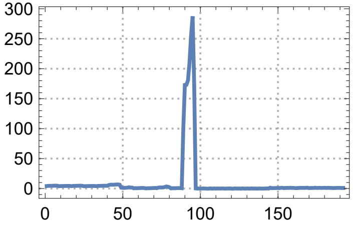

Dataset Index= 126

Molecule Formula= $C_{35}H_{45}N_4O_6S^+$

3D Autocorrelation

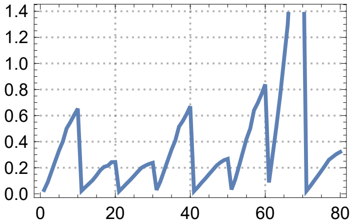

2D Plot

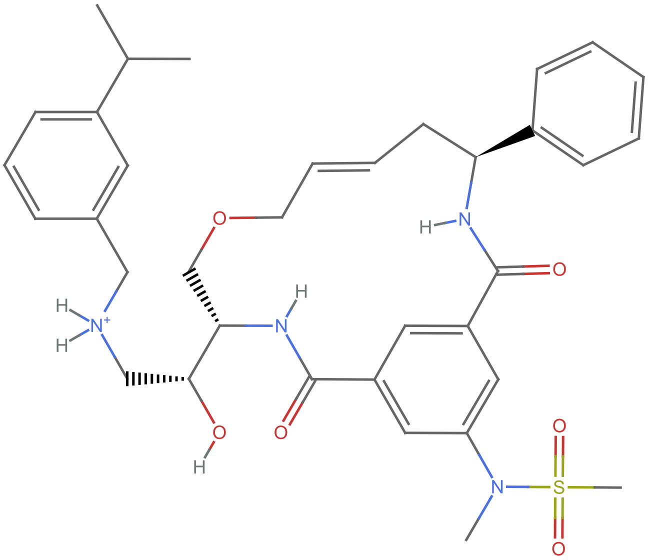

3D Plot

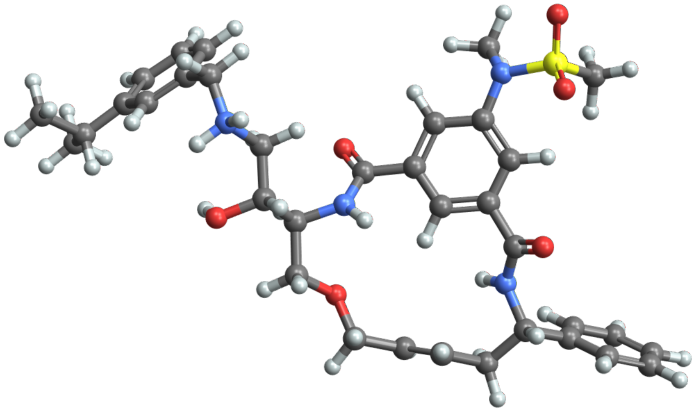

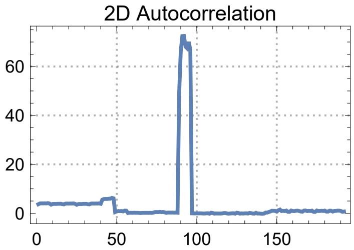

Dataset Index= 127

Molecule Formula= $C_{26}H_{23}ClN_4O$

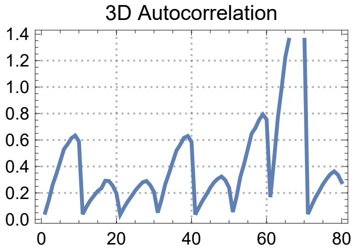

2D Plot

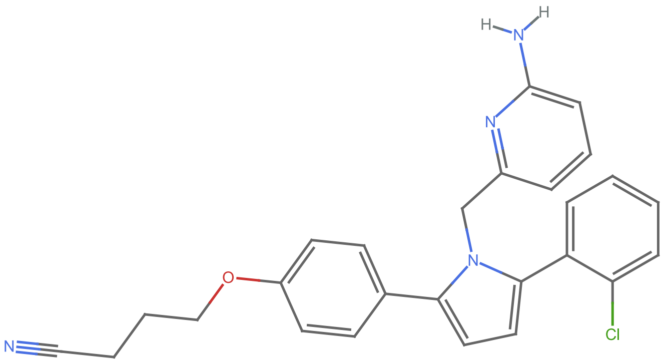

3D Plot

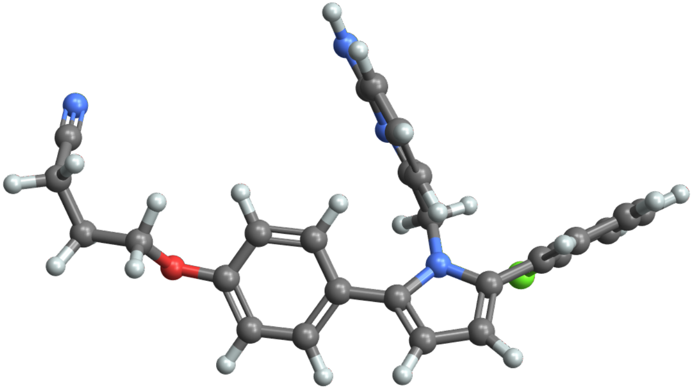

2D Autocorrelation

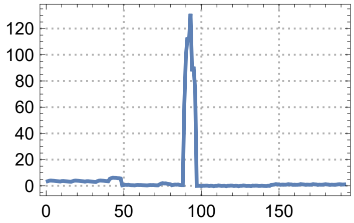

Dataset Index= 128

Molecule Formula= $C_{18}H_{15}F_{2}N_{5}O_{2}$

3D Autocorrelation

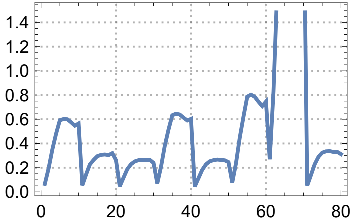

2D Plot

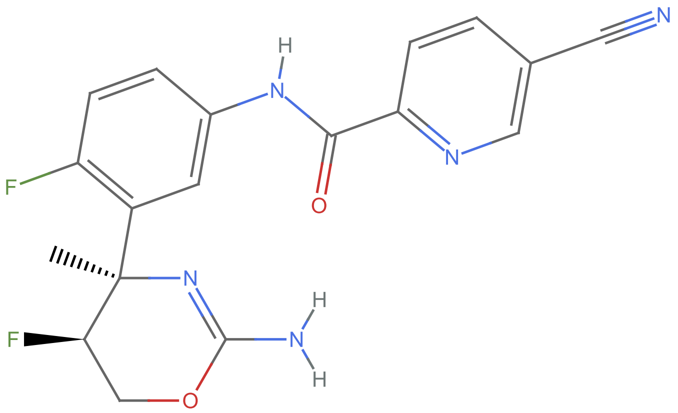

3D Plot

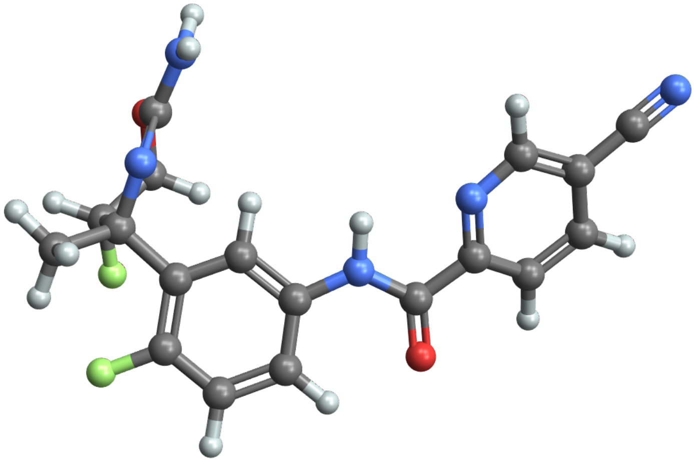

2D Plot

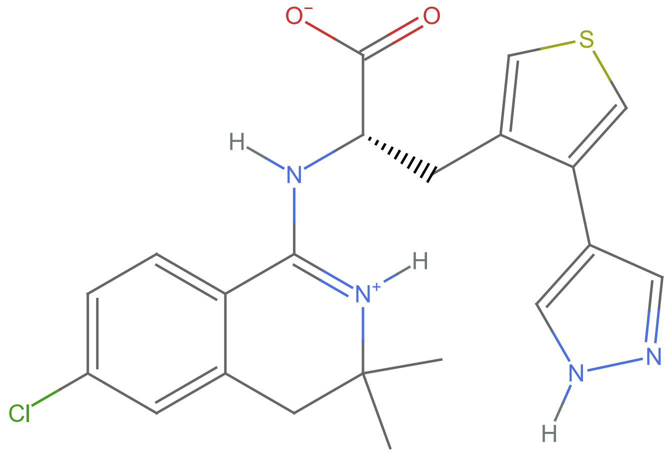

3D Plot

2D Autocorrelation

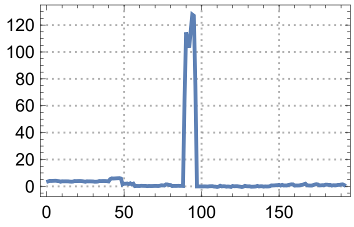

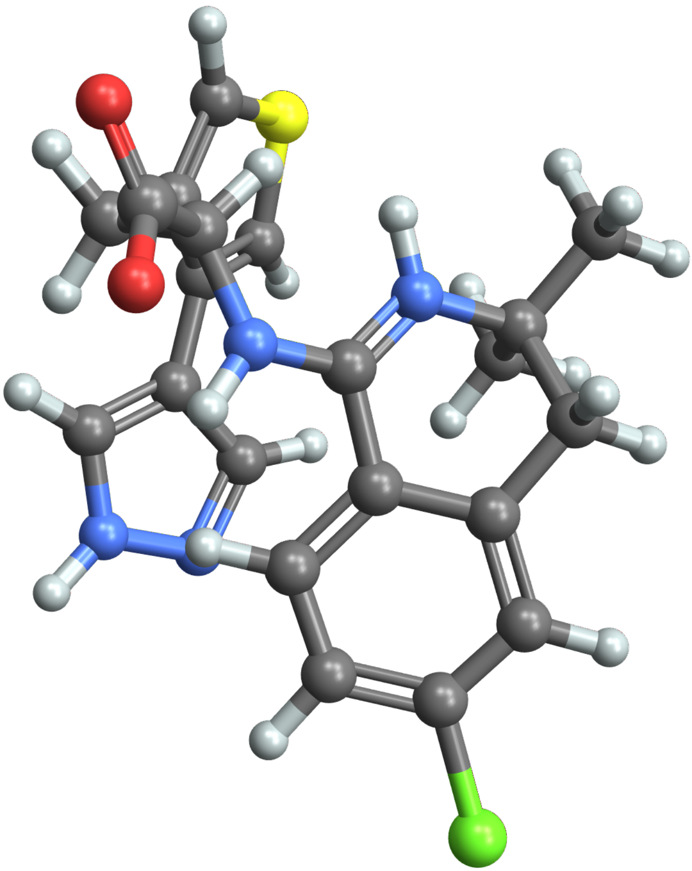

Dataset Index= 129

Molecule Formula= $C_{21}H_{21}ClN_4O_2S$

3D Autocorrelation

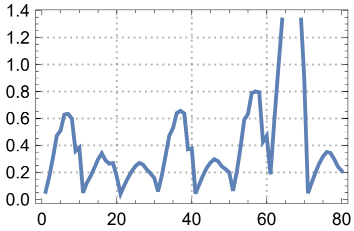

2D Autocorrelation

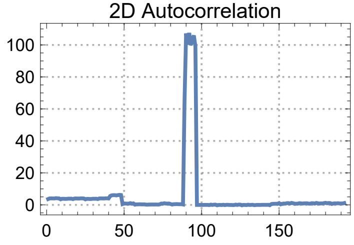

Dataset Index= 130

Molecule Formula= $C_{24}H_{23}ClN_5O^+$

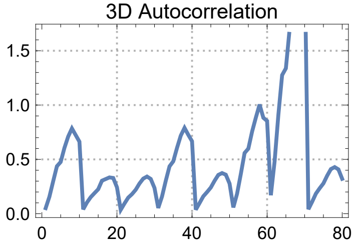

2D Plot

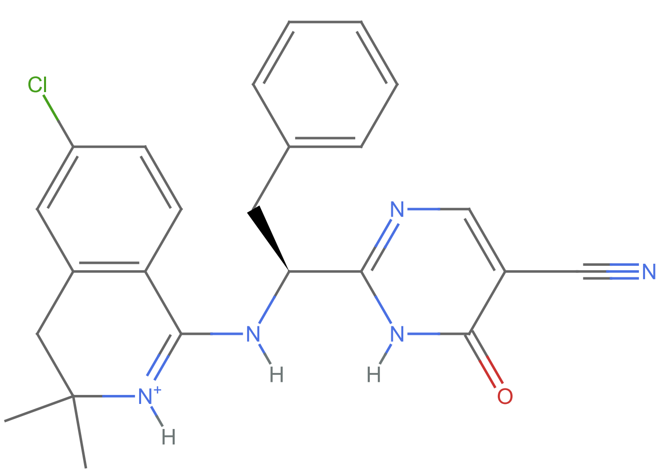

3D Plot

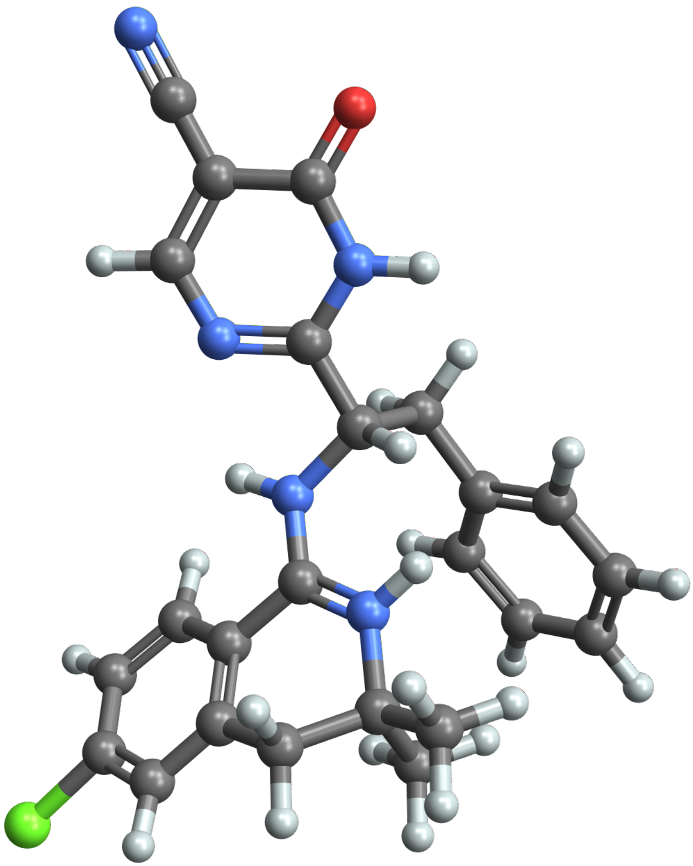

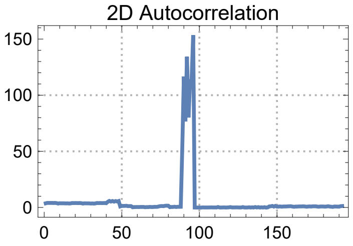

Dataset Index= 131

Molecule Formula= $C_{24}H_{33}F_2N_2O_2S^+$

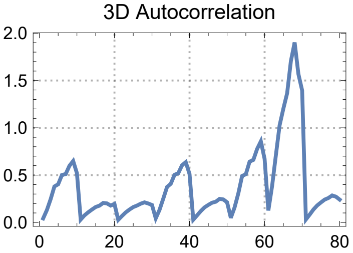

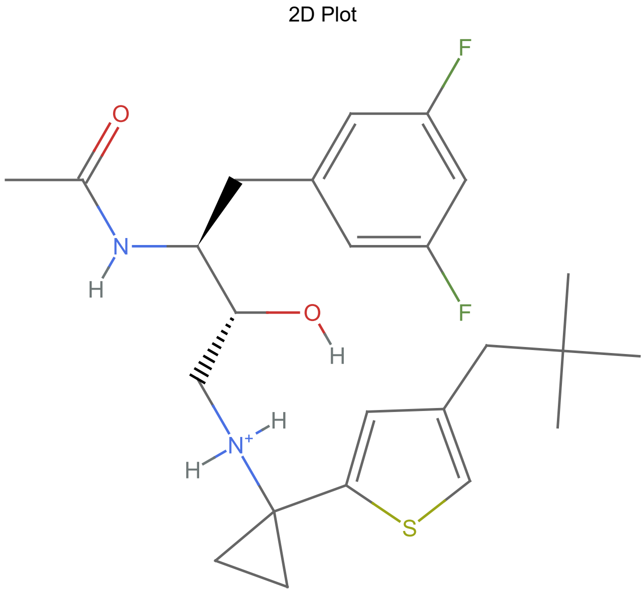

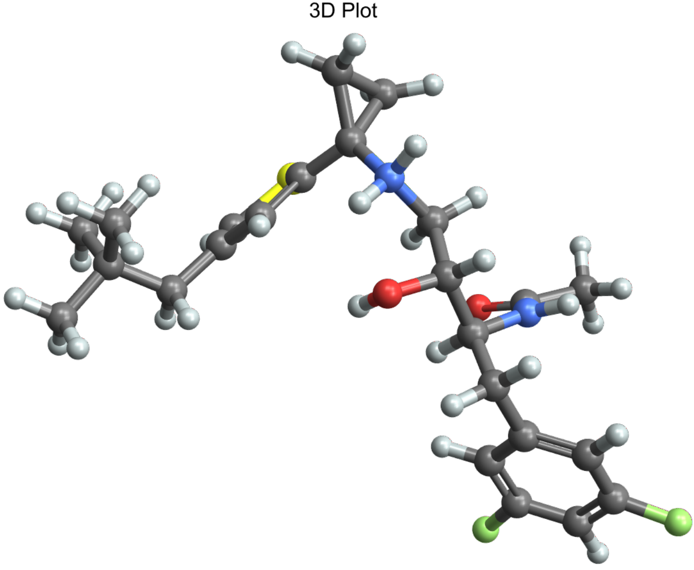

2D Plot

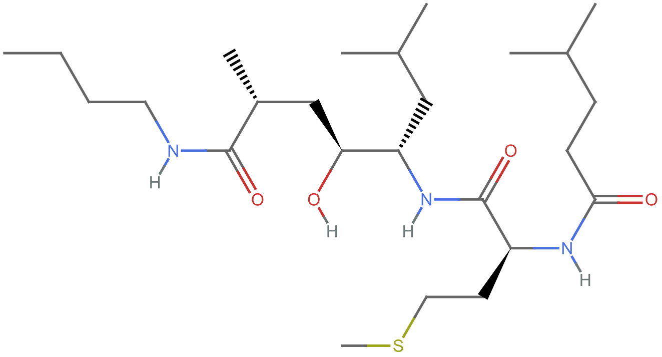

2D Autocorrelation

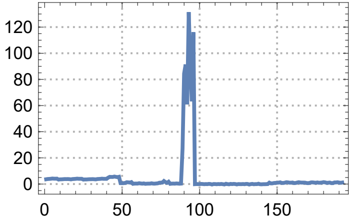

3D Plot

Dataset Index= 132

Molecule Formula= $C_{25}H_{49}N_3O_4S$

3D Autocorrelation

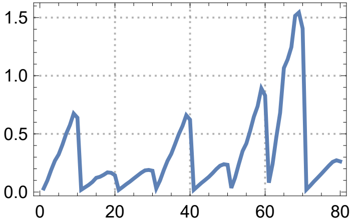

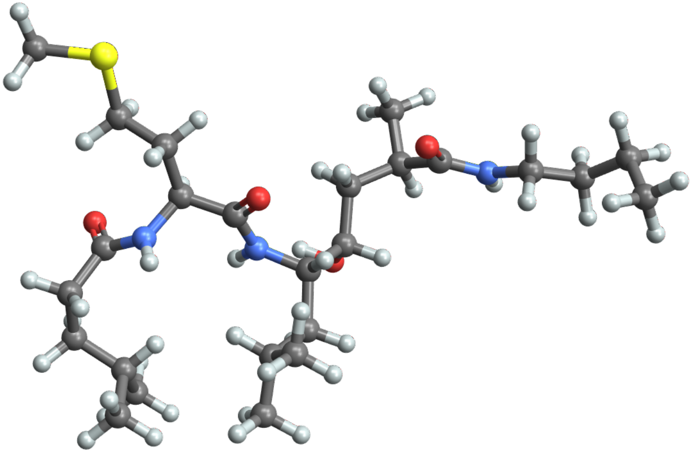

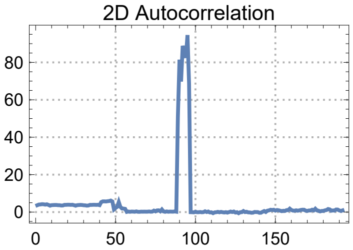

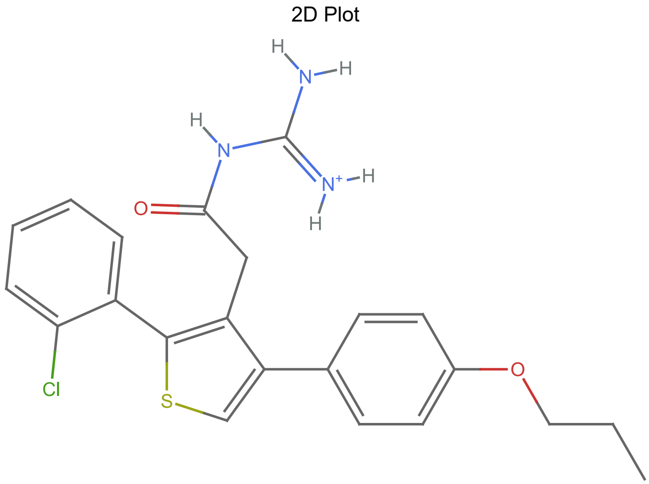

Dataset Index= 133

Molecule Formula= $C_{22}H_{23}ClN_3O_2S^+$

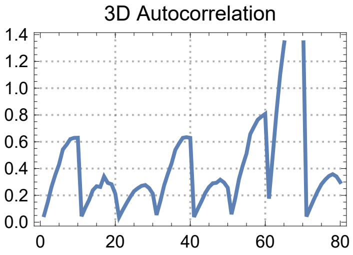

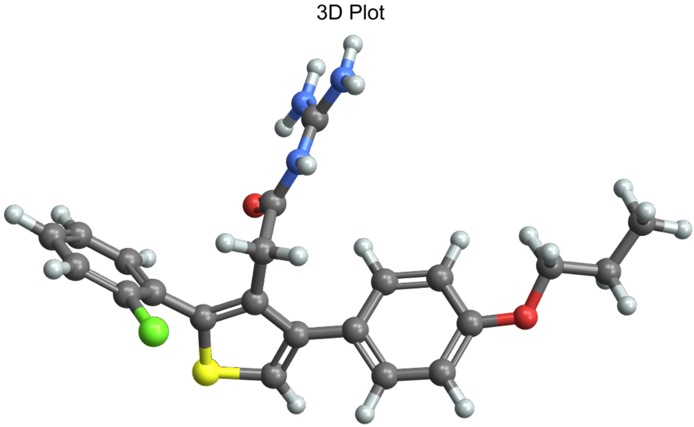

2D Autocorrelation

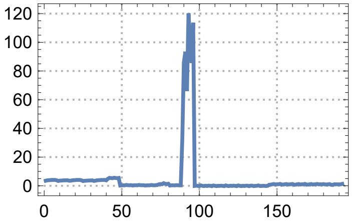

Dataset Index= 134

Molecule Formula= $C_{24}H_{45}N_{3}O_{4}$

3D Autocorrelation

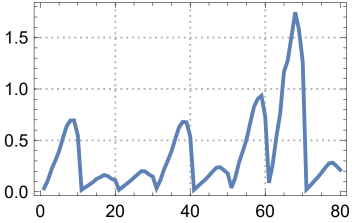

2D Plot

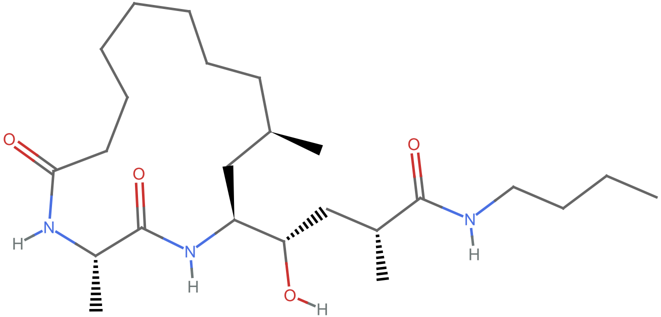

3D Plot

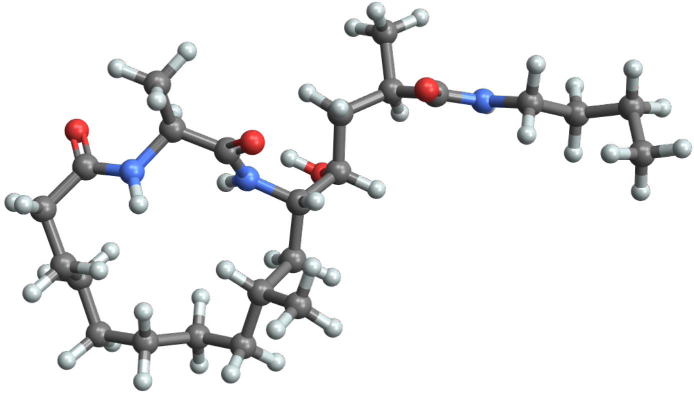

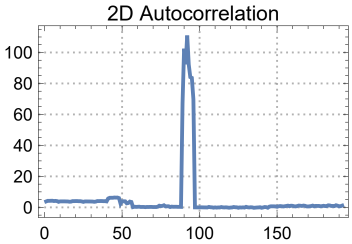

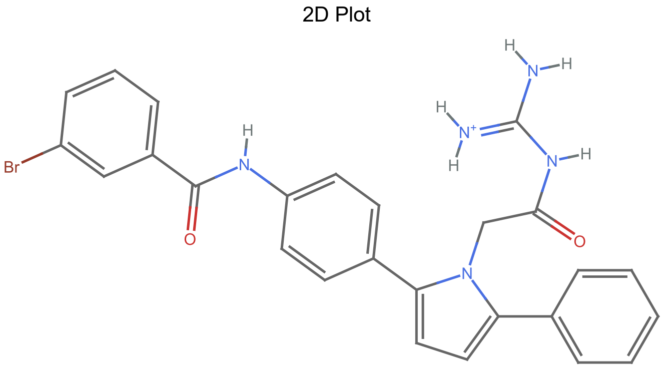

Dataset Index= 135

Molecule Formula= $C_{26}H_{23}BrN_5O_2^+$

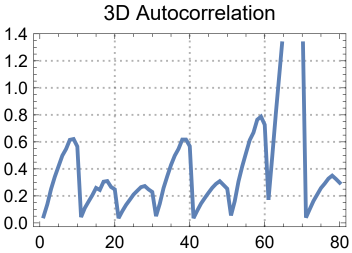

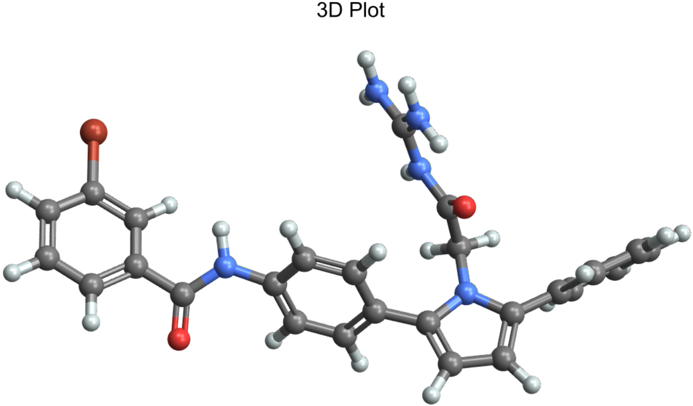

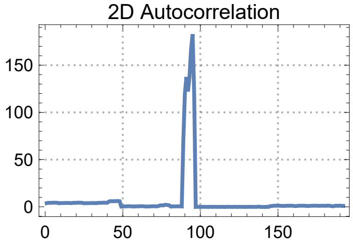

Dataset Index= 136

Molecule Formula= $C_{33}H_{47}N_4O_5^+$

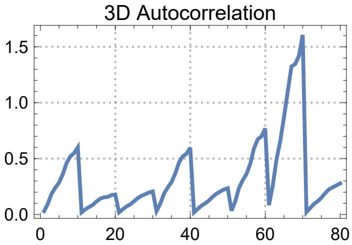

2D Plot

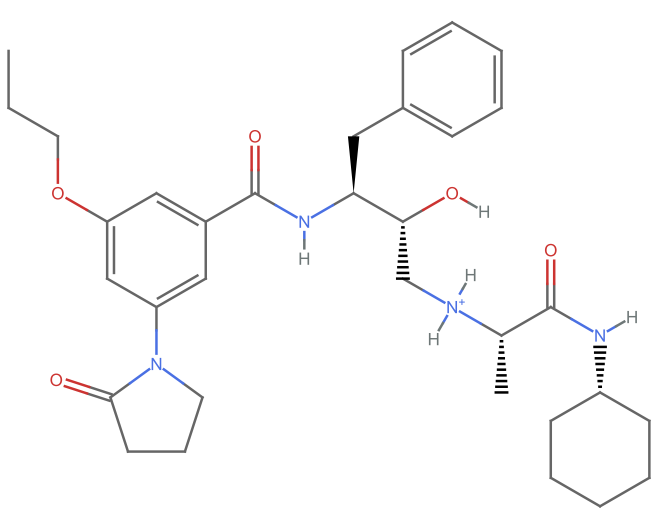

3D Plot

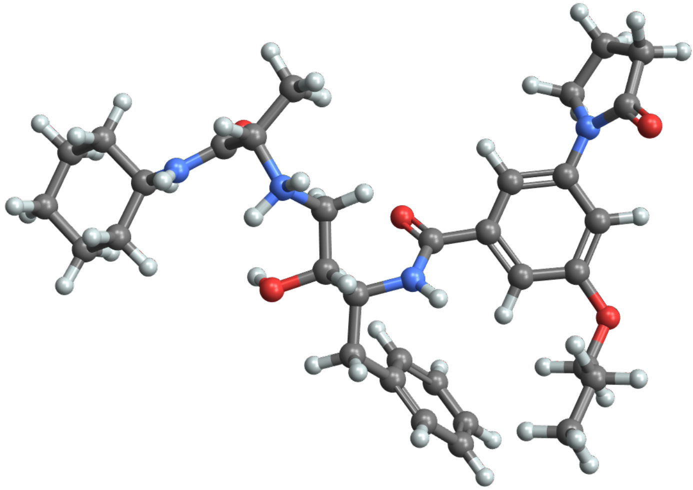

2D Autocorrelation

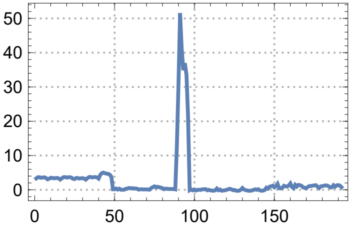

Dataset Index= 137

Molecule Formula= $C_{19}H_{33}N_3O$

3D Autocorrelation

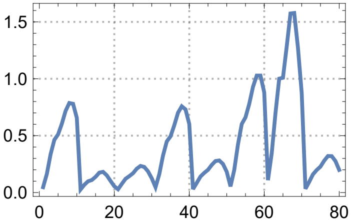

2D Plot

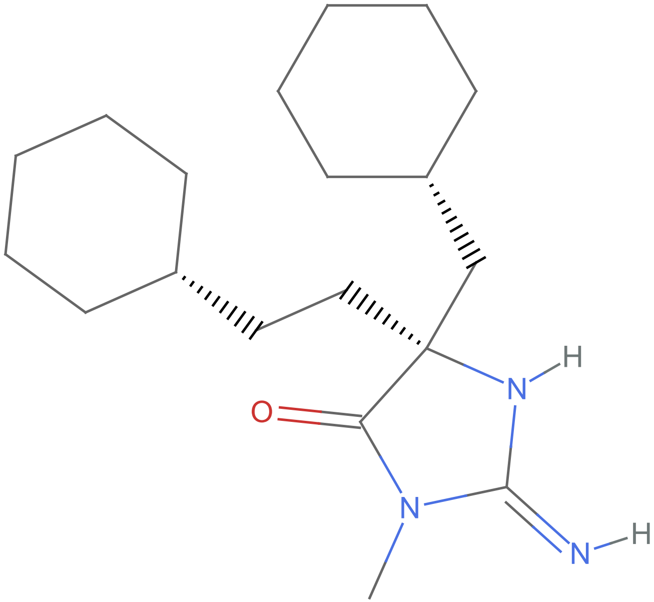

3D Plot

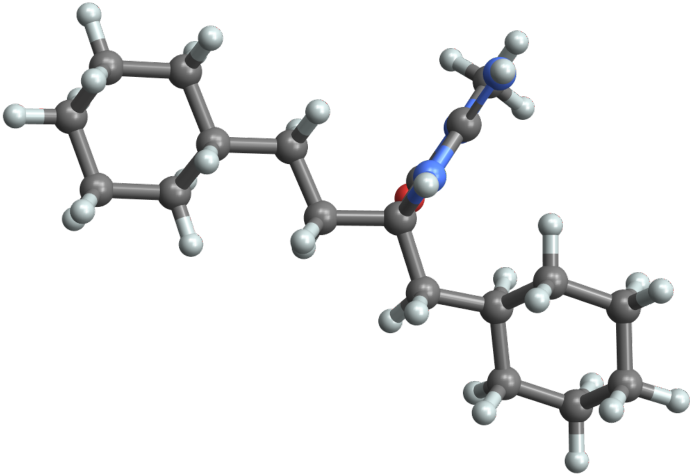

2D Plot

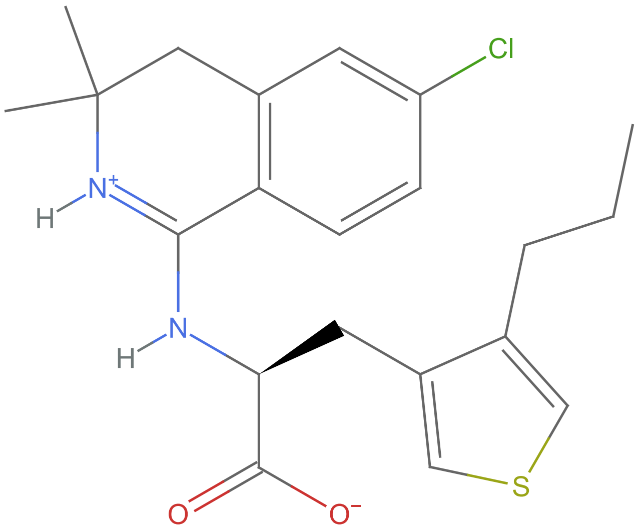

3D Plot

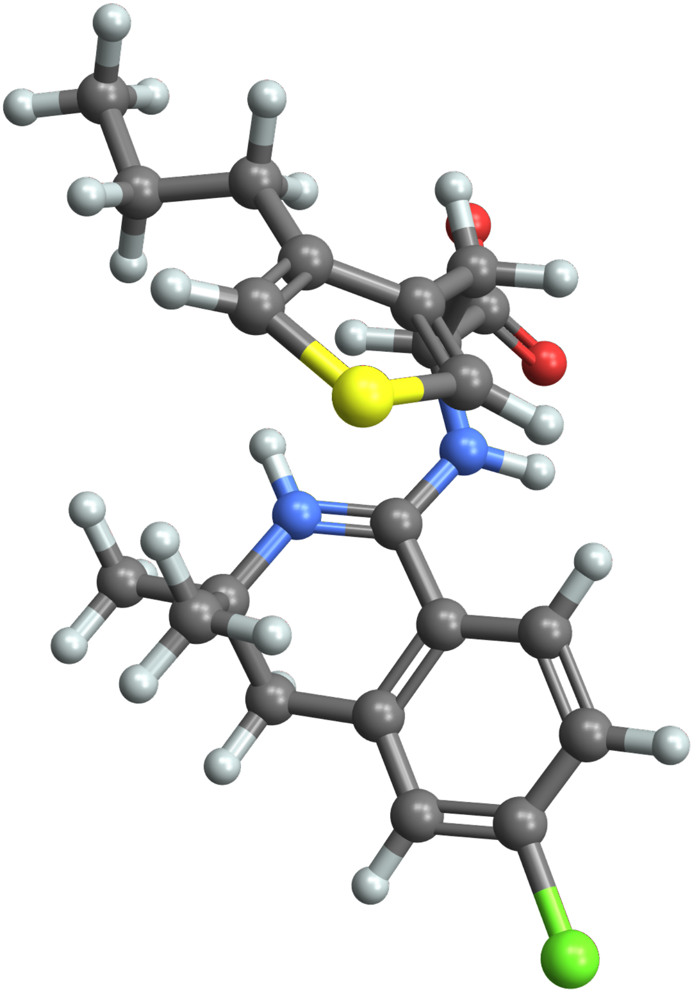

2D Autocorrelation

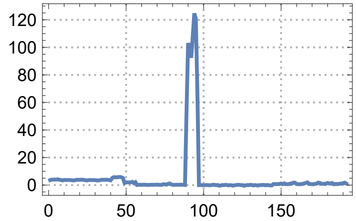

Dataset Index= 138

Molecule Formula= $C_{21}H_{25}ClN_2O_2S$

3D Autocorrelation

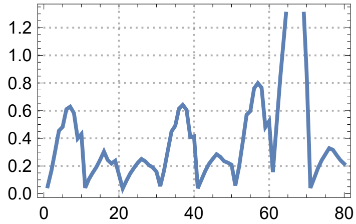

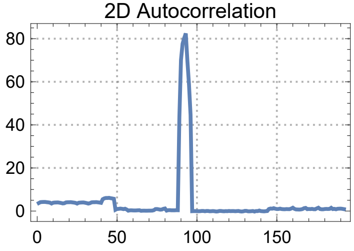

Dataset Index= 139

Molecule Formula= $C_{23}H_{20}N_5OS^+$

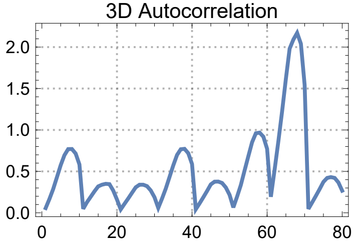

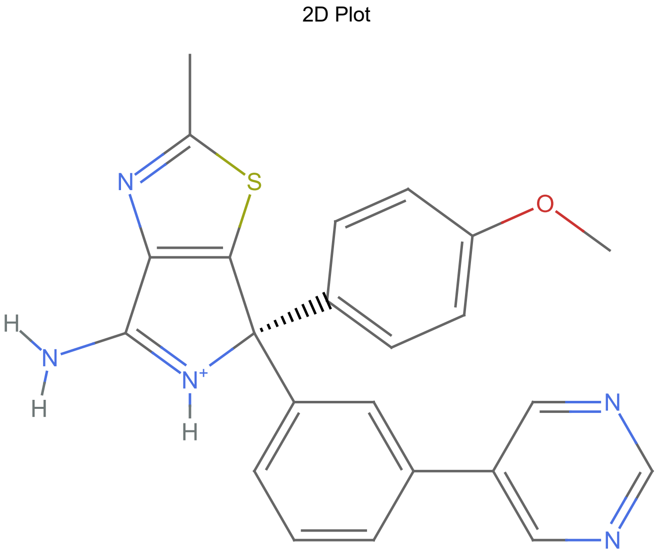

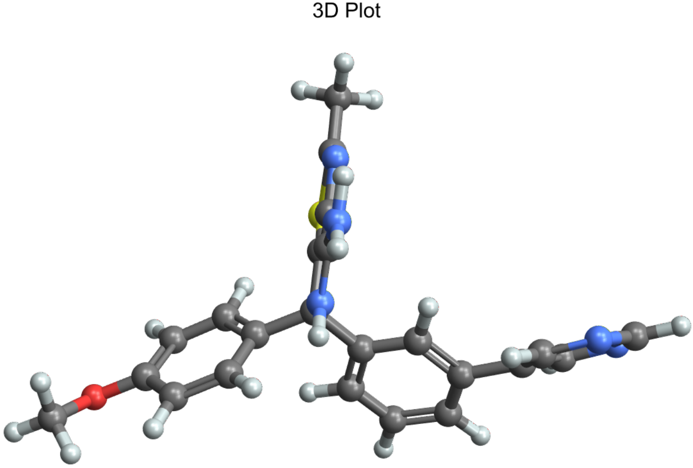

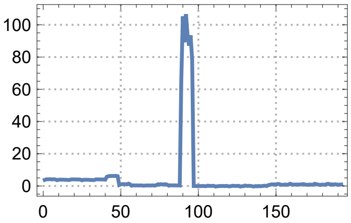

Dataset Index= 140

Molecule Formula= $C_{27}H_{24}ClN_4O_3^+$

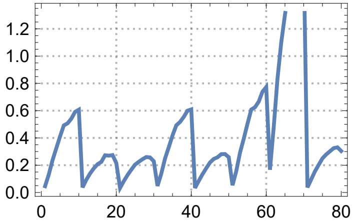

2D Plot

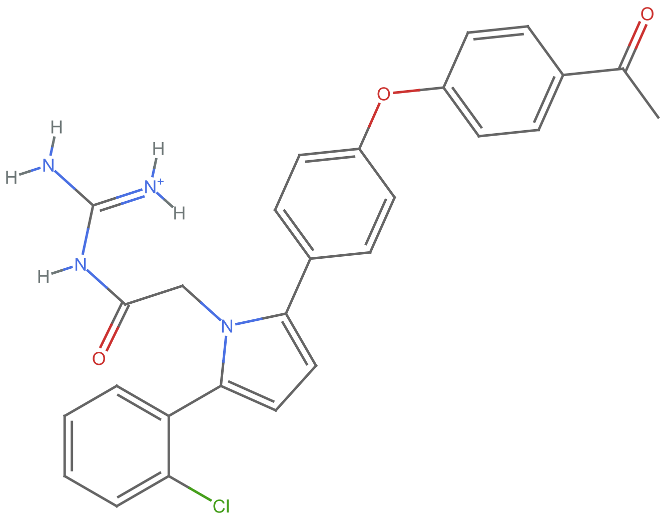

3D Plot

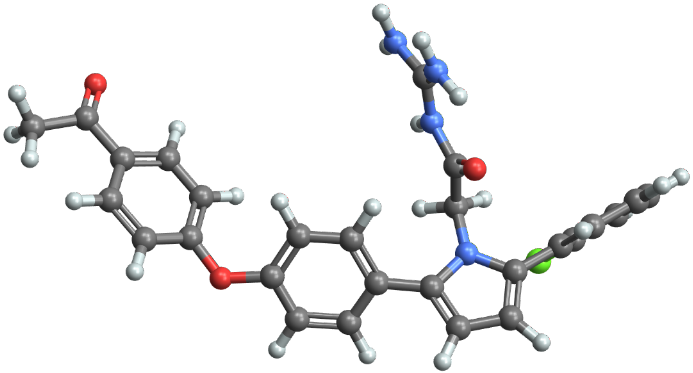

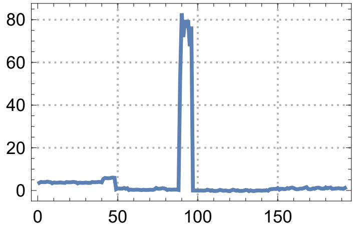

Dataset Index= 141

Molecule Formula= $C_{21}H_{21}ClN_4O_2$

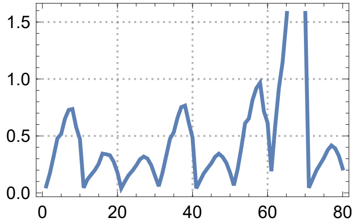

2D Plot

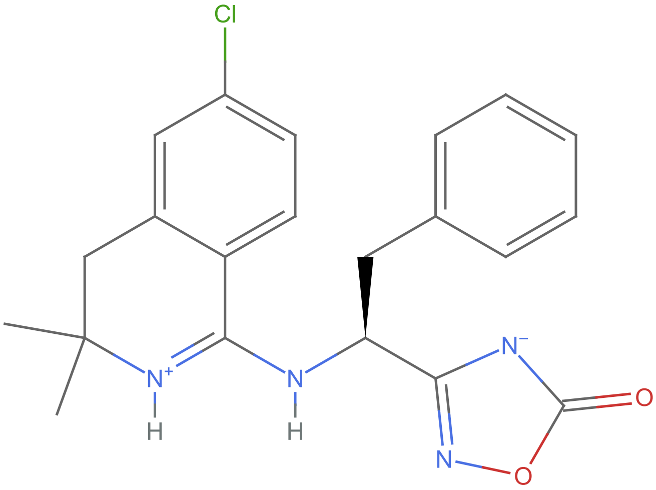

3D Plot

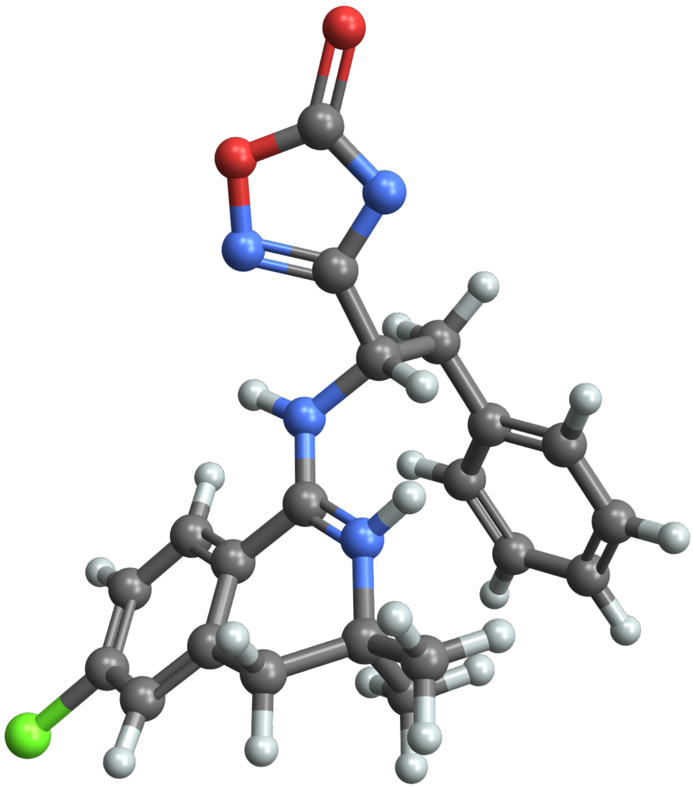

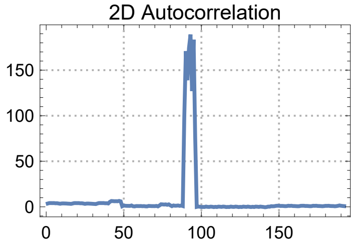

Dataset Index= 142

Molecule Formula= $C_{19}H_{15}F_4N_5O_2$

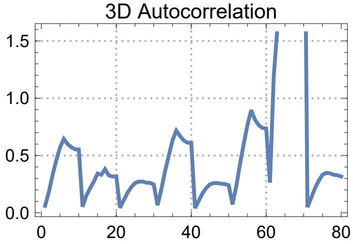

2D Plot

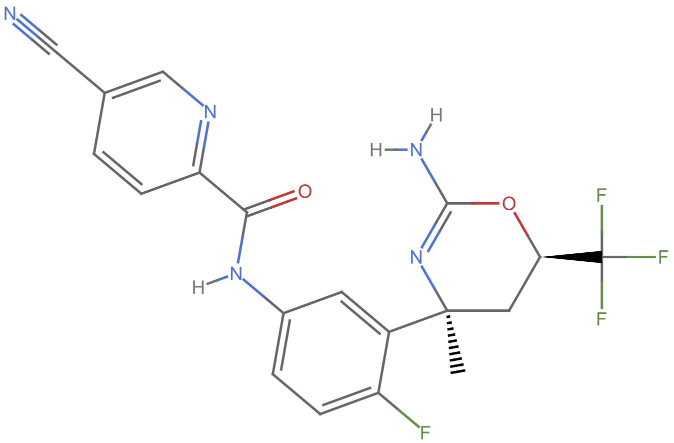

3D Plot

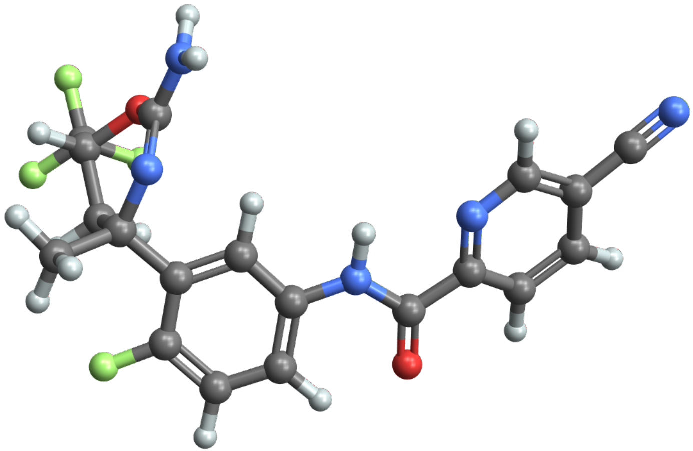

2D Plot

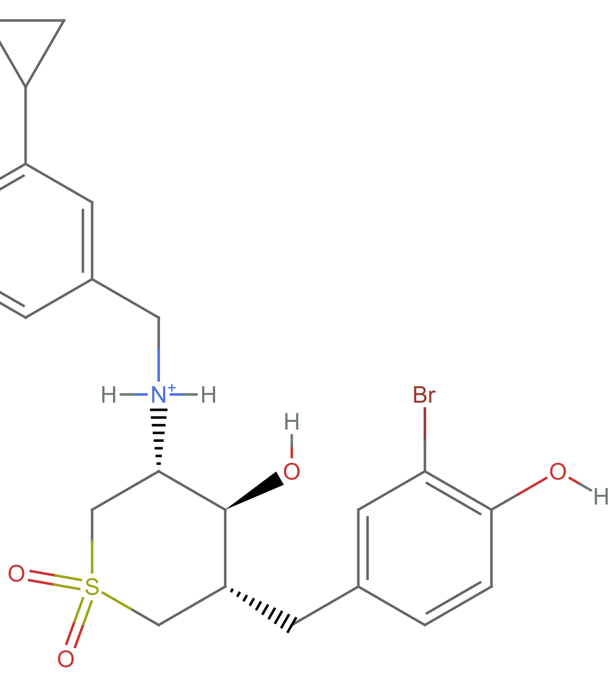

2D Autocorrelation

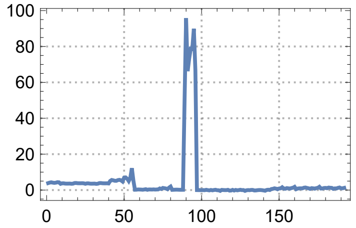

Dataset Index= 143

Molecule Formula= $C_{22}H_{27}BrNO_4S^+$

3D Plot

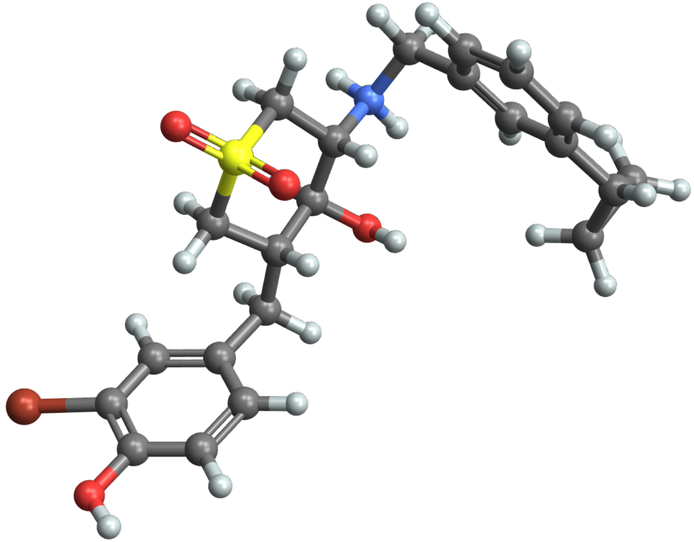

3D Autocorrelation

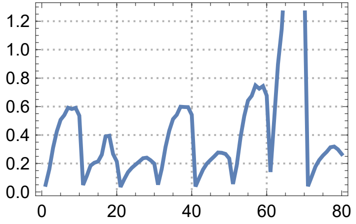

2D Plot

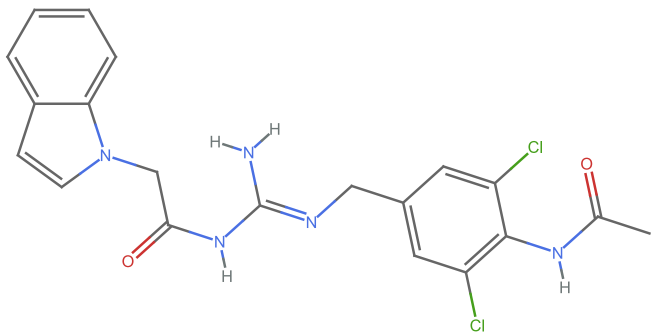

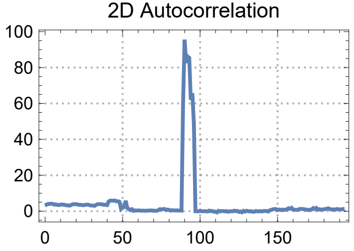

Dataset Index= 144

Molecule Formula= $C_{20}H_{19}Cl_2N_5O_2$

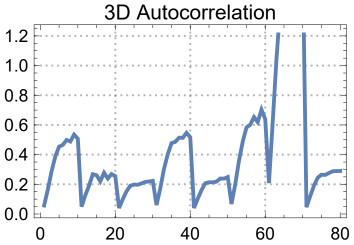

3D Plot

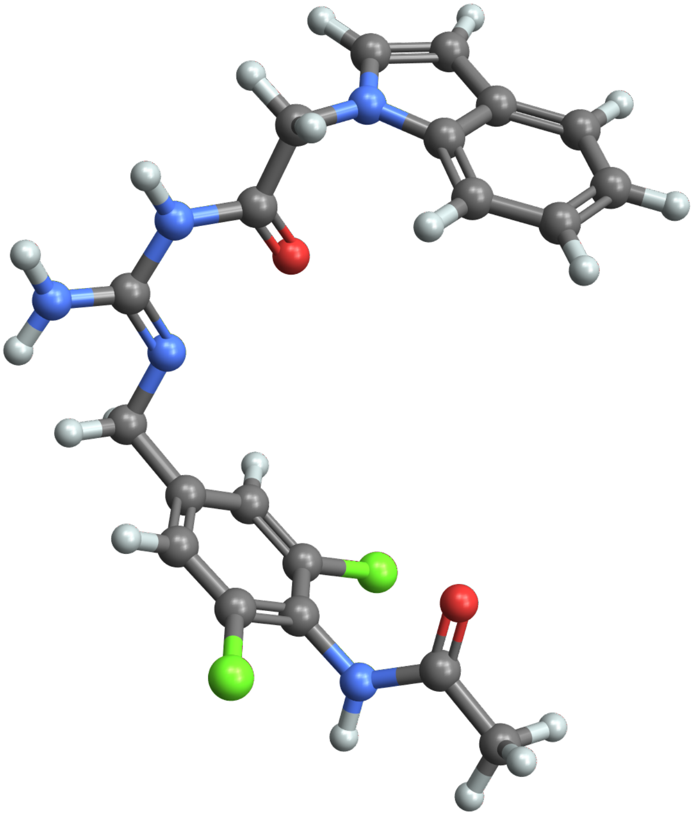

2D Plot

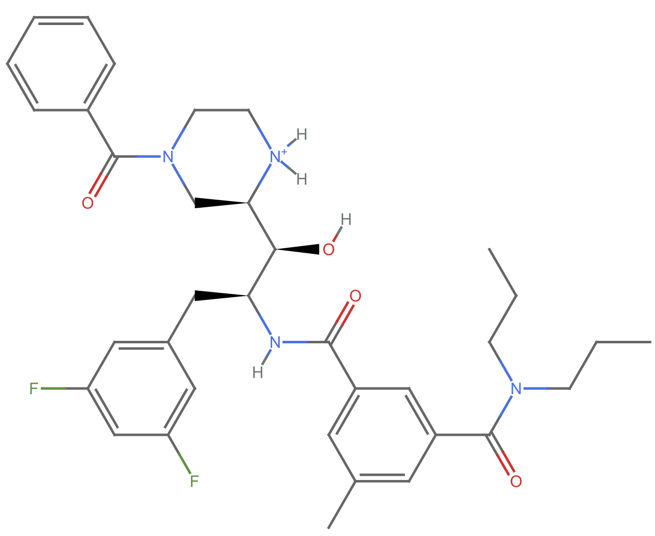

2D Autocorrelation

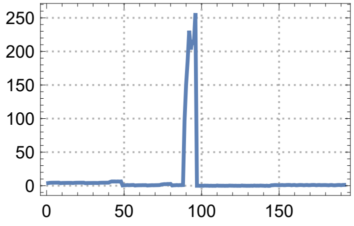

3D Plot

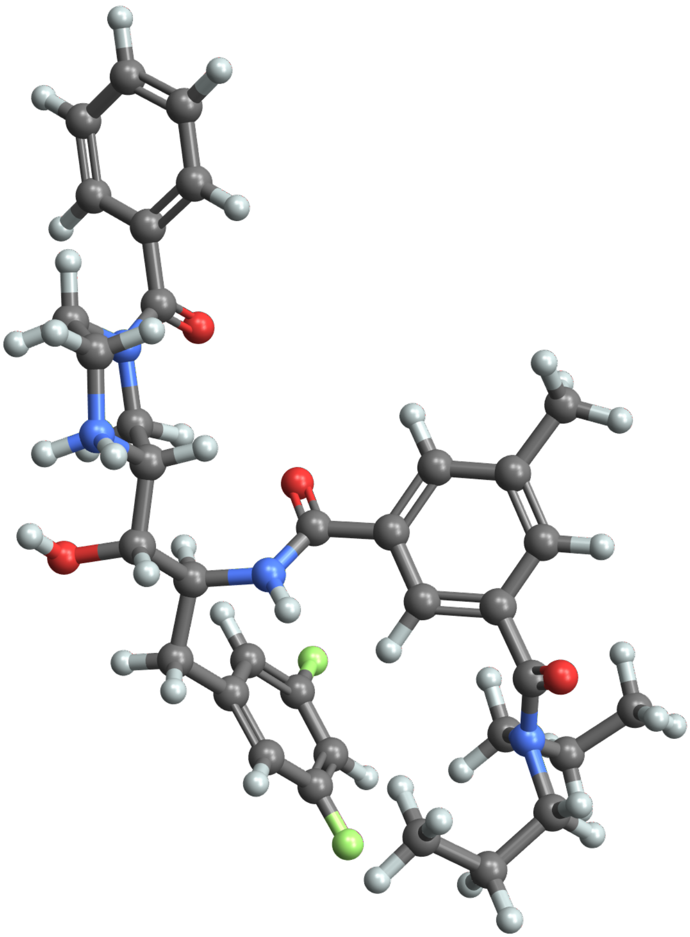

Dataset Index= 145

Molecule Formula= $C_{35}H_{43}F_2N_4O_4^+$

3D Autocorrelation

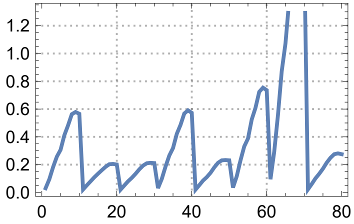

2D Plot

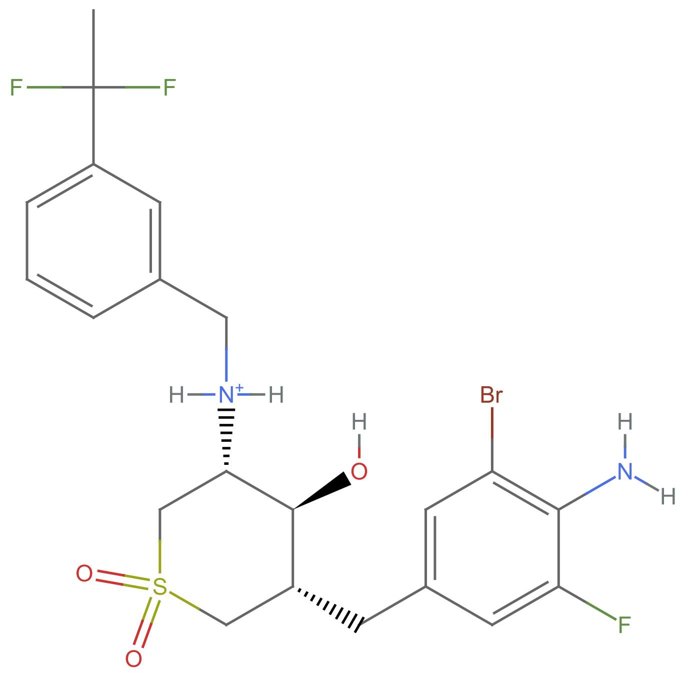

2D Autocorrelation

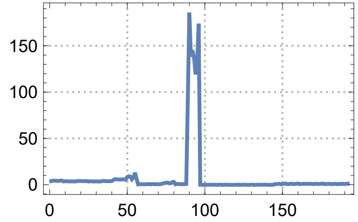

Dataset Index= 146

Molecule Formula= $C_{21}H_{25}BrF_3N_2O_3S^+$

3D Autocorrelation

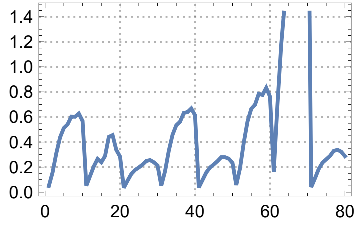

3D Plot

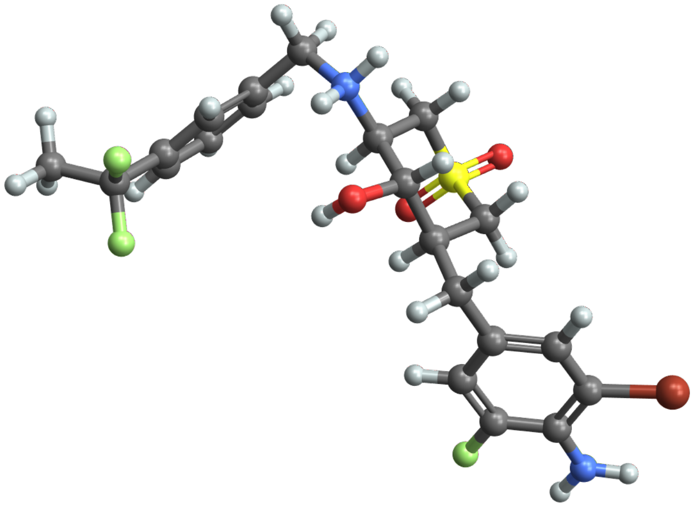

2D Plot

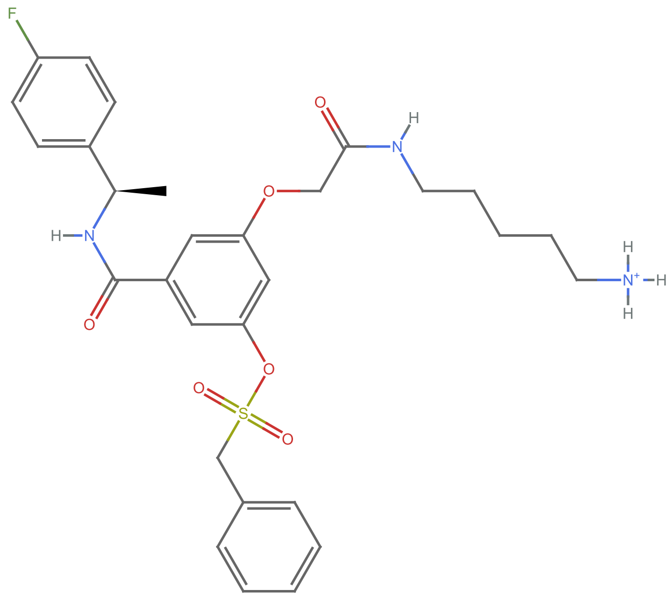

2D Autocorrelation

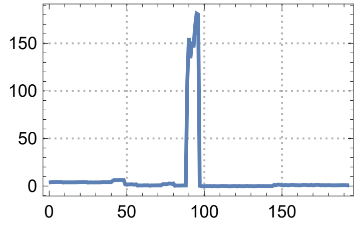

3D Plot

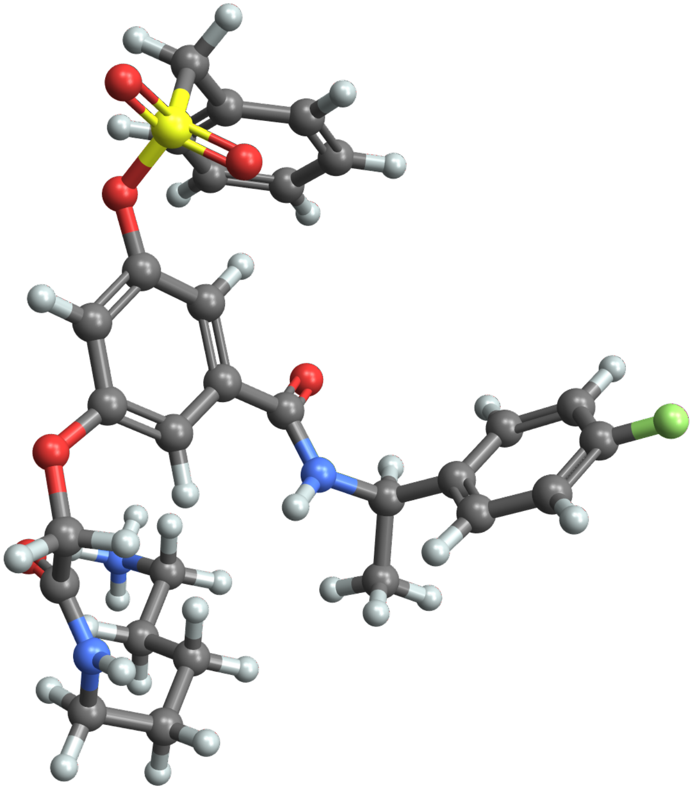

Dataset Index= 147

Molecule Formula= $C_{29}H_{35}FN_3O_6S^+$

3D Autocorrelation

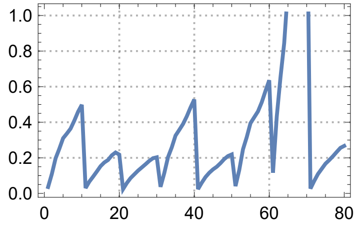

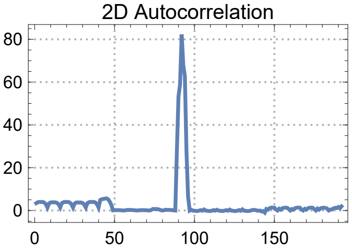

Dataset Index= 148

Molecule Formula= $C_{20}H_{25}N_3O$

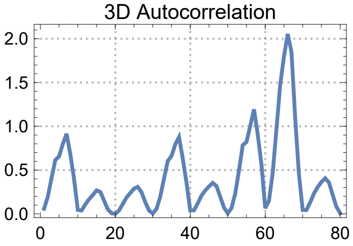

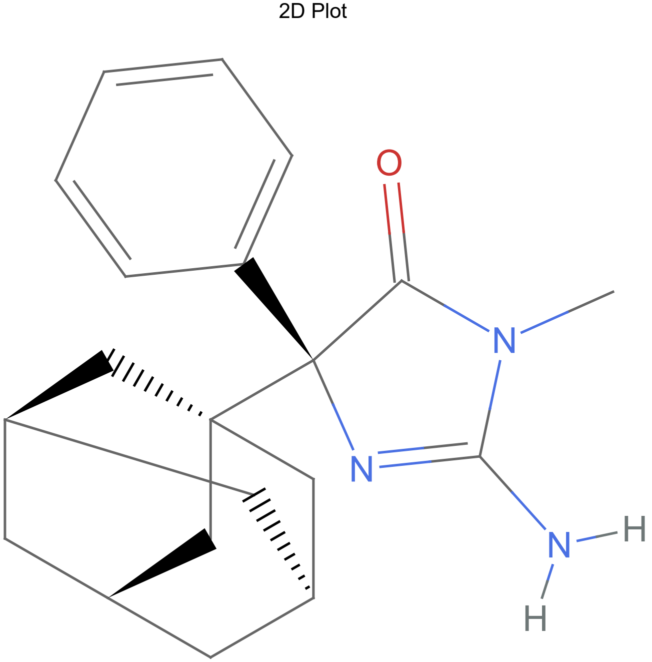

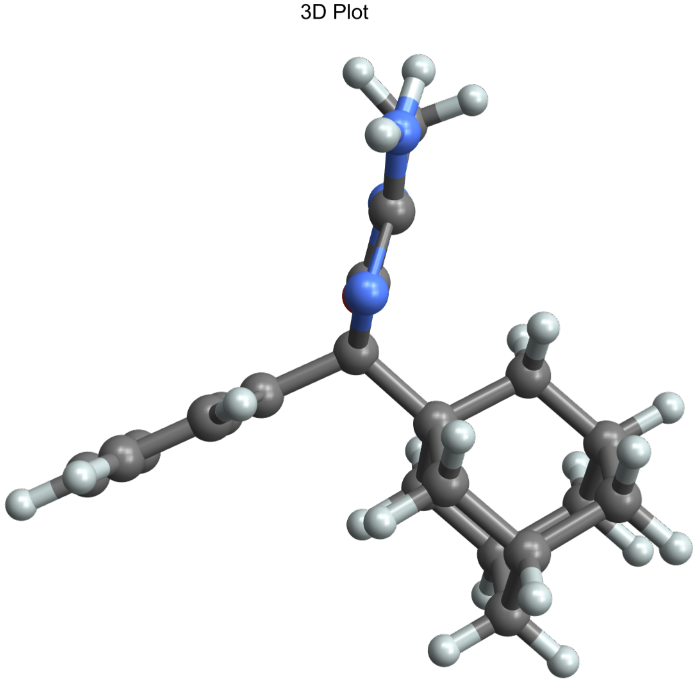

2D Plot

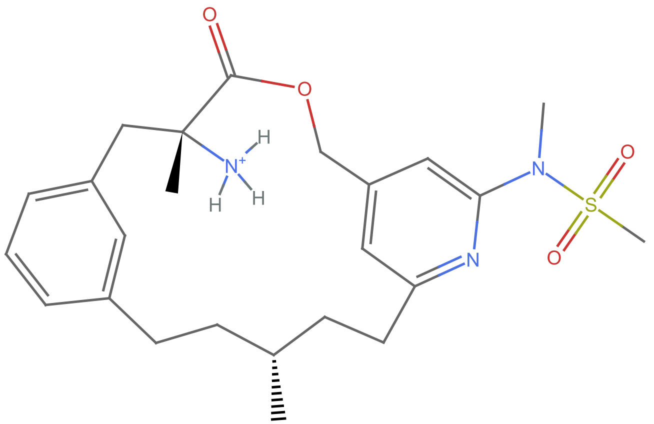

3D Plot

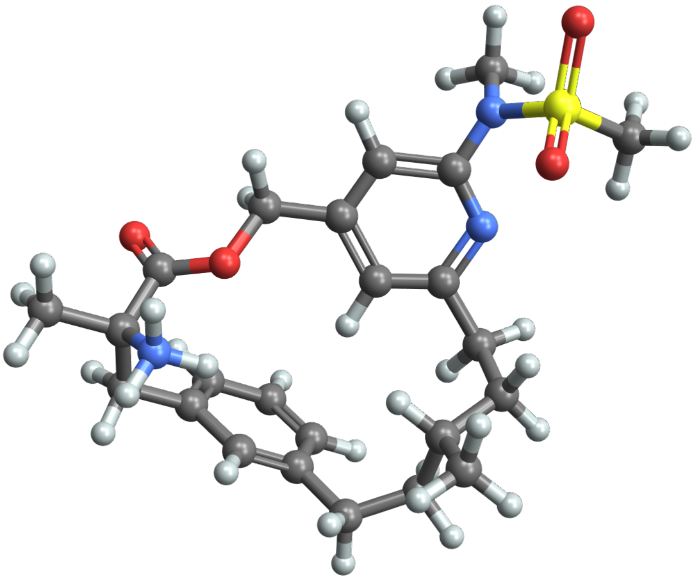

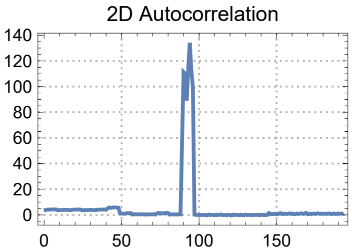

Dataset Index= 149

Molecule Formula= $C_{24}H_{34}N_3O_4S^+$

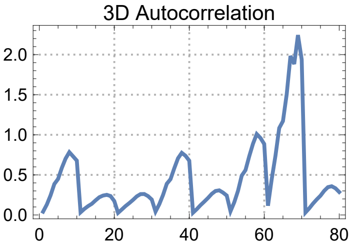

2D Autocorrelation

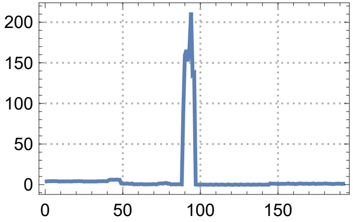

Dataset Index= 150

Molecule Formula= $C_{31}H_{46}N_3O_5S^+$

3D Autocorrelation

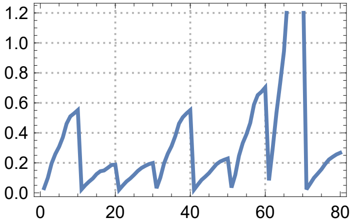

2D Plot

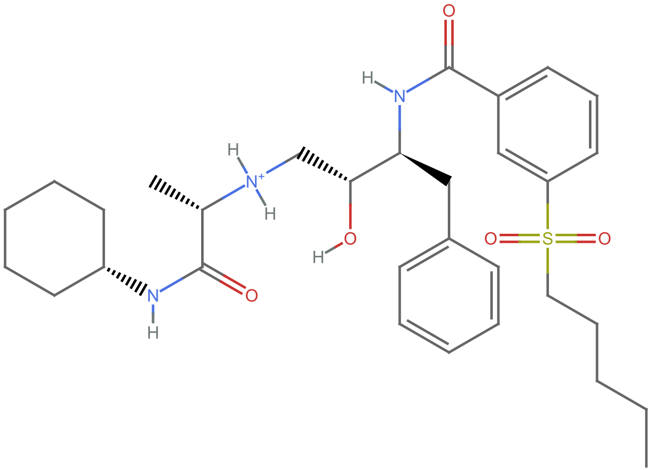

3D Plot

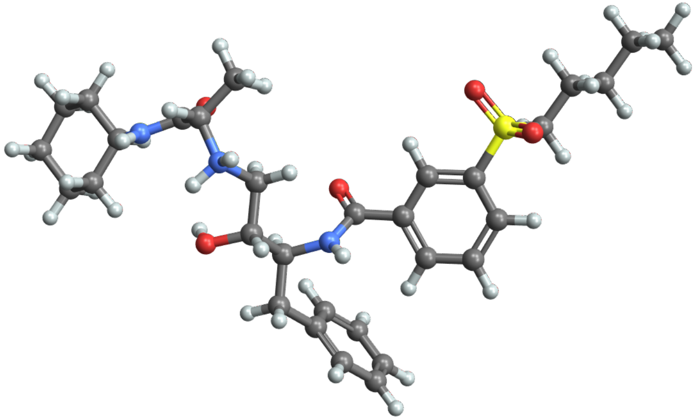

2D Plot

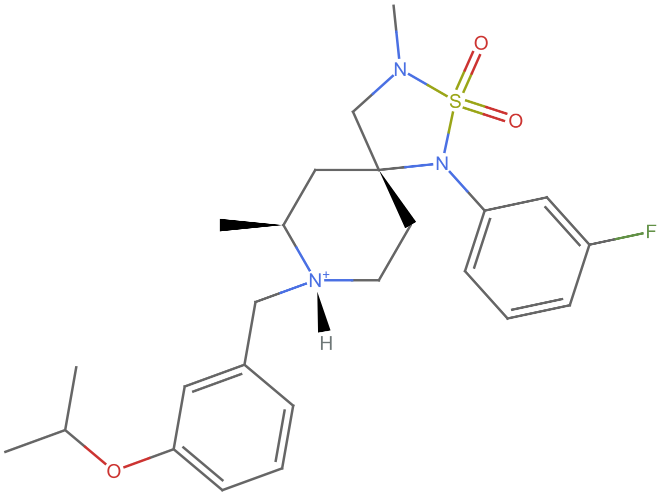

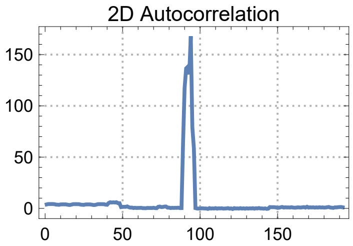

3D Plot

Dataset Index= 151

Molecule Formula= $C_{24}H_{33}FN_3O_3S^+$

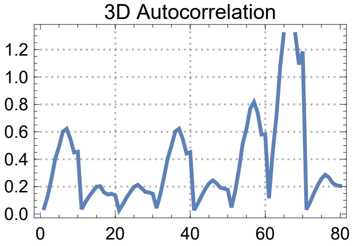

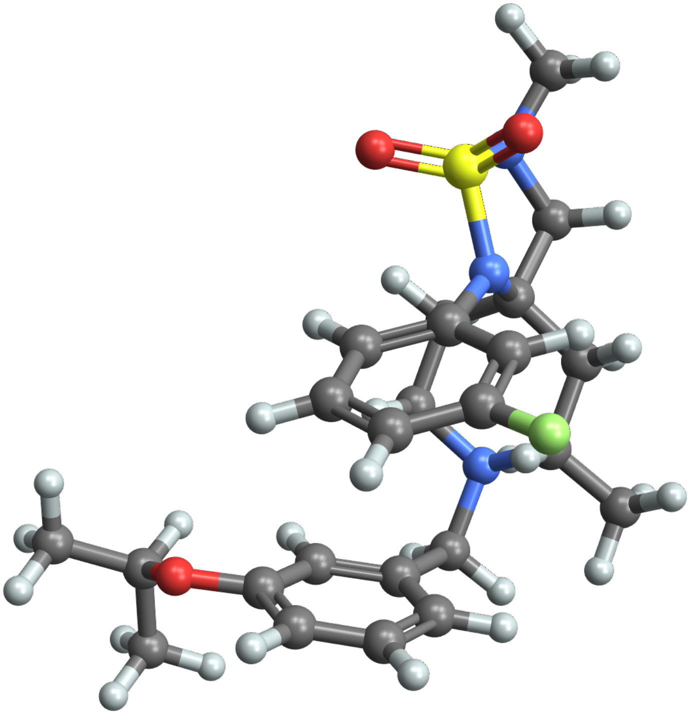

2D Plot

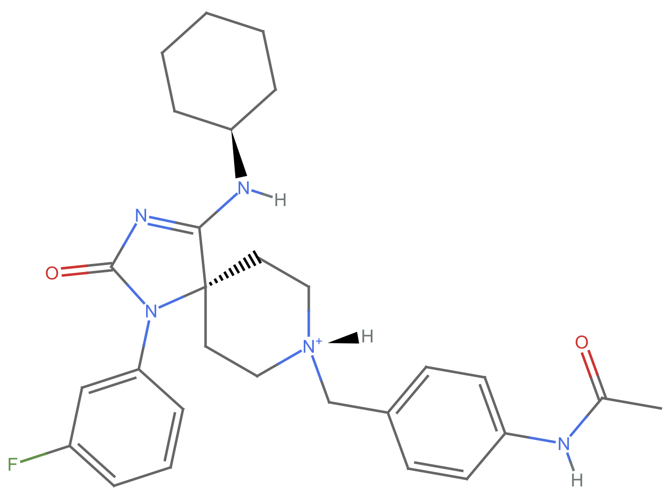

3D Plot

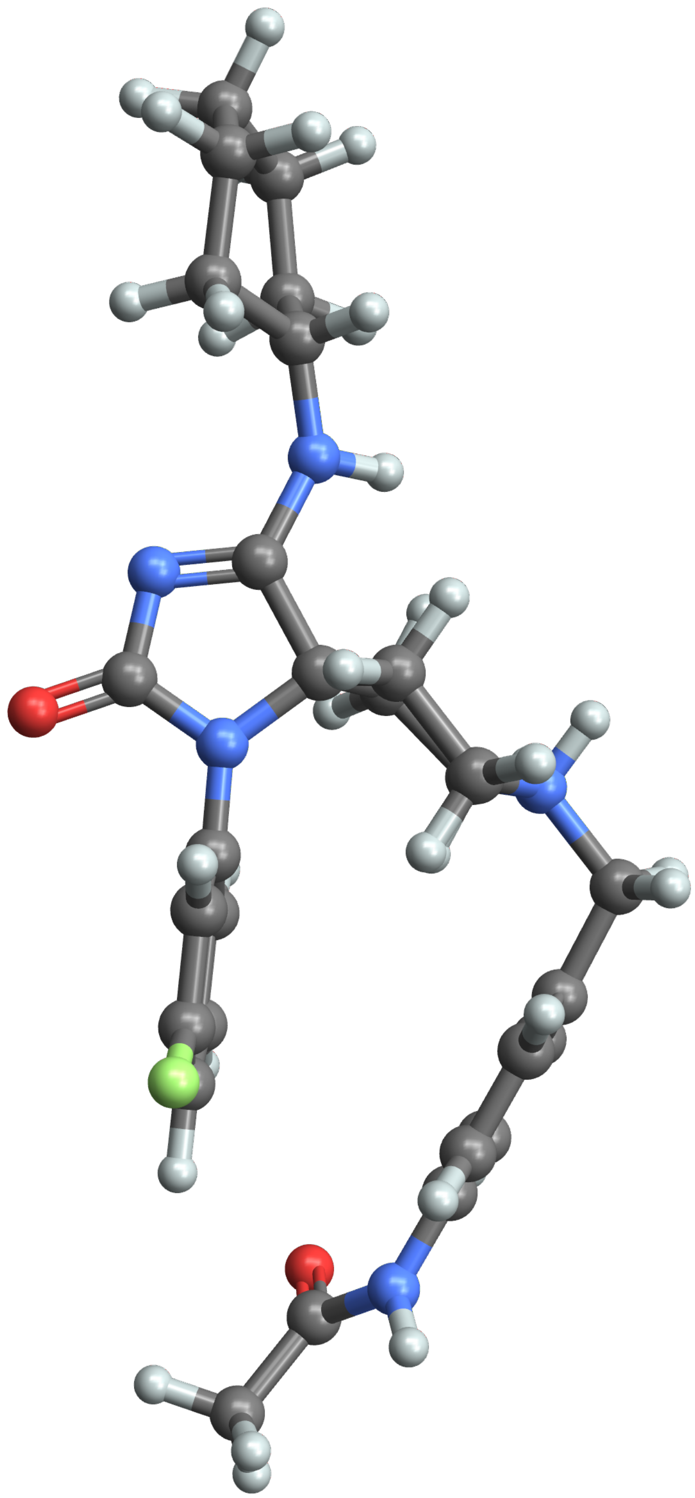

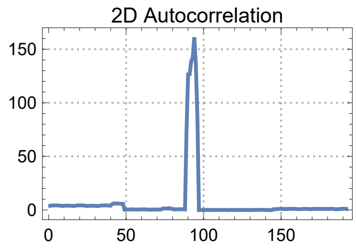

Dataset Index= 152

Molecule Formula= $C_{28}H_{35}FN_5O_2^+$

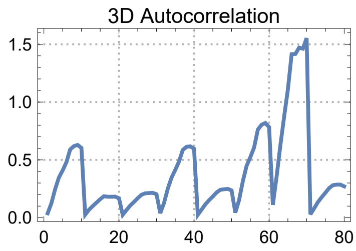

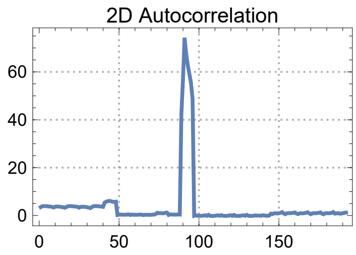

Dataset Index= 153

Molecule Formula= $C_{21}H_{18}FN_3O_2$

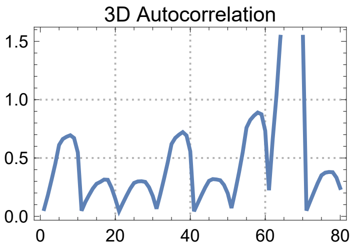

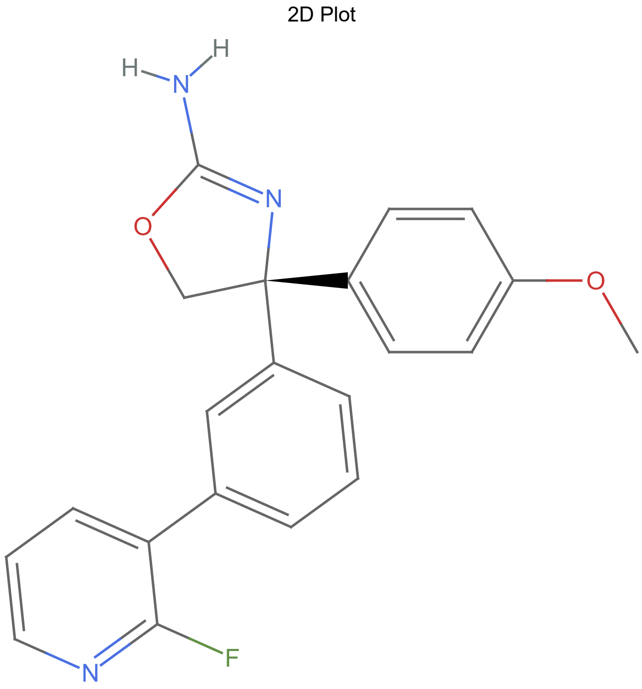

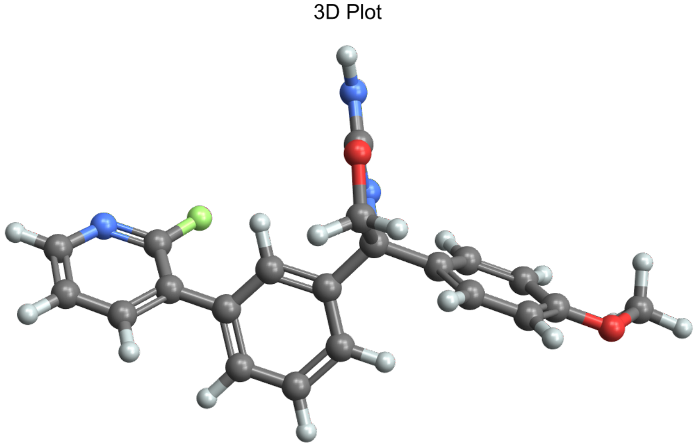

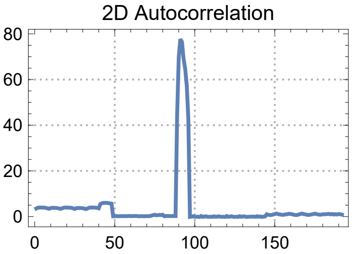

Dataset Index= 154

Molecule Formula= $C_{23}H_{24}N_3O_2^+$

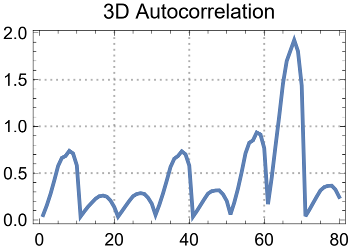

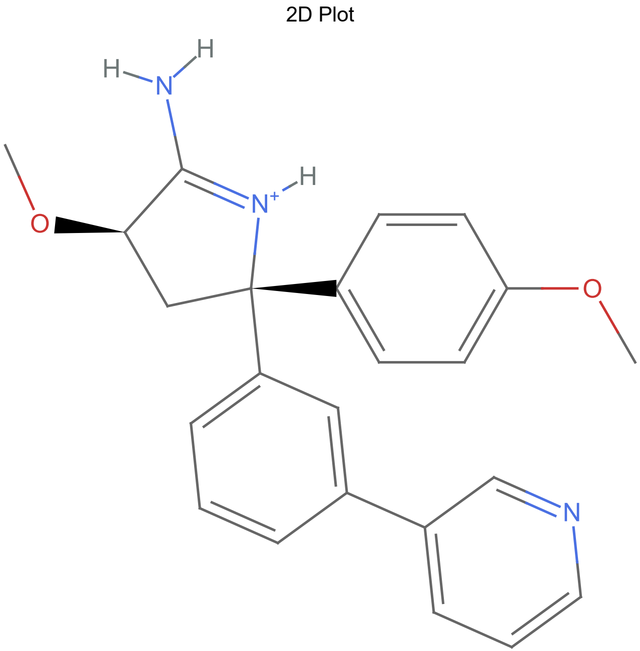

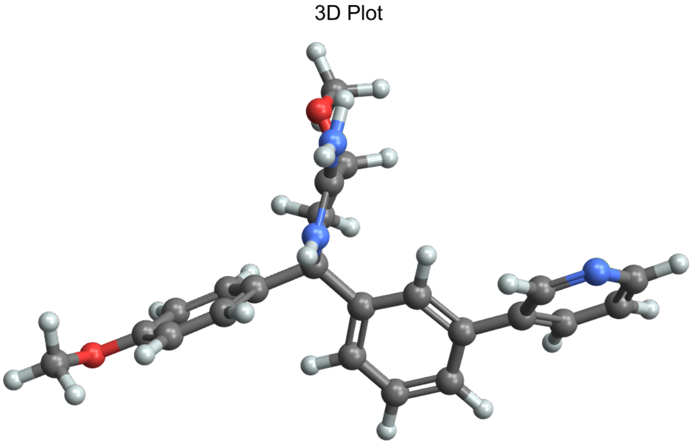

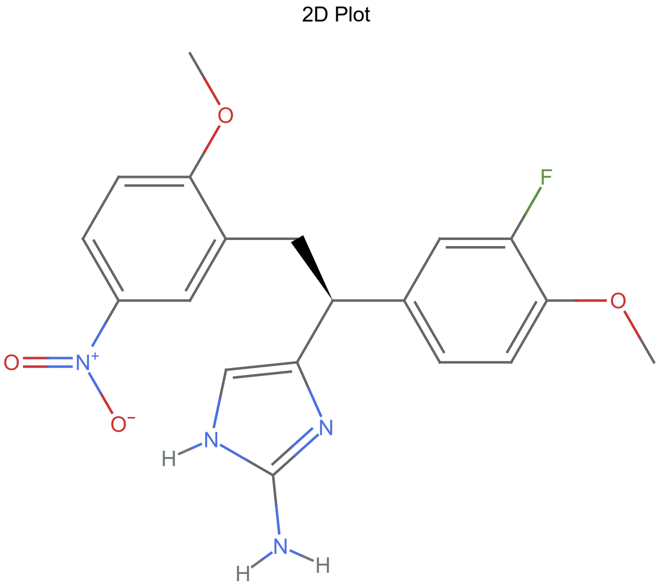

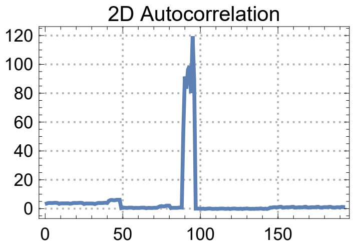

Dataset Index= 155

Molecule Formula= $C_{19}H_{19}FN_4O_4$

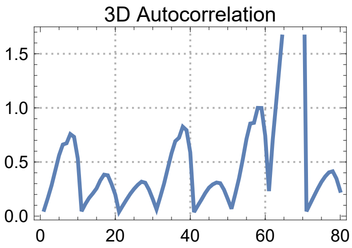

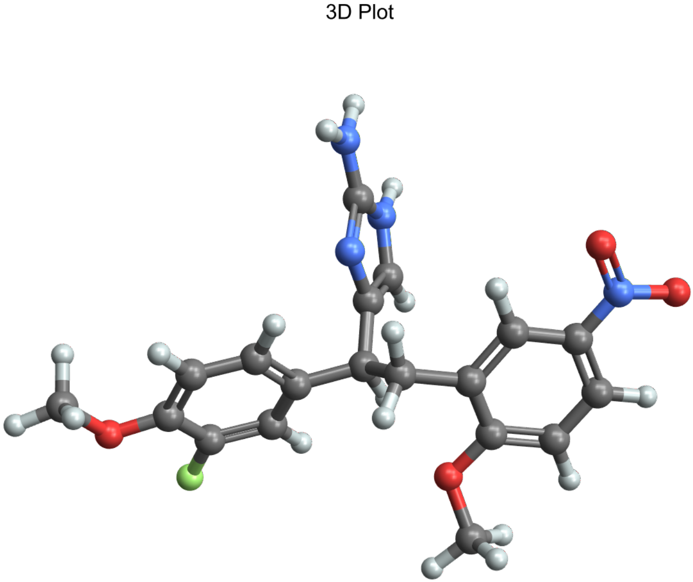

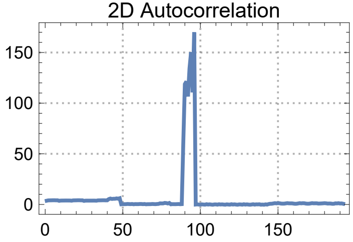

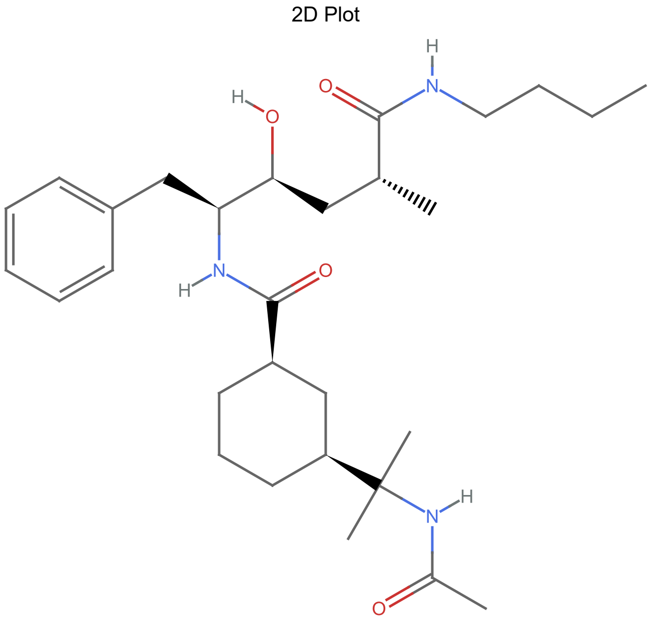

Dataset Index= 156

Molecule Formula= $C_{29}H_{47}N_3O_4$

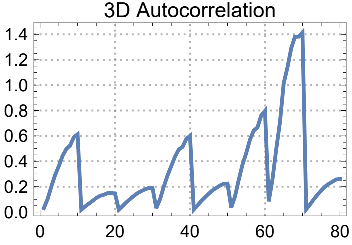

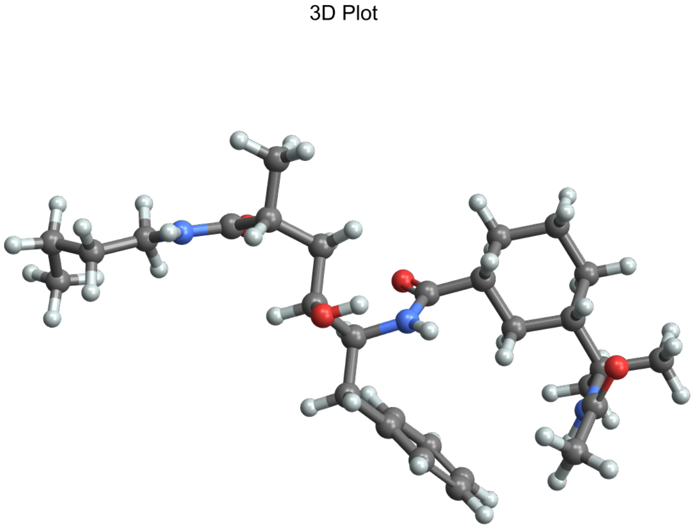

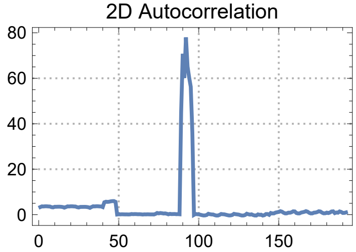

Dataset Index= 157

Molecule Formula= $C_{19}H_{19}N_4O^+$

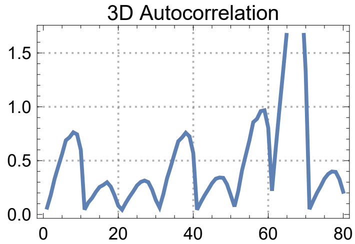

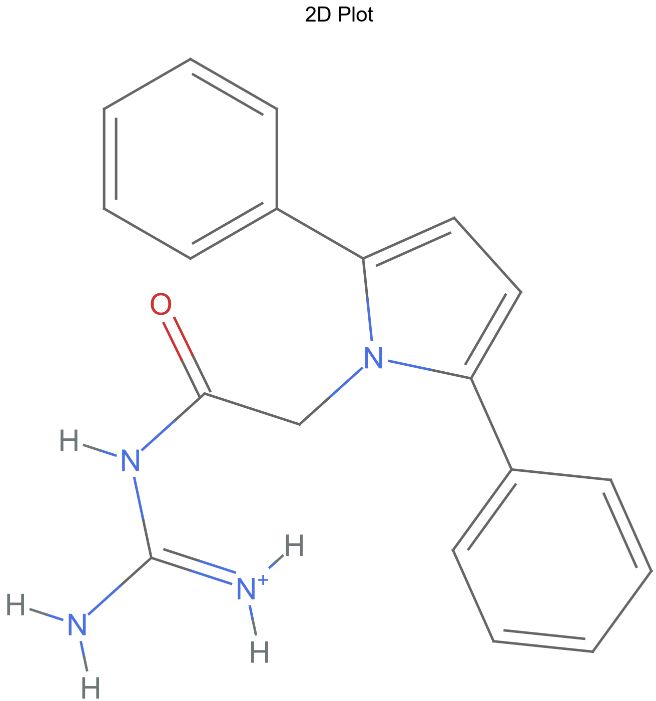

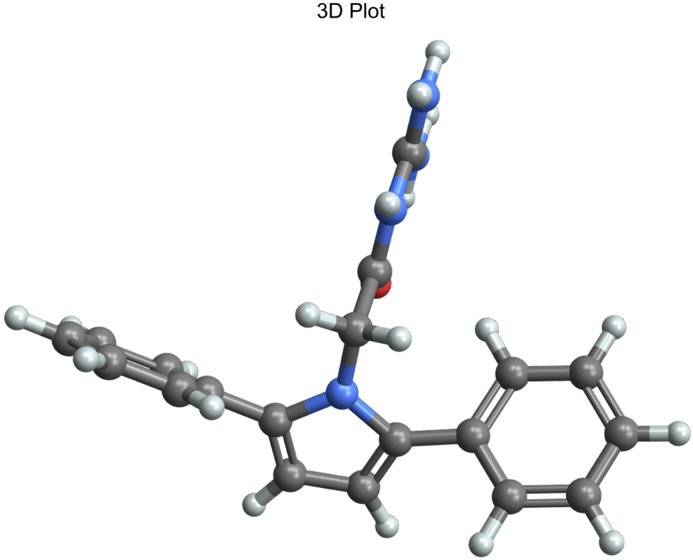

2D Plot

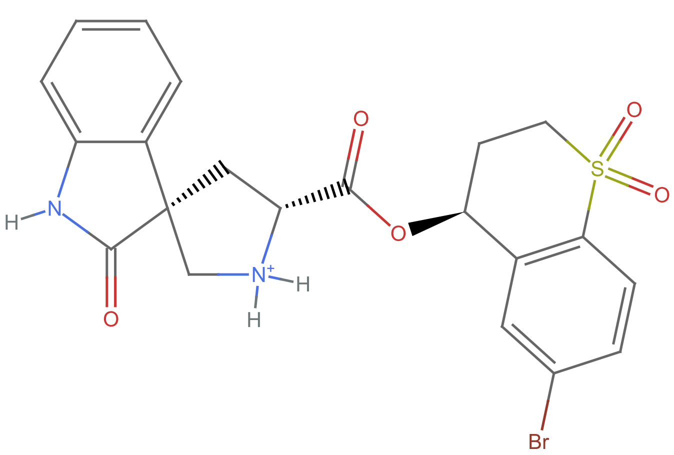

2D Autocorrelation

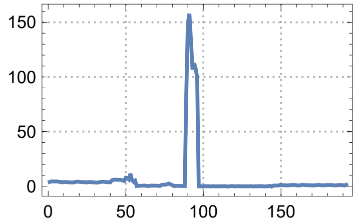

3D Plot

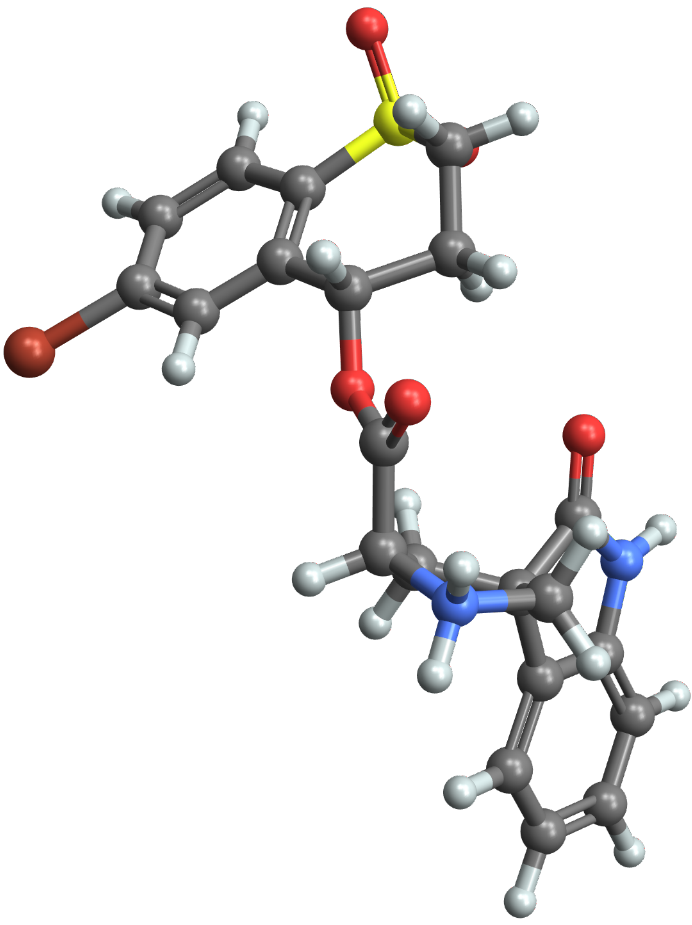

Dataset Index= 158

Molecule Formula= $C_{21}H_{20}BrN_2O_5S^+$

3D Autocorrelation

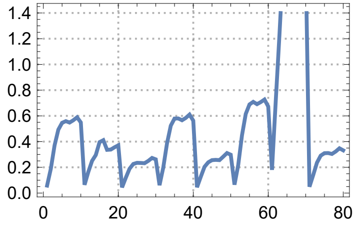

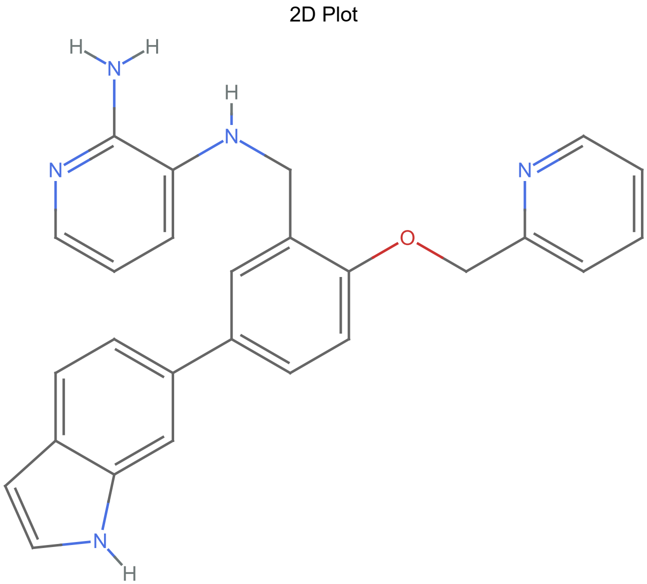

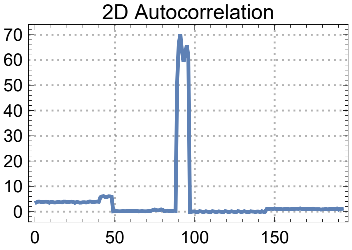

Dataset Index= 159

Molecule Formula= $C_{26}H_{23}N_5O$

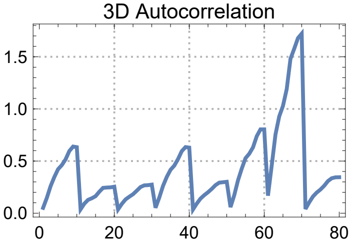

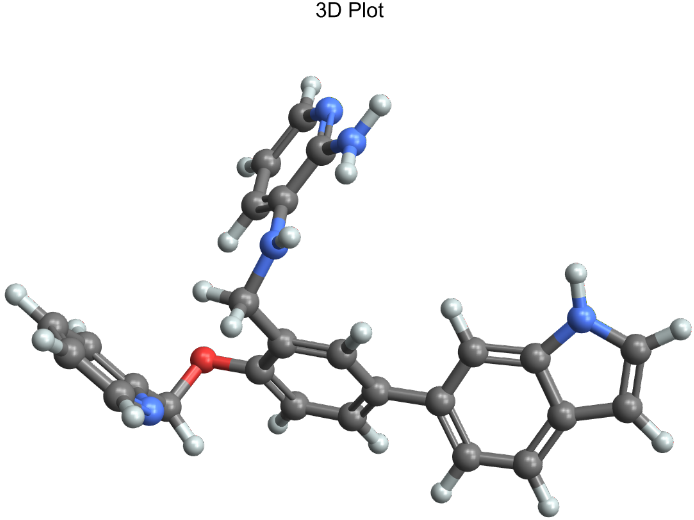

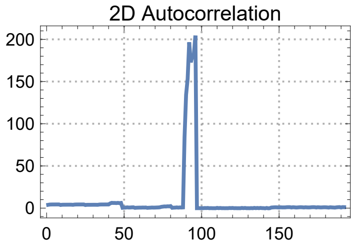

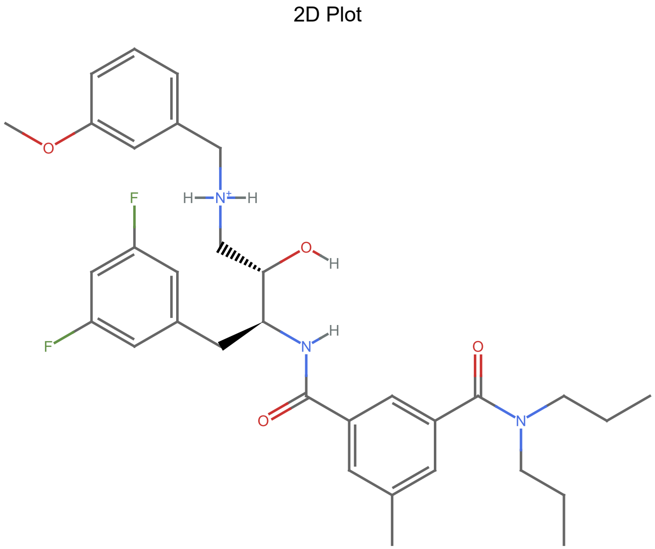

Dataset Index= 160

Molecule Formula= $C_{33}H_{42}F_2N_3O_4^+$

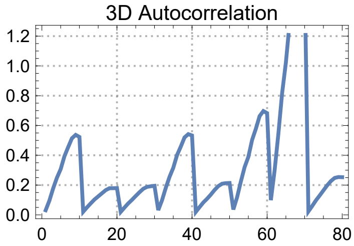

3D Plot

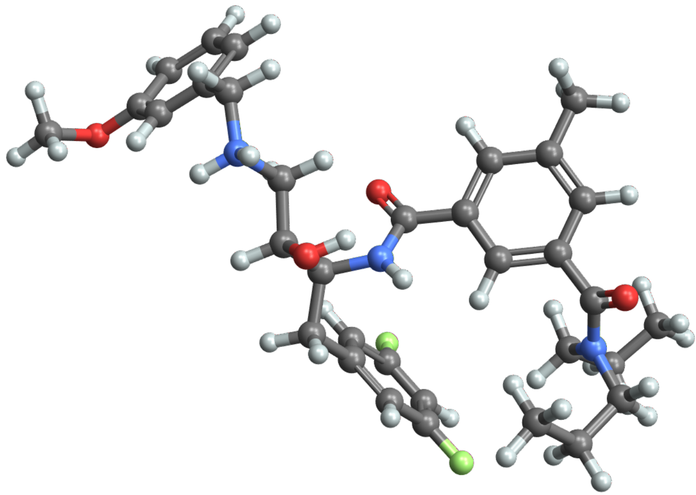

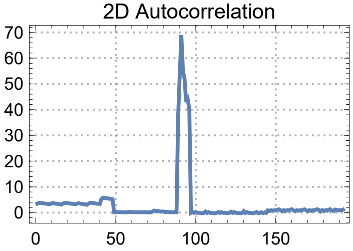

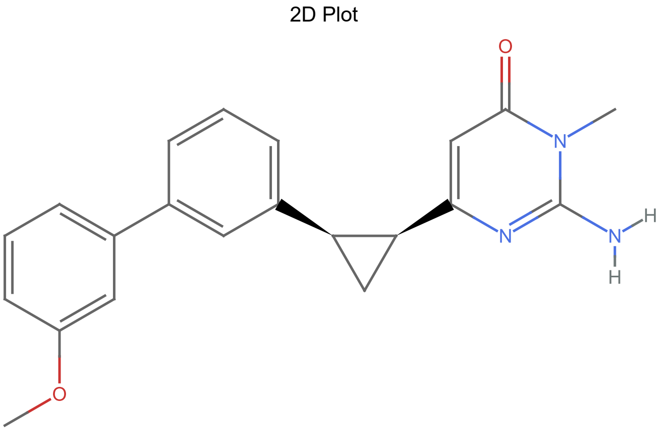

Dataset Index= 161

Molecule Formula= $C_{21}H_{21}N_3O_2$

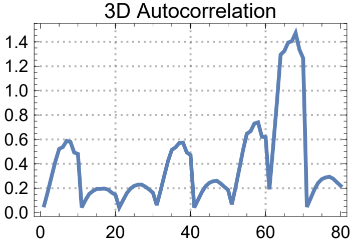

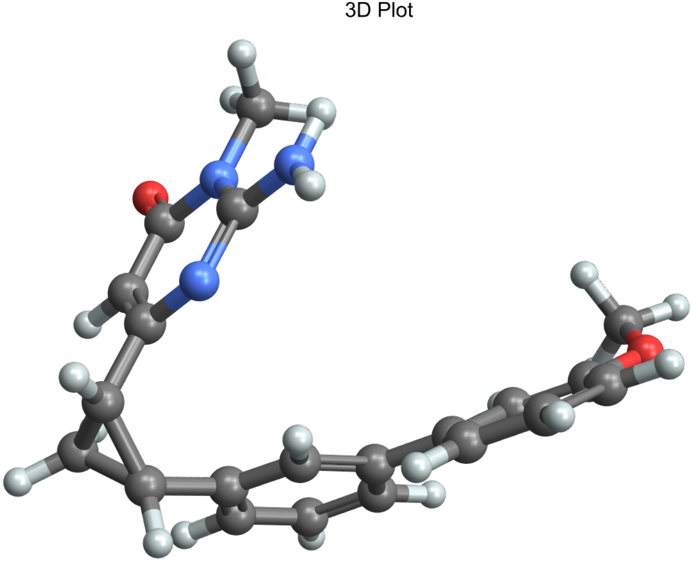

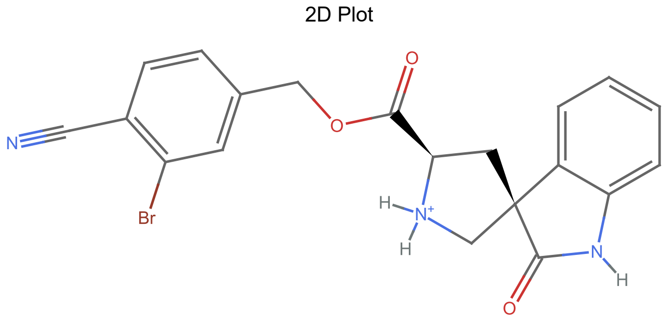

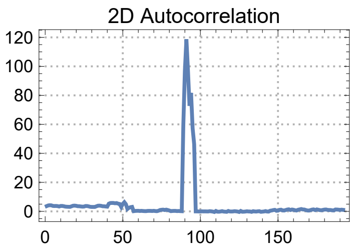

Dataset Index= 162

Molecule Formula= $C_{20}H_{17}BrN_3O_3^+$

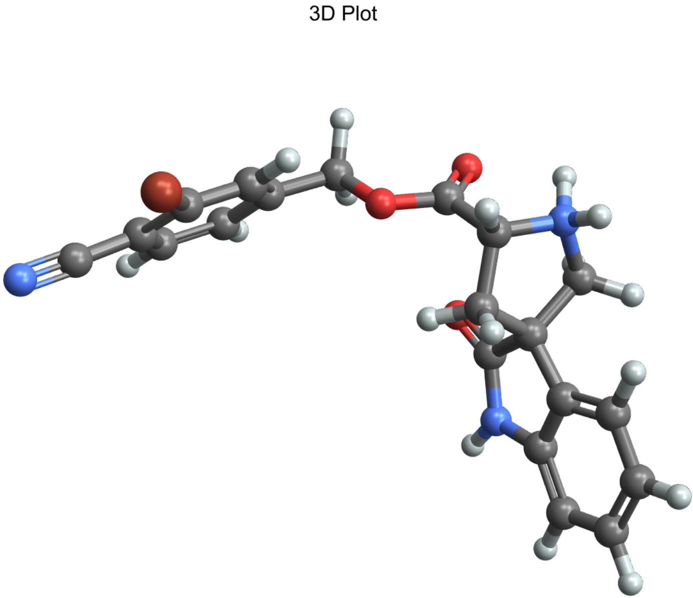

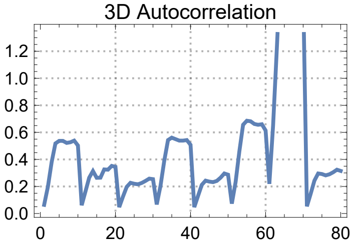

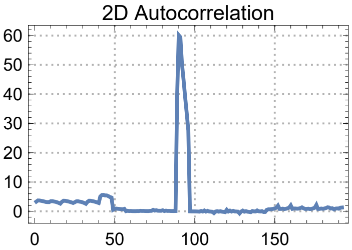

Dataset Index= 163

Molecule Formula= $C_{17}H_{18}ClN_2O^+$

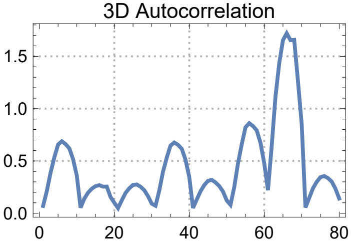

2D Plot

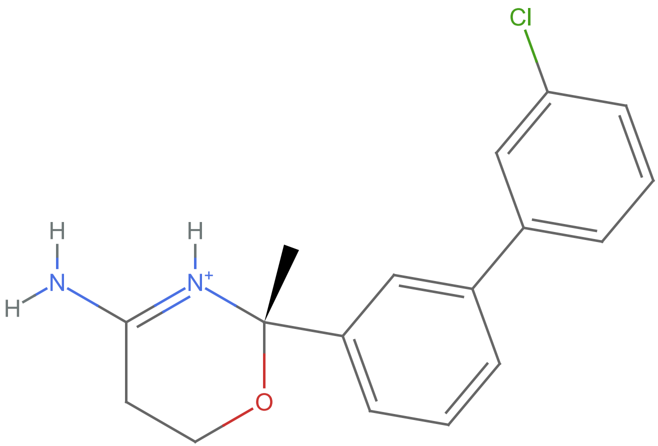

3D Plot

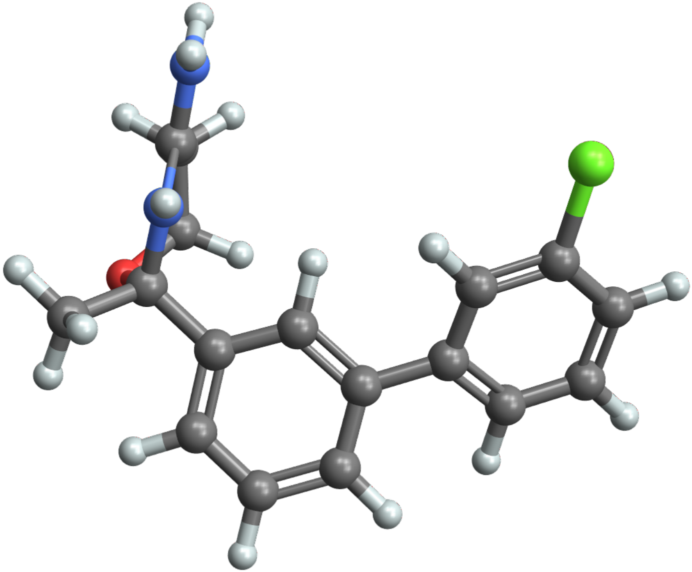

2D Plot

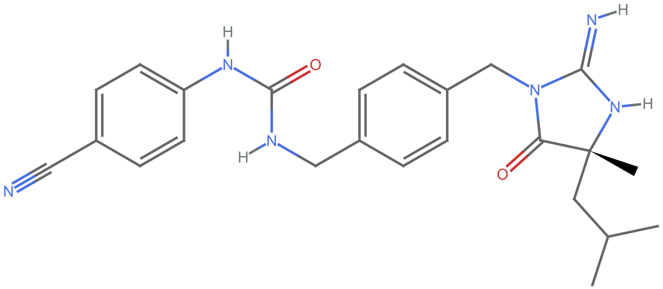

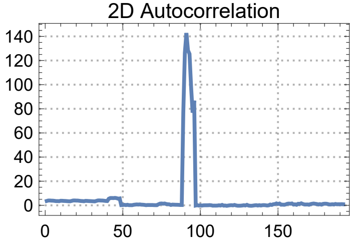

3D Plot

Dataset Index= 164

Molecule Formula= $C_{24}H_{28}N_6O_2$

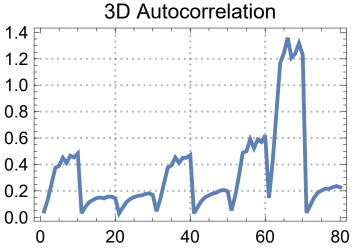

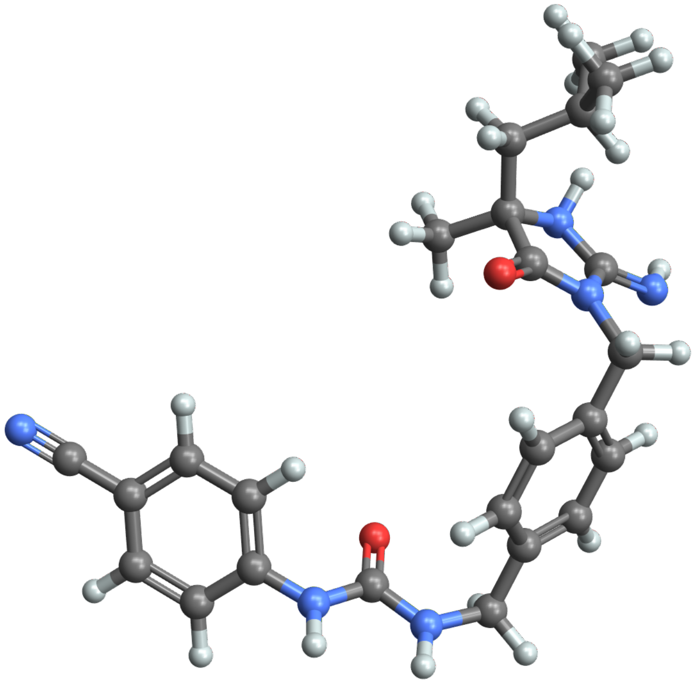

2D Plot

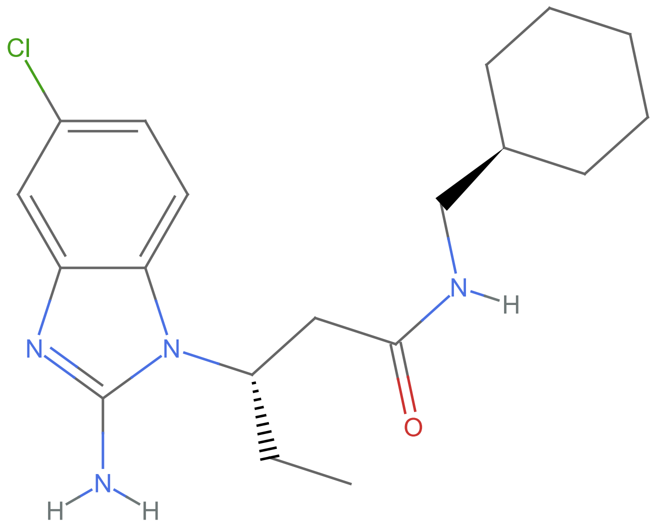

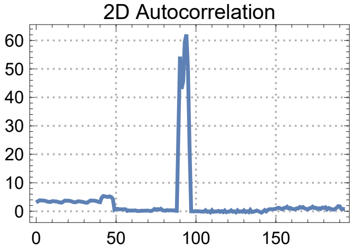

3D Plot

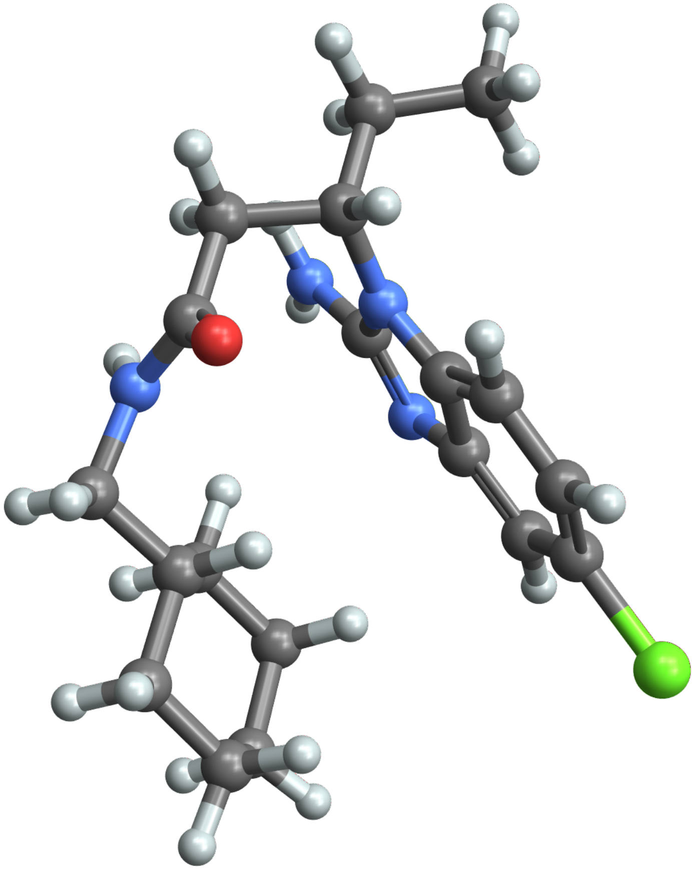

Dataset Index= 165

Molecule Formula= $C_{19}H_{27}ClN_4O$

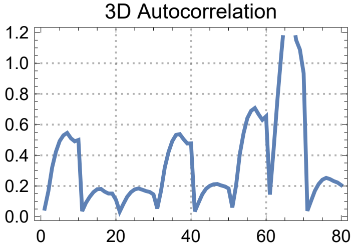

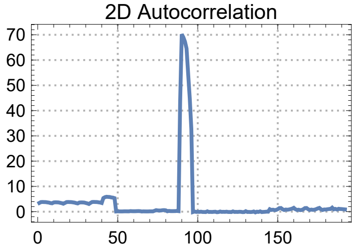

Dataset Index= 166

Molecule Formula= $C_{21}H_{21}N_4O^+$

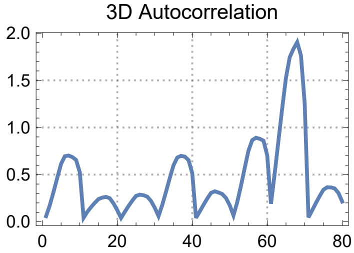

2D Plot

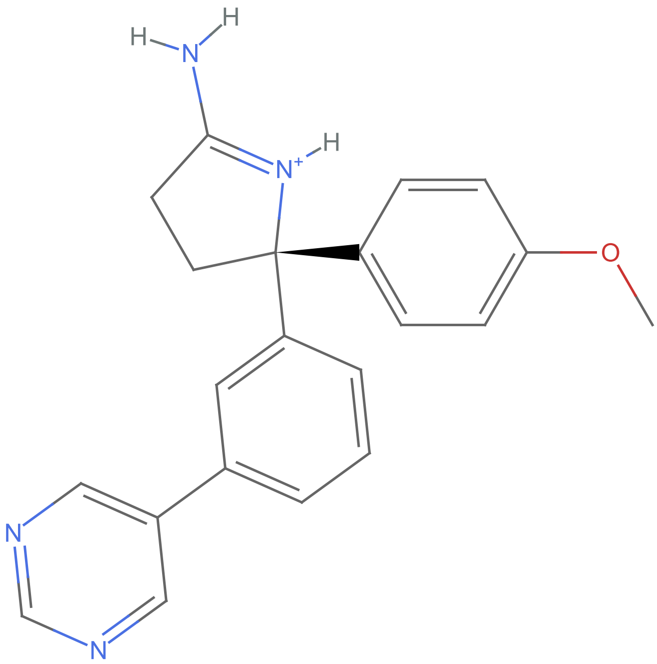

3D Plot

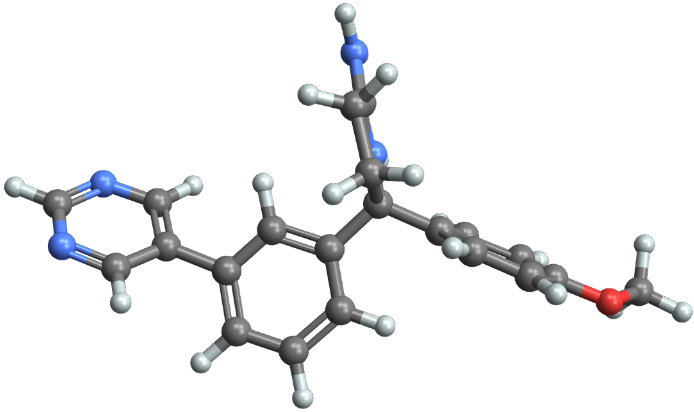

2D Plot

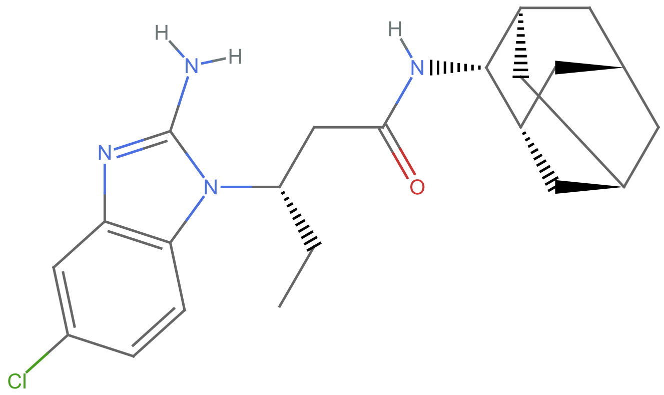

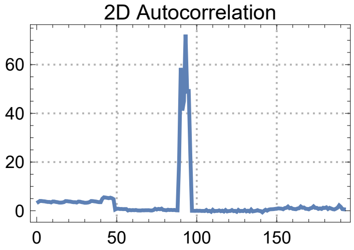

3D Plot

Dataset Index= 167

Molecule Formula= $C_{22}H_{29}ClN_4O$

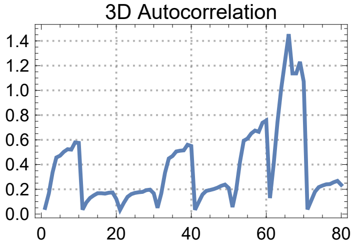

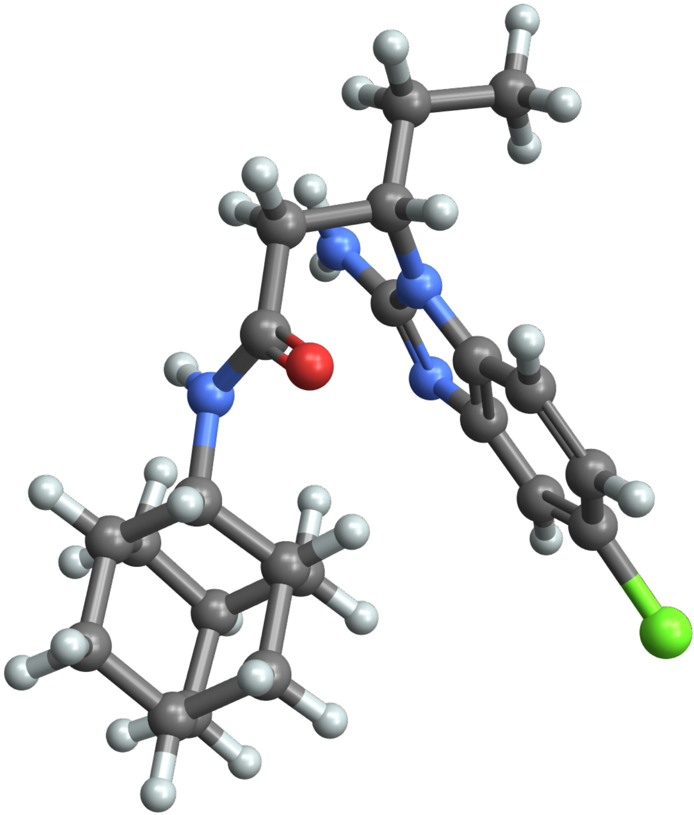

2D Plot

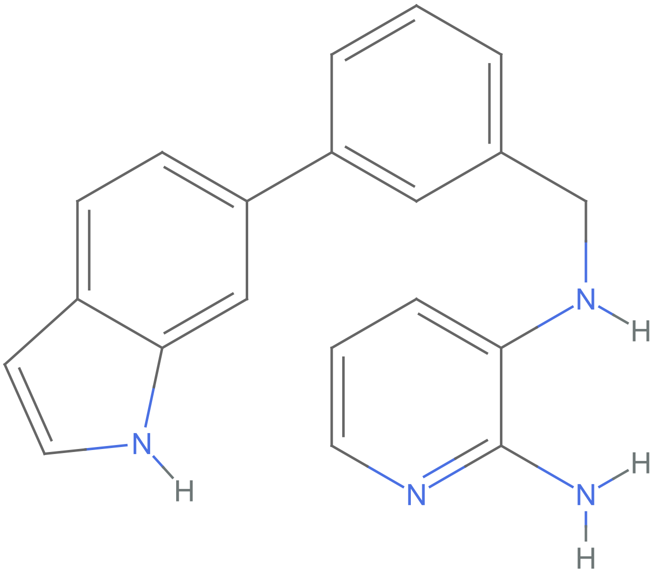

2D Autocorrelation

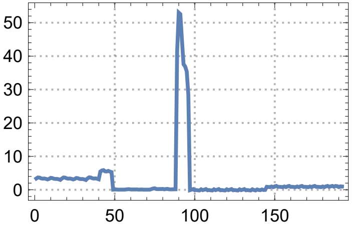

Dataset Index= 168

Molecule Formula= $C_{20}H_{18}N_4$

3D Plot

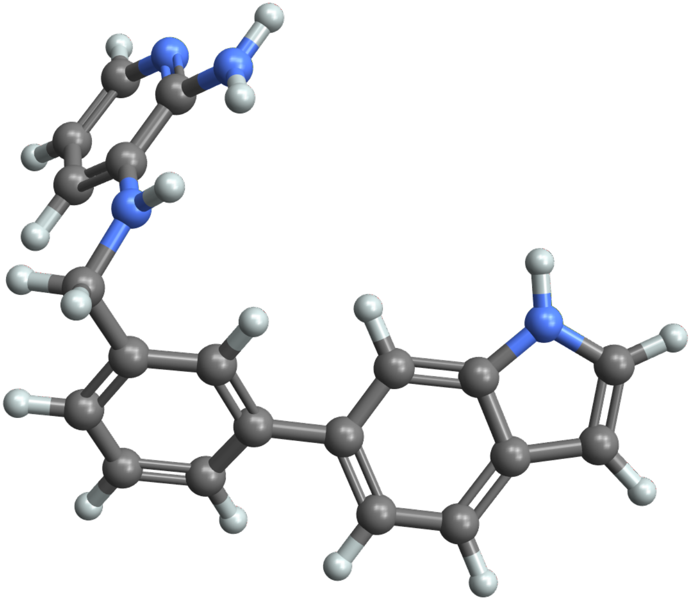

3D Autocorrelation

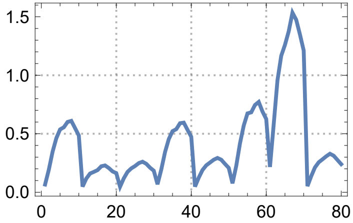

2D Plot

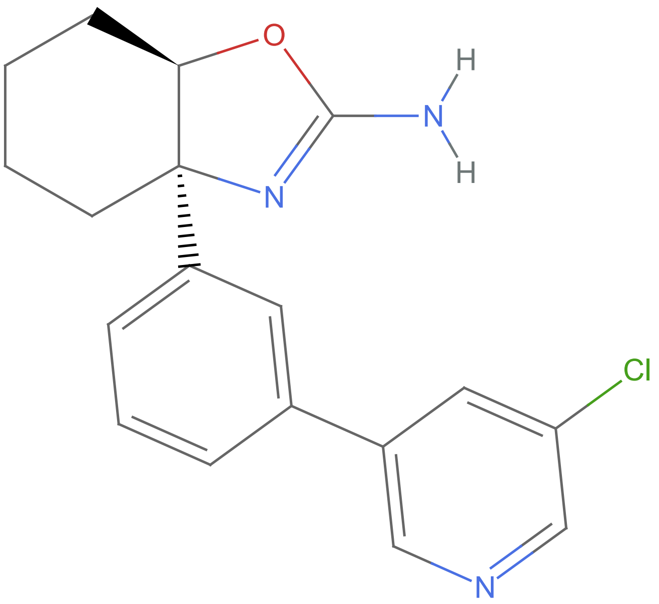

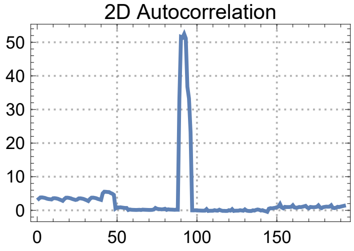

Dataset Index= 169

Molecule Formula= $C_{18}H_{18}ClN_{3}O$

3D Plot

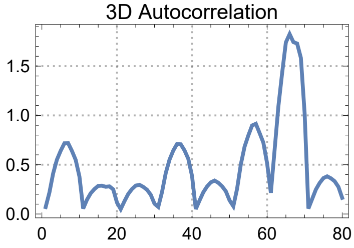

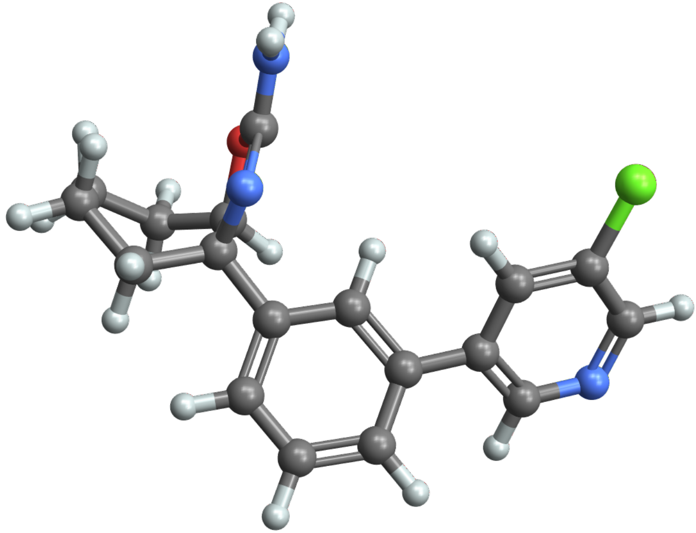

2D Autocorrelation

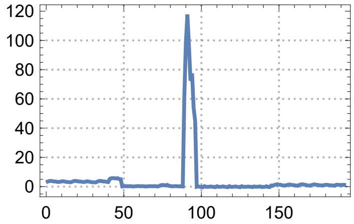

Dataset Index= 170

Molecule Formula= $C_{20}H_{18}N_3O_3^+$

3D Autocorrelation

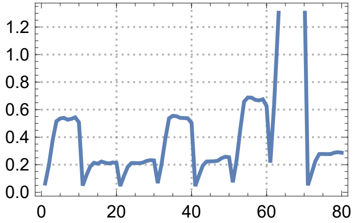

2D Plot

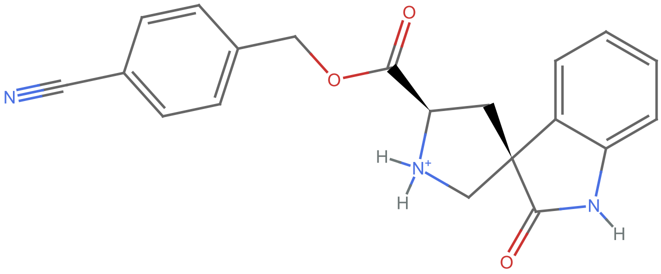

3D Plot

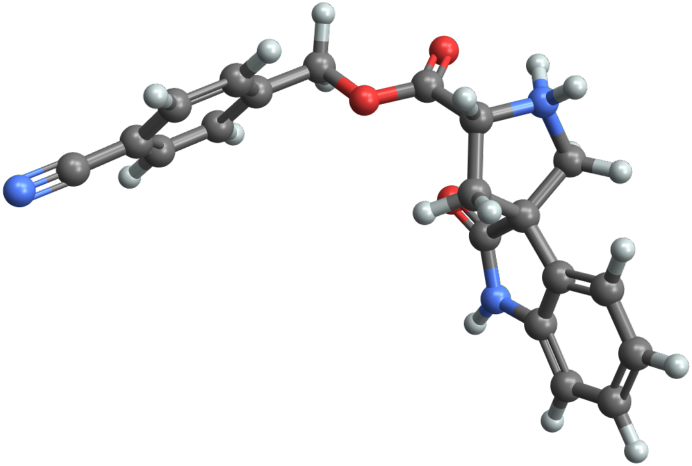

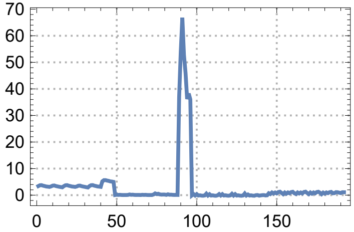

Dataset Index= 171

Molecule Formula= $C_{21}H_{21}N_3O$

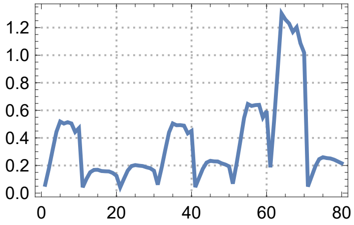

2D Plot

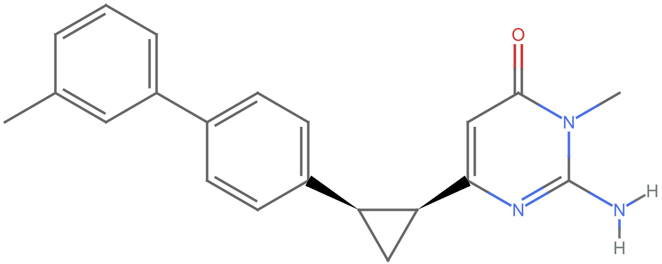

3D Plot

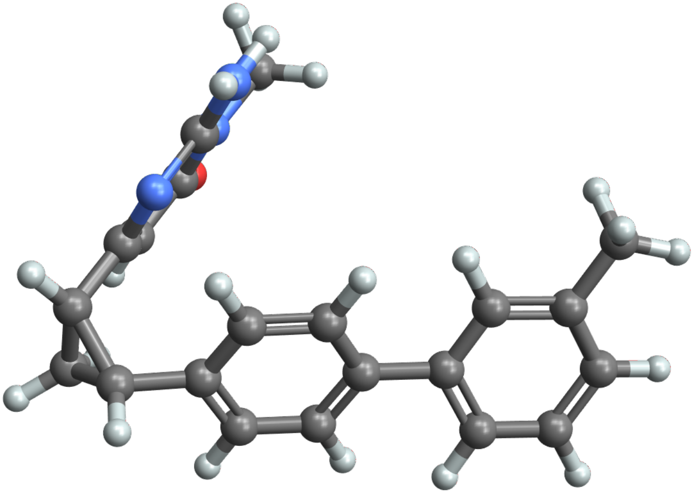

2D Plot

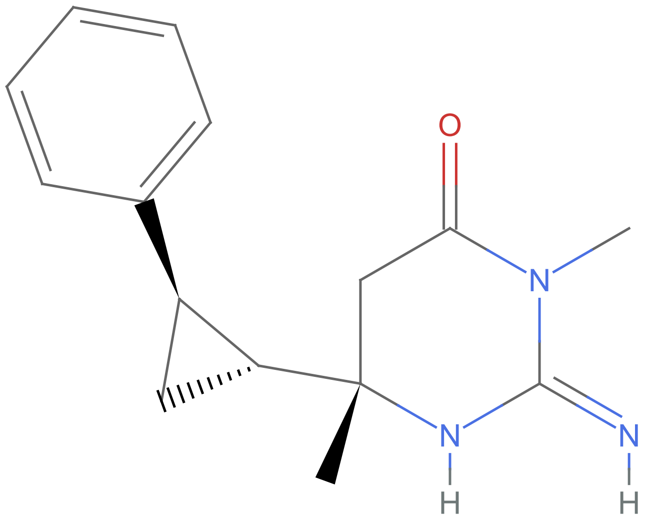

2D Autocorrelation

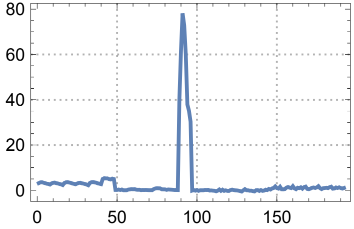

3D Plot

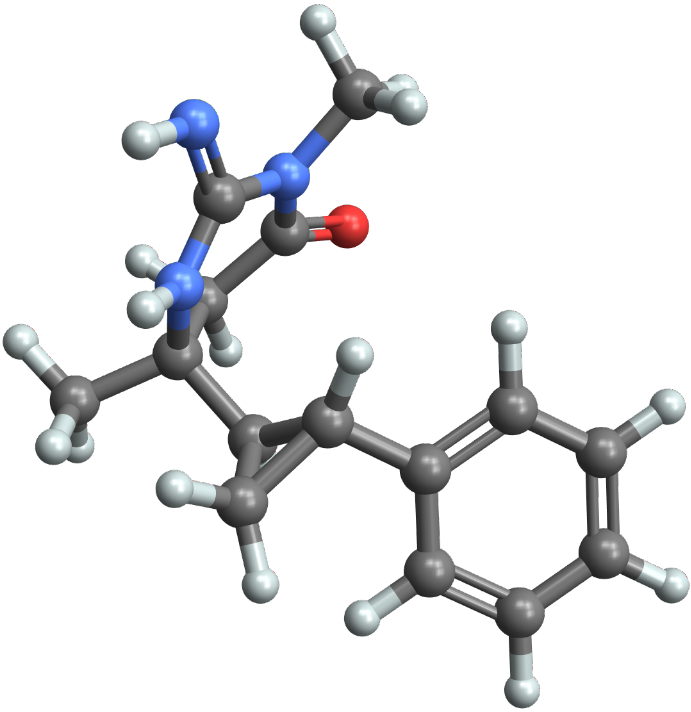

Dataset Index= 172

Molecule Formula= $C_{15}H_{19}N_3O$

3D Autocorrelation

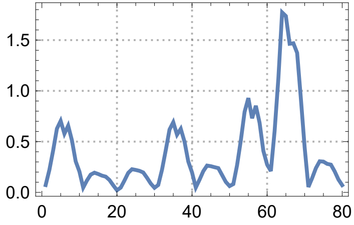

2D Plot

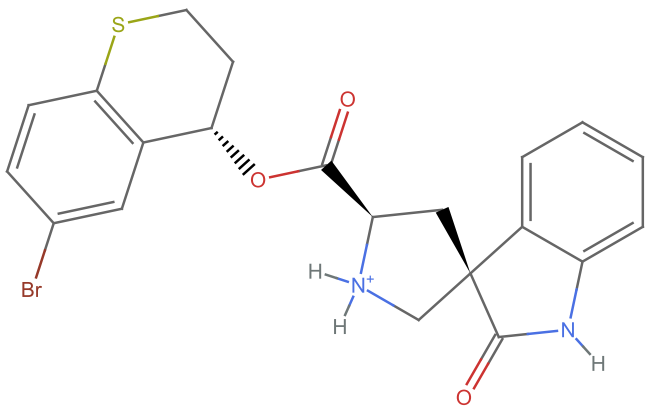

2D Autocorrelation

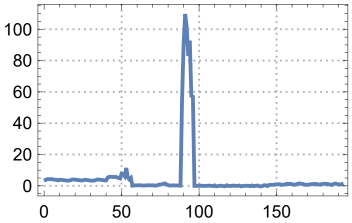

Dataset Index= 173

Molecule Formula= $C_{21}H_{20}BrN_2O_3S^+$

3D Plot

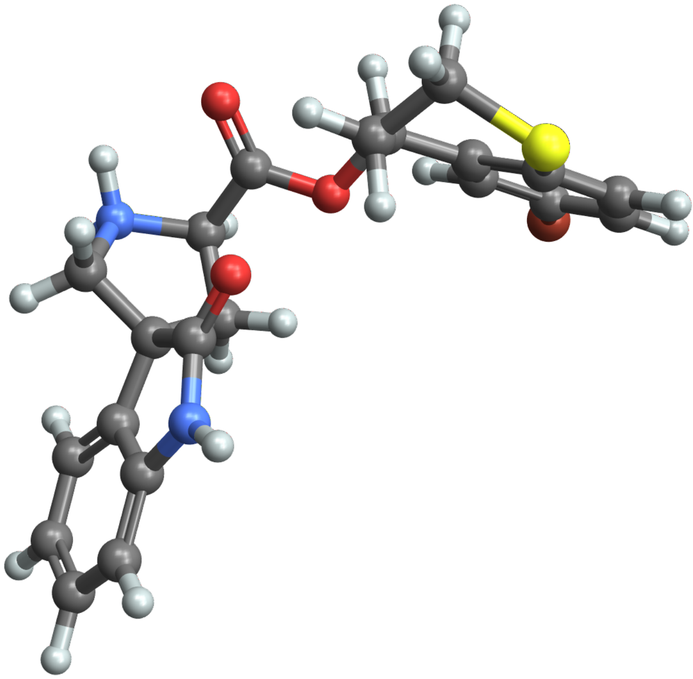

3D Autocorrelation

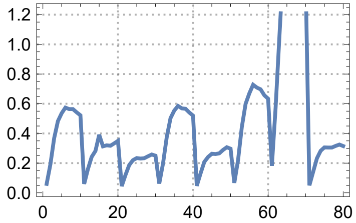

2D Plot

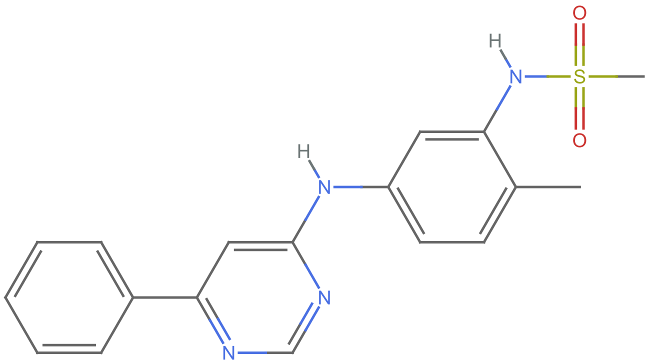

2D Autocorrelation

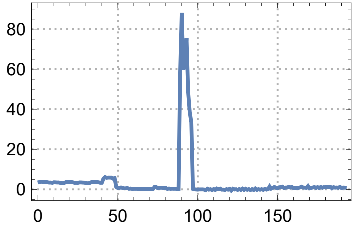

3D Plot

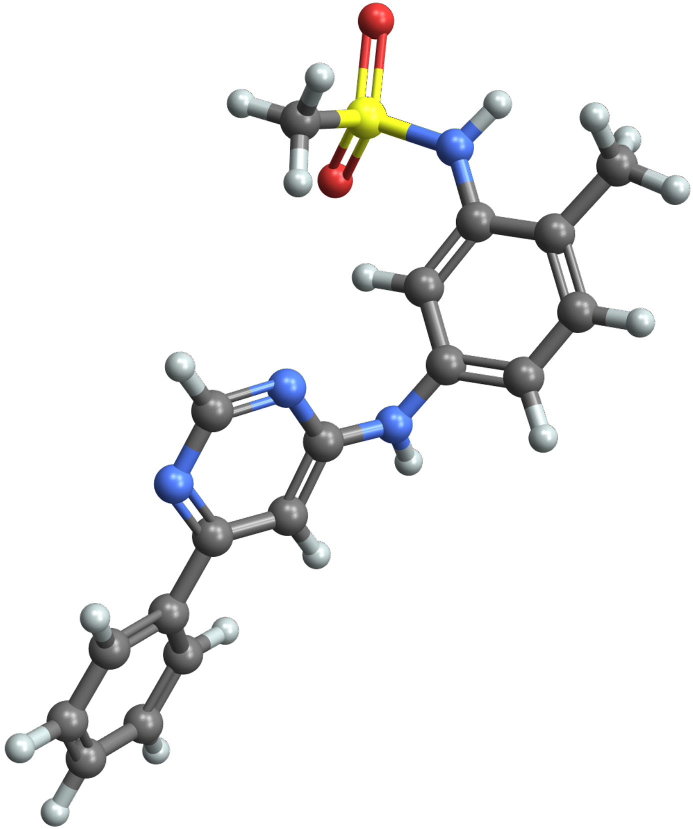

Dataset Index= 174

Molecule Formula= $C_{18}H_{18}N_4O_2S$

3D Autocorrelation

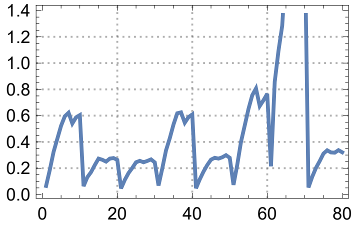

2D Plot

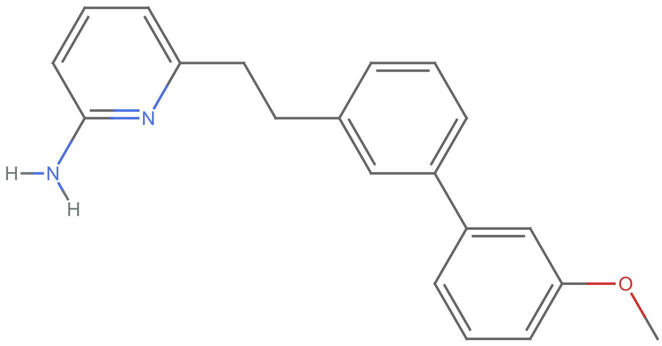

2D Autocorrelation

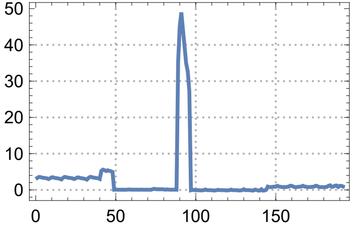

3D Plot

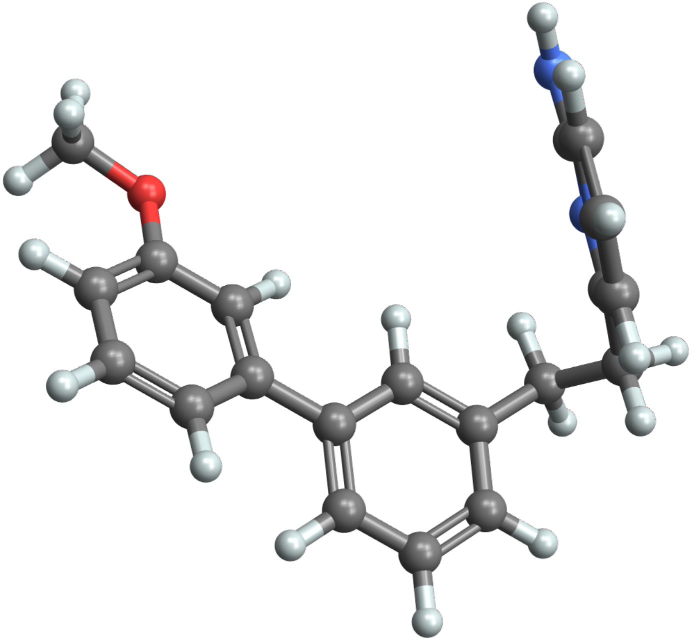

Dataset Index= 175

Molecule Formula= $C_{20}H_{20}N_2O$

3D Autocorrelation

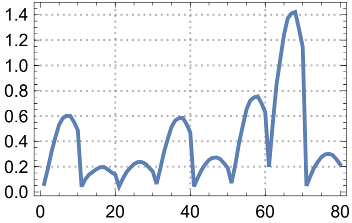

2D Plot

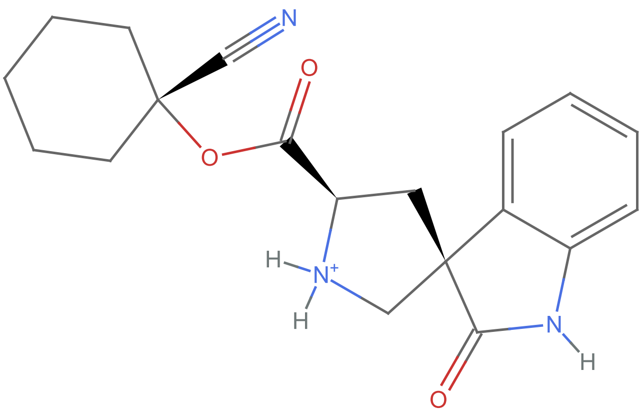

3D Plot

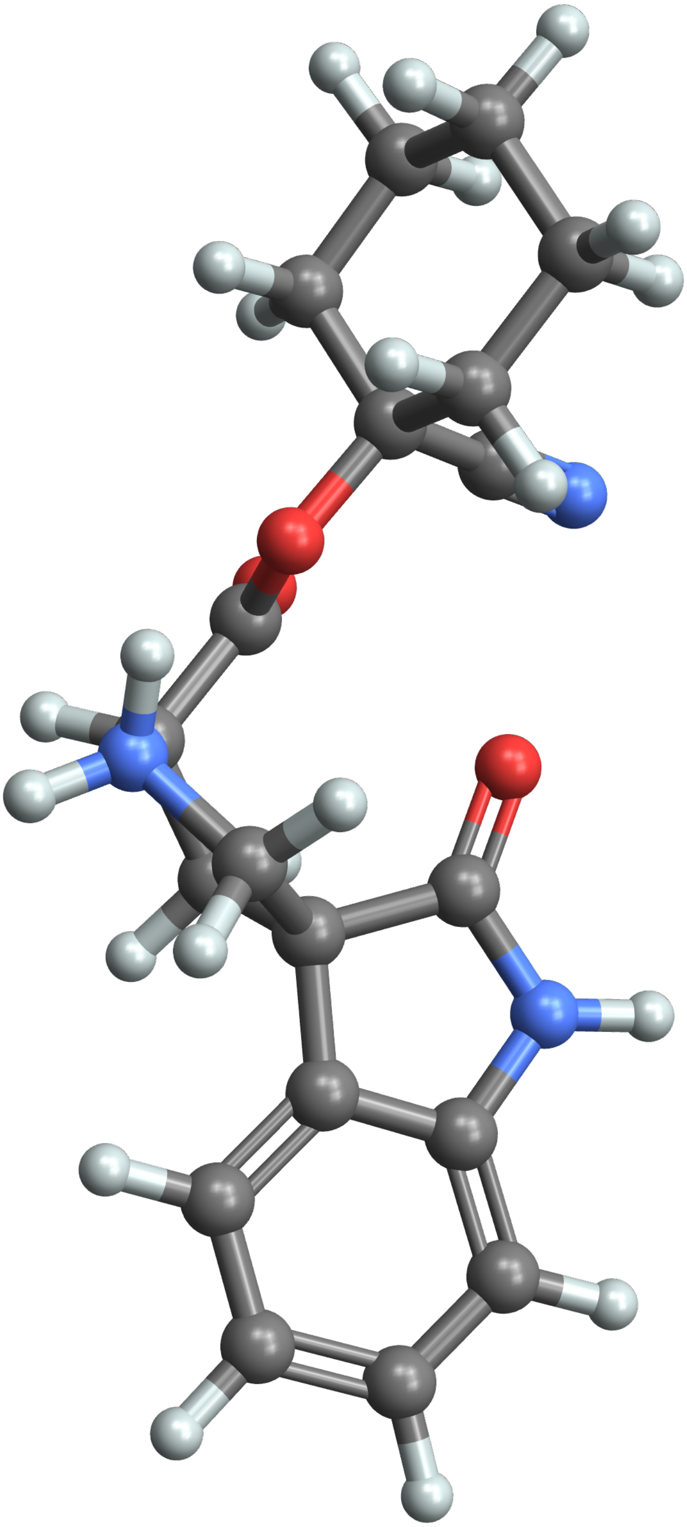

2D Autocorrelation

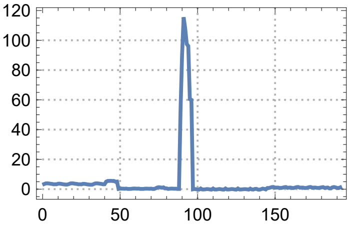

Dataset Index= 176

Molecule Formula= $C_{19}H_{22}N_3O_3^+$

3D Autocorrelation

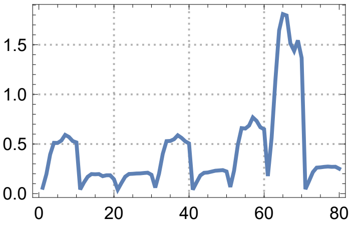

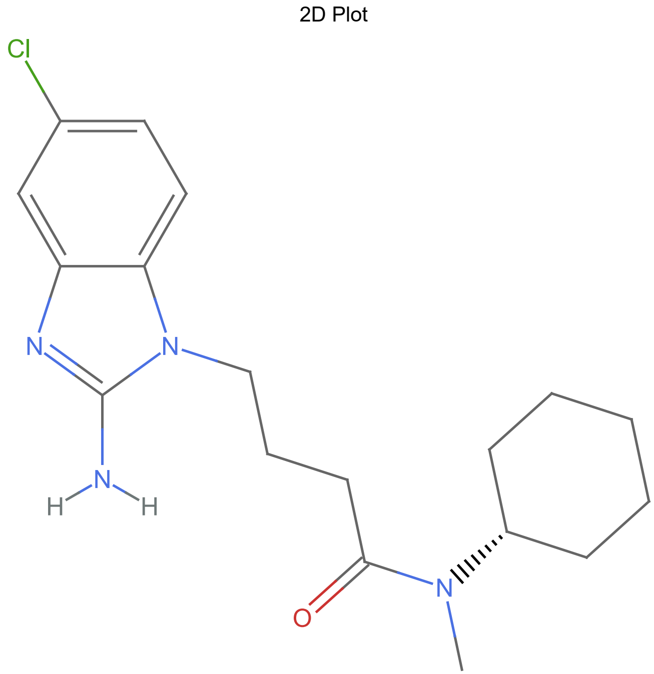

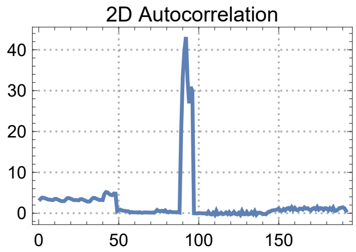

Dataset Index= 177

Molecule Formula= $C_{18}H_{25}ClN_4O$

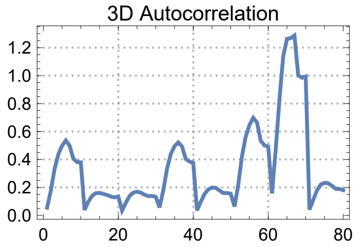

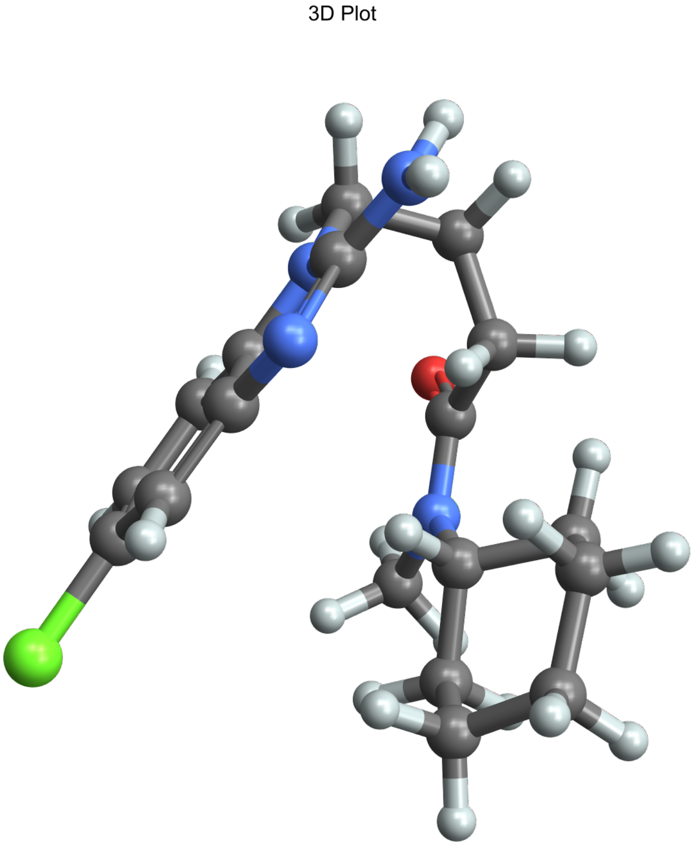

2D Plot

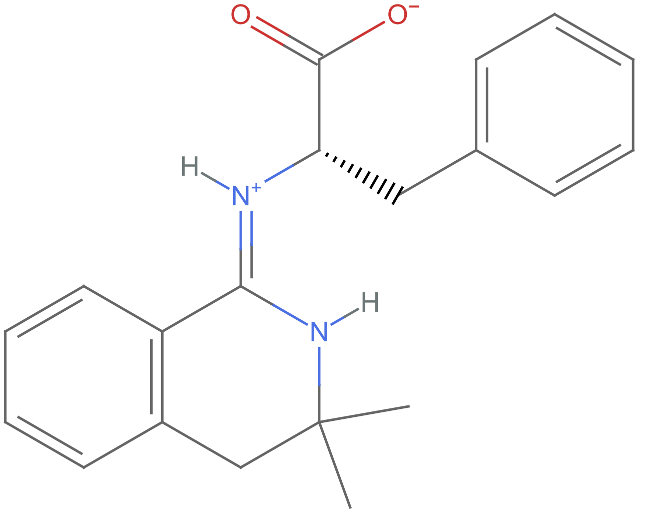

2D Autocorrelation

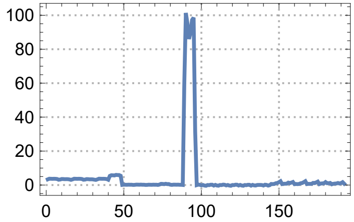

3D Plot

Dataset Index= 178

Molecule Formula= $C_{20}H_{22}N_2O_2$

3D Autocorrelation

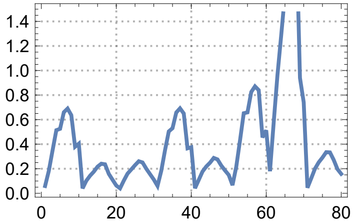

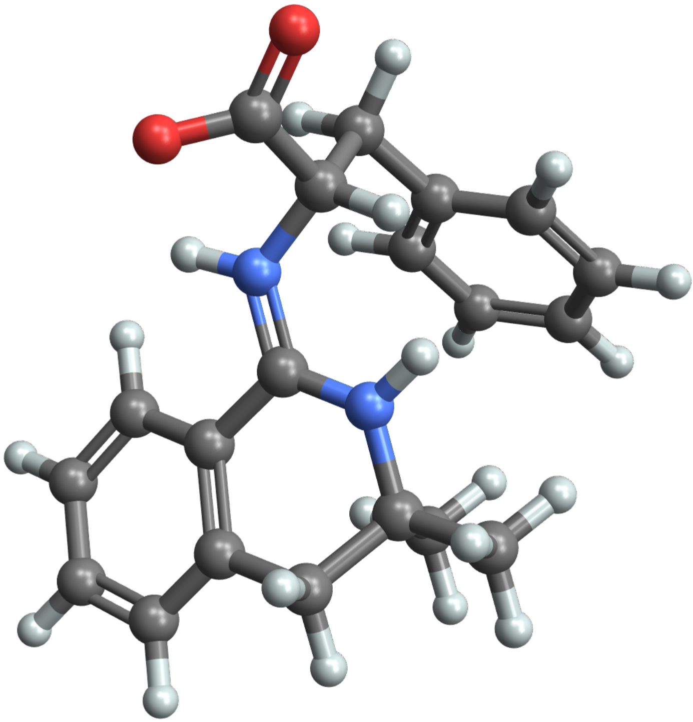

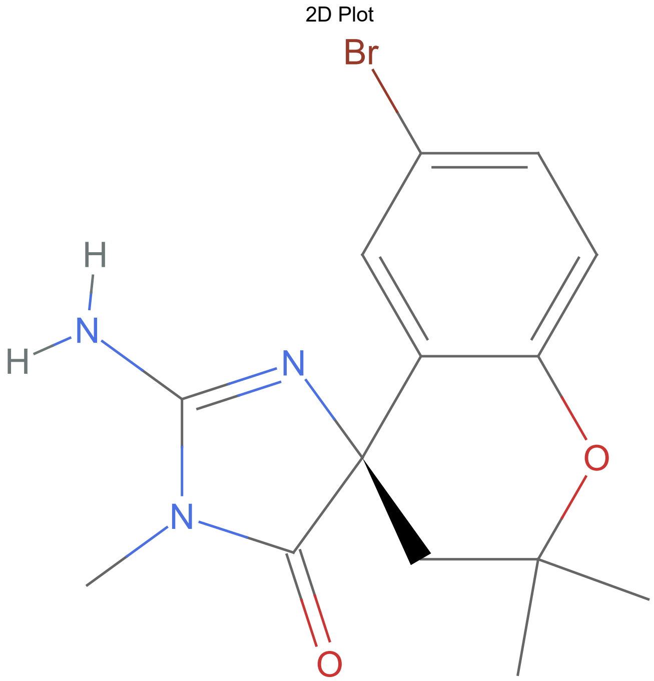

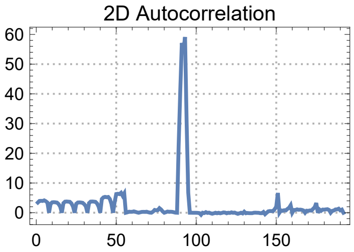

Dataset Index= 179

Molecule Formula= $C_{14}H_{16}BrN_3O_2$

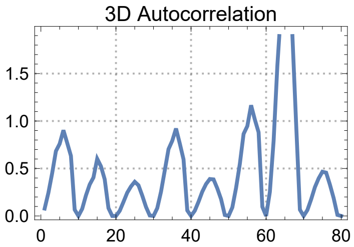

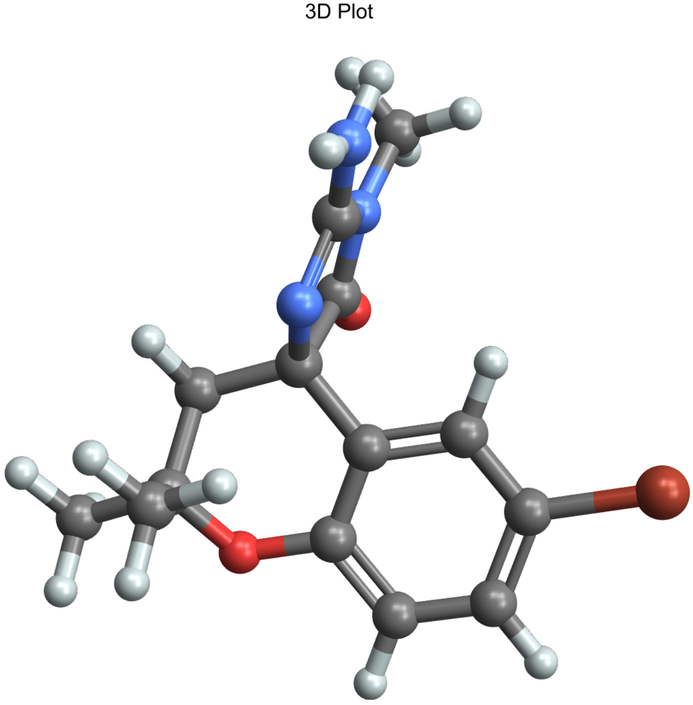

2D Plot

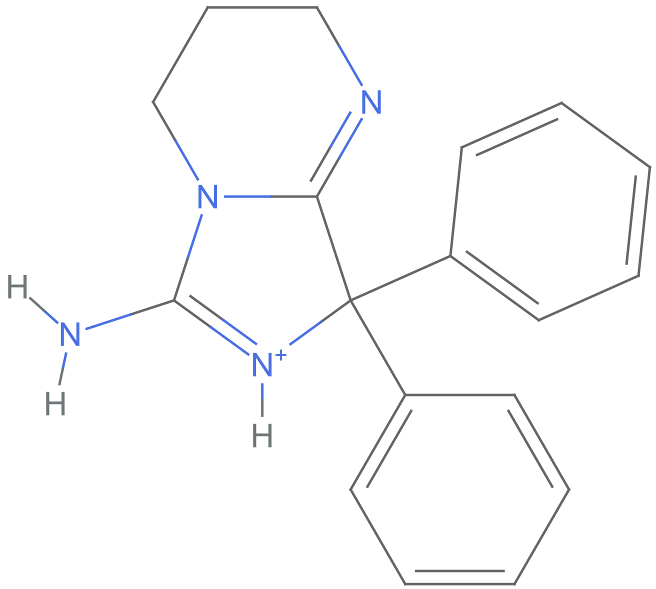

2D Autocorrelation

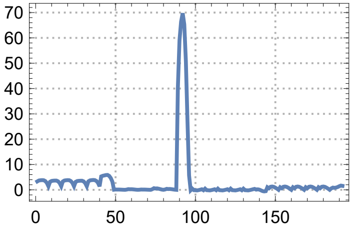

Dataset Index= 180

Molecule Formula= $C_{18}H_{19}N_4^+$

3D Autocorrelation

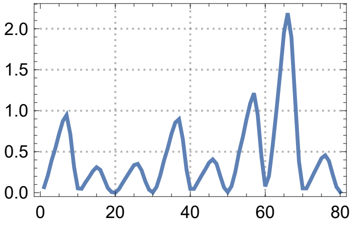

3D Plot

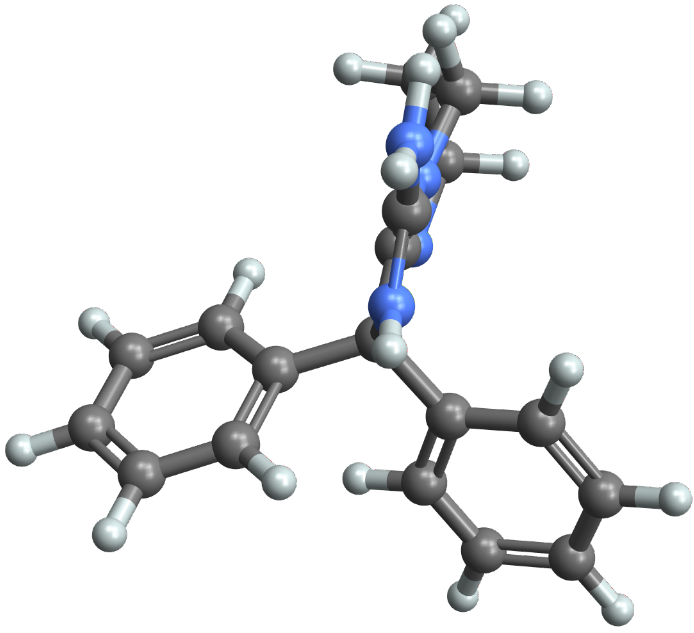

2D Plot

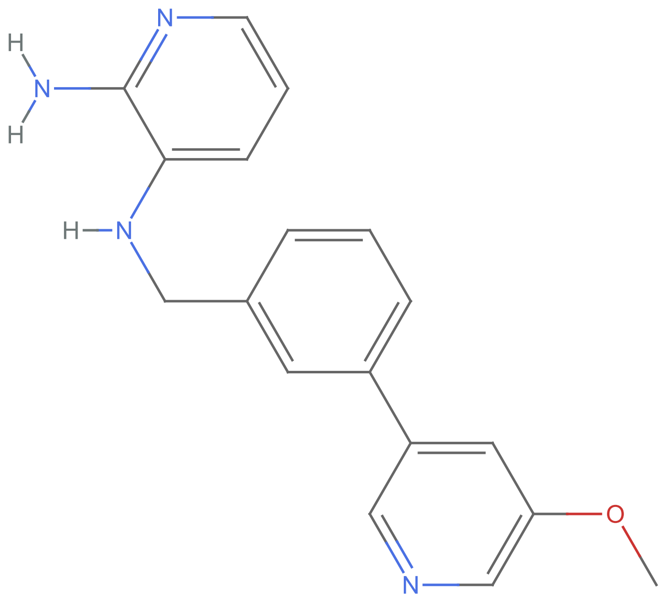

2D Autocorrelation

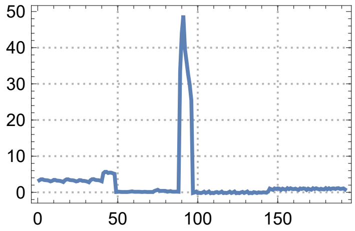

Dataset Index= 181

Molecule Formula= $C_{18}H_{18}N_4O$

3D Plot

3D Autocorrelation

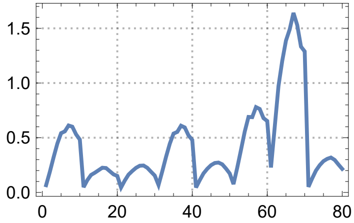

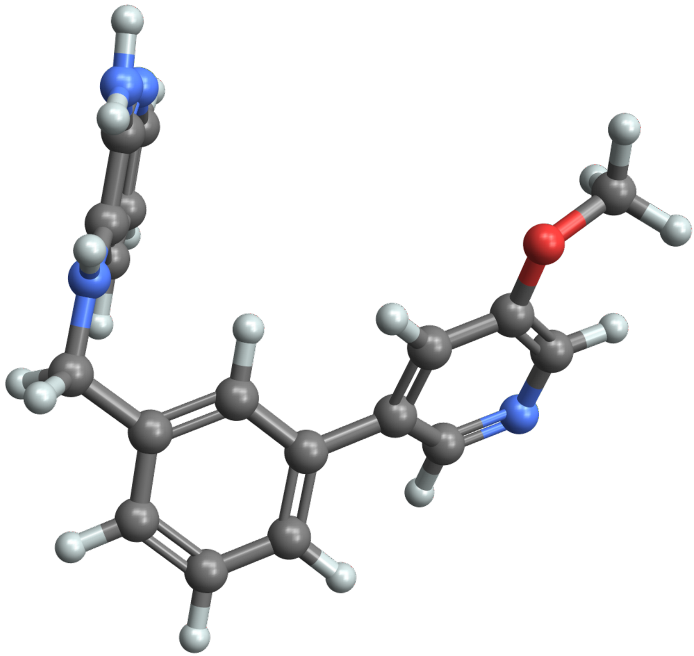

2D Autocorrelation

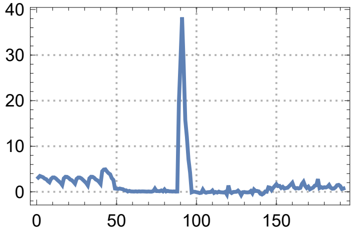

Dataset Index= 182

Molecule Formula= $C_{12}H_{16}N_2OS$

3D Autocorrelation

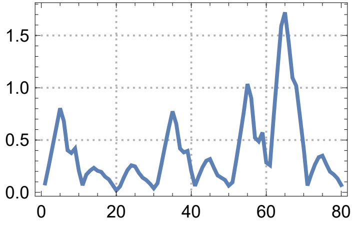

2D Plot

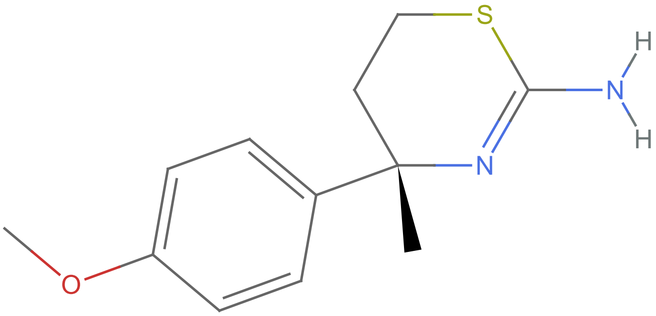

3D Plot

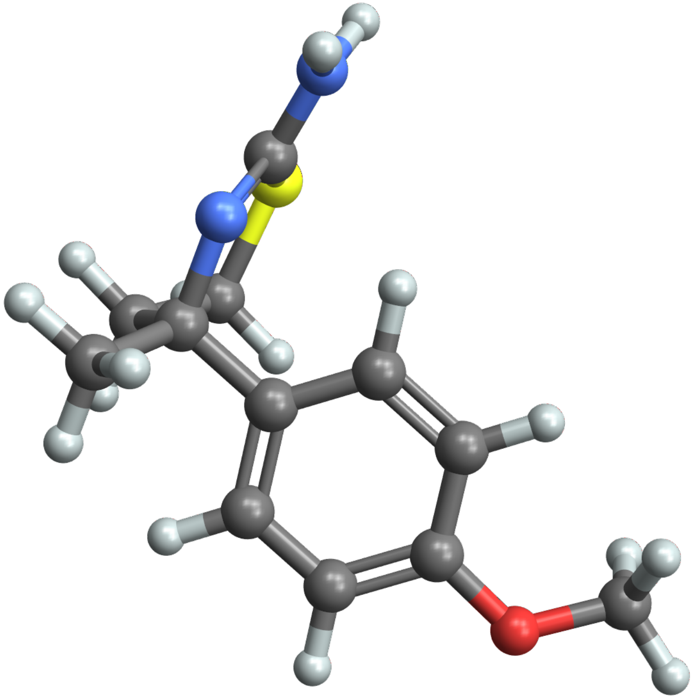

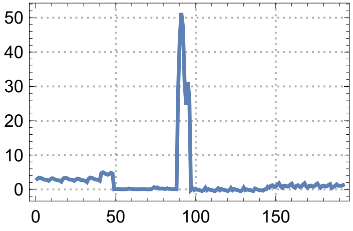

Dataset Index= 183

Molecule Formula= $C_{14}H_{19}N_3O$

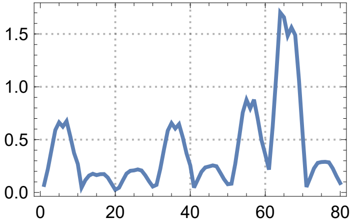

2D Plot

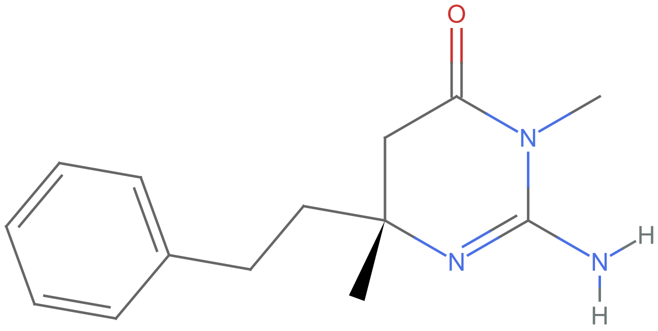

3D Plot

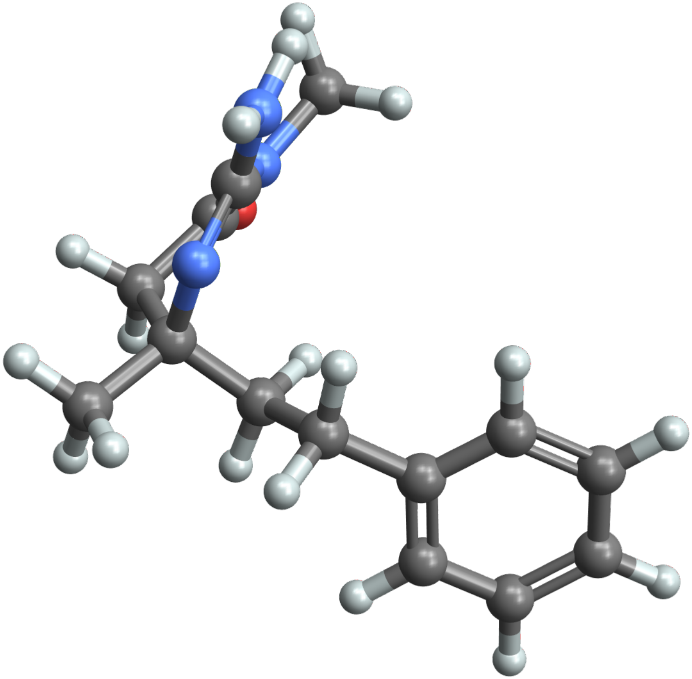

2D Plot

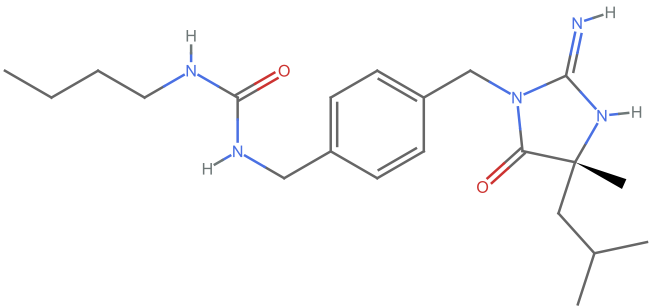

2D Autocorrelation

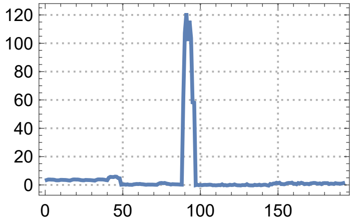

3D Plot

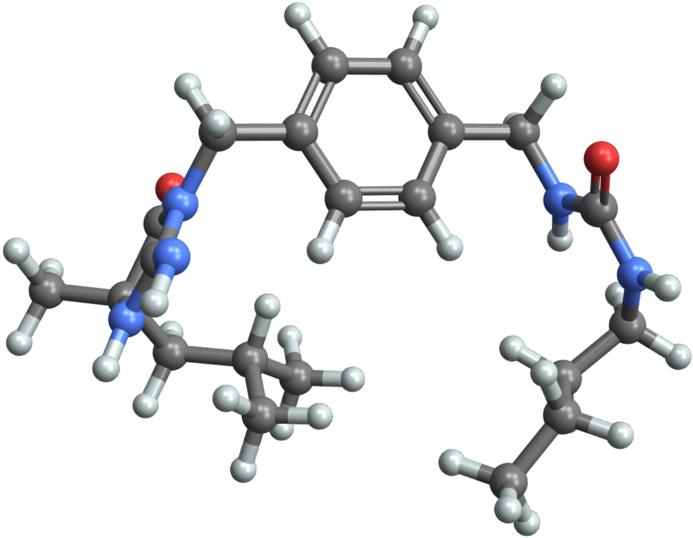

```
Dataset Index=        184

Molecule Formula=     C21H33N5O2
```

3D Autocorrelation

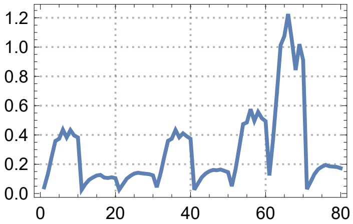

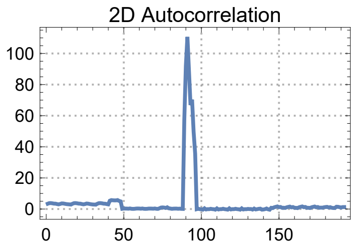

Dataset Index= 185

Molecule Formula= $C_{19}H_{19}N_2O_3^+$

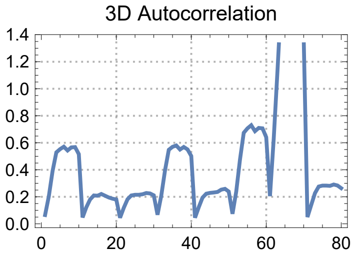

2D Plot

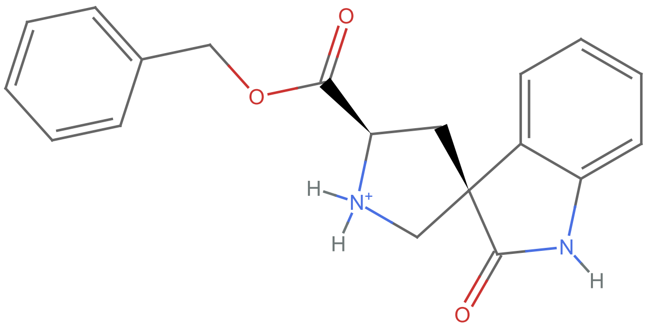

3D Plot

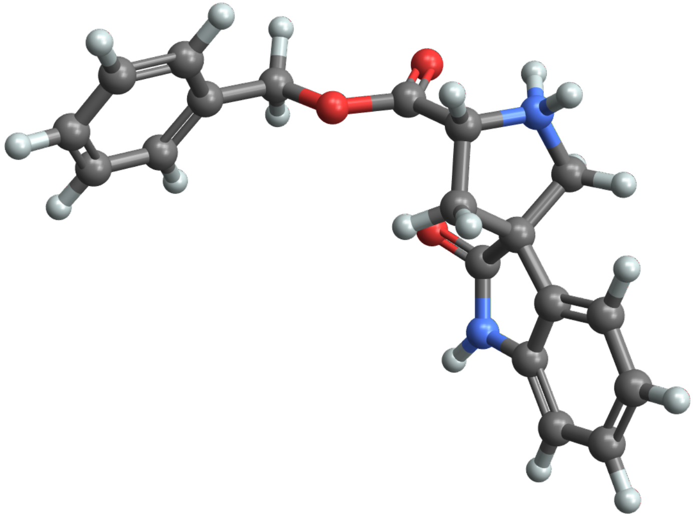

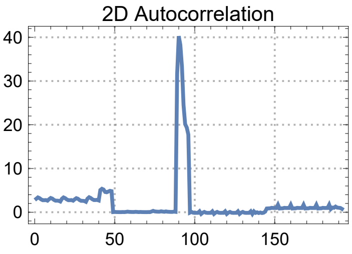

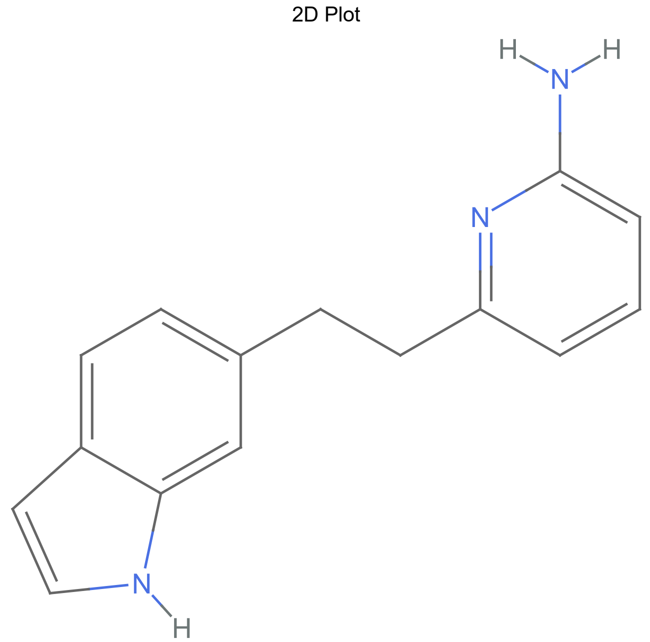

Dataset Index= 186

Molecule Formula= $C_{15}H_{15}N_3$

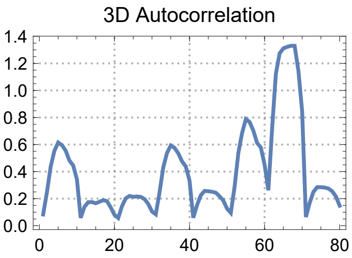

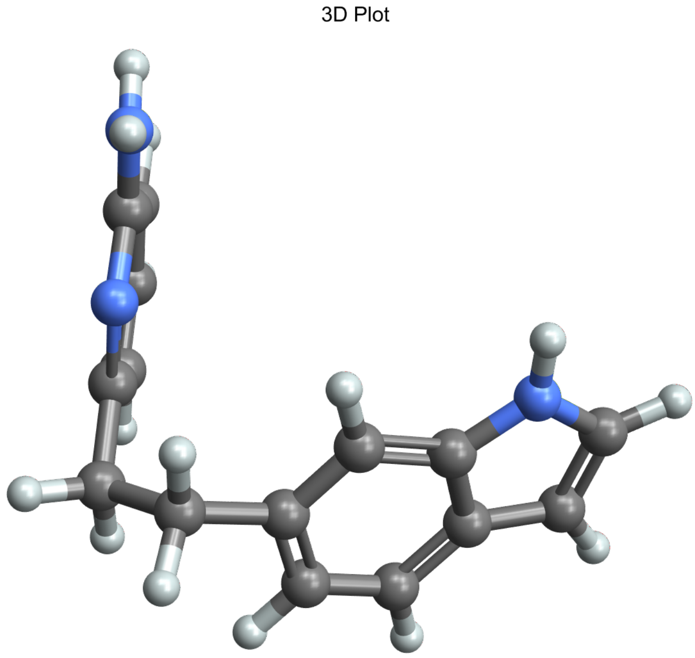

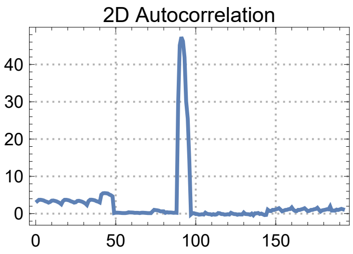

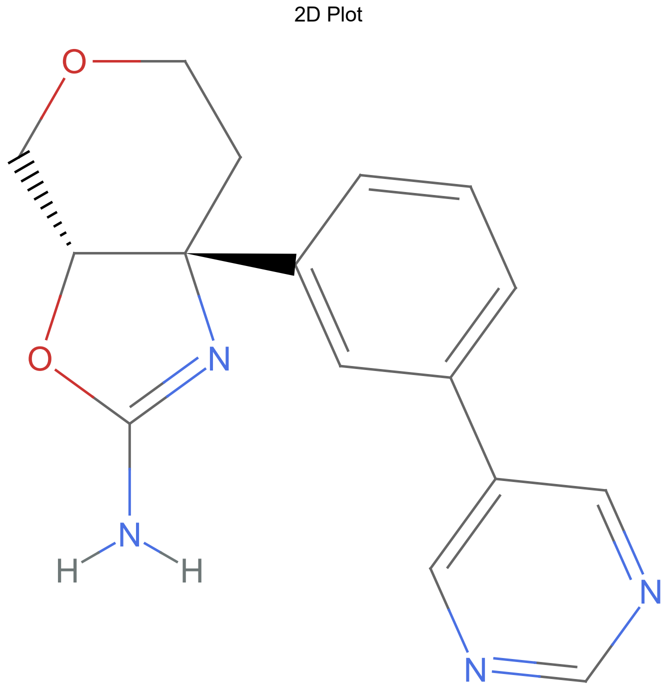

Dataset Index= 187

Molecule Formula= $C_{16}H_{16}N_4O_2$

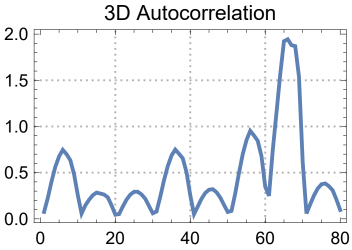

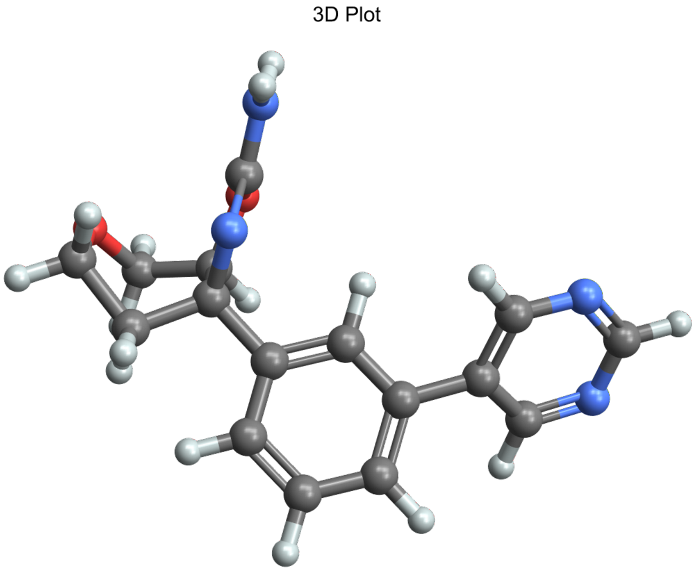

2D Plot

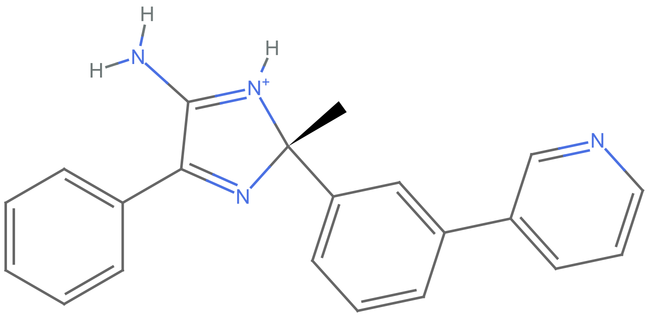

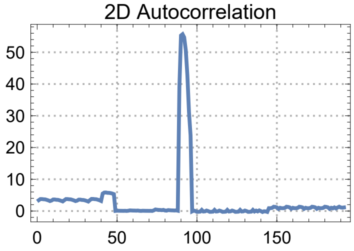

3D Plot

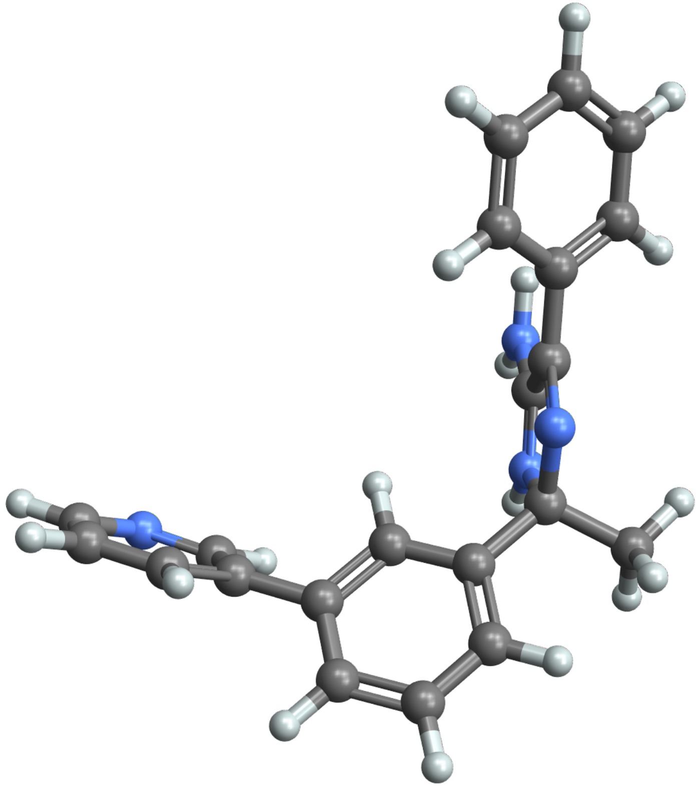

Dataset Index= 188

Molecule Formula= $C_{21}H_{19}N_4^{+}$

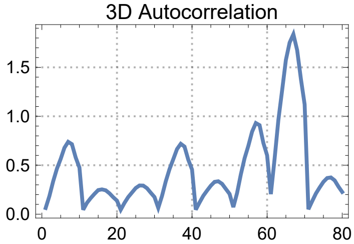

2D Autocorrelation

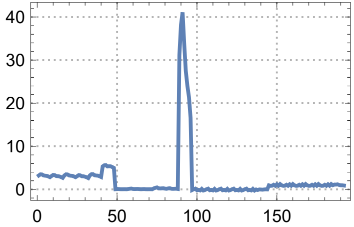

Dataset Index= 189

Molecule Formula= $C_{17}H_{16}N_4$

3D Autocorrelation

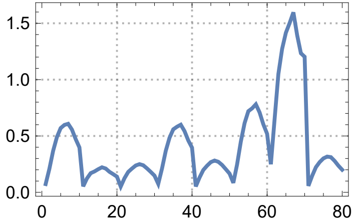

2D Plot

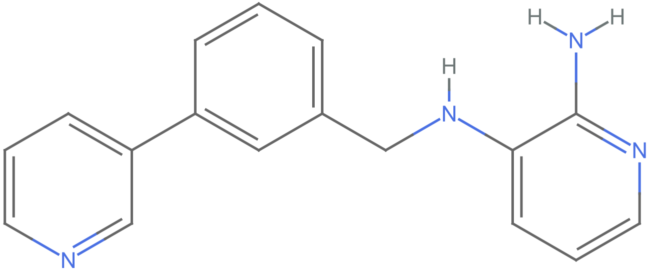

3D Plot

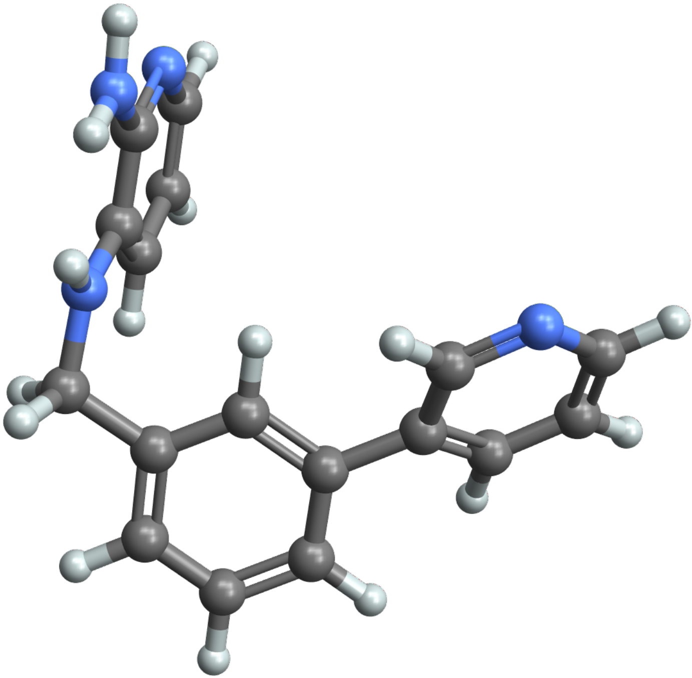

2D Plot

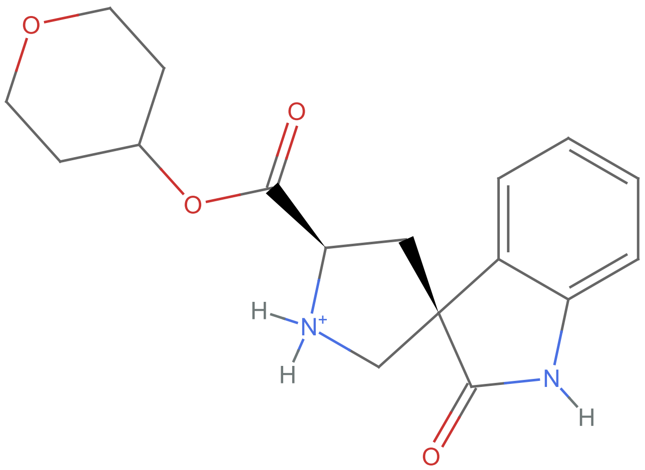

3D Plot

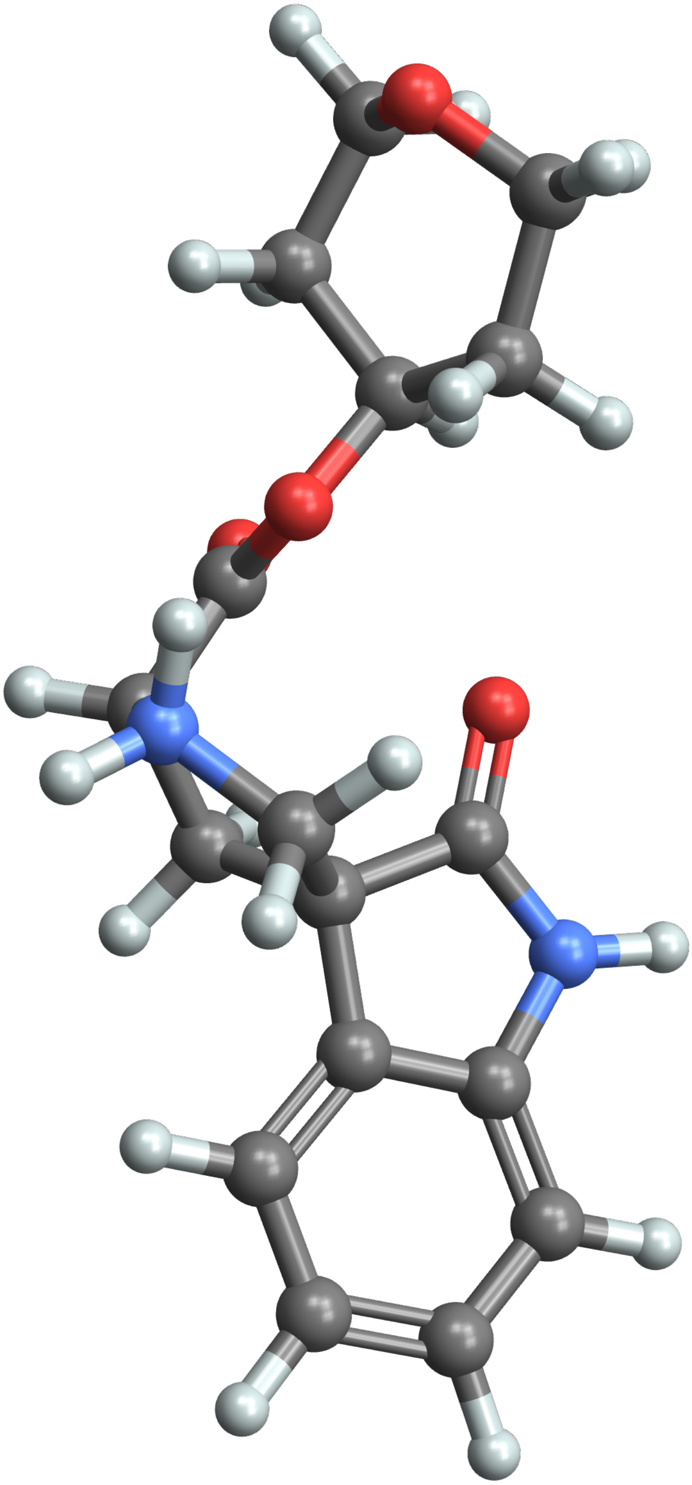

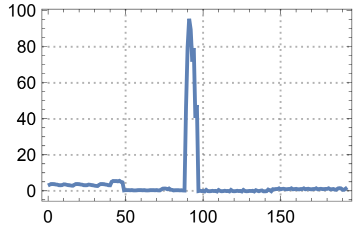

Dataset Index= 190

Molecule Formula= $C_{17}H_{21}N_2O_4^+$

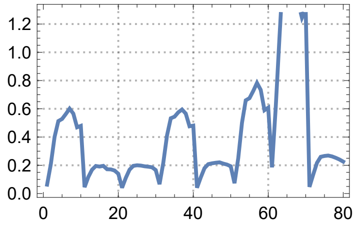

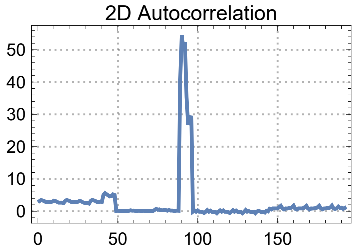

Dataset Index= 191

Molecule Formula= $C_{14}H_{14}N_4O$

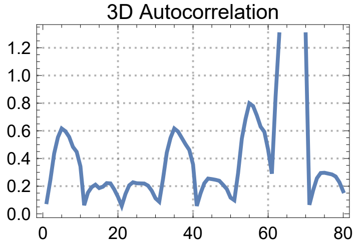

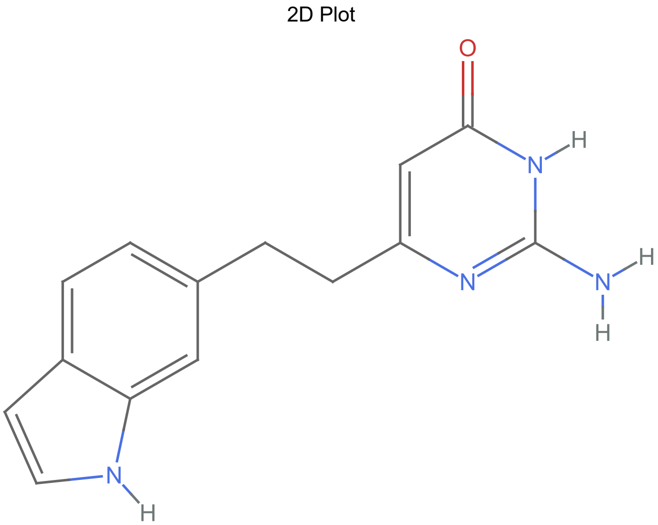

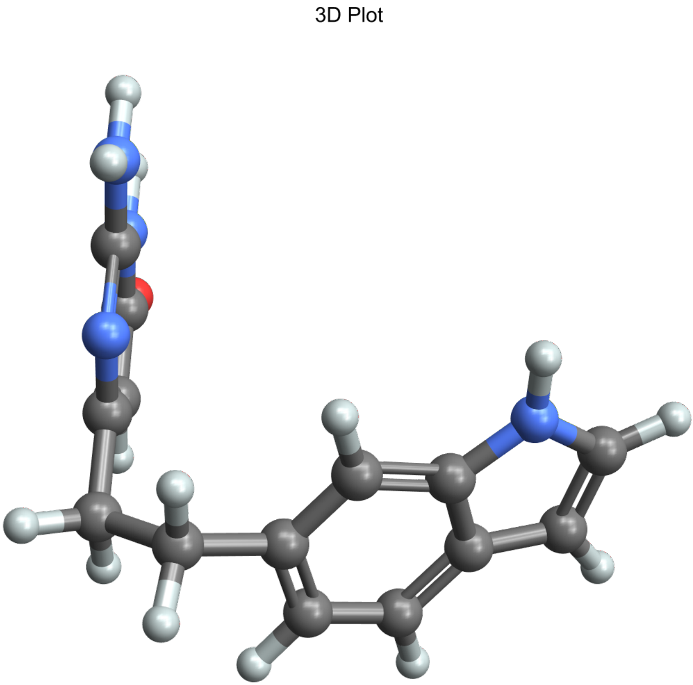

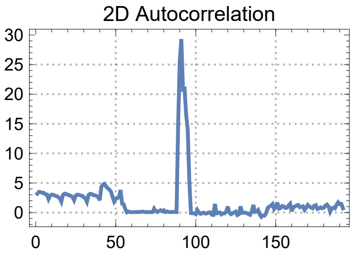

Dataset Index= 192

Molecule Formula= $C_{12}H_{18}ClN_2OS^+$

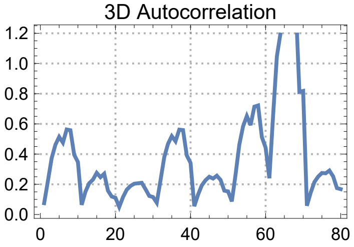

2D Plot

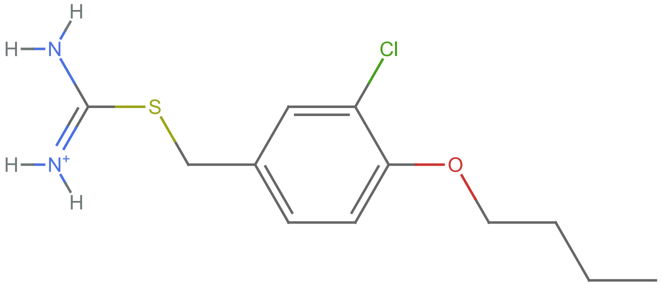

3D Plot

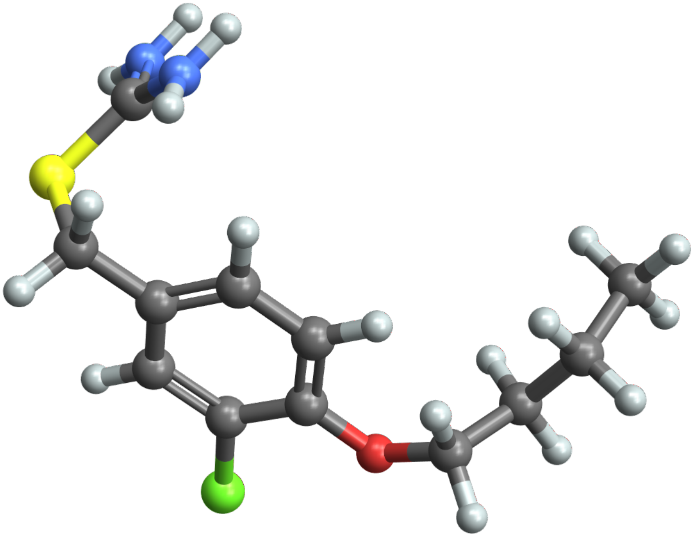

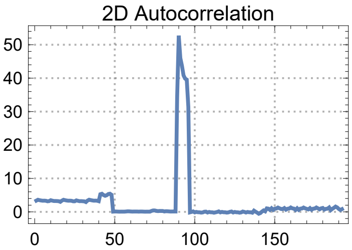

Dataset Index= 193

Molecule Formula= $C_{20}H_{24}N_3O^+$

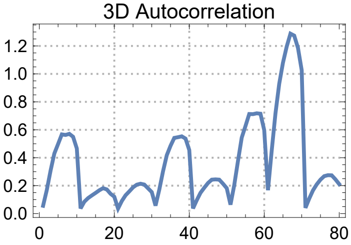

2D Plot

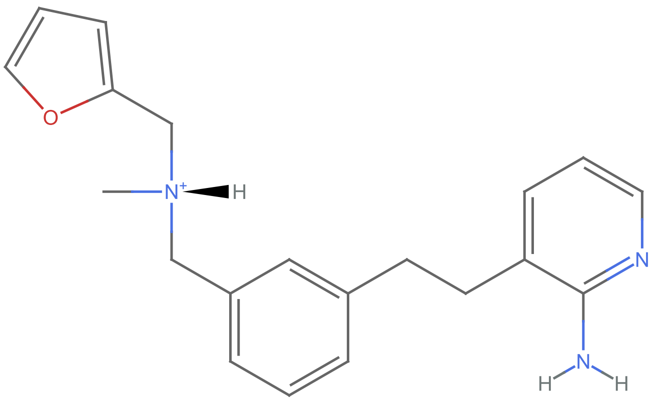

3D Plot

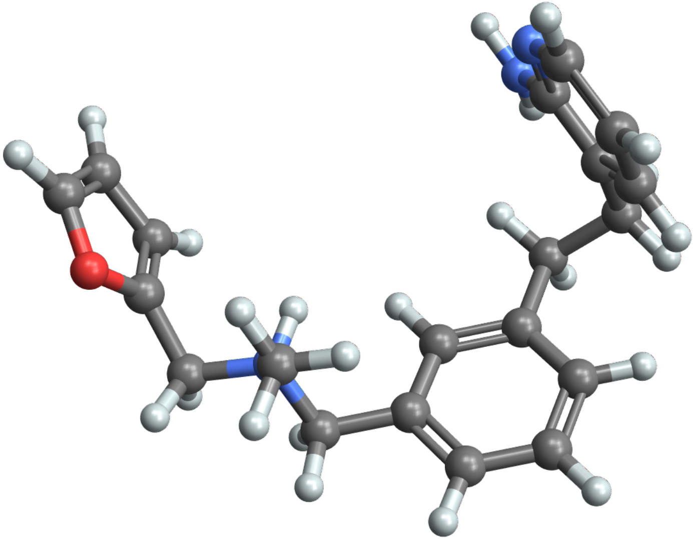

2D Plot

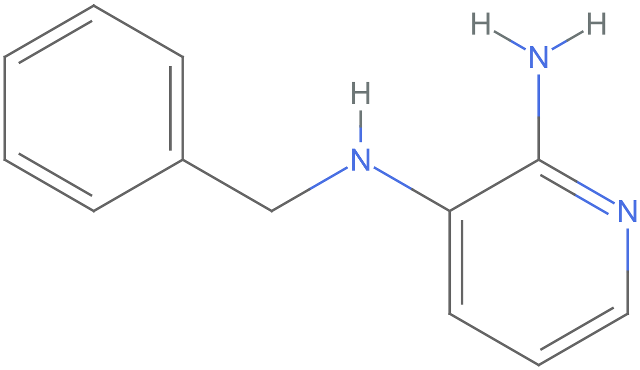

3D Plot

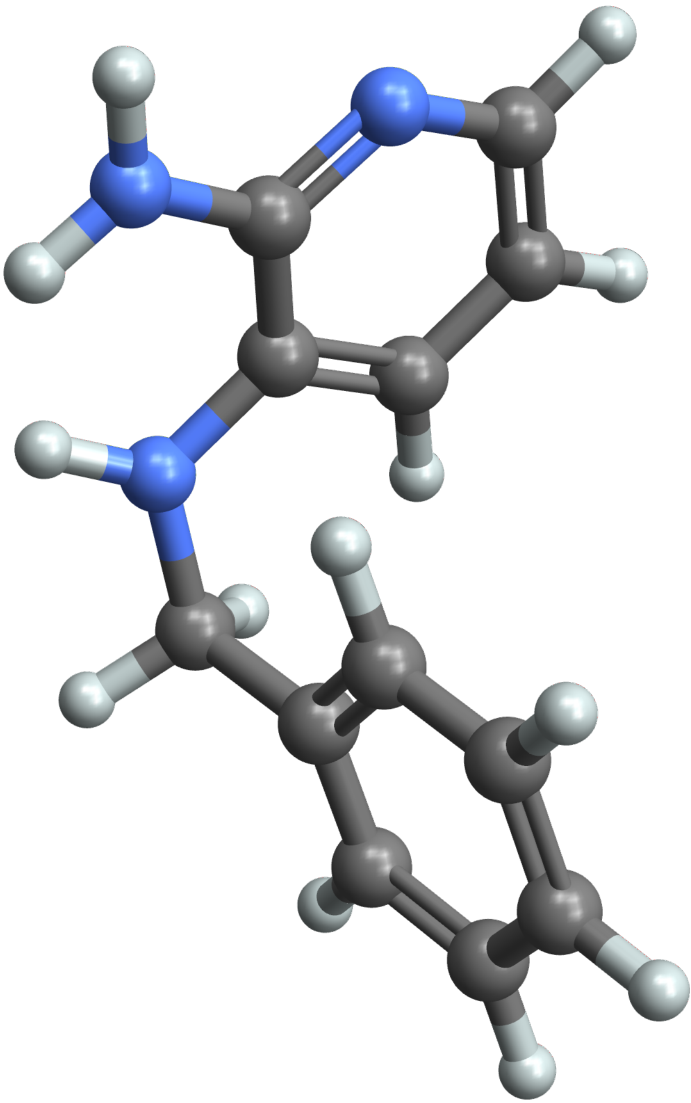

2D Autocorrelation

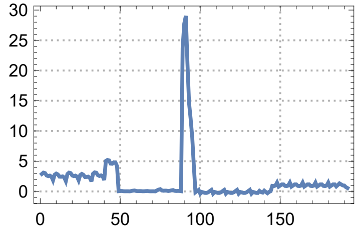

Dataset Index= 194

Molecule Formula= $C_{12}H_{13}N_3$

3D Autocorrelation

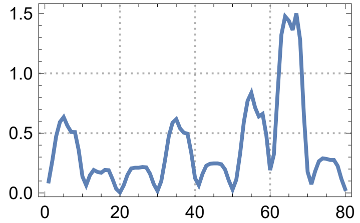

2D Plot

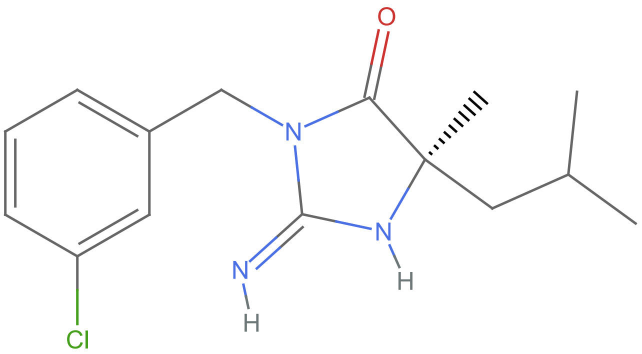

3D Plot

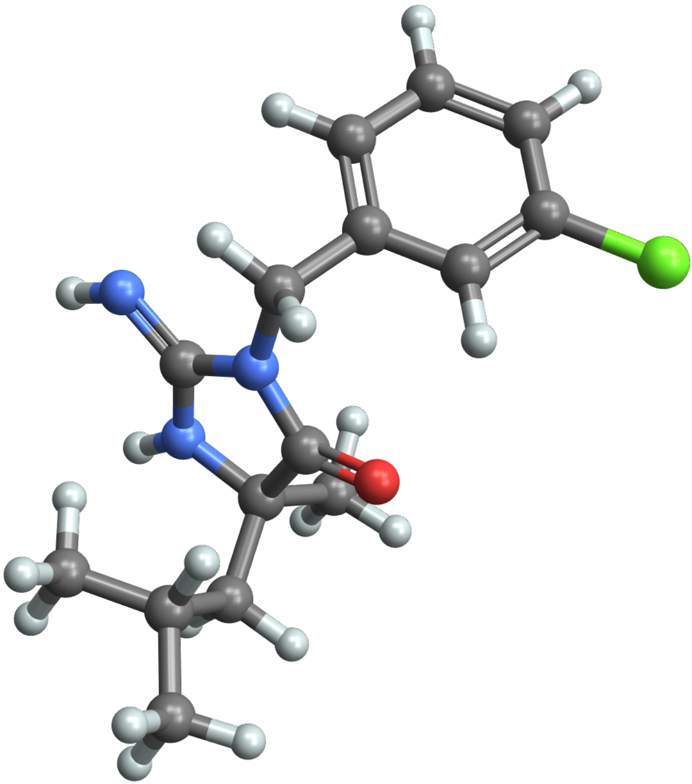

2D Autocorrelation

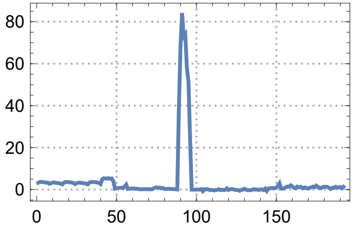

Dataset Index= 195

Molecule Formula= $C_{15}H_{20}ClN_3O$

3D Autocorrelation

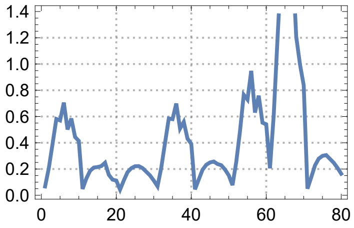

2D Plot

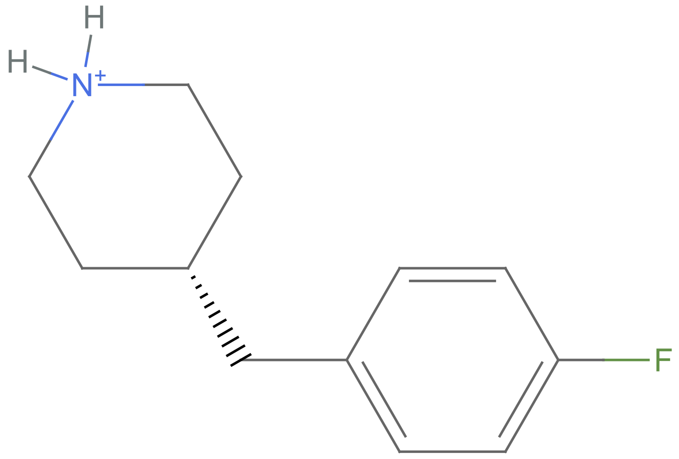

3D Plot

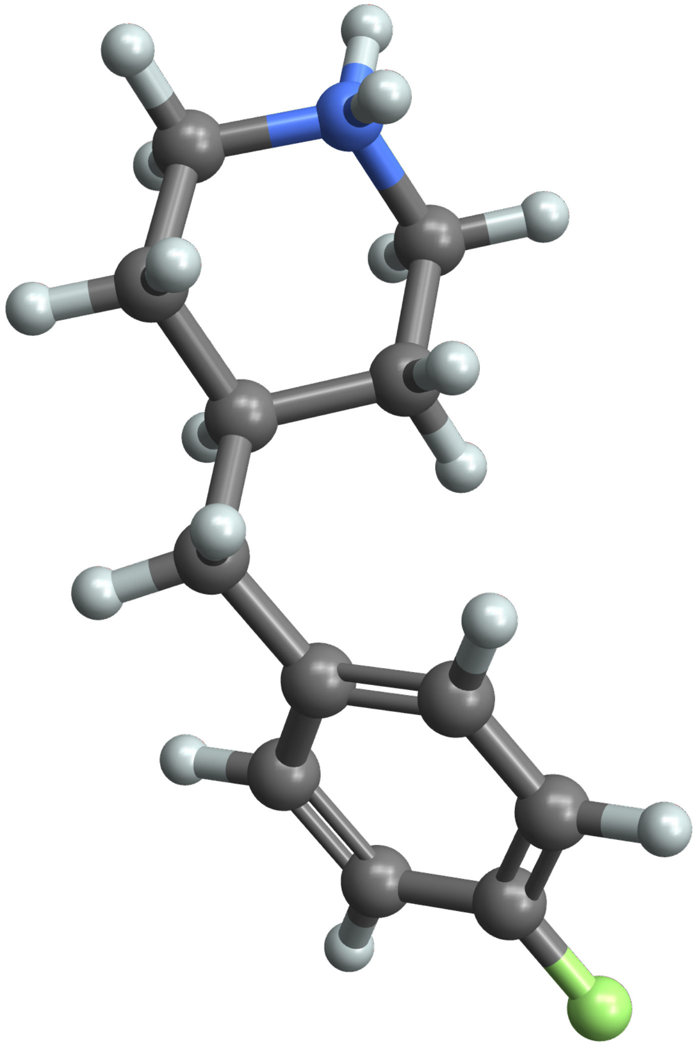

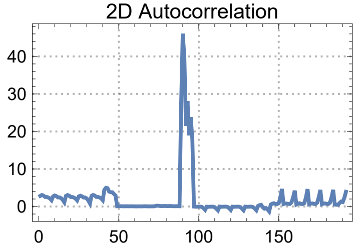

Dataset Index= 196

Molecule Formula= $C_{12}H_{17}FN^{+}$

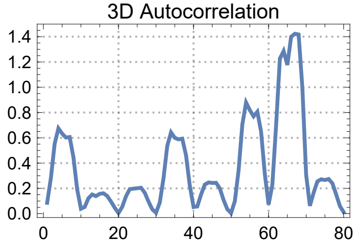

2D Plot

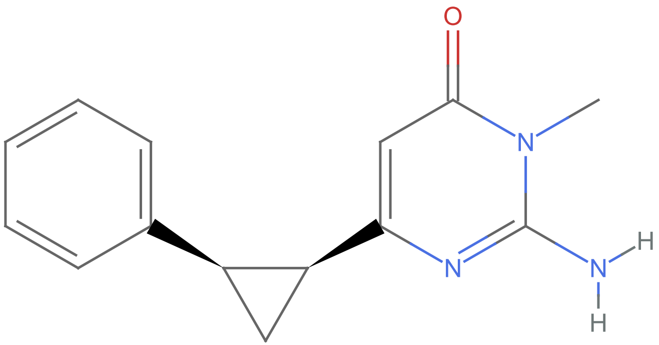

3D Plot

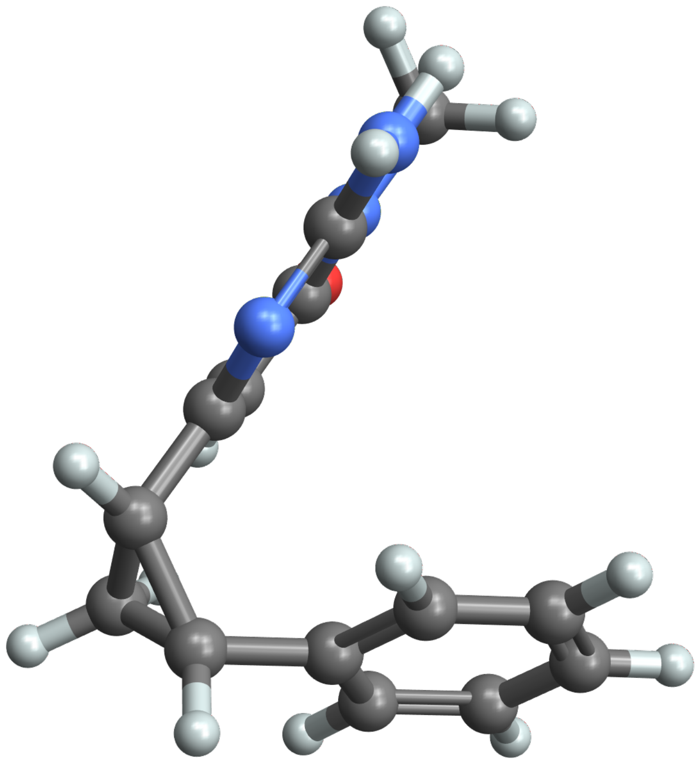

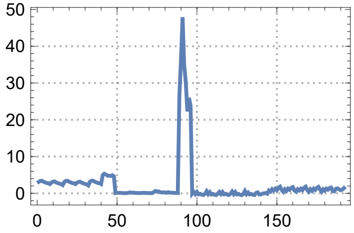

Dataset Index= 197

Molecule Formula= $C_{14}H_{15}N_{3}O$

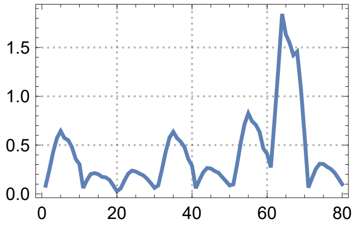

2D Plot

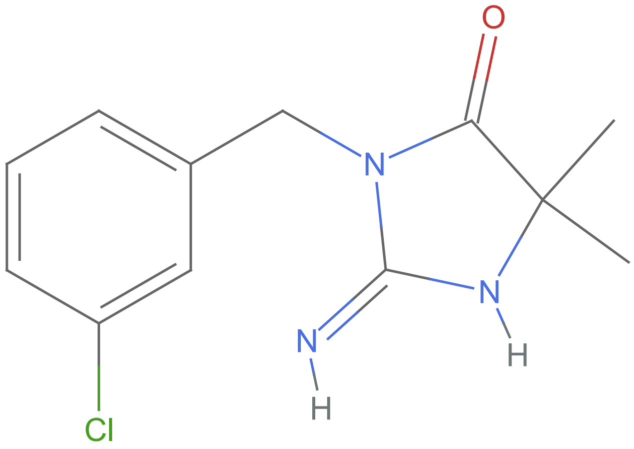

3D Plot

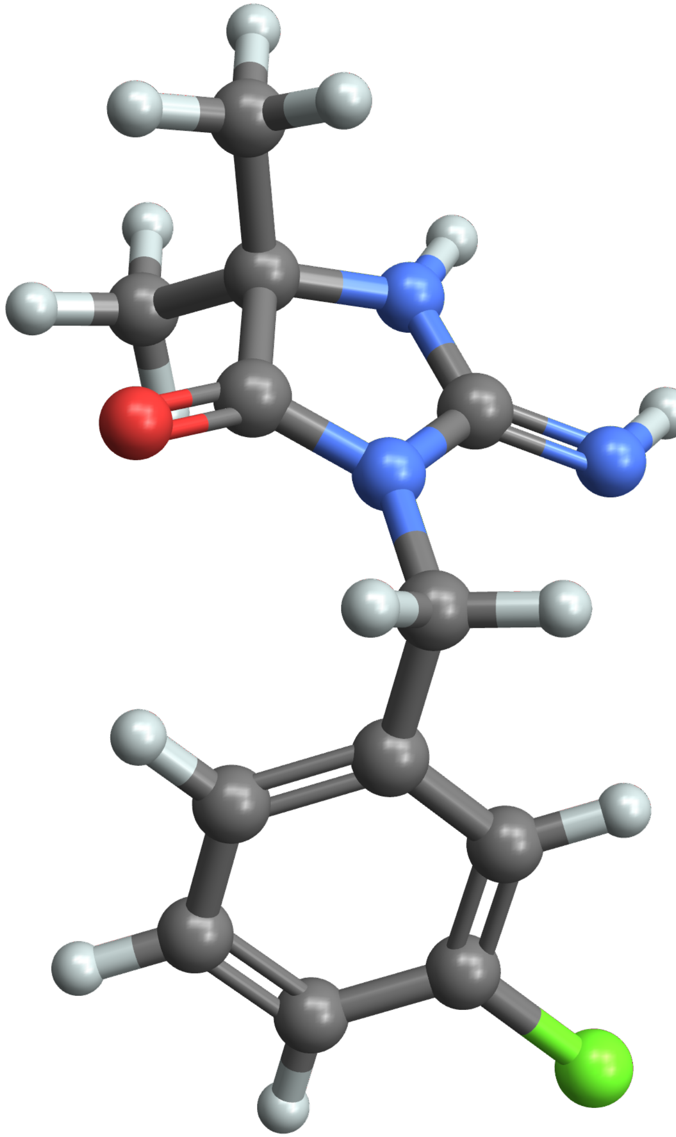

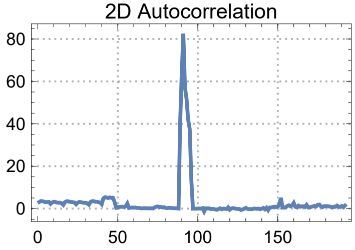

Dataset Index= 198

Molecule Formula= $C_{12}H_{14}ClN_3O$

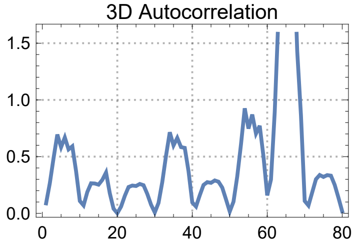

2D Autocorrelation

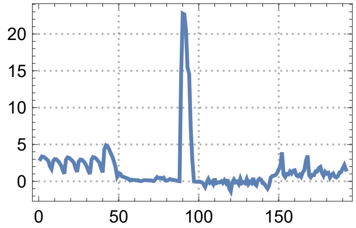

Dataset Index= 199

Molecule Formula= $C_{10}H_{12}ClN_3O$

3D Autocorrelation

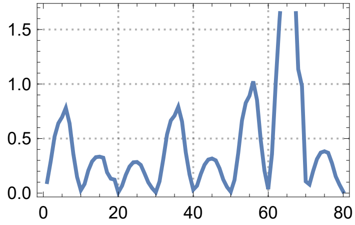

2D Plot

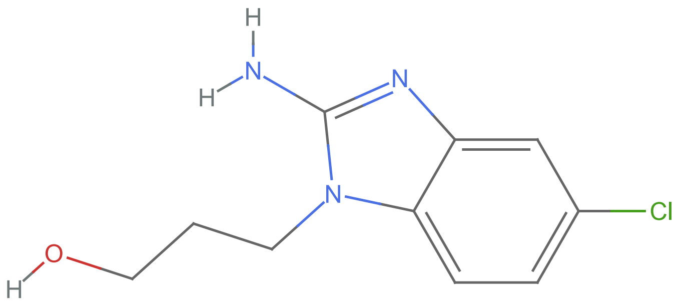

3D Plot

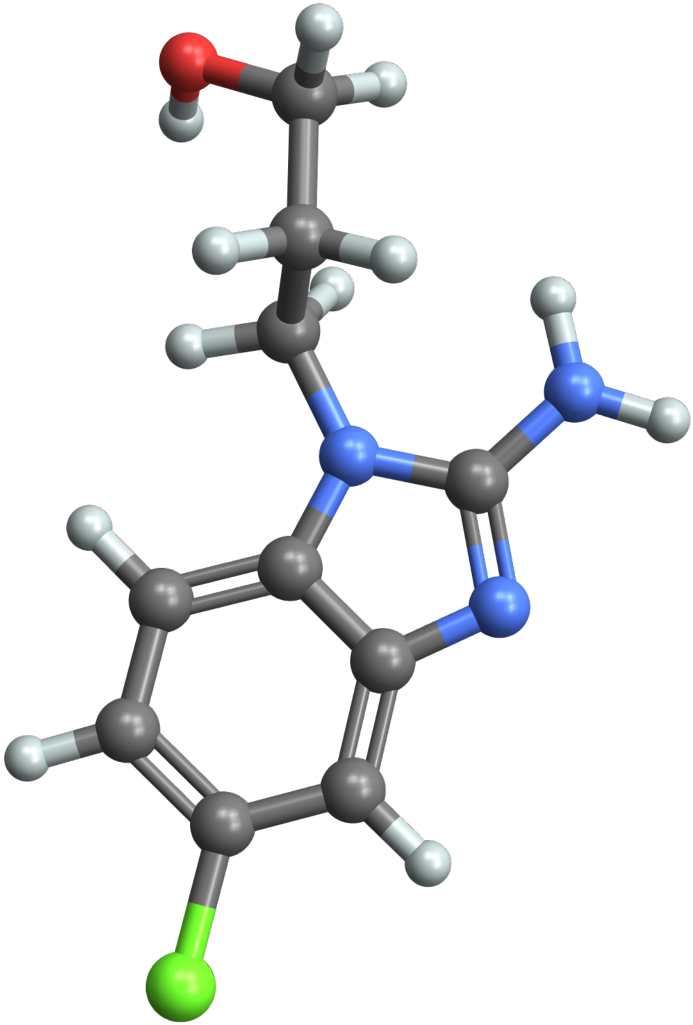

2D Plot

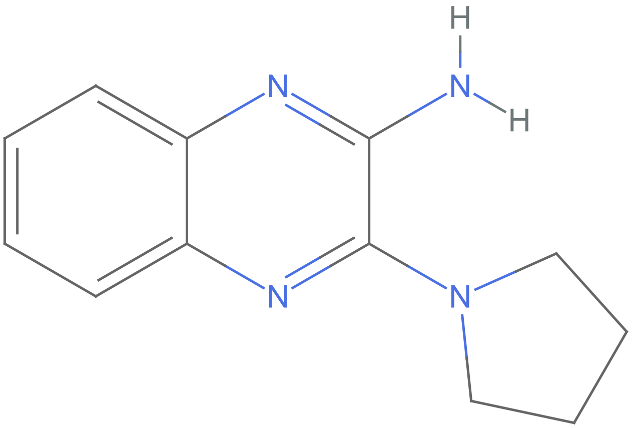

2D Autocorrelation

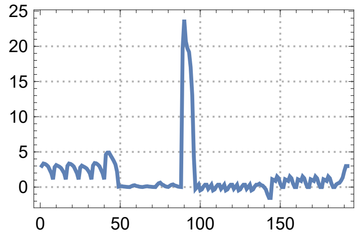

3D Plot

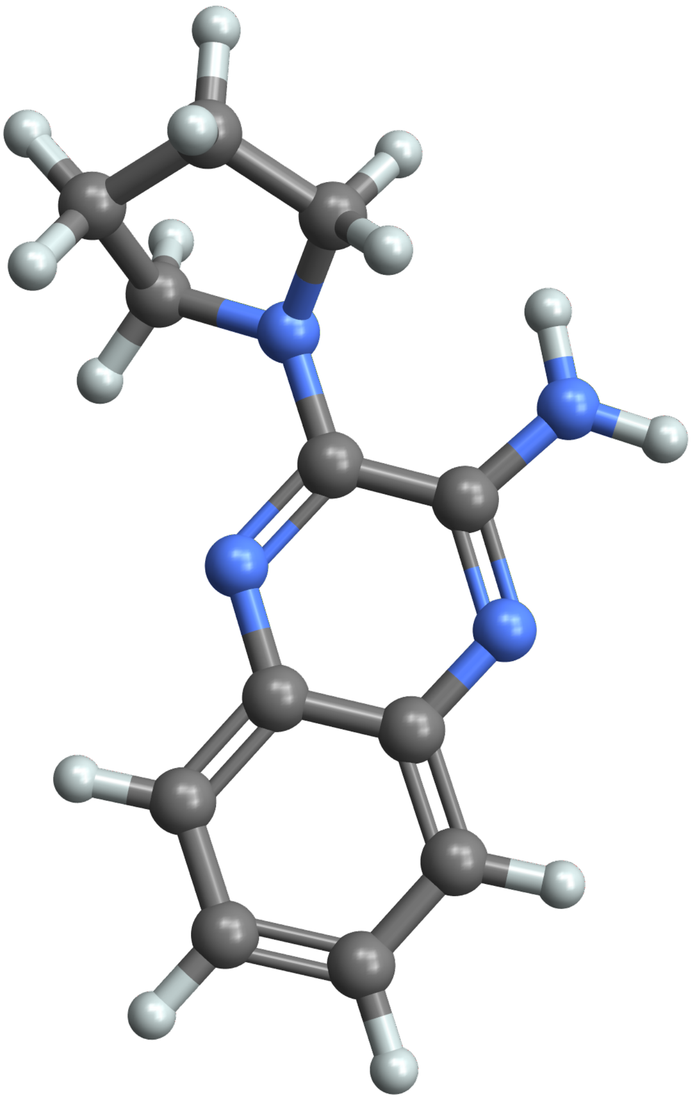

Dataset Index= 200

Molecule Formula= $C_{12}H_{14}N_4$

3D Autocorrelation

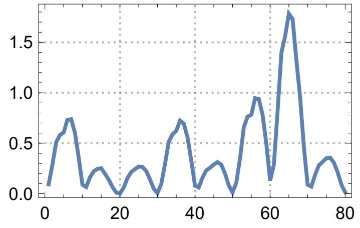

2D Plot

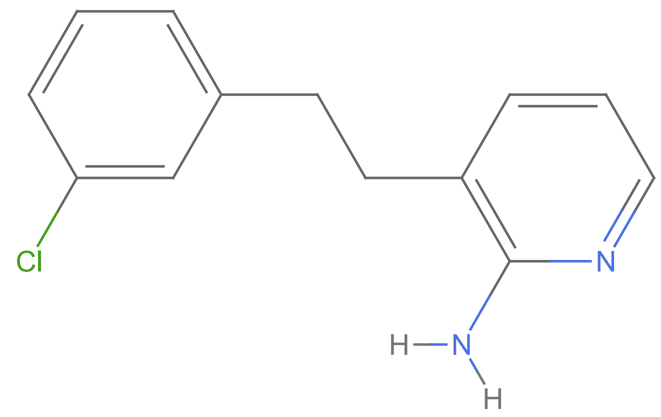

2D Autocorrelation

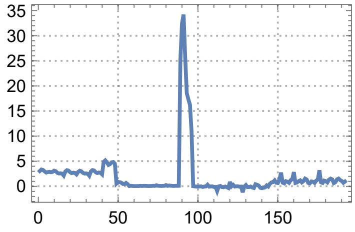

Dataset Index= 201

Molecule Formula= $C_{13}H_{13}ClN_2$

3D Autocorrelation

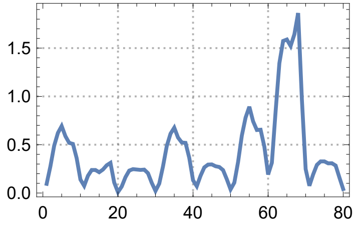

3D Plot

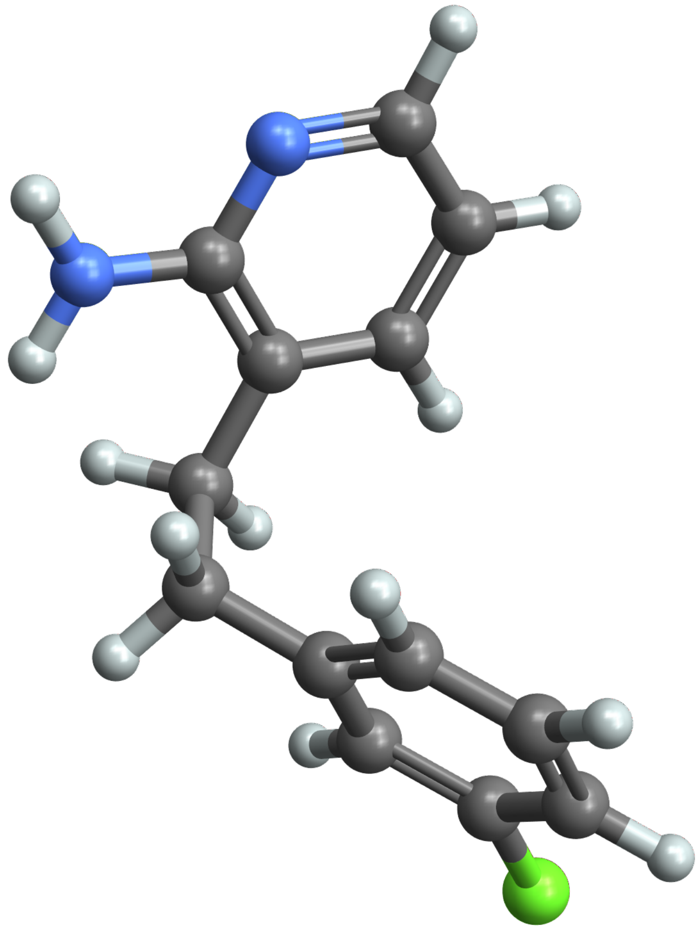

2D Plot

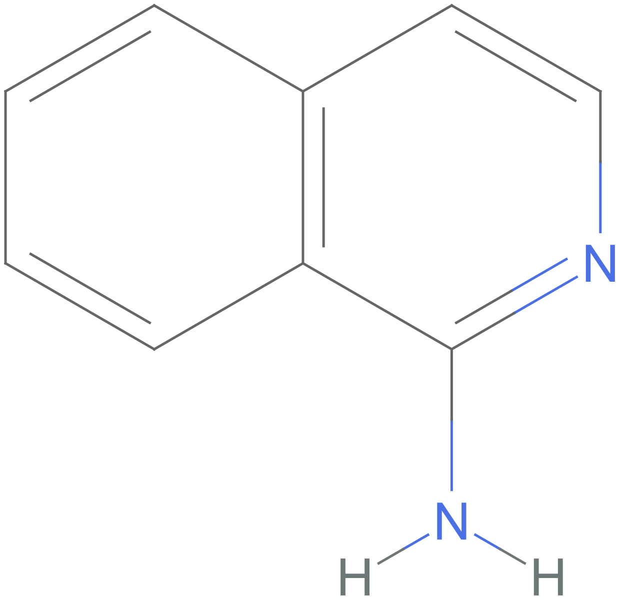

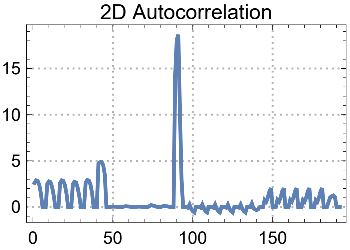

Dataset Index= 202

Molecule Formula= $C_9H_8N_2$

3D Plot

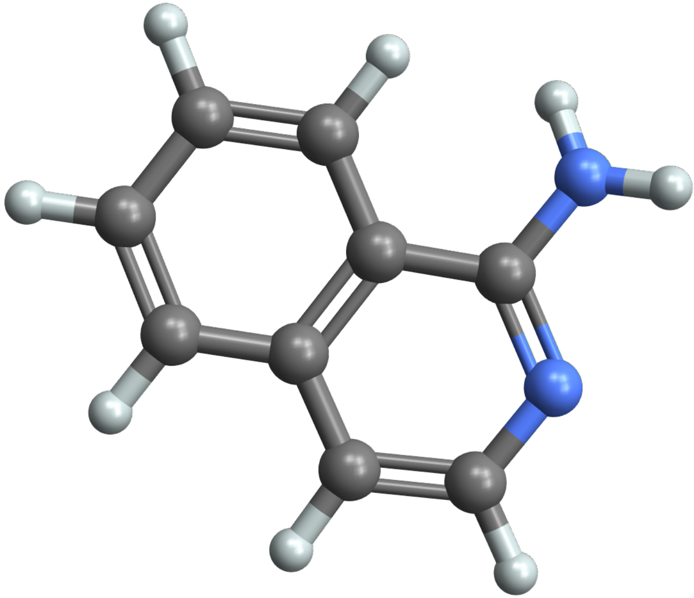

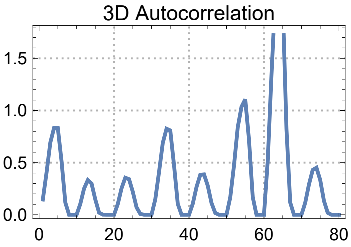

2D Plot

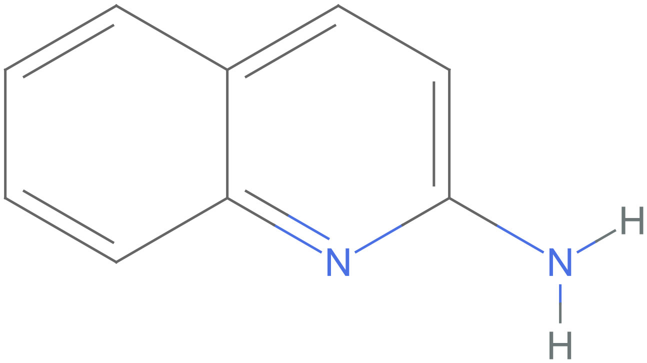

2D Autocorrelation

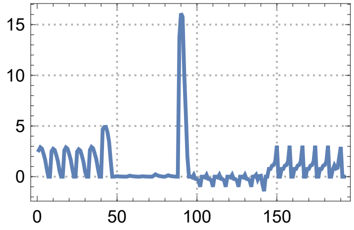

3D Plot

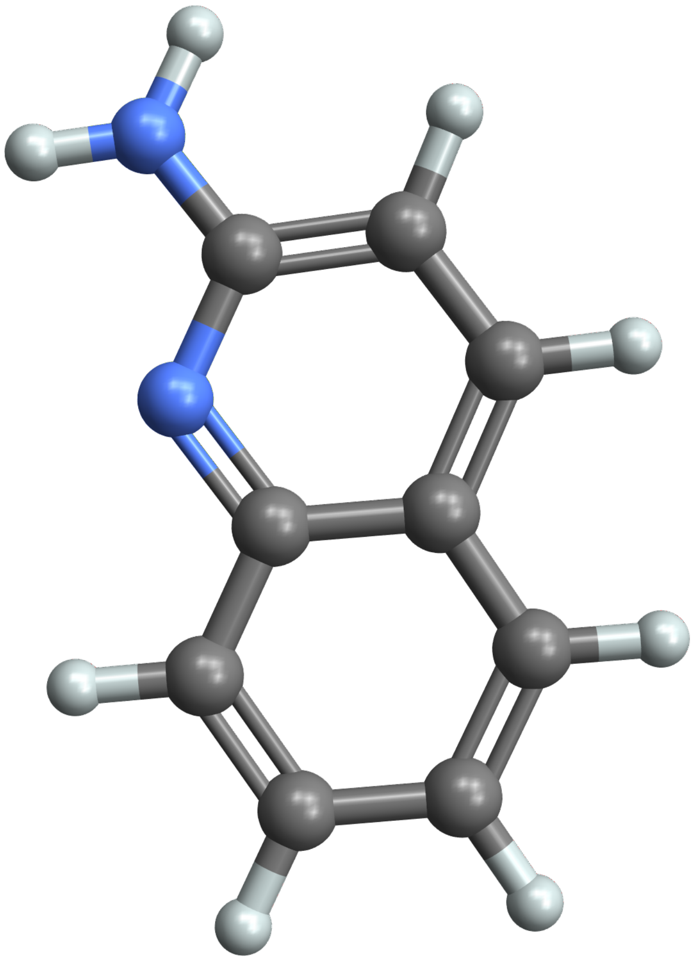

Dataset Index= 203

Molecule Formula= $C_9H_8N_2$

3D Autocorrelation

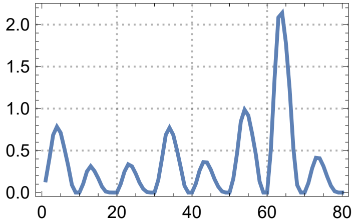

2D Plot

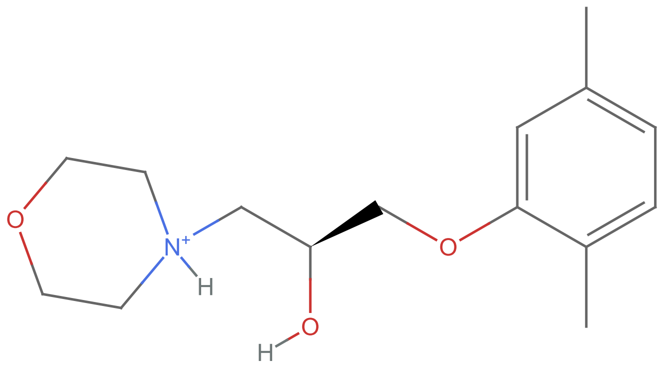

3D Plot

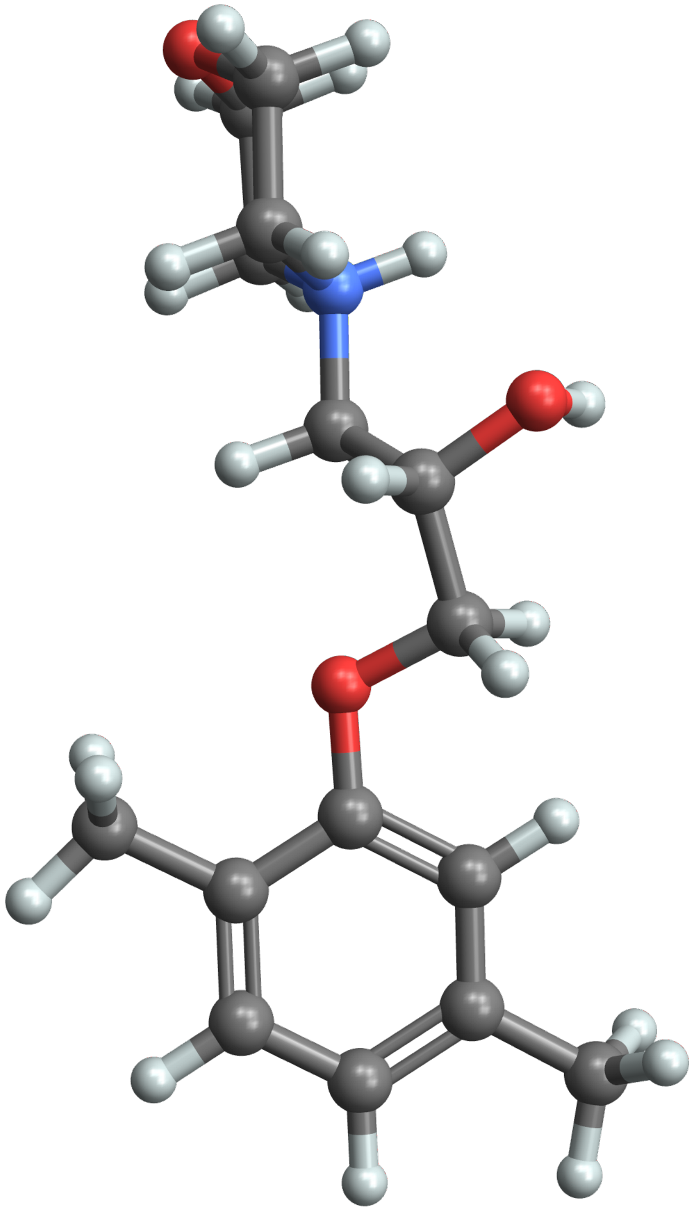

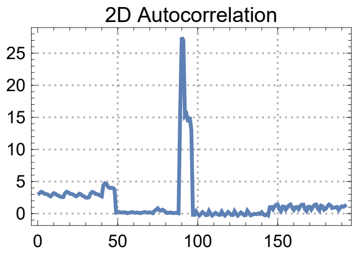

Dataset Index= 204

Molecule Formula= $C_{15}H_{24}NO_3^+$

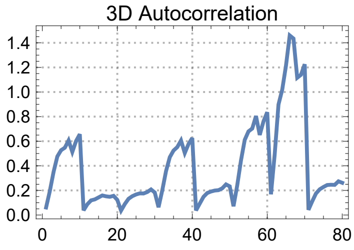

2D Plot

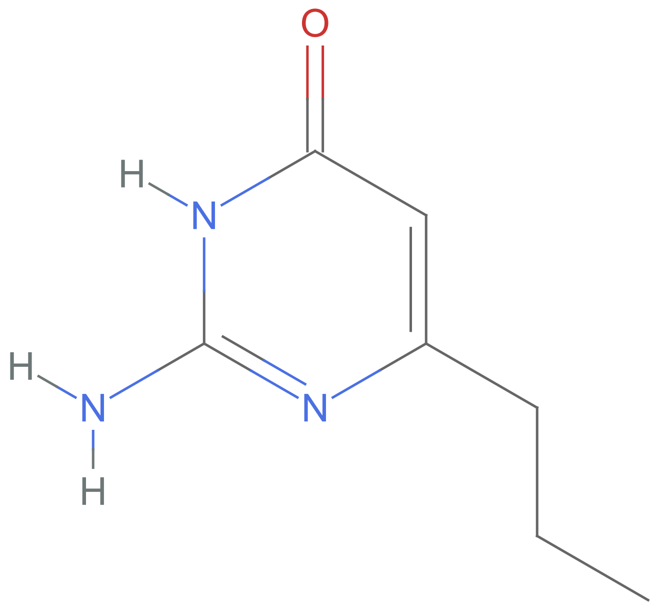

2D Autocorrelation

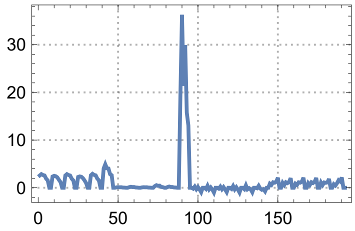

Dataset Index= 205

Molecule Formula= $C_7H_{11}N_3O$

3D Autocorrelation

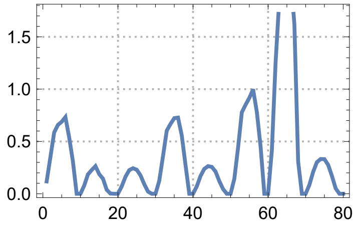

3D Plot

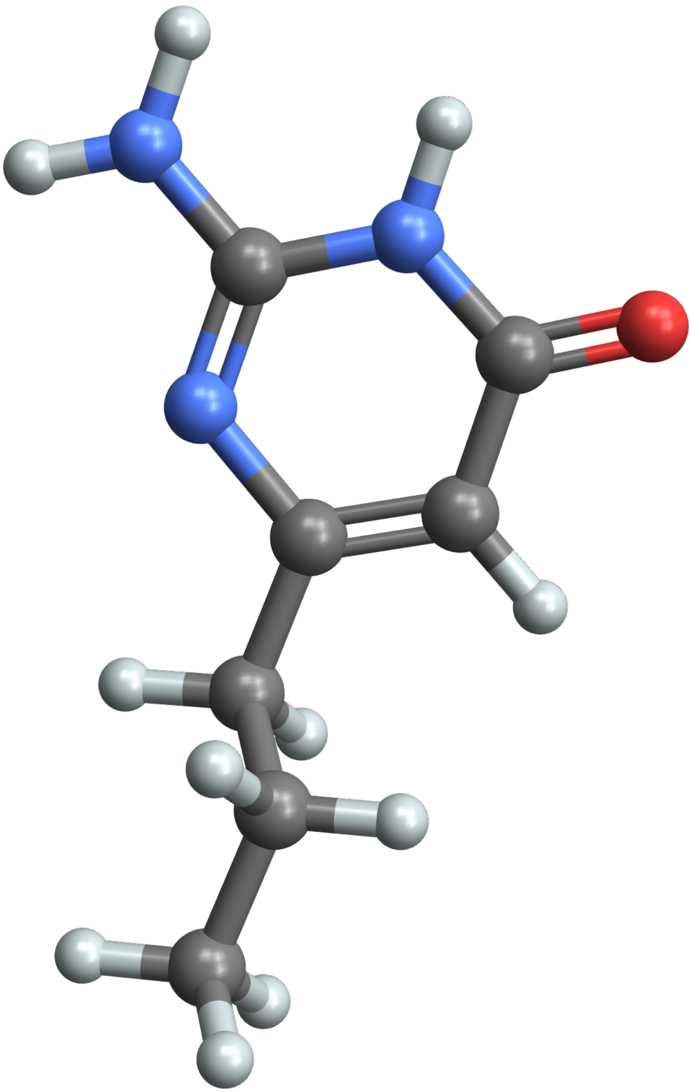

2D Plot

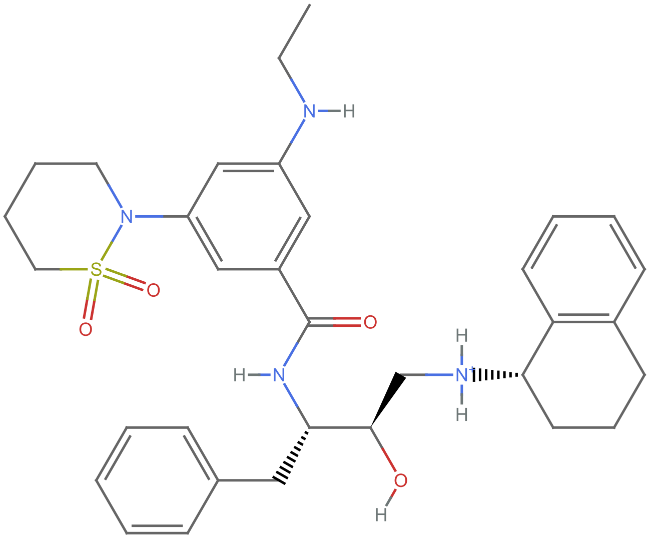

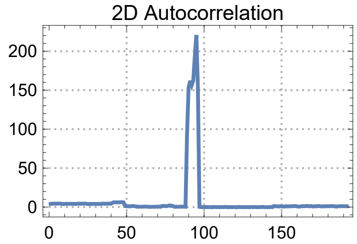

3D Plot

Dataset Index= 206

Molecule Formula= $C_{33}H_{43}N_4O_4S^+$

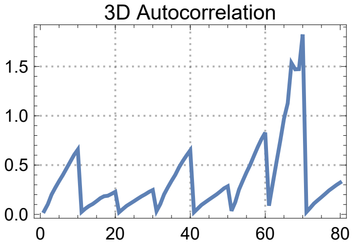

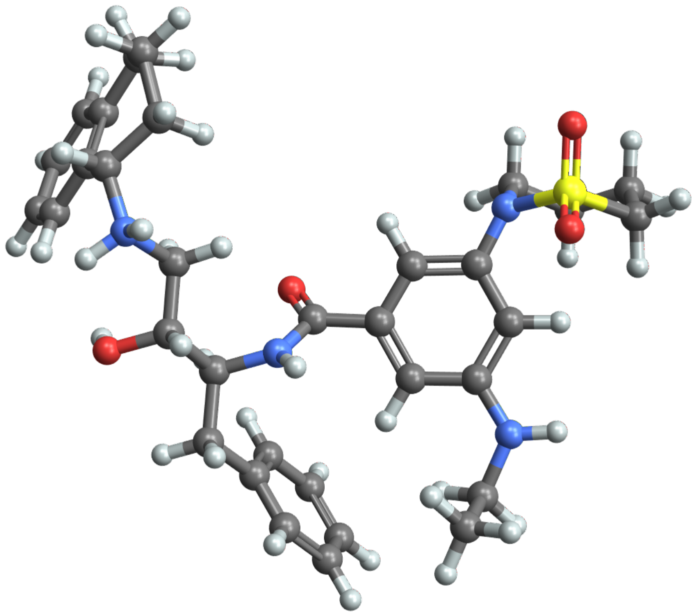

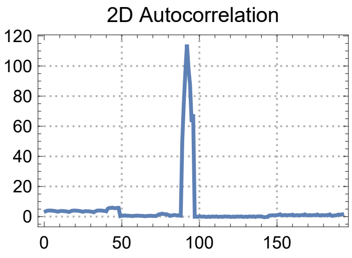

Dataset Index= 207

Molecule Formula= $C_{18}H_{15}F_{2}N_{5}O$

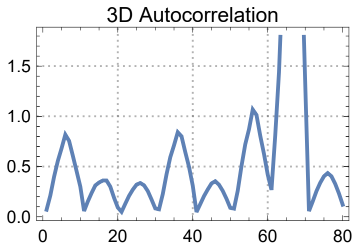

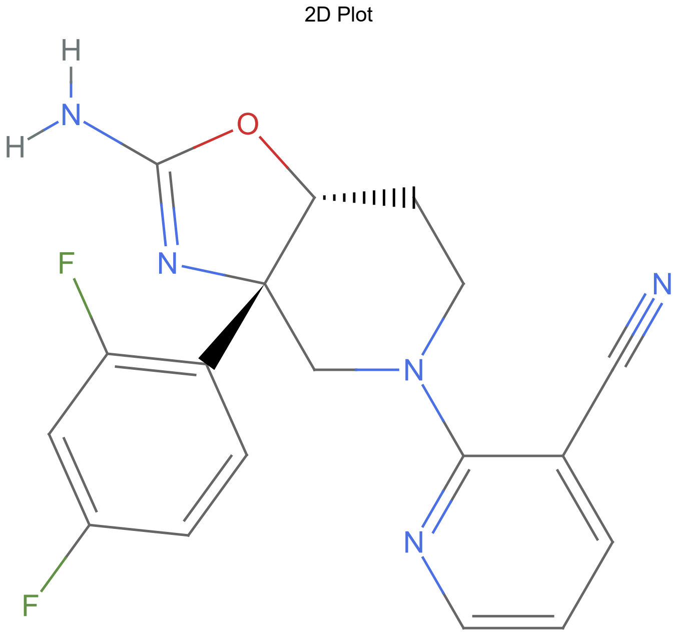

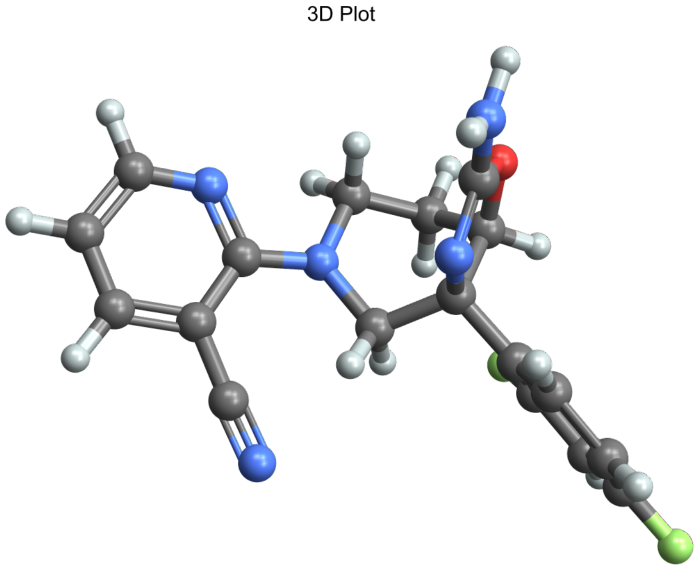

2D Plot

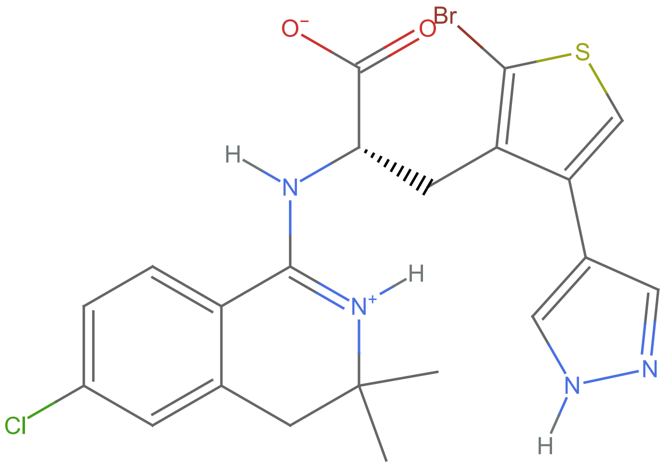

3D Plot

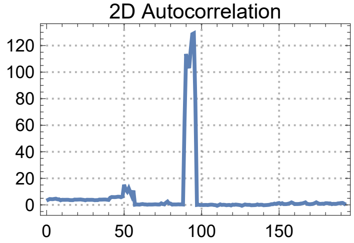

Dataset Index= 208

Molecule Formula= $C_{21}H_{20}BrClN_4O_2S$

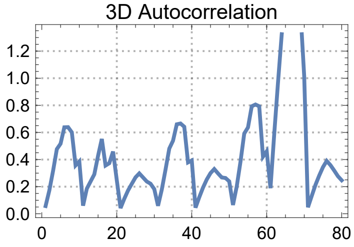

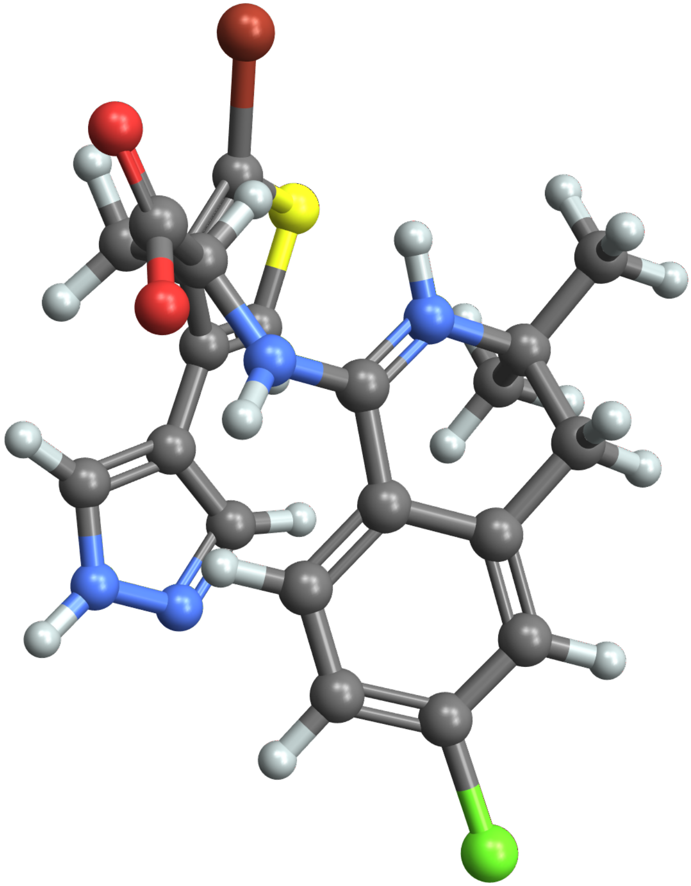

2D Plot

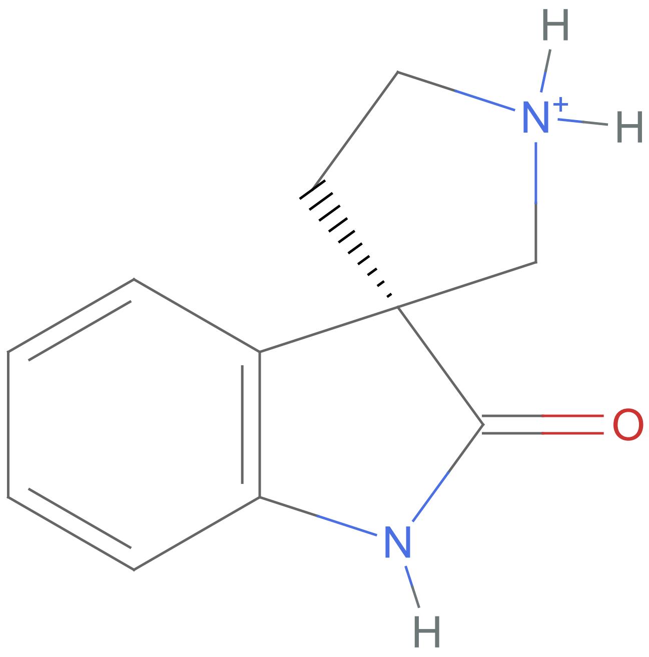

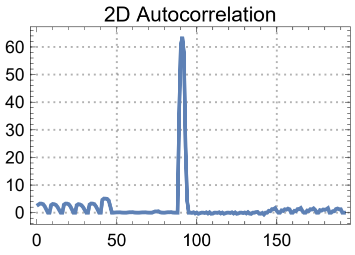

Dataset Index= 209

Molecule Formula= $C_{11}H_{13}N_2O^+$

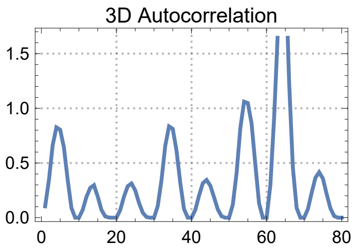

3D Plot

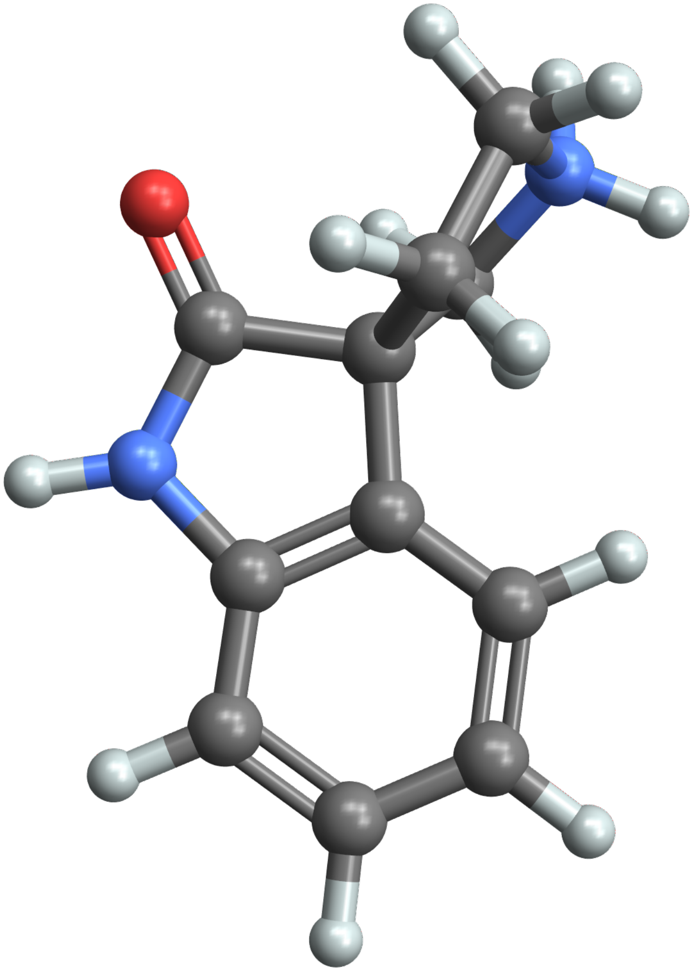

2D Autocorrelation

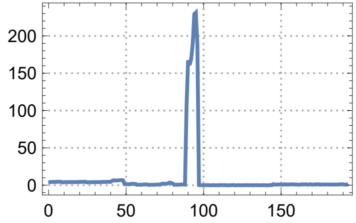

Dataset Index= 210

Molecule Formula= $C_{31}H_{34}FN_5O_5S$

3D Autocorrelation

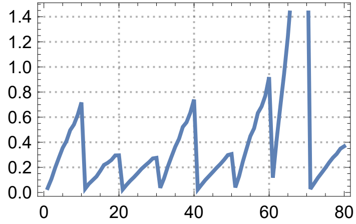

2D Plot

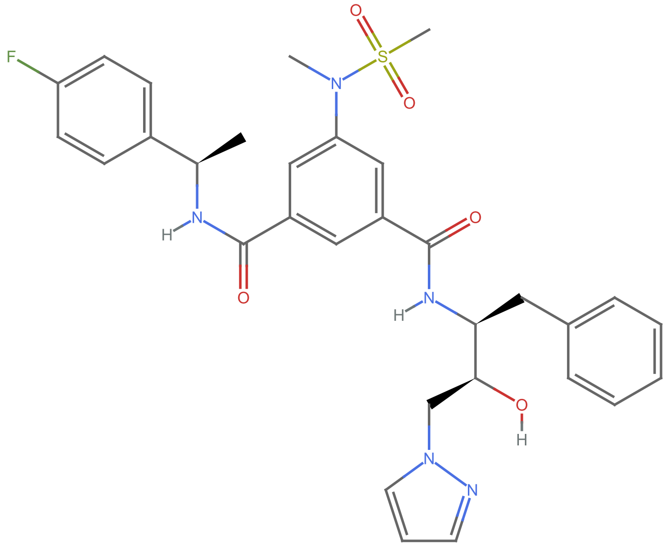

3D Plot

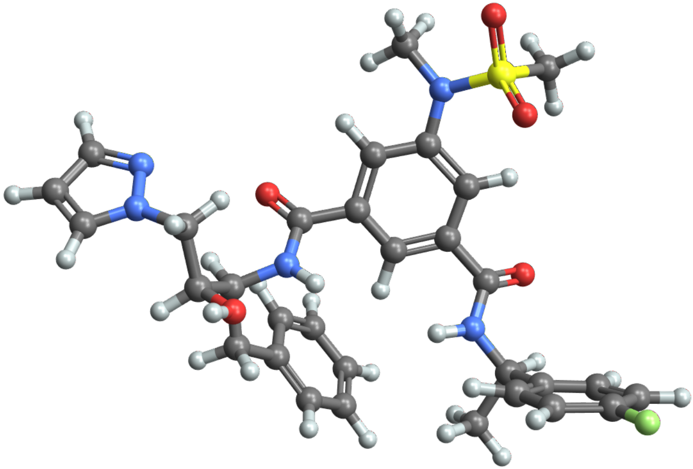

2D Plot

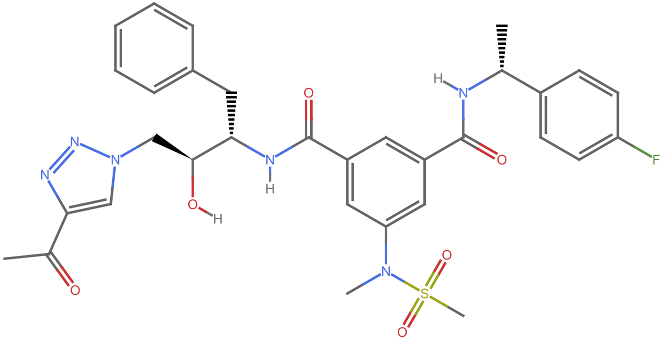

3D Plot

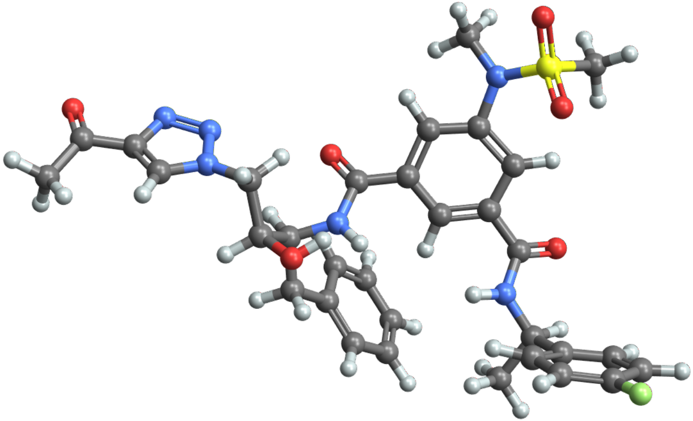

2D Autocorrelation

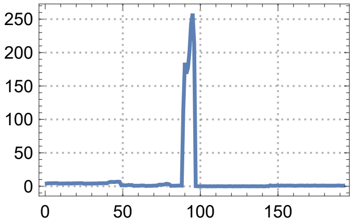

Dataset Index= 211

Molecule Formula= $C_{32}H_{35}FN_6O_6S$

3D Autocorrelation

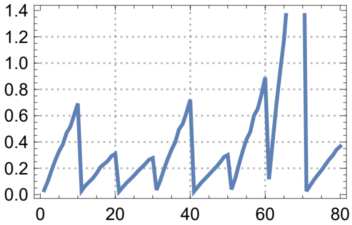

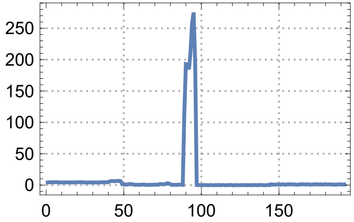

Dataset Index= 212

Molecule Formula= $C_{42}H_{55}N_5O_5S^{2+}$

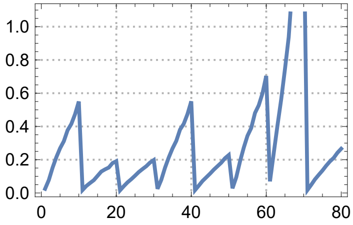

2D Plot

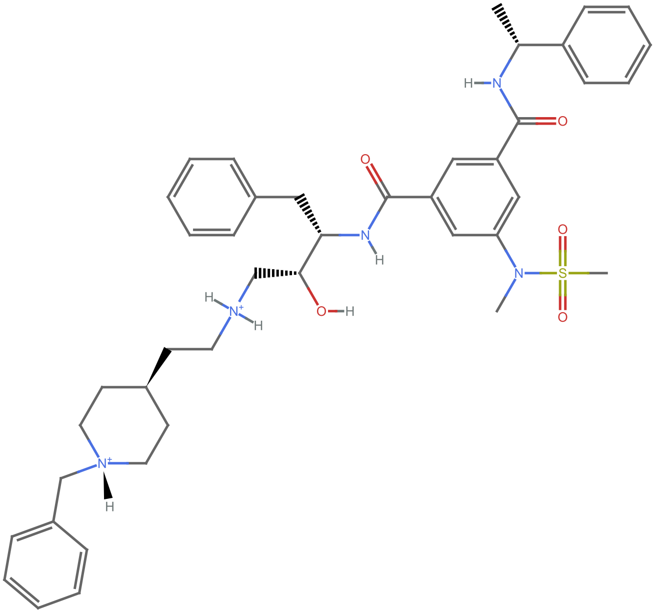

3D Plot

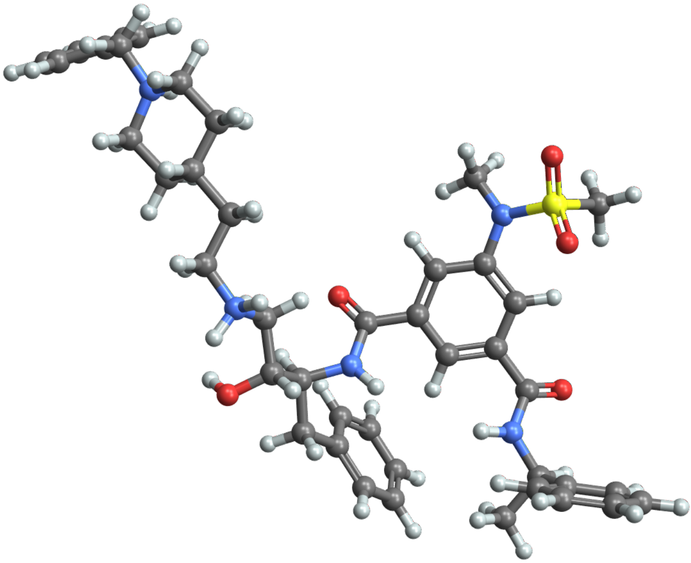

2D Plot

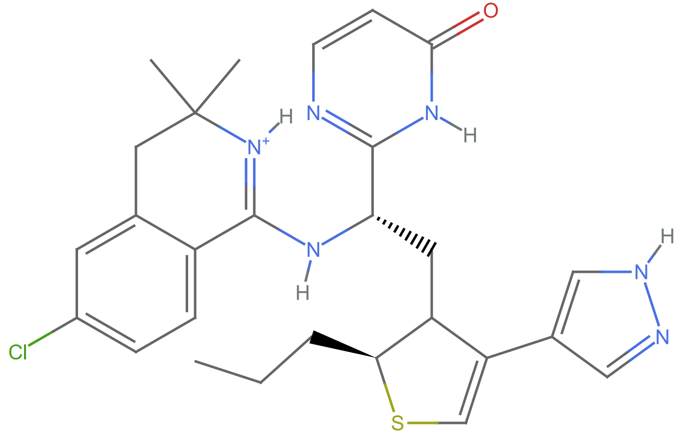

3D Plot

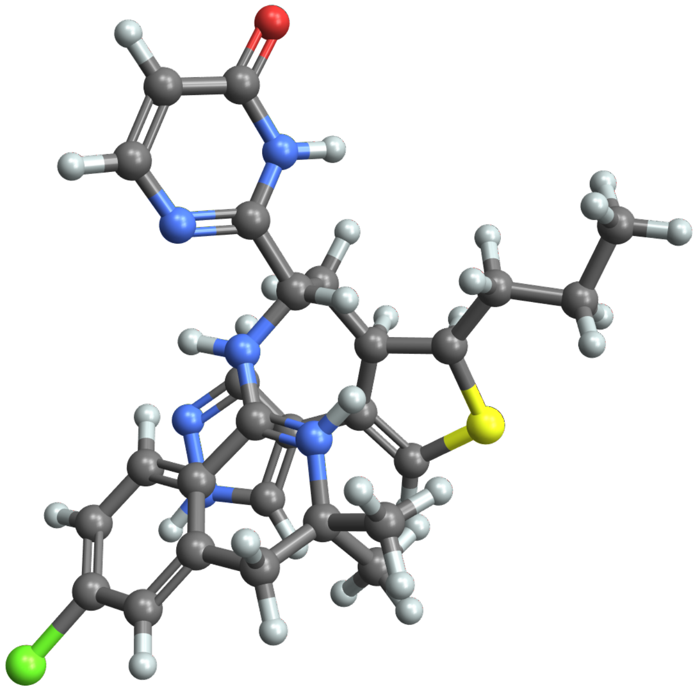

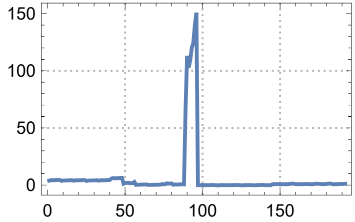

Dataset Index= 213

Molecule Formula= $C_{27}H_{32}ClN_6OS^+$

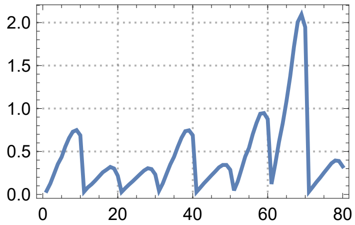

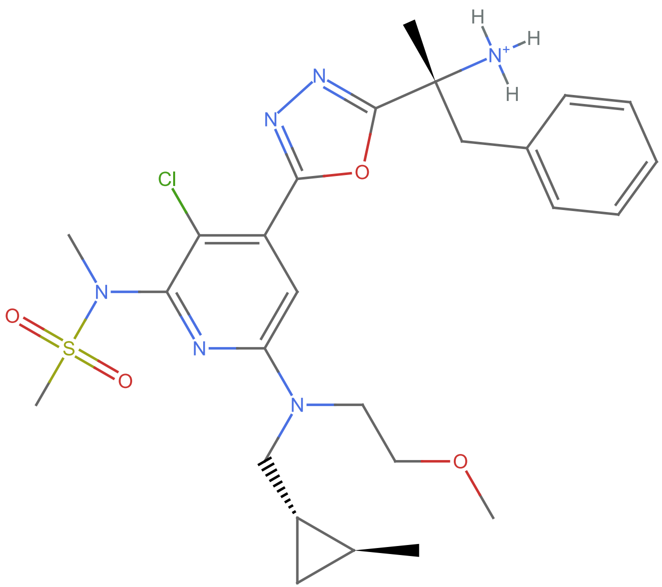

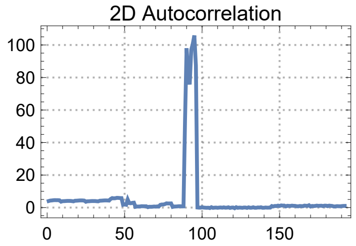

Dataset Index= 214

Molecule Formula= $C_{26}H_{36}ClN_6O_4S^+$

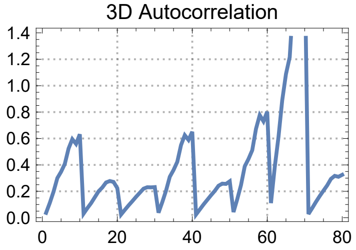

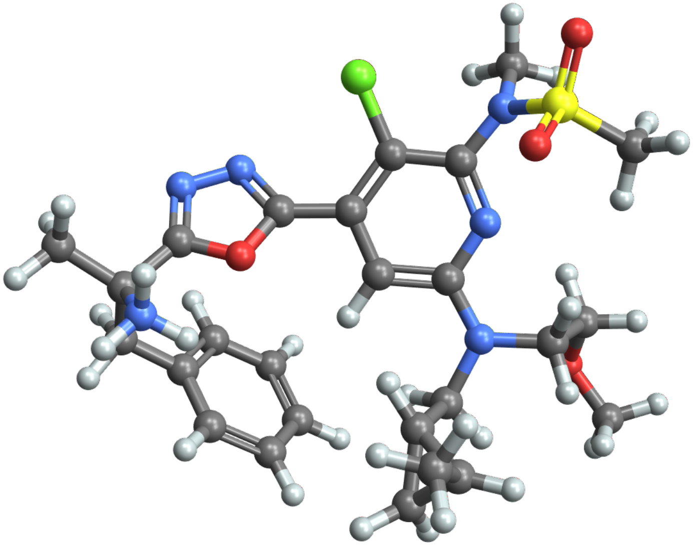

2D Plot

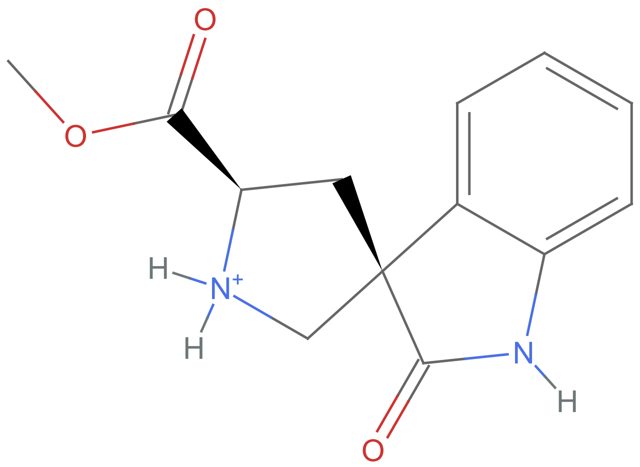

3D Plot

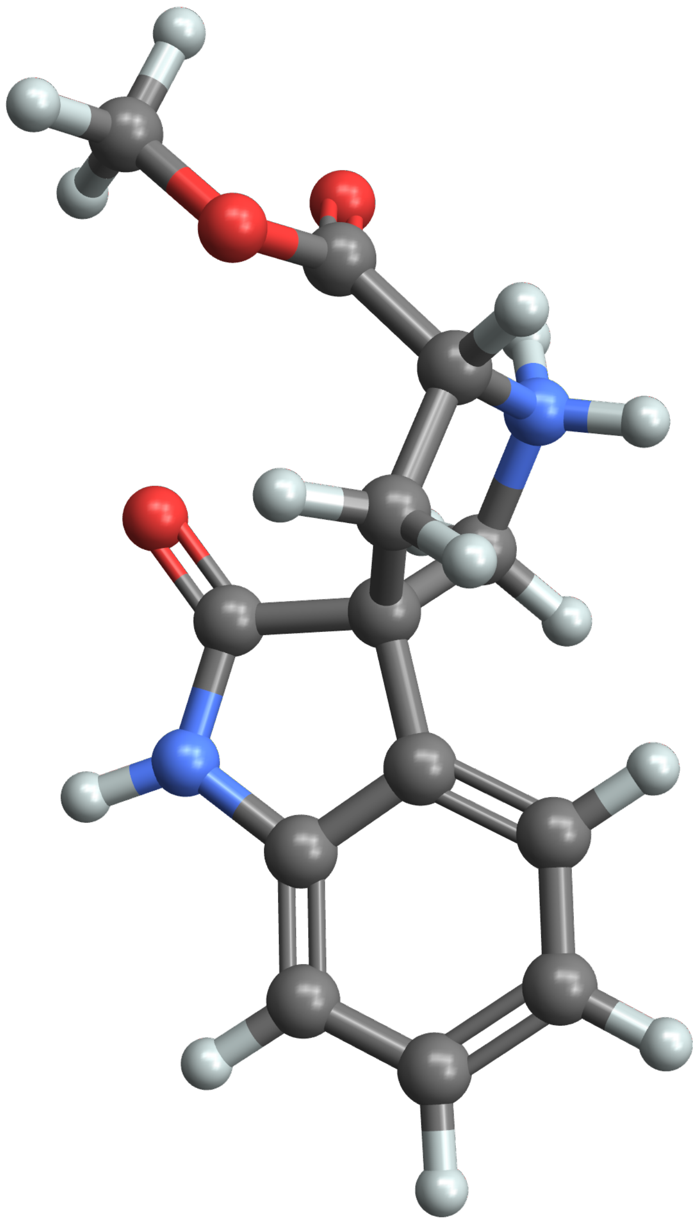

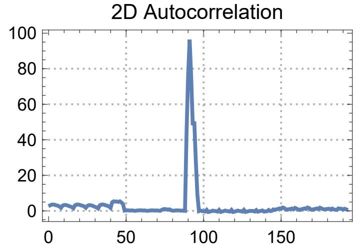

Dataset Index= 215

Molecule Formula= $C_{13}H_{15}N_2O_3^+$

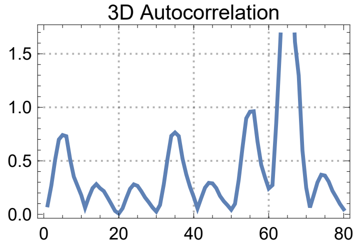

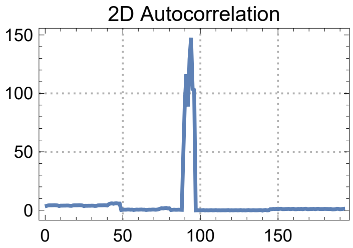

Dataset Index= 216

Molecule Formula= $C_{29}H_{42}N_3O_5^+$

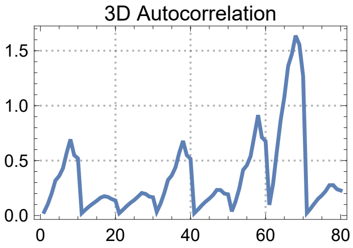

2D Plot

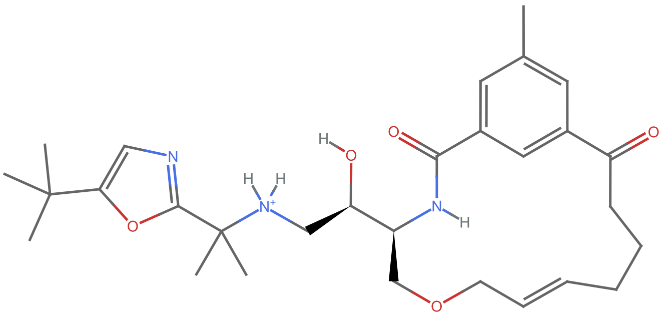

3D Plot

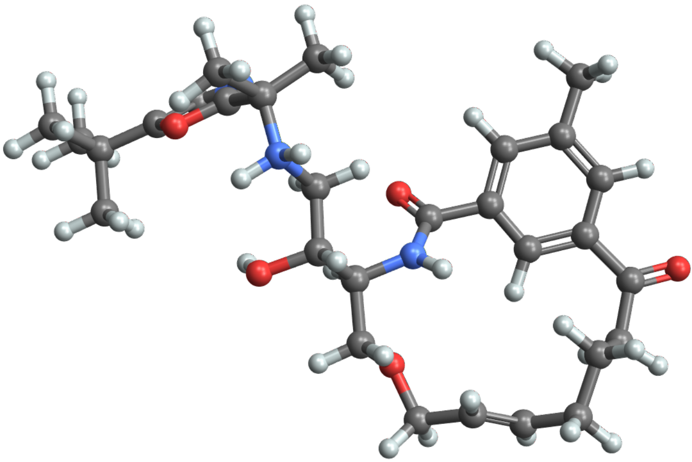

2D Plot

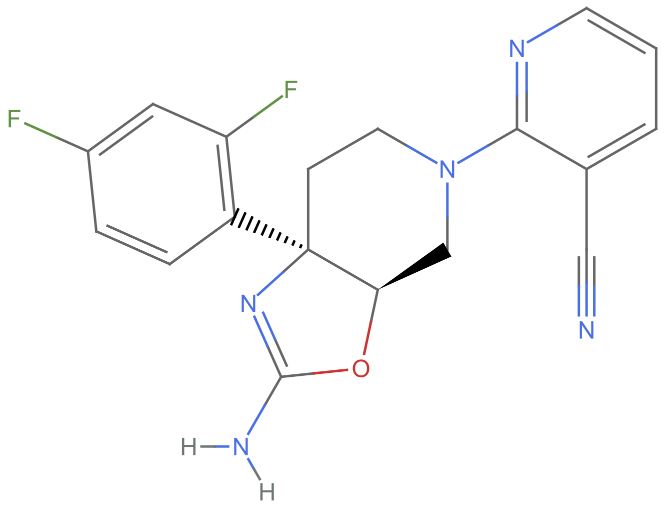

3D Plot

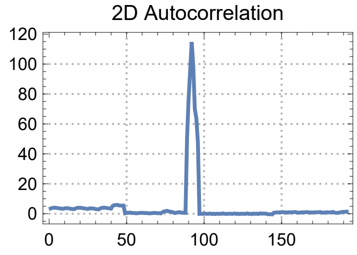

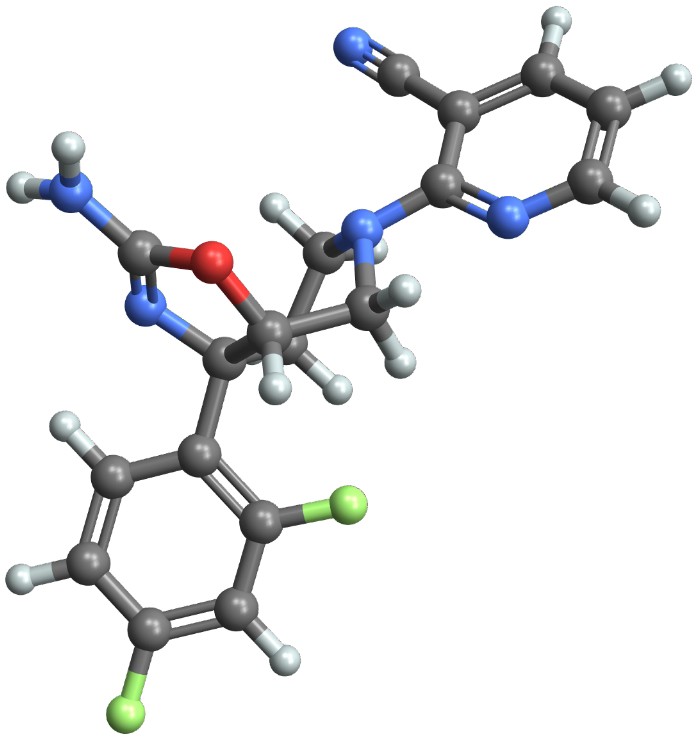

Dataset Index= 217

Molecule Formula= $C_{18}H_{15}F_2N_5O$

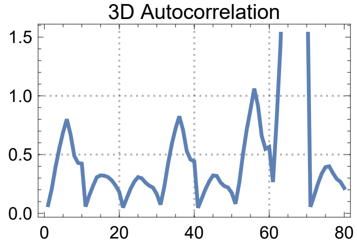

2D Autocorrelation

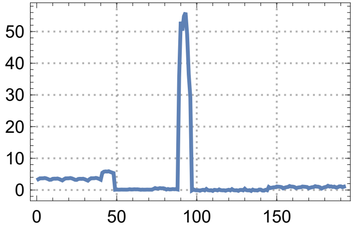

Dataset Index= 218

Molecule Formula= $C_{20}H_{21}N_4O^+$

3D Autocorrelation

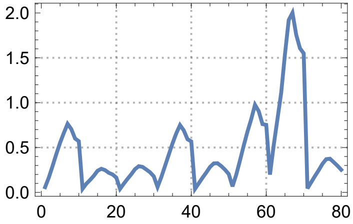

2D Plot

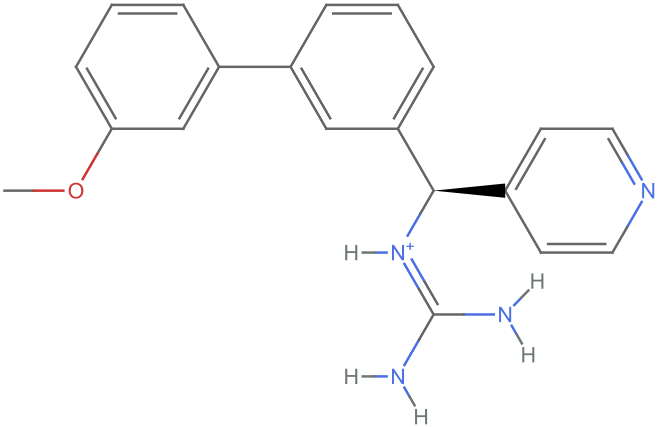

3D Plot

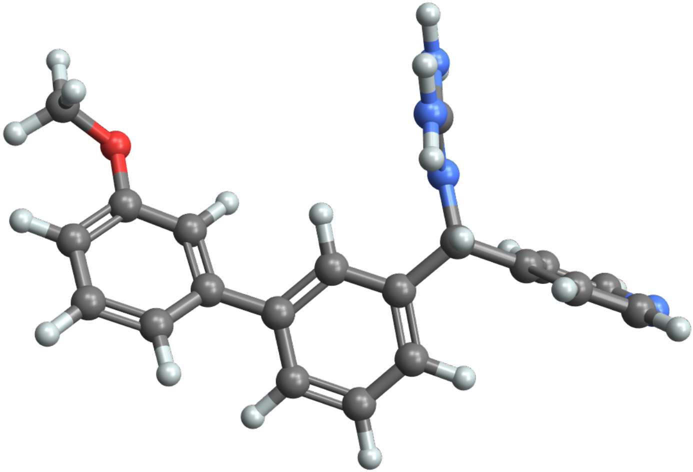

2D Plot

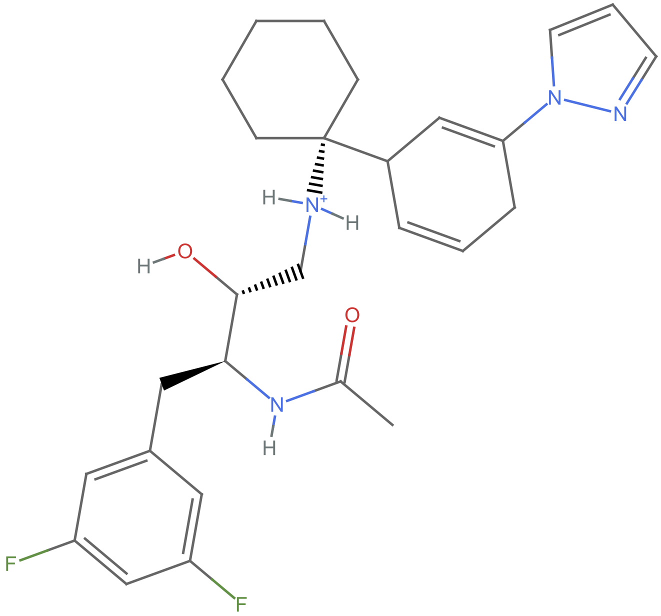

2D Autocorrelation

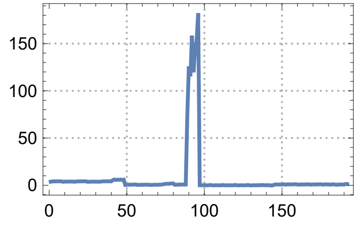

3D Plot

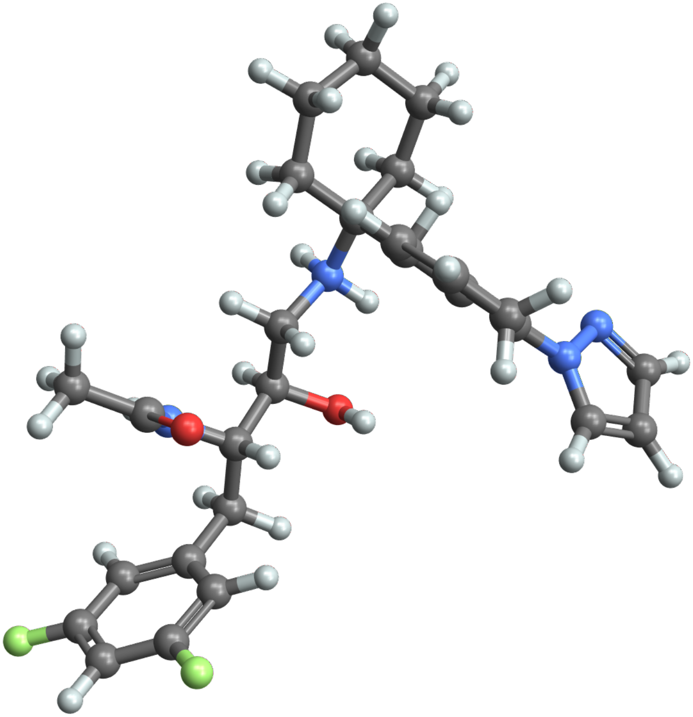

Dataset Index= 219

Molecule Formula= $C_{27}H_{35}F_2N_4O_2^+$

3D Autocorrelation

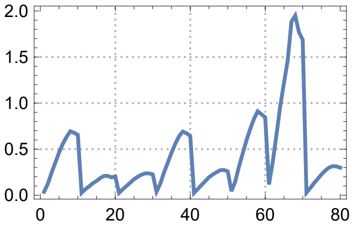

2D Plot

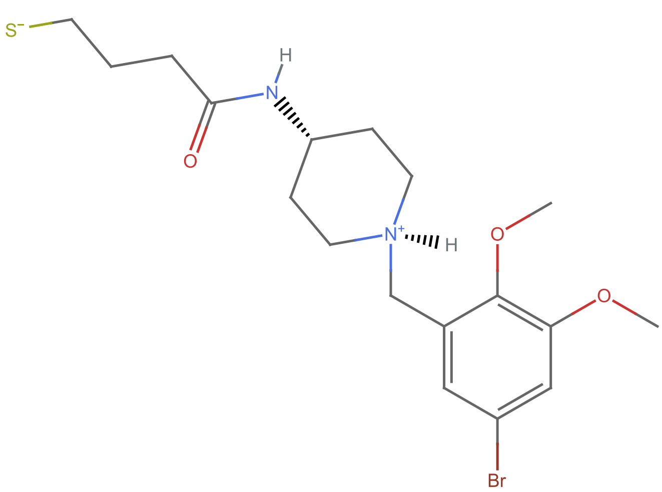

3D Plot

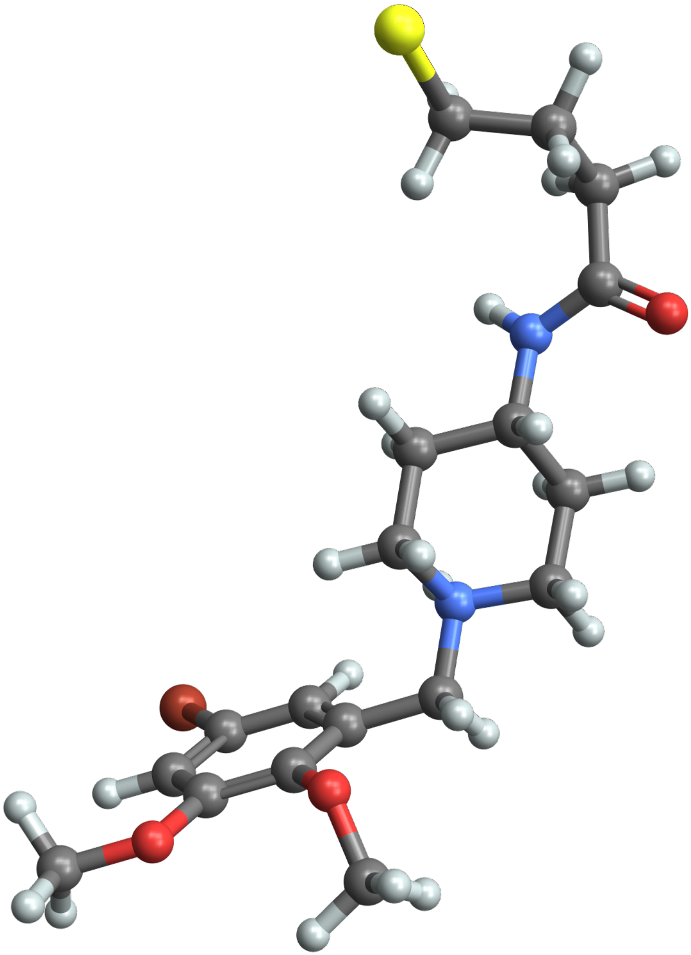

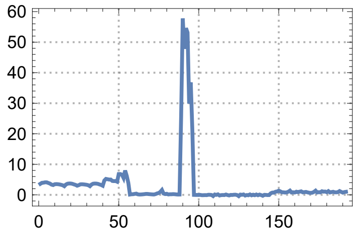

Dataset Index= 220

Molecule Formula= $C_{18}H_{27}BrN_2O_3S$

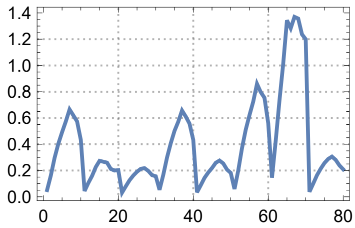

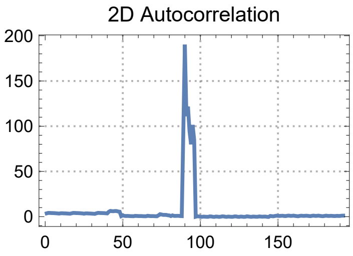

Dataset Index= 221

Molecule Formula= $C_{20}H_{20}F_3N_5O_3$

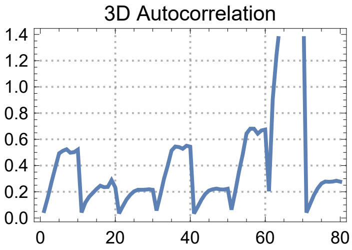

2D Plot

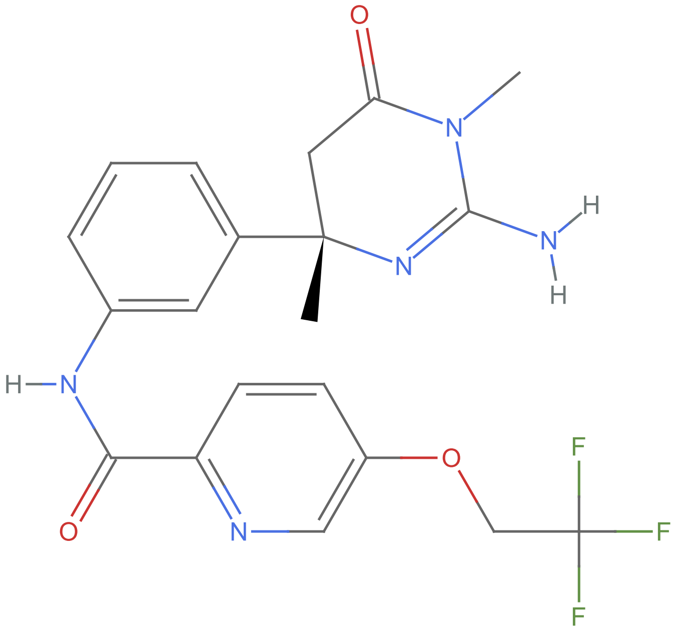

3D Plot

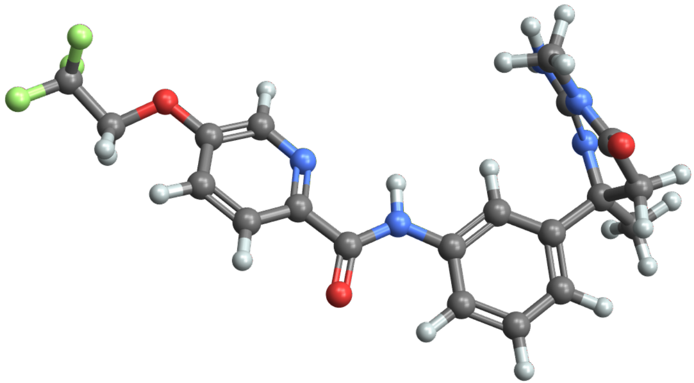

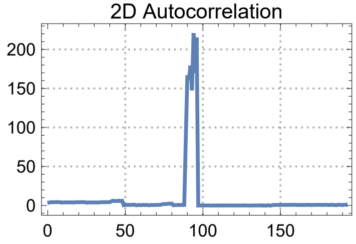

Dataset Index= 222

Molecule Formula= $C_{31}H_{42}F_2N_2O_5$

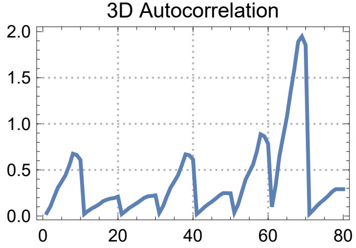

2D Plot

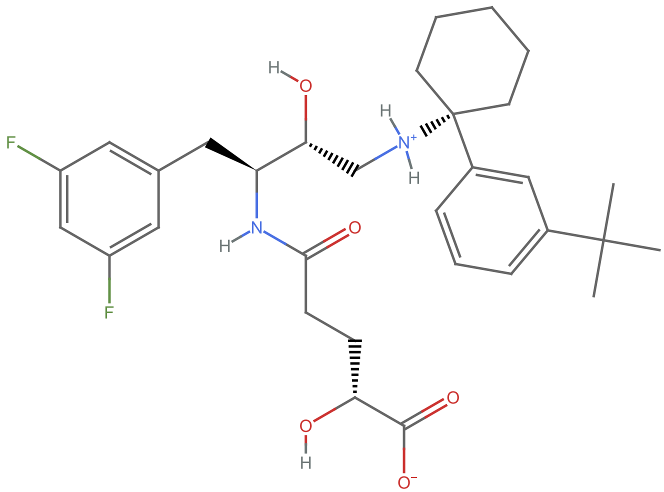

3D Plot

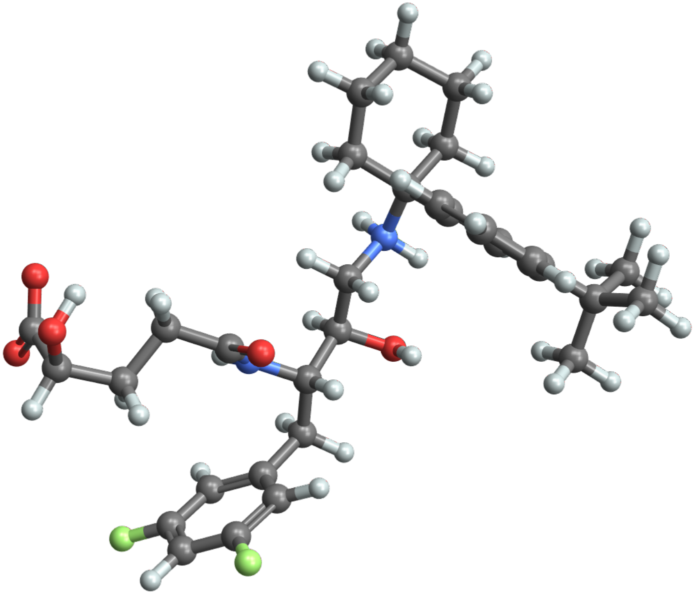

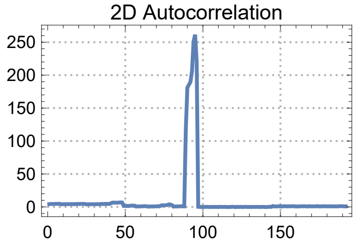

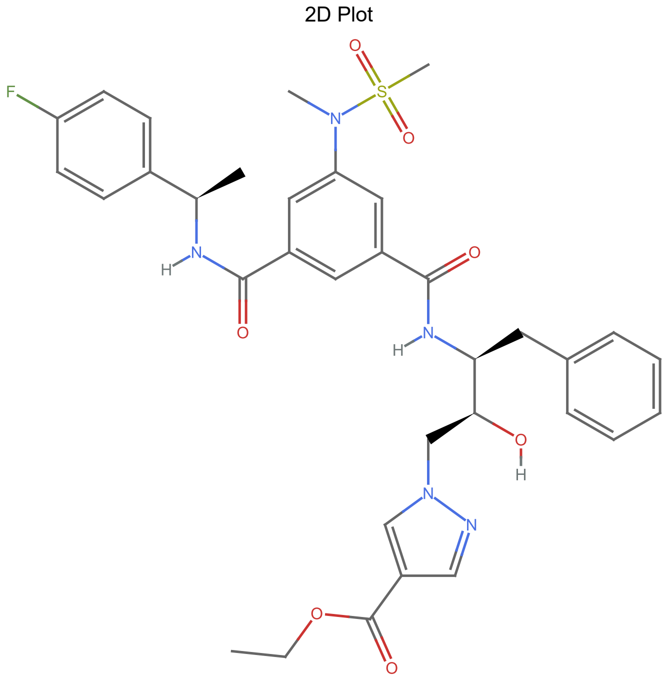

Dataset Index= 223

Molecule Formula= $C_{34}H_{38}FN_5O_7S$

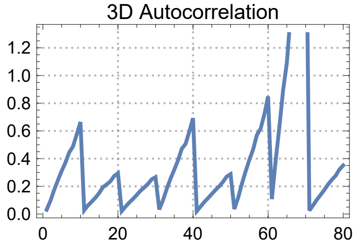

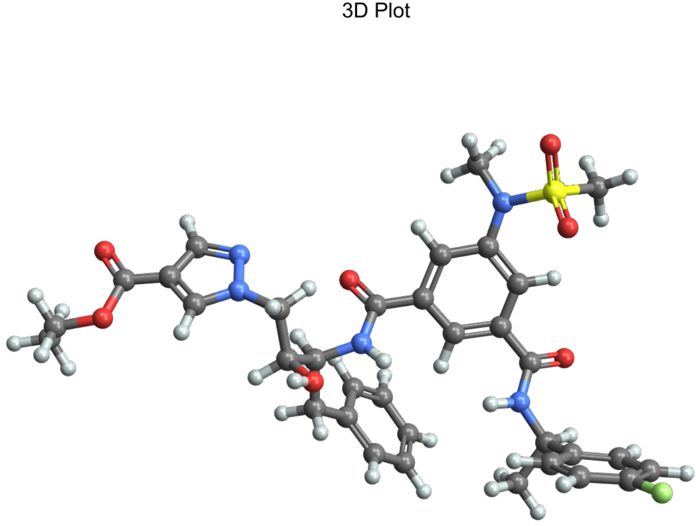

2D Plot

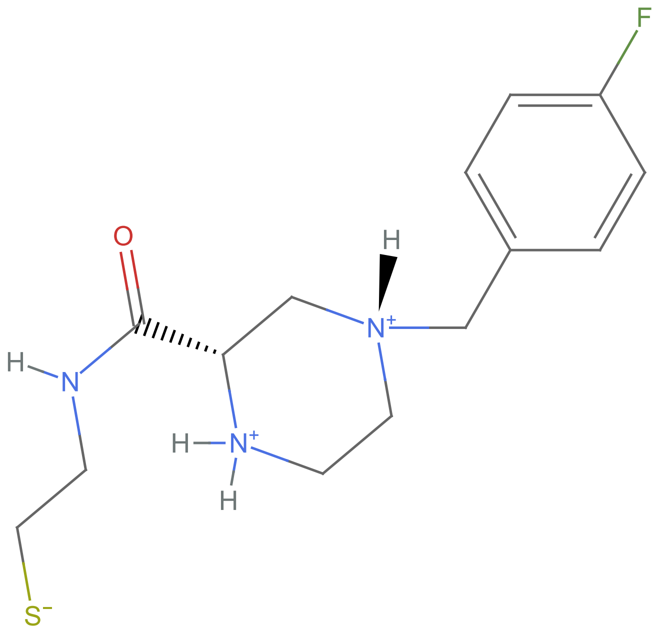

2D Autocorrelation

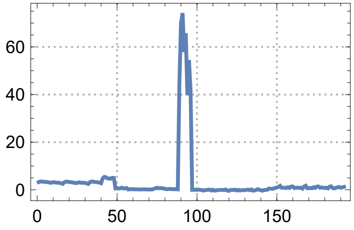

3D Plot

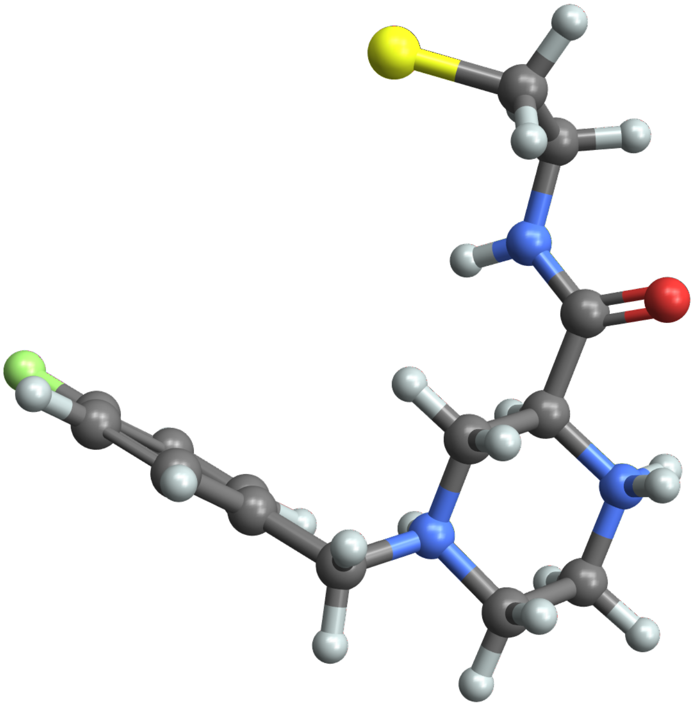

Dataset Index= 224

Molecule Formula= $C_{14}H_{21}FN_3OS^+$

3D Autocorrelation

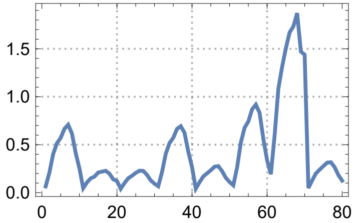

2D Autocorrelation

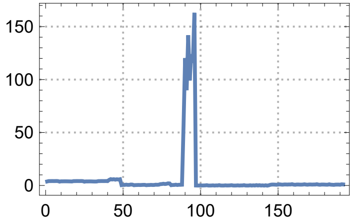


```
Dataset Index=       225

Molecule Formula=    C29H39F2N2O3+
```

3D Autocorrelation

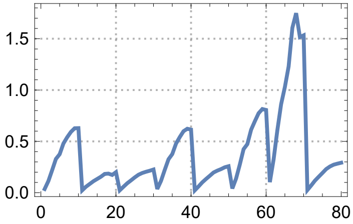

2D Plot

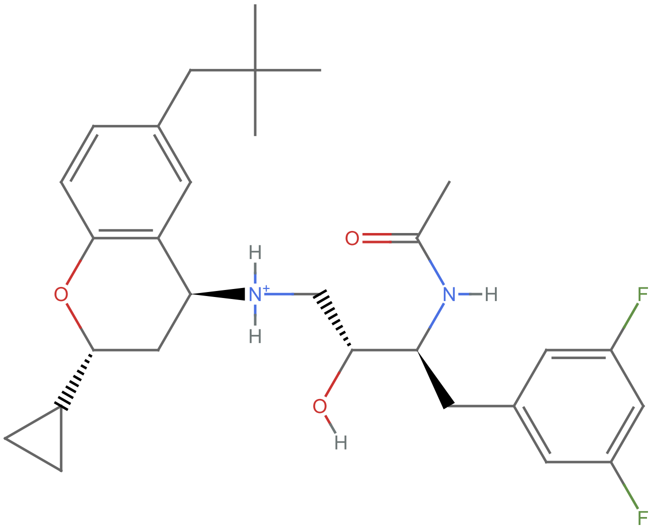

3D Plot

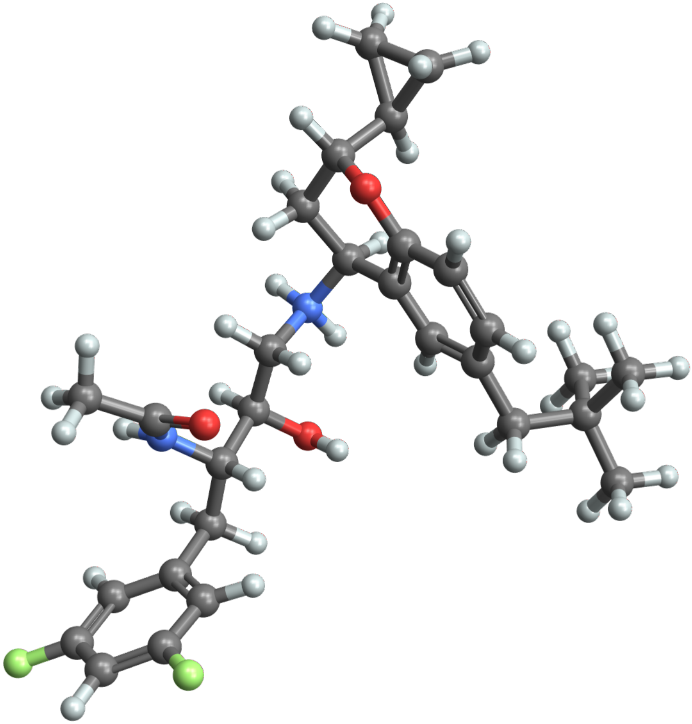

2D Plot

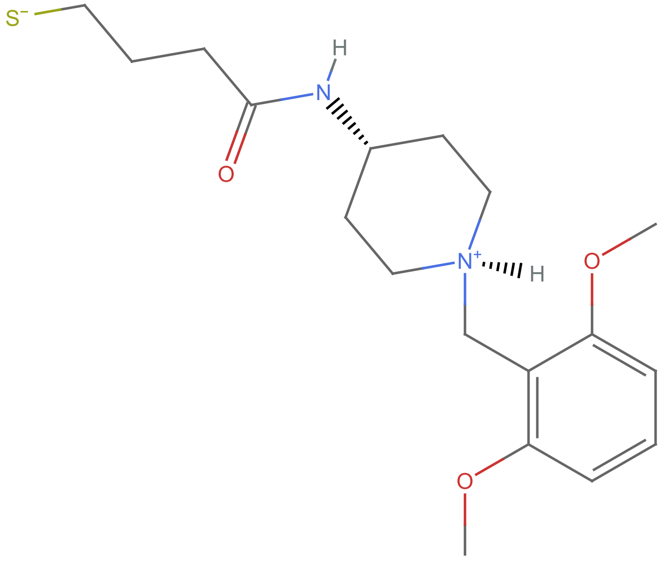

2D Autocorrelation

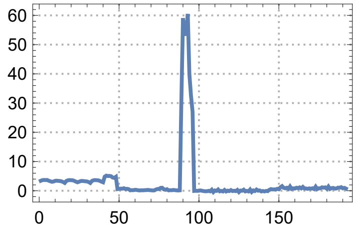

3D Plot

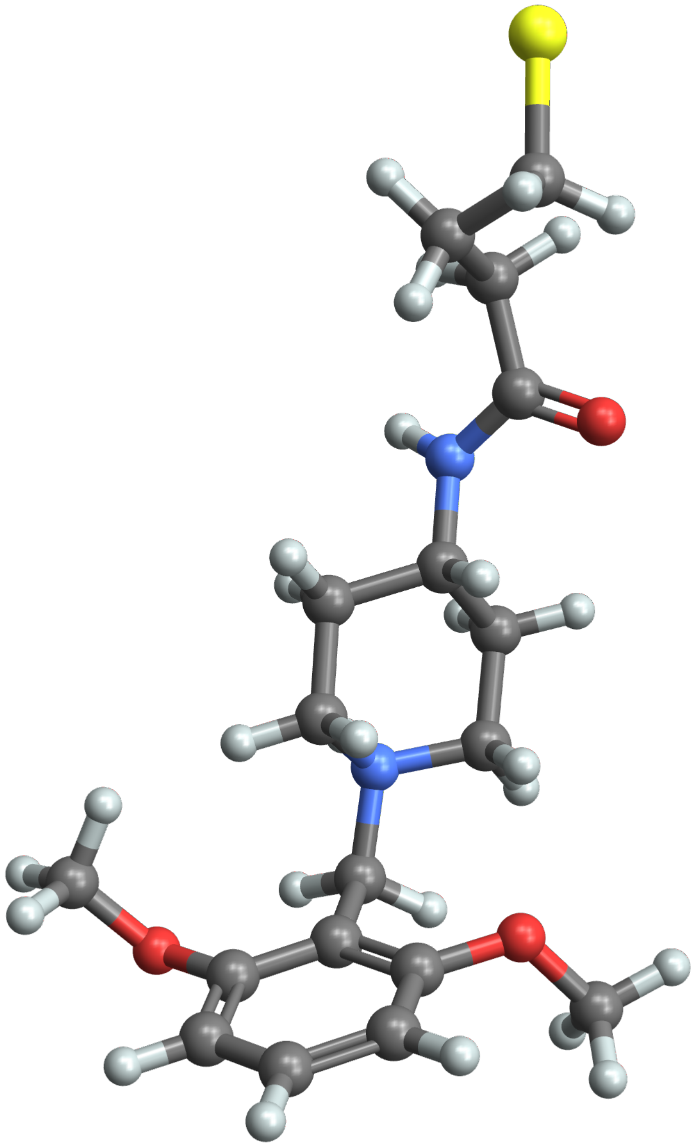

Dataset Index= 226

Molecule Formula= $C_{18}H_{28}N_2O_3S$

3D Autocorrelation

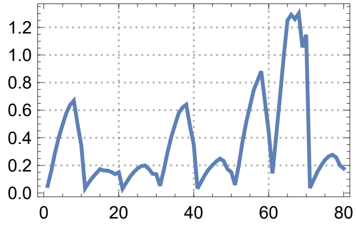

2D Plot

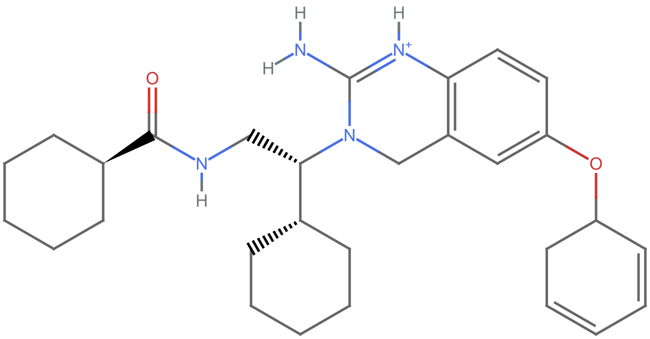

3D Plot

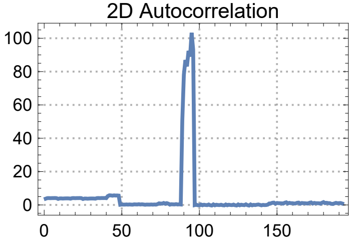

Dataset Index= 227

Molecule Formula= $C_{29}H_{41}N_4O_2^+$

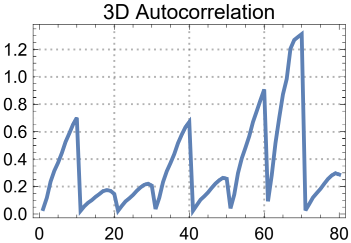

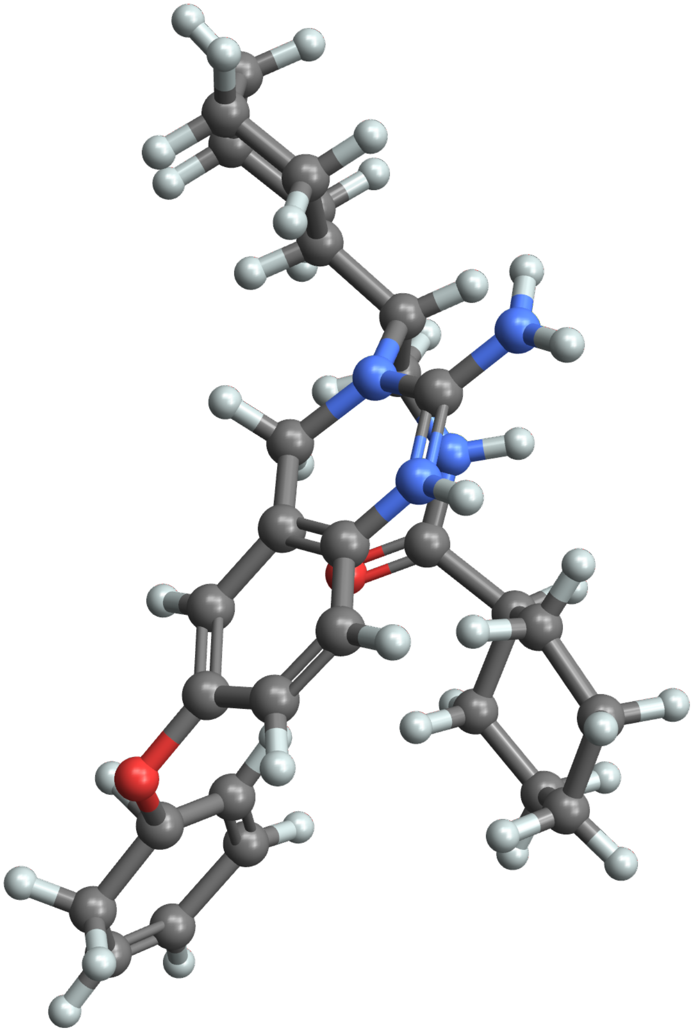

2D Plot

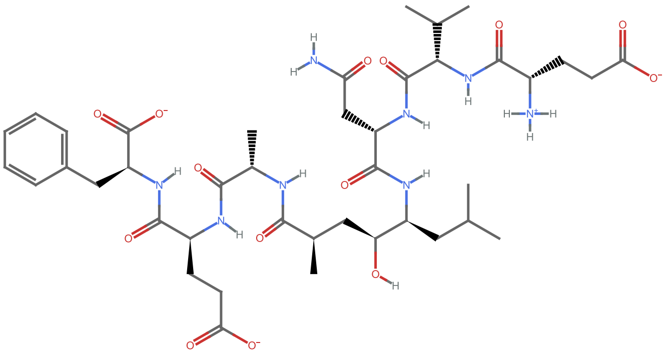

2D Autocorrelation

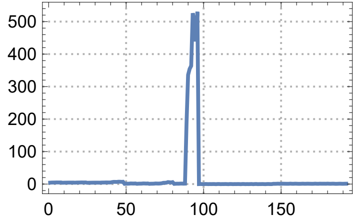

3D Plot

Dataset Index= 228

Molecule Formula= $C_{41}H_{62}N_8O_{14}^{2-}$

3D Autocorrelation

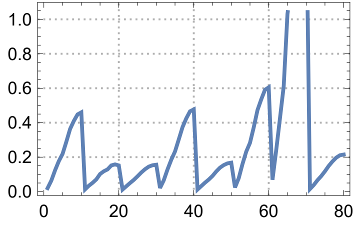

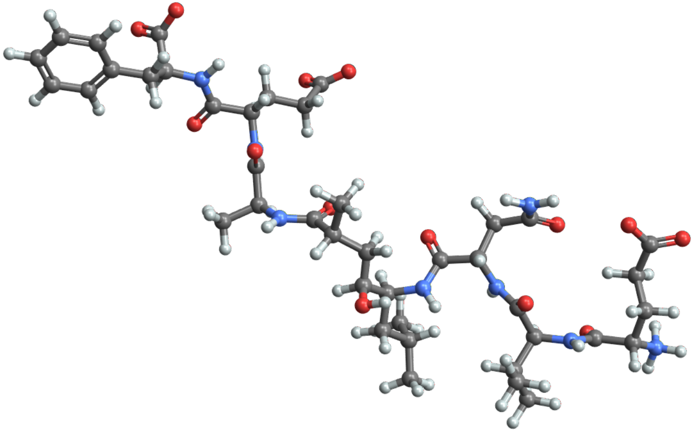

2D Plot

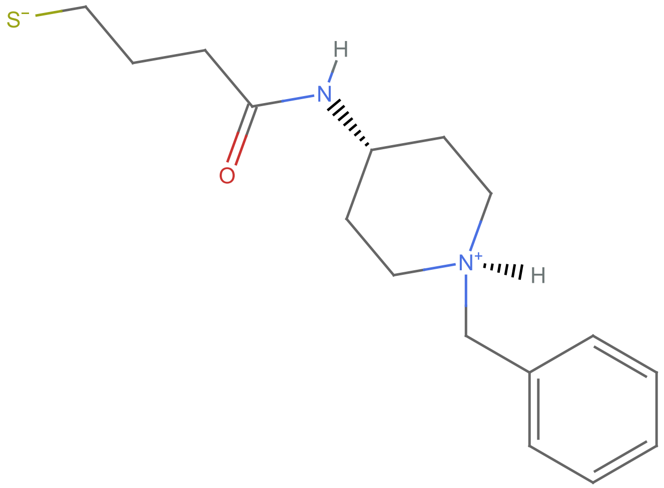

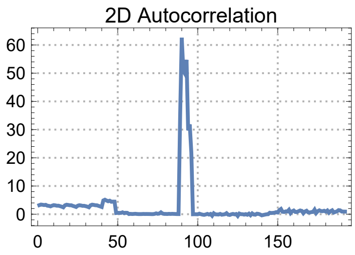

3D Plot

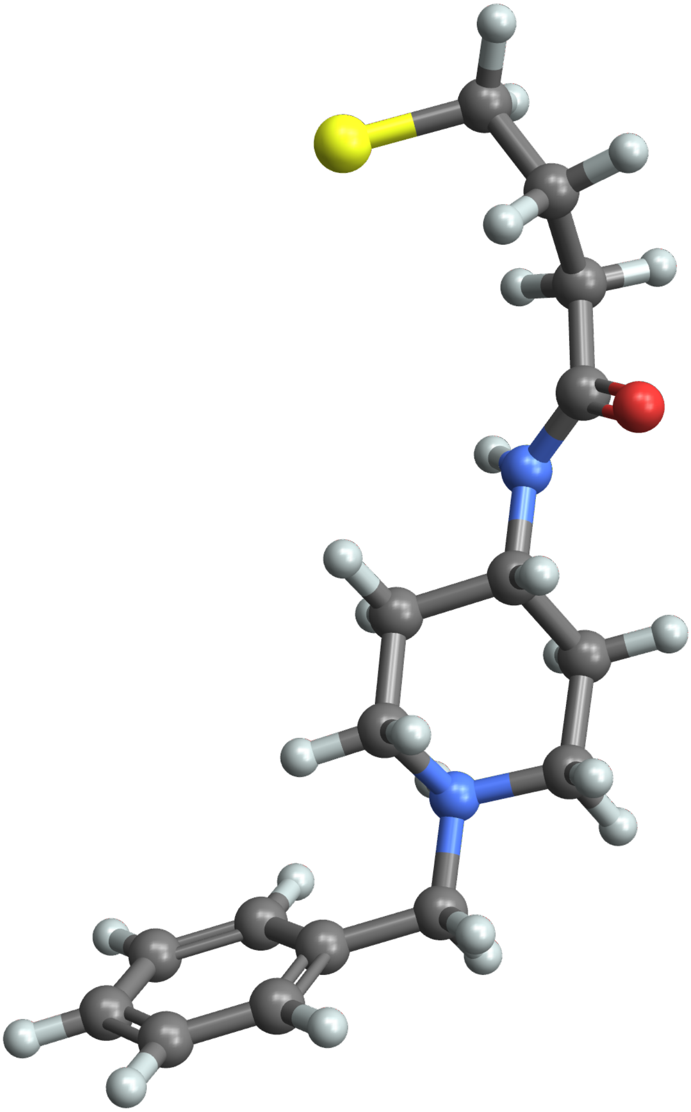

Dataset Index= 229

Molecule Formula= $C_{16}H_{24}N_2OS$

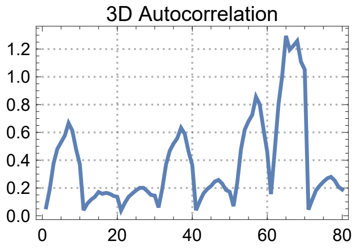

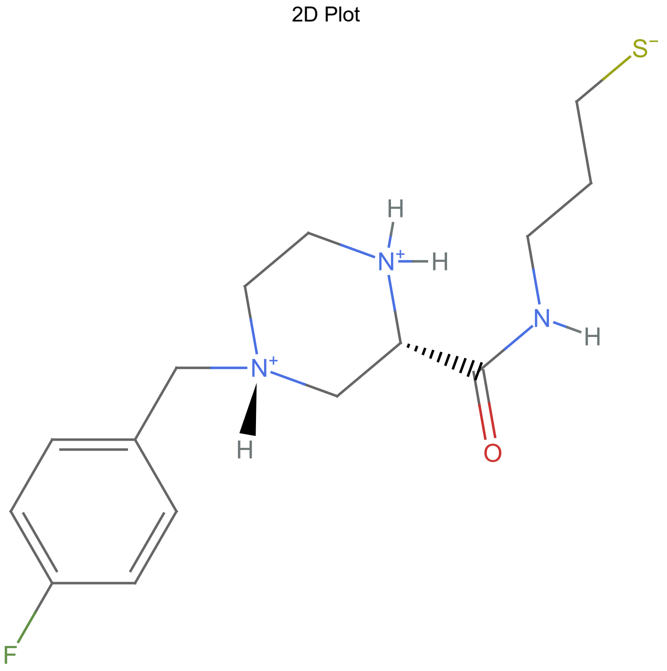

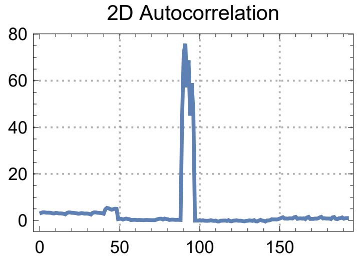

Dataset Index= 230

Molecule Formula= $C_{15}H_{23}FN_3OS^+$

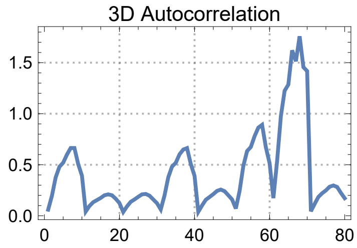

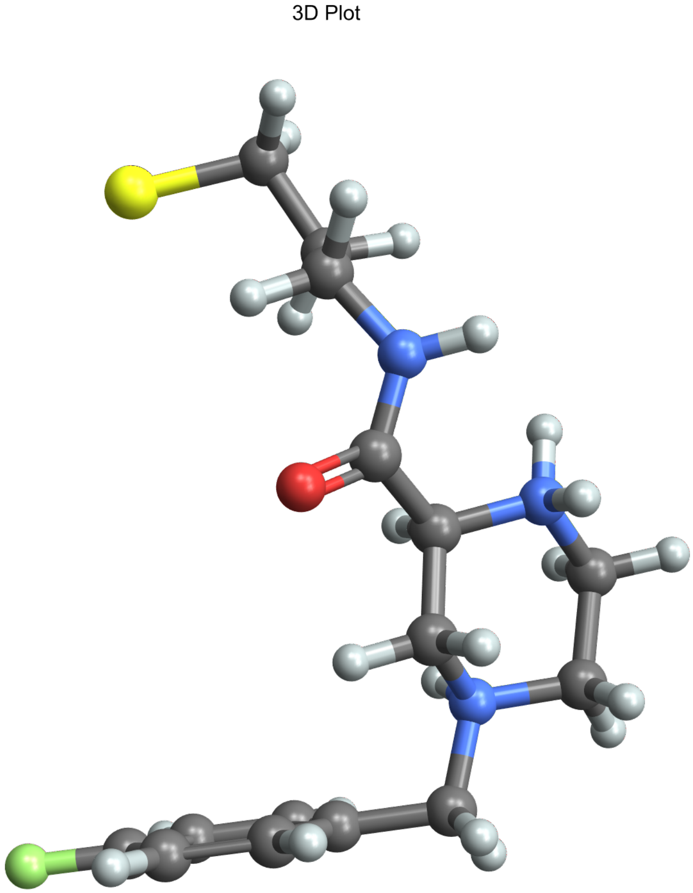

2D Plot

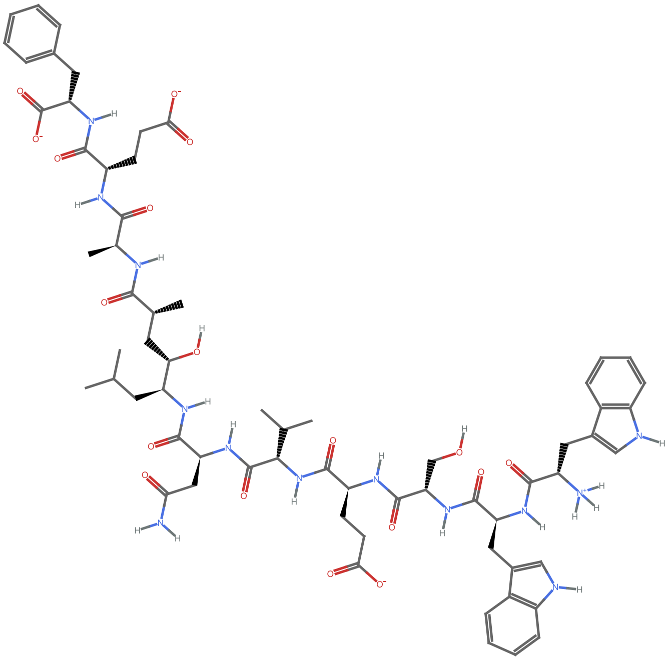

2D Autocorrelation

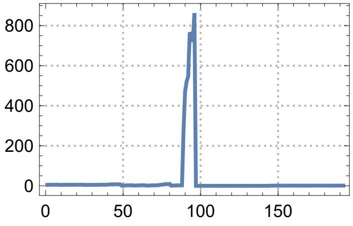

Dataset Index= 231

Molecule Formula= $C_{66}H_{87}N_{13}O_{18}{}^{2-}$

3D Autocorrelation

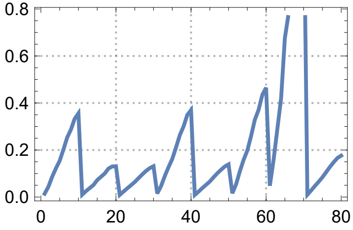

3D Plot

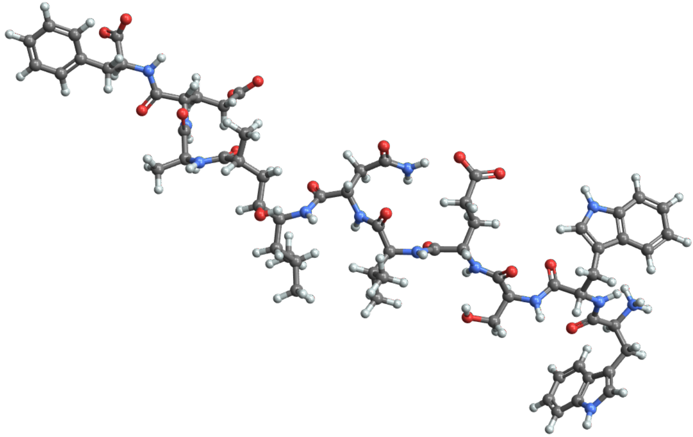

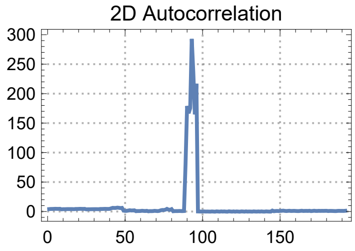

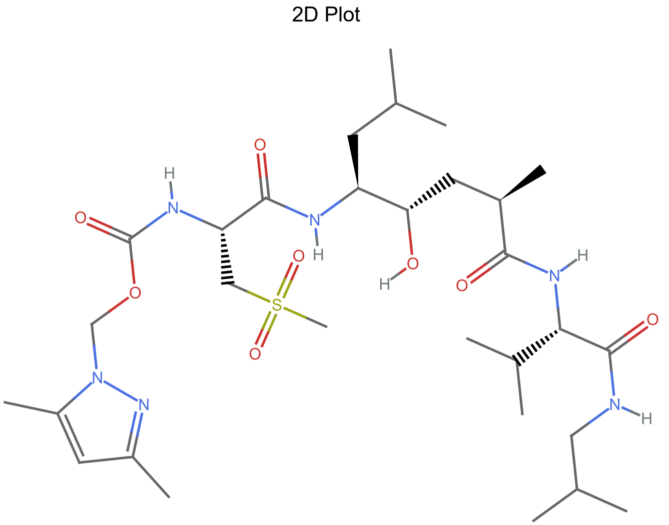

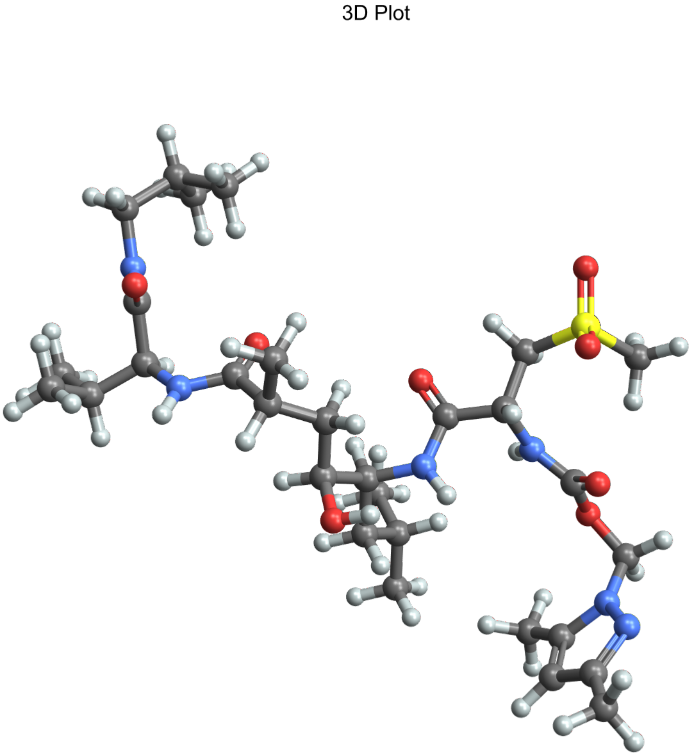

Dataset Index= 232

Molecule Formula= $C_{30}H_{54}N_6O_8S$

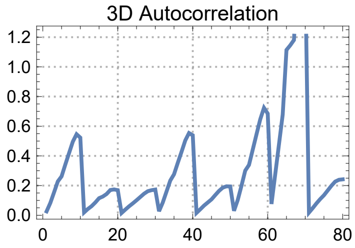

2D Autocorrelation

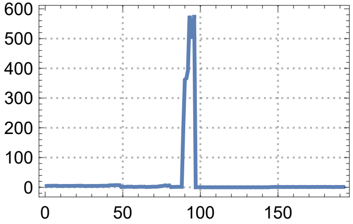

Dataset Index= 233

Molecule Formula= $C_{44}H_{66}N_7O_{15}^{3-}$

3D Autocorrelation

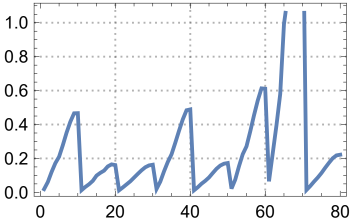

2D Plot

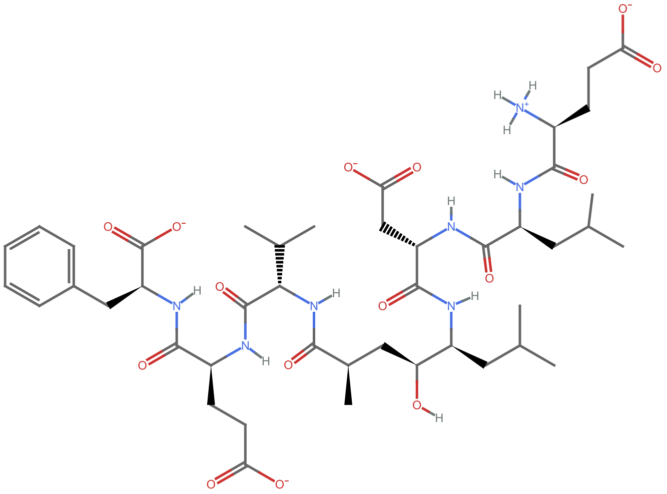

3D Plot

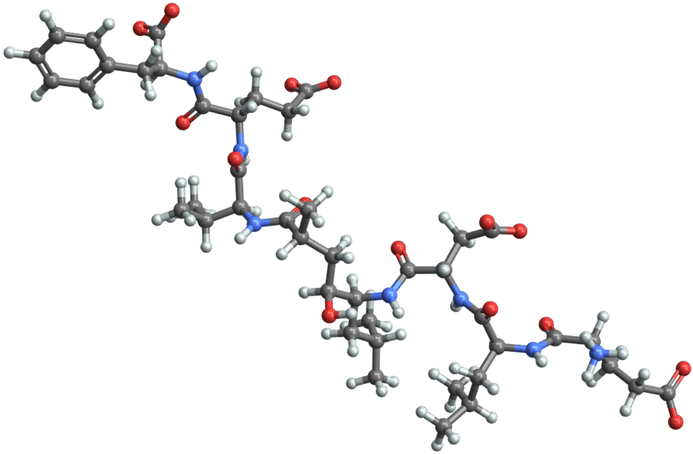

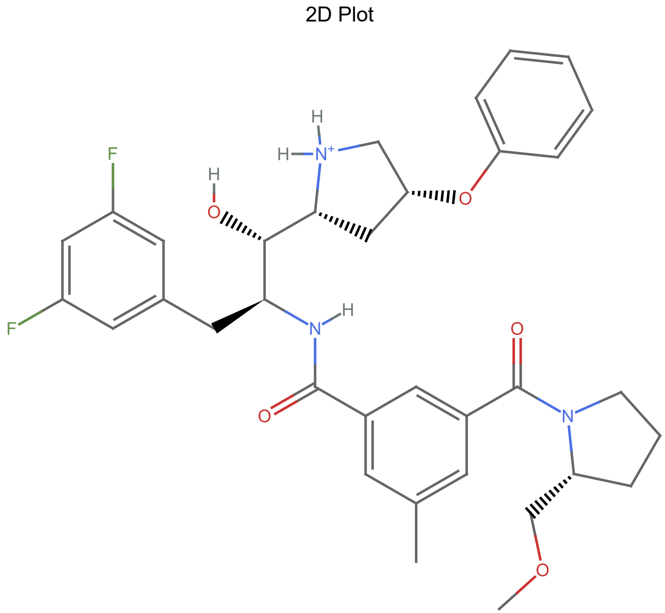

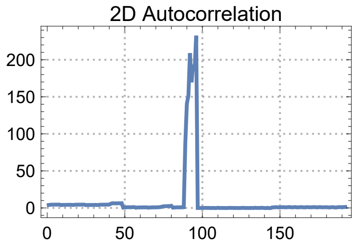

Dataset Index= 234

Molecule Formula= $C_{34}H_{40}F_2N_3O_5^+$

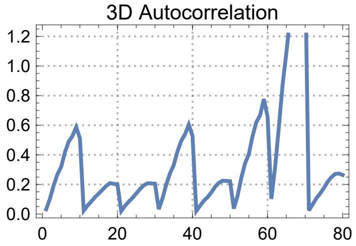

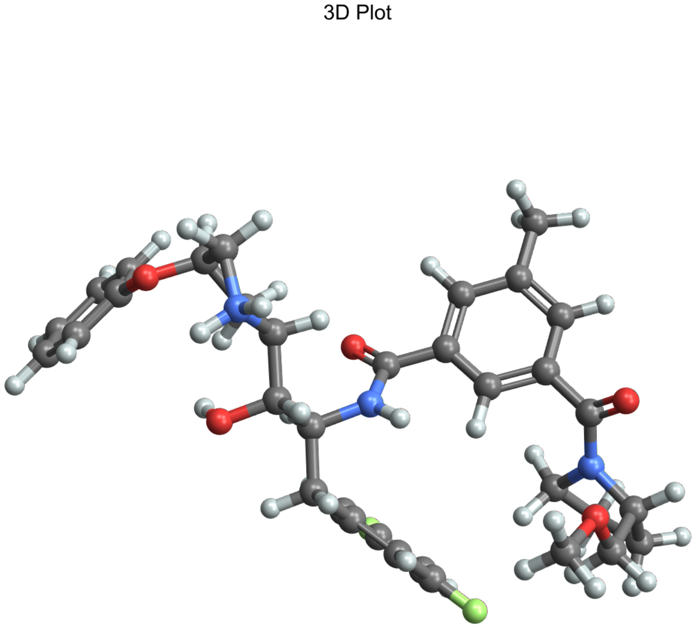

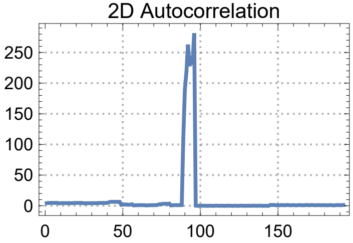

Dataset Index= 235

Molecule Formula= $C_{35}H_{43}F_2N_4O_6S^+$

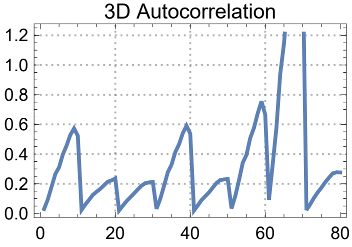

2D Plot

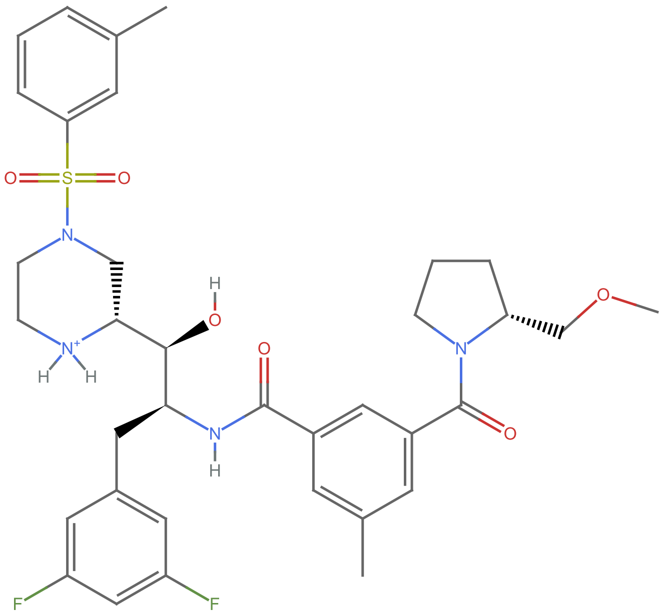

3D Plot

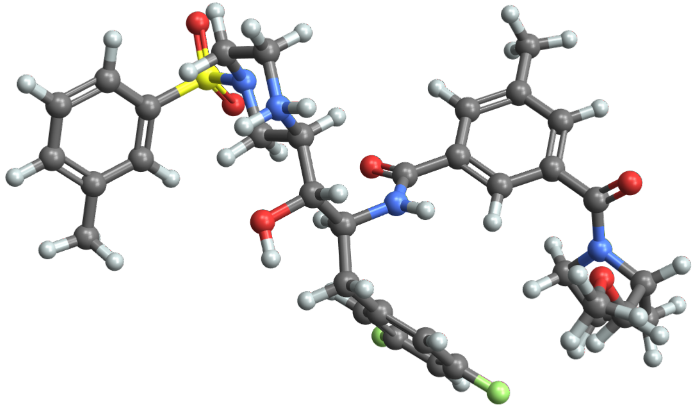

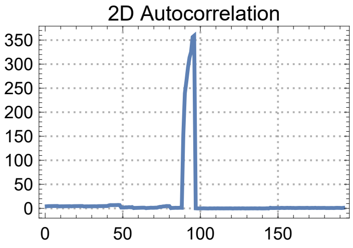

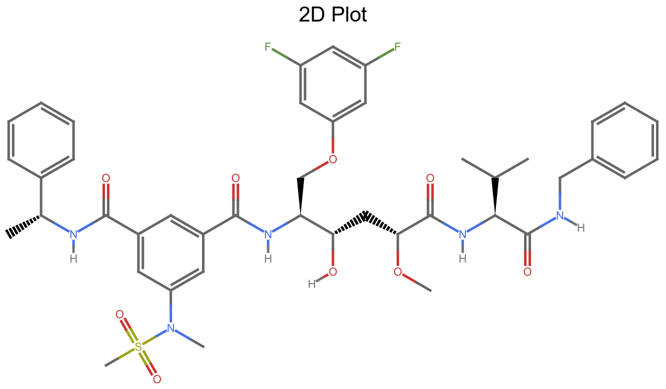

Dataset Index= 236

Molecule Formula= $C_{43}H_{51}F_2N_5O_9S$

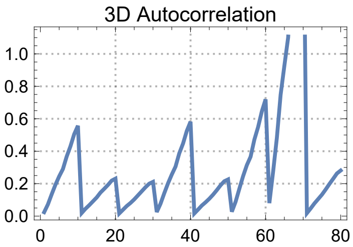

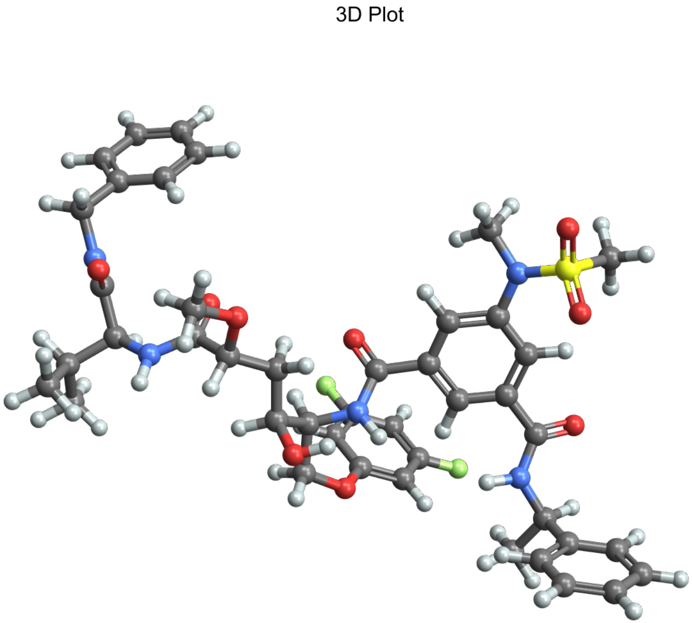

2D Plot

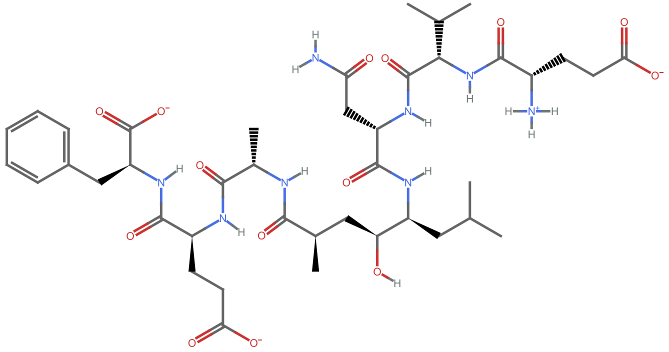

2D Autocorrelation

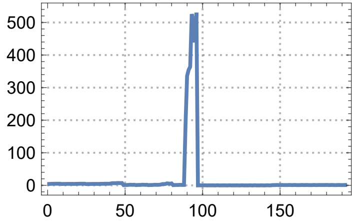

3D Plot

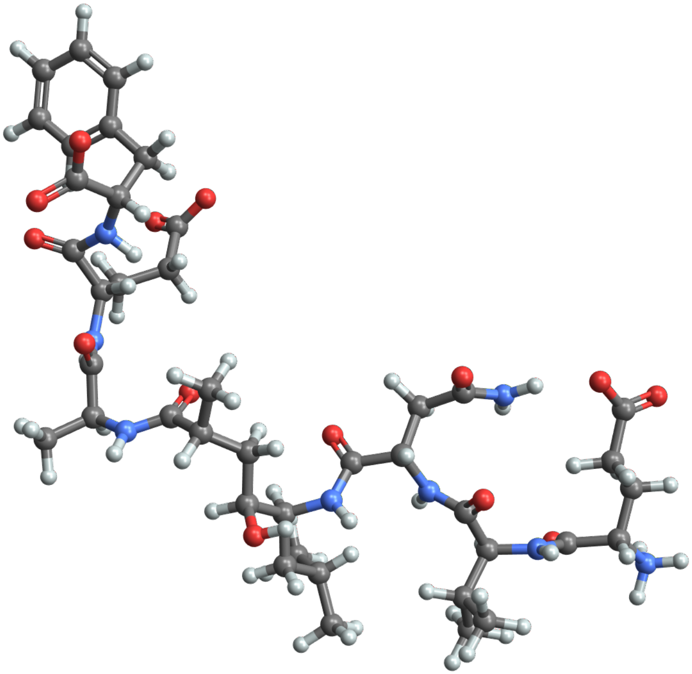

Dataset Index= 237

Molecule Formula= $C_{41}H_{62}N_8O_{14}^{2-}$

3D Autocorrelation

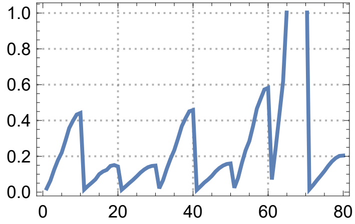

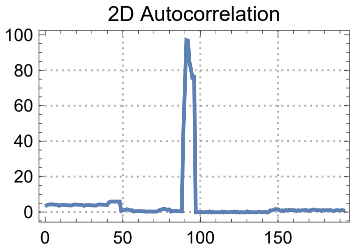

Dataset Index= 238

Molecule Formula= $C_{24}H_{22}N_6O_2S$

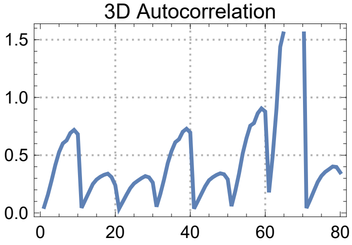

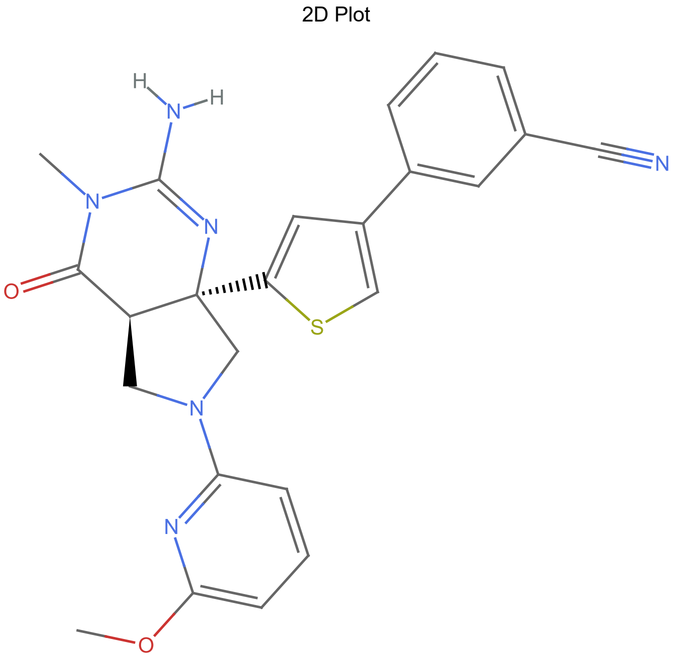

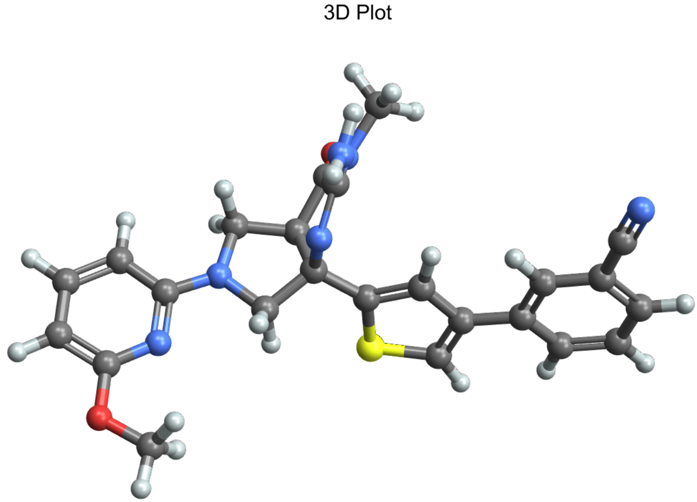

2D Autocorrelation

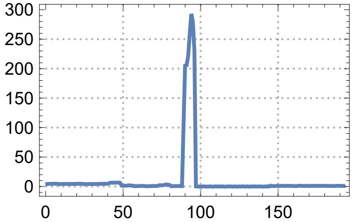

Dataset Index= 239

Molecule Formula= $C_{36}H_{55}N_5O_7S$

3D Autocorrelation

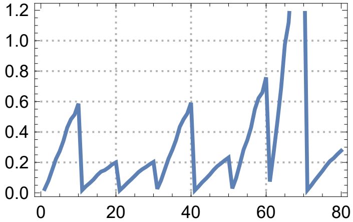

2D Plot

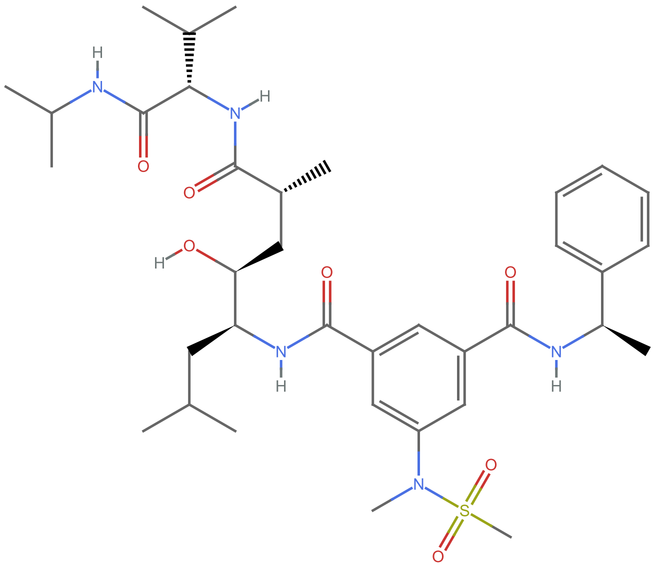

3D Plot

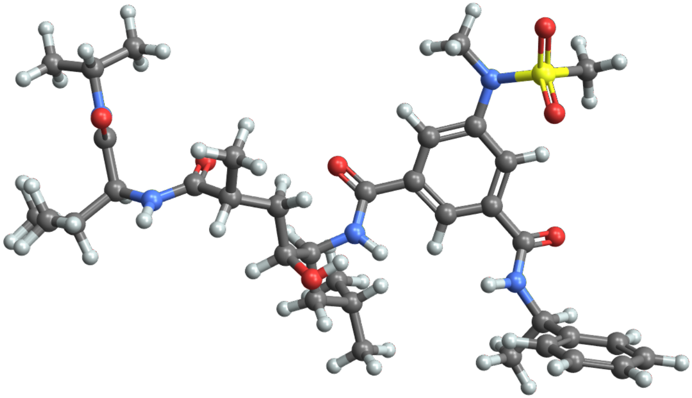

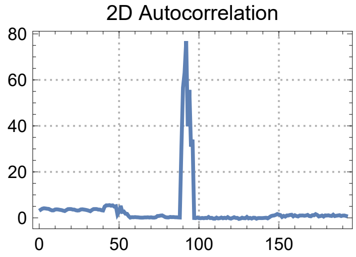

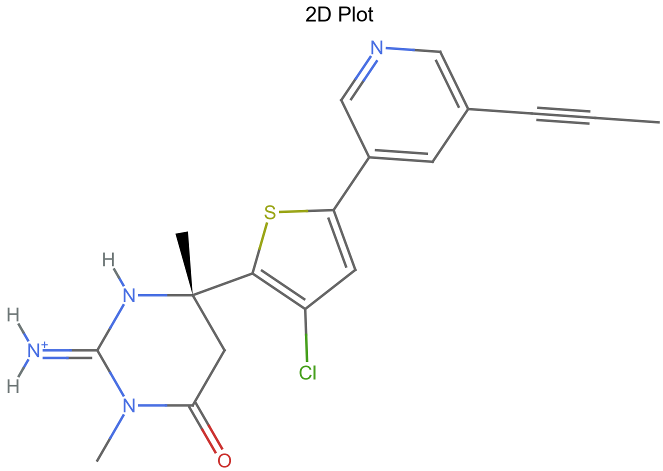

Dataset Index= 240

Molecule Formula= $C_{18}H_{18}ClN_4OS^+$

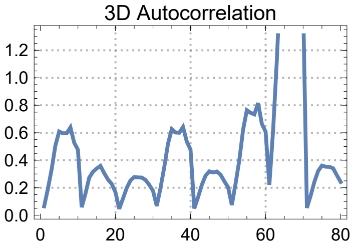

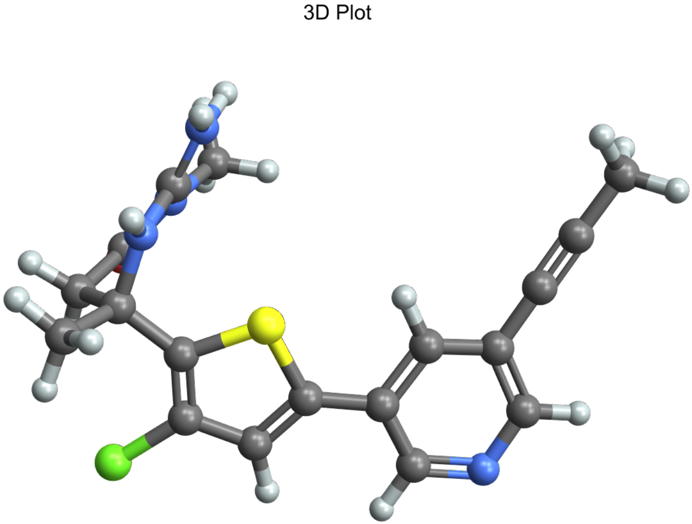

2D Plot

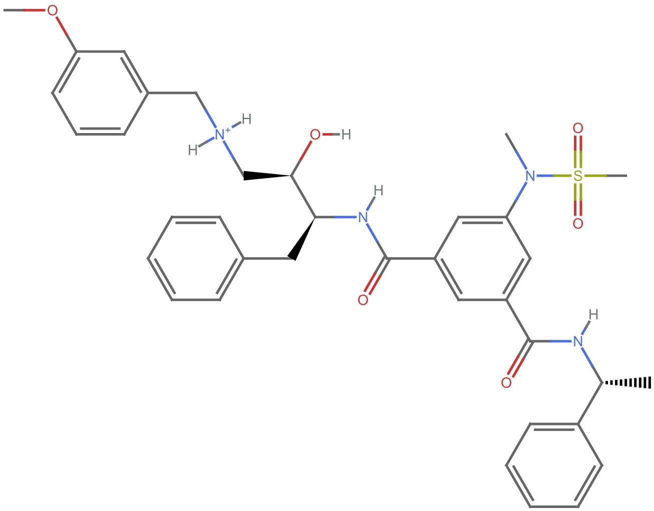

2D Autocorrelation

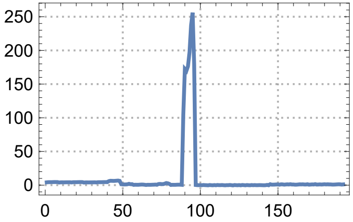

Dataset Index= 241

Molecule Formula= $C_{36}H_{43}N_4O_6S^+$

3D Autocorrelation

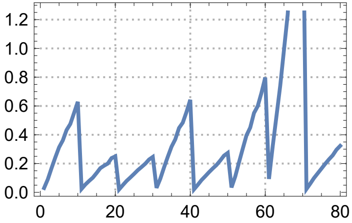

3D Plot

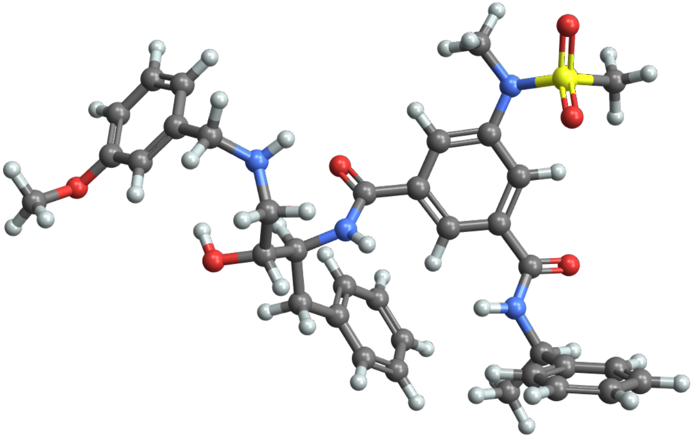

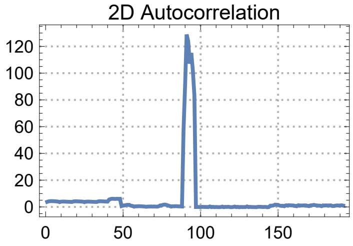

Dataset Index= 242

Molecule Formula= $C_{25}H_{21}N_5O_2S$

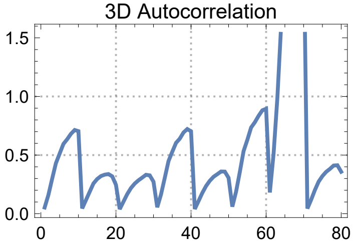

2D Plot

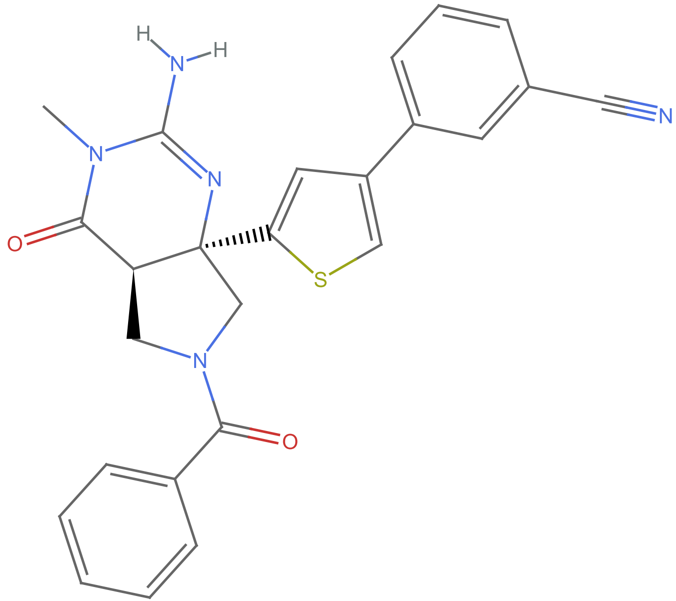

3D Plot

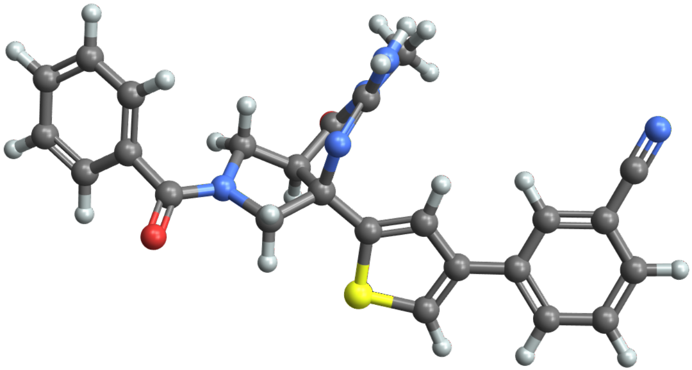

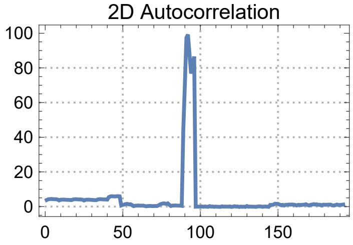

Dataset Index= 243

Molecule Formula= $C_{24}H_{22}N_6O_2S$

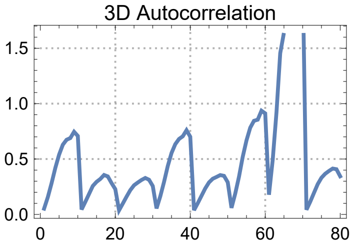

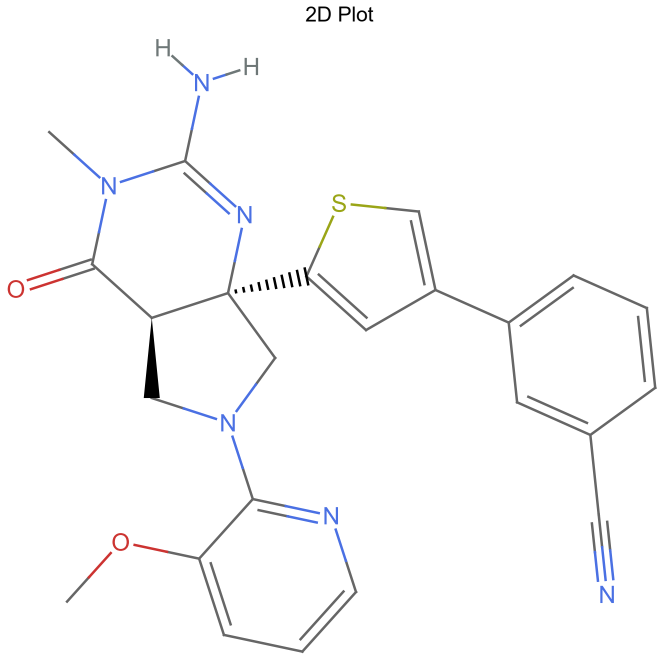

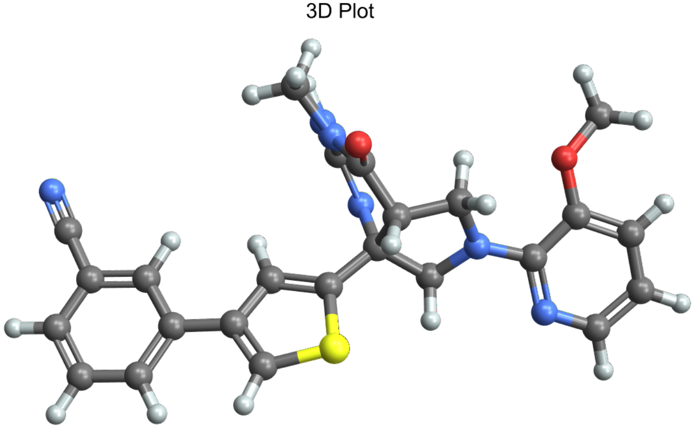

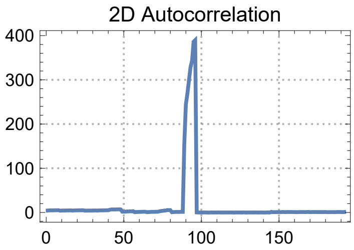

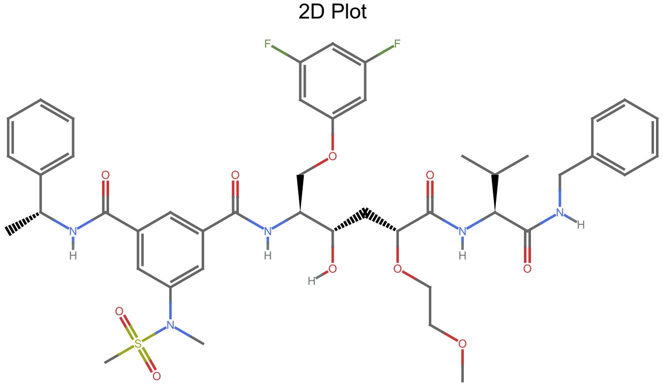

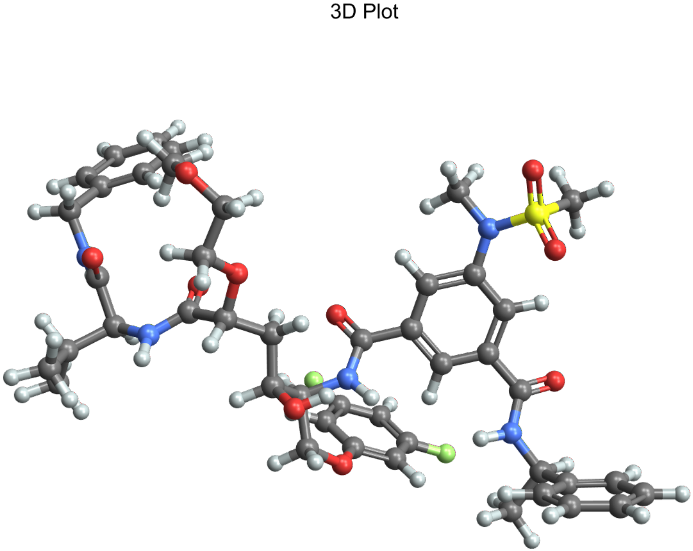

Dataset Index= 244

Molecule Formula= $C_{45}H_{55}F_2N_5O_{10}S$

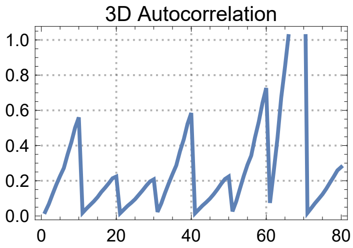

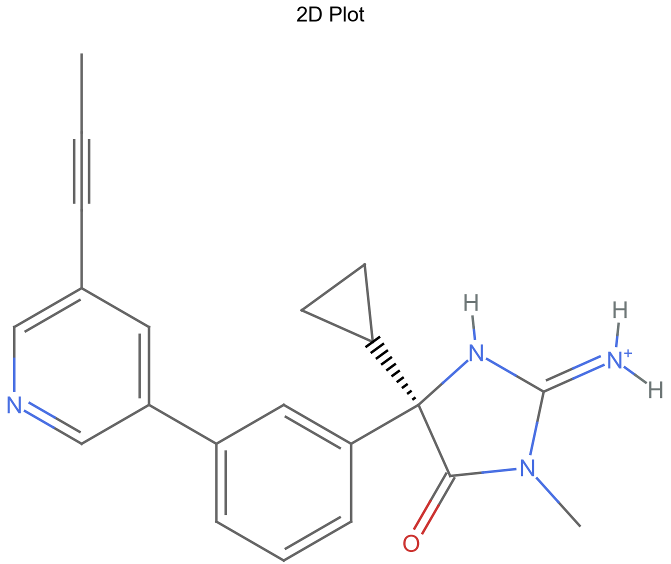

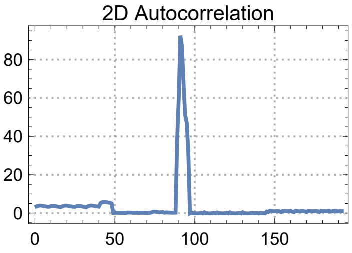

Dataset Index= 245

Molecule Formula= $C_{21}H_{21}N_4O^{+}$

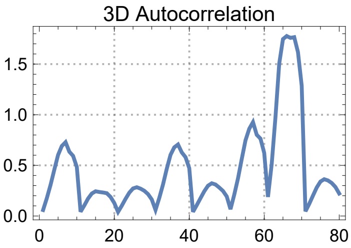

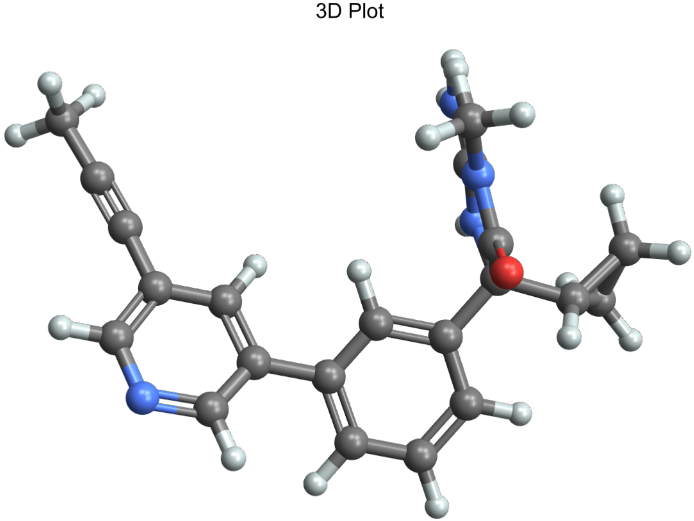

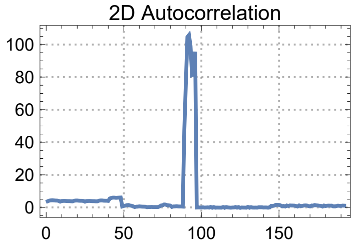

Dataset Index= 246

Molecule Formula= $C_{24}H_{19}N_7OS$

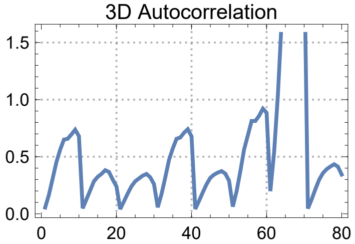

2D Plot

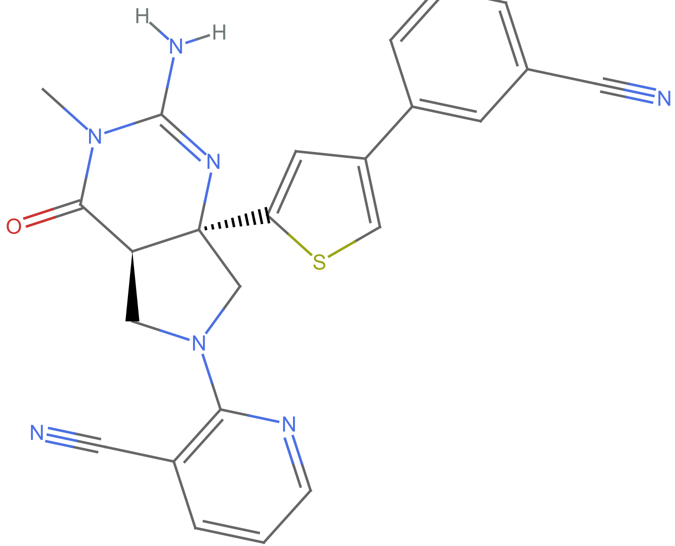

3D Plot

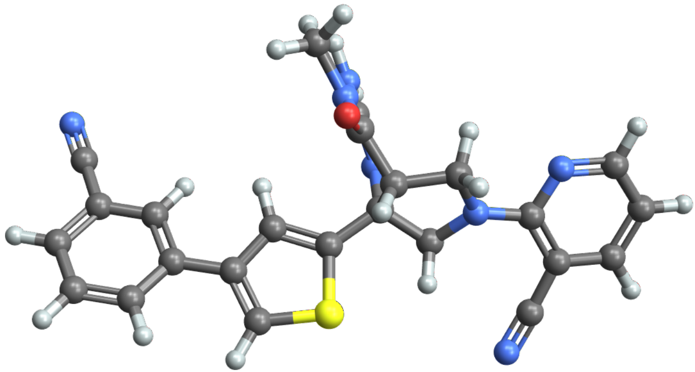

2D Plot

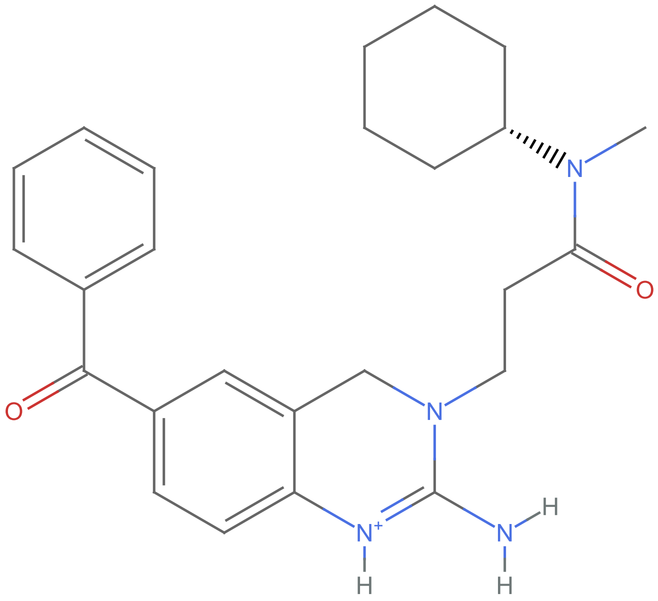

3D Plot

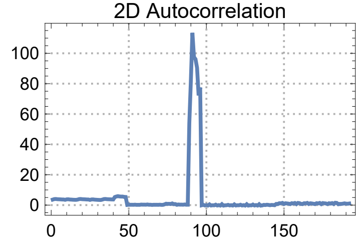

Dataset Index= 247

Molecule Formula= $C_{25}H_{31}N_4O_2^+$

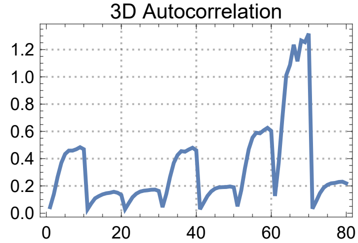

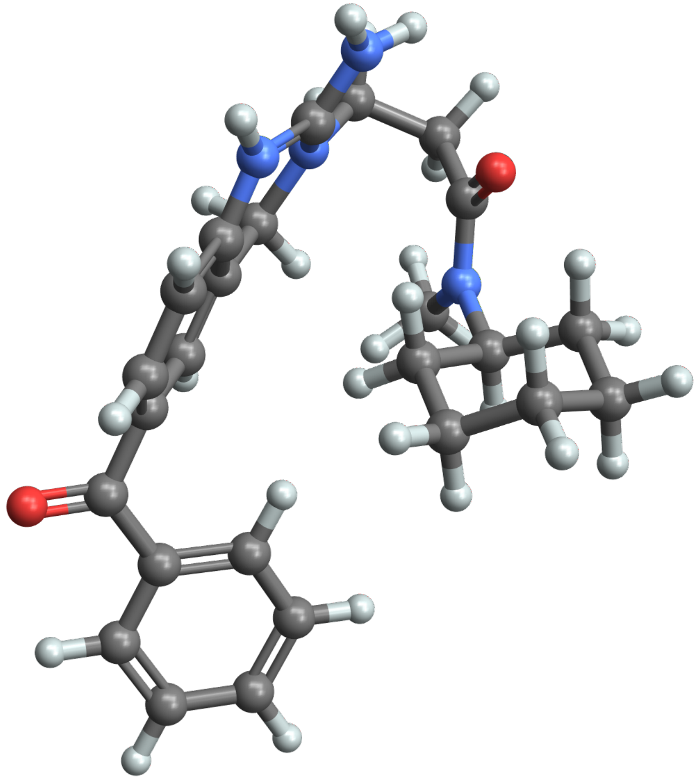

2D Plot

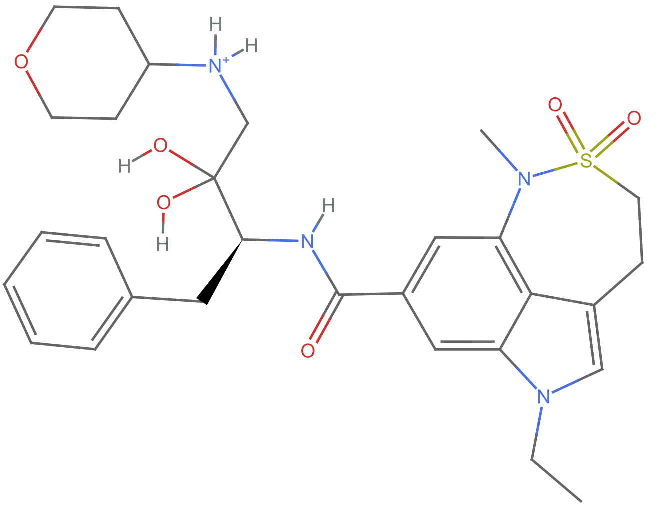

2D Autocorrelation

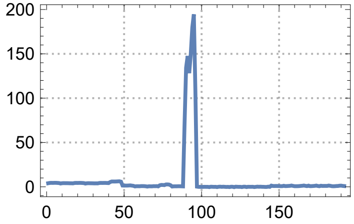

3D Plot

Dataset Index= 248

Molecule Formula= $C_{29}H_{39}N_4O_6S^+$

3D Autocorrelation

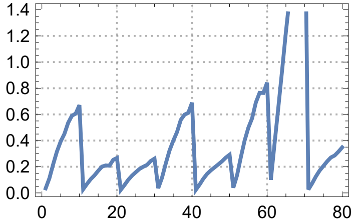

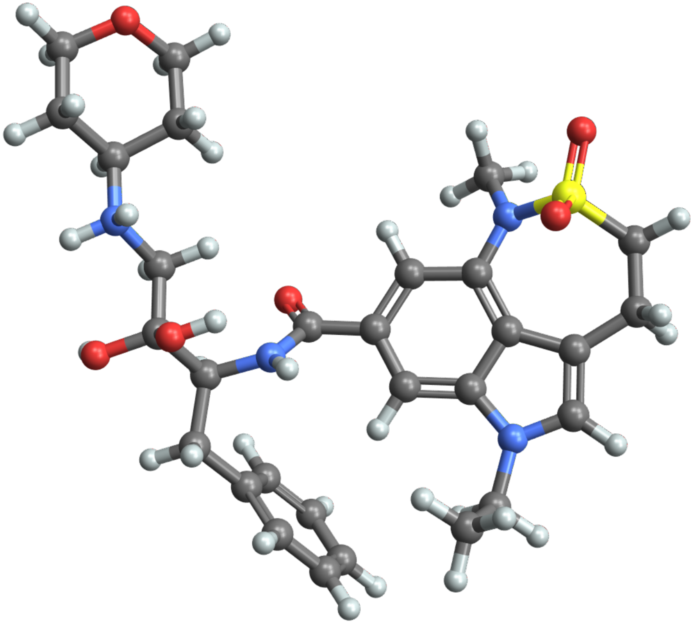

2D Plot

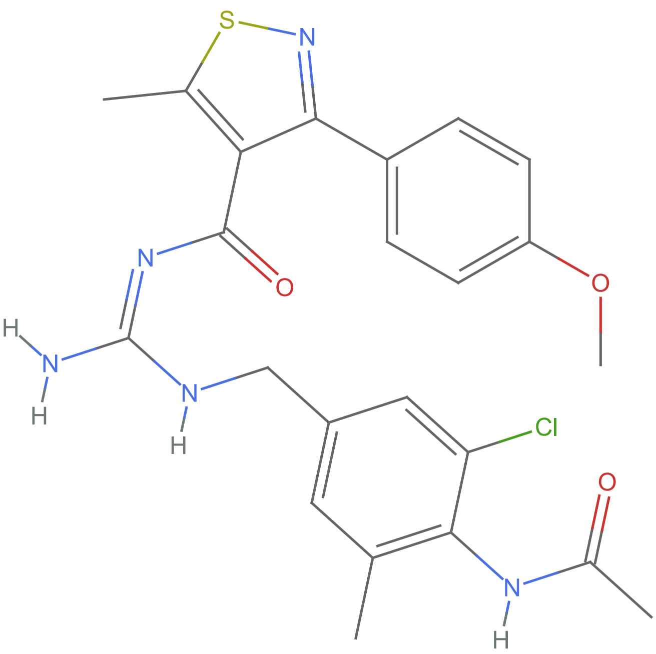

2D Autocorrelation

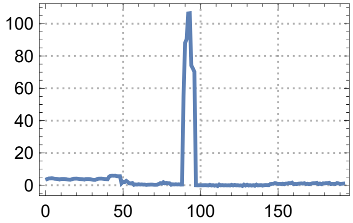

3D Plot

Dataset Index= 249

Molecule Formula= $C_{23}H_{24}ClN_5O_3S$

3D Autocorrelation

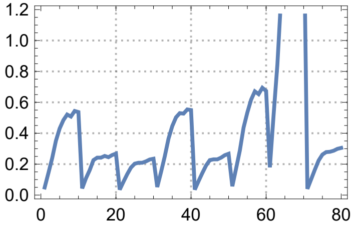

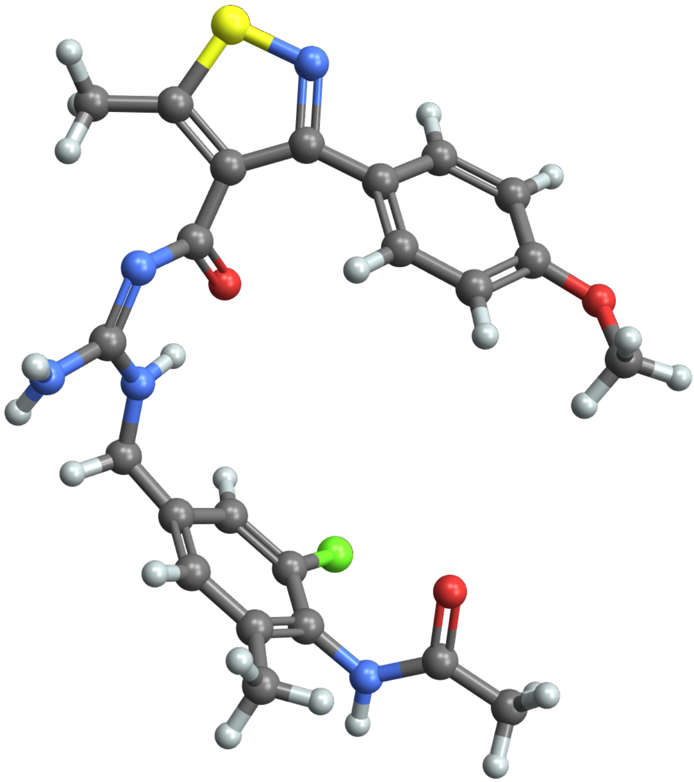

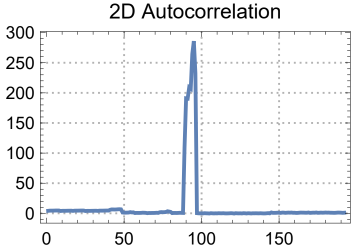

Dataset Index= 250

Molecule Formula= $C_{35}H_{48}N_5O_6S^+$

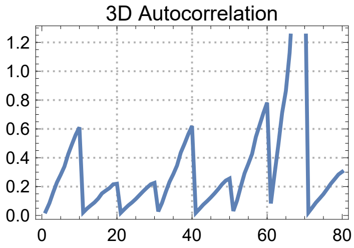

2D Plot

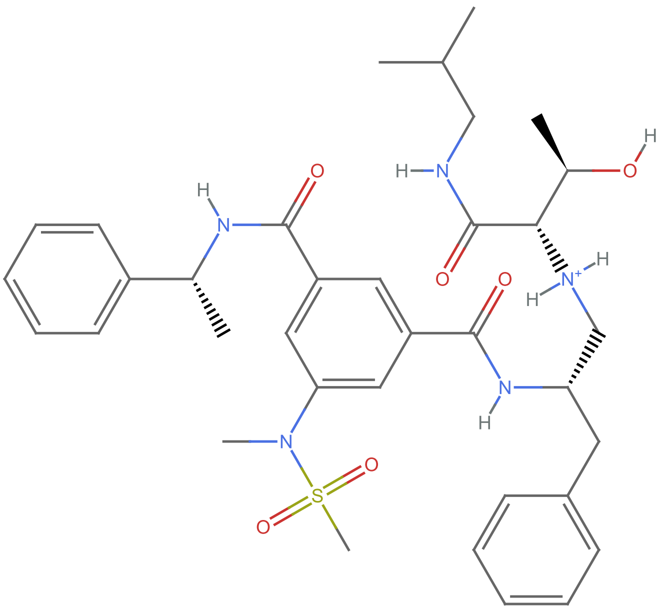

3D Plot

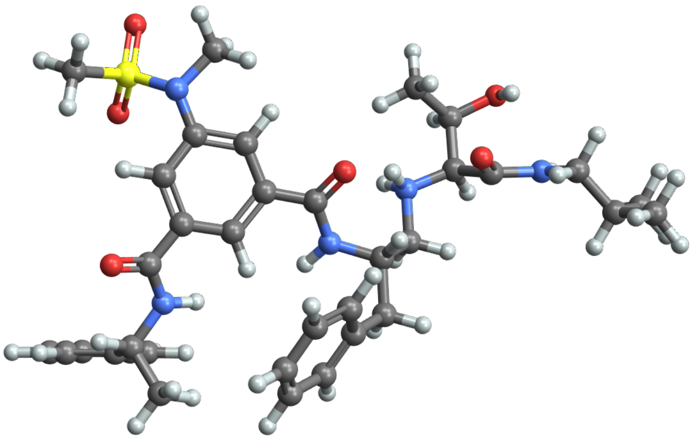

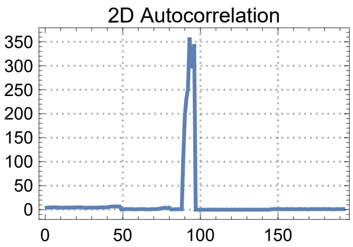

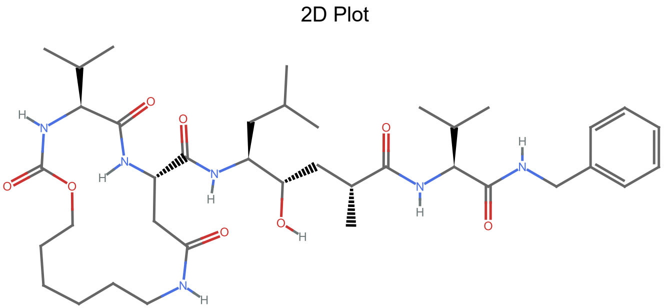

3D Plot

Dataset Index= 251

Molecule Formula= $C_{38}H_{62}N_6O_8$

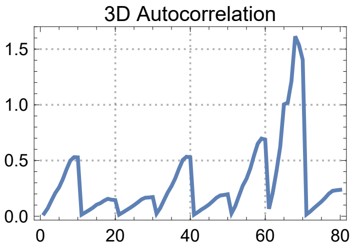

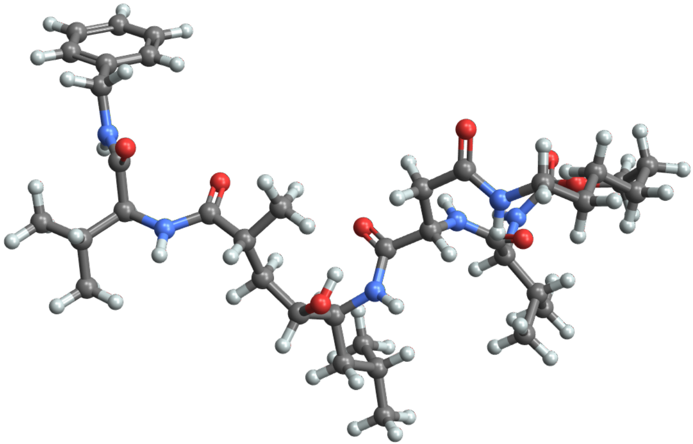

2D Autocorrelation

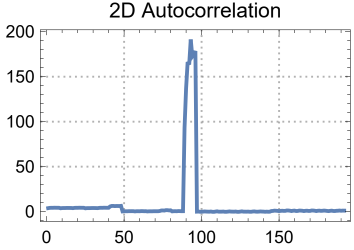

Dataset Index= 252

Molecule Formula= $C_{33}H_{42}FN_4O_3^+$

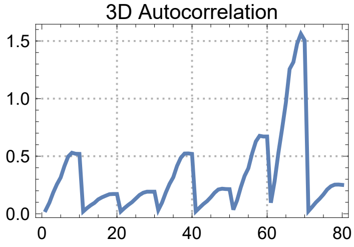

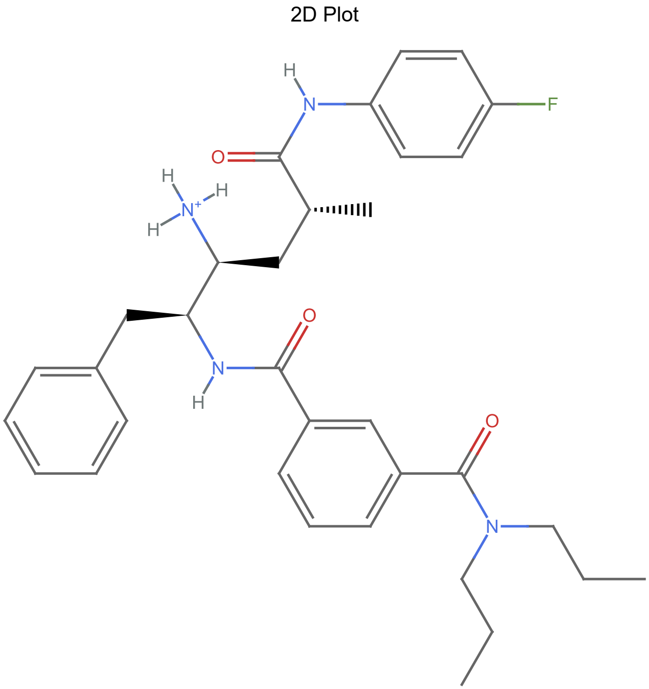

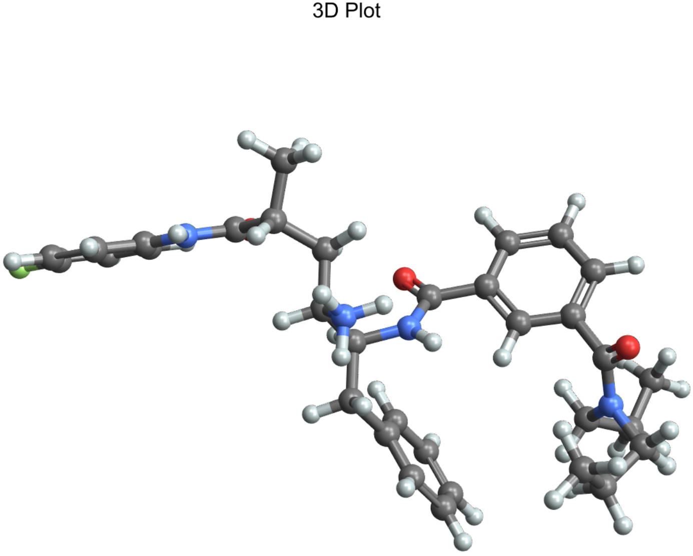

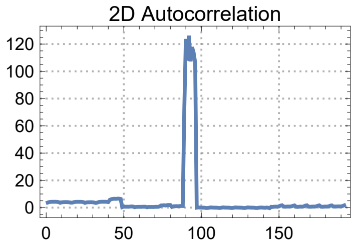

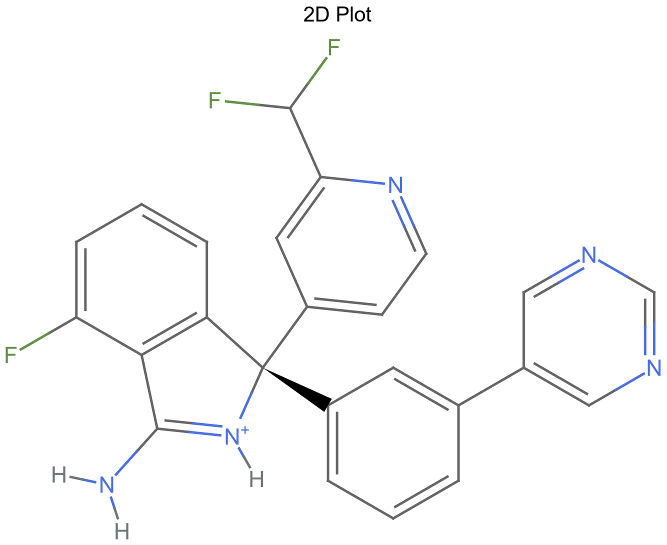

Dataset Index= 253

Molecule Formula= $C_{24}H_{17}F_3N_5^+$

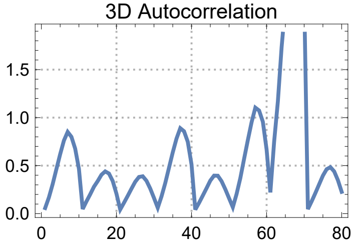

3D Plot

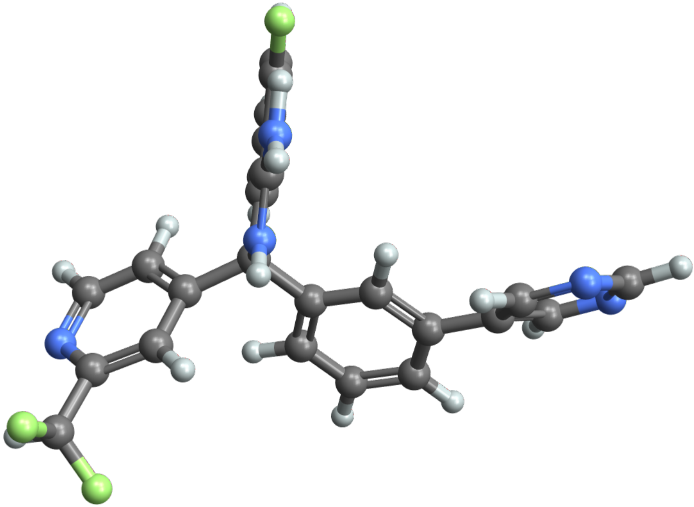

2D Plot

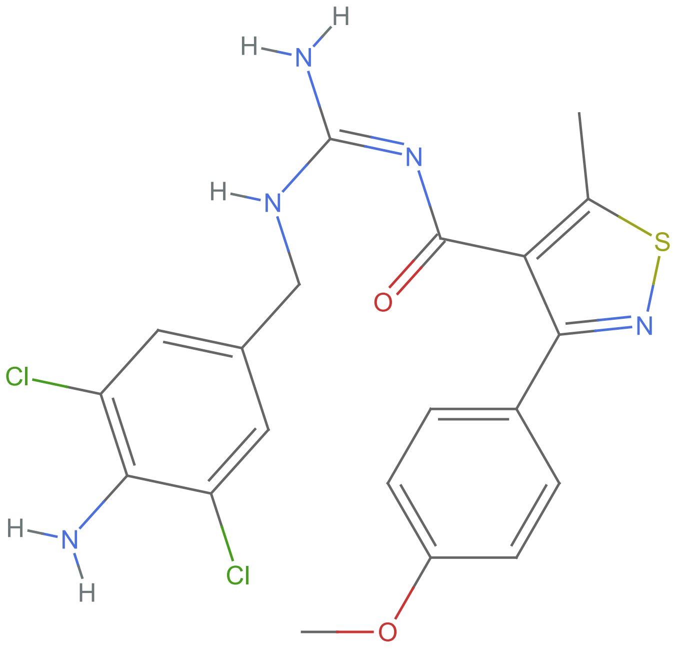

2D Autocorrelation

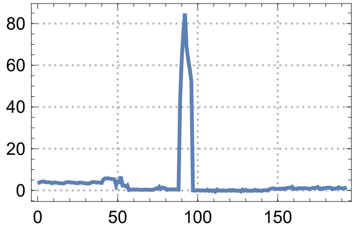

Dataset Index= 254

Molecule Formula= $C_{20}H_{19}Cl_2N_5O_2S$

3D Autocorrelation

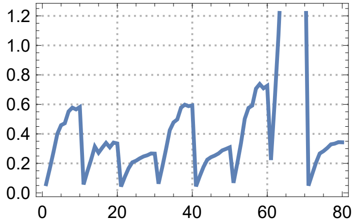

3D Plot

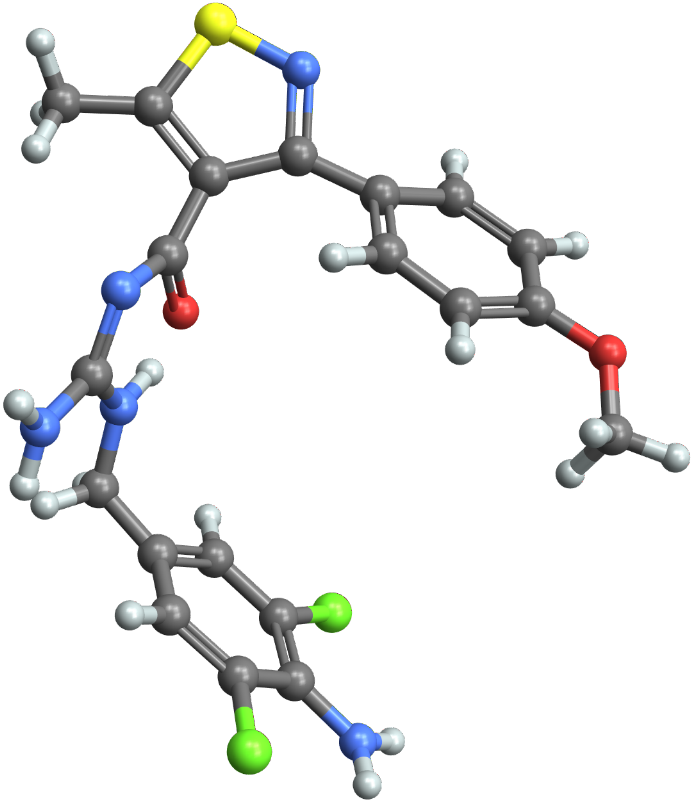

2D Autocorrelation

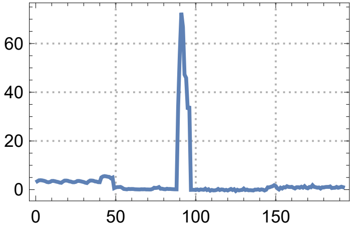

Dataset Index= 255

Molecule Formula= $C_{17}H_{16}N_4OS$

3D Autocorrelation

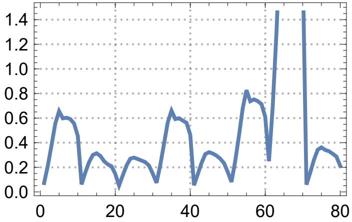

2D Plot

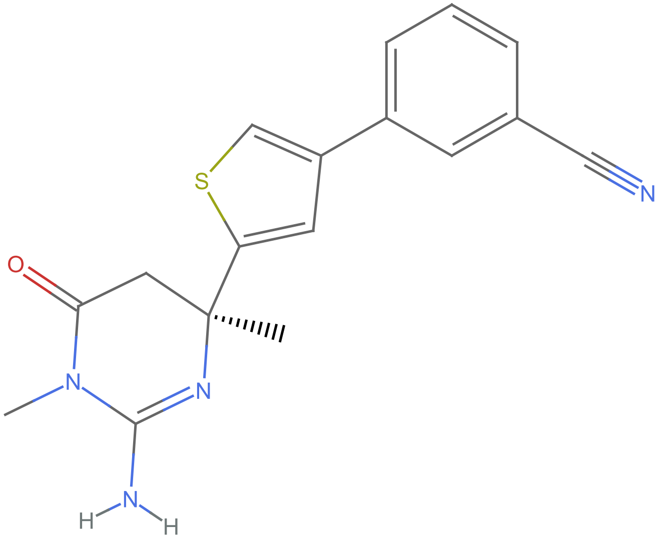

3D Plot

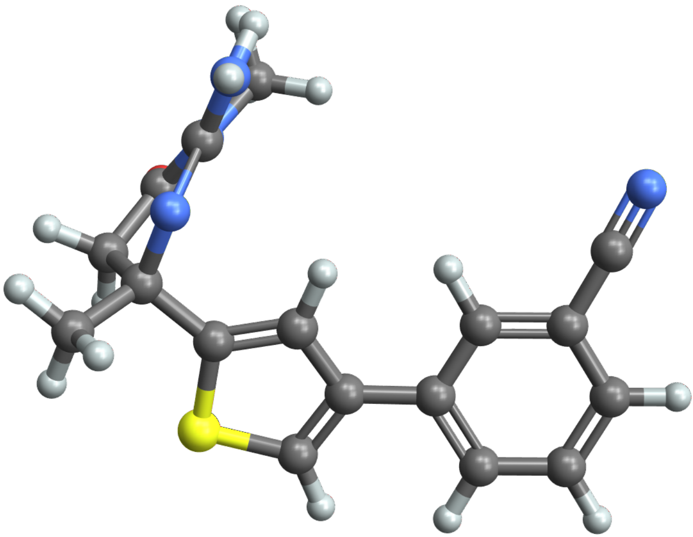

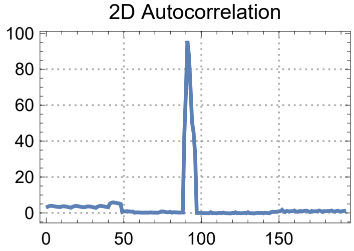

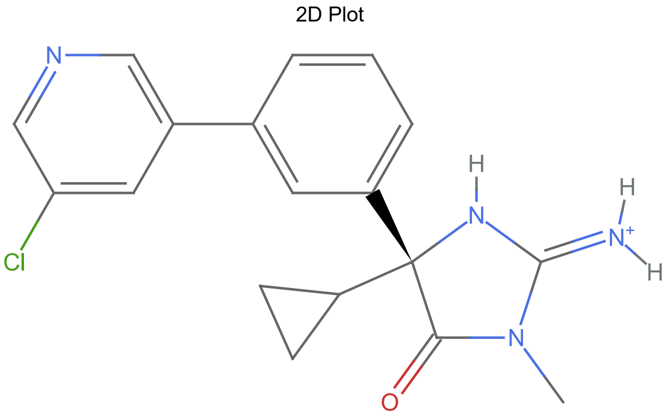

Dataset Index= 256

Molecule Formula= $C_{18}H_{18}ClN_4O^+$

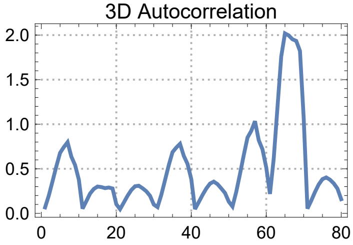

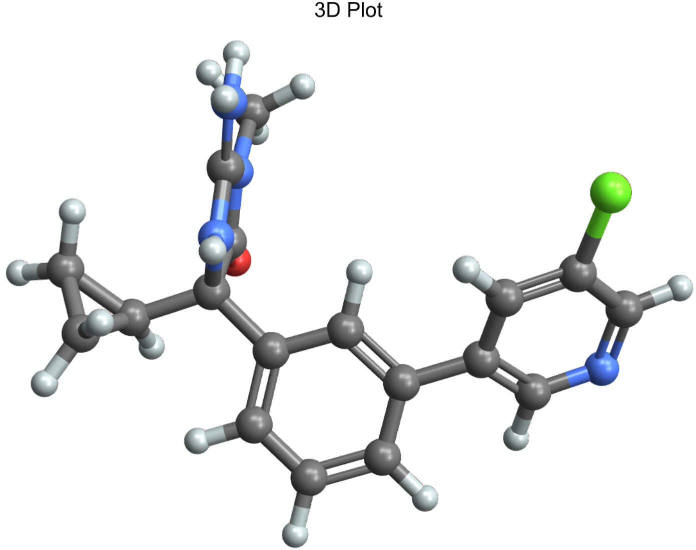

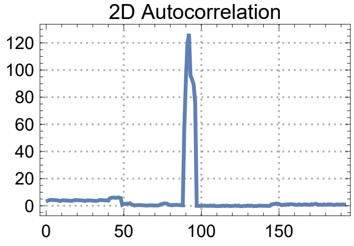

Dataset Index= 257

Molecule Formula= $C_{24}H_{20}FN_5OS$

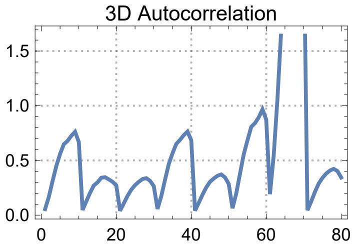

2D Plot

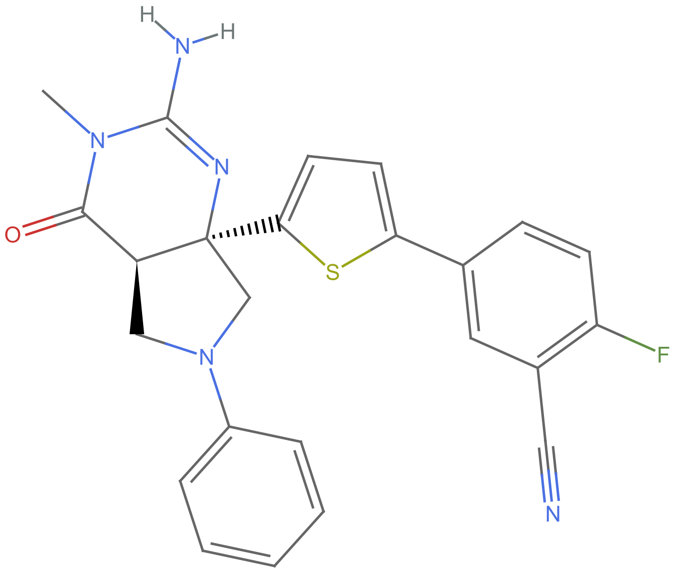

3D Plot

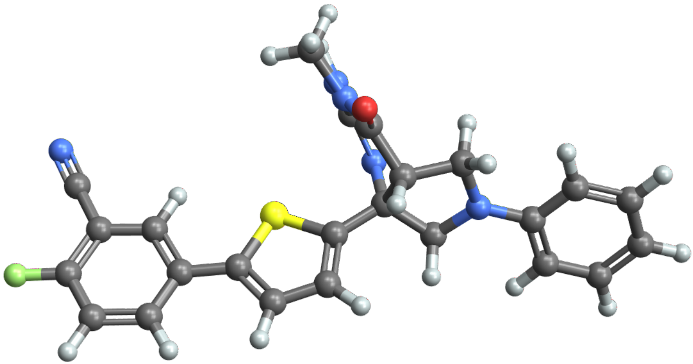

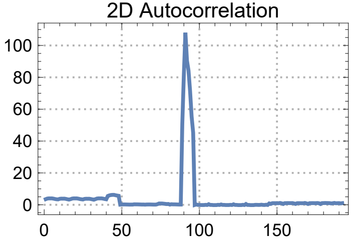

2D Plot

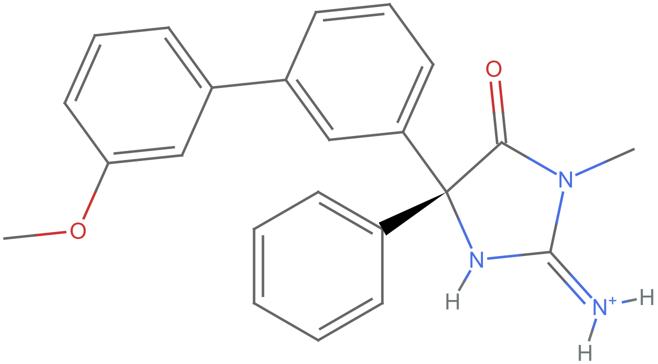

Dataset Index= 258

Molecule Formula= $C_{23}H_{22}N_3O_2^+$

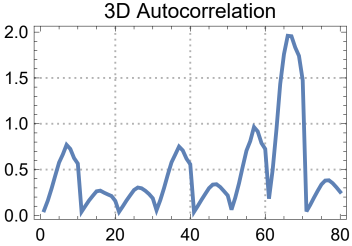

3D Plot

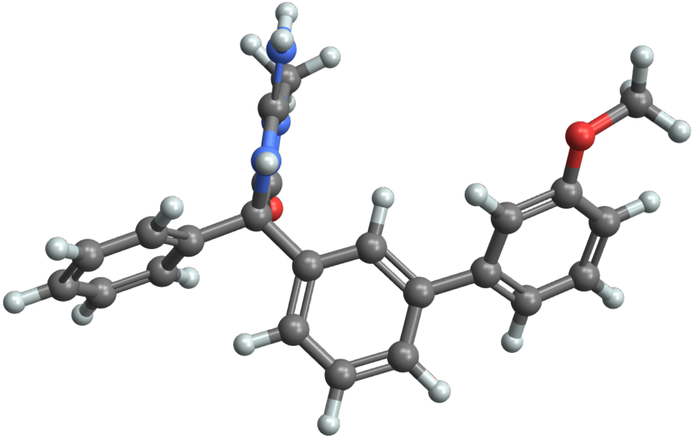

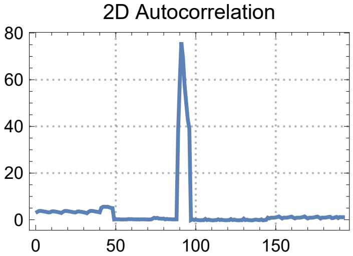

Dataset Index= 259

Molecule Formula= $C_{19}H_{21}N_3O_2$

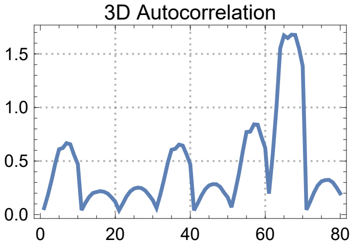

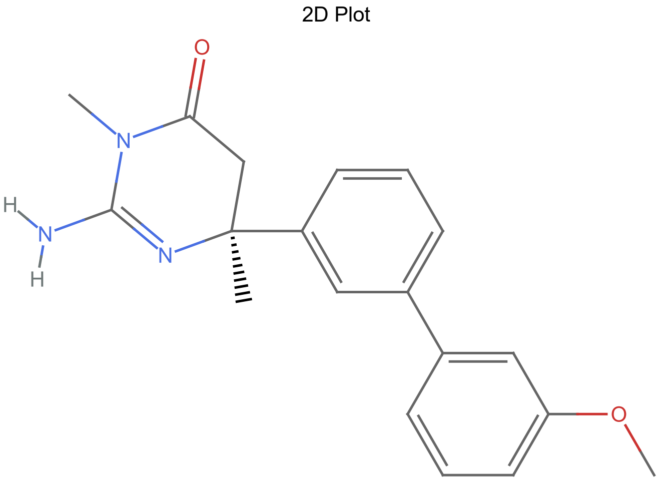

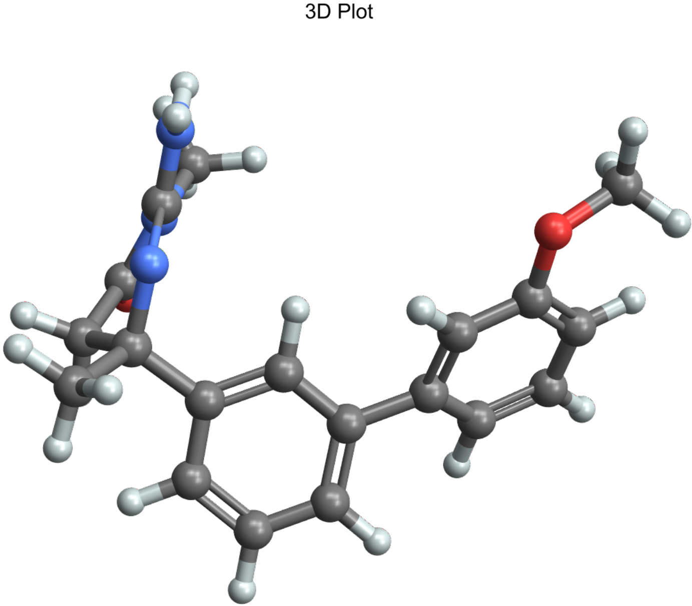

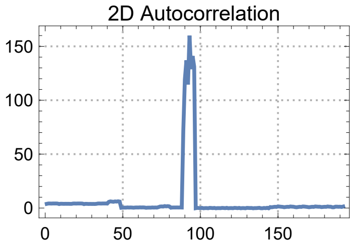

Dataset Index= 260

Molecule Formula= $C_{31}H_{40}N_5O_4^+$

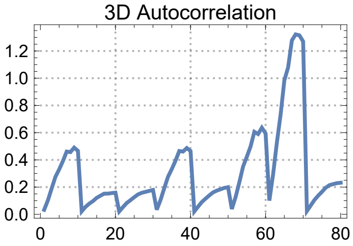

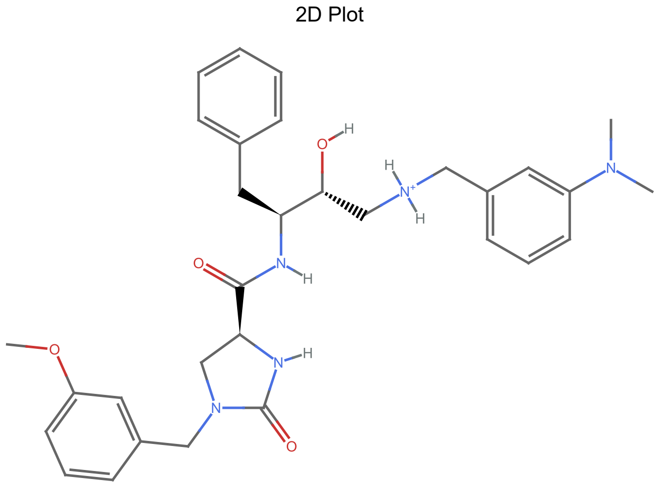

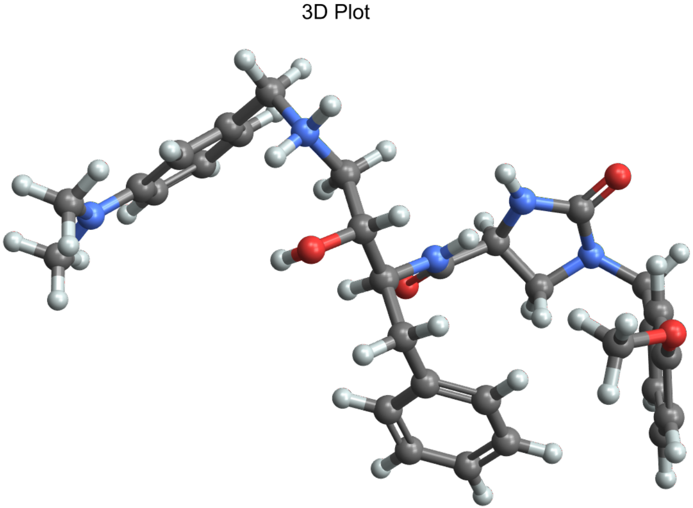

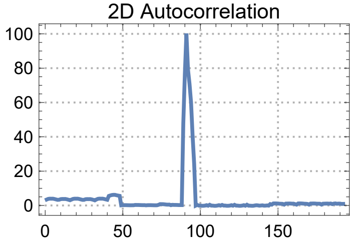

Dataset Index= 261

Molecule Formula= $C_{21}H_{19}N_4O^+$

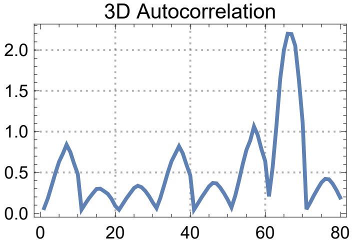

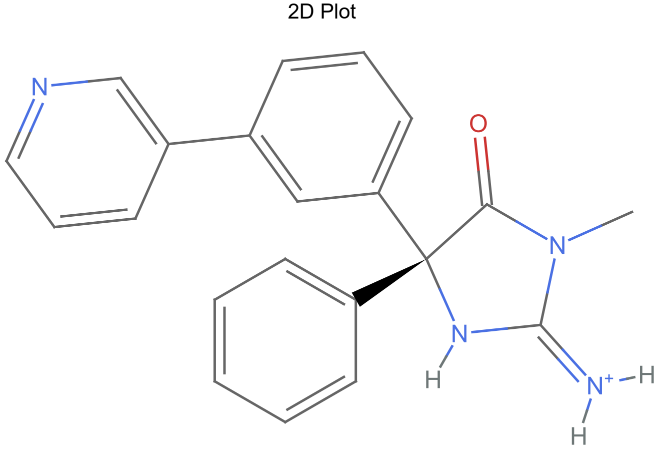

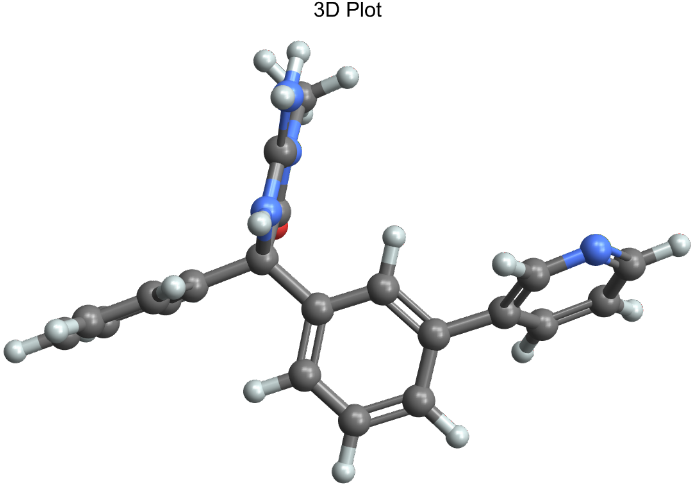

2D Plot

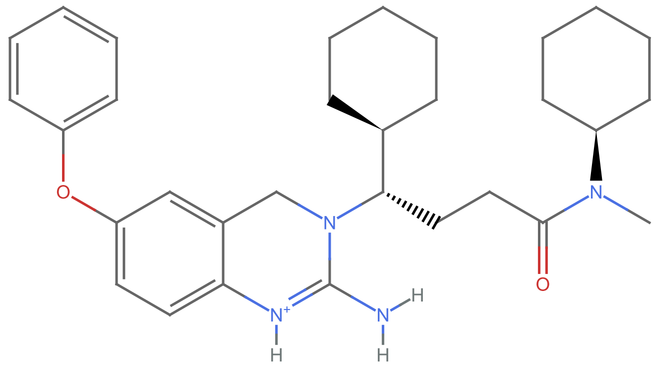

3D Plot

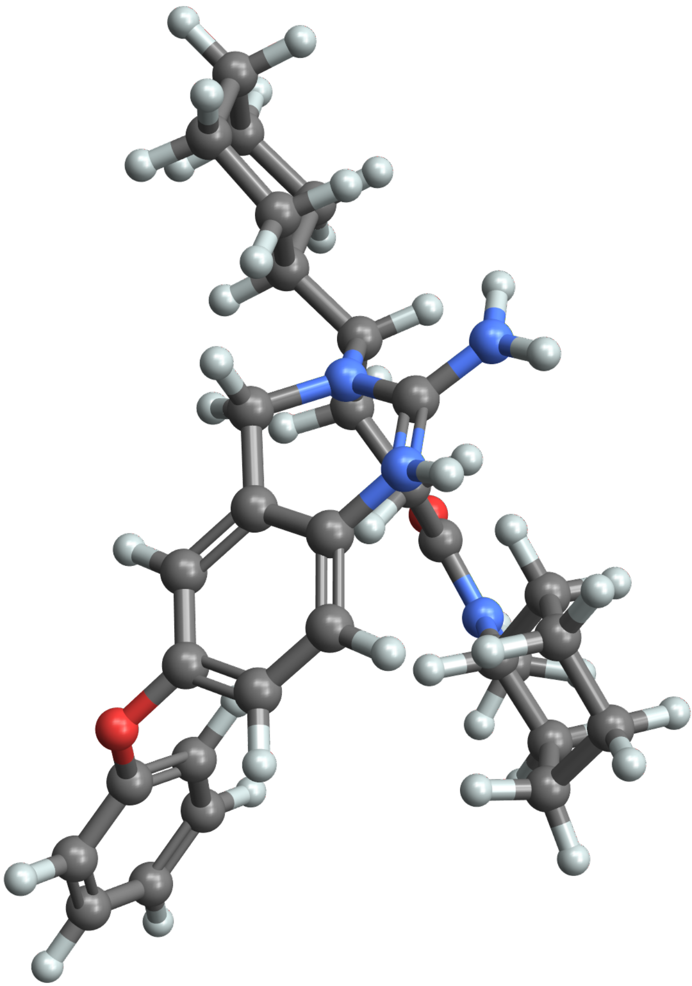

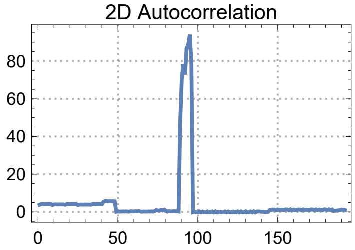

Dataset Index= 262

Molecule Formula= $C_{31}H_{43}N_4O_2^+$

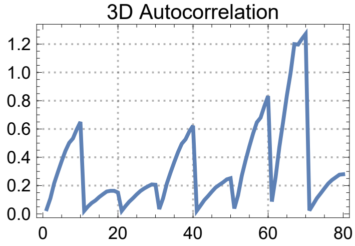

2D Plot

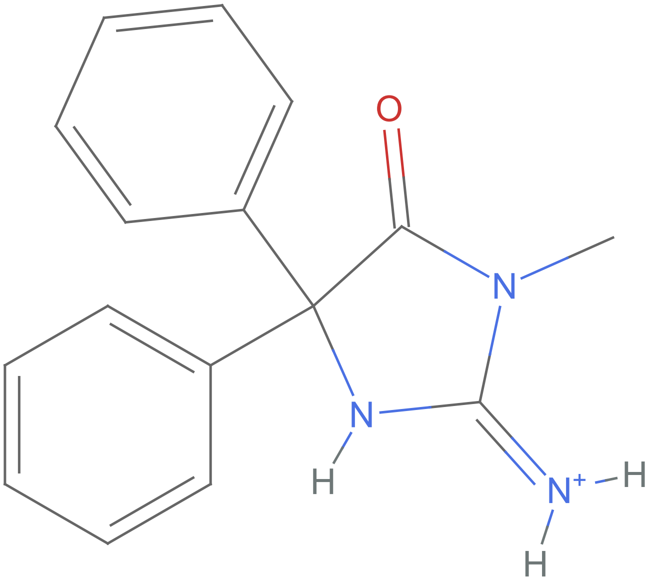

2D Autocorrelation

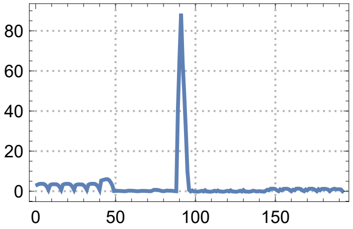

3D Plot

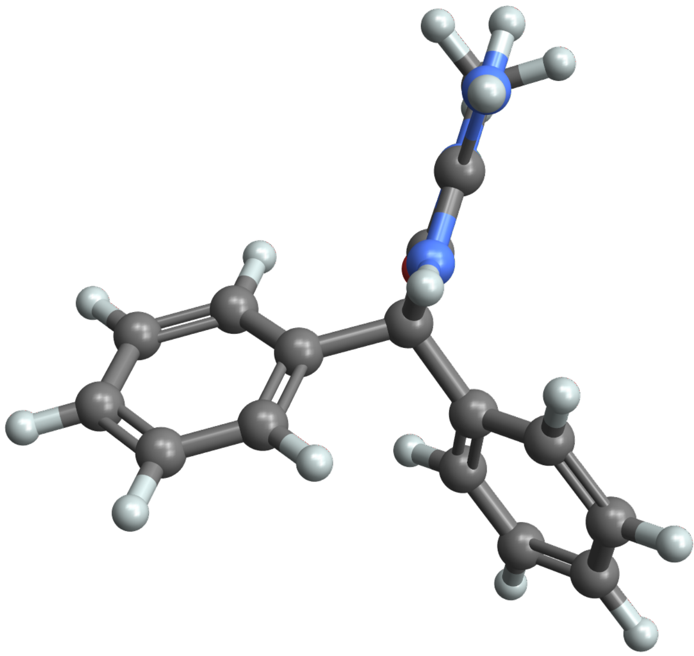

Dataset Index= 263

Molecule Formula= $C_{16}H_{16}N_{3}O^{+}$

3D Autocorrelation

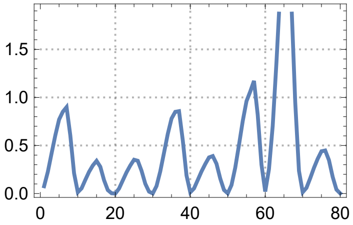

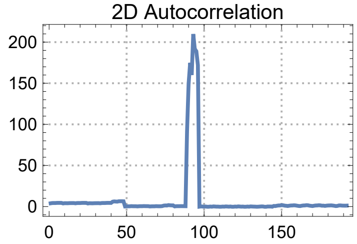

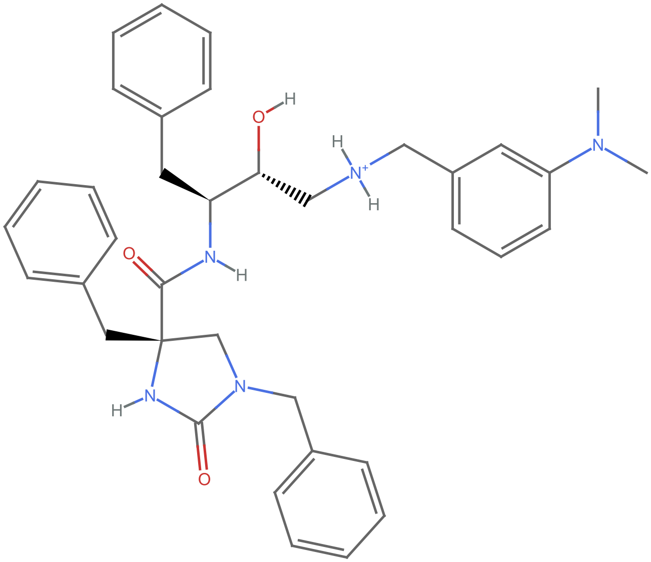

3D Plot

Dataset Index= 264

Molecule Formula= $C_{37}H_{44}N_5O_3^+$

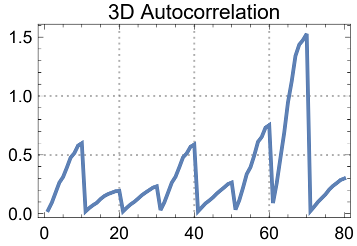

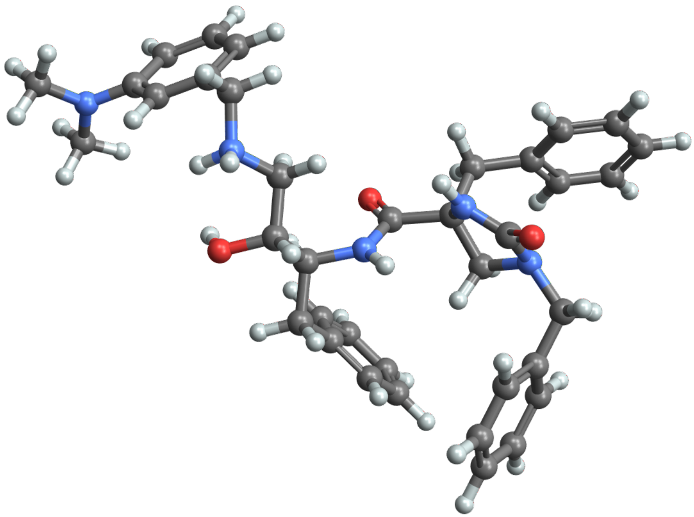

2D Plot

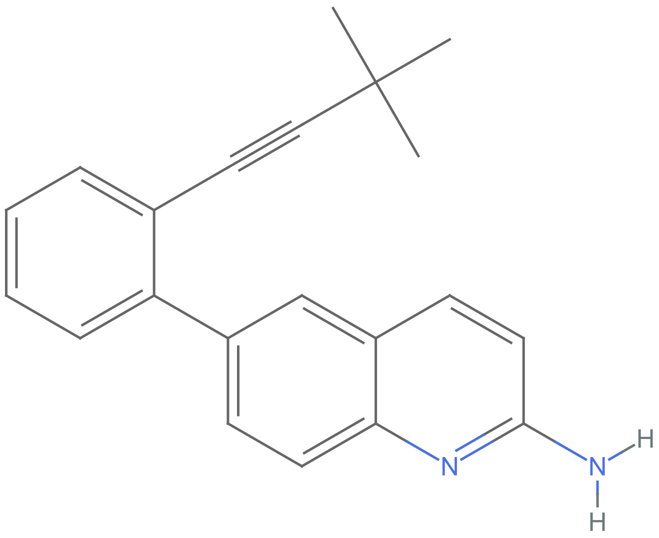

2D Autocorrelation

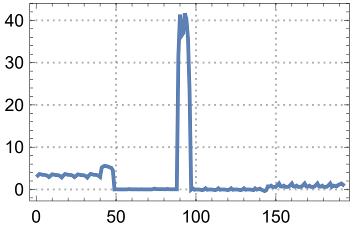

Dataset Index= 265

Molecule Formula= $C_{21}H_{20}N_2$

3D Autocorrelation

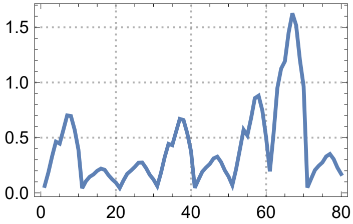

3D Plot

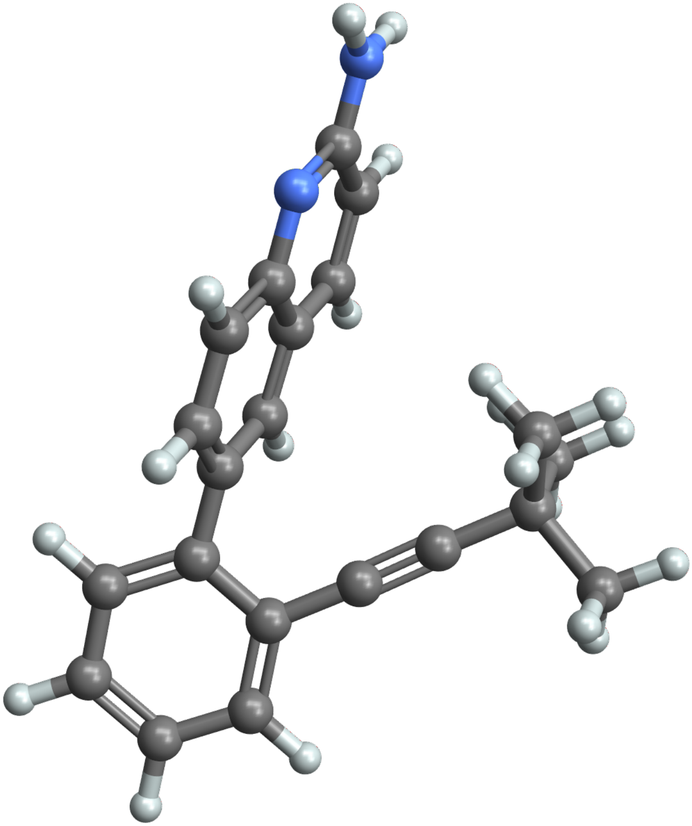

2D Plot

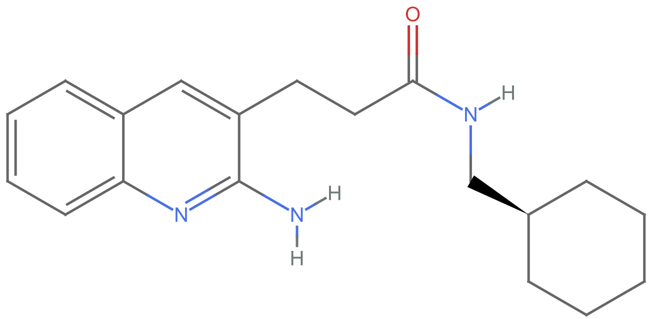

2D Autocorrelation

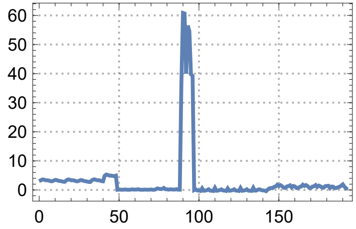

3D Plot

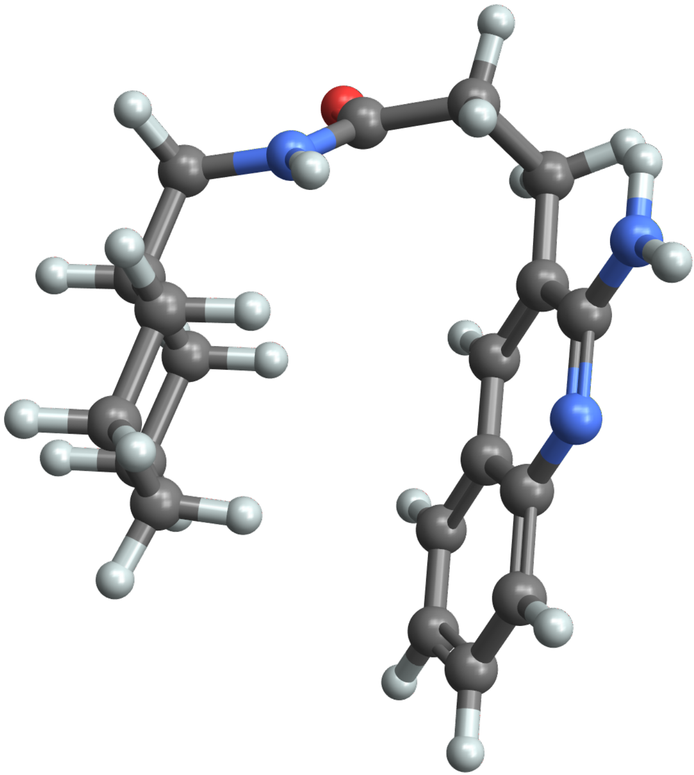

Dataset Index= 266

Molecule Formula= $C_{19}H_{25}N_3O$

3D Autocorrelation

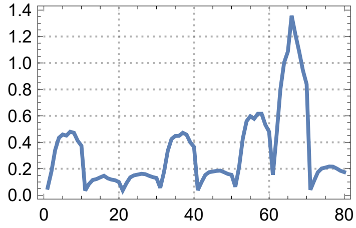

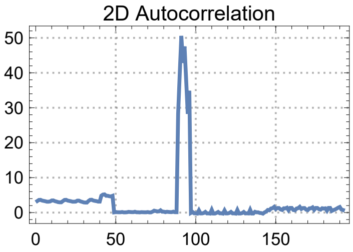

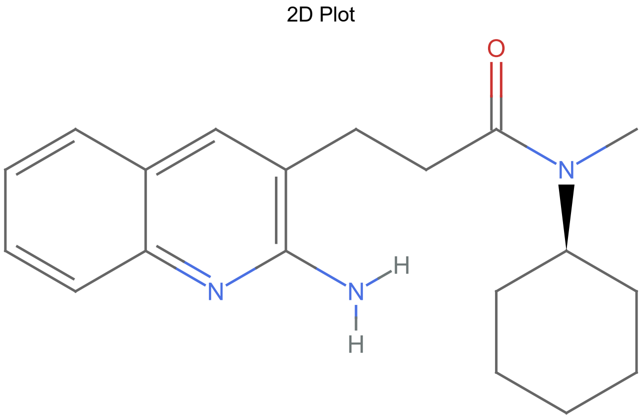

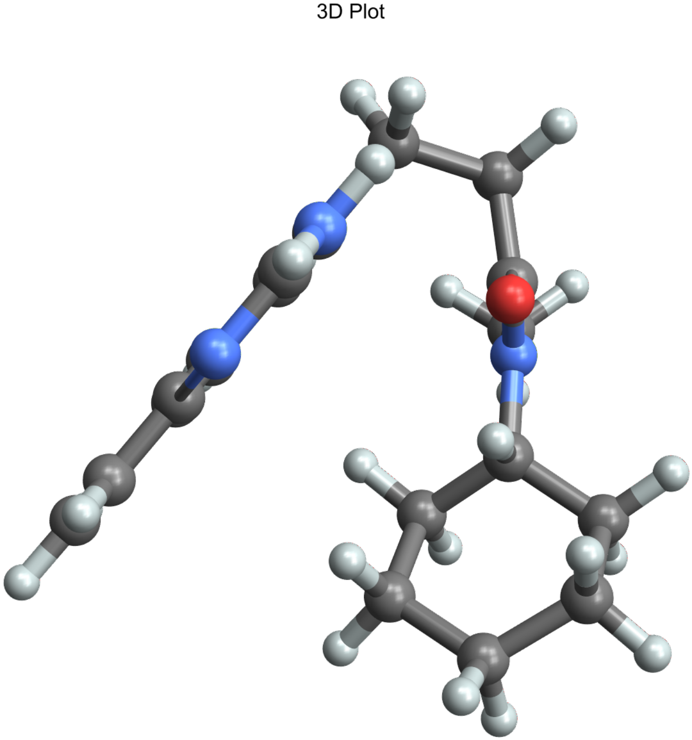

Dataset Index= 267

Molecule Formula= $C_{19}H_{25}N_3O$

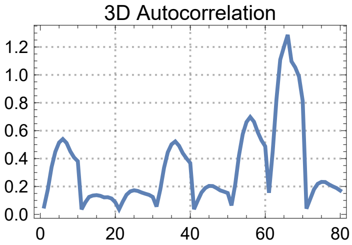

2D Plot

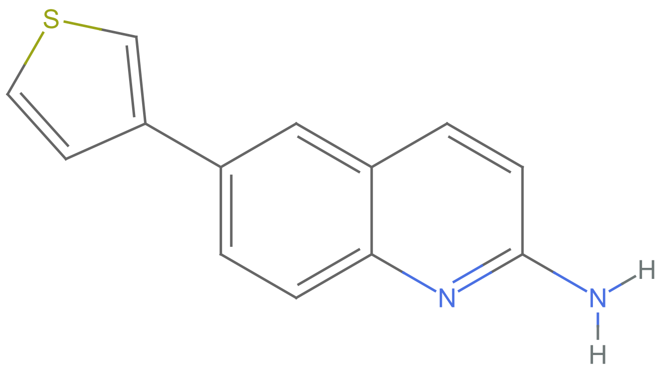

3D Plot

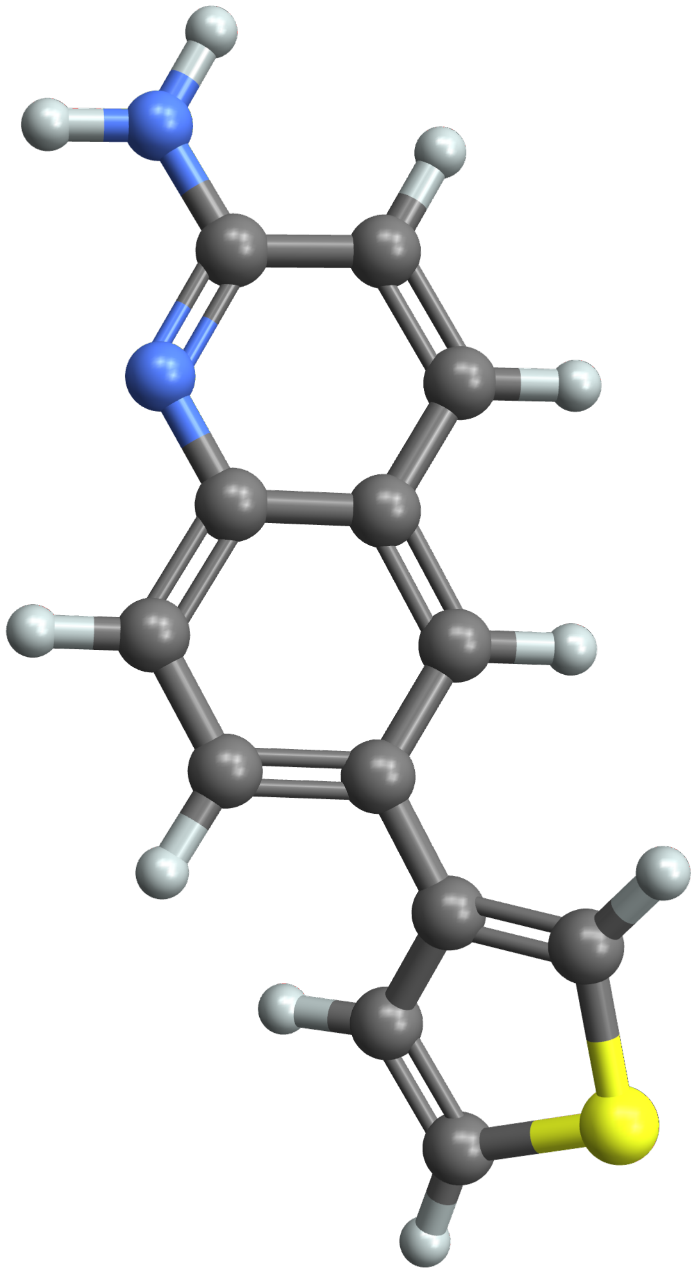

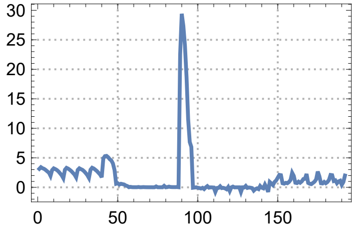

Dataset Index= 268

Molecule Formula= $C_{13}H_{10}N_2S$

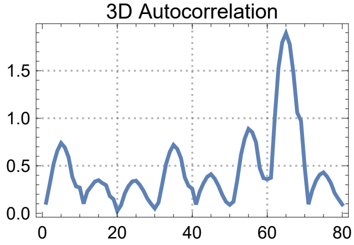

2D Autocorrelation

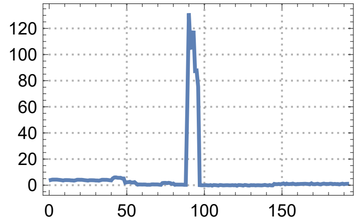

Dataset Index= 269

Molecule Formula= $C_{25}H_{35}ClN_3O_6S^+$

3D Autocorrelation

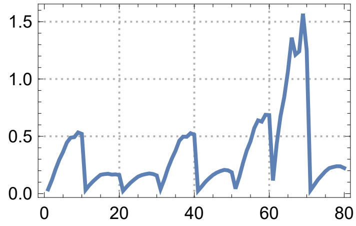

2D Plot

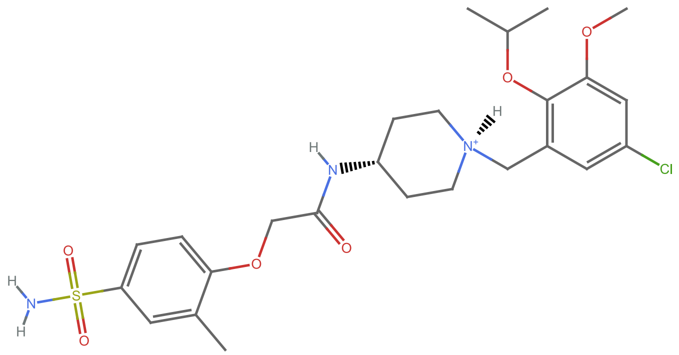

3D Plot

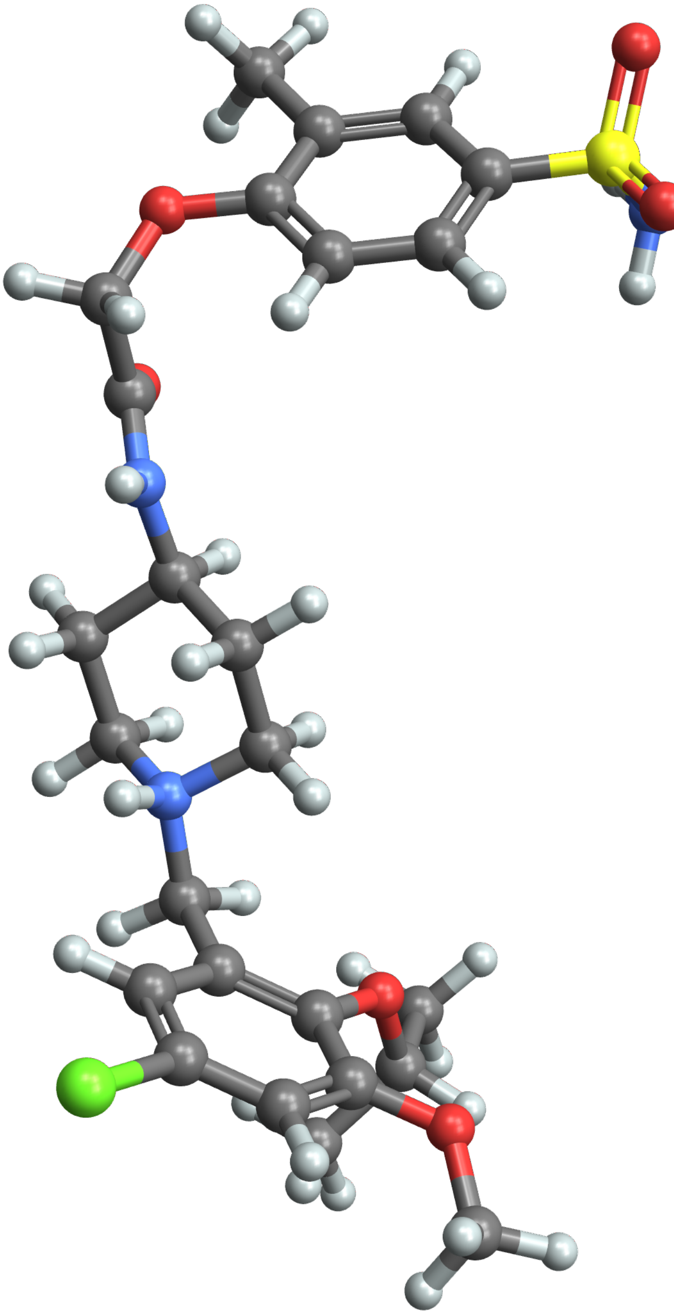

2D Plot

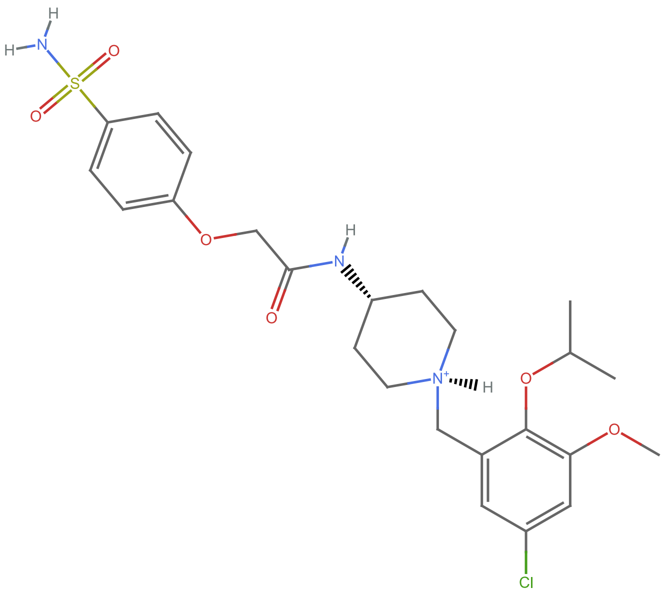

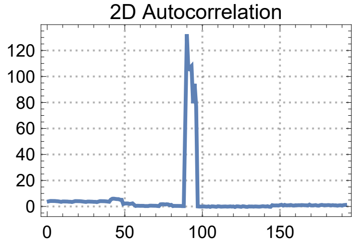

Dataset Index= 270

Molecule Formula= $C_{24}H_{33}ClN_3O_6S^+$

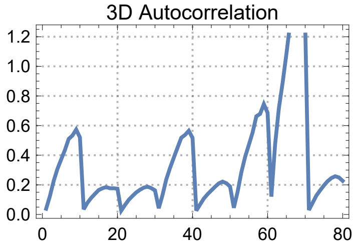

3D Plot

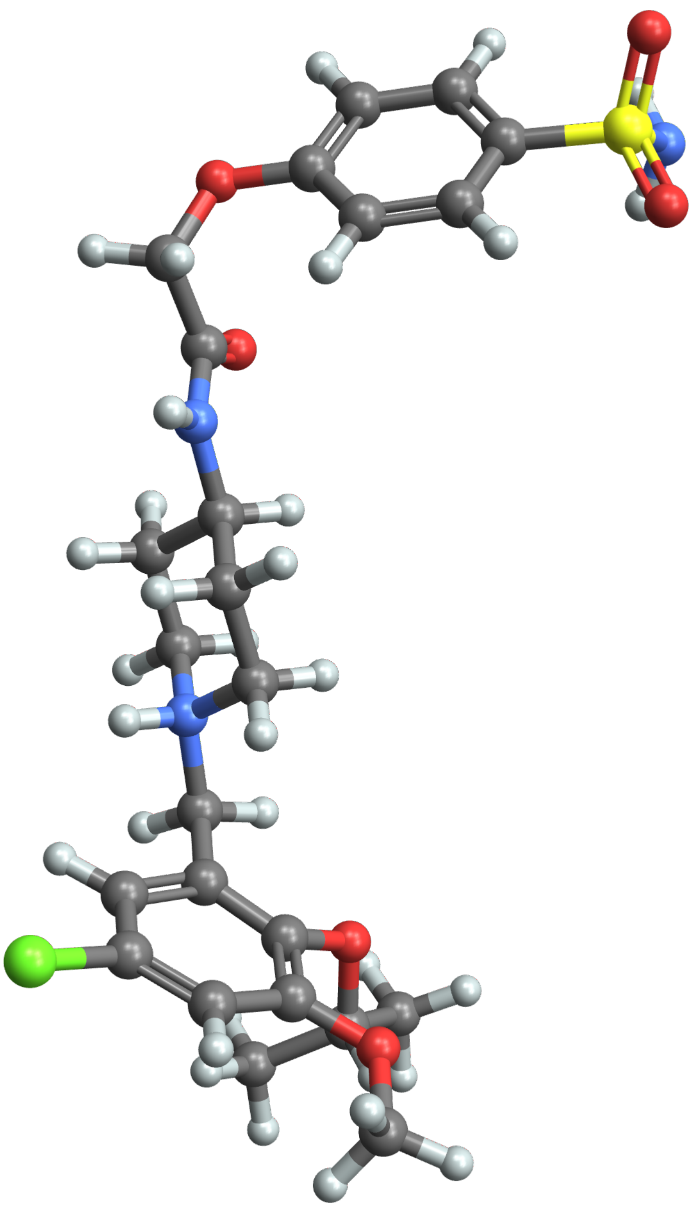

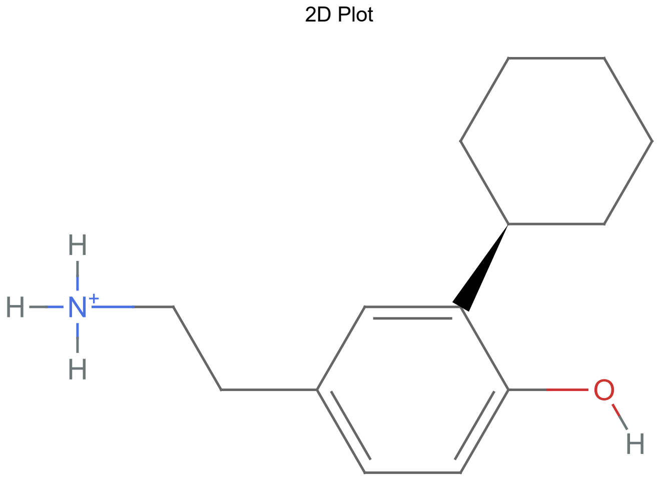

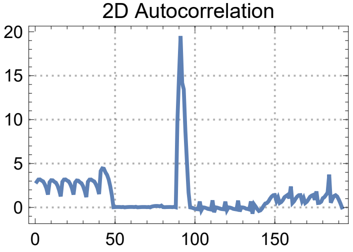

Dataset Index= 271

Molecule Formula= $C_{14}H_{22}NO^{+}$

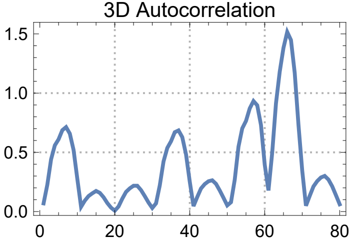

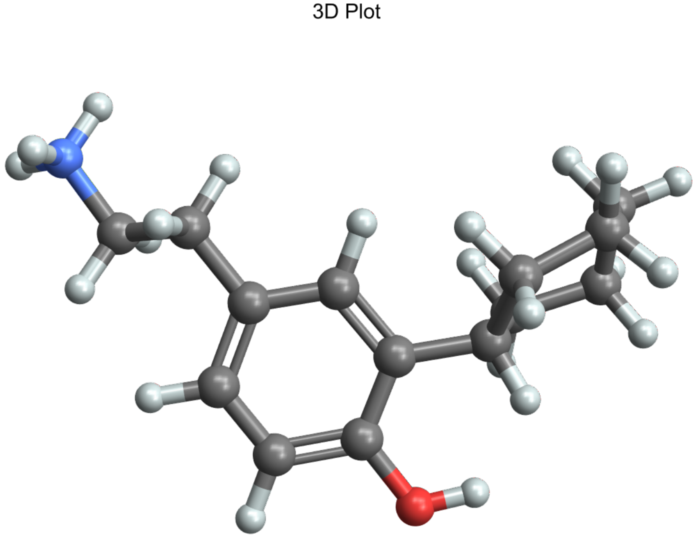

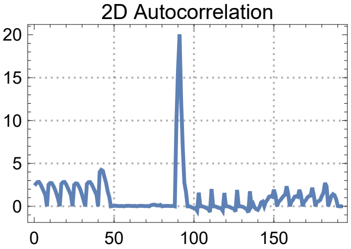

Dataset Index= 272

Molecule Formula= $C_{10}H_{16}NO^{+}$

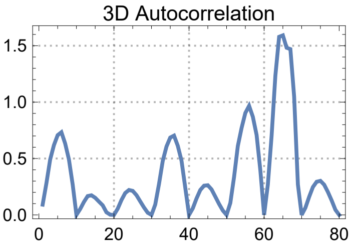

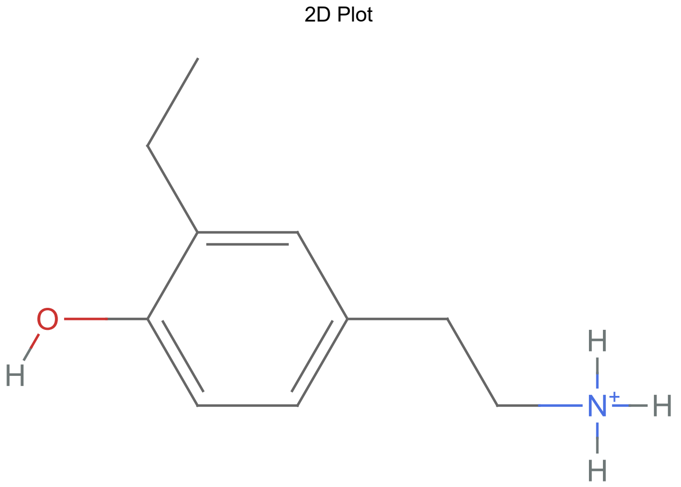

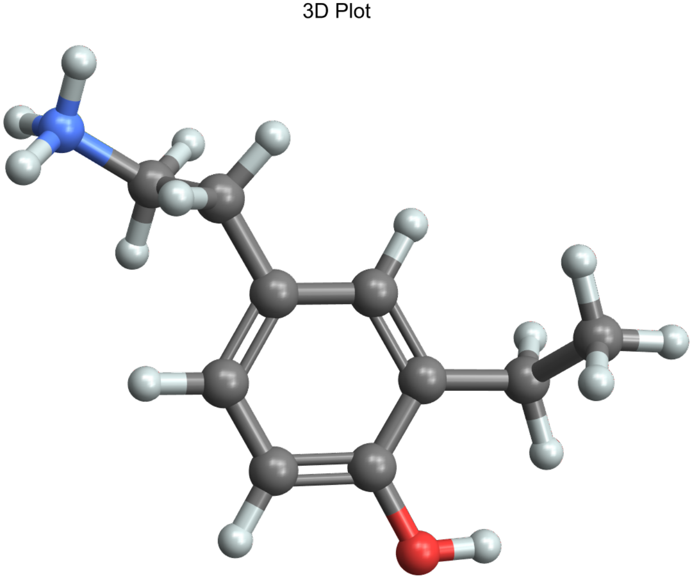

2D Plot

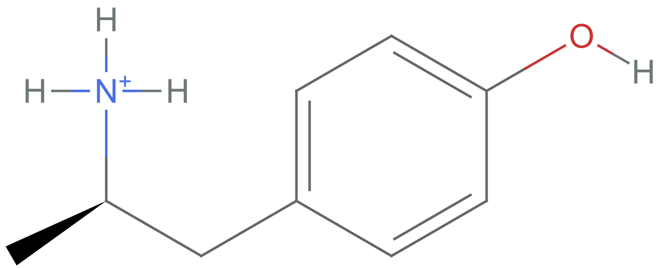

3D Plot

2D Autocorrelation

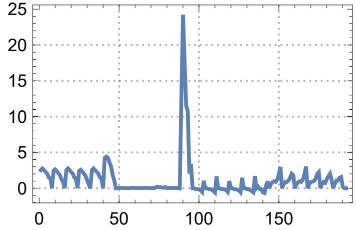

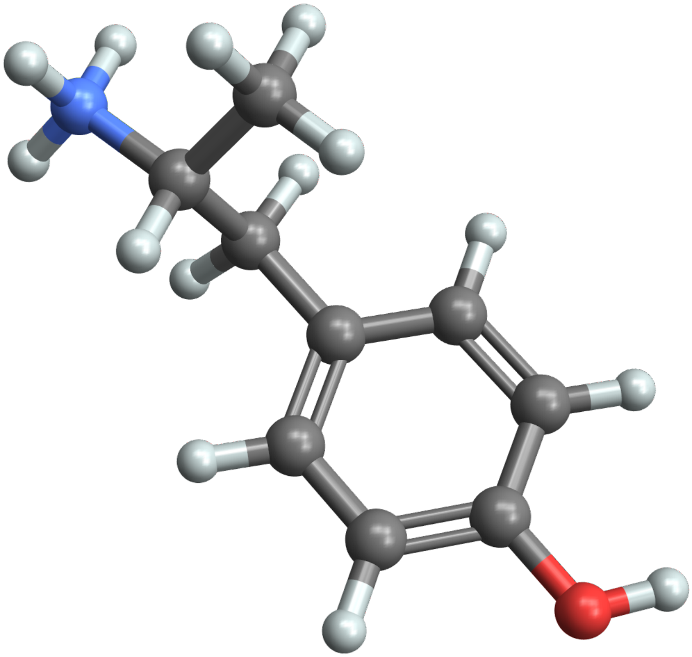

Dataset Index= 273

Molecule Formula= $C_9H_{14}NO^+$

3D Autocorrelation

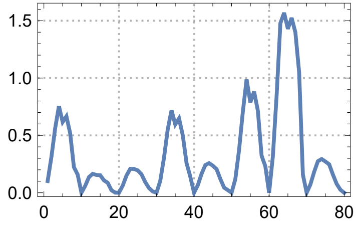

2D Plot

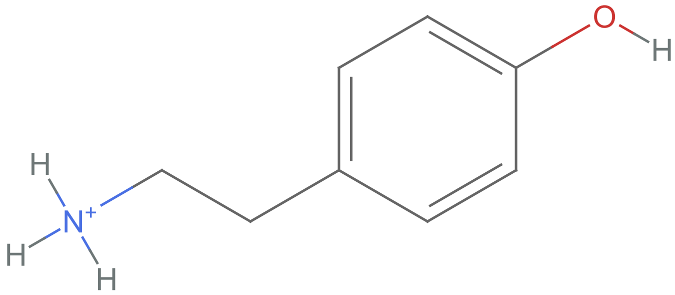

3D Plot

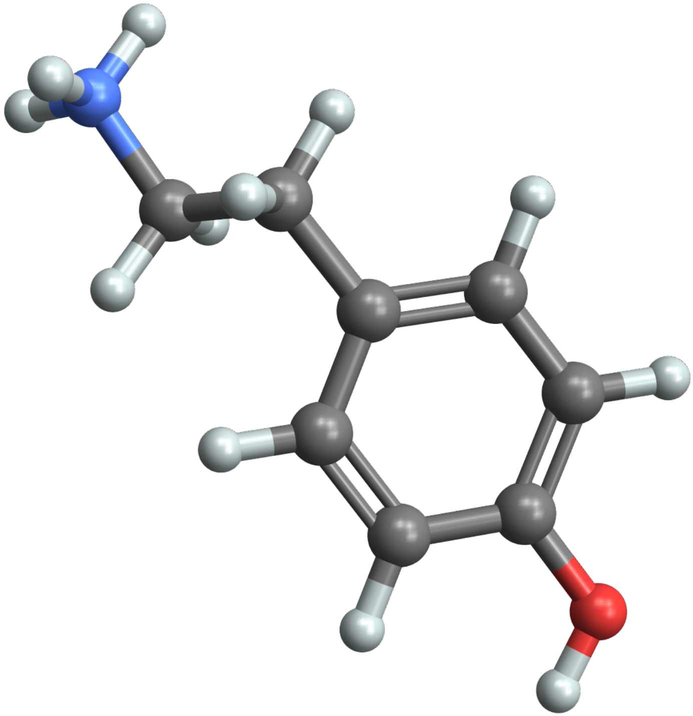

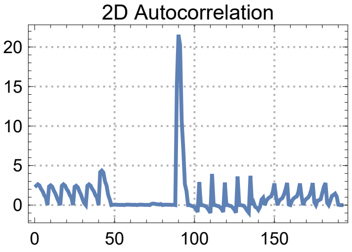

Dataset Index= 274

Molecule Formula= $C_8H_{12}NO^+$

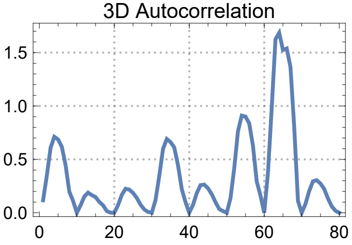

2D Plot

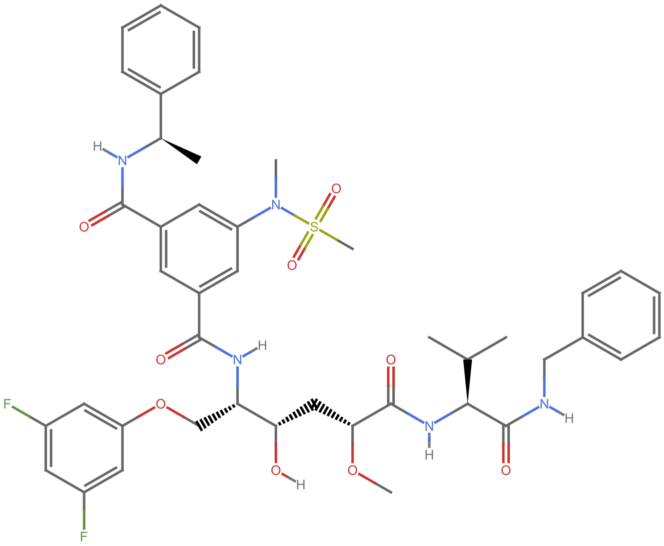

2D Autocorrelation

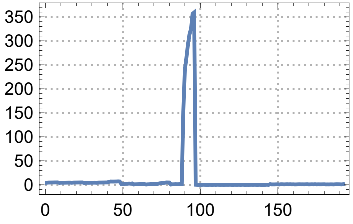

Dataset Index= 275

Molecule Formula= $C_{43}H_{51}F_2N_5O_9S$

3D Plot

3D Autocorrelation

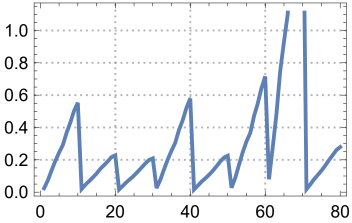

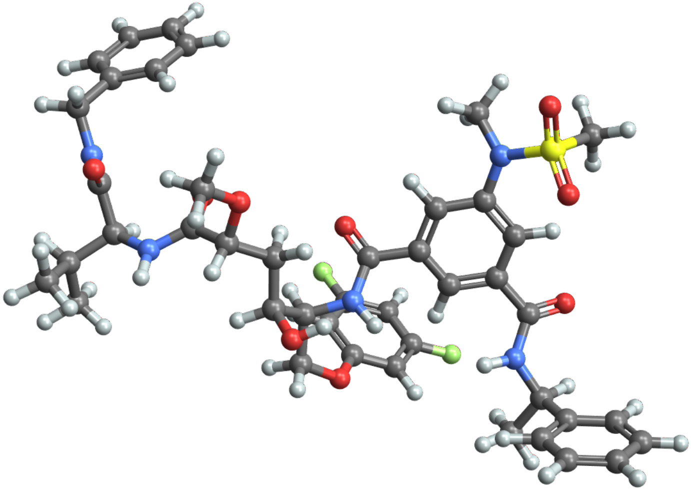

2D Plot

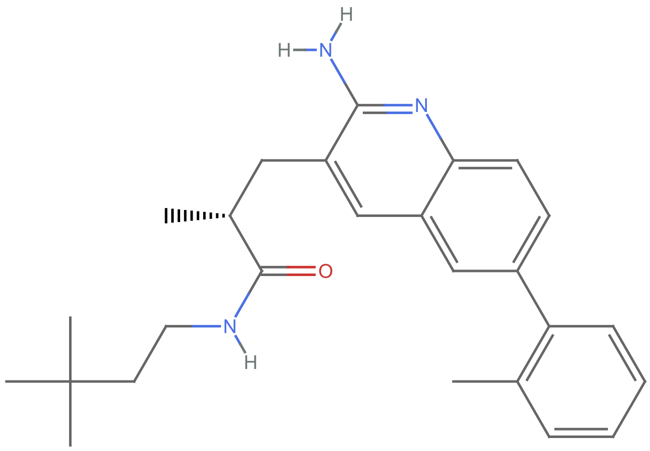

2D Autocorrelation

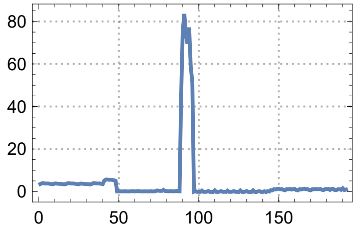

Dataset Index= 276

Molecule Formula= $C_{26}H_{33}N_3O$

3D Autocorrelation

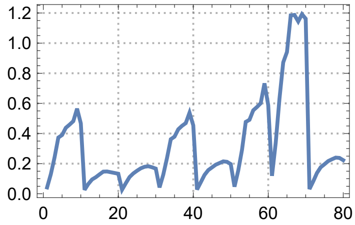

3D Plot

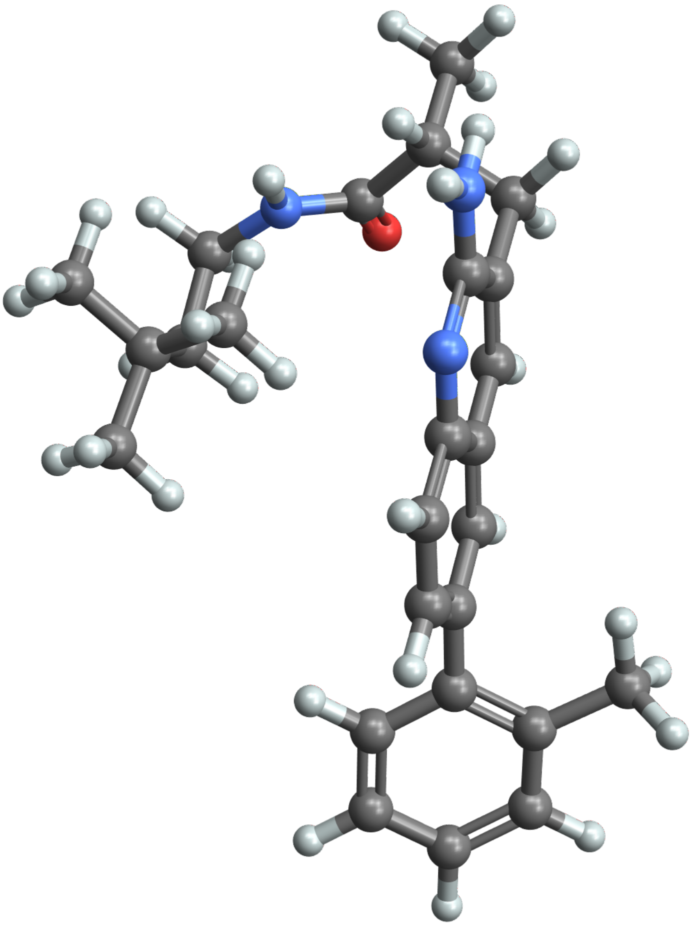

2D Plot

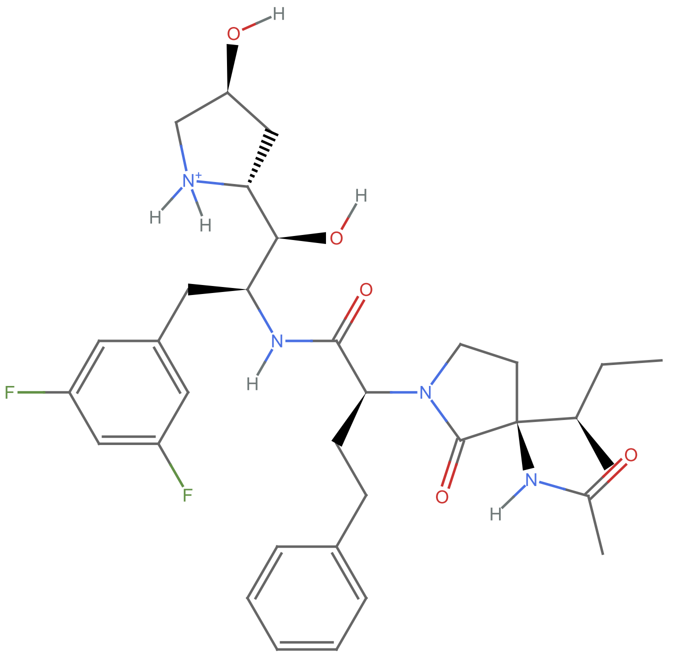

2D Autocorrelation

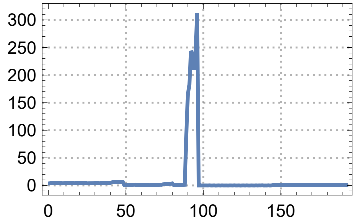

3D Plot

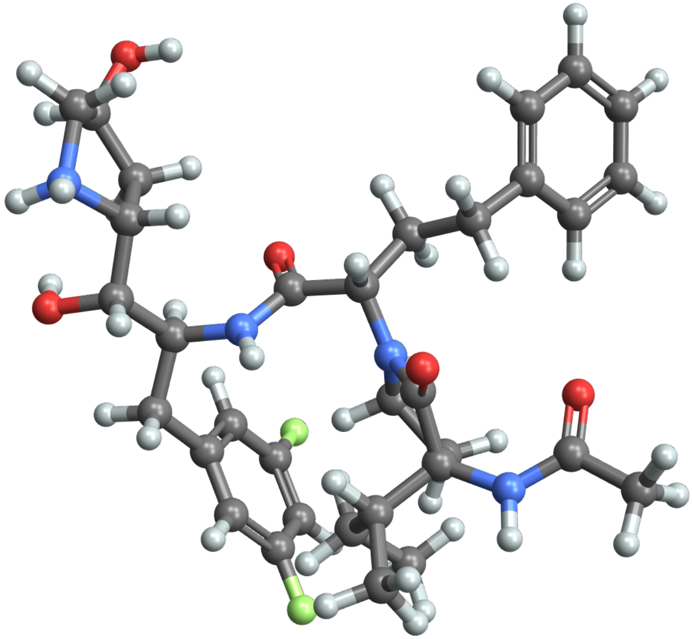

Dataset Index= 277

Molecule Formula= $C_{33}H_{45}F_2N_4O_5^+$

3D Autocorrelation

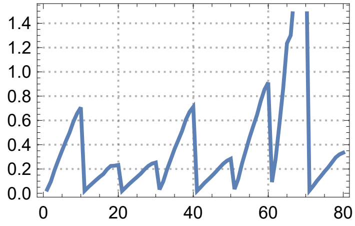

2D Plot

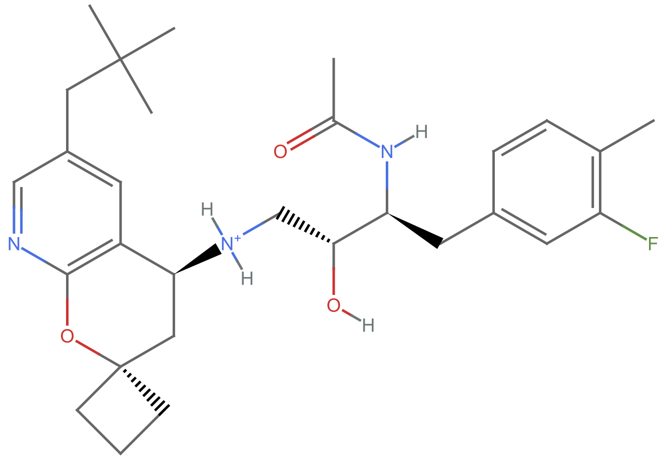

2D Autocorrelation

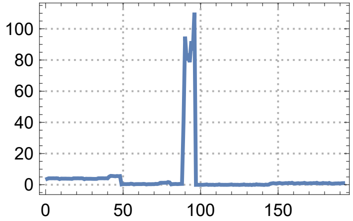

3D Plot

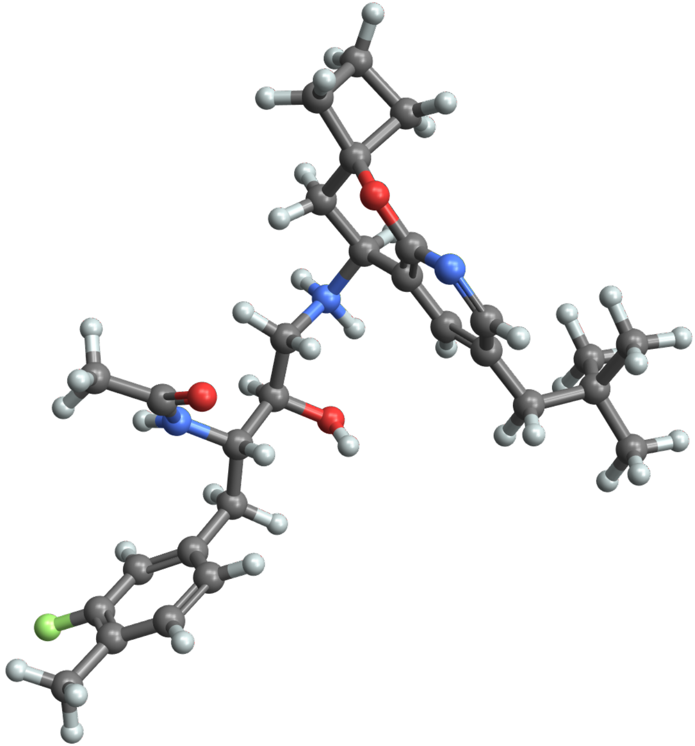

Dataset Index= 278

Molecule Formula= $C_{29}H_{41}FN_3O_3^+$

3D Autocorrelation

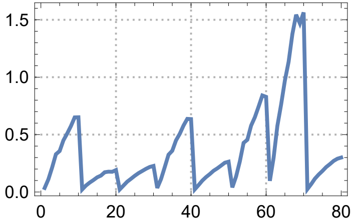

2D Plot

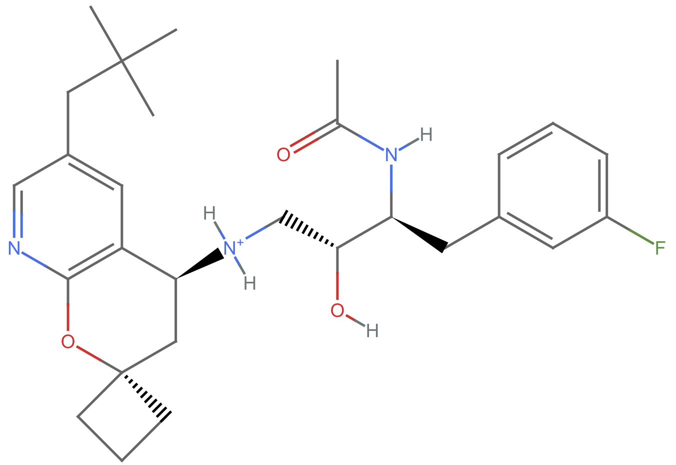

2D Autocorrelation

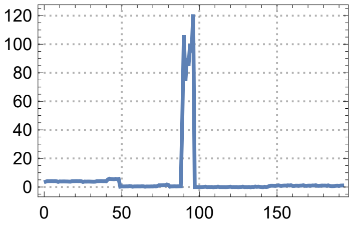

3D Plot

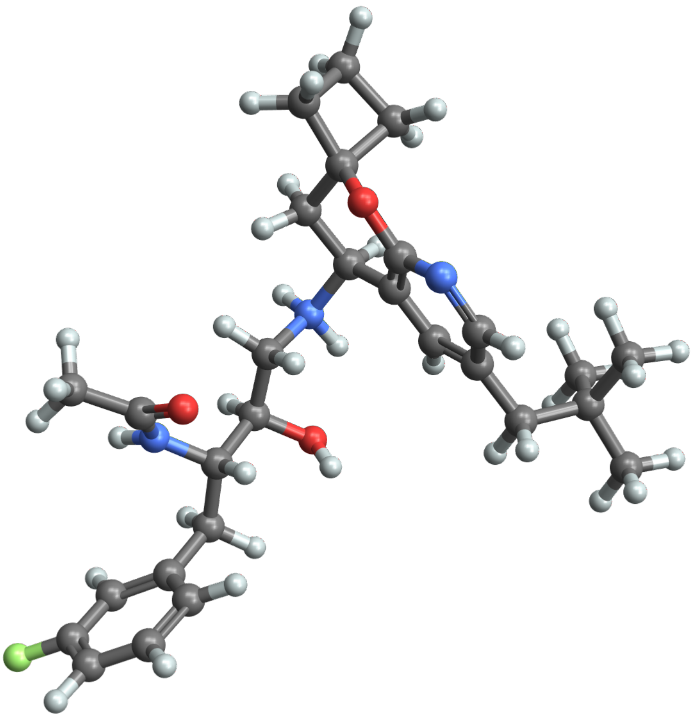

Dataset Index= 279

Molecule Formula= $C_{28}H_{39}FN_3O_3^+$

3D Autocorrelation

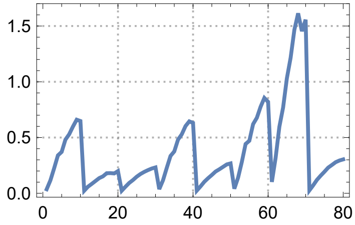

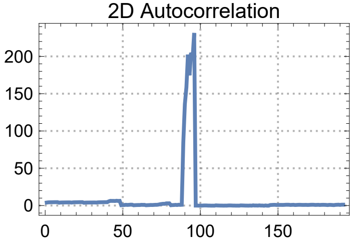

Dataset Index= 280

Molecule Formula= $C_{35}H_{45}F_2N_4O_5^+$

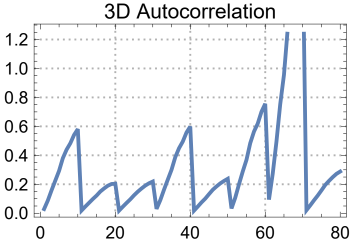

2D Plot

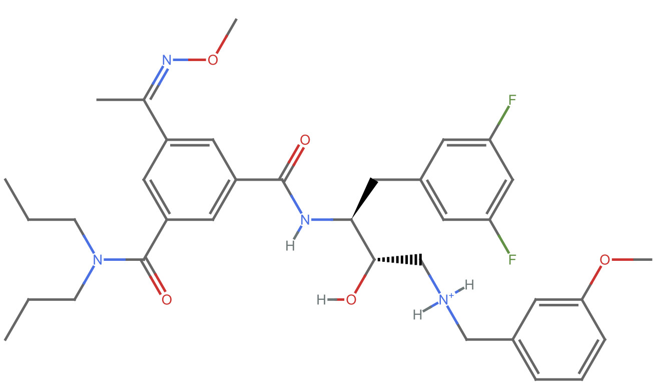

3D Plot

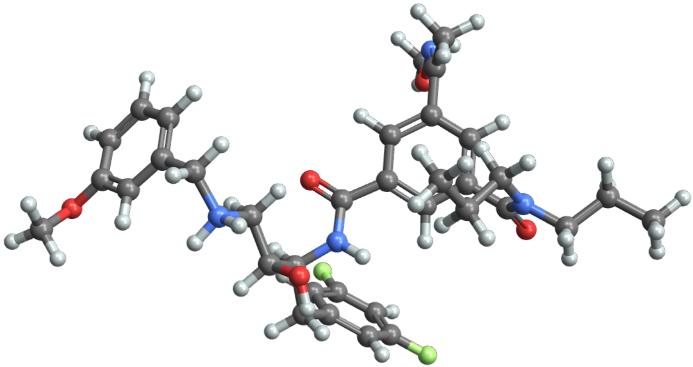

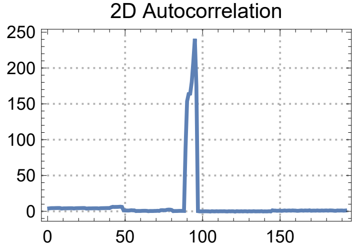

2D Plot

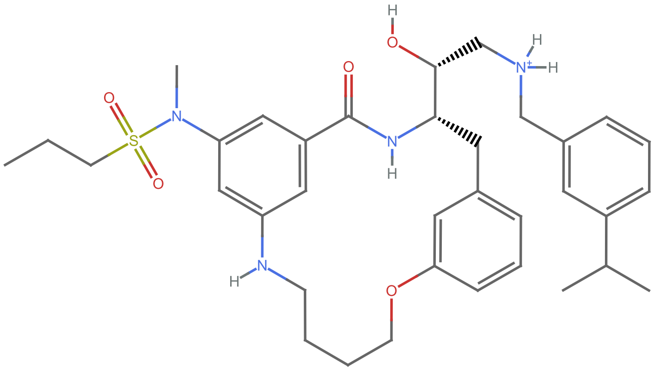

3D Plot

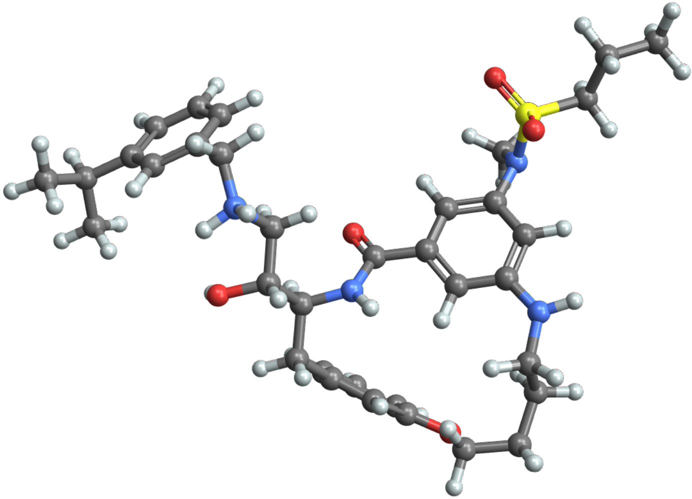

Dataset Index= 281

Molecule Formula= $C_{35}H_{49}N_4O_5S^+$

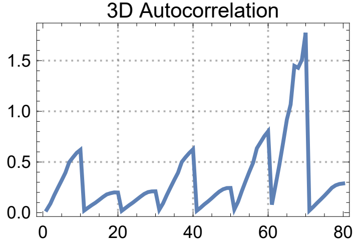

2D Plot

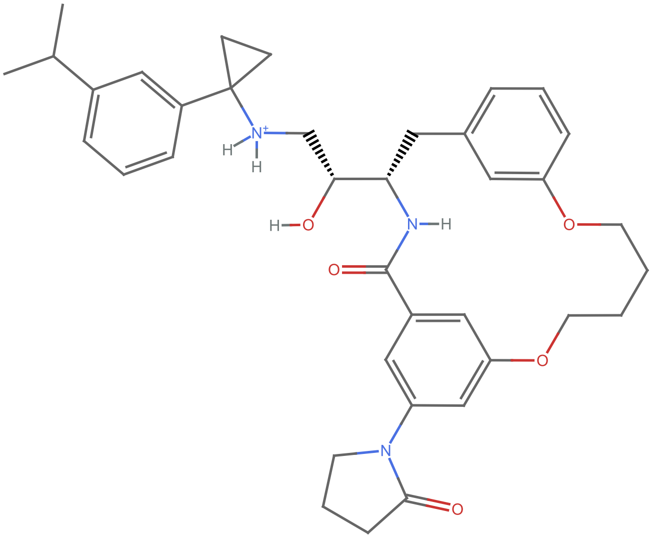

2D Autocorrelation

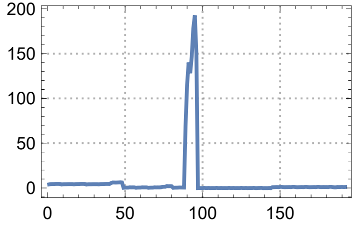

3D Plot

Dataset Index= 282

Molecule Formula= $C_{37}H_{46}N_3O_5^+$

3D Autocorrelation

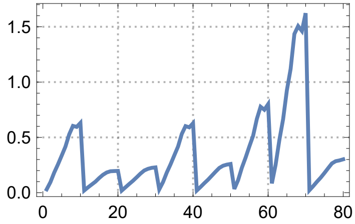

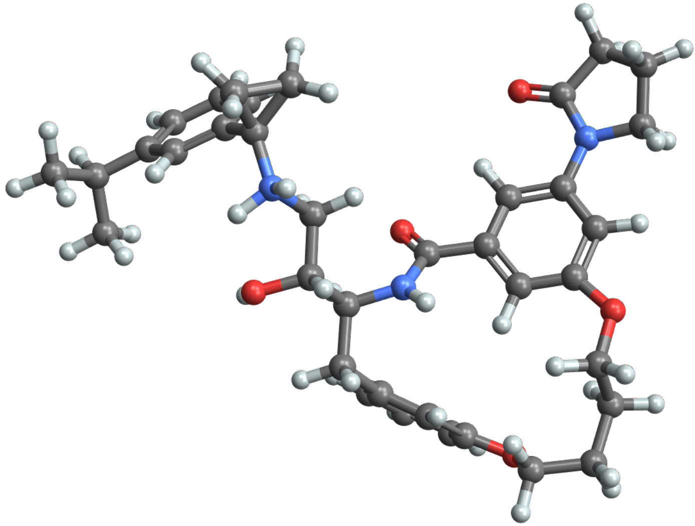

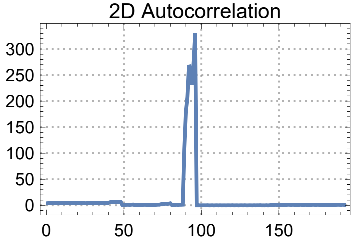

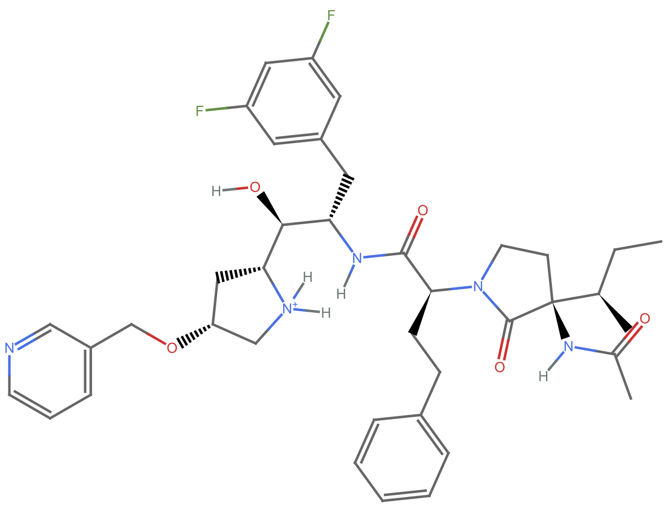

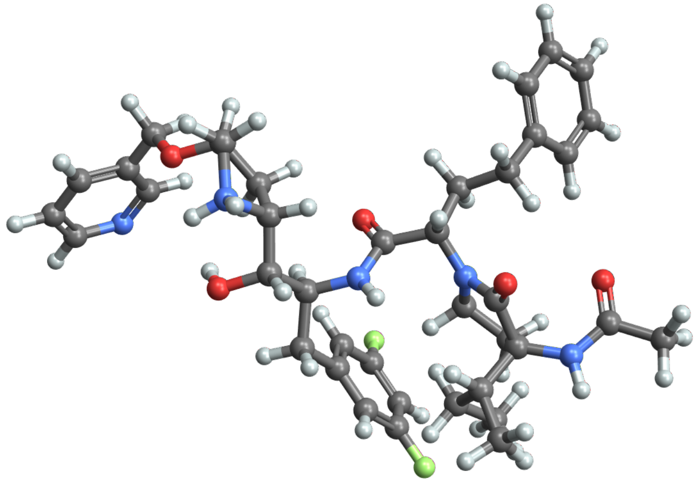

Dataset Index= 283

Molecule Formula= $C_{39}H_{50}F_2N_5O_5^+$

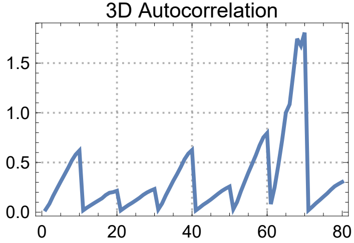

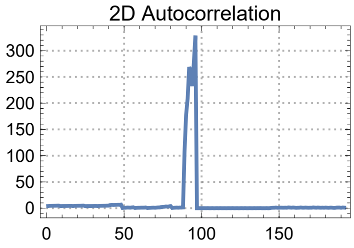

2D Plot

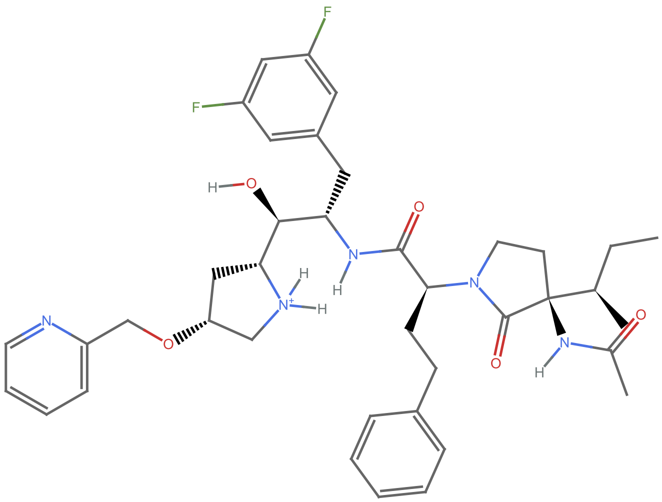

3D Plot

Dataset Index= 284

Molecule Formula= $C_{39}H_{50}F_2N_5O_5^+$

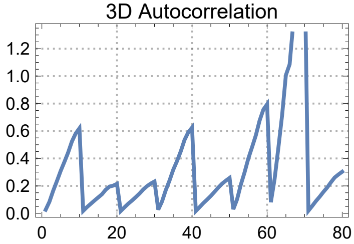

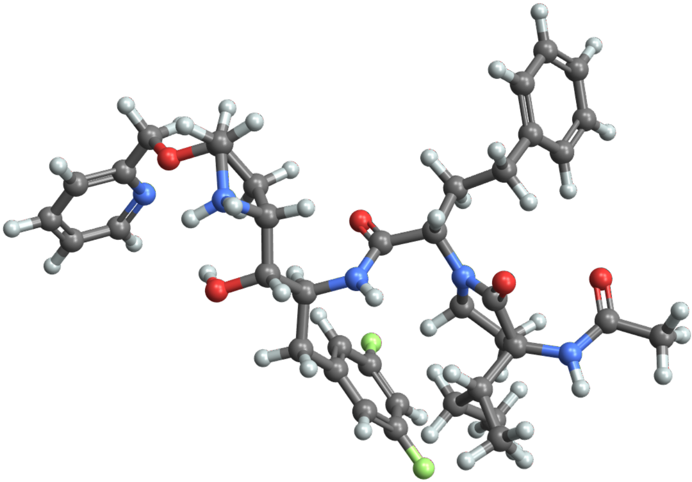

2D Plot

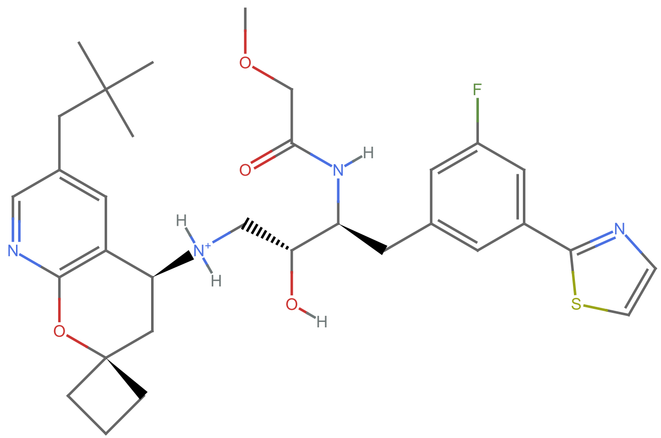

2D Autocorrelation

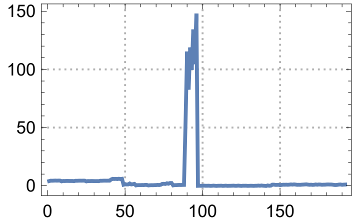

3D Plot

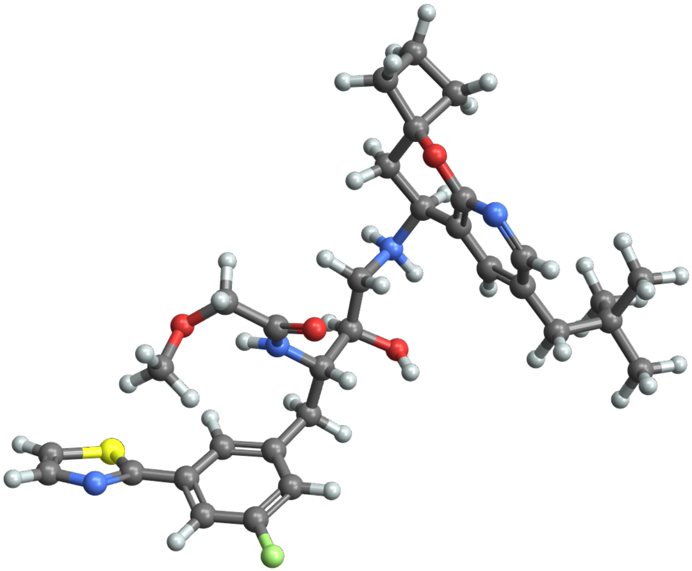

Dataset Index= 285

Molecule Formula= $C_{32}H_{42}FN_4O_4S^+$

3D Autocorrelation

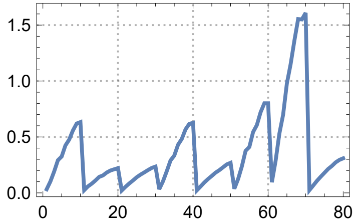

2D Autocorrelation

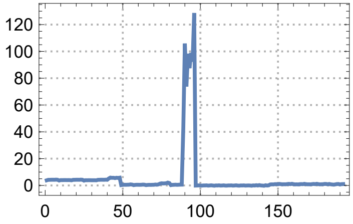

Dataset Index= 286

Molecule Formula= $C_{31}H_{41}FN_3O_4^+$

3D Autocorrelation

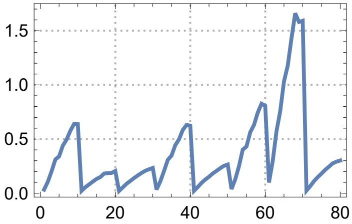

2D Plot

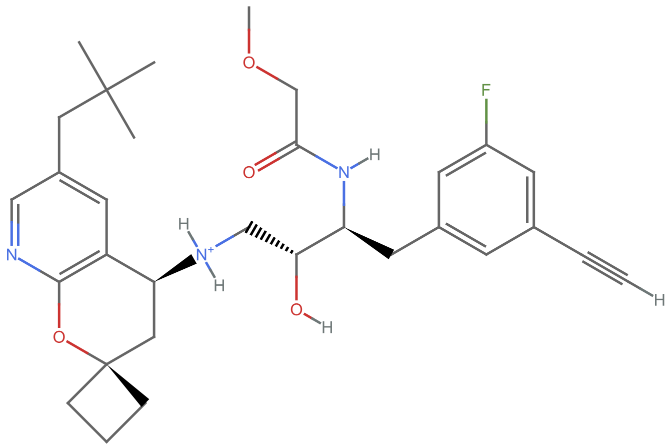

3D Plot

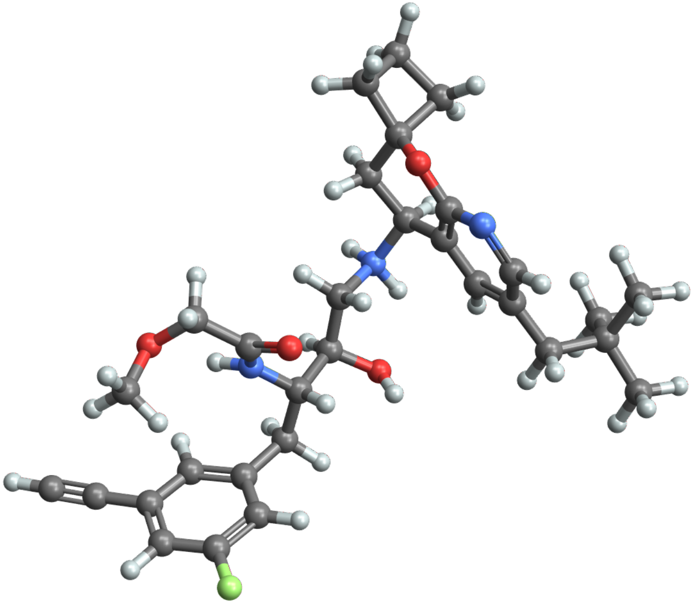

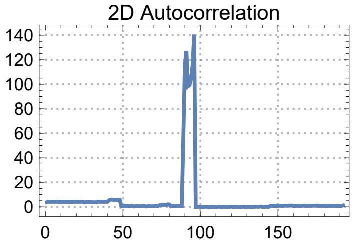

Dataset Index= 287

Molecule Formula= $C_{28}H_{38}F_2N_3O_3^+$

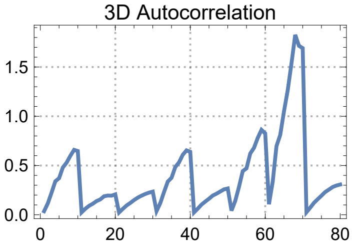

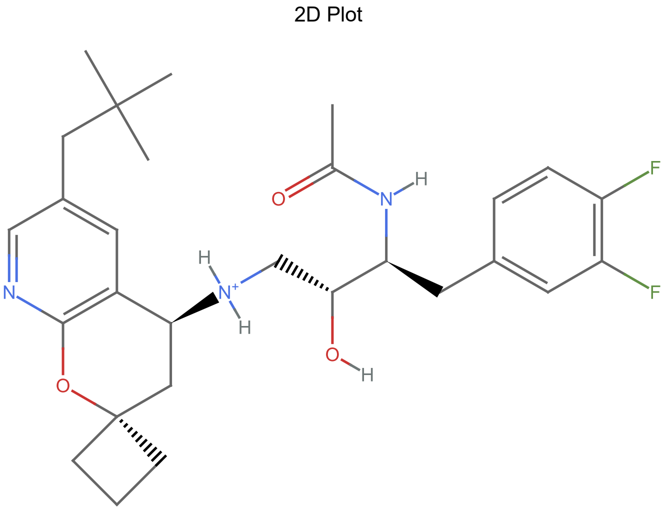

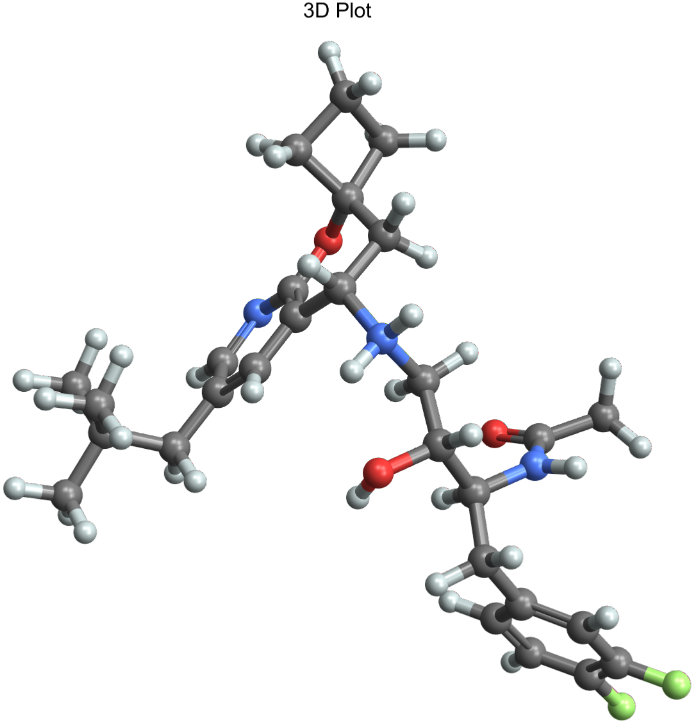

2D Plot

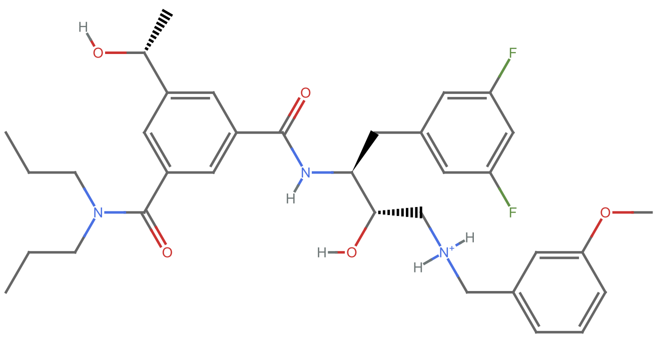

3D Plot

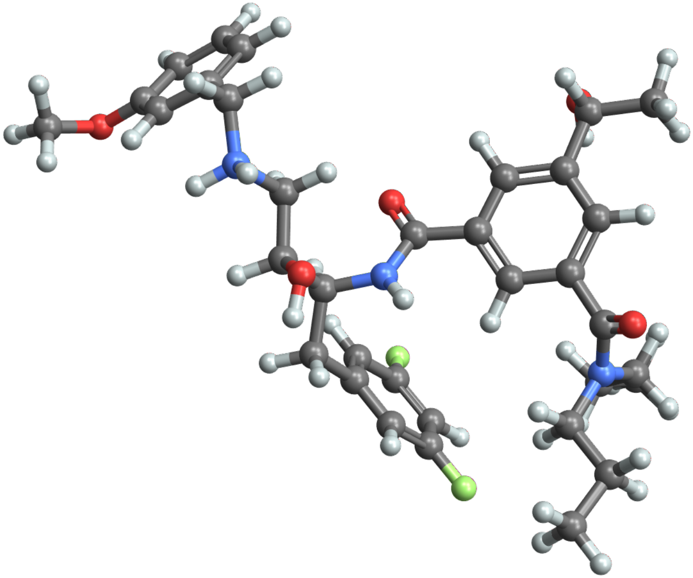

2D Autocorrelation

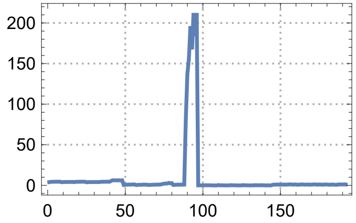

Dataset Index= 288

Molecule Formula= $C_{34}H_{44}F_2N_3O_5^+$

3D Autocorrelation

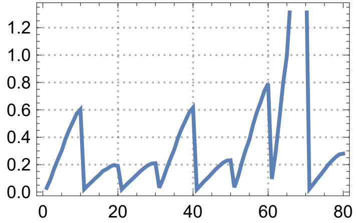

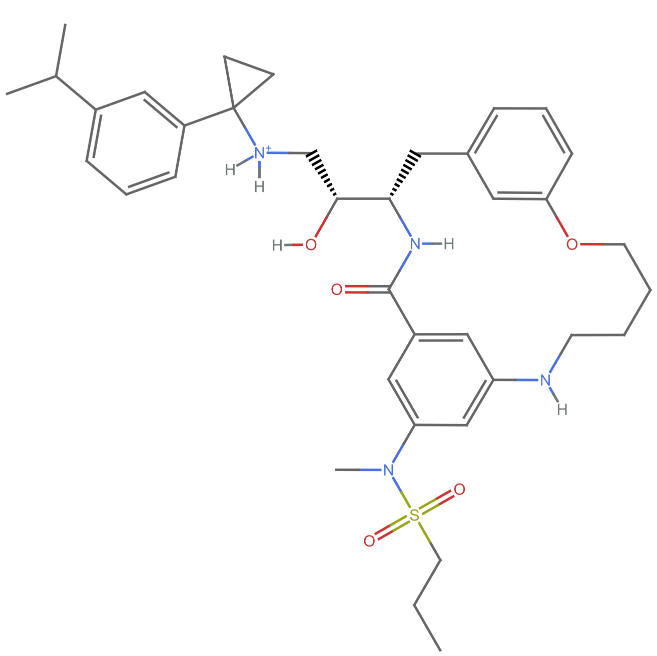

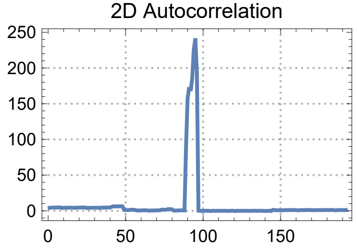

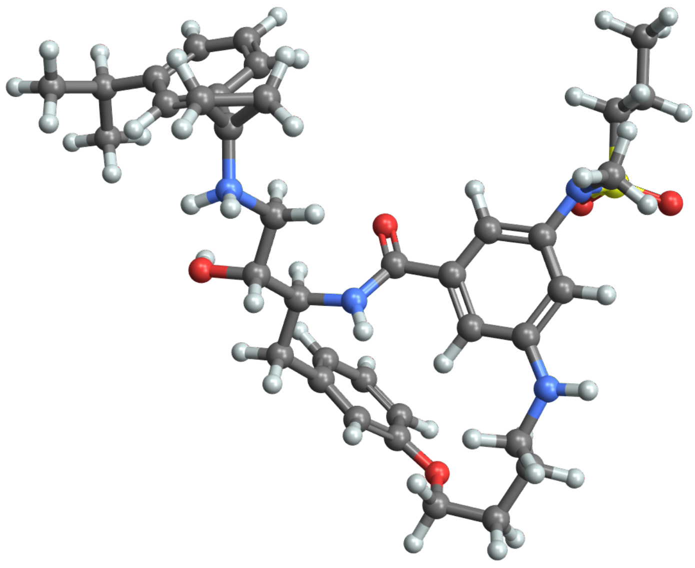

Dataset Index= 289

Molecule Formula= $C_{37}H_{51}N_4O_5S^+$

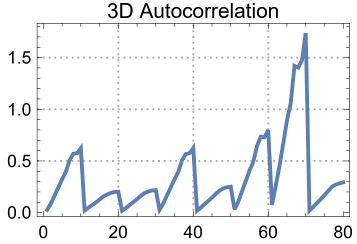

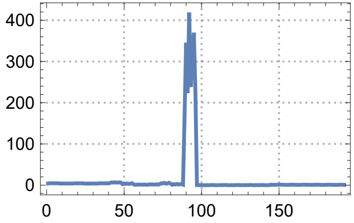

Dataset Index= 290

Molecule Formula= $C_{26}H_{32}F_7N_2O_4S^+$

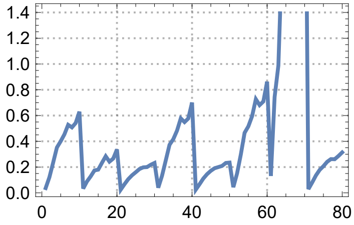

2D Plot

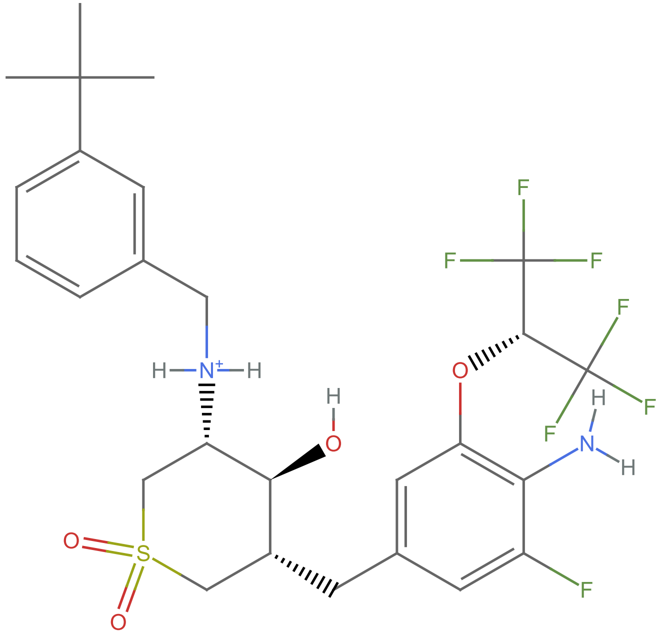

3D Plot

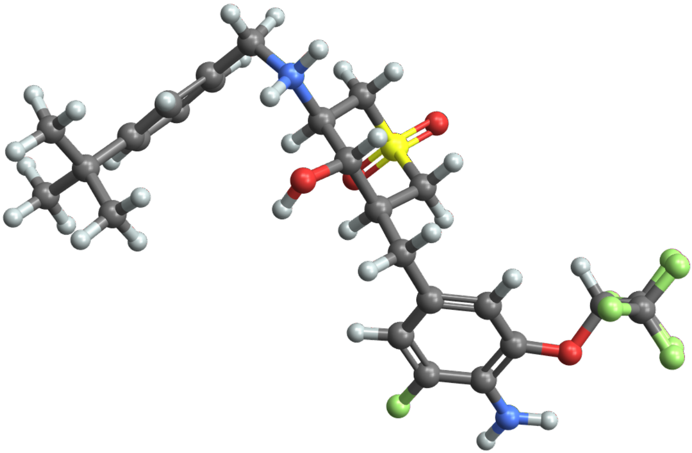

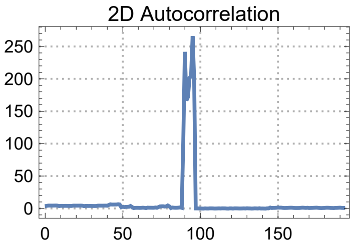

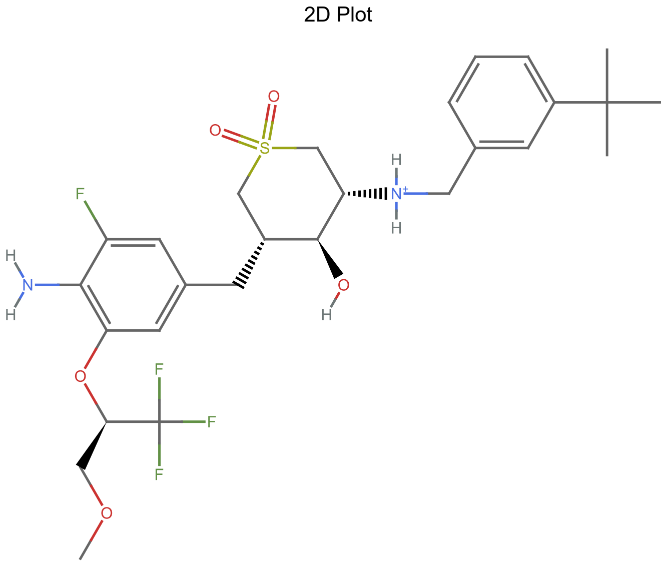

Dataset Index= 291

Molecule Formula= $C_{27}H_{37}F_4N_2O_5S^+$

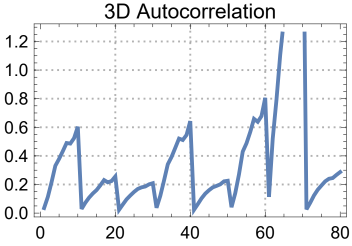

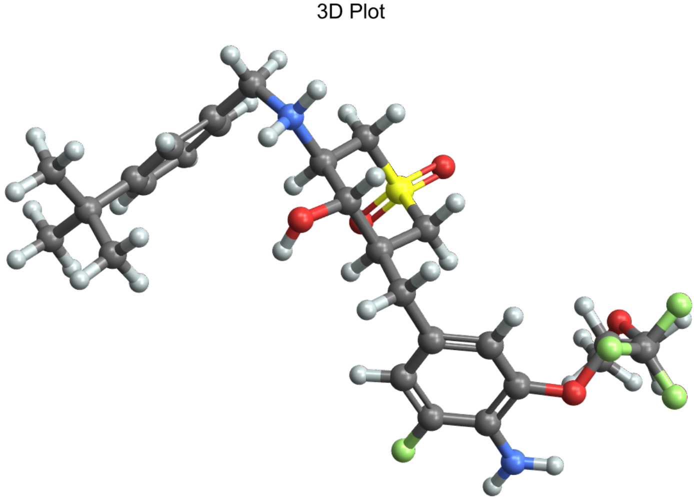

2D Plot

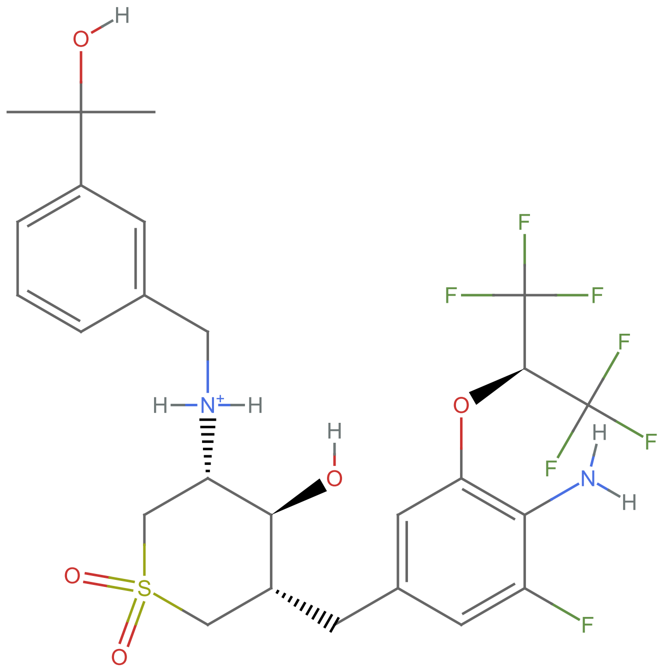

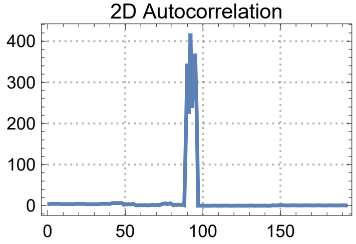

Dataset Index= 292

Molecule Formula= $C_{25}H_{30}F_7N_2O_5S^+$

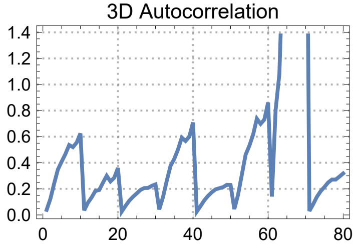

3D Plot

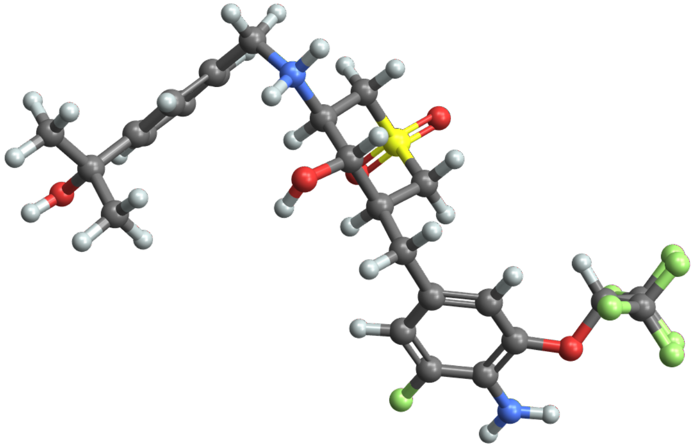

2D Autocorrelation

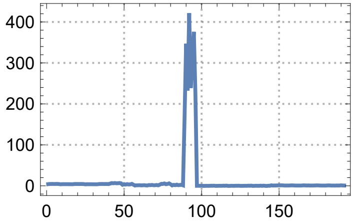

Dataset Index= 293

Molecule Formula= $C_{26}H_{32}F_7N_2O_5S^+$

3D Autocorrelation

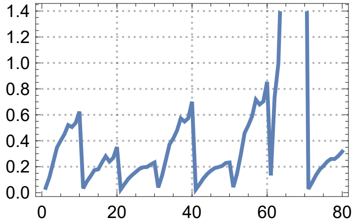

2D Plot

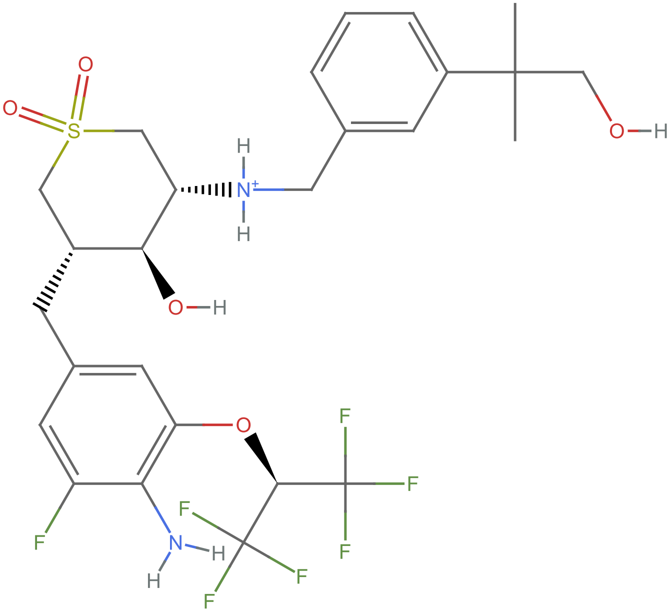

3D Plot

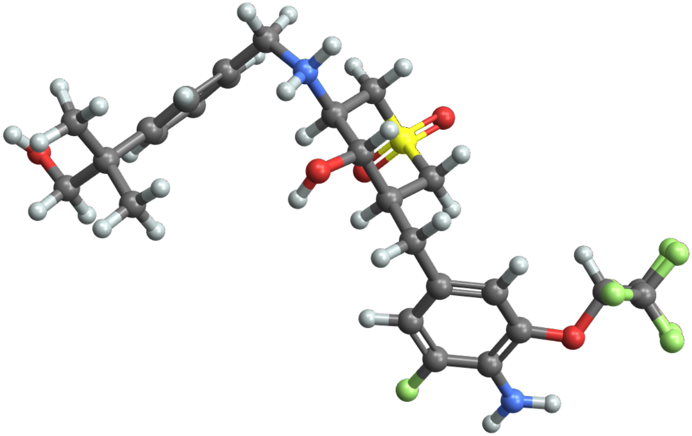

2D Plot

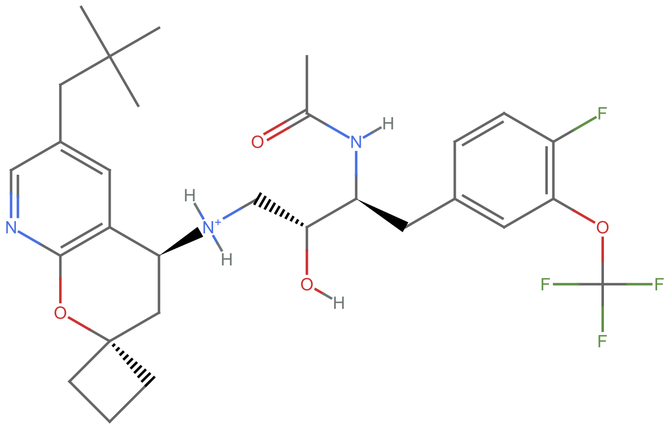

2D Autocorrelation

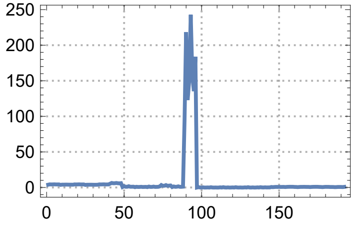

3D Plot

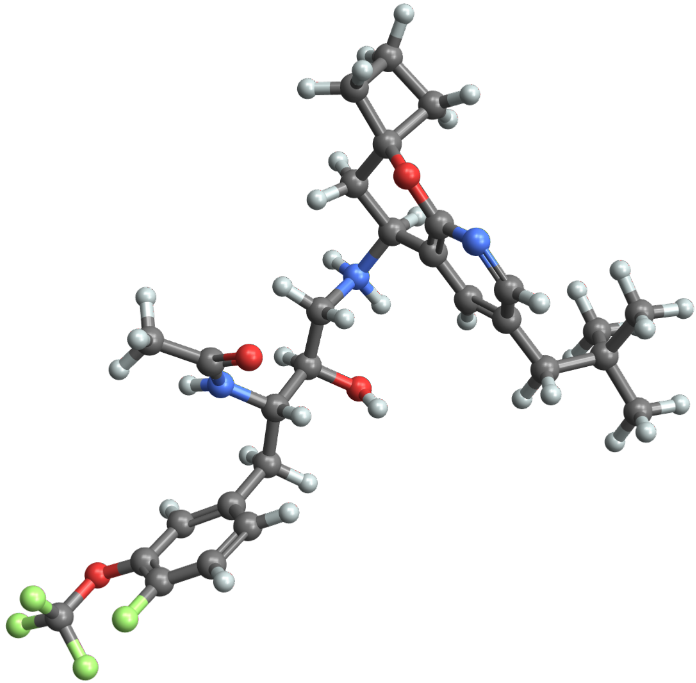

Dataset Index= 294

Molecule Formula= $C_{29}H_{38}F_4N_3O_4^+$

3D Autocorrelation

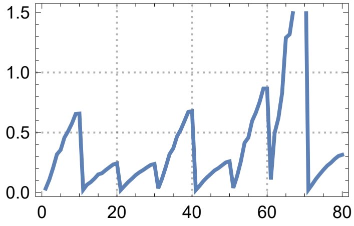

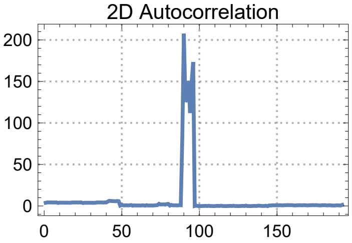

Dataset Index= 295

Molecule Formula= $C_{29}H_{39}F_3N_3O_3^+$

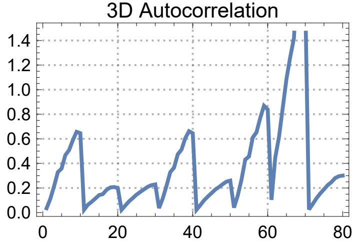

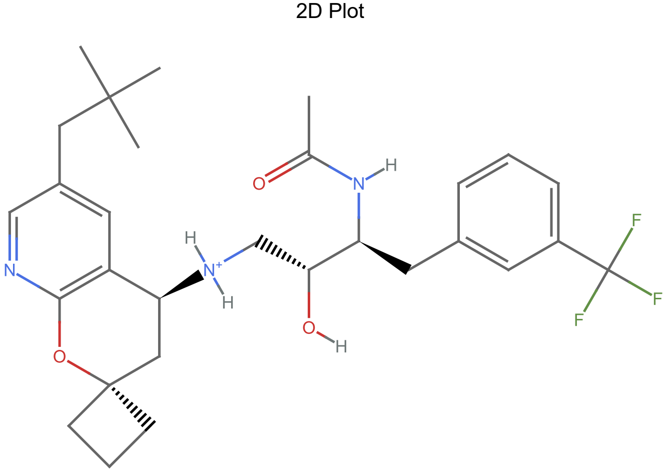

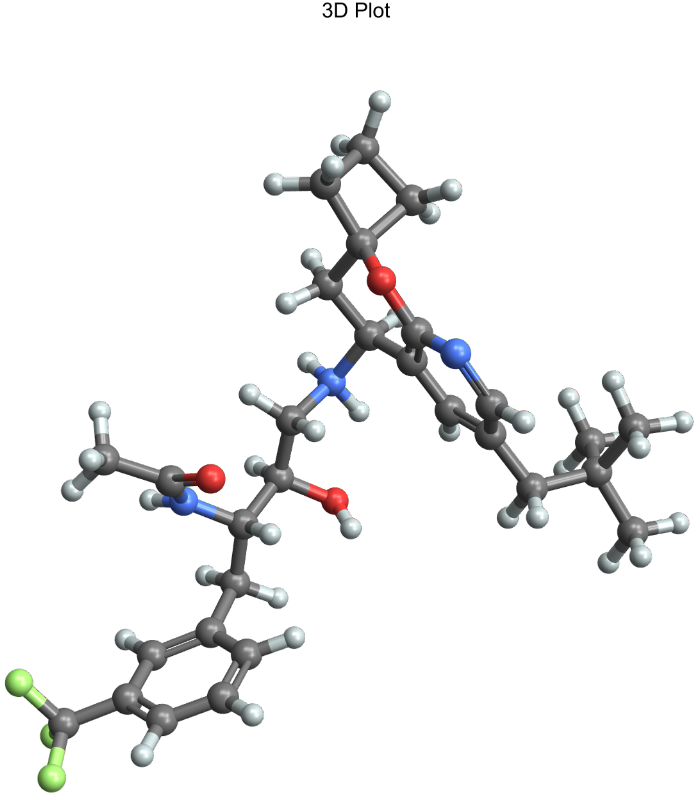

2D Autocorrelation

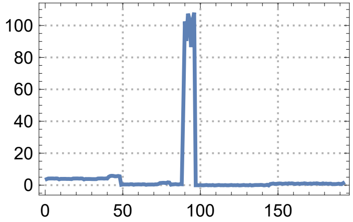

Dataset Index= 296

Molecule Formula= $C_{29}H_{38}FN_4O_3^+$

3D Autocorrelation

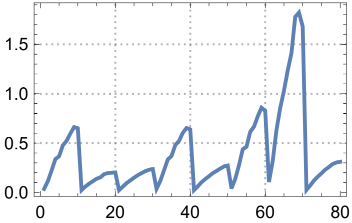

2D Plot

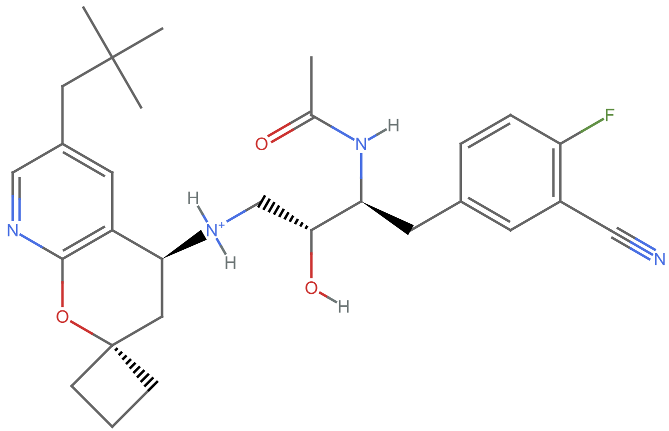

3D Plot

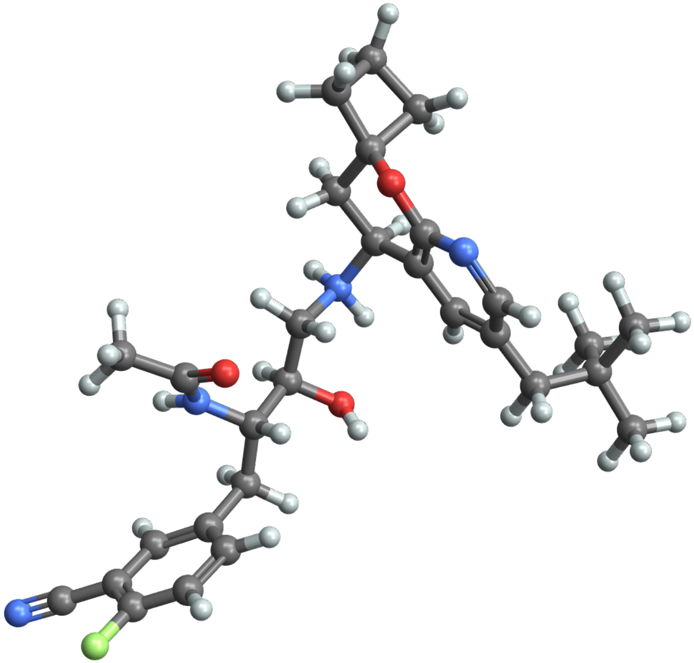

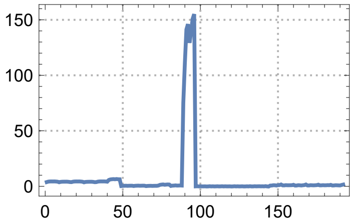

Dataset Index= 297

Molecule Formula= $C_{28}H_{24}FN_5O_2$

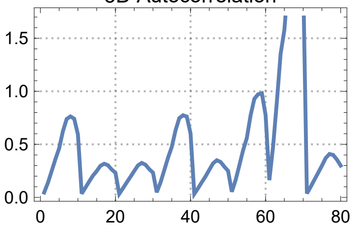

2D Plot

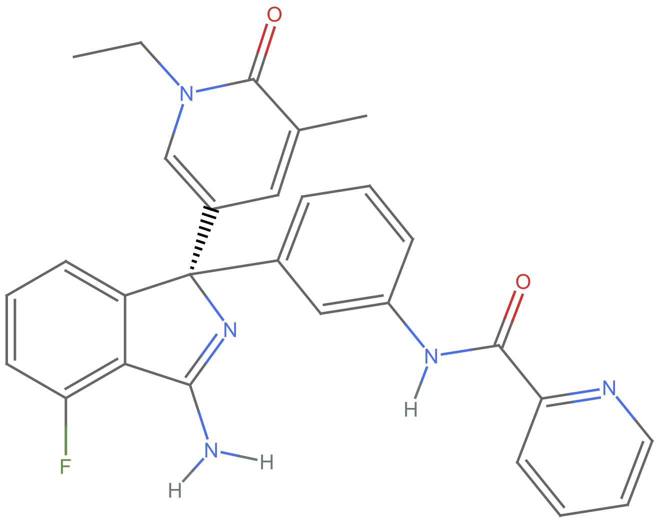

3D Plot

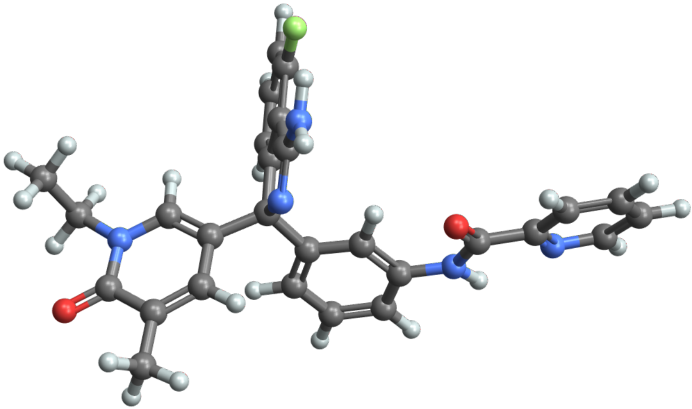

2D Plot

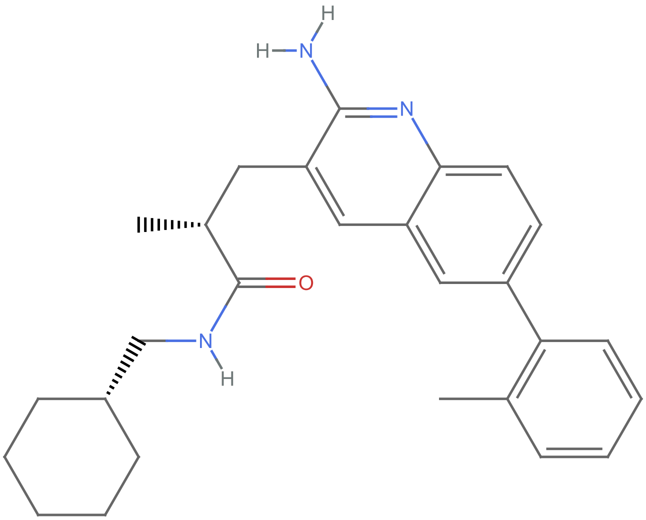

2D Autocorrelation

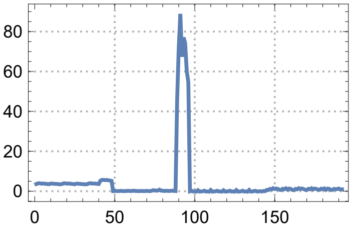

Dataset Index= 298

Molecule Formula= $C_{27}H_{33}N_3O$

3D Autocorrelation

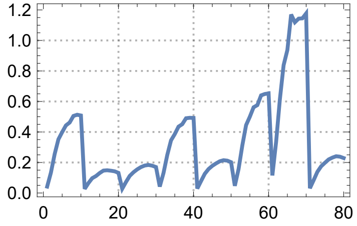

3D Plot

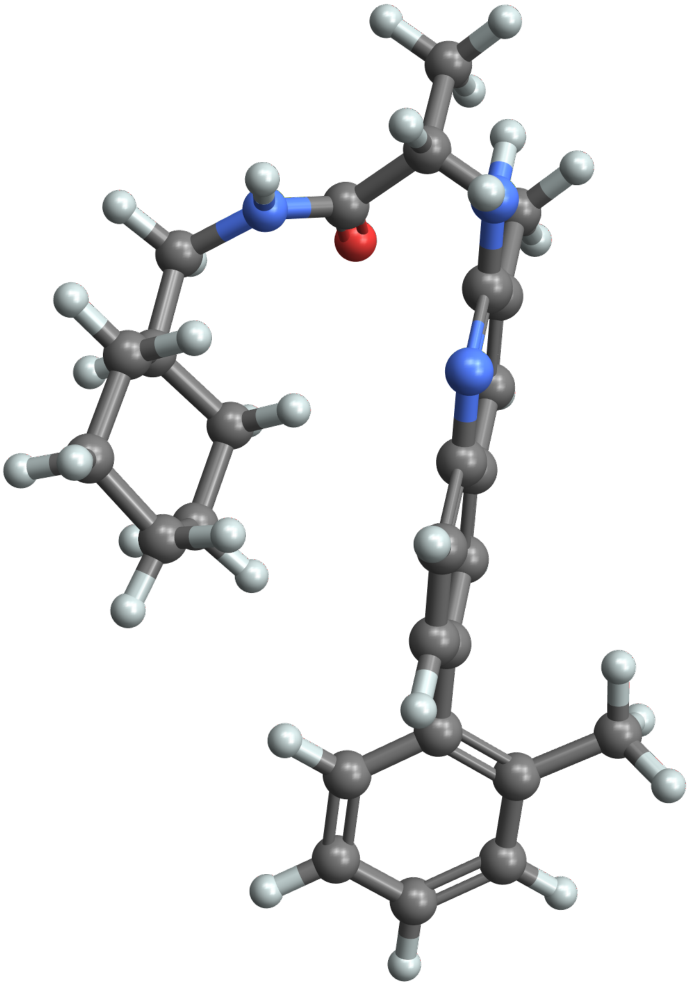

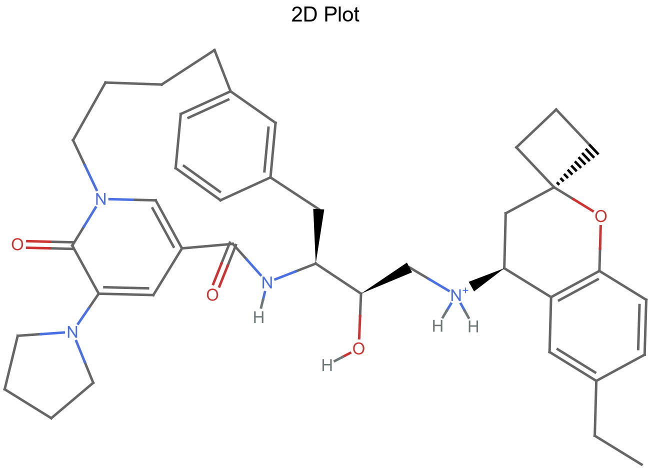

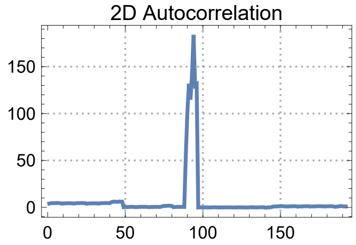

Dataset Index= 299

Molecule Formula= $C_{38}H_{49}N_4O_4^+$

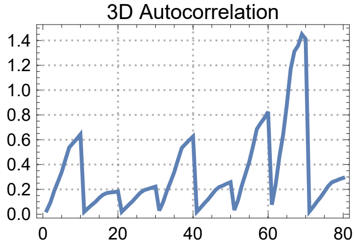

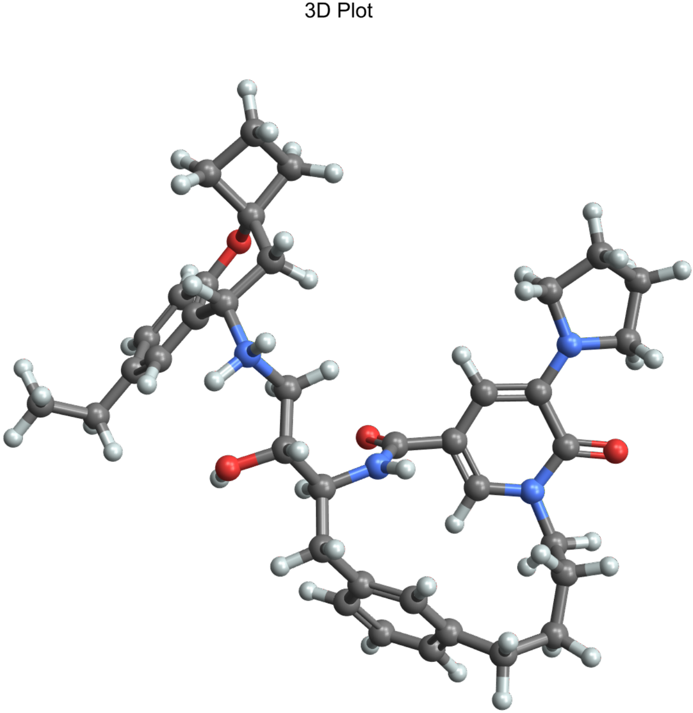

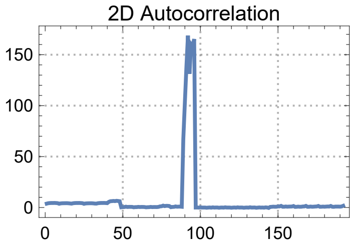

Dataset Index= 300

Molecule Formula= $C_{30}H_{24}F_2N_4O$

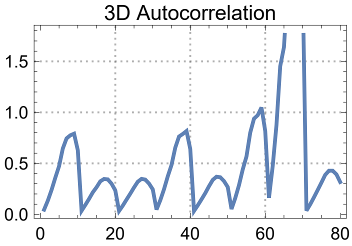

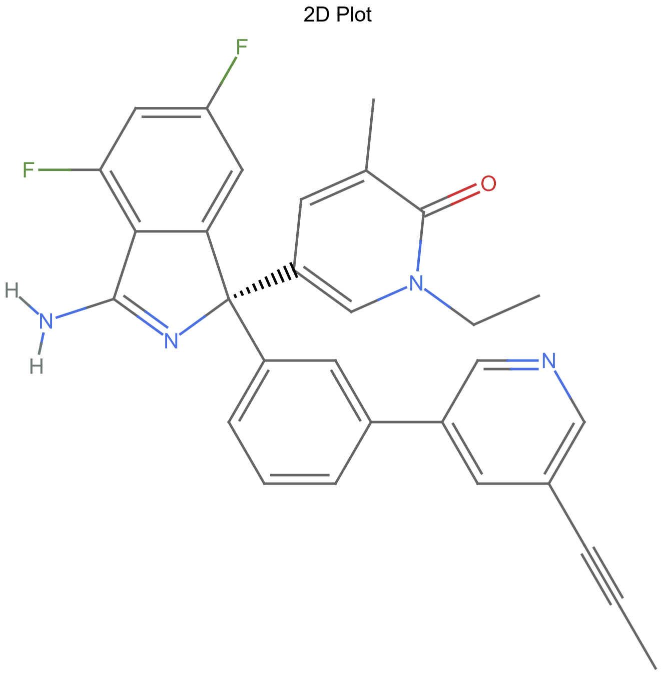

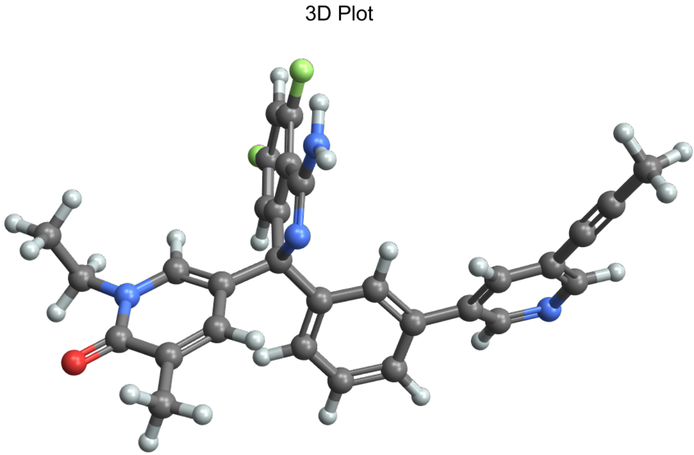

2D Plot

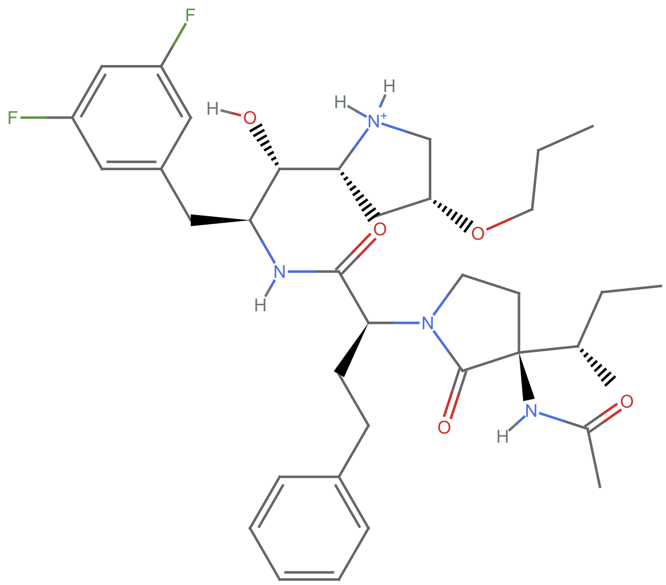

2D Autocorrelation

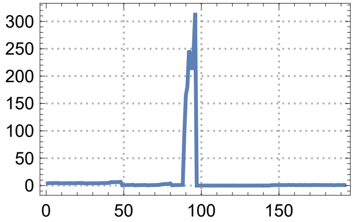

3D Plot

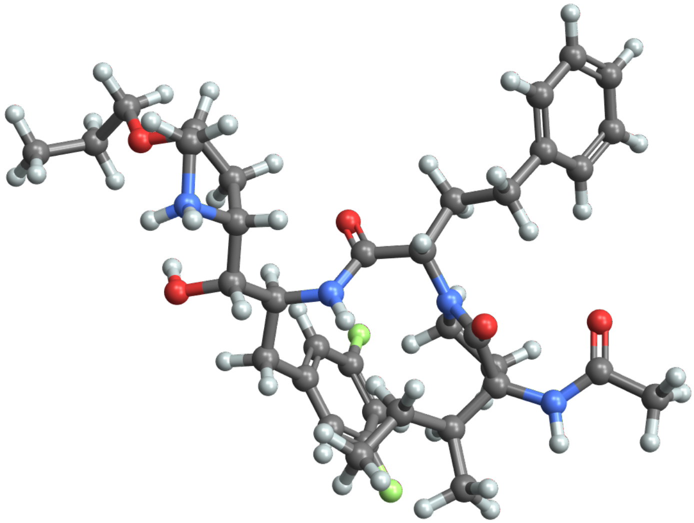

Dataset Index= 301

Molecule Formula= $C_{36}H_{51}F_2N_4O_5^+$

3D Autocorrelation

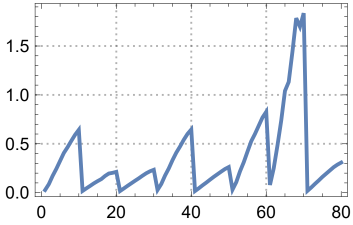

2D Plot

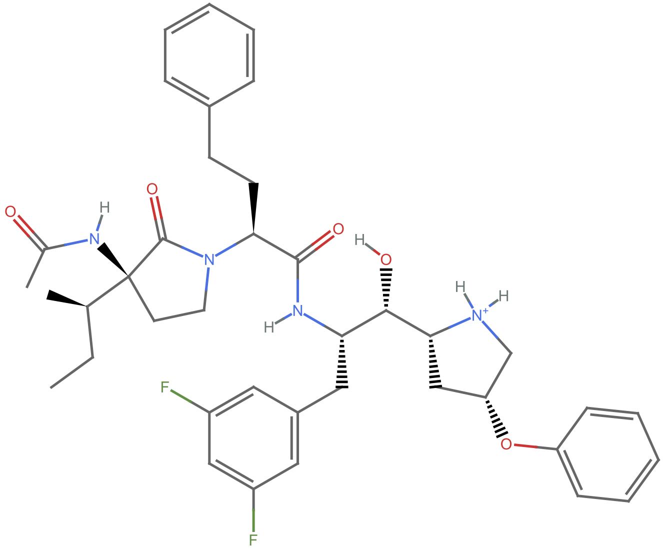

2D Autocorrelation

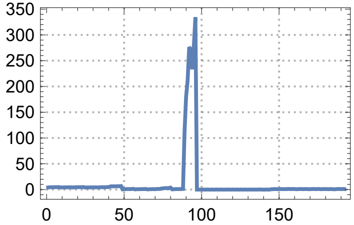

Dataset Index= 302

Molecule Formula= $C_{39}H_{49}F_2N_4O_5^+$

3D Plot

3D Autocorrelation

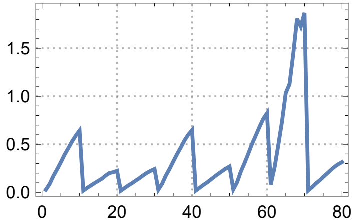

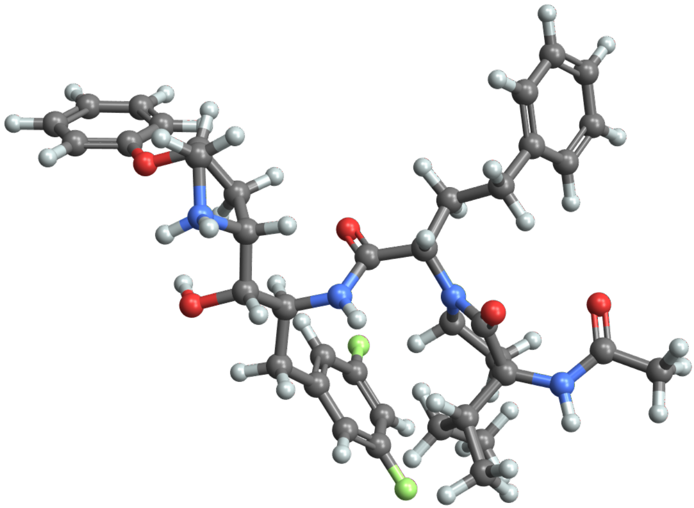

2D Plot

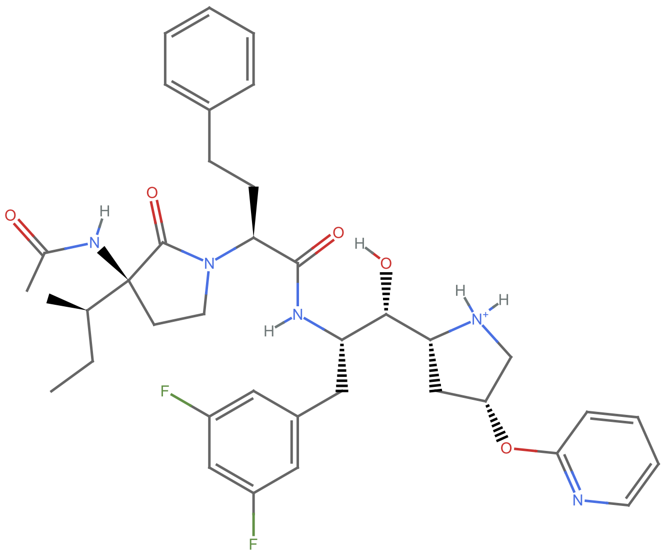

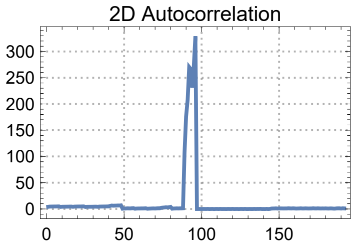

3D Plot

Dataset Index= 303

Molecule Formula= $C_{38}H_{48}F_2N_5O_5^+$

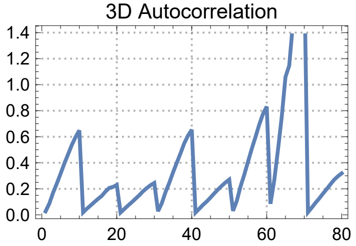

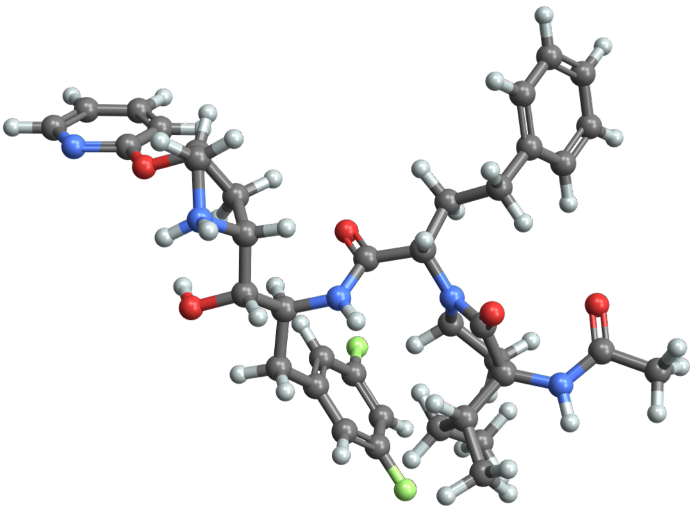

2D Plot

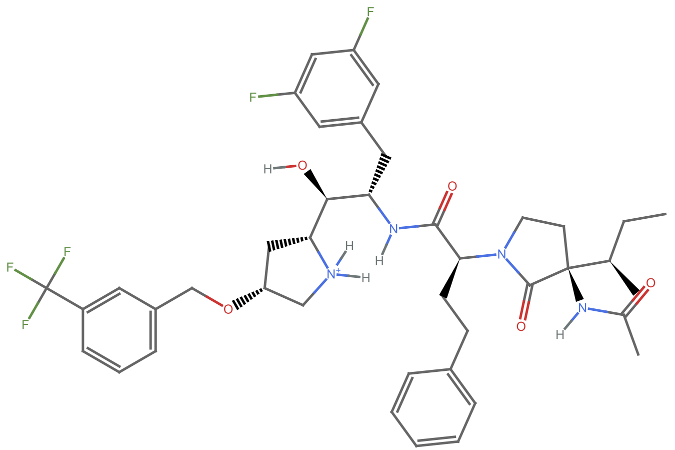

2D Autocorrelation

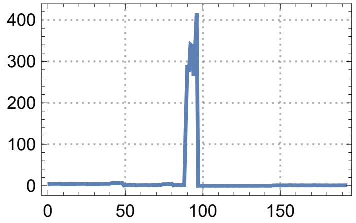

3D Plot

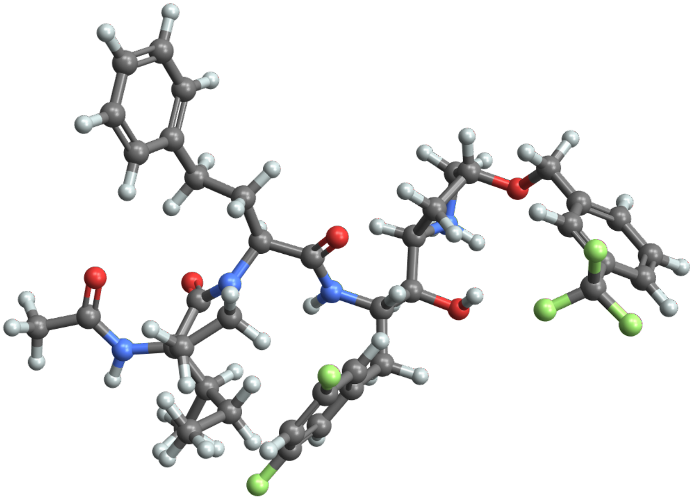

Dataset Index= 304

Molecule Formula= $C_{41}H_{50}F_5N_4O_5^+$

3D Autocorrelation

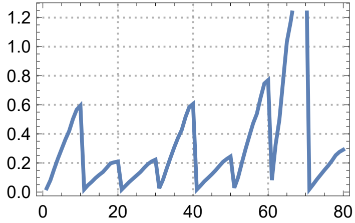

2D Plot

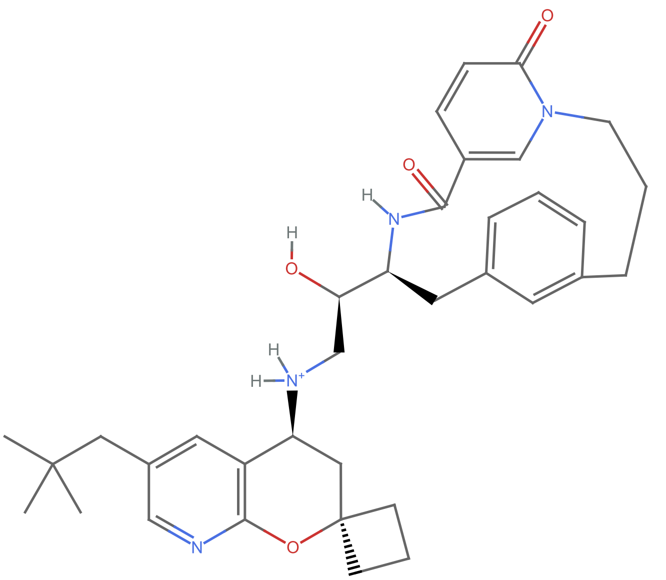

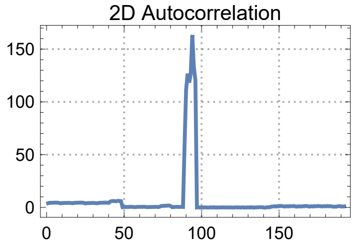

3D Plot

Dataset Index= 305

Molecule Formula= $C_{35}H_{45}N_4O_4^+$

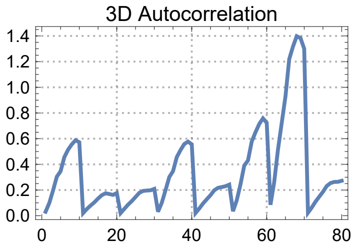

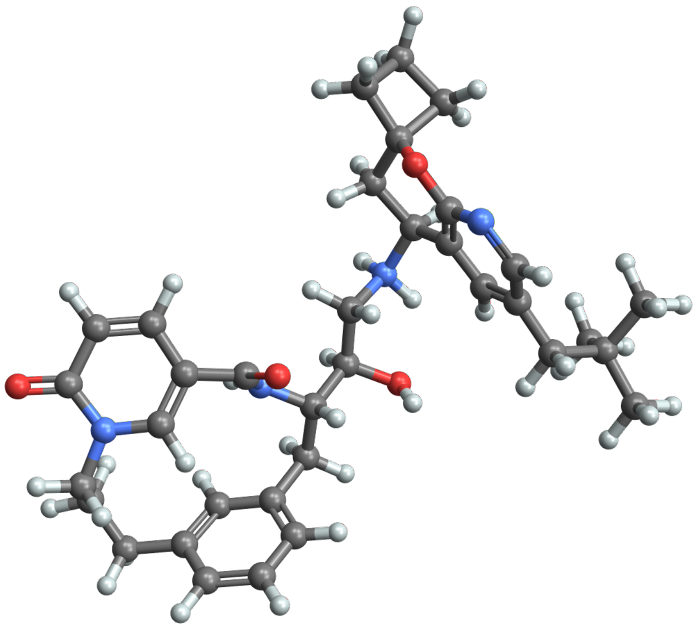

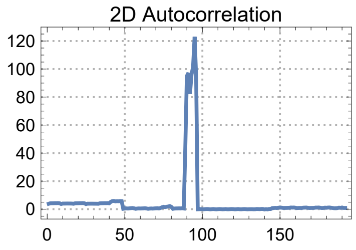

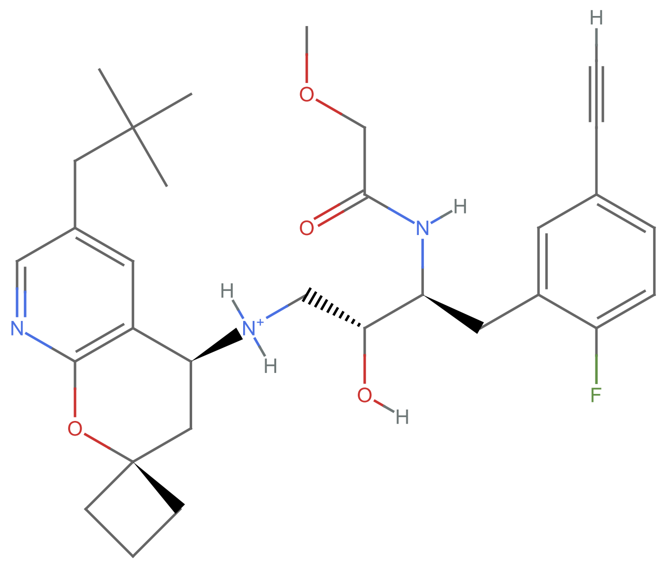

Dataset Index= 306

Molecule Formula= $C_{31}H_{41}FN_3O_4^+$

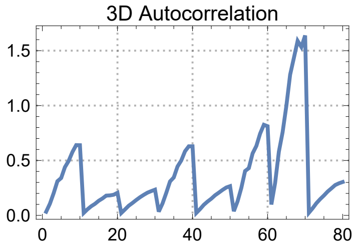

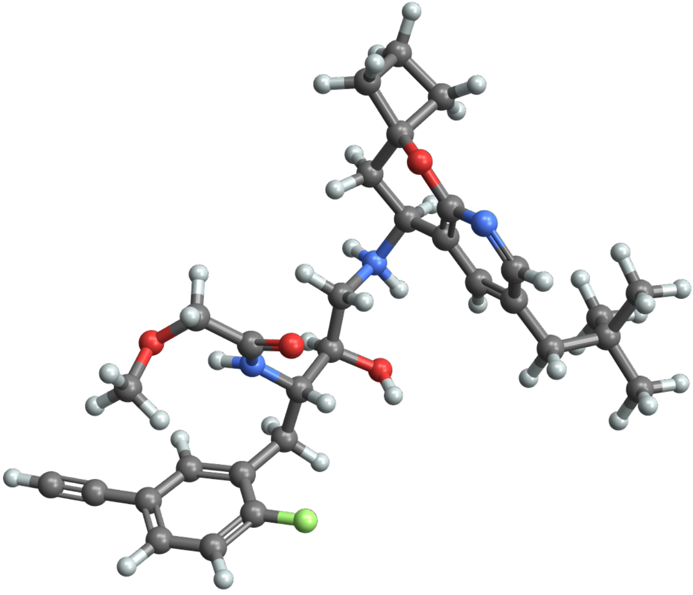

2D Plot

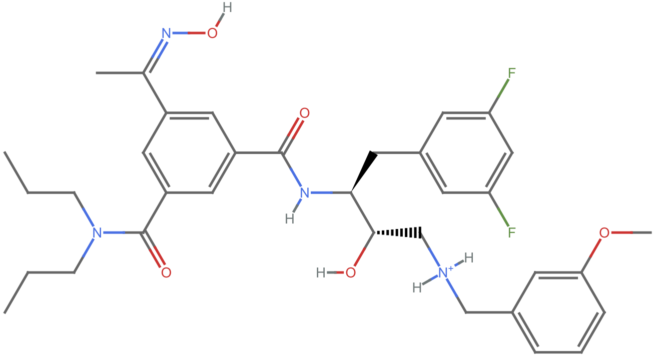

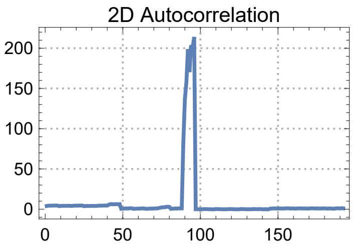

3D Plot

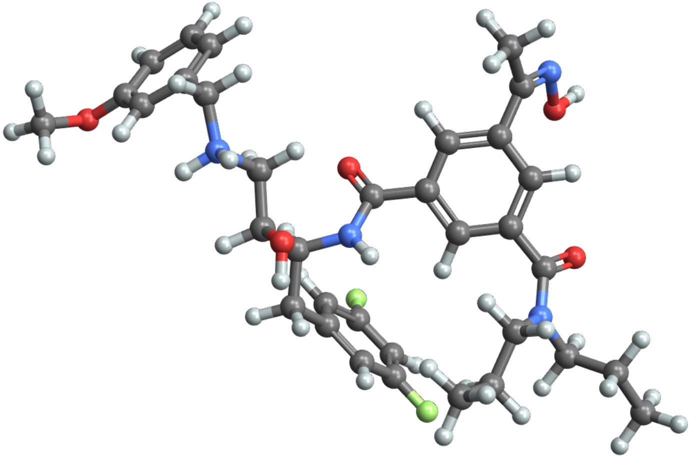

Dataset Index= 307

Molecule Formula= $C_{34}H_{43}F_2N_4O_5^{+}$

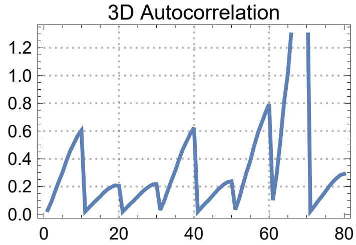

2D Plot

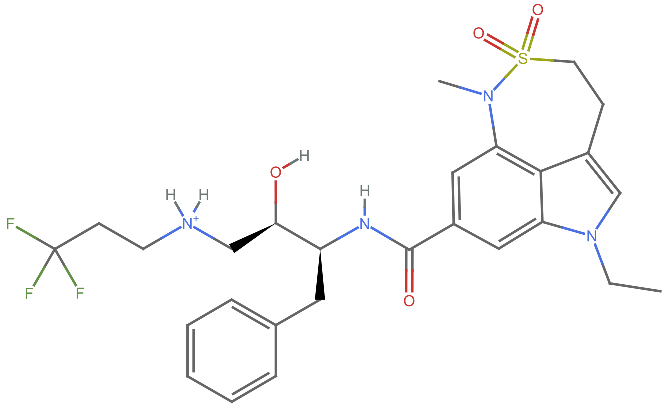

2D Autocorrelation

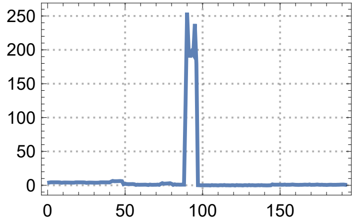

3D Plot

Dataset Index= 308

Molecule Formula= $C_{27}H_{34}F_3N_4O_4S^+$

3D Autocorrelation

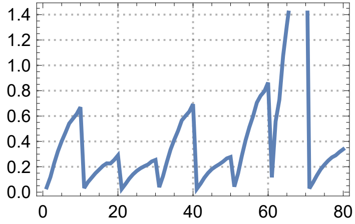

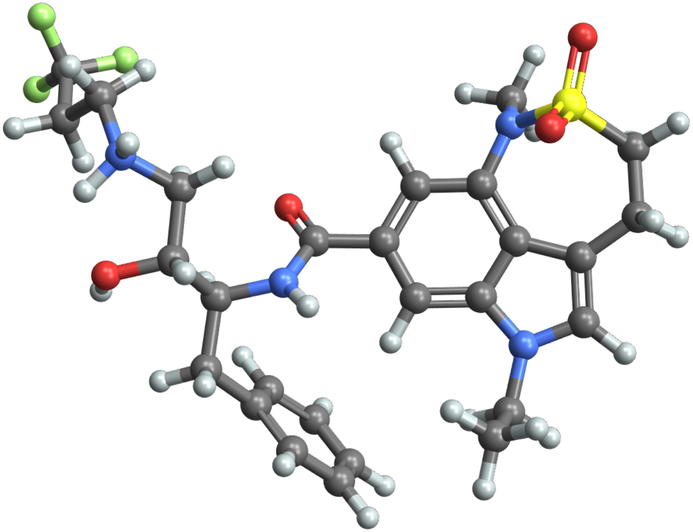

2D Plot

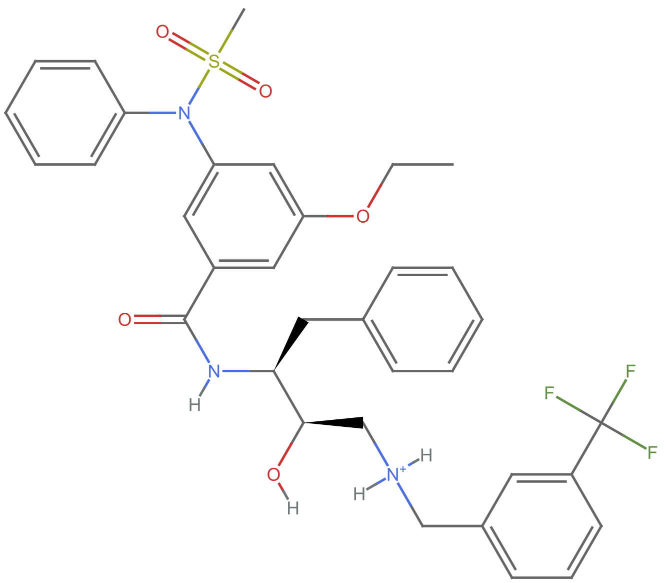

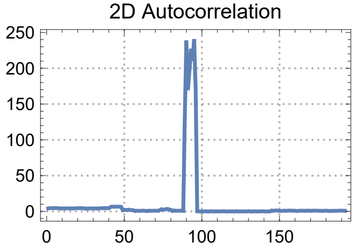

Dataset Index= 309

Molecule Formula= $C_{34}H_{37}F_3N_3O_5S^+$

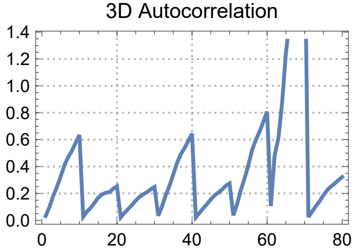

3D Plot

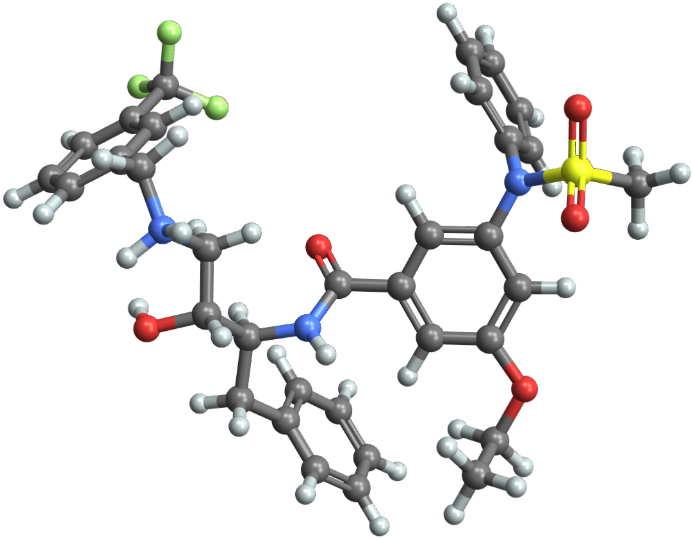

2D Plot

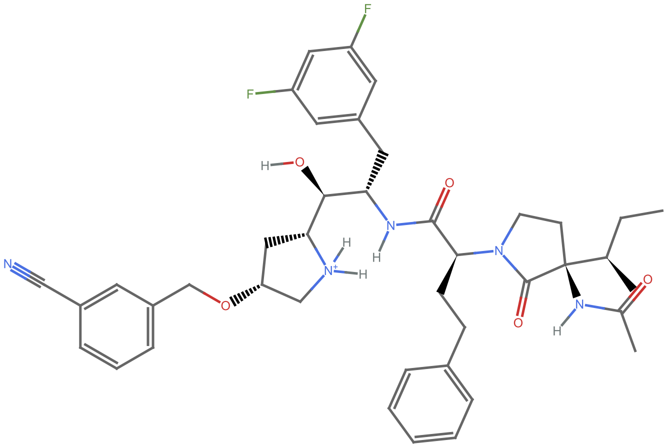

2D Autocorrelation

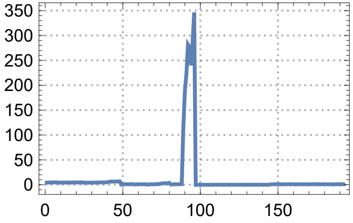

3D Plot

Dataset Index= 310

Molecule Formula= $C_{41}H_{50}F_2N_5O_5^+$

3D Autocorrelation

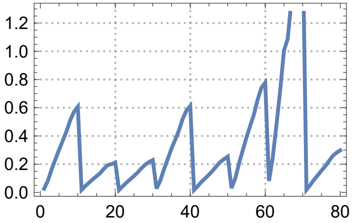

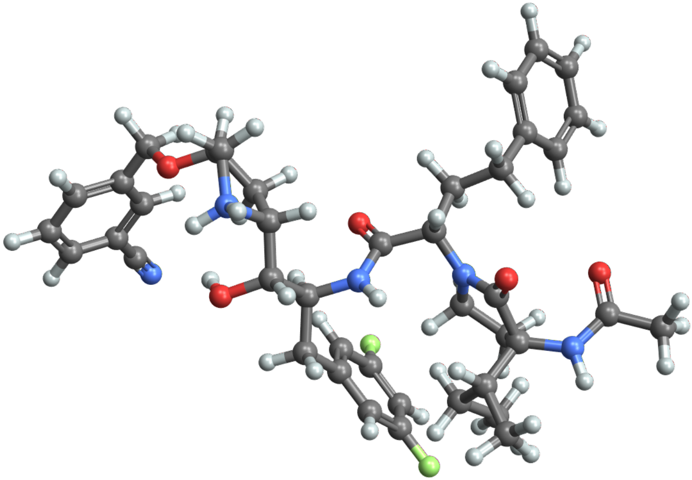

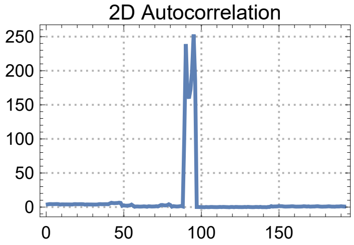

Dataset Index= 311

Molecule Formula= $C_{26}H_{35}F_4N_2O_4S^+$

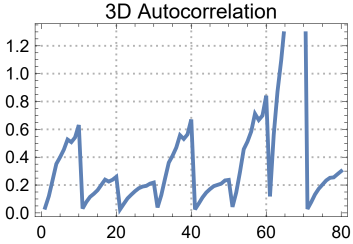

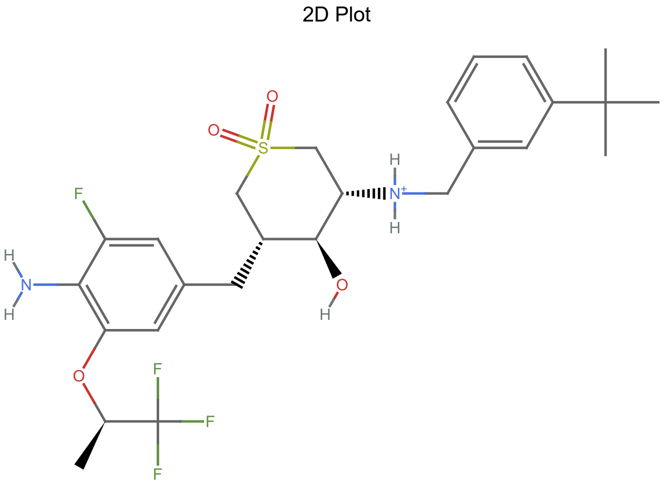

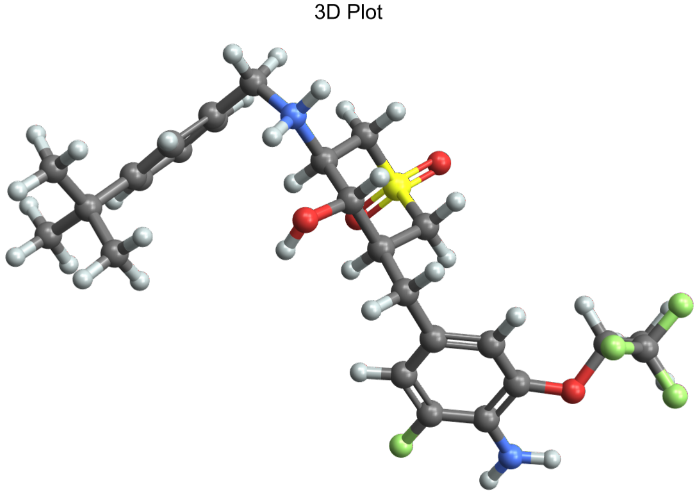

2D Autocorrelation

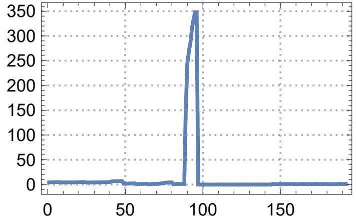

Dataset Index= 312

Molecule Formula= $C_{43}H_{51}F_2N_5O_8S$

3D Autocorrelation

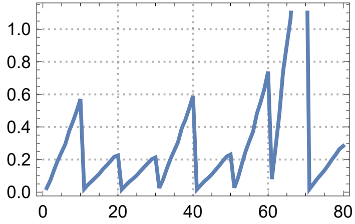

2D Plot

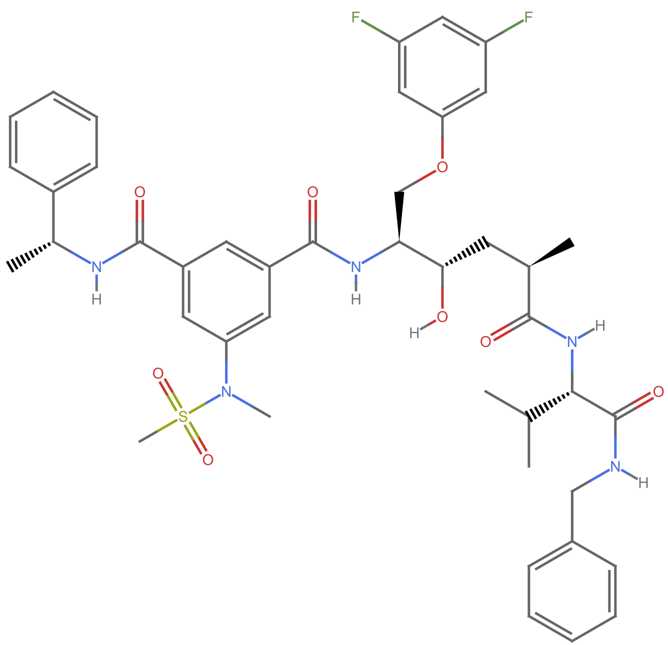

3D Plot

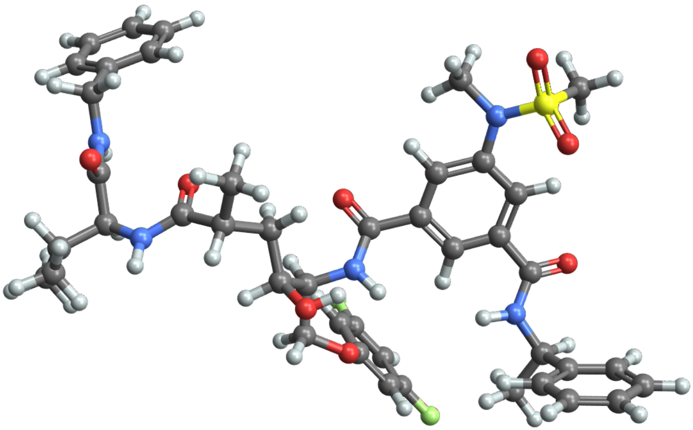

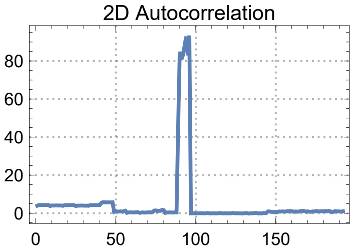

Dataset Index= 313

Molecule Formula= $C_{32}H_{43}N_4O_4S^+$

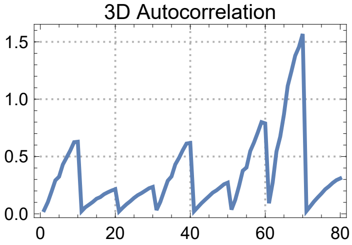

2D Plot

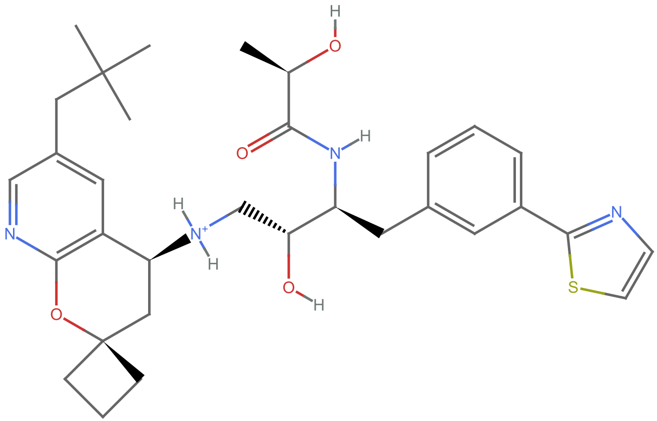

3D Plot

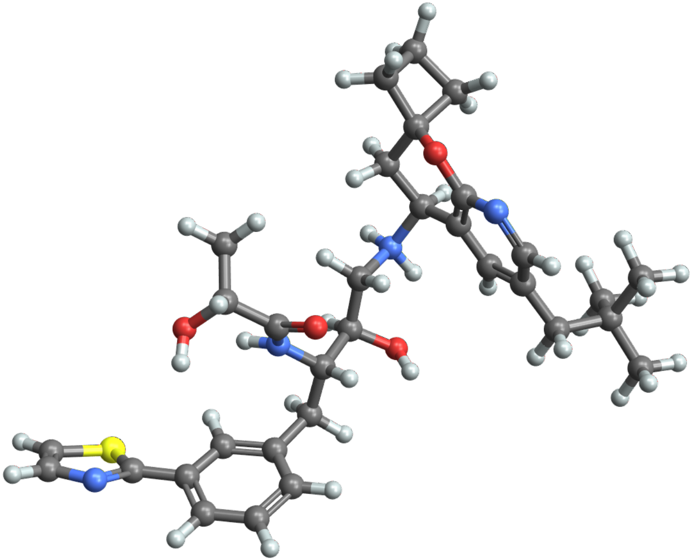

2D Plot

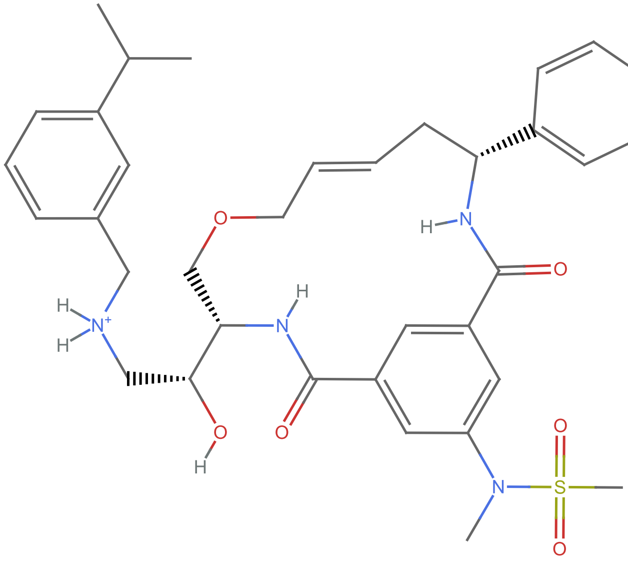

2D Autocorrelation

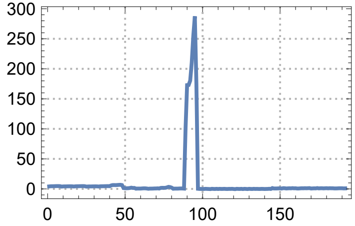

Dataset Index= 314

Molecule Formula= $C_{35}H_{45}N_4O_6S^+$

3D Plot

3D Autocorrelation

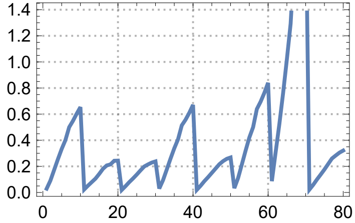

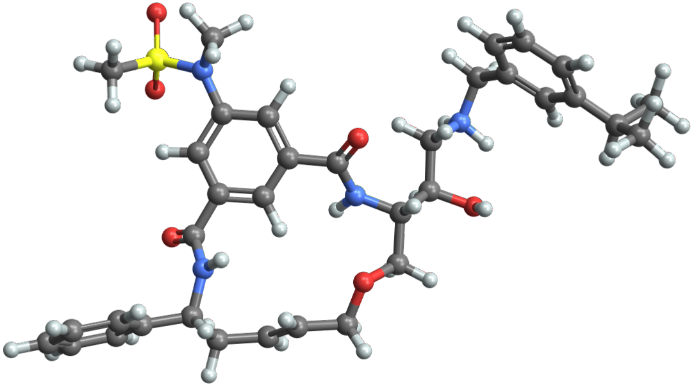

2D Plot

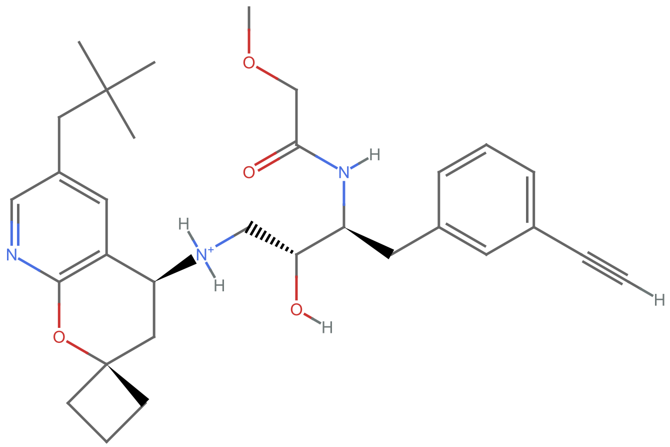

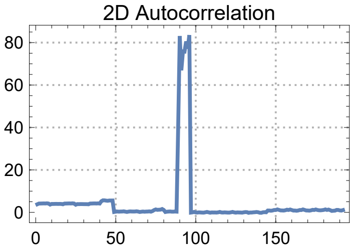

3D Plot

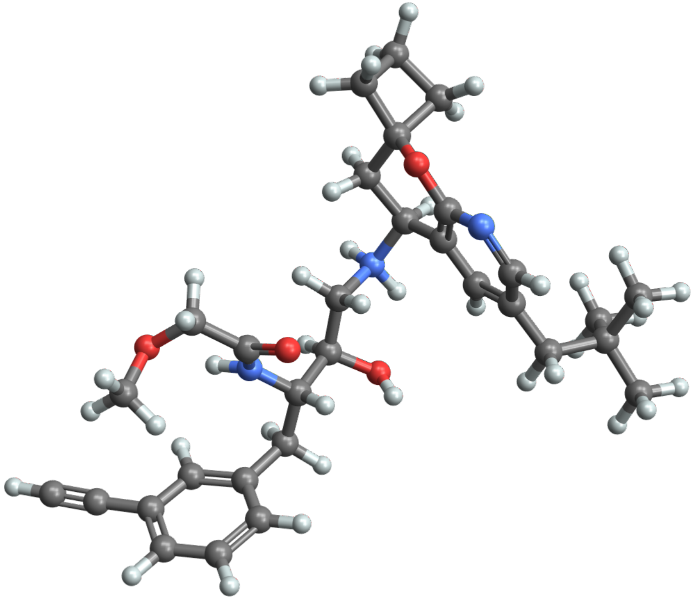

Dataset Index= 315

Molecule Formula= $C_{31}H_{42}N_3O_4^+$

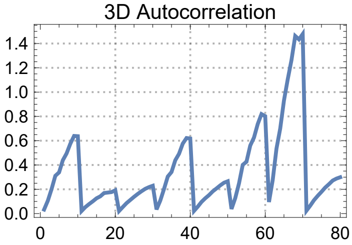

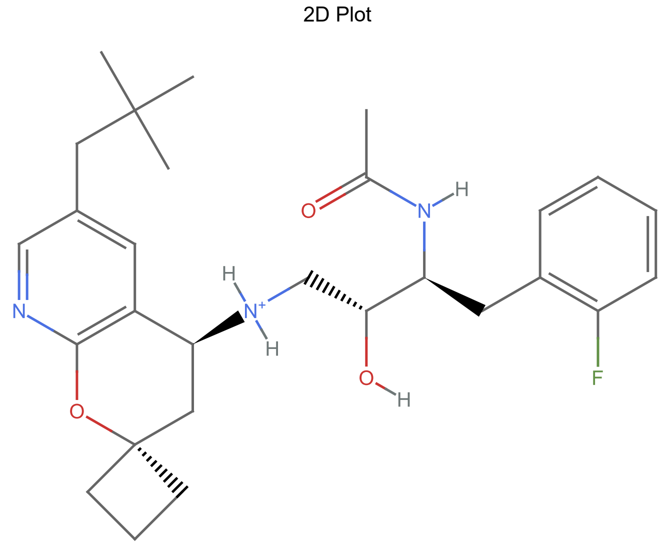

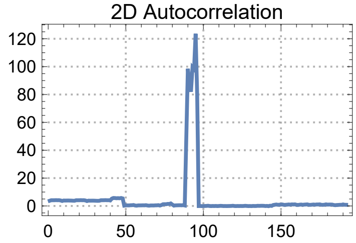

Dataset Index= 316

Molecule Formula= $C_{28}H_{39}FN_3O_3^+$

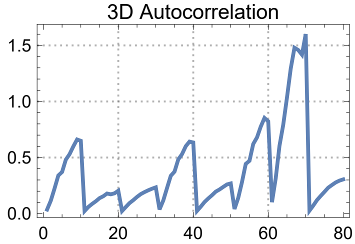

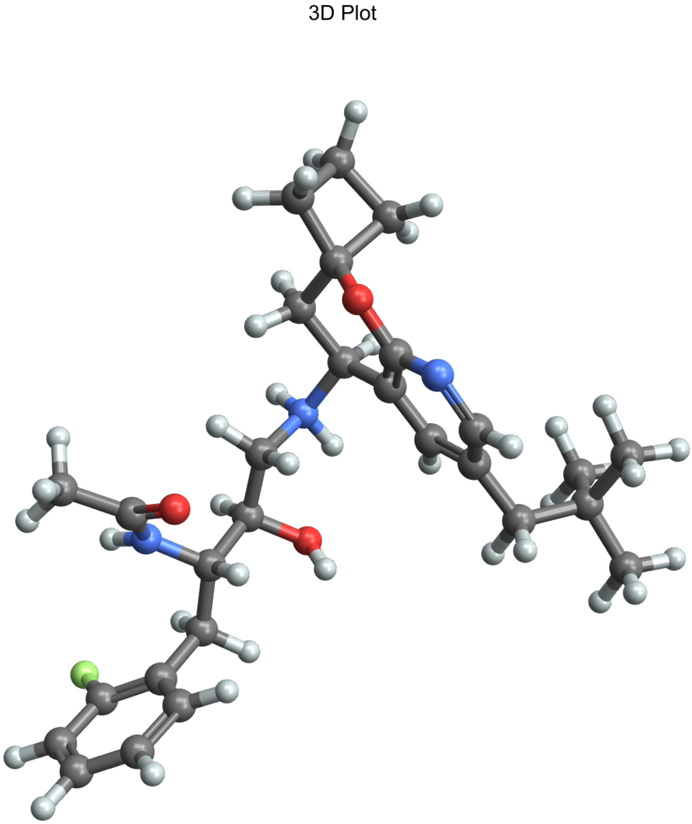

2D Plot

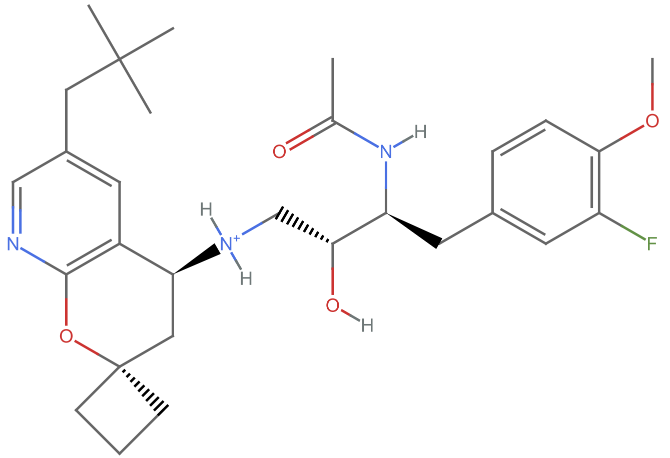

3D Plot

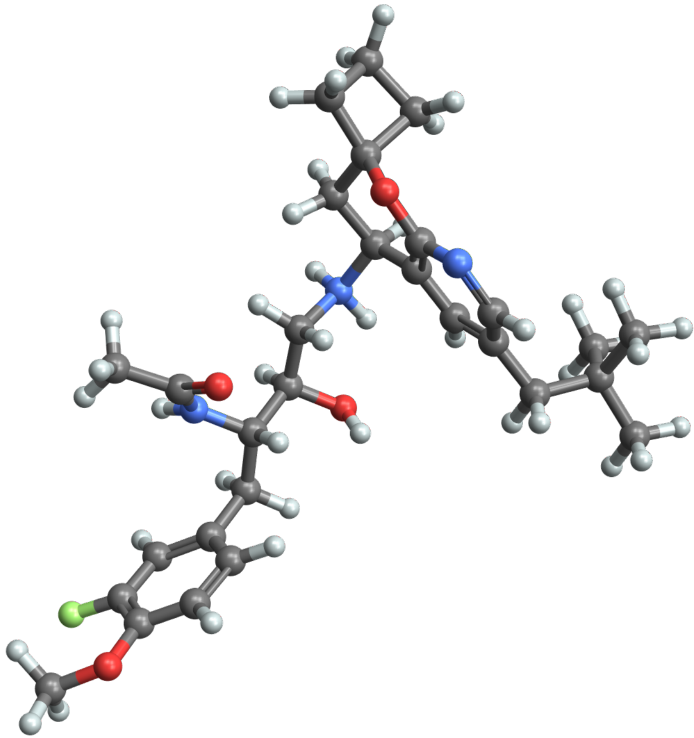

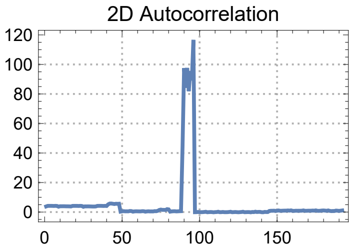

Dataset Index= 317

Molecule Formula= $C_{29}H_{41}FN_3O_4^+$

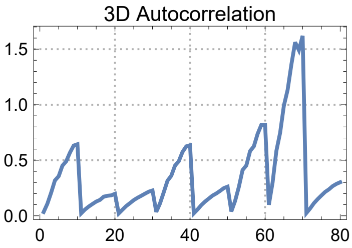

2D Plot

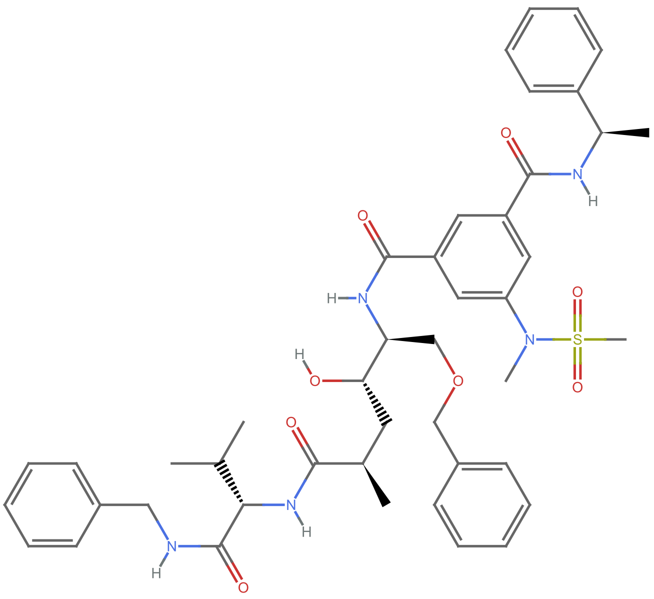

2D Autocorrelation

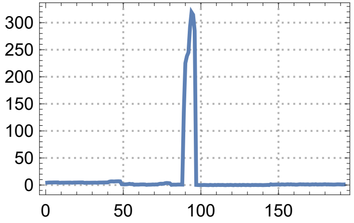

Dataset Index= 318

Molecule Formula= $C_{44}H_{55}N_5O_8S$

3D Autocorrelation

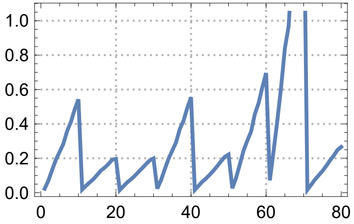

3D Plot

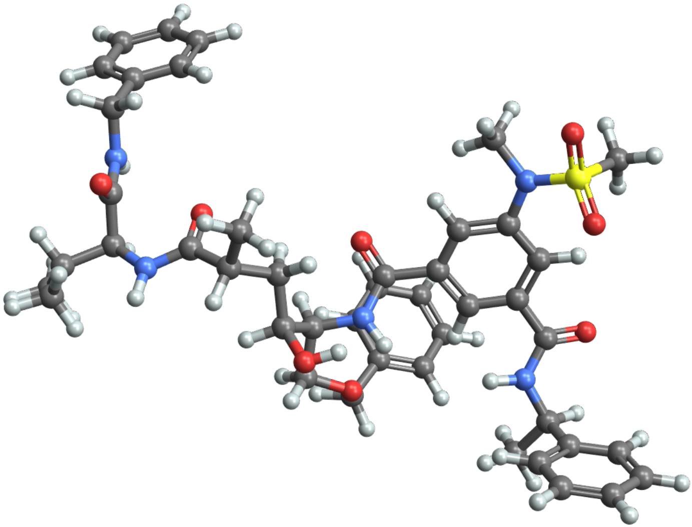

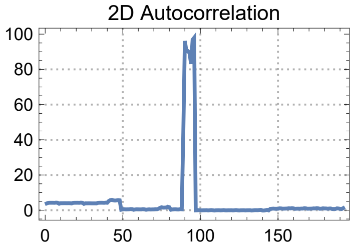

Dataset Index= 319

Molecule Formula= $C_{31}H_{41}FN_3O_4^+$

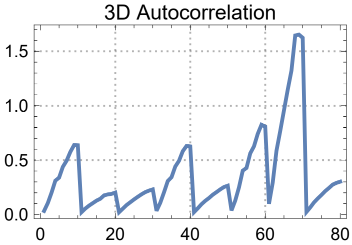

2D Plot

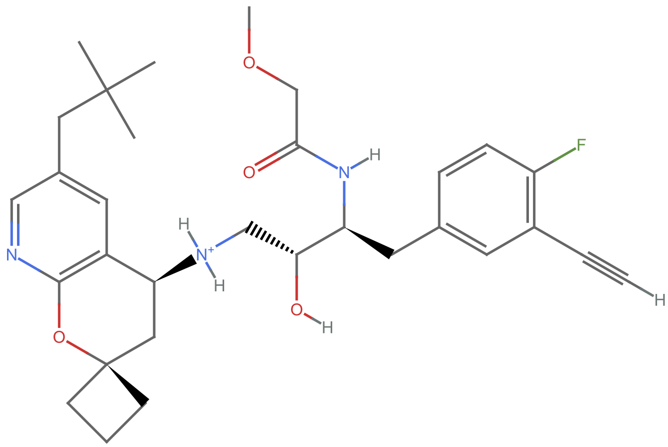

3D Plot

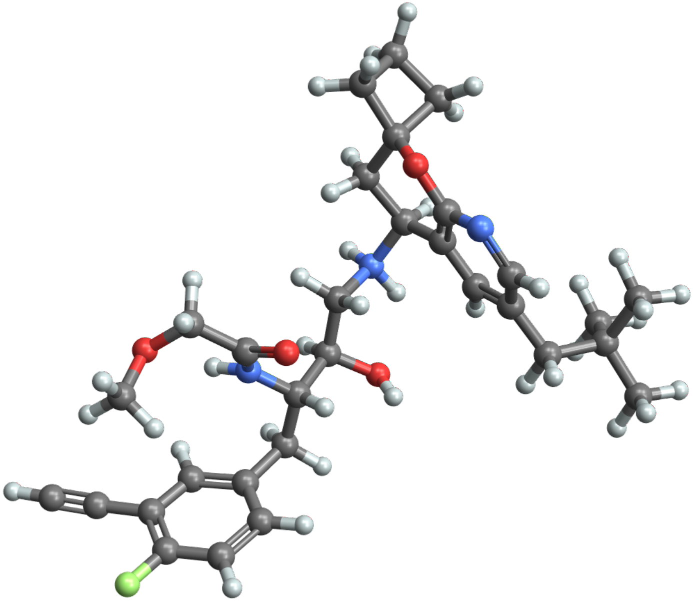

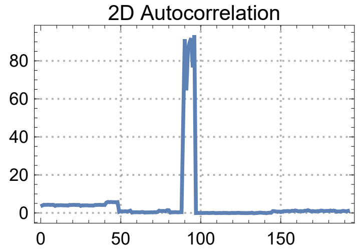

Dataset Index= 320

Molecule Formula= $C_{31}H_{41}N_4O_3S^+$

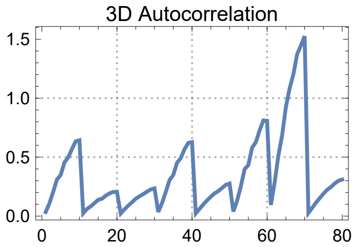

2D Plot

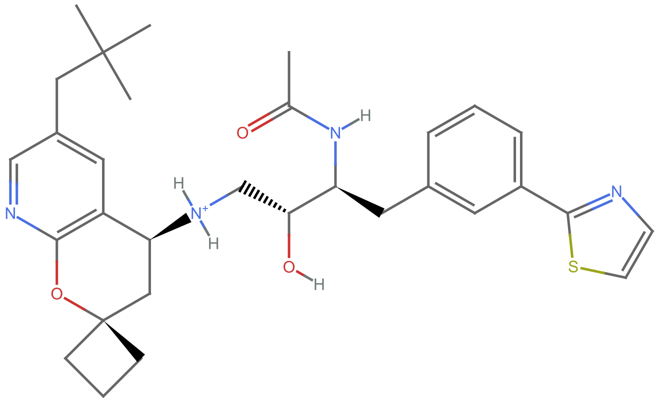

3D Plot

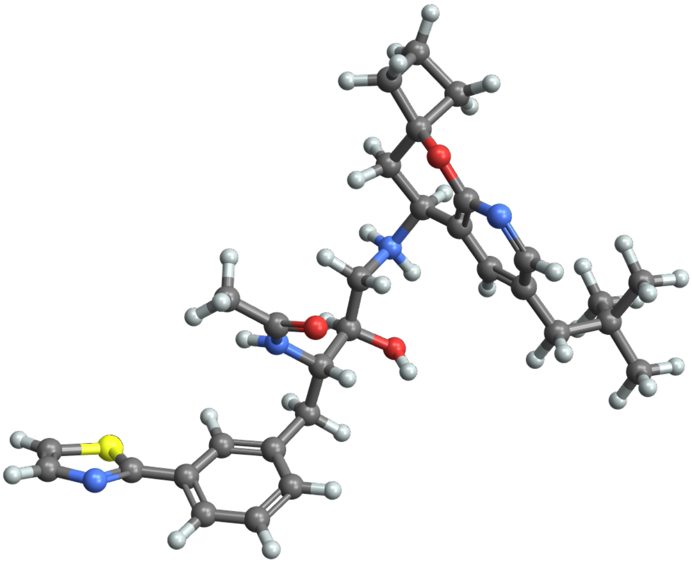

2D Plot

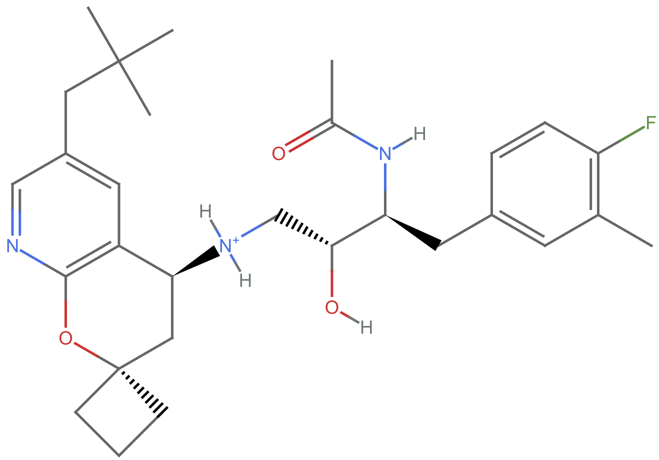

2D Autocorrelation

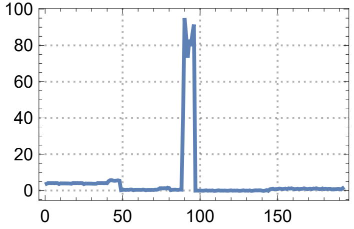

3D Plot

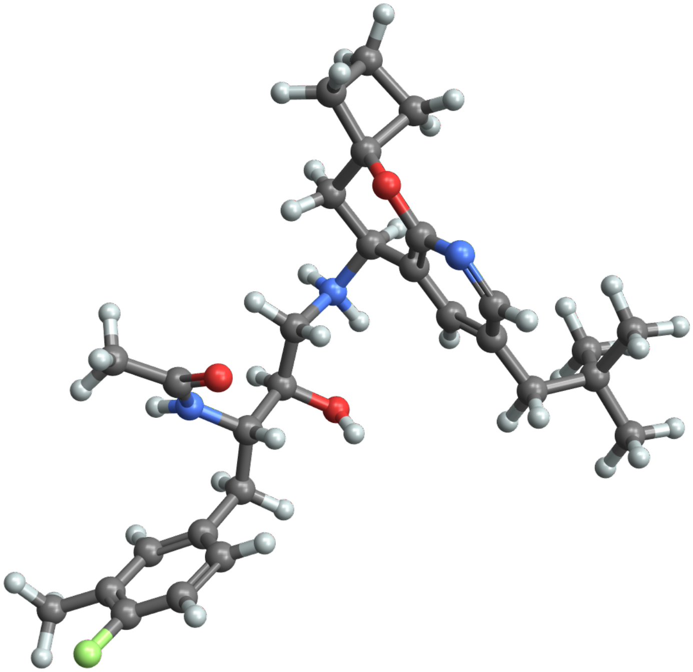

Dataset Index= 321

Molecule Formula= $C_{29}H_{41}FN_3O_3^+$

3D Autocorrelation

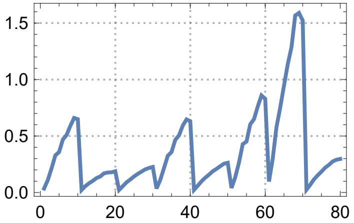

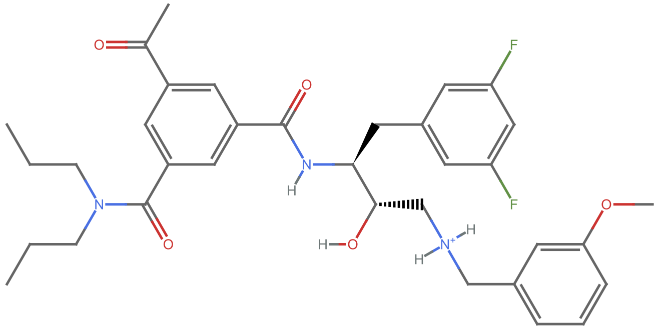

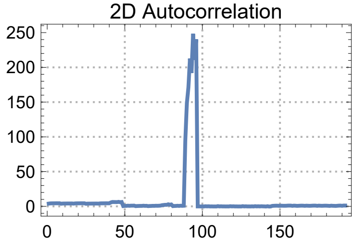

Dataset Index= 322

Molecule Formula= $C_{34}H_{42}F_2N_3O_5^+$

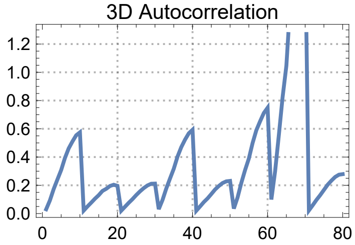

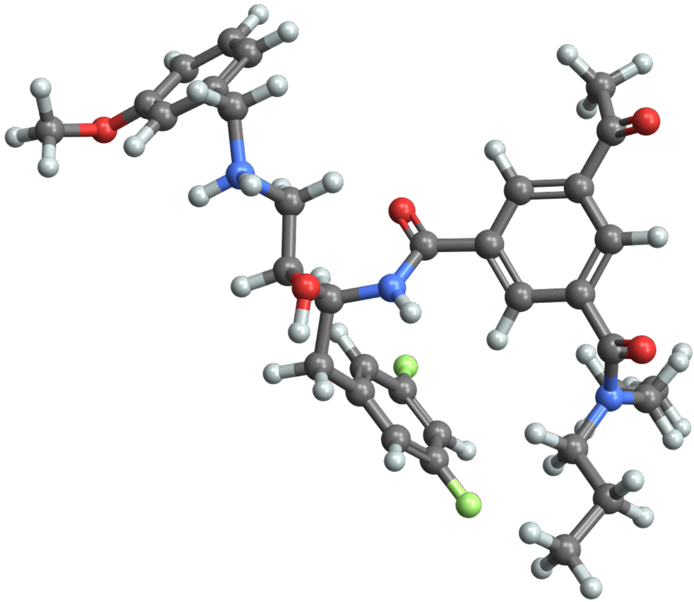

2D Plot

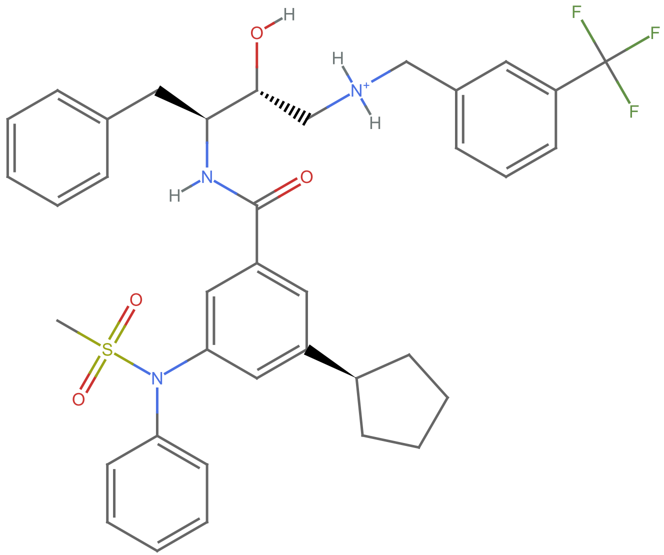

2D Autocorrelation

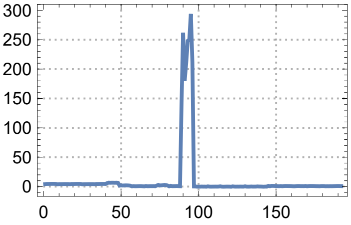

3D Plot

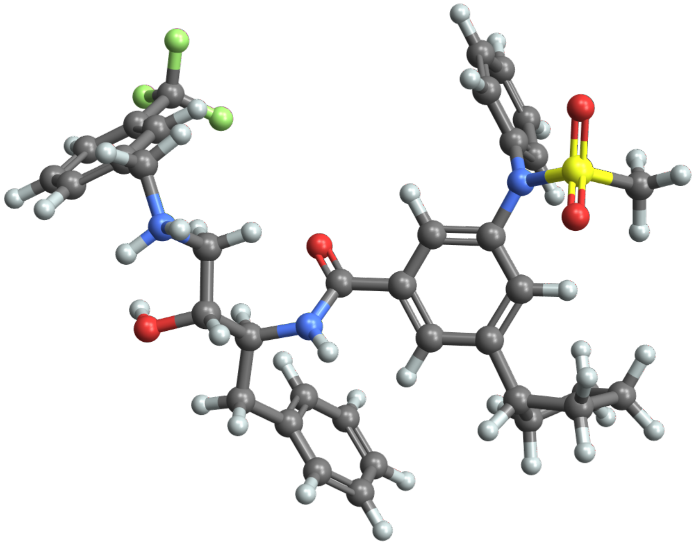

Dataset Index= 323

Molecule Formula= $C_{37}H_{41}F_3N_3O_4S^+$

3D Autocorrelation

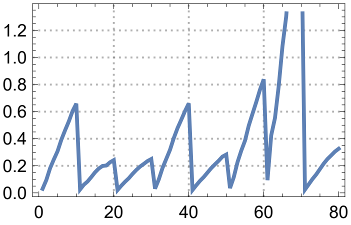

2D Plot

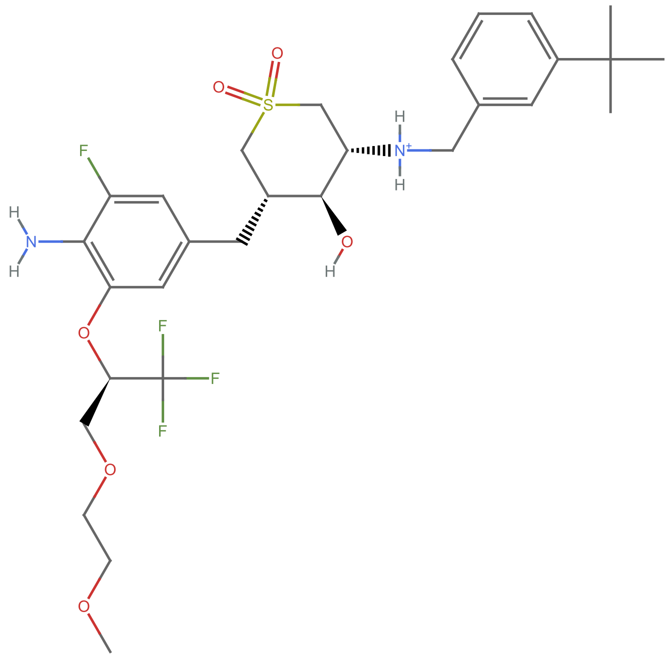

2D Autocorrelation

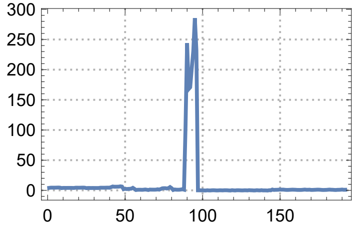

Dataset Index= 324

Molecule Formula= $C_{29}H_{41}F_4N_2O_6S^+$

3D Autocorrelation

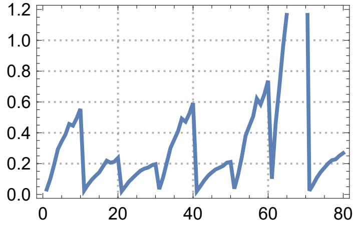

3D Plot

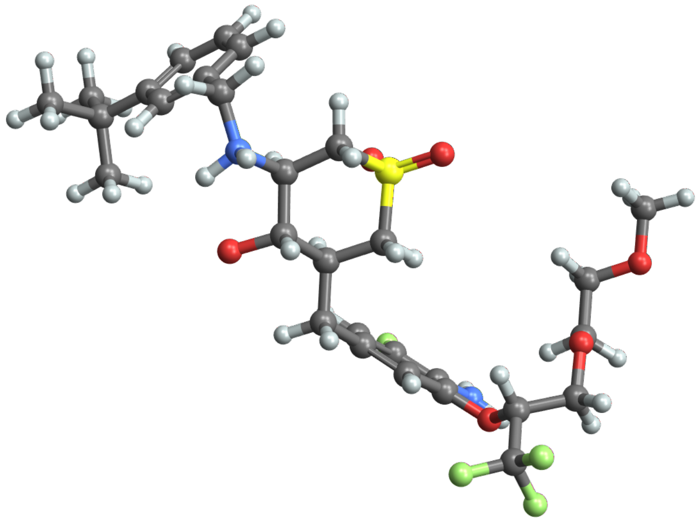

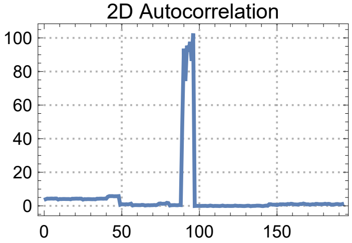

Dataset Index= 325

Molecule Formula= $C_{32}H_{43}N_4O_4S^+$

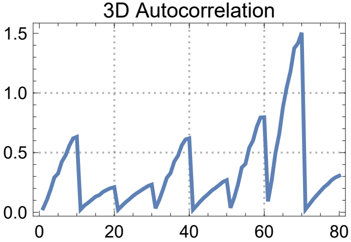

2D Plot

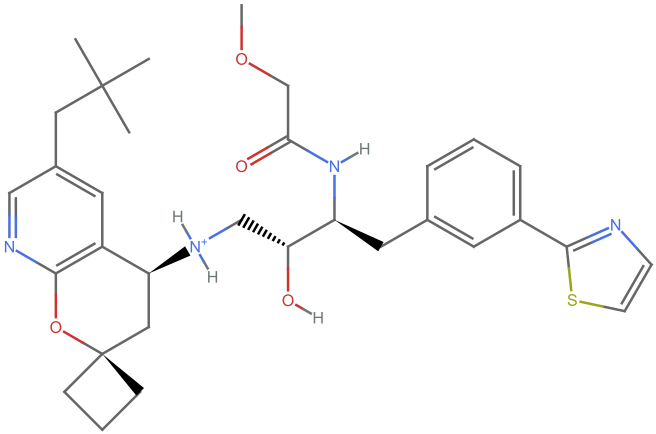

3D Plot

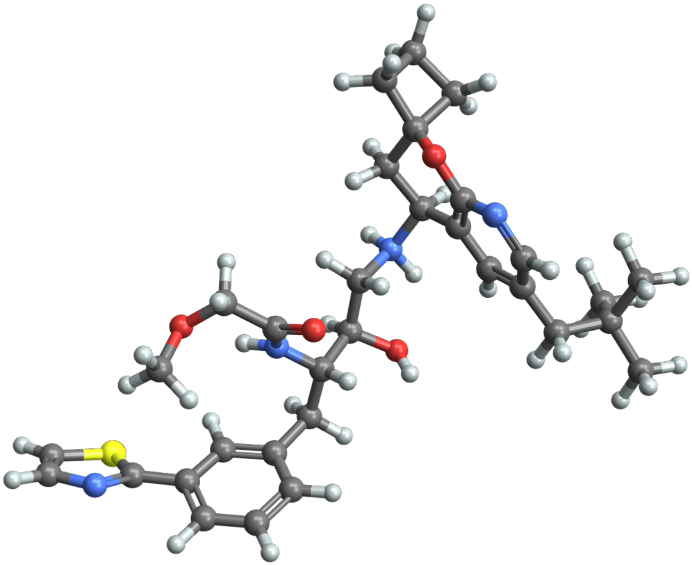

2D Plot

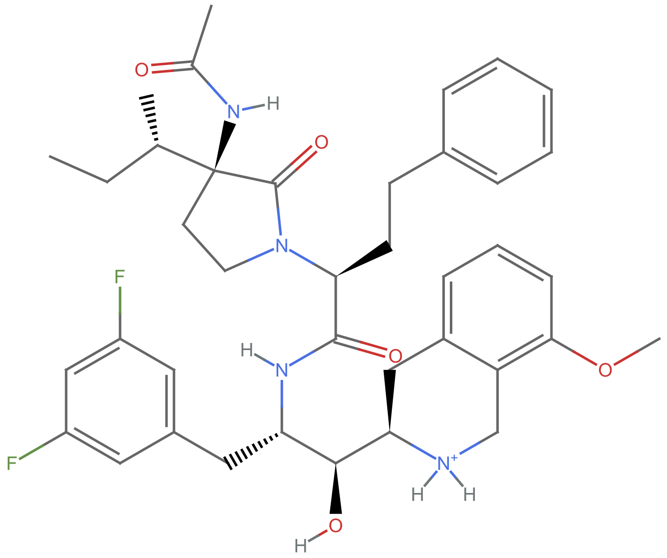

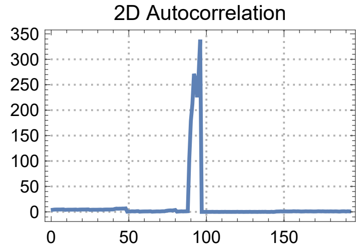

3D Plot

Dataset Index= 326

Molecule Formula= $C_{39}H_{49}F_2N_4O_5^+$

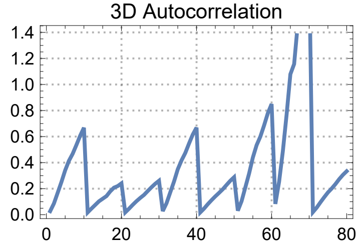

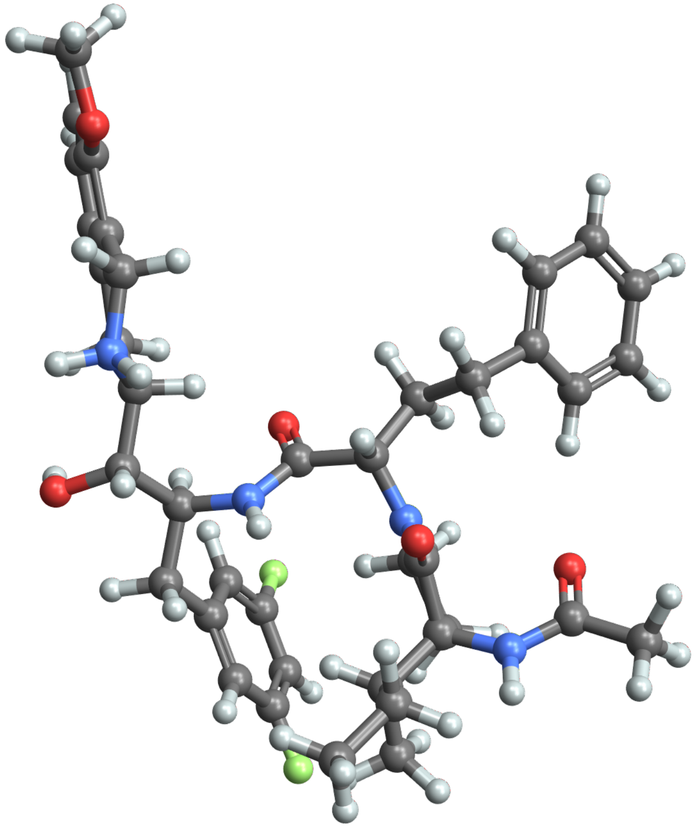

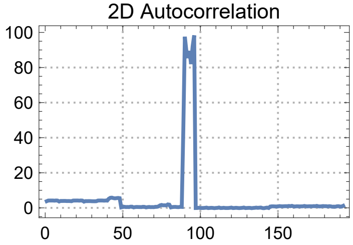

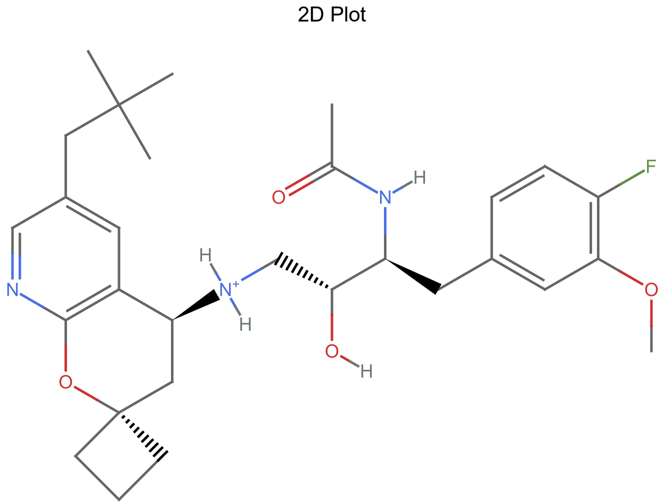

Dataset Index= 327

Molecule Formula= $C_{29}H_{41}FN_3O_4^{+}$

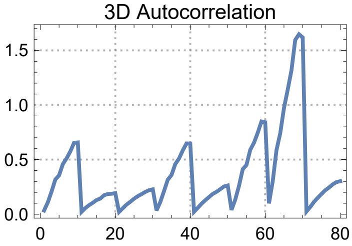

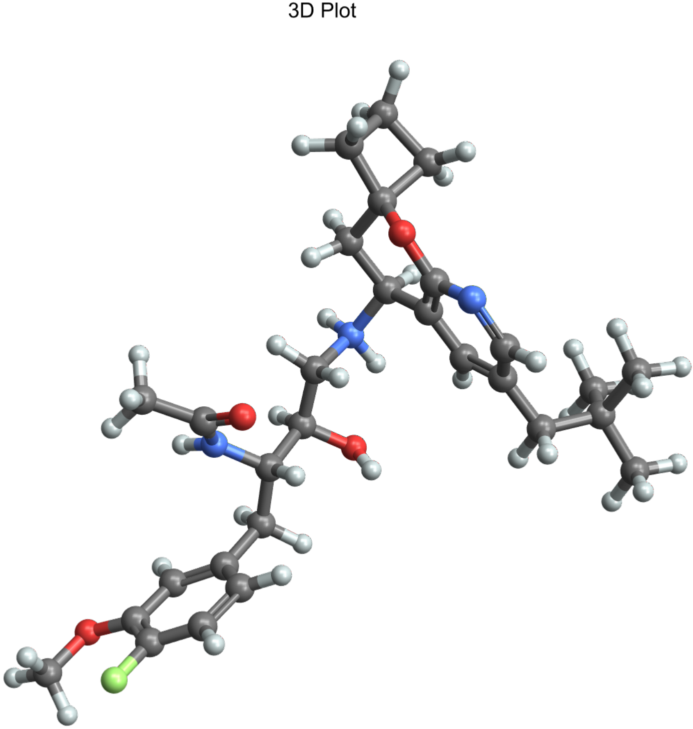

2D Plot

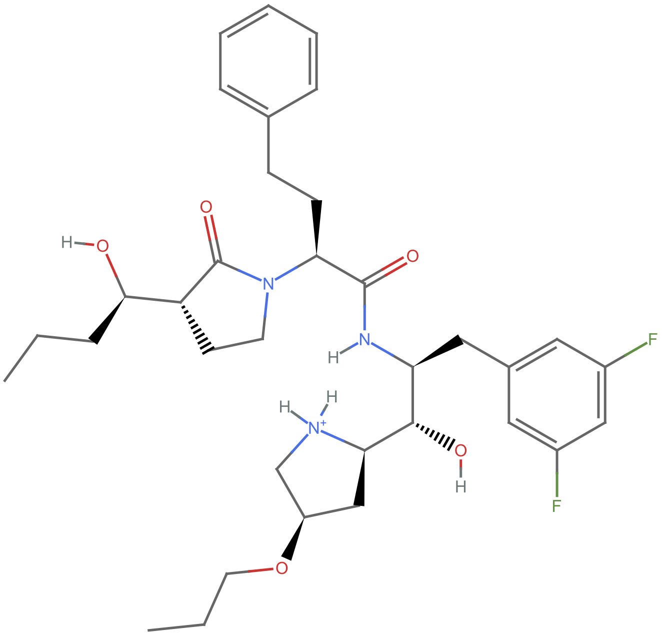

2D Autocorrelation

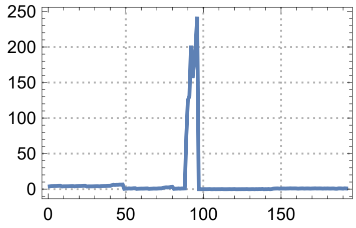

3D Plot

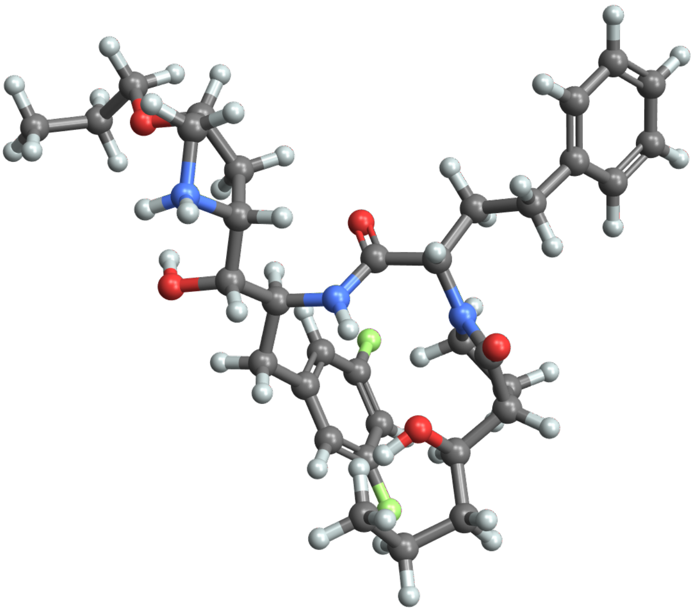

Dataset Index= 328

Molecule Formula= $C_{34}H_{48}F_2N_3O_5^+$

3D Autocorrelation

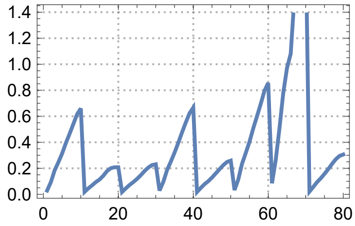

2D Plot

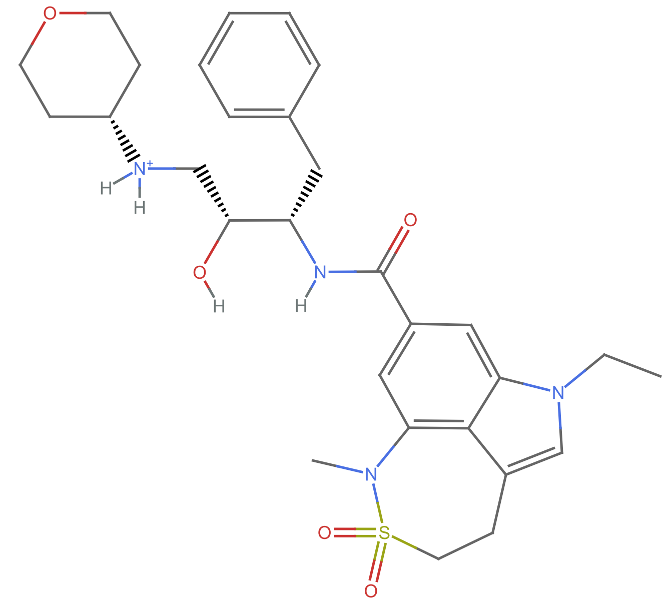

2D Autocorrelation

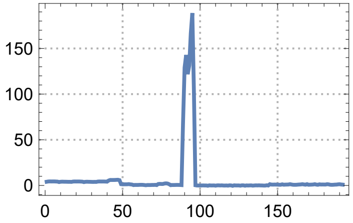

Dataset Index= 329

Molecule Formula= $C_{29}H_{39}N_4O_5S^+$

3D Autocorrelation

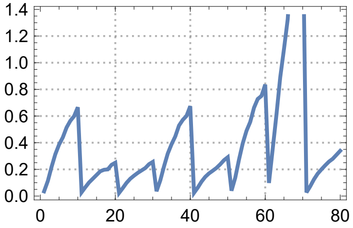

3D Plot

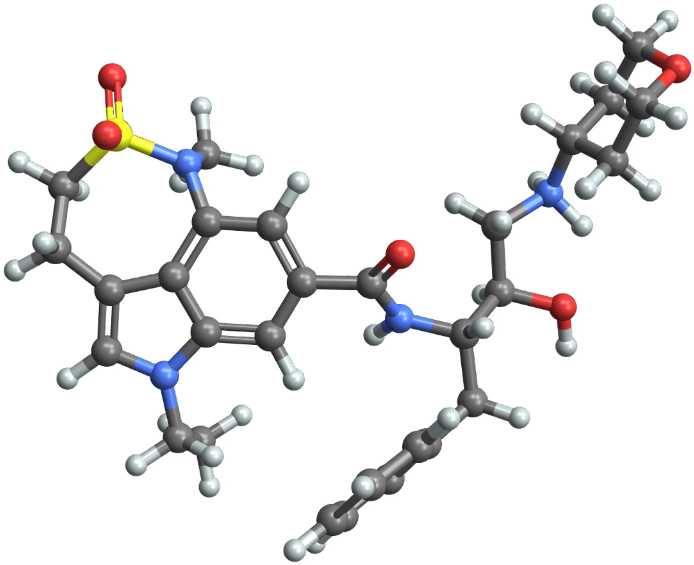

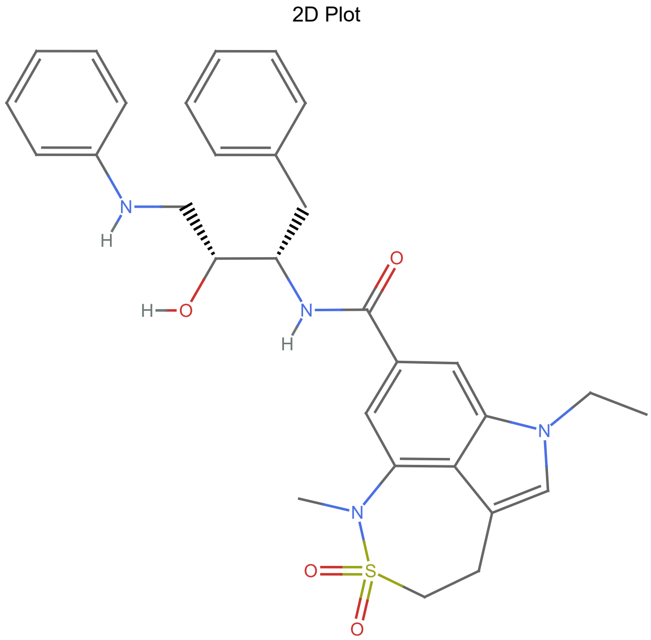

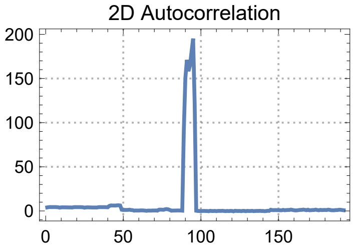

Dataset Index= 330

Molecule Formula= $C_{30}H_{34}N_4O_4S$

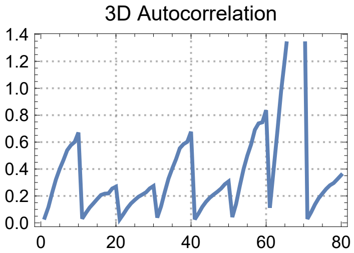

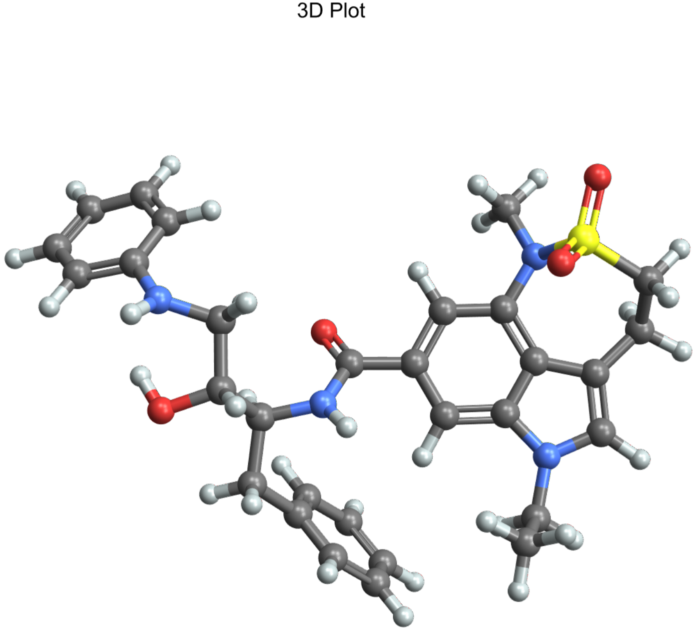

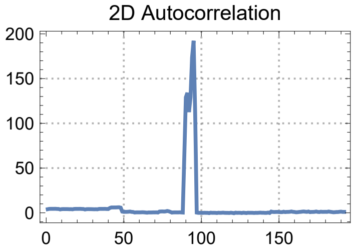

Dataset Index= 331

Molecule Formula= $C_{33}H_{49}N_4O_4S^+$

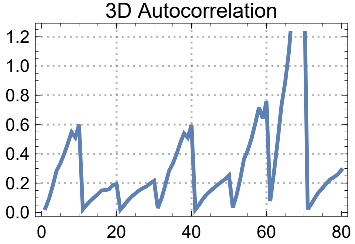

2D Plot

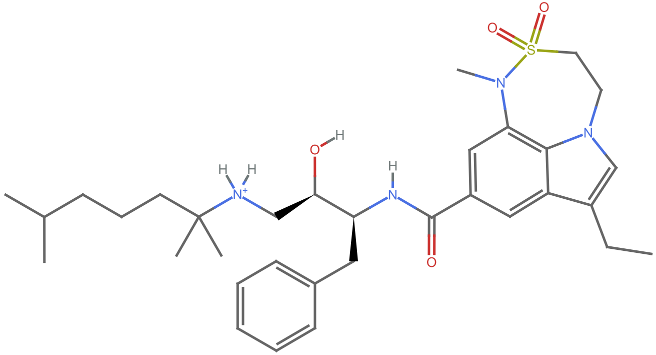

3D Plot

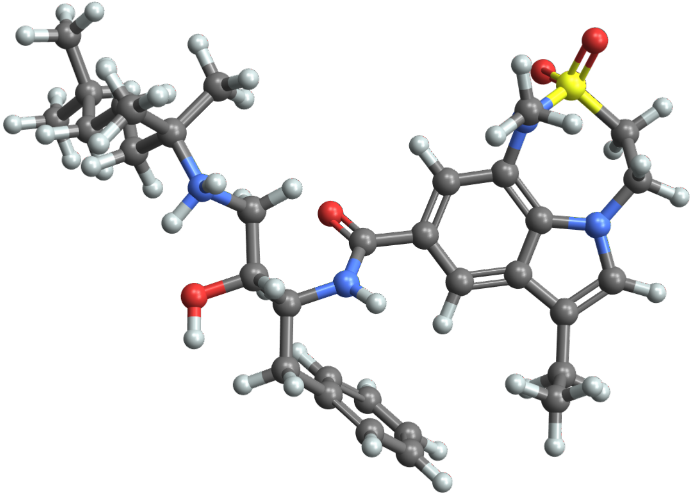

2D Plot

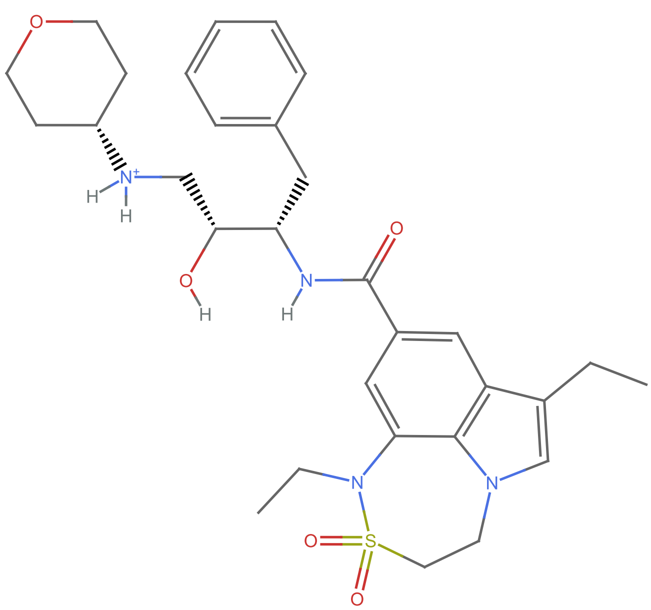

2D Autocorrelation

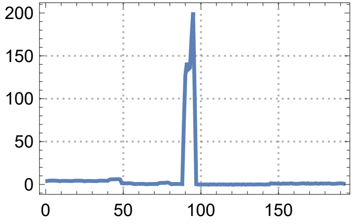

Dataset Index= 332

Molecule Formula= $C_{30}H_{41}N_4O_5S^+$

3D Plot

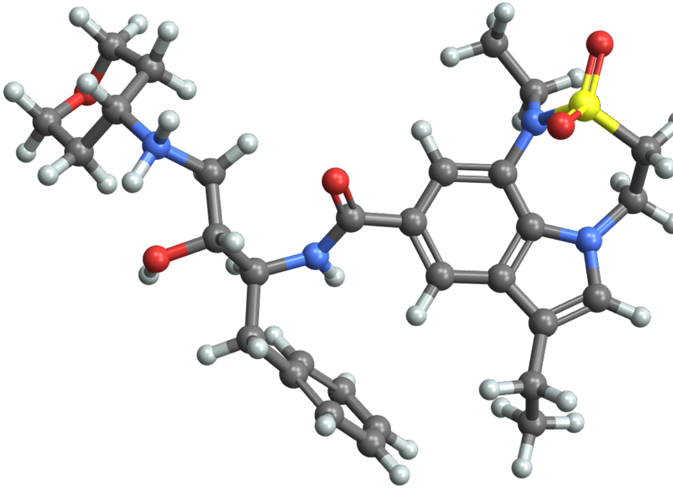

3D Autocorrelation

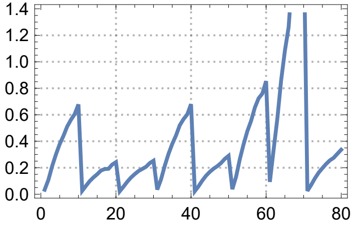

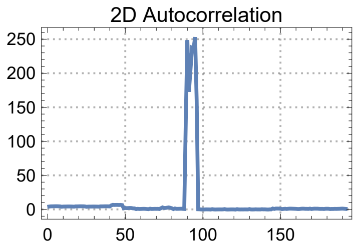

Dataset Index= 333

Molecule Formula= $C_{35}H_{39}F_3N_3O_4S^+$

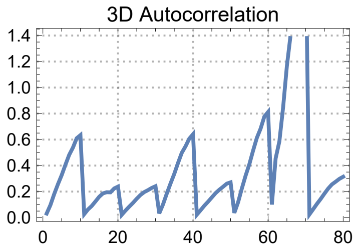

2D Plot

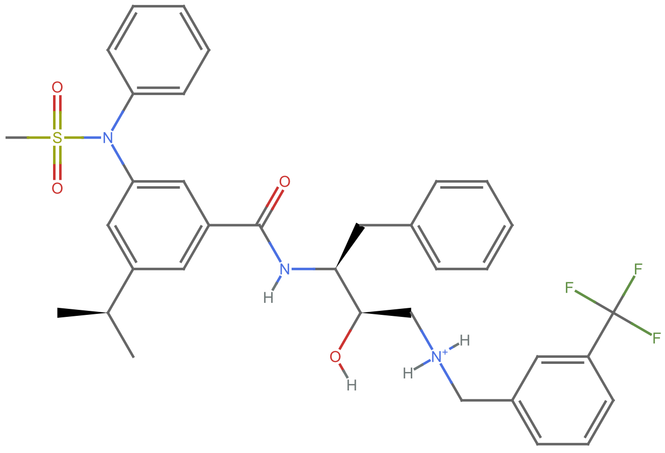

3D Plot

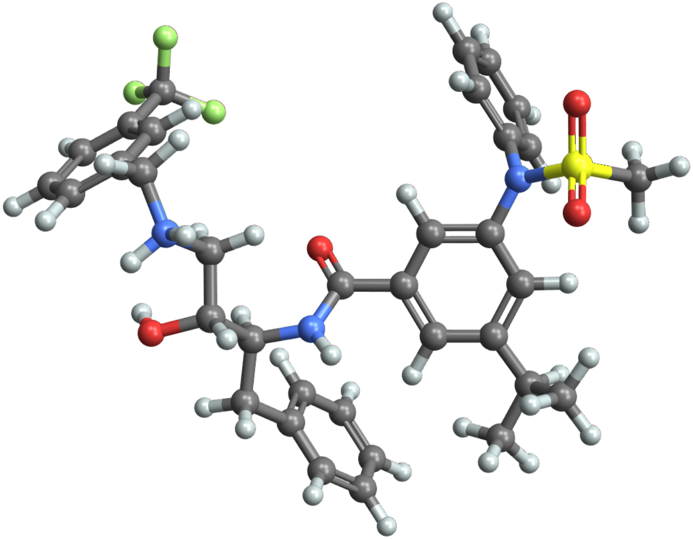

2D Plot

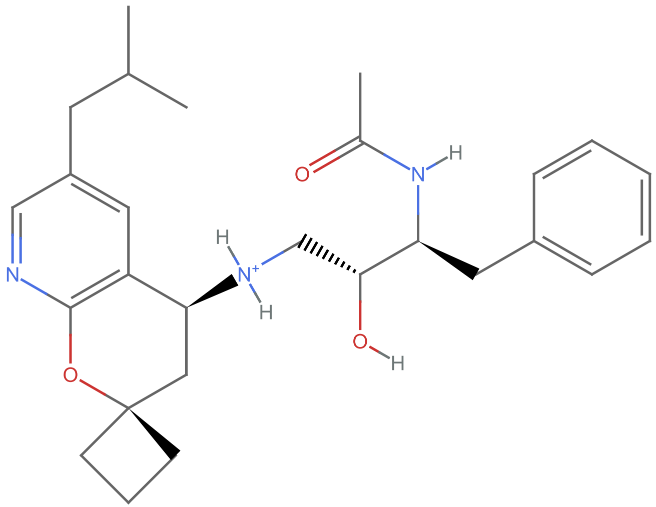

2D Autocorrelation

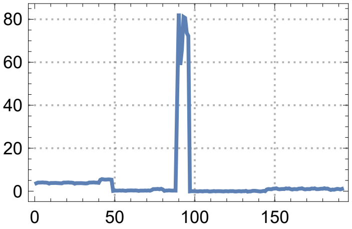

3D Plot

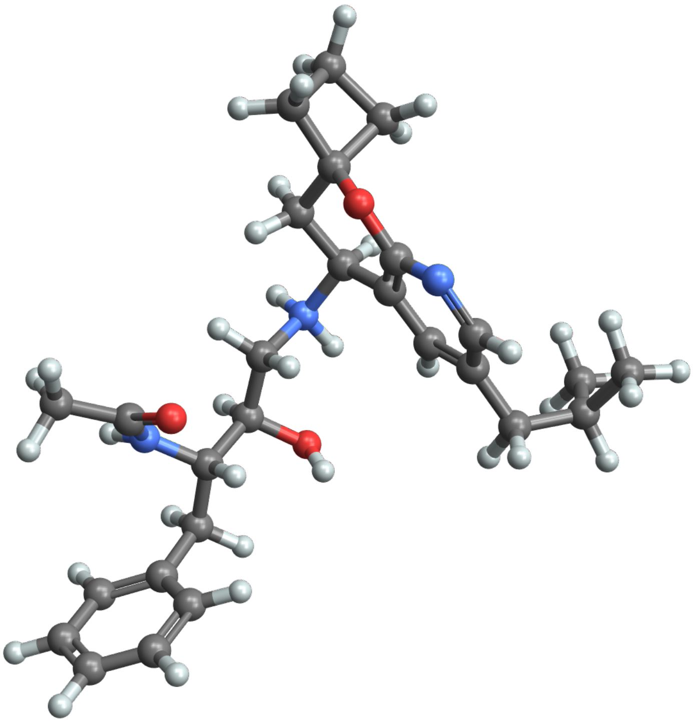

Dataset Index= 334

Molecule Formula= $C_{27}H_{38}N_3O_3^+$

3D Autocorrelation

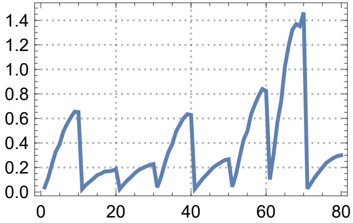

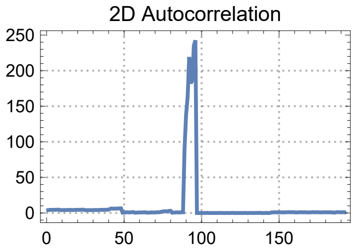

2D Plot

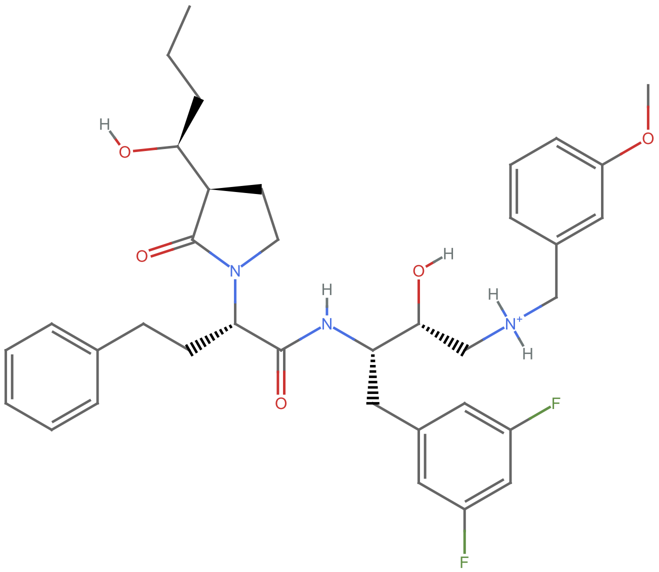

3D Plot

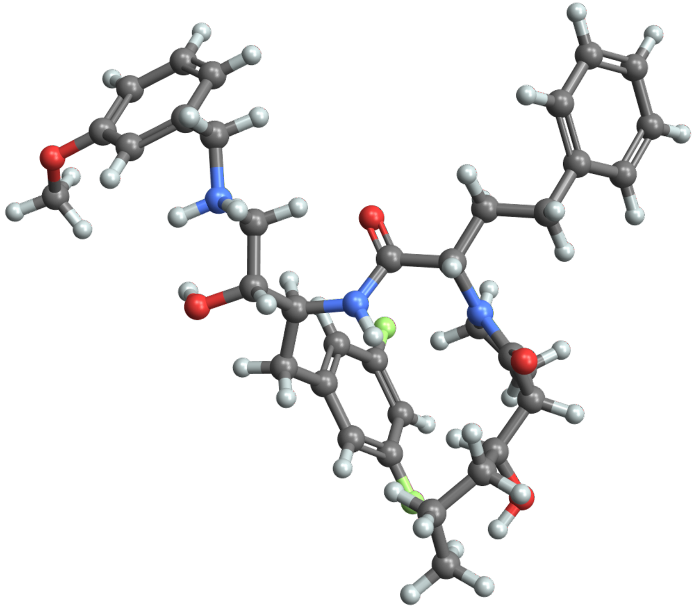

Dataset Index= 335

Molecule Formula= $C_{36}H_{46}F_{2}N_{3}O_{5}^{+}$

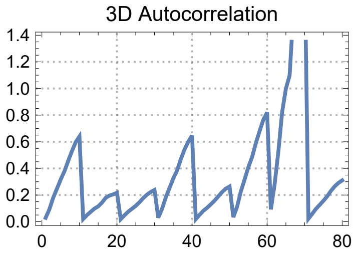

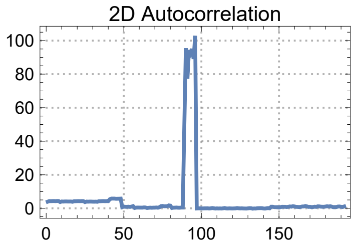

Dataset Index= 336

Molecule Formula= $C_{32}H_{43}N_4O_4S^+$

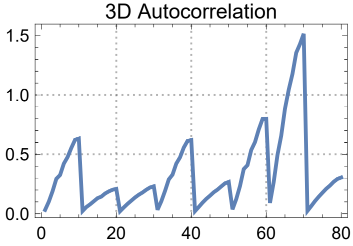

2D Plot

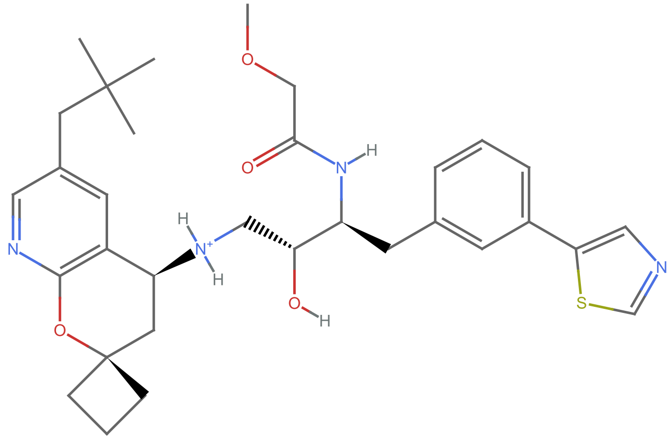

3D Plot

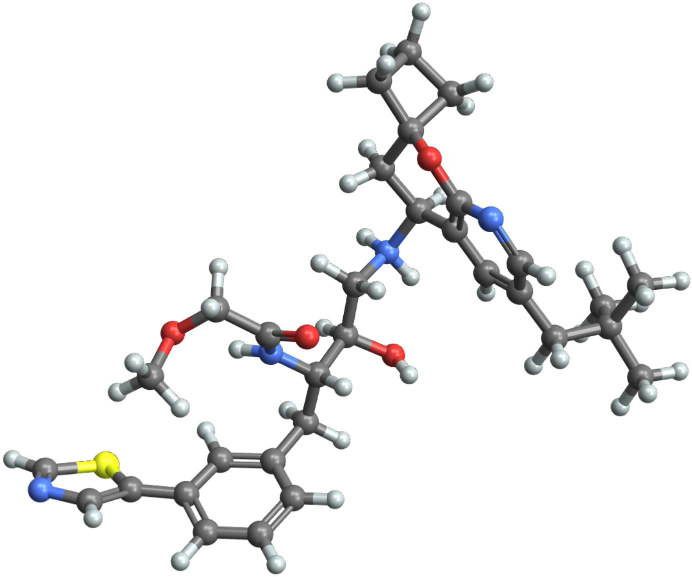

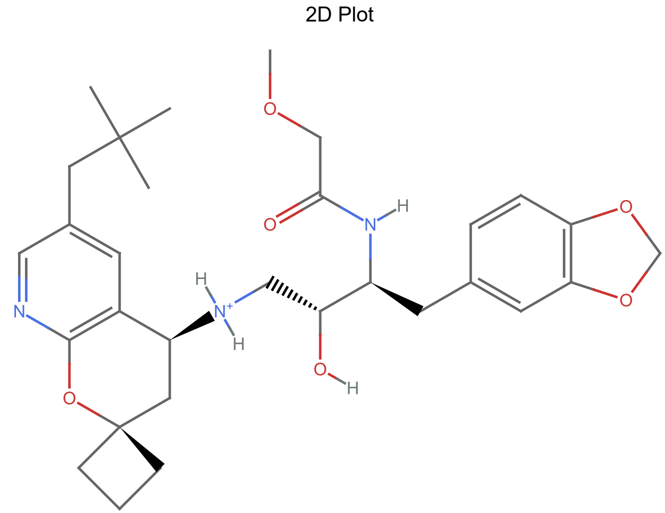

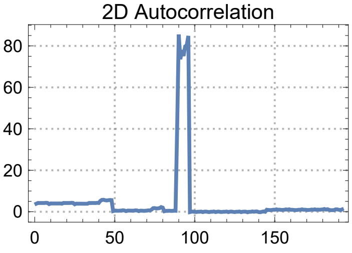

Dataset Index= 337

Molecule Formula= $C_{30}H_{42}N_3O_6^+$

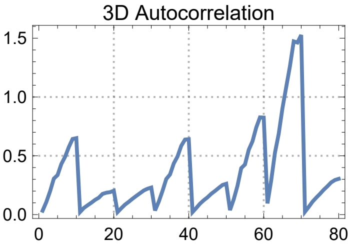

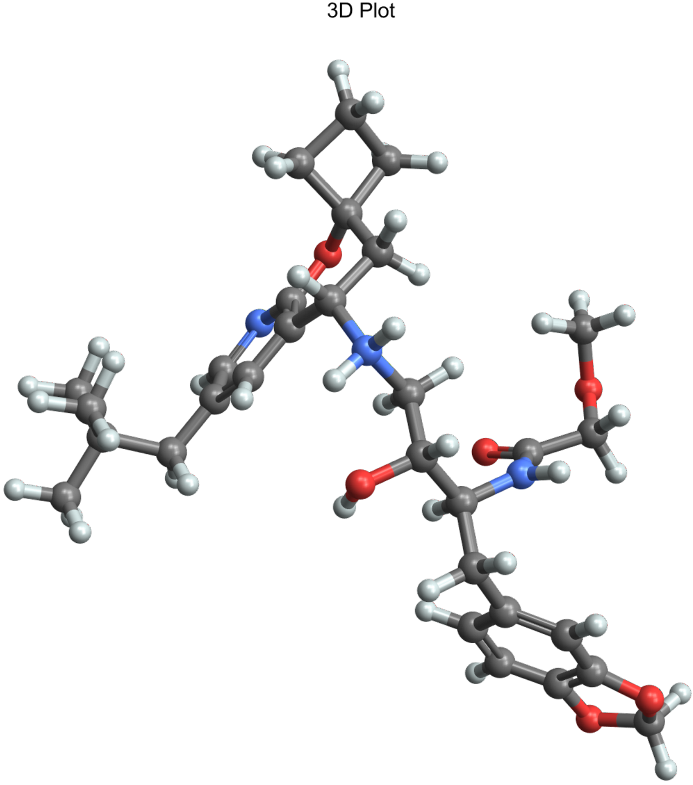

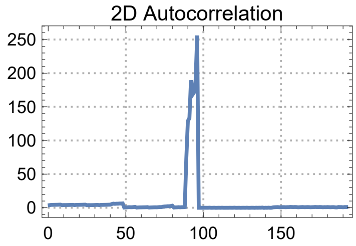

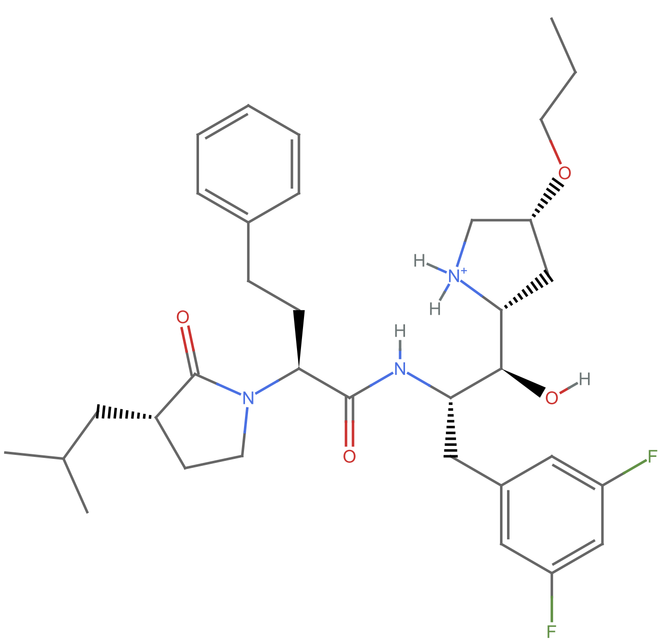

3D Plot

Dataset Index= 338

Molecule Formula= $C_{34}H_{48}F_2N_3O_4^+$

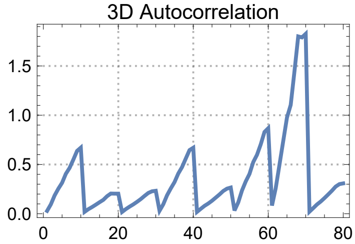

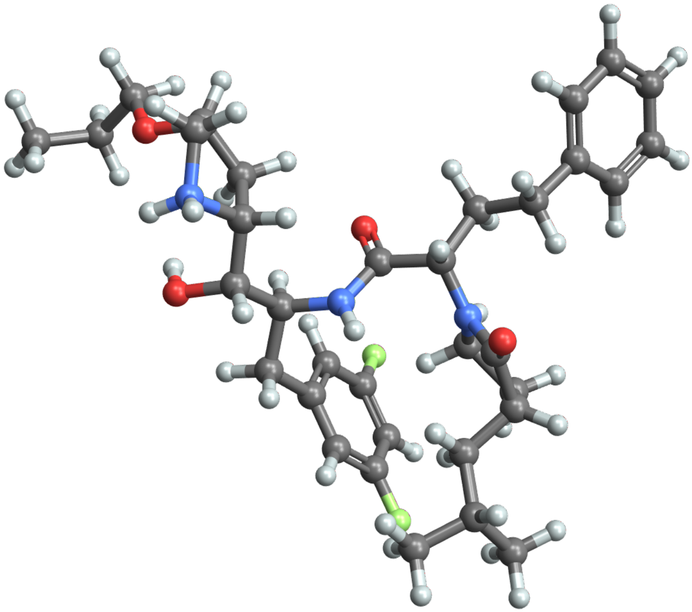

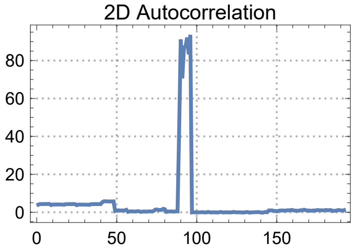

Dataset Index= 339

Molecule Formula= $C_{33}H_{45}N_4O_4S^+$

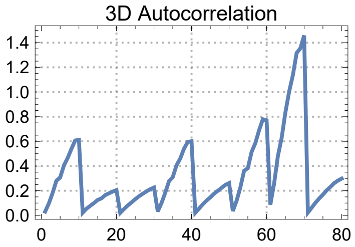

2D Plot

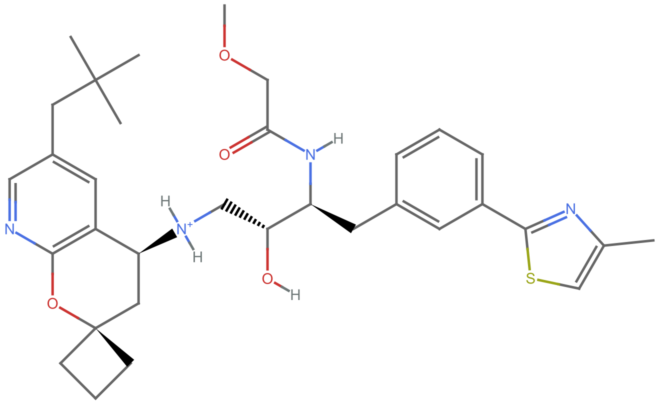

3D Plot

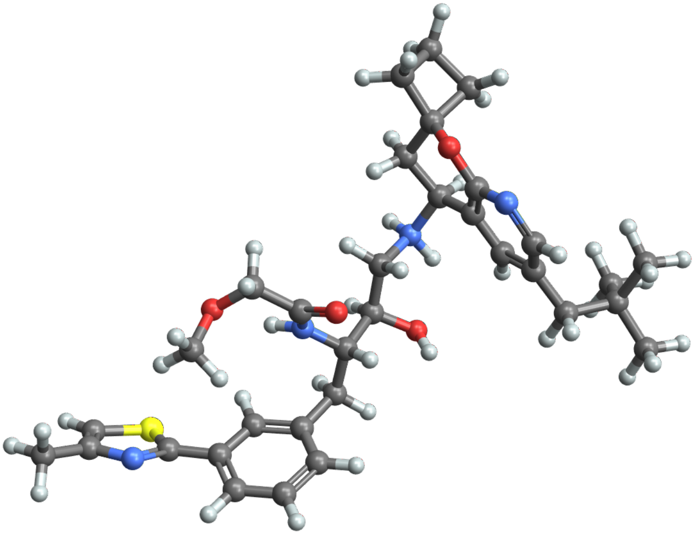

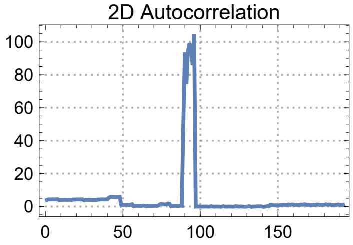

Dataset Index= 340

Molecule Formula= $C_{33}H_{45}N_4O_4S^+$

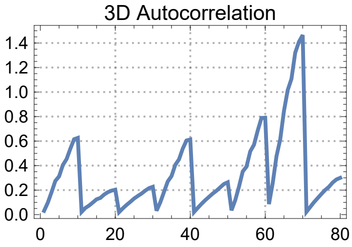

2D Plot

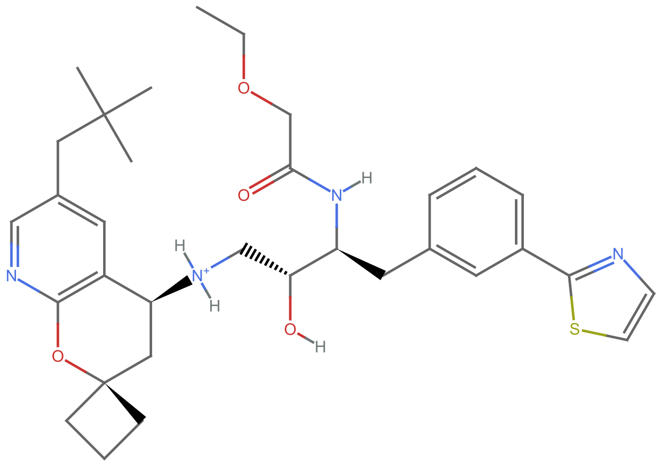

3D Plot

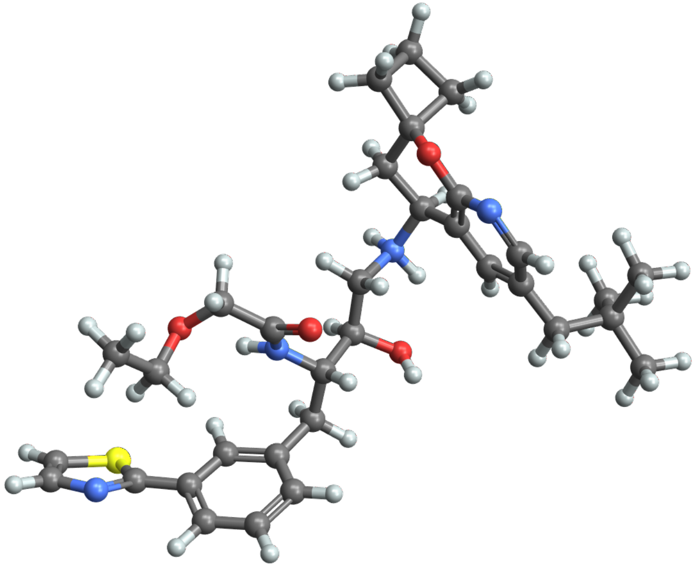

2D Plot

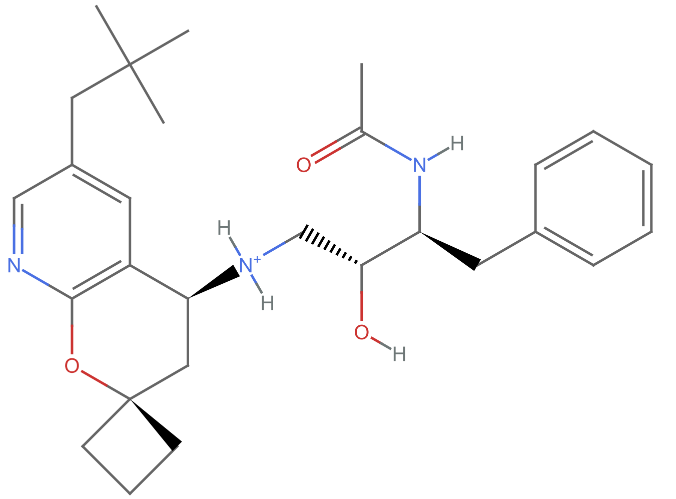

3D Plot

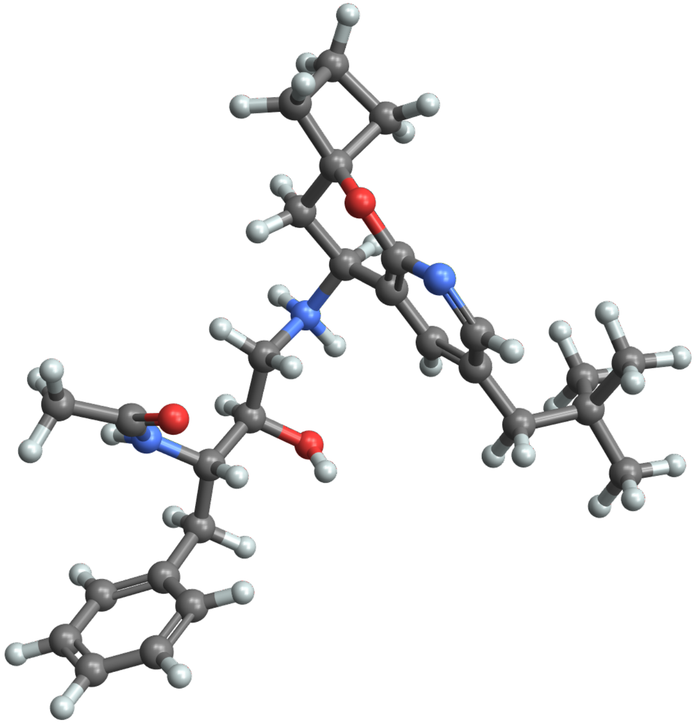

2D Autocorrelation

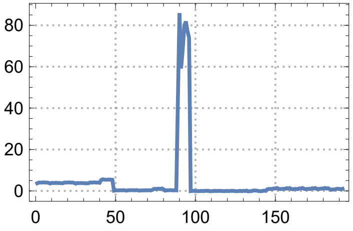

Dataset Index= 341

Molecule Formula= $C_{28}H_{40}N_3O_3^+$

3D Autocorrelation

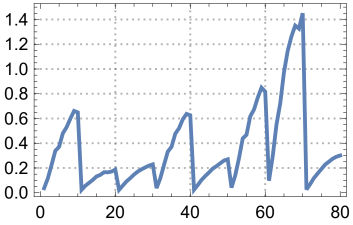

2D Plot

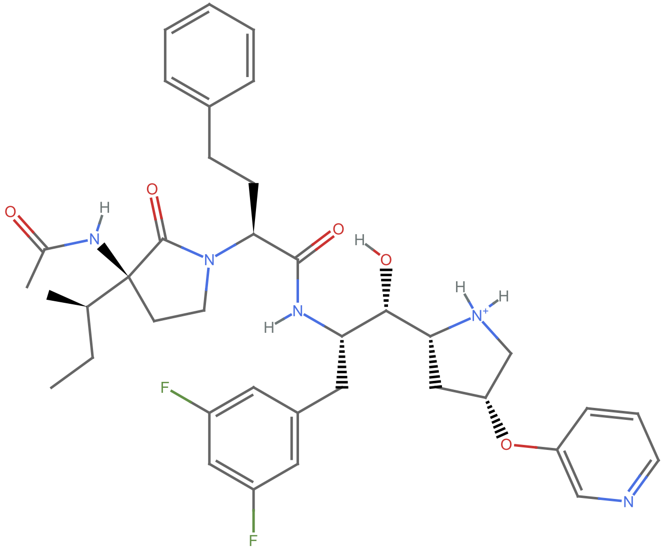

2D Autocorrelation

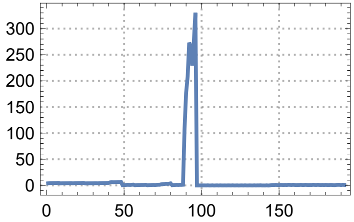

3D Plot

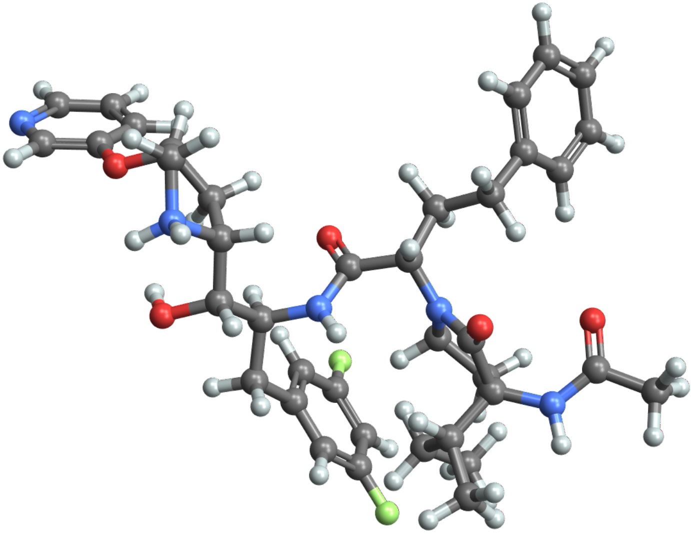

Dataset Index= 342

Molecule Formula= $C_{38}H_{48}F_2N_5O_5^+$

3D Autocorrelation

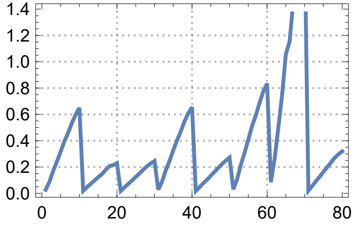

2D Plot

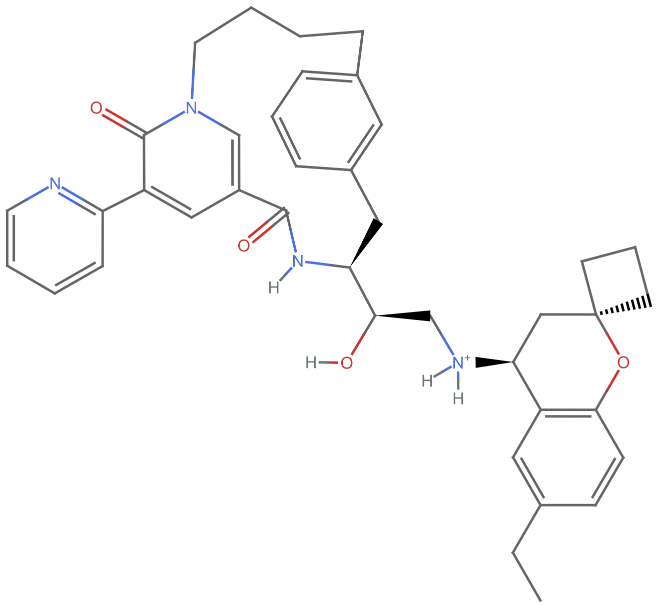

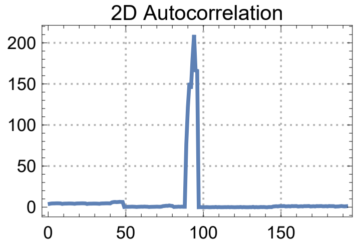

Dataset Index= 343

Molecule Formula= $C_{39}H_{45}N_4O_4^+$

3D Plot

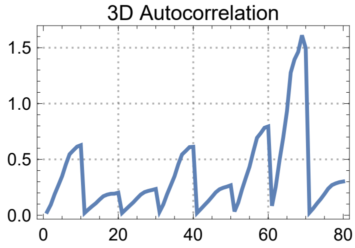

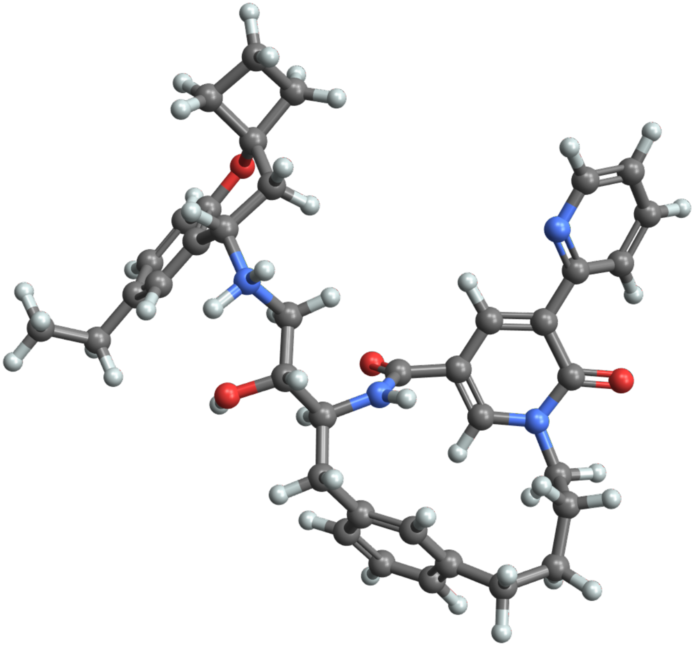

2D Plot

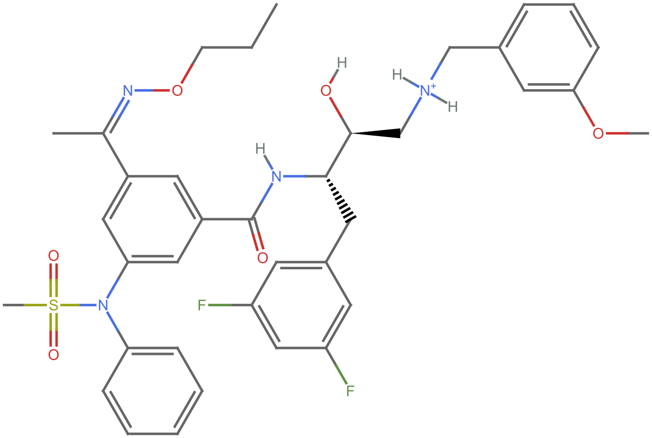

2D Autocorrelation

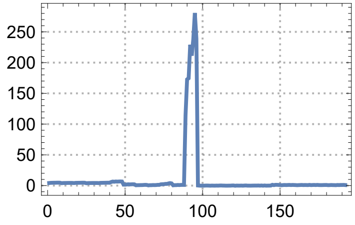

3D Plot

Dataset Index= 344

Molecule Formula= $C_{37}H_{43}F_2N_4O_6S^+$

3D Autocorrelation

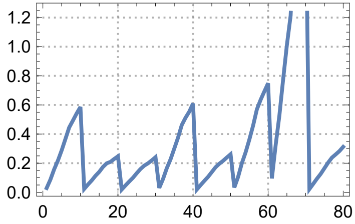

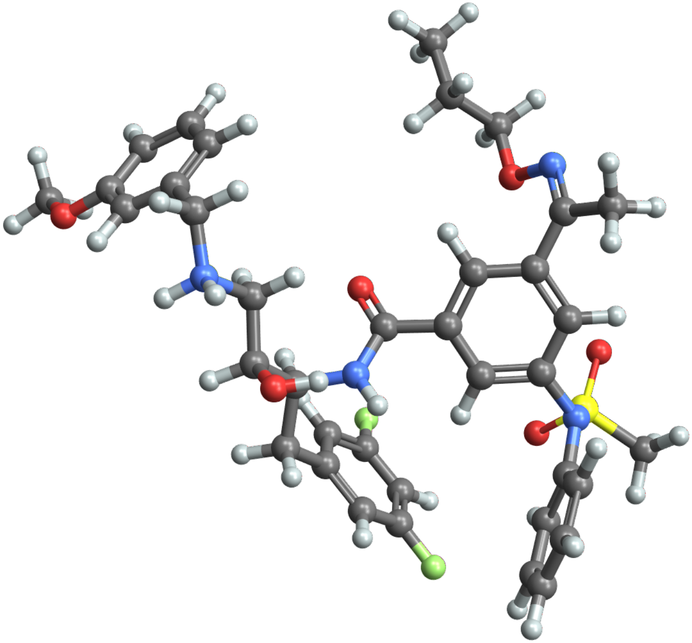

2D Plot

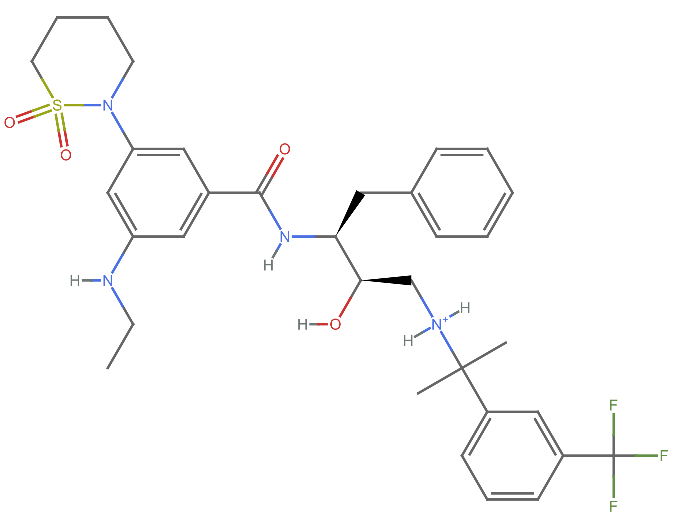

2D Autocorrelation

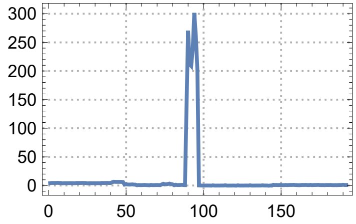

Dataset Index= 345

Molecule Formula= $C_{33}H_{42}F_3N_4O_4S^+$

3D Plot

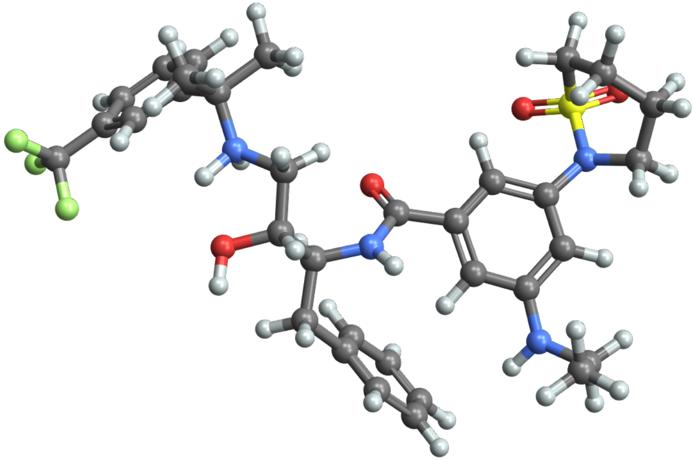

3D Autocorrelation

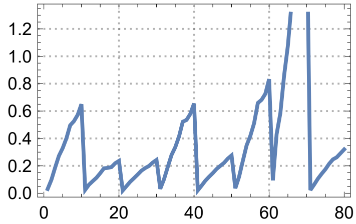

2D Plot

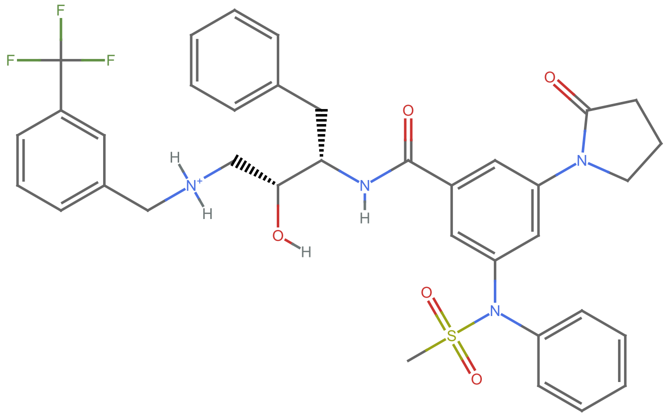

3D Plot

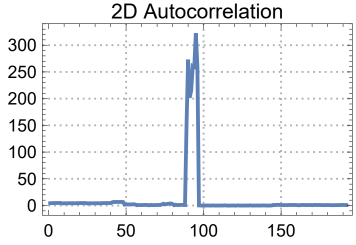

Dataset Index= 346

Molecule Formula= $C_{36}H_{38}F_{3}N_{4}O_{5}S^{+}$

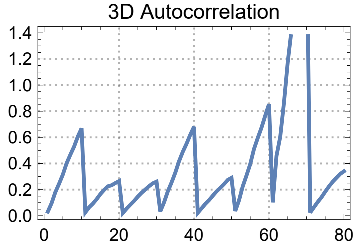

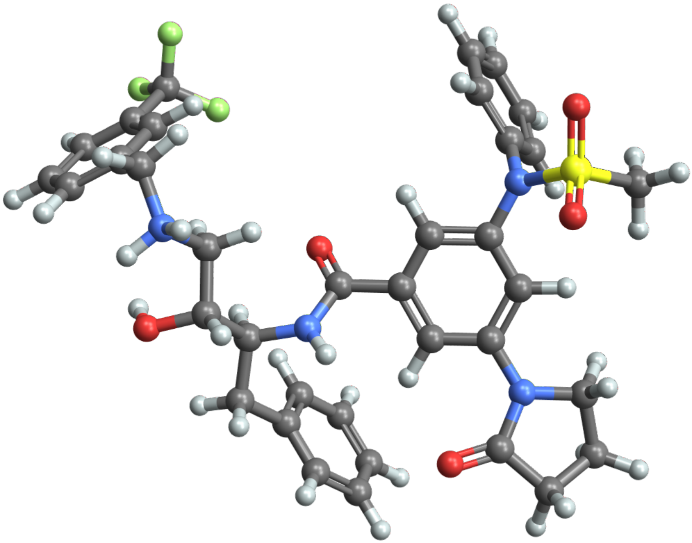

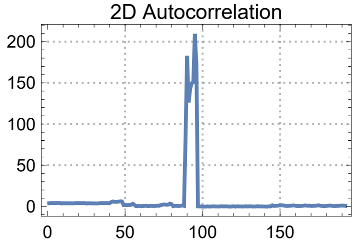

Dataset Index= 347

Molecule Formula= $C_{25}H_{34}F_3N_2O_4S^+$

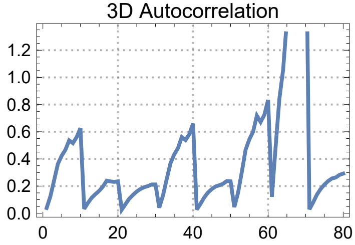

2D Plot

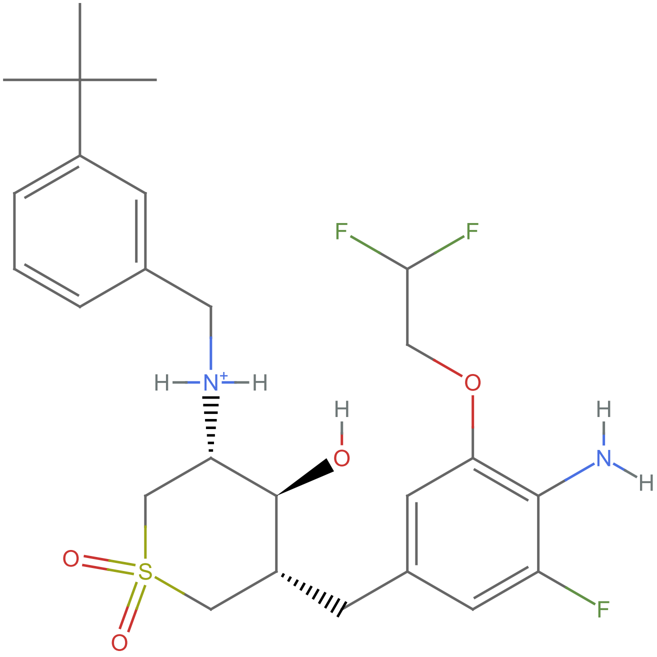

3D Plot

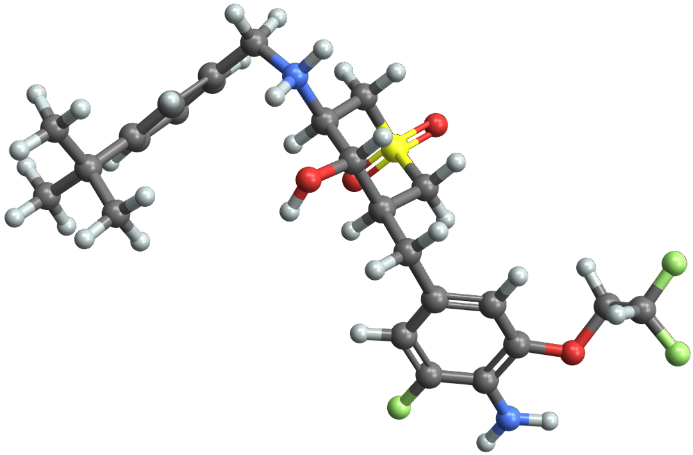

2D Plot

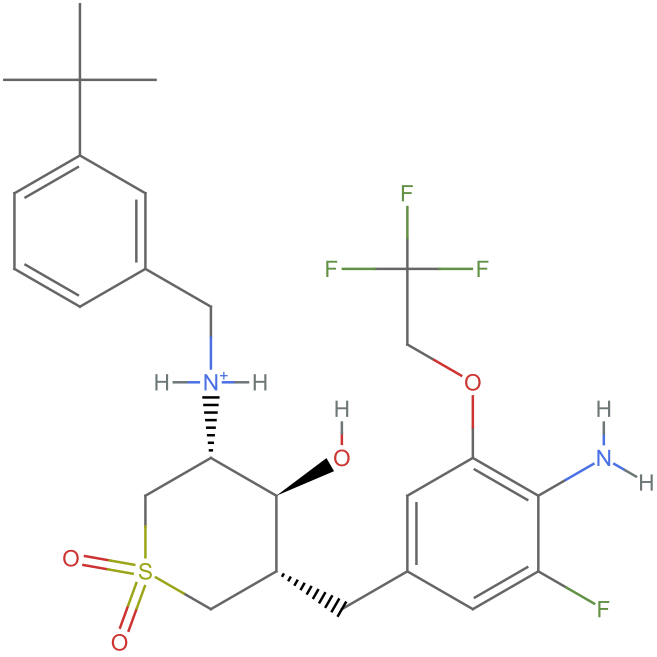

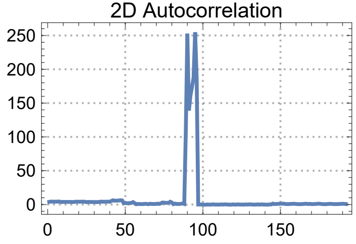

Dataset Index= 348

Molecule Formula= $C_{25}H_{33}F_4N_2O_4S^+$

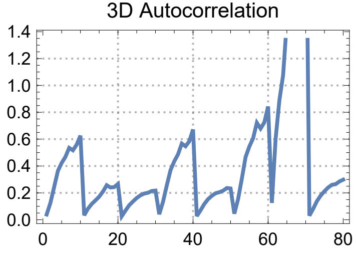

3D Plot

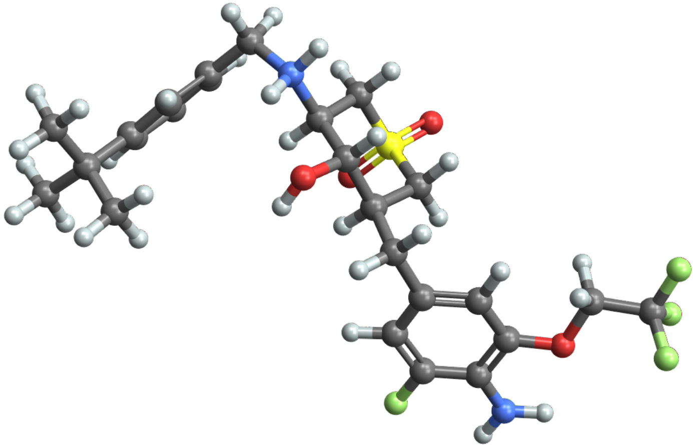

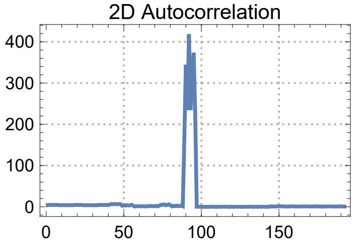

Dataset Index= 349

Molecule Formula= $C_{26}H_{30}F_7N_2O_5S^+$

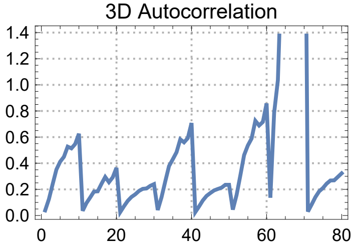

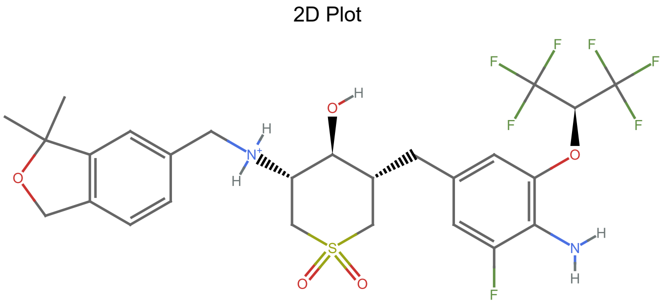

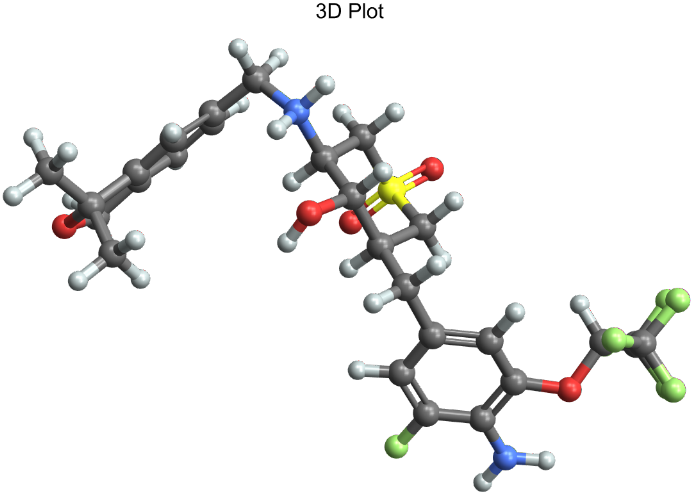

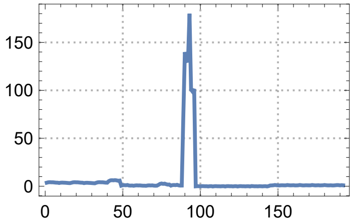

Dataset Index= 350

Molecule Formula= $C_{20}H_{18}F_3N_5O_3$

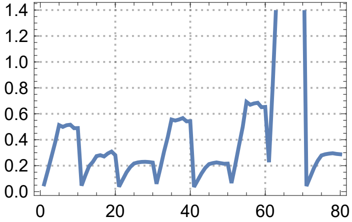

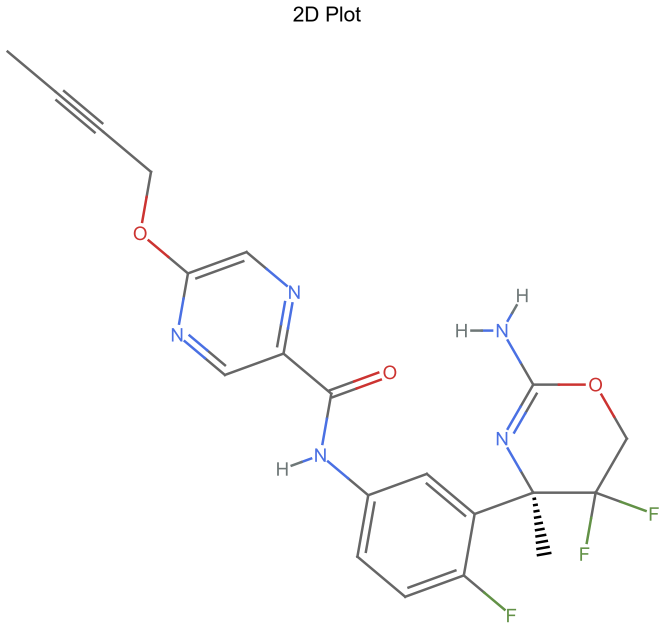

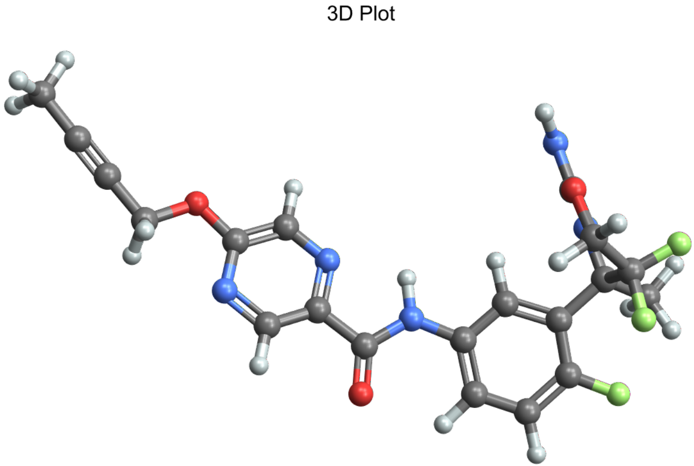

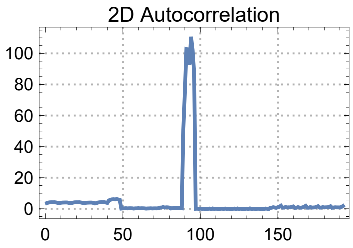

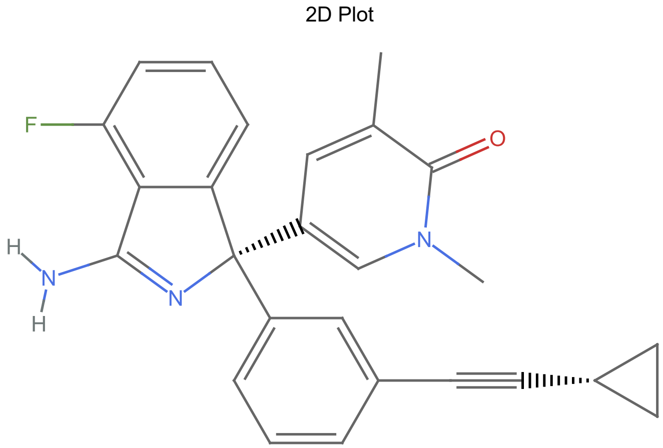

Dataset Index= 351

Molecule Formula= $C_{26}H_{22}FN_3O$

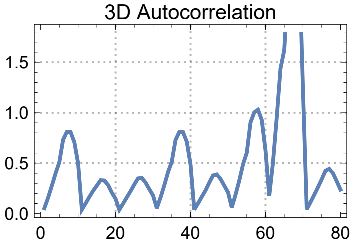

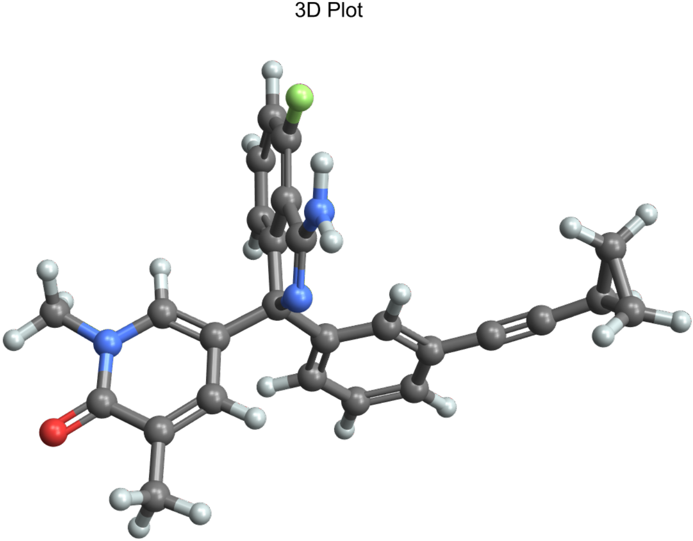

2D Autocorrelation

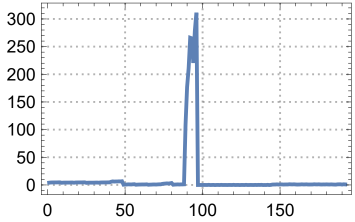

Dataset Index= 352

Molecule Formula= $C_{38}H_{49}F_2N_4O_5^+$

3D Autocorrelation

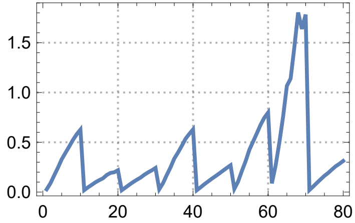

2D Plot

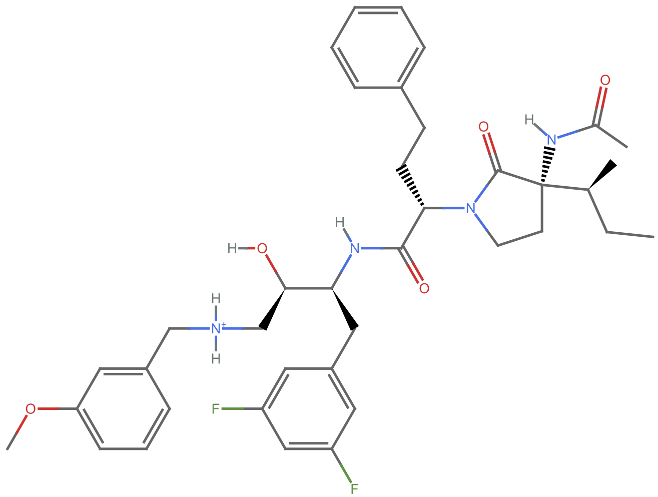

3D Plot

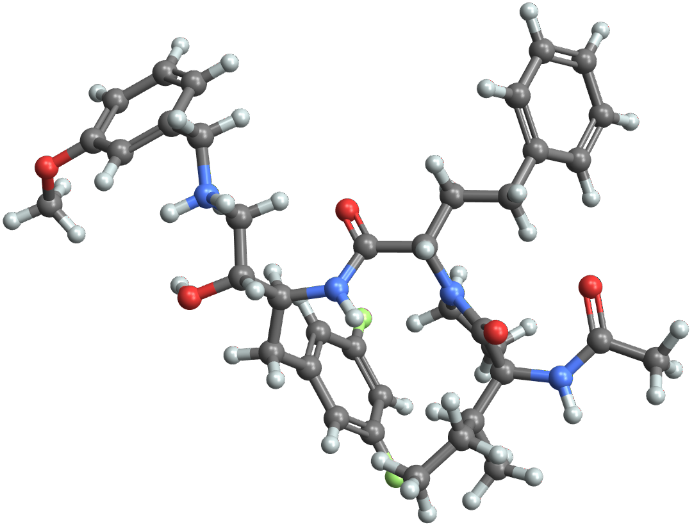

2D Autocorrelation

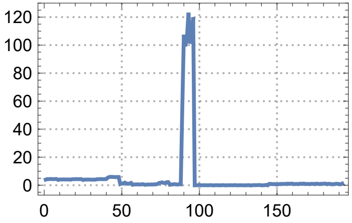

Dataset Index= 353

Molecule Formula= $C_{32}H_{42}FN_4O_4S^+$

3D Autocorrelation

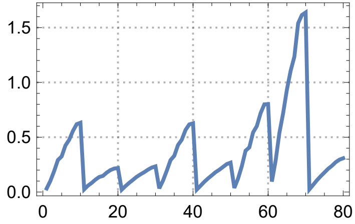

2D Plot

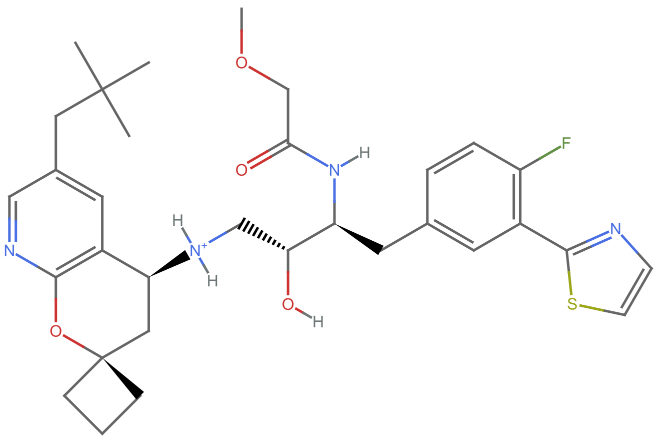

3D Plot

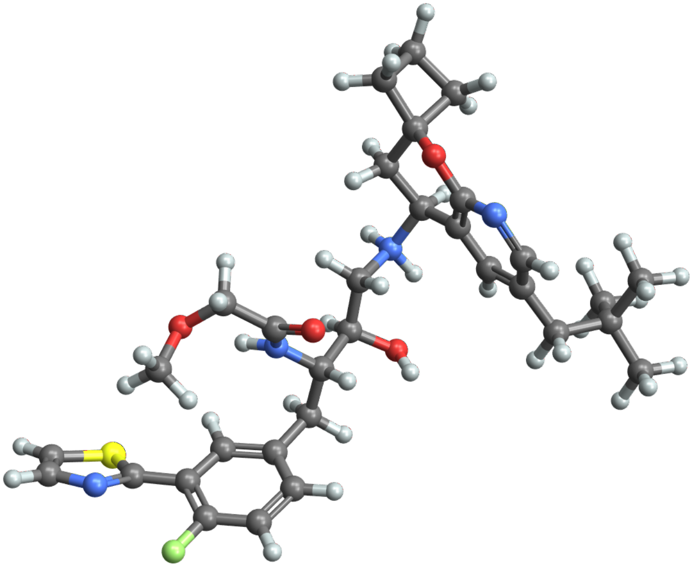

2D Plot

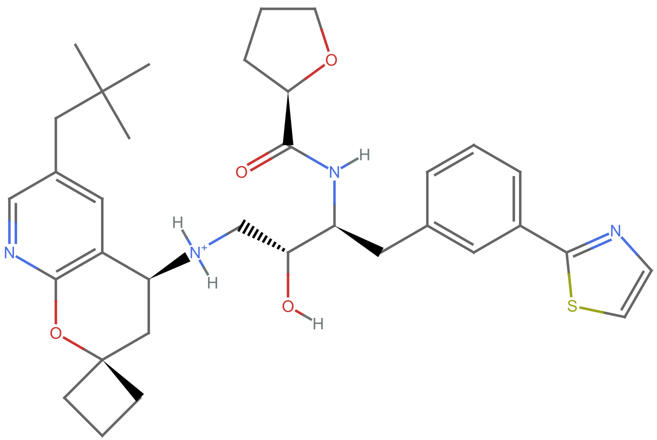

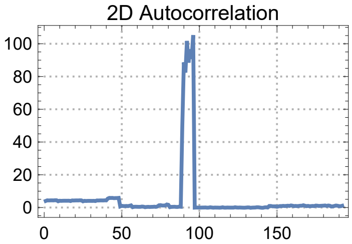

3D Plot

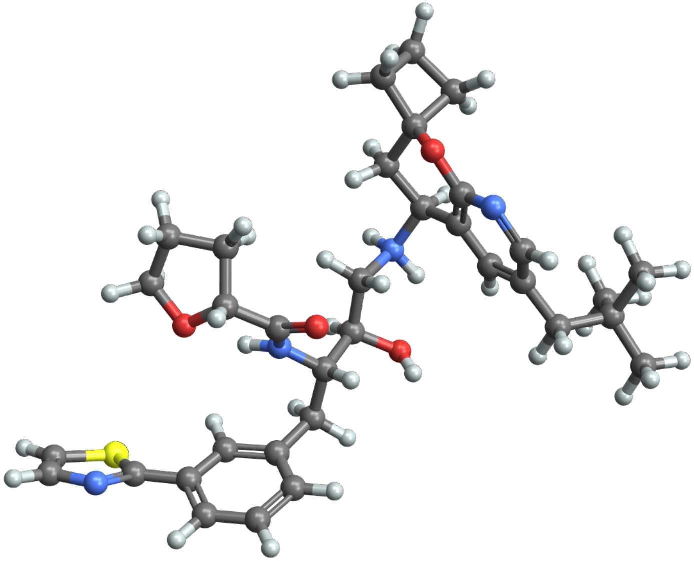

Dataset Index= 354

Molecule Formula= $C_{34}H_{45}N_4O_4S^+$

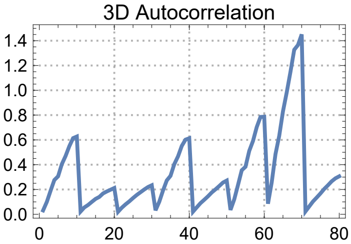

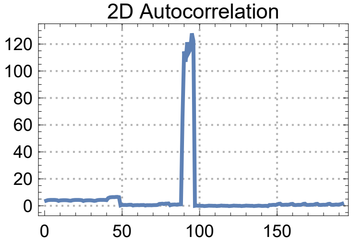

Dataset Index= 355

Molecule Formula= $C_{28}H_{19}F_3N_4$

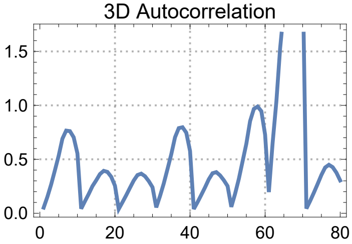

2D Plot

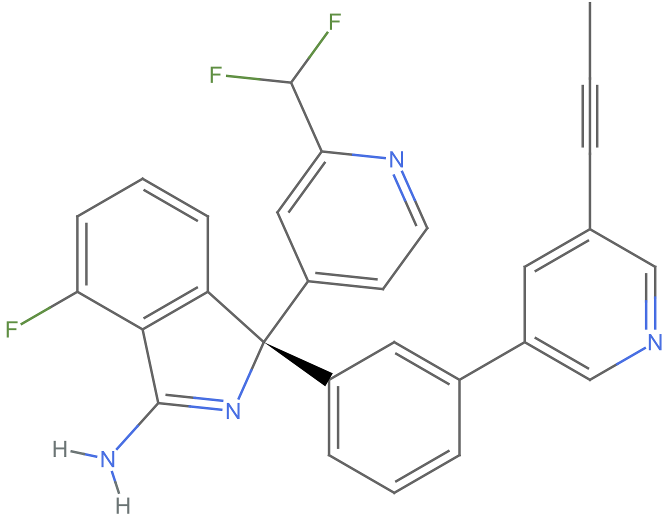

3D Plot

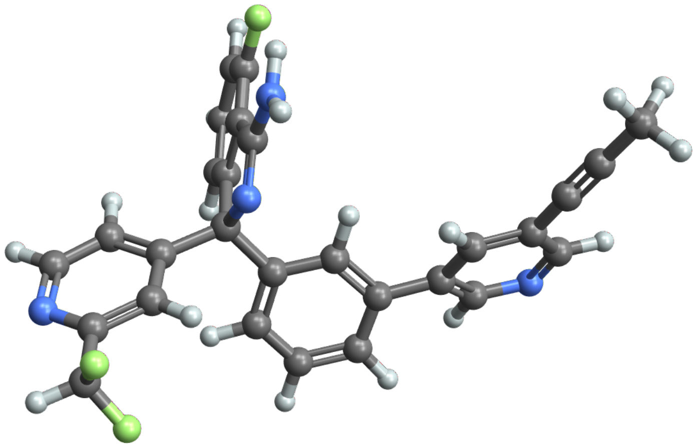

2D Plot

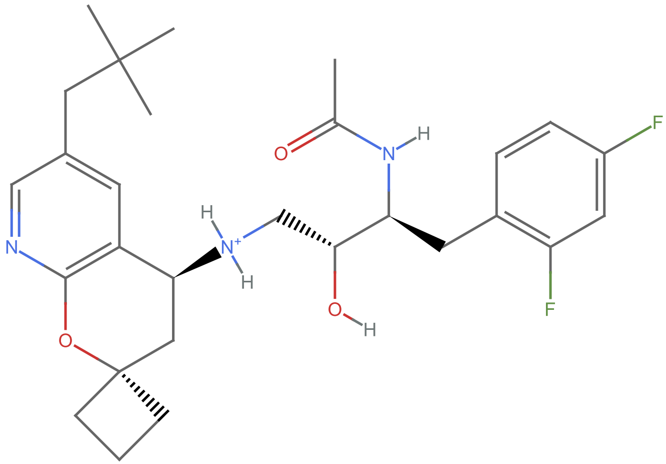

2D Autocorrelation

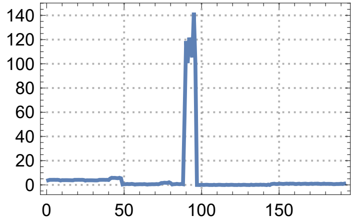

Dataset Index= 356

Molecule Formula= $C_{28}H_{38}F_{2}N_{3}O_{3}^{+}$

3D Autocorrelation

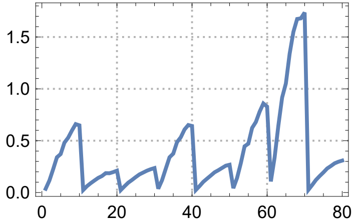

3D Plot

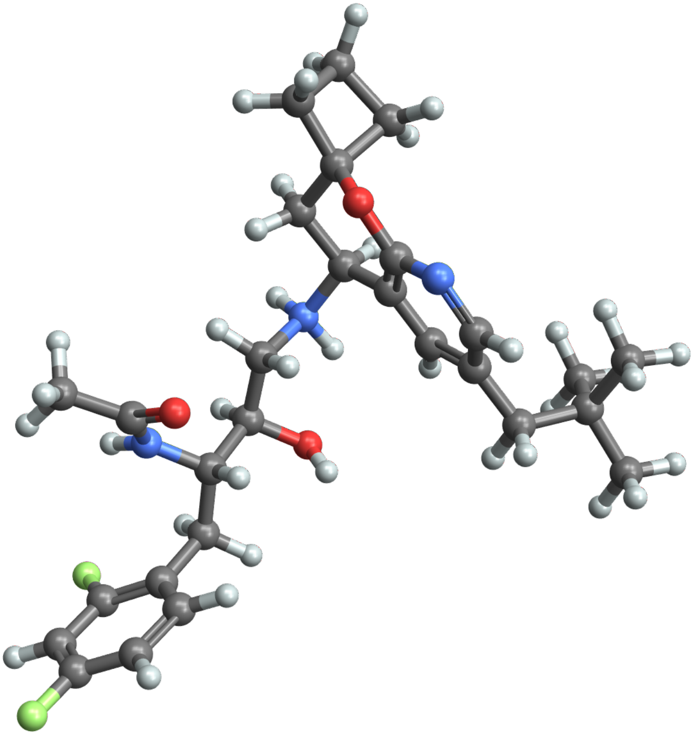

2D Plot

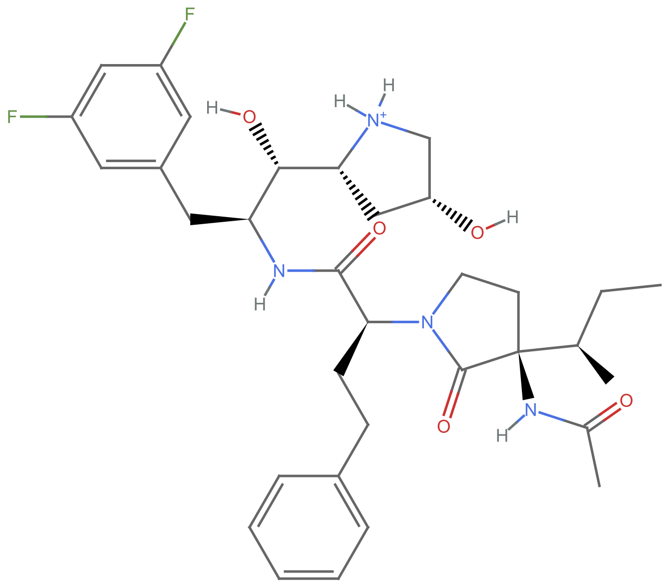

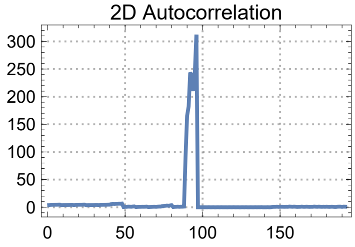

3D Plot

Dataset Index= 357

Molecule Formula= $C_{33}H_{45}F_2N_4O_5^+$

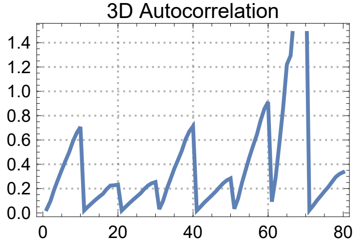

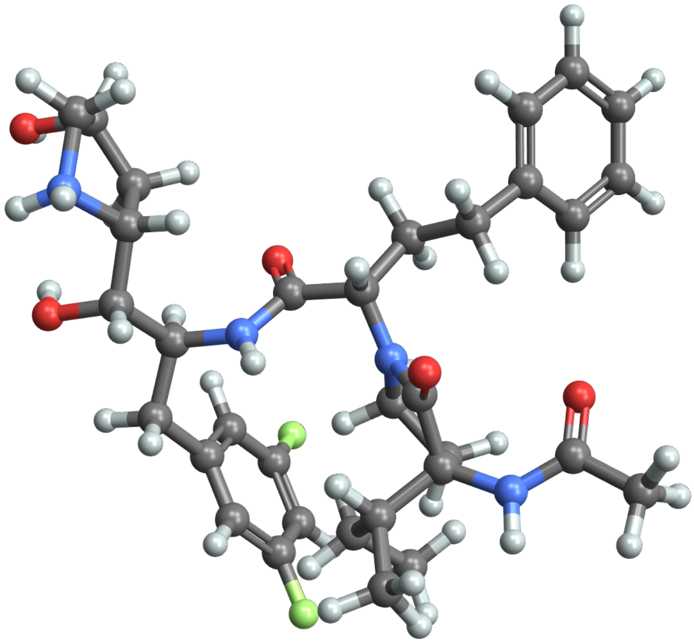

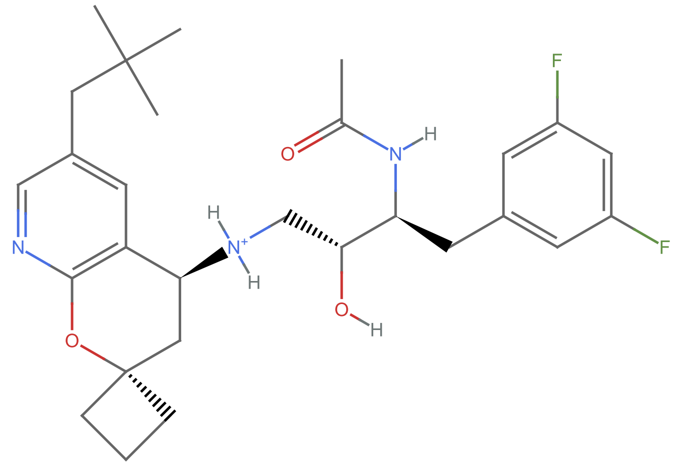

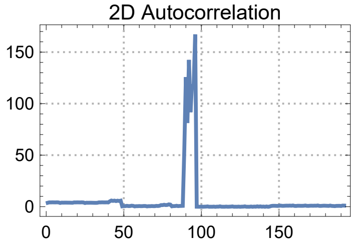

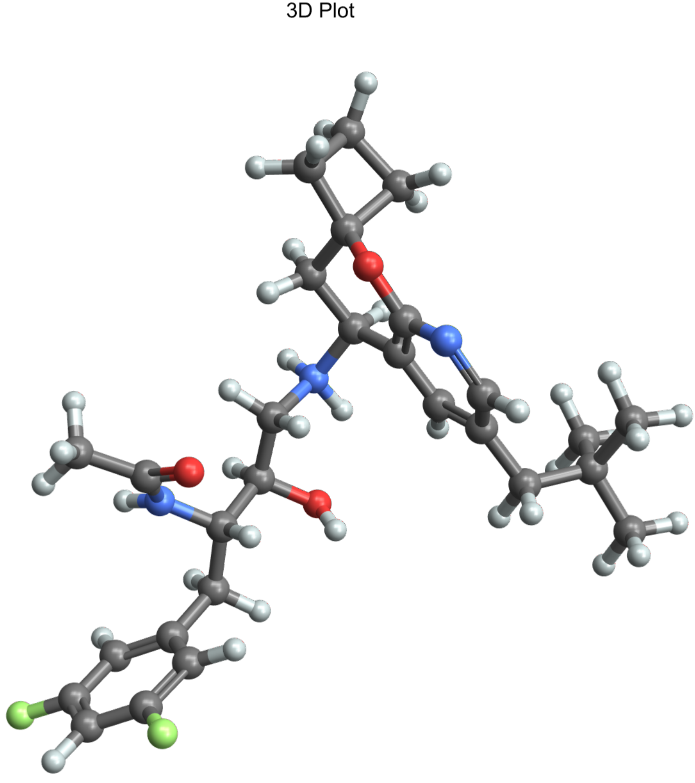

Dataset Index= 358

Molecule Formula= $C_{28}H_{38}F_2N_3O_3^+$

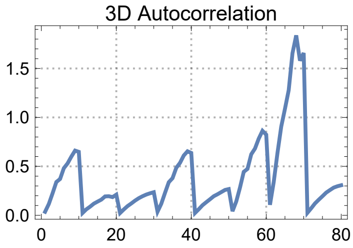

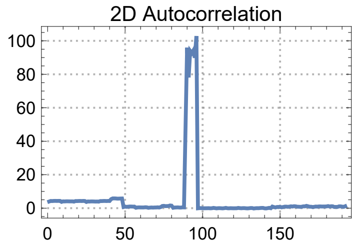

Dataset Index= 359

Molecule Formula= $C_{32}H_{43}N_4O_4S^+$

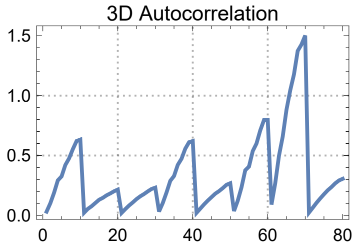

2D Plot

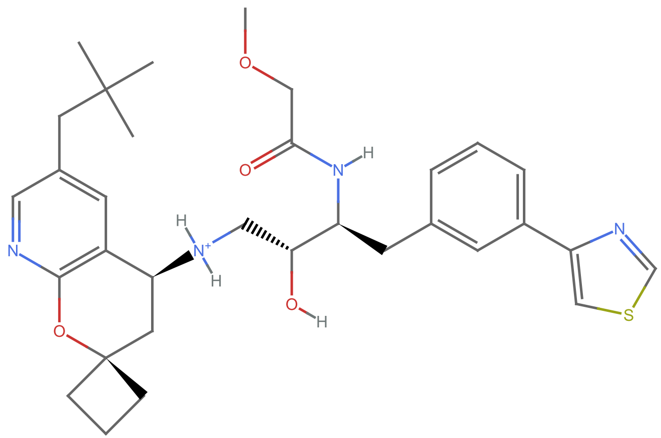

3D Plot

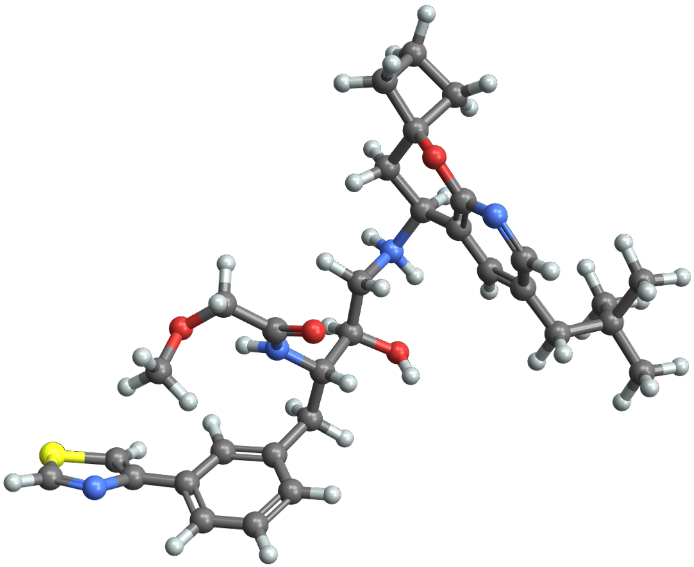

2D Plot

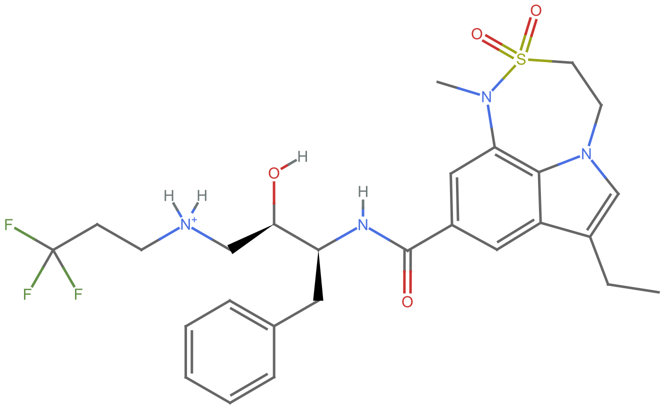

2D Autocorrelation

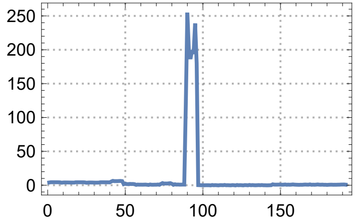

3D Plot

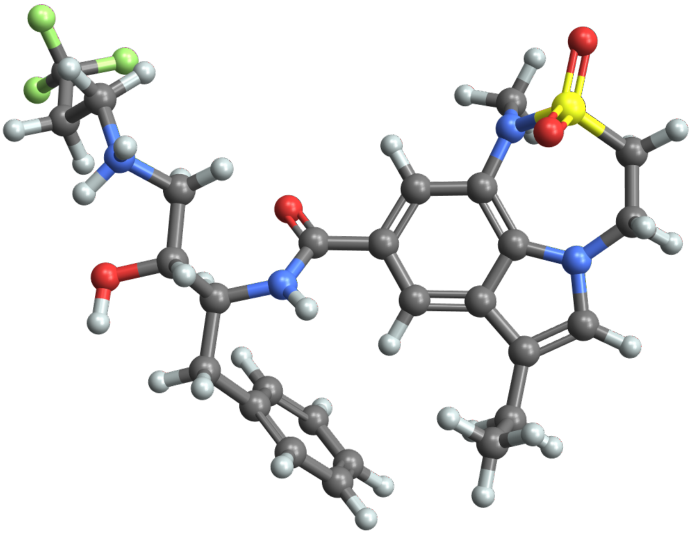

Dataset Index= 360

Molecule Formula= $C_{27}H_{34}F_3N_4O_4S^+$

3D Autocorrelation

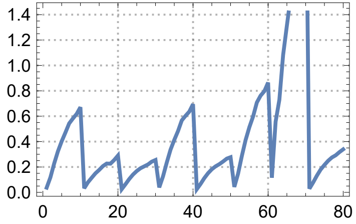

2D Autocorrelation

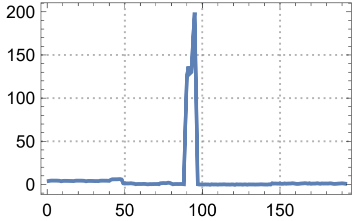

Dataset Index= 361

Molecule Formula= $C_{31}H_{43}N_4O_4S^+$

3D Autocorrelation

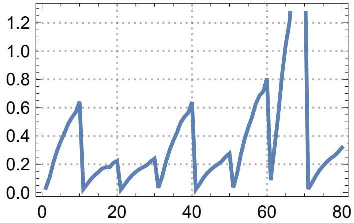

2D Plot

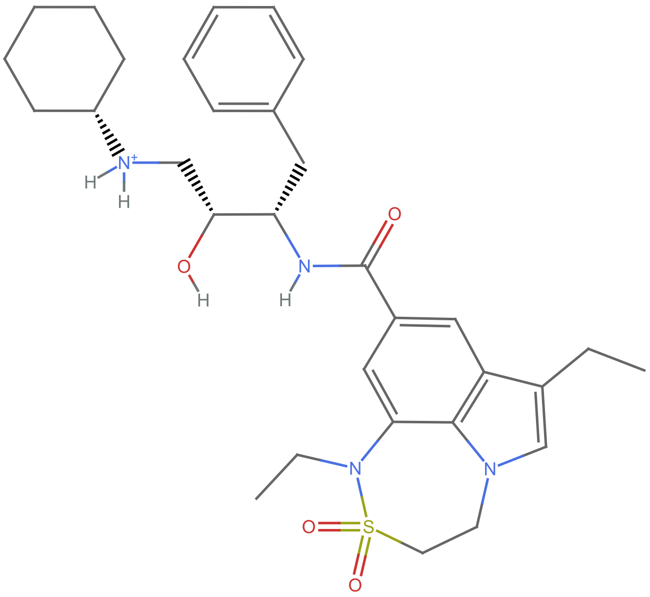

3D Plot

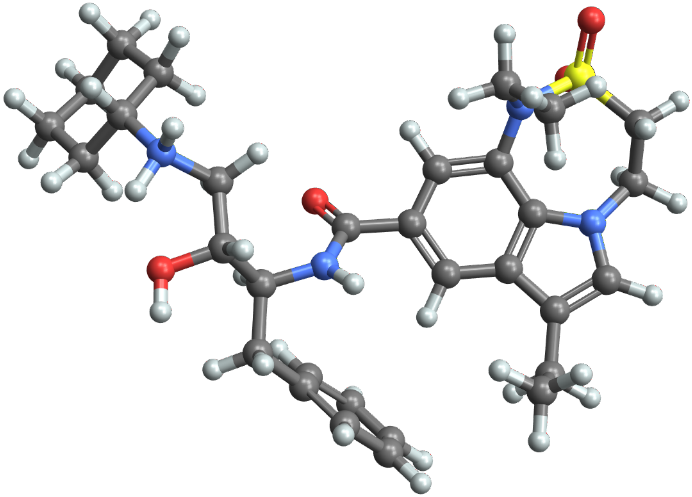

2D Plot

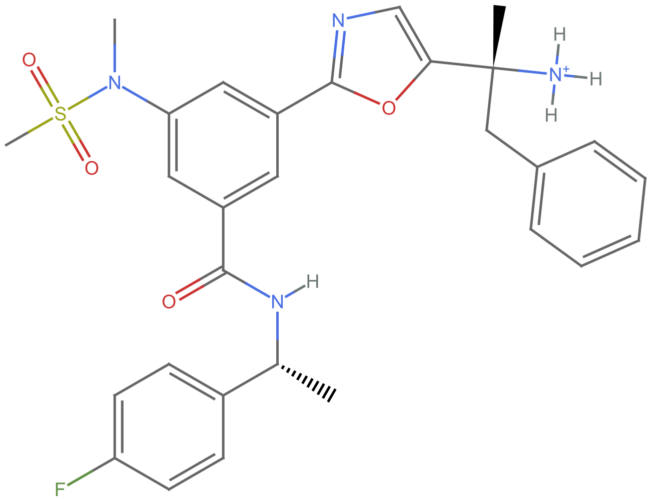

2D Autocorrelation

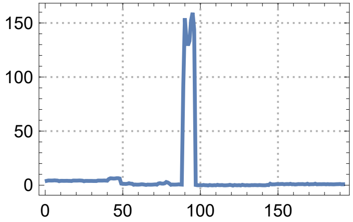

Dataset Index= 362

Molecule Formula= $C_{29}H_{32}FN_4O_4S^+$

3D Plot

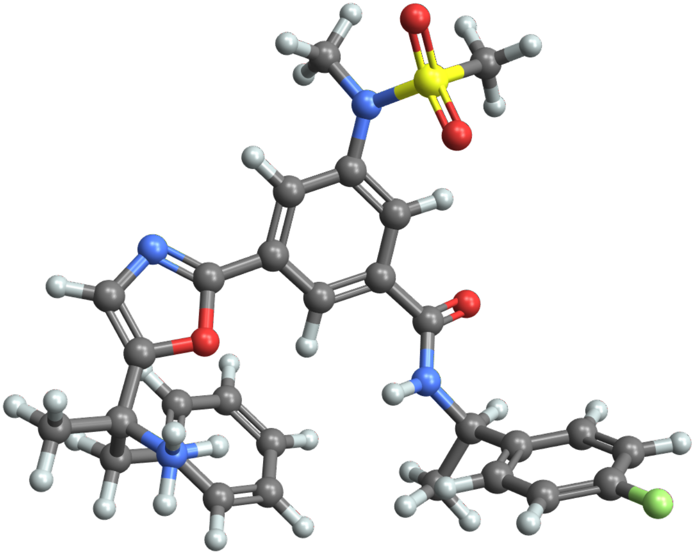

3D Autocorrelation

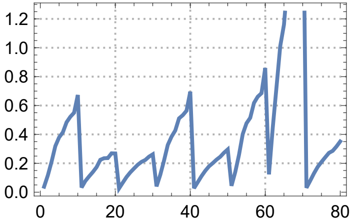

2D Autocorrelation

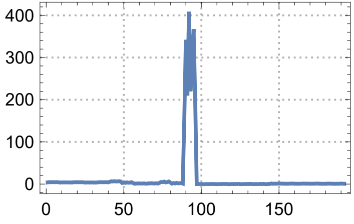

Dataset Index= 363

Molecule Formula= $C_{24}H_{31}F_7N_3O_5S^+$

3D Autocorrelation

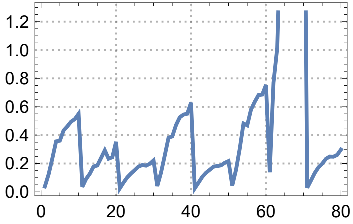

2D Plot

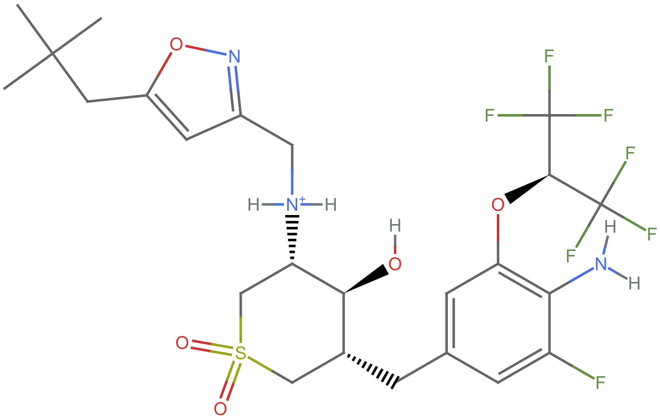

3D Plot

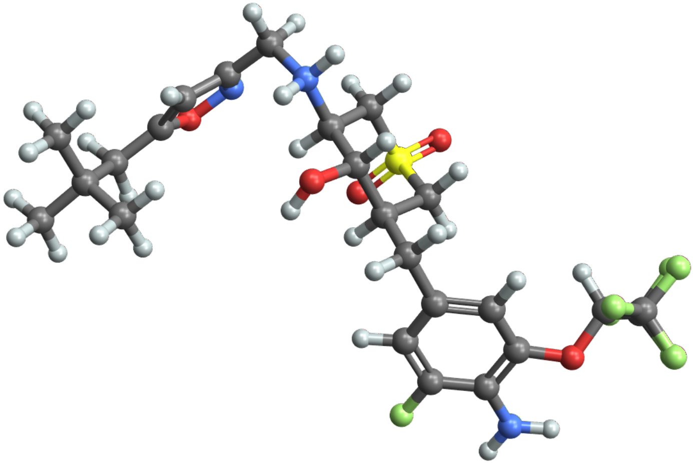

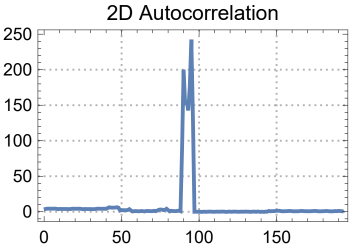

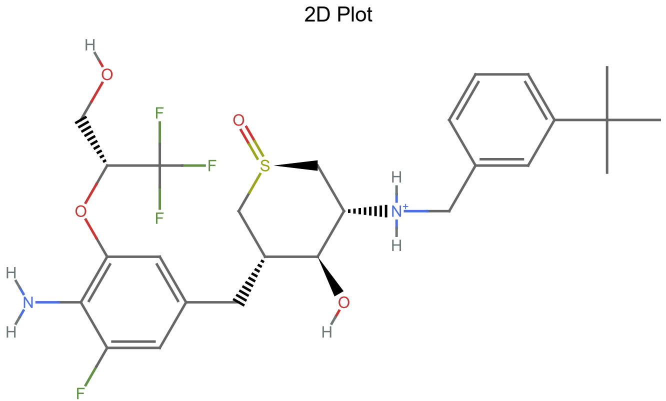

Dataset Index= 364

Molecule Formula= $C_{26}H_{35}F_4N_2O_4S^+$

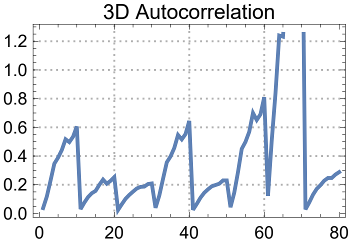

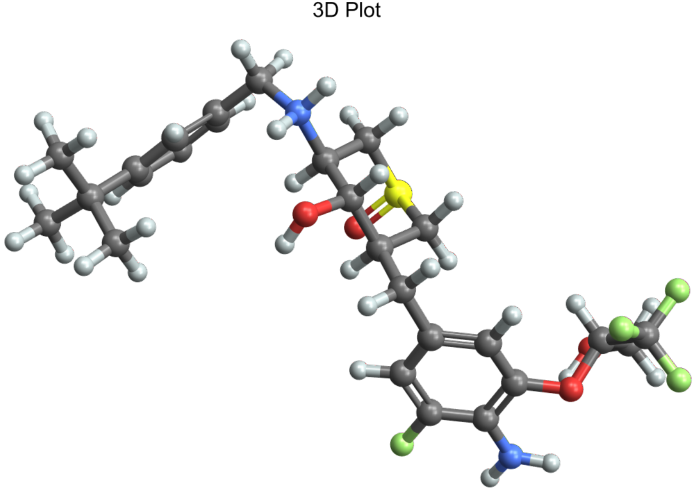

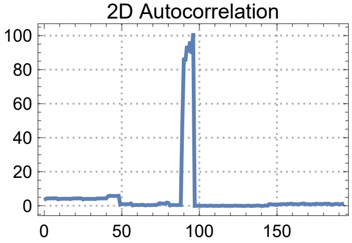

Dataset Index= 365

Molecule Formula= $C_{33}H_{45}N_4O_4S^+$

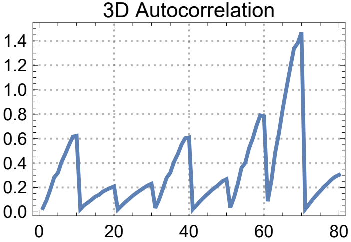

2D Plot

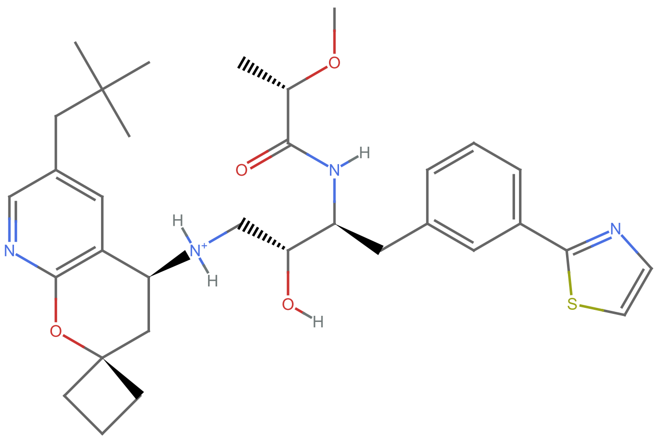

3D Plot

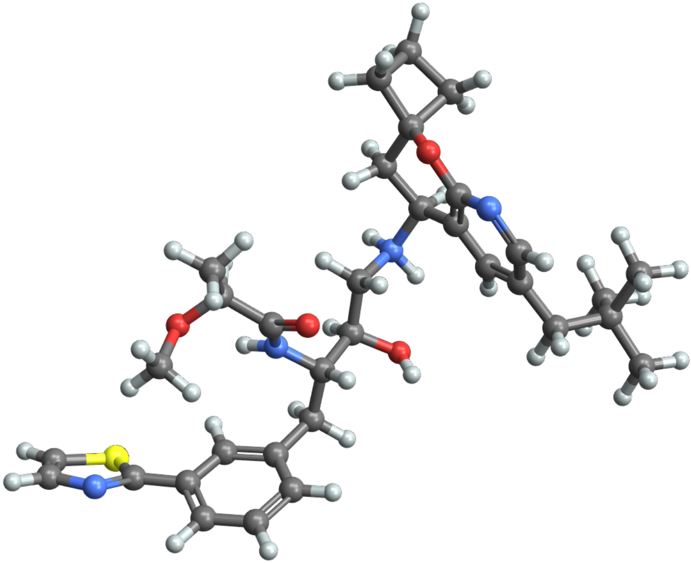

2D Plot

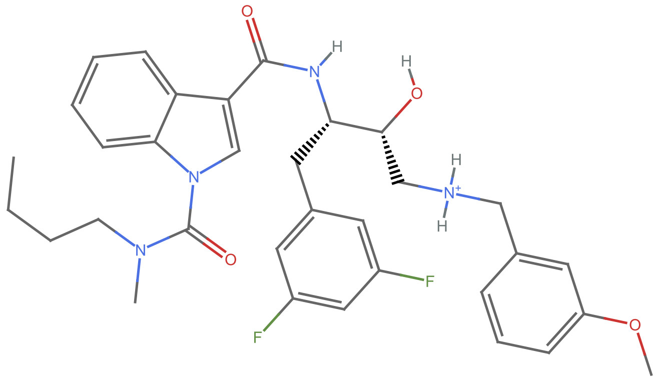

2D Autocorrelation

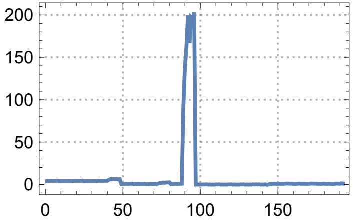

3D Plot

Dataset Index= 366

Molecule Formula= $C_{33}H_{39}F_2N_4O_4^+$

3D Autocorrelation

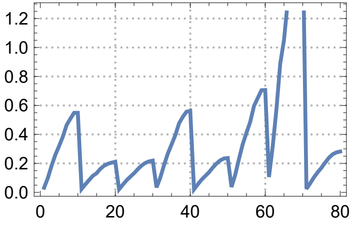

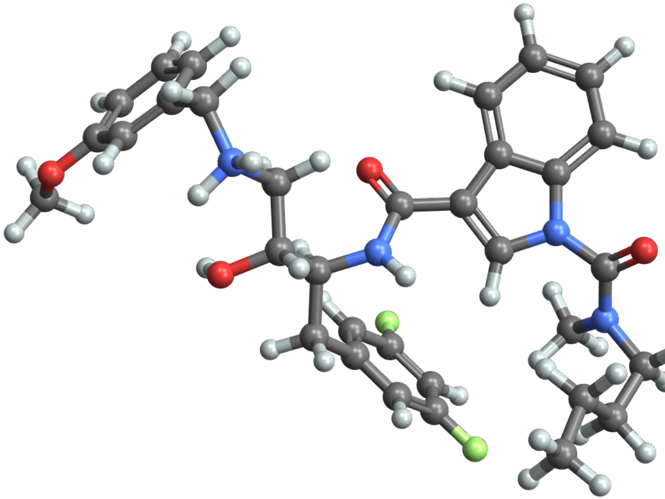

2D Plot

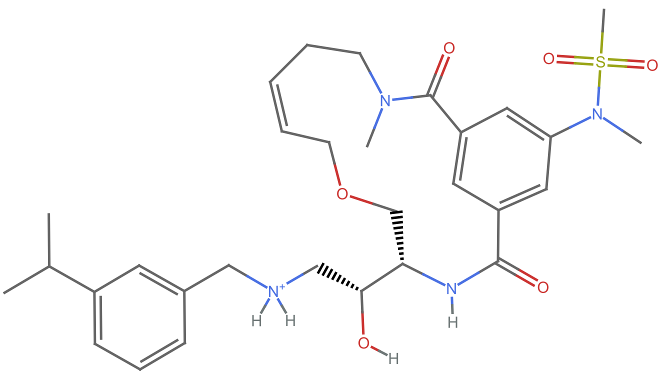

2D Autocorrelation

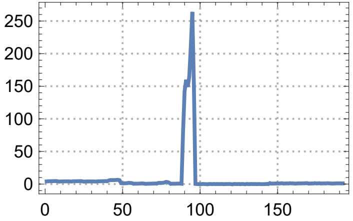

3D Plot

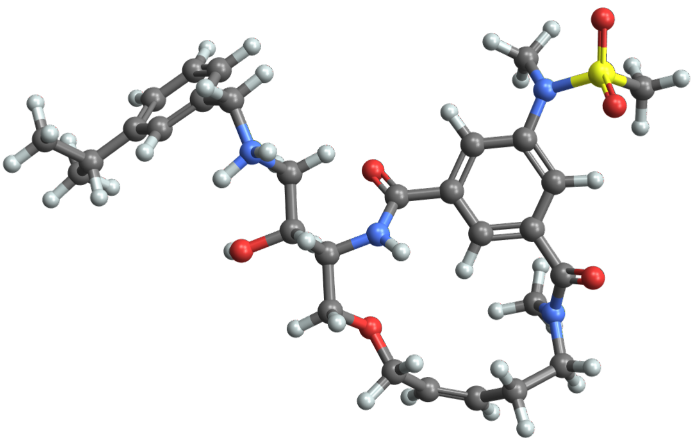

Dataset Index= 367

Molecule Formula= $C_{30}H_{43}N_4O_6S^+$

3D Autocorrelation

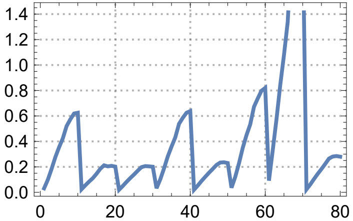

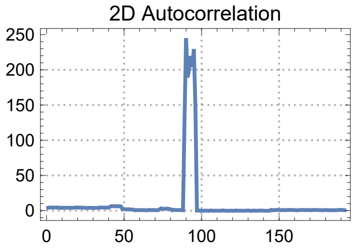

Dataset Index= 368

Molecule Formula= $C_{31}H_{33}F_3N_3O_5S^+$

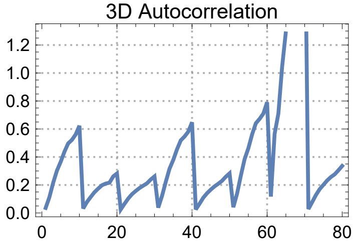

2D Plot

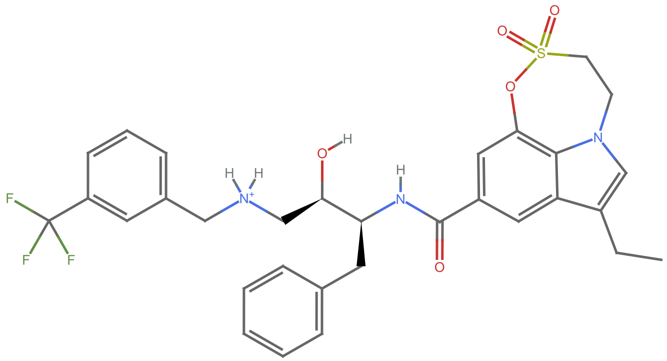

3D Plot

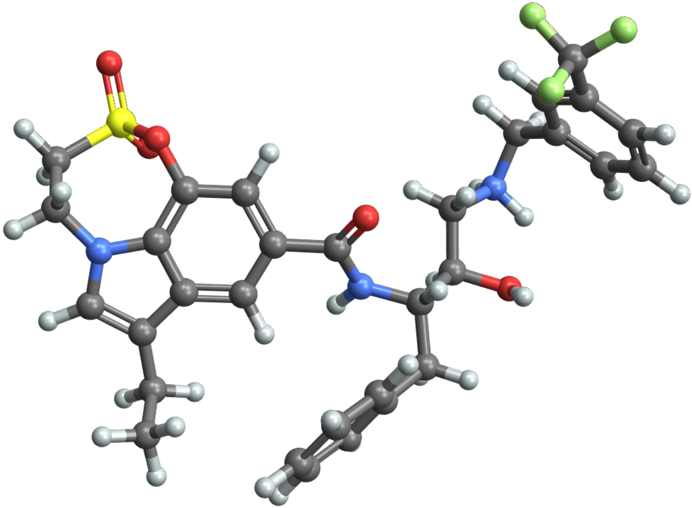

2D Autocorrelation

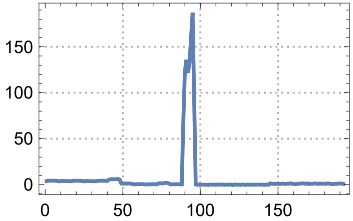

Dataset Index= 369

Molecule Formula= $C_{27}H_{37}N_4O_4S^+$

3D Autocorrelation

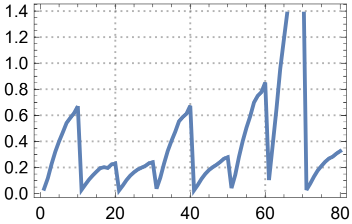

2D Plot

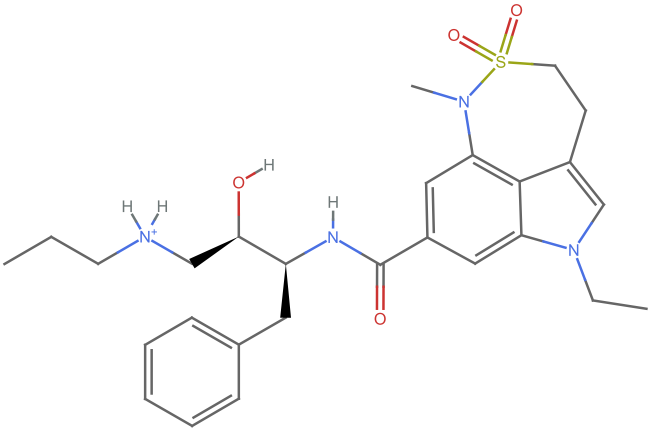

3D Plot

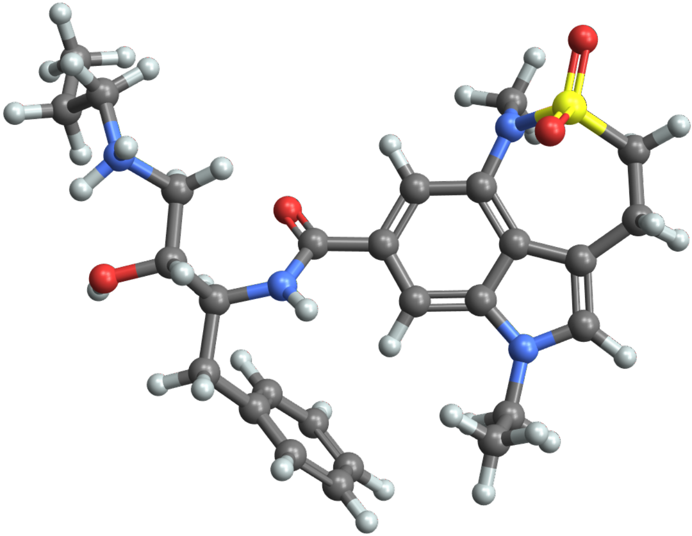

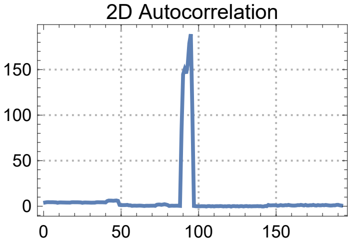

Dataset Index= 370

Molecule Formula= $C_{31}H_{37}N_4O_5S^+$

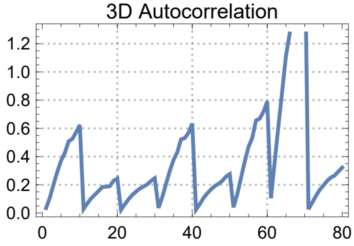

2D Plot

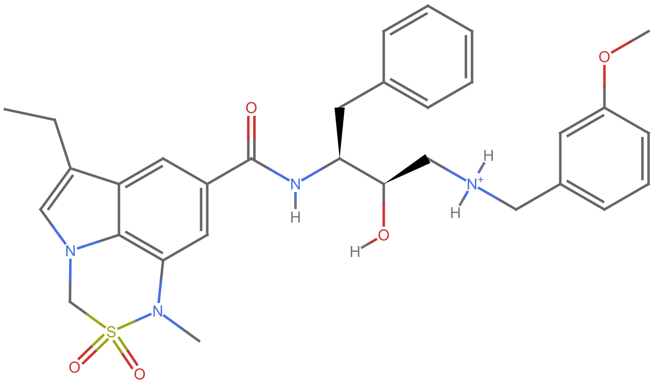

3D Plot

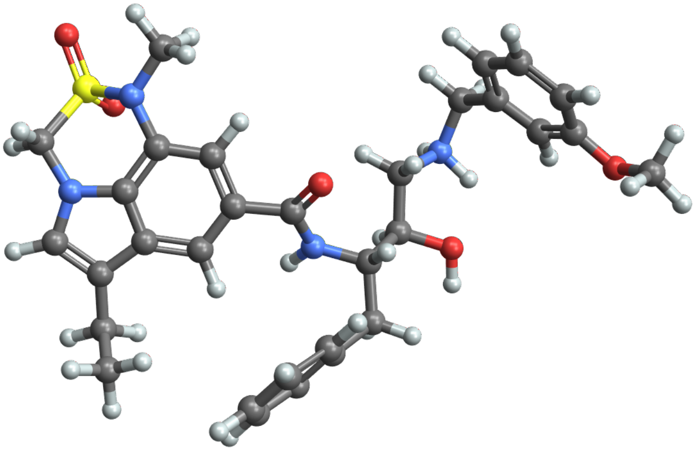

2D Plot

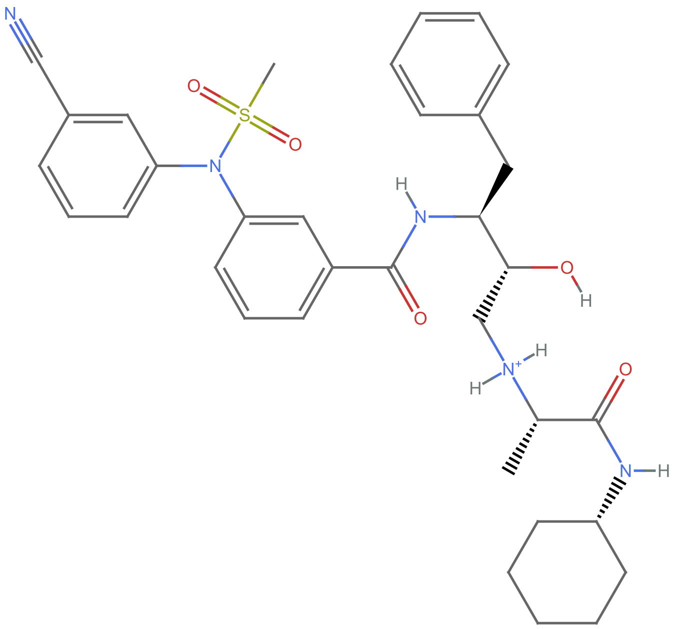

2D Autocorrelation

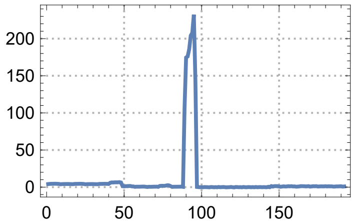

Dataset Index= 371

Molecule Formula= $C_{34}H_{42}N_5O_5S^+$

3D Autocorrelation

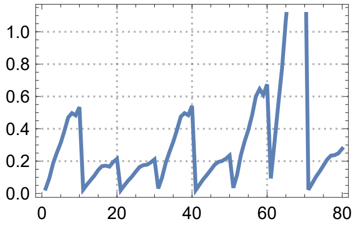

3D Plot

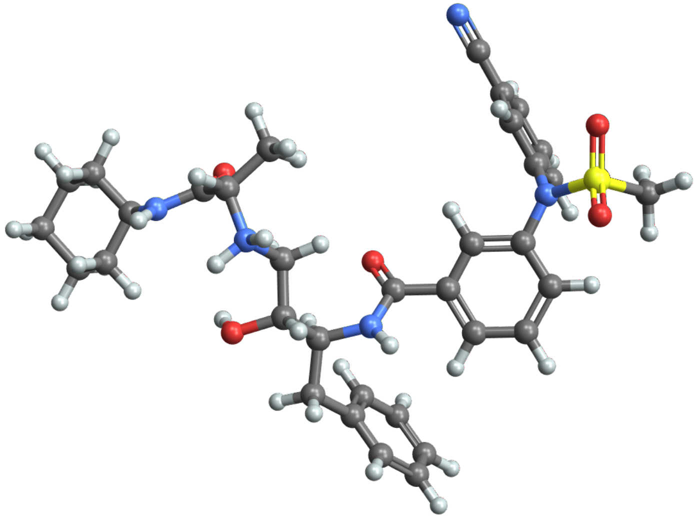

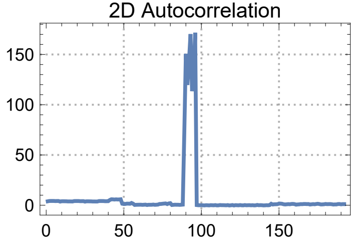

Dataset Index= 372

Molecule Formula= $C_{27}H_{38}FN_2O_4S^+$

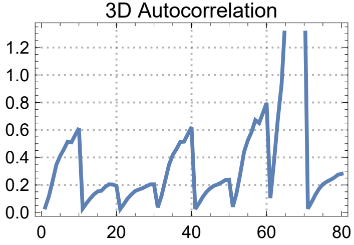

2D Plot

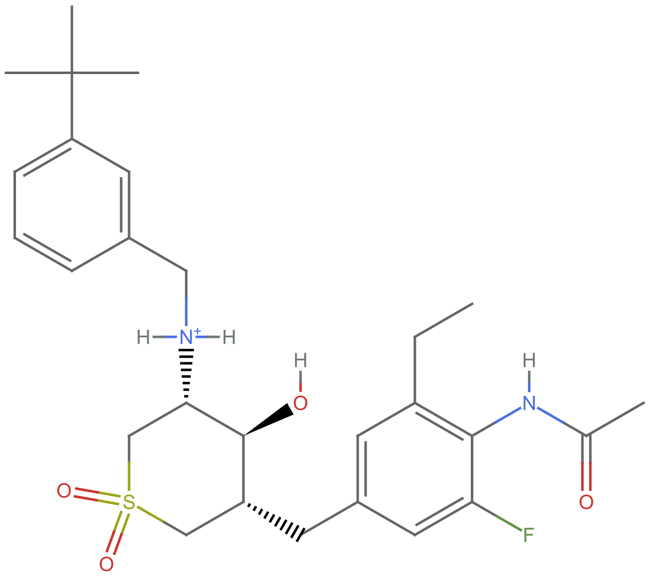

3D Plot

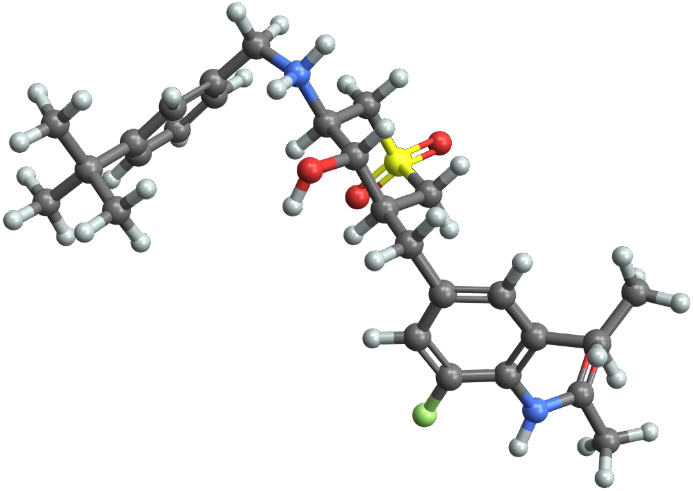

2D Plot

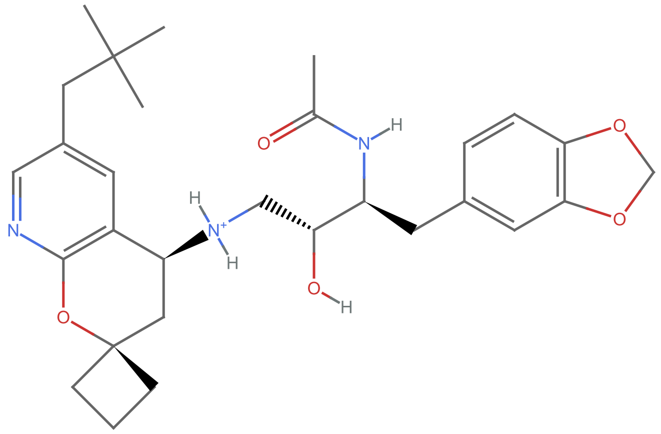

2D Autocorrelation

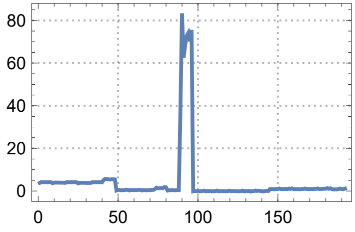

Dataset Index= 373

Molecule Formula= $C_{29}H_{40}N_3O_5^+$

3D Autocorrelation

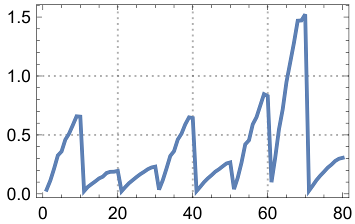

3D Plot

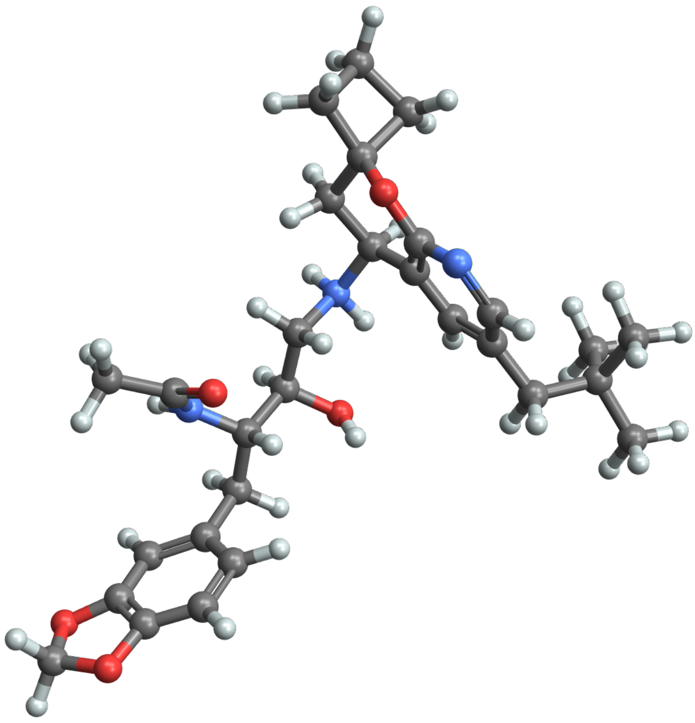

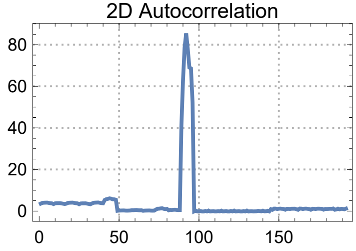

Dataset Index= 374

Molecule Formula= $C_{21}H_{23}N_7O$

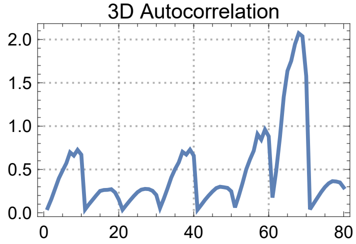

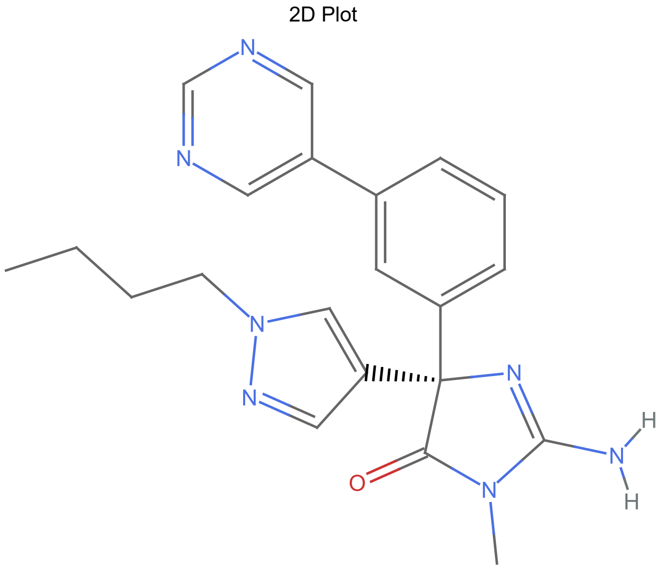

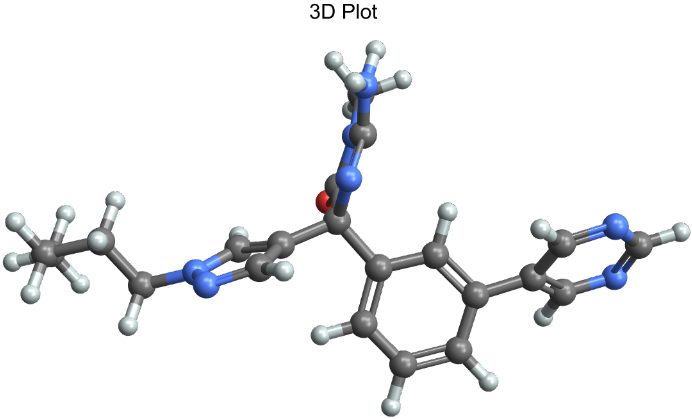

2D Plot

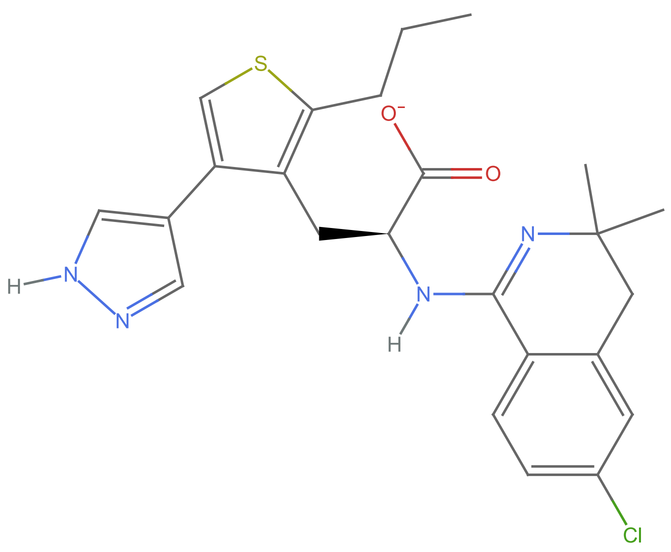

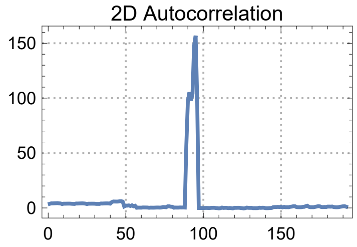

Dataset Index= 375

Molecule Formula= $C_{24}H_{26}ClN_4O_2S^-$

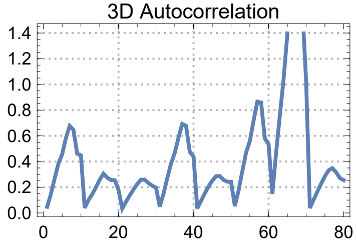

3D Plot

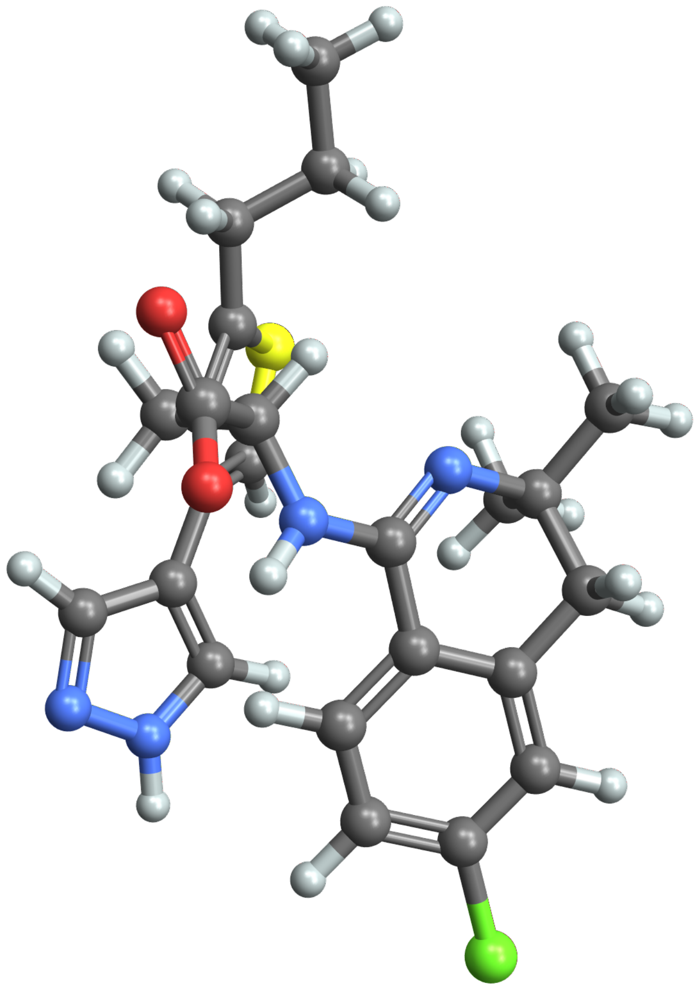

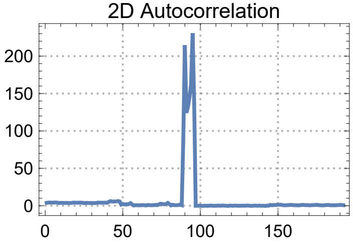

Dataset Index= 376

Molecule Formula= $C_{25}H_{33}F_{4}N_{2}O_{3}S^{+}$

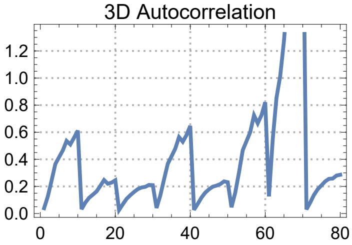

2D Plot

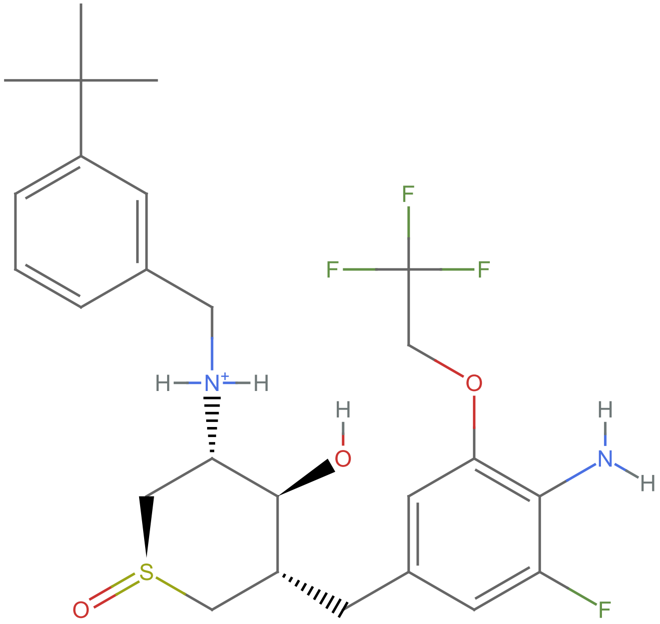

3D Plot

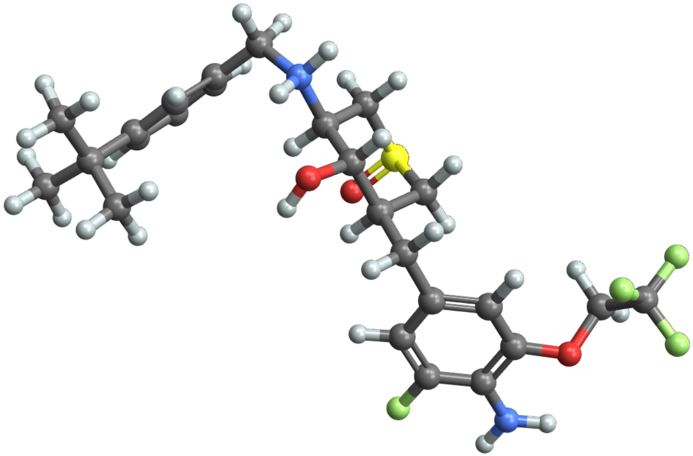

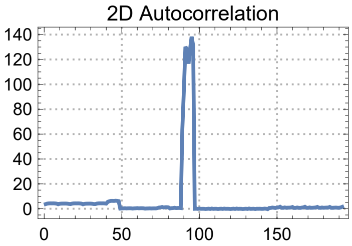

Dataset Index= 377

Molecule Formula= $C_{28}H_{22}FN_5O$

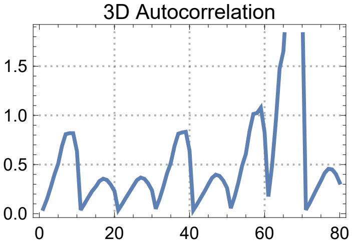

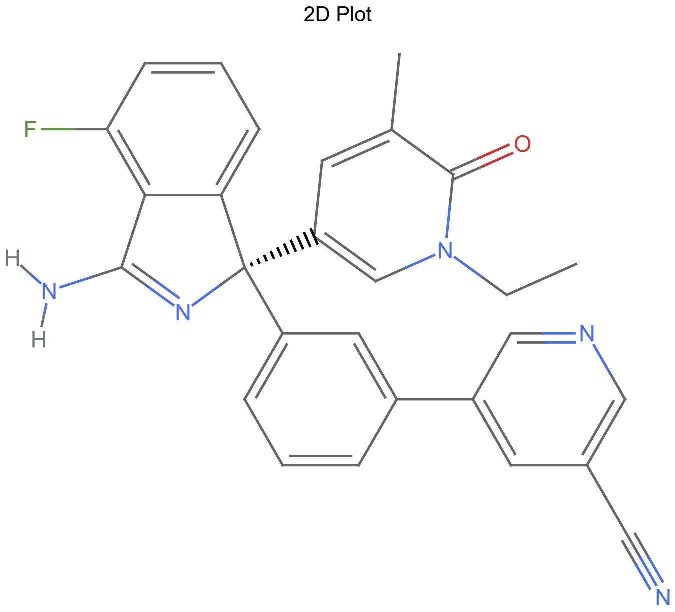

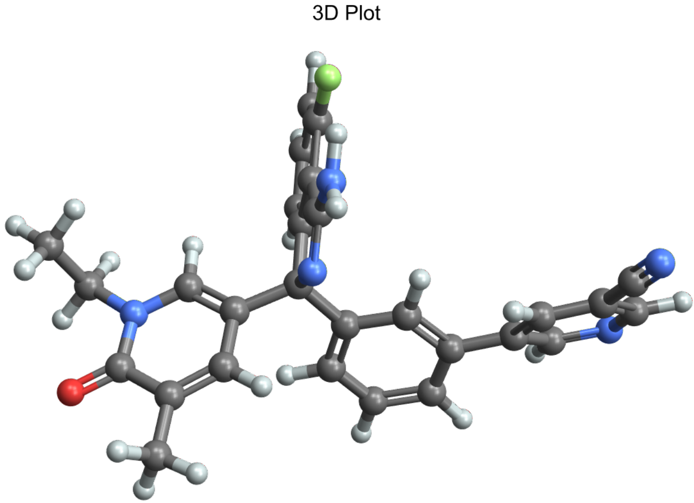

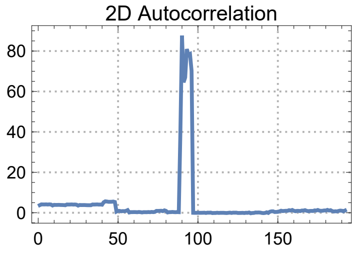

Dataset Index= 378

Molecule Formula= $C_{28}H_{39}ClN_3O_3^+$

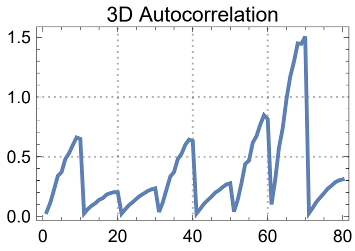

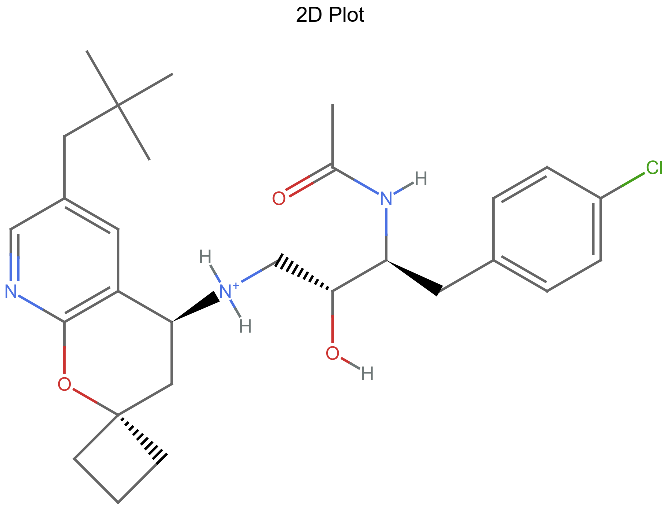

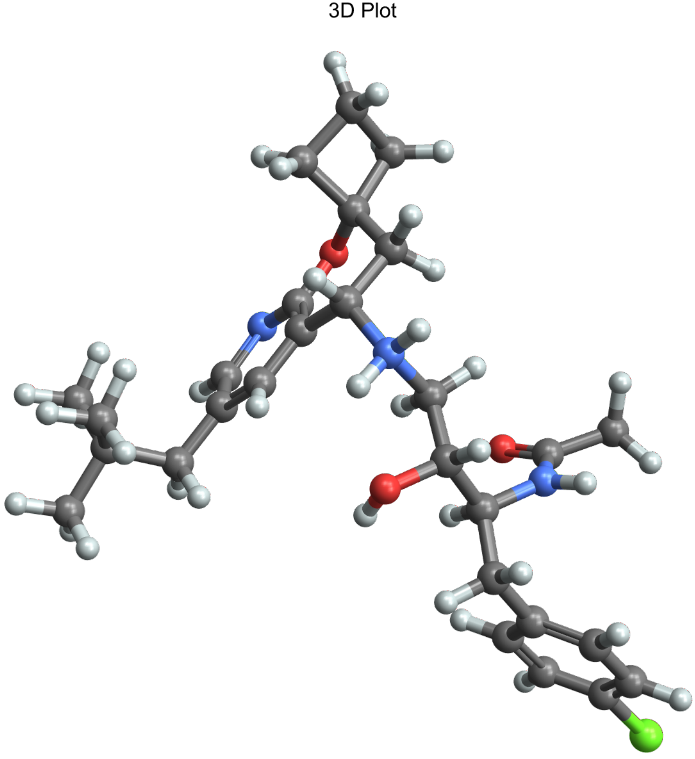

2D Plot

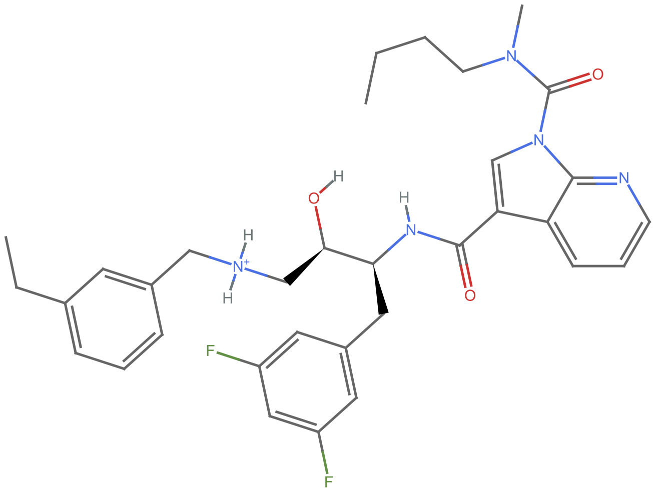

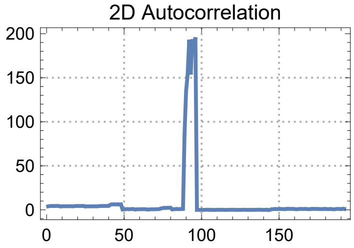

3D Plot

Dataset Index= 379

Molecule Formula= $C_{33}H_{40}F_2N_5O_3^+$

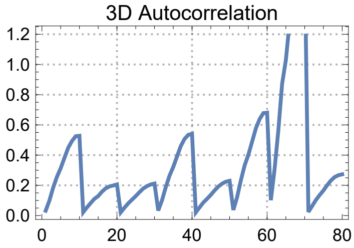

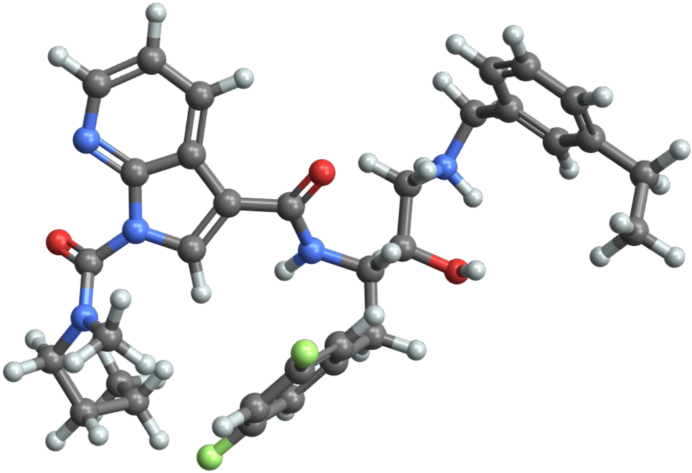

2D Plot

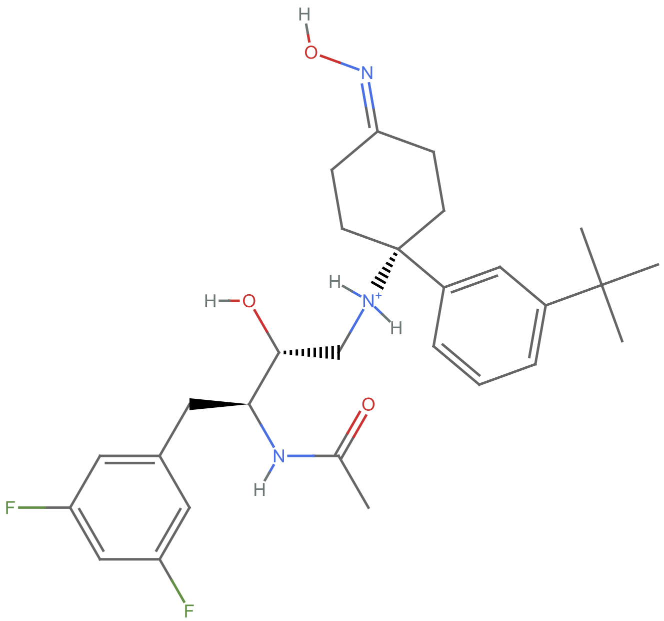

2D Autocorrelation

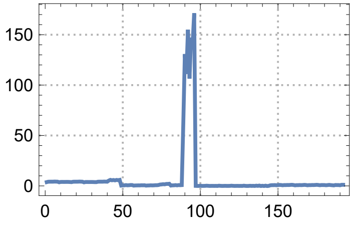

3D Plot

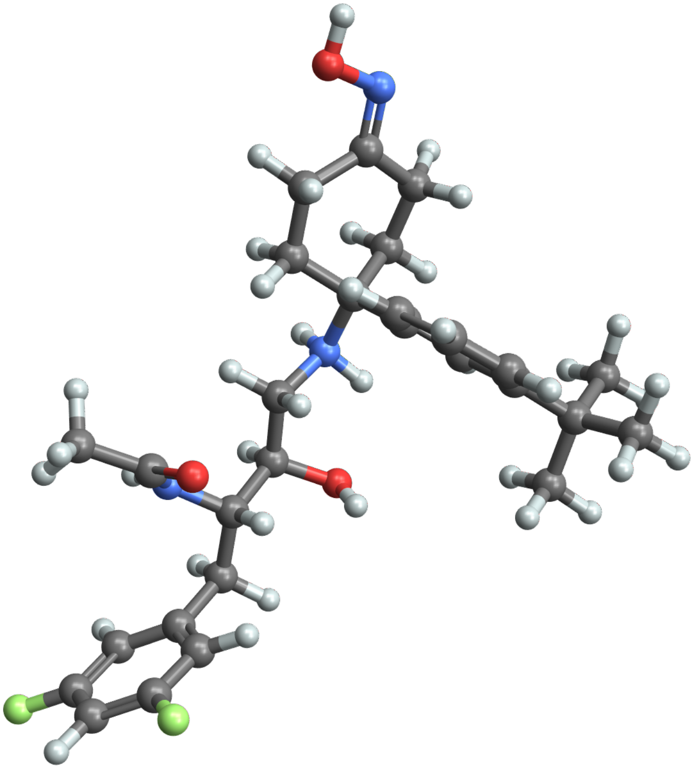

Dataset Index= 380

Molecule Formula= $C_{28}H_{38}F_2N_3O_3^+$

3D Autocorrelation

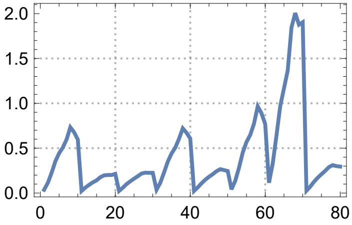

2D Autocorrelation

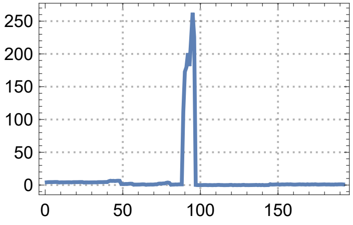

Dataset Index= 381

Molecule Formula= $C_{36}H_{41}F_2N_4O_6S^+$

3D Autocorrelation

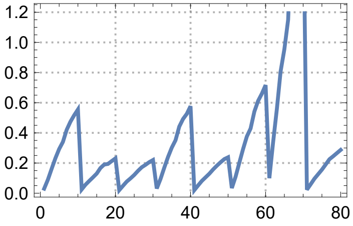

2D Plot

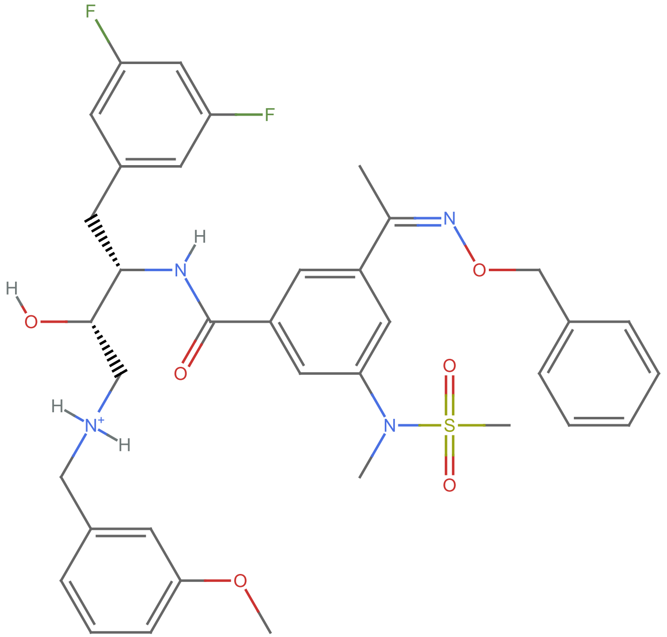

3D Plot

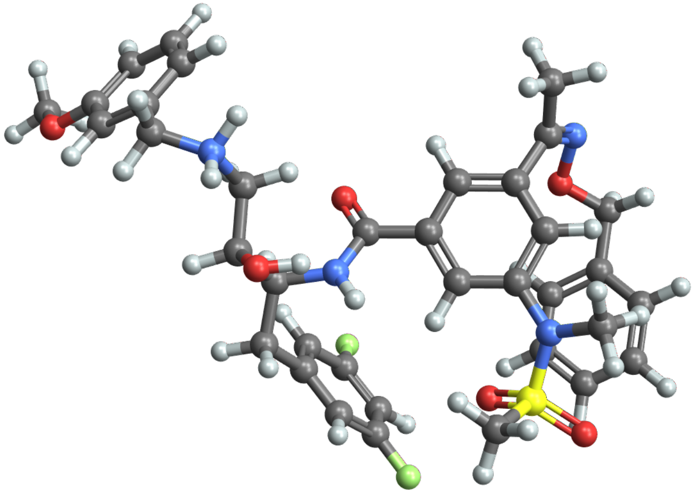

2D Plot

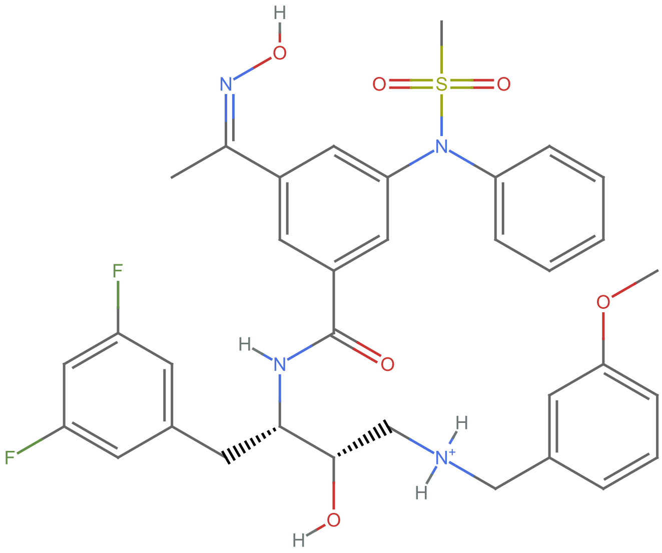

2D Autocorrelation

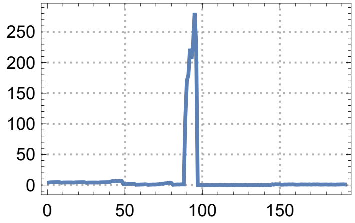

3D Plot

Dataset Index= 382

Molecule Formula= $C_{34}H_{37}F_2N_4O_6S^+$

3D Autocorrelation

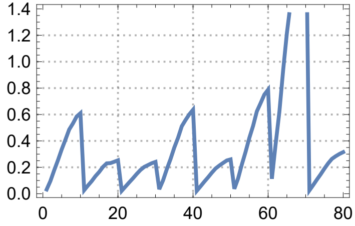

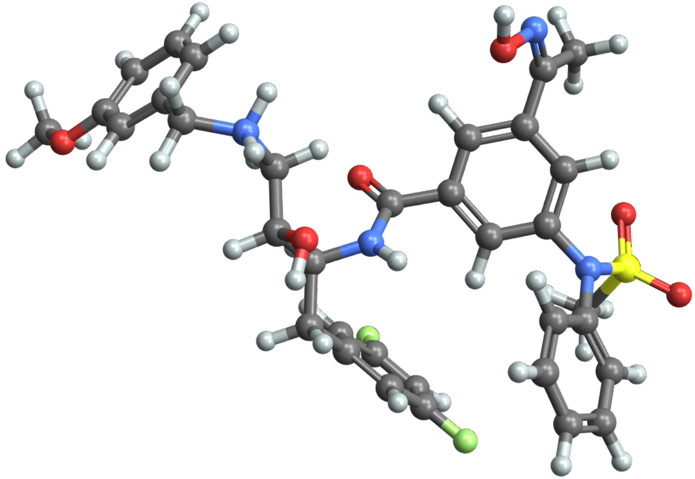

2D Plot

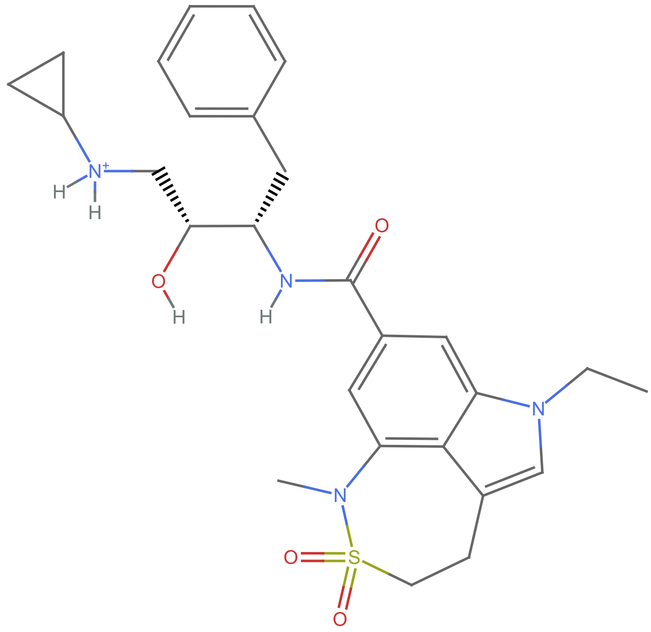

2D Autocorrelation

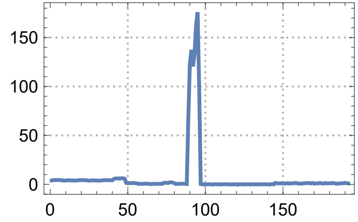

Dataset Index= 383

Molecule Formula= $C_{27}H_{35}N_4O_4S^+$

3D Autocorrelation

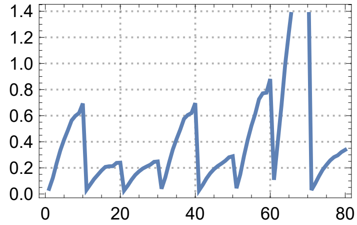

3D Plot

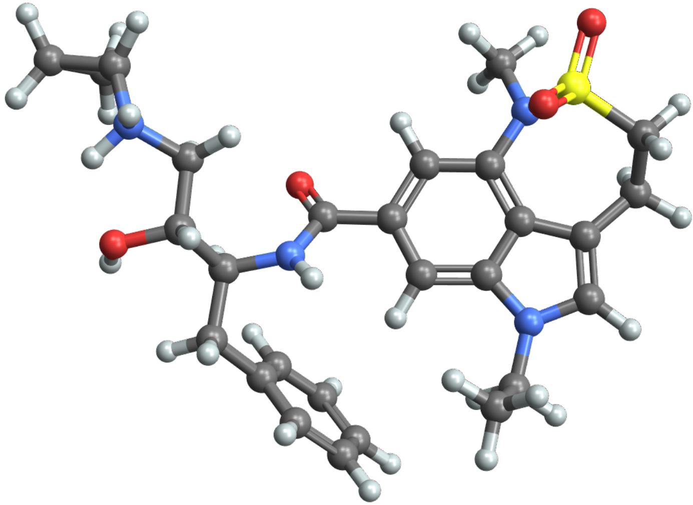

2D Plot

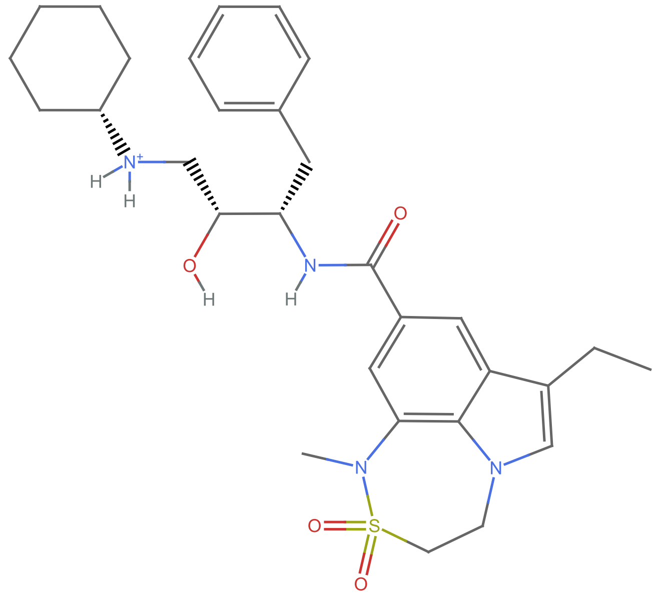

2D Autocorrelation

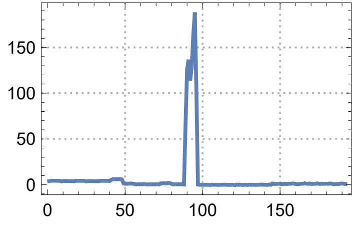

Dataset Index= 384

Molecule Formula= $C_{30}H_{41}N_4O_4S^+$

3D Plot

3D Autocorrelation

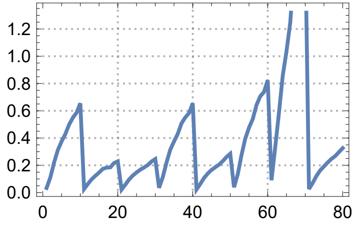

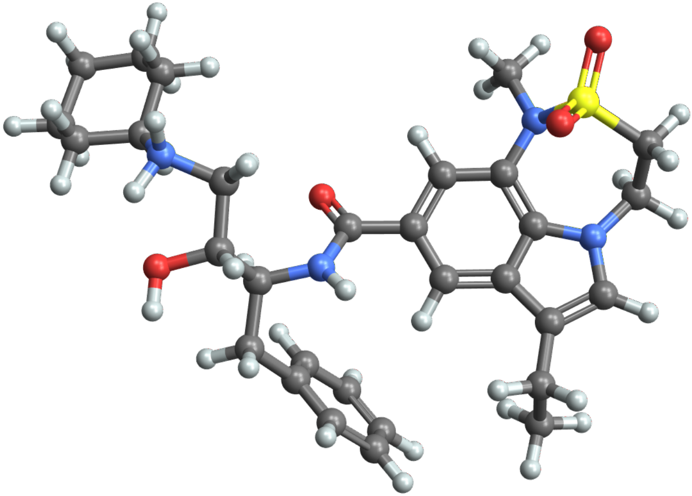

2D Plot

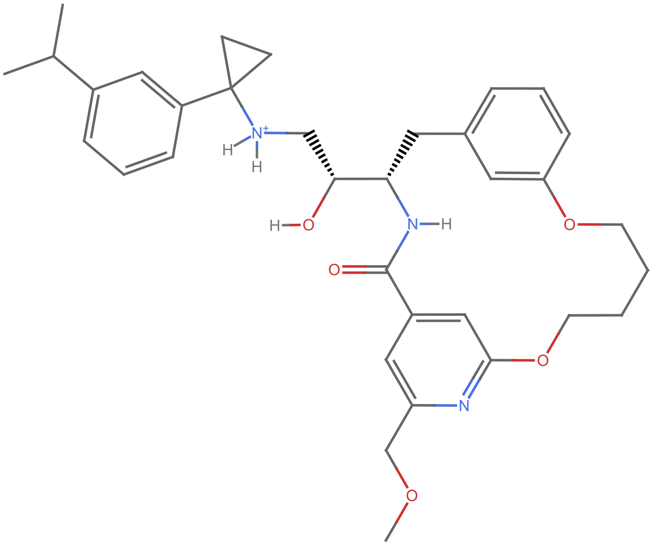

2D Autocorrelation

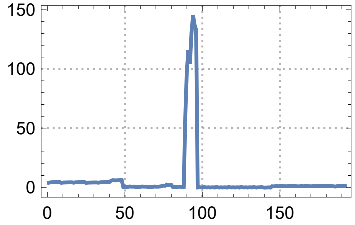

Dataset Index= 385

Molecule Formula= $C_{34}H_{44}N_3O_5^+$

3D Plot

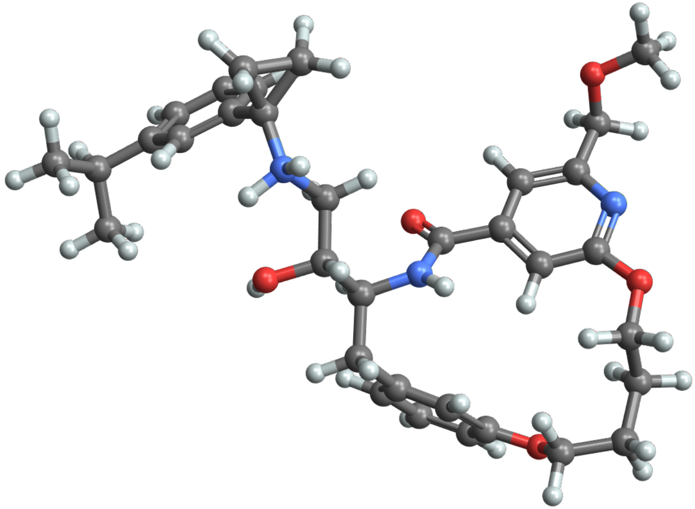

3D Autocorrelation

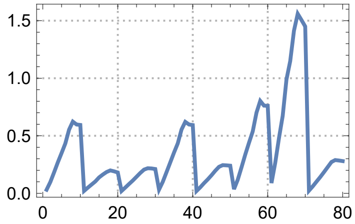

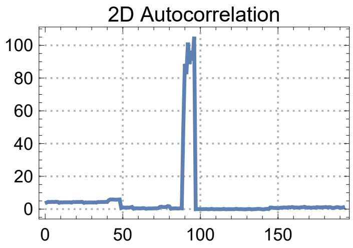

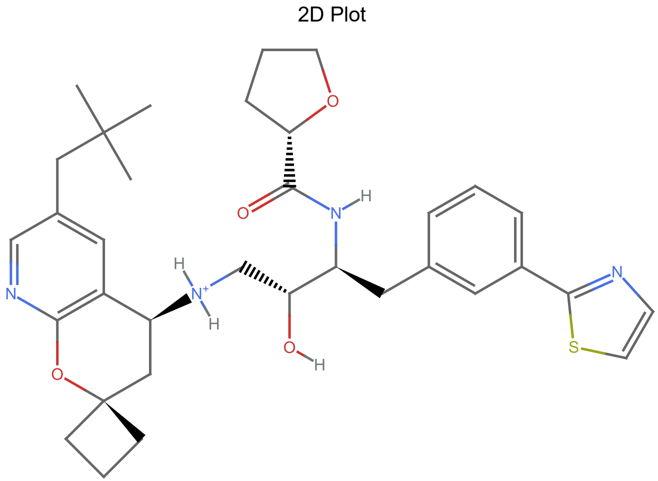

Dataset Index= 386

Molecule Formula= $C_{34}H_{45}N_4O_4S^+$

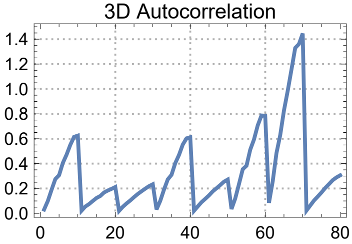

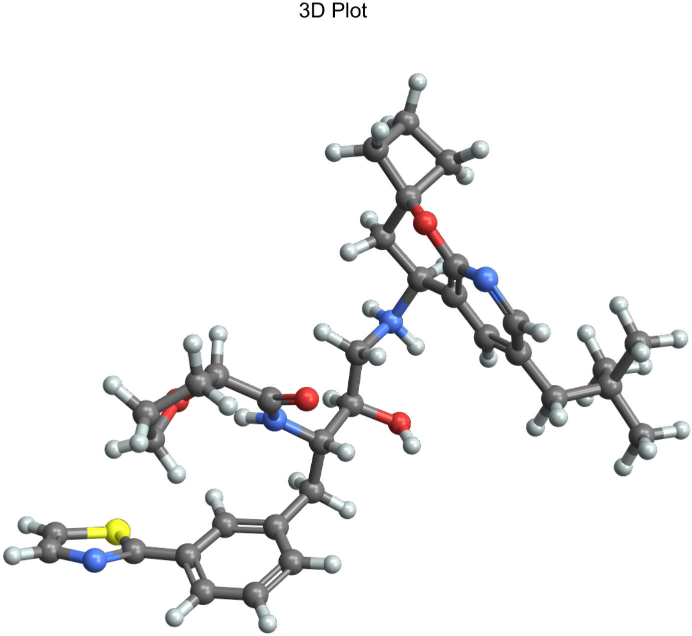

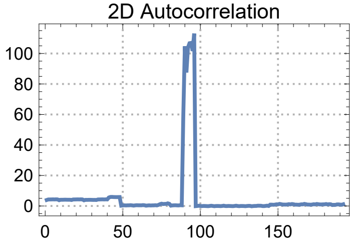

Dataset Index= 387

Molecule Formula= $C_{34}H_{45}N_4O_4^+$

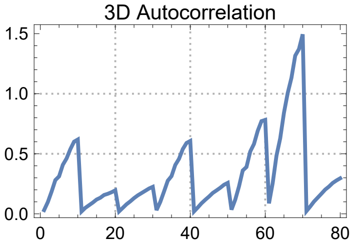

2D Plot

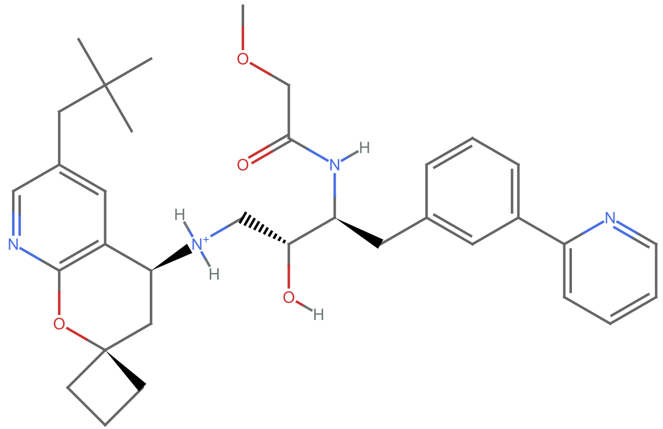

3D Plot

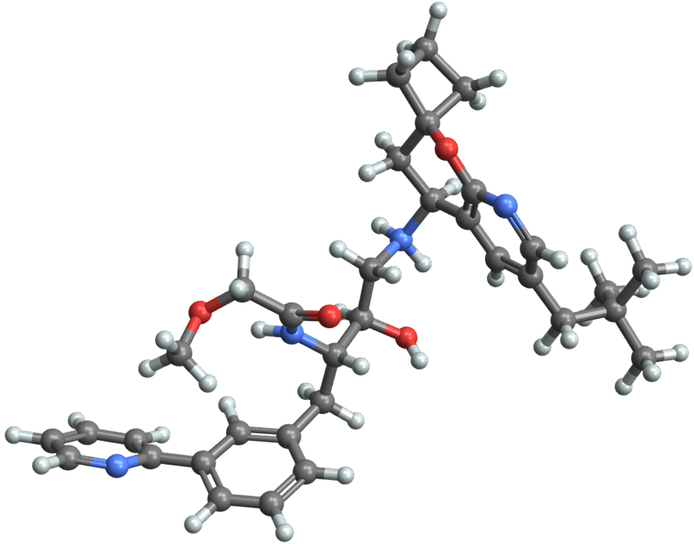

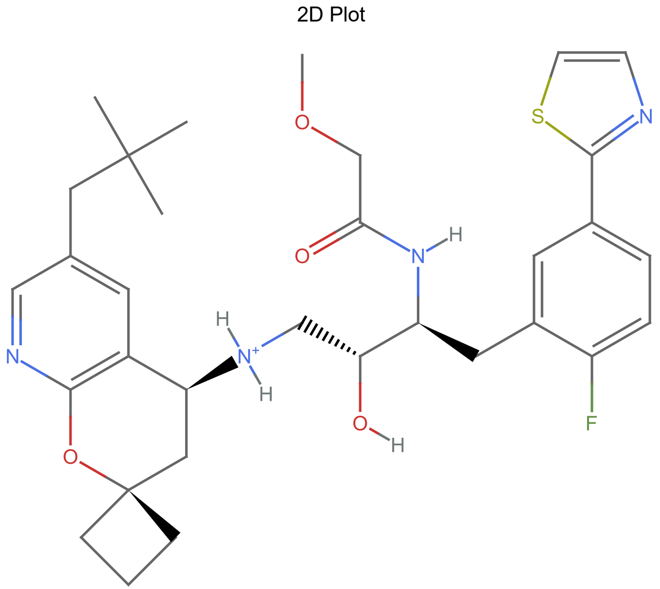

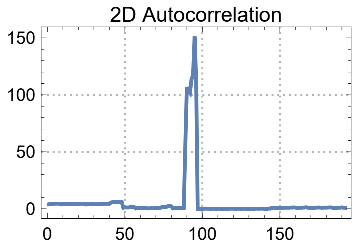

Dataset Index= 388

Molecule Formula= $C_{32}H_{42}FN_4O_4S^+$

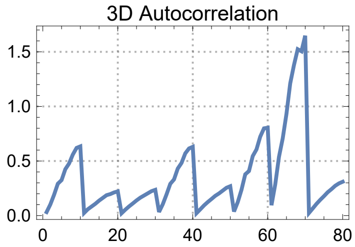

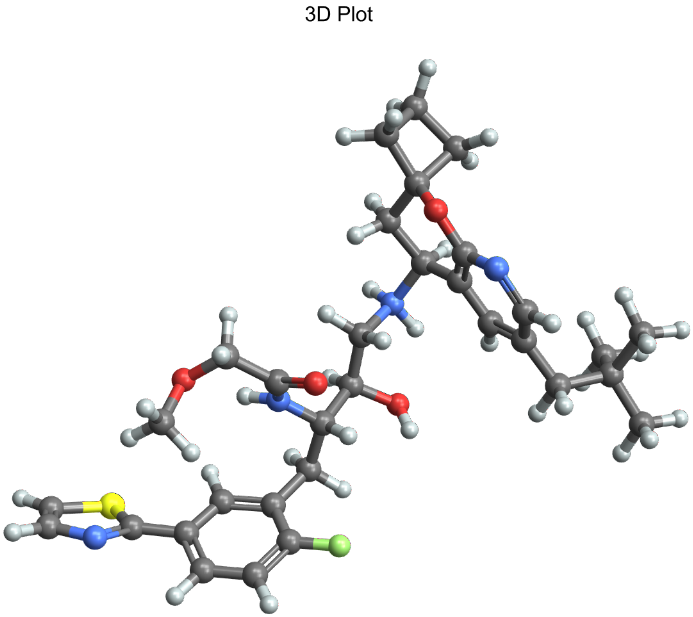

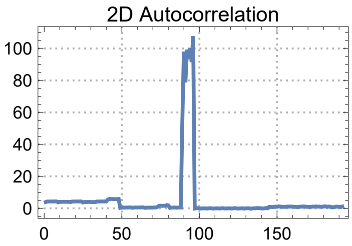

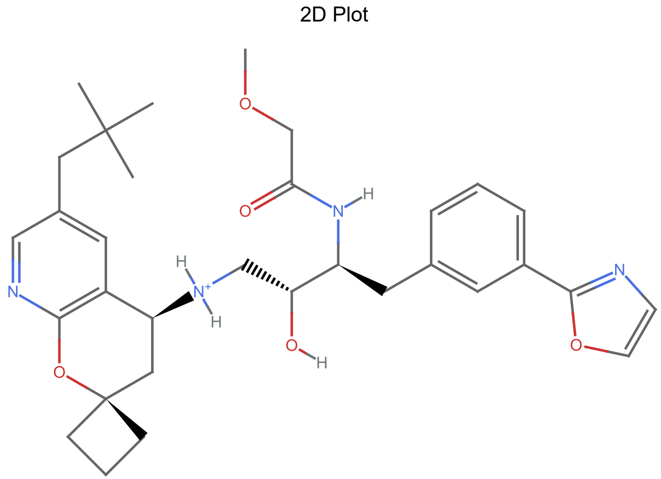

Dataset Index= 389

Molecule Formula= $C_{32}H_{43}N_4O_5^+$

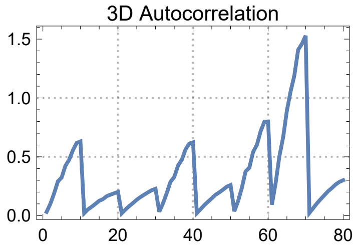

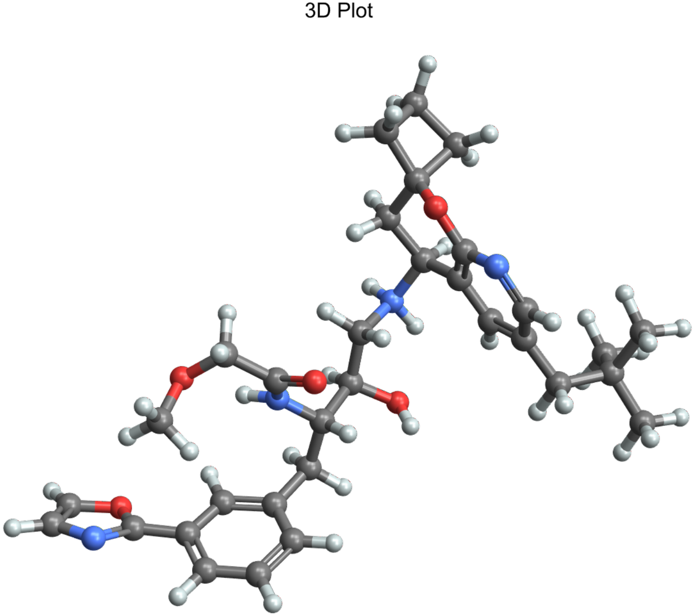

2D Plot

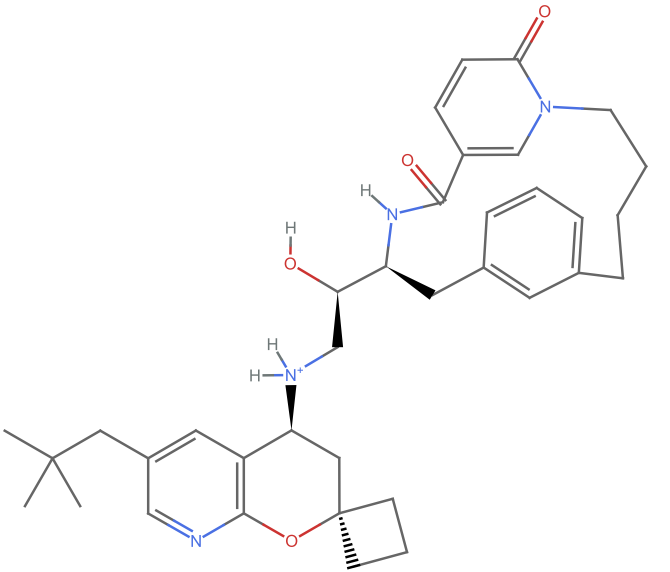

2D Autocorrelation

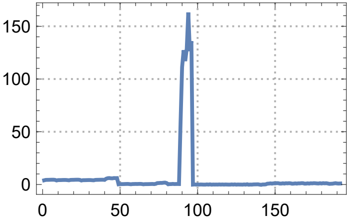

Dataset Index= 390

Molecule Formula= $C_{36}H_{47}N_4O_4^+$

3D Autocorrelation

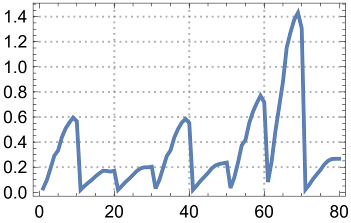

3D Plot

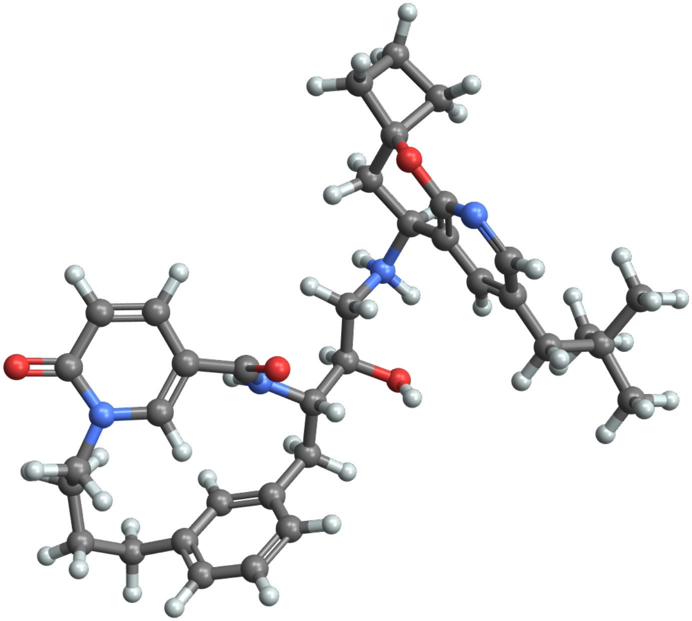

2D Autocorrelation

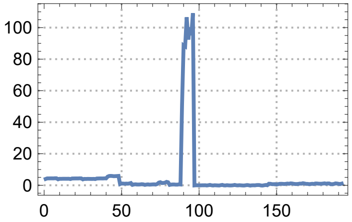

Dataset Index= 391

Molecule Formula= $C_{33}H_{45}N_4O_5S^+$

3D Autocorrelation

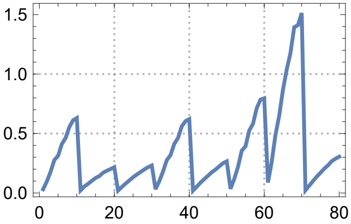

2D Plot

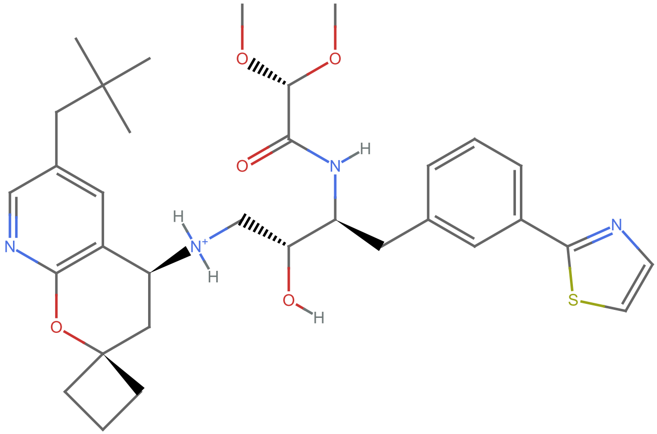

3D Plot

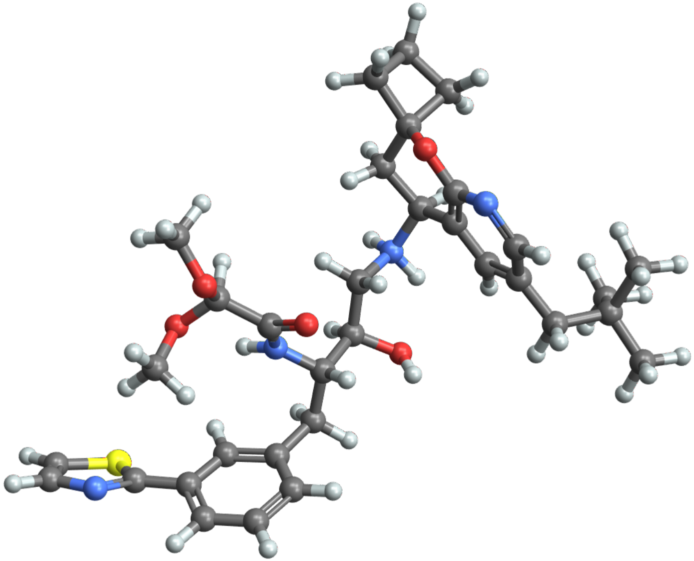

2D Plot

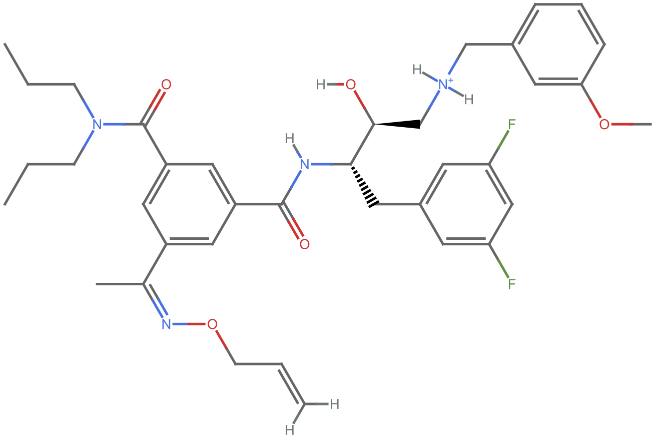

2D Autocorrelation

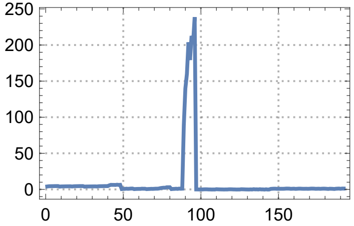

3D Plot

Dataset Index= 392

Molecule Formula= $C_{37}H_{47}F_2N_4O_5^+$

3D Autocorrelation

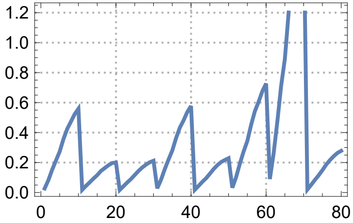

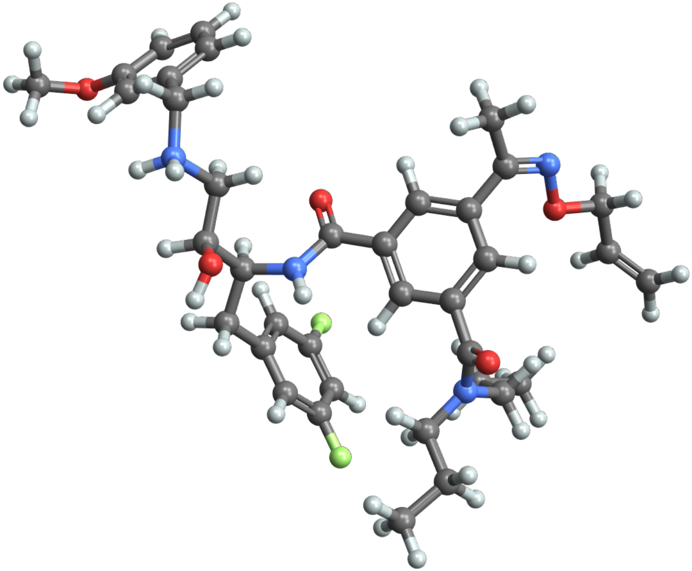

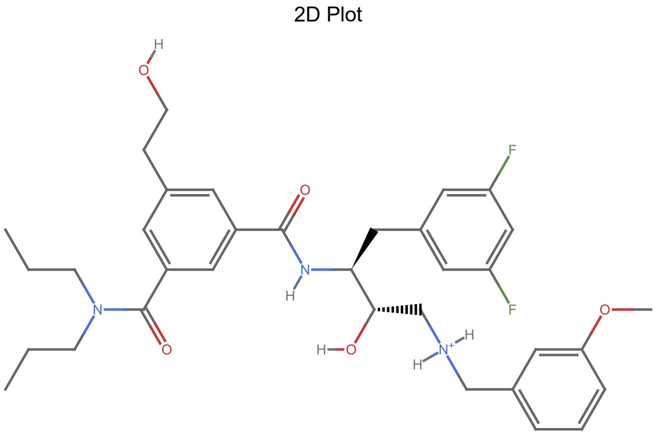

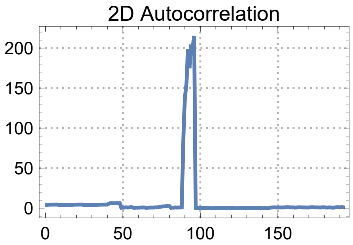

Dataset Index= 393

Molecule Formula= $C_{34}H_{44}F_2N_3O_5^+$

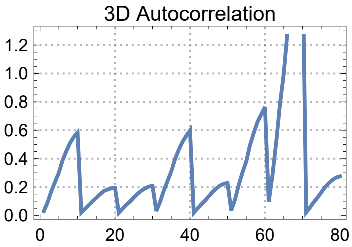

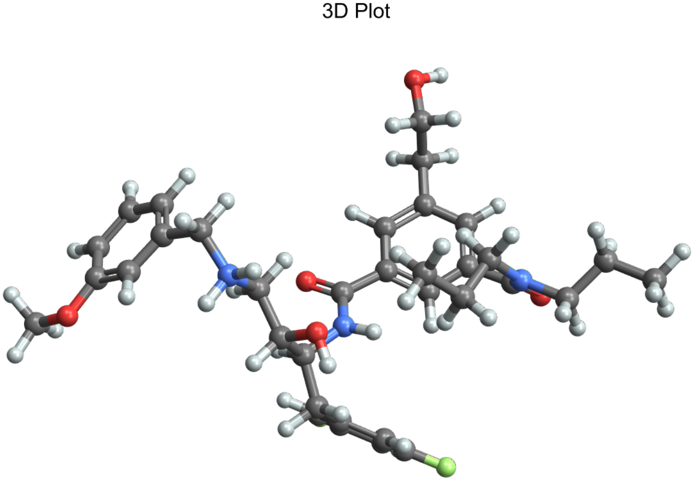

2D Plot

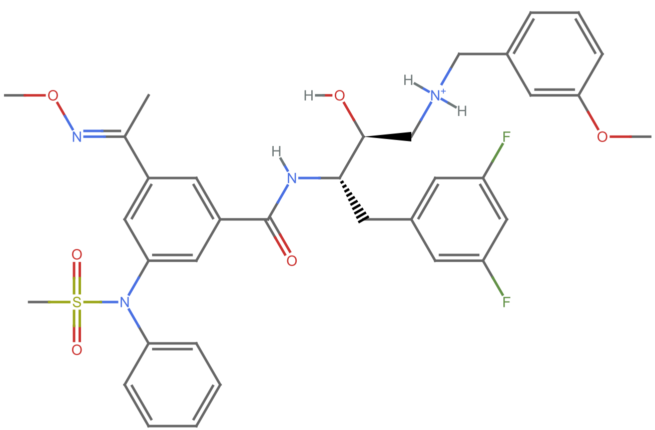

2D Autocorrelation

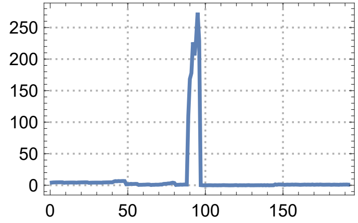

Dataset Index= 394

Molecule Formula= $C_{35}H_{39}F_2N_4O_6S^+$

3D Plot

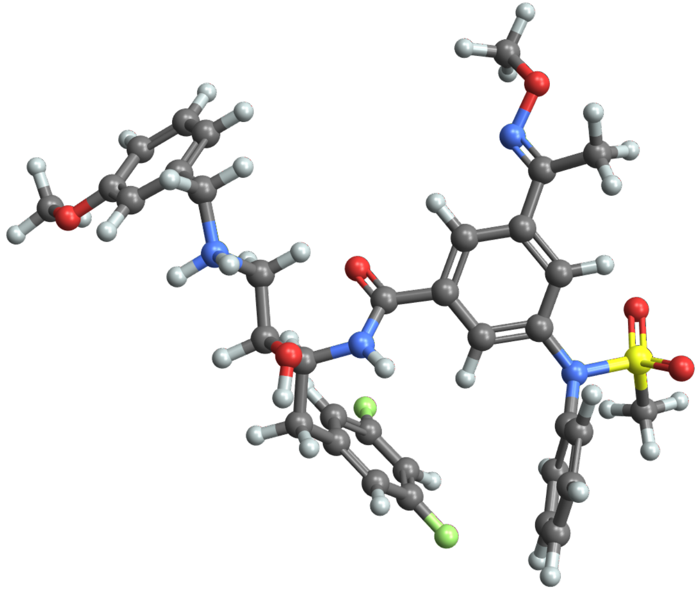

3D Autocorrelation

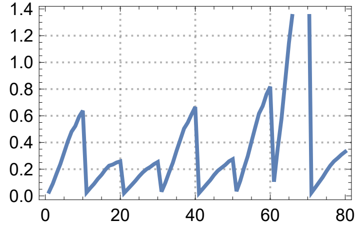

2D Plot

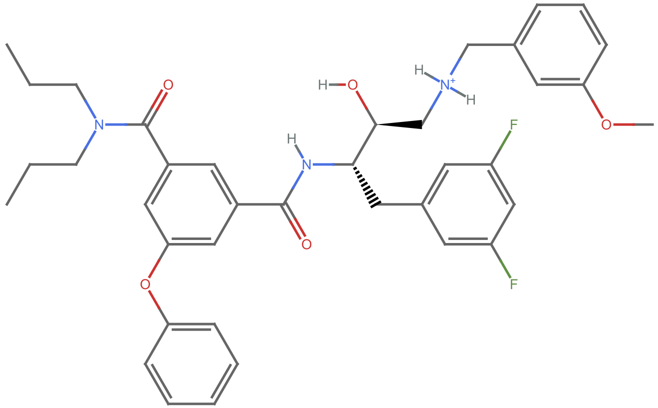

3D Plot

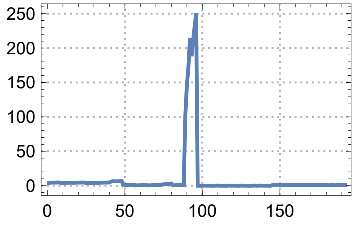

Dataset Index= 395

Molecule Formula= $C_{38}H_{44}F_{2}N_{3}O_{5}^{+}$

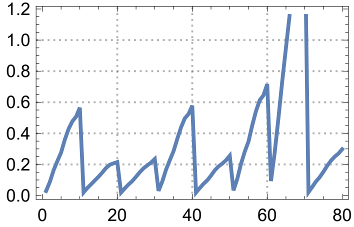

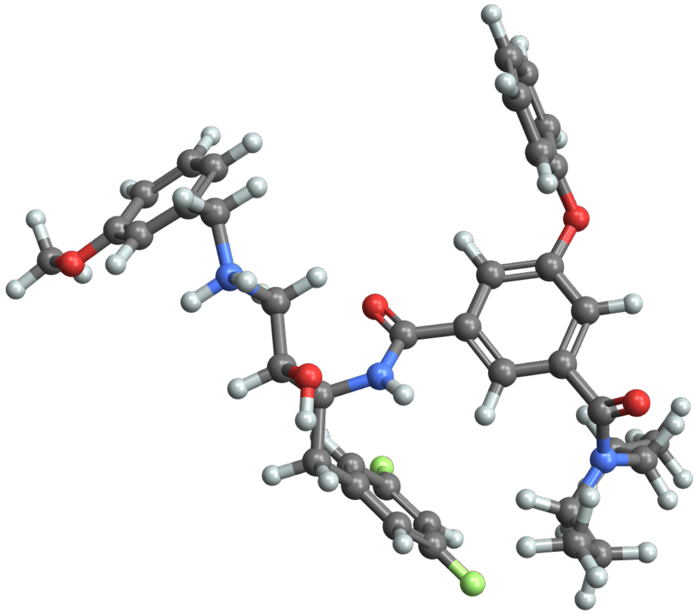

2D Plot

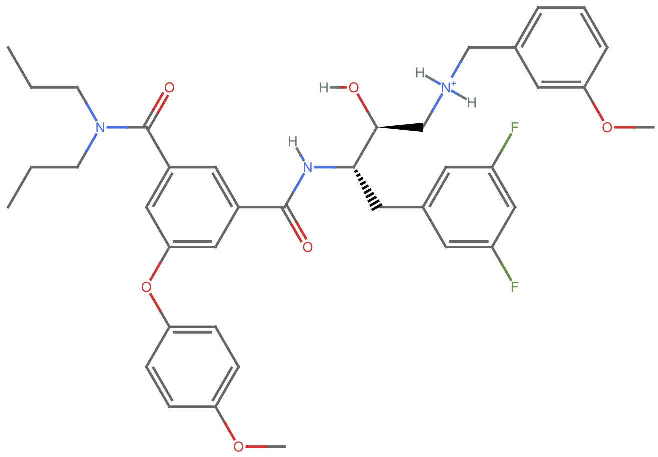

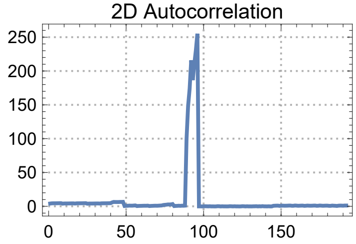

3D Plot

Dataset Index= 396

Molecule Formula= $C_{39}H_{46}F_2N_3O_6^+$

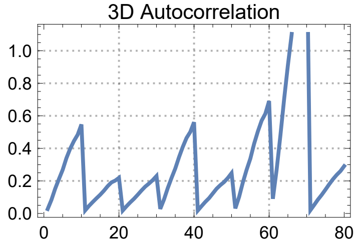

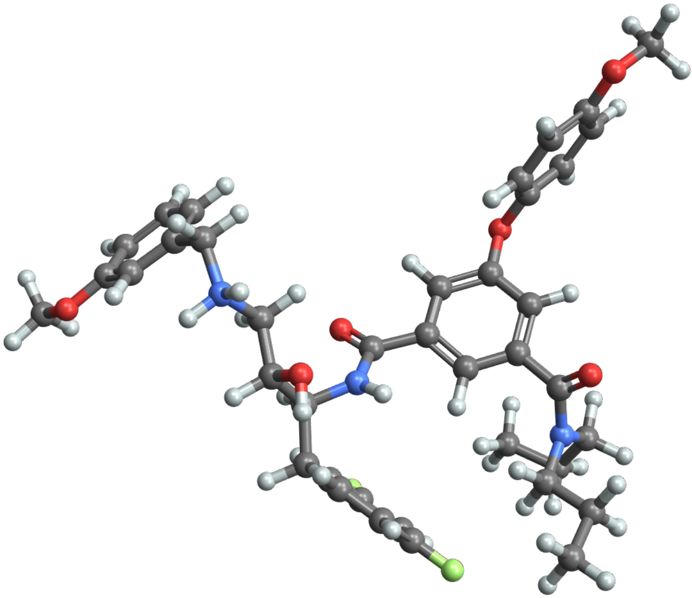

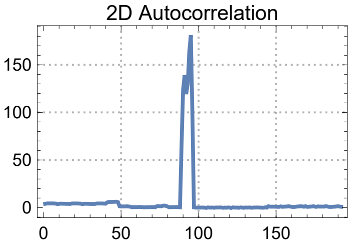

Dataset Index= 397

Molecule Formula= $C_{28}H_{39}N_4O_4S^+$

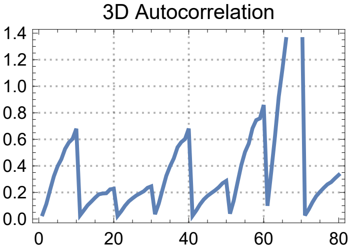

2D Plot

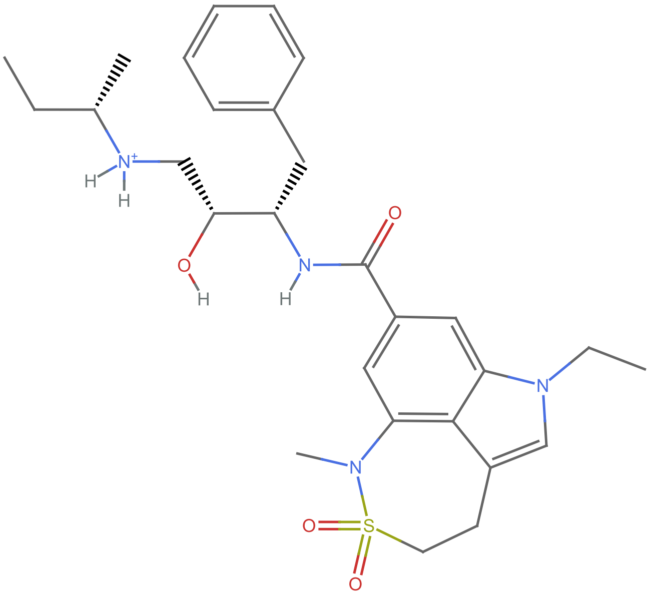

3D Plot

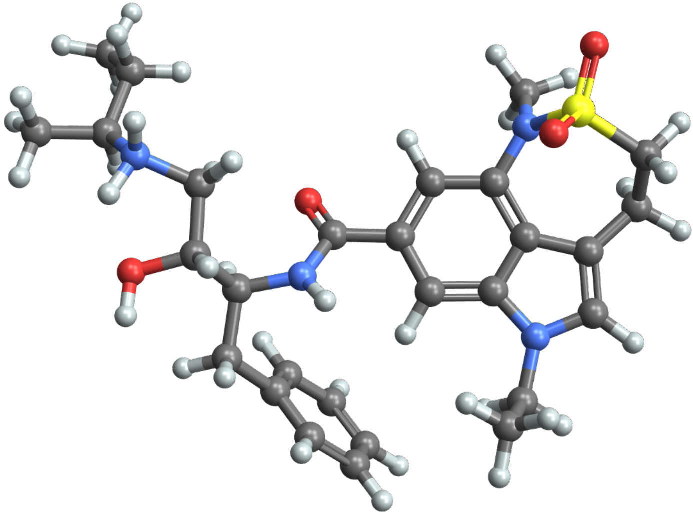

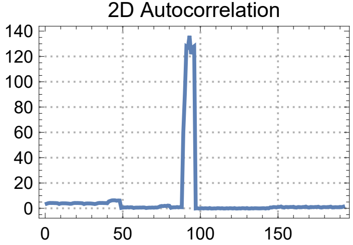

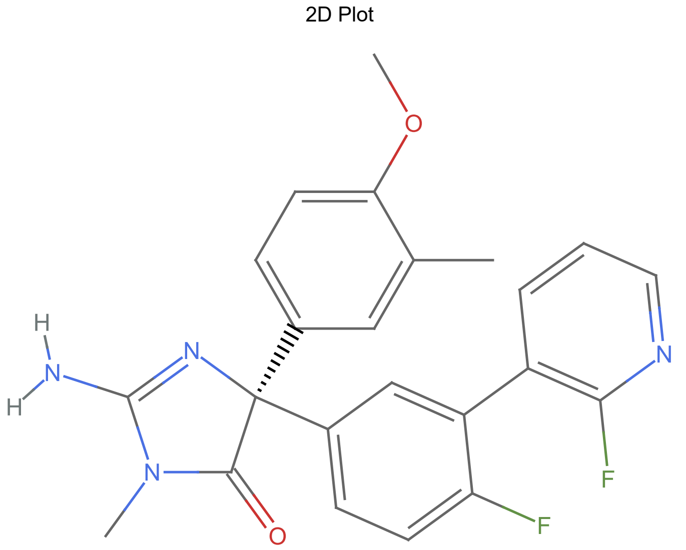

Dataset Index= 398

Molecule Formula= $C_{23}H_{20}F_2N_4O_2$

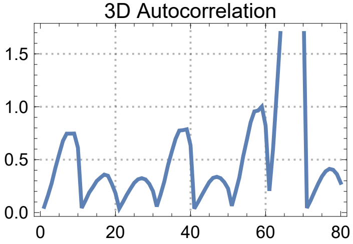

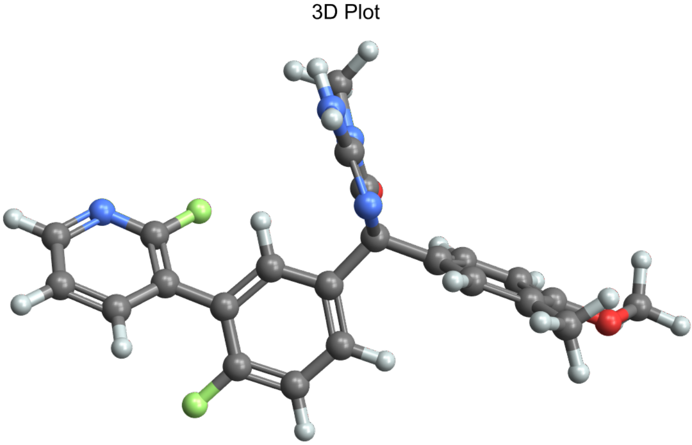

2D Plot

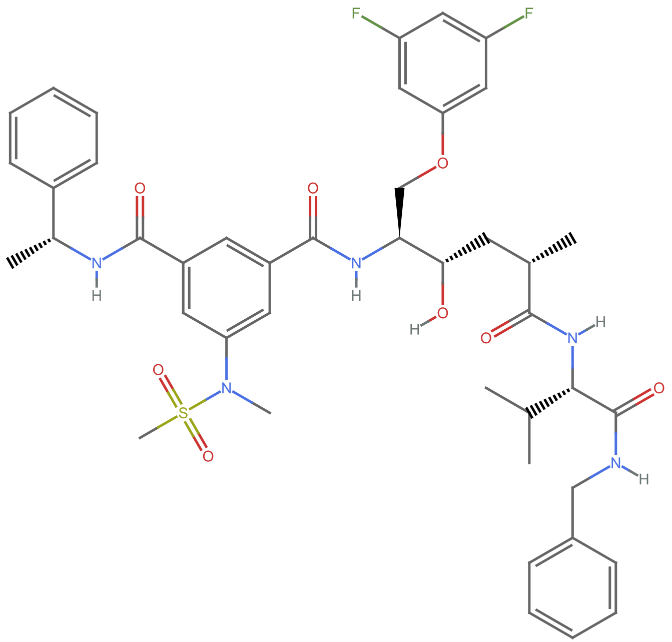

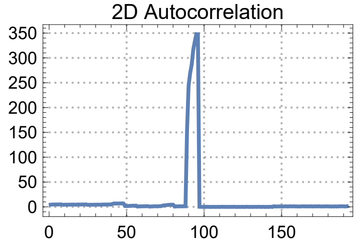

Dataset Index= 399

Molecule Formula= $C_{43}H_{51}F_2N_5O_8S$

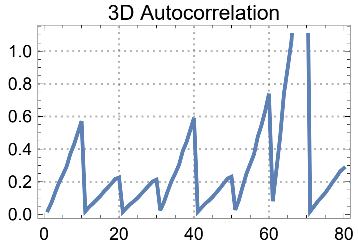

3D Plot

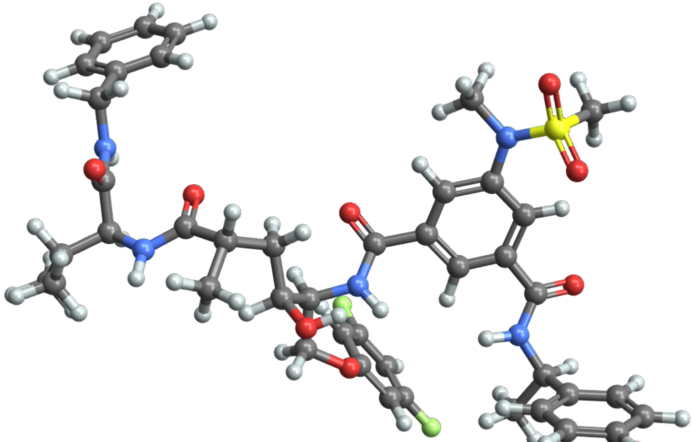

2D Plot

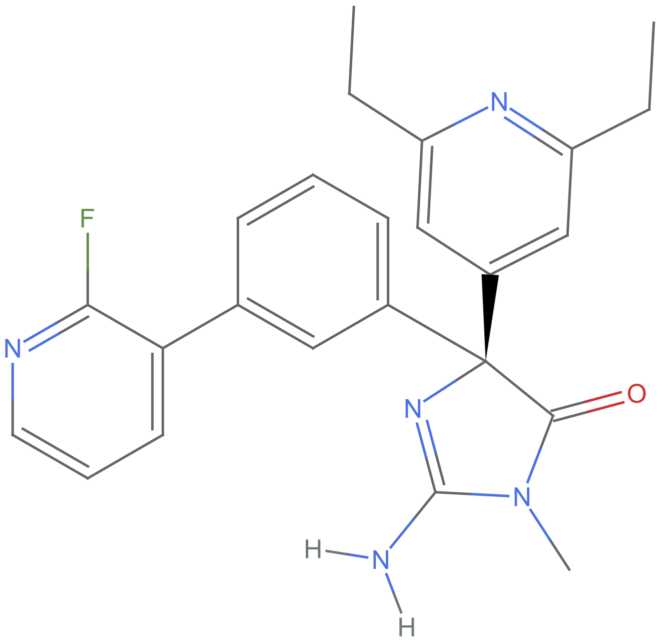

2D Autocorrelation

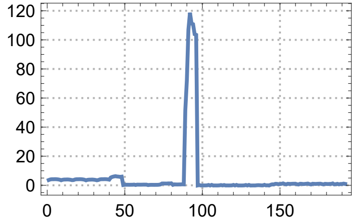

Dataset Index= 400

Molecule Formula= $C_{24}H_{24}FN_5O$

3D Autocorrelation

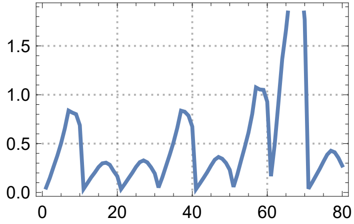

3D Plot

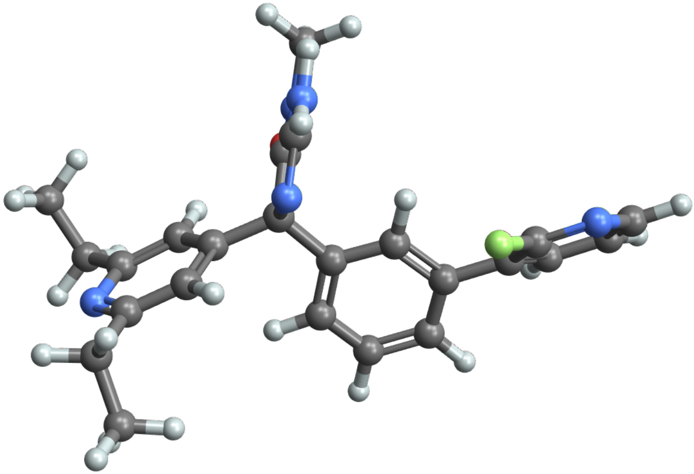

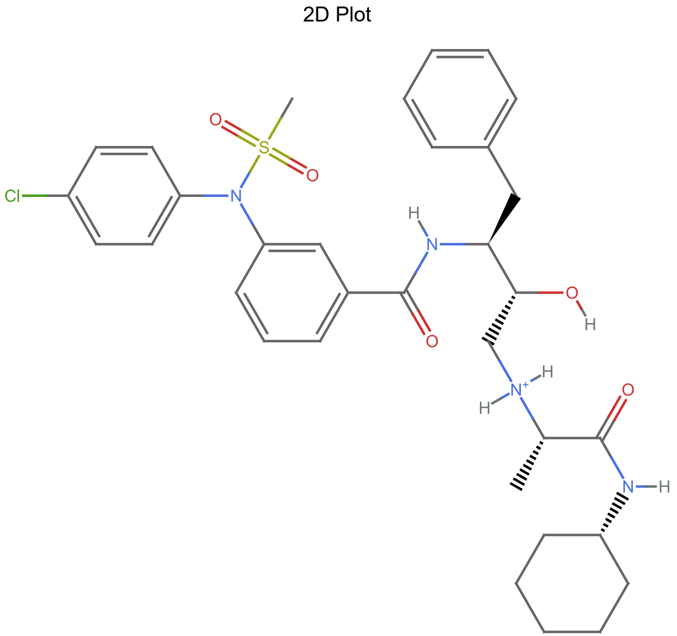

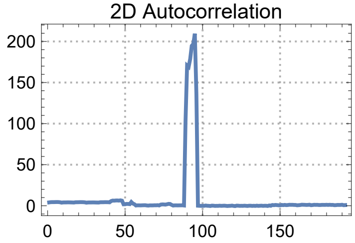

Dataset Index= 401

Molecule Formula= $C_{33}H_{42}ClN_4O_5S^+$

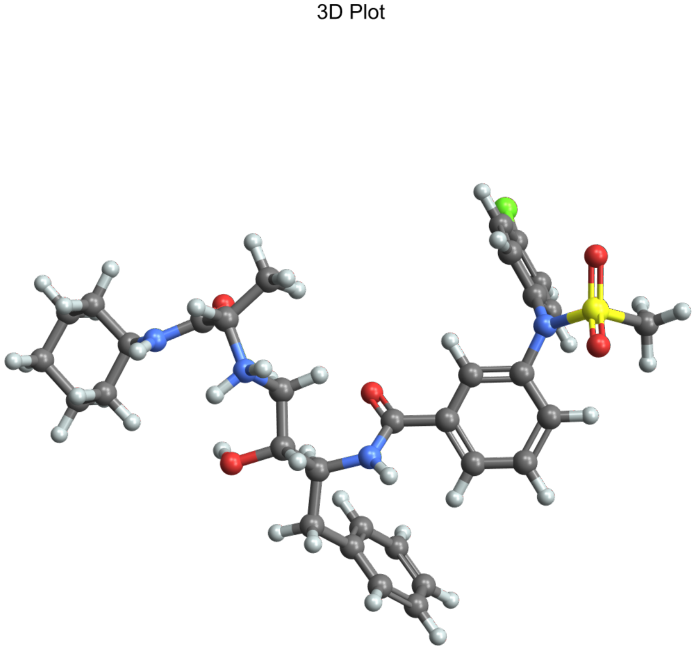

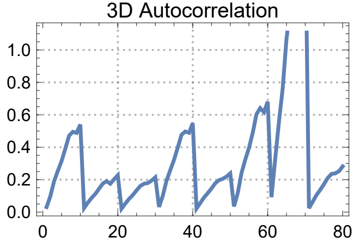

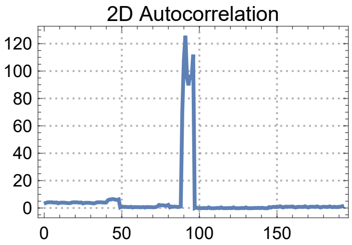

Dataset Index= 402

Molecule Formula= $C_{22}H_{17}F_3N_4O_2$

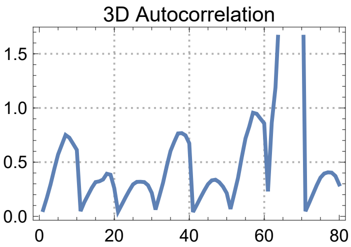

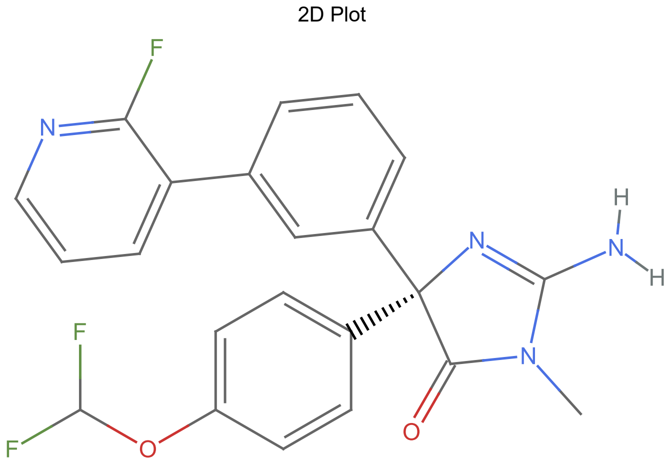

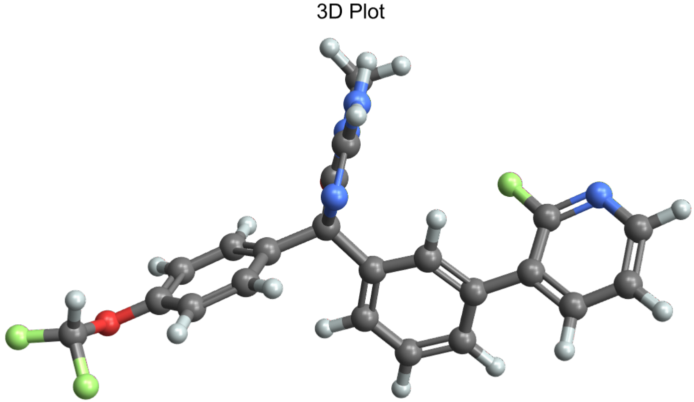

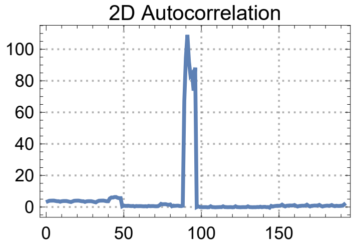

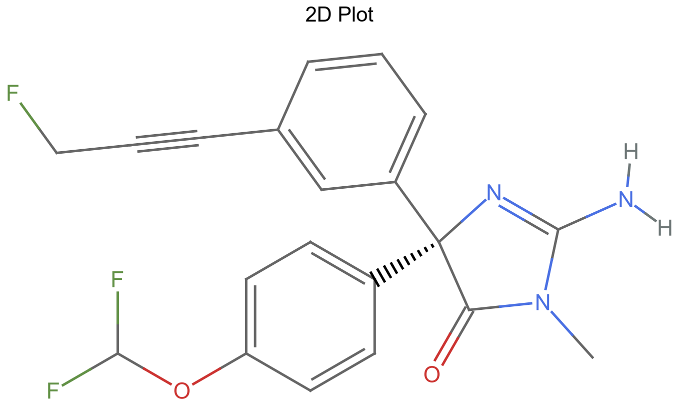

Dataset Index= 403

Molecule Formula= $C_{20}H_{16}F_3N_3O_2$

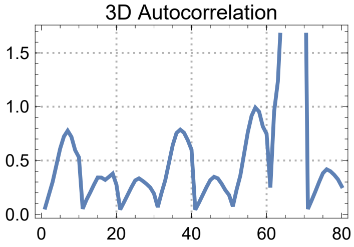

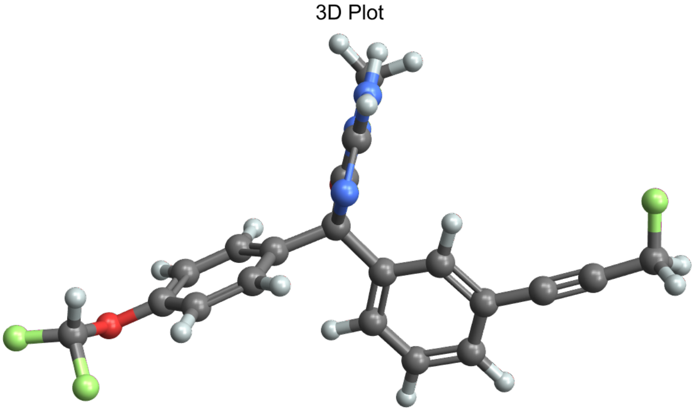

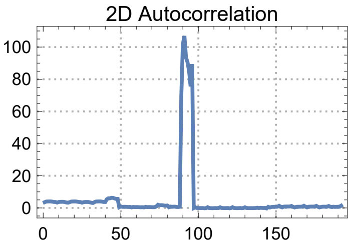

Dataset Index= 404

Molecule Formula= $C_{21}H_{18}F_3N_3O_2$

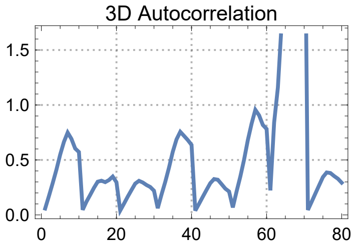

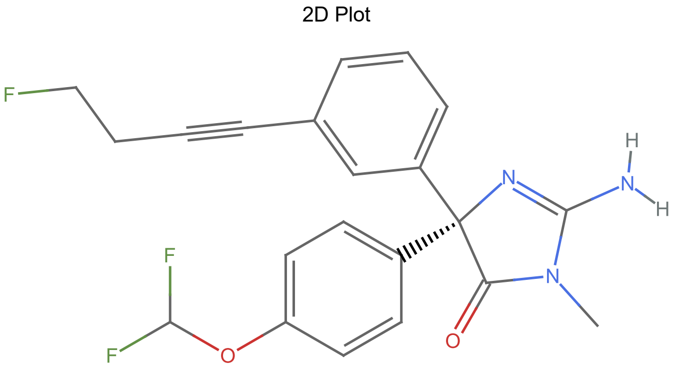

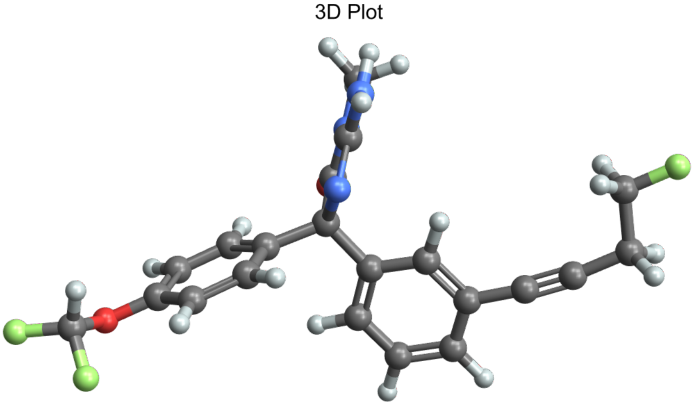

2D Autocorrelation

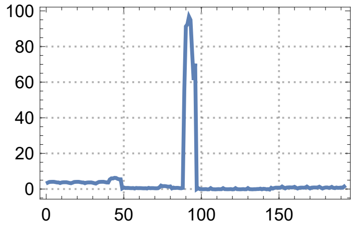

Dataset Index= 405

Molecule Formula= $C_{21}H_{19}F_2N_3O_3$

3D Autocorrelation

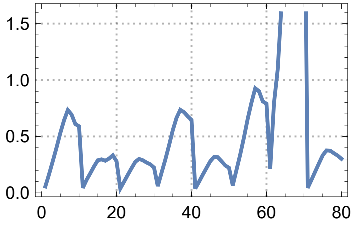

2D Plot

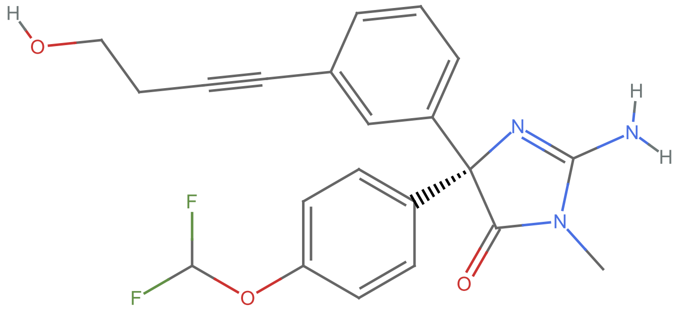

3D Plot

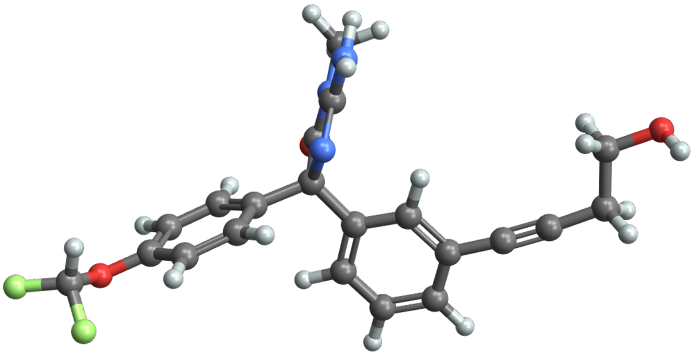

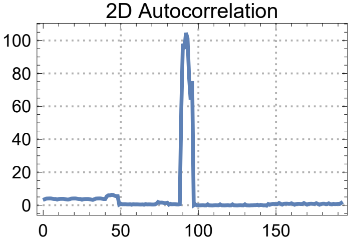

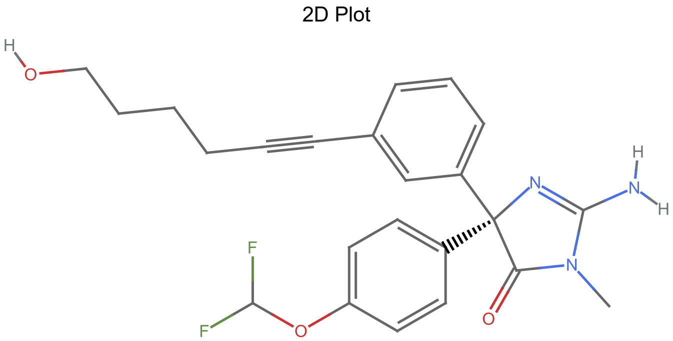

Dataset Index= 406

Molecule Formula= $C_{23}H_{23}F_2N_3O_3$

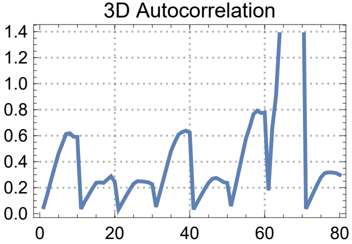

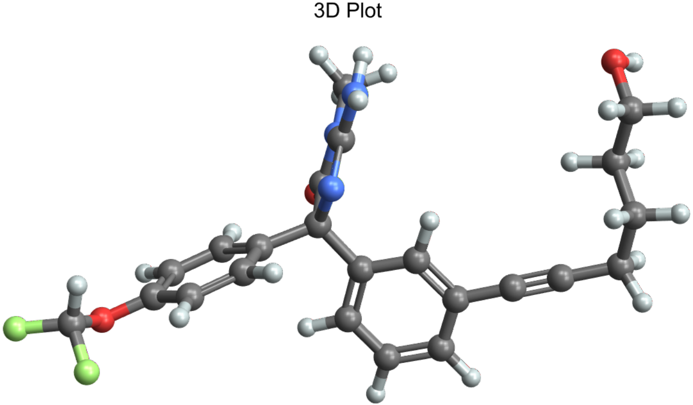

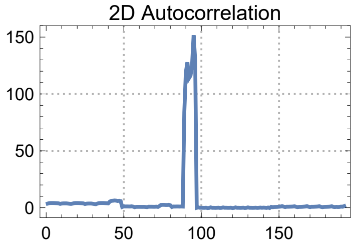

Dataset Index= 407

Molecule Formula= $C_{20}H_{19}F_4N_3O_3$

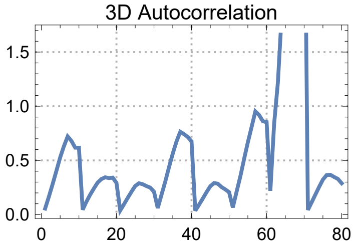

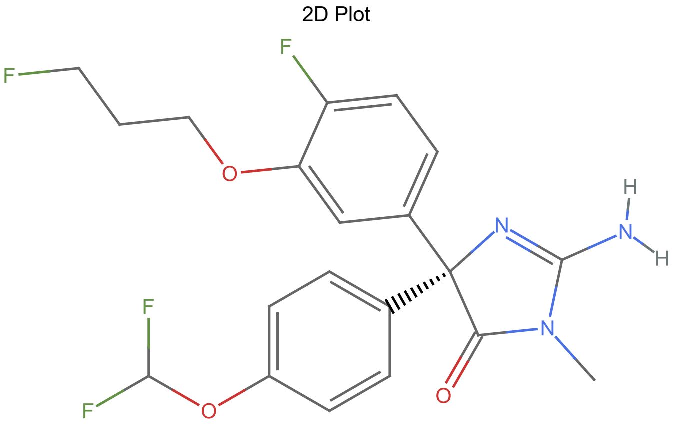

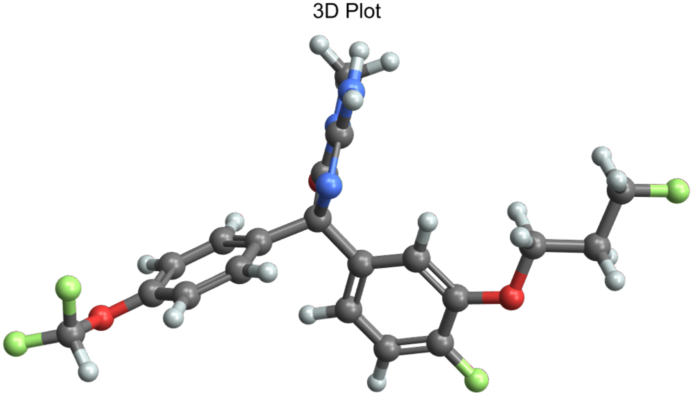

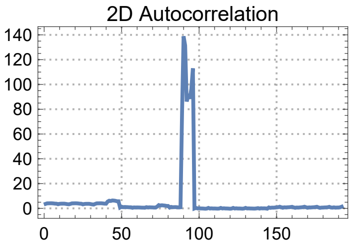

Dataset Index= 408

Molecule Formula= $C_{20}H_{19}F_4N_3O_3$

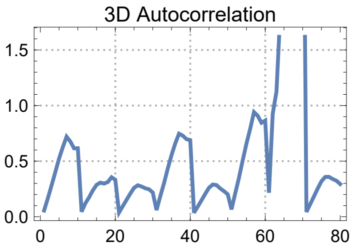

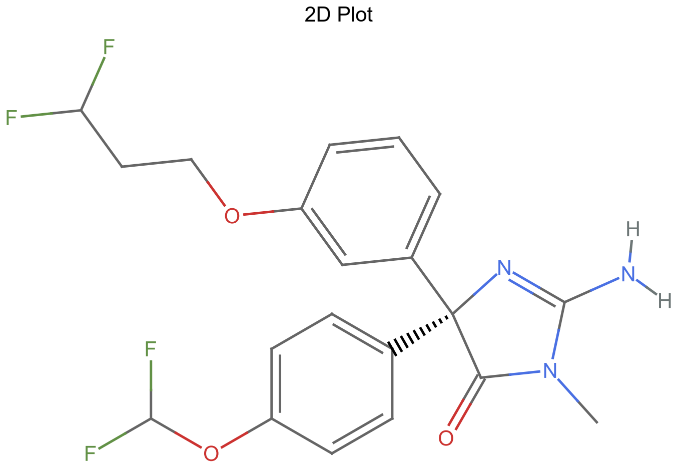

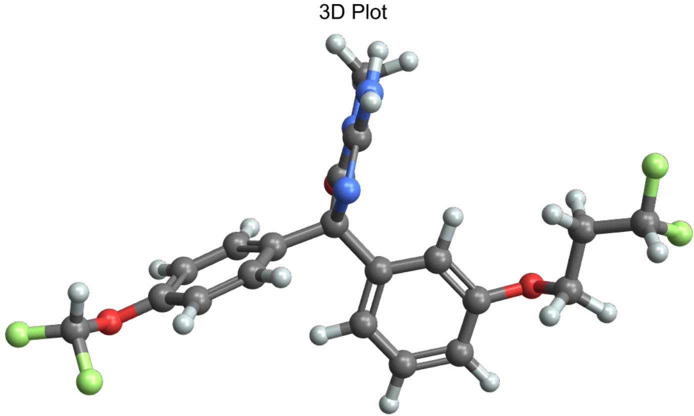

2D Plot

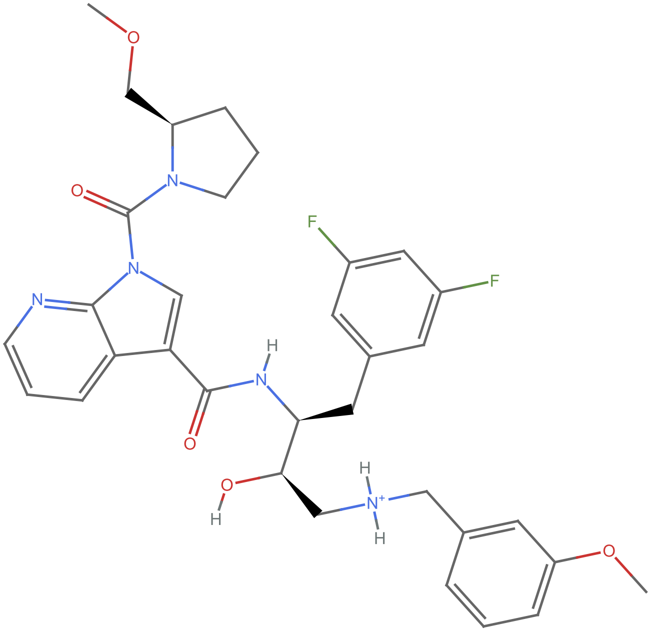

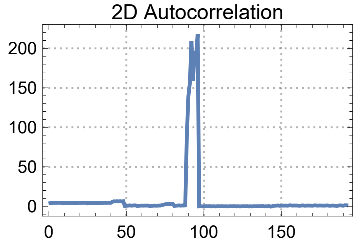

Dataset Index= 409

Molecule Formula= $C_{33}H_{38}F_2N_5O_5^+$

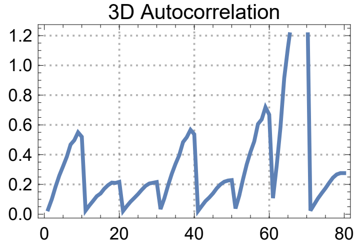

3D Plot

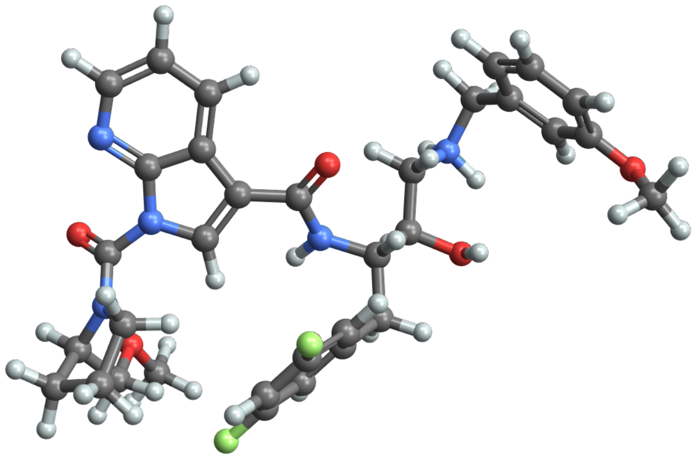

2D Plot

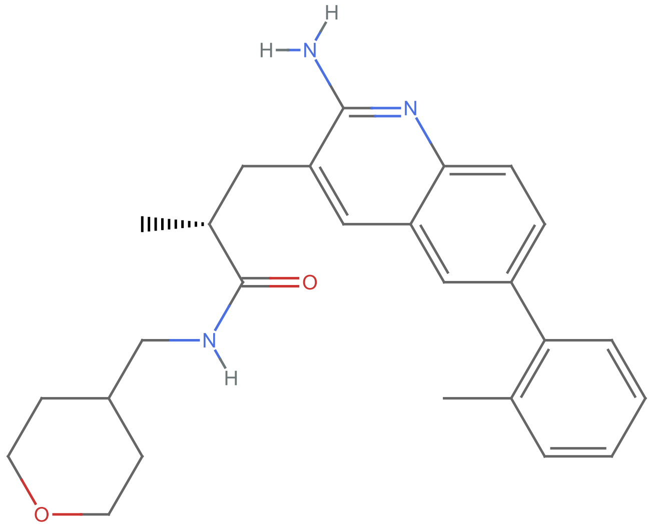

2D Autocorrelation

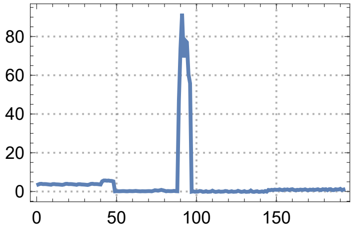

3D Plot

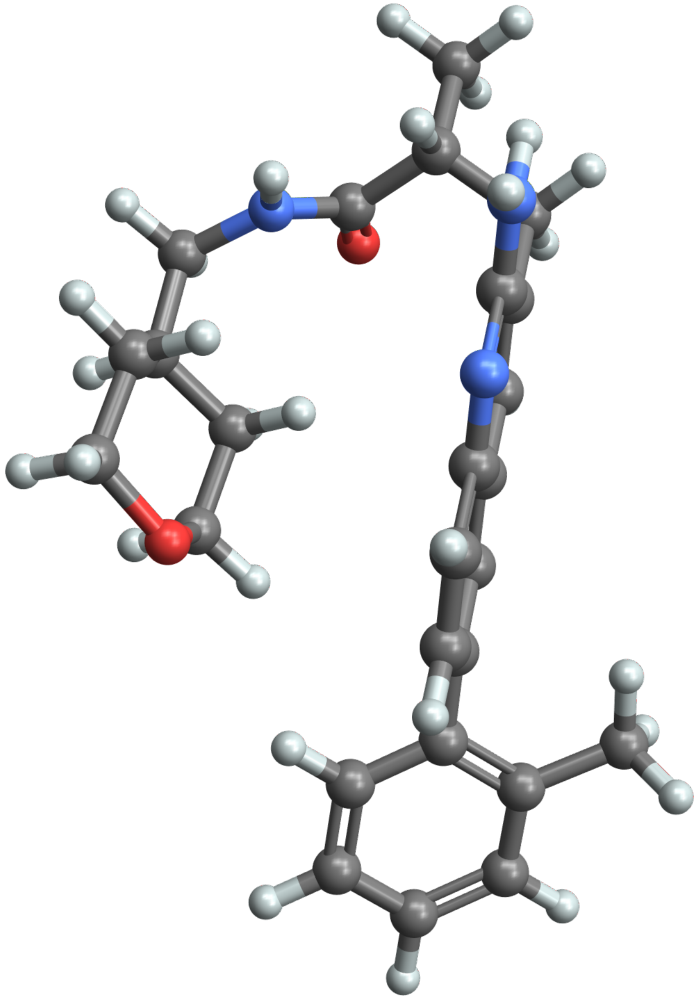

Dataset Index= 410

Molecule Formula= $C_{26}H_{31}N_{3}O_{2}$

3D Autocorrelation

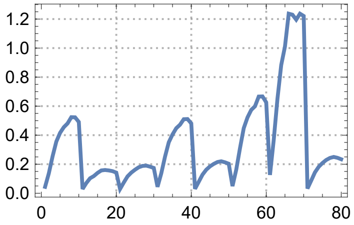

2D Plot

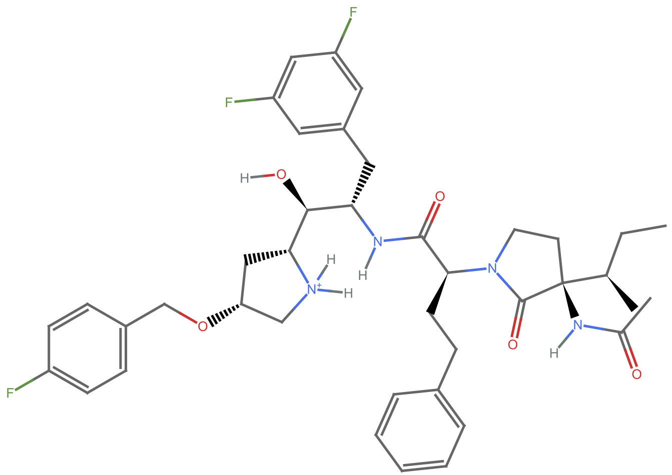

3D Plot

2D Autocorrelation

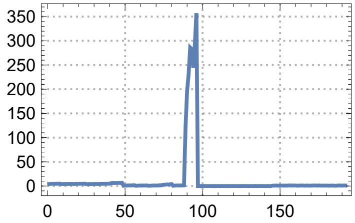

Dataset Index= 411

Molecule Formula= $C_{40}H_{50}F_3N_4O_5^+$

3D Autocorrelation

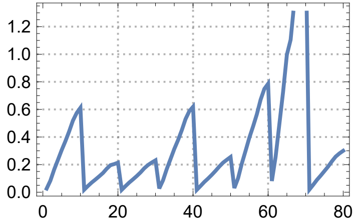

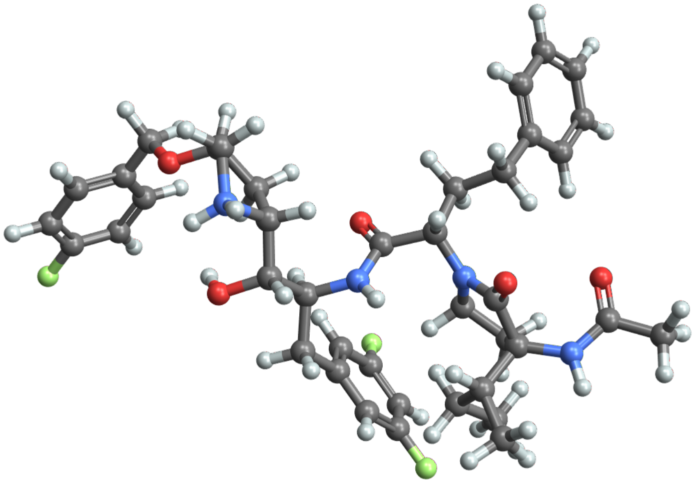

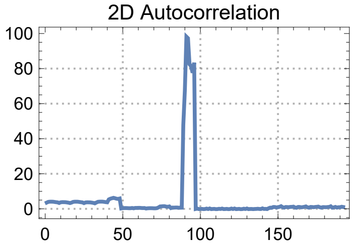

Dataset Index= 412

Molecule Formula= $C_{20}H_{19}FN_6O$

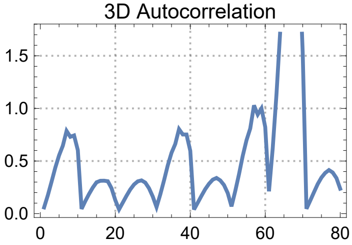

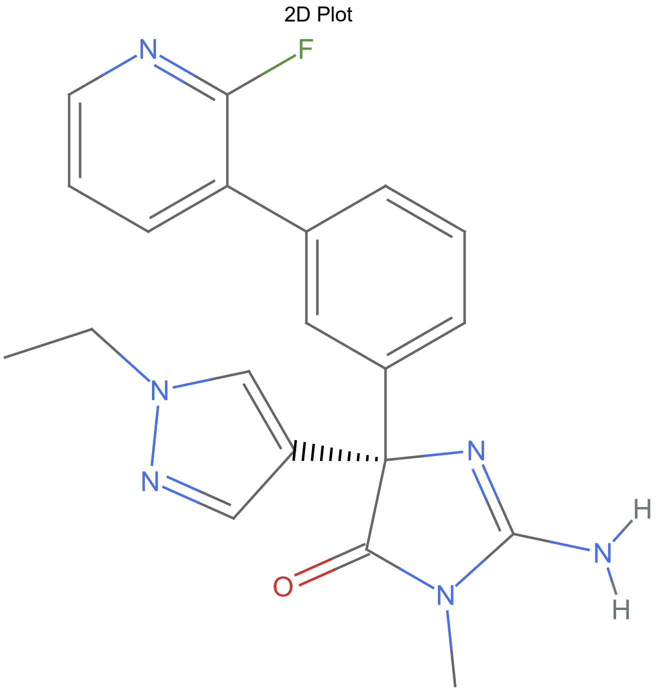

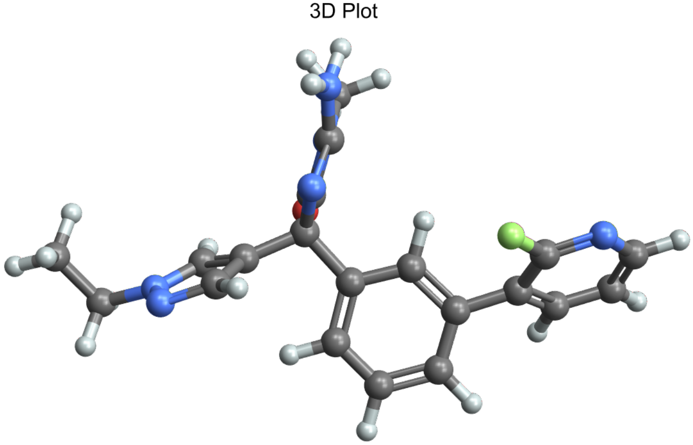

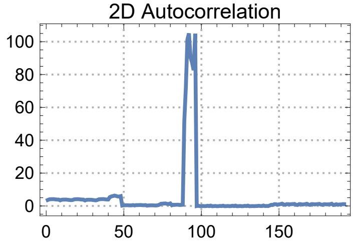

Dataset Index= 413

Molecule Formula= $C_{22}H_{23}FN_6O$

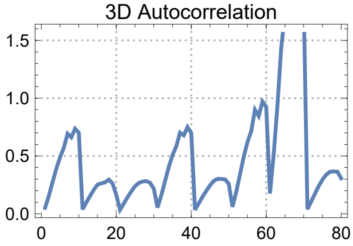

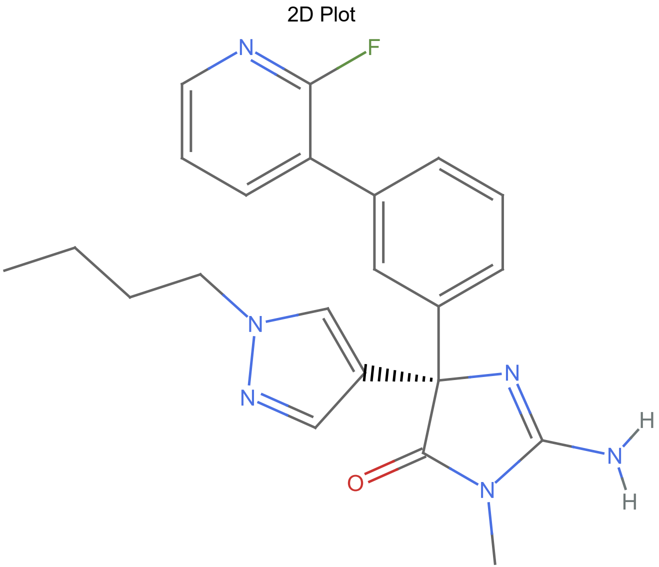

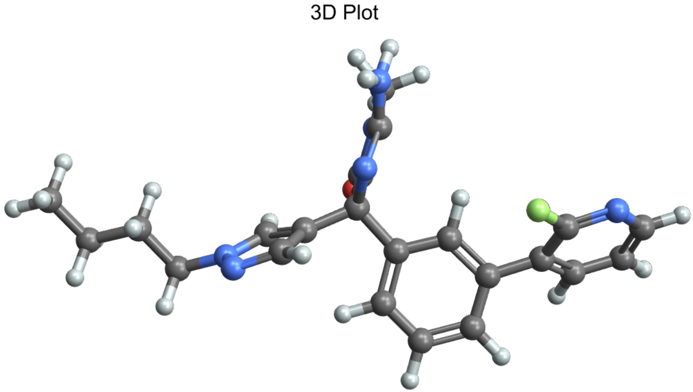

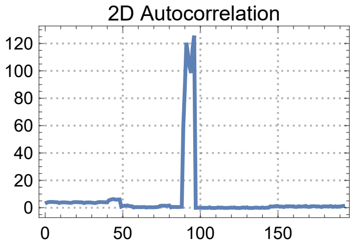

Dataset Index= 414

Molecule Formula= $C_{22}H_{19}FN_4O_2S$

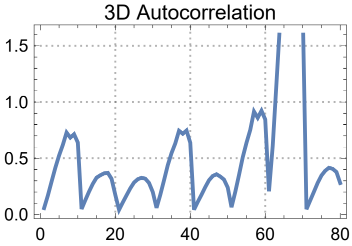

2D Plot

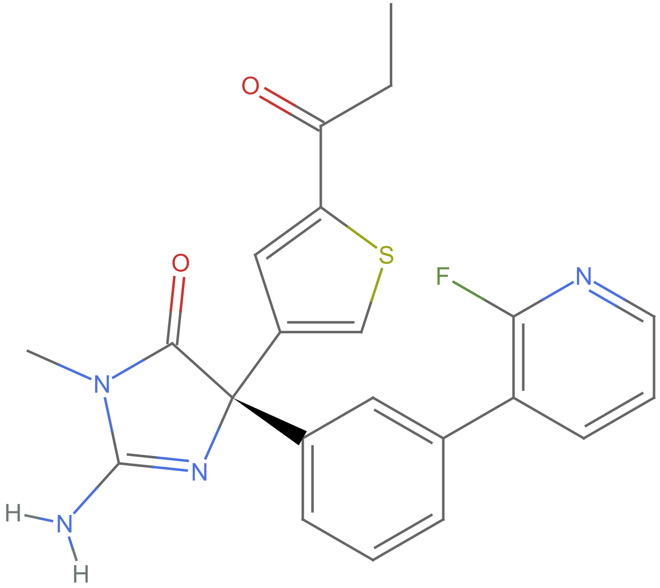

3D Plot

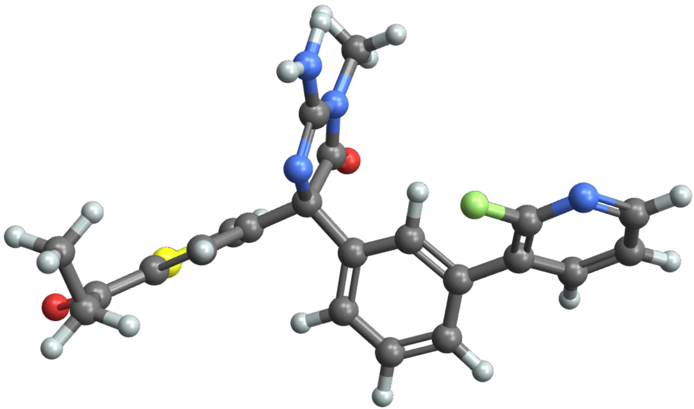

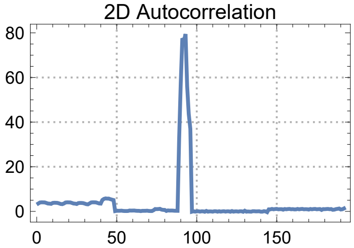

Dataset Index= 415

Molecule Formula= $C_{22}H_{22}N_4O_2$

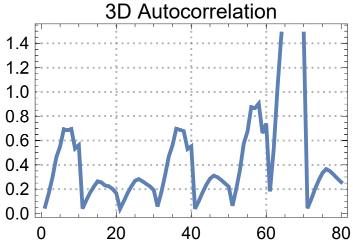

2D Plot

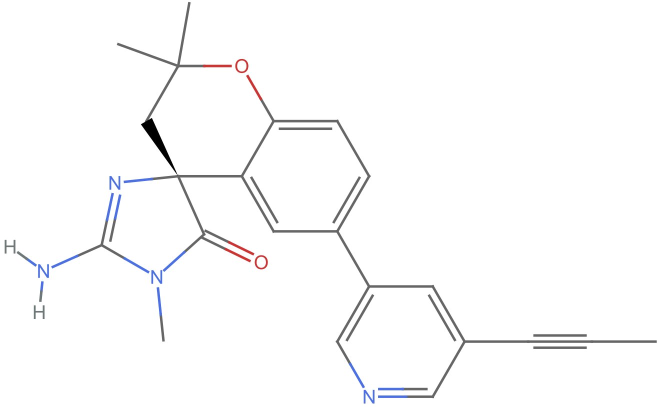

3D Plot

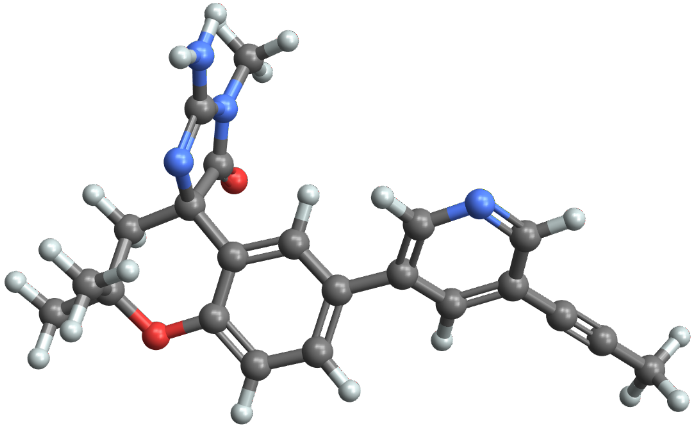

2D Autocorrelation

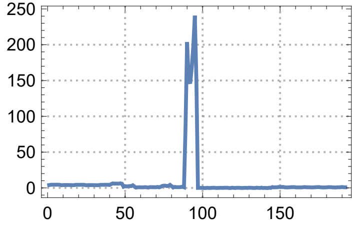

Dataset Index= 416

Molecule Formula= $C_{28}H_{39}F_4N_2O_4S^+$

3D Autocorrelation

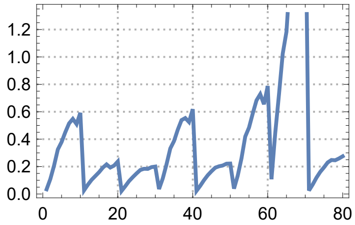

2D Plot

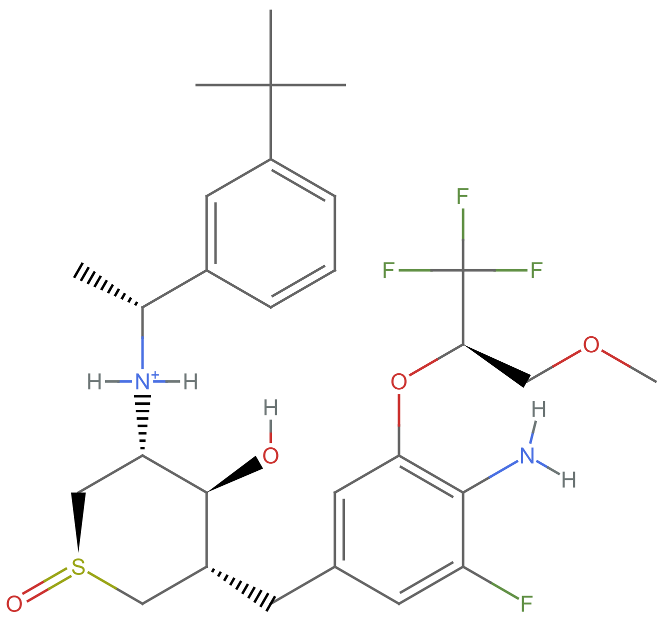

3D Plot

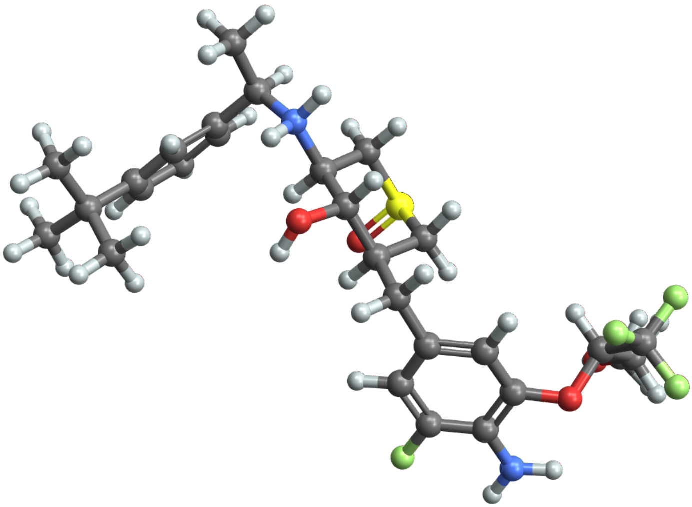

2D Autocorrelation

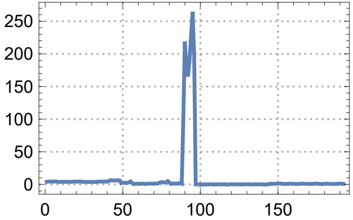

2D Plot

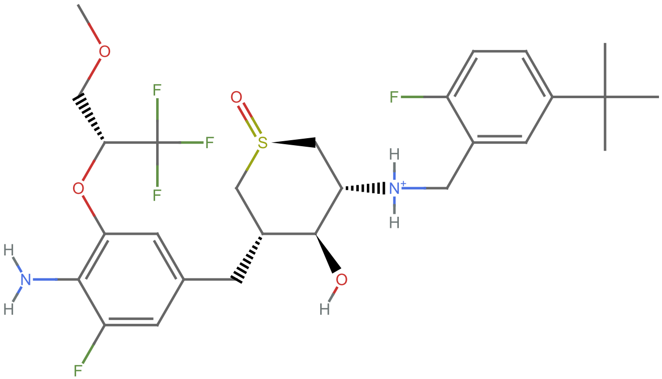

Dataset Index= 417

Molecule Formula= $C_{27}H_{36}F_{5}N_{2}O_{4}S^{+}$

3D Autocorrelation

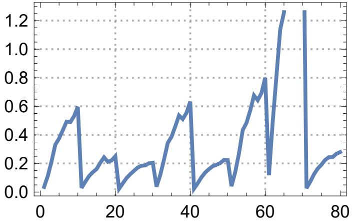

3D Plot

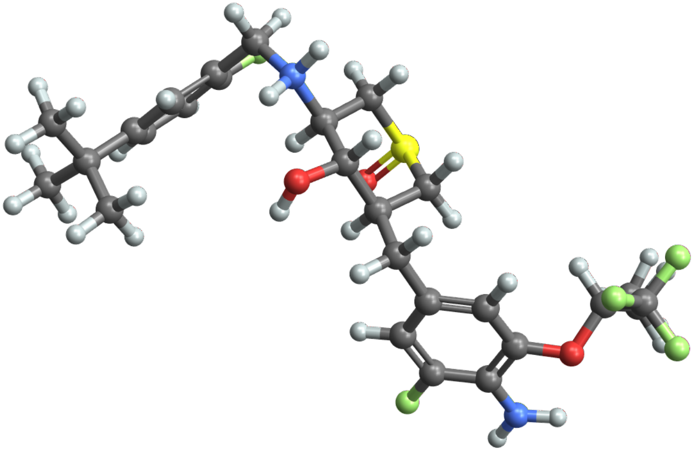

2D Plot

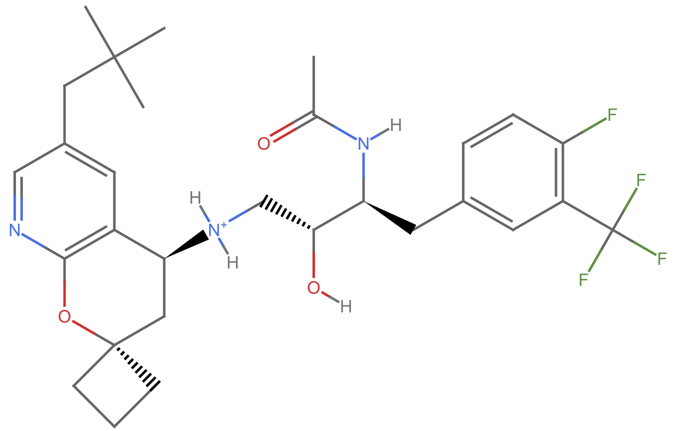

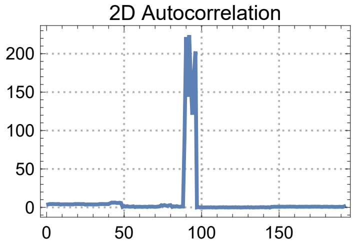

3D Plot

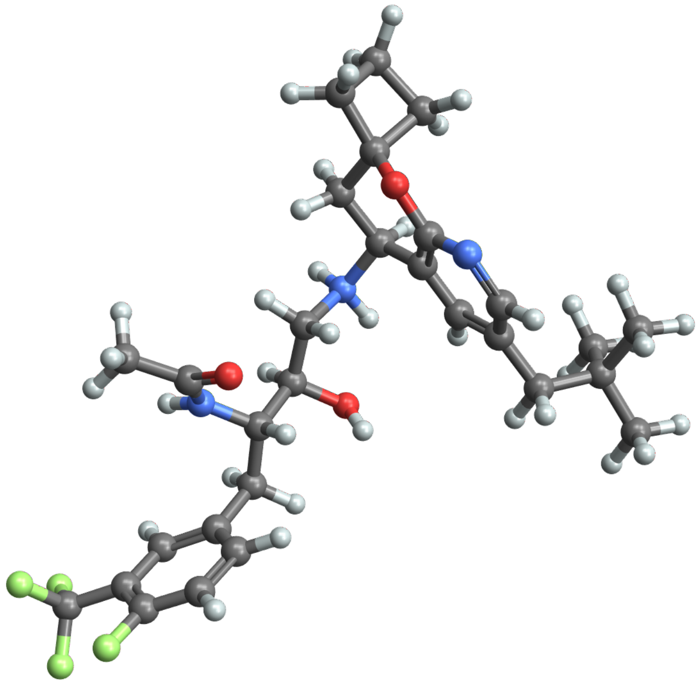

Dataset Index= 418

Molecule Formula= $C_{29}H_{38}F_4N_3O_3^+$

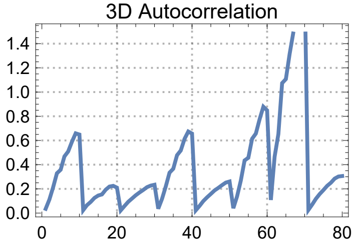

2D Plot

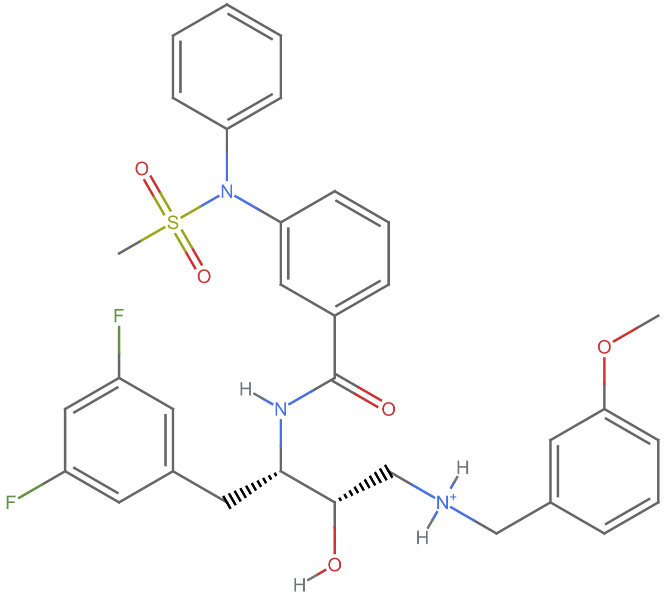

2D Autocorrelation

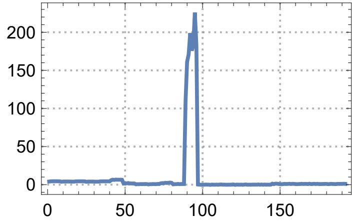

Dataset Index= 419

Molecule Formula= $C_{32}H_{34}F_2N_3O_5S^+$

3D Autocorrelation

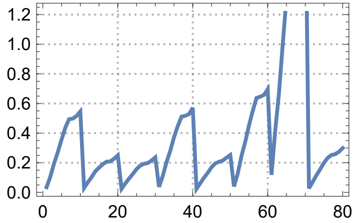

3D Plot

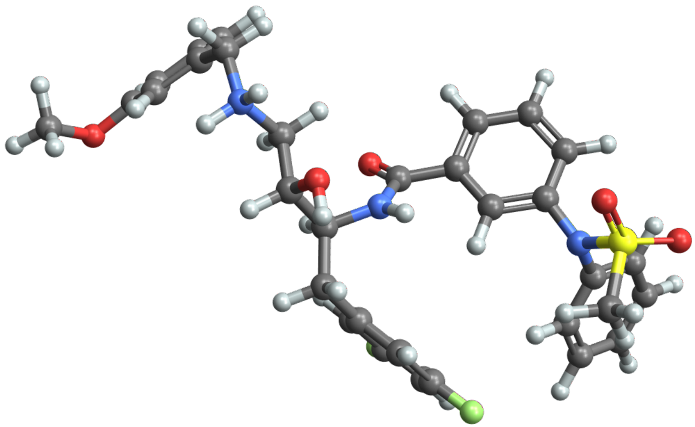

2D Plot

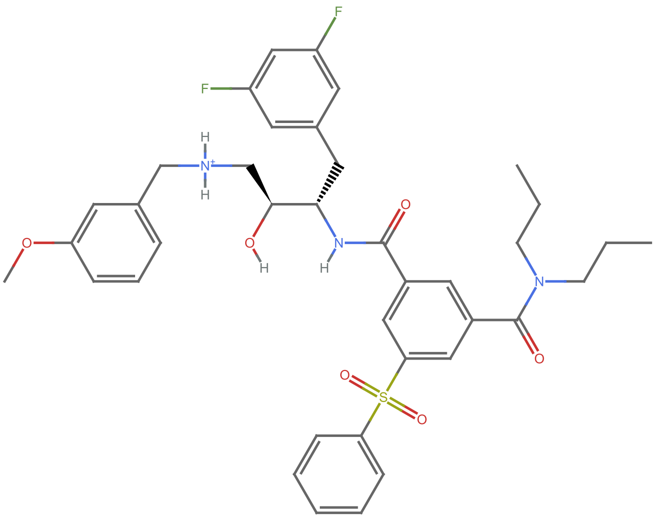

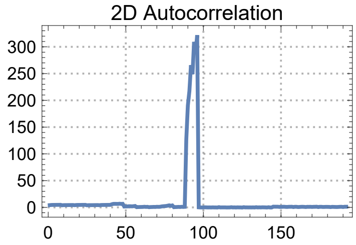

3D Plot

Dataset Index= 420

Molecule Formula= $C_{38}H_{44}F_2N_3O_6S^+$

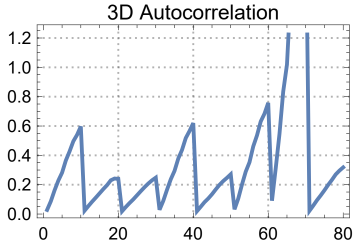

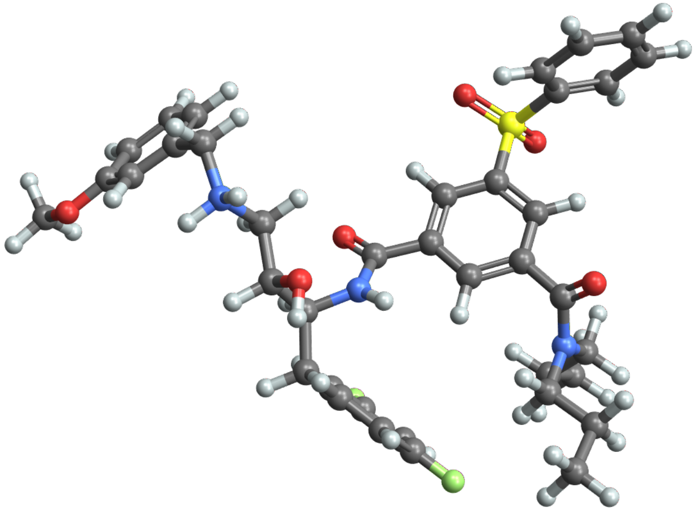

2D Plot

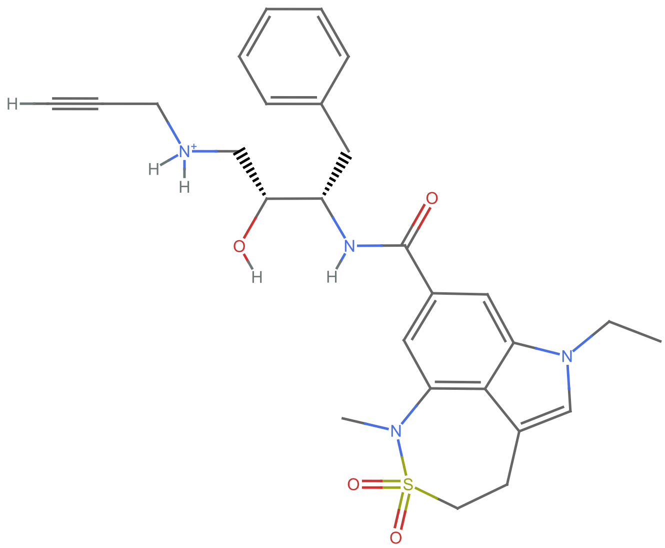

2D Autocorrelation

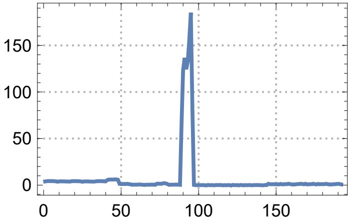

Dataset Index= 421

Molecule Formula= $C_{27}H_{33}N_4O_4S^+$

3D Autocorrelation

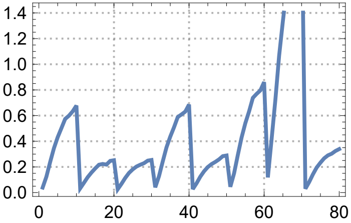

3D Plot

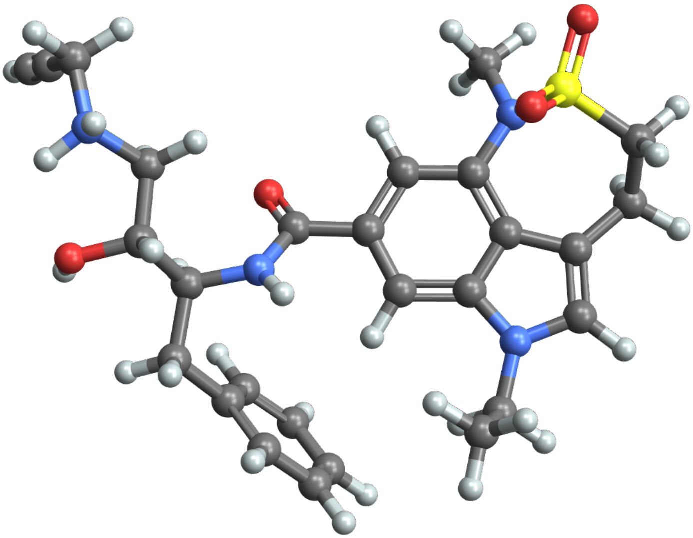

2D Plot

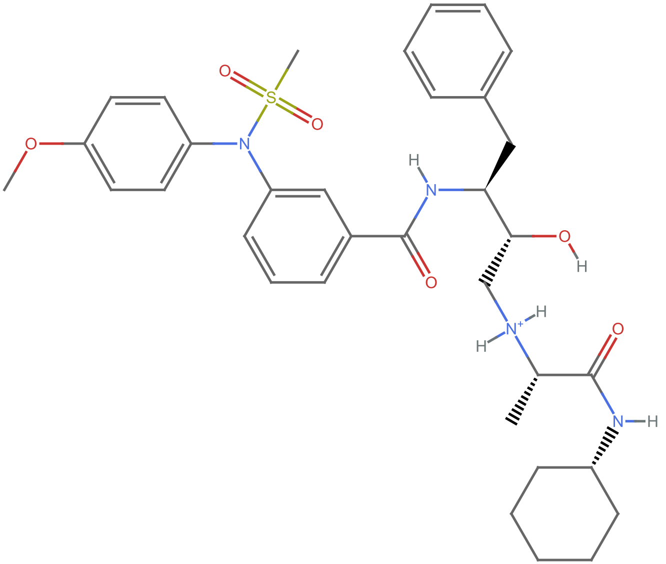

2D Autocorrelation

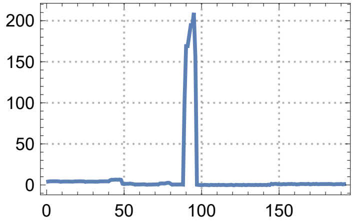

Dataset Index= 422

Molecule Formula= $C_{34}H_{45}N_4O_6S^+$

3D Autocorrelation

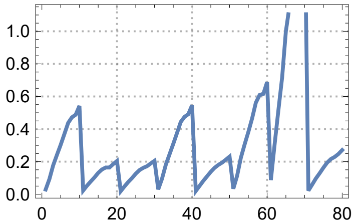

3D Plot

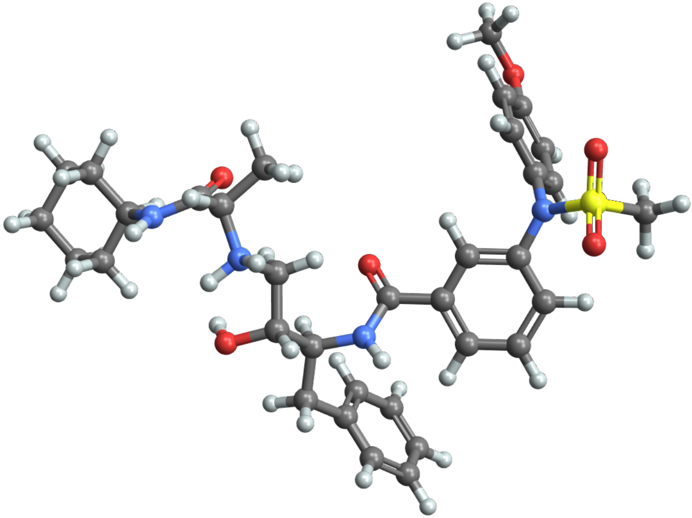

2D Plot

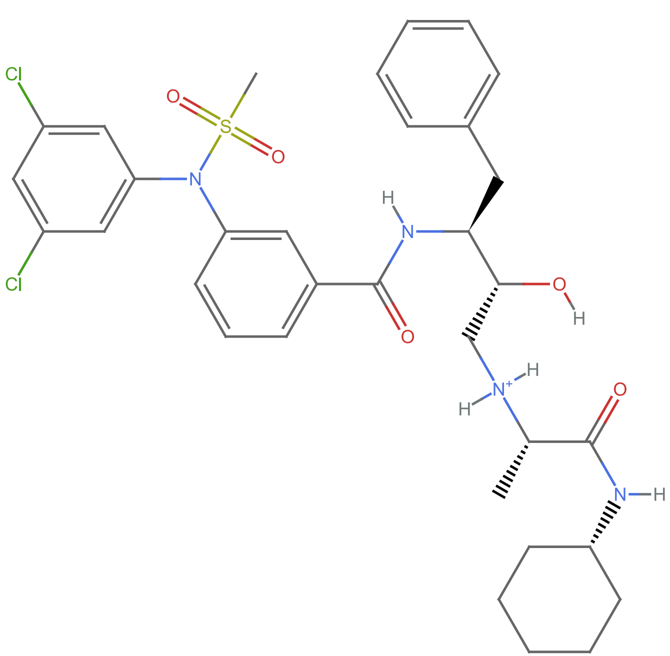

2D Autocorrelation

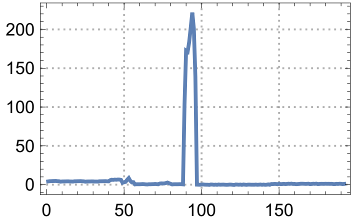

Dataset Index= 423

Molecule Formula= $C_{33}H_{41}Cl_2N_4O_5S^+$

3D Autocorrelation

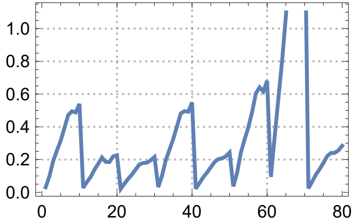

3D Plot

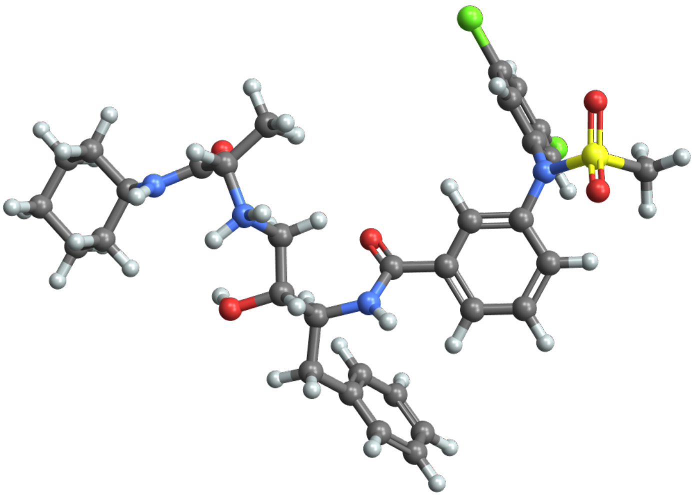

2D Plot

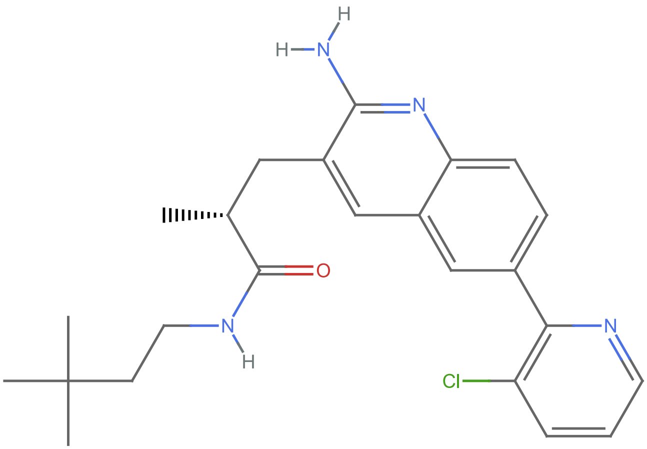

3D Plot

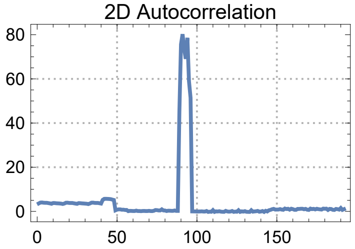

Dataset Index= 424

Molecule Formula= $C_{24}H_{29}ClN_4O$

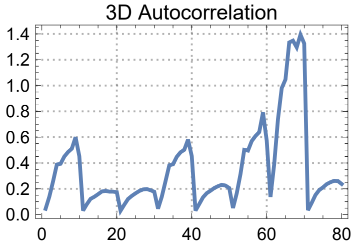

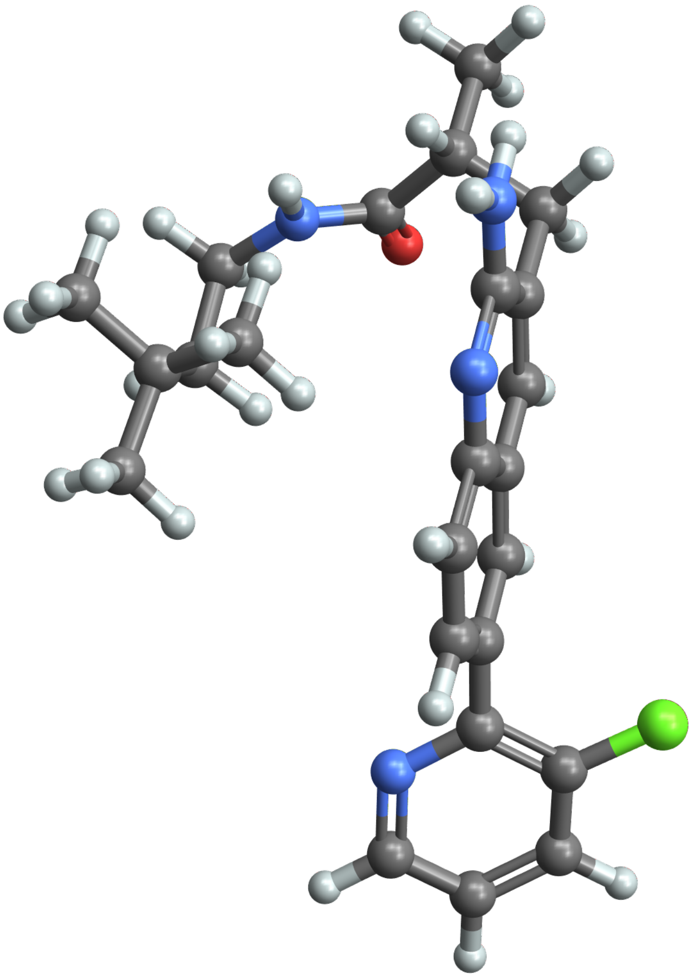

2D Plot

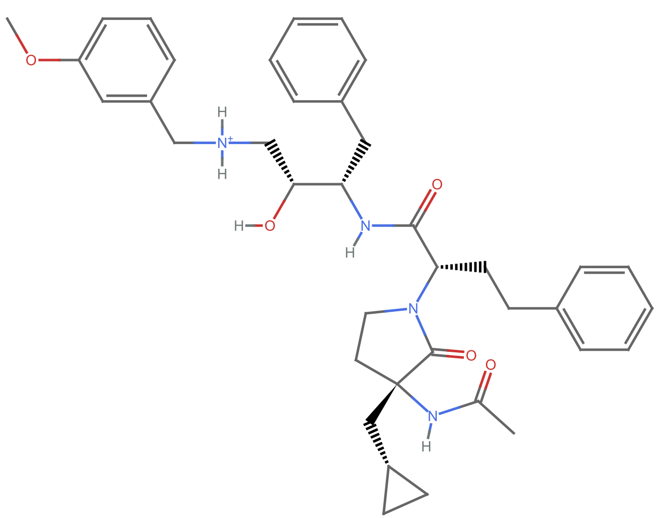

2D Autocorrelation

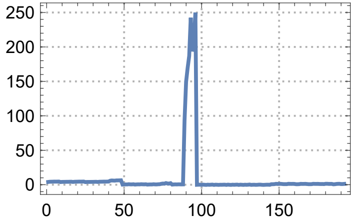

3D Plot

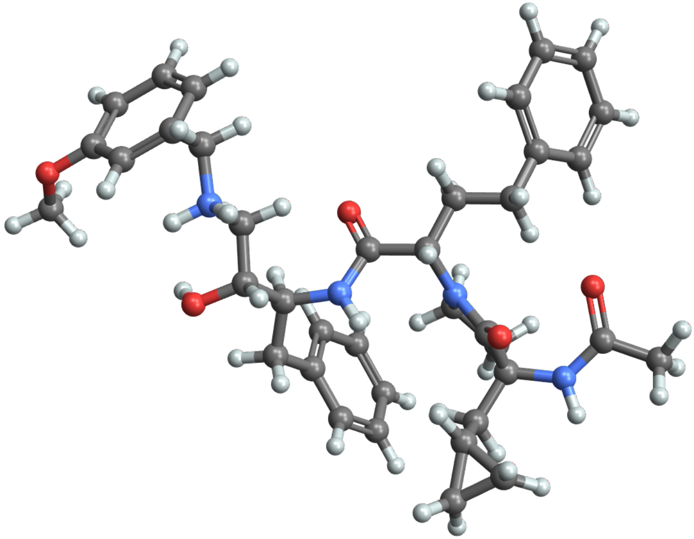

Dataset Index= 425

Molecule Formula= $C_{38}H_{49}N_4O_5{}^+$

3D Autocorrelation

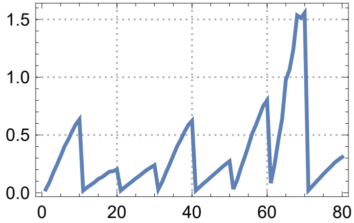

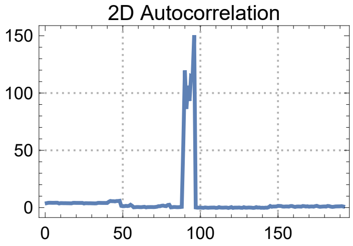

Dataset Index= 426

Molecule Formula= $C_{27}H_{38}FN_2O_4S^+$

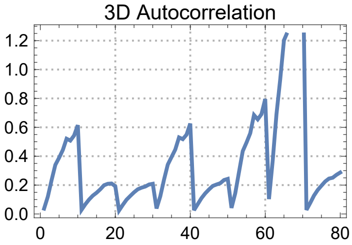

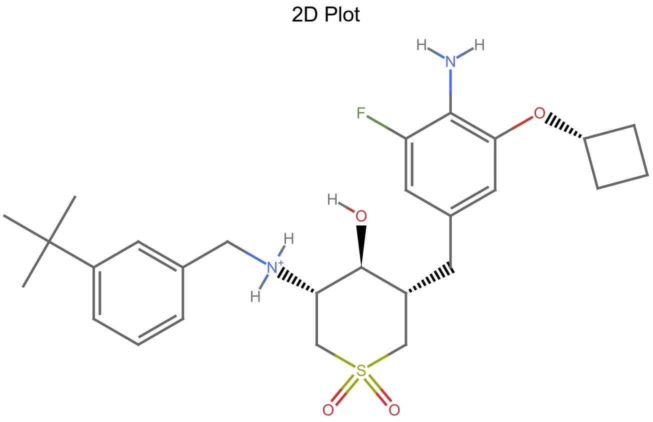

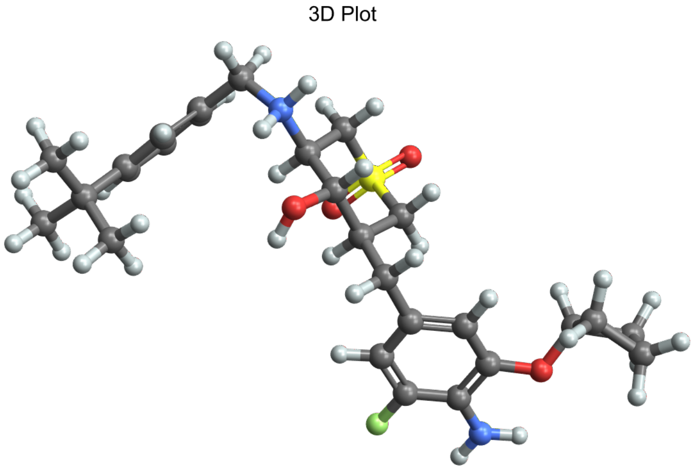

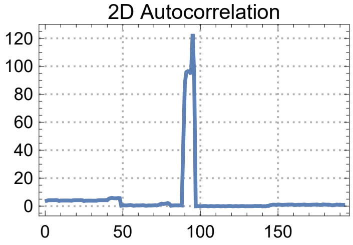

Dataset Index= 427

Molecule Formula= $C_{31}H_{41}FN_3O_4^+$

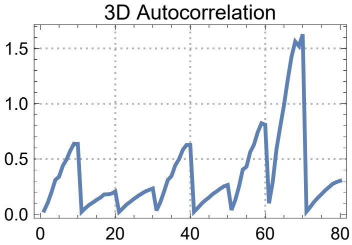

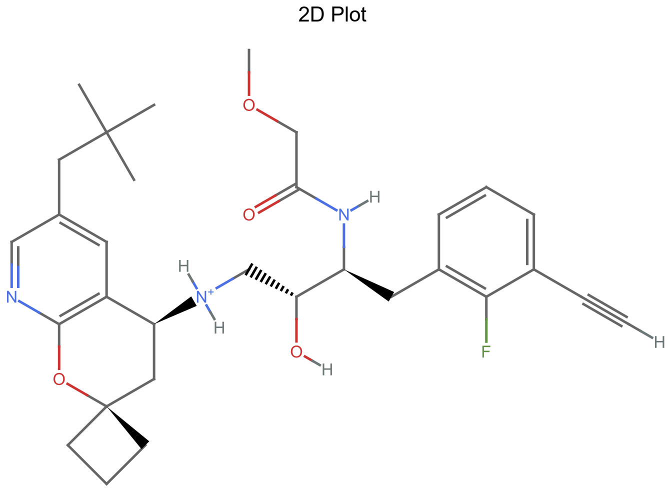

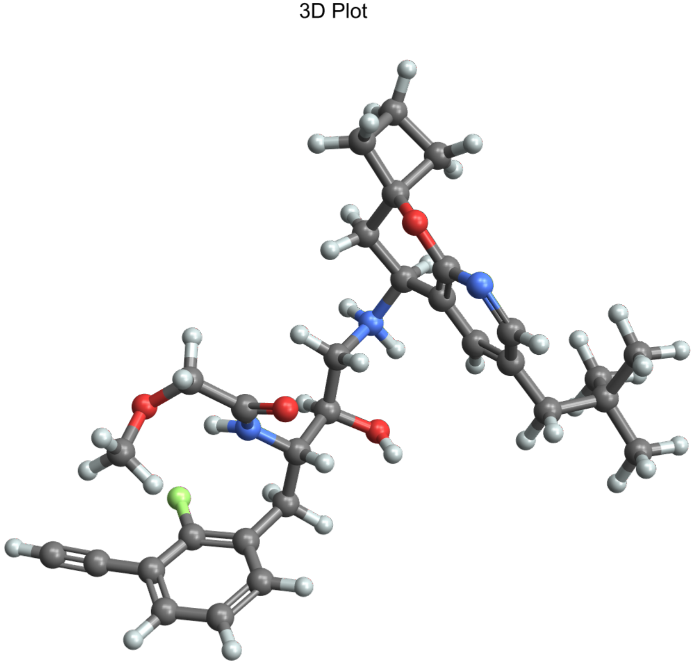

2D Autocorrelation

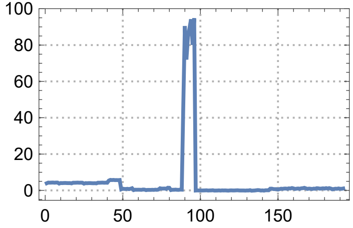

Dataset Index= 428

Molecule Formula= $C_{32}H_{43}N_{4}O_{3}S^{+}$

3D Autocorrelation

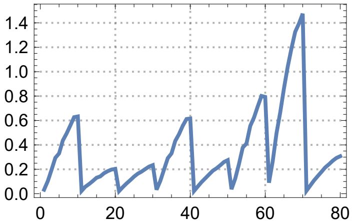

2D Plot

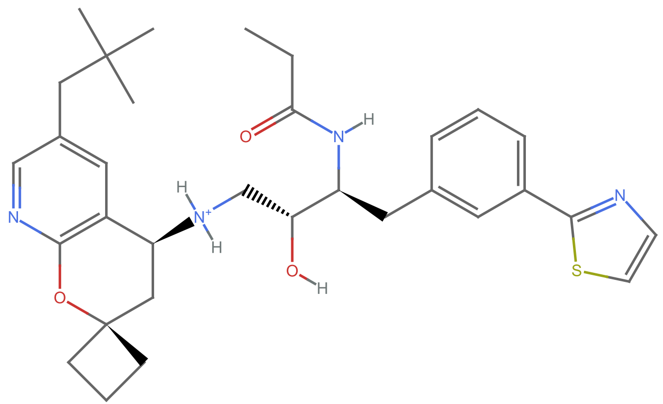

3D Plot

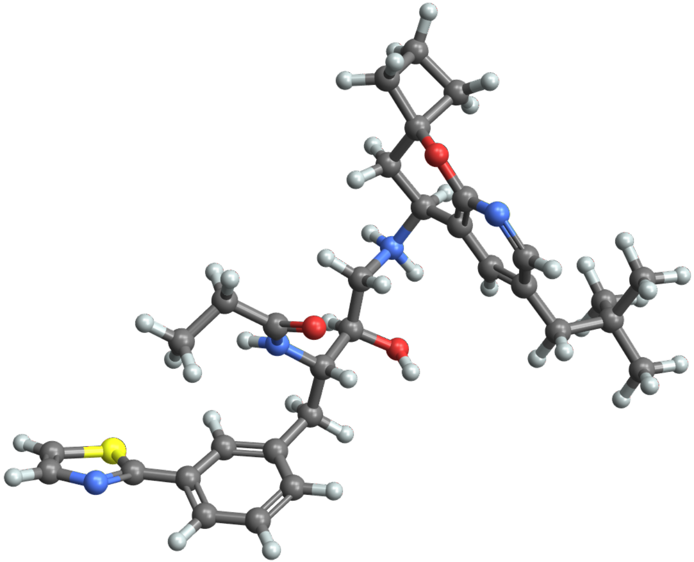

2D Autocorrelation

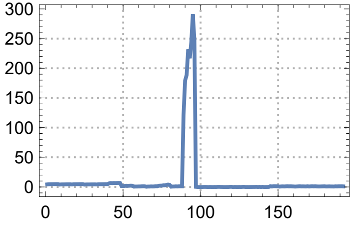

Dataset Index= 429

Molecule Formula= $C_{41}H_{43}F_2N_4O_6S^+$

3D Autocorrelation

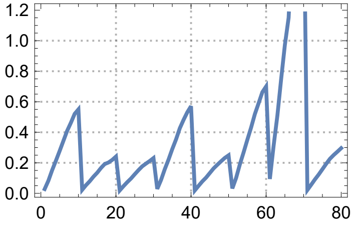

2D Plot

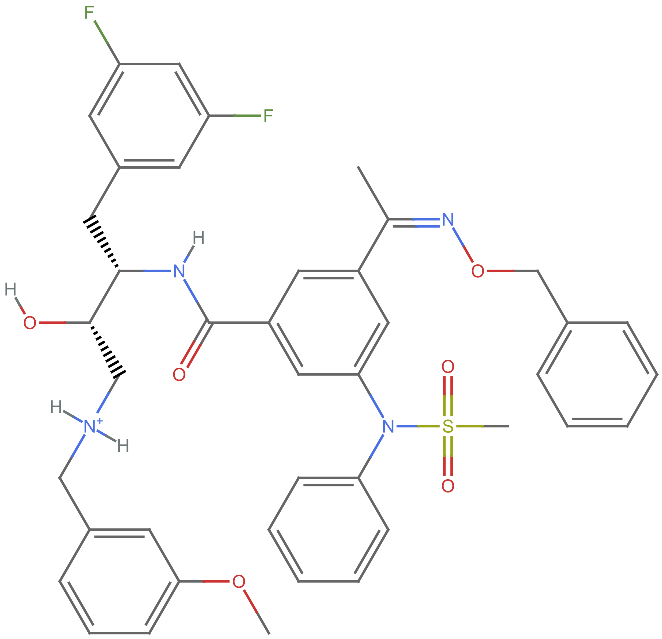

3D Plot

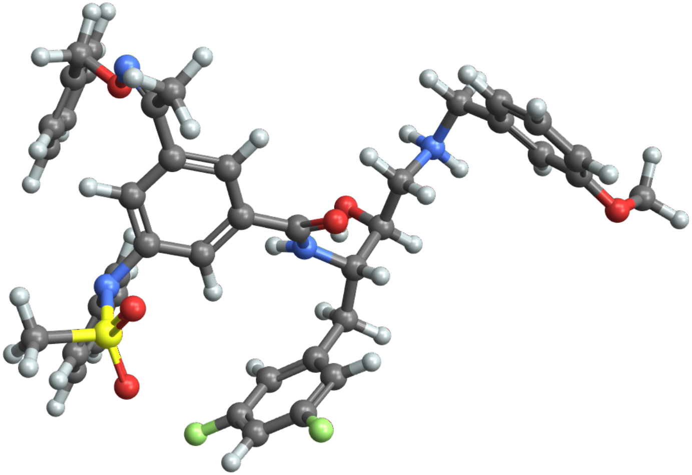

2D Plot

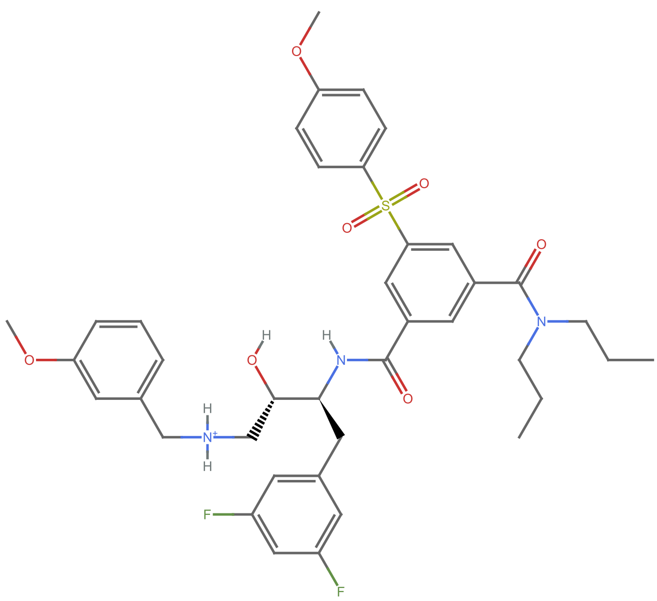

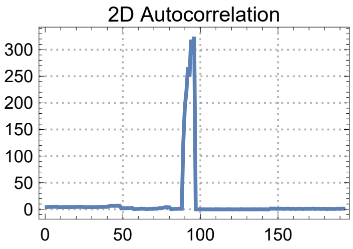

3D Plot

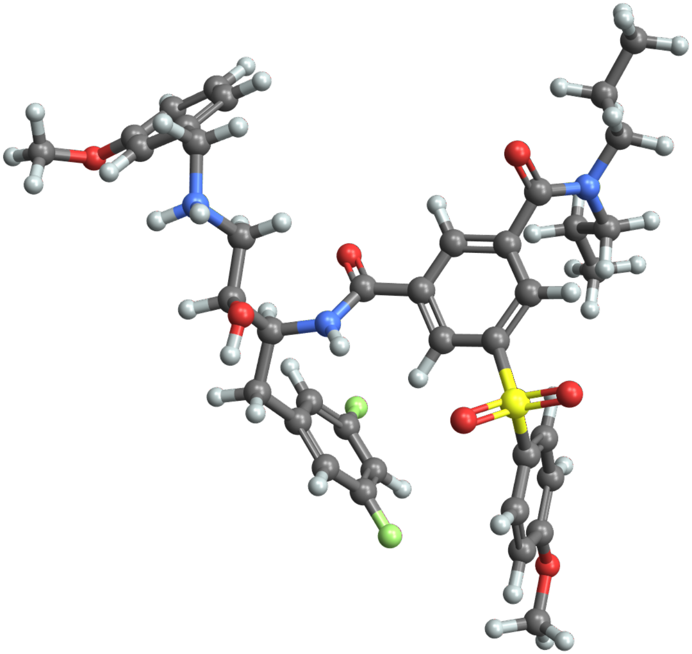

Dataset Index= 430

Molecule Formula= $C_{39}H_{46}F_2N_3O_7S^+$

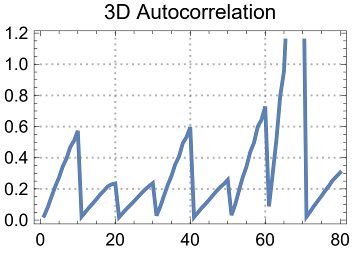

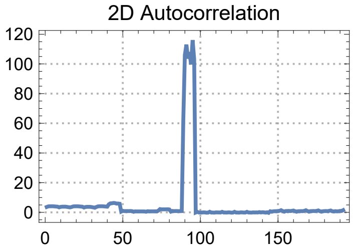

Dataset Index= 431

Molecule Formula= $C_{21}H_{18}F_3N_3O_3$

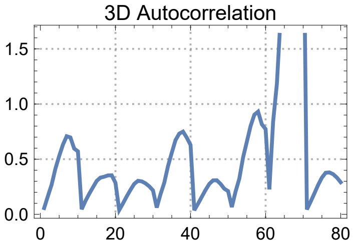

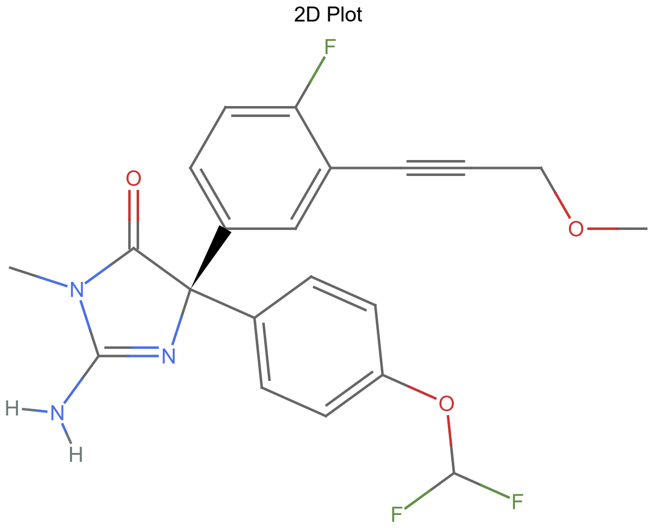

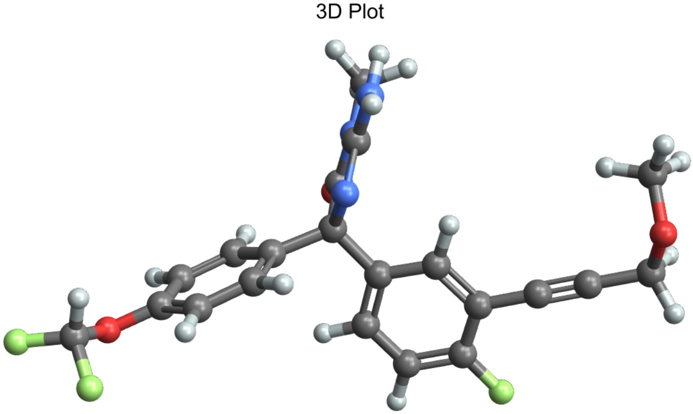

2D Plot

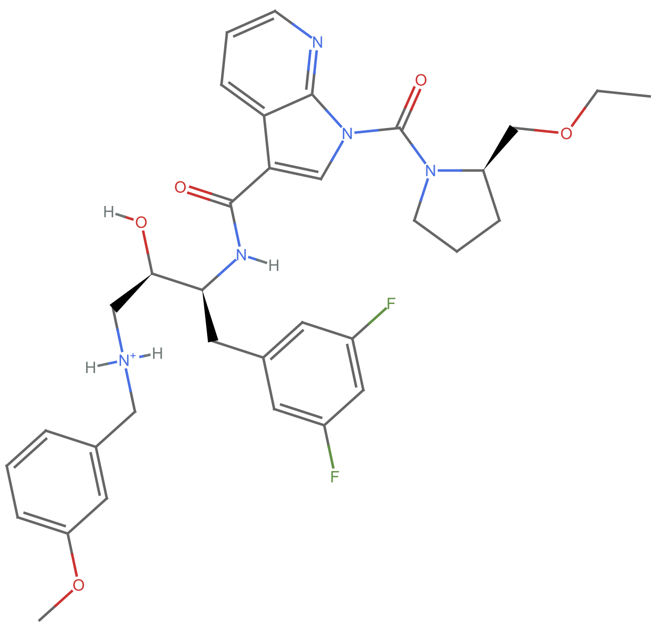

2D Autocorrelation

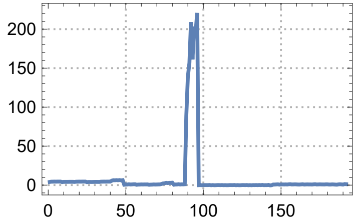

Dataset Index= 432

Molecule Formula= $C_{34}H_{40}F_2N_5O_5^+$

3D Plot

3D Autocorrelation

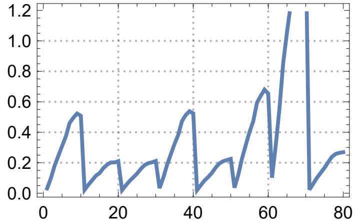

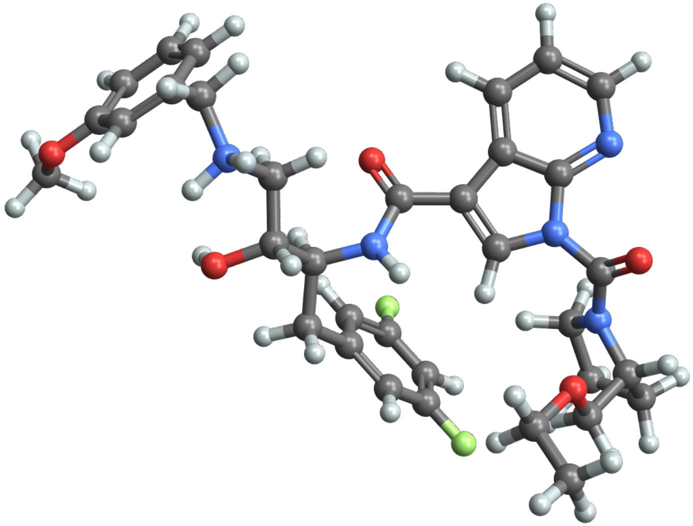

2D Plot

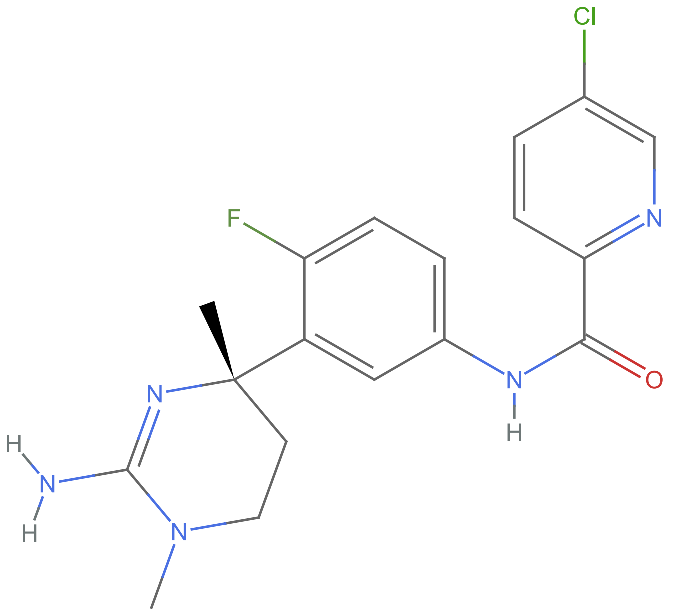

2D Autocorrelation

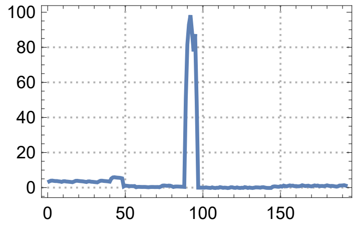

Dataset Index= 433

Molecule Formula= $C_{18}H_{19}ClFN_5O$

3D Autocorrelation

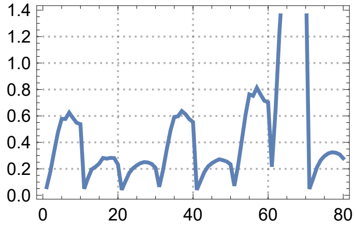

3D Plot

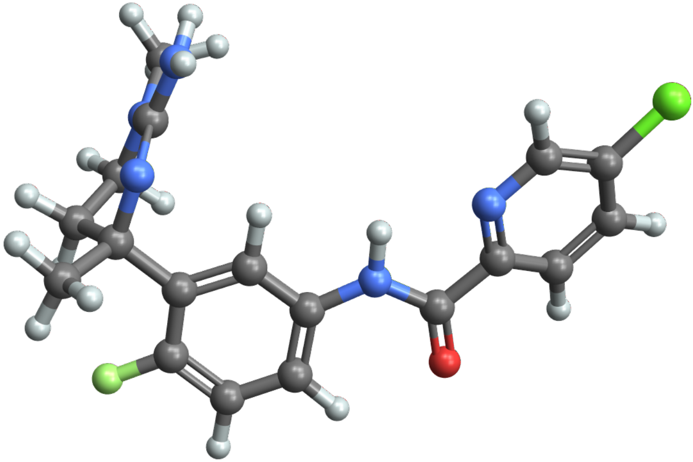

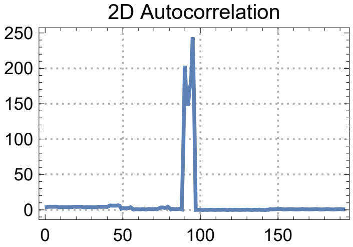

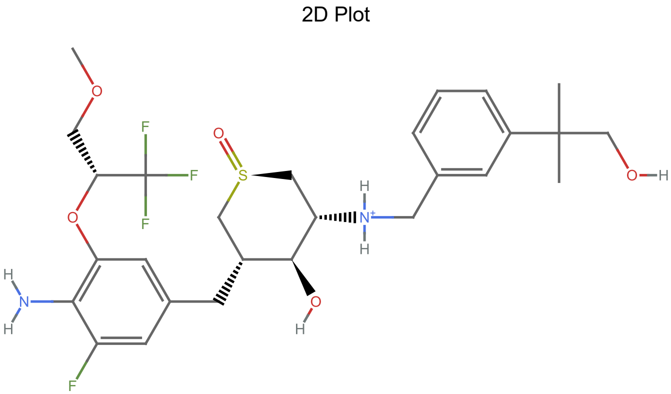

Dataset Index= 434

Molecule Formula= $C_{27}H_{37}F_4N_2O_5S^+$

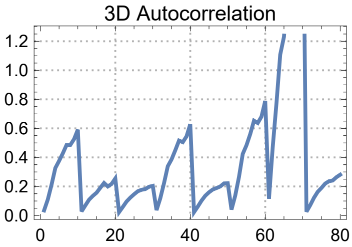

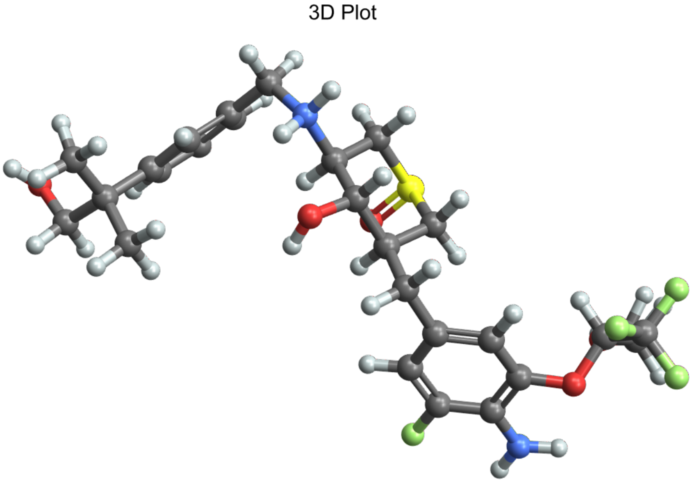

2D Plot

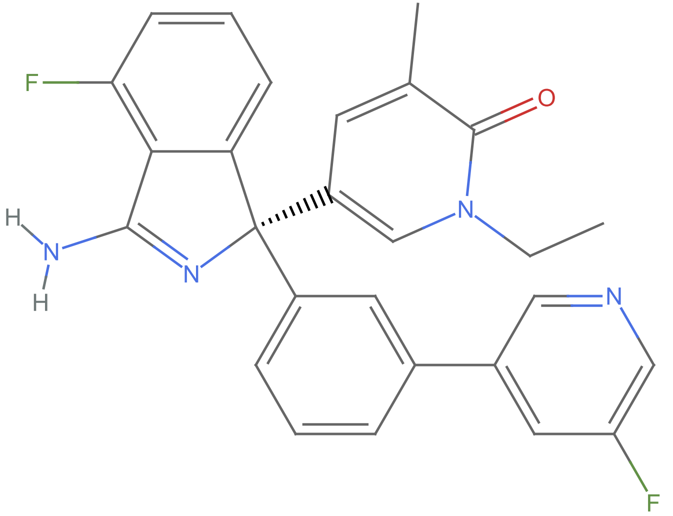

2D Autocorrelation

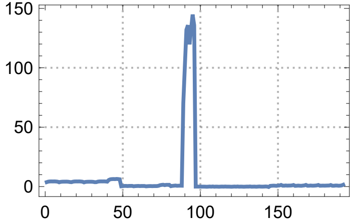

Dataset Index= 435

Molecule Formula= $C_{27}H_{22}F_2N_4O$

3D Plot

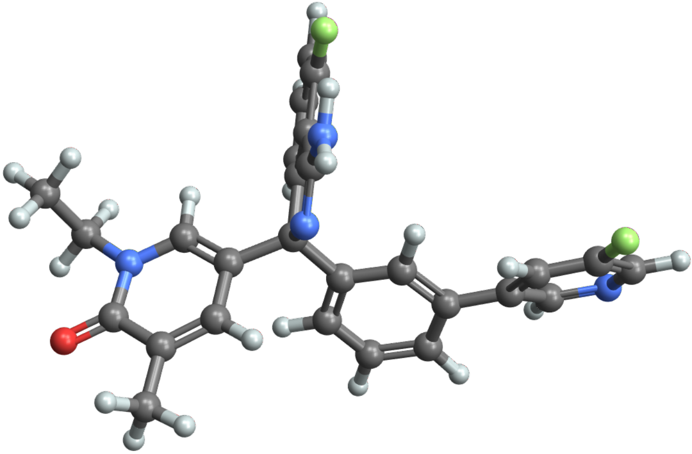

3D Autocorrelation

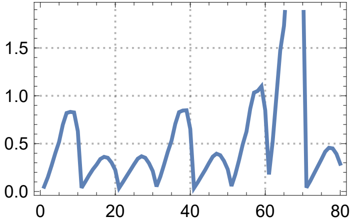

2D Plot

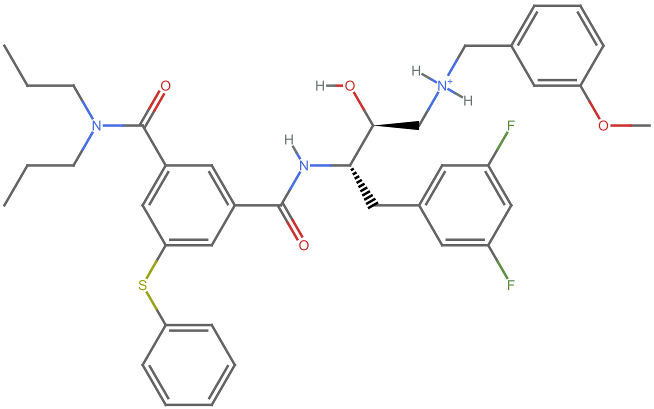

2D Autocorrelation

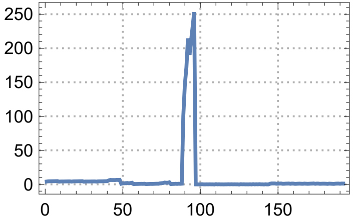

3D Plot

Dataset Index= 436

Molecule Formula= $C_{38}H_{44}F_2N_3O_4S^+$

3D Autocorrelation

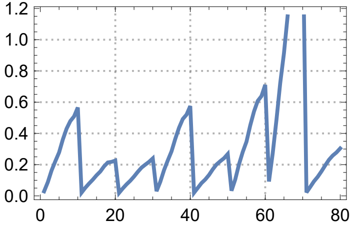

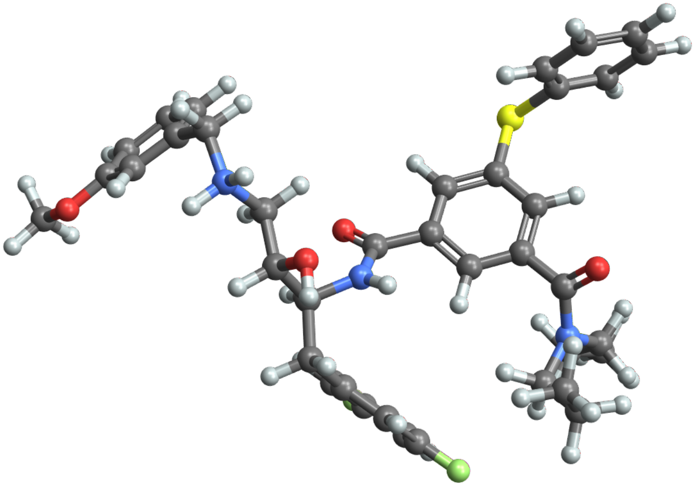

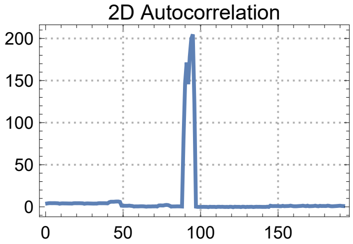

2D Plot

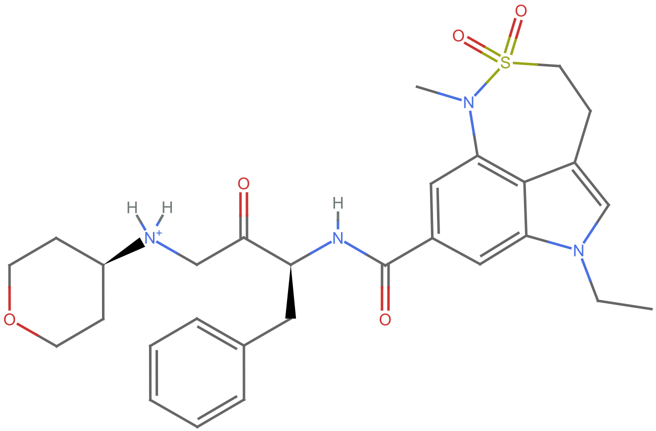

3D Plot

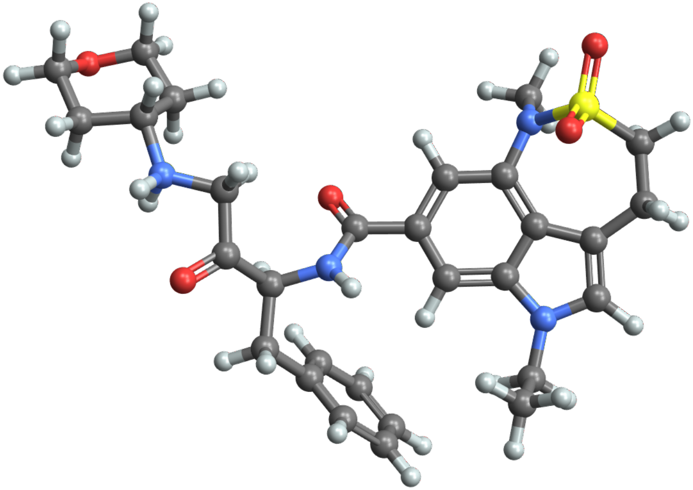

Dataset Index= 437

Molecule Formula= $C_{29}H_{37}N_4O_5S^+$

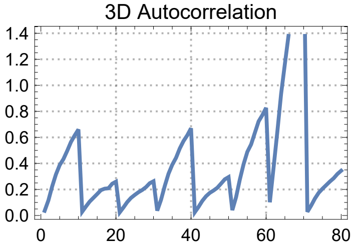

2D Plot

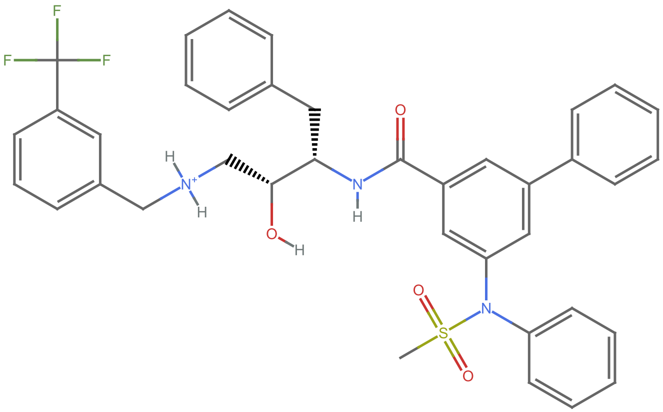

2D Autocorrelation

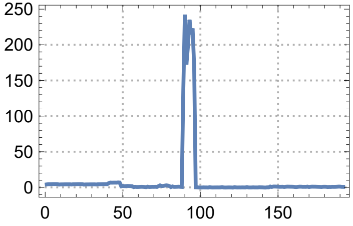

3D Plot

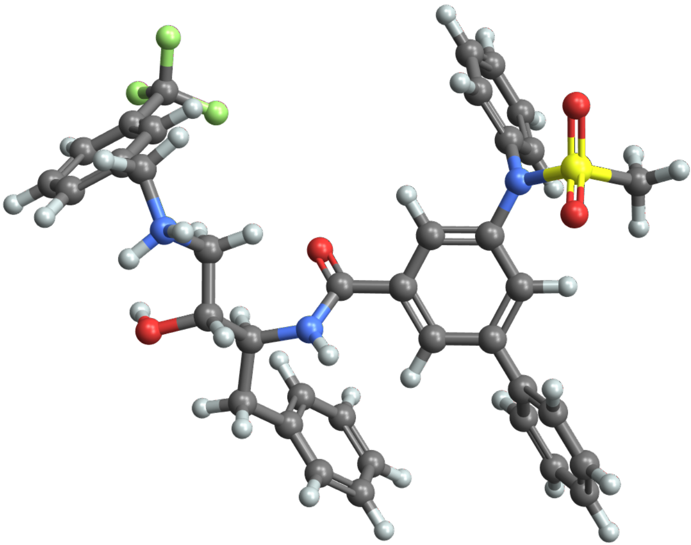

Dataset Index= 438

Molecule Formula= $C_{38}H_{37}F_3N_3O_4S^+$

3D Autocorrelation

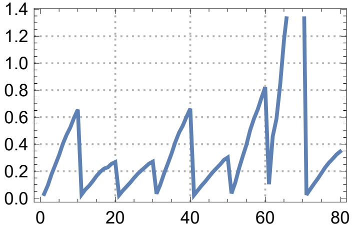

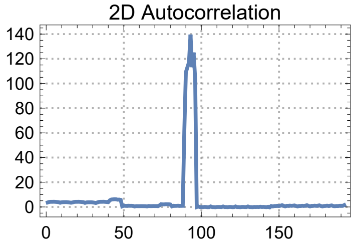

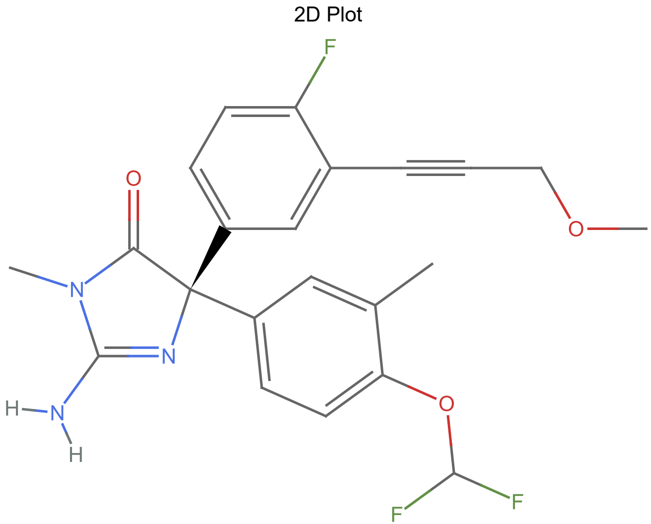

Dataset Index= 439

Molecule Formula= $C_{22}H_{20}F_3N_3O_3$

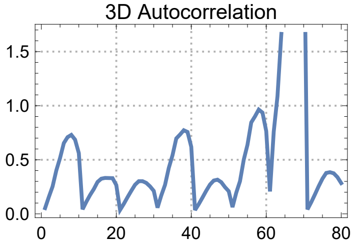

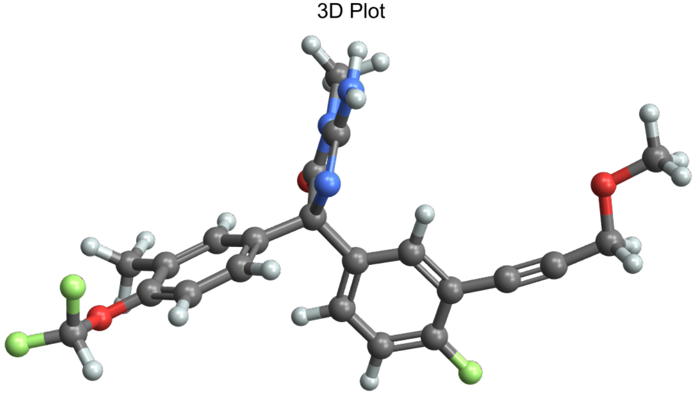

2D Autocorrelation

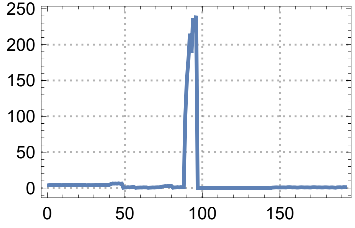

Dataset Index= 440

Molecule Formula= $C_{33}H_{38}F_3N_4O_4^+$

3D Autocorrelation

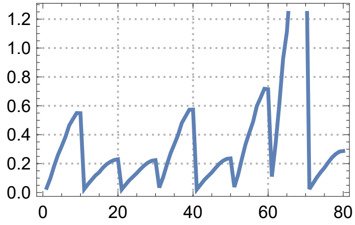

2D Plot

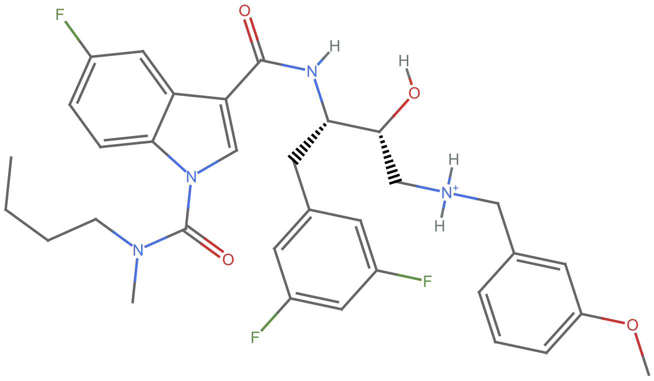

3D Plot

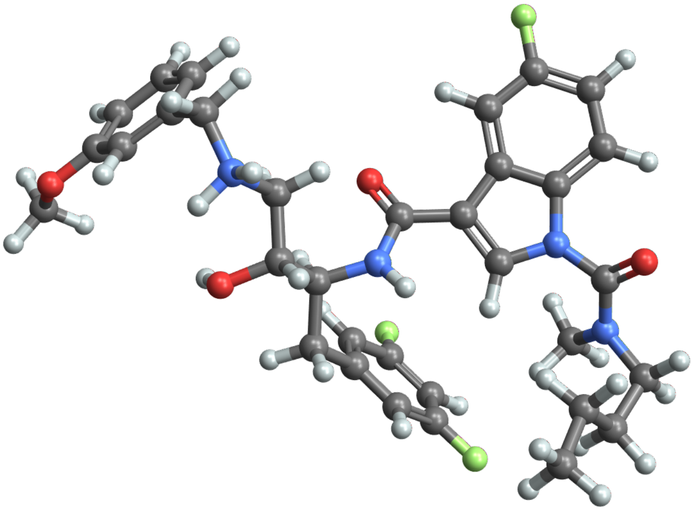

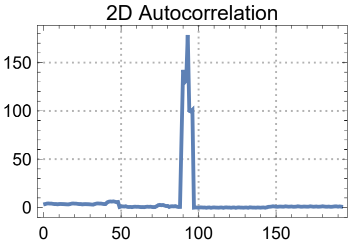

Dataset Index= 441

Molecule Formula= $C_{19}H_{16}F_3N_5O_3$

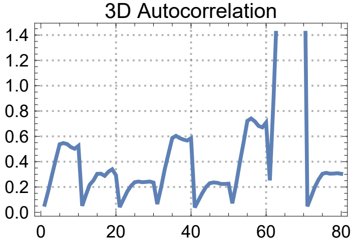

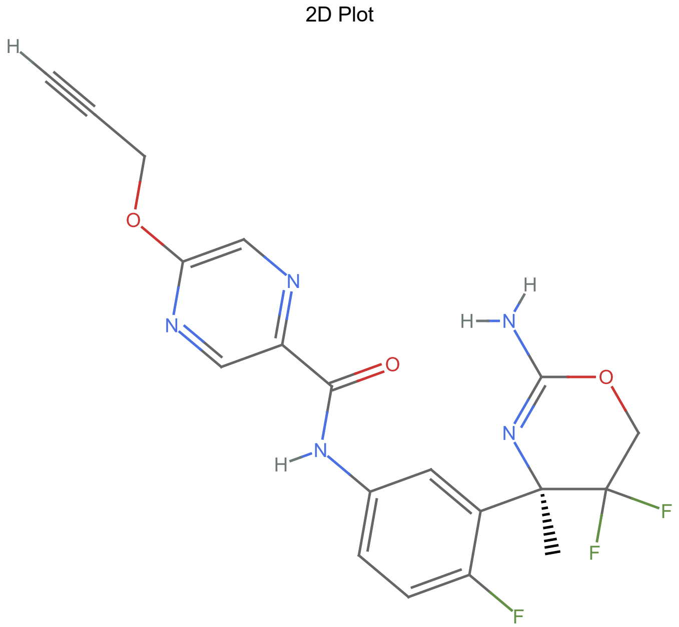

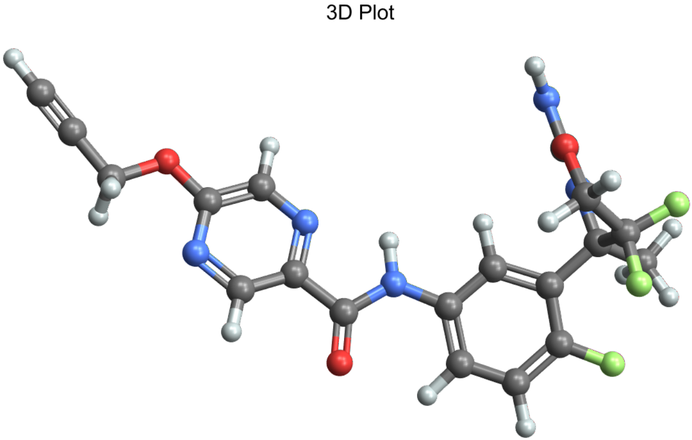

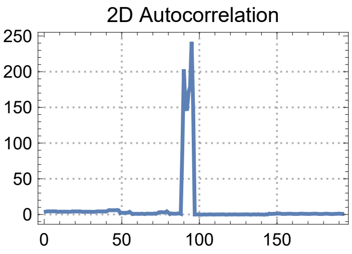

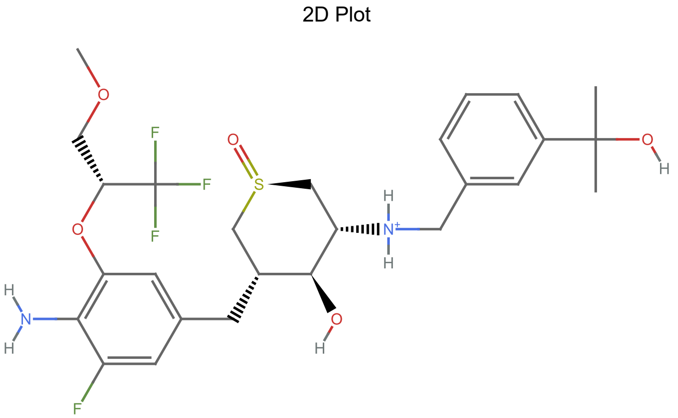

Dataset Index= 442

Molecule Formula= $C_{26}H_{35}F_4N_2O_5S^+$

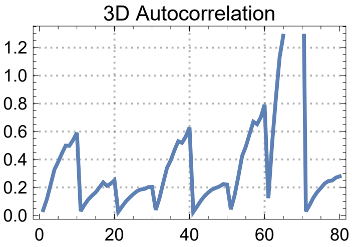

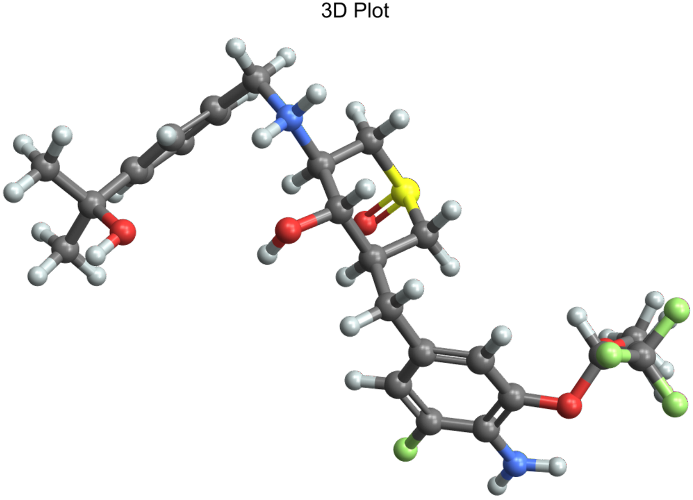

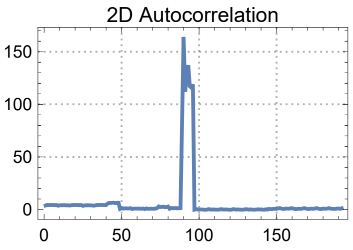

Dataset Index= 443

Molecule Formula= $C_{25}H_{21}F_4N_5O_2$

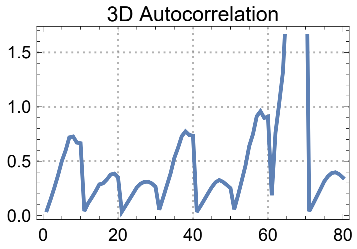

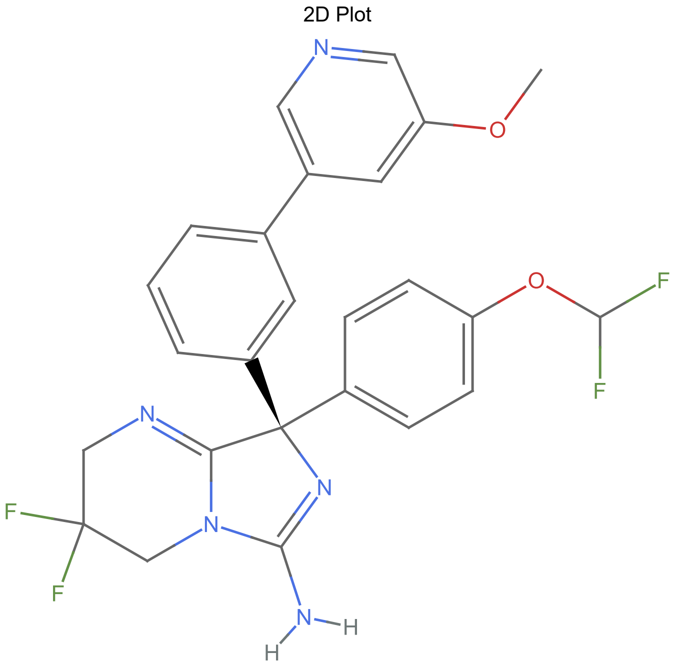

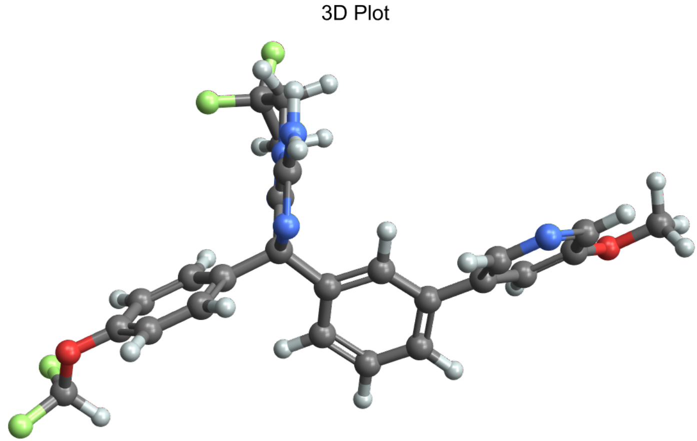

2D Autocorrelation

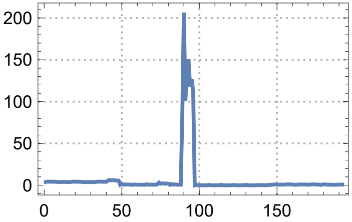

Dataset Index= 444

Molecule Formula= $C_{29}H_{39}F_3N_3O_4^+$

3D Autocorrelation

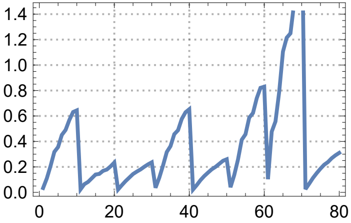

2D Plot

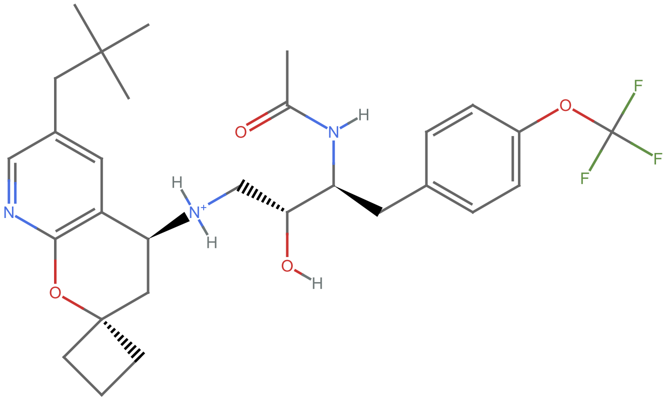

3D Plot

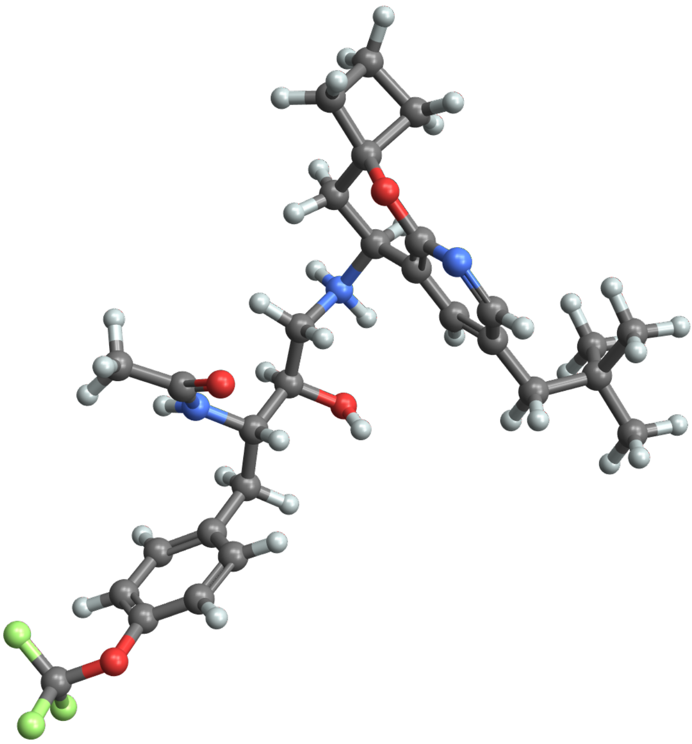

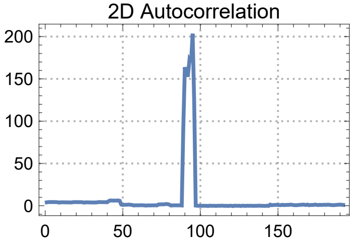

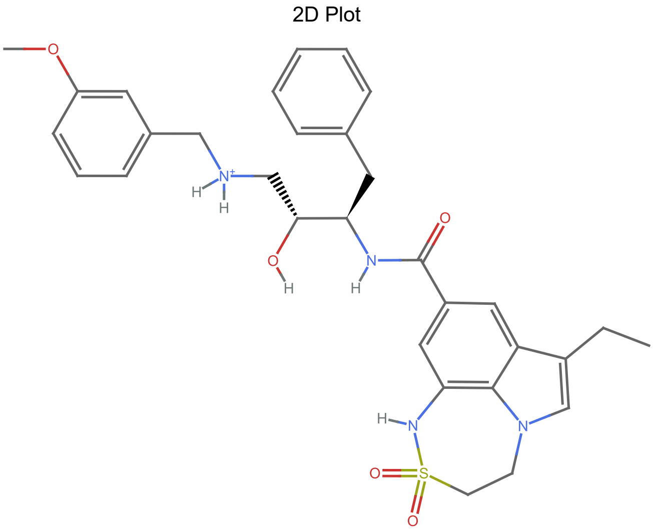

Dataset Index= 445

Molecule Formula= $C_{31}H_{37}N_4O_5S^+$

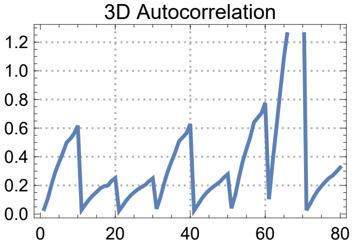

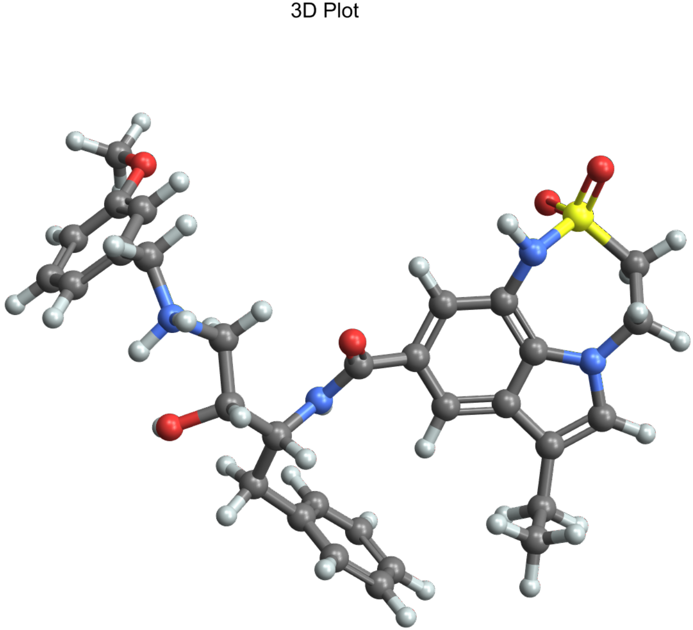

2D Plot

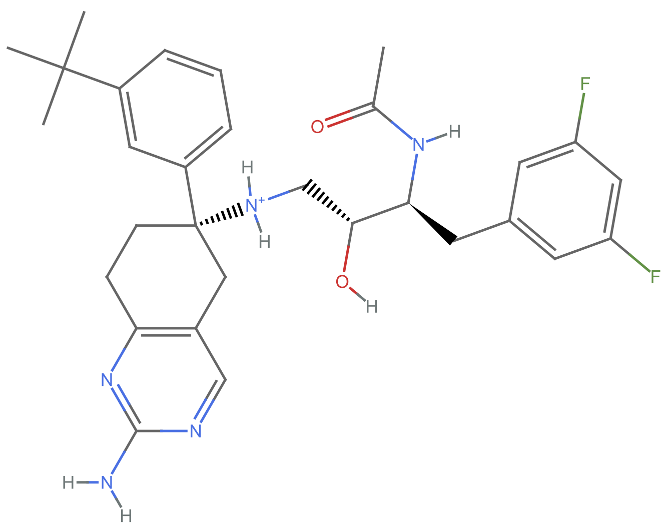

2D Autocorrelation

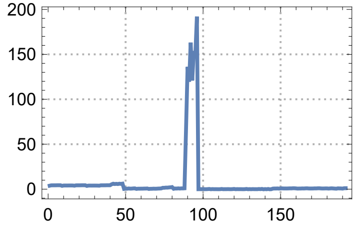

Dataset Index= 446

Molecule Formula= $C_{30}H_{38}F_2N_5O_2^+$

3D Autocorrelation

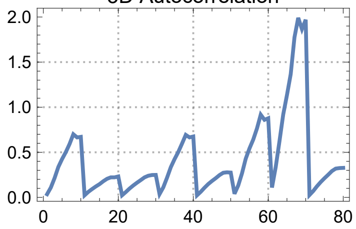

3D Plot

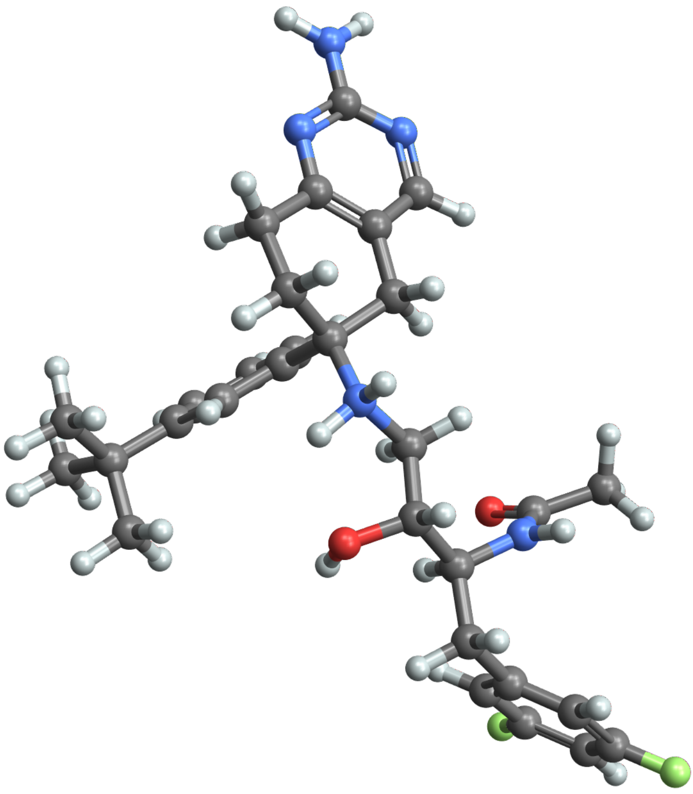

2D Plot

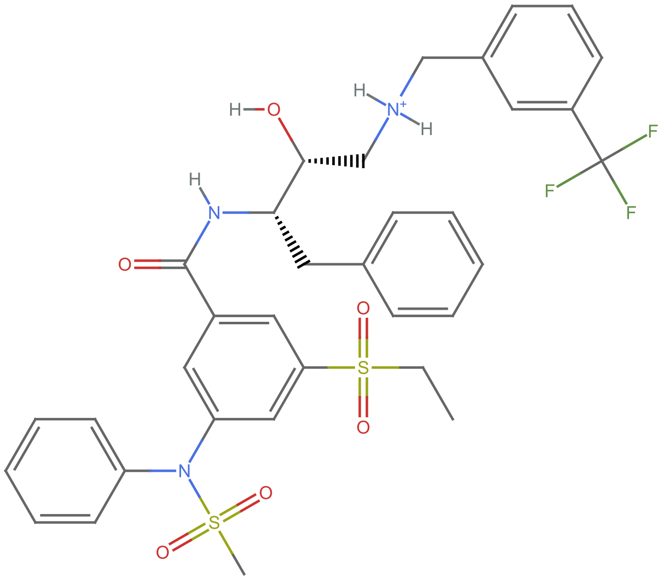

2D Autocorrelation

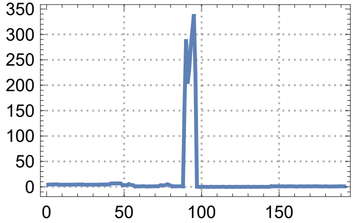

Dataset Index= 447

Molecule Formula= $C_{34}H_{37}F_3N_3O_6S_2^+$

3D Plot

3D Autocorrelation

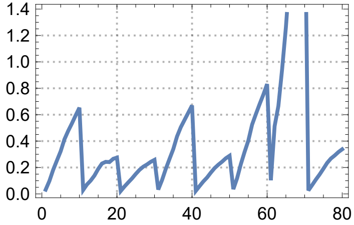

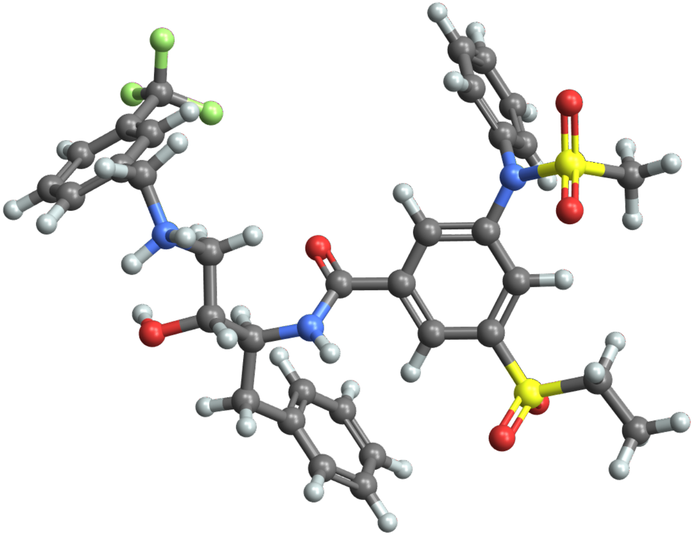

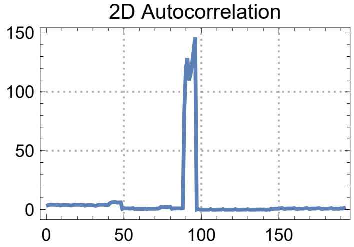

Dataset Index= 448

Molecule Formula= $C_{22}H_{19}F_4N_3O_2$

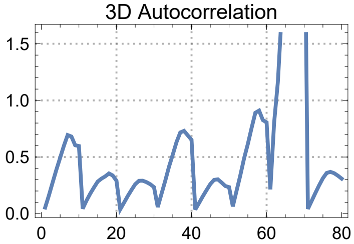

2D Plot

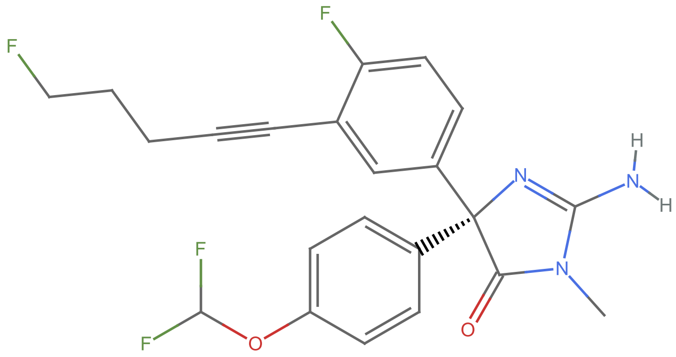

3D Plot

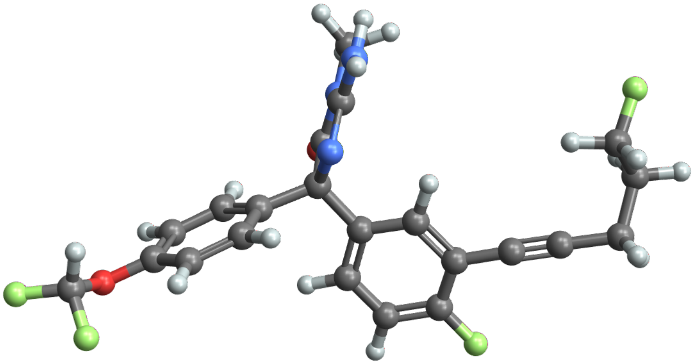

2D Plot

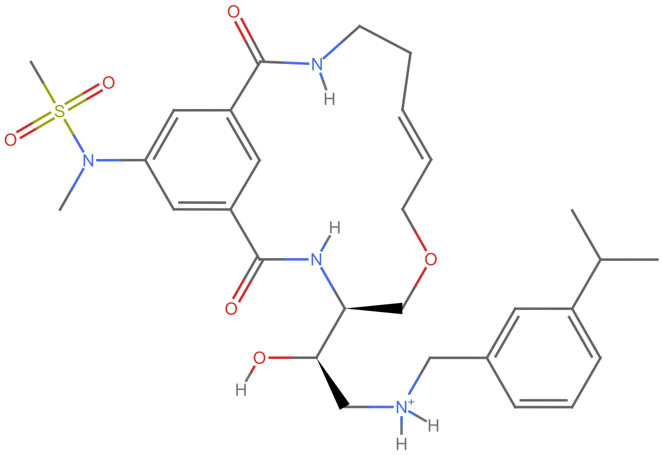

2D Autocorrelation

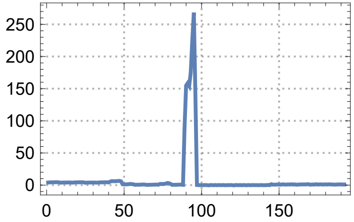

3D Plot

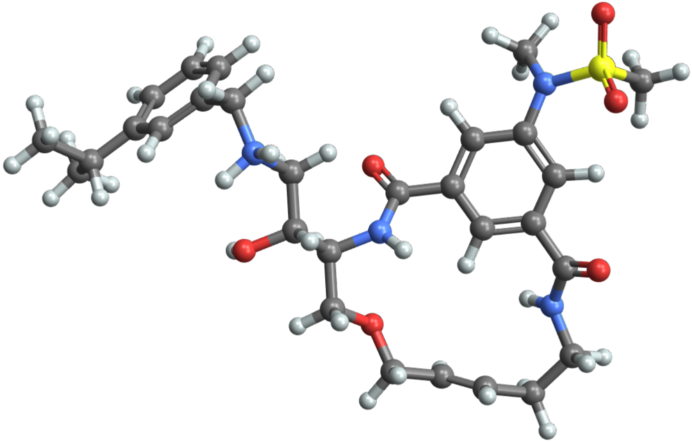

Dataset Index= 449

Molecule Formula= $C_{29}H_{41}N_4O_6S^+$

3D Autocorrelation

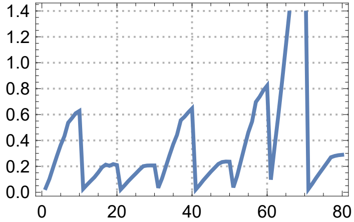

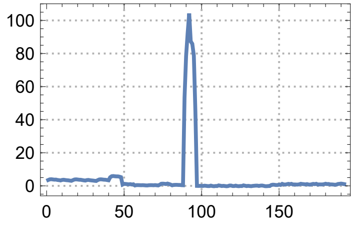

Dataset Index= 450

Molecule Formula= $C_{18}H_{18}ClFN_4O_2$

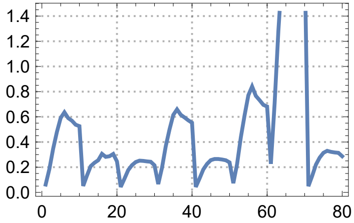

2D Plot

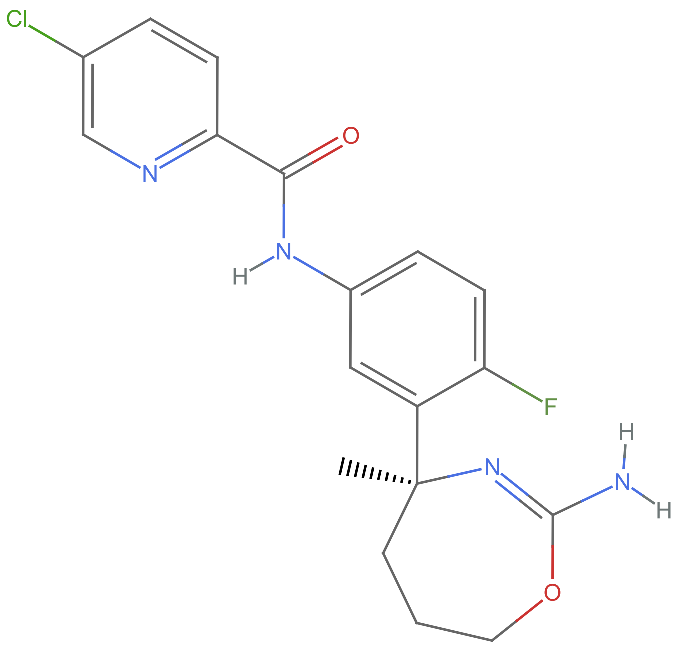

3D Plot

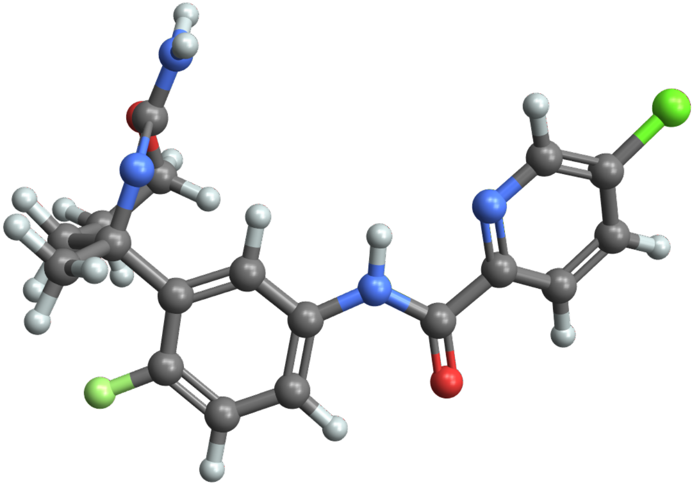

2D Plot

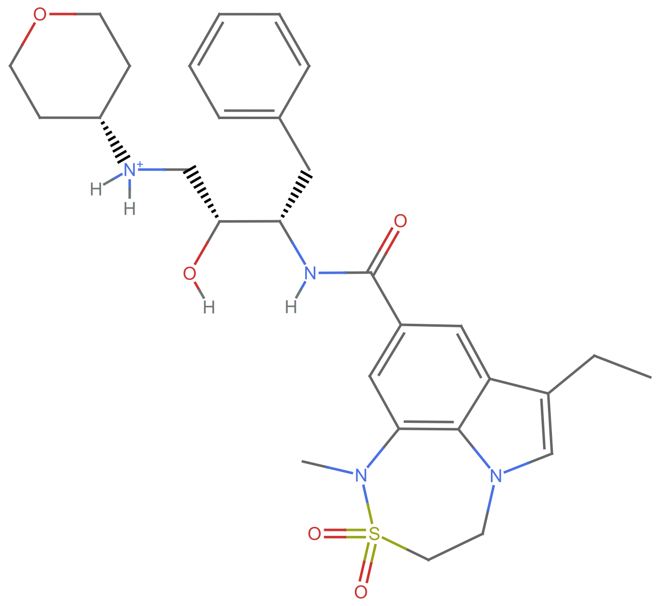

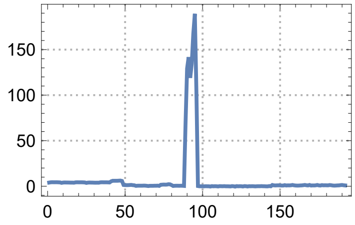

Dataset Index= 451

Molecule Formula= $C_{29}H_{39}N_4O_5S^+$

3D Plot

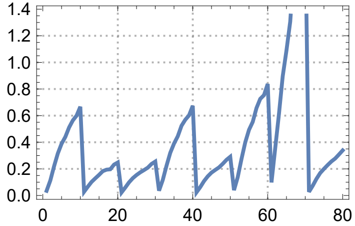

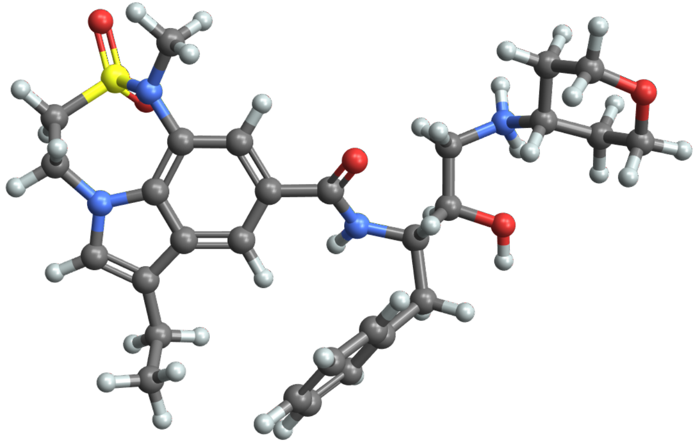

2D Plot

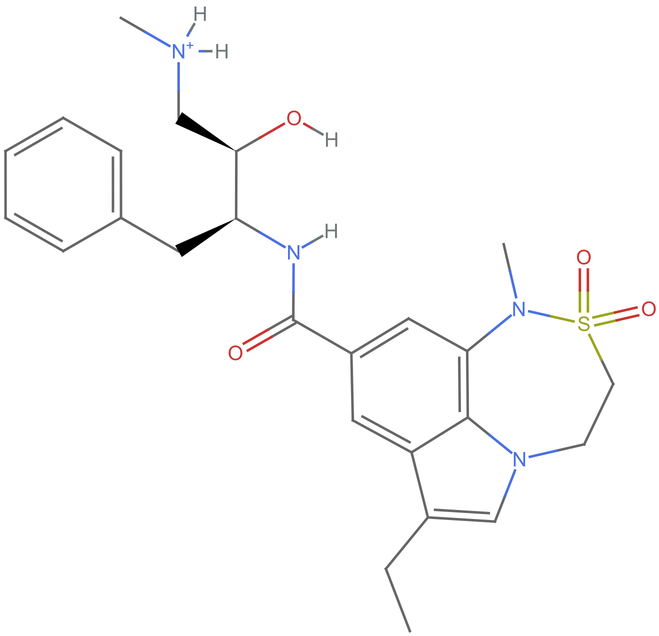

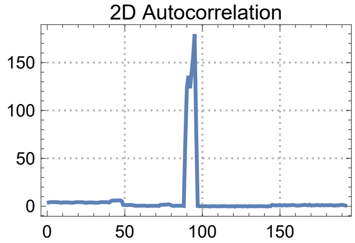

Dataset Index= 452

Molecule Formula= $C_{25}H_{33}N_4O_4S^+$

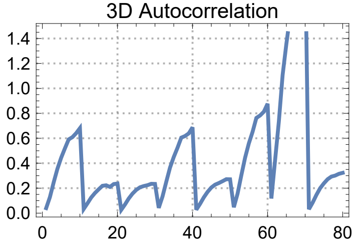

3D Plot

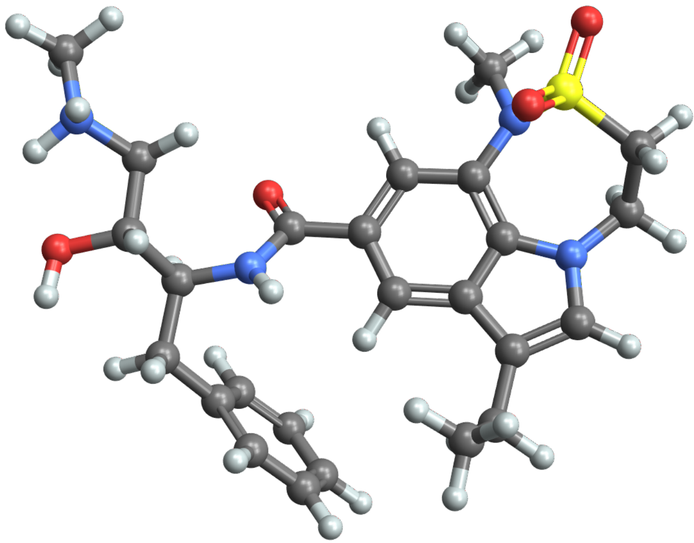

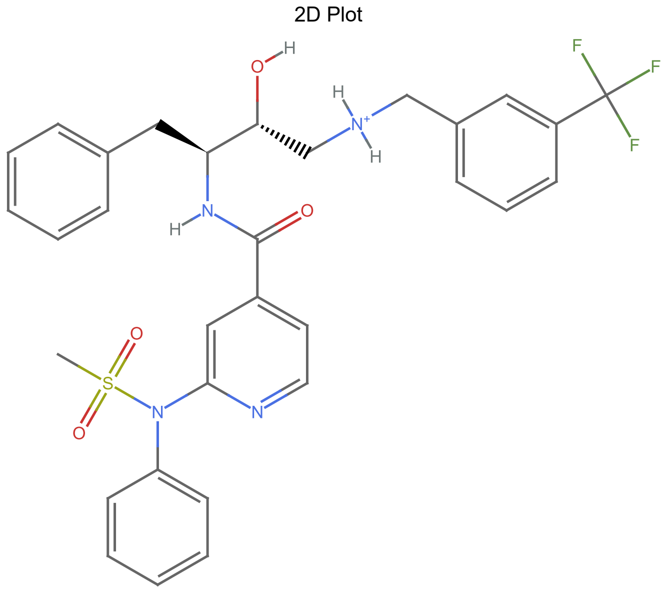

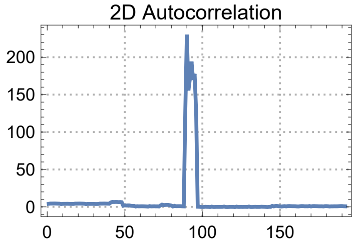

Dataset Index= 453

Molecule Formula= $C_{31}H_{32}F_3N_4O_4S^+$

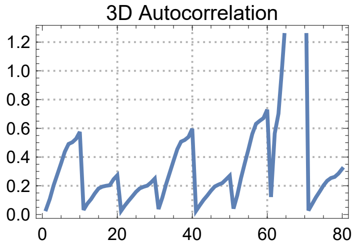

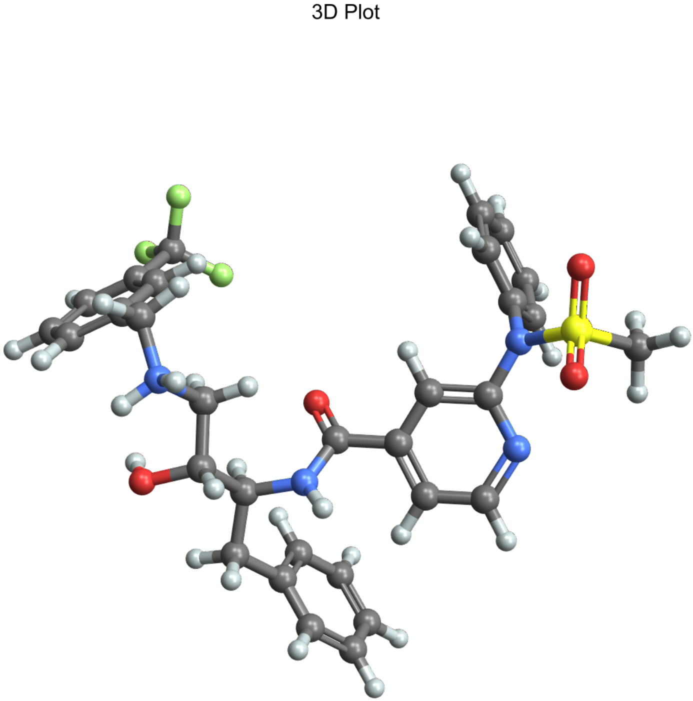

2D Plot

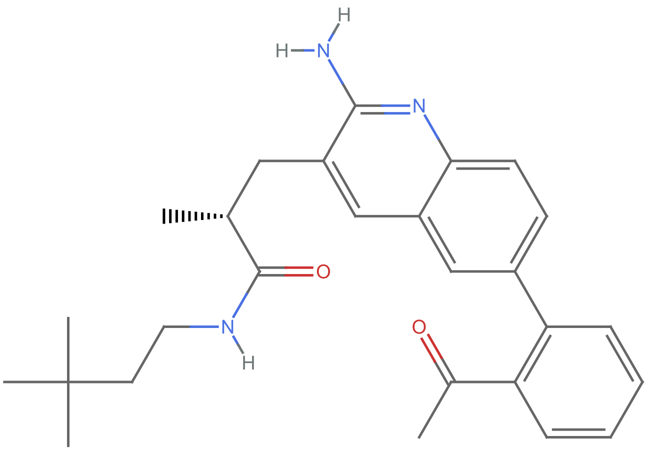

2D Autocorrelation

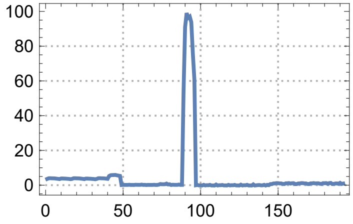

3D Plot

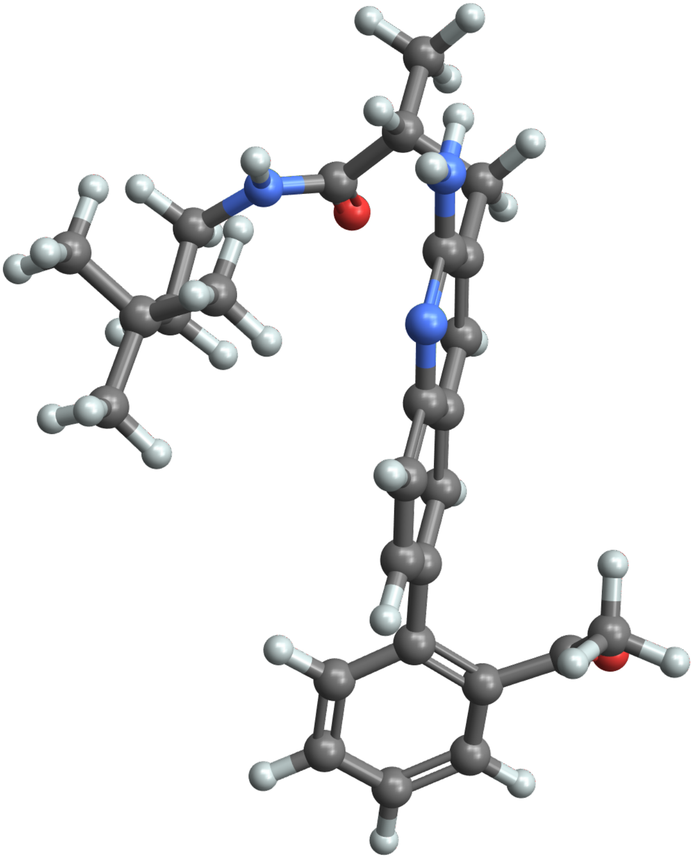

```
Dataset Index=        454

Molecule Formula=     C27H33N3O2
```

3D Autocorrelation

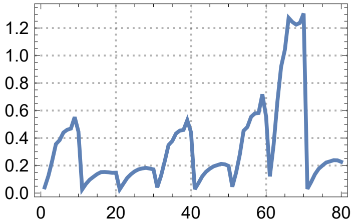

2D Plot

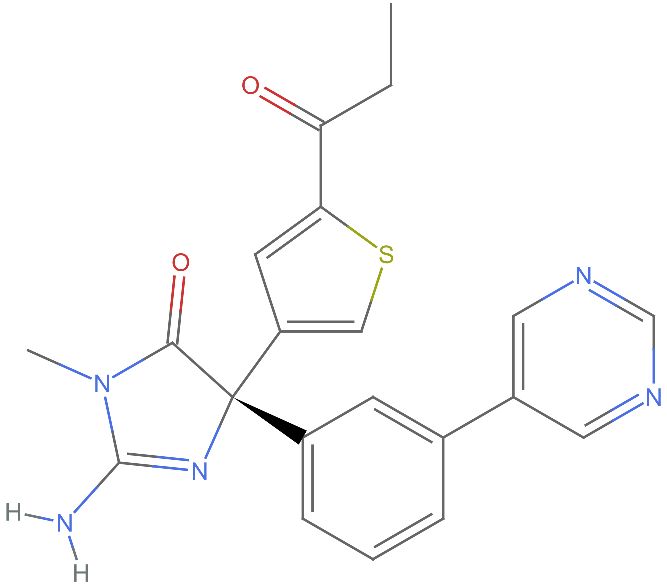

2D Autocorrelation

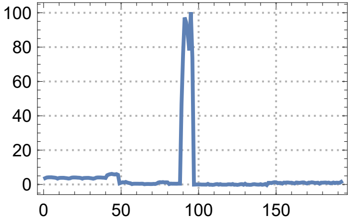

Dataset Index= 455

Molecule Formula= $C_{21}H_{19}N_5O_2S$

3D Autocorrelation

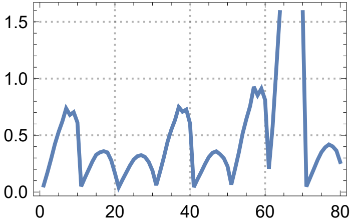

3D Plot

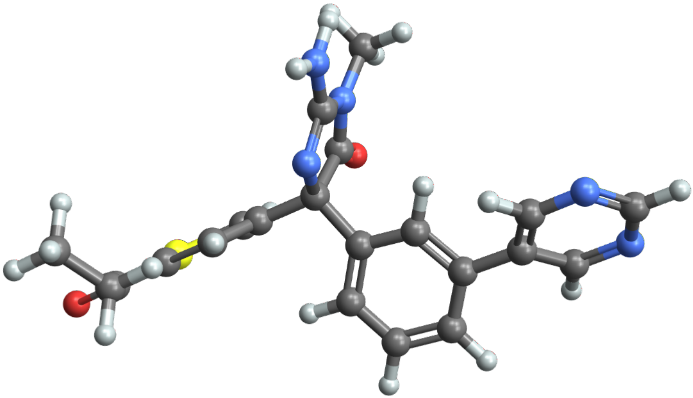

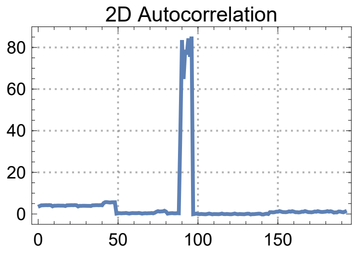

Dataset Index= 456

Molecule Formula= $C_{32}H_{44}N_3O_4^+$

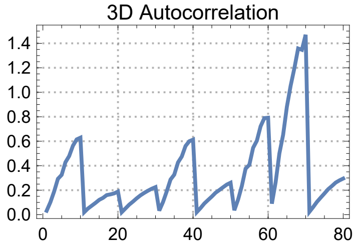

2D Plot

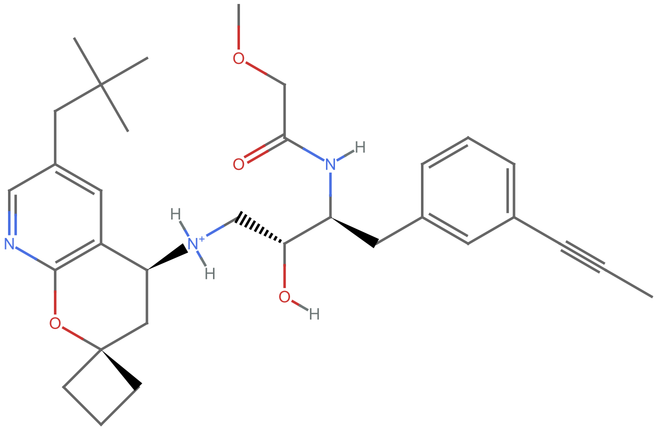

3D Plot

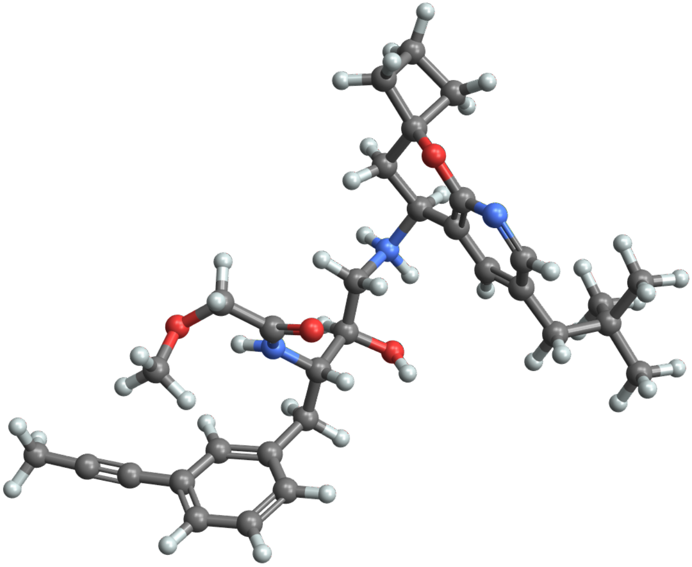

2D Plot

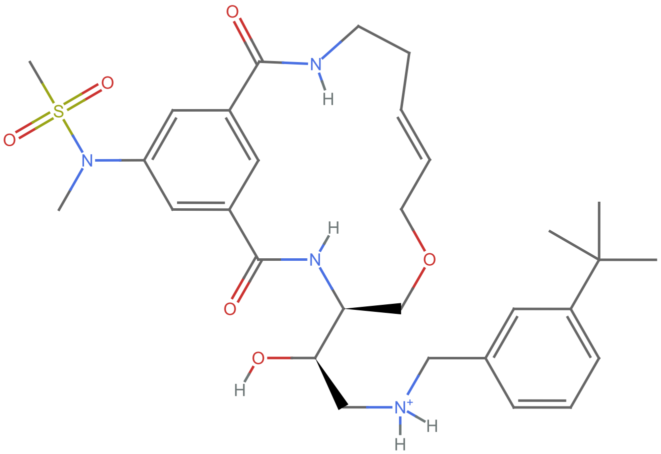

2D Autocorrelation

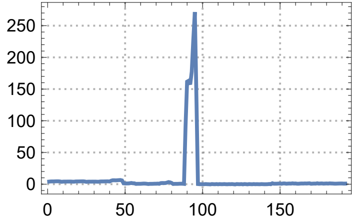

3D Plot

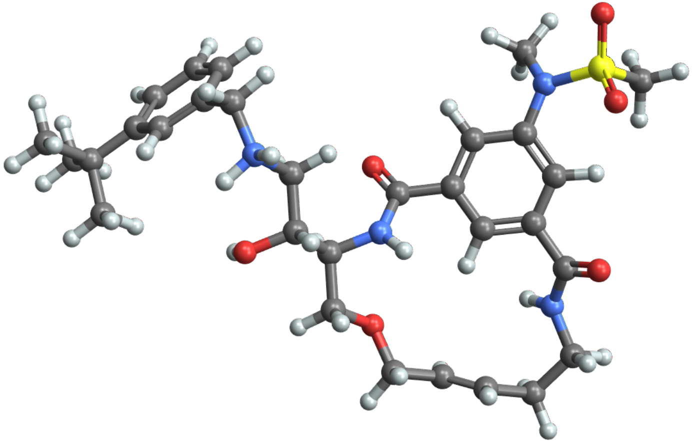

Dataset Index= 457

Molecule Formula= $C_{30}H_{43}N_4O_6S^+$

3D Autocorrelation

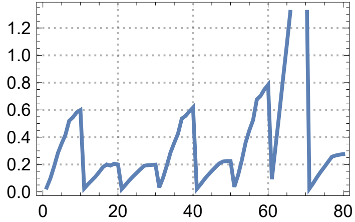

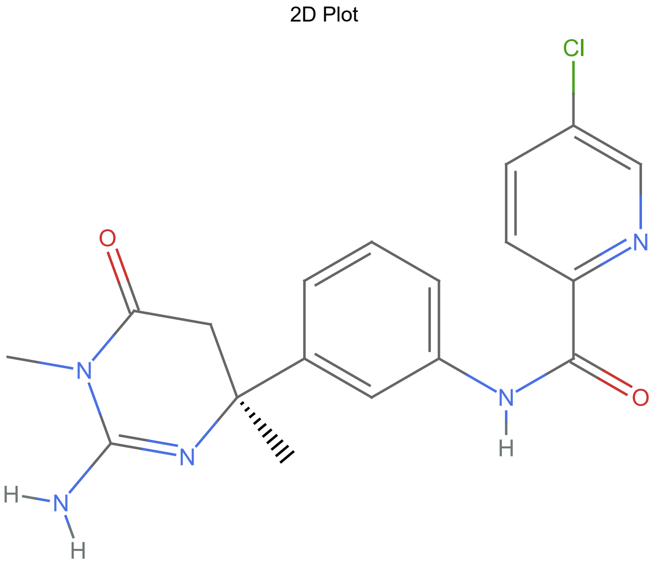

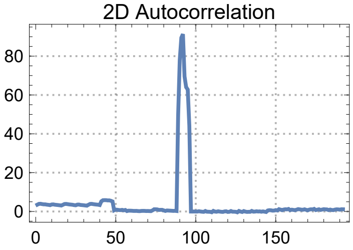

Dataset Index= 458

Molecule Formula= $C_{18}H_{18}ClN_5O_2$

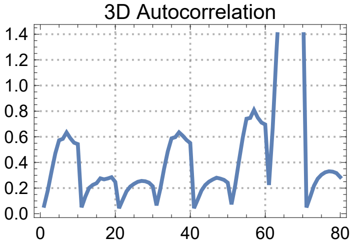

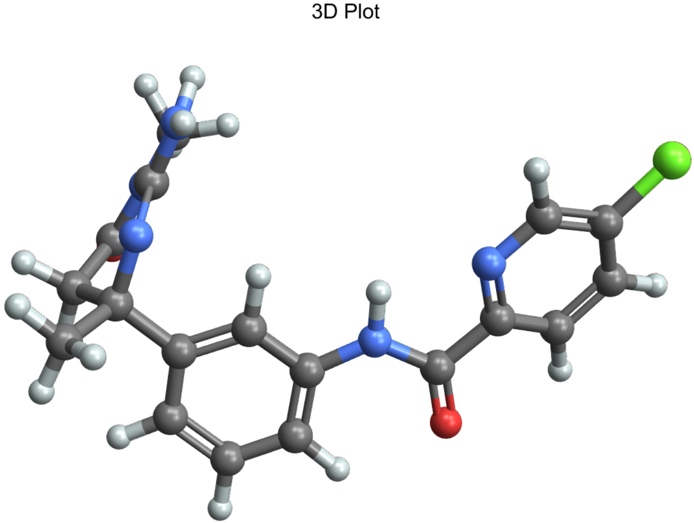

2D Plot

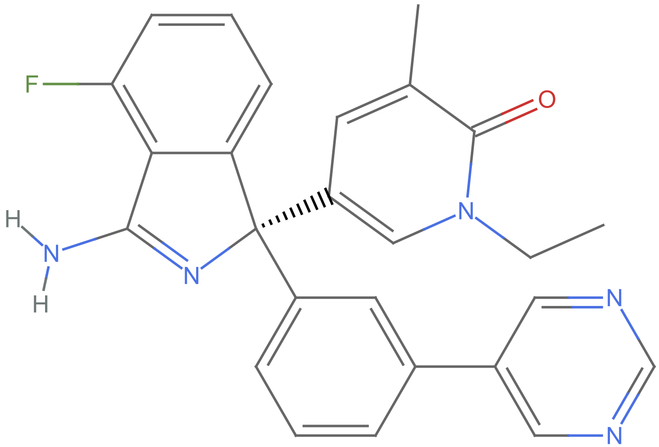

2D Autocorrelation

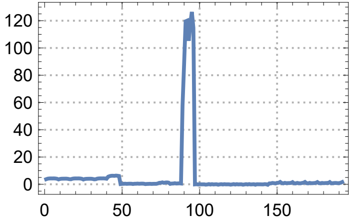

Dataset Index= 459

Molecule Formula= $C_{26}H_{22}FN_5O$

3D Plot

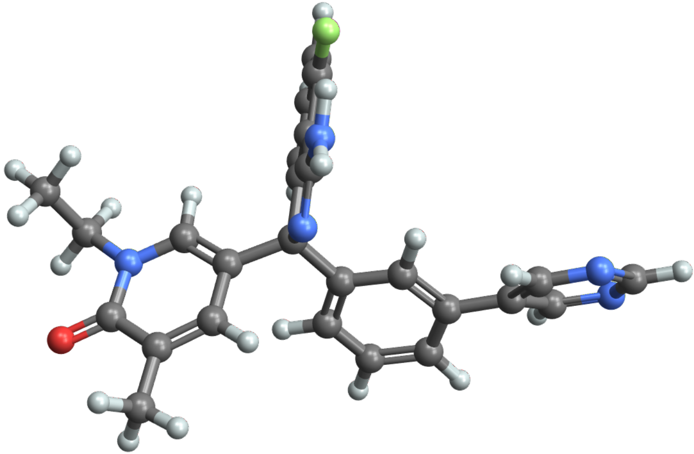

3D Autocorrelation

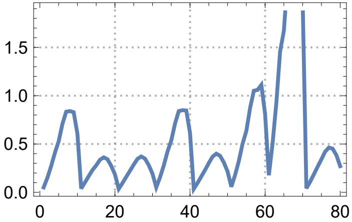

2D Plot

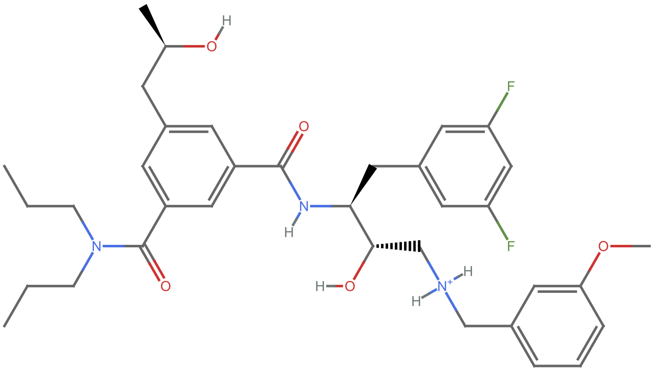

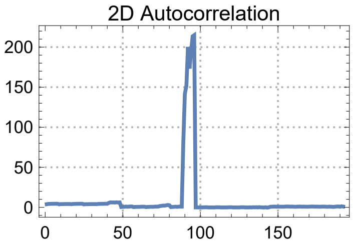

3D Plot

Dataset Index= 460

Molecule Formula= $C_{35}H_{46}F_2N_3O_5^+$

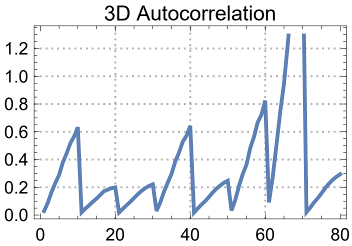

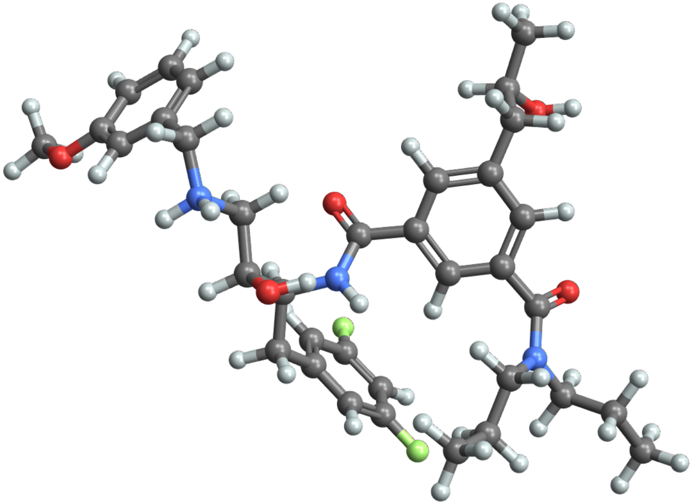

2D Plot

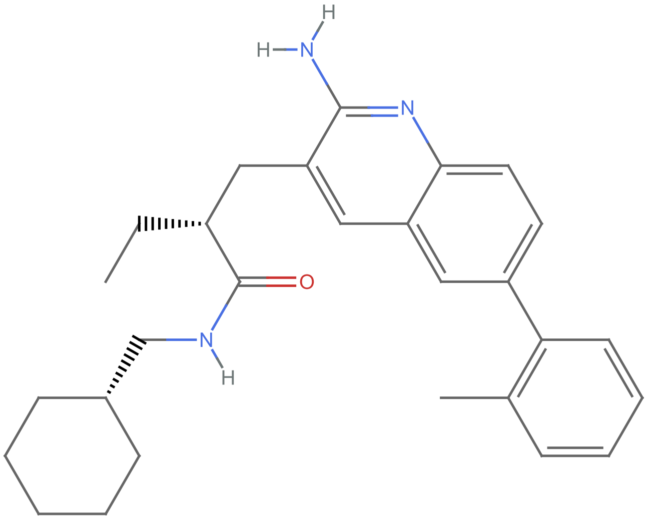

2D Autocorrelation

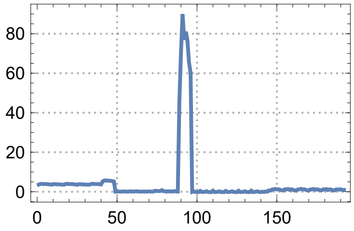

3D Plot

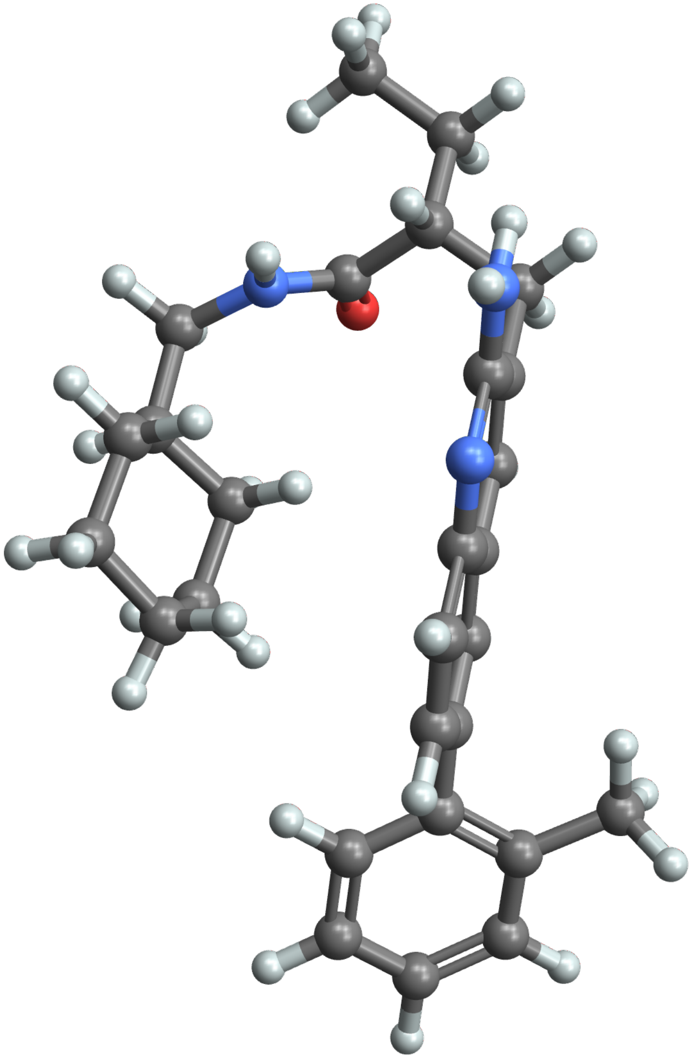

Dataset Index= 461

Molecule Formula= $C_{28}H_{35}N_3O$

3D Autocorrelation

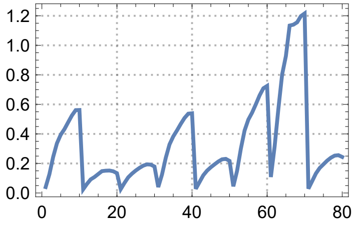

2D Plot

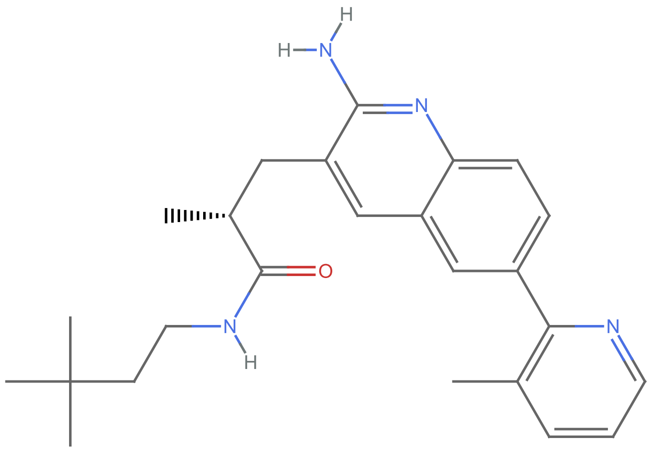

3D Plot

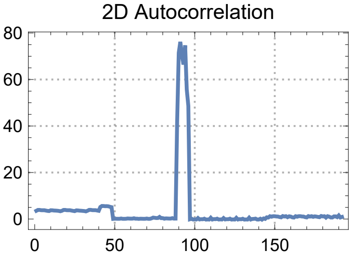

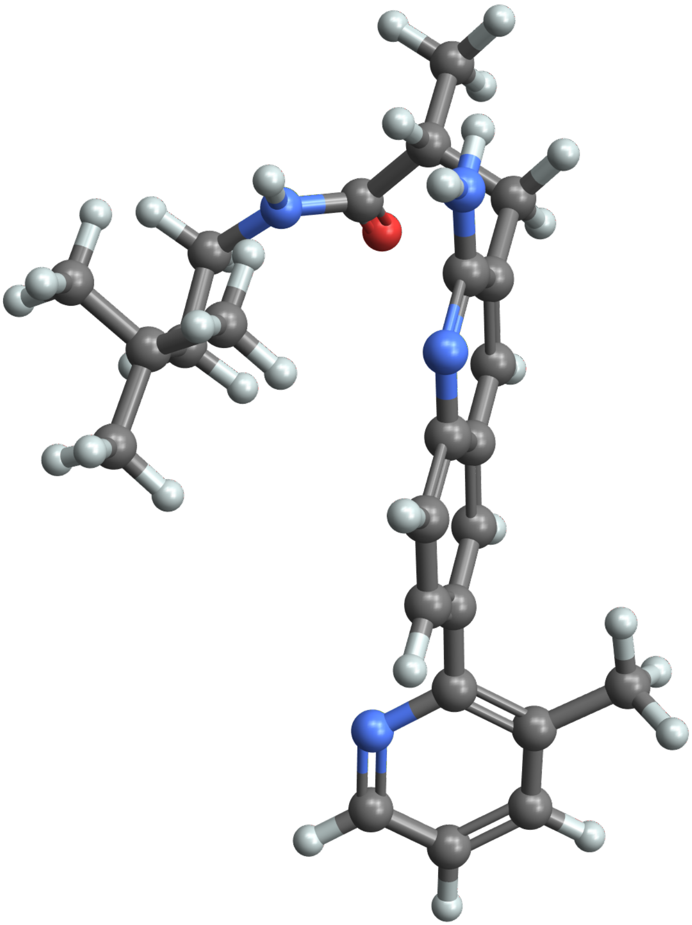

Dataset Index= 462

Molecule Formula= $C_{25}H_{32}N_4O$

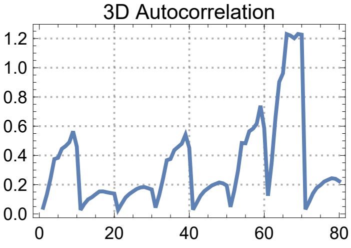

2D Plot

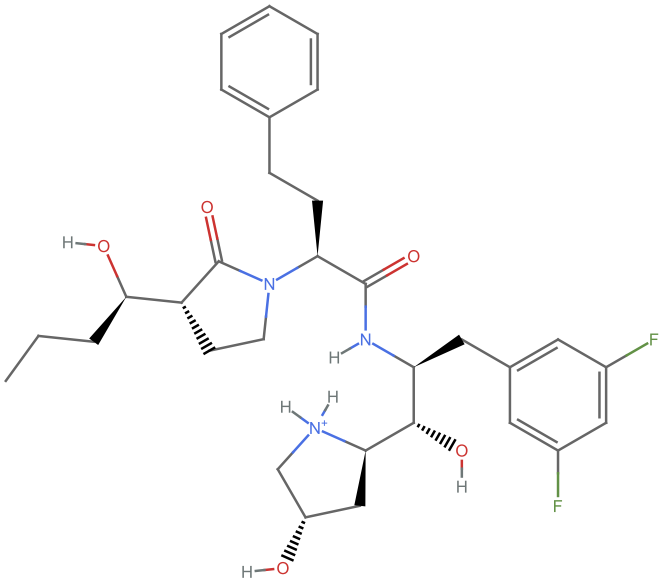

3D Plot

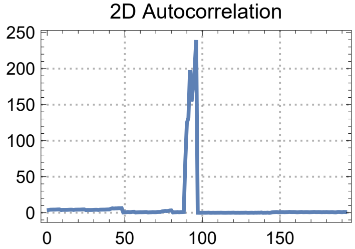

Dataset Index= 463

Molecule Formula= $C_{31}H_{42}F_2N_3O_5^+$

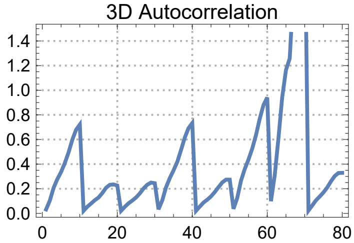

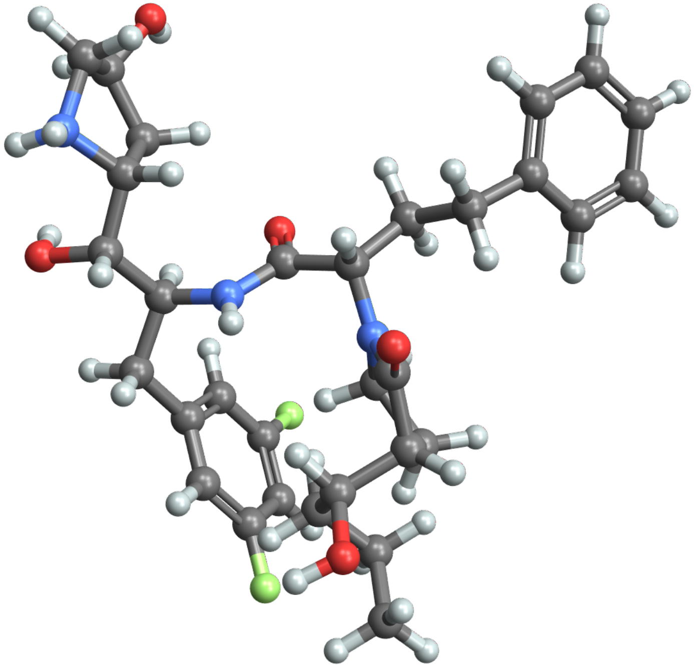

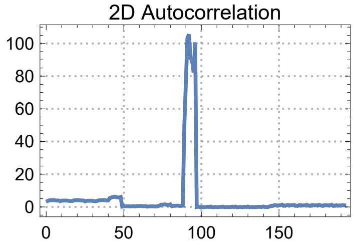

Dataset Index= 464

Molecule Formula= $C_{23}H_{25}FN_6O$

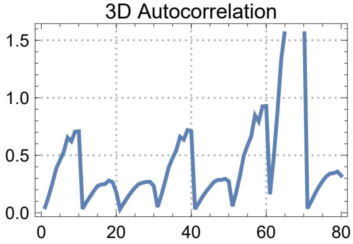

2D Plot

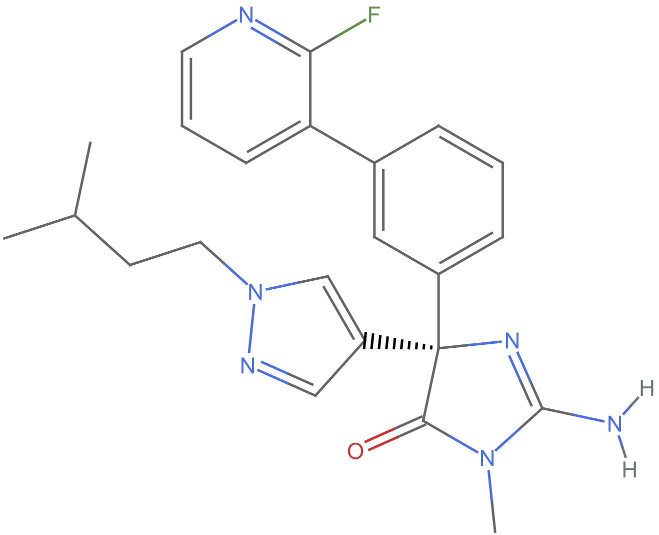

3D Plot

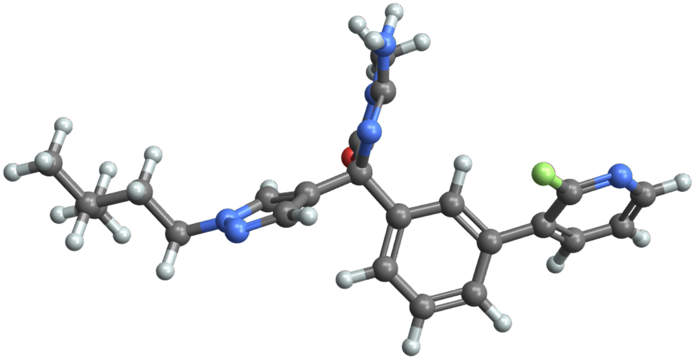

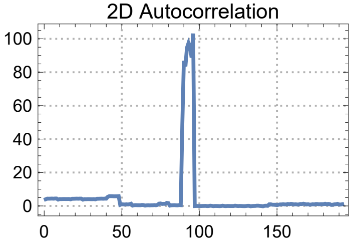

Dataset Index= 465

Molecule Formula= $C_{34}H_{47}N_4O_4S^+$

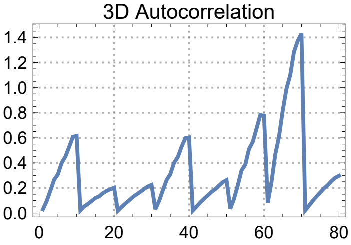

2D Plot

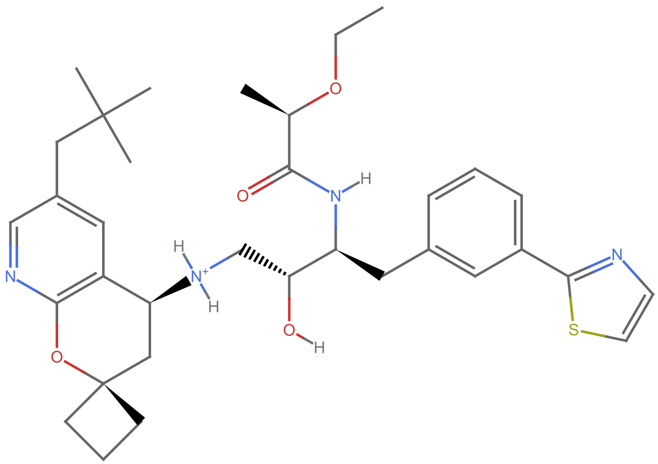

3D Plot

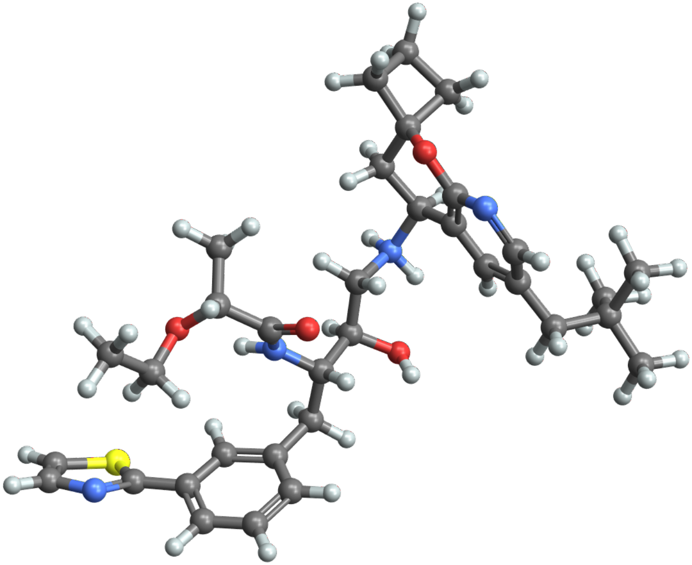

2D Plot

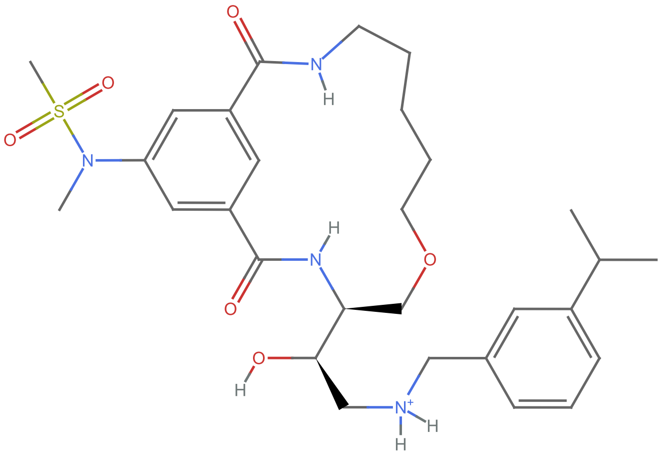

2D Autocorrelation

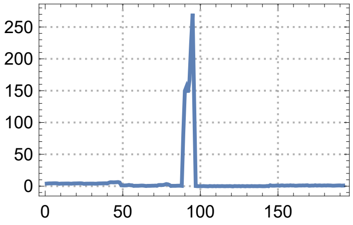

3D Plot

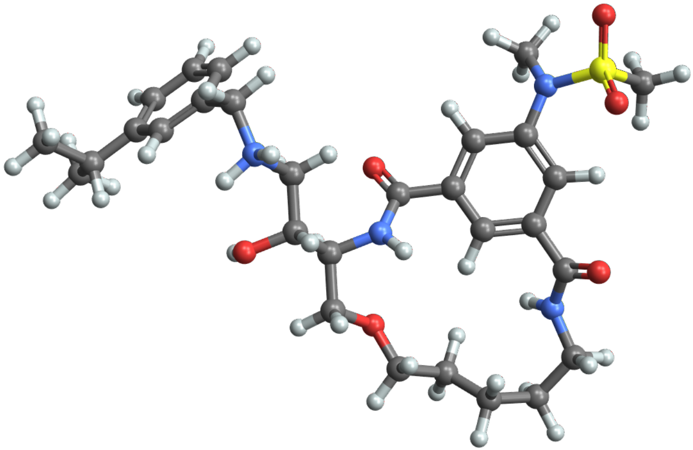

Dataset Index= 466

Molecule Formula= $C_{29}H_{43}N_4O_6S^+$

3D Autocorrelation

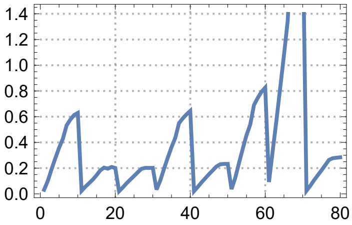

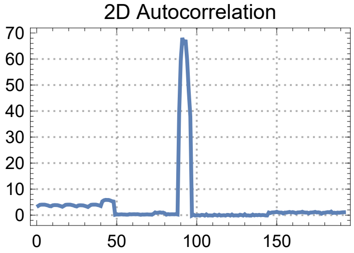

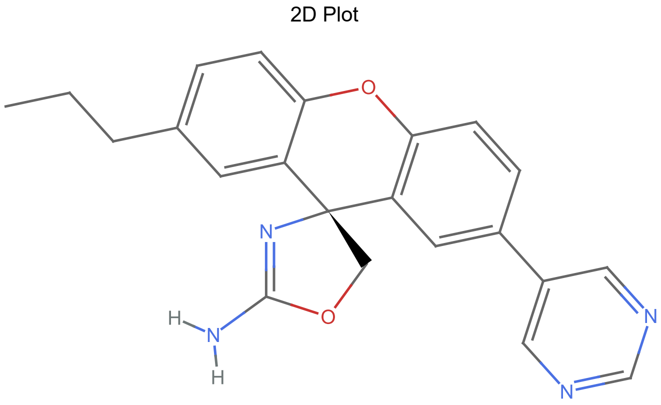

Dataset Index= 467

Molecule Formula= $C_{22}H_{20}N_4O_2$

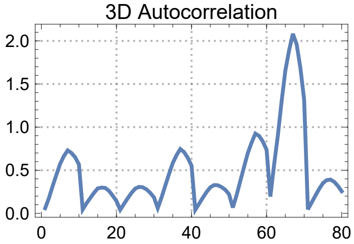

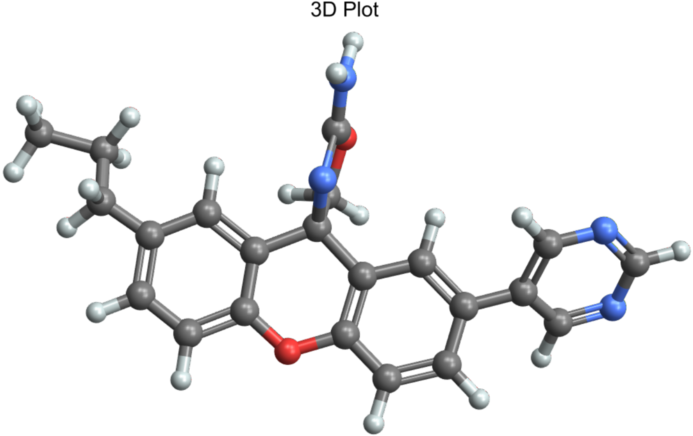

2D Plot

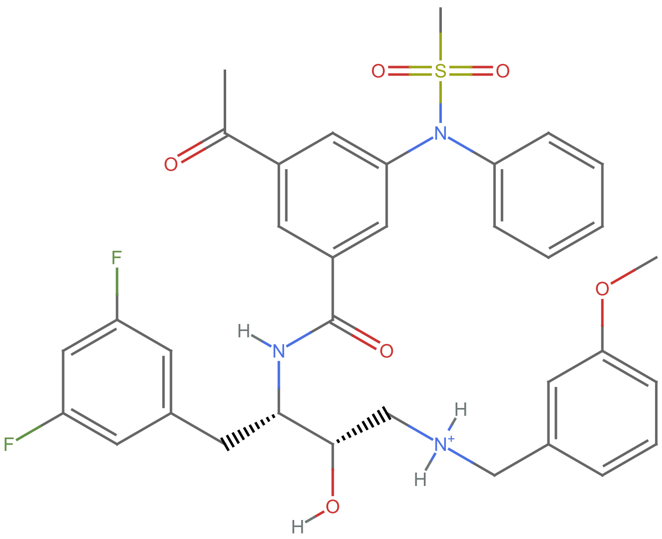

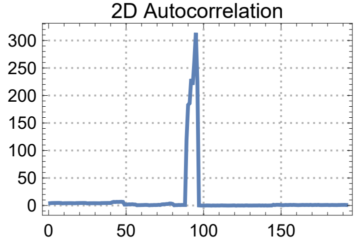

Dataset Index= 468

Molecule Formula= $C_{34}H_{36}F_2N_3O_6S^+$

3D Plot

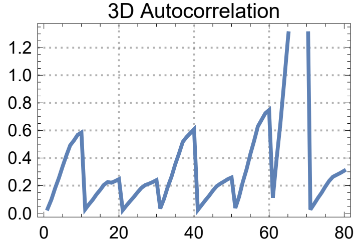

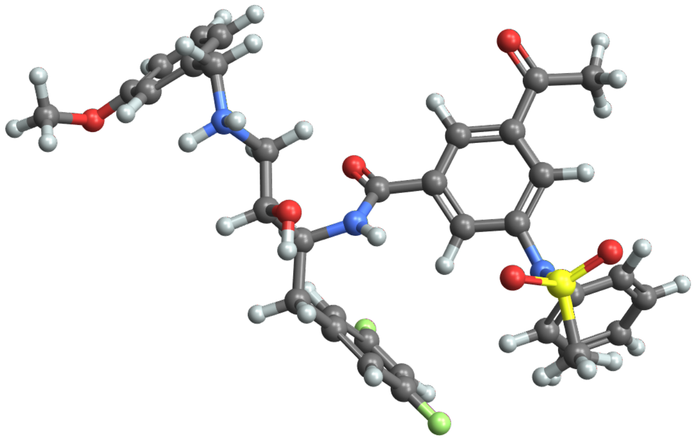

2D Plot

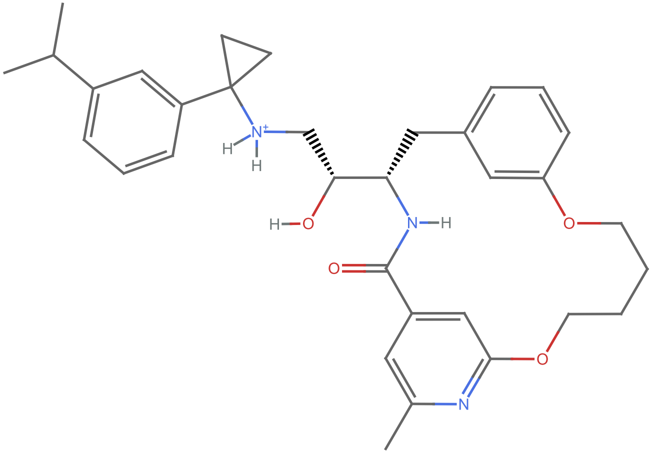

2D Autocorrelation

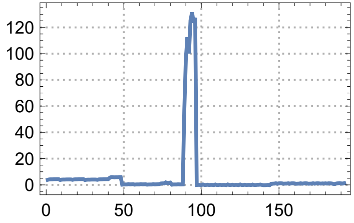

3D Plot

Dataset Index= 469

Molecule Formula= $C_{33}H_{42}N_3O_4^+$

3D Autocorrelation

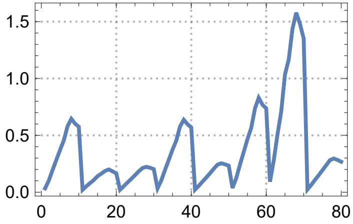

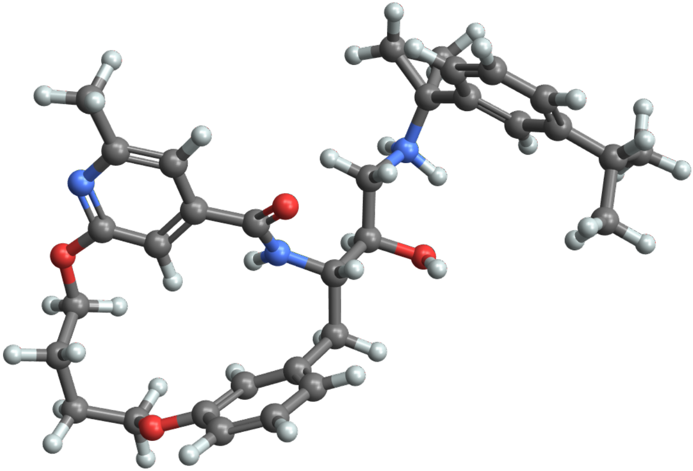

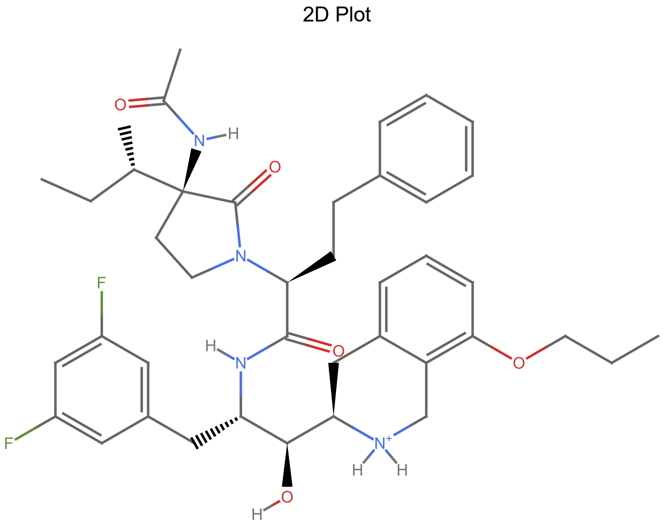

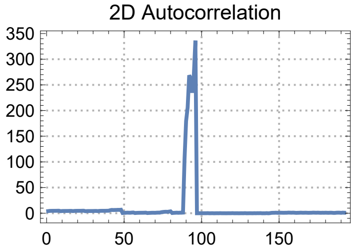

Dataset Index= 470

Molecule Formula= $C_{41}H_{53}F_{2}N_{4}O_{5}^{+}$

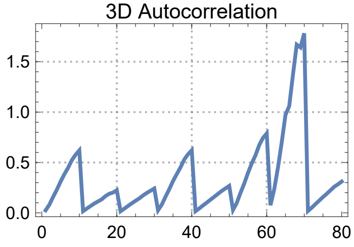

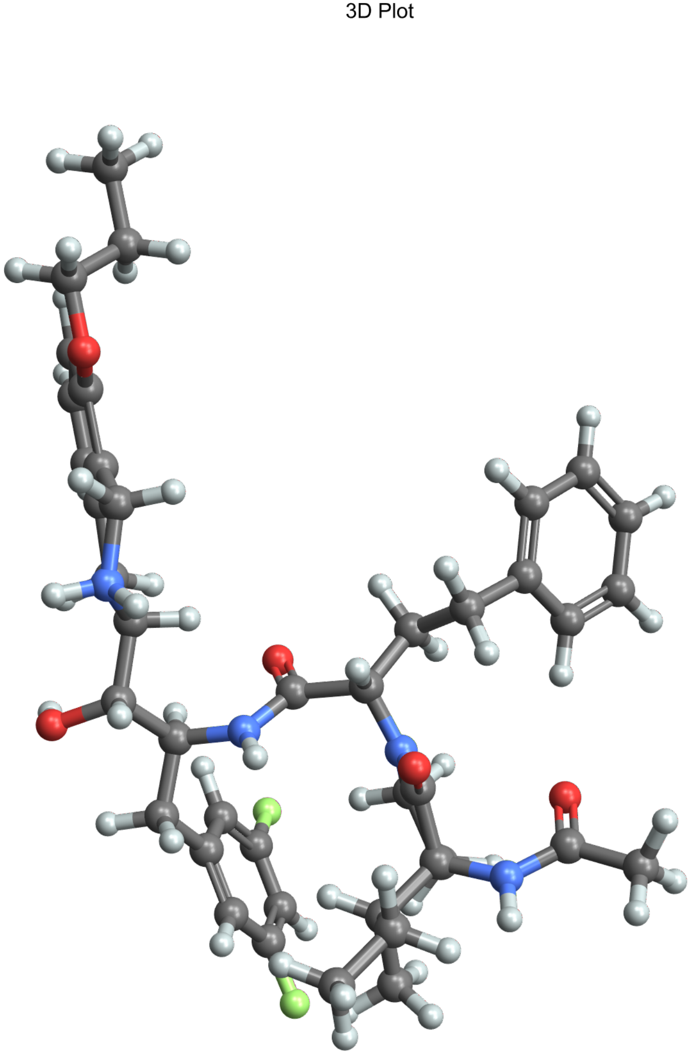

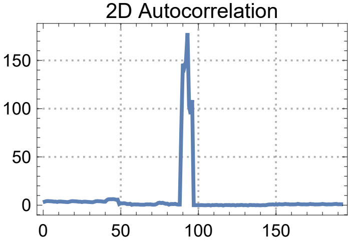

Dataset Index= 471

Molecule Formula= $C_{18}H_{16}ClF_3N_4O_2$

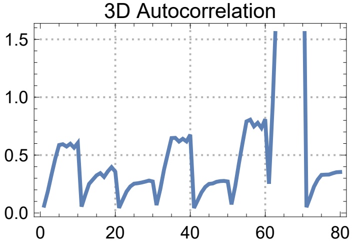

2D Plot

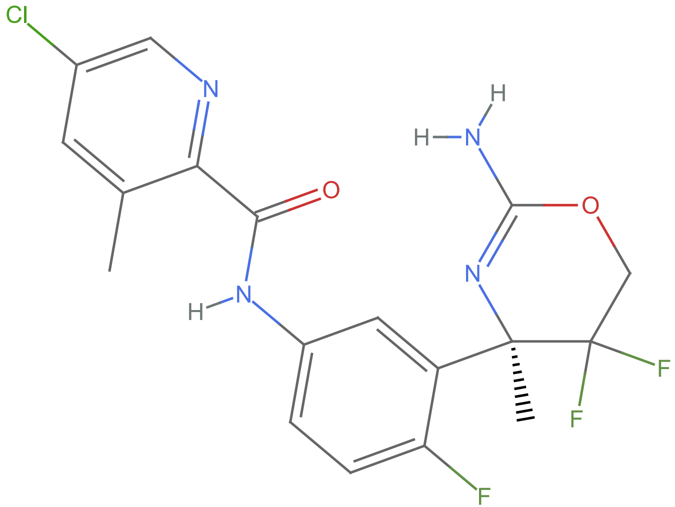

3D Plot

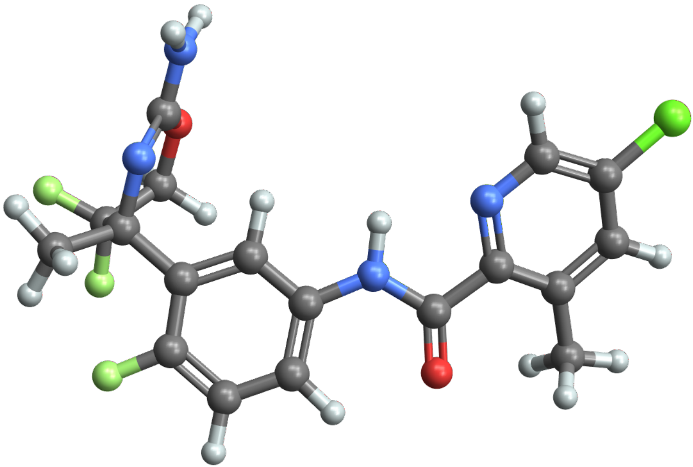

2D Plot

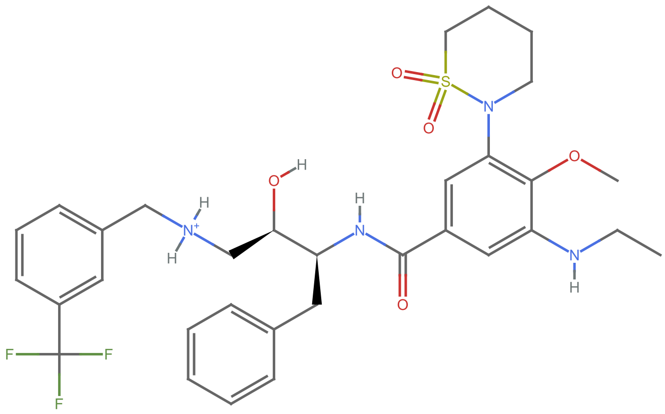

2D Autocorrelation

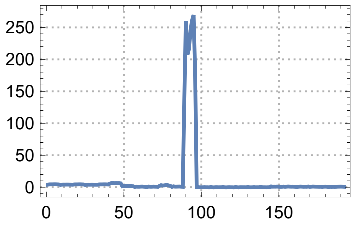

3D Plot

Dataset Index= 472

Molecule Formula= $C_{32}H_{40}F_3N_4O_5S^+$

3D Autocorrelation

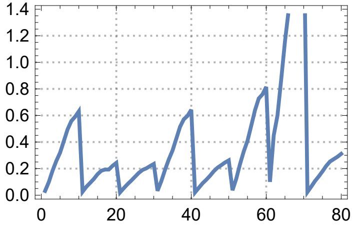

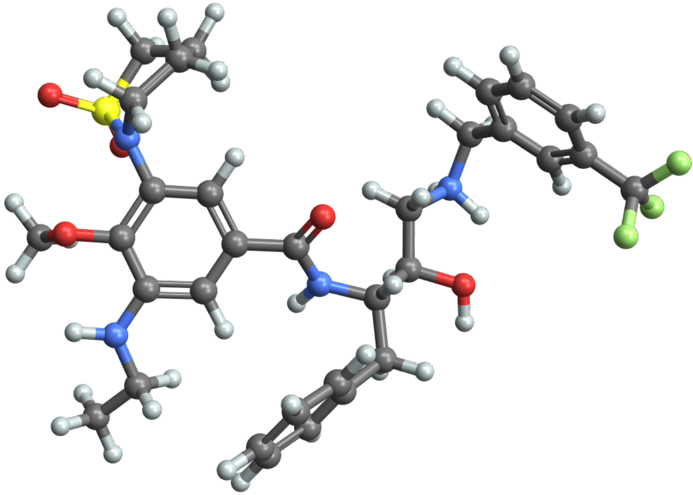

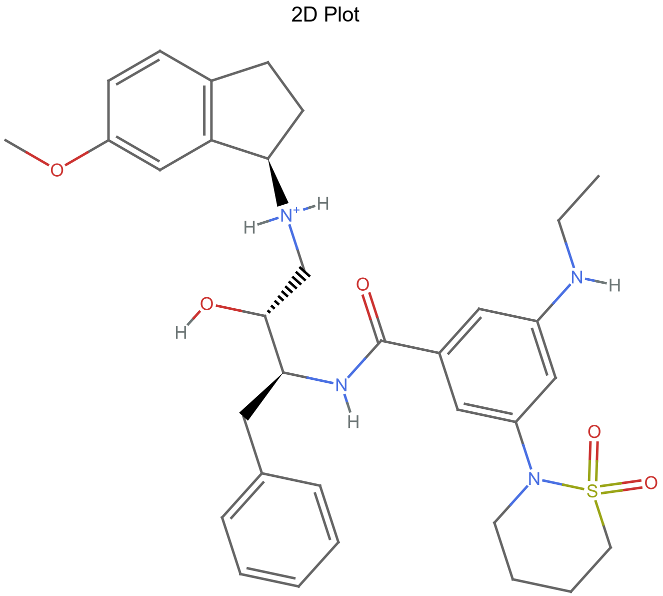

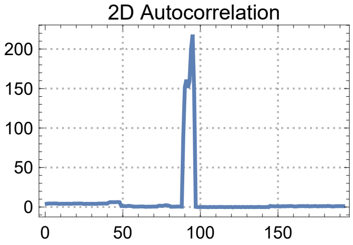

Dataset Index= 473

Molecule Formula= $C_{33}H_{43}N_4O_5S^+$

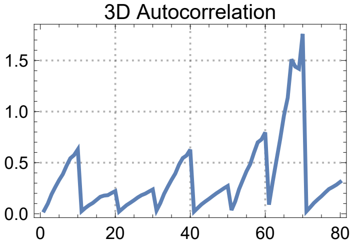

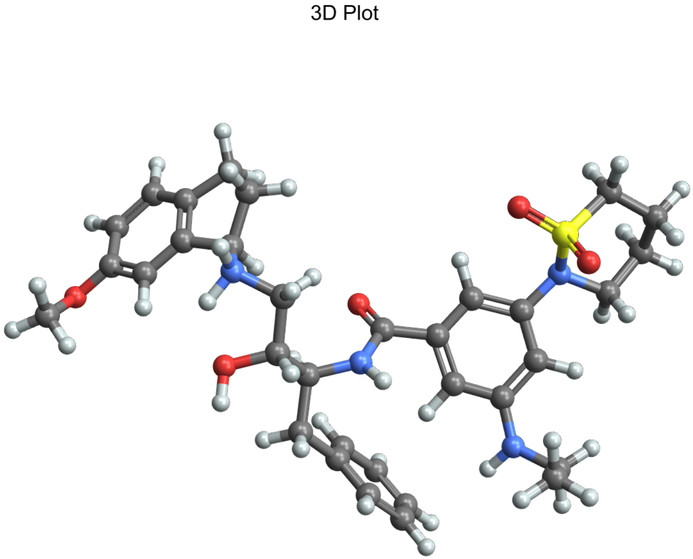

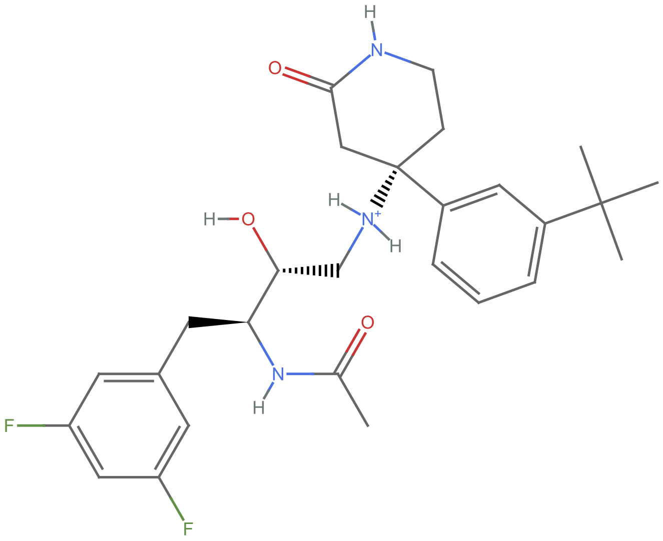

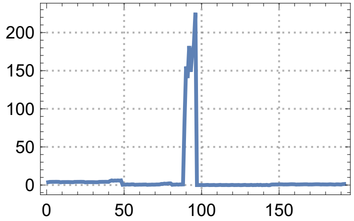

3D Plot

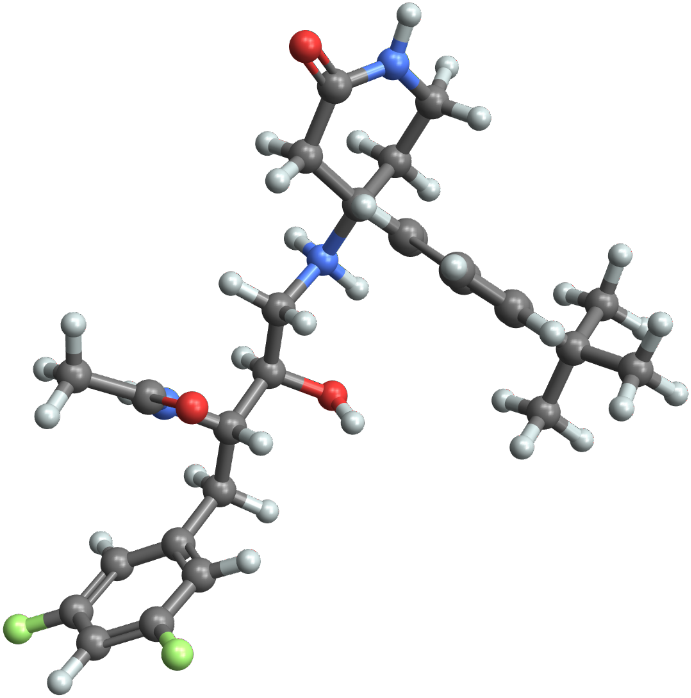

Dataset Index= 474

Molecule Formula= $C_{27}H_{36}F_2N_3O_3^+$

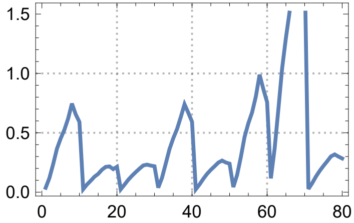

2D Plot

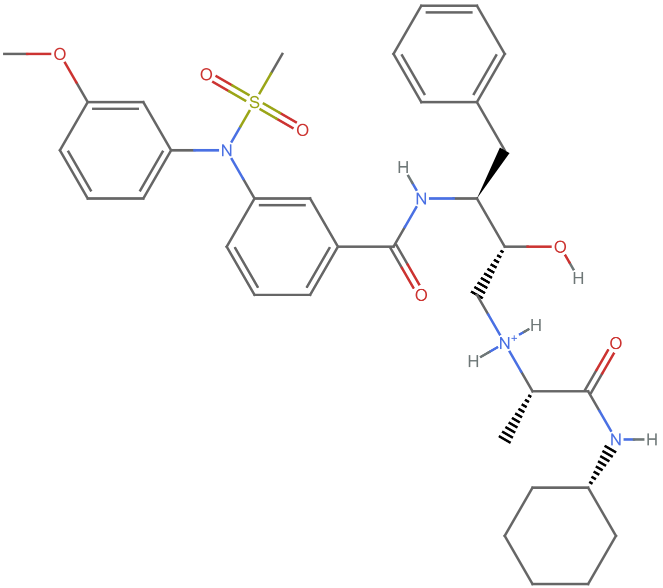

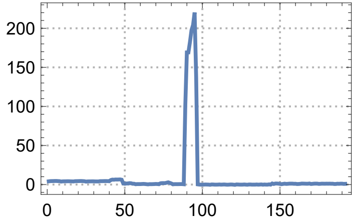

Dataset Index= 475

Molecule Formula= $C_{34}H_{45}N_4O_6S^+$

3D Plot

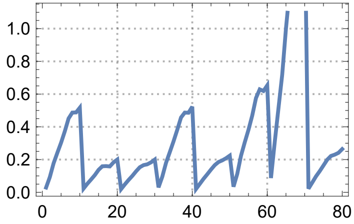

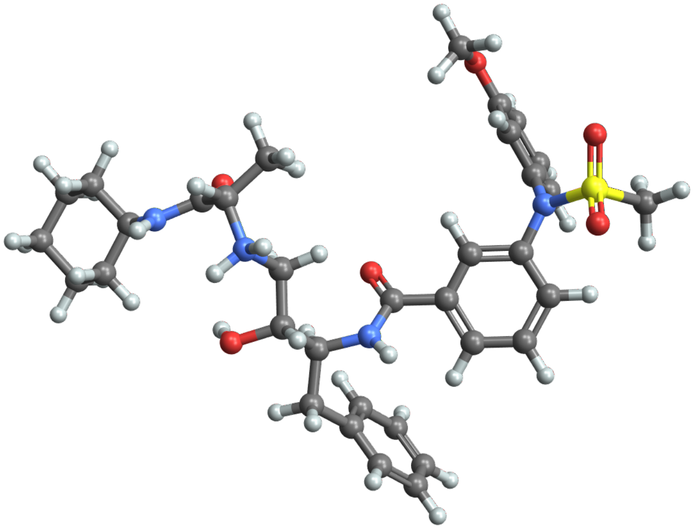

2D Plot

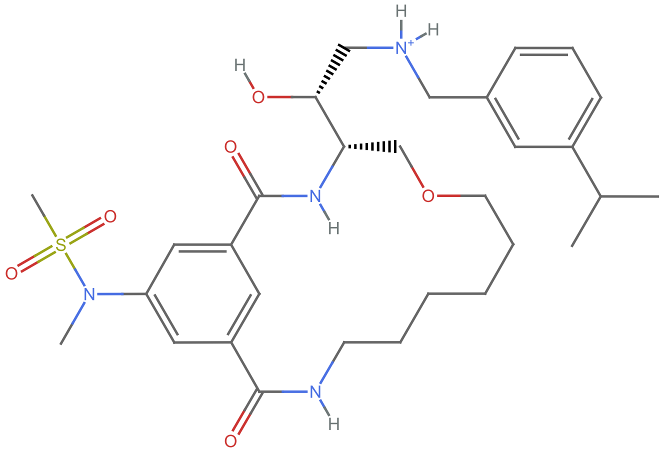

2D Autocorrelation

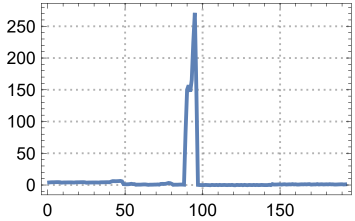

3D Plot

Dataset Index= 476

Molecule Formula= $C_{30}H_{45}N_4O_6S^+$

3D Autocorrelation

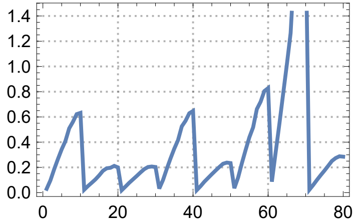

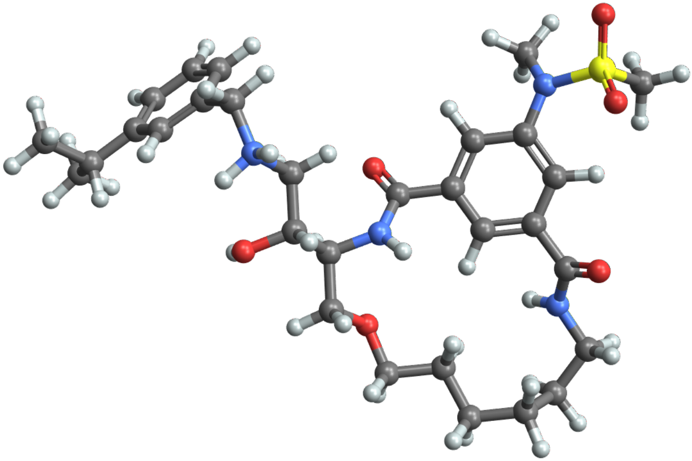

2D Autocorrelation

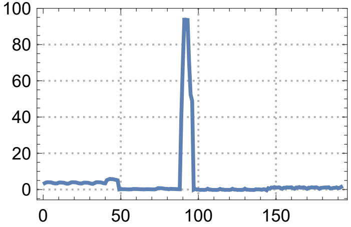

Dataset Index= 477

Molecule Formula= $C_{22}H_{22}N_4O$

3D Autocorrelation

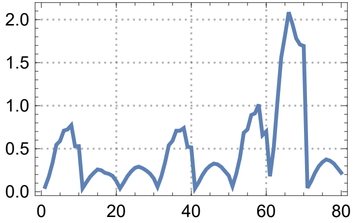

2D Plot

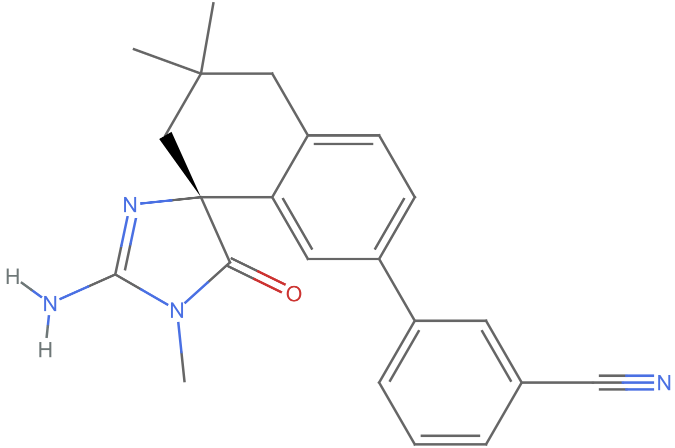

3D Plot

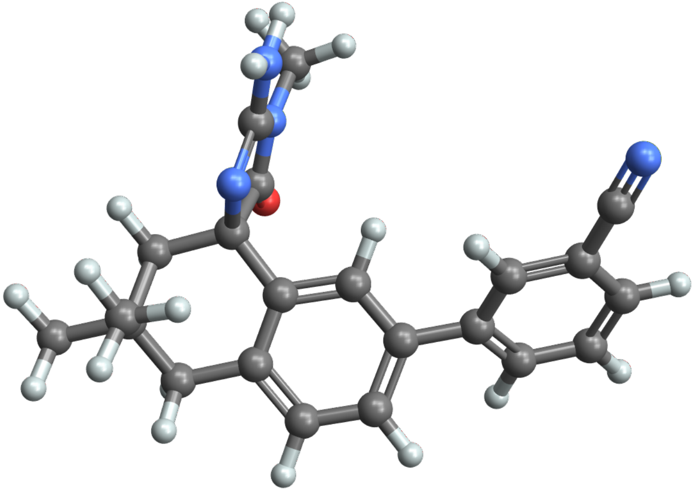

2D Plot

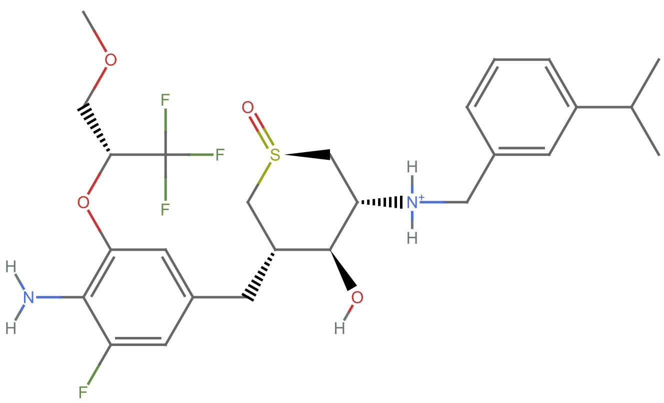

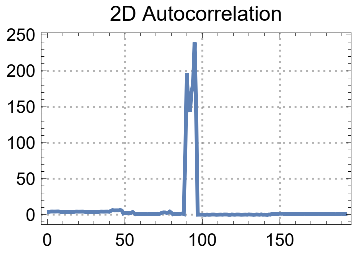

Dataset Index= 478

Molecule Formula= $C_{26}H_{35}F_4N_2O_4S^+$

3D Plot

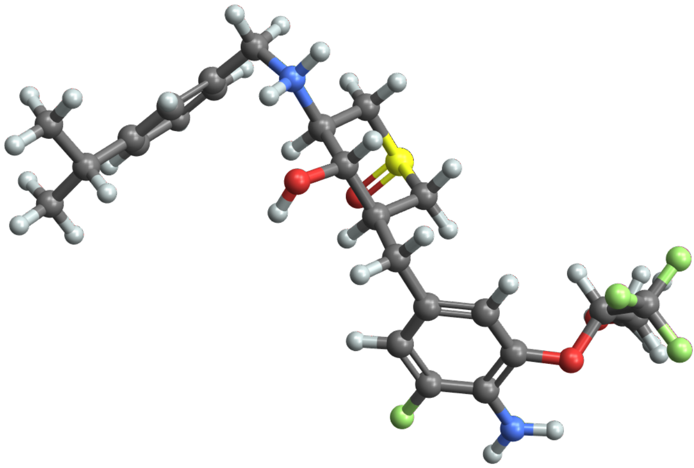

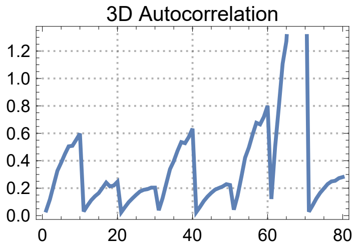

2D Plot

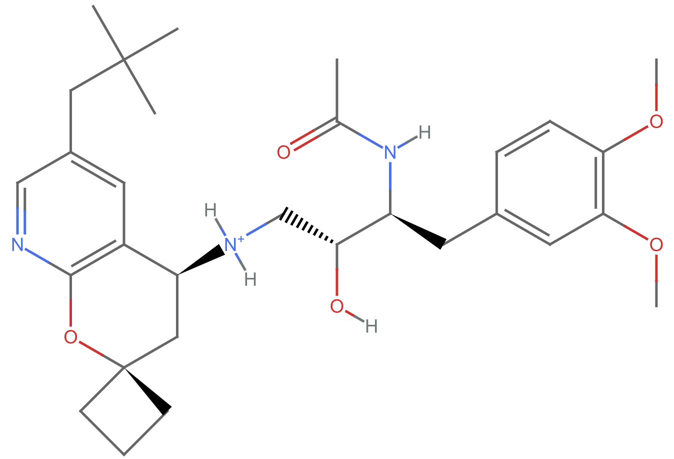

2D Autocorrelation

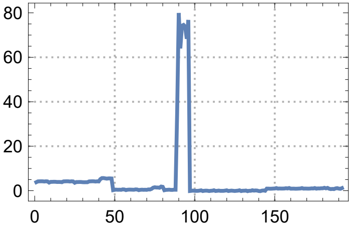

3D Plot

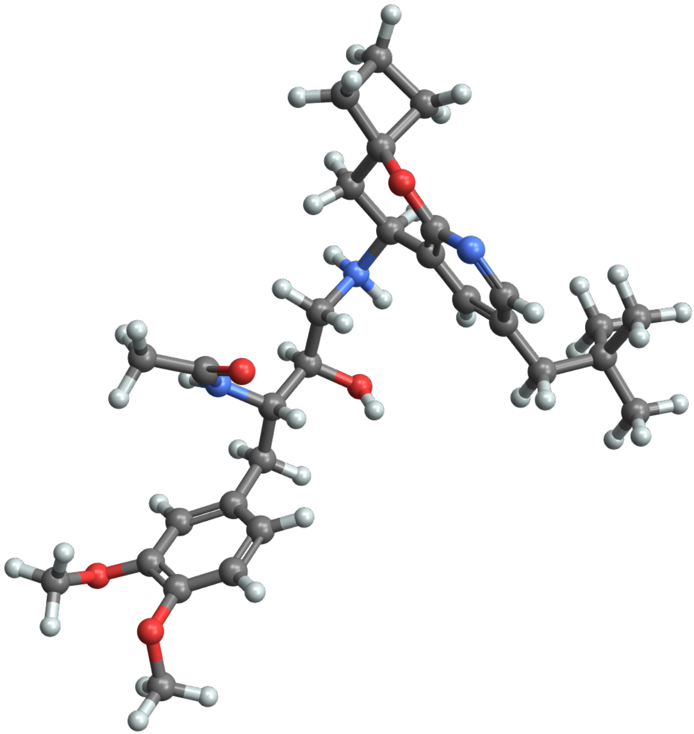

Dataset Index= 479

Molecule Formula= $C_{30}H_{44}N_{3}O_{5}^{+}$

3D Autocorrelation

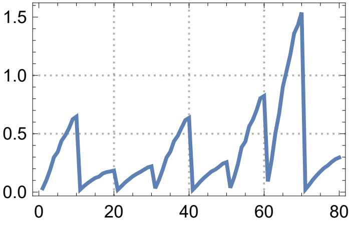

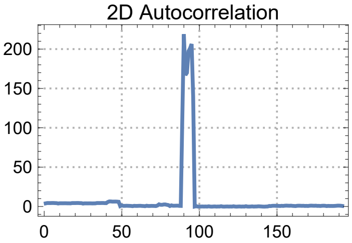

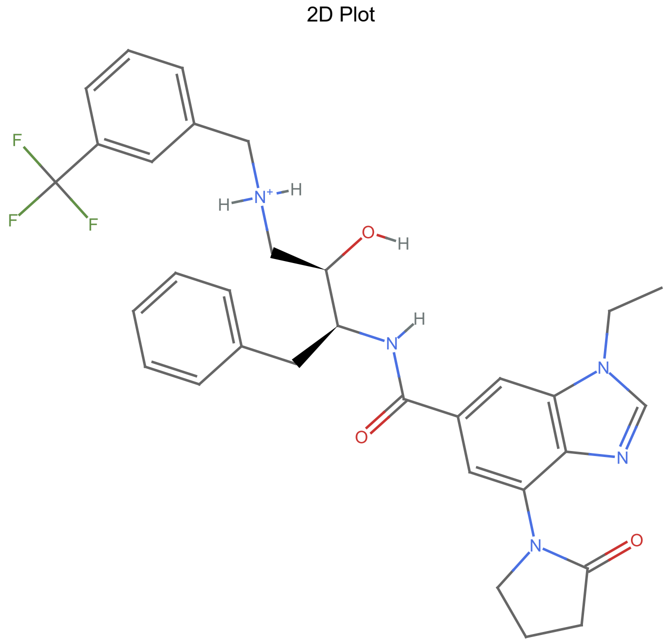

Dataset Index= 480

Molecule Formula= $C_{32}H_{35}F_3N_5O_3^+$

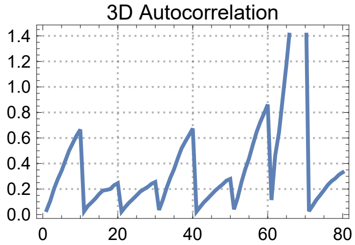

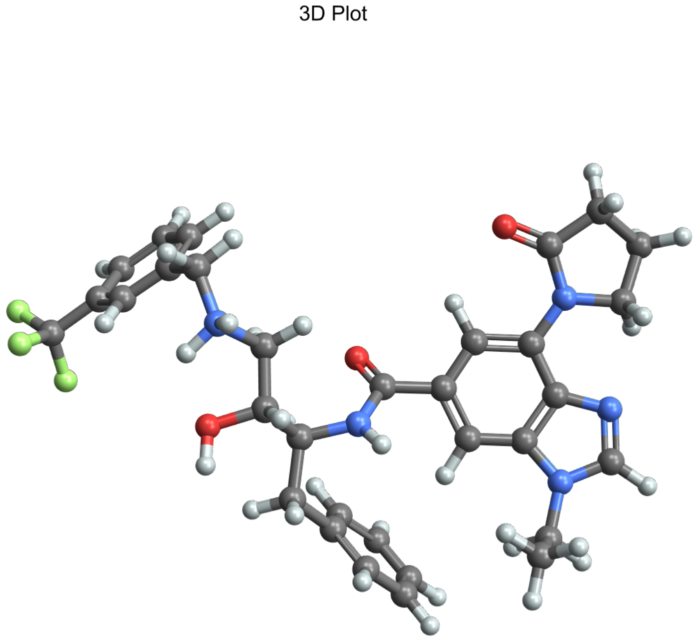

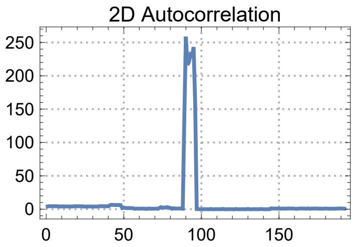

2D Plot

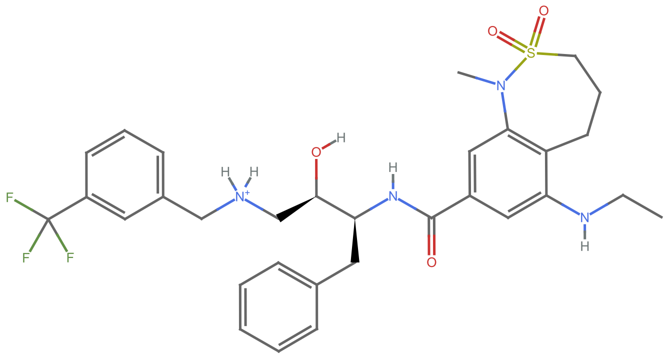

3D Plot

Dataset Index= 481

Molecule Formula= $C_{31}H_{38}F_{3}N_{4}O_{4}S^{+}$

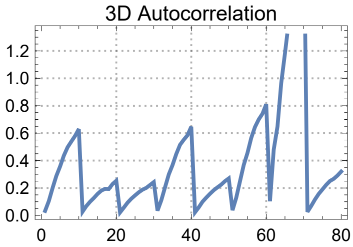

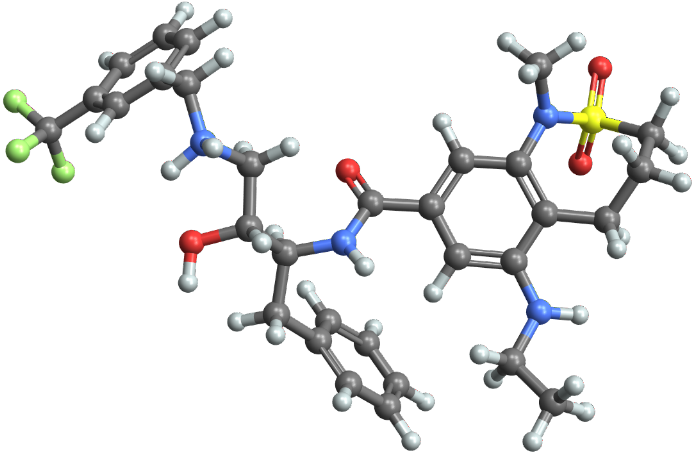

2D Plot

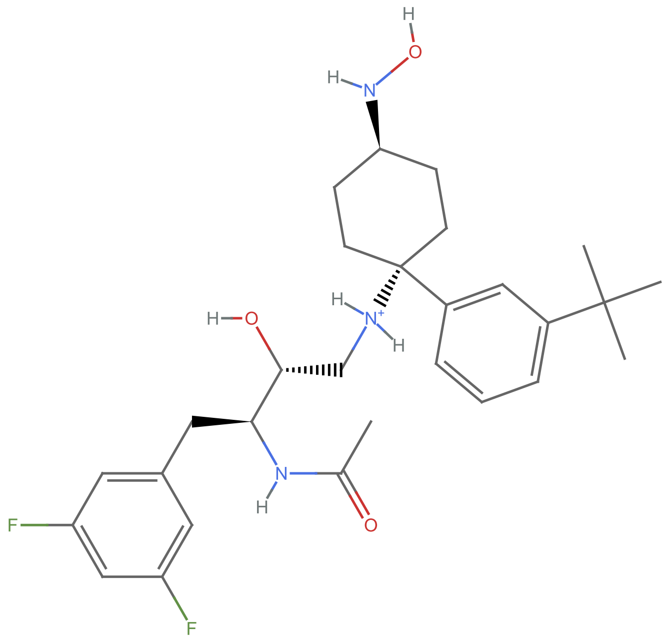

2D Autocorrelation

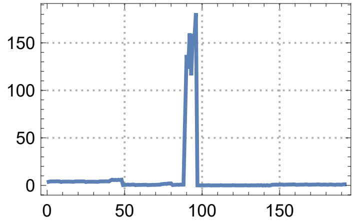

3D Plot

Dataset Index= 482

Molecule Formula= $C_{28}H_{40}F_2N_3O_3^+$

3D Autocorrelation

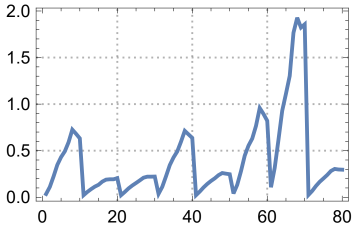

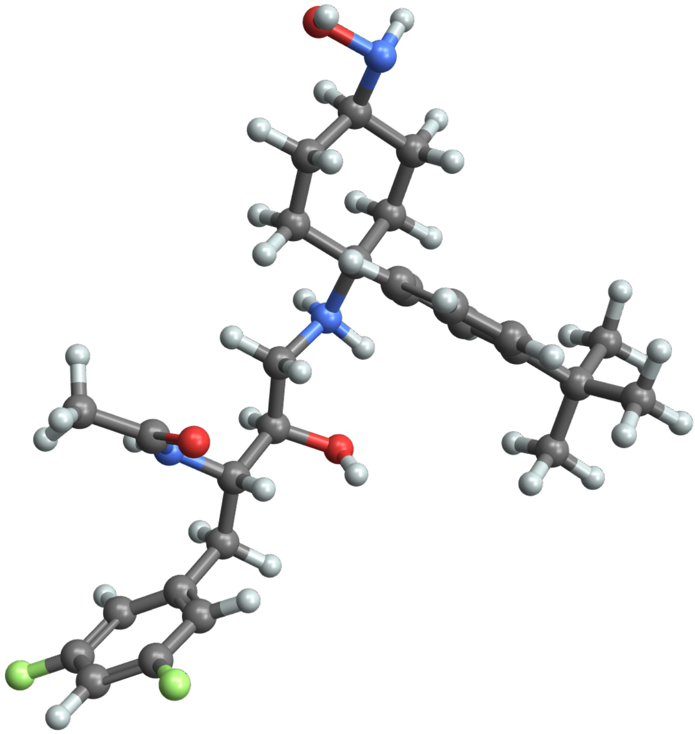

2D Plot

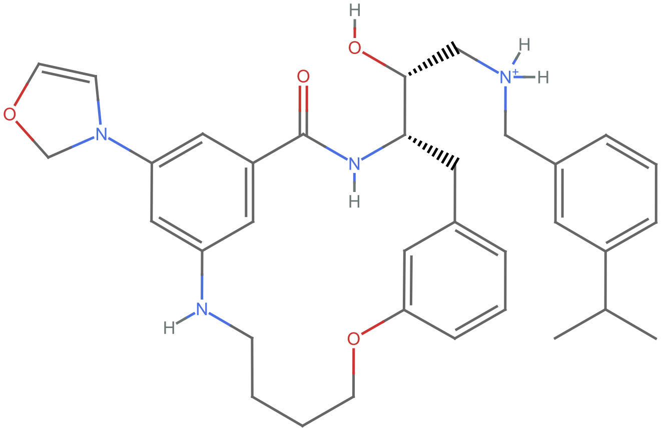

2D Autocorrelation

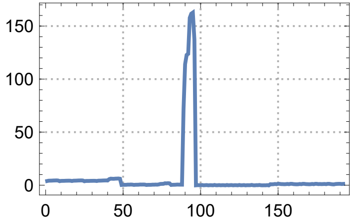

3D Plot

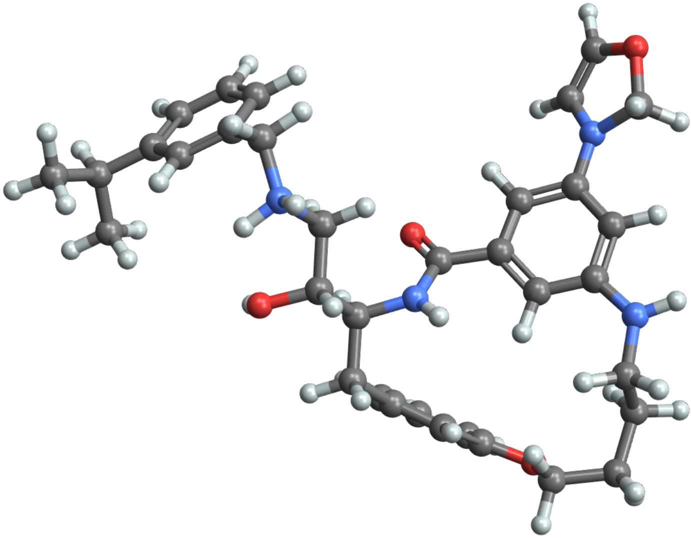

Dataset Index= 483

Molecule Formula= $C_{34}H_{43}N_4O_4^+$

3D Autocorrelation

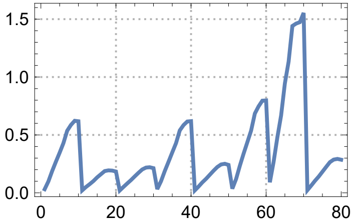

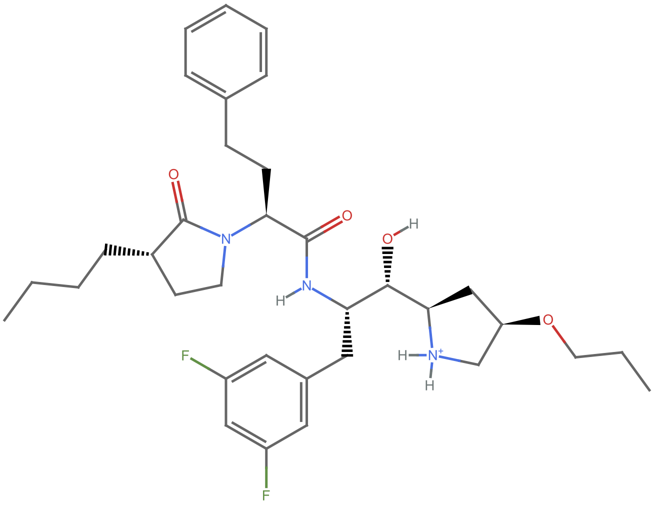

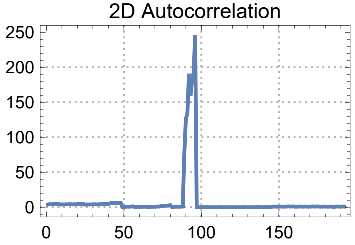

Dataset Index= 484

Molecule Formula= $C_{34}H_{48}F_{2}N_{3}O_{4}^{+}$

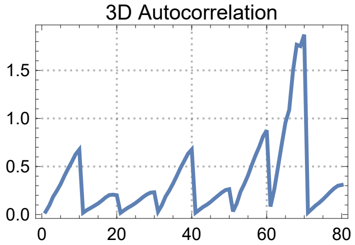

3D Plot

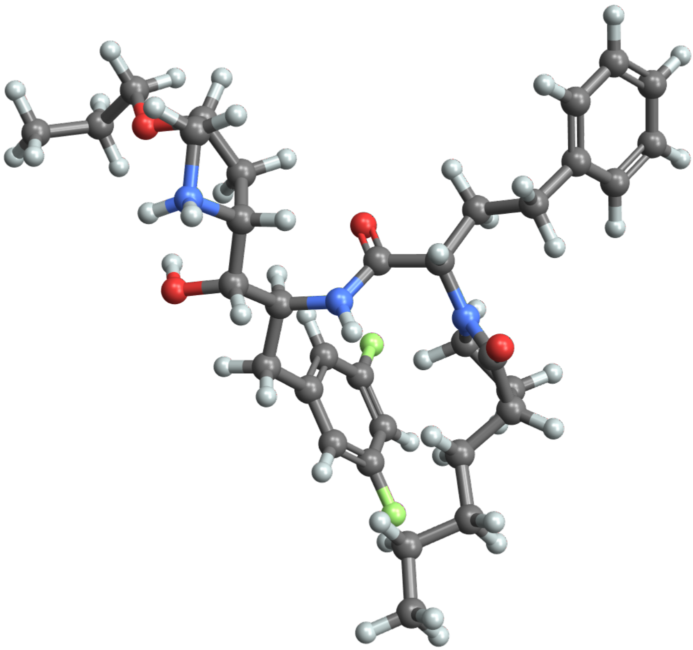

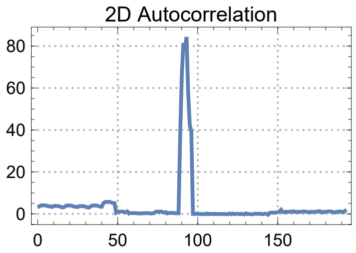

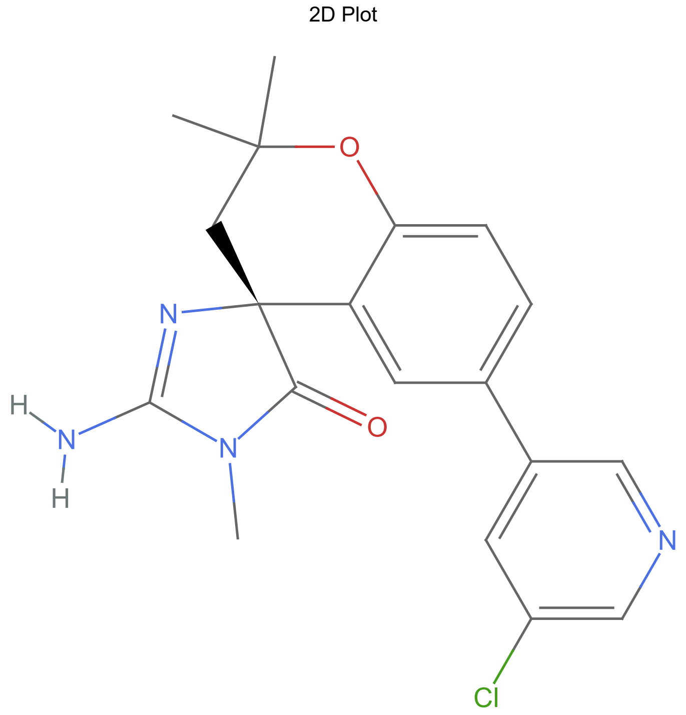

Dataset Index= 485

Molecule Formula= $C_{19}H_{19}ClN_4O_2$

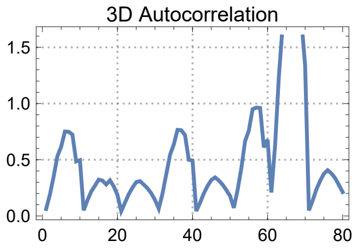

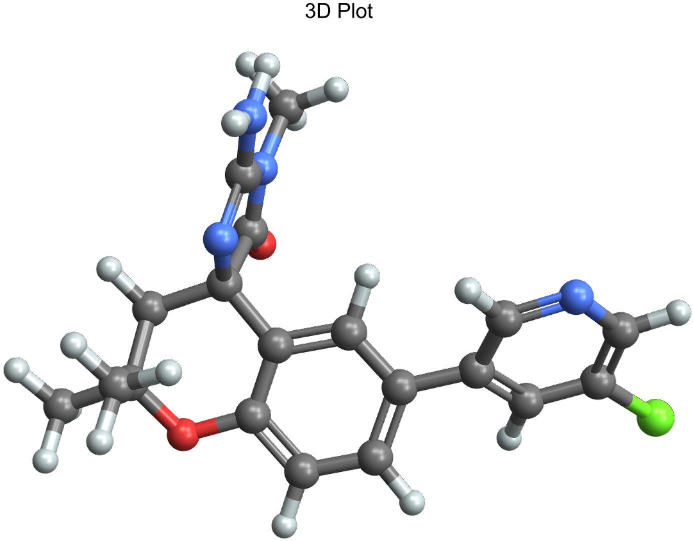

2D Autocorrelation

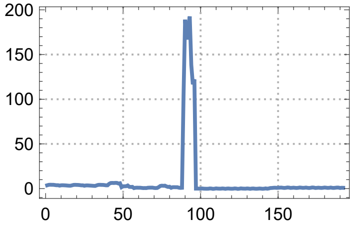

Dataset Index= 486

Molecule Formula= $C_{16}H_{13}ClF_5N_5O_2$

3D Autocorrelation

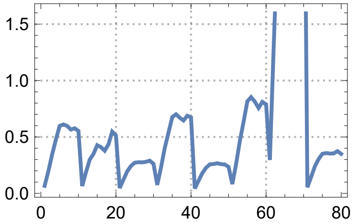

2D Plot

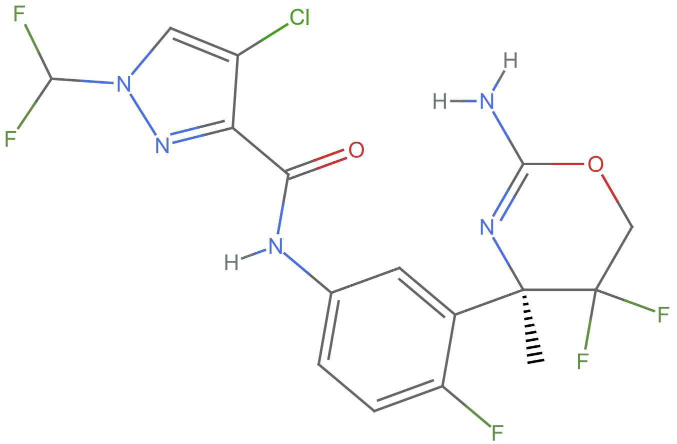

3D Plot

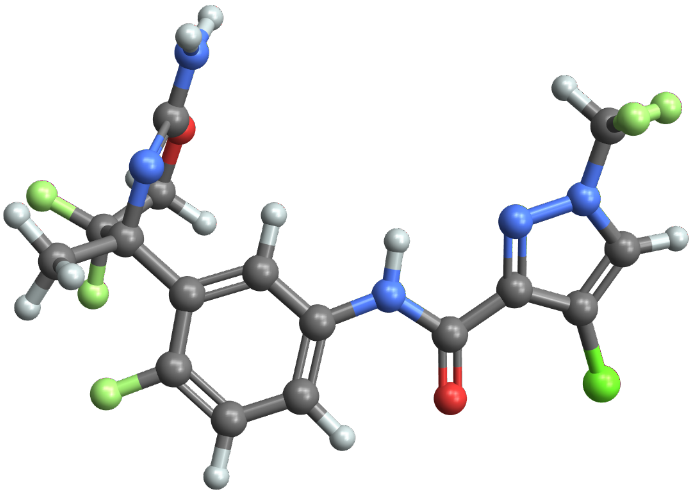

2D Autocorrelation

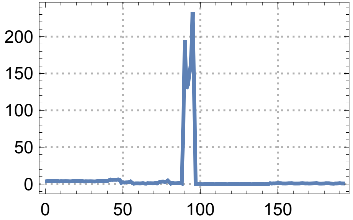

2D Plot

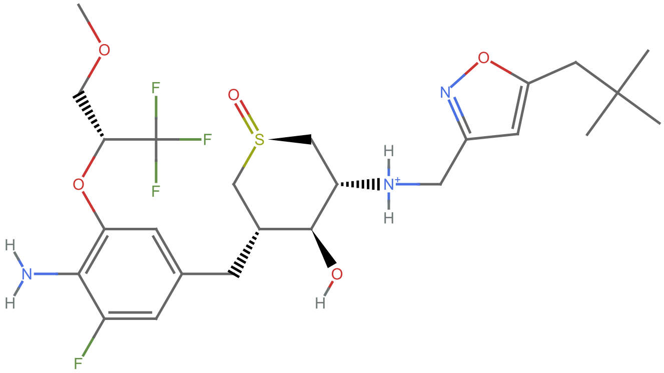

Dataset Index= 487

Molecule Formula= $C_{25}H_{36}F_4N_3O_5S^+$

3D Autocorrelation

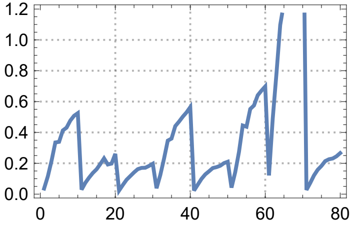

3D Plot

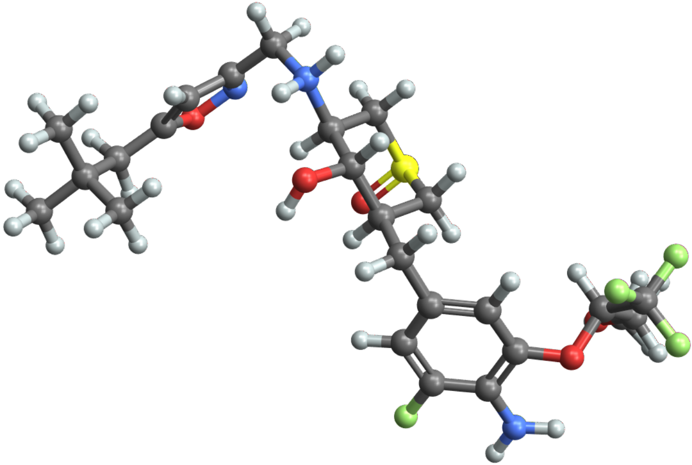

2D Plot

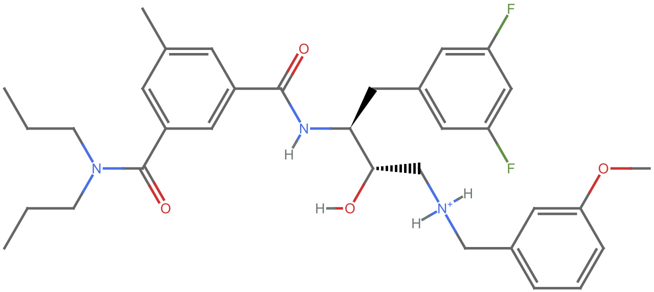

2D Autocorrelation

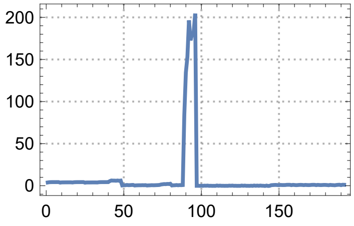

3D Plot

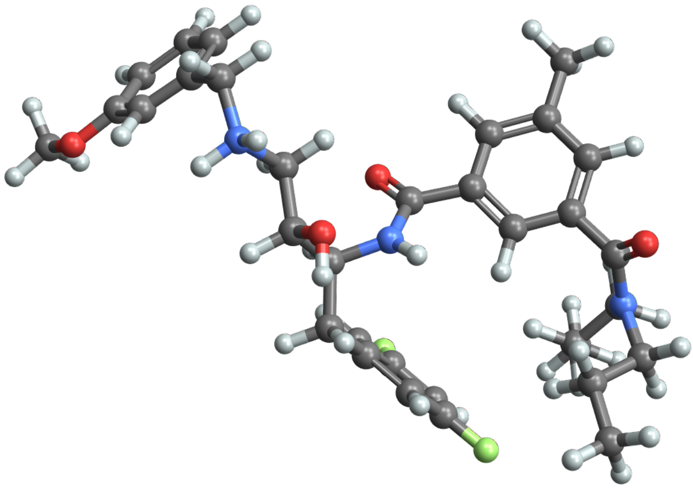

Dataset Index= 488

Molecule Formula= $C_{33}H_{42}F_2N_3O_4^+$

3D Autocorrelation

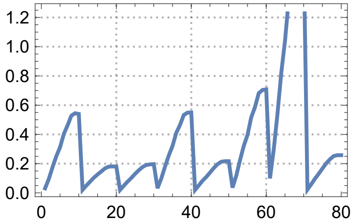

2D Plot

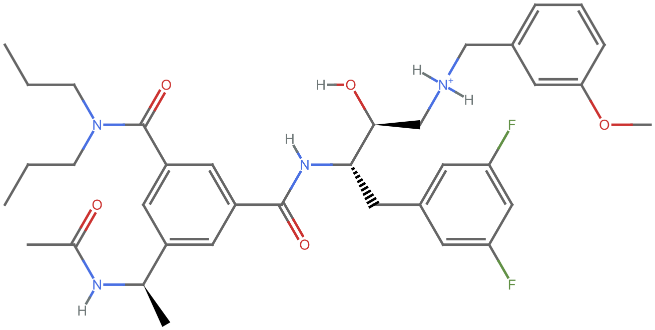

3D Plot

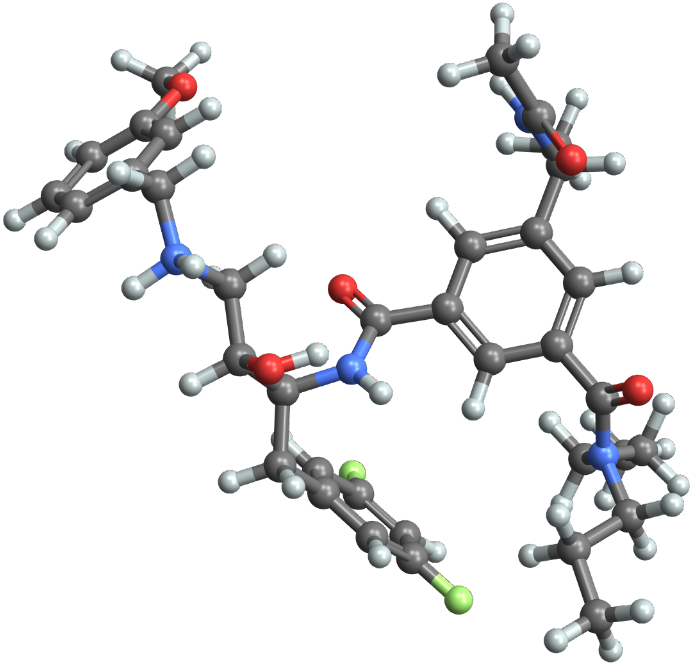

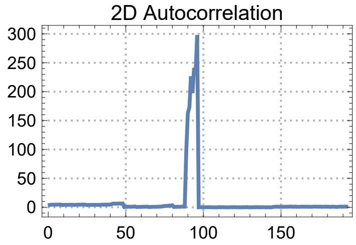

Dataset Index= 489

Molecule Formula= $C_{36}H_{47}F_2N_4O_5^+$

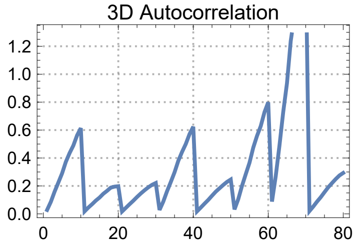

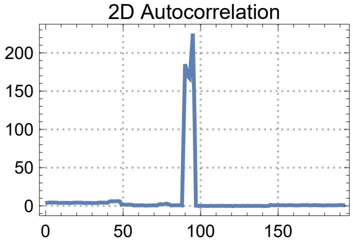

2D Plot

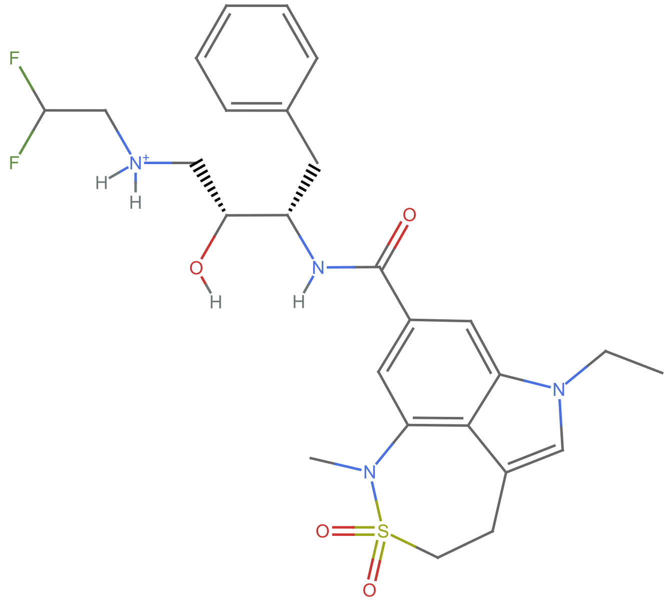

Dataset Index= 490

Molecule Formula= $C_{26}H_{33}F_2N_4O_4S^+$

3D Plot

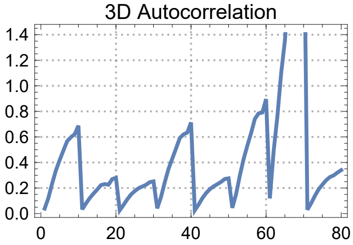

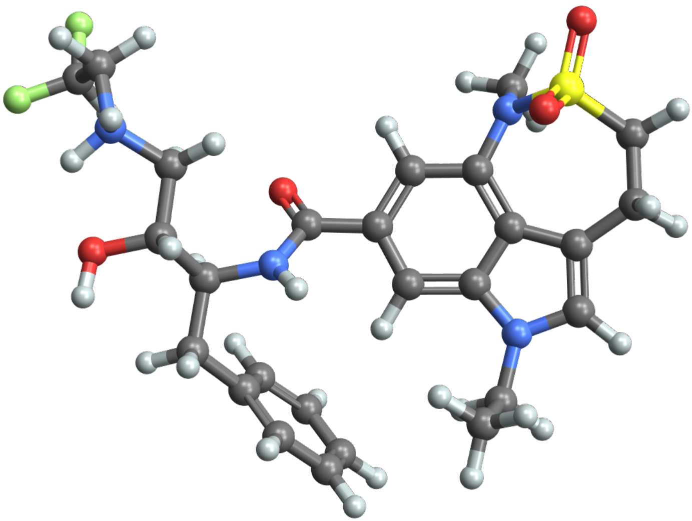

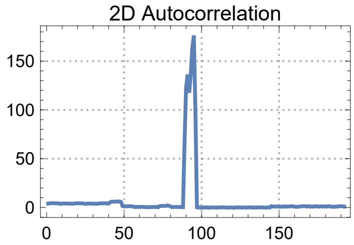

Dataset Index= 491

Molecule Formula= $C_{27}H_{35}N_4O_4S^+$

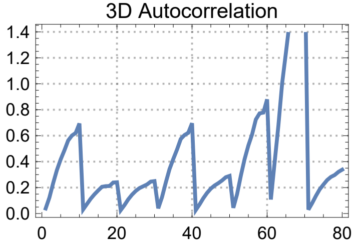

2D Plot

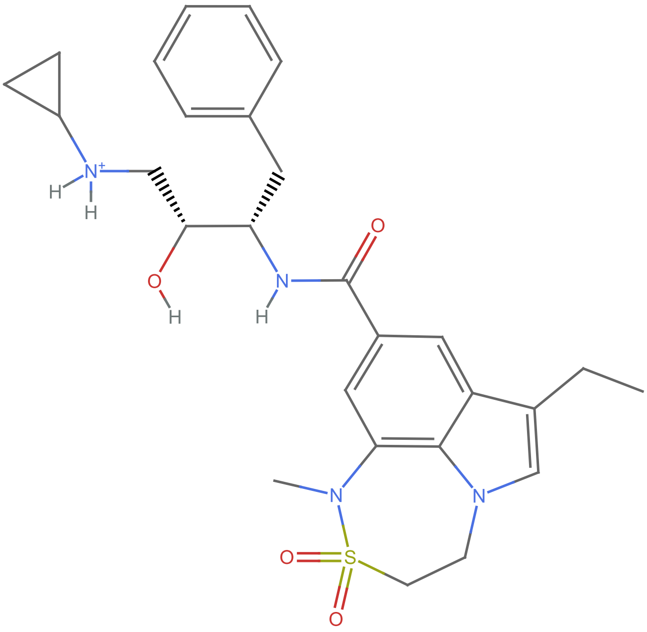

3D Plot

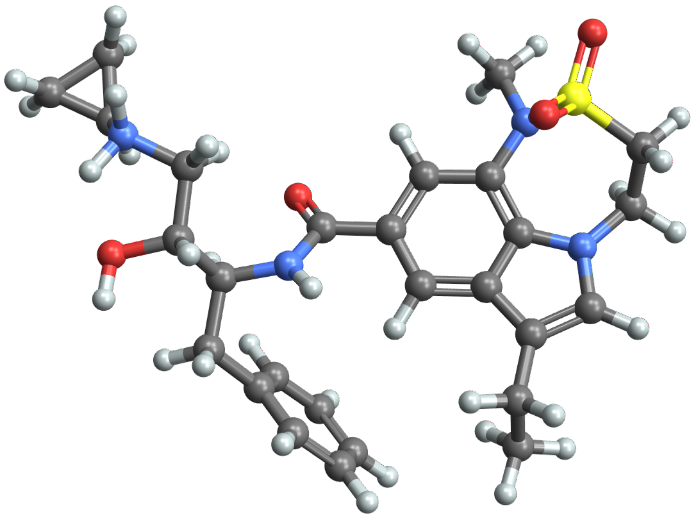

2D Plot

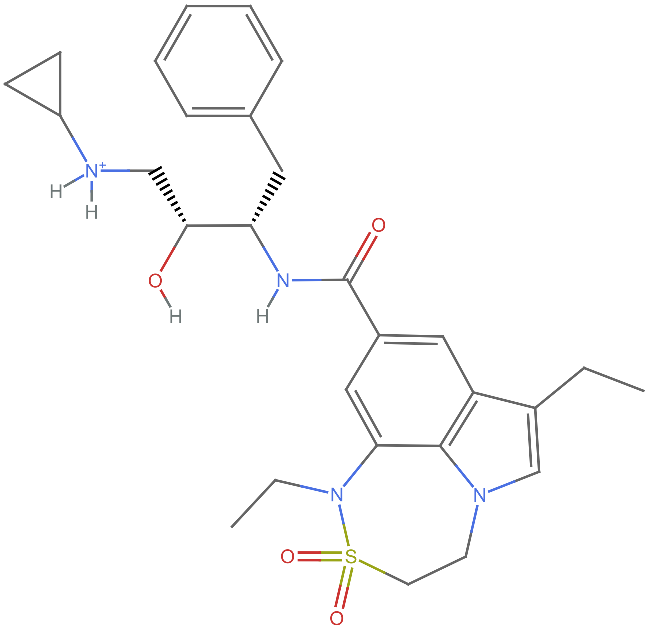

2D Autocorrelation

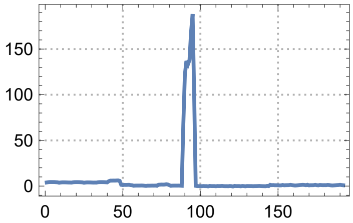

Dataset Index= 492

Molecule Formula= $C_{28}H_{37}N_4O_4S^+$

3D Plot

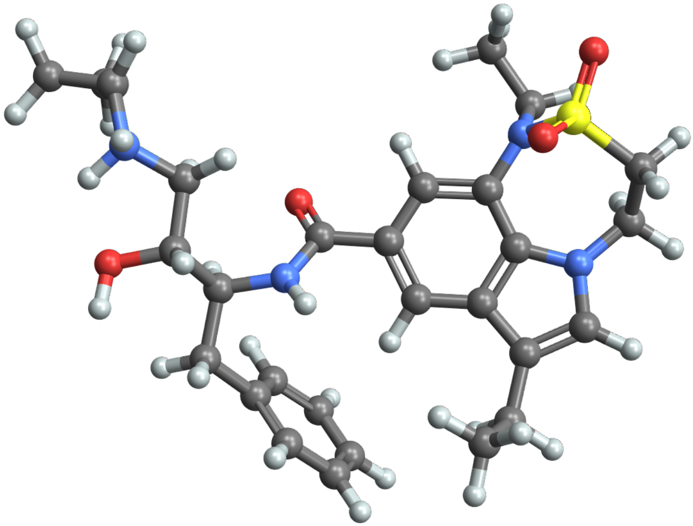

3D Autocorrelation

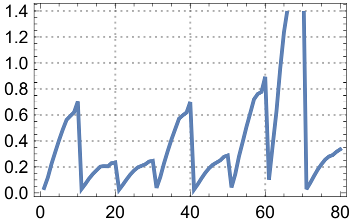

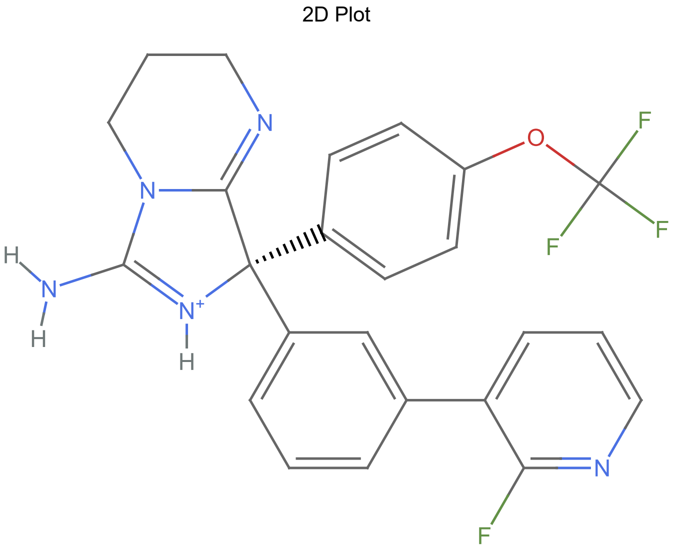

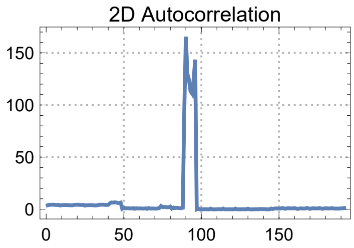

Dataset Index= 493

Molecule Formula= $C_{24}H_{20}F_4N_5O^+$

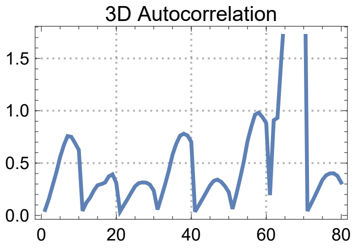

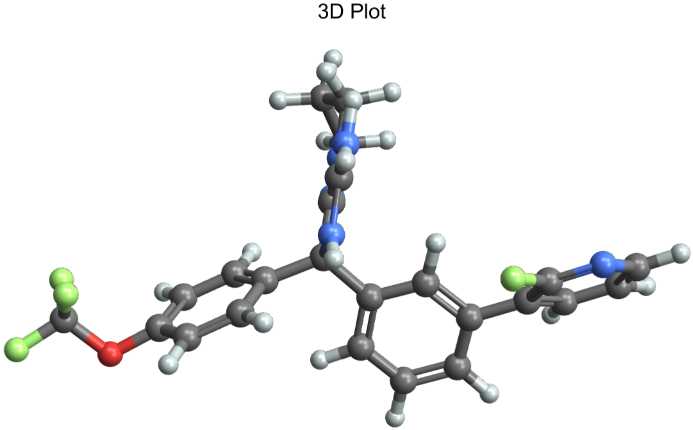

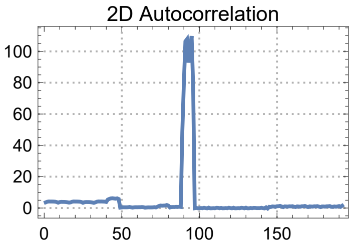

Dataset Index= 494

Molecule Formula= $C_{22}H_{20}FN_5O_2$

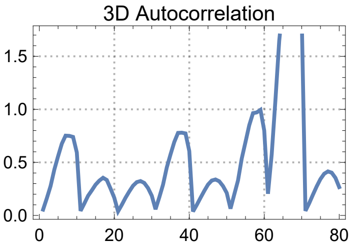

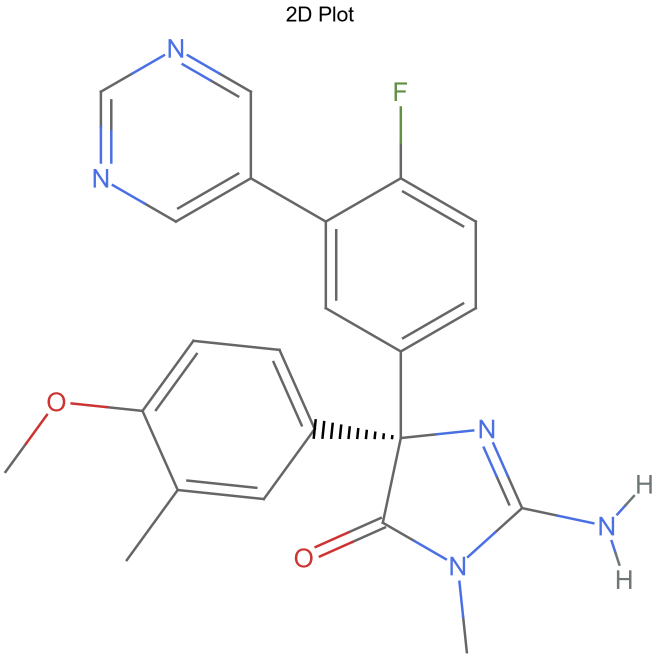

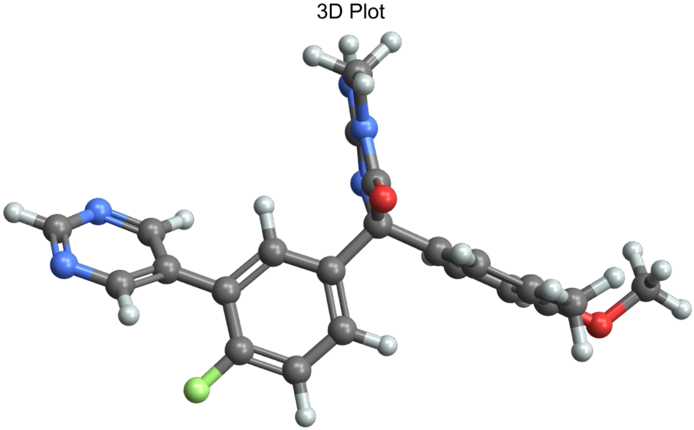

2D Plot

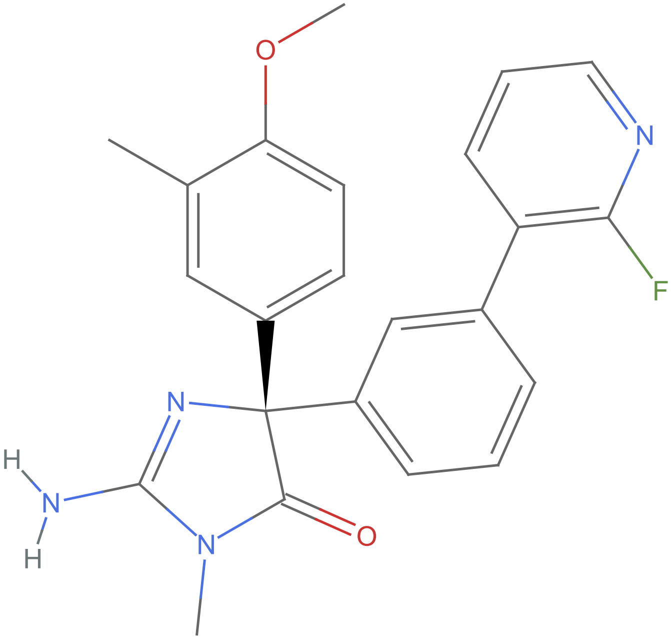

2D Autocorrelation

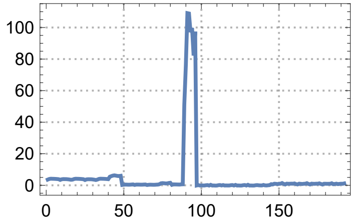

Dataset Index= 495

Molecule Formula= $C_{23}H_{21}FN_4O_2$

3D Autocorrelation

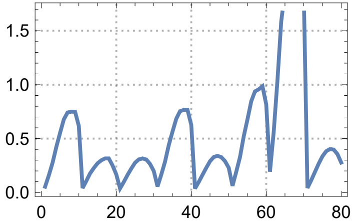

3D Plot

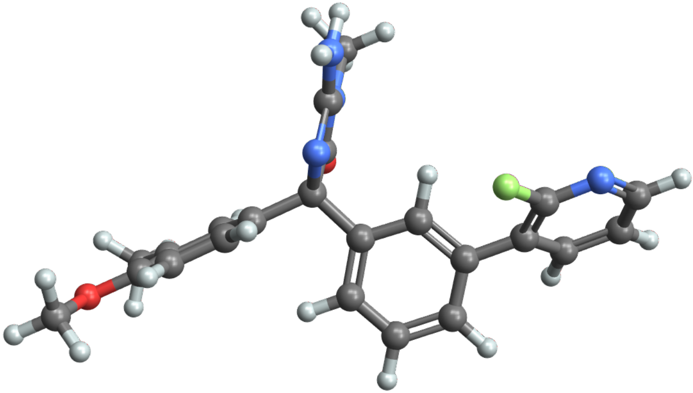

2D Plot

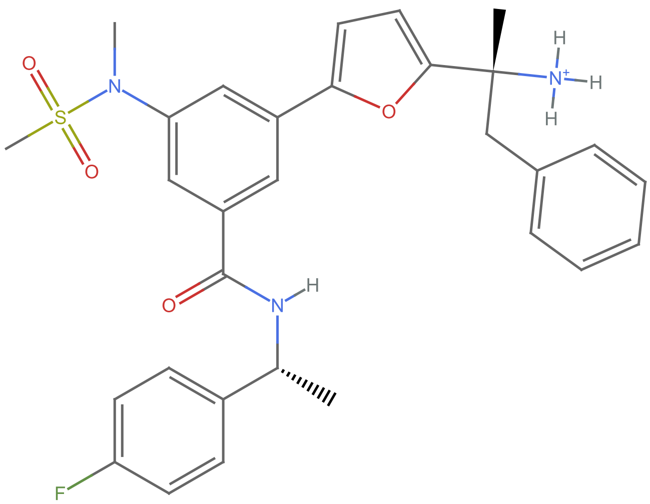

2D Autocorrelation

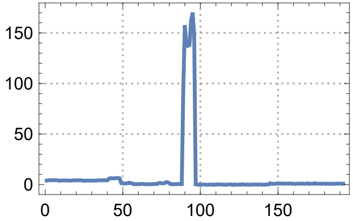

Dataset Index= 496

Molecule Formula= $C_{30}H_{33}FN_3O_4S^+$

3D Autocorrelation

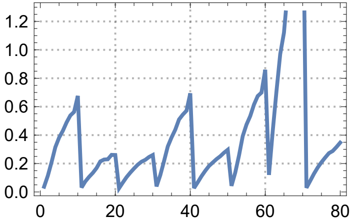

3D Plot

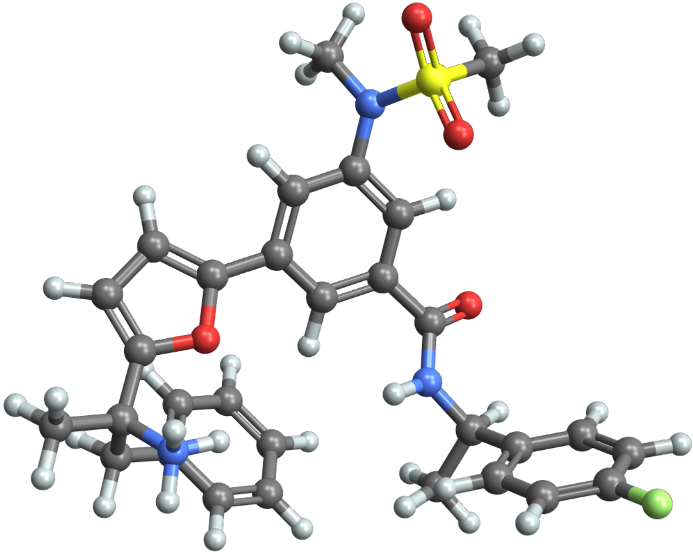

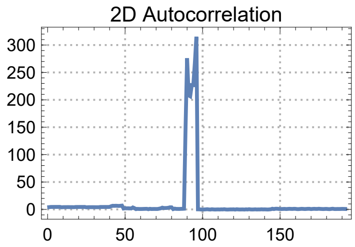

Dataset Index= 497

Molecule Formula= $C_{34}H_{42}F_3N_4O_5S^+$

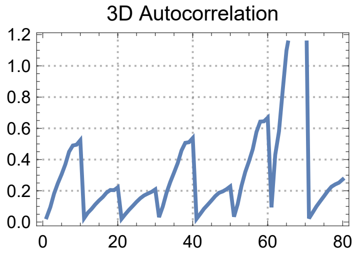

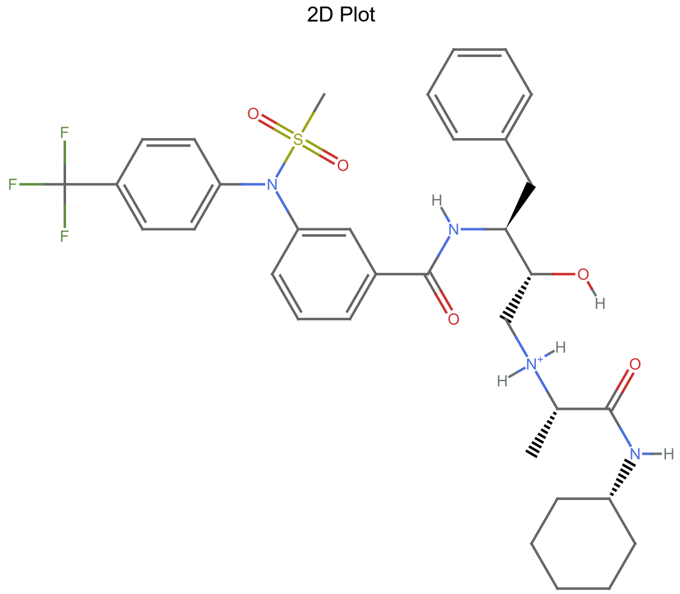

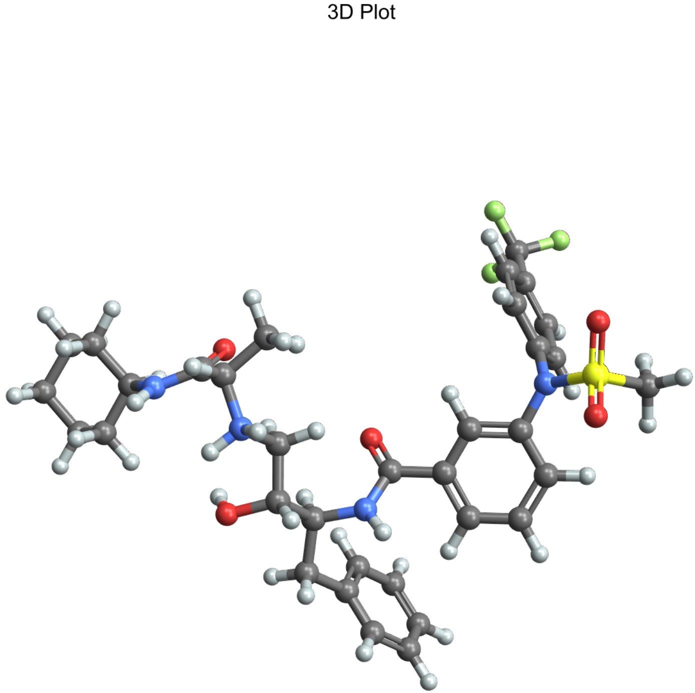

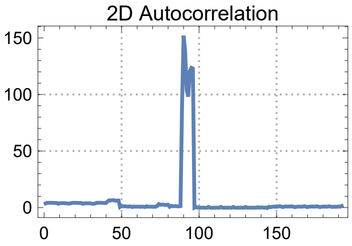

Dataset Index= 498

Molecule Formula= $C_{21}H_{15}F_4N_5O_2$

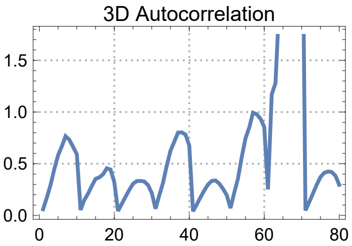

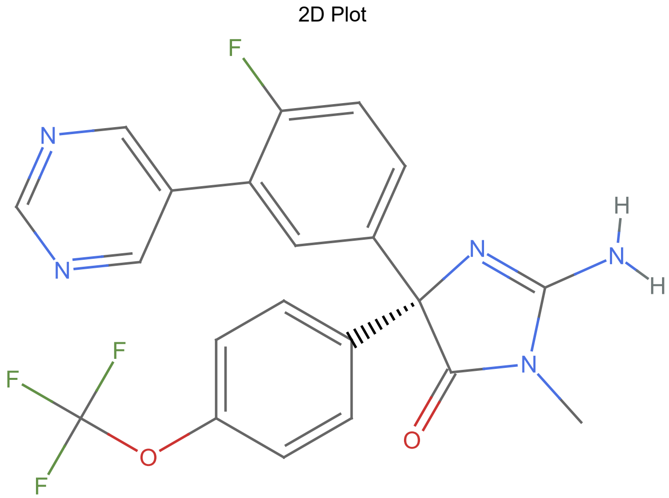

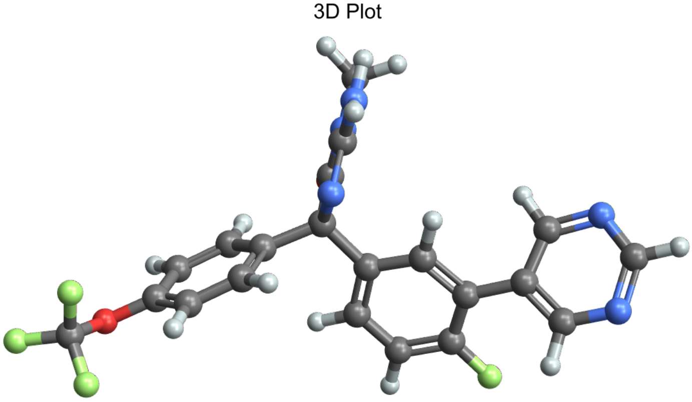

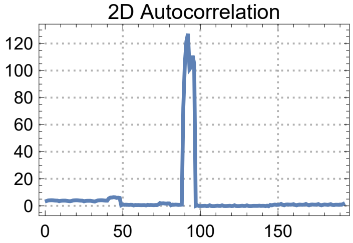

Dataset Index= 499

Molecule Formula= $C_{22}H_{20}F_3N_3O_2$

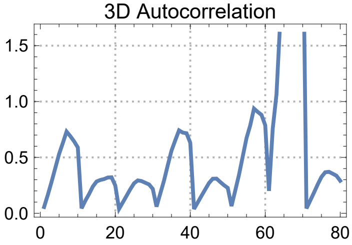

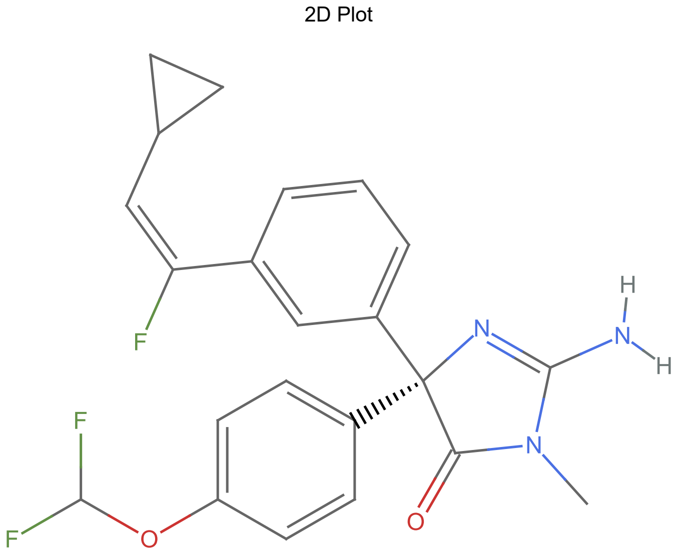

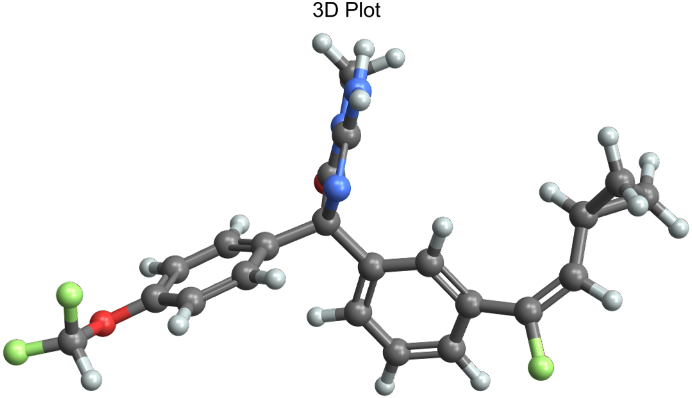

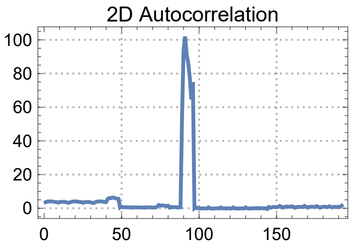

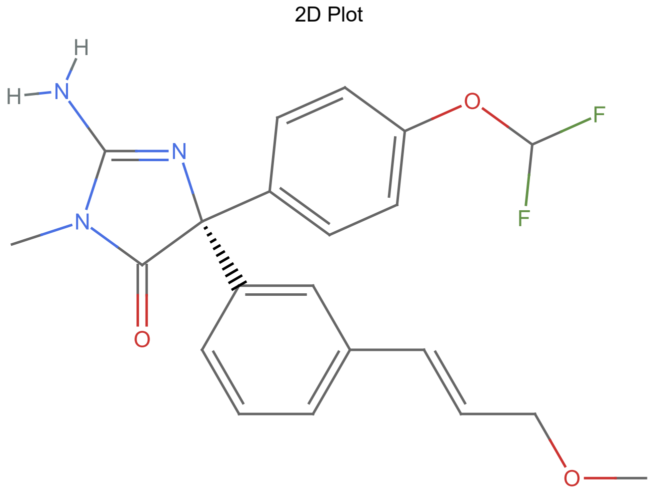

Dataset Index= 500

Molecule Formula= $C_{21}H_{21}F_2N_3O_3$

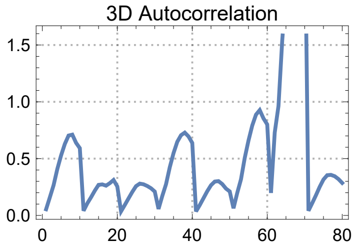

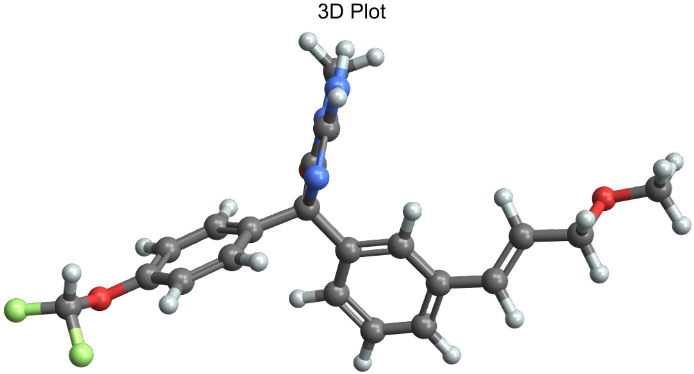

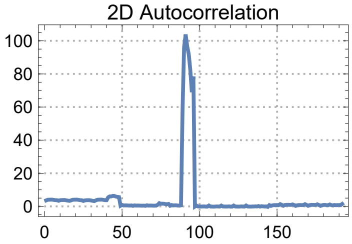

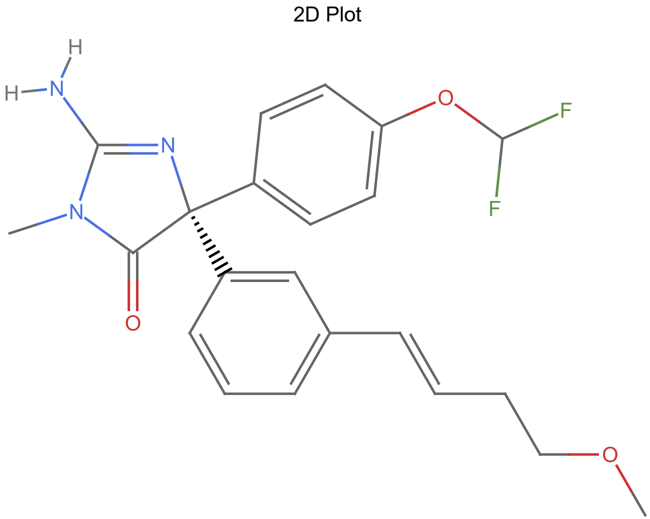

Dataset Index= 501

Molecule Formula= $C_{22}H_{23}F_2N_3O_3$

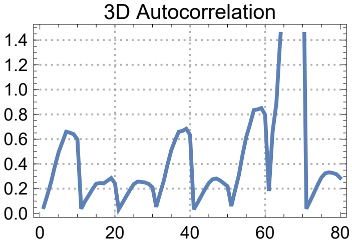

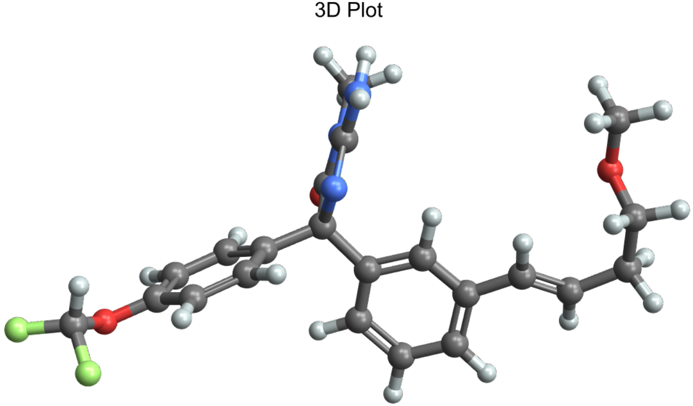

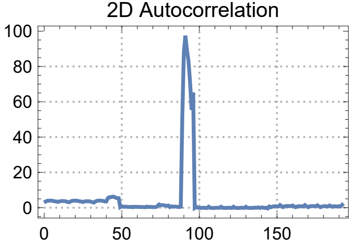

Dataset Index= 502

Molecule Formula= $C_{20}H_{17}F_2N_3O_2$

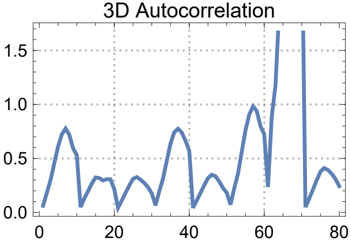

2D Plot

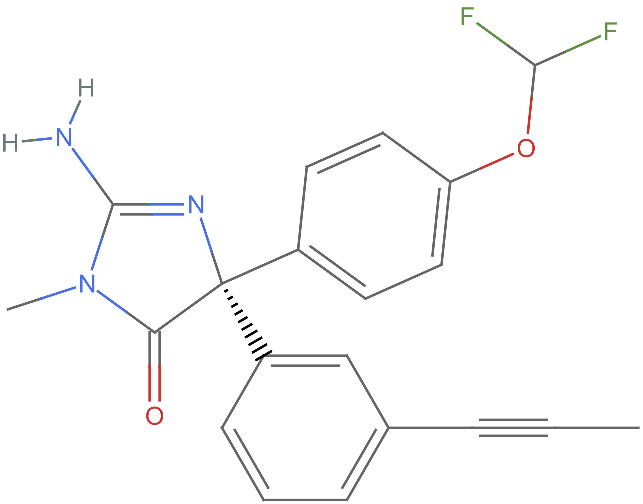

3D Plot

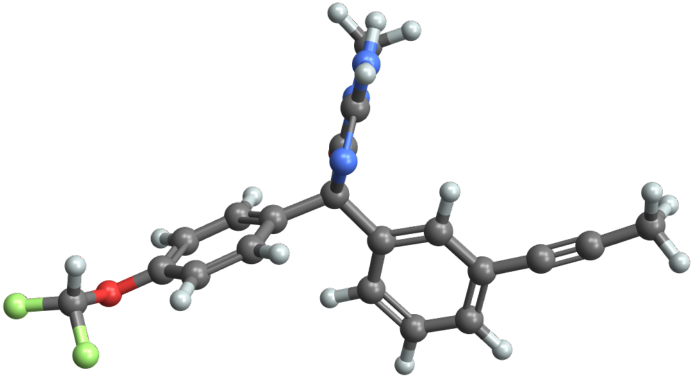

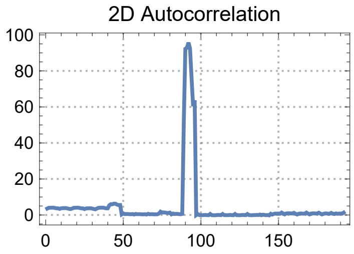

Dataset Index= 503

Molecule Formula= $C_{22}H_{21}F_2N_3O_2$

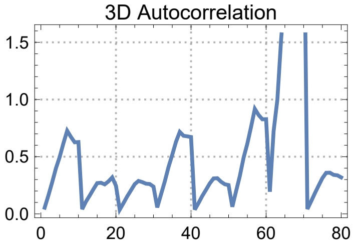

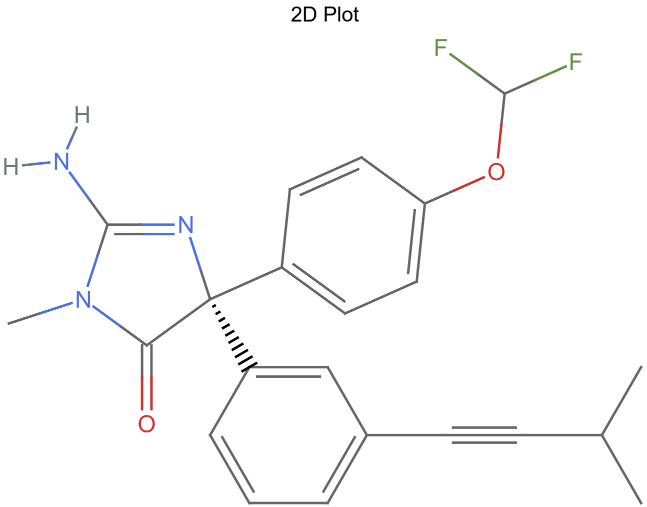

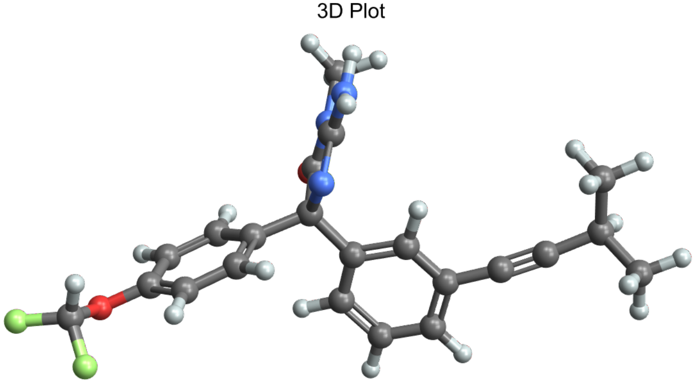

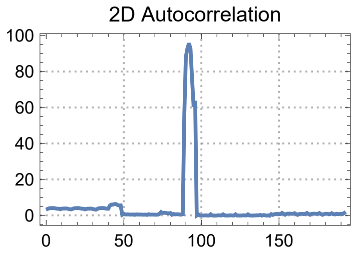

Dataset Index= 504

Molecule Formula= $C_{22}H_{19}F_2N_3O_2$

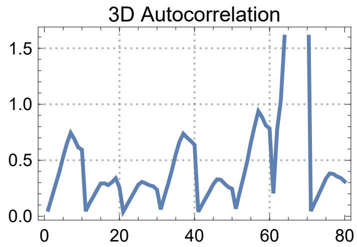

2D Plot

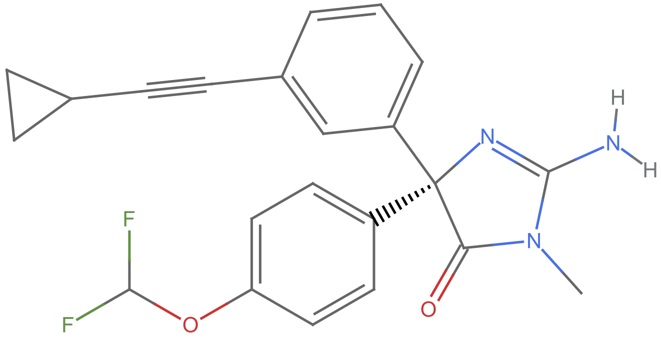

3D Plot

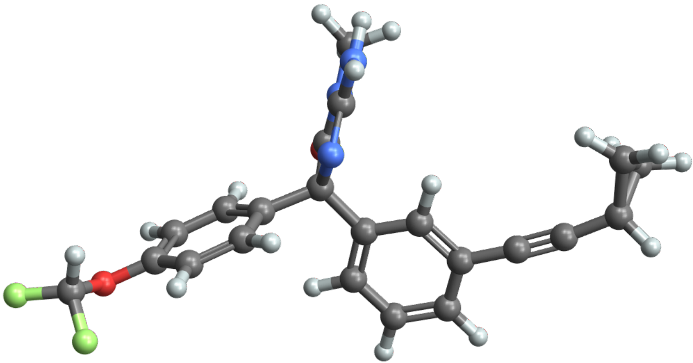

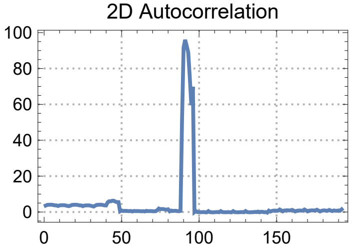

Dataset Index= 505

Molecule Formula= $C_{21}H_{19}F_2N_3O_3$

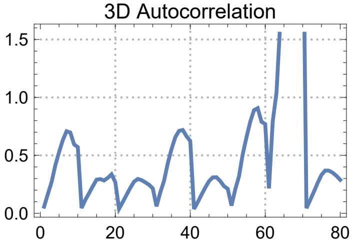

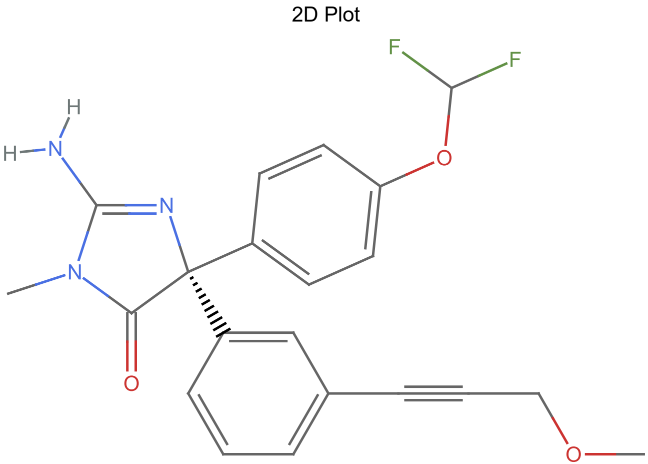

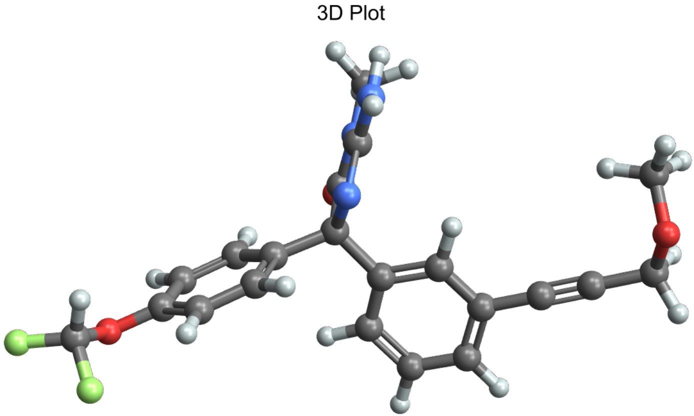

2D Plot

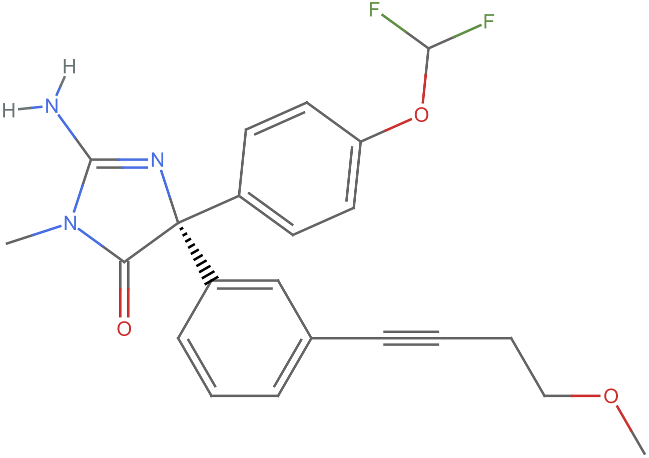

2D Autocorrelation

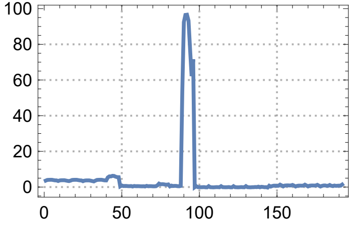

Dataset Index= 506

Molecule Formula= $C_{22}H_{21}F_2N_3O_3$

3D Plot

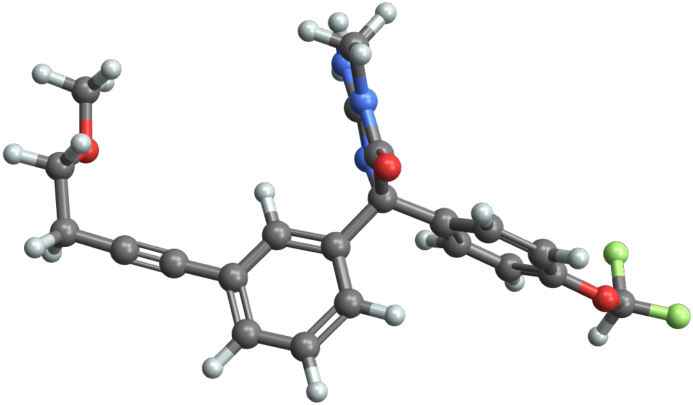

3D Autocorrelation

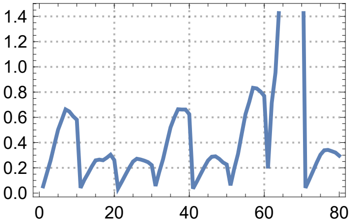

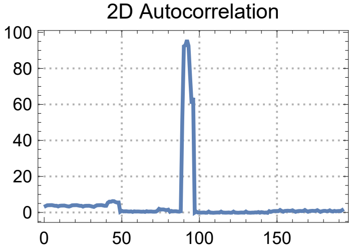

Dataset Index= 507

Molecule Formula= $C_{21}H_{19}F_2N_3O_3$

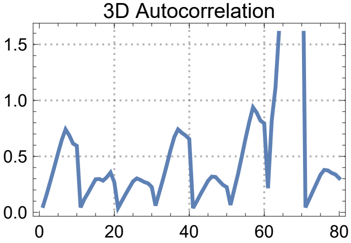

2D Plot

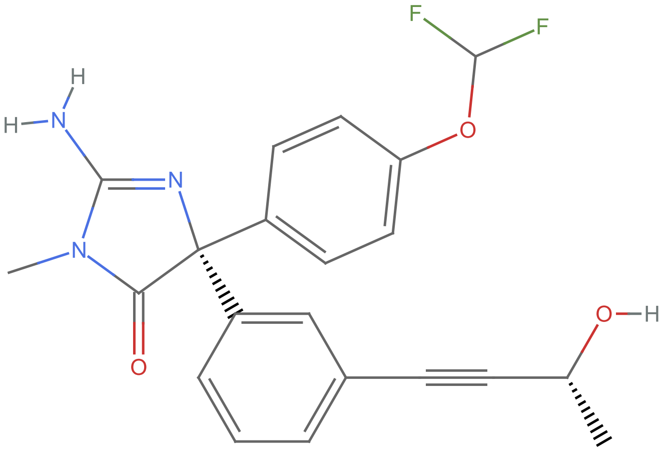

3D Plot

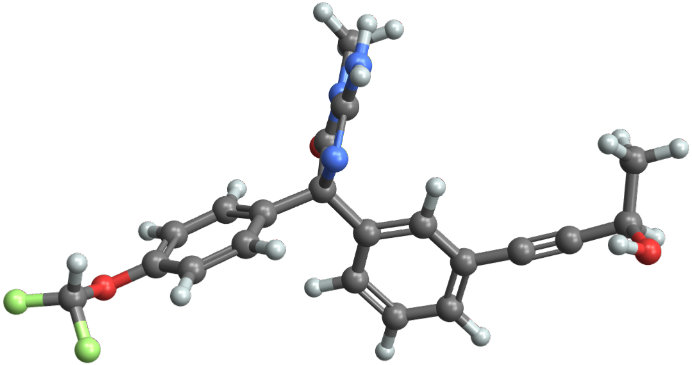

2D Autocorrelation

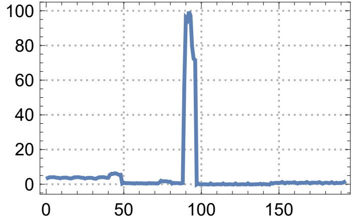

Dataset Index= 508

Molecule Formula= $C_{21}H_{21}F_2N_3O_3$

3D Autocorrelation

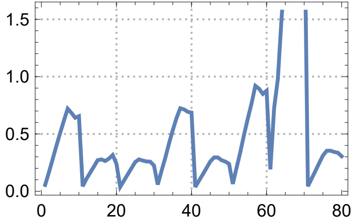

2D Plot

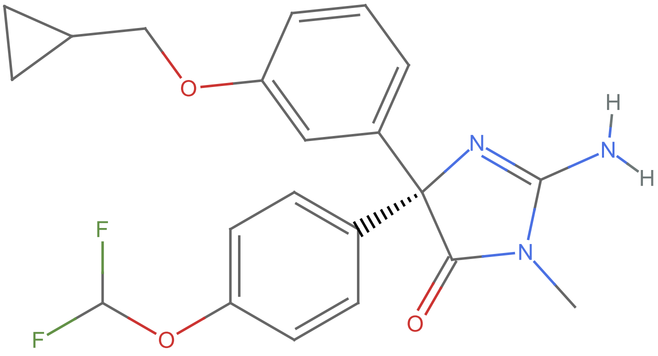

3D Plot

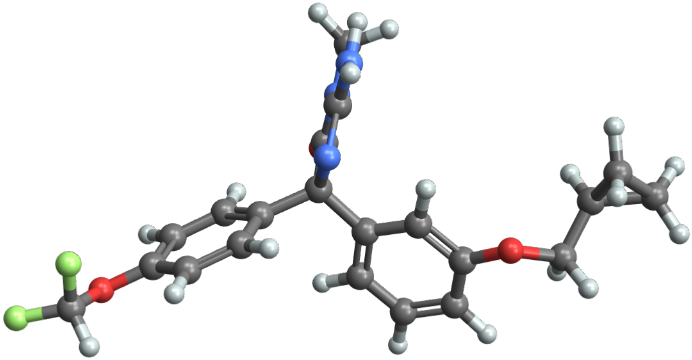

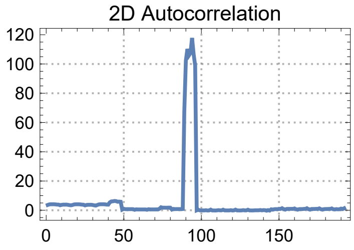

Dataset Index= 509

Molecule Formula= $C_{22}H_{18}F_3N_3O_2$

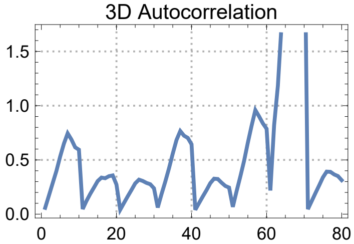

2D Plot

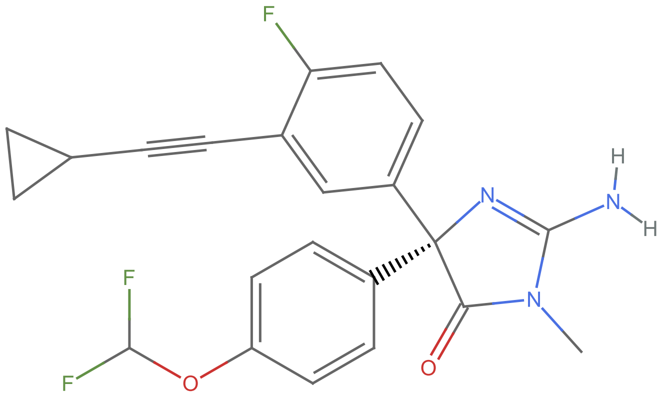

3D Plot

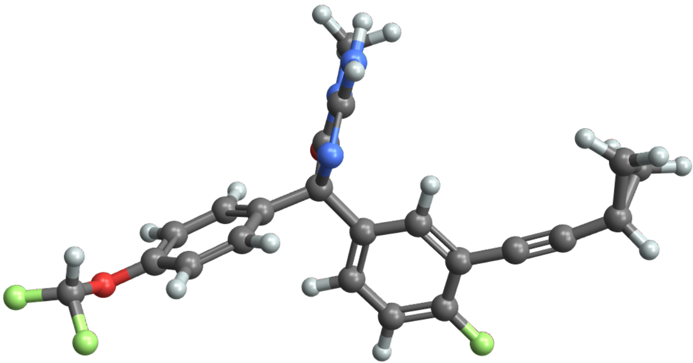

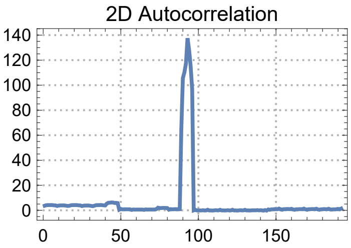

Dataset Index= 510

Molecule Formula= $C_{23}H_{20}F_3N_3O_2$

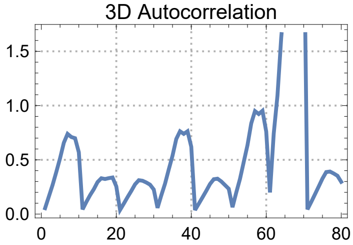

2D Plot

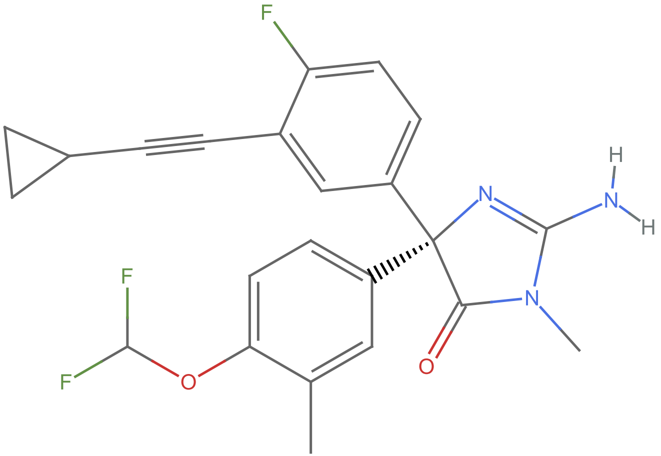

3D Plot

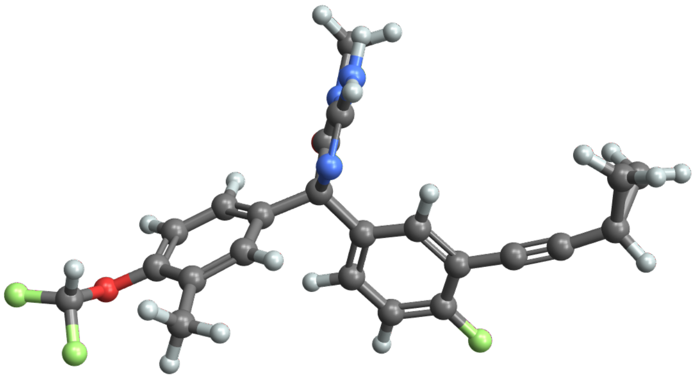

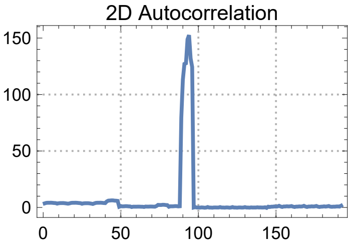

Dataset Index= 511

Molecule Formula= $C_{21}H_{17}F_4N_3O_2$

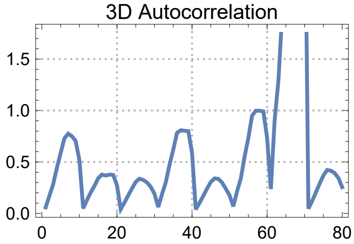

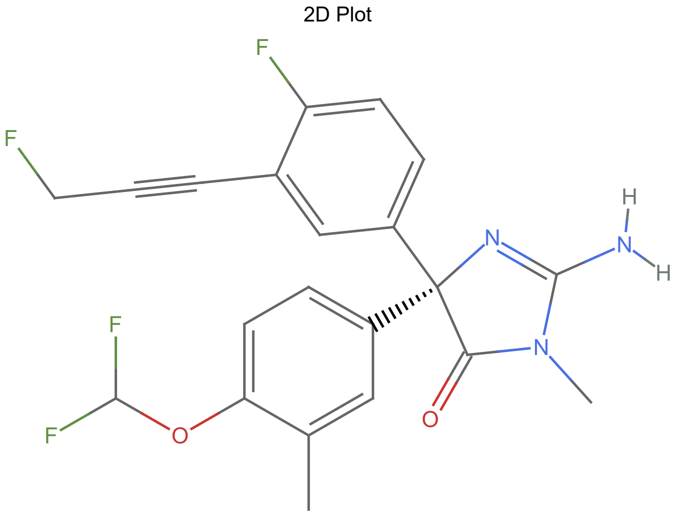

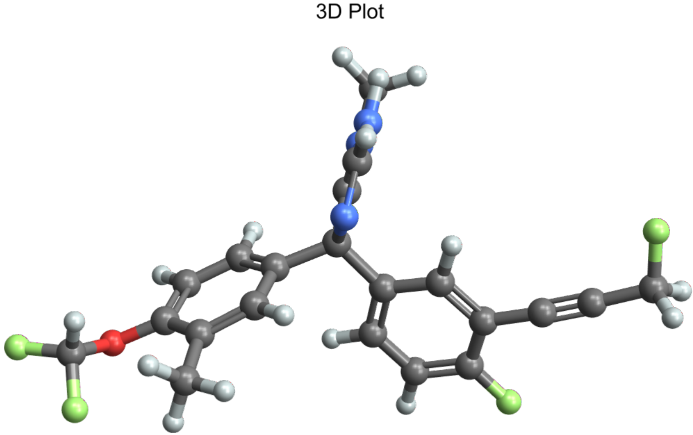

2D Plot

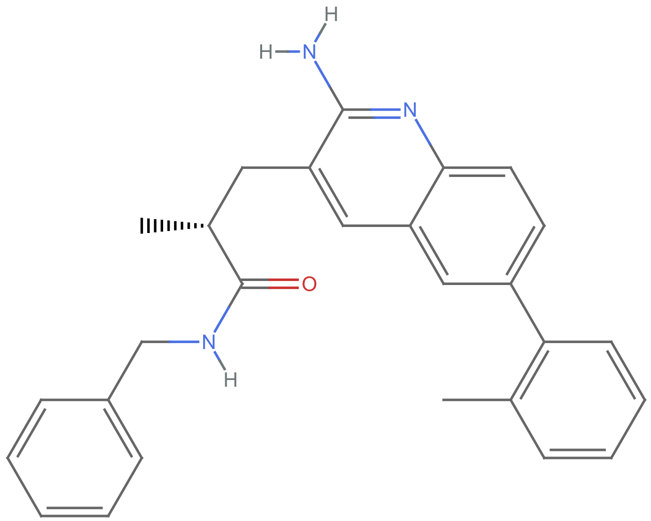

2D Autocorrelation

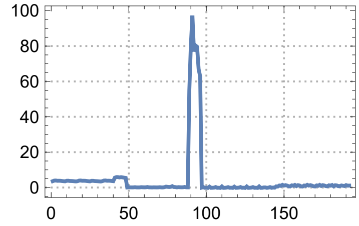

3D Plot

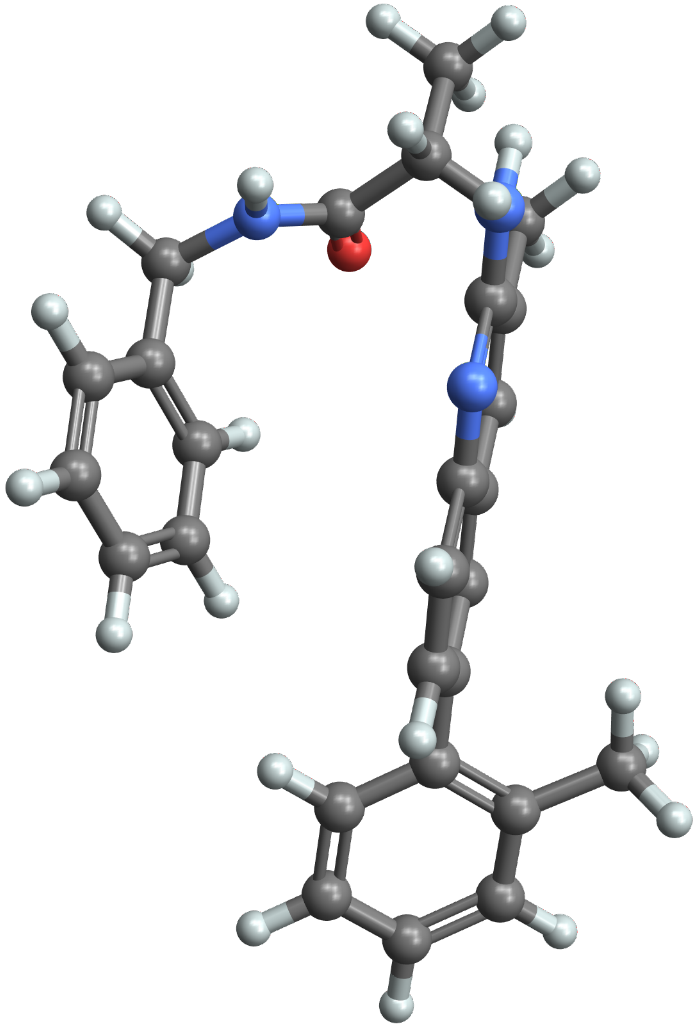

Dataset Index= 512

Molecule Formula= $C_{27}H_{27}N_3O$

3D Autocorrelation

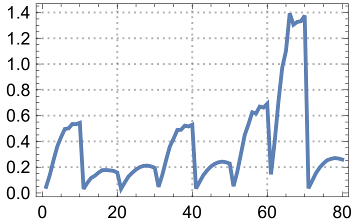

2D Plot

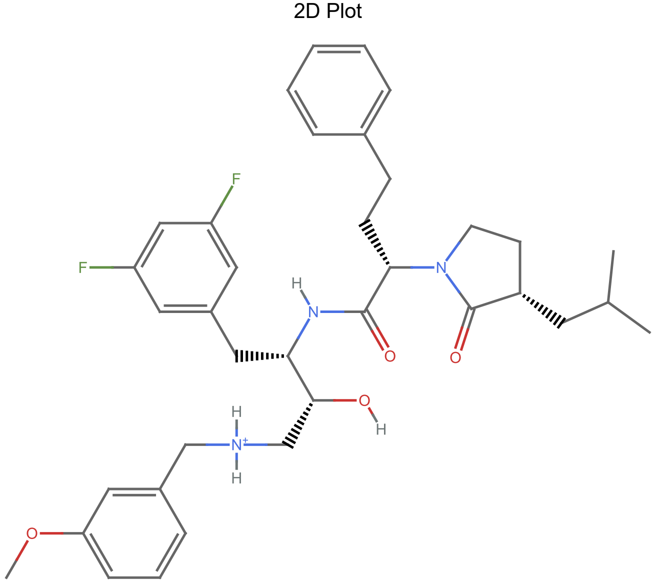

2D Autocorrelation

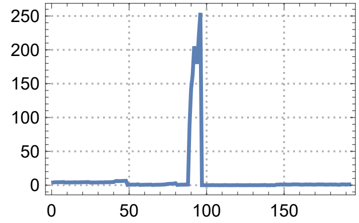

3D Plot

Dataset Index= 513

Molecule Formula= $C_{36}H_{46}F_2N_3O_4^+$

3D Autocorrelation

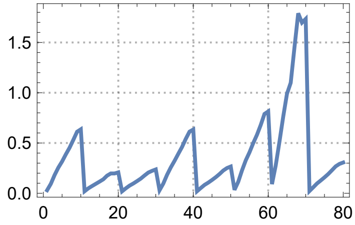

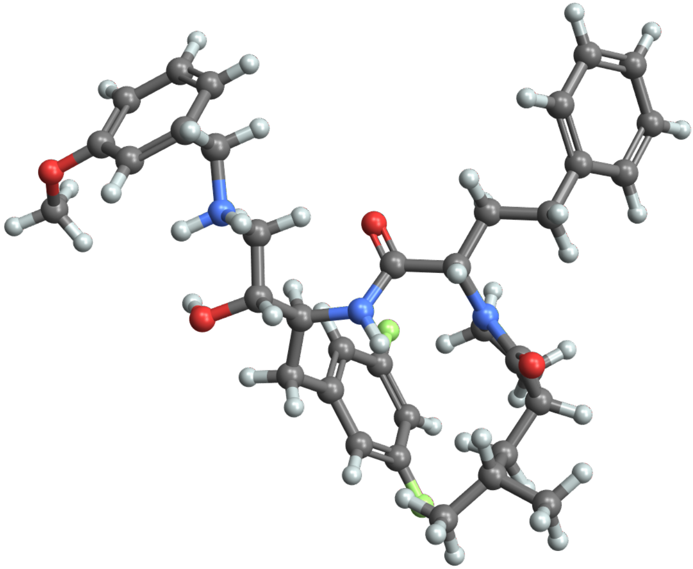

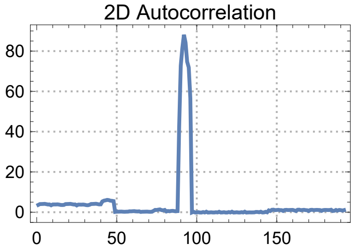

Dataset Index= 514

Molecule Formula= $C_{23}H_{27}N_7O$

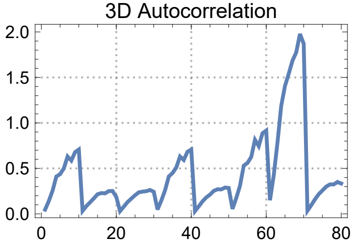

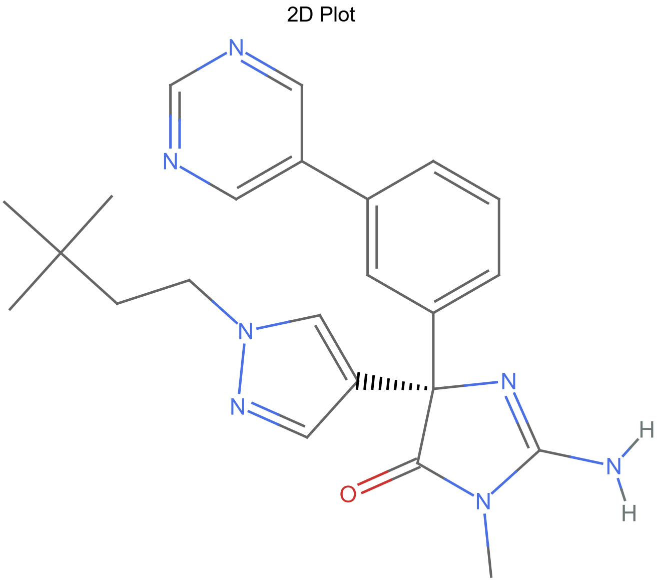

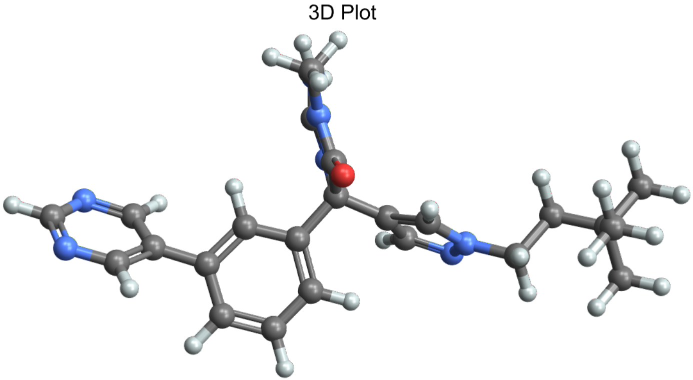

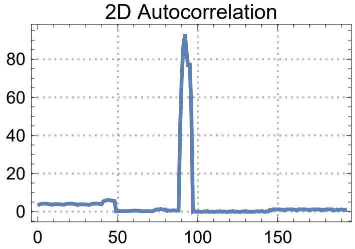

Dataset Index= 515

Molecule Formula= $C_{25}H_{29}N_7O$

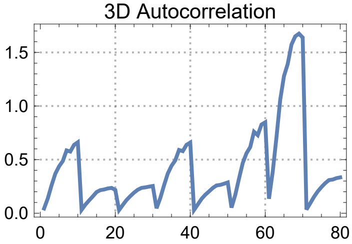

2D Plot

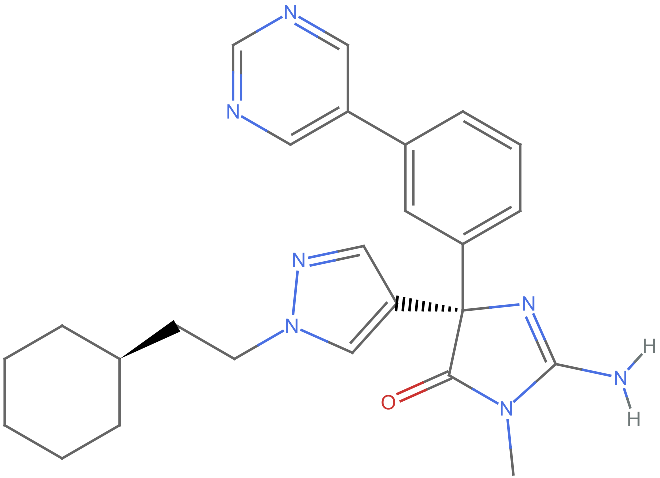

3D Plot

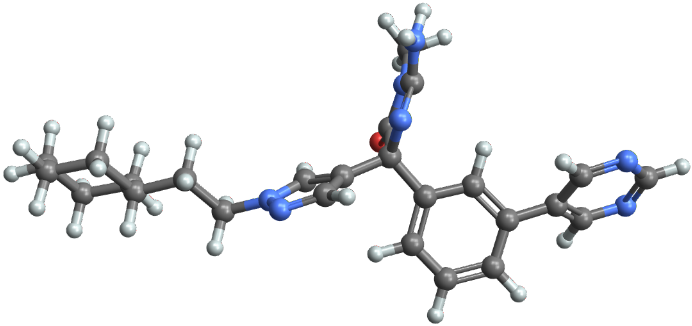

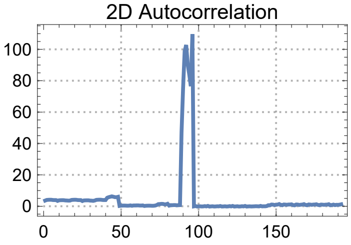

Dataset Index= 516

Molecule Formula= $C_{22}H_{23}FN_6O$

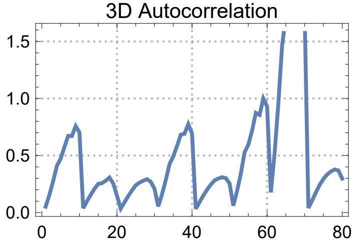

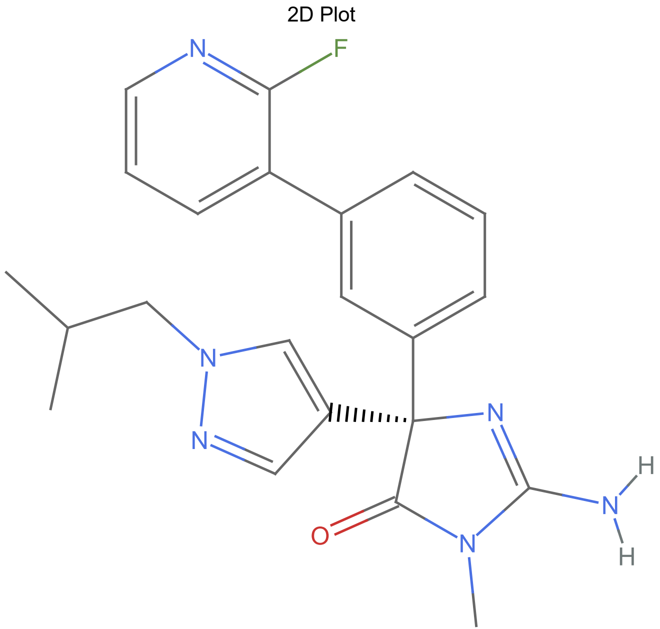

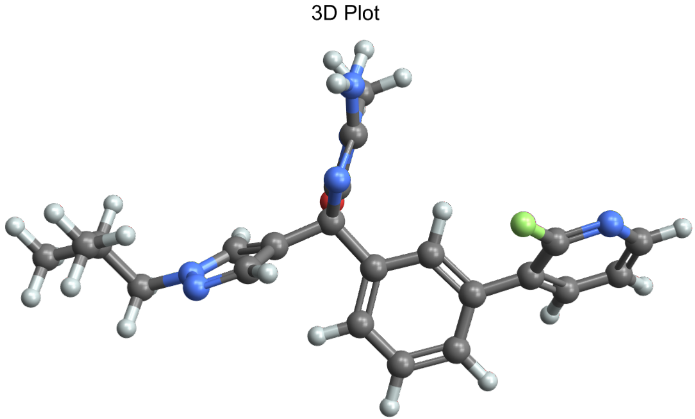

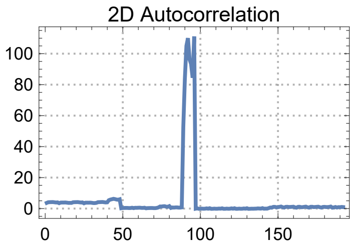

Dataset Index= 517

Molecule Formula= $C_{24}H_{27}FN_6O$

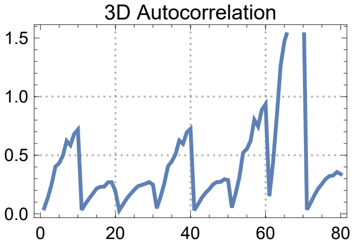

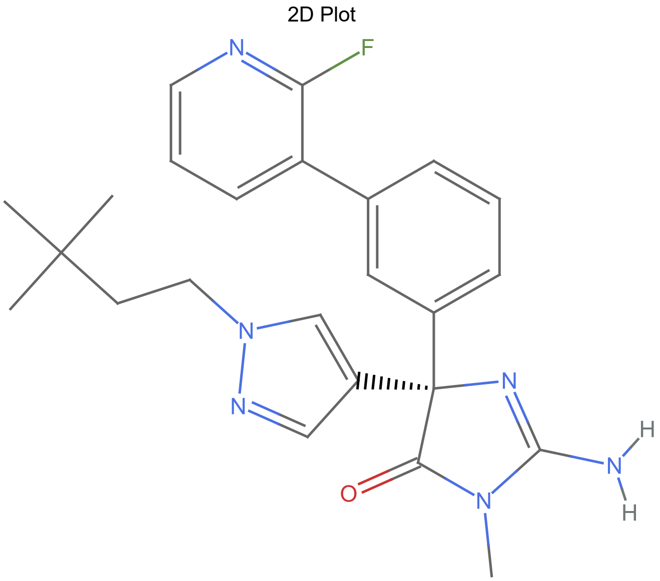

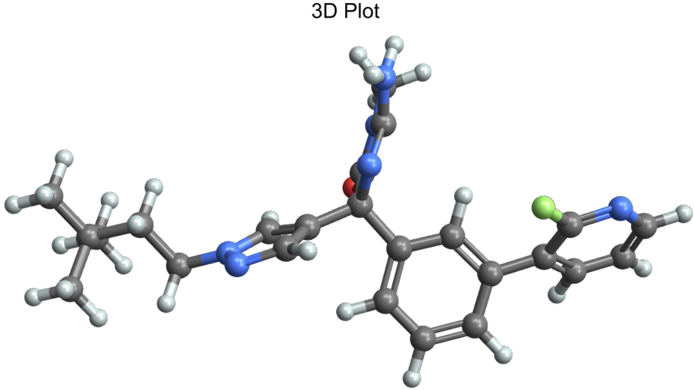

2D Autocorrelation

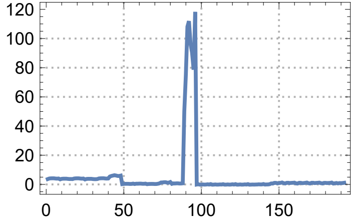

Dataset Index= 518

Molecule Formula= $C_{24}H_{27}FN_6O$

3D Autocorrelation

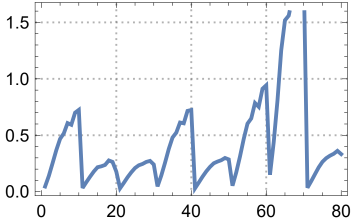

2D Plot

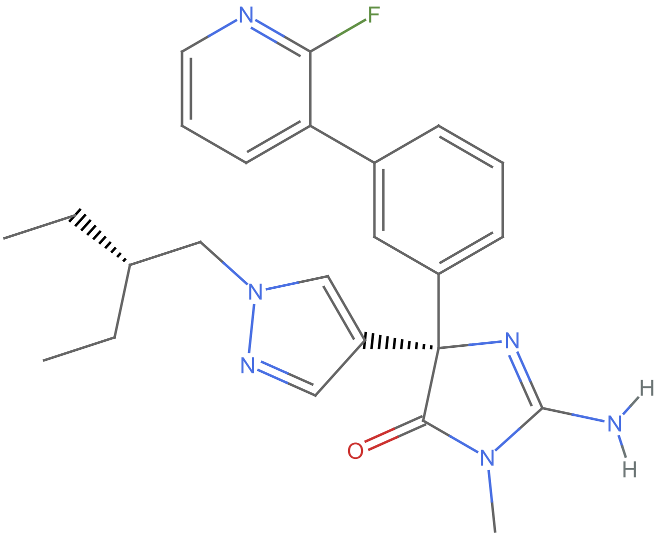

3D Plot

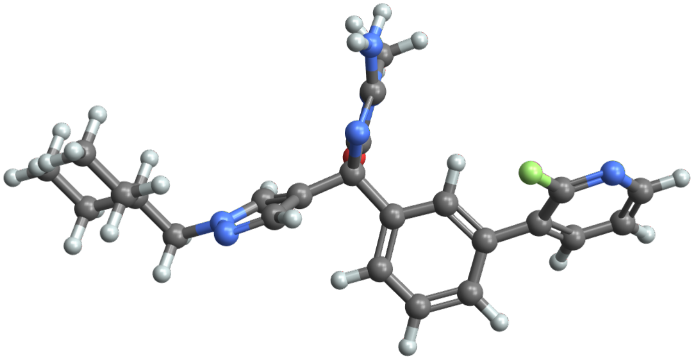

2D Plot

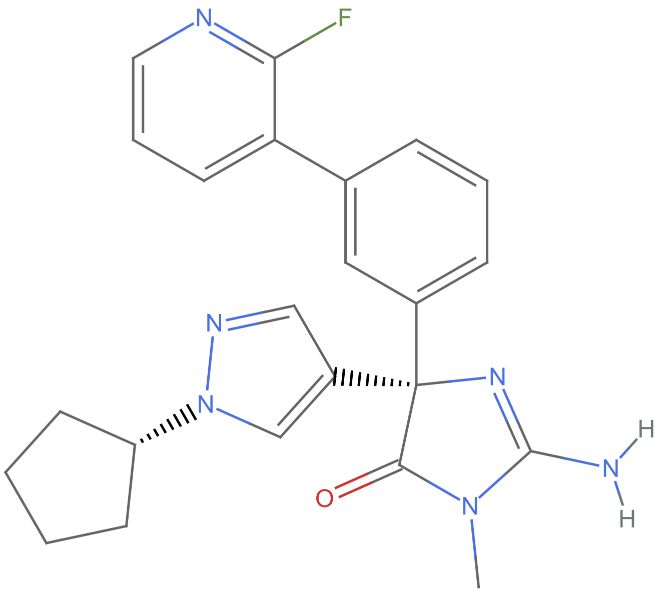

2D Autocorrelation

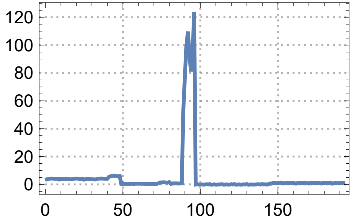

Dataset Index= 519

Molecule Formula= $C_{23}H_{23}FN_6O$

3D Autocorrelation

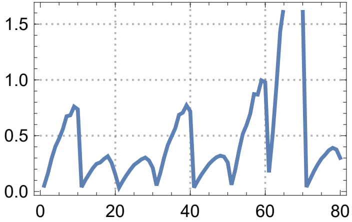

3D Plot

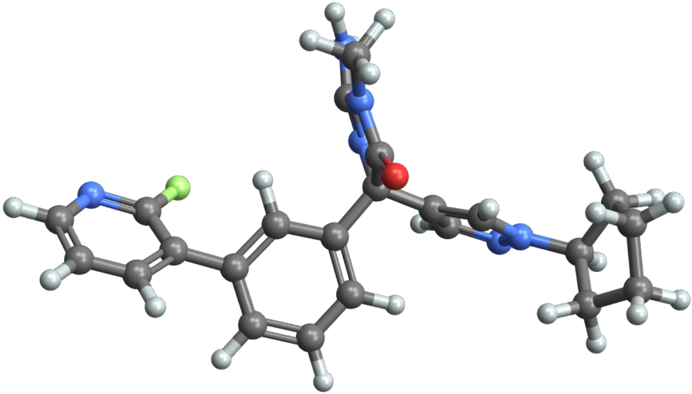

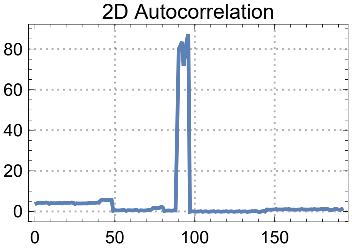

Dataset Index= 520

Molecule Formula= $C_{32}H_{44}N_3O_6^+$

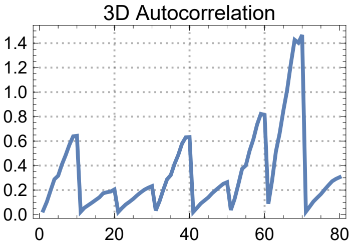

2D Plot

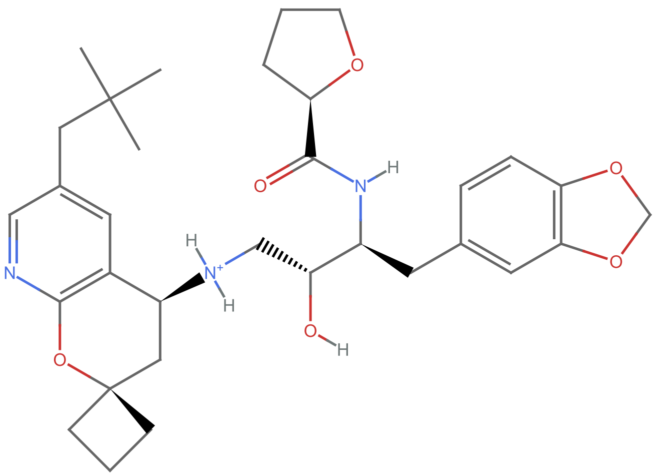

3D Plot

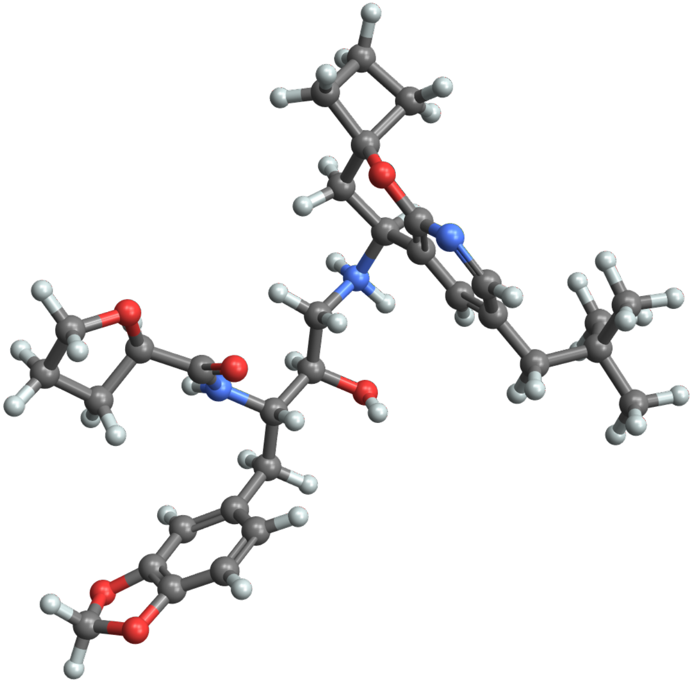

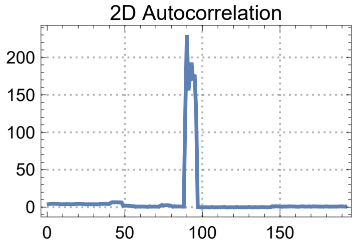

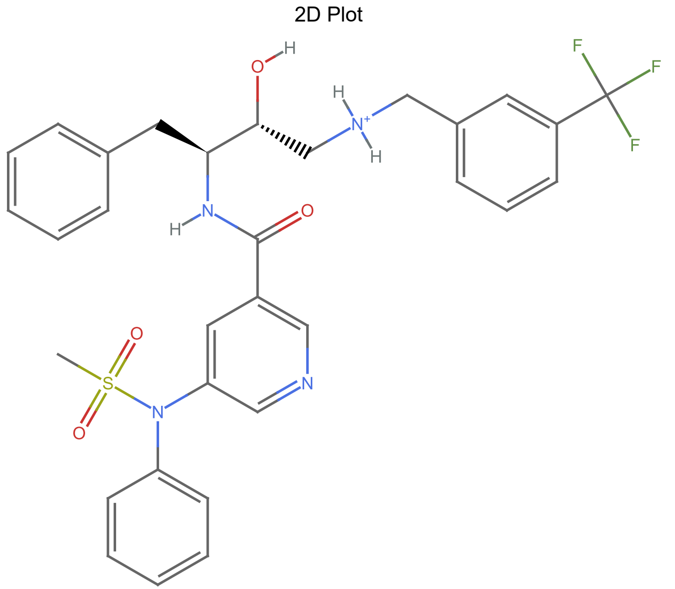

Dataset Index= 521

Molecule Formula= $C_{31}H_{32}F_3N_4O_4S^+$

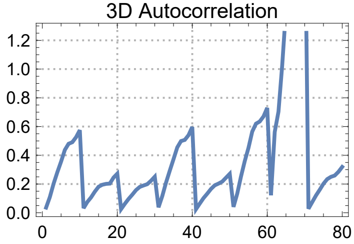

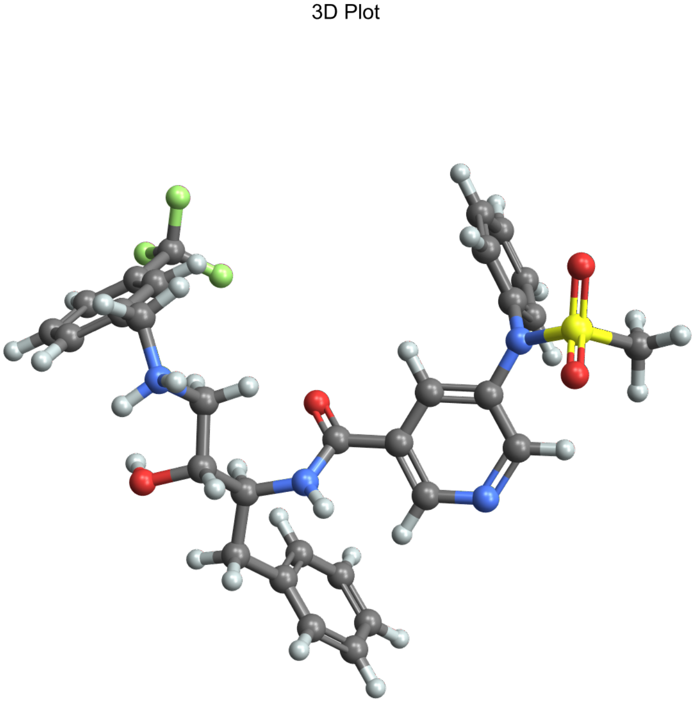

2D Plot

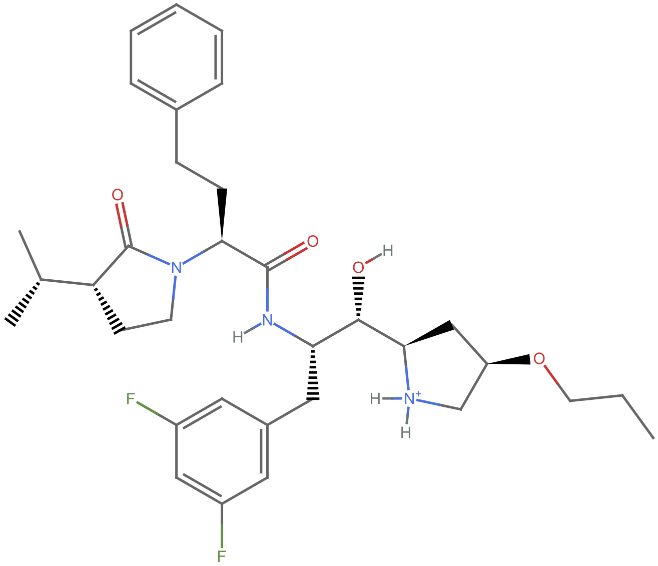

2D Autocorrelation

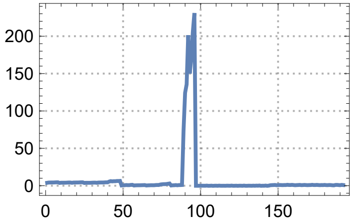

Dataset Index= 522

Molecule Formula= $C_{33}H_{46}F_2N_3O_4^+$

3D Autocorrelation

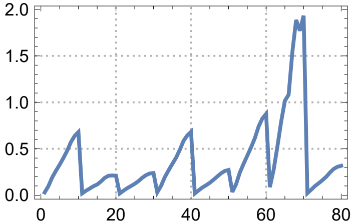

3D Plot

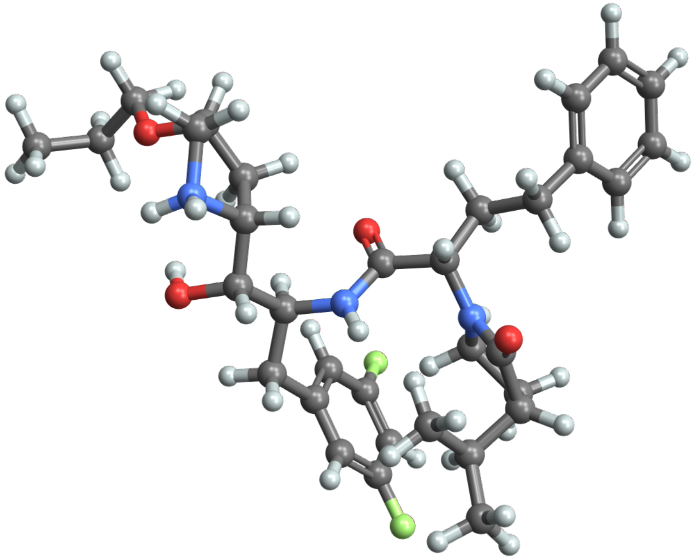

2D Autocorrelation

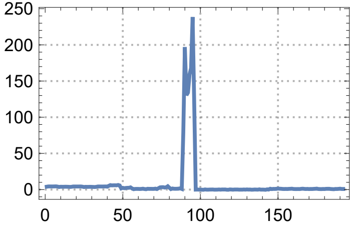

Dataset Index= 523

Molecule Formula= $C_{25}H_{37}F_4N_4O_4S^+$

3D Autocorrelation

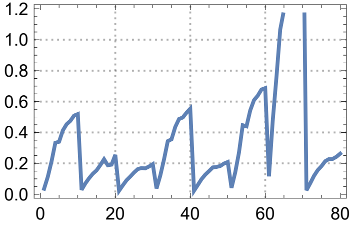

2D Plot

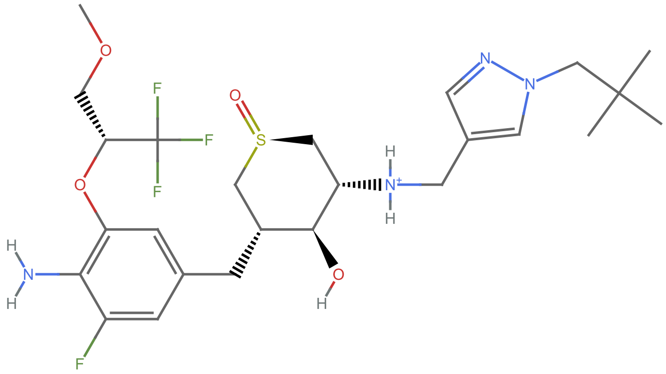

3D Plot

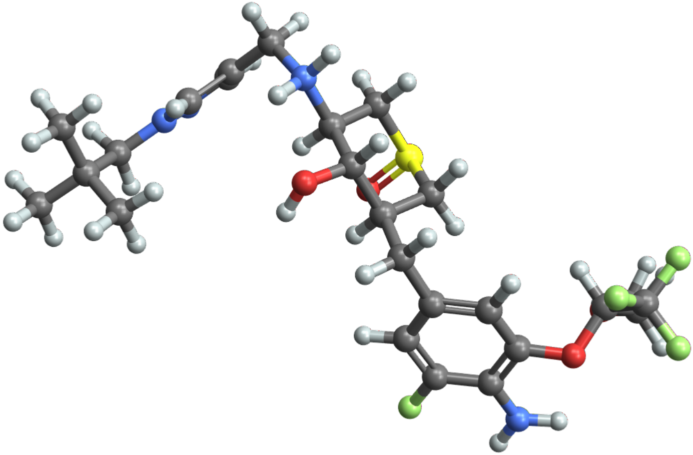

2D Plot

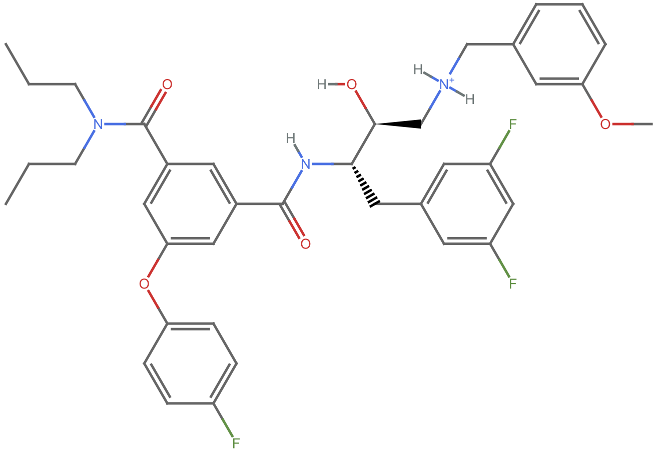

2D Autocorrelation

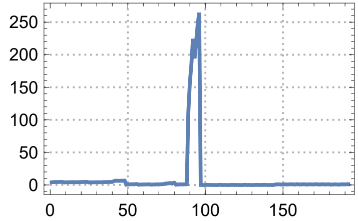

Dataset Index= 524

Molecule Formula= $C_{38}H_{43}F_3N_3O_5^+$

3D Autocorrelation

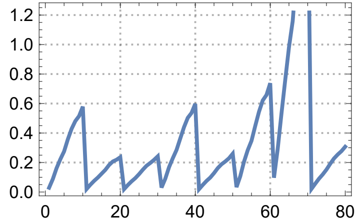

3D Plot

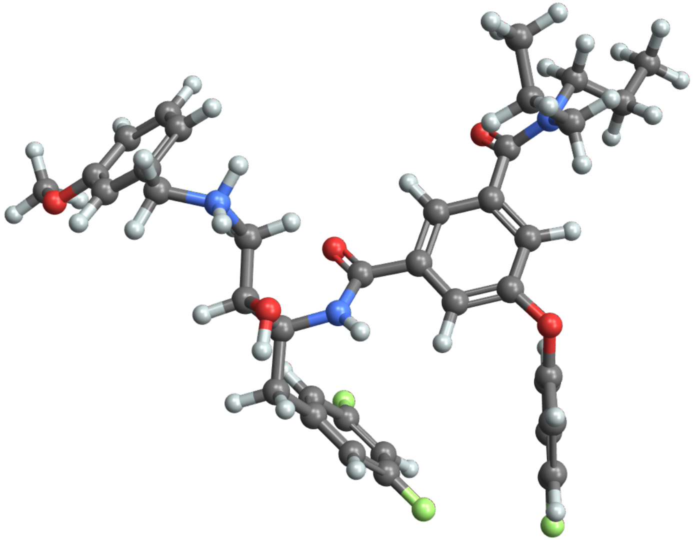

2D Plot

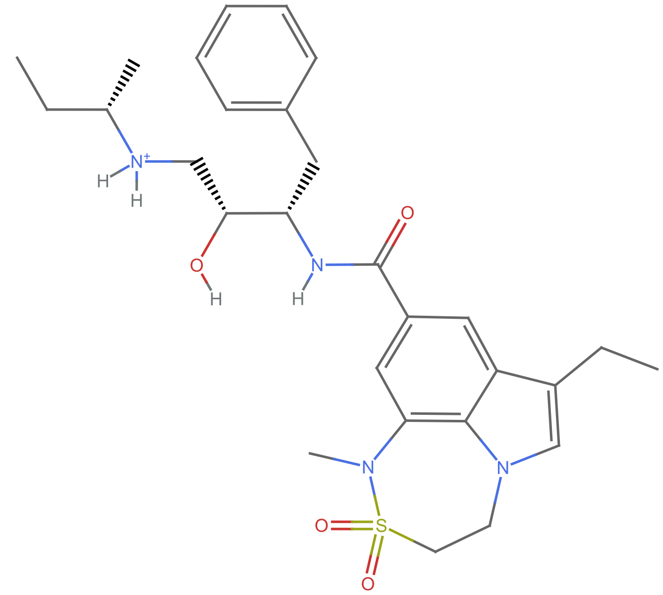

2D Autocorrelation

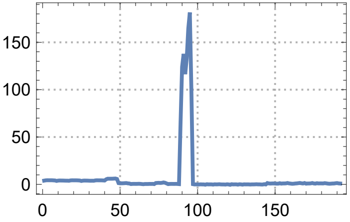

Dataset Index= 525

Molecule Formula= $C_{28}H_{39}N_4O_4S^+$

3D Plot

3D Autocorrelation

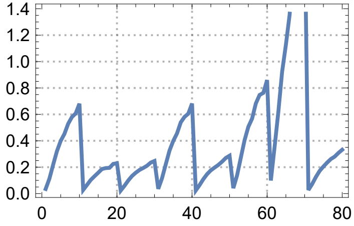

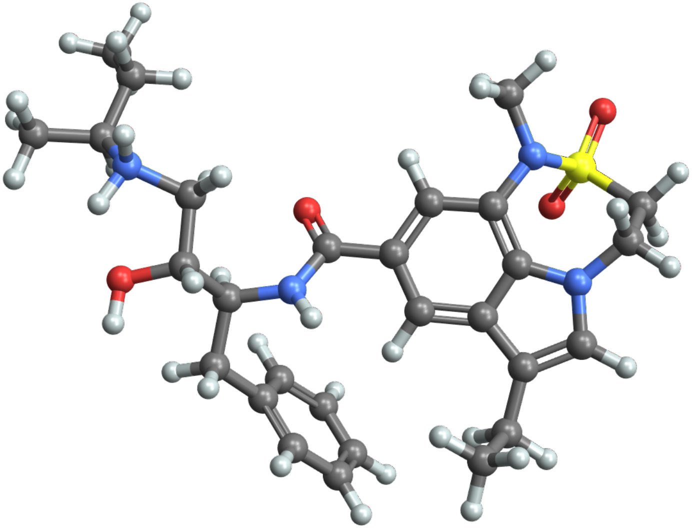

2D Plot

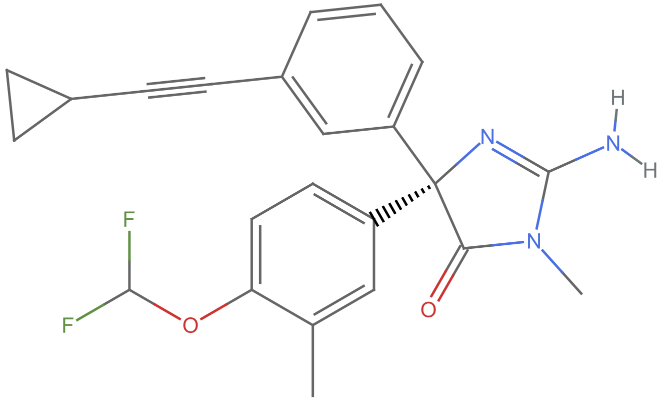

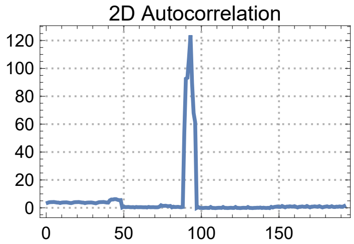

Dataset Index= 526

Molecule Formula= $C_{23}H_{21}F_2N_3O_2$

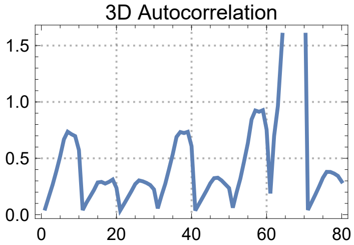

3D Plot

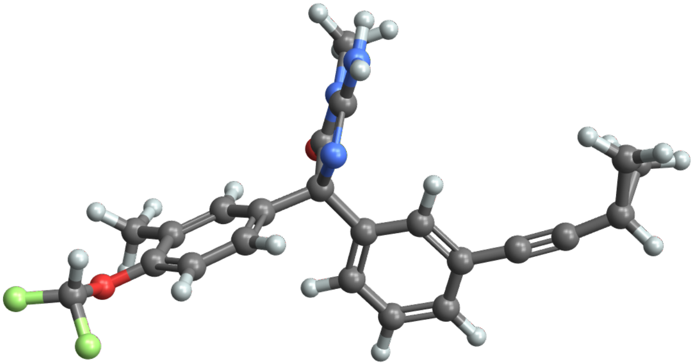

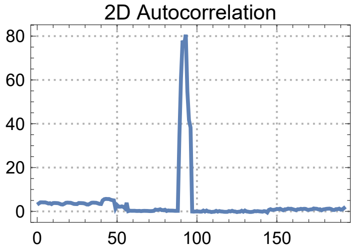

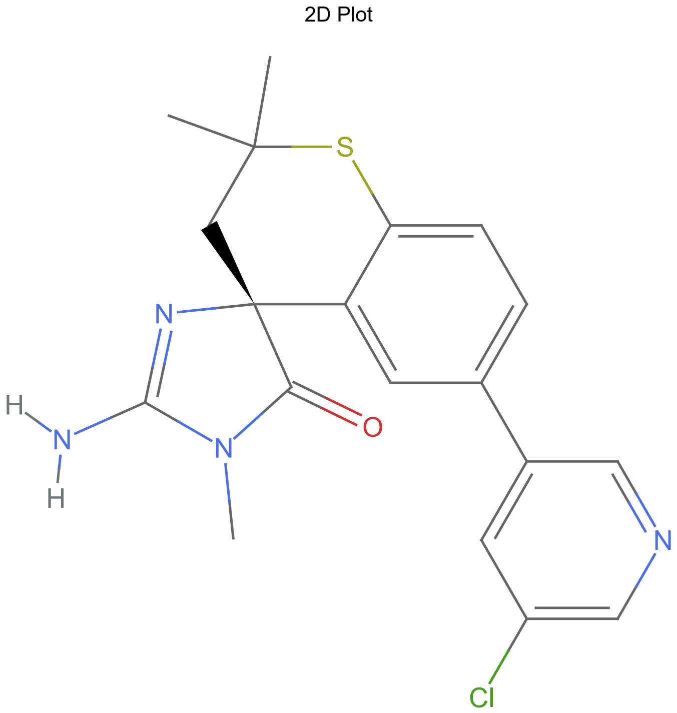

Dataset Index= 527

Molecule Formula= $C_{19}H_{19}ClN_4OS$

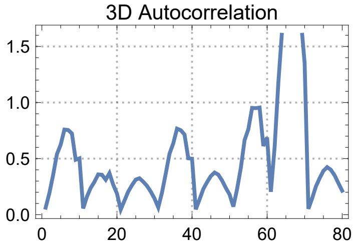

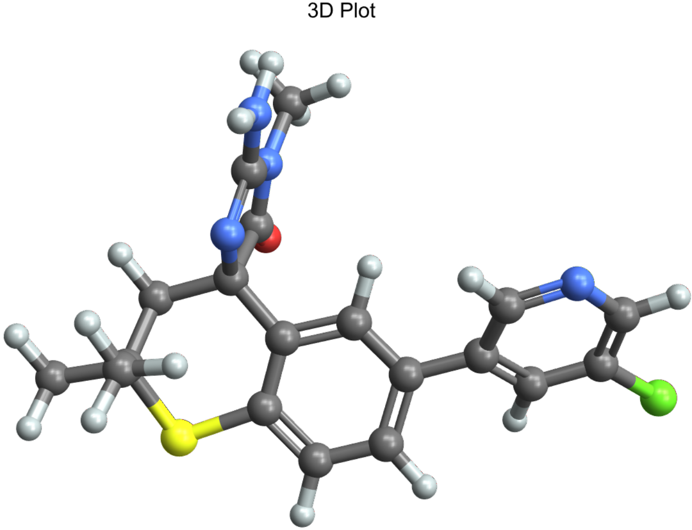

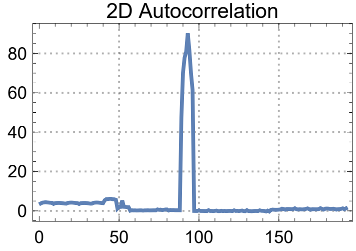

Dataset Index= 528

Molecule Formula= $C_{25}H_{22}Cl_2N_4O$

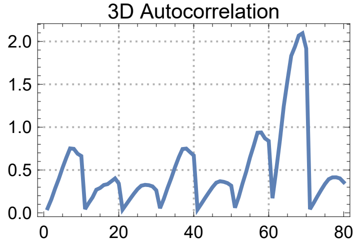

2D Plot

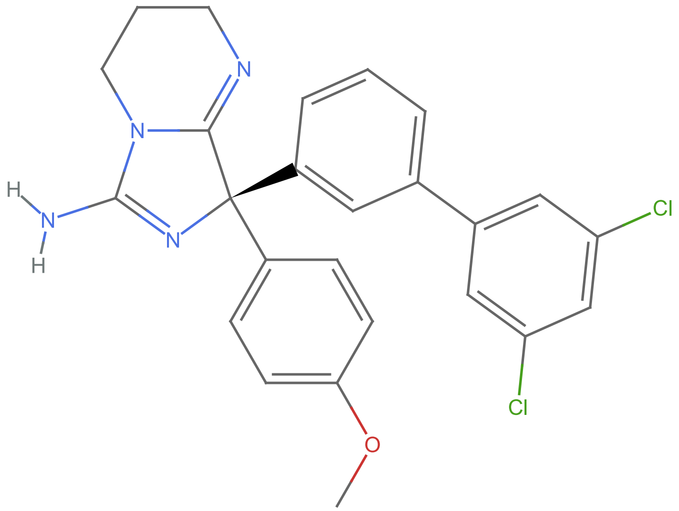

3D Plot

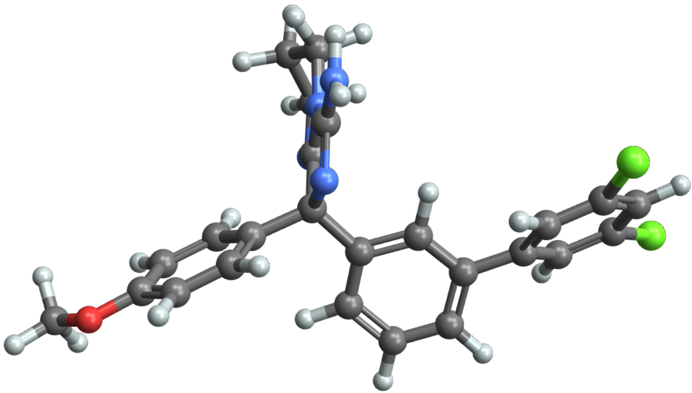

2D Plot

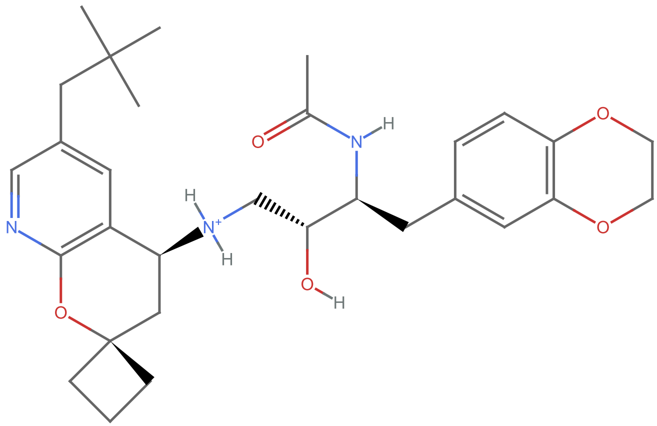

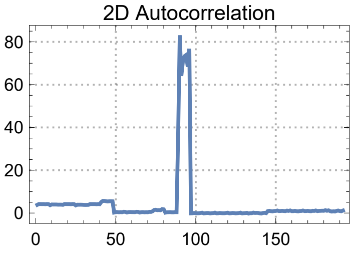

Dataset Index= 529

Molecule Formula= $C_{30}H_{42}N_3O_5^+$

3D Plot

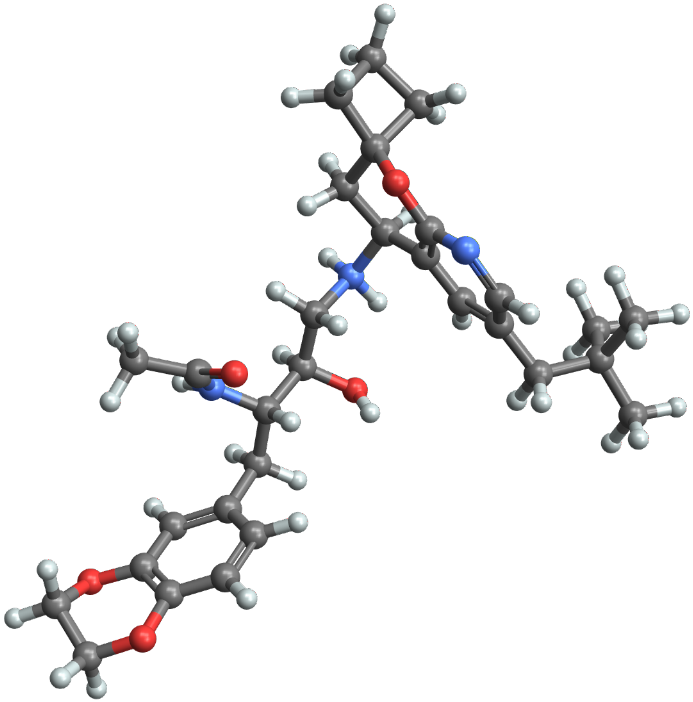

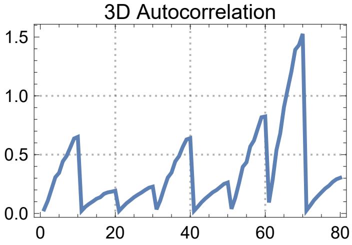

2D Plot

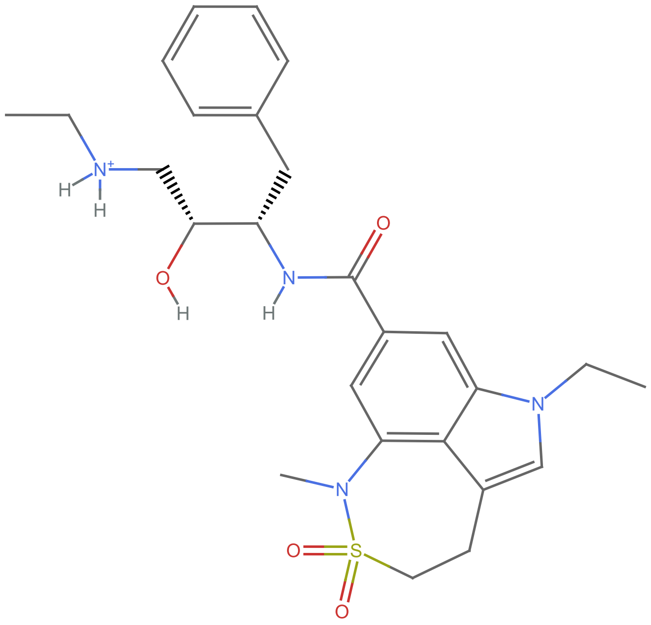

2D Autocorrelation

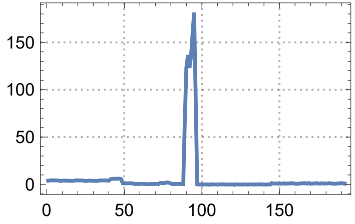

Dataset Index= 530

Molecule Formula= $C_{26}H_{35}N_4O_4S^+$

3D Plot

3D Autocorrelation

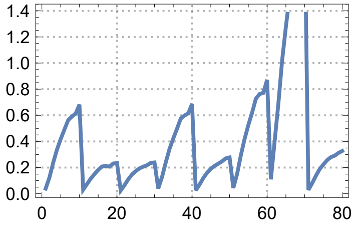

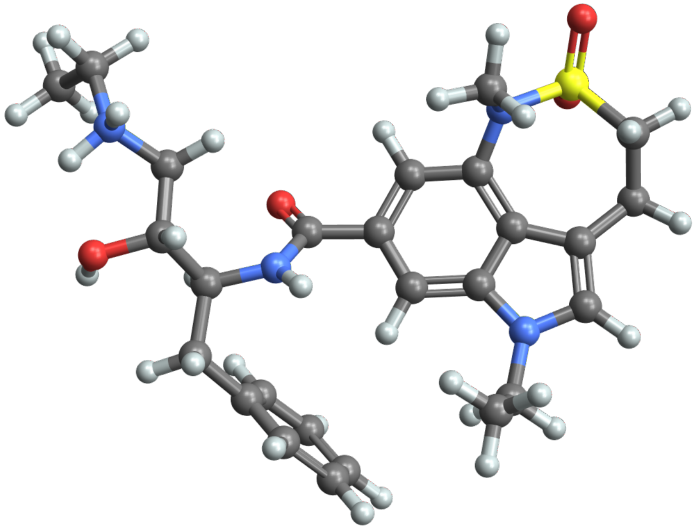

2D Plot

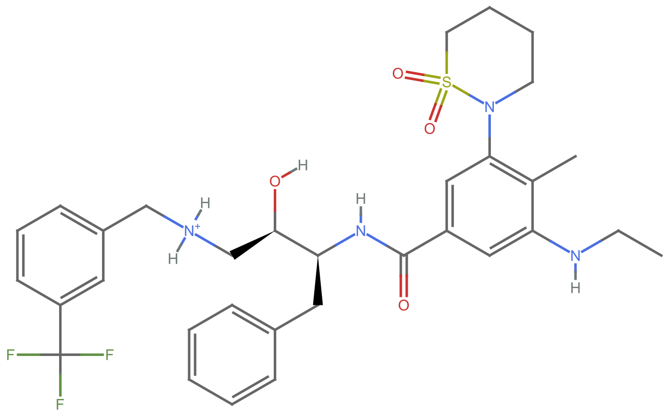

2D Autocorrelation

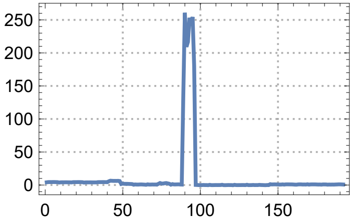

3D Plot

Dataset Index= 531

Molecule Formula= $C_{32}H_{40}F_3N_4O_4S^+$

3D Autocorrelation

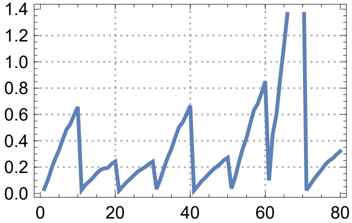

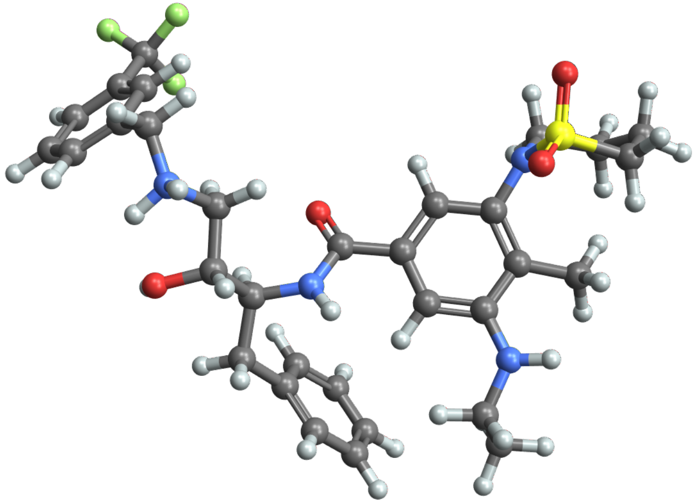

2D Plot

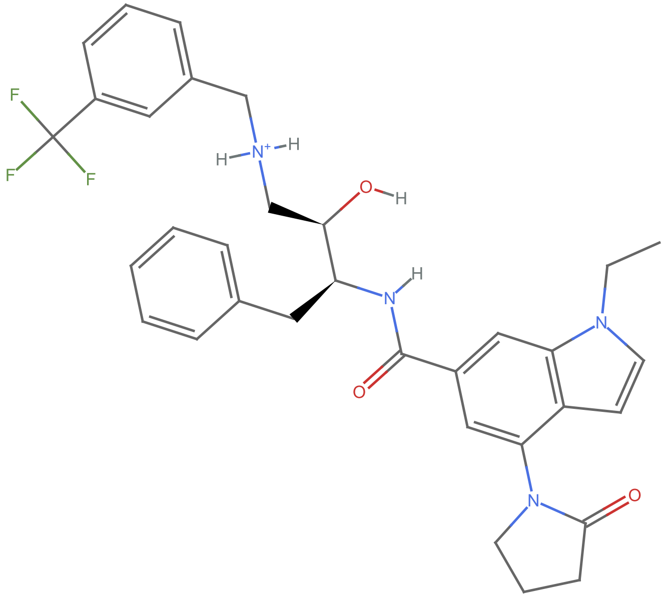

2D Autocorrelation

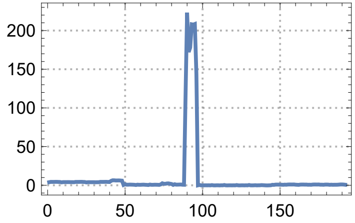

Dataset Index= 532

Molecule Formula= $C_{33}H_{36}F_3N_4O_3^+$

3D Autocorrelation

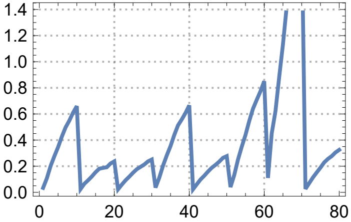

3D Plot

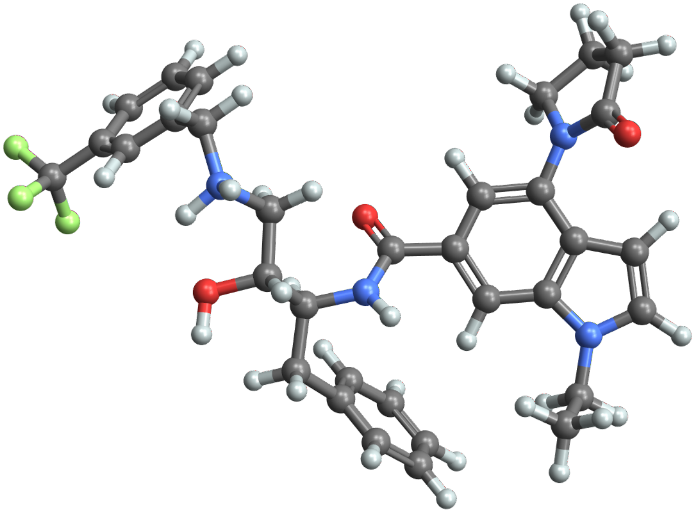

2D Plot

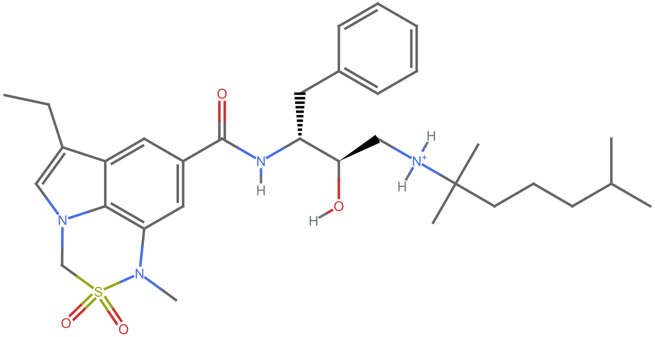

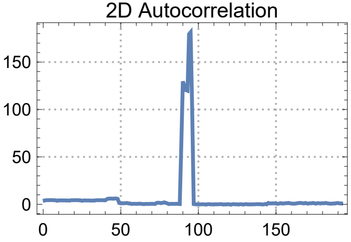

3D Plot

Dataset Index= 533

Molecule Formula= $C_{32}H_{47}N_4O_4S^+$

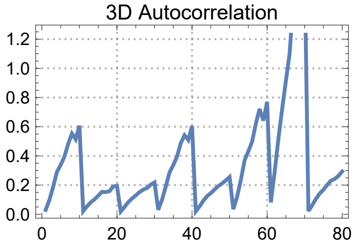

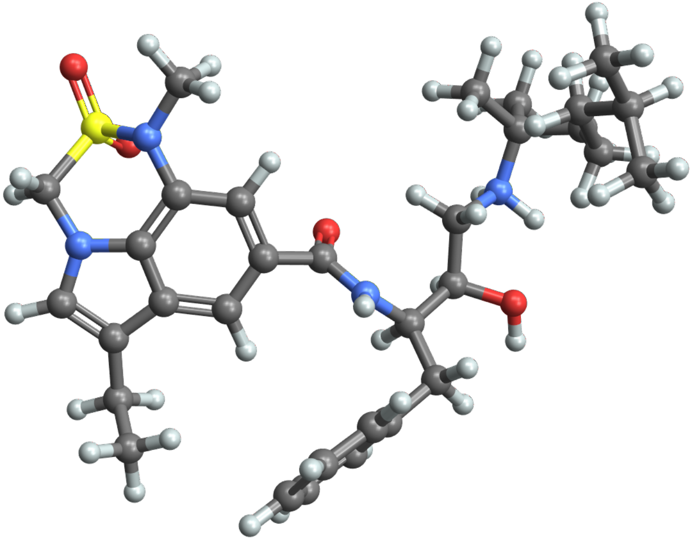

2D Plot

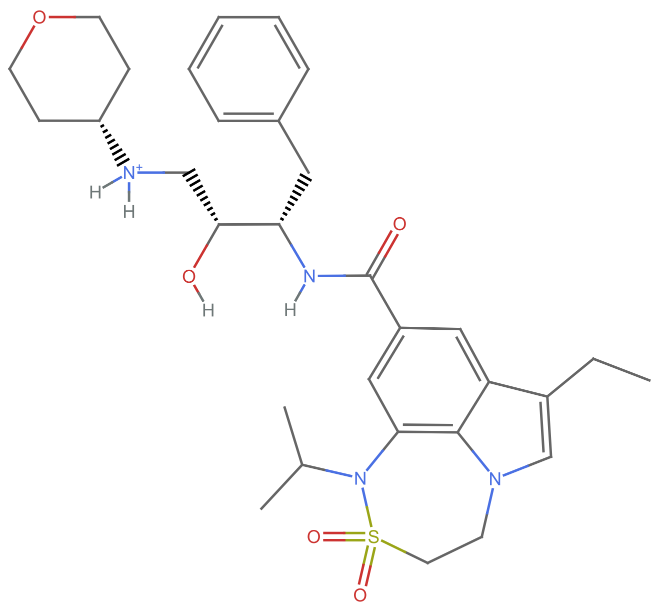

2D Autocorrelation

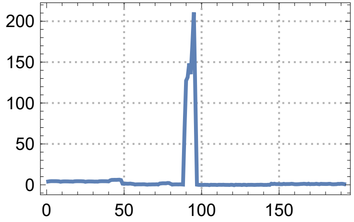

Dataset Index= 534

Molecule Formula= $C_{31}H_{43}N_4O_5S^+$

3D Plot

3D Autocorrelation

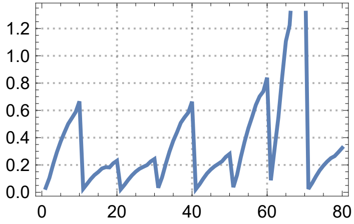

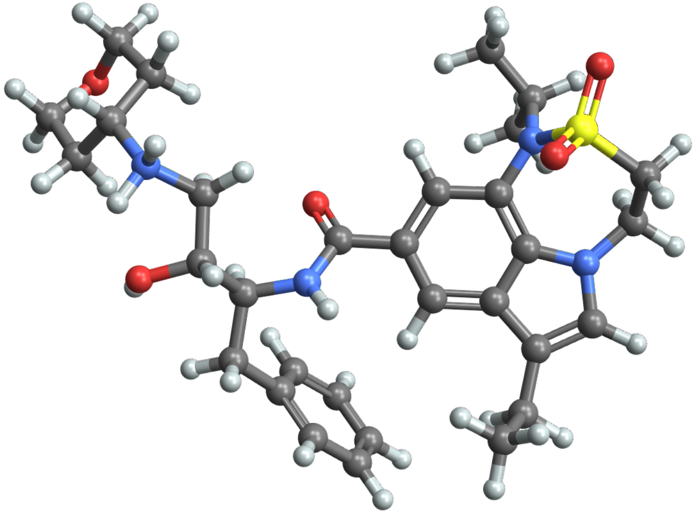

2D Plot

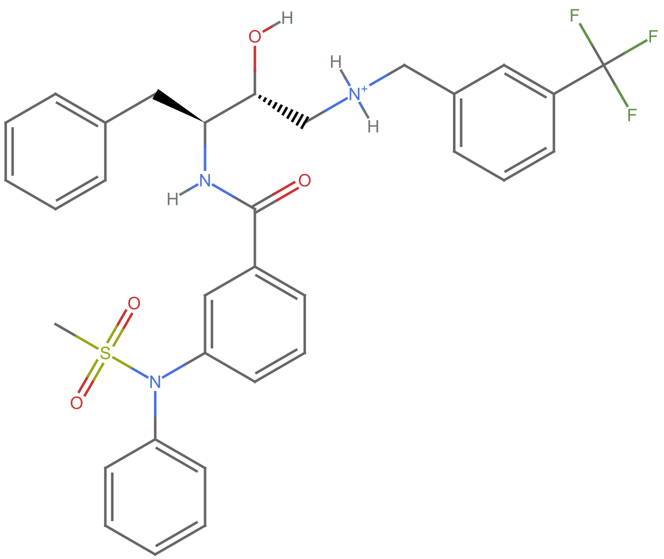

2D Autocorrelation

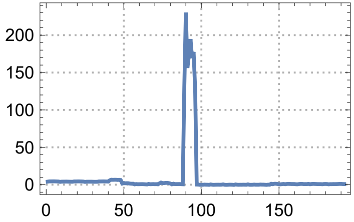

Dataset Index= 535

Molecule Formula= $C_{32}H_{33}F_3N_3O_4S^+$

3D Autocorrelation

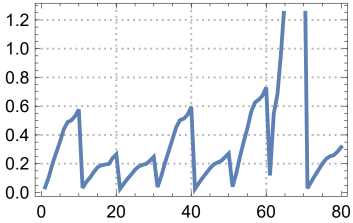

3D Plot

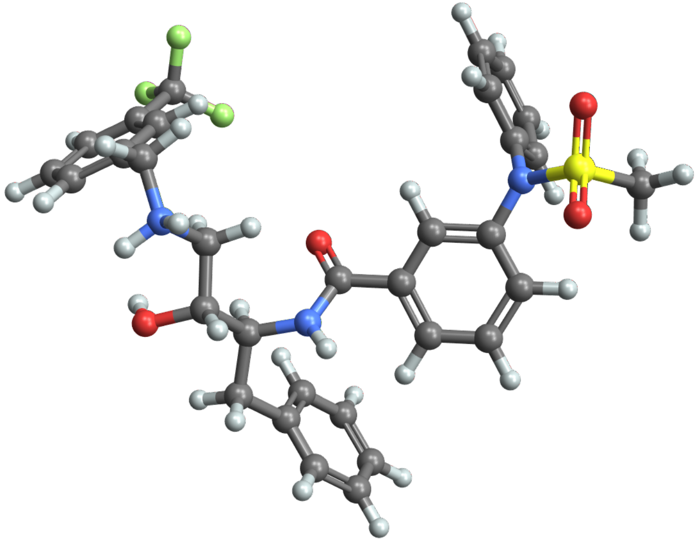

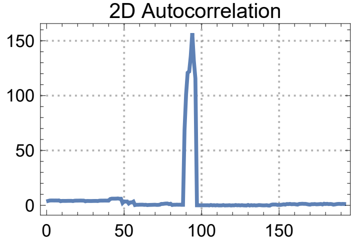

2D Plot

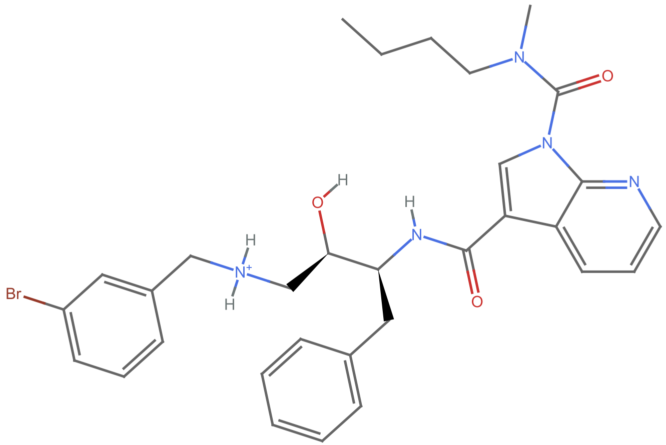

3D Plot

Dataset Index= 536

Molecule Formula= $C_{31}H_{37}BrN_5O_3^+$

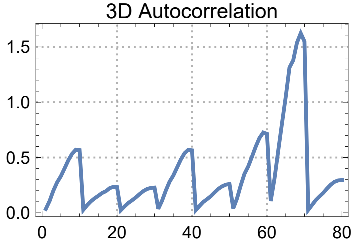

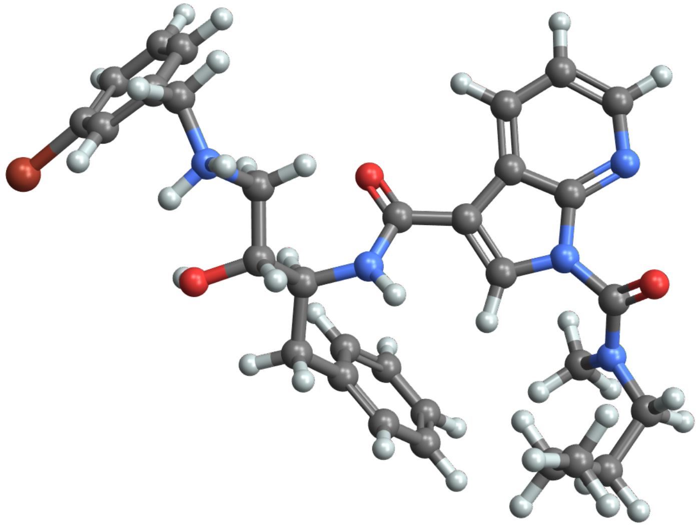

2D Plot

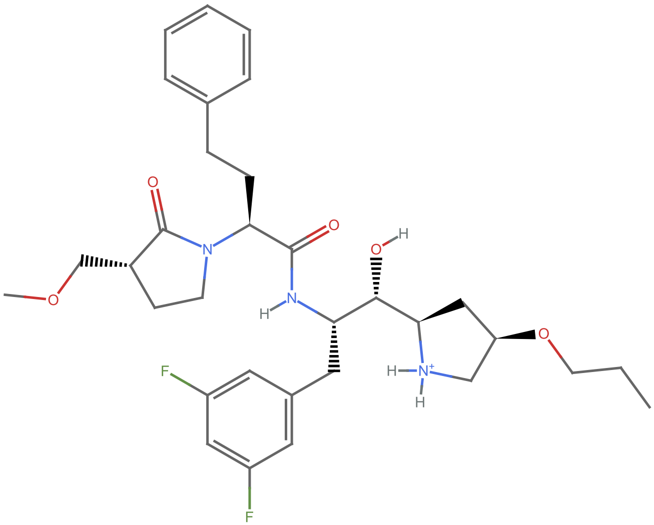

2D Autocorrelation

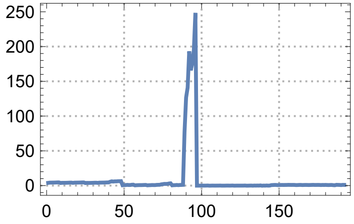

3D Plot

Dataset Index= 537

Molecule Formula= $C_{32}H_{44}F_2N_3O_5^+$

3D Autocorrelation

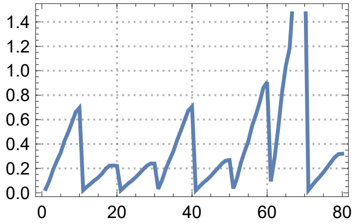

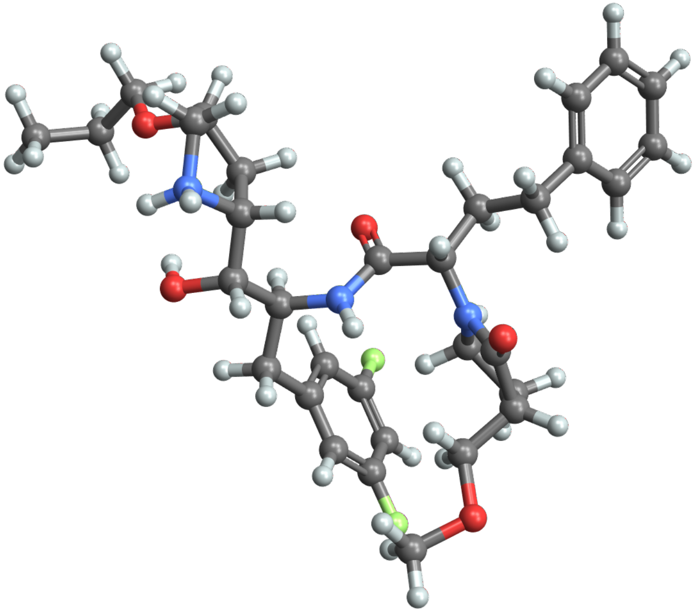

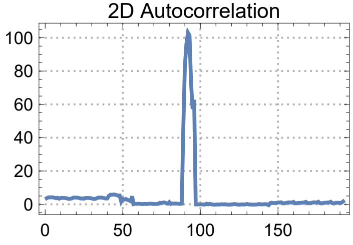

Dataset Index= 538

Molecule Formula= $C_{20}H_{19}ClFN_3OS$

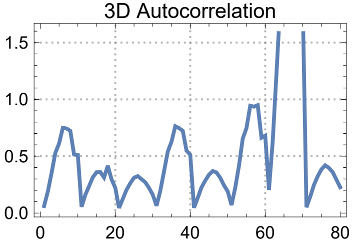

2D Plot

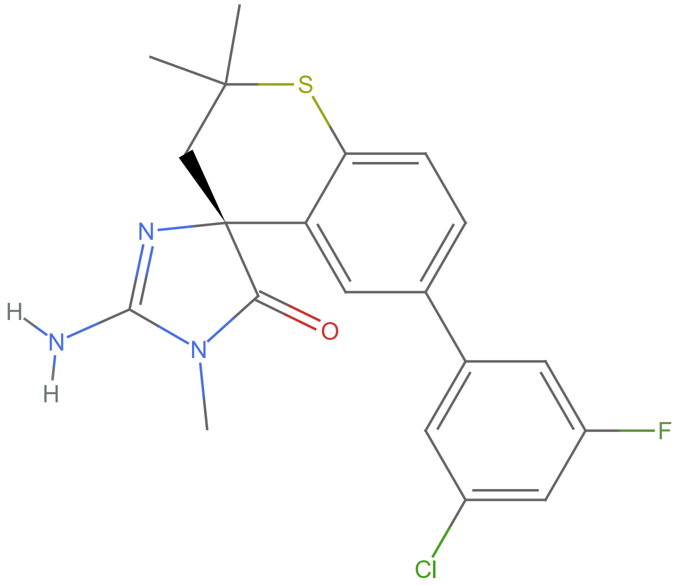

3D Plot

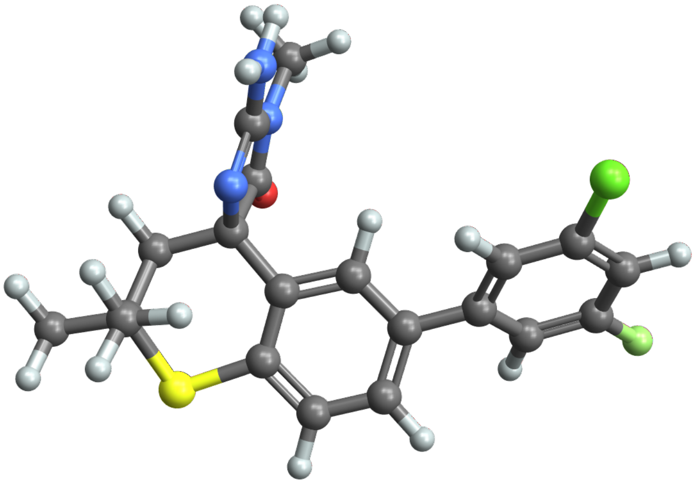

2D Plot

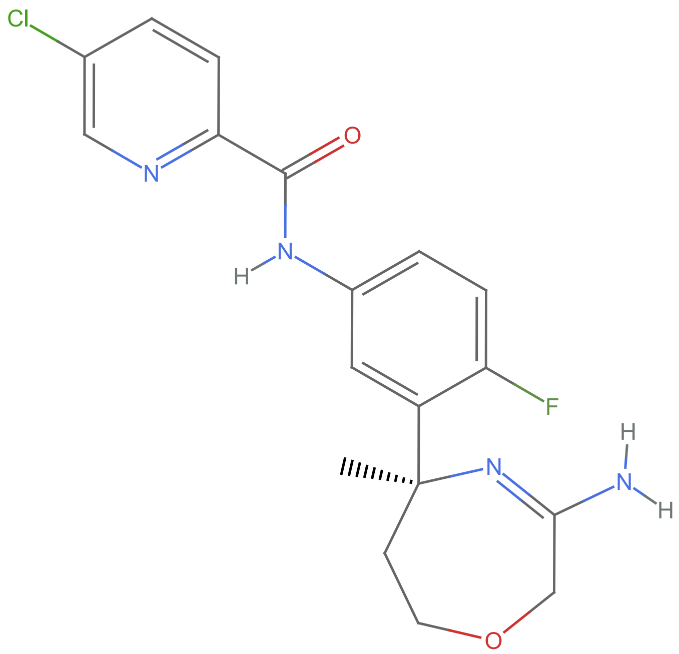

2D Autocorrelation

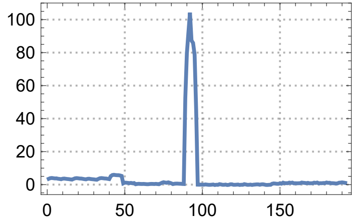

Dataset Index= 539

Molecule Formula= $C_{18}H_{18}ClFN_4O_2$

3D Autocorrelation

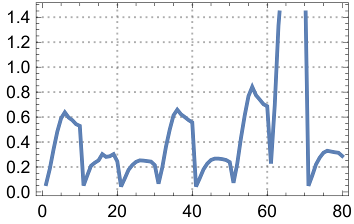

3D Plot

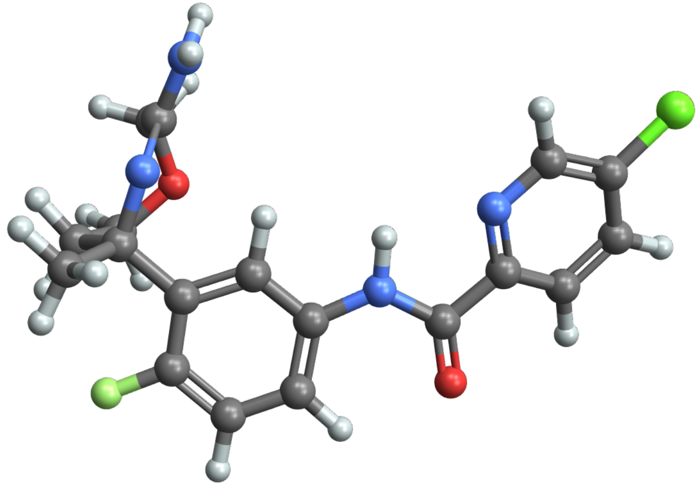

2D Autocorrelation

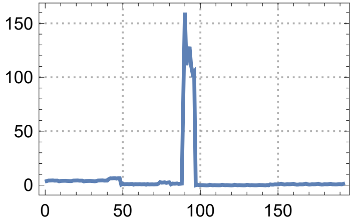

Dataset Index= 540

Molecule Formula= $C_{23}H_{18}F_4N_6O$

3D Autocorrelation

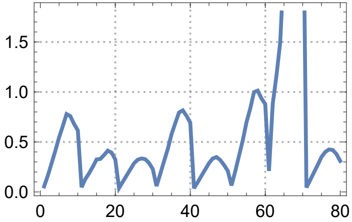

2D Plot

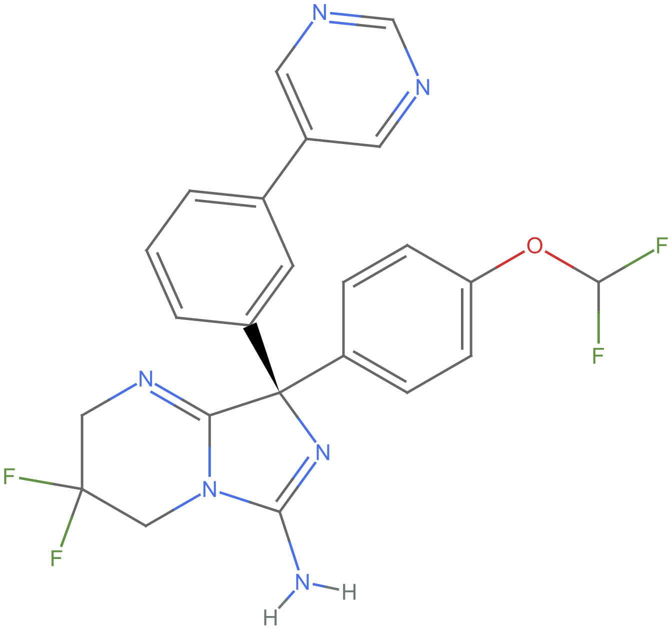

3D Plot

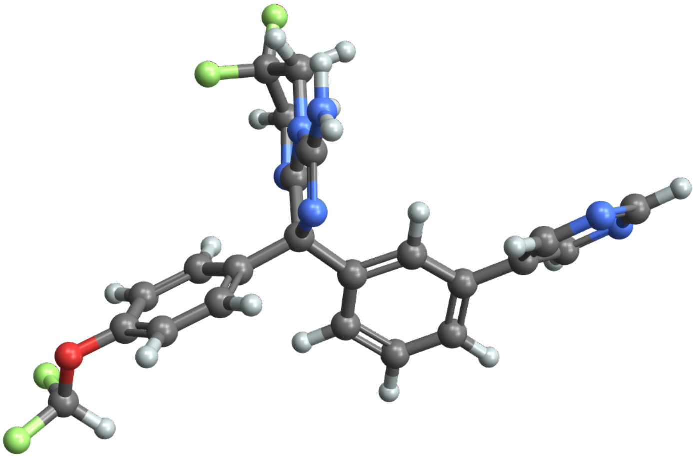

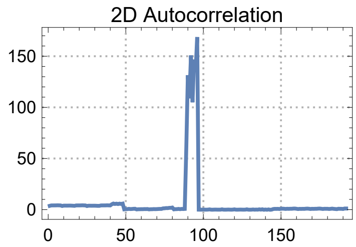

Dataset Index= 541

Molecule Formula= $C_{28}H_{39}F_2N_2O_3^+$

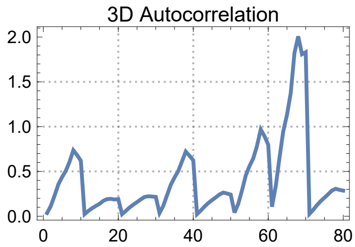

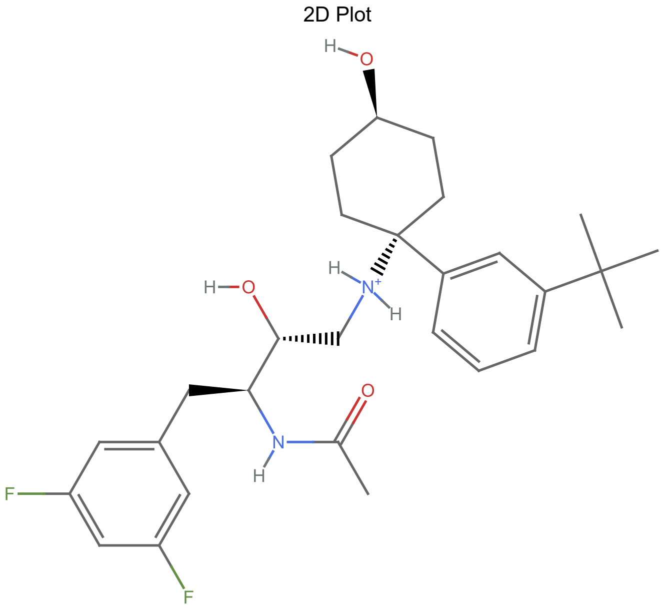

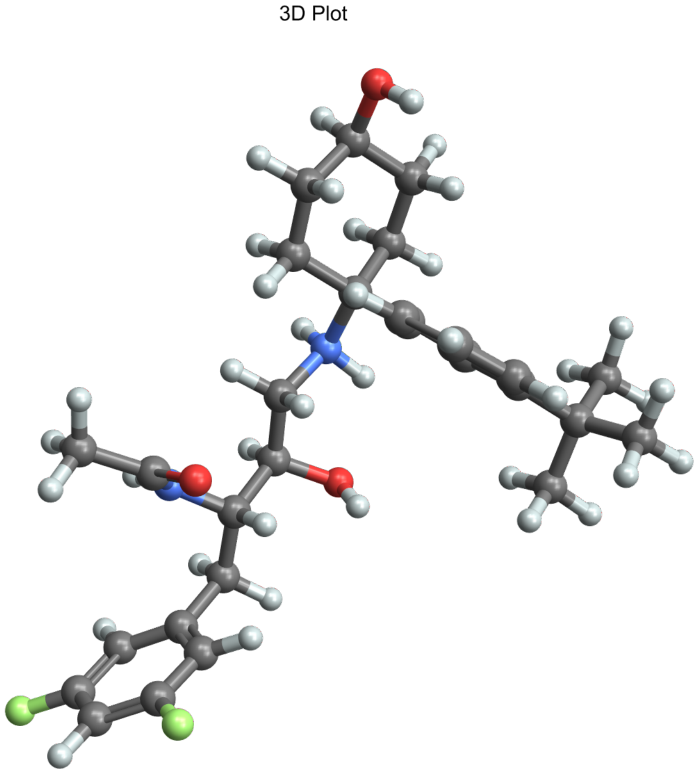

2D Plot

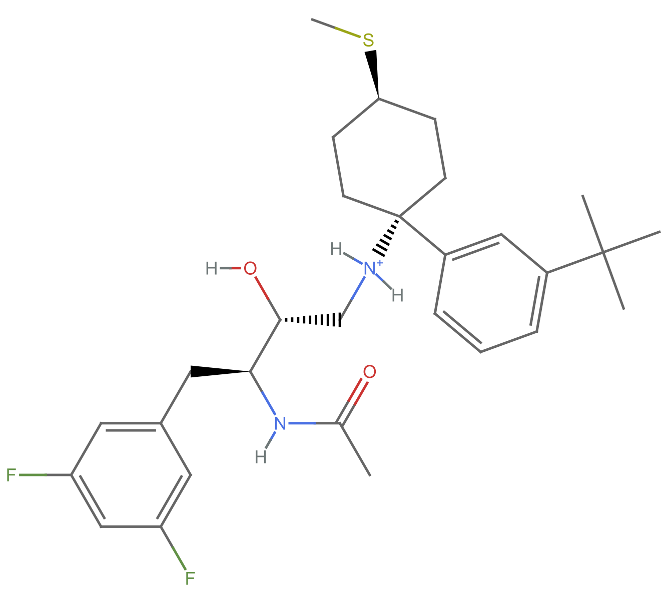

2D Autocorrelation

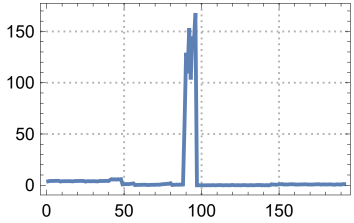

3D Plot

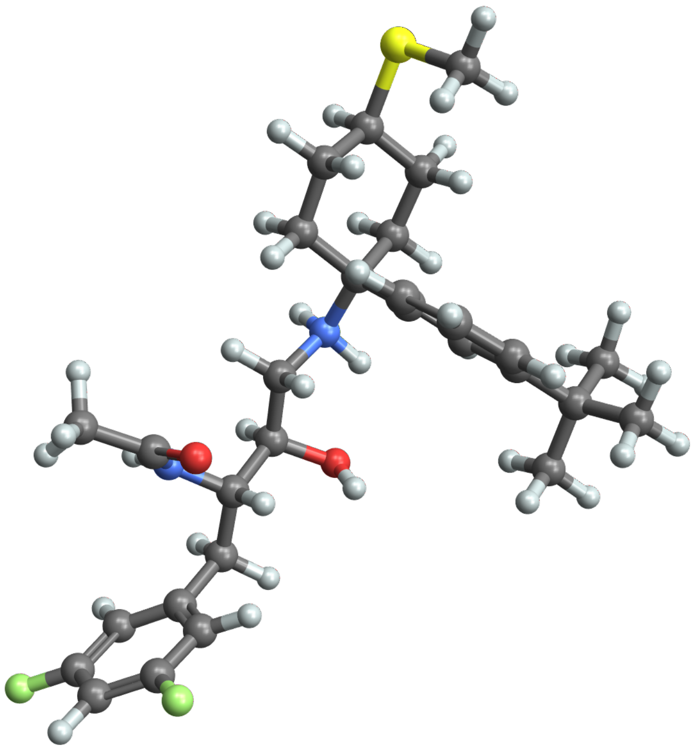

Dataset Index= 542

Molecule Formula= $C_{29}H_{41}F_2N_2O_2S^+$

3D Autocorrelation

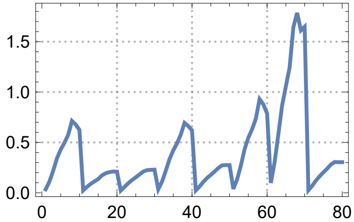

2D Plot

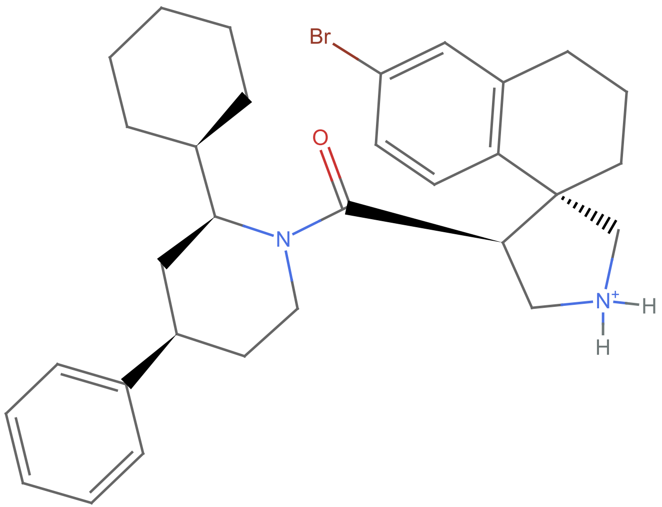

2D Autocorrelation

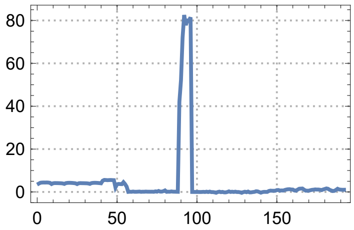

3D Plot

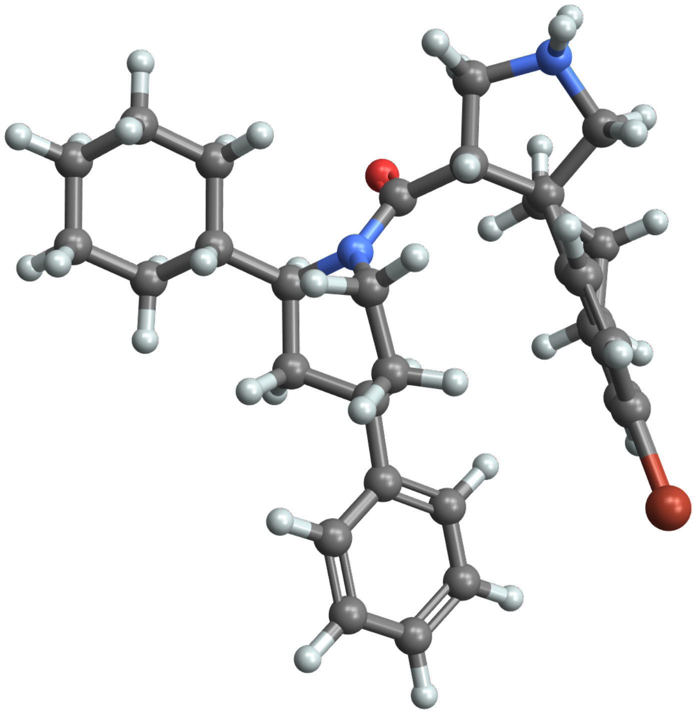

Dataset Index= 543

Molecule Formula= $C_{31}H_{40}BrN_2O^+$

3D Autocorrelation

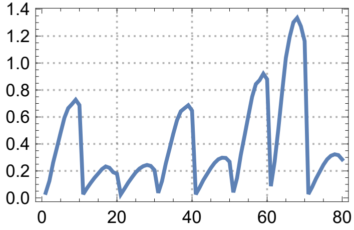

2D Autocorrelation

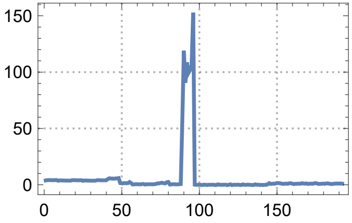

Dataset Index= 544

Molecule Formula= $C_{25}H_{36}FN_2O_4S^+$

3D Autocorrelation

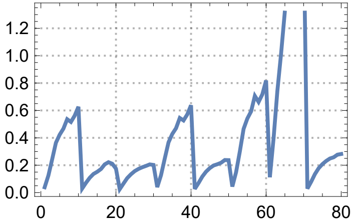

2D Plot

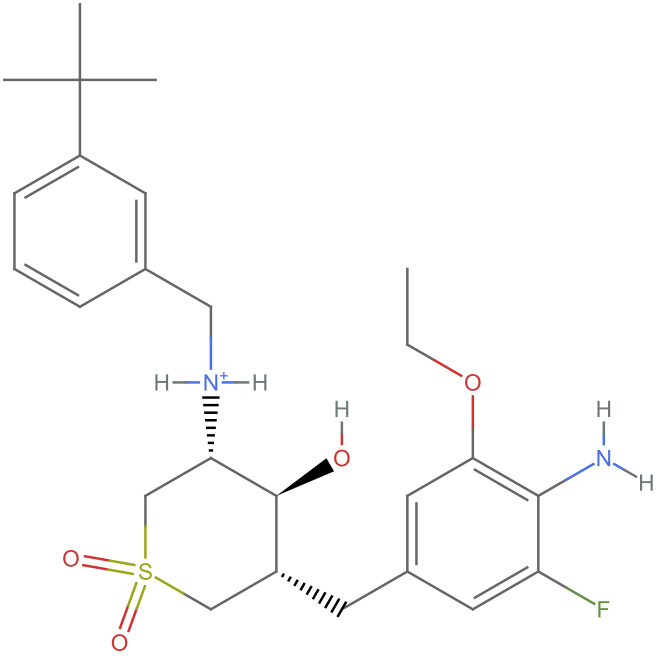

3D Plot

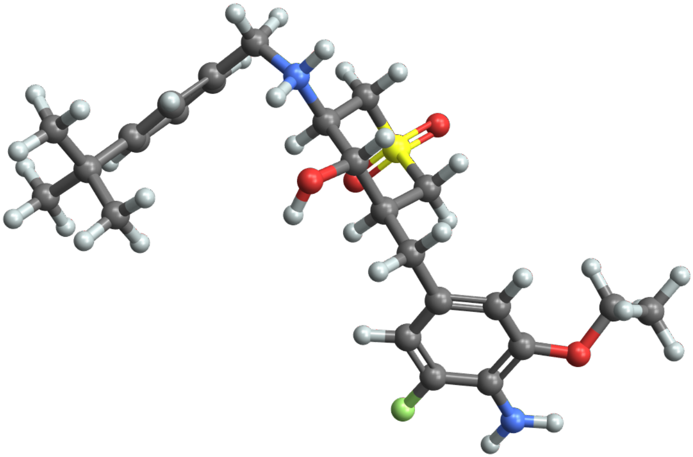

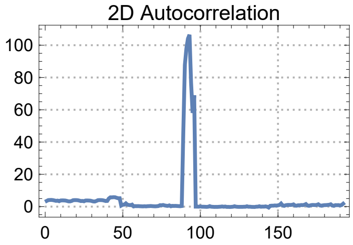

Dataset Index= 545

Molecule Formula= $C_{21}H_{21}ClFN_3O$

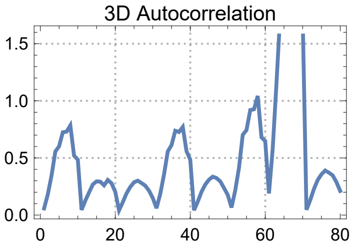

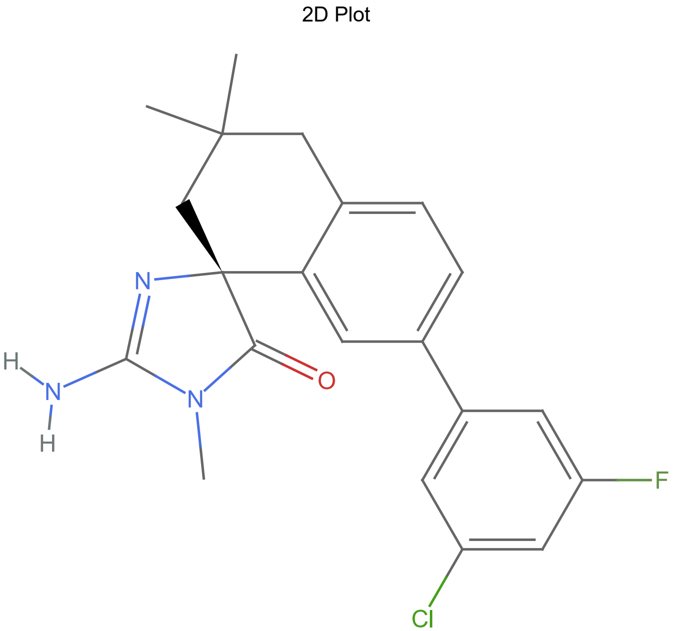

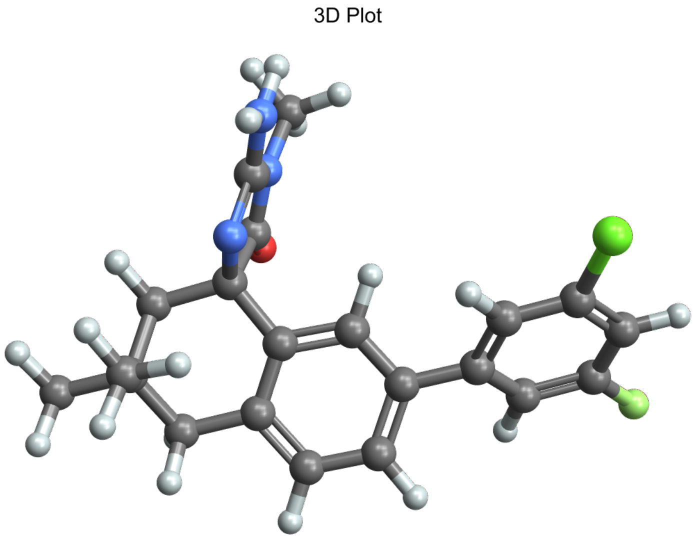

2D Plot

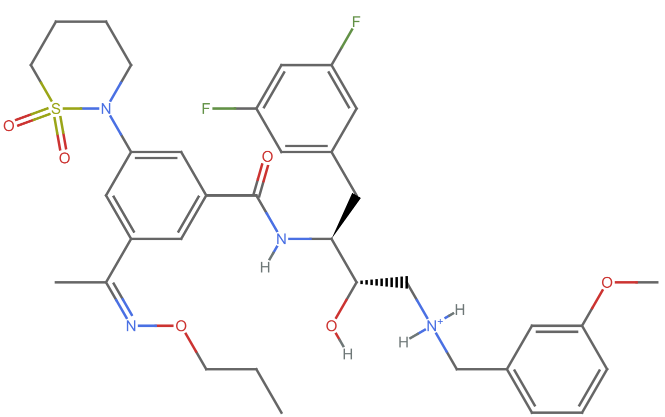

2D Autocorrelation

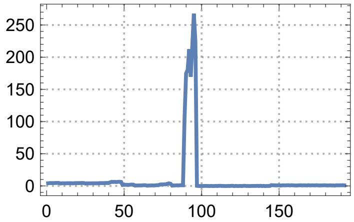

3D Plot

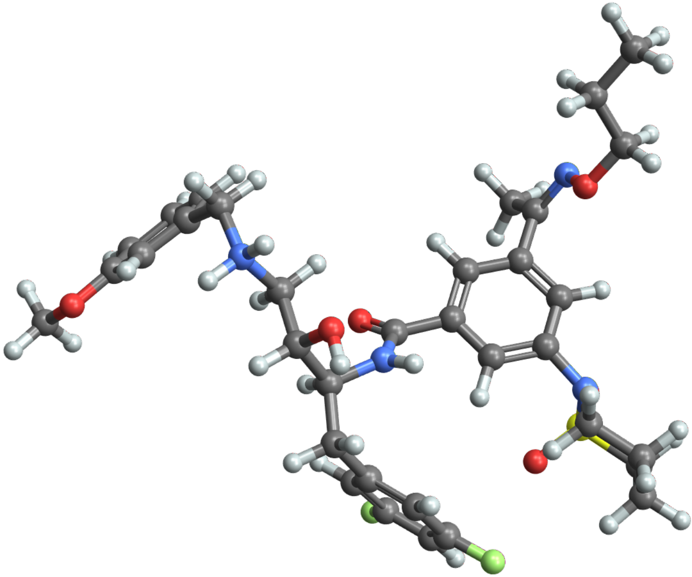

Dataset Index= 546

Molecule Formula= $C_{34}H_{43}F_2N_4O_6S^+$

3D Autocorrelation

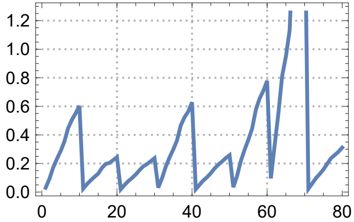

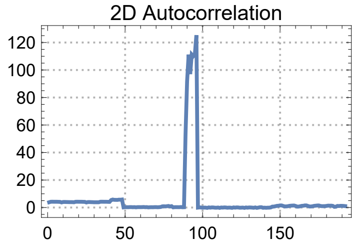

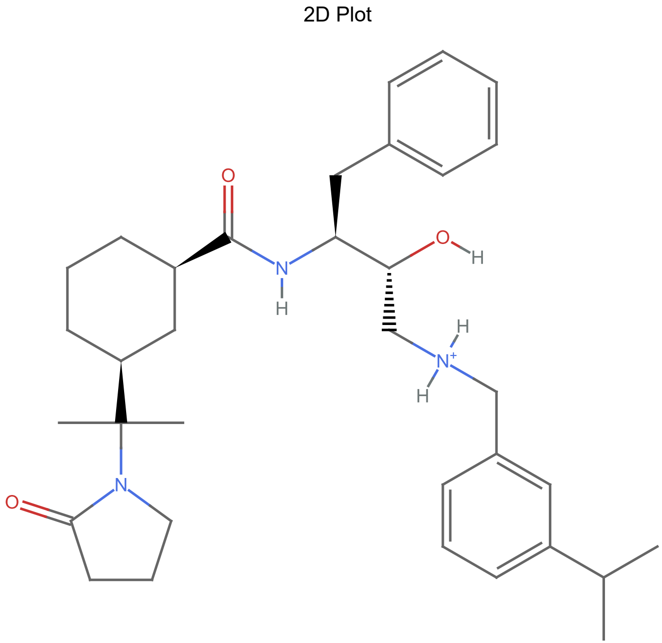

Dataset Index= 547

Molecule Formula= $C_{34}H_{50}N_3O_3^+$

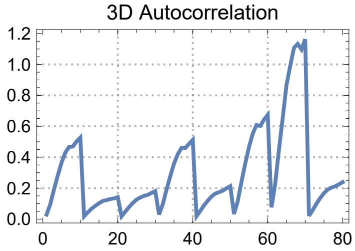

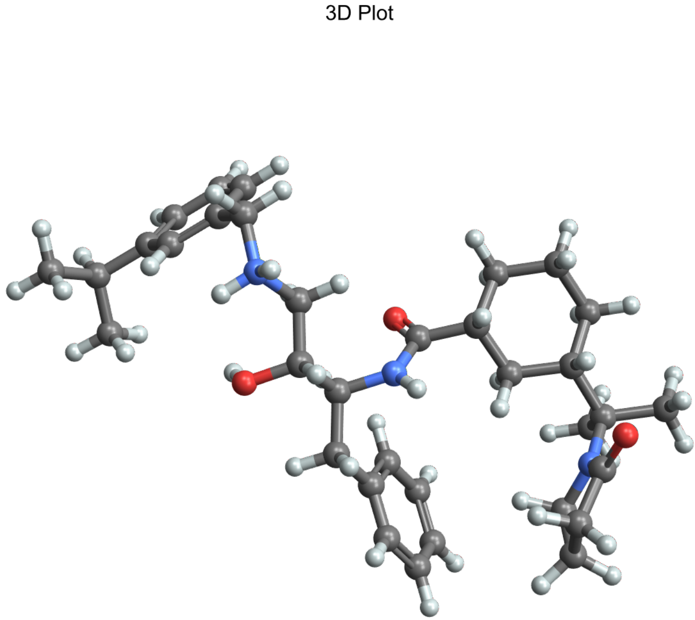

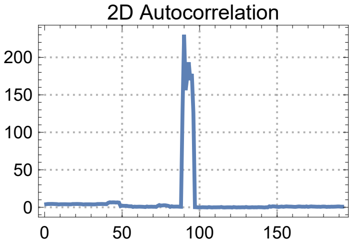

Dataset Index= 548

Molecule Formula= $C_{31}H_{32}F_3N_4O_4S^+$

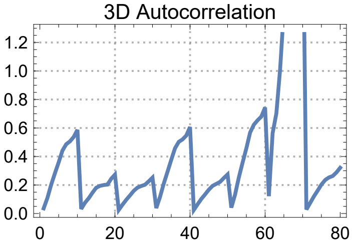

2D Plot

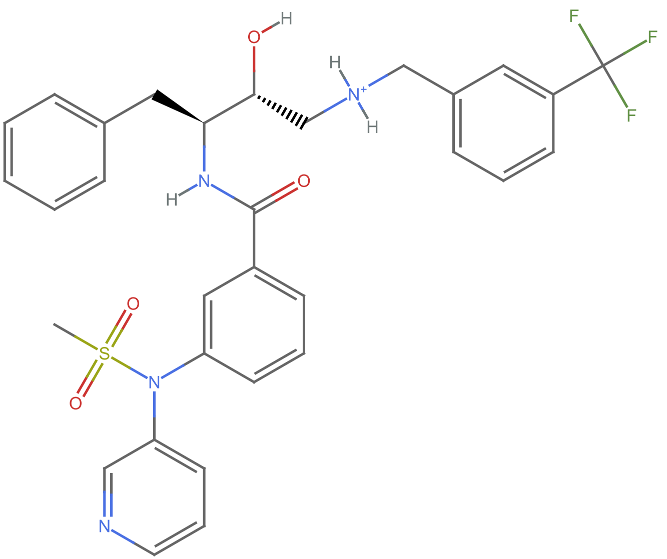

3D Plot

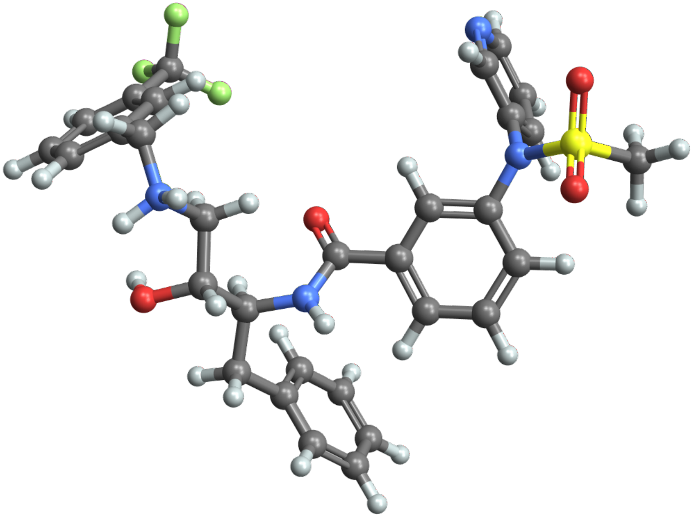

2D Plot

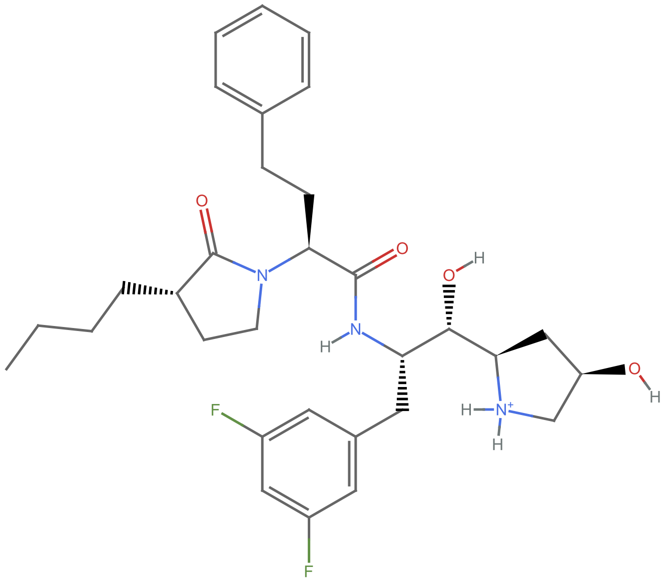

2D Autocorrelation

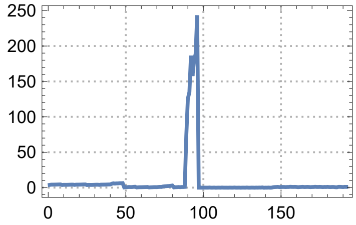

3D Plot

Dataset Index= 549

Molecule Formula= $C_{31}H_{42}F_2N_3O_4^+$

3D Autocorrelation

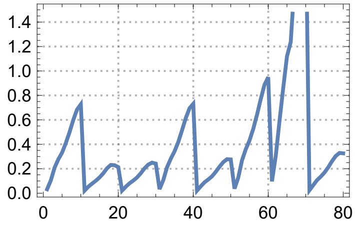

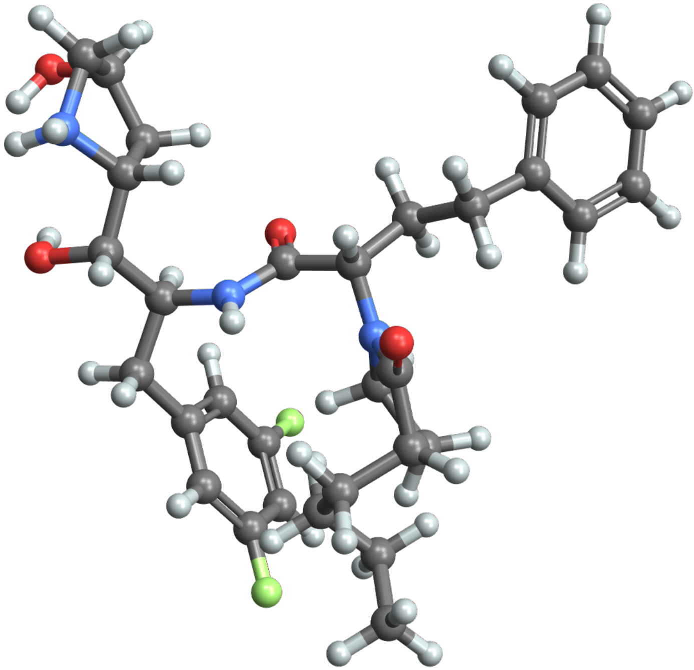

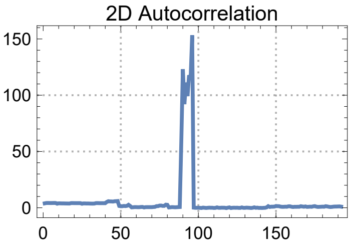

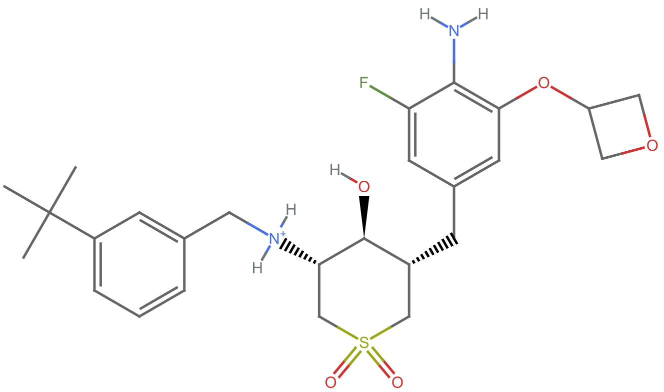

Dataset Index= 550

Molecule Formula= $C_{26}H_{36}FN_2O_5S^+$

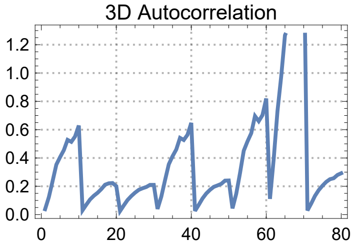

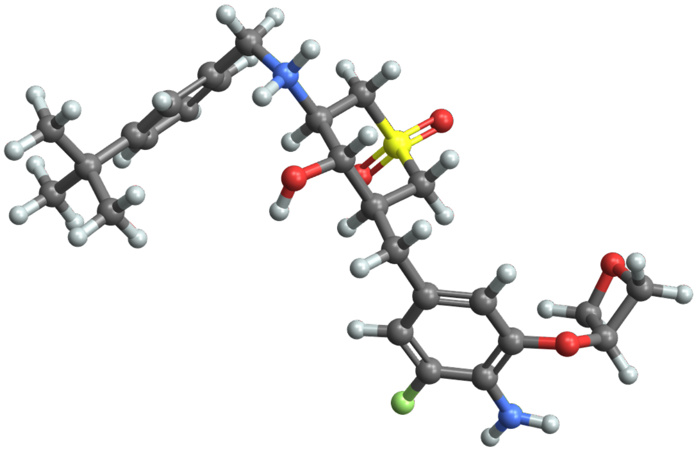

2D Autocorrelation

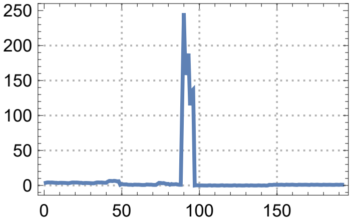

Dataset Index= 551

Molecule Formula= $C_{18}H_{15}F_{6}N_{5}O_{3}$

3D Autocorrelation

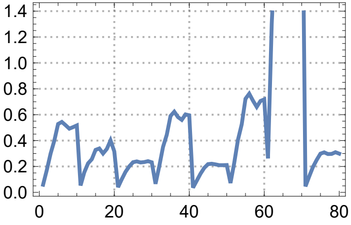

2D Plot

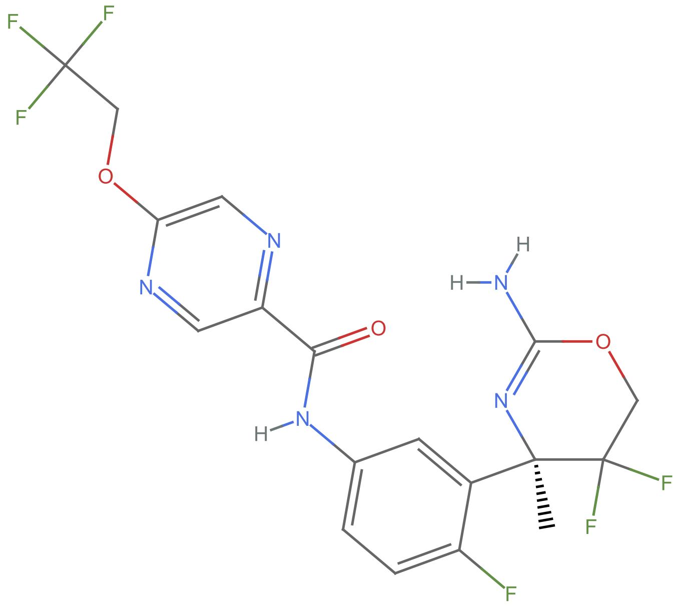

3D Plot

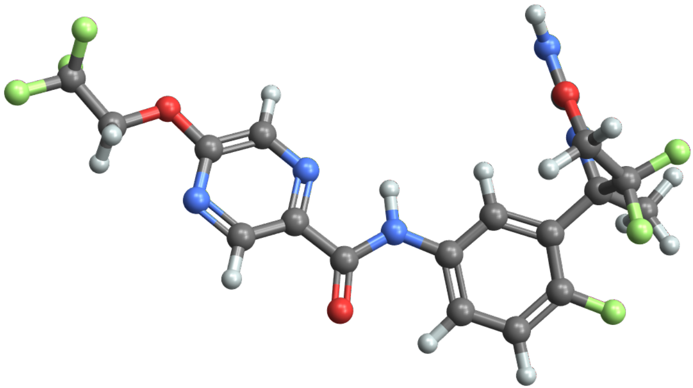

2D Plot

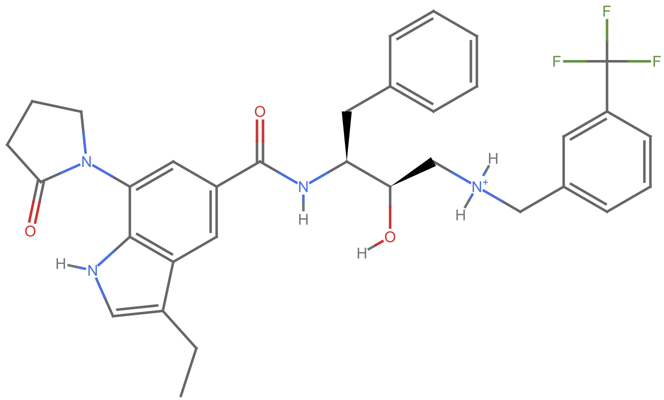

3D Plot

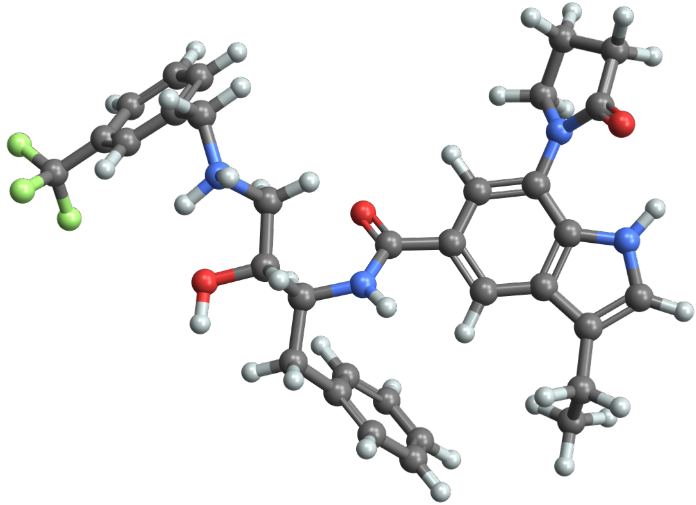

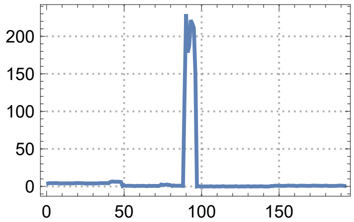

Dataset Index= 552

Molecule Formula= $C_{33}H_{36}F_3N_4O_3^+$

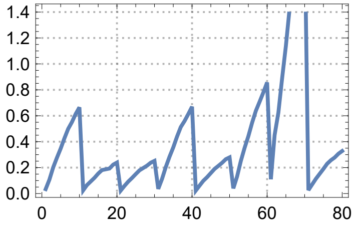

2D Plot

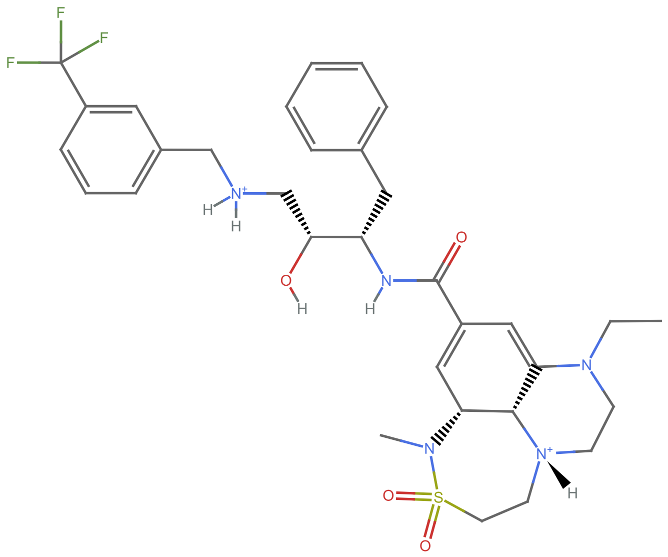

2D Autocorrelation

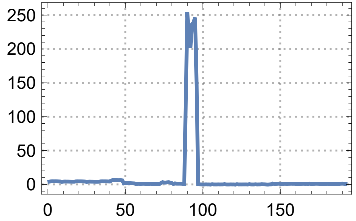

Dataset Index= 553

Molecule Formula= $C_{32}H_{42}F_3N_5O_4S^{2+}$

3D Plot

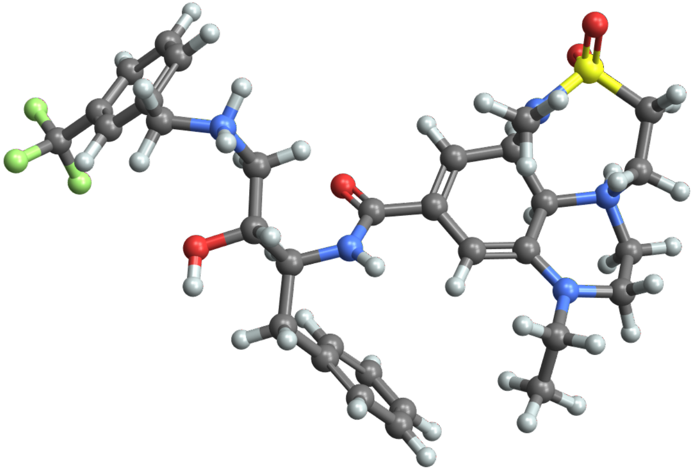

3D Autocorrelation

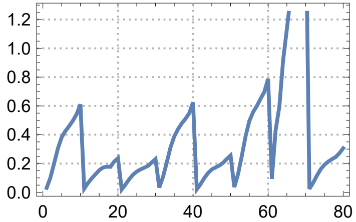

2D Plot

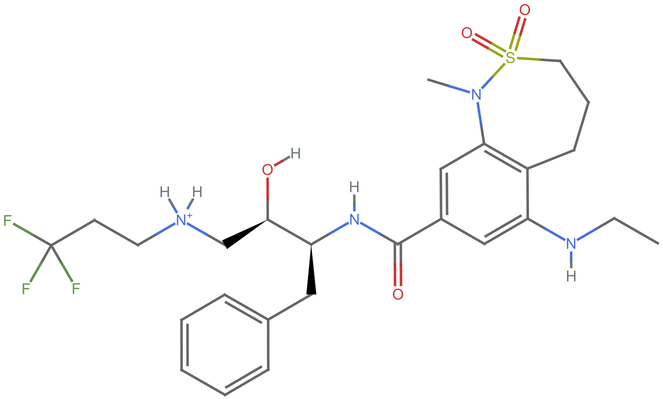

2D Autocorrelation

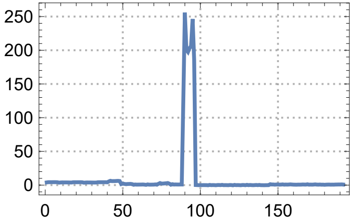

3D Plot

Dataset Index= 554

Molecule Formula= $C_{26}H_{36}F_3N_4O_4S^+$

3D Autocorrelation

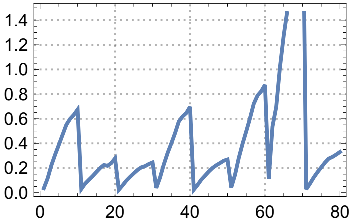

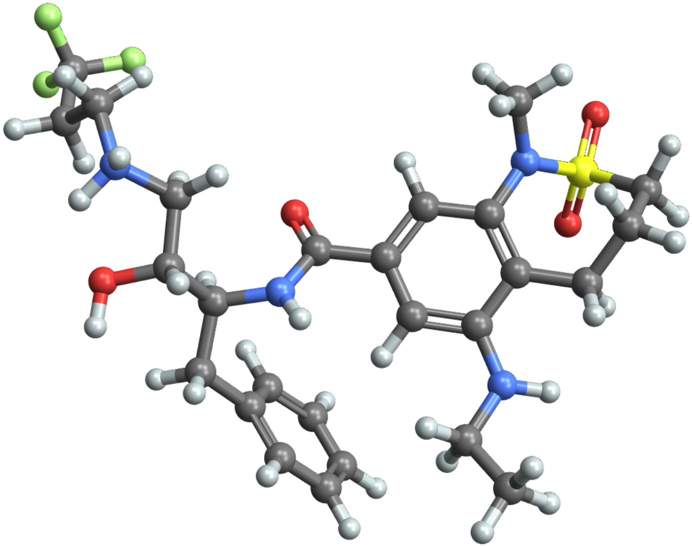

2D Plot

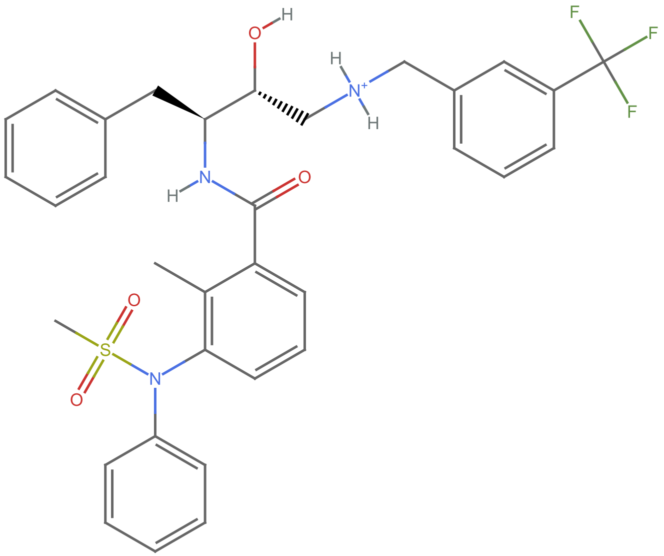

2D Autocorrelation

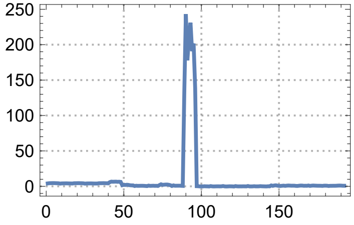

Dataset Index= 555

Molecule Formula= $C_{33}H_{35}F_3N_3O_4S^+$

3D Autocorrelation

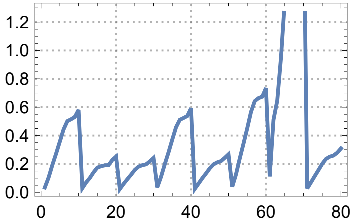

3D Plot

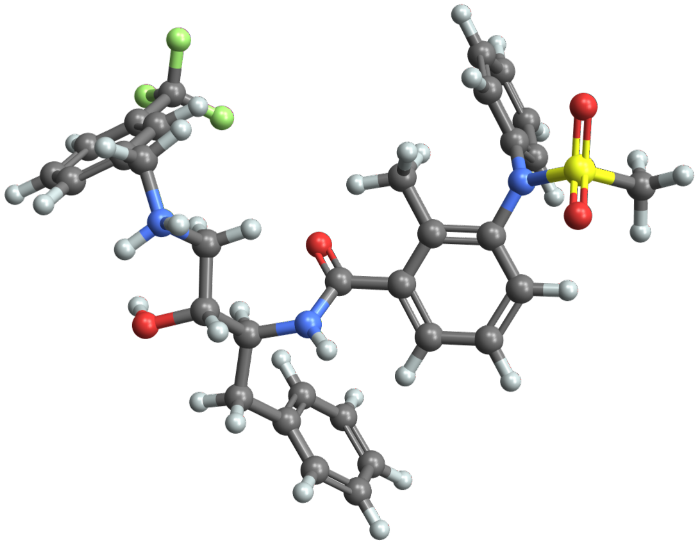

2D Plot

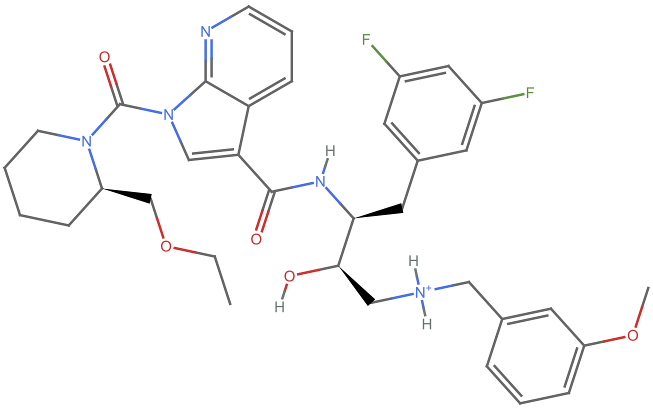

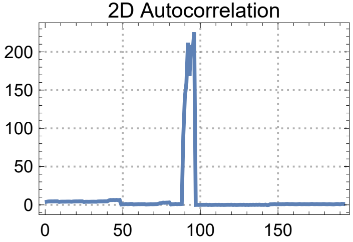

3D Plot

Dataset Index= 556

Molecule Formula= $C_{35}H_{42}F_{2}N_{5}O_{5}^{+}$

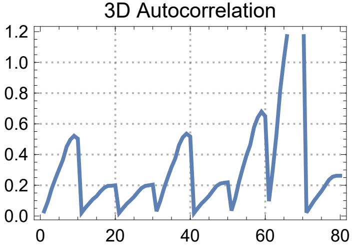

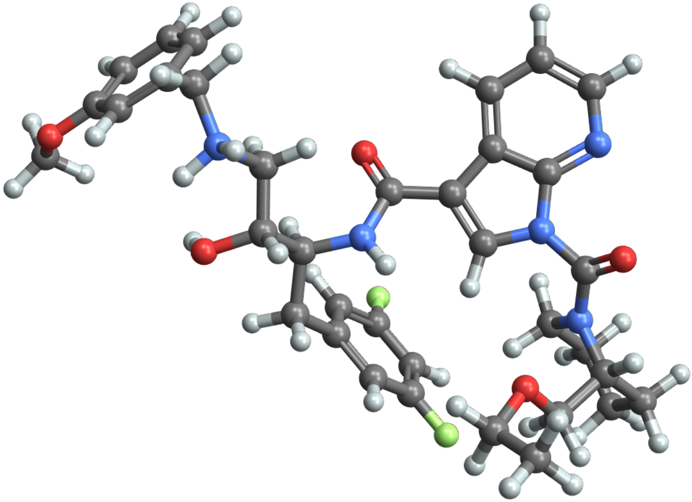

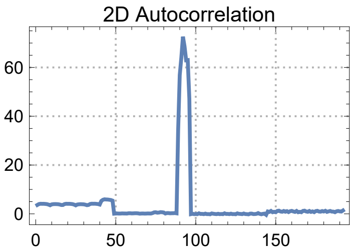

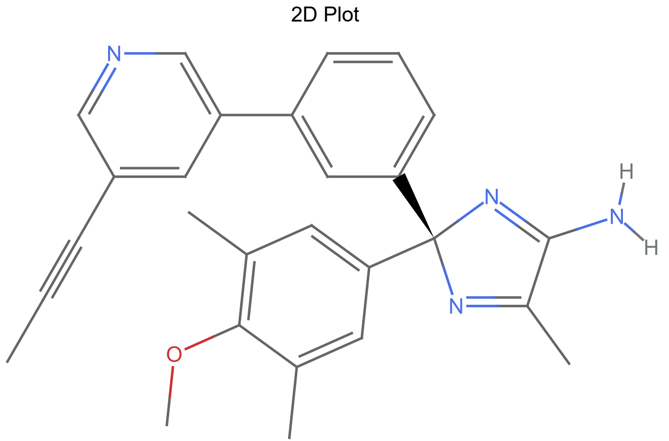

Dataset Index= 557

Molecule Formula= $C_{27}H_{26}N_4O$

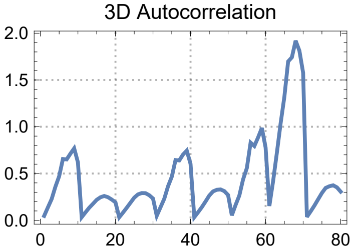

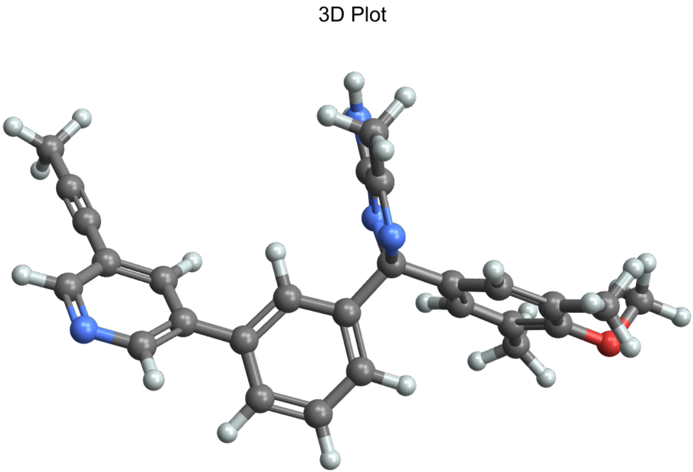

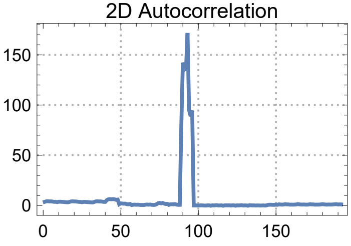

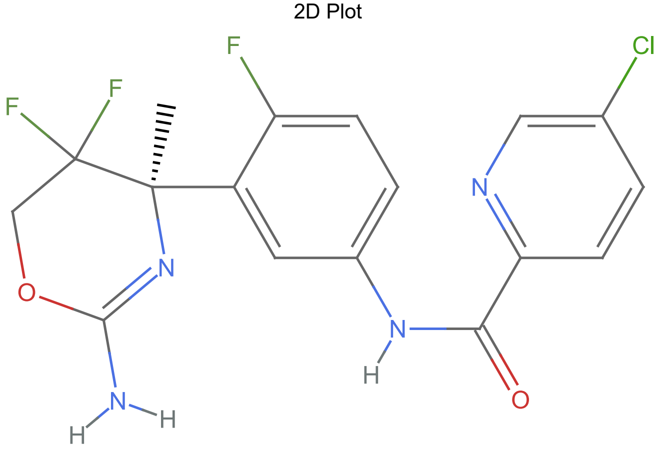

Dataset Index= 558

Molecule Formula= $C_{17}H_{14}ClF_3N_4O_2$

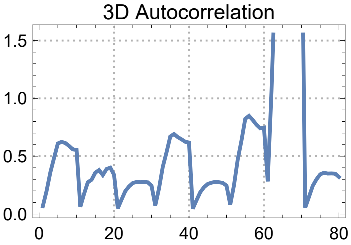

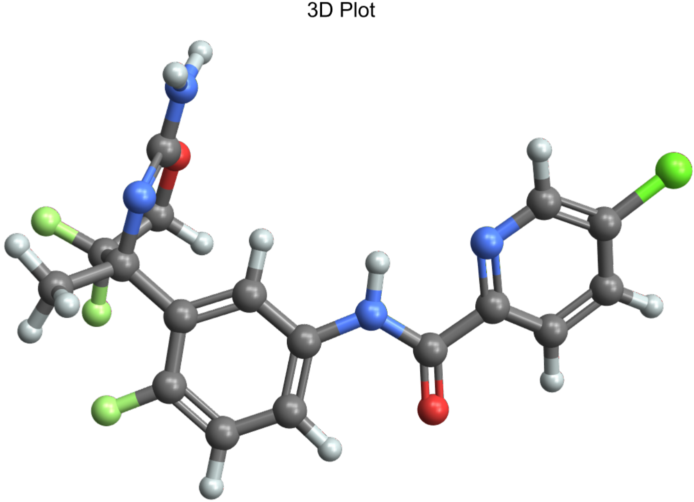

2D Plot

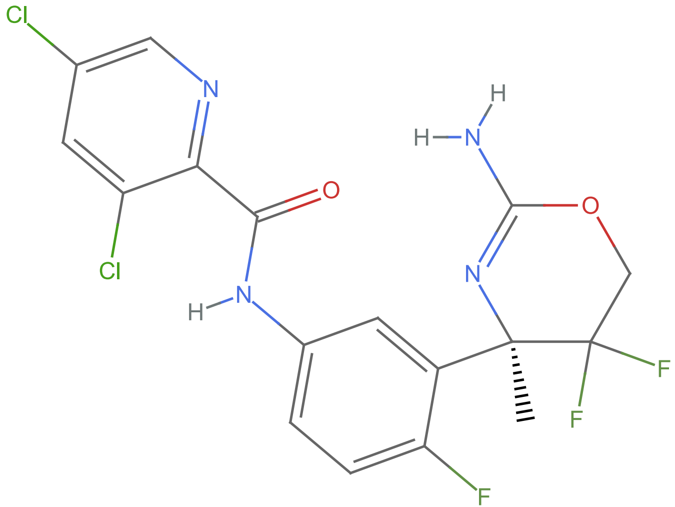

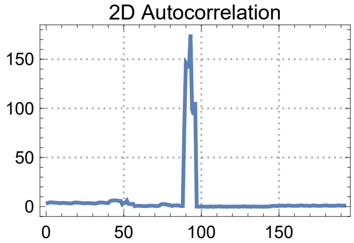

Dataset Index= 559

Molecule Formula= $C_{17}H_{13}Cl_2F_3N_4O_2$

3D Plot

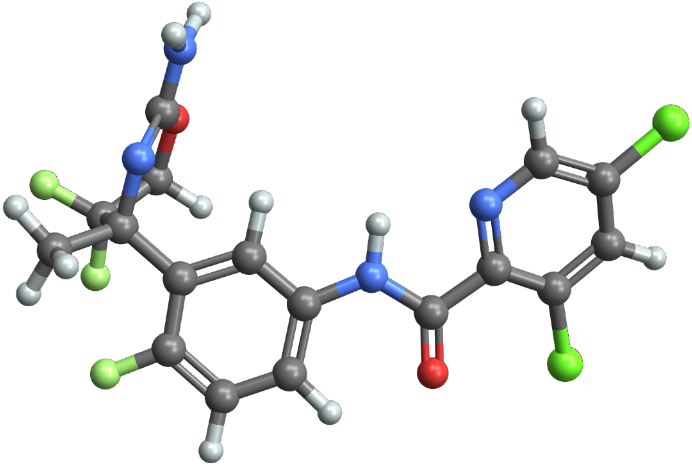

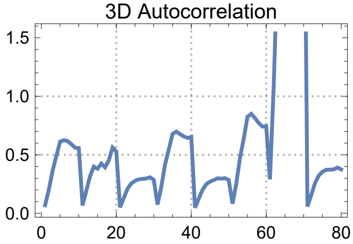

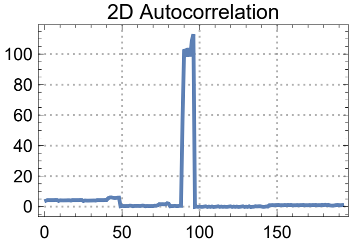

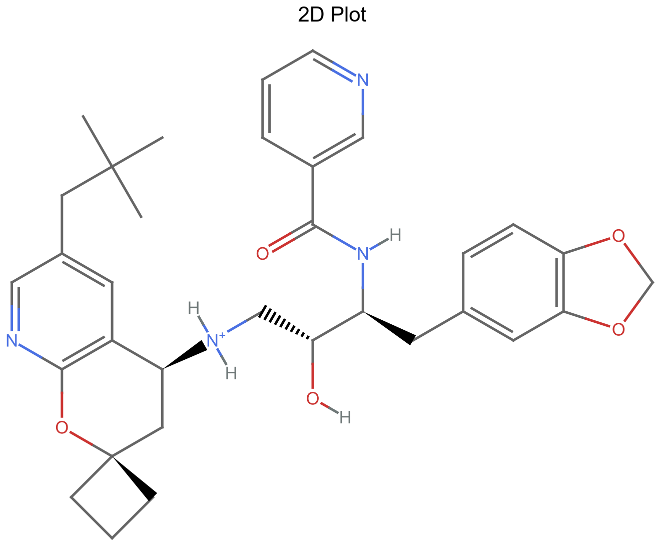

Dataset Index= 560

Molecule Formula= $C_{33}H_{41}N_4O_5^+$

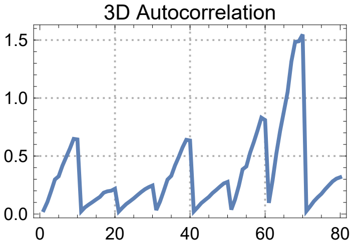

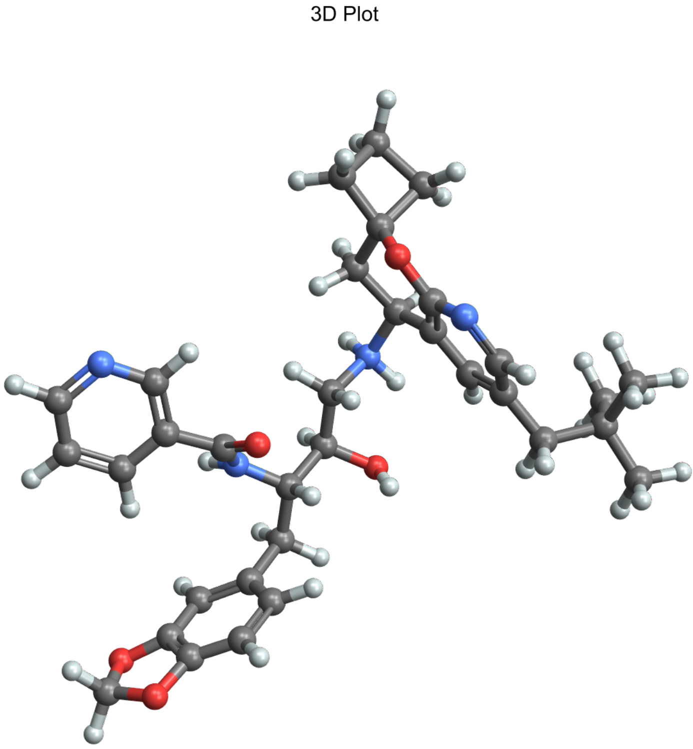

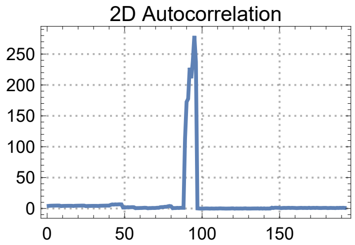

Dataset Index= 561

Molecule Formula= $C_{37}H_{41}F_{2}N_{4}O_{6}S^{+}$

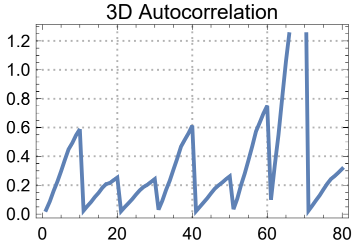

2D Plot

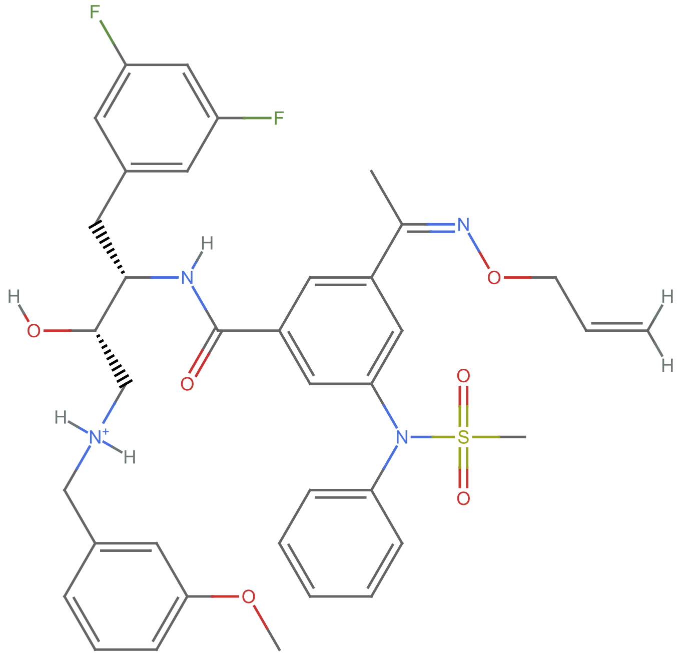

3D Plot

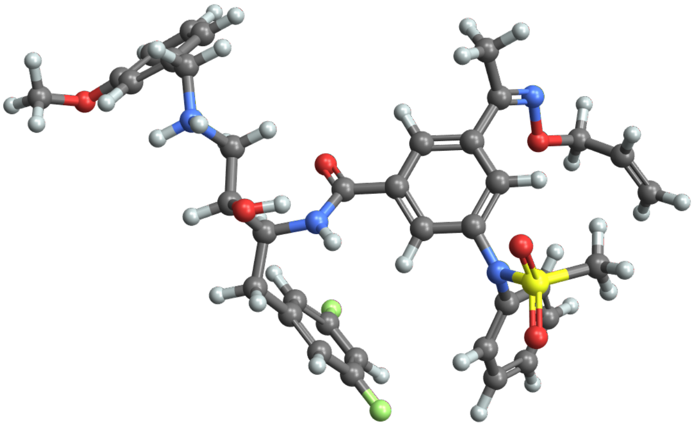

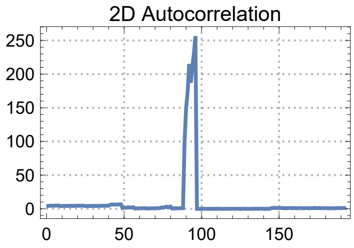

Dataset Index= 562

Molecule Formula= $C_{39}H_{46}F_2N_3O_5S^+$

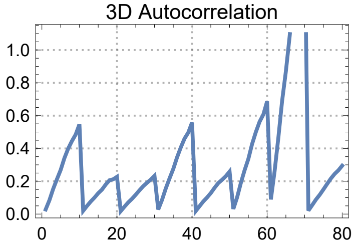

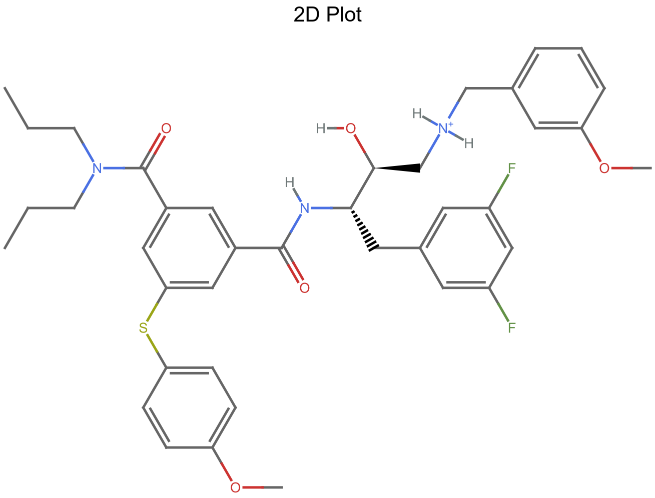

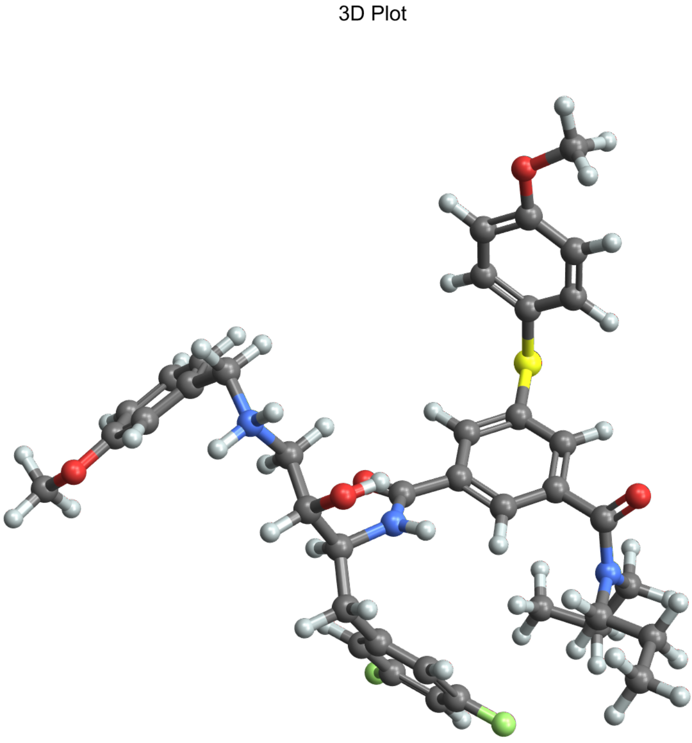

2D Plot

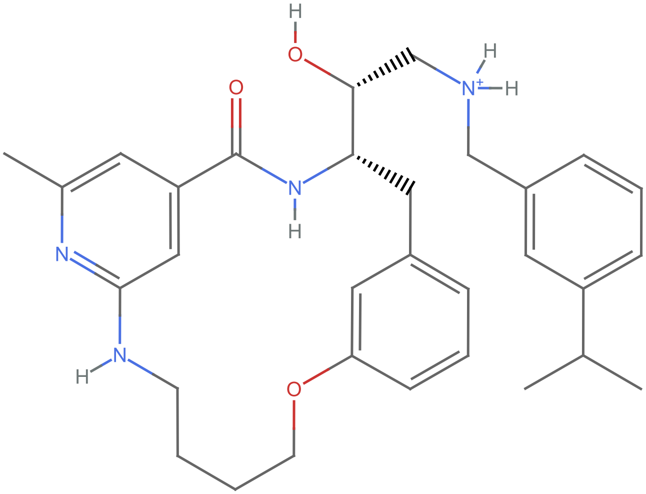

2D Autocorrelation

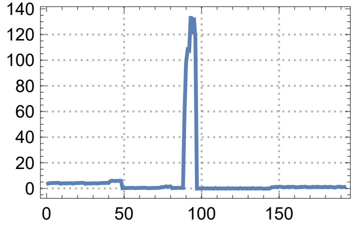

3D Plot

Dataset Index= 563

Molecule Formula= $C_{31}H_{41}N_4O_3^+$

3D Autocorrelation

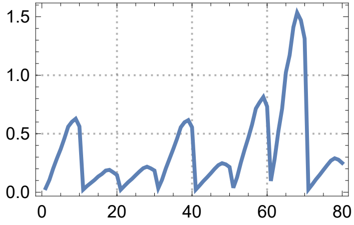

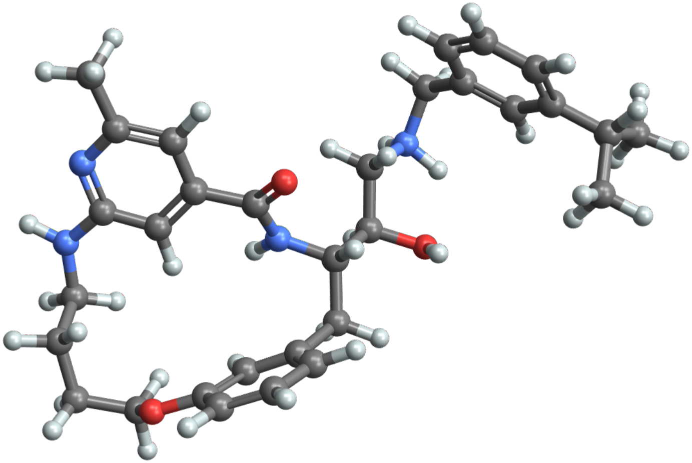

2D Plot

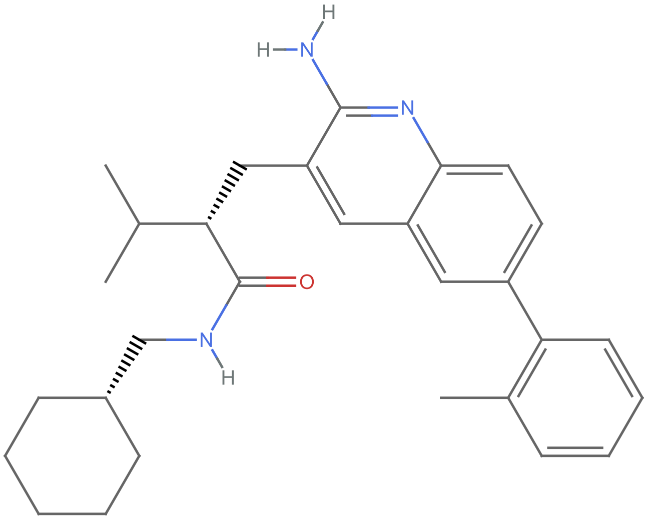

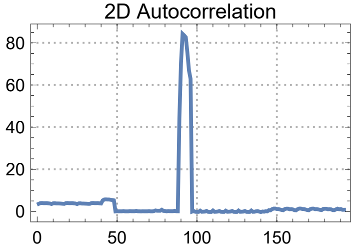

3D Plot

Dataset Index= 564

Molecule Formula= $C_{29}H_{37}N_3O$

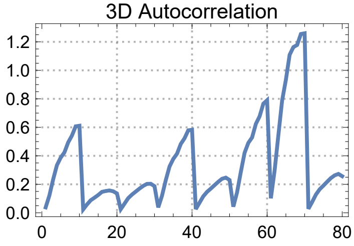

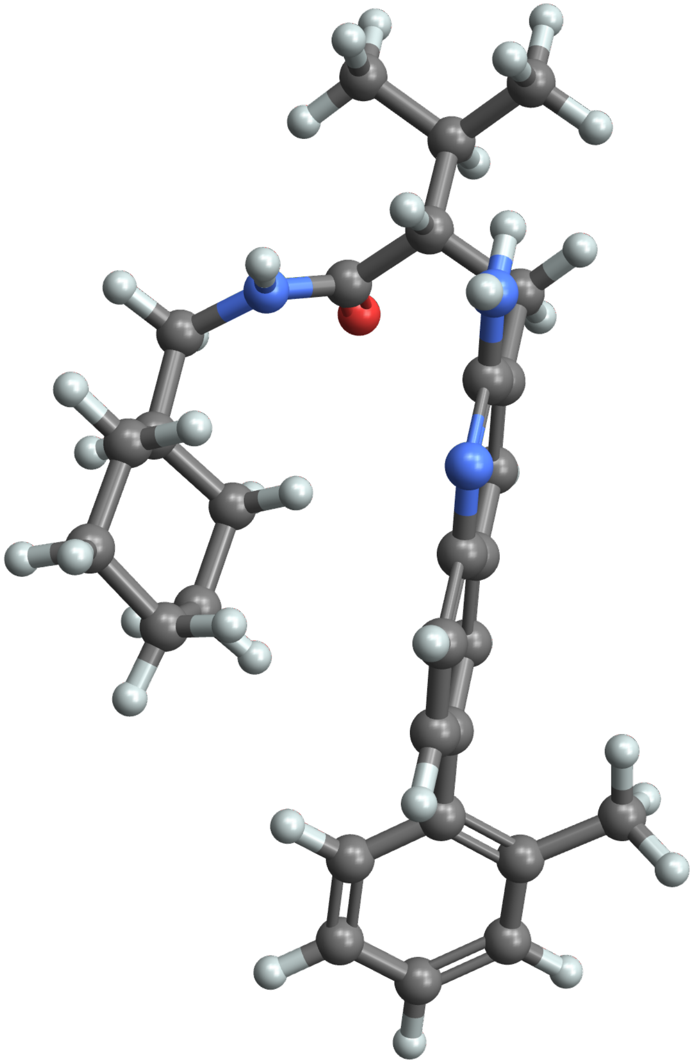

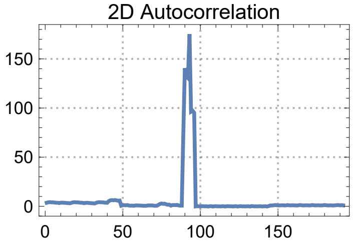

Dataset Index= 565

Molecule Formula= $C_{17}H_{16}F_3N_5O_3$

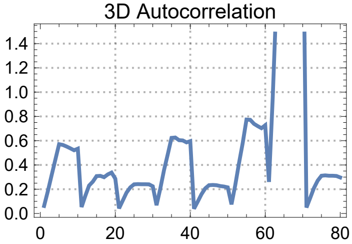

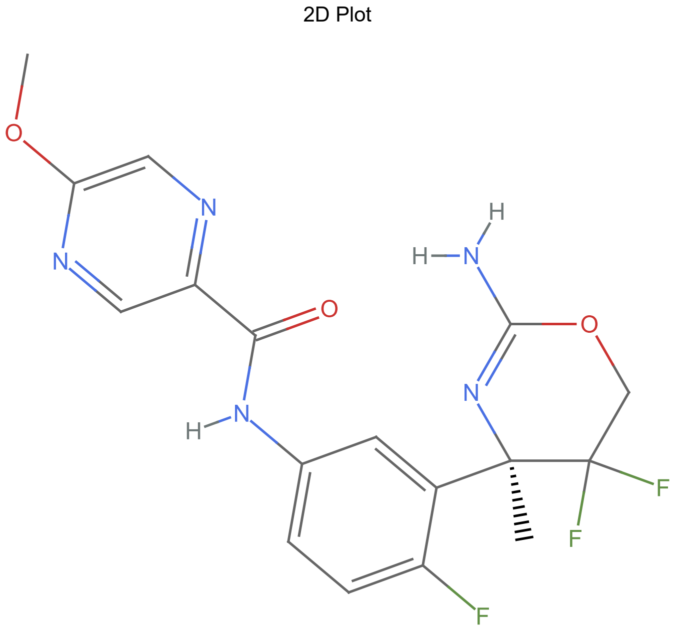

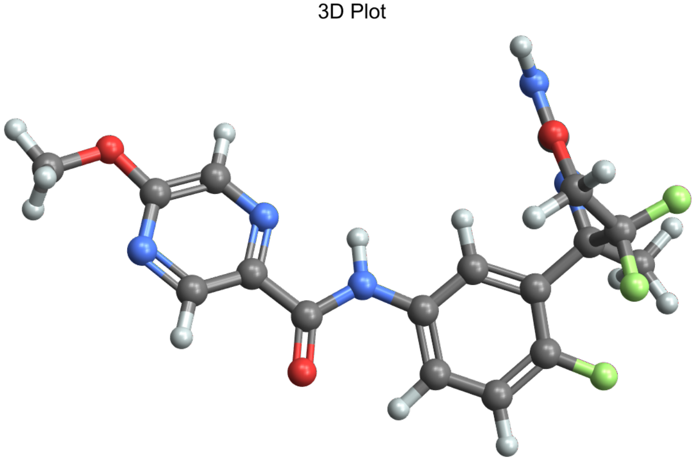

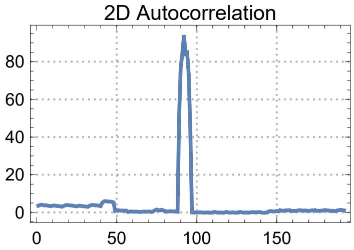

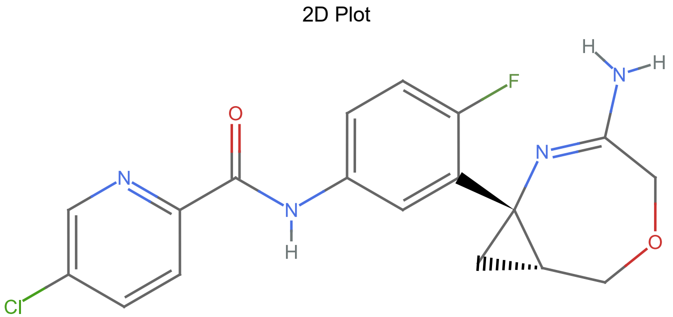

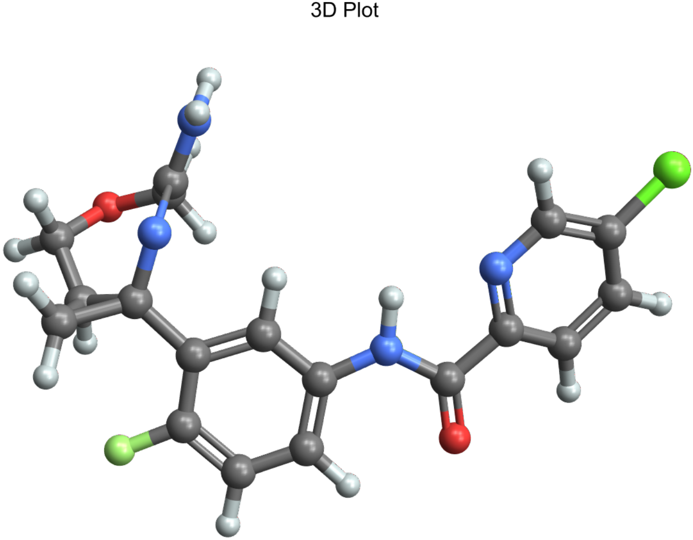

Dataset Index= 566

Molecule Formula= $C_{18}H_{16}ClFN_4O_2$

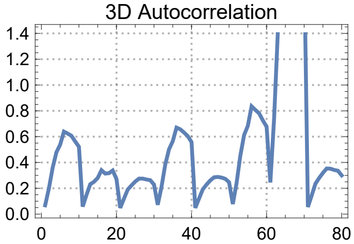

2D Plot

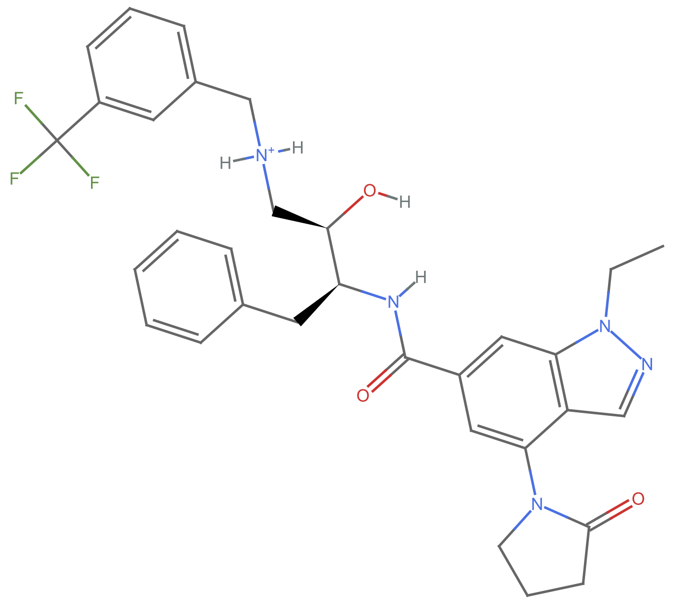

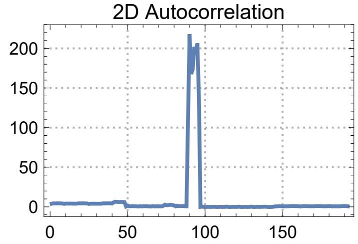

Dataset Index= 567

Molecule Formula= $C_{32}H_{35}F_3N_5O_3^+$

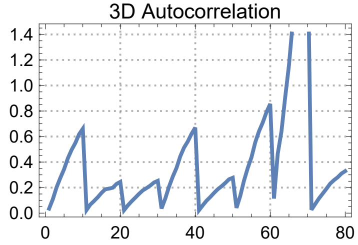

3D Plot

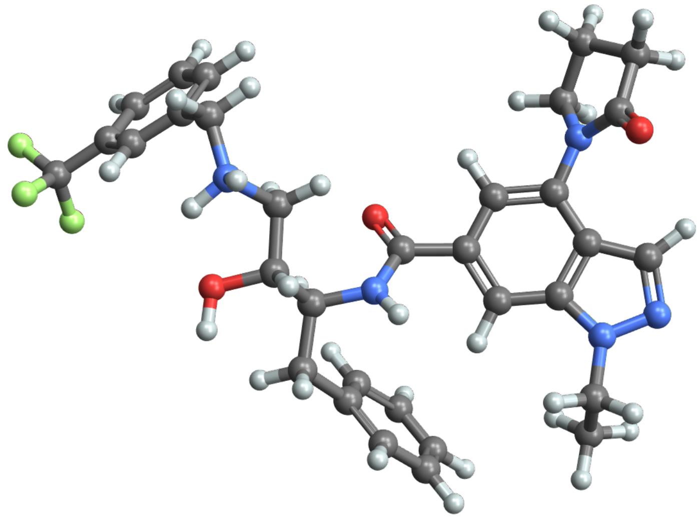

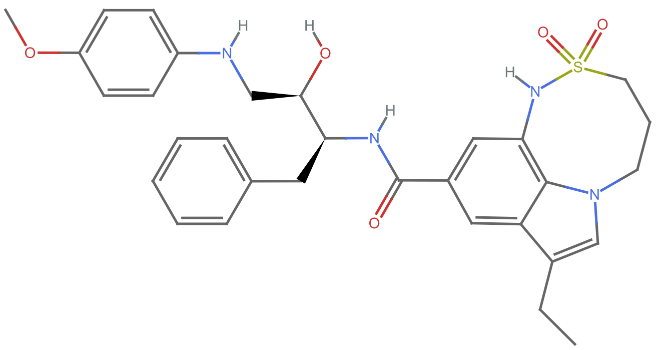

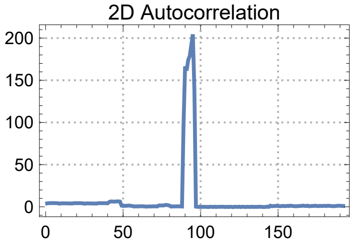

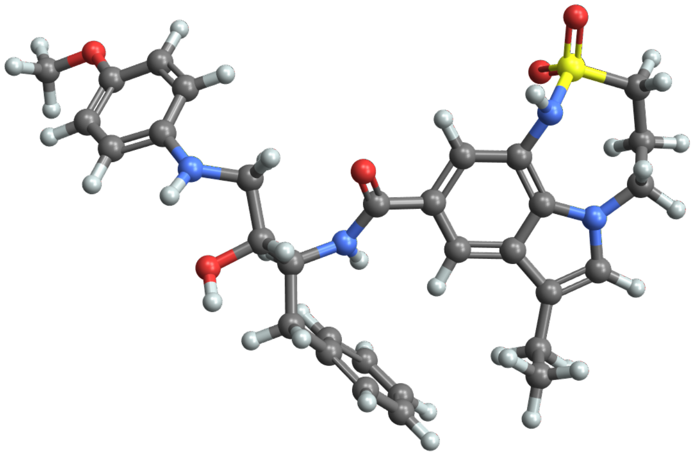

Dataset Index= 568

Molecule Formula= $C_{31}H_{36}N_4O_5S$

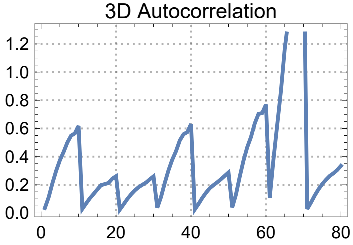

2D Plot

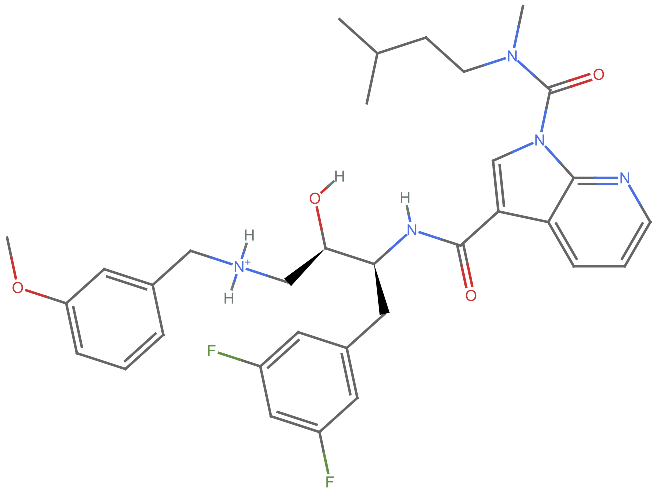

2D Autocorrelation

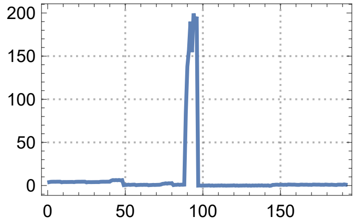

3D Plot

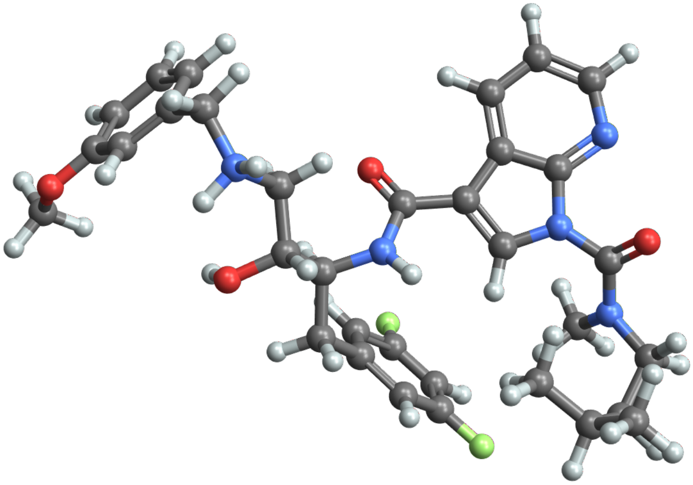

Dataset Index= 569

Molecule Formula= $C_{33}H_{40}F_2N_5O_4^+$

3D Autocorrelation

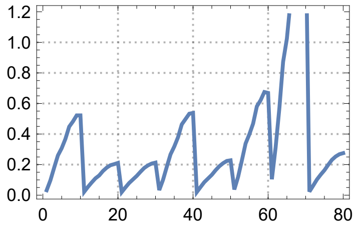

2D Autocorrelation

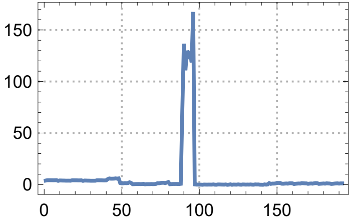

Dataset Index= 570

Molecule Formula= $C_{26}H_{34}FN_4O_3S^+$

3D Autocorrelation

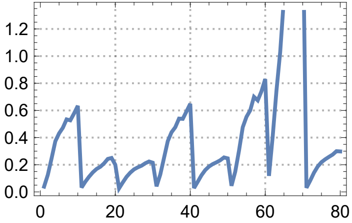

2D Plot

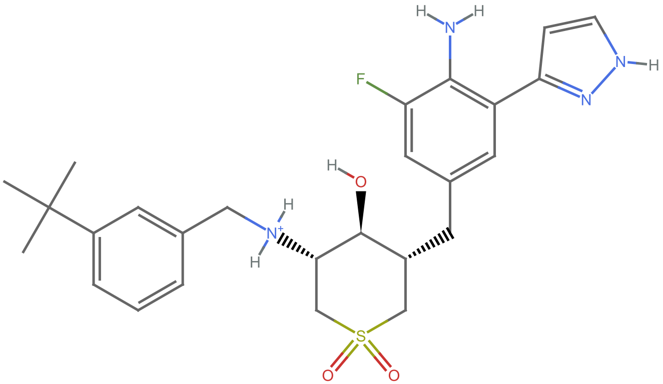

3D Plot

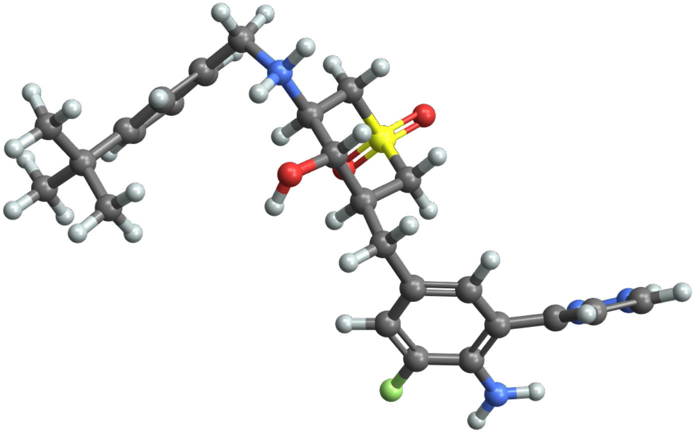

2D Autocorrelation

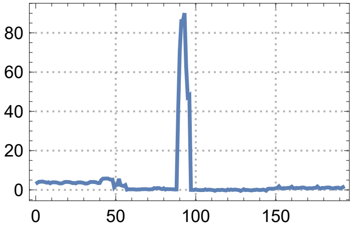

Dataset Index= 571

Molecule Formula= $C_{20}H_{19}Cl_2N_3O_2$

3D Autocorrelation

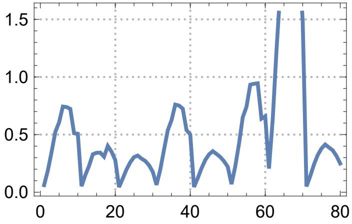

2D Plot

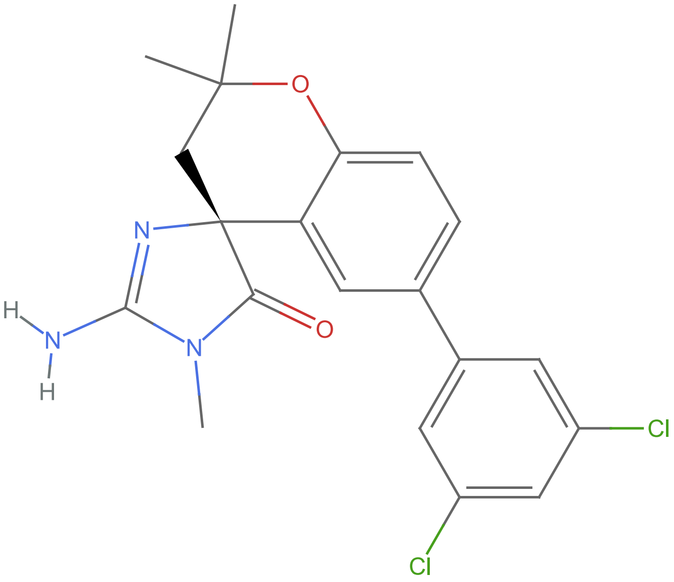

3D Plot

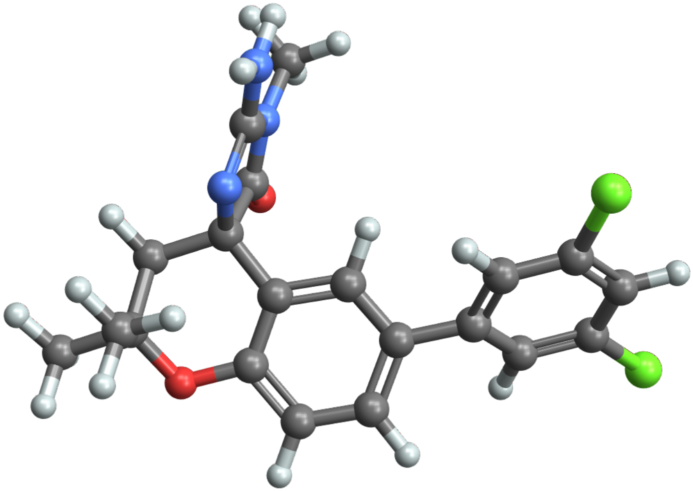

2D Plot

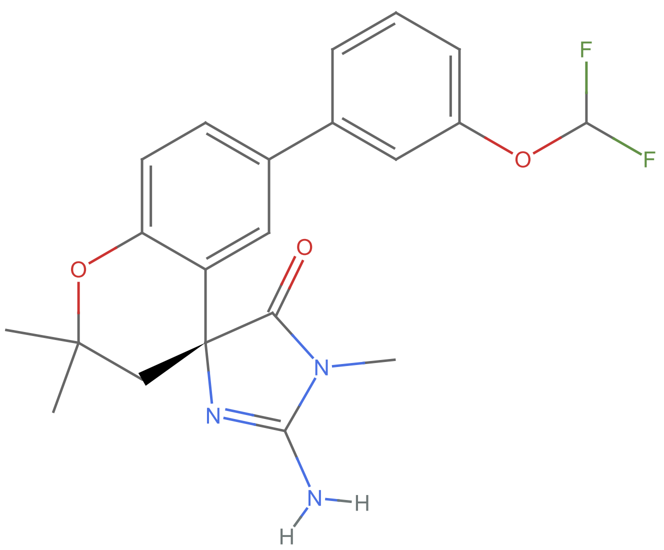

2D Autocorrelation

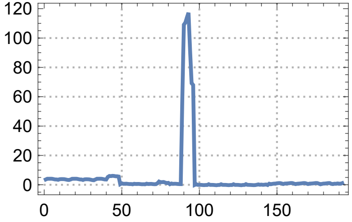

Dataset Index= 572

Molecule Formula= $C_{21}H_{21}F_2N_3O_3$

3D Plot

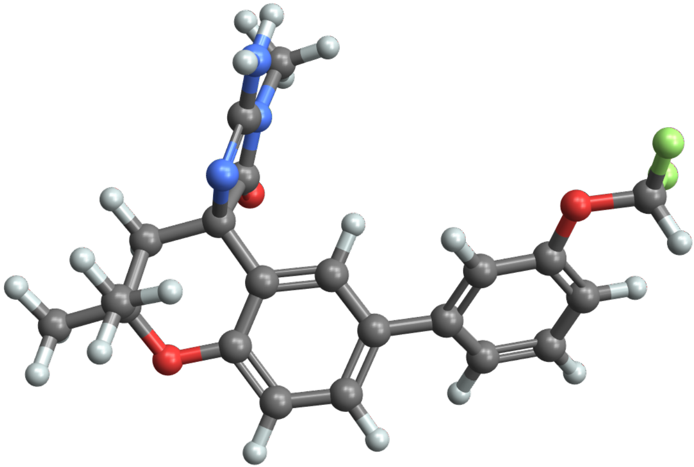

3D Autocorrelation

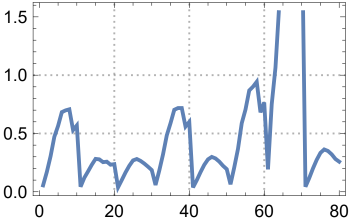

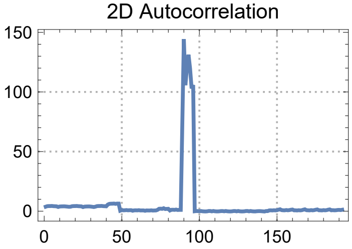

Dataset Index= 573

Molecule Formula= $C_{24}H_{20}F_3N_5O$

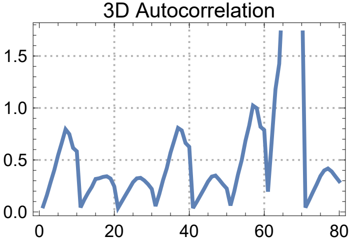

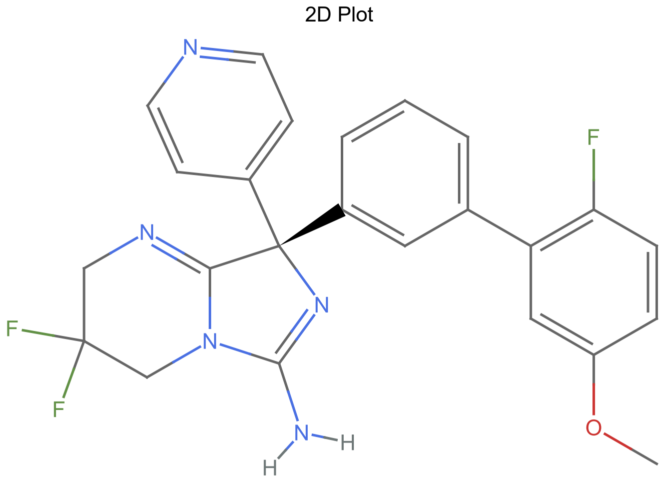

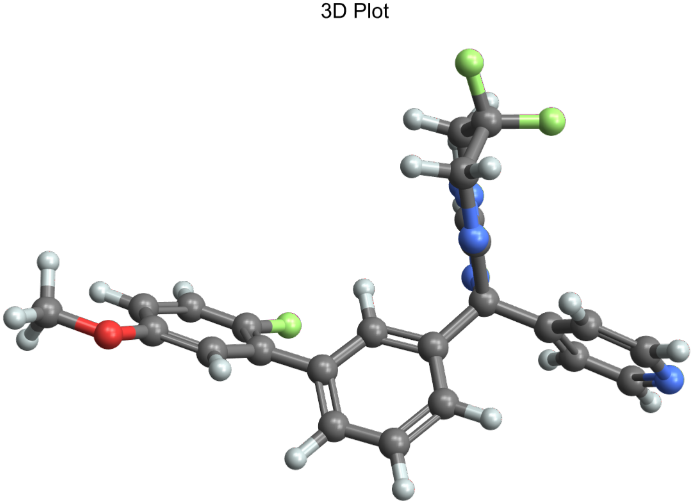

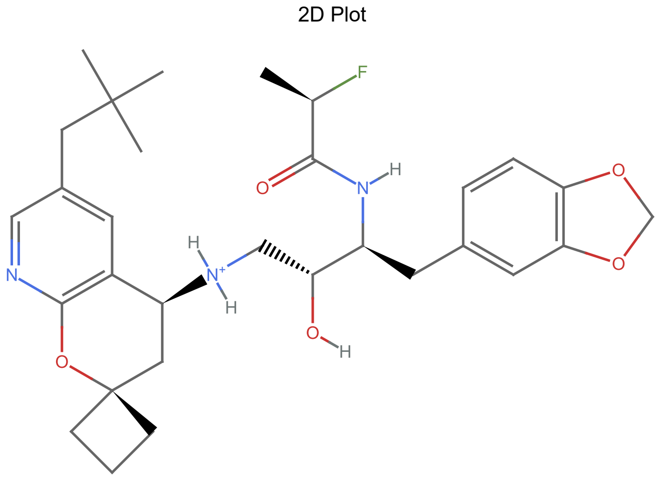

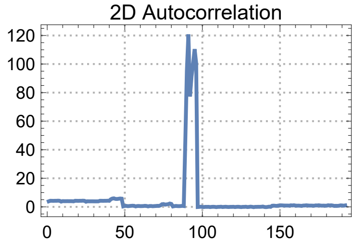

Dataset Index= 574

Molecule Formula= $C_{30}H_{41}FN_3O_5^+$

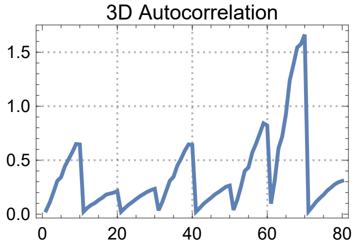

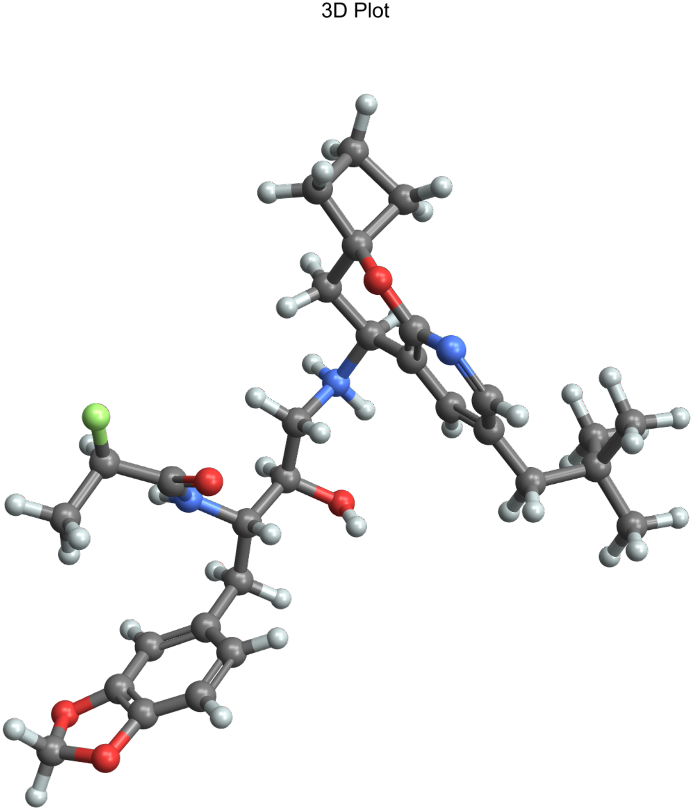

2D Plot

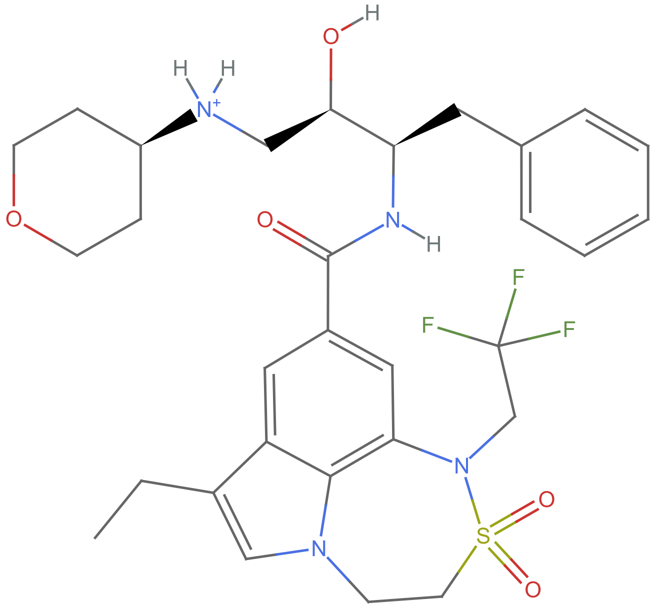

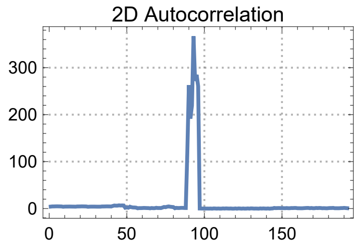

Dataset Index= 575

Molecule Formula= $C_{30}H_{38}F_3N_4O_5S^+$

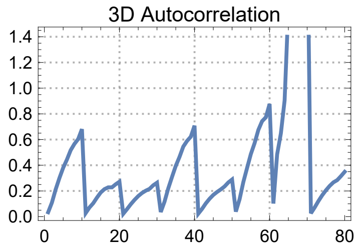

3D Plot

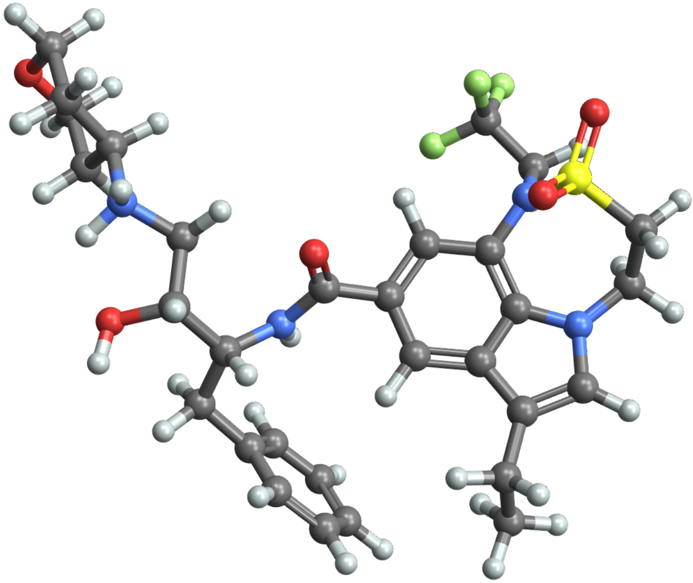

2D Plot

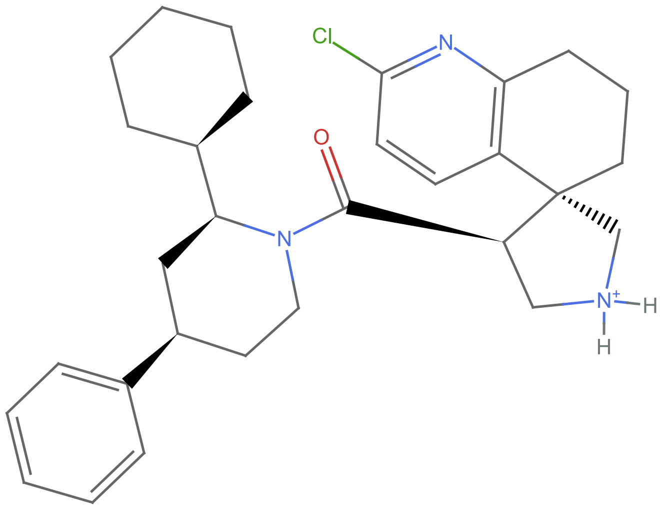

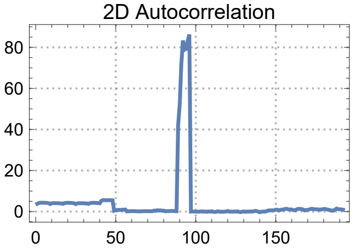

3D Plot

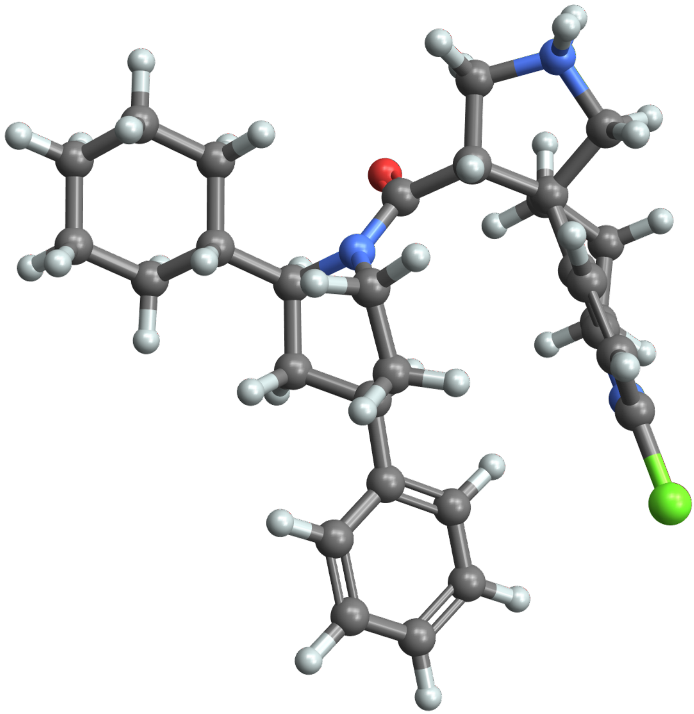

Dataset Index= 576

Molecule Formula= $C_{30}H_{39}ClN_3O^+$

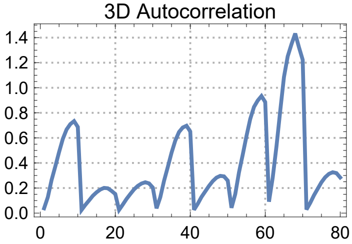

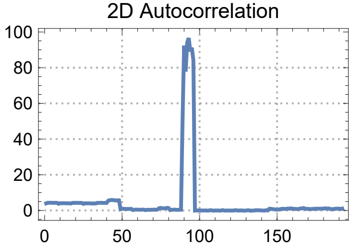

Dataset Index= 577

Molecule Formula= $C_{32}H_{43}N_4O_4S^+$

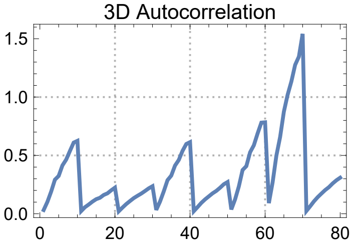

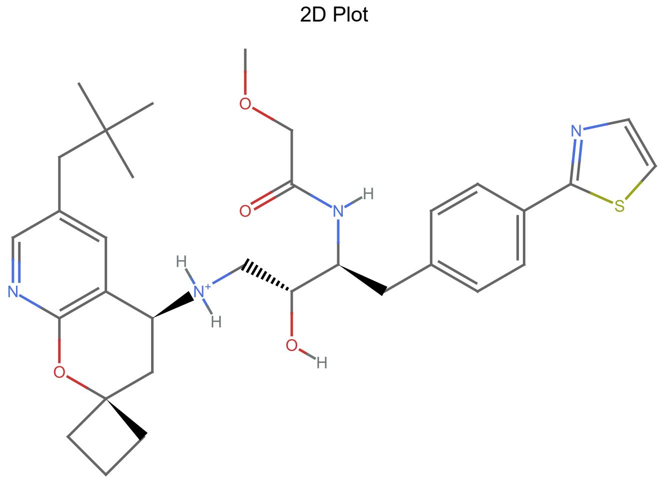

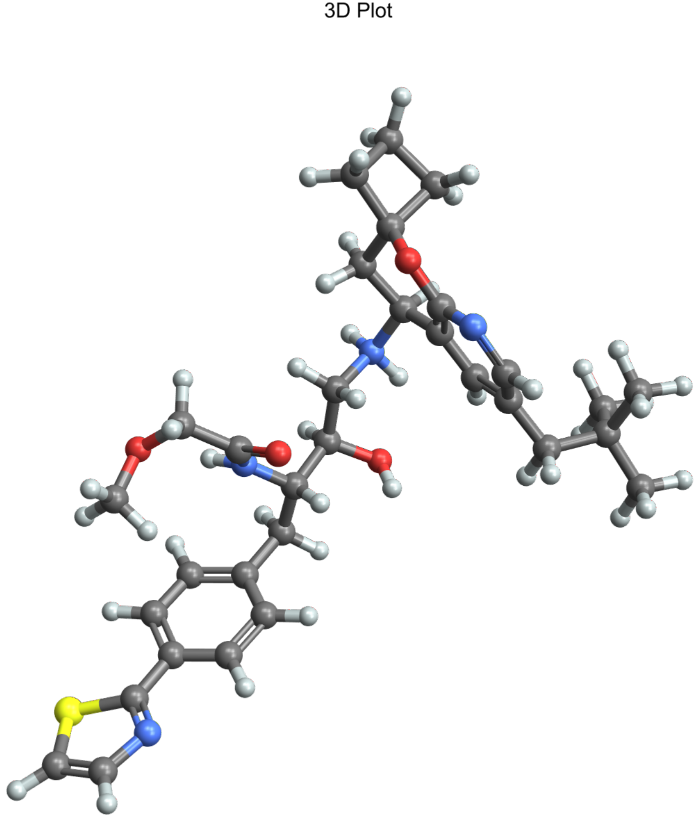

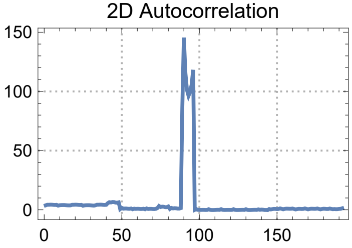

Dataset Index= 578

Molecule Formula= $C_{23}H_{17}F_4N_5O_2$

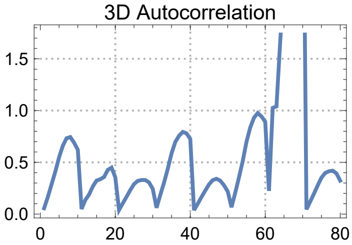

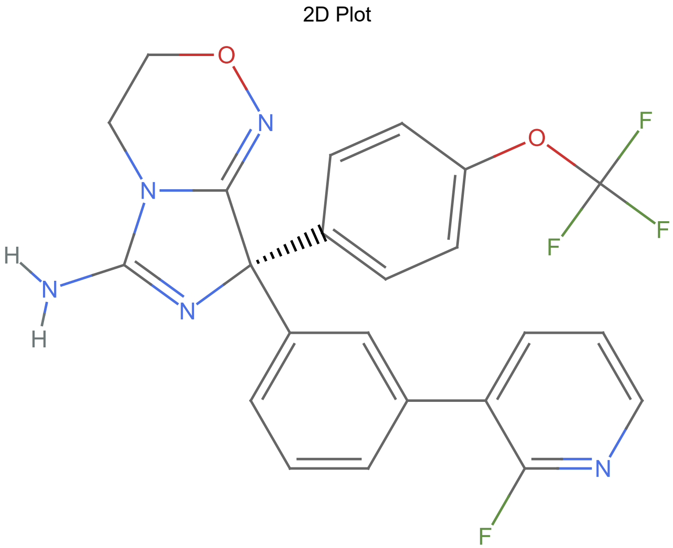

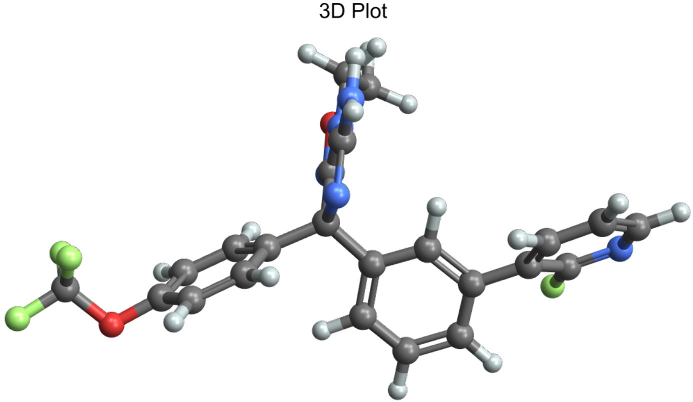

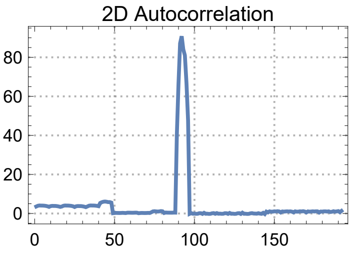

Dataset Index= 579

Molecule Formula= $C_{22}H_{21}N_5O_2$

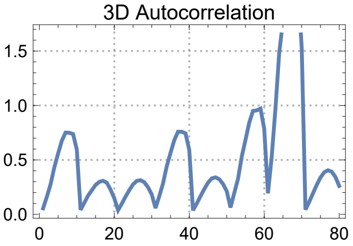

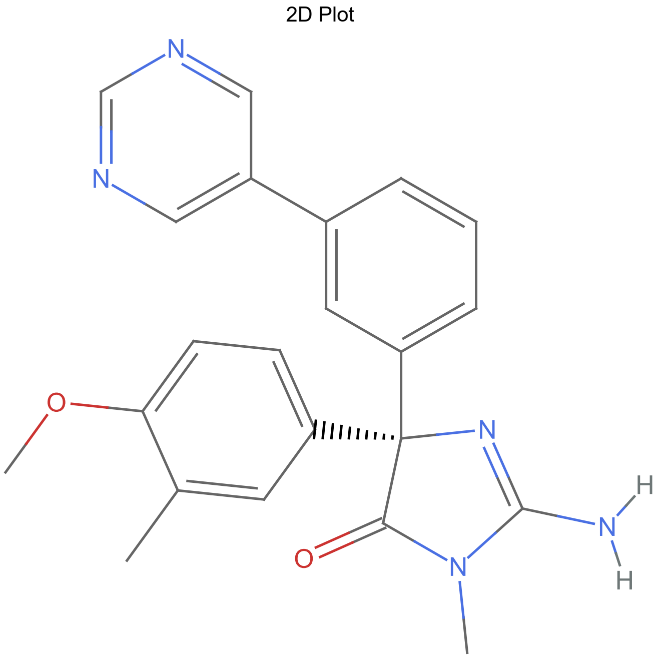

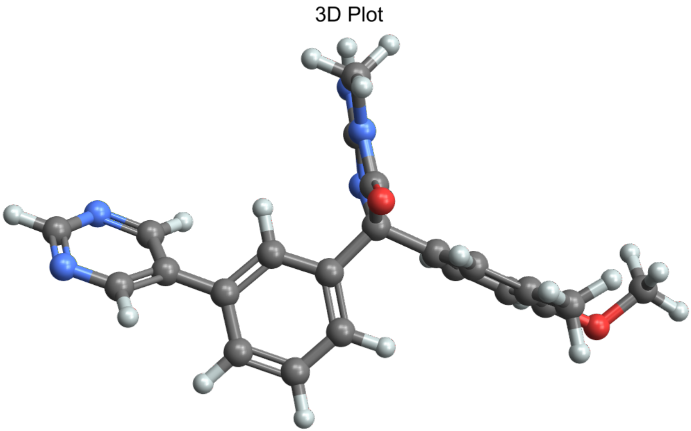

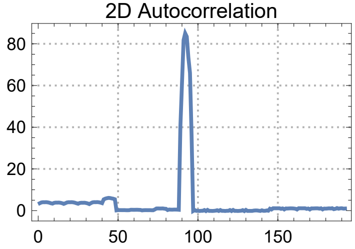

Dataset Index= 580

Molecule Formula= $C_{21}H_{20}N_6O$

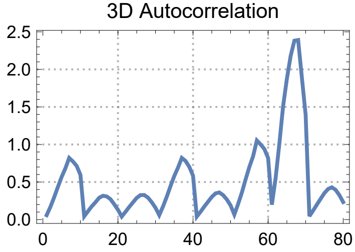

2D Plot

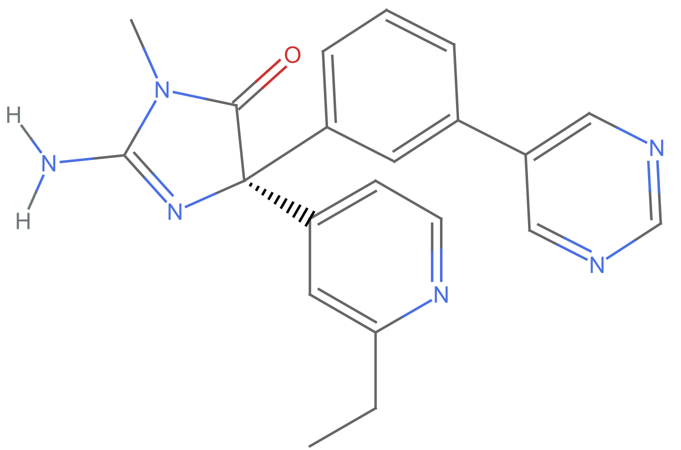

3D Plot

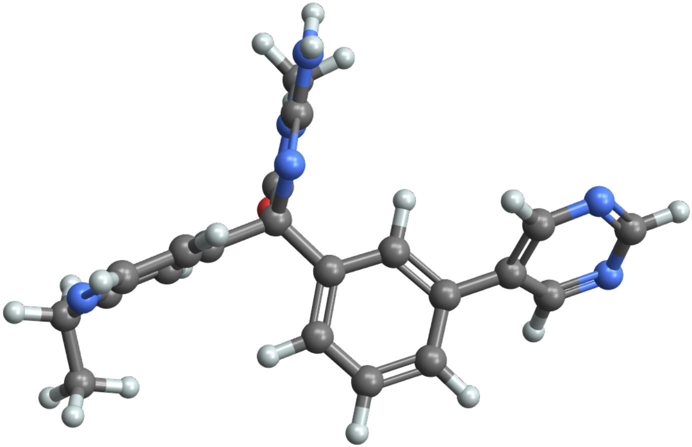

2D Plot

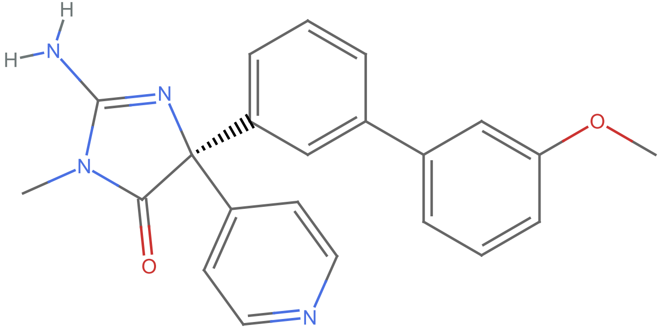

2D Autocorrelation

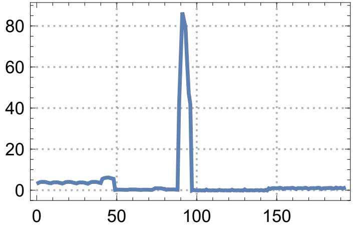

Dataset Index= 581

Molecule Formula= $C_{22}H_{20}N_4O_2$

3D Autocorrelation

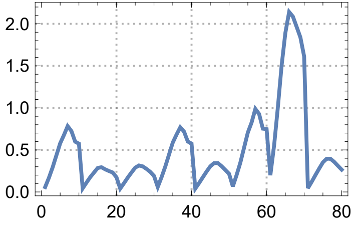

3D Plot

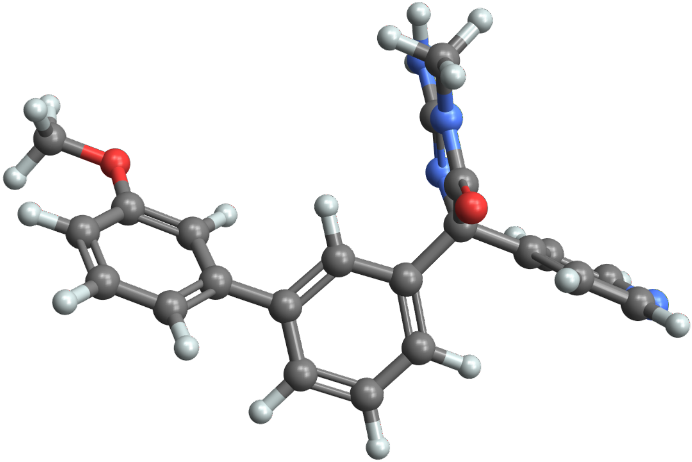

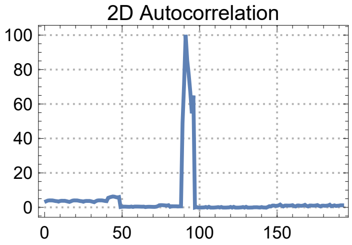

Dataset Index= 582

Molecule Formula= $C_{20}H_{16}FN_5O$

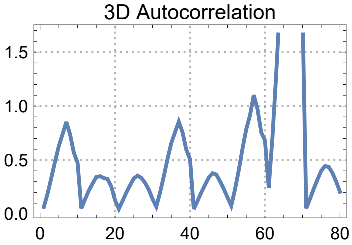

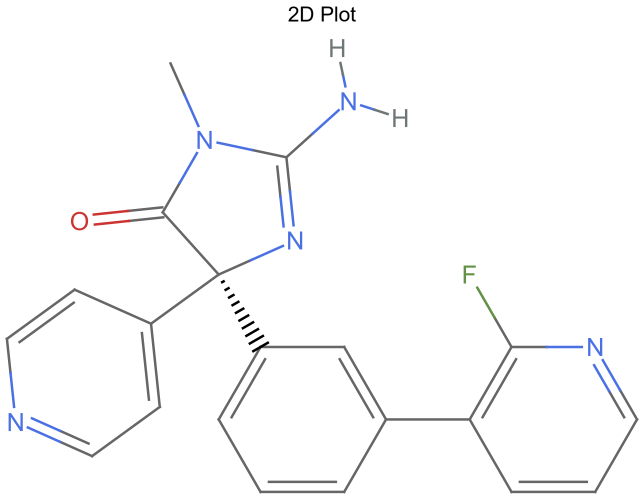

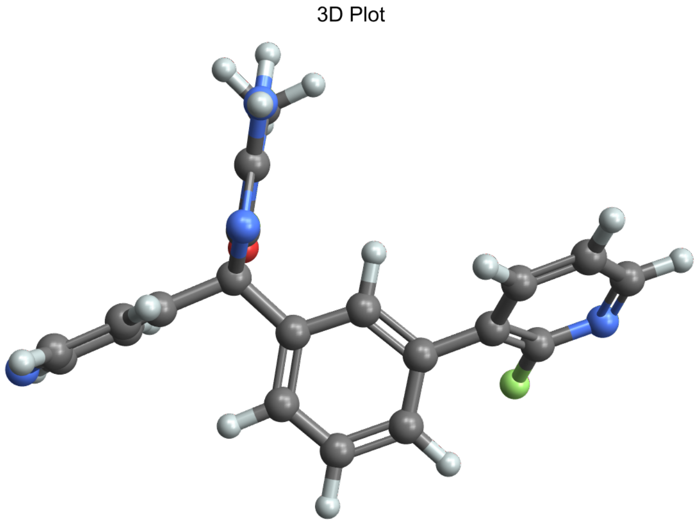

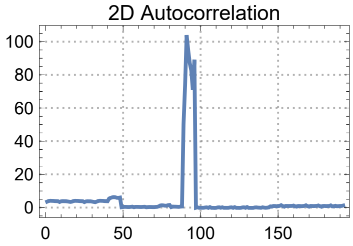

Dataset Index= 583

Molecule Formula= $C_{22}H_{19}FN_4O_2$

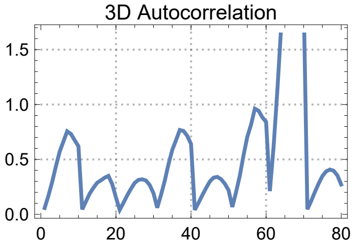

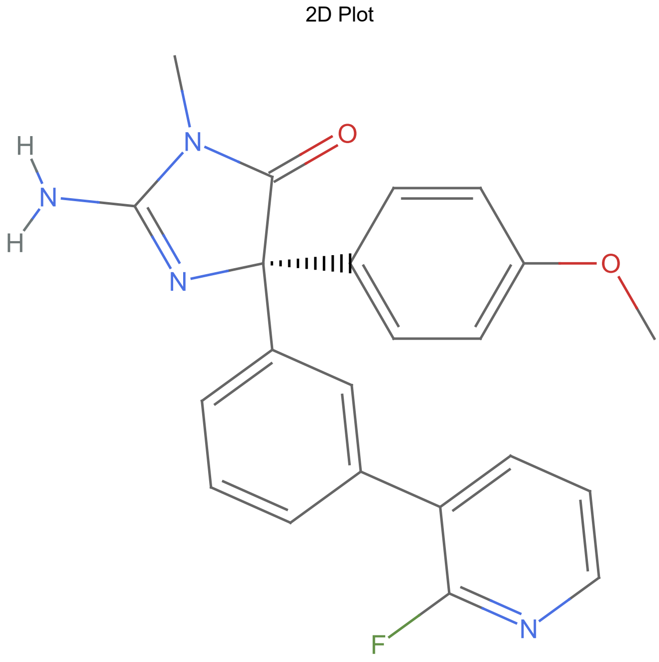

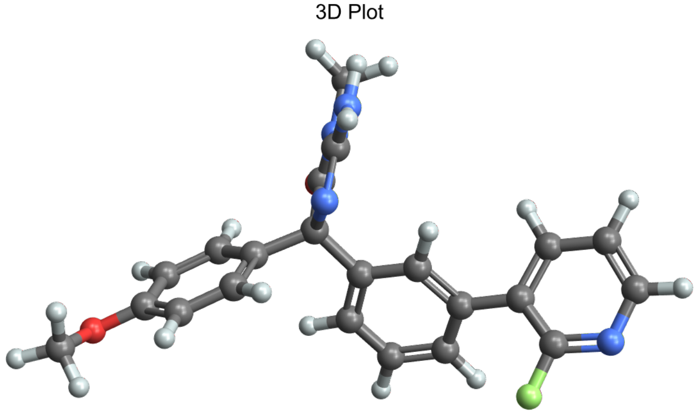

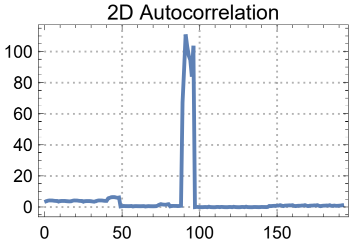

Dataset Index= 584

Molecule Formula= $C_{23}H_{20}F_2N_4O_2$

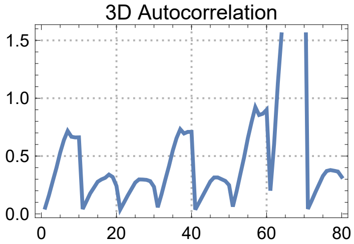

2D Plot

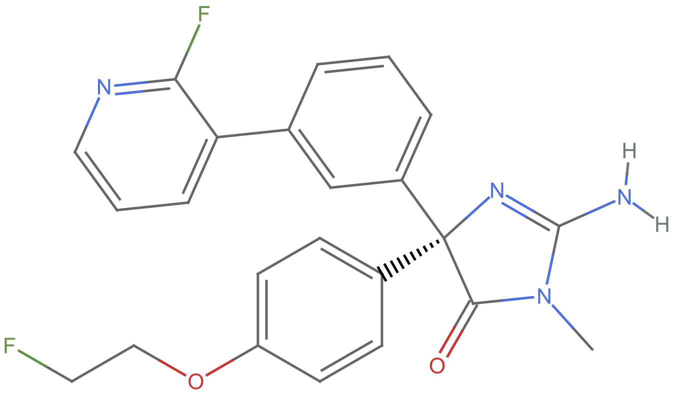

3D Plot

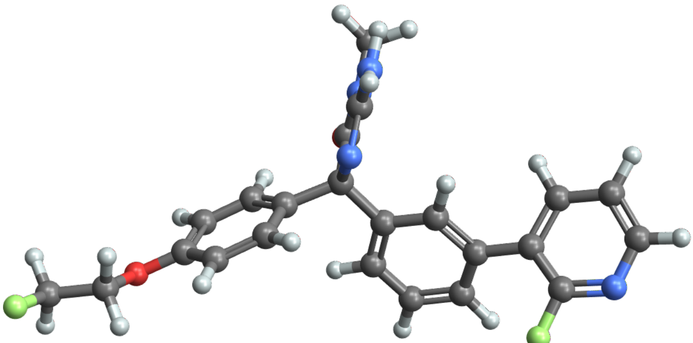

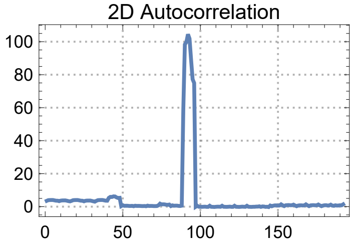

Dataset Index= 585

Molecule Formula= $C_{21}H_{23}F_2N_3O_2$

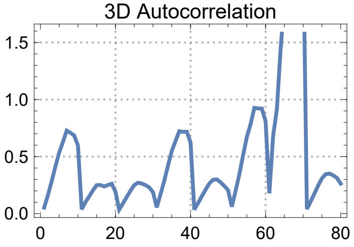

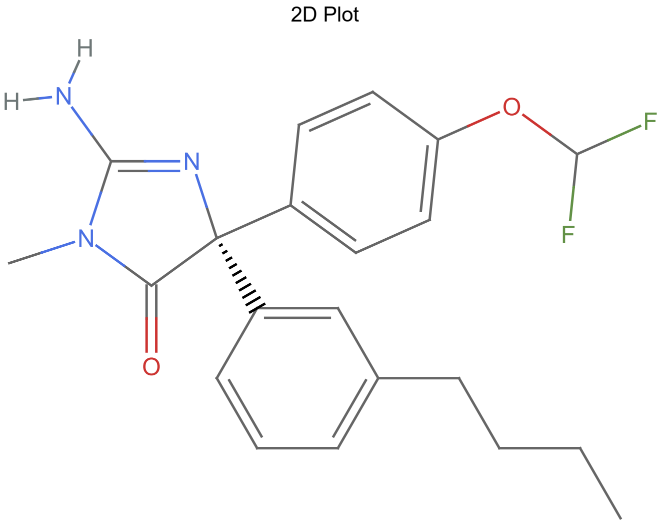

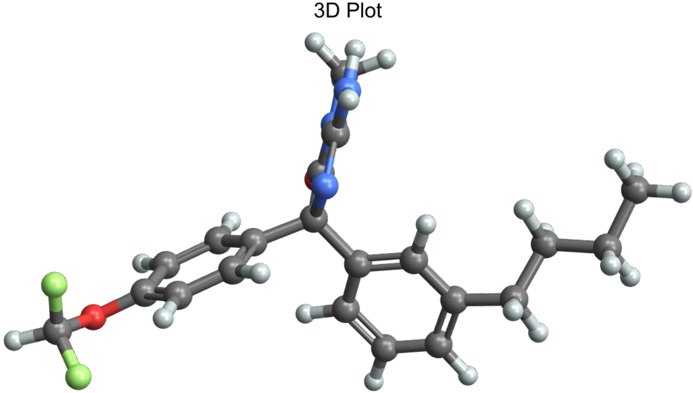

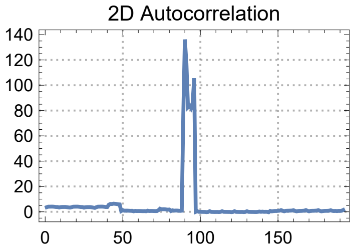

Dataset Index= 586

Molecule Formula= $C_{21}H_{19}F_4N_3O_2$

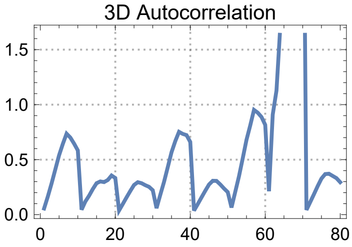

2D Plot

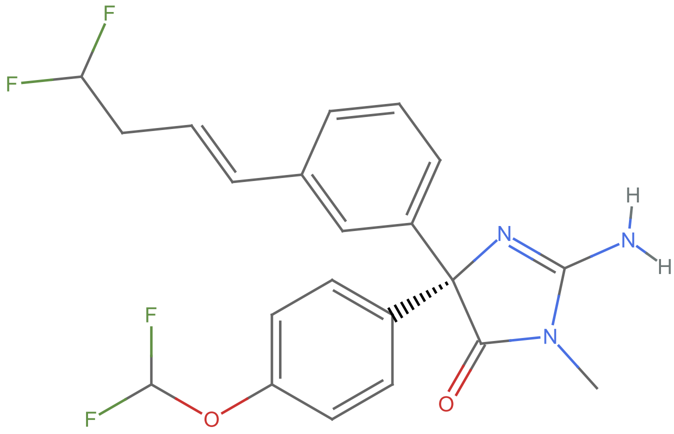

3D Plot

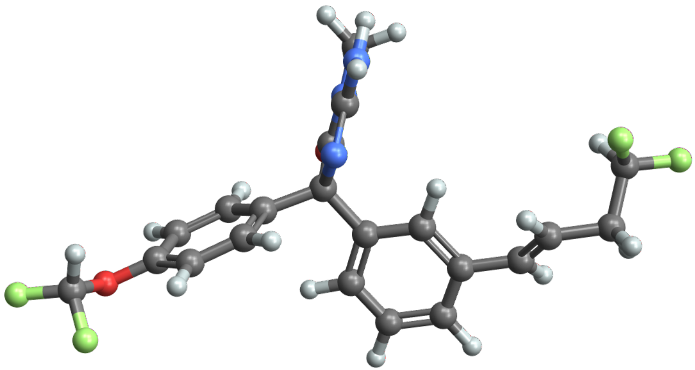

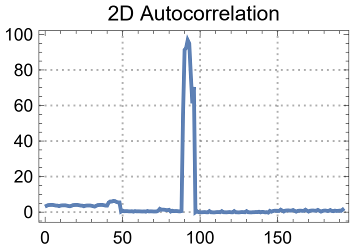

Dataset Index= 587

Molecule Formula= $C_{22}H_{21}F_2N_3O_2$

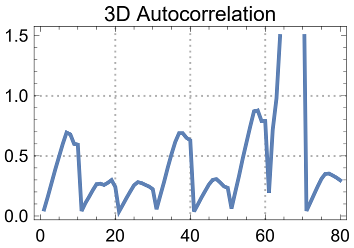

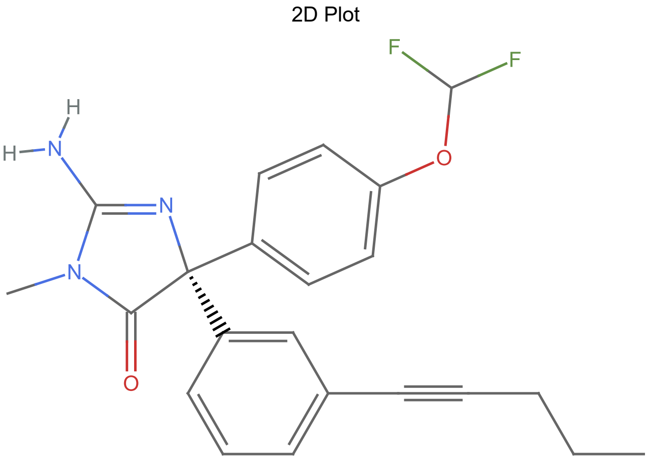

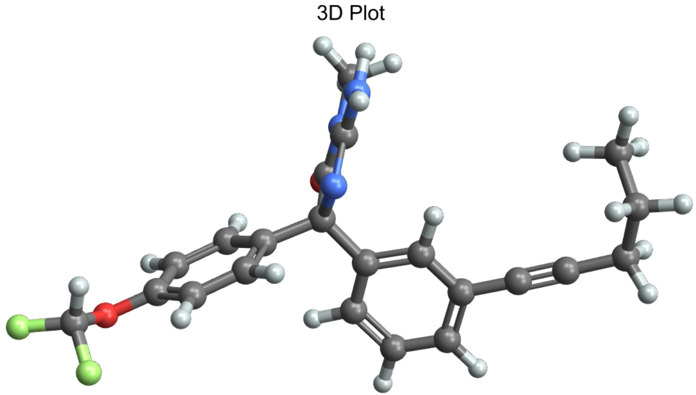

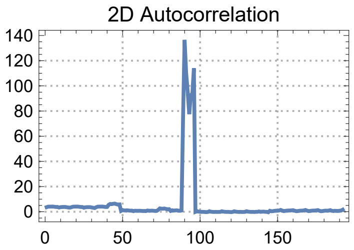

Dataset Index= 588

Molecule Formula= $C_{19}H_{17}F_4N_3O_3$

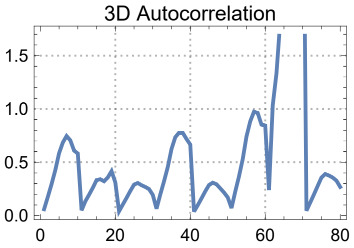

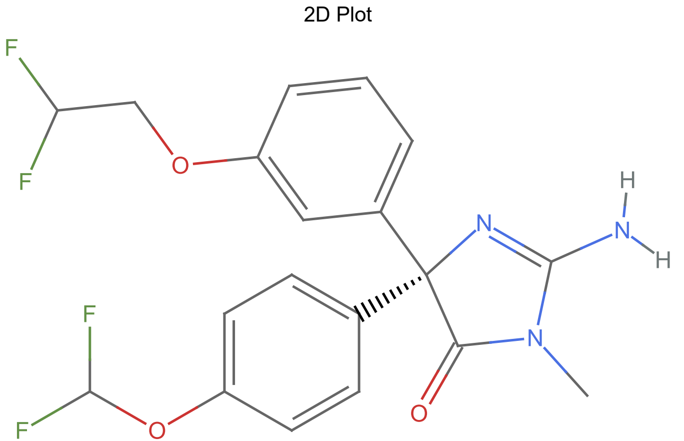

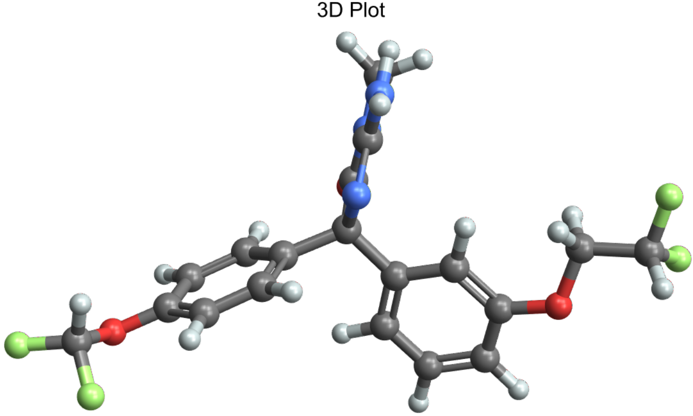

2D Autocorrelation

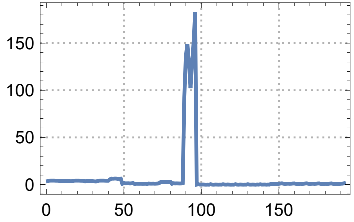

Dataset Index= 589

Molecule Formula= $C_{21}H_{18}F_5N_3O_3$

3D Autocorrelation

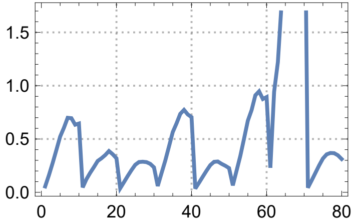

2D Plot

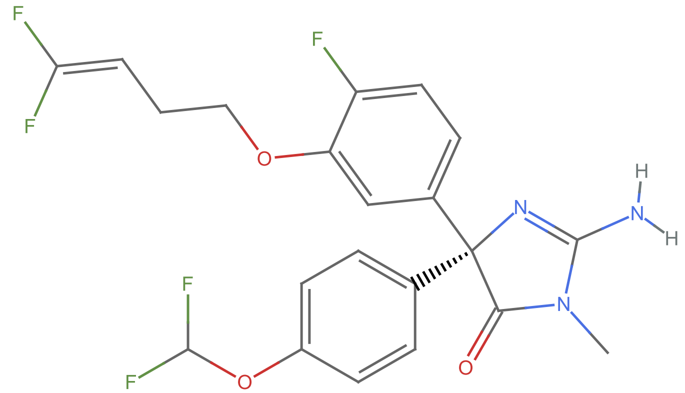

3D Plot

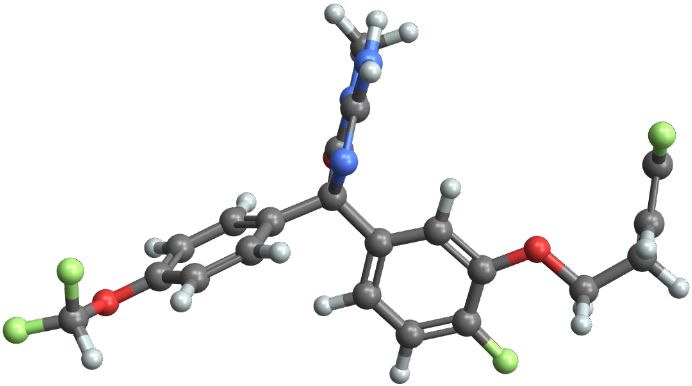

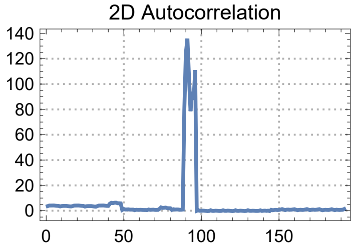

Dataset Index= 590

Molecule Formula= $C_{21}H_{19}F_4N_3O_3$

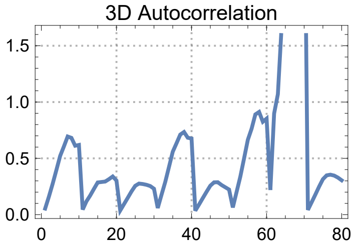

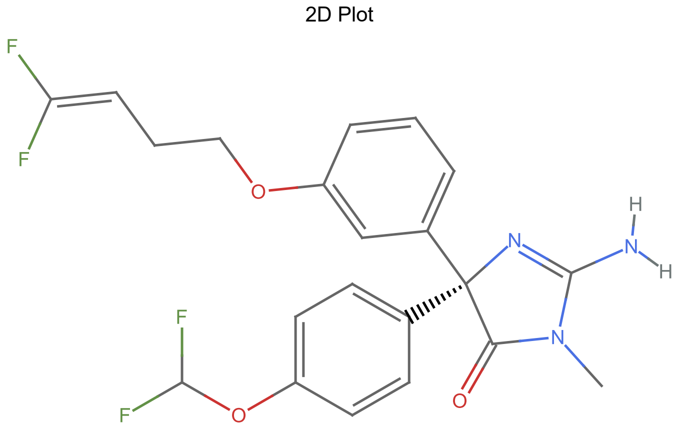

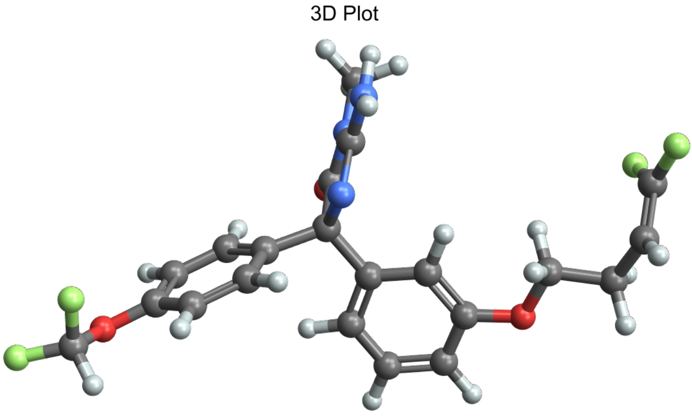

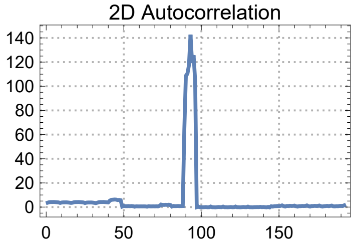

Dataset Index= 591

Molecule Formula= $C_{23}H_{22}F_3N_3O_2$

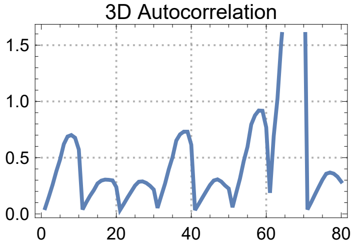

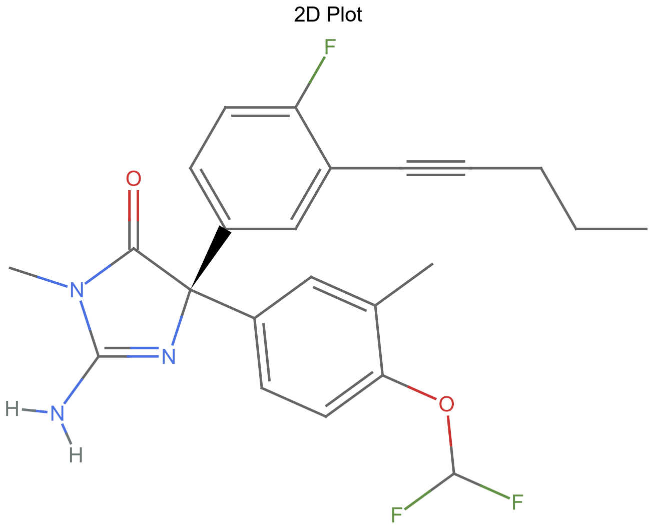

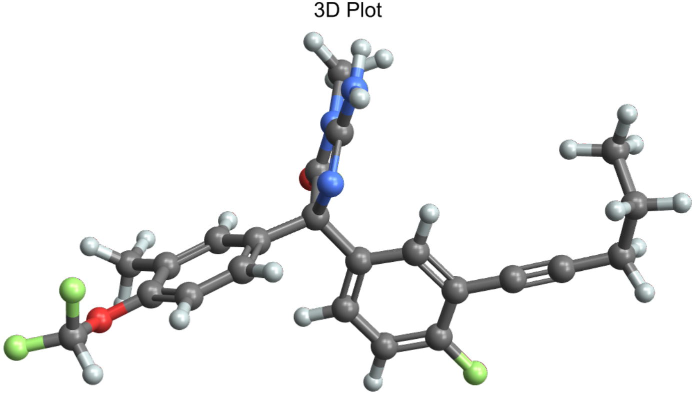

2D Plot

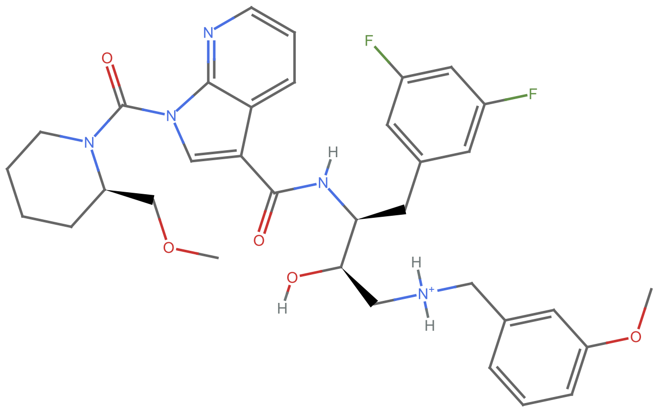

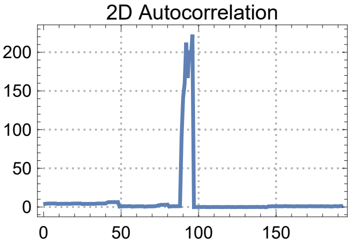

3D Plot

Dataset Index= 592

Molecule Formula= $C_{34}H_{40}F_2N_5O_5^+$

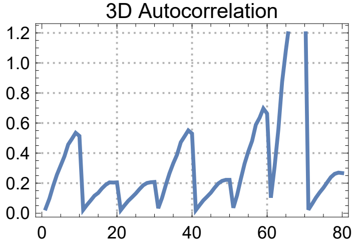

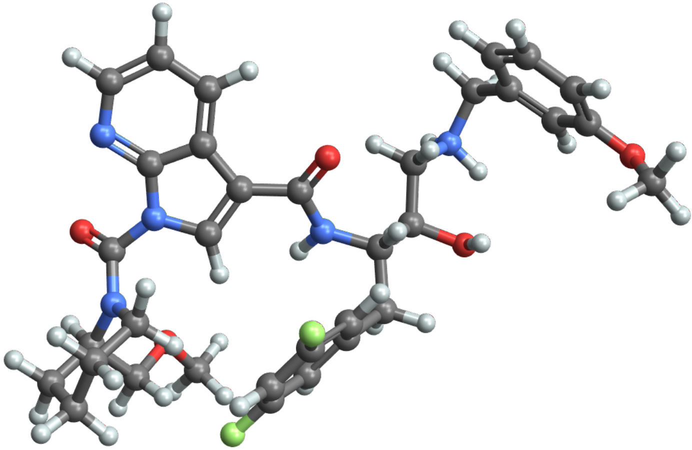

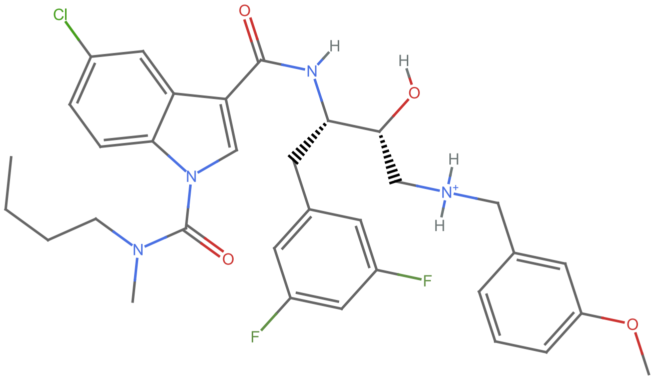

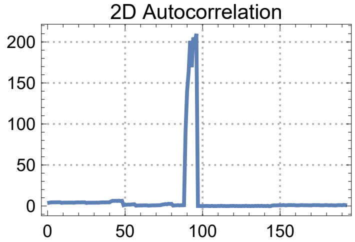

3D Plot

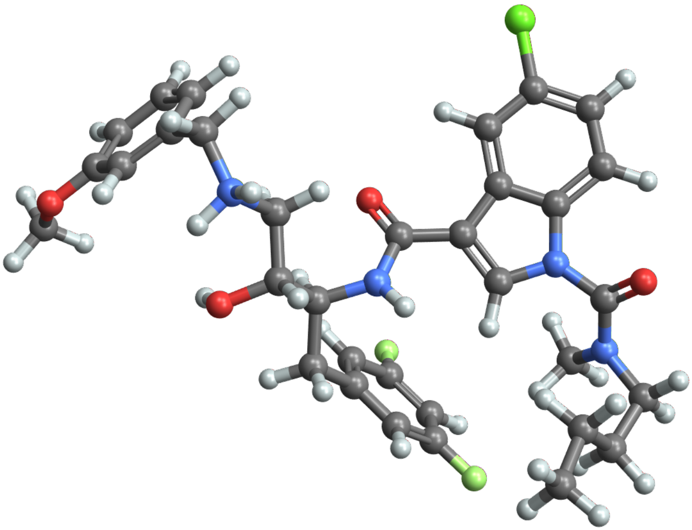

Dataset Index= 593

Molecule Formula= $C_{33}H_{38}ClF_2N_4O_4^+$

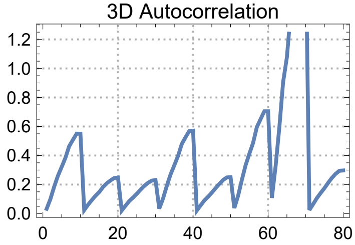

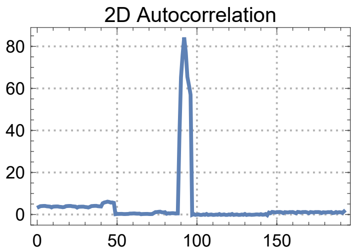

Dataset Index= 594

Molecule Formula= $C_{21}H_{23}N_7O$

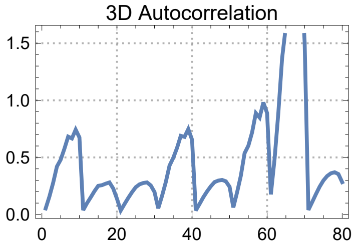

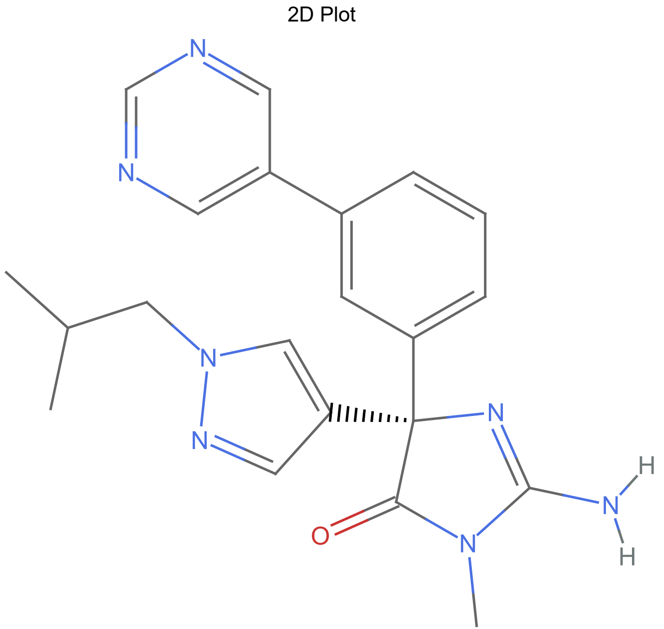

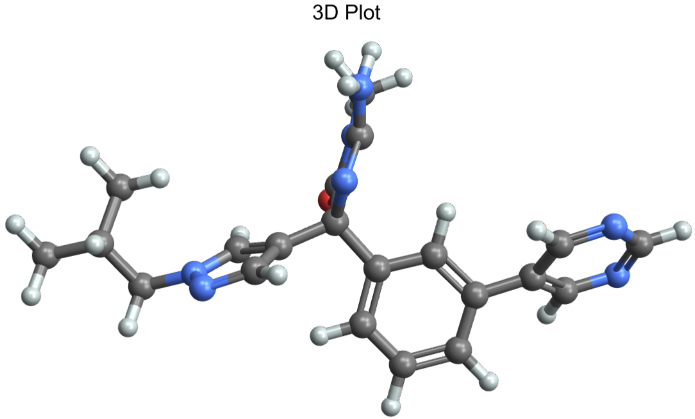

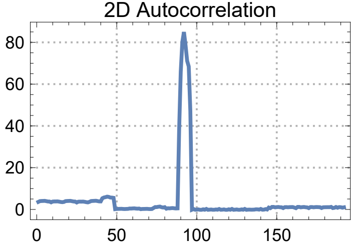

Dataset Index= 595

Molecule Formula= $C_{22}H_{25}N_7O$

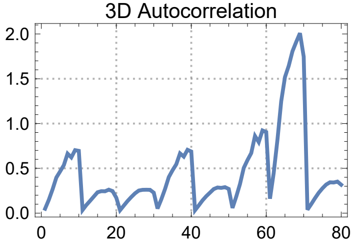

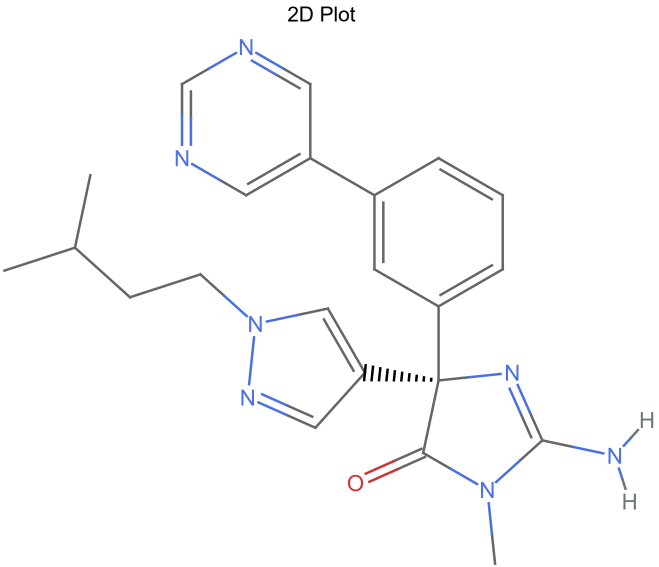

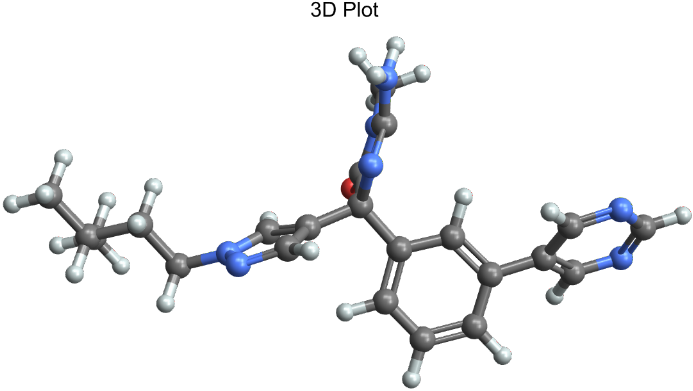

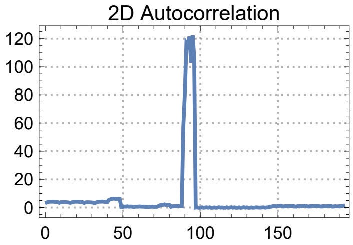

Dataset Index= 596

Molecule Formula= $C_{20}H_{18}F_2N_6O$

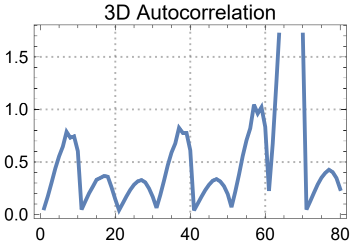

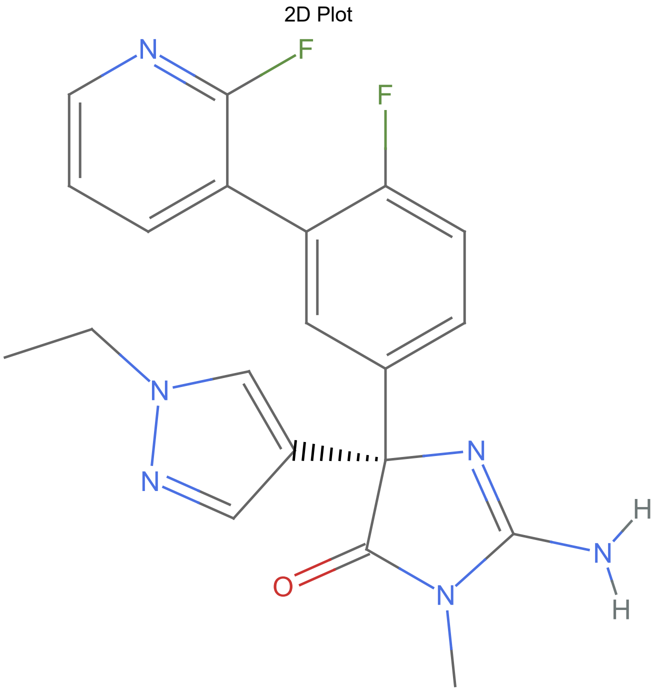

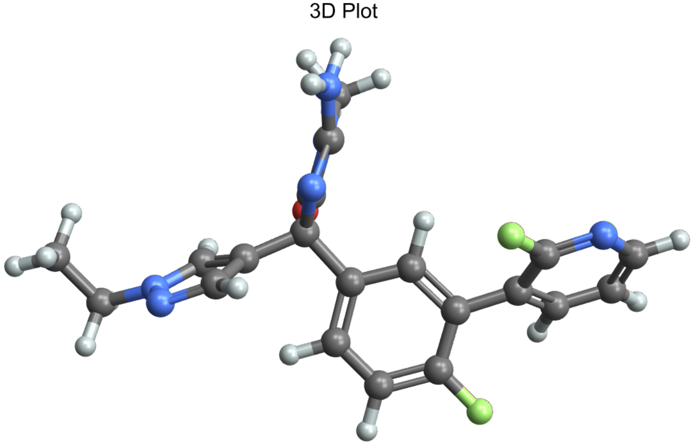

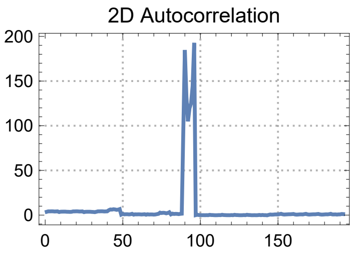

Dataset Index= 597

Molecule Formula= $C_{20}H_{16}F_4N_6O$

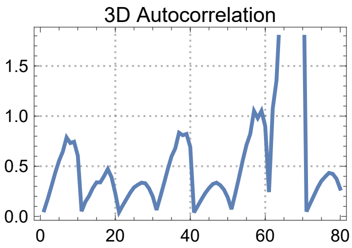

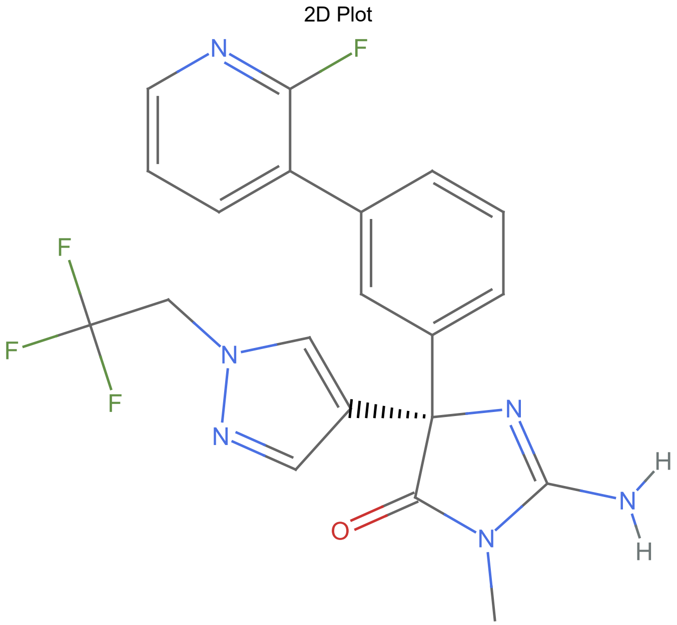

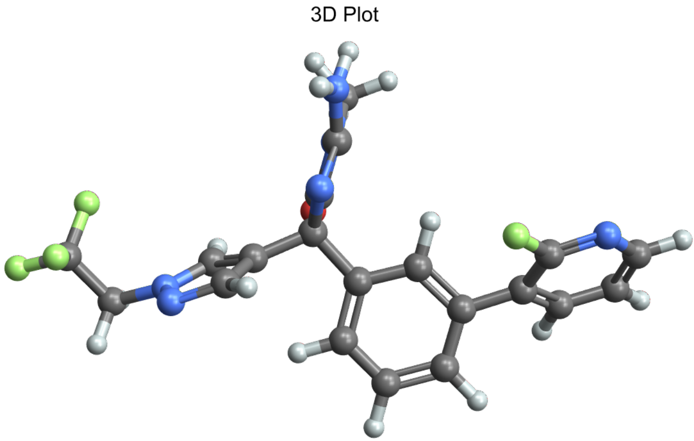

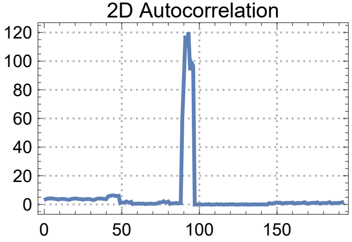

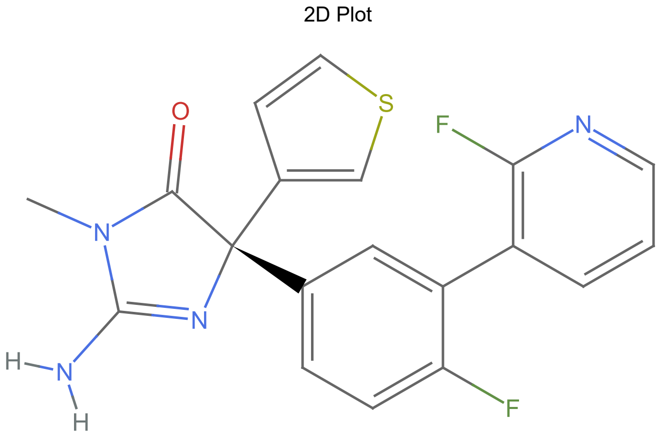

Dataset Index= 598

Molecule Formula= $C_{19}H_{14}F_2N_4OS$

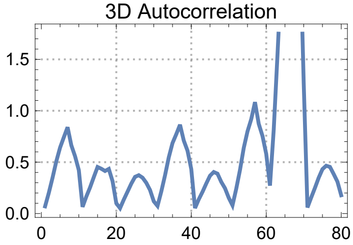

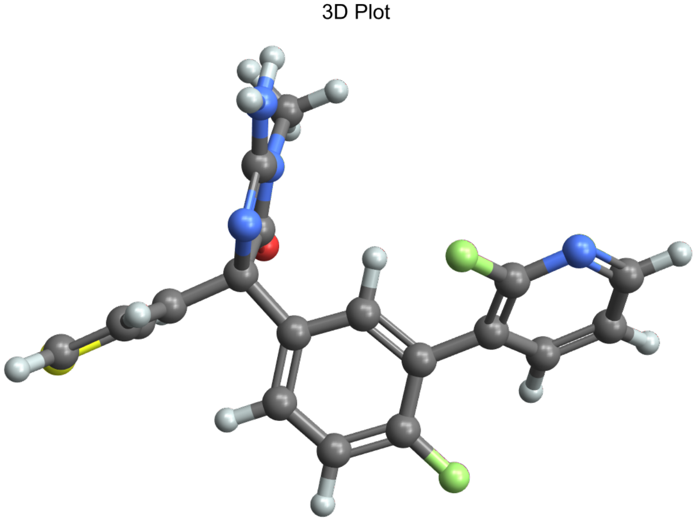

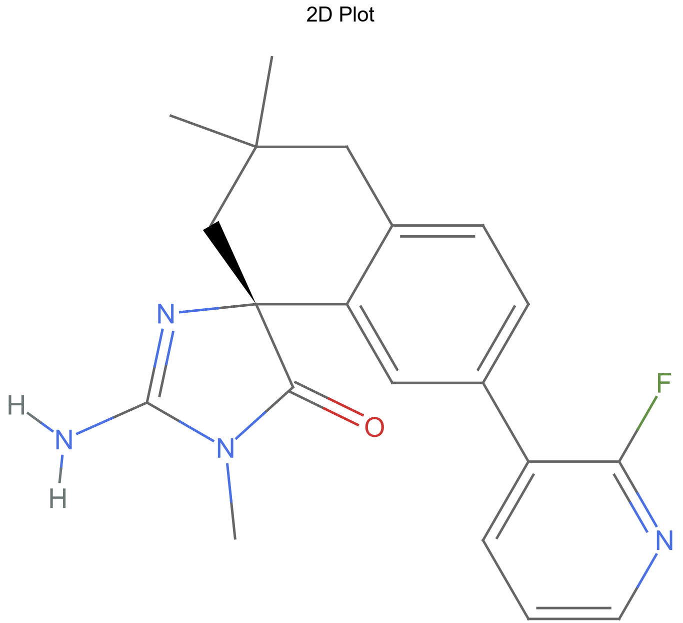

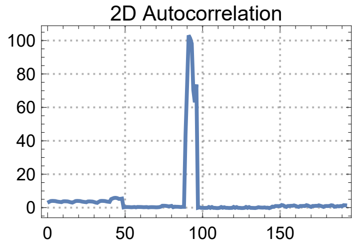

Dataset Index= 599

Molecule Formula= $C_{20}H_{21}FN_4O$

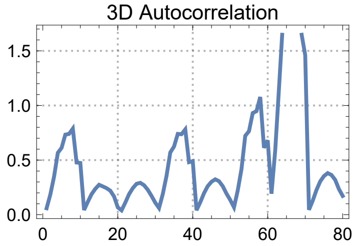

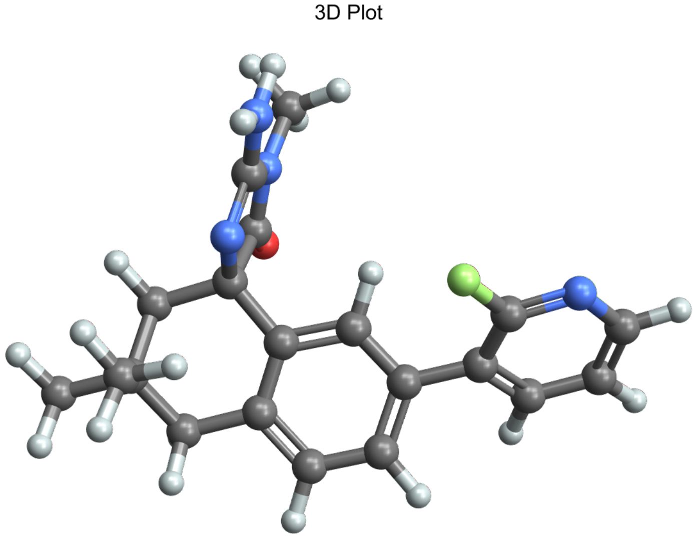

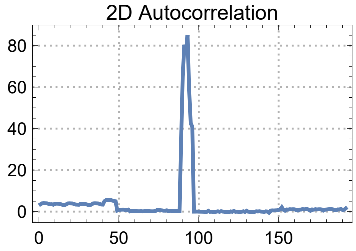

Dataset Index= 600

Molecule Formula= $C_{20}H_{21}ClN_4O$

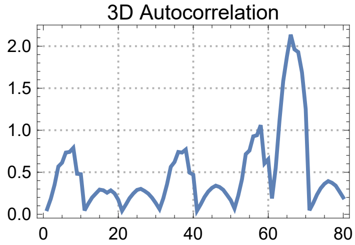

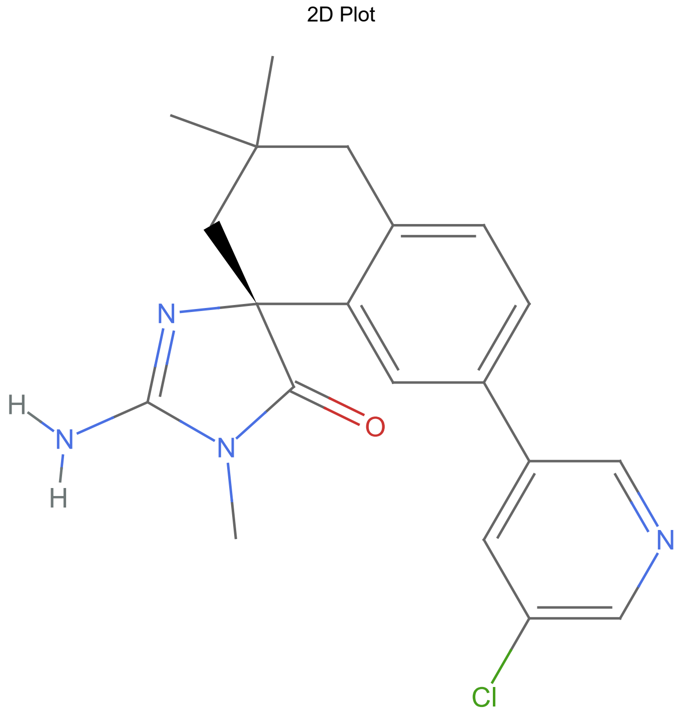

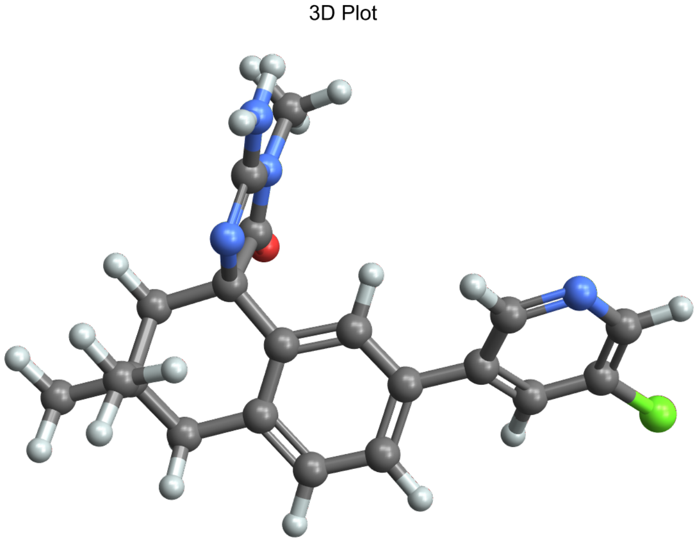

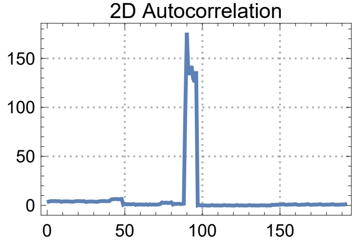

Dataset Index= 601

Molecule Formula= $C_{24}H_{18}F_5N_5O$

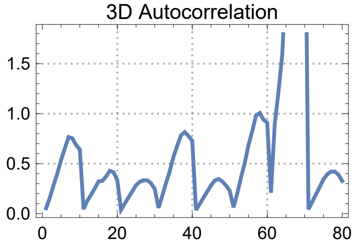

2D Plot

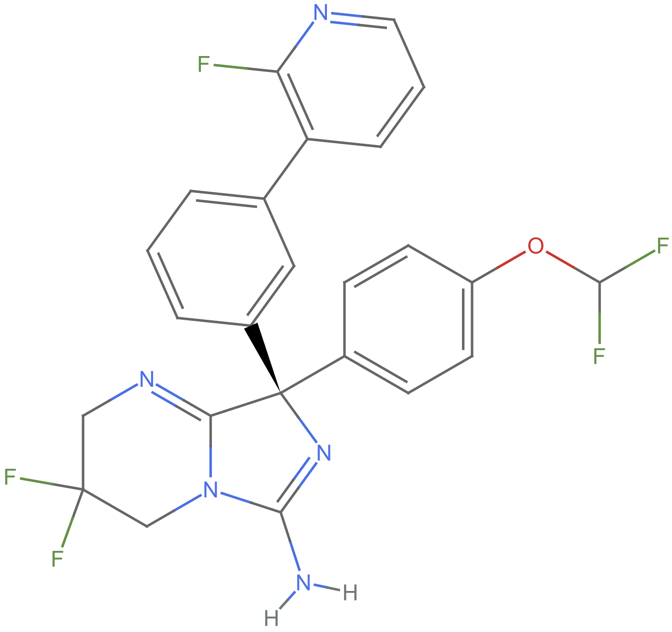

3D Plot

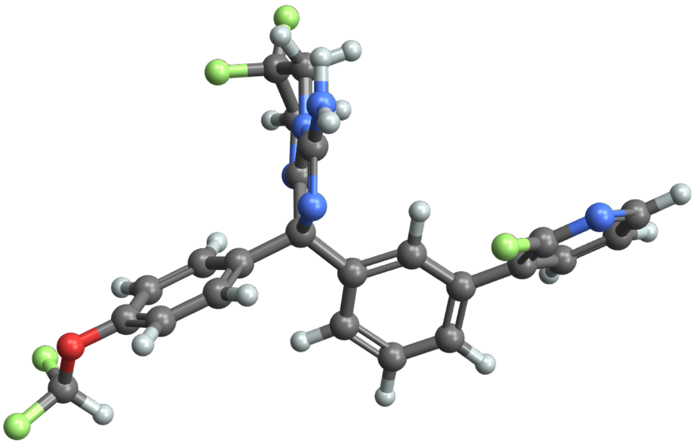

2D Autocorrelation

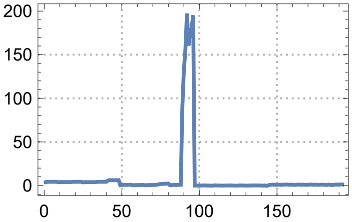

Dataset Index= 602

Molecule Formula= $C_{32}H_{40}F_2N_3O_4^+$

3D Autocorrelation

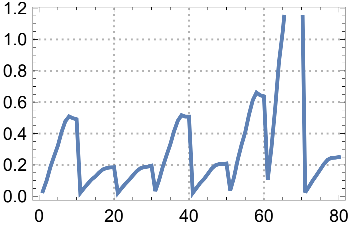

2D Plot

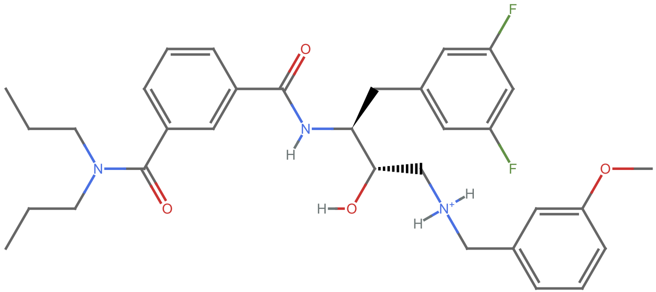

3D Plot

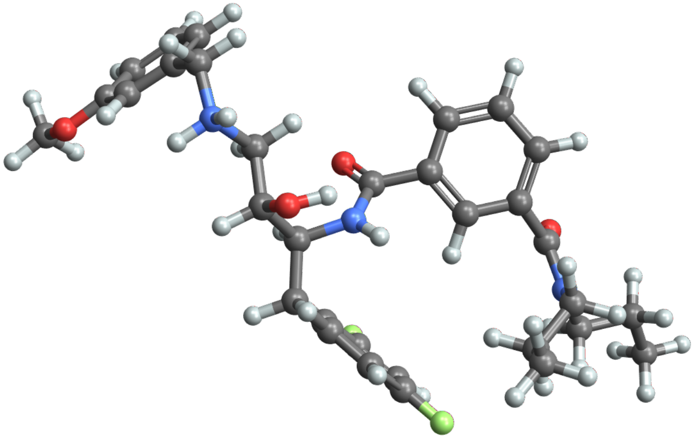

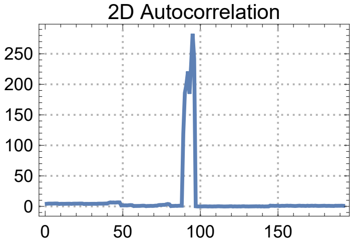

Dataset Index= 603

Molecule Formula= $C_{38}H_{43}F_2N_4O_6S^+$

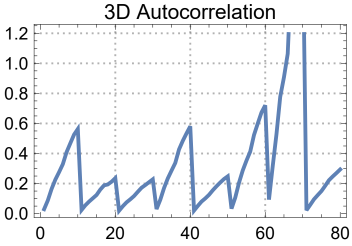

2D Plot

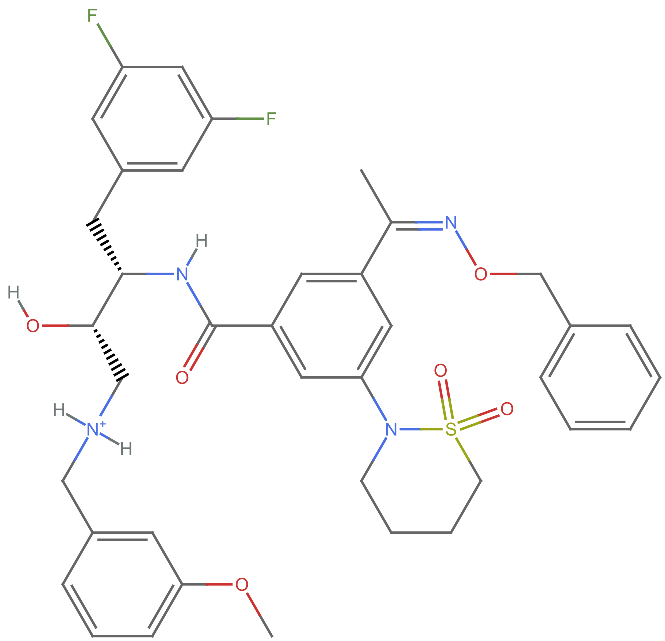

3D Plot

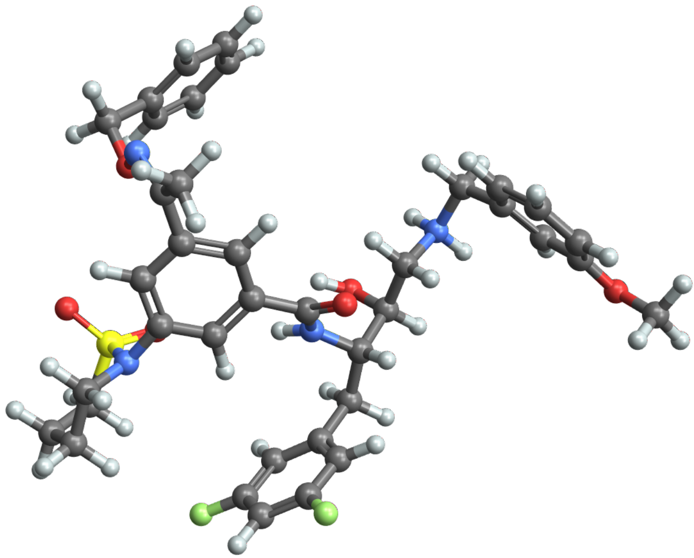

2D Plot

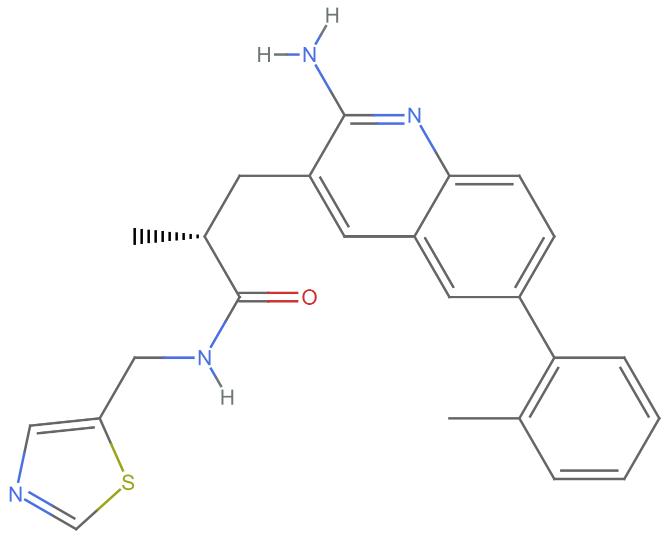

3D Plot

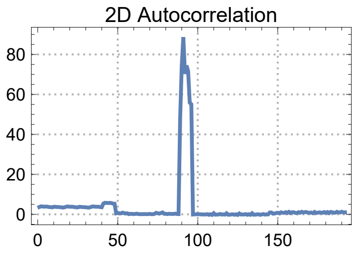

Dataset Index= 604

Molecule Formula= $C_{24}H_{24}N_4OS$

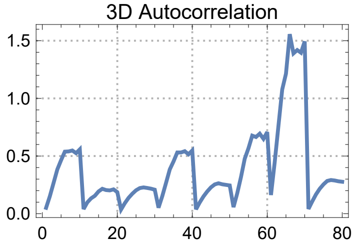

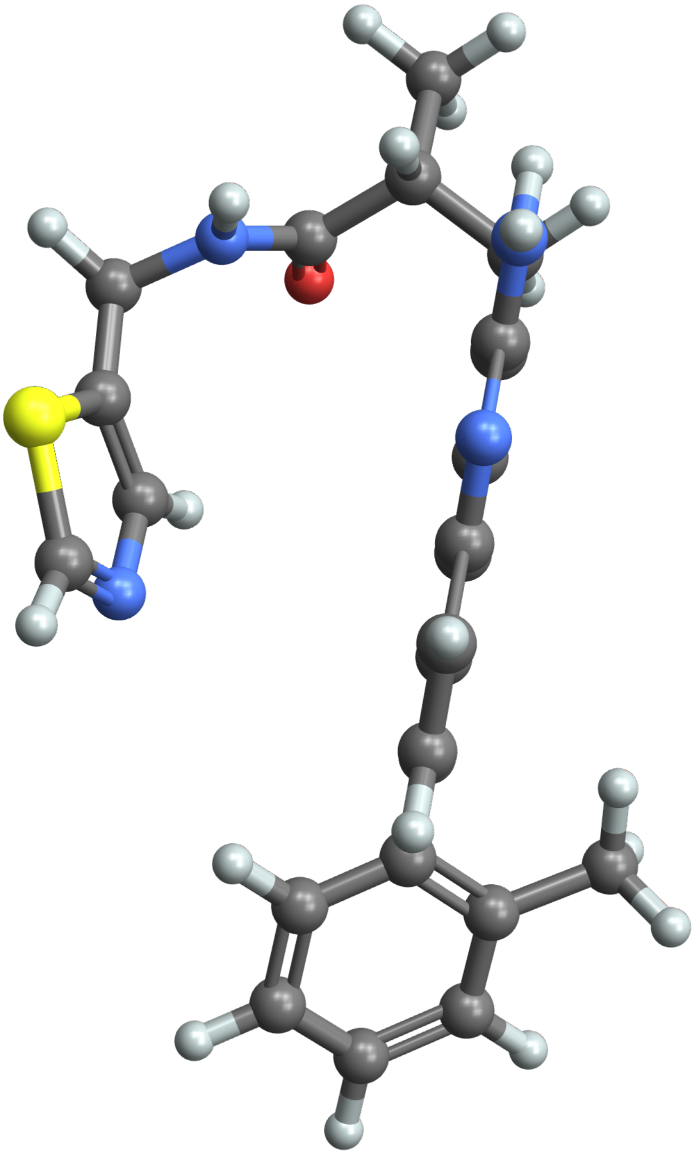

2D Plot

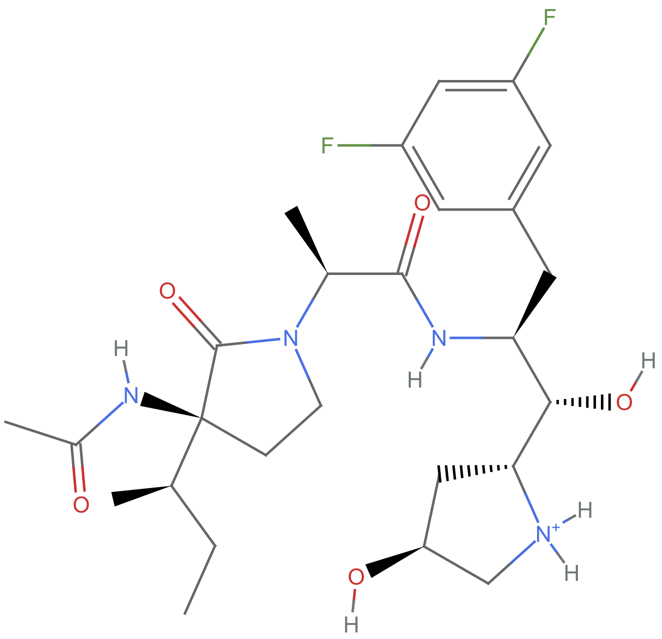

2D Autocorrelation

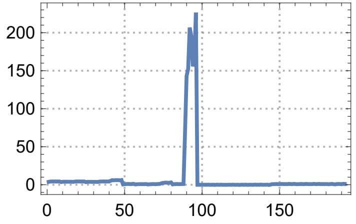

Dataset Index= 605

Molecule Formula= $C_{26}H_{39}F_2N_4O_5^+$

3D Plot

3D Autocorrelation

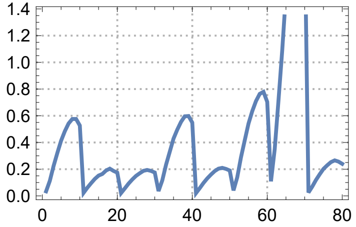

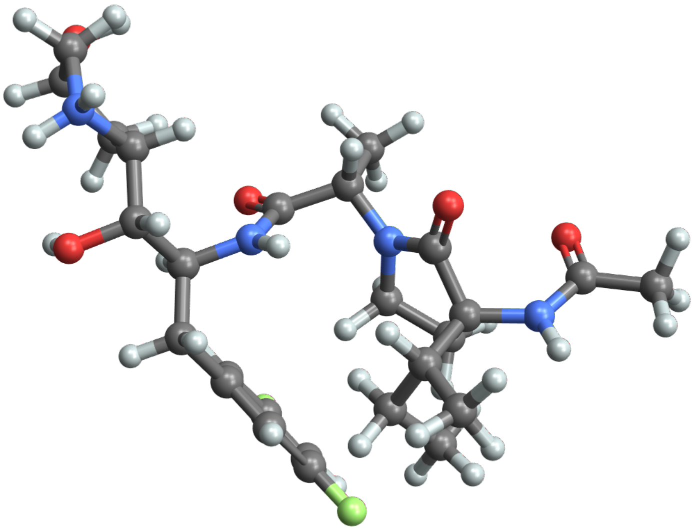

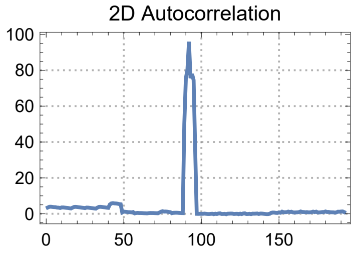

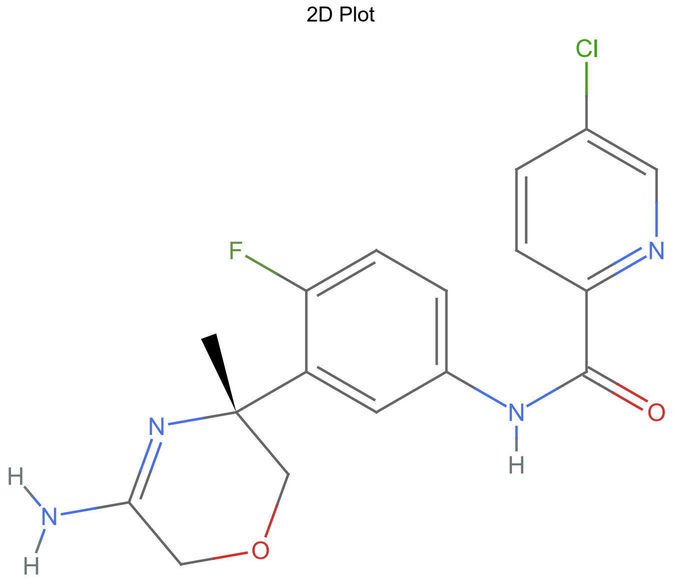

Dataset Index= 606

Molecule Formula= $C_{17}H_{16}ClFN_4O_2$

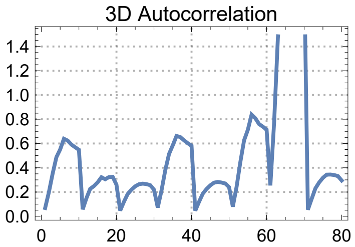

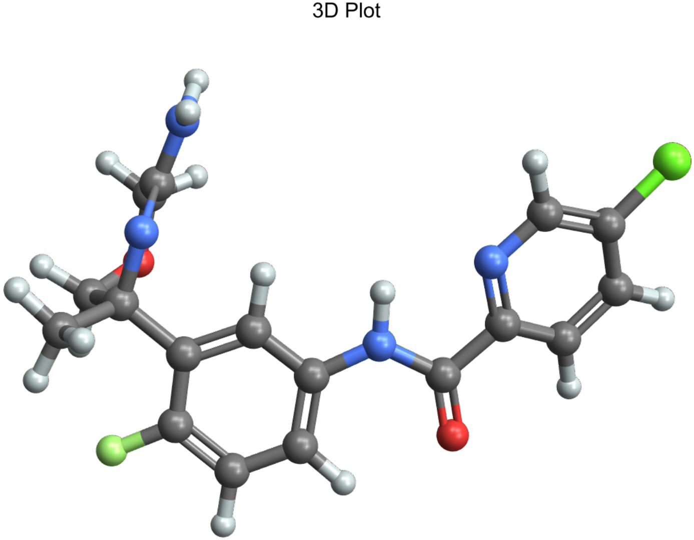

2D Plot

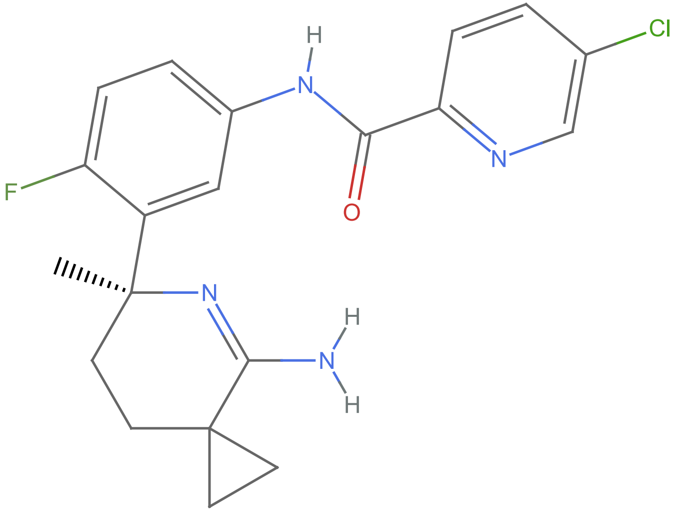

2D Autocorrelation

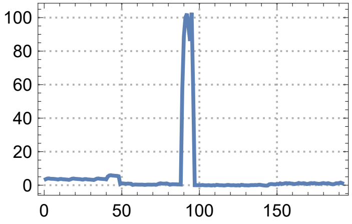

Dataset Index= 607

Molecule Formula= $C_{20}H_{20}ClFN_4O$

3D Autocorrelation

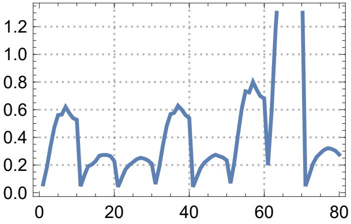

3D Plot

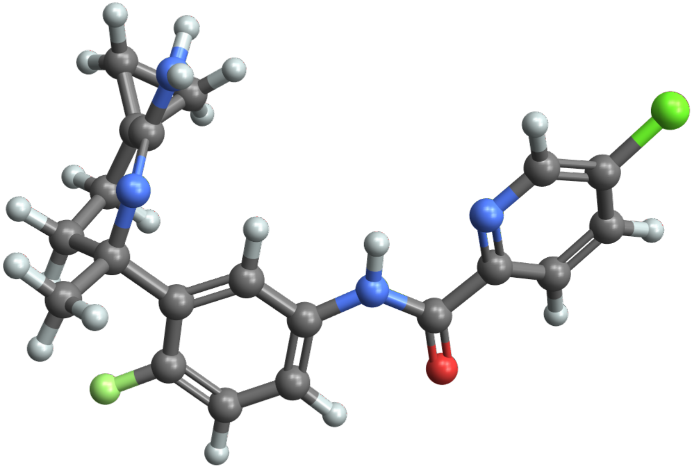

2D Plot

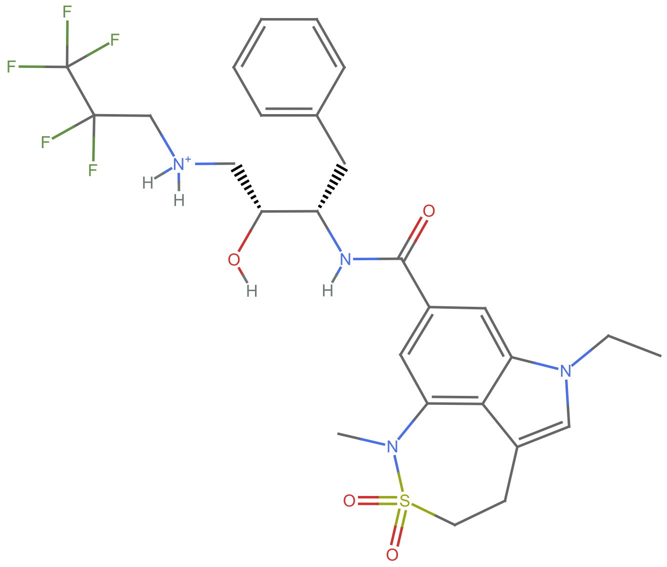

2D Autocorrelation

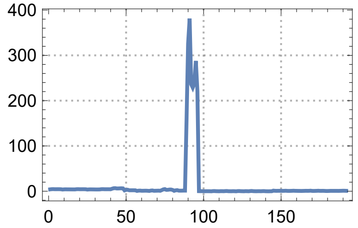

Dataset Index= 608

Molecule Formula= $C_{27}H_{32}F_5N_4O_4S^+$

3D Plot

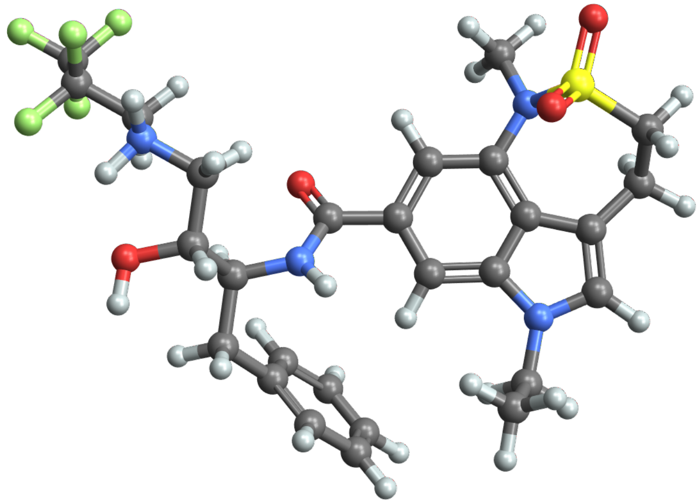

3D Autocorrelation

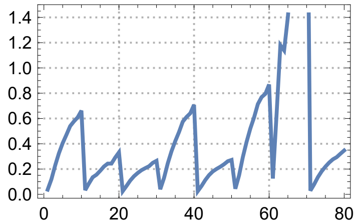

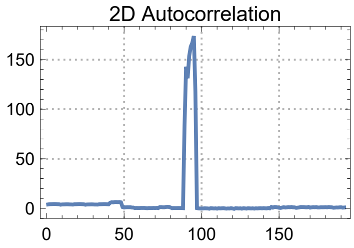

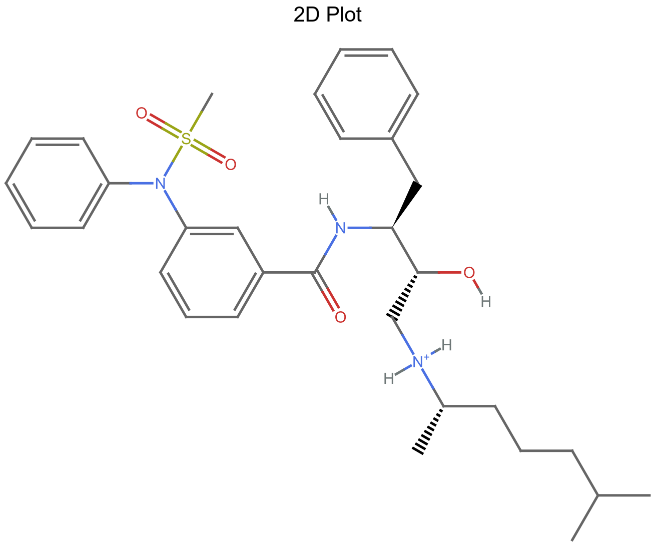

Dataset Index= 609

Molecule Formula= $C_{32}H_{44}N_3O_4S^+$

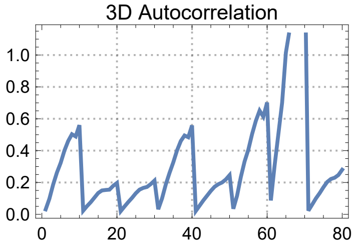

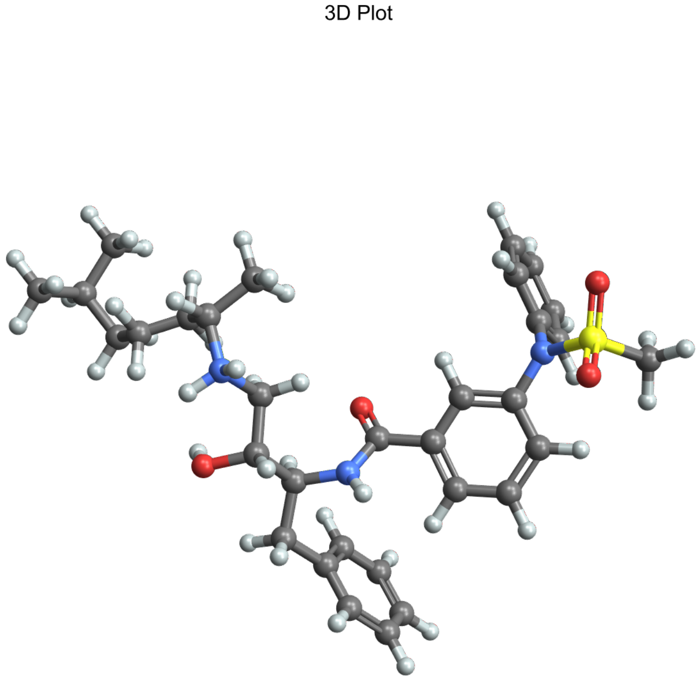

2D Plot

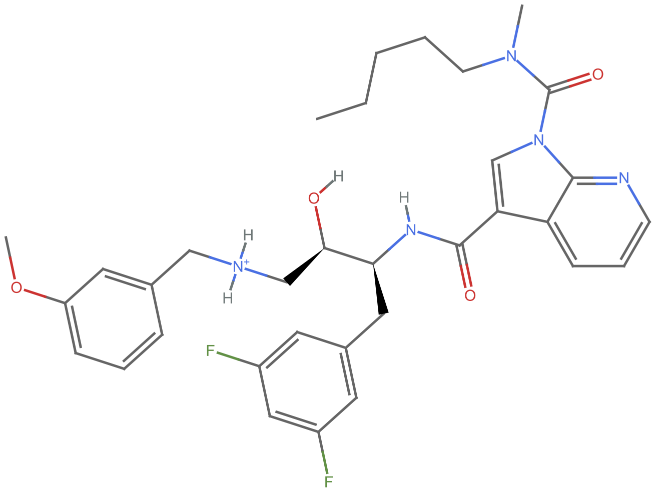

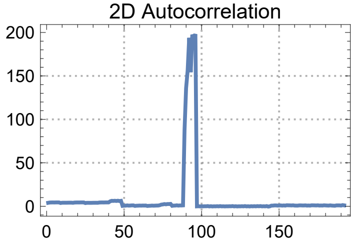

3D Plot

Dataset Index= 610

Molecule Formula= $C_{33}H_{40}F_2N_5O_4^+$

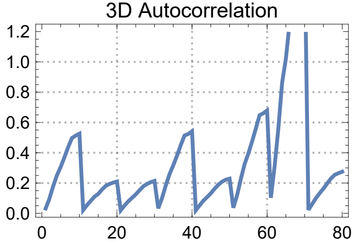

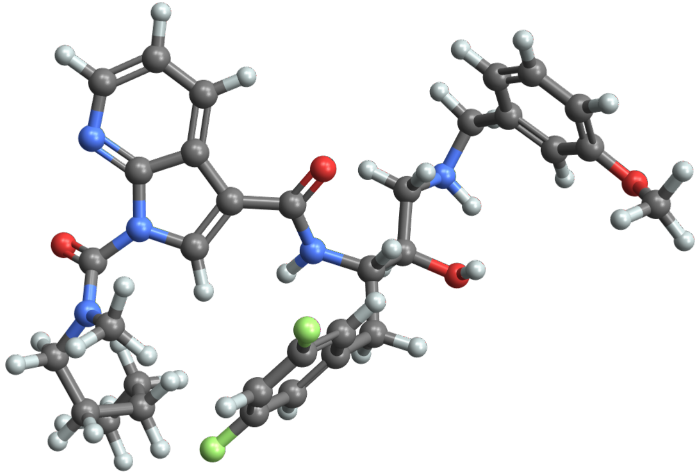

2D Plot

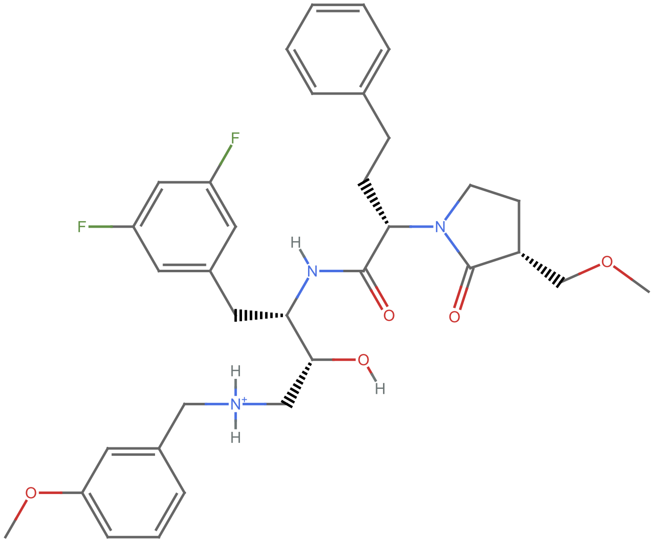

2D Autocorrelation

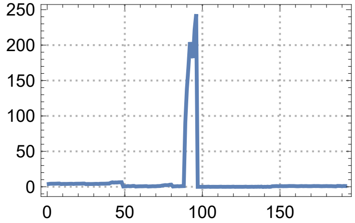

3D Plot

Dataset Index= 611

Molecule Formula= $C_{34}H_{42}F_2N_3O_5^+$

3D Autocorrelation

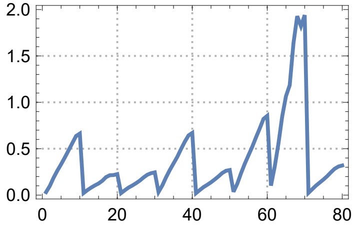

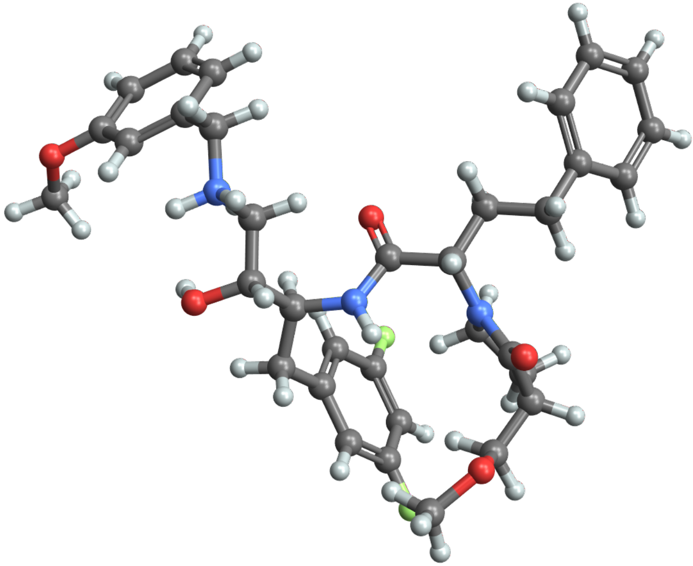

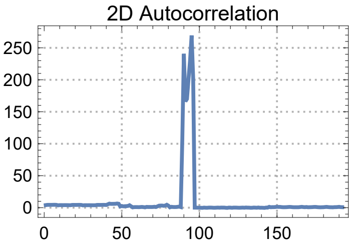

Dataset Index= 612

Molecule Formula= $C_{28}H_{39}F_4N_2O_5S^+$

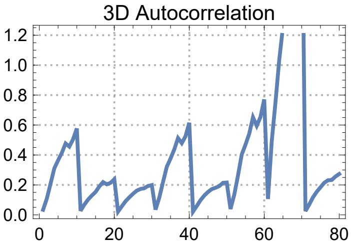

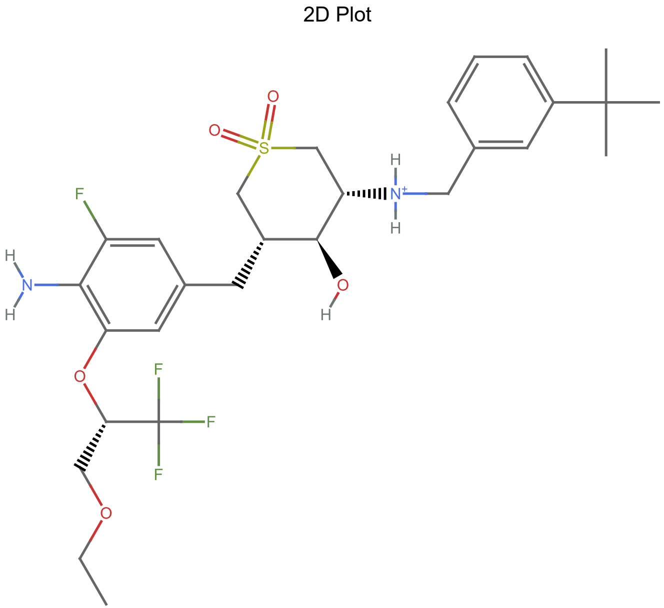

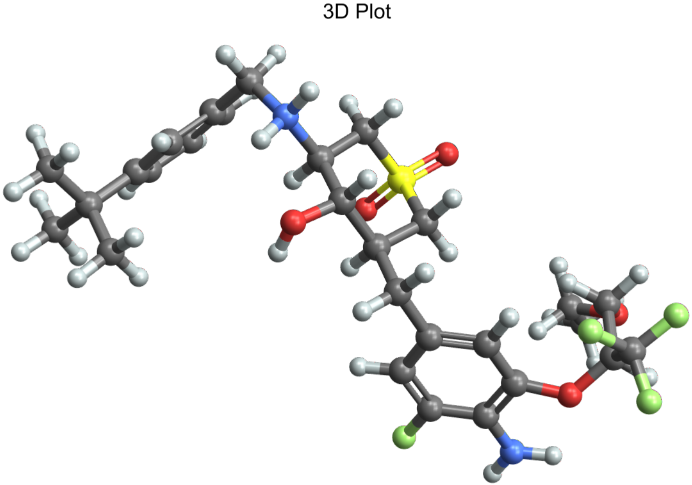

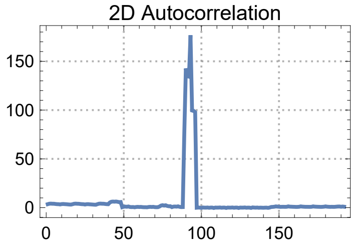

Dataset Index= 613

Molecule Formula= $C_{19}H_{15}F_3N_4O_2$

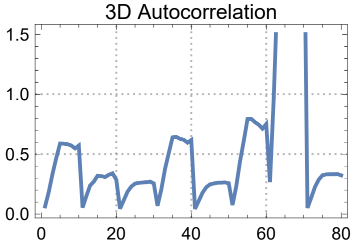

2D Plot

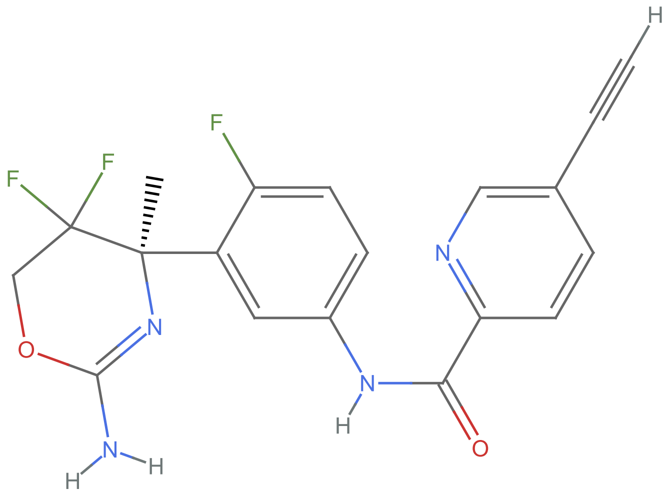

3D Plot

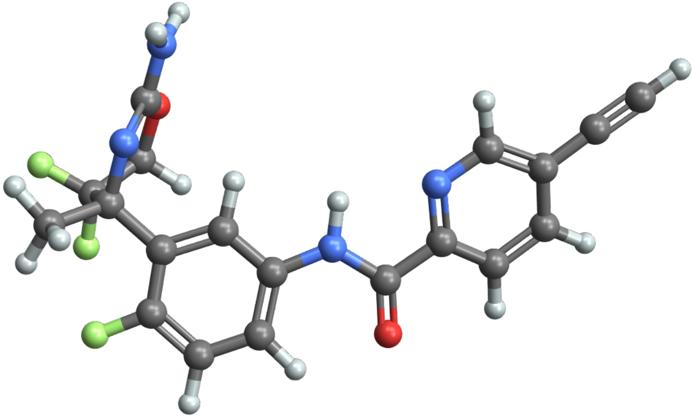

2D Autocorrelation

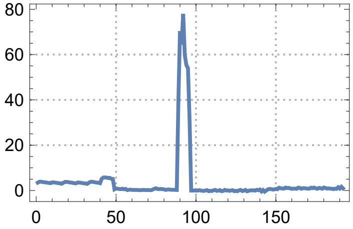

Dataset Index= 614

Molecule Formula= $C_{18}H_{21}ClN_5O^+$

3D Autocorrelation

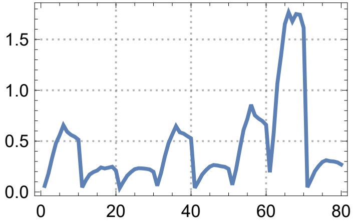

2D Plot

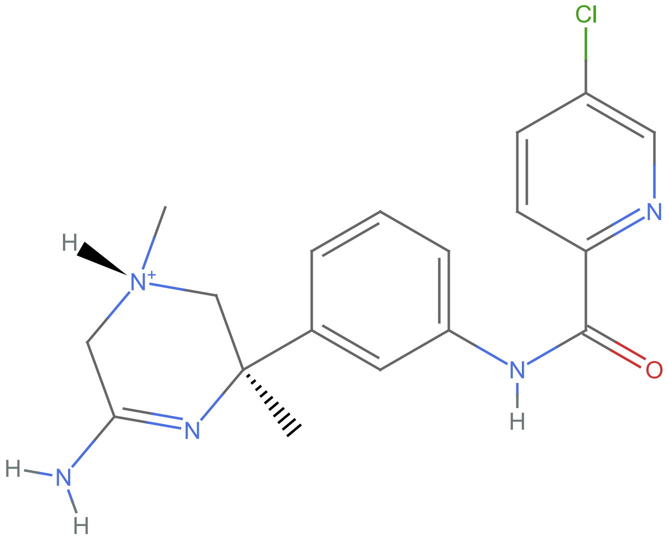

3D Plot

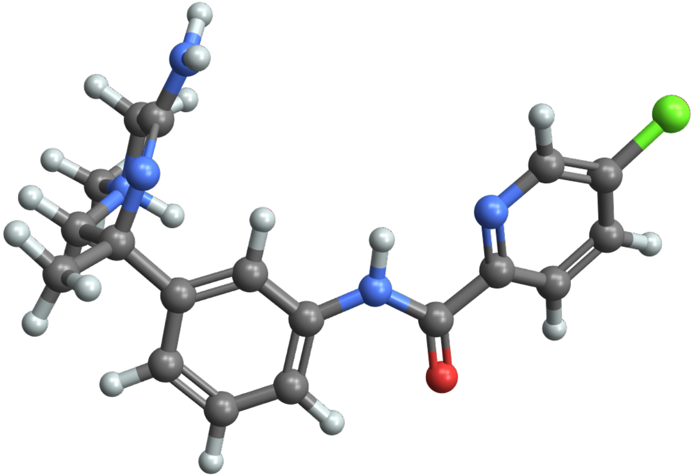

2D Plot

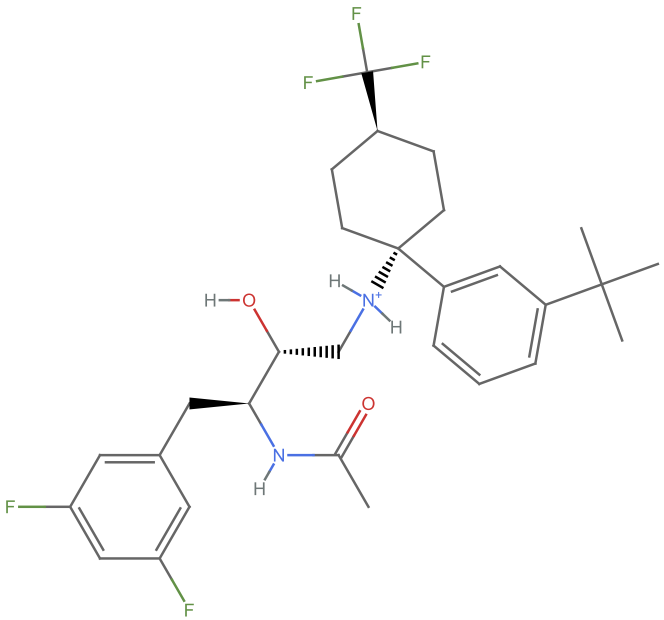

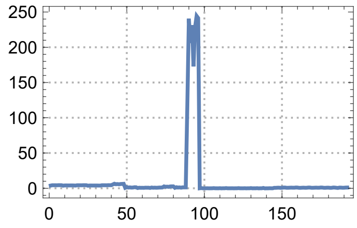

3D Plot

Dataset Index= 615

Molecule Formula= $C_{29}H_{38}F_5N_2O_2^+$

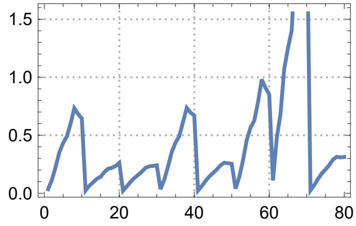

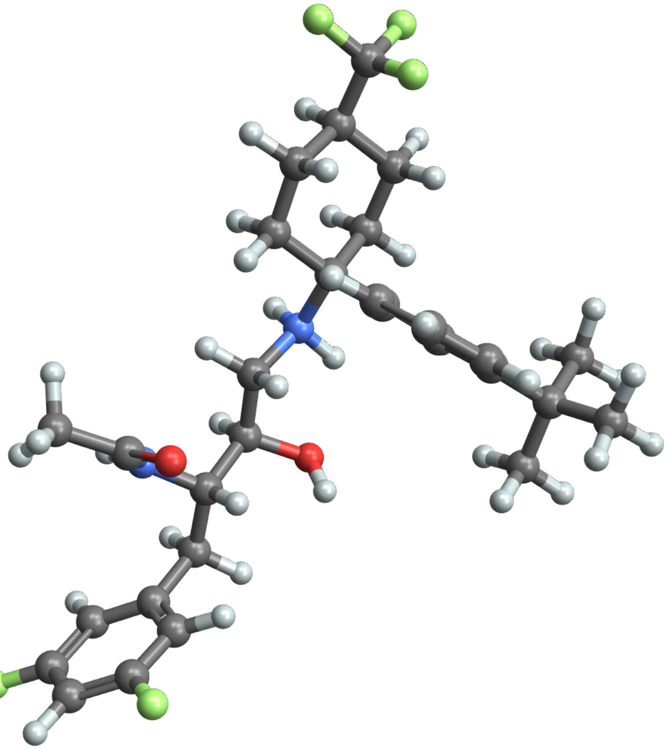

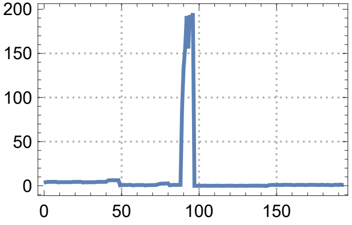

Dataset Index= 616

Molecule Formula= $C_{32}H_{38}F_2N_5O_4^+$

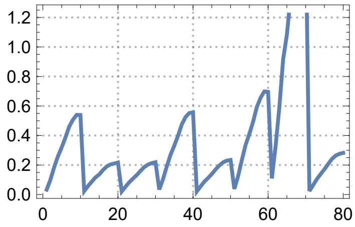

2D Plot

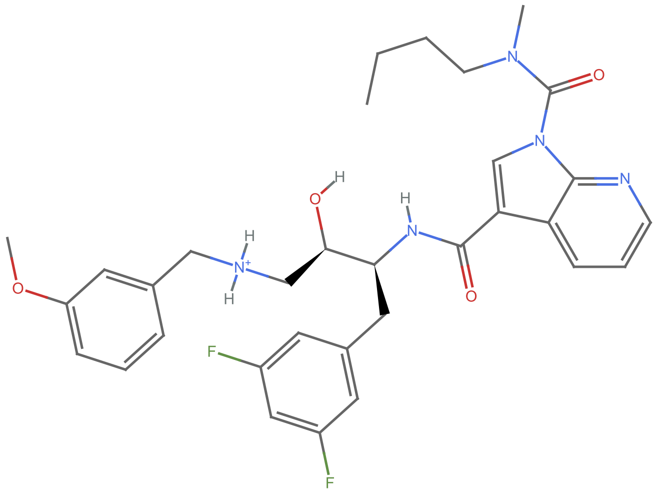

3D Plot

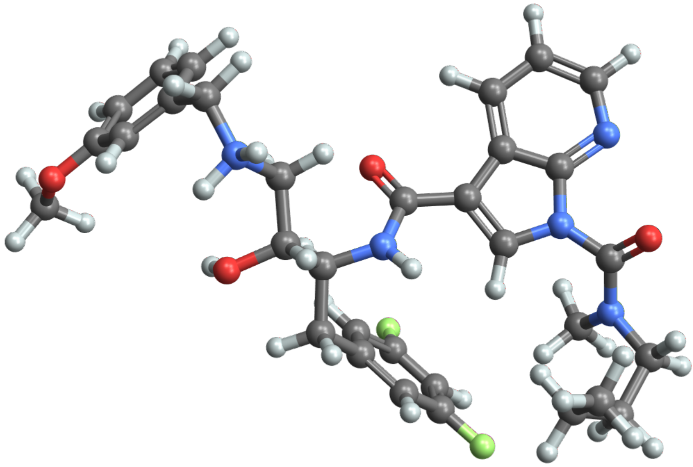

2D Plot

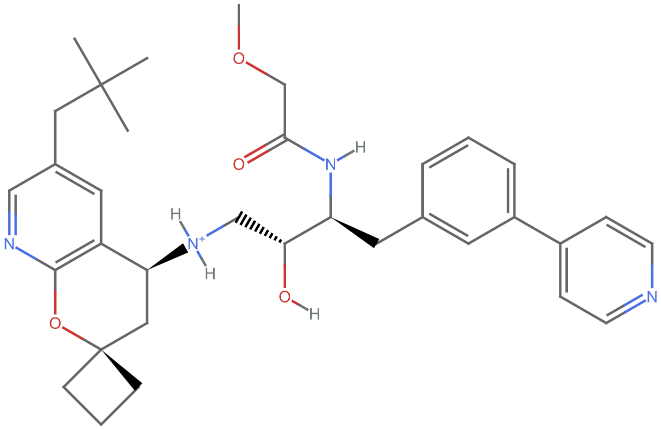

2D Autocorrelation

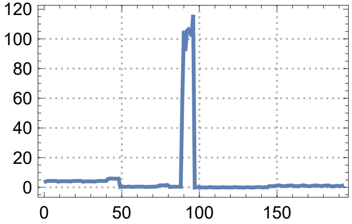

Dataset Index= 617

Molecule Formula= $C_{34}H_{45}N_4O_4^+$

3D Plot

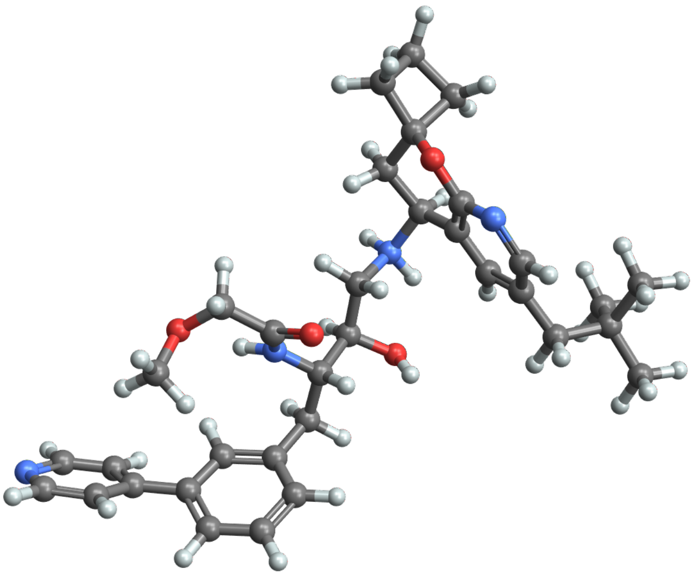

3D Autocorrelation

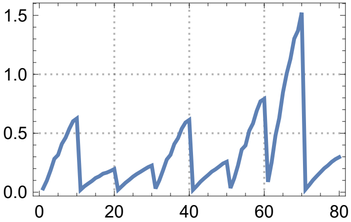

2D Plot

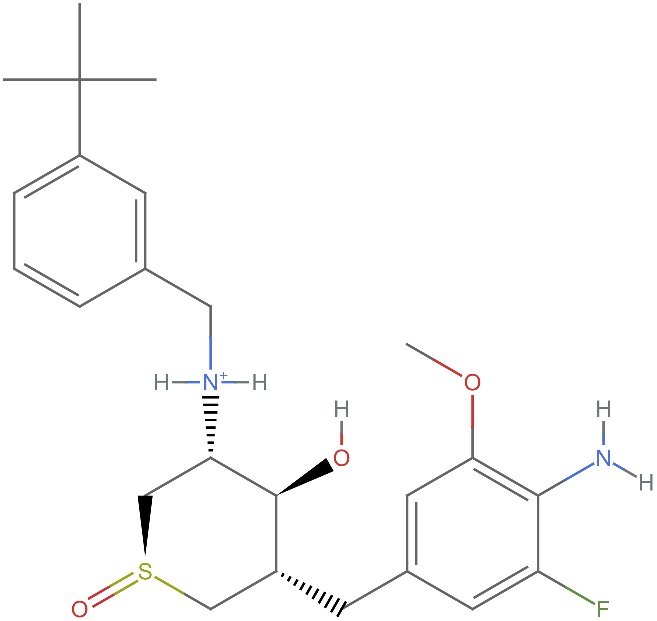

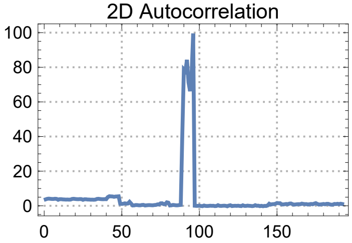

Dataset Index= 618

Molecule Formula= $C_{24}H_{34}FN_2O_3S^+$

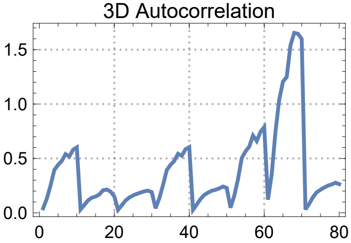

3D Plot

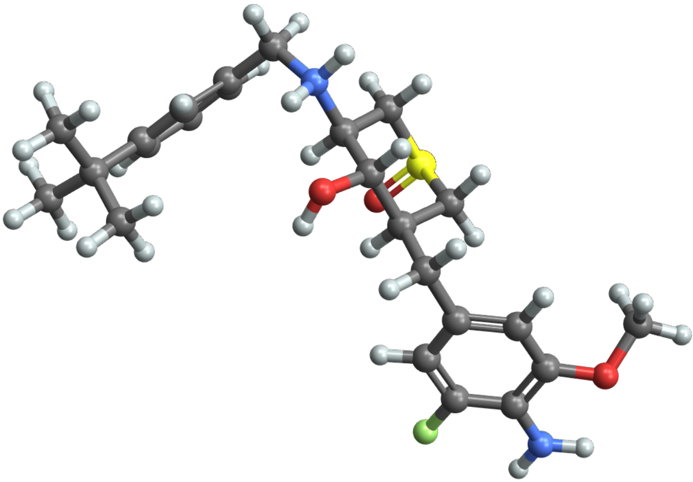

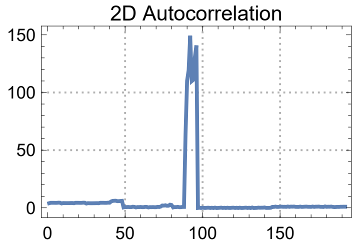

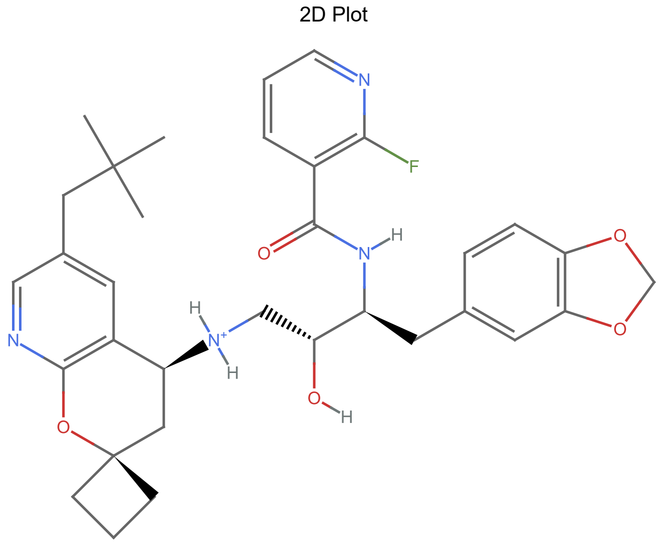

Dataset Index= 619

Molecule Formula= $C_{33}H_{40}FN_4O_5^+$

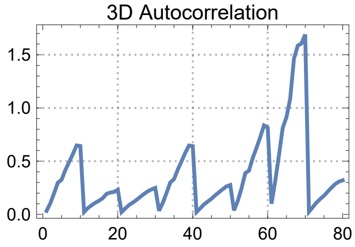

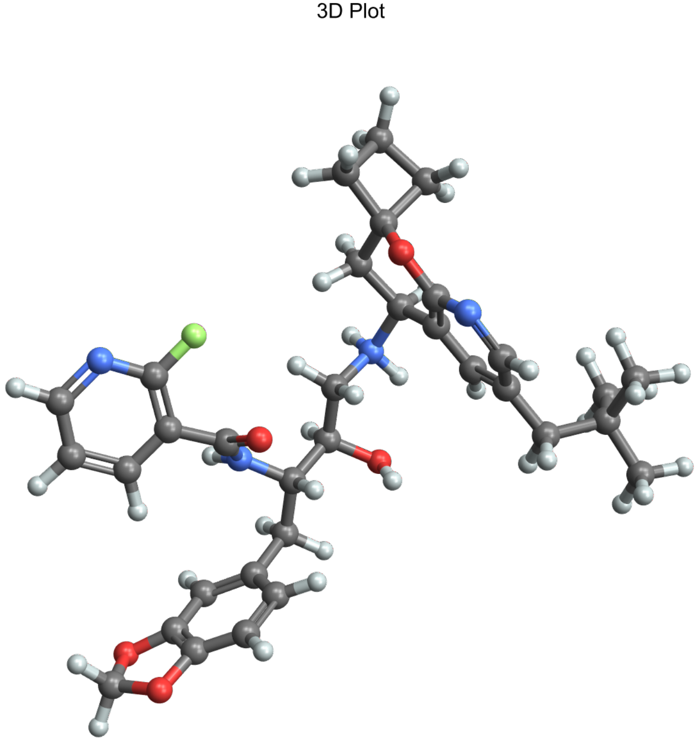

2D Plot

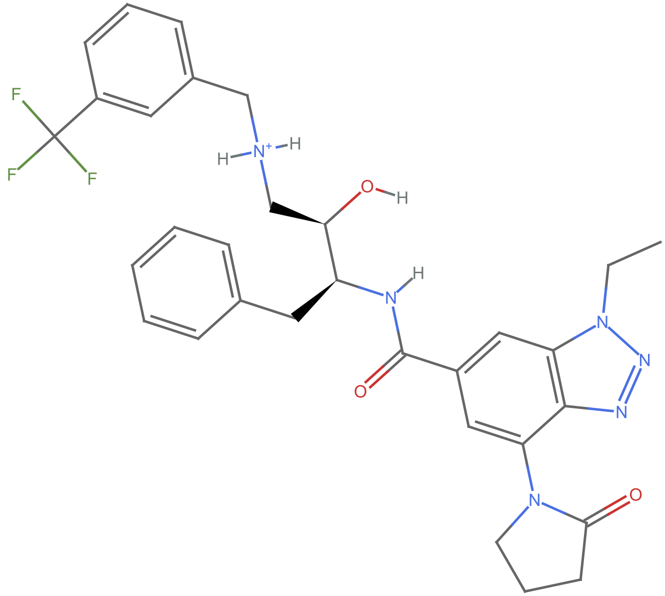

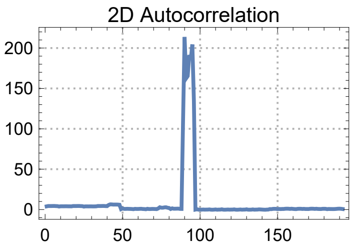

Dataset Index= 620

Molecule Formula= $C_{31}H_{34}F_3N_6O_3^+$

3D Plot

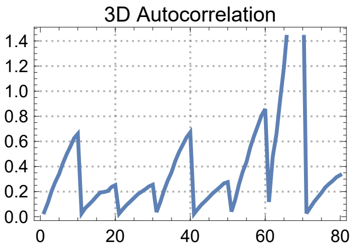

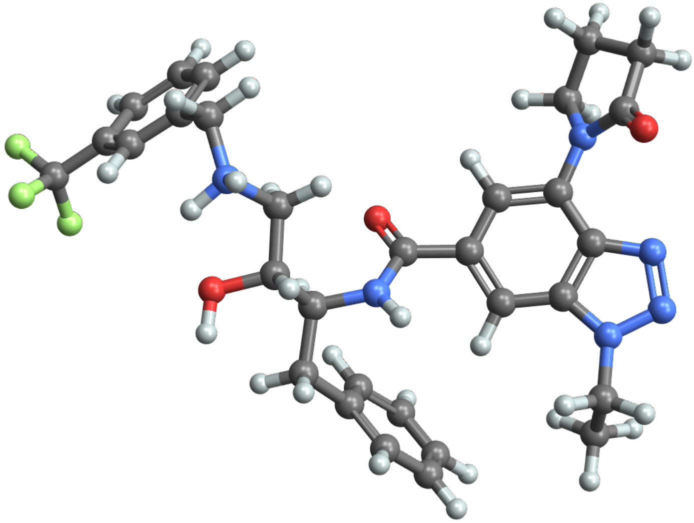

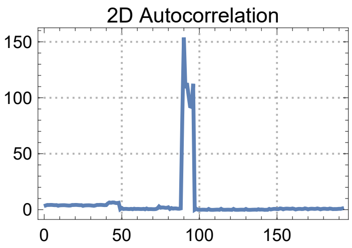

Dataset Index= 621

Molecule Formula= $C_{23}H_{20}F_3N_6O^+$

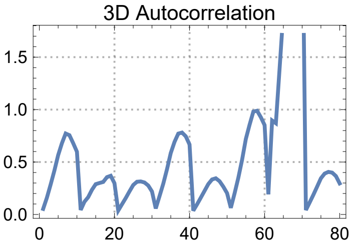

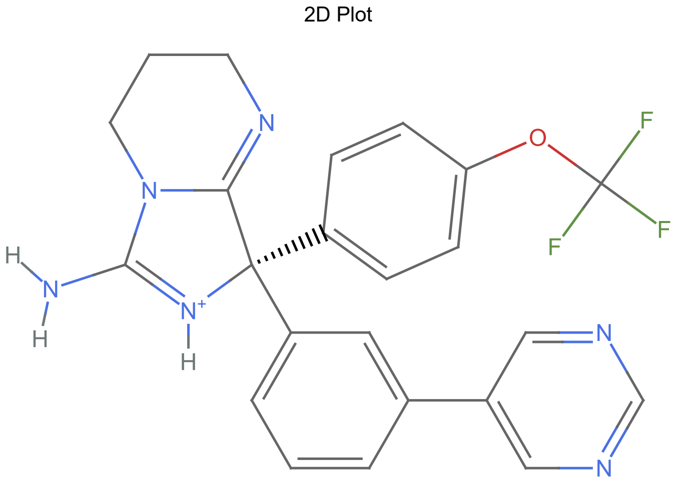

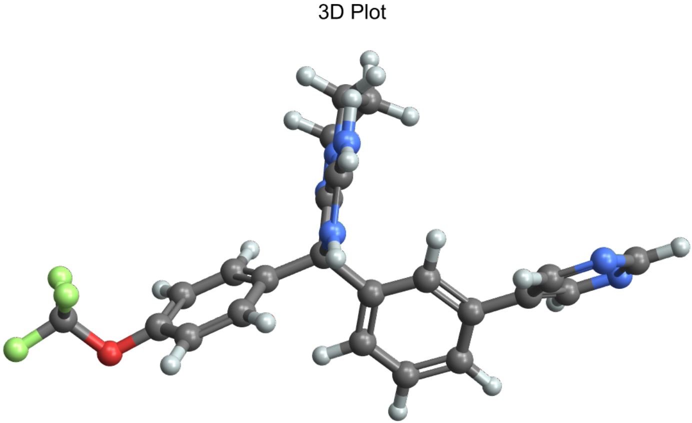

2D Plot

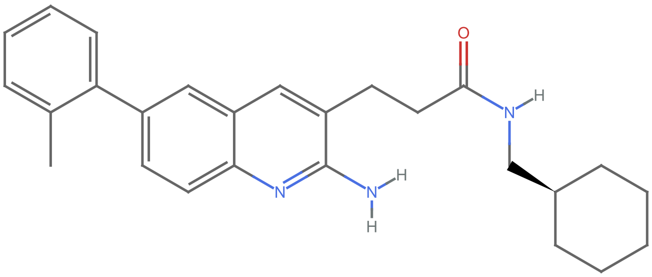

3D Plot

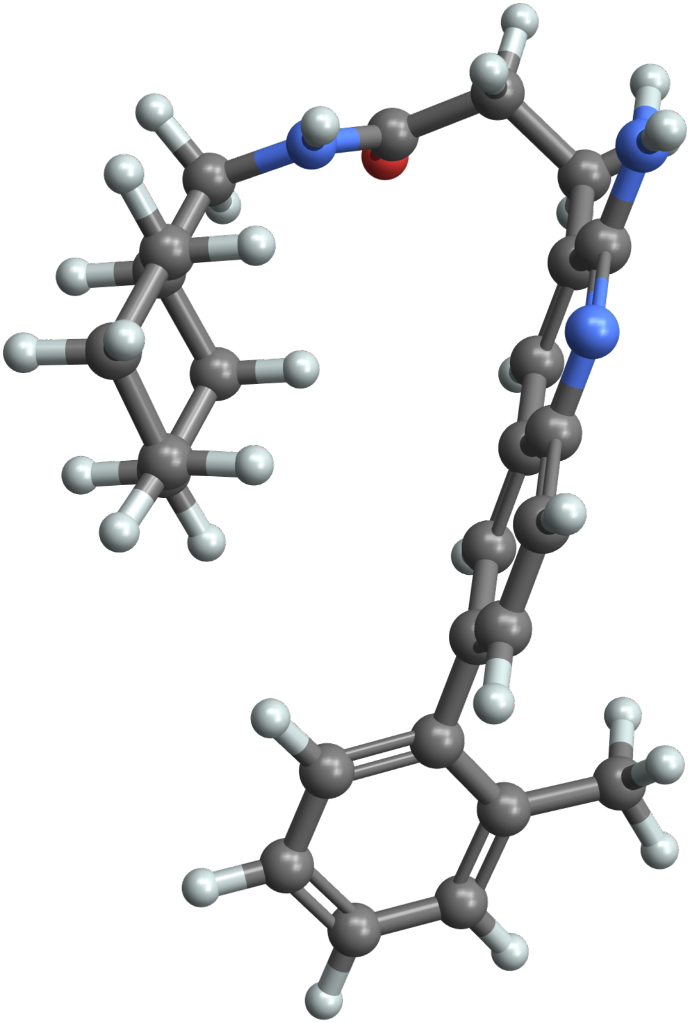

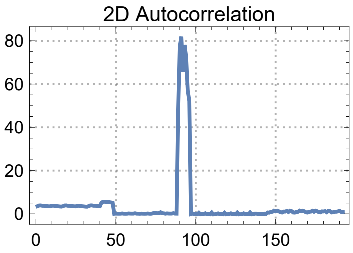

Dataset Index= 622

Molecule Formula= $C_{26}H_{31}N_3O$

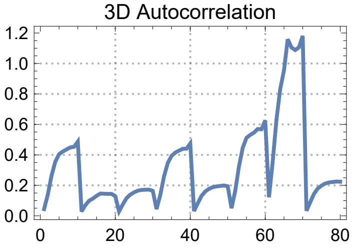

2D Plot

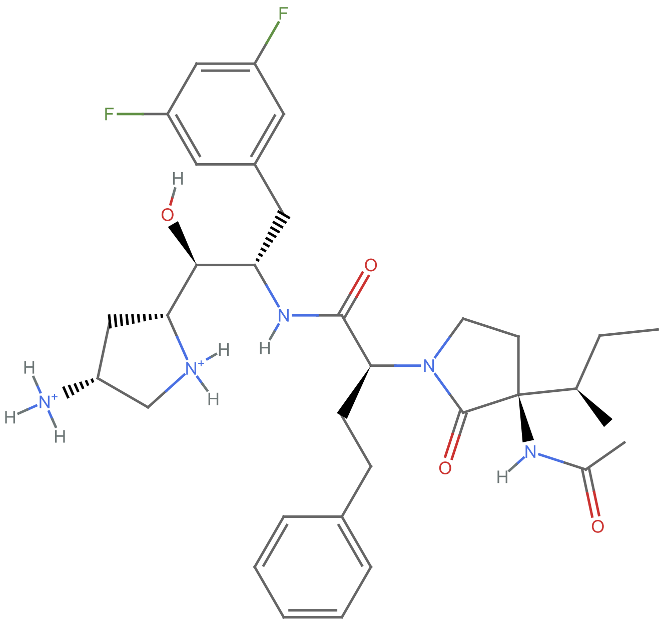

3D Plot

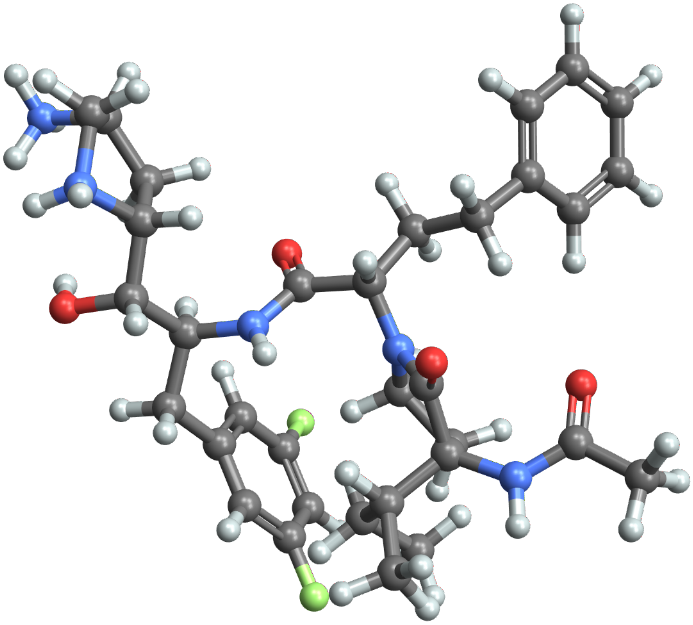

2D Autocorrelation

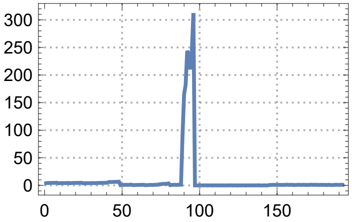

Dataset Index= 623

Molecule Formula= $C_{33}H_{47}F_{2}N_{5}O_{4}^{2+}$

3D Autocorrelation

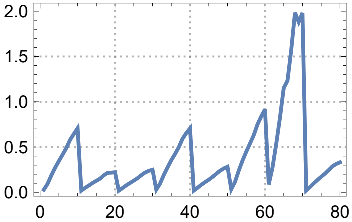

2D Plot

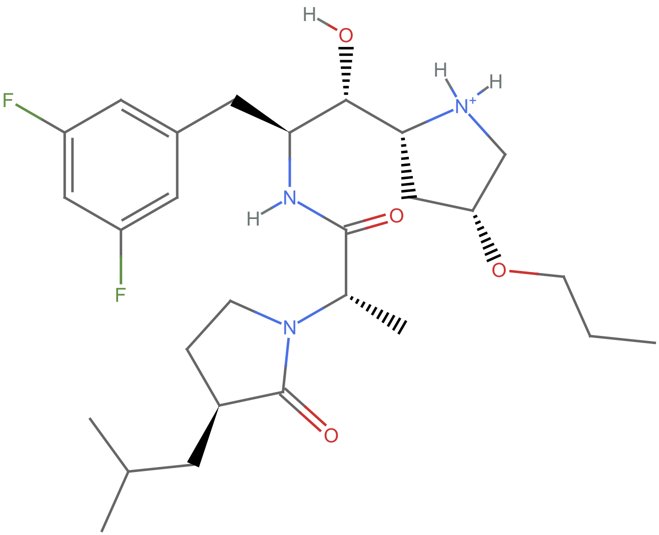

2D Autocorrelation

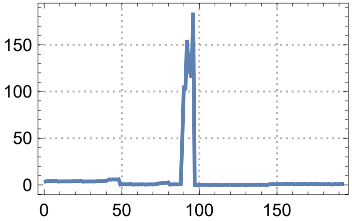

Dataset Index= 624

Molecule Formula= $C_{27}H_{42}F_2N_3O_4^+$

3D Autocorrelation

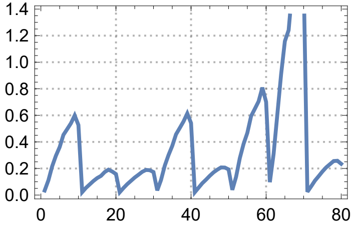

3D Plot

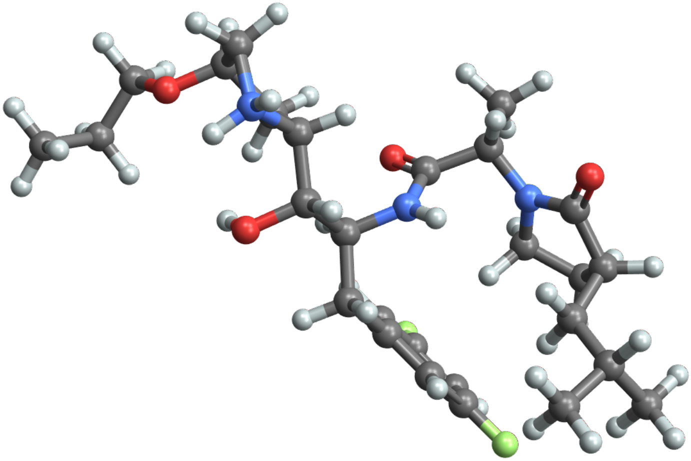

2D Autocorrelation

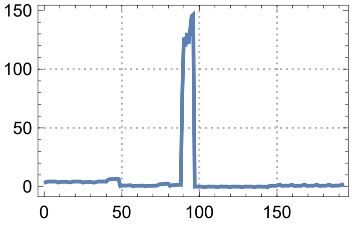

Dataset Index= 625

Molecule Formula= $C_{24}H_{15}F_4N_5$

3D Autocorrelation

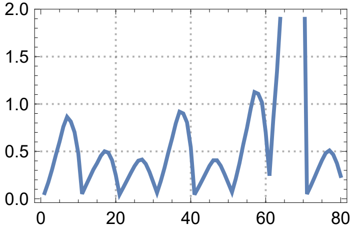

2D Plot

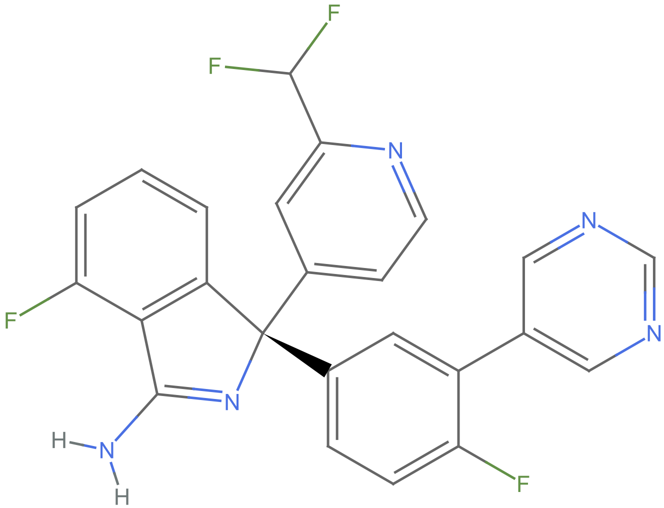

3D Plot

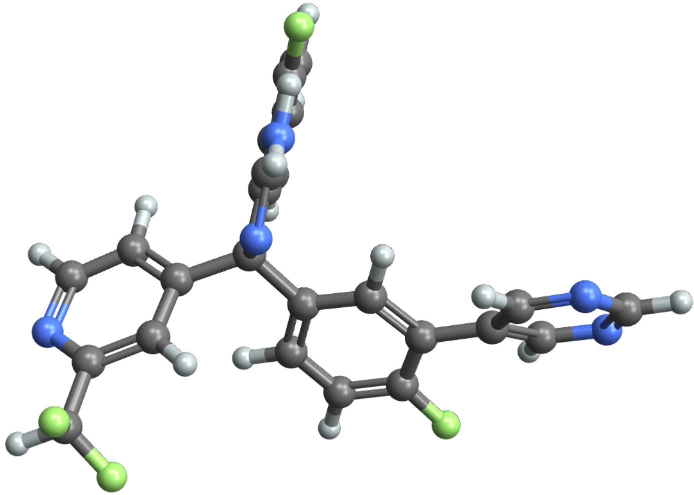

2D Plot

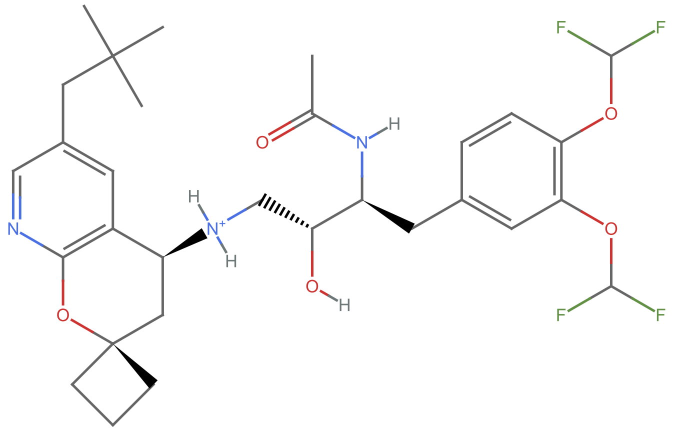

2D Autocorrelation

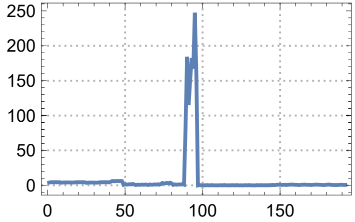


```
Dataset Index=       626

Molecule Formula=    C30H40F4N3O5+
```

3D Autocorrelation

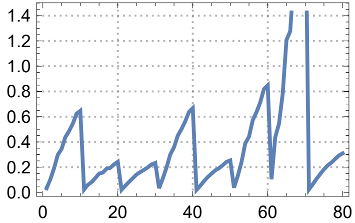

3D Plot

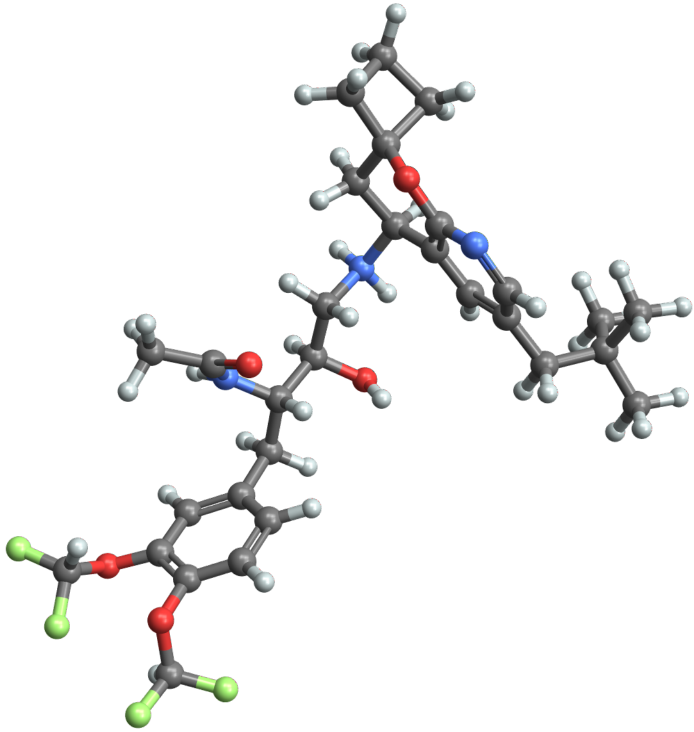

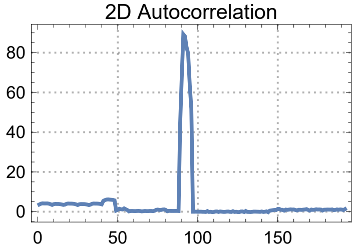

Dataset Index= 627

Molecule Formula= $C_{22}H_{19}ClN_4O_2$

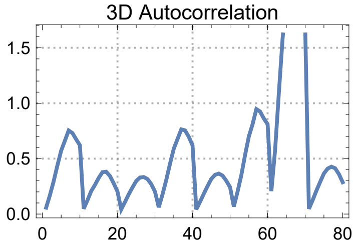

2D Plot

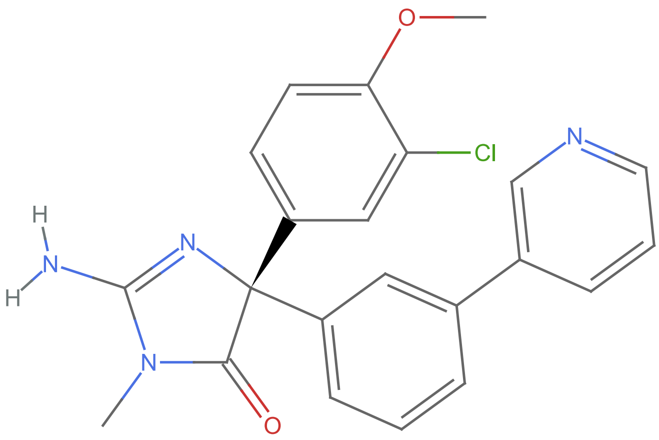

3D Plot

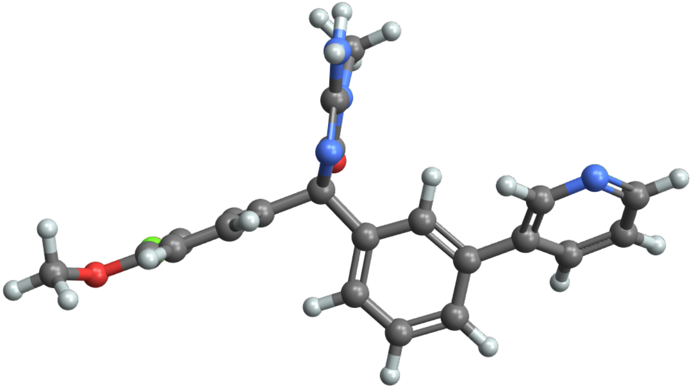

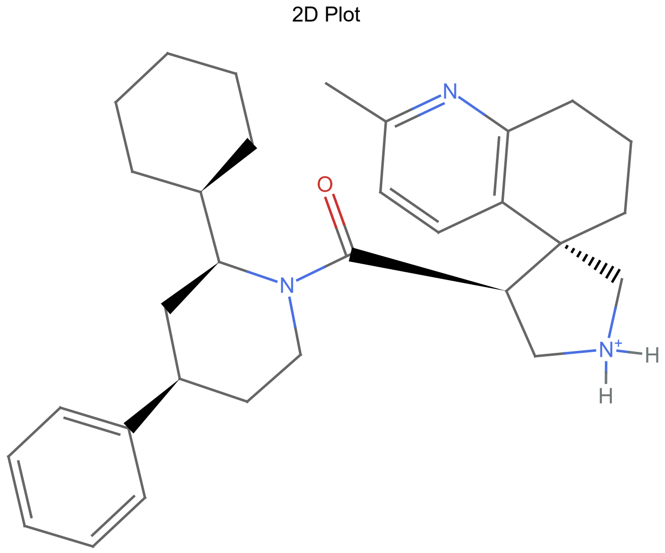

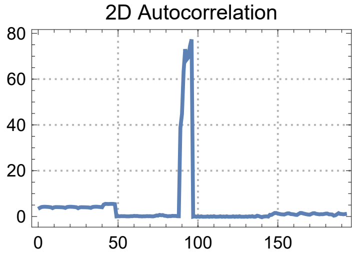

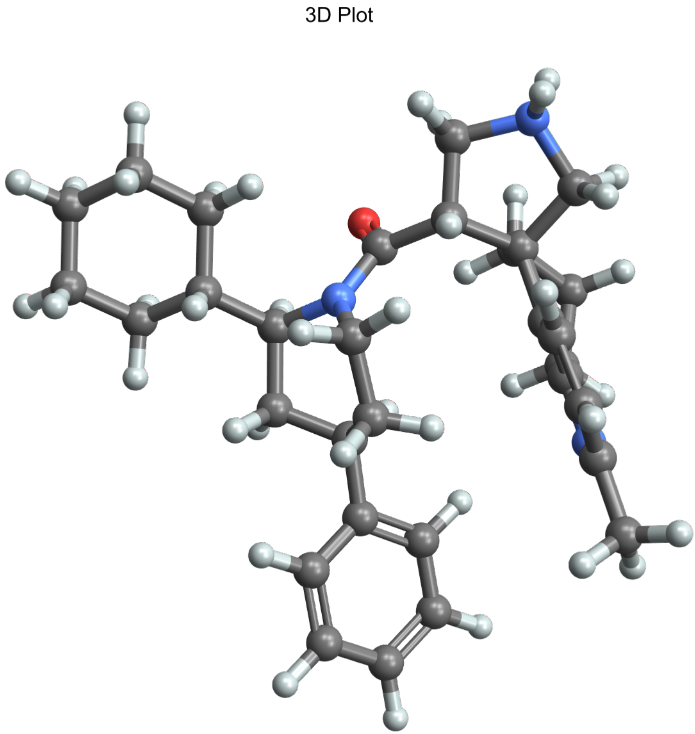

Dataset Index= 628

Molecule Formula= $C_{31}H_{42}N_3O^+$

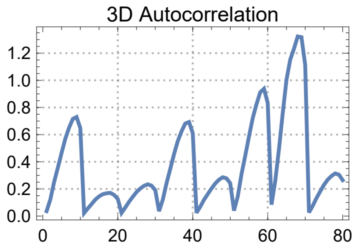

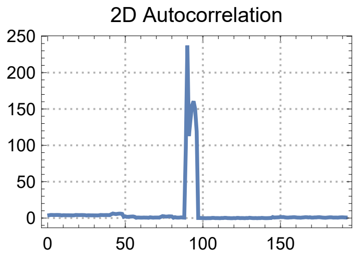

Dataset Index= 629

Molecule Formula= $C_{25}H_{34}F_3N_2O_4S^+$

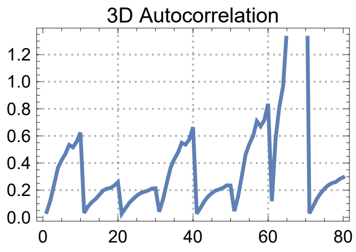

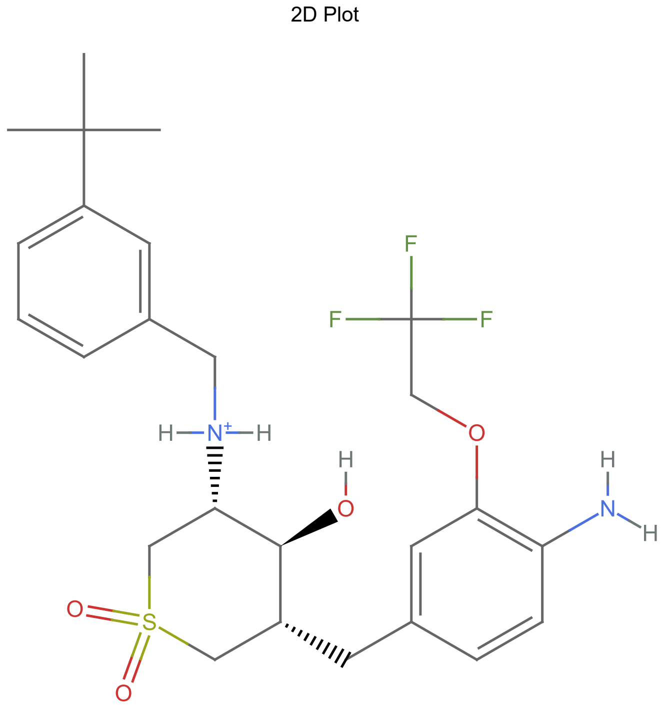

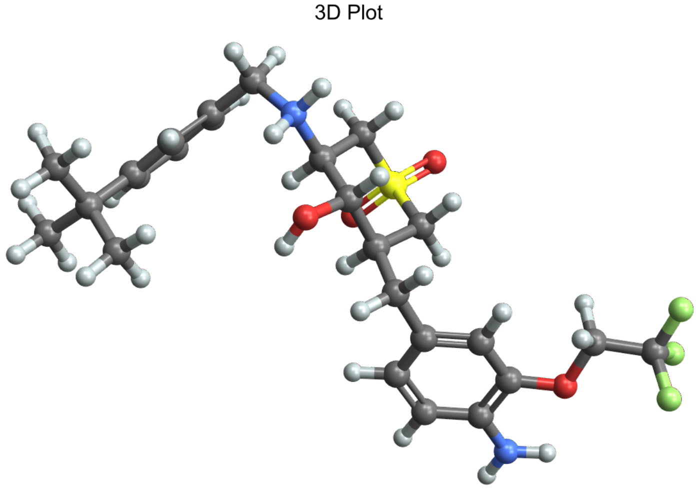

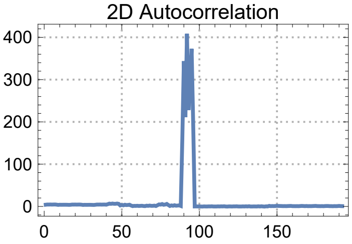

Dataset Index= 630

Molecule Formula= $C_{24}H_{32}F_7N_4O_4S^+$

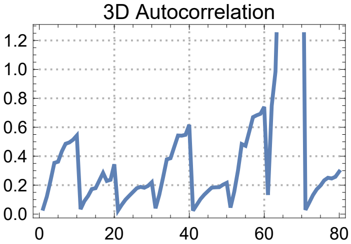

2D Plot

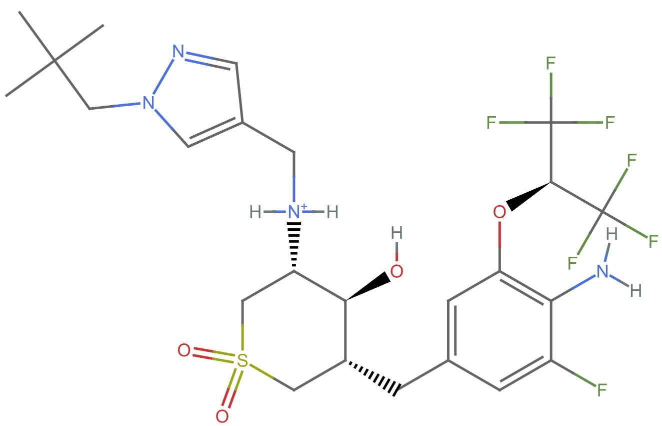

3D Plot

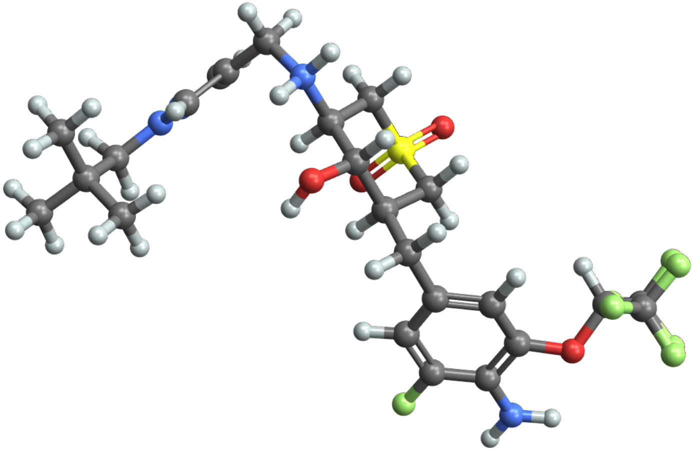

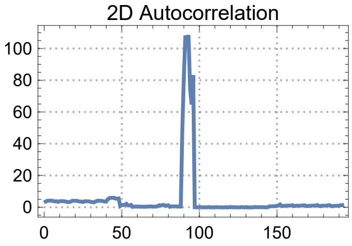

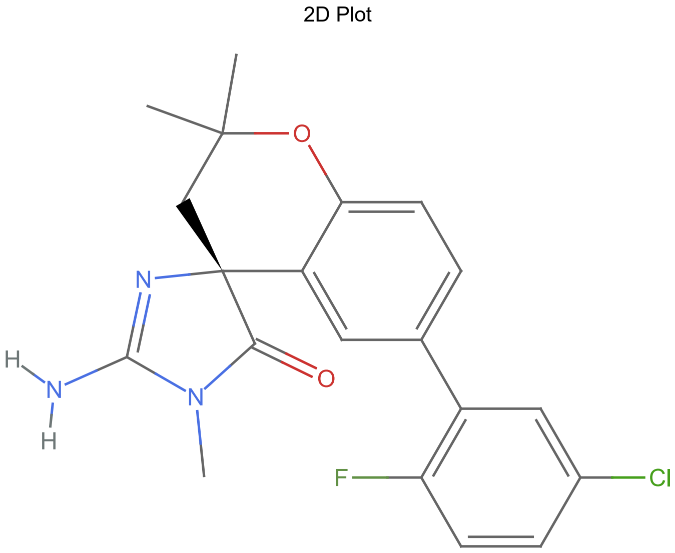

Dataset Index= 631

Molecule Formula= $C_{20}H_{19}ClFN_3O_2$

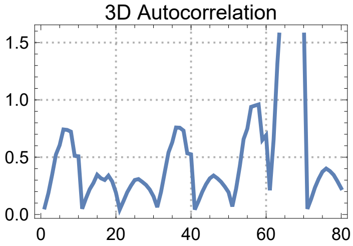

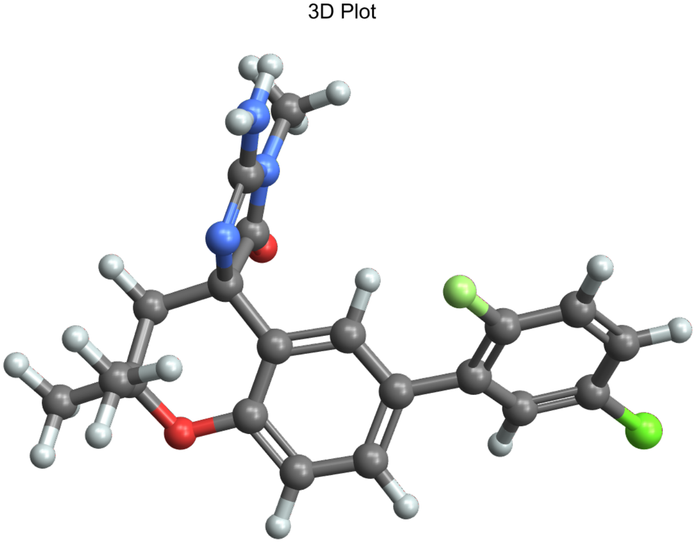

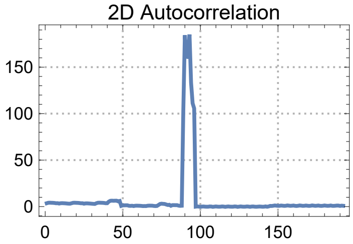

Dataset Index= 632

Molecule Formula= $C_{16}H_{14}F_5N_5O_2$

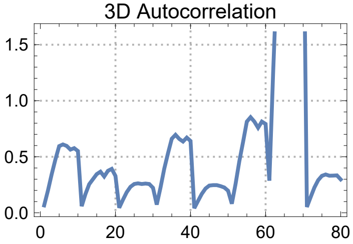

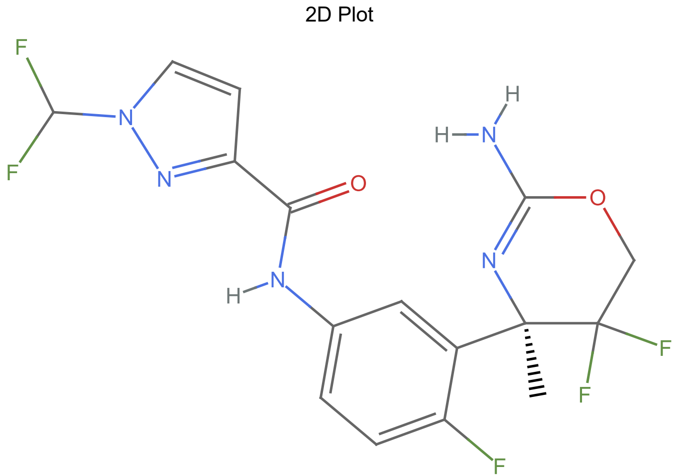

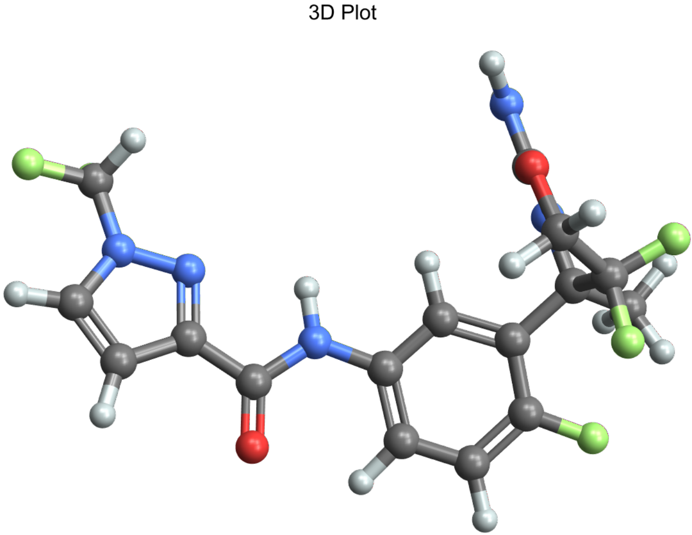

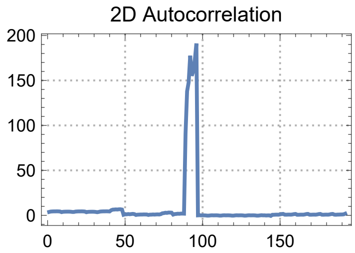

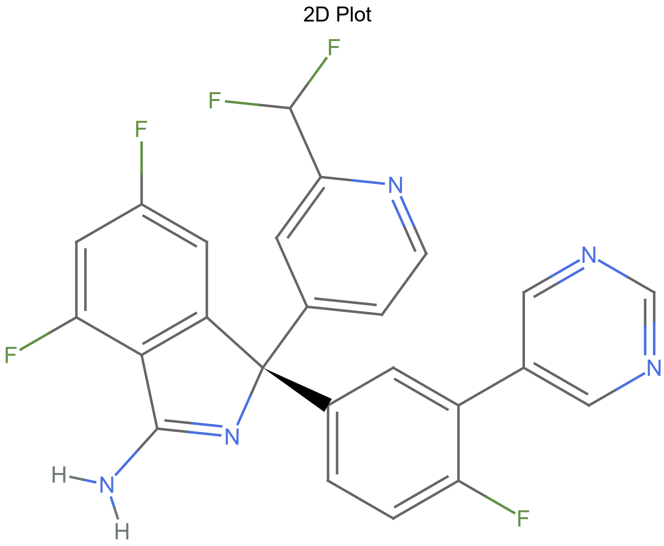

Dataset Index= 633

Molecule Formula= $C_{24}H_{14}F_5N_5$

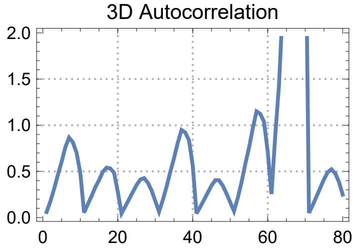

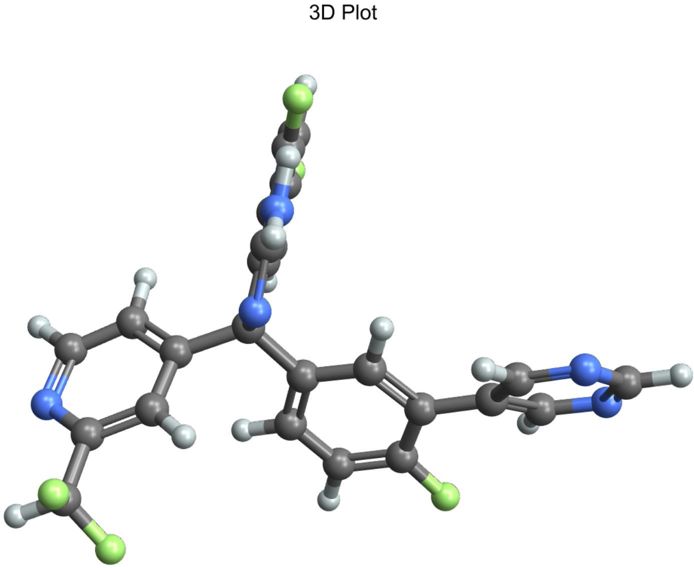

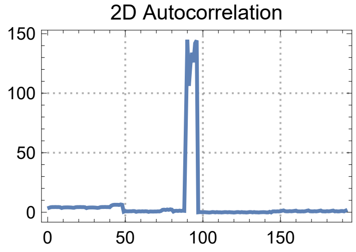

Dataset Index= 634

Molecule Formula= $C_{25}H_{22}F_3N_5O$

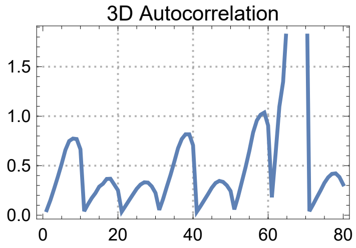

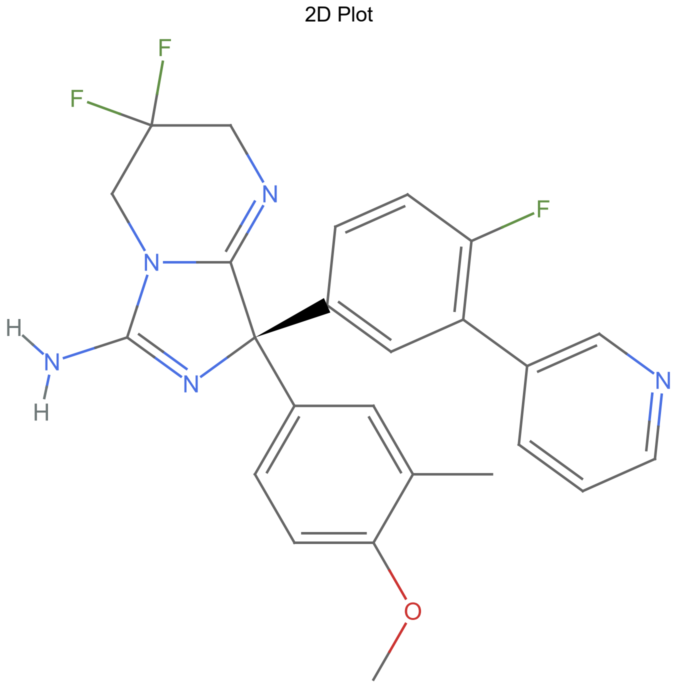

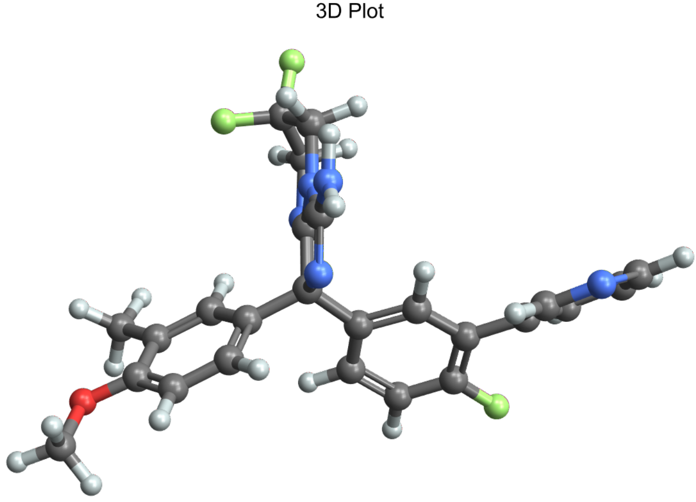

2D Plot

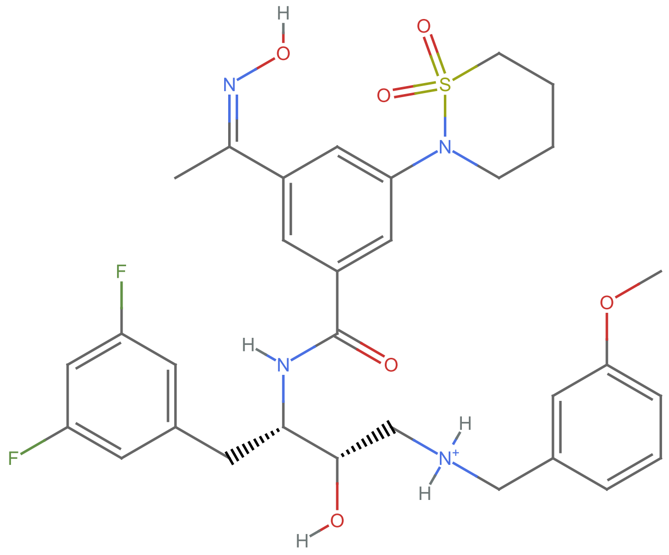

2D Autocorrelation

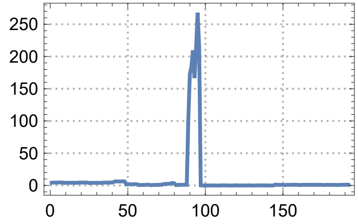

Dataset Index= 635

Molecule Formula= $C_{31}H_{37}F_2N_4O_6S^+$

3D Plot

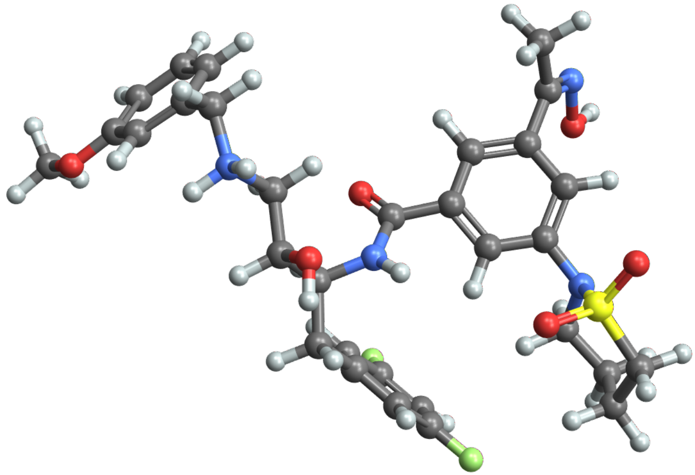

3D Autocorrelation

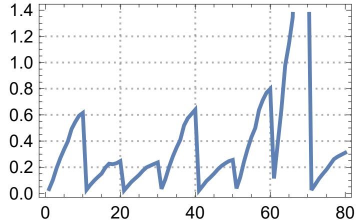

2D Plot

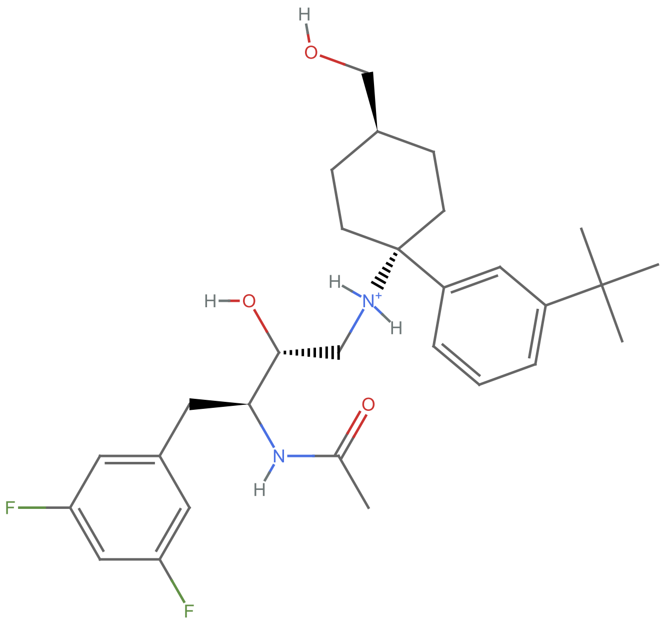

2D Autocorrelation

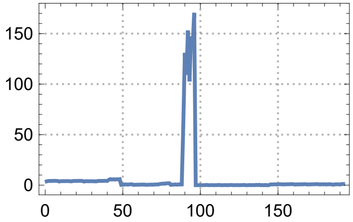

3D Plot

Dataset Index= 636

Molecule Formula= $C_{29}H_{41}F_2N_2O_3^+$

3D Autocorrelation

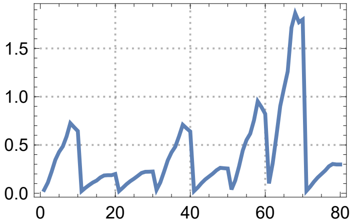

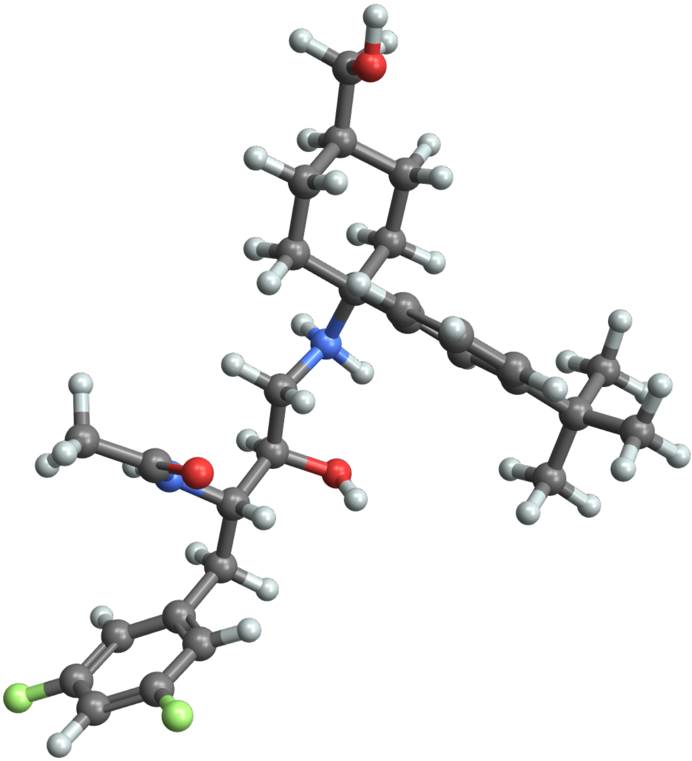

2D Plot

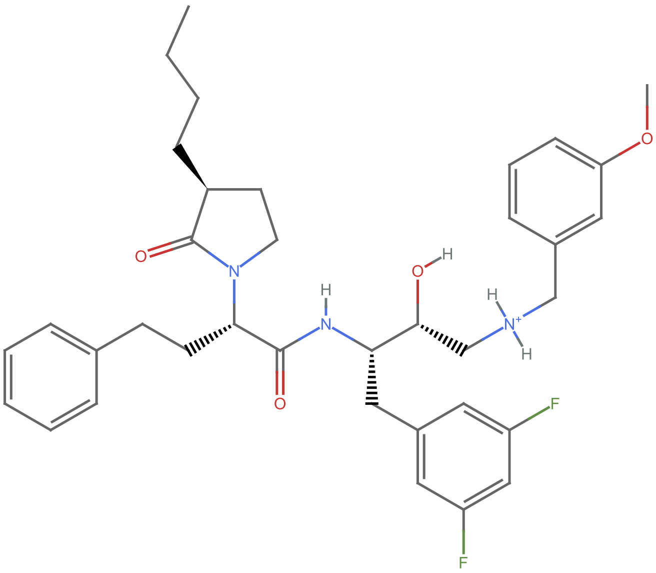

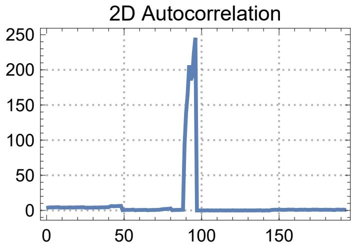

3D Plot

Dataset Index= 637

Molecule Formula= $C_{36}H_{46}F_2N_3O_4^+$

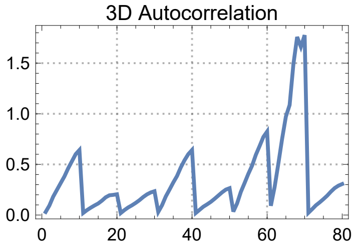

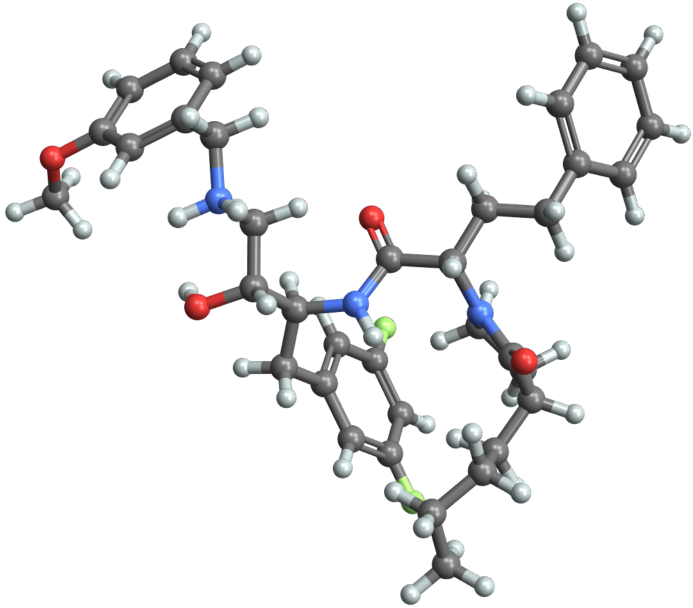

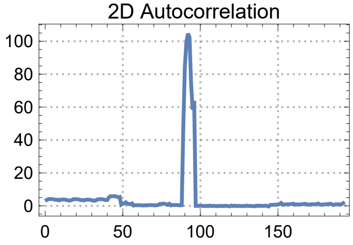

Dataset Index= 638

Molecule Formula= $C_{20}H_{19}ClFN_3O_2$

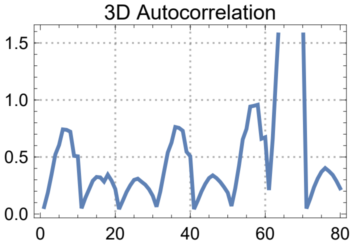

2D Plot

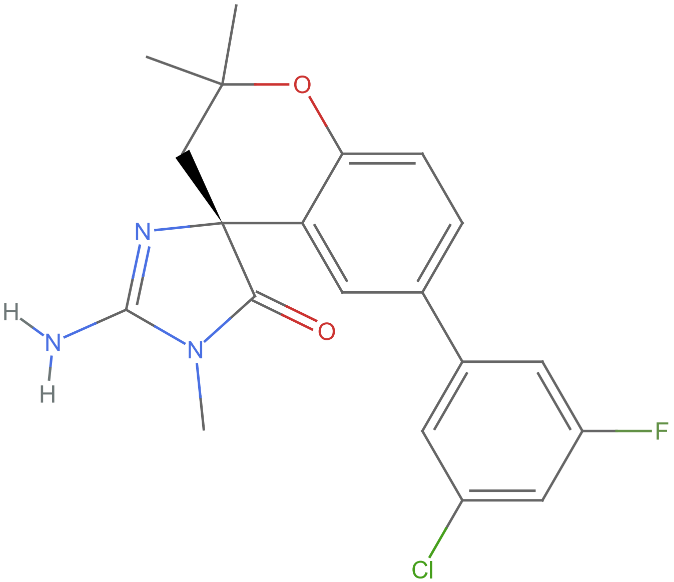

3D Plot

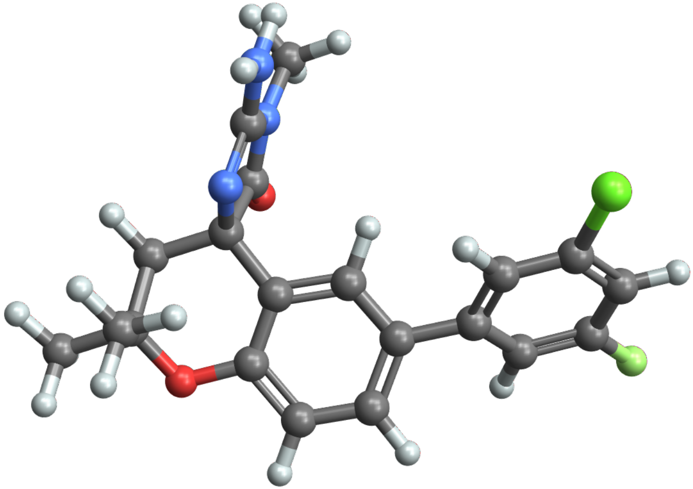

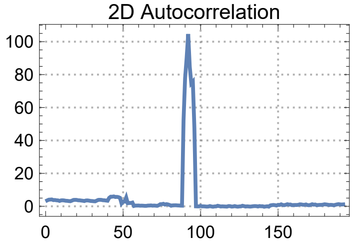

Dataset Index= 639

Molecule Formula= $C_{17}H_{15}Cl_2FN_4O_2$

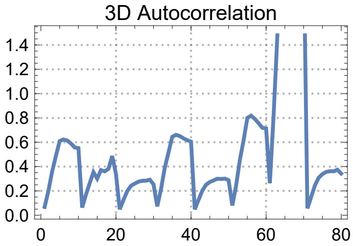

2D Plot

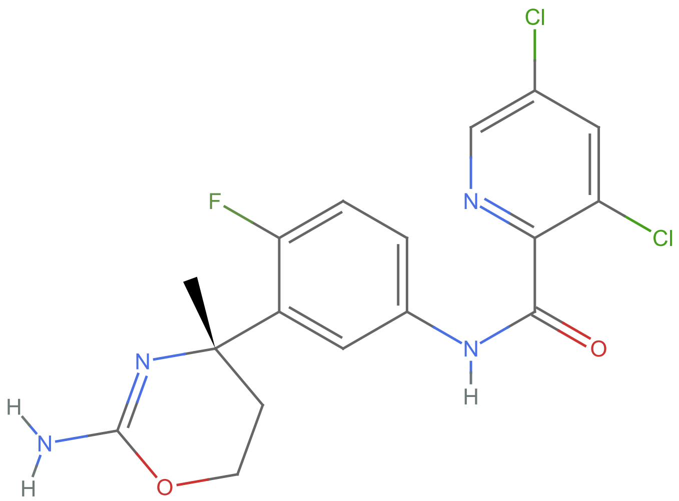

3D Plot

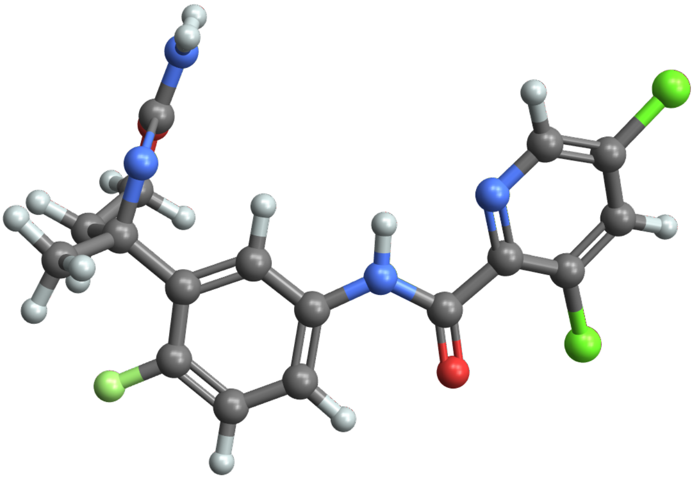

2D Plot

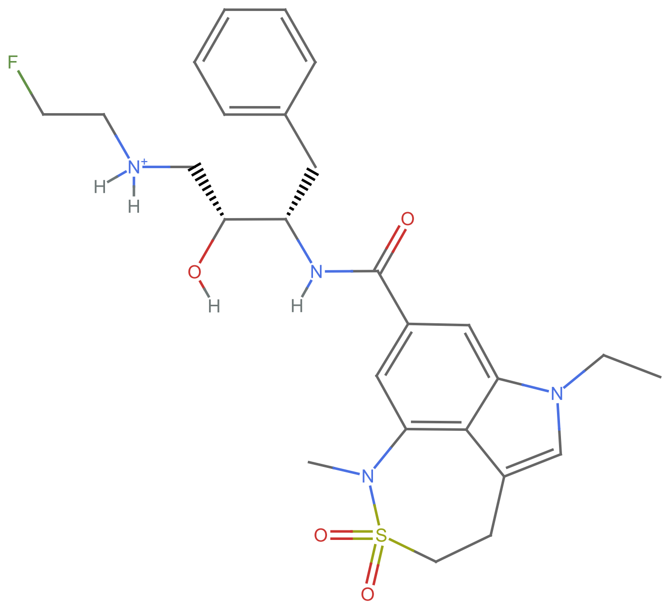

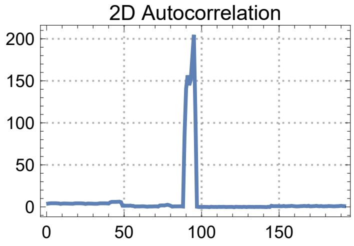


```
Dataset Index=      640

Molecule Formula=   C26H34FN4O4S+
```

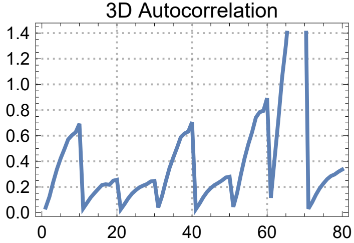

3D Plot

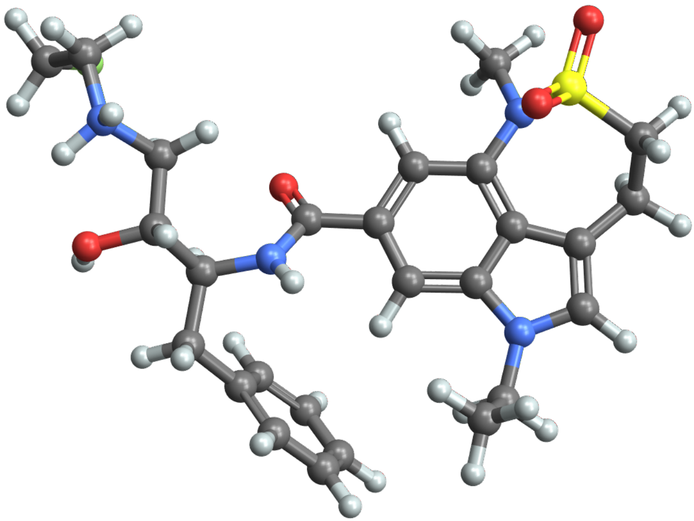

2D Plot

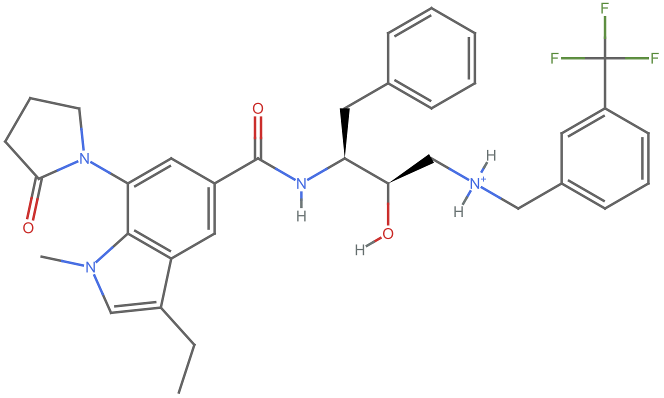

2D Autocorrelation

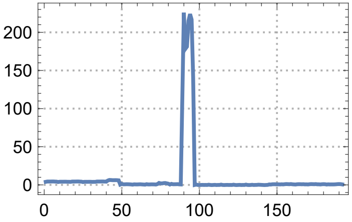

3D Plot

Dataset Index= 641

Molecule Formula= $C_{34}H_{38}F_{3}N_{4}O_{3}^{+}$

3D Autocorrelation

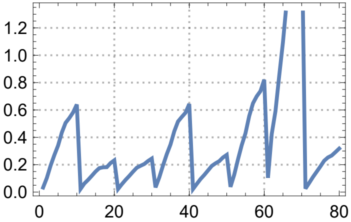

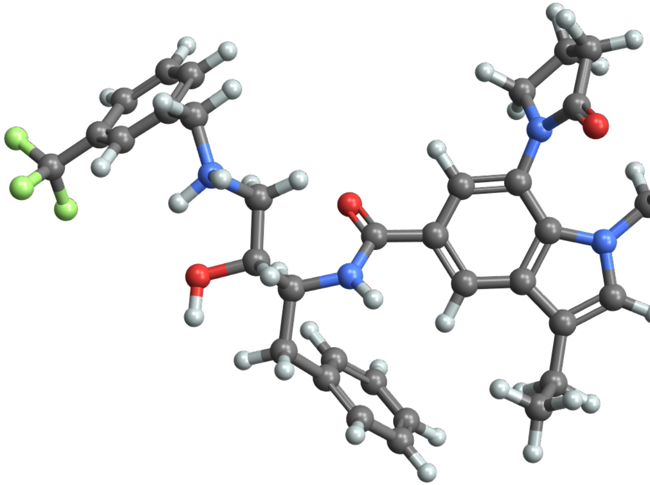

2D Plot

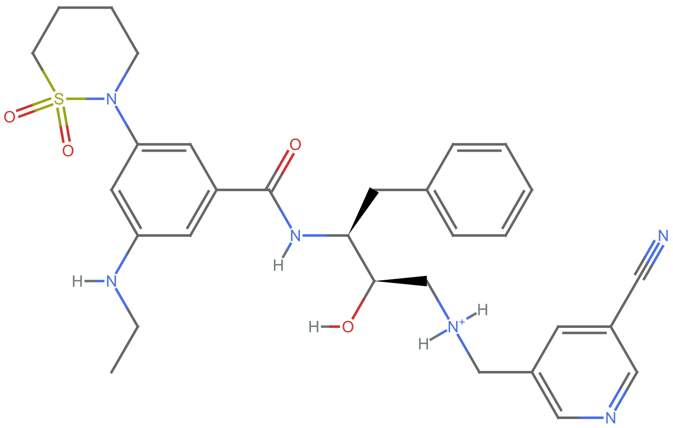

2D Autocorrelation

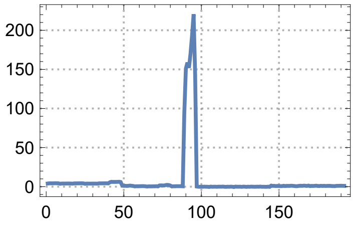

3D Plot

Dataset Index= 642

Molecule Formula= $C_{30}H_{37}N_6O_4S^+$

3D Autocorrelation

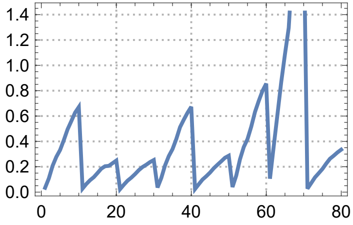

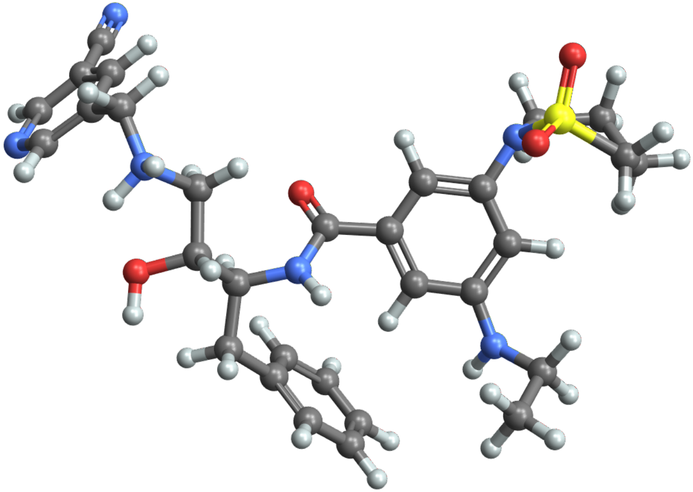

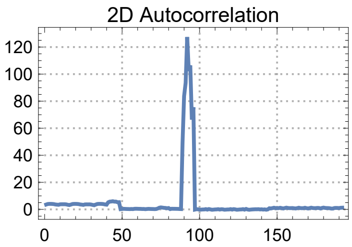

Dataset Index= 643

Molecule Formula= $C_{22}H_{26}N_4O_3$

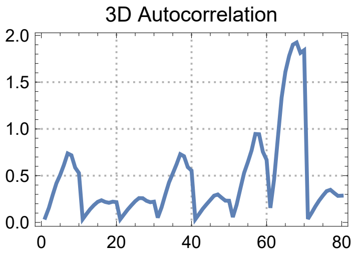

2D Plot

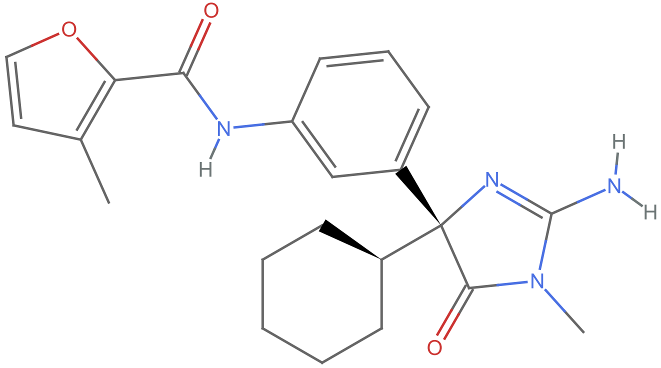

3D Plot

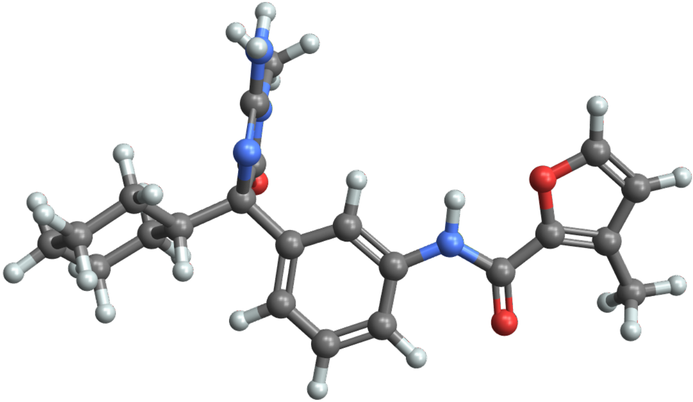

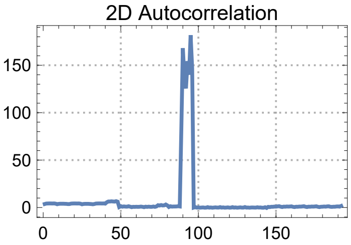

Dataset Index= 644

Molecule Formula= $C_{23}H_{19}F_3N_4O_2$

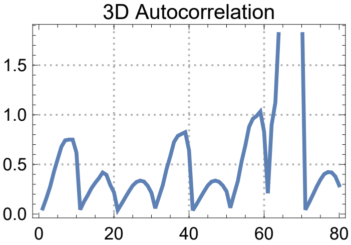

2D Plot

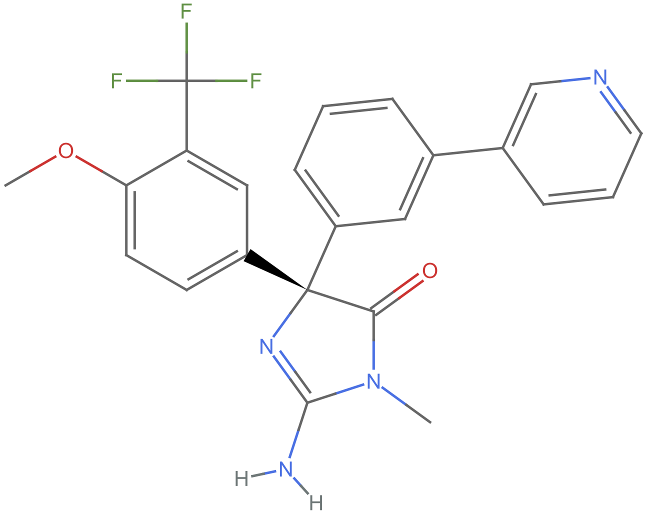

3D Plot

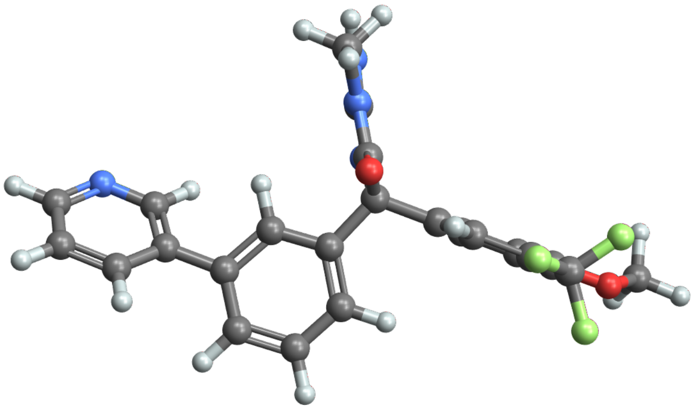

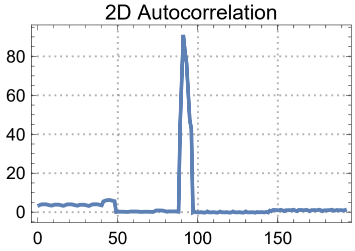

Dataset Index= 645

Molecule Formula= $C_{22}H_{17}N_5O$

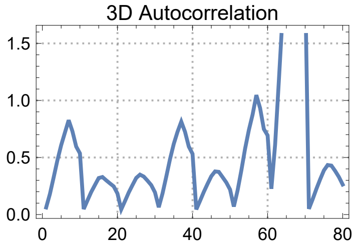

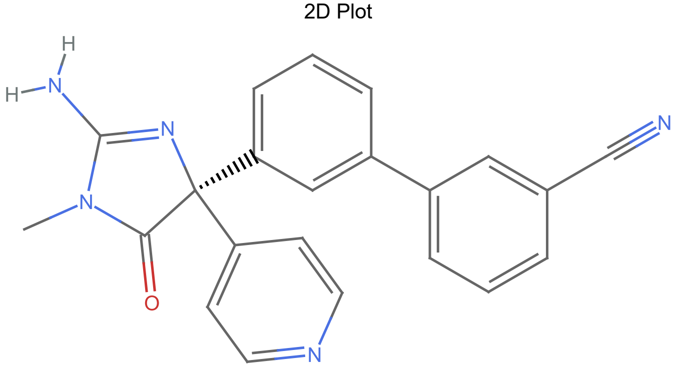

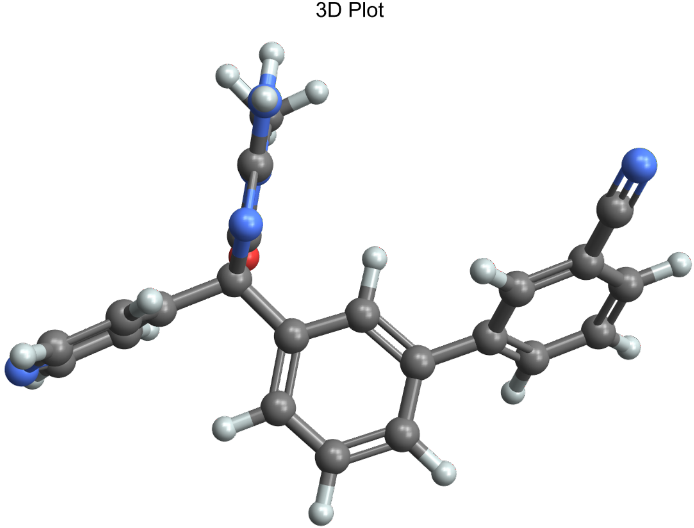

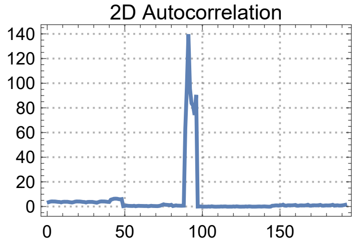

Dataset Index= 646

Molecule Formula= $C_{21}H_{16}F_2N_4O$

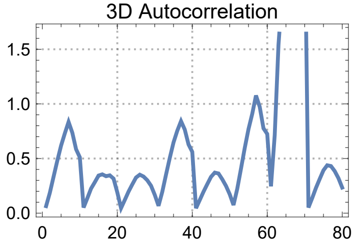

2D Plot

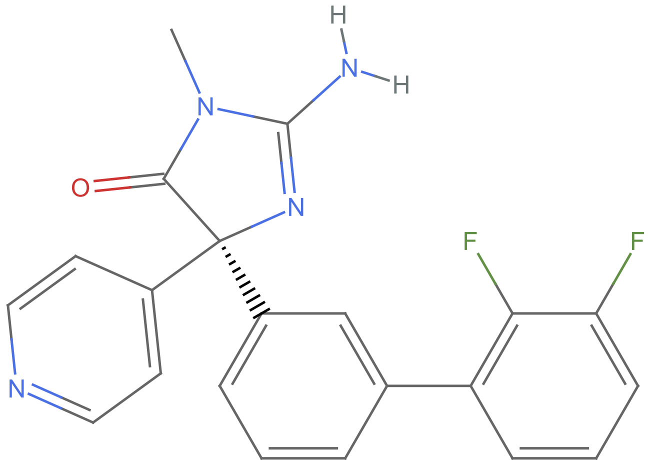

3D Plot

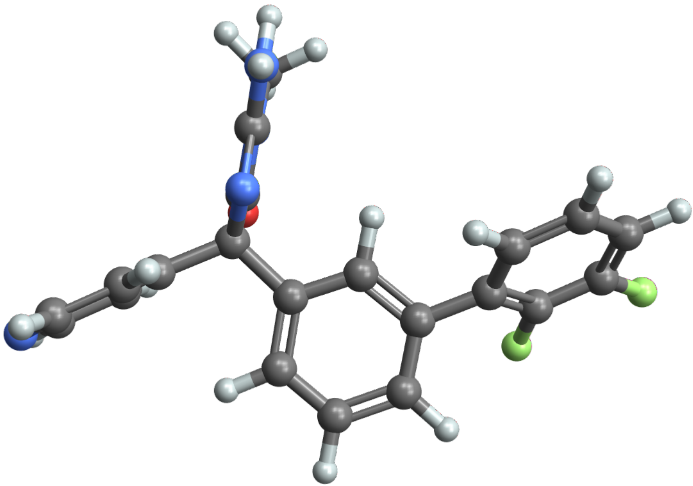

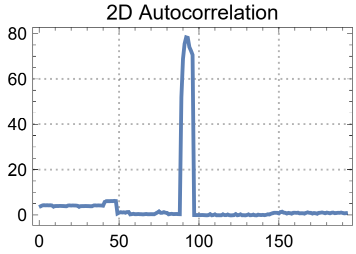

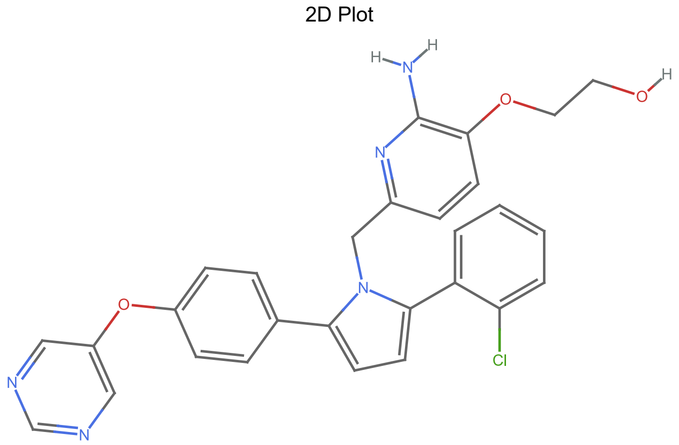

Dataset Index= 647

Molecule Formula= $C_{28}H_{24}ClN_5O_3$

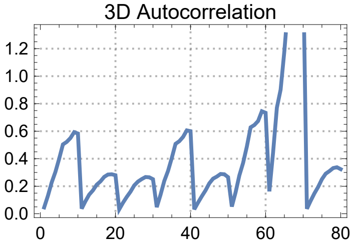

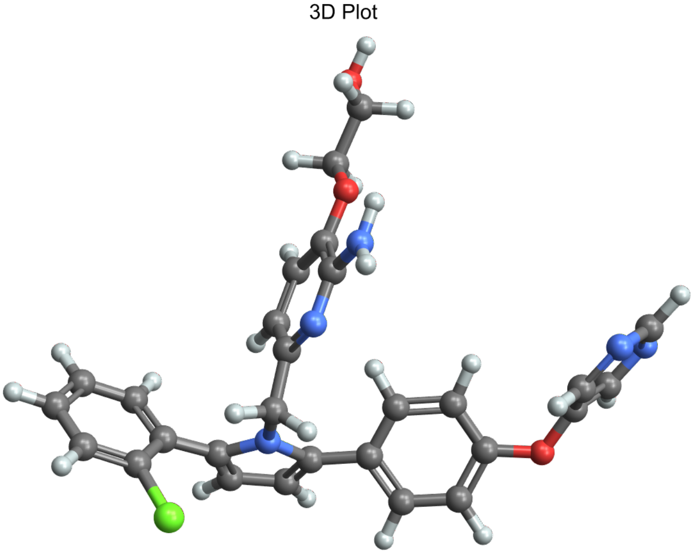

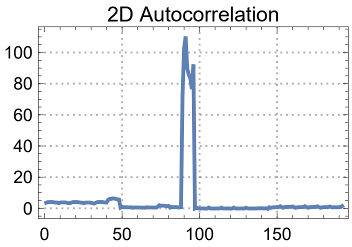

Dataset Index= 648

Molecule Formula= $C_{21}H_{20}F_3N_3O_2$

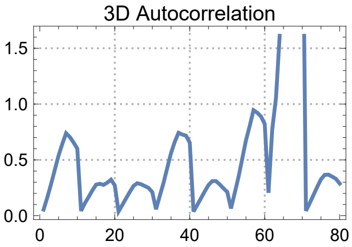

2D Plot

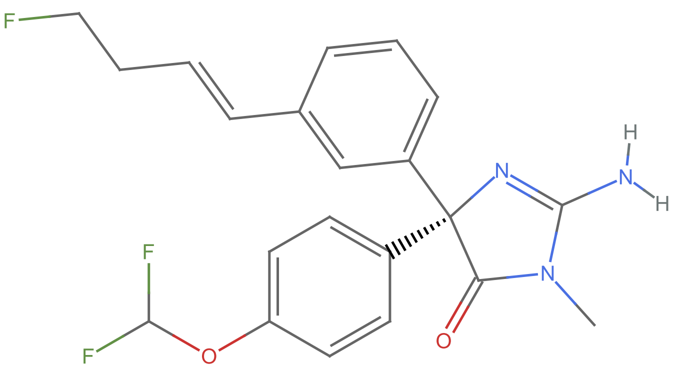

3D Plot

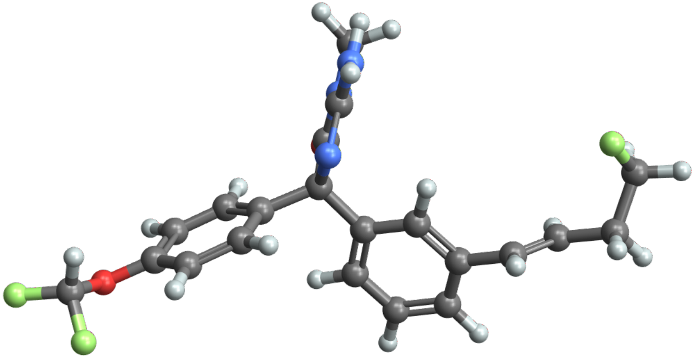

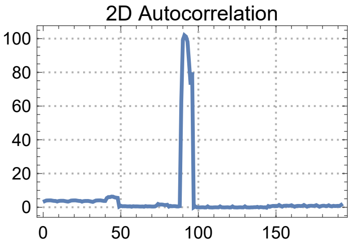

Dataset Index= 649

Molecule Formula= $C_{22}H_{23}F_2N_3O_3$

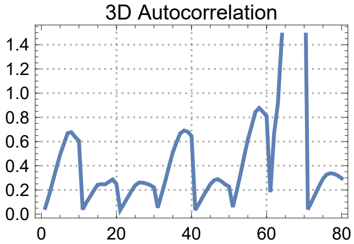

2D Plot

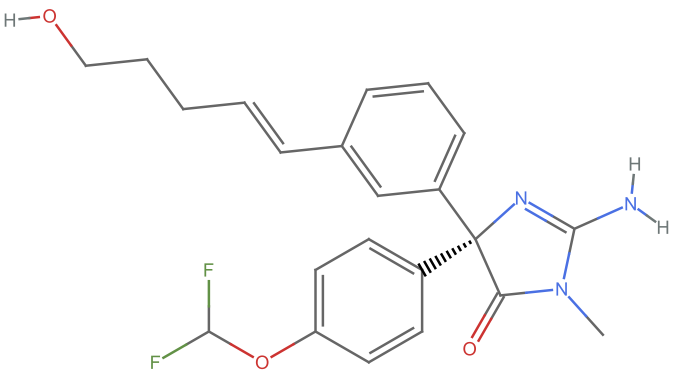

3D Plot

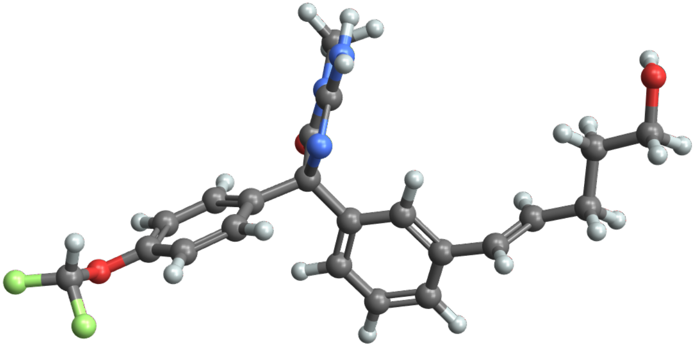

2D Autocorrelation

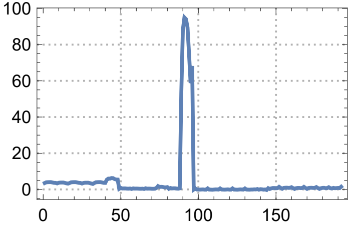

Dataset Index= 650

Molecule Formula= $C_{21}H_{19}F_2N_3O_2$

3D Autocorrelation

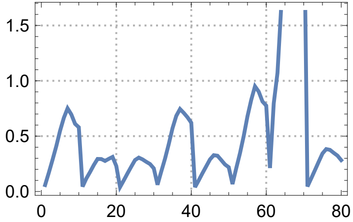

2D Plot

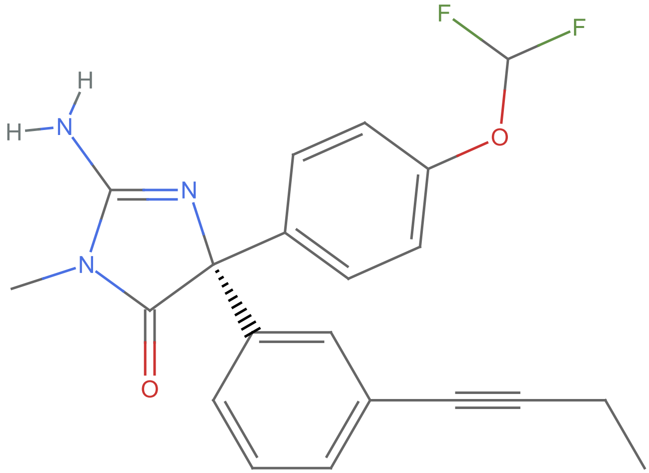

3D Plot

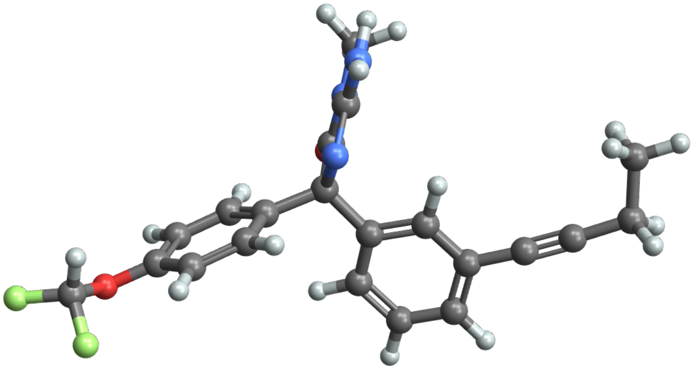

2D Autocorrelation

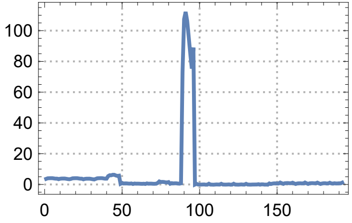

2D Plot

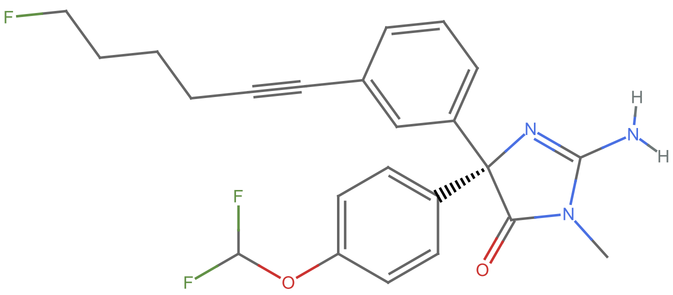

Dataset Index= 651

Molecule Formula= $C_{23}H_{22}F_{3}N_{3}O_{2}$

3D Autocorrelation

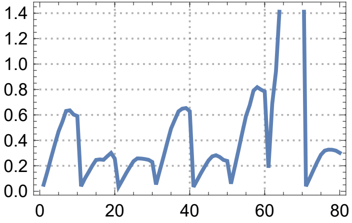

3D Plot

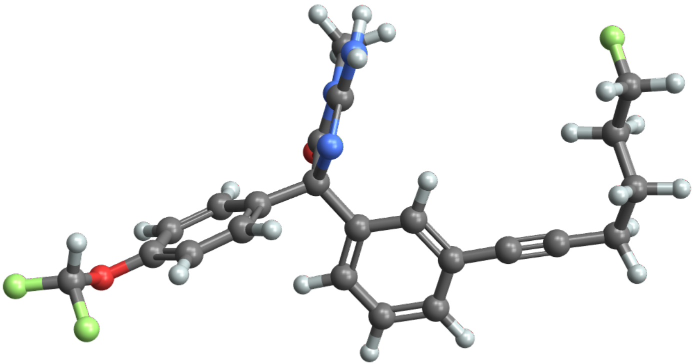

2D Plot

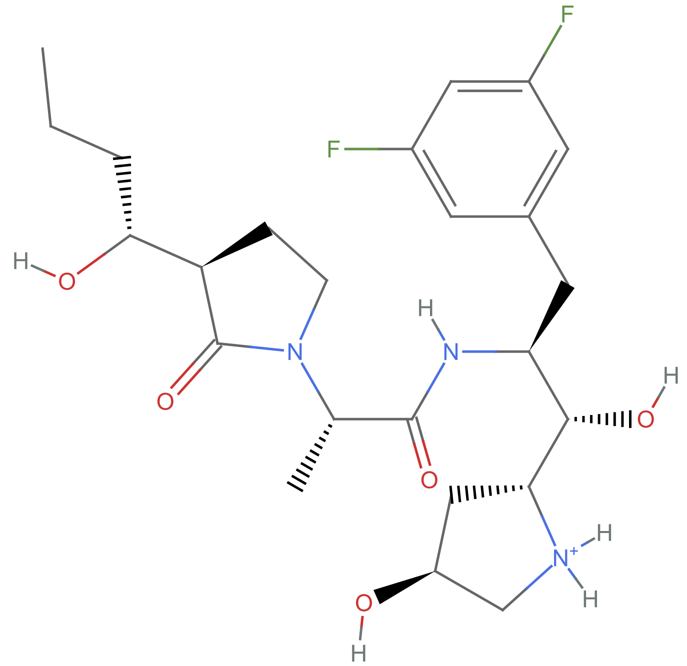

2D Autocorrelation

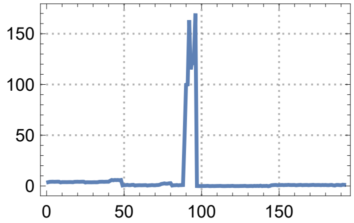

3D Plot

Dataset Index= 652

Molecule Formula= $C_{24}H_{36}F_2N_3O_5^+$

3D Autocorrelation

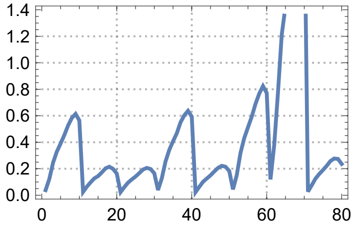

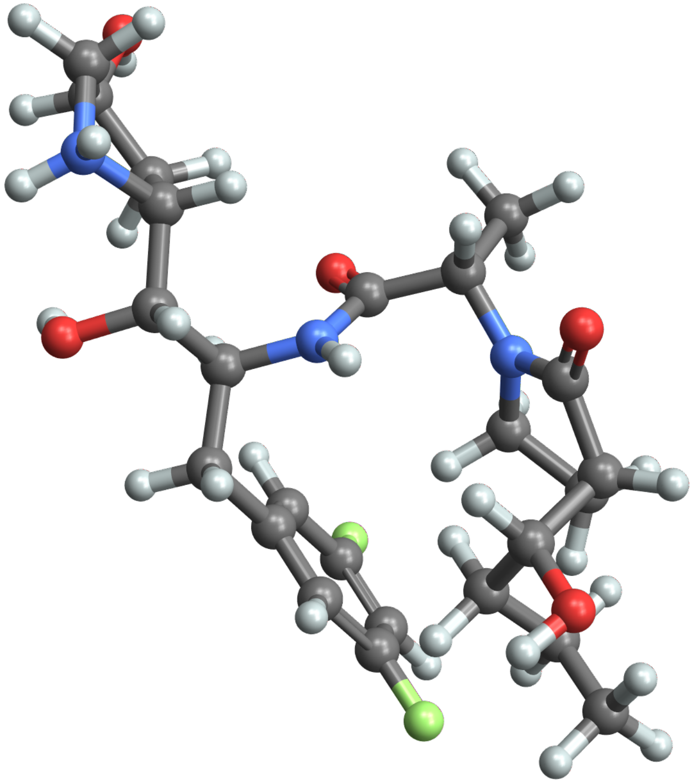

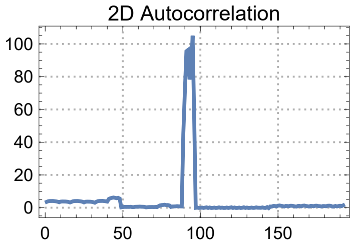

Dataset Index= 653

Molecule Formula= $C_{19}H_{18}FN_7O$

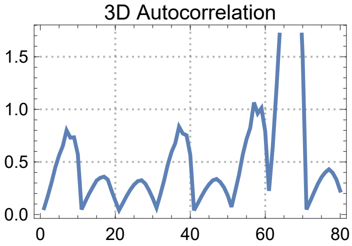

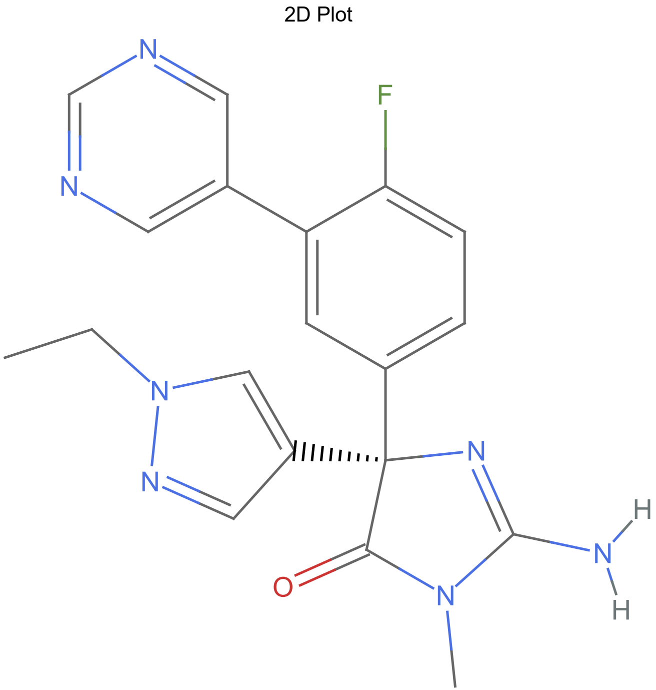

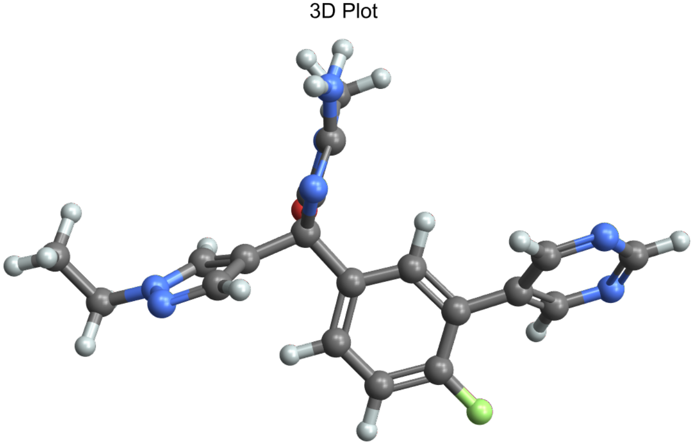

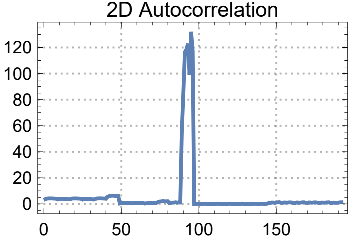

Dataset Index= 654

Molecule Formula= $C_{21}H_{20}F_2N_6O$

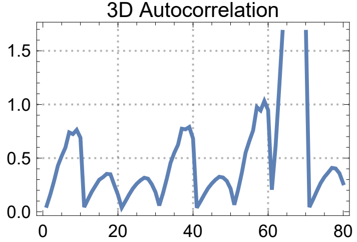

2D Plot

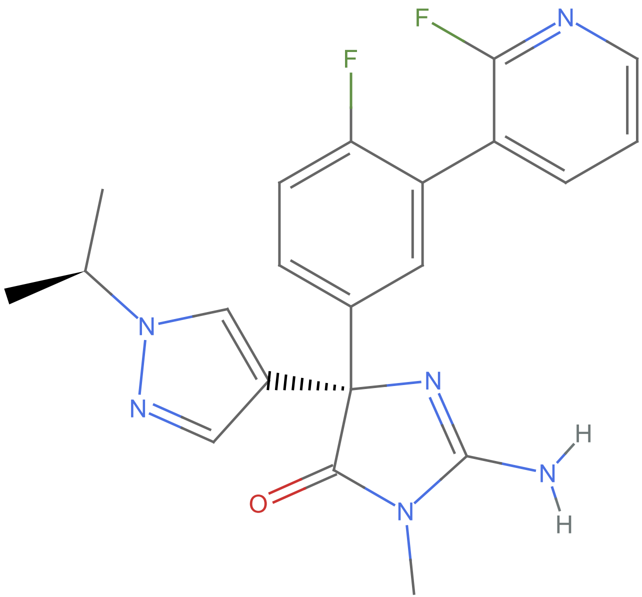

3D Plot

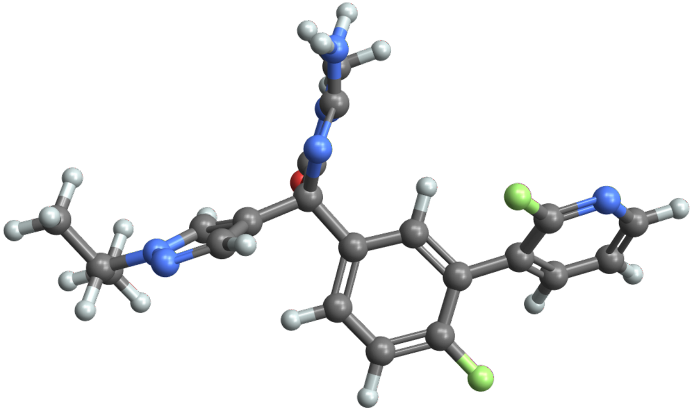

2D Plot

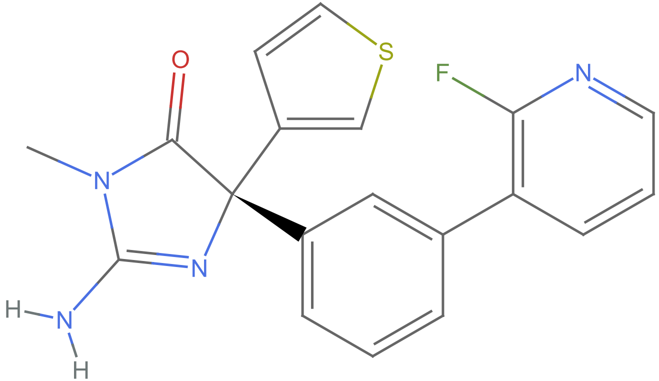

2D Autocorrelation

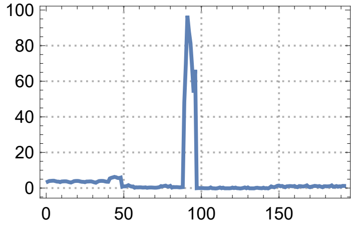

Dataset Index= 655

Molecule Formula= $C_{19}H_{15}FN_4OS$

3D Plot

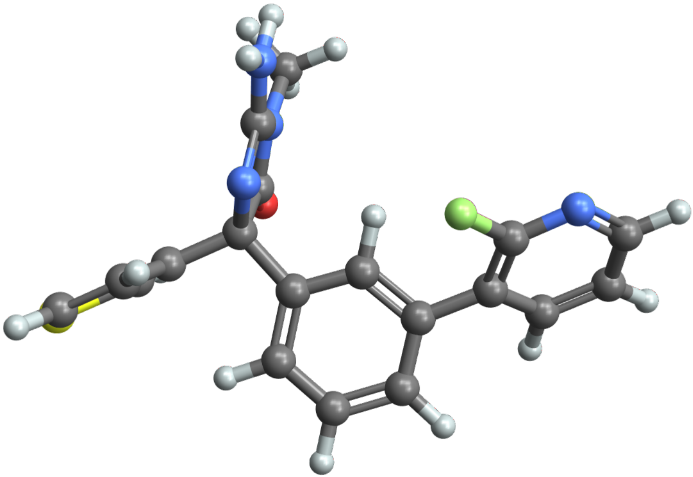

3D Autocorrelation

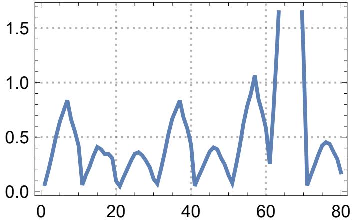

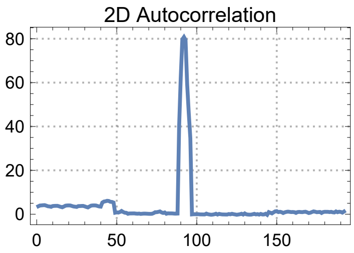

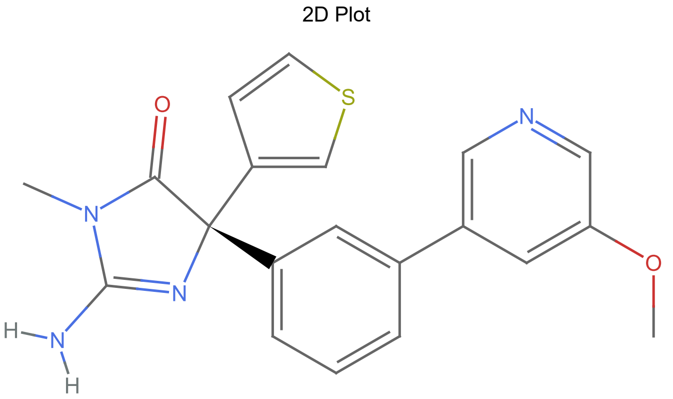

Dataset Index= 656

Molecule Formula= $C_{20}H_{18}N_4O_2S$

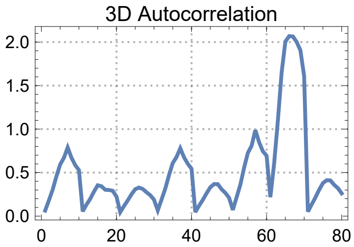

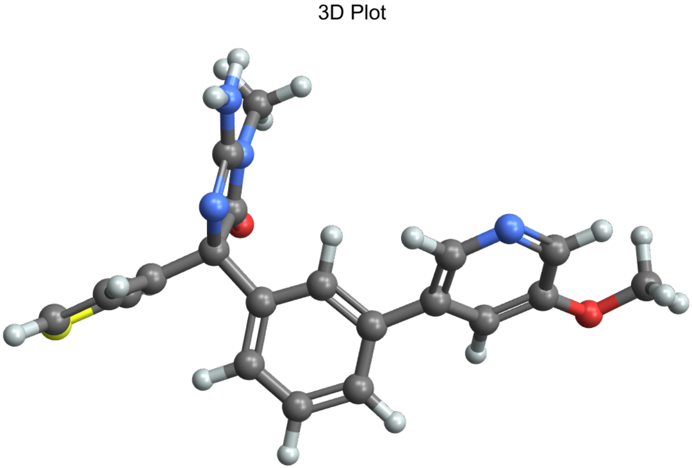

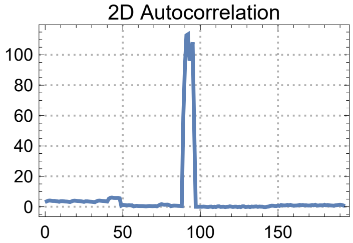

Dataset Index= 657

Molecule Formula= $C_{18}H_{17}ClFN_5O_2$

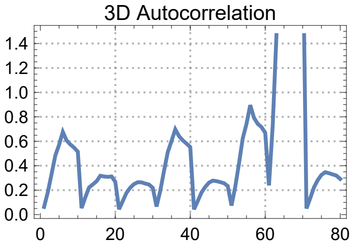

2D Plot

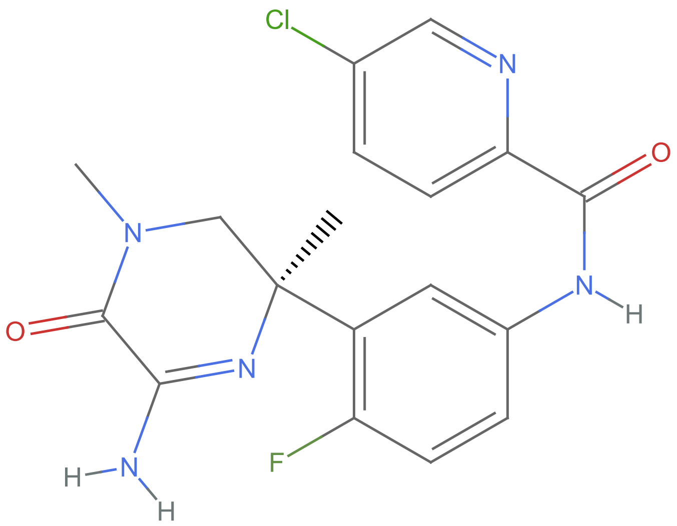

3D Plot

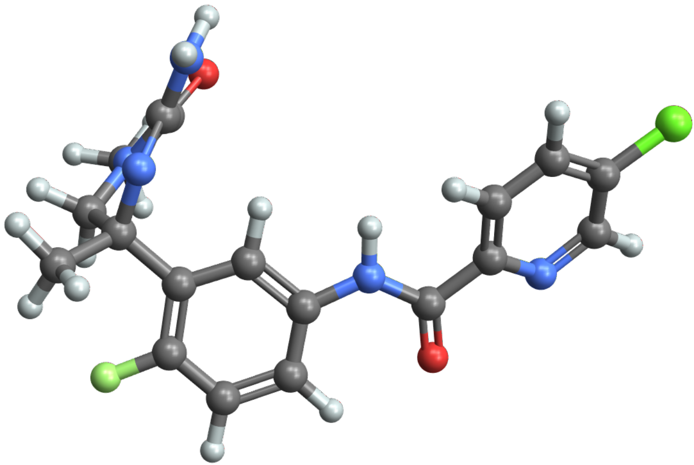

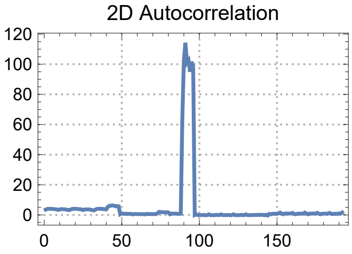

Dataset Index= 658

Molecule Formula= $C_{20}H_{16}F_3N_3O_2$

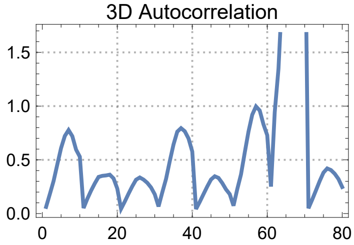

2D Plot

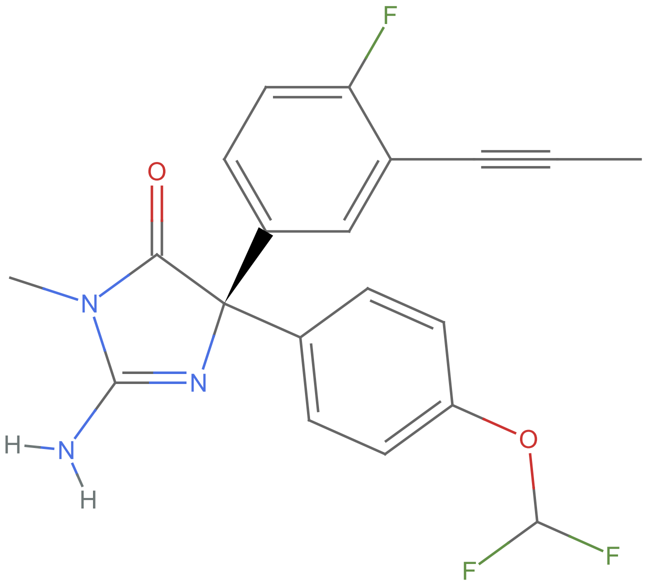

3D Plot

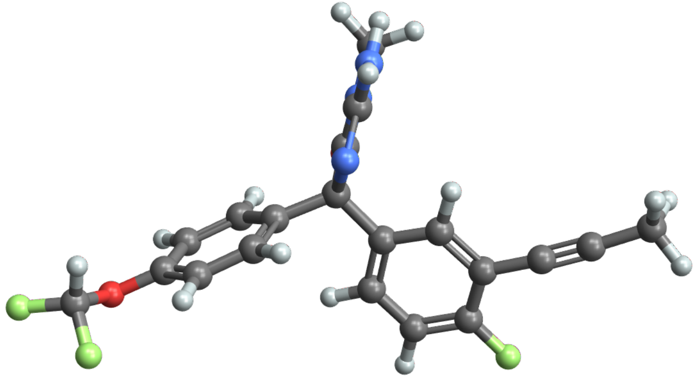

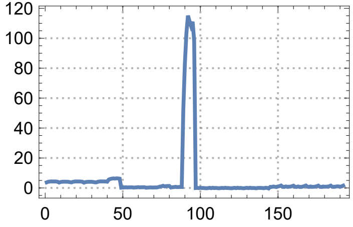

Dataset Index= 659

Molecule Formula= $C_{27}H_{20}FN_5O$

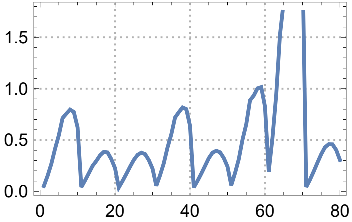

2D Plot

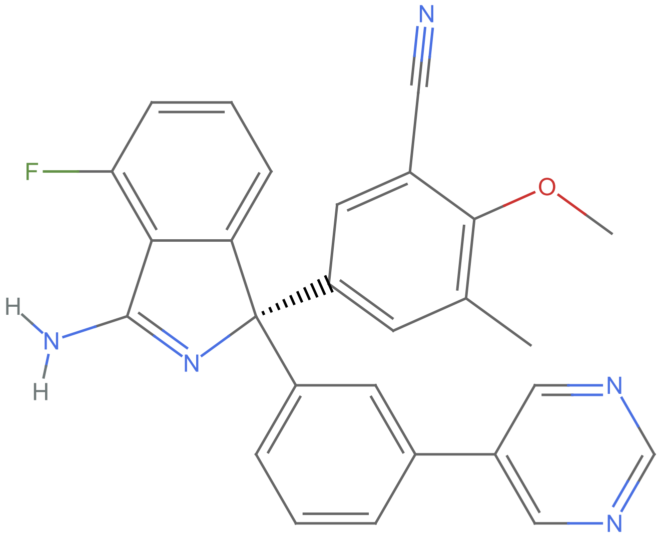

3D Plot

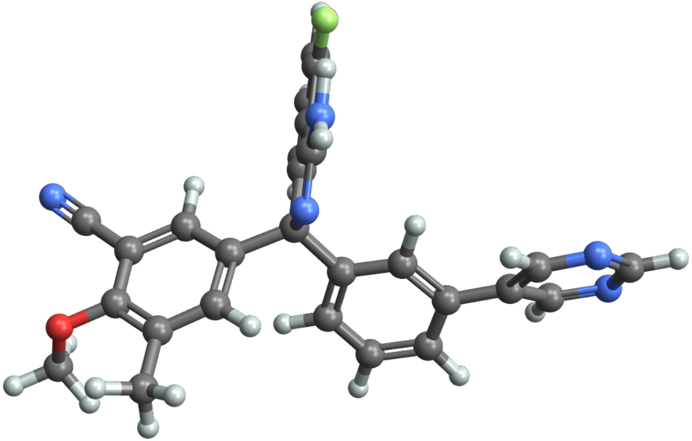

2D Plot

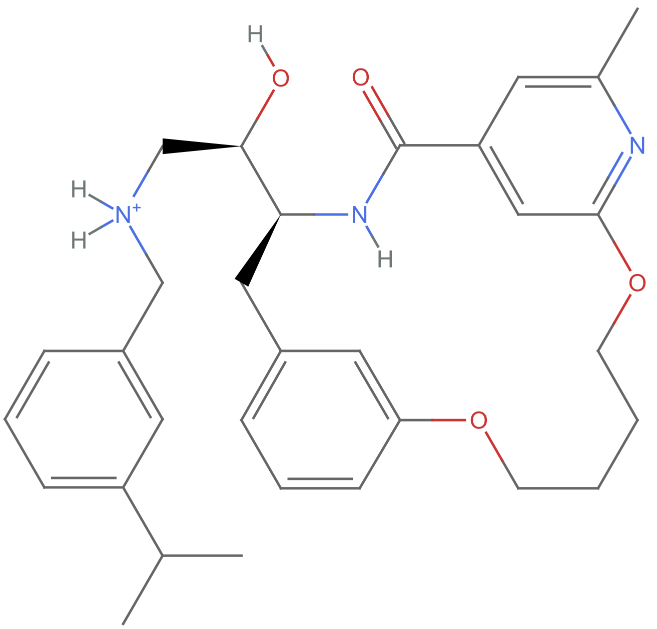

2D Autocorrelation

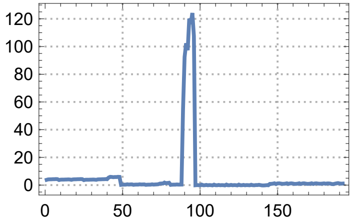

Dataset Index= 660

Molecule Formula= $C_{31}H_{40}N_3O_4^+$

3D Autocorrelation

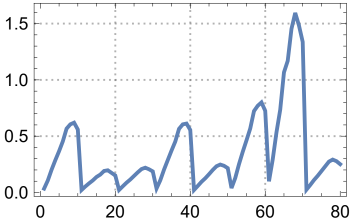

3D Plot

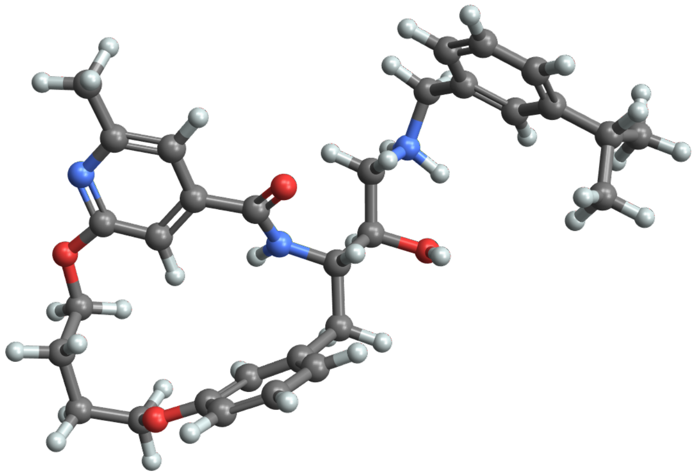

2D Autocorrelation

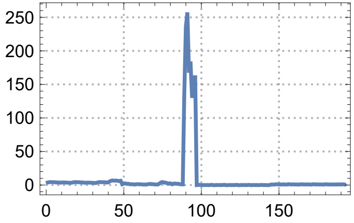

Dataset Index= 661

Molecule Formula= $C_{19}H_{15}F_{7}N_{4}O_{3}$

3D Autocorrelation

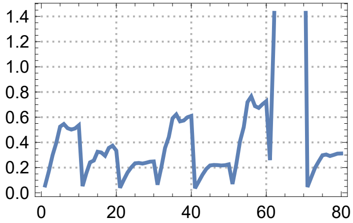

2D Plot

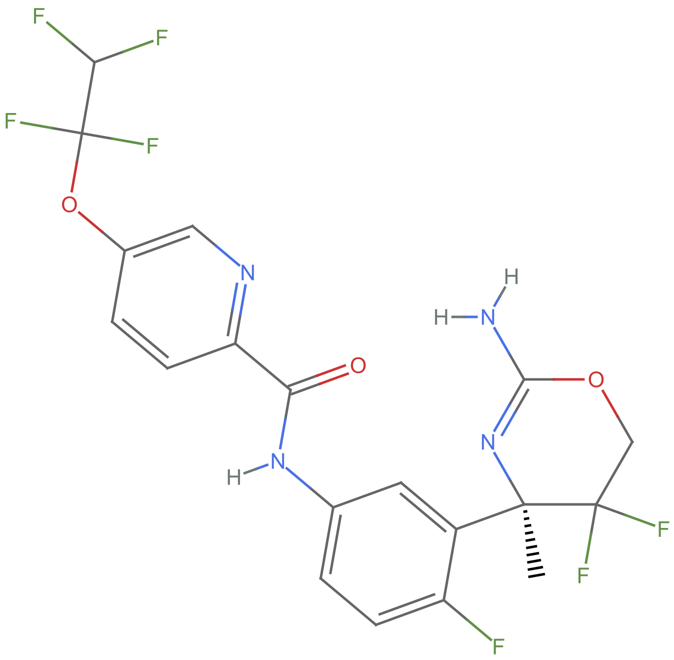

3D Plot

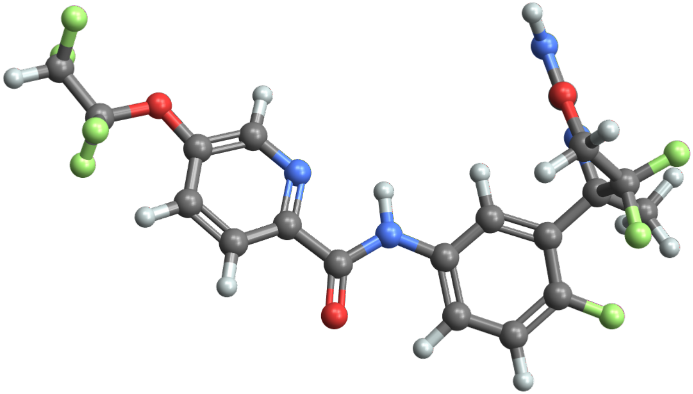

2D Plot

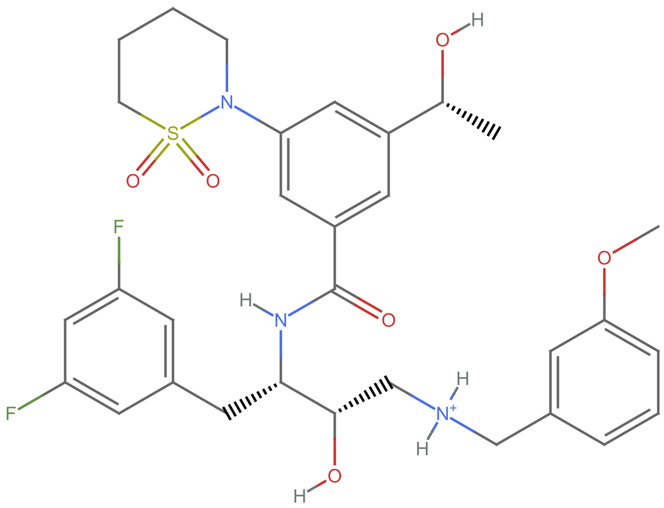

2D Autocorrelation

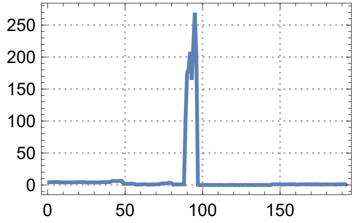

3D Plot

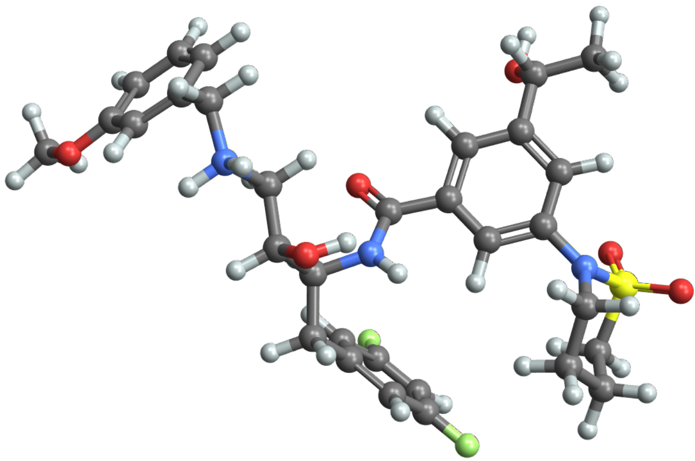

Dataset Index= 662

Molecule Formula= $C_{31}H_{38}F_2N_3O_6S^+$

3D Autocorrelation

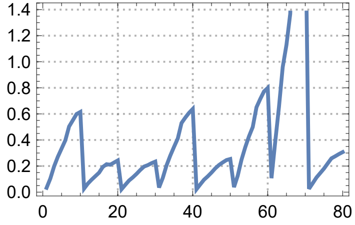

2D Plot

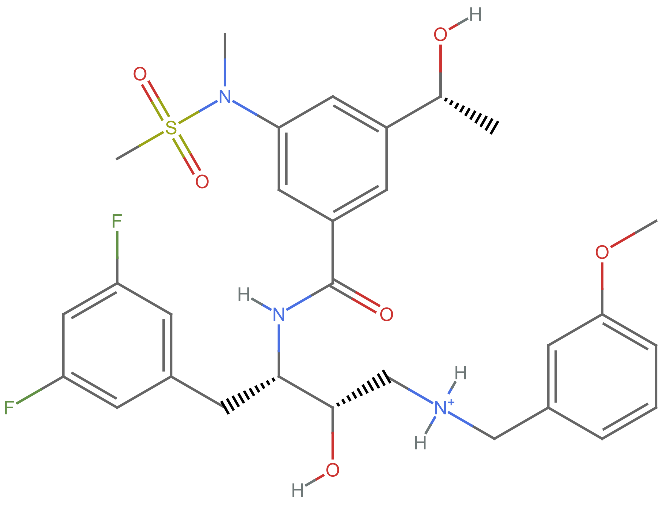

2D Autocorrelation

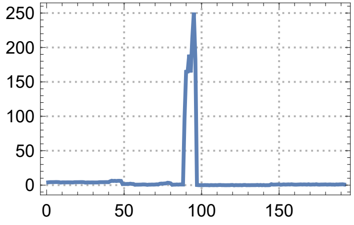

3D Plot

Dataset Index= 663

Molecule Formula= $C_{29}H_{36}F_2N_3O_6S^+$

3D Autocorrelation

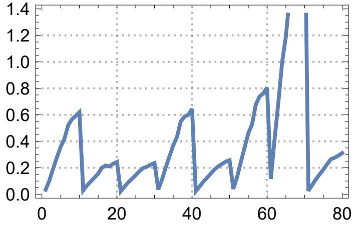

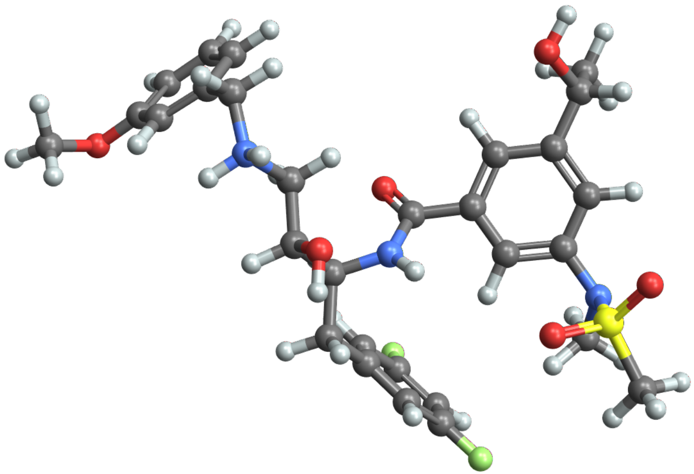

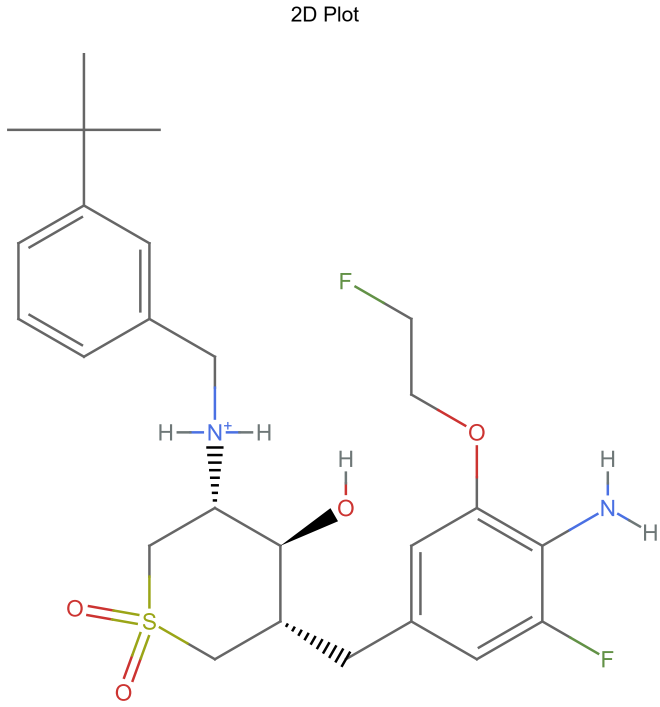

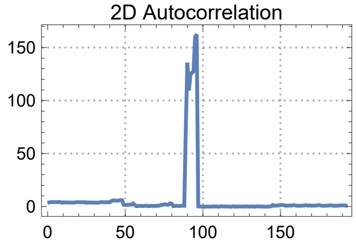

Dataset Index= 664

Molecule Formula= $C_{25}H_{35}F_2N_2O_4S^+$

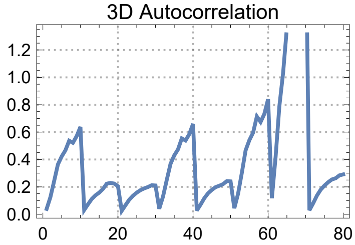

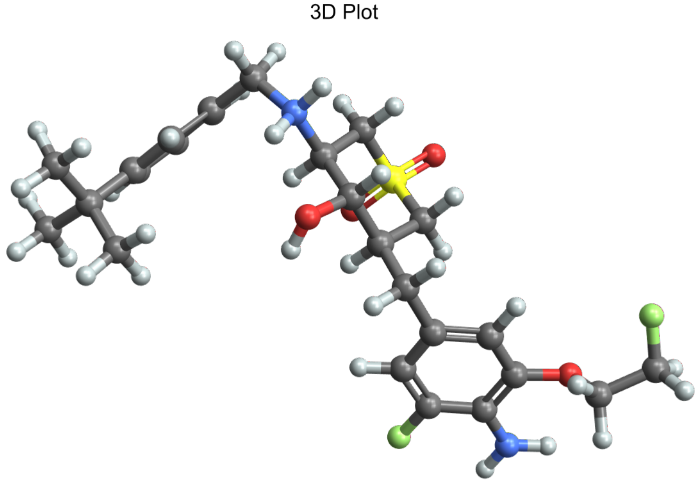

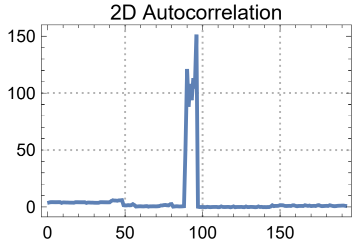

Dataset Index= 665

Molecule Formula= $C_{26}H_{38}FN_2O_4S^+$

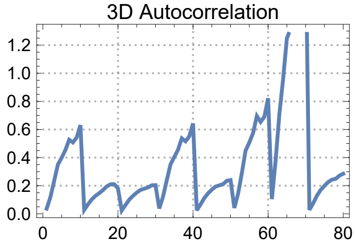

2D Plot

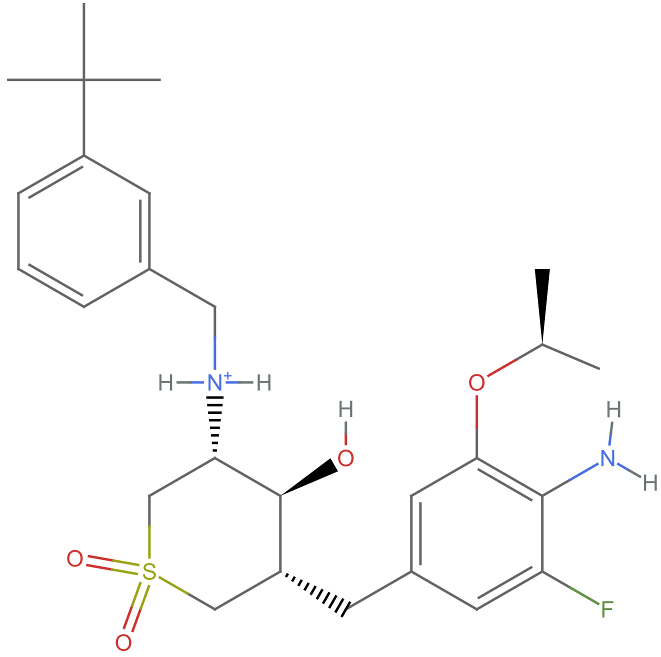

3D Plot

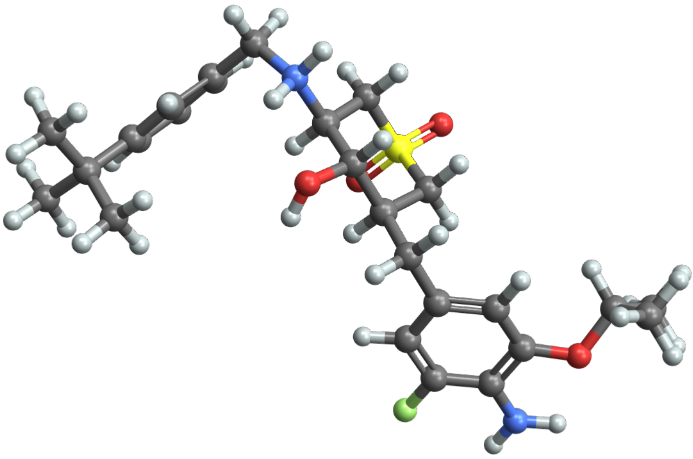

2D Plot

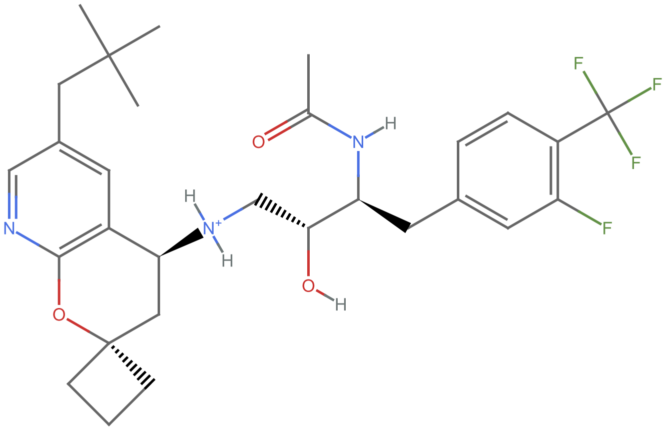

2D Autocorrelation

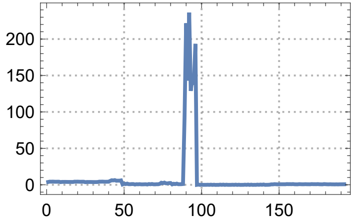

Dataset Index= 666

Molecule Formula= $C_{29}H_{38}F_4N_3O_3^+$

3D Autocorrelation

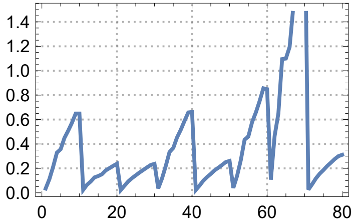

3D Plot

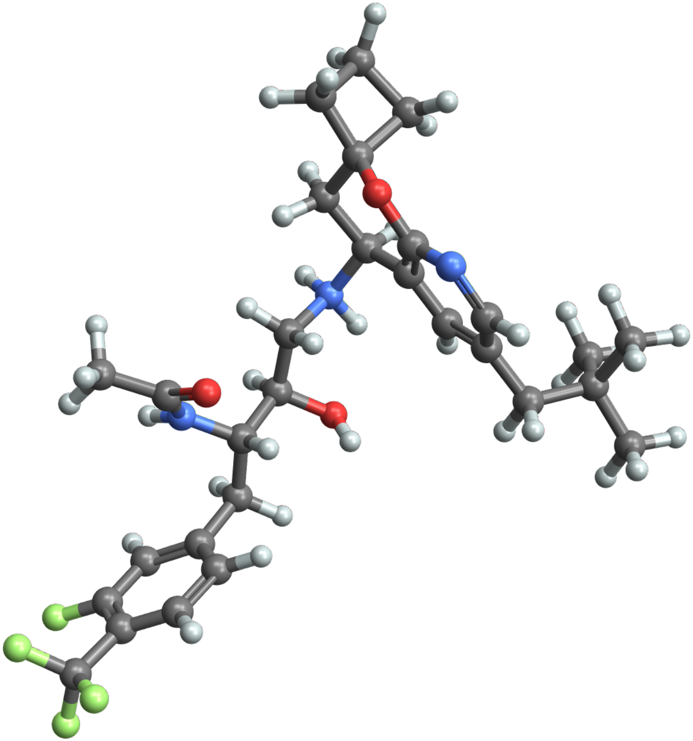

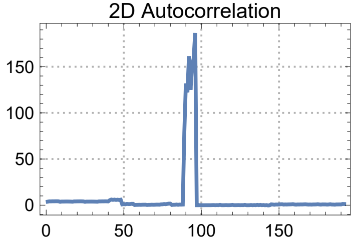

Dataset Index= 667

Molecule Formula= $C_{28}H_{33}F_{2}N_{2}O_{2}S^{+}$

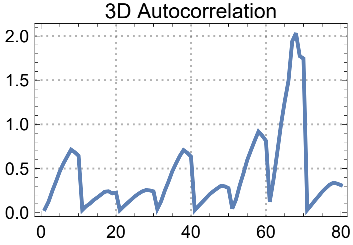

2D Plot

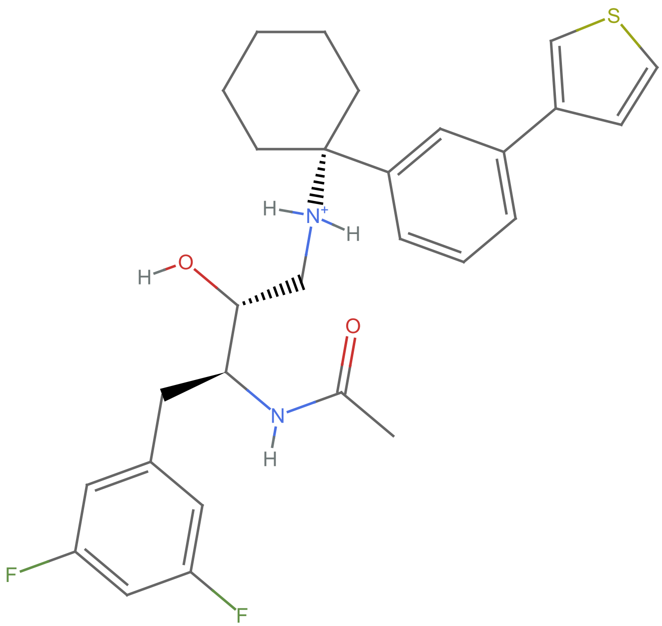

3D Plot

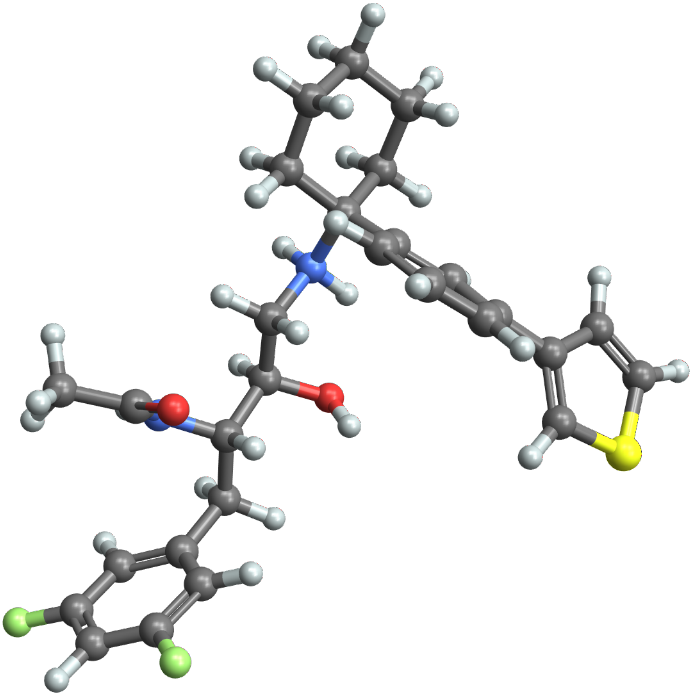

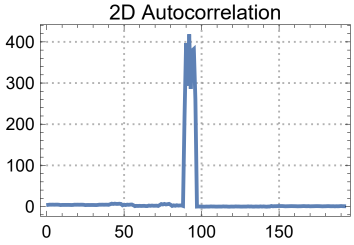

Dataset Index=   668

Molecule Formula= $C_{23}H_{23}F_{10}N_2O_5S^+$

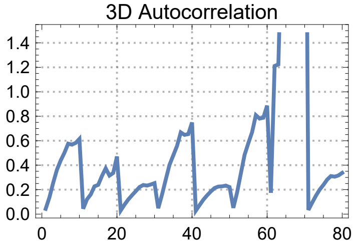

2D Plot

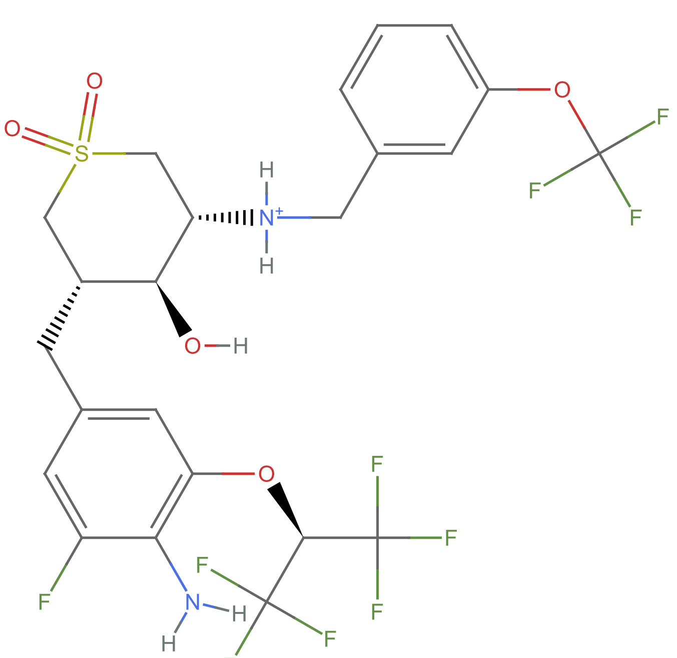

3D Plot

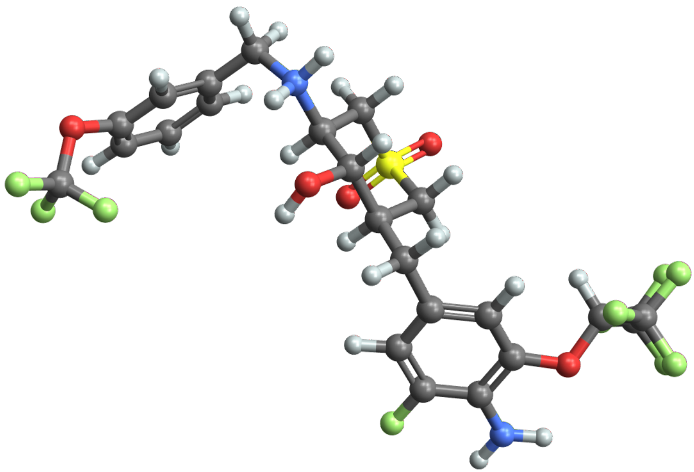

2D Plot

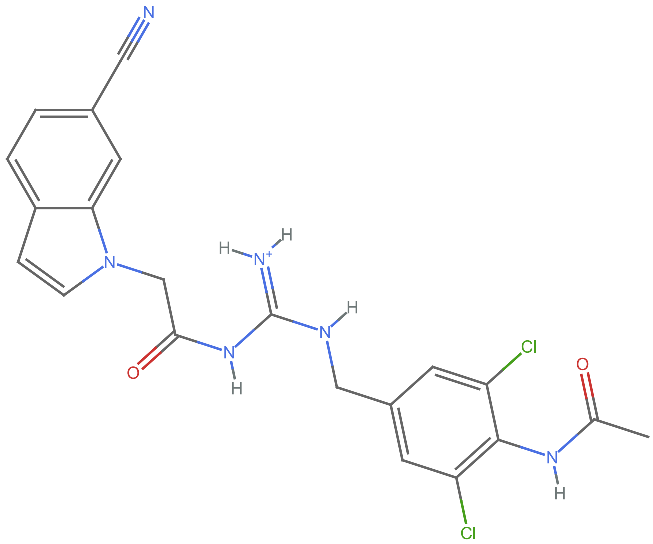

3D Plot

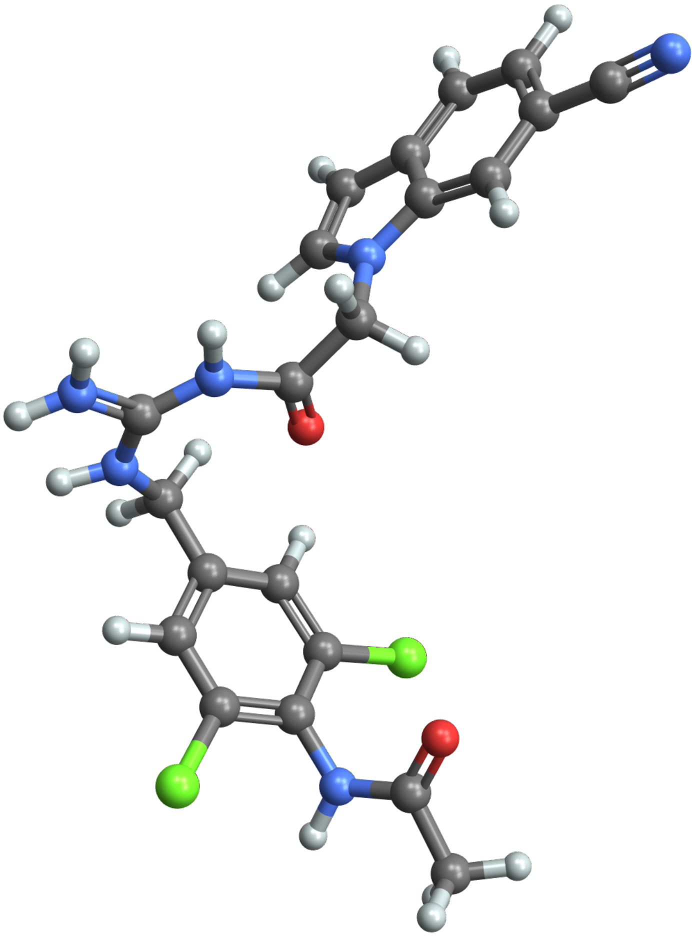

2D Autocorrelation

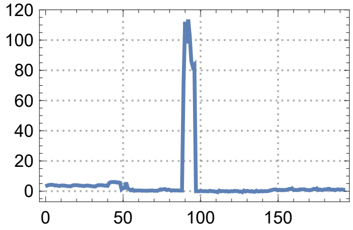

Dataset Index= 669

Molecule Formula= $C_{21}H_{19}Cl_2N_6O_2^+$

3D Autocorrelation

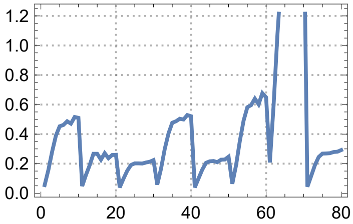

2D Plot

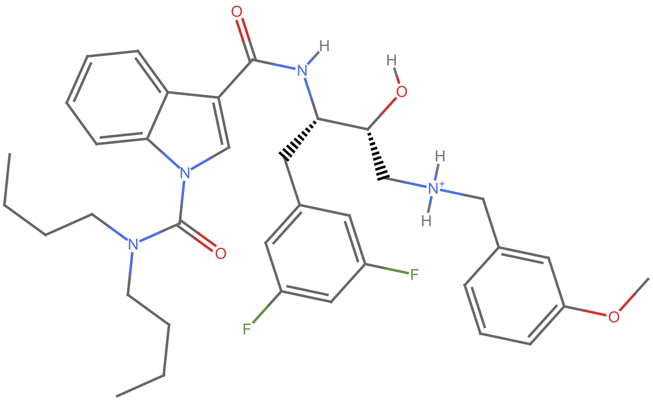

2D Autocorrelation

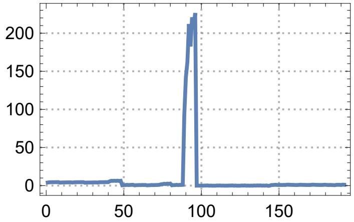

Dataset Index= 670

Molecule Formula= $C_{36}H_{45}F_{2}N_{4}O_{4}^{+}$

3D Autocorrelation

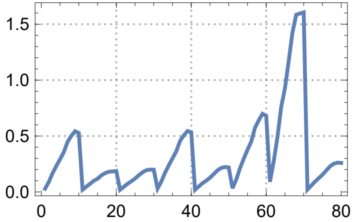

3D Plot

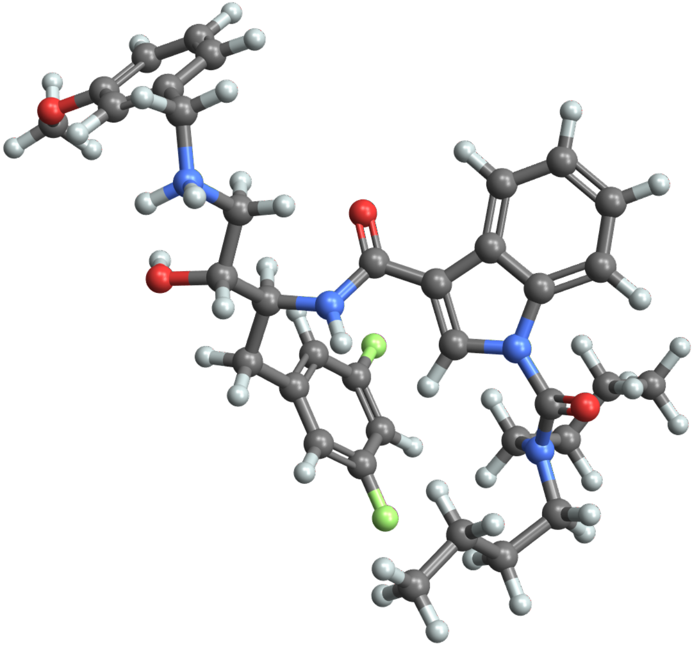

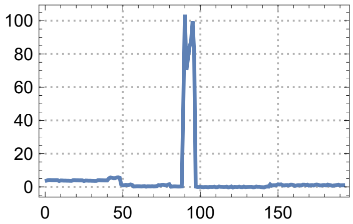

Dataset Index= 671

Molecule Formula= $C_{26}H_{39}N_2O_4S^+$

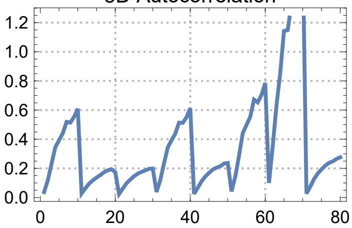

2D Plot

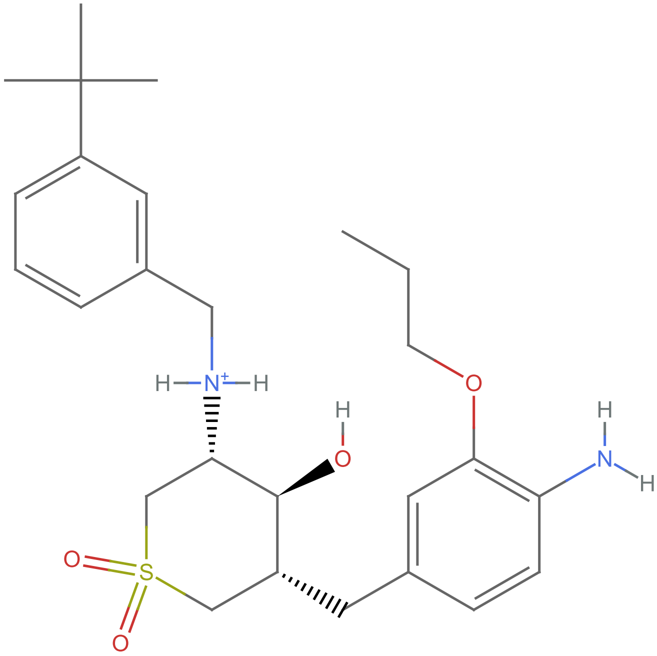

3D Plot

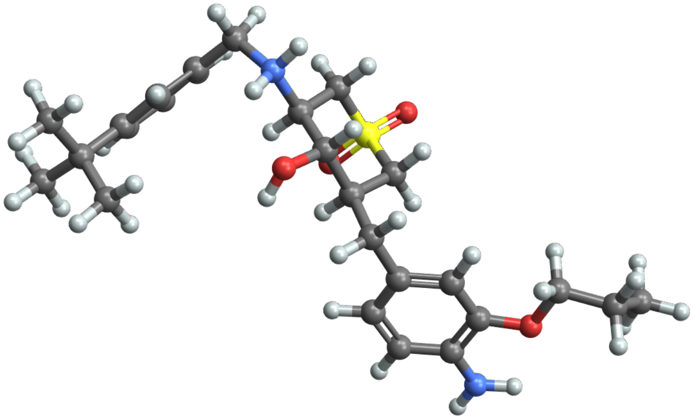

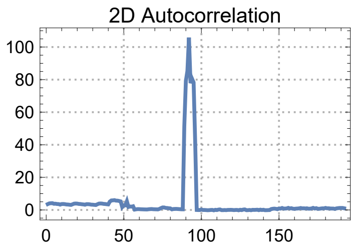

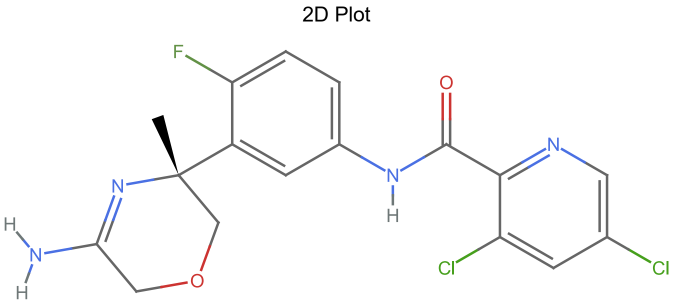

Dataset Index= 672

Molecule Formula= $C_{17}H_{15}Cl_2FN_4O_2$

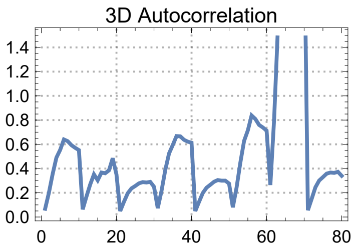

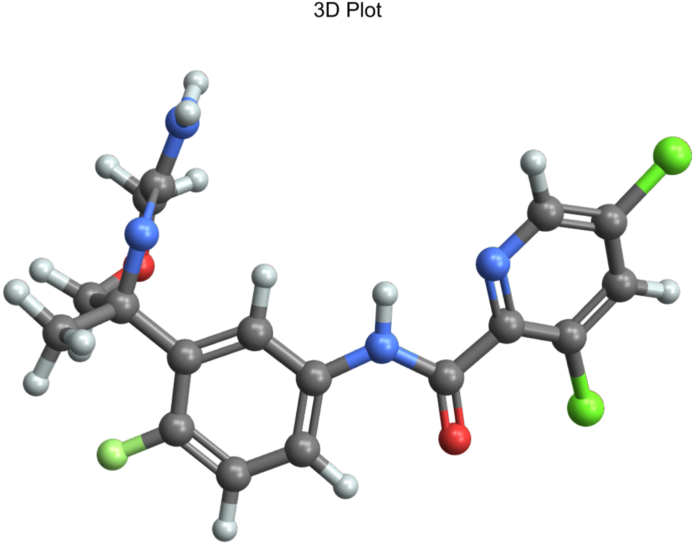

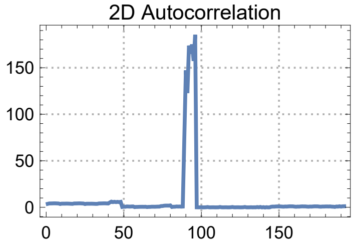

Dataset Index= 673

Molecule Formula= $C_{28}H_{38}F_{3}N_{2}O_{2}^{+}$

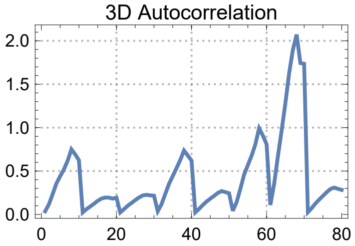

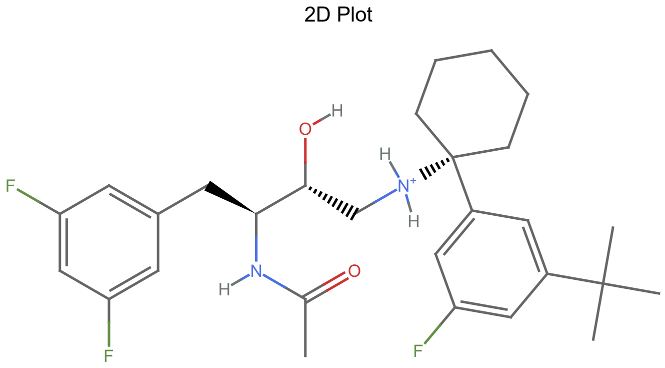

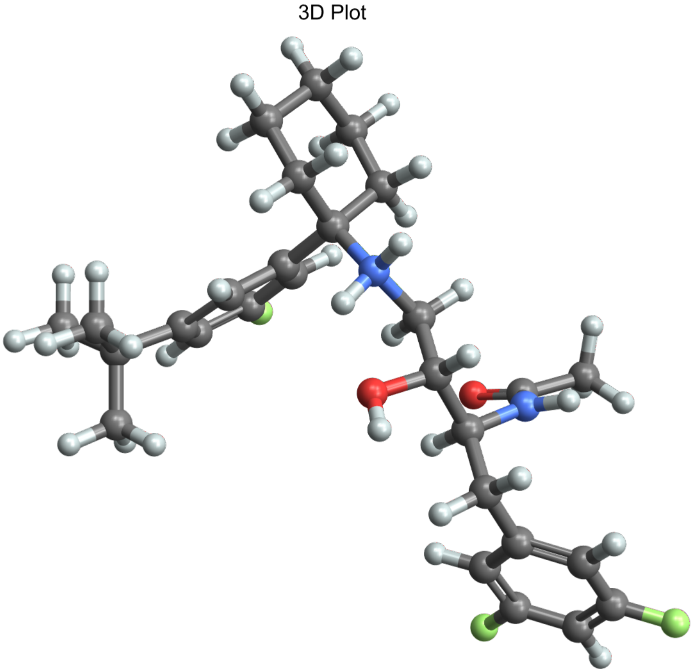

2D Plot

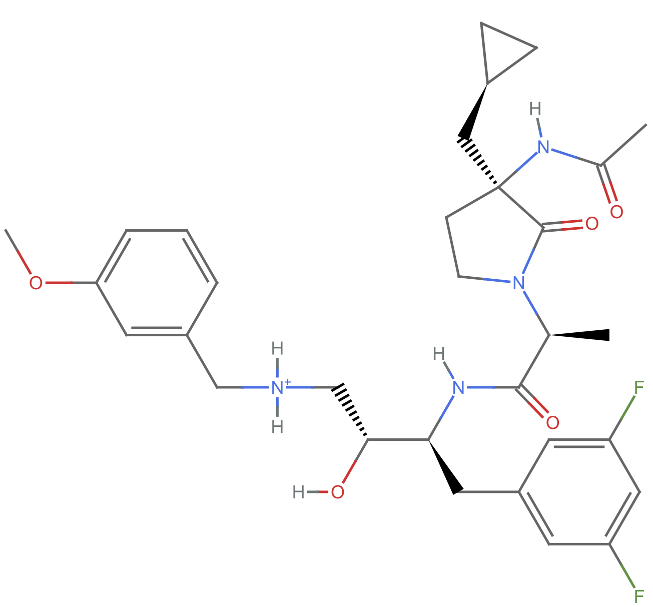

2D Autocorrelation

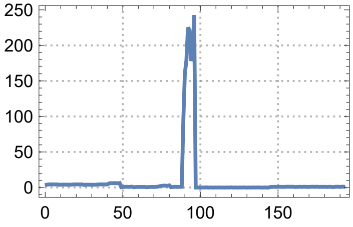

Dataset Index= 674

Molecule Formula= $C_{31}H_{41}F_2N_4O_5^+$

3D Autocorrelation

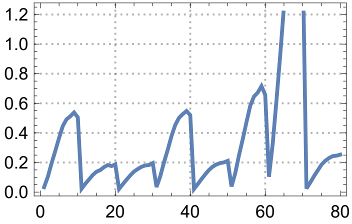

3D Plot

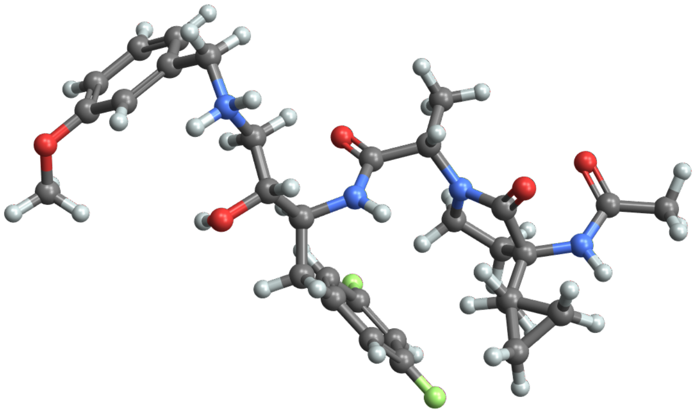

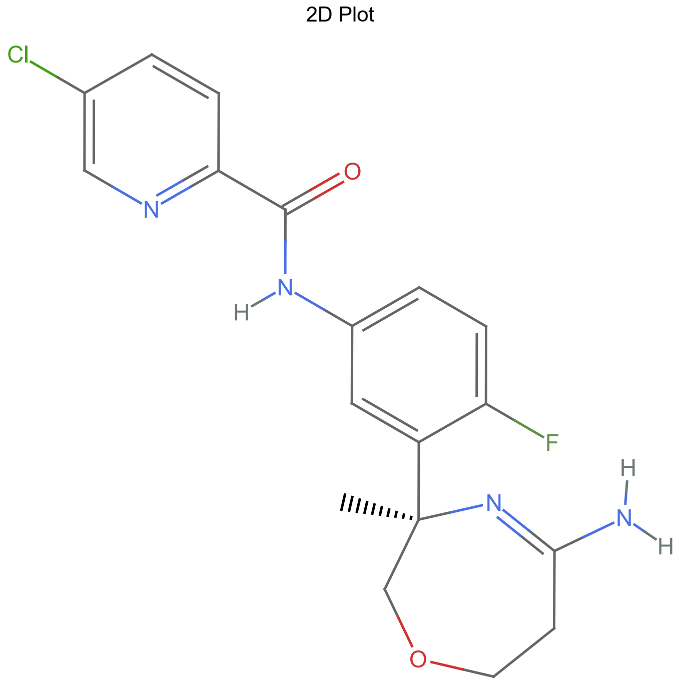

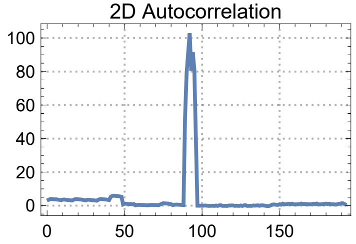

Dataset Index= 675

Molecule Formula= $C_{18}H_{18}ClFN_4O_2$

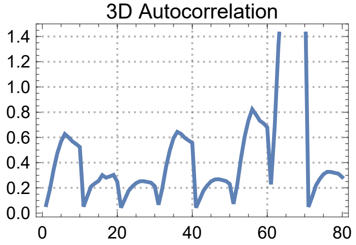

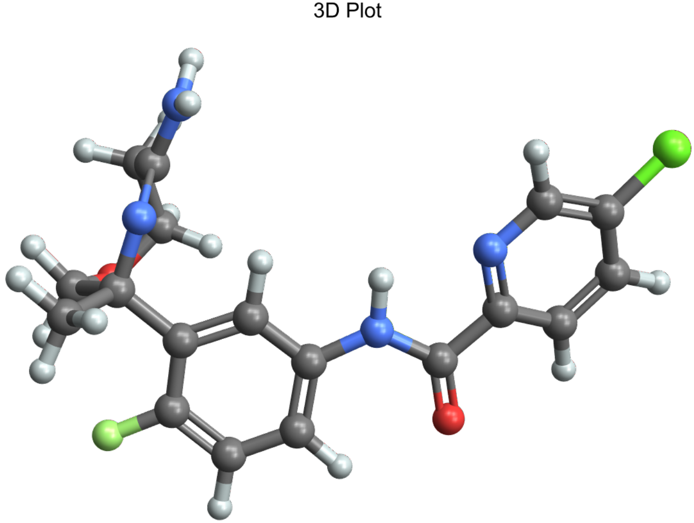

2D Plot

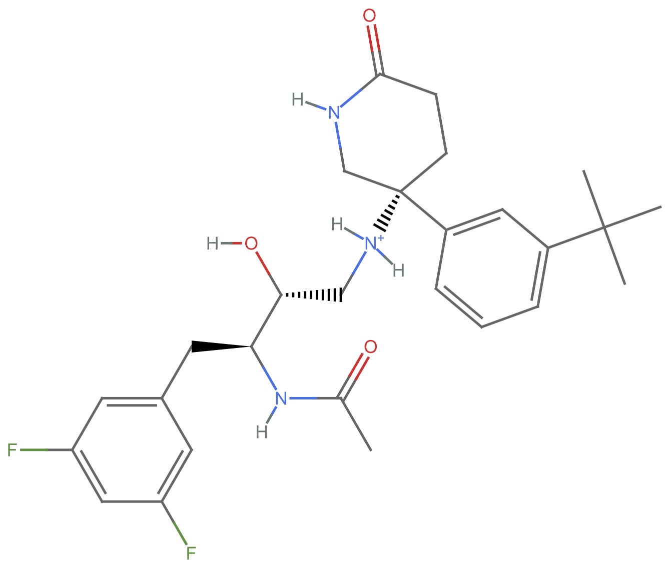

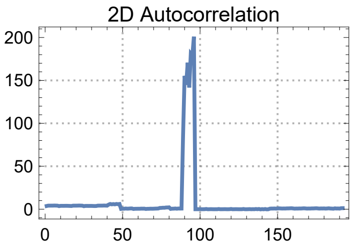

Dataset Index= 676

Molecule Formula= $C_{27}H_{36}F_2N_3O_3^+$

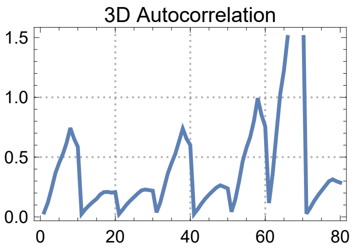

3D Plot

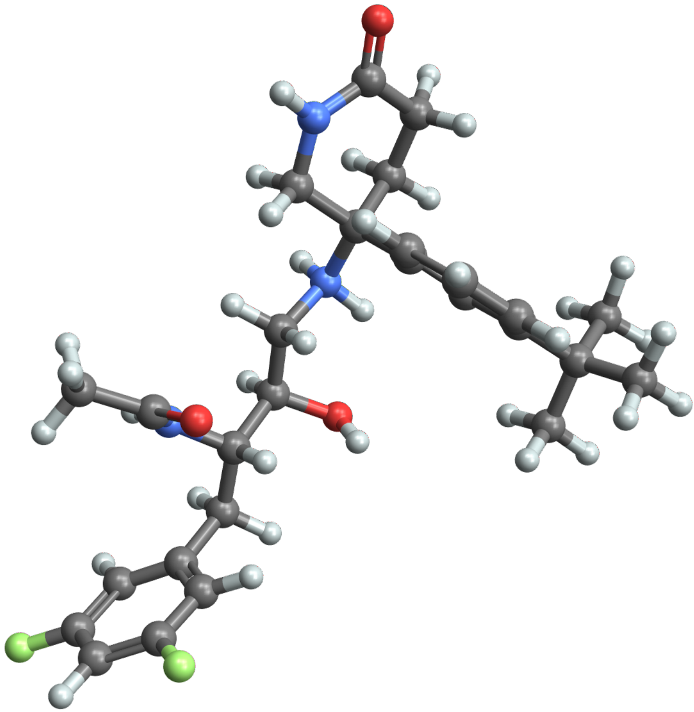

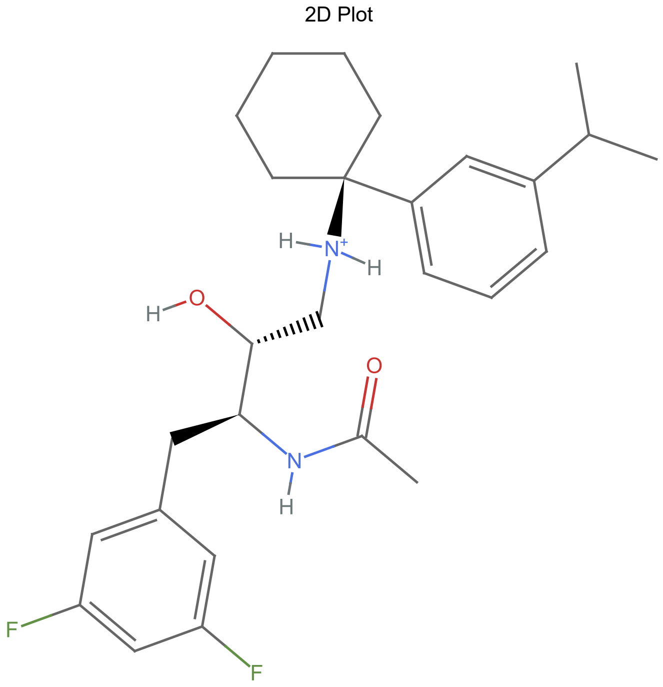

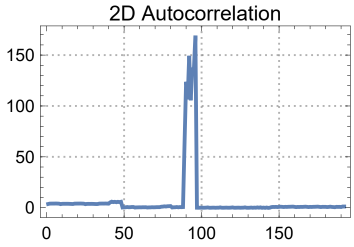

Dataset Index= 677

Molecule Formula= $C_{27}H_{37}F_2N_2O_2^+$

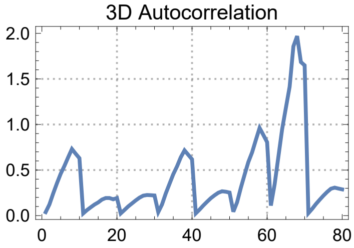

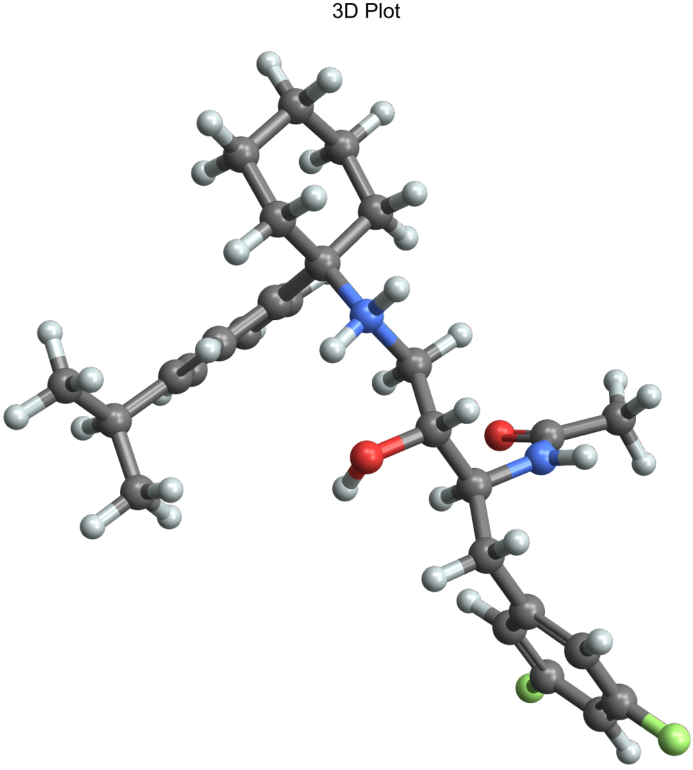

2D Autocorrelation

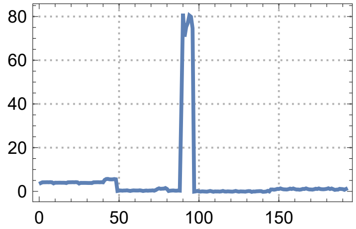

Dataset Index= 678

Molecule Formula= $C_{31}H_{42}N_3O_4^+$

3D Autocorrelation

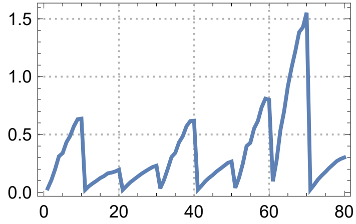

2D Plot

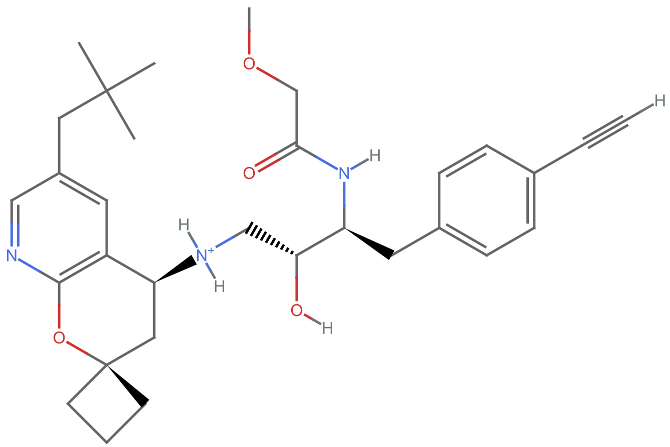

3D Plot

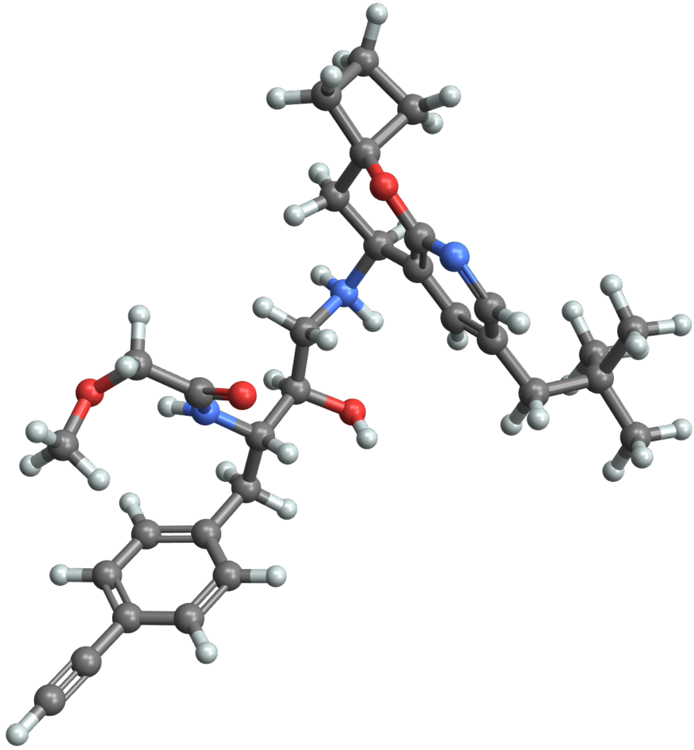

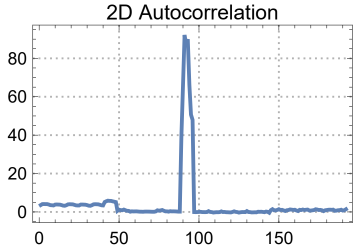

Dataset Index= 679

Molecule Formula= $C_{21}H_{20}N_4OS$

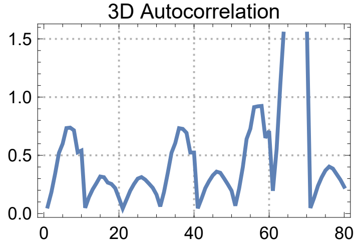

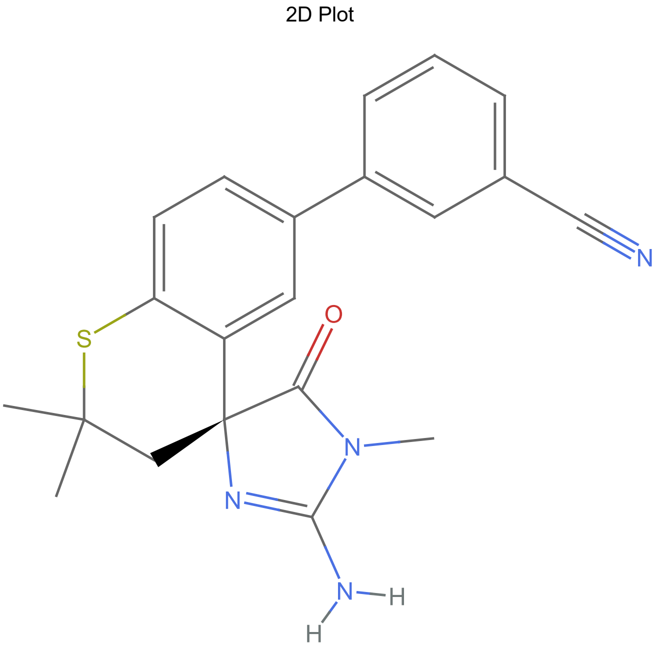

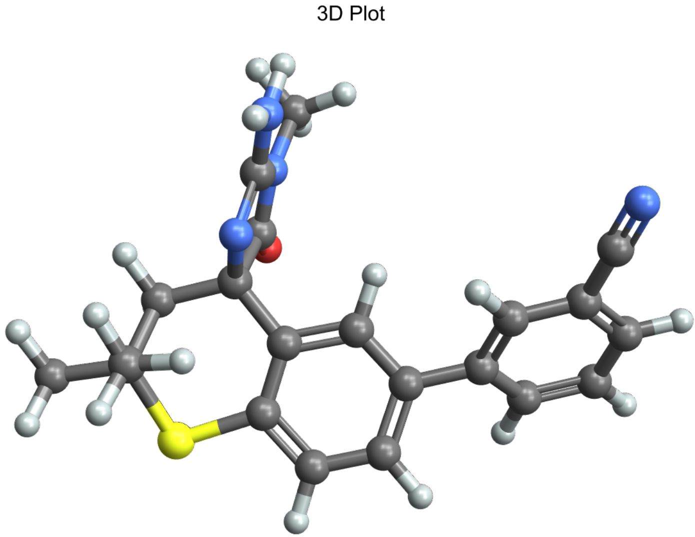

2D Plot

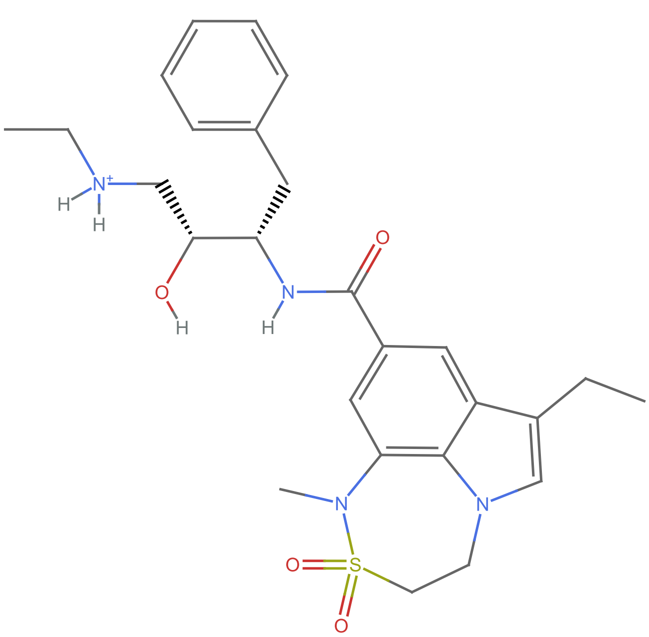

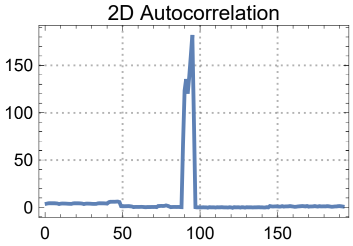

Dataset Index= 680

Molecule Formula= $C_{26}H_{35}N_4O_4S^+$

3D Plot

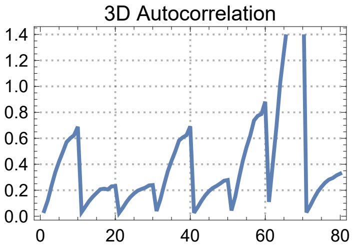

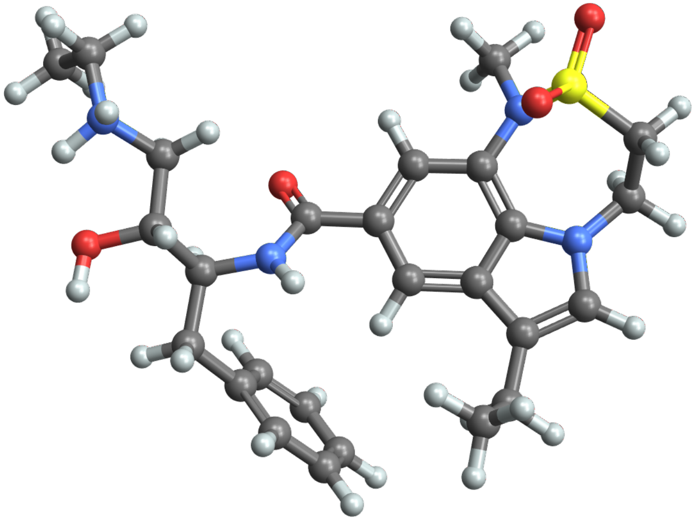

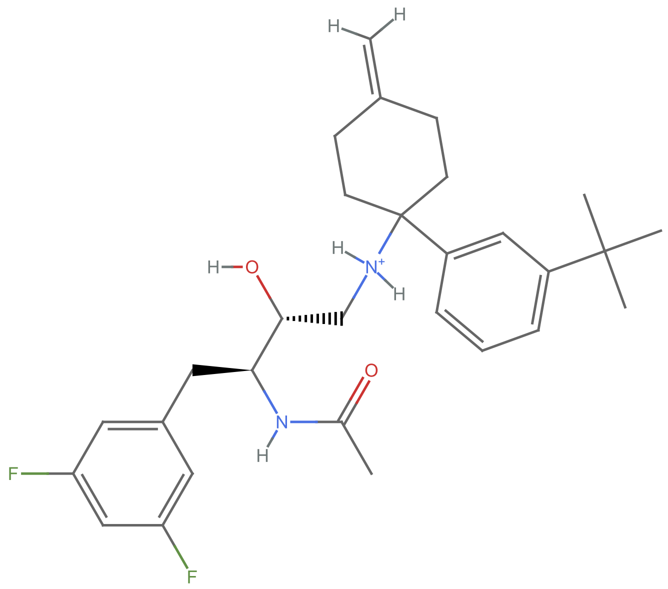

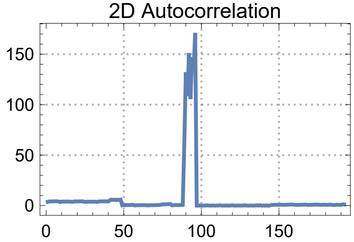

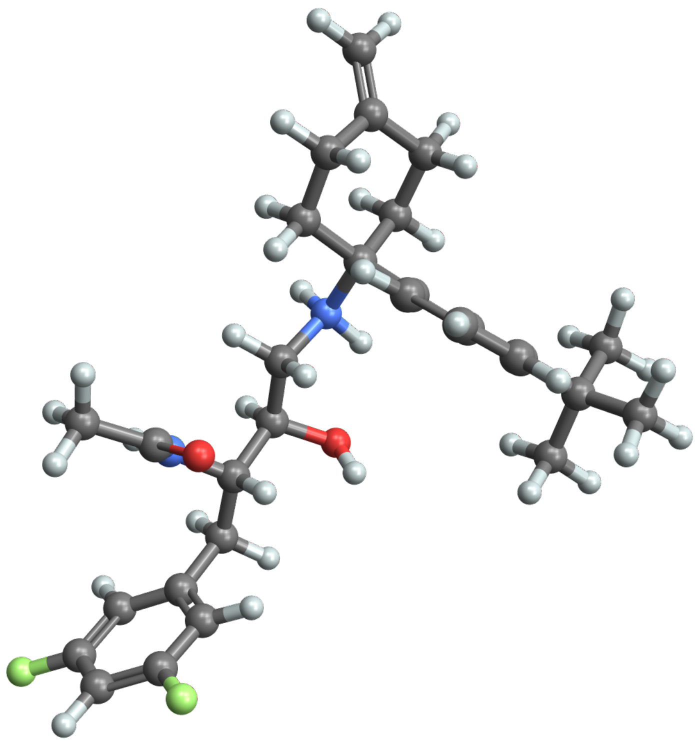

Dataset Index= 681

Molecule Formula= $C_{29}H_{39}F_2N_2O_2^+$

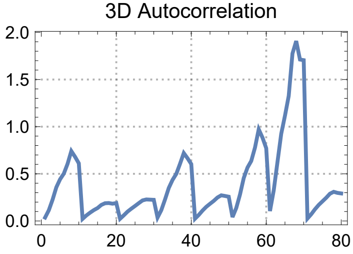

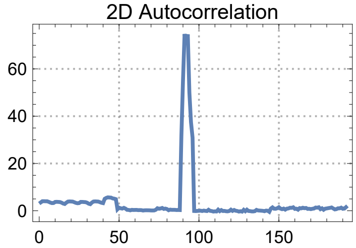

Dataset Index= 682

Molecule Formula= $C_{18}H_{19}N_5OS$

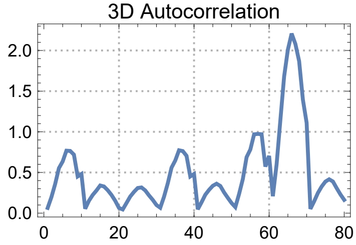

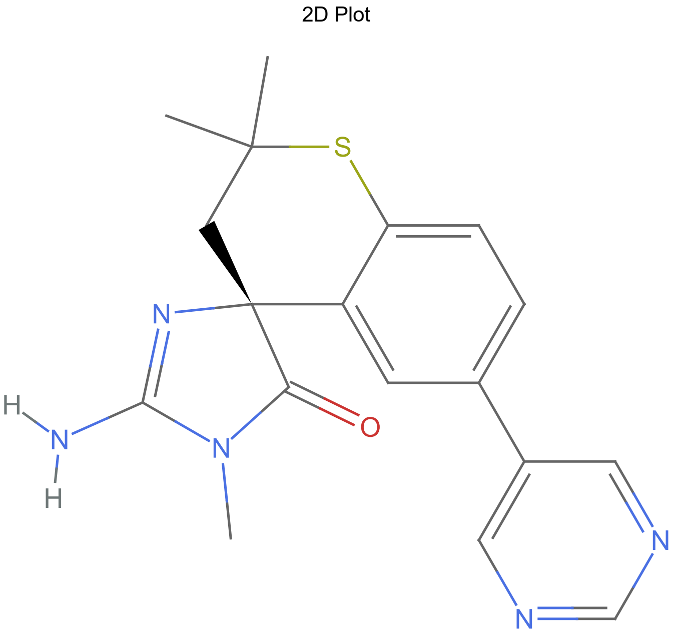

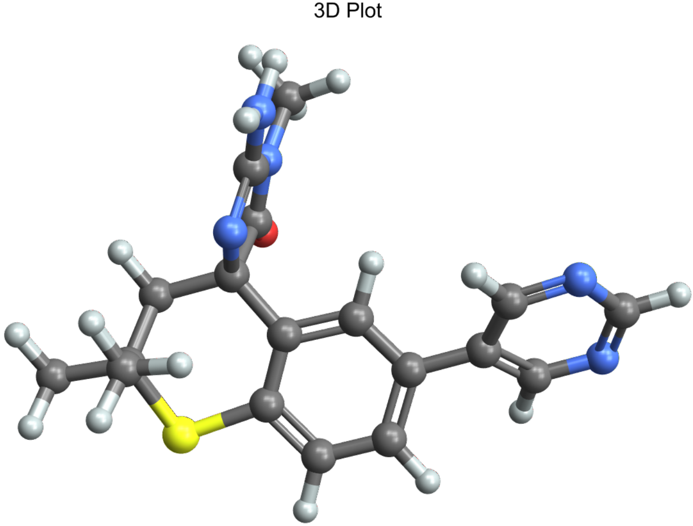

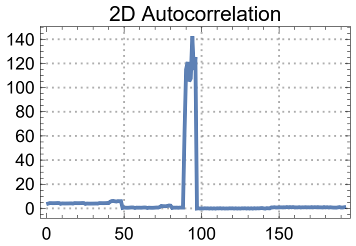

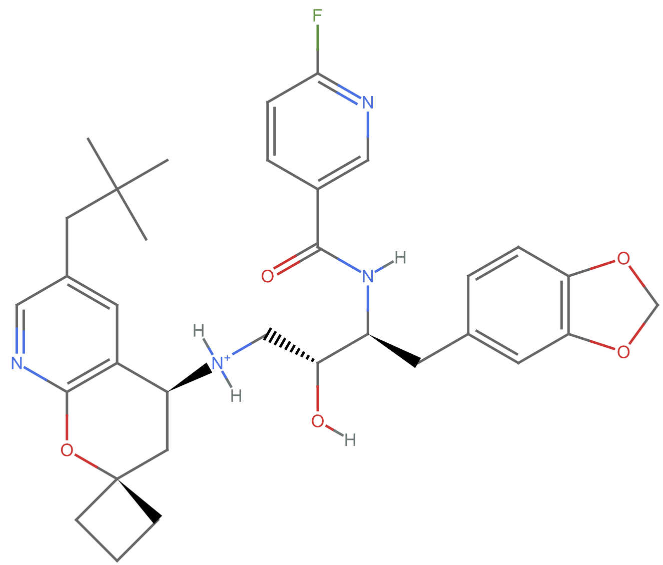

3D Plot

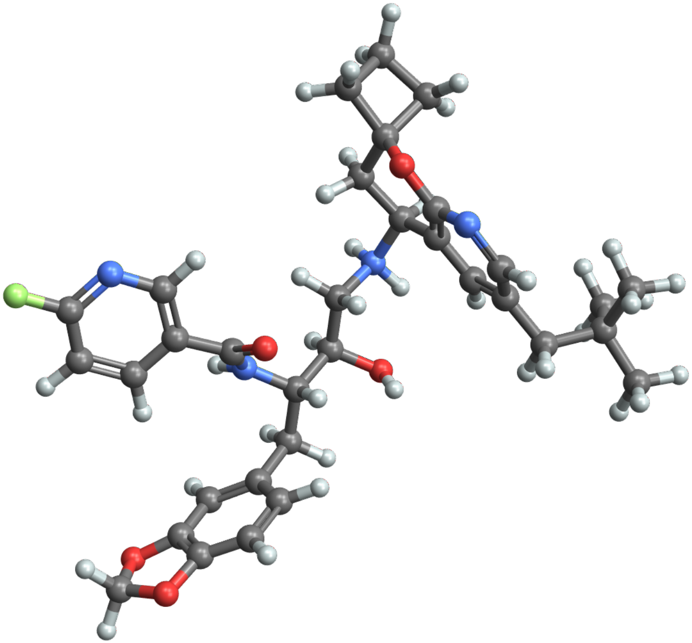

Dataset Index= 683

Molecule Formula= $C_{33}H_{40}FN_4O_5^+$

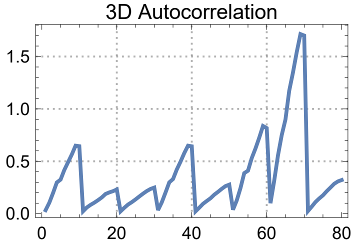

2D Plot

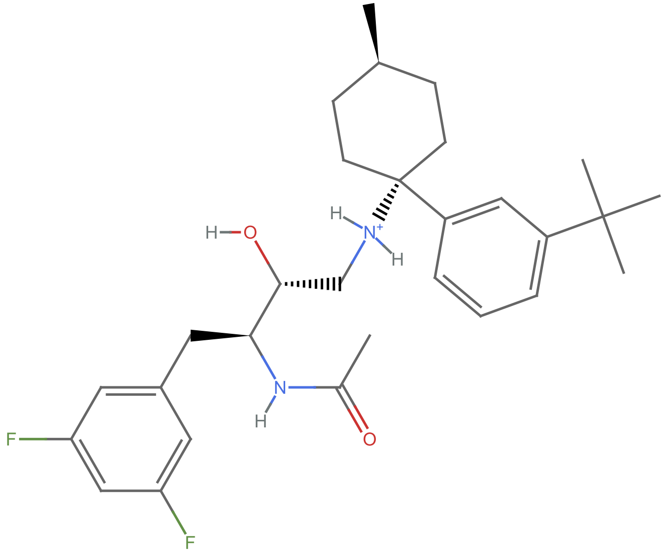

2D Autocorrelation

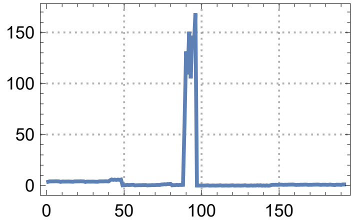

3D Plot

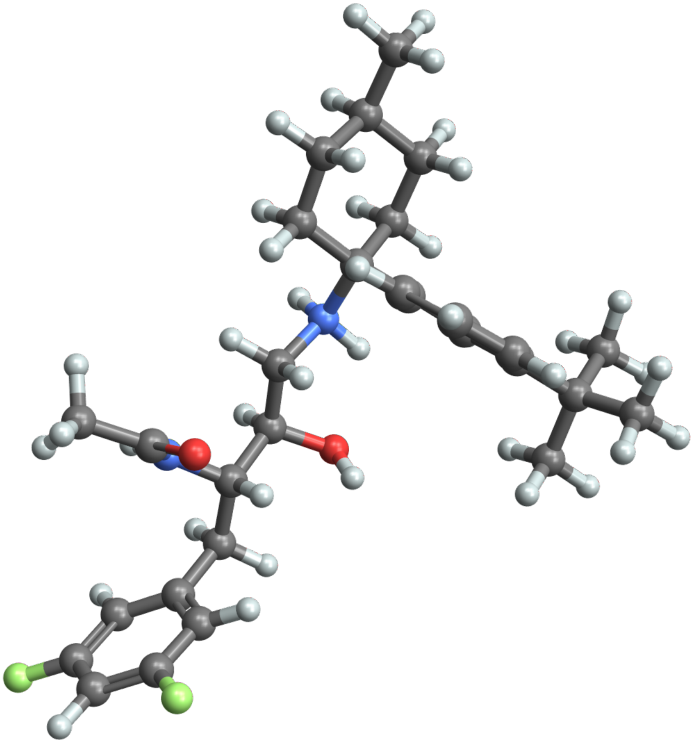

Dataset Index= 684

Molecule Formula= $C_{29}H_{41}F_2N_2O_2^+$

3D Autocorrelation

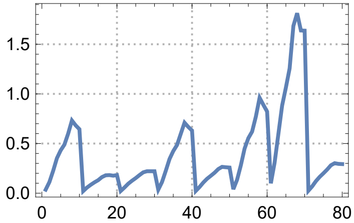

2D Plot

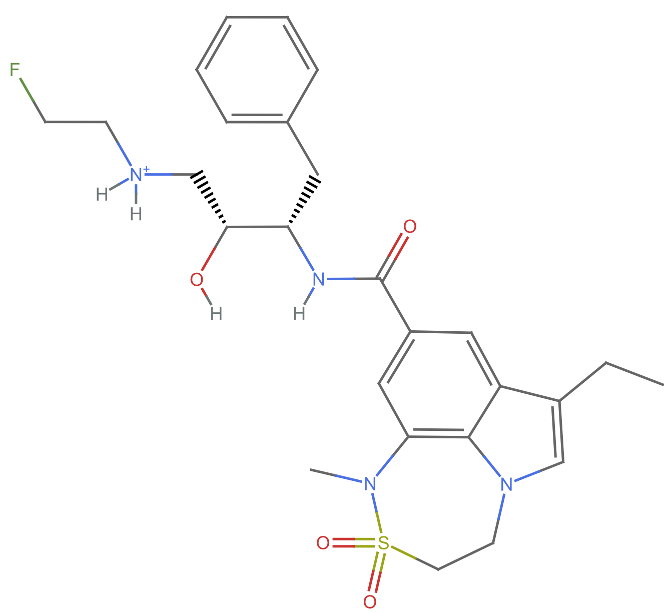

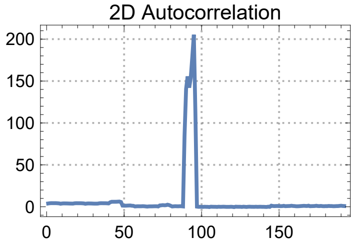

Dataset Index= 685

Molecule Formula= $C_{26}H_{34}FN_4O_4S^+$

3D Plot

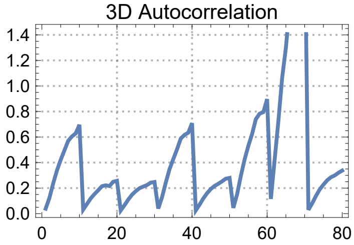

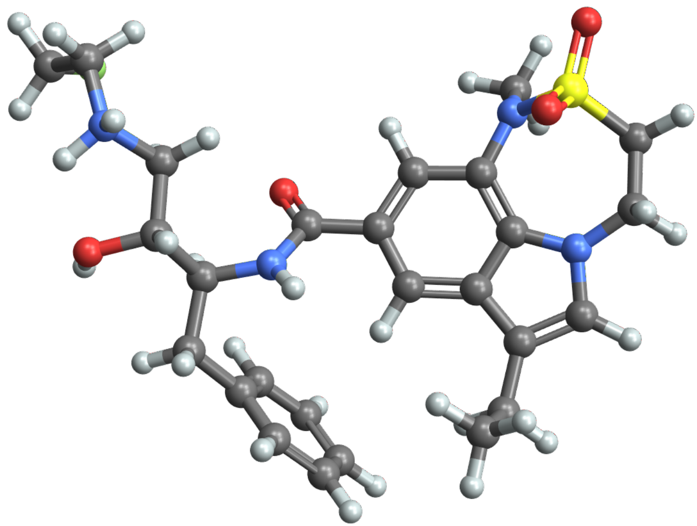

2D Plot

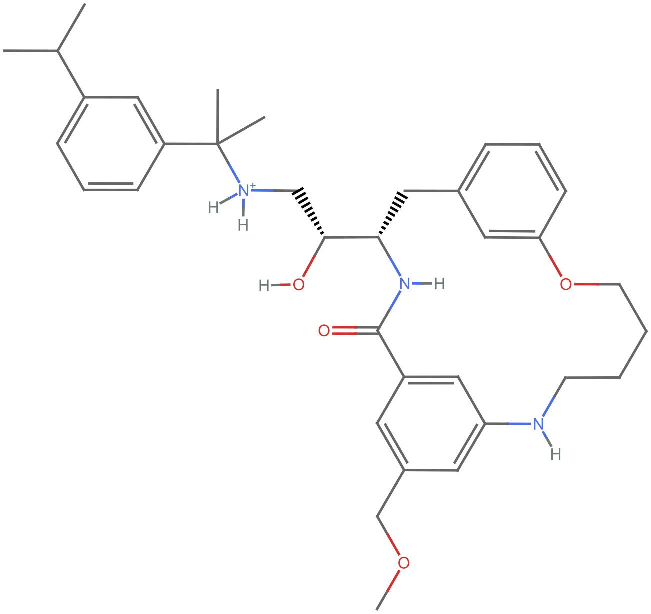

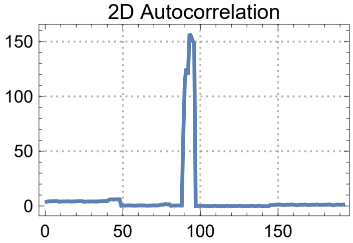

Dataset Index= 686

Molecule Formula= $C_{35}H_{48}N_3O_4^+$

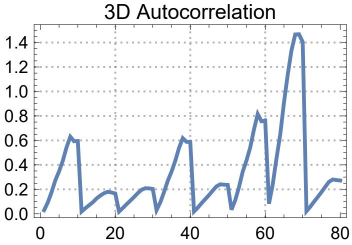

3D Plot

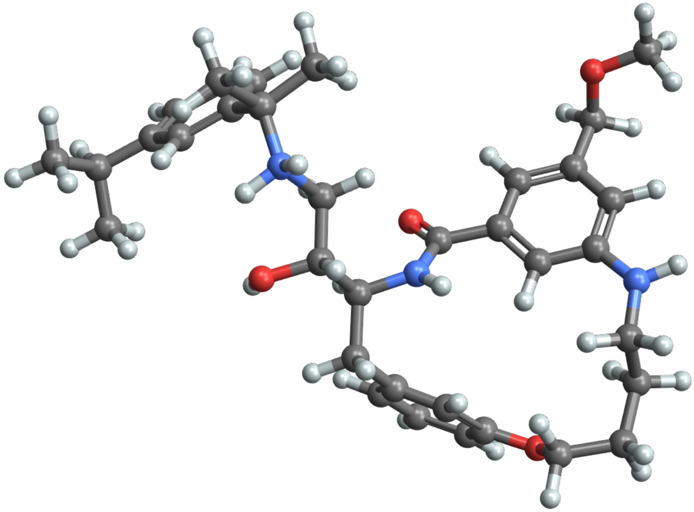

2D Plot

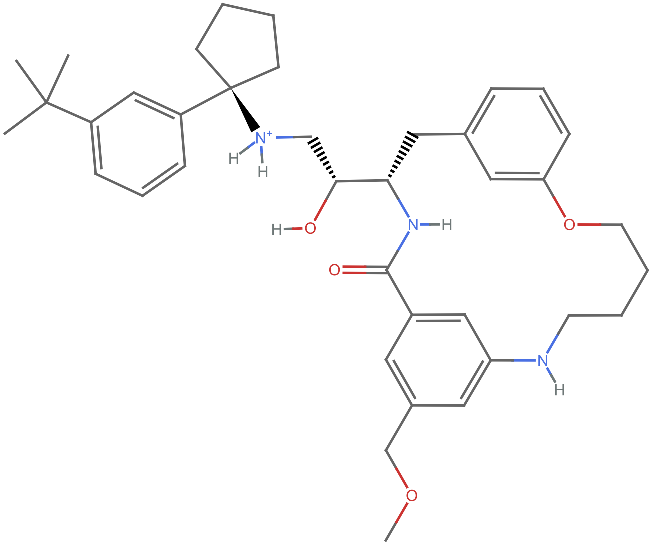

2D Autocorrelation

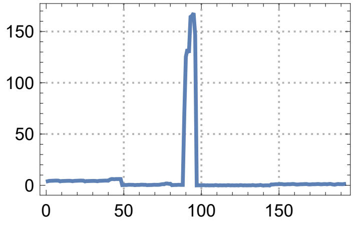

3D Plot

Dataset Index= 687

Molecule Formula= $C_{38}H_{52}N_3O_4^+$

3D Autocorrelation

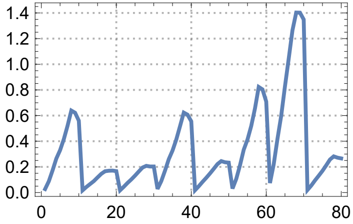

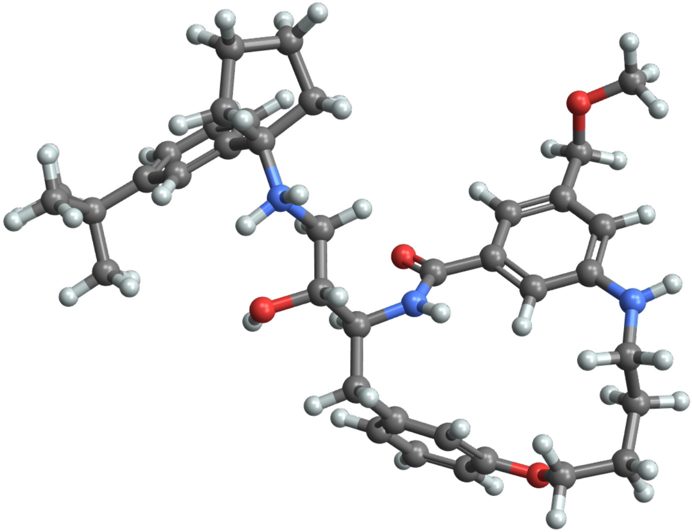

2D Autocorrelation

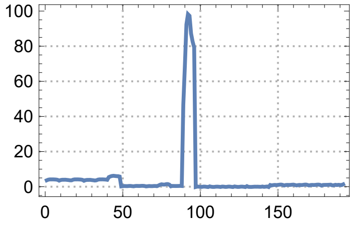

Dataset Index= 688

Molecule Formula= $C_{24}H_{24}N_4O_3$

3D Autocorrelation

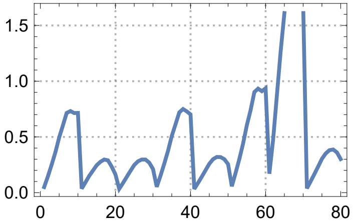

2D Plot

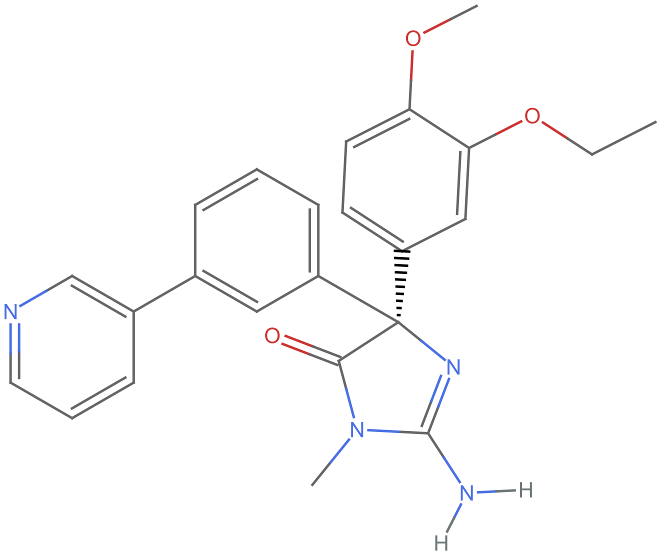

3D Plot

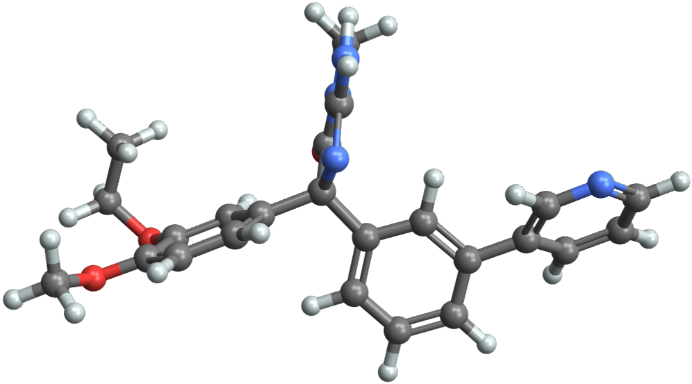

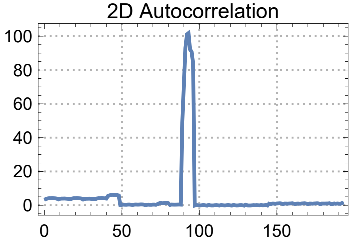

Dataset Index= 689

Molecule Formula= $C_{25}H_{26}N_4O_3$

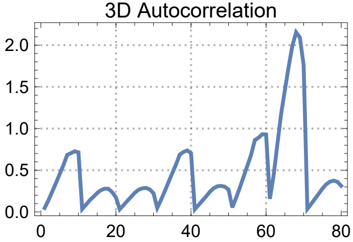

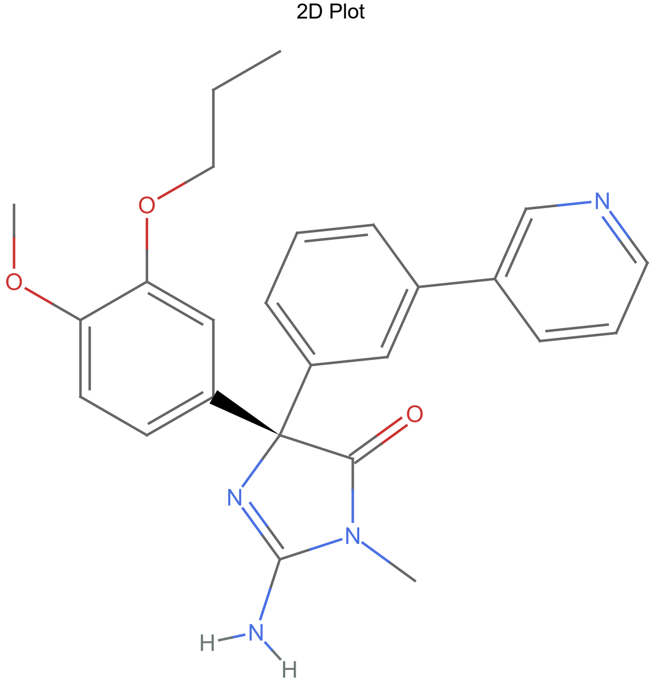

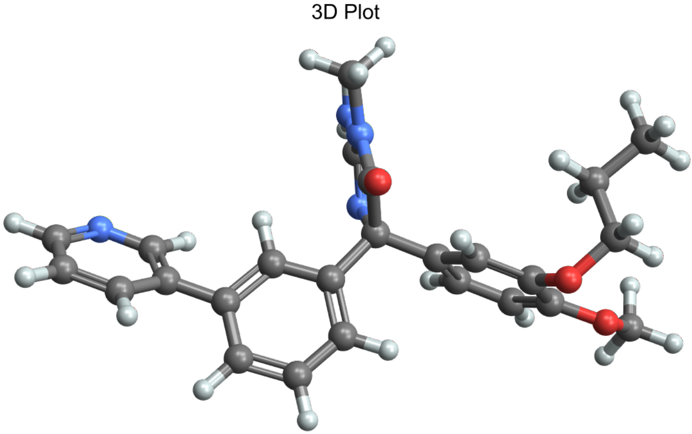

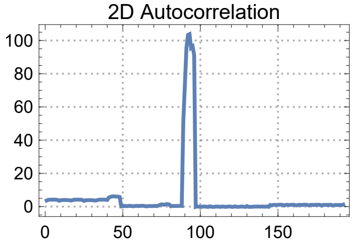

Dataset Index= 690

Molecule Formula= $C_{26}H_{28}N_4O_3$

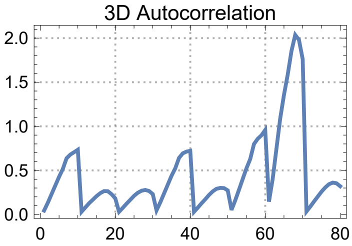

2D Plot

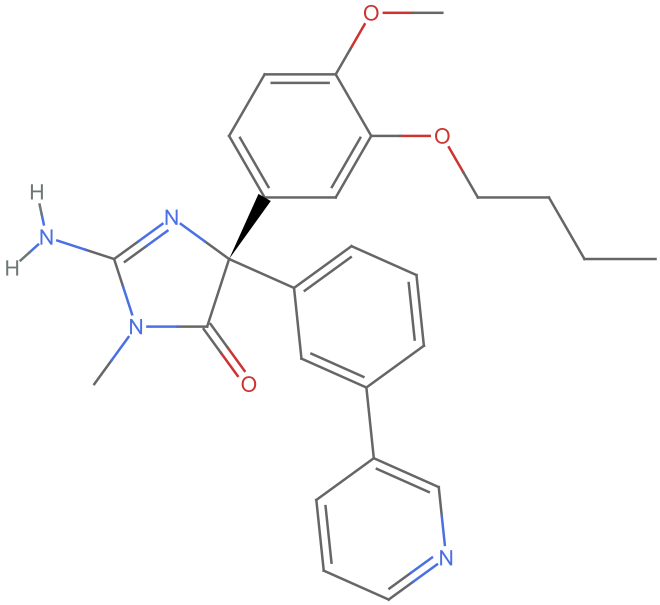

3D Plot

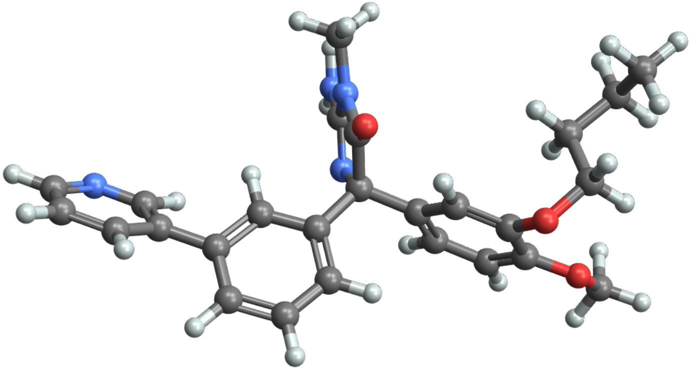

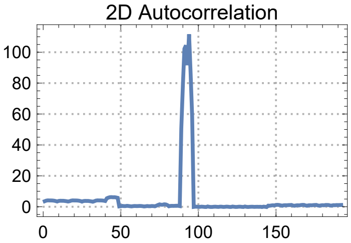

Dataset Index= 691

Molecule Formula= $C_{22}H_{19}FN_4O_2$

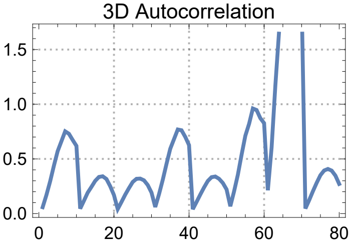

2D Plot

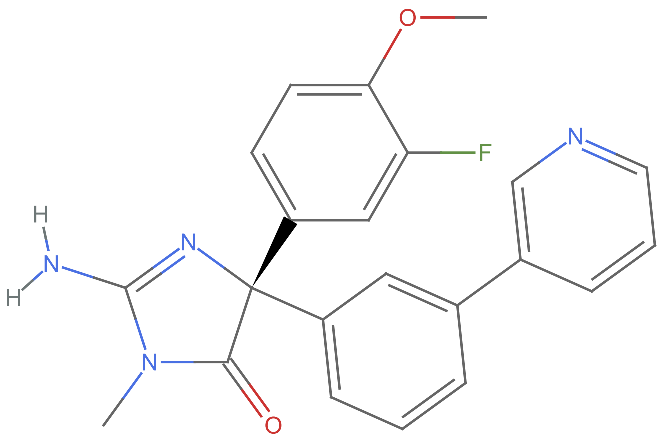

3D Plot

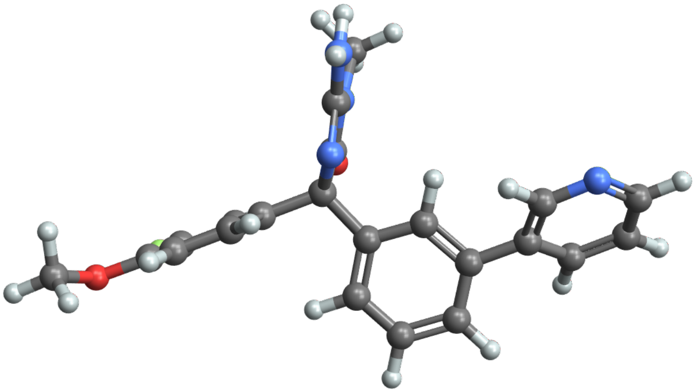

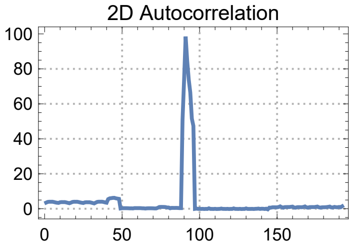

Dataset Index= 692

Molecule Formula= $C_{21}H_{17}FN_4O$

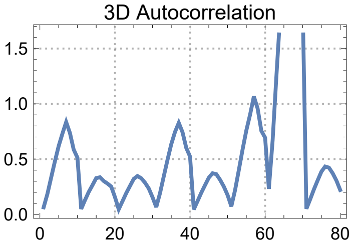

2D Plot

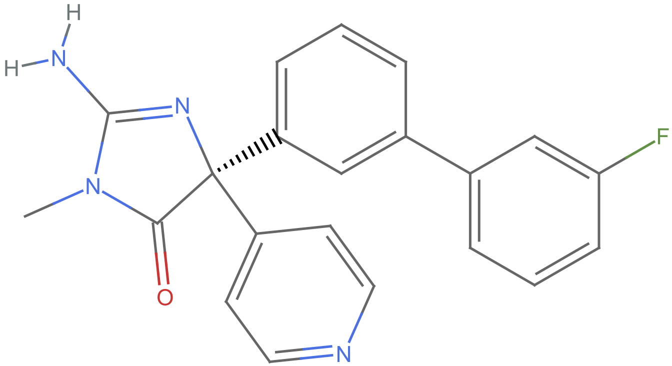

3D Plot

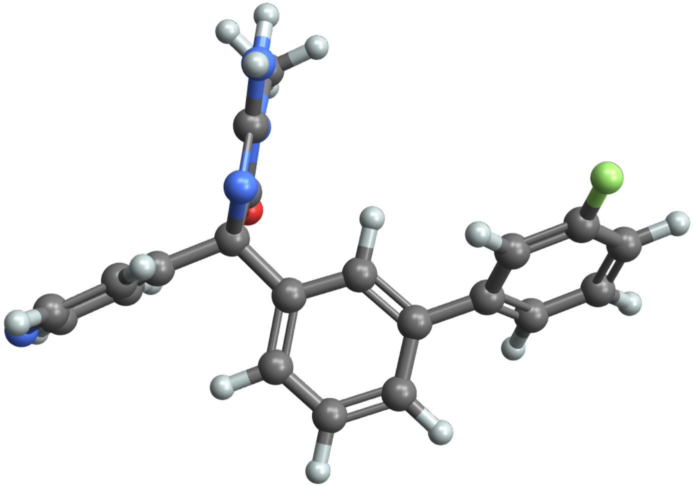

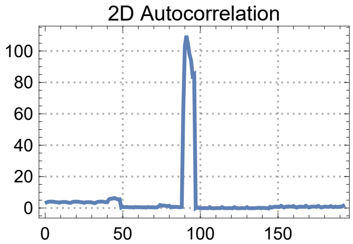

Dataset Index= 693

Molecule Formula= $C_{21}H_{20}F_2N_4O_2$

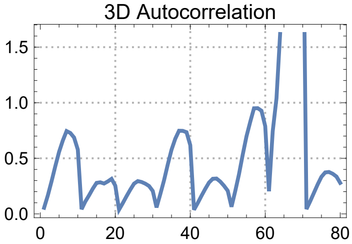

2D Plot

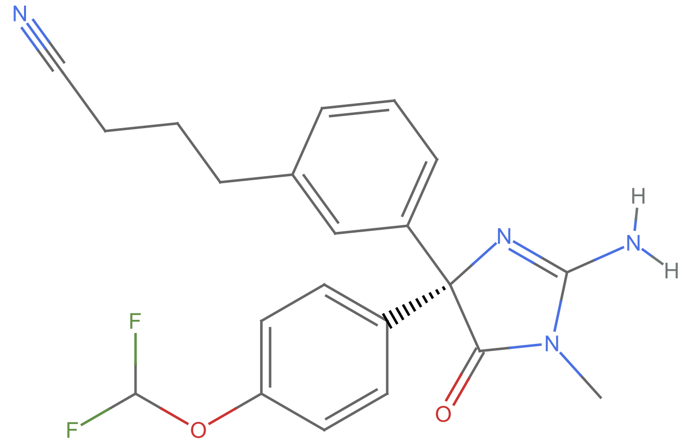

3D Plot

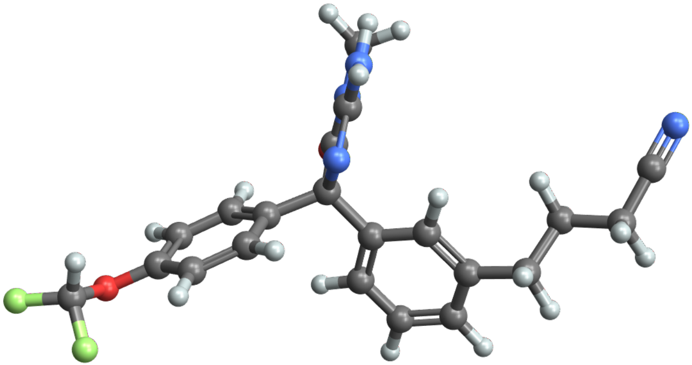

2D Plot

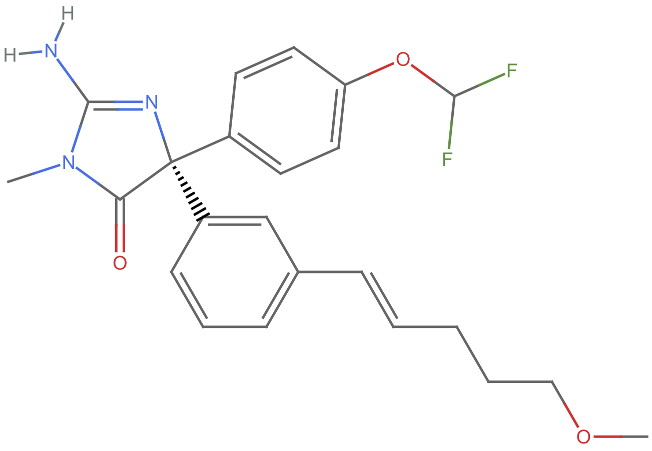

2D Autocorrelation

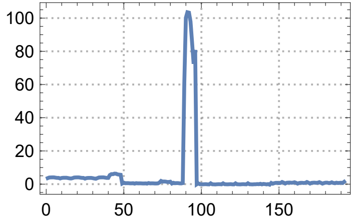

Dataset Index= 694

Molecule Formula= $C_{23}H_{25}F_2N_3O_3$

3D Autocorrelation

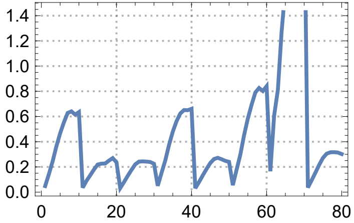

3D Plot

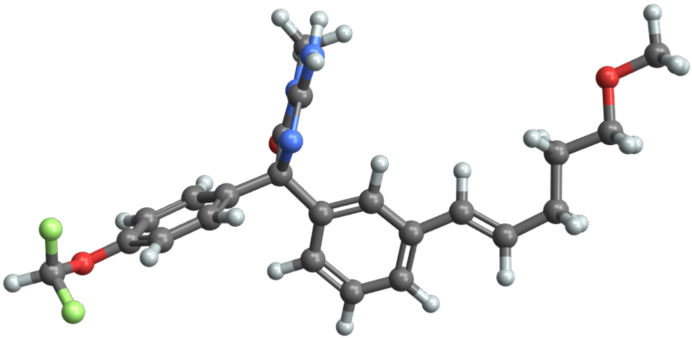

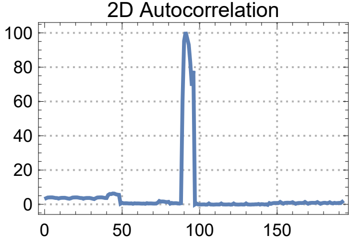

Dataset Index= 695

Molecule Formula= $C_{21}H_{21}F_2N_3O_3$

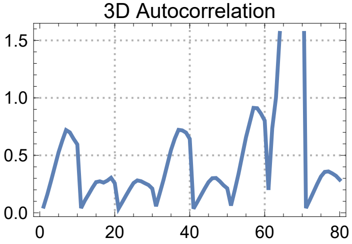

2D Plot

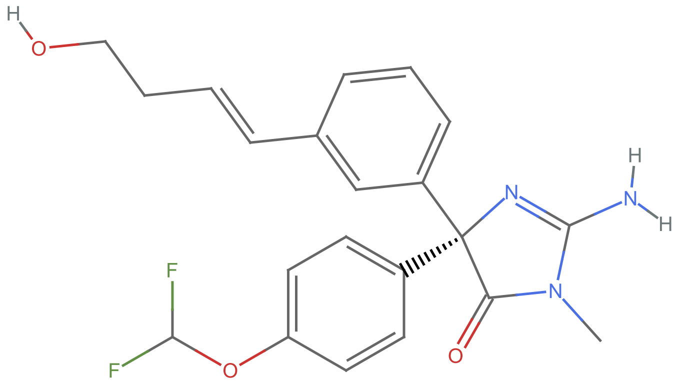

3D Plot

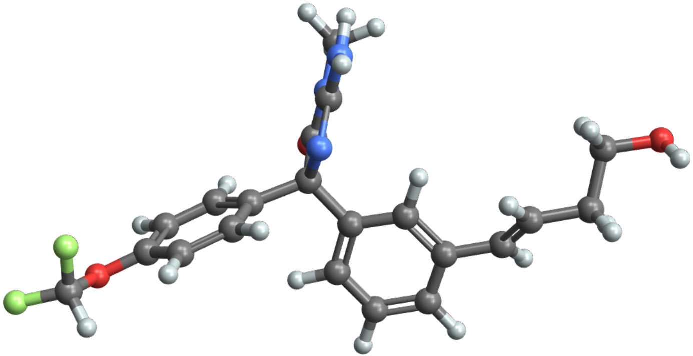

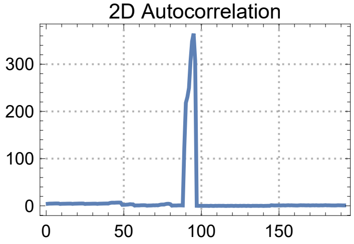

Dataset Index= 696

Molecule Formula= $C_{40}H_{53}N_5O_8S_2$

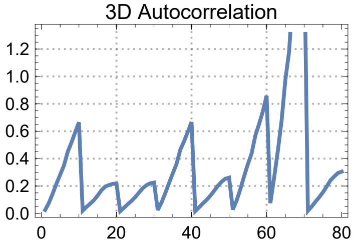

2D Plot

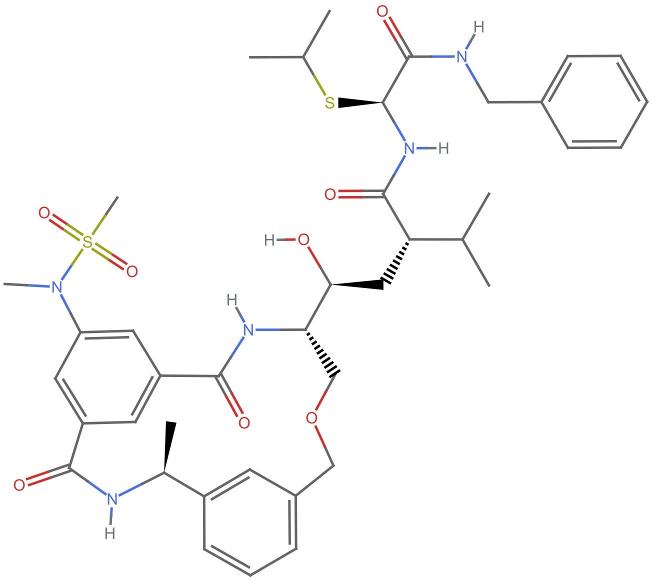

3D Plot

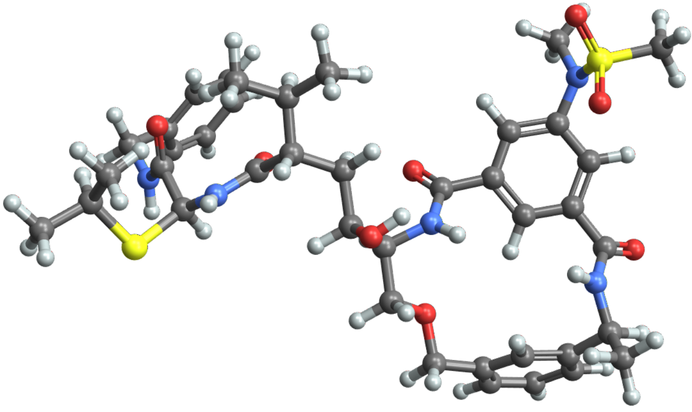

2D Plot

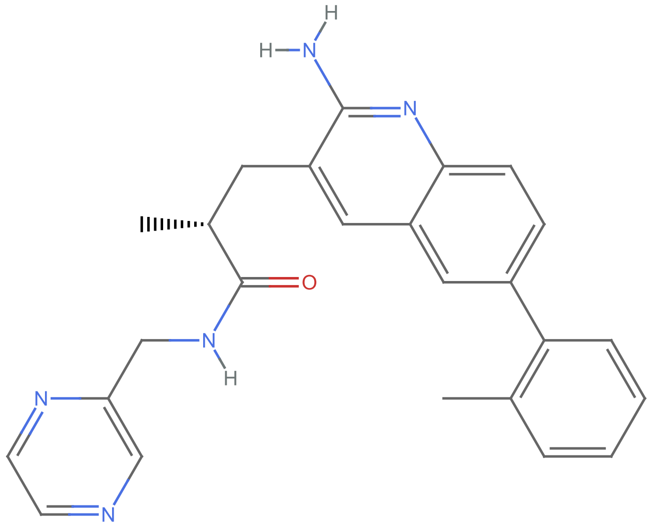

2D Autocorrelation

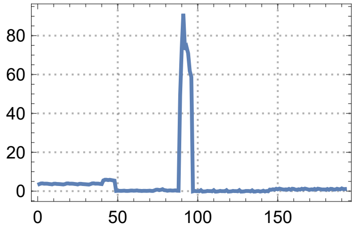

3D Plot

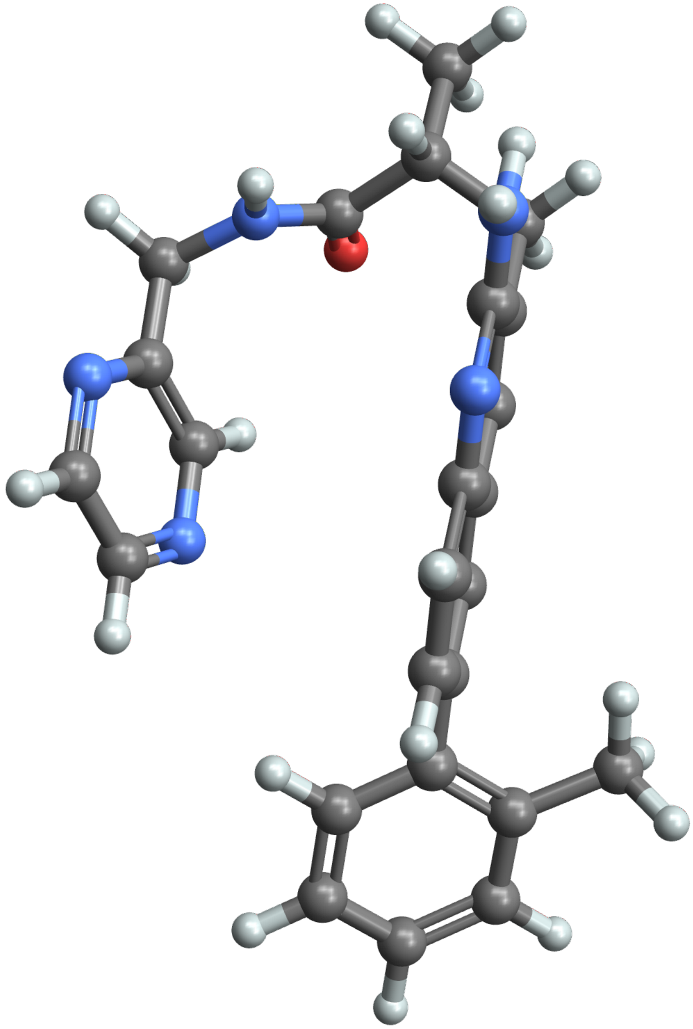

Dataset Index= 697

Molecule Formula= $C_{25}H_{25}N_5O$

3D Autocorrelation

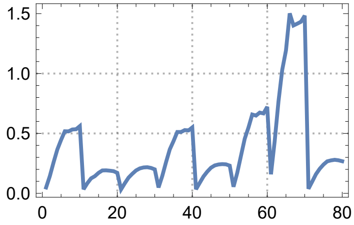

2D Plot

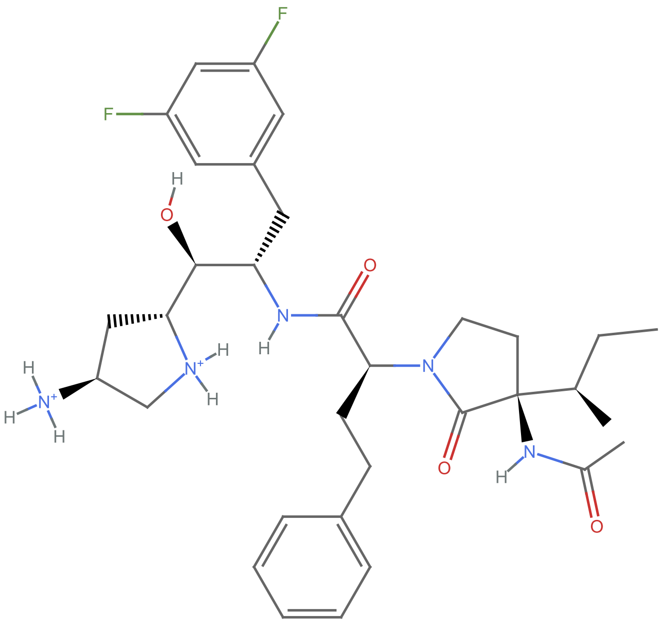

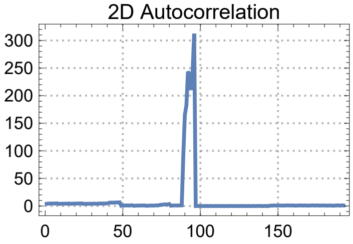

3D Plot

Dataset Index= 698

Molecule Formula= $C_{33}H_{47}F_2N_5O_4^{2+}$

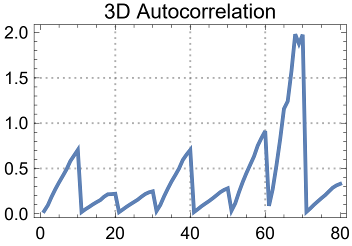

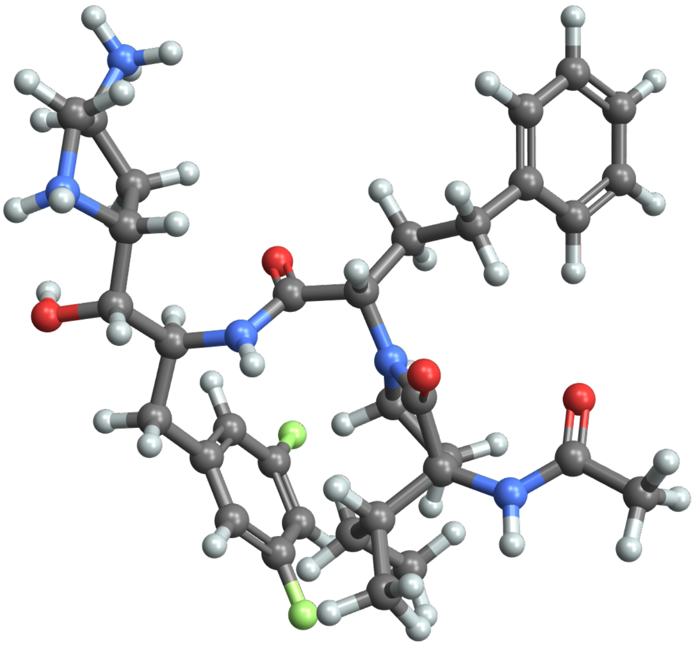

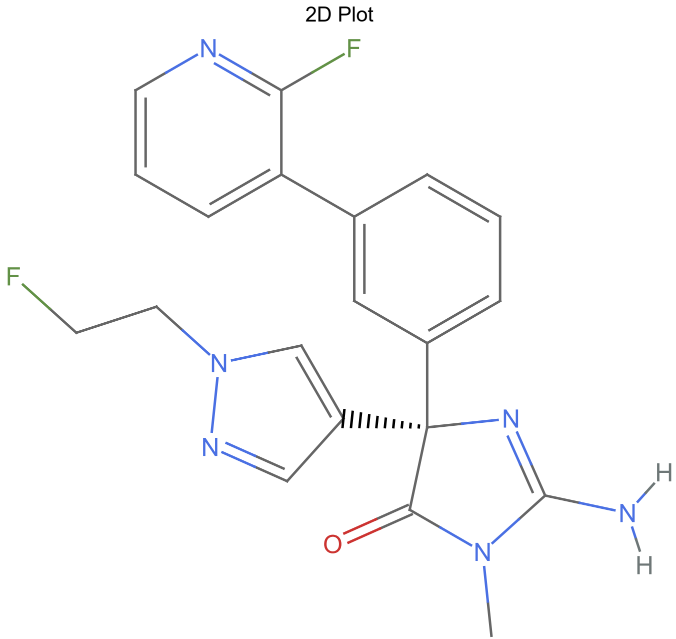

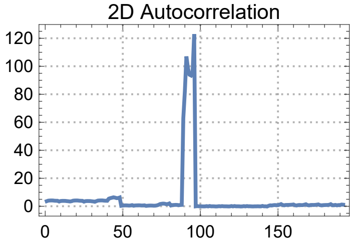

Dataset Index= 699

Molecule Formula= $C_{20}H_{18}F_2N_6O$

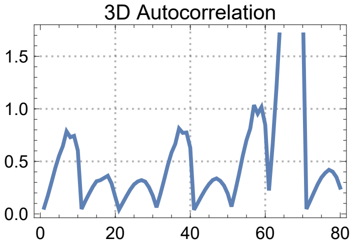

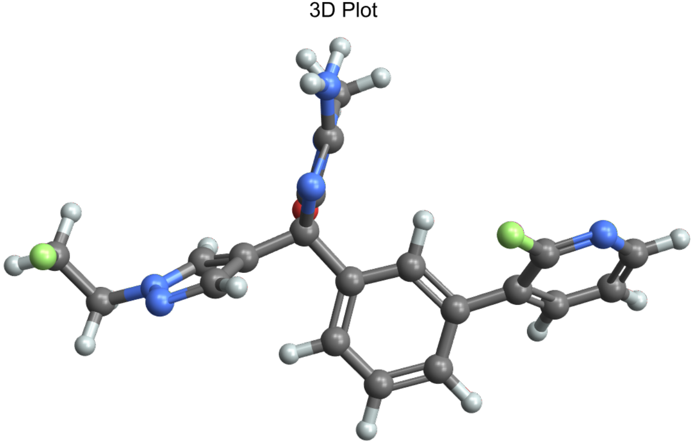

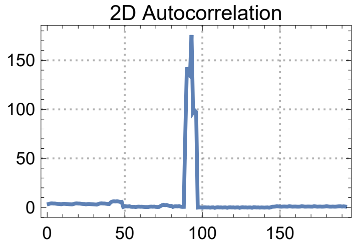

Dataset Index= 700

Molecule Formula= $C_{18}H_{17}F_3N_4O_3$

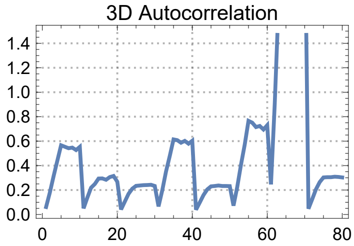

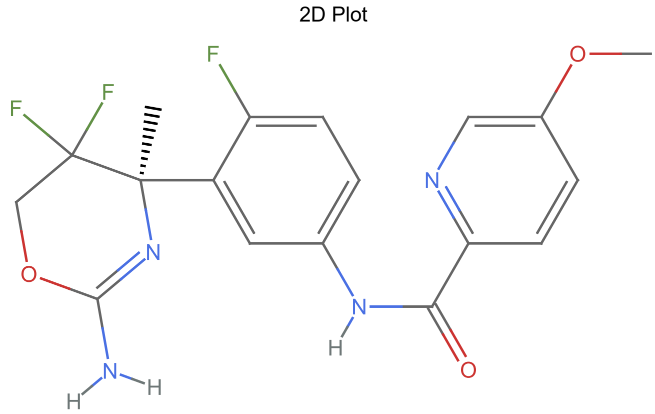

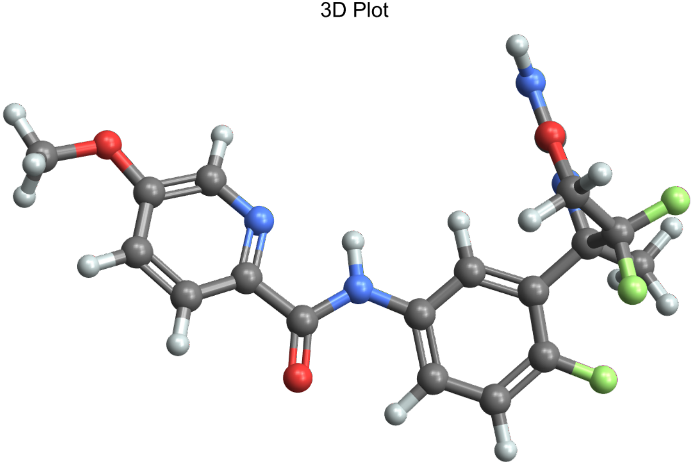

2D Plot

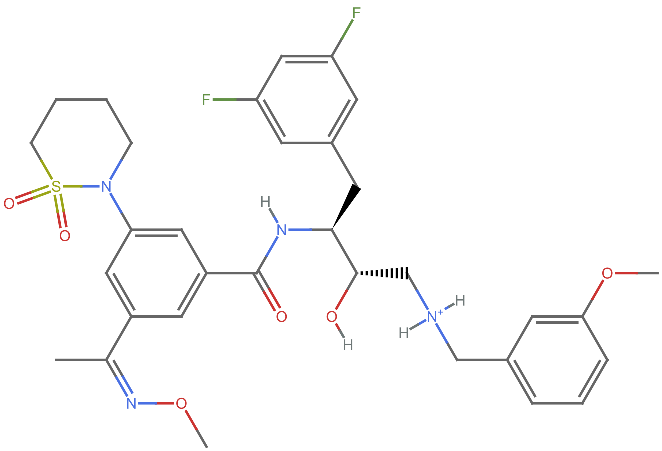

2D Autocorrelation

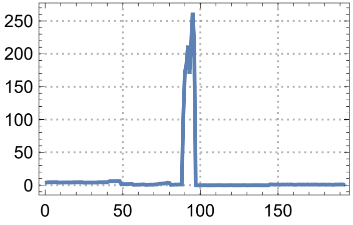

3D Plot

Dataset Index= 701

Molecule Formula= $C_{32}H_{39}F_2N_4O_6S^+$

3D Autocorrelation

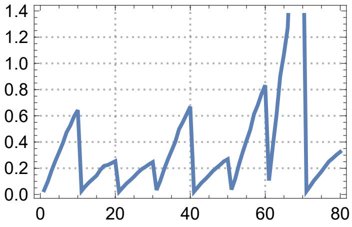

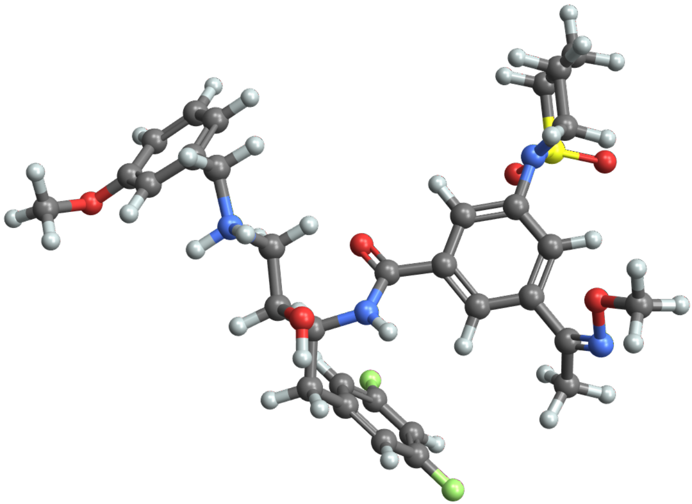

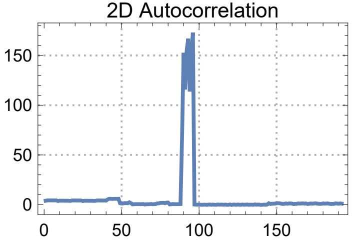

Dataset Index= 702

Molecule Formula= $C_{29}H_{44}FN_3O_4S^{2+}$

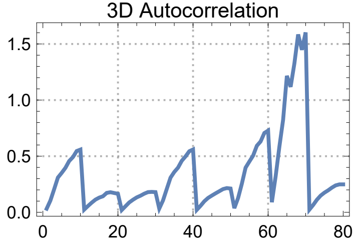

2D Plot

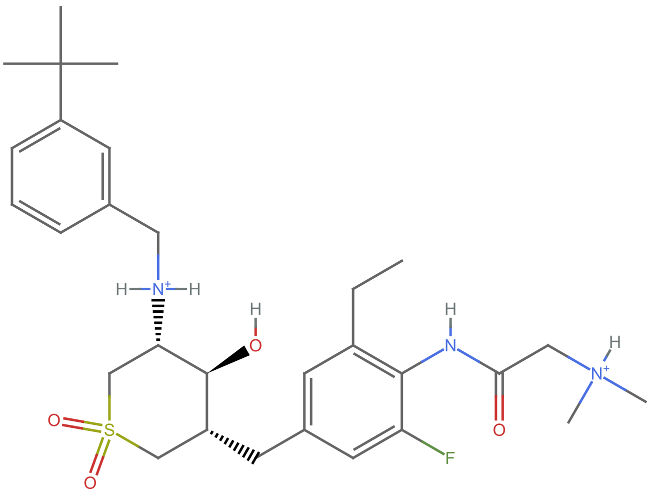

3D Plot

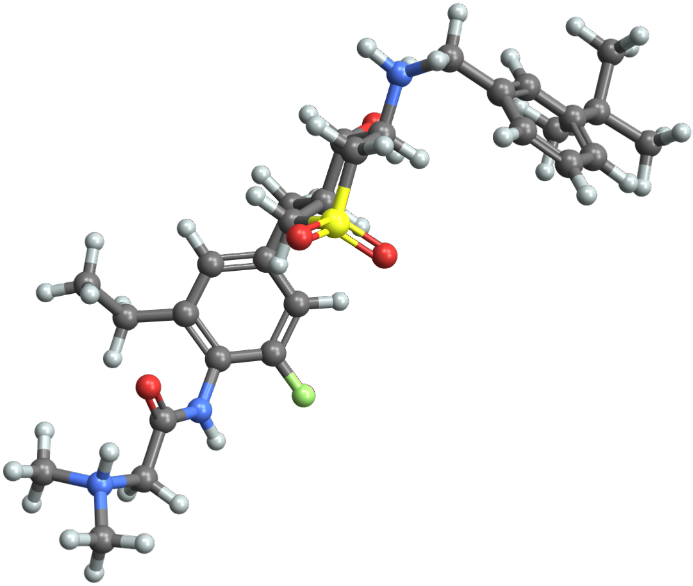

2D Plot

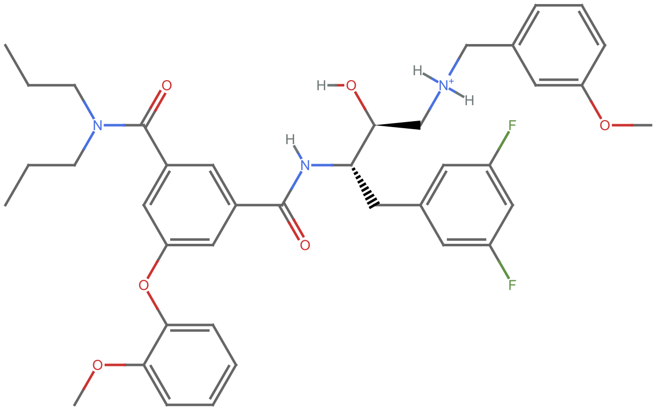

2D Autocorrelation

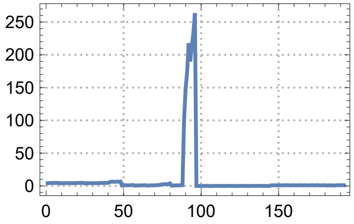

3D Plot

Dataset Index= 703

Molecule Formula= $C_{39}H_{46}F_2N_3O_6^+$

3D Autocorrelation

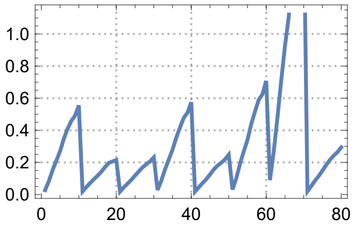

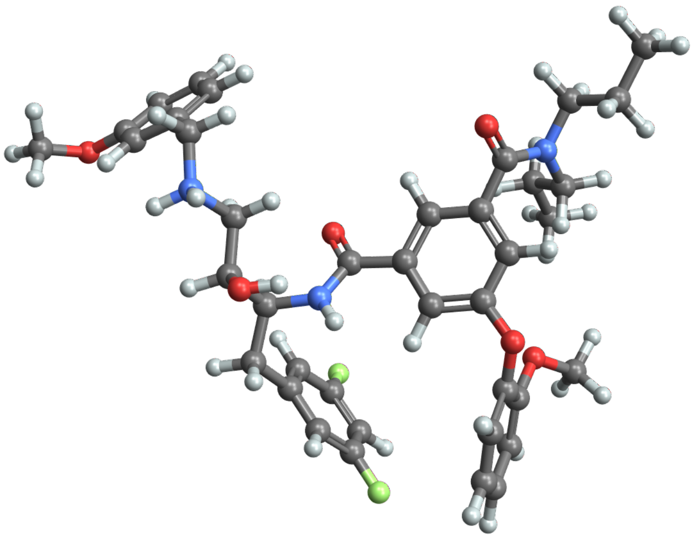

2D Plot

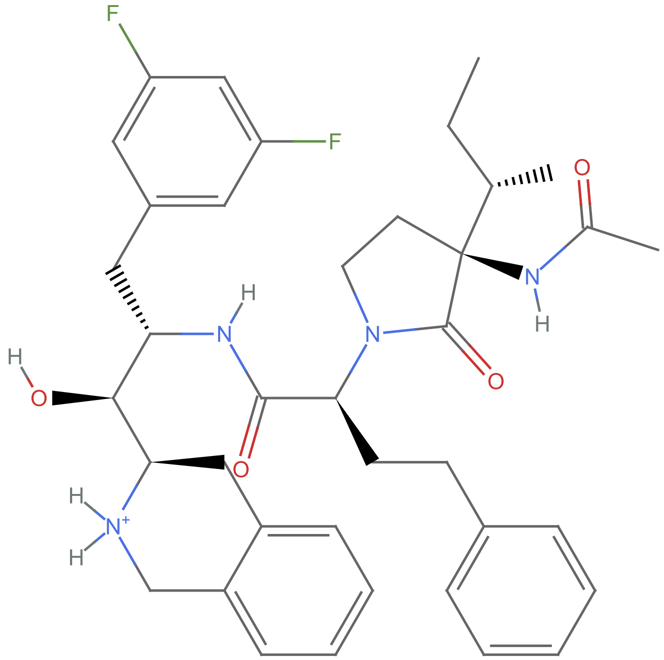

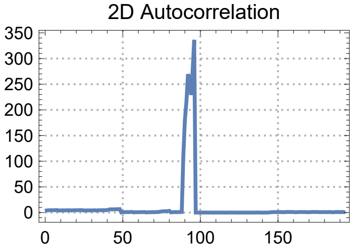

3D Plot

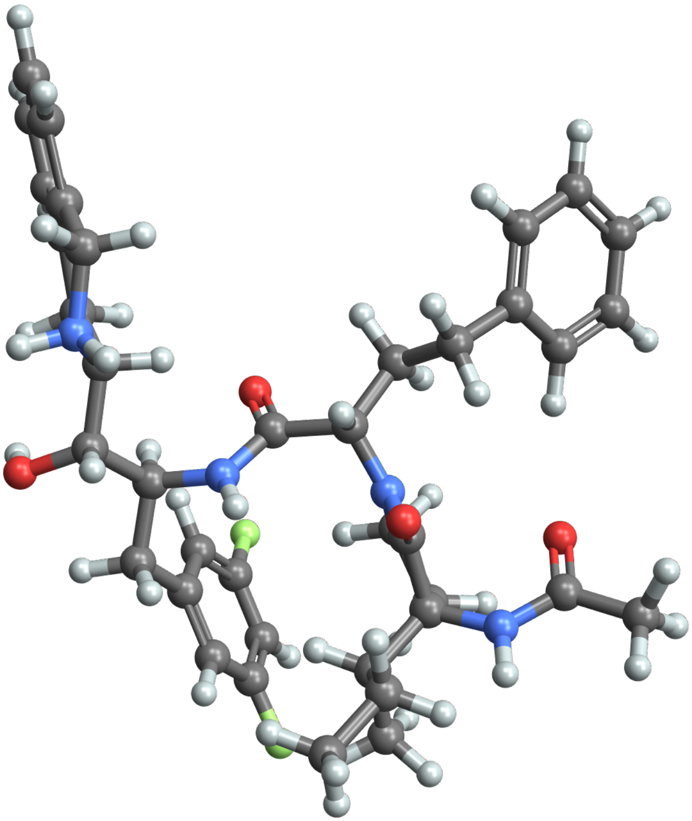

Dataset Index= 704

Molecule Formula= $C_{38}H_{47}F_2N_4O_4^+$

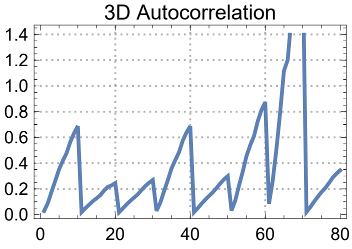

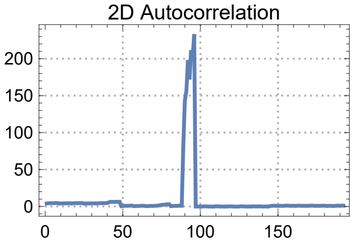

Dataset Index= 705

Molecule Formula= $C_{38}H_{51}F_{2}N_{4}O_{5}^{+}$

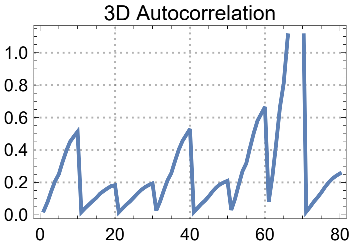

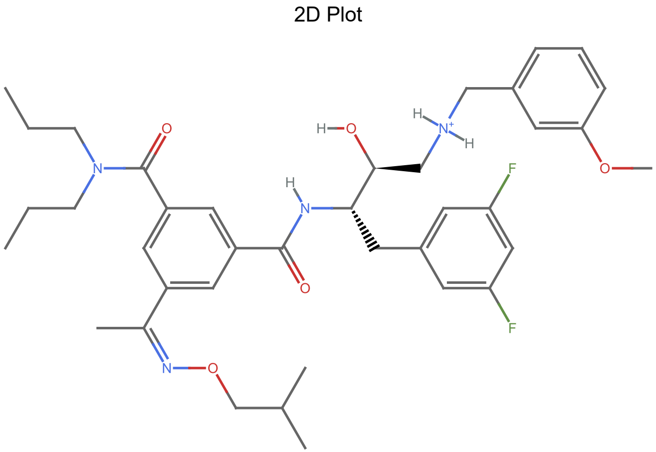

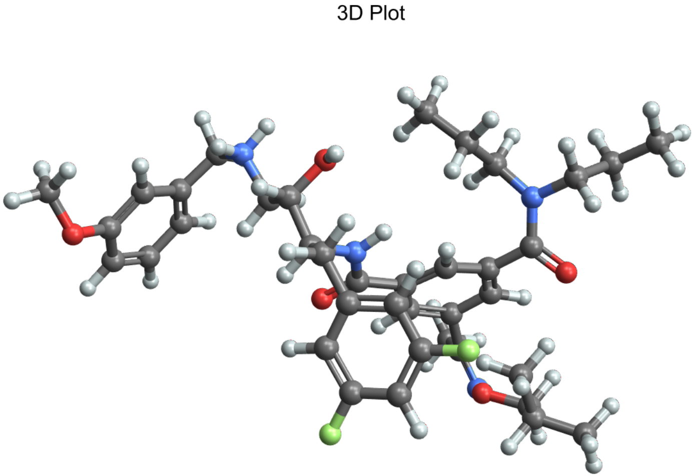

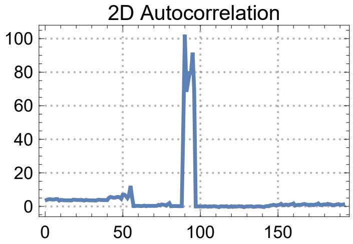

Dataset Index= 706

Molecule Formula= $C_{23}H_{31}BrNO_4S^+$

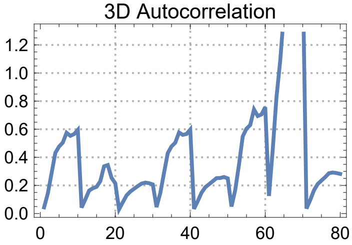

2D Plot

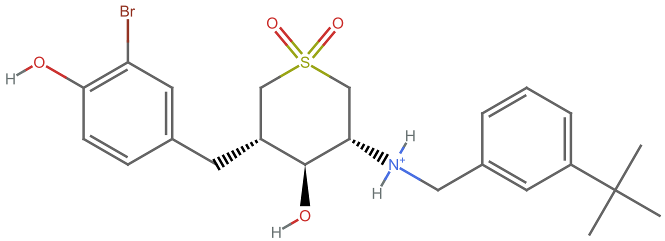

3D Plot

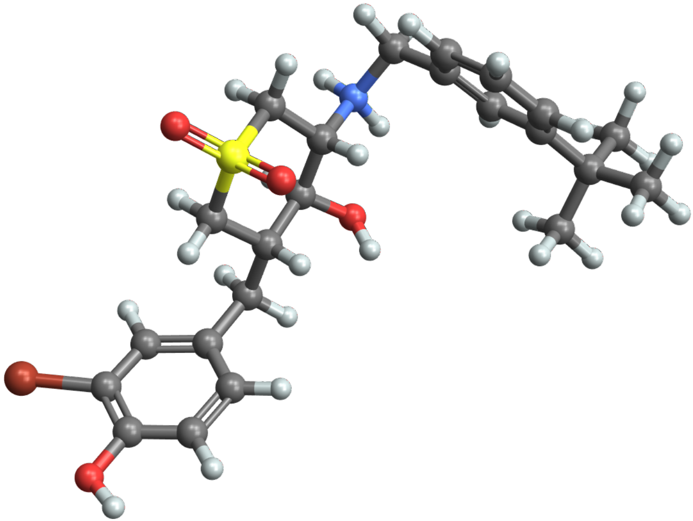

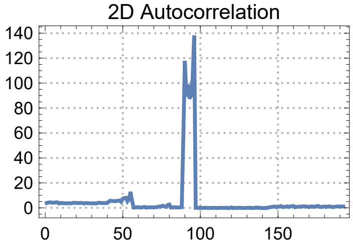

Dataset Index= 707

Molecule Formula= $C_{23}H_{31}BrFN_2O_3S^+$

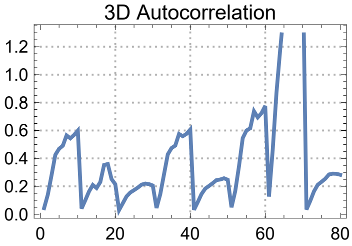

2D Plot

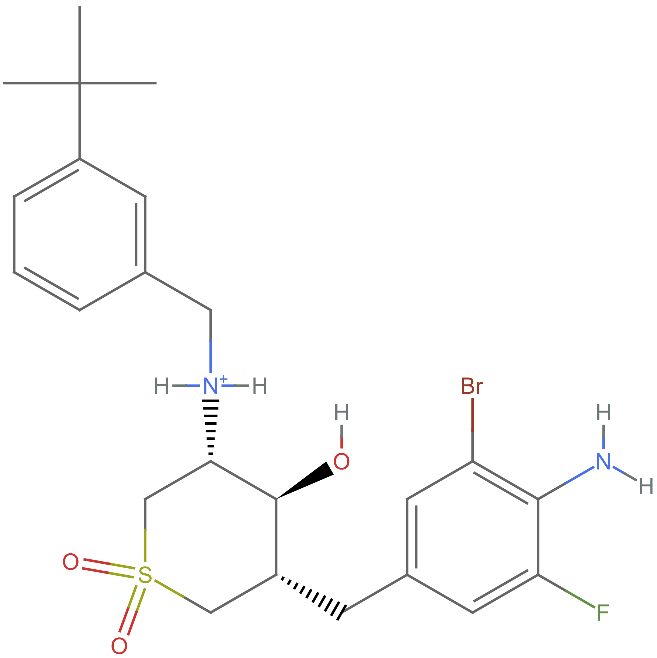

3D Plot

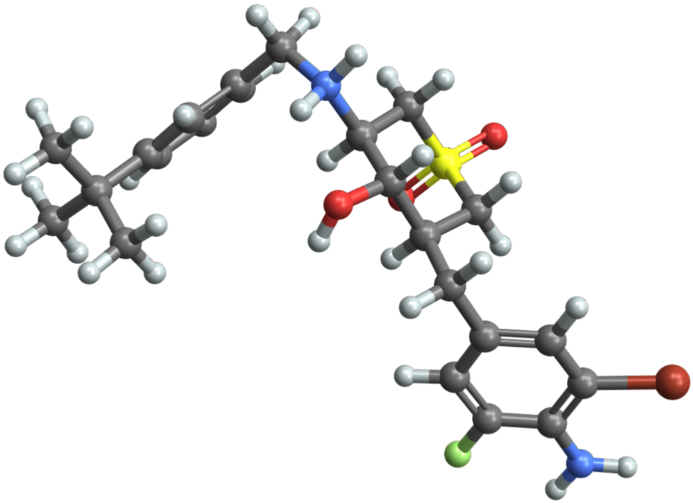

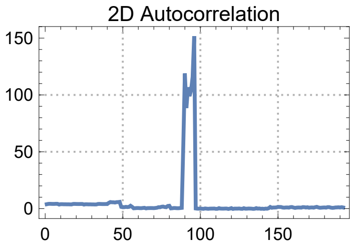

Dataset Index= 708

Molecule Formula= $C_{26}H_{38}FN_2O_4S^+$

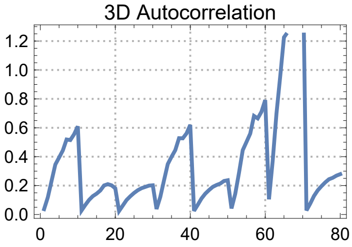

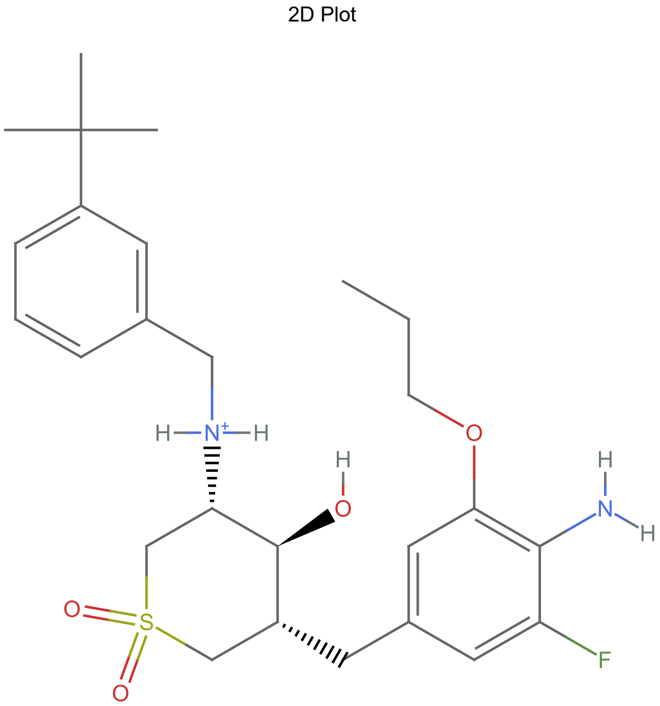

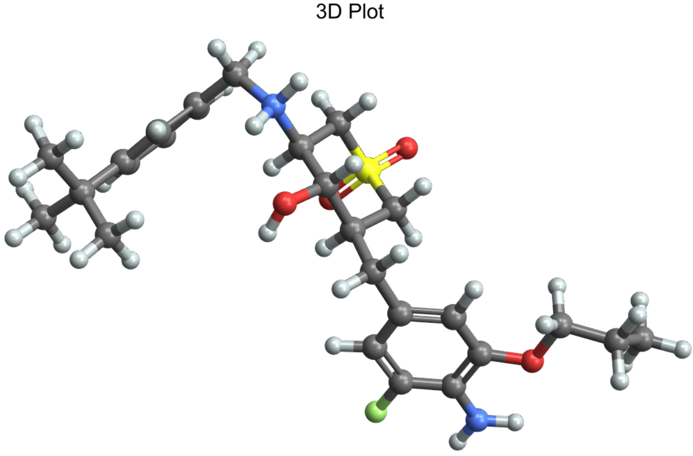

2D Autocorrelation

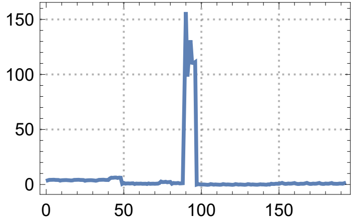

Dataset Index= 709

Molecule Formula= $C_{24}H_{22}F_4N_4O_2$

3D Autocorrelation

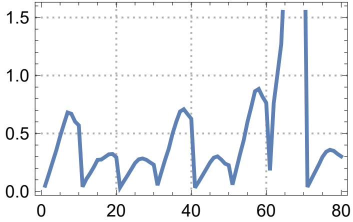

2D Plot

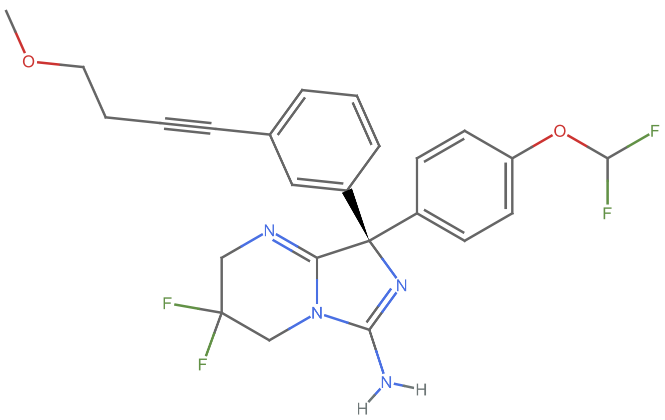

3D Plot

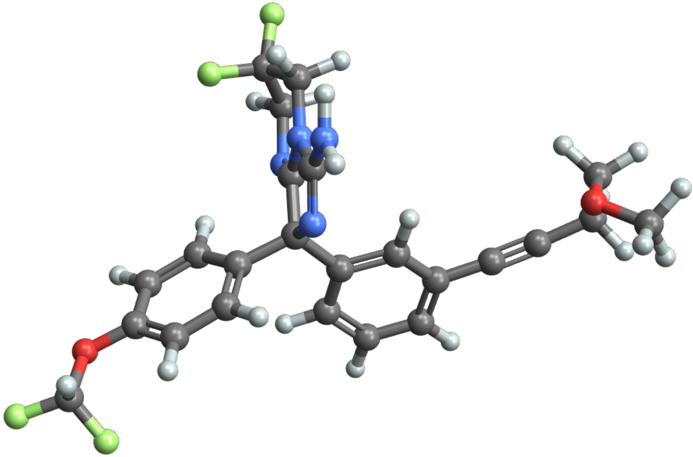

2D Autocorrelation

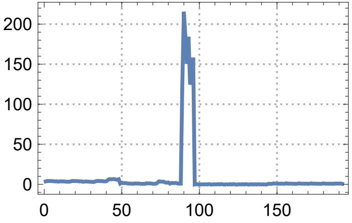

Dataset Index= 710

Molecule Formula= $C_{18}H_{14}F_6N_4O_3$

3D Autocorrelation

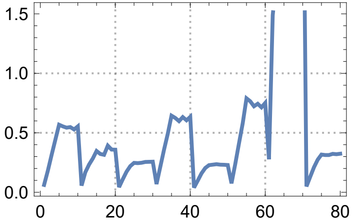

2D Plot

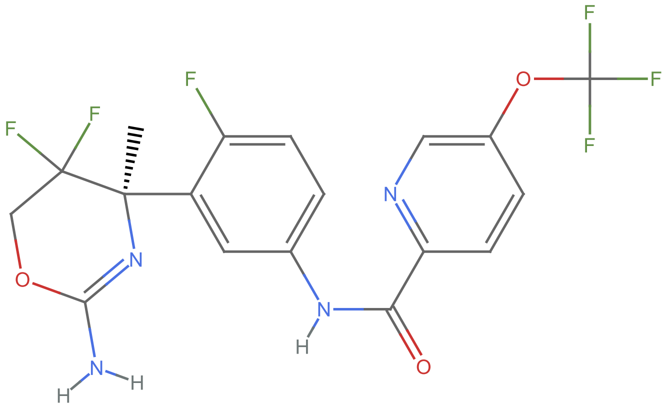

3D Plot

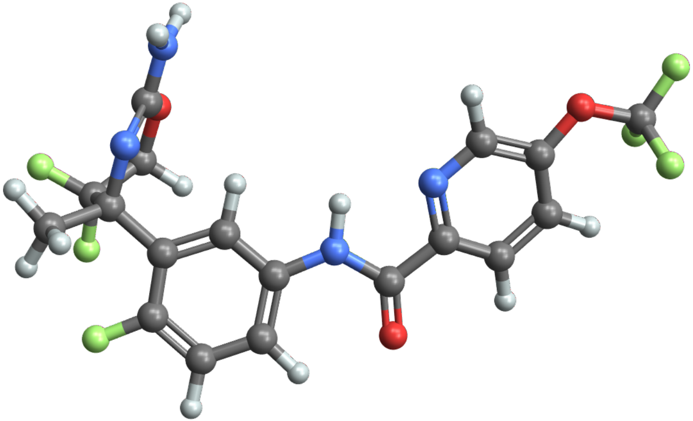

2D Plot

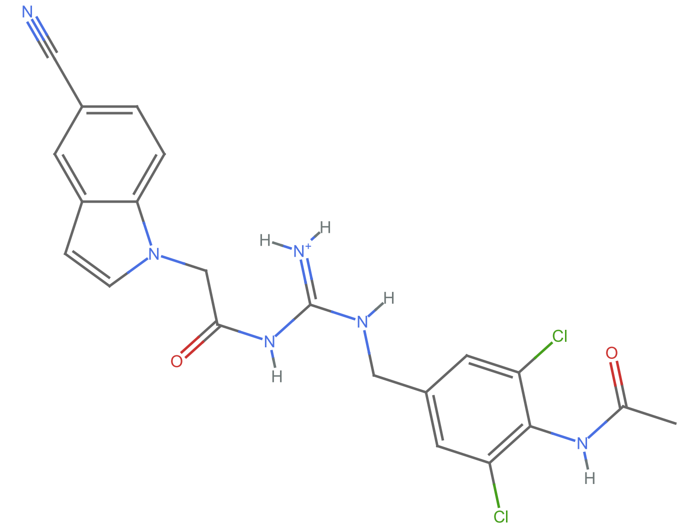

3D Plot

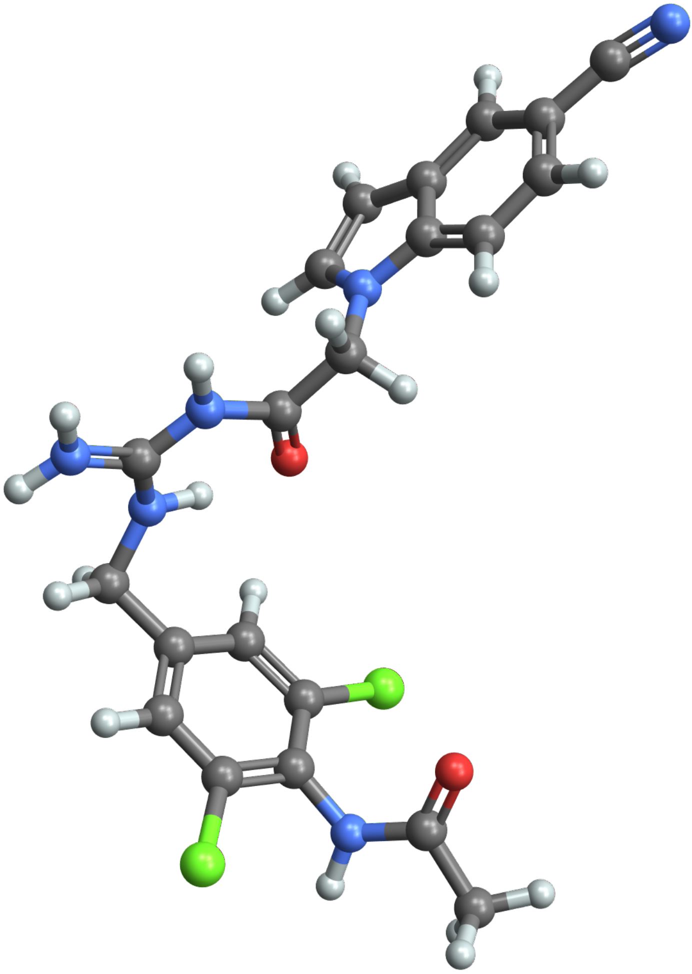

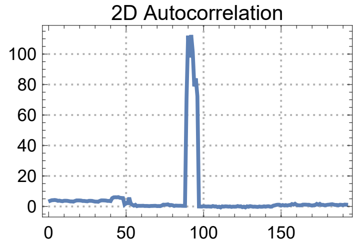

Dataset Index= 711

Molecule Formula= $C_{21}H_{19}Cl_2N_6O_2^+$

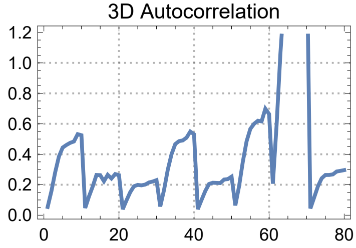

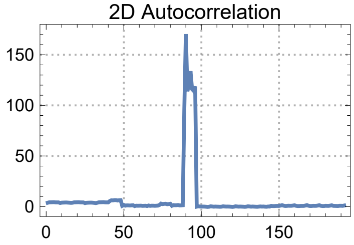

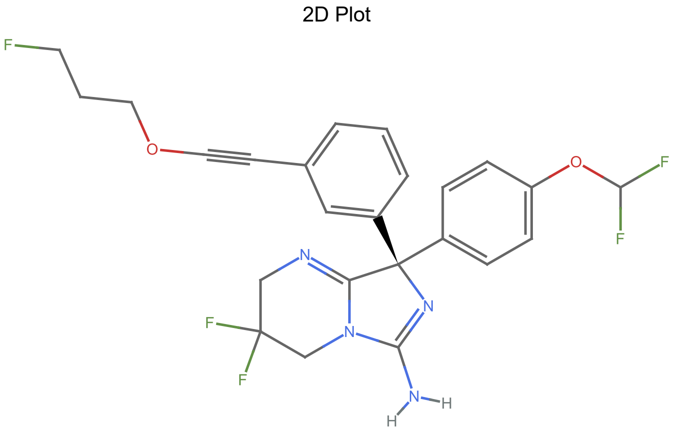

Dataset Index= 712

Molecule Formula= $C_{24}H_{21}F_5N_4O_2$

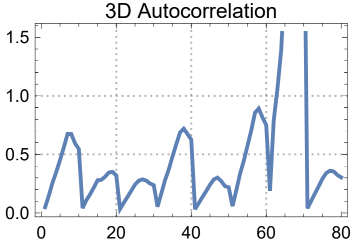

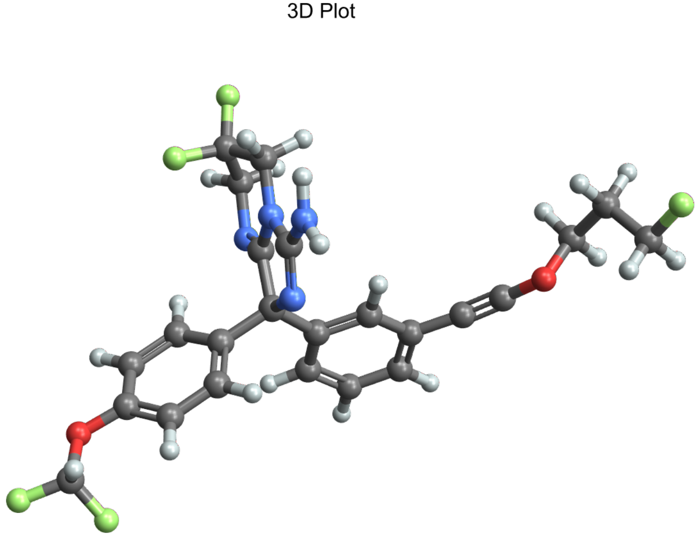

2D Plot

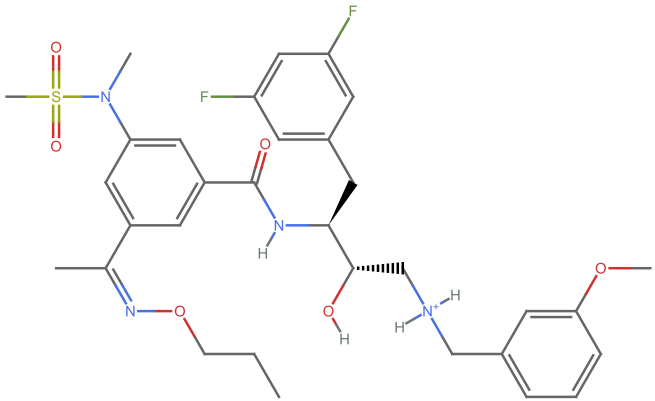

2D Autocorrelation

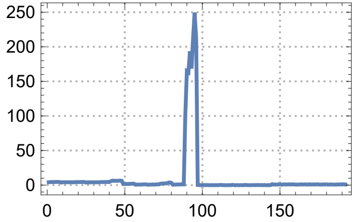

Dataset Index= 713

Molecule Formula= $C_{32}H_{41}F_2N_4O_6S^+$

3D Autocorrelation

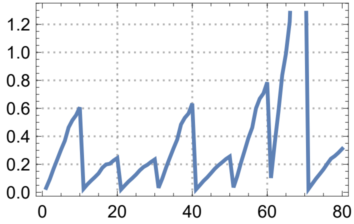

3D Plot

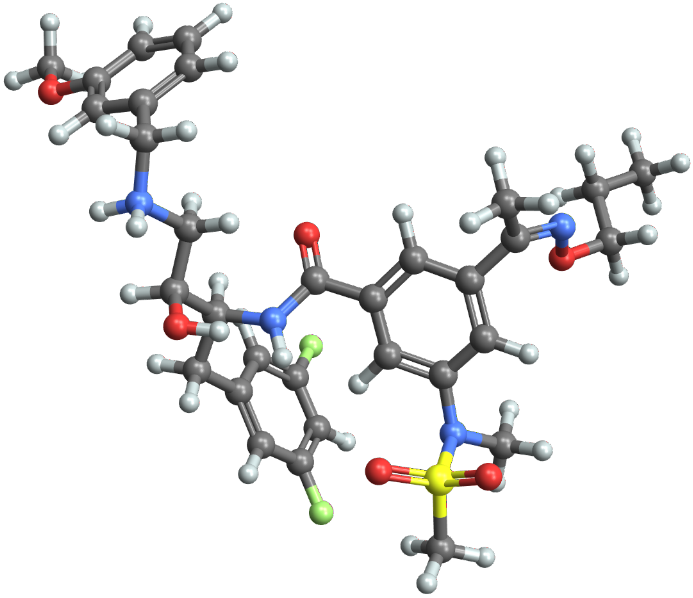

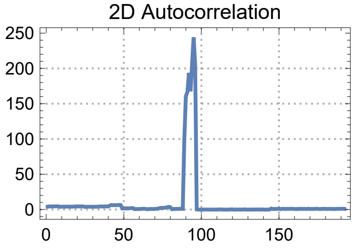

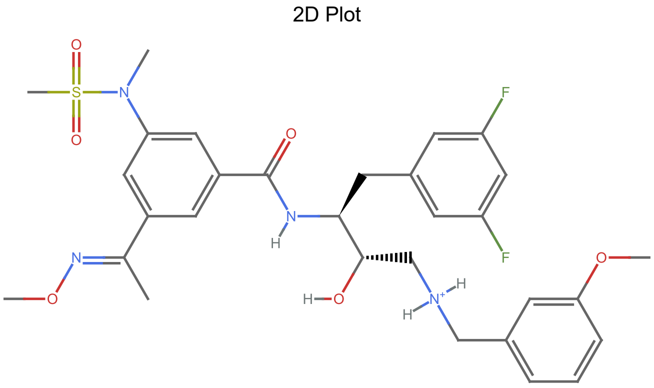

Dataset Index= 714

Molecule Formula= $C_{30}H_{37}F_2N_4O_6S^+$

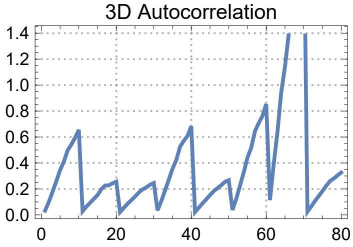

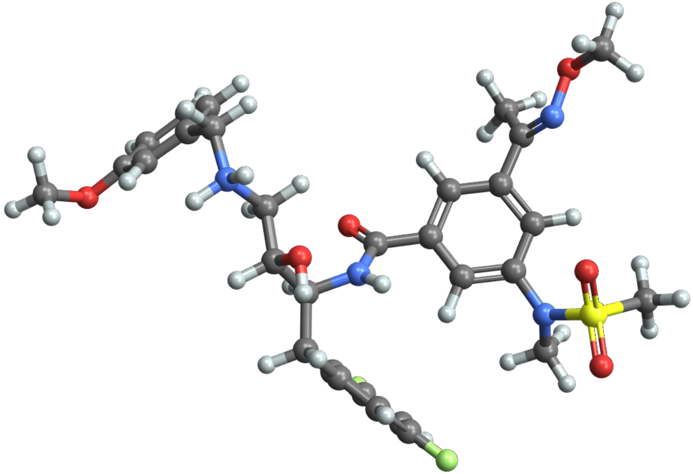

2D Plot

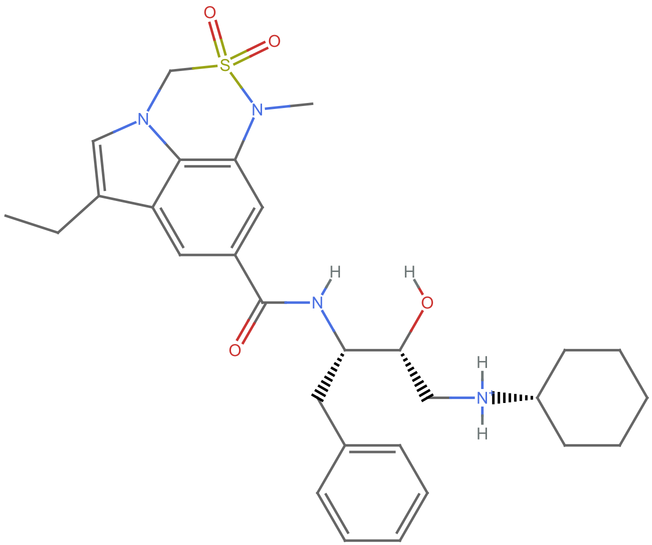

2D Autocorrelation

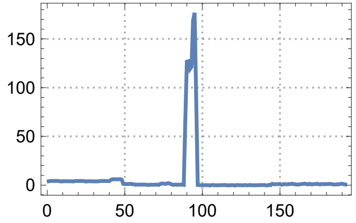

Dataset Index= 715

Molecule Formula= $C_{29}H_{39}N_4O_4S^+$

3D Autocorrelation

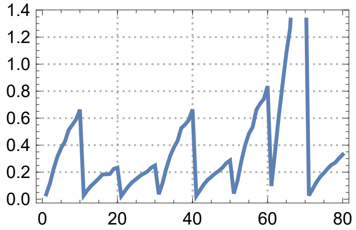

3D Plot

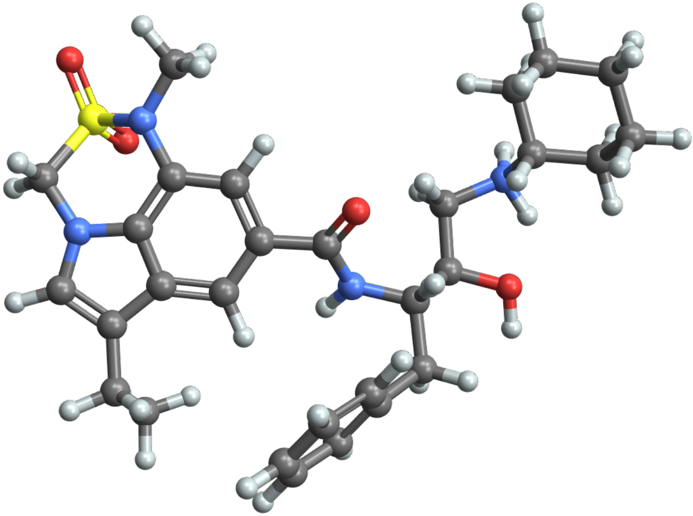

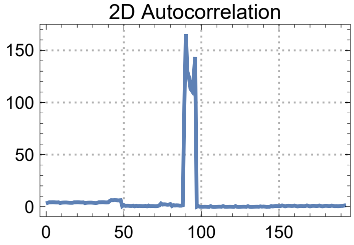

Dataset Index= 716

Molecule Formula= $C_{24}H_{20}F_4N_5O^+$

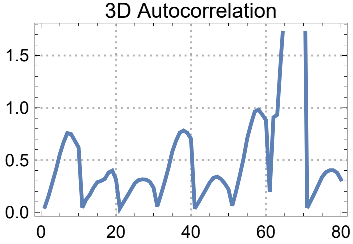

2D Plot

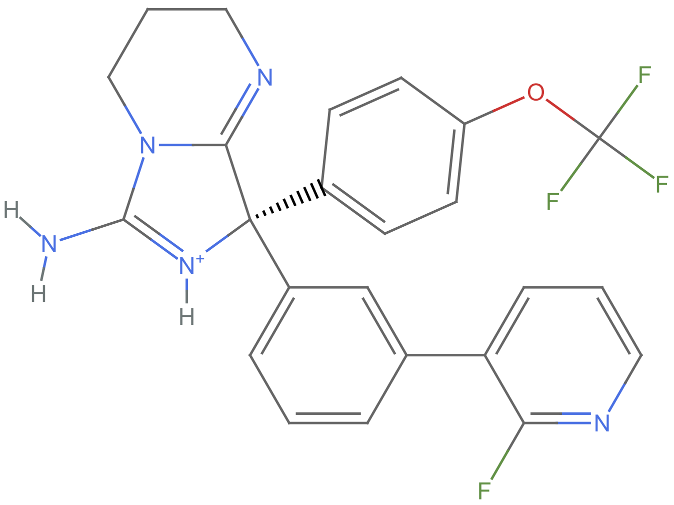

3D Plot

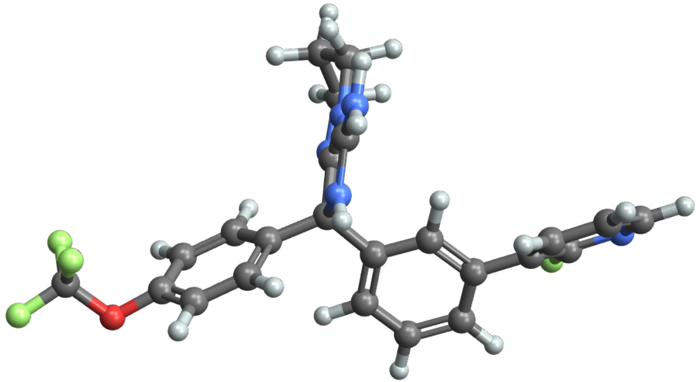

2D Plot

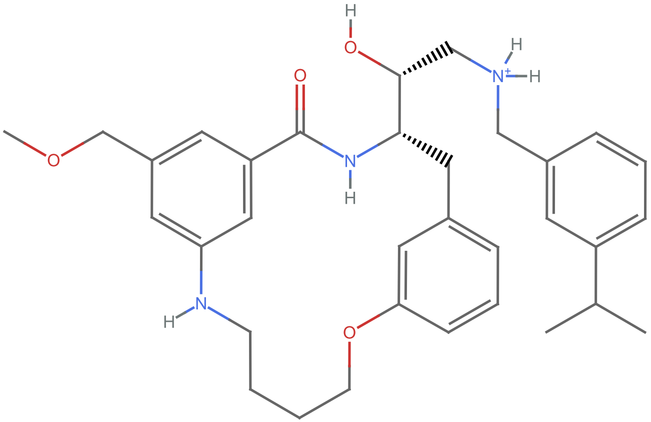

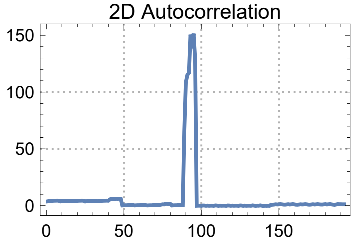

3D Plot

Dataset Index= 717

Molecule Formula= $C_{33}H_{44}N_3O_4^+$

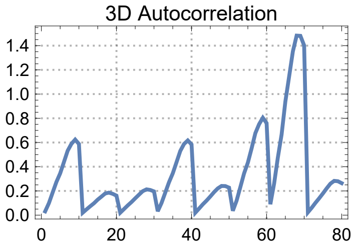

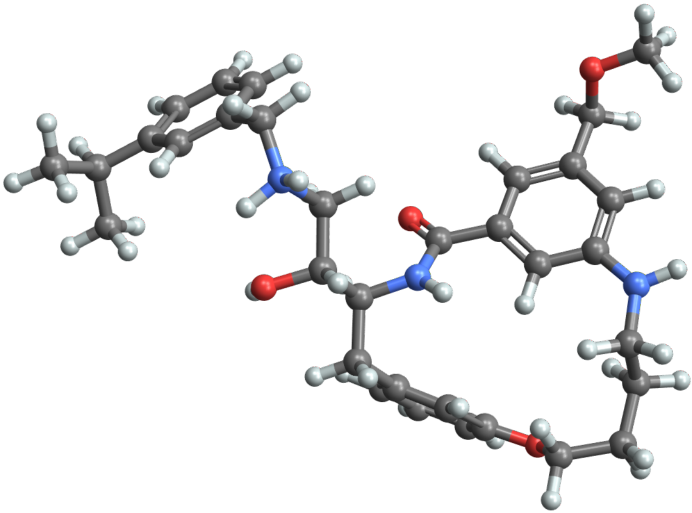

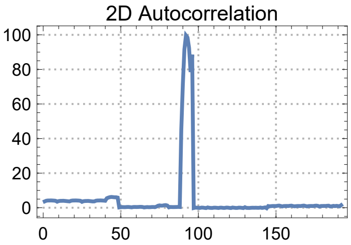

Dataset Index= 718

Molecule Formula= $C_{25}H_{26}N_4O_3$

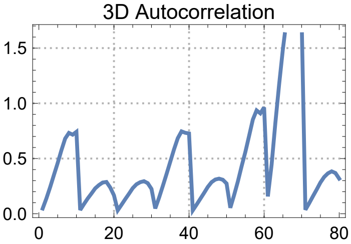

2D Plot

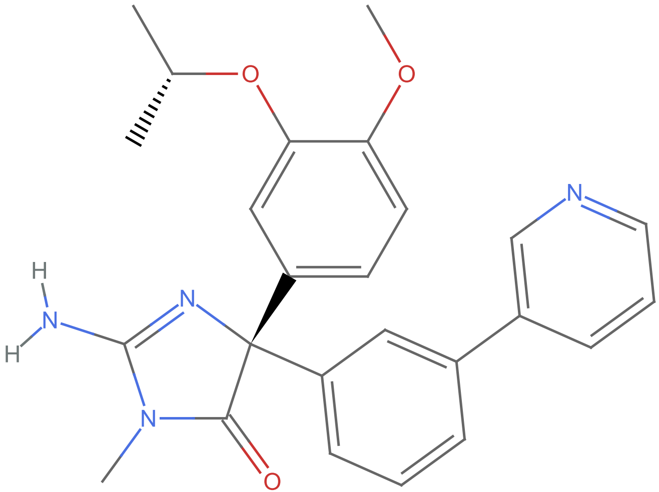

3D Plot

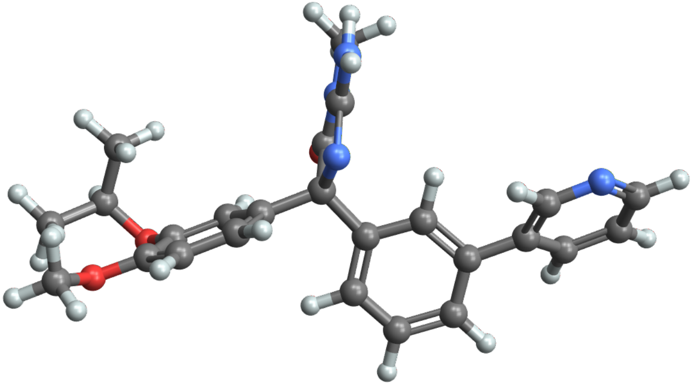

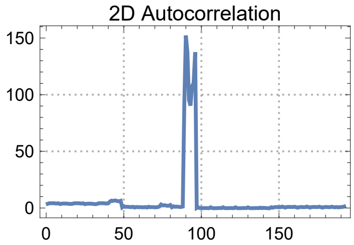

Dataset Index= 719

Molecule Formula= $C_{22}H_{16}F_4N_4O_2$

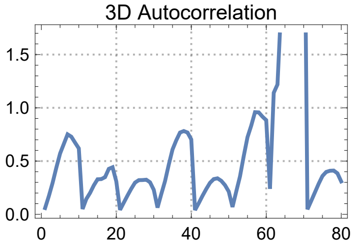

2D Plot

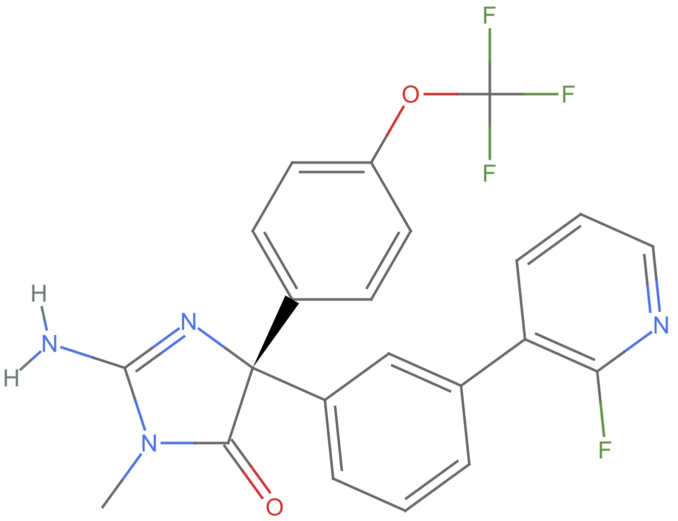

3D Plot

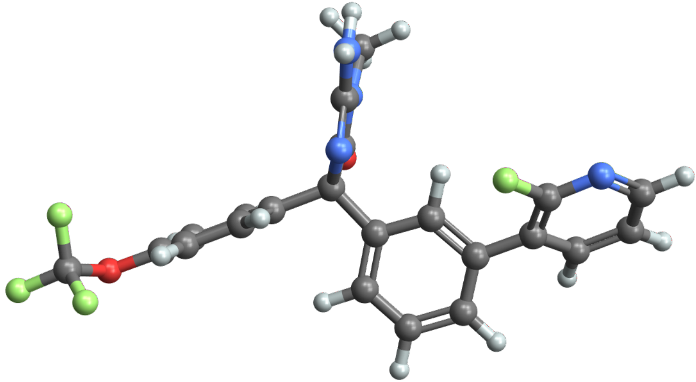

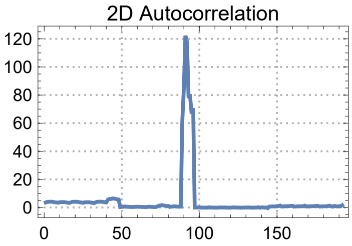

Dataset Index= 720

Molecule Formula= $C_{21}H_{16}F_2N_4O$

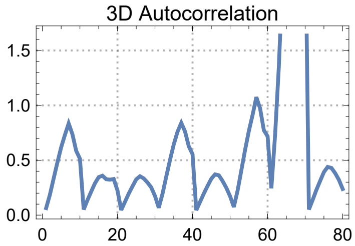

2D Plot

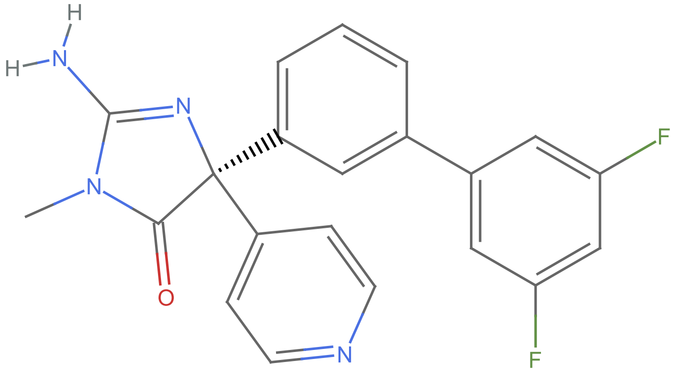

3D Plot

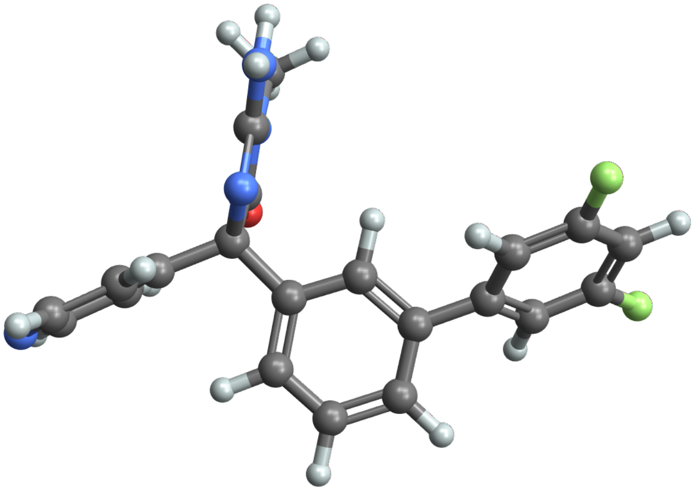

2D Autocorrelation

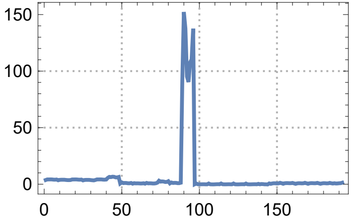

Dataset Index= 721

Molecule Formula= $C_{22}H_{16}F_4N_4O_2$

3D Autocorrelation

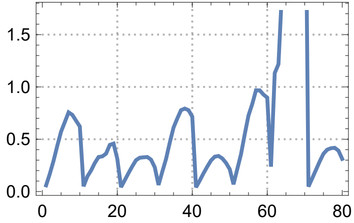

2D Plot

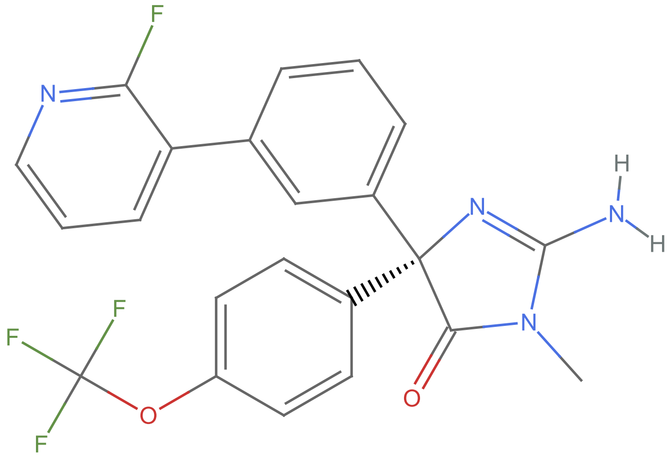

3D Plot

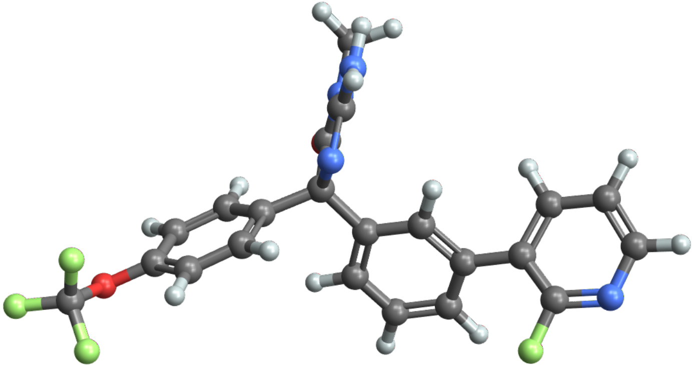

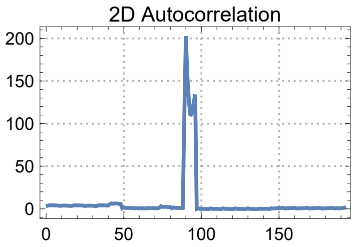

Dataset Index= 722

Molecule Formula= $C_{21}H_{20}F_5N_3O_2$

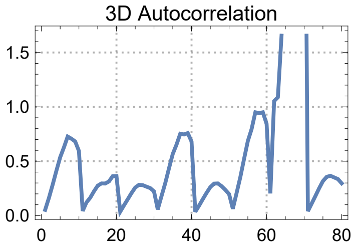

2D Plot

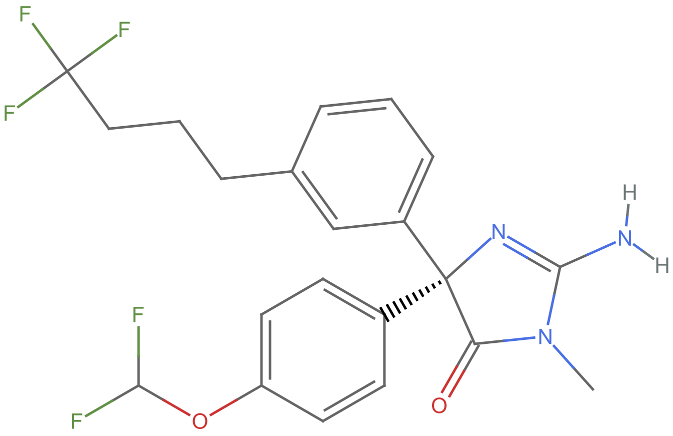

3D Plot

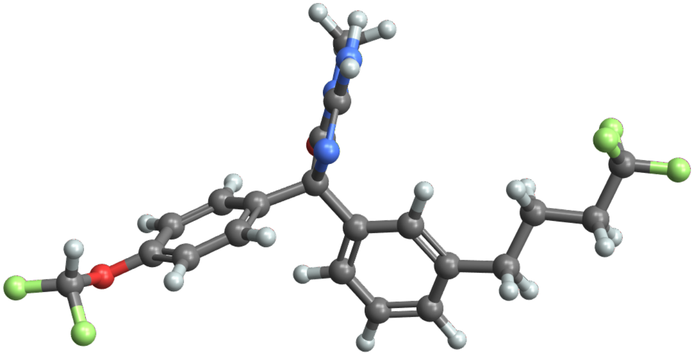

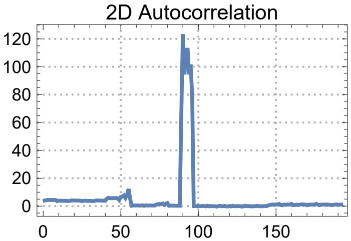

Dataset Index= 723

Molecule Formula= $C_{24}H_{32}BrN_2O_5S^+$

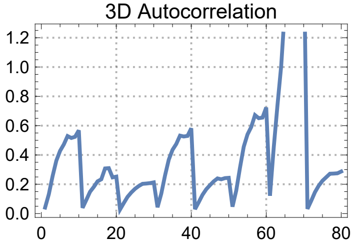

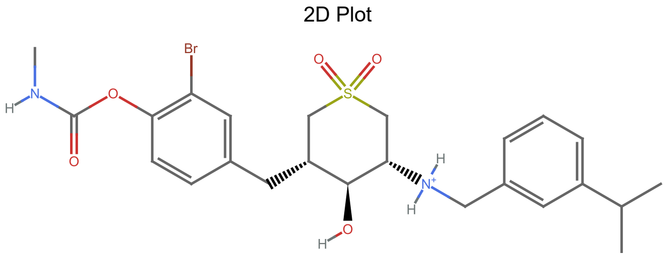

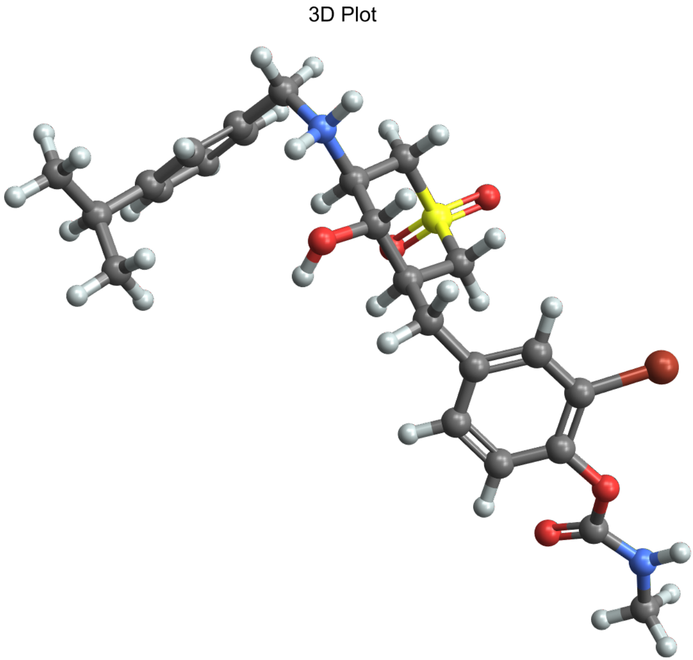

2D Plot

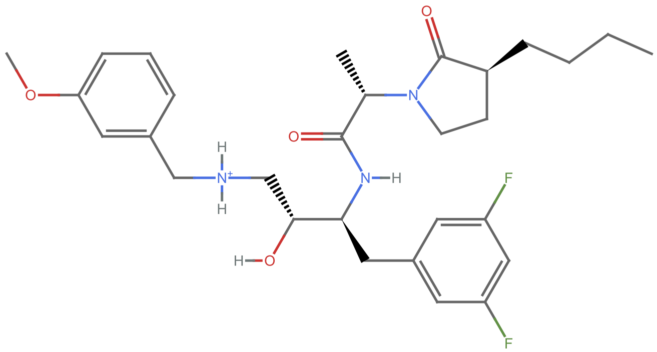

2D Autocorrelation

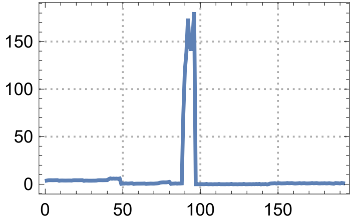

3D Plot

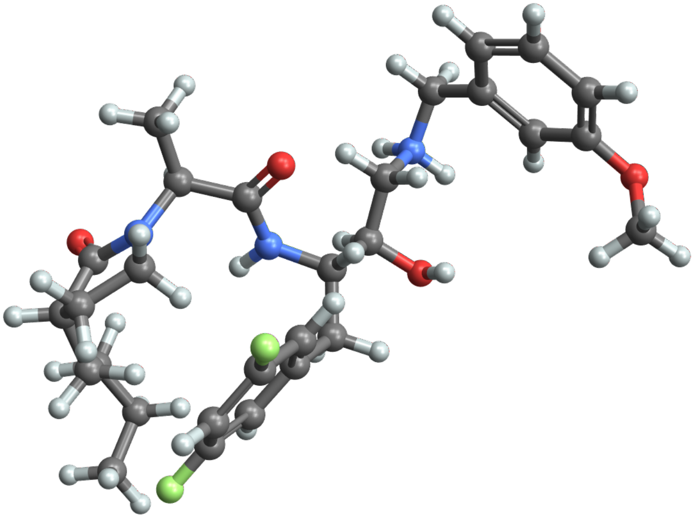

Dataset Index= 724

Molecule Formula= $C_{29}H_{40}F_2N_3O_4^+$

3D Autocorrelation

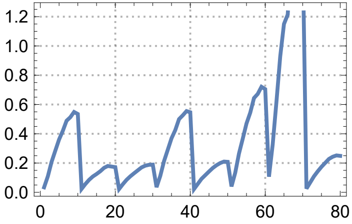

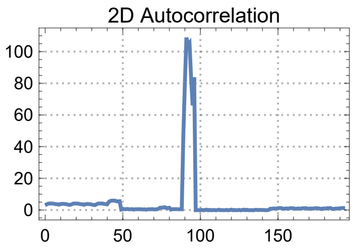

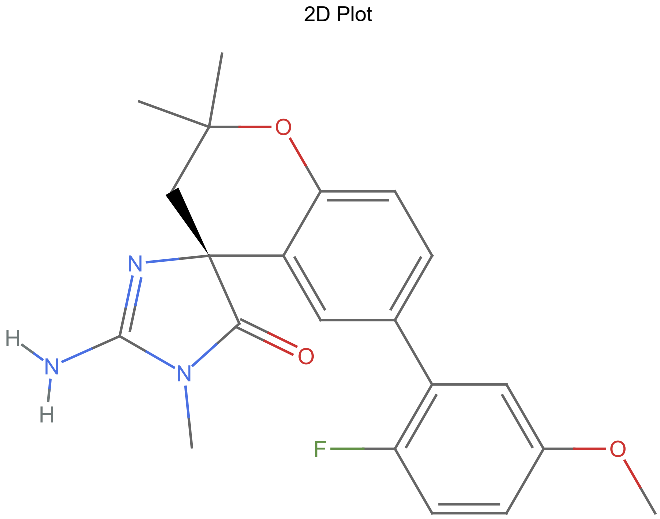

Dataset Index= 725

Molecule Formula= $C_{21}H_{22}FN_3O_3$

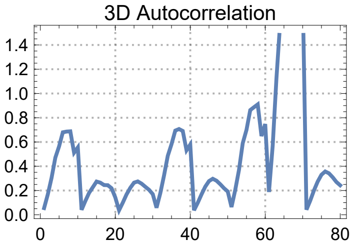

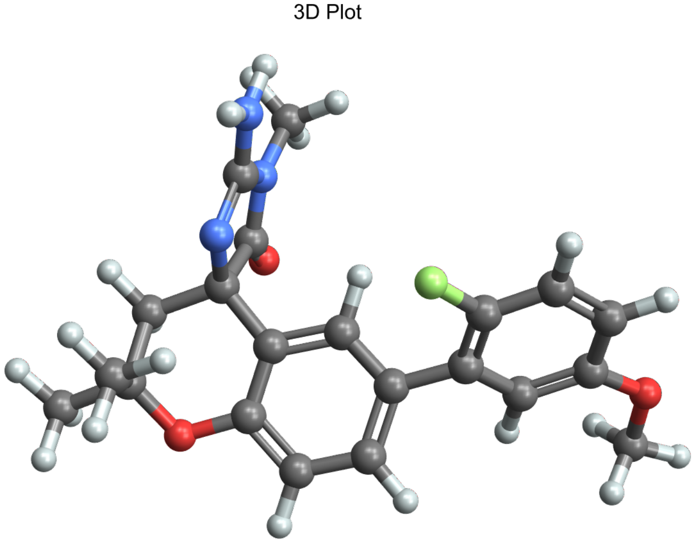

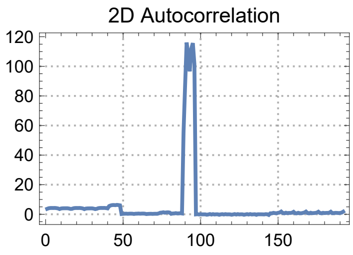

Dataset Index= 726

Molecule Formula= $C_{25}H_{20}FN_5O$

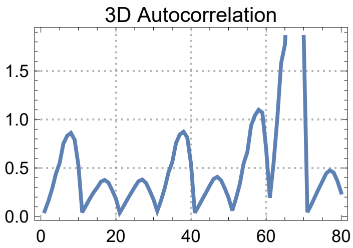

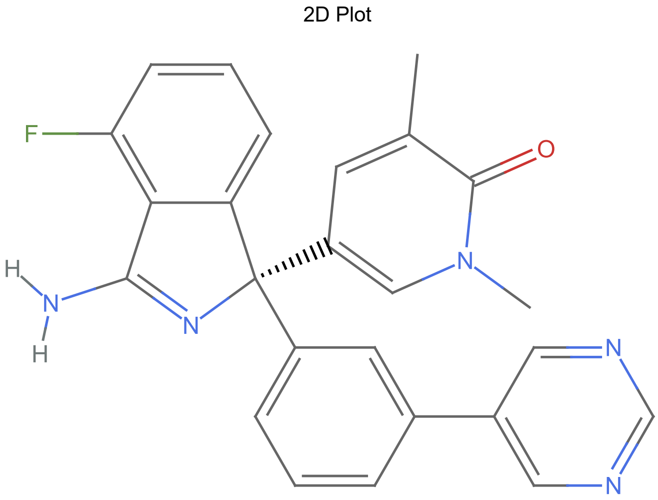

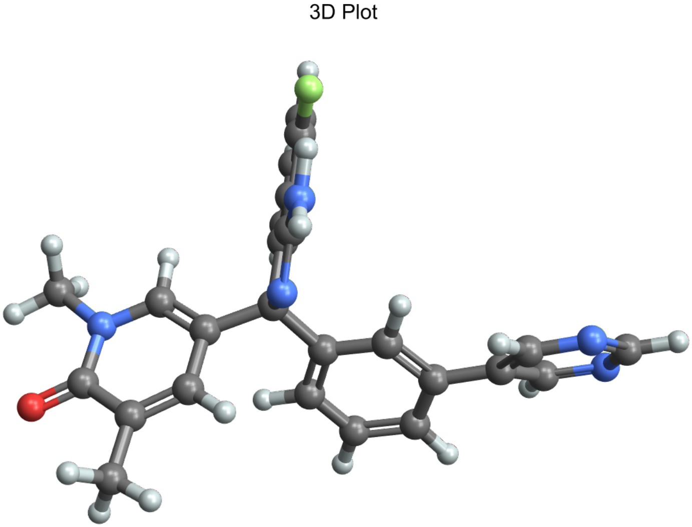

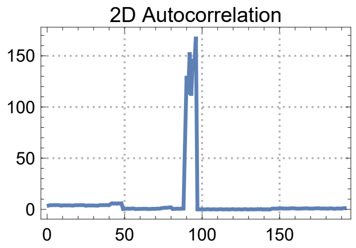

Dataset Index= 727

Molecule Formula= $C_{27}H_{37}F_2N_2O_3^+$

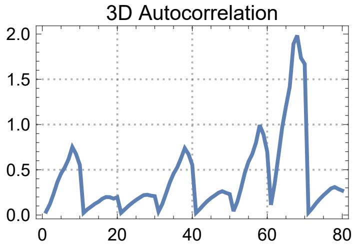

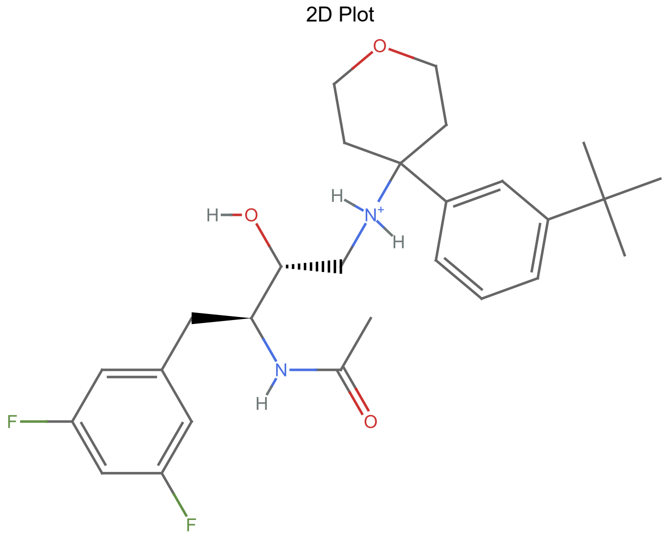

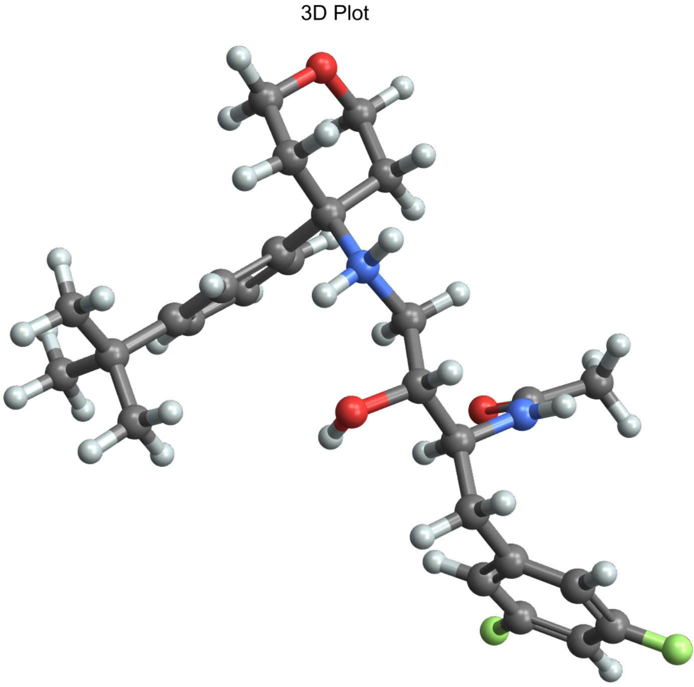

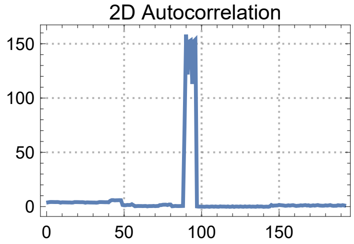

Dataset Index= 728

Molecule Formula= $C_{27}H_{40}FN_3O_4S^{2+}$

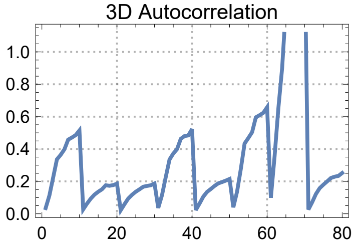

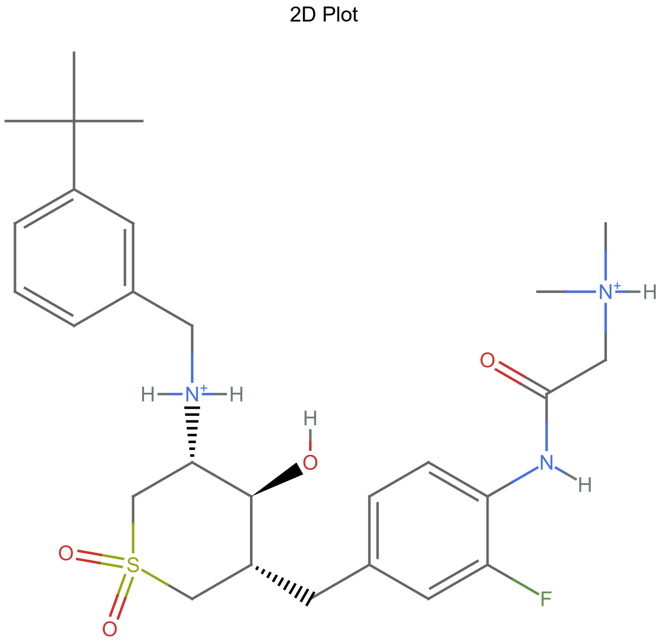

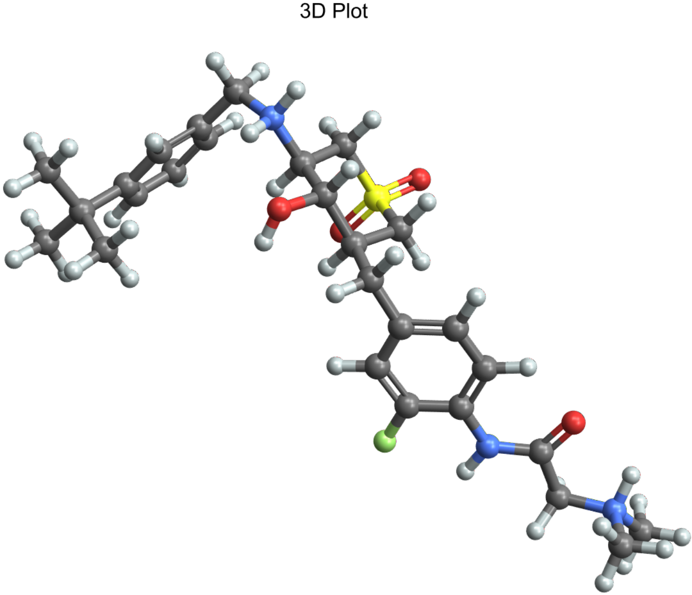

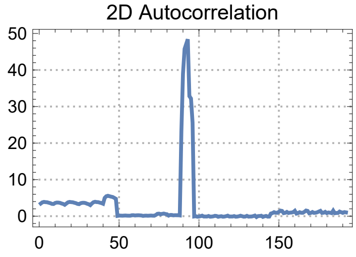

Dataset Index= 729

Molecule Formula= $C_{21}H_{20}N_4O$

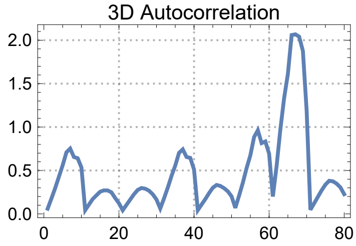

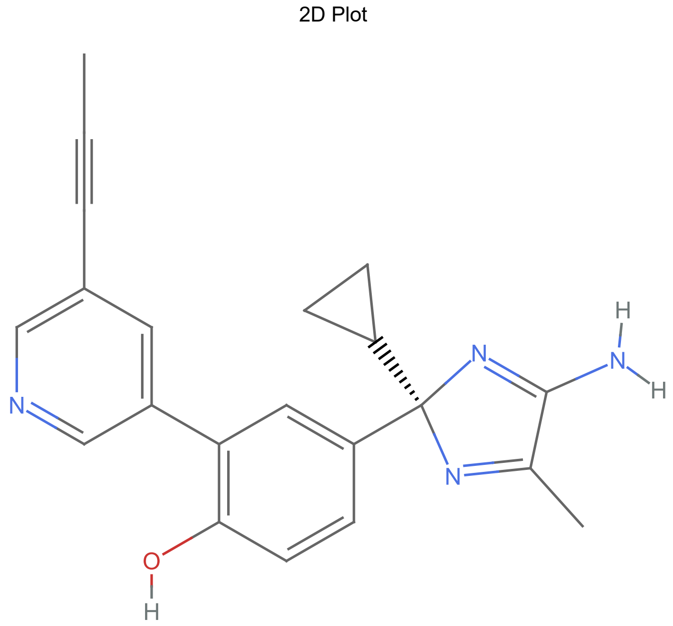

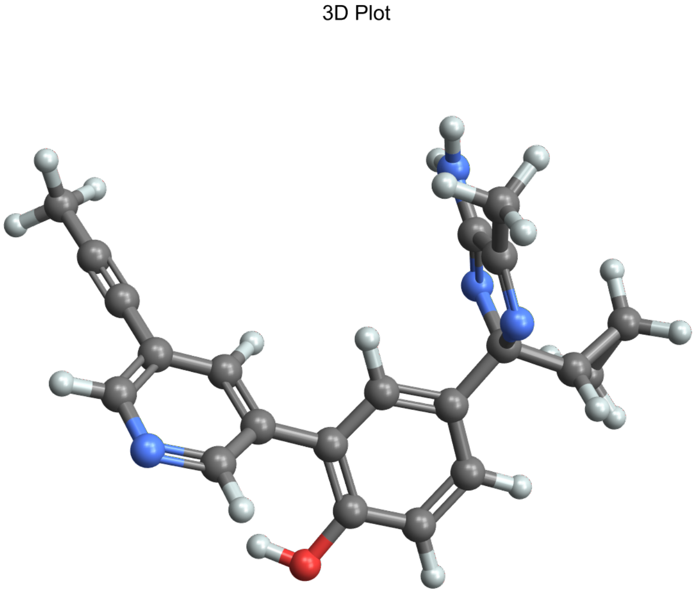

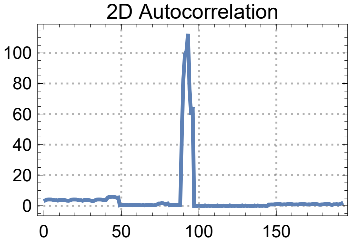

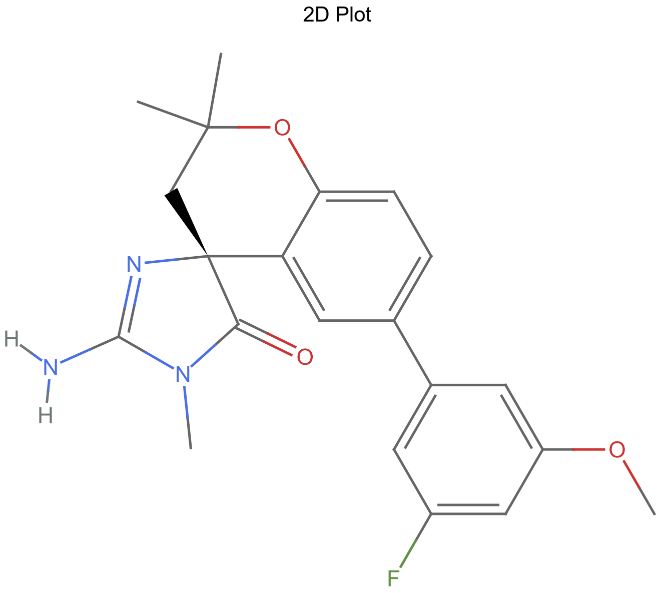

Dataset Index= 730

Molecule Formula= $C_{21}H_{22}FN_3O_3$

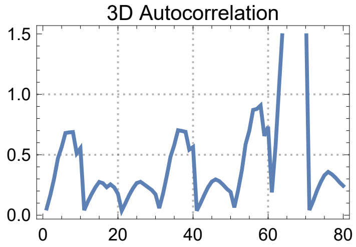

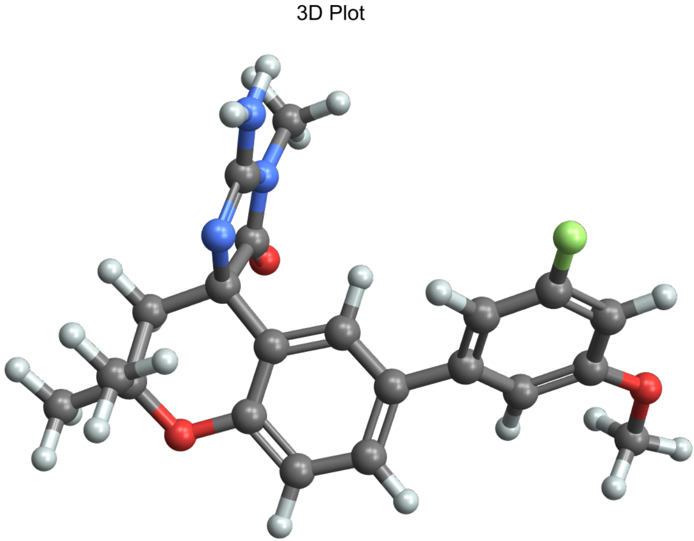

2D Autocorrelation

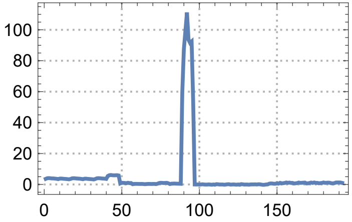

Dataset Index= 731

Molecule Formula= $C_{22}H_{18}ClFN_4O$

3D Autocorrelation

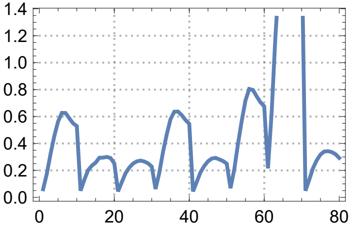

2D Plot

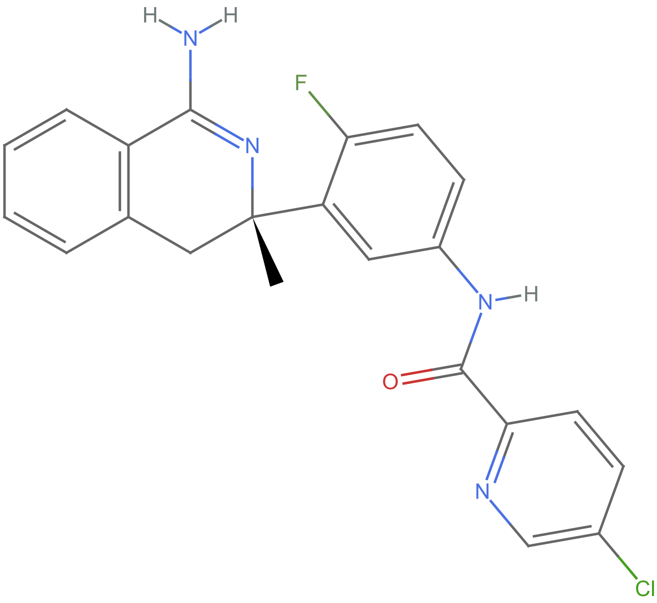

3D Plot

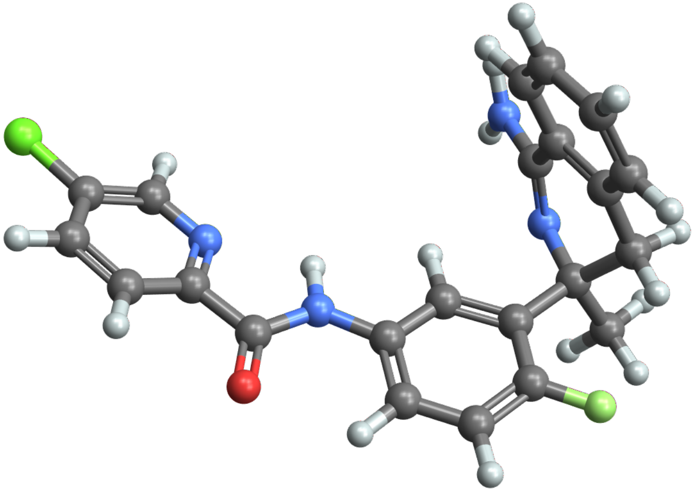

2D Plot

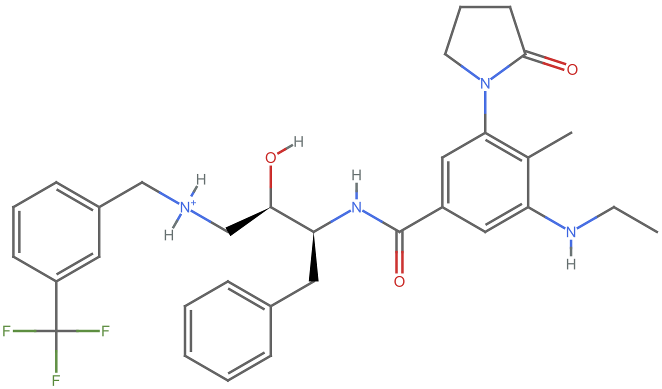

2D Autocorrelation

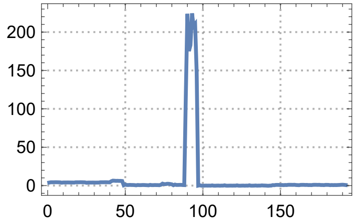

3D Plot

Dataset Index= 732

Molecule Formula= $C_{32}H_{38}F_3N_4O_3^+$

3D Autocorrelation

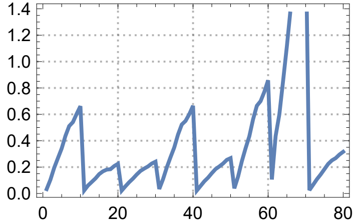

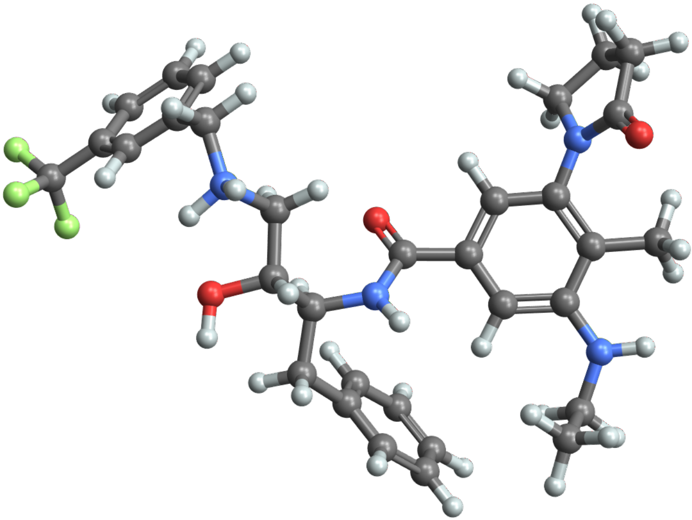

2D Autocorrelation

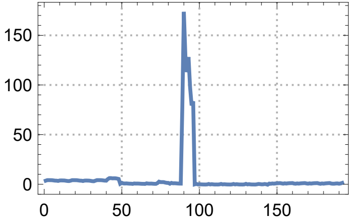

Dataset Index= 733

Molecule Formula= $C_{20}H_{19}F_3N_4O_2$

3D Autocorrelation

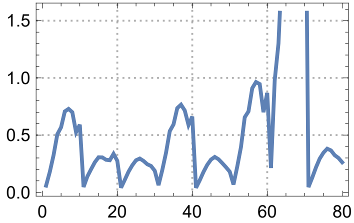

2D Plot

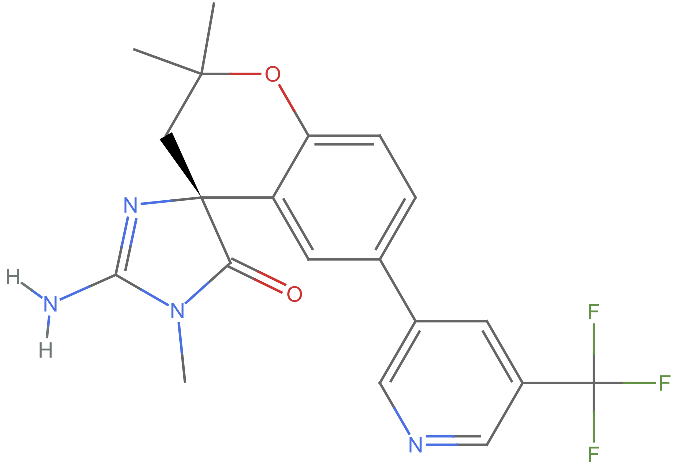

3D Plot

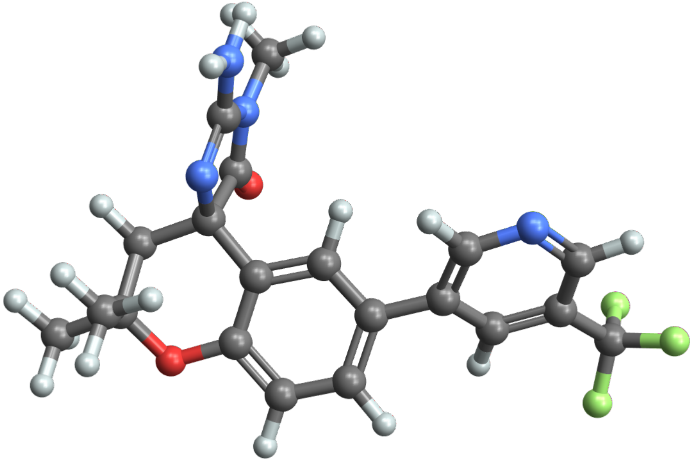

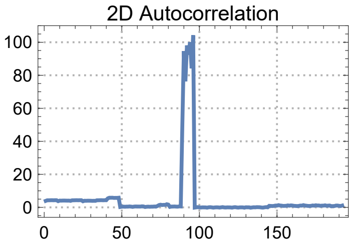

Dataset Index= 734

Molecule Formula= $C_{33}H_{46}N_5O_4^+$

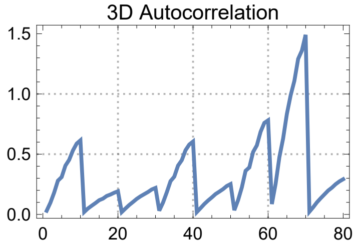

2D Plot

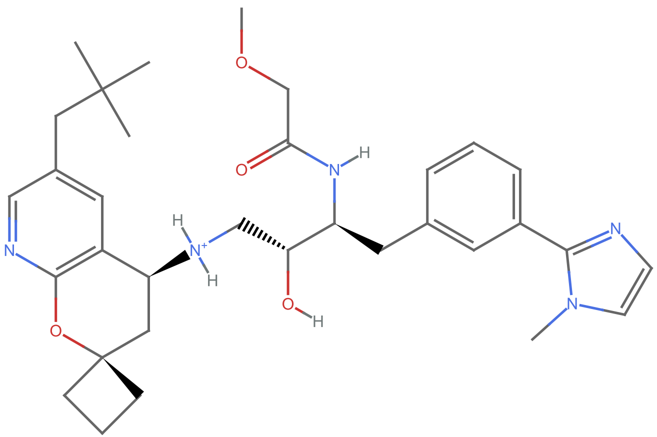

3D Plot

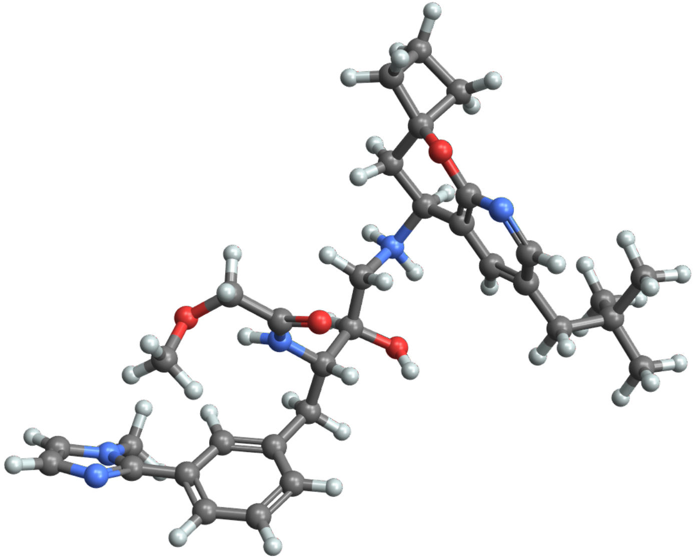

2D Plot

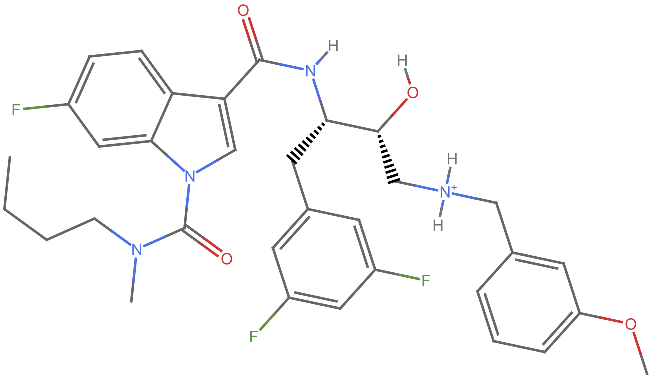

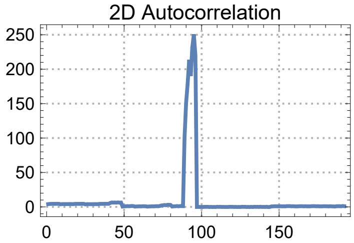

3D Plot

Dataset Index= 735

Molecule Formula= $C_{33}H_{38}F_3N_4O_4^+$

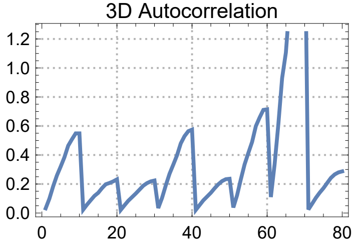

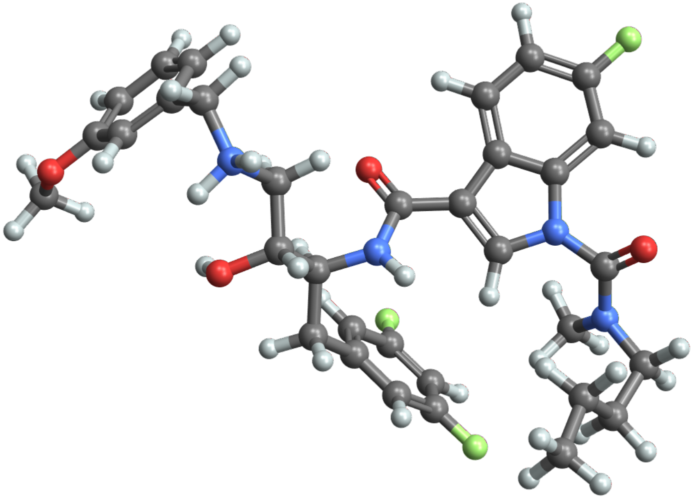

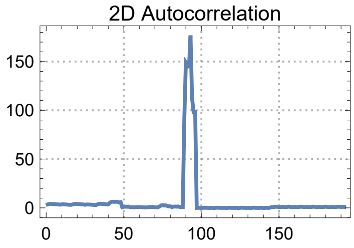

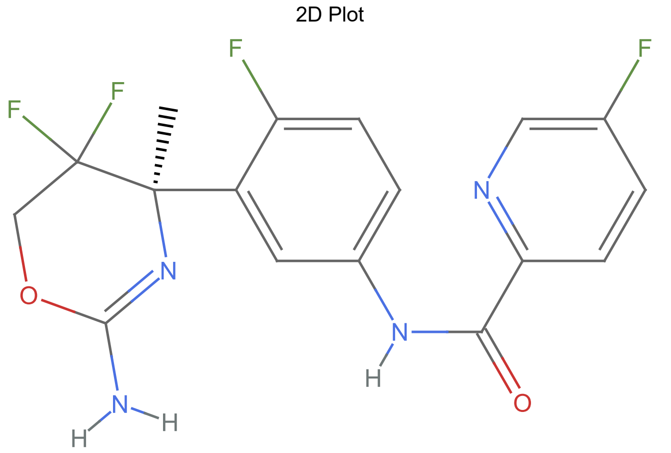

Dataset Index= 736

Molecule Formula= $C_{17}H_{14}F_4N_4O_2$

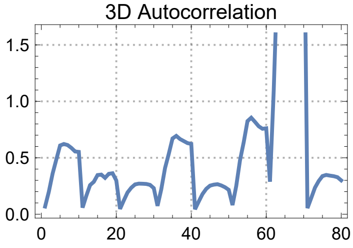

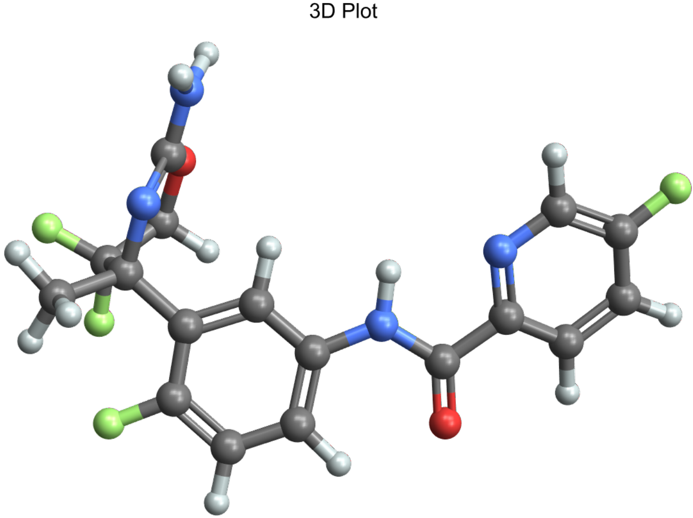

2D Plot

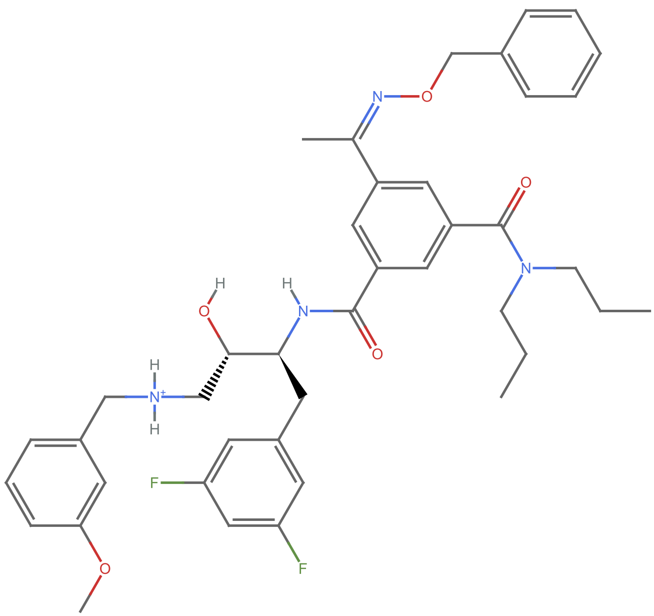

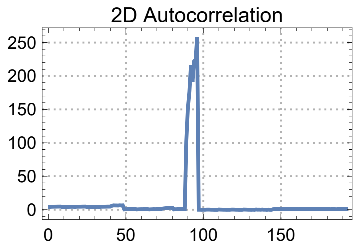

Dataset Index= 737

Molecule Formula= $C_{41}H_{49}F_{2}N_{4}O_{5}^{+}$

3D Plot

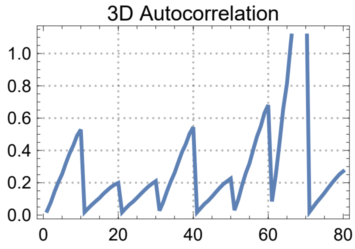

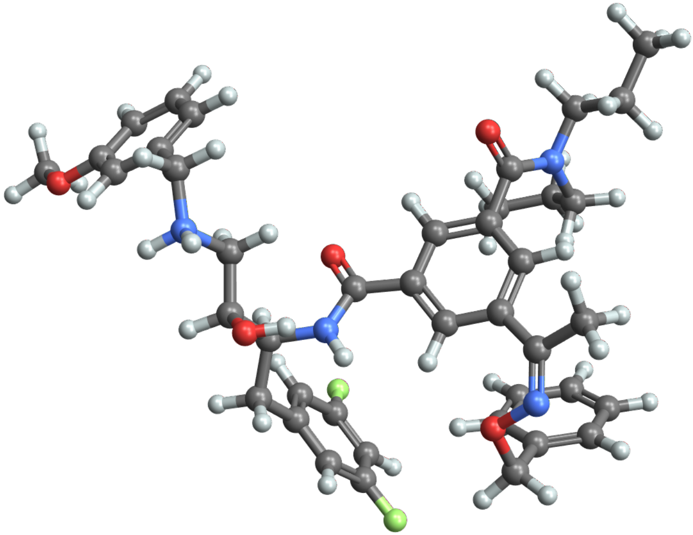

2D Plot

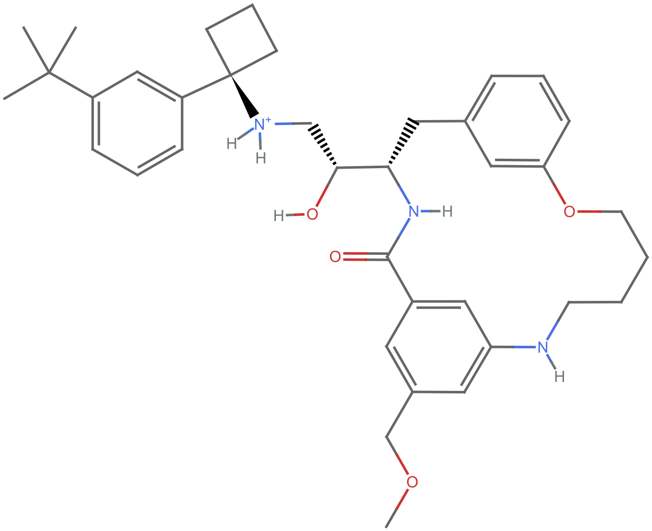

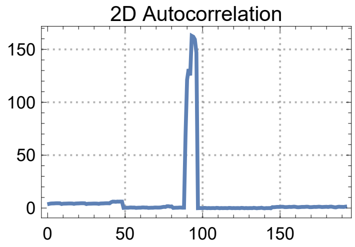

Dataset Index= 738

Molecule Formula= $C_{37}H_{50}N_3O_4^+$

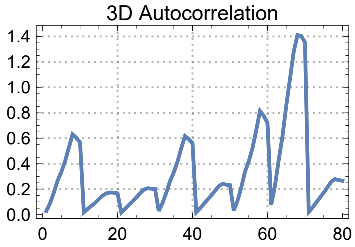

3D Plot

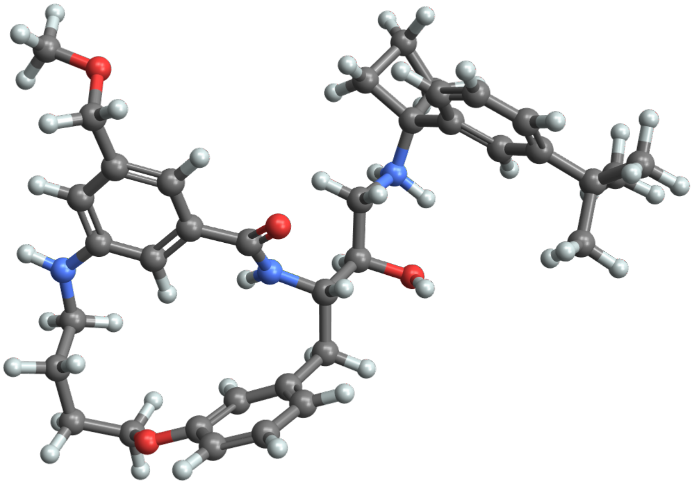

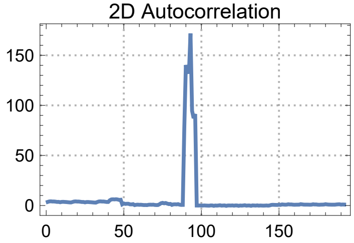

Dataset Index= 739

Molecule Formula= $C_{16}H_{13}ClF_3N_5O_2$

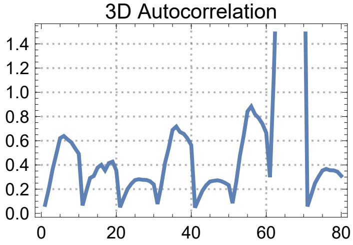

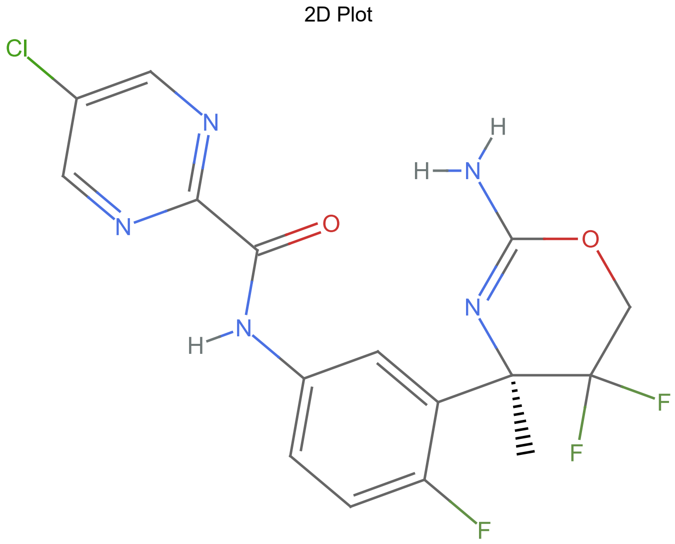

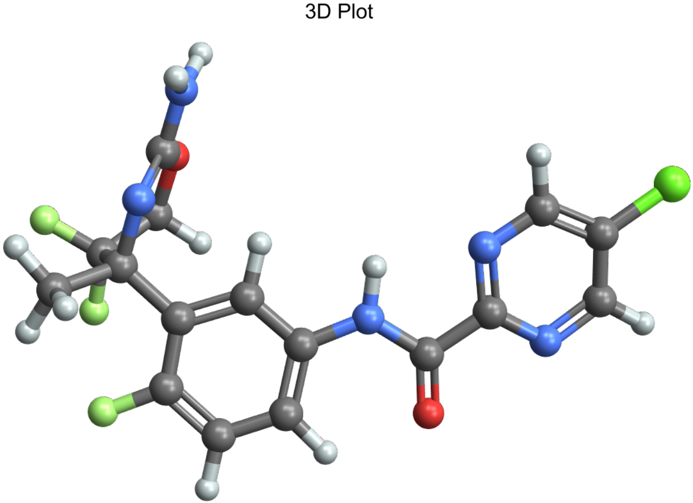

2D Plot

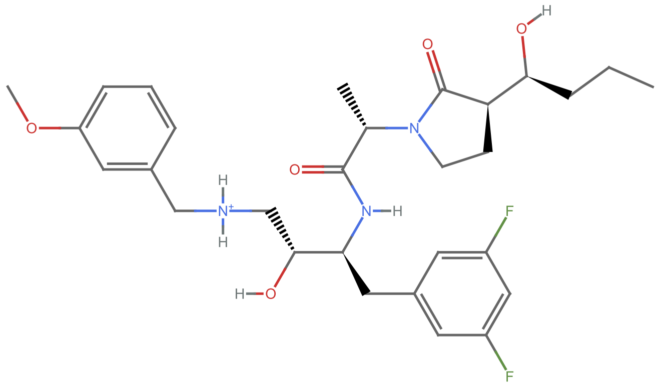

3D Plot

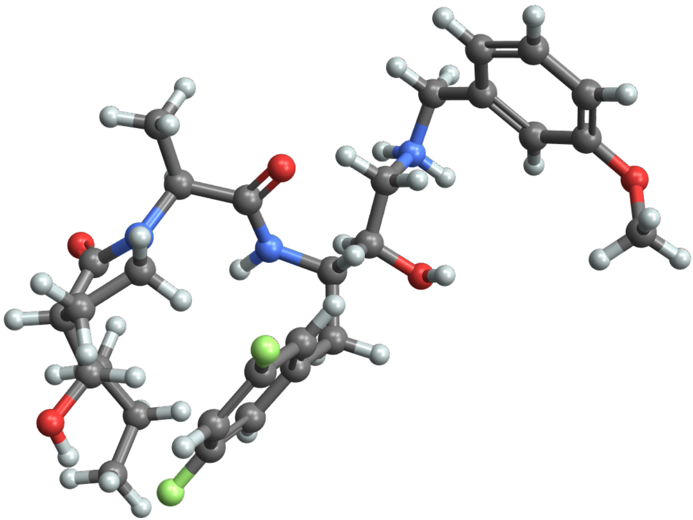

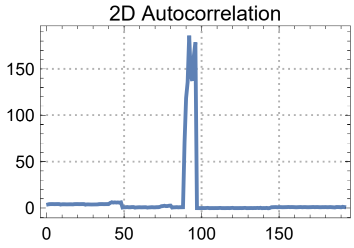

Dataset Index= 740

Molecule Formula= $C_{29}H_{40}F_2N_3O_5^+$

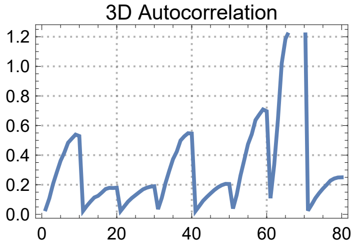

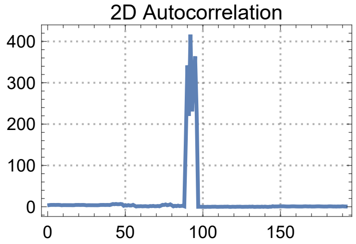

Dataset Index= 741

Molecule Formula= $C_{25}H_{31}F_7N_3O_4S^+$

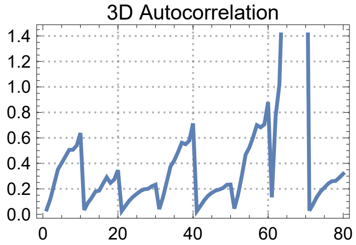

2D Plot

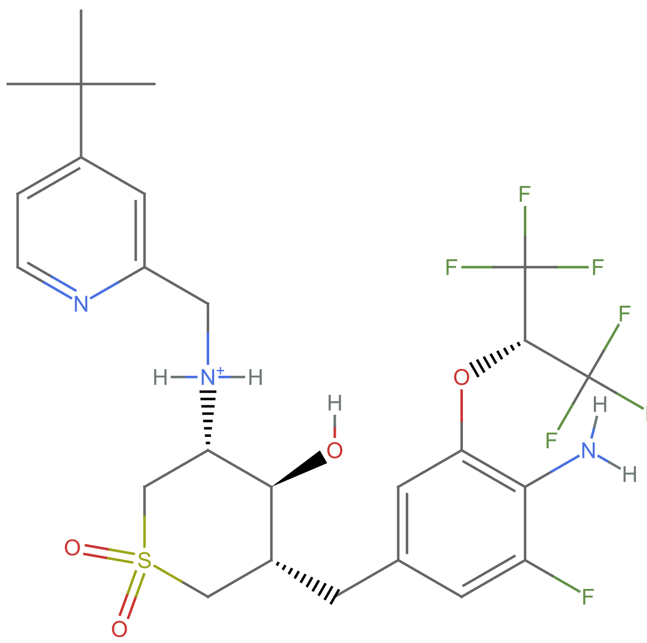

3D Plot

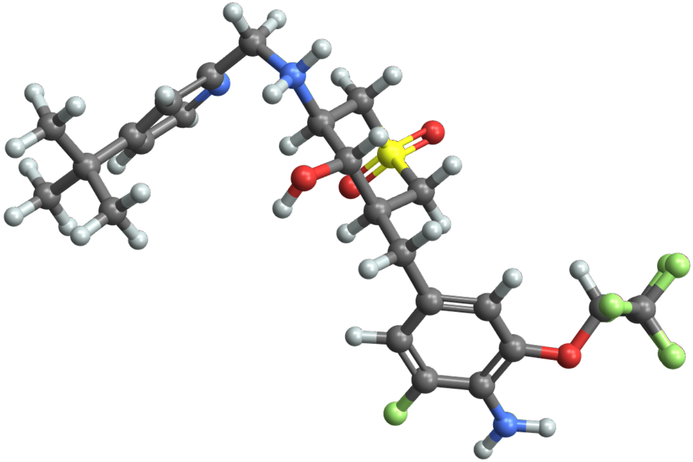

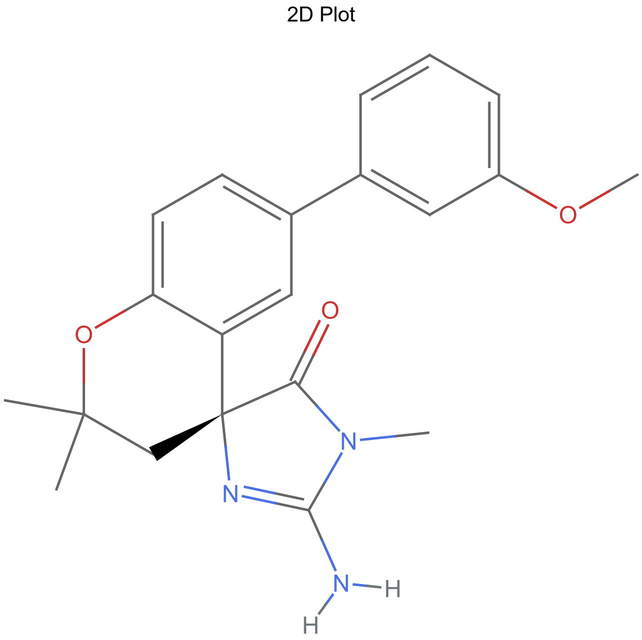

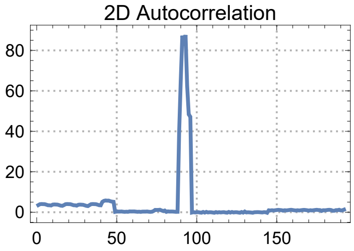

Dataset Index= 742

Molecule Formula= $C_{21}H_{23}N_3O_3$

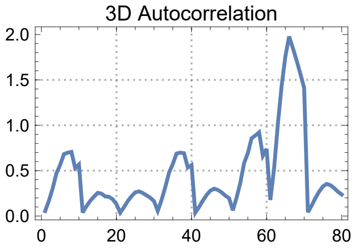

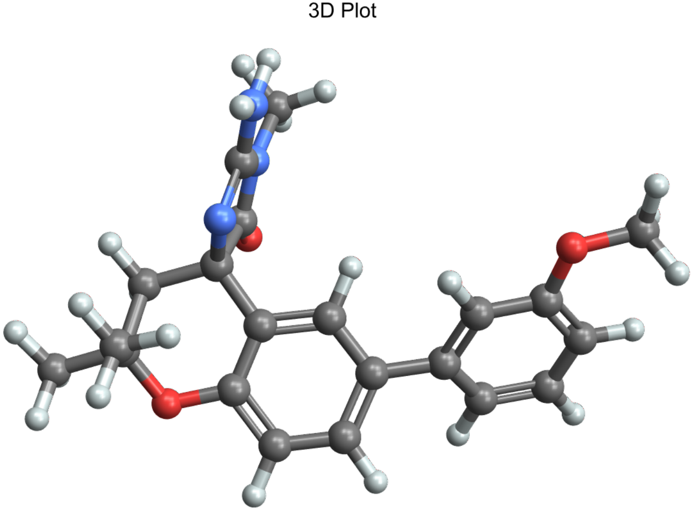

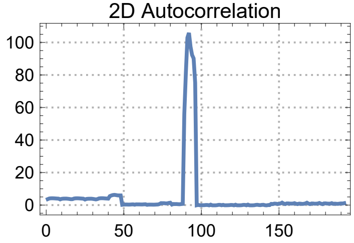

Dataset Index= 743

Molecule Formula= $C_{24}H_{23}FN_5O^+$

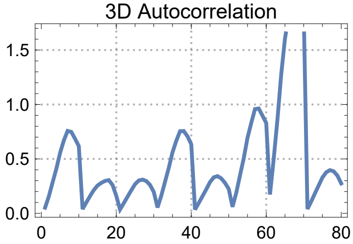

2D Plot

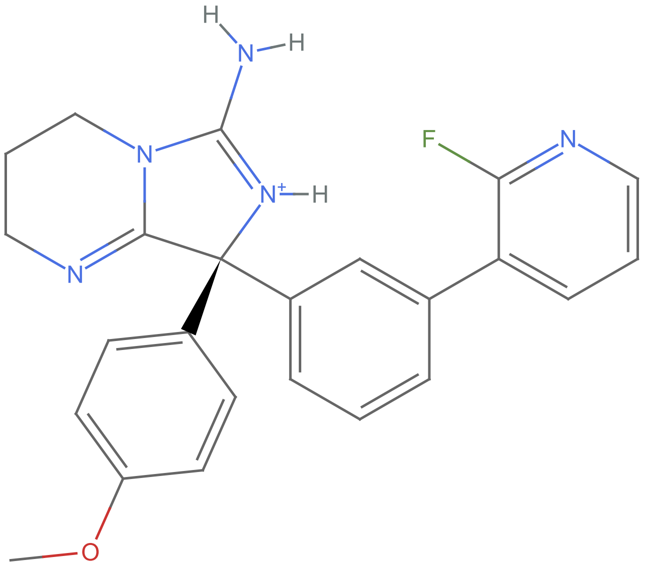

3D Plot

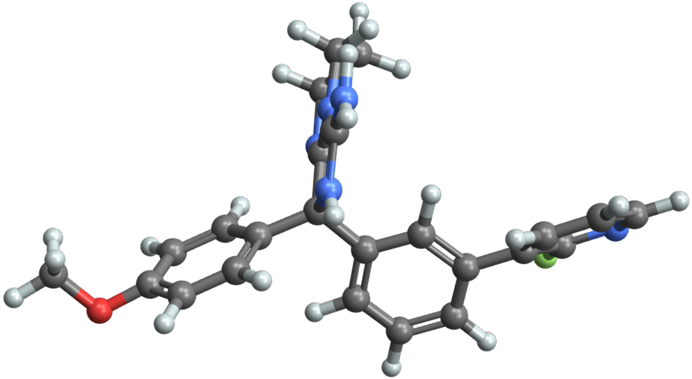

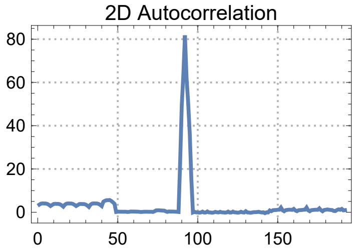

Dataset Index= 744

Molecule Formula= $C_{21}H_{27}N_3O_2$

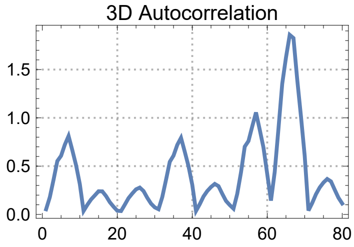

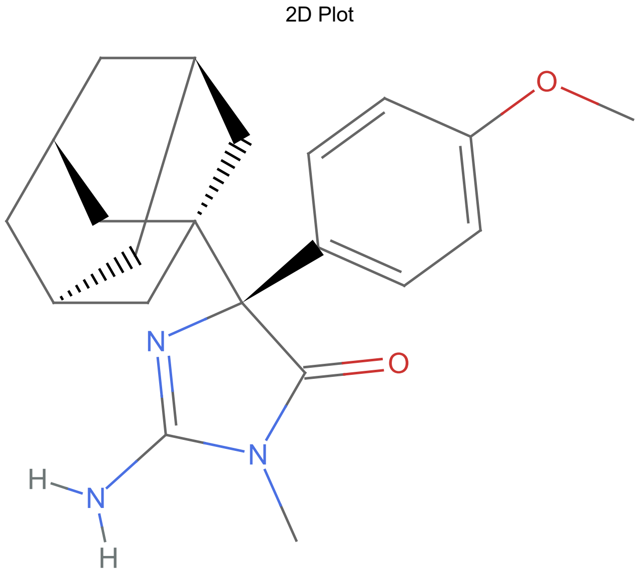

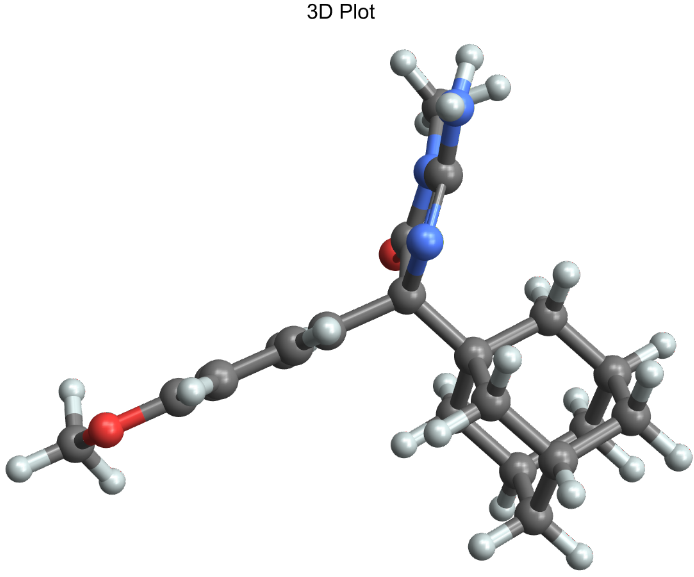

2D Autocorrelation

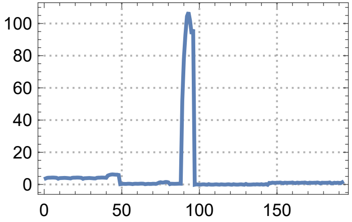

Dataset Index= 745

Molecule Formula= $C_{27}H_{28}N_4O_3$

3D Autocorrelation

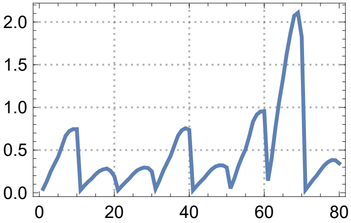

2D Plot

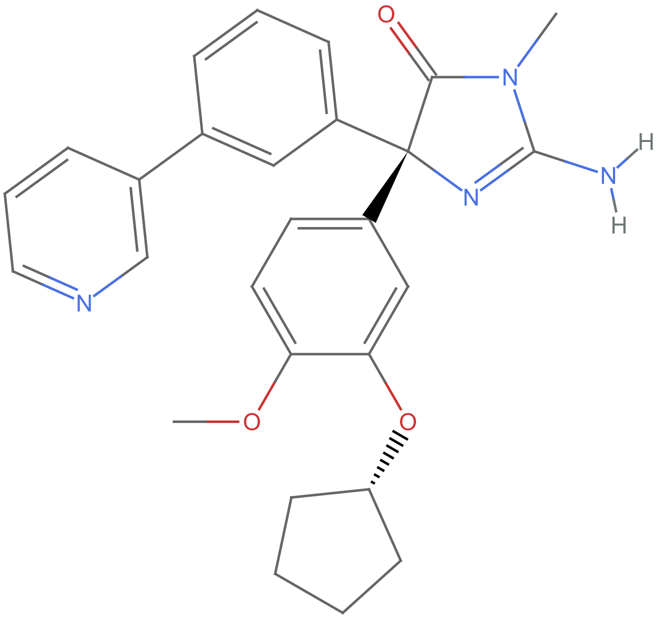

3D Plot

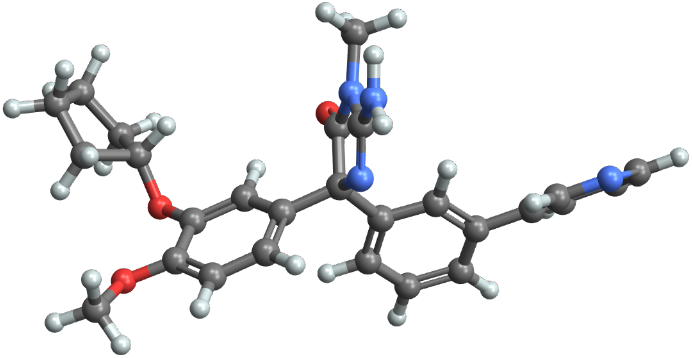

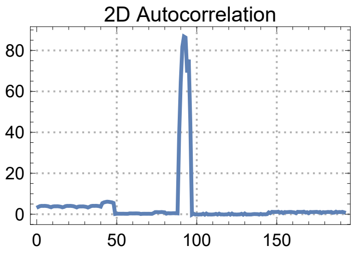

Dataset Index= 746

Molecule Formula= $C_{22}H_{22}N_6O$

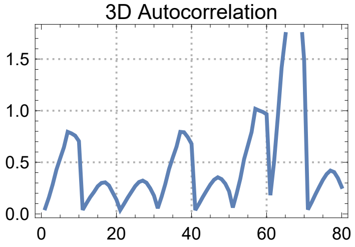

2D Plot

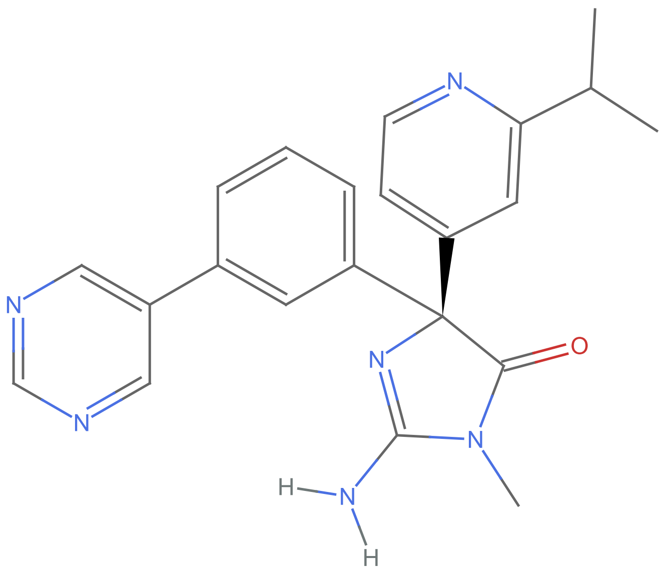

3D Plot

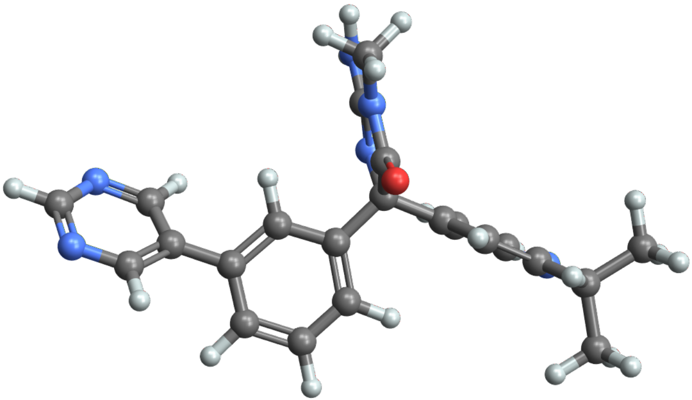

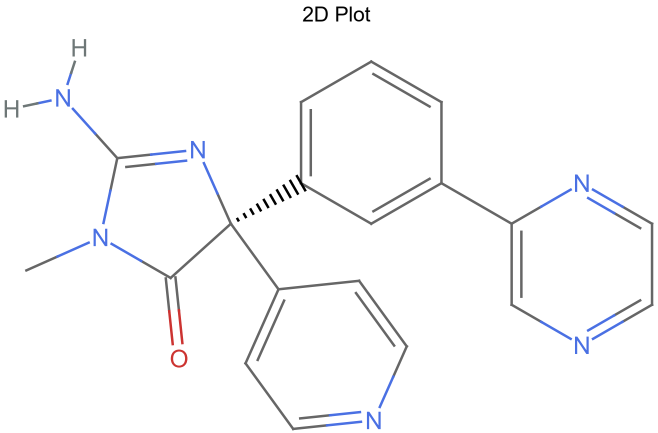

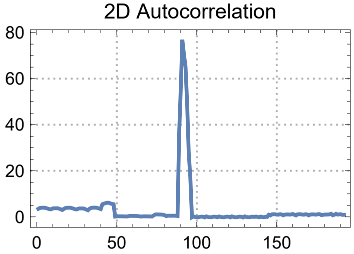

Dataset Index= 747

Molecule Formula= $C_{19}H_{16}N_6O$

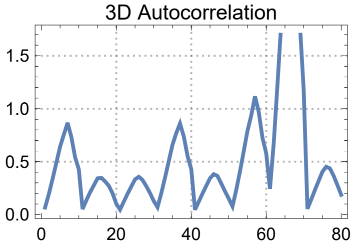

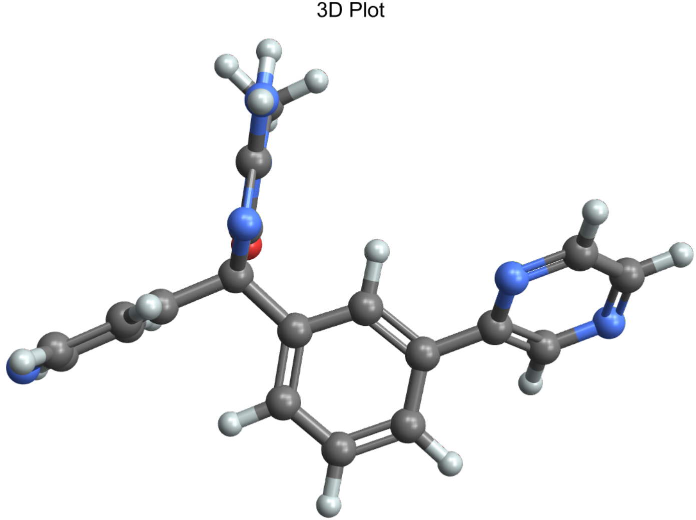

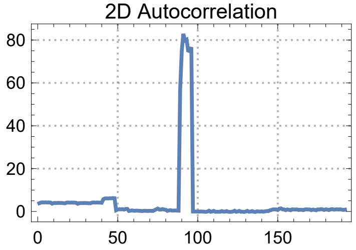

Dataset Index= 748

Molecule Formula= $C_{28}H_{25}ClN_6O_2$

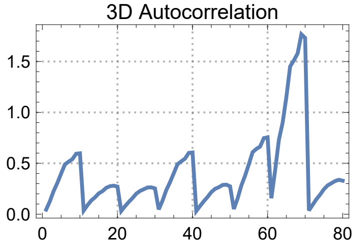

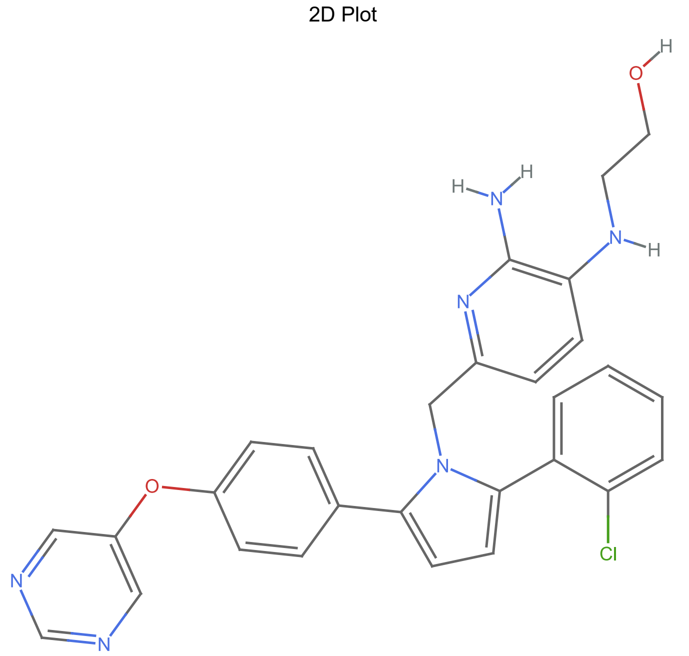

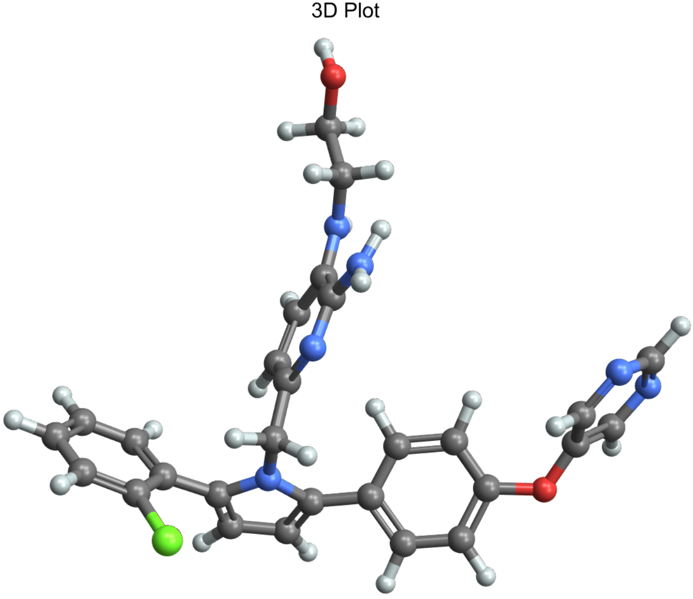

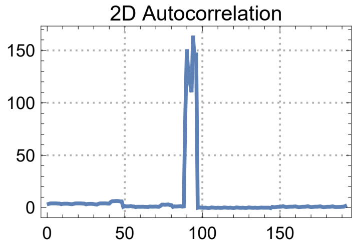

Dataset Index= 749

Molecule Formula= $C_{19}H_{16}F_5N_3O_3$

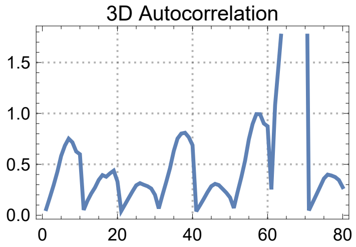

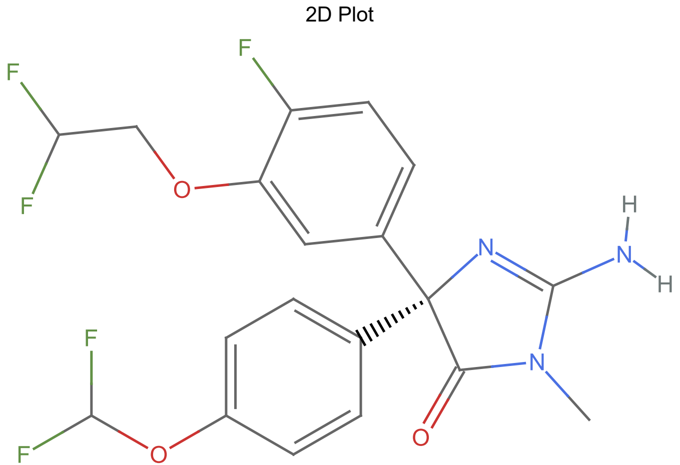

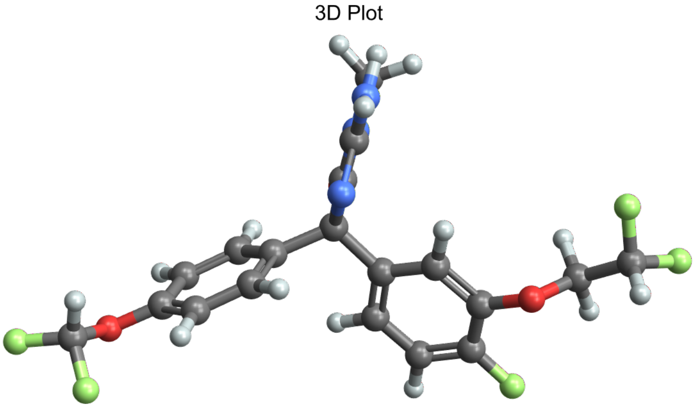

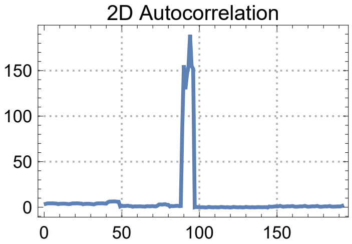

Dataset Index= 750

Molecule Formula= $C_{20}H_{18}F_5N_3O_3$

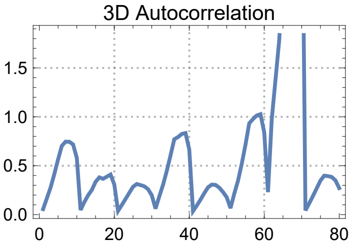

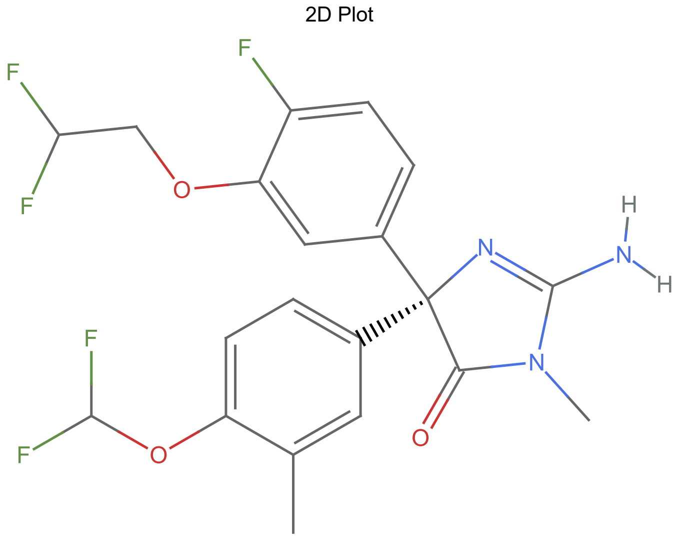

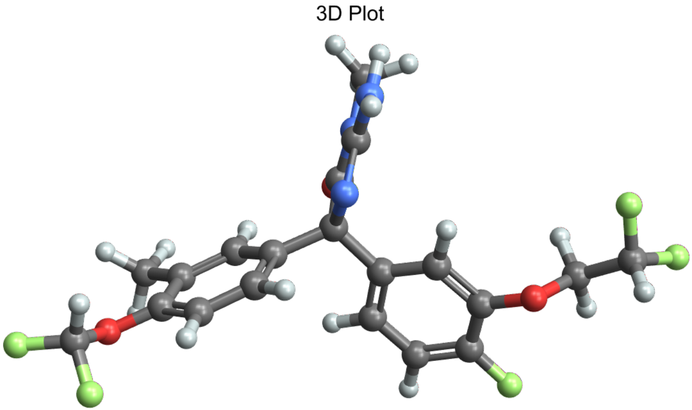

2D Plot

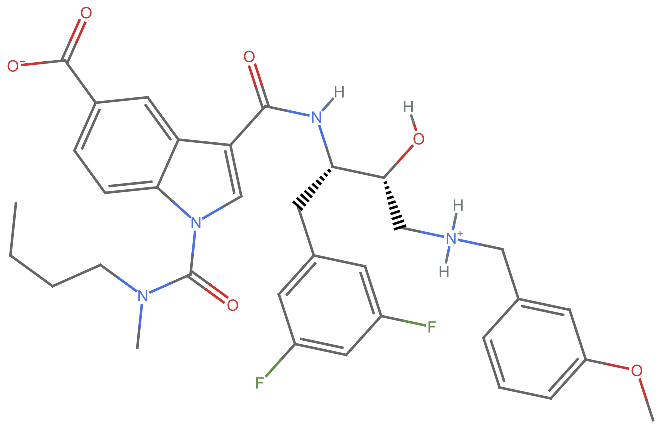

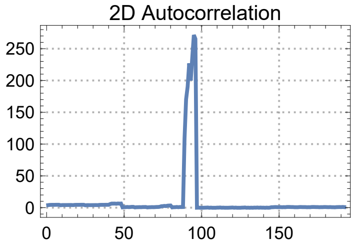

3D Plot

Dataset Index= 751

Molecule Formula= $C_{34}H_{38}F_2N_4O_6$

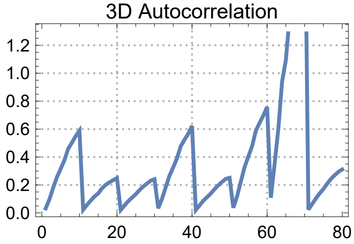

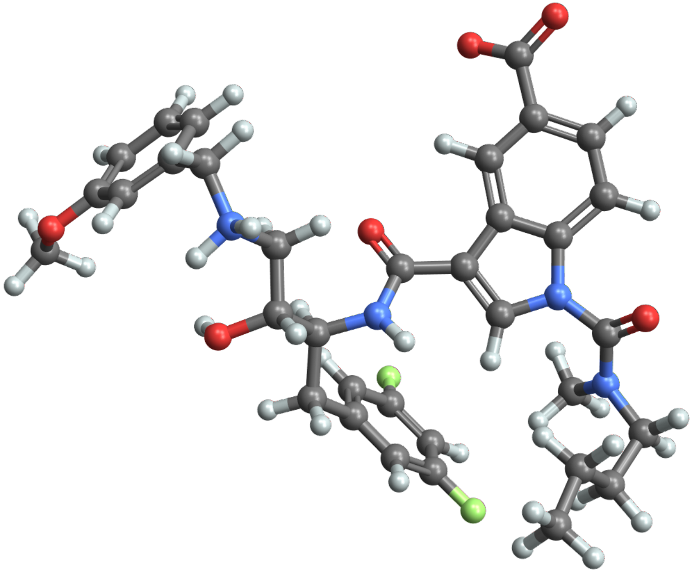

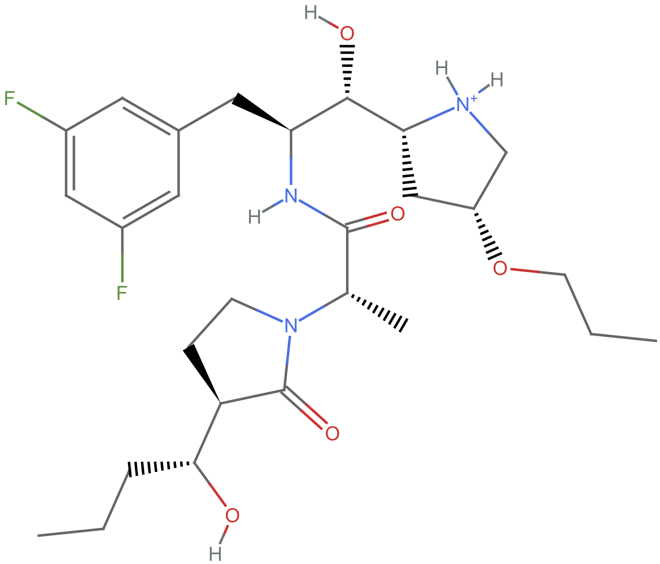

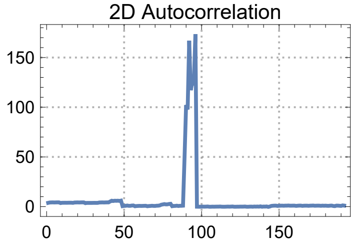

3D Plot

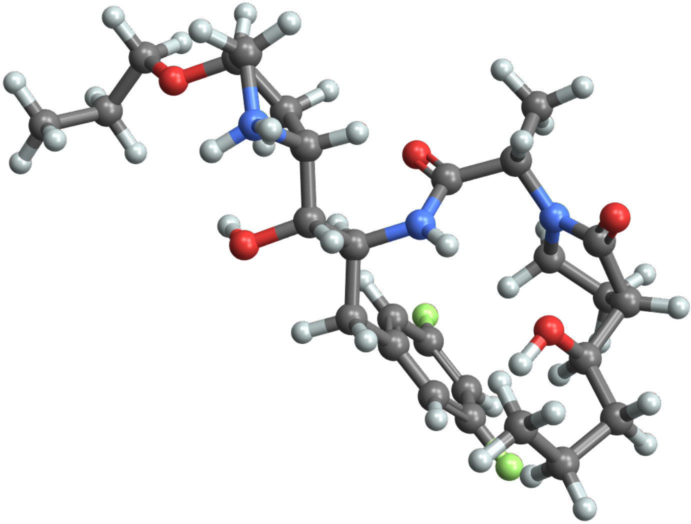

Dataset Index= 752

Molecule Formula= $C_{27}H_{42}F_2N_3O_5^+$

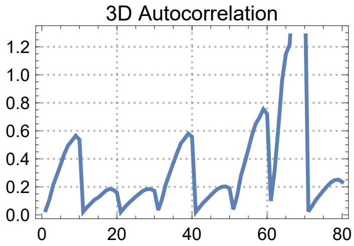

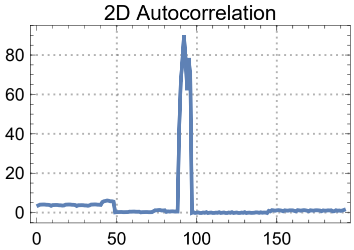

Dataset Index= 753

Molecule Formula= $C_{22}H_{23}N_7O$

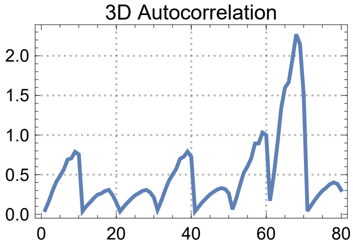

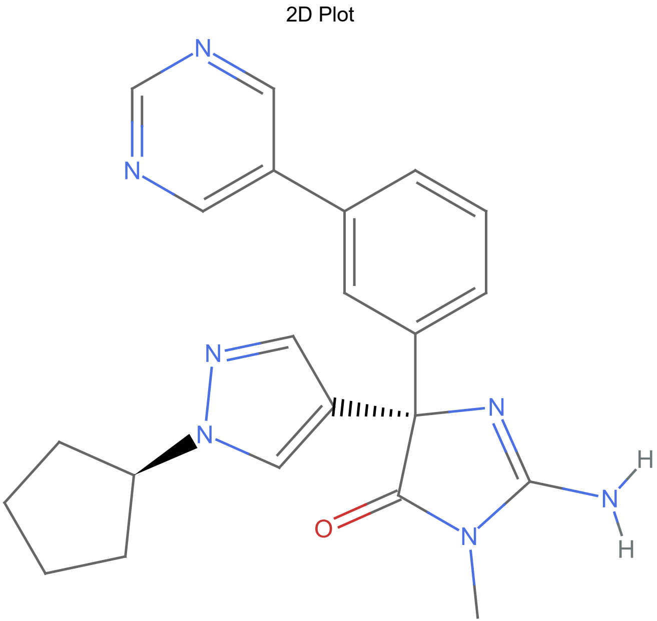

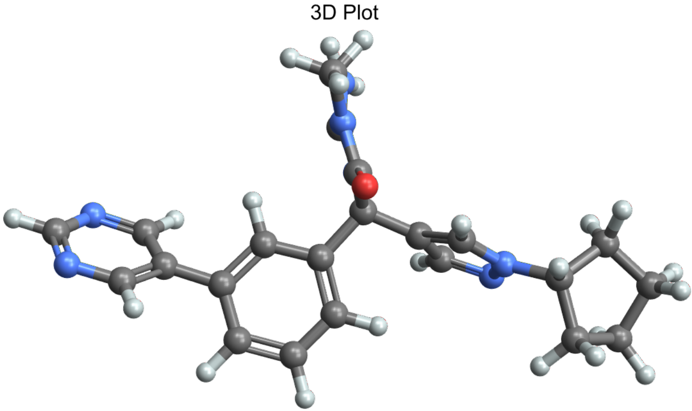

2D Plot

2D Autocorrelation

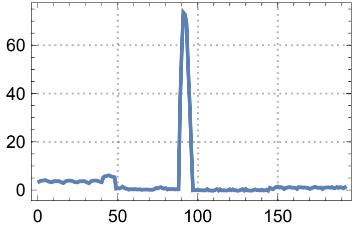

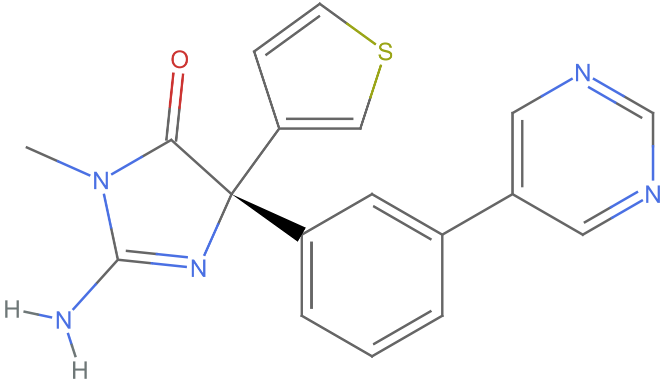

Dataset Index= 754

Molecule Formula= $C_{18}H_{15}N_5OS$

3D Plot

3D Autocorrelation

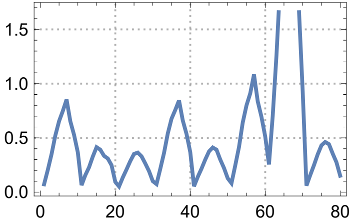

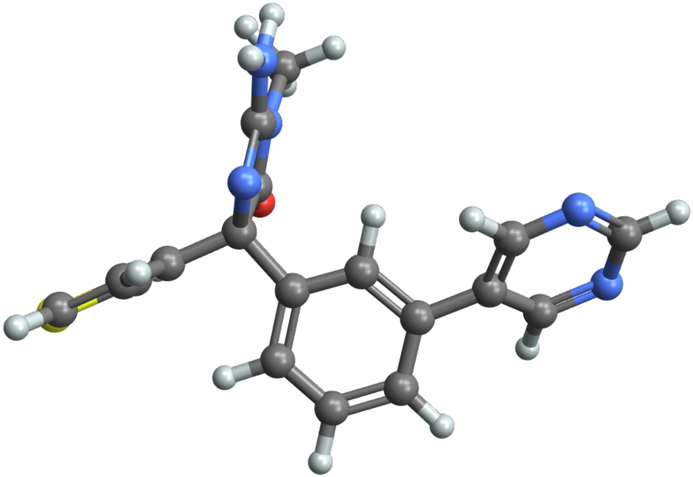

2D Plot

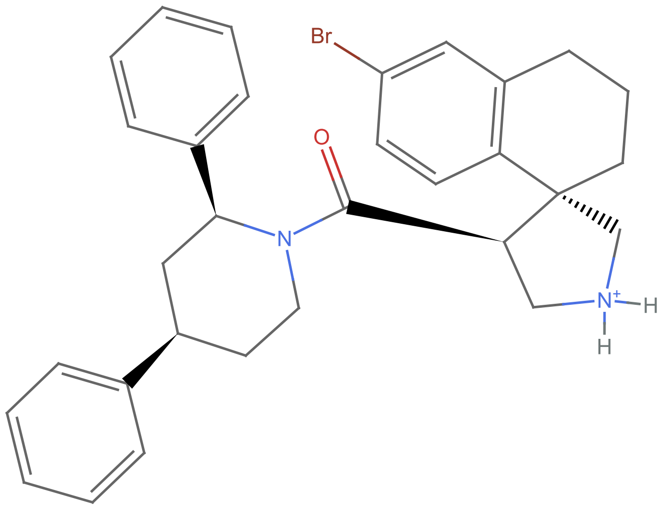

2D Autocorrelation

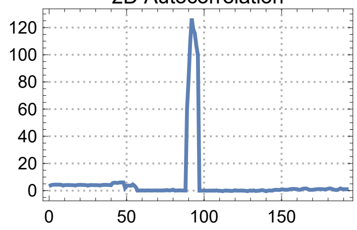

3D Plot

Dataset Index= 755

Molecule Formula= $C_{31}H_{34}BrN_2O^+$

3D Autocorrelation

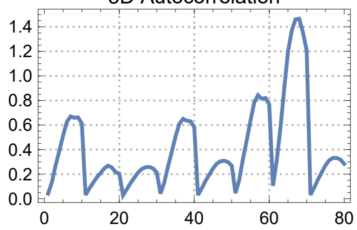

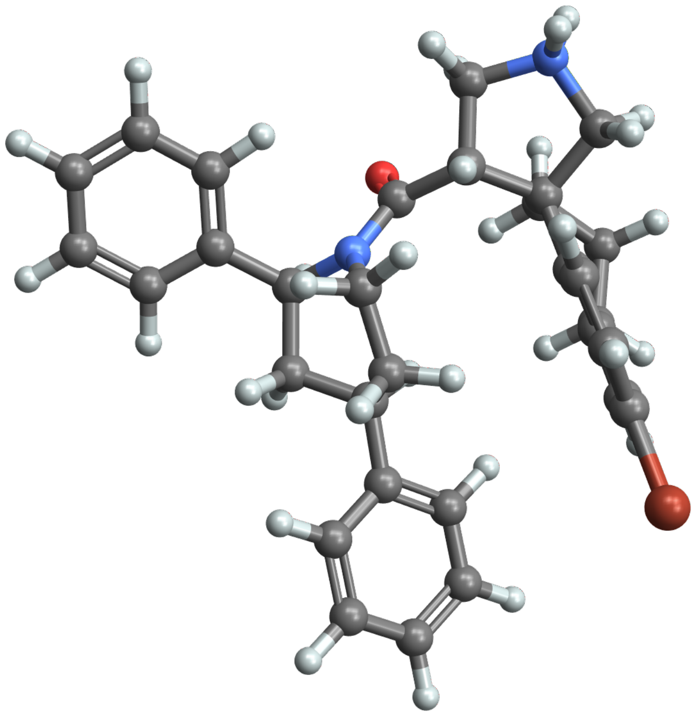

2D Autocorrelation

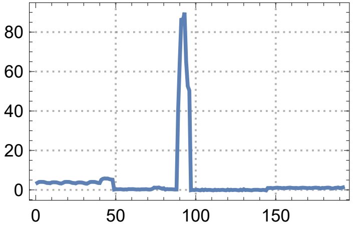

Dataset Index= 756

Molecule Formula= $C_{22}H_{25}N_3O_3$

3D Autocorrelation

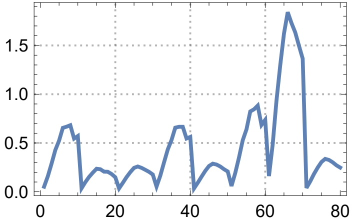

2D Plot

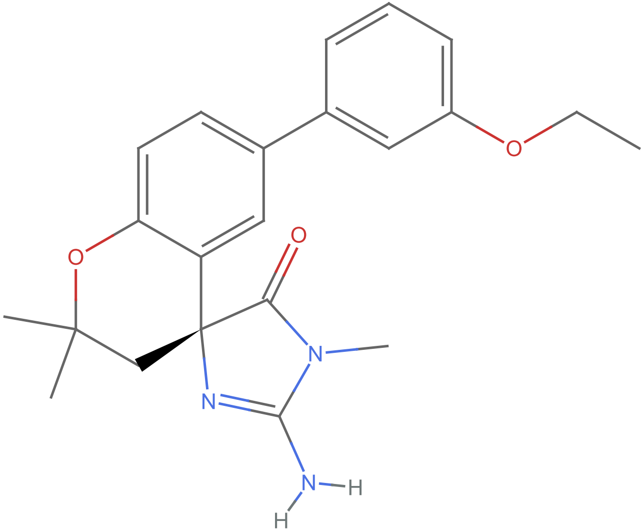

3D Plot

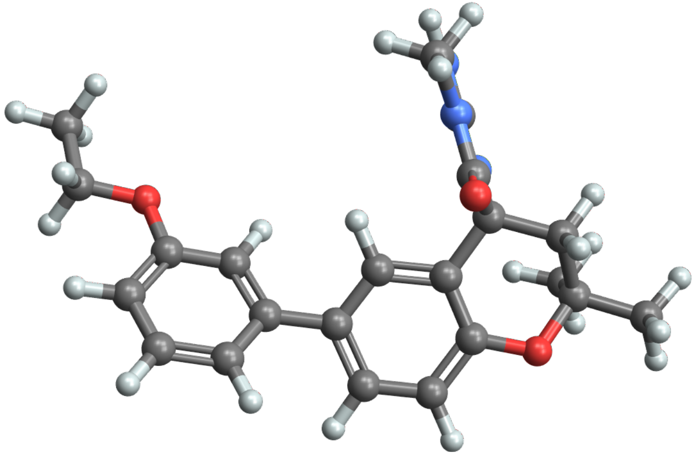

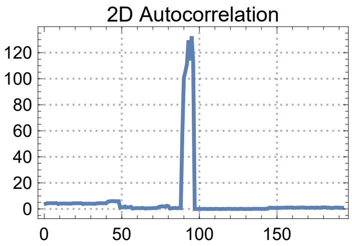

2D Plot

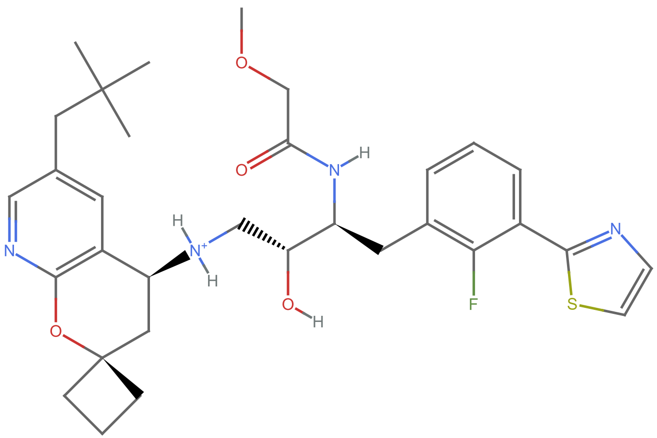

3D Plot

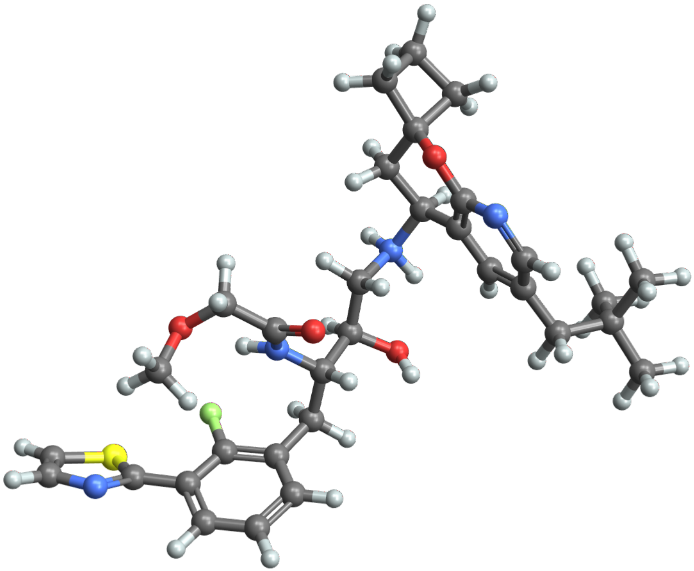

Dataset Index= 757

Molecule Formula= $C_{32}H_{42}FN_4O_4S^+$

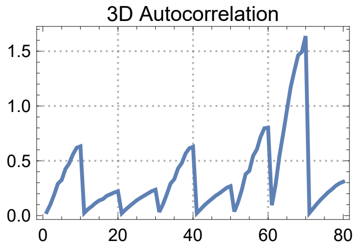

2D Plot

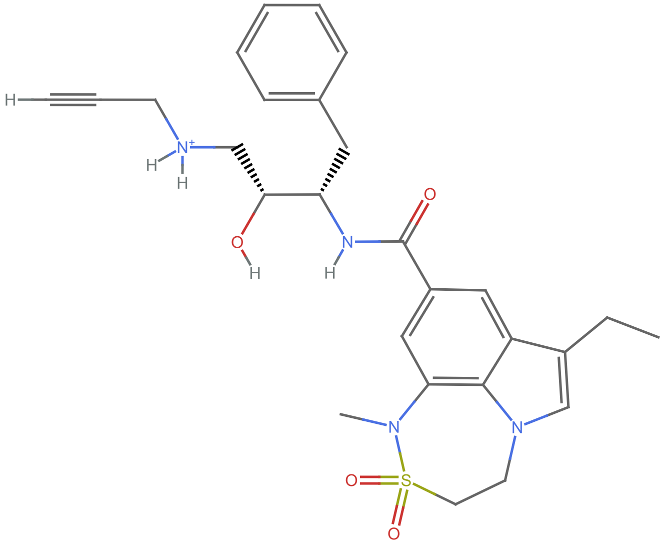

2D Autocorrelation

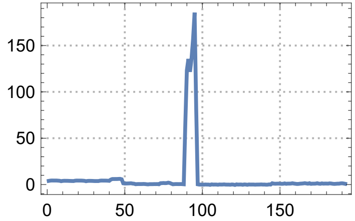

Dataset Index= 758

Molecule Formula= $C_{27}H_{33}N_4O_4S^+$

3D Autocorrelation

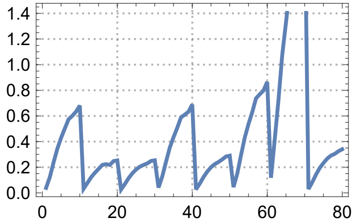

3D Plot

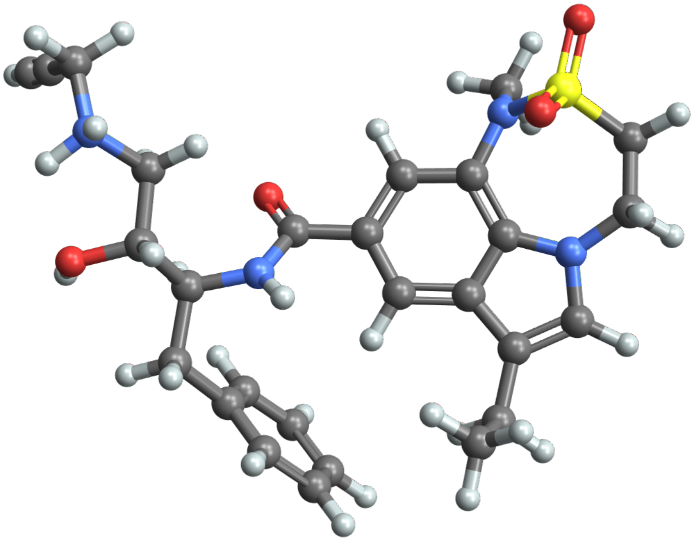

2D Autocorrelation

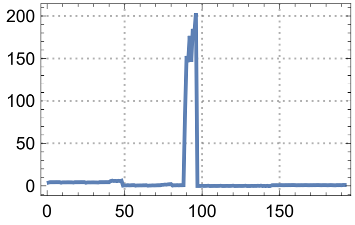

Dataset Index= 759

Molecule Formula= $C_{33}H_{43}F_2N_4O_2^+$

3D Autocorrelation

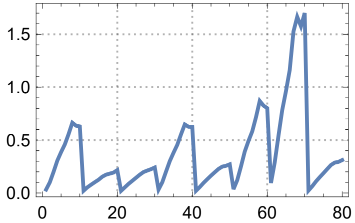

2D Plot

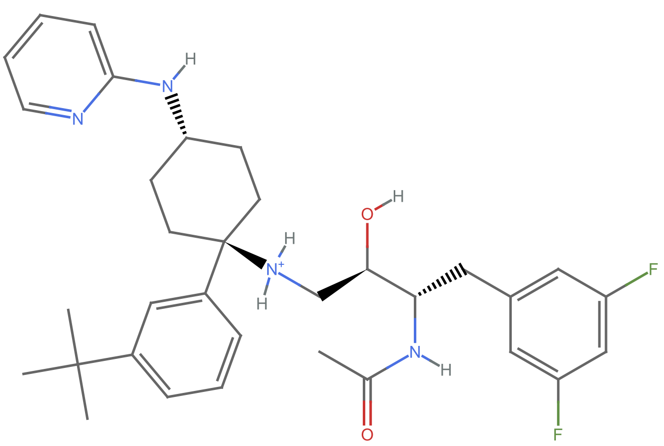

3D Plot

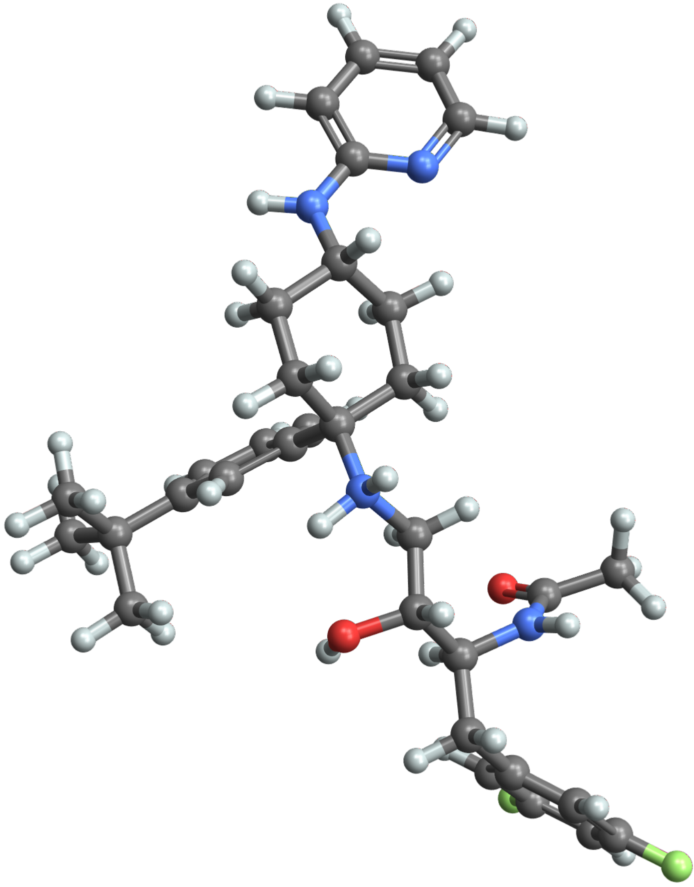

2D Autocorrelation

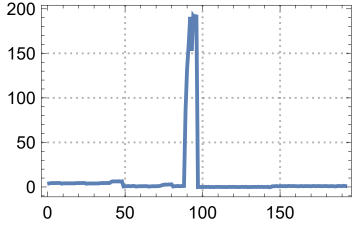

Dataset Index= 760

Molecule Formula= $C_{31}H_{36}F_2N_5O_5^+$

3D Autocorrelation

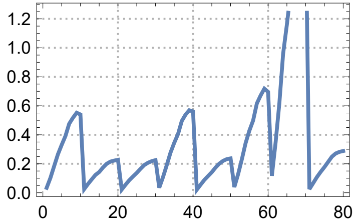

2D Plot

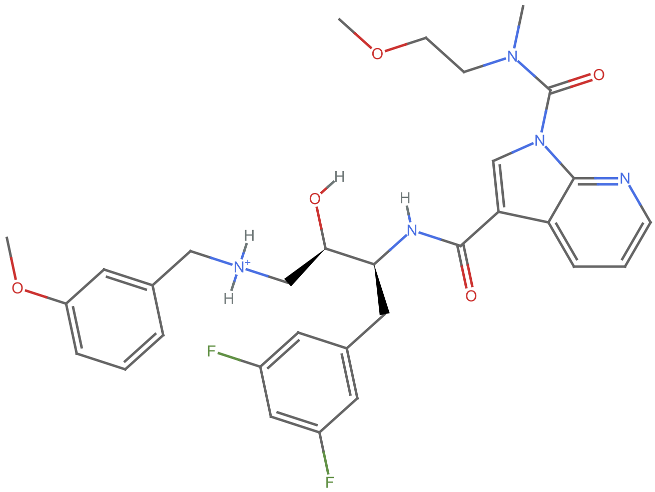

3D Plot

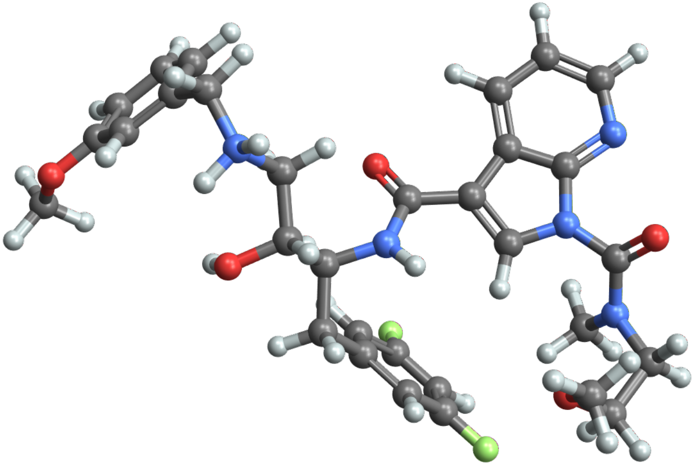

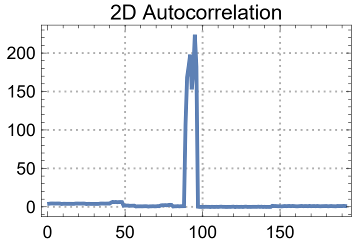

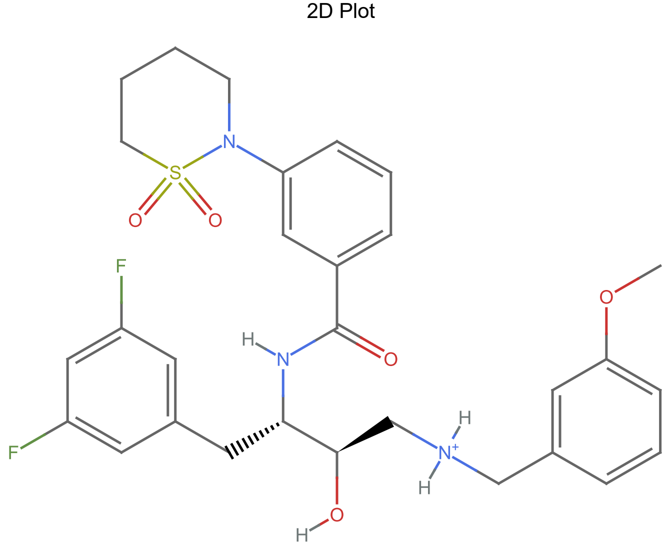

3D Plot

Dataset Index= 761

Molecule Formula= $C_{29}H_{34}F_2N_3O_5S^+$

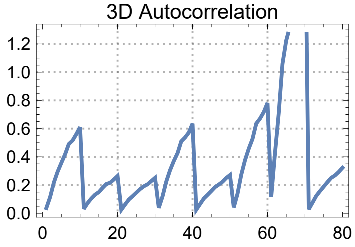

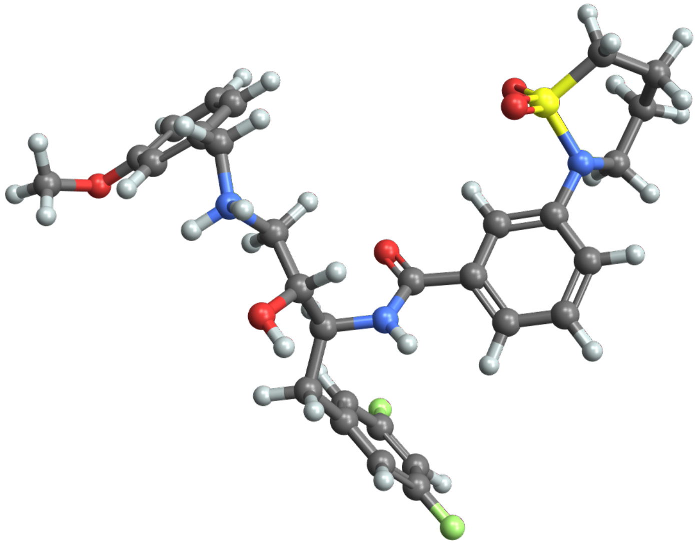

2D Plot

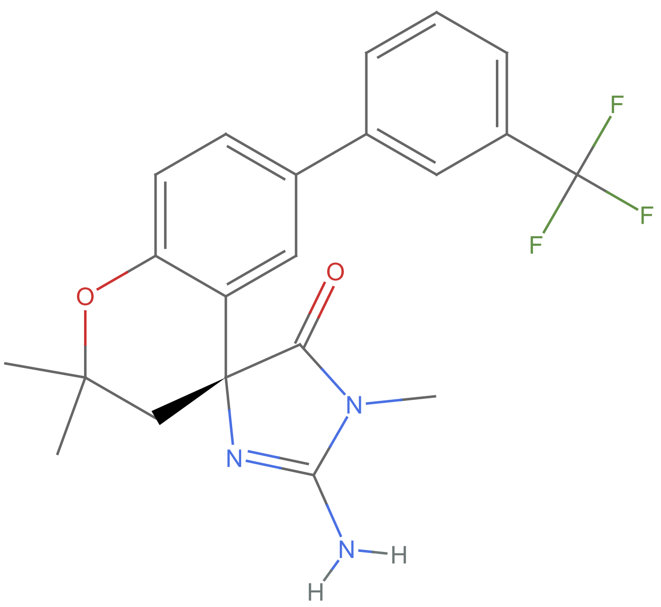

2D Autocorrelation

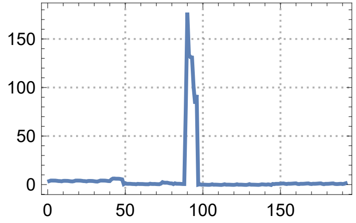

Dataset Index= 762

Molecule Formula= $C_{21}H_{20}F_3N_3O_2$

3D Autocorrelation

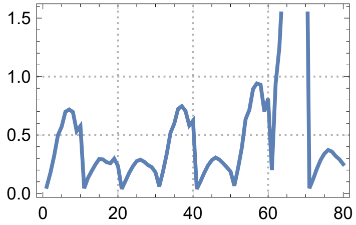

3D Plot

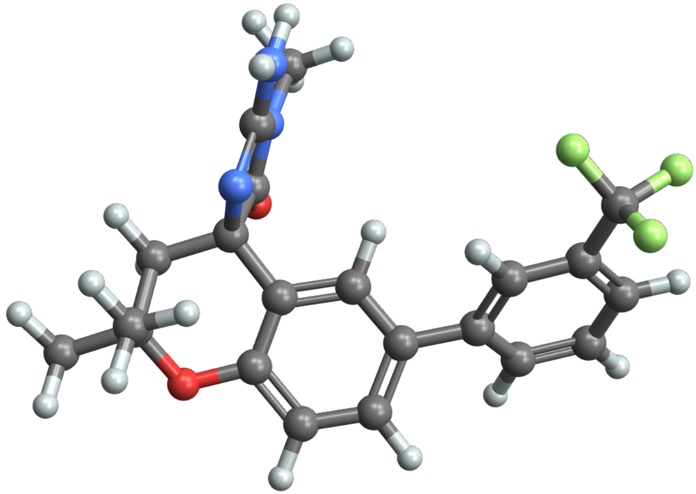

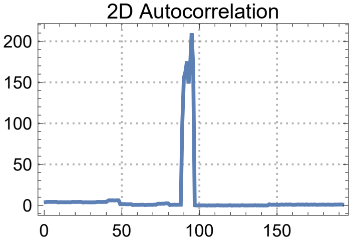

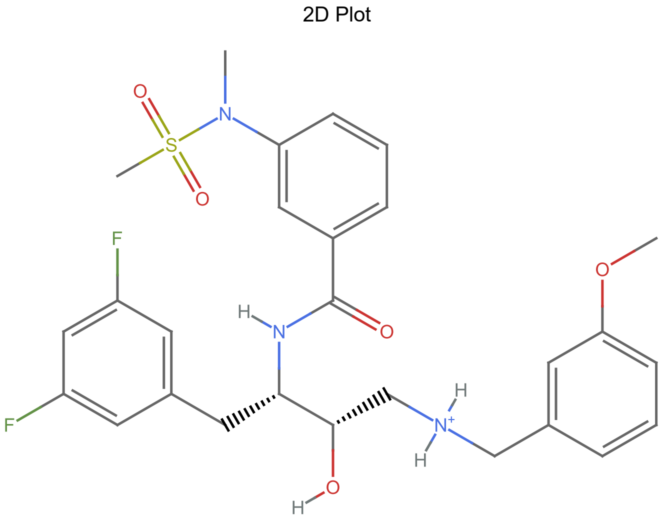

Dataset Index= 763

Molecule Formula= $C_{27}H_{32}F_2N_3O_5S^+$

3D Plot

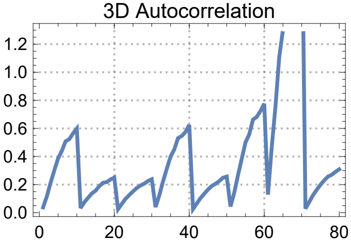

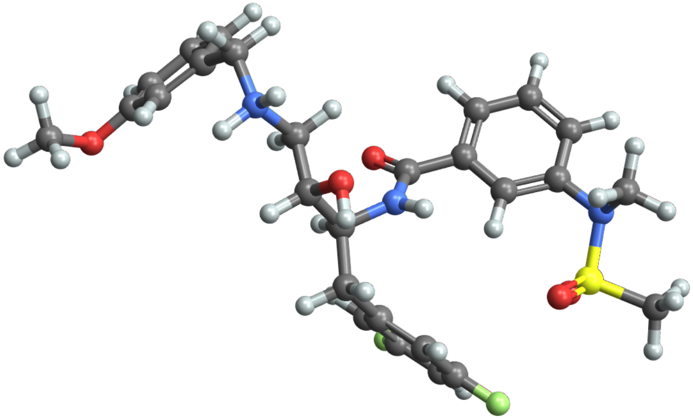

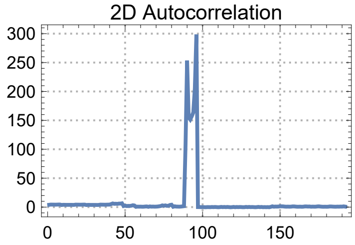

Dataset Index= 764

Molecule Formula= $C_{26}H_{35}F_4N_2O_4S^+$

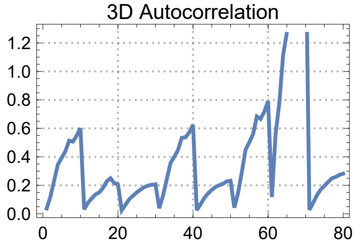

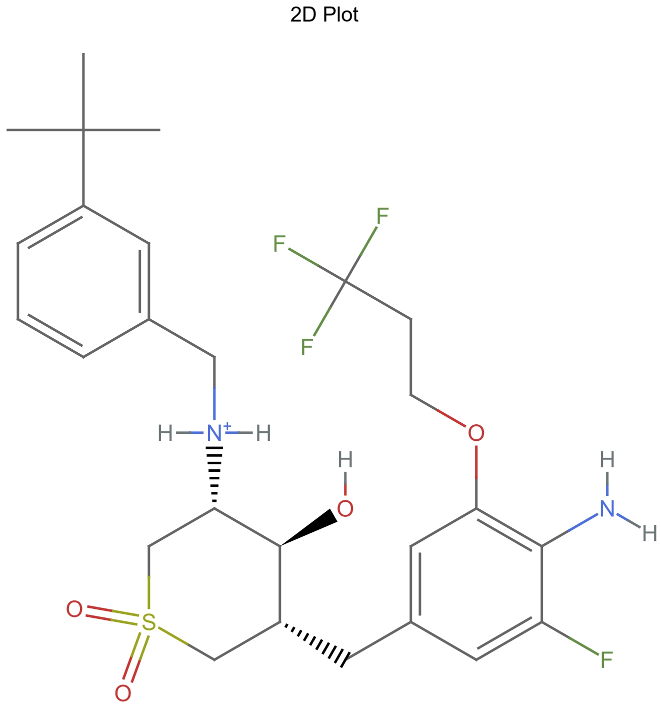

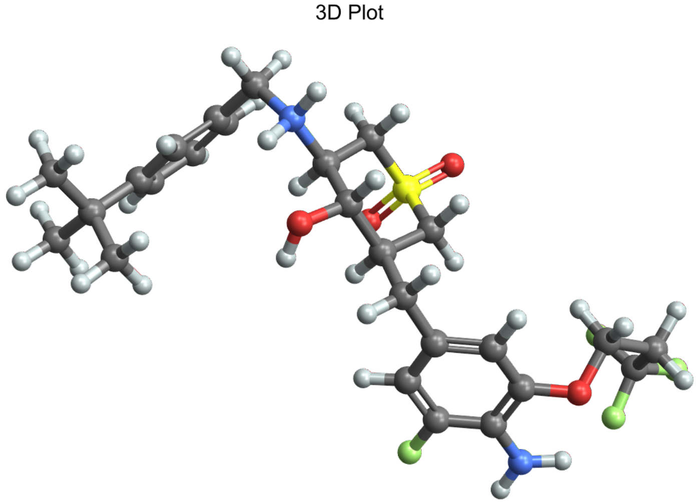

2D Plot

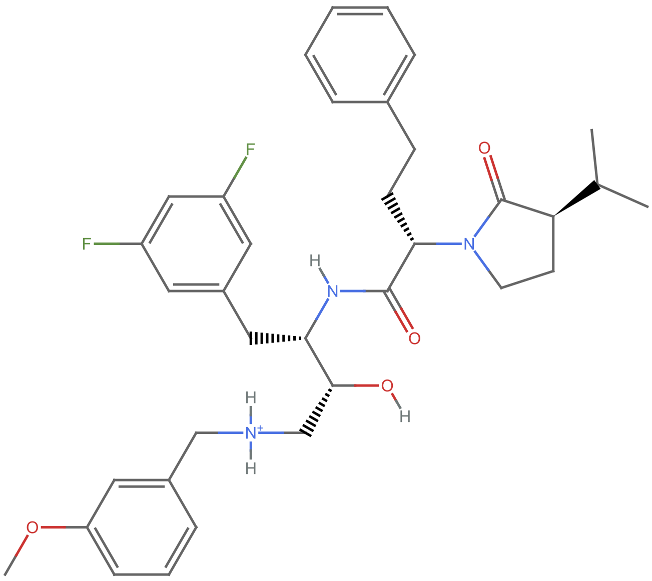

2D Autocorrelation

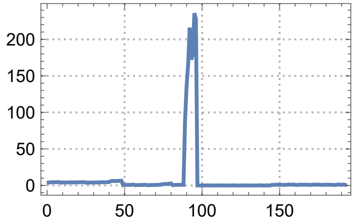

3D Plot

Dataset Index= 765

Molecule Formula= $C_{35}H_{44}F_2N_3O_4^+$

3D Autocorrelation

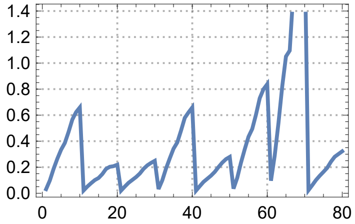

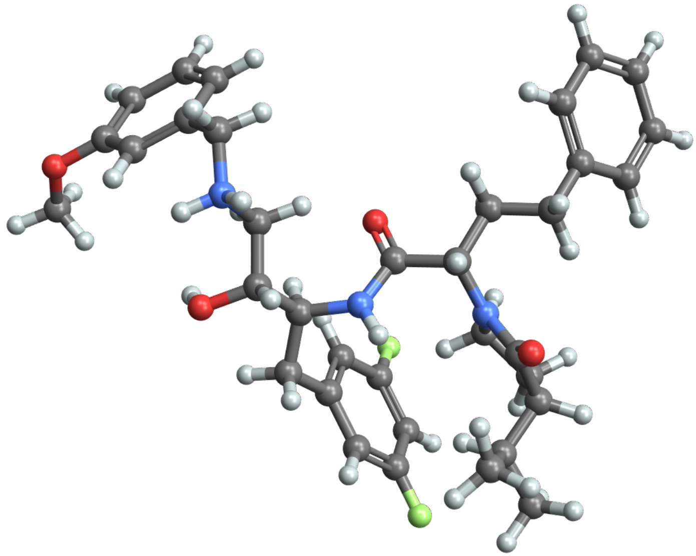

2D Plot

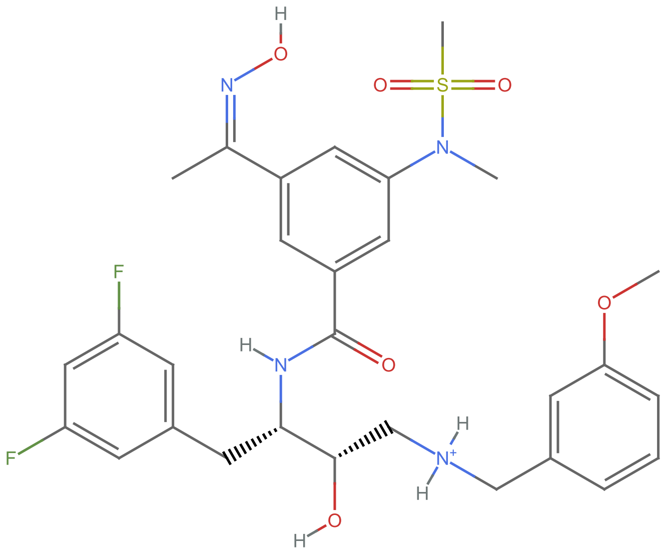

2D Autocorrelation

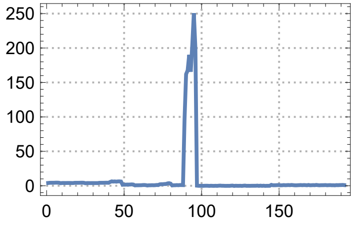

Dataset Index= 766

Molecule Formula= $C_{29}H_{35}F_2N_4O_6S^+$

3D Autocorrelation

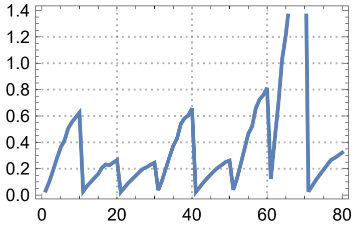

3D Plot

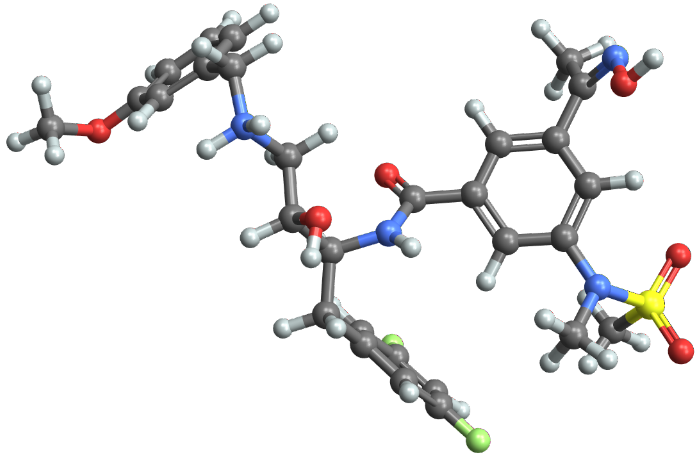

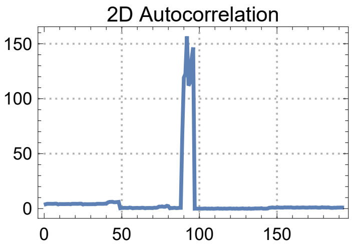

Dataset Index= 767

Molecule Formula= $C_{34}H_{41}FN_3O_5^+$

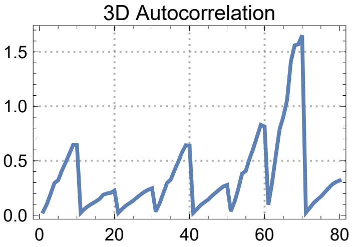

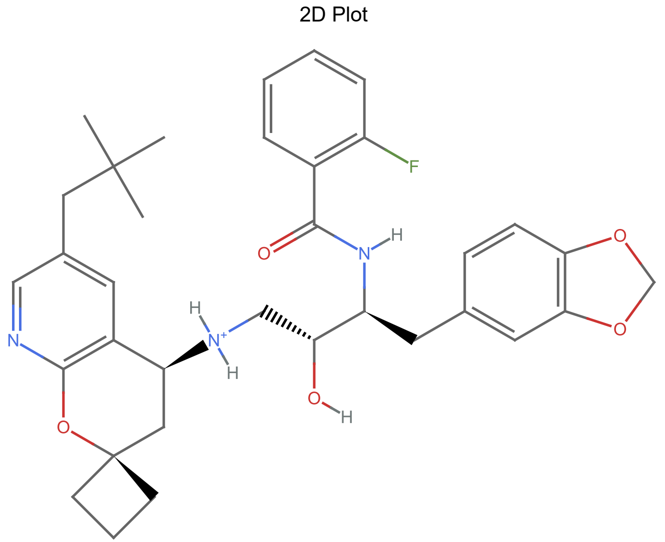

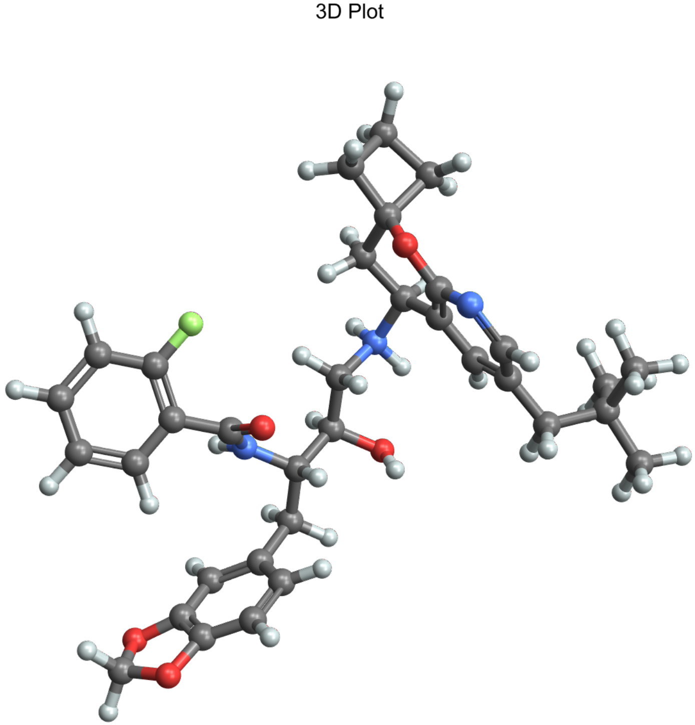

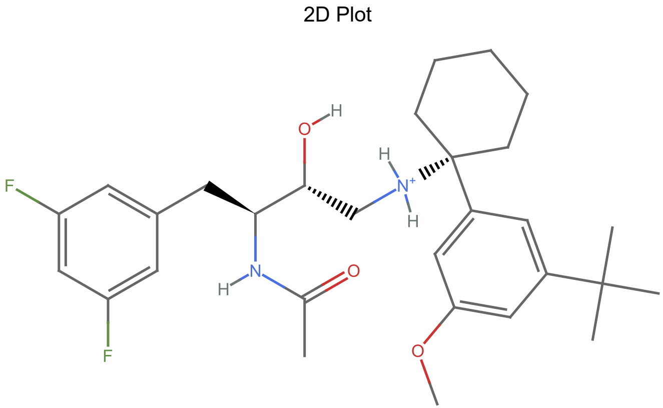

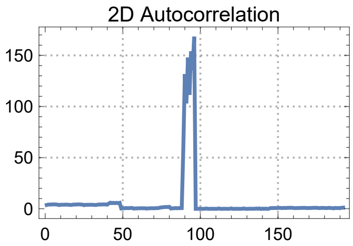

Dataset Index= 768

Molecule Formula= $C_{29}H_{41}F_2N_2O_3^+$

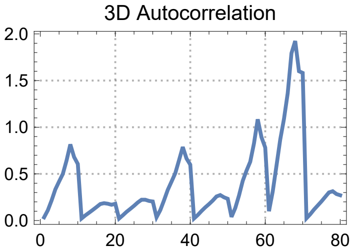

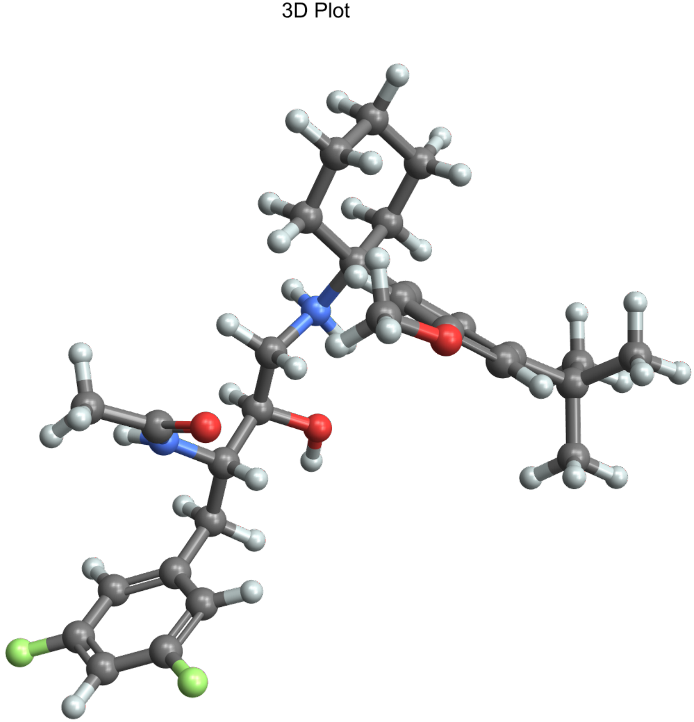

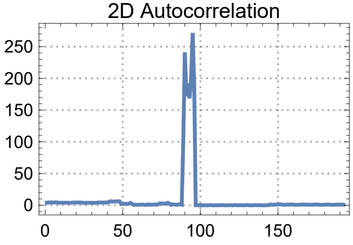

Dataset Index= 769

Molecule Formula= $C_{27}H_{37}F_4N_2O_4S^+$

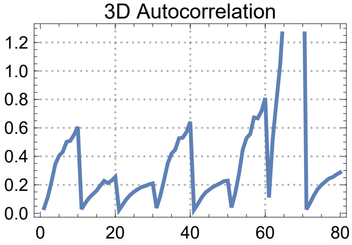

2D Plot

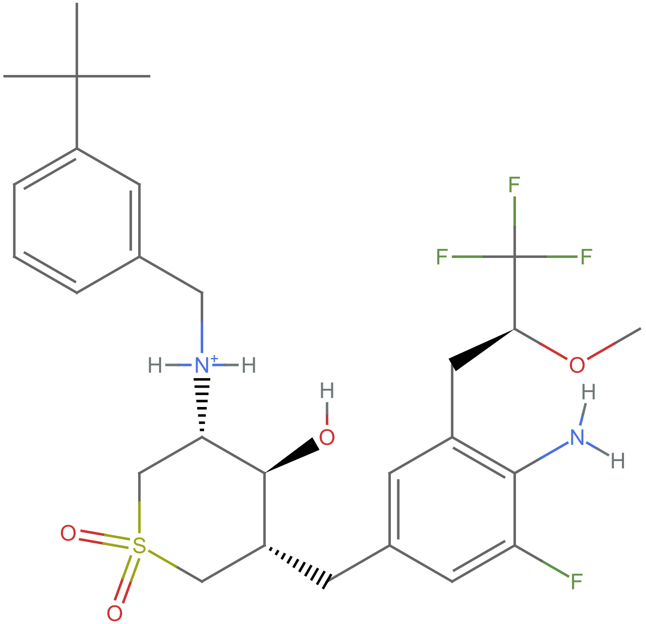

3D Plot

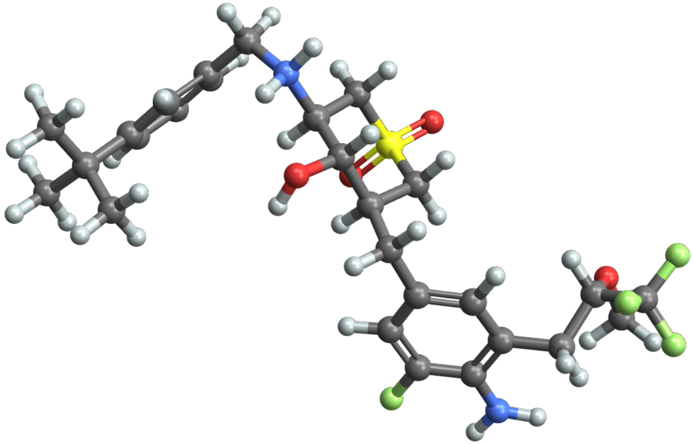

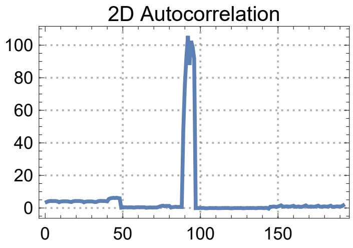

Dataset Index= 770

Molecule Formula= $C_{26}H_{22}FN_5O$

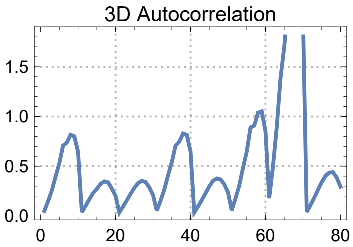

2D Plot

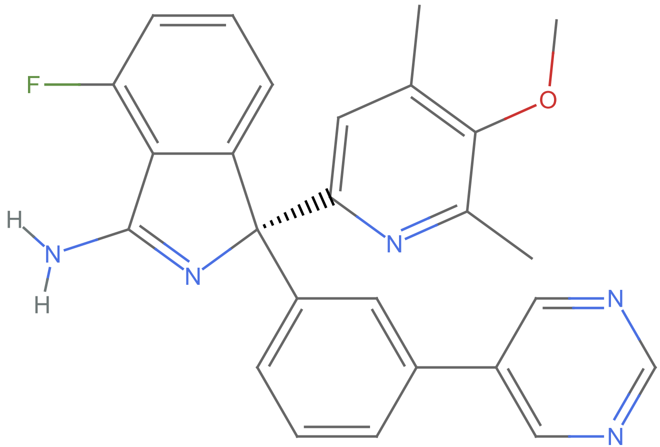

3D Plot

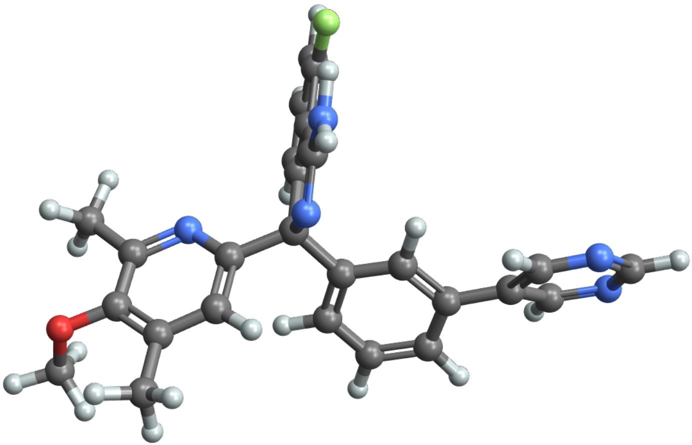

2D Plot

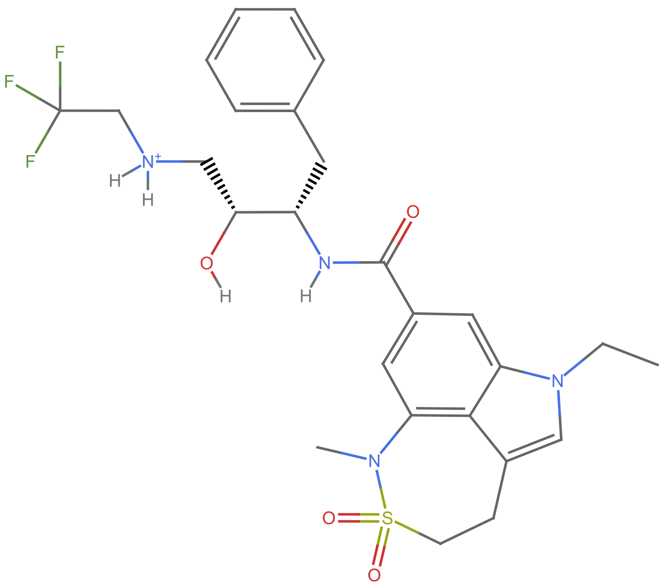

2D Autocorrelation

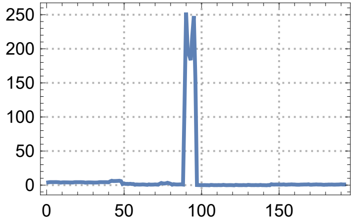

Dataset Index= 771

Molecule Formula= $C_{26}H_{32}F_{3}N_{4}O_{4}S^{+}$

3D Autocorrelation

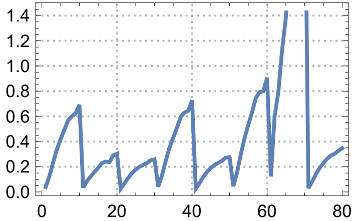

3D Plot

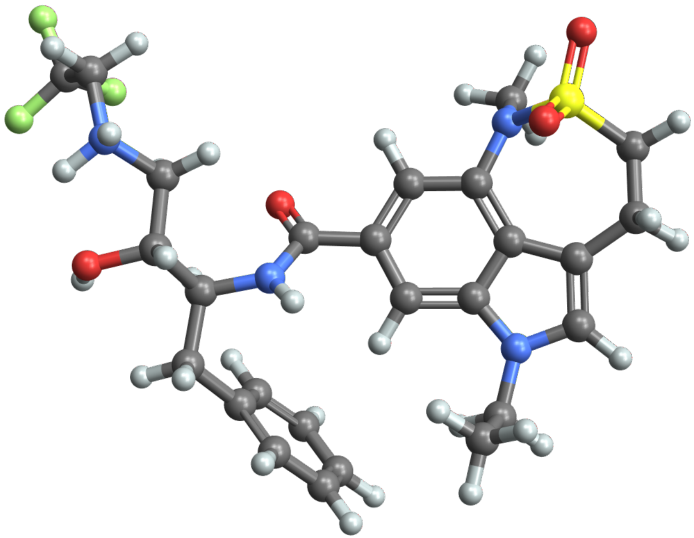

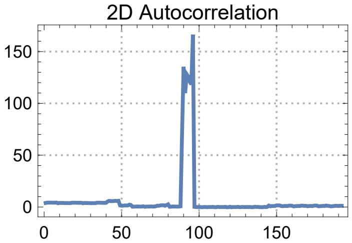

Dataset Index= 772

Molecule Formula= $C_{27}H_{34}FN_2O_4S^+$

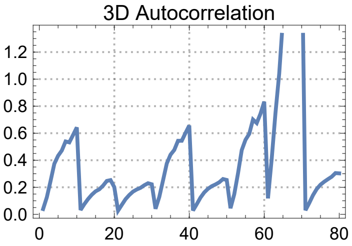

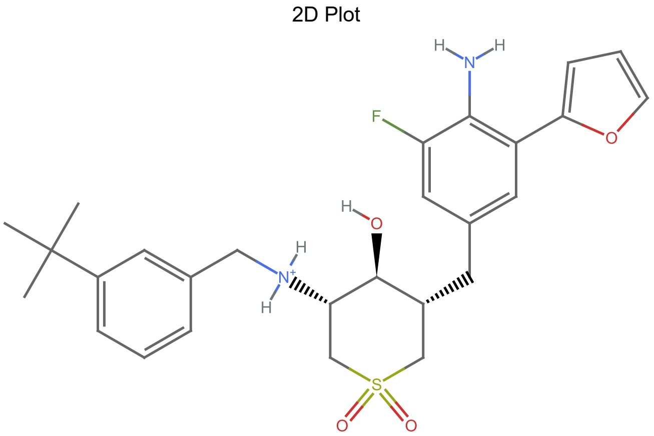

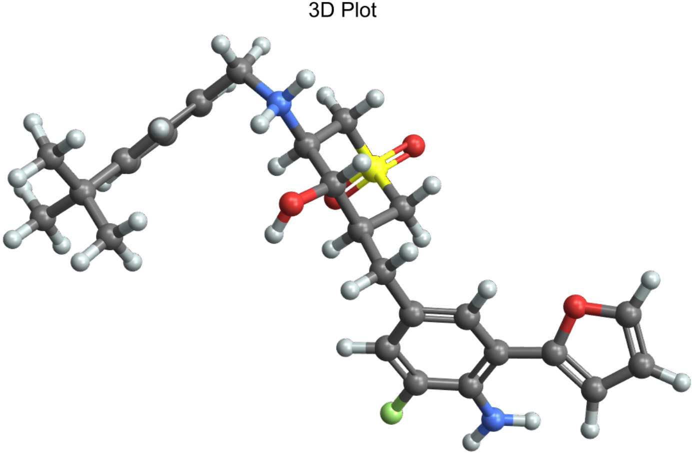

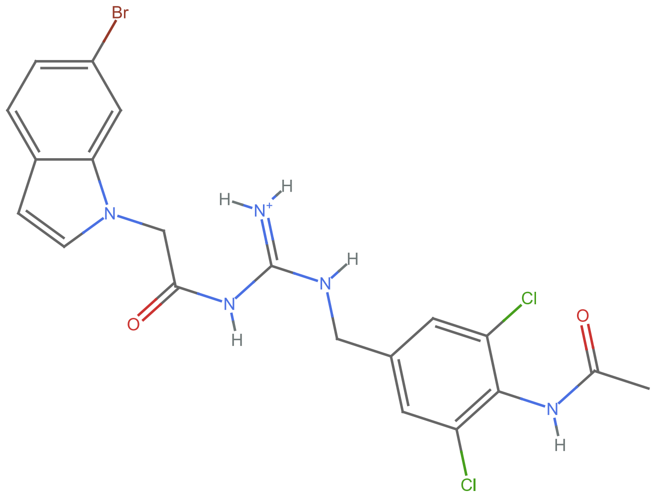

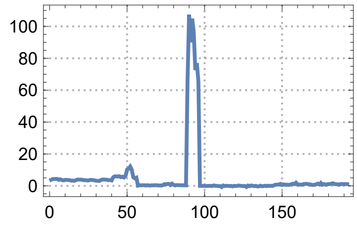

Dataset Index= 773

Molecule Formula= $C_{20}H_{19}BrCl_2N_5O_2^+$

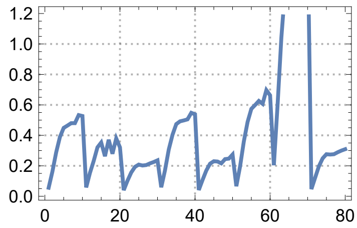

3D Plot

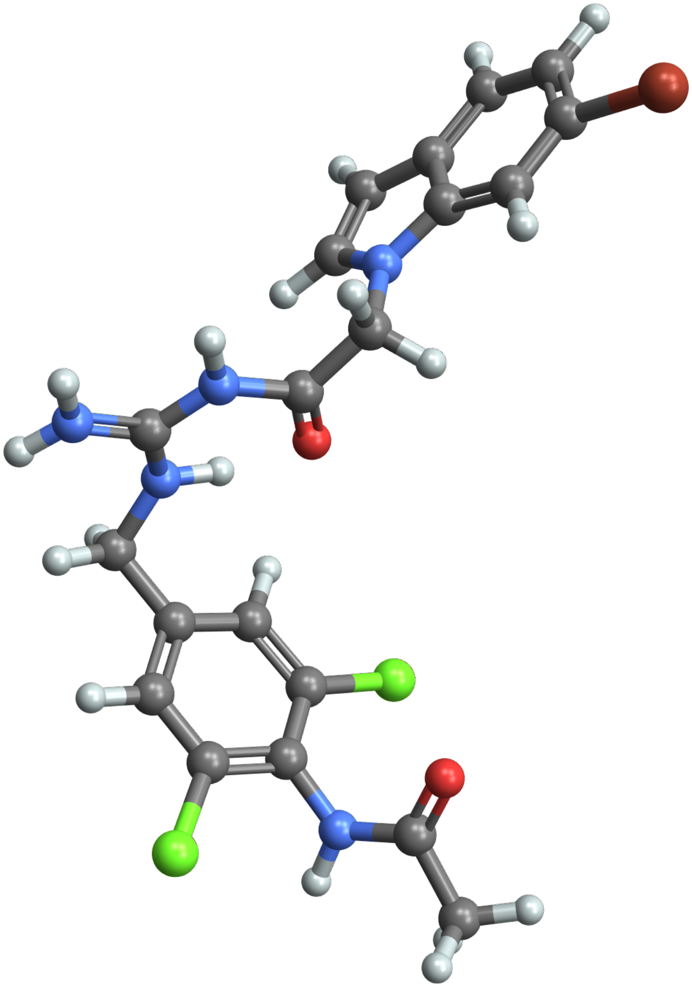

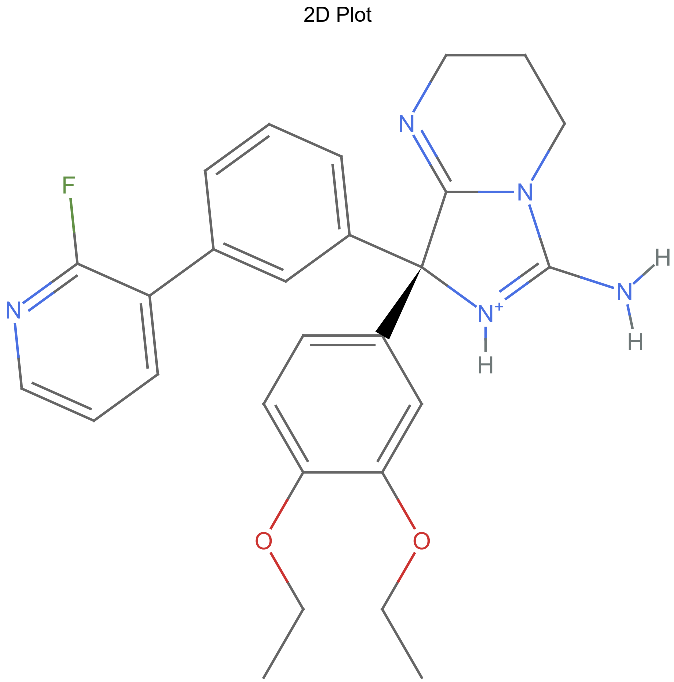

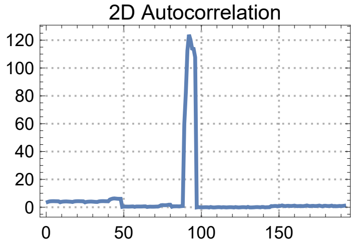

Dataset Index= 774

Molecule Formula= $C_{27}H_{29}FN_5O_2^+$

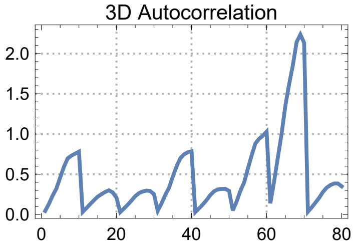

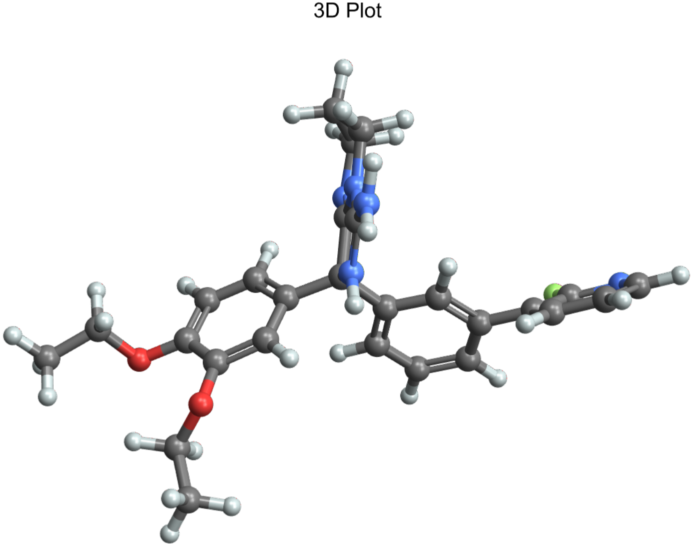

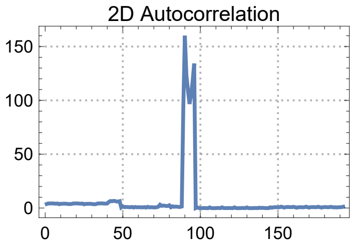

Dataset Index= 775

Molecule Formula= $C_{23}H_{18}F_4N_5O^+$

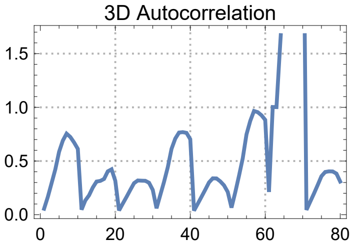

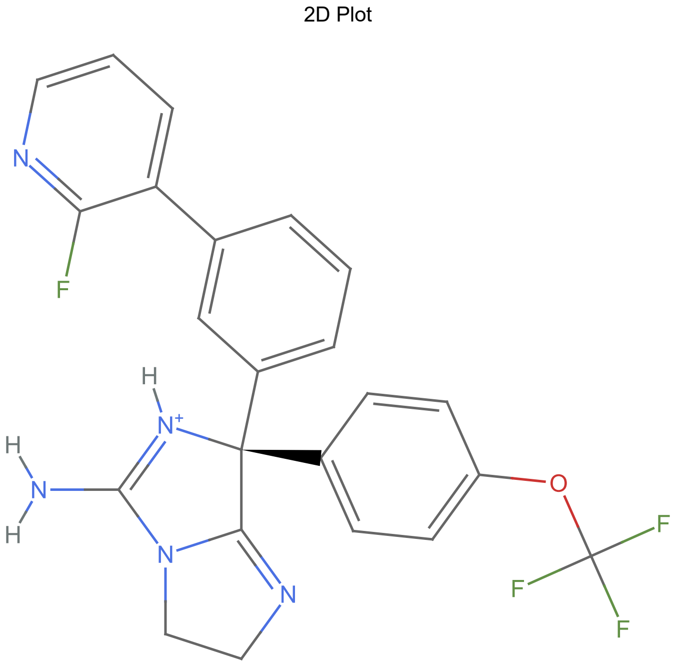

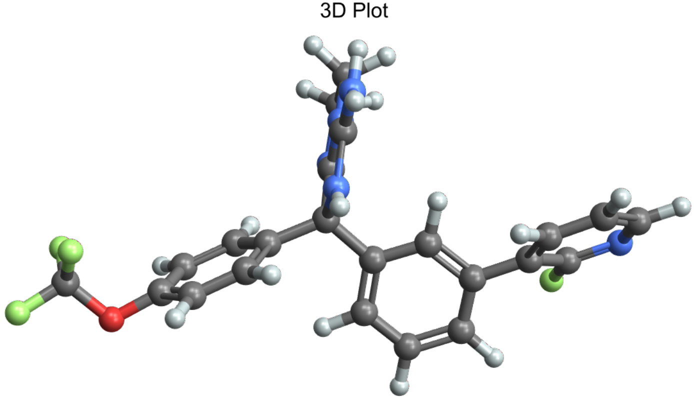

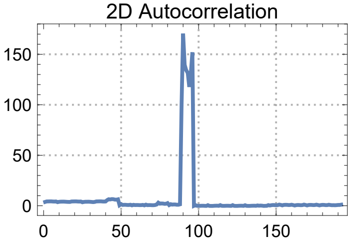

Dataset Index= 776

Molecule Formula= $C_{25}H_{22}F_4N_5O^+$

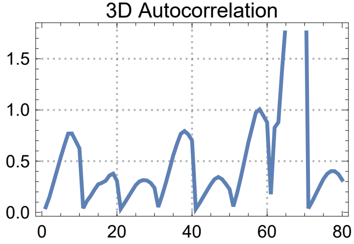

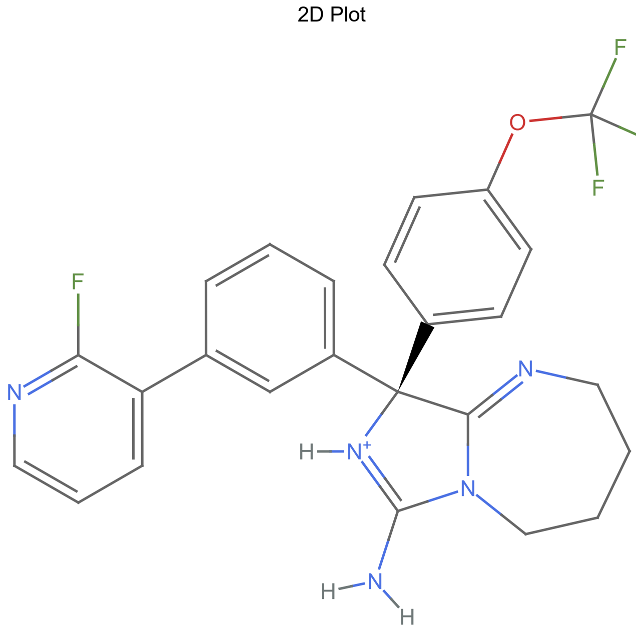

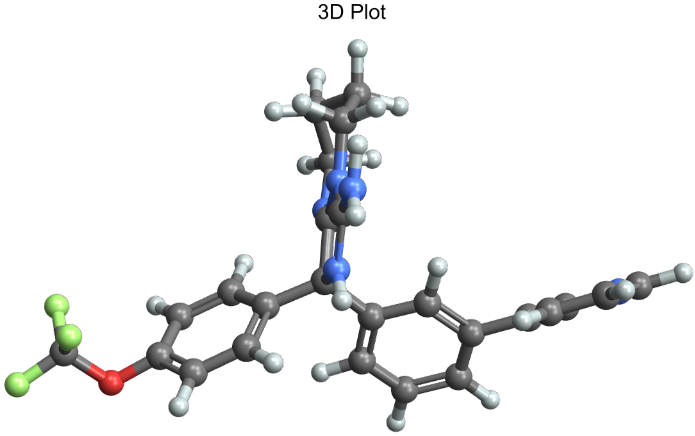

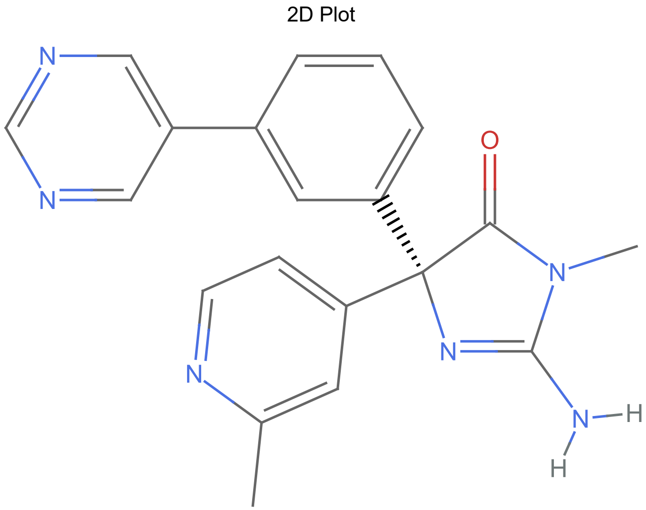

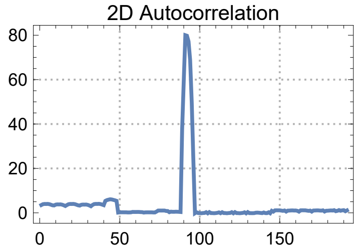

Dataset Index= 777

Molecule Formula= $C_{20}H_{18}N_6O$

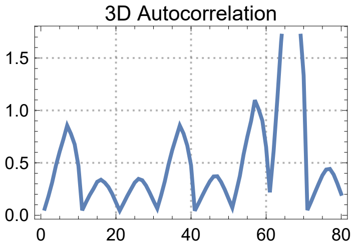

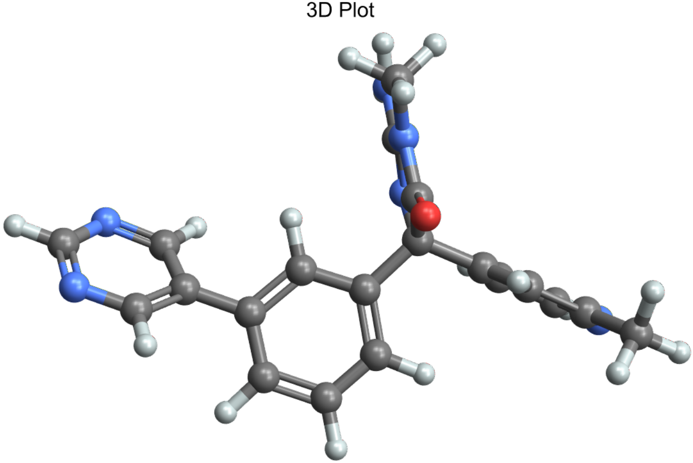

2D Autocorrelation

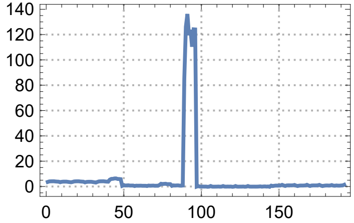

Dataset Index= 778

Molecule Formula= $C_{21}H_{20}F_3N_3O_3$

3D Autocorrelation

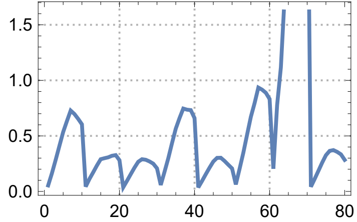

2D Plot

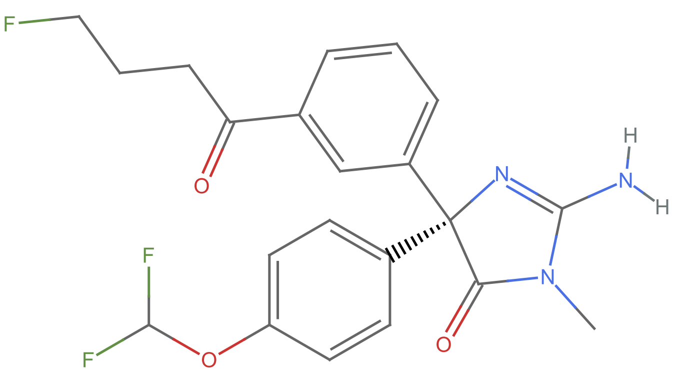

3D Plot

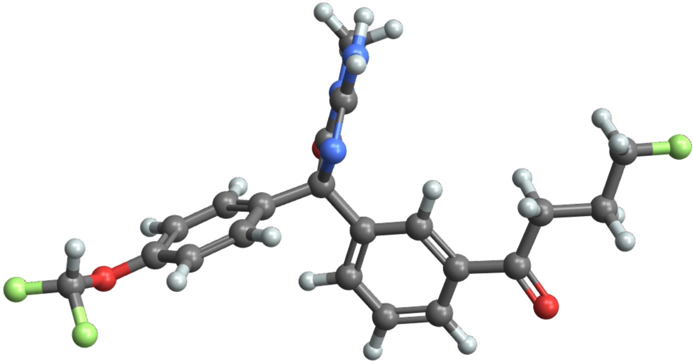

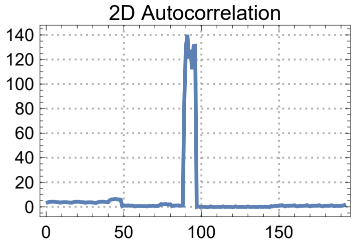

Dataset Index= 779

Molecule Formula= $C_{21}H_{21}F_4N_3O_2$

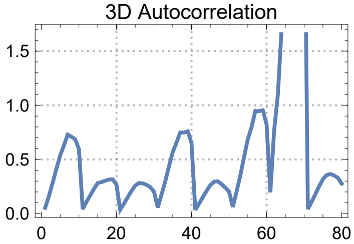

2D Plot

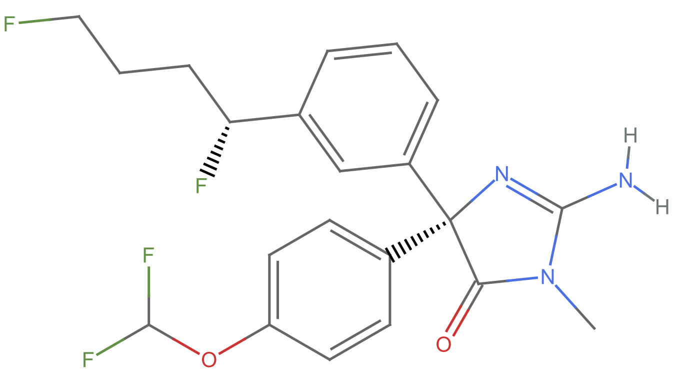

3D Plot

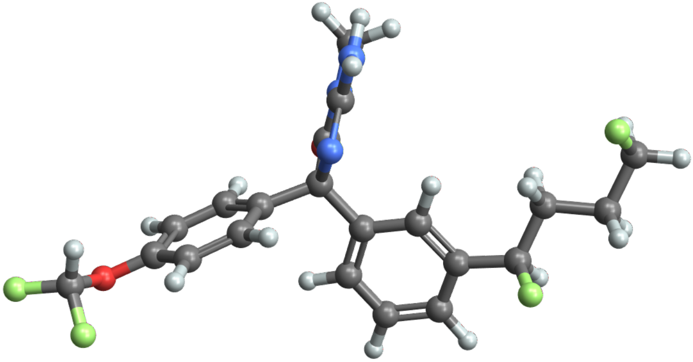

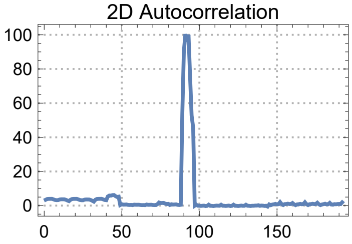

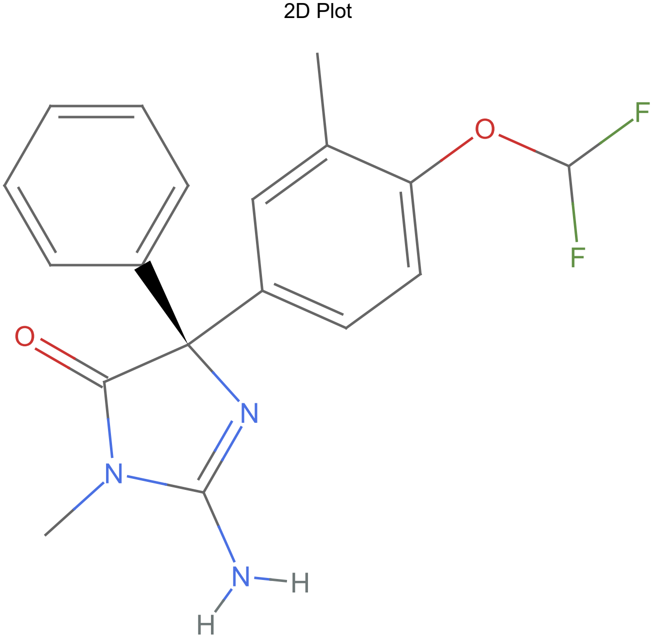

Dataset Index= 780

Molecule Formula= $C_{18}H_{17}F_2N_3O_2$

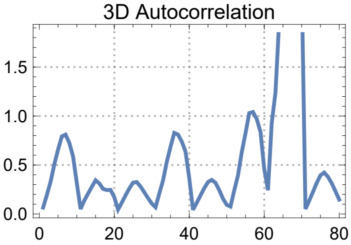

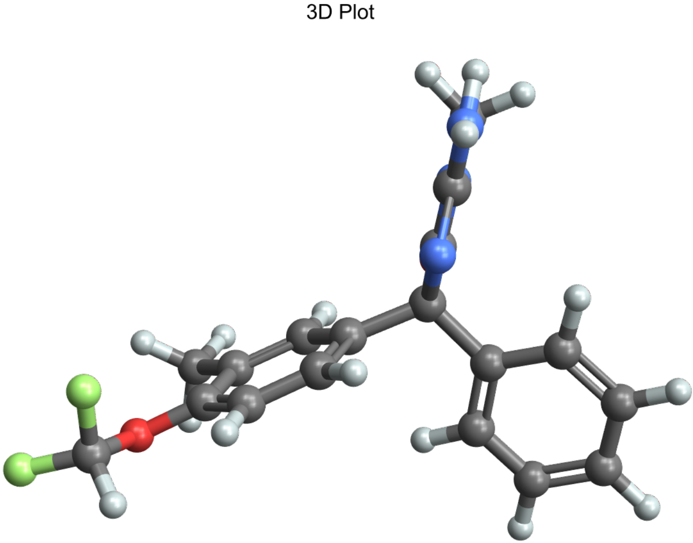

2D Autocorrelation

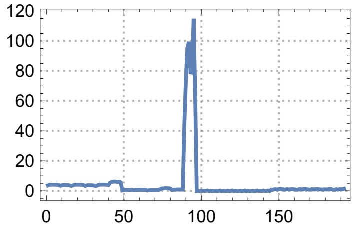

Dataset Index= 781

Molecule Formula= $C_{20}H_{20}FN_7O$

3D Autocorrelation

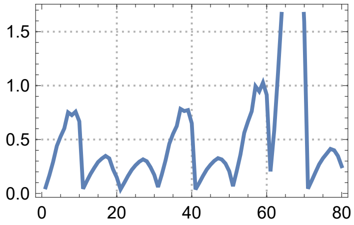

2D Plot

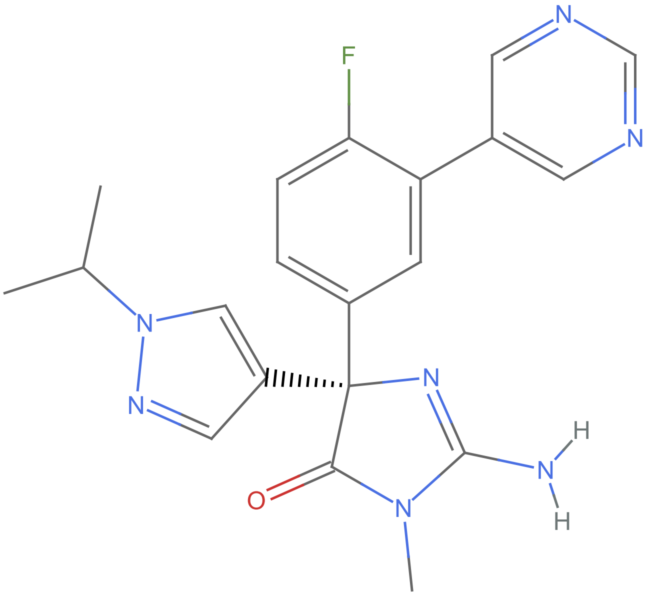

3D Plot

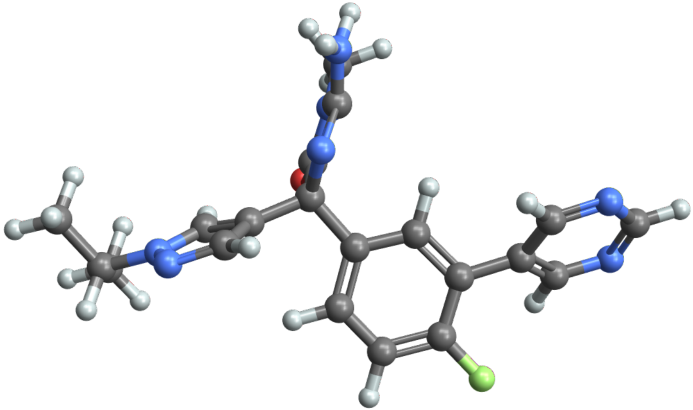

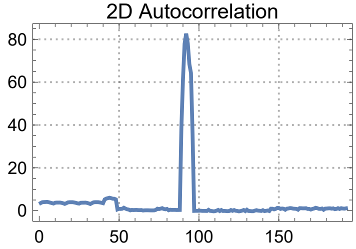

Dataset Index= 782

Molecule Formula= $C_{20}H_{19}N_5OS$

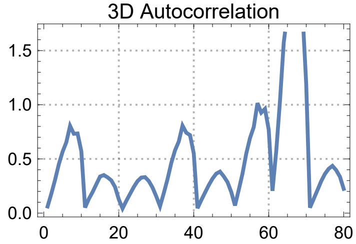

2D Plot

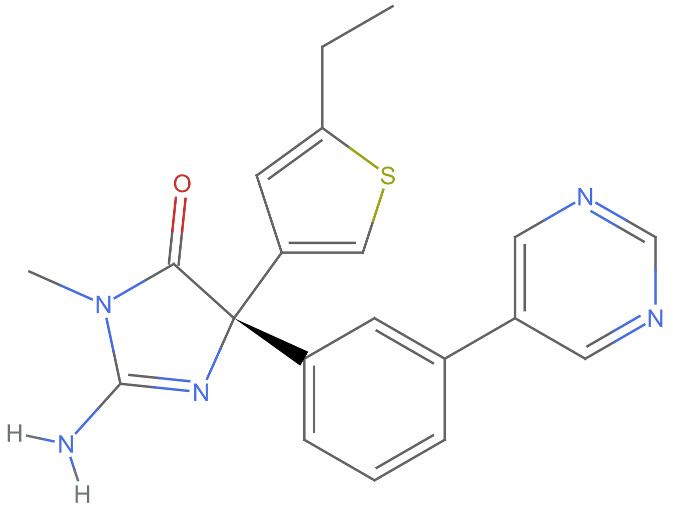

3D Plot

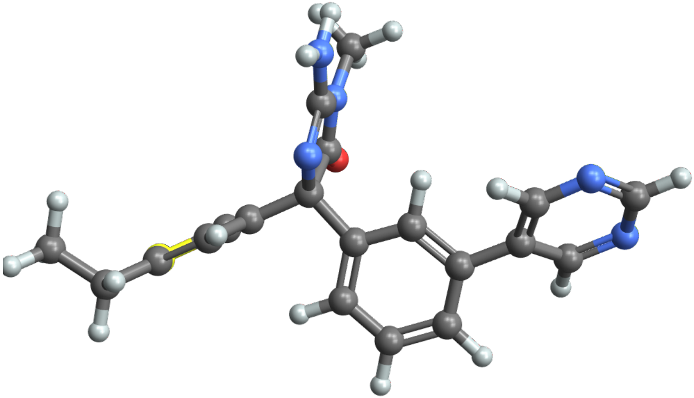

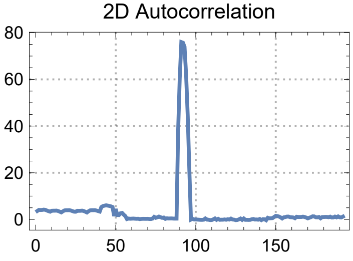

Dataset Index= 783

Molecule Formula= $C_{18}H_{14}ClN_5OS$

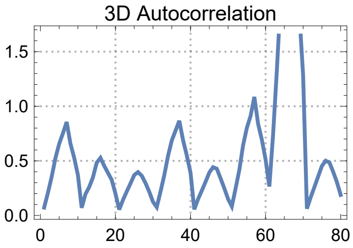

2D Plot

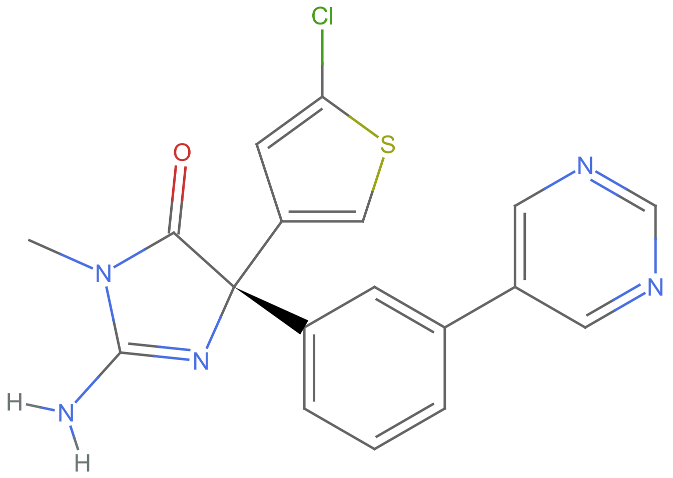

3D Plot

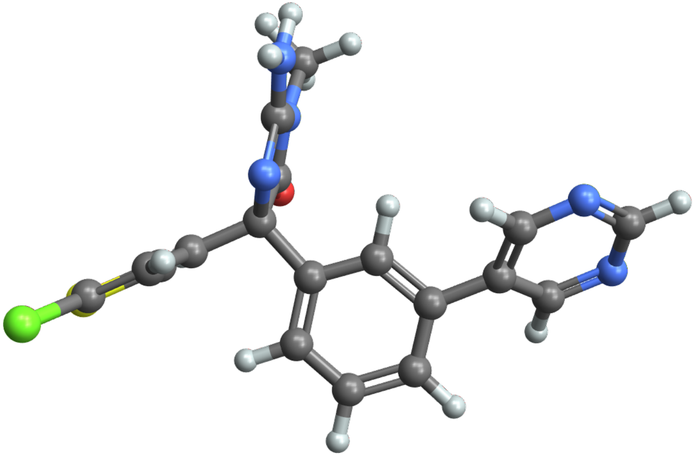

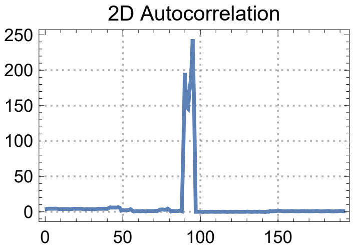

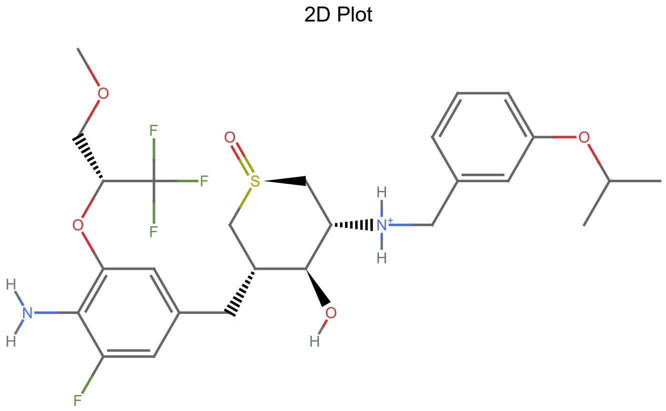

Dataset Index= 784

Molecule Formula= $C_{26}H_{35}F_4N_2O_5S^+$

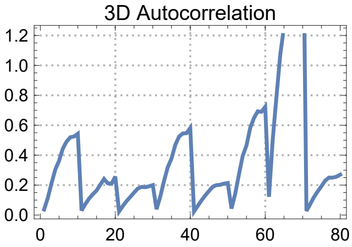

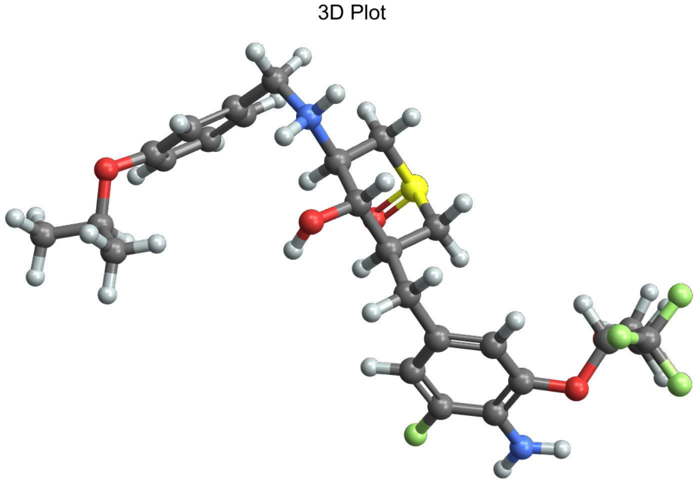

2D Plot

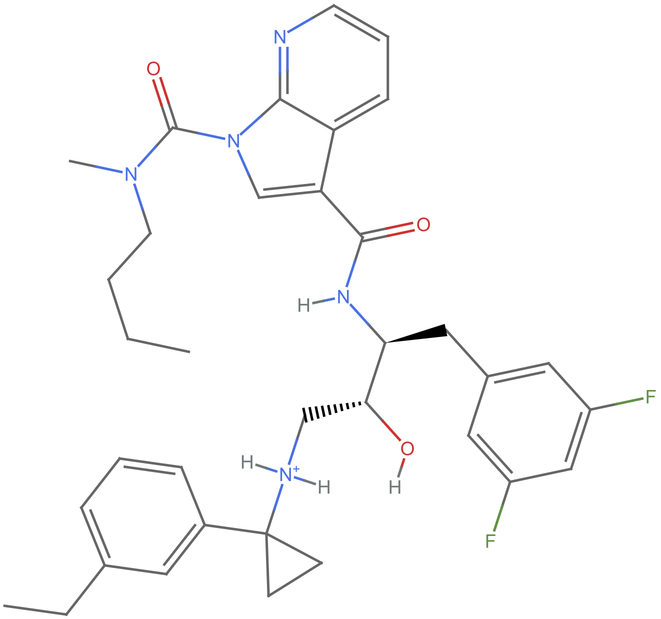

2D Autocorrelation

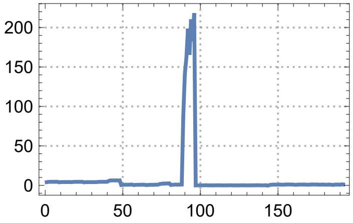

Dataset Index= 785

Molecule Formula= $C_{35}H_{42}F_2N_5O_3^+$

3D Autocorrelation

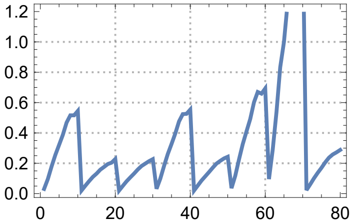

3D Plot

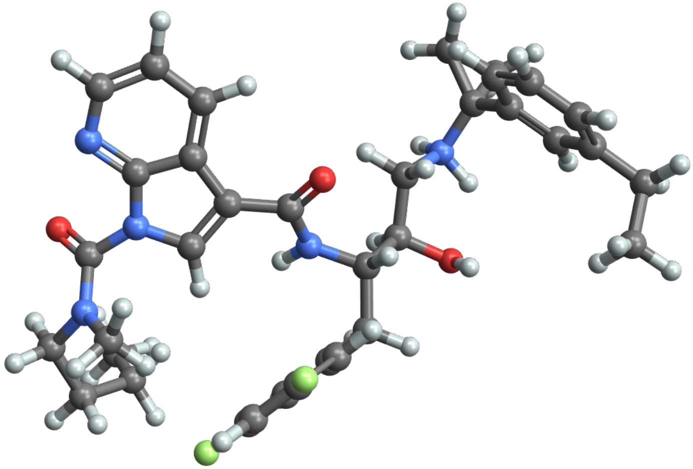

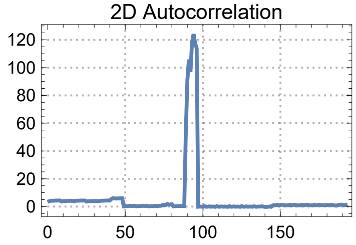

Dataset Index= 786

Molecule Formula= $C_{32}H_{42}N_3O_4^+$

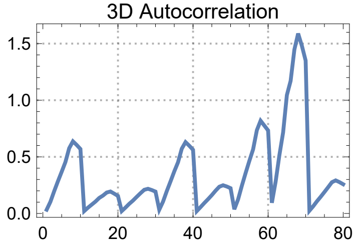

2D Plot

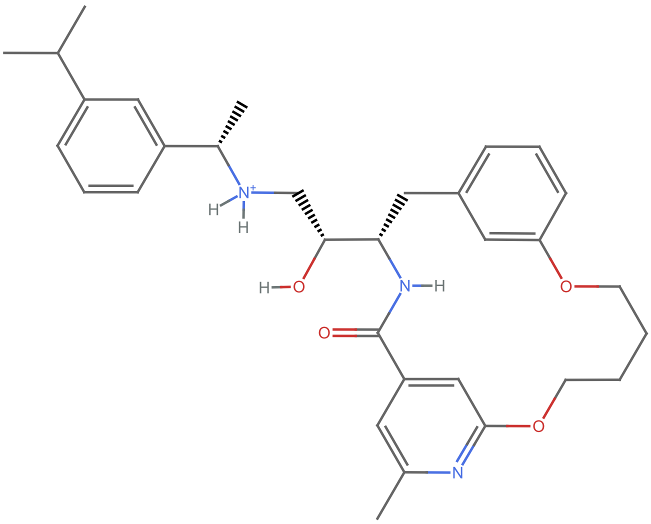

3D Plot

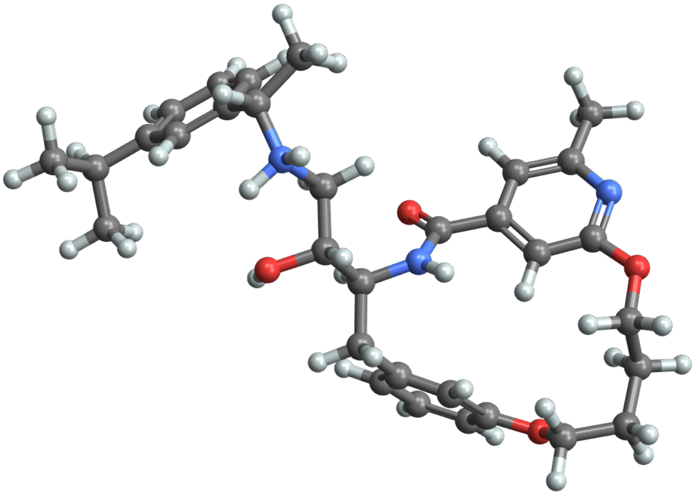

2D Autocorrelation

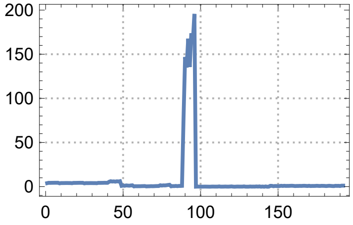

Dataset Index= 787

Molecule Formula= $C_{31}H_{41}F_2N_4O_2S^+$

3D Autocorrelation

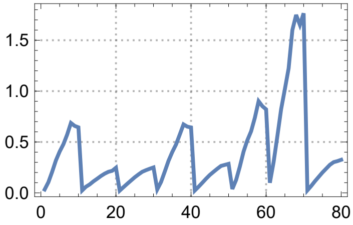

2D Plot

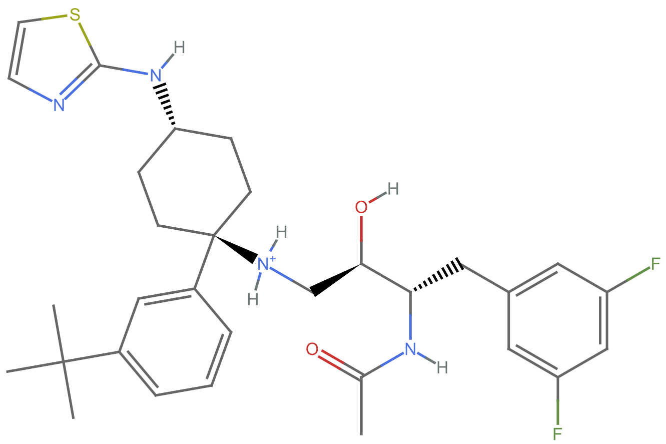

3D Plot

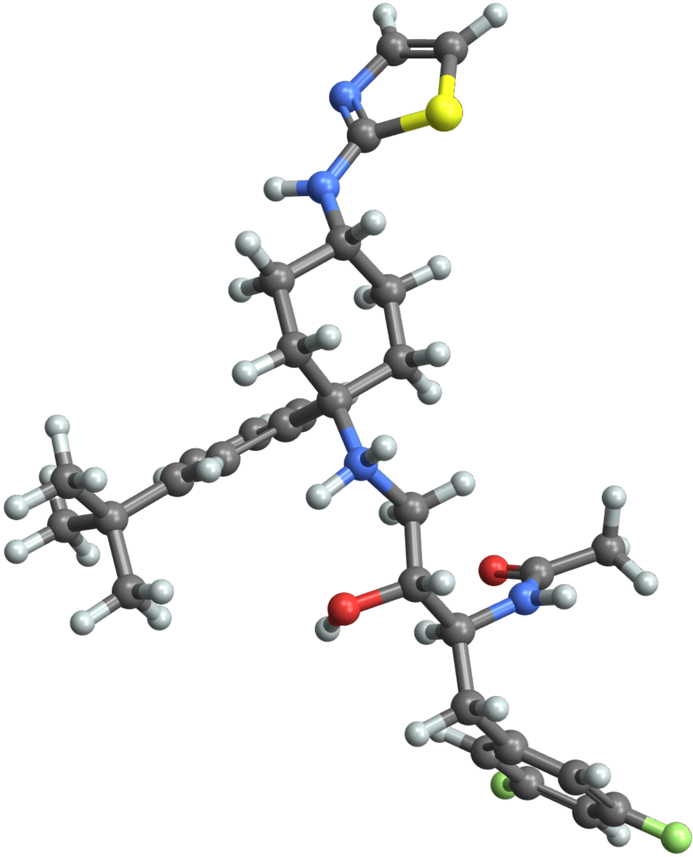

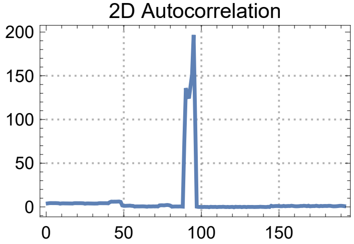

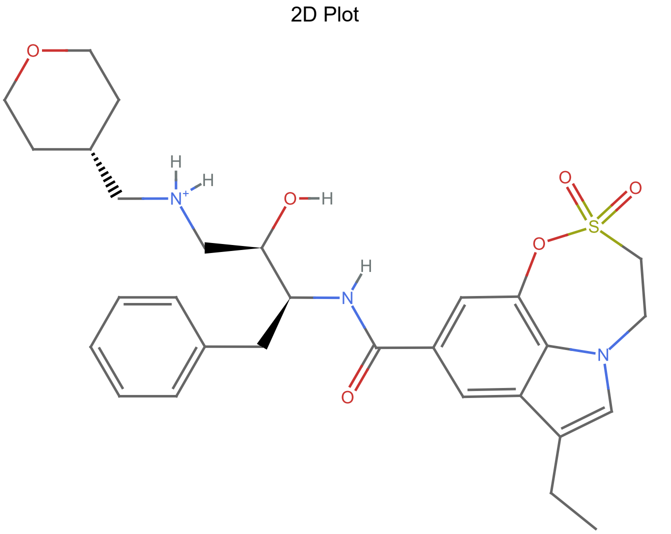

Dataset Index= 788

Molecule Formula= $C_{29}H_{38}N_3O_6S^+$

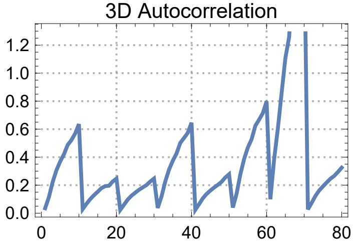

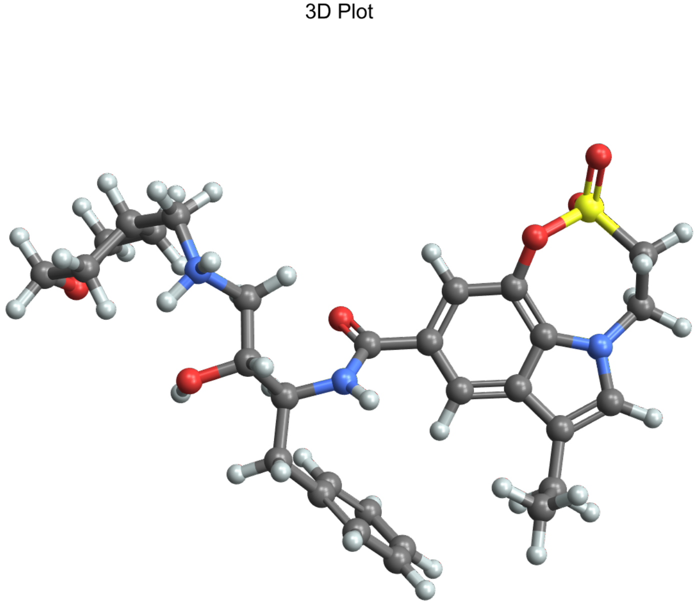

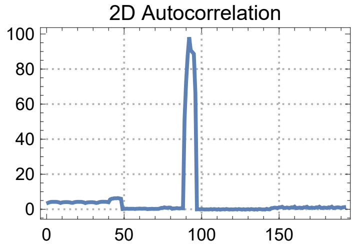

Dataset Index= 789

Molecule Formula= $C_{26}H_{20}FN_5$

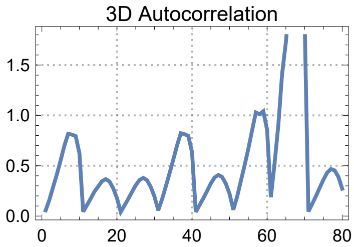

2D Plot

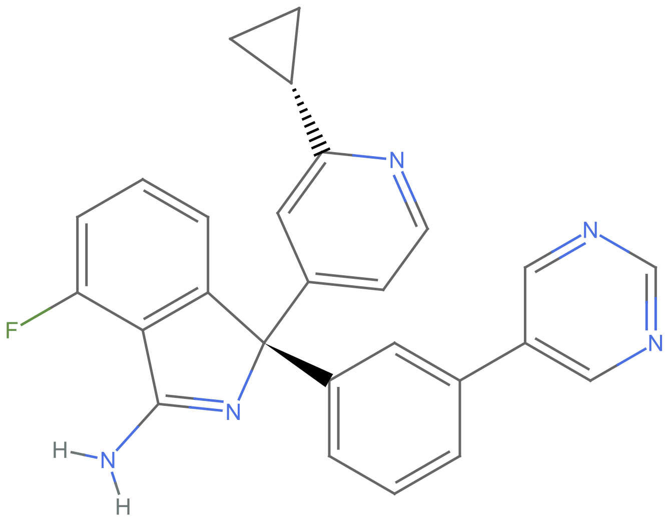

3D Plot

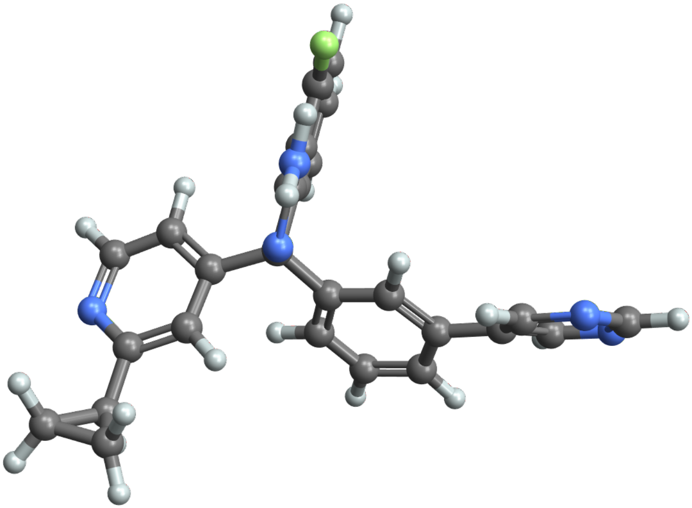

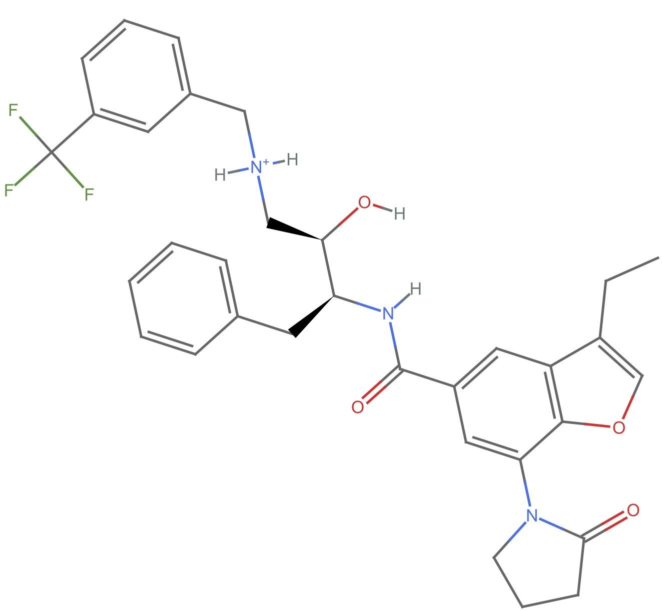

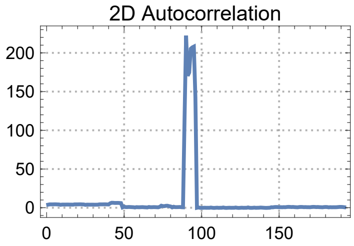

Dataset Index= 790

Molecule Formula= $C_{33}H_{35}F_3N_3O_4^+$

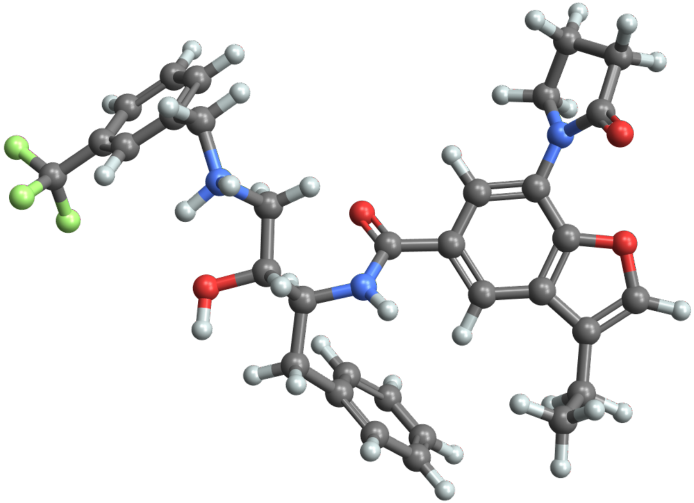

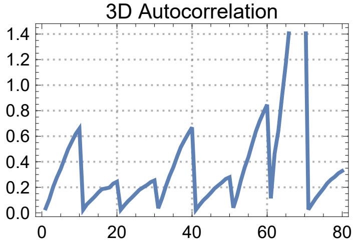

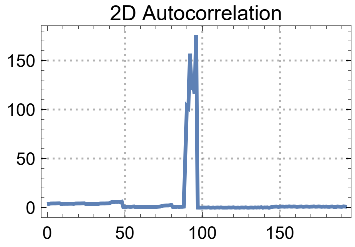

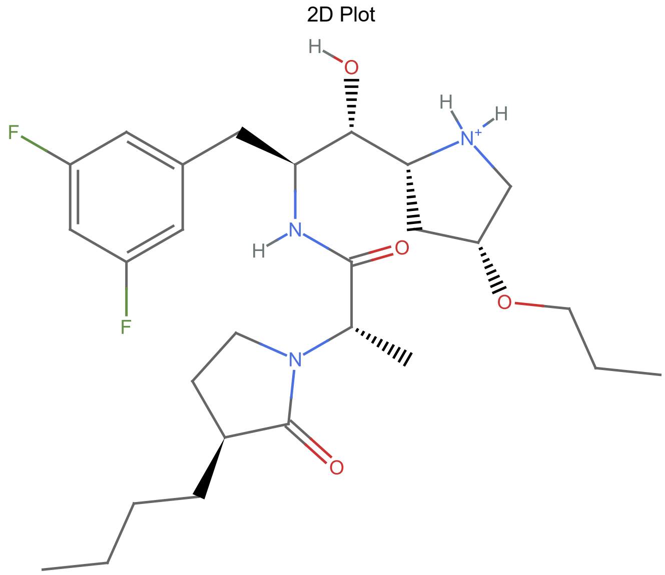

Dataset Index= 791

Molecule Formula= $C_{27}H_{42}F_{2}N_{3}O_{4}^{+}$

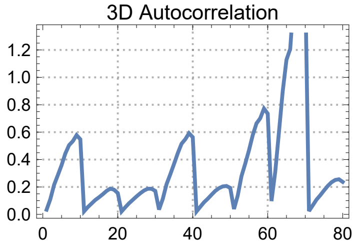

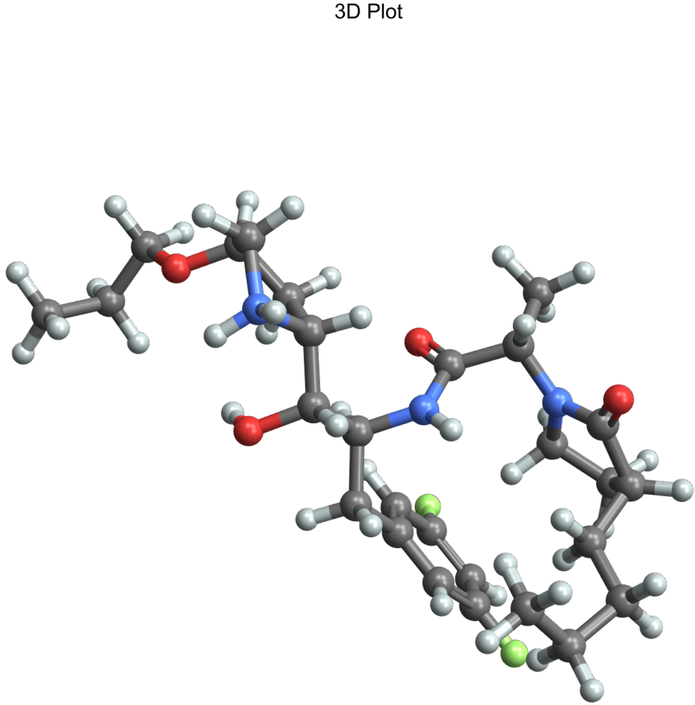

2D Autocorrelation

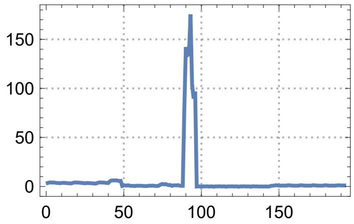

2D Plot

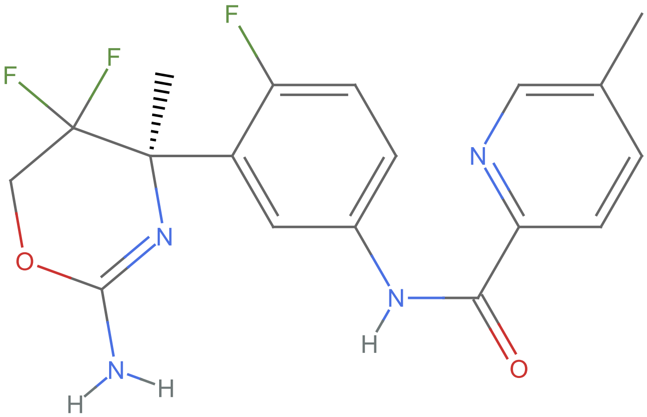

Dataset Index= 792

Molecule Formula= $C_{18}H_{17}F_3N_4O_2$

3D Autocorrelation

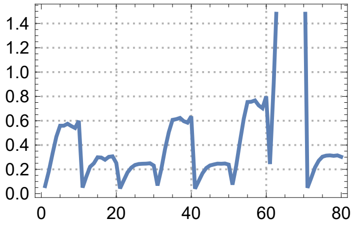

3D Plot

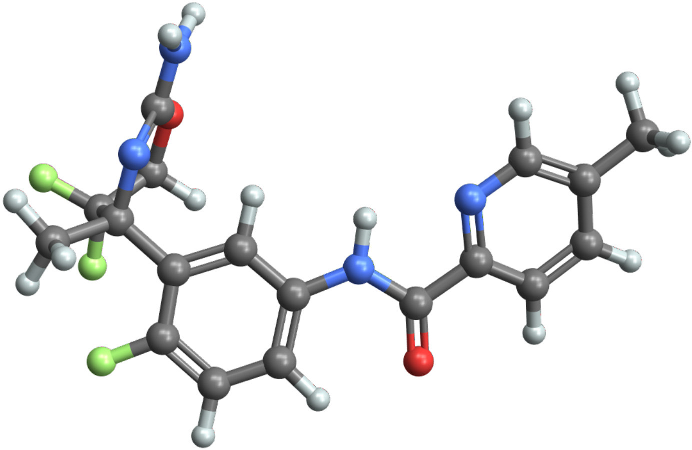

2D Autocorrelation

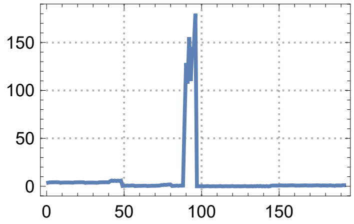

2D Plot

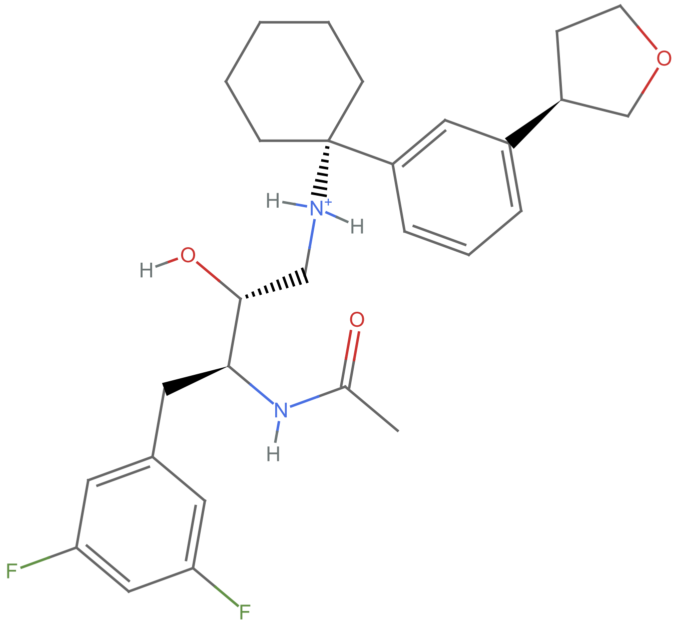

Dataset Index= 793

Molecule Formula= $C_{28}H_{37}F_2N_2O_3^+$

3D Autocorrelation

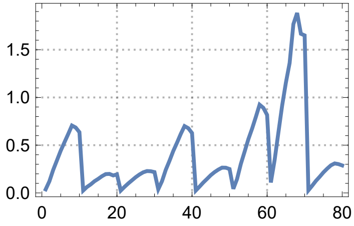

3D Plot

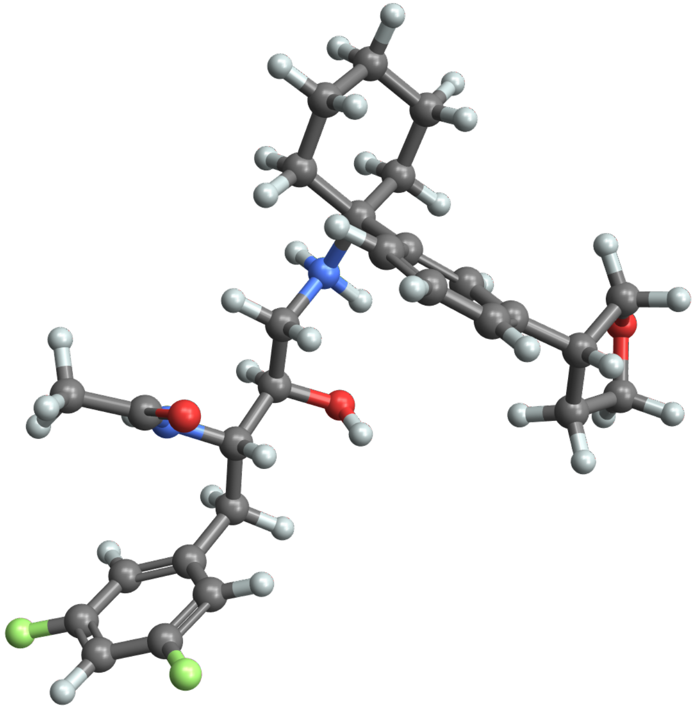

2D Plot

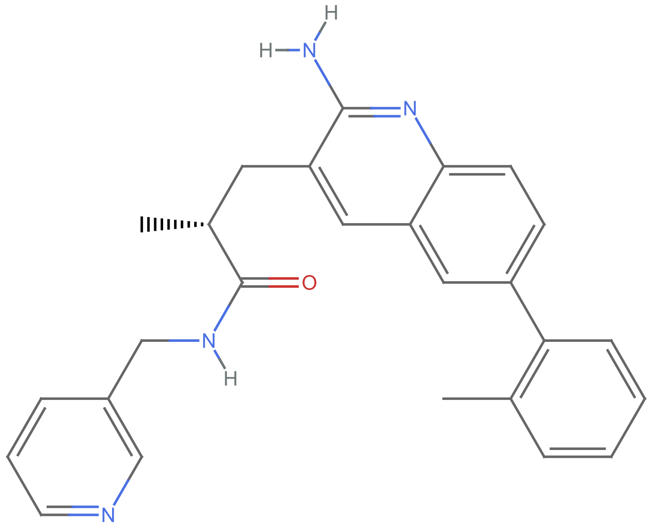

3D Plot

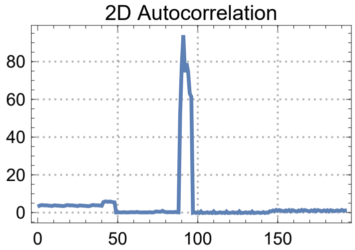

Dataset Index= 794

Molecule Formula= $C_{26}H_{26}N_4O$

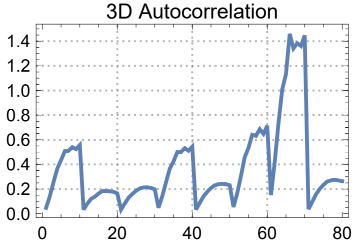

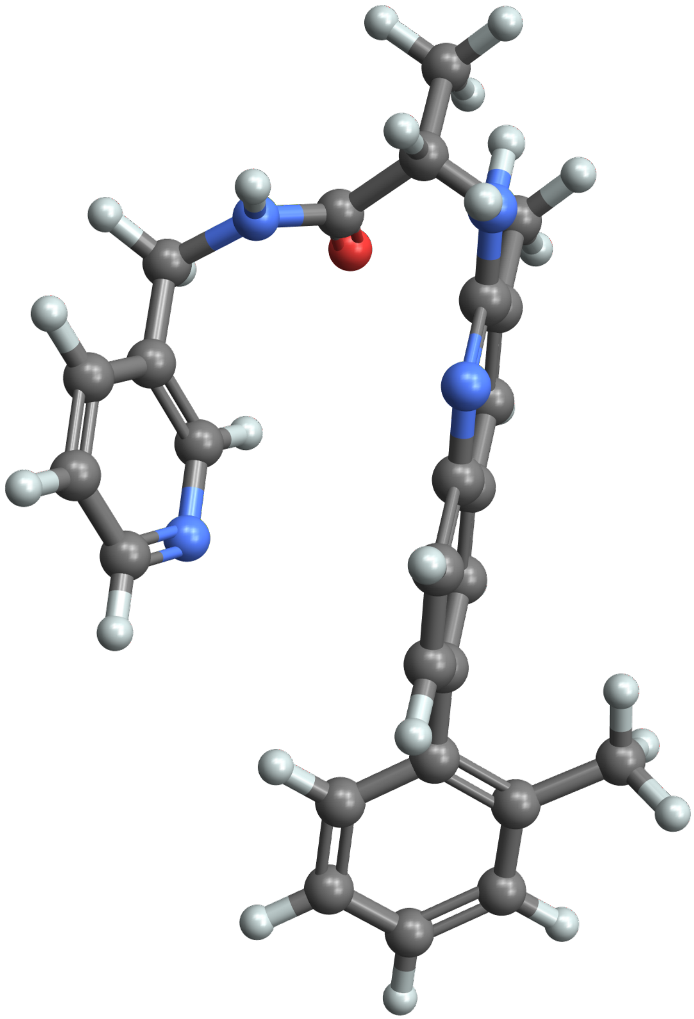

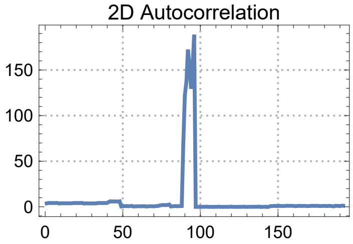

Dataset Index= 795

Molecule Formula= $C_{29}H_{40}F_2N_3O_4^+$

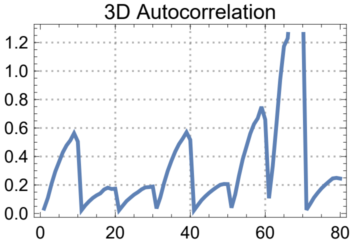

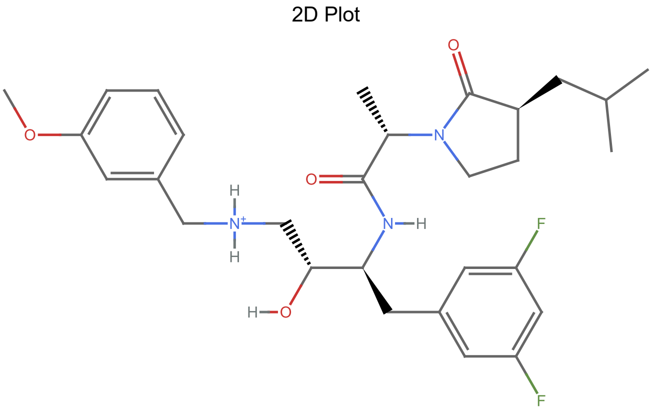

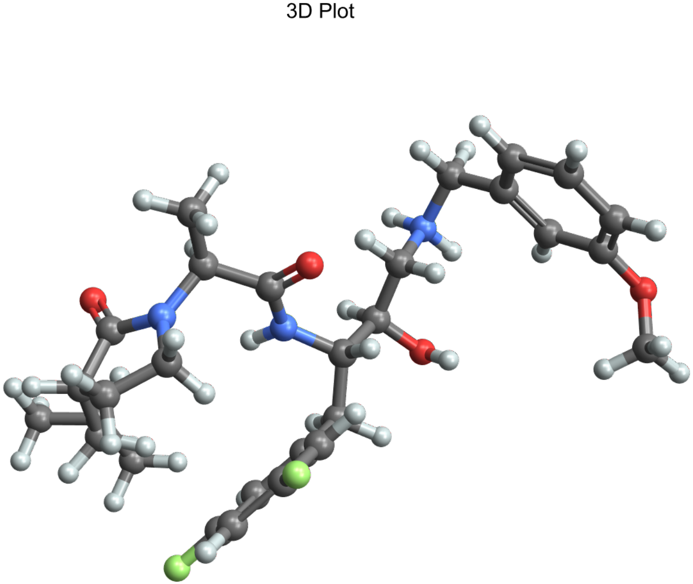

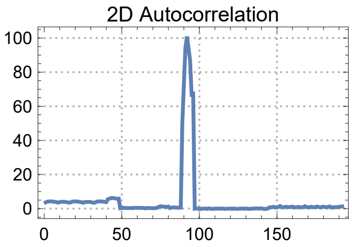

Dataset Index= 796

Molecule Formula= $C_{24}H_{22}FN_5O$

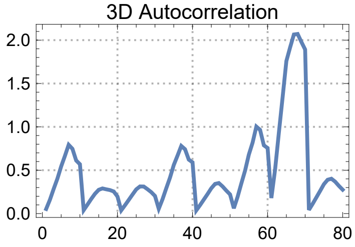

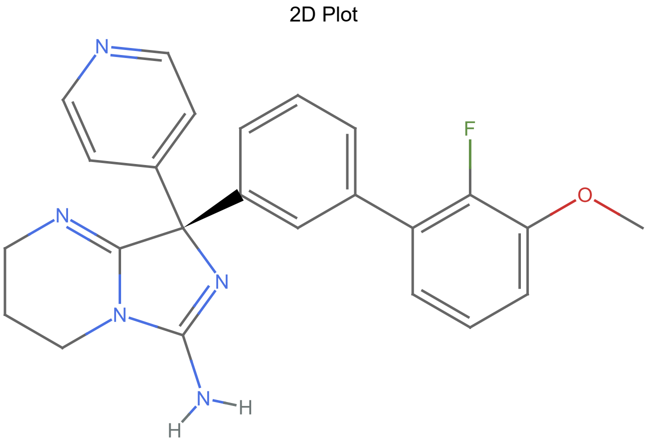

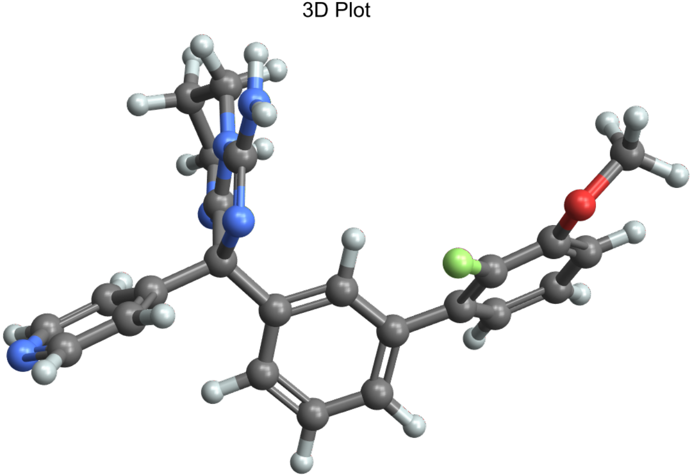

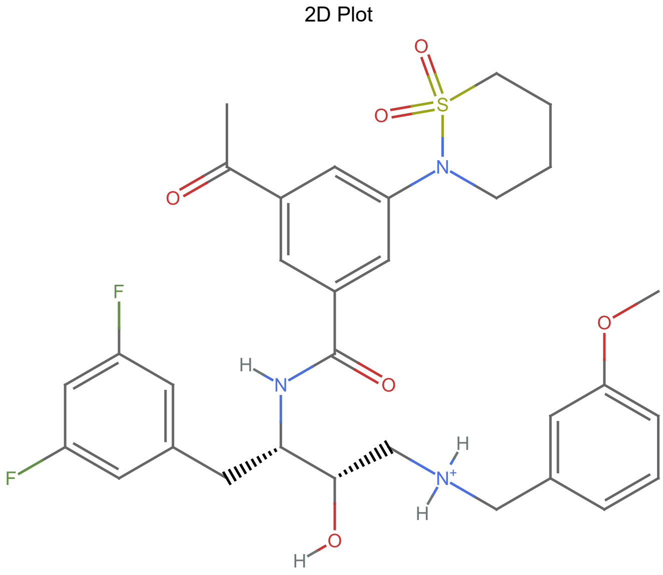

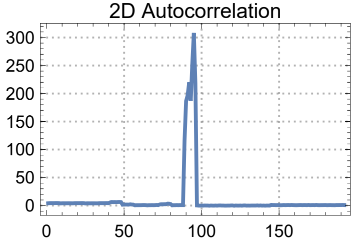

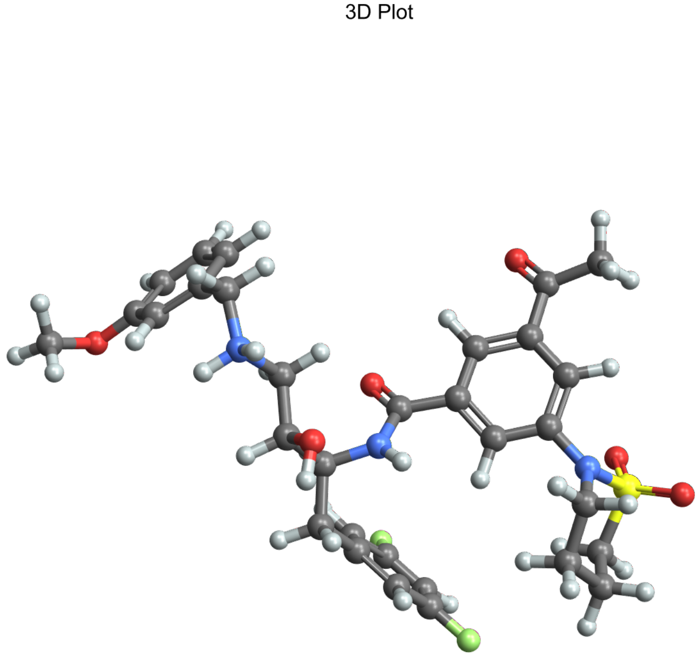

Dataset Index= 797

Molecule Formula= $C_{31}H_{36}F_2N_3O_6S^+$

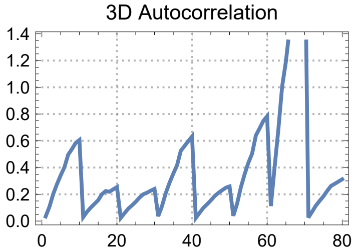

2D Autocorrelation

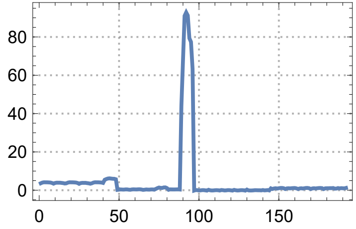

Dataset Index= 798

Molecule Formula= $C_{23}H_{22}N_4O_3$

3D Autocorrelation

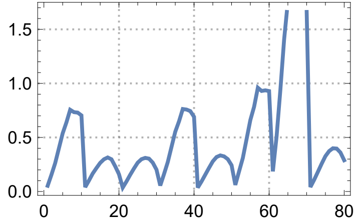

2D Plot

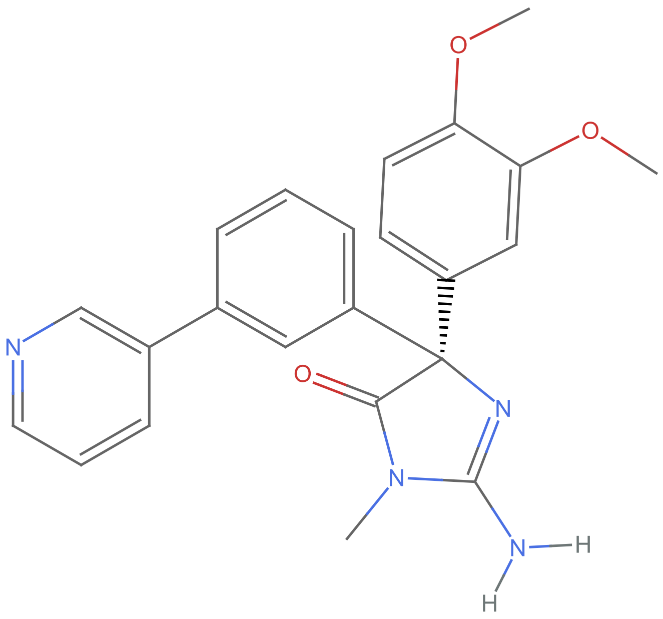

3D Plot

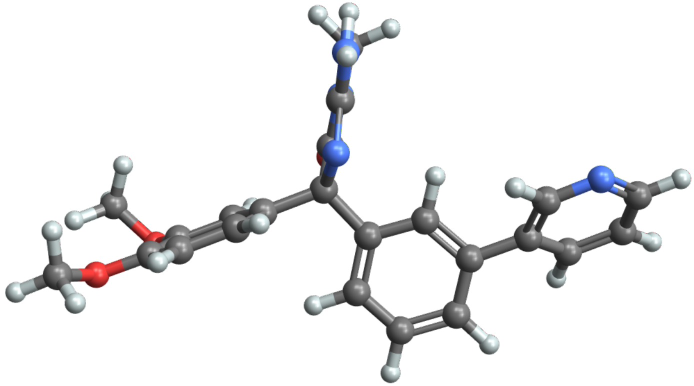

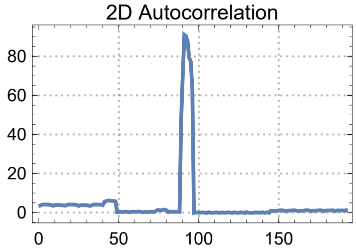

Dataset Index= 799

Molecule Formula= $C_{23}H_{20}N_4O_3$

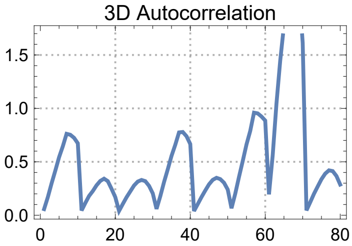

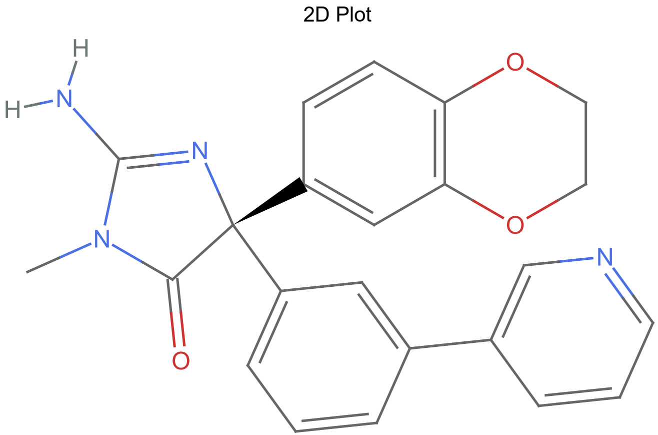

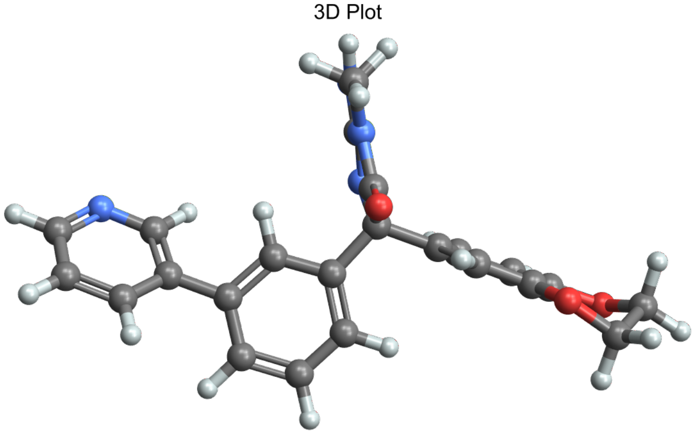

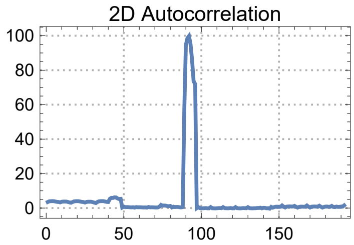

Dataset Index= 800

Molecule Formula= $C_{20}H_{21}F_2N_3O_2$

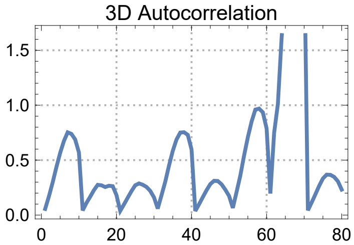

2D Plot

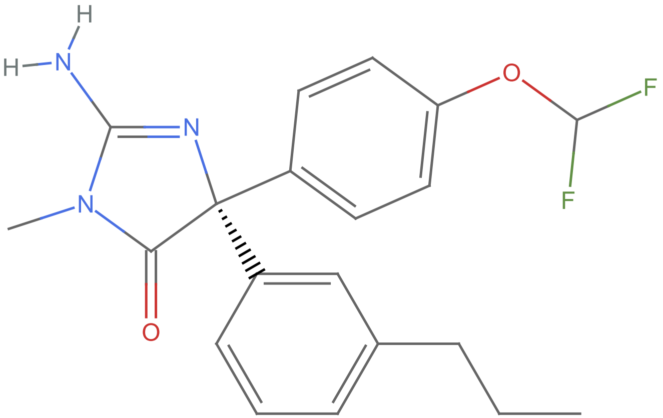

3D Plot

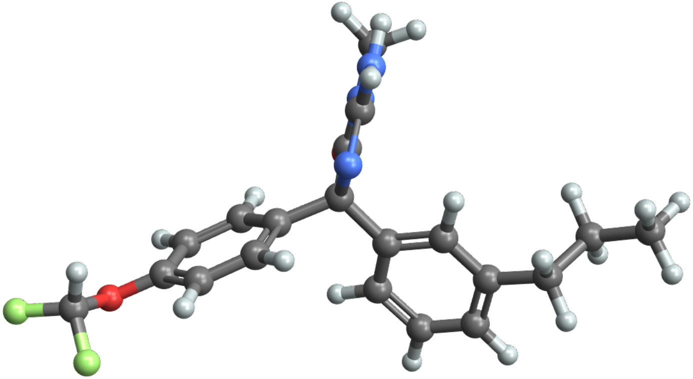

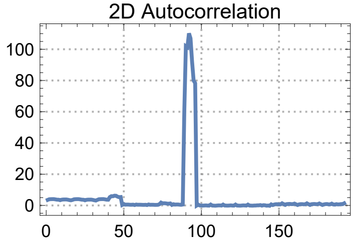

Dataset Index= 801

Molecule Formula= $C_{22}H_{25}F_2N_3O_2$

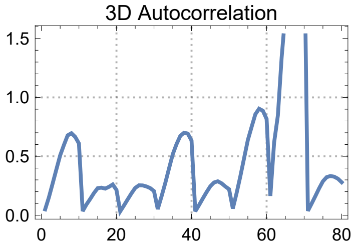

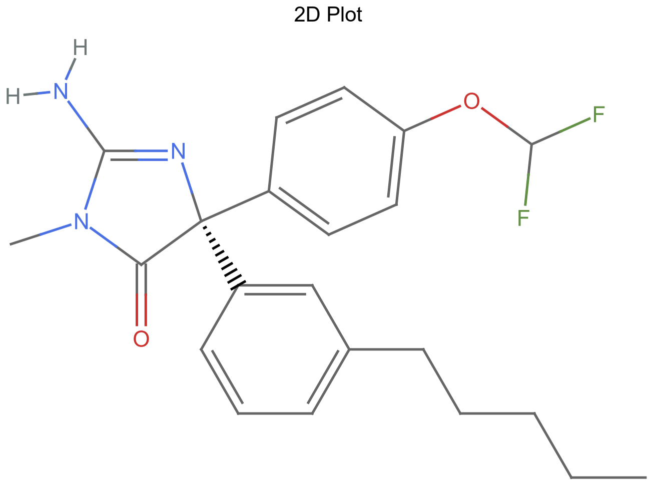

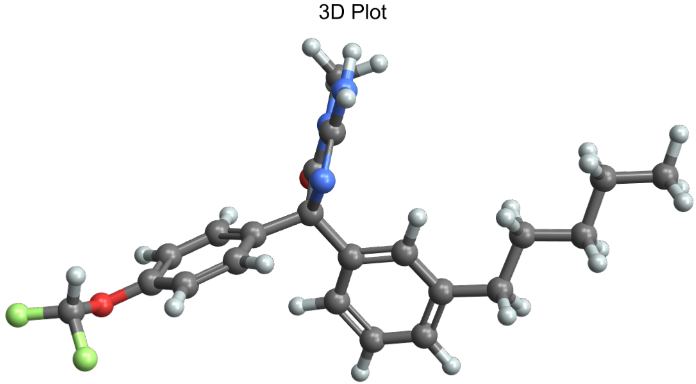

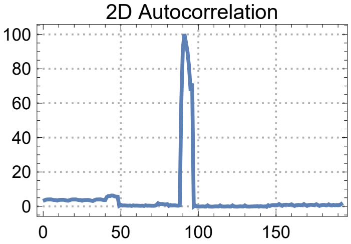

Dataset Index= 802

Molecule Formula= $C_{22}H_{21}F_2N_3O_2$

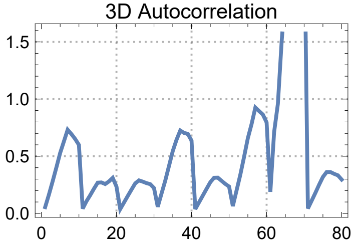

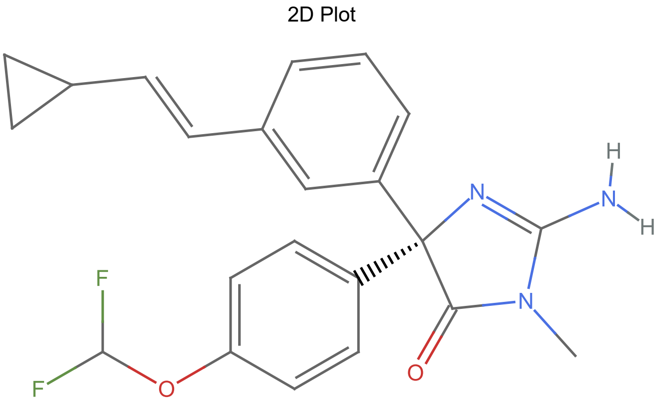

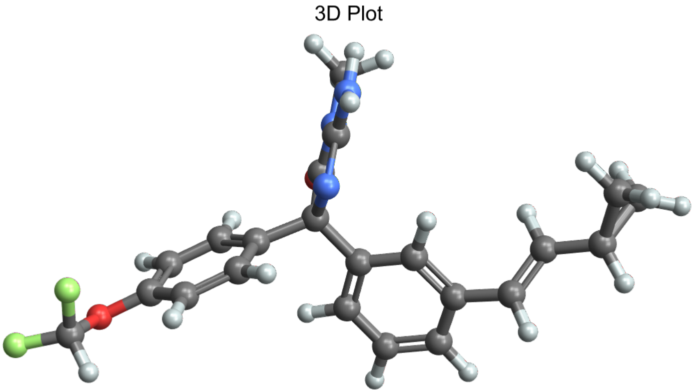

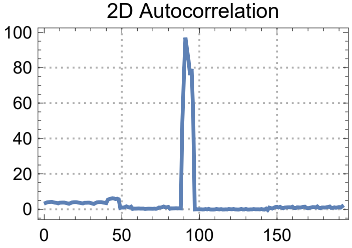

Dataset Index= 803

Molecule Formula= $C_{19}H_{15}FN_4OS$

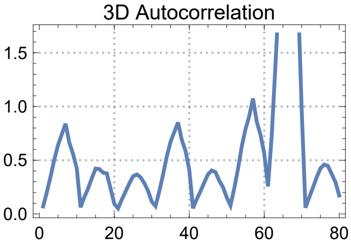

2D Plot

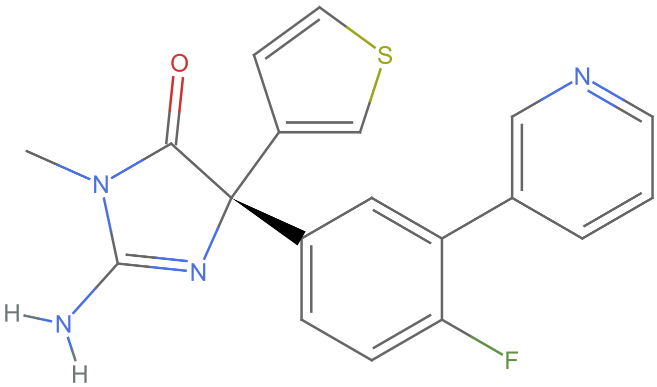

3D Plot

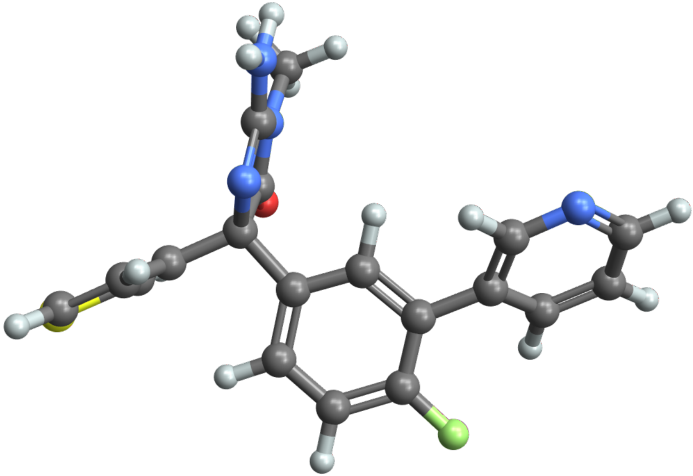

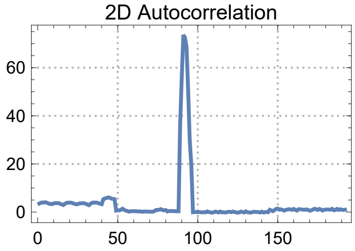

Dataset Index= 804

Molecule Formula= $C_{18}H_{15}N_5OS$

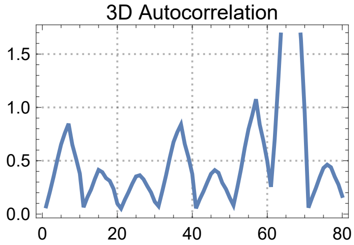

2D Plot

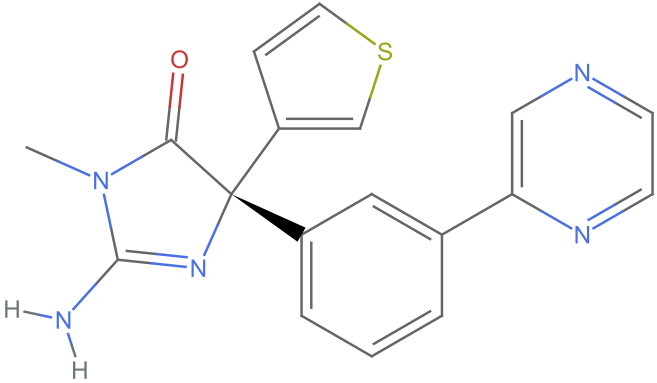

3D Plot

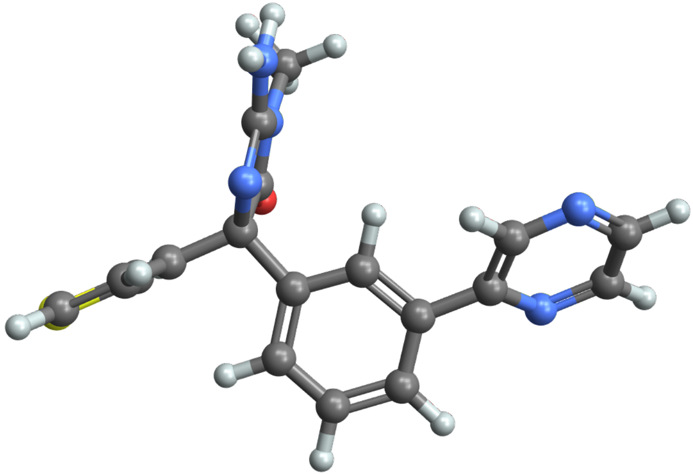

2D Plot

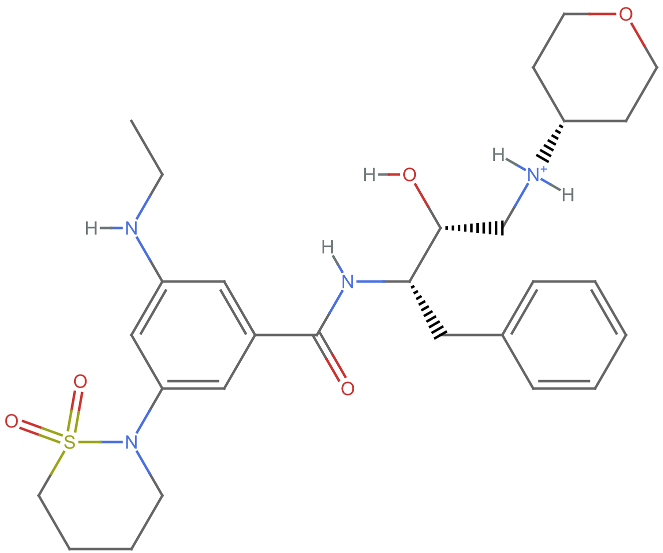

2D Autocorrelation

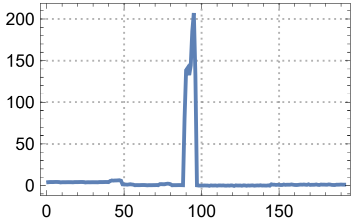

3D Plot

Dataset Index= 805

Molecule Formula= $C_{28}H_{41}N_4O_5S^+$

3D Autocorrelation

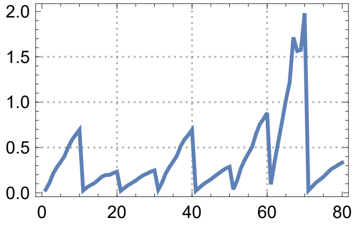

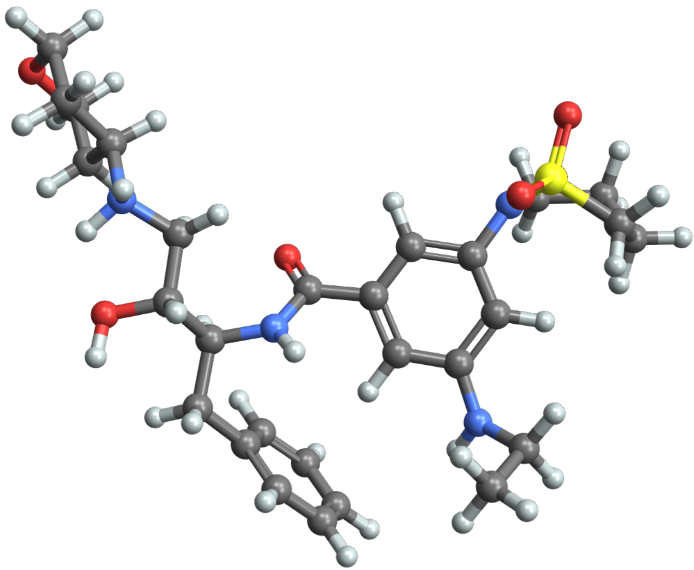

2D Plot

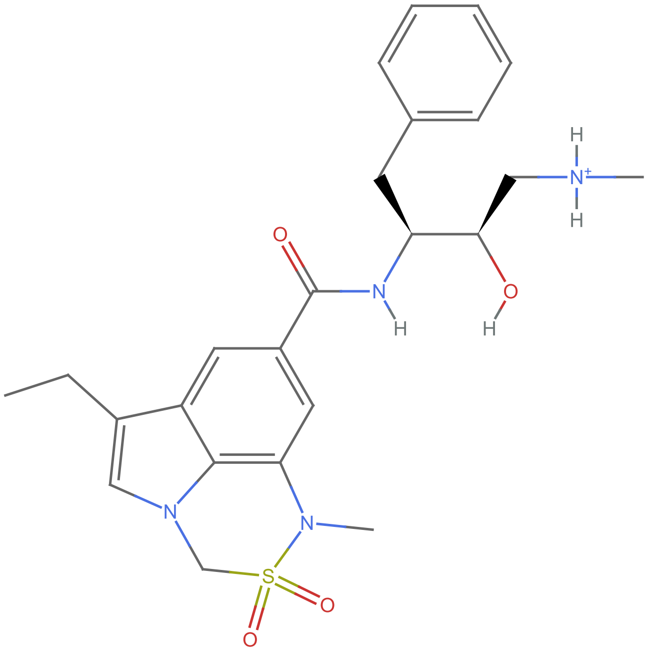

2D Autocorrelation

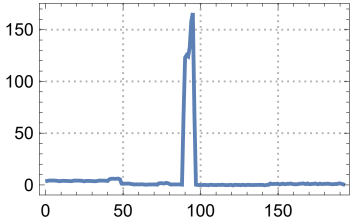

Dataset Index= 806

Molecule Formula= $C_{24}H_{31}N_4O_4S^+$

3D Plot

3D Autocorrelation

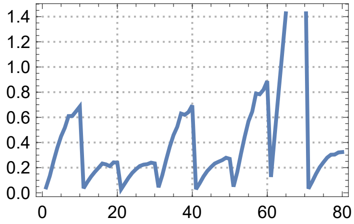

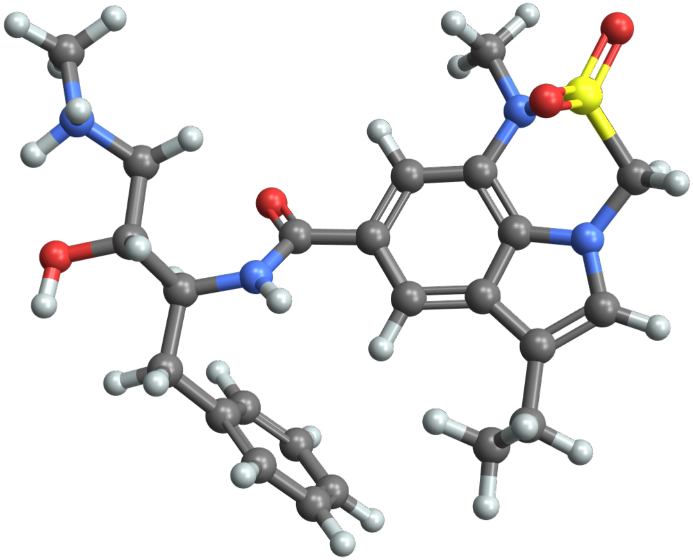

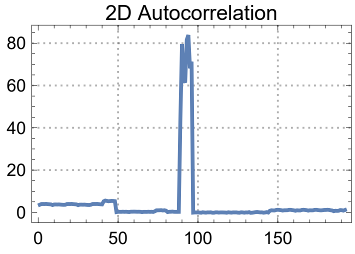

Dataset Index= 807

Molecule Formula= $C_{25}H_{34}N_3O_3^+$

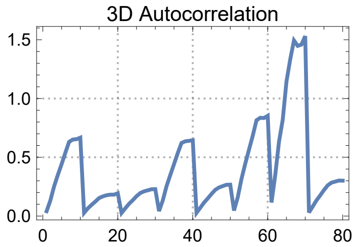

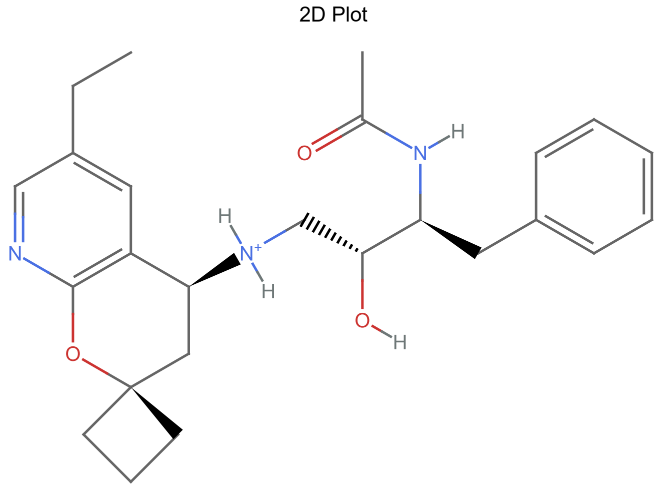

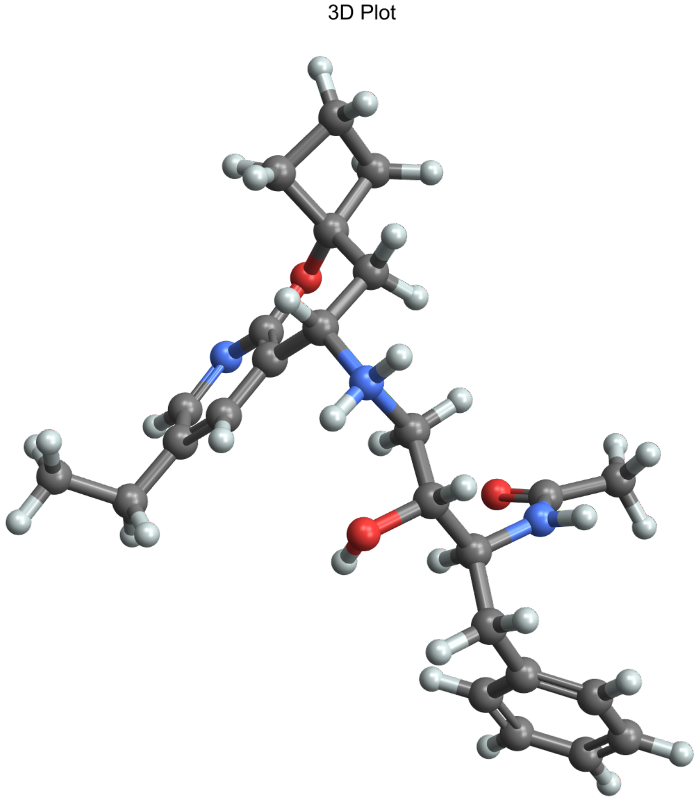

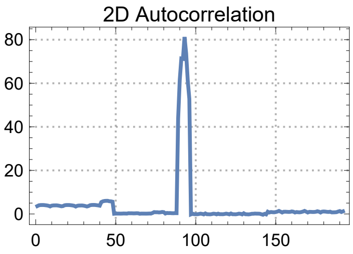

Dataset Index= 808

Molecule Formula= $C_{24}H_{23}N_5O$

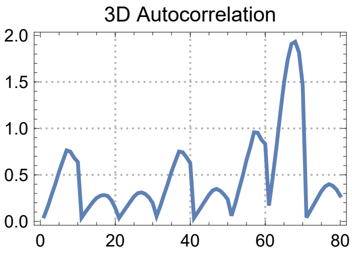

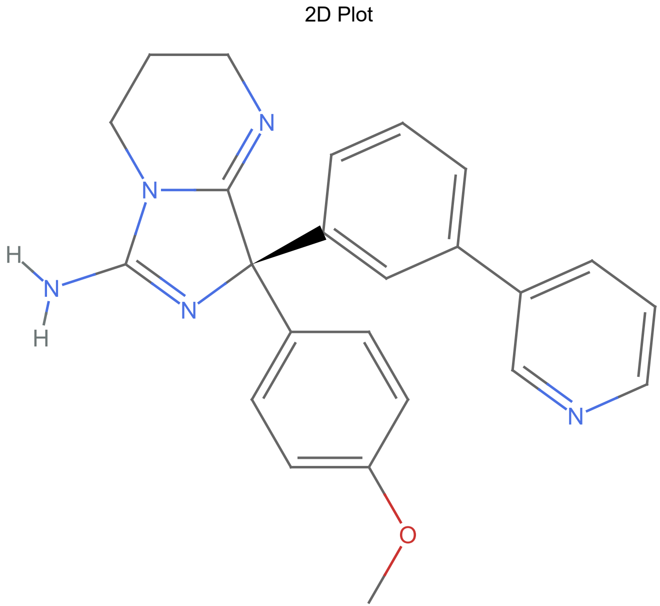

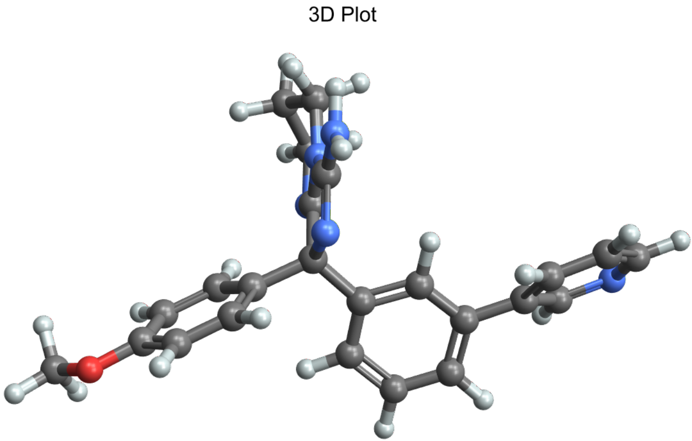

2D Plot

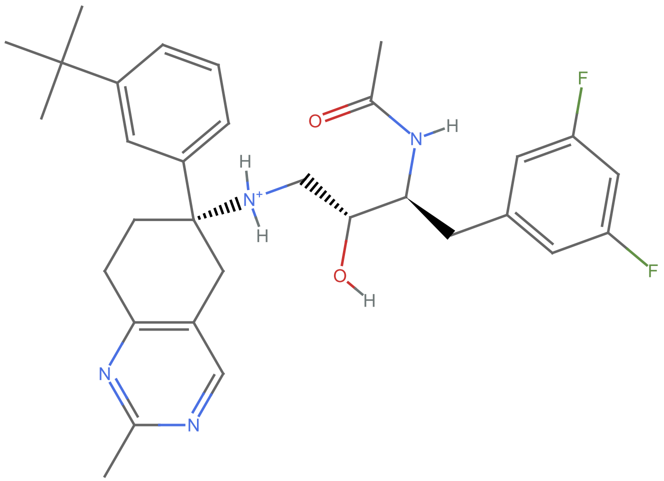

2D Autocorrelation

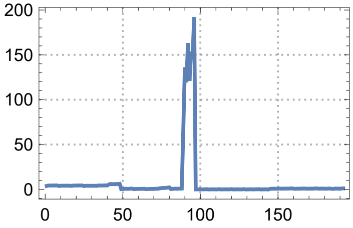


```
Dataset Index=       809

Molecule Formula=    C31H39F2N4O2+
```

3D Autocorrelation

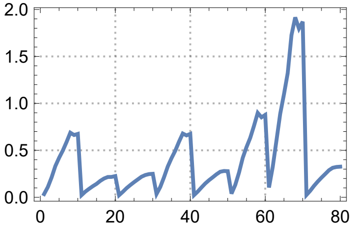

3D Plot

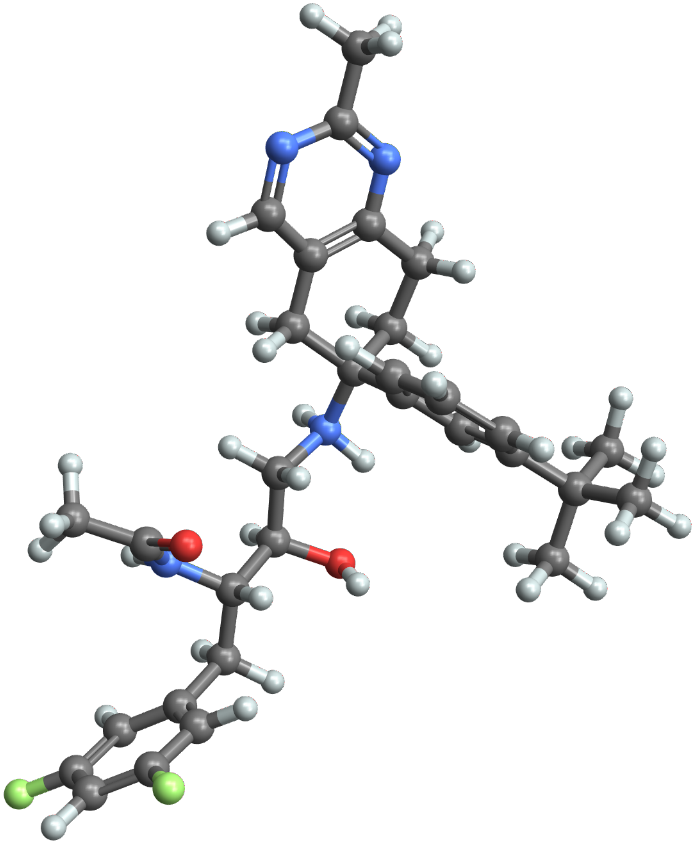

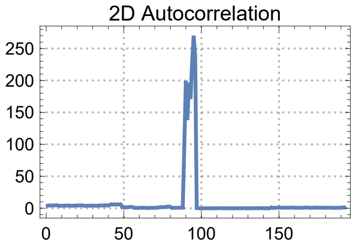

Dataset Index= 810

Molecule Formula= $C_{29}H_{42}F_2N_3O_4S^+$

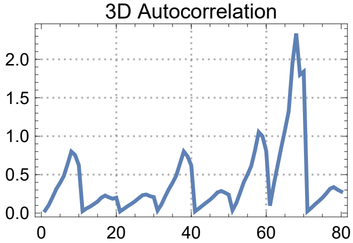

2D Plot

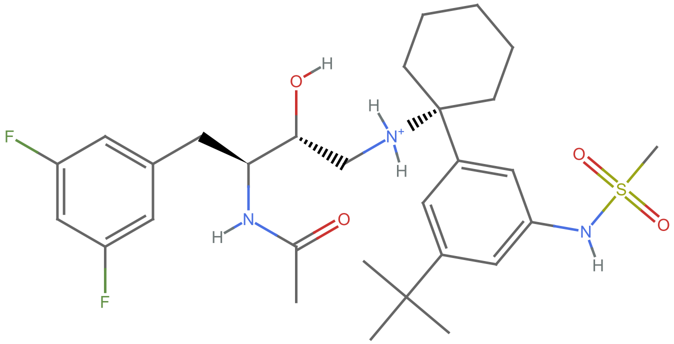

3D Plot

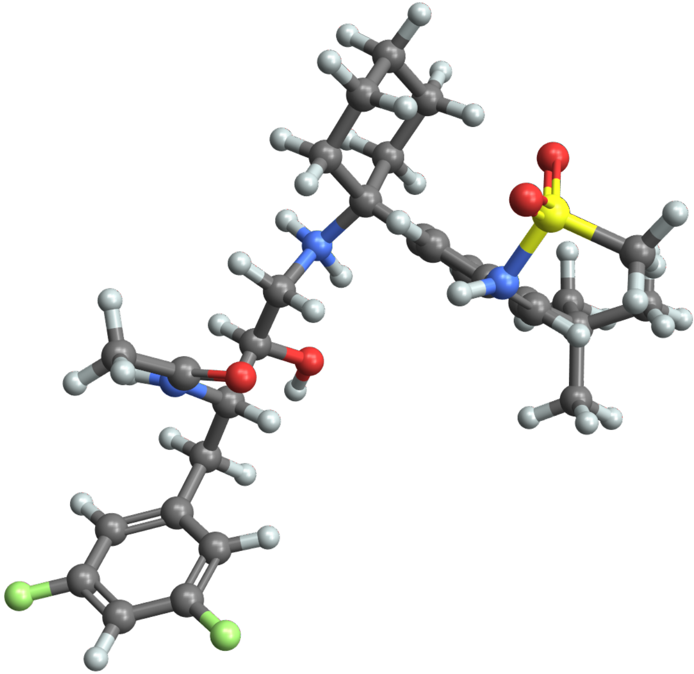

2D Autocorrelation

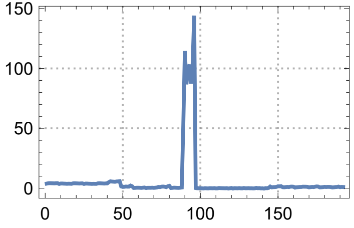

Dataset Index= 811

Molecule Formula= $C_{26}H_{36}FN_2O_3S^+$

3D Autocorrelation

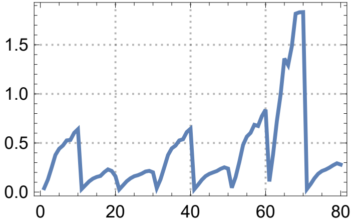

2D Plot

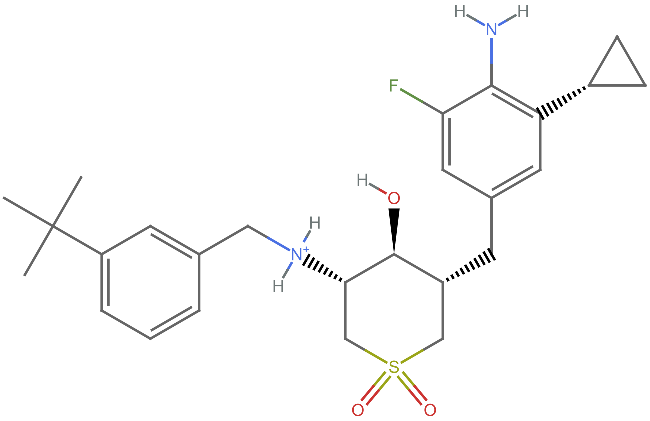

3D Plot

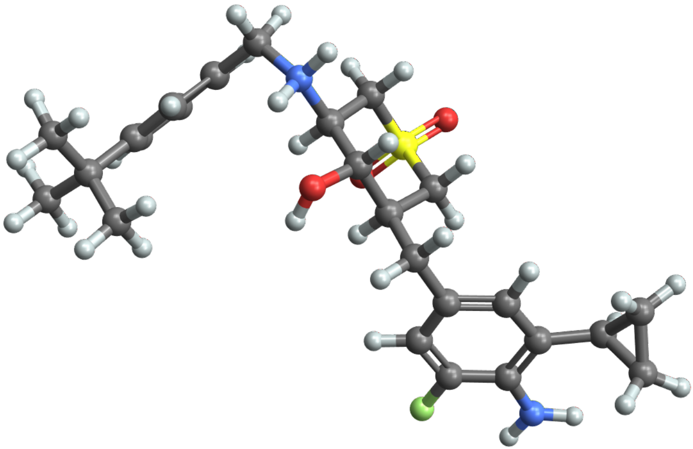

2D Plot

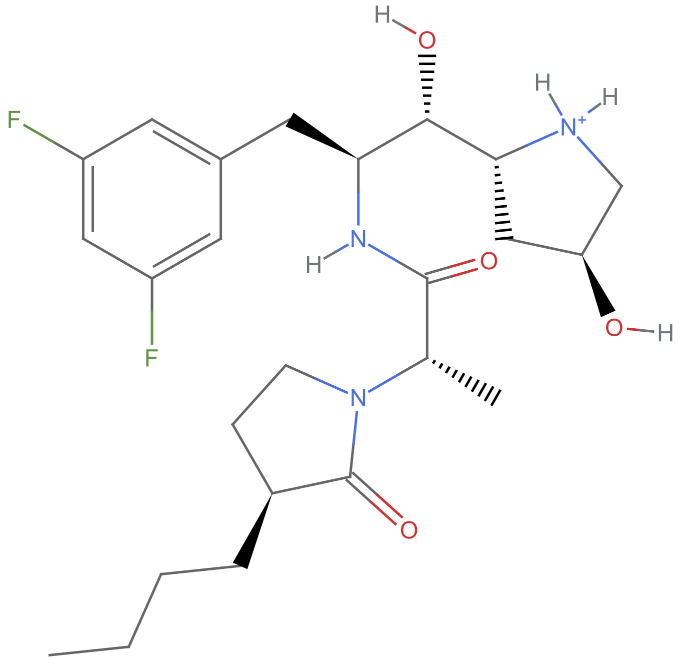

2D Autocorrelation

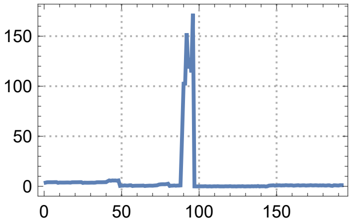

3D Plot

Dataset Index= 812

Molecule Formula= $C_{24}H_{36}F_2N_3O_4^+$

3D Autocorrelation

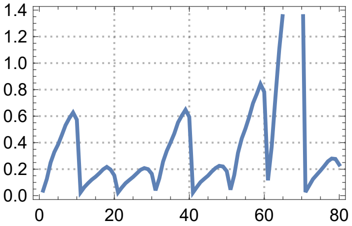

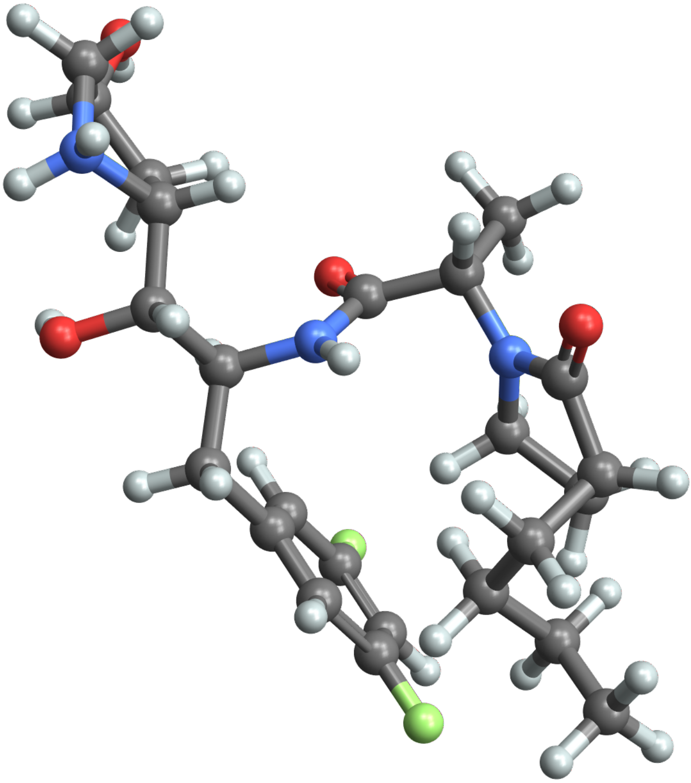

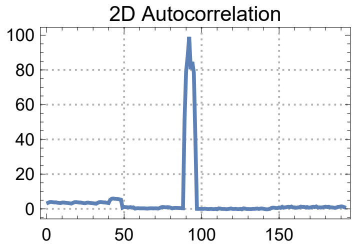

Dataset Index= 813

Molecule Formula= $C_{19}H_{18}ClFN_4O$

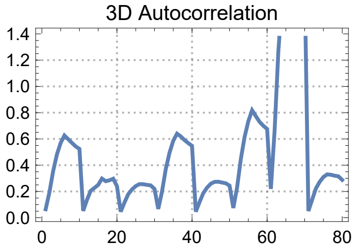

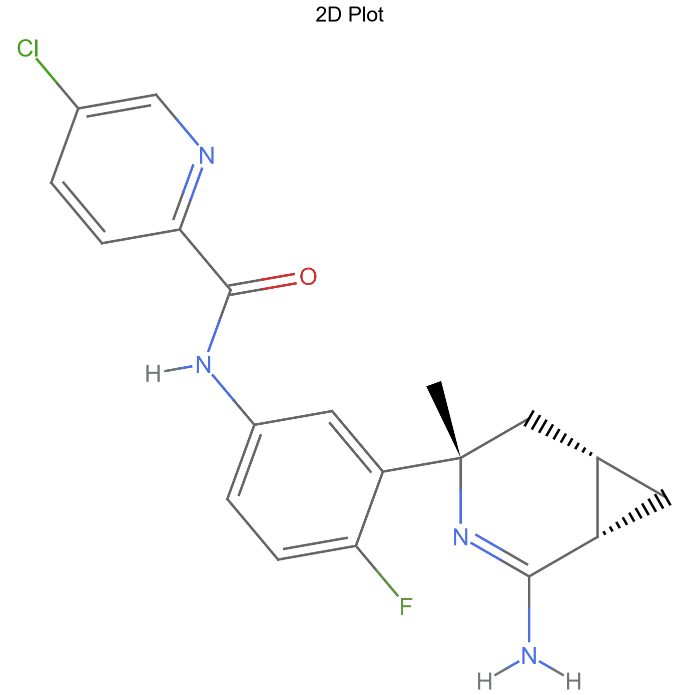

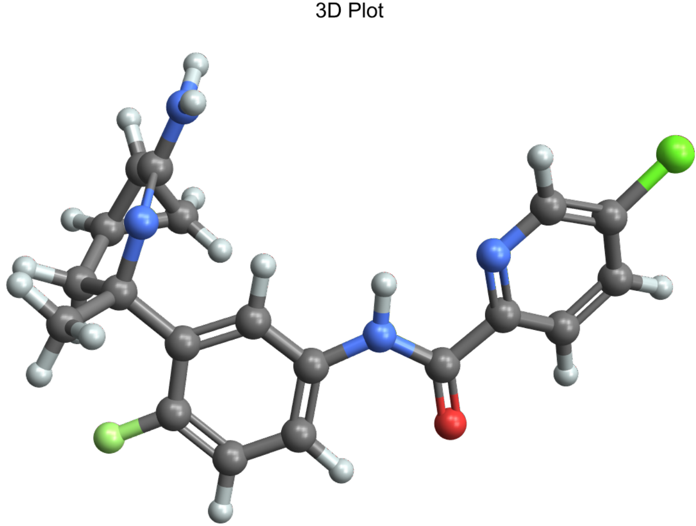

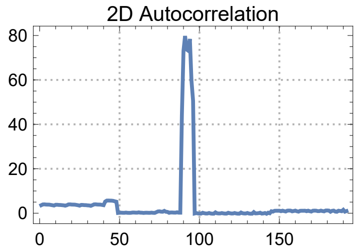

Dataset Index= 814

Molecule Formula= $C_{25}H_{32}N_4O_2$

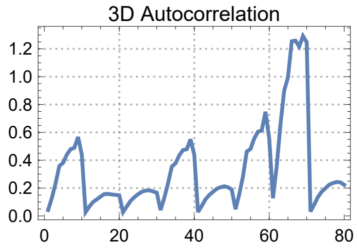

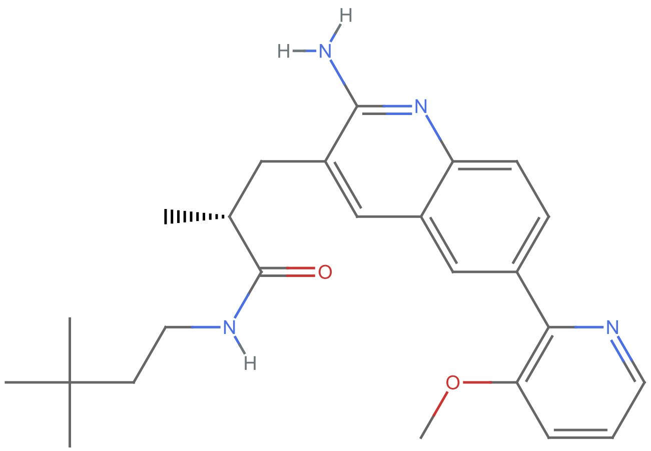

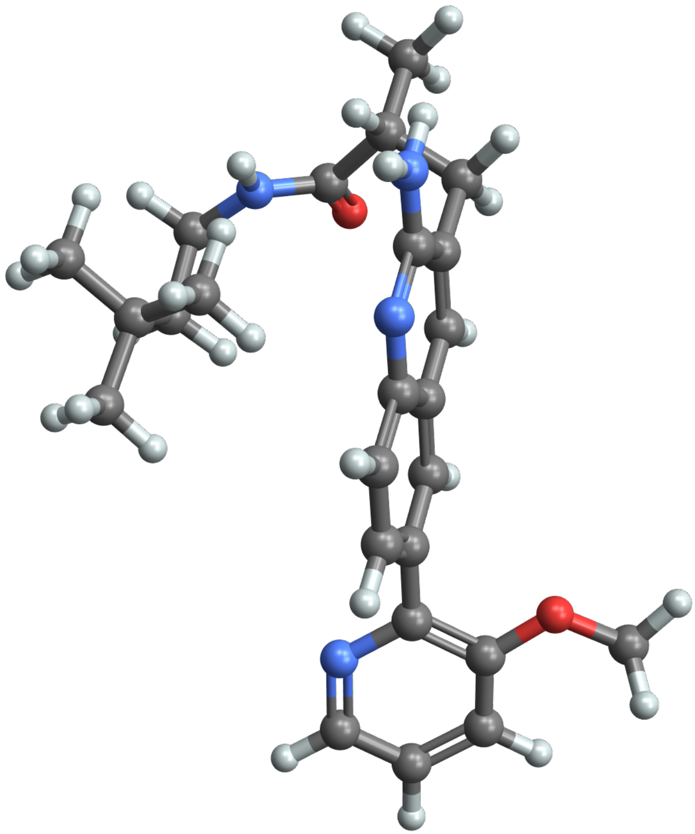

2D Autocorrelation

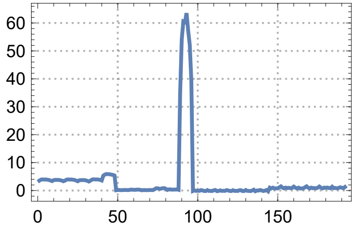

Dataset Index= 815

Molecule Formula= $C_{22}H_{21}N_5O$

3D Autocorrelation

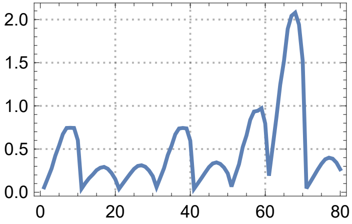

2D Plot

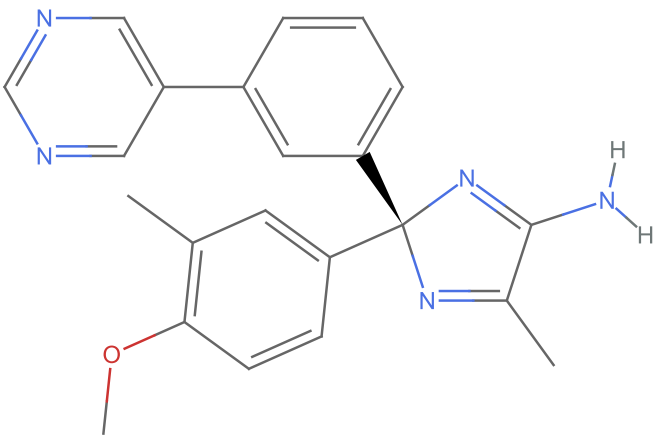

3D Plot

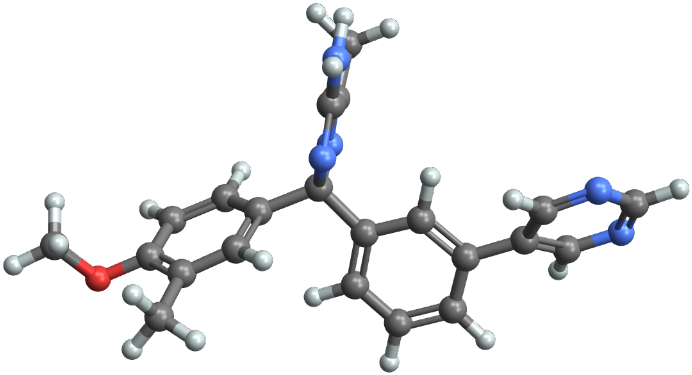

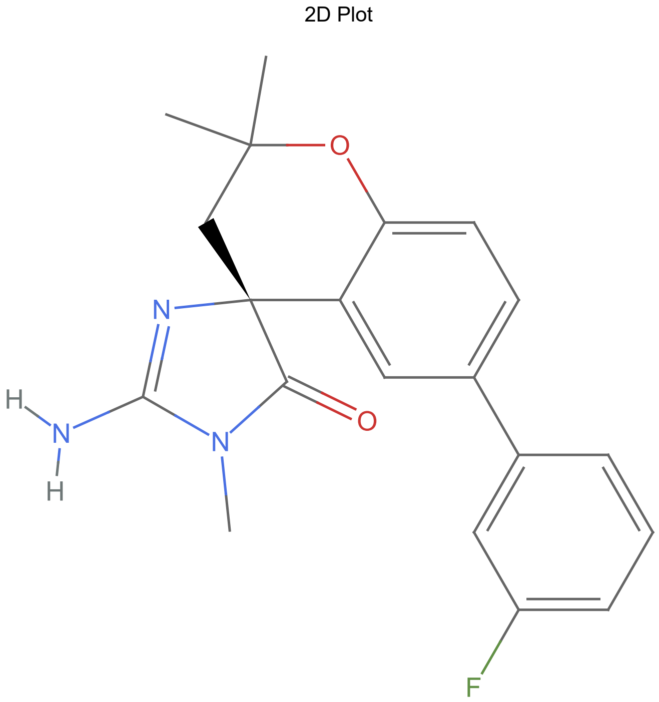

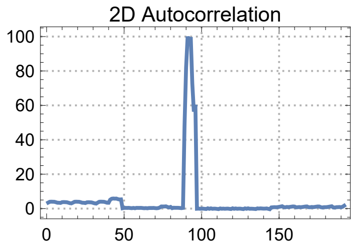

Dataset Index= 816

Molecule Formula= $C_{20}H_{20}FN_3O_2$

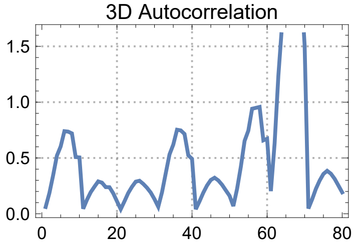

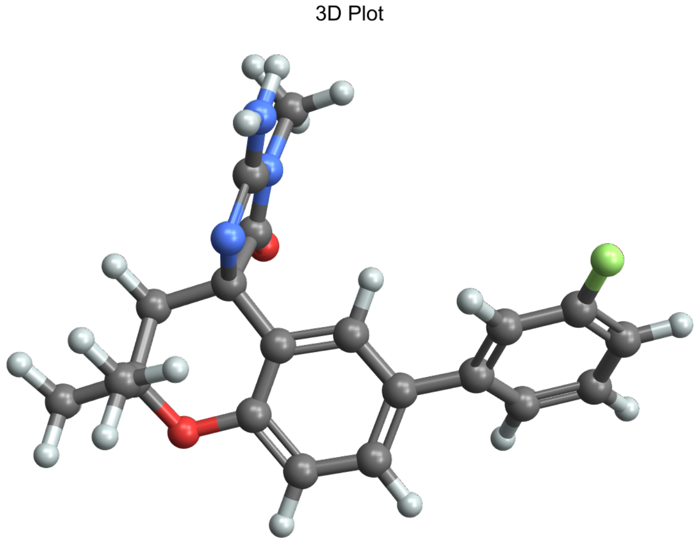

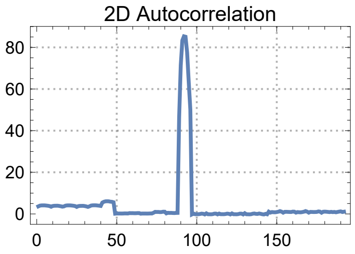

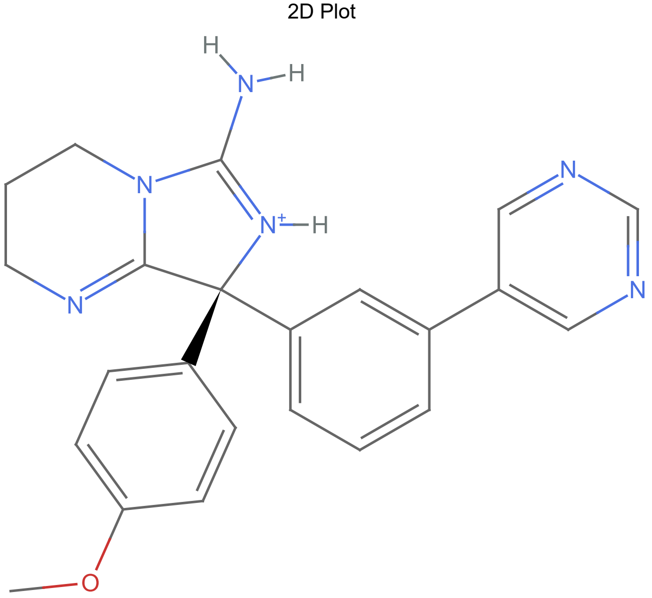

Dataset Index= 817

Molecule Formula= $C_{23}H_{23}N_6O^+$

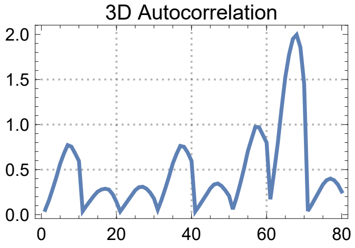

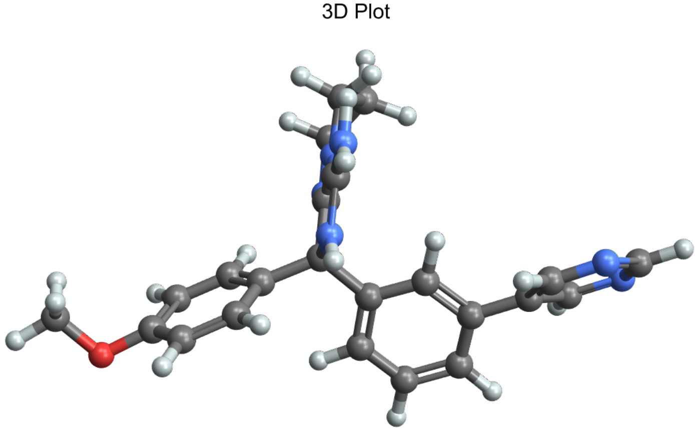

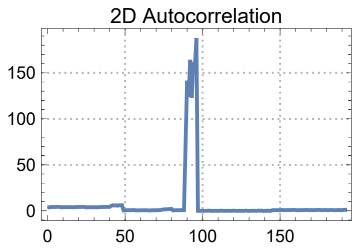

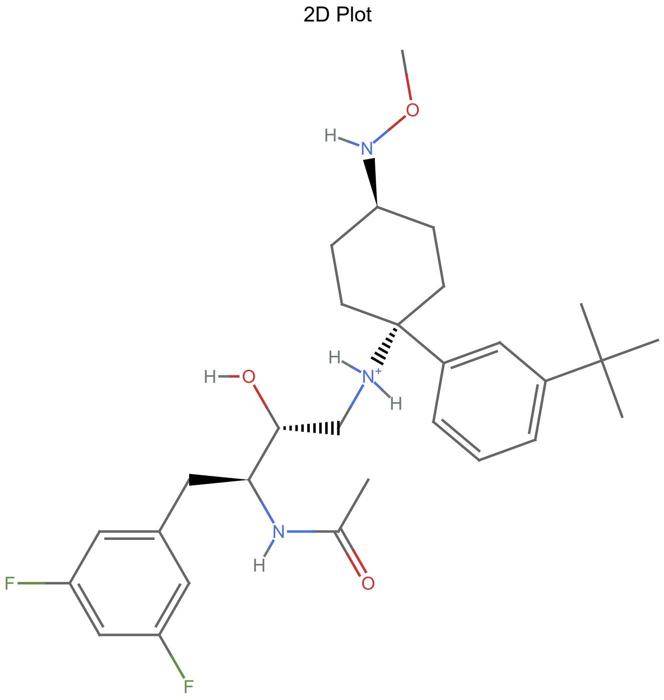

Dataset Index= 818

Molecule Formula= $C_{29}H_{42}F_2N_3O_3^+$

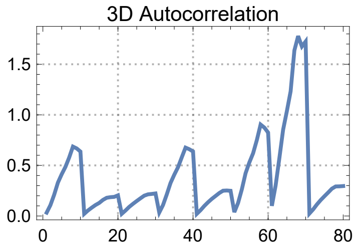

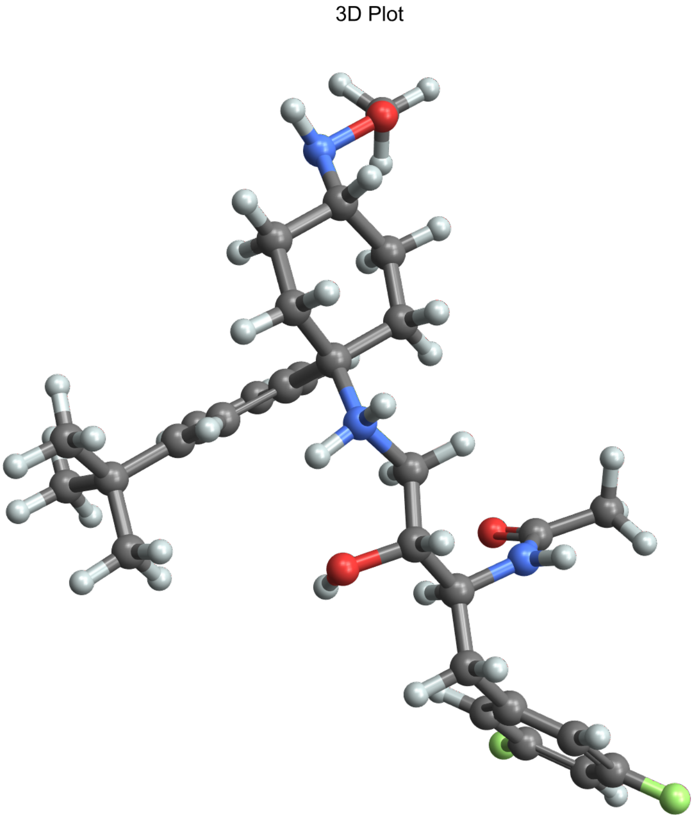

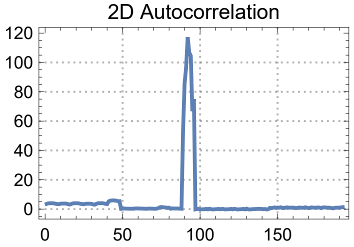

Dataset Index= 819

Molecule Formula= $C_{21}H_{24}N_4O_3$

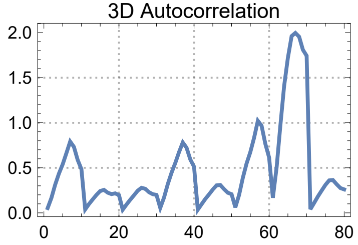

2D Plot

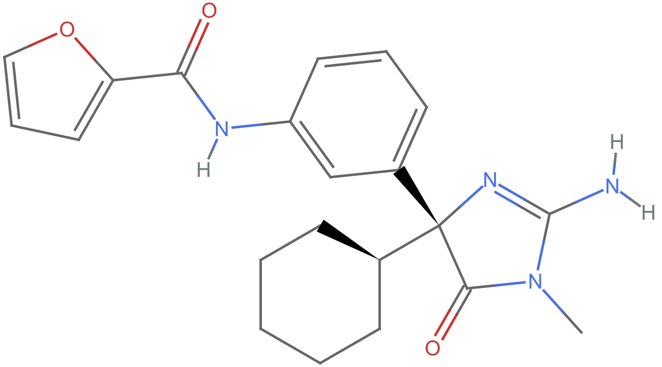

3D Plot

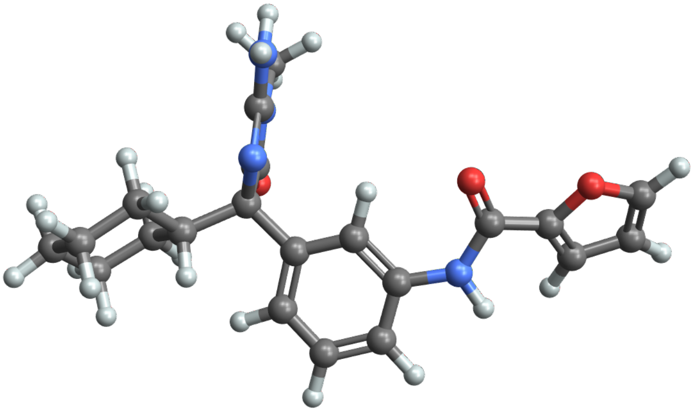

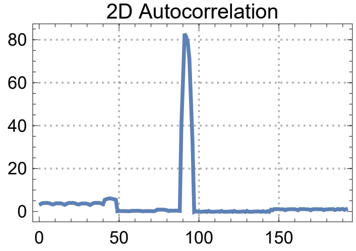

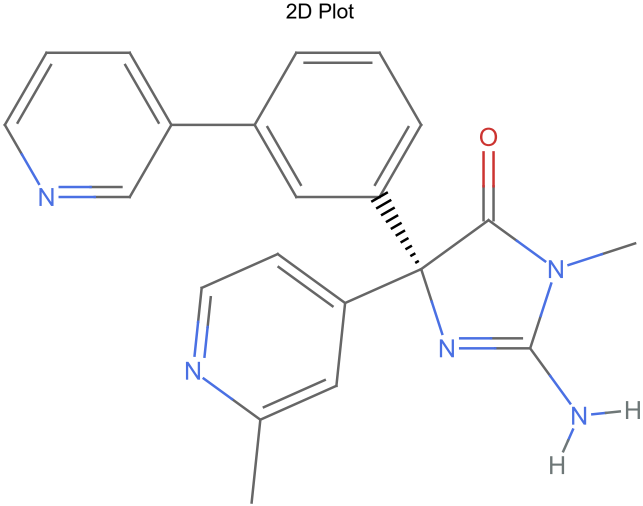

Dataset Index= 820

Molecule Formula= $C_{21}H_{19}N_5O$

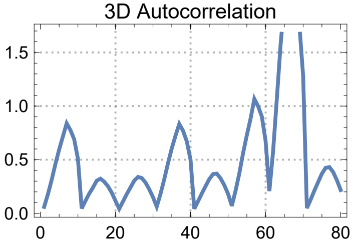

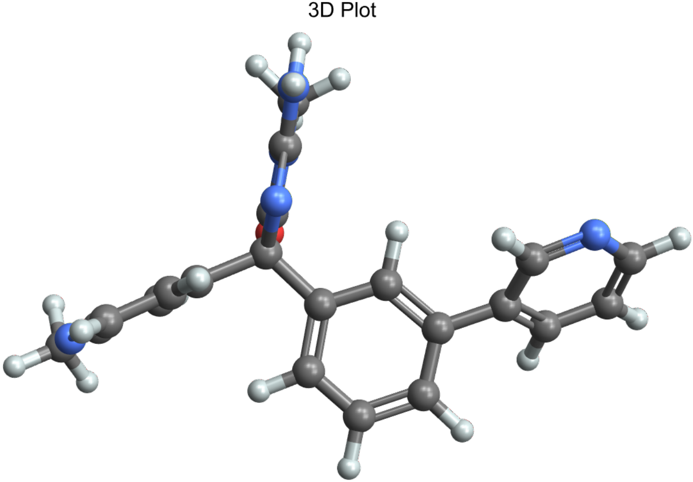

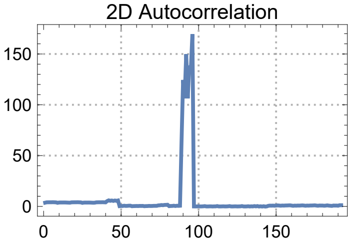

Dataset Index= 821

Molecule Formula= $C_{27}H_{35}F_2N_2O_2^+$

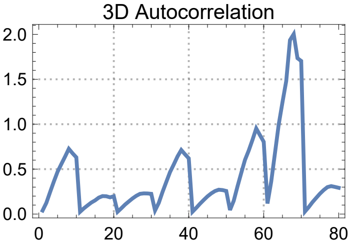

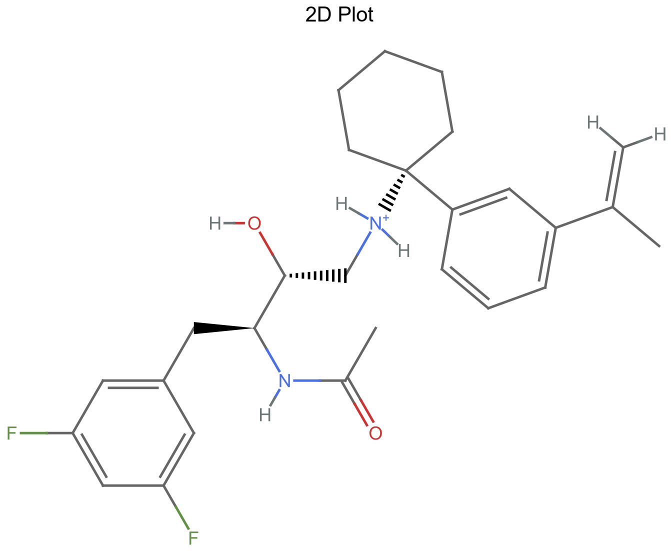

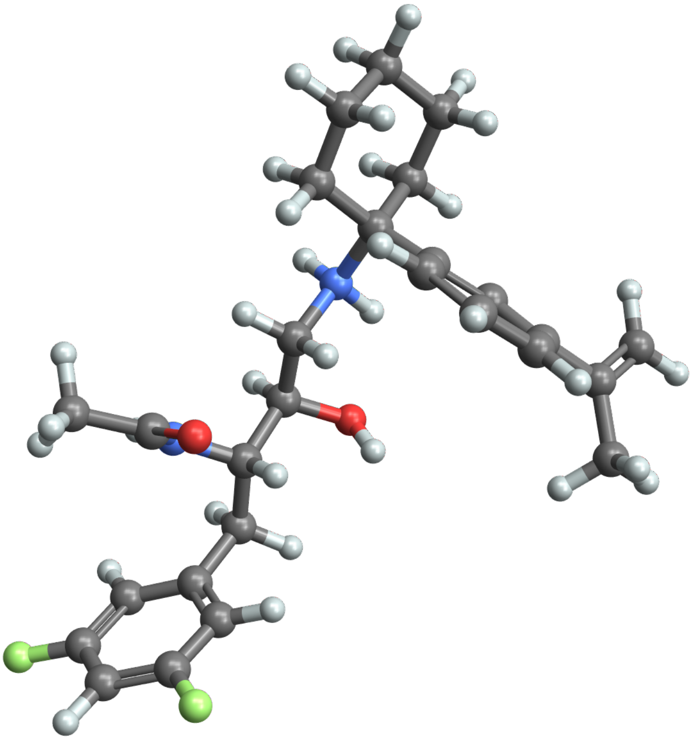

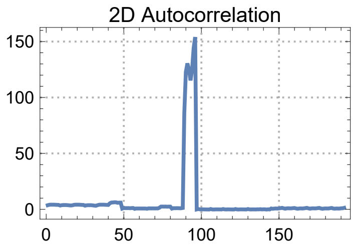

Dataset Index= 822

Molecule Formula= $C_{21}H_{21}F_4N_3O_3$

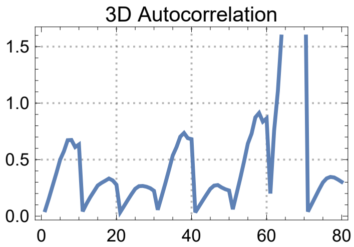

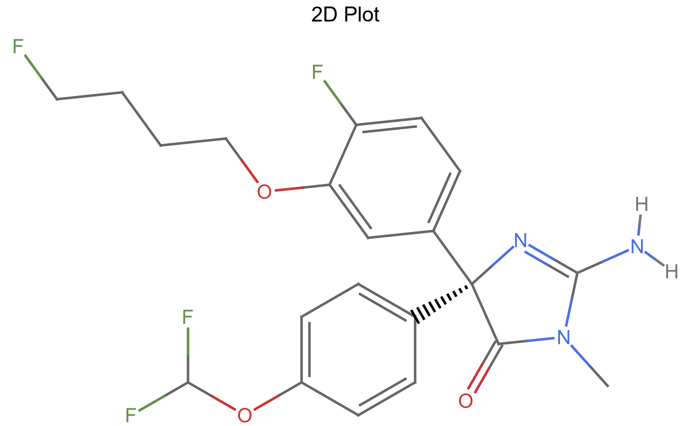

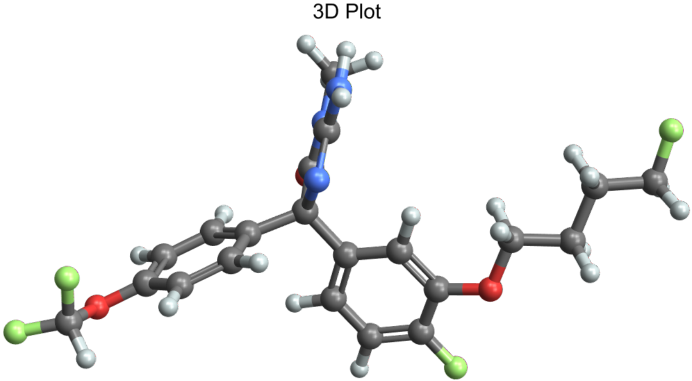

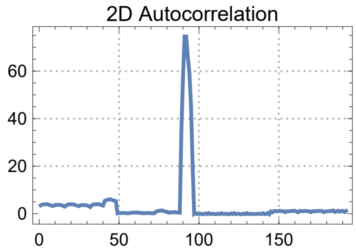

Dataset Index= 823

Molecule Formula= $C_{18}H_{17}N_7O$

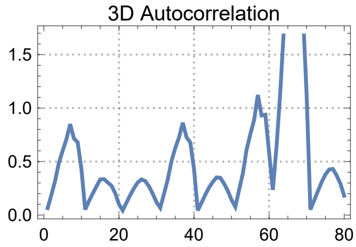

2D Plot

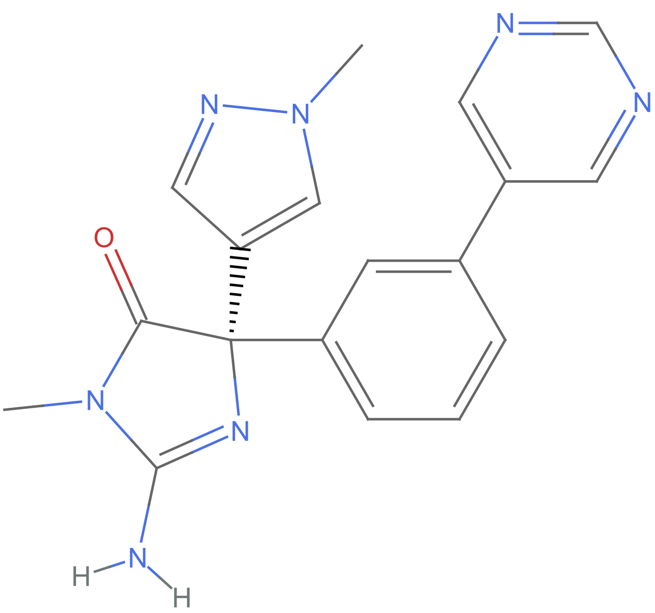

3D Plot

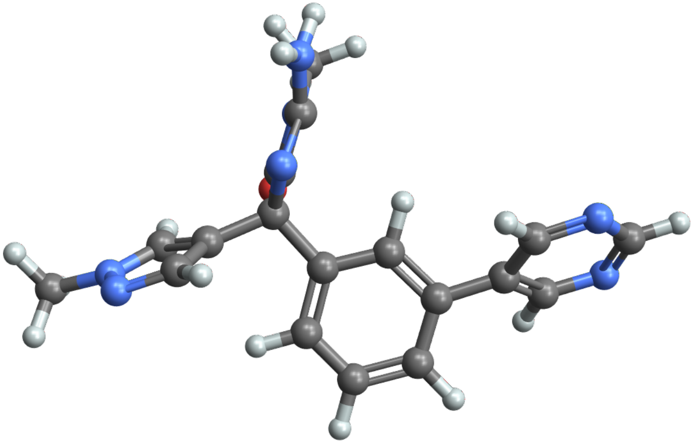

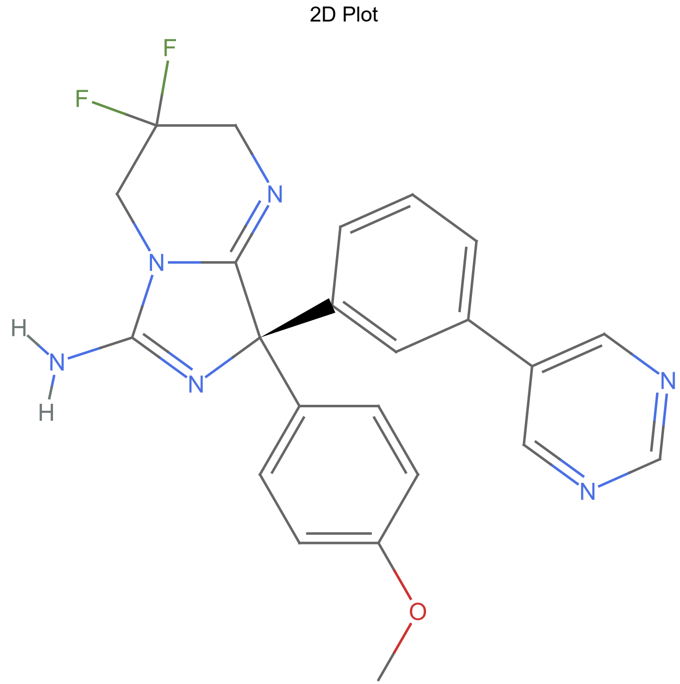

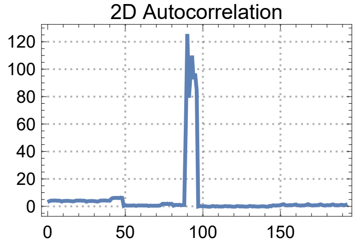

Dataset Index= 824

Molecule Formula= $C_{23}H_{20}F_2N_6O$

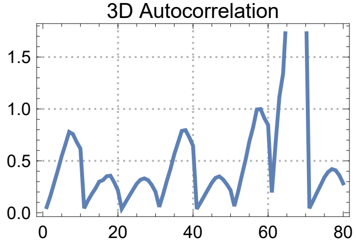

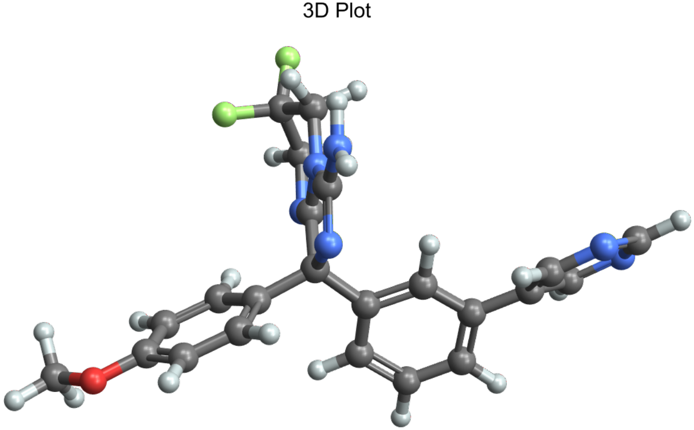

2D Autocorrelation

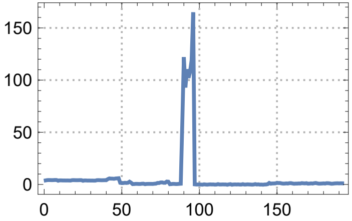

Dataset Index= 825

Molecule Formula= $C_{26}H_{38}FN_2O_5S^+$

3D Autocorrelation

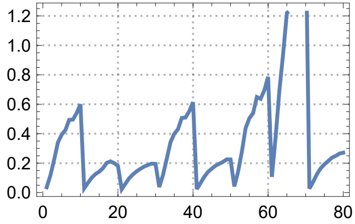

2D Plot

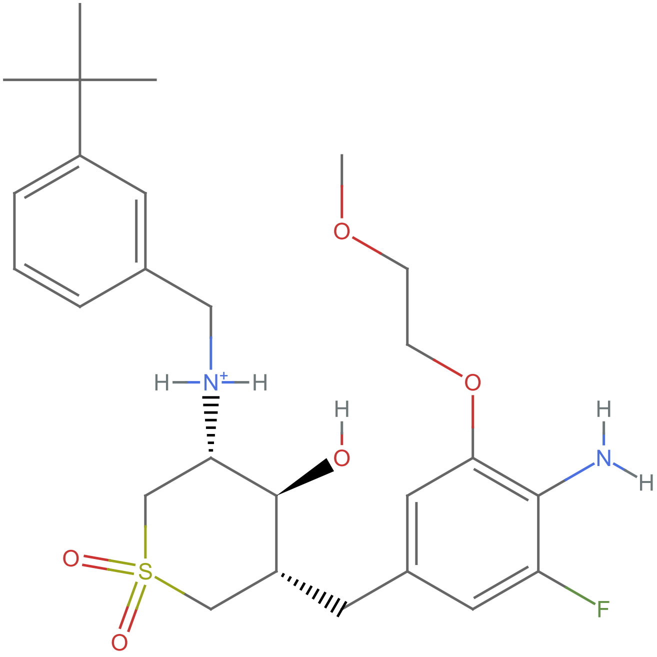

3D Plot

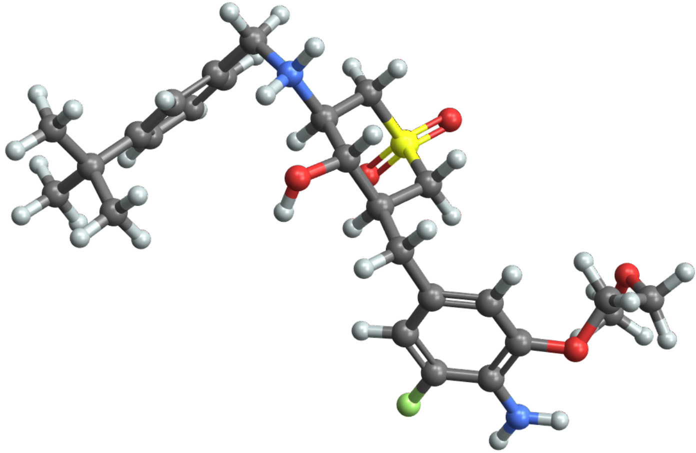

2D Autocorrelation

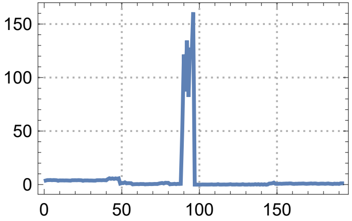

Dataset Index= 826

Molecule Formula= $C_{26}H_{37}F_2N_2O_2S^+$

3D Autocorrelation

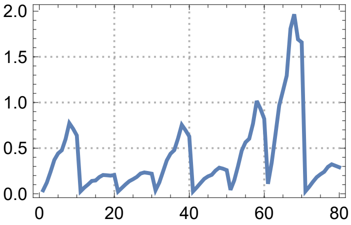

2D Plot

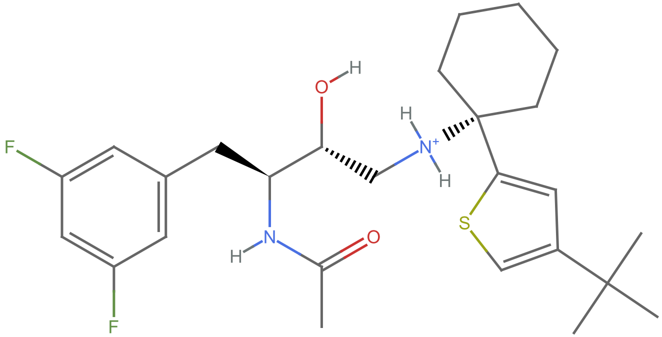

3D Plot

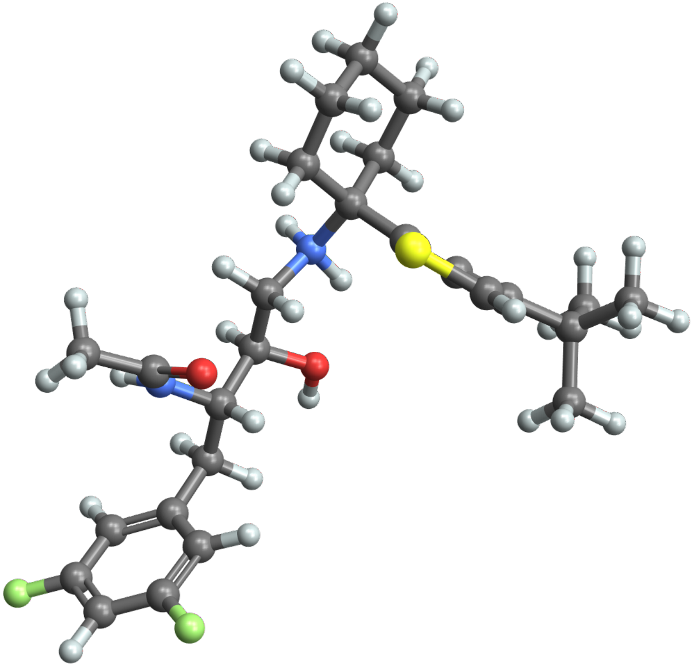

2D Plot

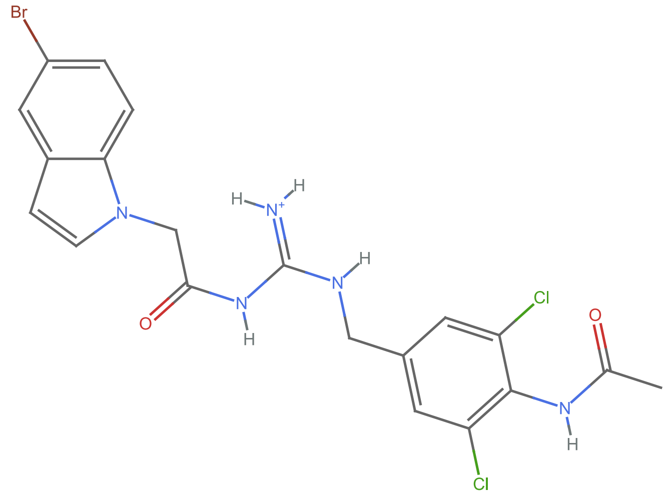

2D Autocorrelation

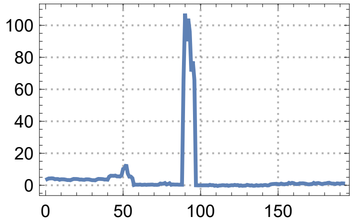

3D Plot

Dataset Index= 827

Molecule Formula= $C_{20}H_{19}BrCl_2N_5O_2^+$

3D Autocorrelation

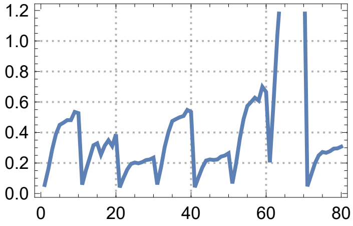

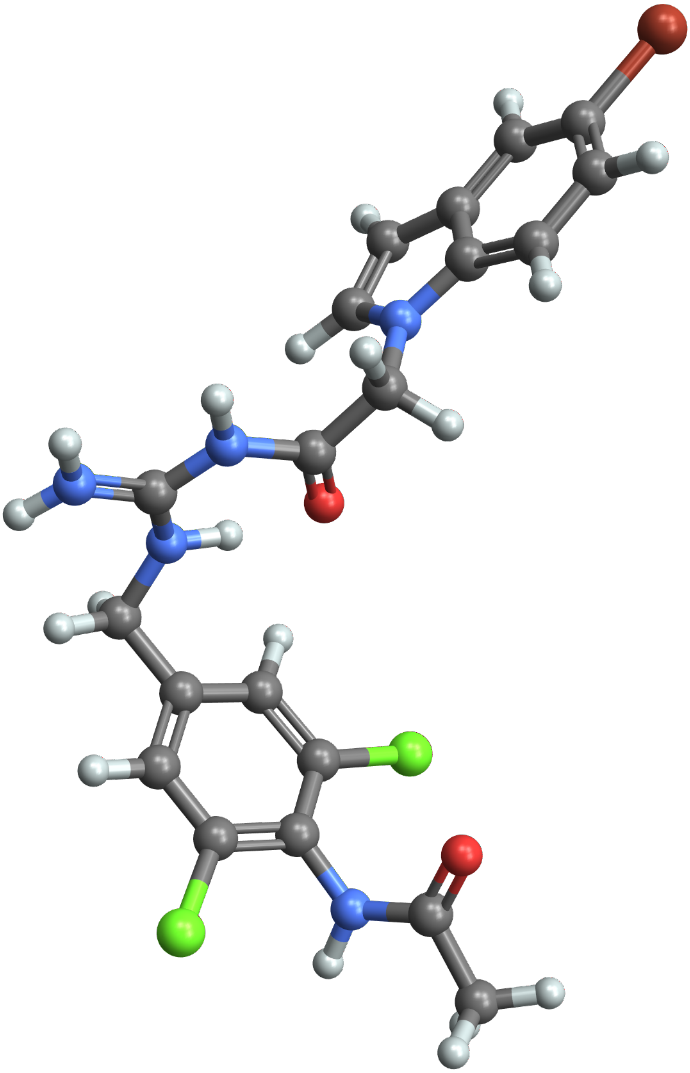

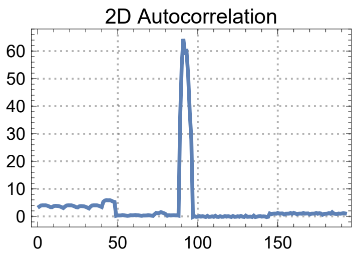

Dataset Index= 828

Molecule Formula= $C_{20}H_{16}N_4O_3$

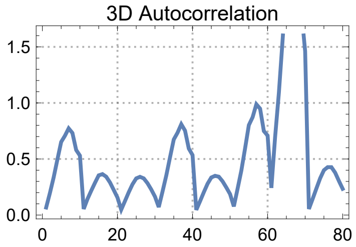

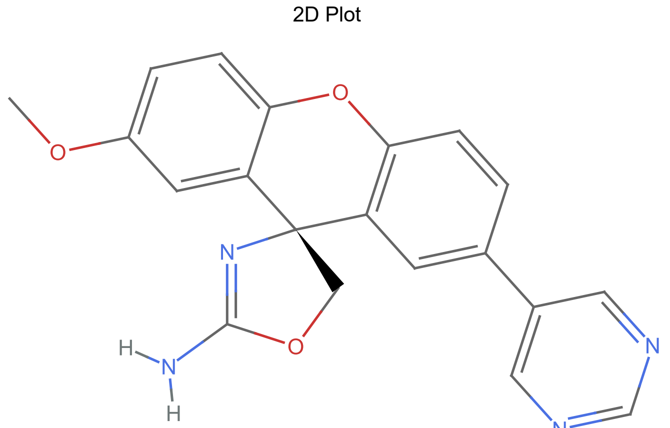

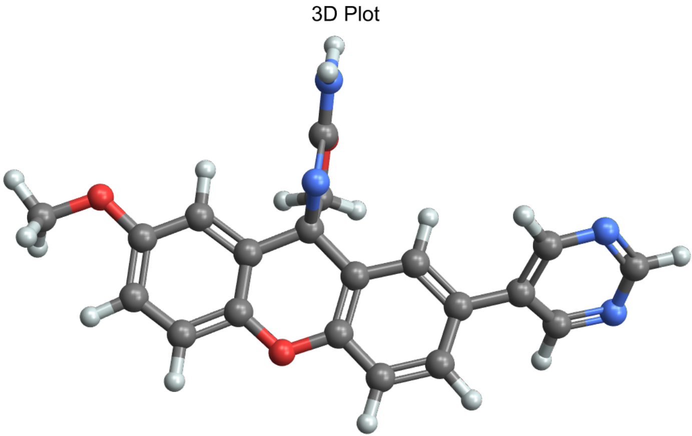

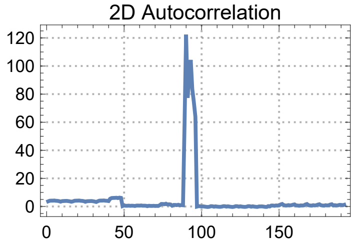

Dataset Index= 829

Molecule Formula= $C_{21}H_{17}F_2N_7$

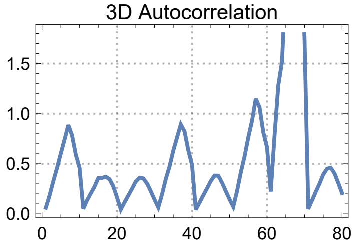

2D Plot

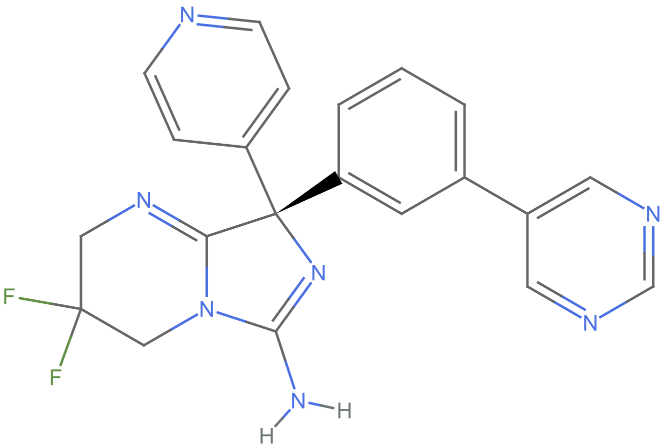

3D Plot

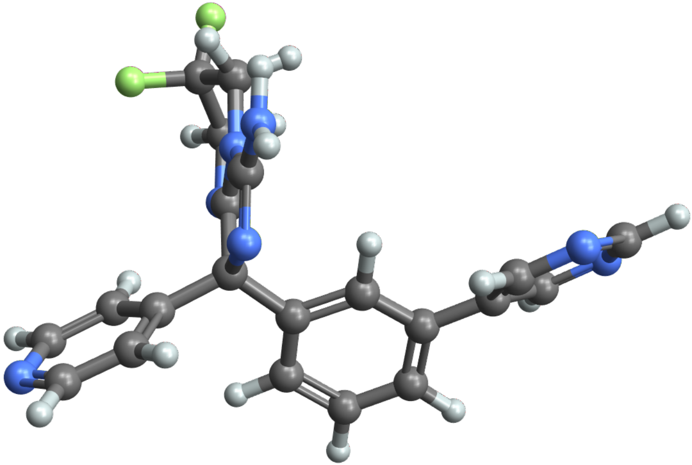

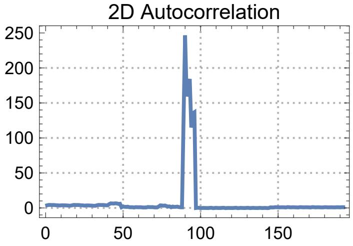

Dataset Index= 830

Molecule Formula= $C_{19}H_{16}F_6N_4O_3$

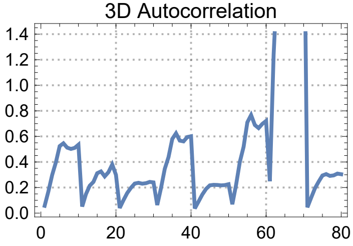

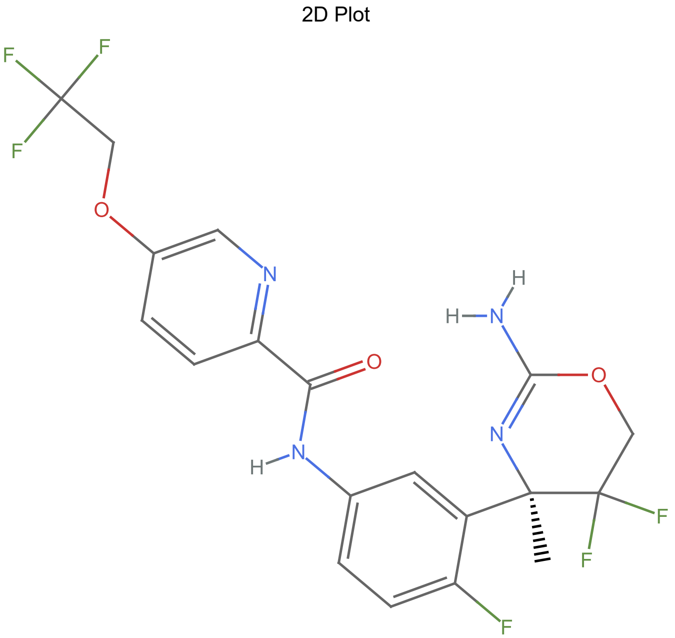

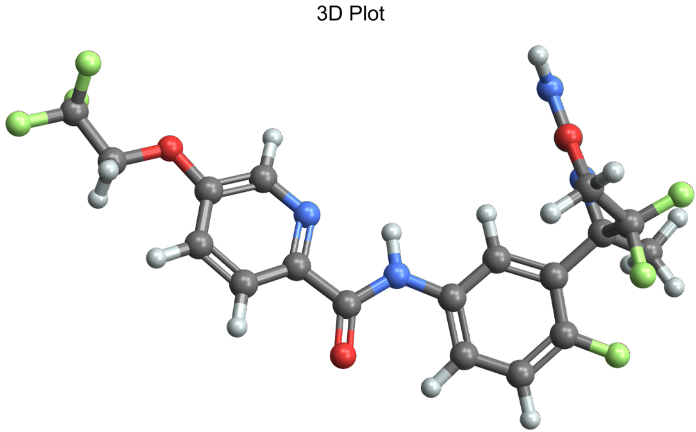

2D Autocorrelation

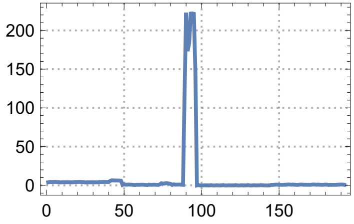

2D Plot

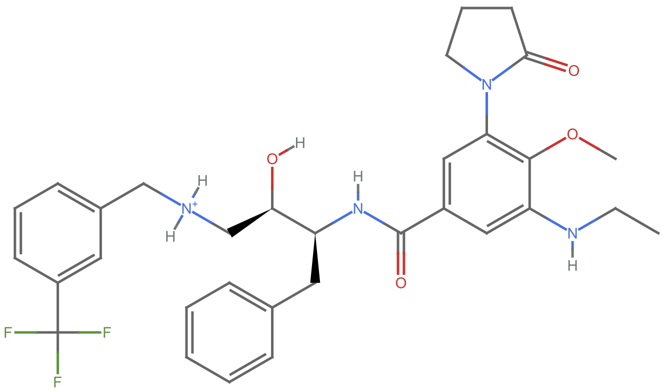

3D Plot

Dataset Index= 831

Molecule Formula= $C_{32}H_{38}F_3N_4O_4^+$

3D Autocorrelation

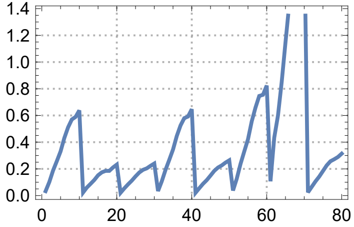

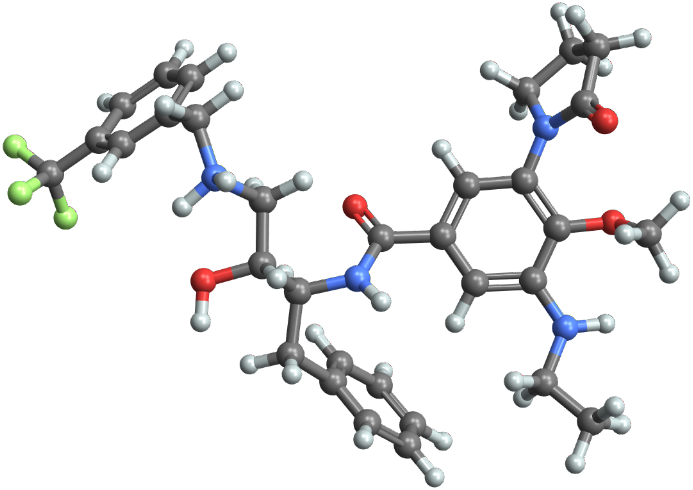

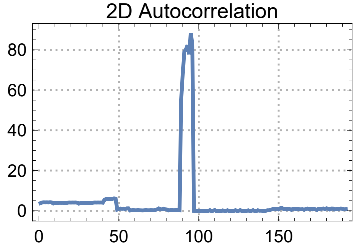

Dataset Index= 832

Molecule Formula= $C_{29}H_{32}ClN_3O_3$

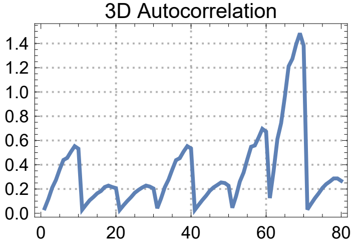

2D Plot

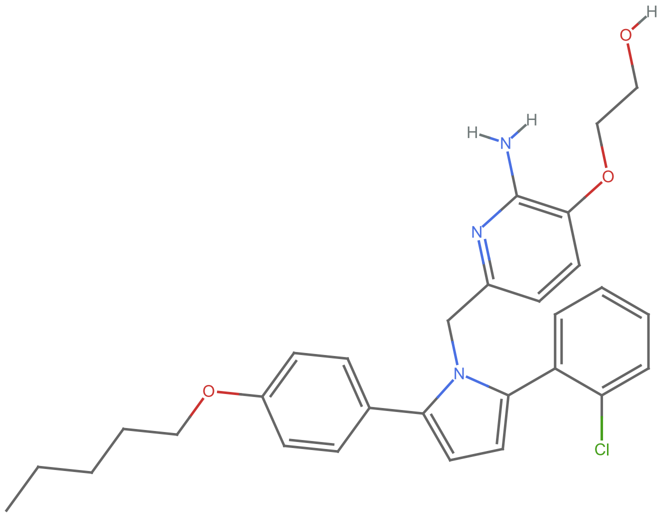

3D Plot

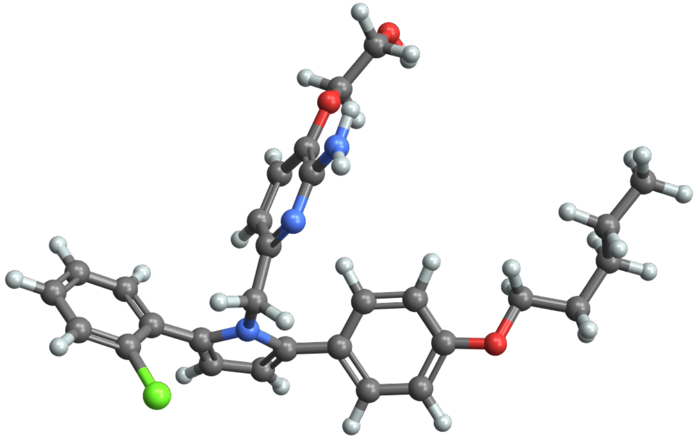

2D Plot

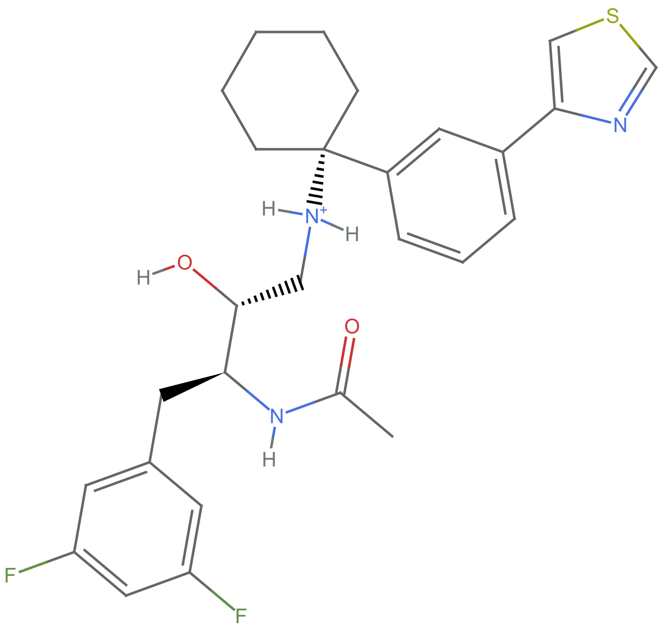

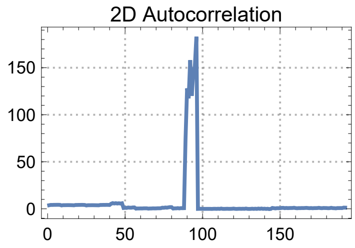

3D Plot

Dataset Index= 833

Molecule Formula= $C_{27}H_{32}F_2N_3O_2S^+$

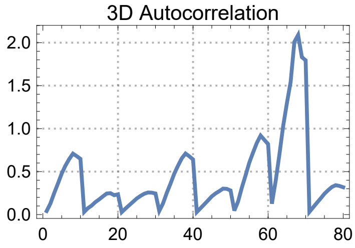

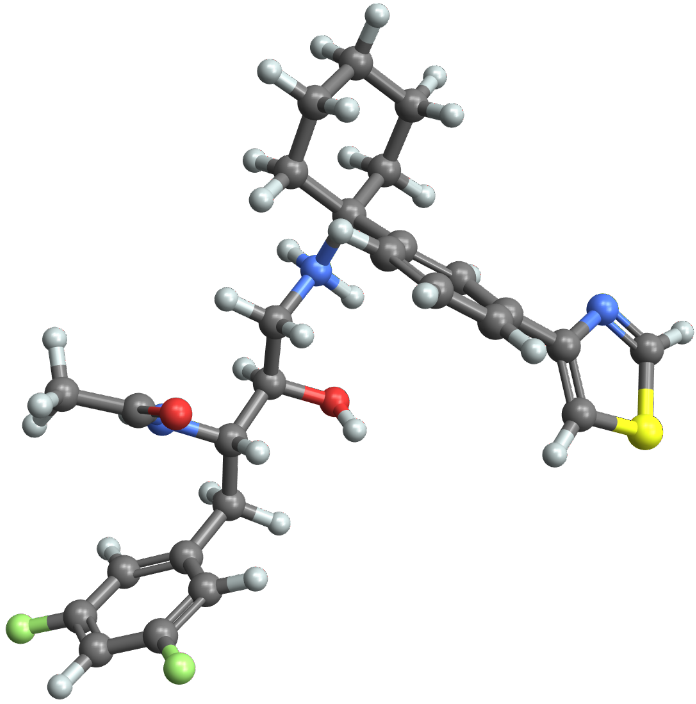

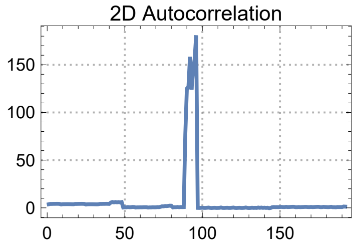

2D Plot

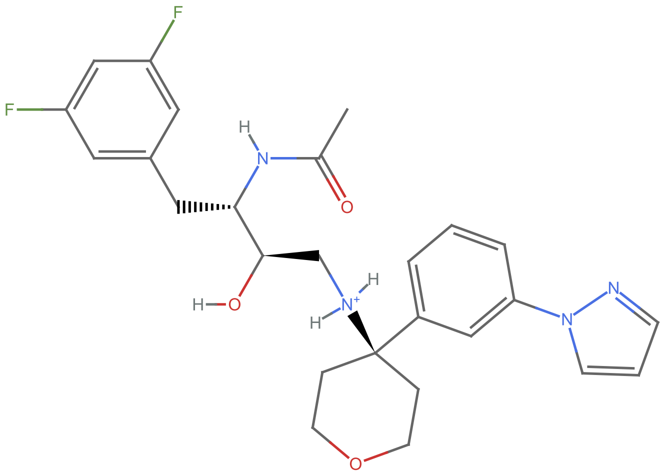

3D Plot

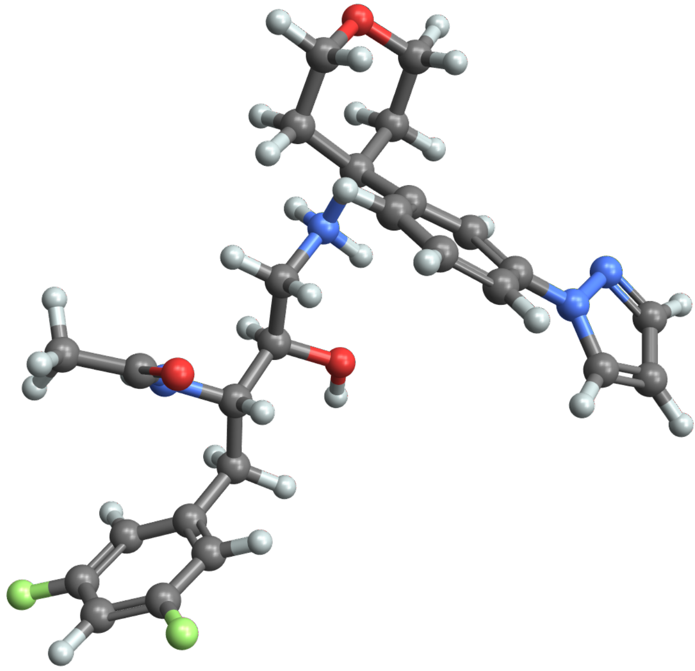

Dataset Index= 834

Molecule Formula= $C_{26}H_{31}F_2N_4O_3^+$

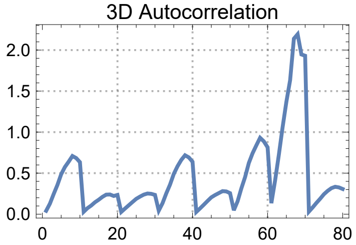

2D Autocorrelation

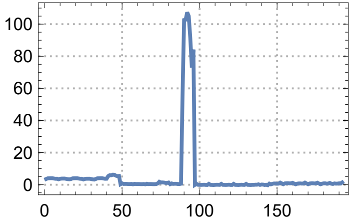

Dataset Index= 835

Molecule Formula= $C_{22}H_{25}F_2N_3O_2$

3D Autocorrelation

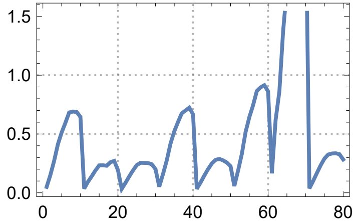

2D Plot

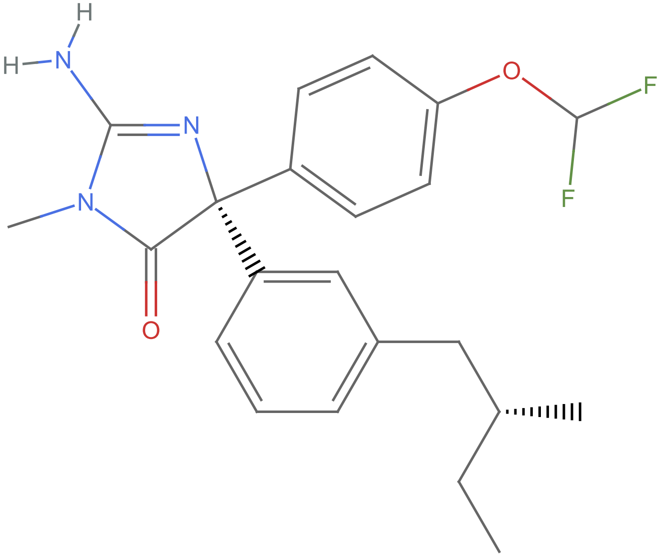

3D Plot

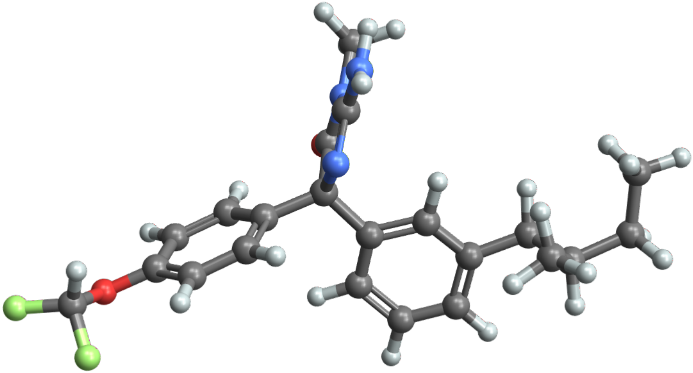

2D Plot

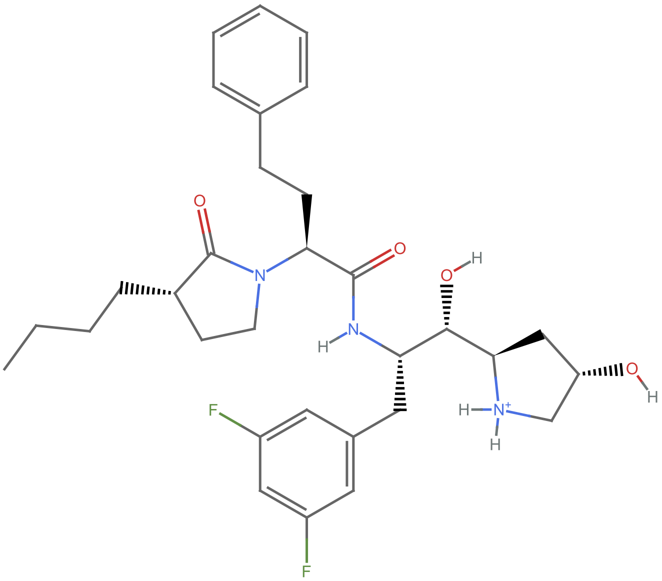

2D Autocorrelation

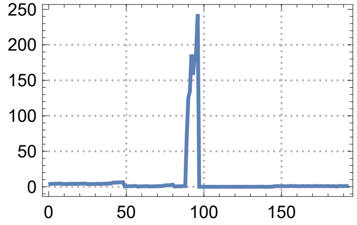

3D Plot

Dataset Index= 836

Molecule Formula= $C_{31}H_{42}F_2N_3O_4^+$

3D Autocorrelation

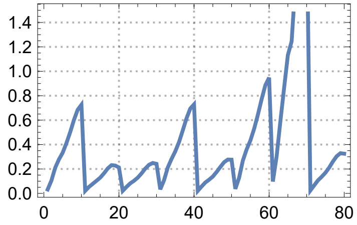

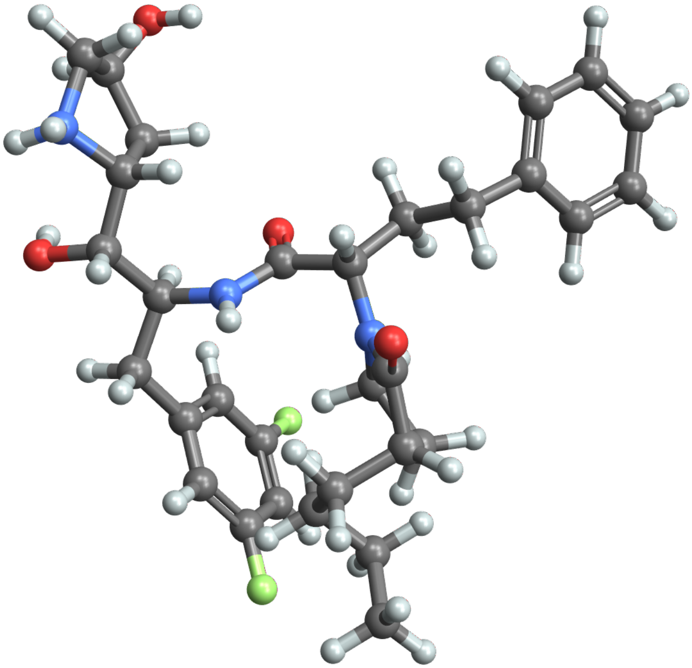

2D Plot

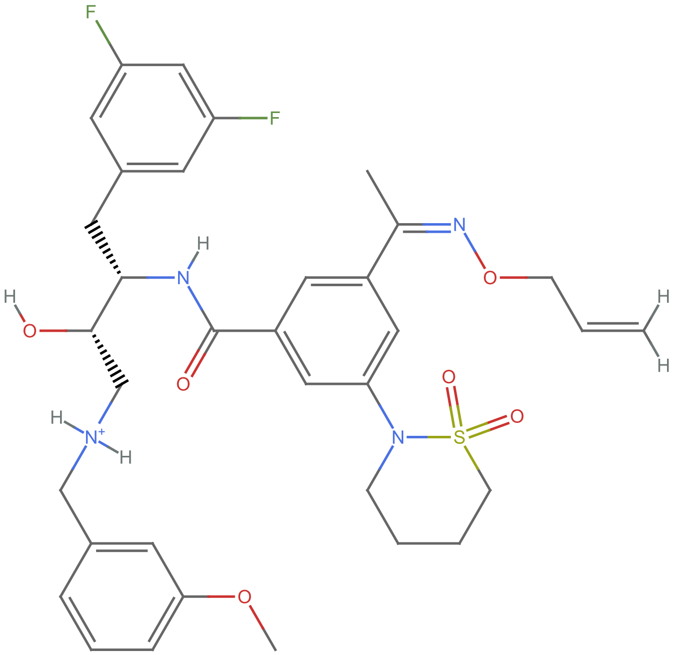

2D Autocorrelation

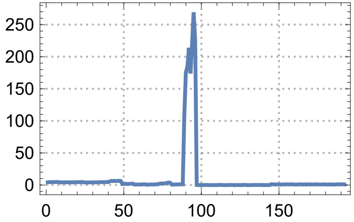

Dataset Index= 837

Molecule Formula= $C_{34}H_{41}F_2N_4O_6S^+$

3D Autocorrelation

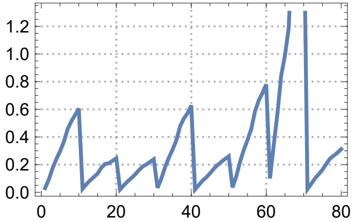

3D Plot

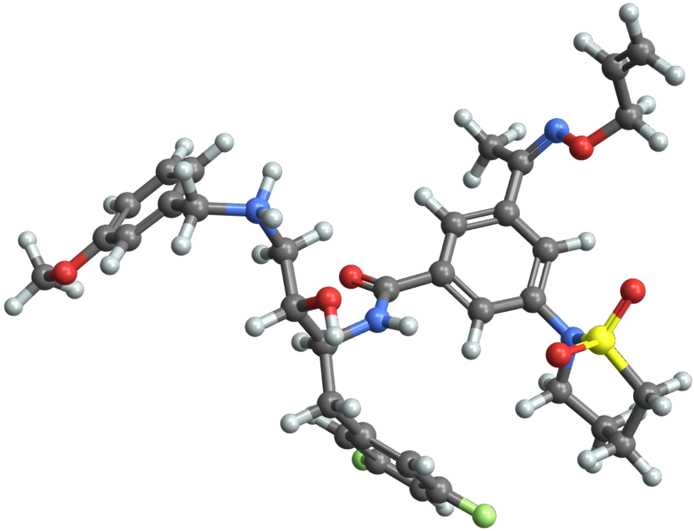

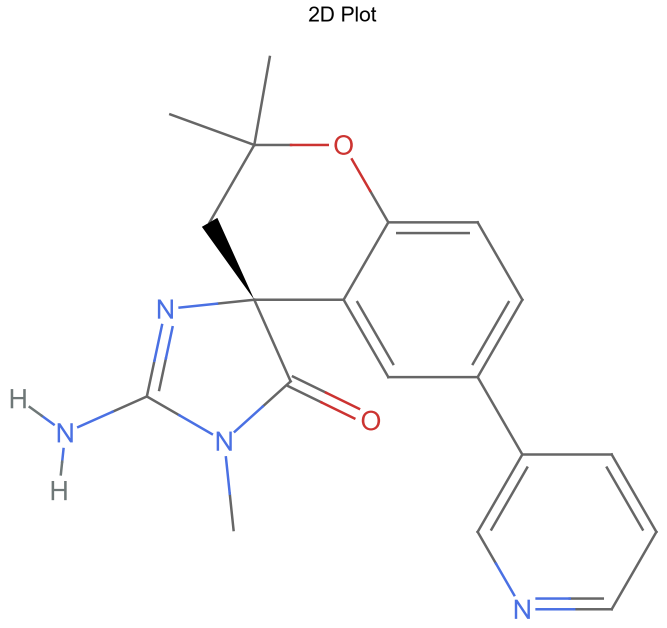

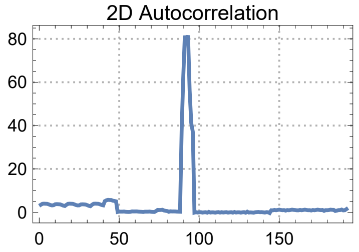

Dataset Index= 838

Molecule Formula= $C_{19}H_{20}N_4O_2$

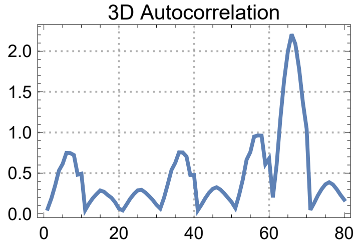

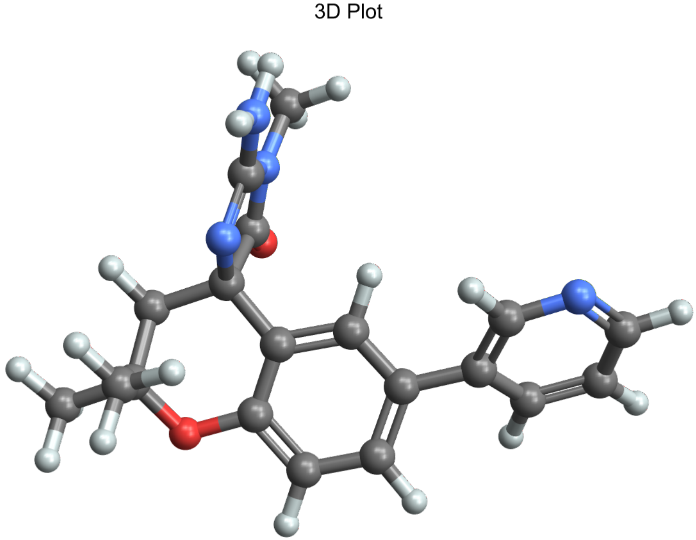

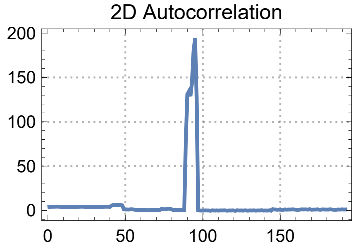

2D Plot

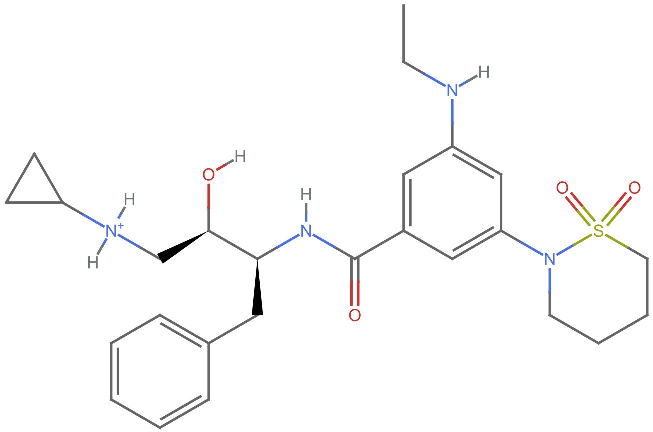

3D Plot

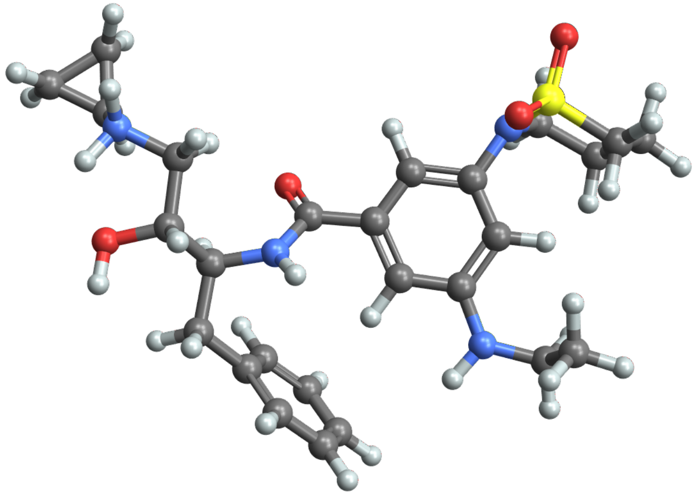

Dataset Index= 839

Molecule Formula= $C_{26}H_{37}N_4O_4S^+$

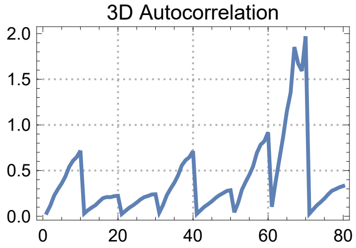

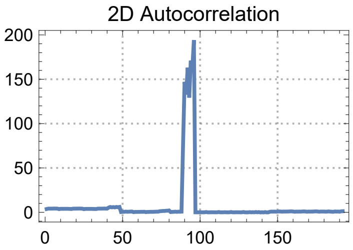

Dataset Index= 840

Molecule Formula= $C_{28}H_{37}F_2N_2O_3^+$

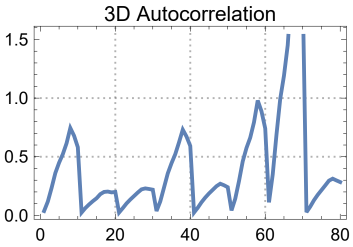

2D Plot

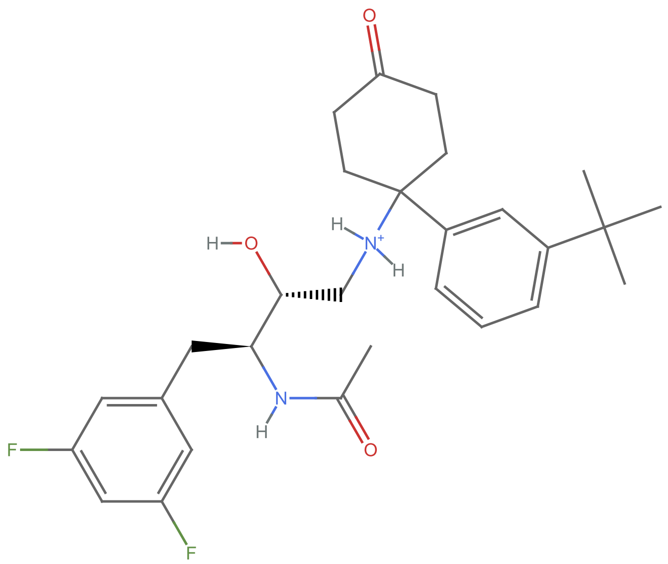

3D Plot

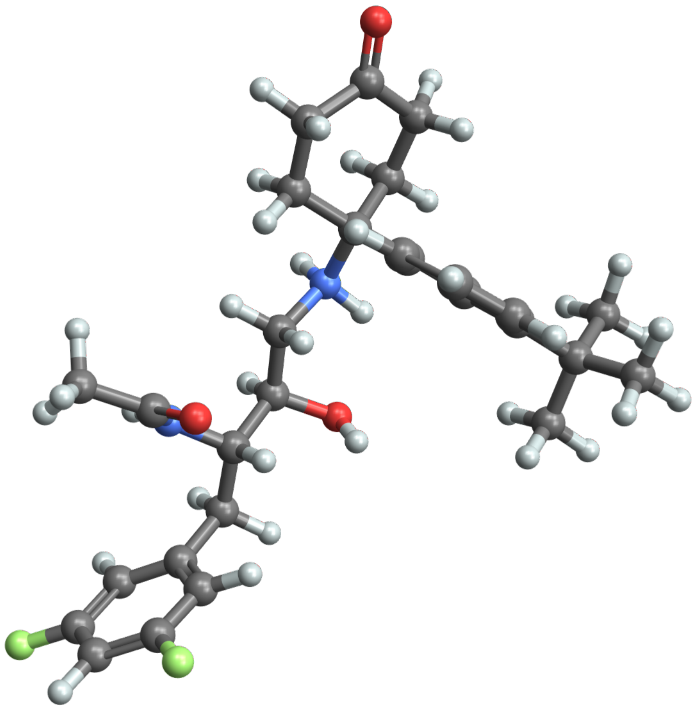

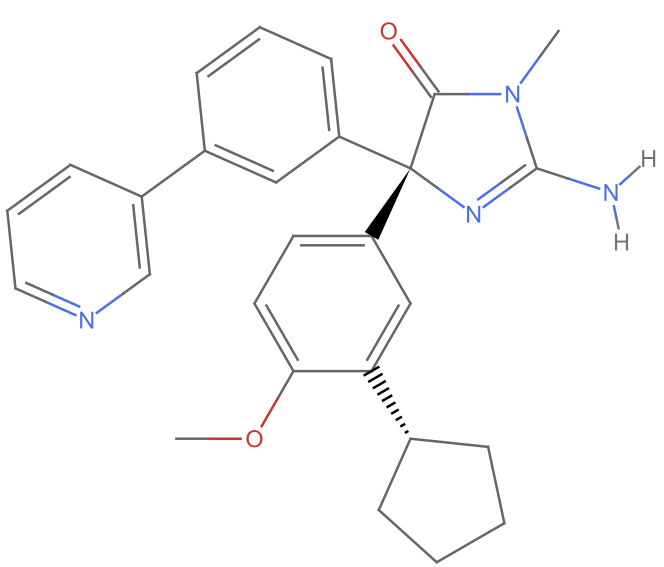

2D Autocorrelation

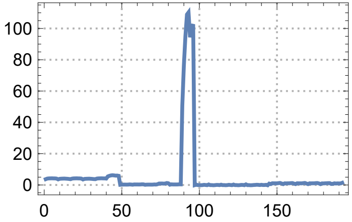

Dataset Index= 841

Molecule Formula= $C_{27}H_{28}N_4O_2$

3D Autocorrelation

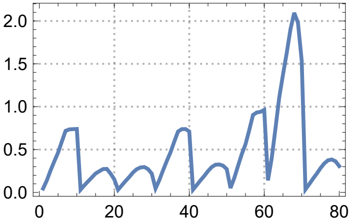

3D Plot

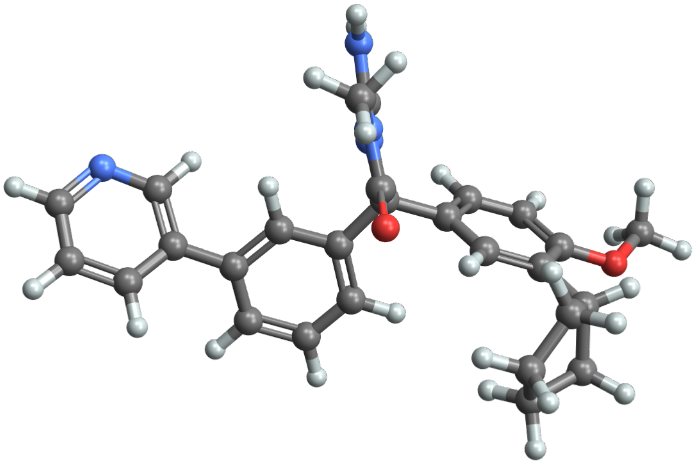

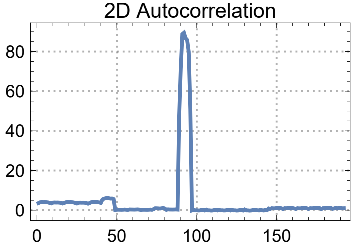

Dataset Index= 842

Molecule Formula= $C_{23}H_{20}N_4O_2$

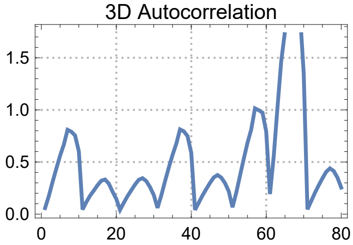

2D Plot

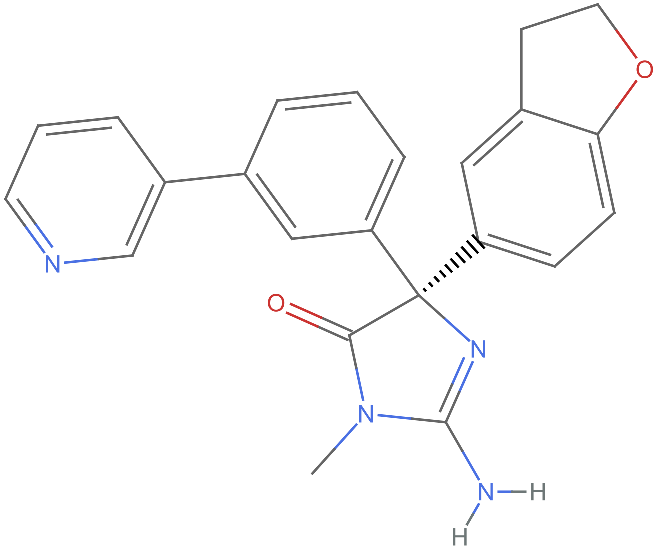

3D Plot

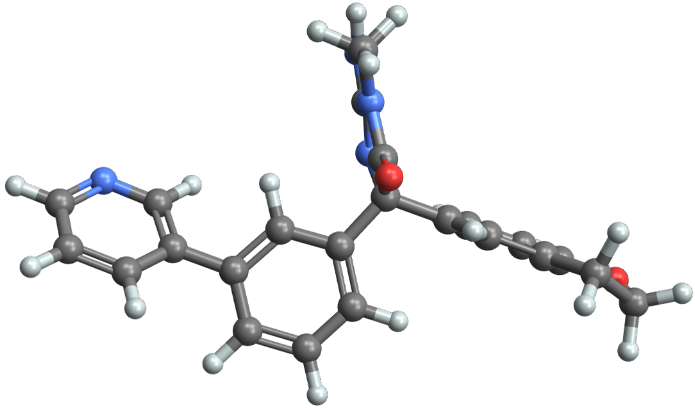

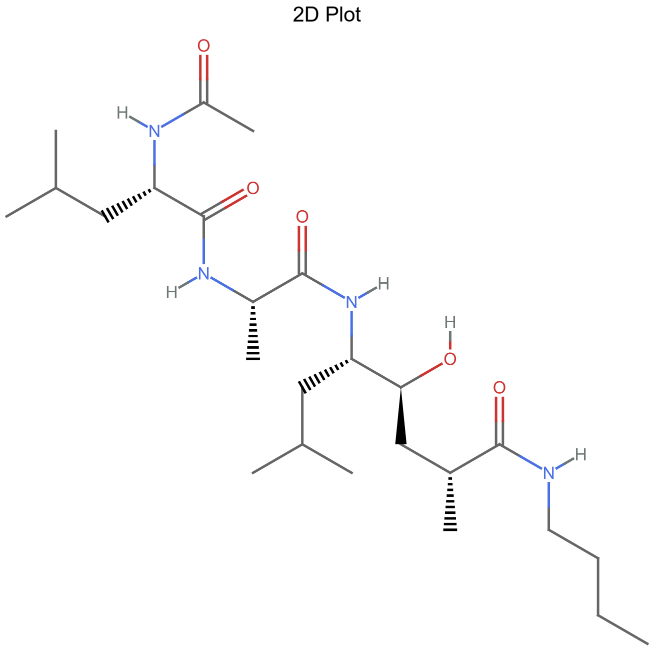

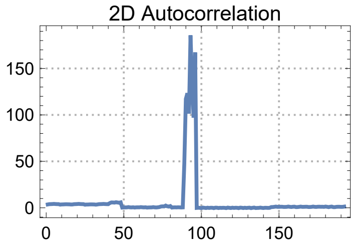

Dataset Index= 843

Molecule Formula= $C_{25}H_{48}N_4O_5$

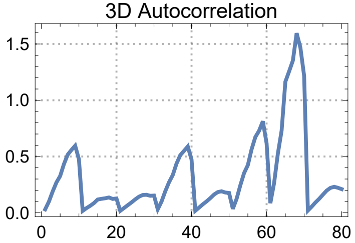

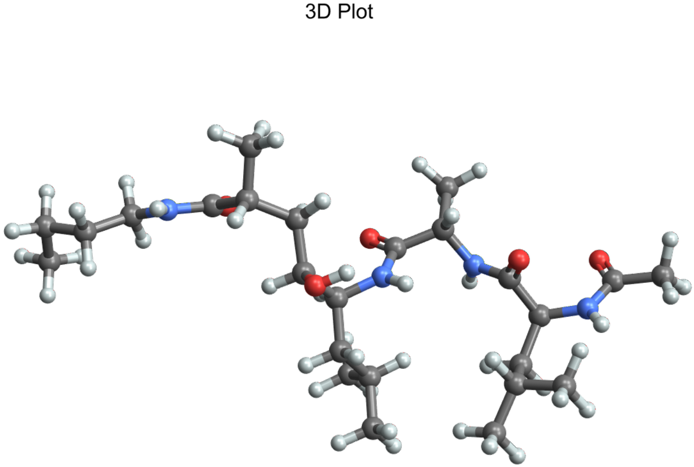

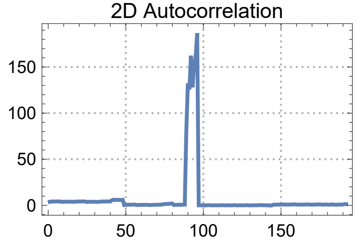

Dataset Index= 844

Molecule Formula= $C_{28}H_{33}F_2N_2O_3^+$

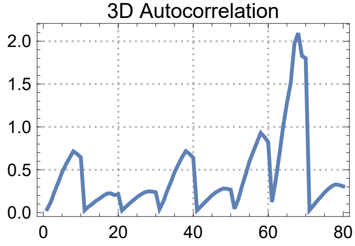

2D Plot

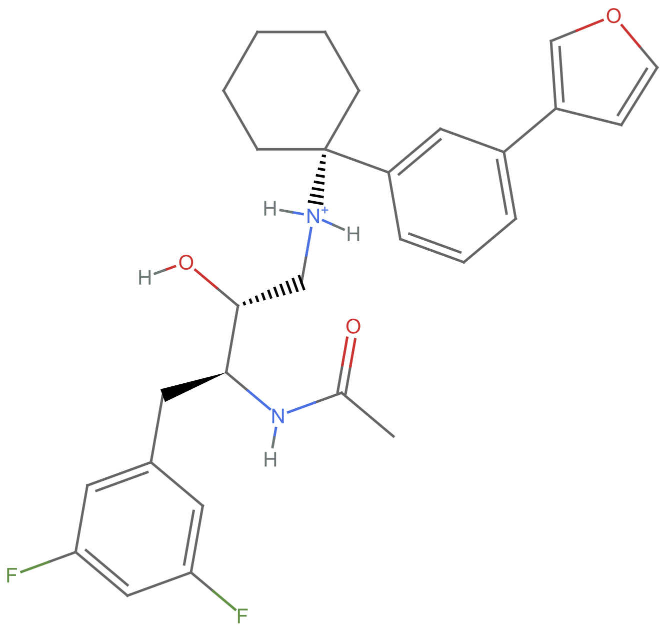

3D Plot

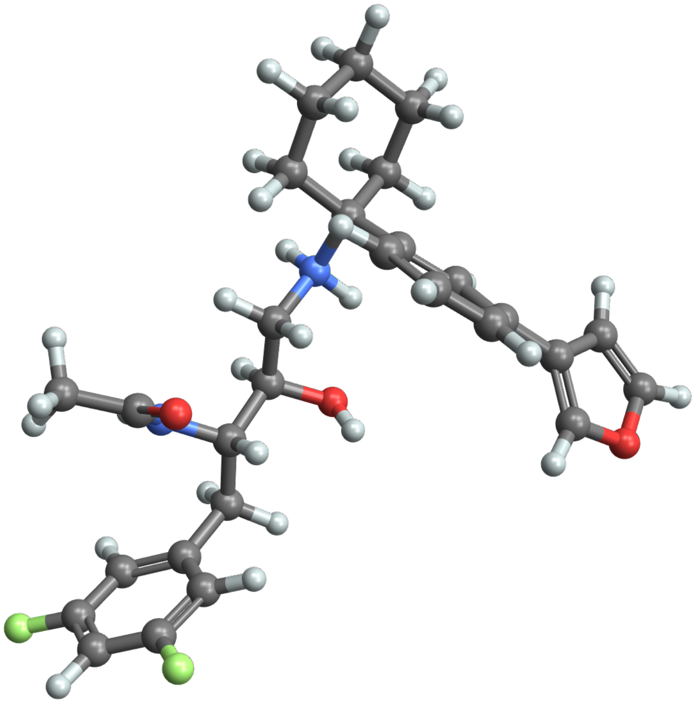

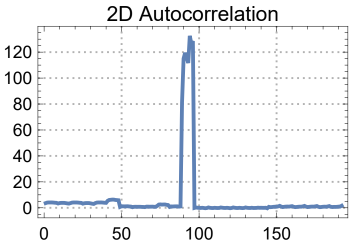

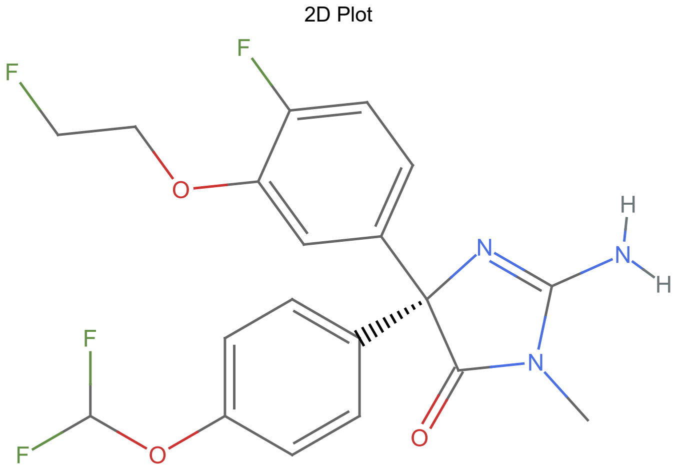

Dataset Index= 845

Molecule Formula= $C_{19}H_{17}F_4N_3O_3$

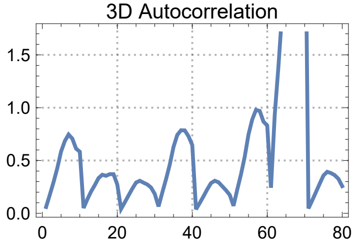

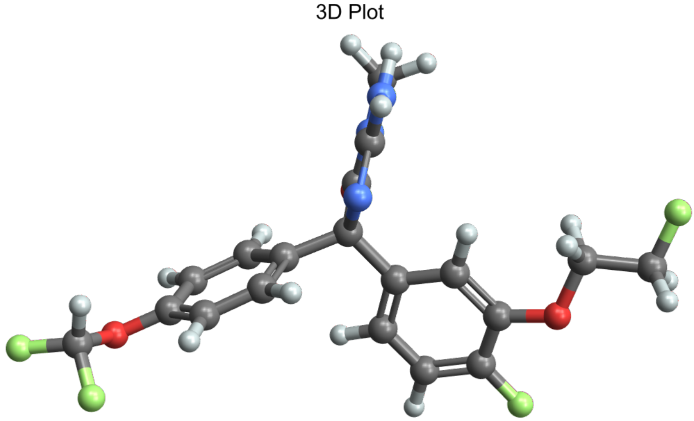

2D Plot

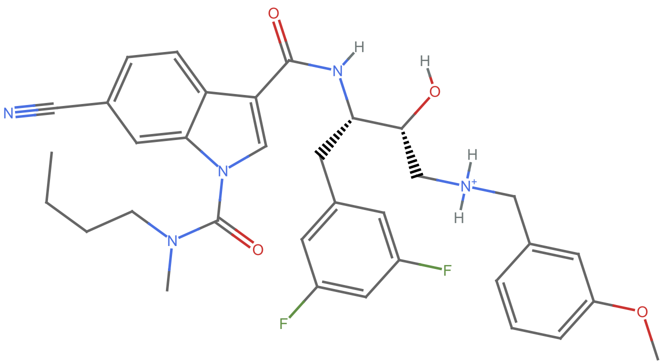

3D Plot

2D Autocorrelation

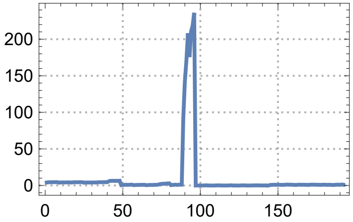

Dataset Index= 846

Molecule Formula= $C_{34}H_{38}F_2N_5O_4^+$

3D Autocorrelation

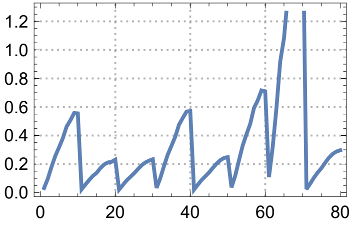

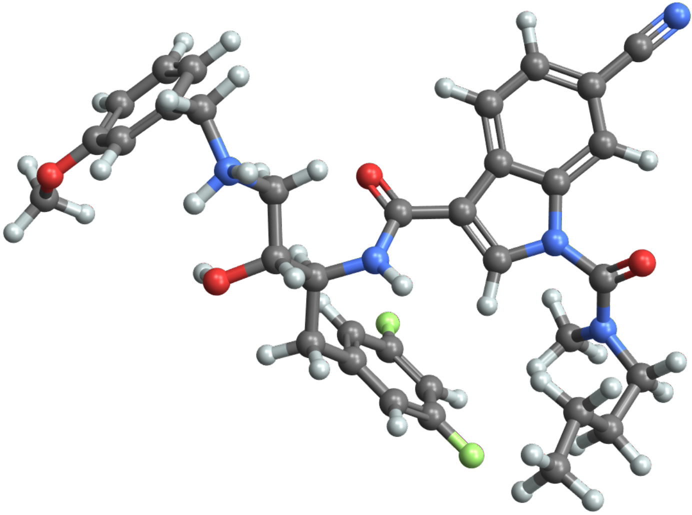

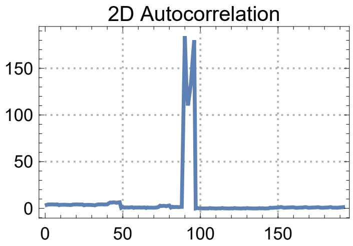

Dataset Index= 847

Molecule Formula= $C_{19}H_{15}F_4N_7O$

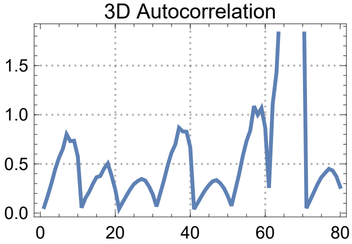

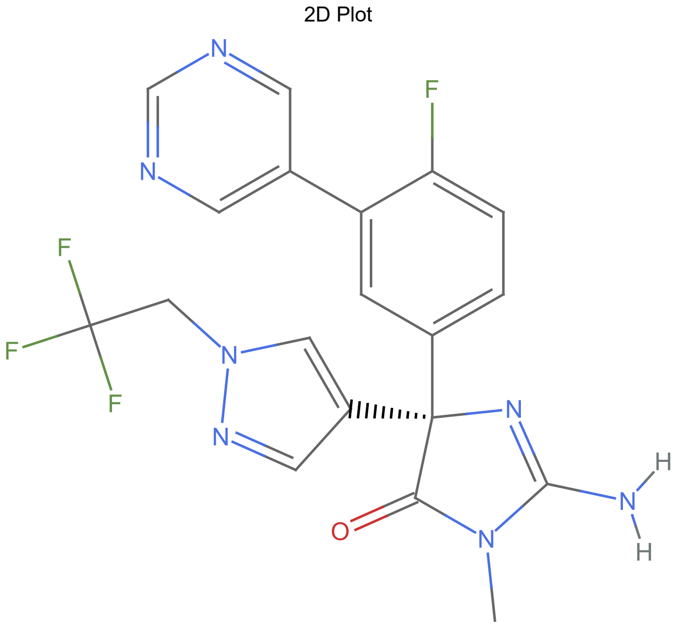

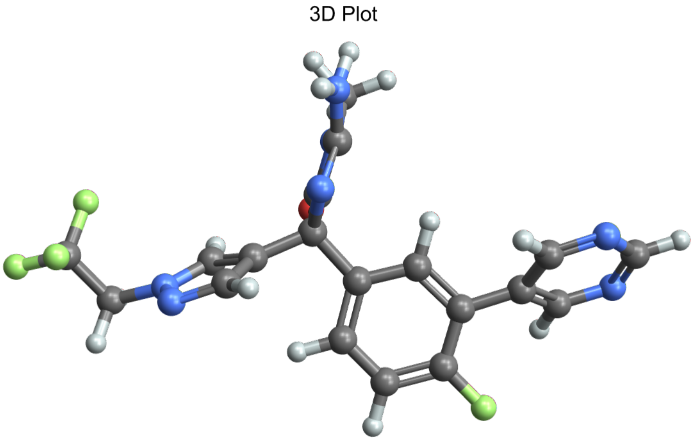

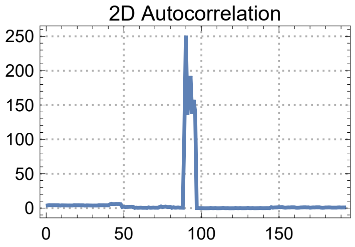

Dataset Index= 848

Molecule Formula= $C_{27}H_{34}F_3N_2O_3S^+$

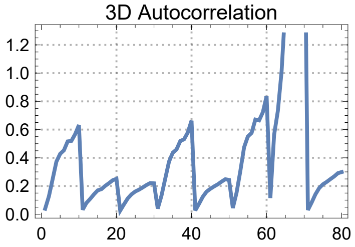

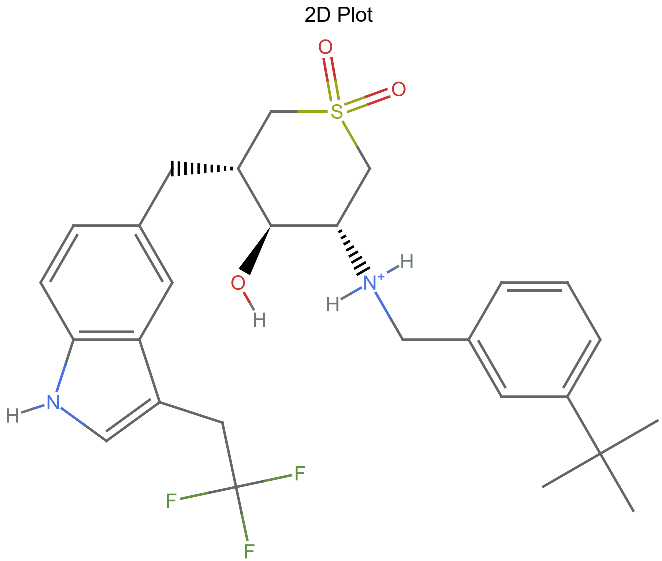

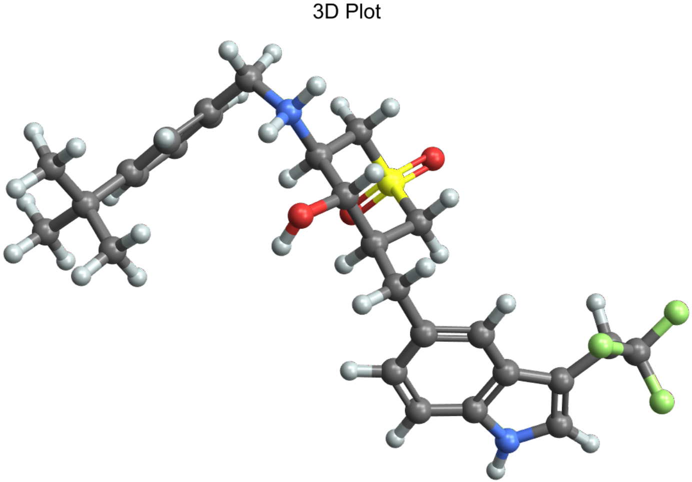

2D Plot

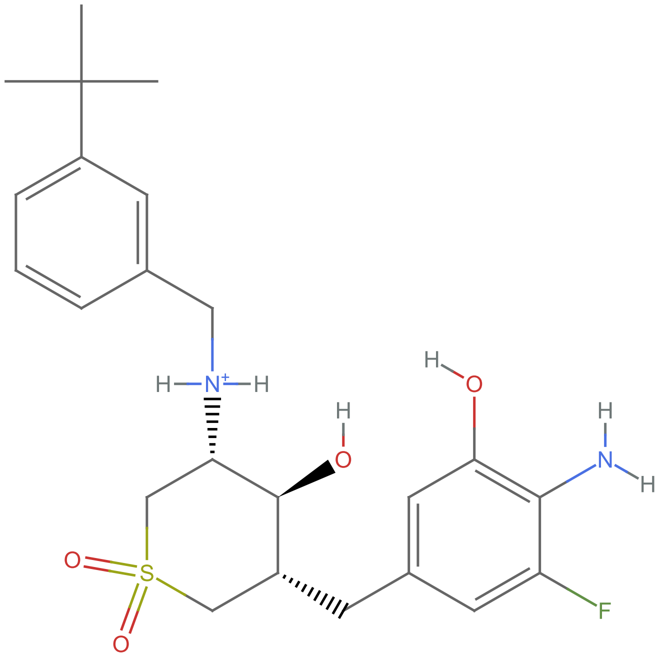

2D Autocorrelation

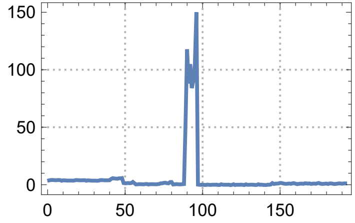

Dataset Index= 849

Molecule Formula= $C_{23}H_{32}FN_2O_4S^+$

3D Autocorrelation

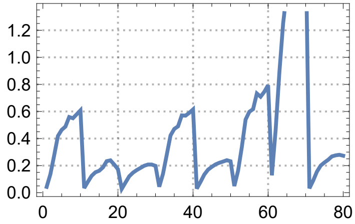

3D Plot

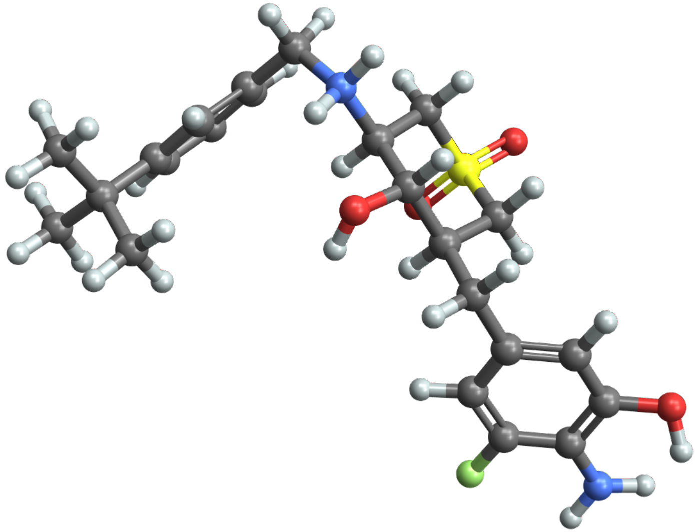

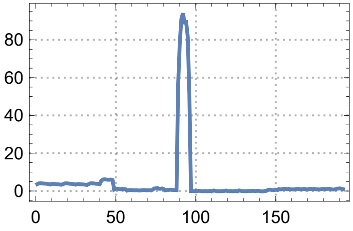

Dataset Index= 850

Molecule Formula= $C_{21}H_{16}ClFN_4O_2$

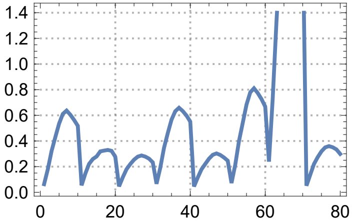

2D Plot

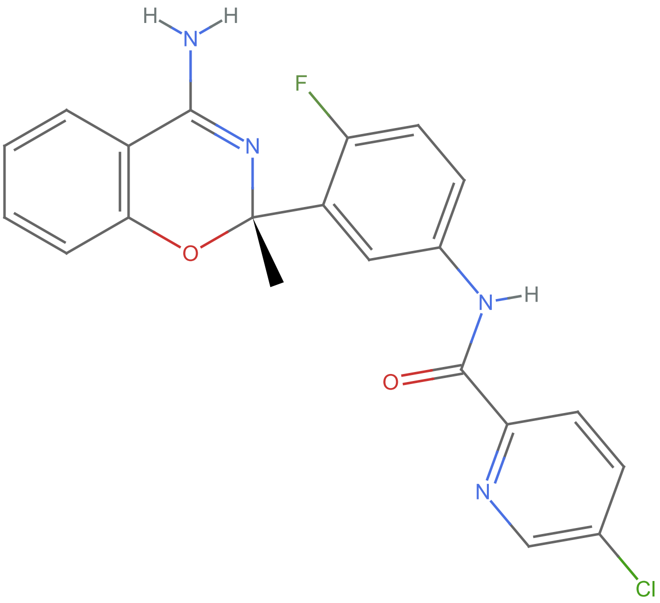

3D Plot

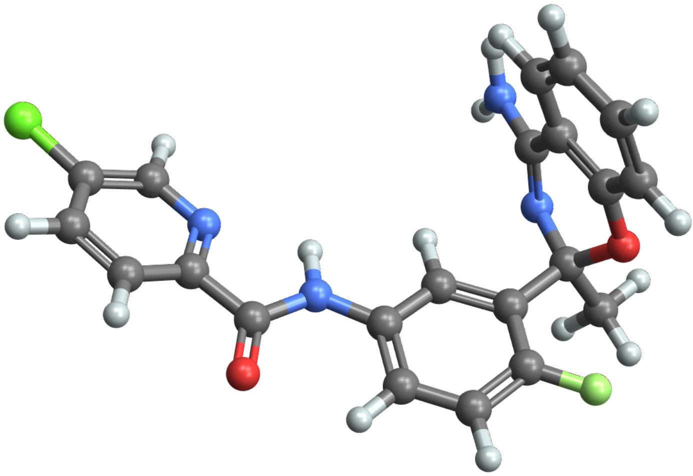

2D Plot

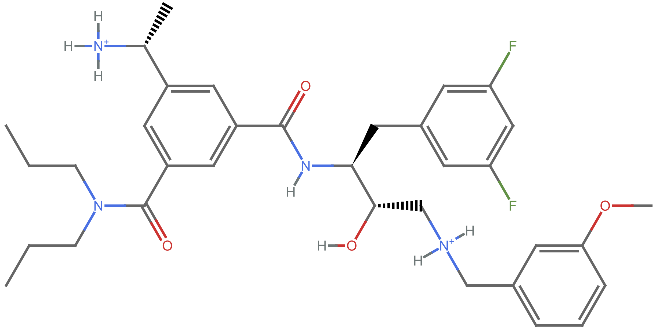

3D Plot

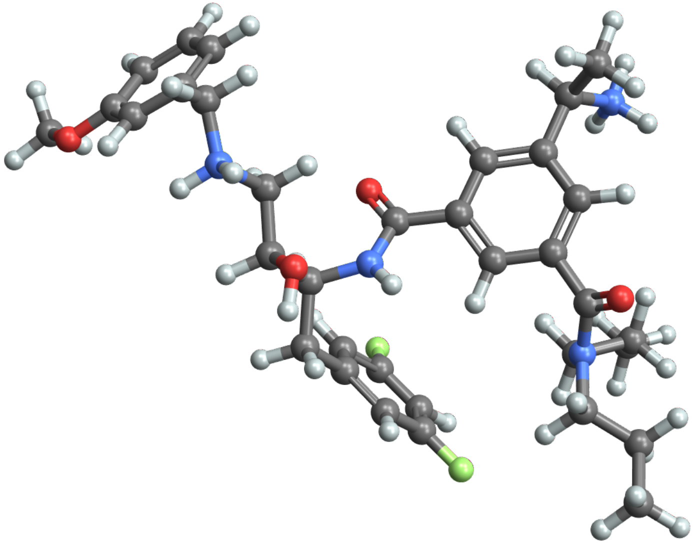

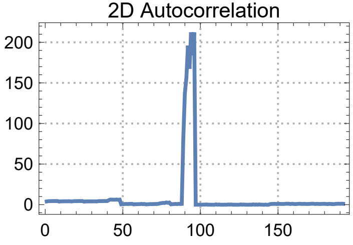

Dataset Index= 851

Molecule Formula= $C_{34}H_{46}F_2N_4O_4^{2+}$

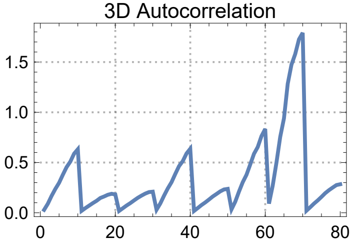

2D Plot

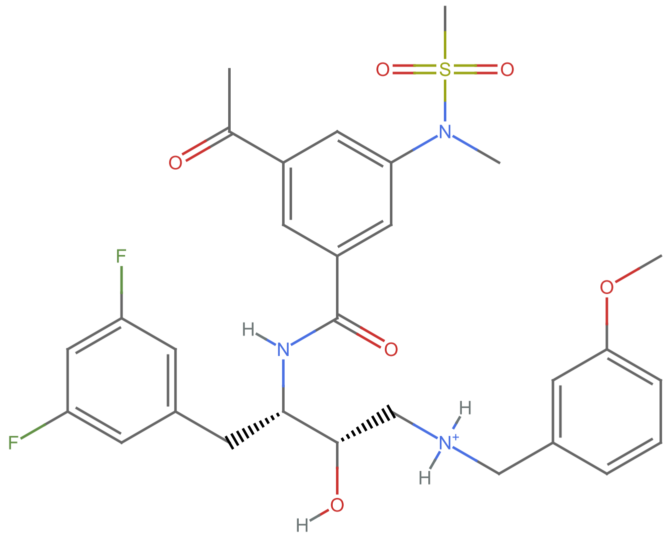

2D Autocorrelation

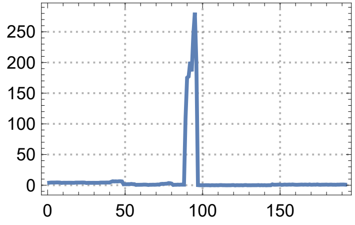

3D Plot

Dataset Index= 852

Molecule Formula= $C_{29}H_{34}F_2N_3O_6S^+$

3D Autocorrelation

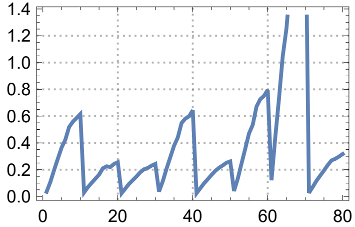

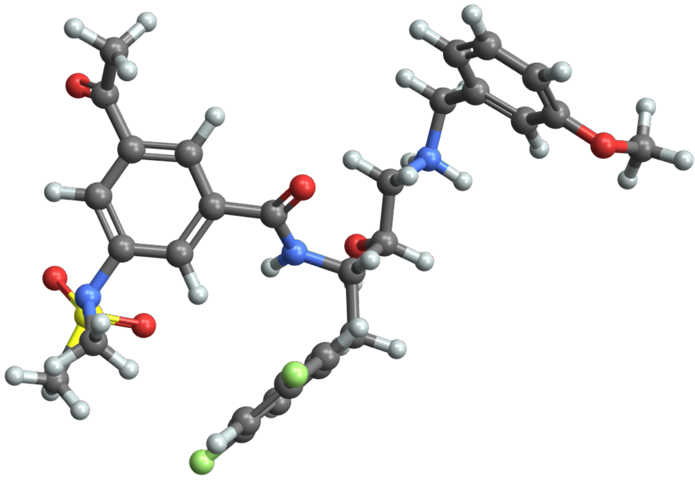

2D Autocorrelation

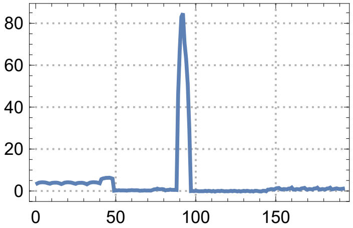

Dataset Index= 853

Molecule Formula= $C_{23}H_{16}FN_5$

3D Autocorrelation

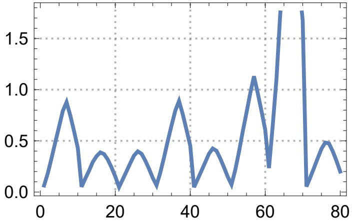

2D Plot

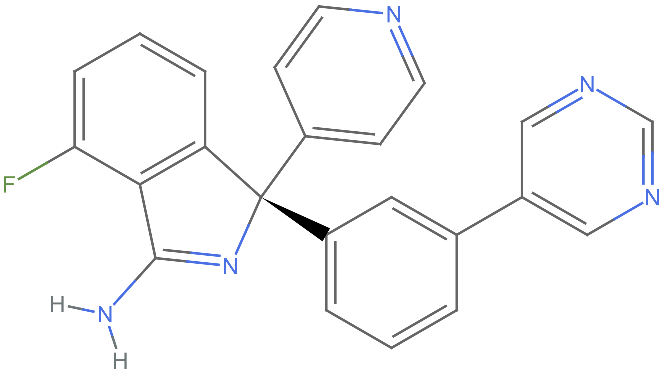

3D Plot

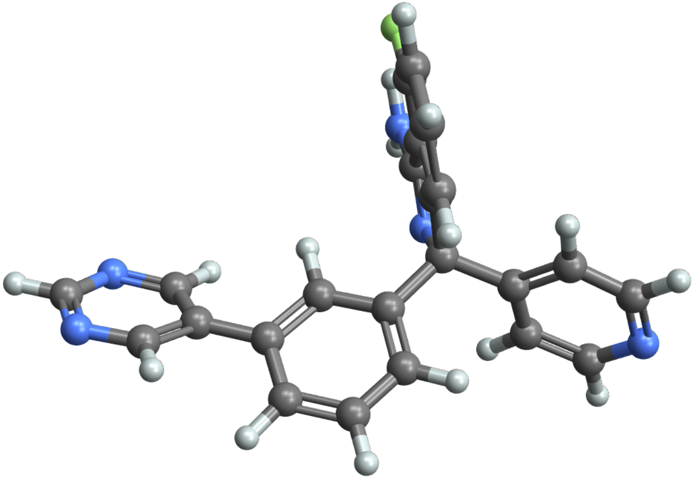

2D Plot

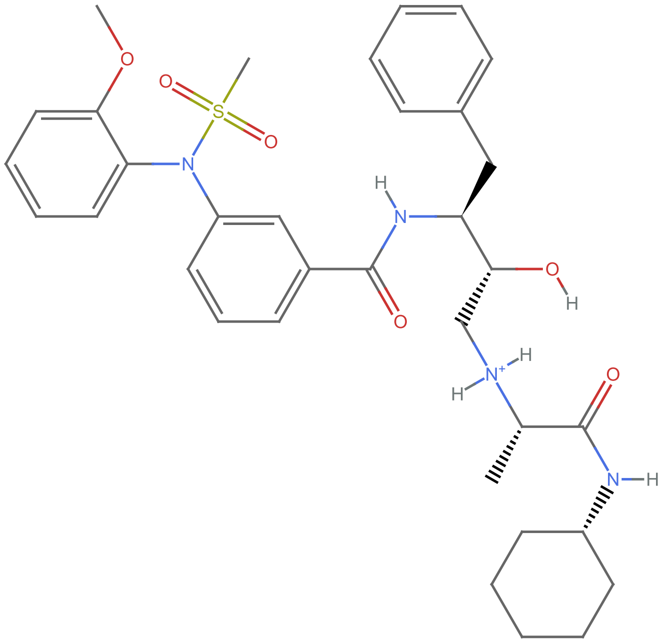

2D Autocorrelation

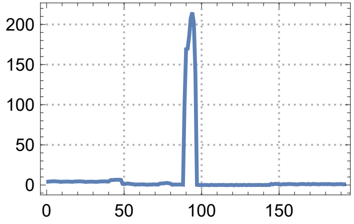

Dataset Index= 854

Molecule Formula= $C_{34}H_{45}N_4O_6S^+$

3D Autocorrelation

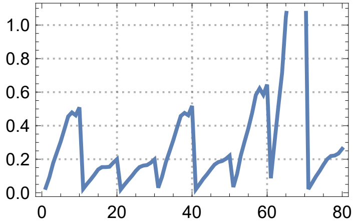

3D Plot

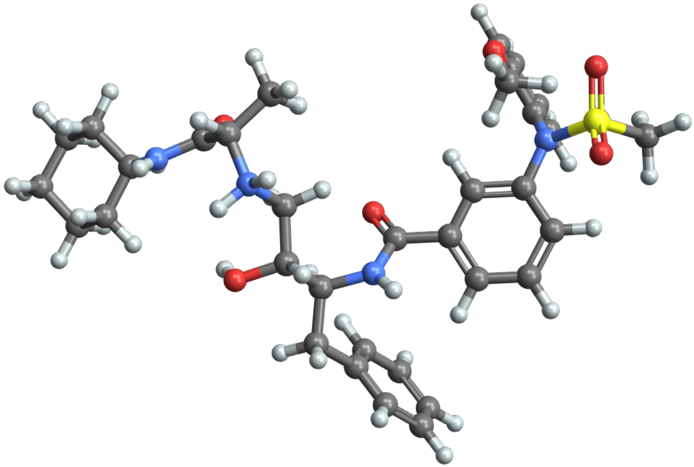

2D Plot

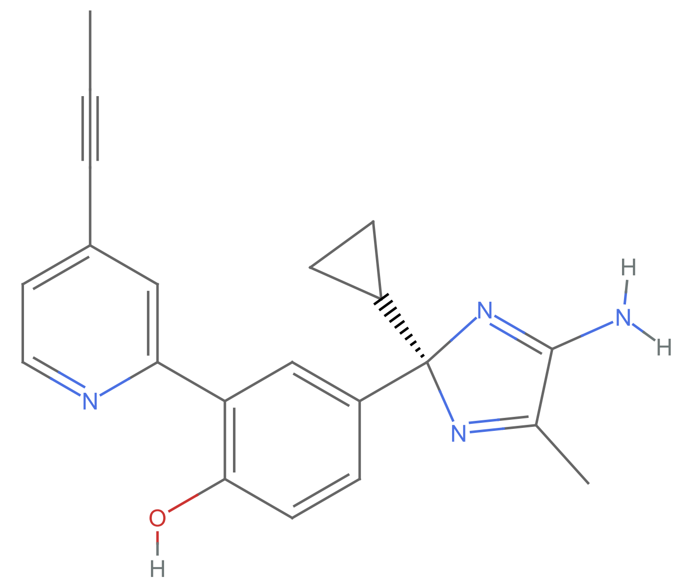

2D Autocorrelation

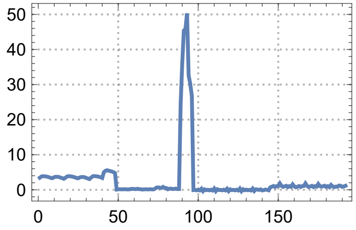

Dataset Index= 855

Molecule Formula= $C_{21}H_{20}N_4O$

3D Plot

3D Autocorrelation

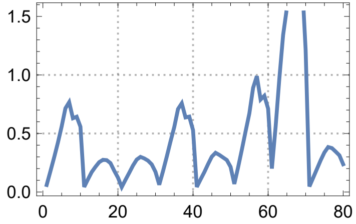

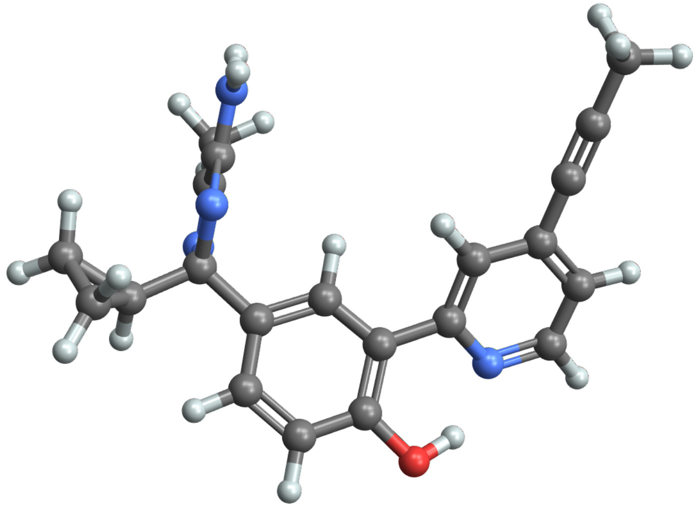

2D Autocorrelation

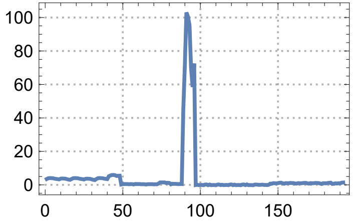

Dataset Index= 856

Molecule Formula= $C_{19}H_{19}FN_4O_2$

3D Autocorrelation

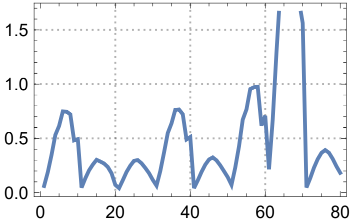

2D Plot

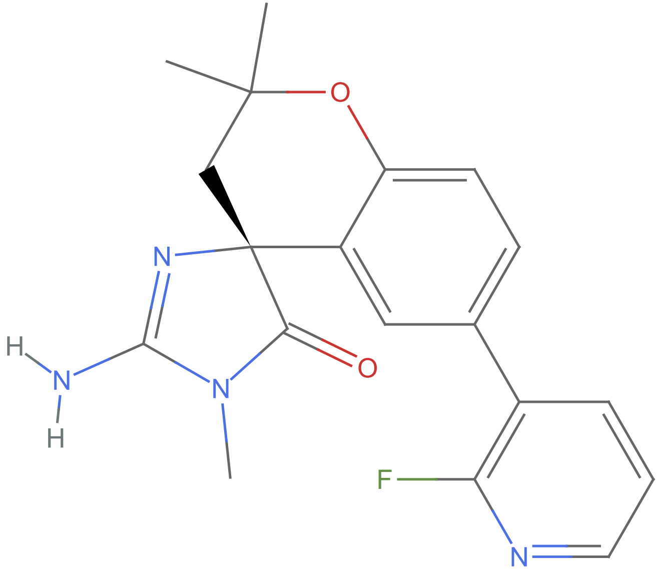

3D Plot

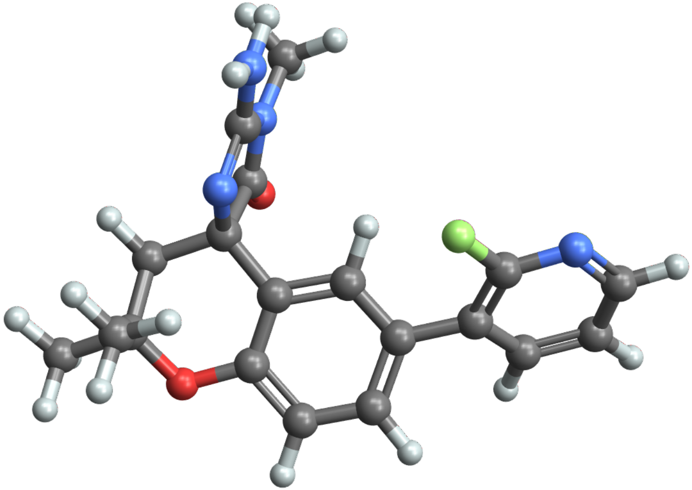

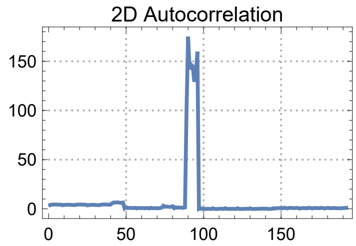

Dataset Index= 857

Molecule Formula= $C_{26}H_{24}F_4N_5O^+$

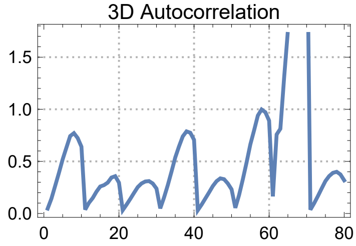

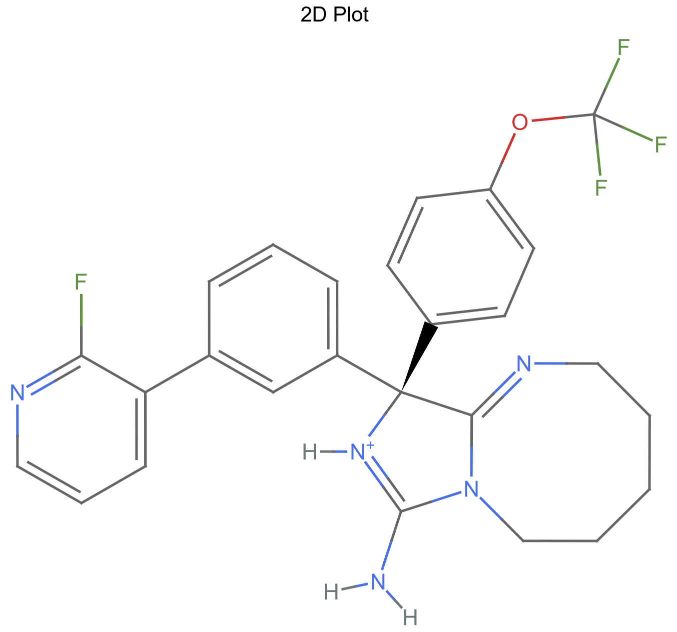

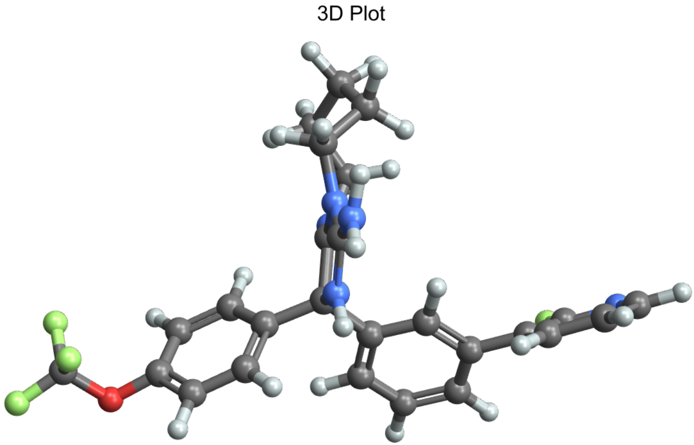

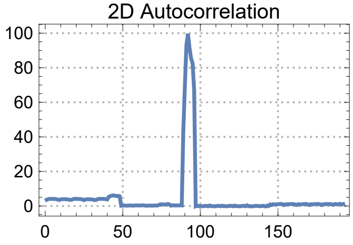

Dataset Index= 858

Molecule Formula= $C_{24}H_{24}N_4O_2$

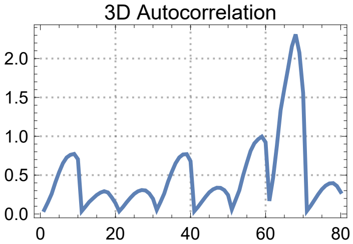

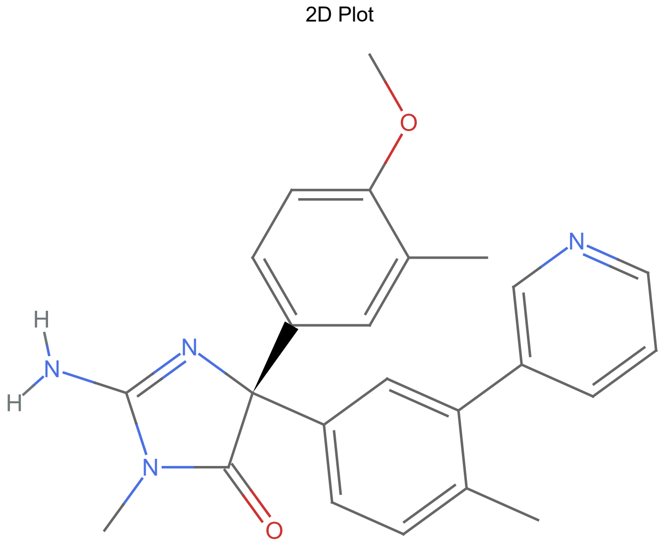

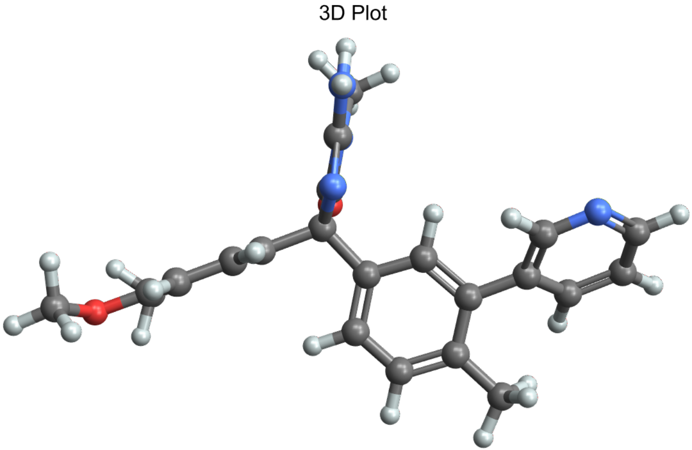

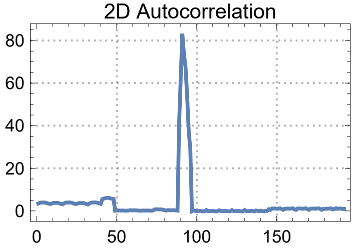

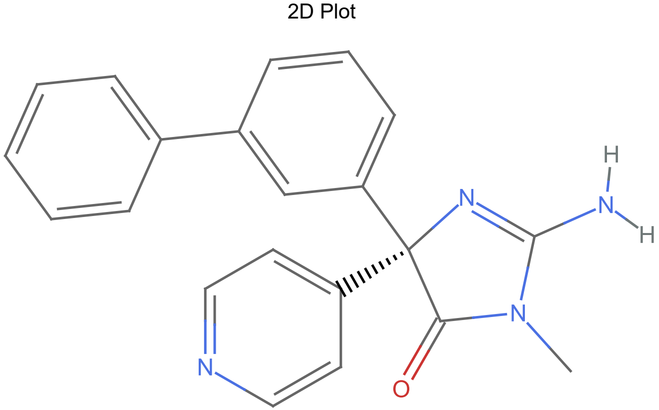

Dataset Index= 859

Molecule Formula= $C_{21}H_{18}N_4O$

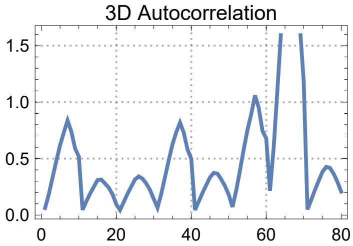

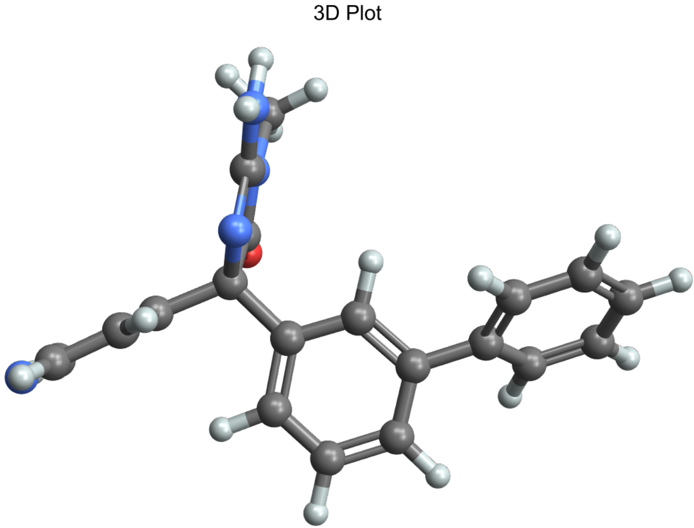

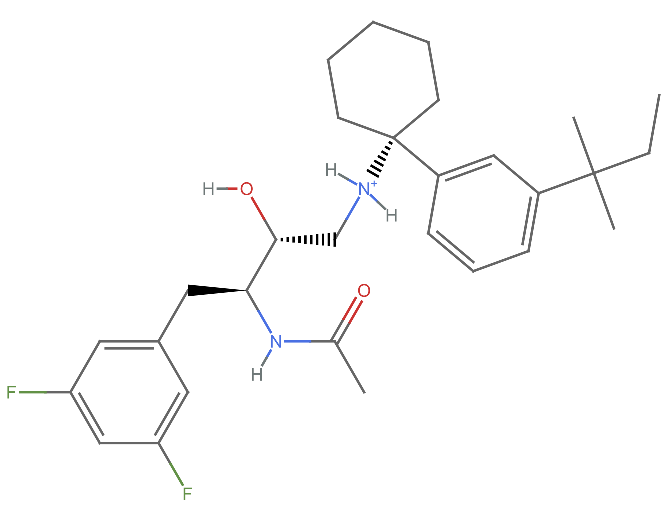

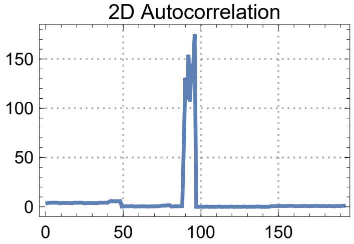

3D Plot

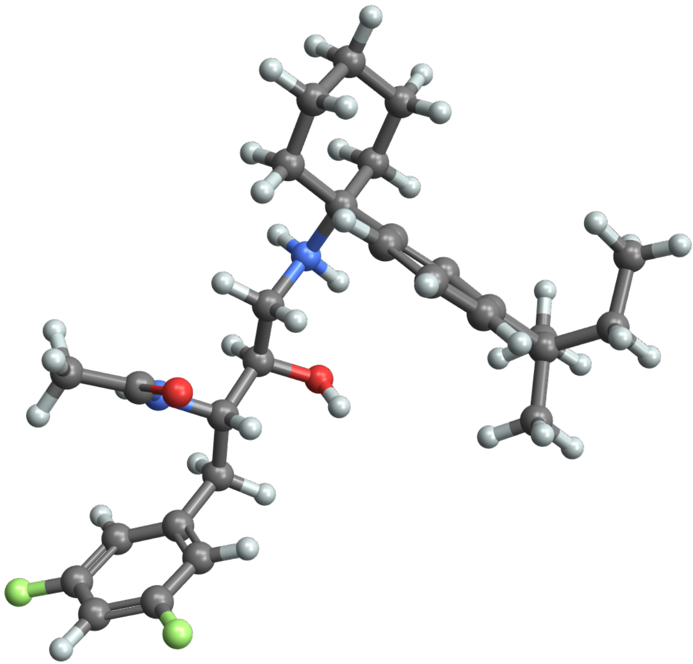

Dataset Index= 860

Molecule Formula= $C_{29}H_{41}F_2N_2O_2^+$

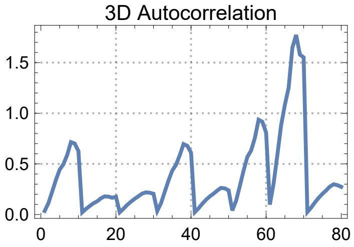

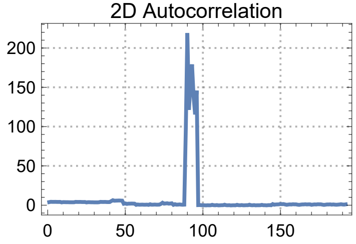

Dataset Index= 861

Molecule Formula= $C_{24}H_{32}F_3N_2O_4S^+$

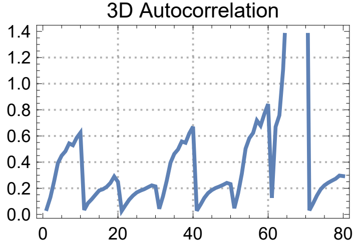

2D Plot

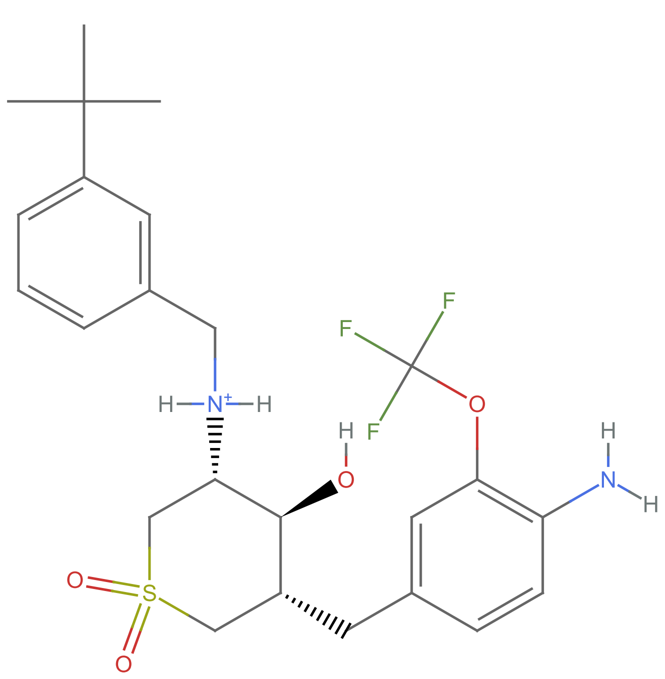

3D Plot

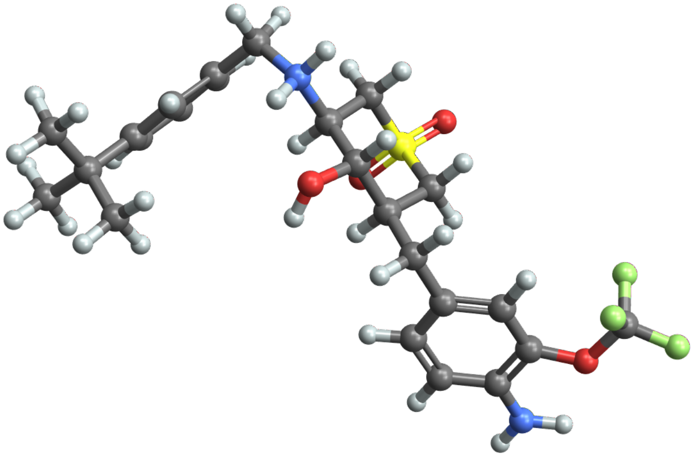

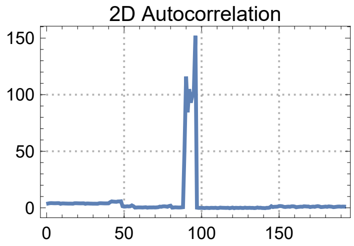

Dataset Index= 862

Molecule Formula= $C_{26}H_{38}FN_2O_3S^+$

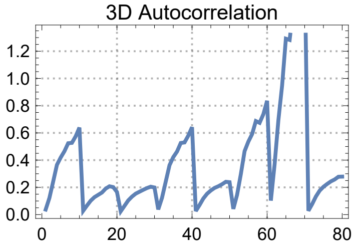

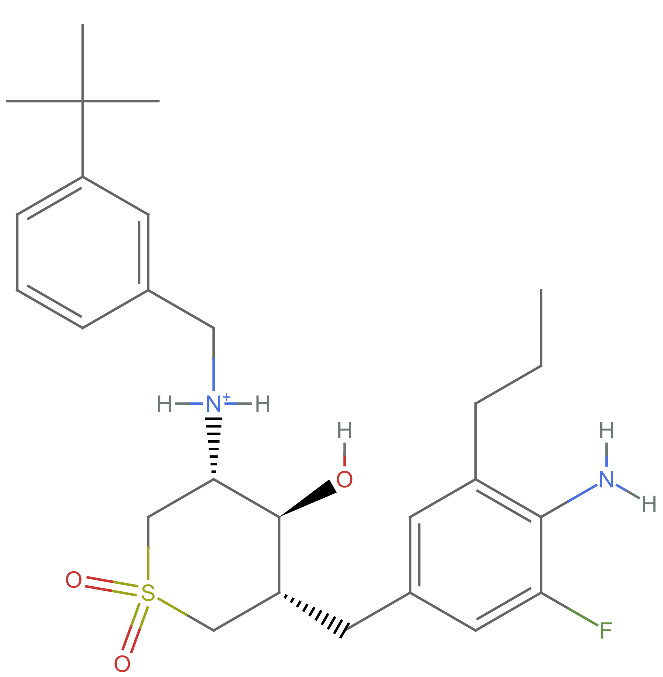

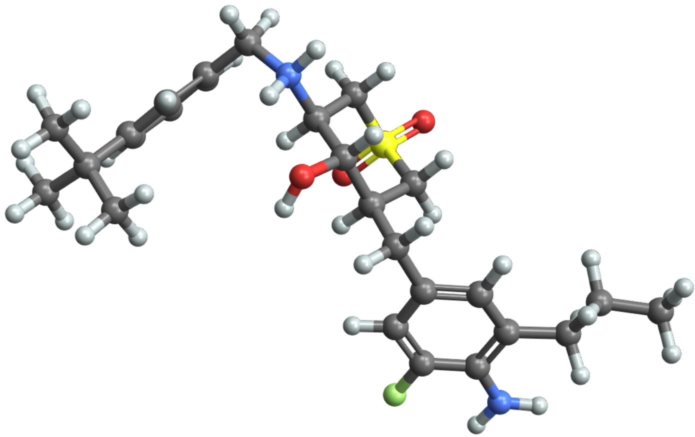

2D Autocorrelation

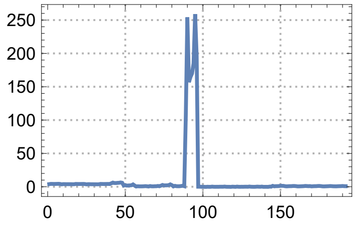

Dataset Index= 863

Molecule Formula= $C_{26}H_{35}F_4N_2O_3S^+$

3D Autocorrelation

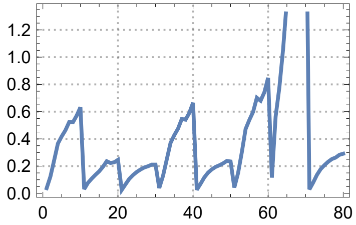

2D Plot

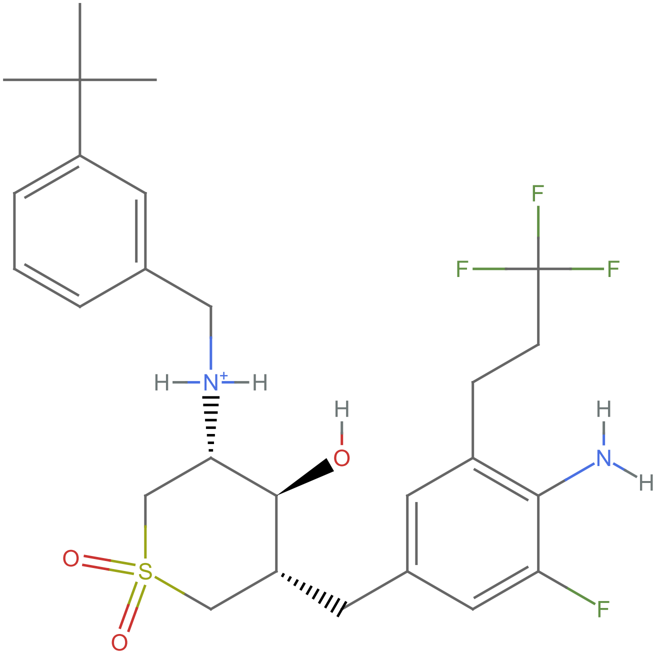

3D Plot

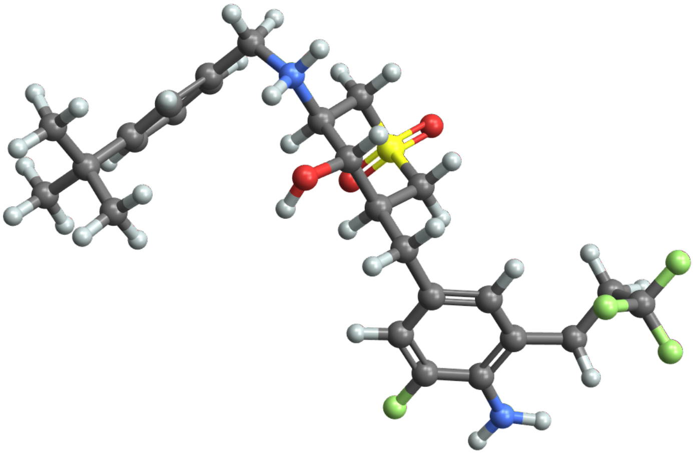

2D Autocorrelation

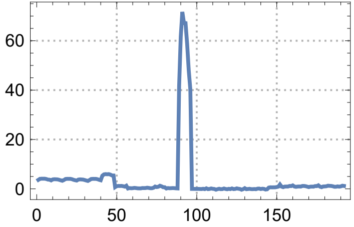

2D Plot

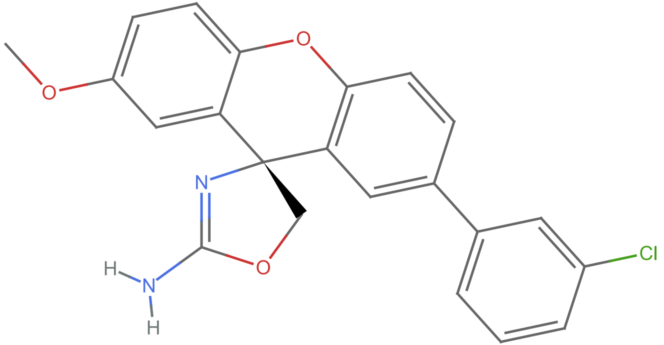

Dataset Index= 864

Molecule Formula= $C_{22}H_{17}ClN_2O_3$

3D Autocorrelation

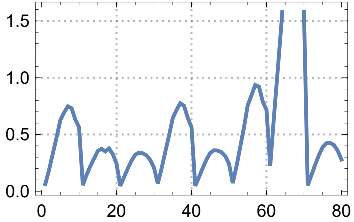

3D Plot

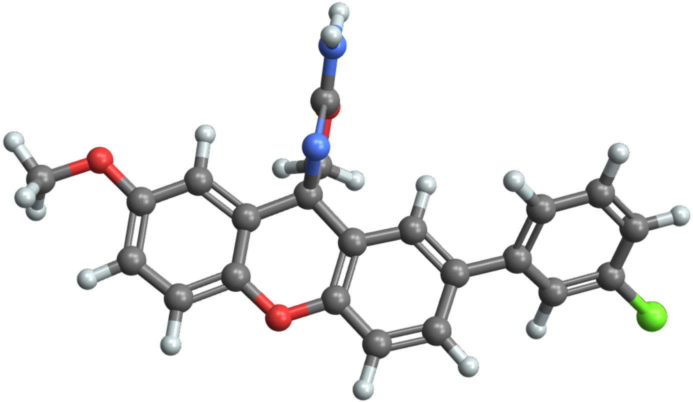

2D Autocorrelation

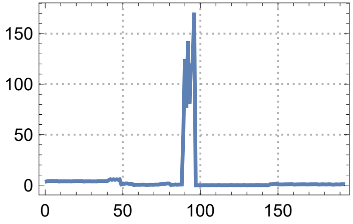

Dataset Index= 865

Molecule Formula= $C_{27}H_{39}F_2N_2O_2S^+$

3D Autocorrelation

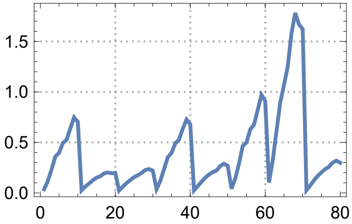

2D Plot

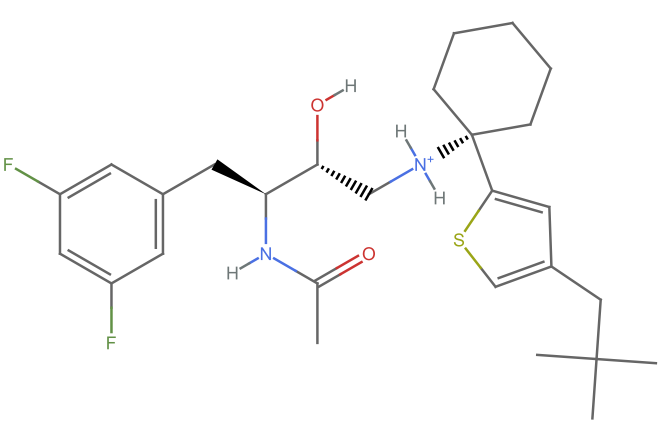

3D Plot

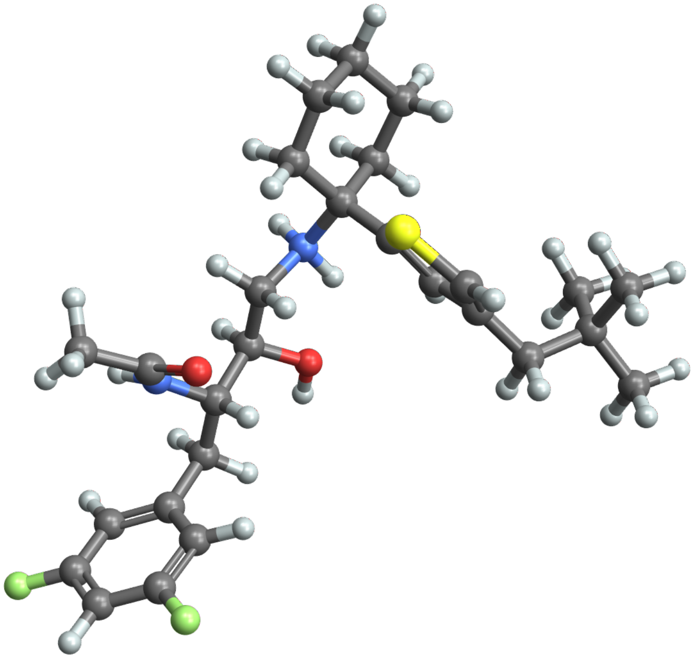

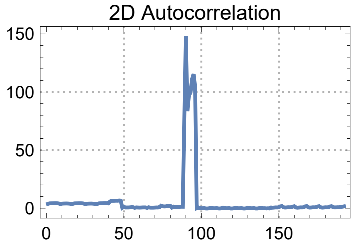

Dataset Index= 866

Molecule Formula= $C_{24}H_{16}F_3N_5$

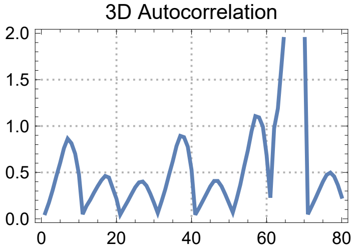

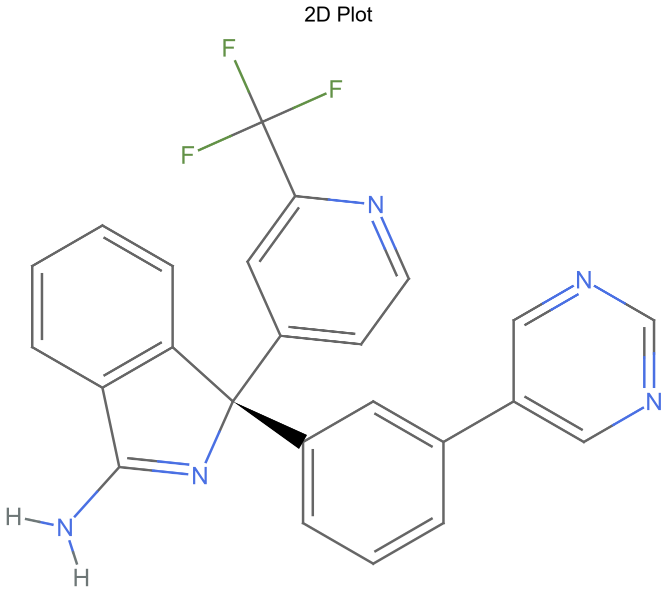

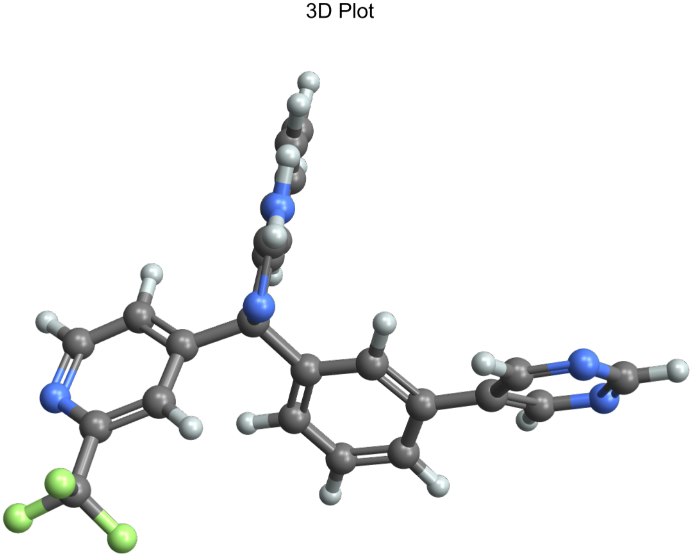

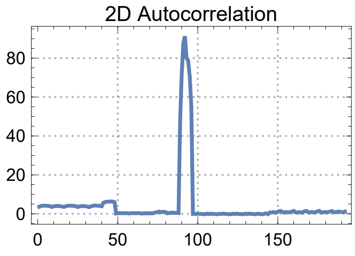

Dataset Index= 867

Molecule Formula= $C_{25}H_{19}FN_4O$

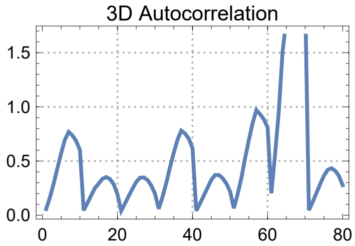

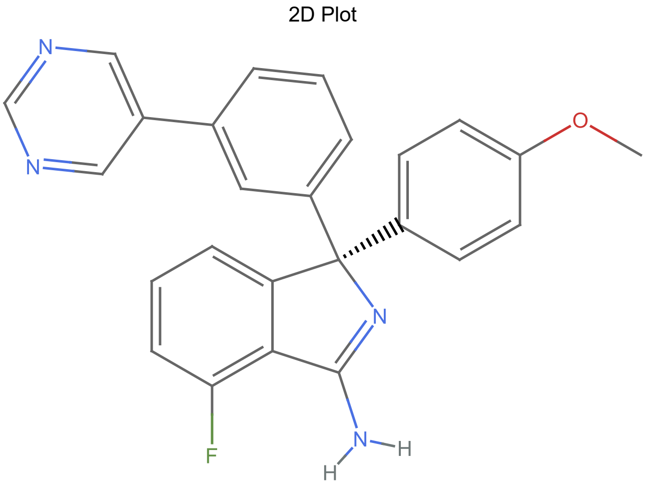

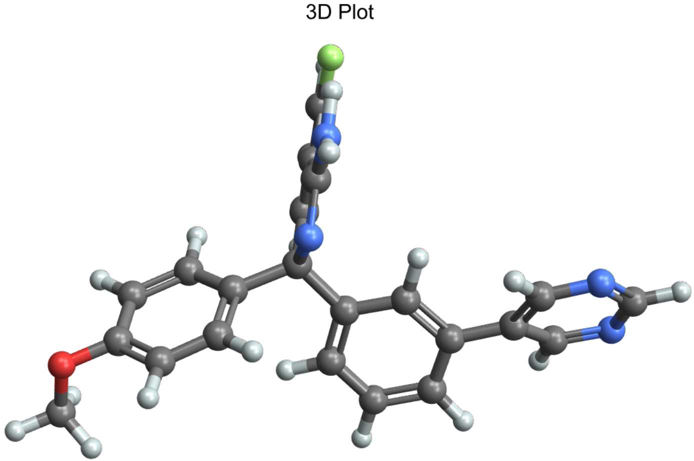

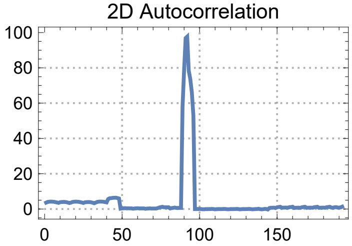

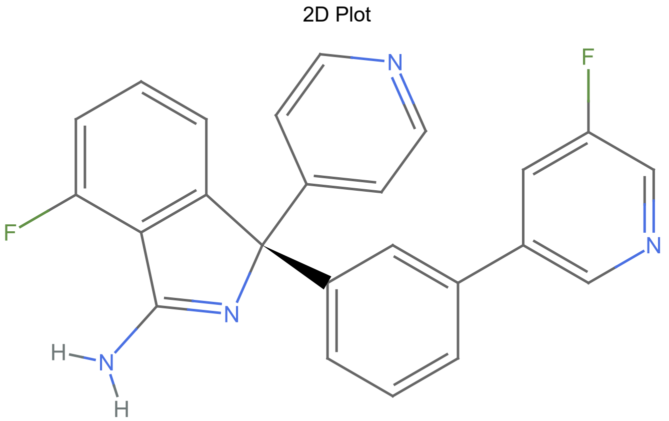

Dataset Index= 868

Molecule Formula= $C_{24}H_{16}F_2N_4$

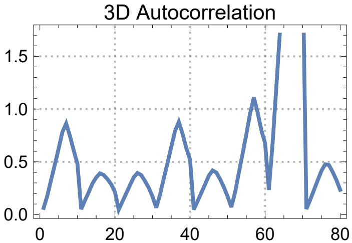

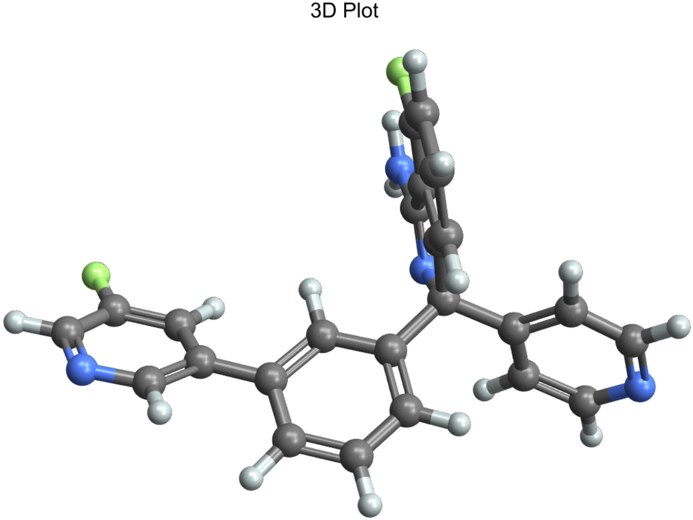

2D Plot

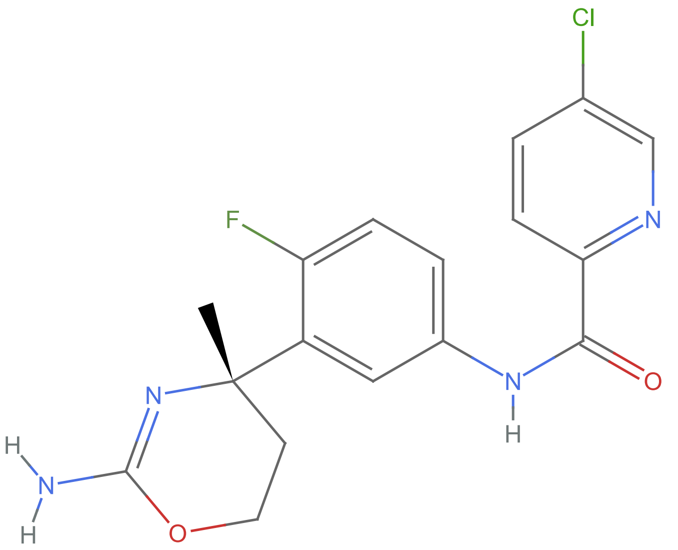

2D Autocorrelation

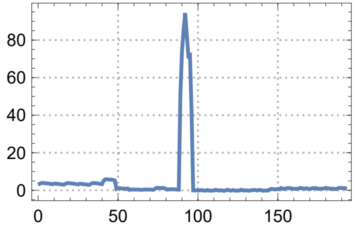

Dataset Index= 869

Molecule Formula= $C_{17}H_{16}ClFN_4O_2$

3D Plot

3D Autocorrelation

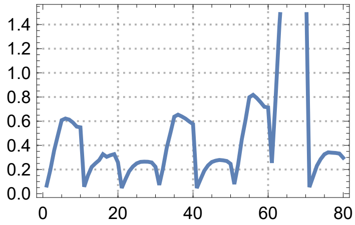

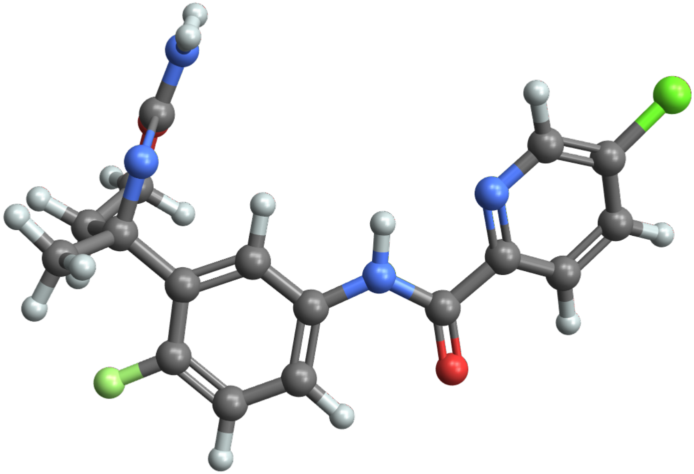

2D Autocorrelation

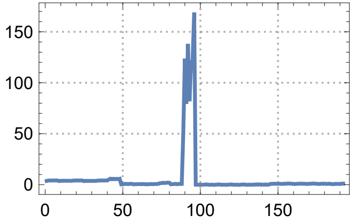

Dataset Index= 870

Molecule Formula= $C_{26}H_{38}F_2N_3O_3^+$

3D Autocorrelation

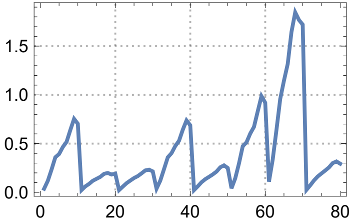

2D Plot

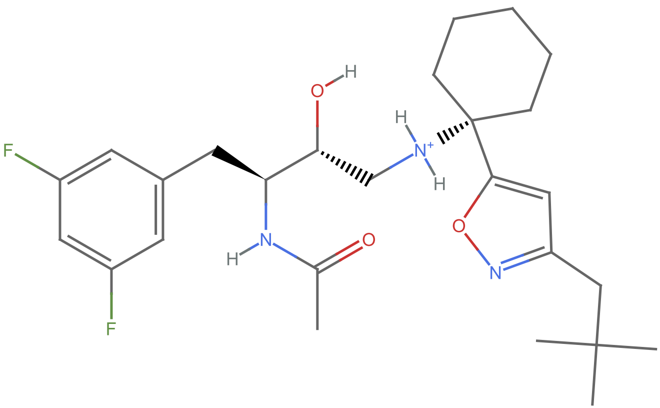

3D Plot

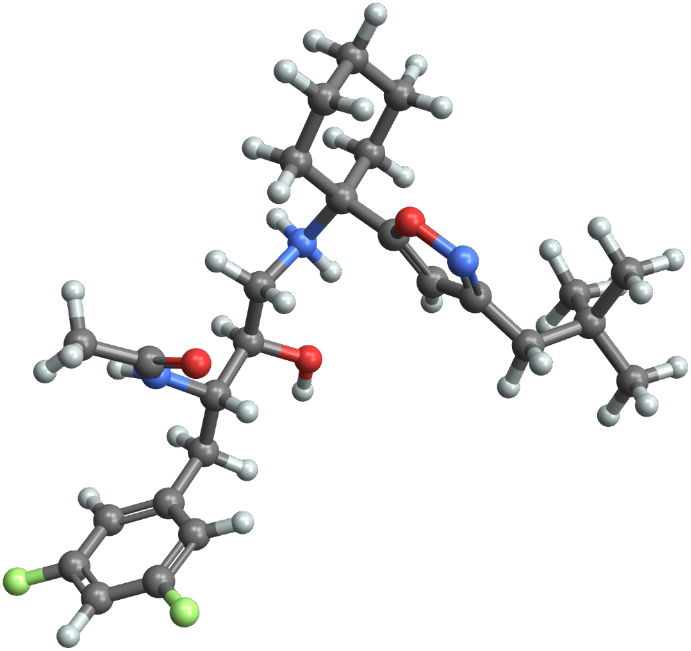

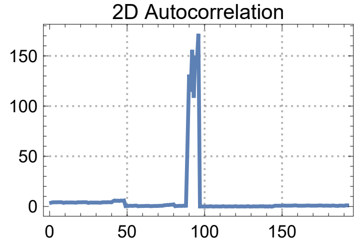

Dataset Index= 871

Molecule Formula= $C_{29}H_{41}F_2N_2O_3^+$

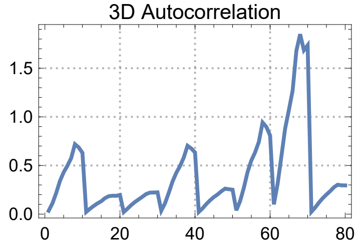

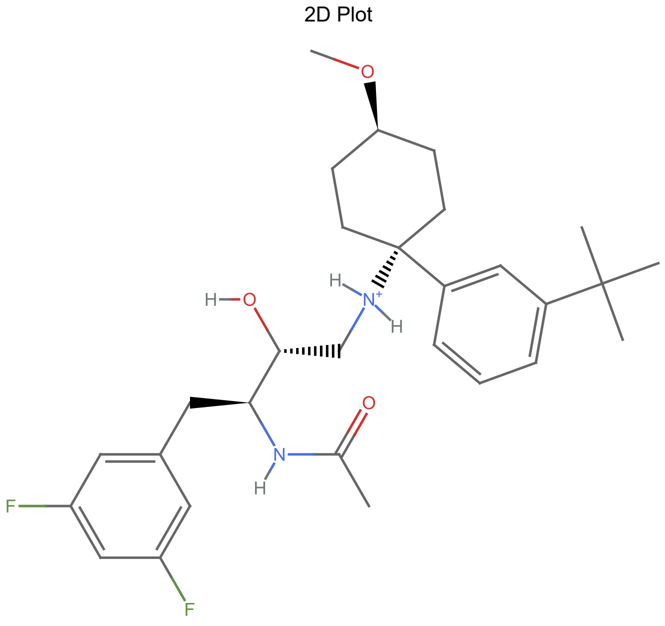

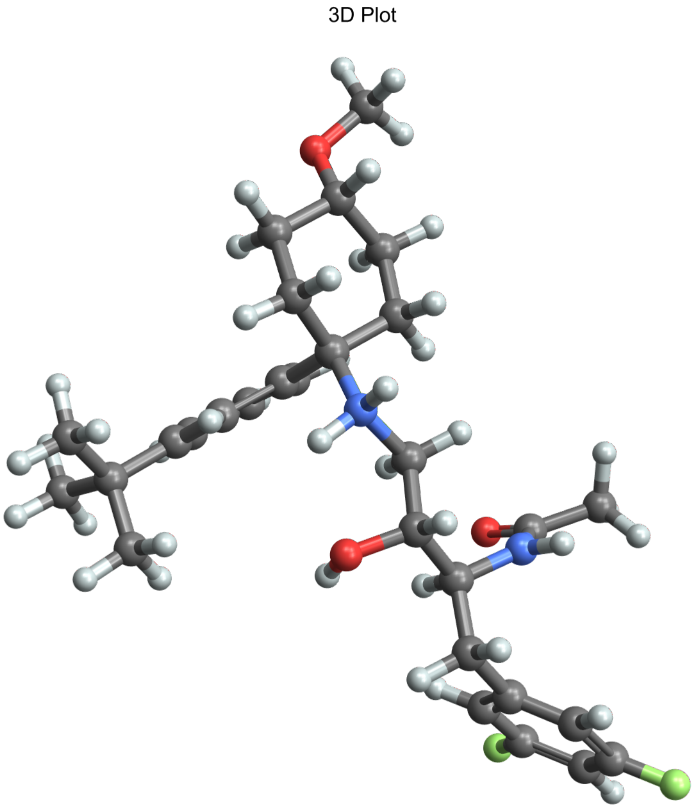

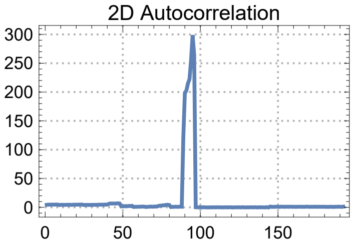

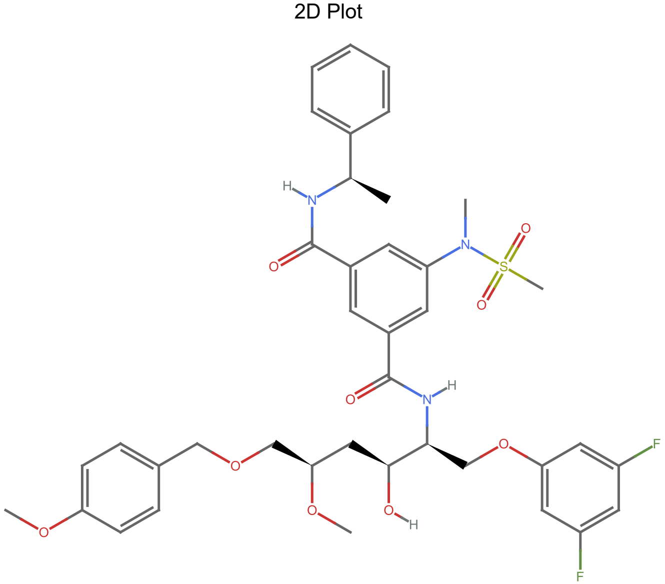

Dataset Index= 872

Molecule Formula= $C_{39}H_{45}F_2N_3O_9S$

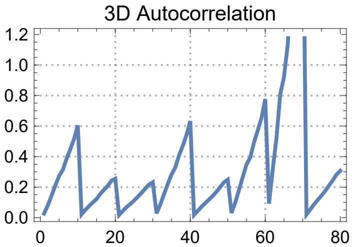

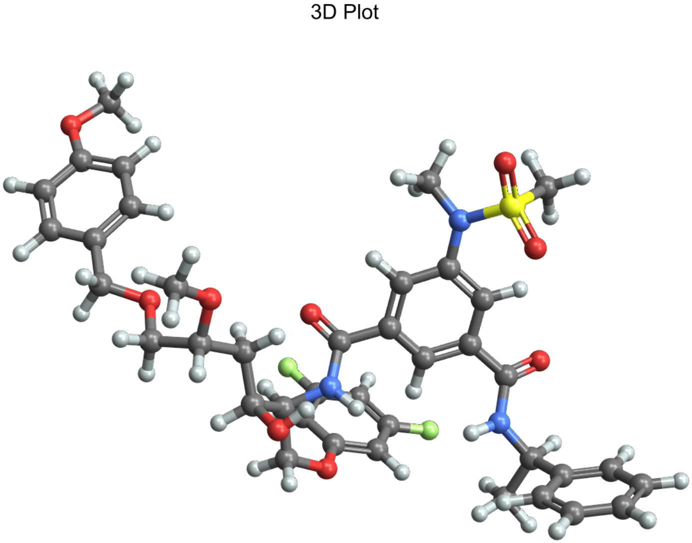

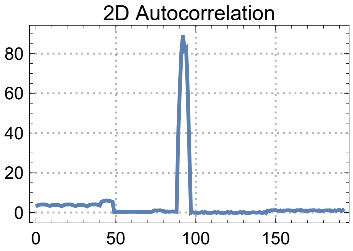

Dataset Index= 873

Molecule Formula= $C_{21}H_{20}N_6O$

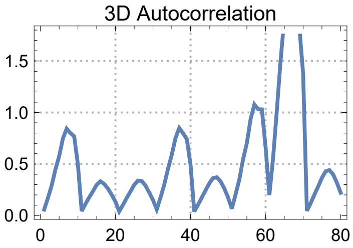

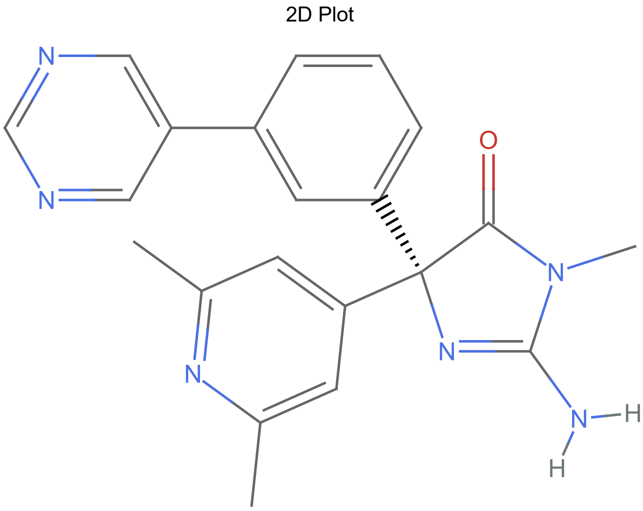

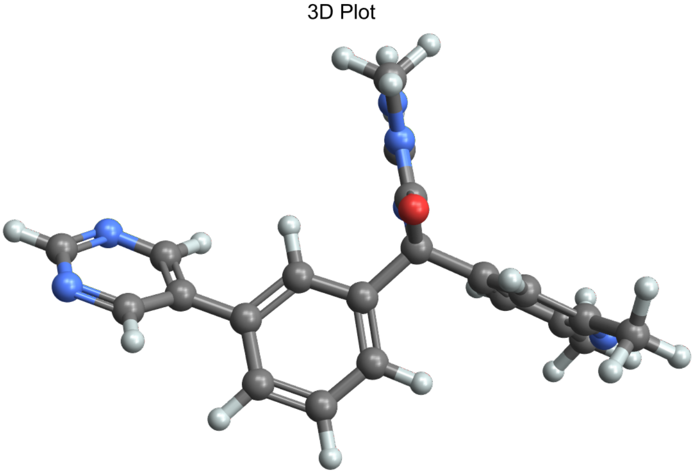

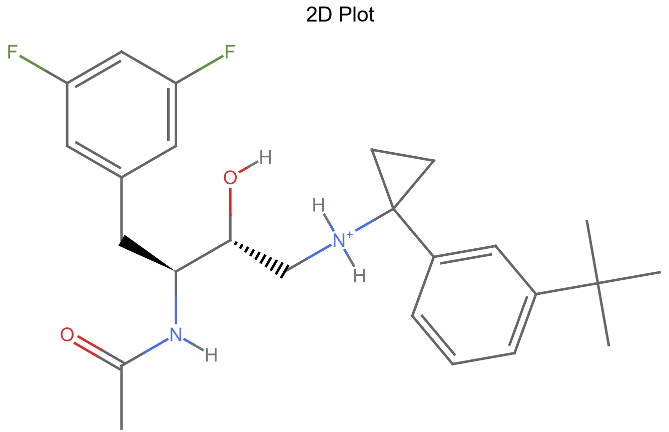

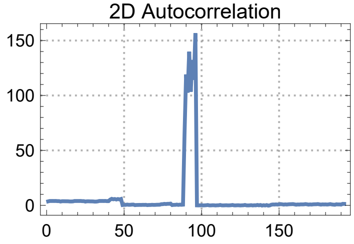

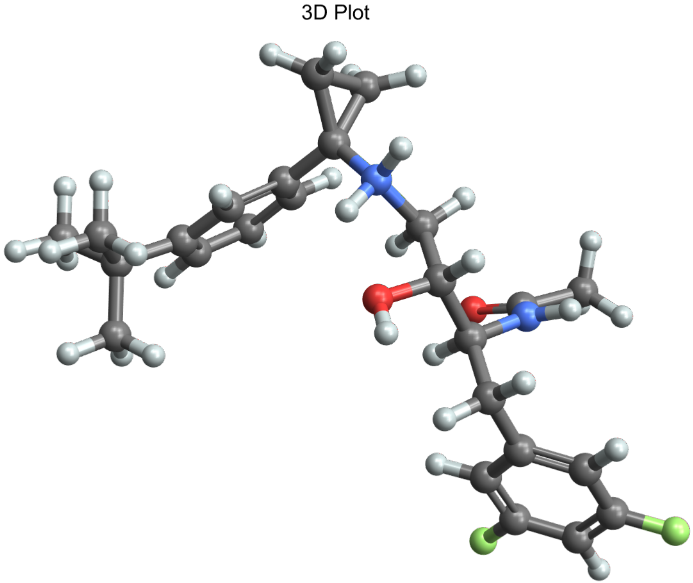

Dataset Index= 874

Molecule Formula= $C_{25}H_{33}F_2N_2O_2^+$

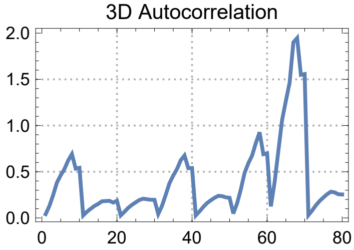

2D Autocorrelation

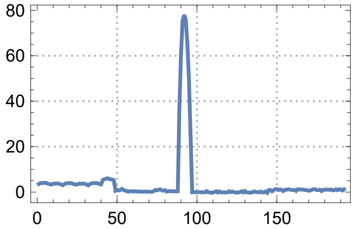

Dataset Index= 875

Molecule Formula= $C_{19}H_{17}N_5OS$

3D Autocorrelation

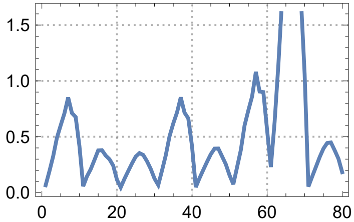

2D Plot

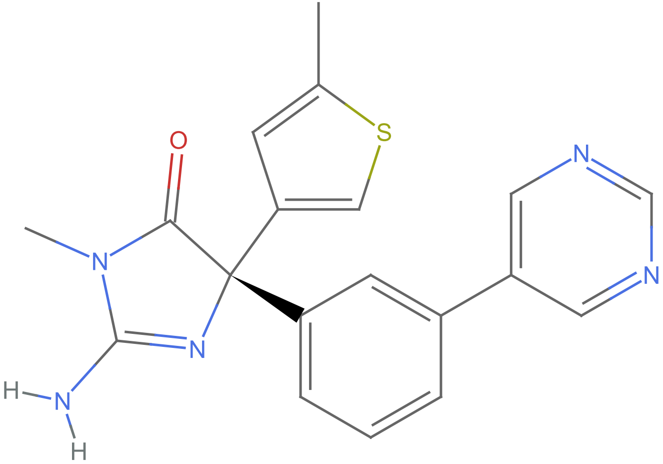

3D Plot

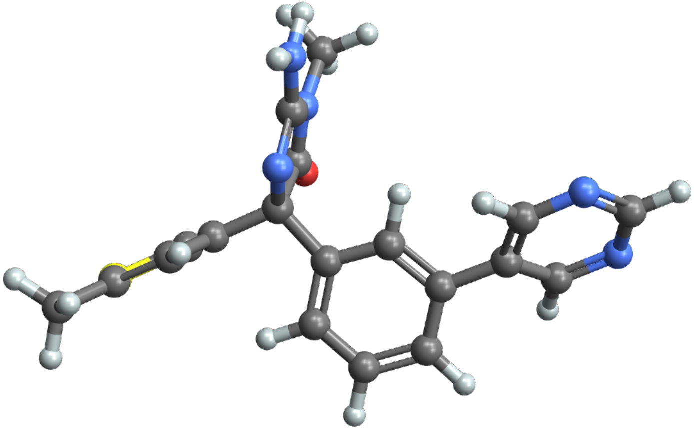

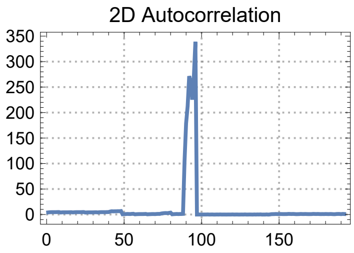

2D Plot

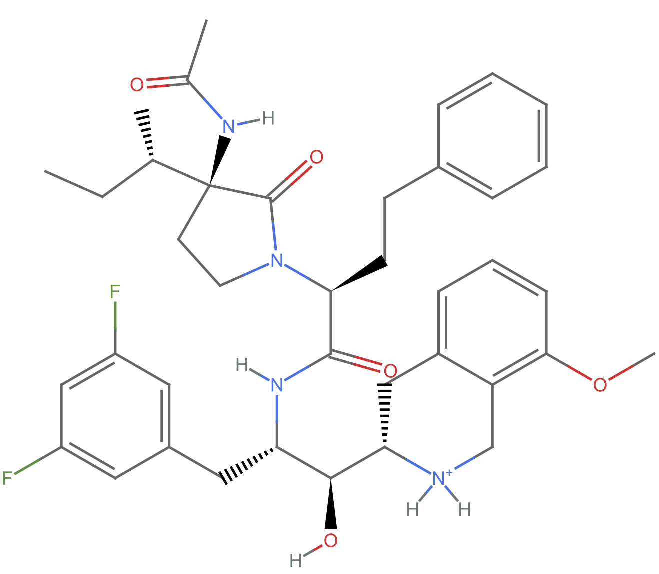

3D Plot

Dataset Index= 876

Molecule Formula= $C_{39}H_{49}F_2N_4O_5^+$

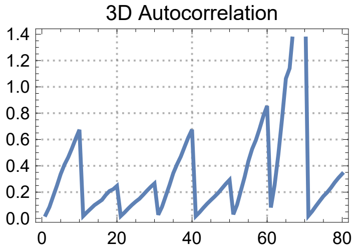

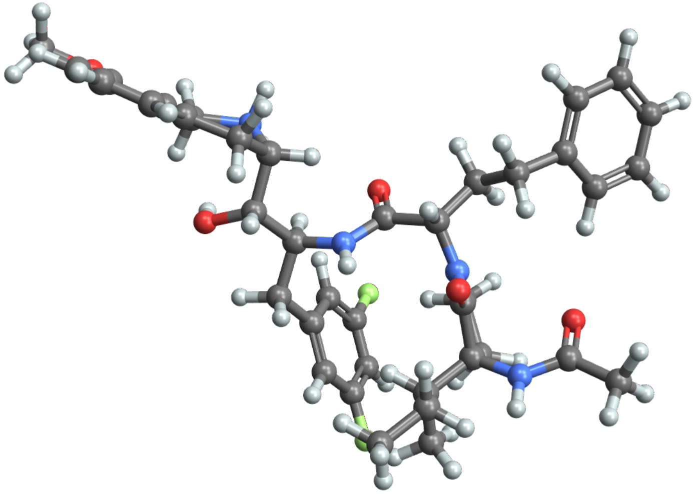

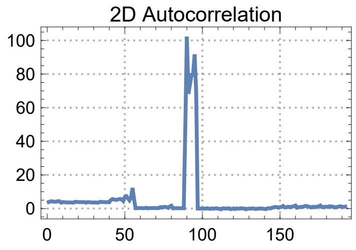

Dataset Index= 877

Molecule Formula= $C_{23}H_{32}BrN_2O_3S^+$

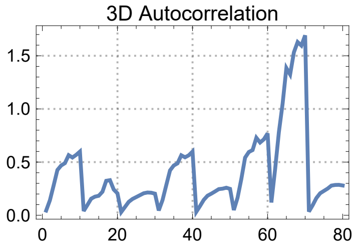

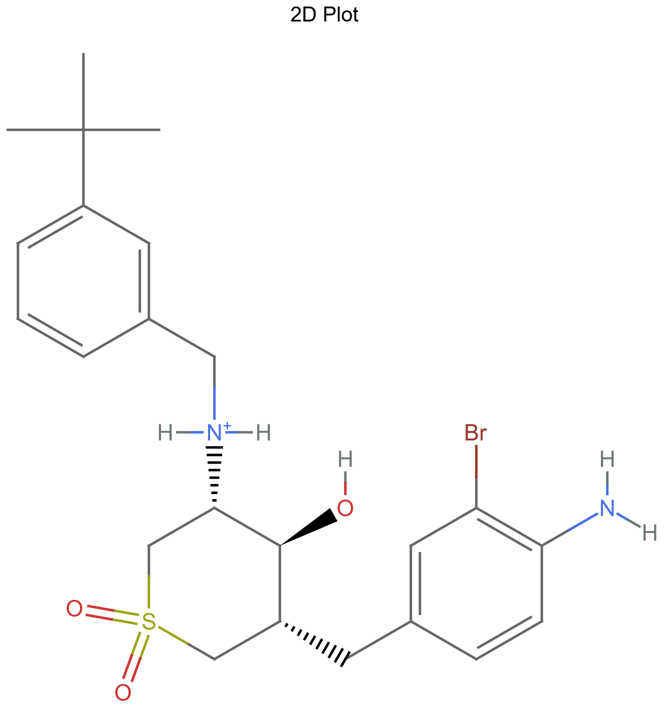

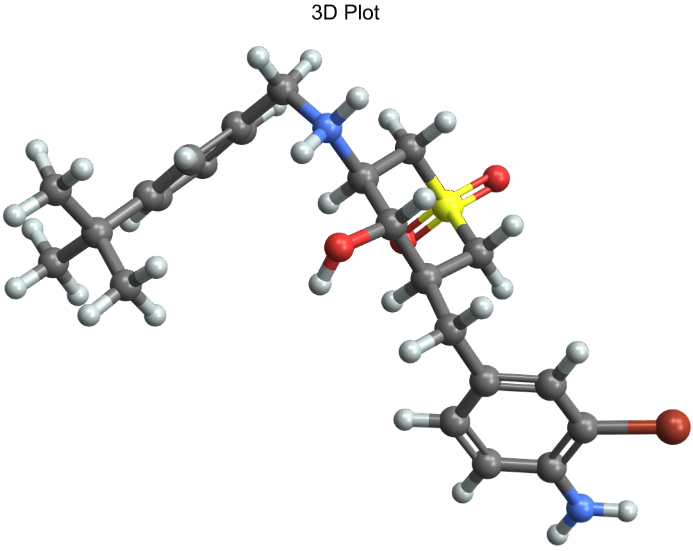

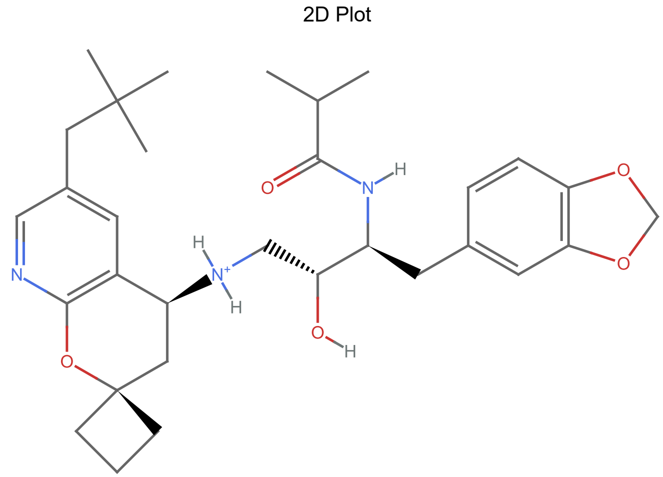

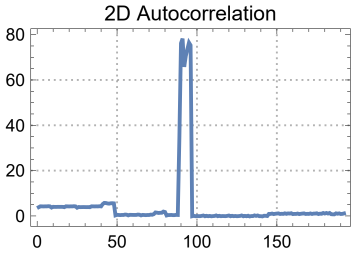

Dataset Index= 878

Molecule Formula= $C_{31}H_{44}N_3O_5^+$

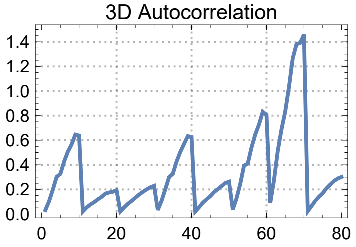

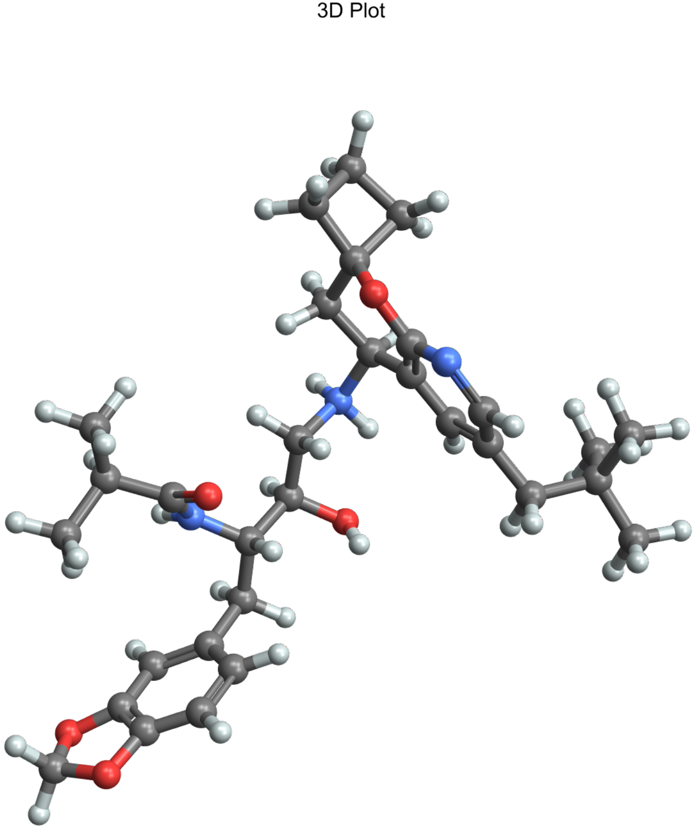

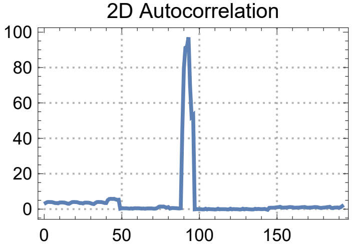

Dataset Index= 879

Molecule Formula= $C_{19}H_{19}FN_4O_2$

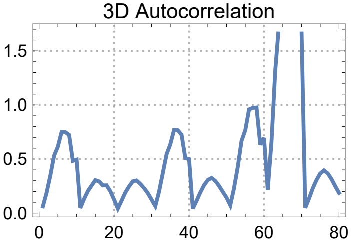

2D Plot

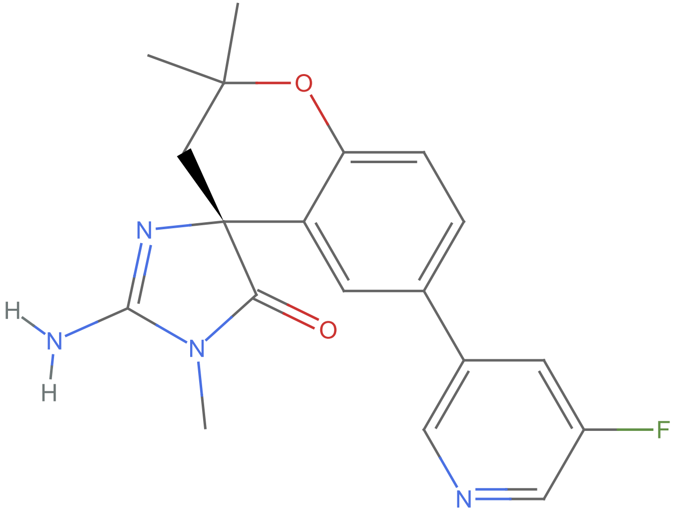

3D Plot

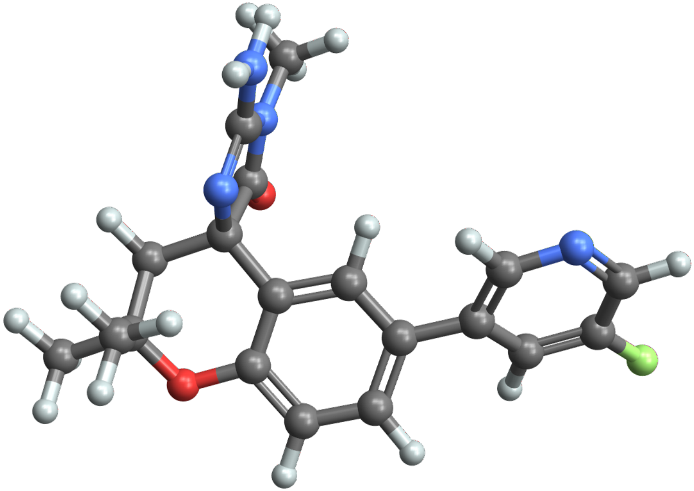

2D Plot

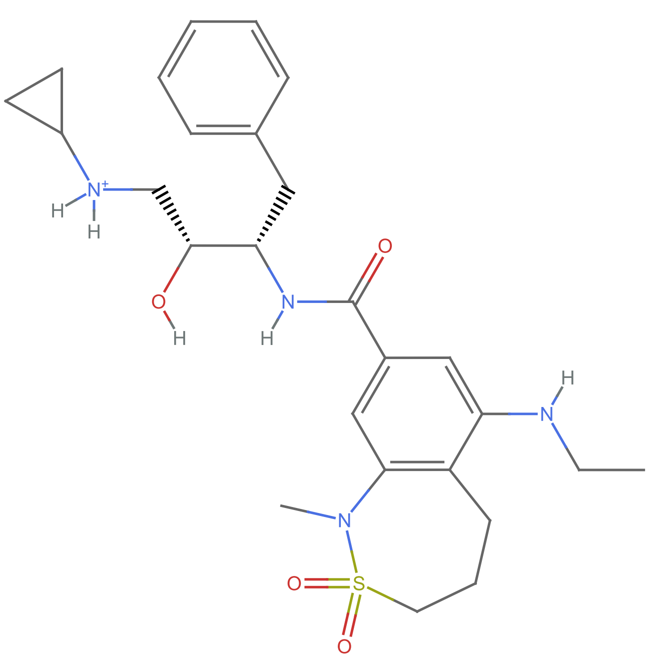

2D Autocorrelation

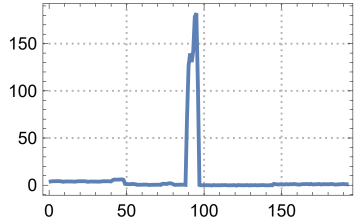

Dataset Index= 880

Molecule Formula= $C_{26}H_{37}N_4O_4S^+$

3D Autocorrelation

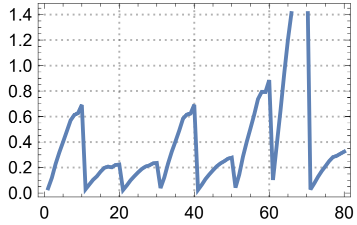

3D Plot

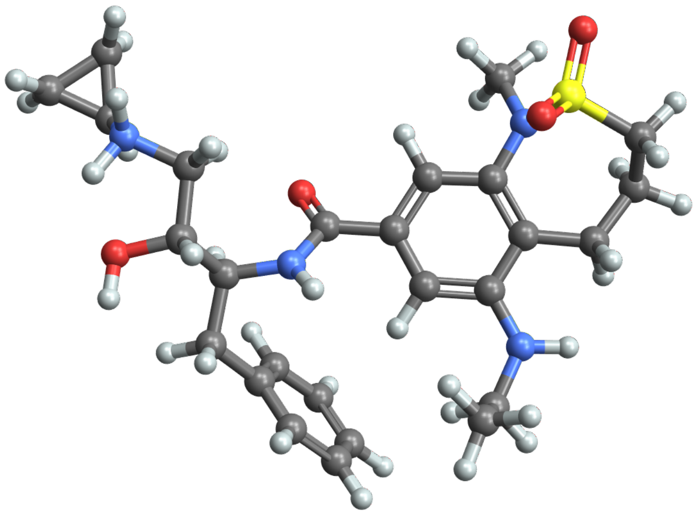

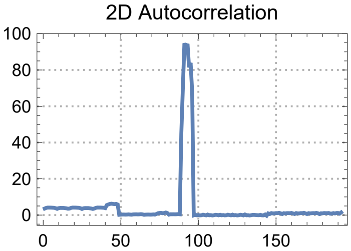

Dataset Index= 881

Molecule Formula= $C_{23}H_{19}N_5O_2$

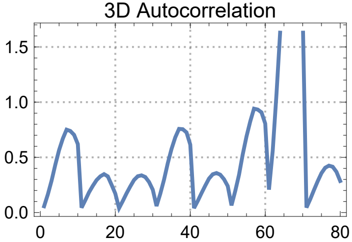

2D Plot

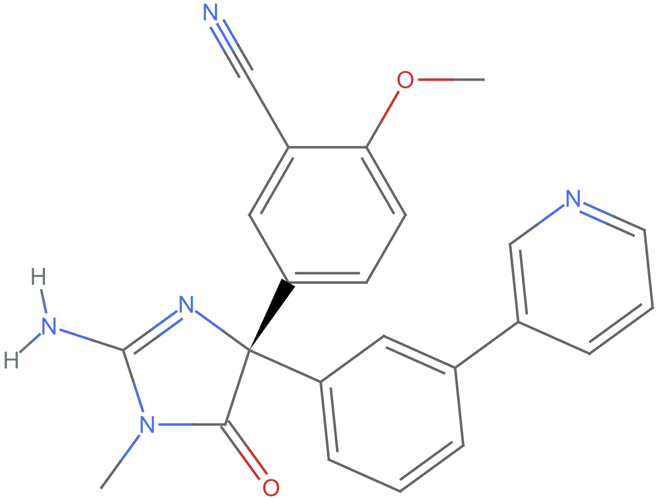

3D Plot

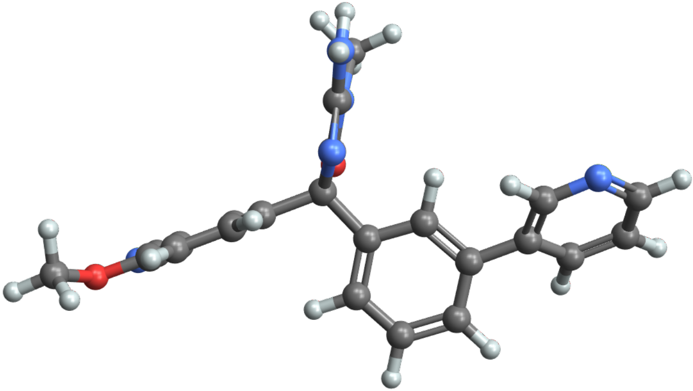

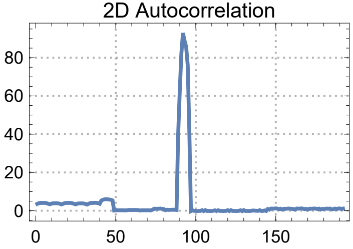

Dataset Index= 882

Molecule Formula= $C_{22}H_{22}N_6O$

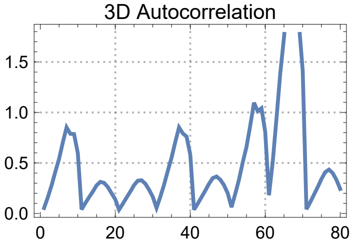

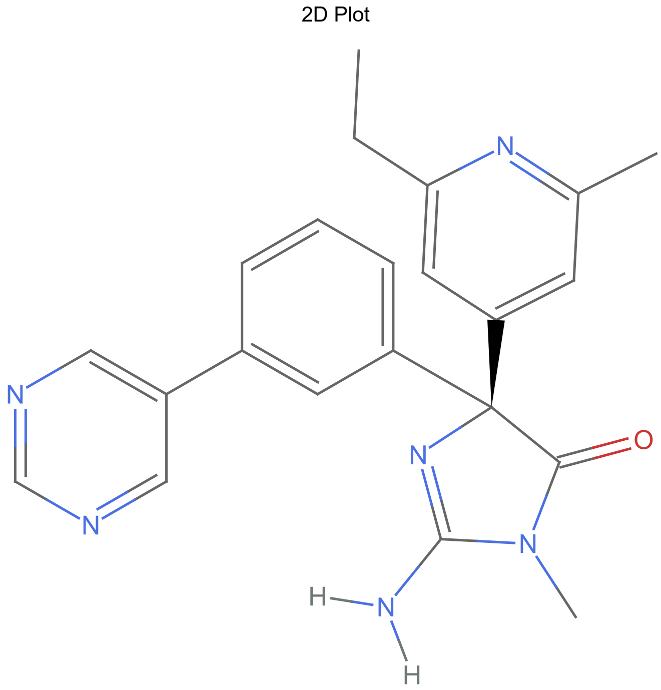

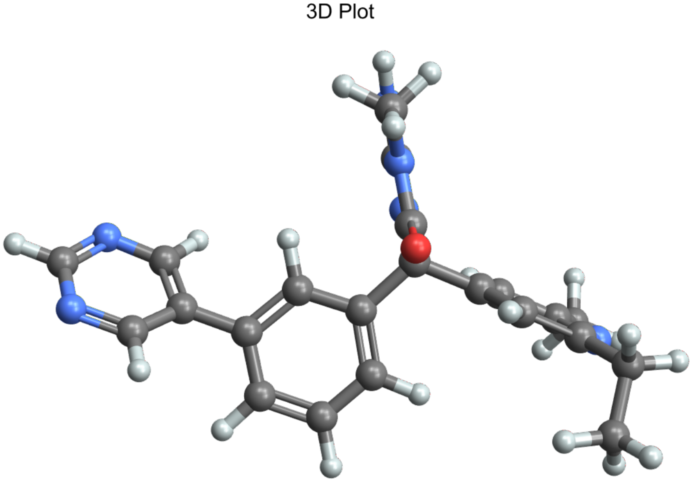

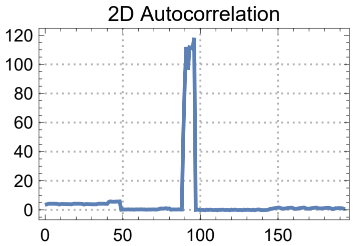

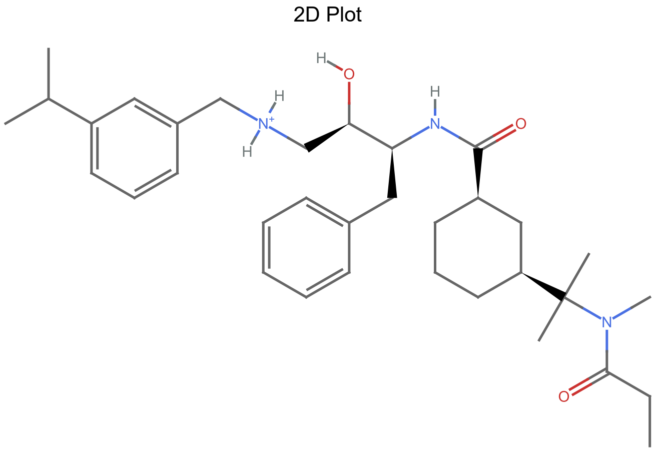

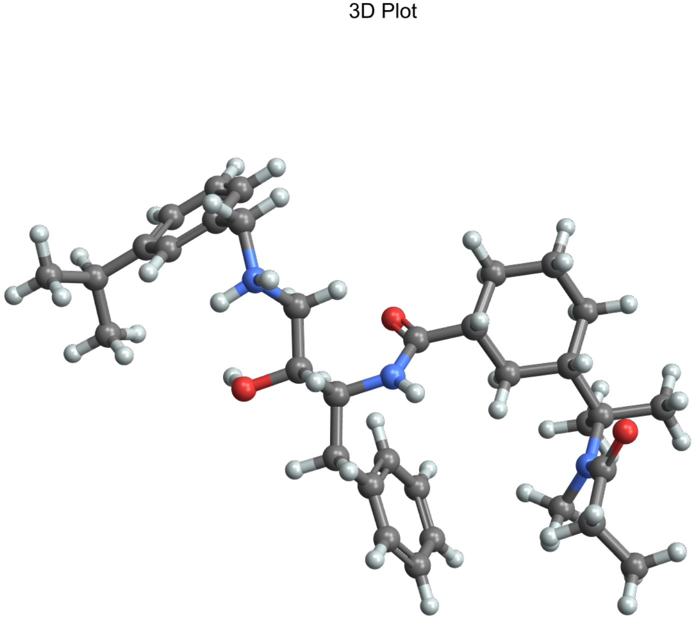

Dataset Index= 883

Molecule Formula= $C_{34}H_{52}N_3O_3^+$

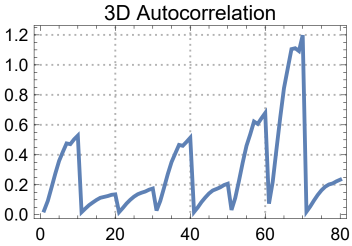

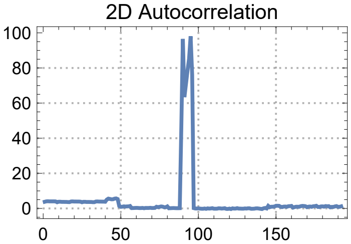

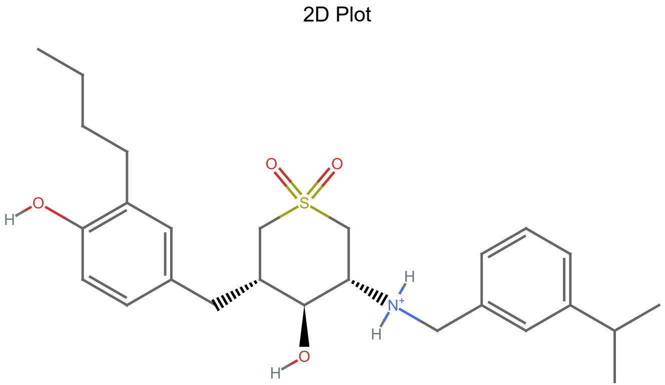

Dataset Index= 884

Molecule Formula= $C_{26}H_{38}NO_4S^+$

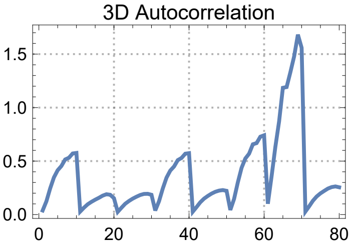

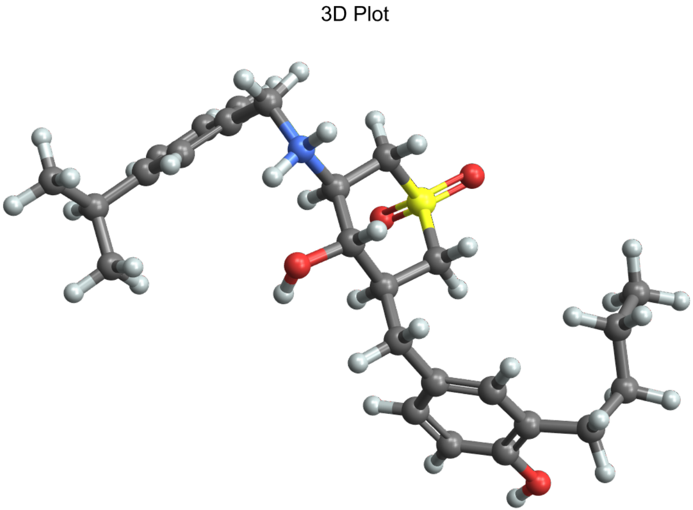

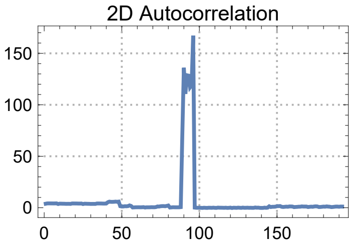

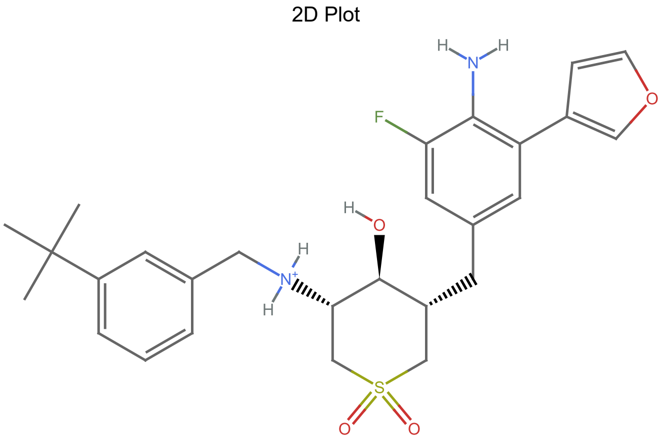

Dataset Index= 885

Molecule Formula= $C_{27}H_{34}FN_2O_4S^+$

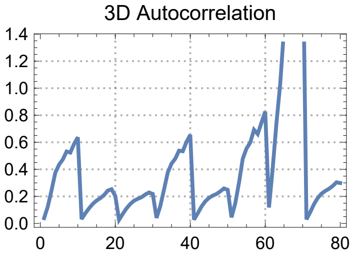

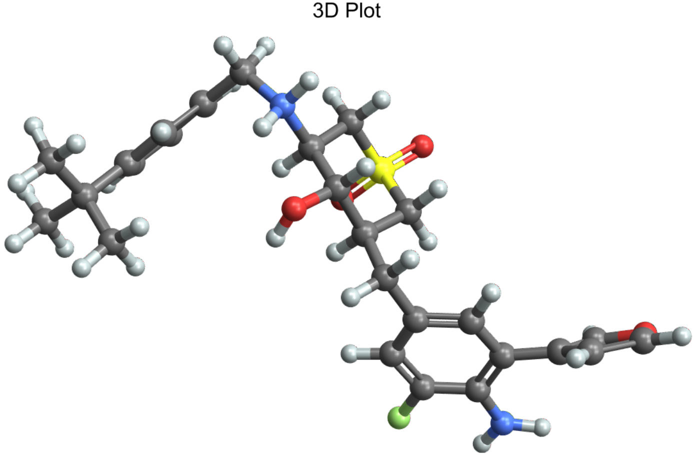

2D Plot

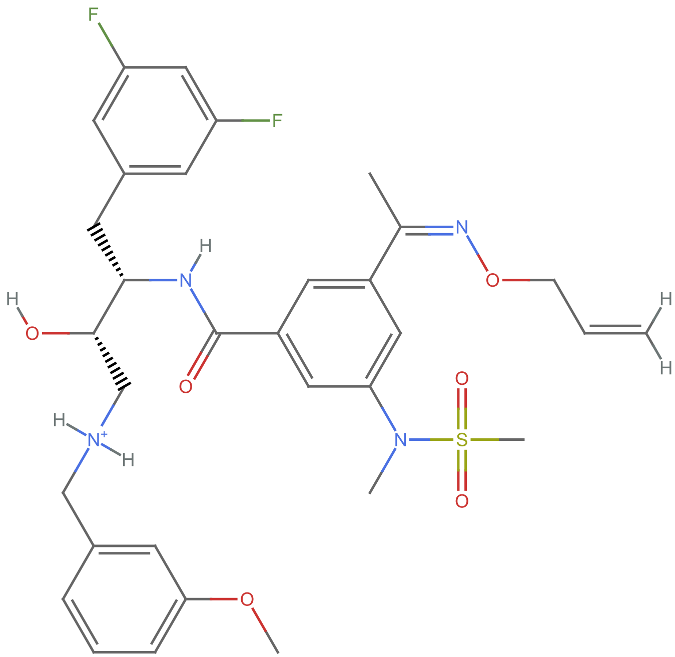

2D Autocorrelation

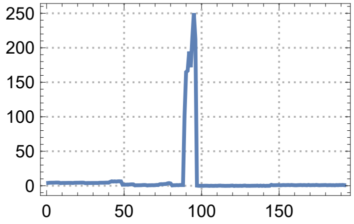

Dataset Index= 886

Molecule Formula= $C_{32}H_{39}F_{2}N_{4}O_{6}S^{+}$

3D Autocorrelation

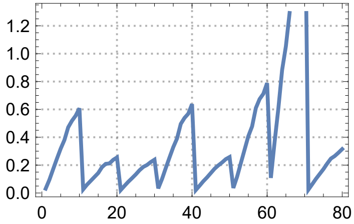

3D Plot

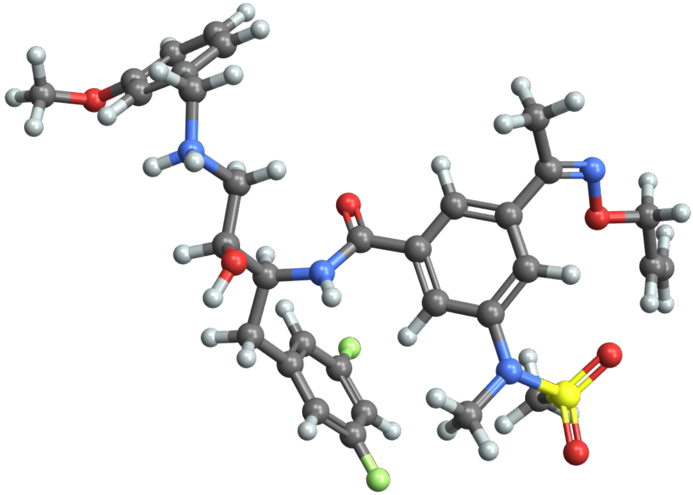

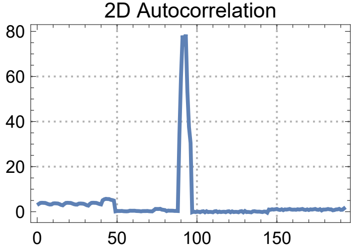

Dataset Index= 887

Molecule Formula= $C_{18}H_{19}N_5O_2$

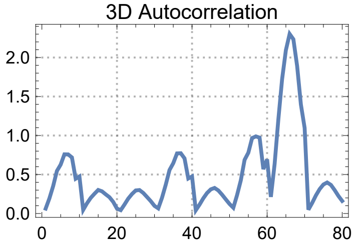

2D Plot

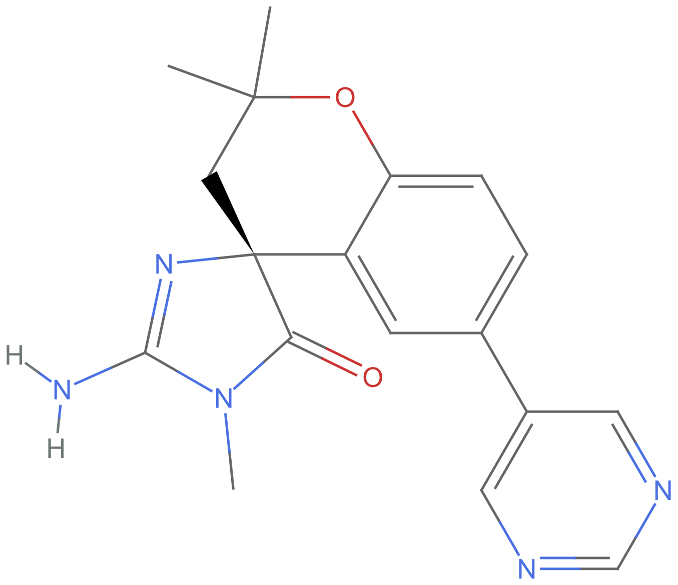

3D Plot

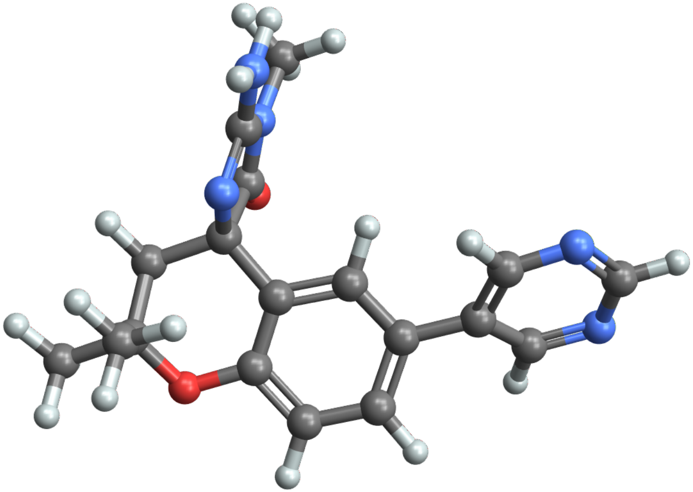

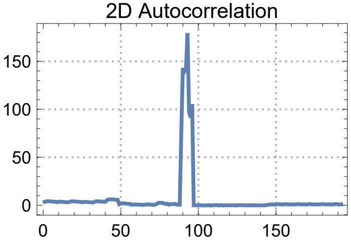

Dataset Index= 888

Molecule Formula= $C_{16}H_{15}ClF_3N_5O_2$

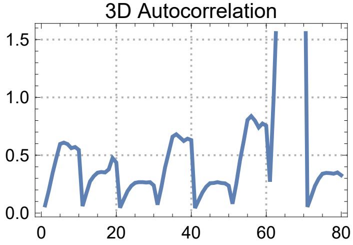

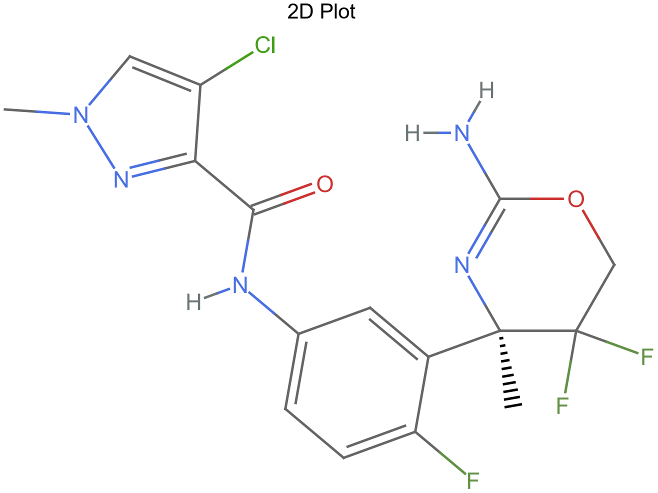

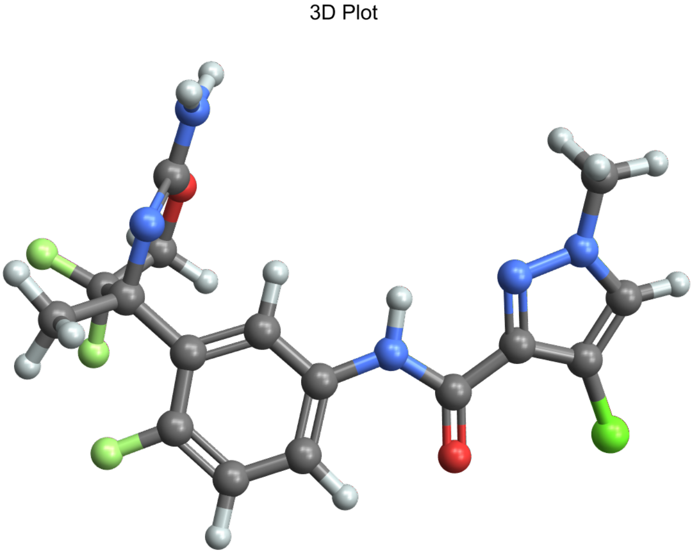

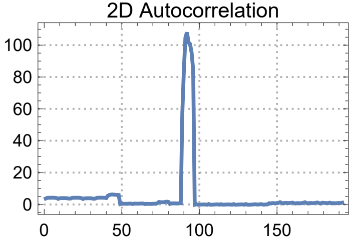

Dataset Index= 889

Molecule Formula= $C_{24}H_{21}FN_5O_2^+$

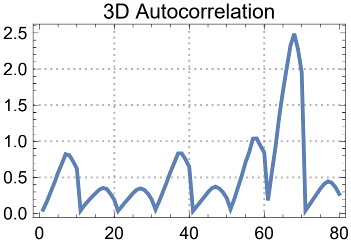

2D Plot

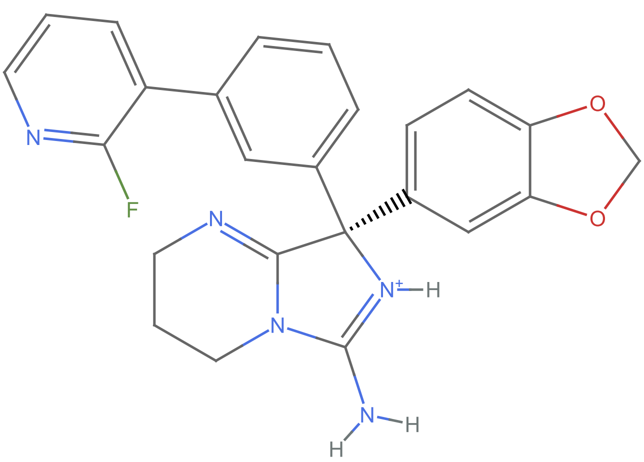

3D Plot

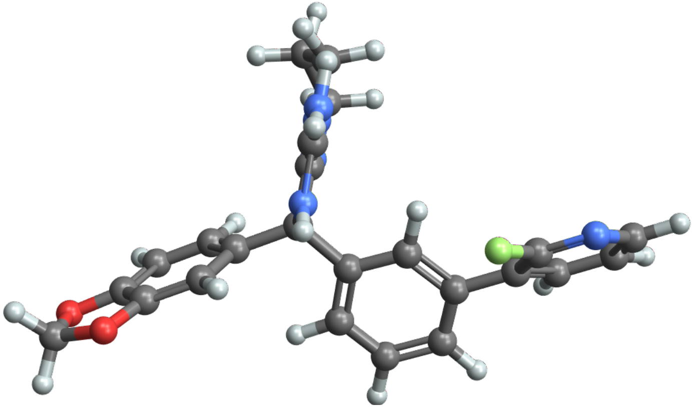

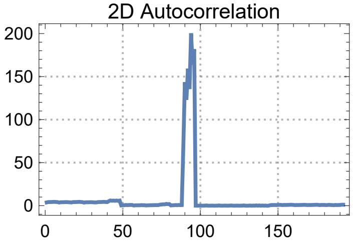

Dataset Index= 890

Molecule Formula= $C_{30}H_{41}F_2N_2O_3^+$

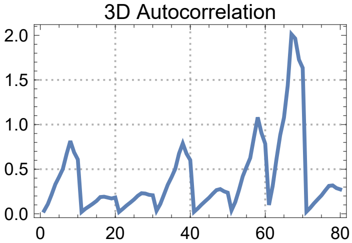

2D Plot

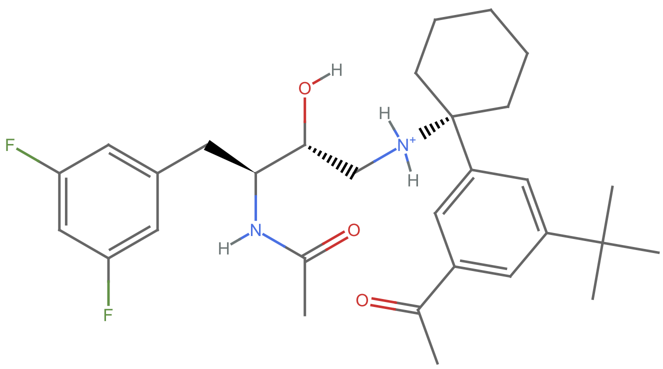

3D Plot

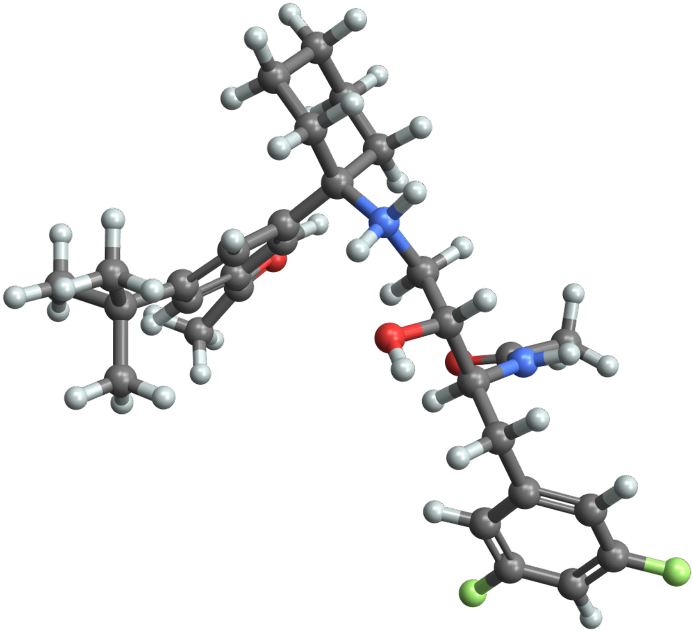

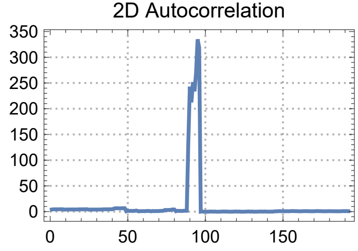

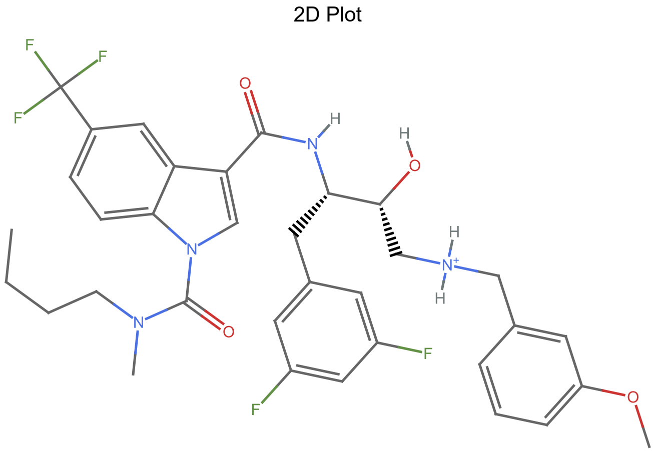

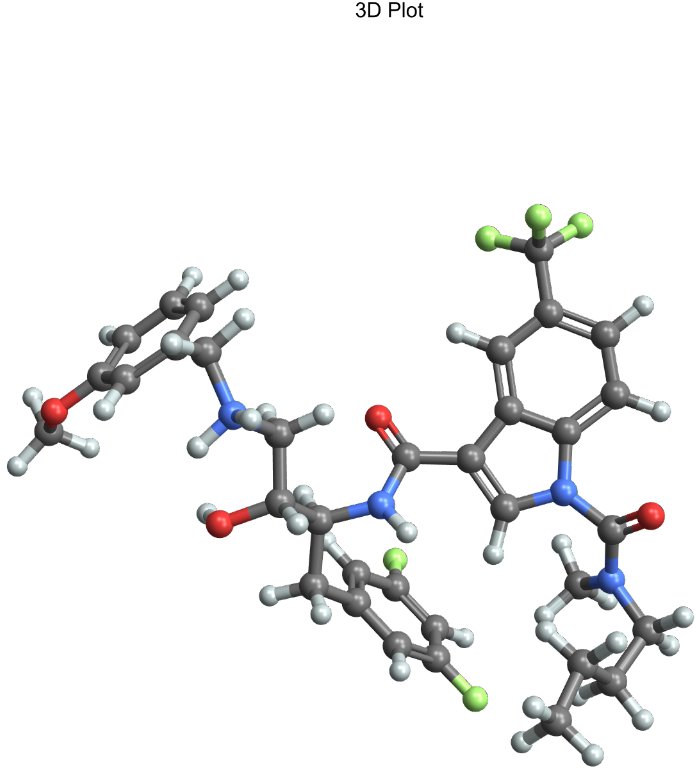

Dataset Index= 891

Molecule Formula= $C_{34}H_{38}F_5N_4O_4^+$

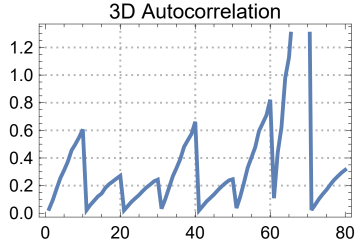

2D Autocorrelation

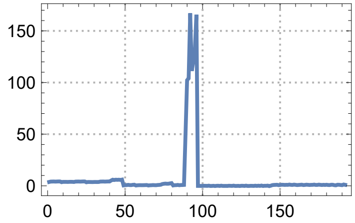

2D Plot

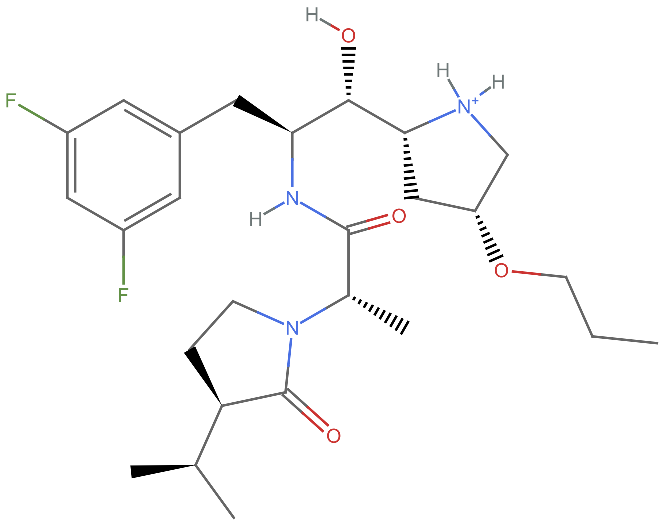

Dataset Index= 892

Molecule Formula= $C_{26}H_{40}F_2N_3O_4^+$

3D Autocorrelation

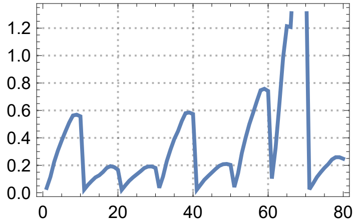

3D Plot

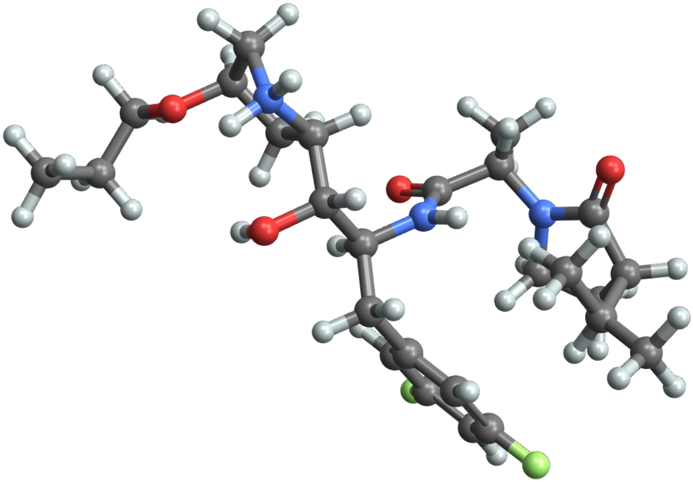

2D Plot

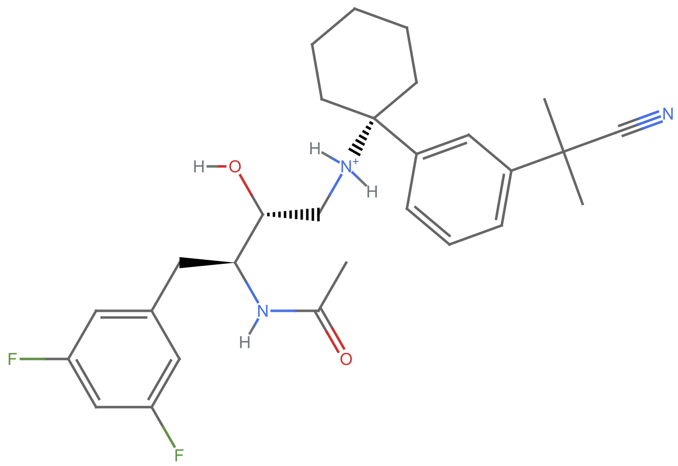

2D Autocorrelation

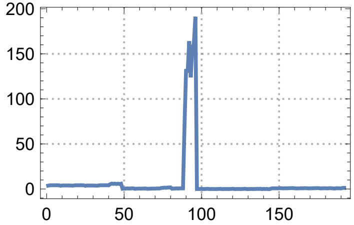

3D Plot

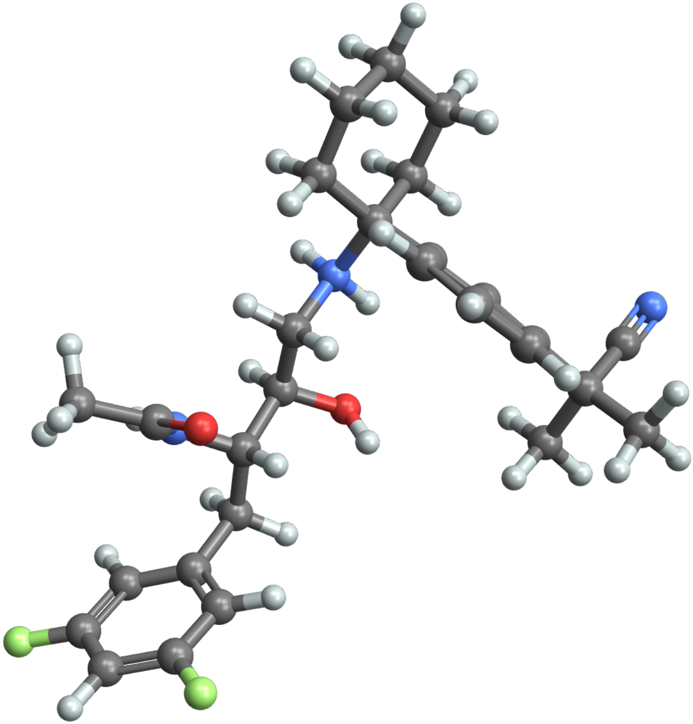

Dataset Index= 893

Molecule Formula= $C_{28}H_{36}F_2N_3O_2^+$

3D Autocorrelation

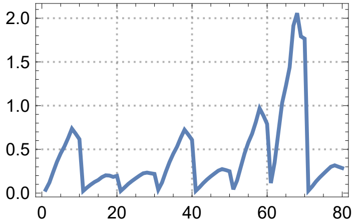

2D Autocorrelation

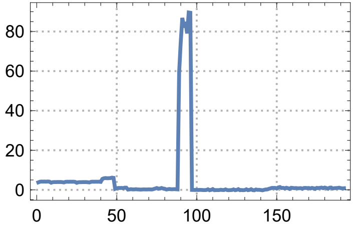

2D Plot

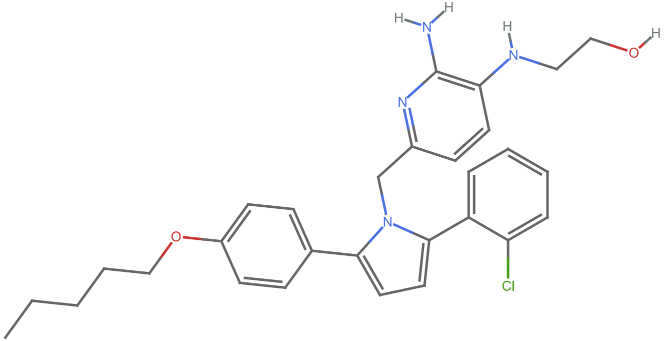

Dataset Index= 894

Molecule Formula= $C_{29}H_{33}ClN_4O_2$

3D Autocorrelation

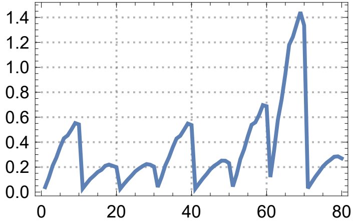

3D Plot

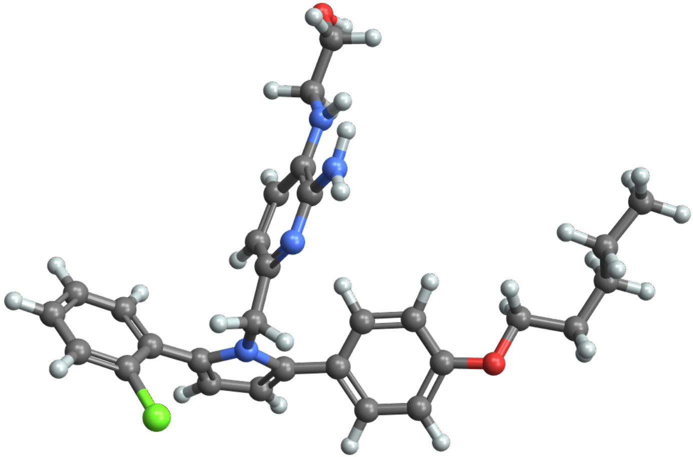

2D Plot

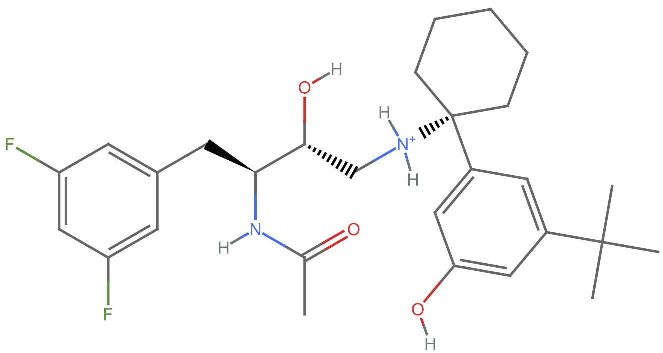

2D Autocorrelation

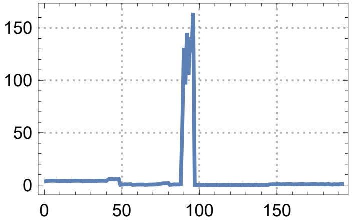

3D Plot

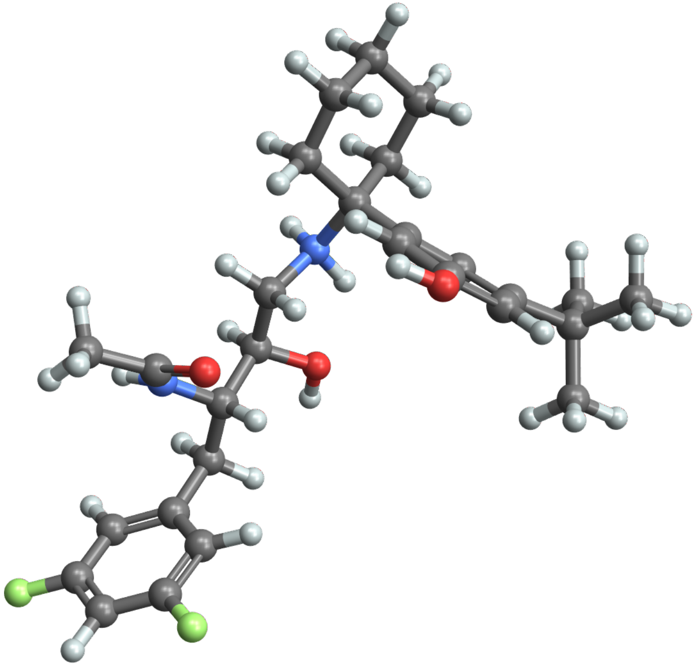

Dataset Index= 895

Molecule Formula= $C_{28}H_{39}F_2N_2O_3^+$

3D Autocorrelation

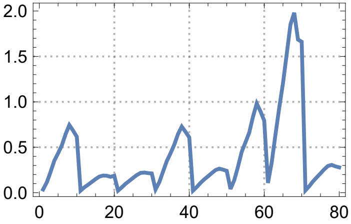

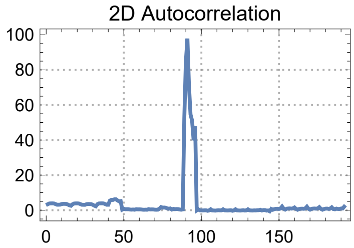

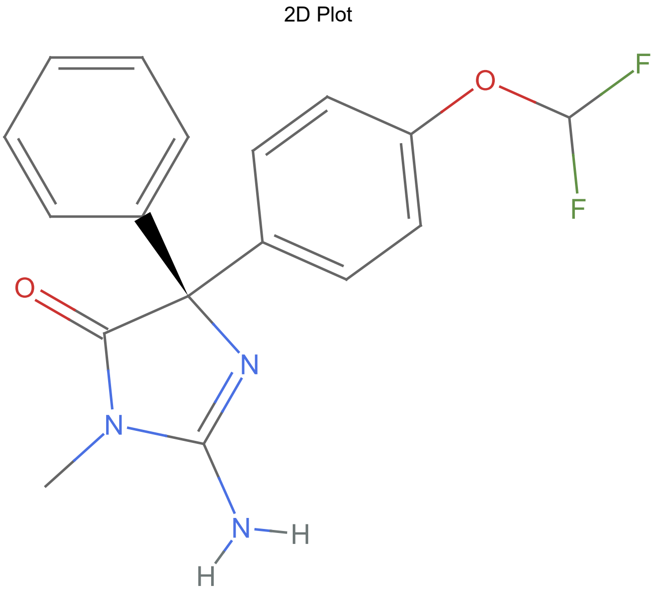

Dataset Index= 896

Molecule Formula= $C_{17}H_{15}F_2N_3O_2$

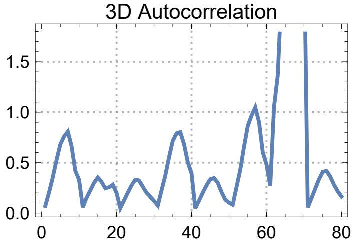

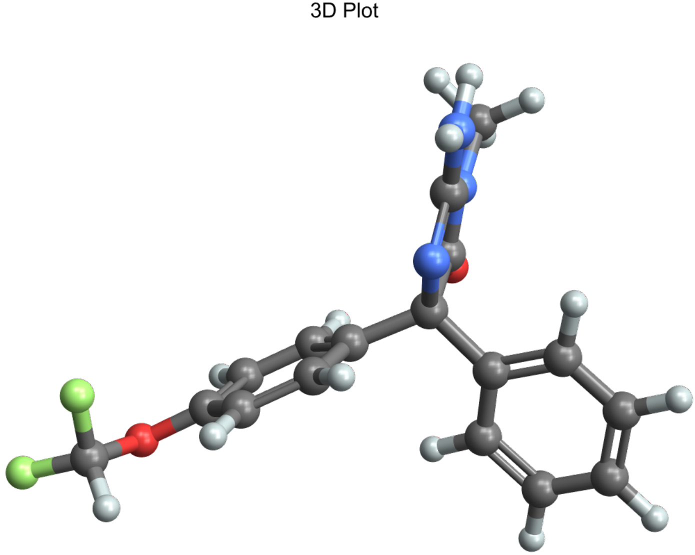

2D Plot

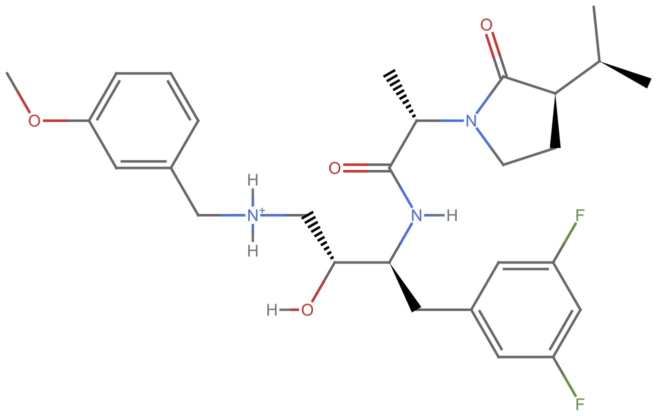

2D Autocorrelation

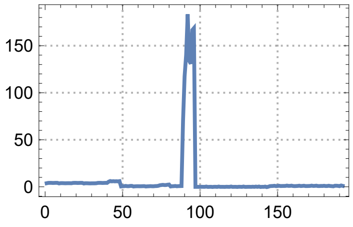

Dataset Index= 897

Molecule Formula= $C_{28}H_{38}F_2N_3O_4^+$

3D Autocorrelation

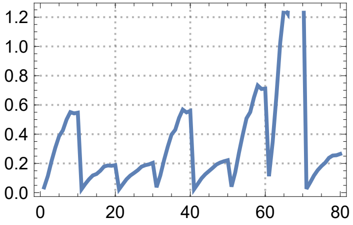

3D Plot

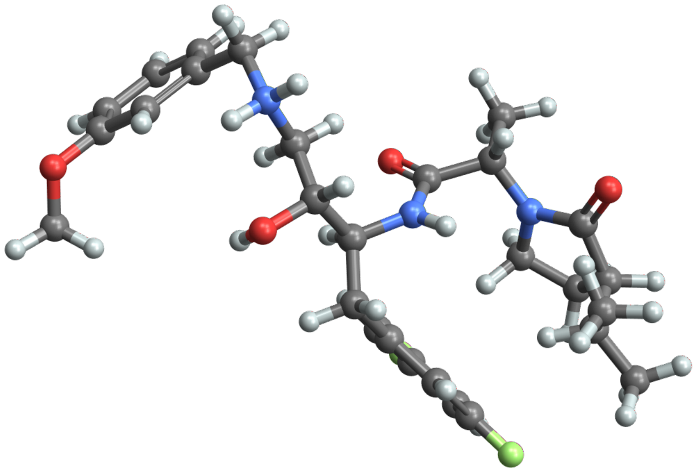

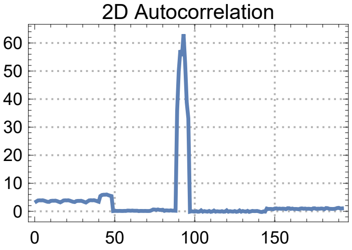

Dataset Index= 898

Molecule Formula= $C_{22}H_{20}N_4O$

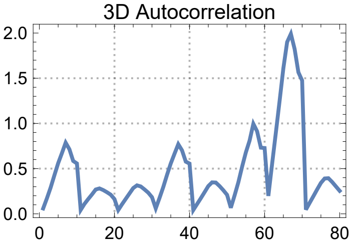

2D Plot

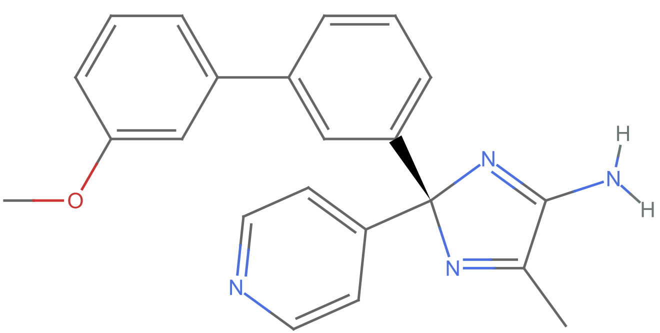

3D Plot

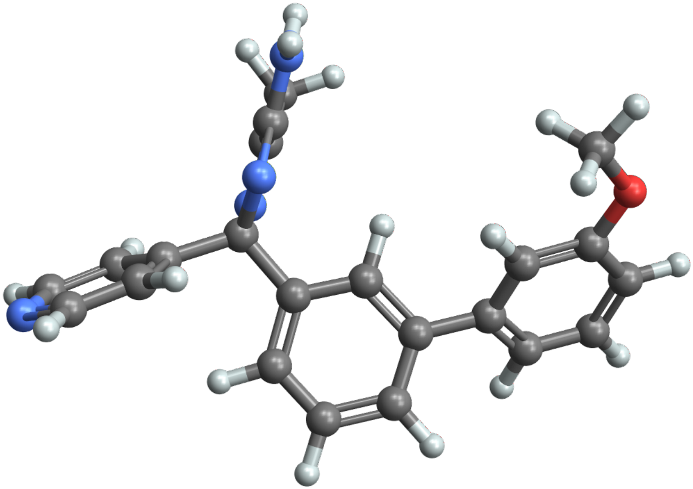

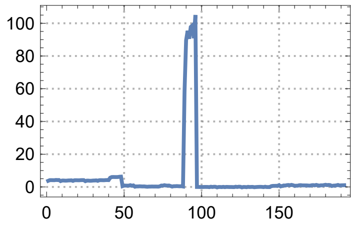

Dataset Index= 899

Molecule Formula= $C_{26}H_{24}ClN_5O$

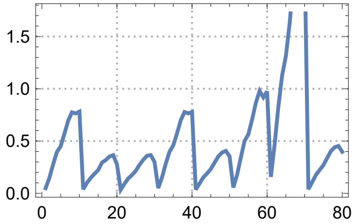

2D Plot

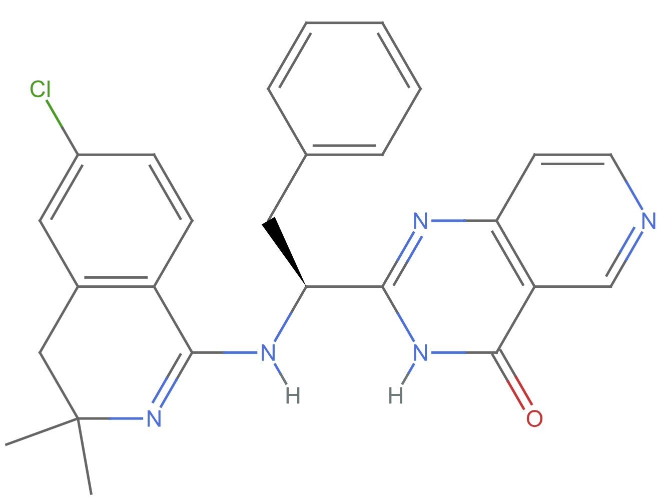

3D Plot

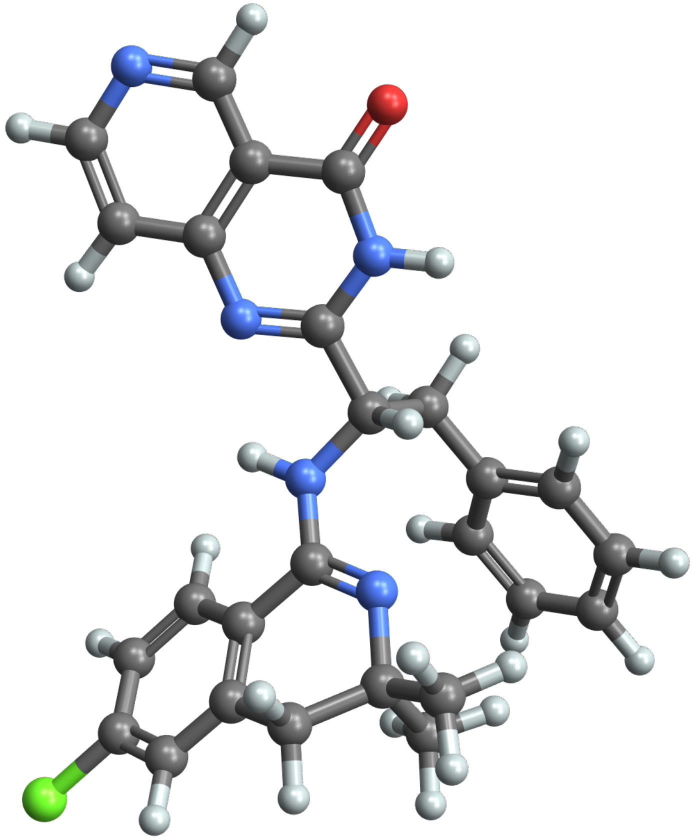

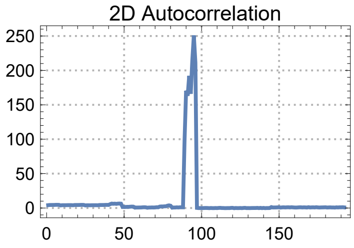

Dataset Index= 900

Molecule Formula= $C_{33}H_{43}F_2N_4O_6S^+$

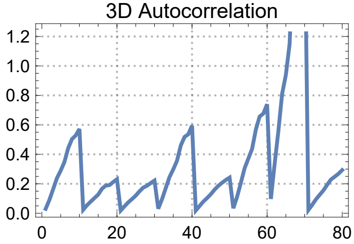

2D Plot

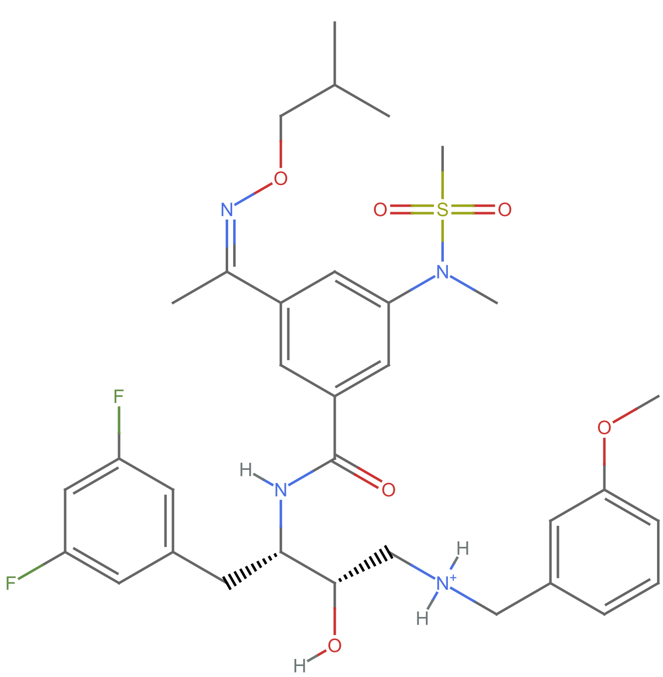

3D Plot

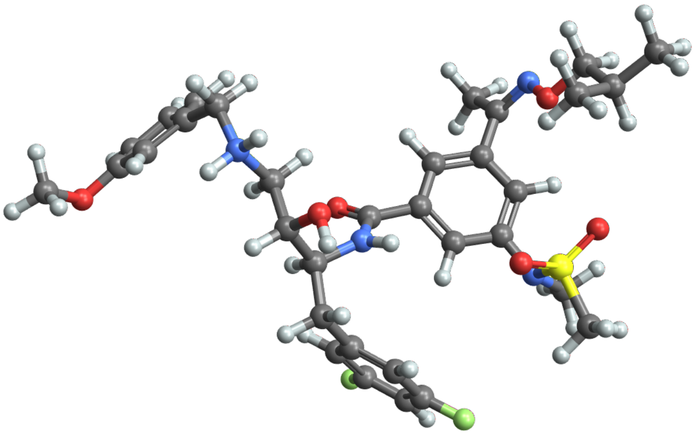

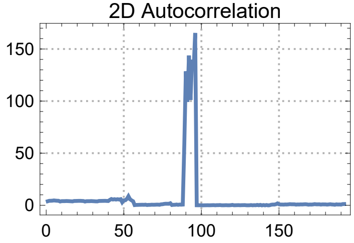

Dataset Index= 901

Molecule Formula= $C_{28}H_{38}BrF_2N_2O_2^+$

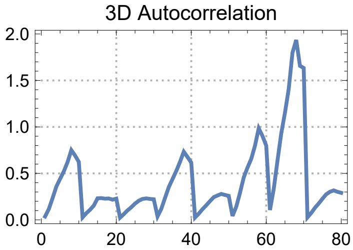

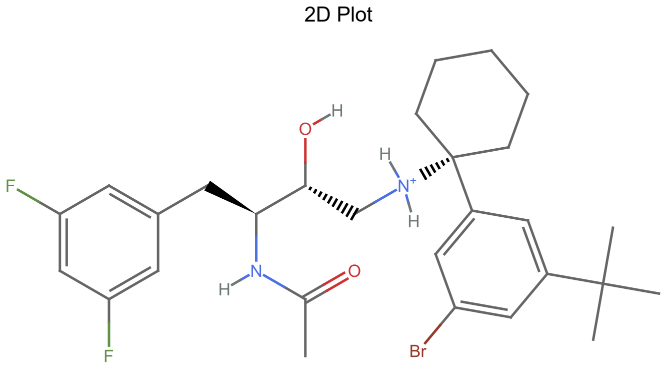

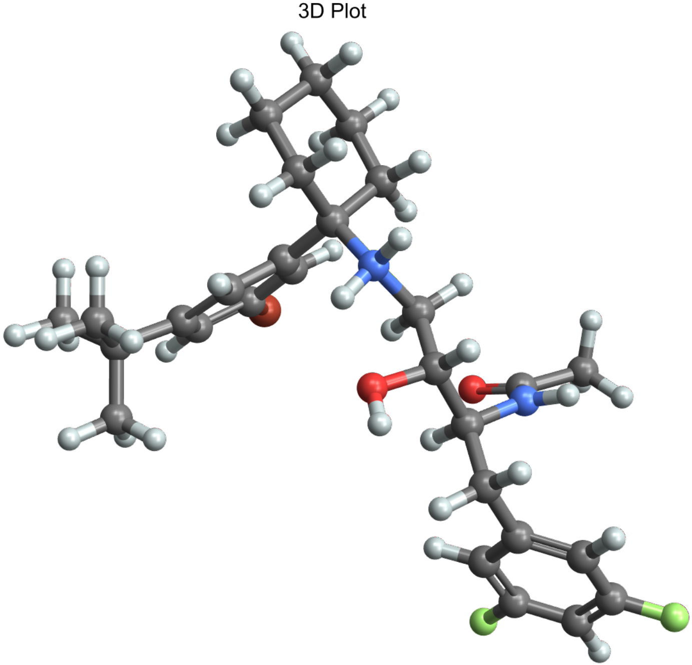

2D Plot

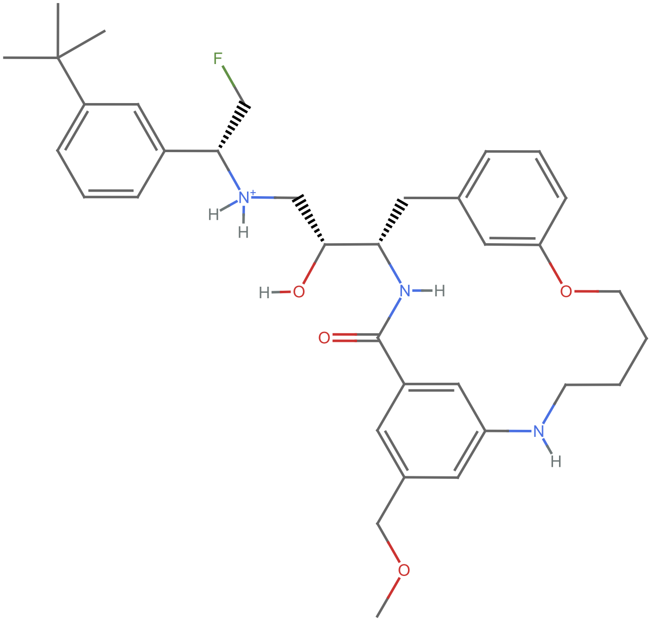

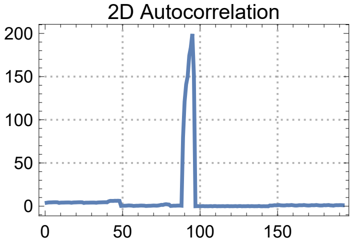

Dataset Index= 902

Molecule Formula= $C_{35}H_{47}FN_3O_4^+$

3D Plot

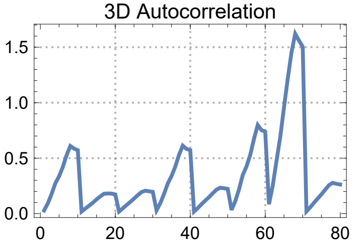

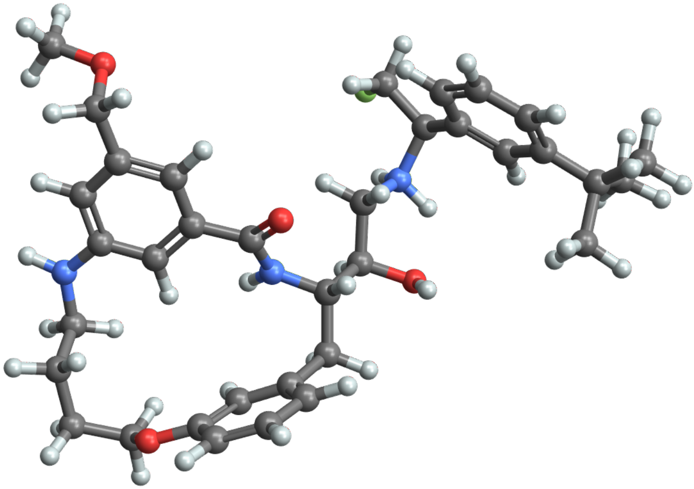

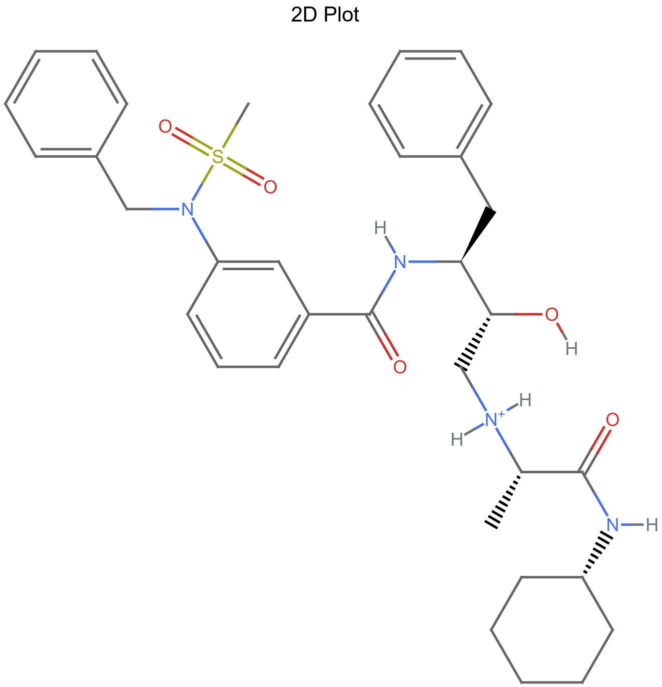

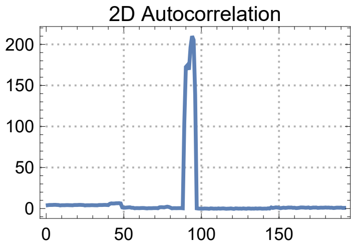

Dataset Index= 903

Molecule Formula= $C_{34}H_{45}N_4O_5S^+$

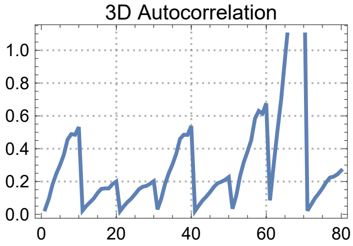

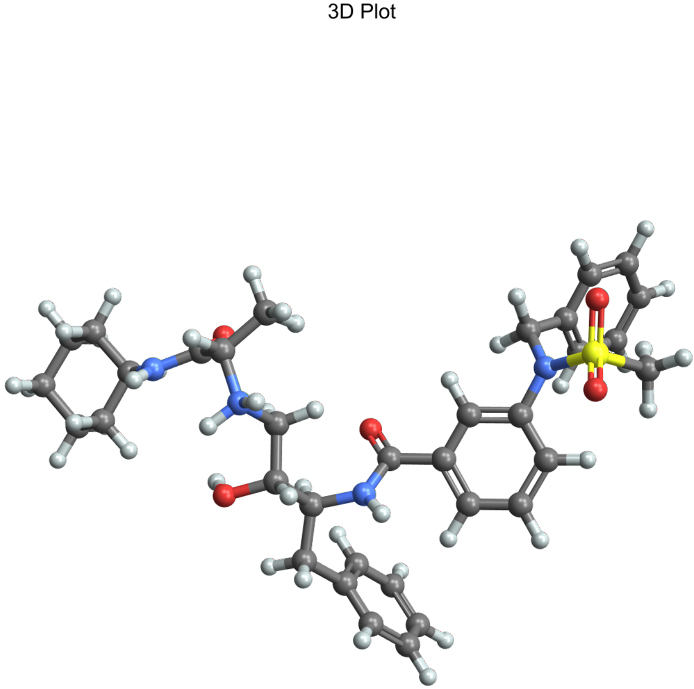

2D Plot

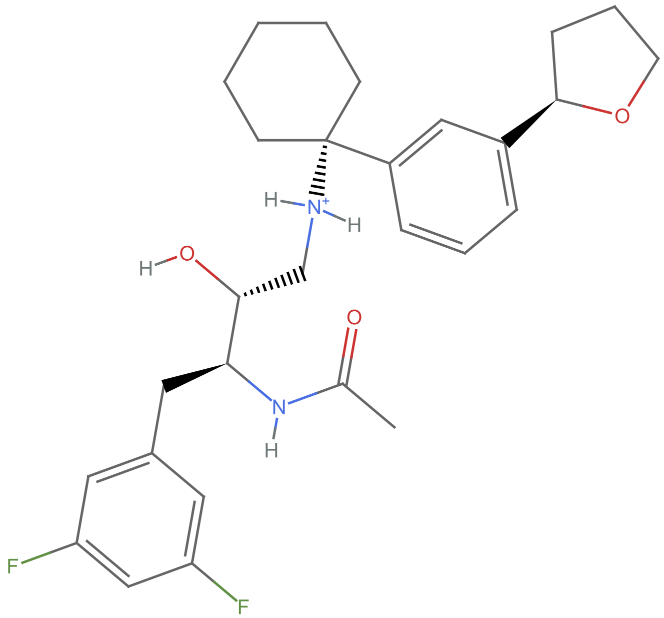

2D Autocorrelation

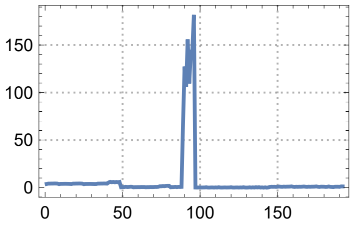

Dataset Index= 904

Molecule Formula= $C_{28}H_{37}F_2N_2O_3^+$

3D Plot

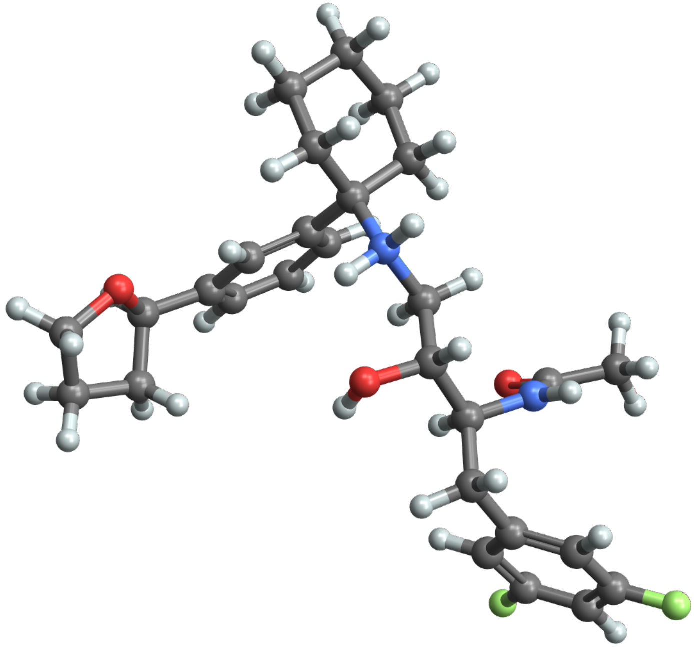

3D Autocorrelation

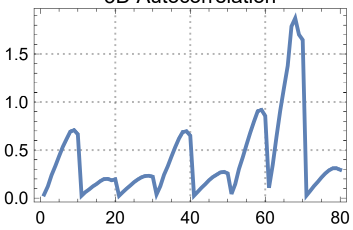

2D Autocorrelation

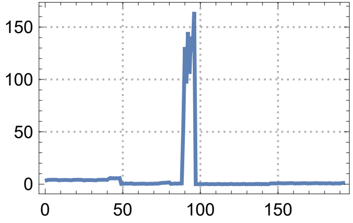

Dataset Index= 905

Molecule Formula= $C_{29}H_{41}F_2N_2O_2^+$

3D Autocorrelation

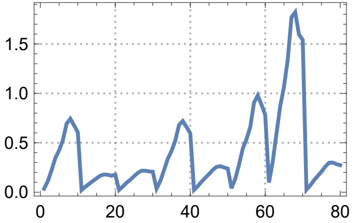

2D Plot

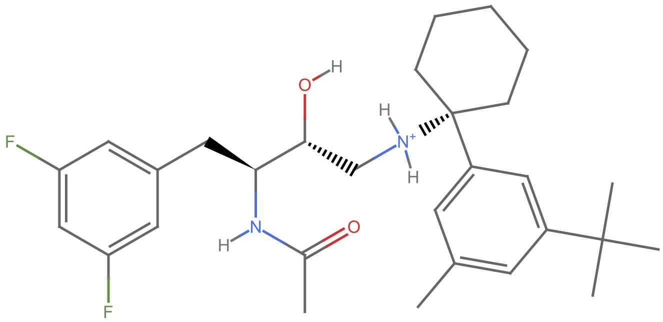

3D Plot

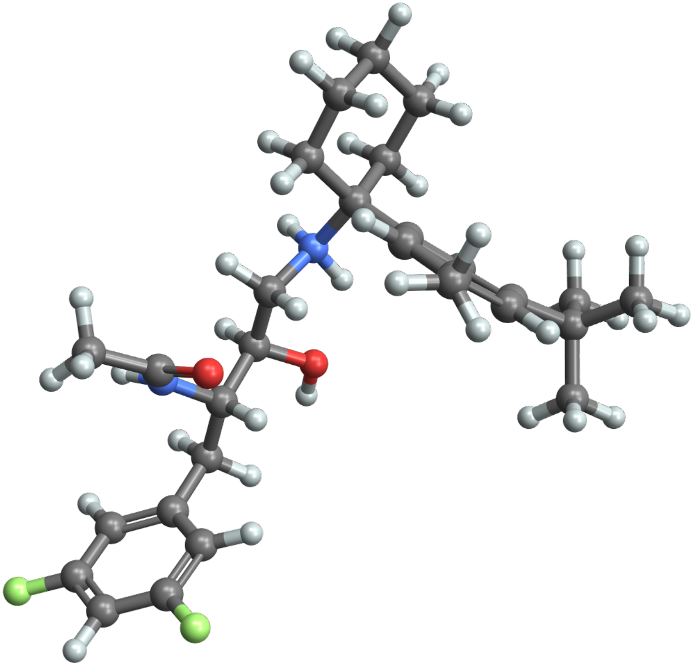

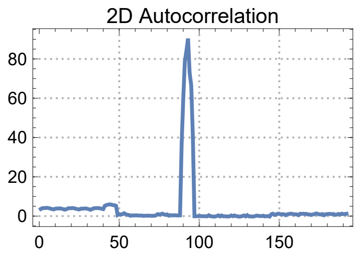

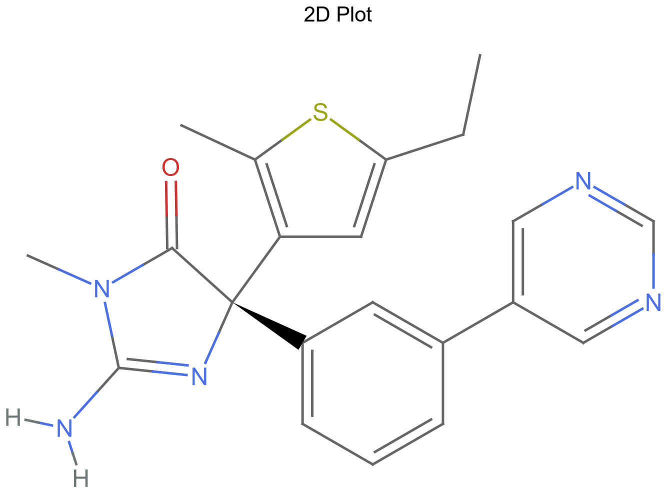

Dataset Index= 906

Molecule Formula= $C_{21}H_{21}N_5OS$

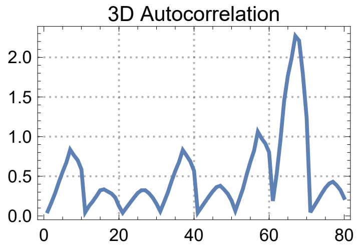

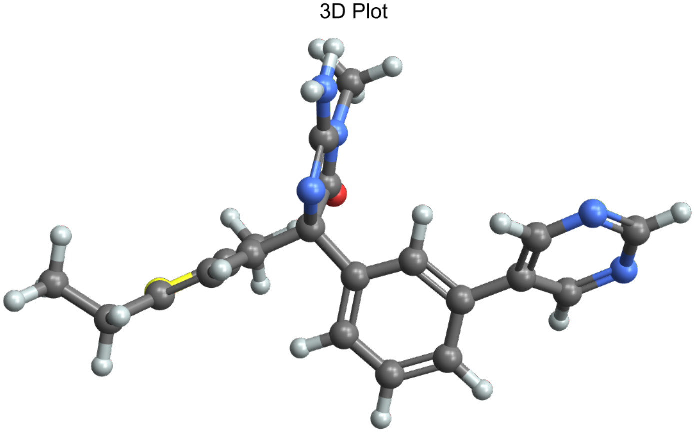

2D Plot

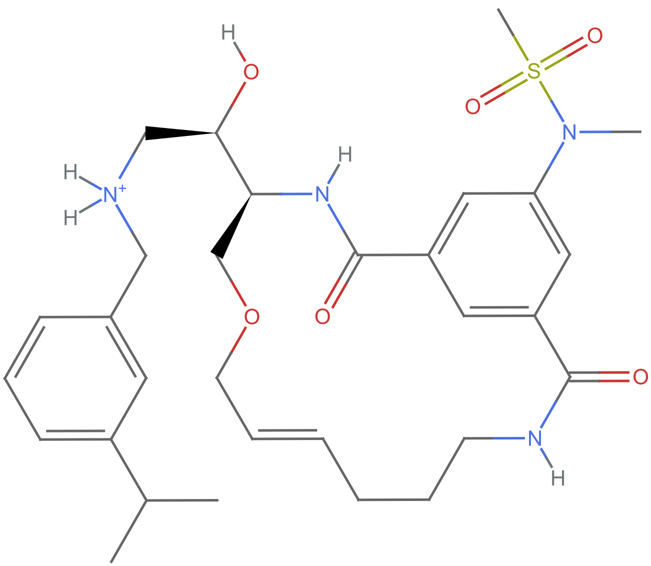

2D Autocorrelation

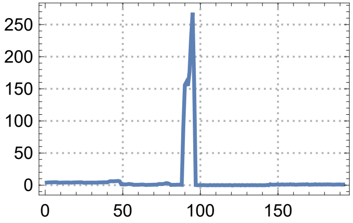

Dataset Index= 907

Molecule Formula= $C_{30}H_{43}N_4O_6S^+$

3D Autocorrelation

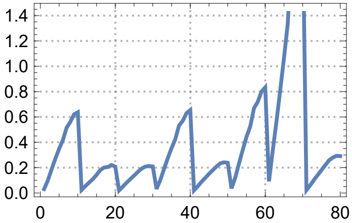

3D Plot

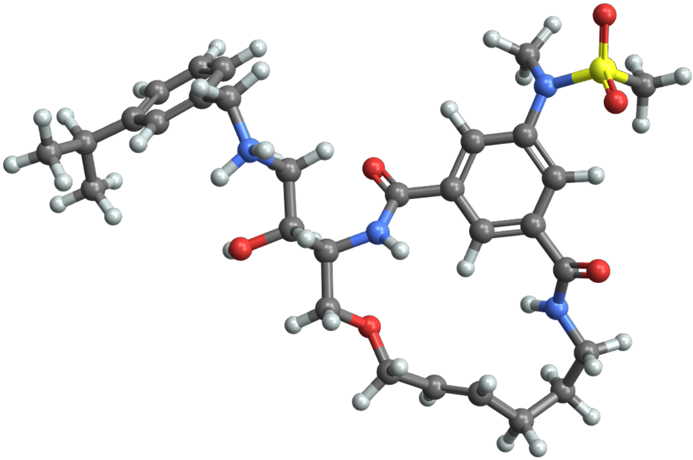

2D Plot

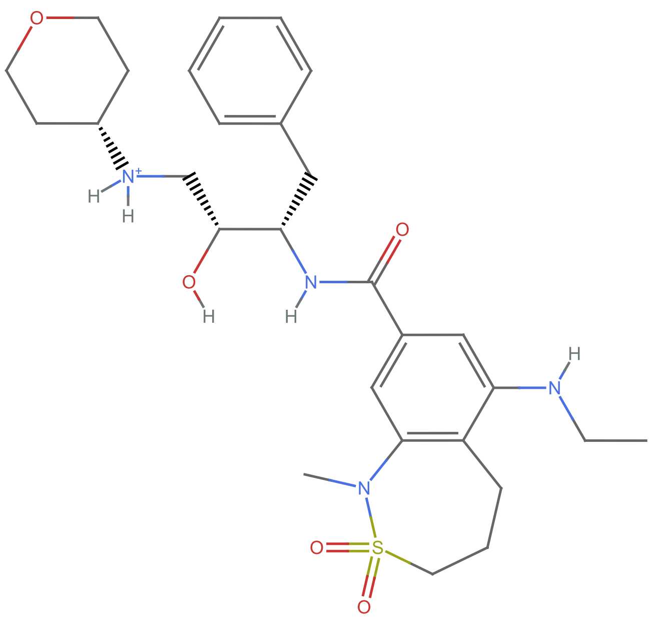

2D Autocorrelation

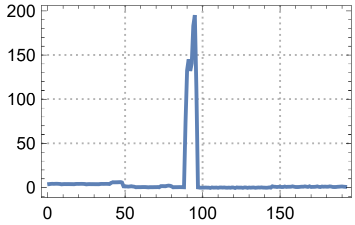

Dataset Index= 908

Molecule Formula= $C_{28}H_{41}N_4O_5S^+$

3D Plot

3D Autocorrelation

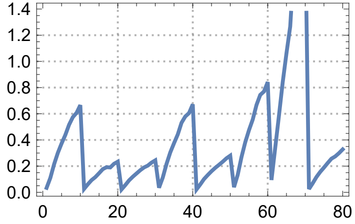

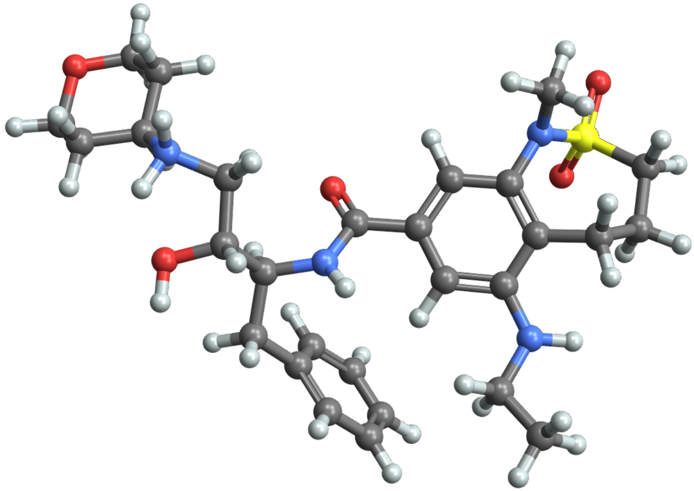

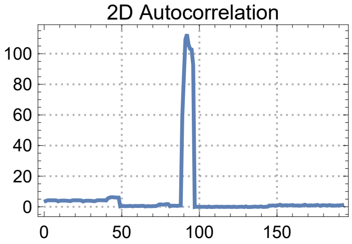

Dataset Index= 909

Molecule Formula= $C_{25}H_{23}FN_5O_2^+$

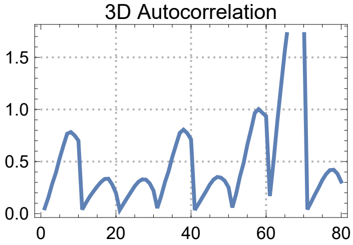

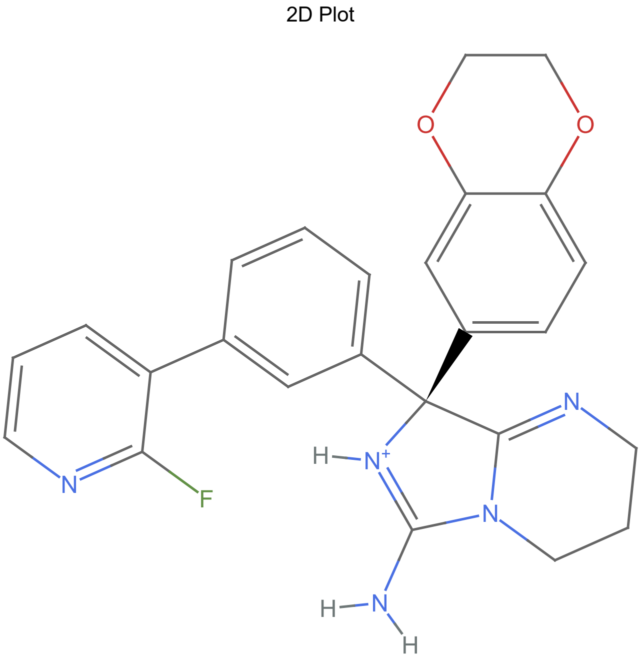

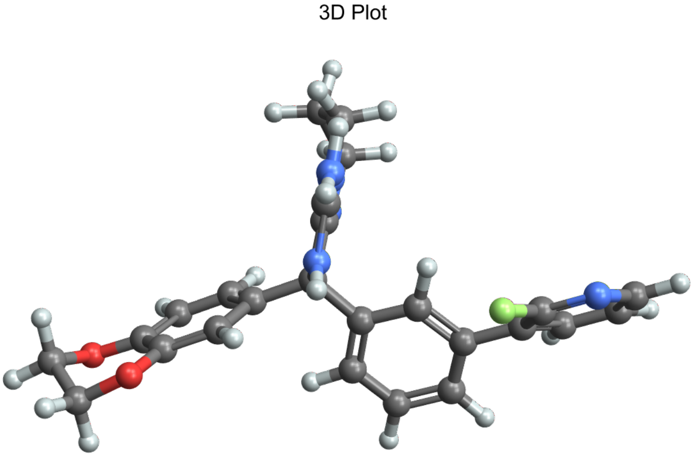

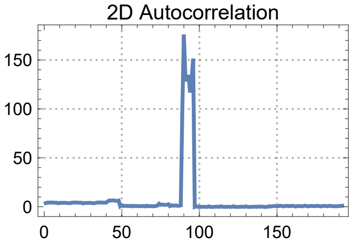

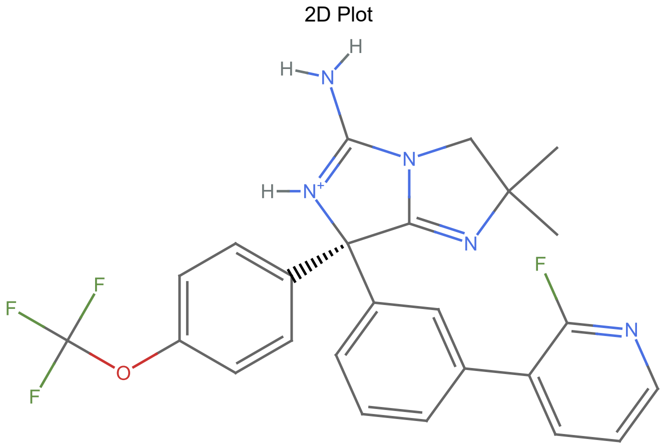

Dataset Index= 910

Molecule Formula= $C_{25}H_{22}F_4N_5O^+$

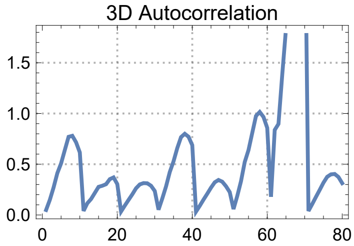

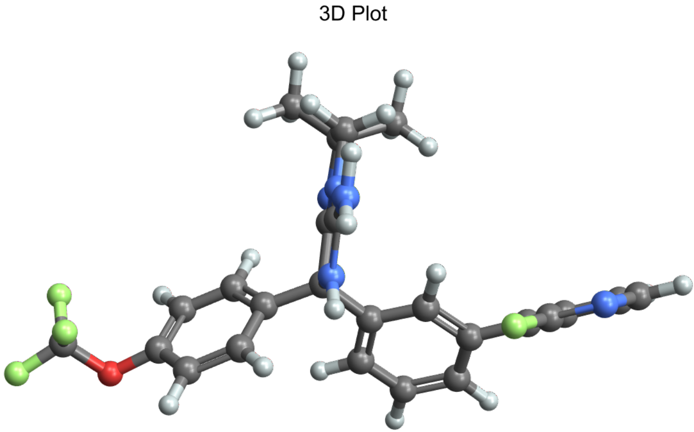

2D Plot

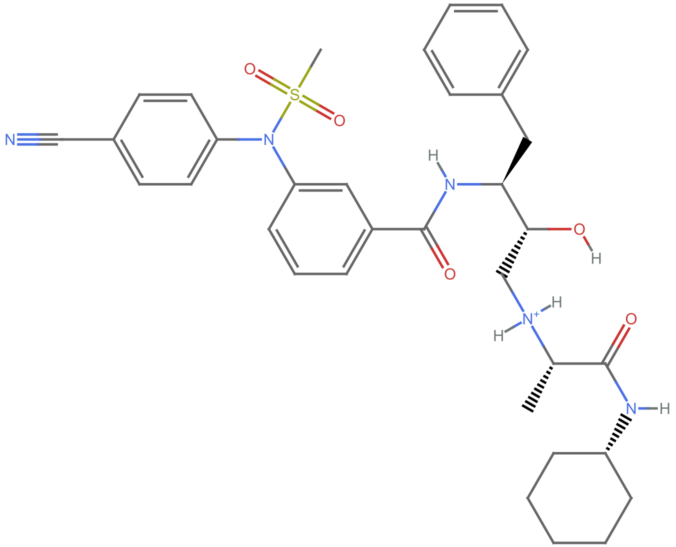

2D Autocorrelation

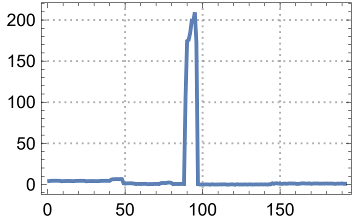

Dataset Index= 911

Molecule Formula= $C_{34}H_{42}N_5O_5S^+$

3D Plot

3D Autocorrelation

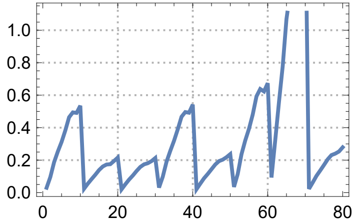

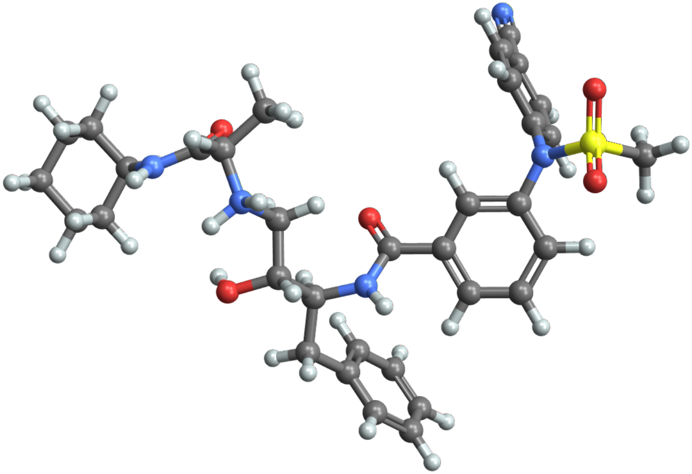

2D Plot

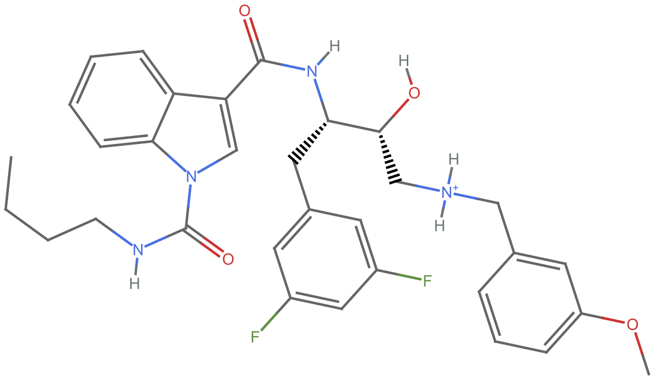

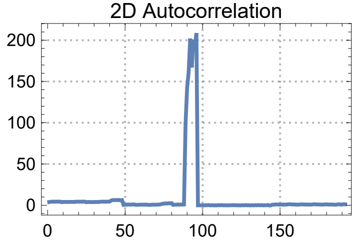

Dataset Index= 912

Molecule Formula= $C_{32}H_{37}F_2N_4O_4^+$

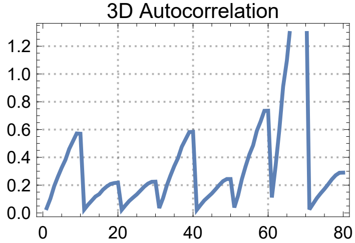

3D Plot

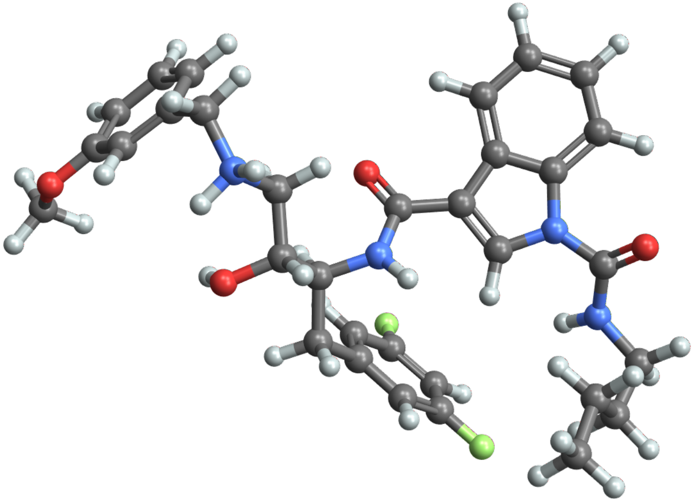

2D Autocorrelation

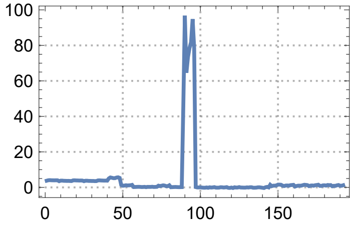

Dataset Index= 913

Molecule Formula= $C_{25}H_{36}NO_4S^+$

3D Autocorrelation

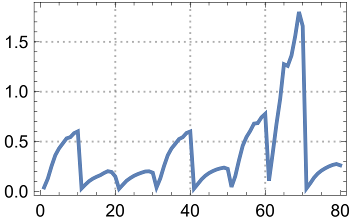

2D Plot

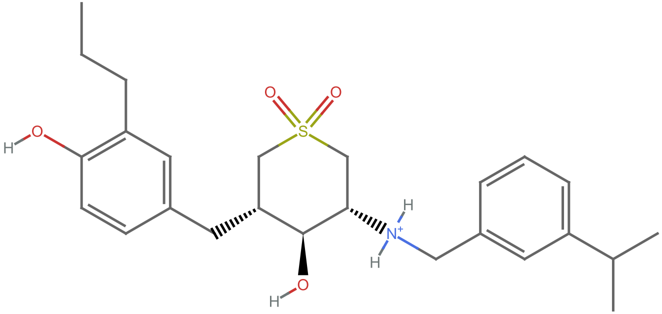

3D Plot

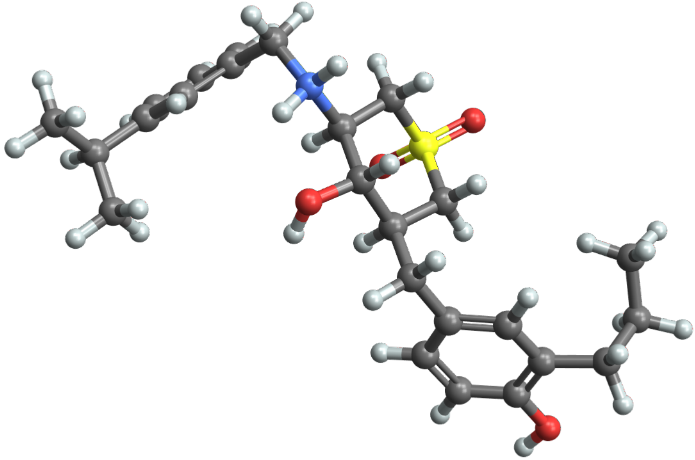

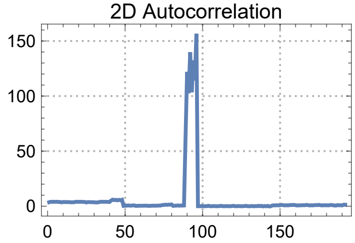

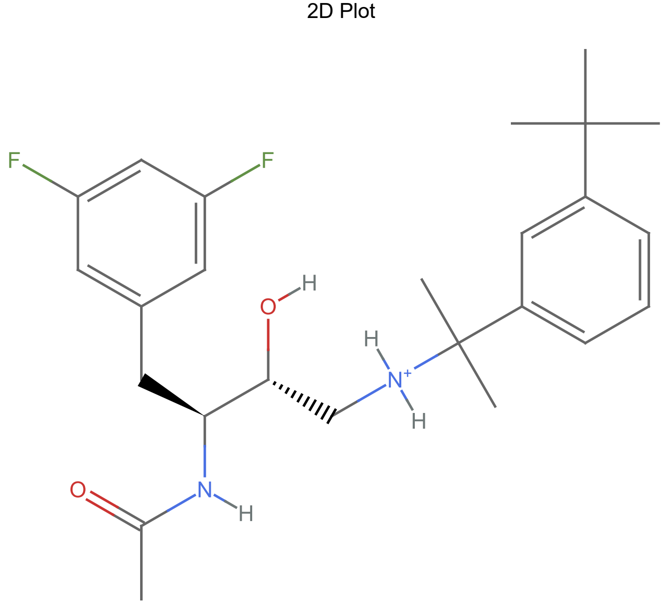

Dataset Index= 914

Molecule Formula= $C_{25}H_{35}F_2N_2O_2^+$

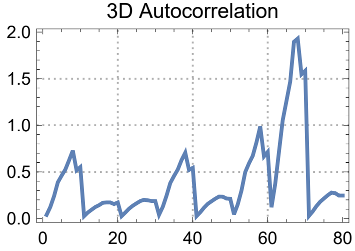

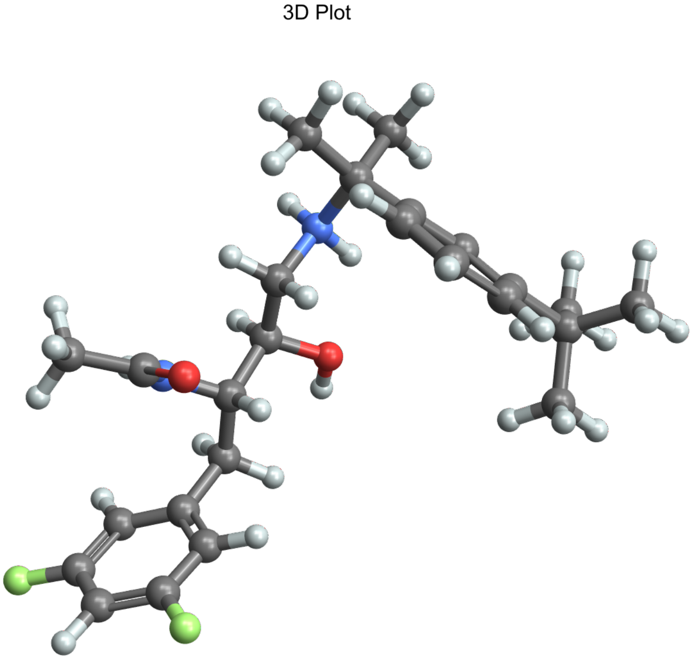

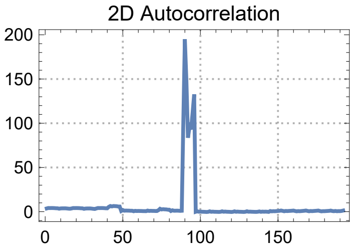

Dataset Index= 915

Molecule Formula= $C_{20}H_{18}F_5N_3O_3$

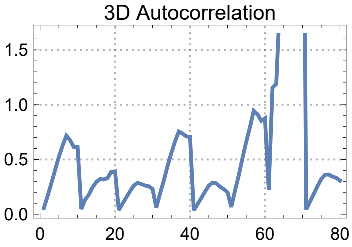

2D Plot

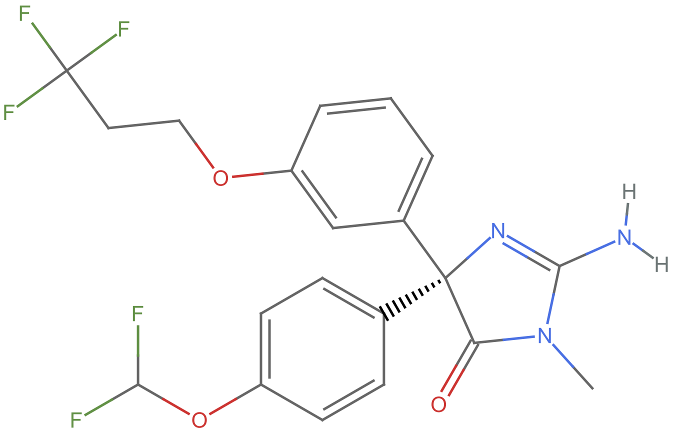

3D Plot

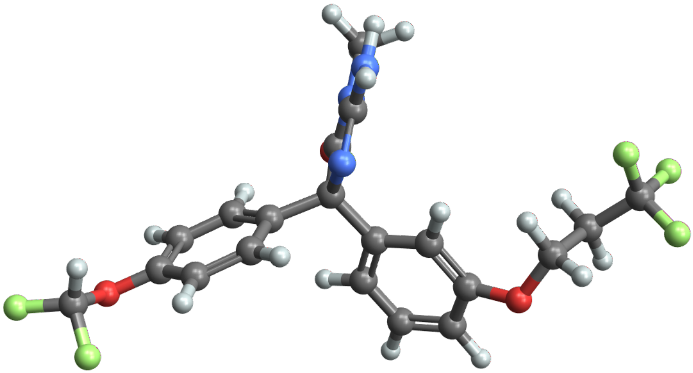

2D Autocorrelation

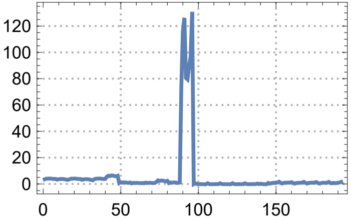

Dataset Index= 916

Molecule Formula= $C_{18}H_{15}F_4N_3O_3$

3D Autocorrelation

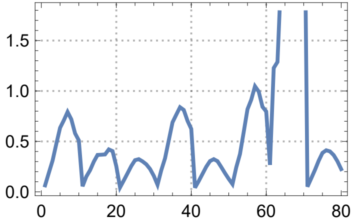

2D Plot

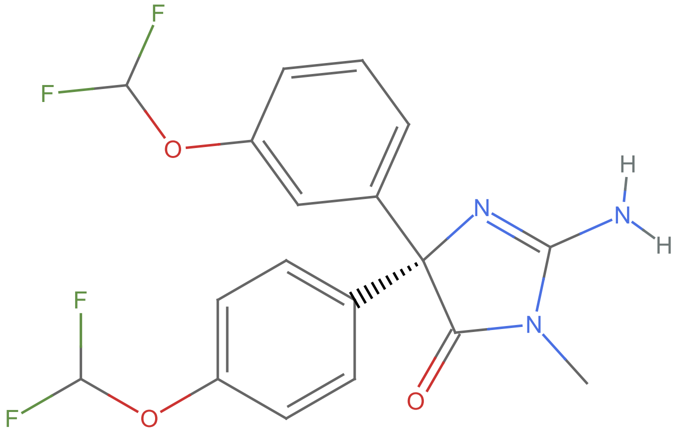

3D Plot

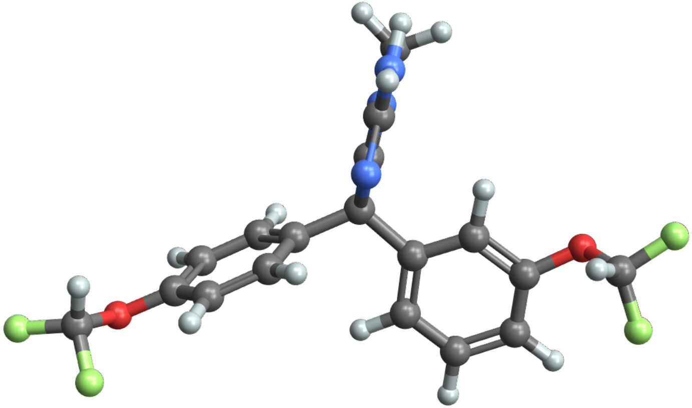

2D Plot

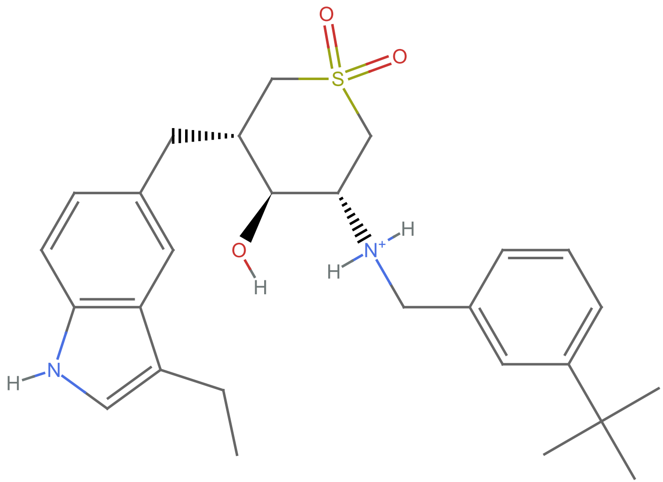

2D Autocorrelation

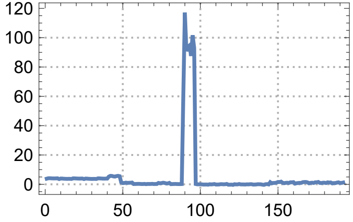

Dataset Index= 917

Molecule Formula= $C_{27}H_{37}N_2O_3S^+$

3D Plot

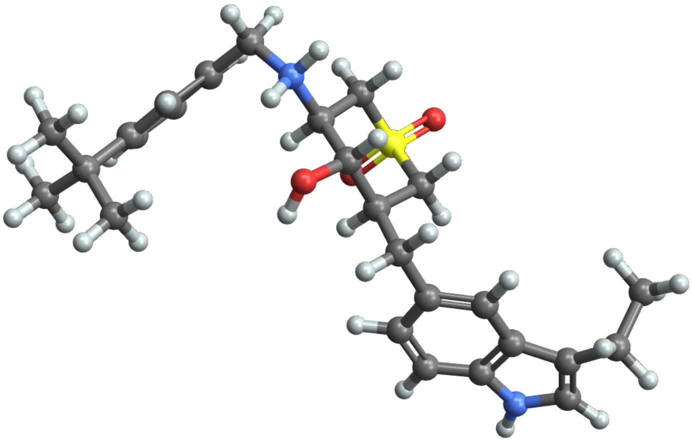

3D Autocorrelation

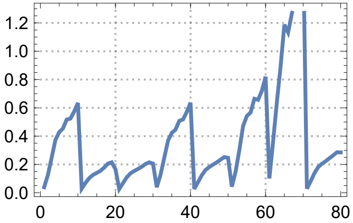

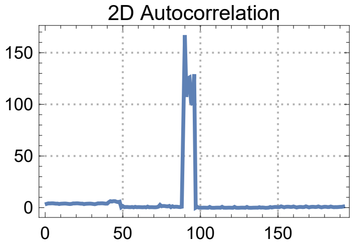

Dataset Index= 918

Molecule Formula= $C_{24}H_{26}F_3N_4O^+$

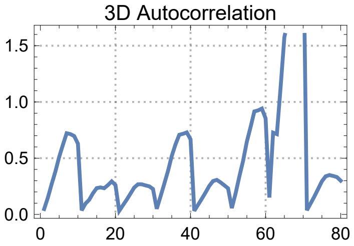

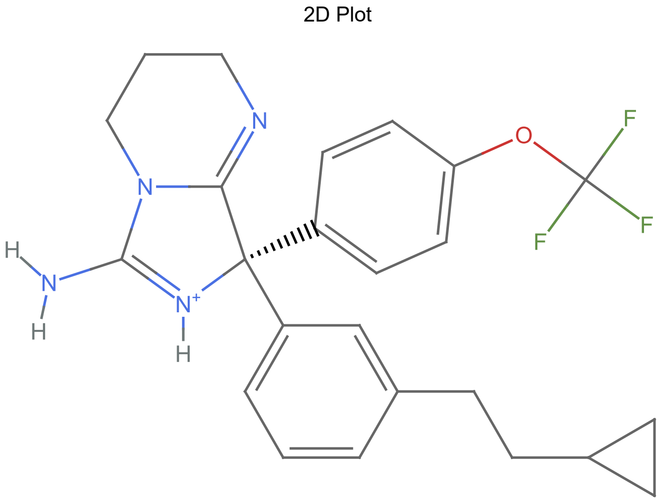

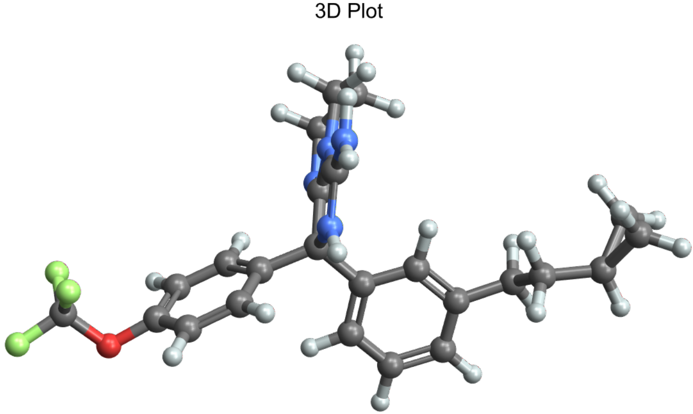

2D Autocorrelation

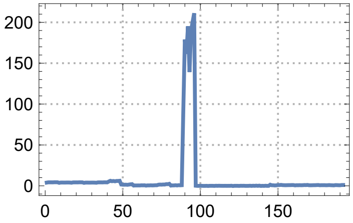

Dataset Index= 919

Molecule Formula= $C_{29}H_{41}F_2N_2O_4S^+$

3D Autocorrelation

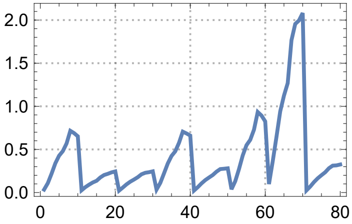

2D Plot

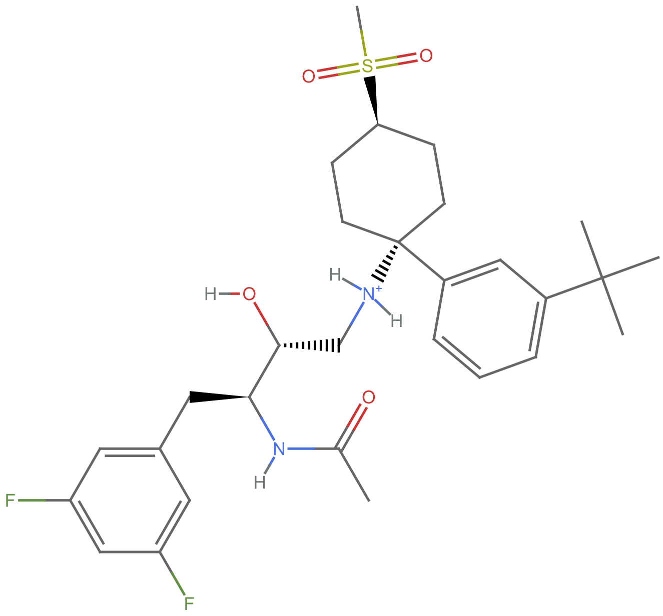

3D Plot

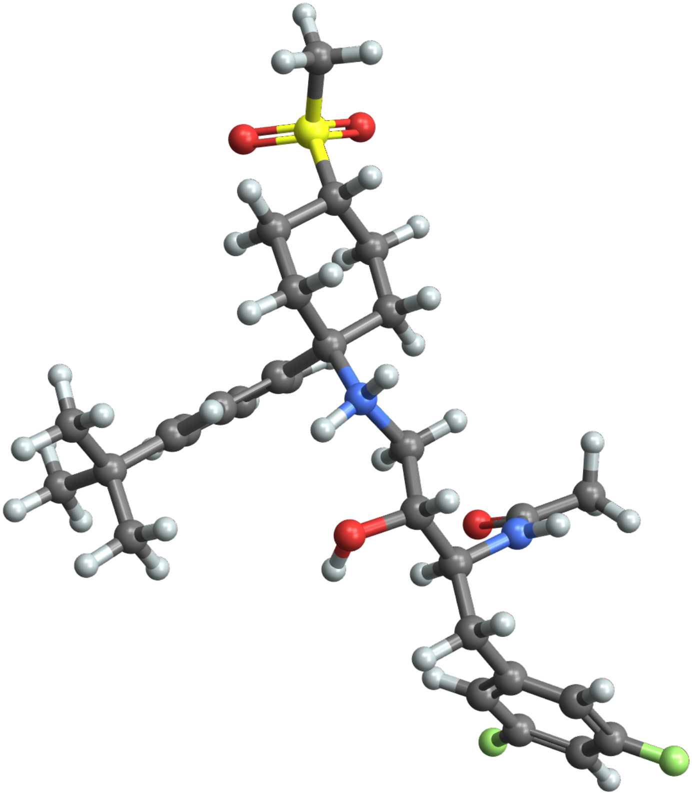

2D Plot

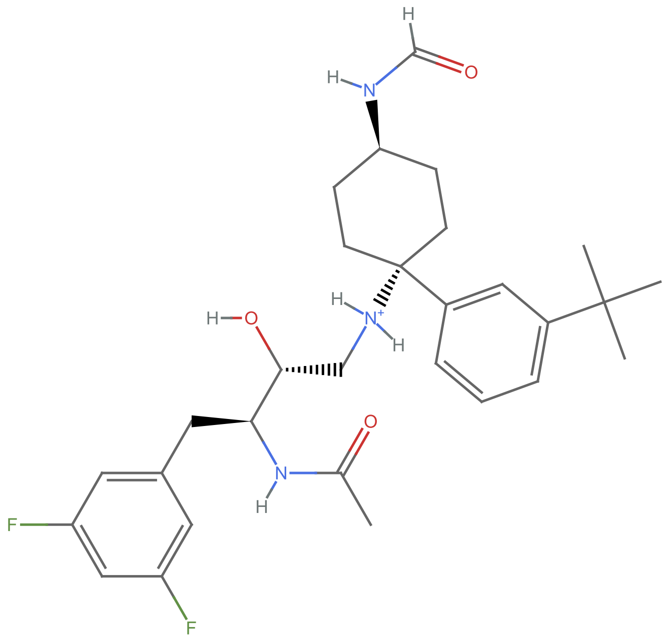

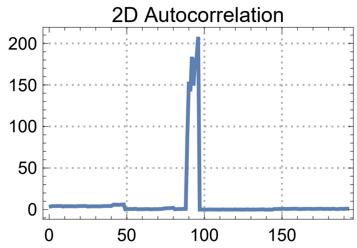

3D Plot

Dataset Index= 920

Molecule Formula= $C_{29}H_{40}F_2N_3O_3^+$

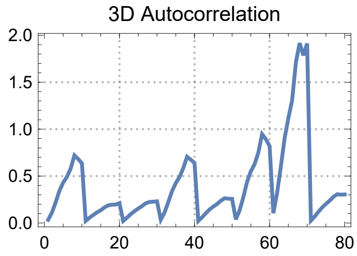

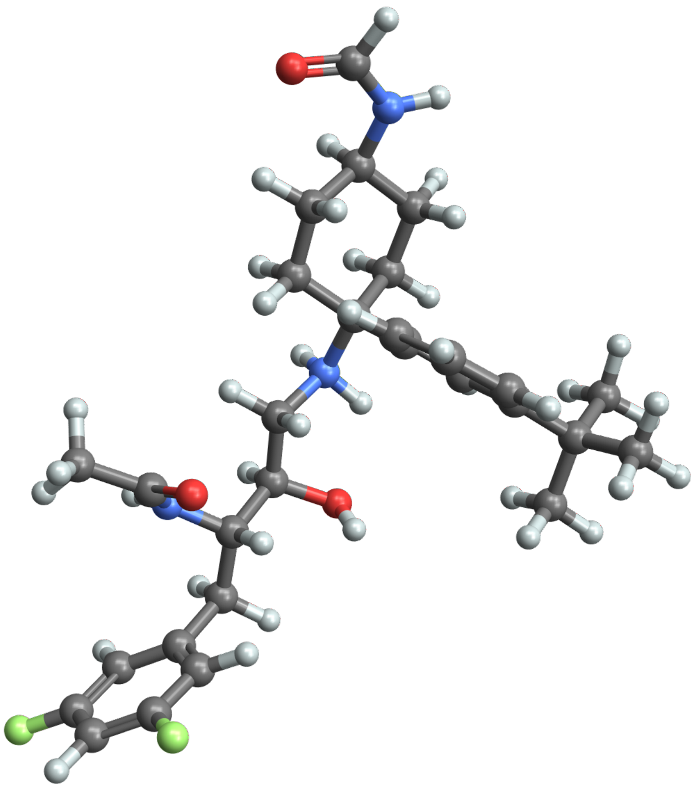

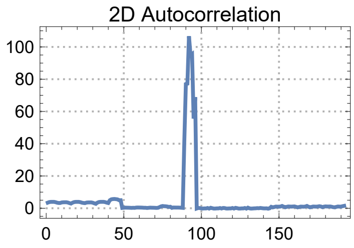

Dataset Index= 921

Molecule Formula= $C_{19}H_{26}N_4O_3$

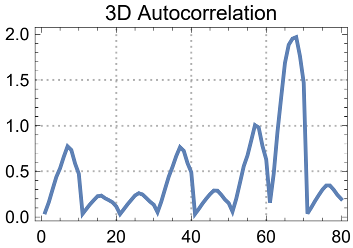

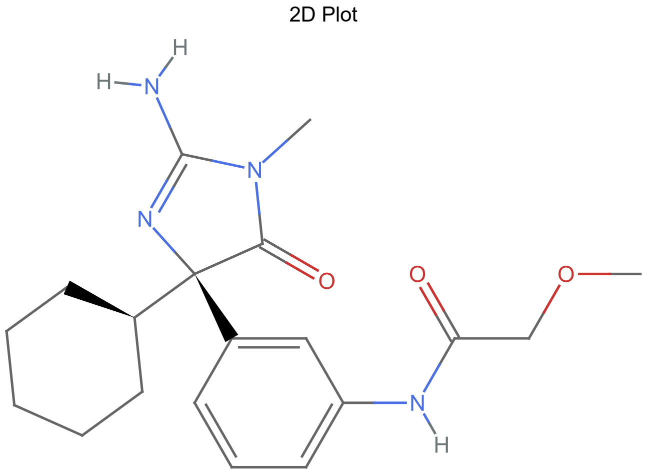

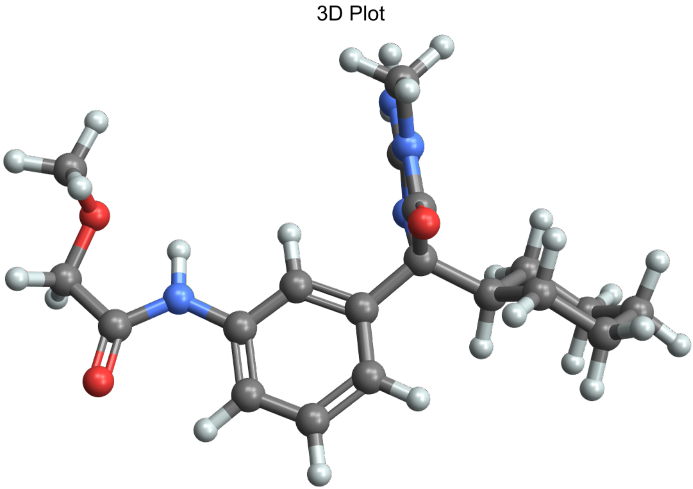

2D Plot

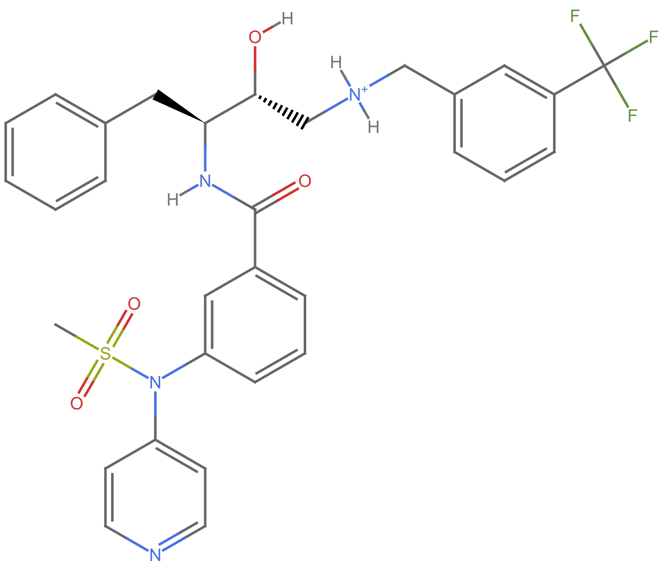

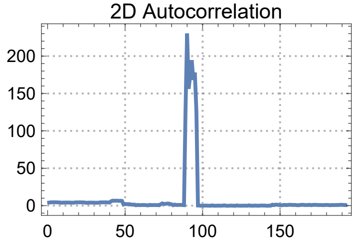

Dataset Index= 922

Molecule Formula= $C_{31}H_{32}F_3N_4O_4S^+$

3D Plot

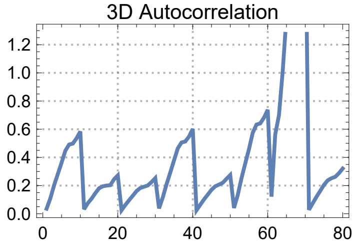

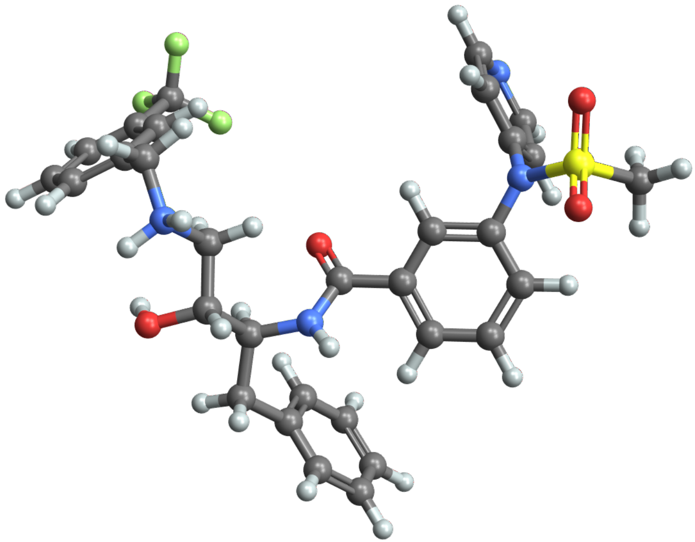

2D Plot

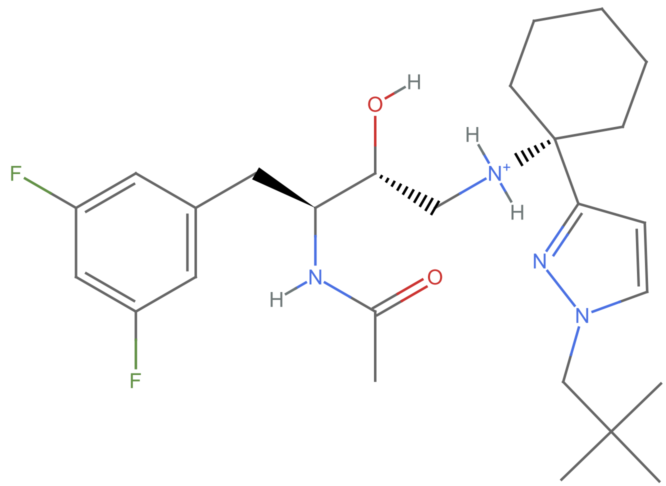

2D Autocorrelation

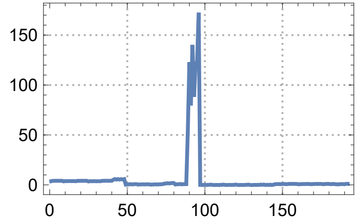

3D Plot

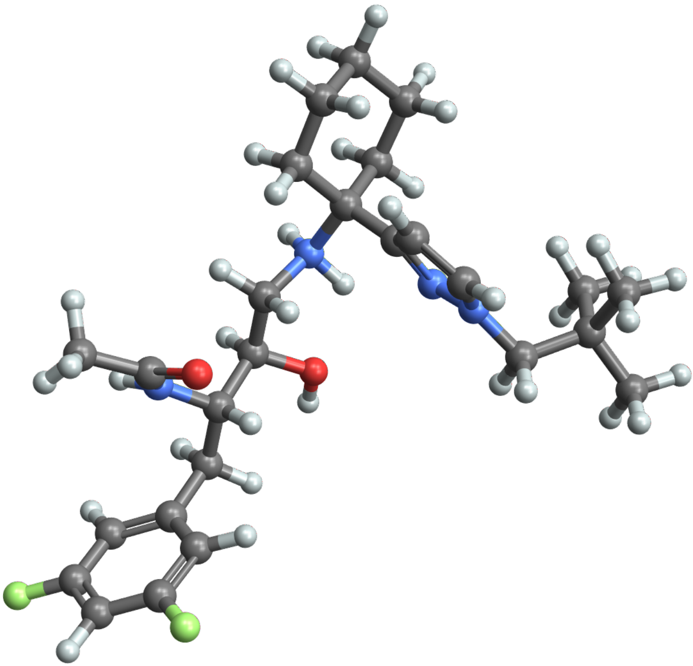

Dataset Index= 923

Molecule Formula= $C_{26}H_{39}F_2N_4O_2^+$

3D Autocorrelation

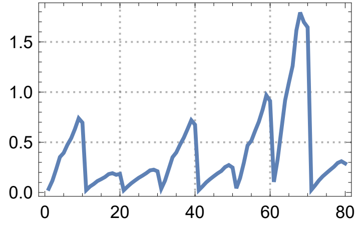

2D Plot

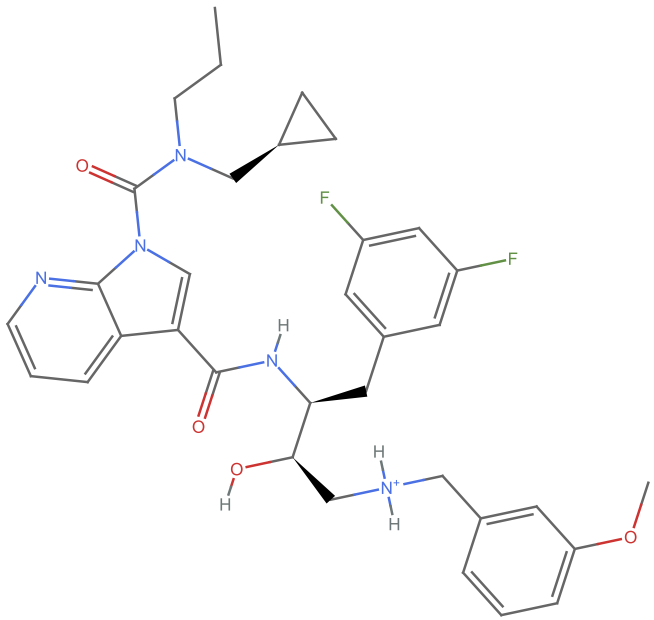

2D Autocorrelation

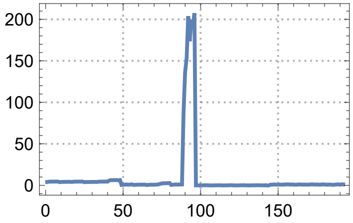


```
Dataset Index=      924

Molecule Formula=   C34H40F2N5O4+
```

3D Plot

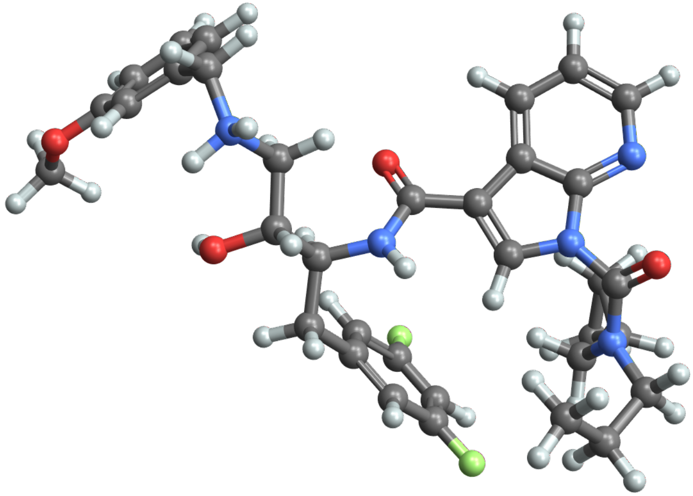

3D Autocorrelation

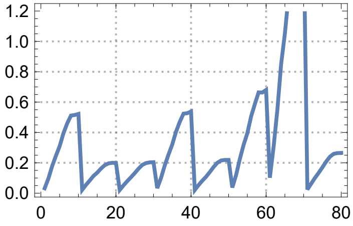

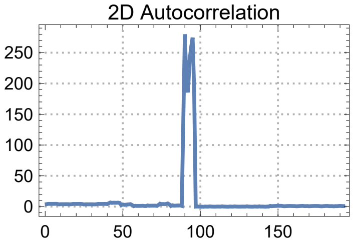

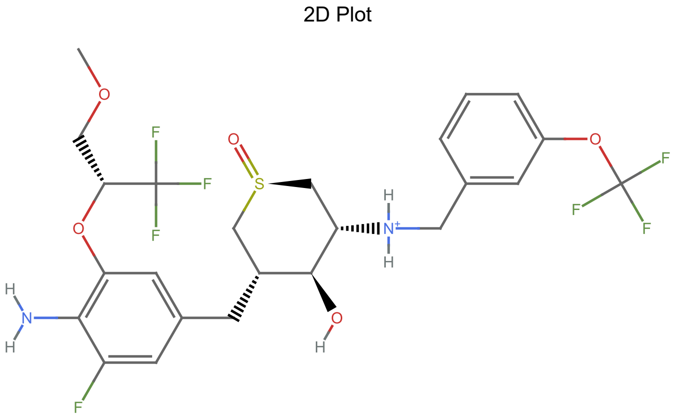

Dataset Index= 925

Molecule Formula= $C_{24}H_{28}F_7N_2O_5S^+$

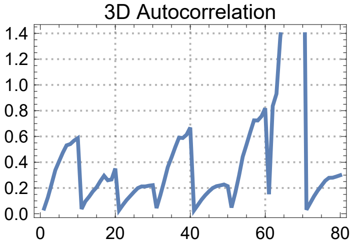

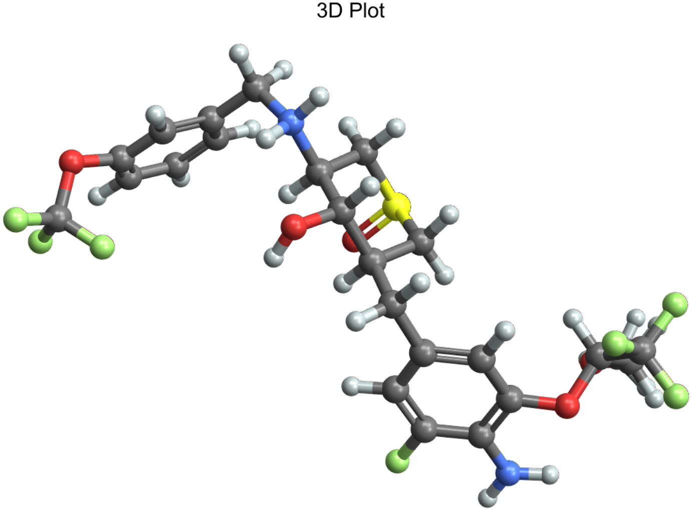

2D Plot

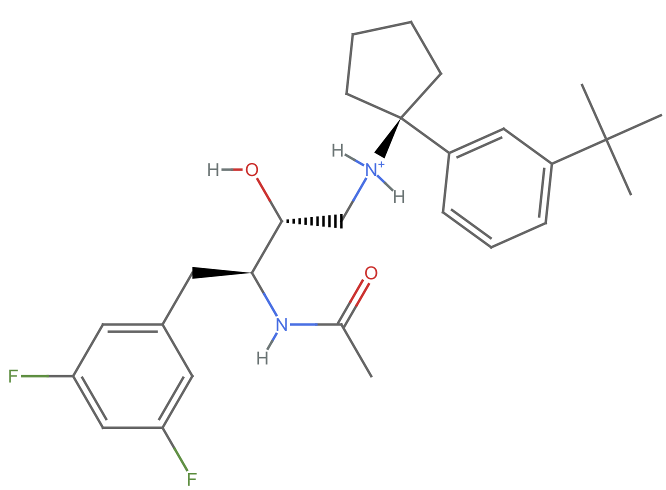

2D Autocorrelation

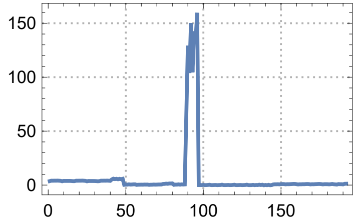

3D Plot

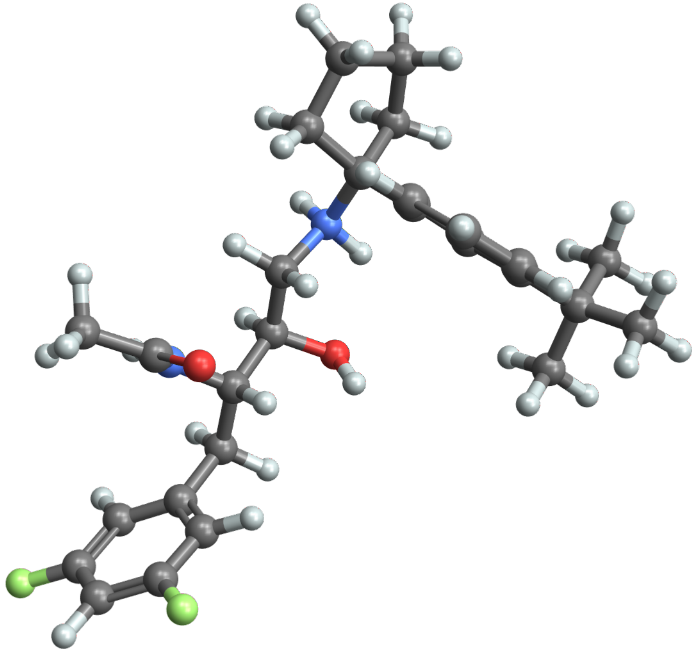

Dataset Index= 926

Molecule Formula= $C_{27}H_{37}F_2N_2O_2^+$

3D Autocorrelation

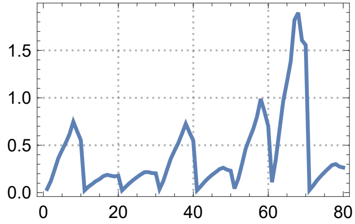

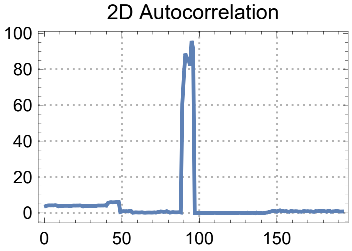

Dataset Index= 927

Molecule Formula= $C_{30}H_{35}ClN_4O_2$

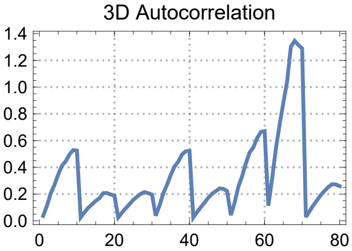

2D Plot

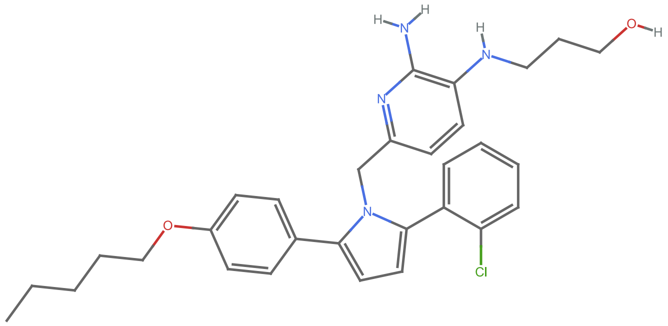

3D Plot

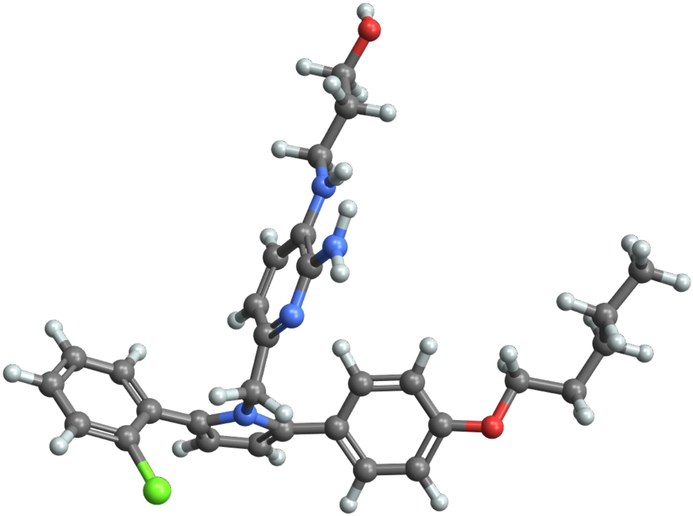

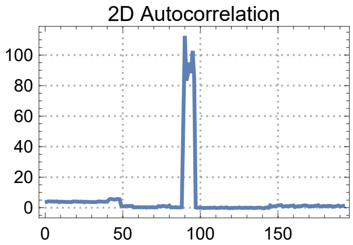

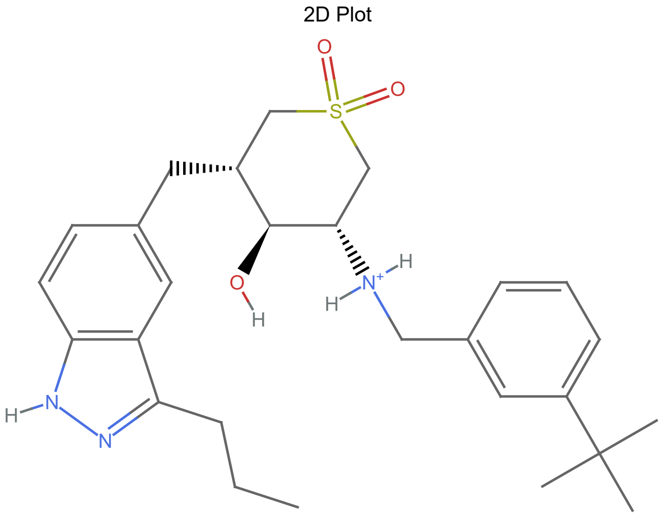

Dataset Index= 928

Molecule Formula= $C_{27}H_{38}N_3O_3S^+$

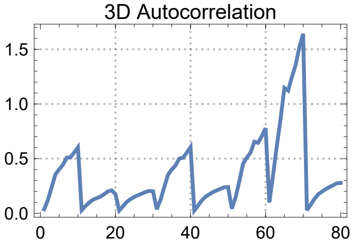

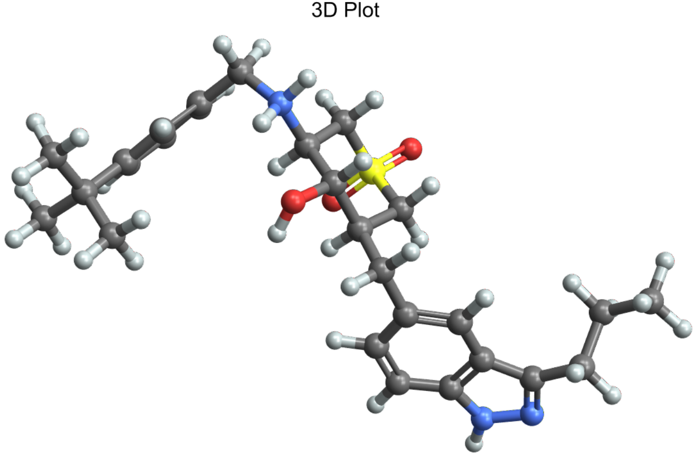

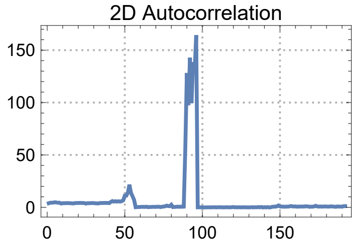

Dataset Index= 929

Molecule Formula= $C_{28}H_{38}F_2IN_2O_2^+$

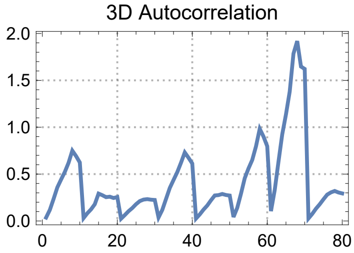

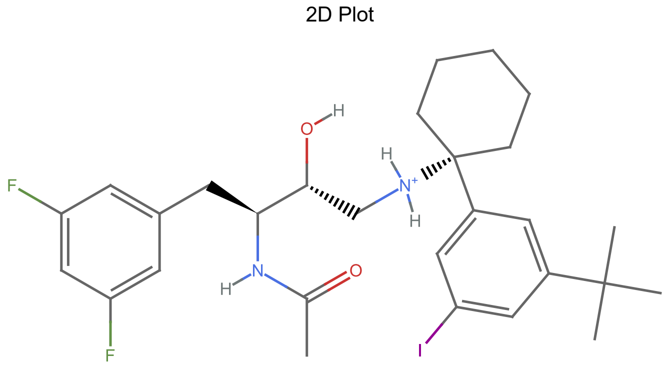

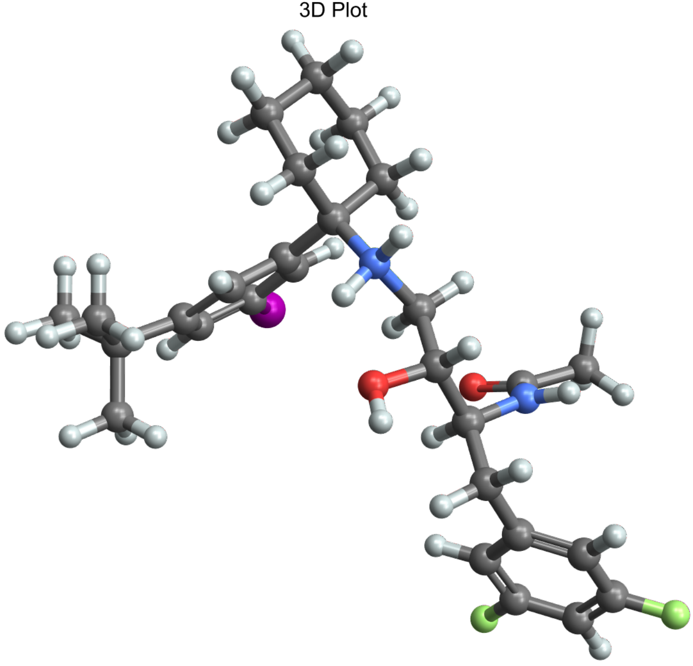

2D Plot

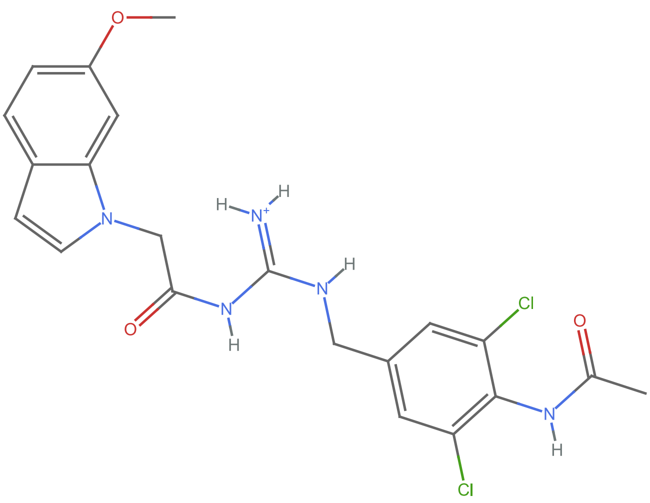

2D Autocorrelation

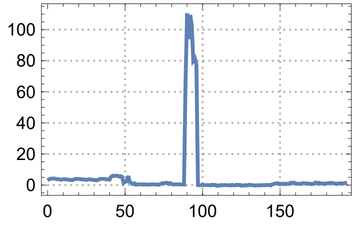

3D Plot

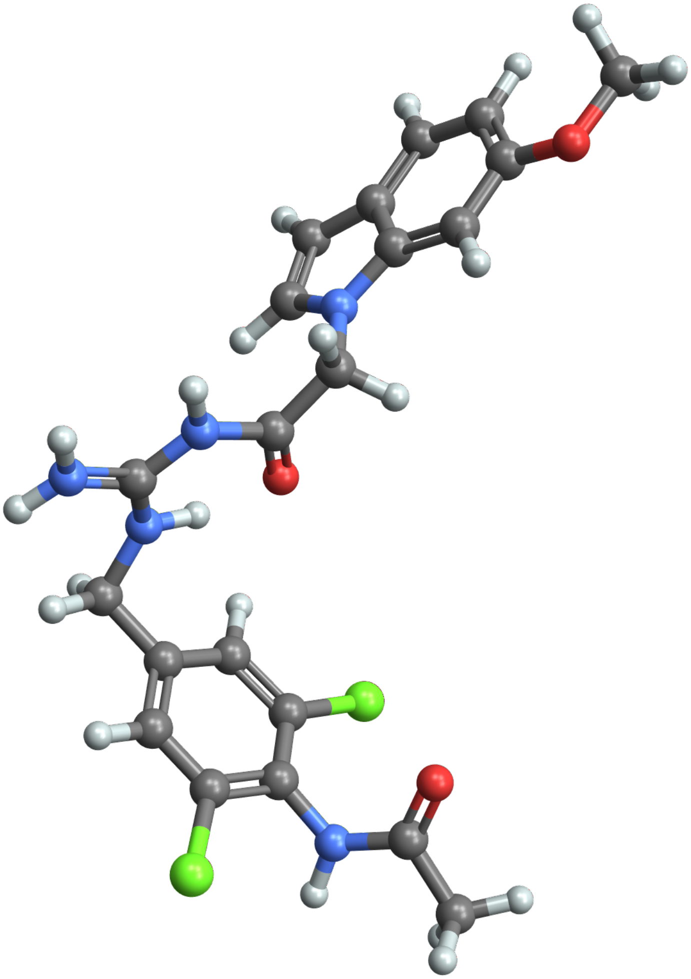

Dataset Index= 930

Molecule Formula= $C_{21}H_{22}Cl_2N_5O_3^+$

3D Autocorrelation

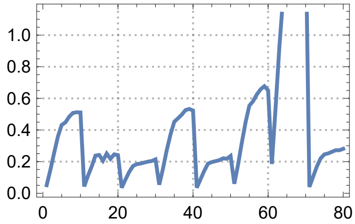

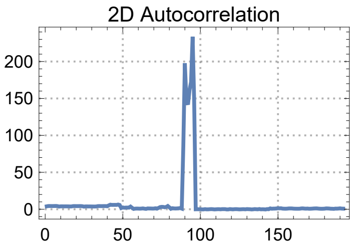

Dataset Index= 931

Molecule Formula= $C_{26}H_{36}F_4N_3O_4S^+$

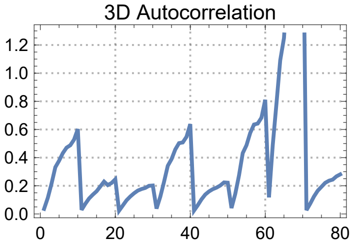

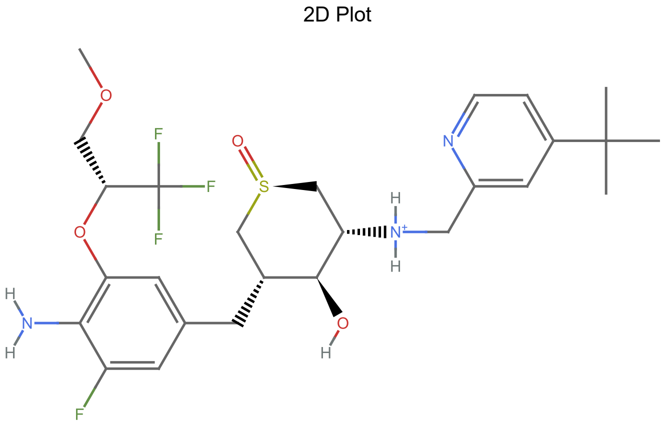

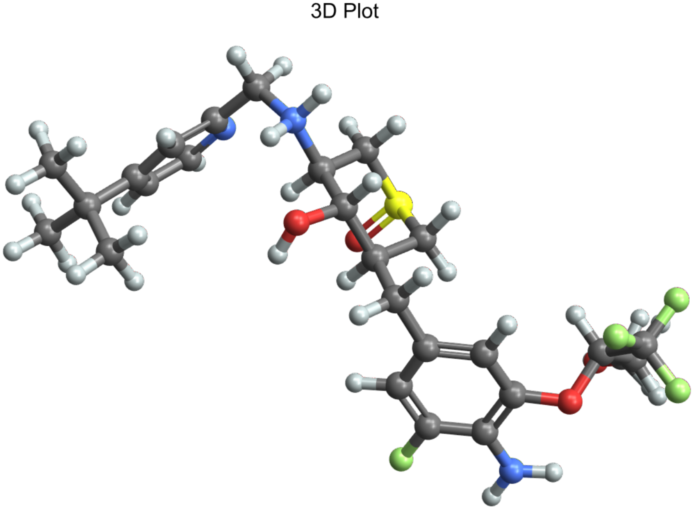

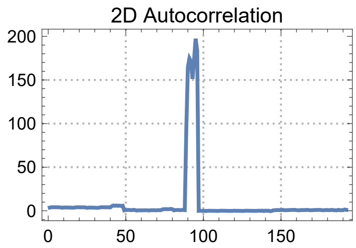

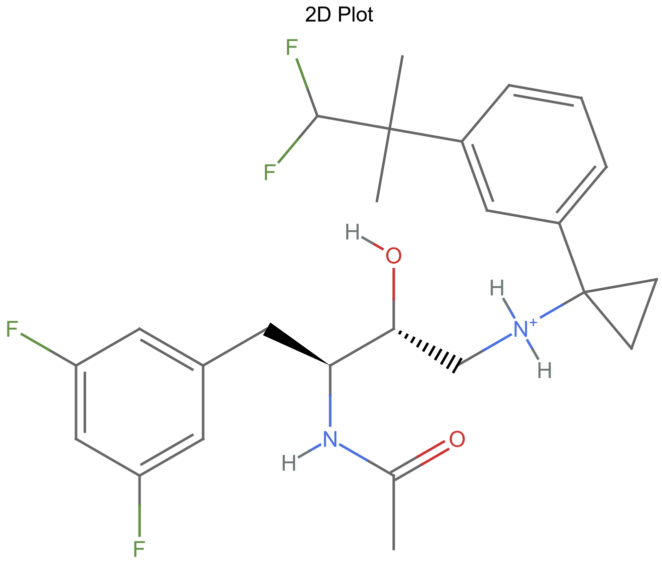

Dataset Index= 932

Molecule Formula= $C_{25}H_{31}F_4N_2O_2^+$

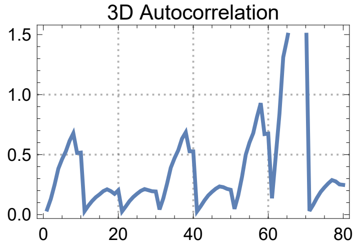

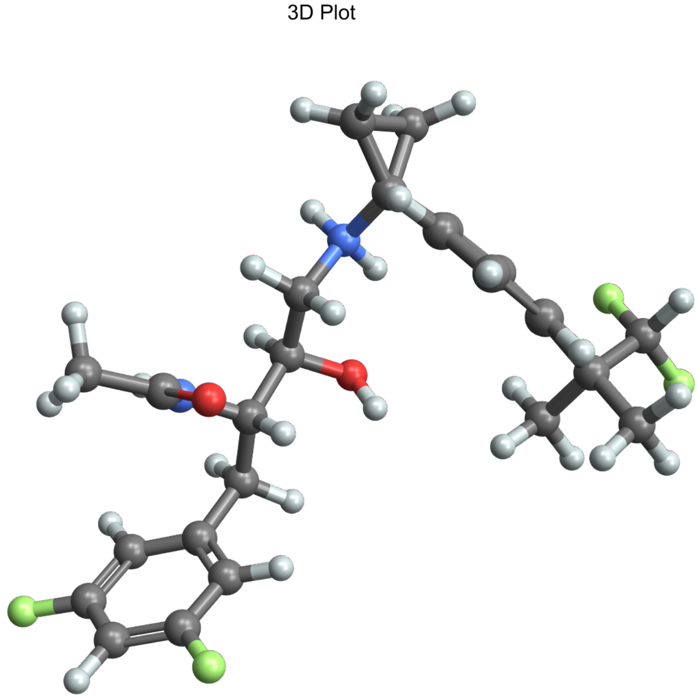

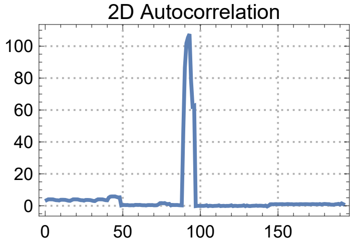

Dataset Index= 933

Molecule Formula= $C_{20}H_{22}N_4O_4$

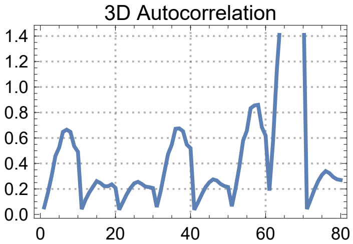

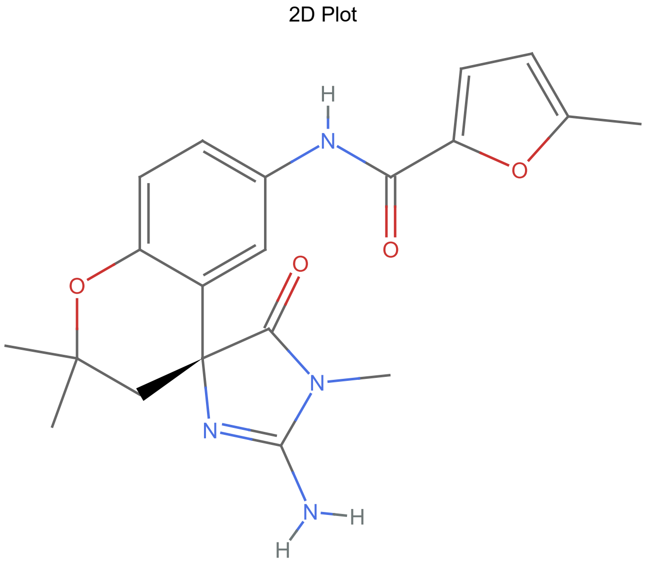

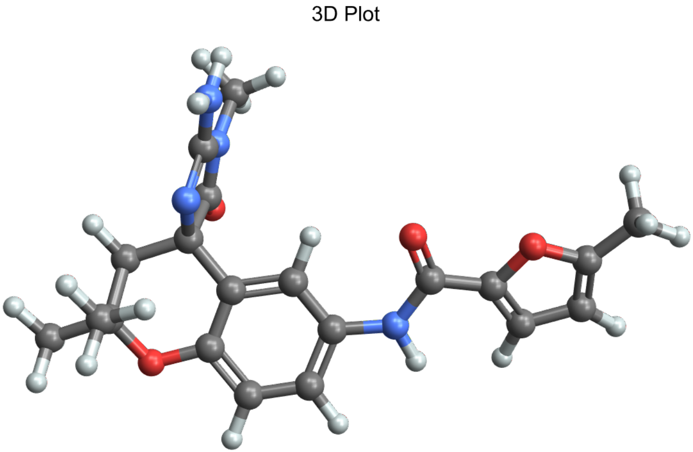

2D Autocorrelation

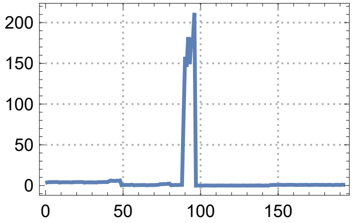

Dataset Index= 934

Molecule Formula= $C_{30}H_{42}F_2N_3O_4^+$

3D Autocorrelation

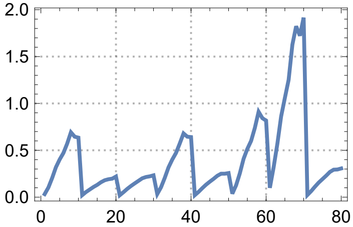

2D Plot

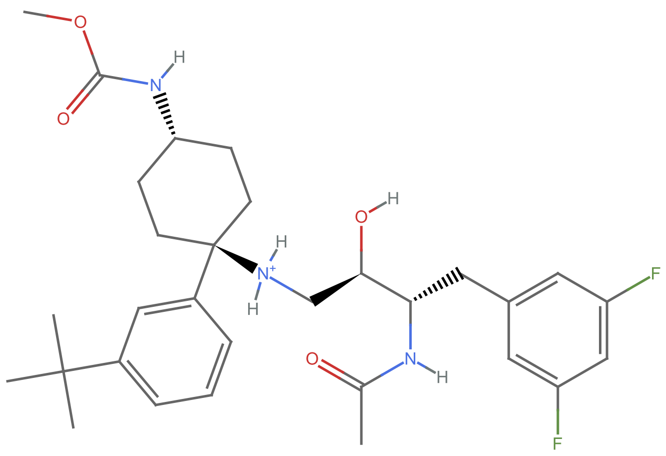

3D Plot

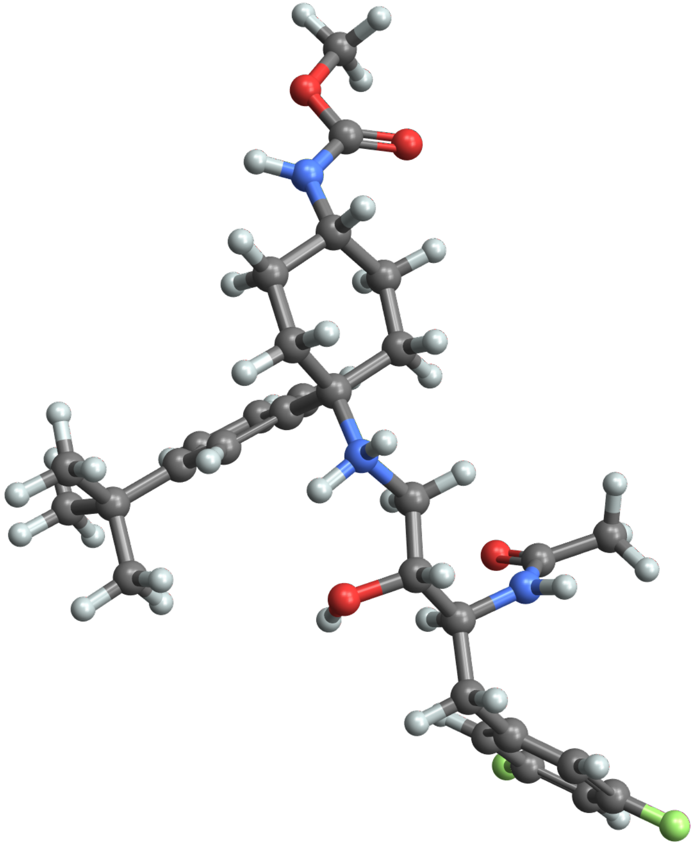

2D Plot

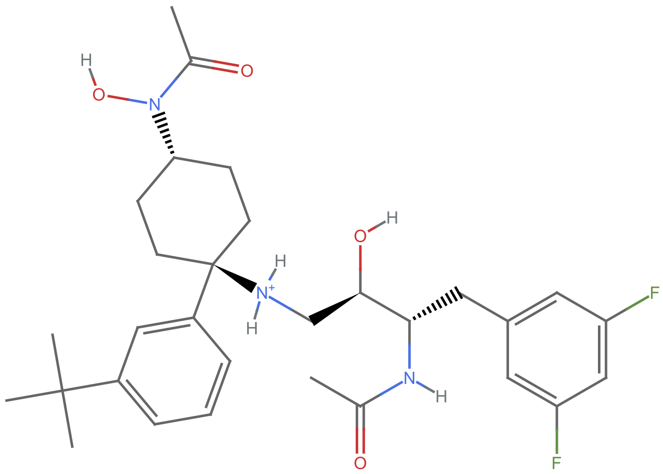

3D Plot

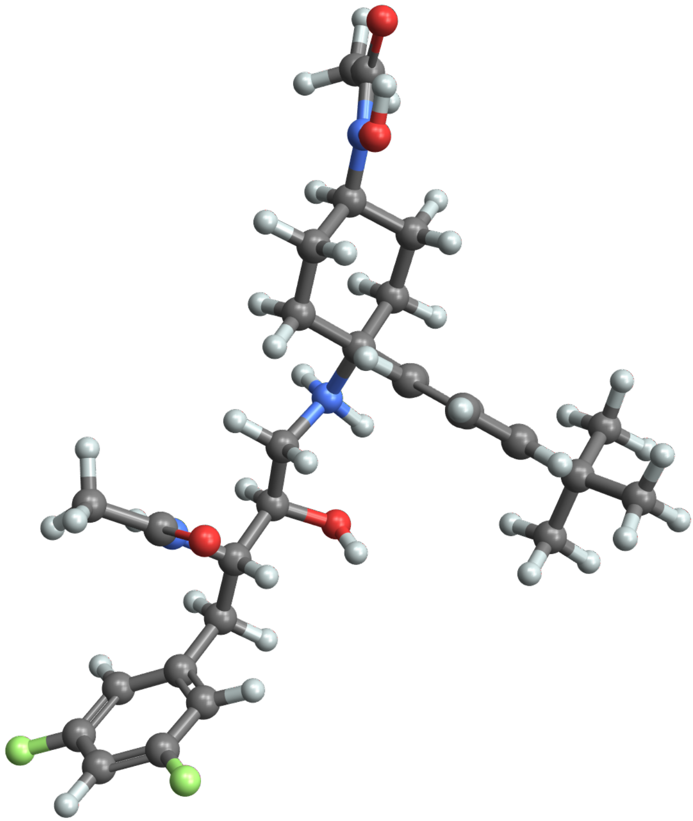

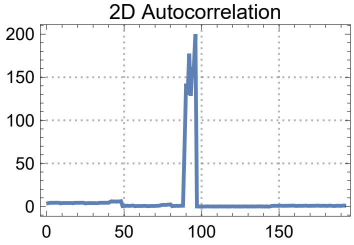

Dataset Index= 935

Molecule Formula= $C_{30}H_{42}F_2N_3O_4^+$

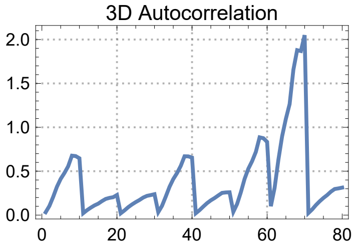

2D Autocorrelation

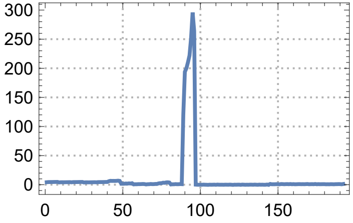

Dataset Index= 936

Molecule Formula= $C_{38}H_{43}F_2N_3O_8S$

3D Autocorrelation

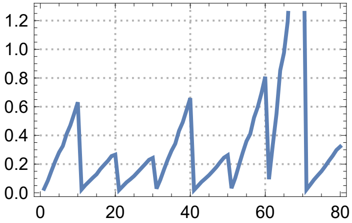

2D Plot

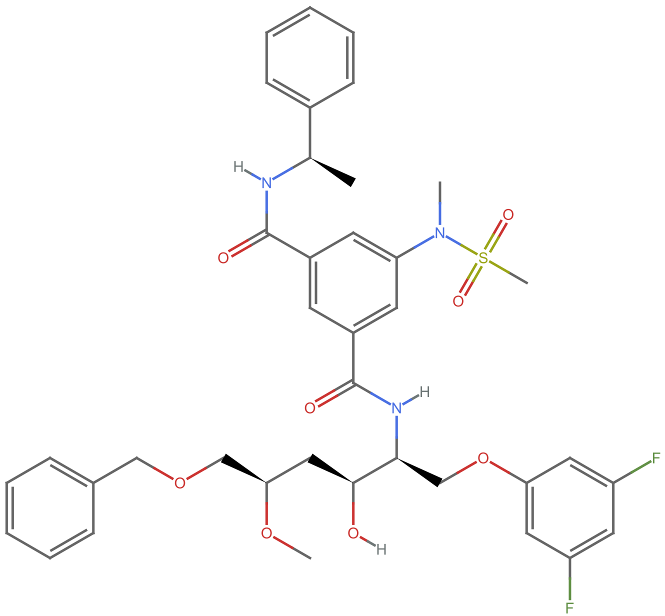

3D Plot

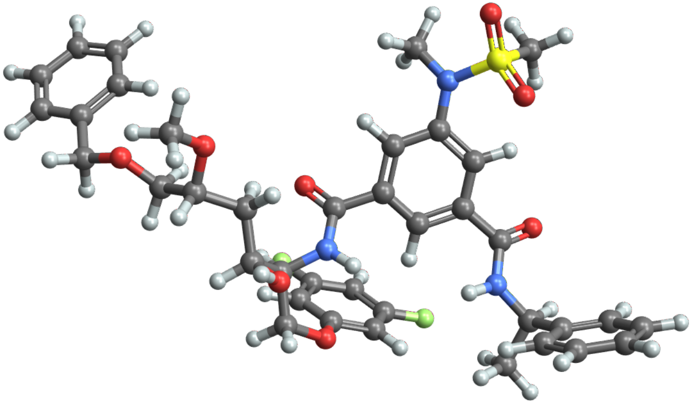

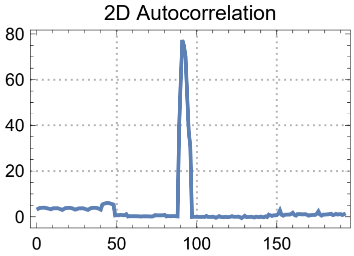

Dataset Index= 937

Molecule Formula= $C_{19}H_{16}N_4OS$

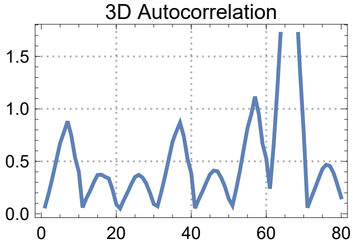

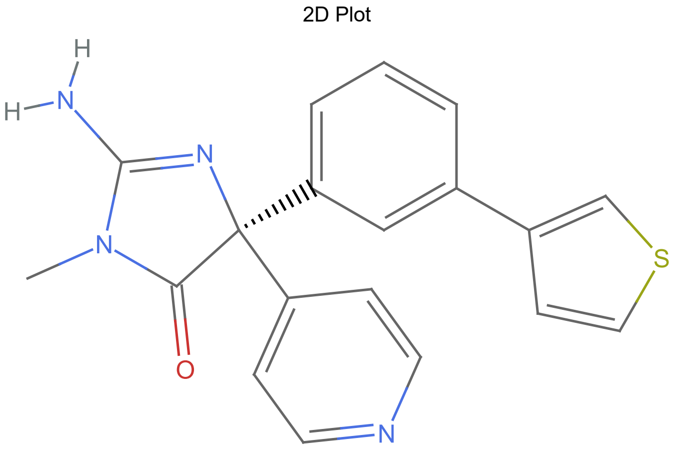

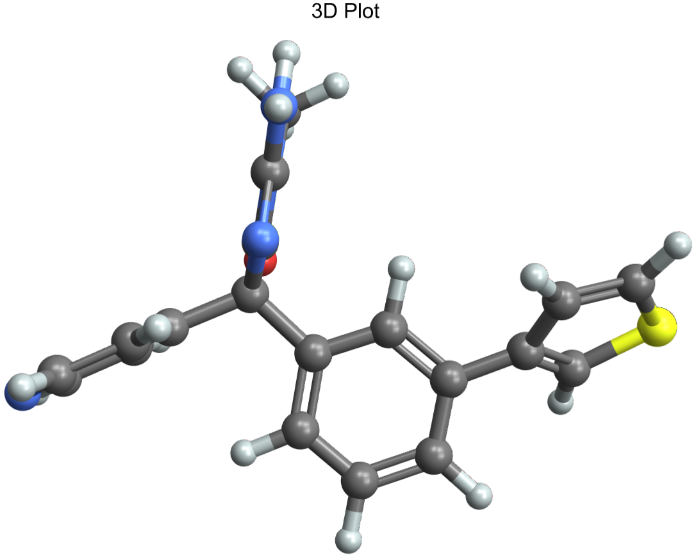

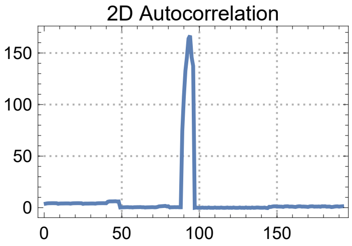

Dataset Index= 938

Molecule Formula= $C_{34}H_{43}N_4O_4^+$

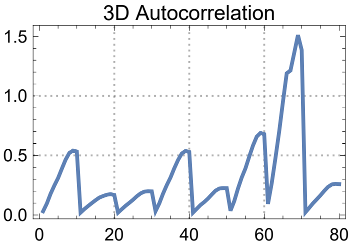

2D Plot

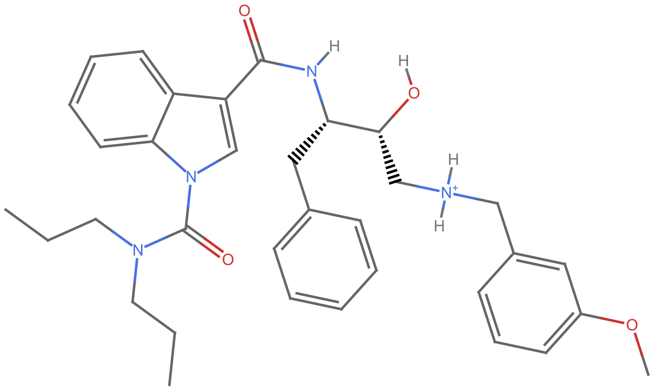

3D Plot

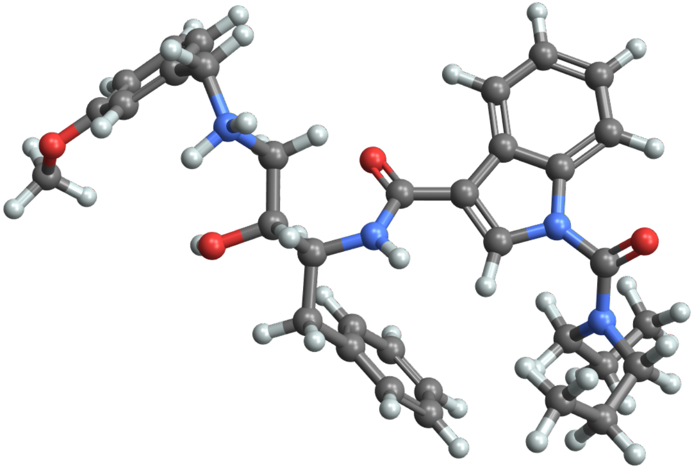

2D Plot

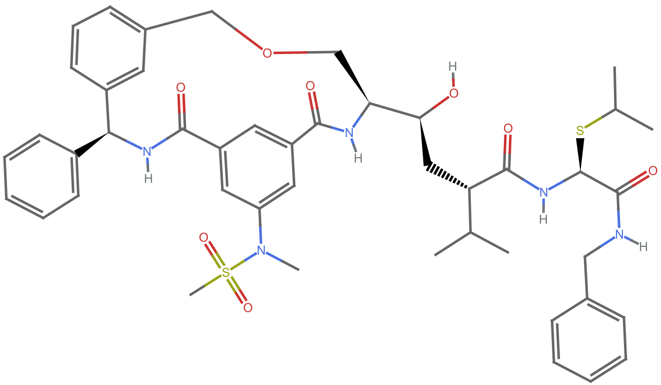

2D Autocorrelation

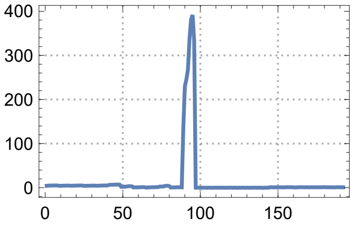

3D Plot

Dataset Index= 939

Molecule Formula= $C_{45}H_{55}N_5O_8S_2$

3D Autocorrelation

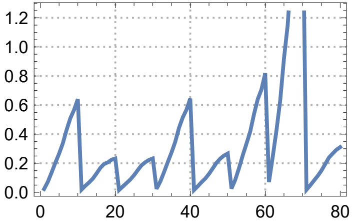

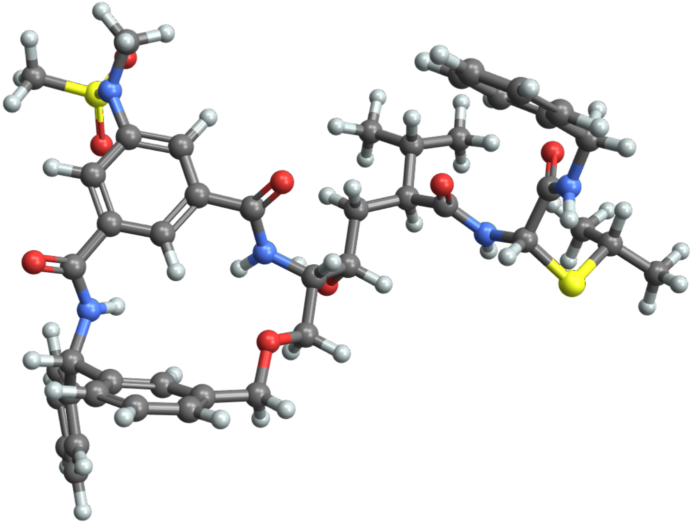

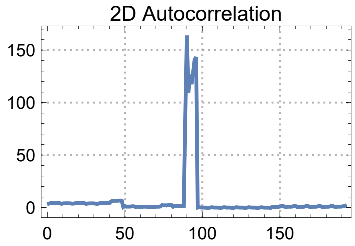

Dataset Index= 940

Molecule Formula= $C_{24}H_{15}F_4N_5$

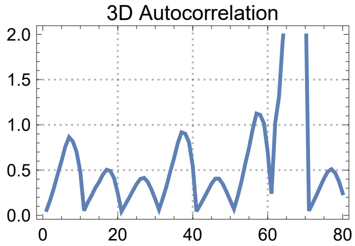

2D Plot

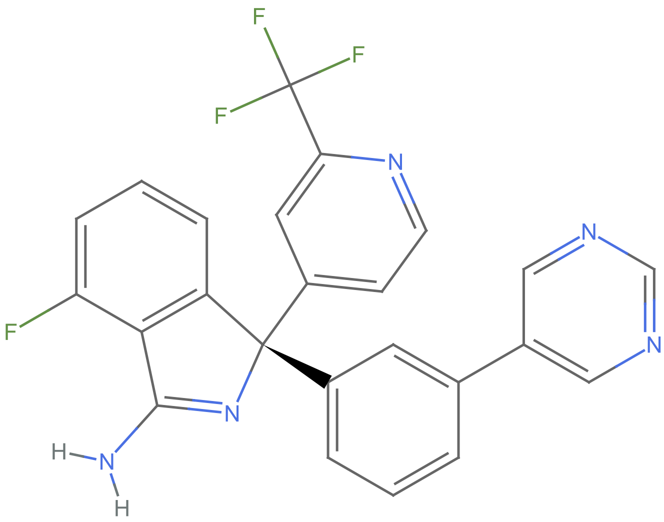

3D Plot

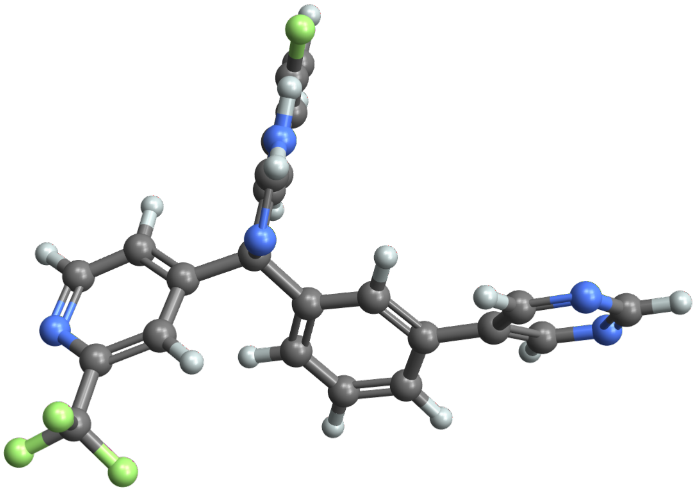

2D Plot

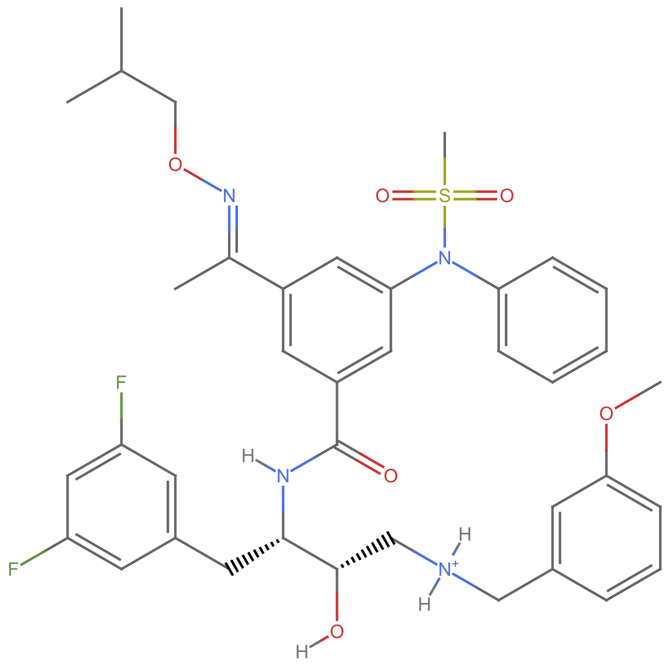

2D Autocorrelation

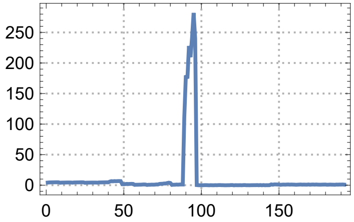

Dataset Index= 941

Molecule Formula= $C_{38}H_{45}F_2N_4O_6S^+$

3D Plot

3D Autocorrelation

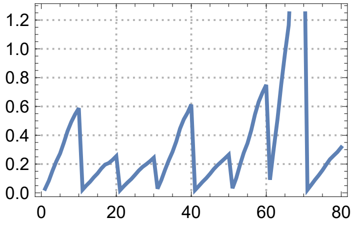

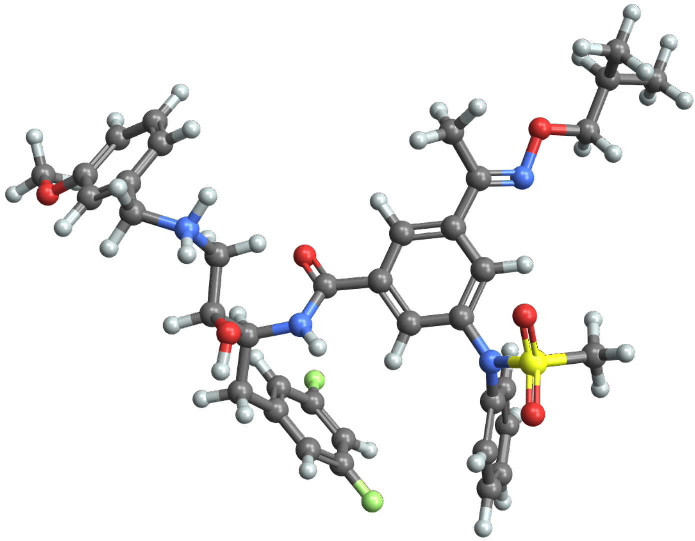

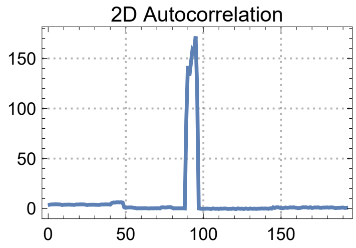

Dataset Index= 942

Molecule Formula= $C_{30}H_{38}N_3O_4S^+$

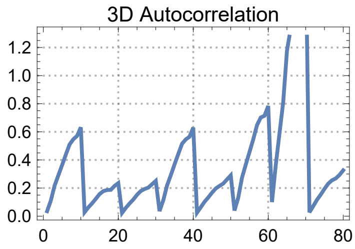

2D Plot

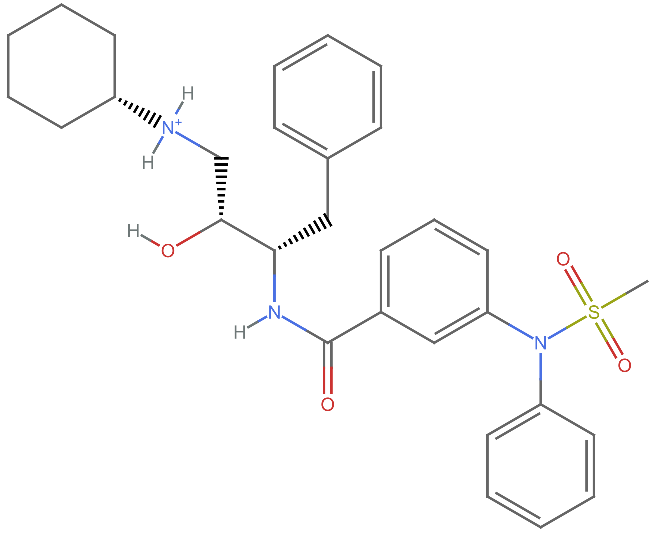

3D Plot

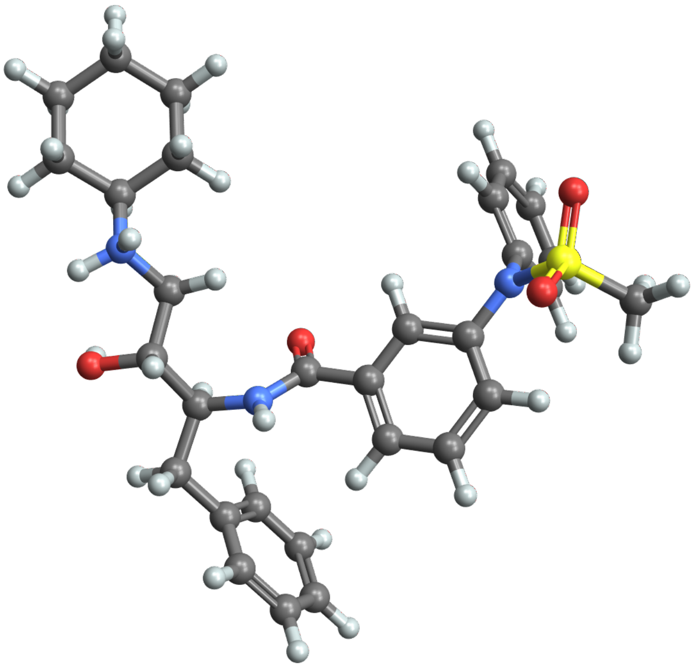

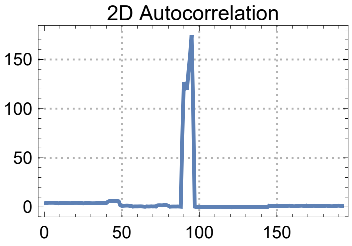

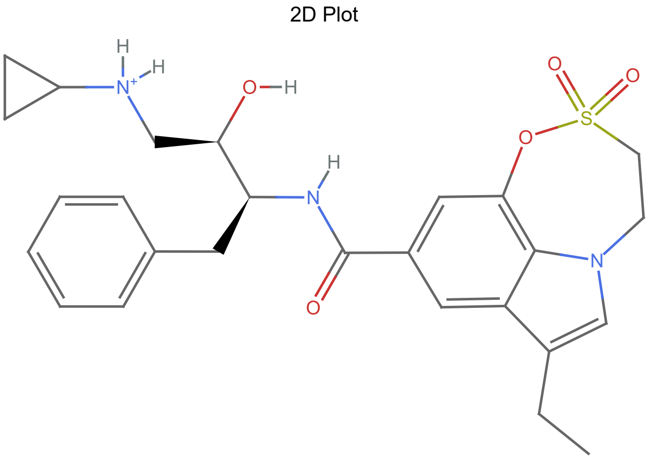

Dataset Index= 943

Molecule Formula= $C_{26}H_{32}N_3O_5S^+$

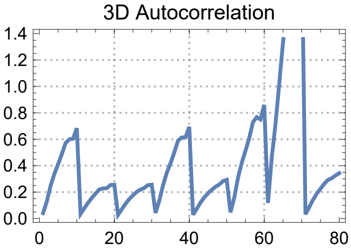

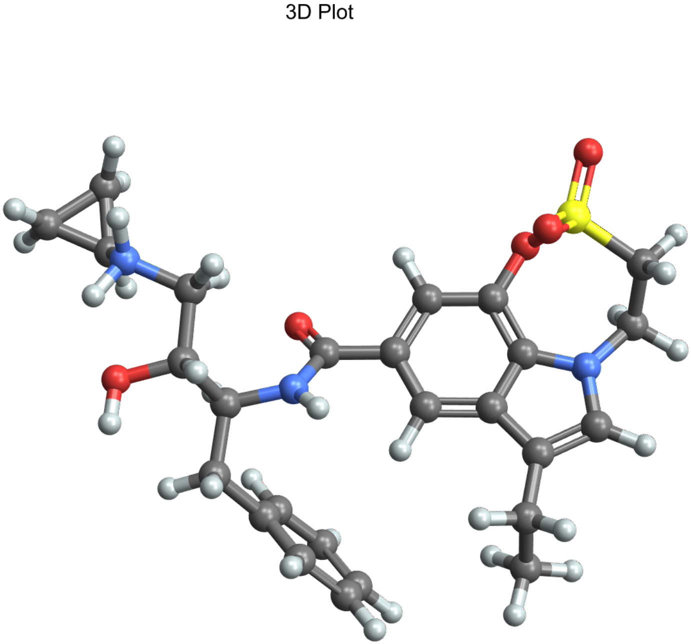

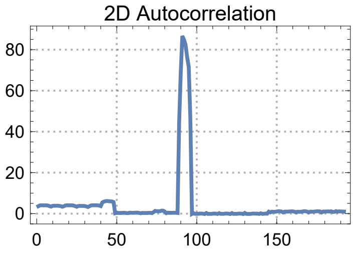

Dataset Index= 944

Molecule Formula= $C_{22}H_{18}N_4O_3$

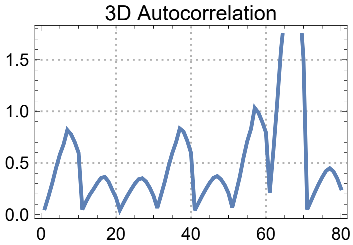

2D Plot

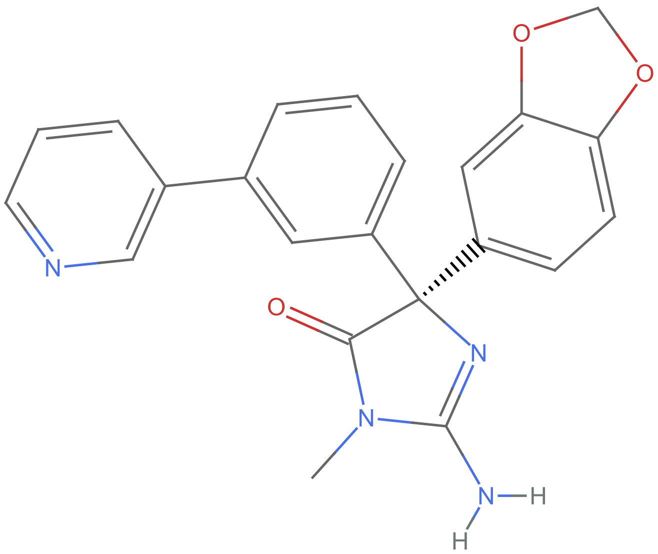

3D Plot

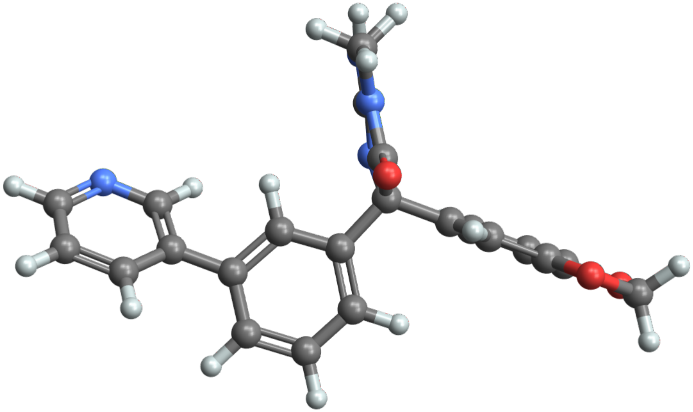

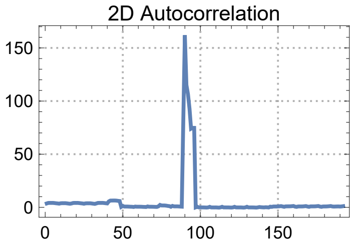

Dataset Index= 945

Molecule Formula= $C_{22}H_{17}F_3N_4O$

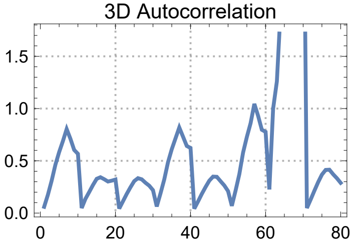

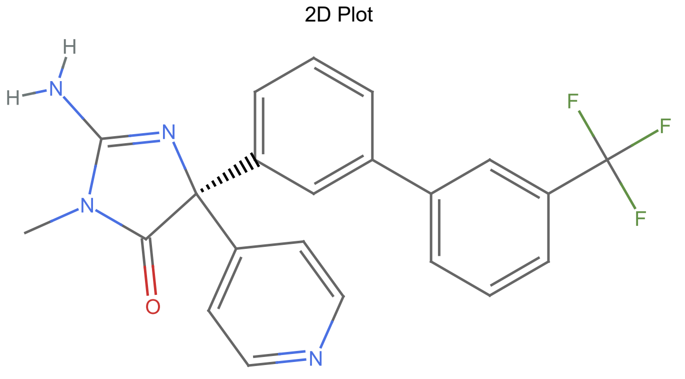

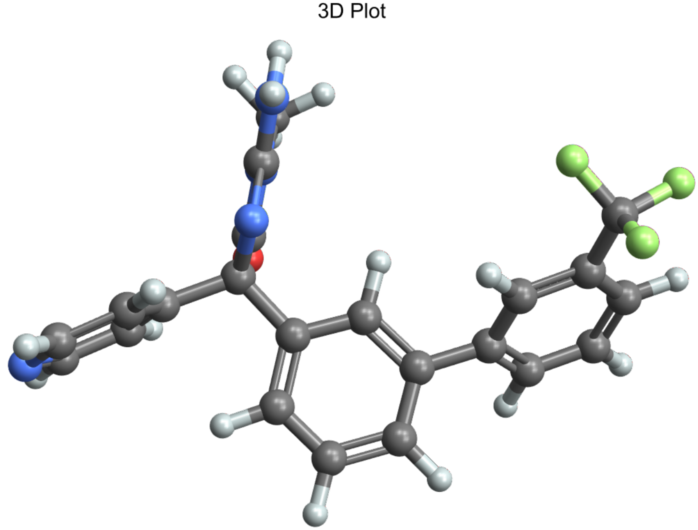

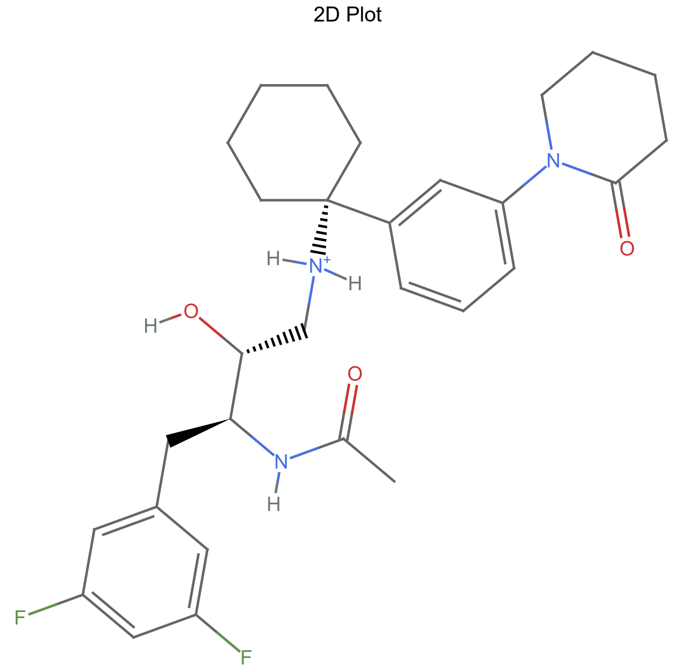

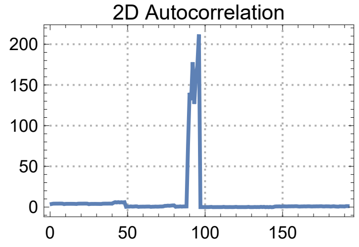

Dataset Index= 946

Molecule Formula= $C_{29}H_{38}F_2N_3O_3^+$

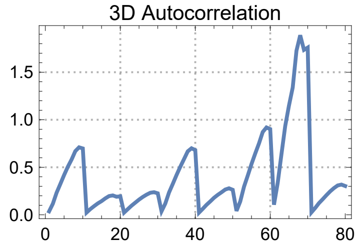

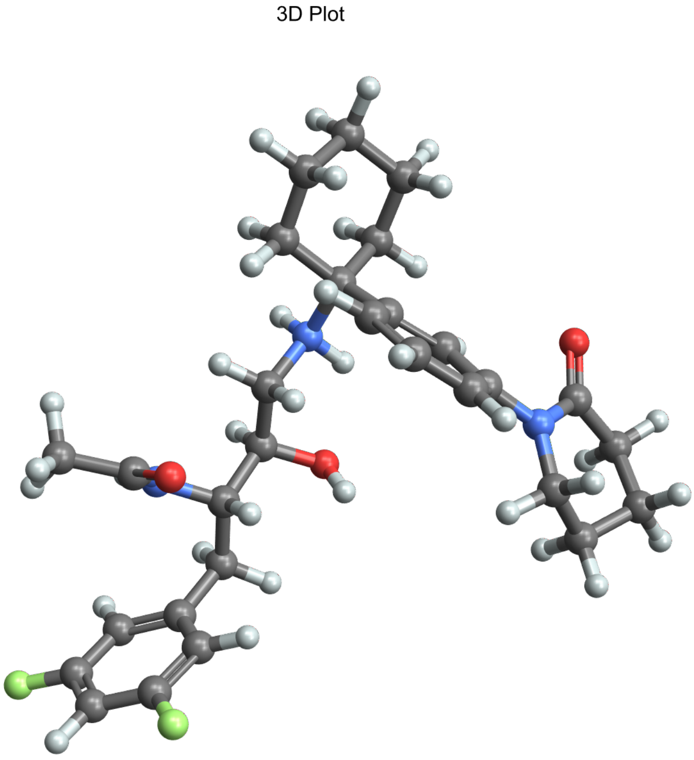

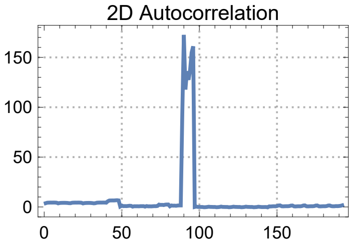

Dataset Index= 947

Molecule Formula= $C_{26}H_{15}F_4N_5$

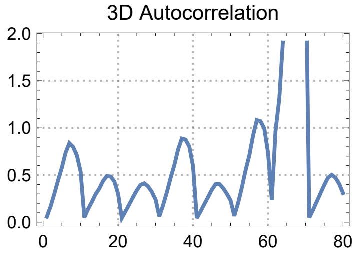

2D Plot

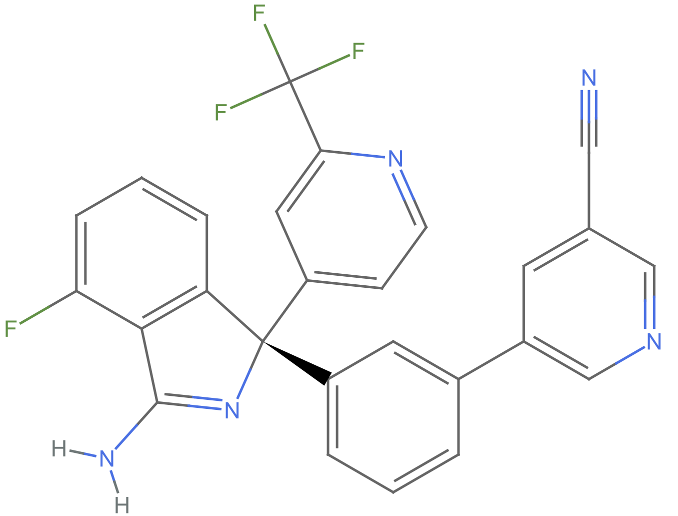

3D Plot

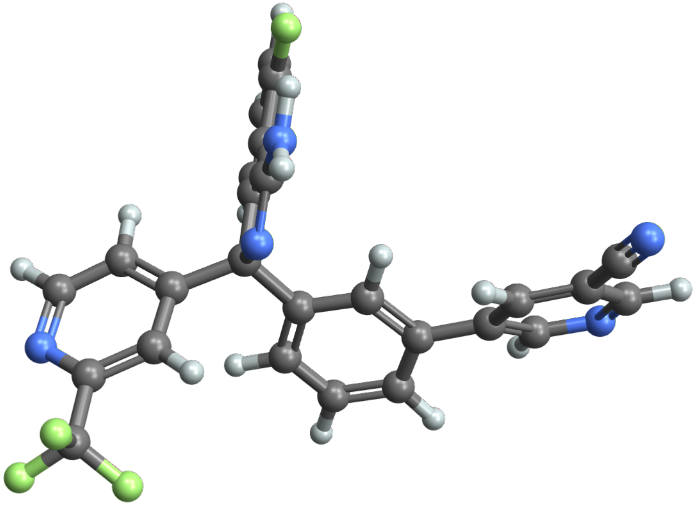

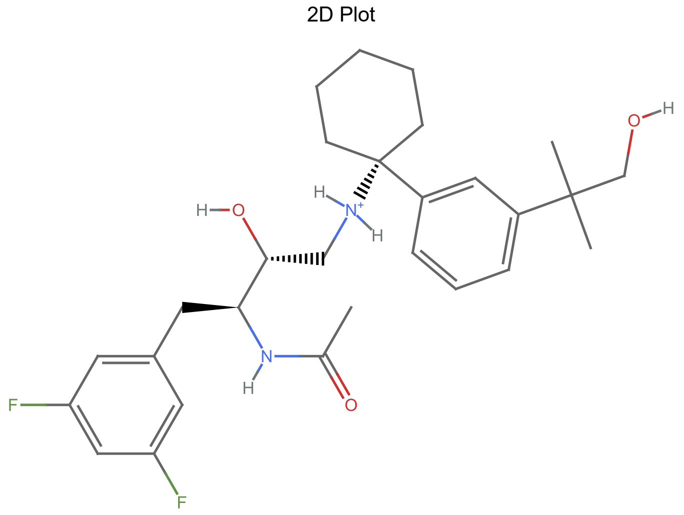

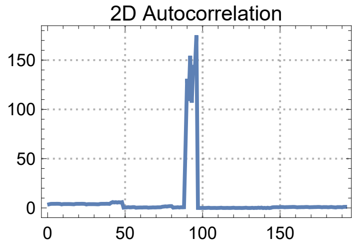

Dataset Index= 948

Molecule Formula= $C_{28}H_{39}F_2N_2O_3^+$

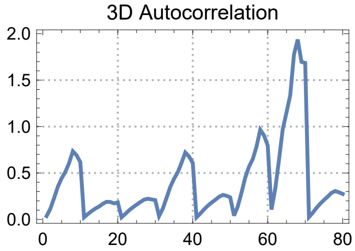

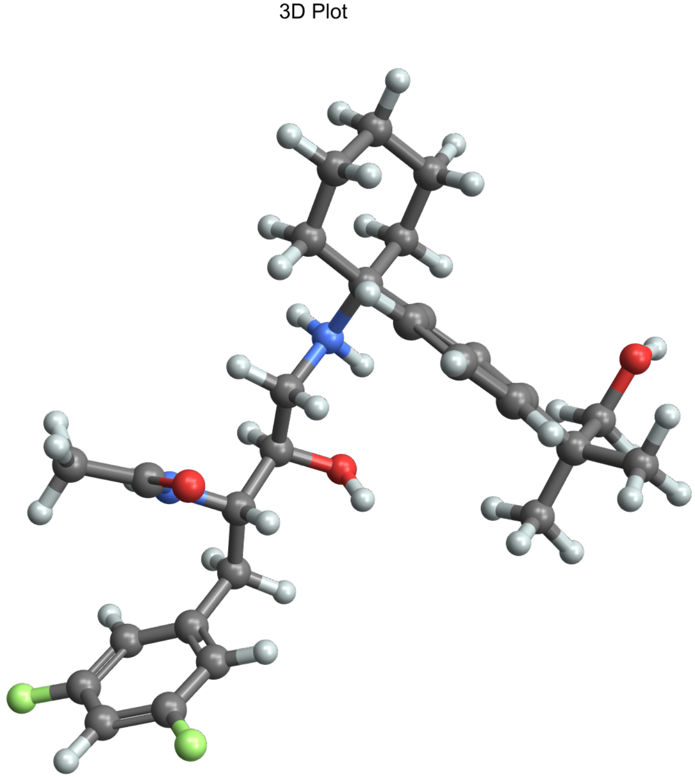

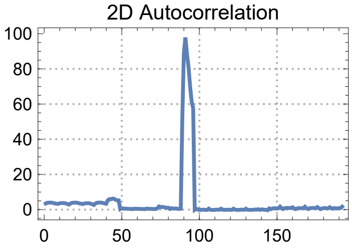

Dataset Index= 949

Molecule Formula= $C_{19}H_{15}F_2N_3O_2$

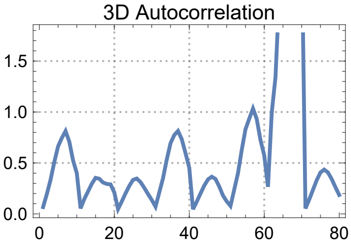

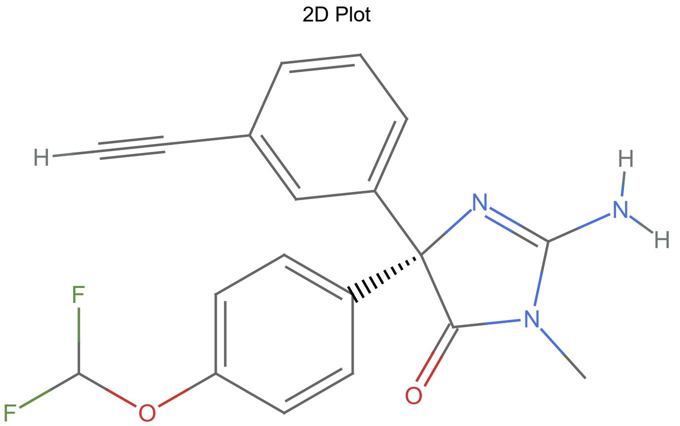

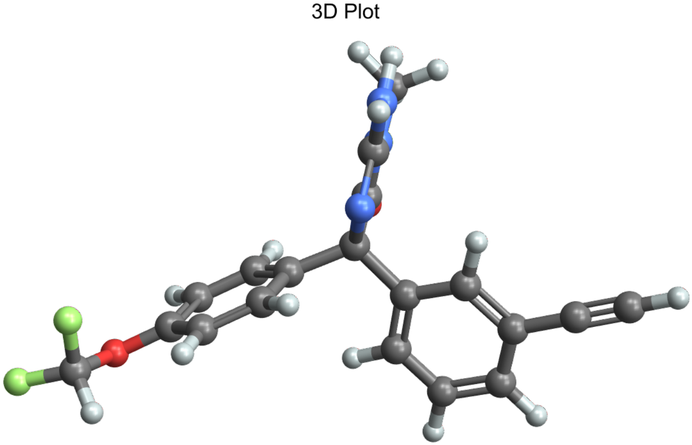

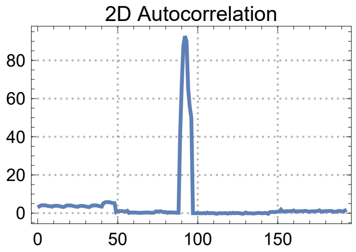

Dataset Index= 950

Molecule Formula= $C_{21}H_{22}ClN_{3}O_{2}$

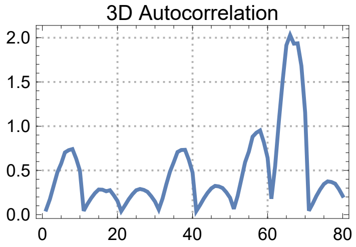

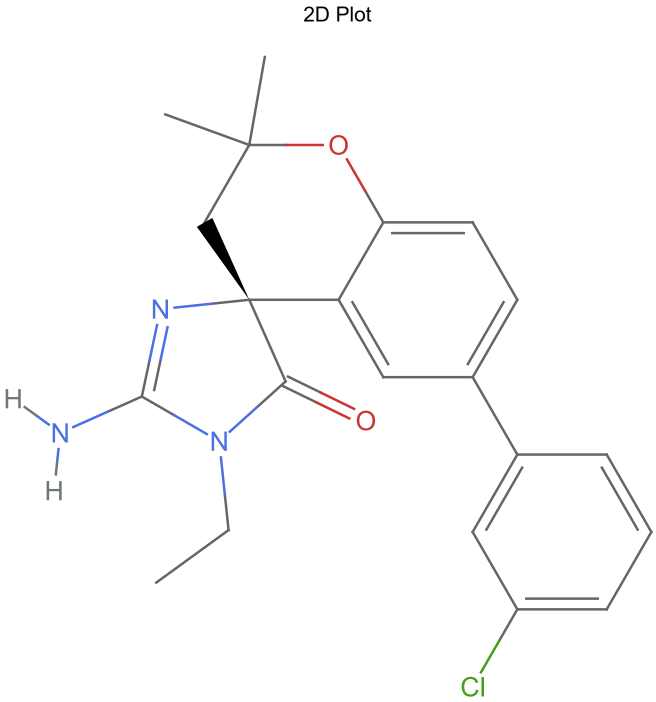

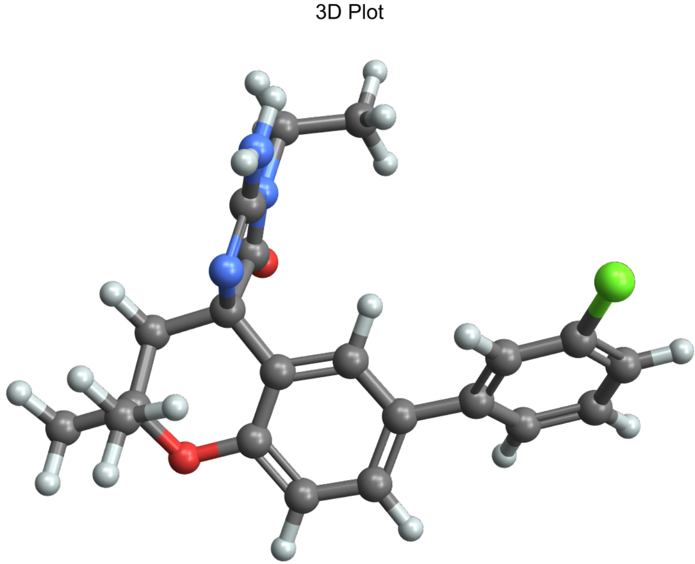

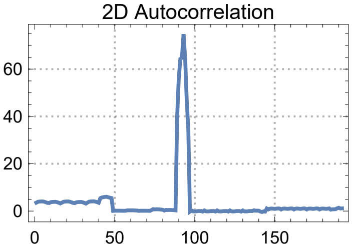

Dataset Index= 951

Molecule Formula= $C_{22}H_{20}N_6$

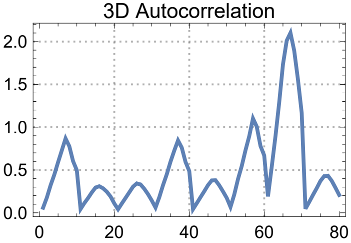

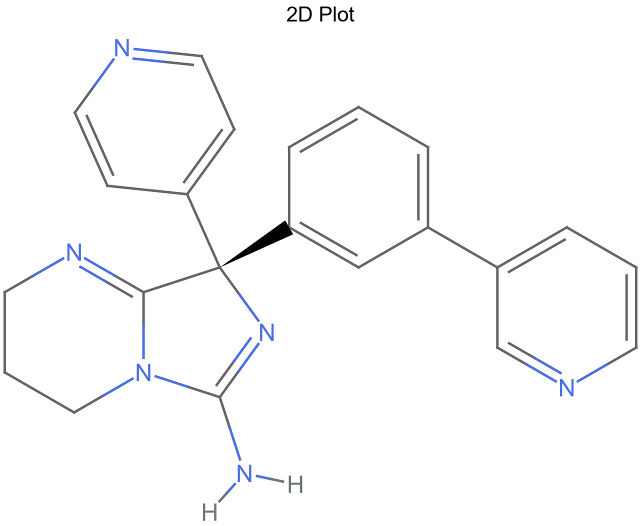

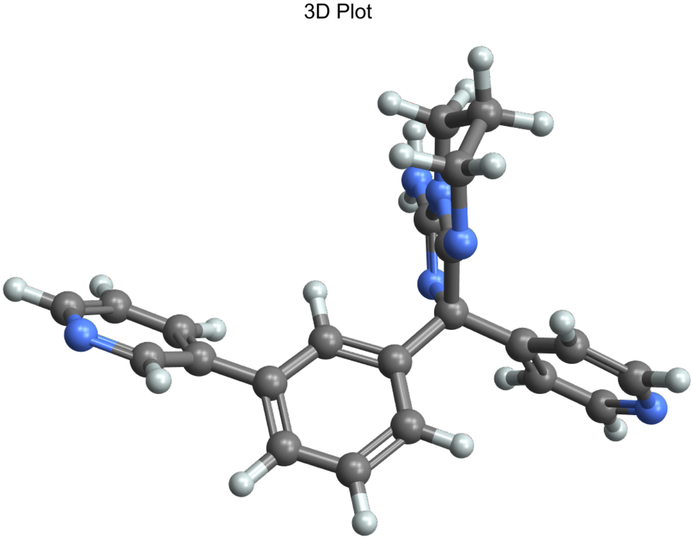

2D Plot

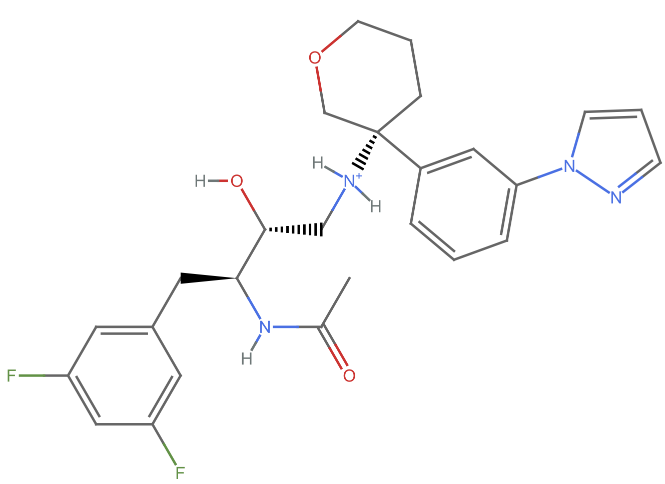

2D Autocorrelation

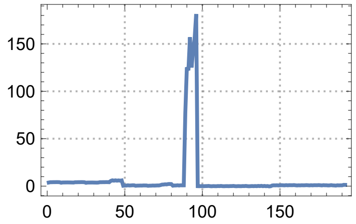

3D Plot

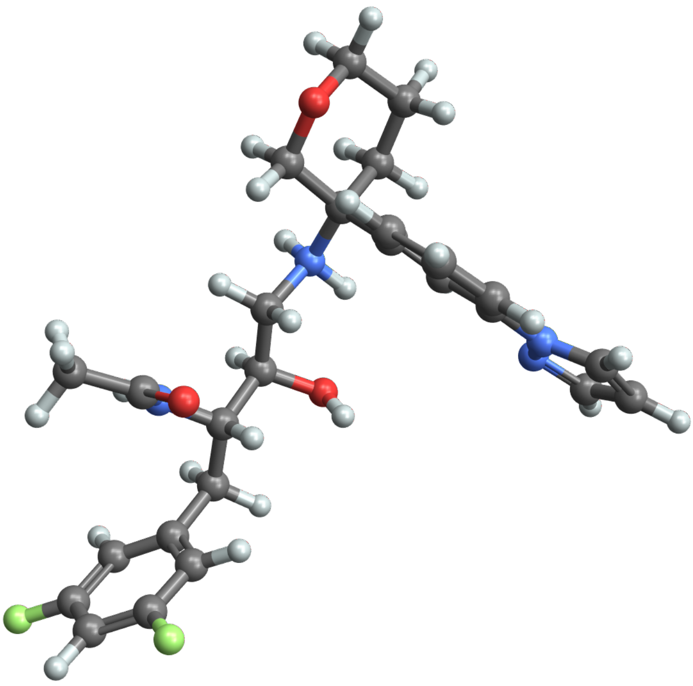

Dataset Index= 952

Molecule Formula= $C_{26}H_{31}F_2N_4O_3^+$

3D Autocorrelation

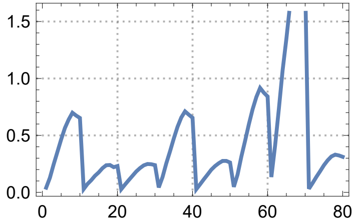

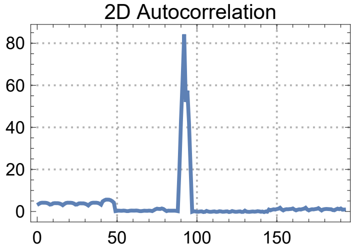

Dataset Index= 953

Molecule Formula= $C_{22}H_{29}N_3O_3$

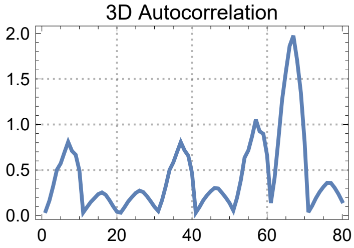

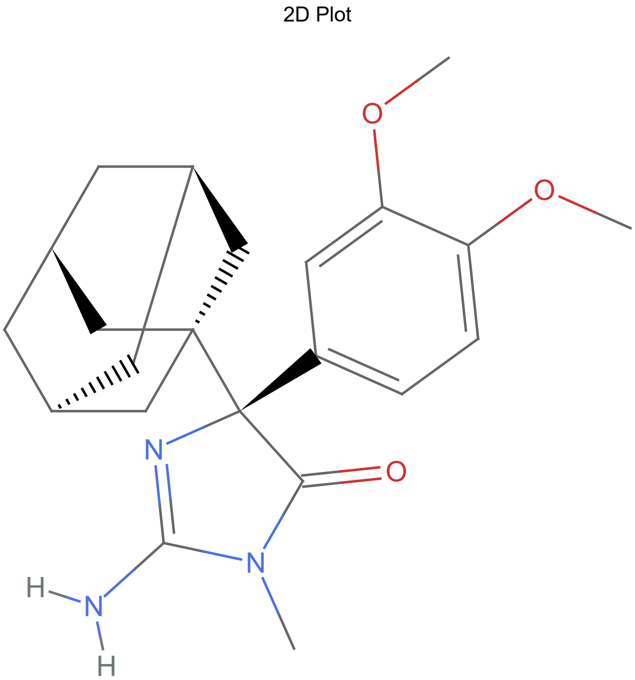

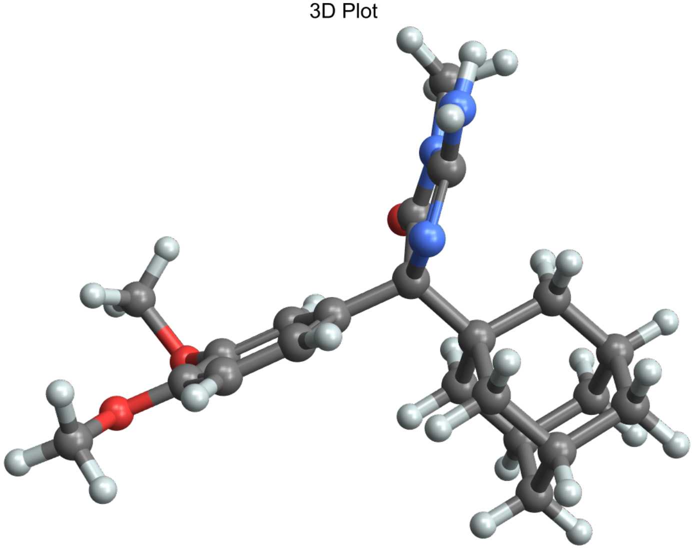

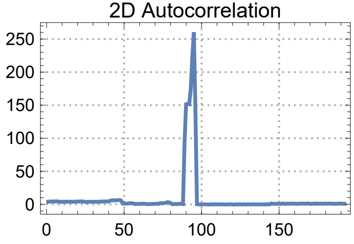

Dataset Index= 954

Molecule Formula= $C_{26}H_{35}N_4O_6S^+$

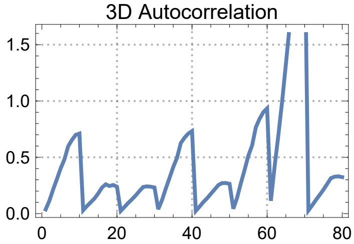

2D Plot

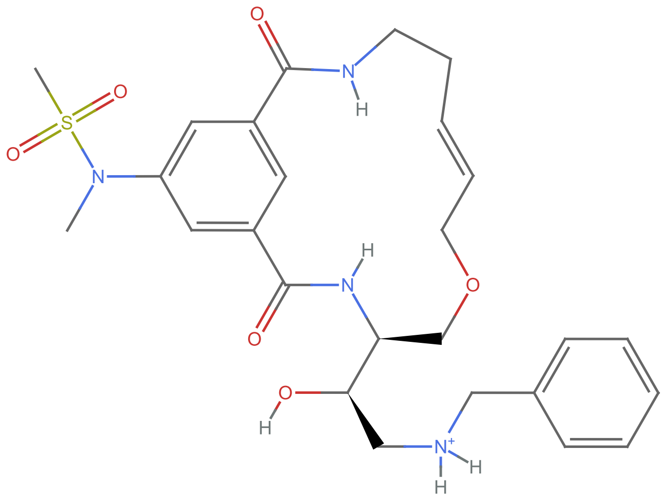

3D Plot

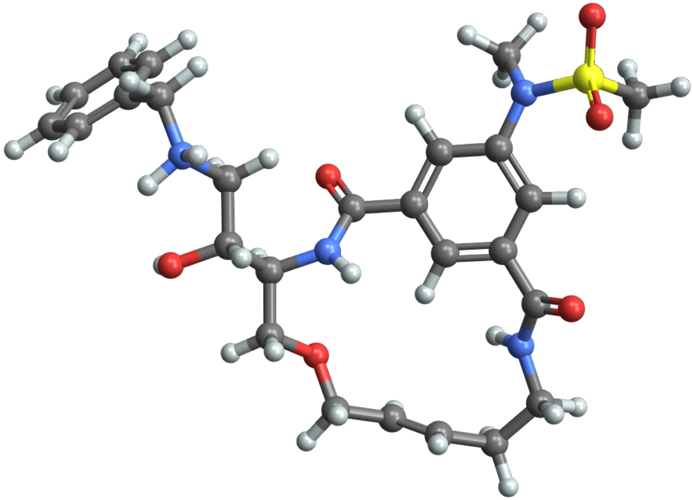

2D Plot

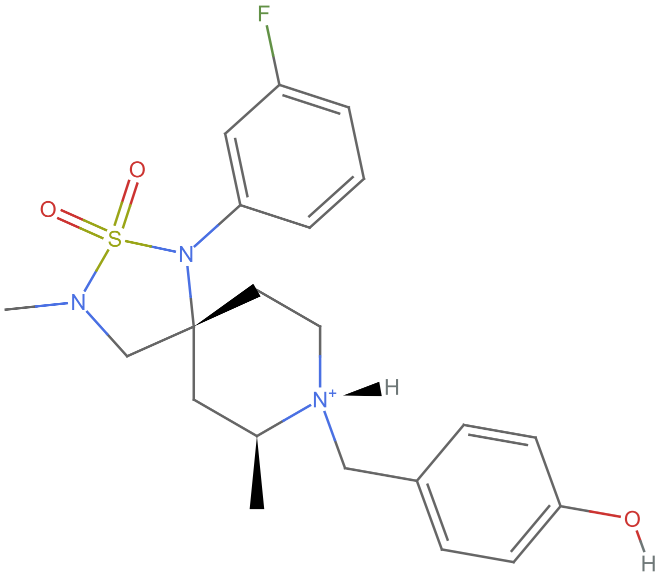

2D Autocorrelation

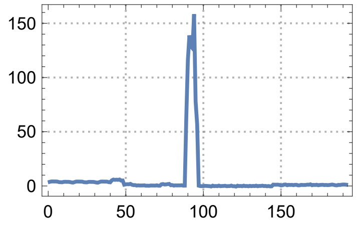

Dataset Index= 955

Molecule Formula= $C_{21}H_{27}FN_3O_3S^+$

3D Autocorrelation

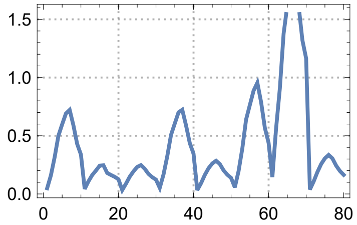

3D Plot

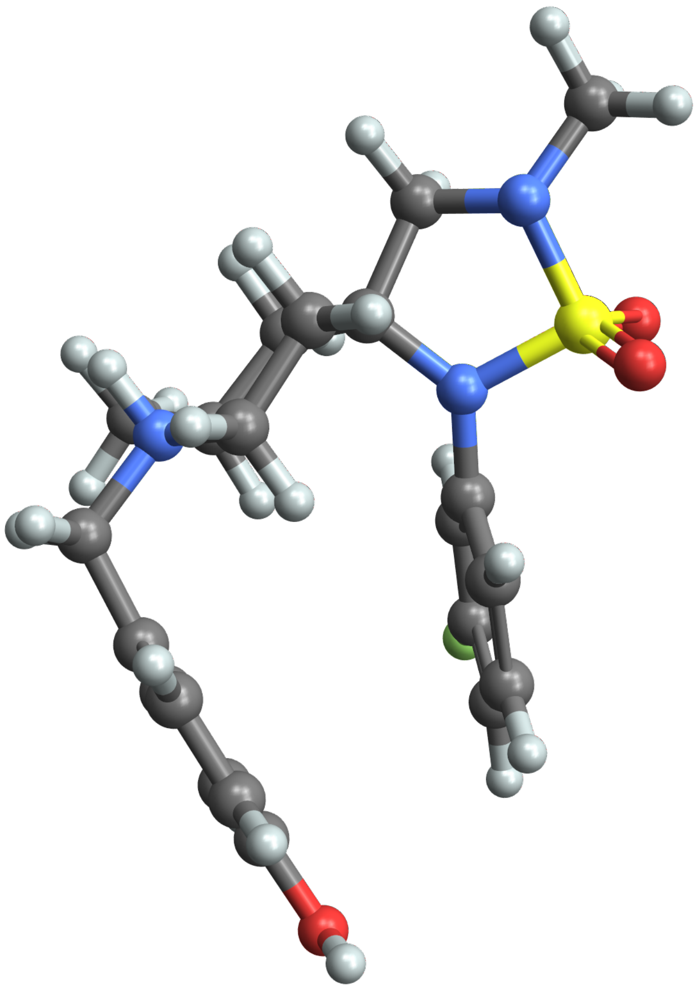

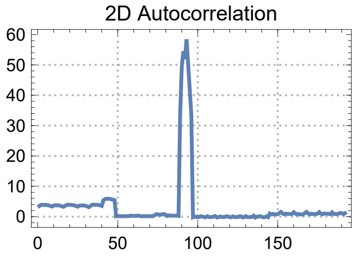

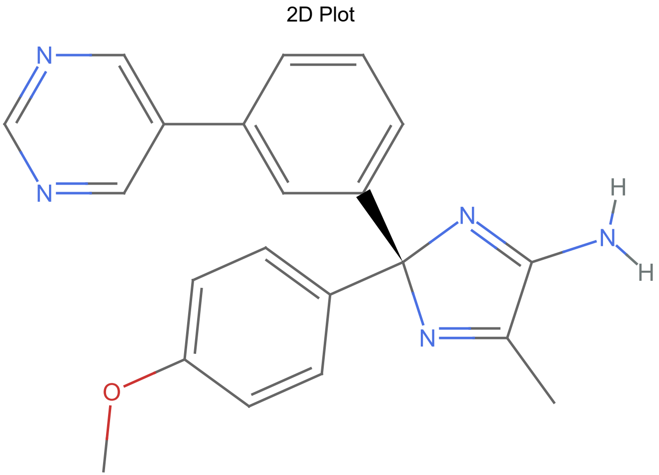

Dataset Index= 956

Molecule Formula= $C_{21}H_{19}N_5O$

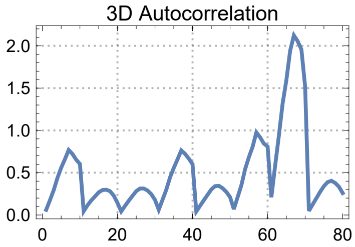

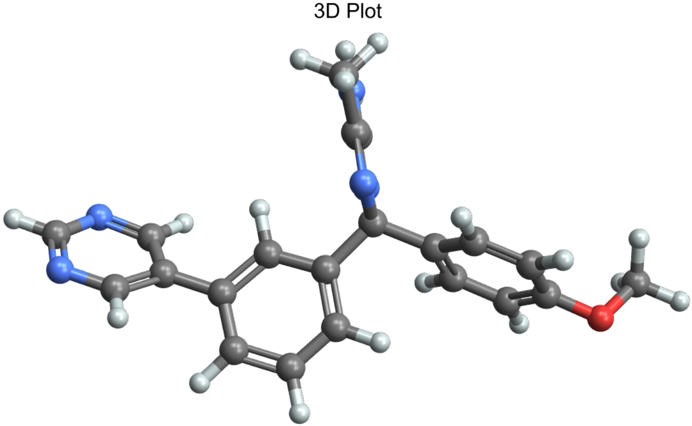

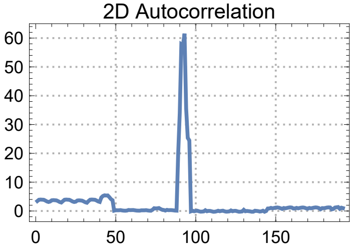

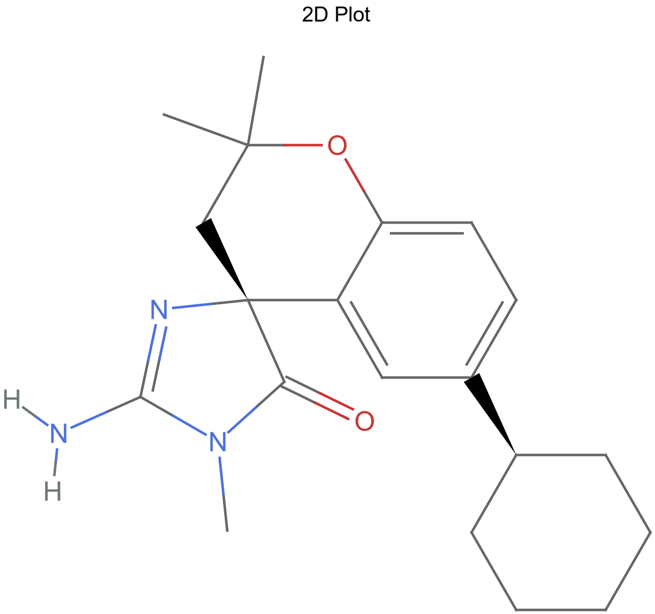

Dataset Index= 957

Molecule Formula= $C_{20}H_{27}N_3O_2$

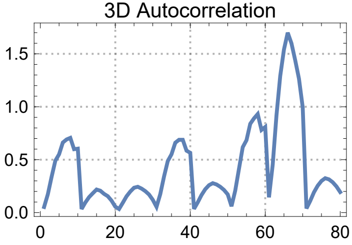

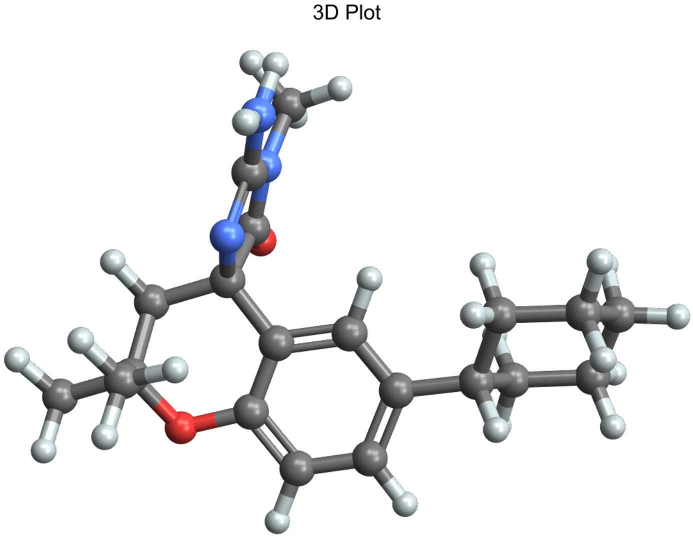

2D Autocorrelation

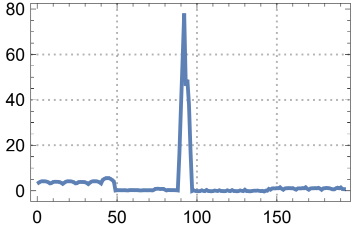

Dataset Index= 958

Molecule Formula= $C_{23}H_{31}N_3O_2$

3D Autocorrelation

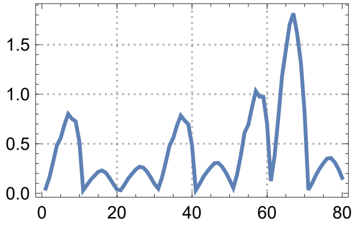

2D Plot

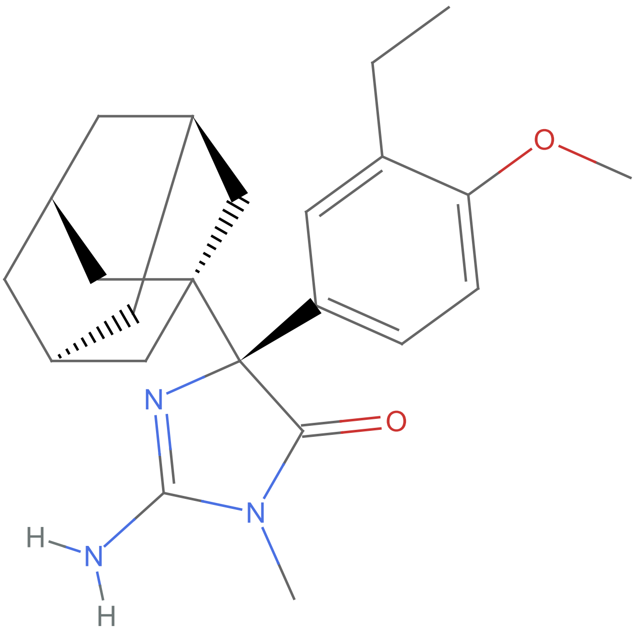

3D Plot

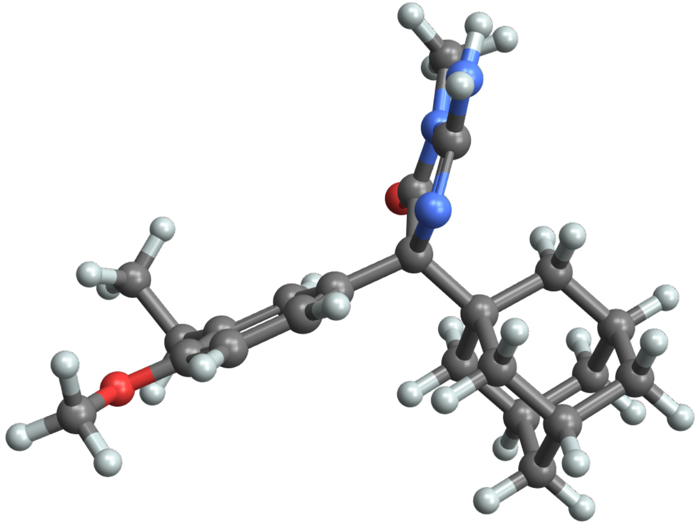

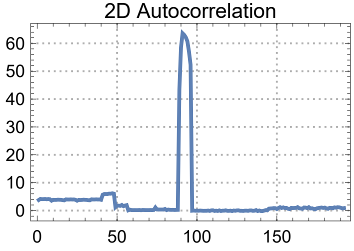

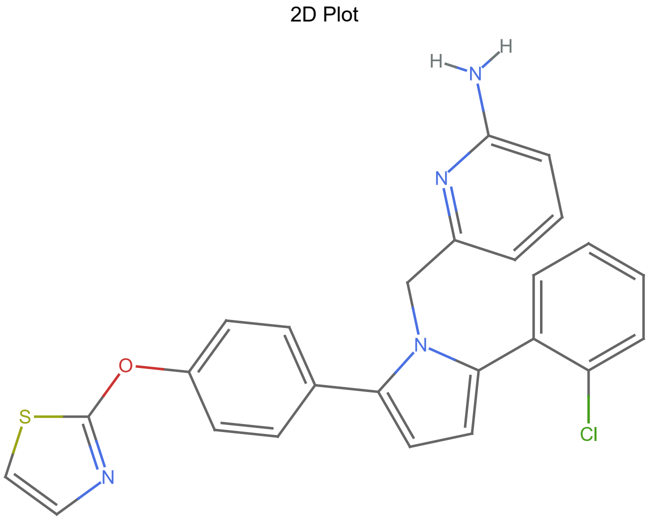

Dataset Index= 959

Molecule Formula= $C_{25}H_{19}ClN_4OS$

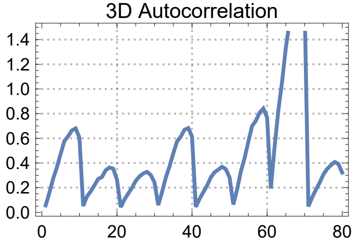

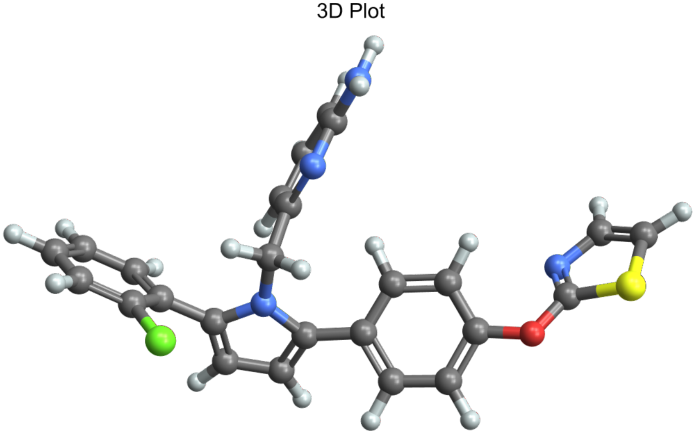

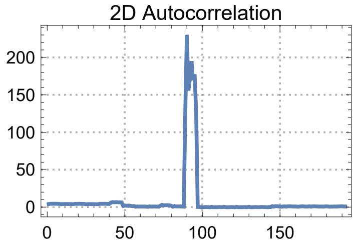

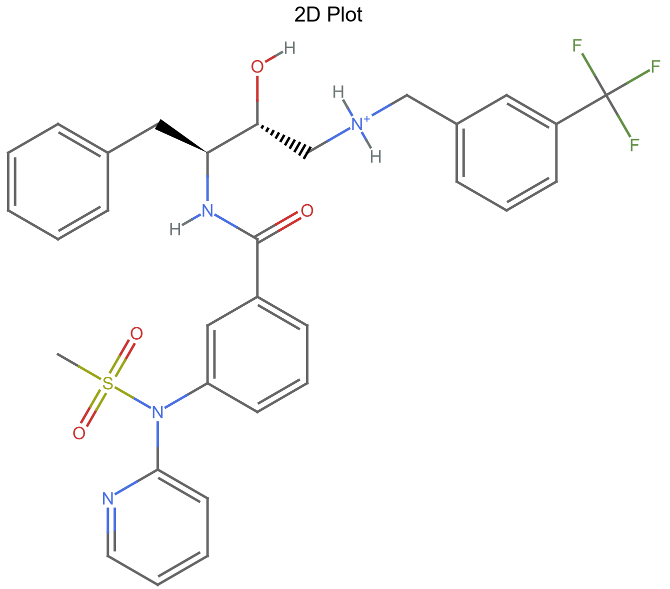

Dataset Index= 960

Molecule Formula= $C_{31}H_{32}F_3N_4O_4S^+$

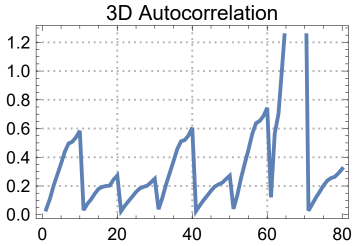

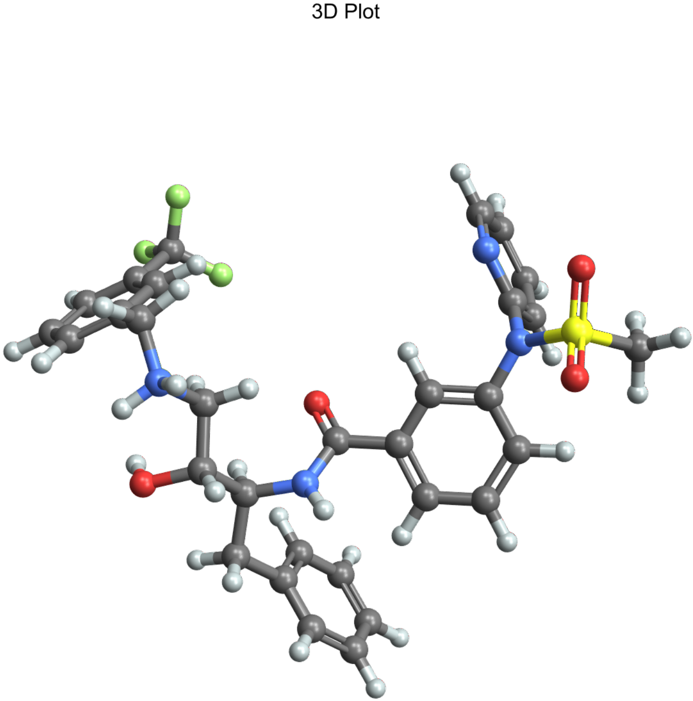

2D Plot

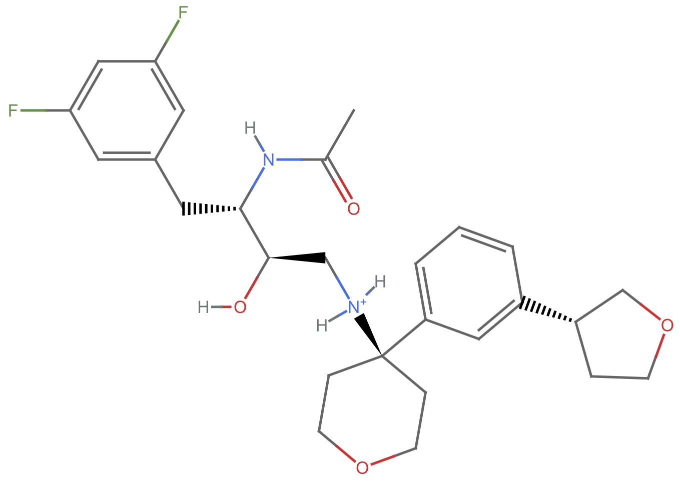

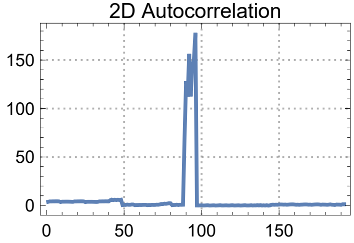

3D Plot

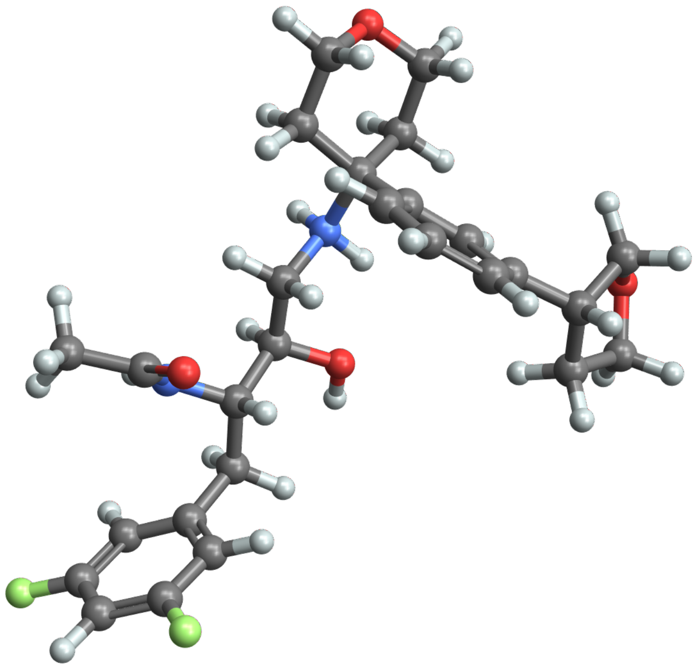

Dataset Index= 961

Molecule Formula= $C_{27}H_{35}F_2N_2O_4^+$

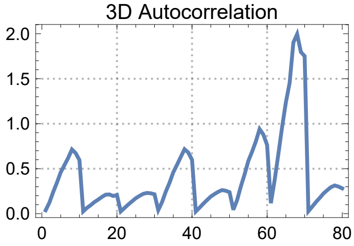

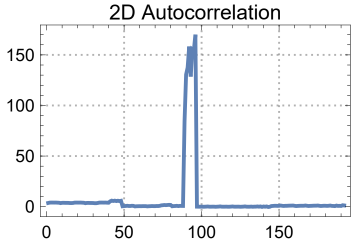

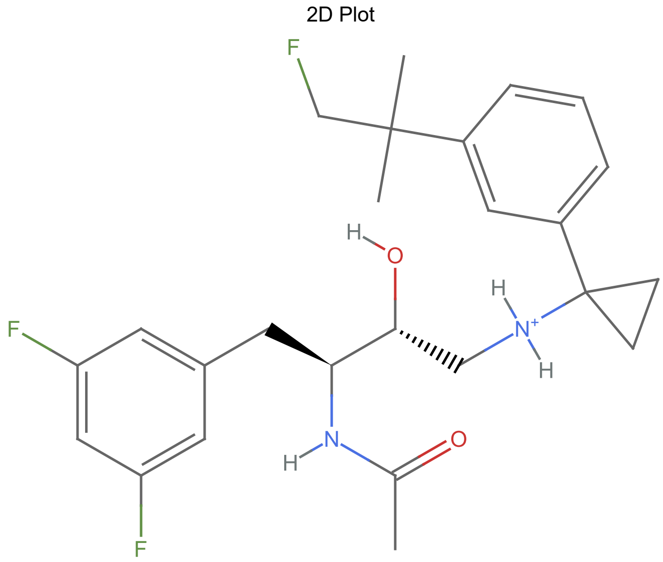

Dataset Index= 962

Molecule Formula= $C_{25}H_{32}F_3N_2O_2^+$

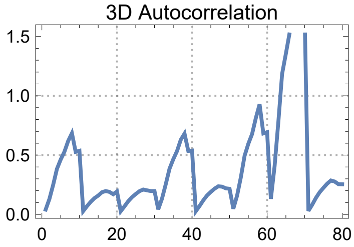

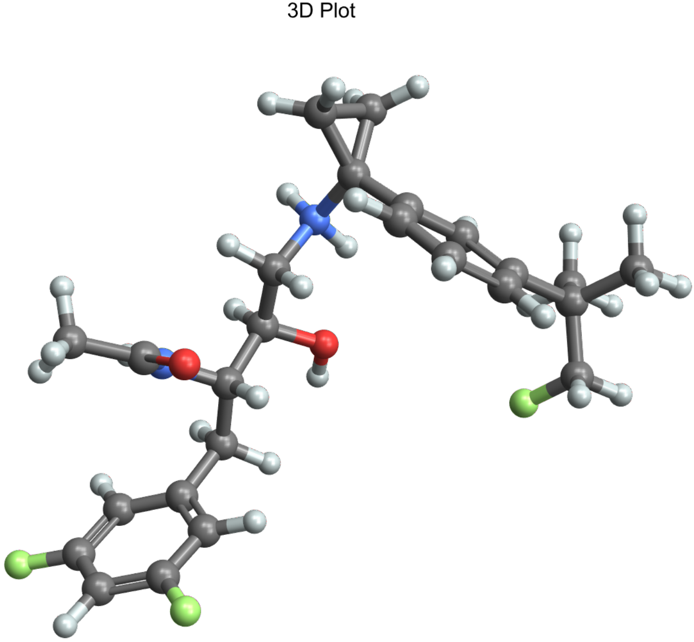

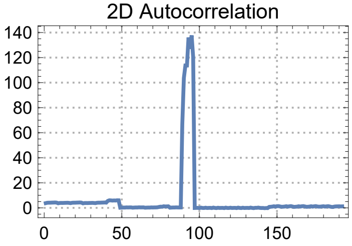

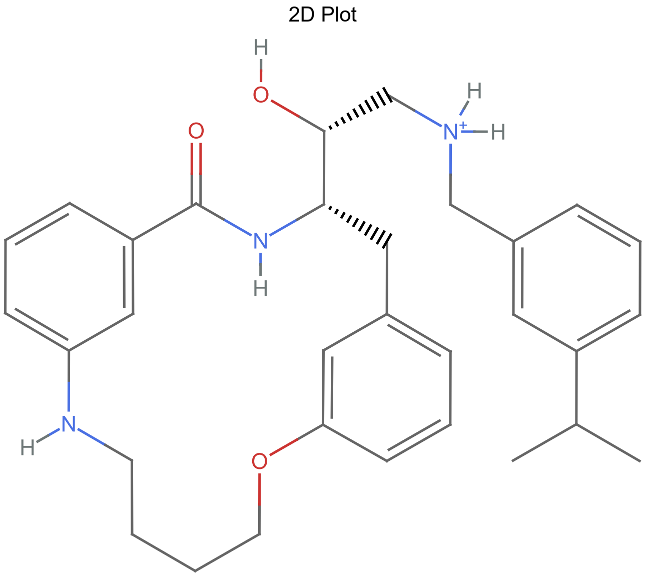

Dataset Index= 963

Molecule Formula= $C_{31}H_{40}N_3O_3^+$

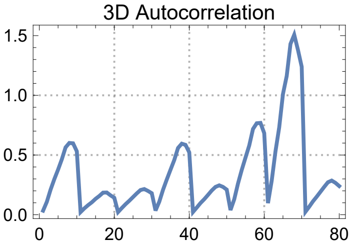

3D Plot

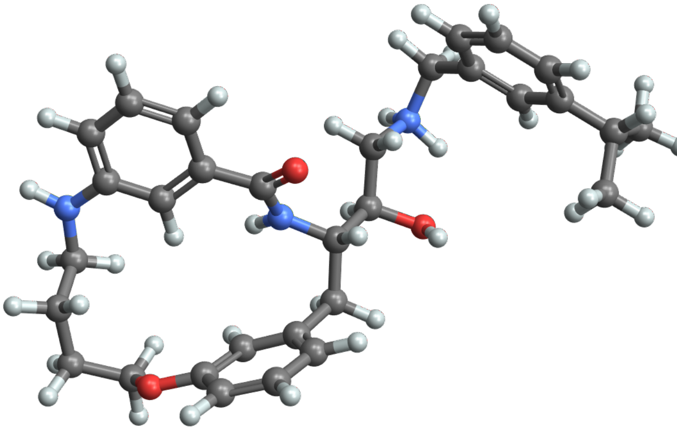

2D Plot

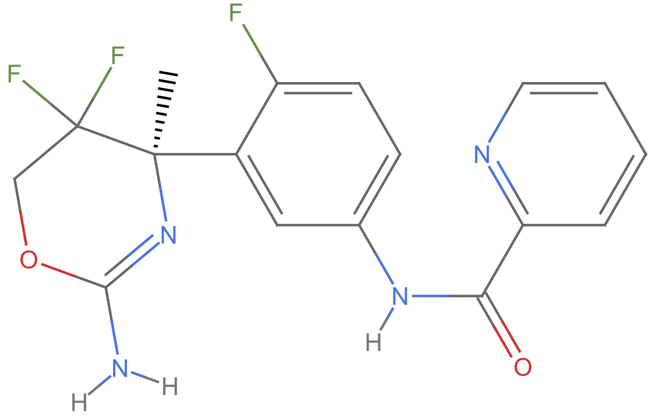

2D Autocorrelation

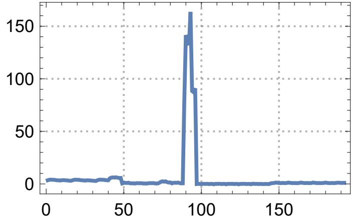

Dataset Index= 964

Molecule Formula= $C_{17}H_{15}F_3N_4O_2$

3D Autocorrelation

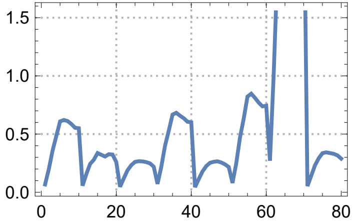

3D Plot

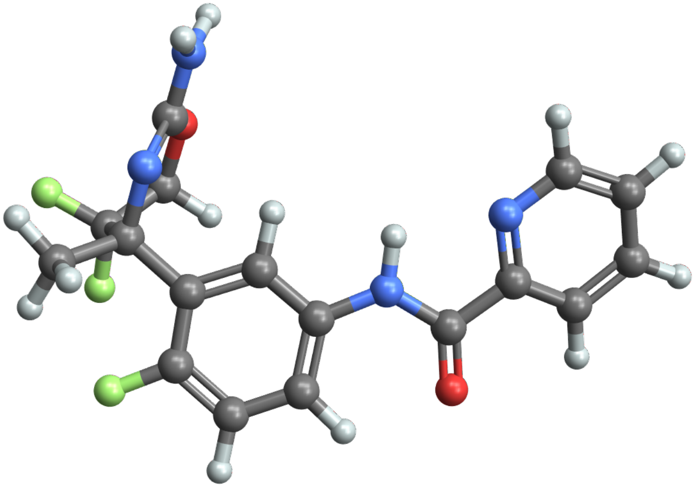

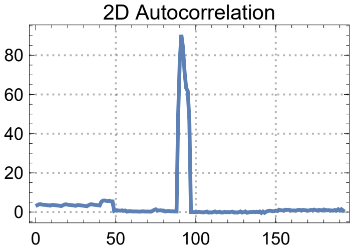

Dataset Index= 965

Molecule Formula= $C_{18}H_{18}ClN_5O_2$

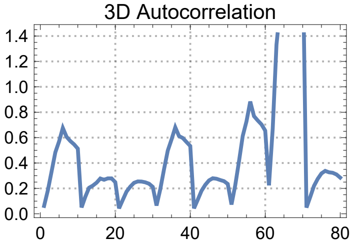

2D Plot

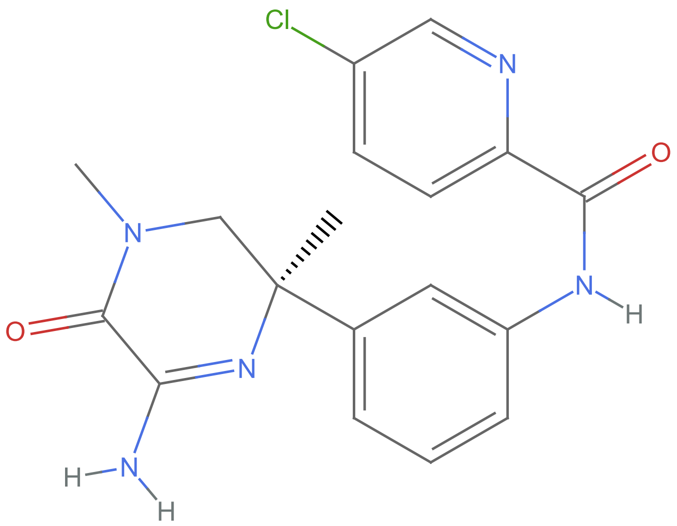

3D Plot

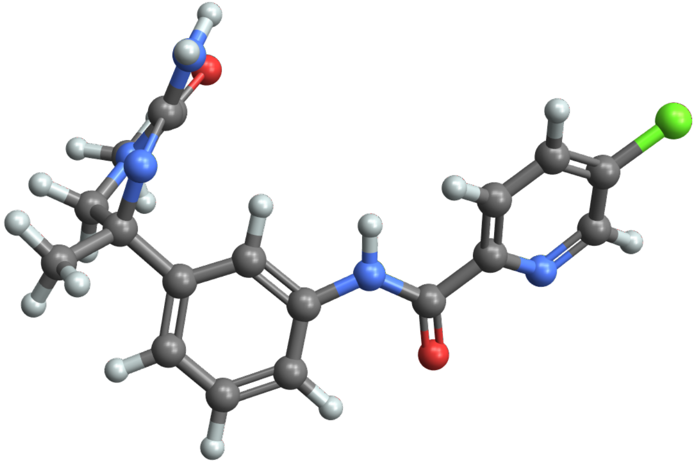

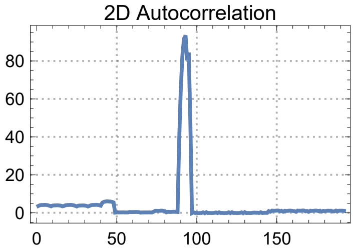

Dataset Index= 966

Molecule Formula= $C_{23}H_{24}N_6O$

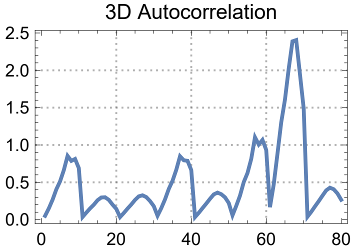

2D Plot

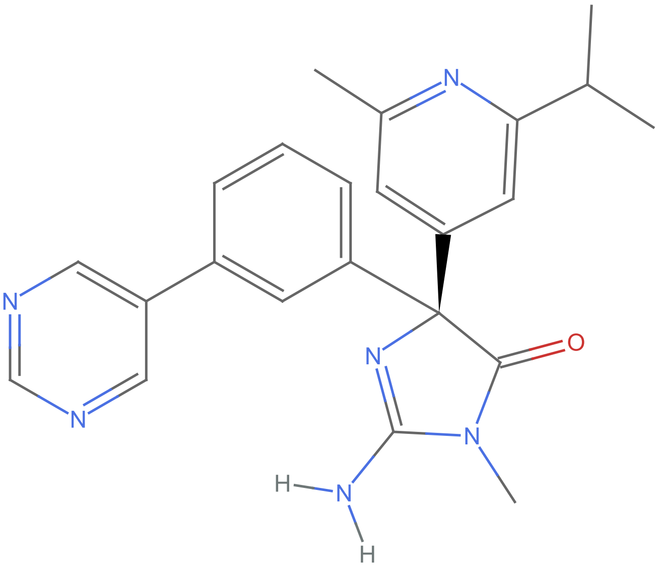

3D Plot

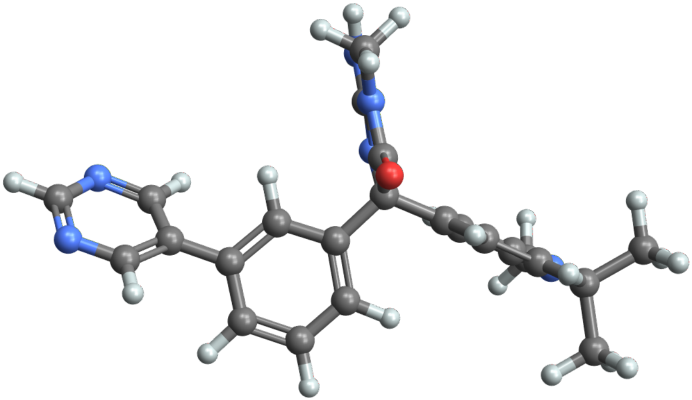

2D Plot

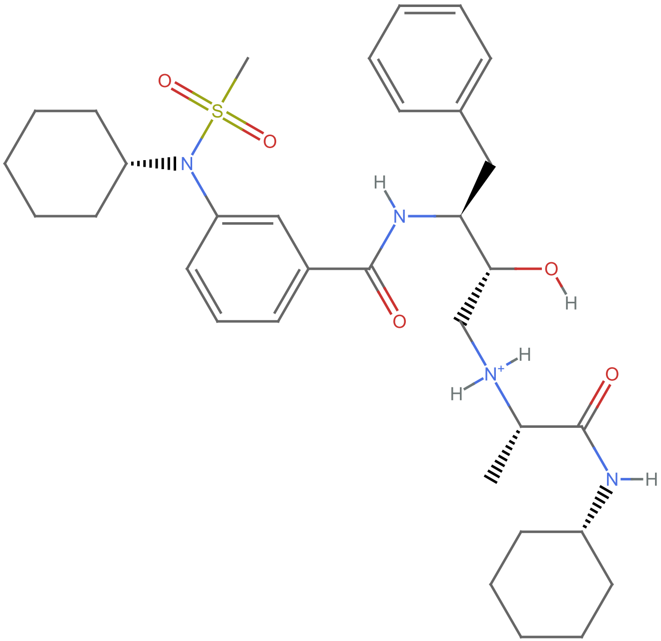

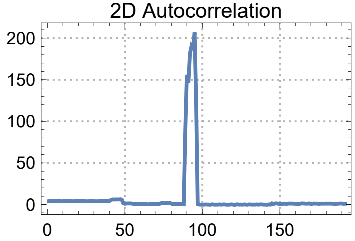

Dataset Index= 967

Molecule Formula= $C_{33}H_{49}N_4O_5S^+$

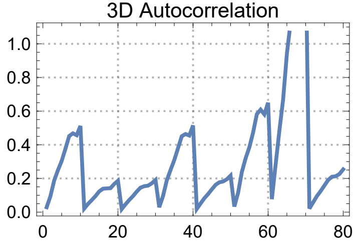

3D Plot

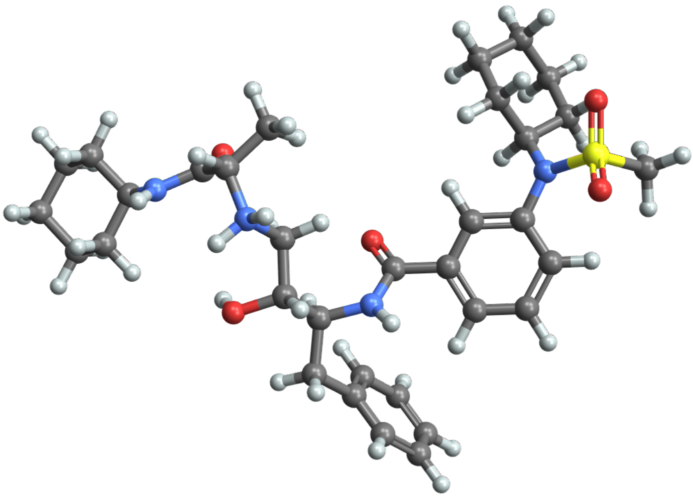

2D Plot

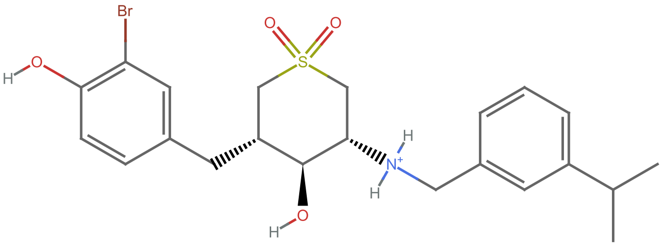

2D Autocorrelation

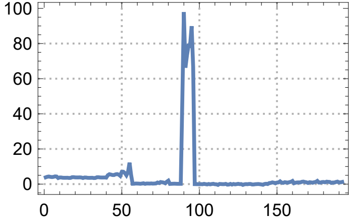

3D Plot

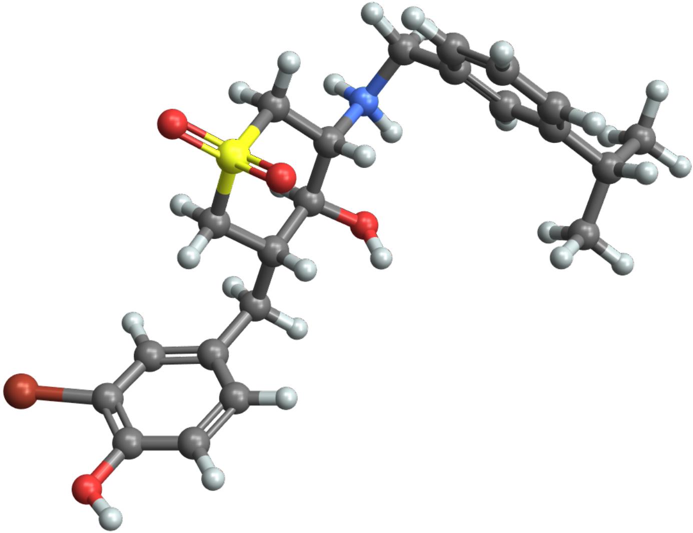

Dataset Index= 968

Molecule Formula= $C_{22}H_{29}BrNO_4S^+$

3D Autocorrelation

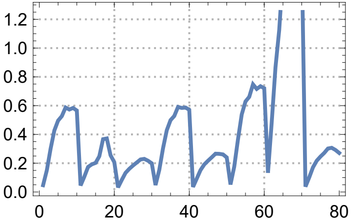

2D Plot

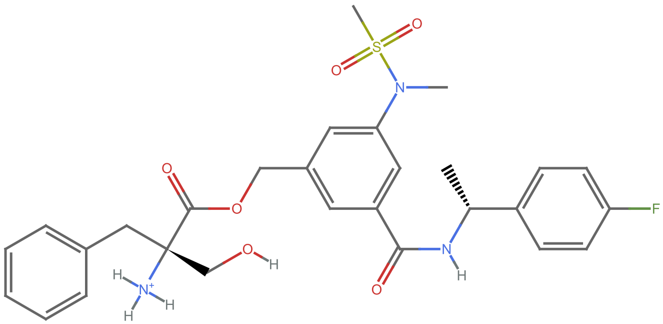

3D Plot

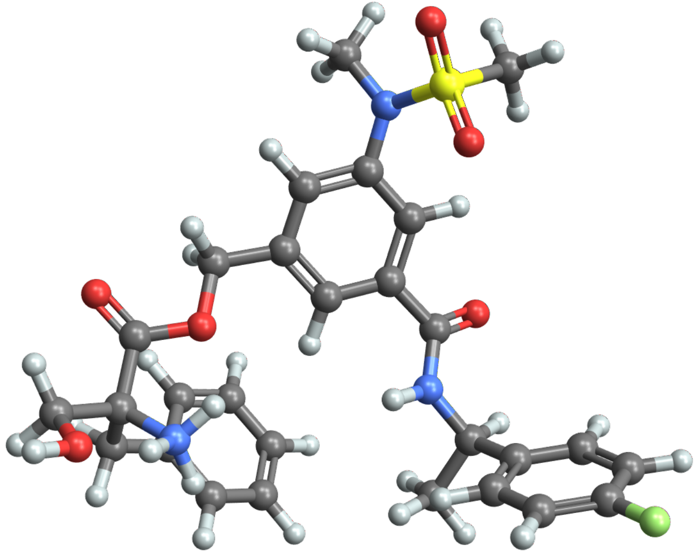

2D Autocorrelation

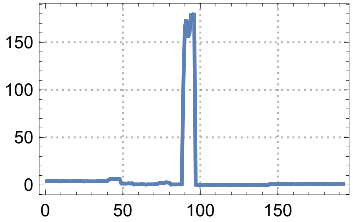

Dataset Index= 969

Molecule Formula= $C_{28}H_{33}FN_3O_6S^+$

3D Autocorrelation

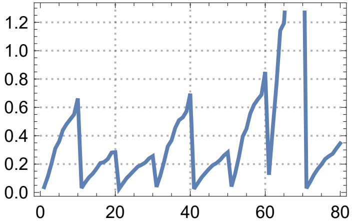

2D Plot

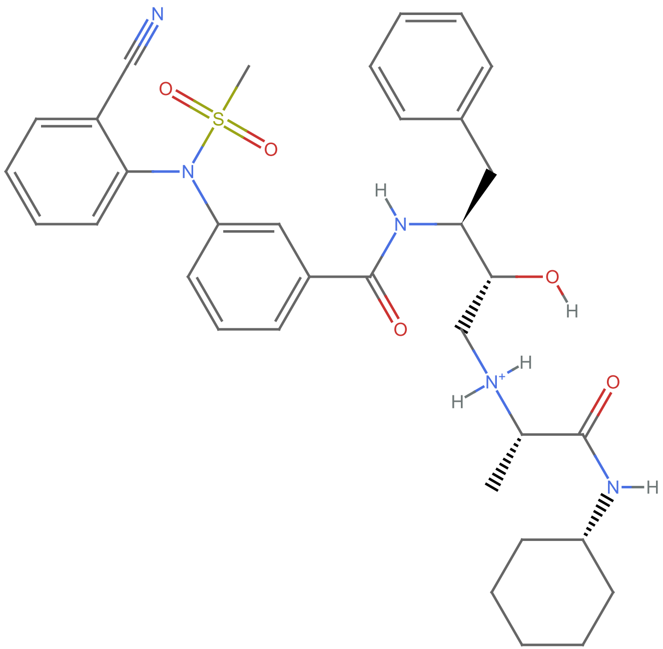

2D Autocorrelation

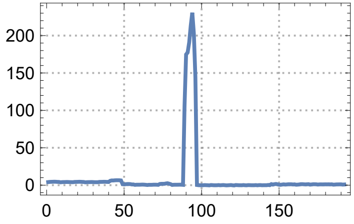

Dataset Index= 970

Molecule Formula= $C_{34}H_{42}N_5O_5S^+$

3D Autocorrelation

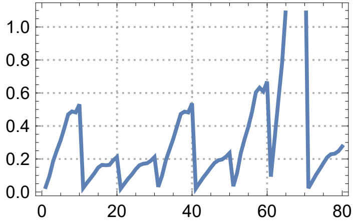

3D Plot

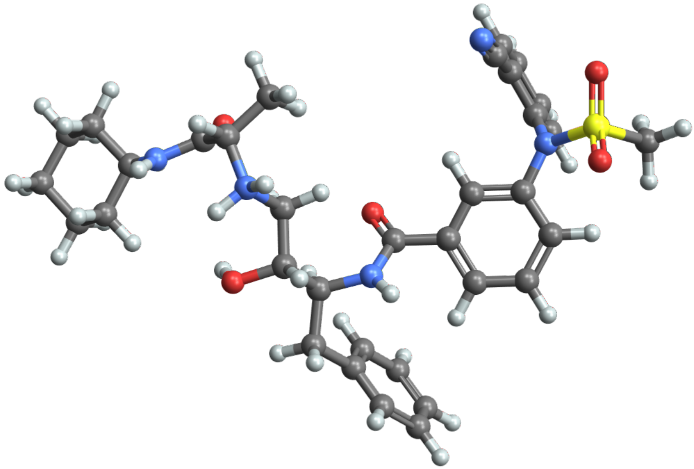

2D Autocorrelation

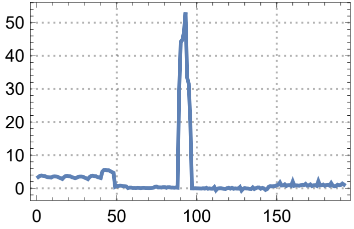

Dataset Index= 971

Molecule Formula= $C_{18}H_{17}ClN_4$

3D Autocorrelation

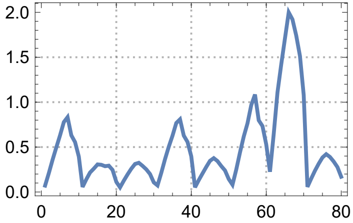

2D Plot

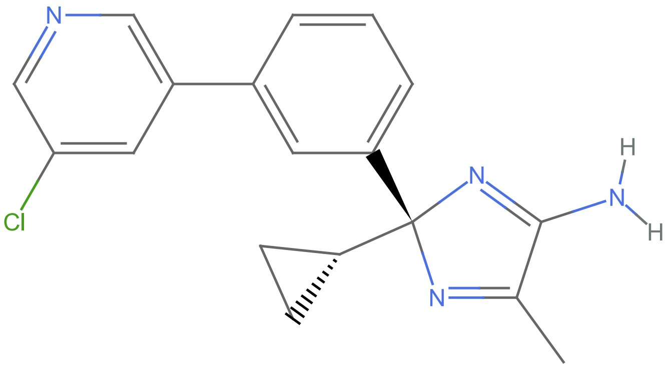

3D Plot

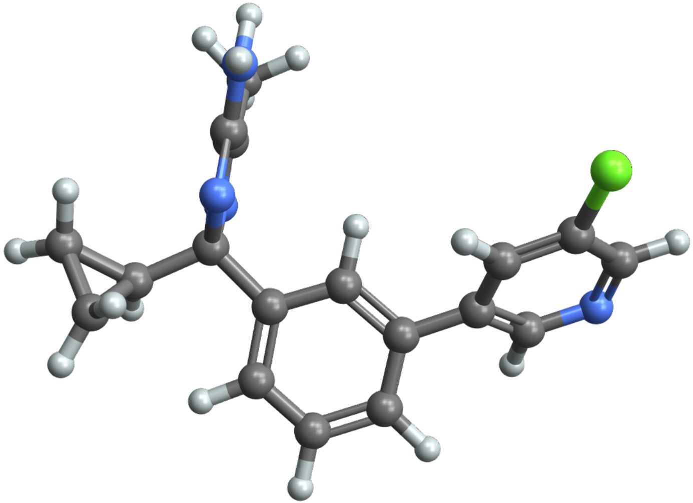

2D Plot

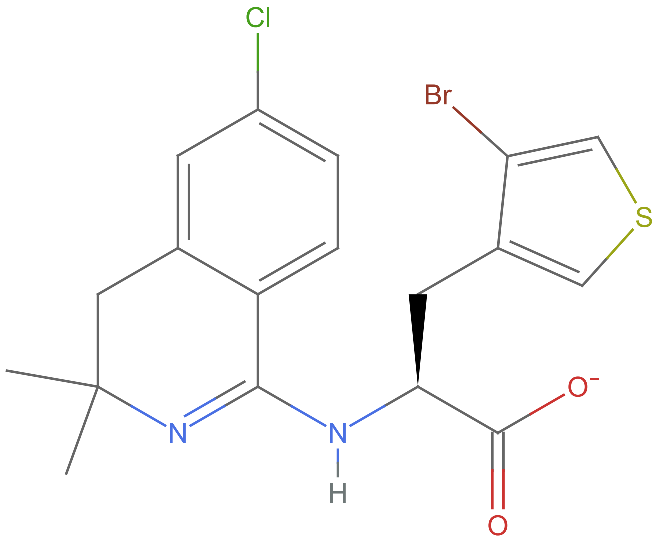

3D Plot

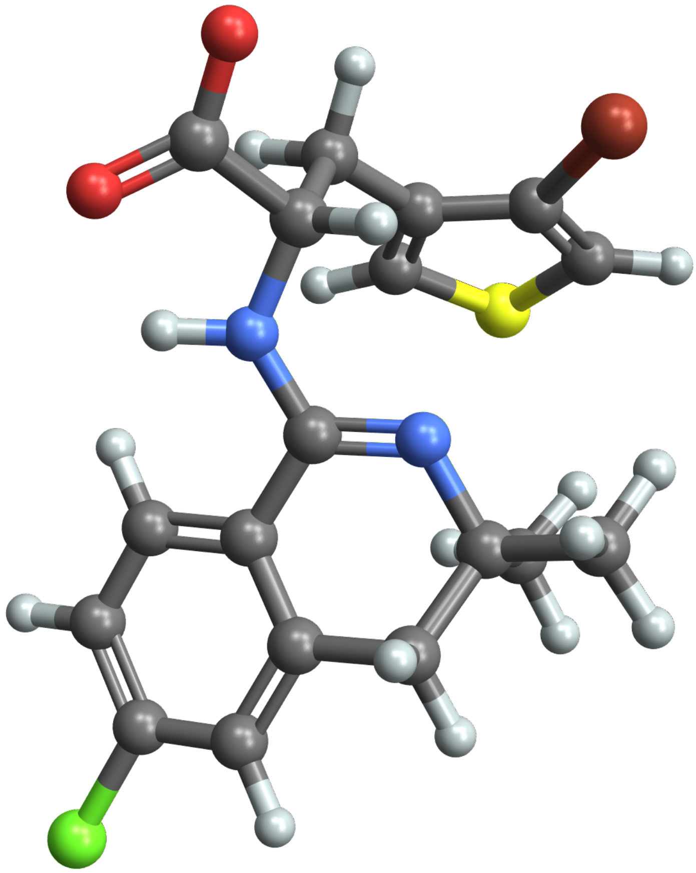

2D Autocorrelation

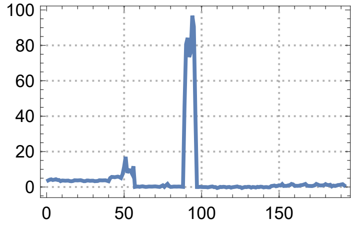

Dataset Index= 972

Molecule Formula= $C_{18}H_{17}BrClN_2O_2S^-$

3D Autocorrelation

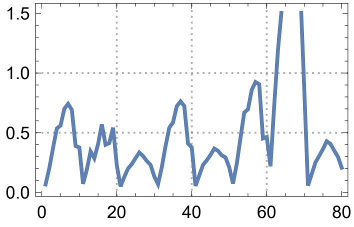

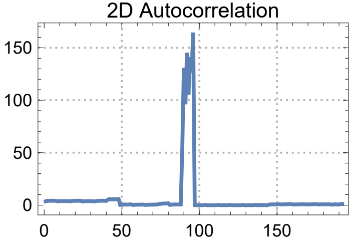

Dataset Index= 973

Molecule Formula= $C_{28}H_{40}F_2N_3O_2^+$

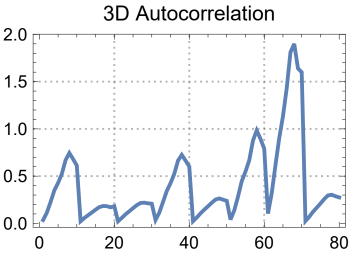

2D Plot

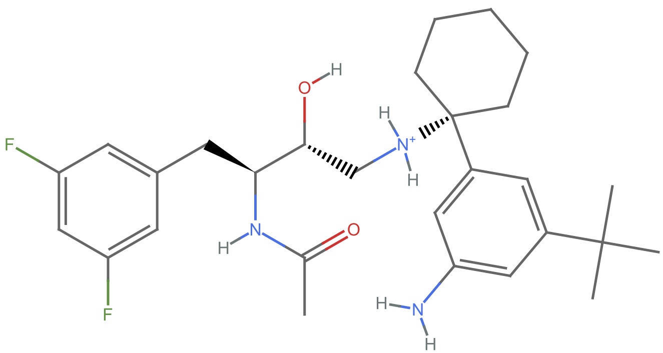

3D Plot

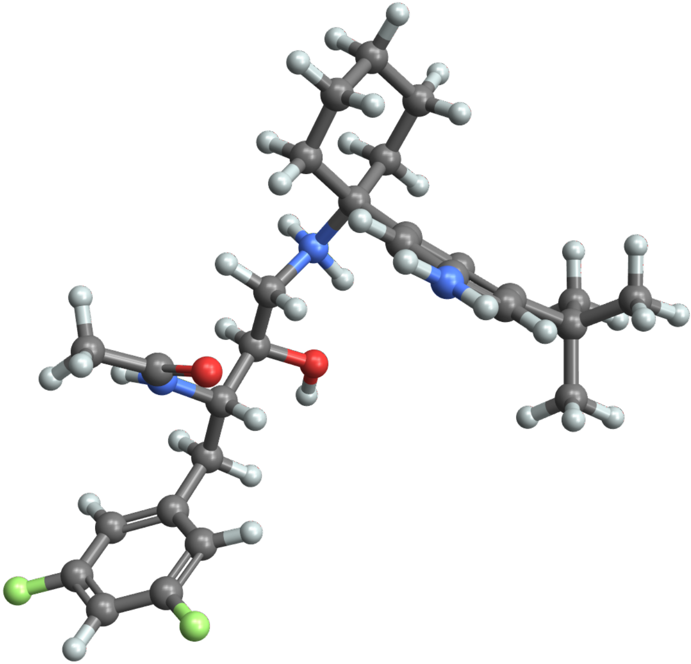

2D Plot

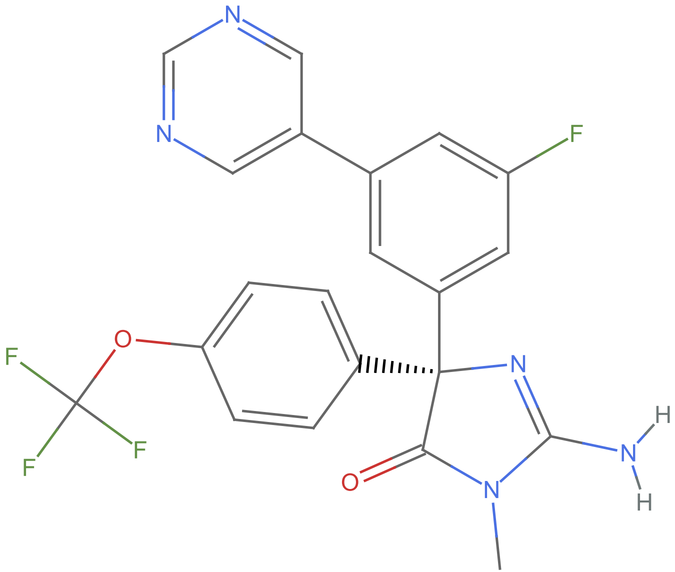

2D Autocorrelation

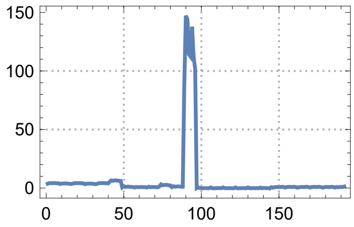

Dataset Index= 974

Molecule Formula= $C_{21}H_{15}F_4N_5O_2$

3D Autocorrelation

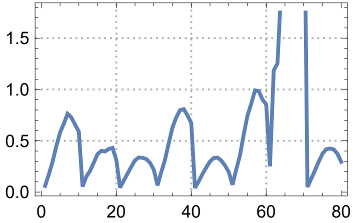

3D Plot

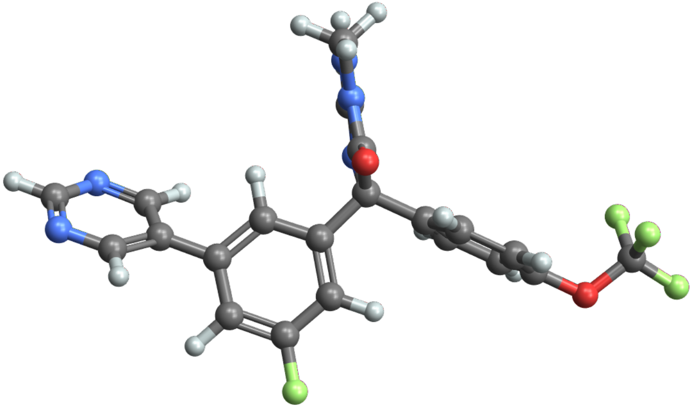

2D Autocorrelation

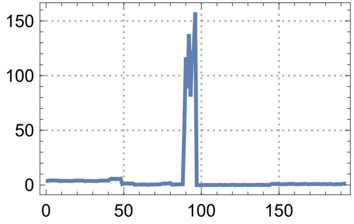

Dataset Index= 975

Molecule Formula= $C_{25}H_{35}F_2N_2O_2S^+$

3D Autocorrelation

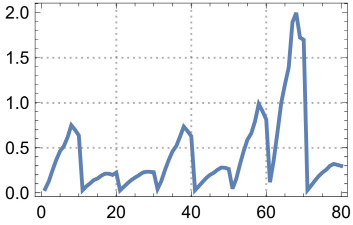

2D Plot

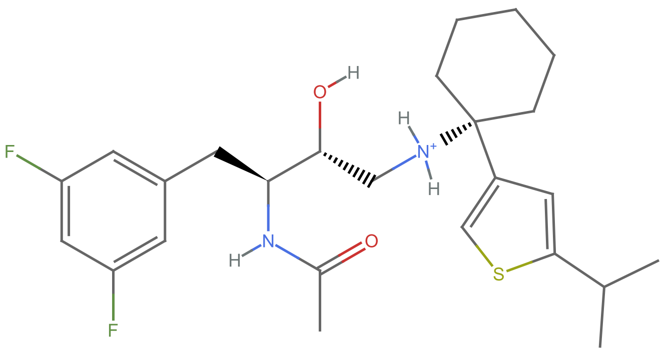

3D Plot

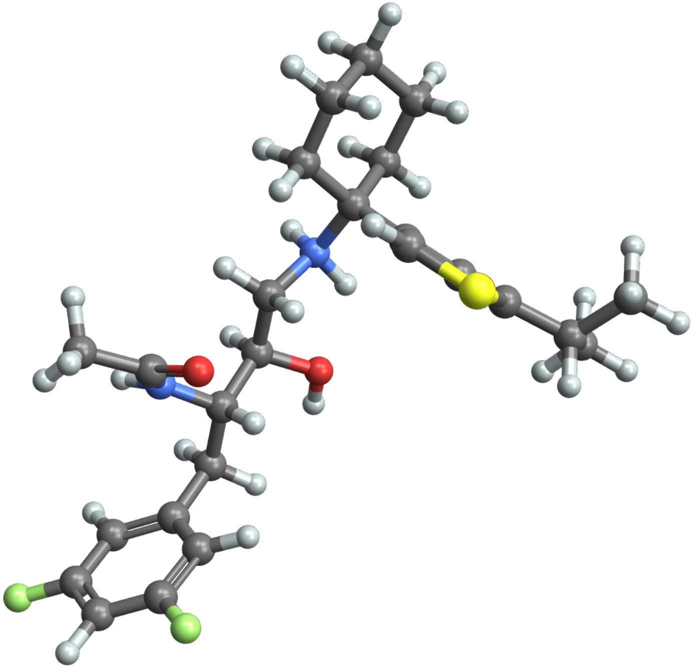

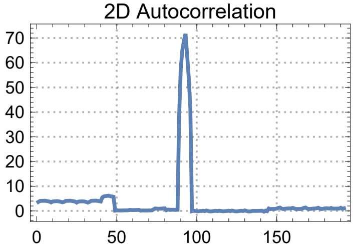

Dataset Index= 976

Molecule Formula= $C_{23}H_{20}N_6O$

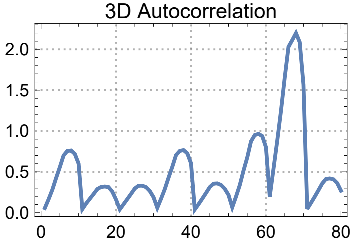

2D Plot

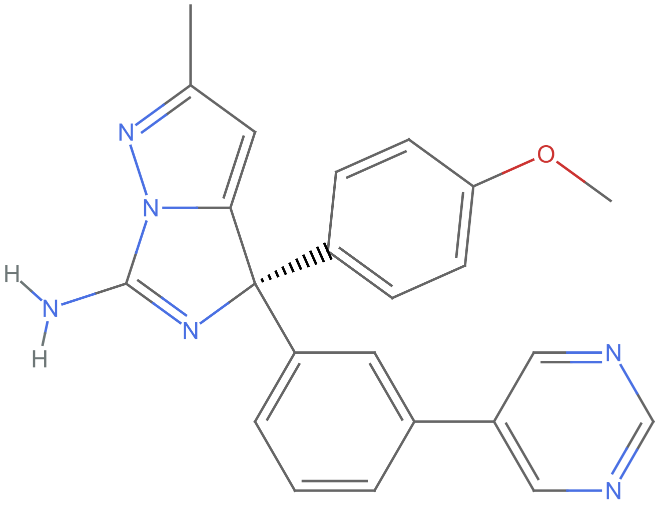

3D Plot

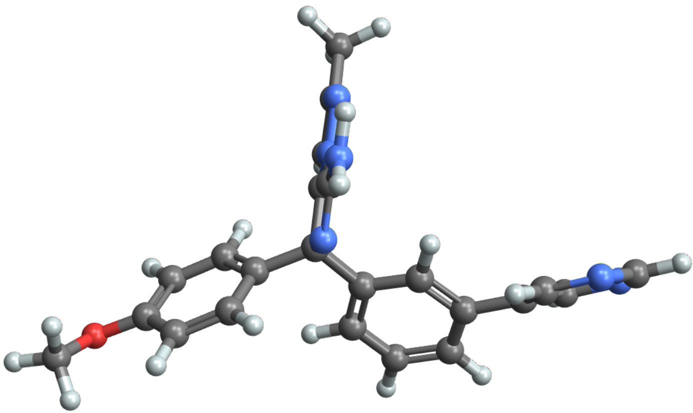

2D Plot

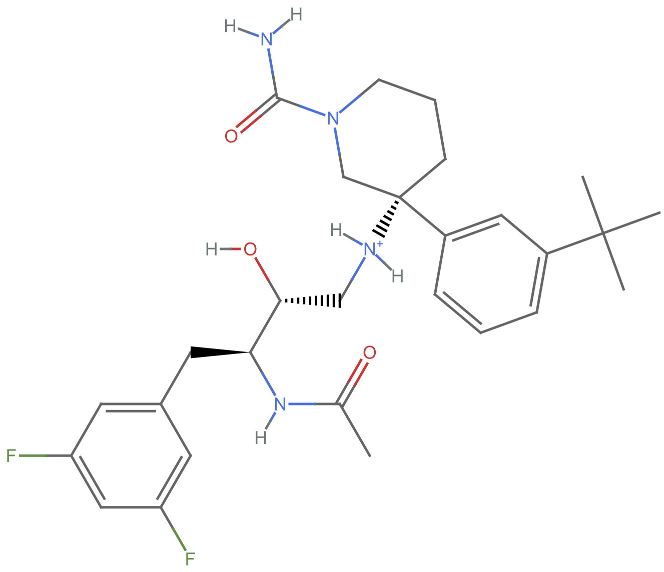

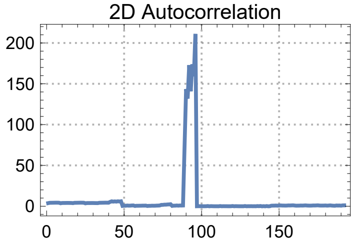

Dataset Index= 977

Molecule Formula= $C_{28}H_{39}F_{2}N_{4}O_{3}^{+}$

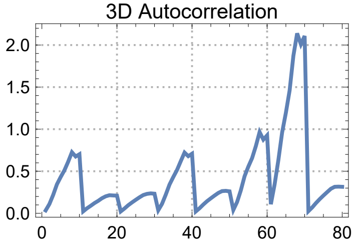

3D Plot

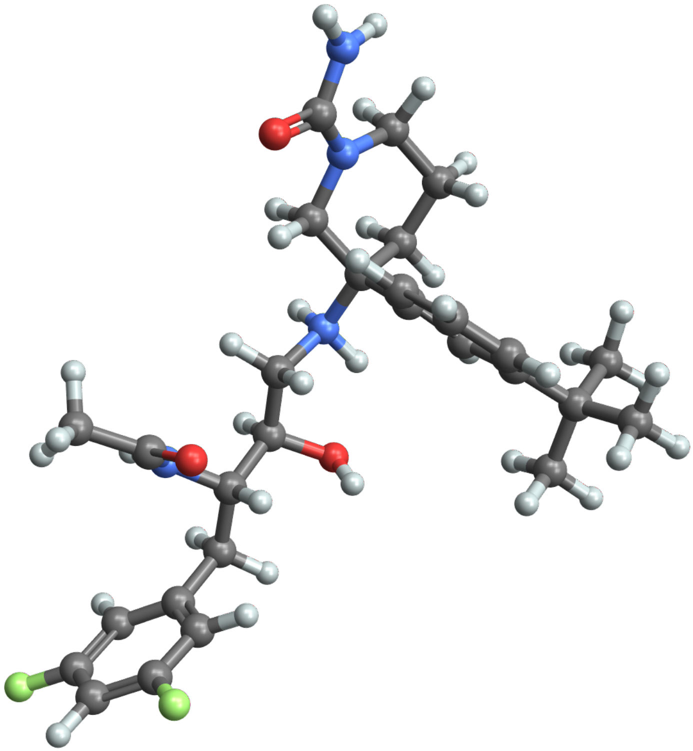

2D Autocorrelation

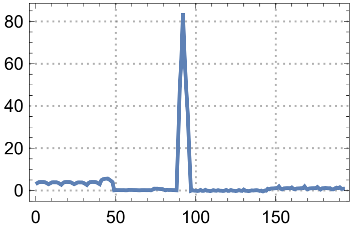

Dataset Index= 978

Molecule Formula= $C_{22}H_{29}N_3O_2$

3D Autocorrelation

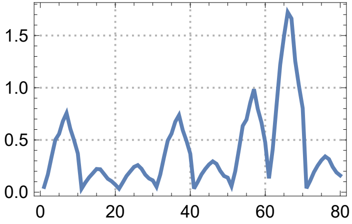

2D Plot

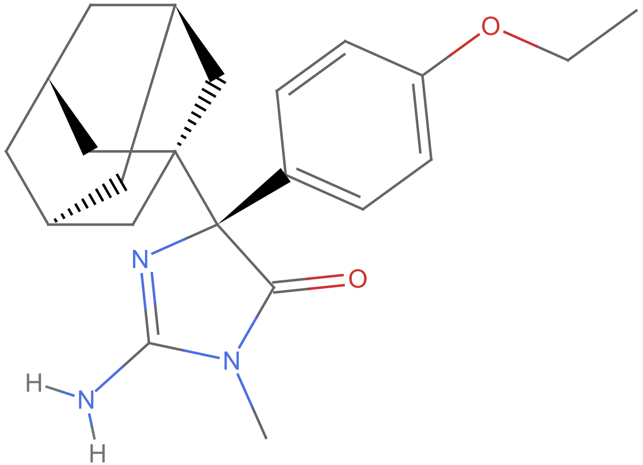

3D Plot

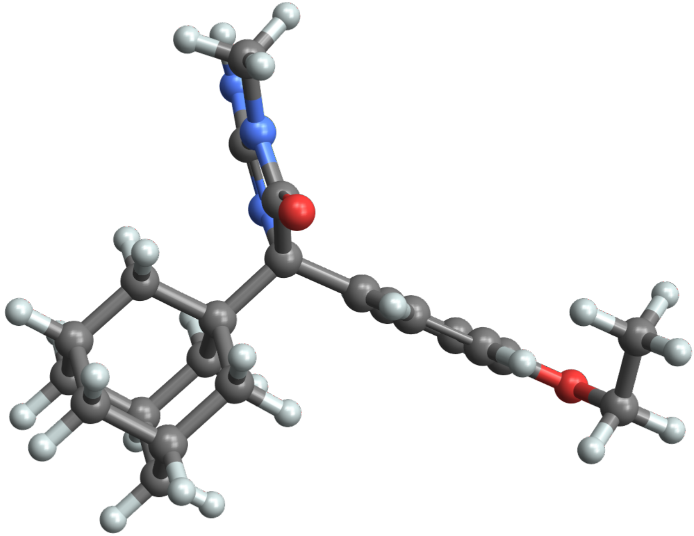

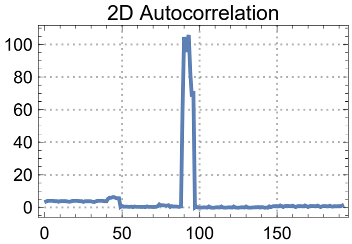

Dataset Index= 979

Molecule Formula= $C_{25}H_{25}F_2N_3O_2$

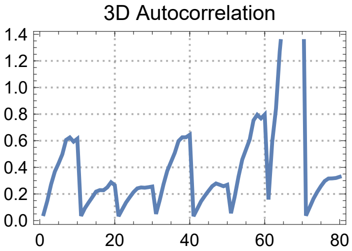

2D Plot

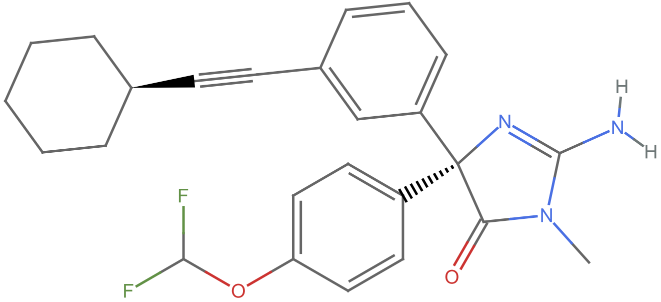

3D Plot

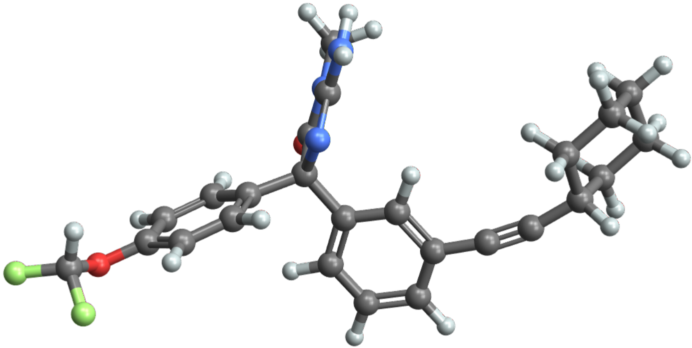

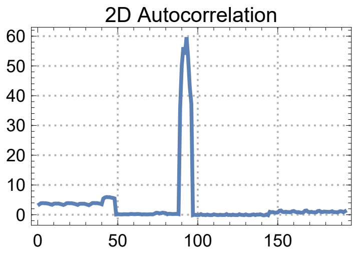

Dataset Index= 980

Molecule Formula= $C_{22}H_{20}N_4O$

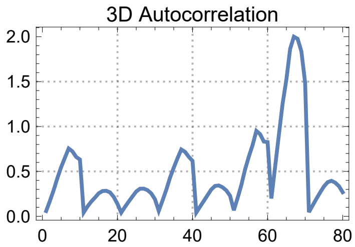

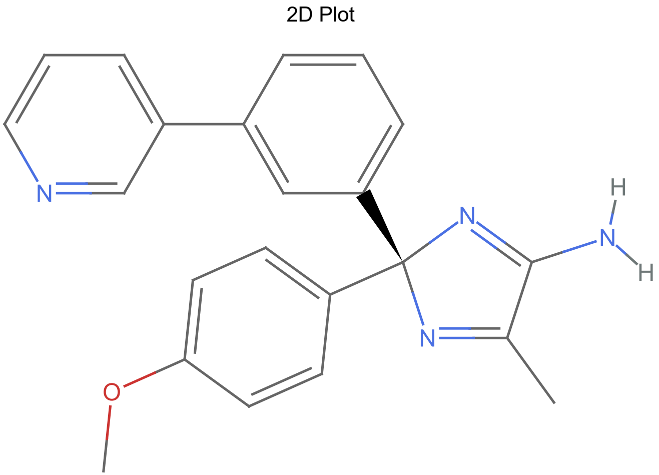

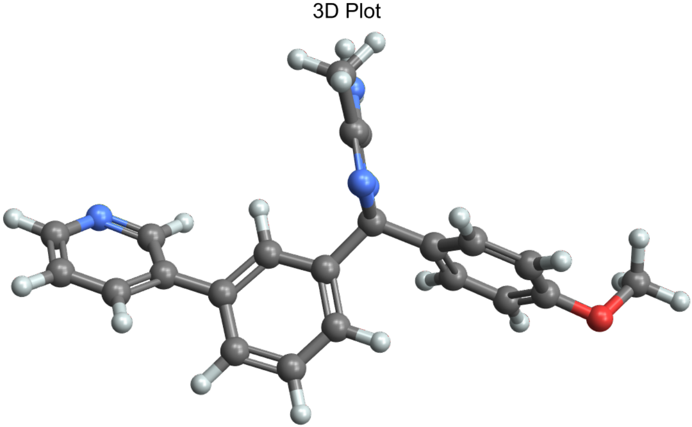

2D Plot

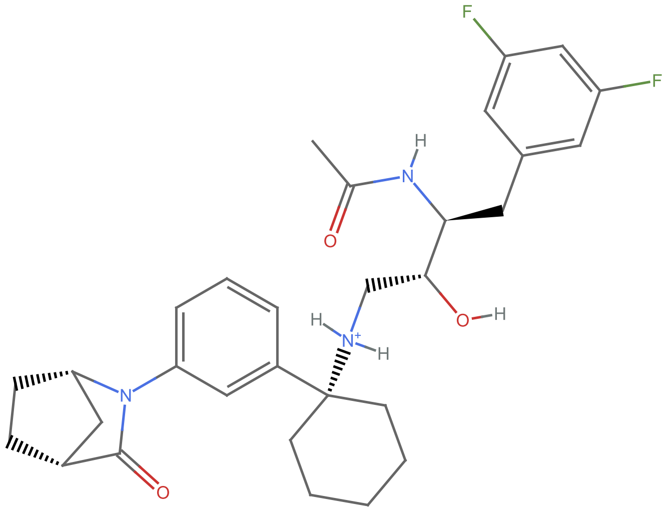

2D Autocorrelation

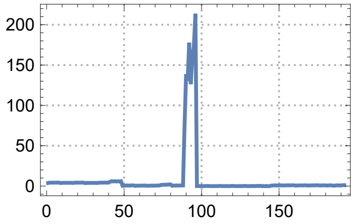

3D Plot

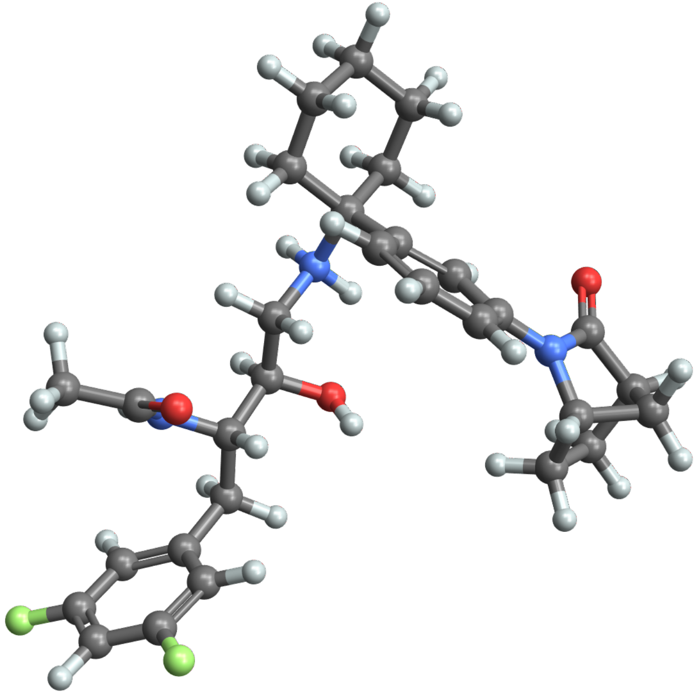

Dataset Index= 981

Molecule Formula= $C_{30}H_{38}F_2N_3O_3^+$

3D Autocorrelation

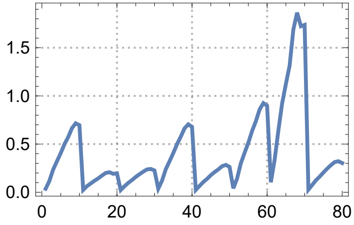

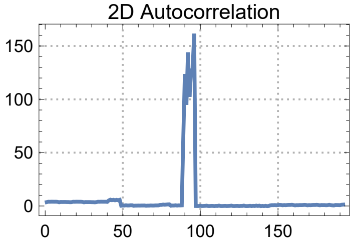

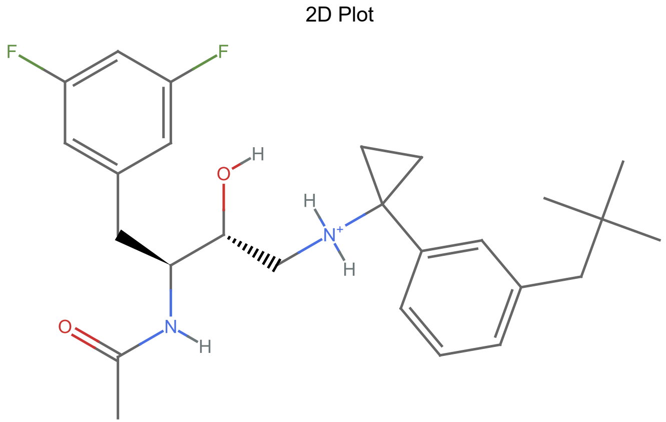

Dataset Index= 982

Molecule Formula= $C_{26}H_{35}F_{2}N_{2}O_{2}^{+}$

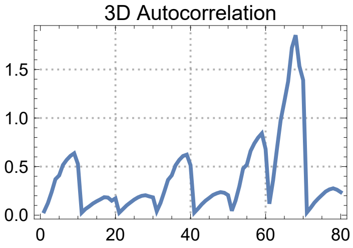

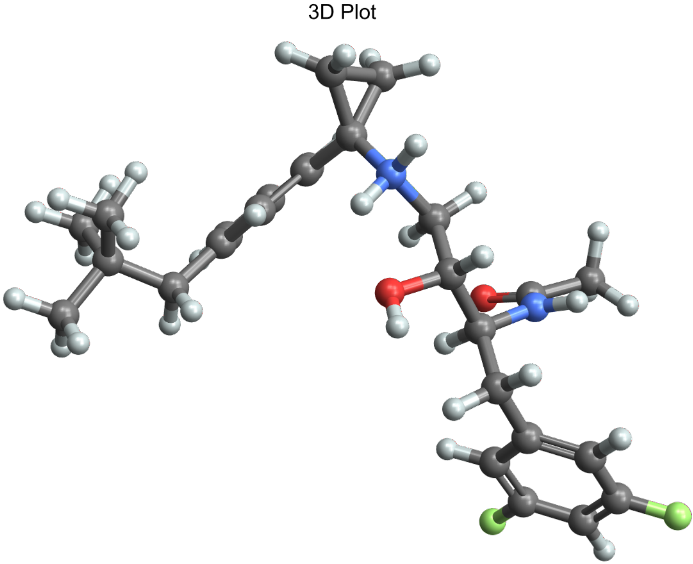

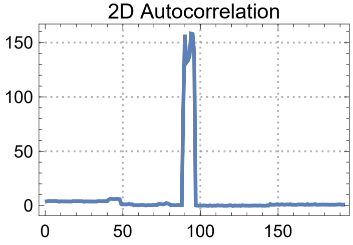

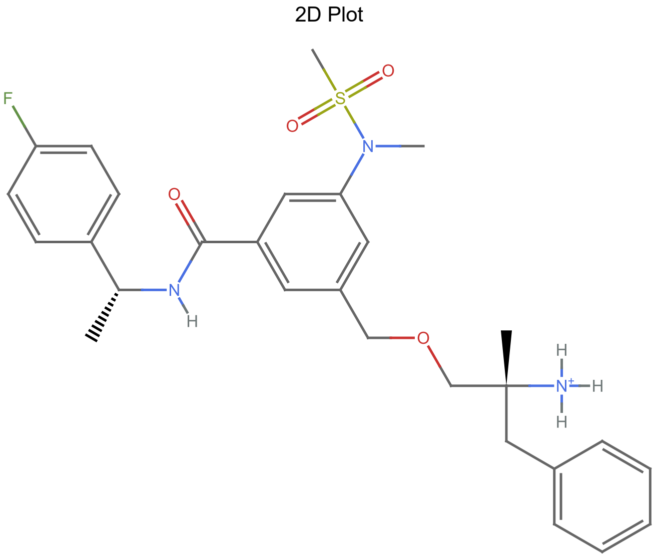

Dataset Index= 983

Molecule Formula= $C_{28}H_{35}FN_3O_4S^+$

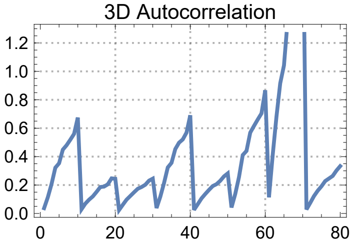

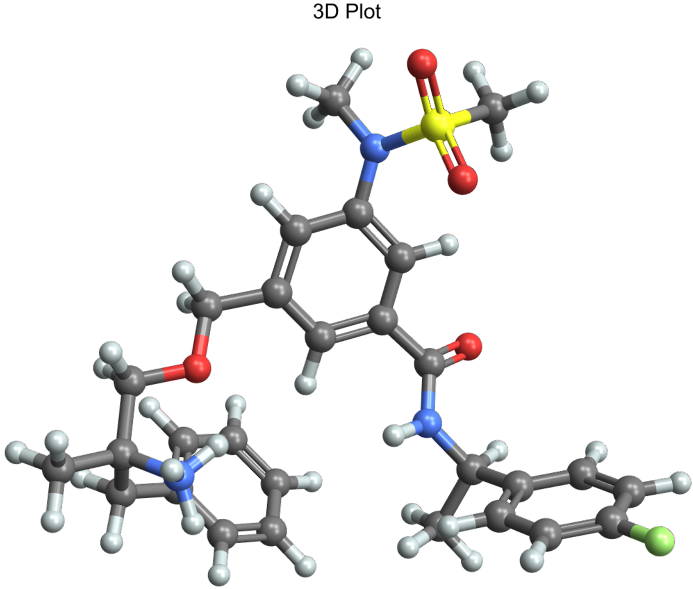

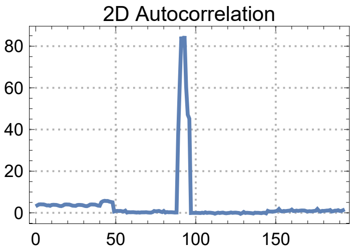

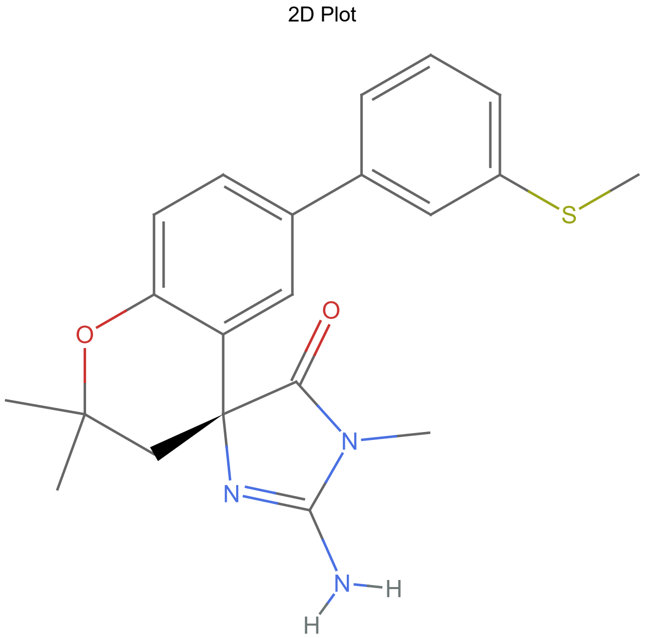

Dataset Index= 984

Molecule Formula= $C_{21}H_{23}N_{3}O_{2}S$

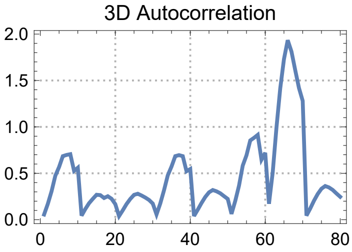

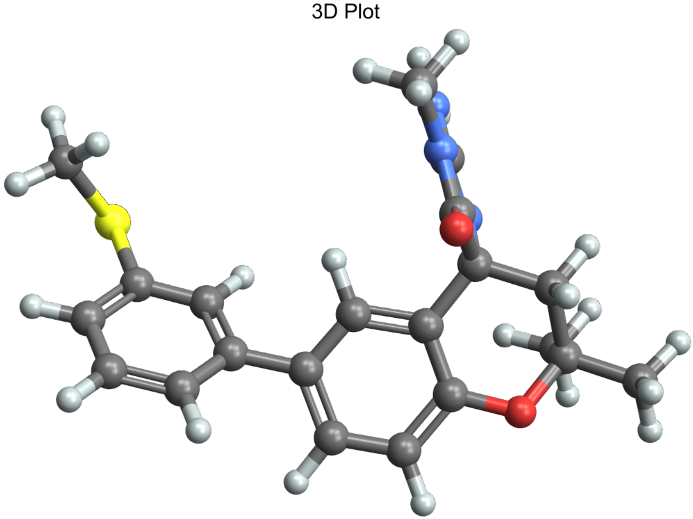

2D Autocorrelation

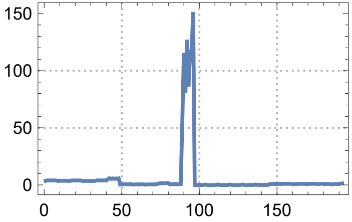

2D Plot

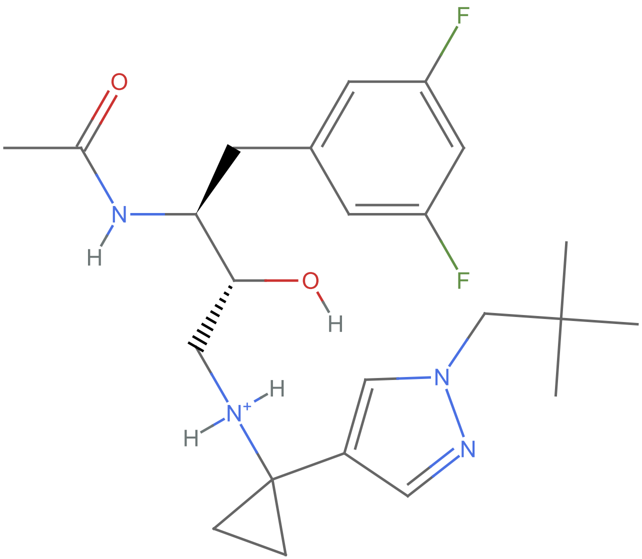

Dataset Index= 985

Molecule Formula= $C_{23}H_{33}F_2N_4O_2^+$

3D Autocorrelation

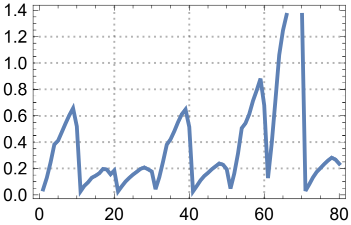

3D Plot

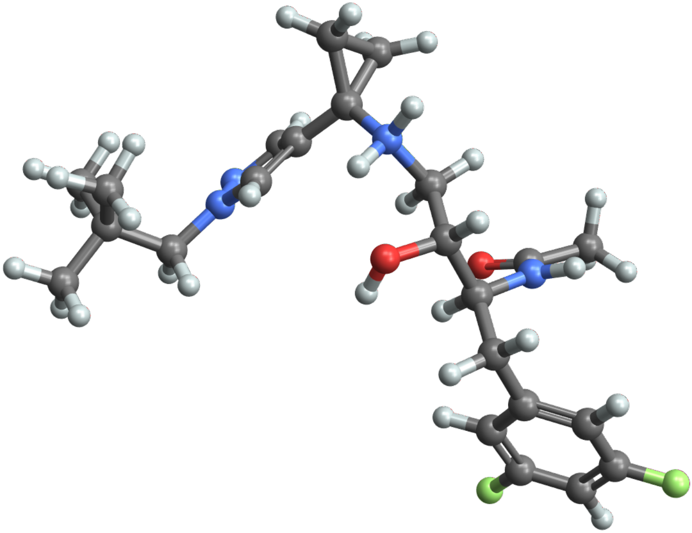

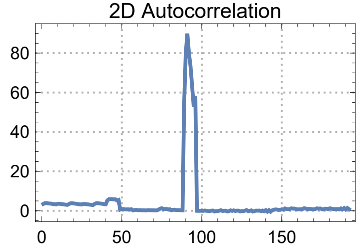

Dataset Index= 986

Molecule Formula= $C_{17}H_{16}ClN_5O_2$

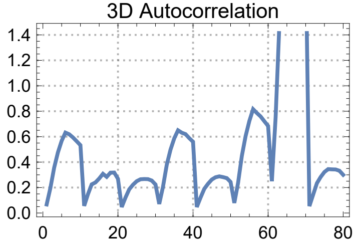

2D Plot

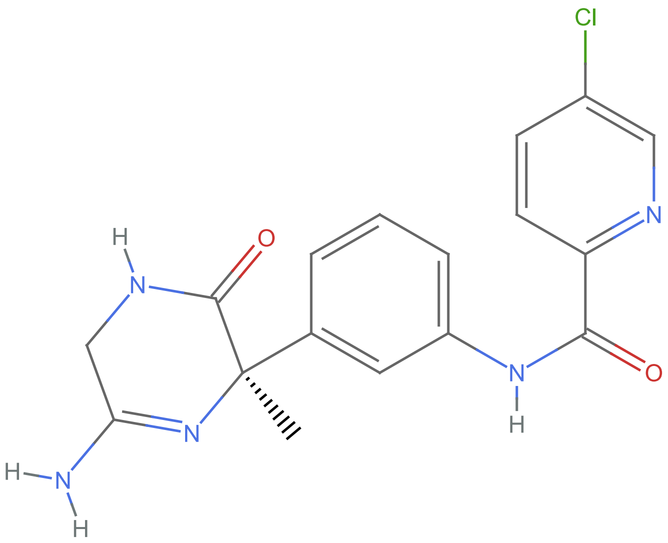

3D Plot

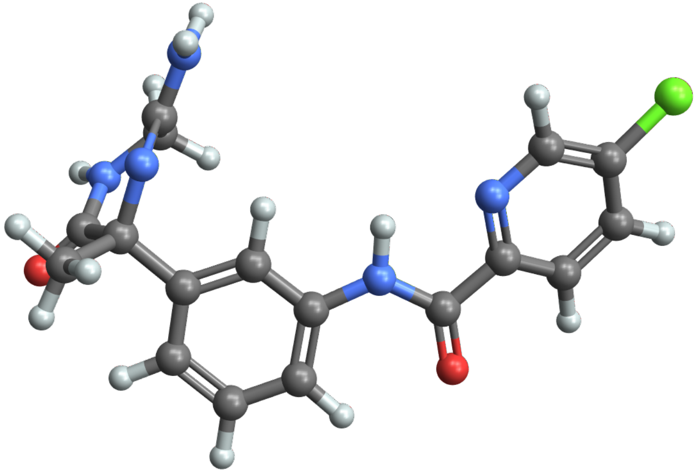

2D Plot

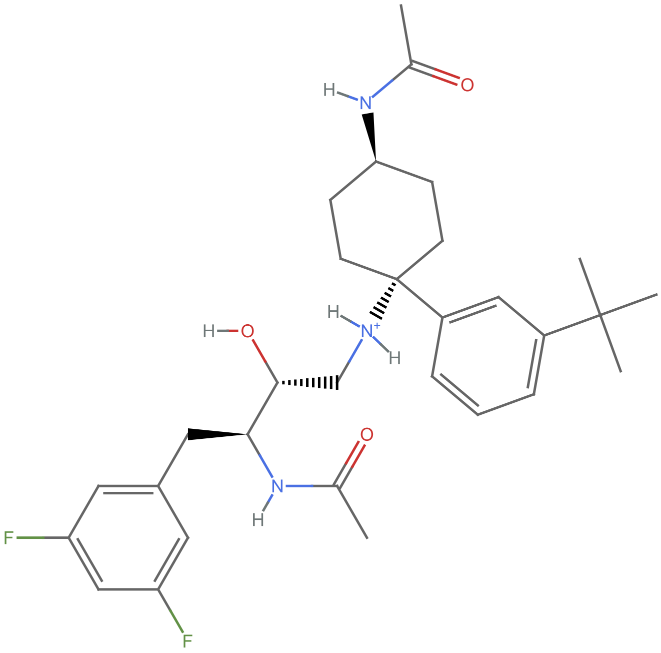

2D Autocorrelation

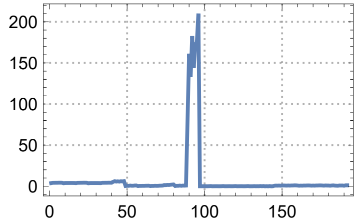

Dataset Index= 987

Molecule Formula= $C_{30}H_{42}F_2N_3O_3^+$

3D Autocorrelation

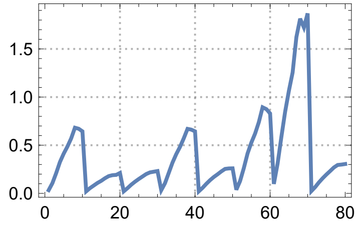

3D Plot

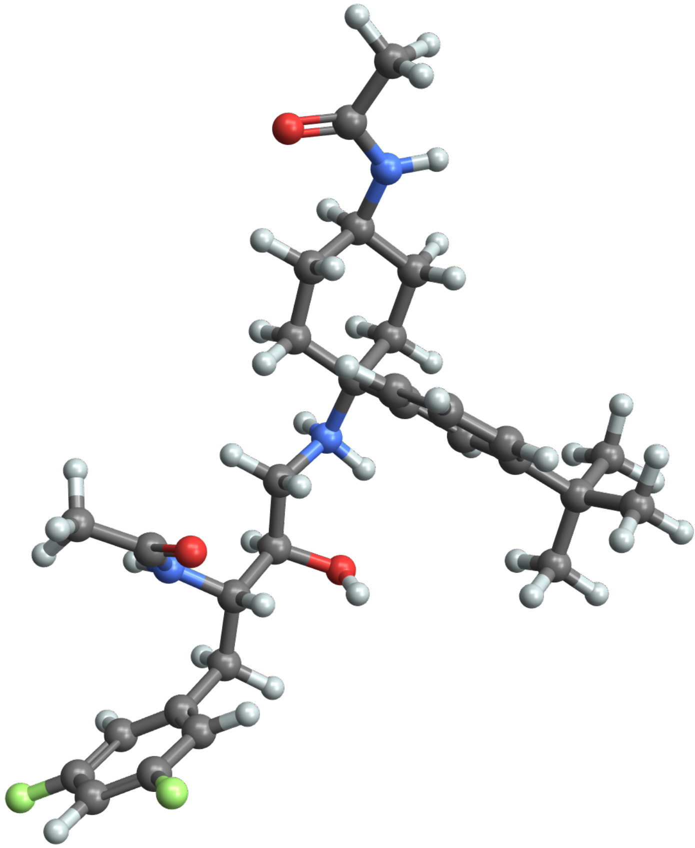

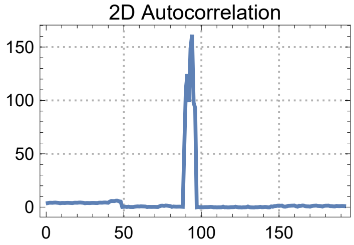

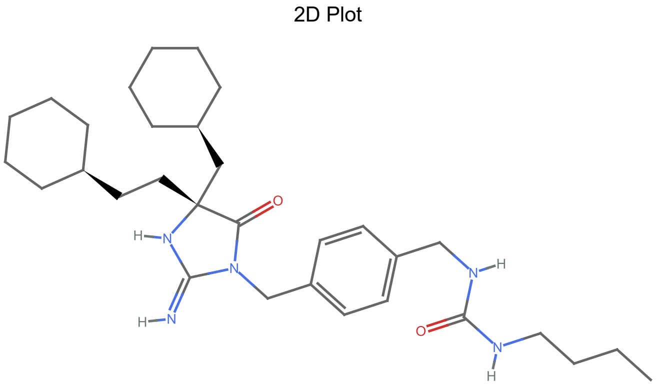

Dataset Index= 988

Molecule Formula= $C_{31}H_{49}N_5O_2$

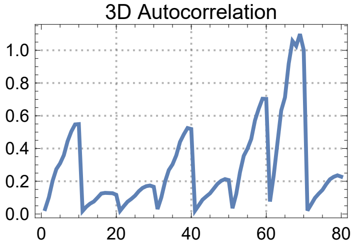

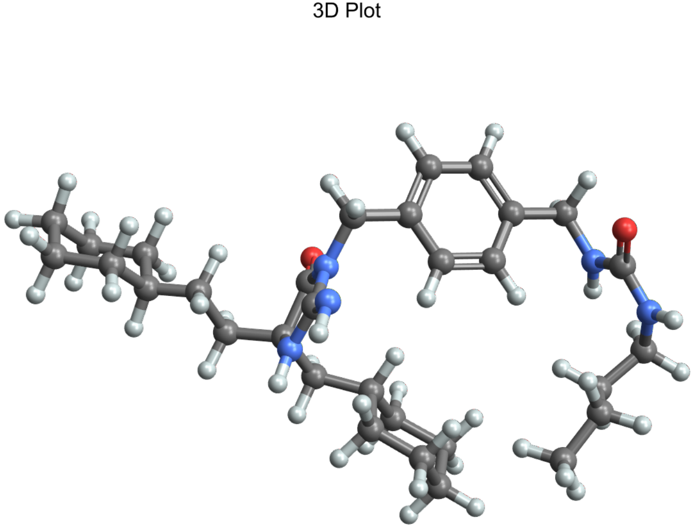

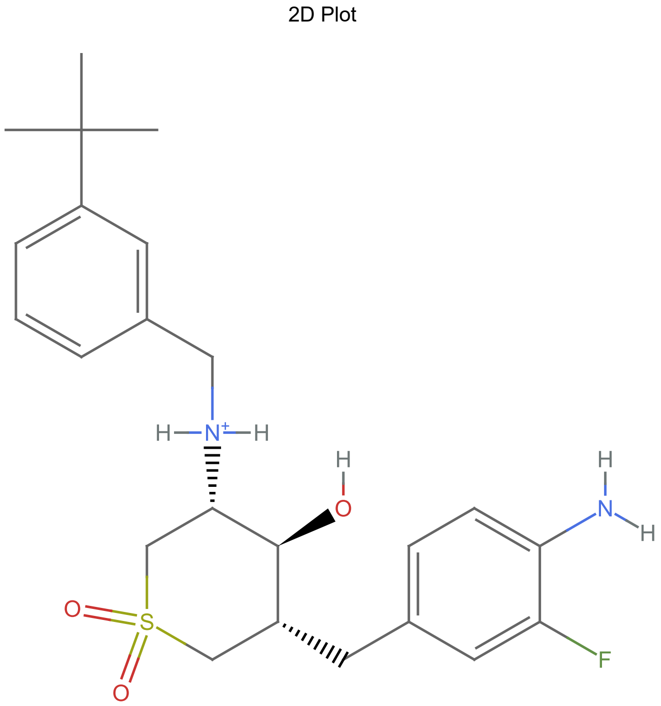

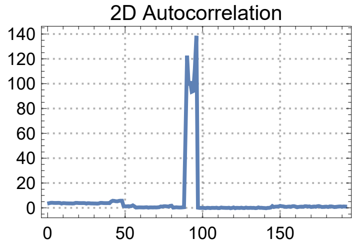

Dataset Index= 989

Molecule Formula= $C_{23}H_{32}FN_2O_3S^+$

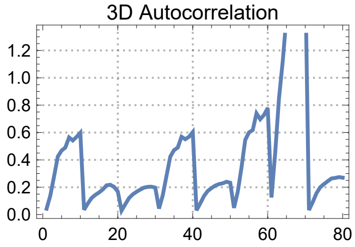

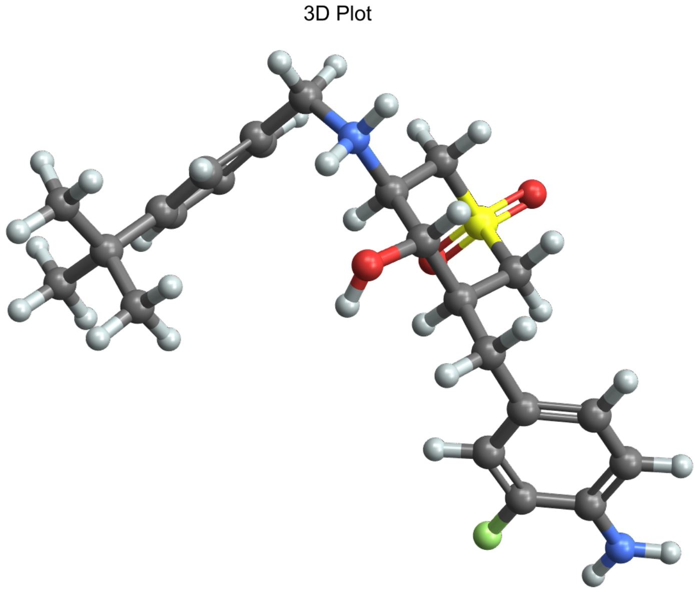

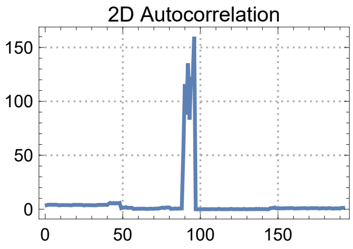

Dataset Index= 990

Molecule Formula= $C_{25}H_{35}F_2N_2O_2S^+$

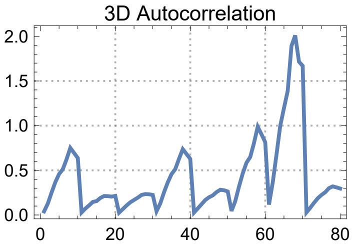

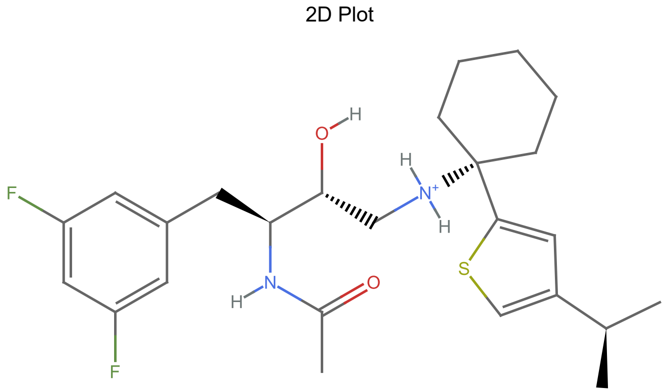

3D Plot

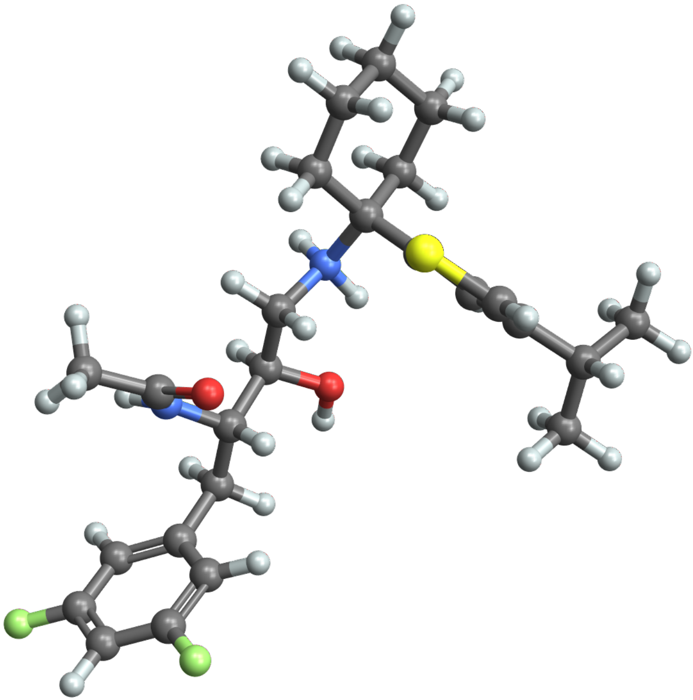

2D Plot

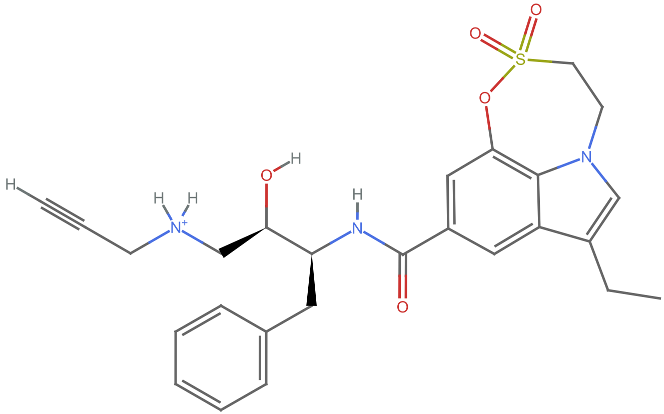

2D Autocorrelation

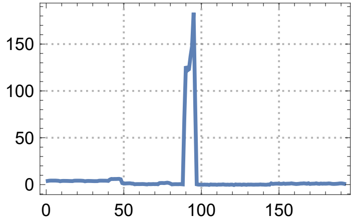

3D Plot

Dataset Index= 991

Molecule Formula= $C_{26}H_{30}N_3O_5S^+$

3D Autocorrelation

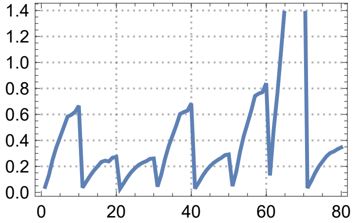

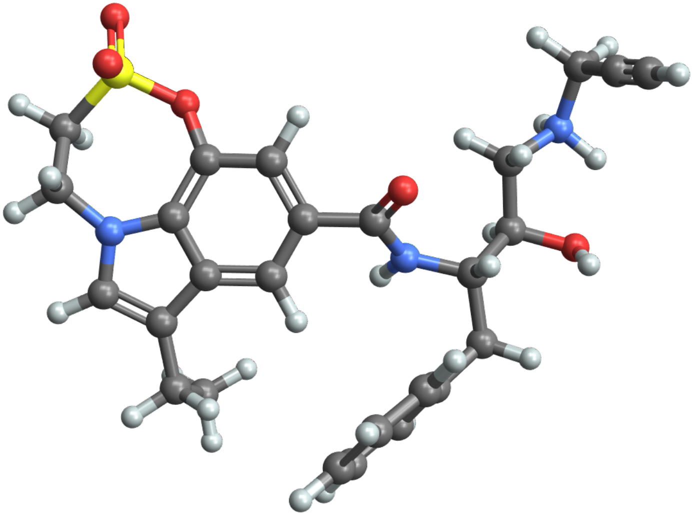

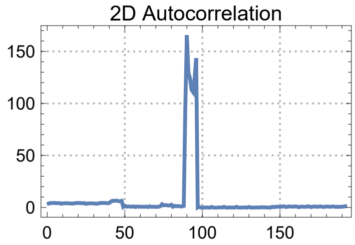

Dataset Index= 992

Molecule Formula= $C_{24}H_{20}F_4N_5O^+$

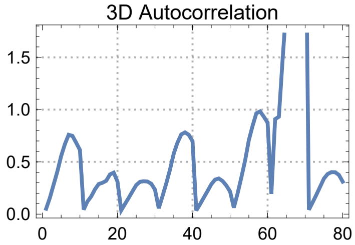

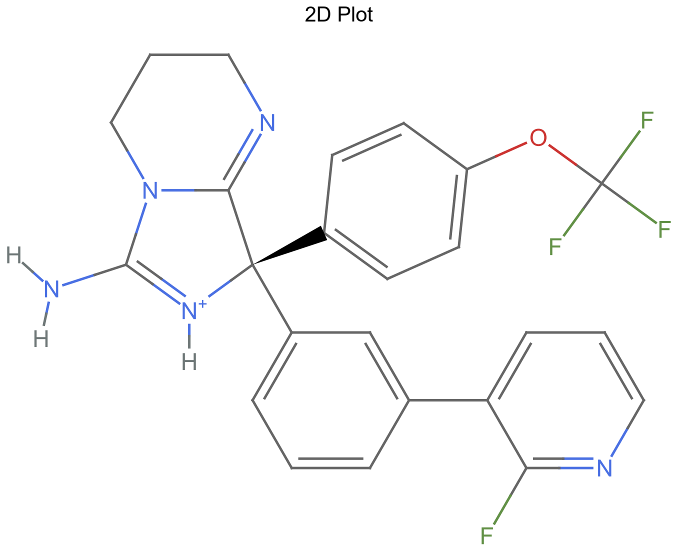

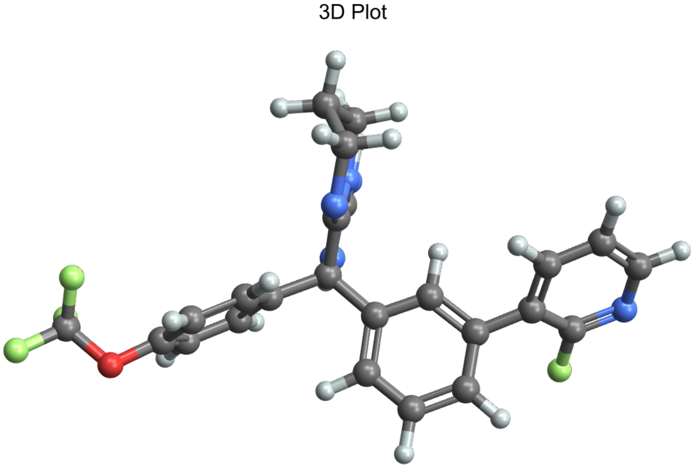

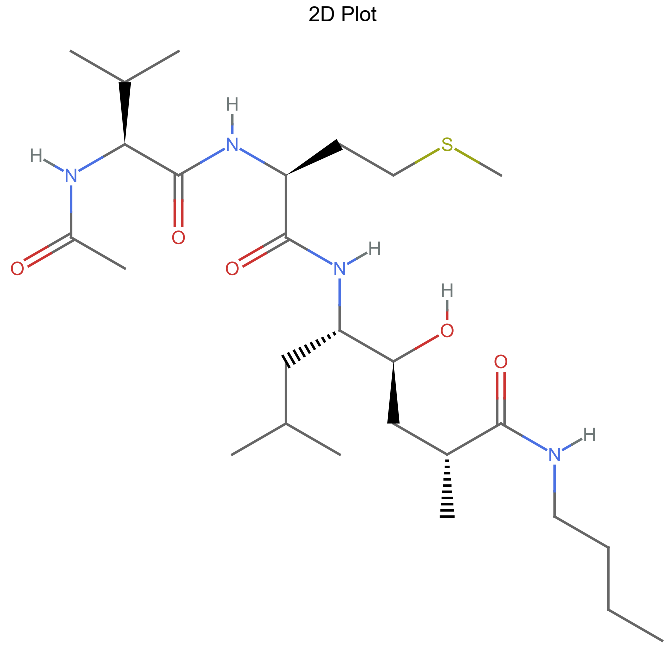

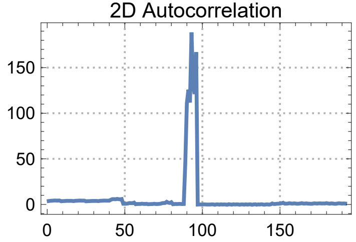

Dataset Index= 993

Molecule Formula= $C_{26}H_{50}N_4O_5S$

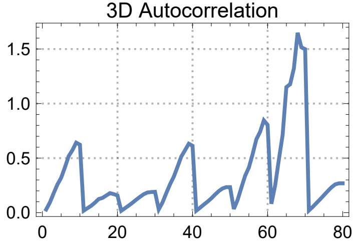

3D Plot

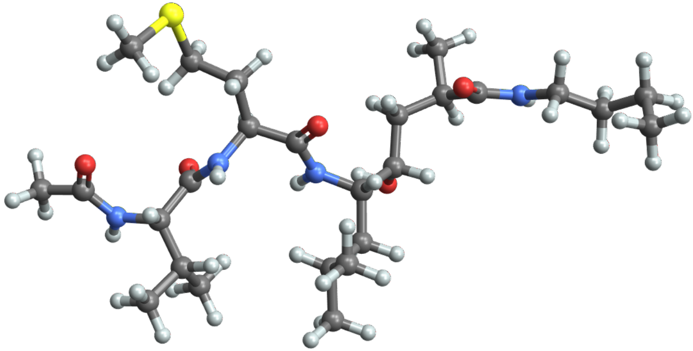

2D Plot

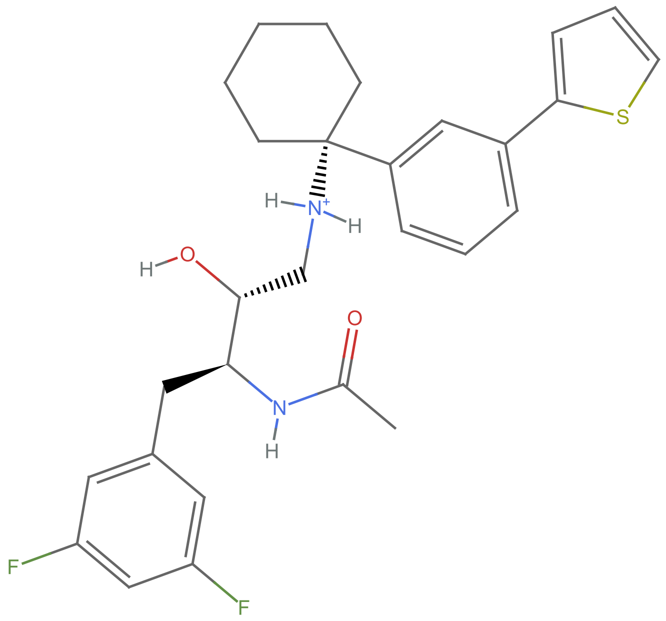

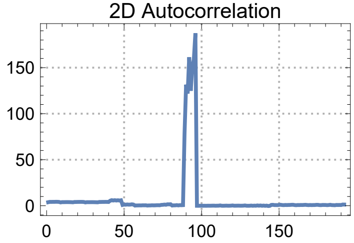

Dataset Index= 994

Molecule Formula= $C_{28}H_{33}F_2N_2O_2S^+$

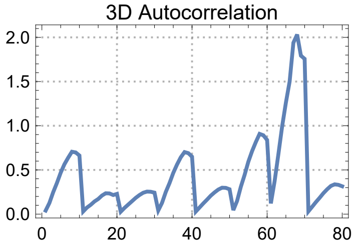

3D Plot

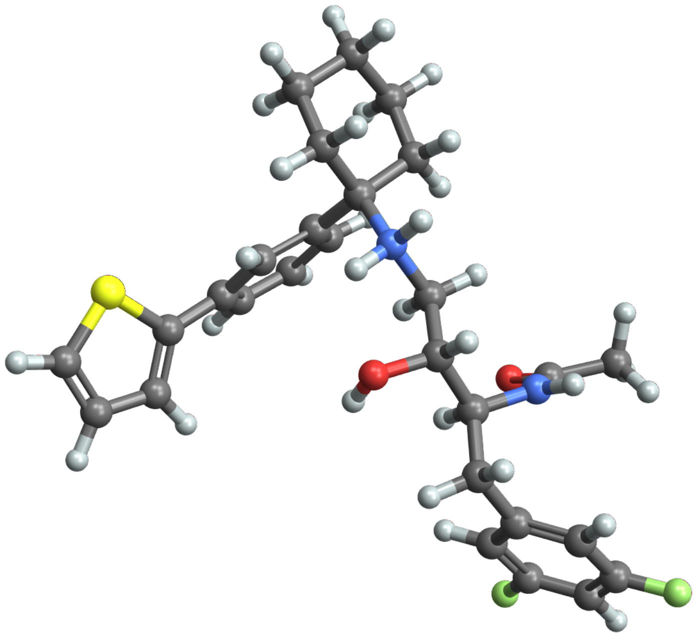

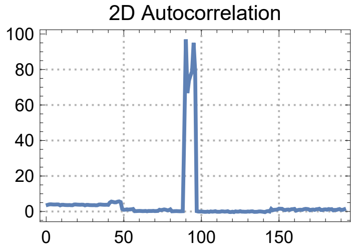

Dataset Index= 995

Molecule Formula= $C_{24}H_{34}NO_4S^+$

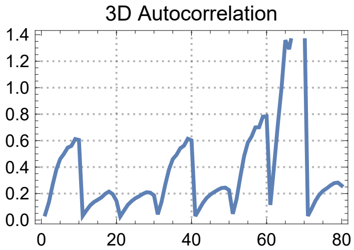

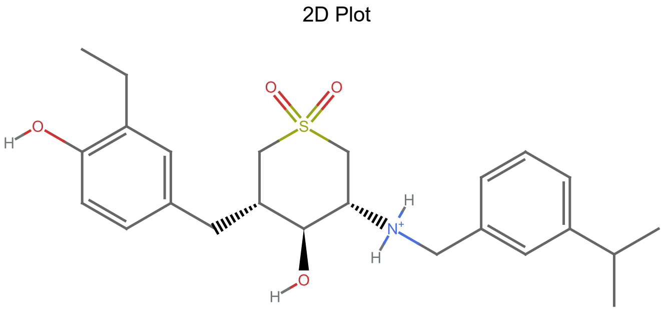

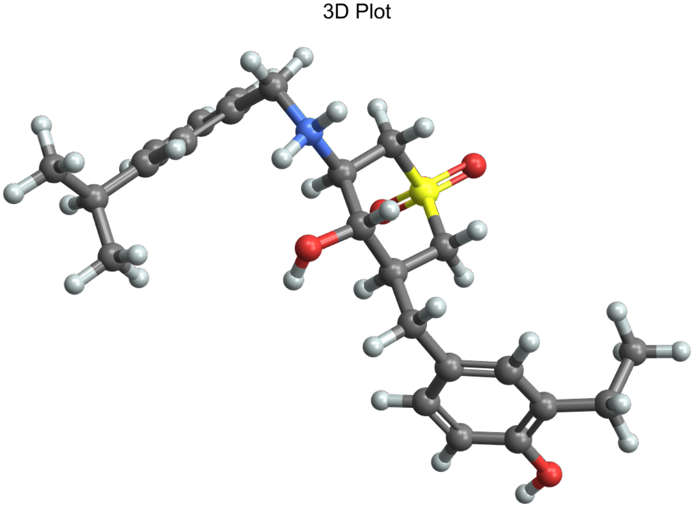

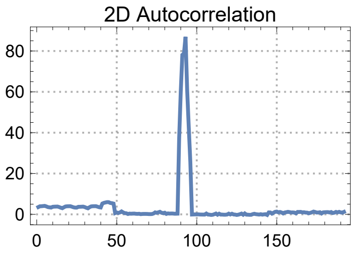

Dataset Index= 996

Molecule Formula= $C_{19}H_{17}N_5OS$

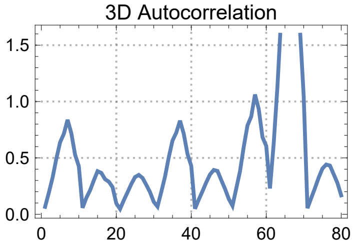

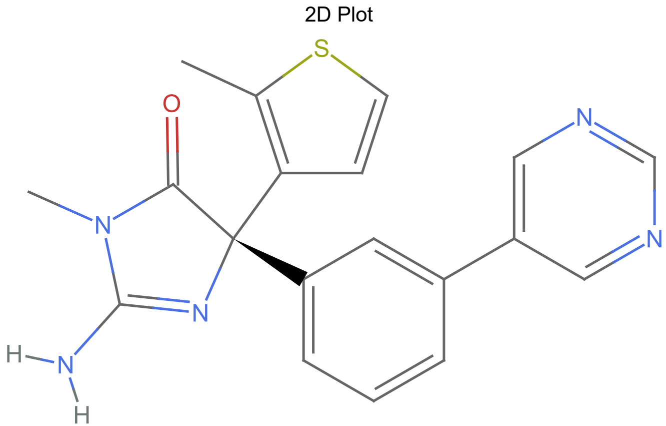

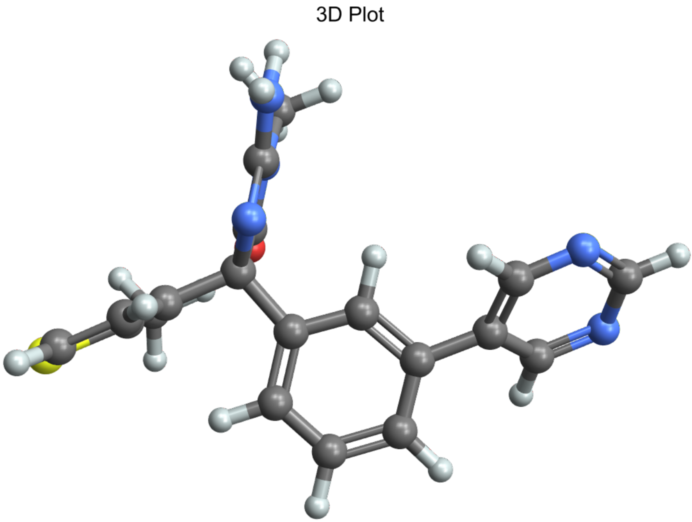

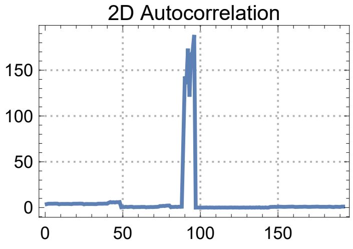

2D Plot

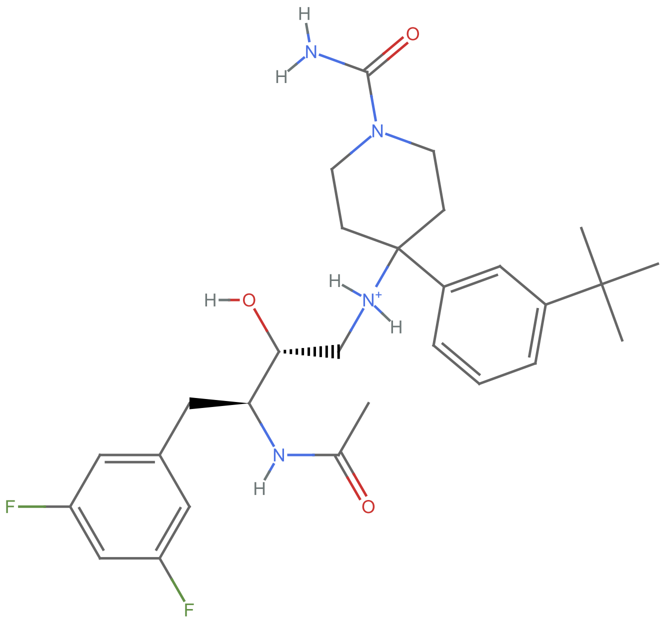

3D Plot

Dataset Index= 997

Molecule Formula= $C_{28}H_{39}F_2N_4O_3^+$

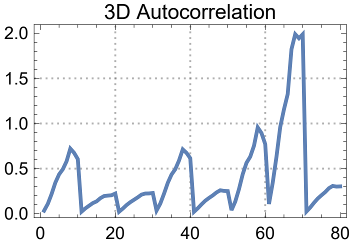

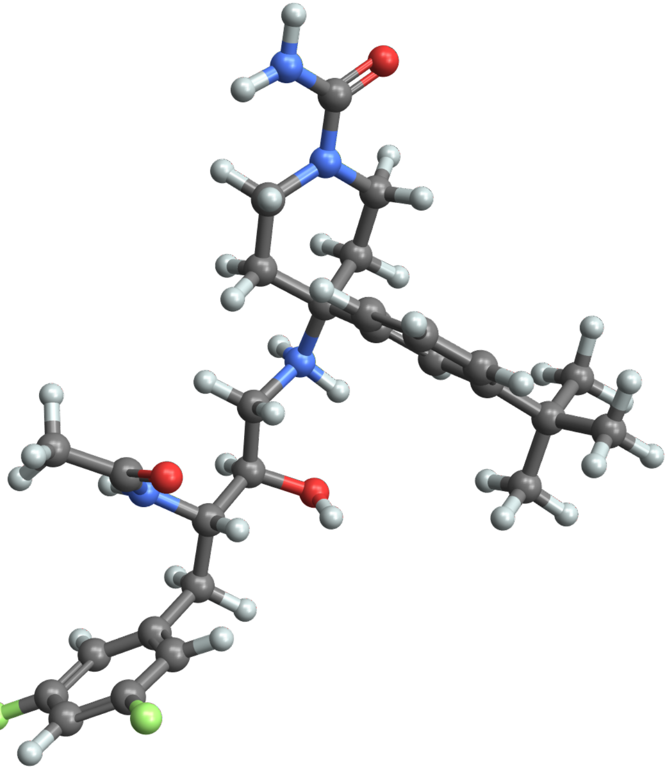

2D Plot

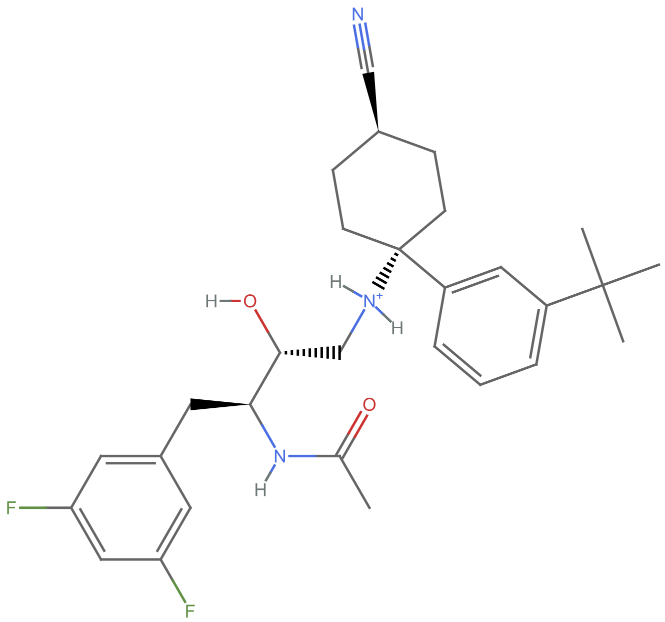

2D Autocorrelation

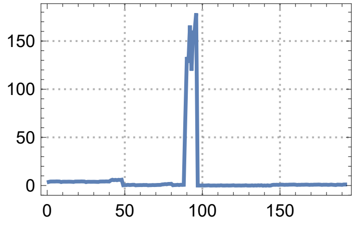

3D Plot

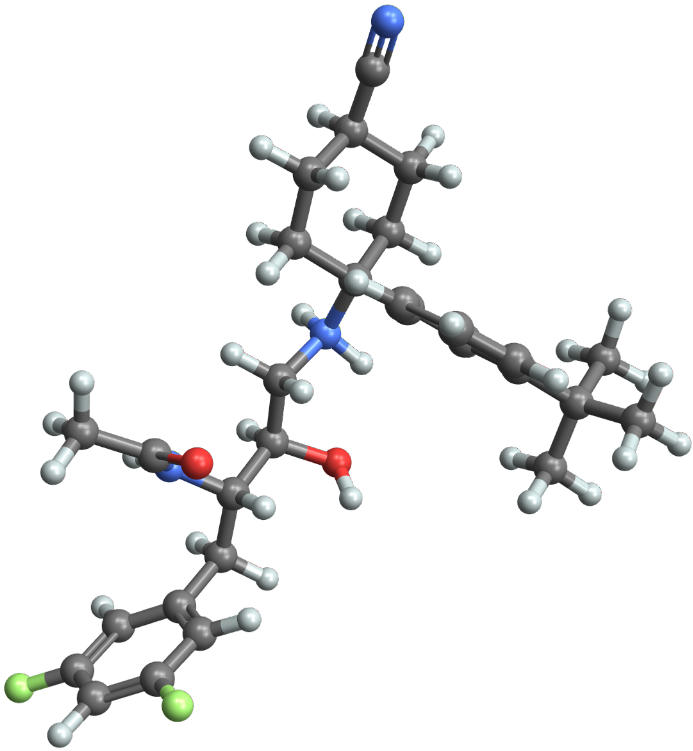

Dataset Index= 998

Molecule Formula= $C_{29}H_{38}F_2N_3O_2^+$

3D Autocorrelation

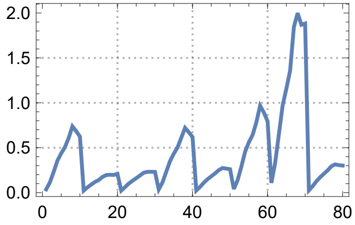

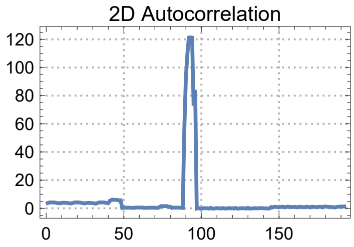

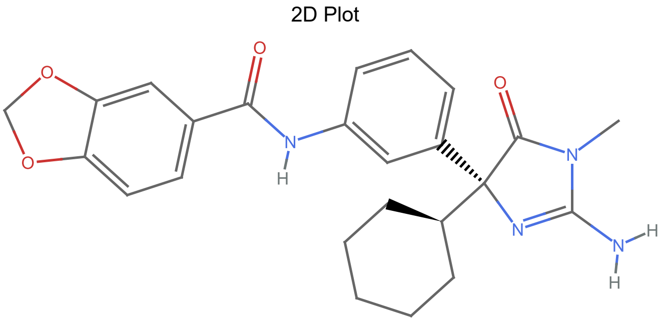

Dataset Index= 999

Molecule Formula= $C_{24}H_{26}N_4O_4$

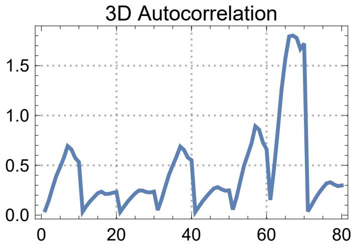

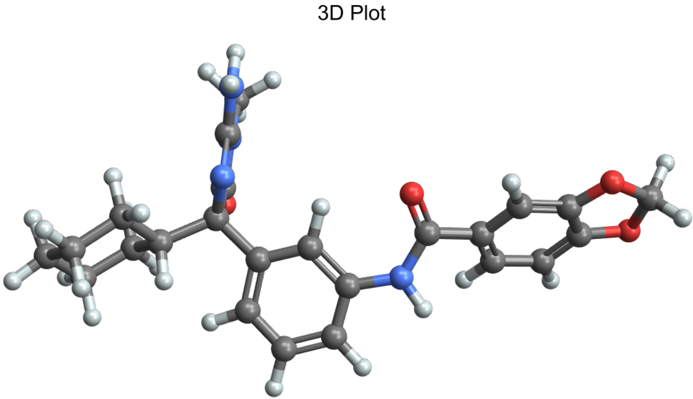

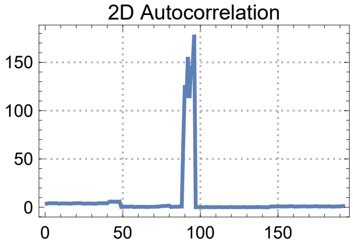

Dataset Index= 1000

Molecule Formula= $C_{28}H_{37}F_2N_2O_2^+$

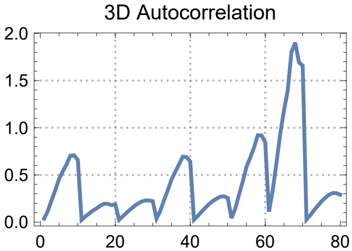

2D Plot

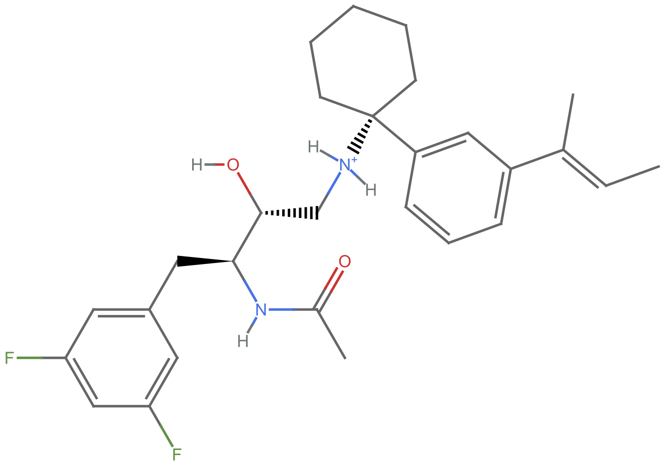

3D Plot

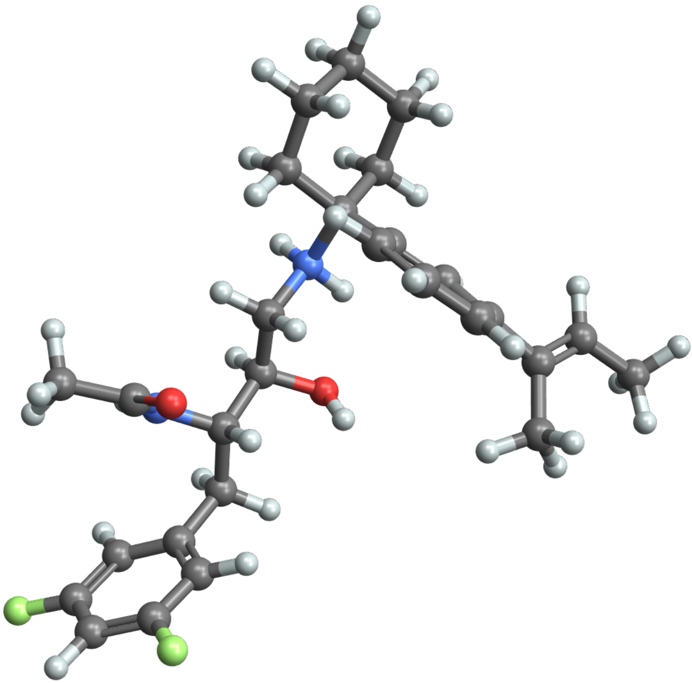

2D Plot

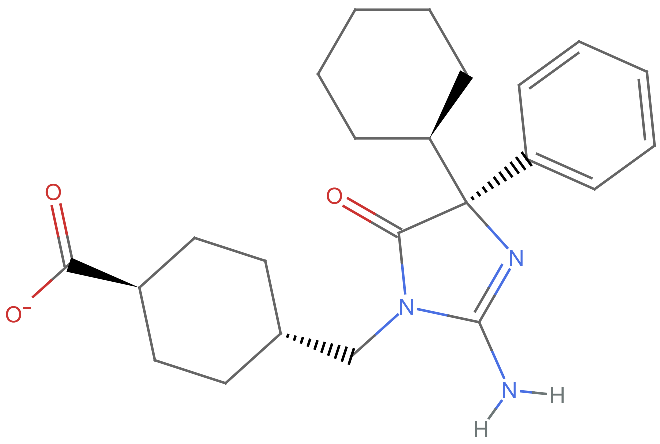

3D Plot

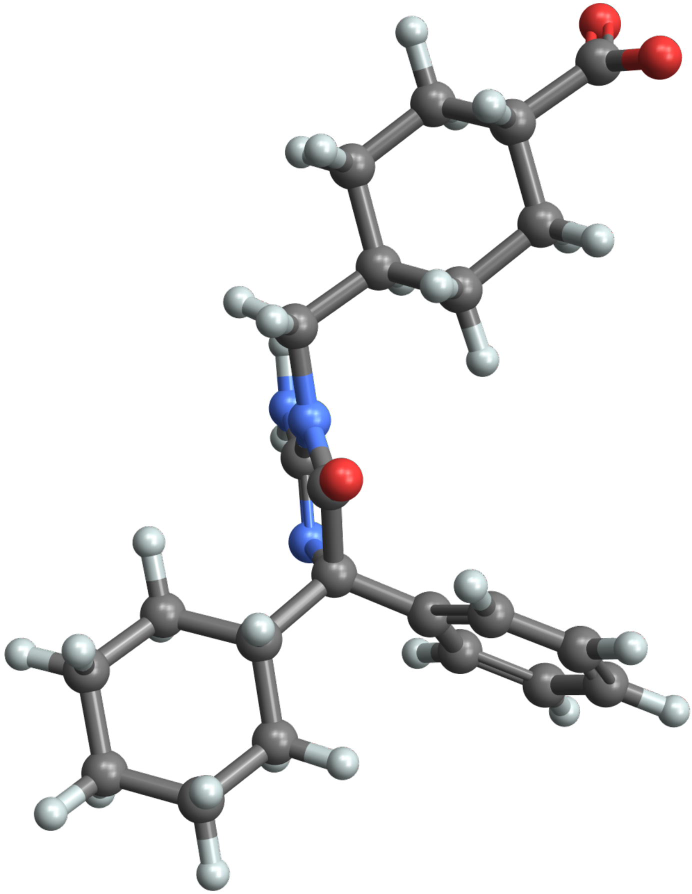

2D Autocorrelation

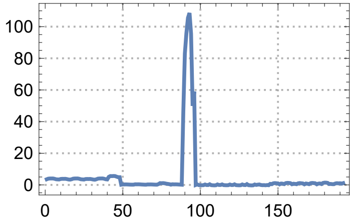

Dataset Index= 1001

Molecule Formula= $C_{23}H_{30}N_{3}O_{3}^{-}$

3D Autocorrelation

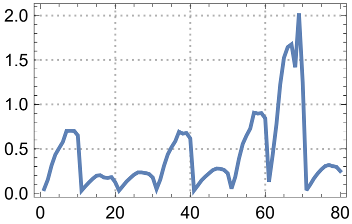

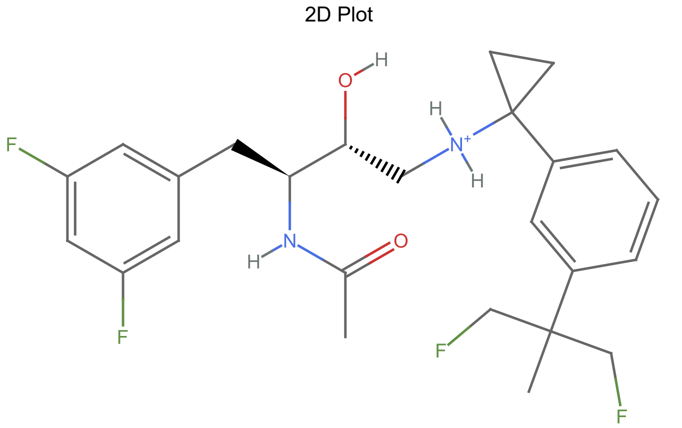

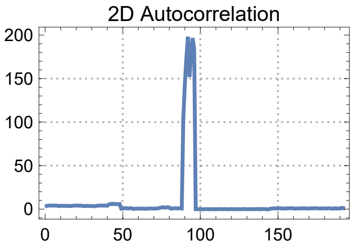

Dataset Index= 1002

Molecule Formula= $C_{25}H_{31}F_4N_2O_2^+$

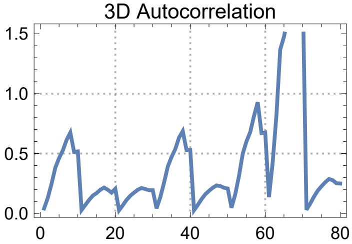

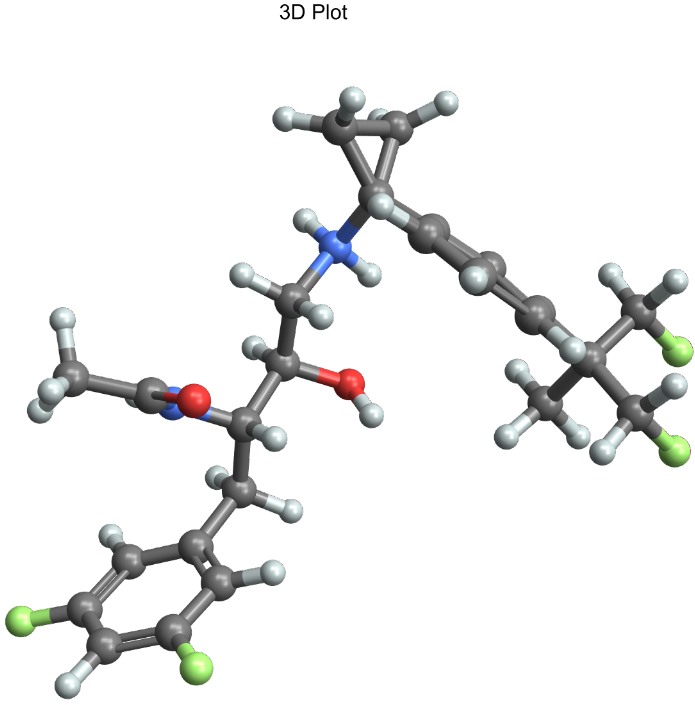

2D Plot

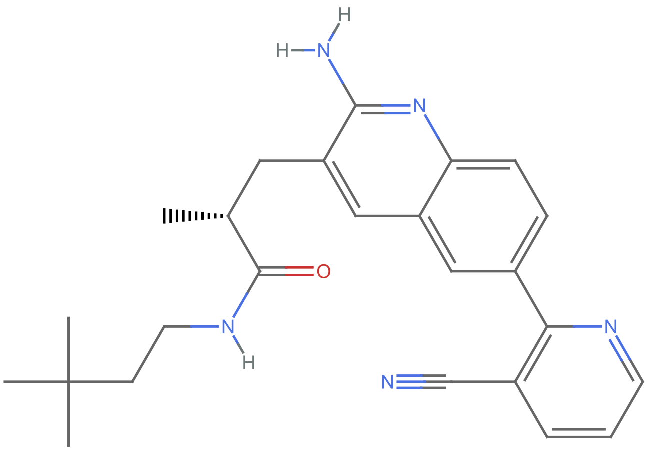

3D Plot

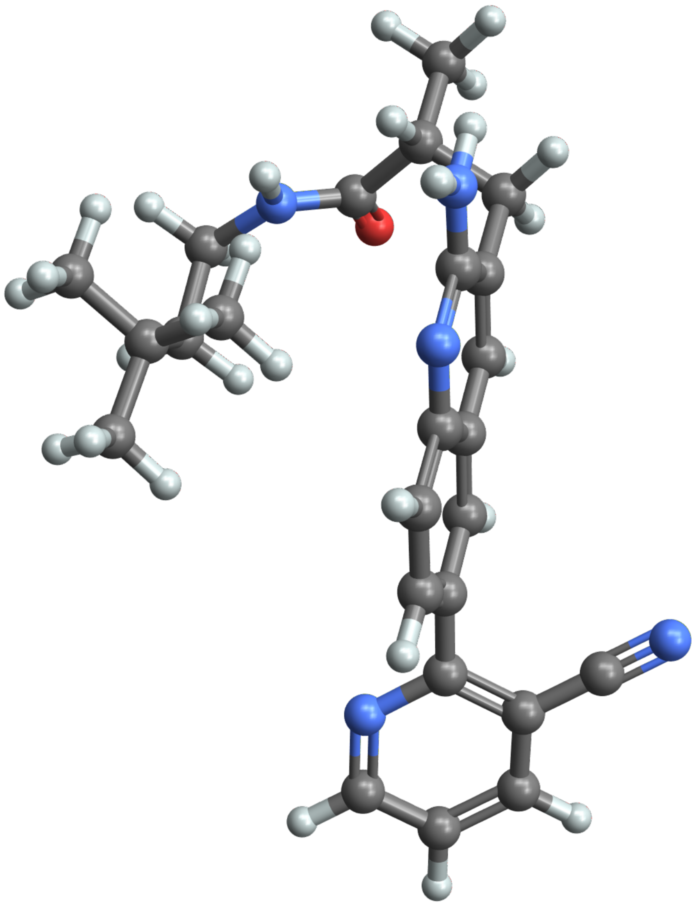

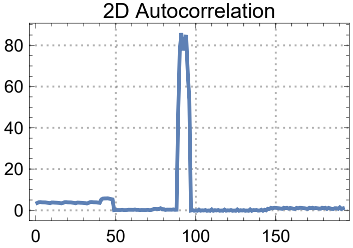

Dataset Index= 1003

Molecule Formula= $C_{25}H_{29}N_5O$

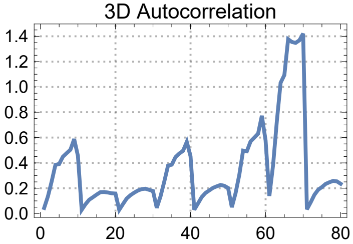

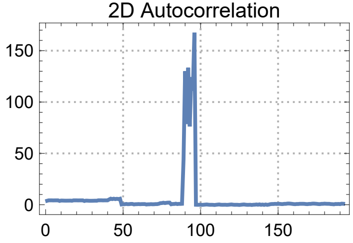

Dataset Index= 1004

Molecule Formula= $C_{30}H_{42}F_2N_5O_2^+$

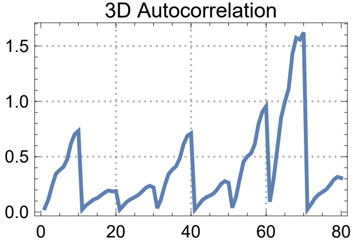

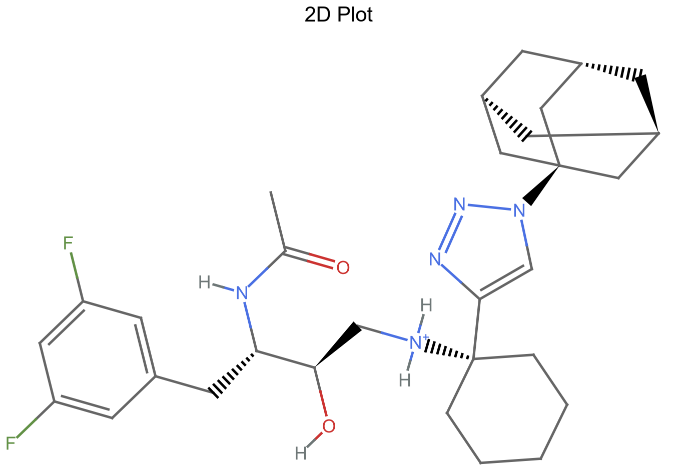

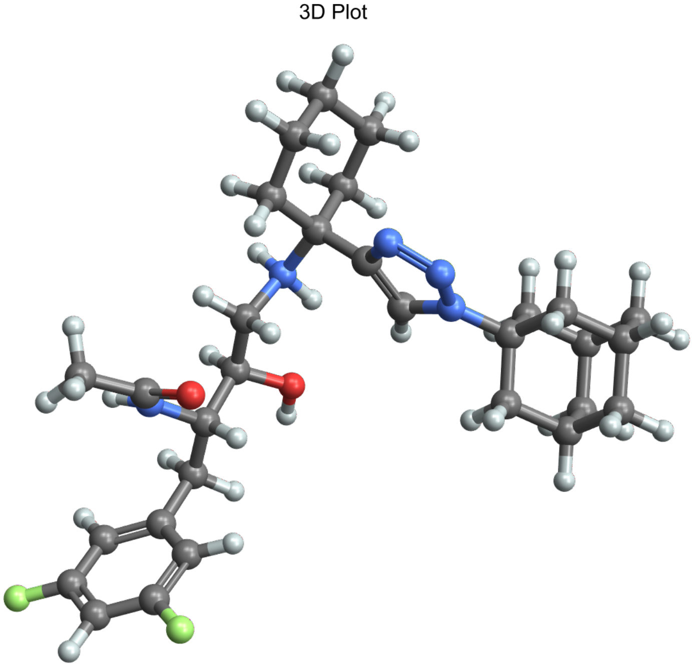

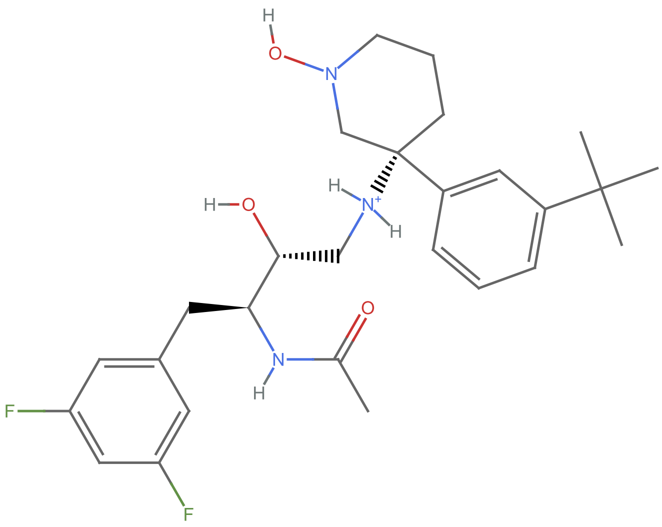

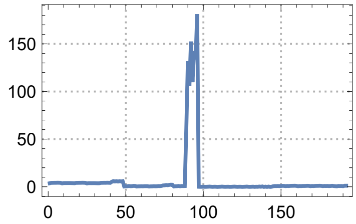

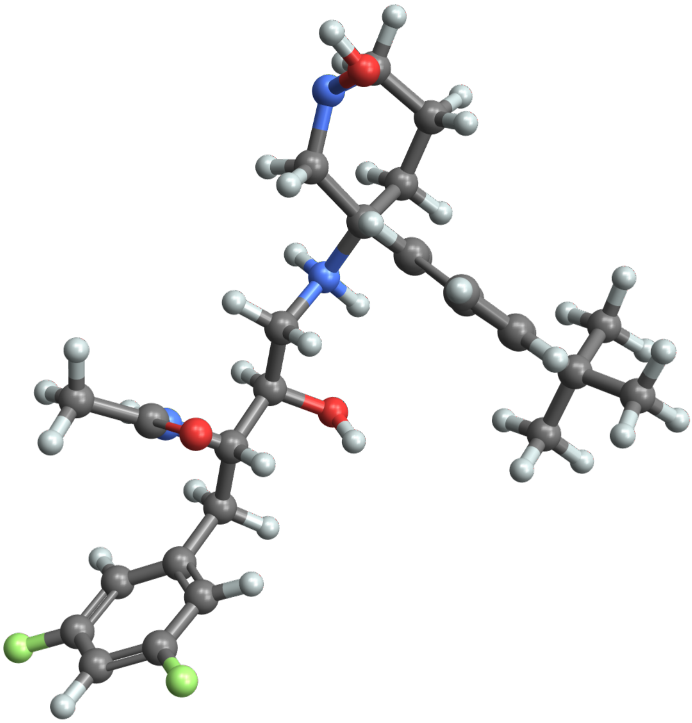

Dataset Index= 1005

Molecule Formula= $C_{27}H_{38}F_2N_3O_3^+$

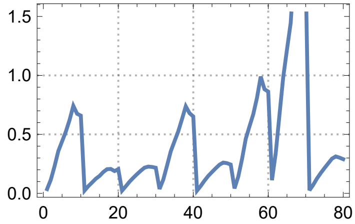

2D Plot

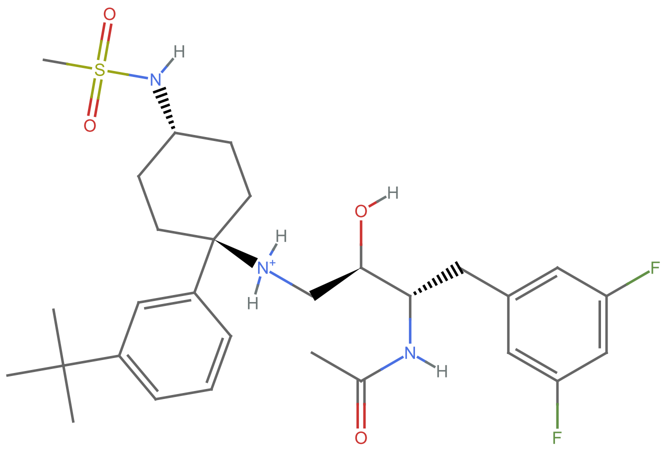

2D Autocorrelation

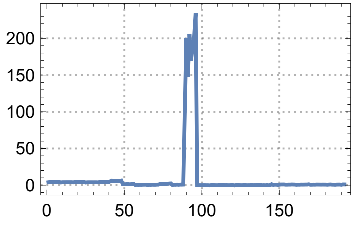

3D Plot

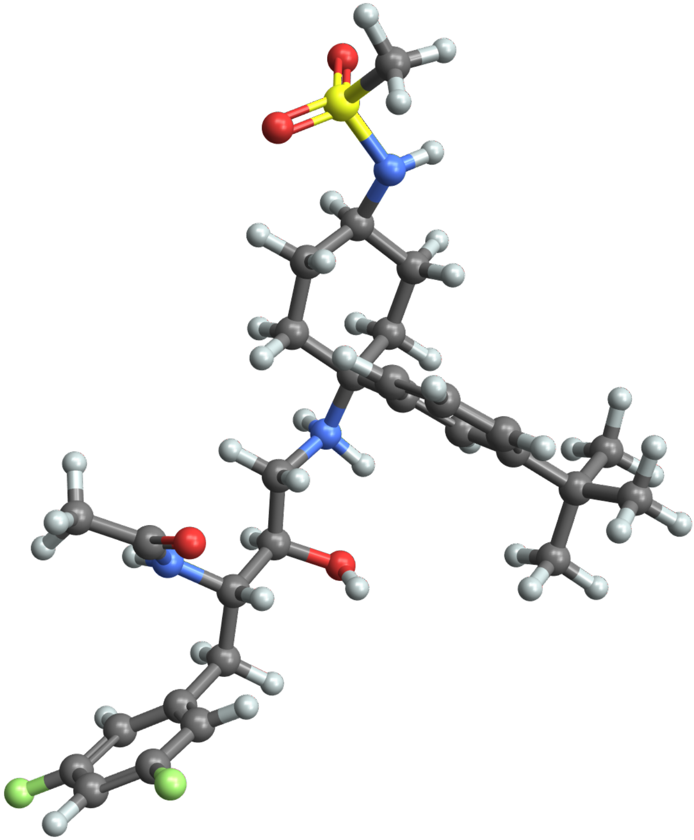

Dataset Index= 1006

Molecule Formula= $C_{29}H_{42}F_{2}N_{3}O_{4}S^{+}$

3D Autocorrelation

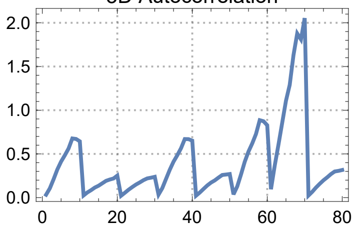

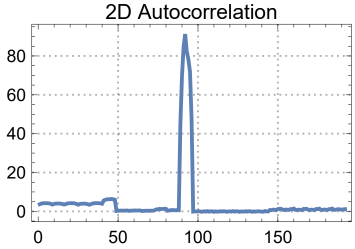

Dataset Index= 1007

Molecule Formula= $C_{24}H_{18}FN_5O$

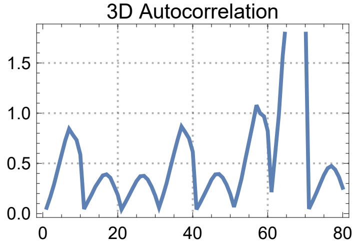

2D Plot

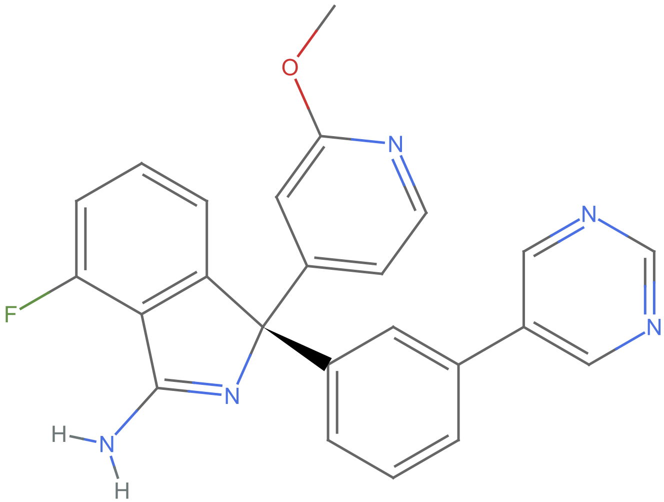

3D Plot

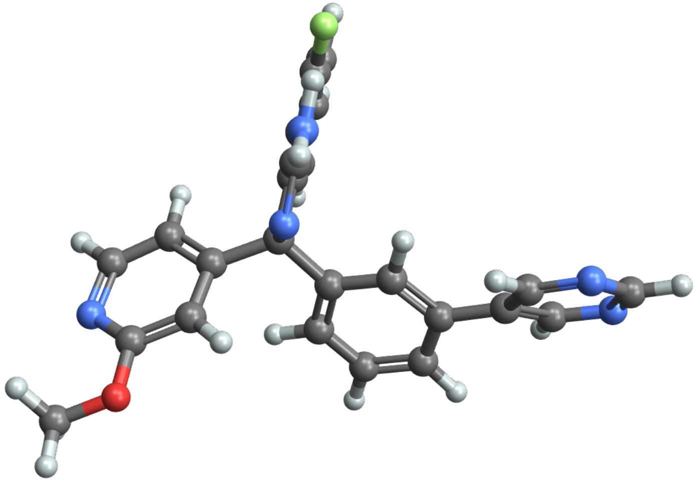

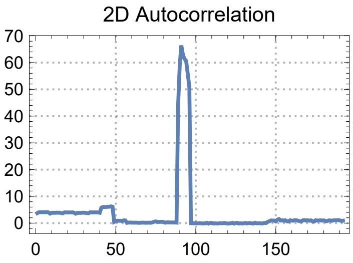

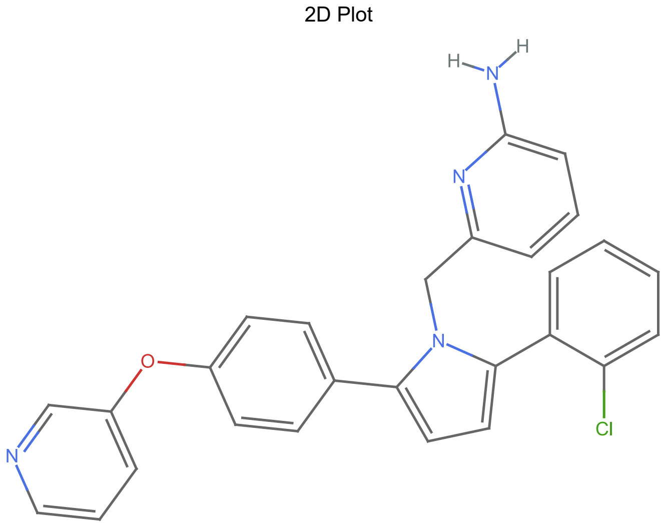

Dataset Index= 1008

Molecule Formula= $C_{27}H_{21}ClN_4O$

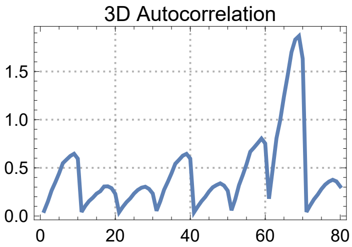

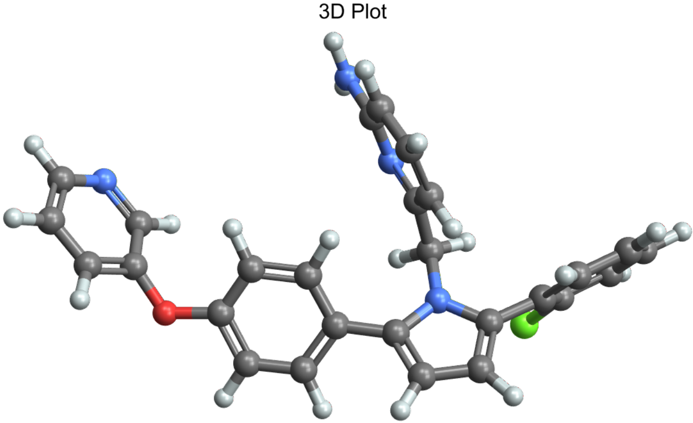

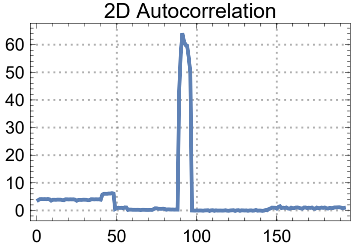

Dataset Index= 1009

Molecule Formula= $C_{26}H_{20}ClN_5O$

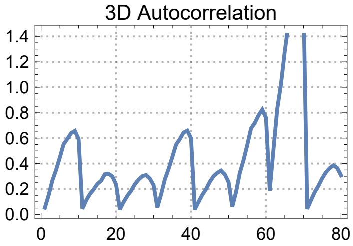

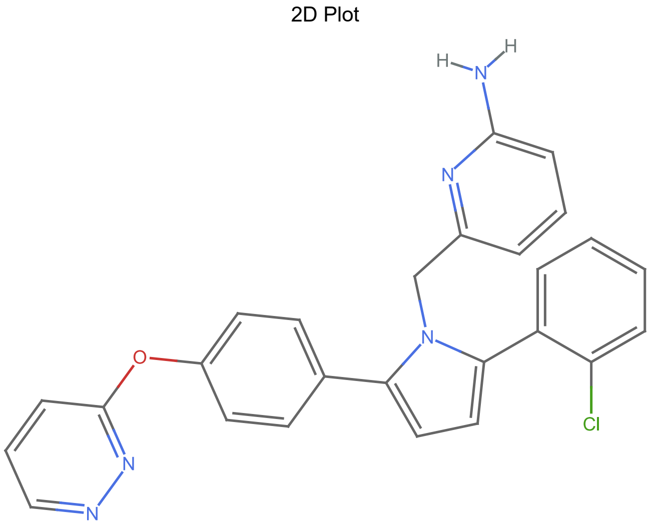

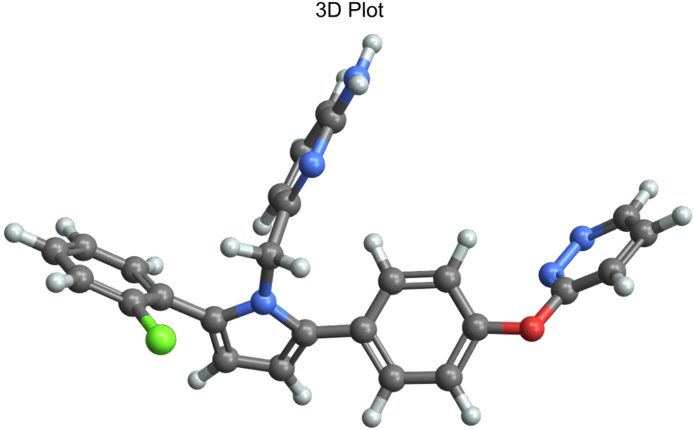

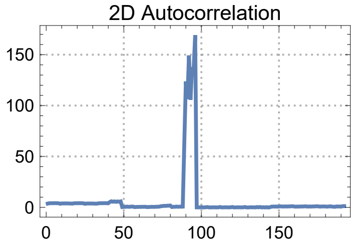

Dataset Index= 1010

Molecule Formula= $C_{26}H_{36}F_2N_3O_2^+$

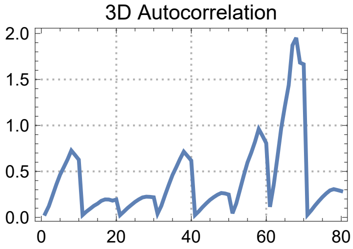

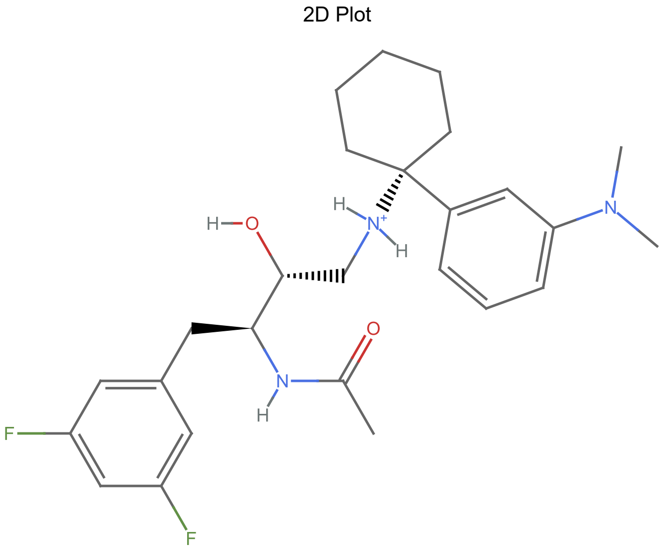

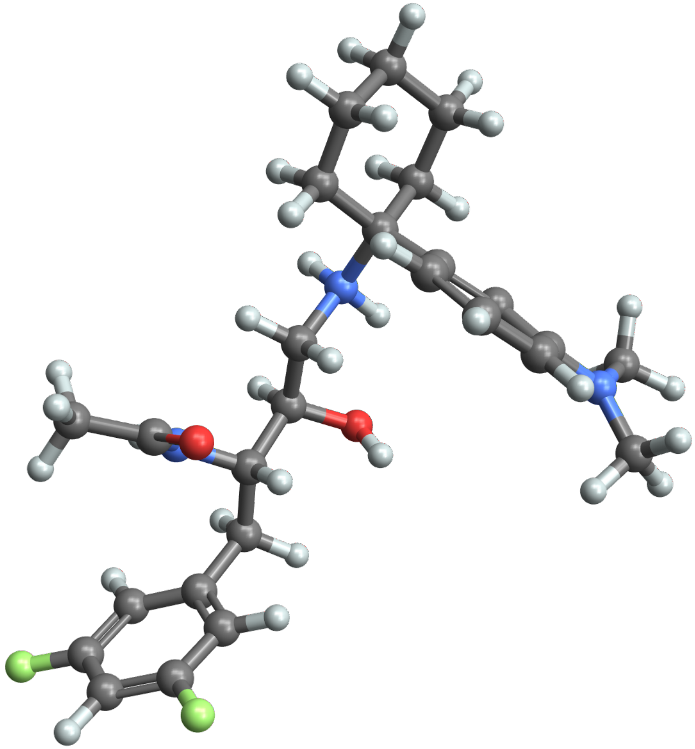

2D Plot

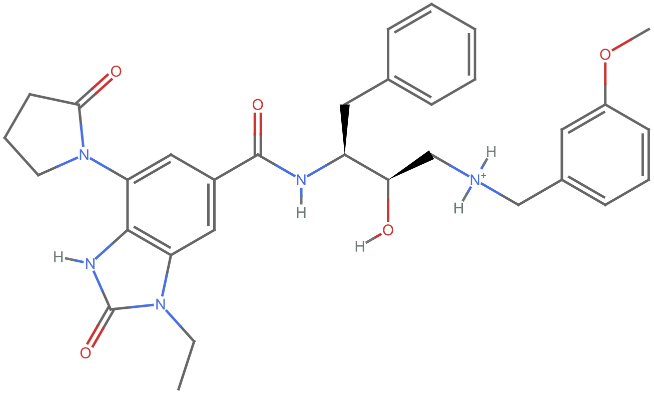

2D Autocorrelation

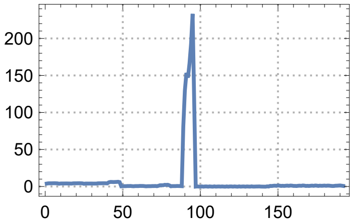

3D Plot

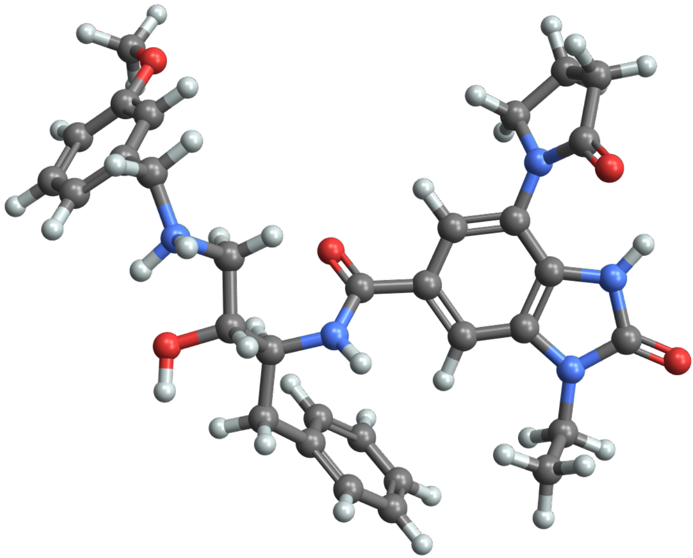

Dataset Index= 1011

Molecule Formula= $C_{32}H_{38}N_5O_5^+$

3D Autocorrelation

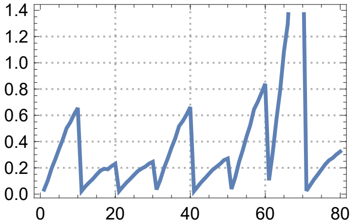

2D Plot

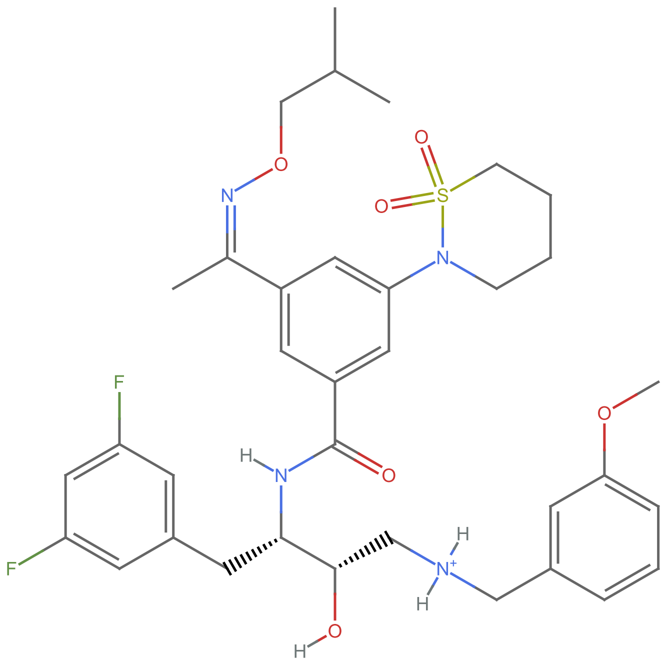

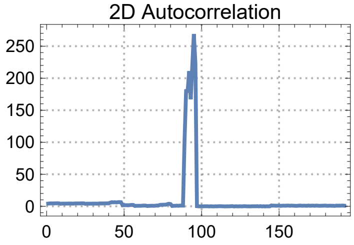

Dataset Index= 1012

Molecule Formula= $C_{35}H_{45}F_2N_4O_6S^+$

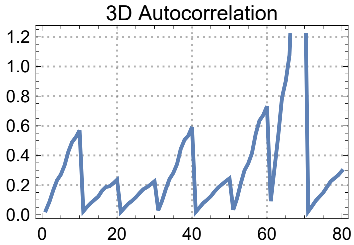

3D Plot

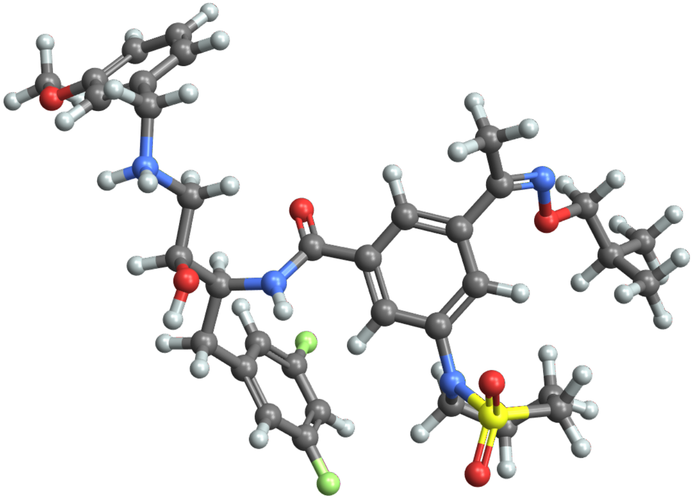

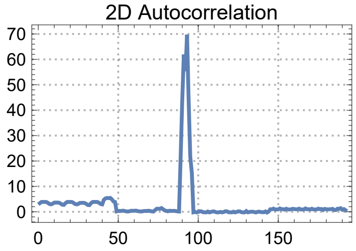

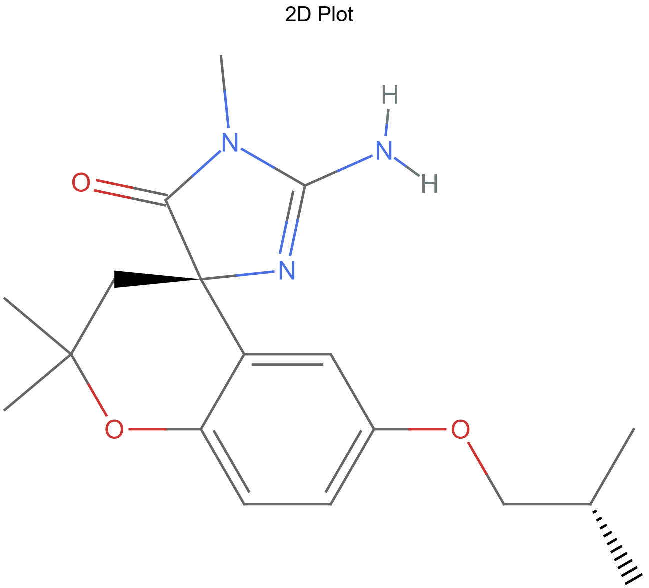

Dataset Index= 1013

Molecule Formula= $C_{18}H_{25}N_3O_3$

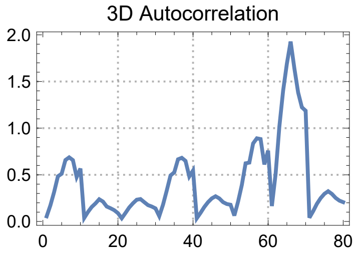

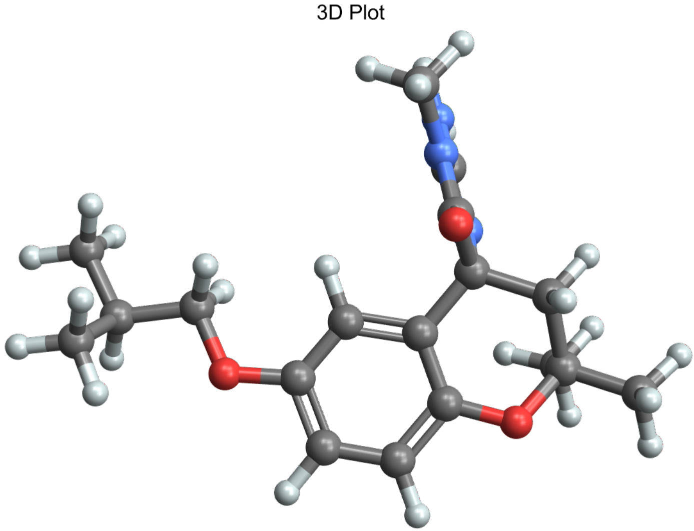

2D Plot

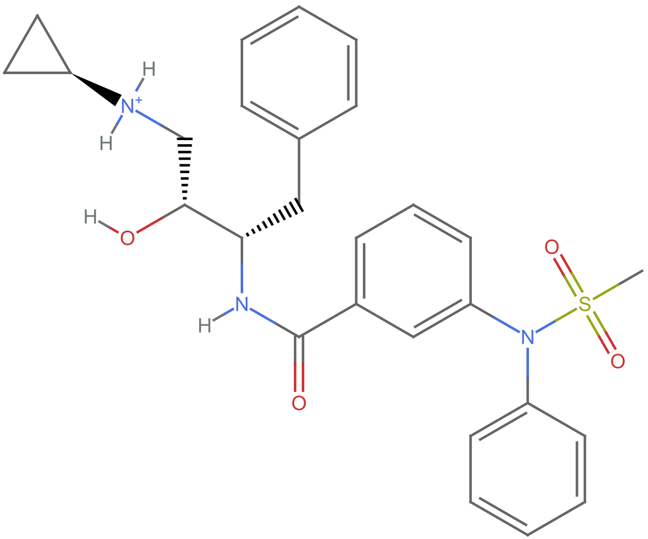

2D Autocorrelation

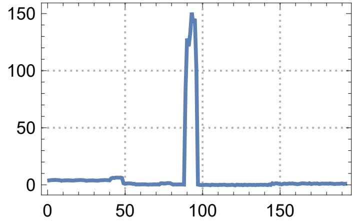

3D Plot

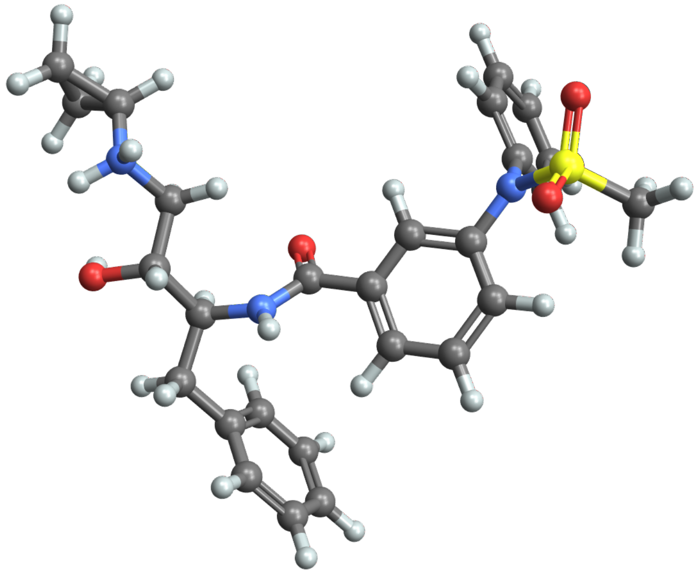

Dataset Index= 1014

Molecule Formula= $C_{27}H_{32}N_3O_4S^+$

3D Autocorrelation

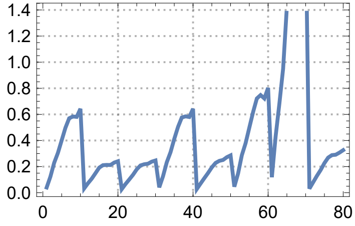

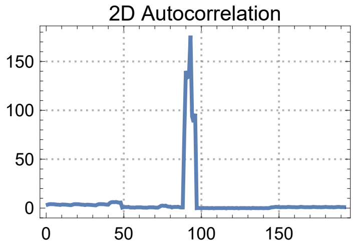

Dataset Index= 1015

Molecule Formula= $C_{16}H_{16}F_3N_5O_2$

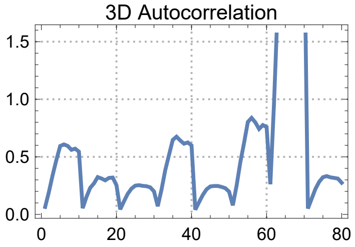

2D Plot

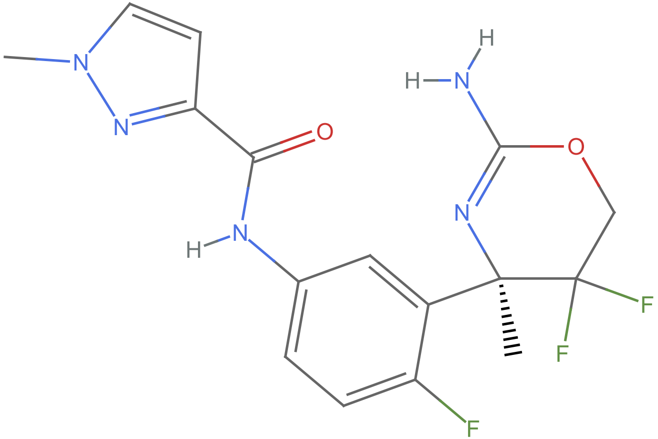

3D Plot

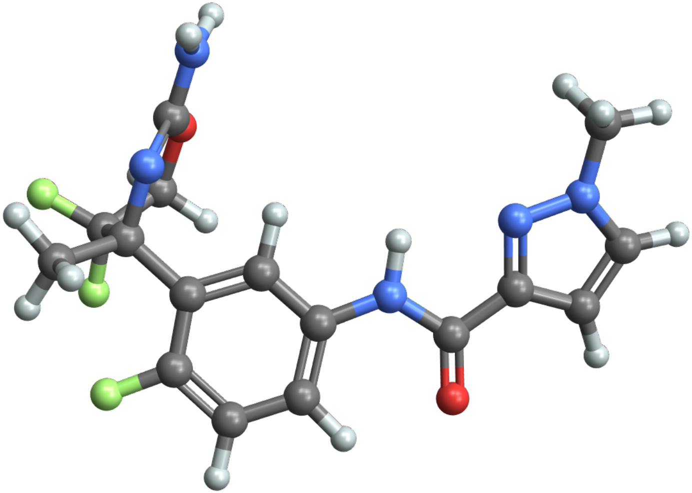

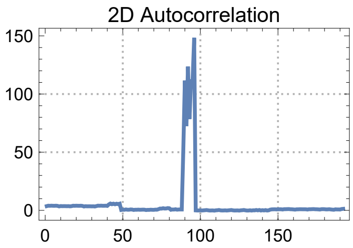

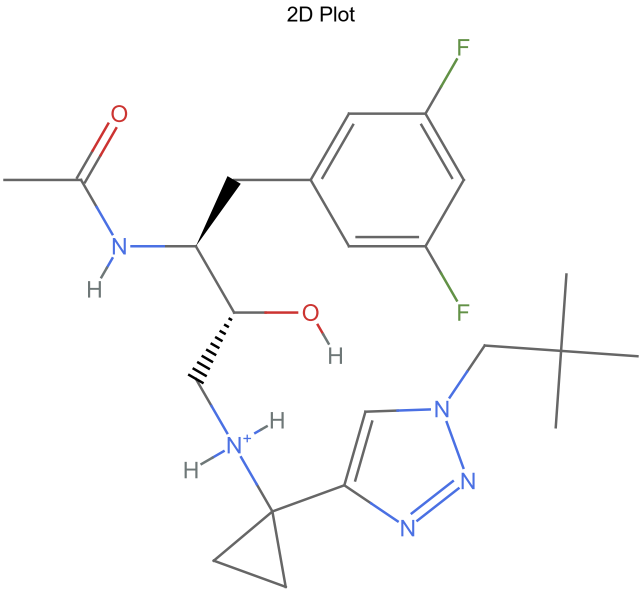

Dataset Index= 1016

Molecule Formula= $C_{22}H_{32}F_2N_5O_2^+$

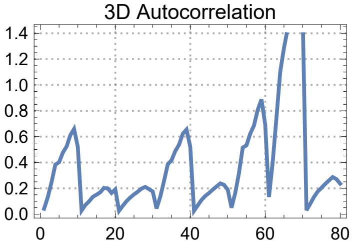

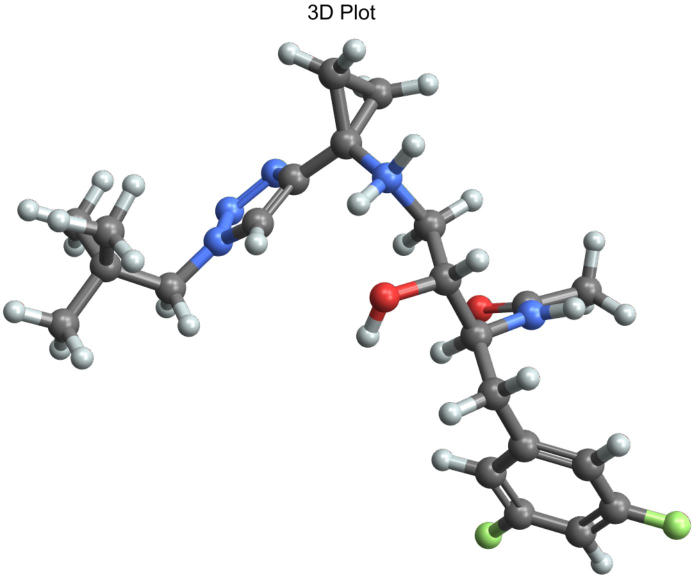

2D Plot

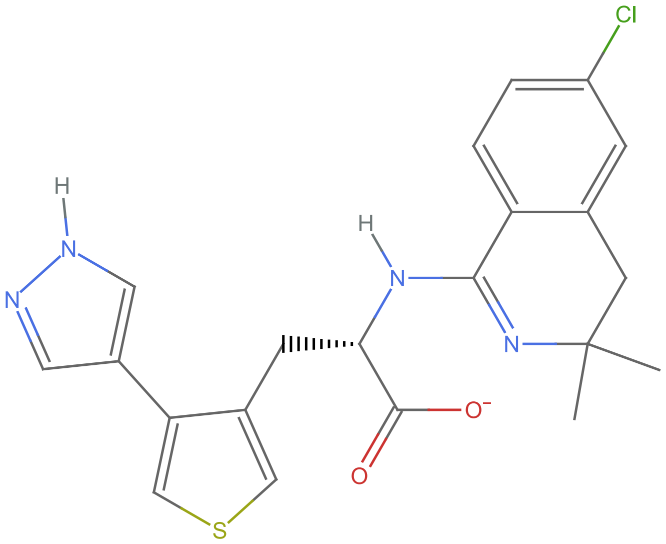

3D Plot

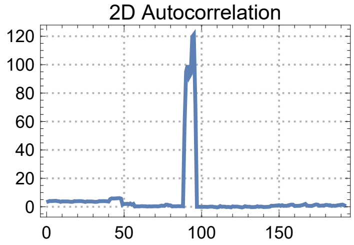

Dataset Index= 1017

Molecule Formula= $C_{21}H_{20}ClN_4O_2S^-$

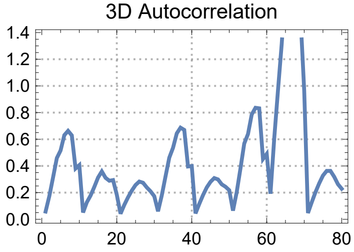

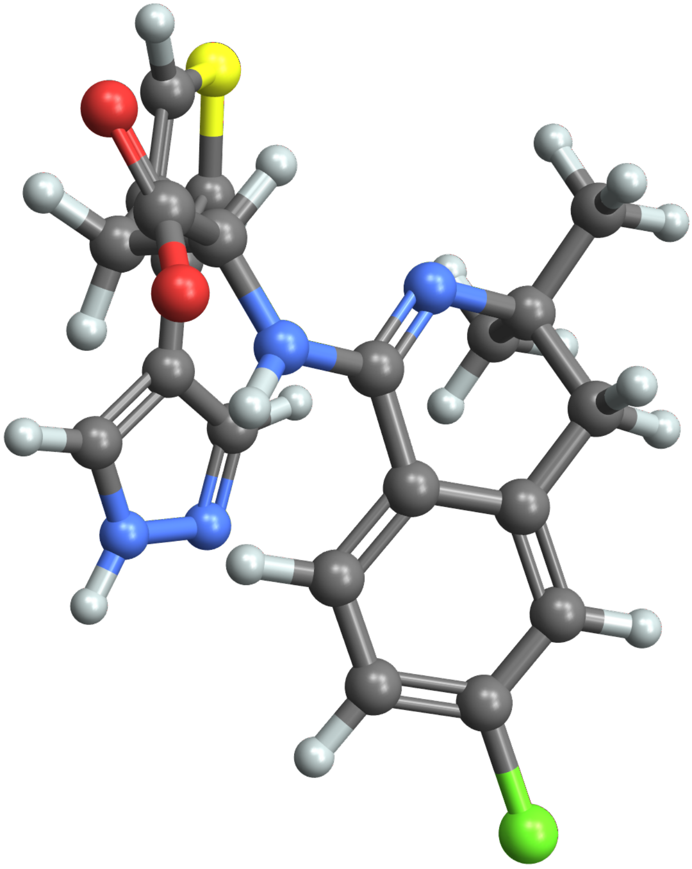

2D Plot

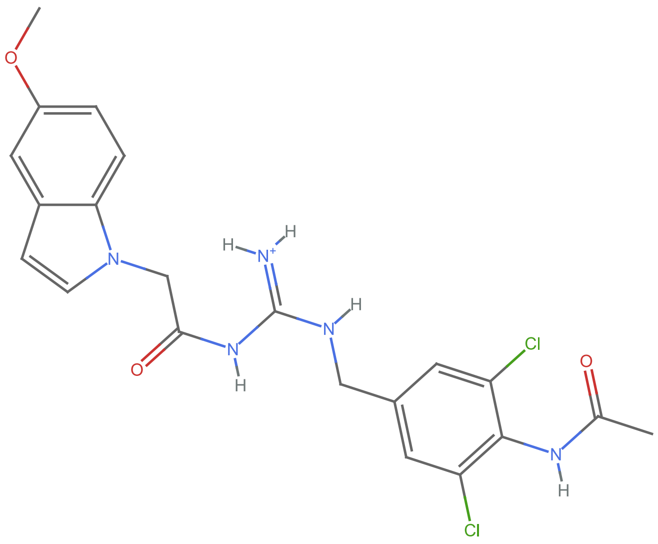

3D Plot

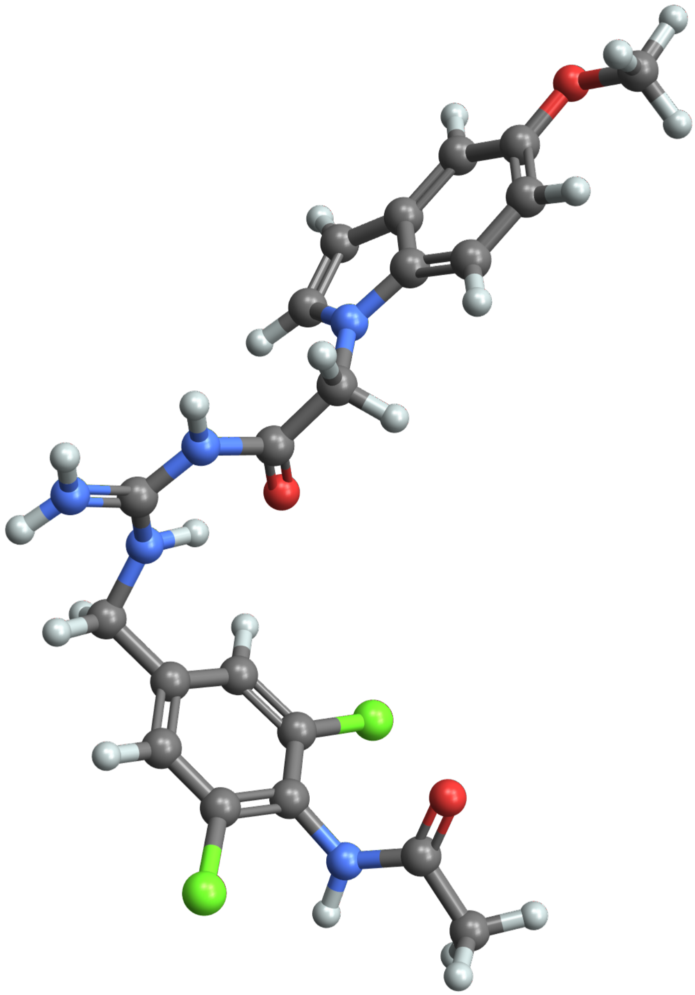

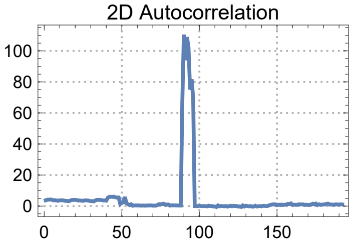

Dataset Index= 1018

Molecule Formula= $C_{21}H_{22}Cl_2N_5O_3^+$

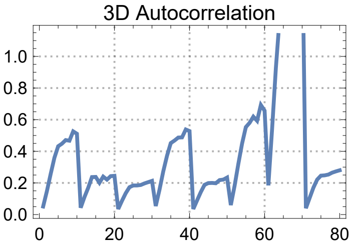

2D Autocorrelation

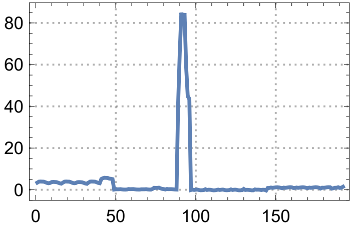

Dataset Index= 1019

Molecule Formula= $C_{20}H_{21}N_3O_2$

3D Autocorrelation

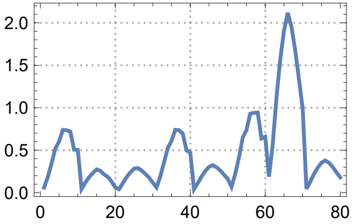

2D Plot

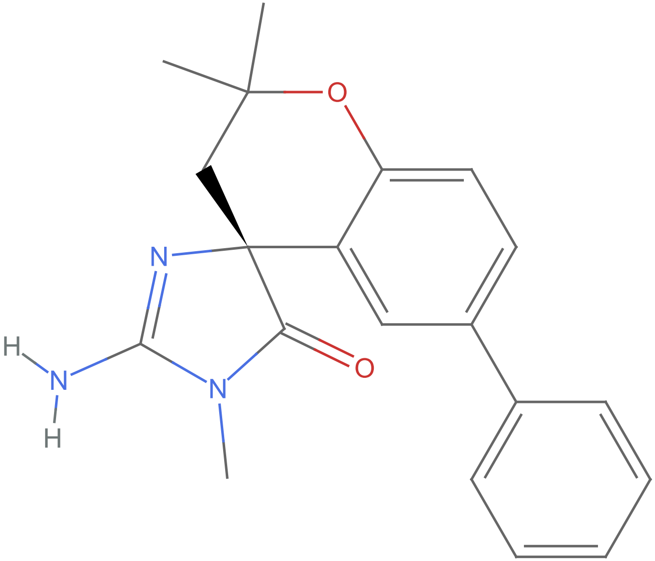

3D Plot

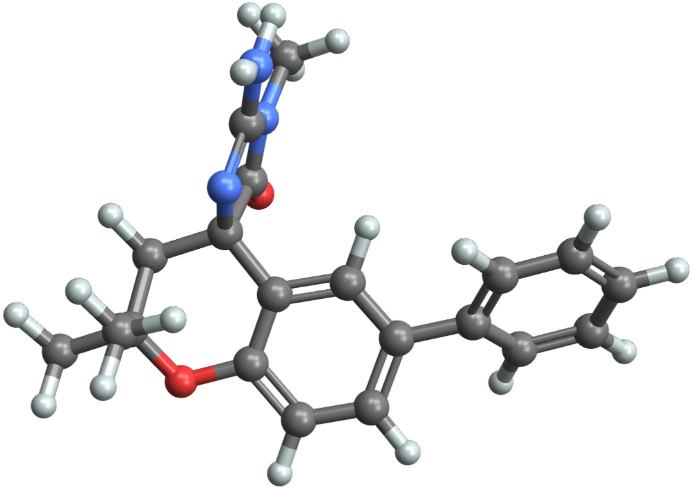

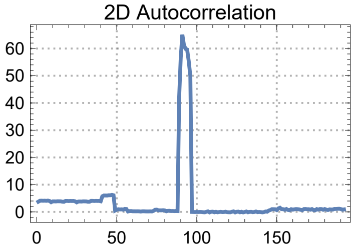

Dataset Index= 1020

Molecule Formula= $C_{26}H_{20}ClN_5O$

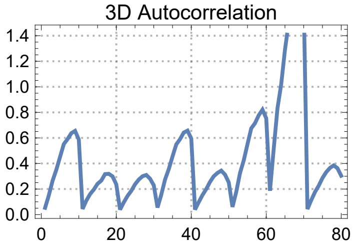

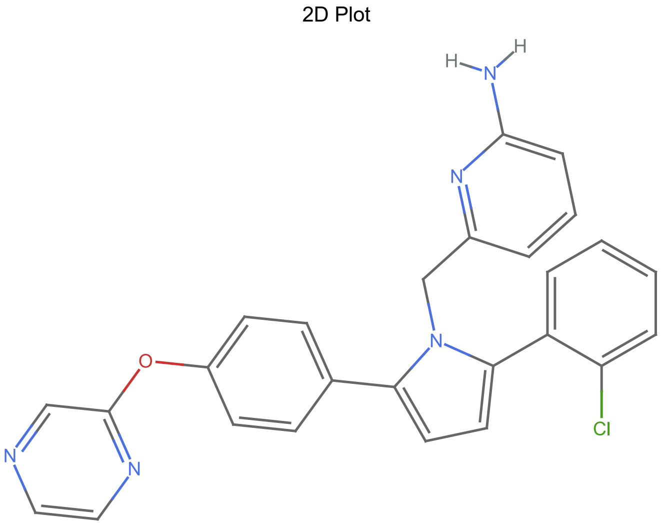

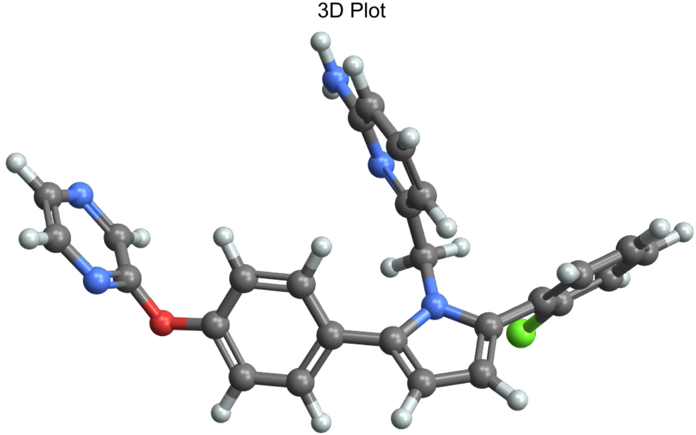

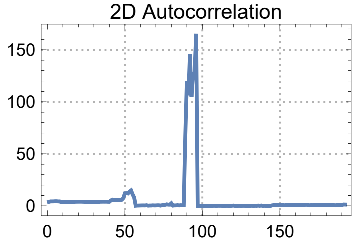

Dataset Index= 1021

Molecule Formula= $C_{24}H_{30}F_2IN_2O_2^+$

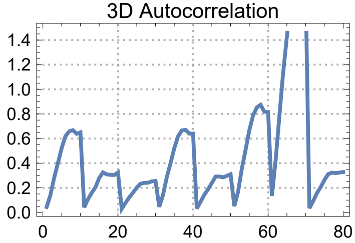

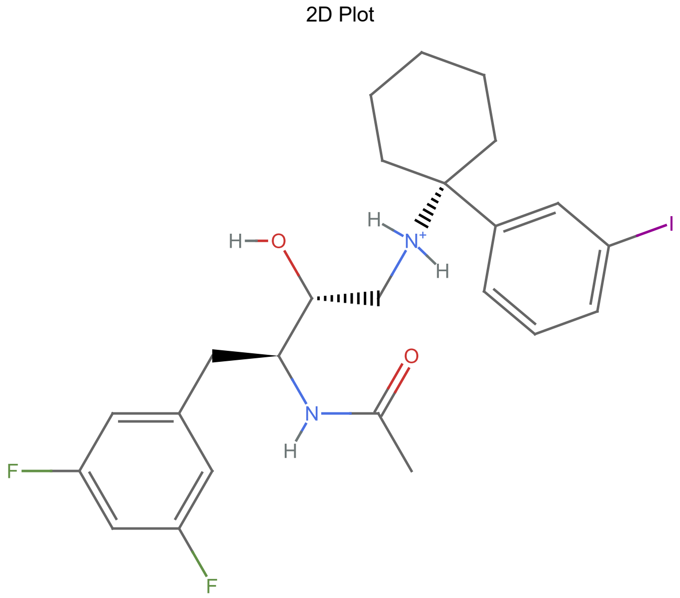

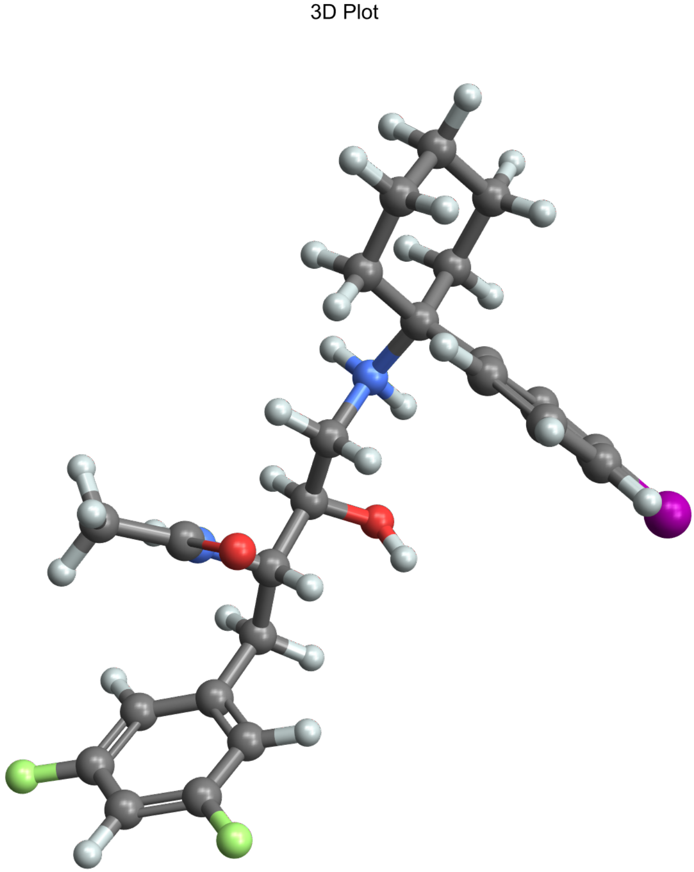

2D Autocorrelation

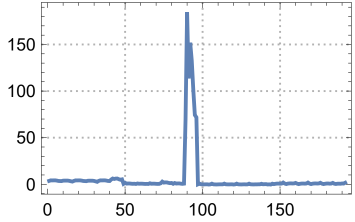

Dataset Index= 1022

Molecule Formula= $C_{21}H_{24}F_3N_3O_2$

3D Autocorrelation

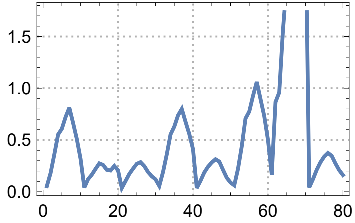

2D Plot

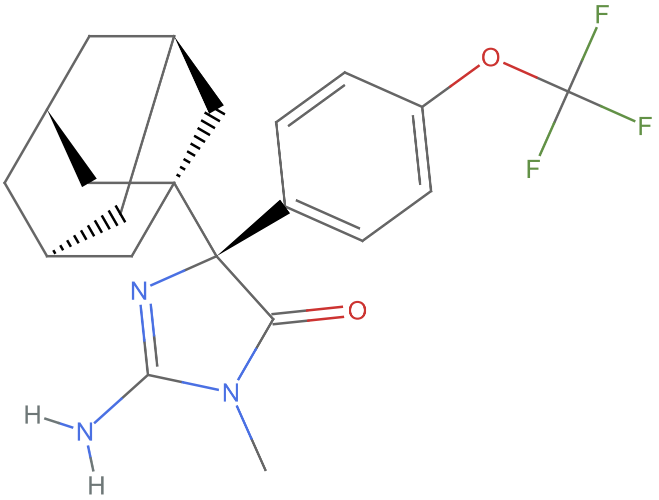

3D Plot

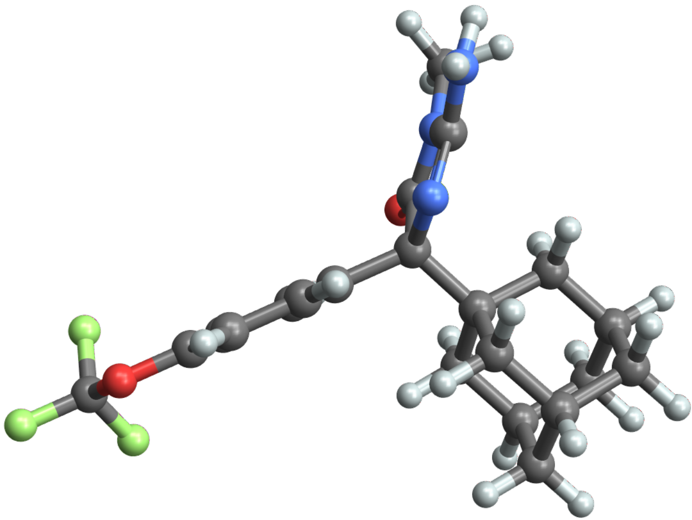

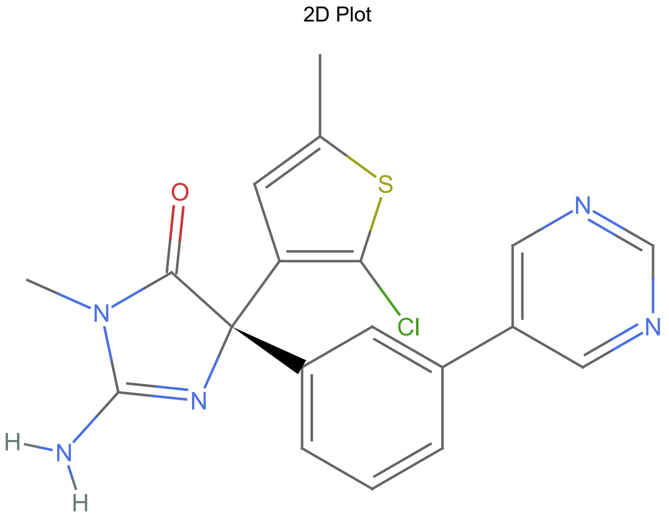

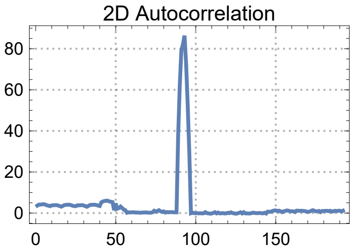

Dataset Index= 1023

Molecule Formula= $C_{19}H_{16}ClN_5OS$

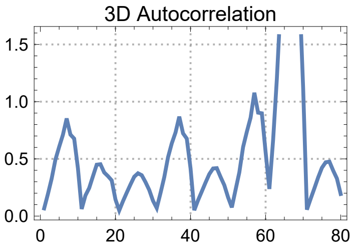

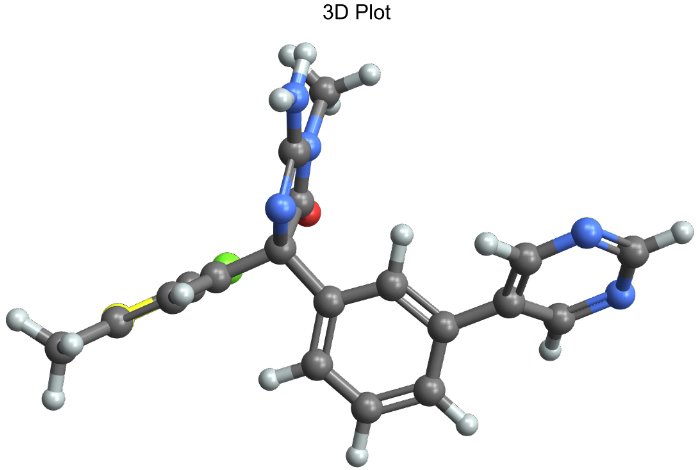

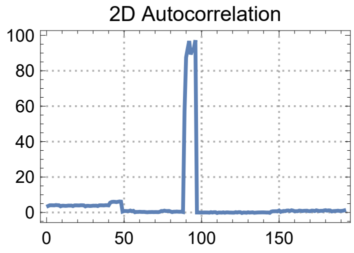

Dataset Index= 1024

Molecule Formula= $C_{24}H_{22}ClN_5O$

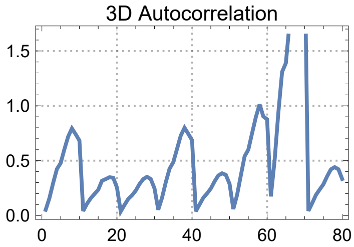

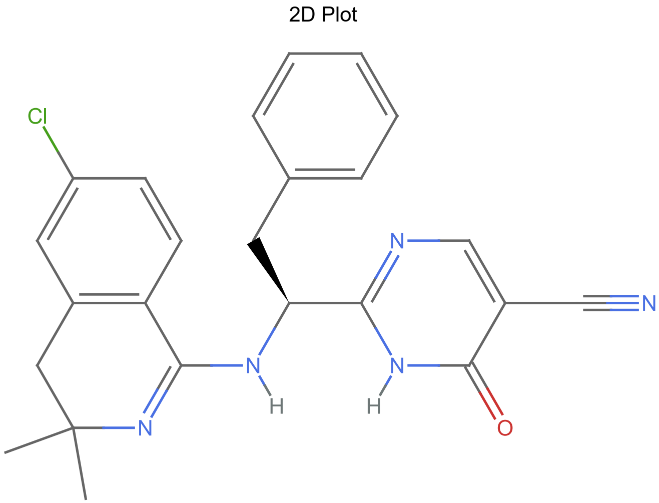

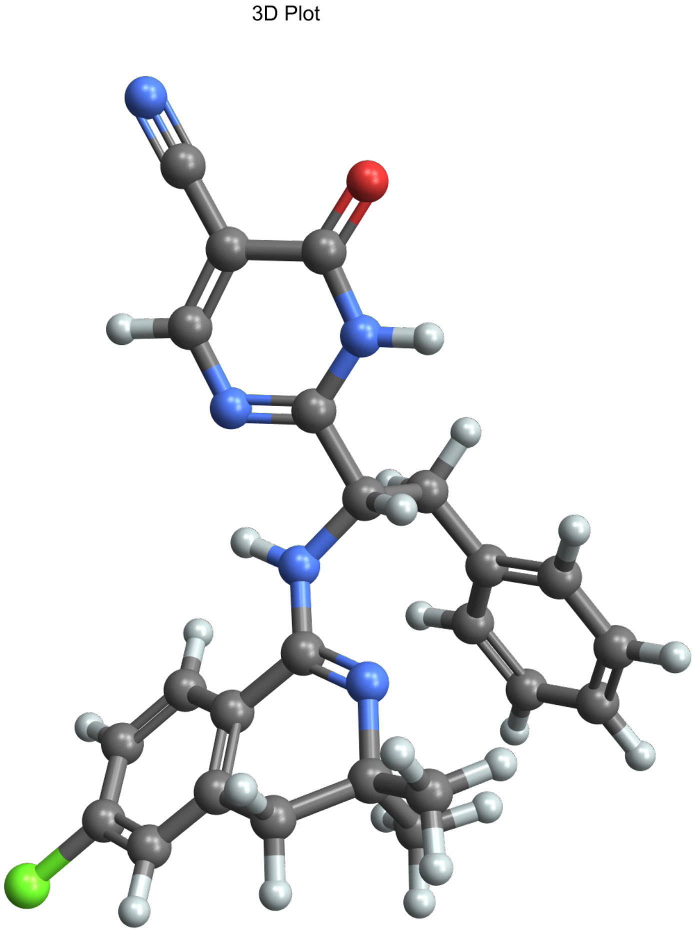

2D Plot

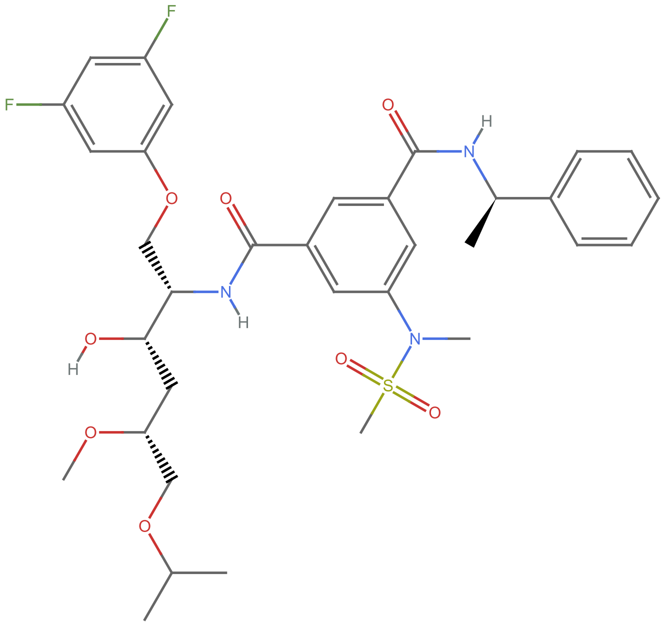

2D Autocorrelation

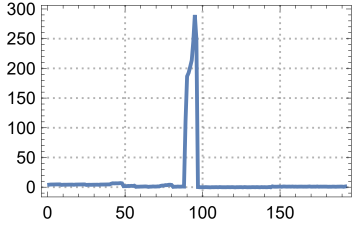

Dataset Index= 1025

Molecule Formula= $C_{34}H_{43}F_2N_3O_8S$

3D Autocorrelation

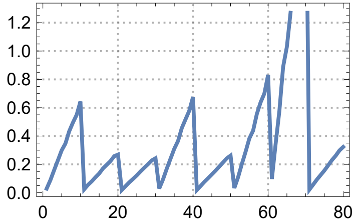

3D Plot

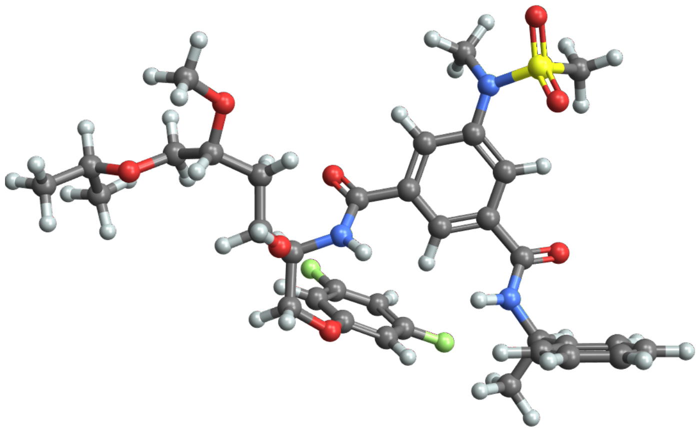

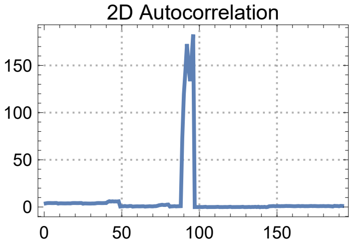

Dataset Index= 1026

Molecule Formula= $C_{27}H_{36}F_2N_3O_5^+$

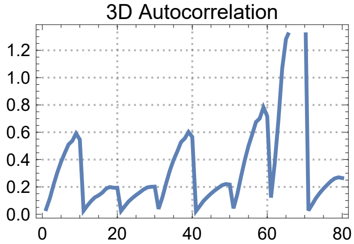

2D Plot

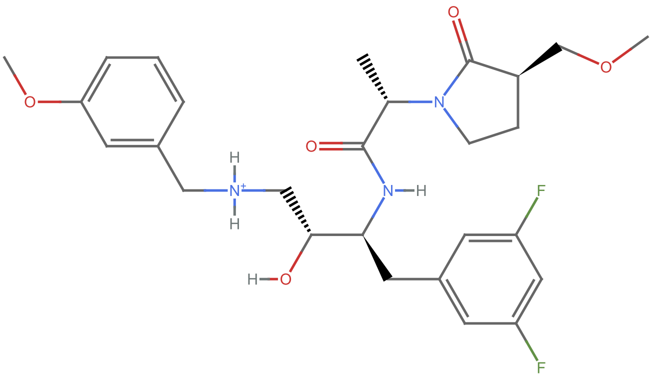

3D Plot

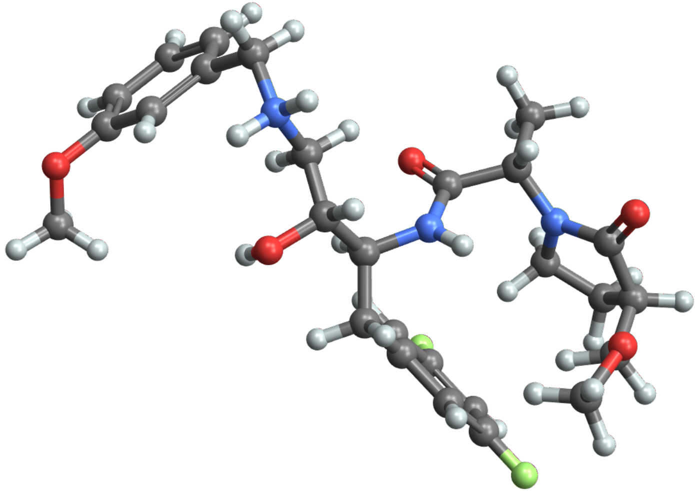

2D Plot

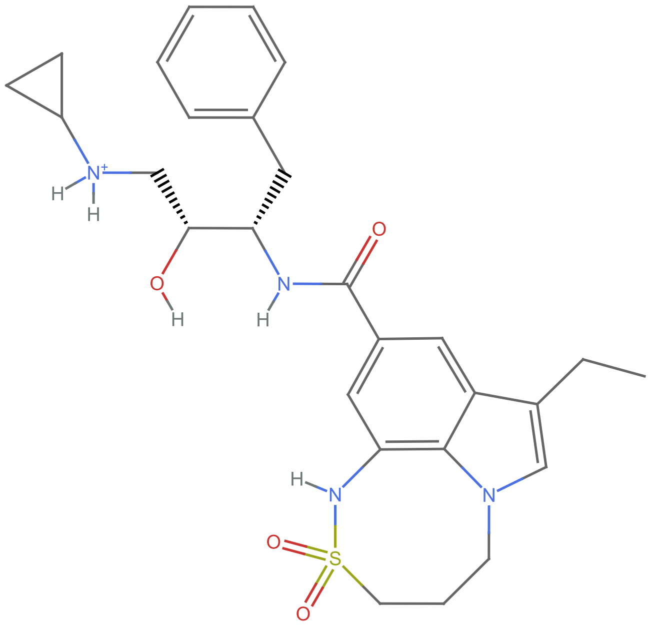

2D Autocorrelation

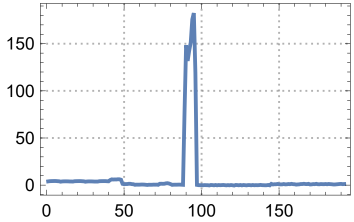

Dataset Index= 1027

Molecule Formula= $C_{27}H_{35}N_4O_4S^+$

3D Plot

3D Autocorrelation

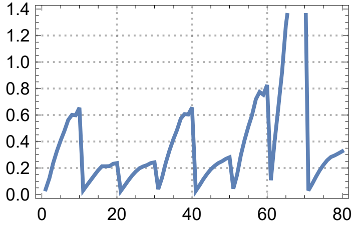

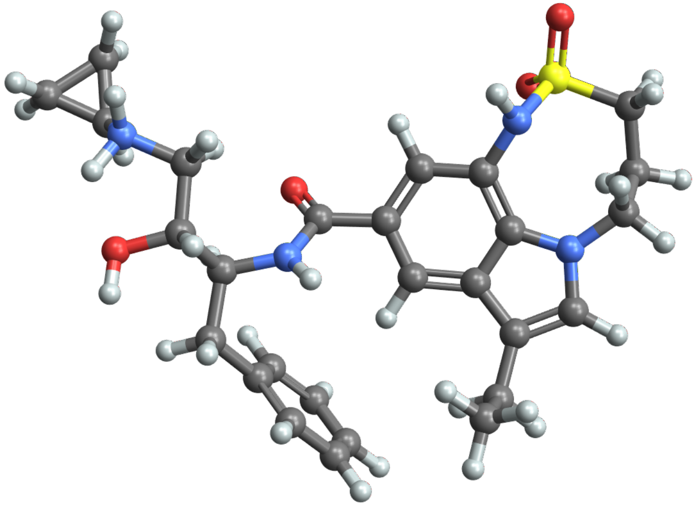

2D Plot

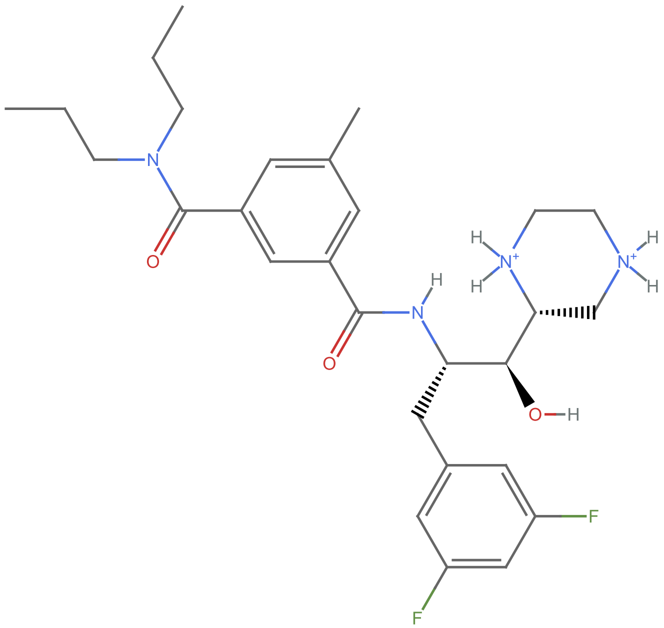

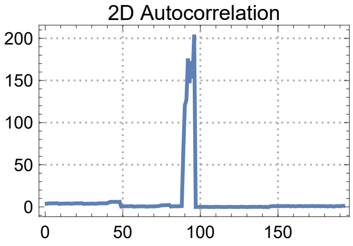

3D Plot

Dataset Index= 1028

Molecule Formula= $C_{28}H_{40}F_2N_4O_3^{2+}$

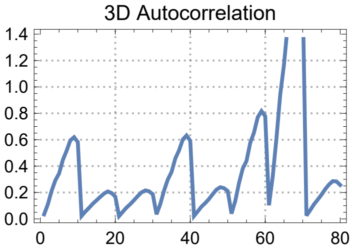

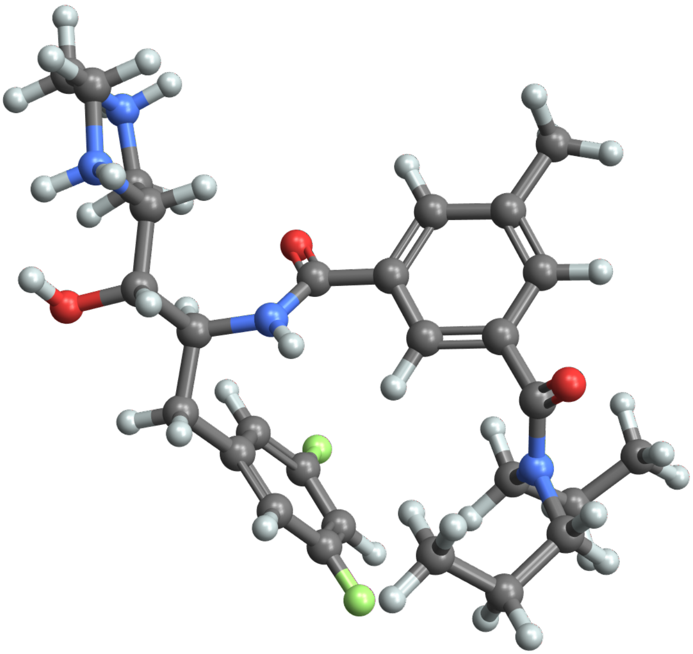

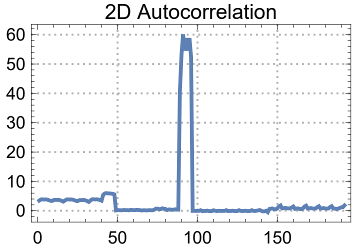

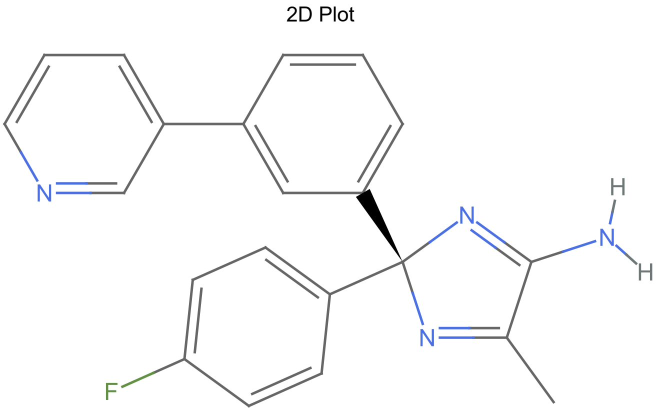

Dataset Index= 1029

Molecule Formula= $C_{21}H_{17}FN_4$

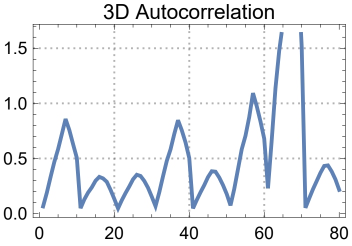

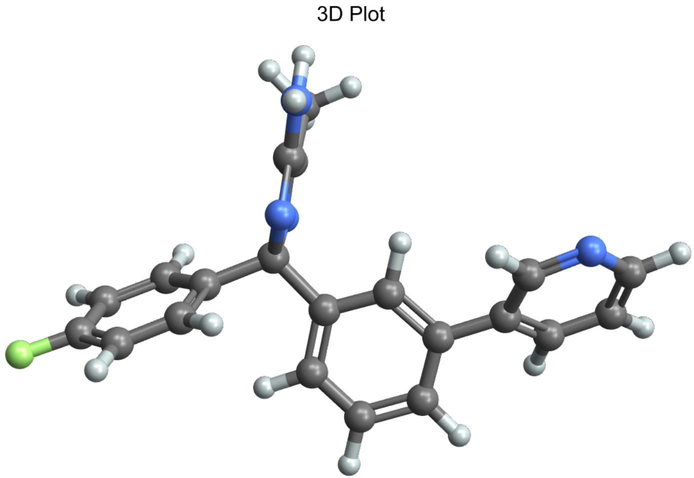

2D Plot

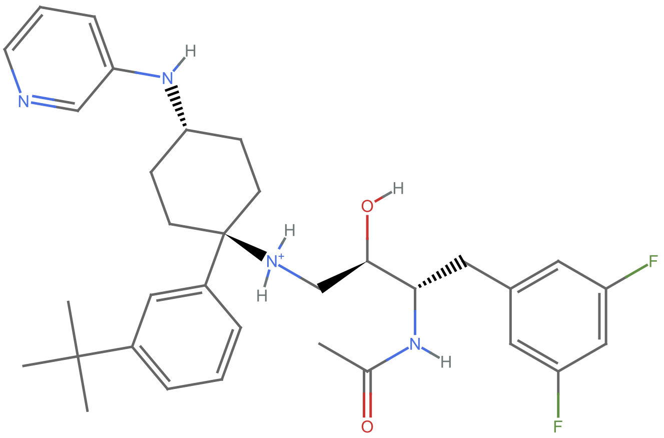

3D Plot

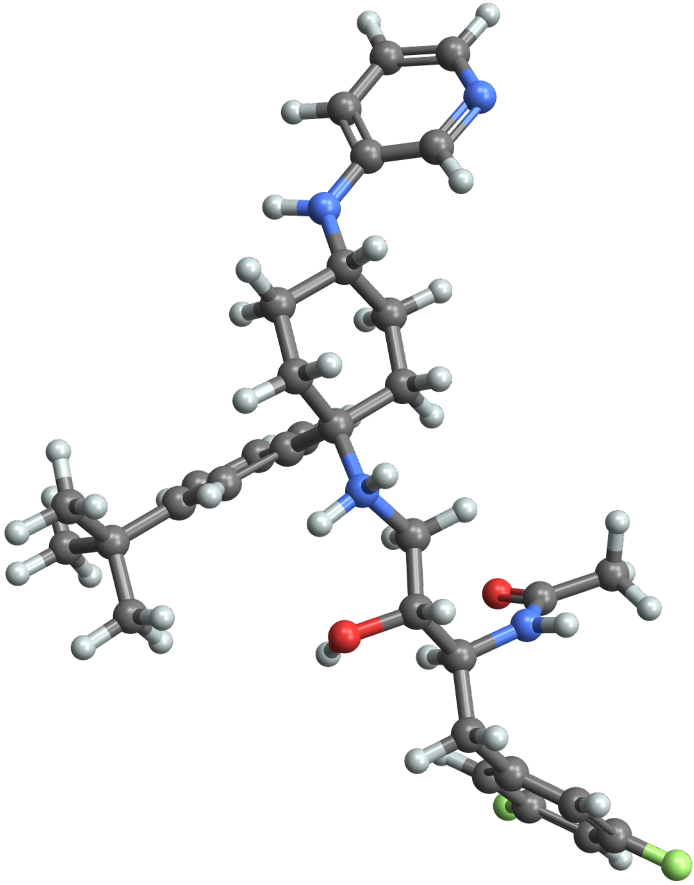

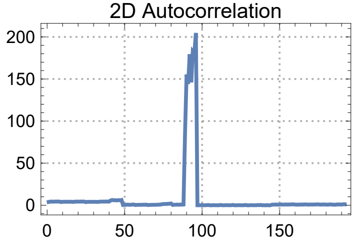

Dataset Index= 1030

Molecule Formula= $C_{33}H_{43}F_2N_4O_2^+$

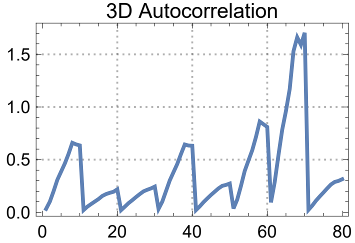

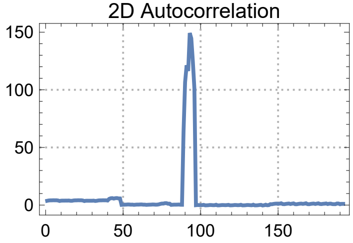

Dataset Index= 1031

Molecule Formula= $C_{28}H_{38}N_3O_4^+$

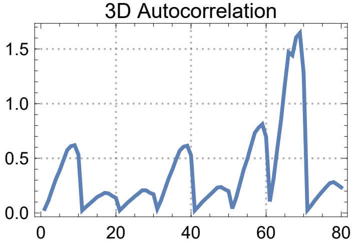

2D Plot

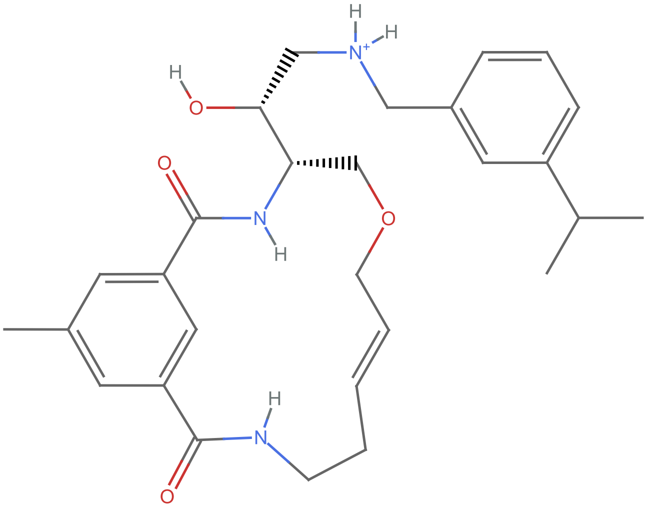

3D Plot

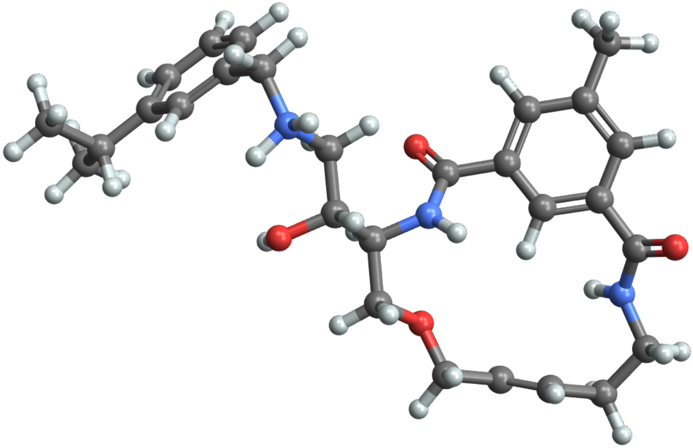

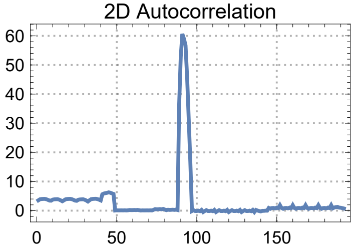

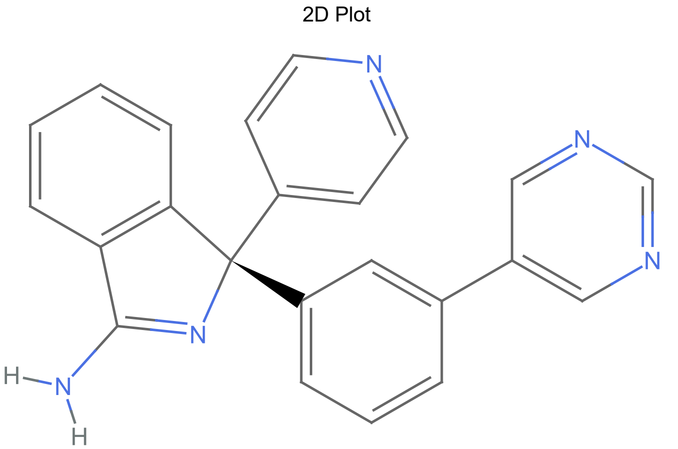

Dataset Index= 1032

Molecule Formula= $C_{23}H_{17}N_5$

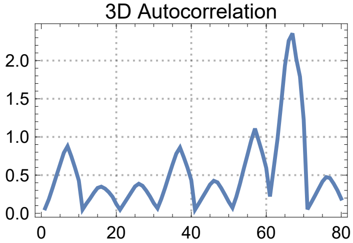

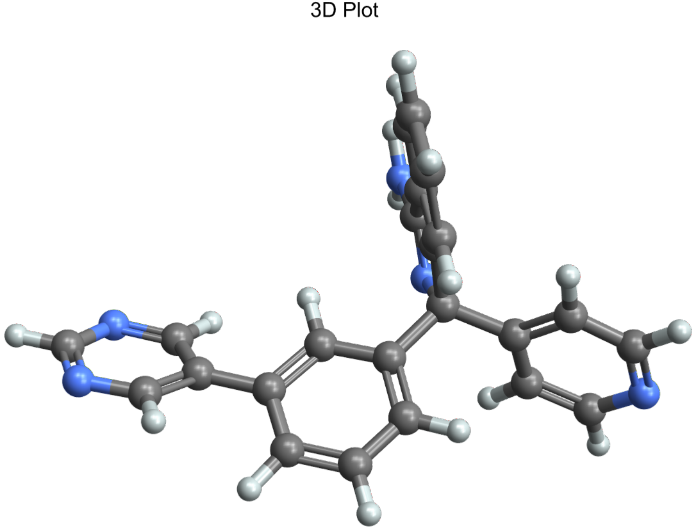

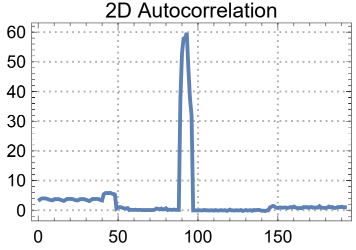

Dataset Index= 1033

Molecule Formula= $C_{21}H_{19}N_3OS$

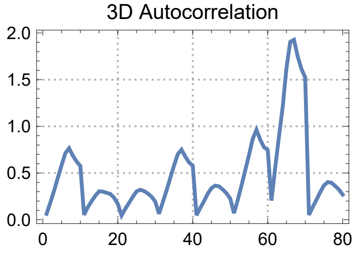

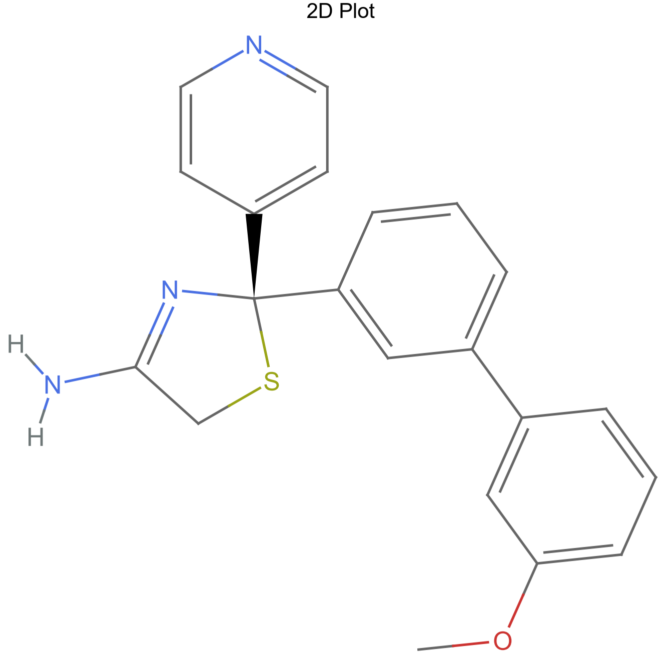

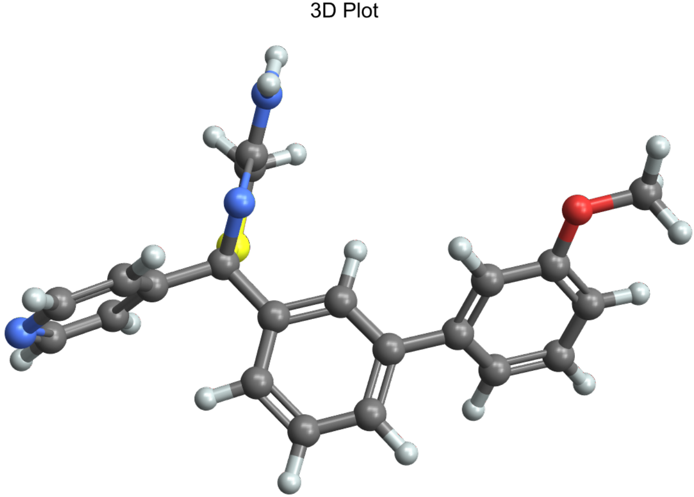

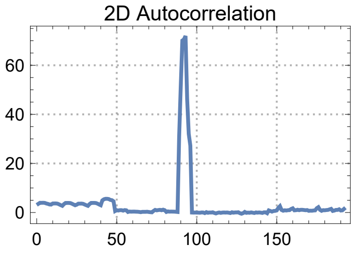

Dataset Index= 1034

Molecule Formula= $C_{17}H_{18}N_4O_2S$

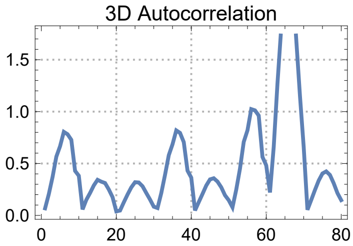

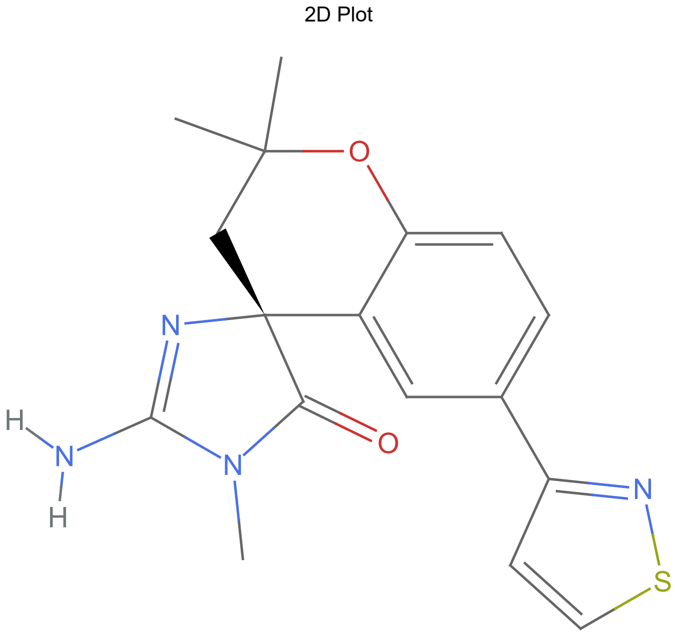

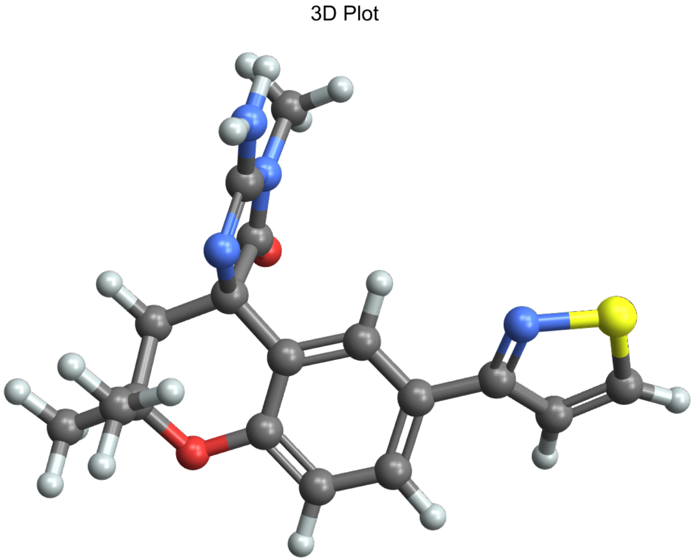

2D Plot

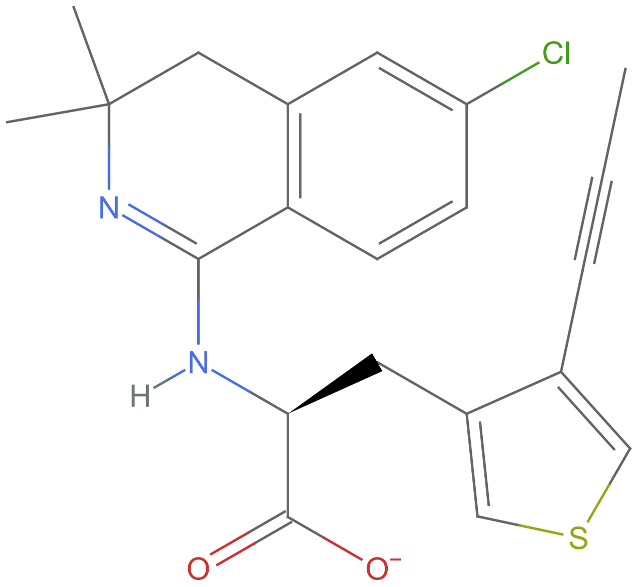

2D Autocorrelation

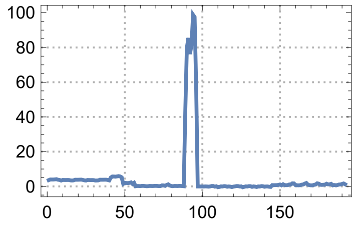

Dataset Index= 1035

Molecule Formula= $C_{21}H_{20}ClN_2O_2S^-$

3D Autocorrelation

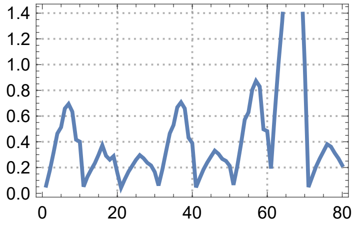

3D Plot

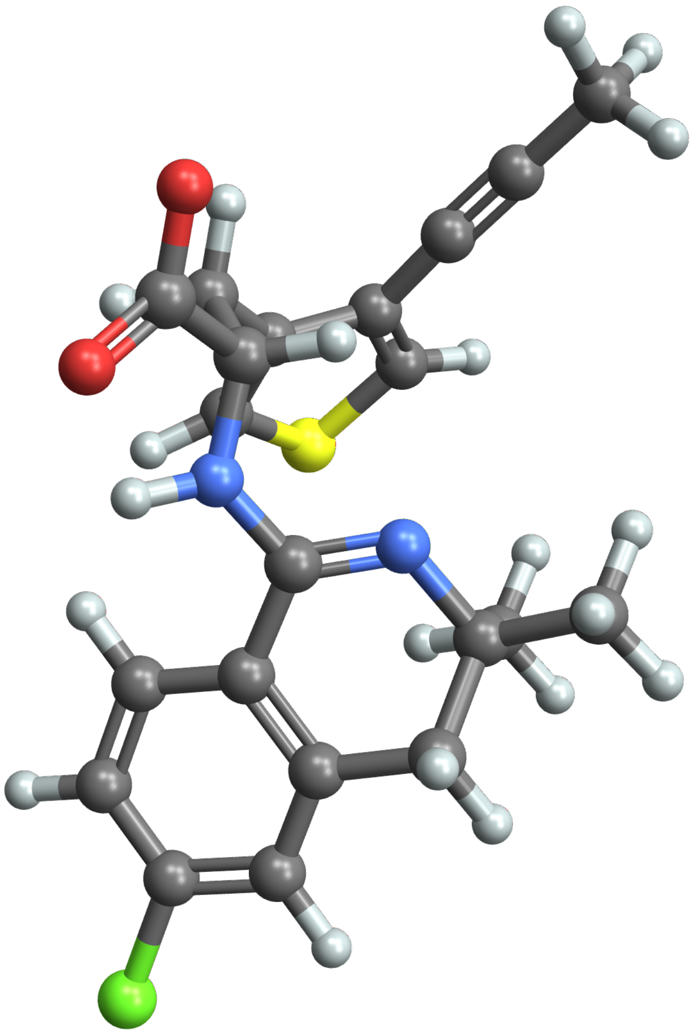

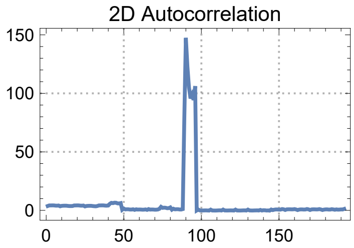

Dataset Index= 1036

Molecule Formula= $C_{23}H_{19}F_3N_4O_3$

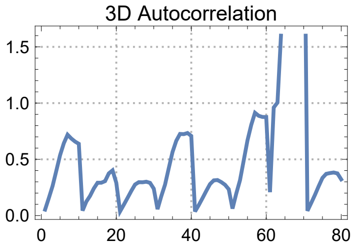

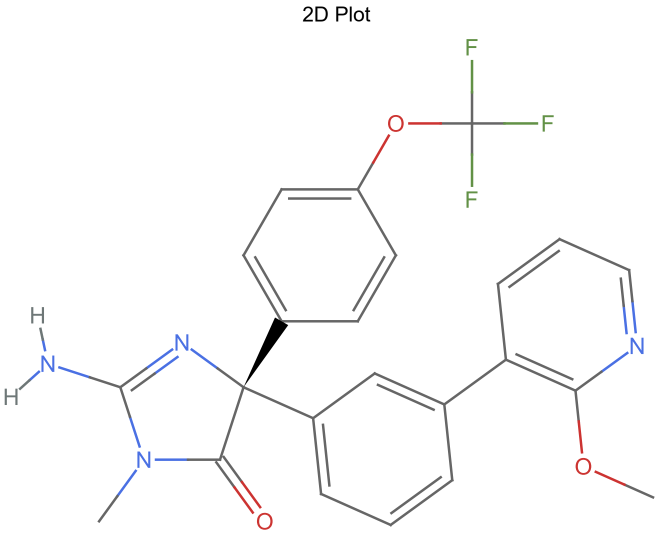

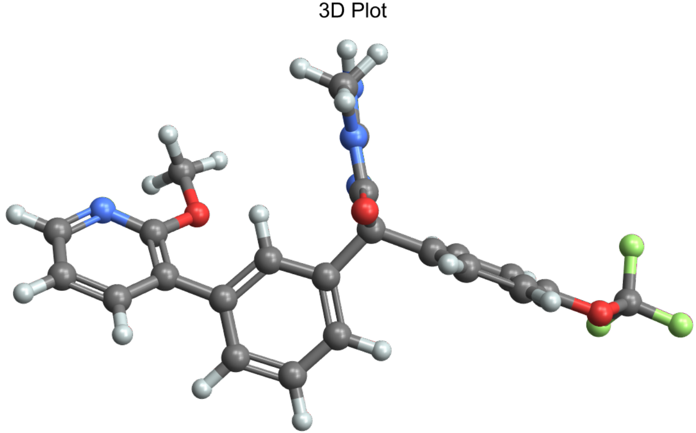

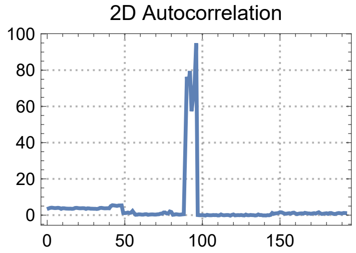

Dataset Index= 1037

Molecule Formula= $C_{23}H_{32}FN_2O_3S^+$

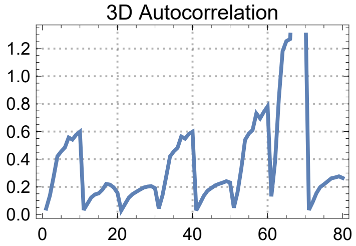

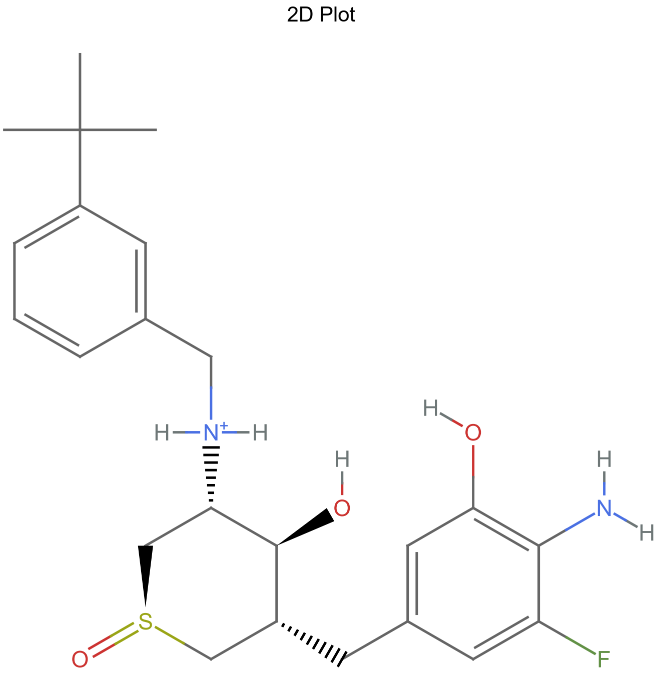

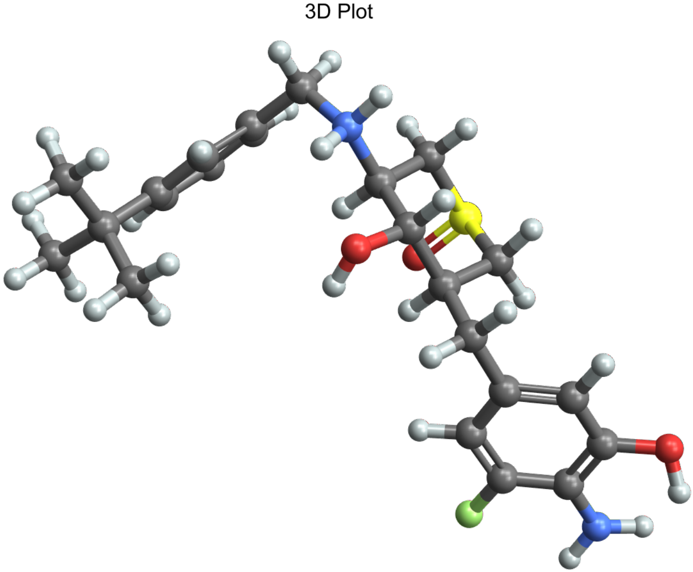

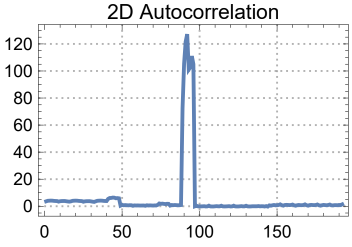

Dataset Index= 1038

Molecule Formula= $C_{22}H_{20}F_3N_3O_2$

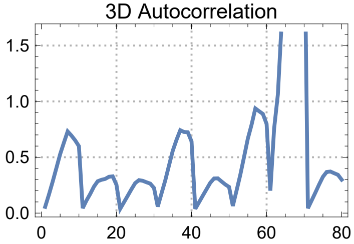

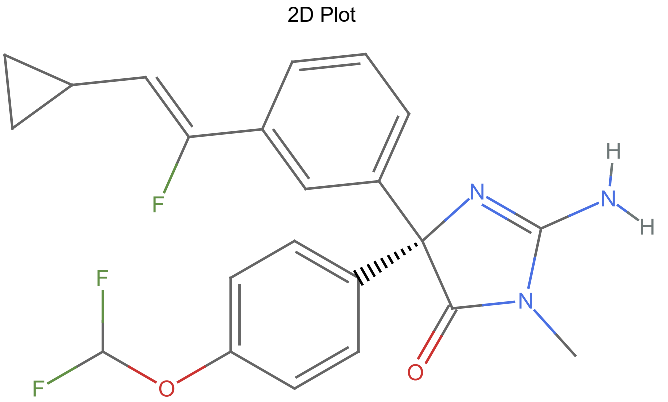

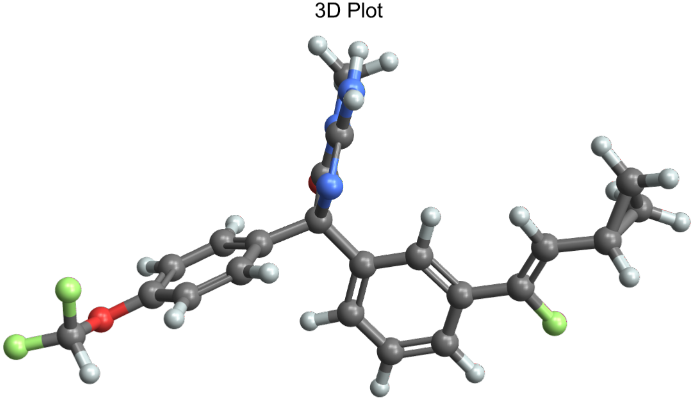

2D Plot

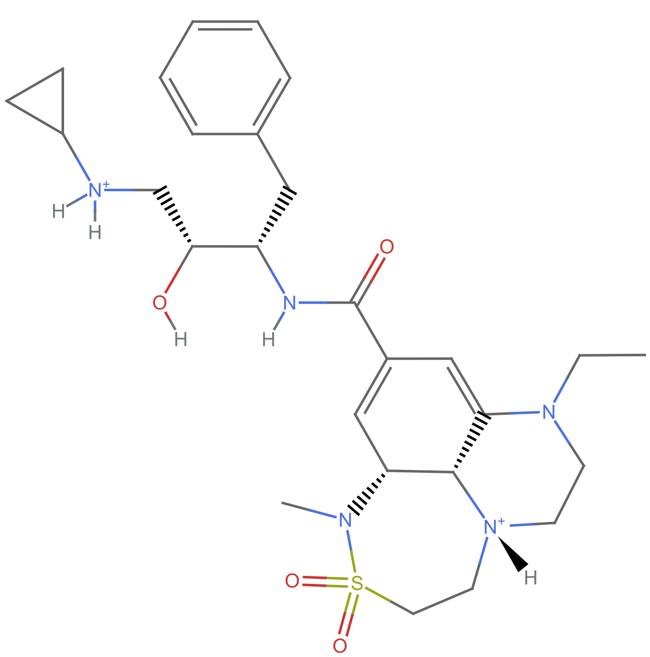

2D Autocorrelation

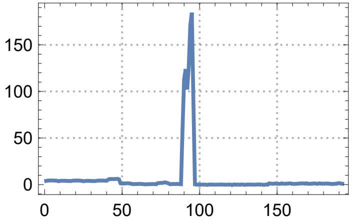

Dataset Index= 1039

Molecule Formula= $C_{27}H_{41}N_5O_4S^{2+}$

3D Plot

3D Autocorrelation

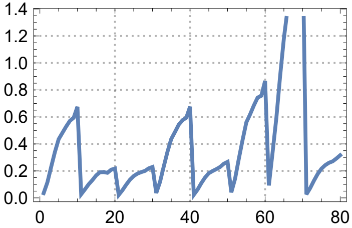

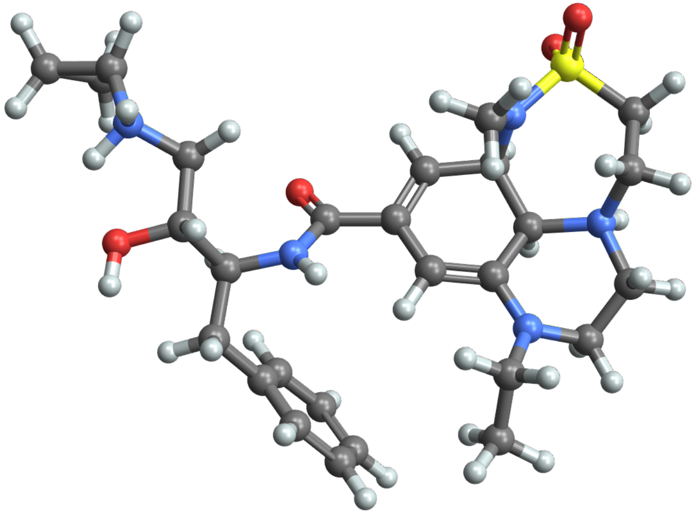

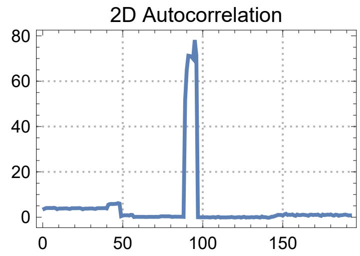

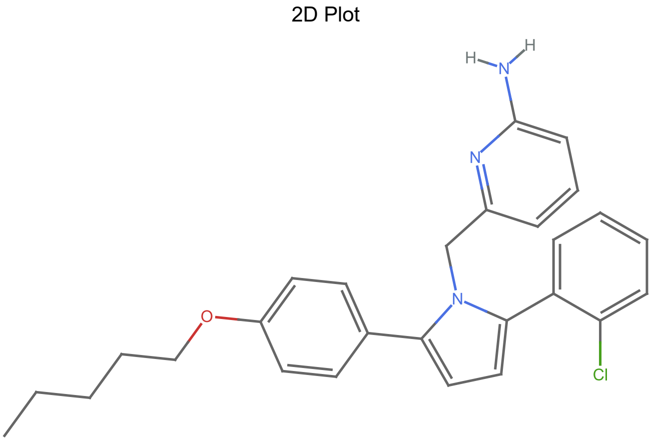

Dataset Index= 1040

Molecule Formula= $C_{27}H_{28}ClN_3O$

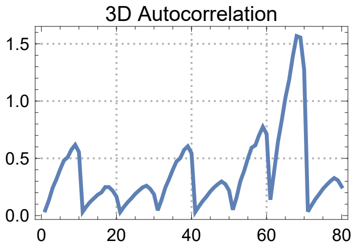

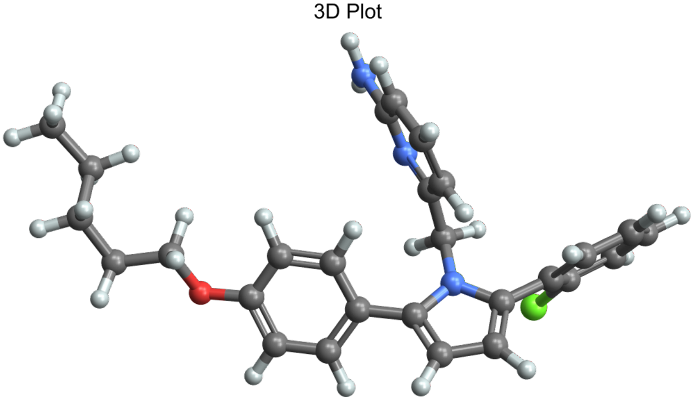

2D Plot

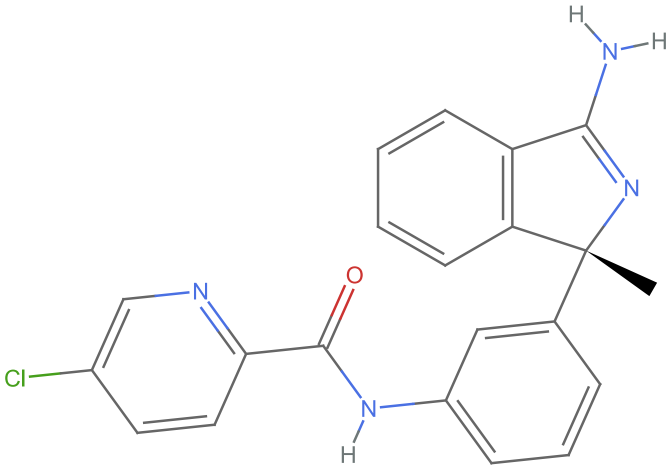

2D Autocorrelation

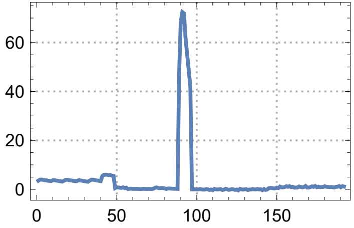

Dataset Index= 1041

Molecule Formula= $C_{21}H_{17}ClN_4O$

3D Plot

3D Autocorrelation

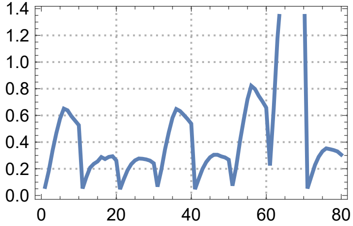

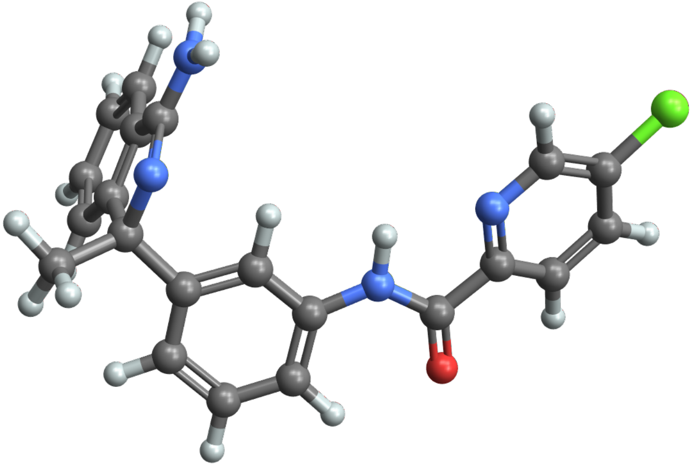

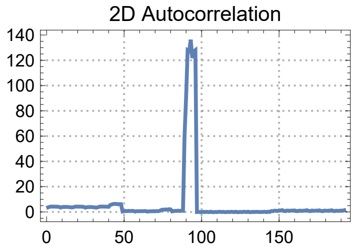

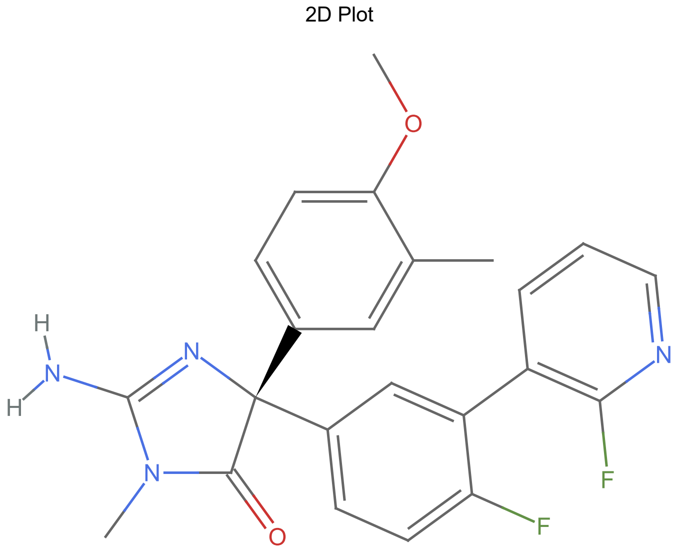

Dataset Index= 1042

Molecule Formula= $C_{23}H_{20}F_2N_4O_2$

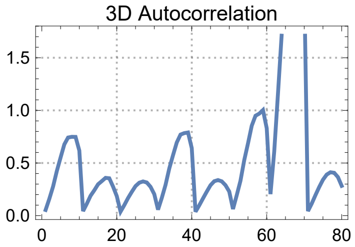

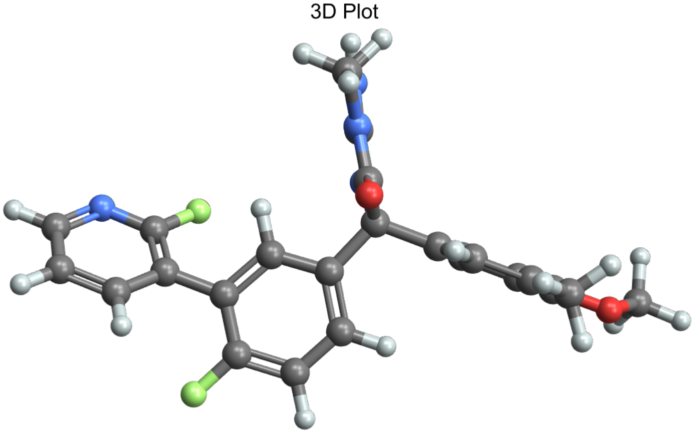

2D Plot

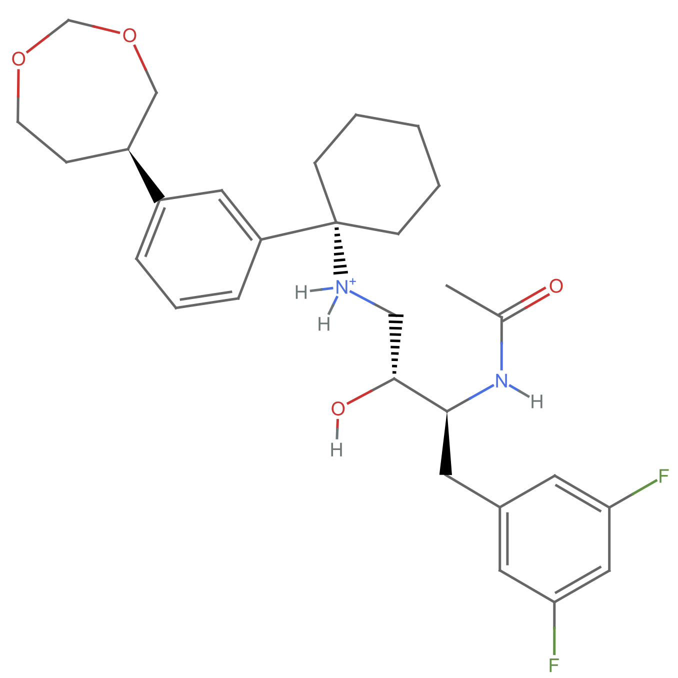

2D Autocorrelation

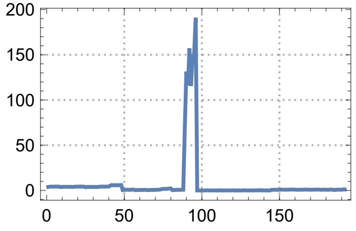

Dataset Index= 1043

Molecule Formula= $C_{29}H_{39}F_2N_2O_4^+$

3D Autocorrelation

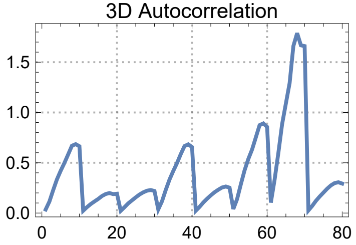

3D Plot

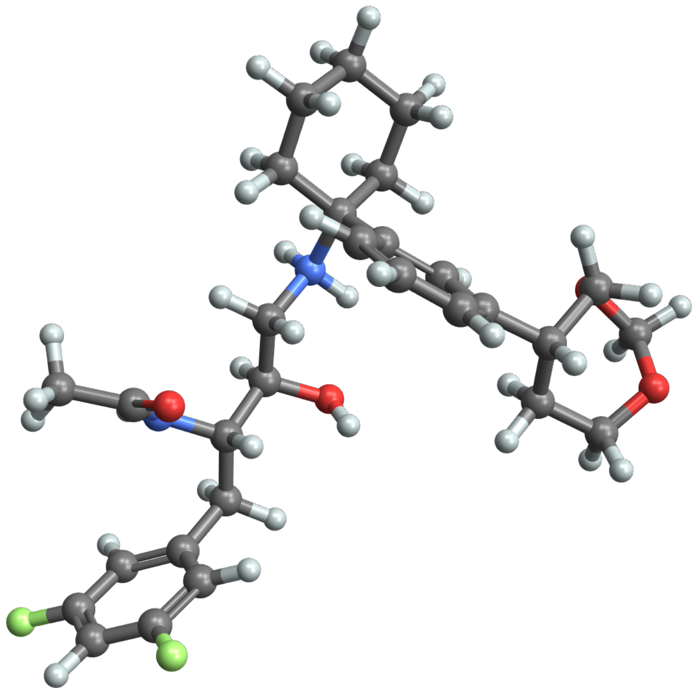

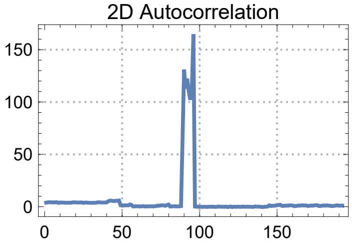

Dataset Index= 1044

Molecule Formula= $C_{28}H_{38}FN_2O_3S^+$

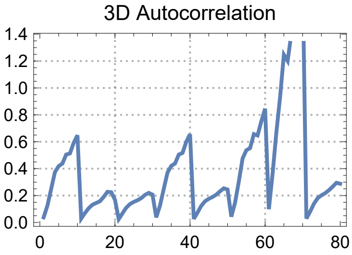

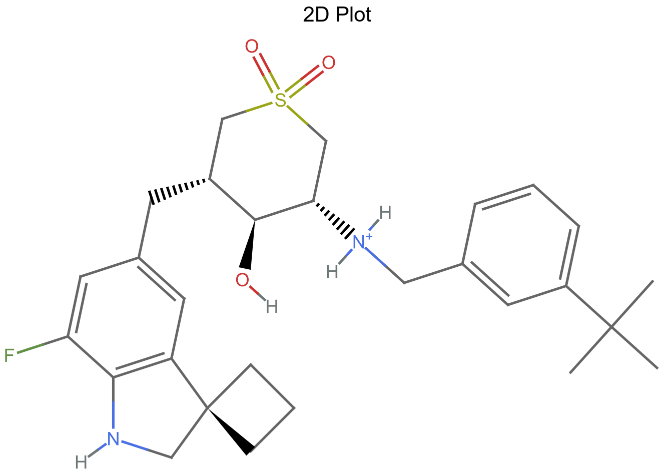

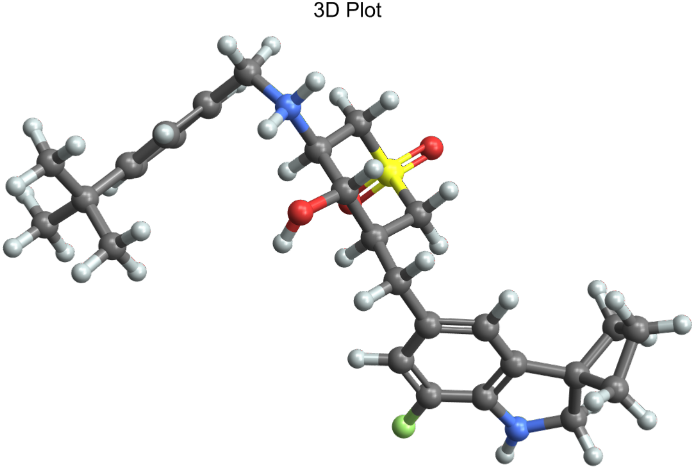

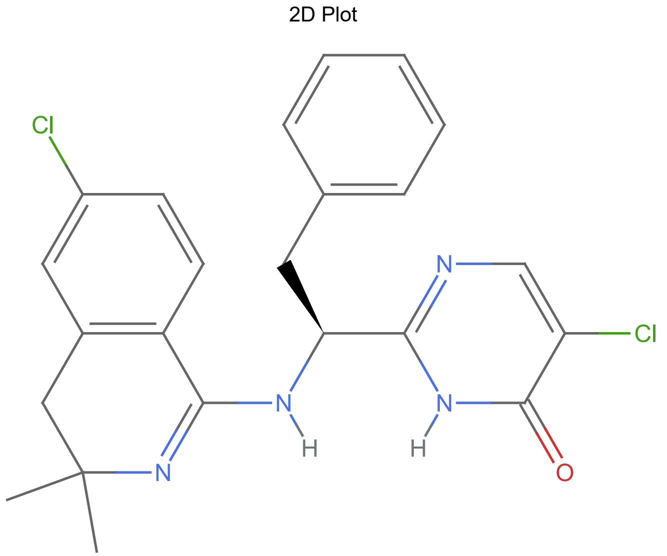

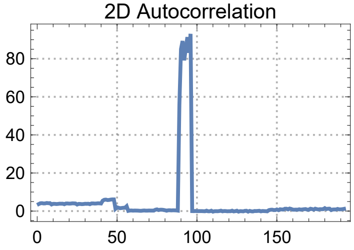

Dataset Index= 1045

Molecule Formula= $C_{23}H_{22}Cl_2N_4O$

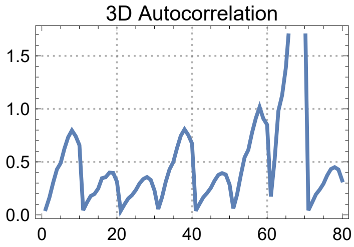

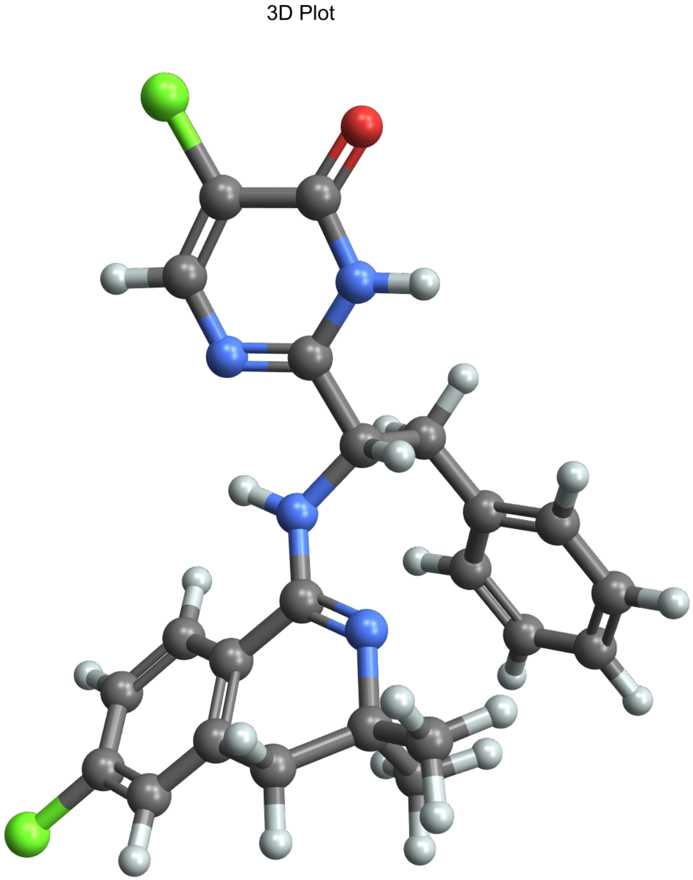

2D Plot

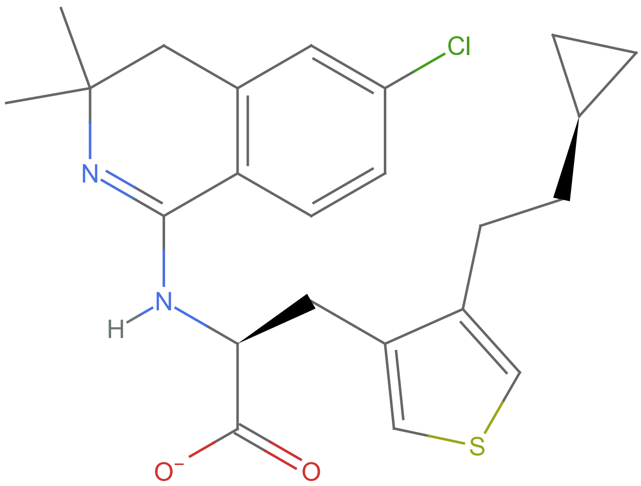

3D Plot

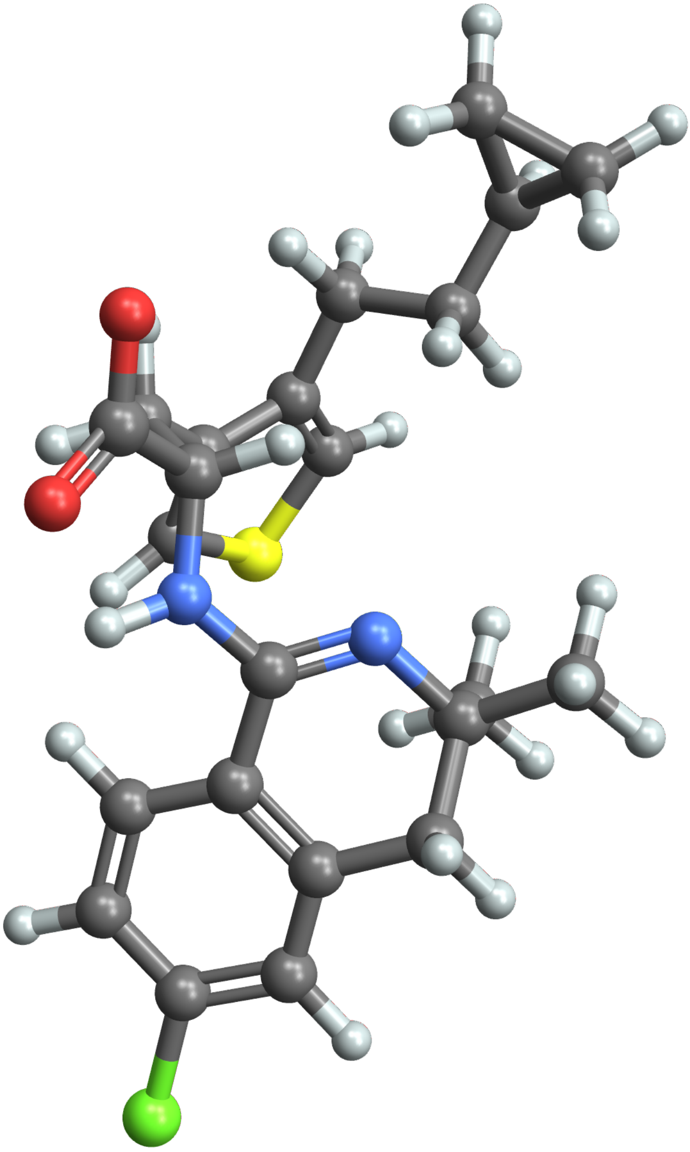

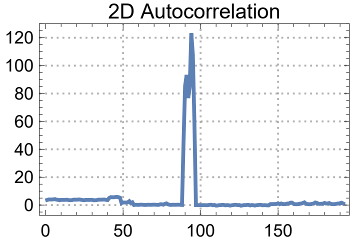

Dataset Index= 1046

Molecule Formula= $C_{23}H_{26}ClN_2O_2S^-$

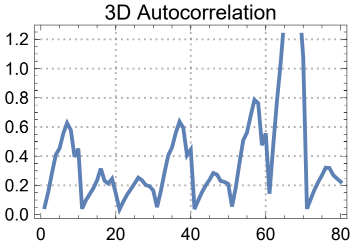

2D Plot

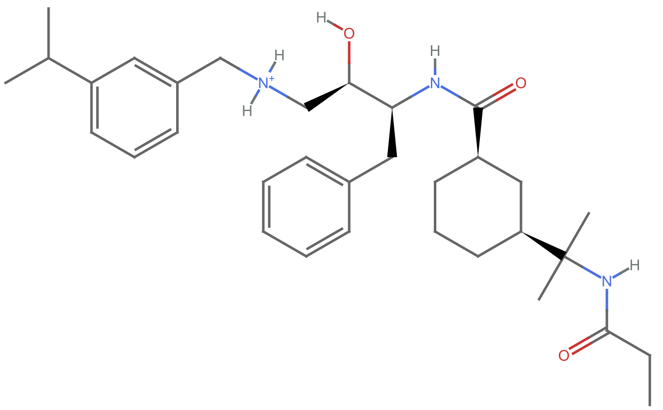

2D Autocorrelation

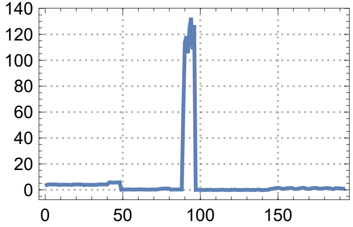

3D Plot

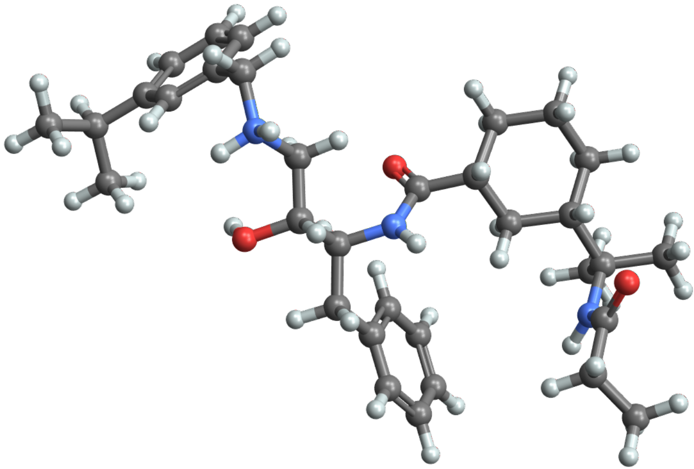

Dataset Index= 1047

Molecule Formula= $C_{33}H_{50}N_3O_3^+$

3D Autocorrelation

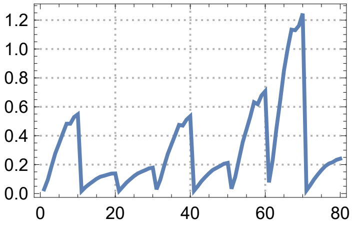

2D Plot

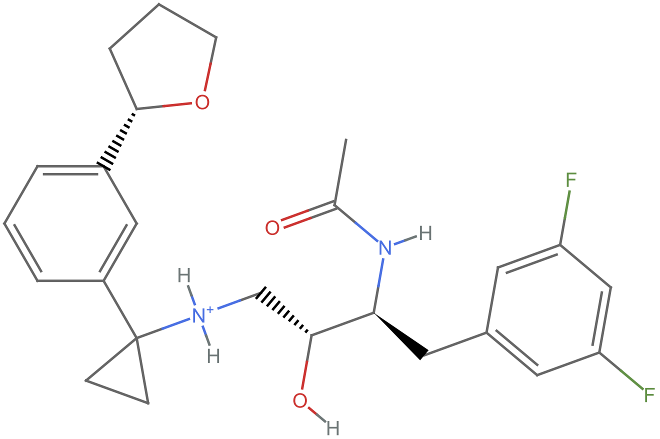

2D Autocorrelation

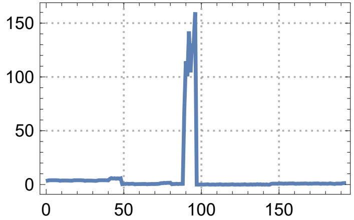

3D Plot

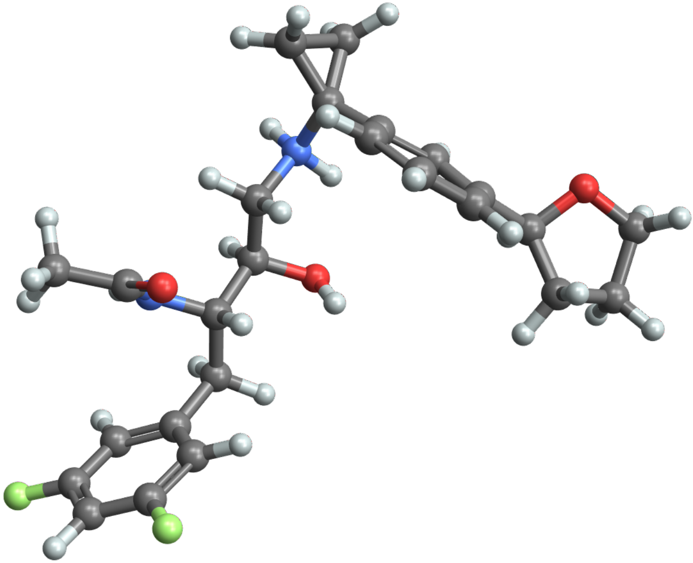

Dataset Index= 1048

Molecule Formula= $C_{25}H_{31}F_2N_2O_3^+$

3D Autocorrelation

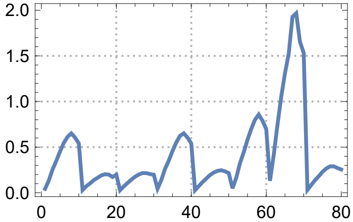

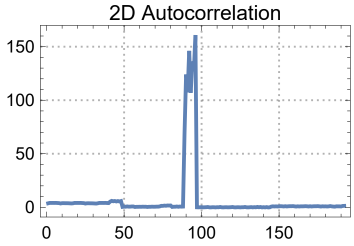

Dataset Index= 1049

Molecule Formula= $C_{26}H_{33}F_2N_2O_3^+$

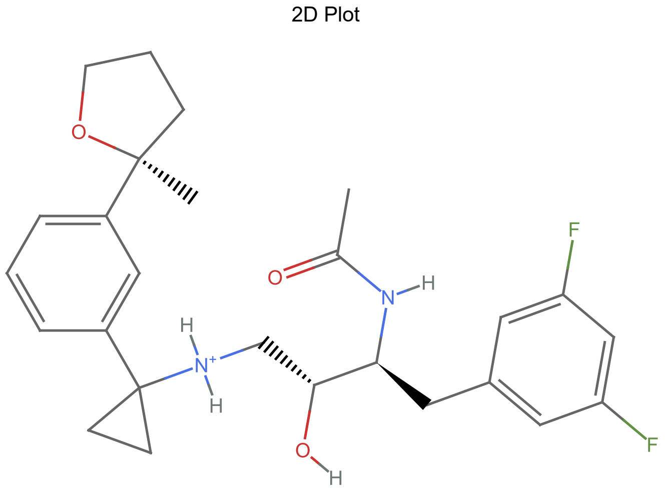

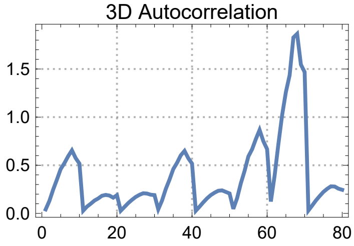

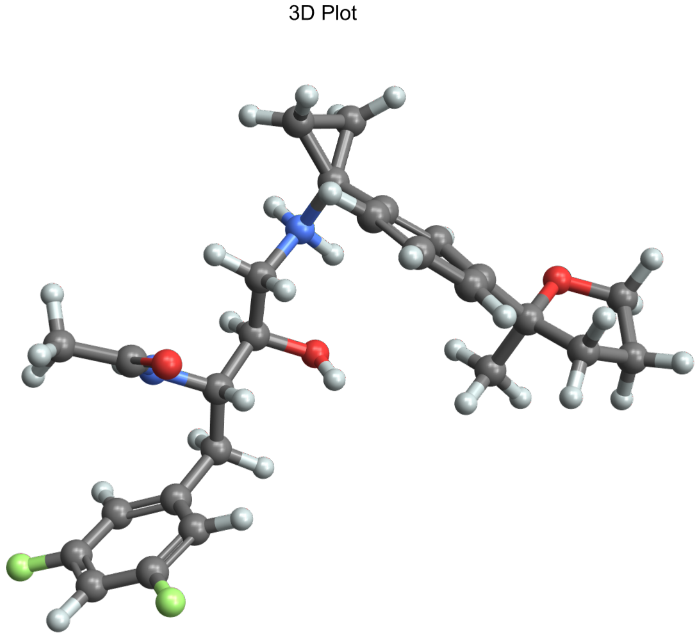

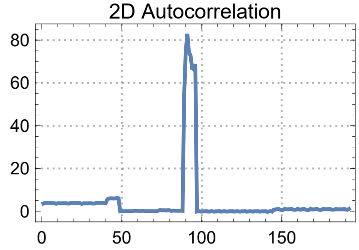

Dataset Index= 1050

Molecule Formula= $C_{26}H_{24}N_4O$

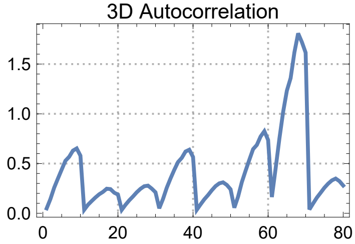

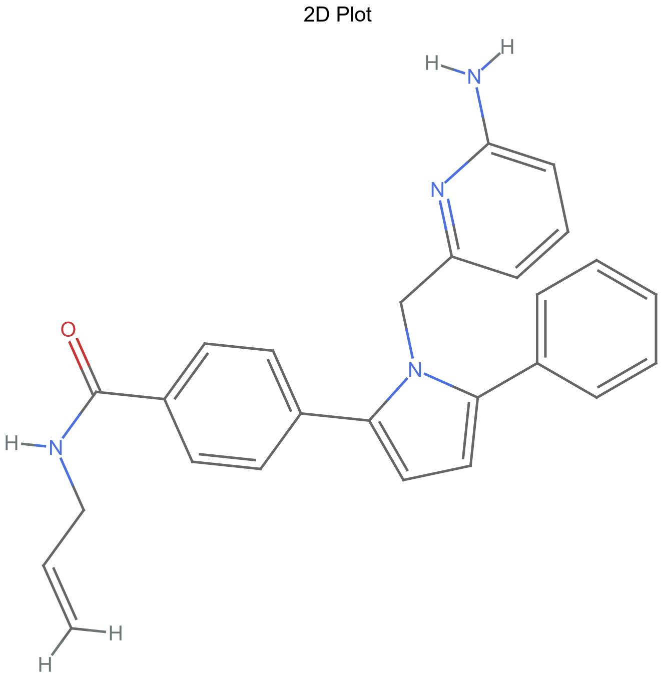

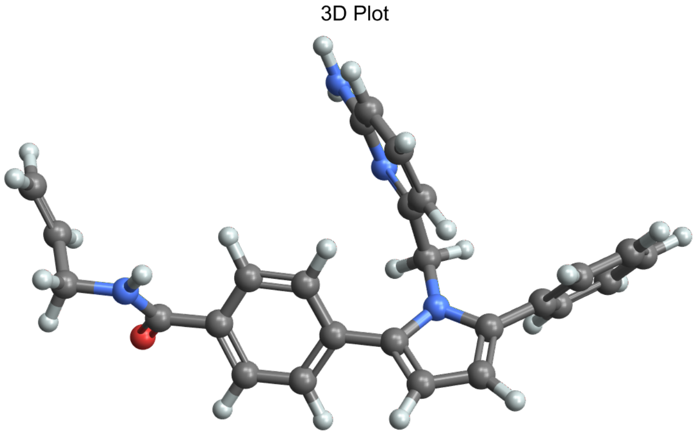

2D Autocorrelation

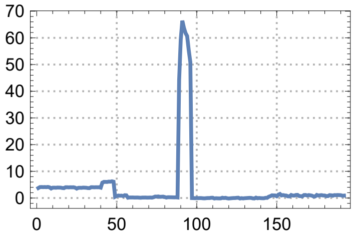

Dataset Index= 1051

Molecule Formula= $C_{27}H_{21}ClN_4O$

3D Autocorrelation

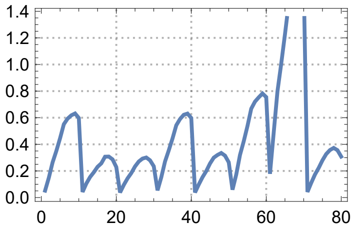

2D Plot

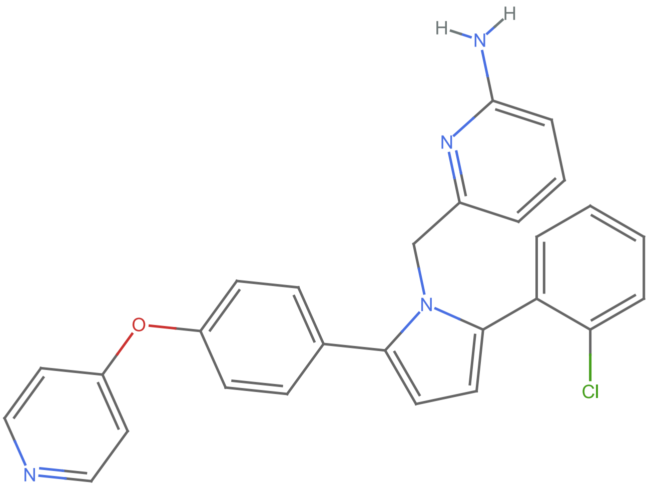

3D Plot

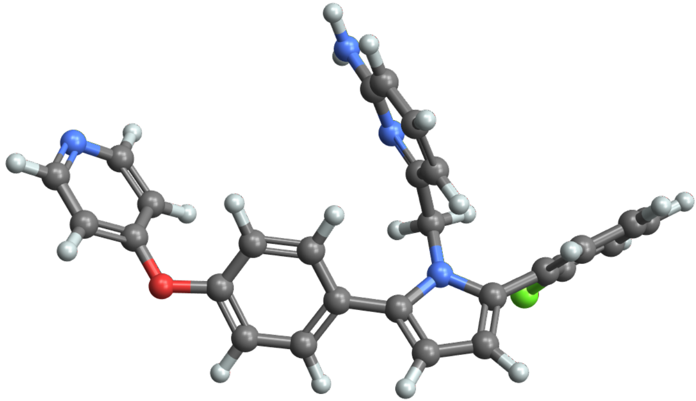

2D Plot

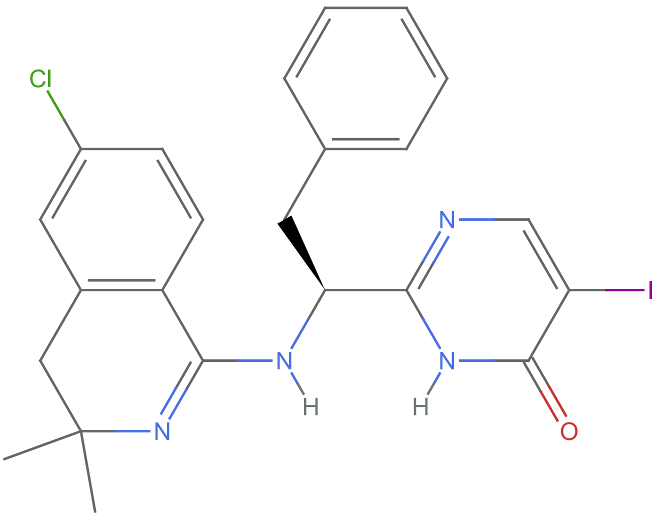

2D Autocorrelation

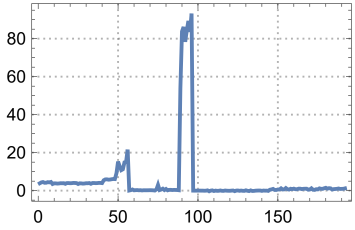

Dataset Index= 1052

Molecule Formula= $C_{23}H_{22}ClIN_4O$

3D Autocorrelation

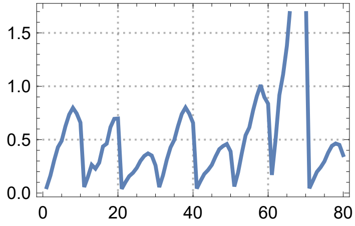

3D Plot

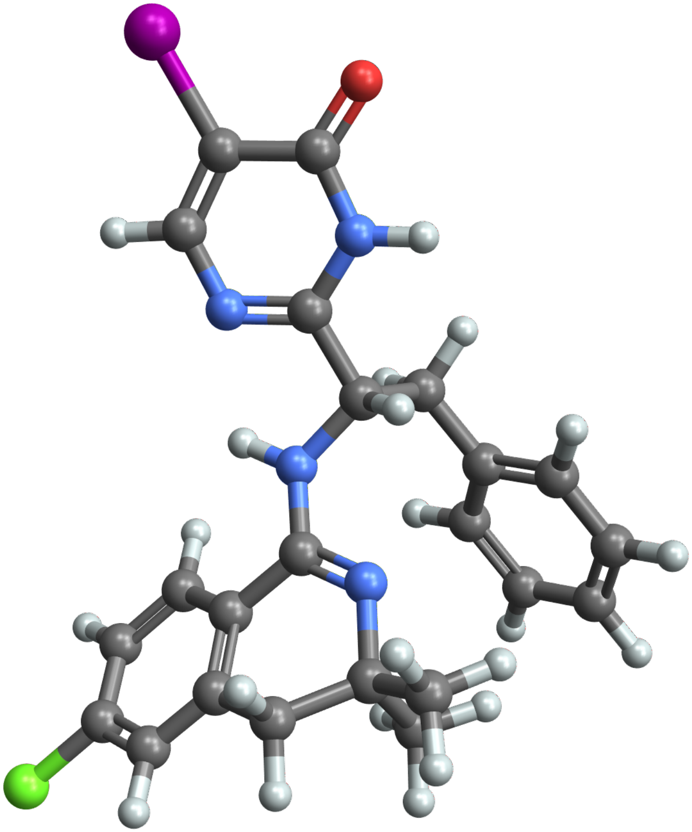

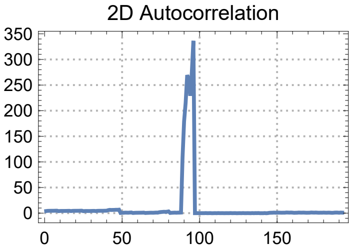

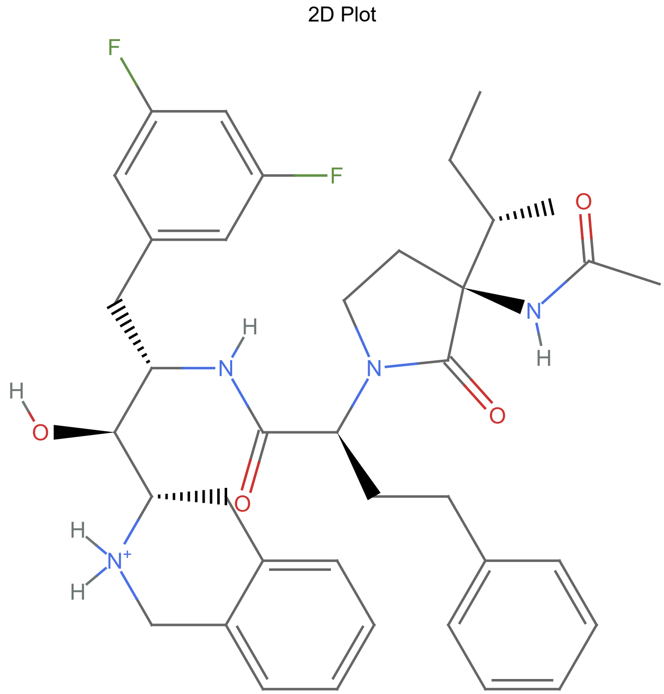

Dataset Index= 1053

Molecule Formula= $C_{38}H_{47}F_2N_4O_4^+$

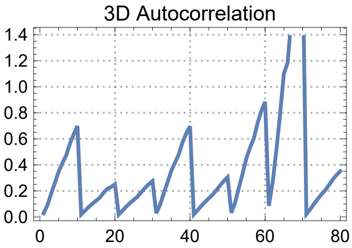

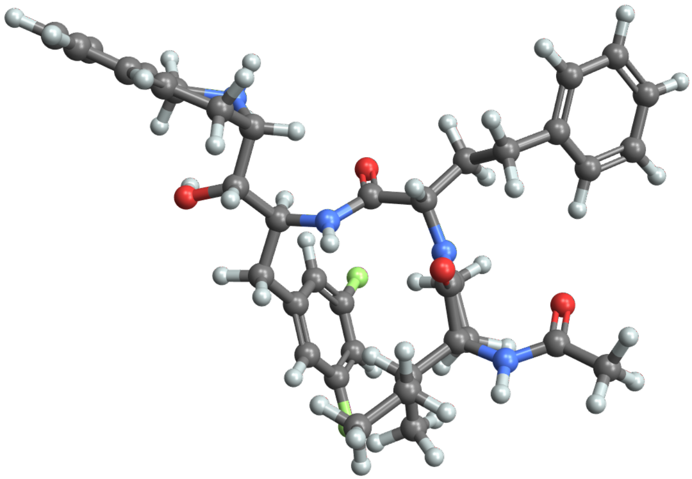

2D Plot

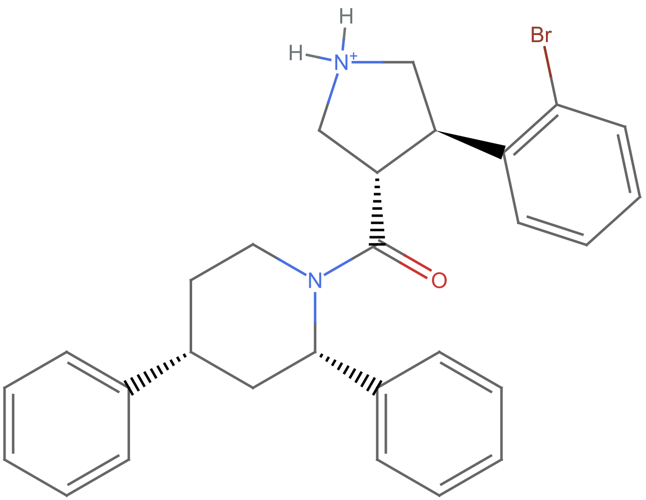

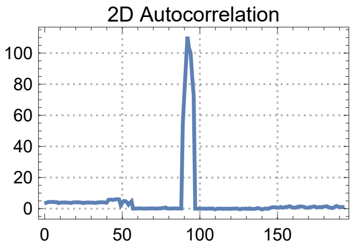

3D Plot

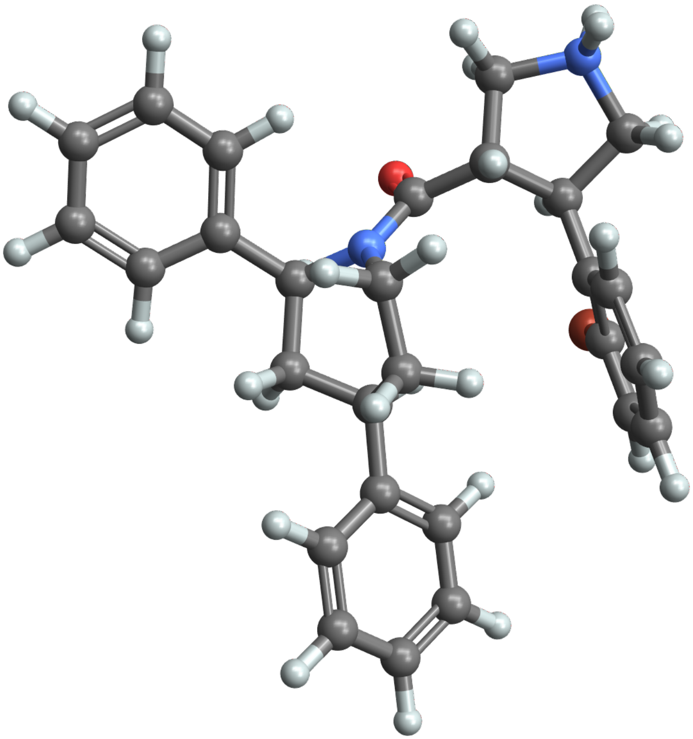

Dataset Index= 1054

Molecule Formula= $C_{28}H_{30}BrN_2O^+$

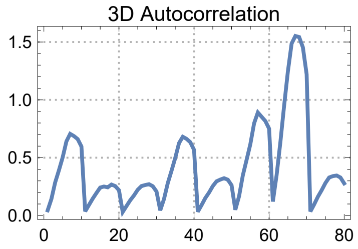

2D Plot

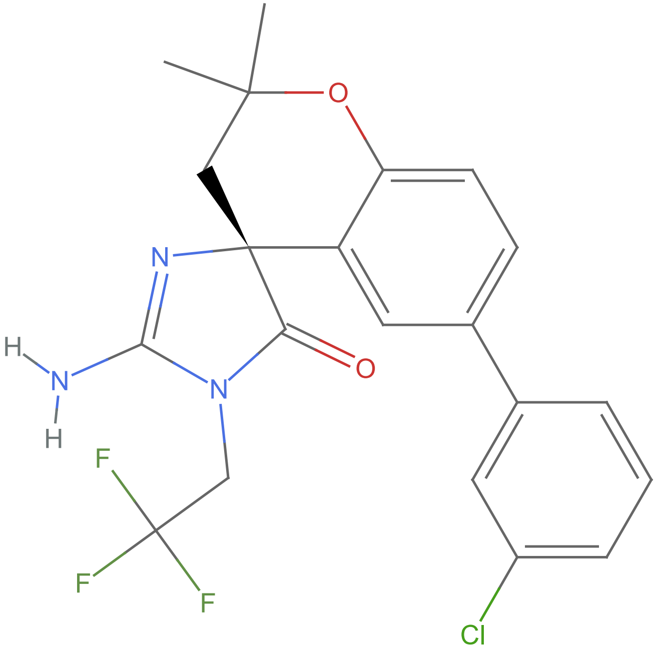

2D Autocorrelation

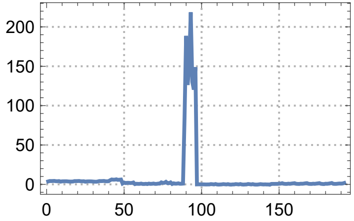

Dataset Index= 1055

Molecule Formula= $C_{21}H_{19}ClF_3N_3O_2$

3D Autocorrelation

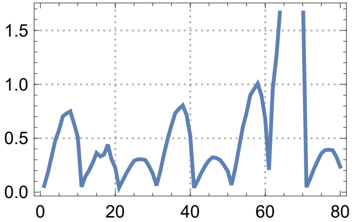

3D Plot

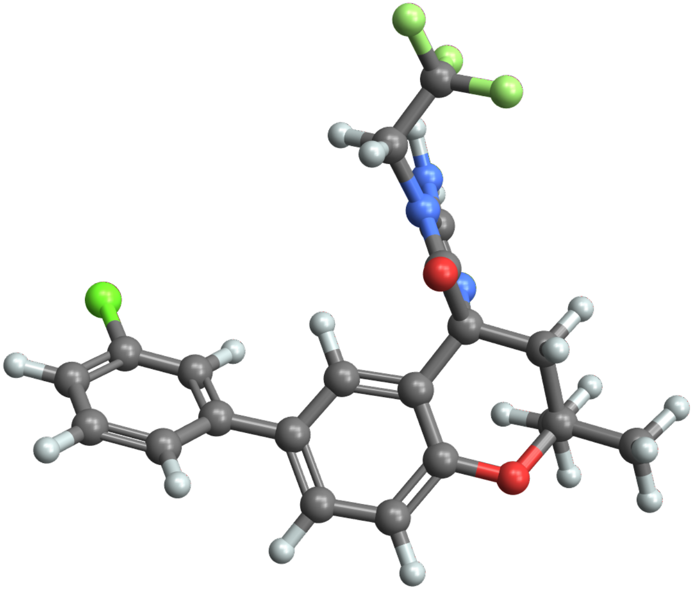

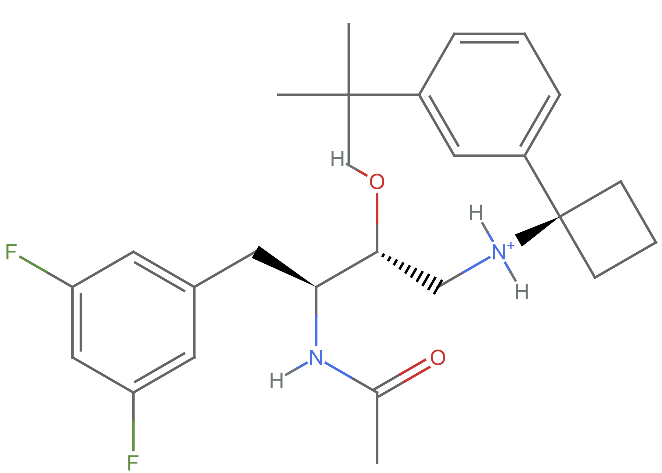

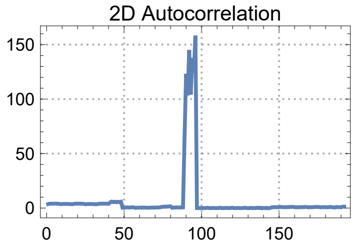

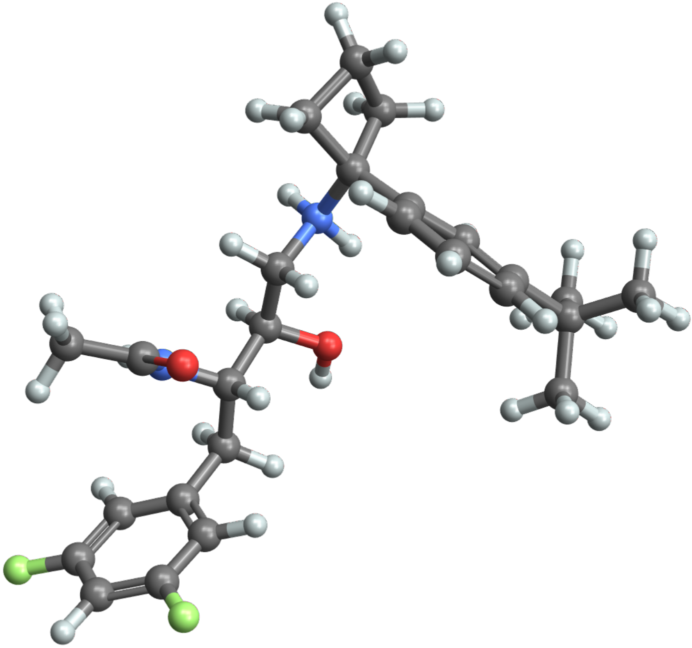

Dataset Index= 1056

Molecule Formula= $C_{26}H_{35}F_2N_2O_2^+$

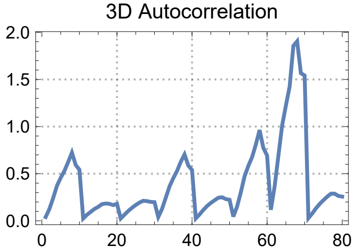

2D Autocorrelation

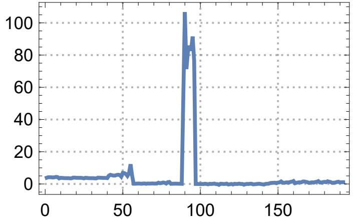

Dataset Index= 1057

Molecule Formula= $C_{23}H_{31}BrNO_3S^+$

3D Autocorrelation

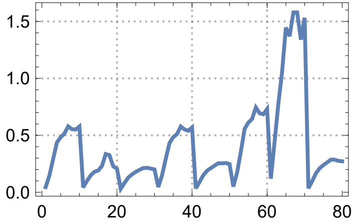

2D Plot

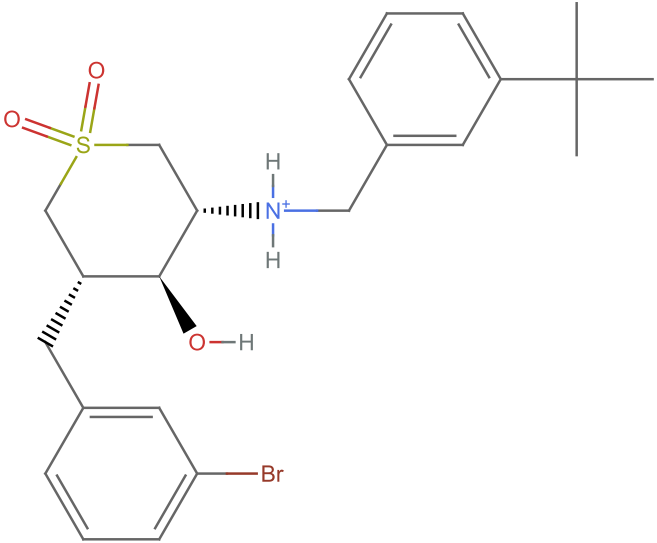

3D Plot

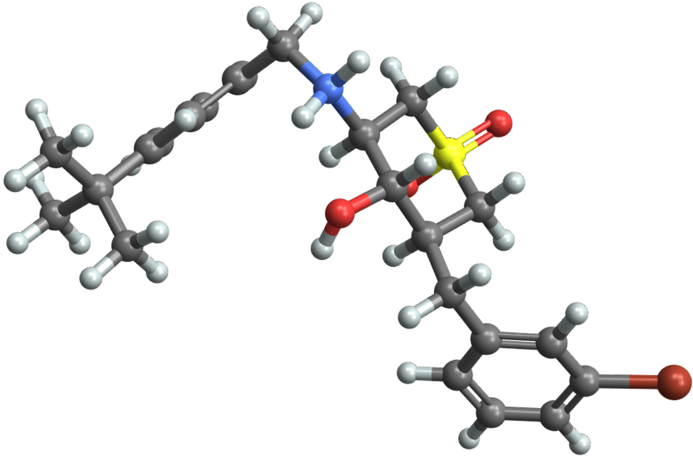

2D Plot

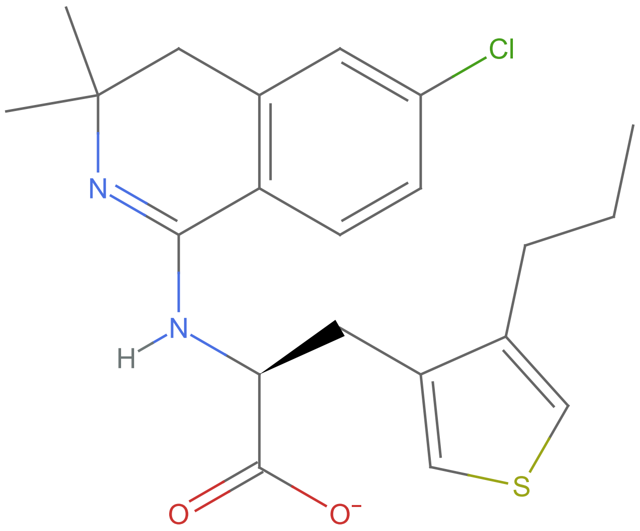

3D Plot

2D Autocorrelation

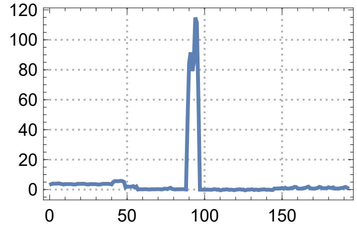

Dataset Index= 1058

Molecule Formula= $C_{21}H_{24}ClN_2O_2S^-$

3D Autocorrelation

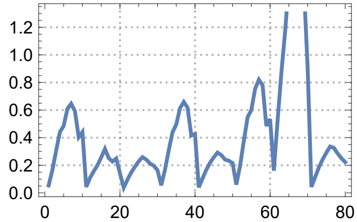

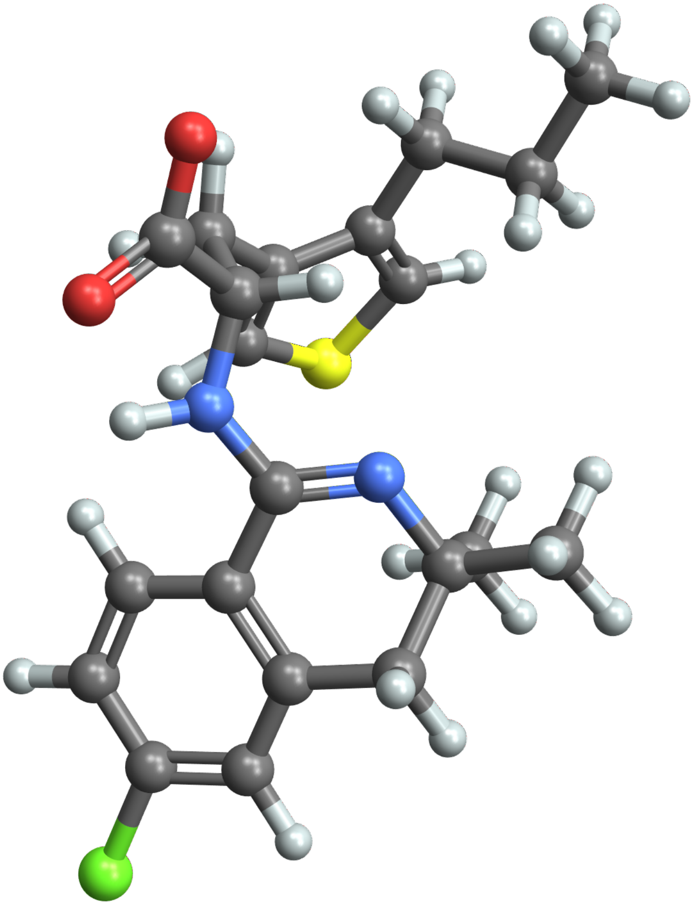

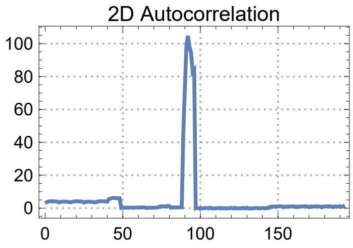

Dataset Index= 1059

Molecule Formula= $C_{24}H_{21}N_5O_2$

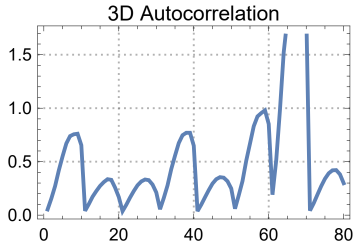

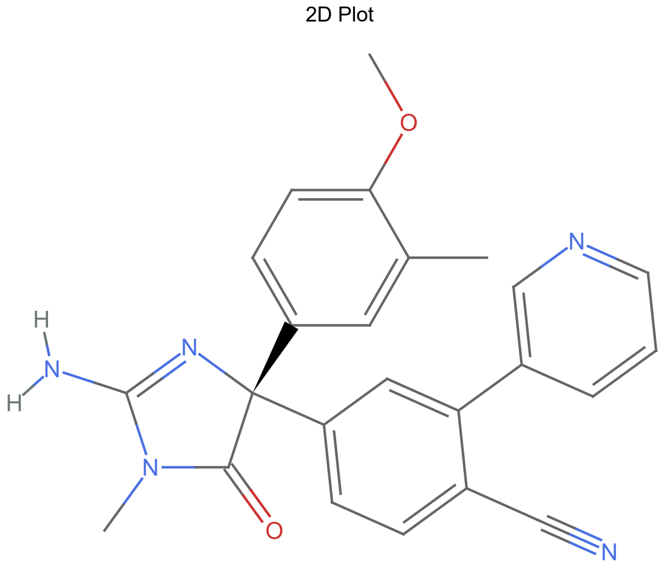

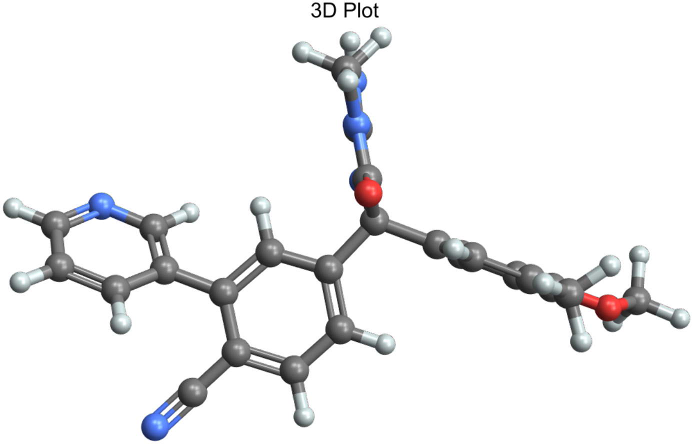

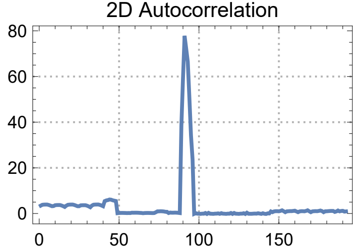

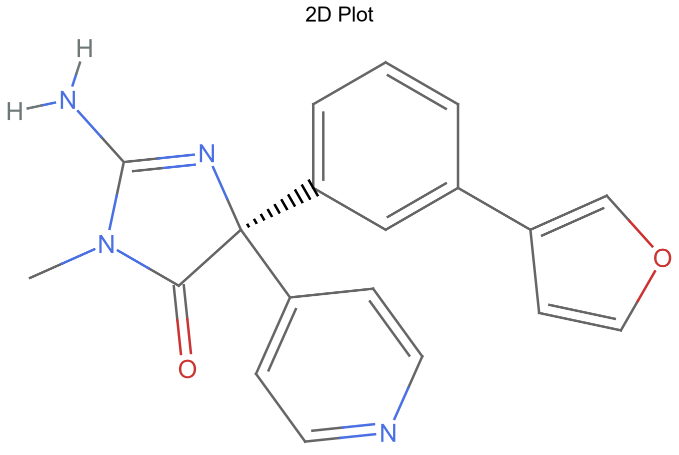

Dataset Index= 1060

Molecule Formula= $C_{19}H_{16}N_4O_2$

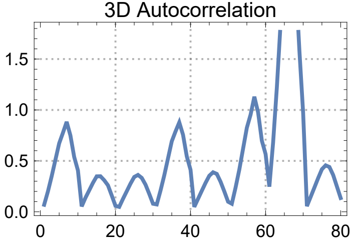

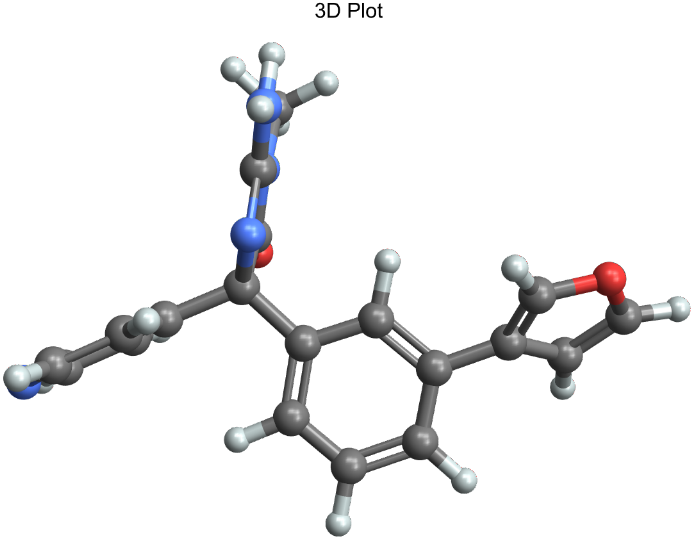

2D Plot

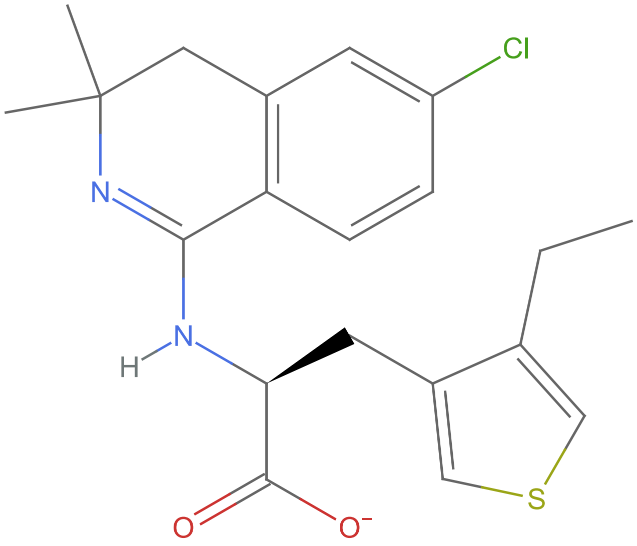

3D Plot

2D Autocorrelation

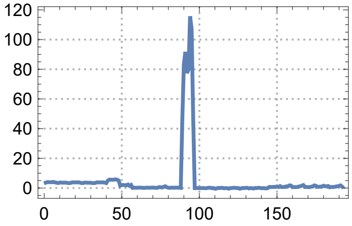

Dataset Index= 1061

Molecule Formula= $C_{20}H_{22}ClN_2O_2S^-$

3D Autocorrelation

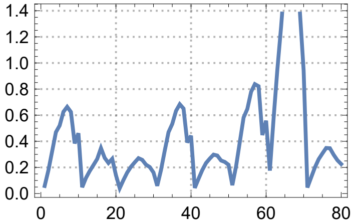

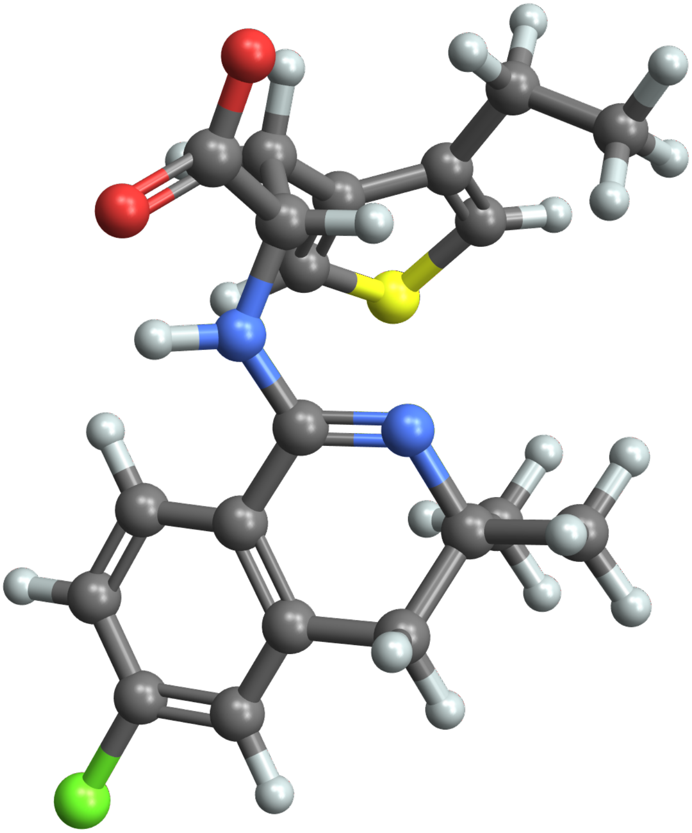

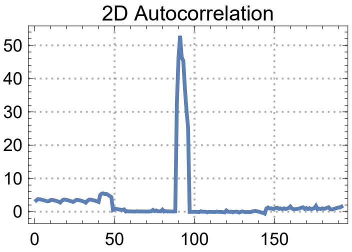

Dataset Index= 1062

Molecule Formula= $C_{18}H_{20}N_2OS$

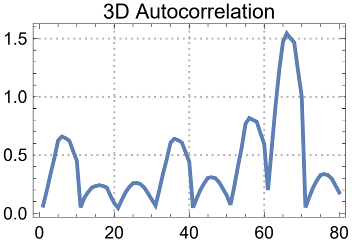

2D Plot

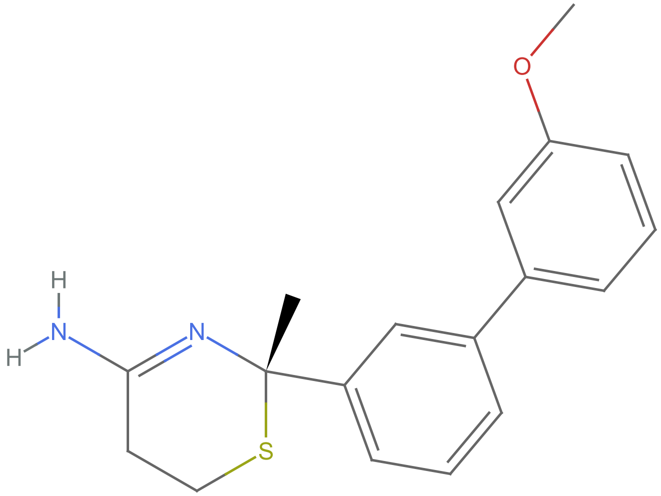

3D Plot

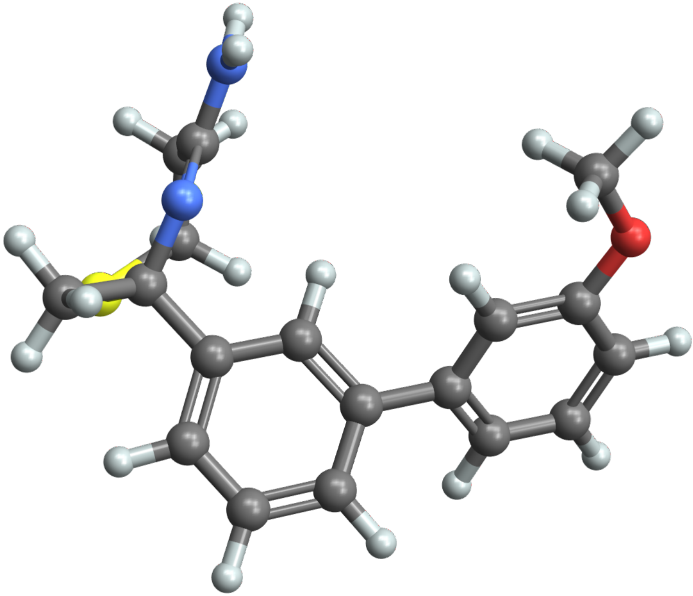

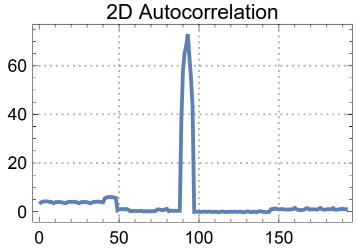

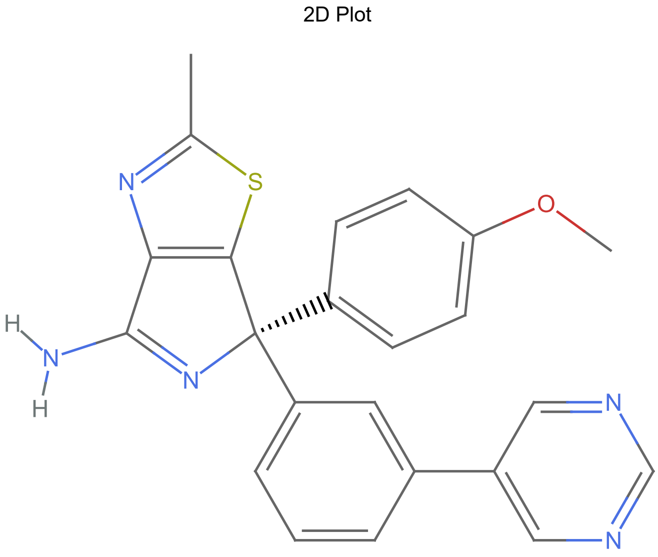

Dataset Index= 1063

Molecule Formula= $C_{23}H_{19}N_5OS$

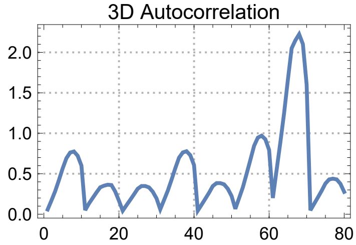

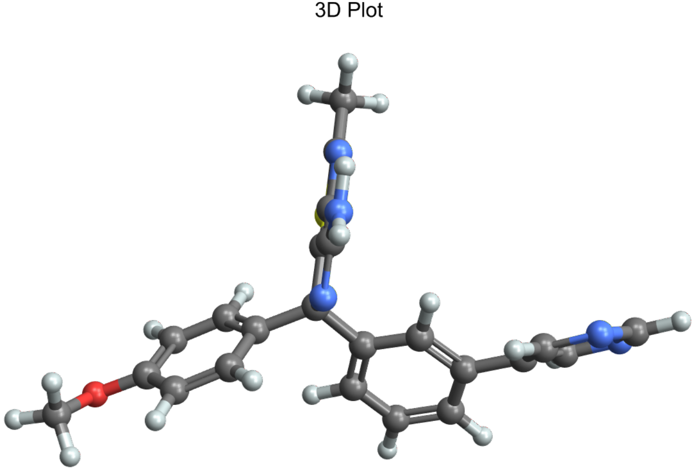

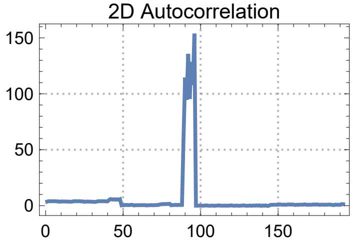

Dataset Index= 1064

Molecule Formula= $C_{24}H_{32}F_2N_3O_2^+$

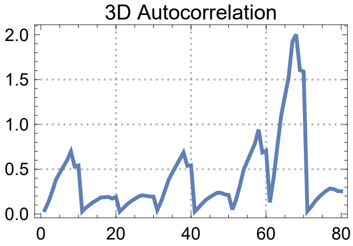

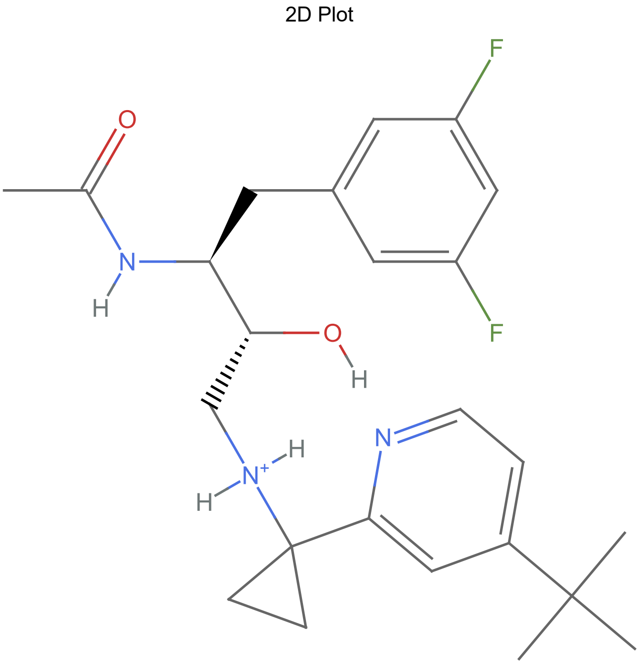

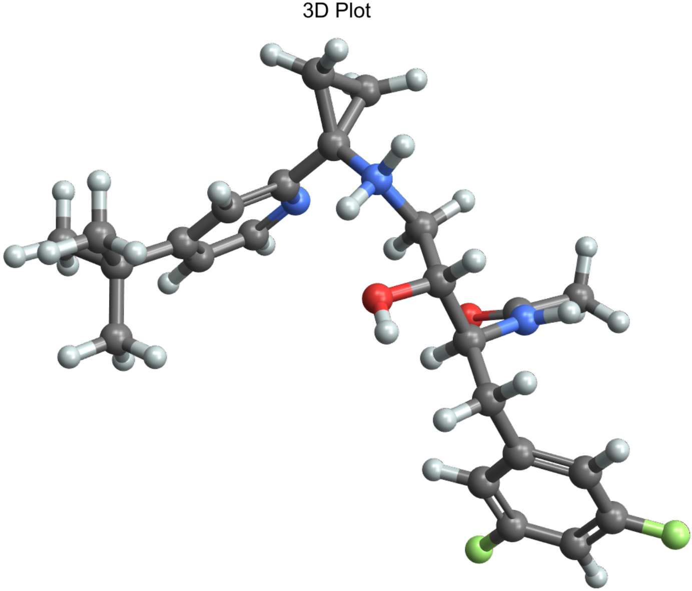

2D Plot

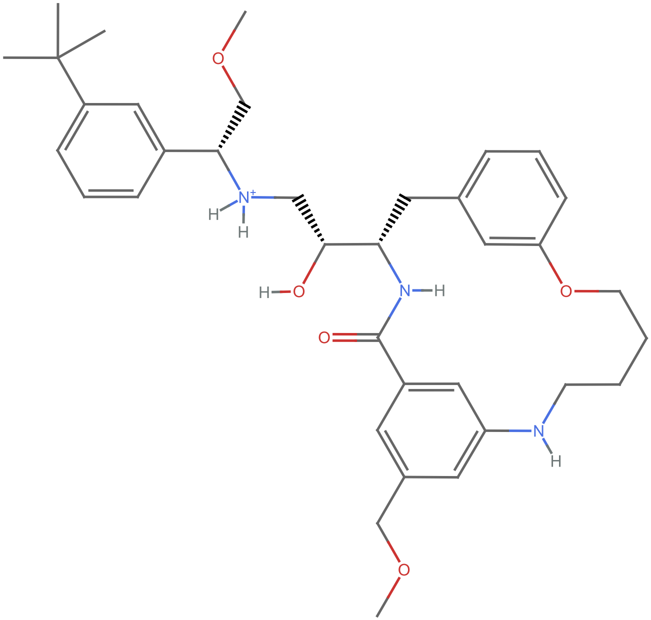

2D Autocorrelation

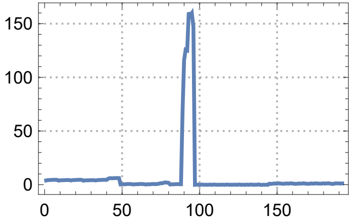

Dataset Index= 1065

Molecule Formula= $C_{36}H_{50}N_{3}O_{5}^{+}$

3D Autocorrelation

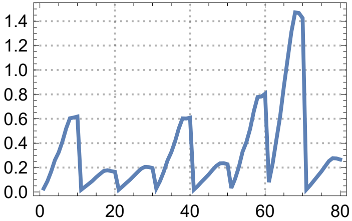

3D Plot

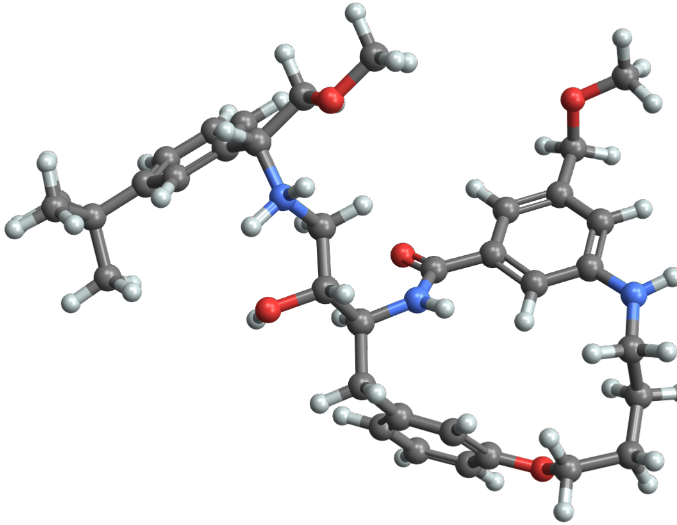

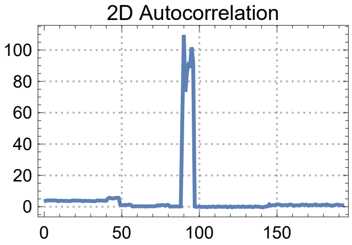

Dataset Index= 1066

Molecule Formula= $C_{26}H_{38}NO_4S^+$

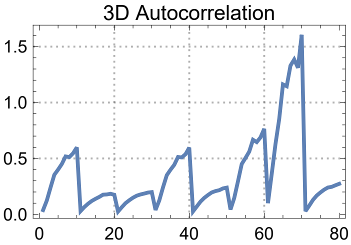

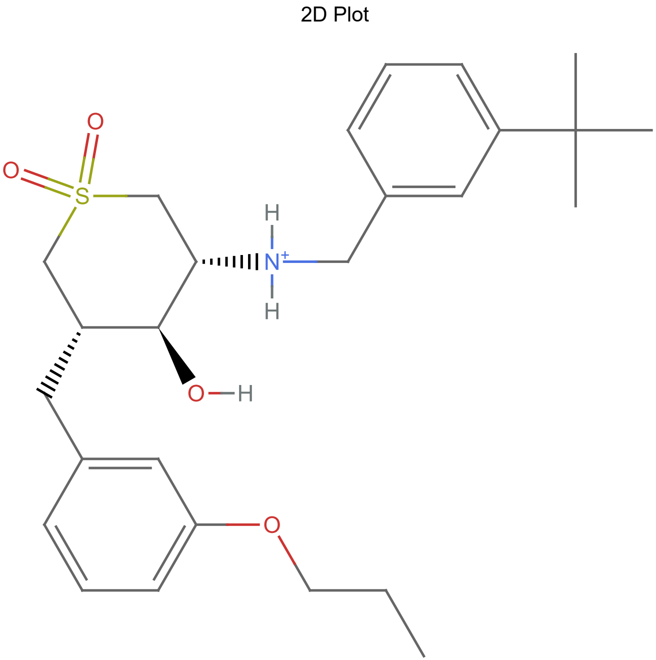

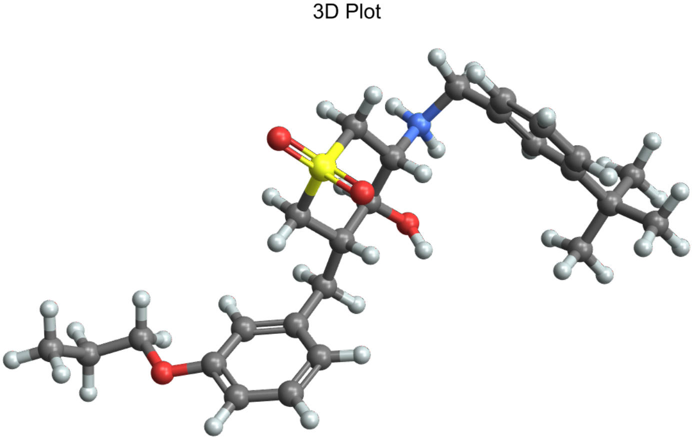

2D Autocorrelation

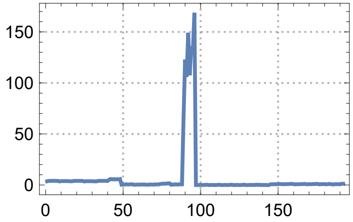

Dataset Index= 1067

Molecule Formula= $C_{26}H_{35}F_2N_2O_2^+$

3D Autocorrelation

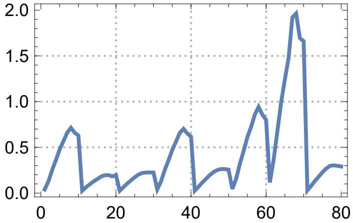

2D Plot

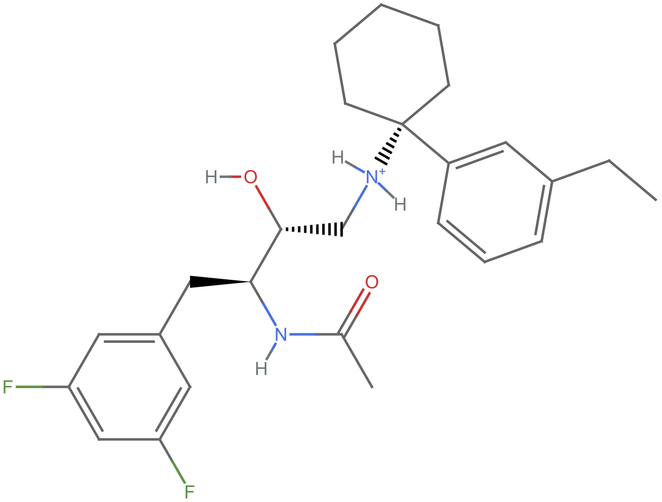

3D Plot

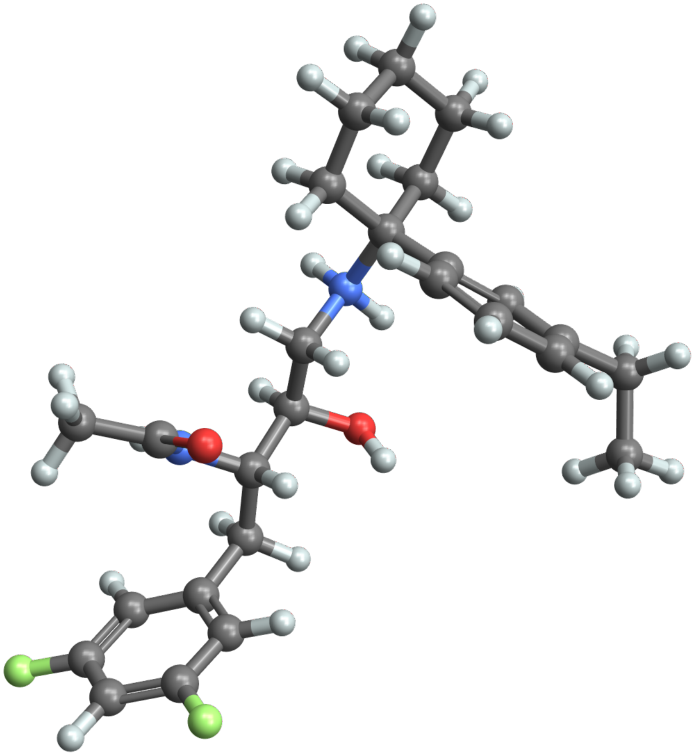

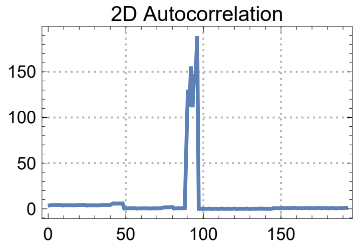

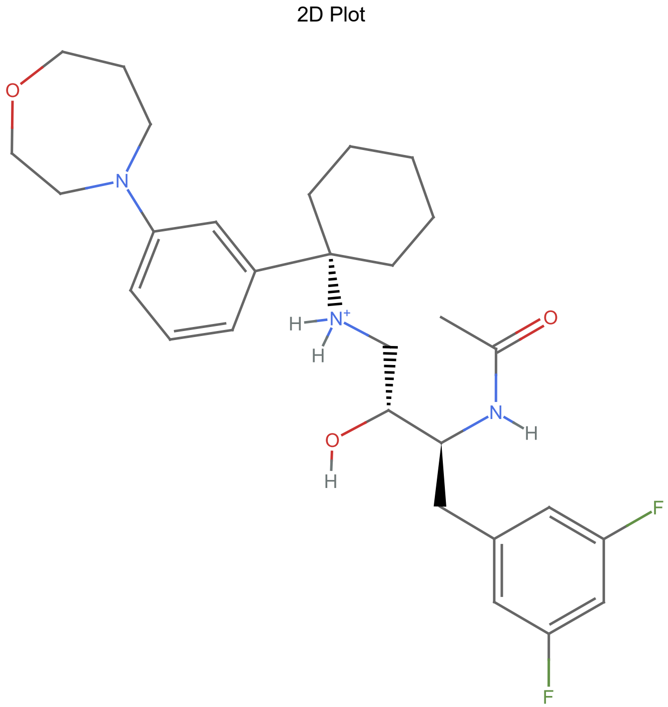

Dataset Index= 1068

Molecule Formula= $C_{29}H_{40}F_2N_3O_3^+$

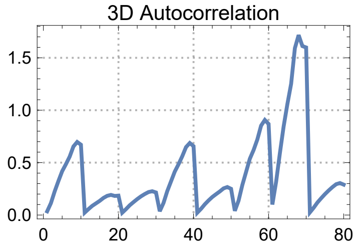

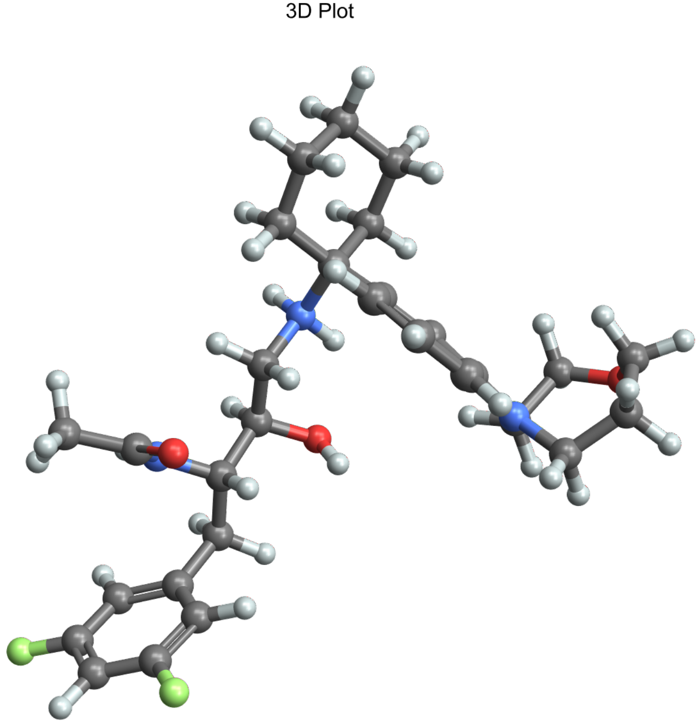

2D Autocorrelation

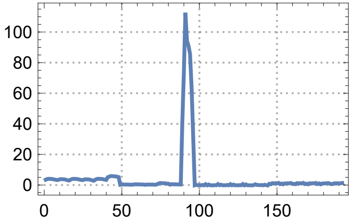

Dataset Index= 1069

Molecule Formula= $C_{19}H_{19}N_5O_2$

3D Autocorrelation

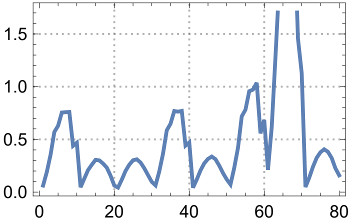

2D Plot

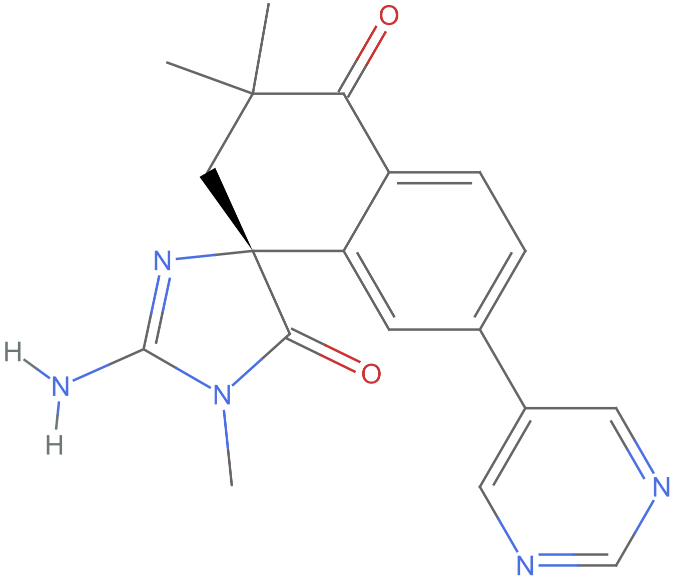

3D Plot

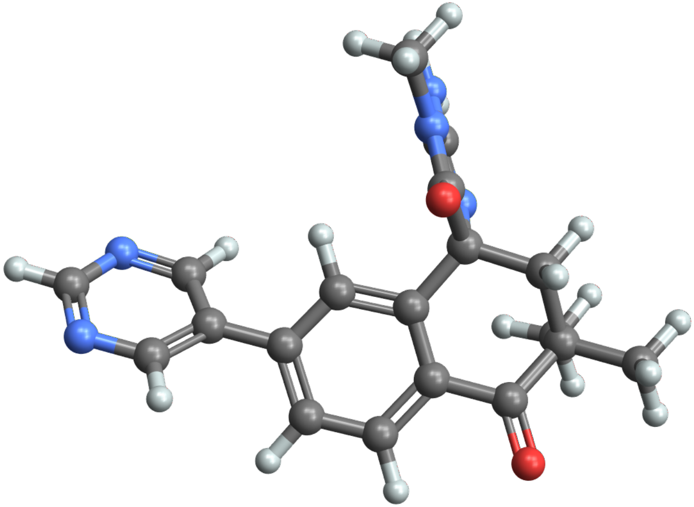

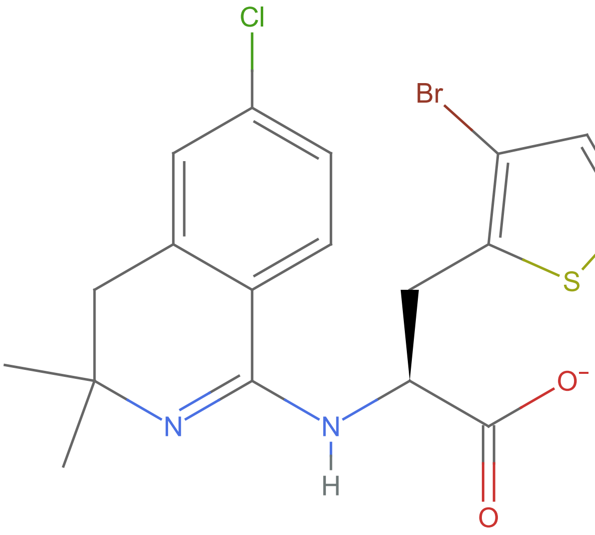

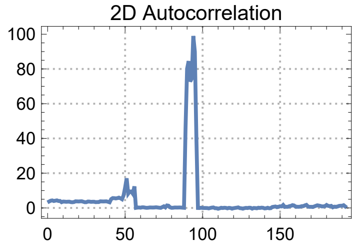

Dataset Index= 1070

Molecule Formula= $C_{18}H_{17}BrClN_2O_2S^-$

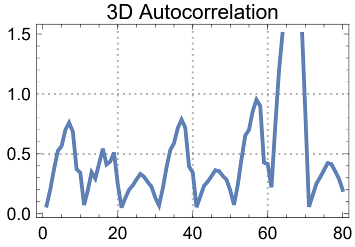

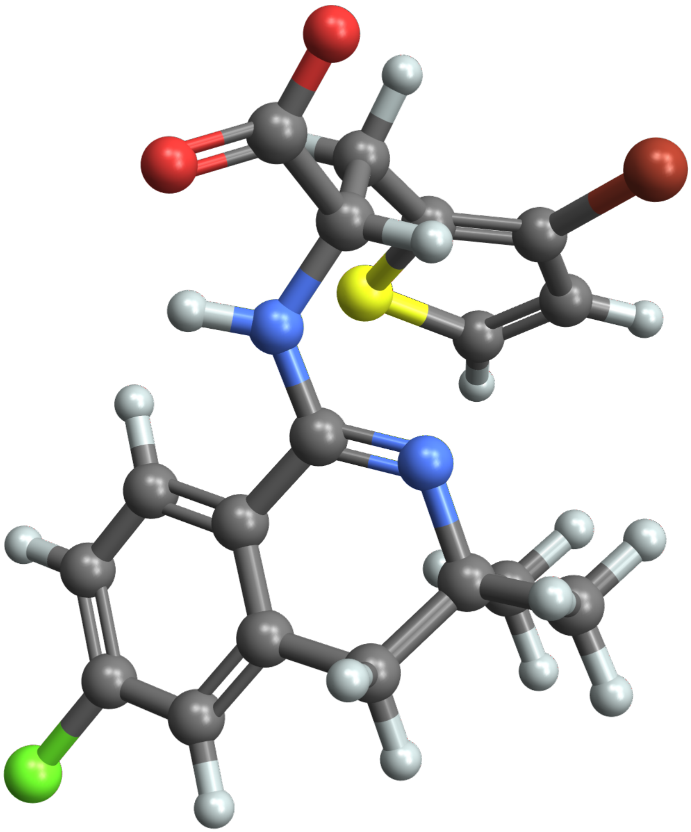

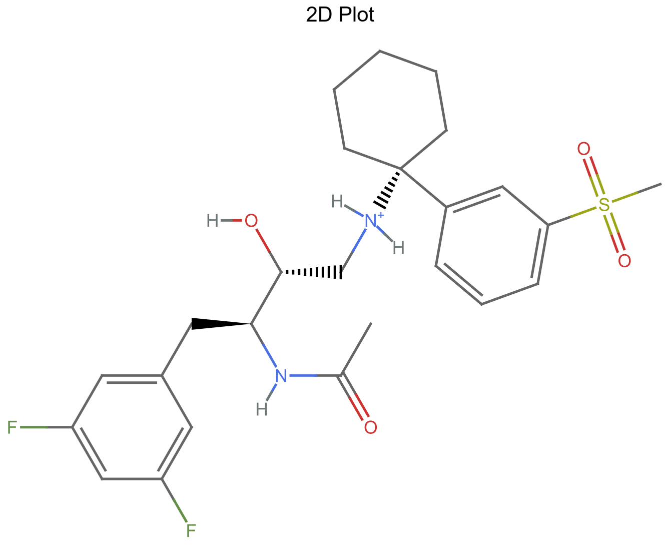

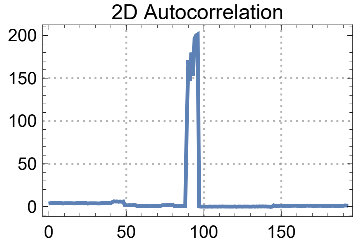

Dataset Index= 1071

Molecule Formula= $C_{25}H_{33}F_2N_2O_4S^+$

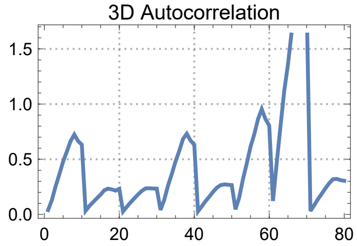

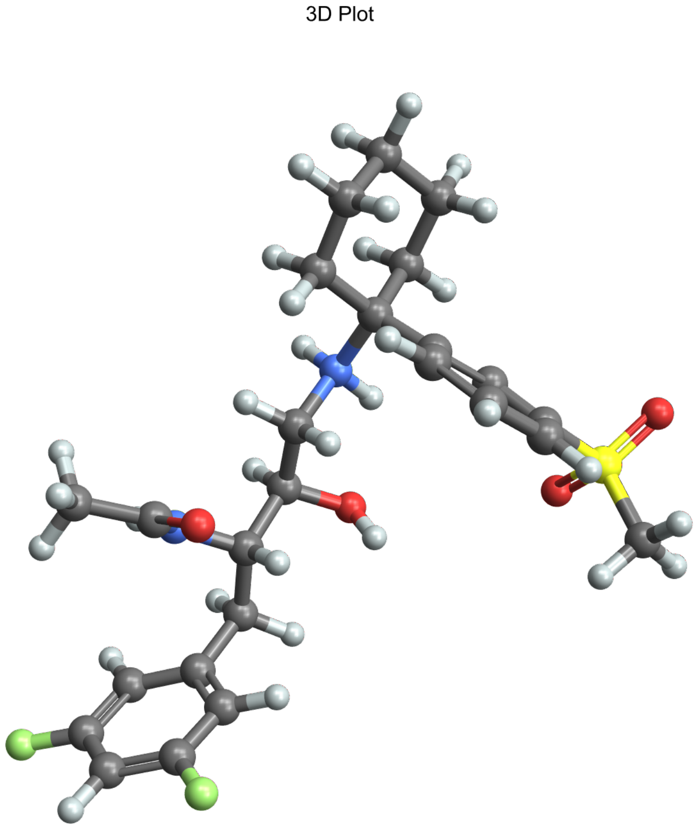

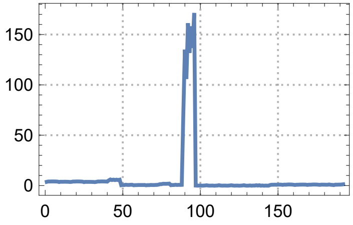

Dataset Index= 1072

Molecule Formula= $C_{25}H_{34}F_2N_3O_3^+$

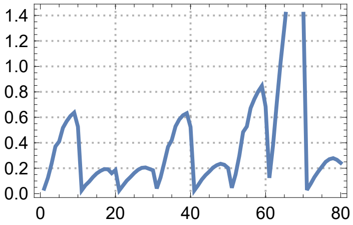

2D Plot

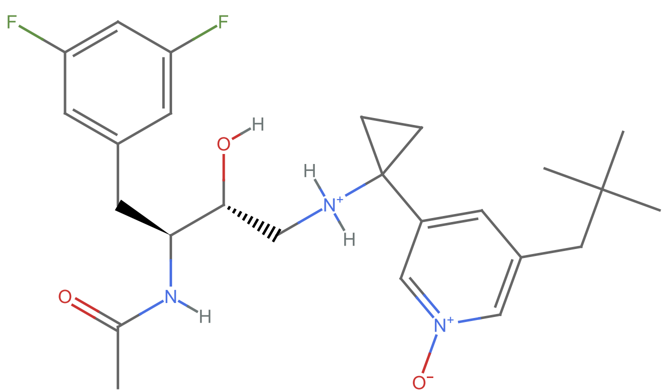

3D Plot

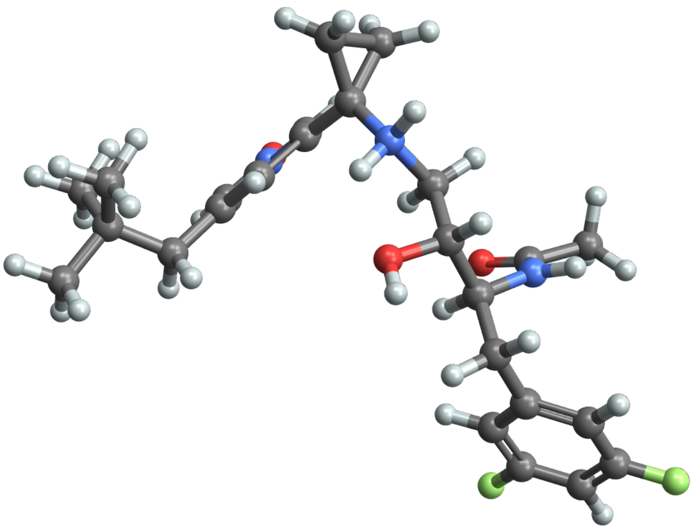

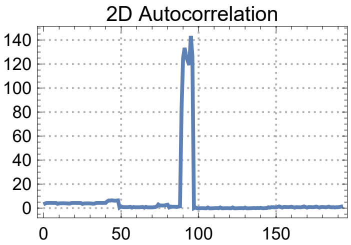

Dataset Index= 1073

Molecule Formula= $C_{24}H_{19}F_3N_5O_2^+$

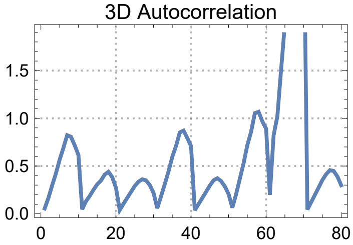

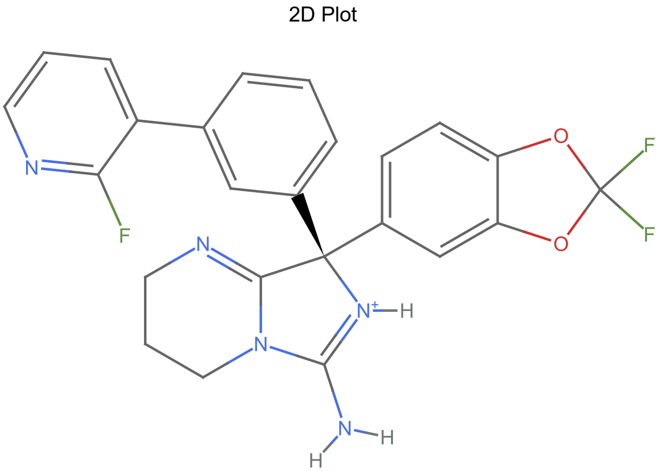

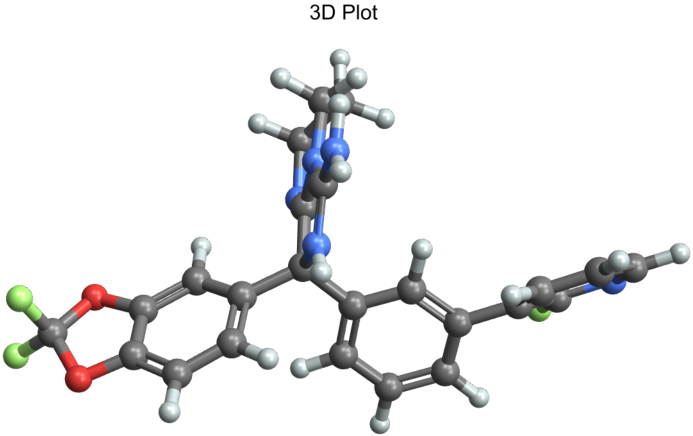

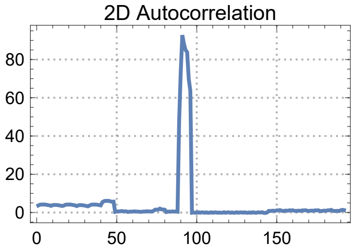

Dataset Index= 1074

Molecule Formula= $C_{23}H_{19}FN_2O_4$

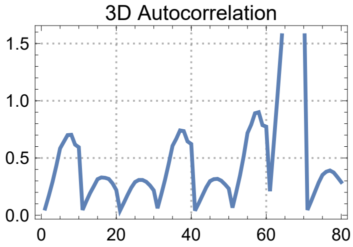

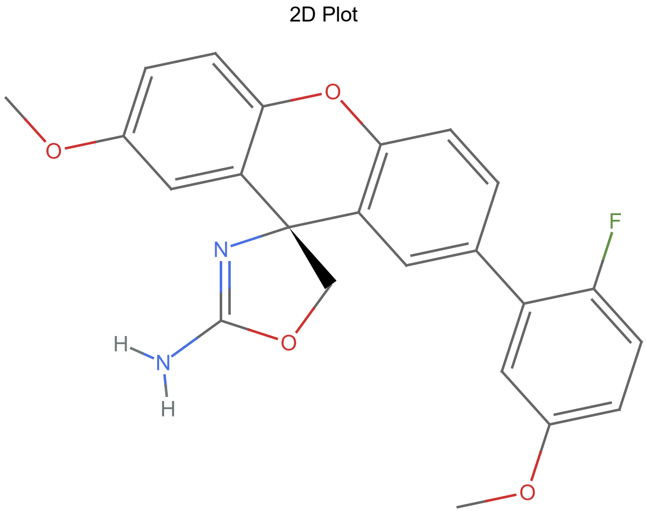

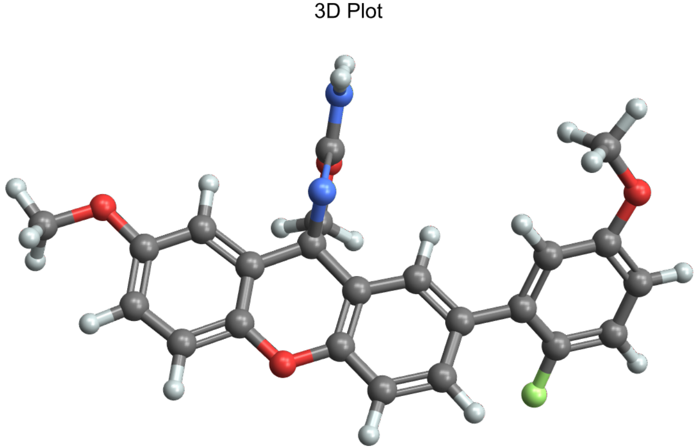

2D Plot

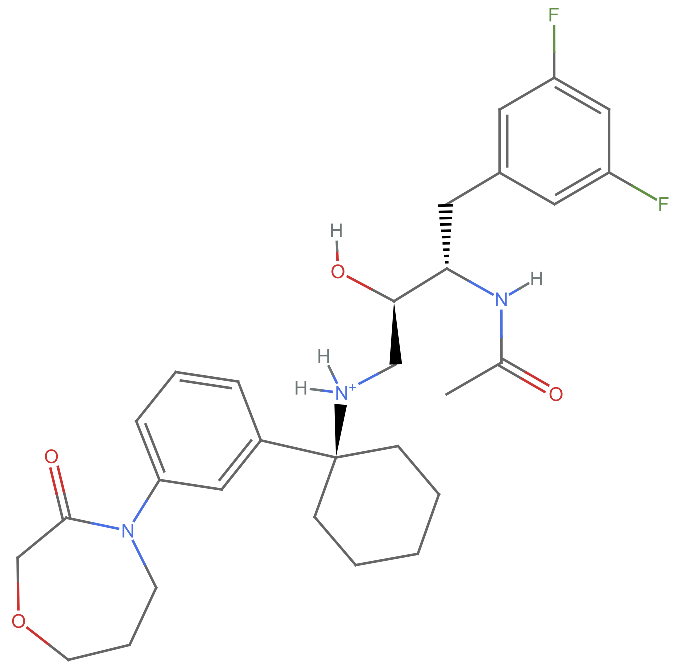

2D Autocorrelation

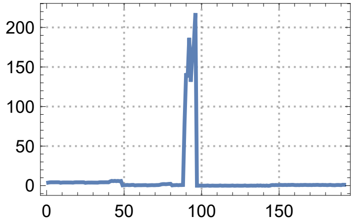

Dataset Index= 1075

Molecule Formula= $C_{29}H_{38}F_2N_3O_4^+$

3D Plot

3D Autocorrelation

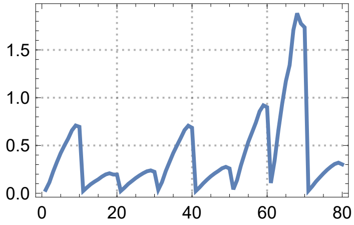

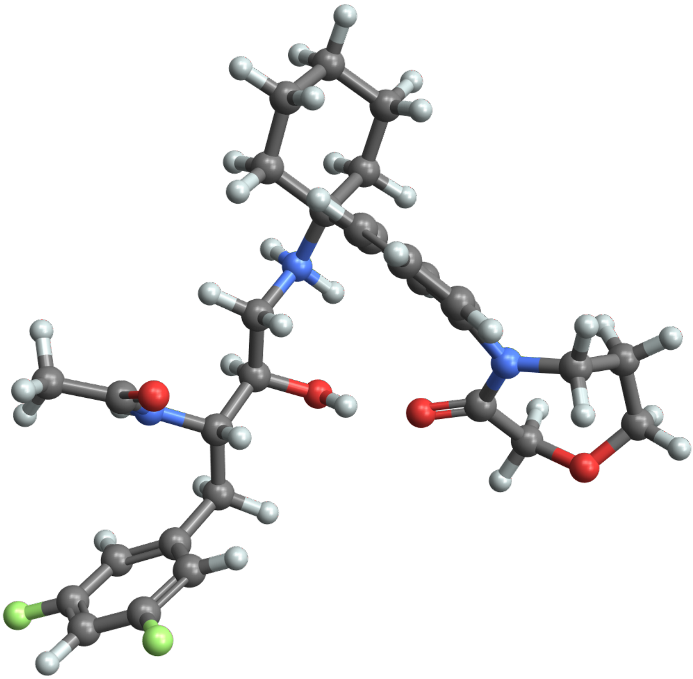

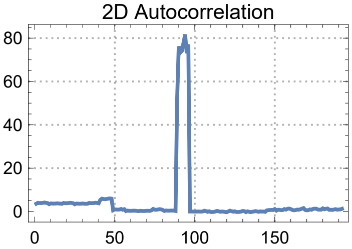

Dataset Index= 1076

Molecule Formula= $C_{21}H_{21}ClN_4O_2$

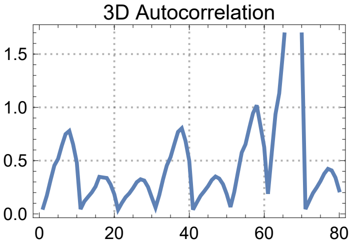

2D Plot

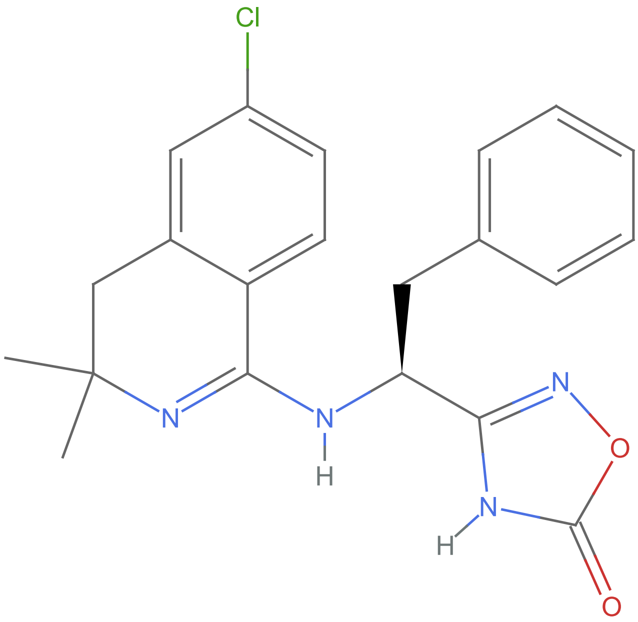

3D Plot

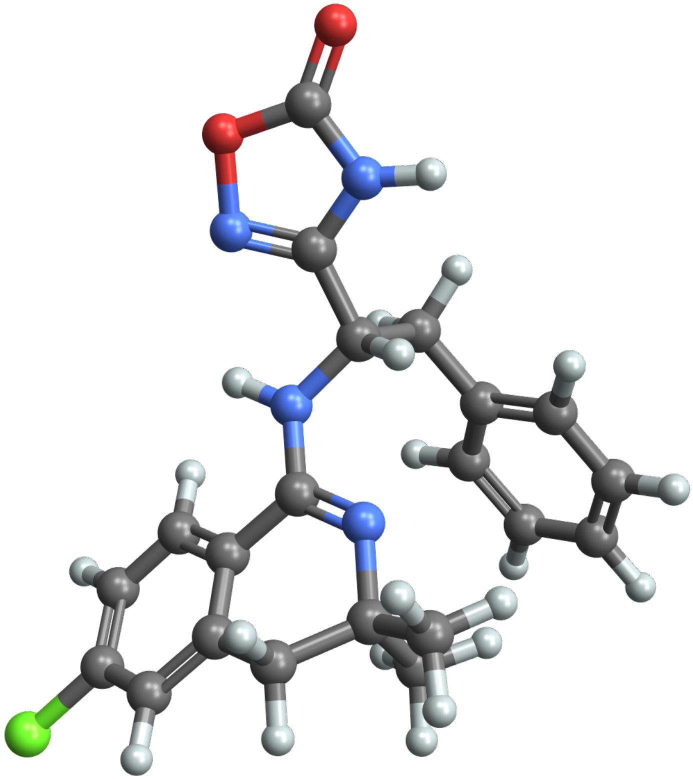

2D Autocorrelation

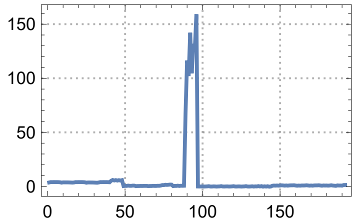

Dataset Index= 1077

Molecule Formula= $C_{25}H_{31}F_{2}N_{2}O_{3}^{+}$

3D Autocorrelation

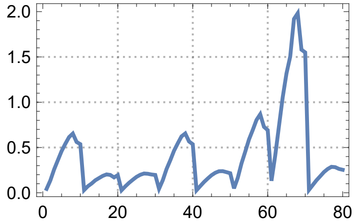

2D Plot

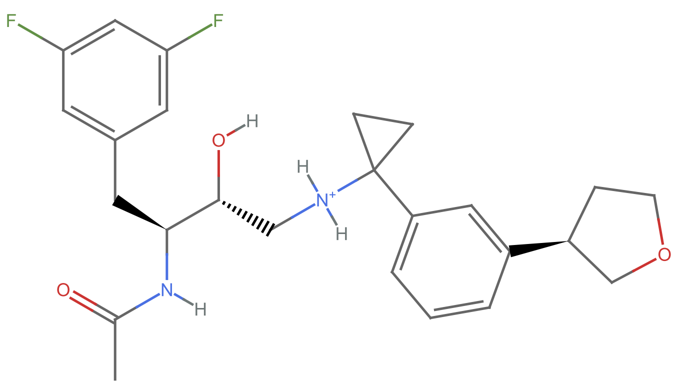

3D Plot

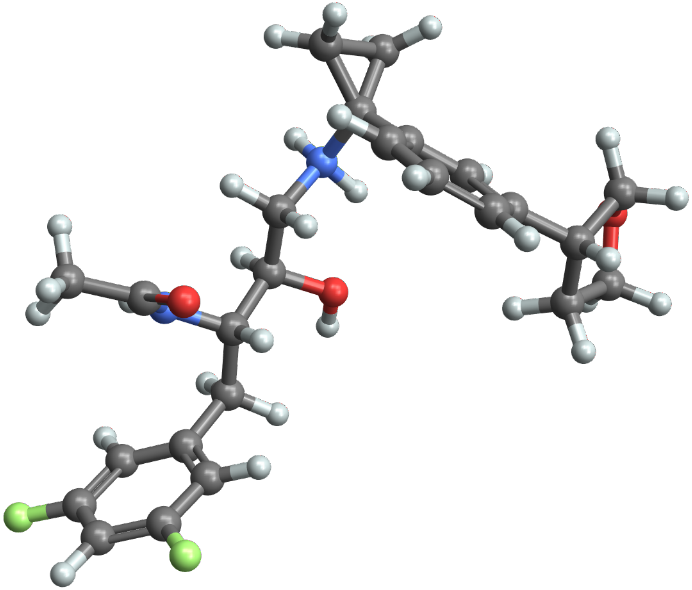

2D Autocorrelation

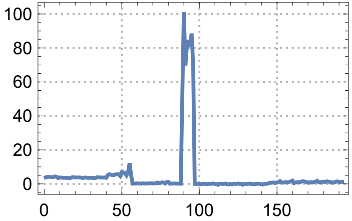

Dataset Index= 1078

Molecule Formula= $C_{22}H_{29}BrNO_3S^+$

3D Autocorrelation

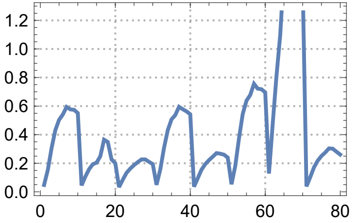

2D Plot

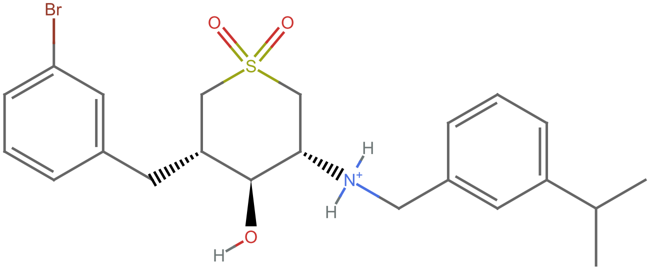

3D Plot

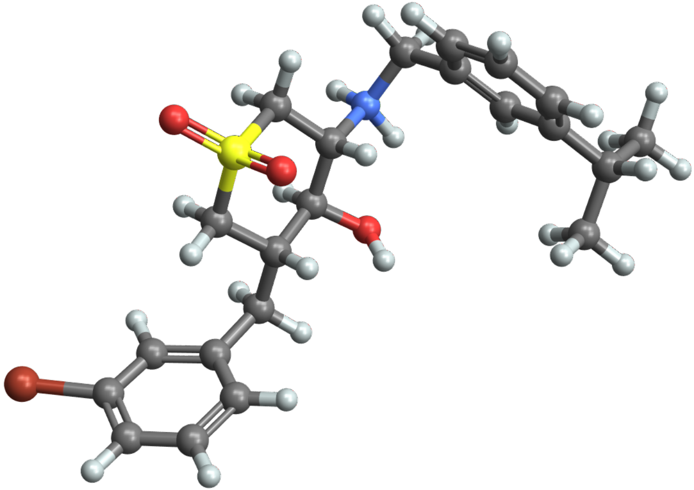

2D Plot

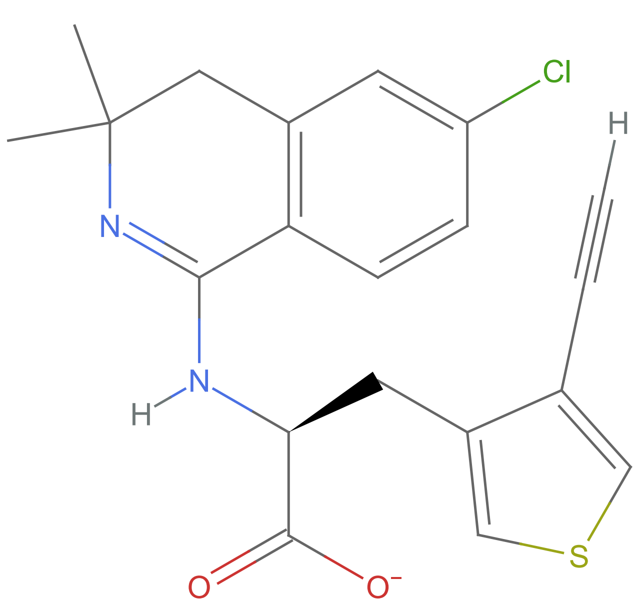

3D Plot

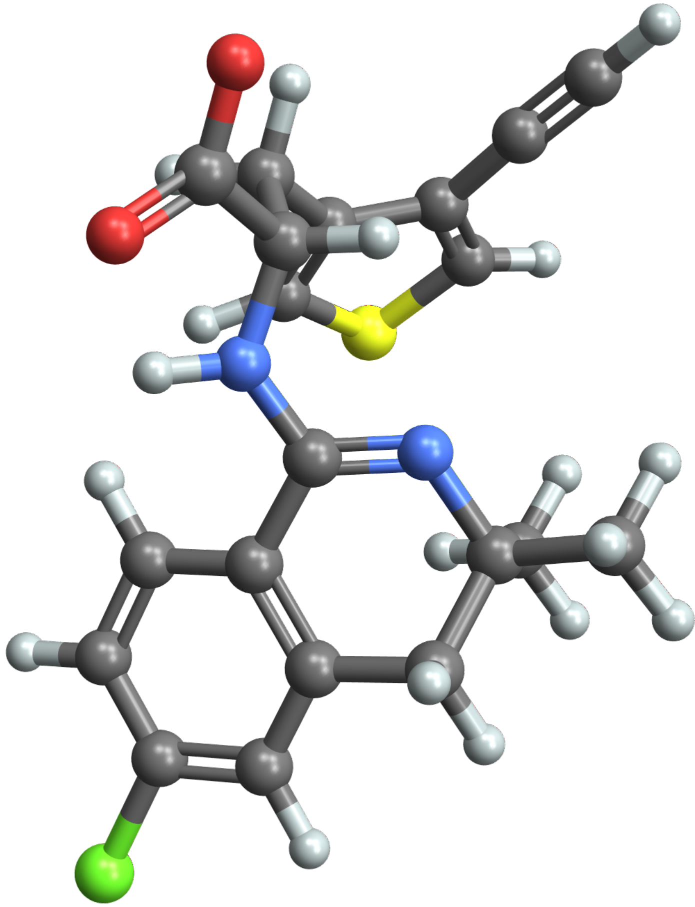

2D Autocorrelation

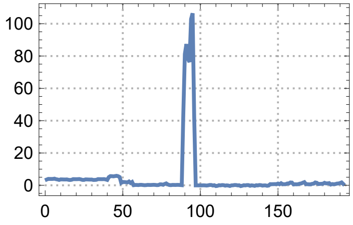

Dataset Index= 1079

Molecule Formula= $C_{20}H_{18}ClN_2O_2S^-$

3D Autocorrelation

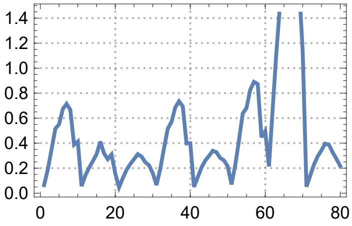

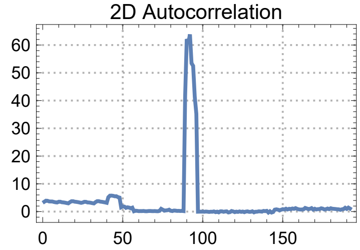

Dataset Index= 1080

Molecule Formula= $C_{17}H_{15}ClN_4OS$

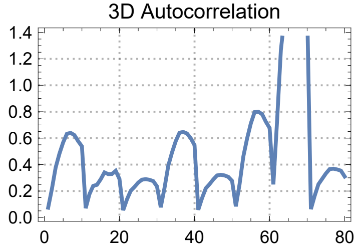

2D Plot

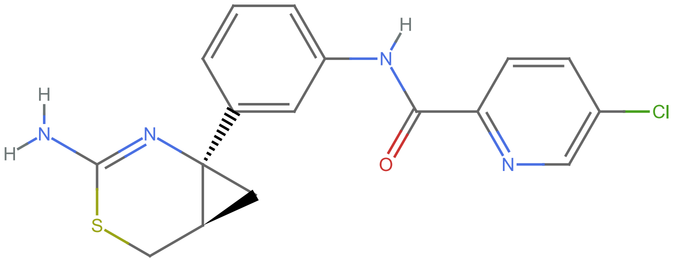

3D Plot

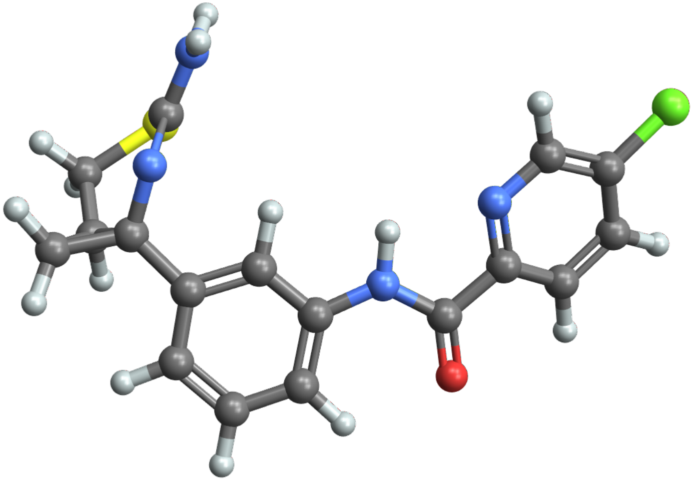

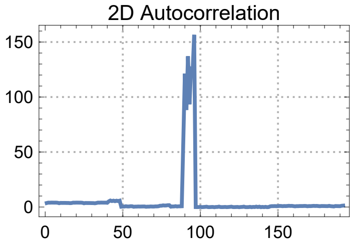

Dataset Index= 1081

Molecule Formula= $C_{25}H_{34}F_2N_3O_2^+$

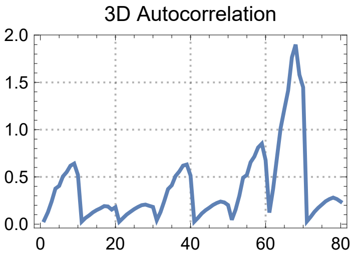

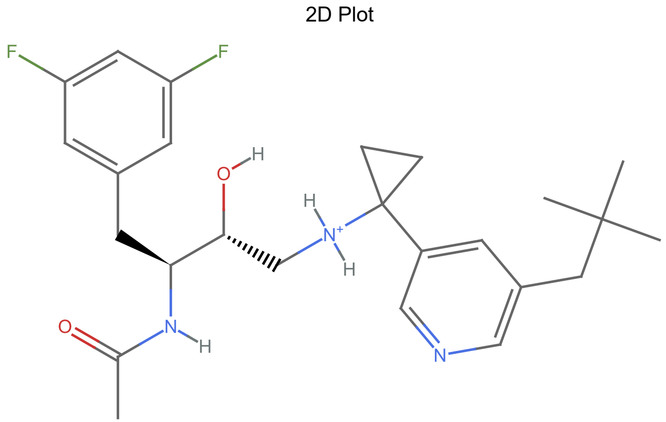

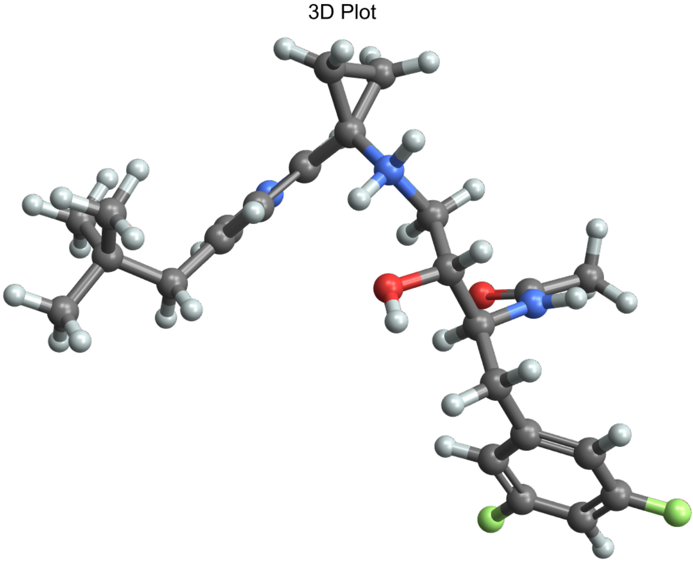

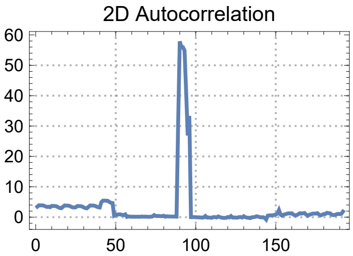

Dataset Index= 1082

Molecule Formula= $C_{19}H_{20}ClN_{3}O$

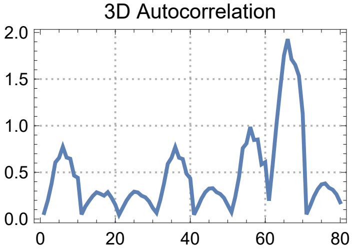

2D Plot

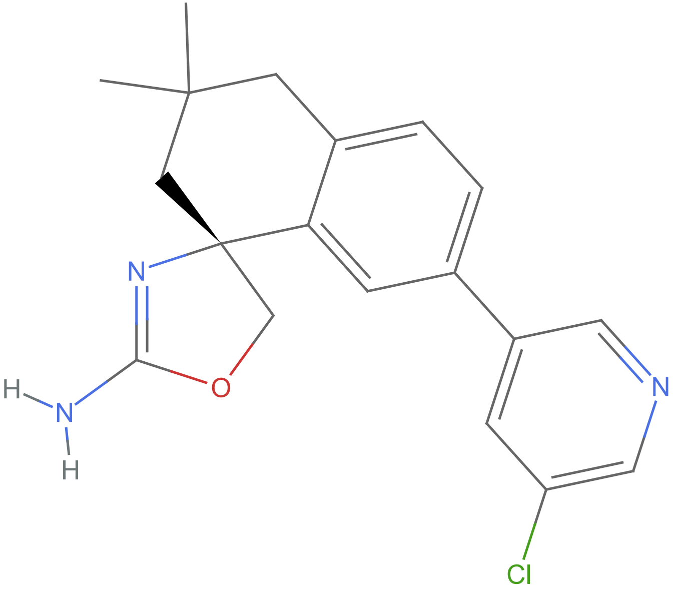

3D Plot

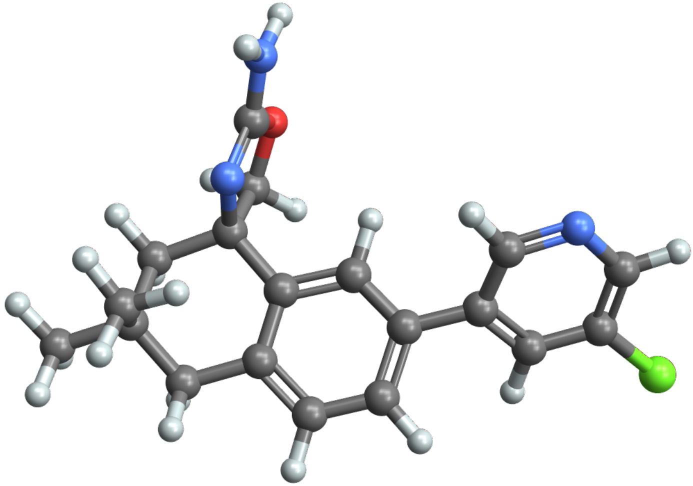

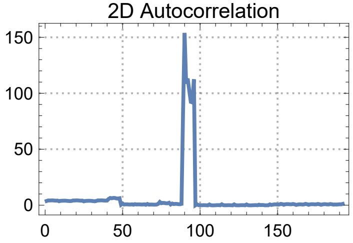

Dataset Index= 1083

Molecule Formula= $C_{23}H_{20}F_3N_6O^+$

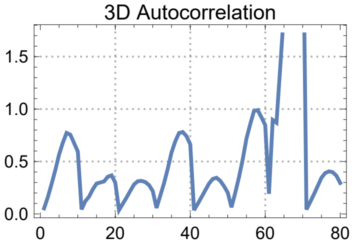

2D Plot

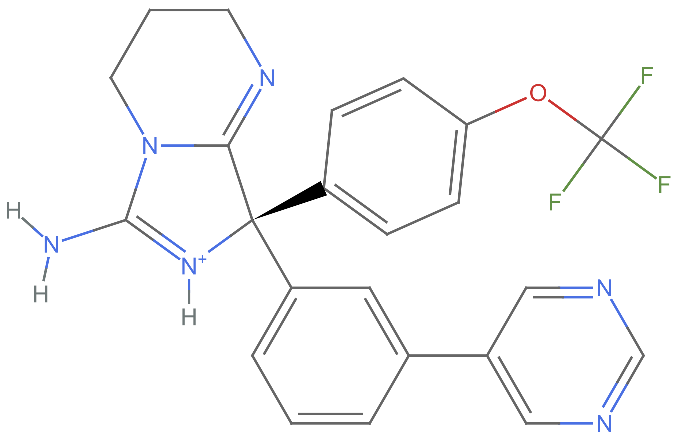

3D Plot

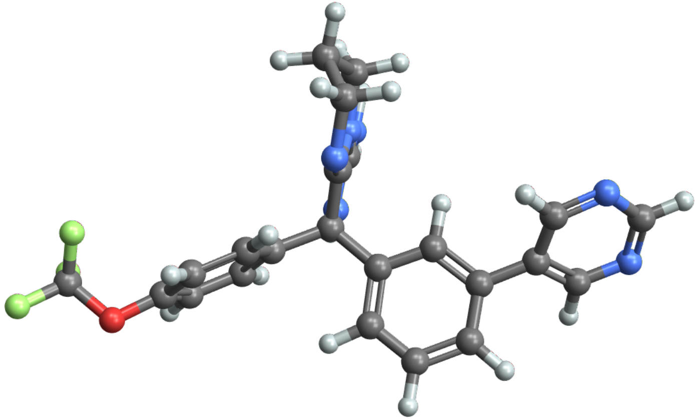

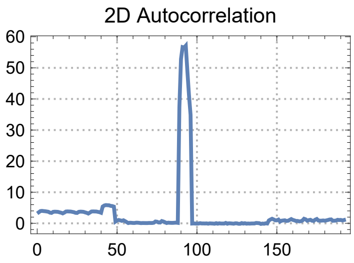

Dataset Index= 1084

Molecule Formula= $C_{21}H_{19}N_3OS$

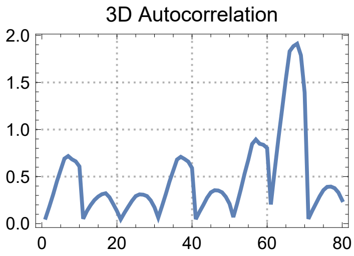

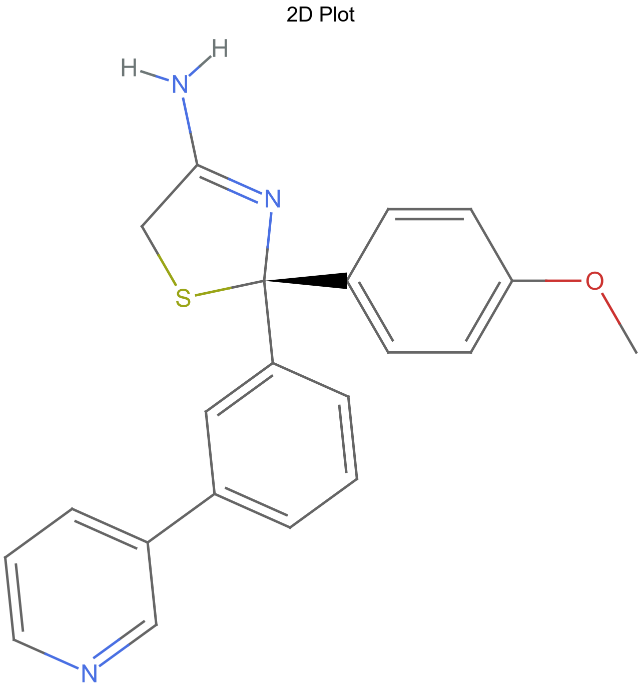

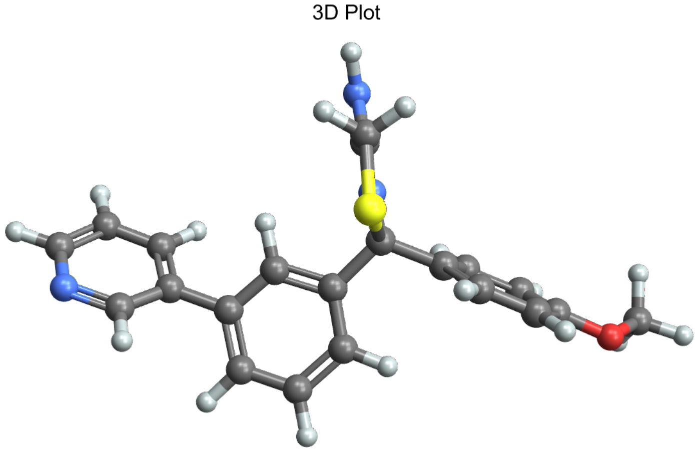

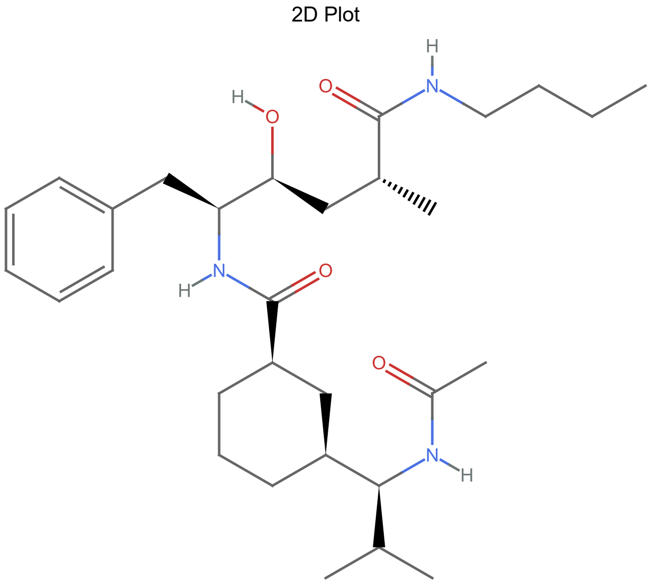

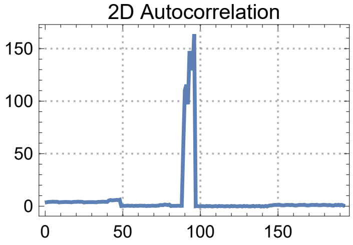

Dataset Index= 1085

Molecule Formula= $C_{30}H_{49}N_3O_4$

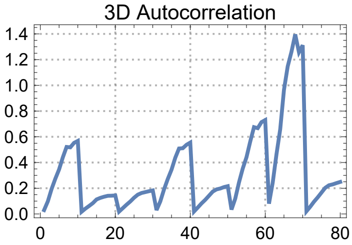

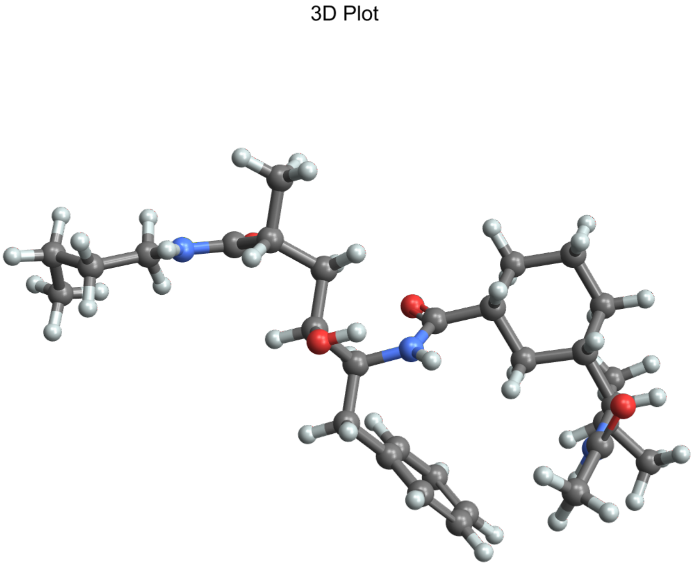

2D Plot

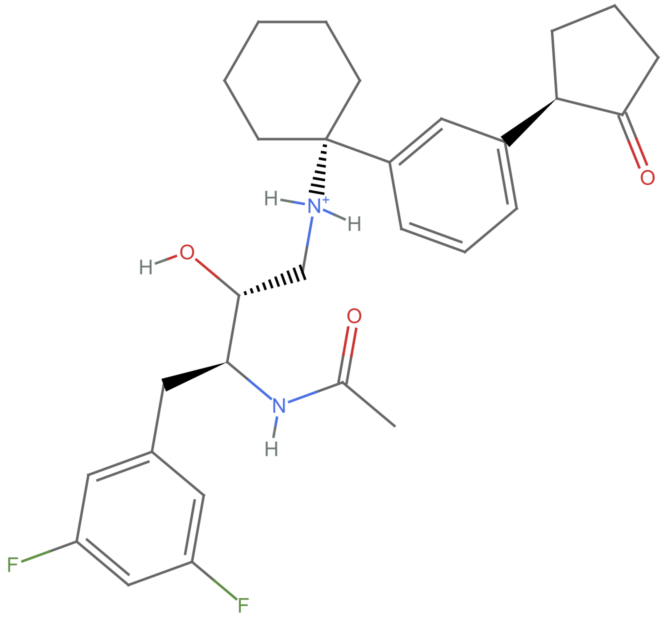

2D Autocorrelation

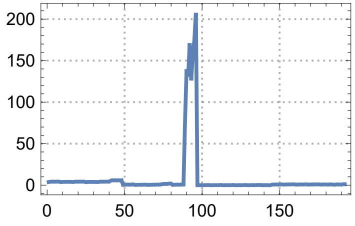

3D Plot

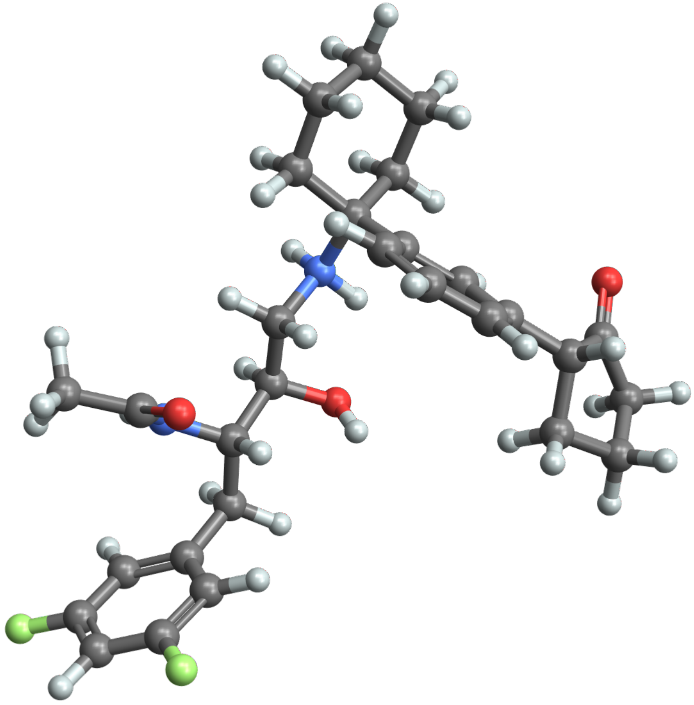

Dataset Index= 1086

Molecule Formula= $C_{29}H_{37}F_2N_2O_3^+$

3D Autocorrelation

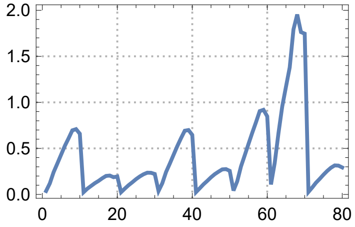

2D Autocorrelation

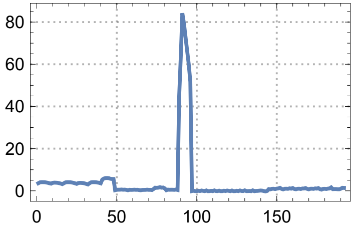

Dataset Index= 1087

Molecule Formula= $C_{21}H_{16}FN_3O_3$

3D Autocorrelation

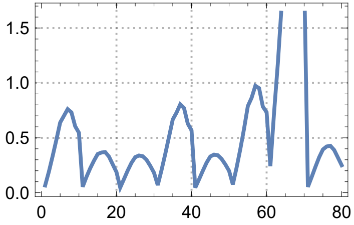

2D Plot

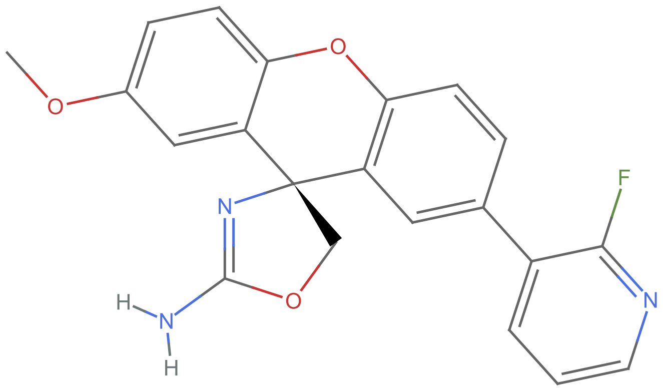

3D Plot

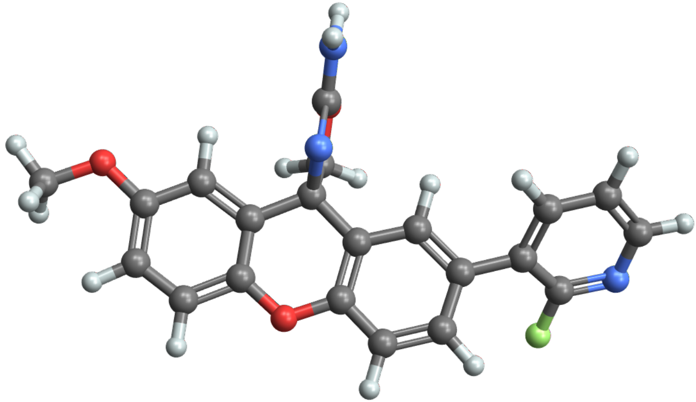

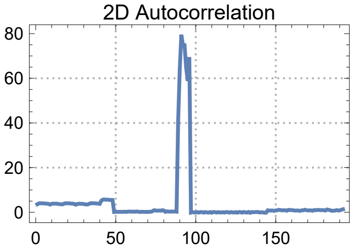

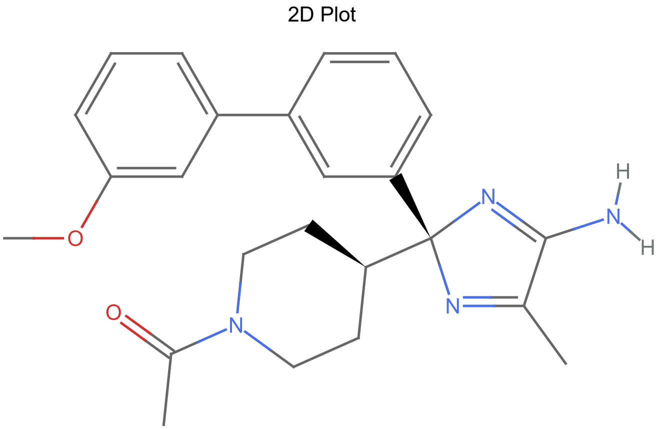

Dataset Index= 1088

Molecule Formula= $C_{24}H_{28}N_4O_2$

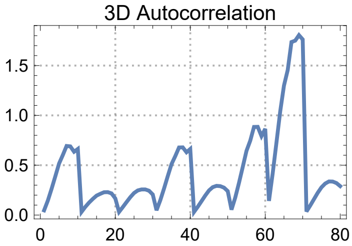

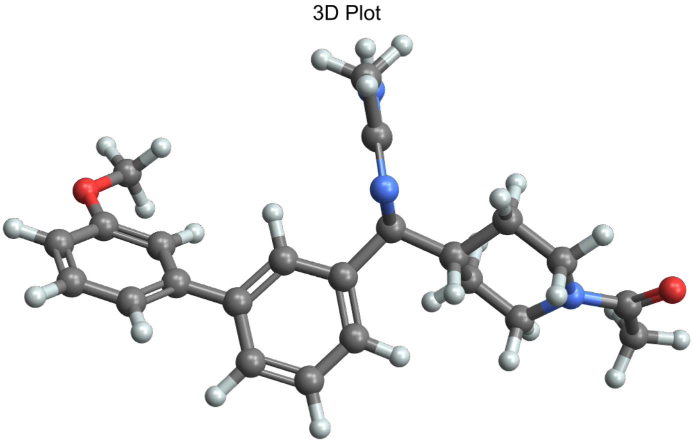

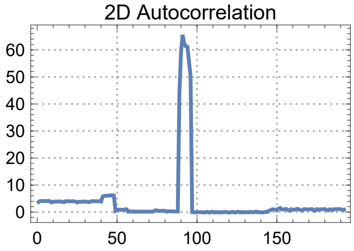

Dataset Index= 1089

Molecule Formula= $C_{27}H_{21}ClN_4O$

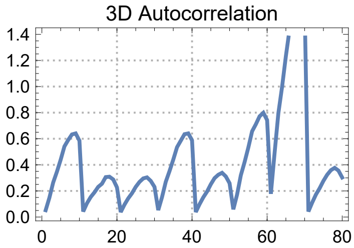

2D Plot

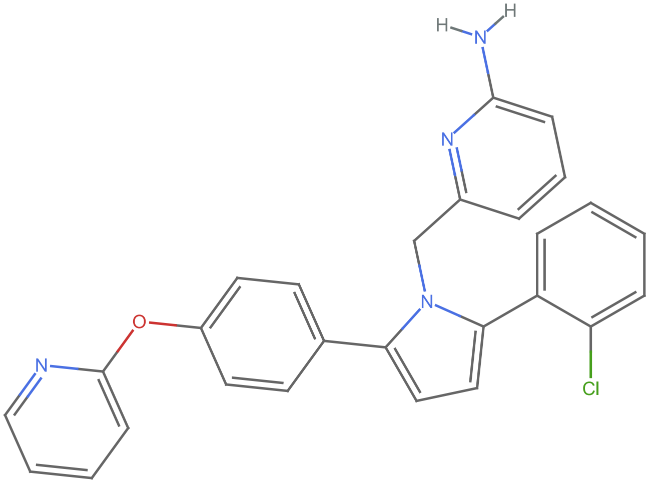

3D Plot

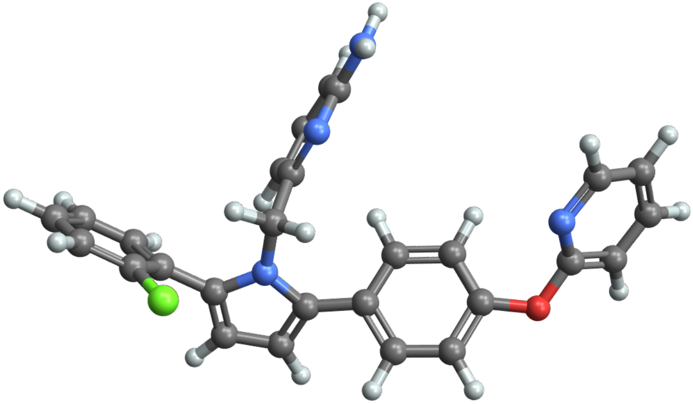

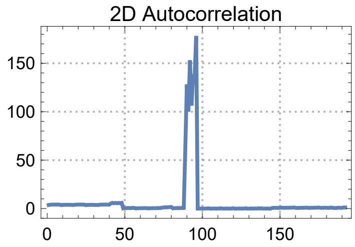

Dataset Index= 1090

Molecule Formula= $C_{28}H_{39}F_2N_2O_2^+$

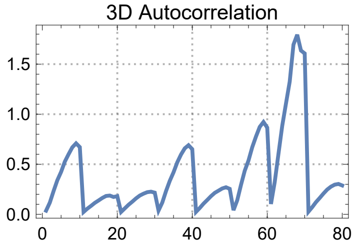

2D Plot

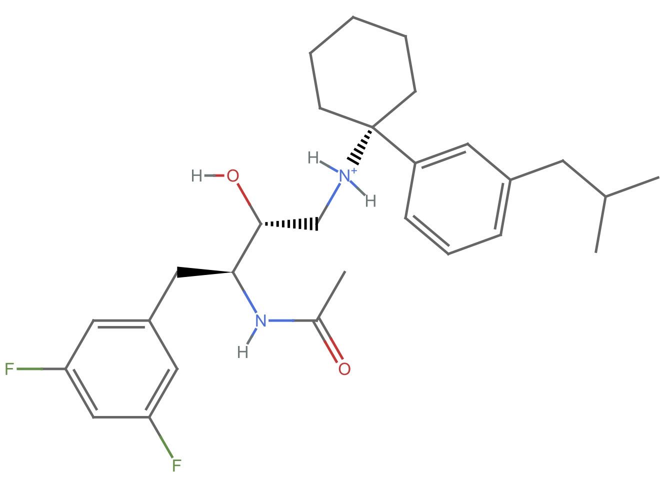

3D Plot

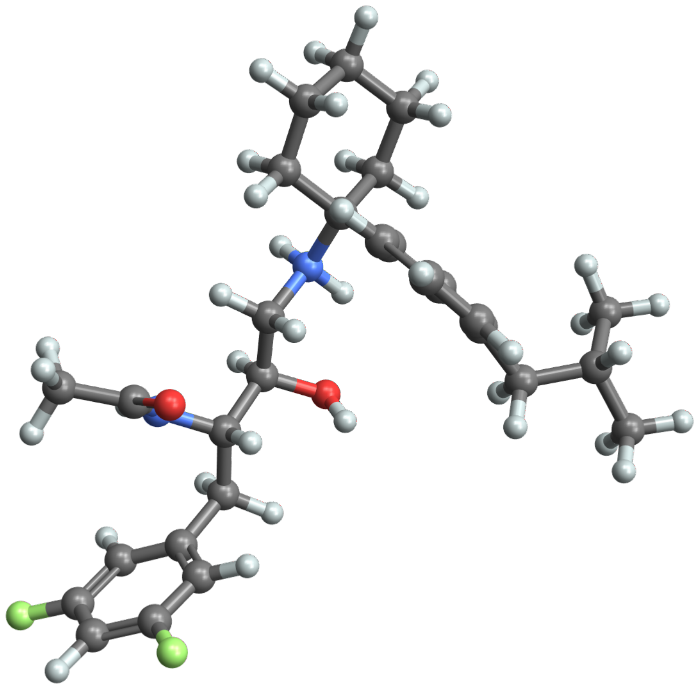

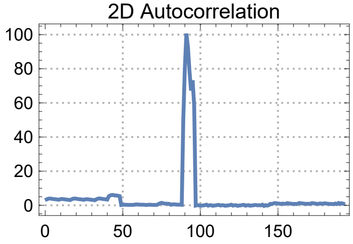

Dataset Index= 1091

Molecule Formula= $C_{19}H_{18}N_6O_2$

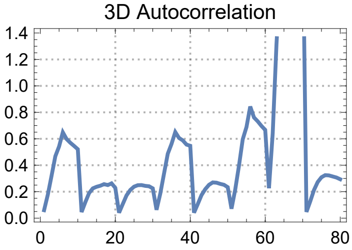

2D Plot

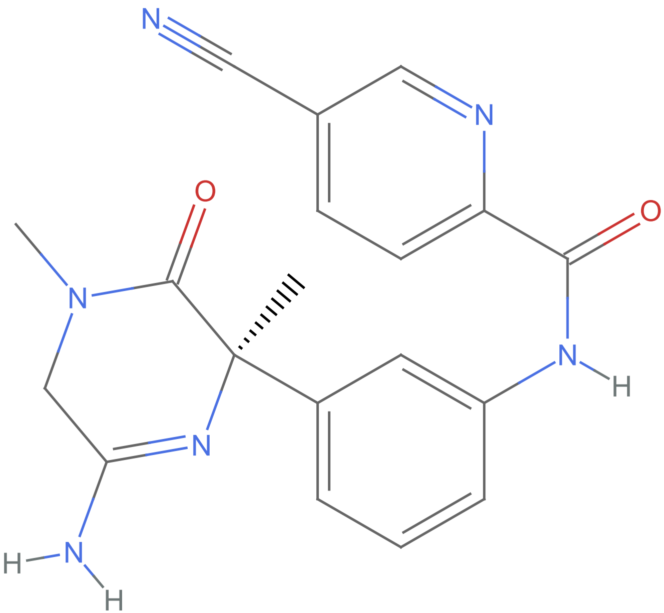

3D Plot

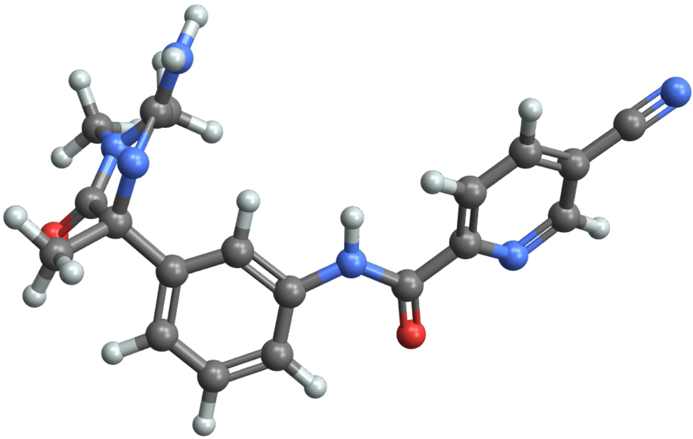

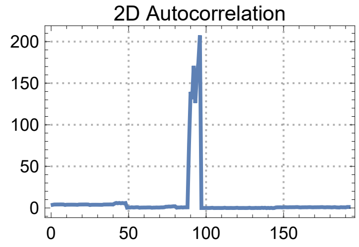

Dataset Index= 1092

Molecule Formula= $C_{28}H_{36}F_2N_3O_3^+$

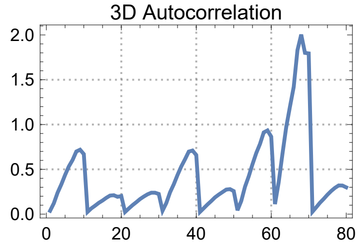

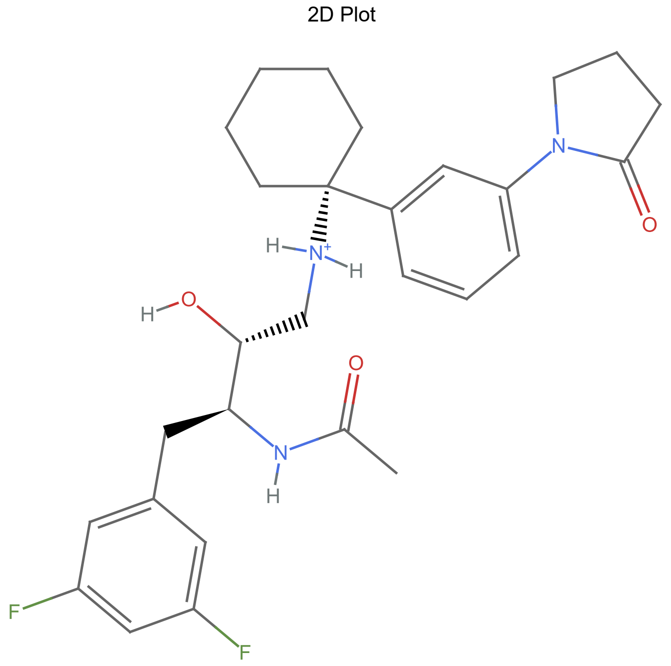

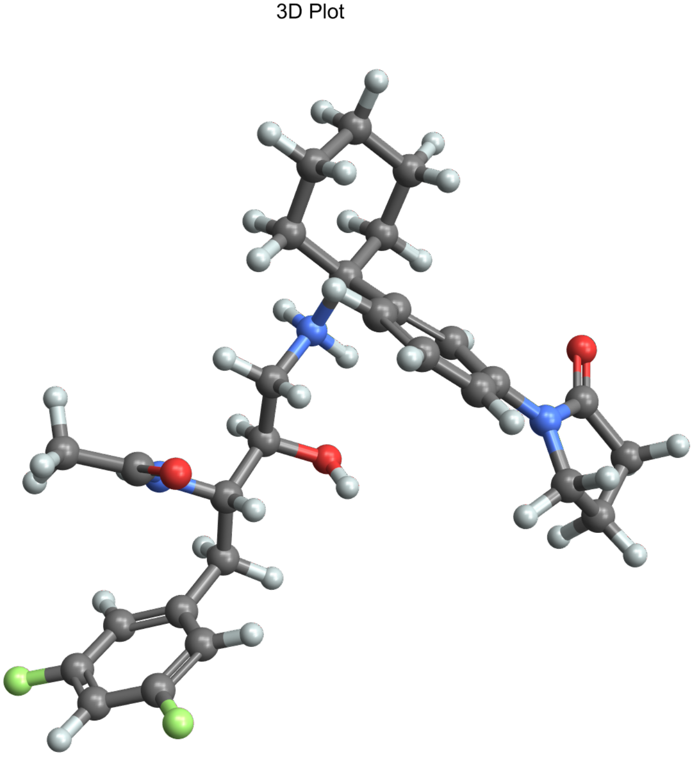

2D Plot

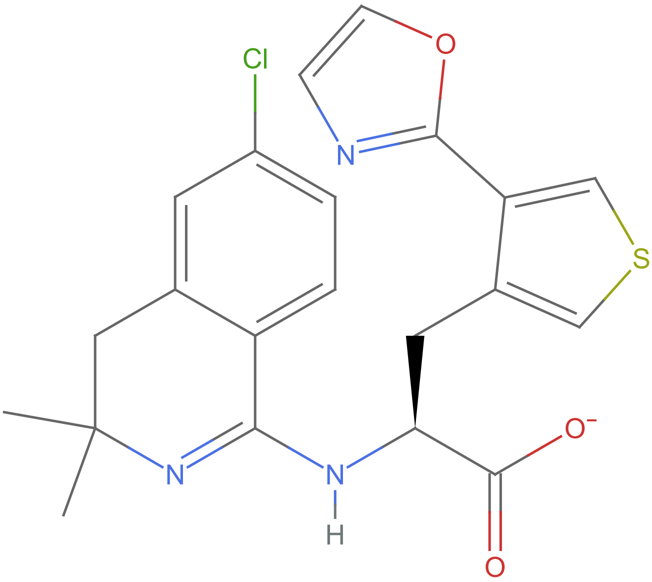

3D Plot

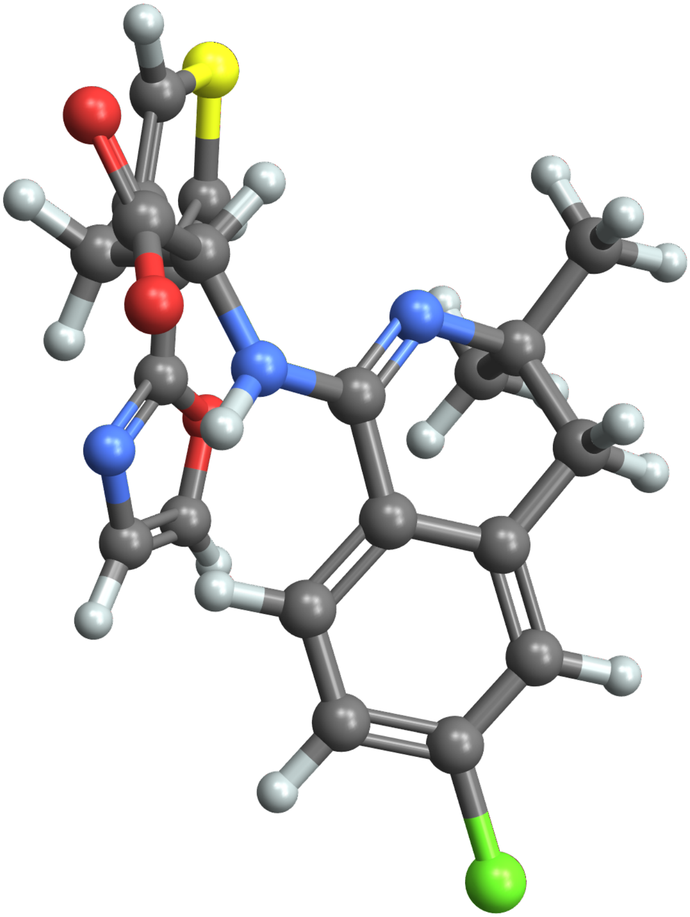

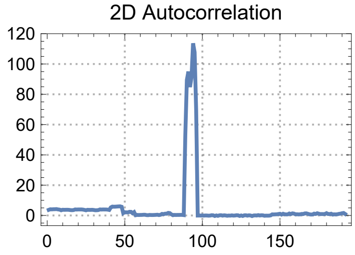

Dataset Index= 1093

Molecule Formula= $C_{21}H_{19}ClN_3O_3S^-$

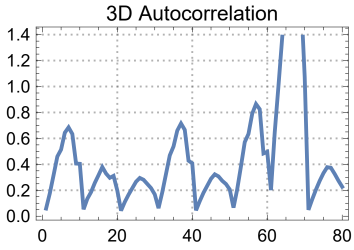

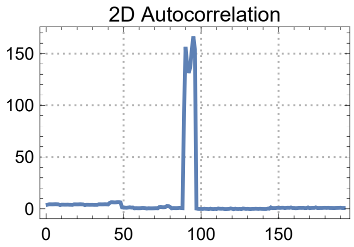

Dataset Index= 1094

Molecule Formula= $C_{28}H_{32}FN_6O_3S^+$

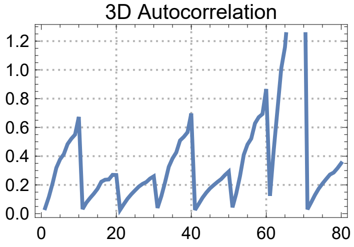

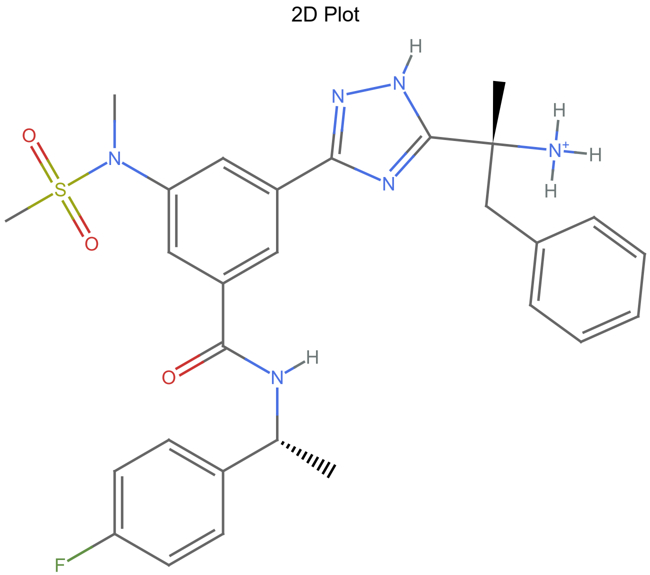

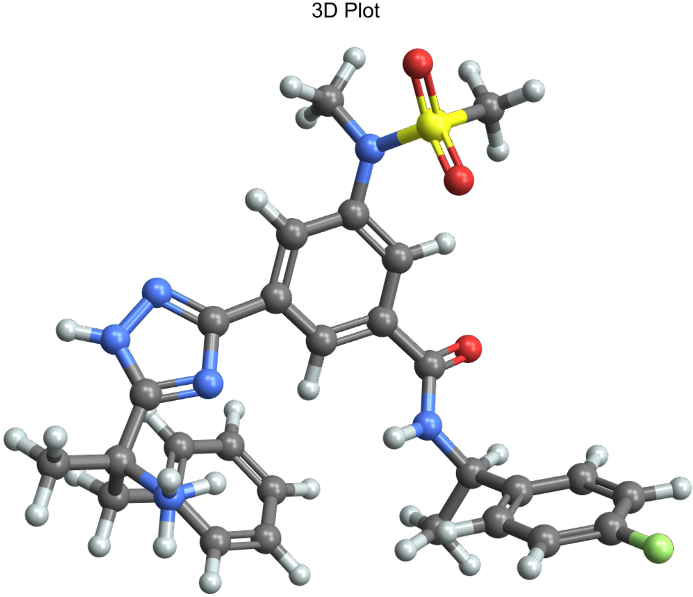

2D Plot

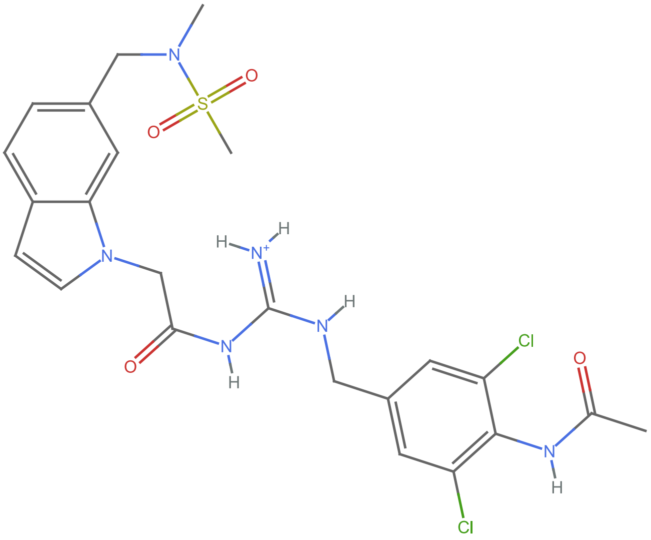

2D Autocorrelation

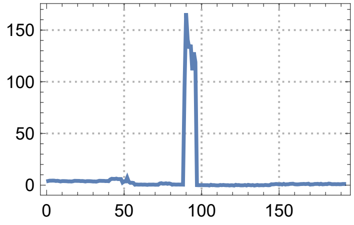

Dataset Index= 1095

Molecule Formula= $C_{23}H_{27}Cl_2N_6O_4S^+$

3D Autocorrelation

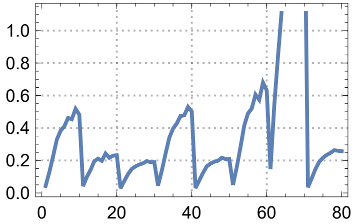

3D Plot

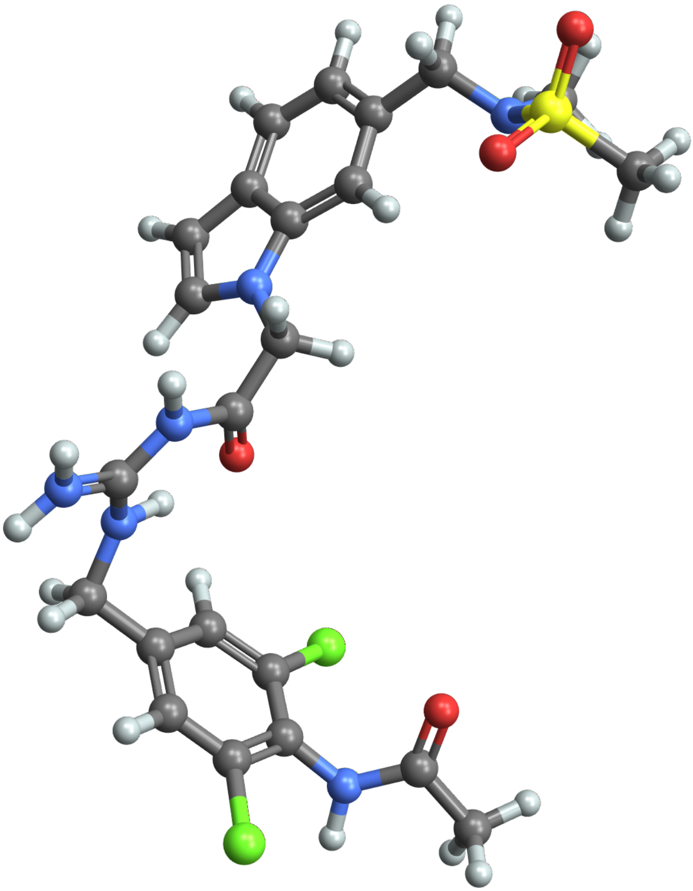

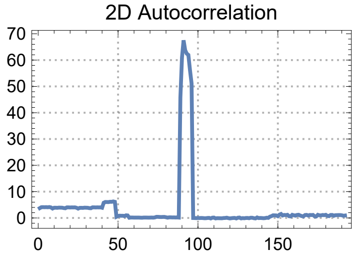

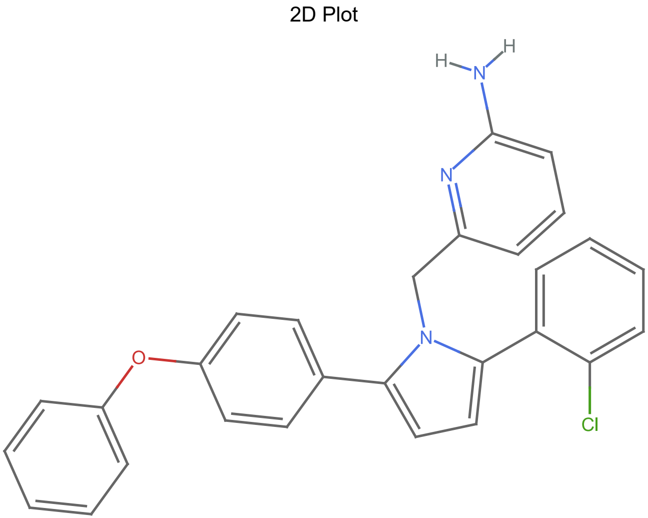

Dataset Index= 1096

Molecule Formula= $C_{28}H_{22}ClN_3O$

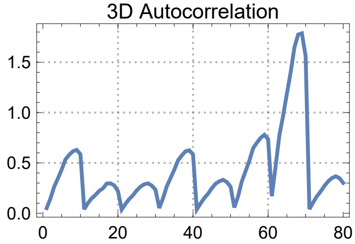

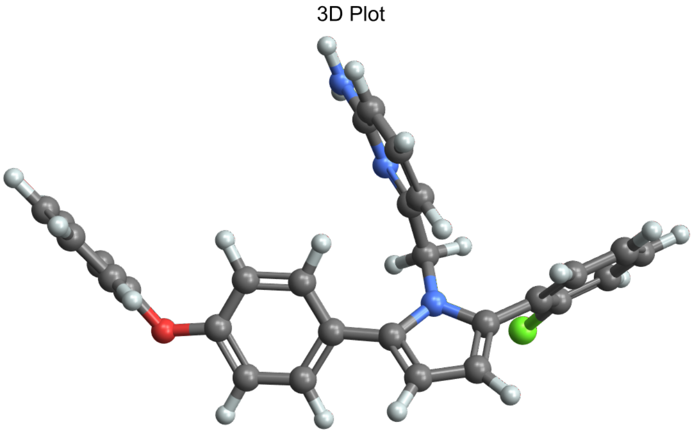

2D Plot

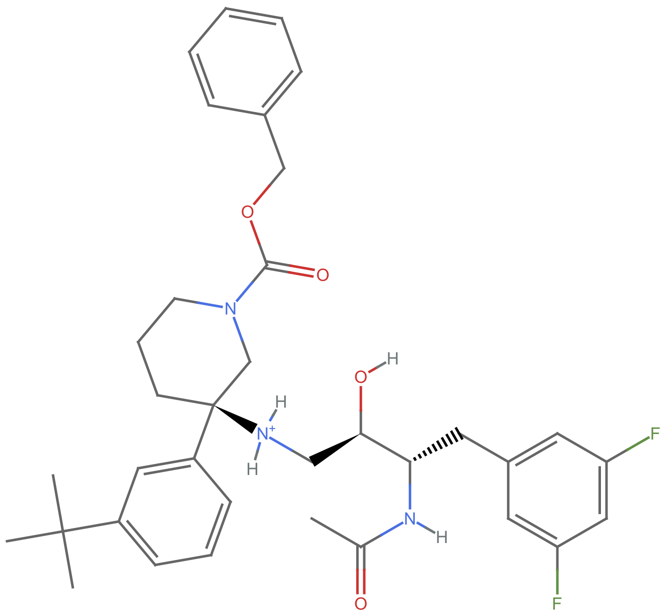

3D Plot

2D Autocorrelation

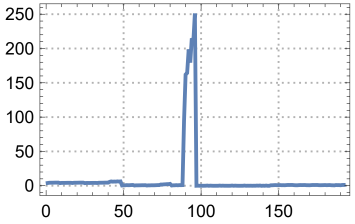

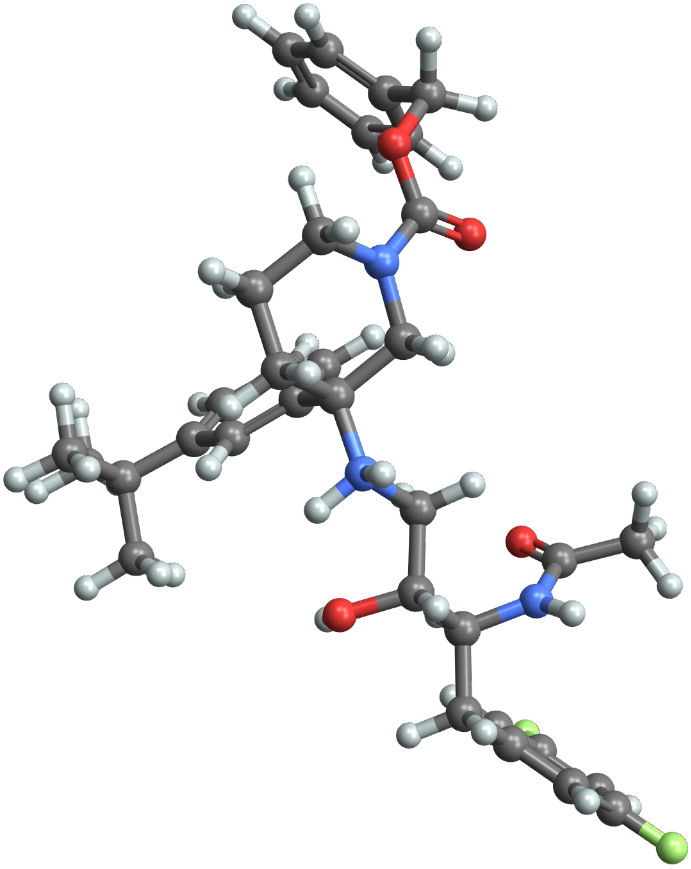

Dataset Index= 1097

Molecule Formula= $C_{35}H_{44}F_2N_3O_4^+$

3D Autocorrelation

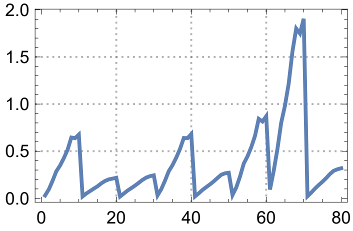

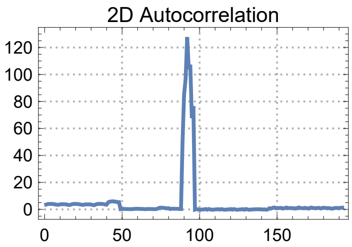

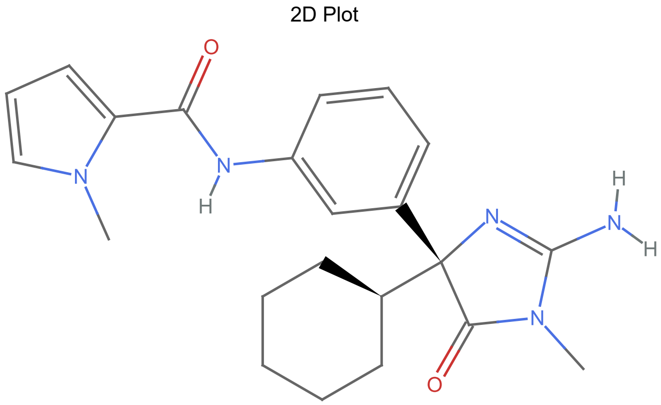

Dataset Index= 1098

Molecule Formula= $C_{22}H_{27}N_5O_2$

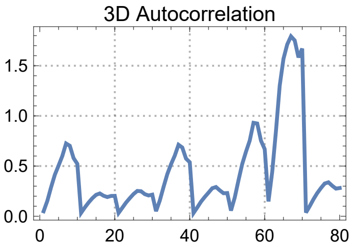

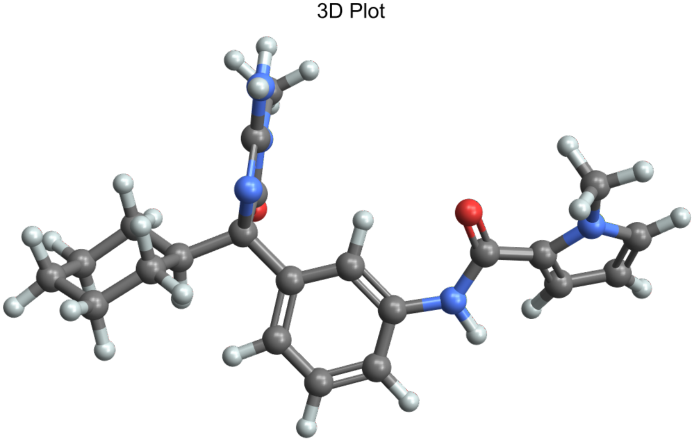

2D Plot

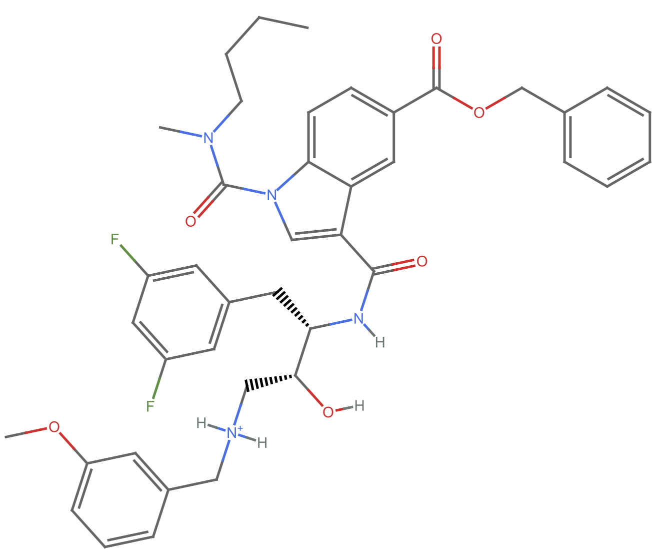

2D Autocorrelation

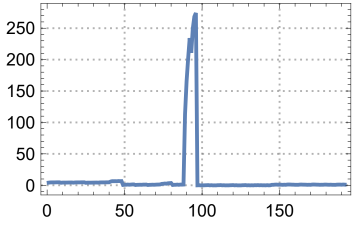

3D Plot

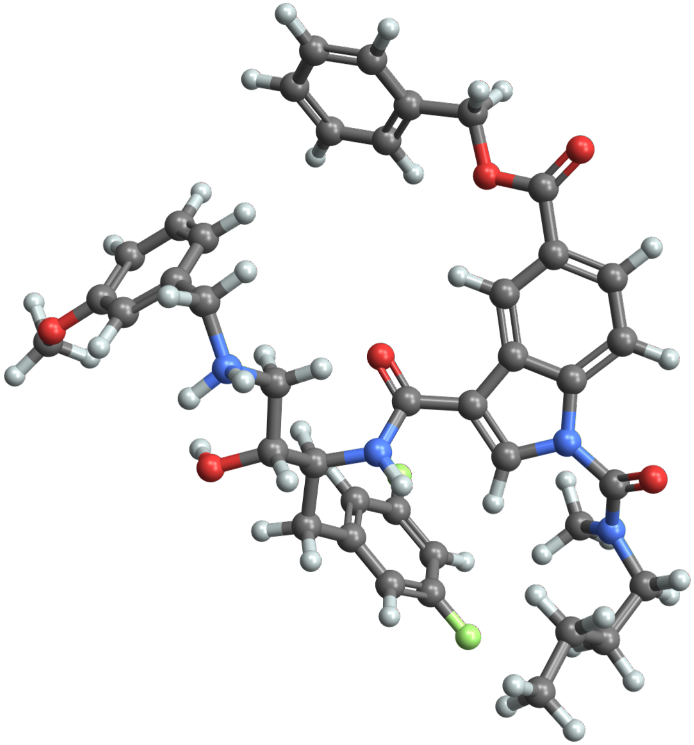

Dataset Index= 1099

Molecule Formula= $C_{41}H_{45}F_2N_4O_6^+$

3D Autocorrelation

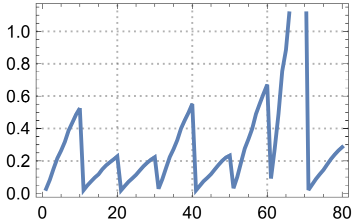

2D Plot

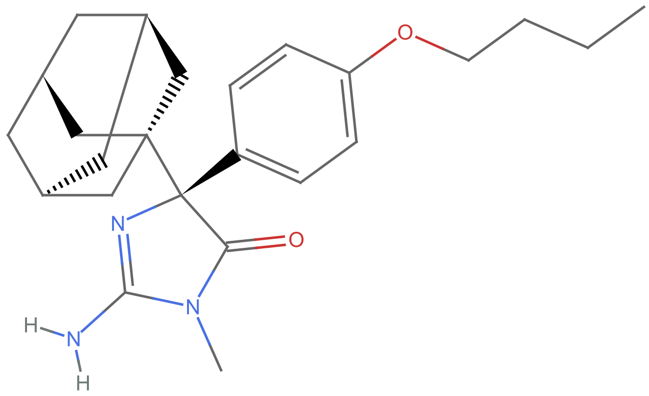

2D Autocorrelation

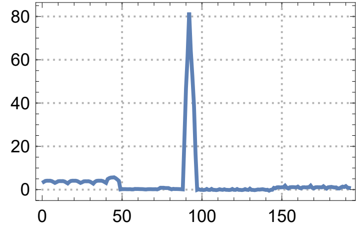

3D Plot

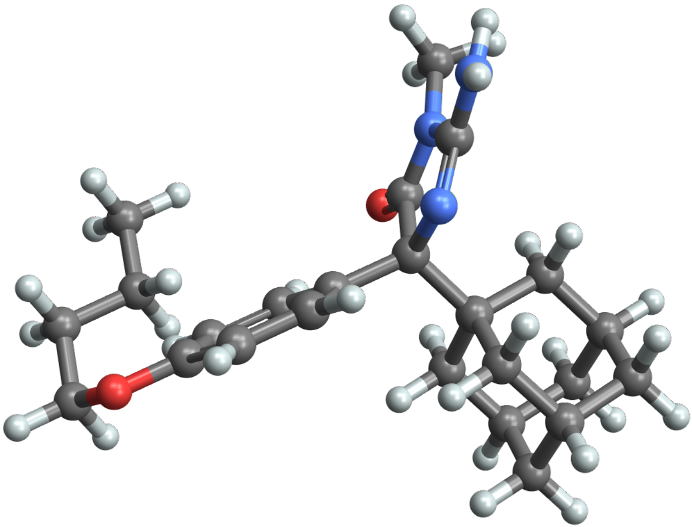

Dataset Index= 1100

Molecule Formula= $C_{24}H_{33}N_3O_2$

3D Autocorrelation

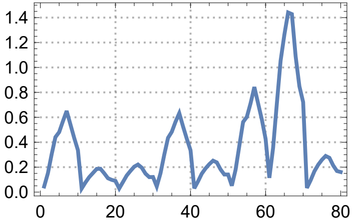

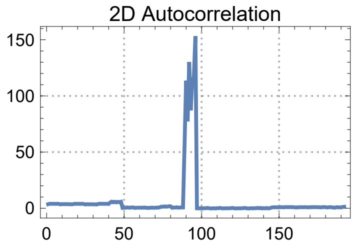

Dataset Index= 1101

Molecule Formula= $C_{23}H_{33}F_2N_4O_2^+$

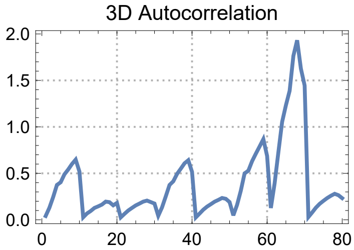

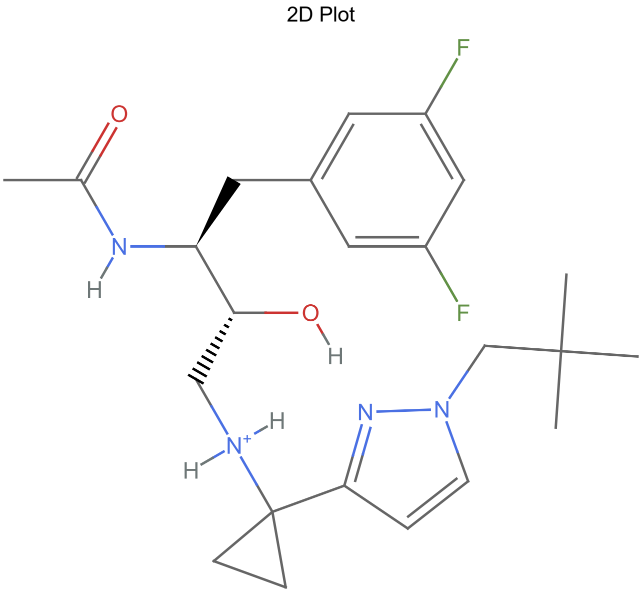

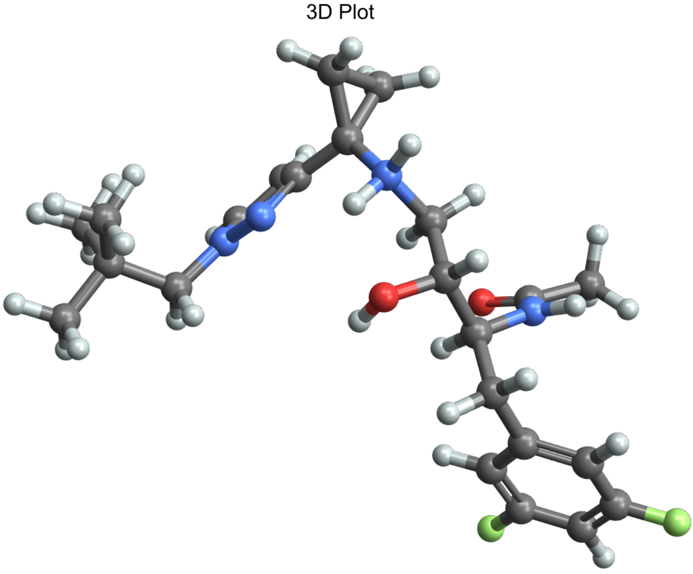

2D Plot

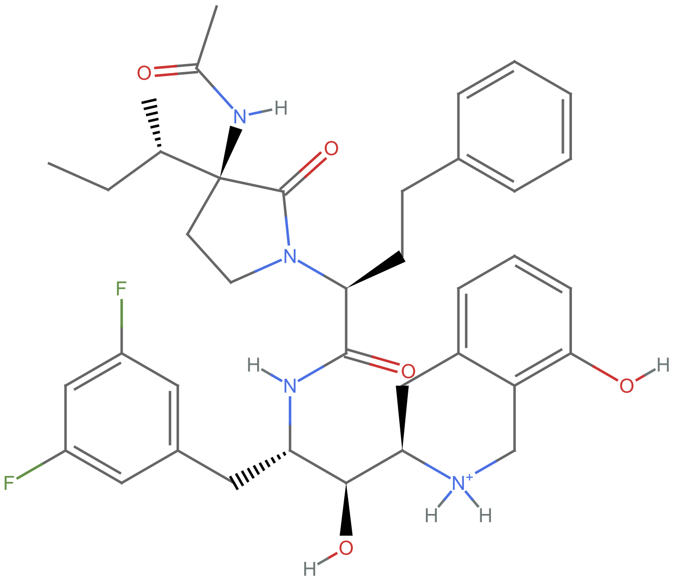

3D Plot

2D Autocorrelation

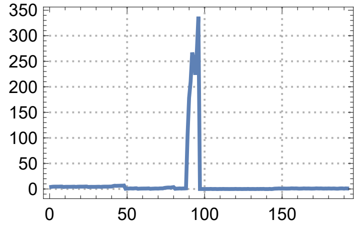

Dataset Index= 1102

Molecule Formula= $C_{38}H_{47}F_2N_4O_5^+$

3D Autocorrelation

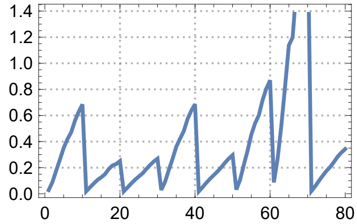

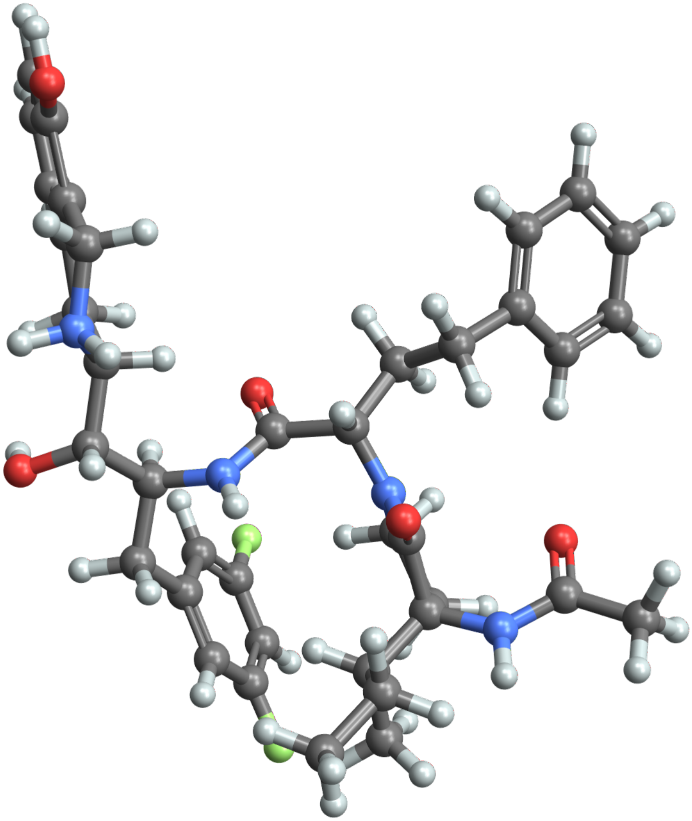

2D Plot

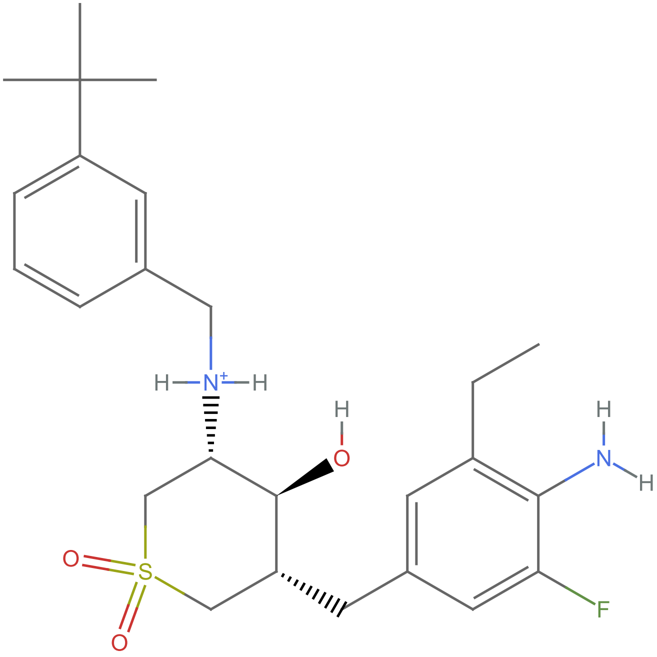

2D Autocorrelation

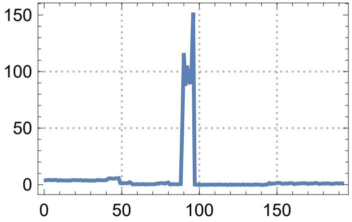

Dataset Index= 1103

Molecule Formula= $C_{25}H_{36}FN_2O_3S^+$

3D Autocorrelation

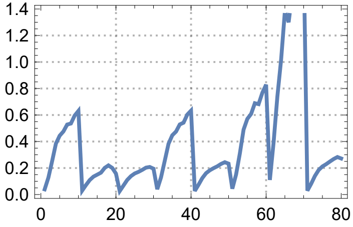

3D Plot

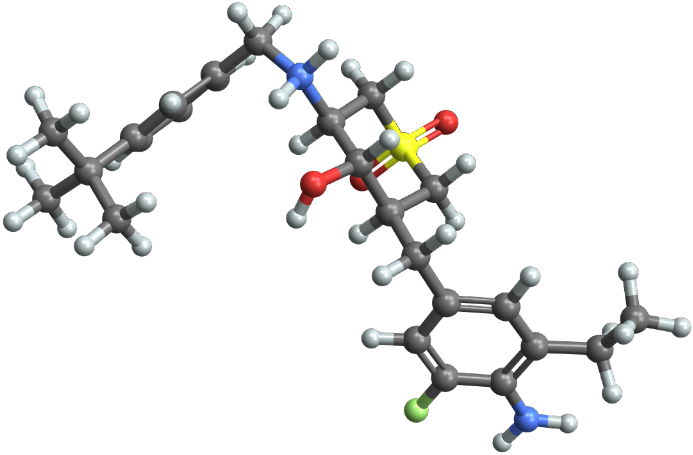

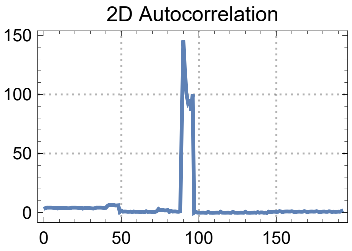

Dataset Index= 1104

Molecule Formula= $C_{23}H_{19}F_3N_4O_3$

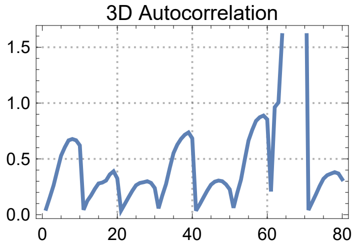

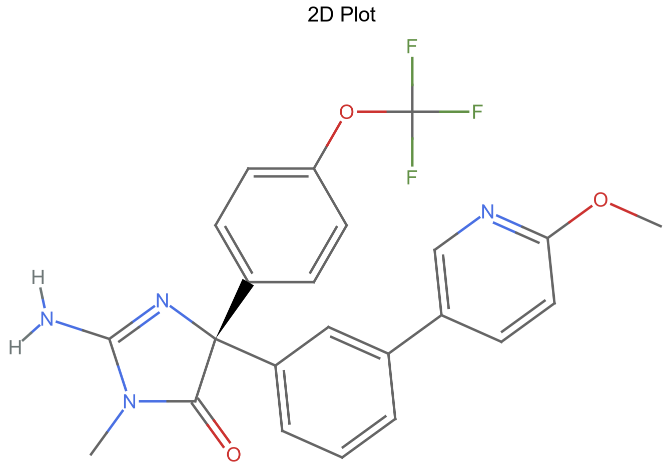

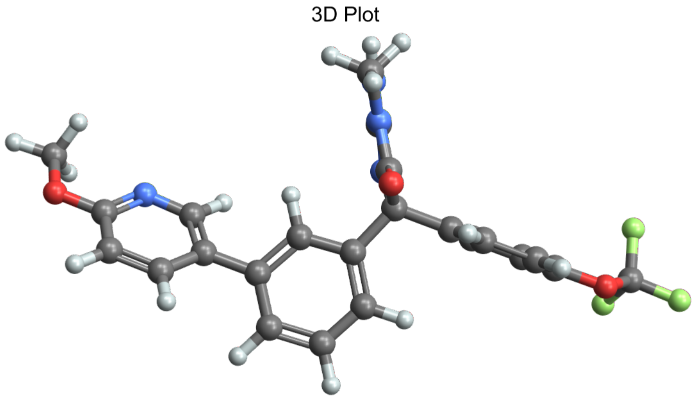

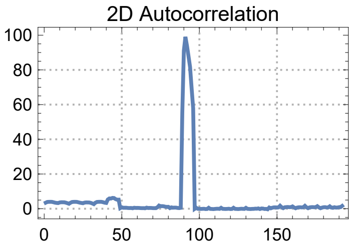

Dataset Index= 1105

Molecule Formula= $C_{19}H_{19}F_2N_3O_2$

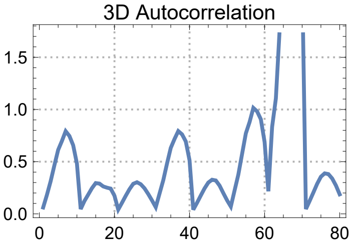

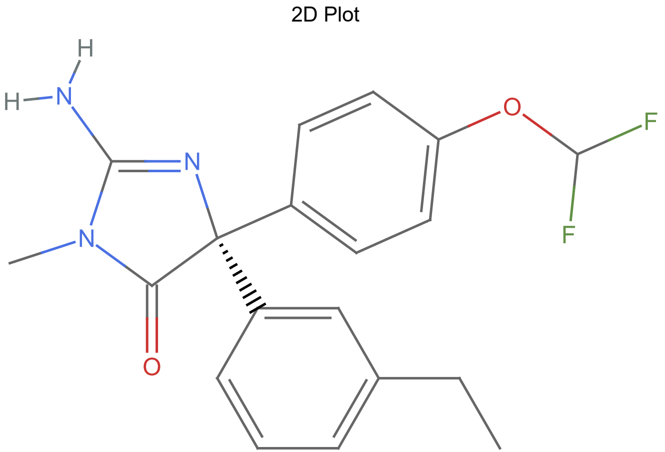

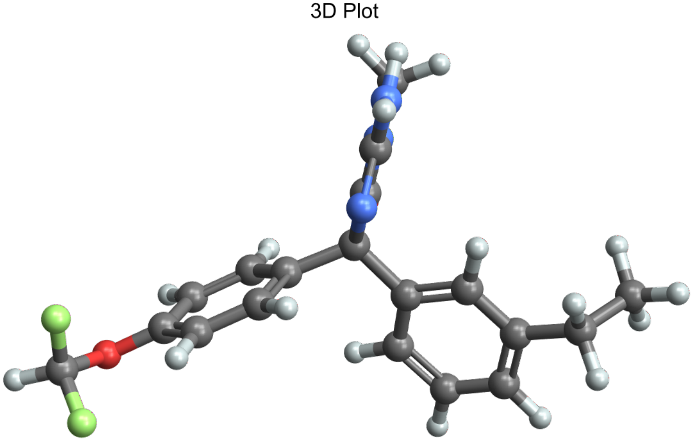

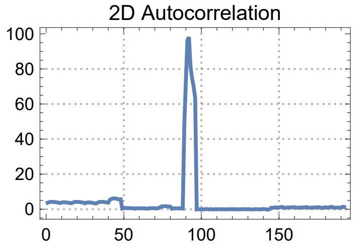

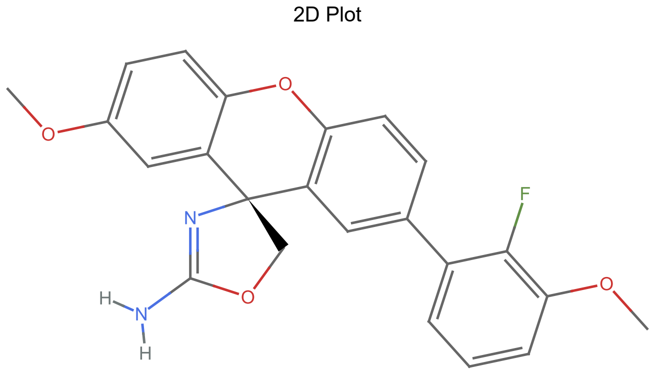

Dataset Index= 1106

Molecule Formula= $C_{23}H_{19}FN_2O_4$

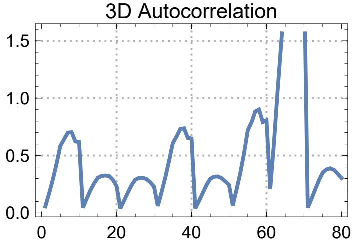

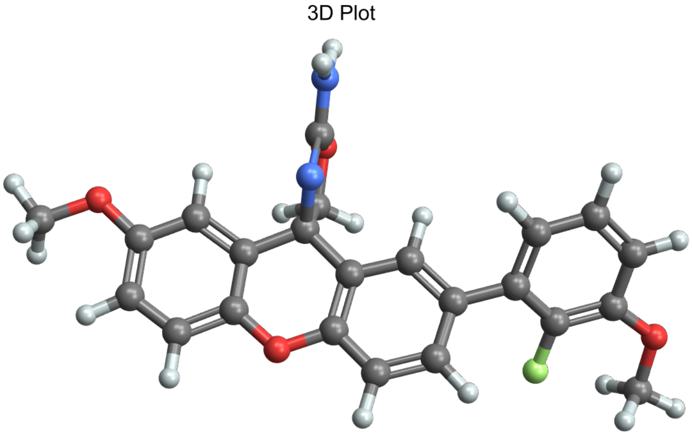

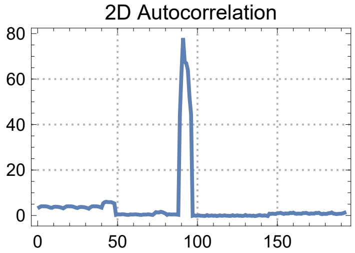

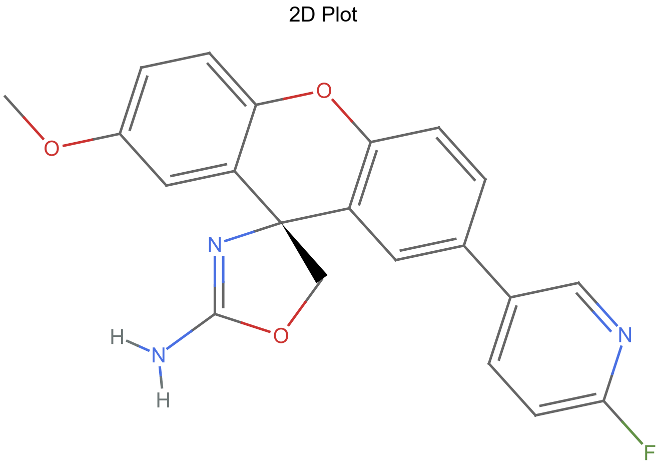

Dataset Index= 1107

Molecule Formula= $C_{21}H_{16}FN_3O_3$

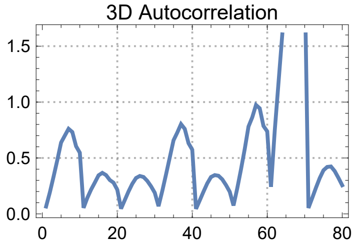

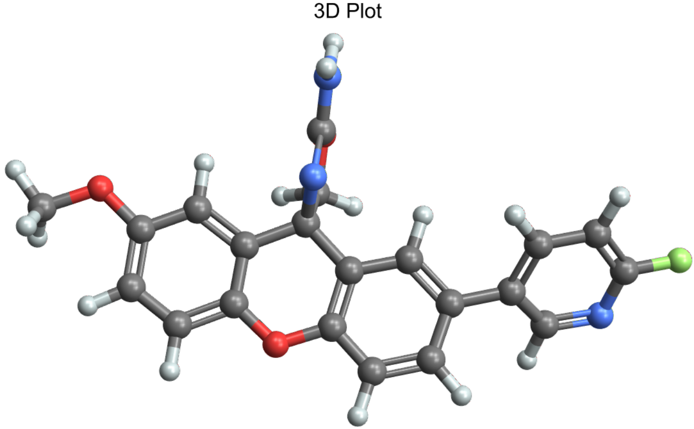

2D Plot

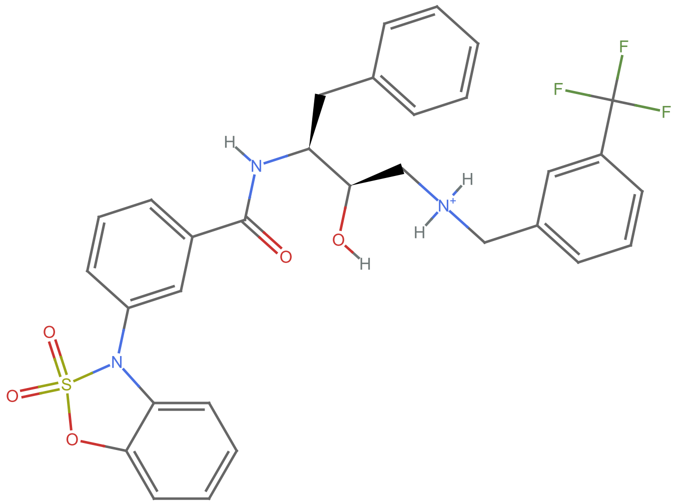

2D Autocorrelation

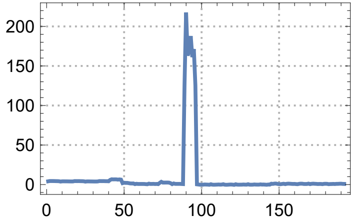

Dataset Index= 1108

Molecule Formula= $C_{31}H_{29}F_3N_3O_5S^+$

3D Plot

3D Autocorrelation

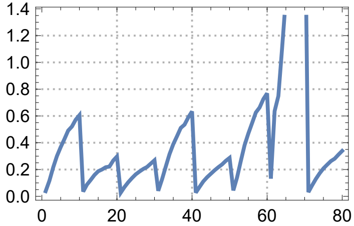

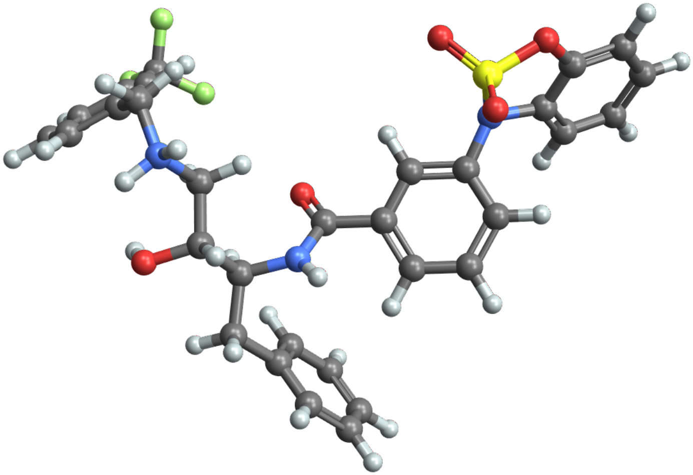

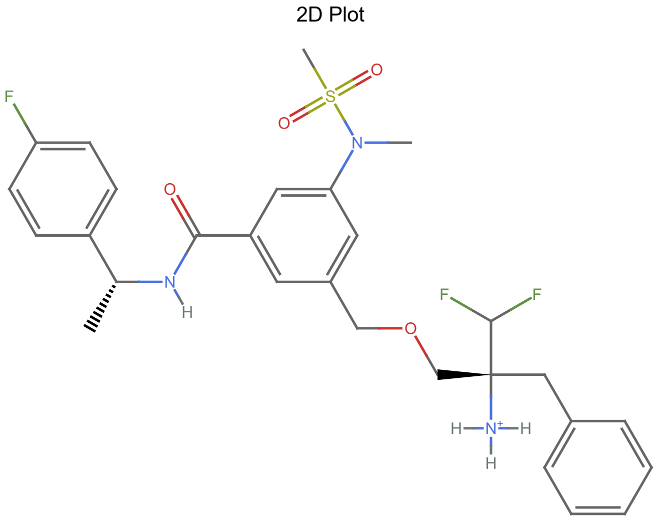

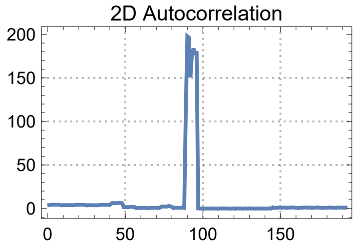

Dataset Index= 1109

Molecule Formula= $C_{28}H_{33}F_{3}N_{3}O_{4}S^{+}$

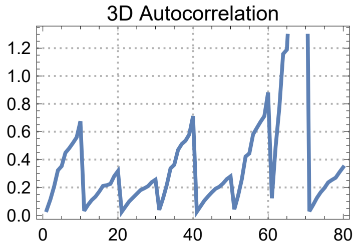

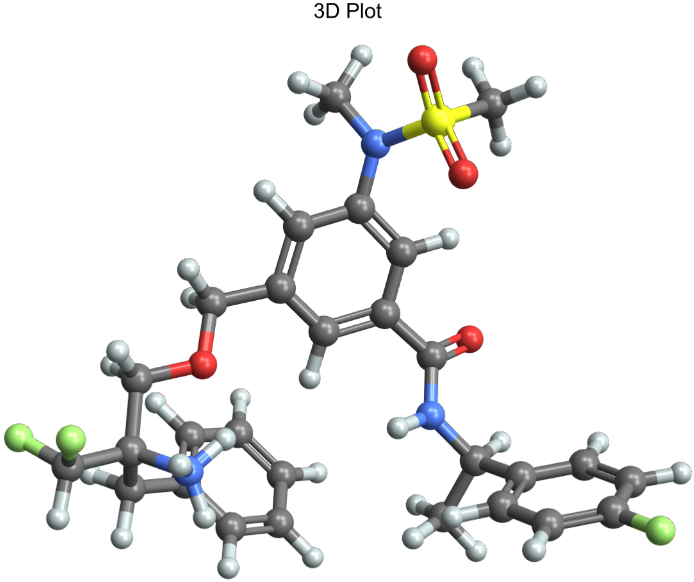

2D Plot

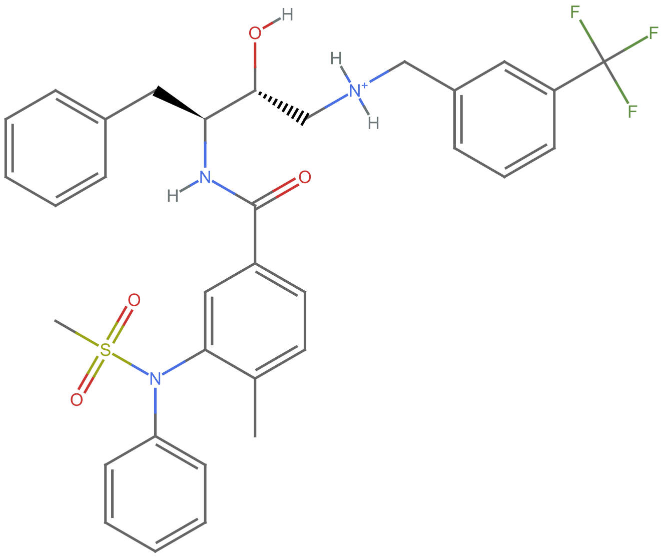

2D Autocorrelation

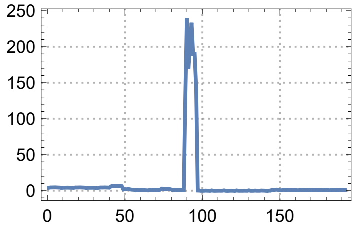

Dataset Index= 1110

Molecule Formula= $C_{33}H_{35}F_3N_3O_4S^+$

3D Autocorrelation

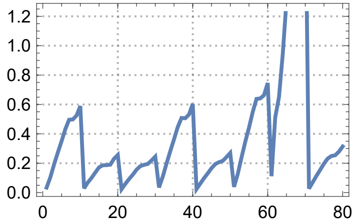

3D Plot

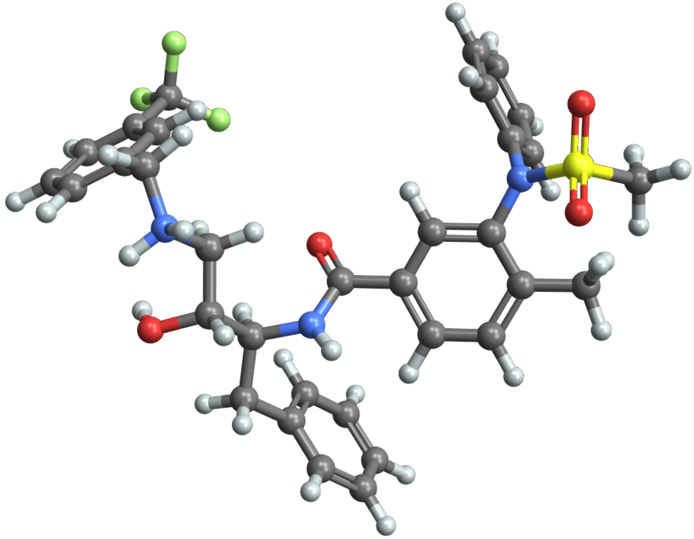

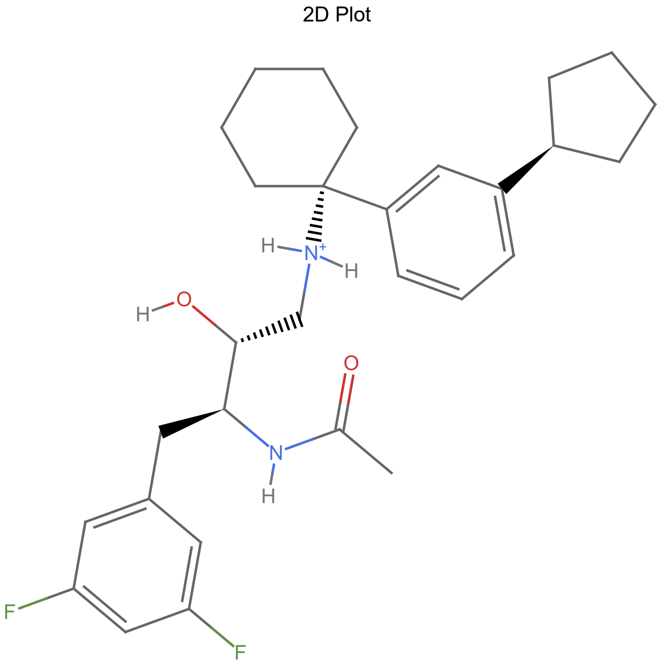

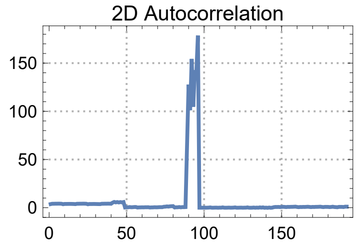

Dataset Index= 1111

Molecule Formula= $C_{29}H_{39}F_2N_2O_2^+$

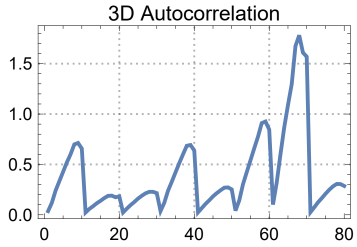

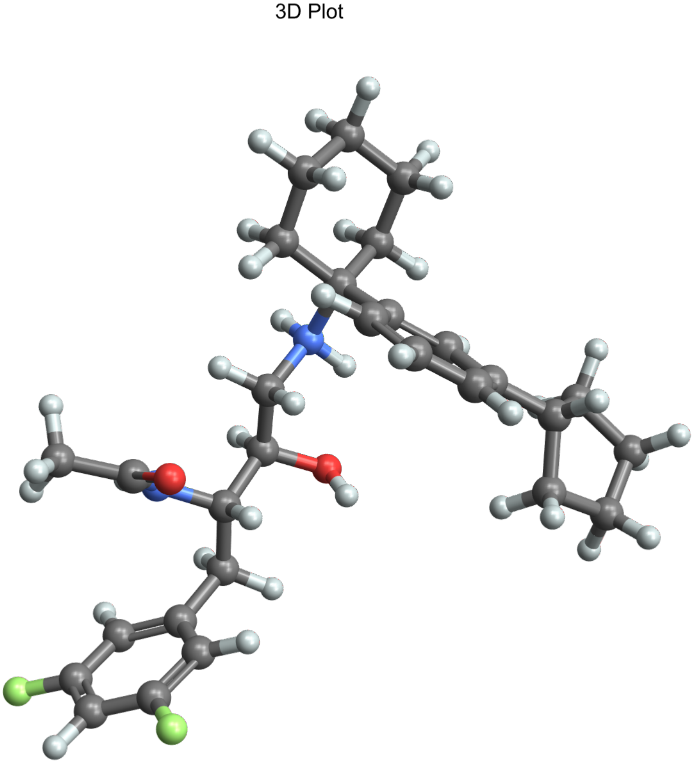

2D Plot

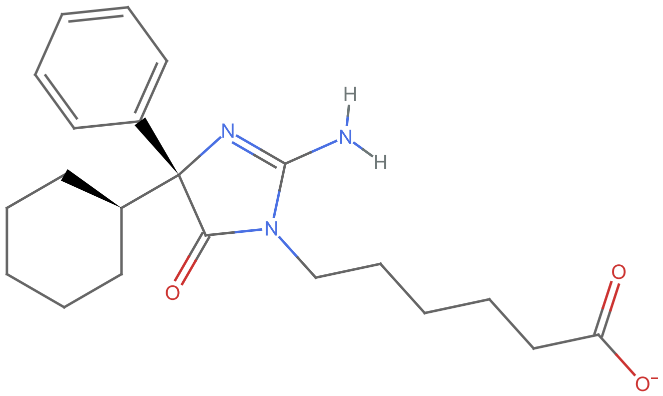

2D Autocorrelation

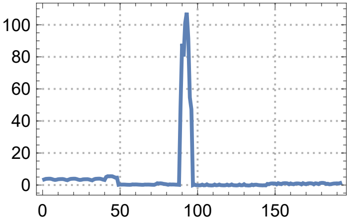

3D Plot

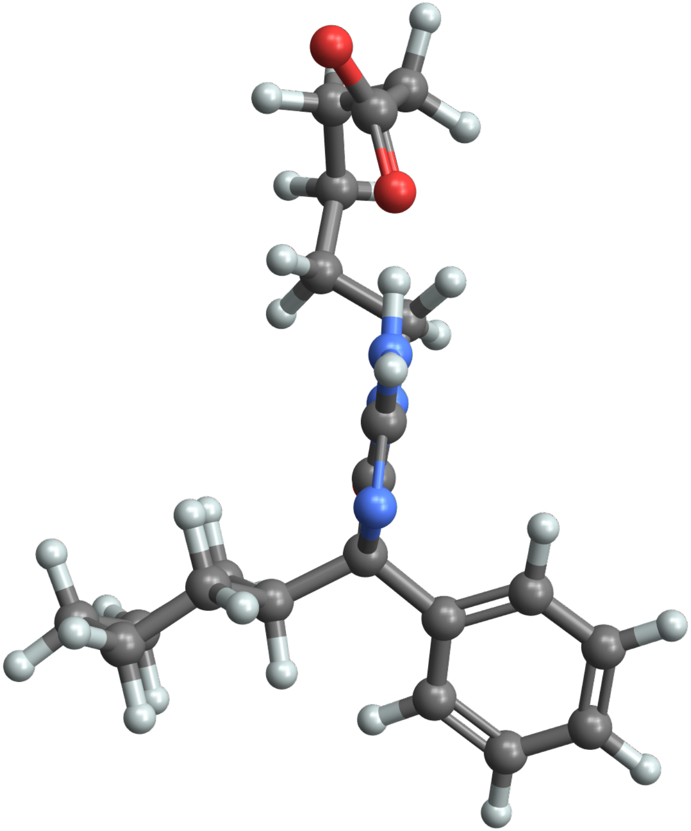

Dataset Index= 1112

Molecule Formula= $C_{21}H_{28}N_3O_3^-$

3D Autocorrelation

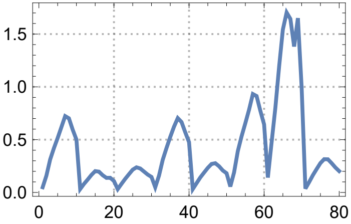

2D Plot

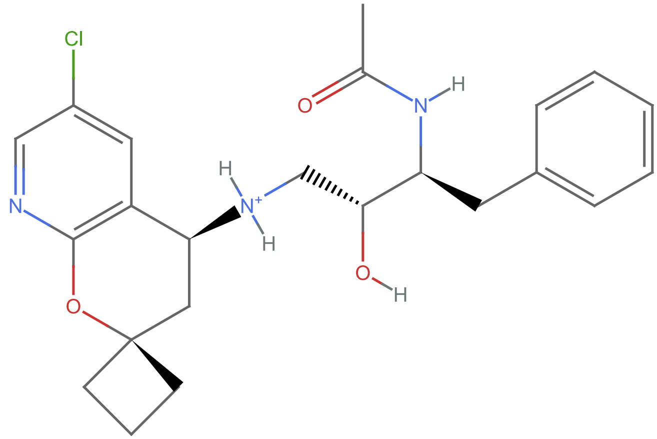

3D Plot

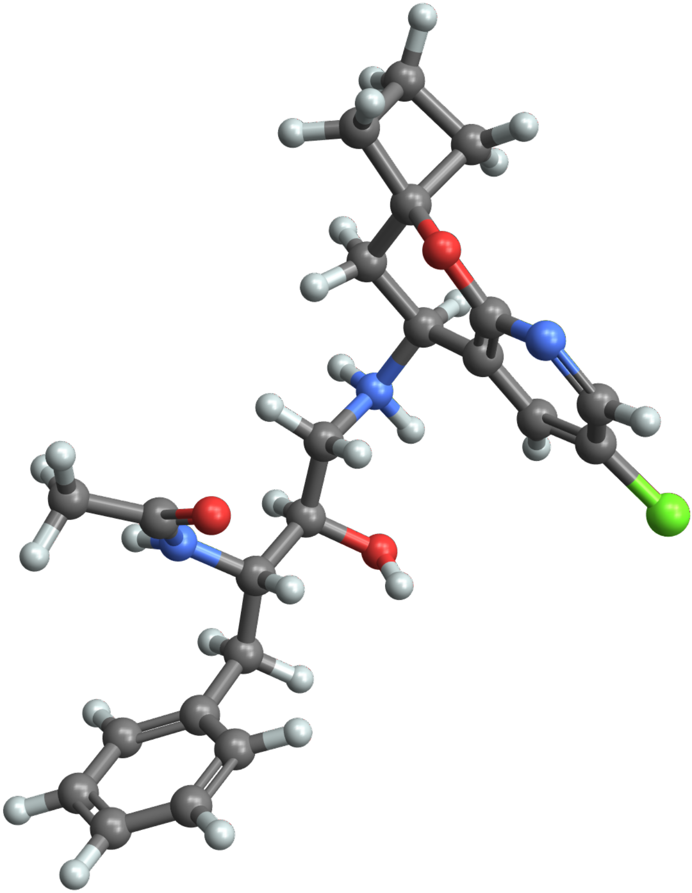

2D Autocorrelation

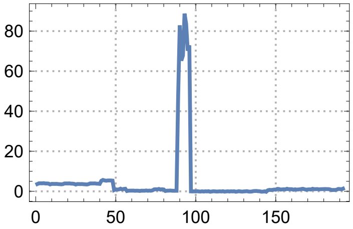

Dataset Index= 1113

Molecule Formula= $C_{23}H_{29}ClN_3O_3^+$

3D Autocorrelation

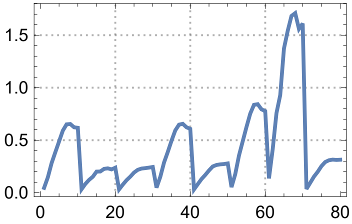

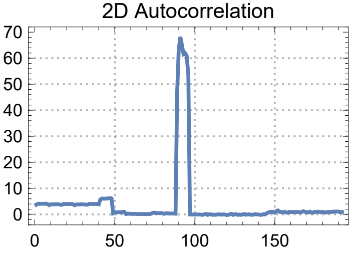

Dataset Index= 1114

Molecule Formula= $C_{26}H_{21}ClN_6$

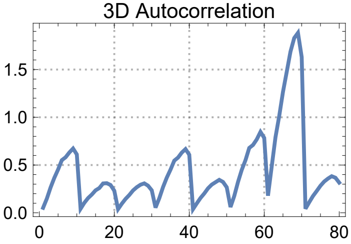

2D Plot

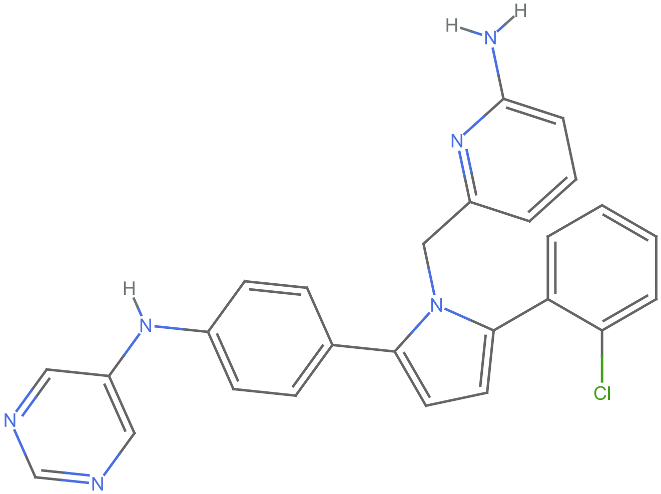

3D Plot

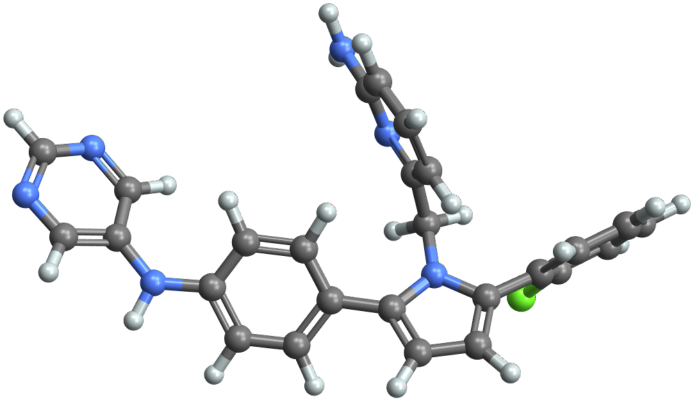

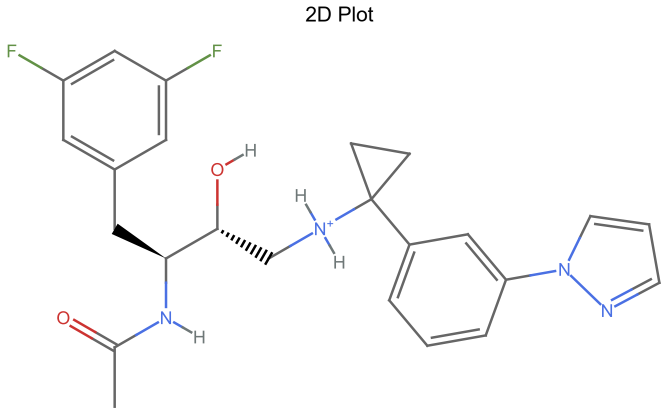

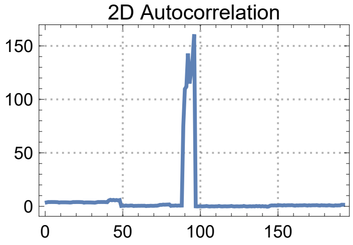

Dataset Index= 1115

Molecule Formula= $C_{24}H_{27}F_2N_4O_2^+$

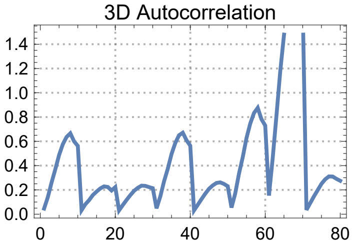

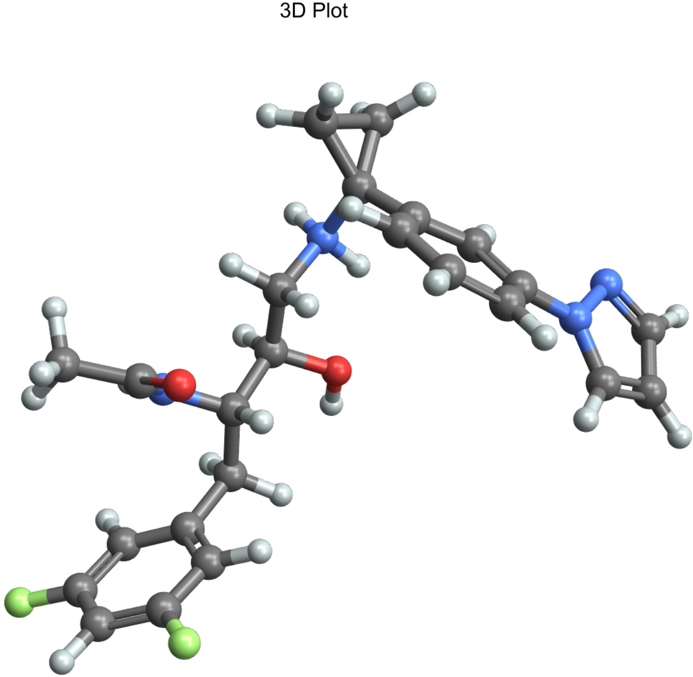

2D Plot

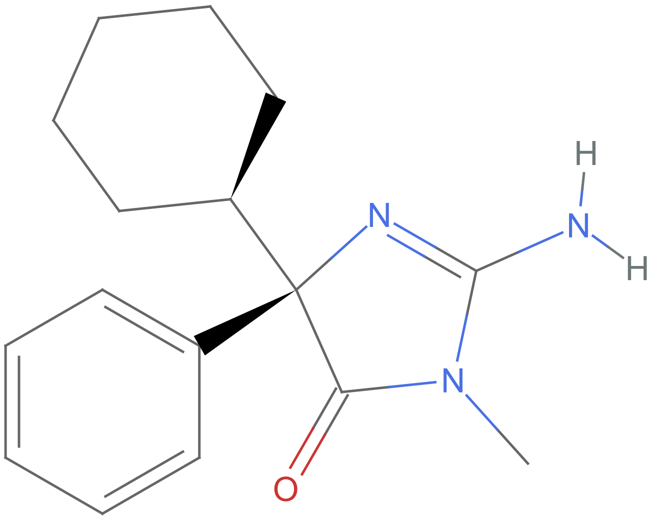

2D Autocorrelation

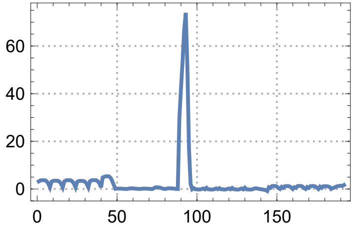

Dataset Index= 1116

Molecule Formula= $C_{16}H_{21}N_3O$

3D Plot

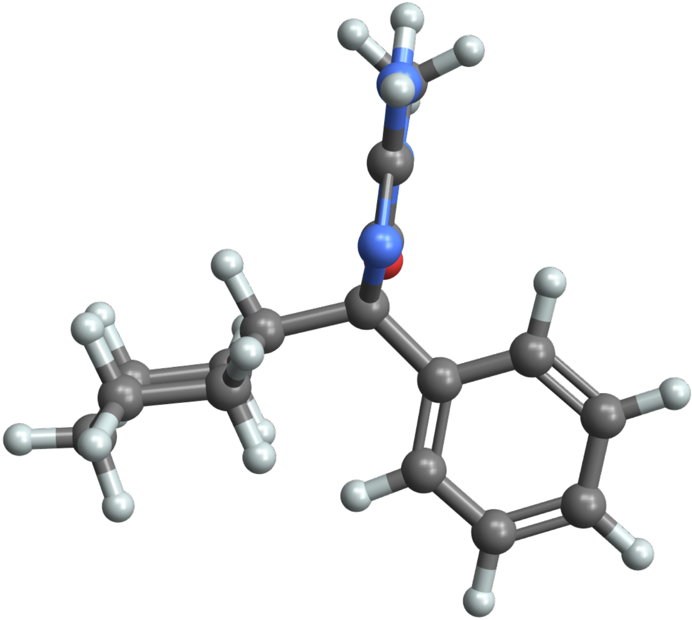

3D Autocorrelation

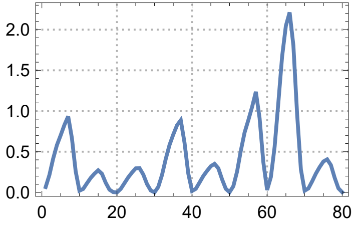

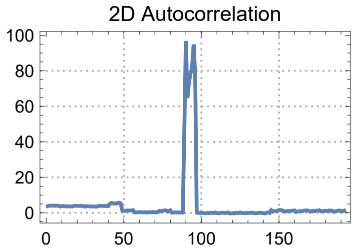

Dataset Index= 1117

Molecule Formula= $C_{24}H_{34}NO_5S^+$

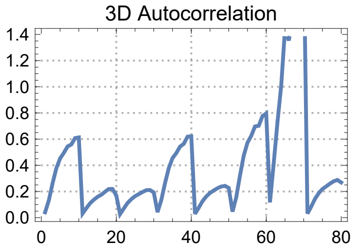

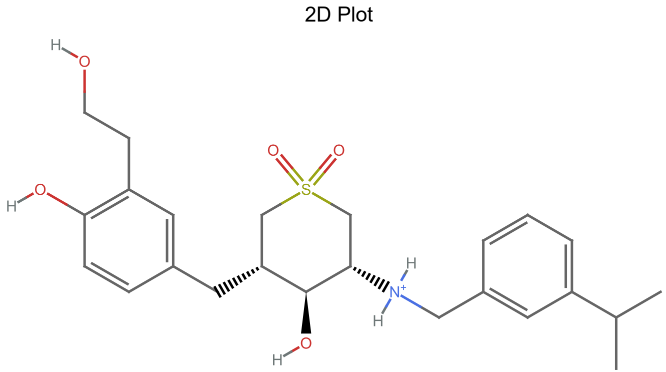

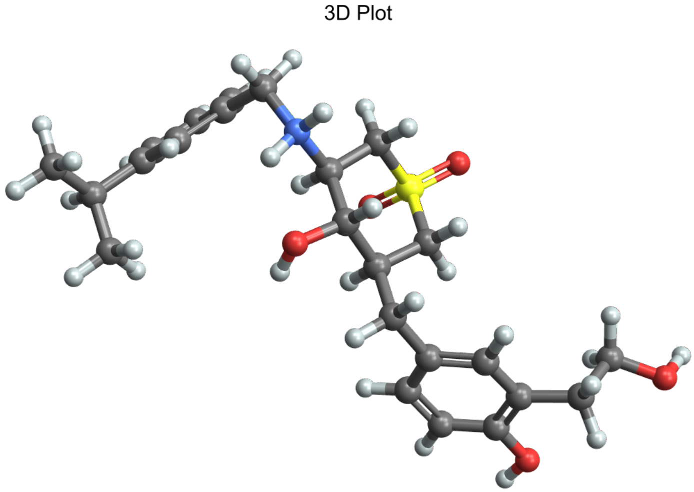

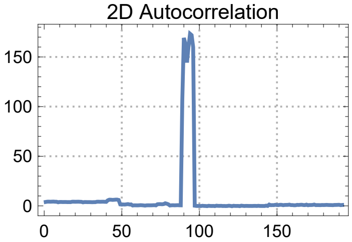

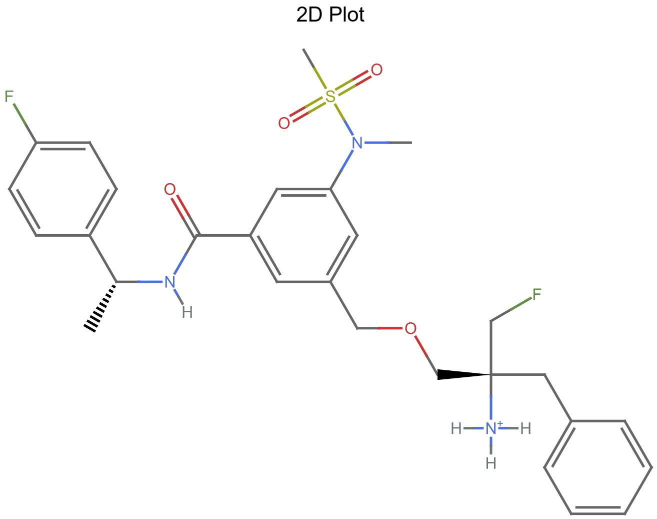

Dataset Index= 1118

Molecule Formula= $C_{28}H_{34}F_2N_3O_4S^+$

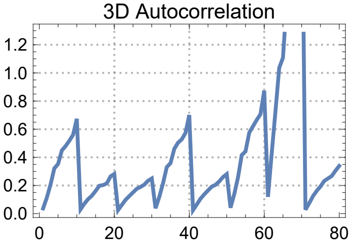

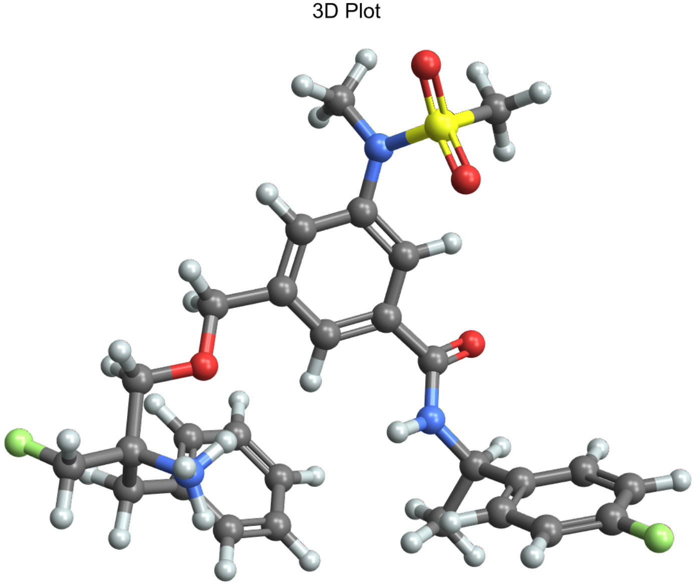

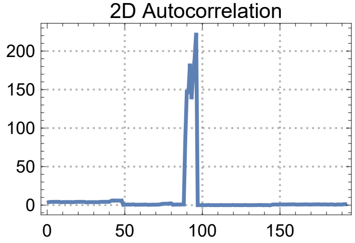

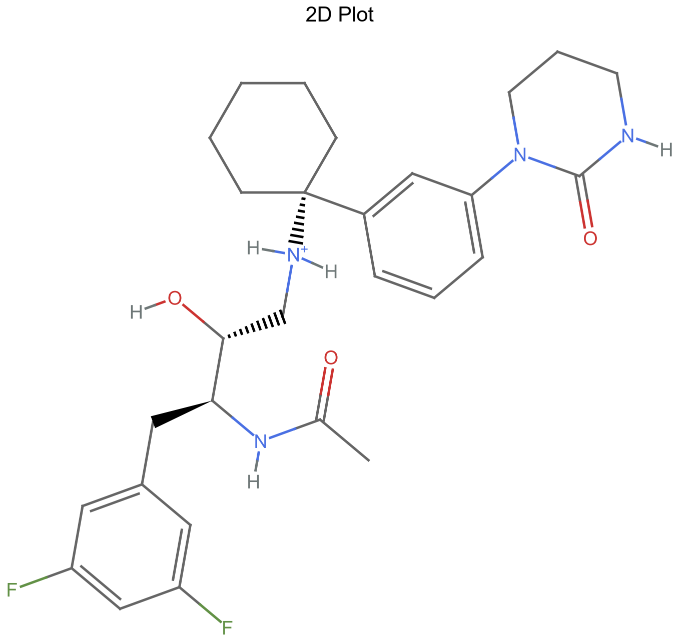

Dataset Index= 1119

Molecule Formula= $C_{28}H_{37}F_2N_4O_3^+$

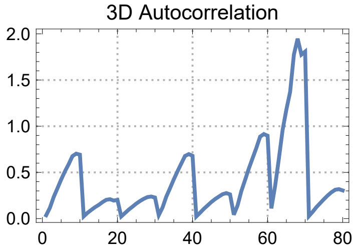

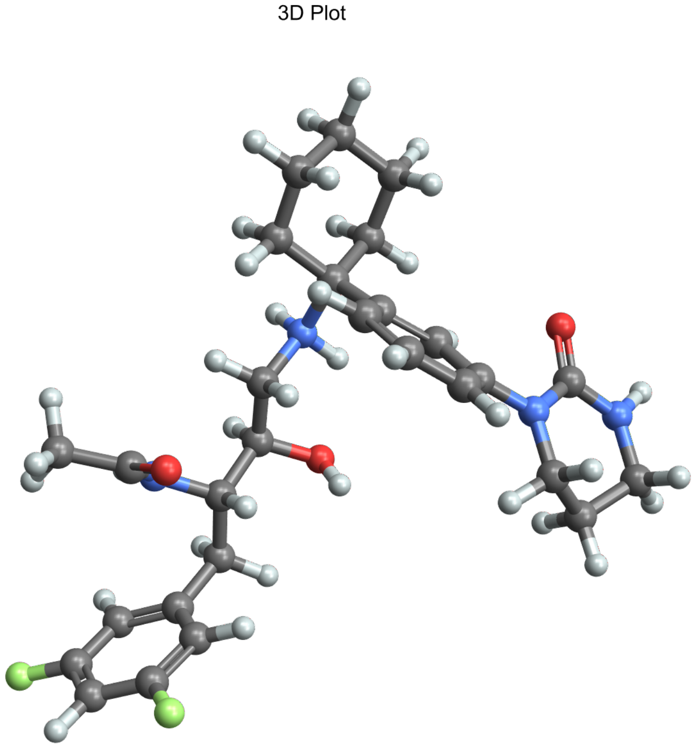

2D Plot

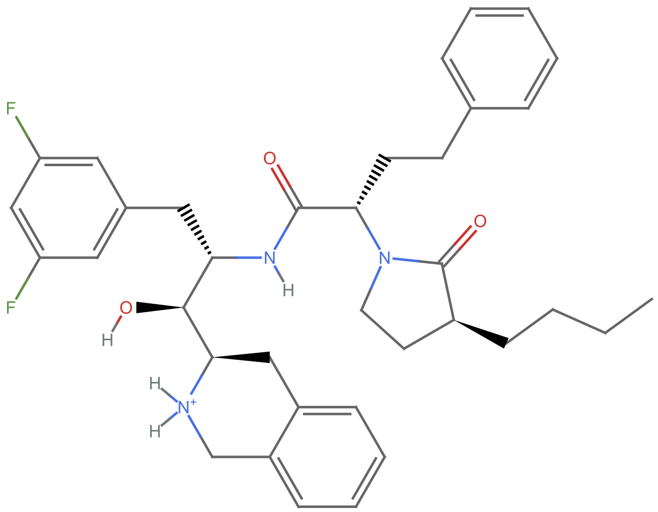

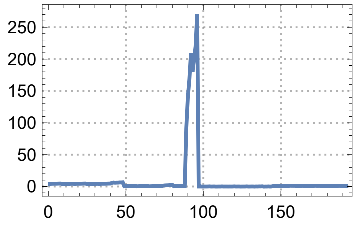

3D Plot

Dataset Index= 1120

Molecule Formula= $C_{36}H_{44}F_2N_3O_3^+$

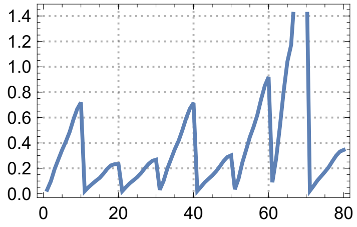

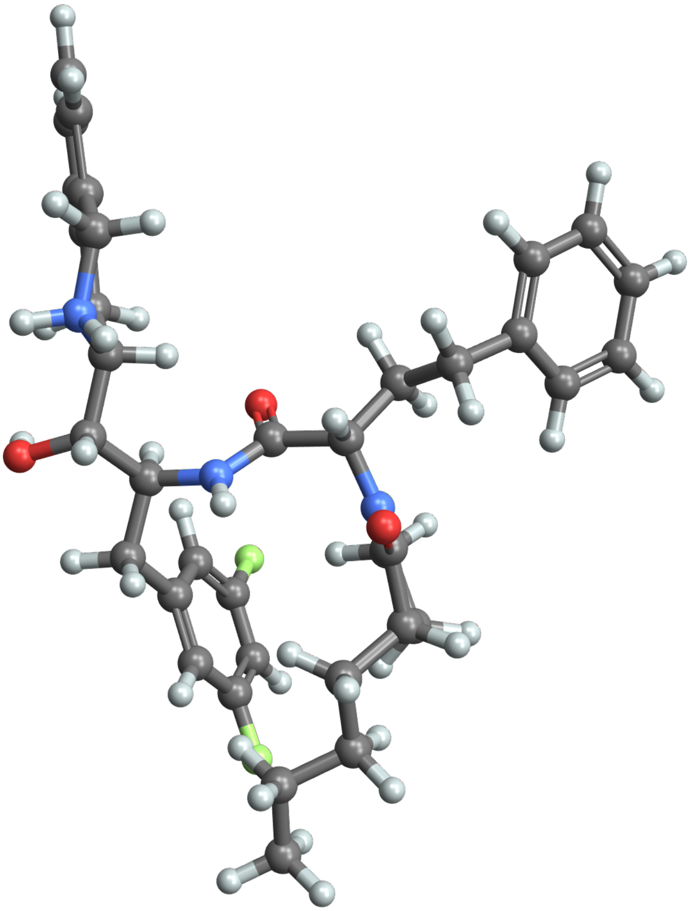

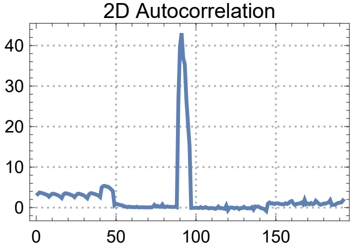

Dataset Index= 1121

Molecule Formula= $C_{15}H_{16}N_4S$

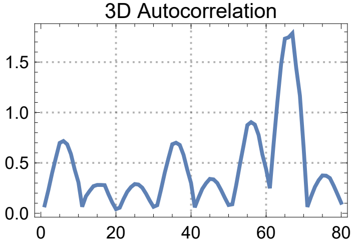

2D Plot

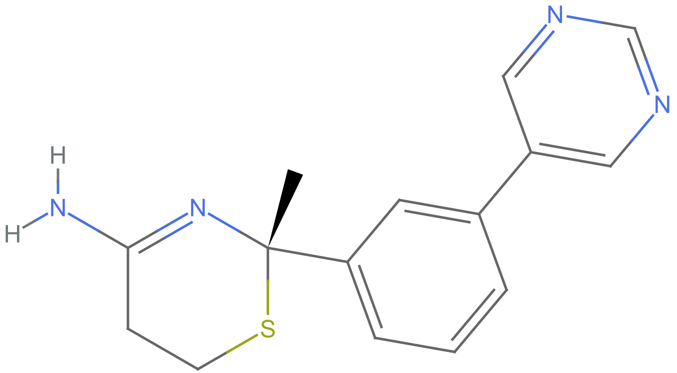

3D Plot

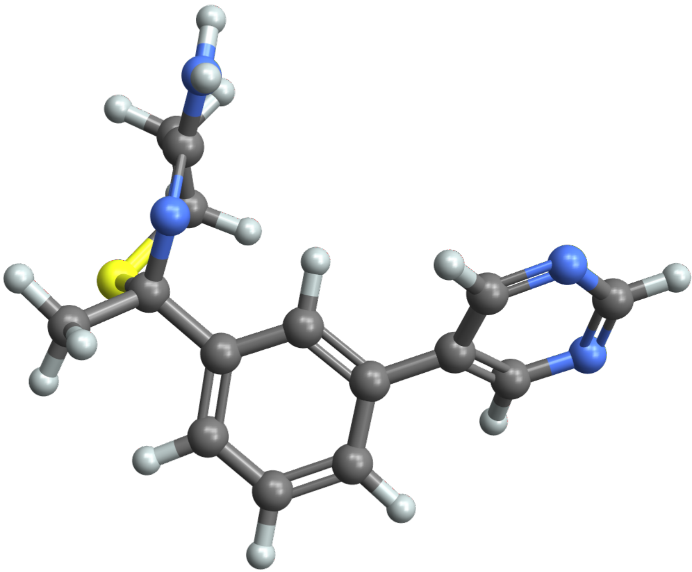

2D Plot

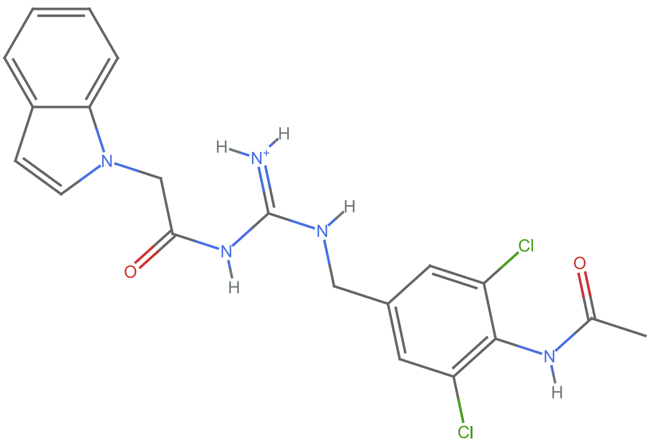

2D Autocorrelation

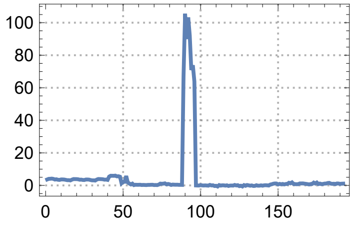

Dataset Index= 1122

Molecule Formula= $C_{20}H_{20}Cl_2N_5O_2^+$

3D Autocorrelation

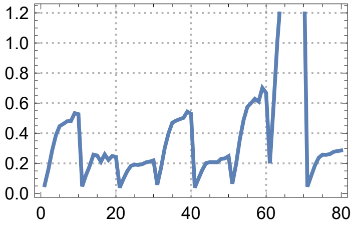

3D Plot

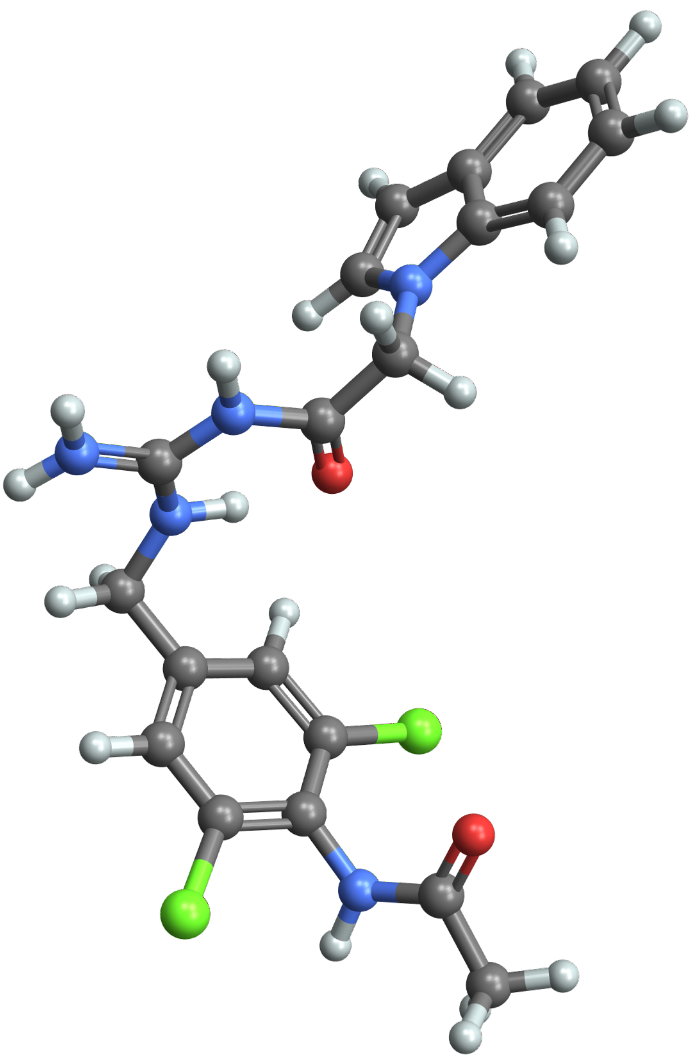

2D Autocorrelation

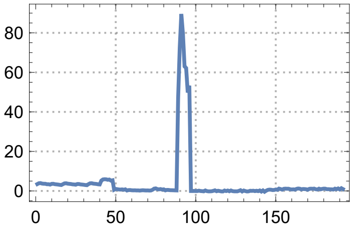

Dataset Index= 1123

Molecule Formula= $C_{17}H_{16}ClN_5O_2$

3D Autocorrelation

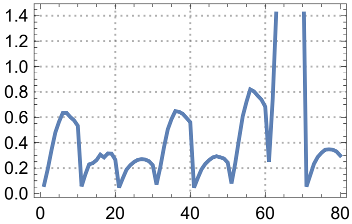

2D Plot

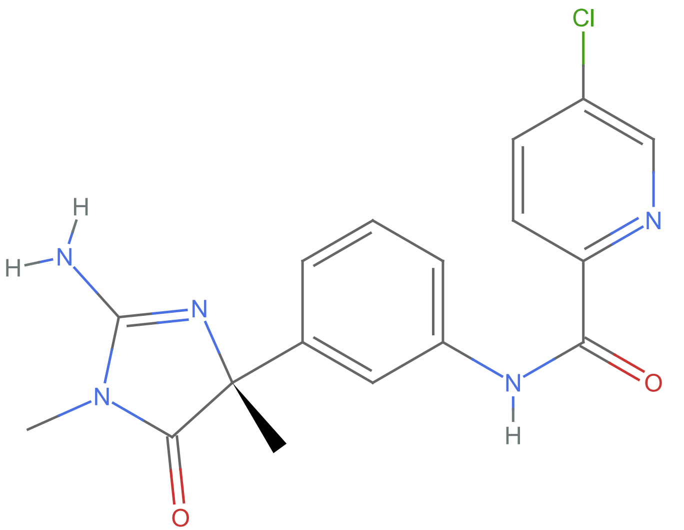

3D Plot

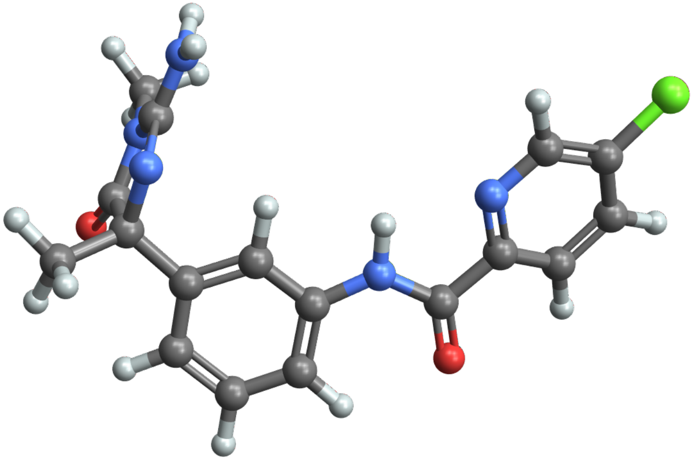

2D Autocorrelation

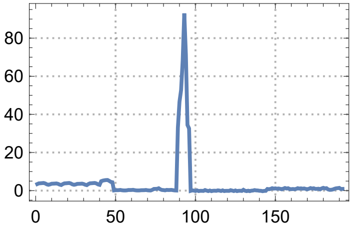

Dataset Index= 1124

Molecule Formula= $C_{20}H_{27}N_3O_2$

3D Autocorrelation

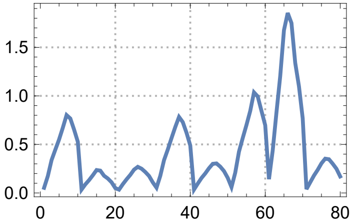

2D Plot

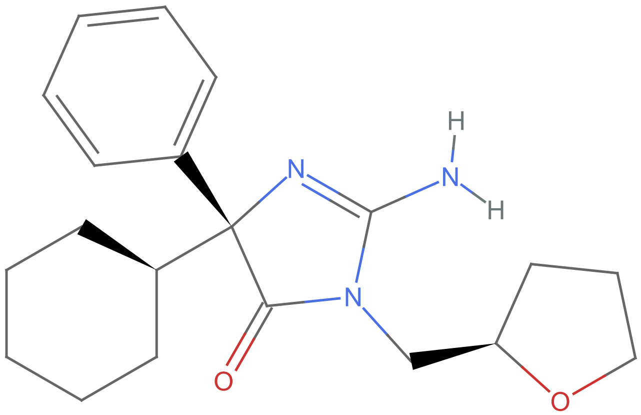

3D Plot

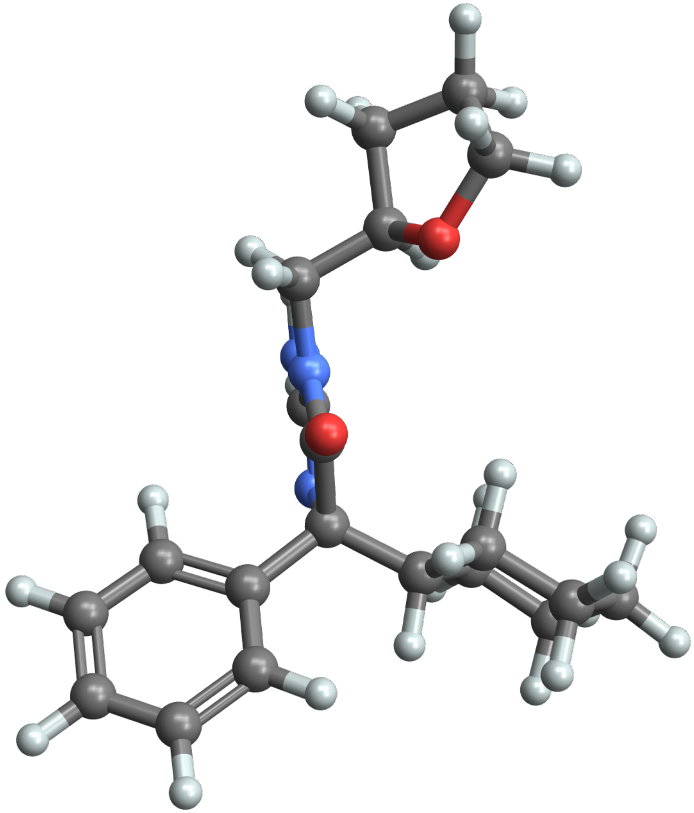

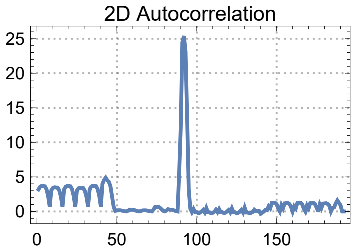

Dataset Index= 1125

Molecule Formula= $C_{16}H_{27}N_3O$

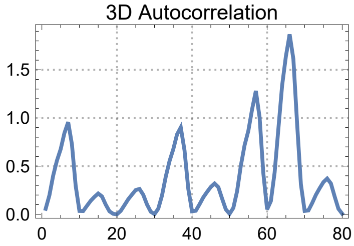

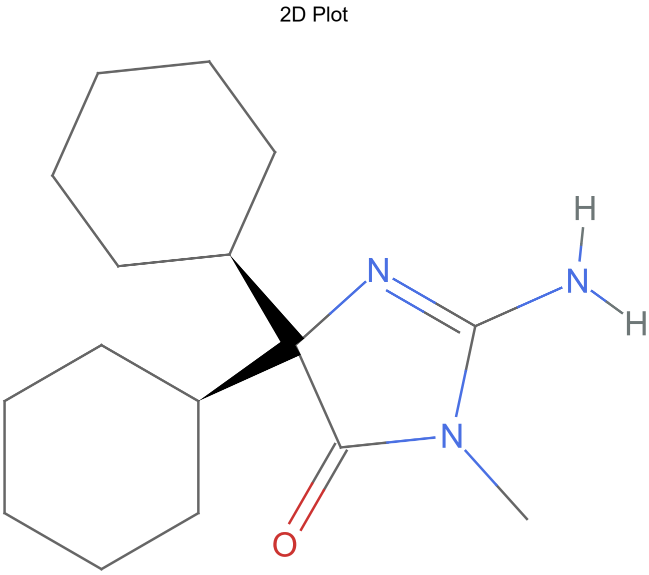

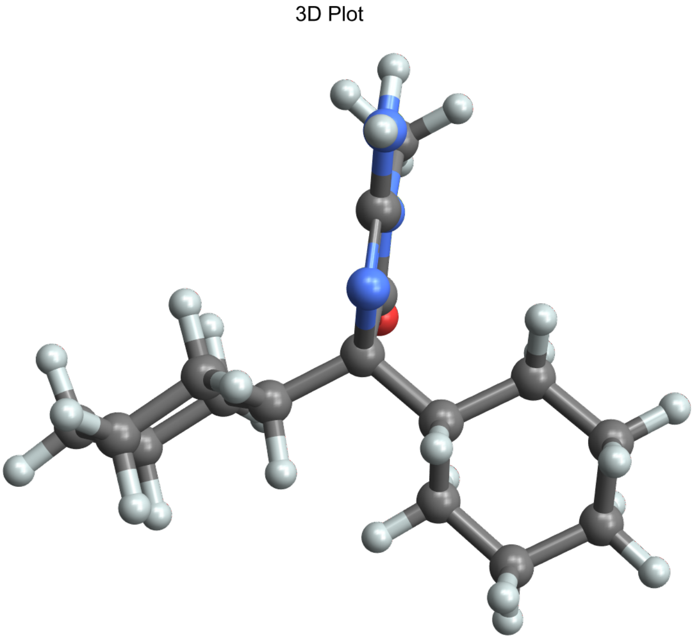

2D Plot

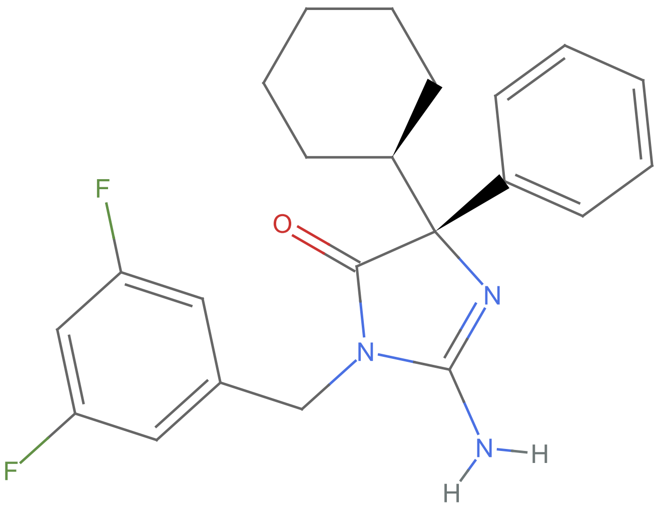

2D Autocorrelation

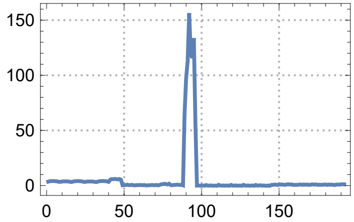

Dataset Index= 1126

Molecule Formula= $C_{22}H_{23}F_2N_3O$

3D Autocorrelation

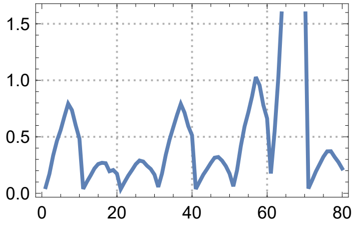

3D Plot

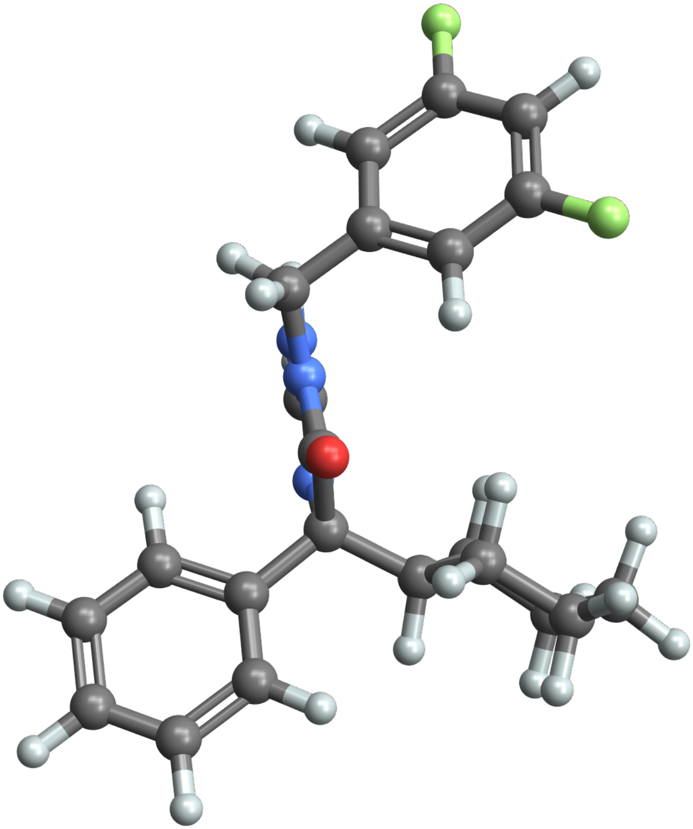

2D Plot

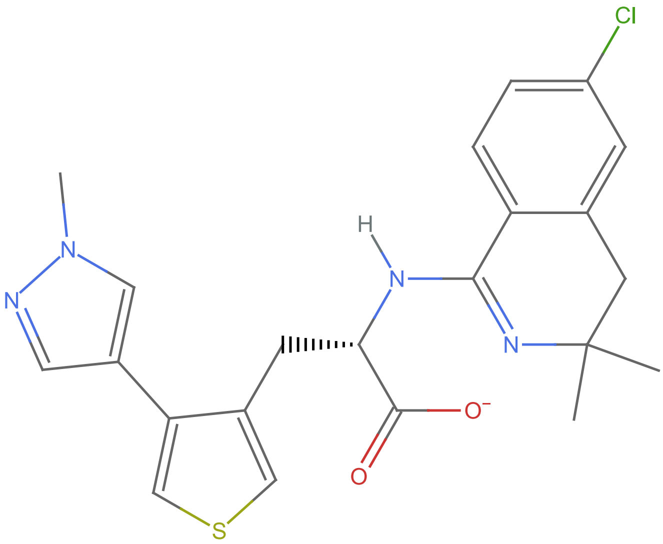

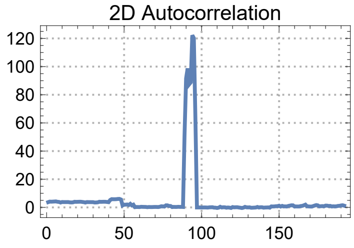

3D Plot

Dataset Index= 1127

Molecule Formula= $C_{22}H_{22}ClN_4O_2S^-$

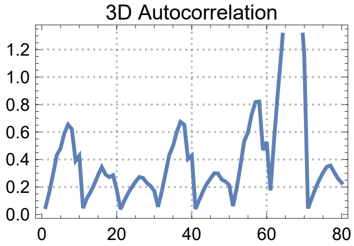

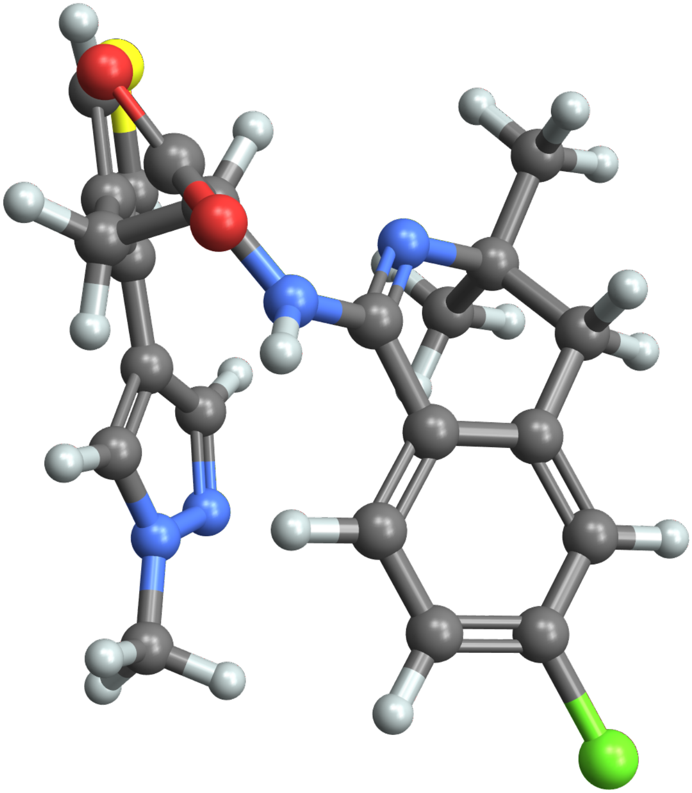

2D Plot

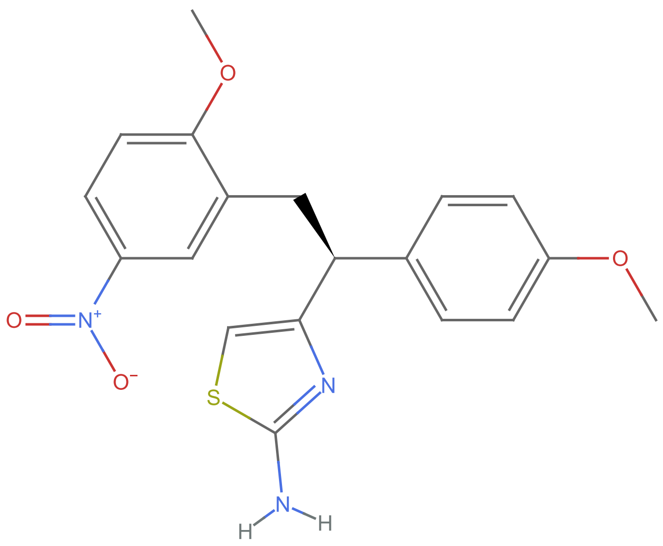

2D Autocorrelation

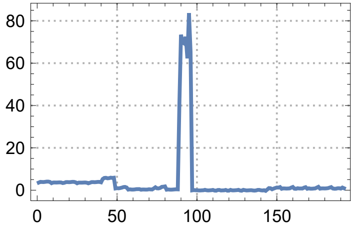

Dataset Index= 1128

Molecule Formula= $C_{19}H_{19}N_3O_4S$

3D Plot

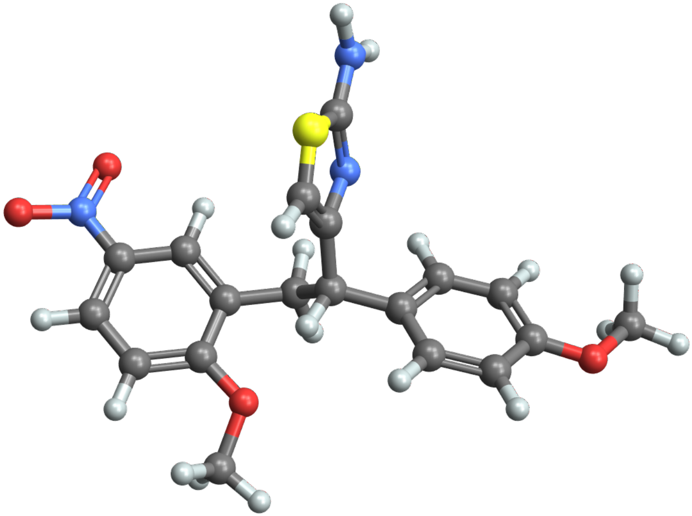

3D Autocorrelation

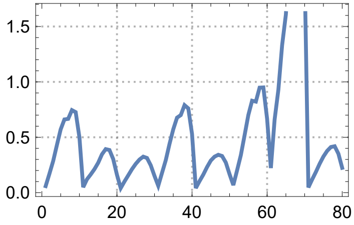

2D Plot

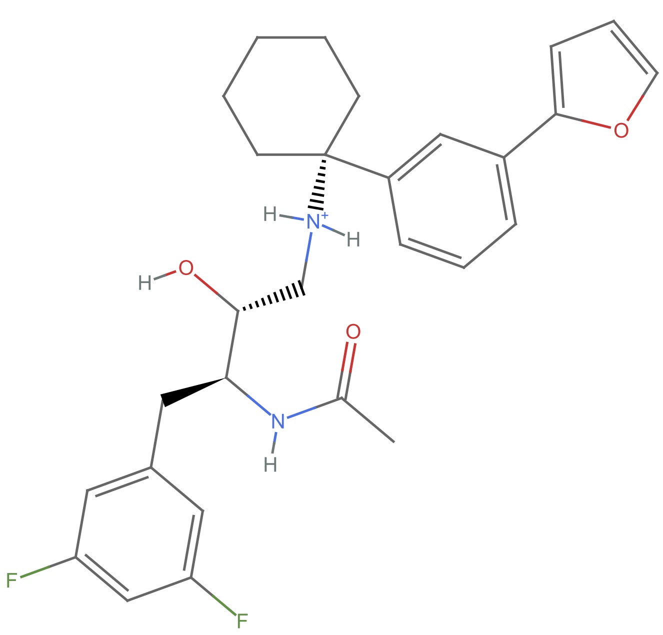

2D Autocorrelation

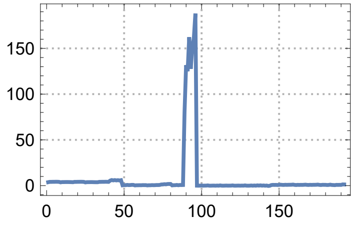

3D Plot

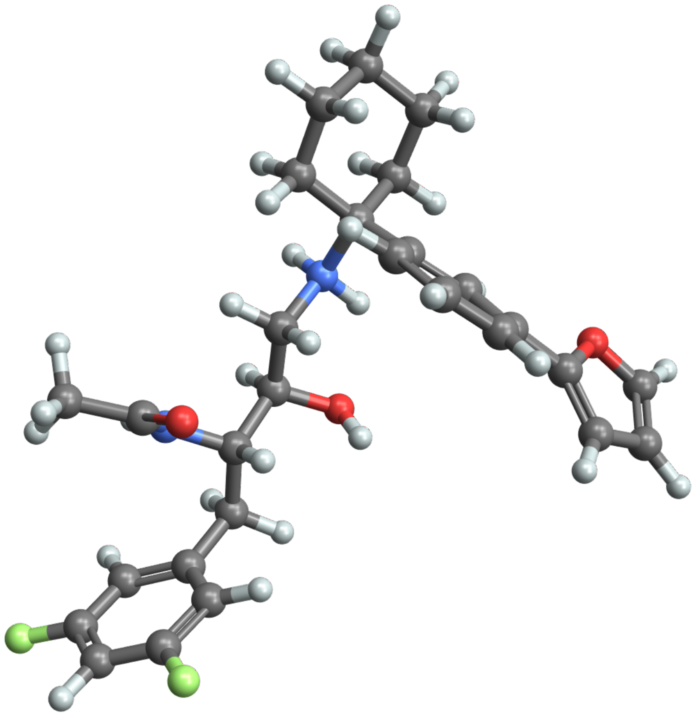

Dataset Index= 1129

Molecule Formula= $C_{28}H_{33}F_2N_2O_3^+$

3D Autocorrelation

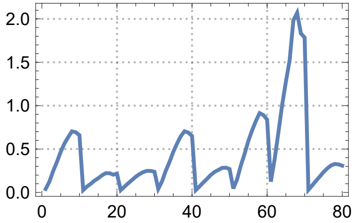

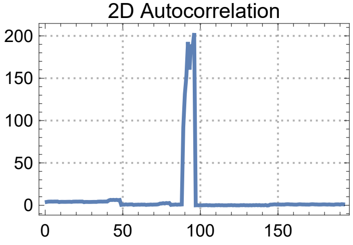

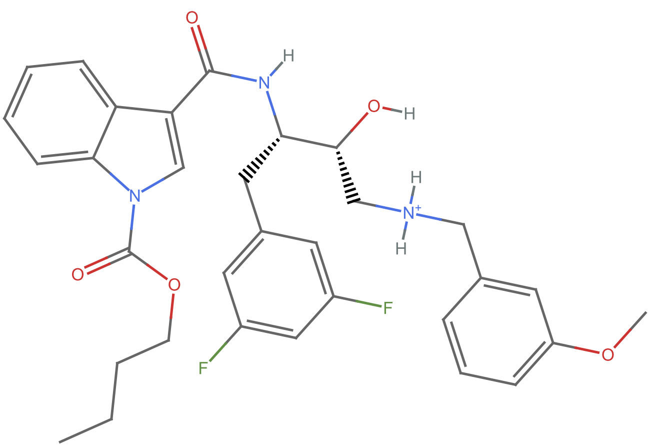

Dataset Index= 1130

Molecule Formula= $C_{32}H_{36}F_2N_3O_5^+$

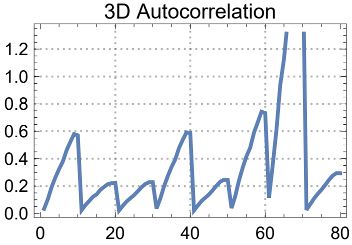

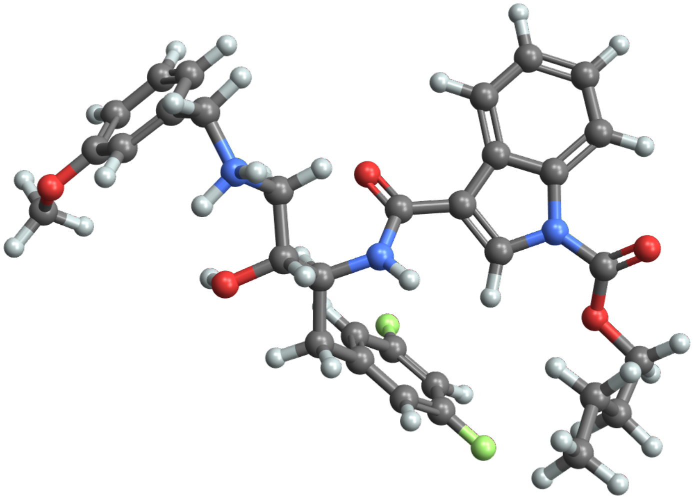

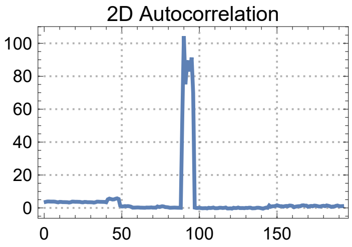

Dataset Index= 1131

Molecule Formula= $C_{22}H_{30}NO_3S^+$

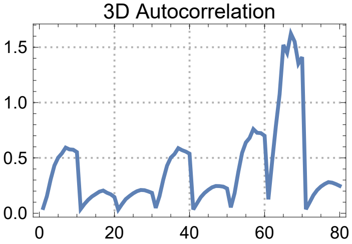

2D Plot

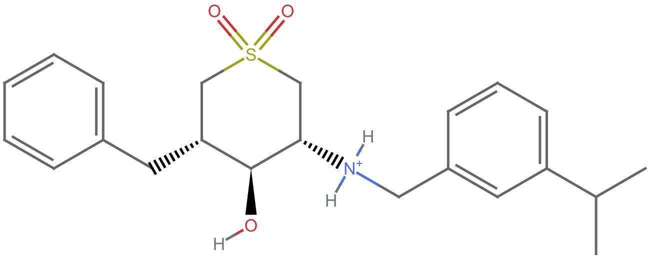

3D Plot

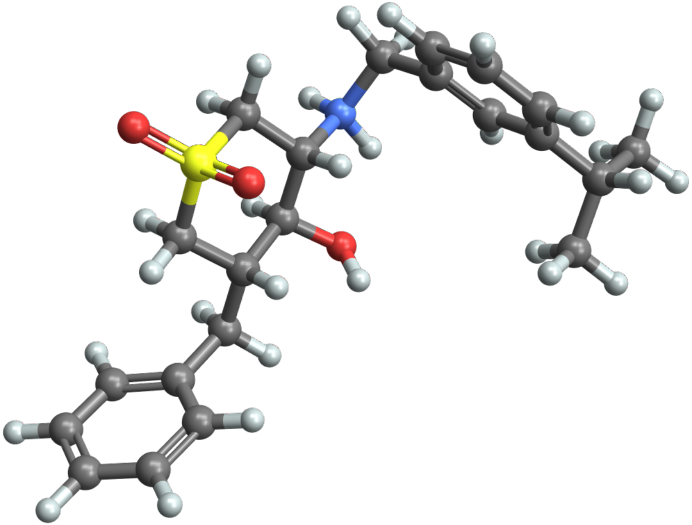

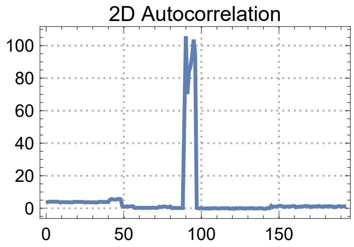

Dataset Index= 1132

Molecule Formula= $C_{26}H_{38}NO_4S^+$

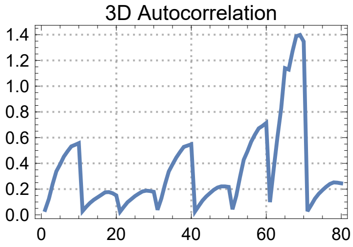

2D Plot

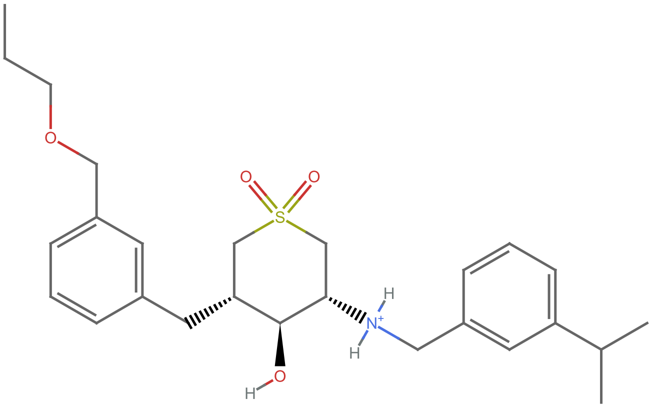

3D Plot

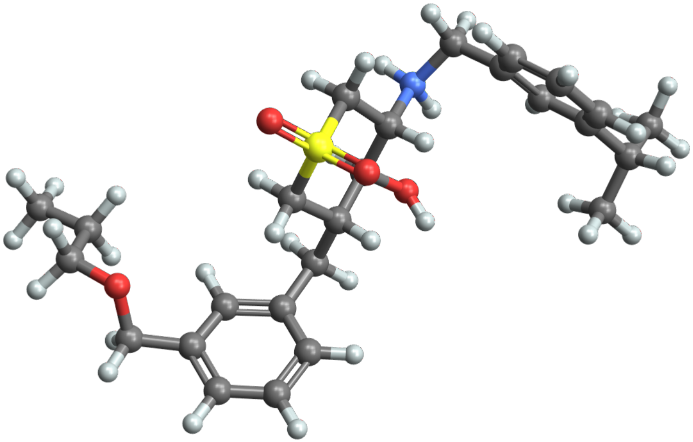

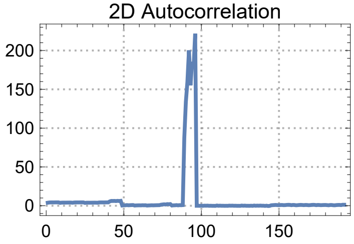

Dataset Index= 1133

Molecule Formula= $C_{32}H_{38}F_2N_3O_3^+$

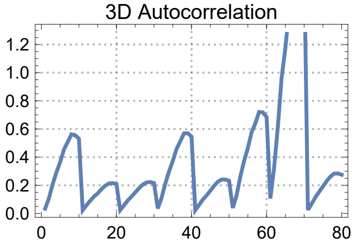

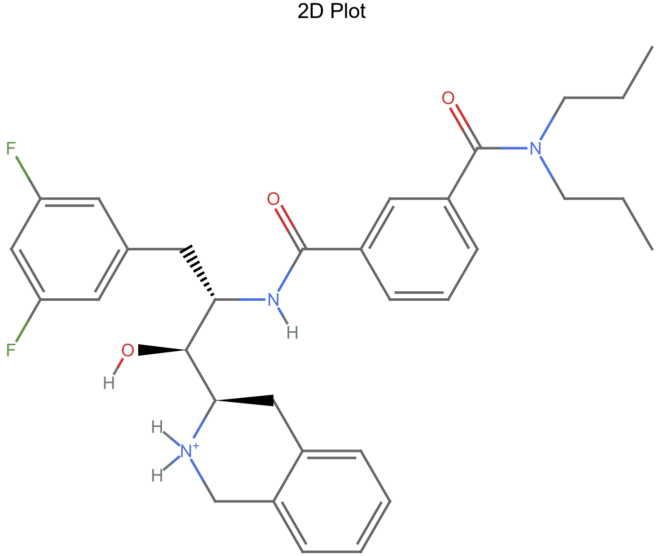

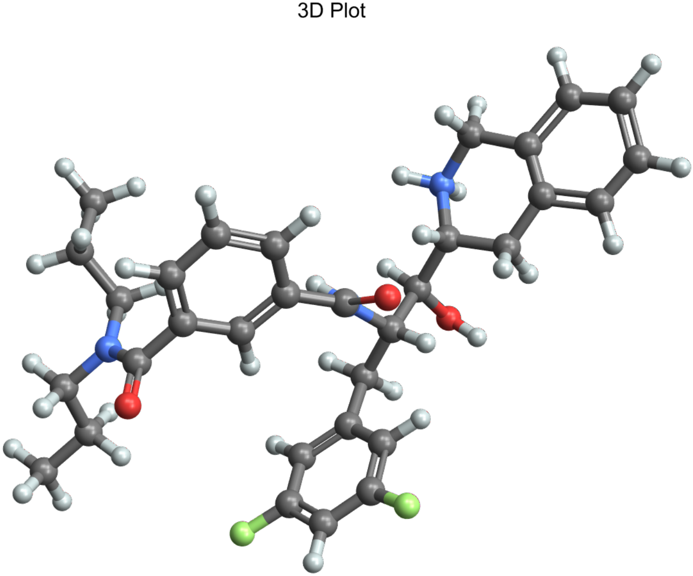

2D Plot

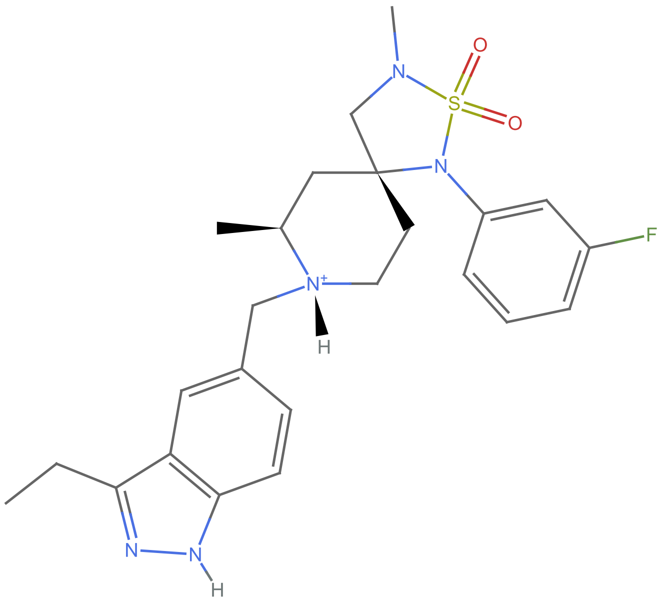

2D Autocorrelation

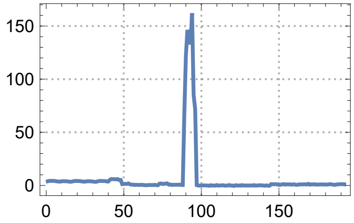

Dataset Index= 1134

Molecule Formula= $C_{24}H_{31}FN_5O_2S^+$

3D Autocorrelation

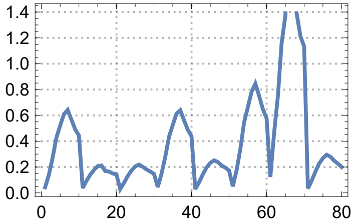

3D Plot

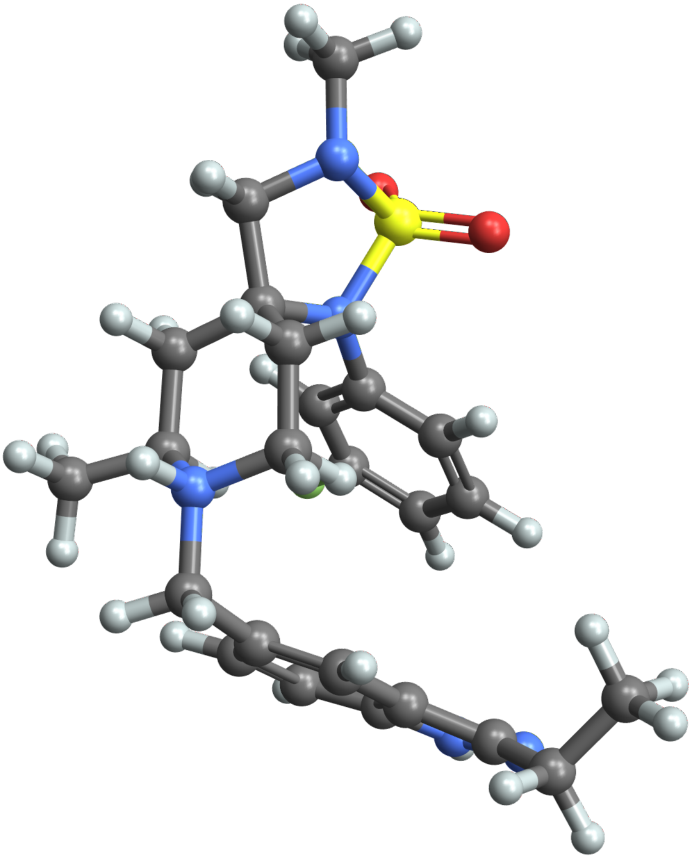

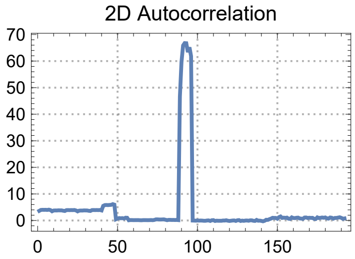

Dataset Index= 1135

Molecule Formula= $C_{25}H_{24}ClN_3O$

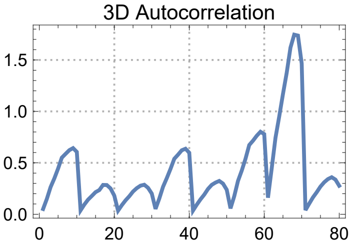

2D Plot

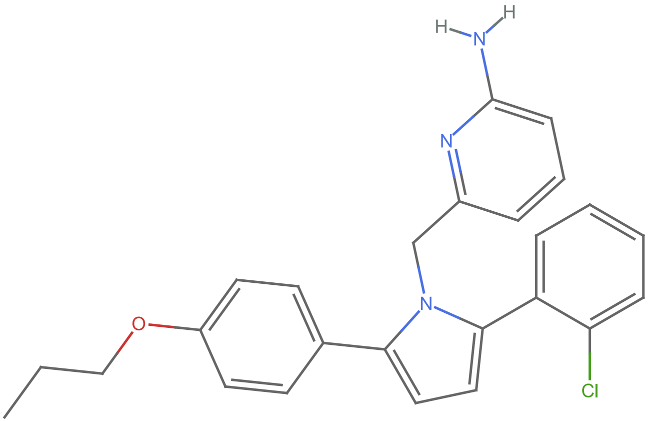

3D Plot

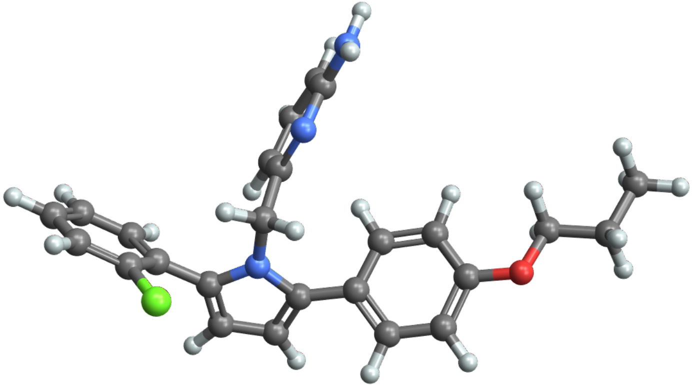

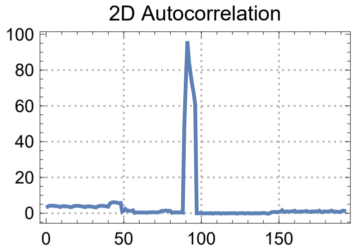

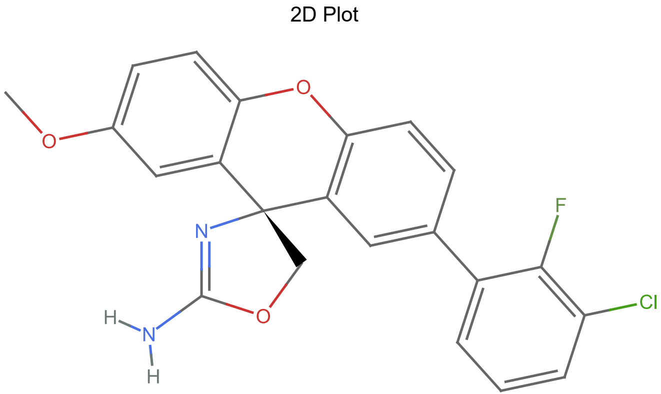

Dataset Index= 1136

Molecule Formula= $C_{22}H_{16}ClFN_2O_3$

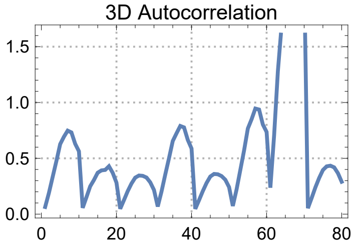

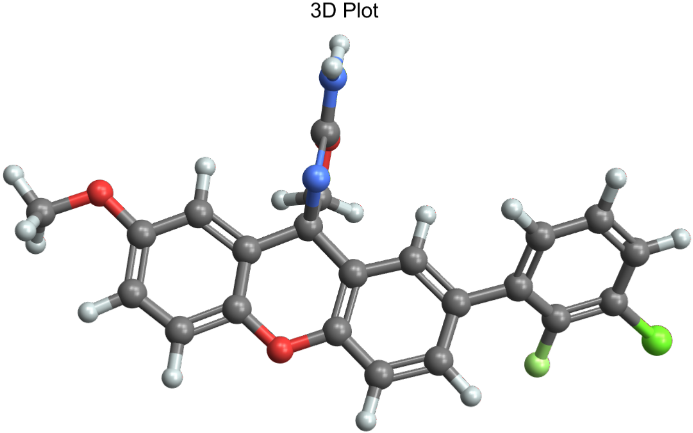

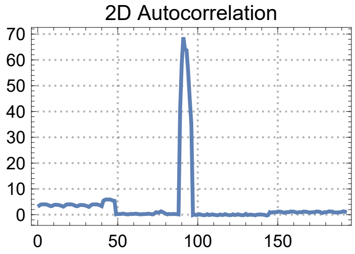

Dataset Index= 1137

Molecule Formula= $C_{22}H_{18}N_2O_3$

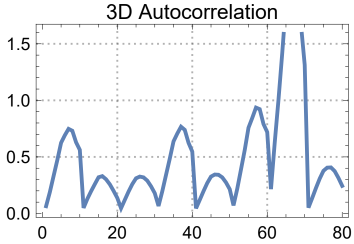

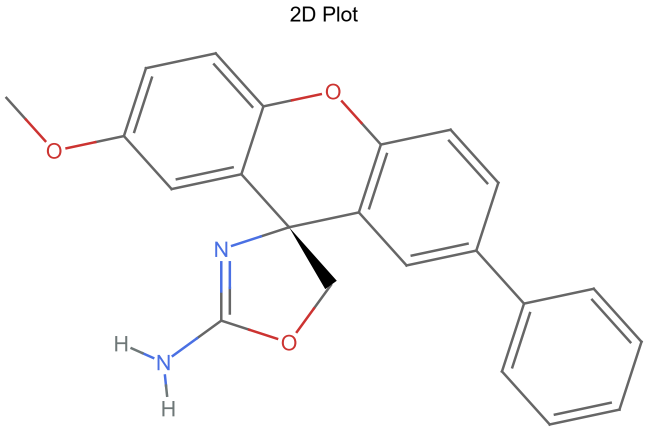

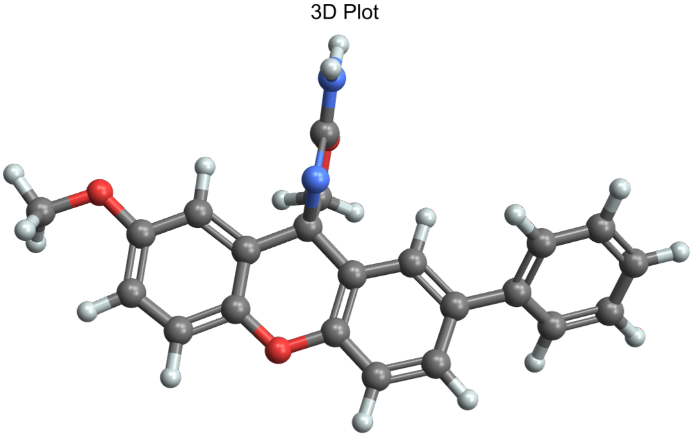

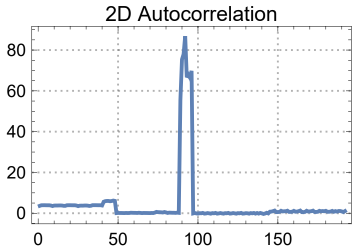

Dataset Index= 1138

Molecule Formula= $C_{26}H_{24}N_4O$

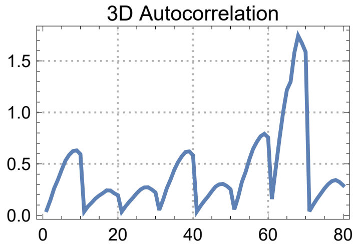

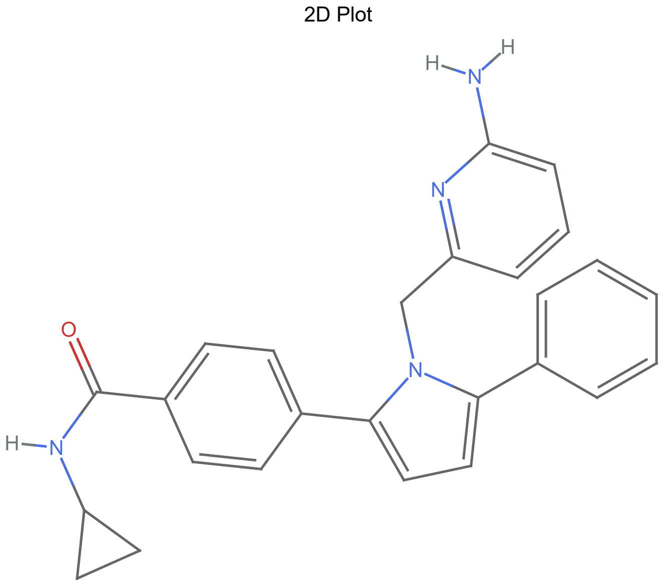

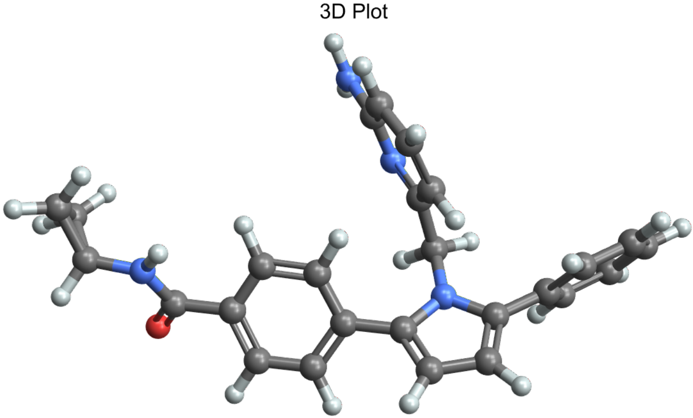

2D Plot

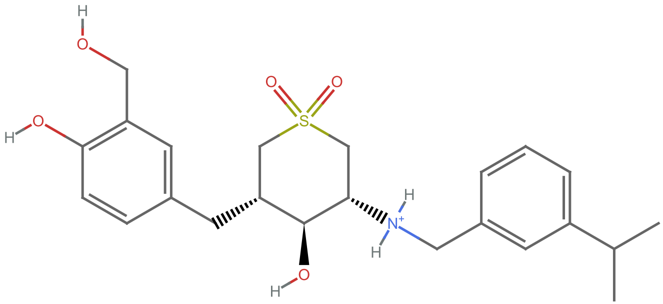

3D Plot

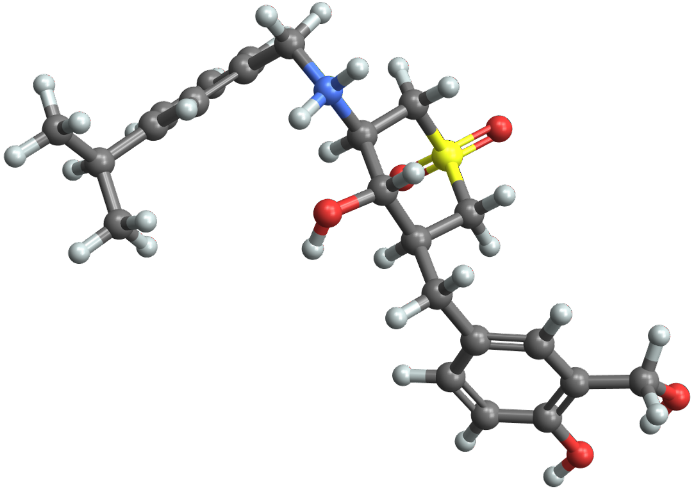

2D Autocorrelation

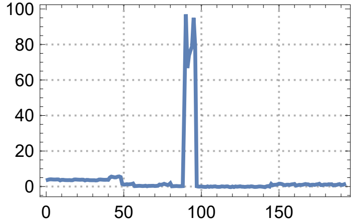

Dataset Index= 1139

Molecule Formula= $C_{23}H_{32}NO_5S^+$

3D Autocorrelation

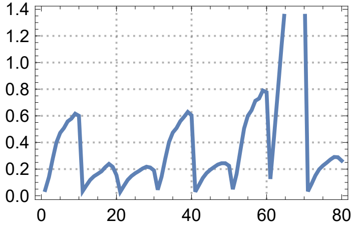

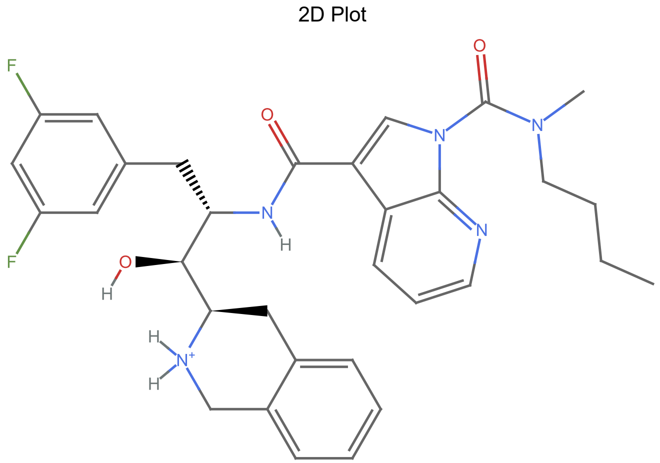

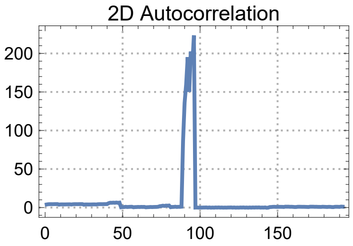

Dataset Index= 1140

Molecule Formula= $C_{32}H_{36}F_2N_5O_3^+$

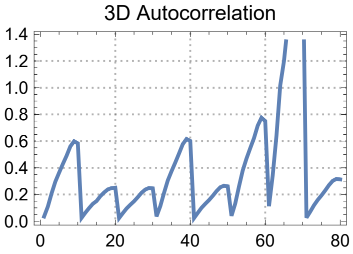

3D Plot

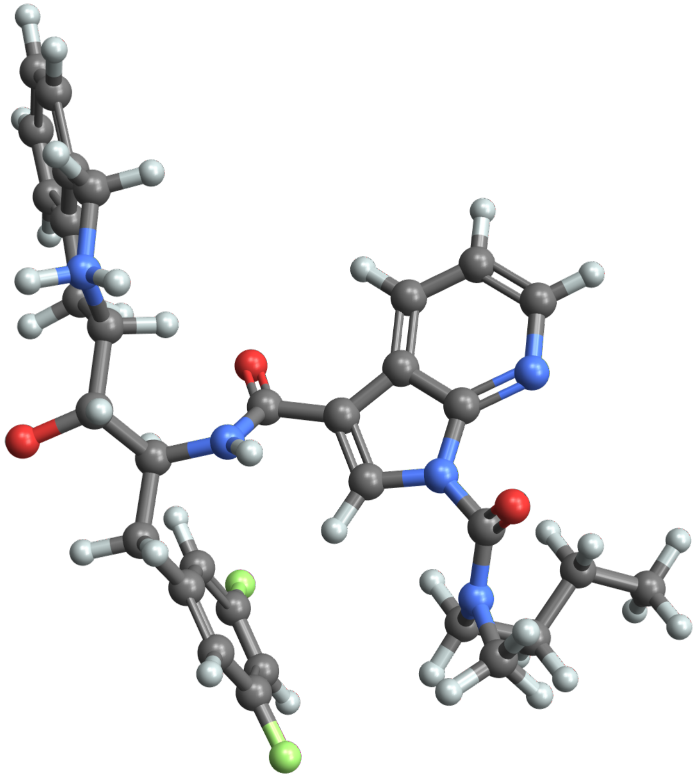

2D Plot

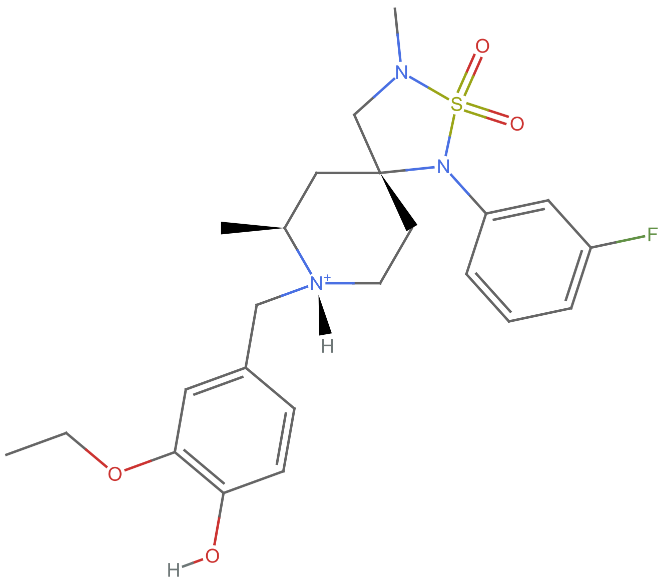

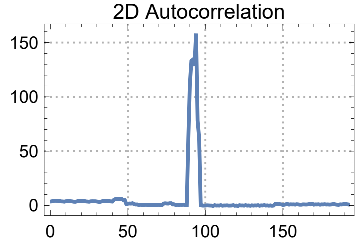

Dataset Index= 1141

Molecule Formula= $C_{23}H_{31}FN_3O_4S^+$

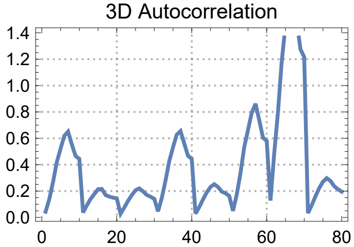

3D Plot

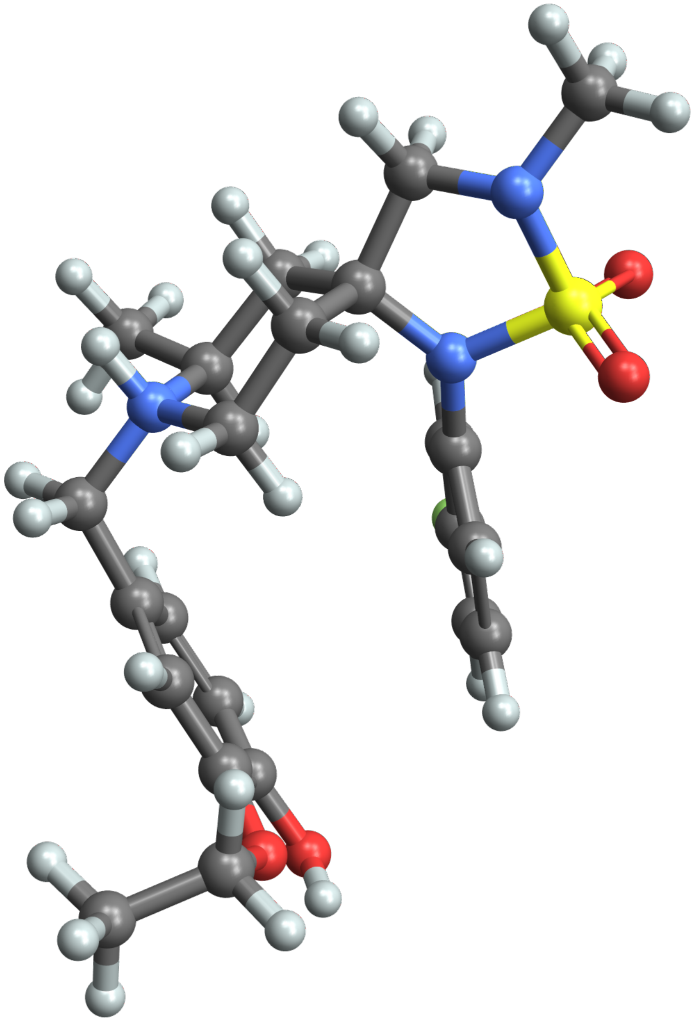

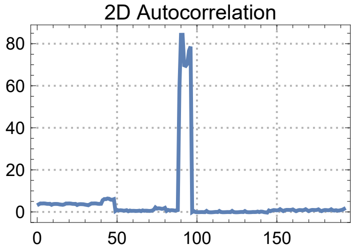

Dataset Index= 1142

Molecule Formula= $C_{21}H_{16}F_3N_3O_2$

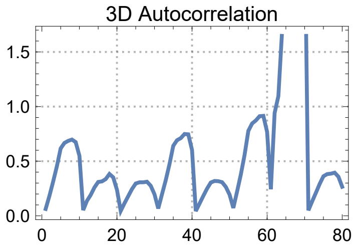

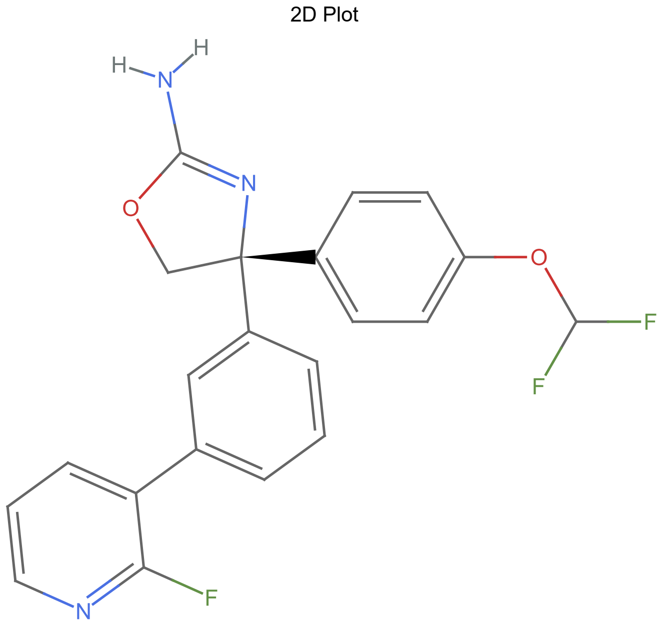

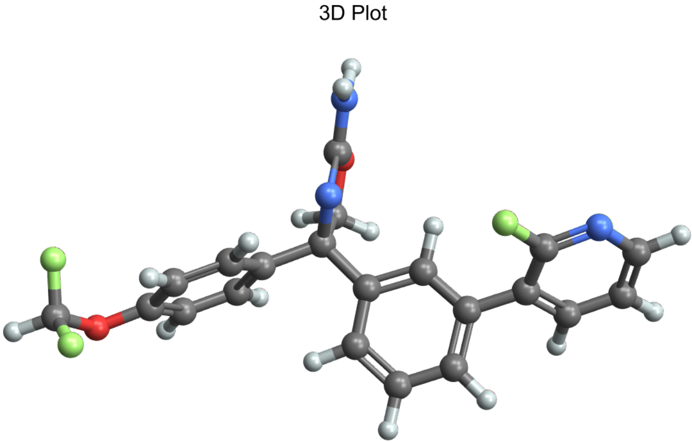

2D Plot

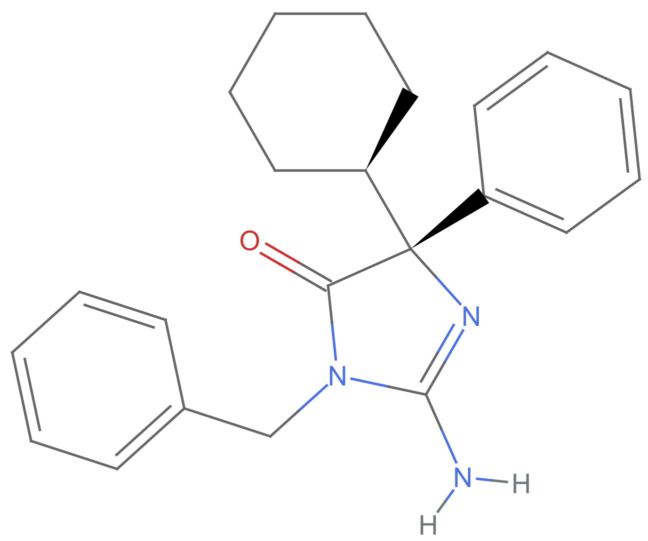

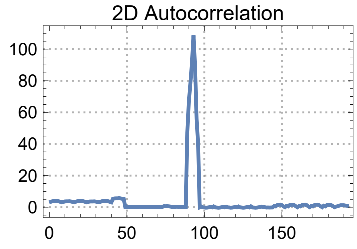

3D Plot

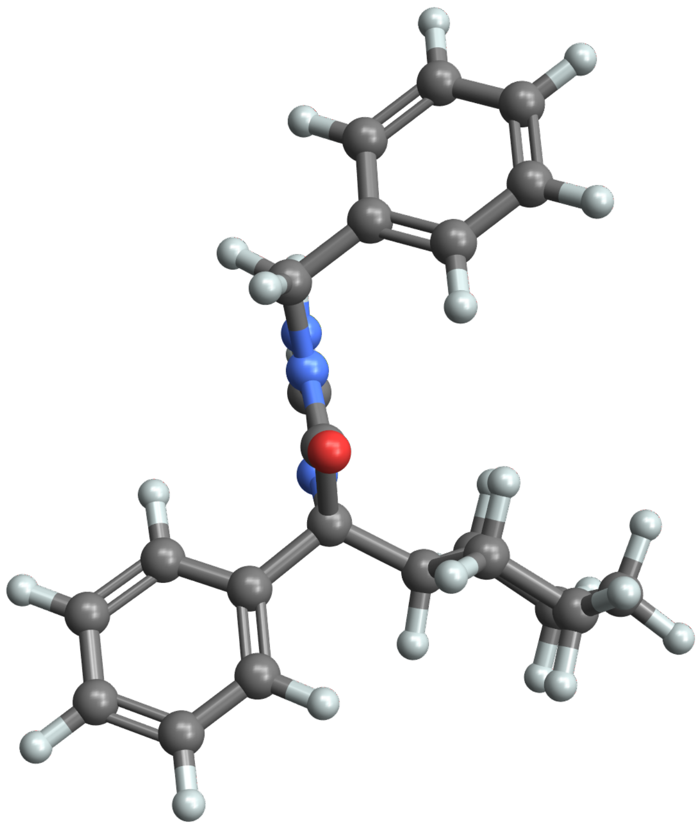

Dataset Index= 1143

Molecule Formula= $C_{22}H_{25}N_3O$

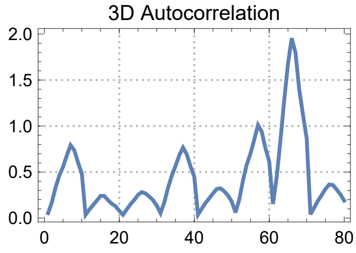

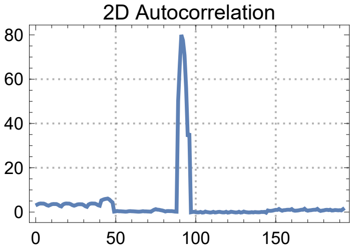

Dataset Index= 1144

Molecule Formula= $C_{18}H_{18}FN_3O_2$

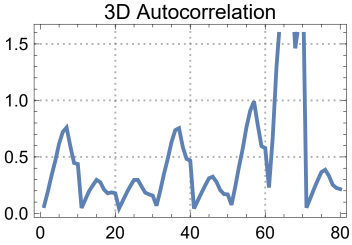

2D Plot

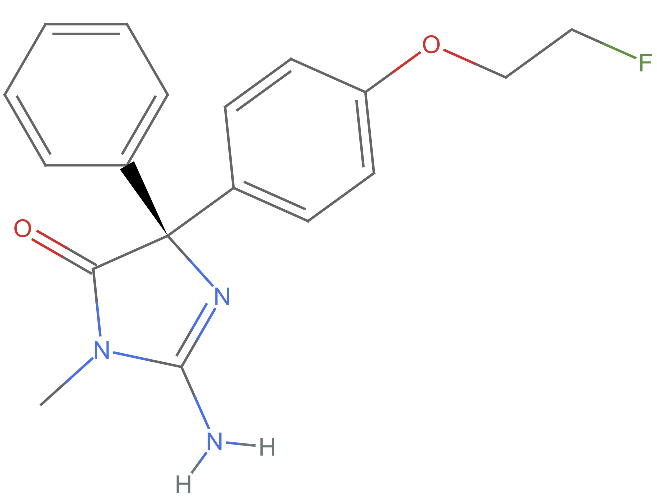

3D Plot

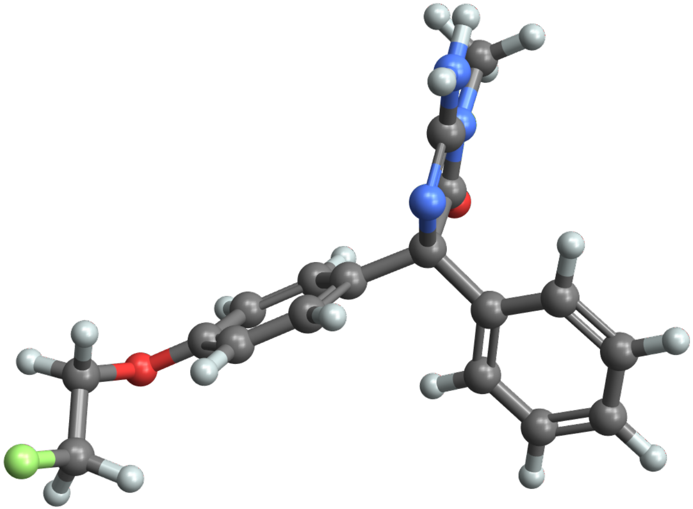

2D Autocorrelation

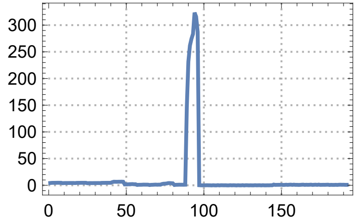

Dataset Index= 1145

Molecule Formula= $C_{37}H_{47}F_2N_5O_8S$

3D Autocorrelation

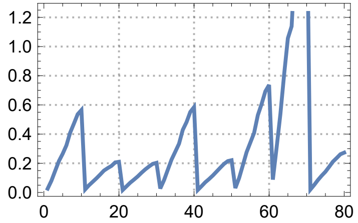

2D Plot

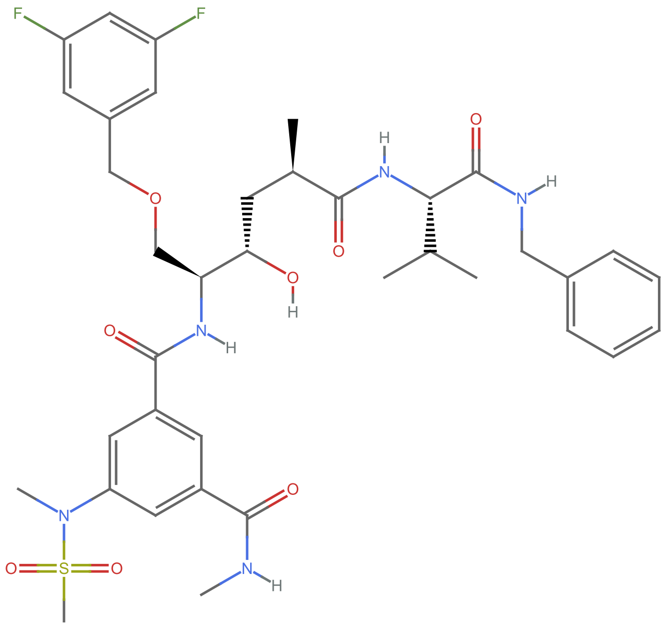

3D Plot

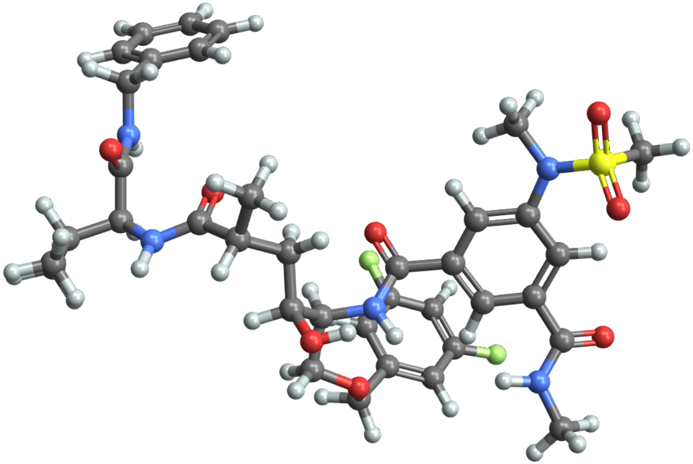

2D Plot

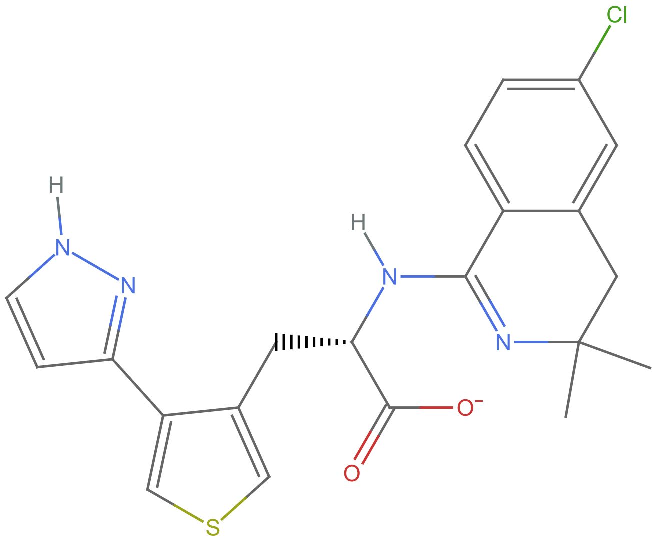

3D Plot

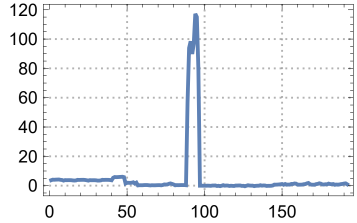

Dataset Index= 1146

Molecule Formula= $C_{21}H_{20}ClN_4O_2S^-$

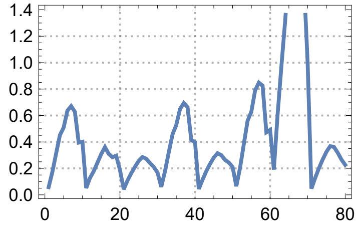

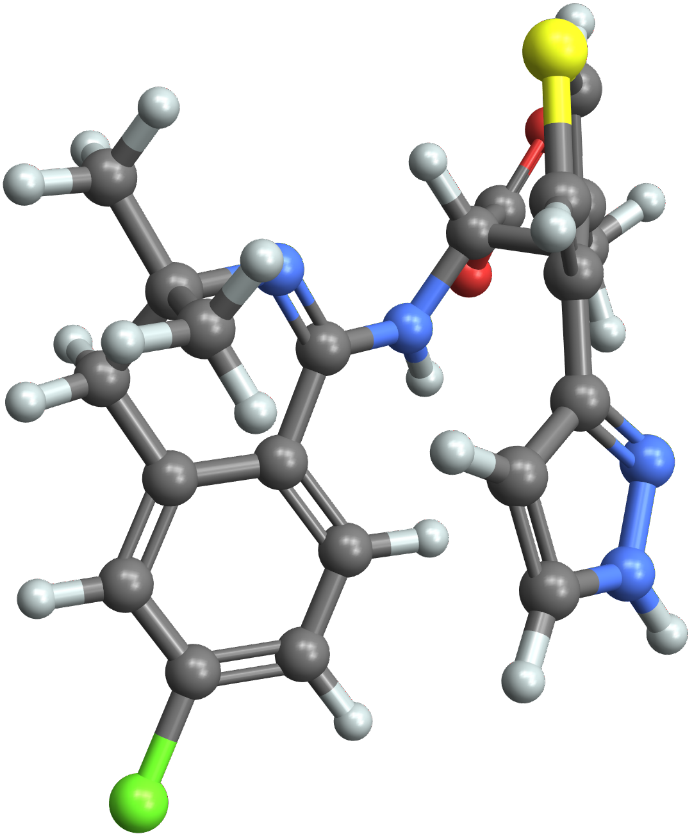

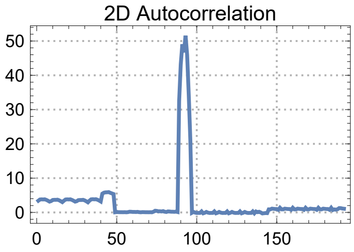

Dataset Index= 1147

Molecule Formula= $C_{21}H_{18}N_4$

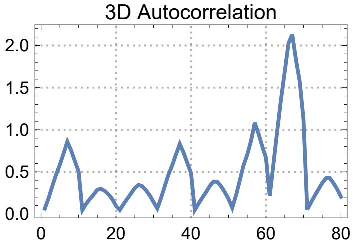

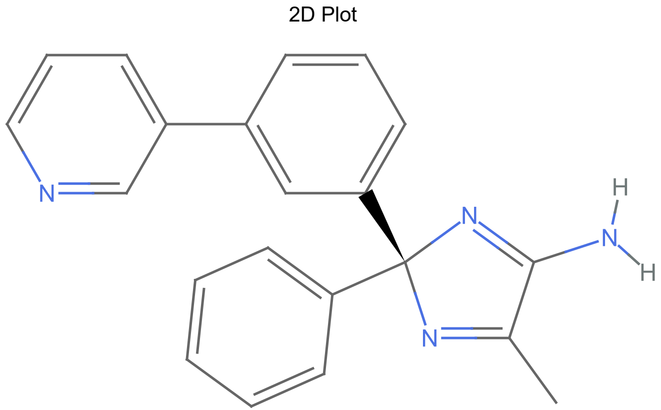

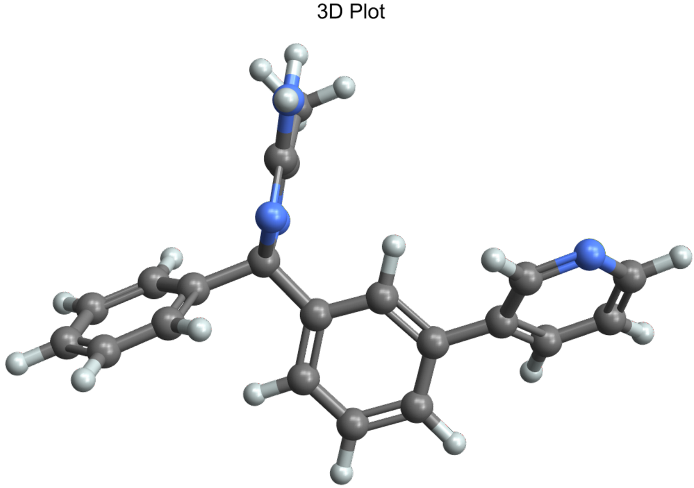

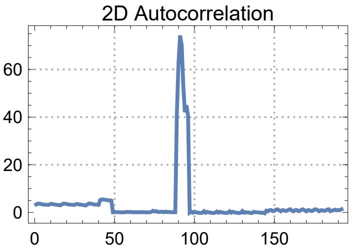

Dataset Index= 1148

Molecule Formula= $C_{20}H_{23}N_3O$

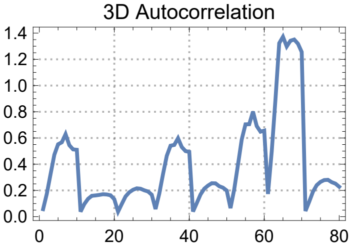

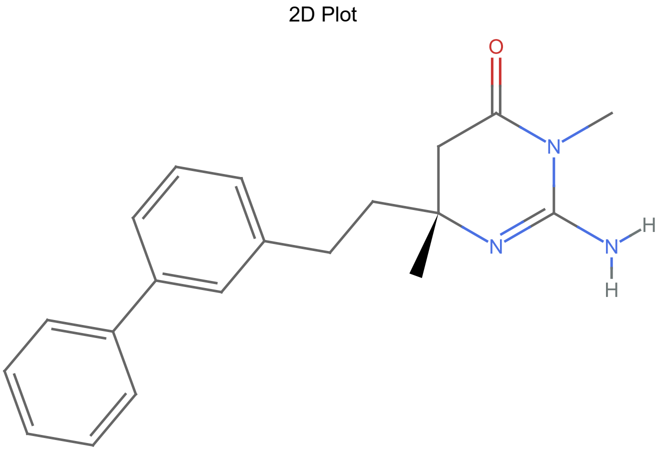

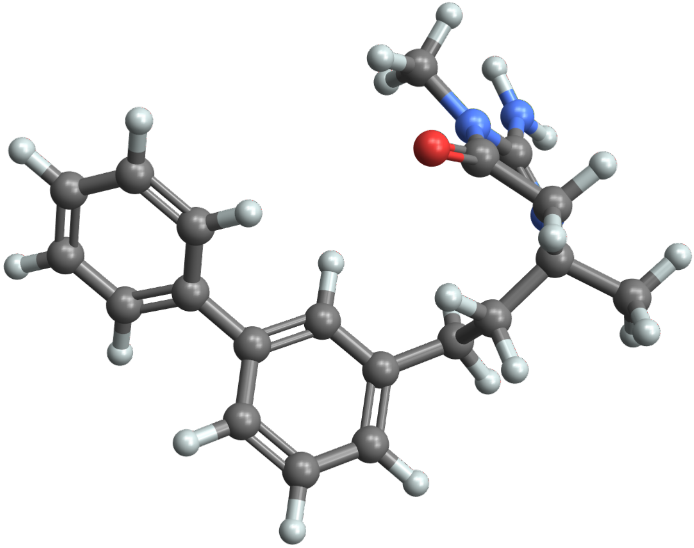

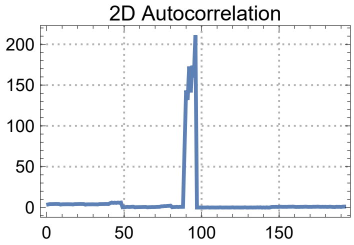

Dataset Index= 1149

Molecule Formula= $C_{29}H_{40}F_2N_3O_3^+$

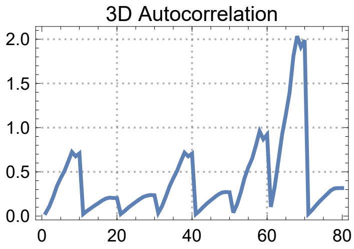

2D Plot

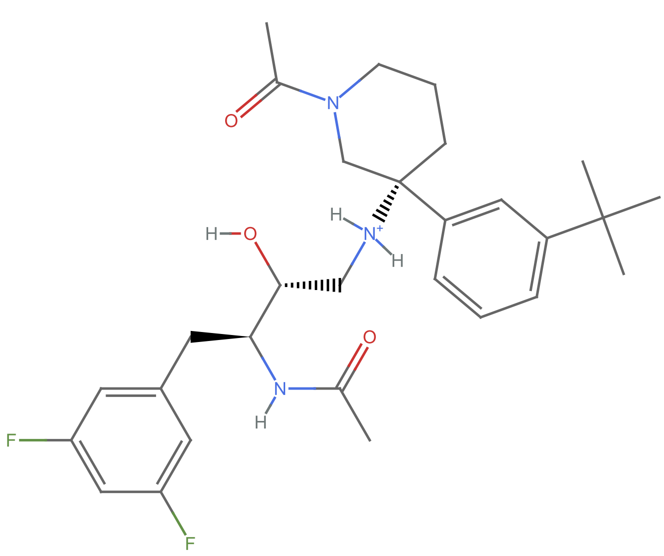

3D Plot

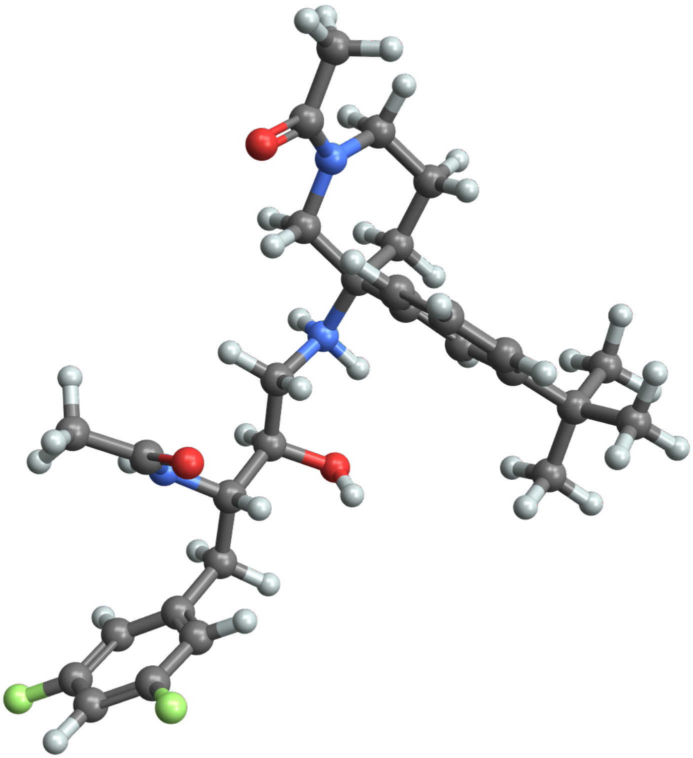

2D Plot

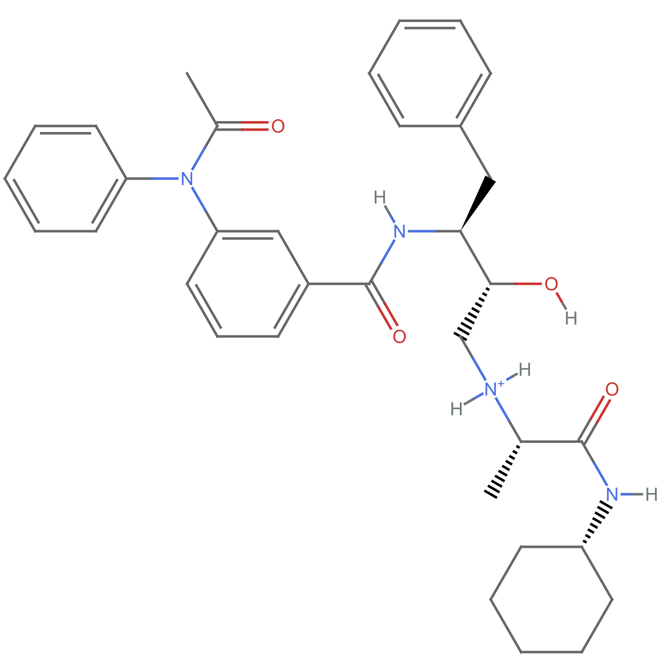

2D Autocorrelation

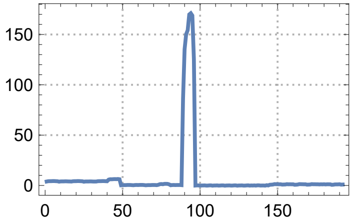

Dataset Index= 1150

Molecule Formula= $C_{34}H_{43}N_4O_4^+$

3D Autocorrelation

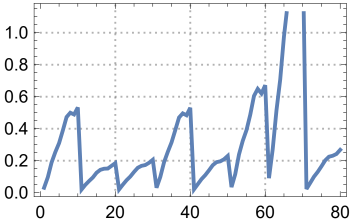

3D Plot

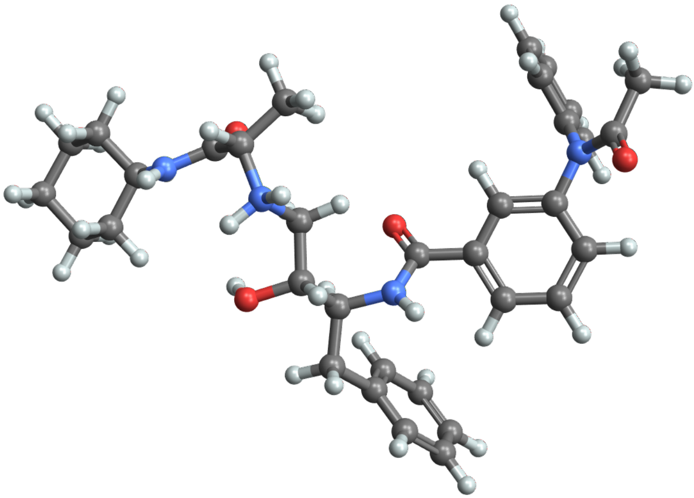

2D Plot

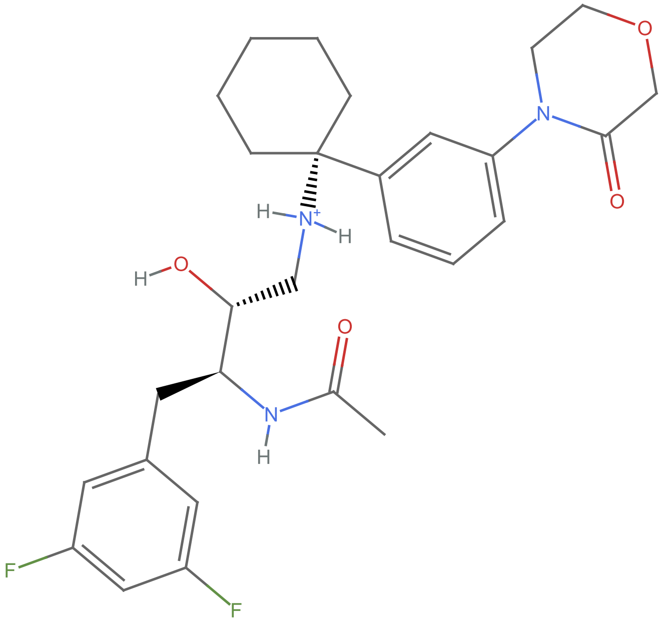

2D Autocorrelation

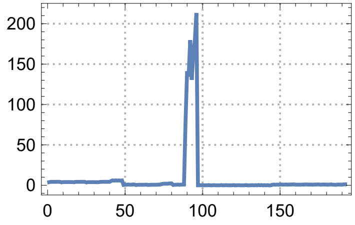

Dataset Index= 1151

Molecule Formula= $C_{28}H_{36}F_2N_3O_4^+$

3D Autocorrelation

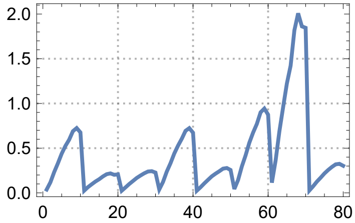

3D Plot

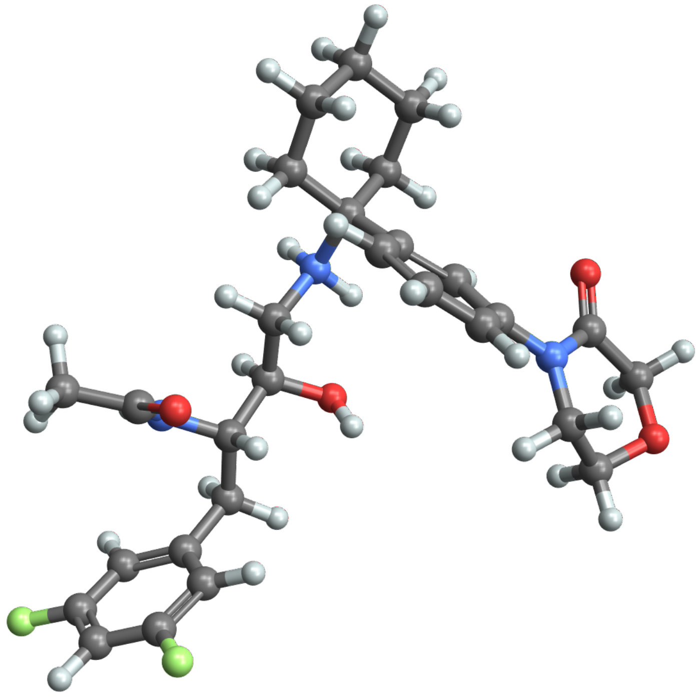

2D Plot

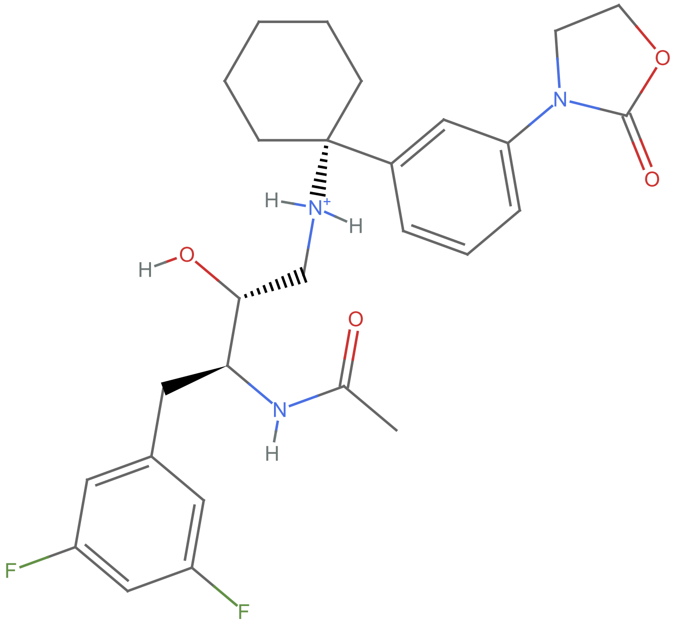

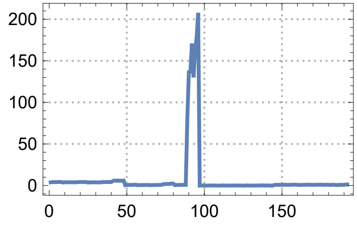

3D Plot

Dataset Index= 1152

Molecule Formula= $C_{27}H_{34}F_2N_3O_4^+$

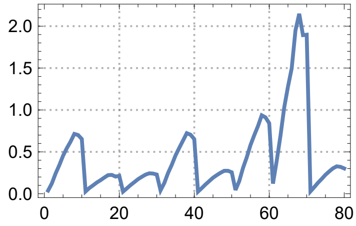

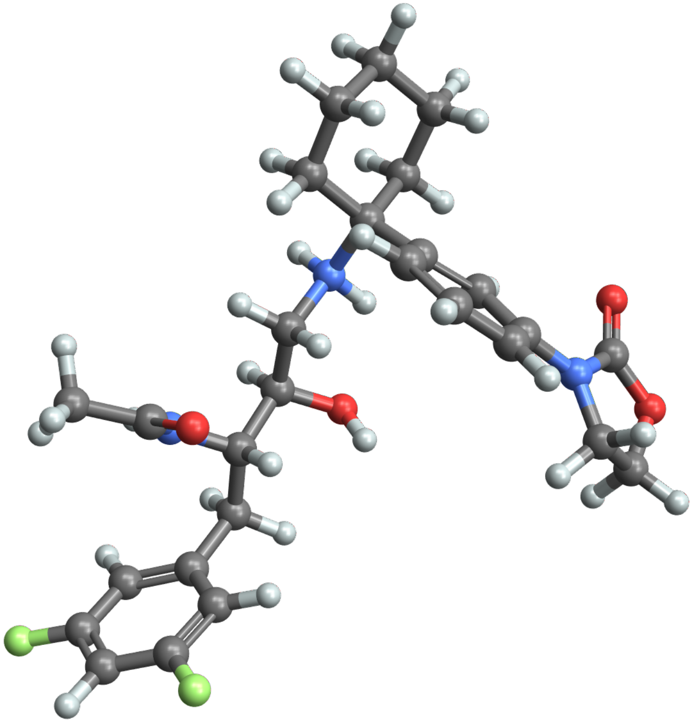

2D Plot

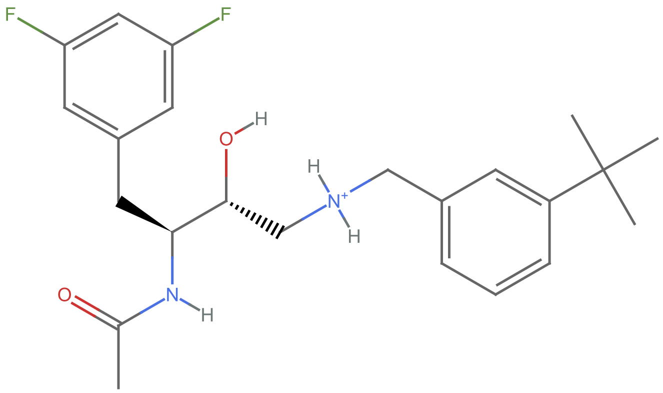

2D Autocorrelation

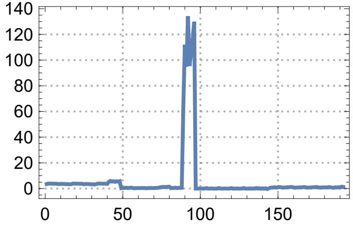

3D Plot

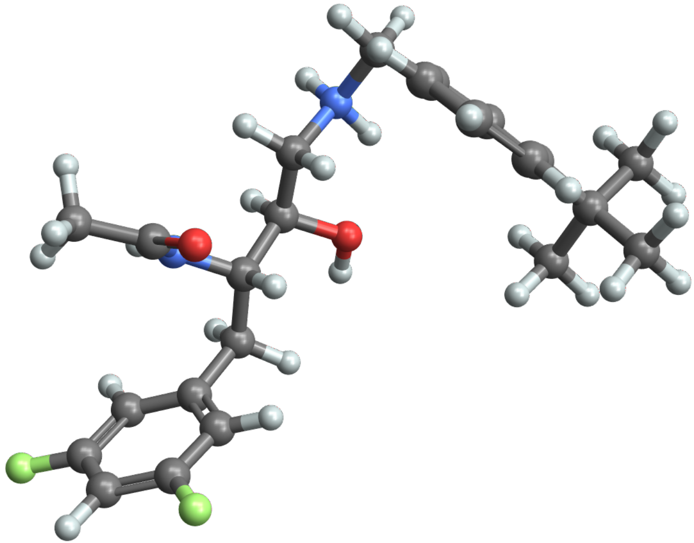

Dataset Index= 1153

Molecule Formula= $C_{23}H_{31}F_2N_2O_2^+$

3D Autocorrelation

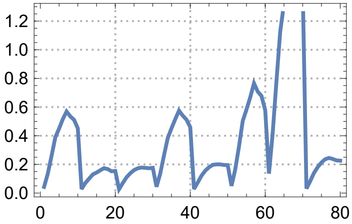

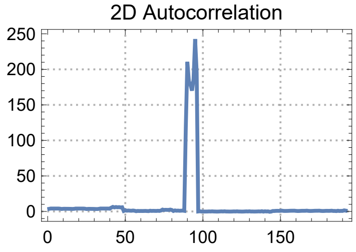

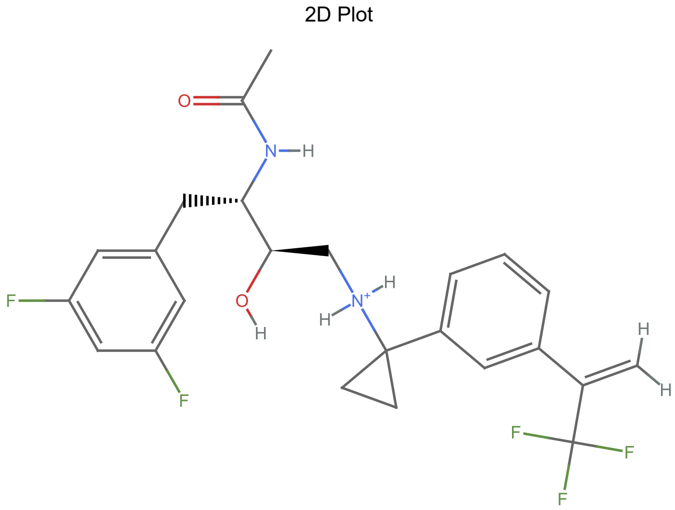

Dataset Index= 1154

Molecule Formula= $C_{24}H_{26}F_5N_2O_2^+$

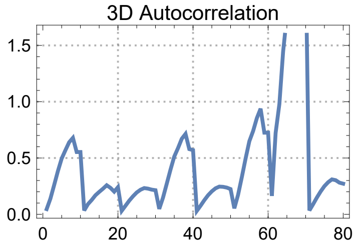

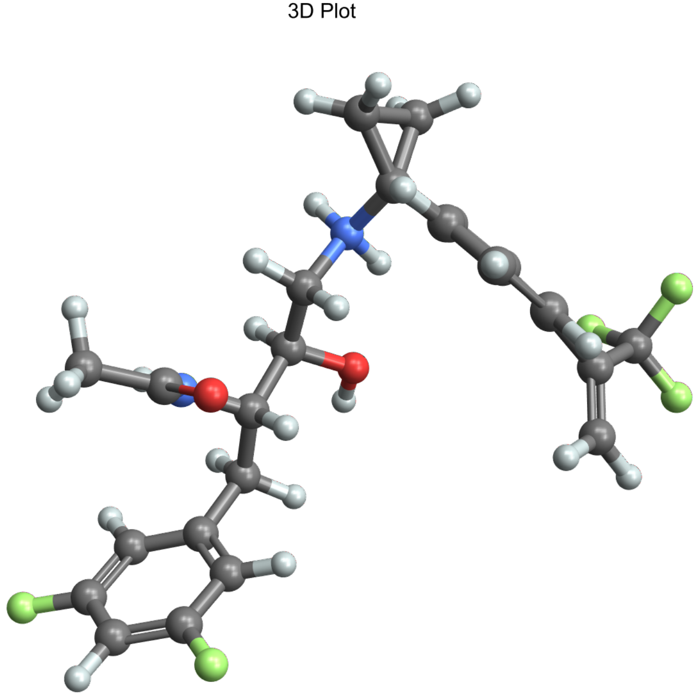

2D Plot

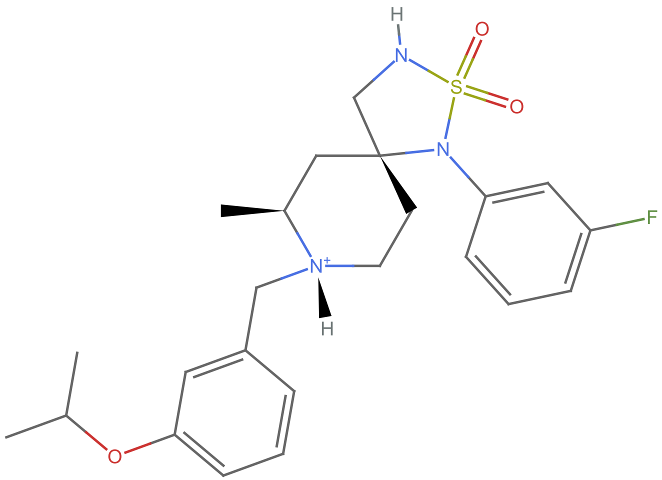

2D Autocorrelation

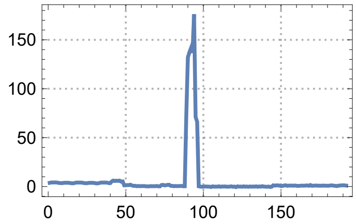

3D Plot

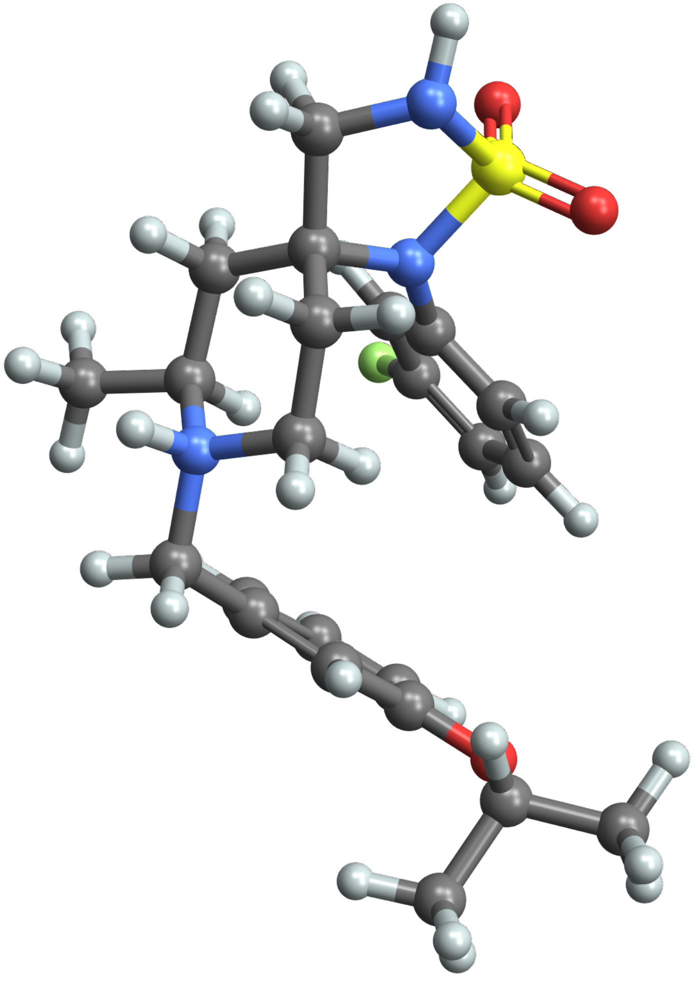

Dataset Index= 1155

Molecule Formula= $C_{23}H_{31}FN_3O_3S^+$

3D Autocorrelation

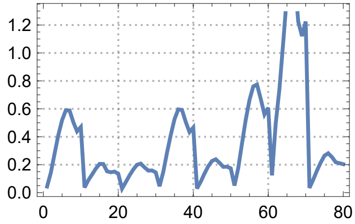

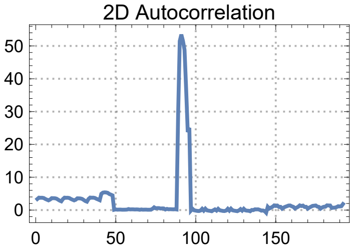

Dataset Index= 1156

Molecule Formula= $C_{18}H_{20}N_4O$

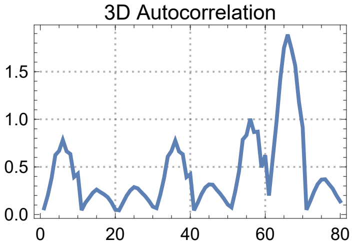

2D Plot

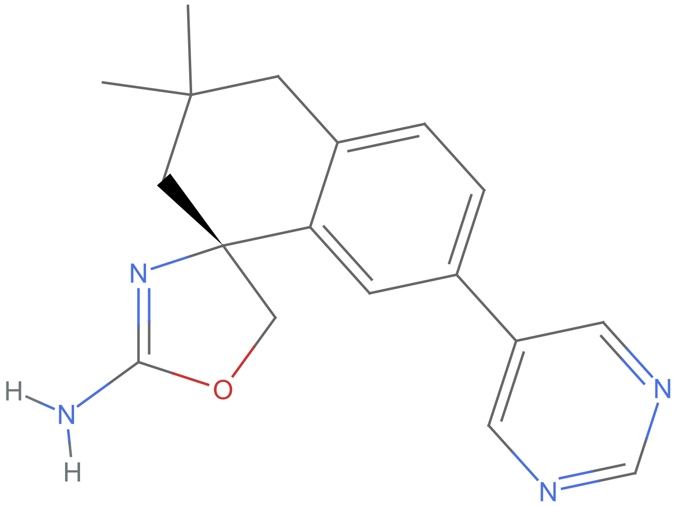

3D Plot

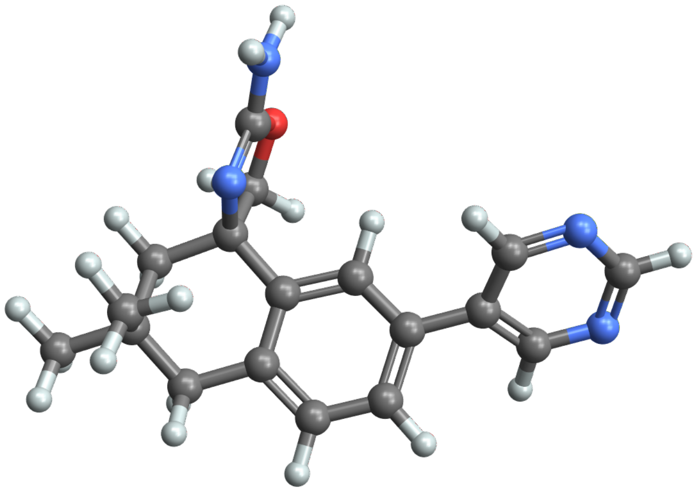

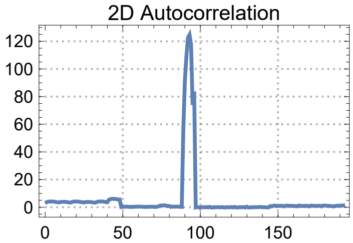

Dataset Index= 1157

Molecule Formula= $C_{24}H_{28}N_4O_3$

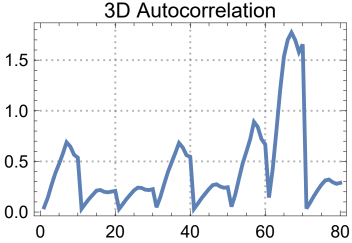

2D Plot

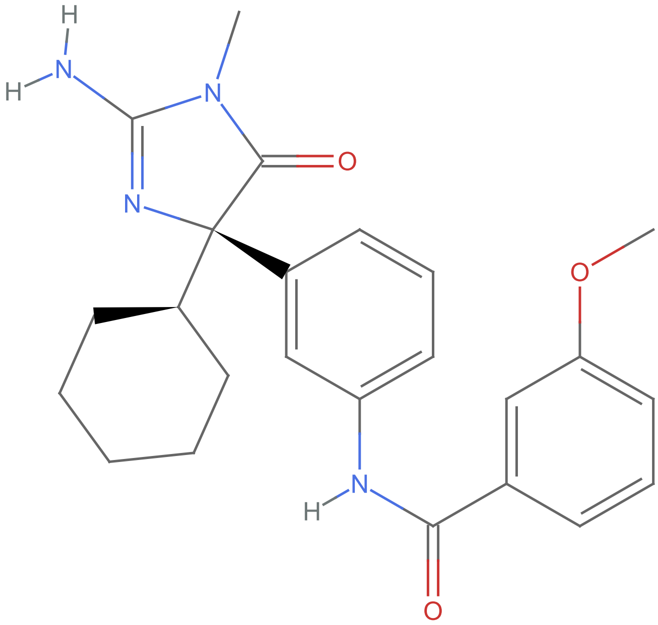

3D Plot

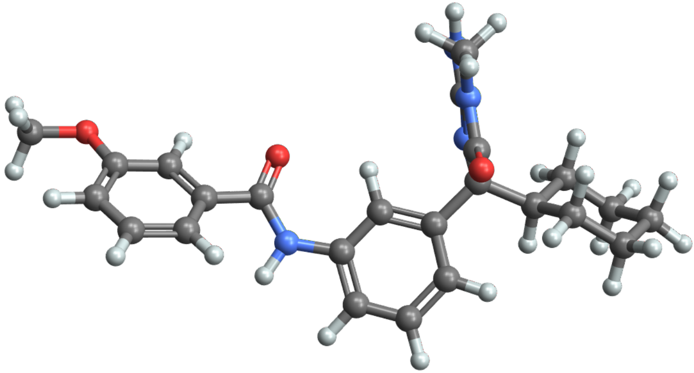

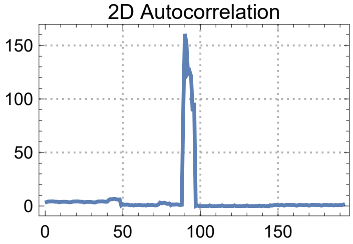

Dataset Index= 1158

Molecule Formula= $C_{21}H_{15}F_4N_5O_2$

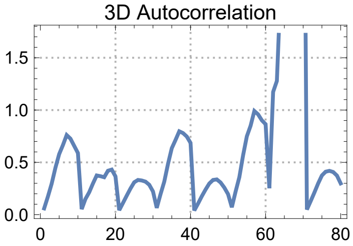

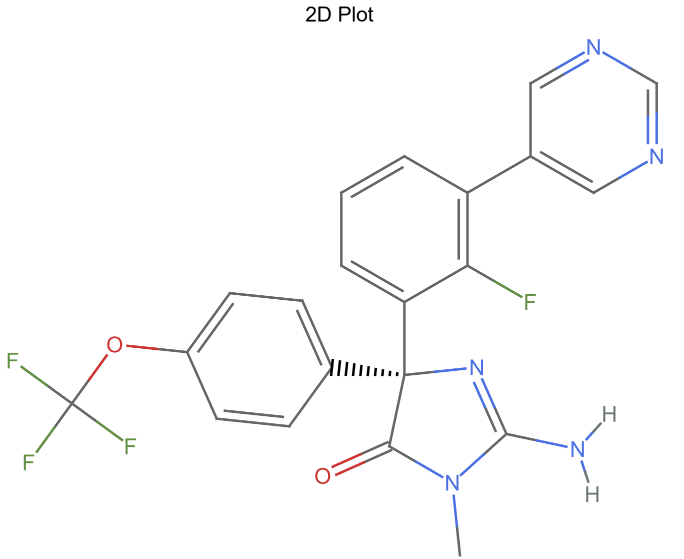

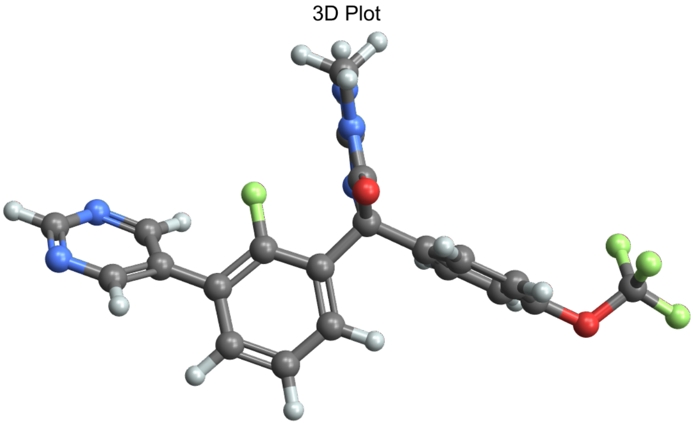

2D Plot

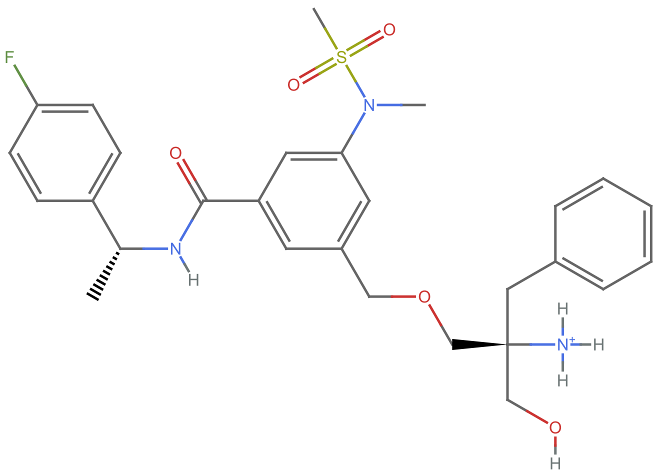

2D Autocorrelation

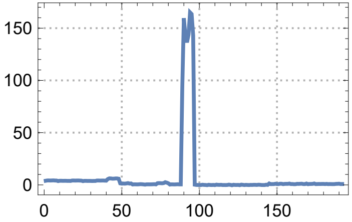

Dataset Index= 1159

Molecule Formula= $C_{28}H_{35}FN_3O_5S^+$

3D Plot

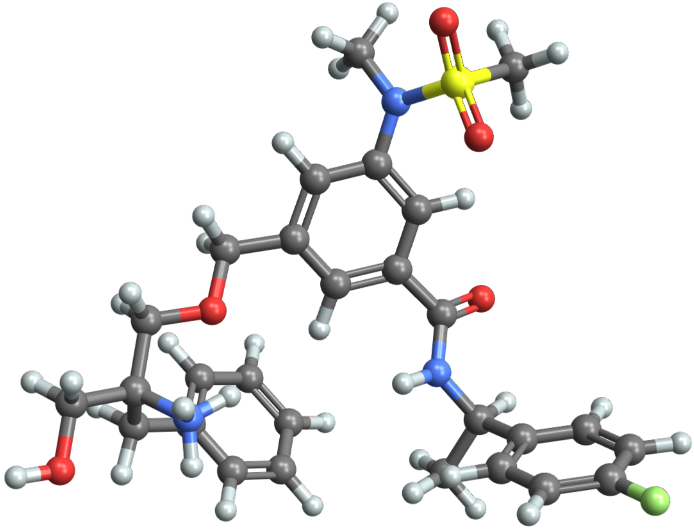

3D Autocorrelation

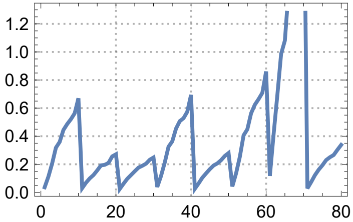

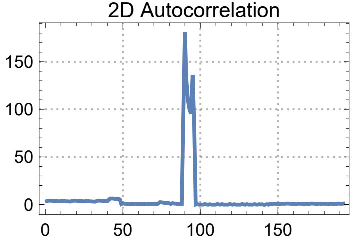

Dataset Index= 1160

Molecule Formula= $C_{19}H_{18}F_3N_5O_2$

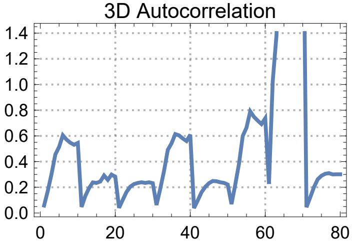

2D Plot

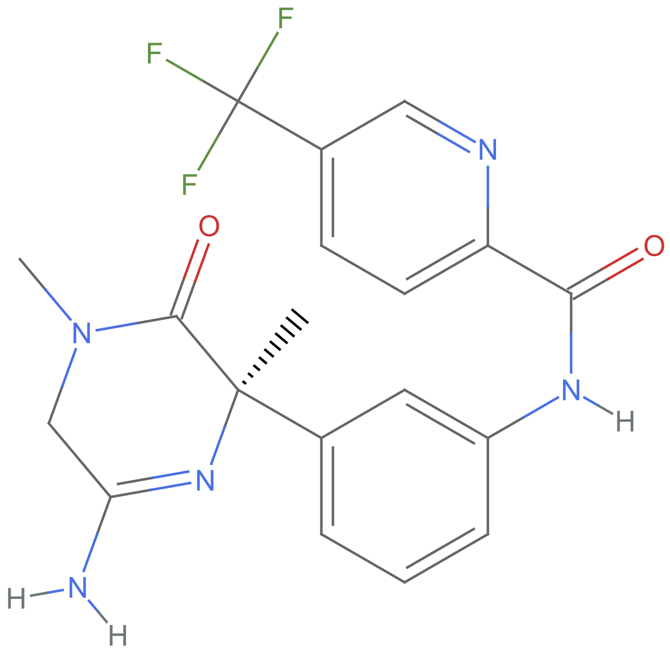

3D Plot

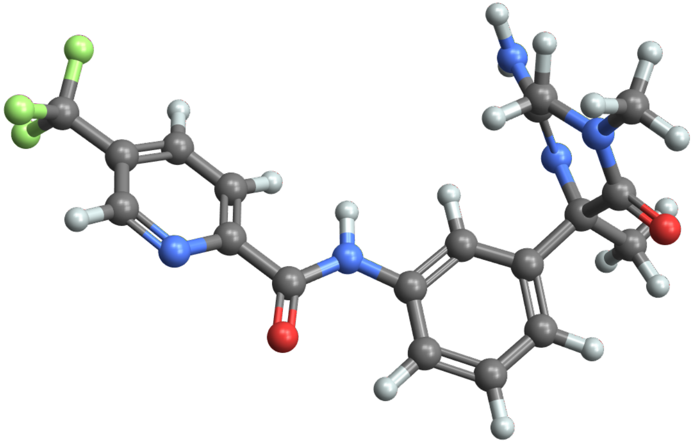

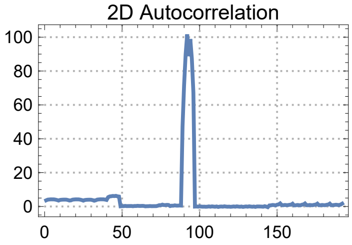

Dataset Index= 1161

Molecule Formula= $C_{25}H_{20}FN_5$

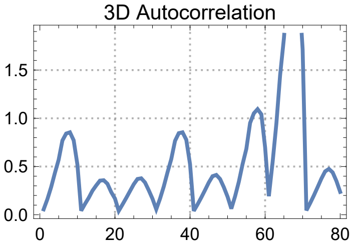

2D Plot

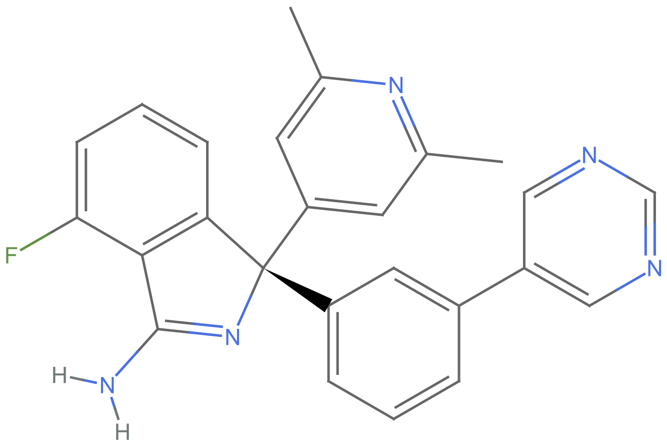

3D Plot

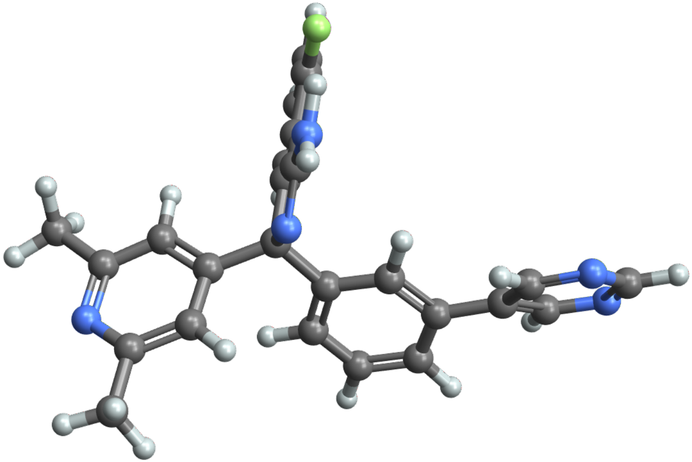

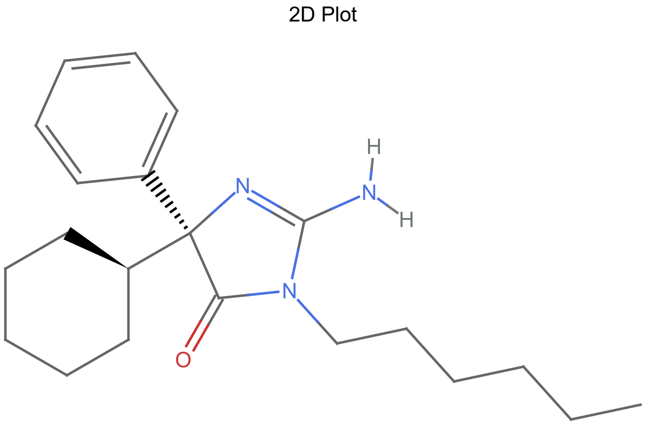

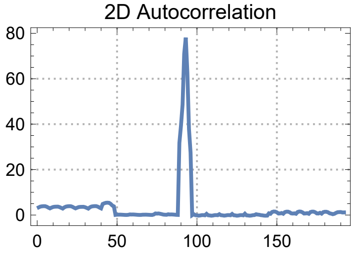

Dataset Index= 1162

Molecule Formula= $C_{21}H_{31}N_3O$

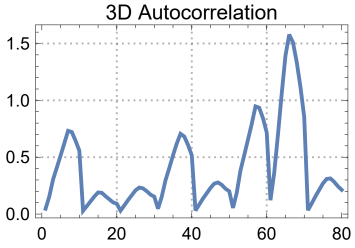

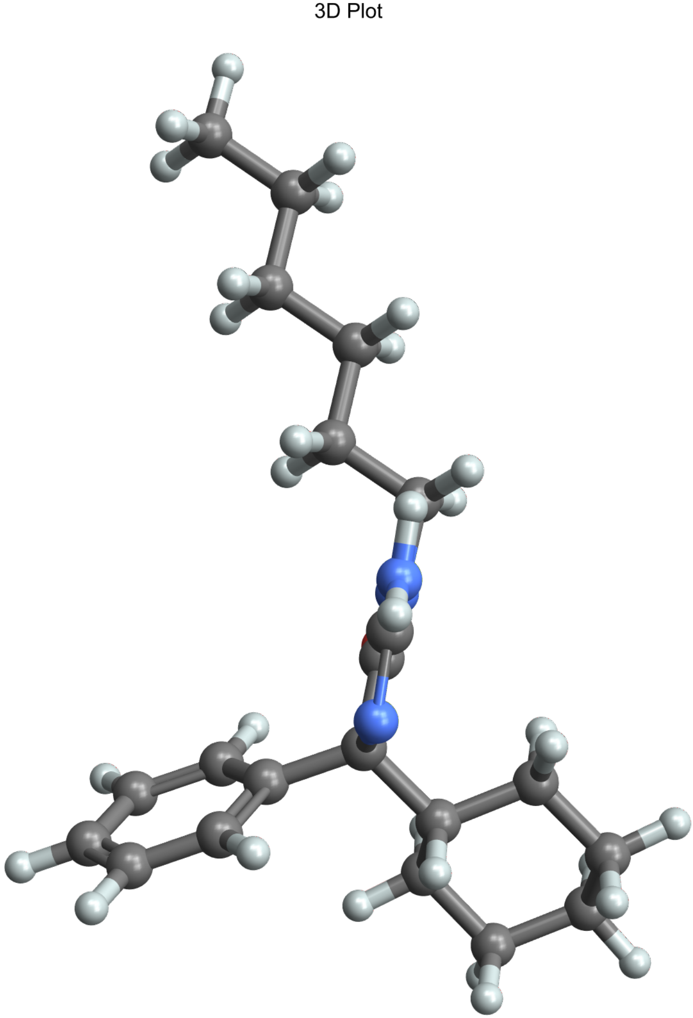

2D Plot

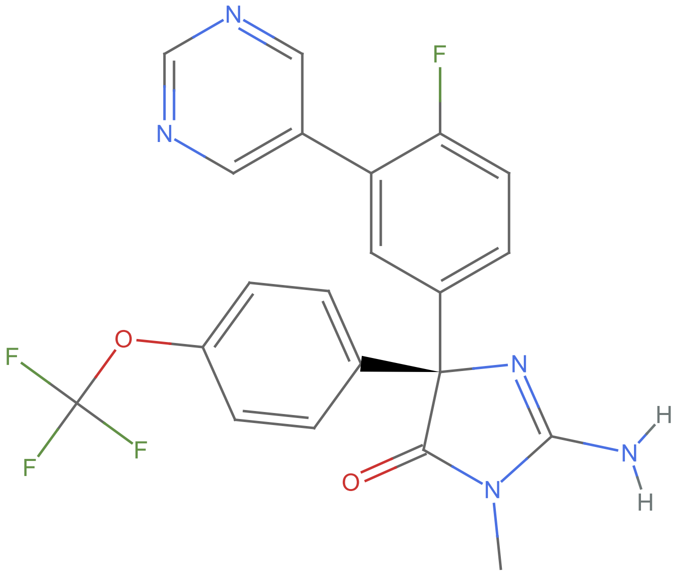

2D Autocorrelation

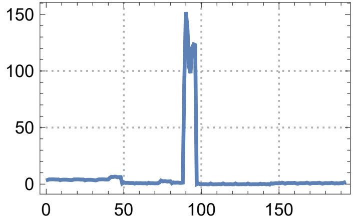

Dataset Index= 1163

Molecule Formula= $C_{21}H_{15}F_4N_5O_2$

3D Autocorrelation

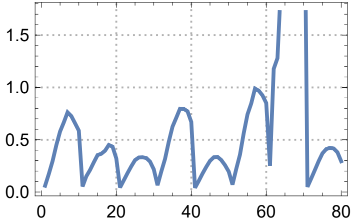

3D Plot

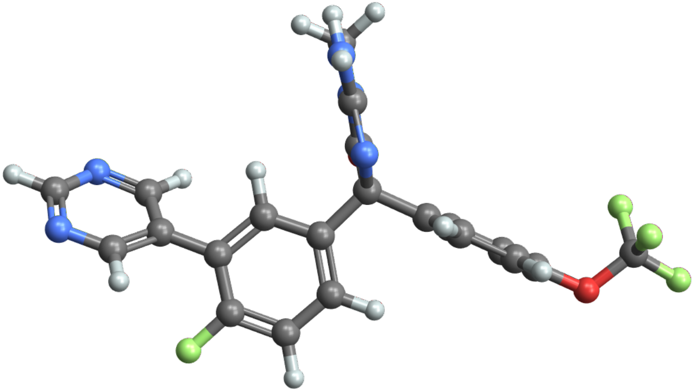

2D Autocorrelation

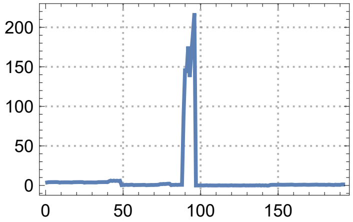

Dataset Index= 1164

Molecule Formula= $C_{27}H_{35}F_{2}N_{4}O_{3}^{+}$

3D Autocorrelation

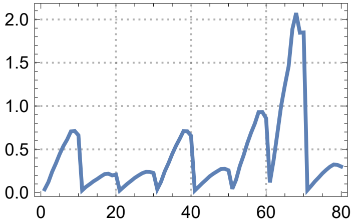

2D Plot

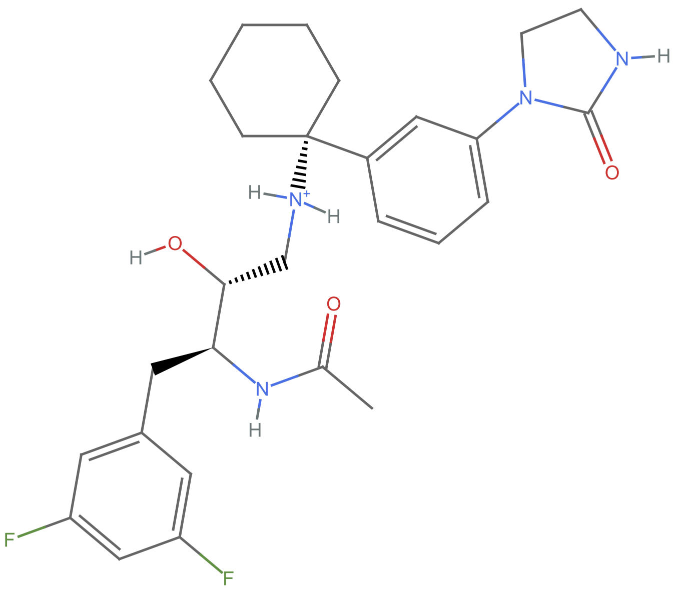

3D Plot

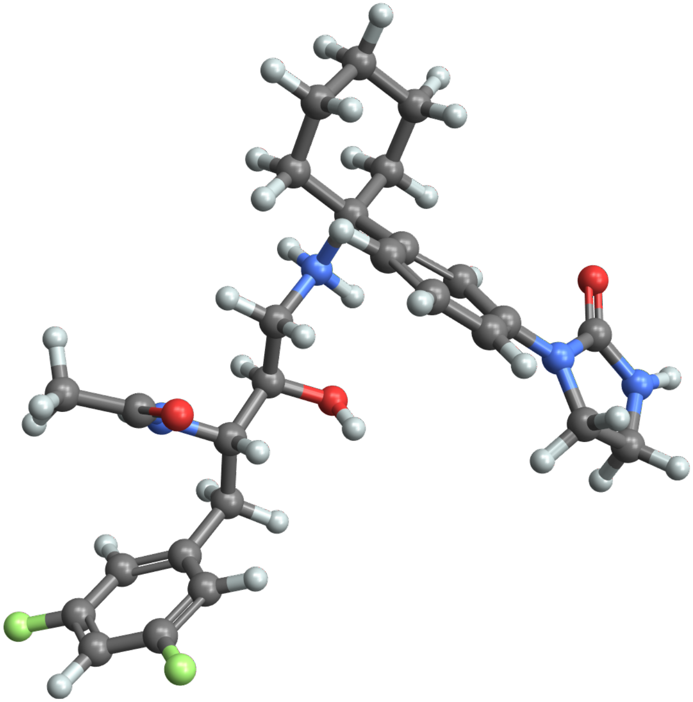

2D Autocorrelation

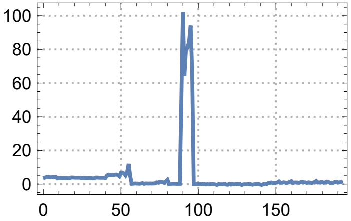

Dataset Index= 1165

Molecule Formula= $C_{22}H_{29}BrNO_5S^+$

3D Autocorrelation

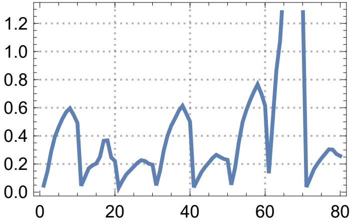

2D Plot

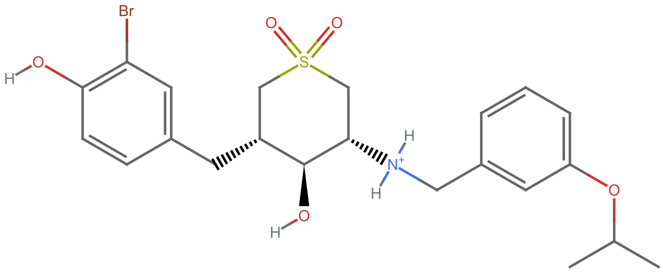

3D Plot

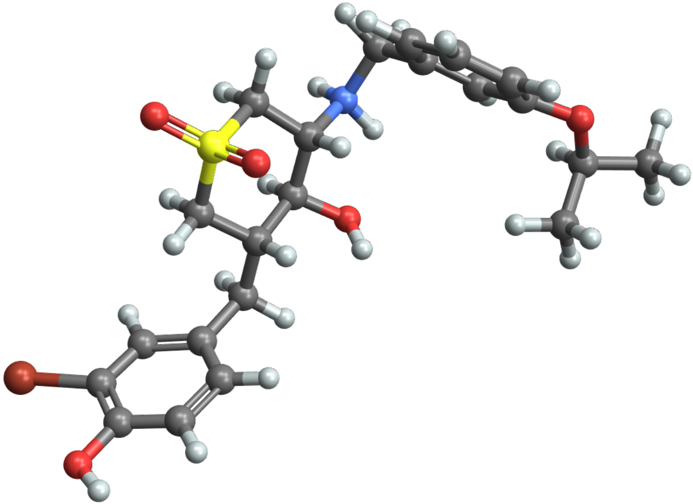

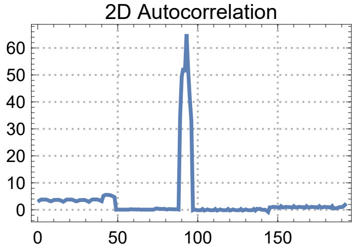

Dataset Index= 1166

Molecule Formula= $C_{21}H_{24}N_4$

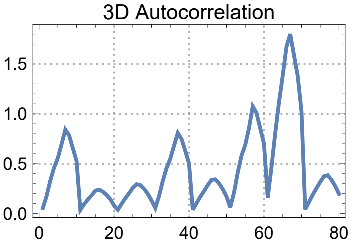

2D Plot

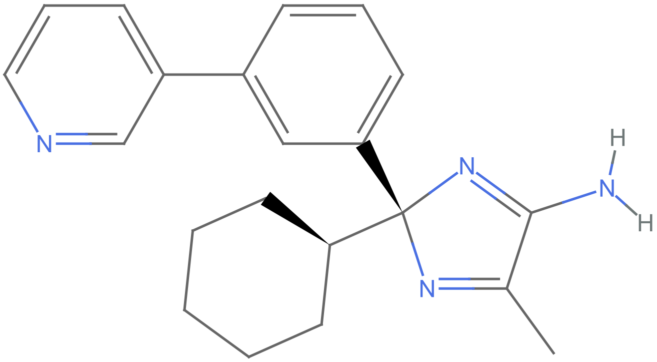

3D Plot

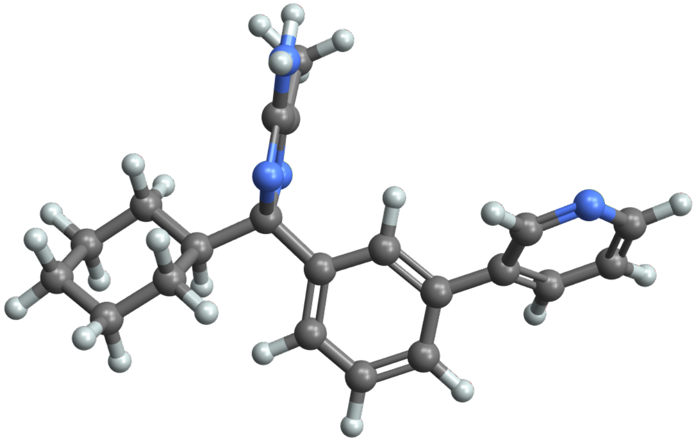

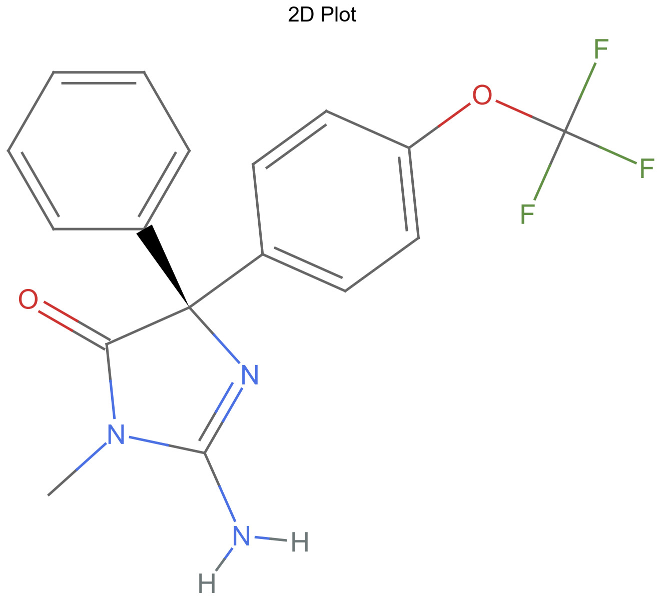

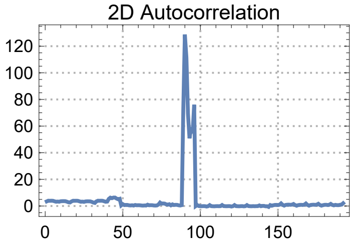

Dataset Index= 1167

Molecule Formula= $C_{17}H_{14}F_3N_3O_2$

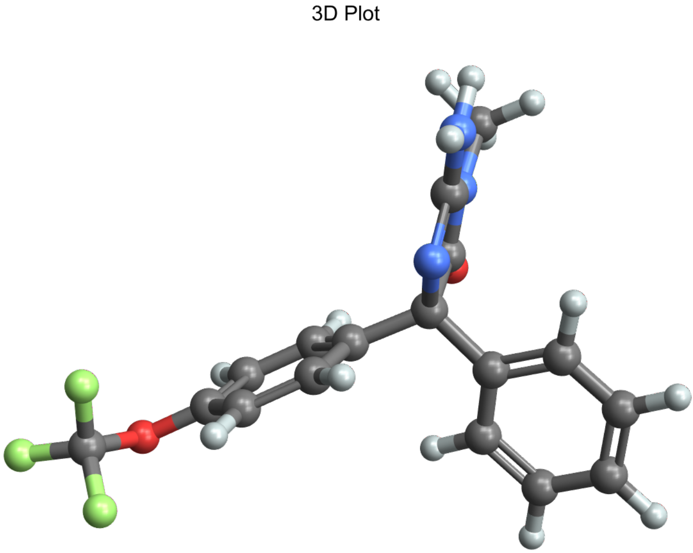

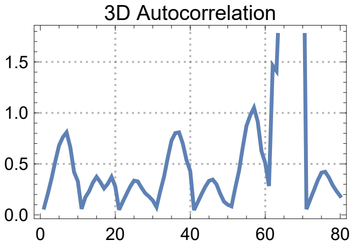

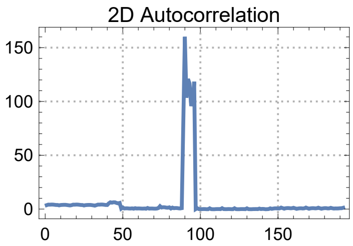

Dataset Index= 1168

Molecule Formula= $C_{22}H_{24}F_3N_4O^+$

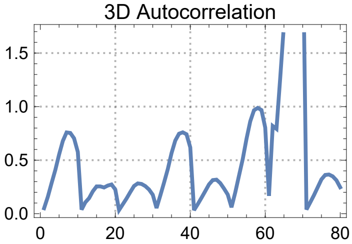

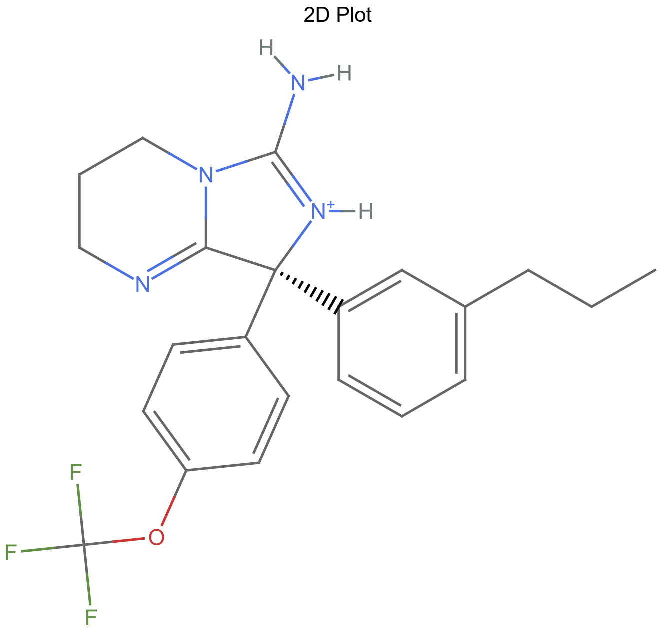

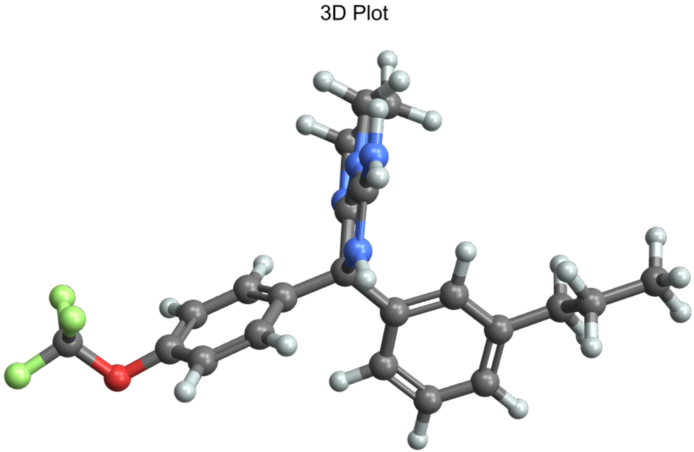

2D Plot

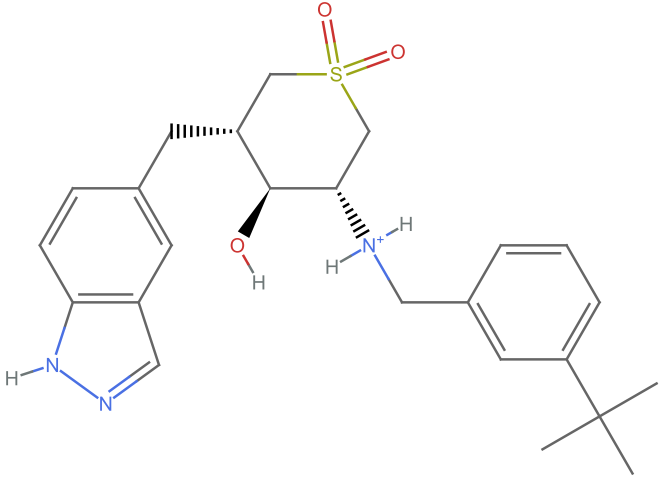

2D Autocorrelation

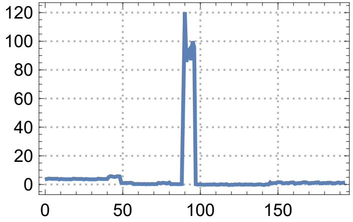

Dataset Index= 1169

Molecule Formula= $C_{24}H_{32}N_3O_3S^+$

3D Plot

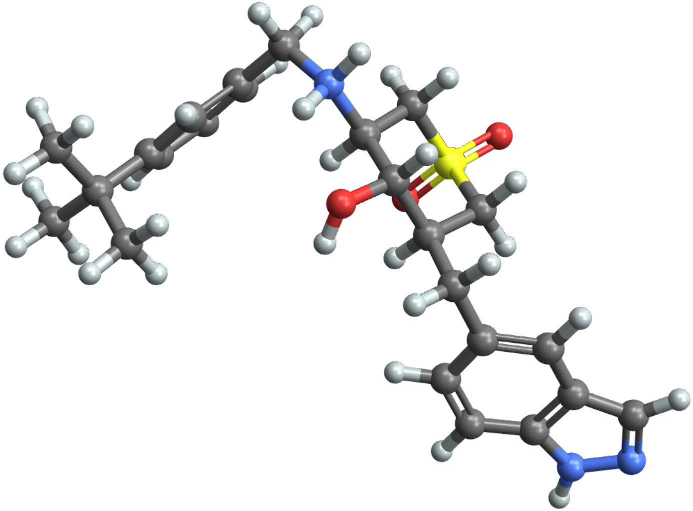

3D Autocorrelation

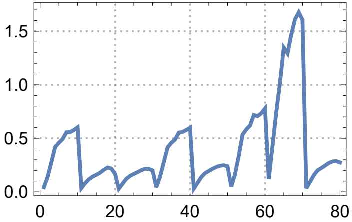

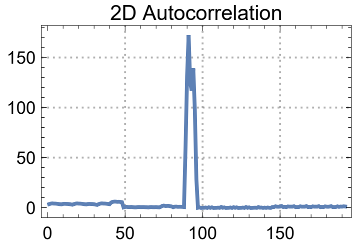

Dataset Index= 1170

Molecule Formula= $C_{19}H_{19}F_2N_5O$

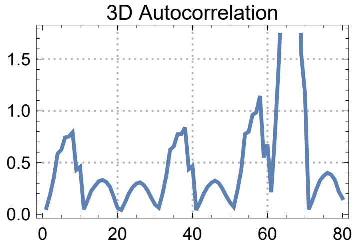

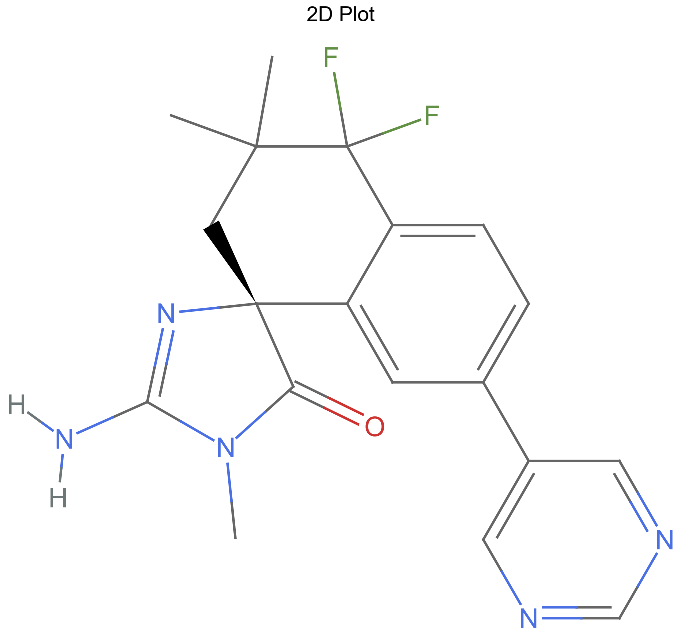

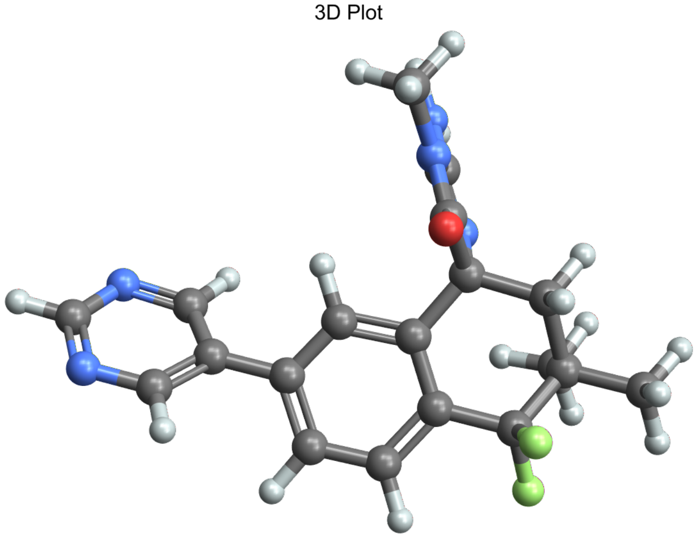

2D Autocorrelation

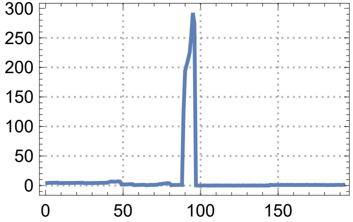

Dataset Index= 1171

Molecule Formula= $C_{37}H_{42}F_2N_4O_7S$

3D Autocorrelation

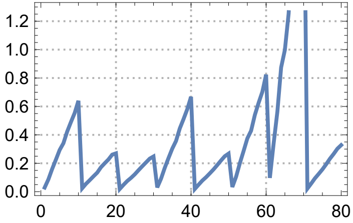

2D Plot

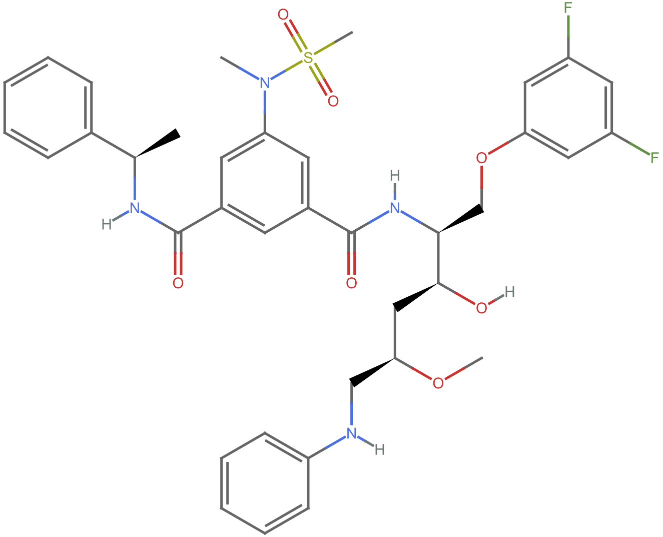

3D Plot

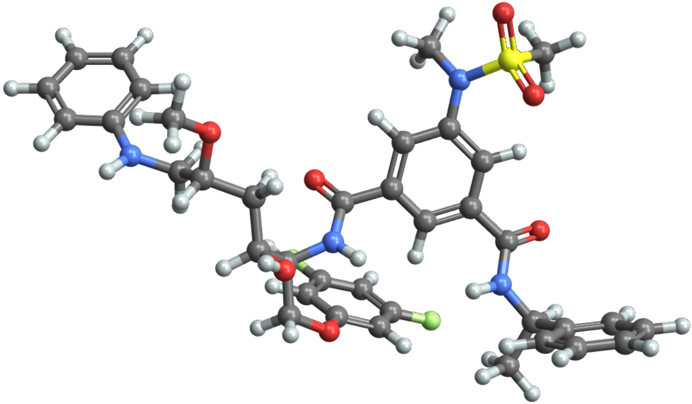

2D Autocorrelation

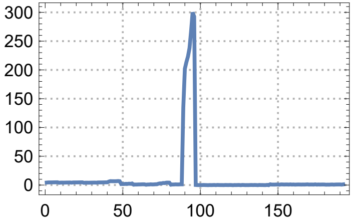

Dataset Index= 1172

Molecule Formula= $C_{37}H_{41}F_3N_4O_7S$

3D Autocorrelation

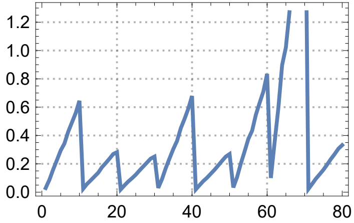

2D Plot

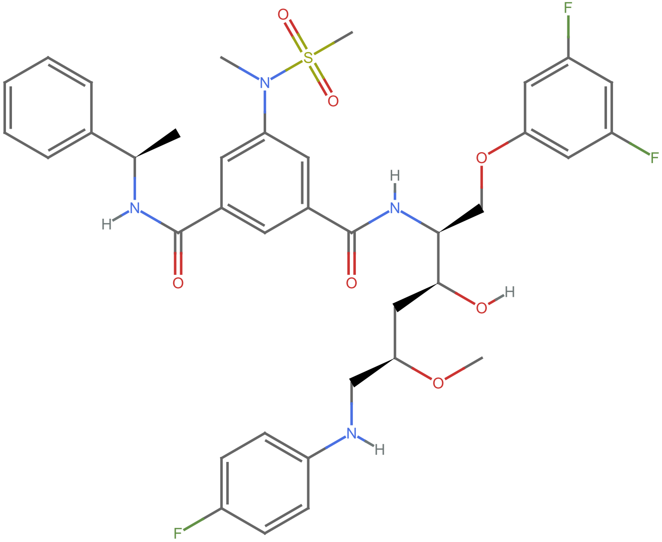

3D Plot

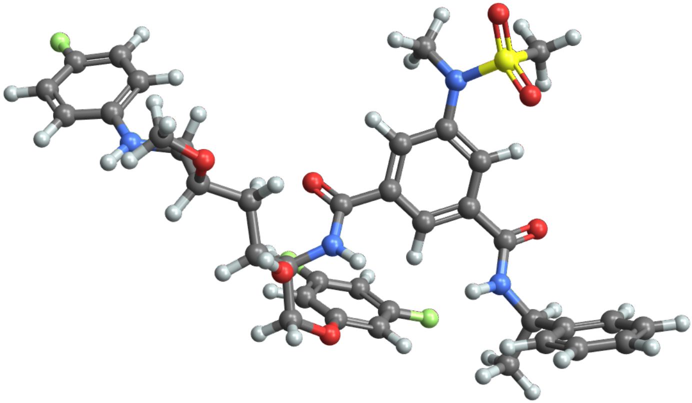

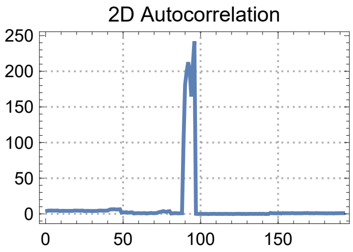

Dataset Index= 1173

Molecule Formula= $C_{35}H_{41}F_2N_3O_8S$

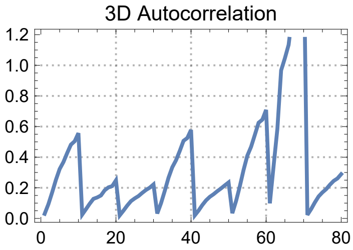

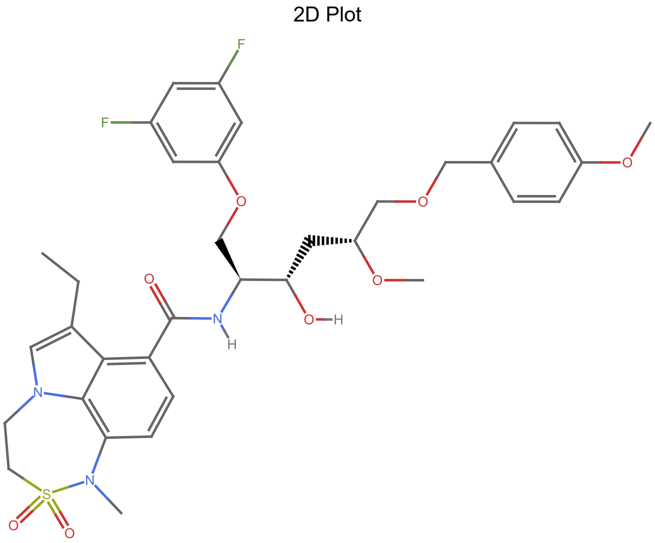

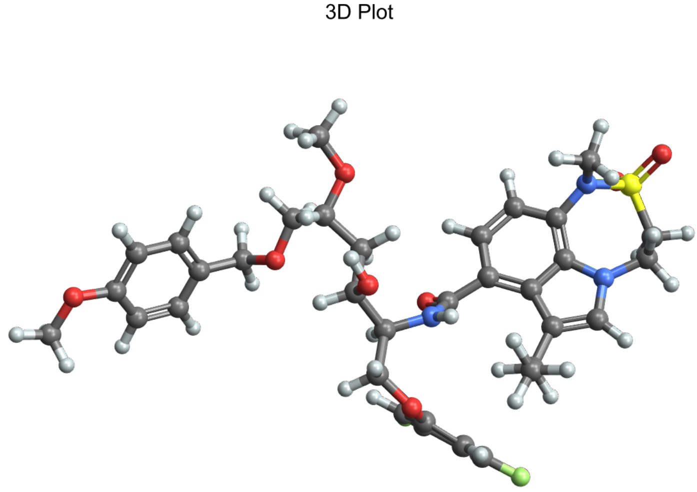

2D Plot

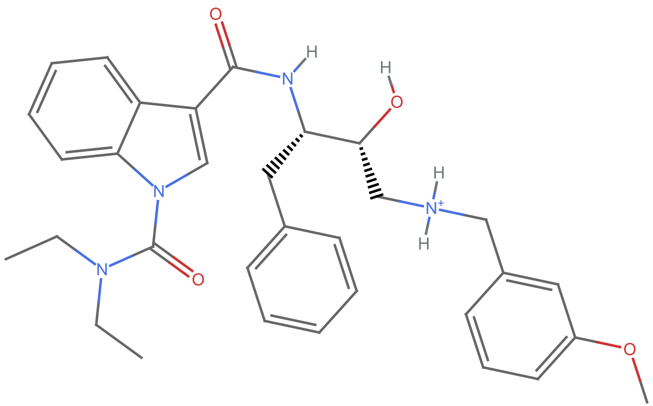

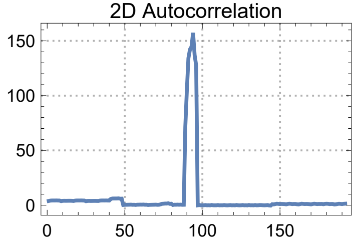

3D Plot

Dataset Index= 1174

Molecule Formula= $C_{32}H_{39}N_4O_4{}^+$

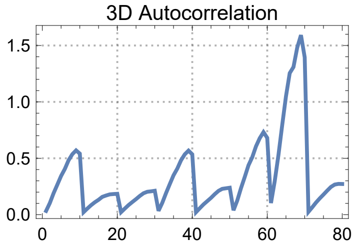

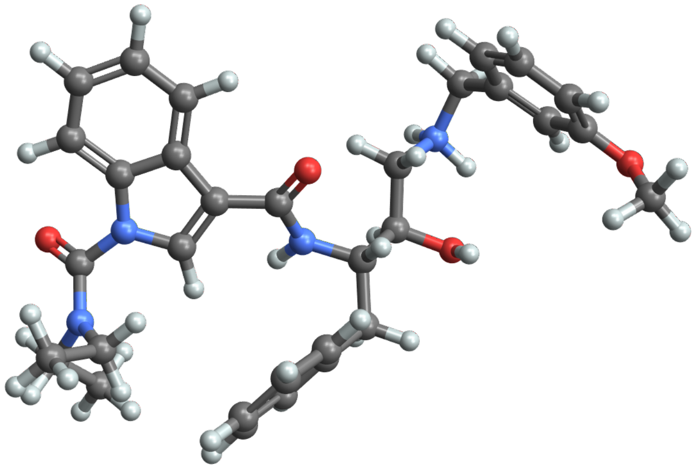

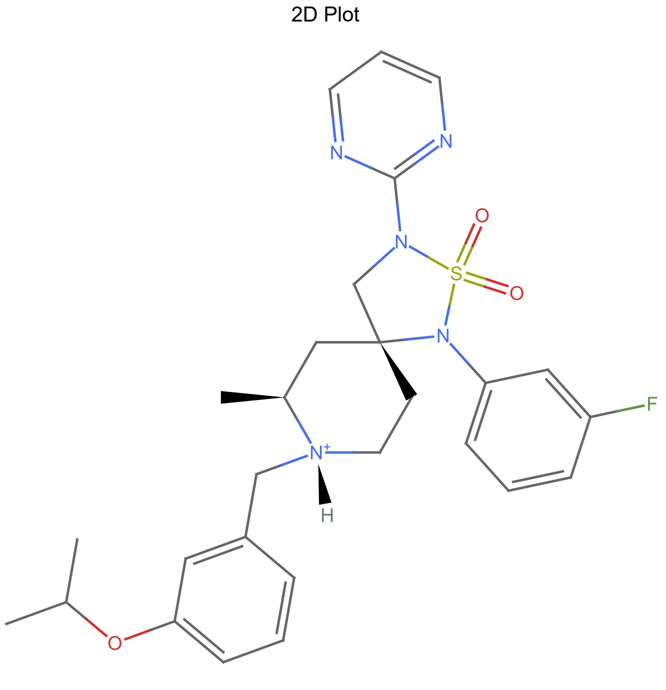

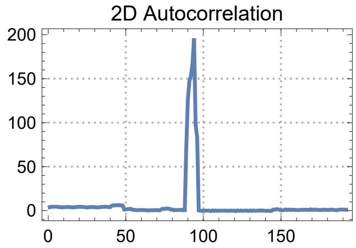

Dataset Index= 1175

Molecule Formula= $C_{27}H_{33}FN_5O_3S^+$

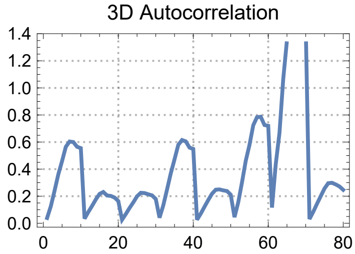

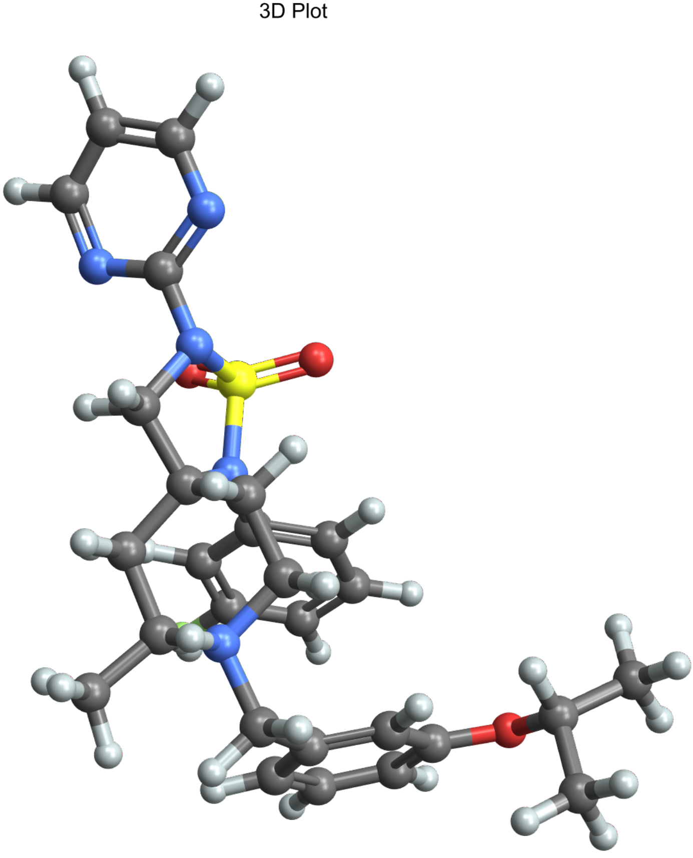

2D Autocorrelation

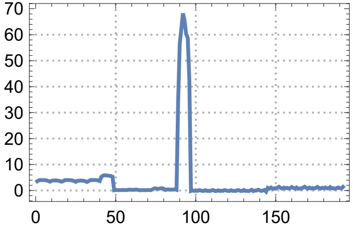

Dataset Index= 1176

Molecule Formula= $C_{23}H_{23}N_5O$

3D Autocorrelation

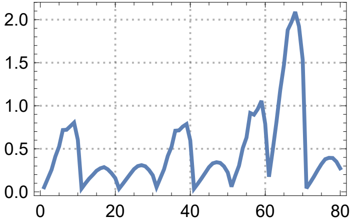

2D Plot

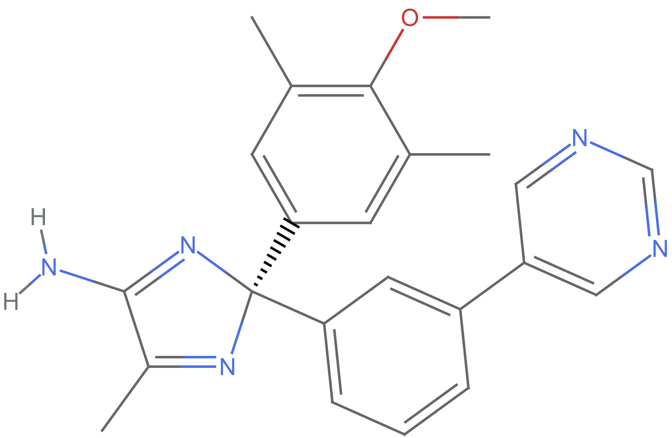

3D Plot

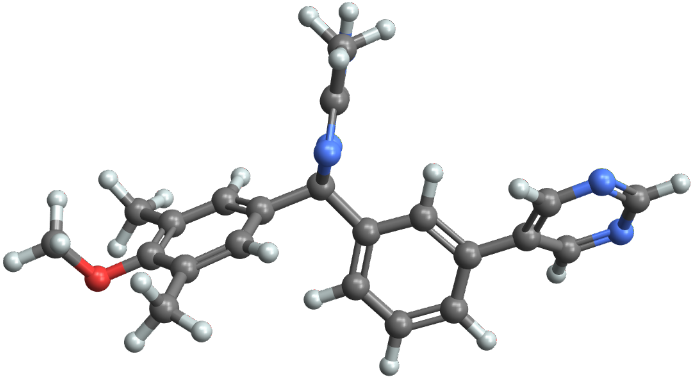

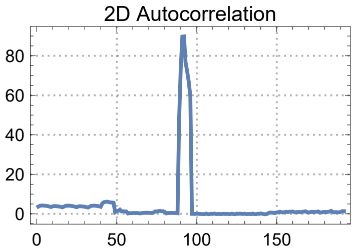

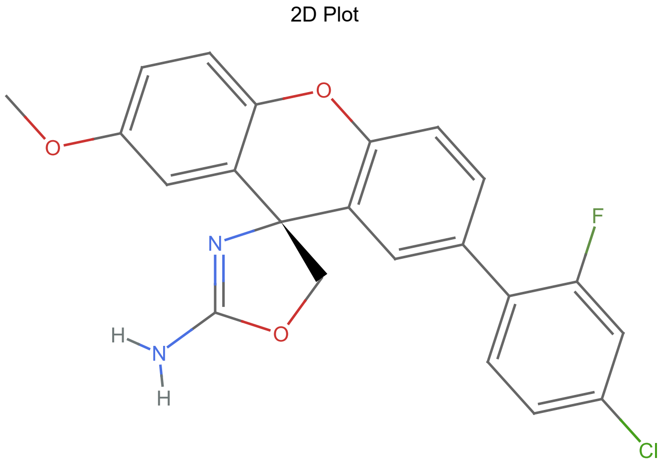

Dataset Index= 1177

Molecule Formula= $C_{22}H_{16}ClFN_2O_3$

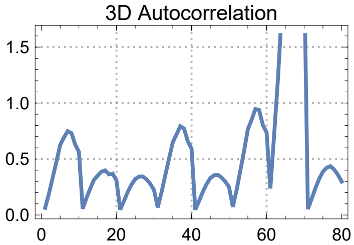

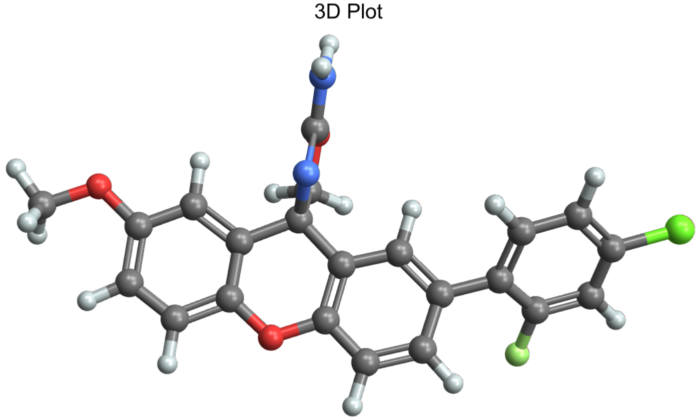

2D Autocorrelation

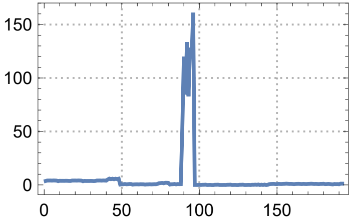

Dataset Index= 1178

Molecule Formula= $C_{25}H_{36}F_2N_3O_3^+$

3D Autocorrelation

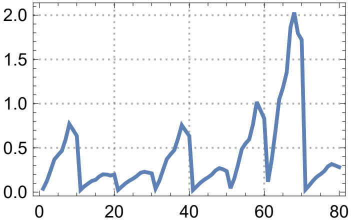

2D Plot

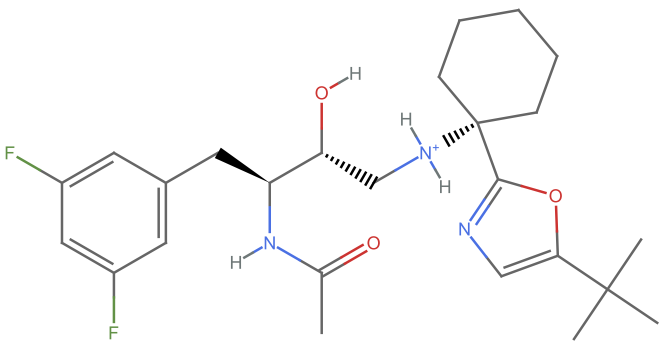

3D Plot

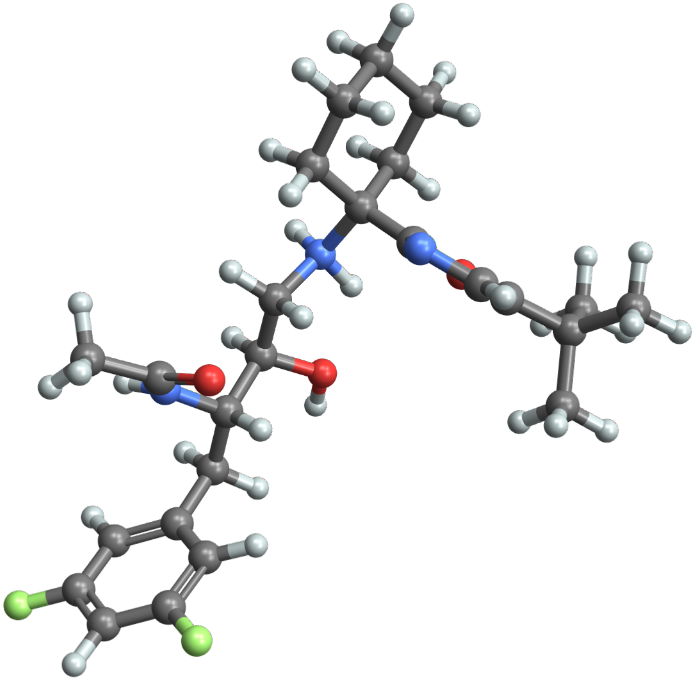

2D Plot

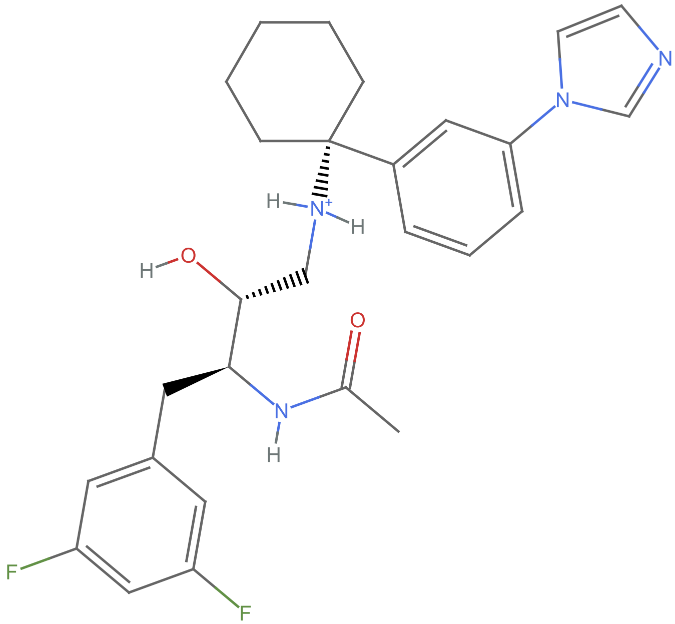

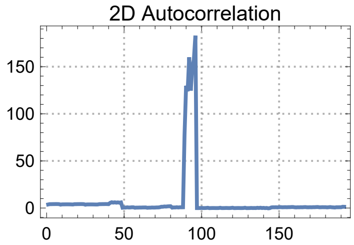

Dataset Index= 1179

Molecule Formula= $C_{27}H_{33}F_{2}N_{4}O_{2}^{+}$

3D Plot

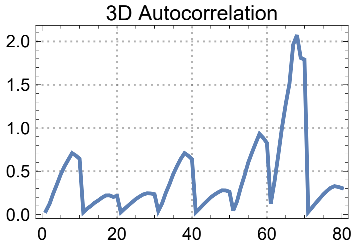

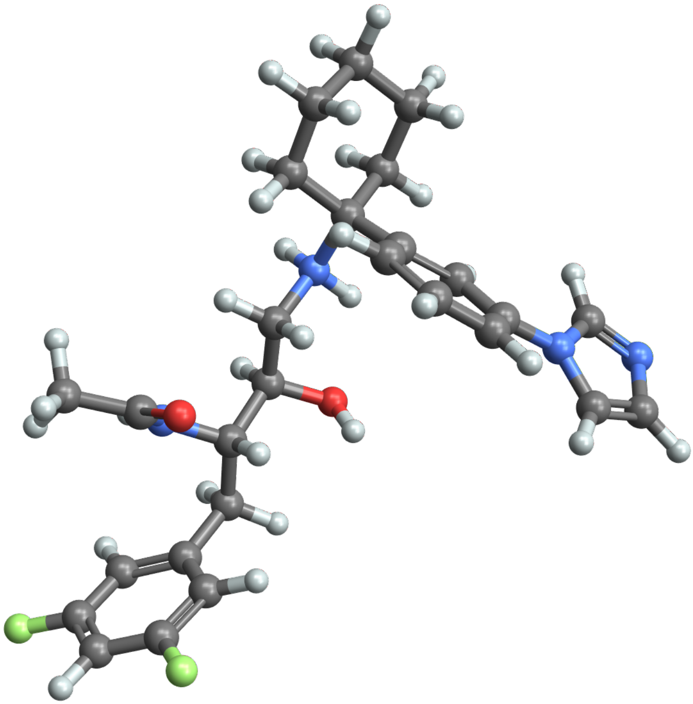

2D Plot

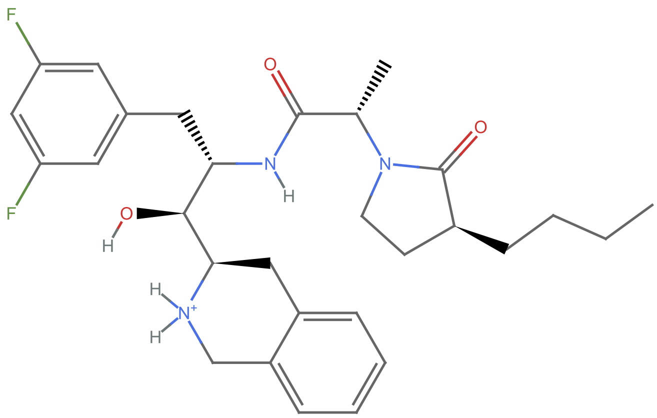

3D Plot

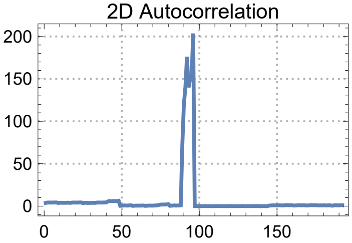

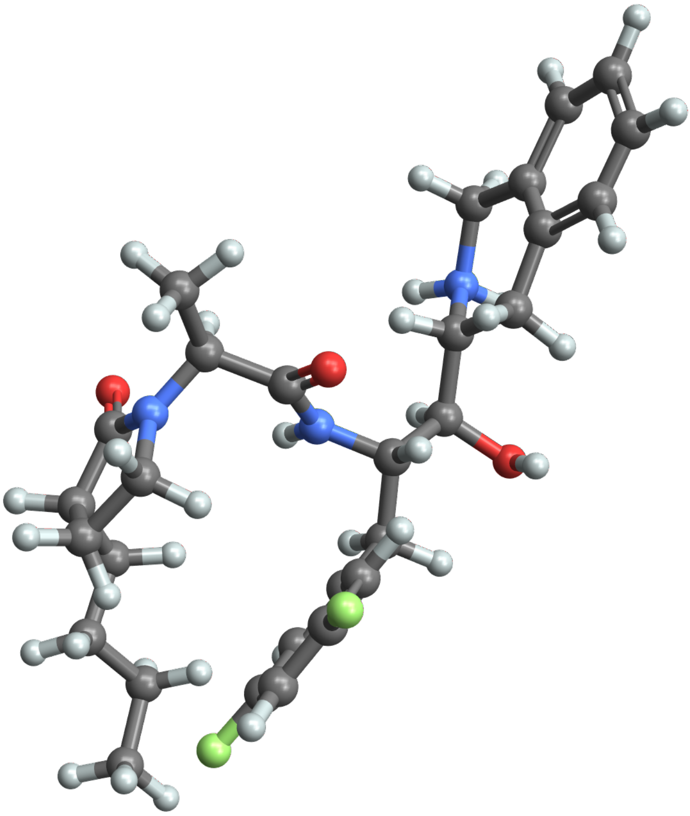

Dataset Index= 1180

Molecule Formula= $C_{29}H_{38}F_2N_3O_3^+$

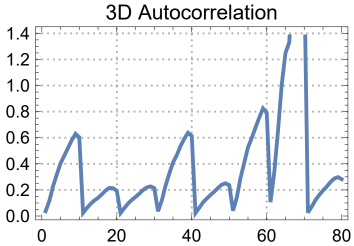

2D Plot

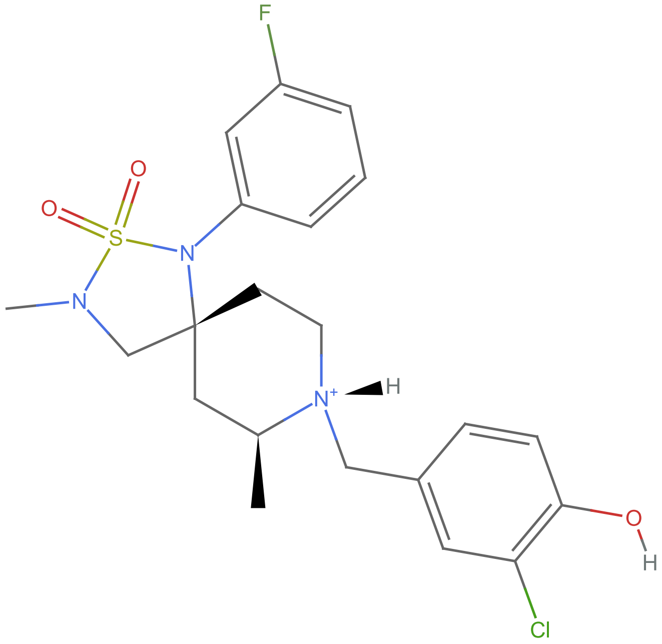

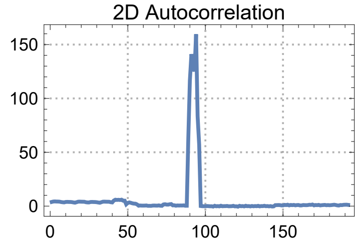

Dataset Index= 1181

Molecule Formula= $C_{21}H_{26}ClFN_3O_3S^+$

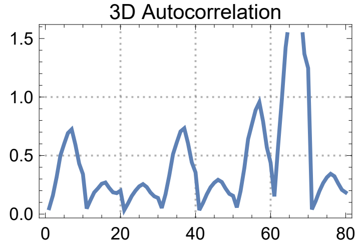

3D Plot

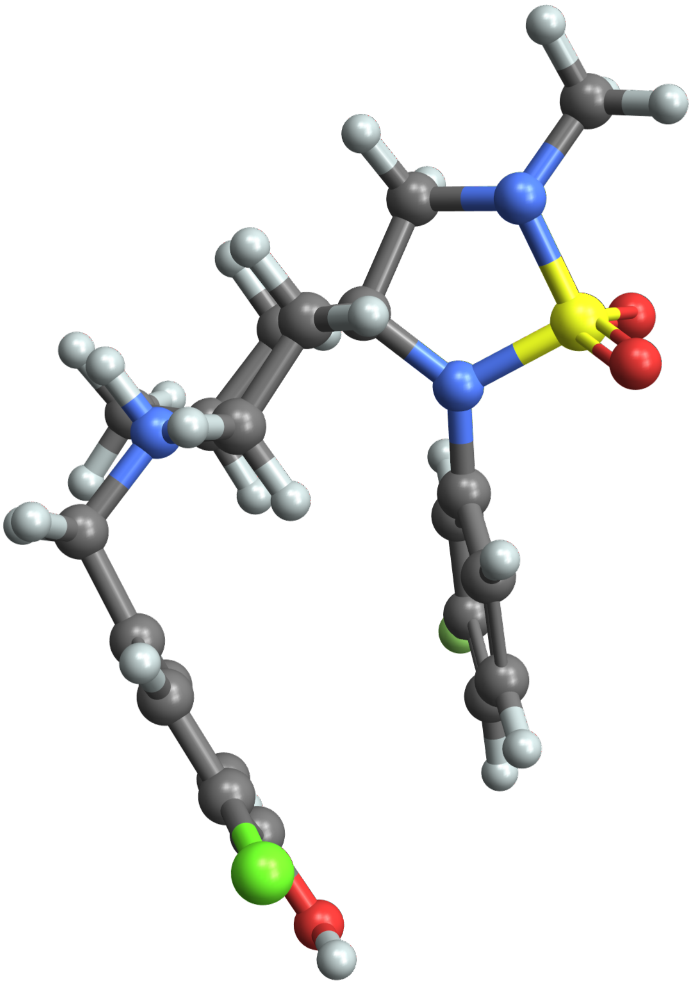

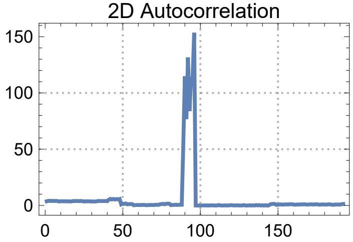

Dataset Index= 1182

Molecule Formula= $C_{24}H_{33}F_2N_2O_2S^+$

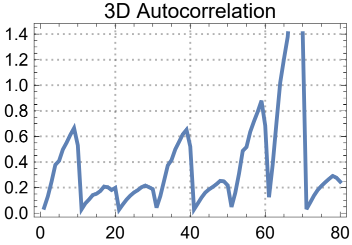

2D Plot

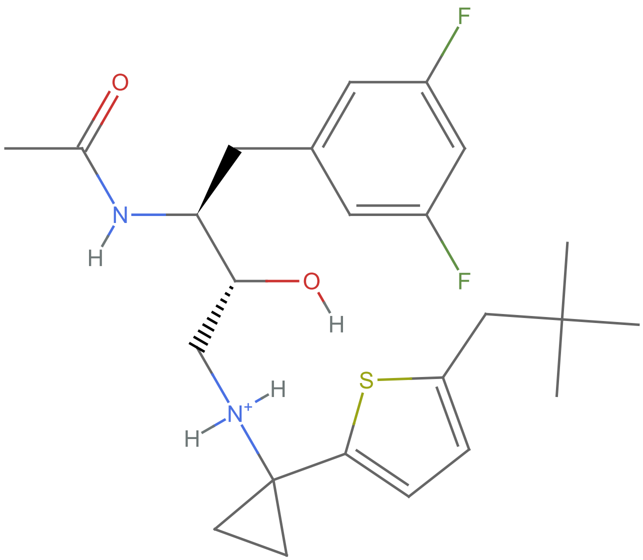

3D Plot

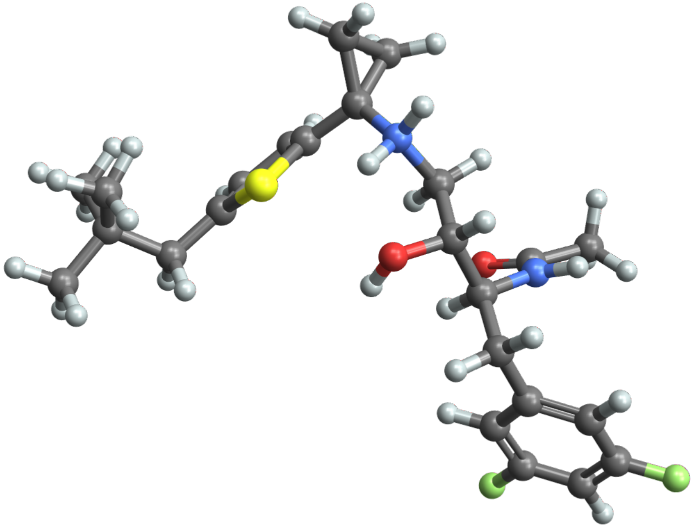

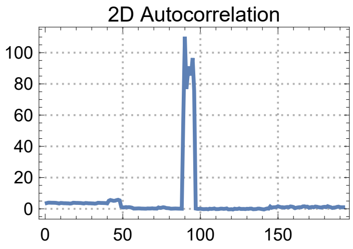

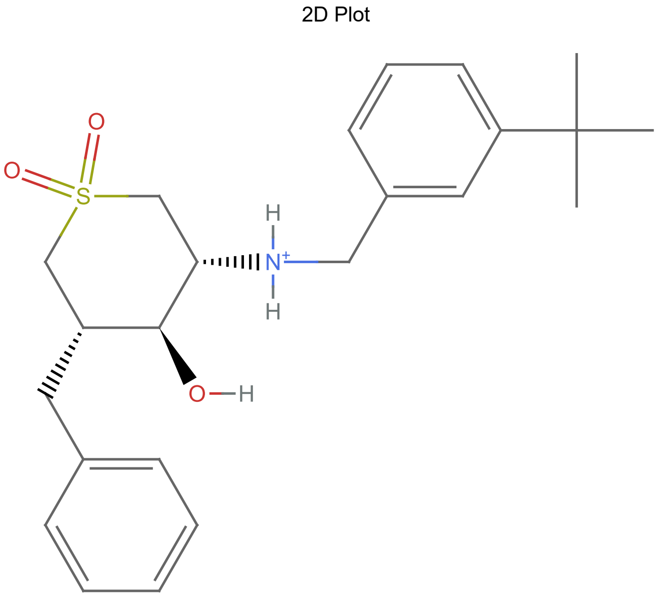

Dataset Index= 1183

Molecule Formula= $C_{23}H_{32}NO_3S^+$

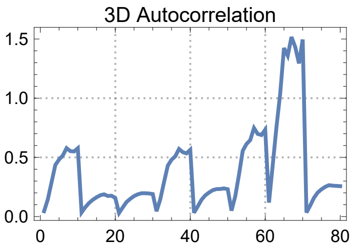

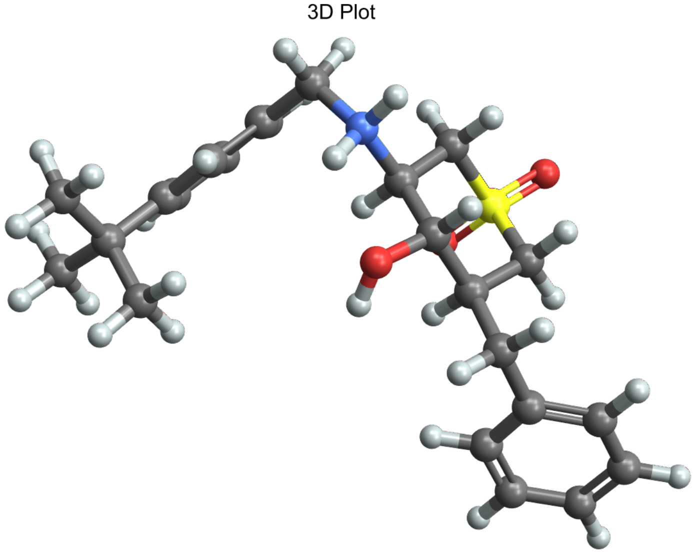

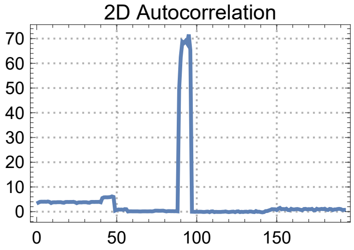

Dataset Index= 1184

Molecule Formula= $C_{26}H_{26}ClN_{3}O$

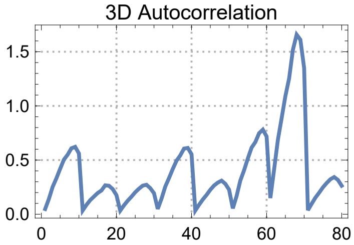

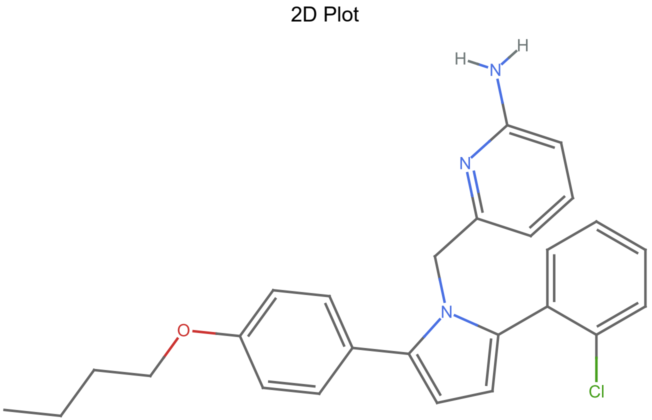

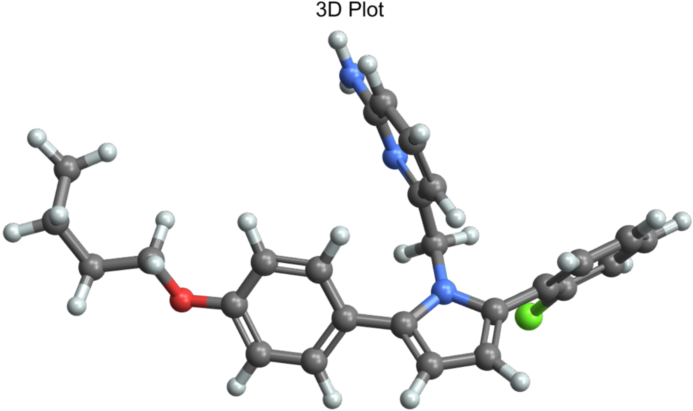

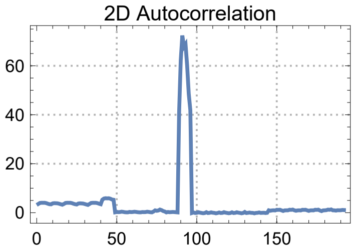

Dataset Index= 1185

Molecule Formula= $C_{23}H_{20}N_2O_3$

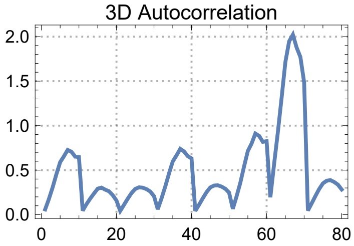

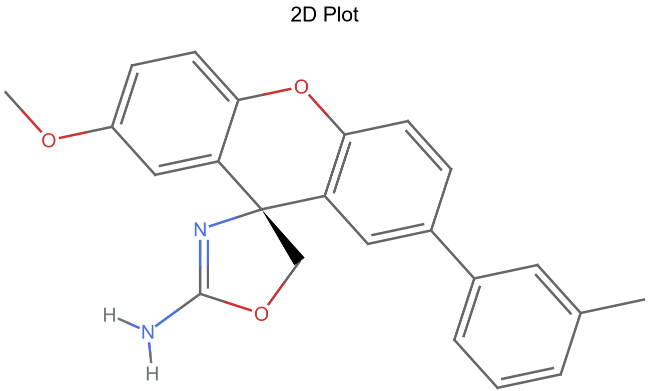

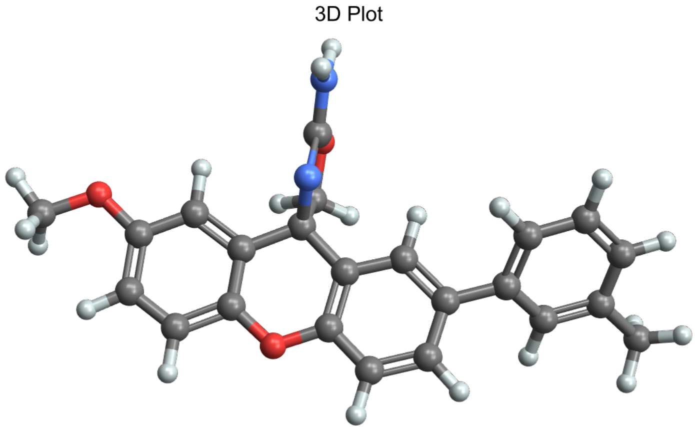

2D Plot

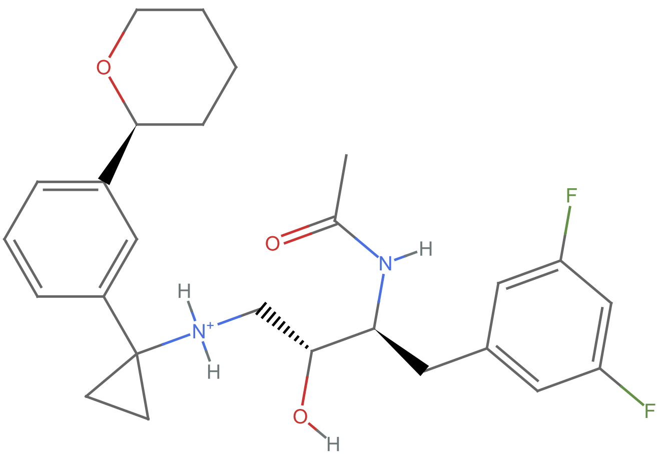

2D Autocorrelation

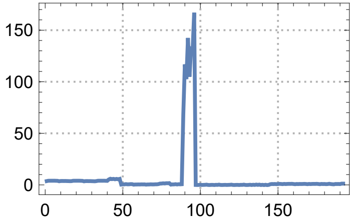

3D Plot

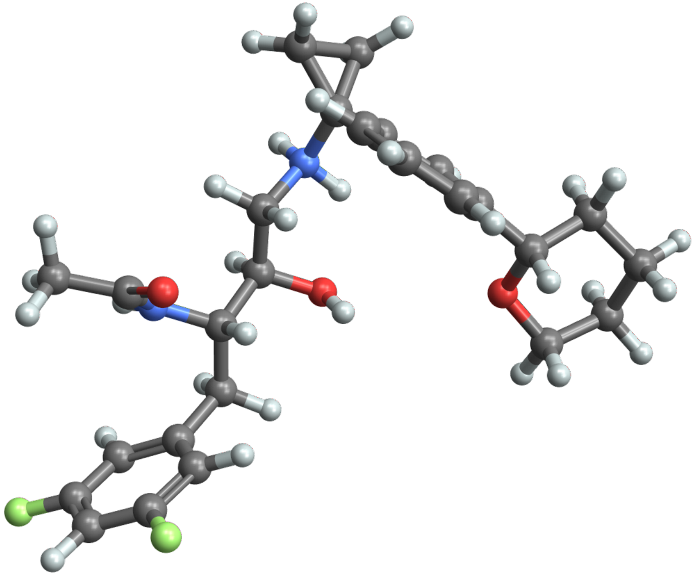

Dataset Index= 1186

Molecule Formula= $C_{26}H_{33}F_2N_2O_3^+$

3D Autocorrelation

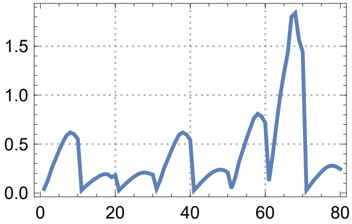

2D Plot

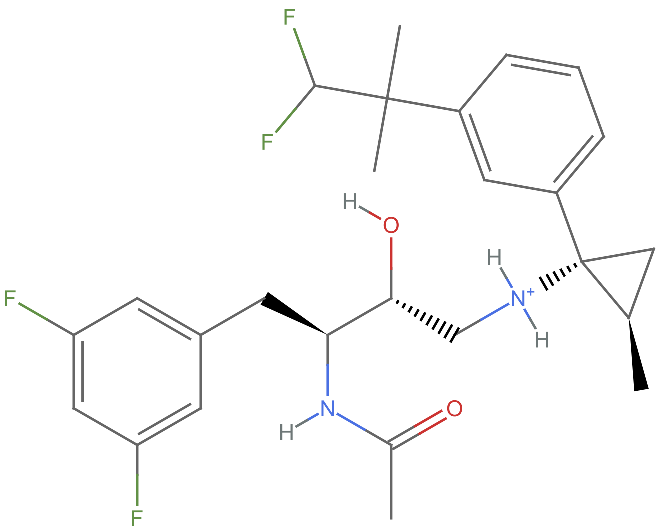

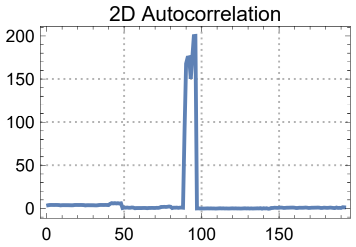

Dataset Index= 1187

Molecule Formula= $C_{26}H_{33}F_4N_2O_2^+$

3D Plot

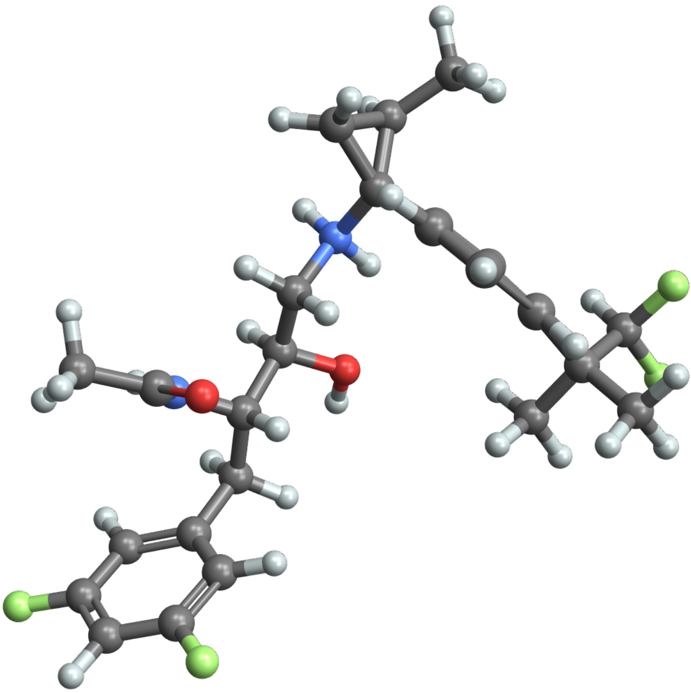

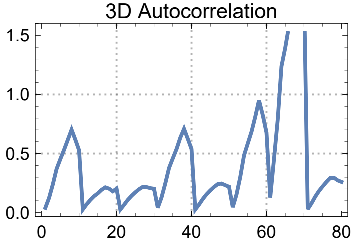

2D Plot

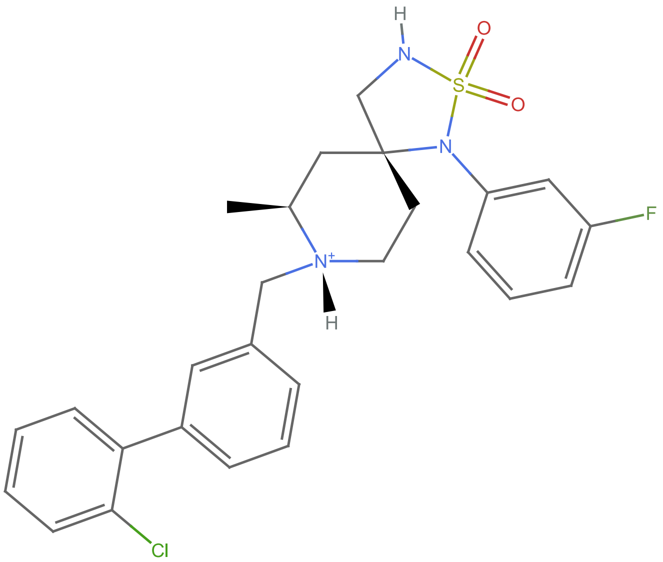

2D Autocorrelation

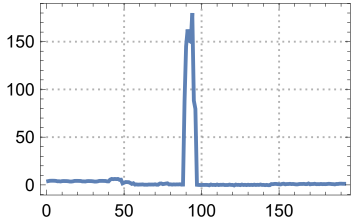

Dataset Index= 1188

Molecule Formula= $C_{26}H_{28}ClFN_3O_2S^+$

3D Autocorrelation

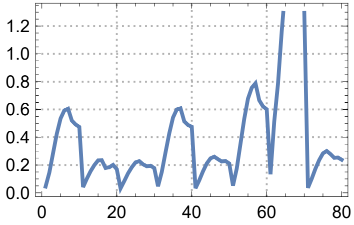

3D Plot

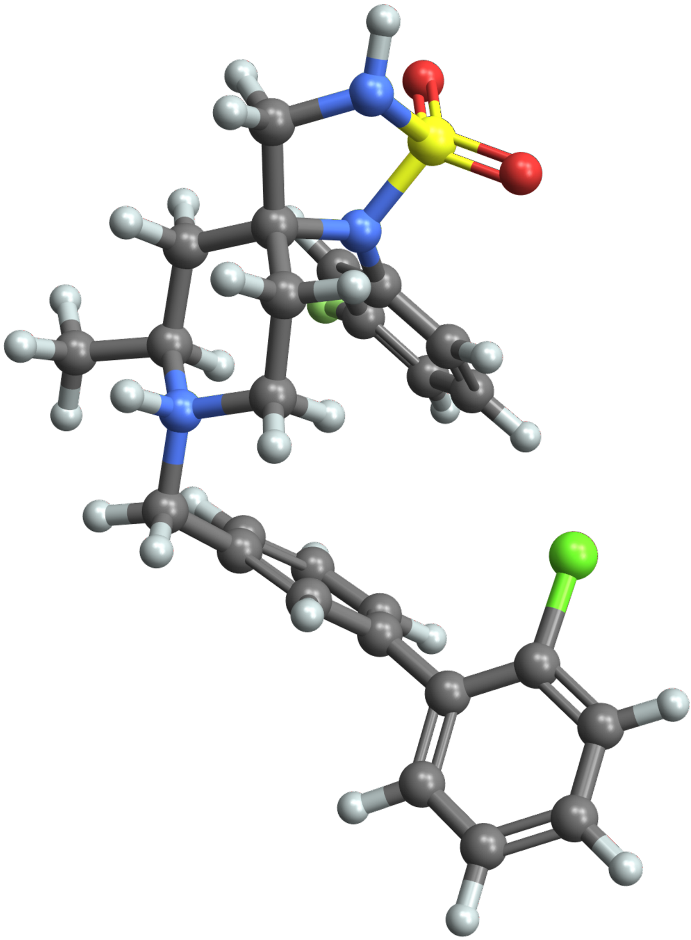

2D Plot

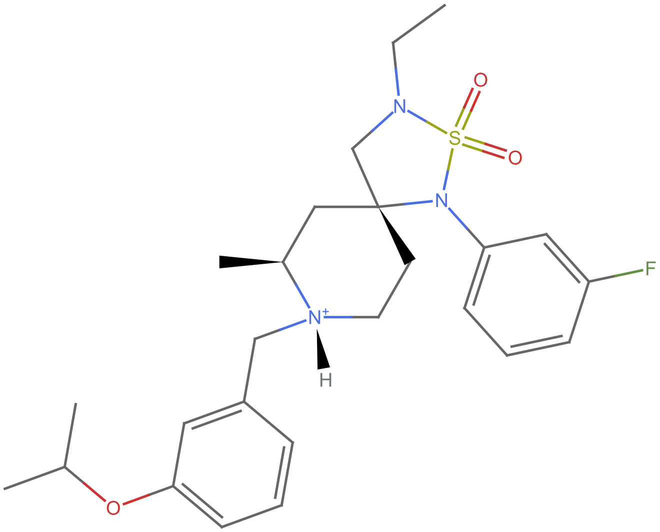

2D Autocorrelation

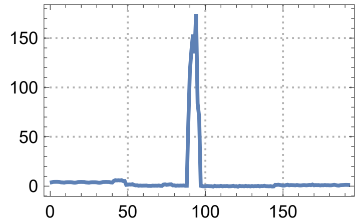

3D Plot

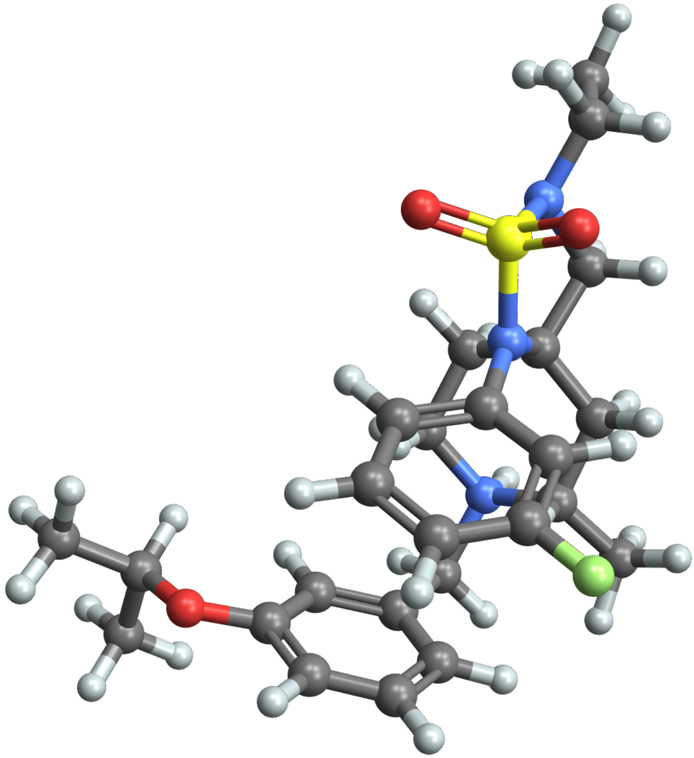

Dataset Index= 1189

Molecule Formula= $C_{25}H_{35}FN_3O_3S^+$

3D Autocorrelation

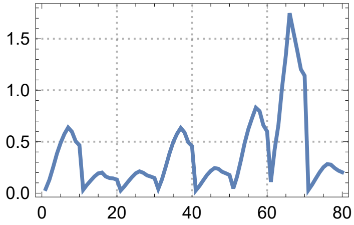

2D Plot

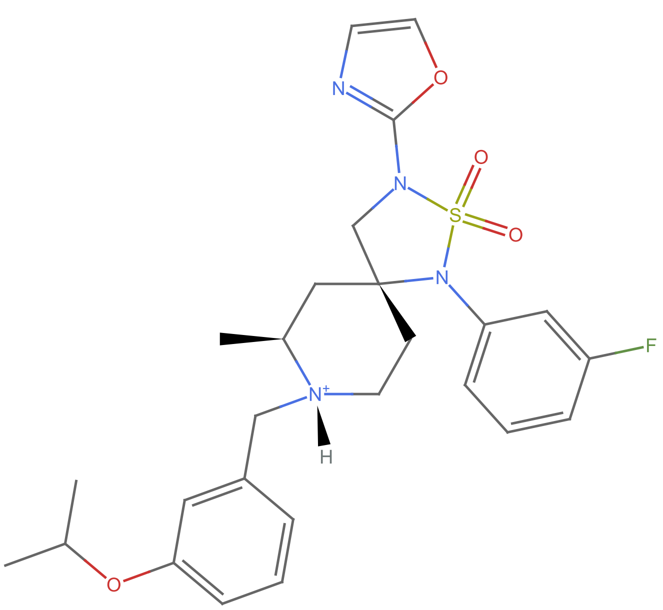

2D Autocorrelation

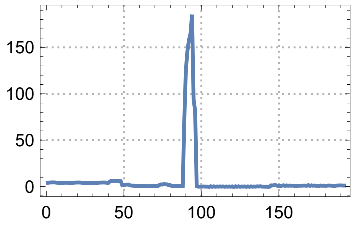

3D Plot

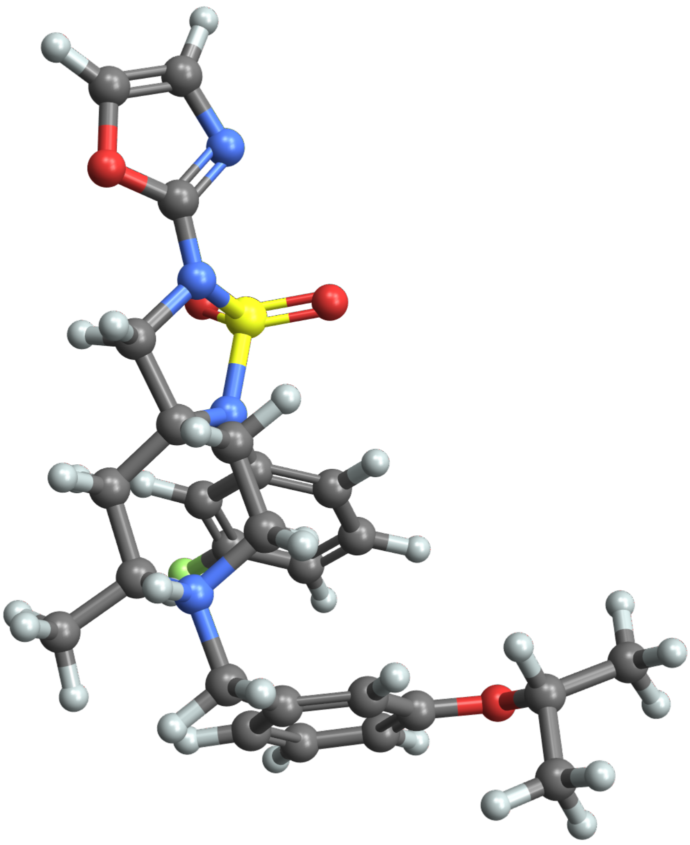

Dataset Index= 1190

Molecule Formula= $C_{26}H_{32}FN_4O_4S^+$

3D Autocorrelation

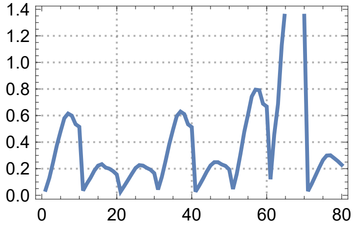

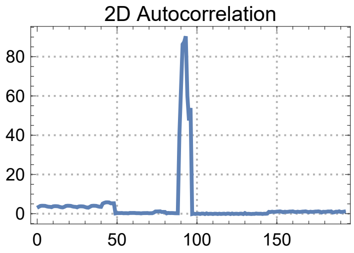

Dataset Index= 1191

Molecule Formula= $C_{21}H_{23}N_3O_3$

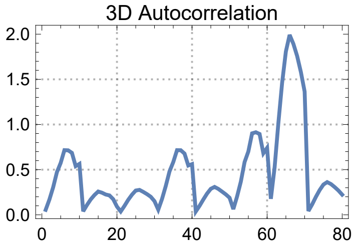

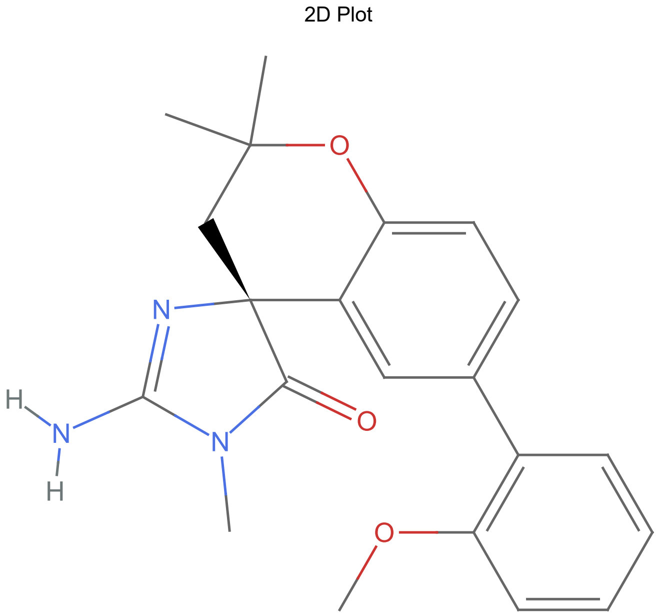

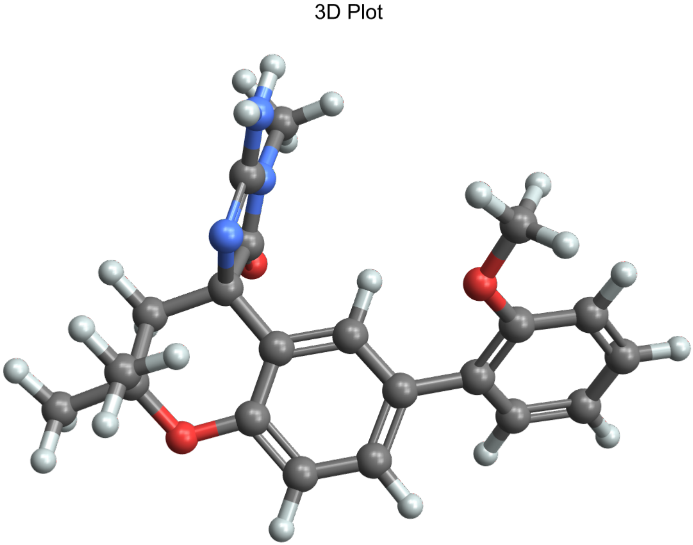

2D Plot

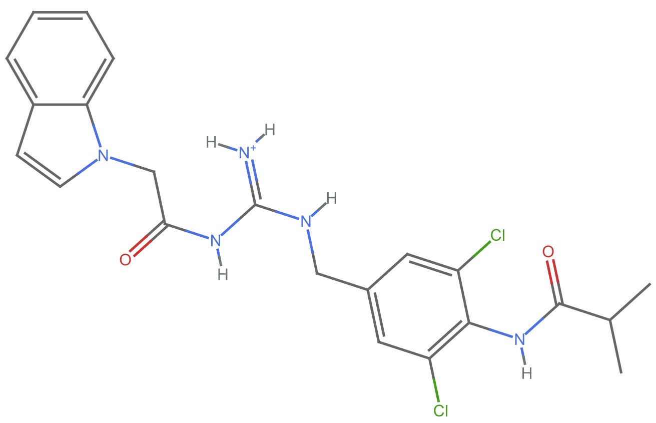

3D Plot

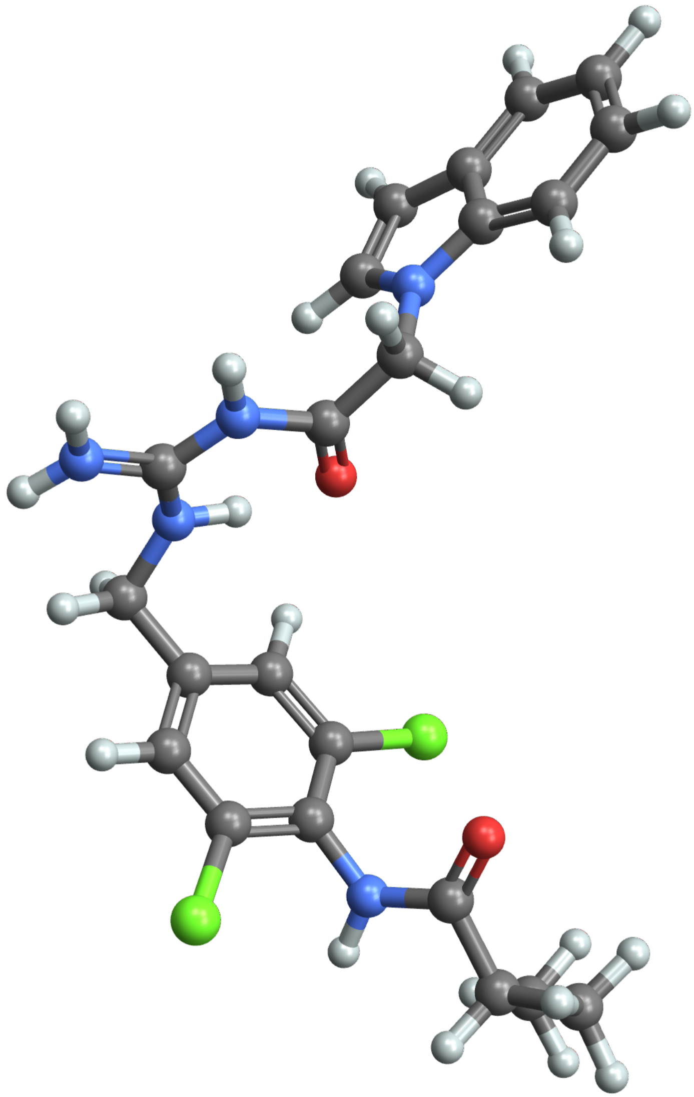

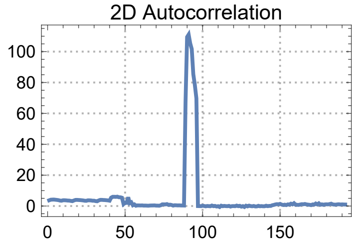

Dataset Index= 1192

Molecule Formula= $C_{22}H_{24}Cl_2N_5O_2^+$

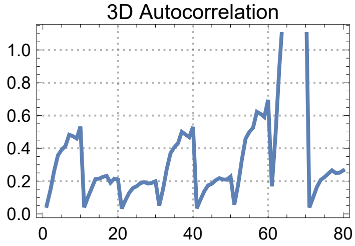

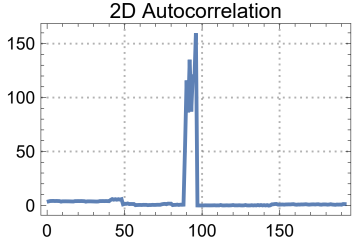

Dataset Index= 1193

Molecule Formula= $C_{24}H_{33}F_2N_2O_2S^+$

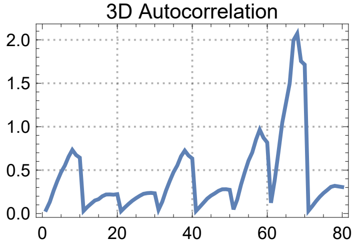

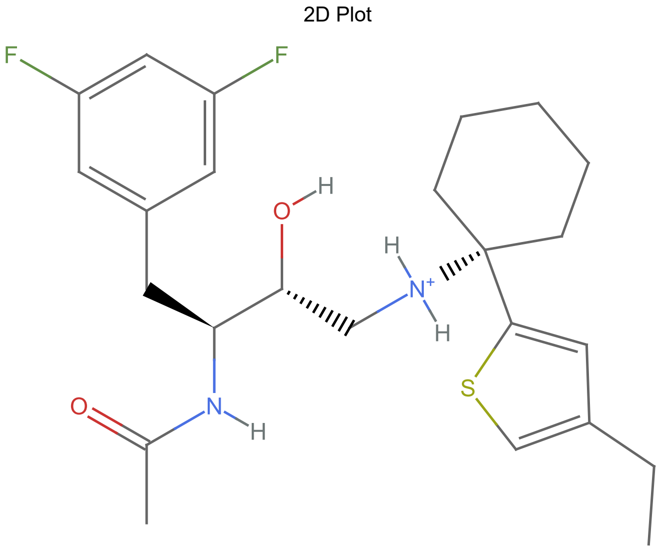

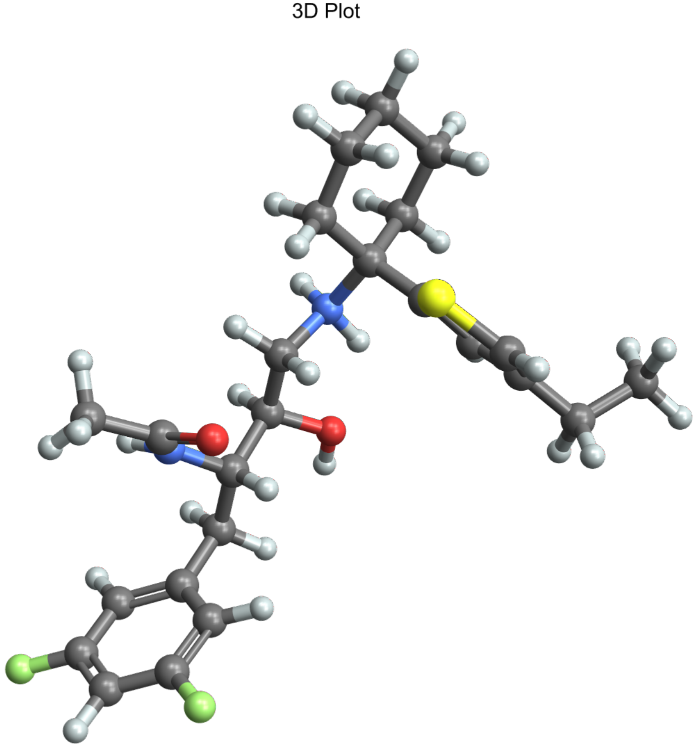

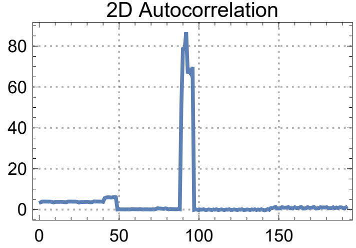

Dataset Index= 1194

Molecule Formula= $C_{26}H_{26}N_4O$

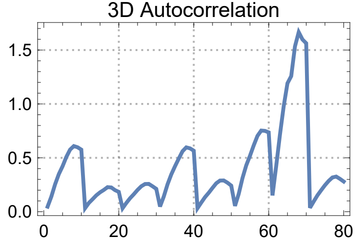

2D Plot

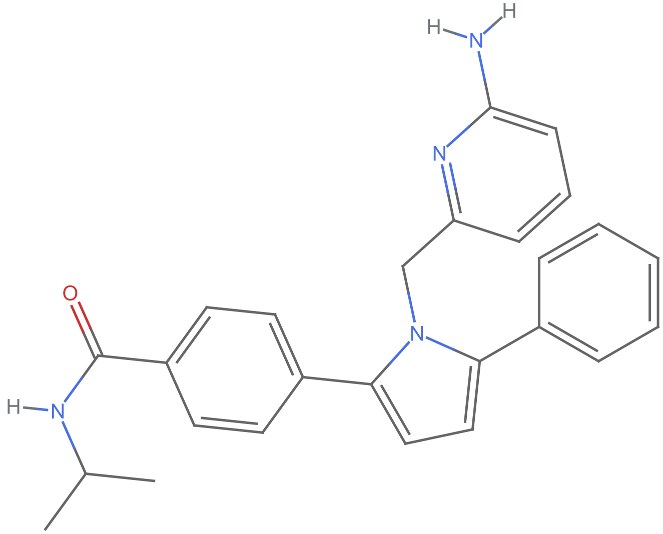

3D Plot

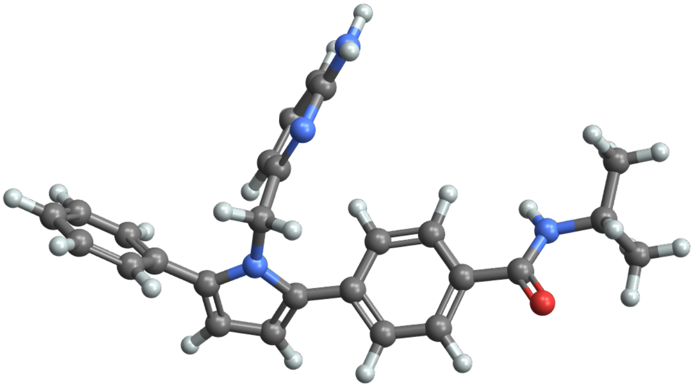

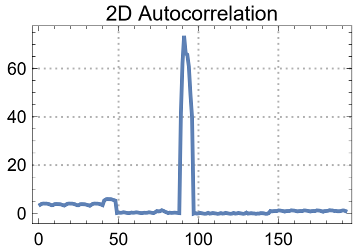

Dataset Index= 1195

Molecule Formula= $C_{23}H_{20}N_2O_3$

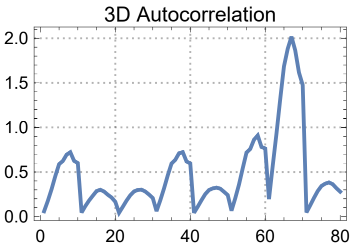

2D Plot

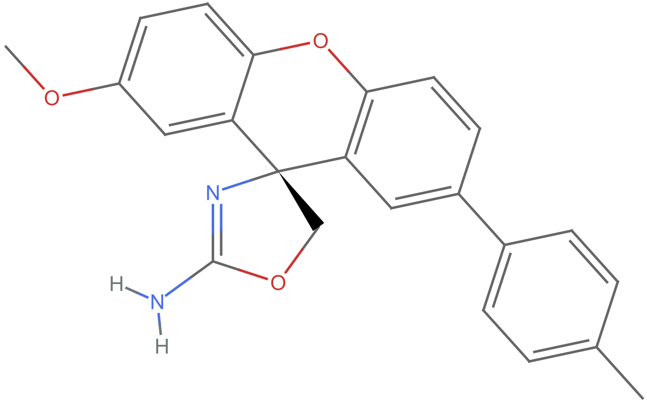

3D Plot

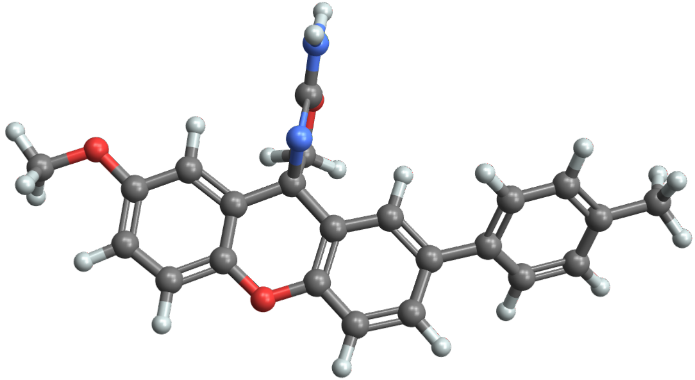

2D Plot

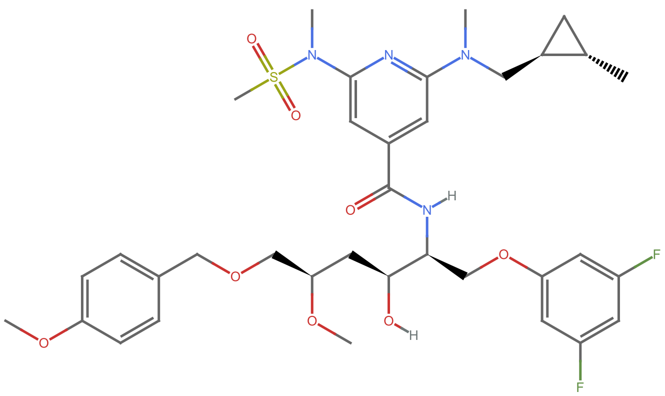

2D Autocorrelation

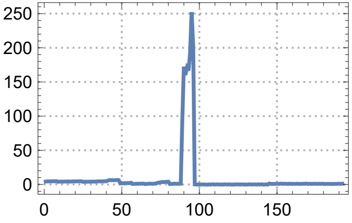

Dataset Index= 1196

Molecule Formula= $C_{35}H_{46}F_2N_4O_8S$

3D Plot

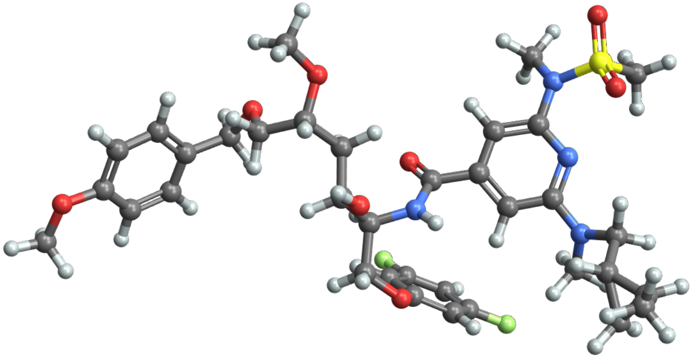

3D Autocorrelation

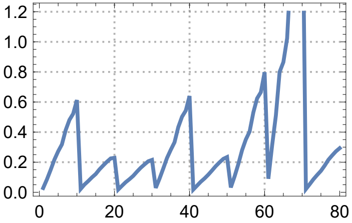

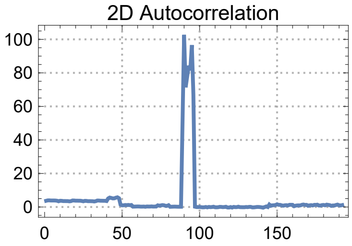

Dataset Index= 1197

Molecule Formula= $C_{22}H_{30}NO_4S^+$

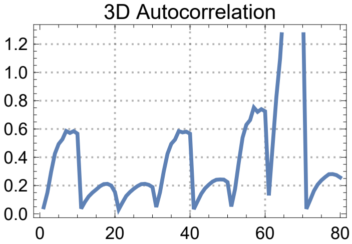

2D Plot

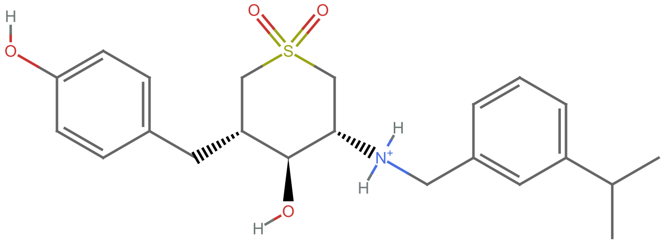

3D Plot

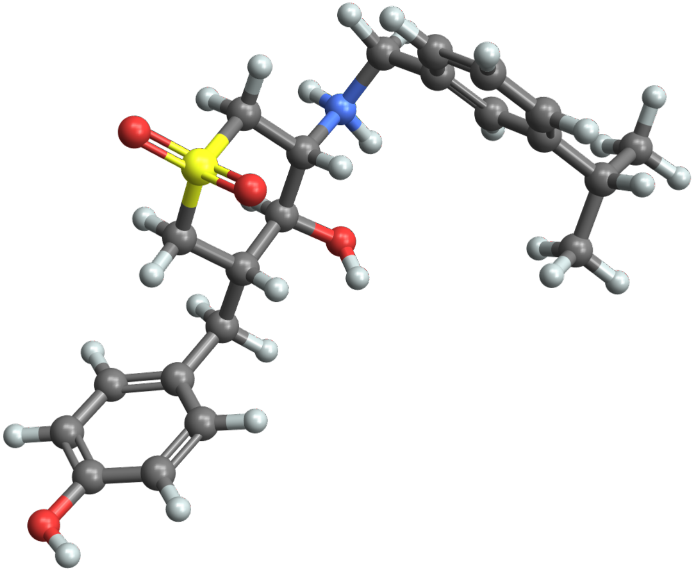

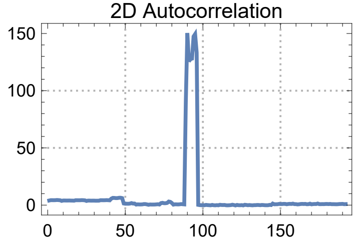

Dataset Index= 1198

Molecule Formula= $C_{28}H_{31}FN_5O_4S^+$

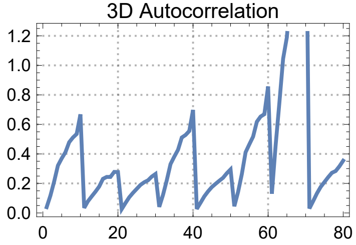

2D Plot

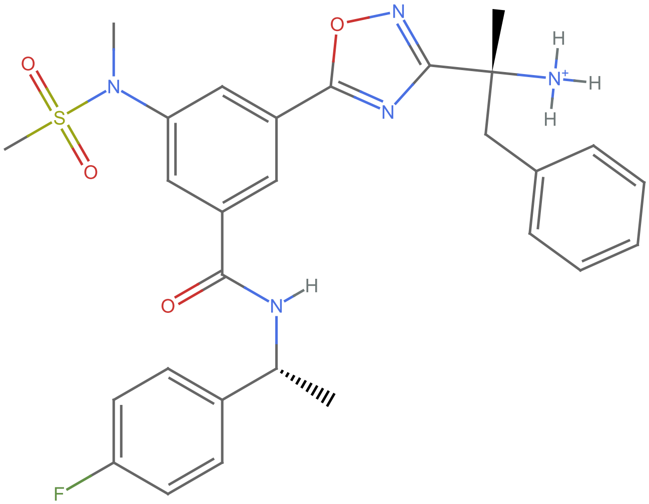

3D Plot

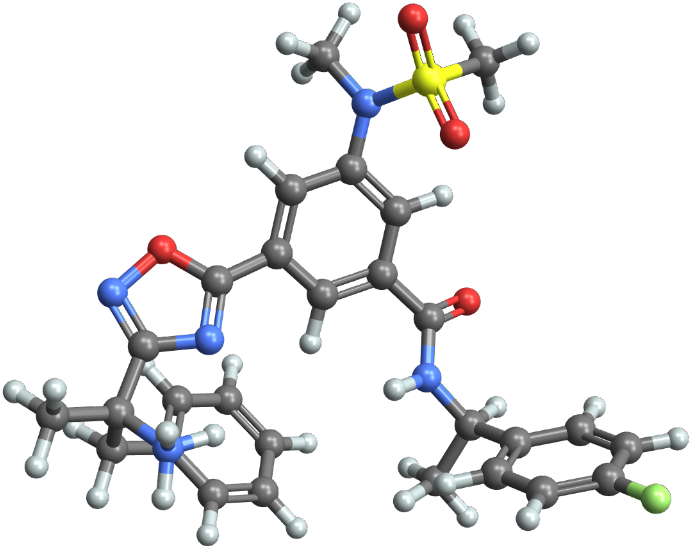

2D Autocorrelation

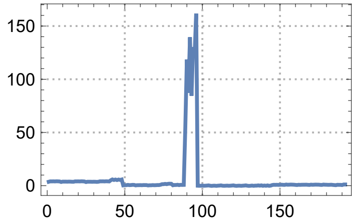

Dataset Index= 1199

Molecule Formula= $C_{25}H_{37}F_2N_4O_2^+$

3D Autocorrelation

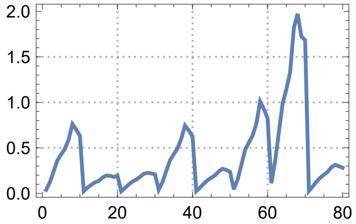

2D Plot

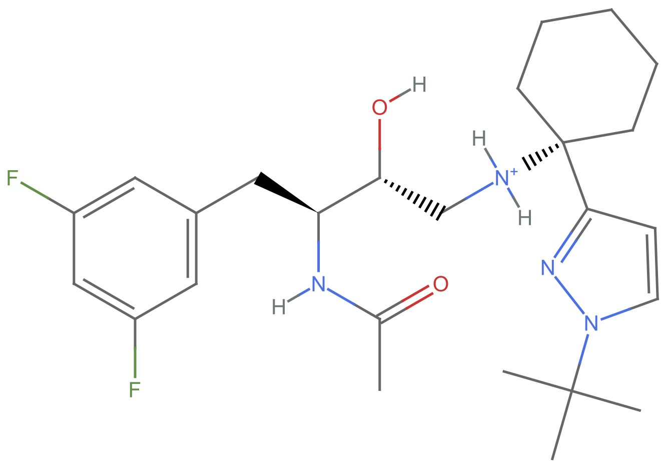

3D Plot

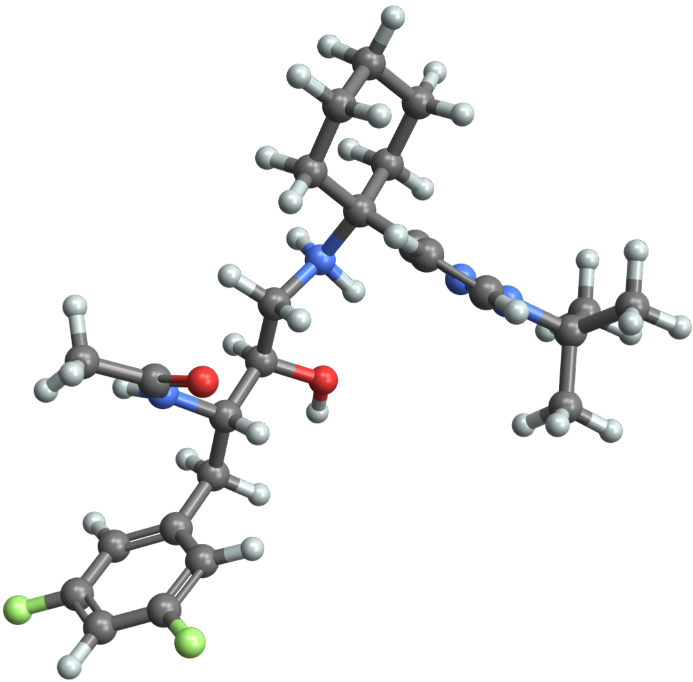

2D Plot

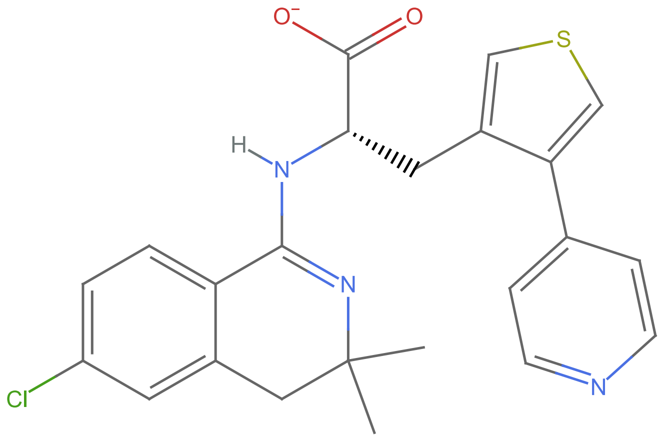

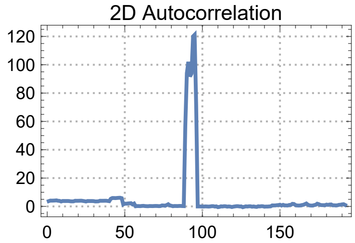

3D Plot

Dataset Index= 1200

Molecule Formula= $C_{23}H_{21}ClN_3O_2S^-$

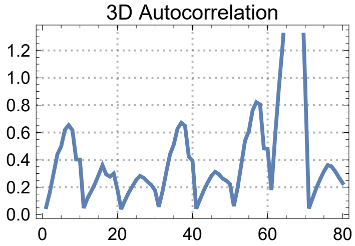

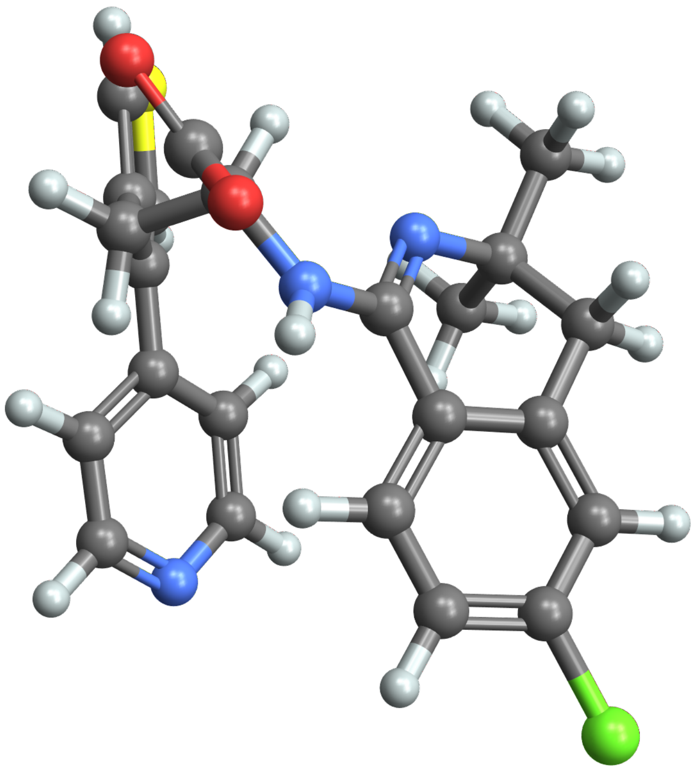

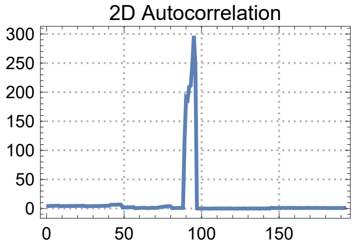

Dataset Index= 1201

Molecule Formula= $C_{35}H_{43}F_2N_3O_8S$

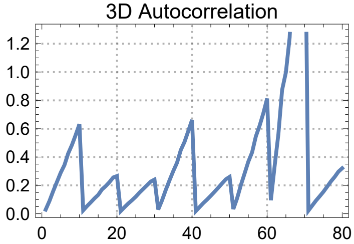

2D Plot

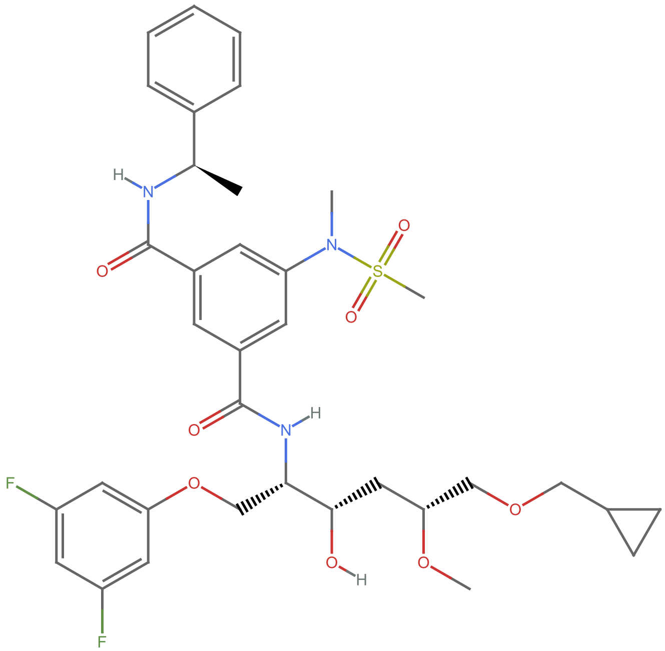

3D Plot

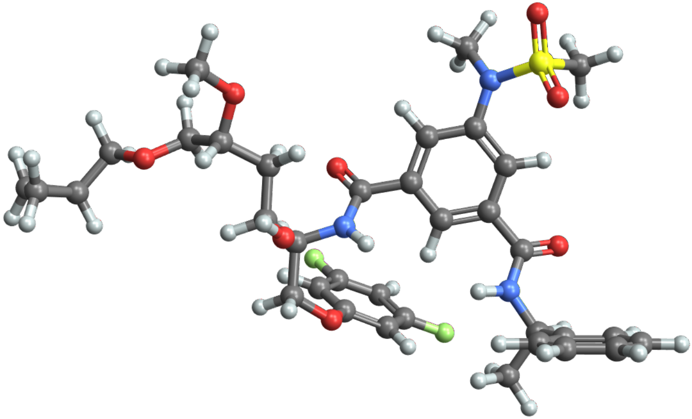

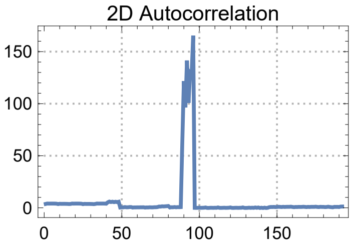

Dataset Index= 1202

Molecule Formula= $C_{27}H_{34}F_2N_3O_2^+$

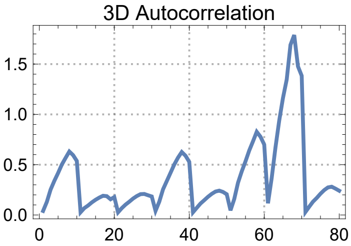

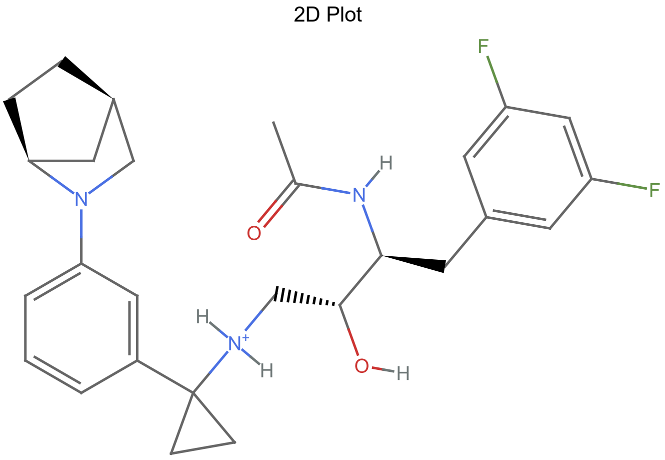

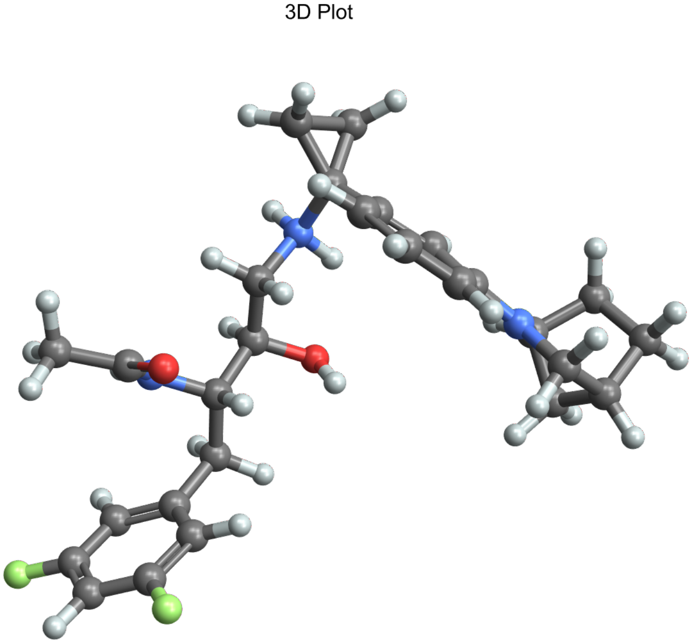

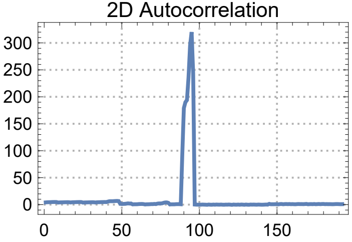

Dataset Index= 1203

Molecule Formula= $C_{35}H_{52}N_4O_8S$

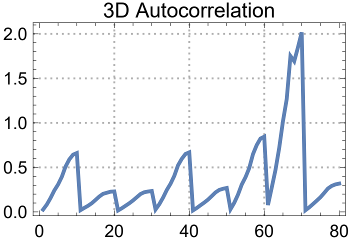

2D Plot

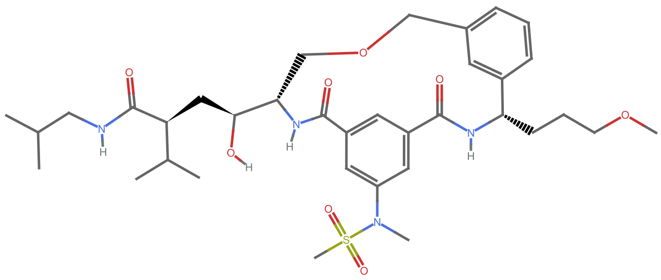

3D Plot

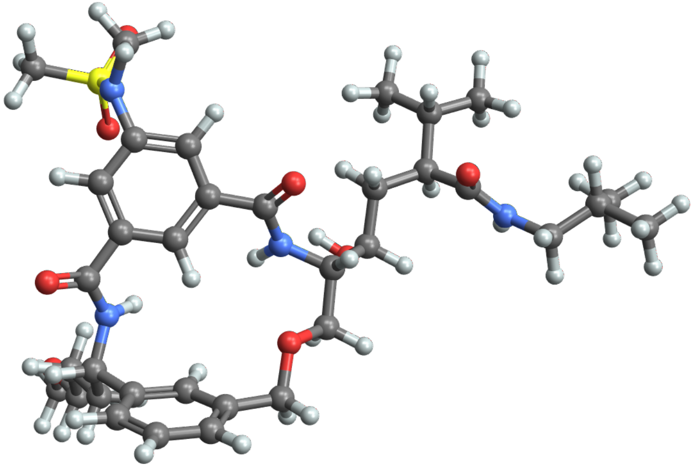

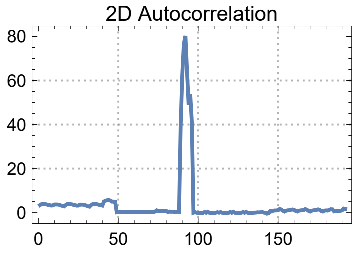

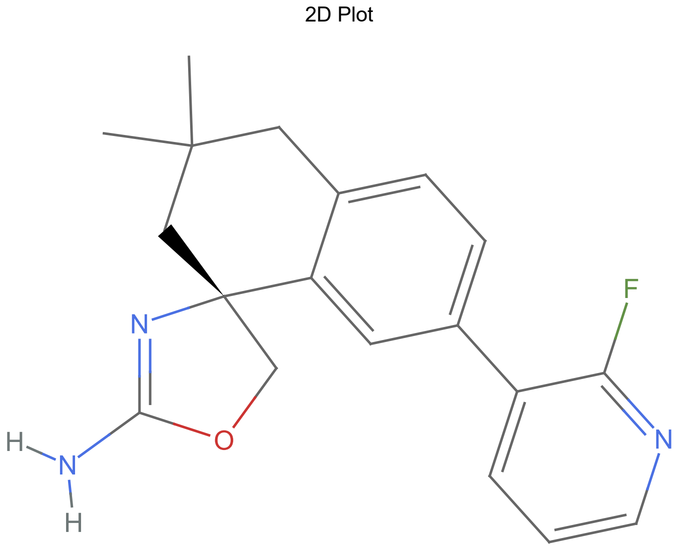

Dataset Index= 1204

Molecule Formula= $C_{19}H_{20}FN_3O$

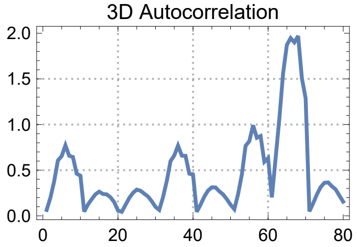

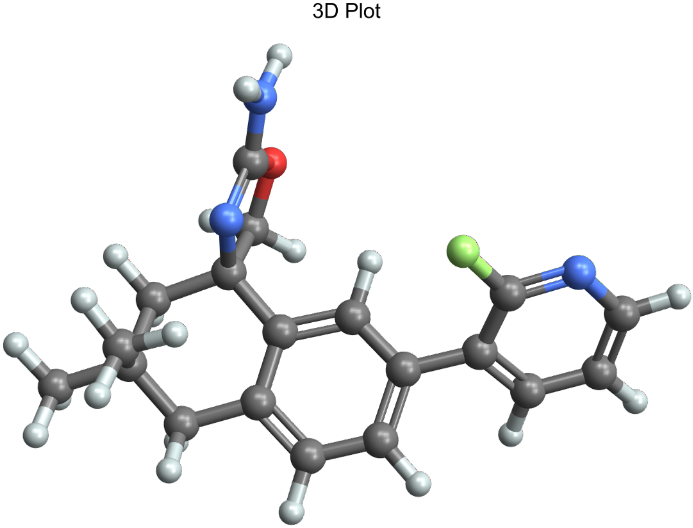

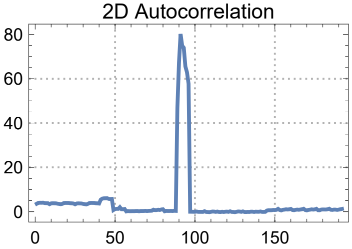

Dataset Index= 1205

Molecule Formula= $C_{22}H_{18}ClFN_2O_2$

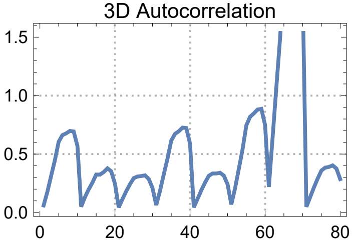

2D Plot

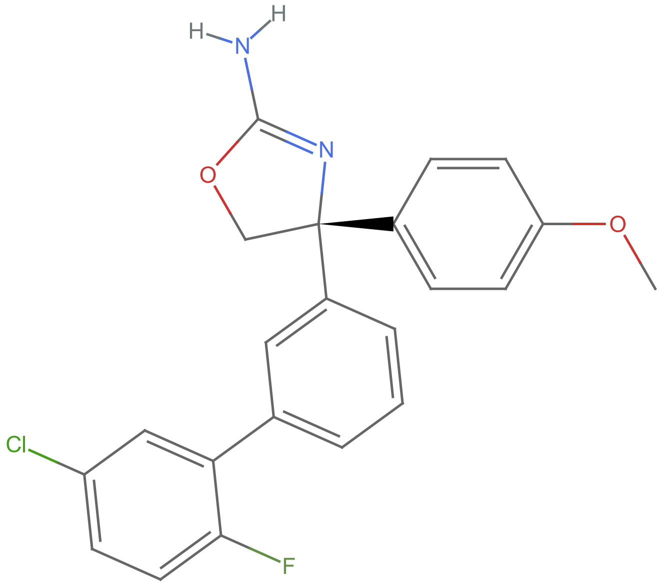

3D Plot

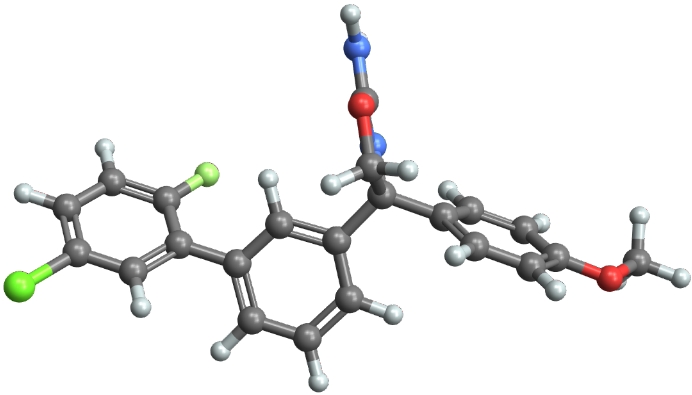

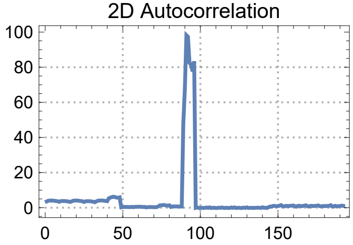

Dataset Index= 1206

Molecule Formula= $C_{20}H_{19}FN_6O$

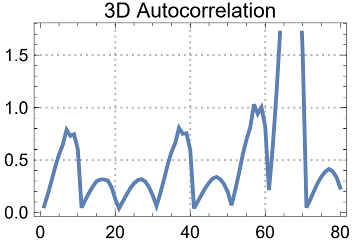

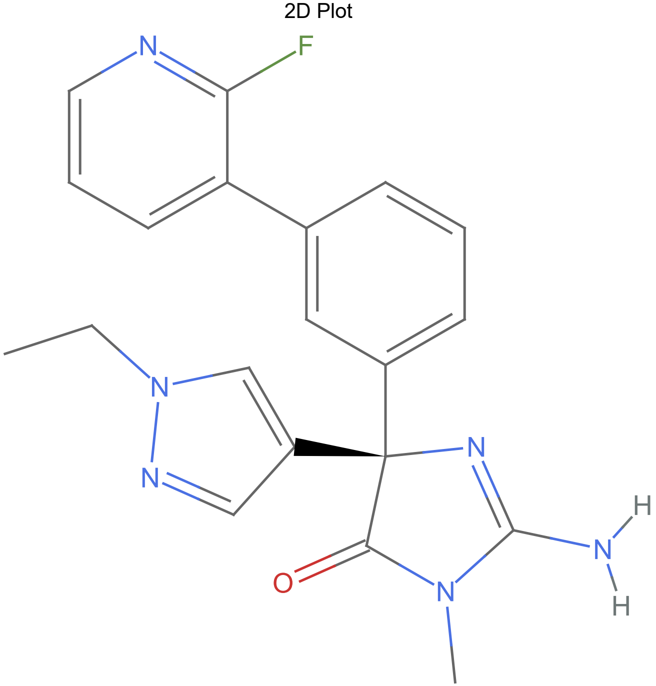

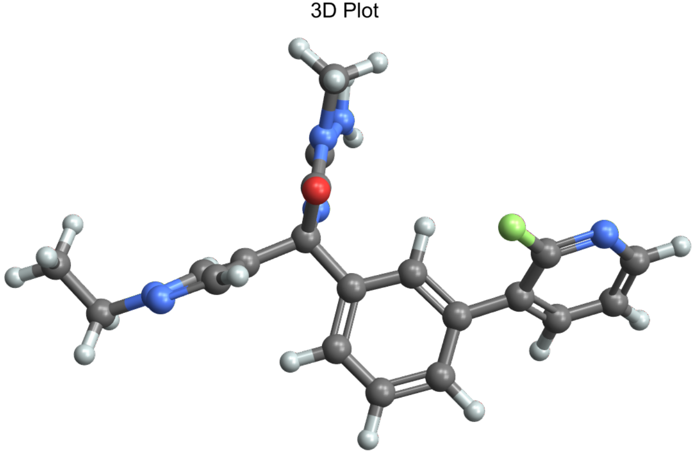

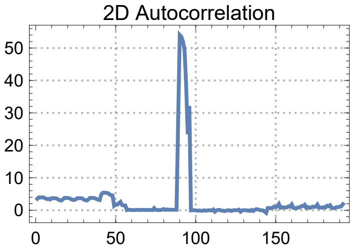

Dataset Index= 1207

Molecule Formula= $C_{19}H_{20}ClN_3S$

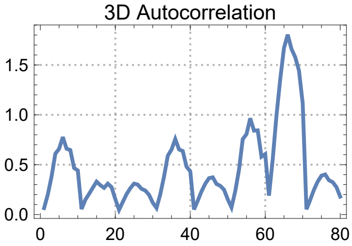

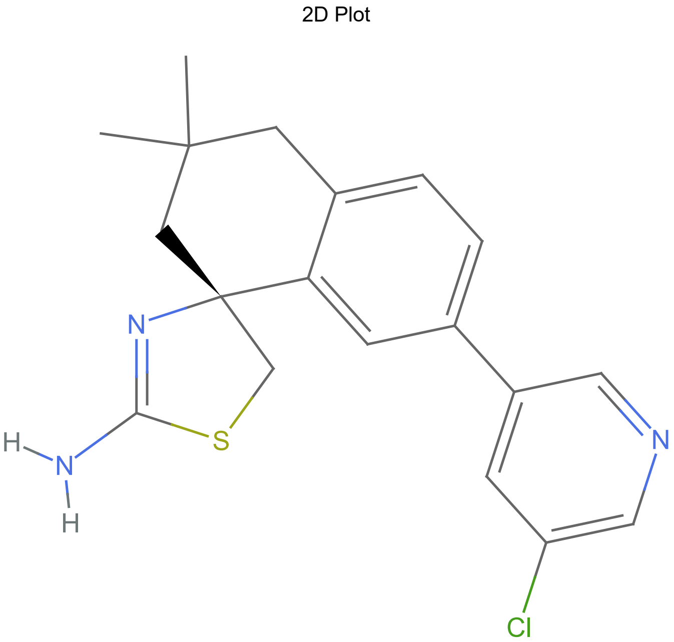

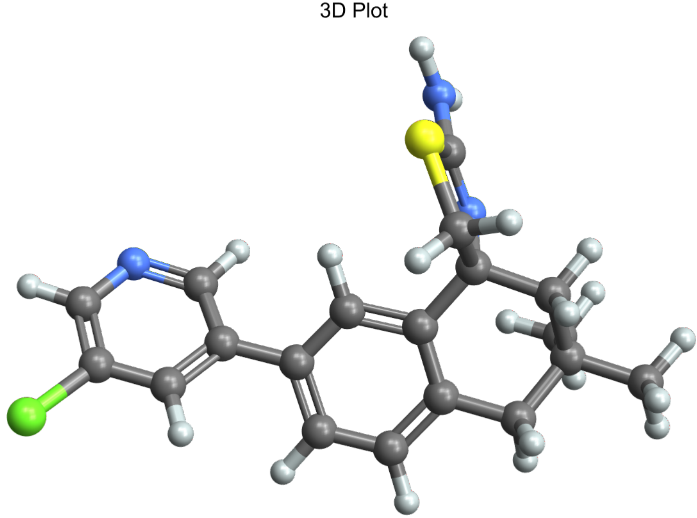

2D Plot

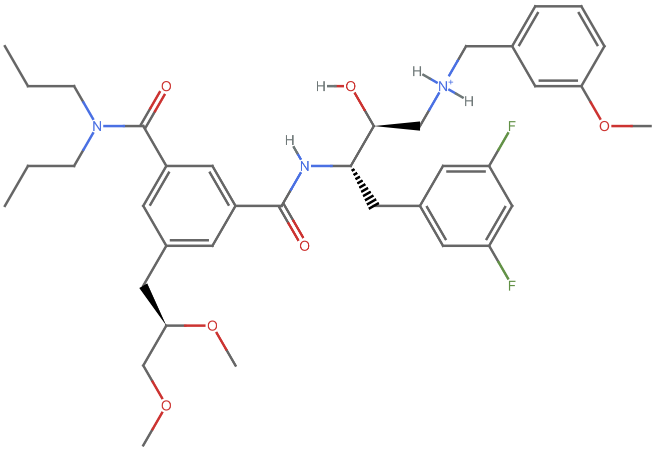

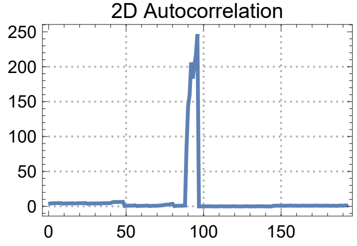

3D Plot

Dataset Index= 1208

Molecule Formula= $C_{37}H_{50}F_2N_3O_6^+$

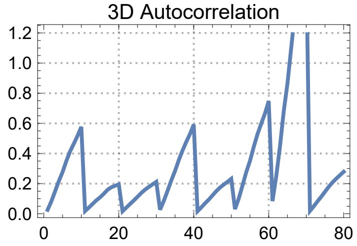

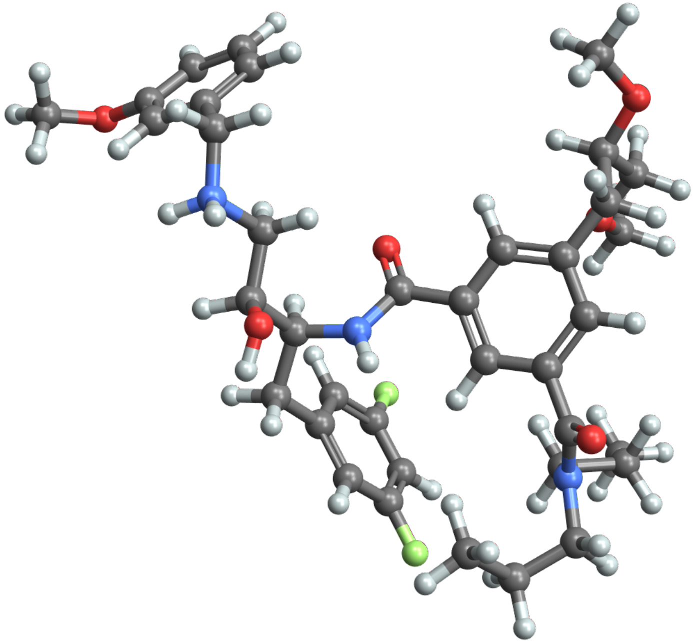

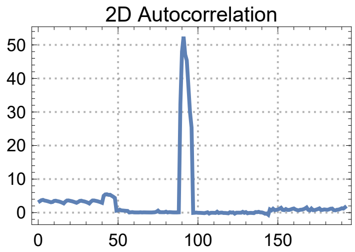

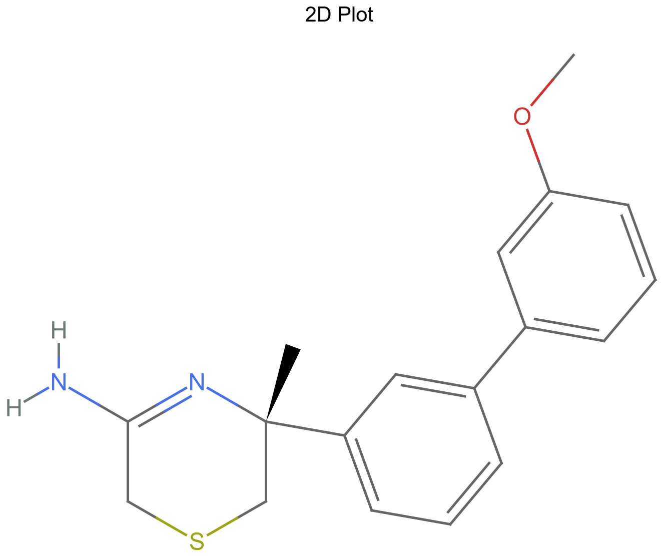

Dataset Index= 1209

Molecule Formula= $C_{18}H_{20}N_2OS$

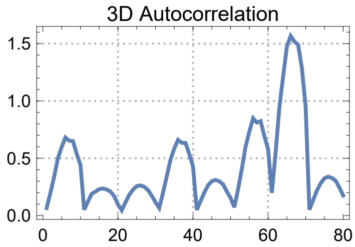

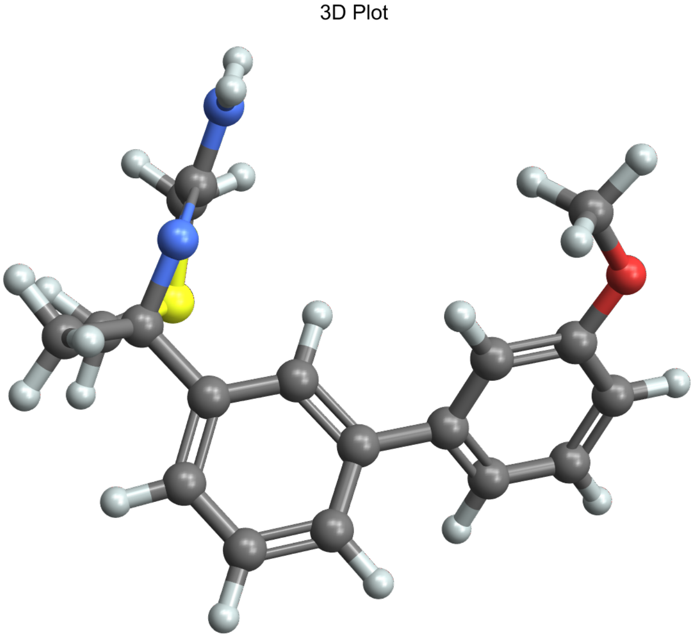

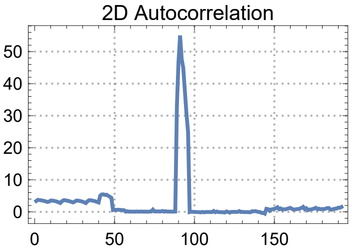

Dataset Index= 1210

Molecule Formula= $C_{18}H_{20}N_2OS$

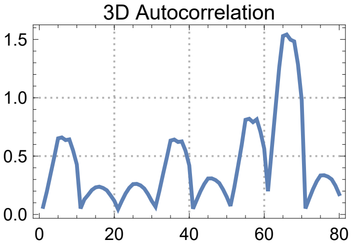

2D Plot

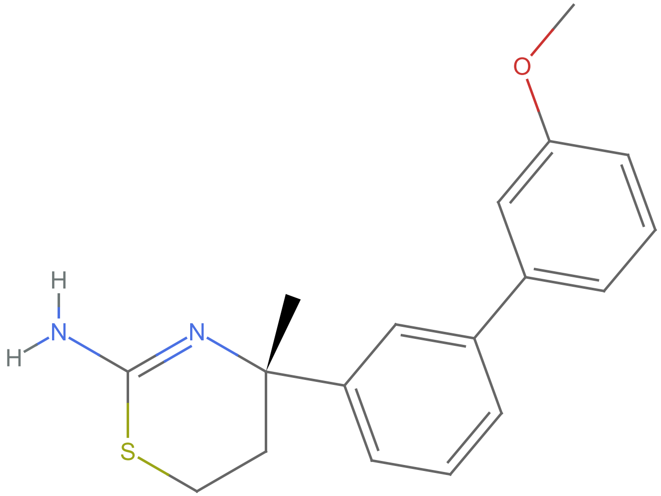

3D Plot

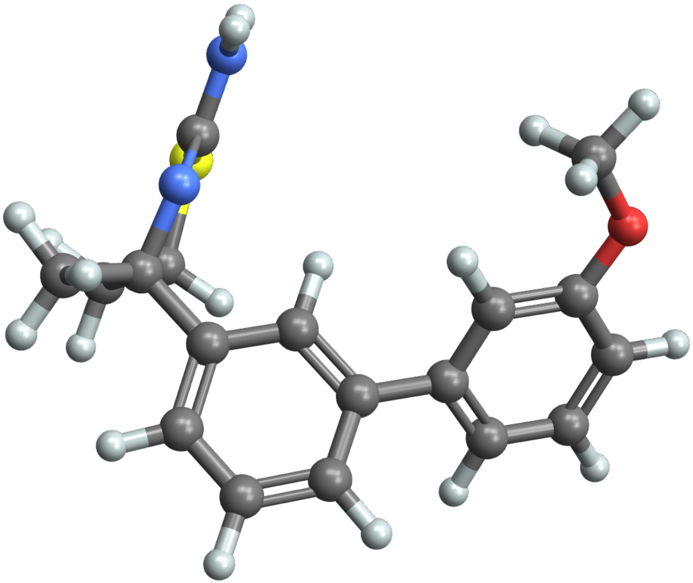

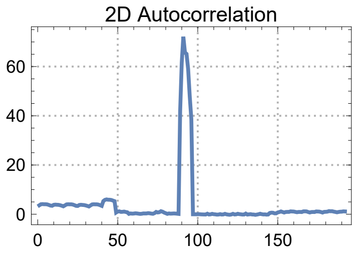

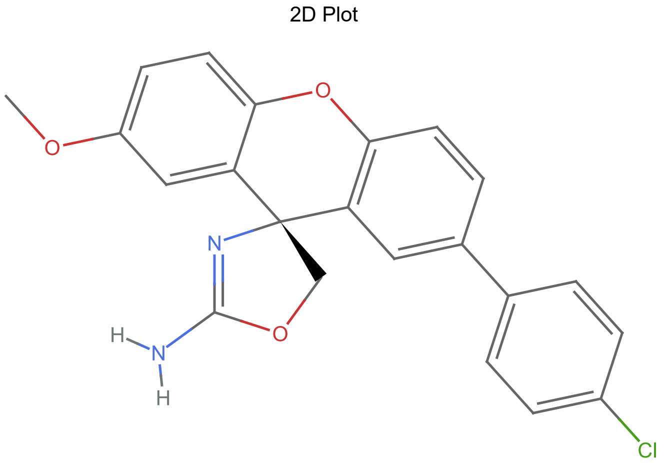

Dataset Index= 1211

Molecule Formula= $C_{22}H_{17}ClN_2O_3$

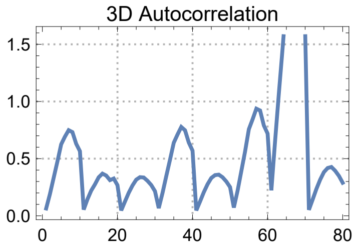

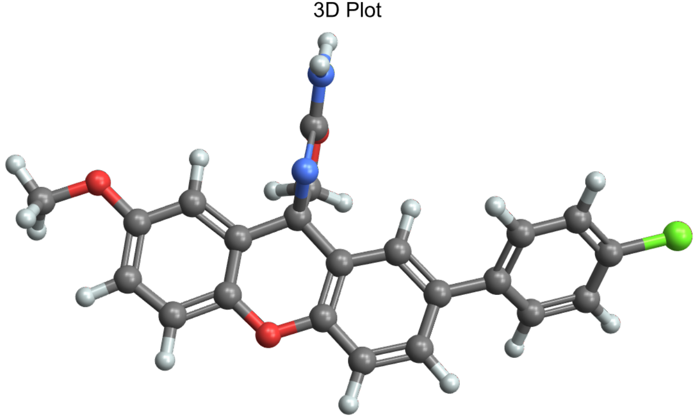

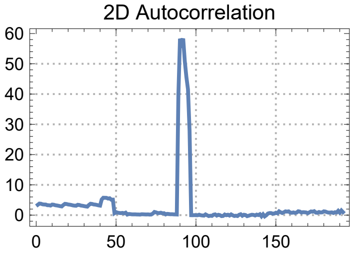

Dataset Index= 1212

Molecule Formula= $C_{16}H_{15}ClN_4O_2$

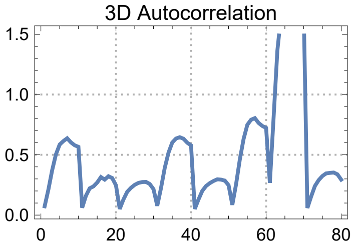

2D Plot

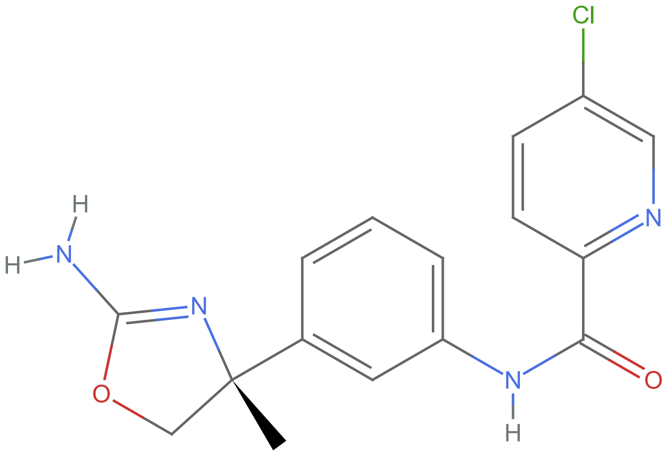

3D Plot

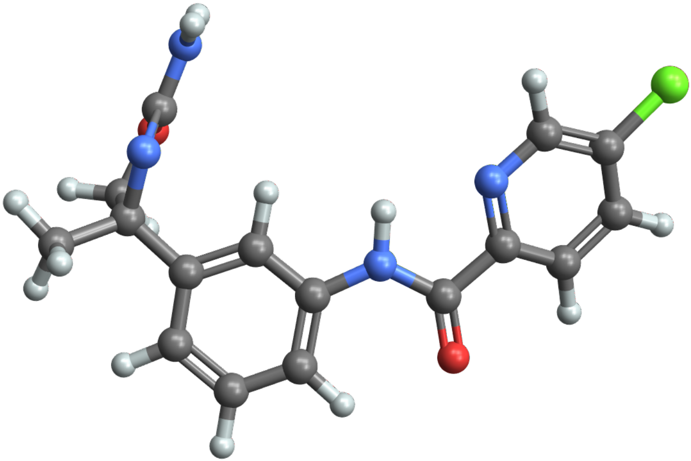

2D Autocorrelation

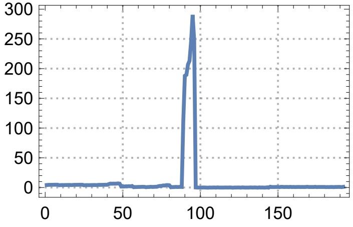

Dataset Index= 1213

Molecule Formula= $C_{34}H_{41}F_2N_3O_8S$

3D Autocorrelation

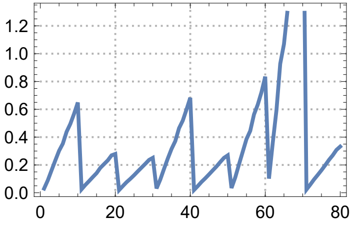

2D Plot

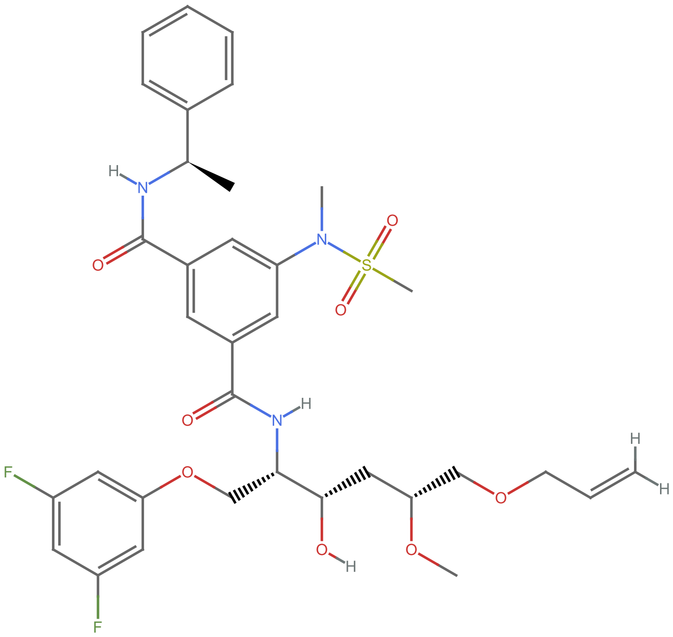

3D Plot

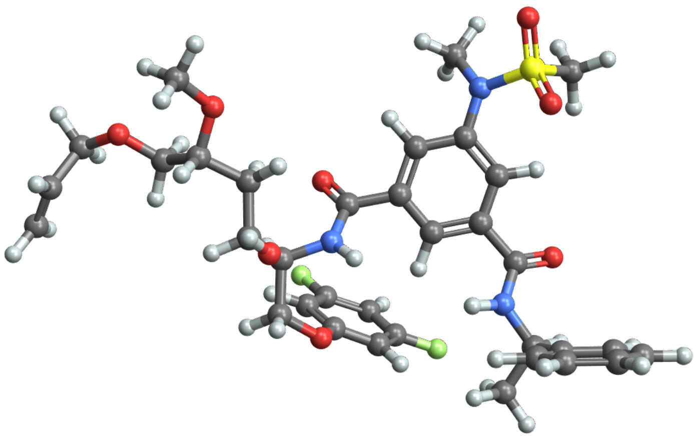

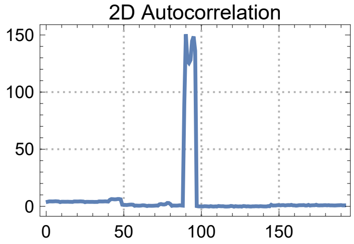

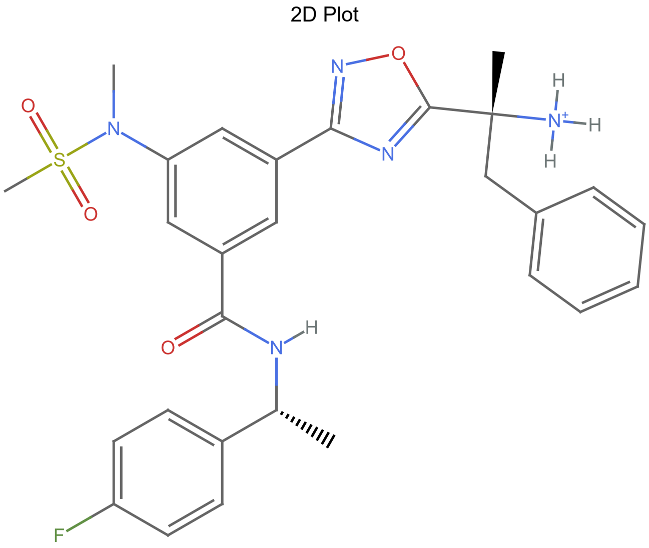

Dataset Index= 1214

Molecule Formula= $C_{28}H_{31}FN_5O_4S^+$

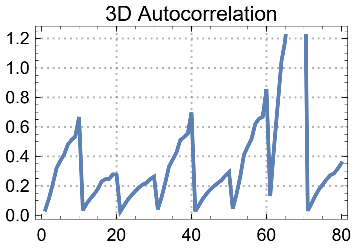

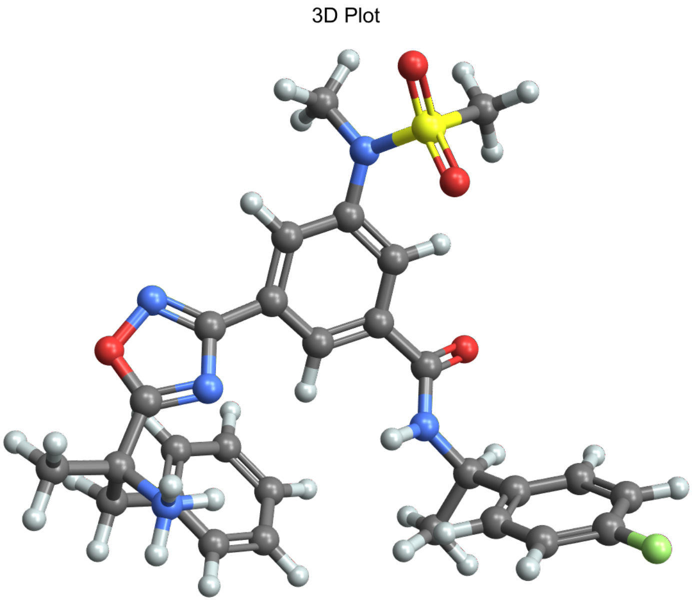

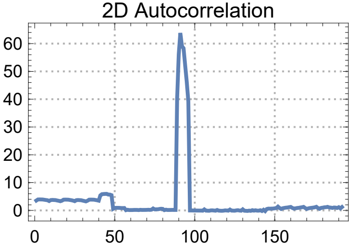

Dataset Index= 1215

Molecule Formula= $C_{22}H_{19}ClN_2O_2$

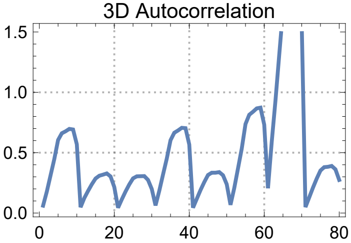

2D Plot

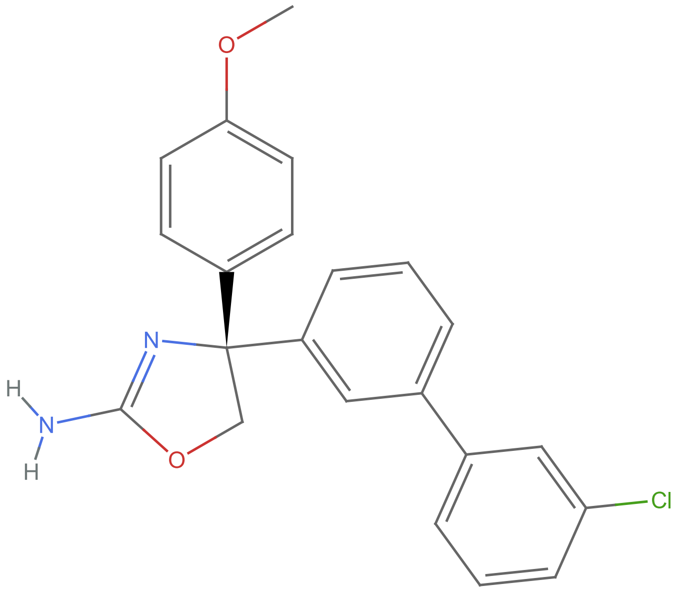

3D Plot

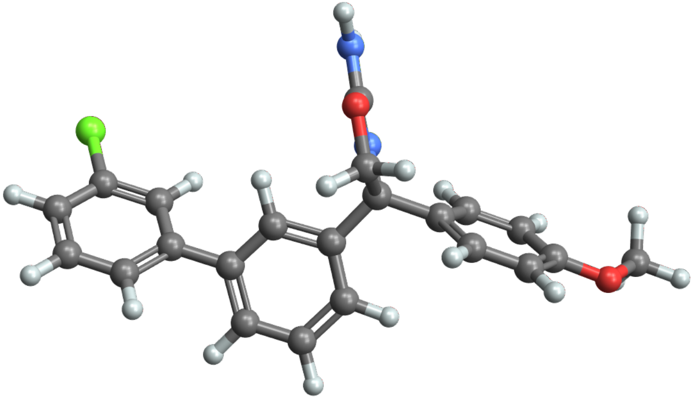

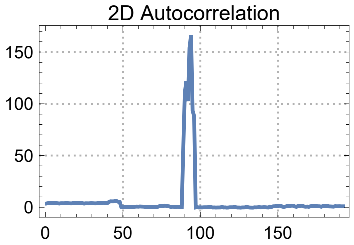

Dataset Index= 1216

Molecule Formula= $C_{30}H_{47}N_5O_2$

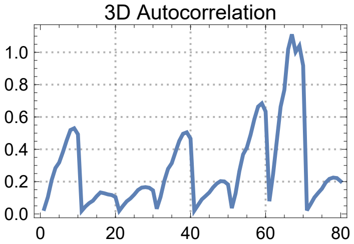

2D Plot

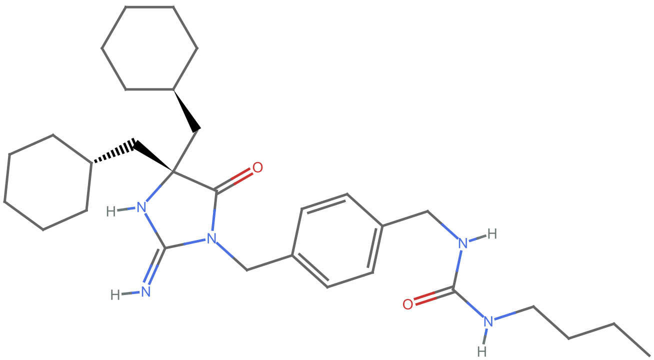

3D Plot

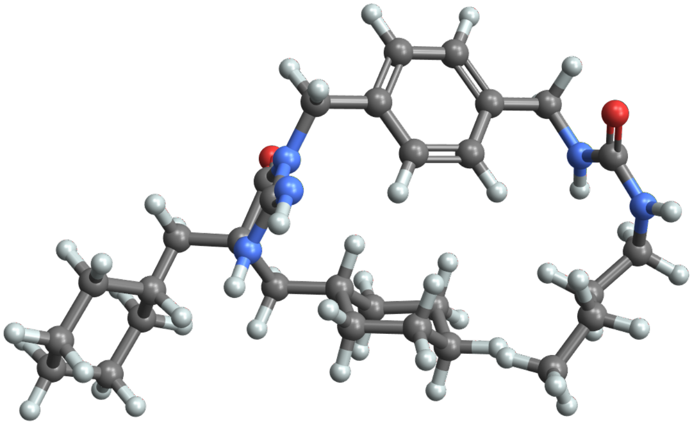

2D Autocorrelation

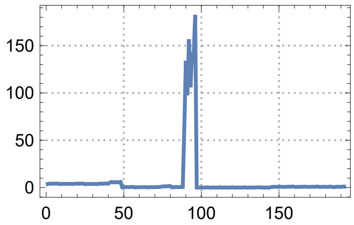

Dataset Index= 1217

Molecule Formula= $C_{29}H_{41}F_2N_2O_2^+$

3D Autocorrelation

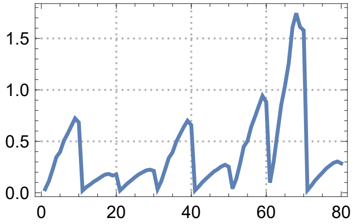

2D Plot

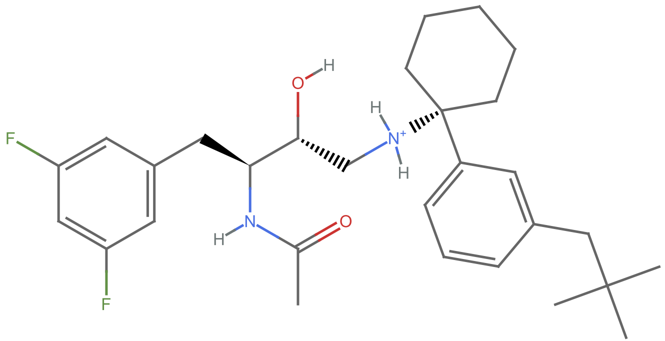

3D Plot

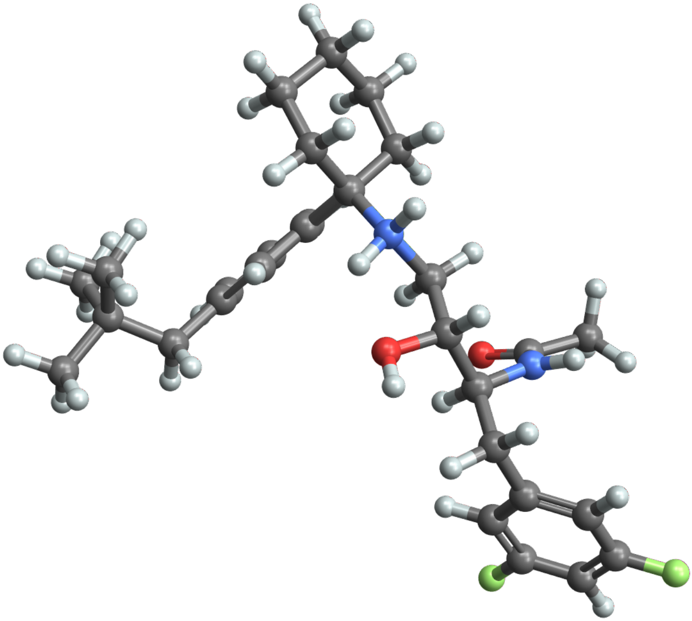

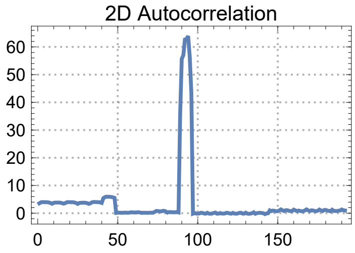

Dataset Index= 1218

Molecule Formula= $C_{23}H_{23}N_5O$

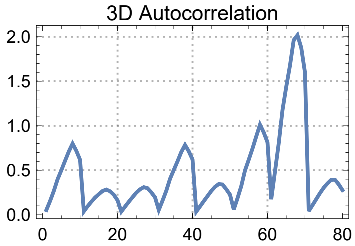

2D Plot

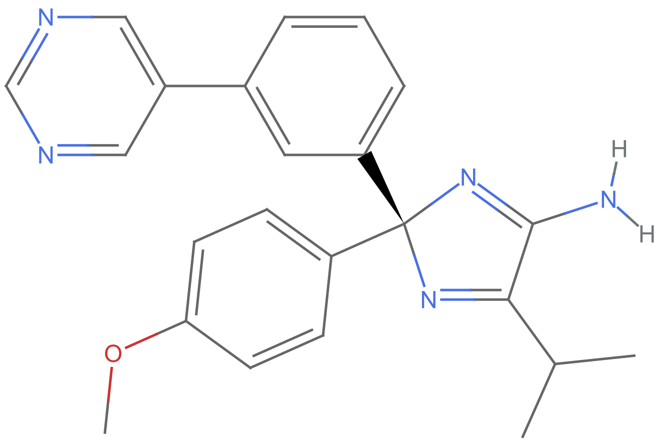

3D Plot

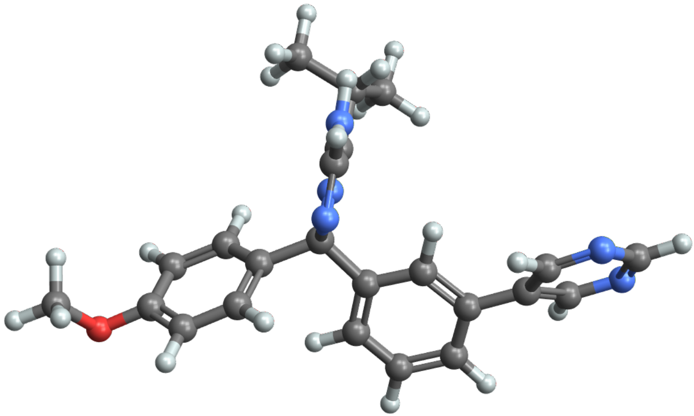

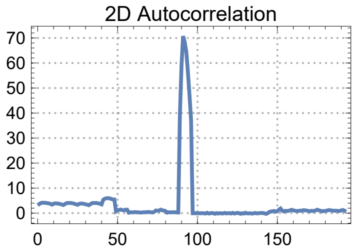

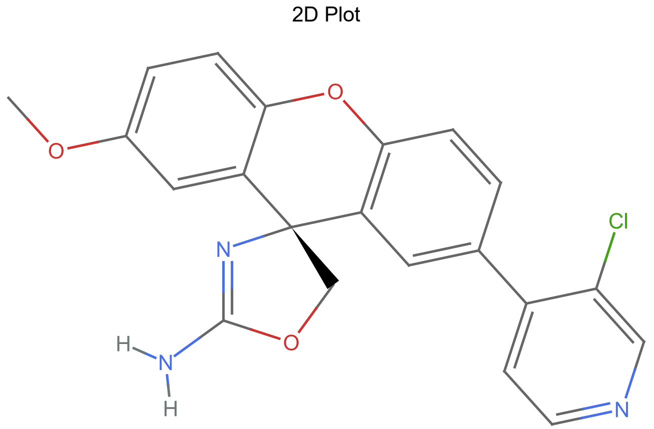

Dataset Index= 1219

Molecule Formula= $C_{21}H_{16}ClN_3O_3$

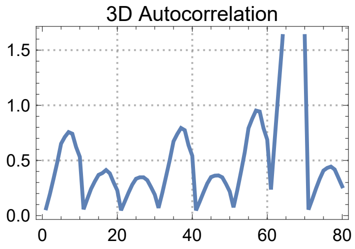

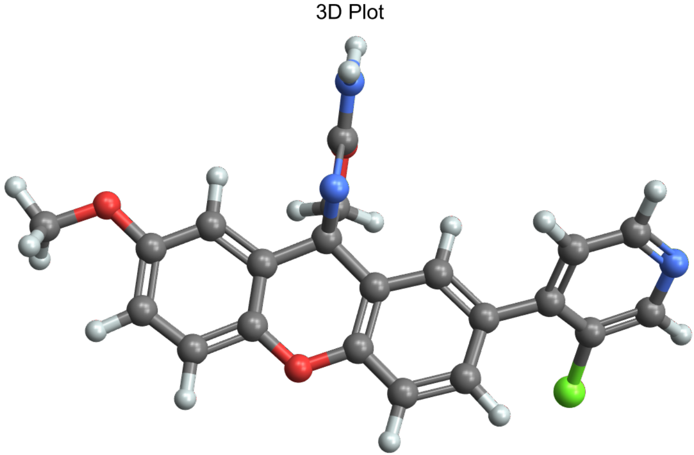

2D Plot

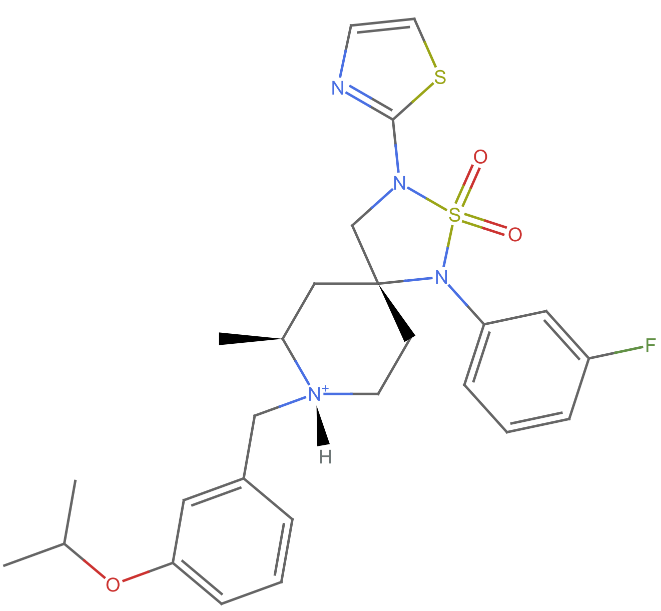

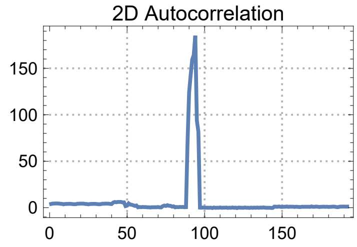

3D Plot

Dataset Index= 1220

Molecule Formula= $C_{26}H_{32}FN_4O_3S_2^+$

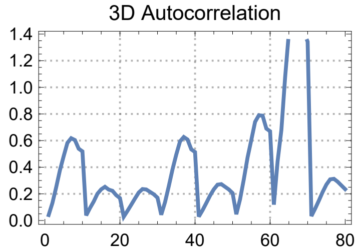

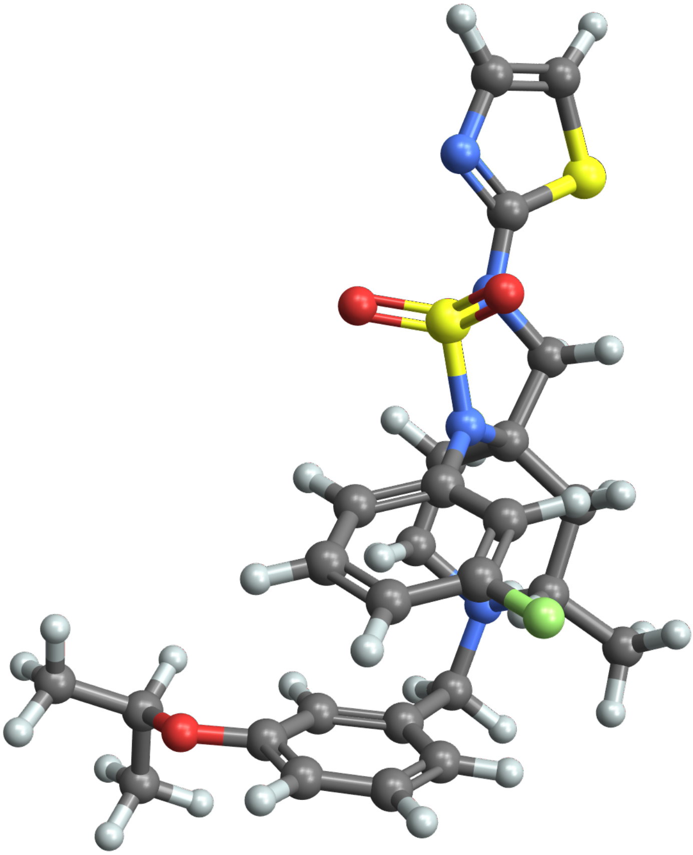

2D Autocorrelation

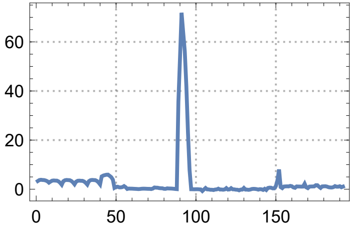

Dataset Index= 1221

Molecule Formula= $C_{16}H_{14}ClN_3O$

3D Autocorrelation

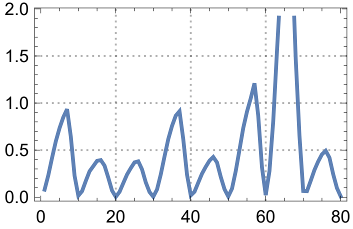

2D Plot

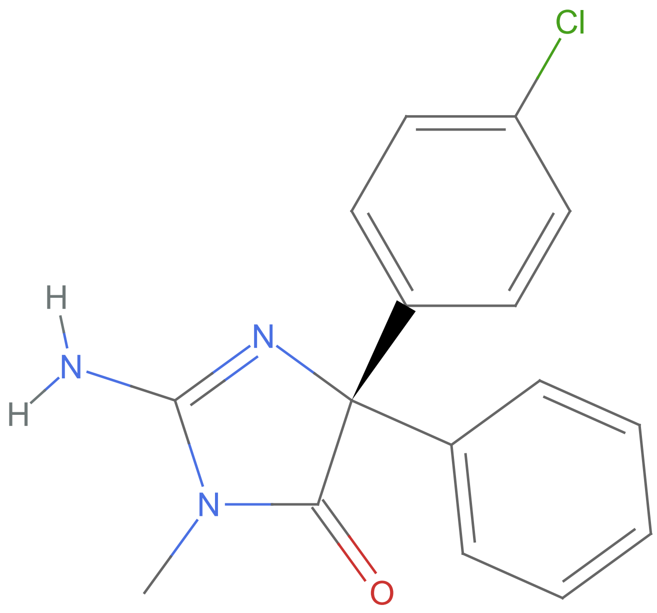

3D Plot

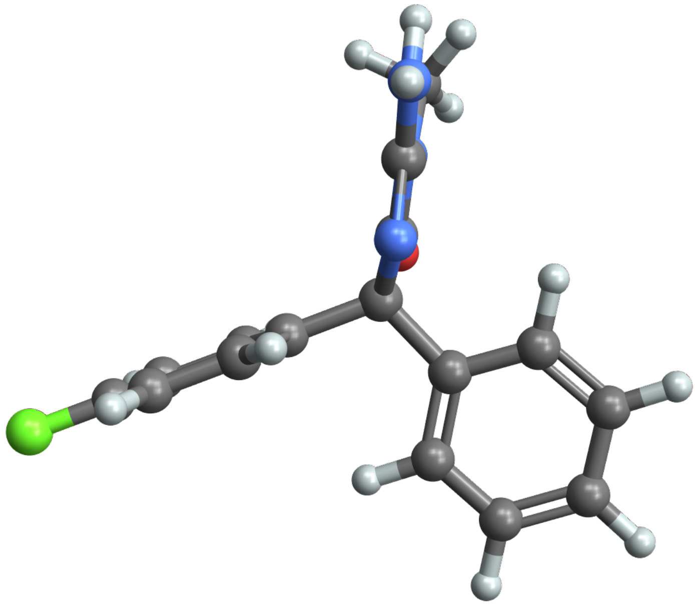

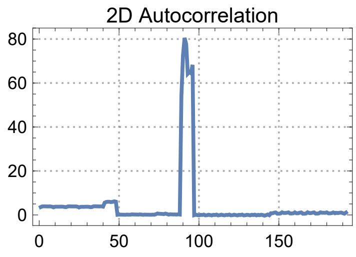

Dataset Index= 1222

Molecule Formula= $C_{25}H_{24}N_4O$

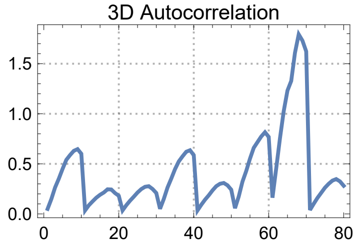

2D Plot

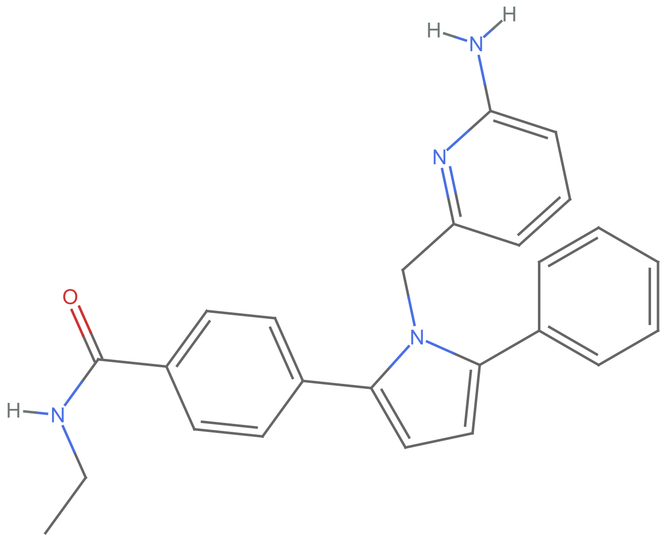

3D Plot

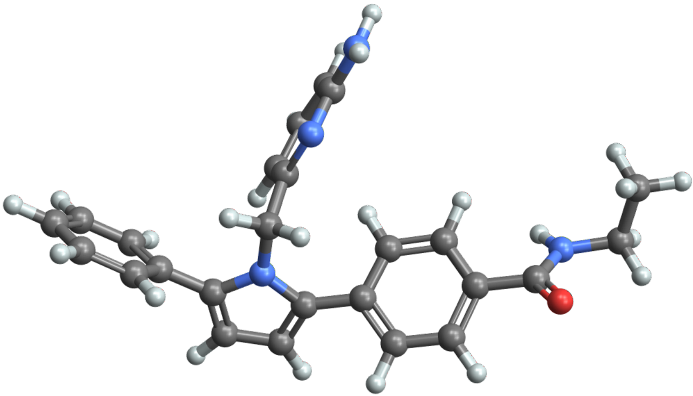

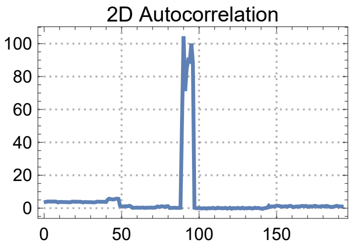

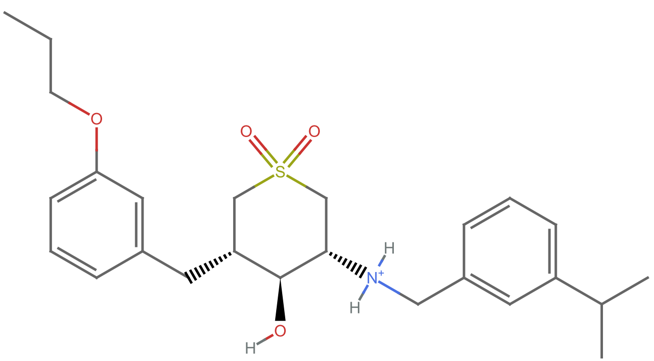

Dataset Index= 1223

Molecule Formula= $C_{25}H_{36}NO_4S^+$

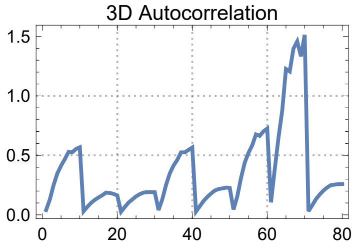

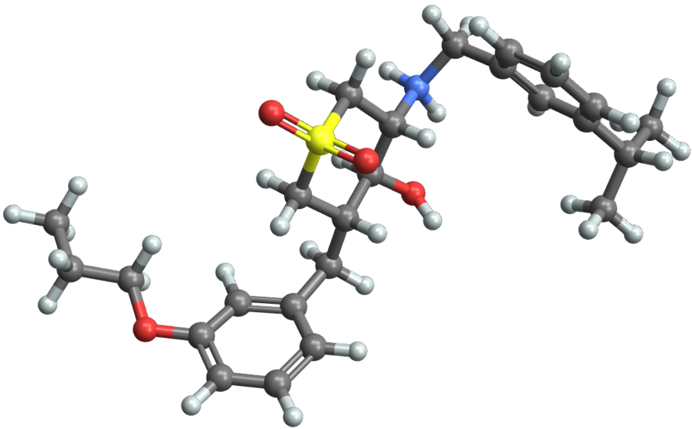

2D Plot

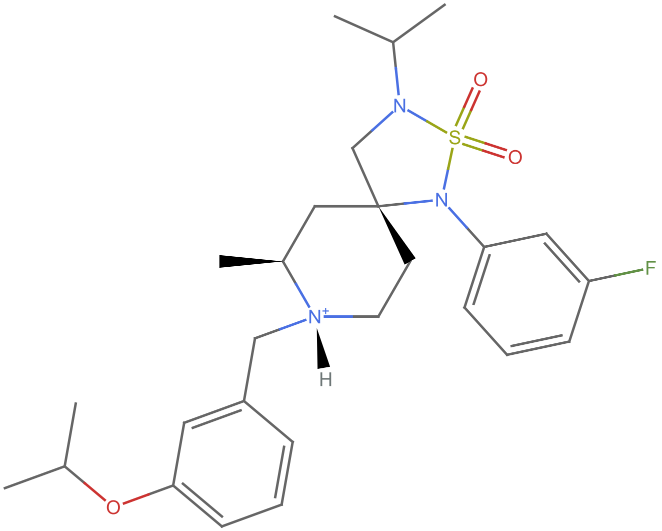

2D Autocorrelation

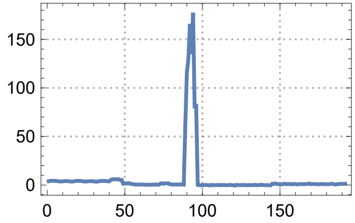

3D Plot

Dataset Index= 1224

Molecule Formula= $C_{26}H_{37}FN_3O_3S^+$

3D Autocorrelation

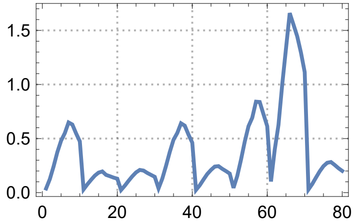

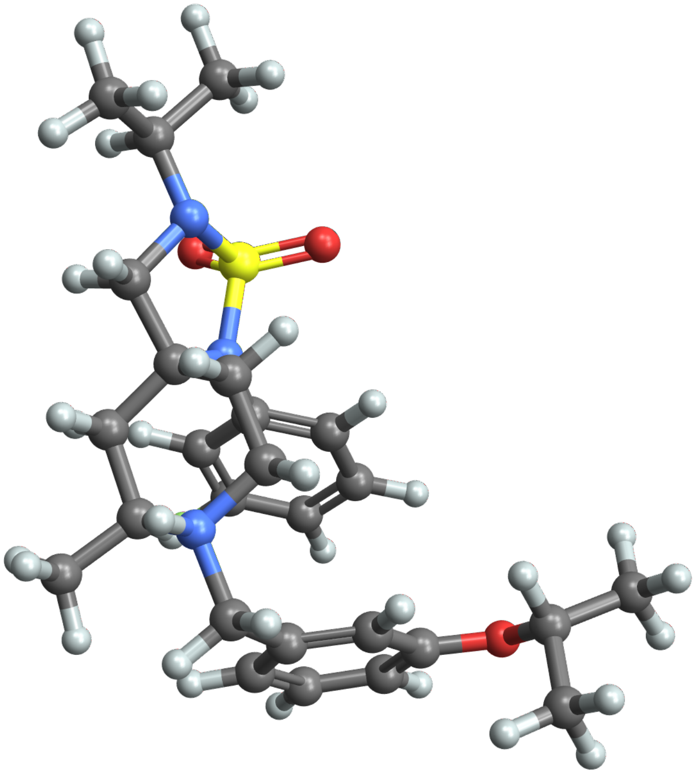

2D Autocorrelation

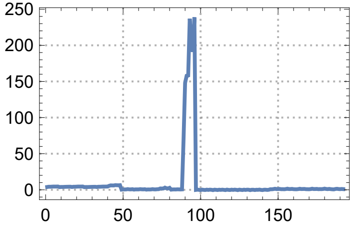

Dataset Index= 1225

Molecule Formula= $C_{34}H_{58}N_4O_6$

3D Autocorrelation

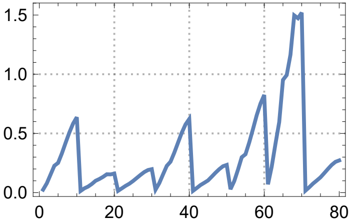

2D Plot

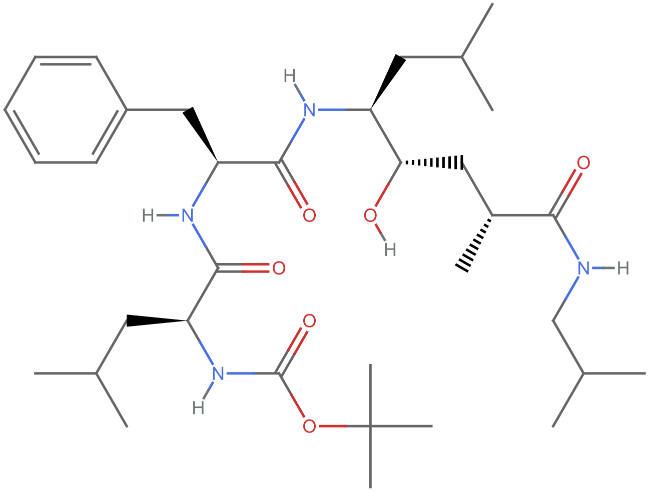

3D Plot

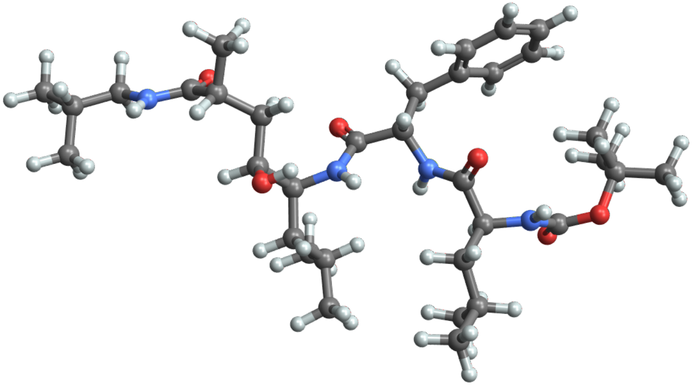

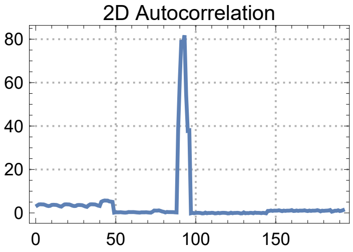

Dataset Index= 1226

Molecule Formula= $C_{19}H_{20}N_4O_2$

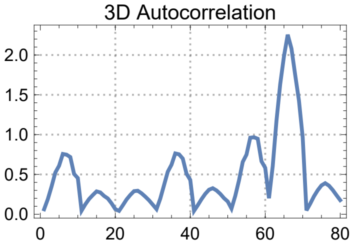

2D Plot

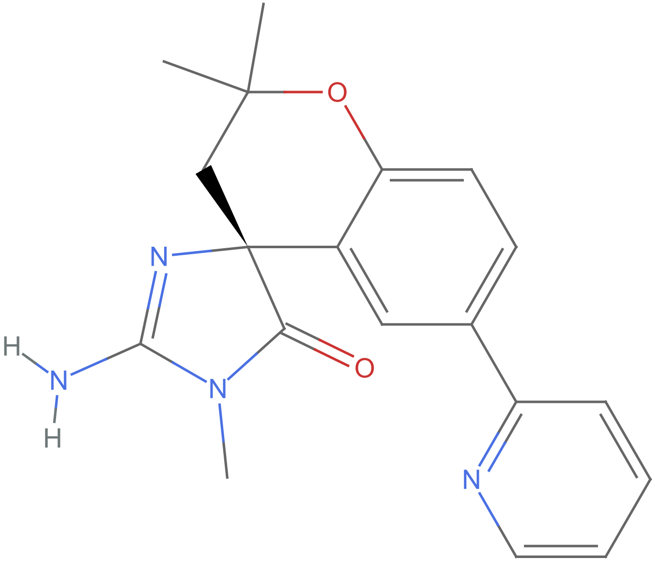

3D Plot

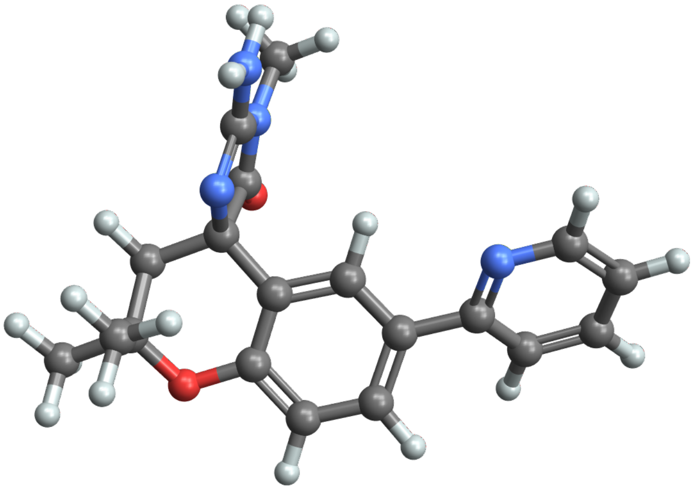

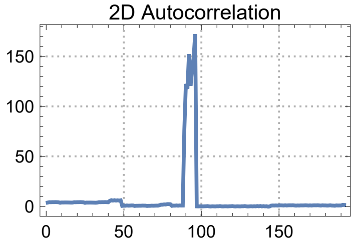

Dataset Index= 1227

Molecule Formula= $C_{25}H_{29}F_2N_4O_3^+$

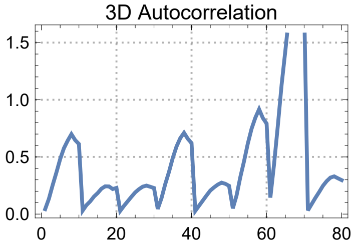

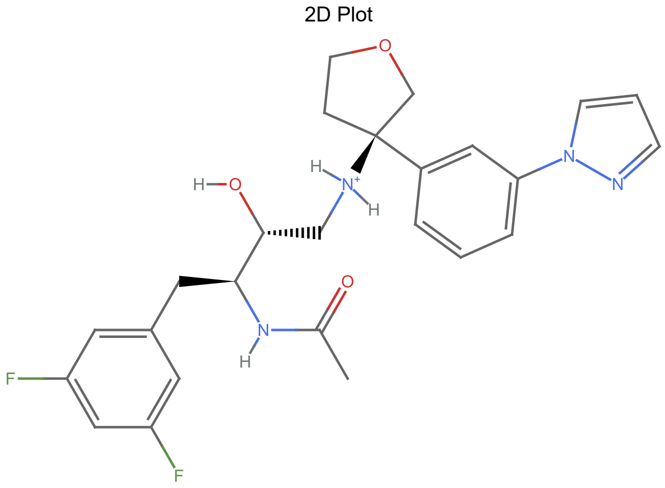

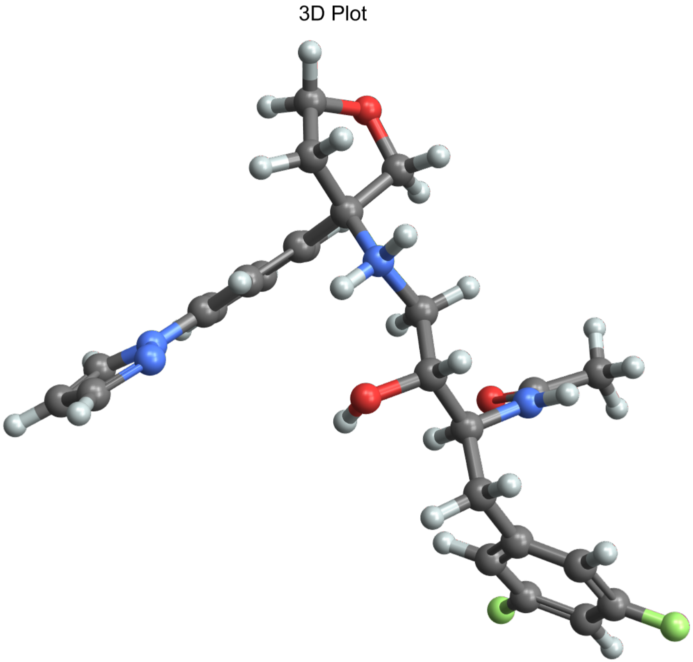

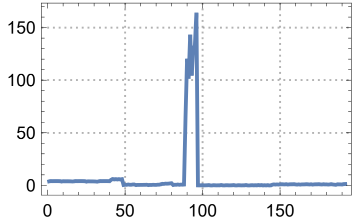

Dataset Index= 1228

Molecule Formula= $C_{26}H_{32}F_2N_3O_3^+$

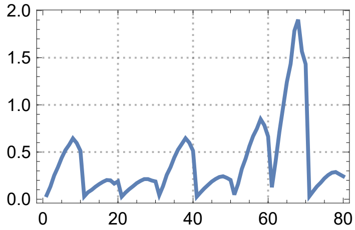

2D Plot

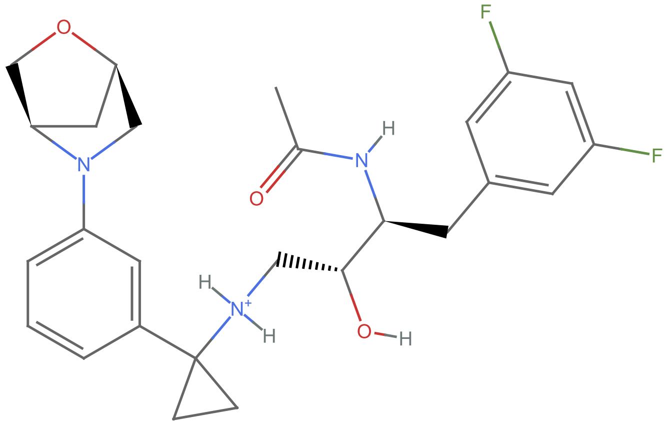

3D Plot

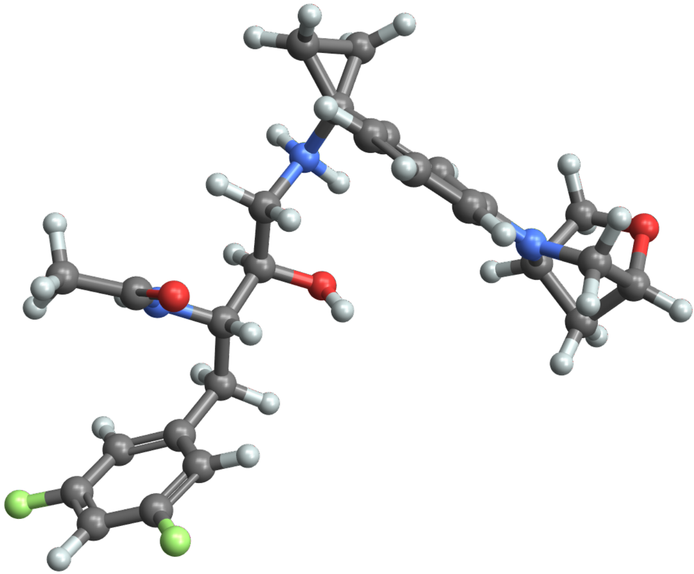

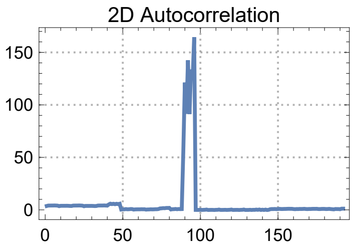

Dataset Index= 1229

Molecule Formula= $C_{26}H_{37}F_2N_4O_2^+$

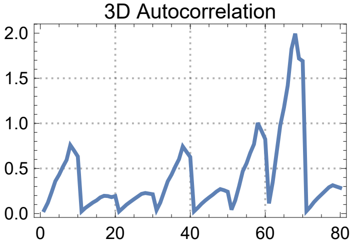

2D Plot

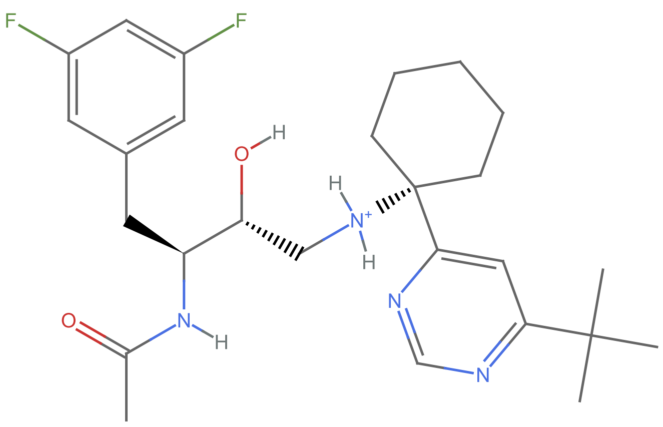

3D Plot

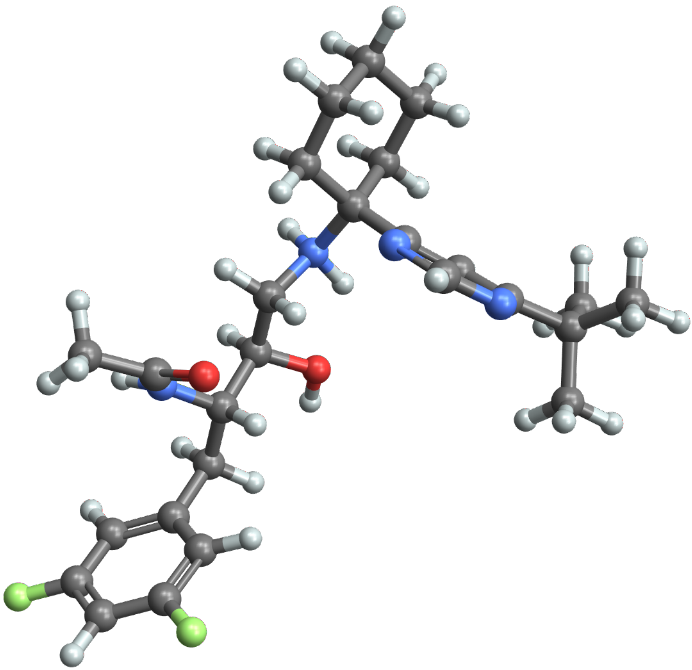

2D Plot

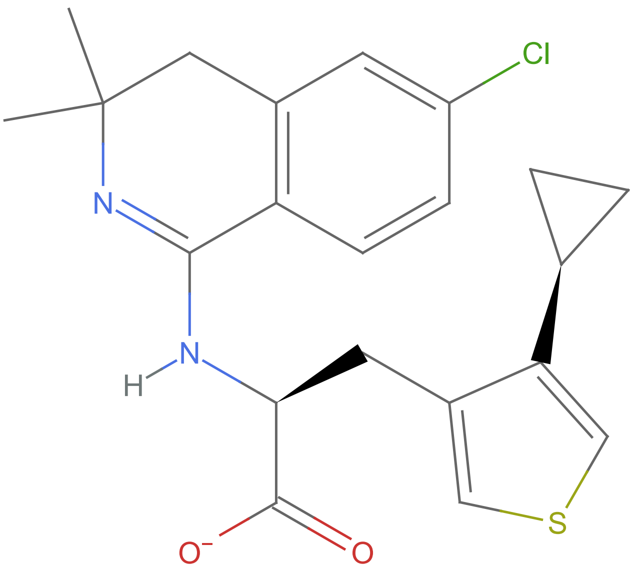

3D Plot

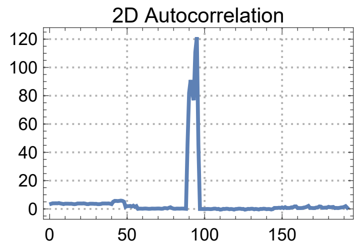

Dataset Index= 1230

Molecule Formula= $C_{21}H_{22}ClN_2O_2S^-$

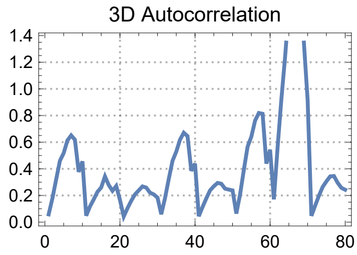

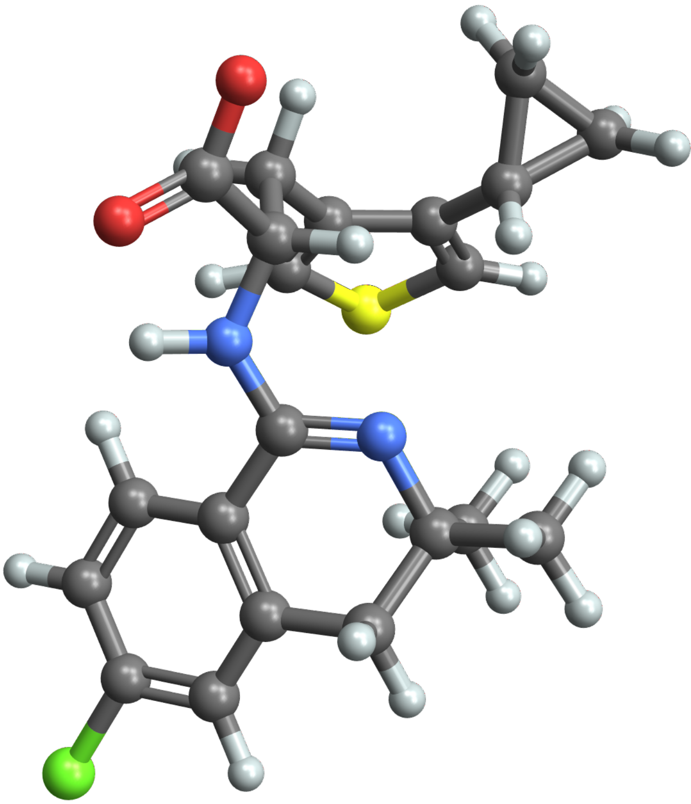

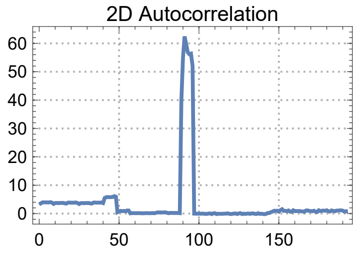

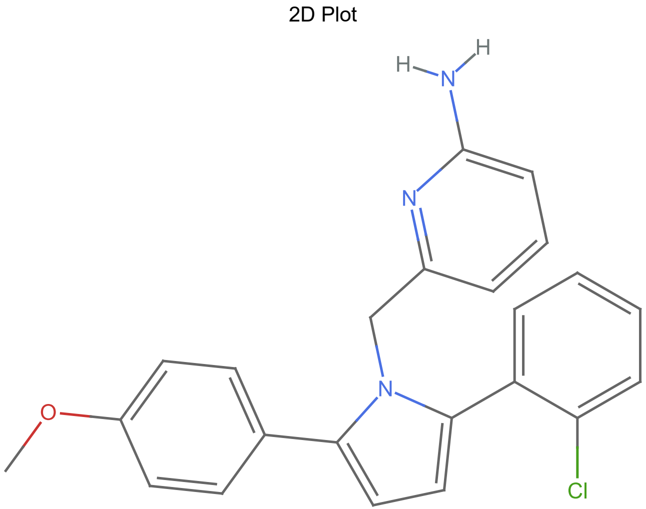

Dataset Index= 1231

Molecule Formula= $C_{23}H_{20}ClN_3O$

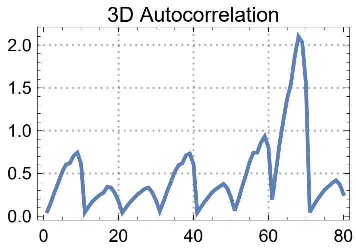

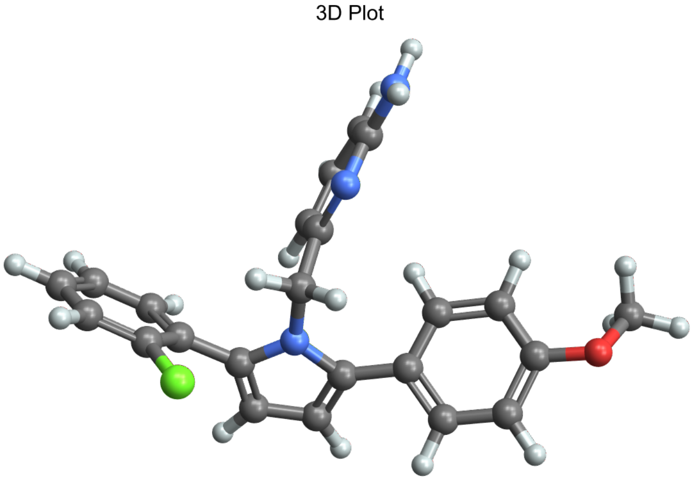

2D Autocorrelation

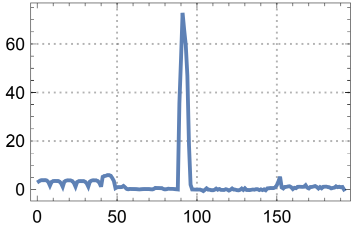

Dataset Index= 1232

Molecule Formula= $C_{16}H_{14}ClN_3O$

3D Autocorrelation

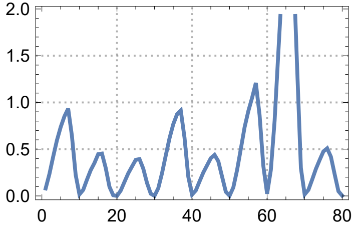

2D Plot

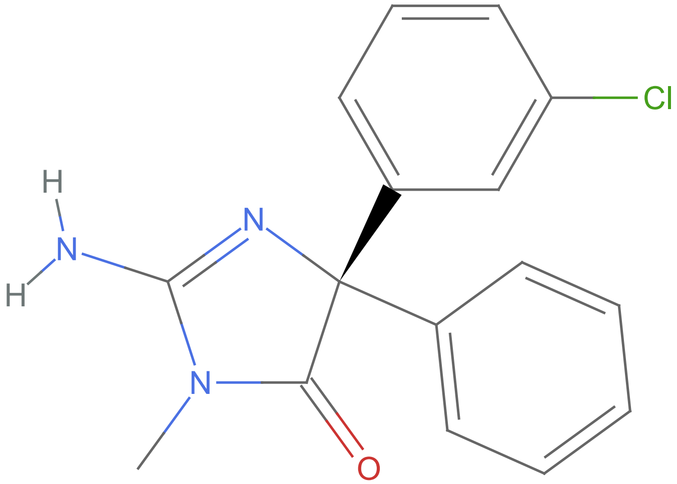

3D Plot

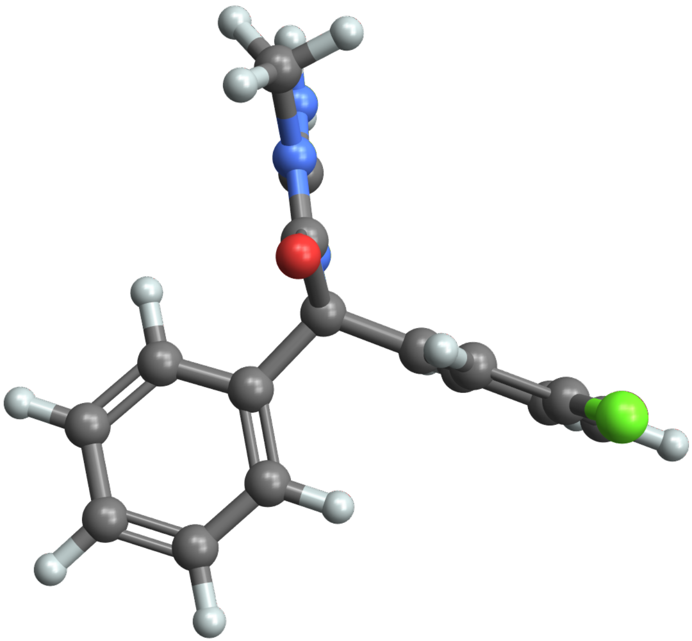

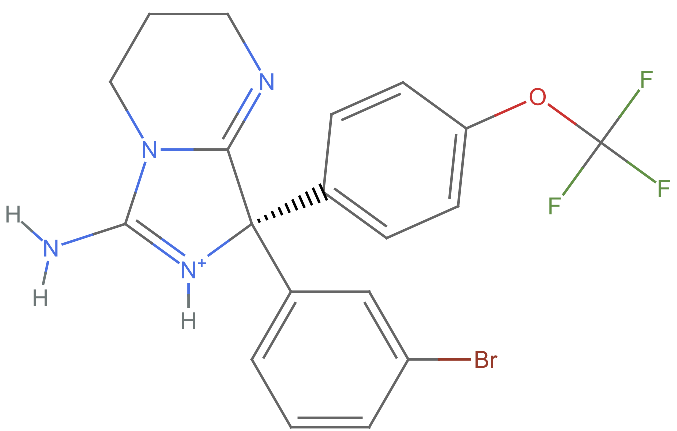

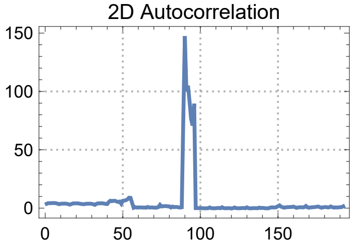

Dataset Index= 1233

Molecule Formula= $C_{19}H_{17}BrF_{3}N_{4}O^{+}$

3D Plot

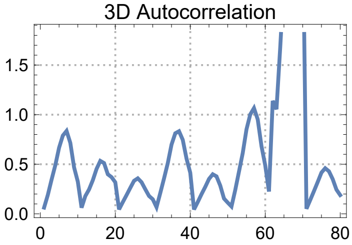

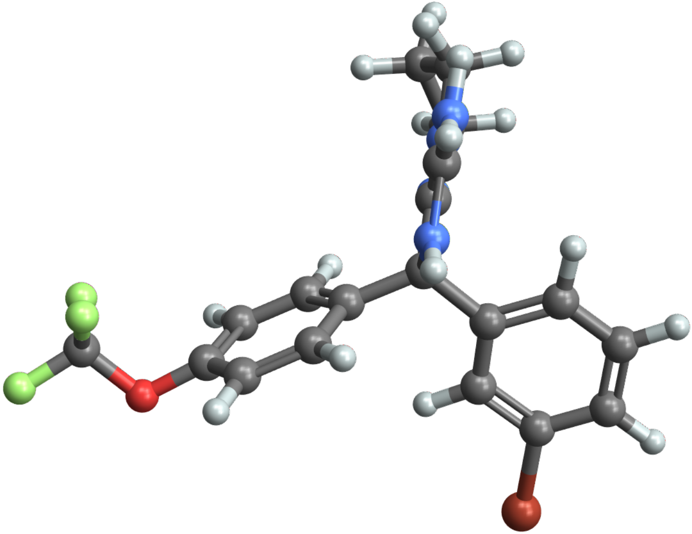

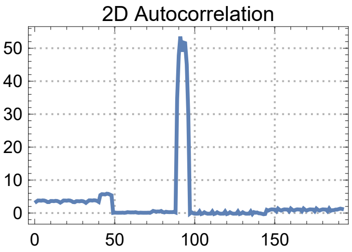

Dataset Index= 1234

Molecule Formula= $C_{21}H_{19}N_5$

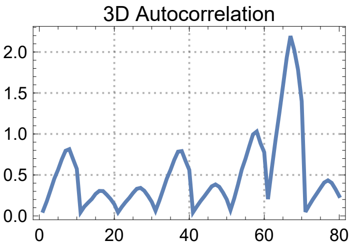

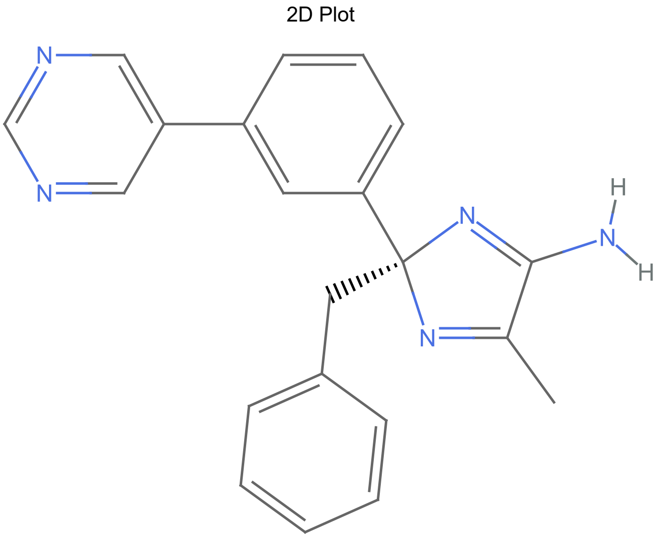

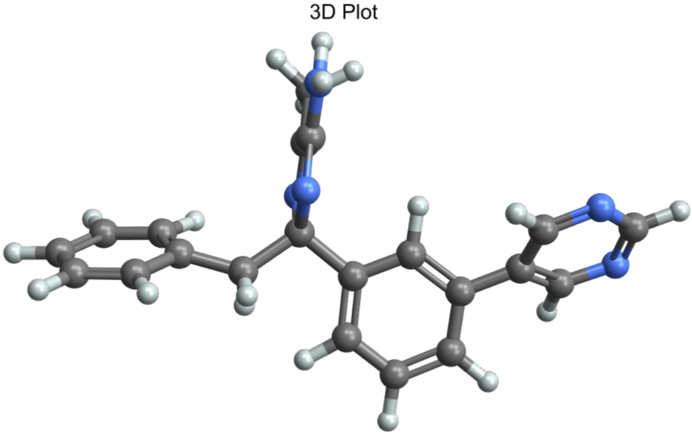

2D Autocorrelation

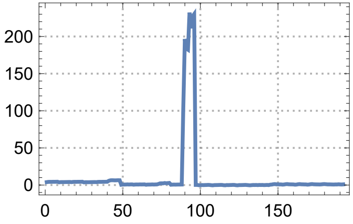

Dataset Index= 1235

Molecule Formula= $C_{30}H_{41}N_5O_8$

3D Autocorrelation

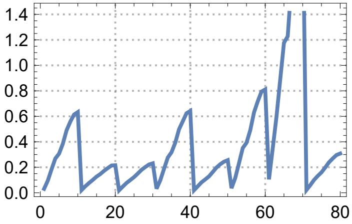

2D Plot

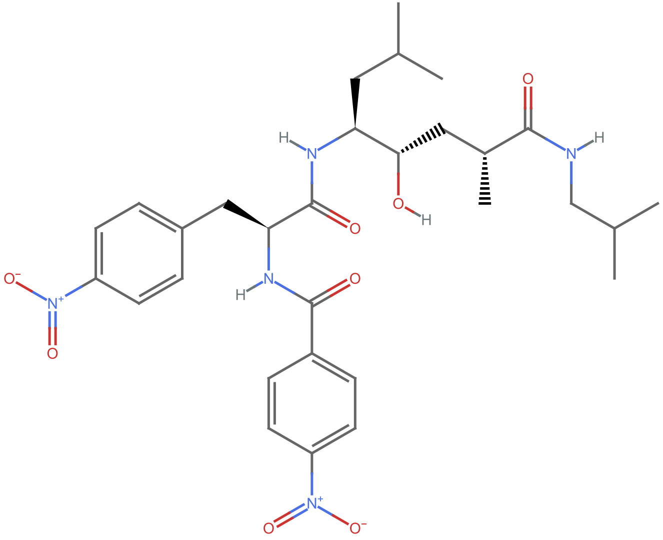

3D Plot

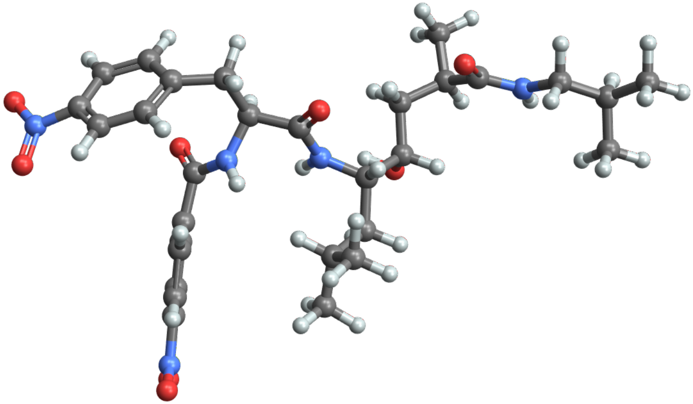

2D Plot

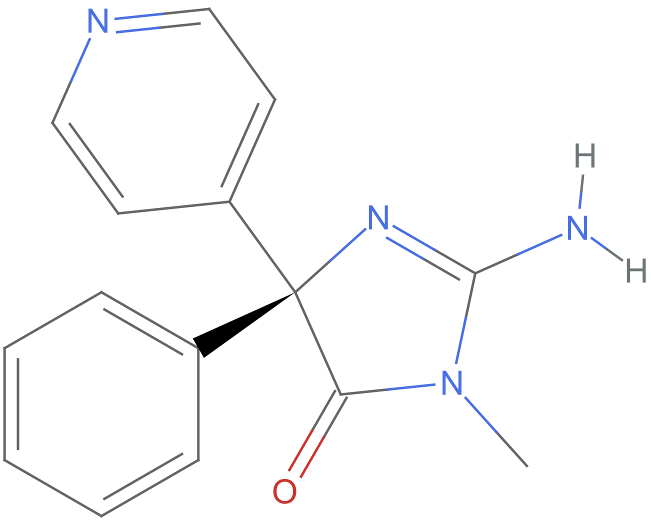

2D Autocorrelation

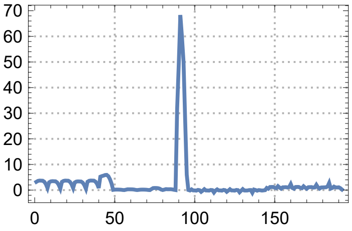

Dataset Index= 1236

Molecule Formula= $C_{15}H_{14}N_4O$

3D Autocorrelation

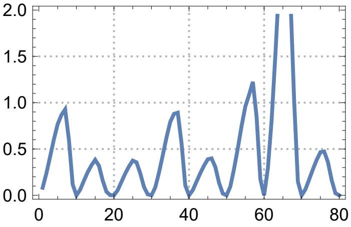

3D Plot

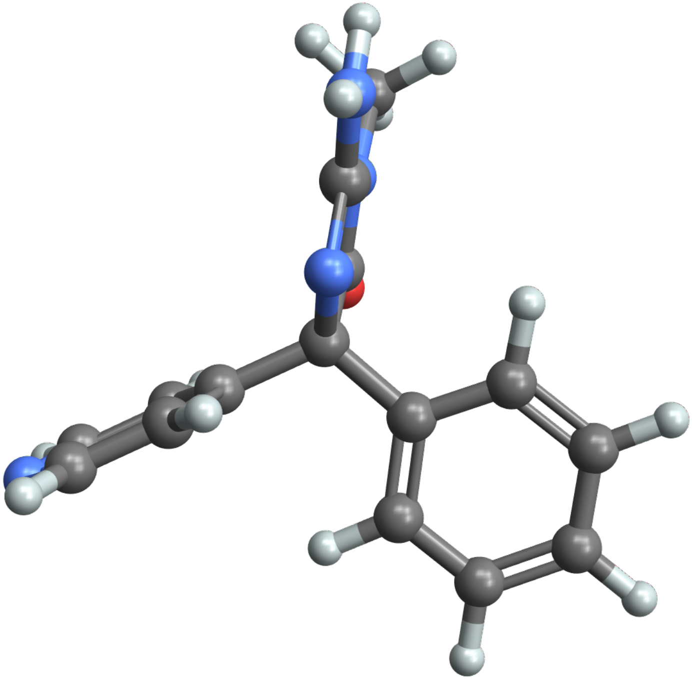

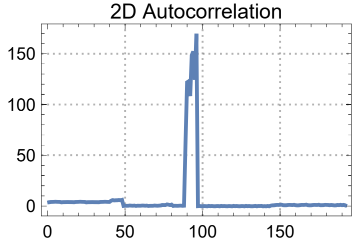

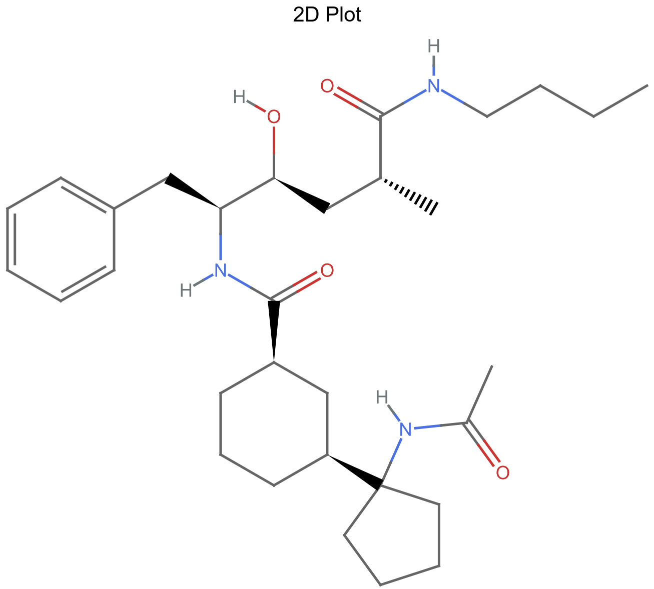

Dataset Index= 1237

Molecule Formula= $C_{31}H_{49}N_3O_4$

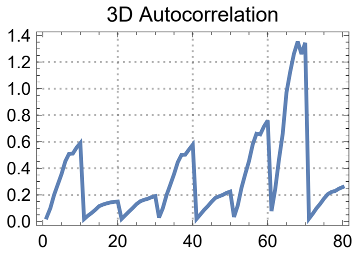

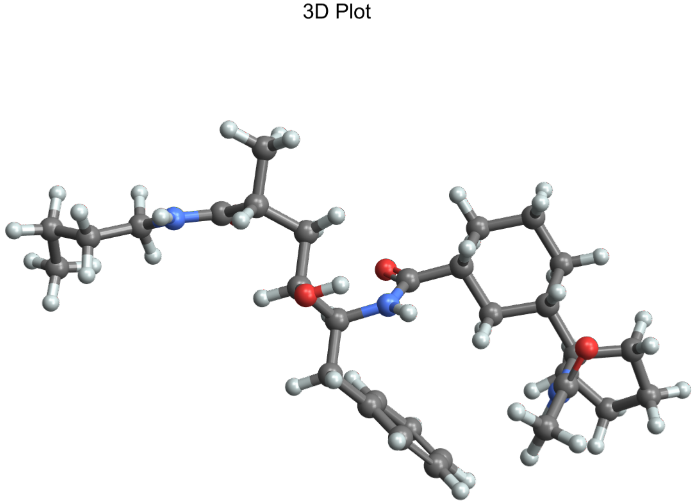

2D Autocorrelation

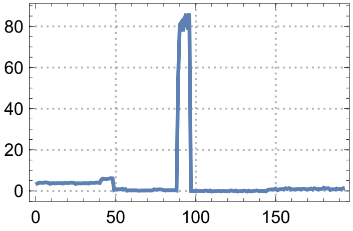

Dataset Index= 1238

Molecule Formula= $C_{23}H_{23}ClN_4O$

3D Autocorrelation

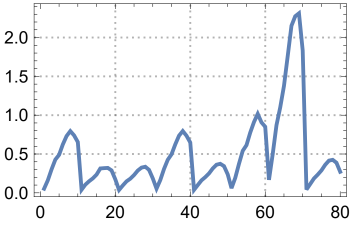

2D Plot

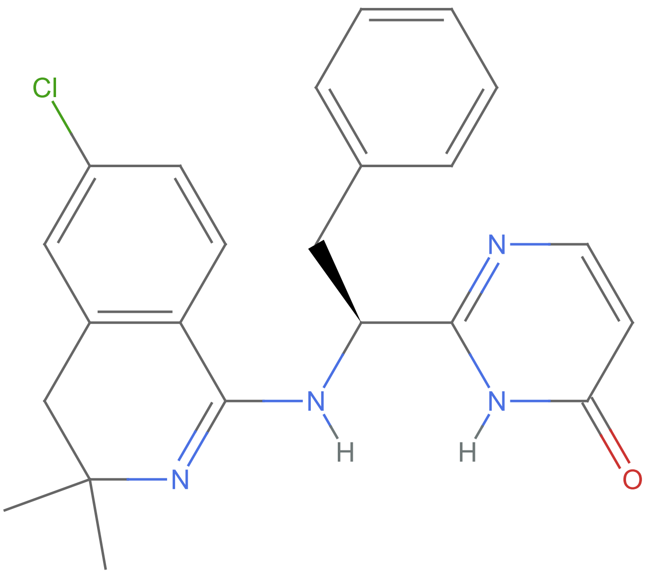

3D Plot

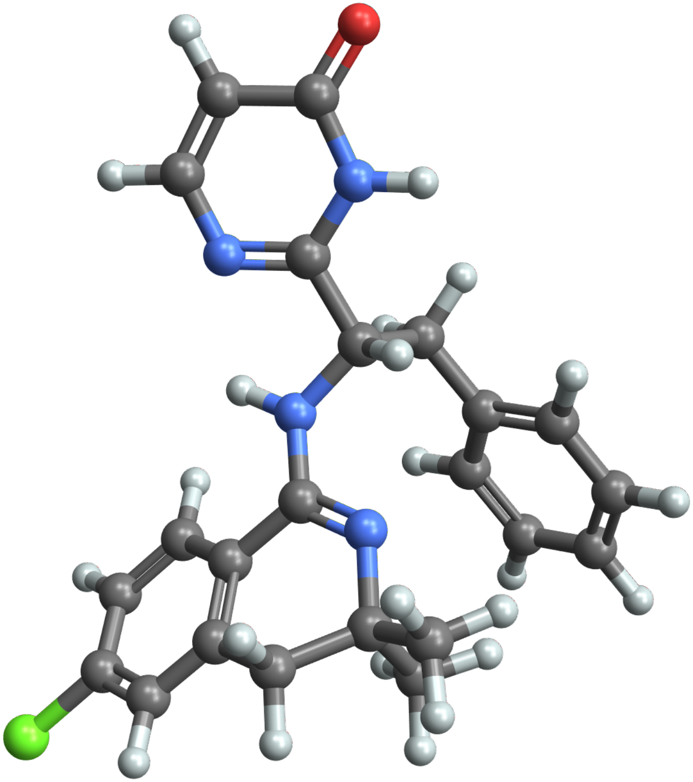

2D Autocorrelation

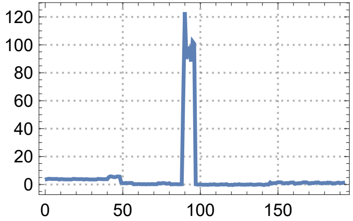

Dataset Index= 1239

Molecule Formula= $C_{25}H_{33}N_2O_3S^+$

3D Autocorrelation

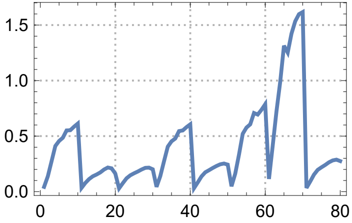

2D Plot

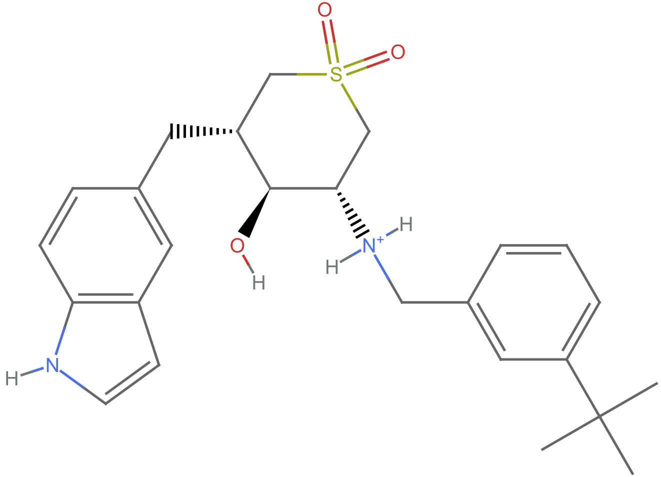

3D Plot

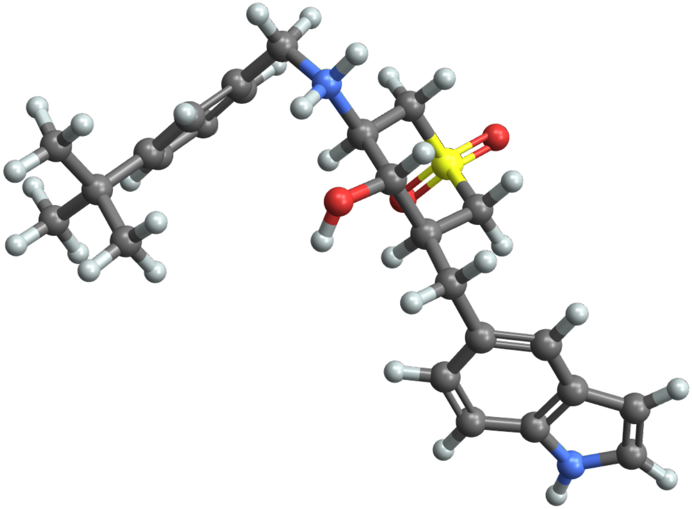

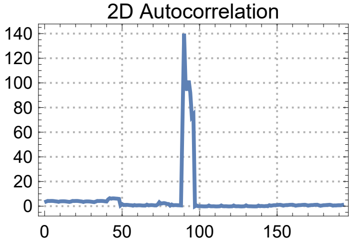

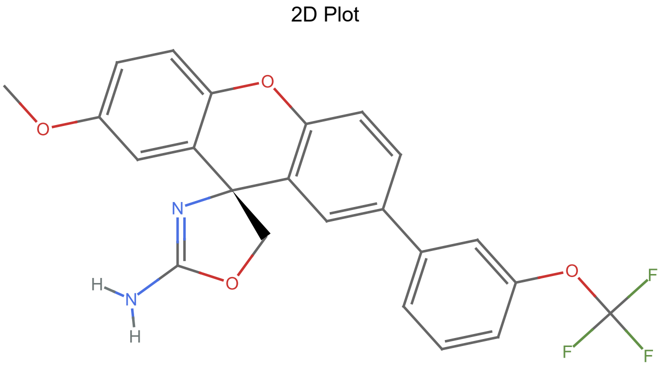

Dataset Index= 1240

Molecule Formula= $C_{23}H_{17}F_3N_2O_4$

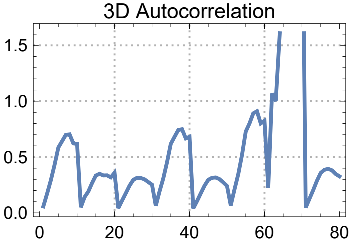

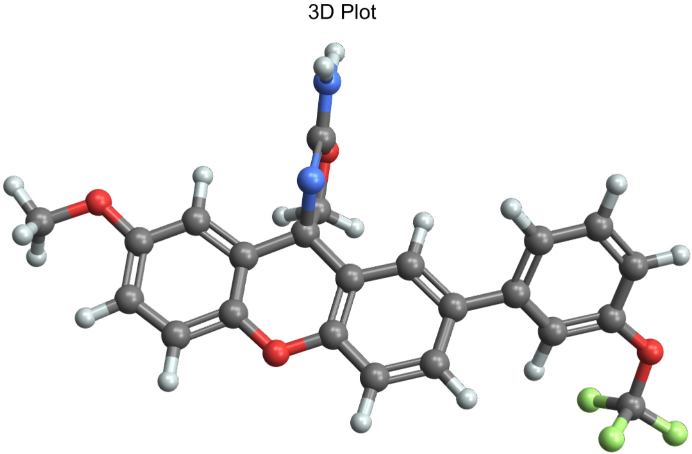

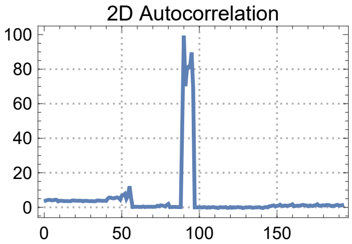

Dataset Index= 1241

Molecule Formula= $C_{23}H_{31}BrNO_4S^+$

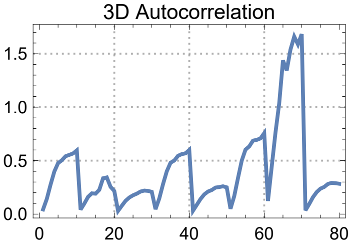

2D Plot

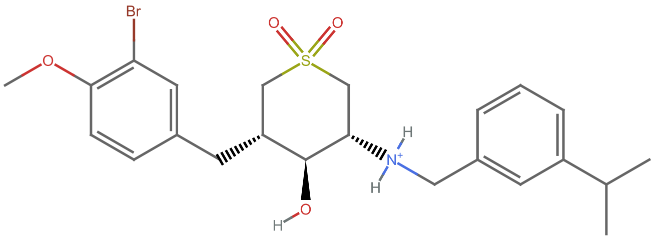

3D Plot

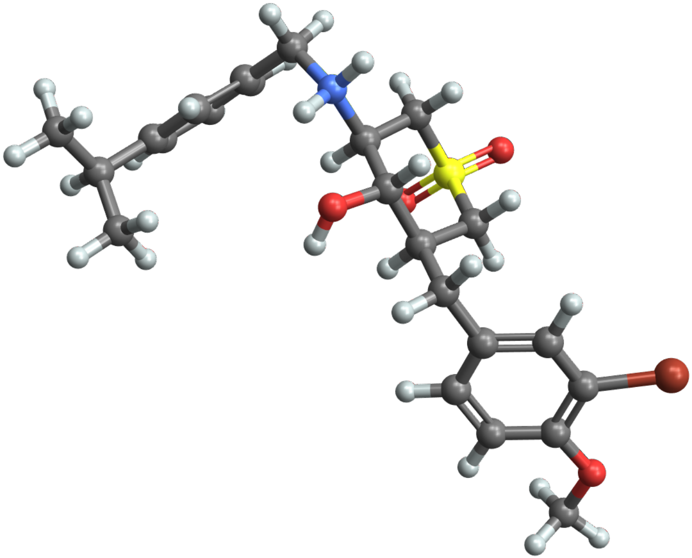

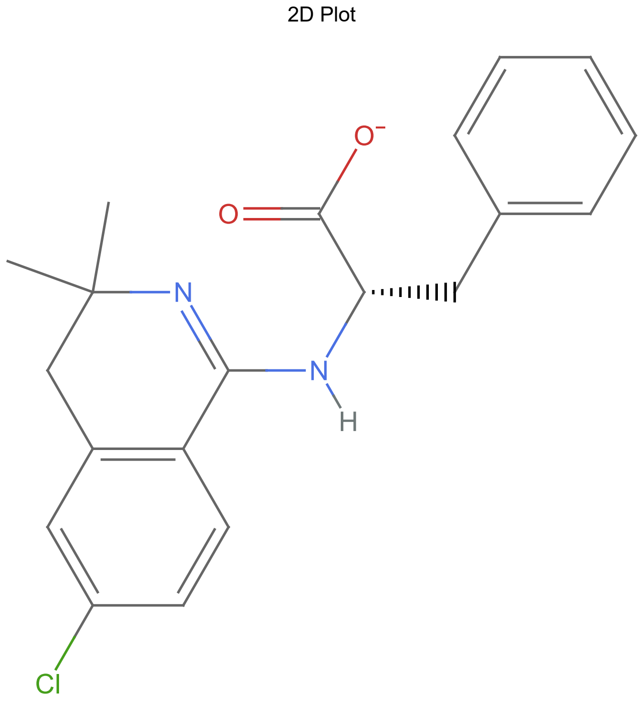

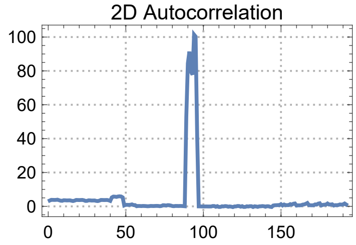

Dataset Index= 1242

Molecule Formula= $C_{20}H_{20}ClN_2O_2^-$

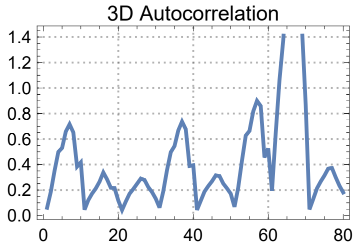

3D Plot

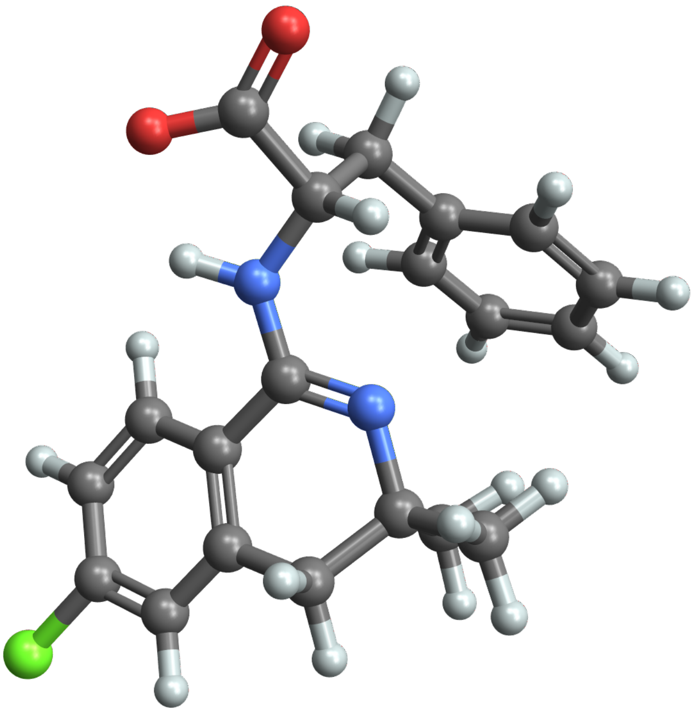

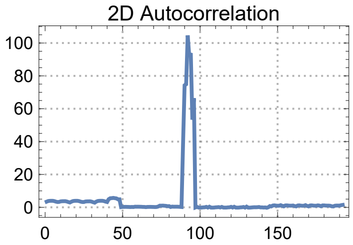

Dataset Index= 1243

Molecule Formula= $C_{20}H_{28}N_4O_2$

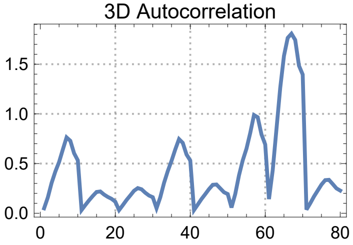

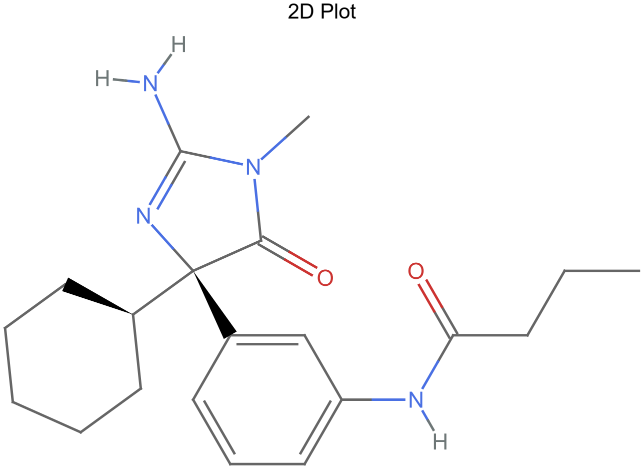

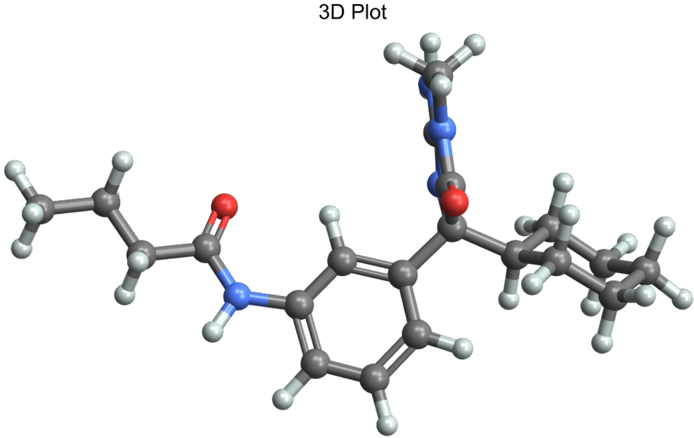

2D Plot

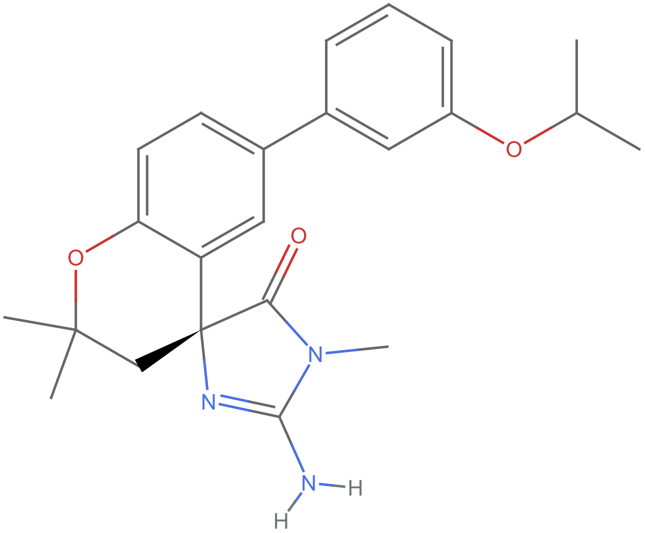

2D Autocorrelation

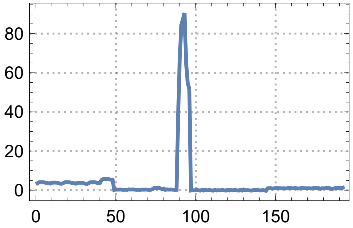

Dataset Index= 1244

Molecule Formula= $C_{23}H_{27}N_3O_3$

3D Autocorrelation

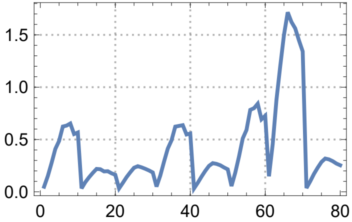

3D Plot

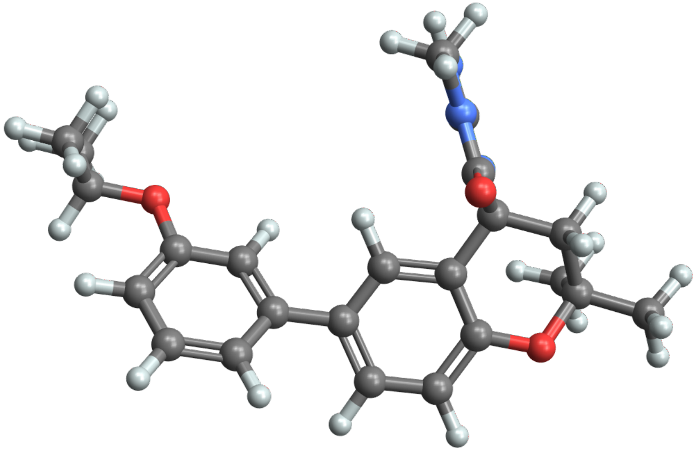

2D Plot

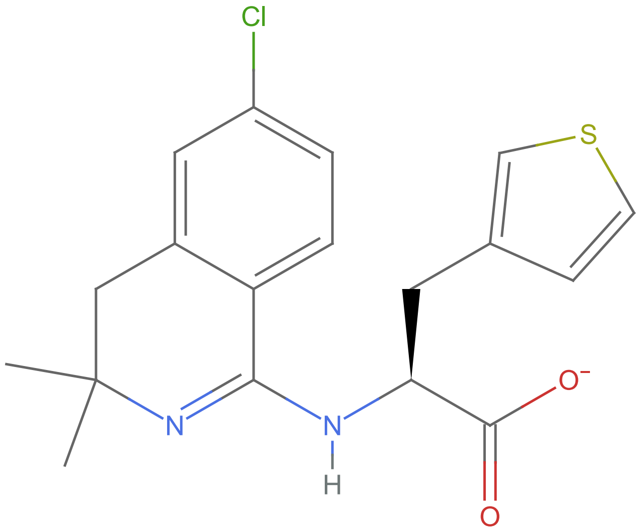

2D Autocorrelation

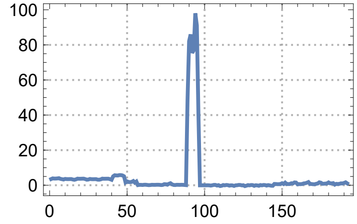

3D Plot

Dataset Index= 1245

Molecule Formula= $C_{18}H_{18}ClN_2O_2S^-$

3D Autocorrelation

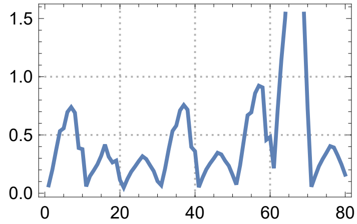

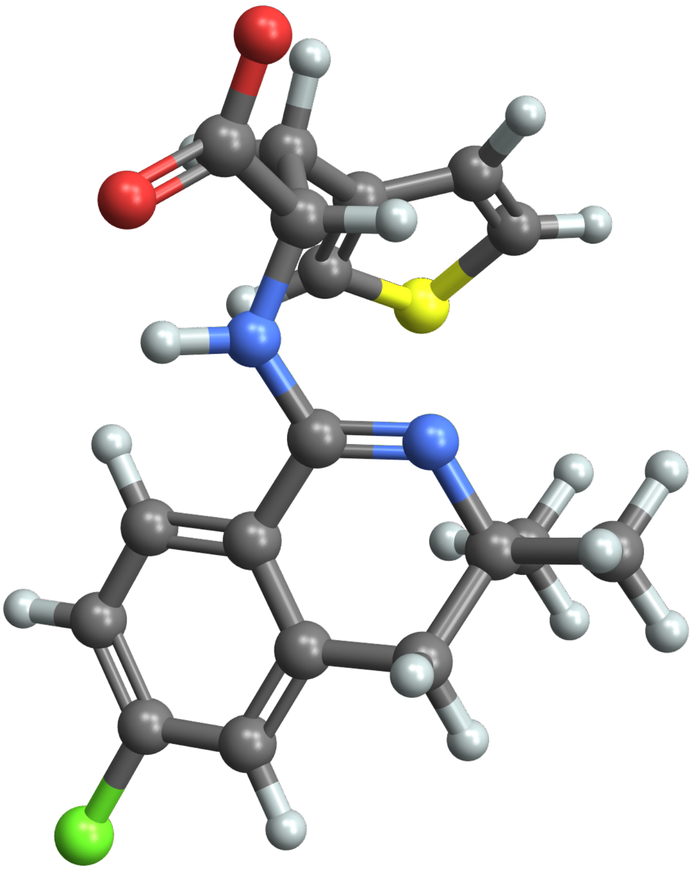

2D Plot

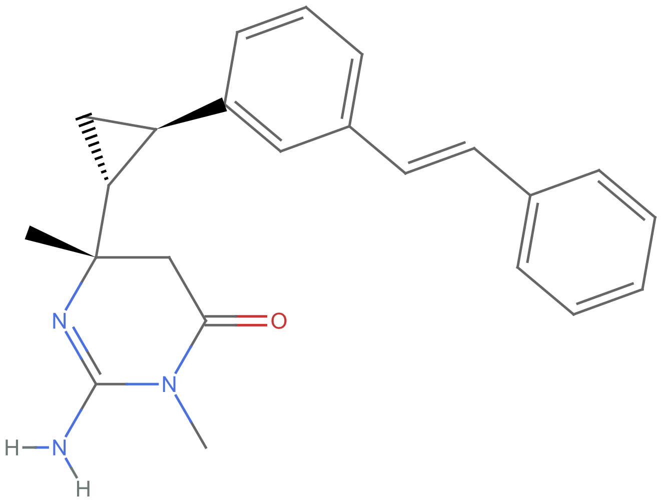

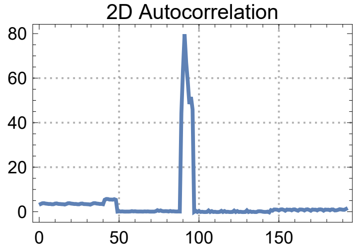

Dataset Index= 1246

Molecule Formula= $C_{23}H_{25}N_3O$

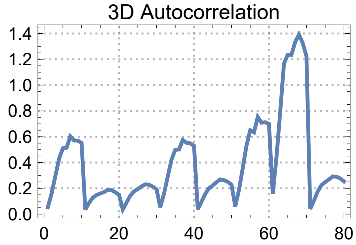

3D Plot

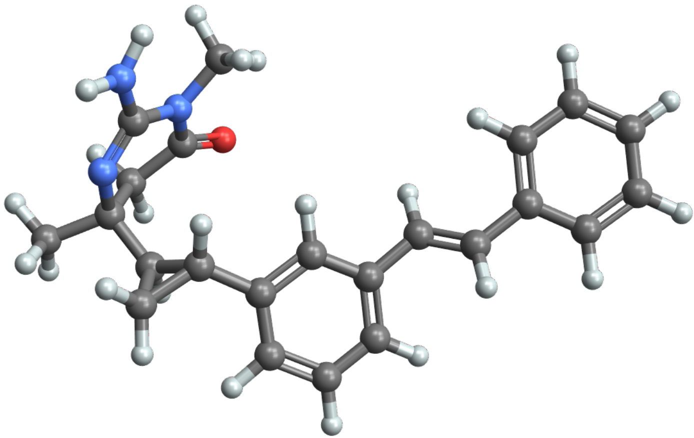

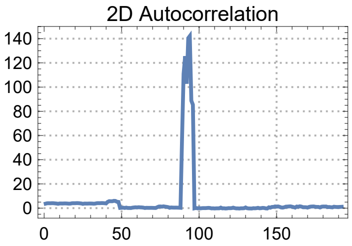

2D Plot

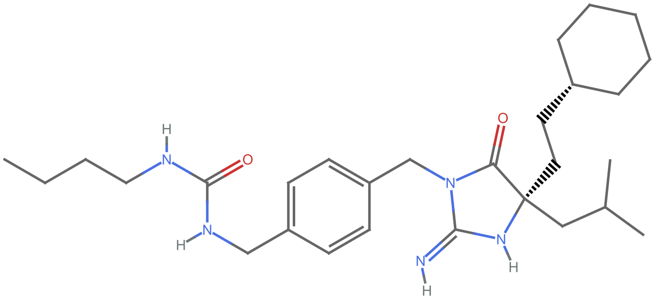

3D Plot

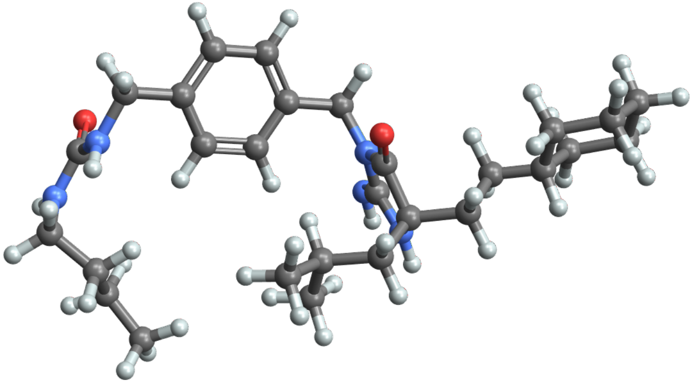

Dataset Index= 1247

Molecule Formula= $C_{28}H_{45}N_5O_2$

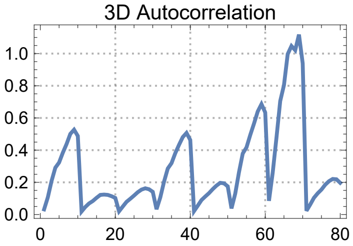

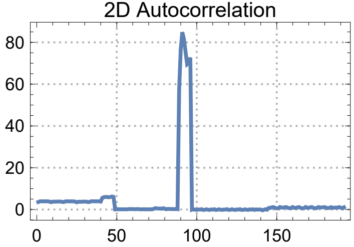

Dataset Index= 1248

Molecule Formula= $C_{26}H_{26}N_4O$

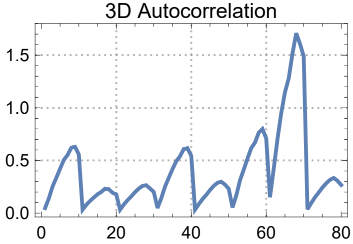

2D Plot

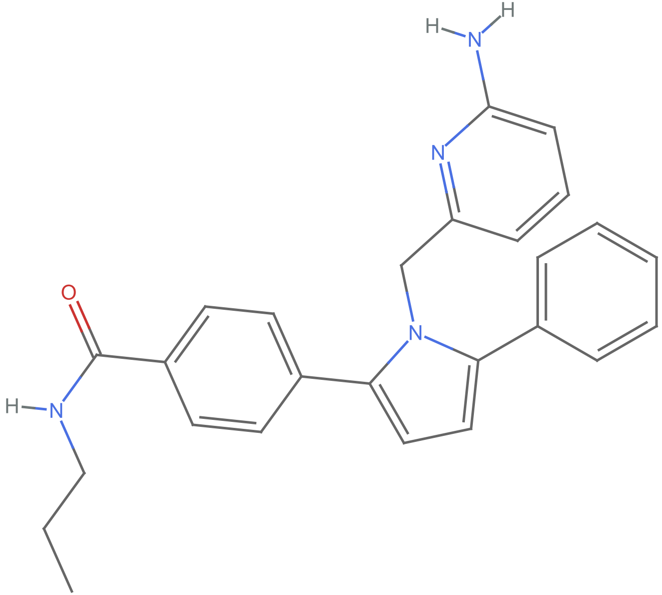

3D Plot

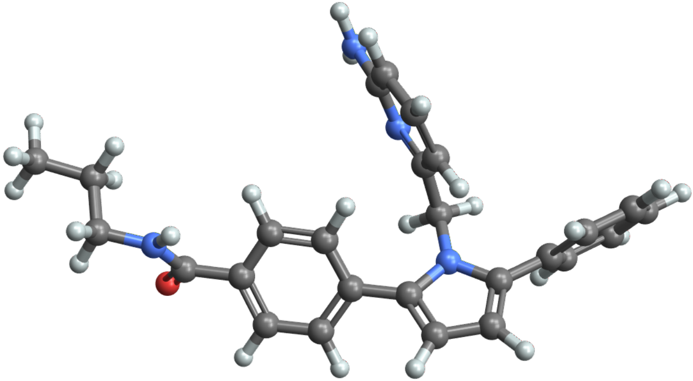

2D Autocorrelation

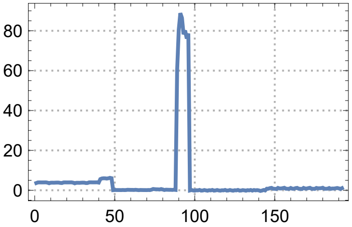

Dataset Index= 1249

Molecule Formula= $C_{27}H_{28}N_4O$

3D Autocorrelation

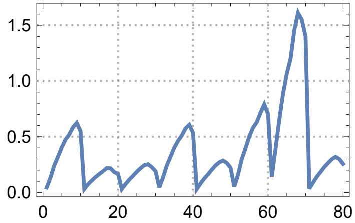

2D Plot

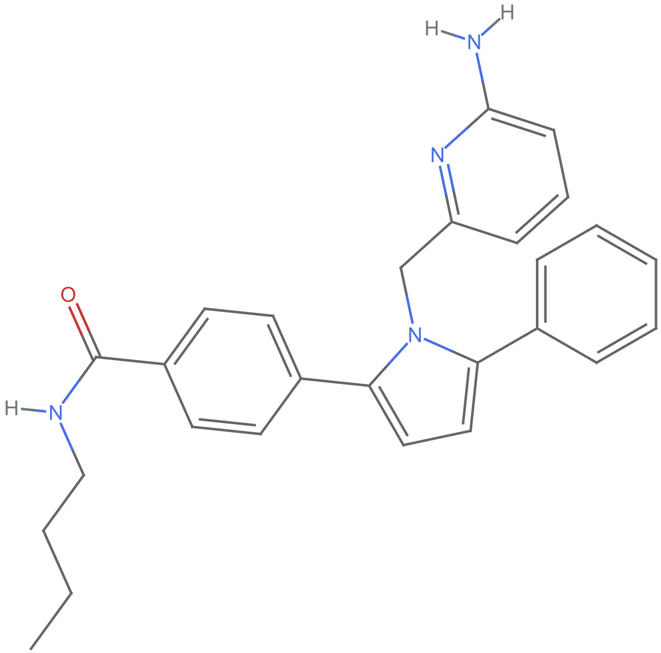

3D Plot

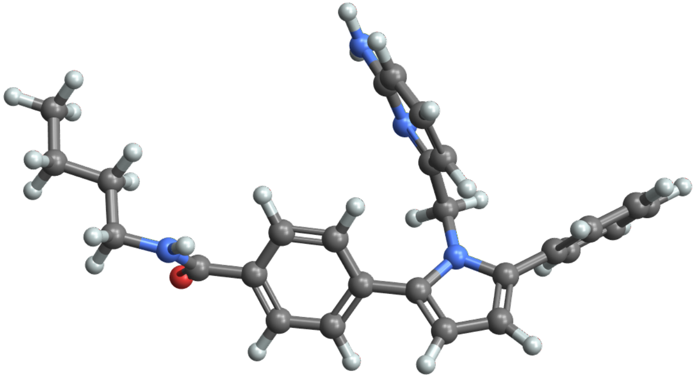

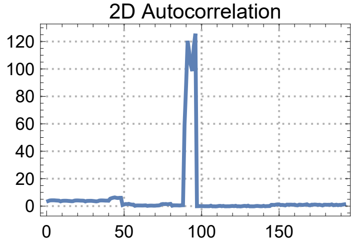

Dataset Index= 1250

Molecule Formula= $C_{22}H_{19}FN_4O_2S$

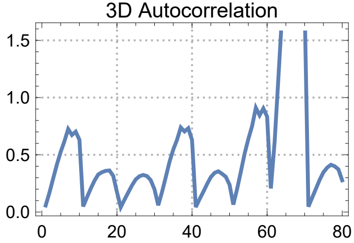

2D Plot

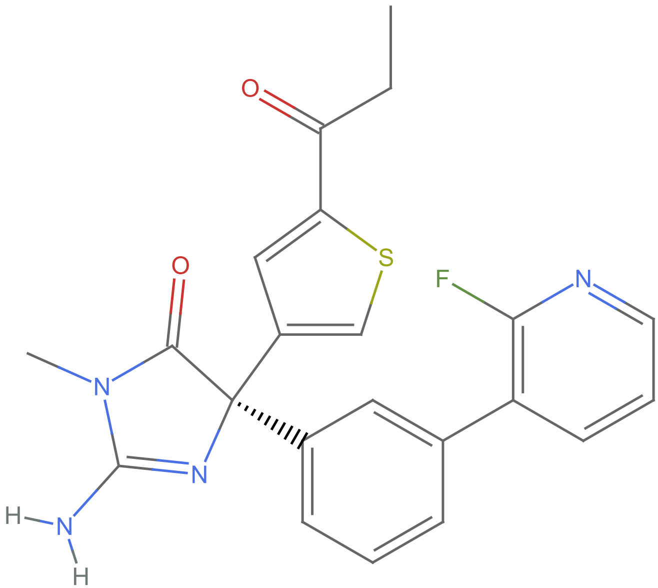

3D Plot

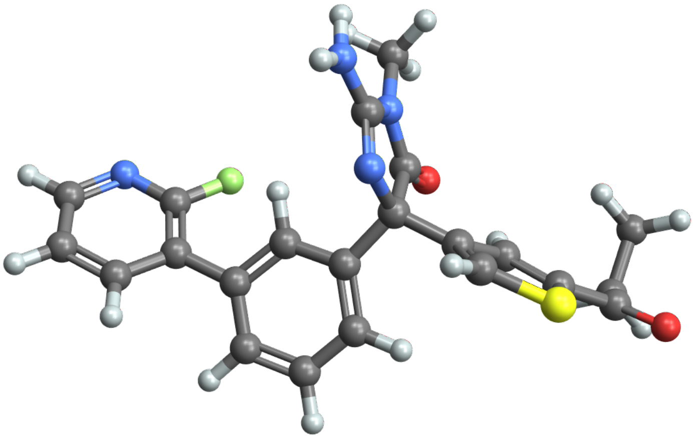

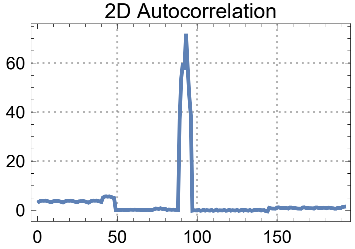

Dataset Index= 1251

Molecule Formula= $C_{22}H_{25}N_3O_2$

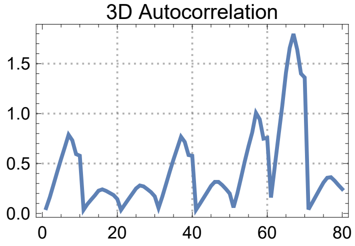

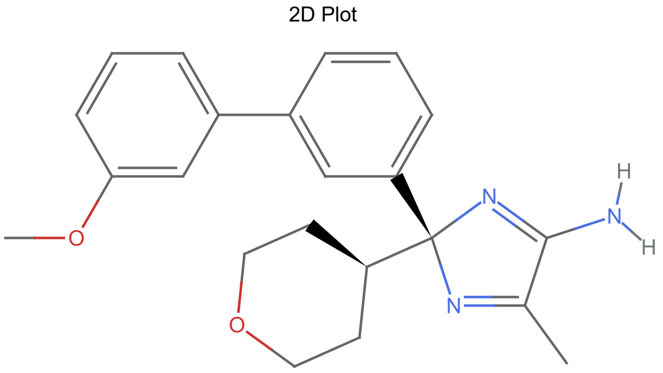

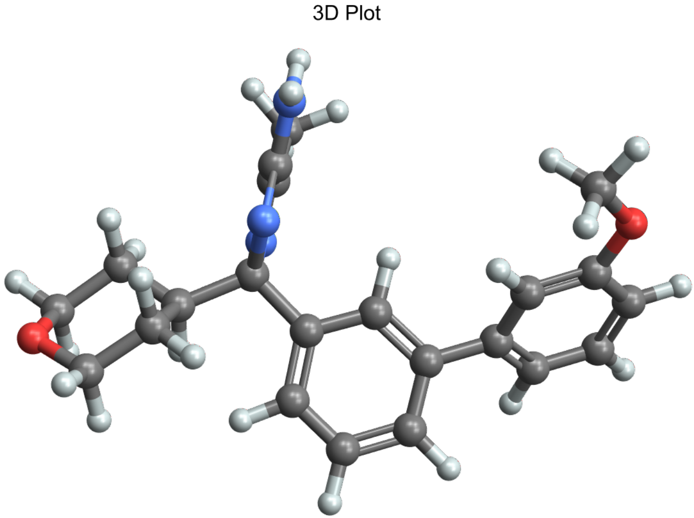

2D Plot

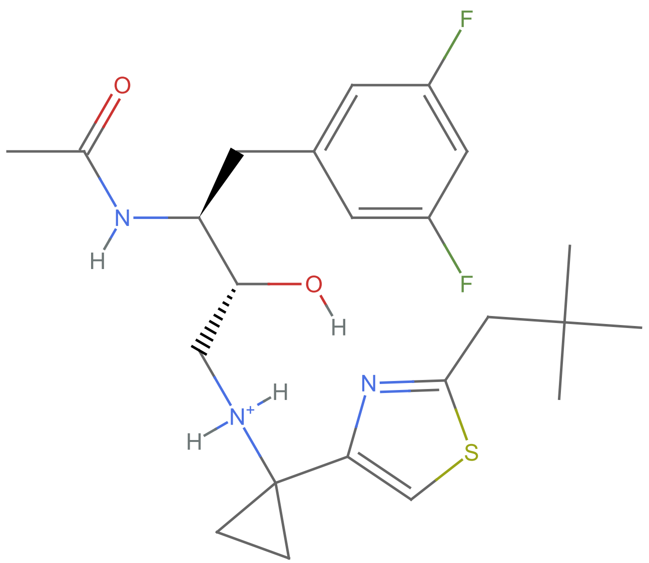

2D Autocorrelation

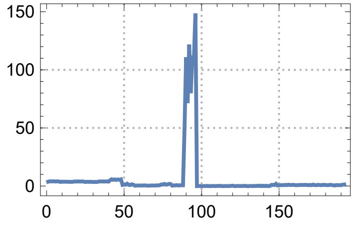

Dataset Index= 1252

Molecule Formula= $C_{23}H_{32}F_2N_3O_2S^+$

3D Plot

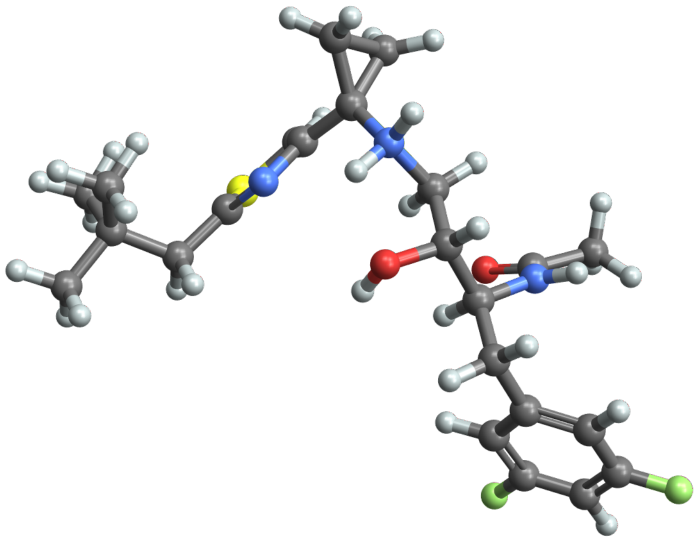

3D Autocorrelation

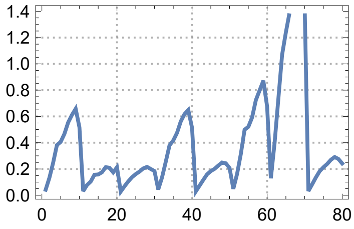

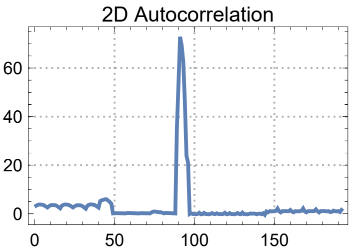

Dataset Index= 1253

Molecule Formula= $C_{17}H_{17}N_3O_2$

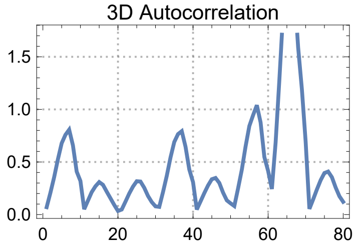

2D Plot

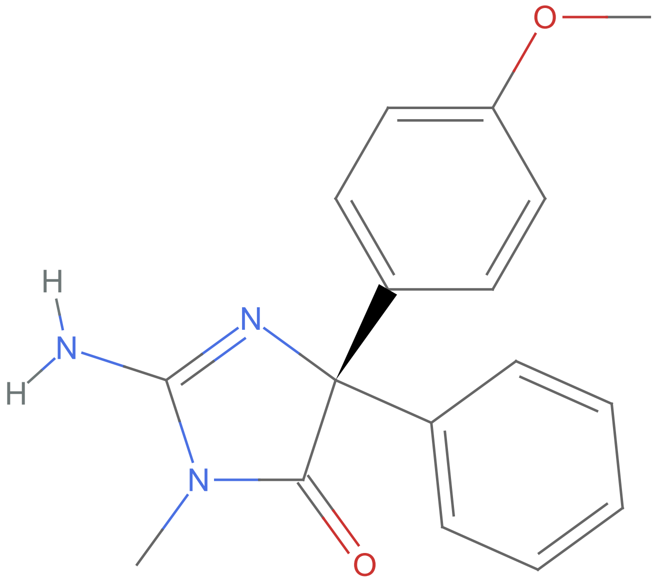

3D Plot

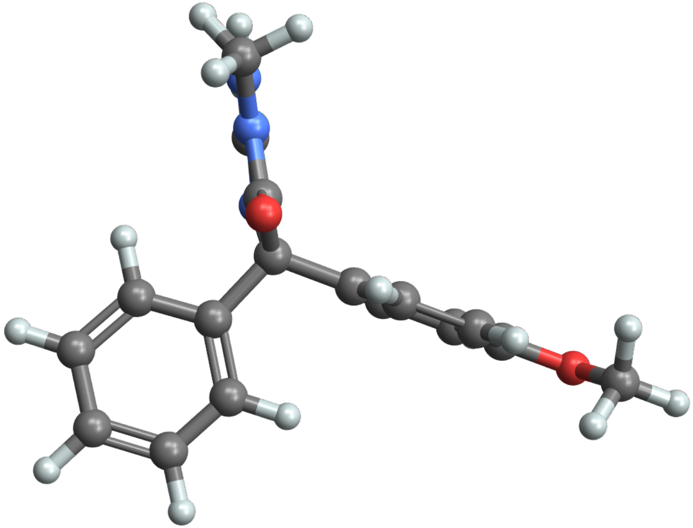

2D Plot

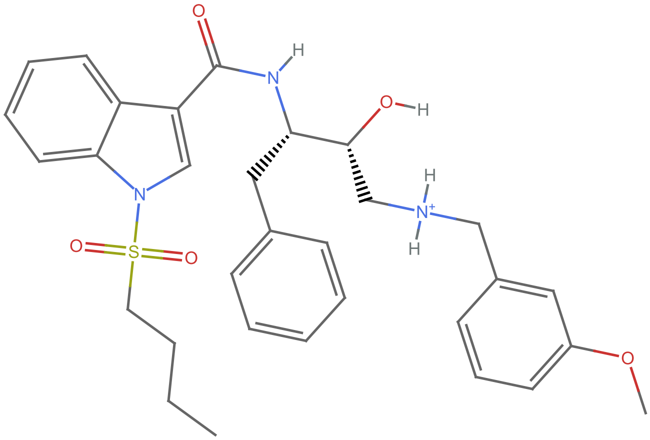

2D Autocorrelation

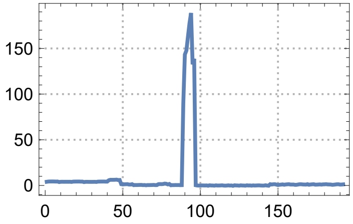

Dataset Index= 1254

Molecule Formula= $C_{31}H_{38}N_{3}O_{5}S^{+}$

3D Autocorrelation

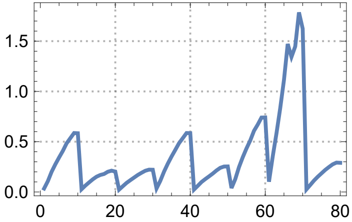

3D Plot

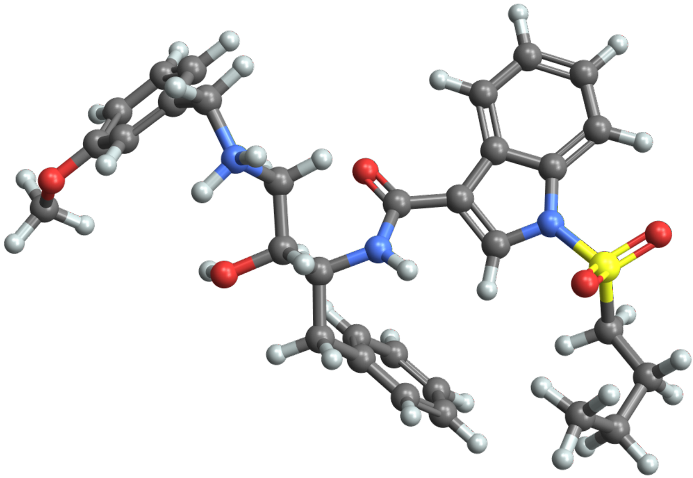

2D Plot

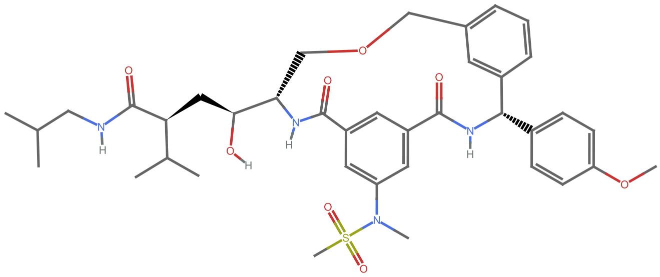

3D Plot

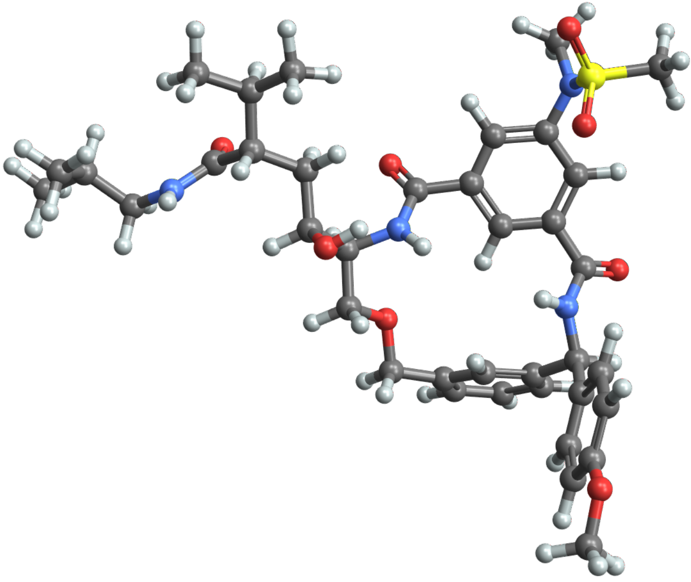

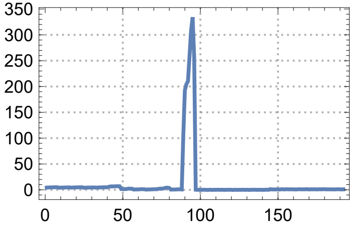

Dataset Index= 1255

Molecule Formula= $C_{38}H_{50}N_{4}O_{8}S$

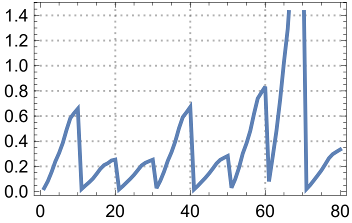

2D Plot

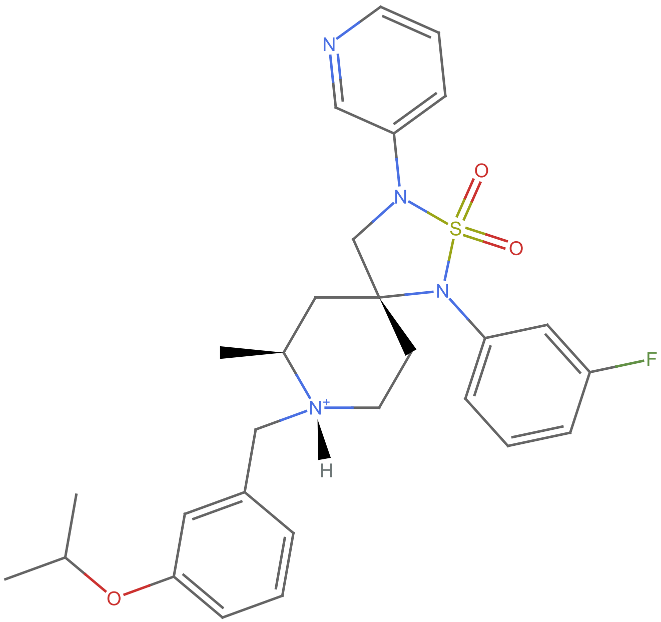

2D Autocorrelation

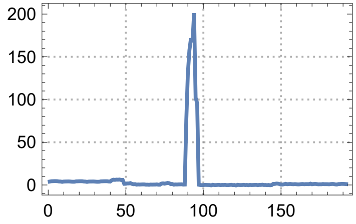

Dataset Index= 1256

Molecule Formula= $C_{28}H_{34}FN_4O_3S^+$

3D Autocorrelation

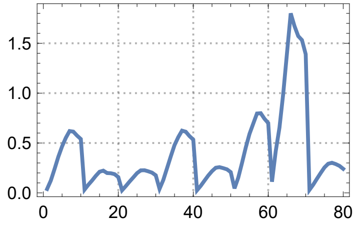

3D Plot

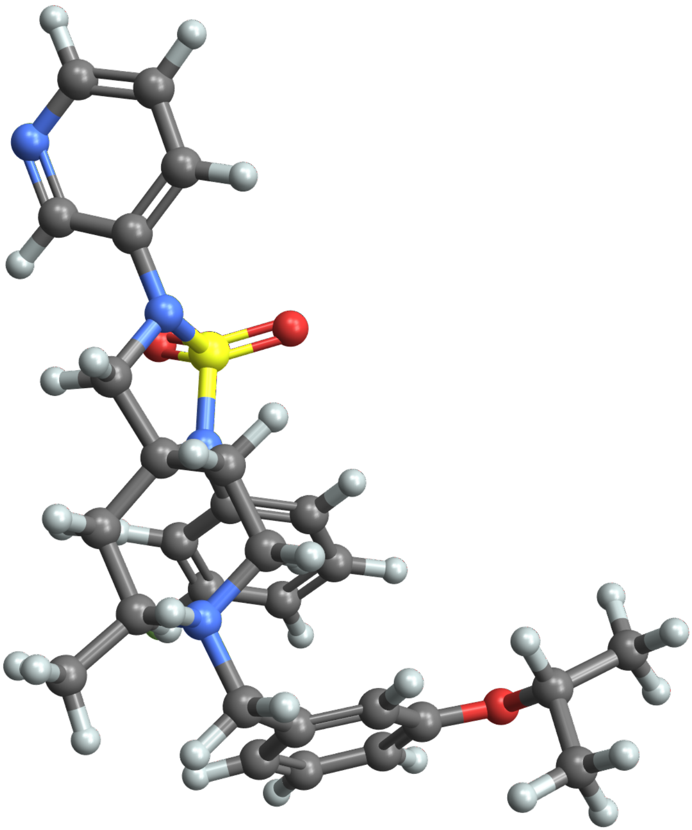

2D Plot

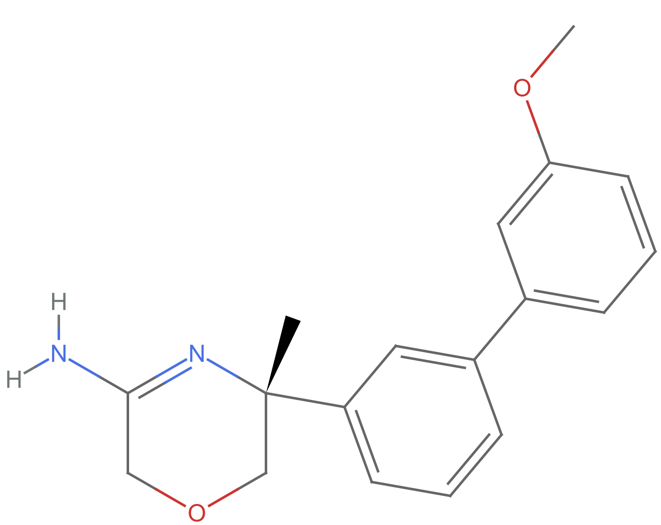

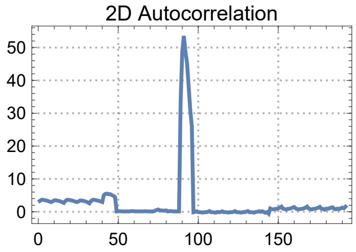

Dataset Index= 1257

Molecule Formula= $C_{18}H_{20}N_2O_2$

3D Plot

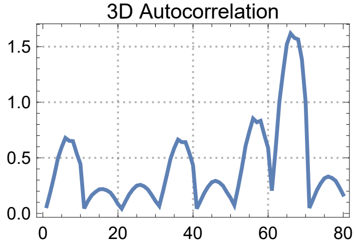

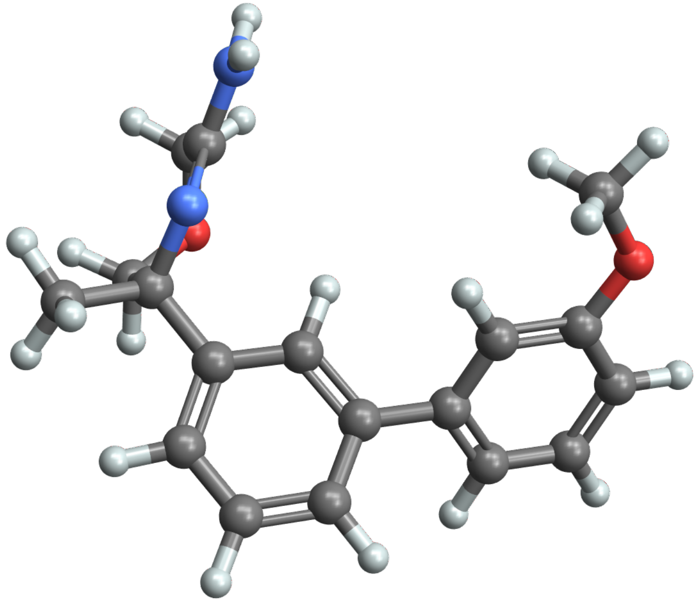

2D Autocorrelation

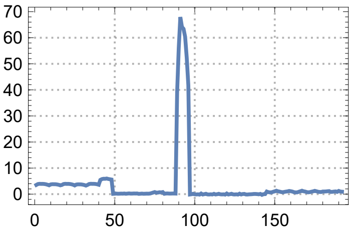

Dataset Index= 1258

Molecule Formula= $C_{23}H_{23}N_3O_2$

3D Autocorrelation

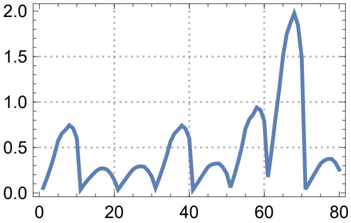

2D Plot

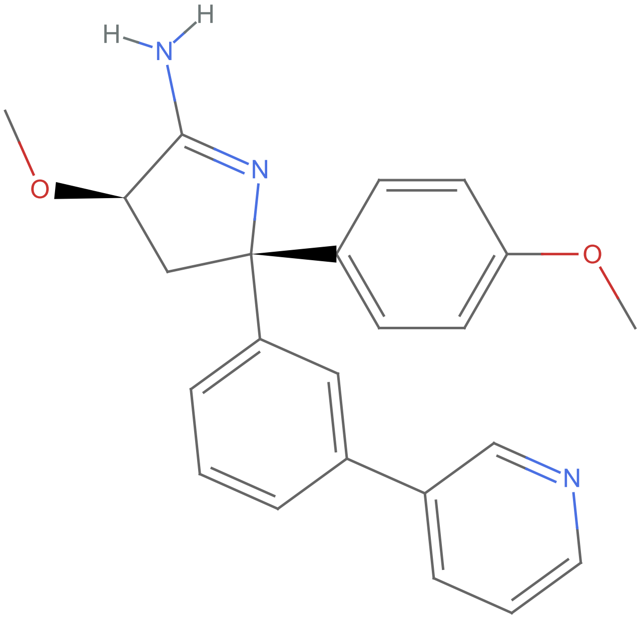

3D Plot

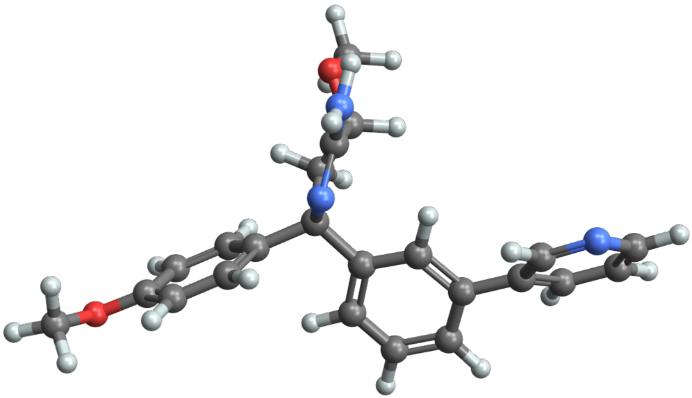

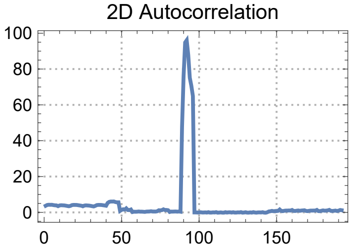

Dataset Index= 1259

Molecule Formula= $C_{23}H_{18}ClFN_2O_3$

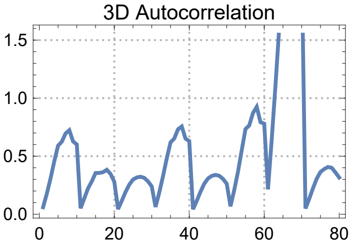

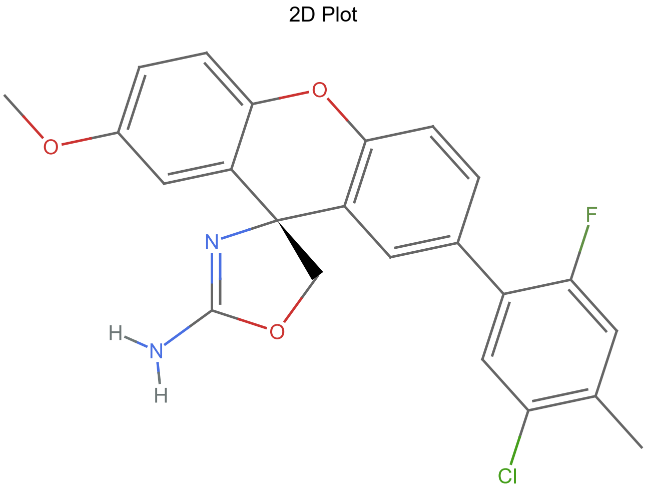

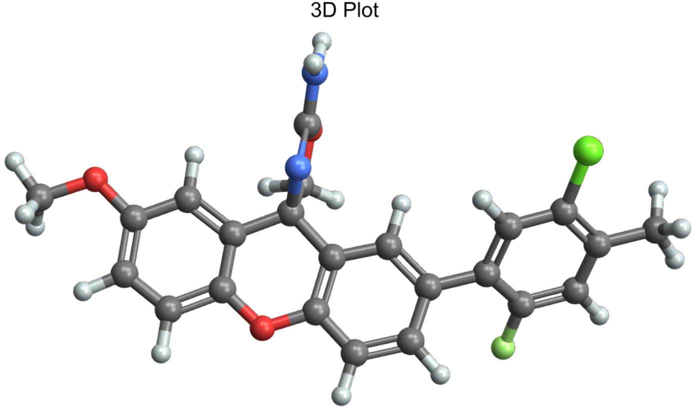

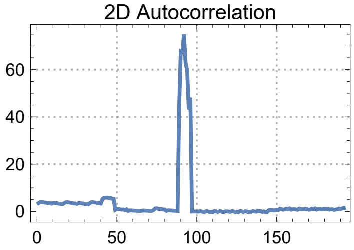

Dataset Index= 1260

Molecule Formula= $C_{18}H_{17}ClN_4O_3$

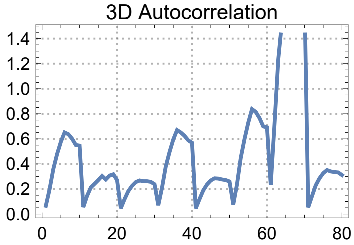

2D Plot

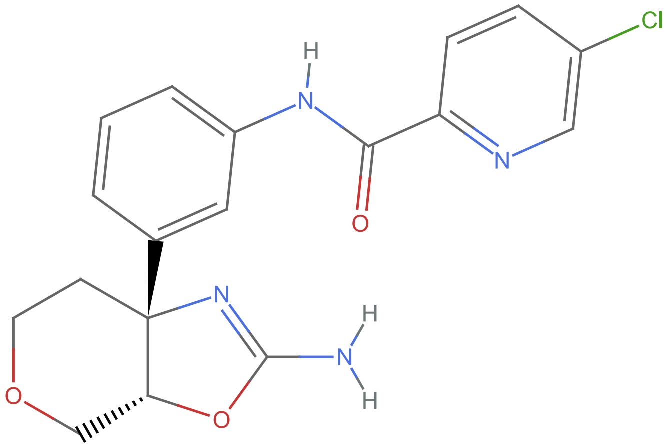

3D Plot

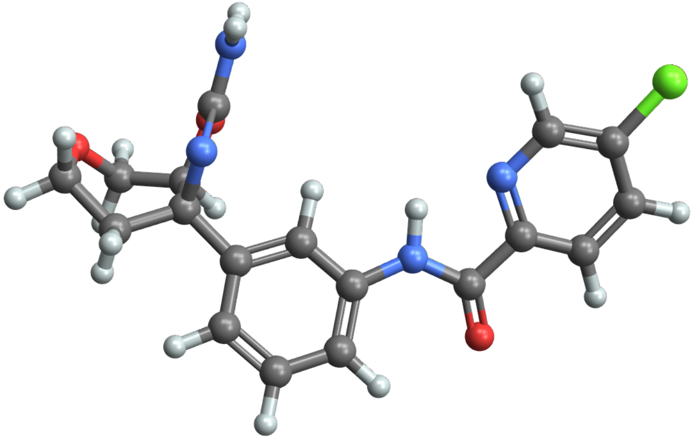

2D Plot

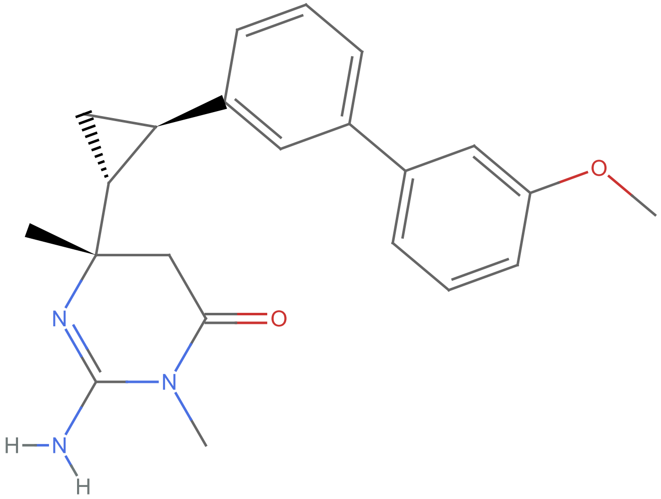

2D Autocorrelation

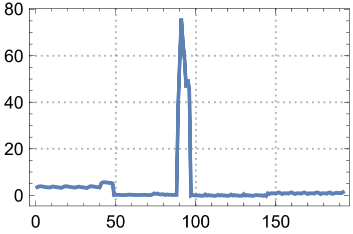

Dataset Index= 1261

Molecule Formula= $C_{22}H_{25}N_3O_2$

3D Plot

3D Autocorrelation

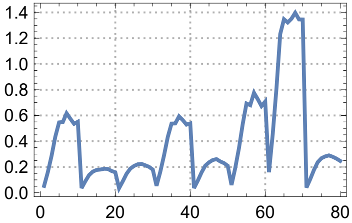

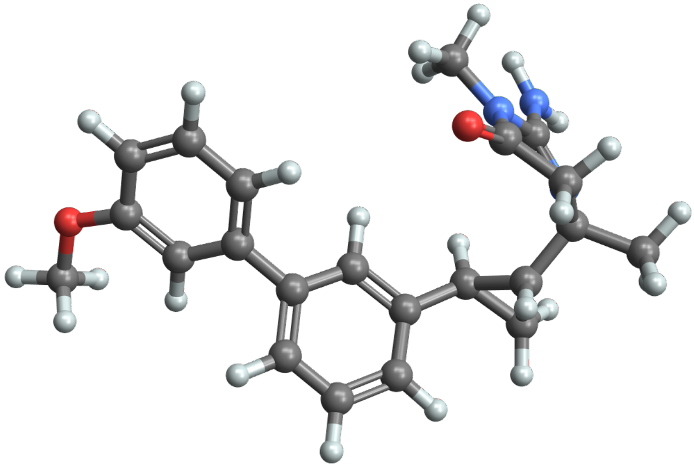

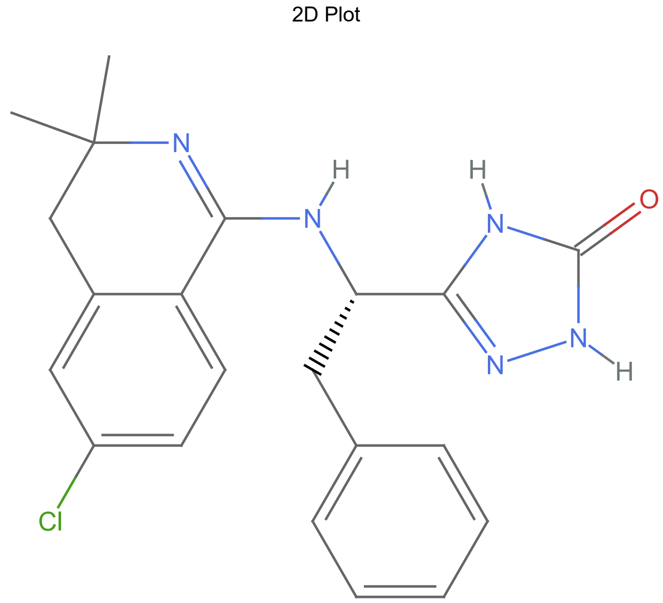

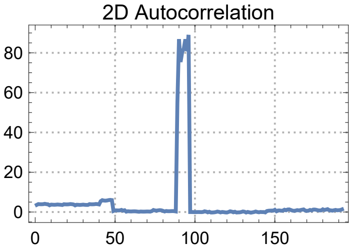

Dataset Index= 1262

Molecule Formula= $C_{21}H_{22}ClN_5O$

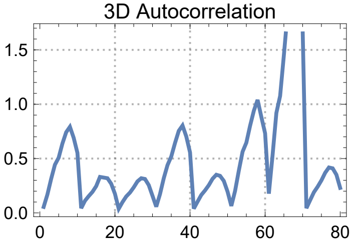

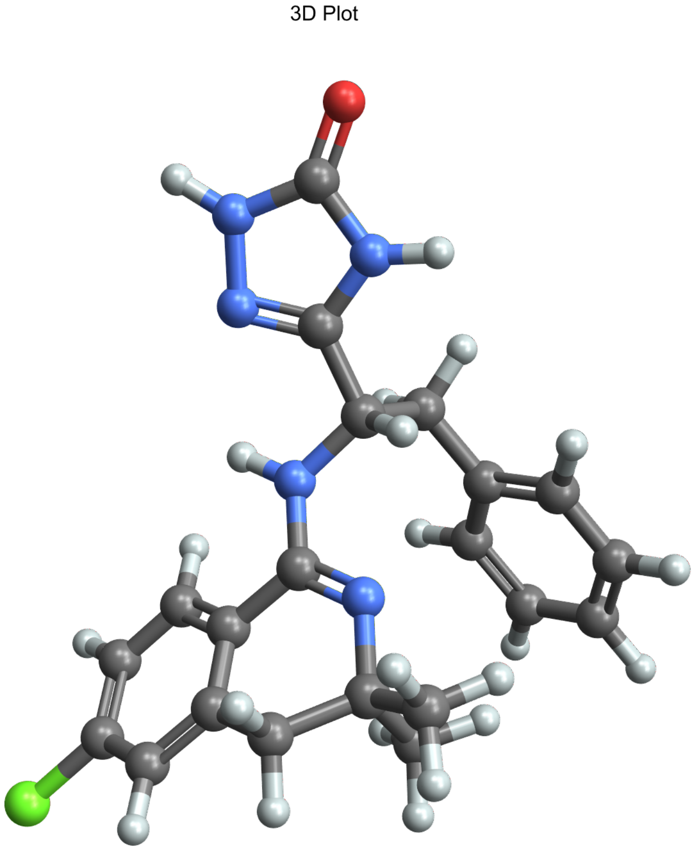

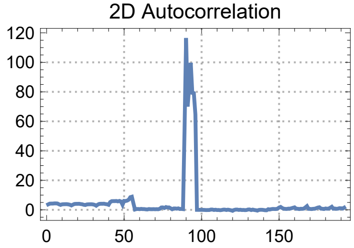

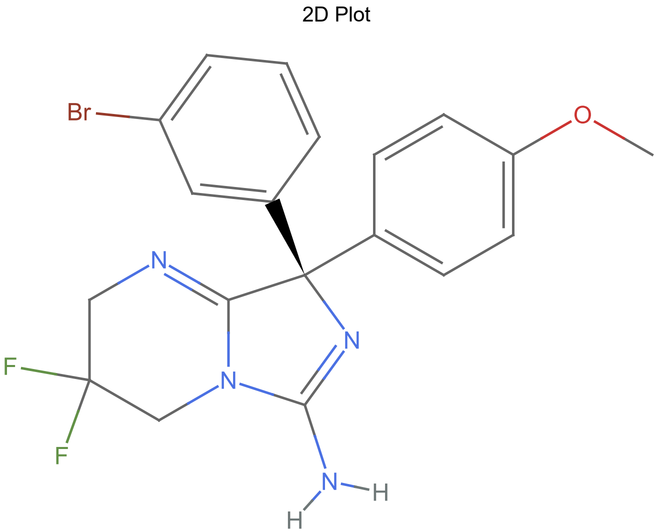

Dataset Index= 1263

Molecule Formula= $C_{19}H_{17}BrF_2N_4O$

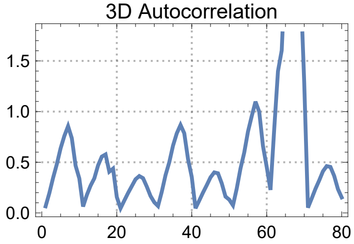

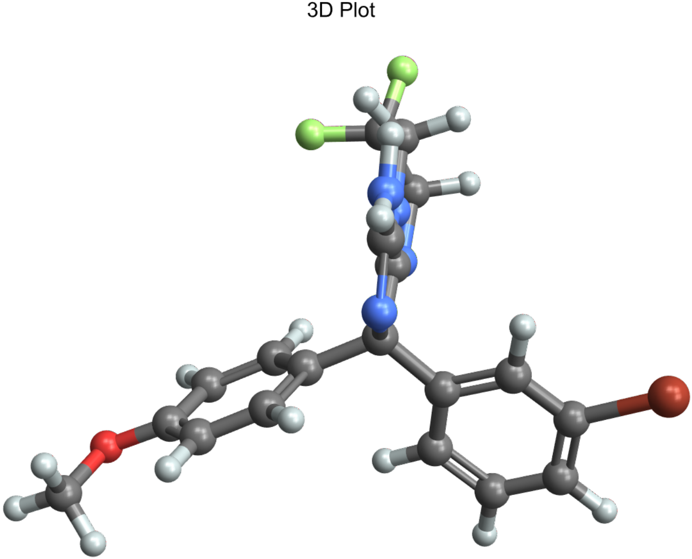

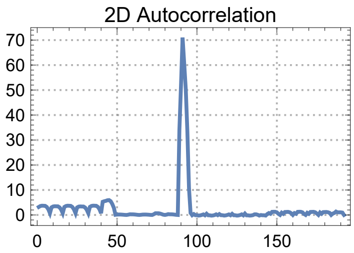

Dataset Index= 1264

Molecule Formula= $C_{16}H_{15}N_3O$

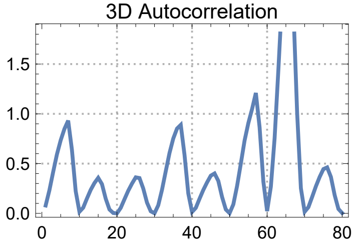

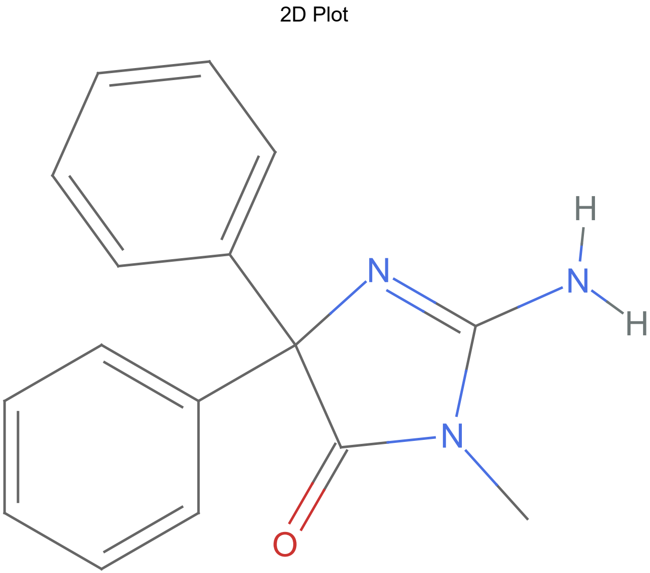

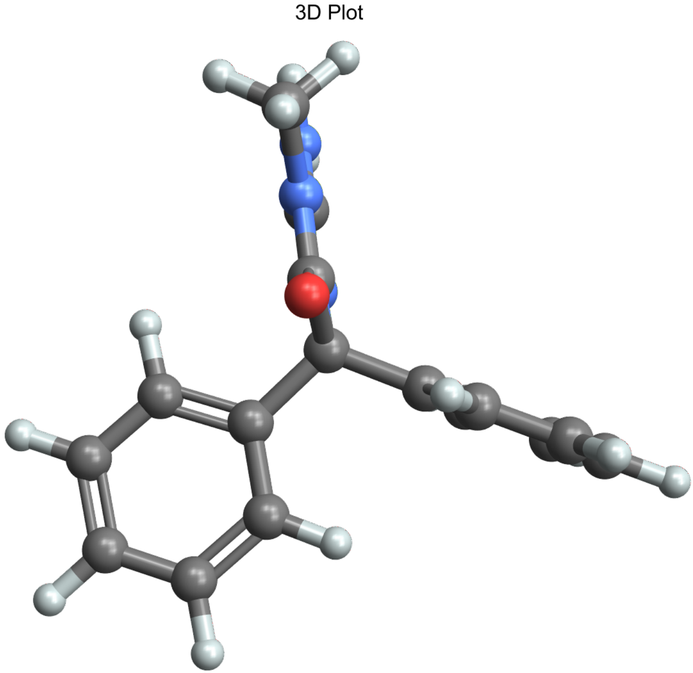

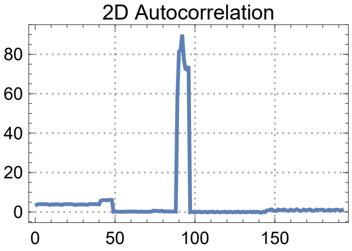

Dataset Index= 1265

Molecule Formula= $C_{27}H_{26}N_4O$

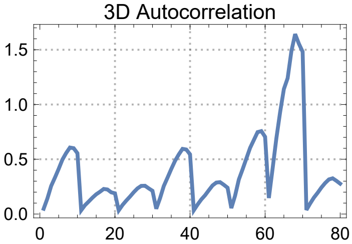

2D Plot

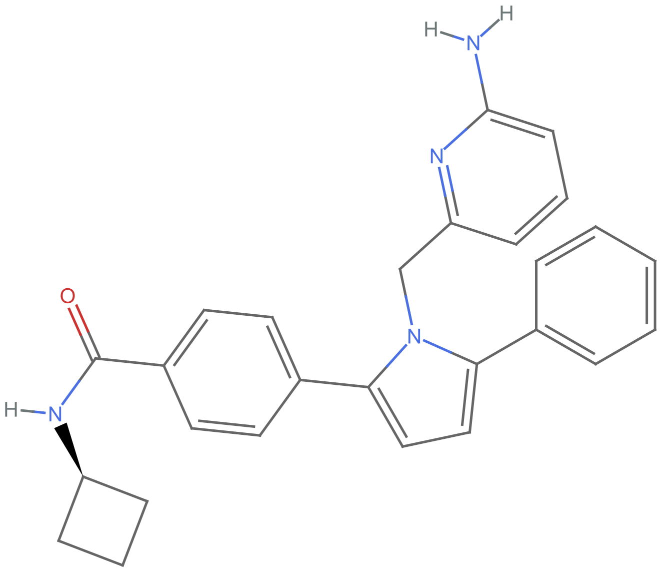

3D Plot

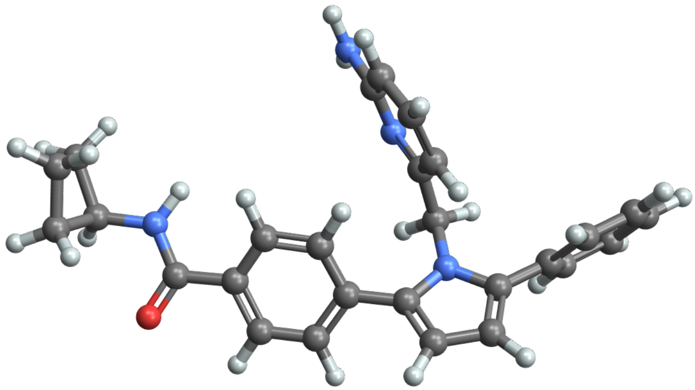

2D Plot

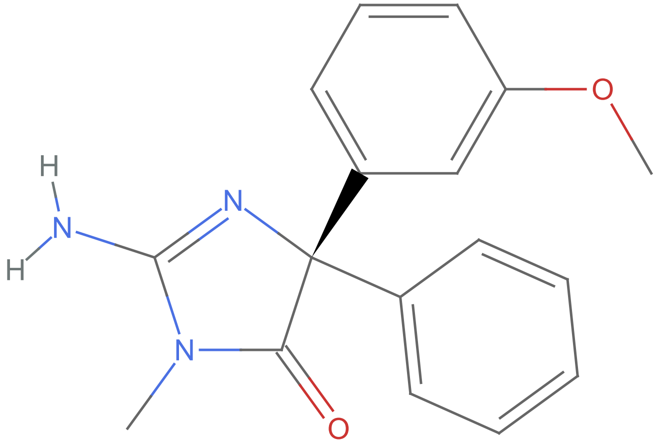

2D Autocorrelation

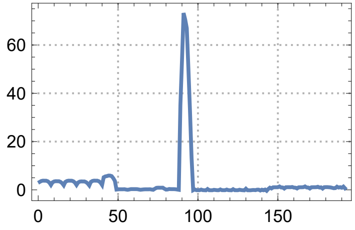

3D Plot

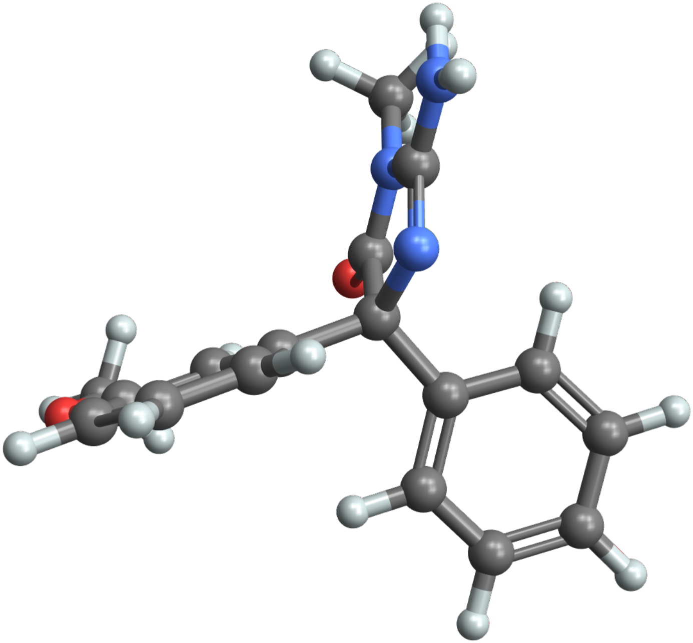

Dataset Index= 1266

Molecule Formula= $C_{17}H_{17}N_3O_2$

3D Autocorrelation

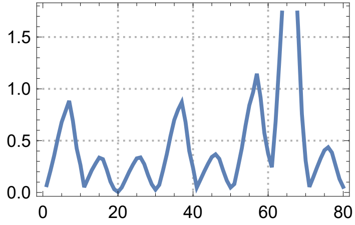

2D Plot

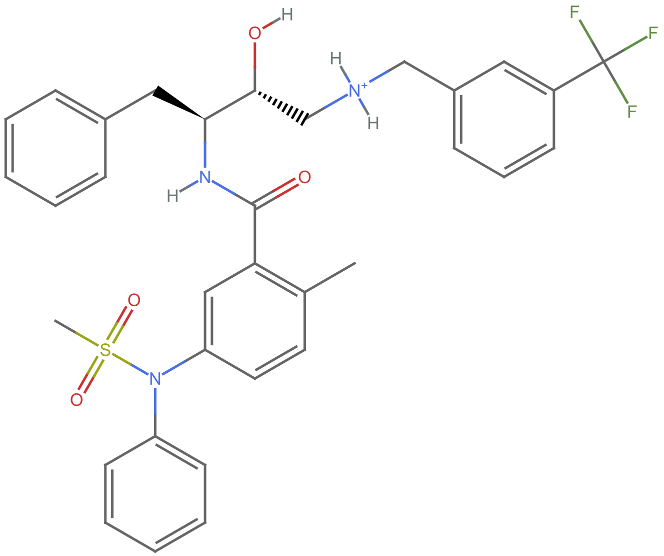

2D Autocorrelation

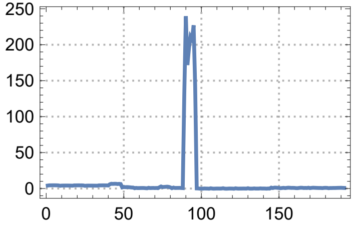

Dataset Index= 1267

Molecule Formula= $C_{33}H_{35}F_3N_3O_4S^+$

3D Plot

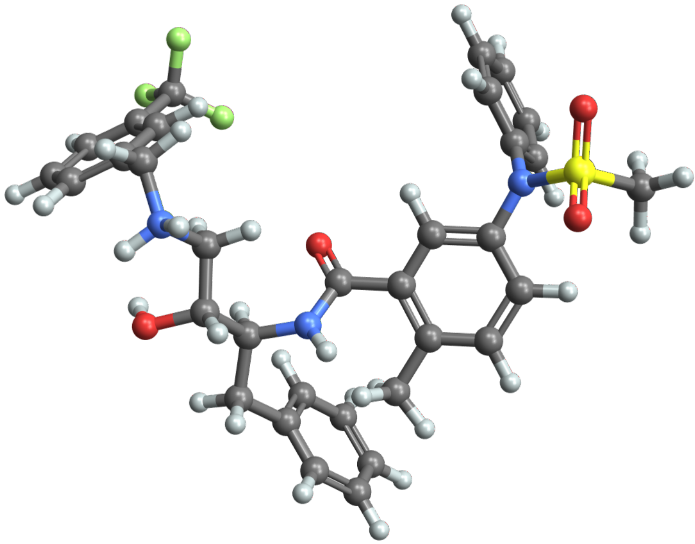

3D Autocorrelation

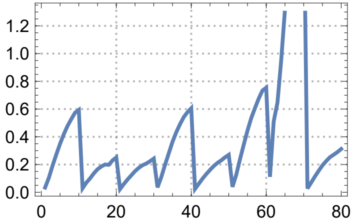

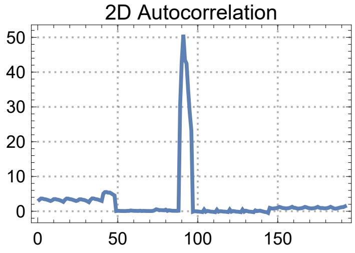

Dataset Index= 1268

Molecule Formula= $C_{18}H_{19}N_3O$

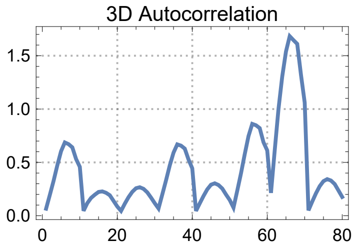

2D Plot

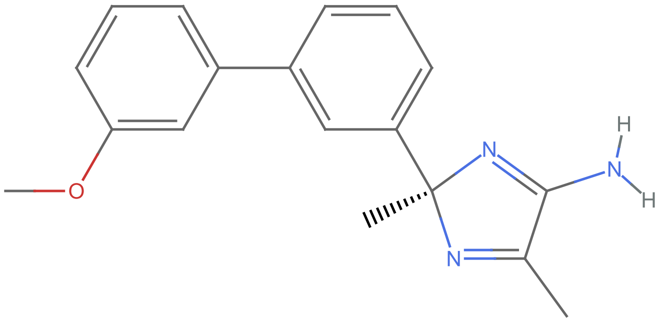

3D Plot

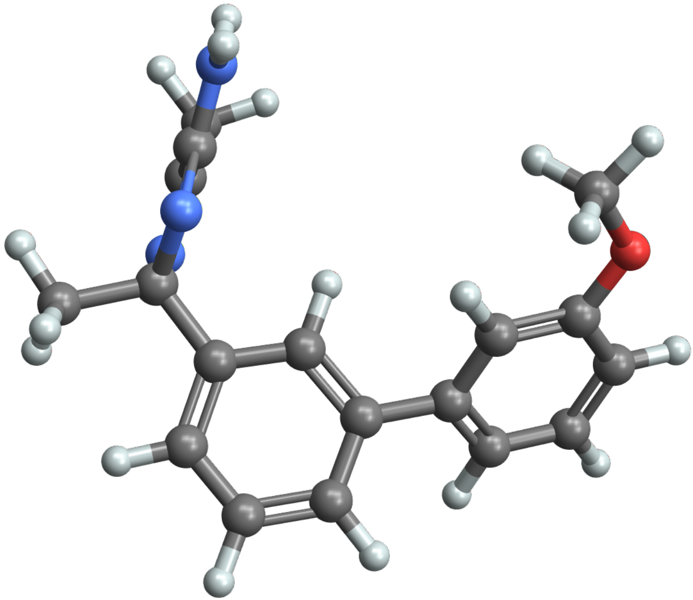

2D Autocorrelation

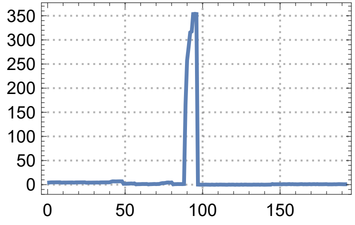

Dataset Index= 1269

Molecule Formula= $C_{44}H_{52}F_3N_5O_8S$

3D Autocorrelation

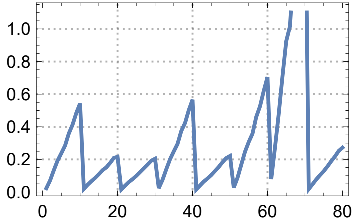

2D Plot

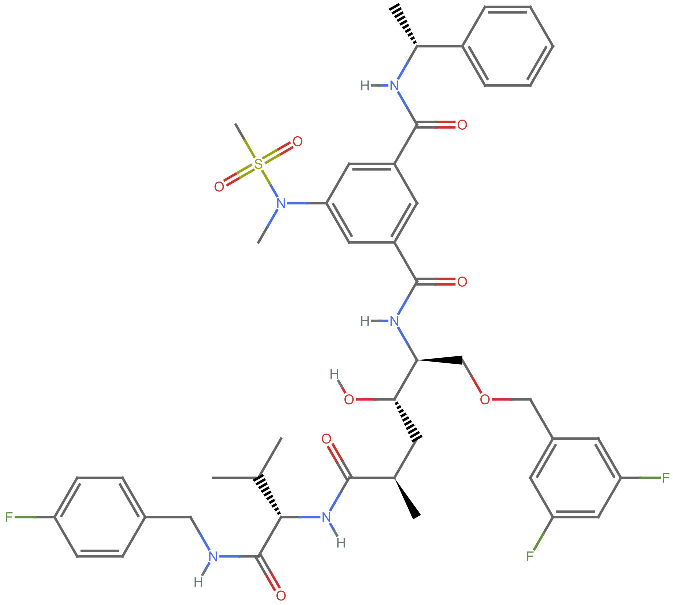

3D Plot

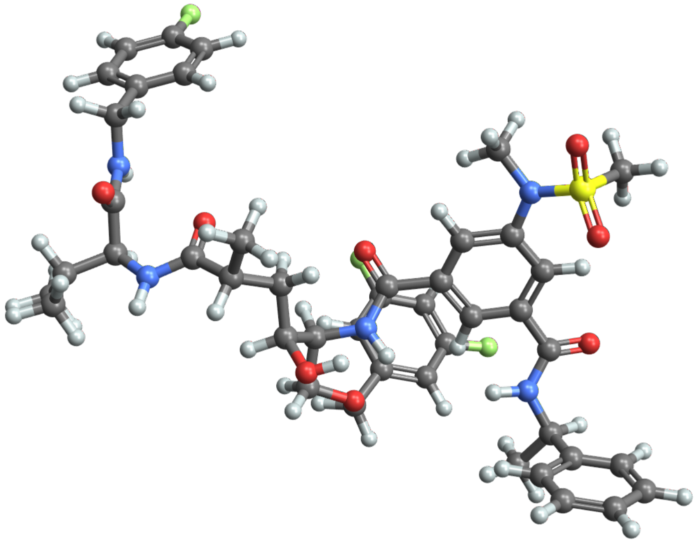

2D Plot

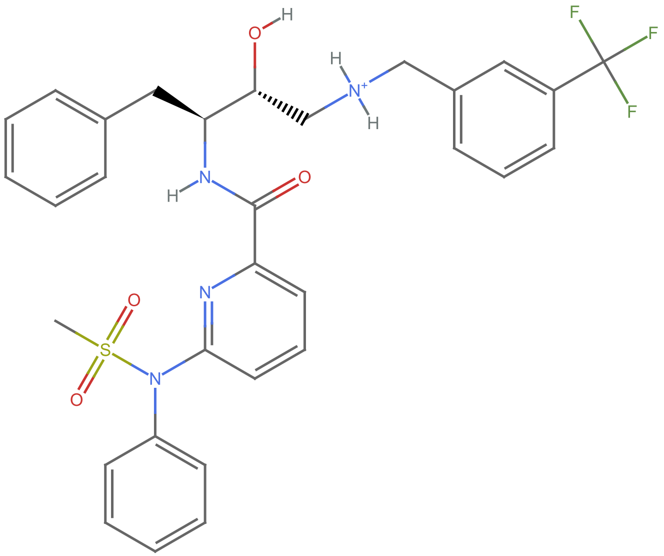

2D Autocorrelation

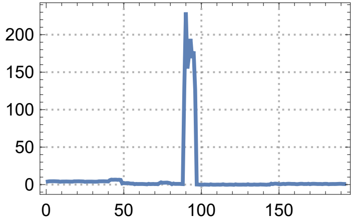

Dataset Index= 1270

Molecule Formula= $C_{31}H_{32}F_3N_4O_4S^+$

3D Autocorrelation

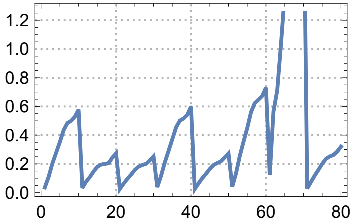

3D Plot

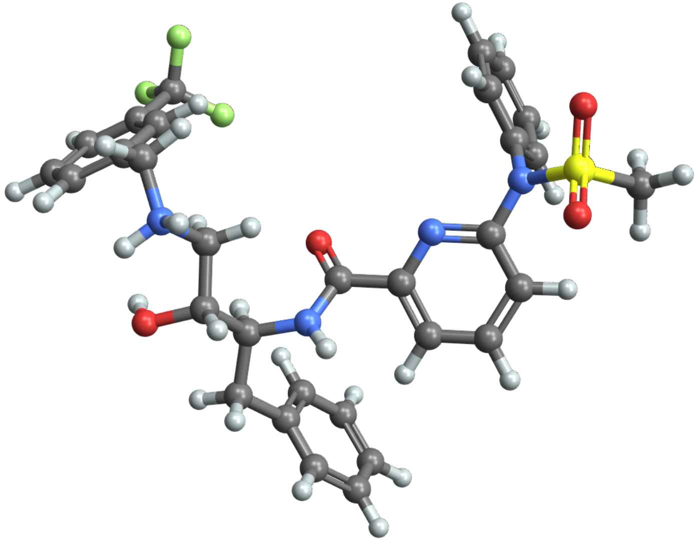

2D Plot

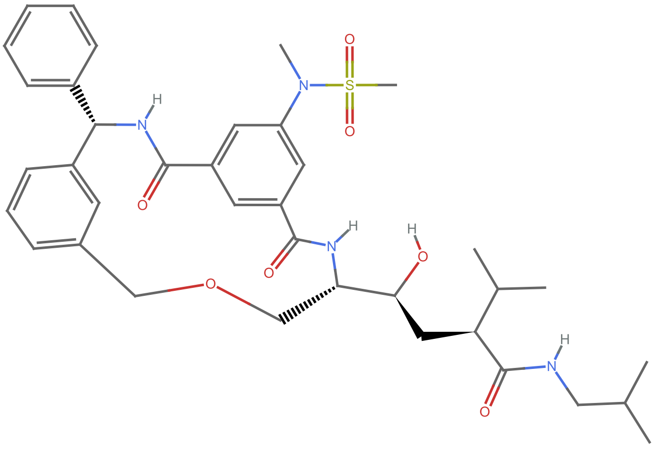

2D Autocorrelation

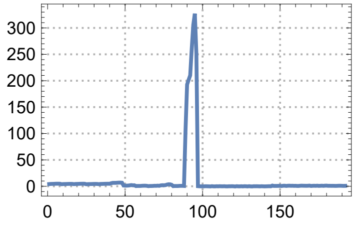

3D Plot

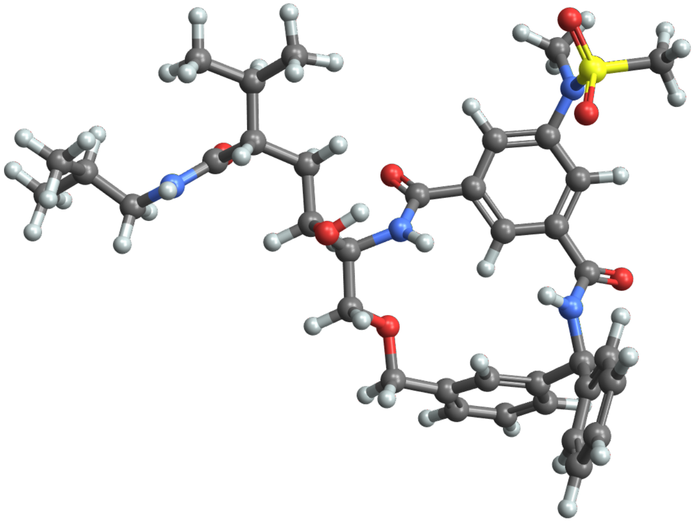

Dataset Index= 1271

Molecule Formula= $C_{37}H_{48}N_4O_7S$

3D Autocorrelation

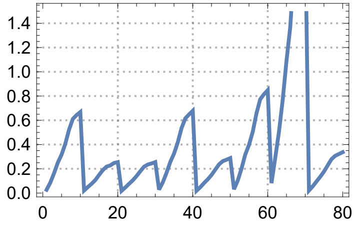

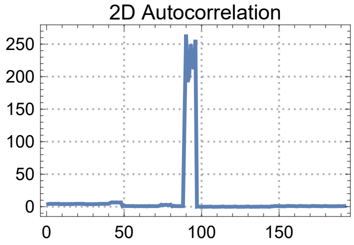

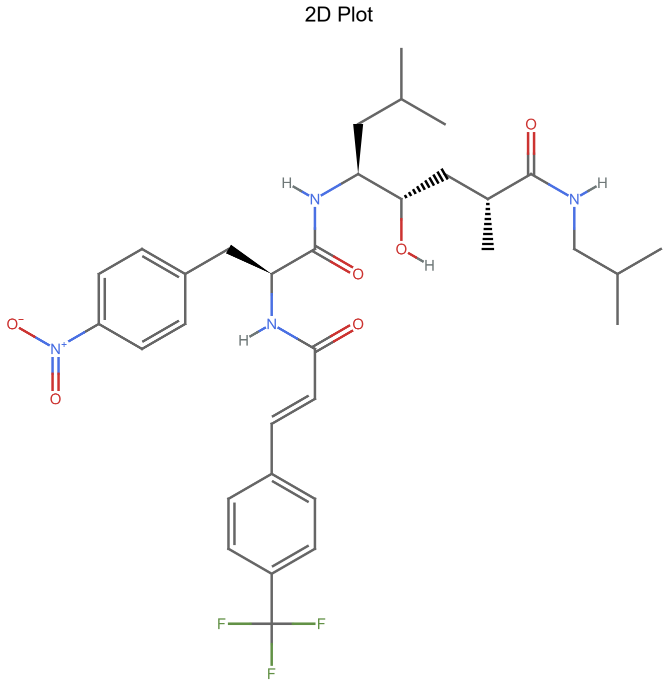

Dataset Index= 1272

Molecule Formula= $C_{33}H_{43}F_3N_4O_6$

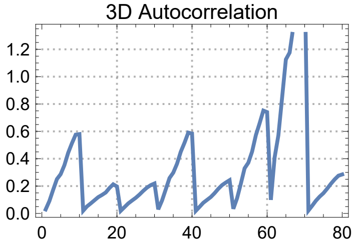

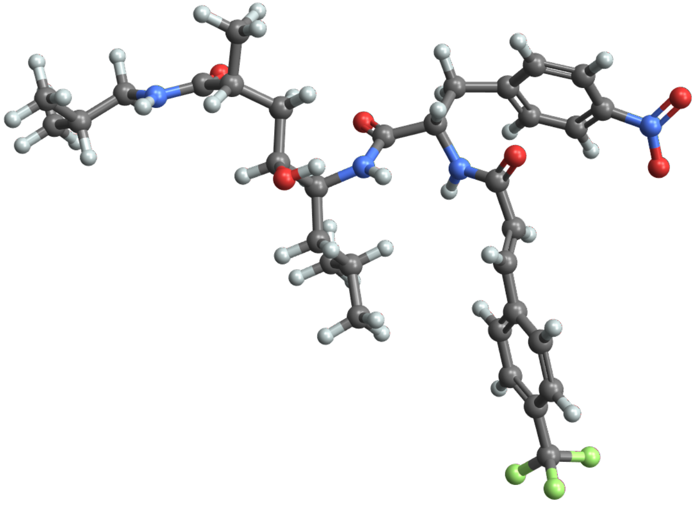

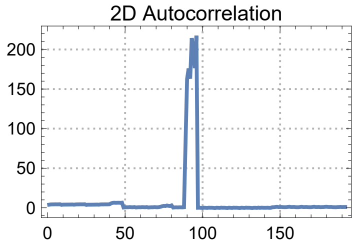

Dataset Index= 1273

Molecule Formula= $C_{32}H_{46}N_4O_8$

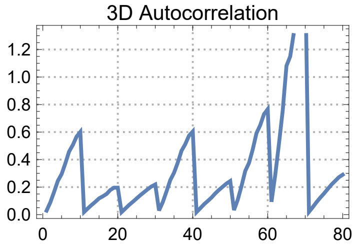

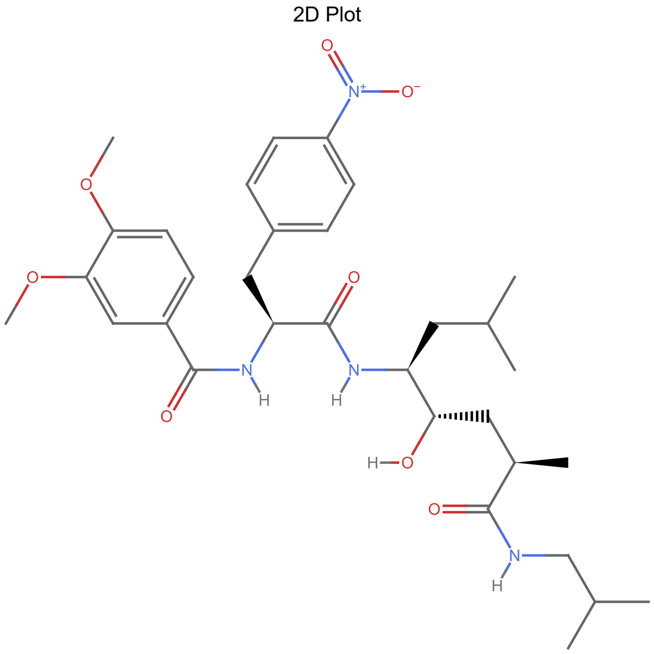

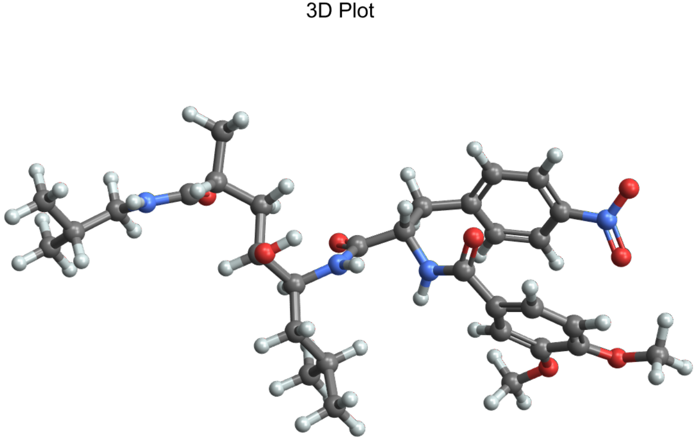

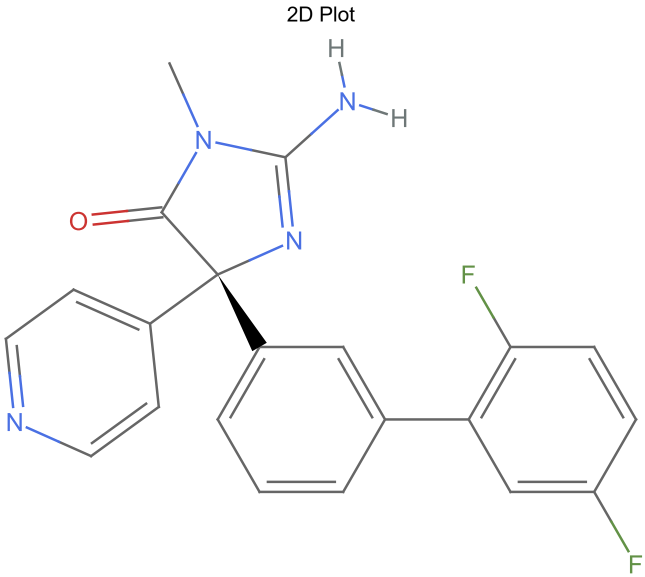

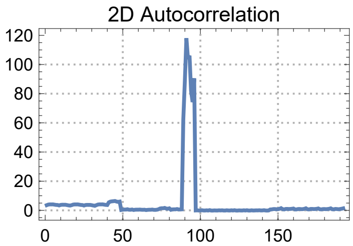

Dataset Index= 1274

Molecule Formula= $C_{21}H_{16}F_2N_4O$

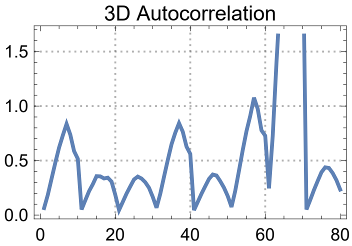

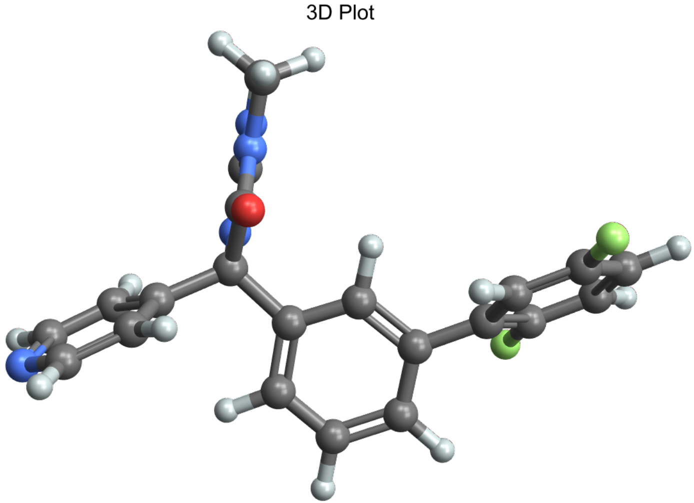

2D Autocorrelation

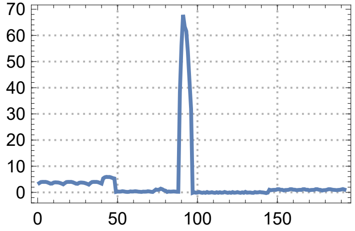

Dataset Index= 1275

Molecule Formula= $C_{21}H_{17}N_3O_3$

3D Autocorrelation

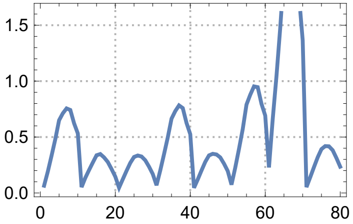

2D Plot

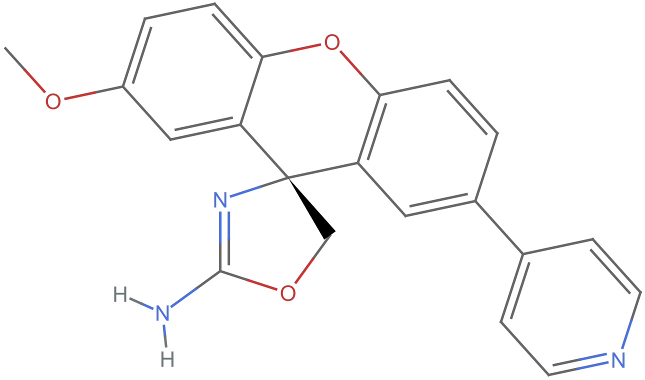

3D Plot

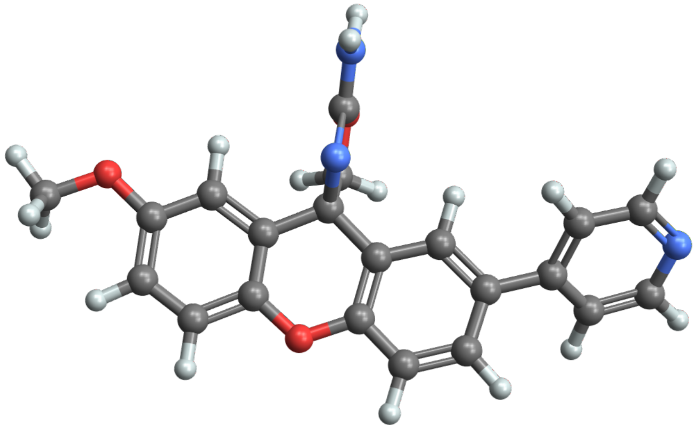

2D Plot

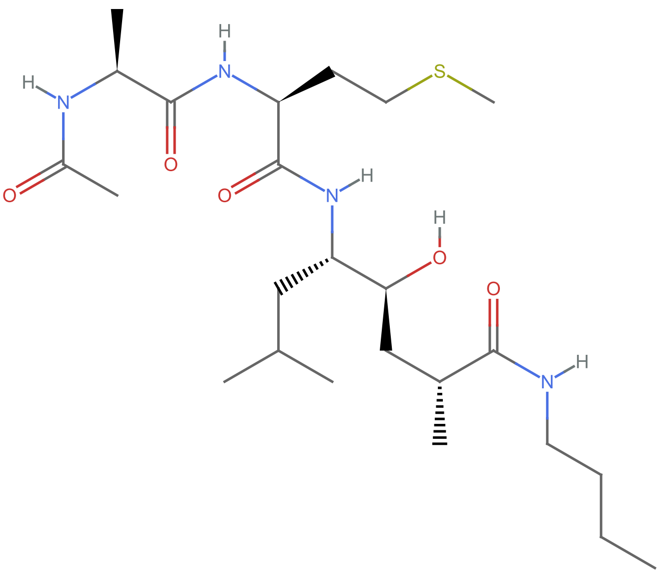

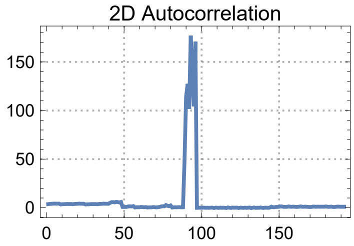

Dataset Index= 1276

Molecule Formula= $C_{24}H_{46}N_4O_5S$

3D Plot

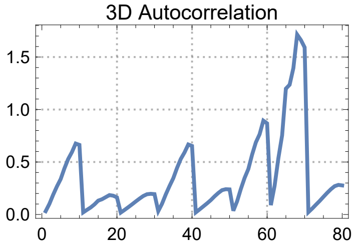

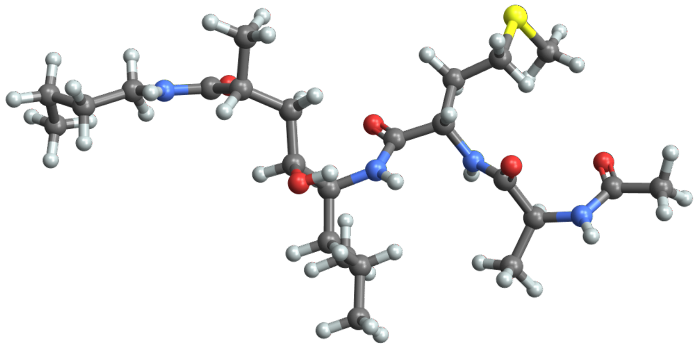

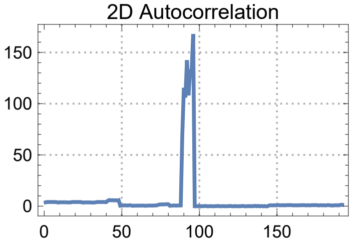

Dataset Index= 1277

Molecule Formula= $C_{25}H_{31}F_2N_2O_4^+$

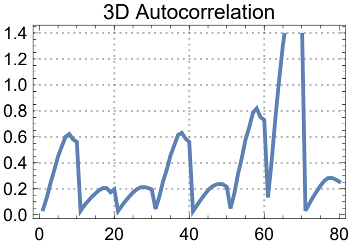

2D Plot

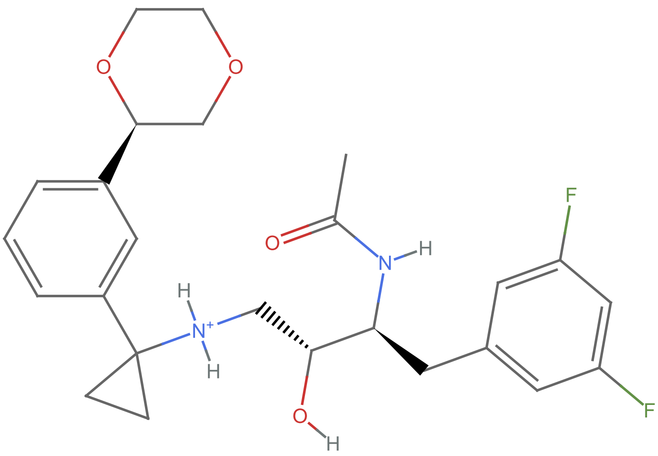

3D Plot

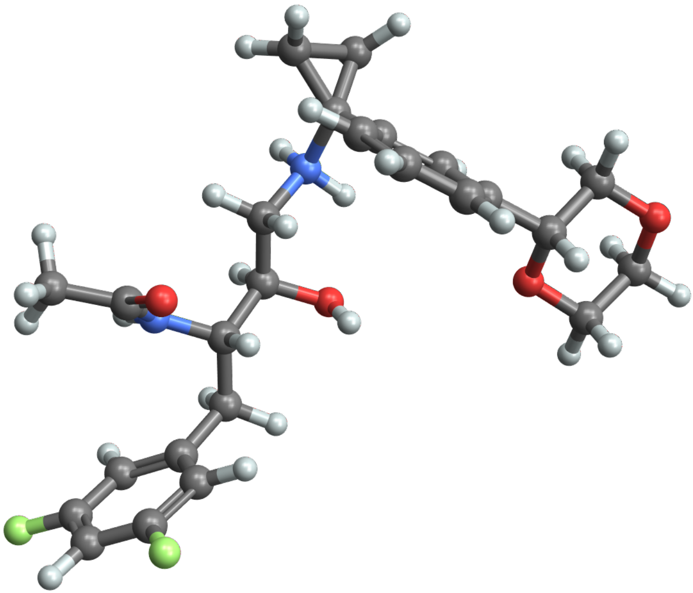

2D Plot

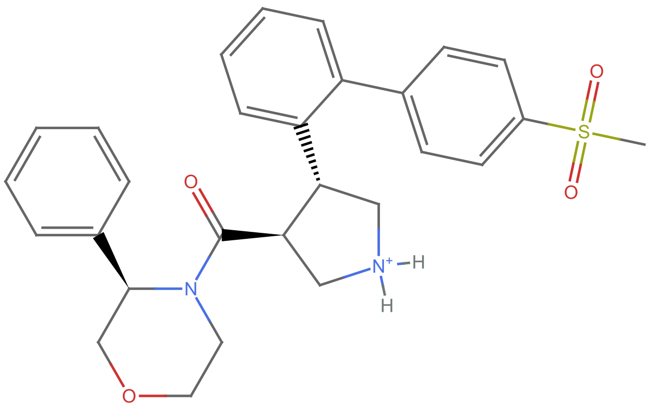

2D Autocorrelation

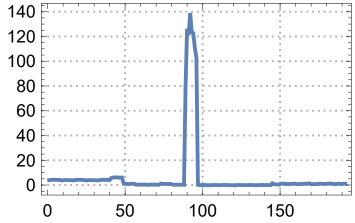

3D Plot

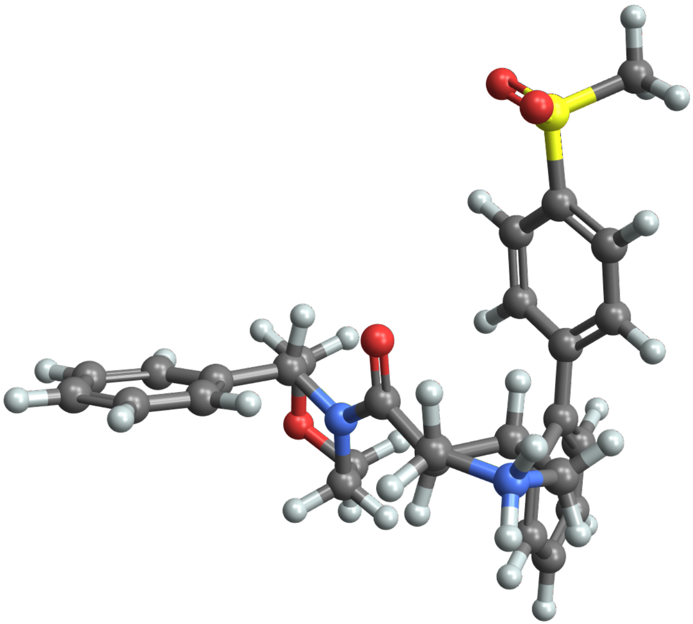

Dataset Index= 1278

Molecule Formula= $C_{28}H_{31}N_2O_4S^+$

3D Autocorrelation

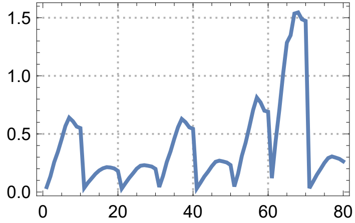

2D Plot

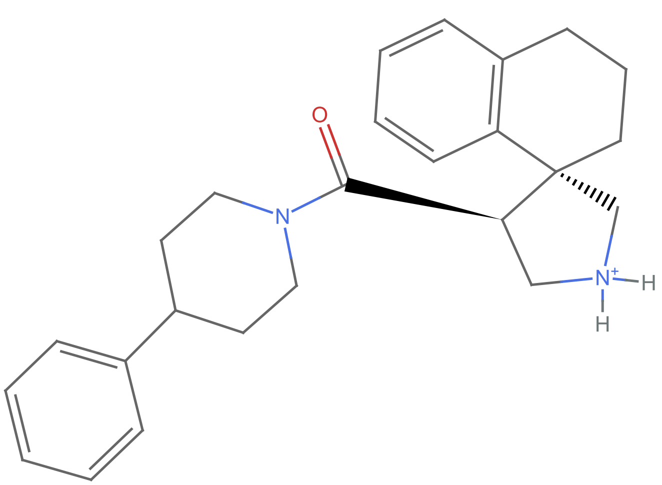

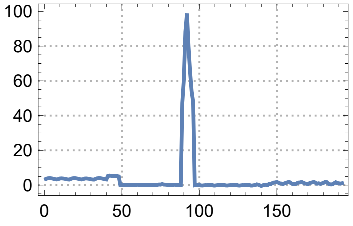

3D Plot

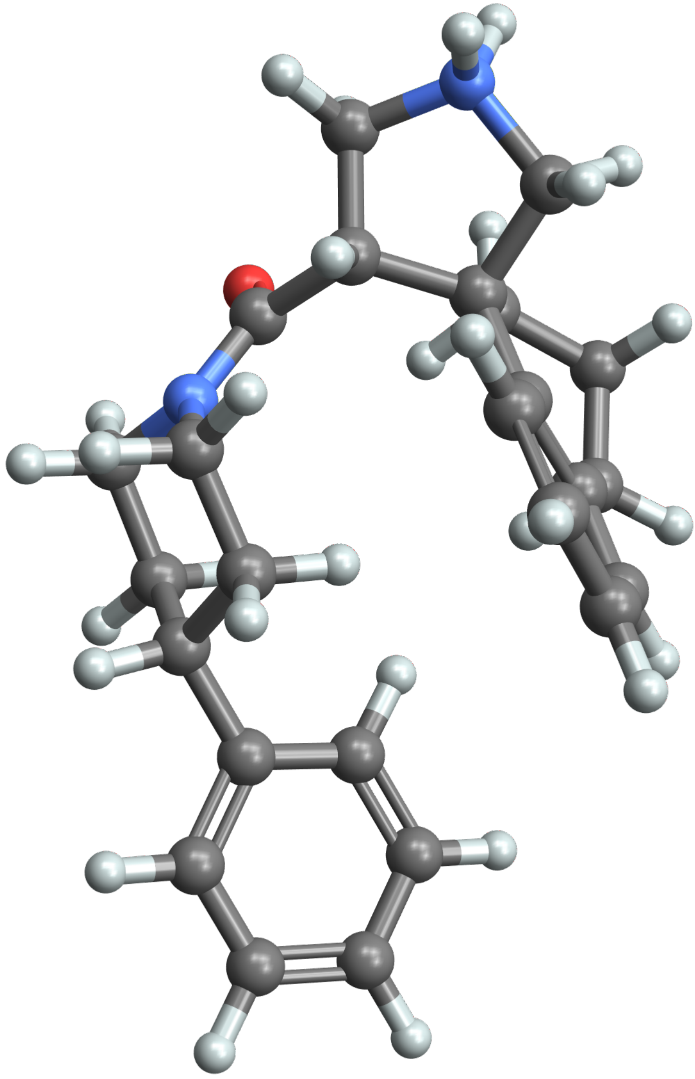

Dataset Index= 1279

Molecule Formula= $C_{25}H_{31}N_2O^+$

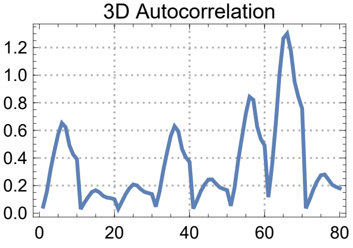

2D Plot

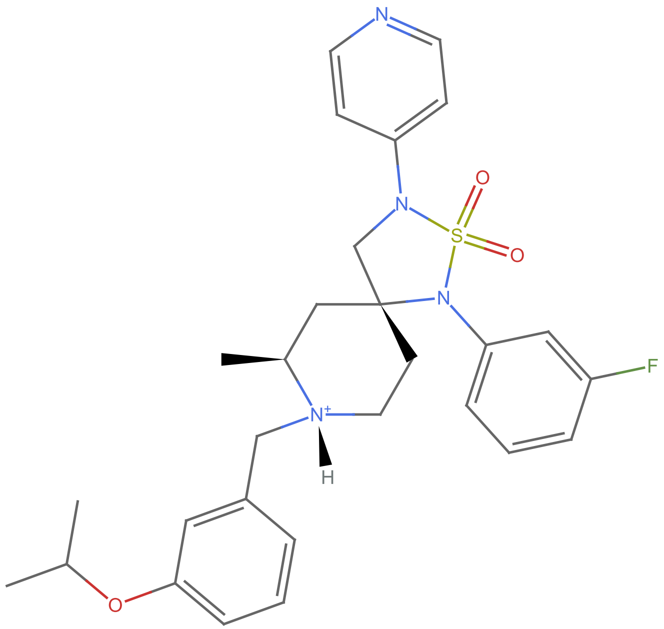

2D Autocorrelation

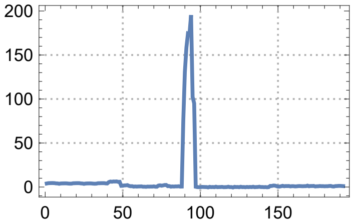

3D Plot

Dataset Index= 1280

Molecule Formula= $C_{28}H_{34}FN_4O_3S^+$

3D Autocorrelation

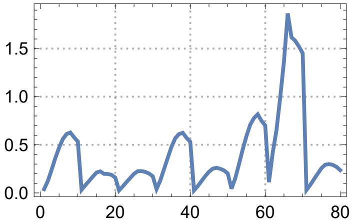

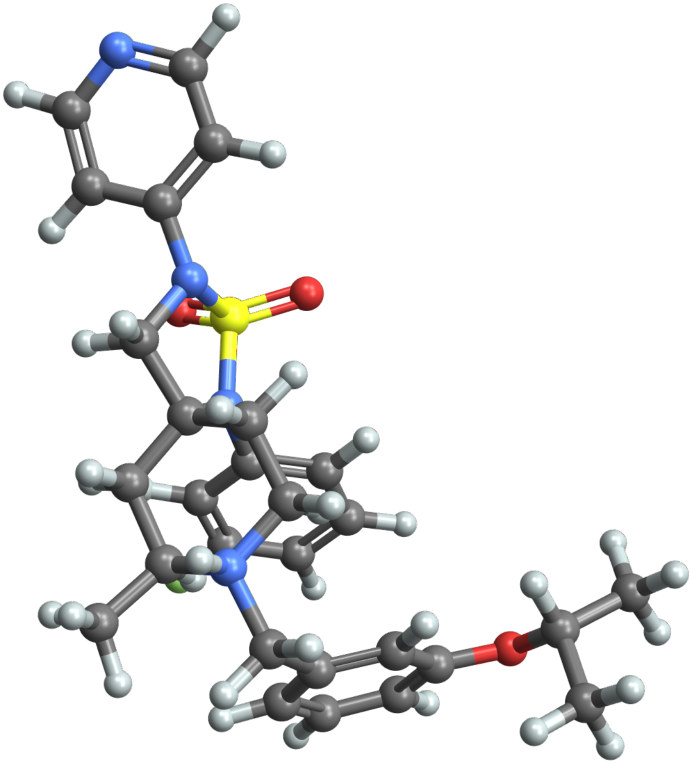

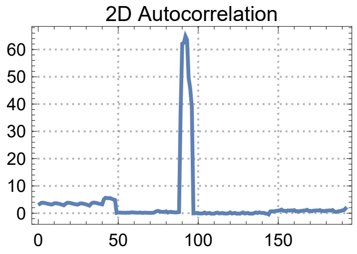

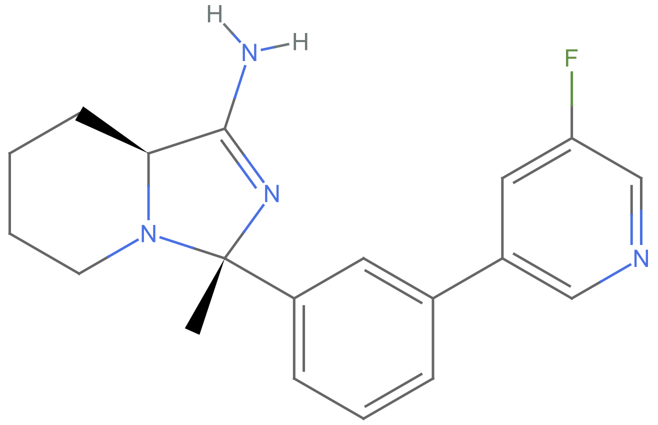

Dataset Index= 1281

Molecule Formula= $C_{19}H_{21}FN_4$

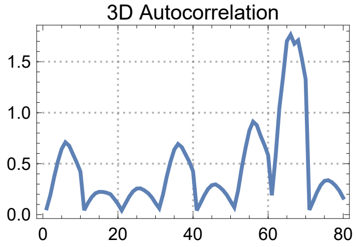

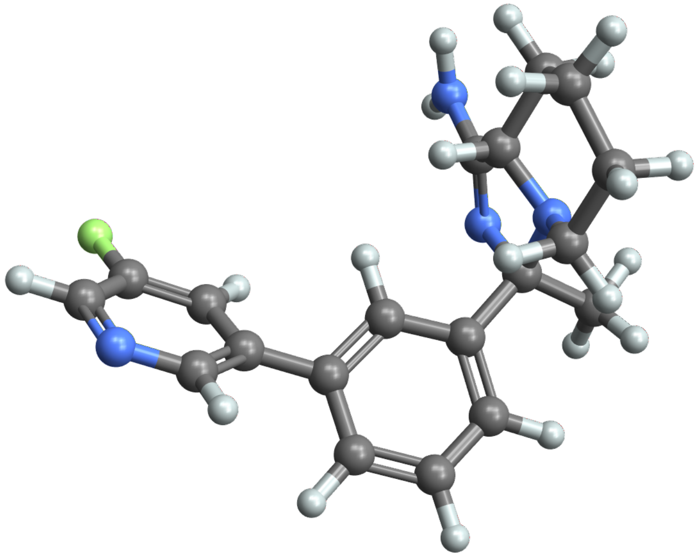

2D Plot

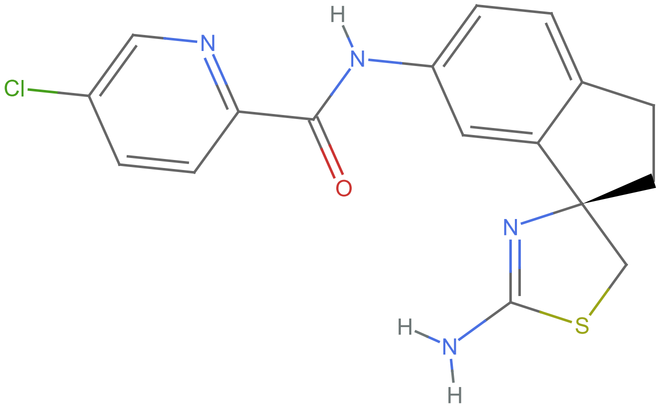

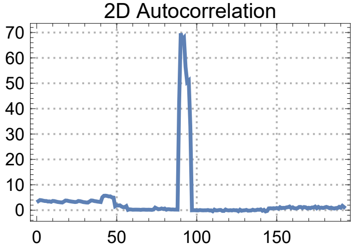

Dataset Index= 1282

Molecule Formula= $C_{17}H_{15}ClN_4OS$

3D Plot

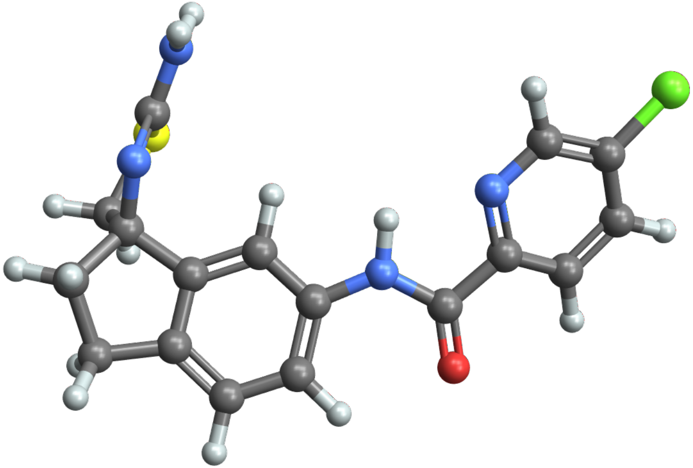

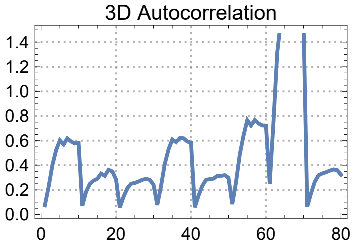

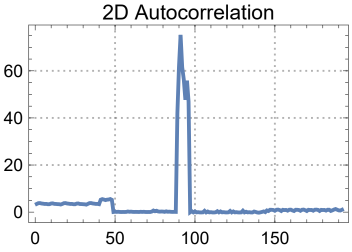

Dataset Index= 1283

Molecule Formula= $C_{23}H_{27}N_3O$

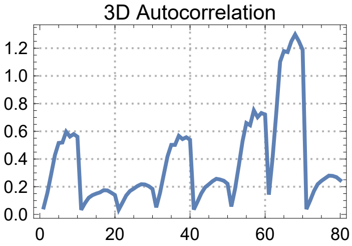

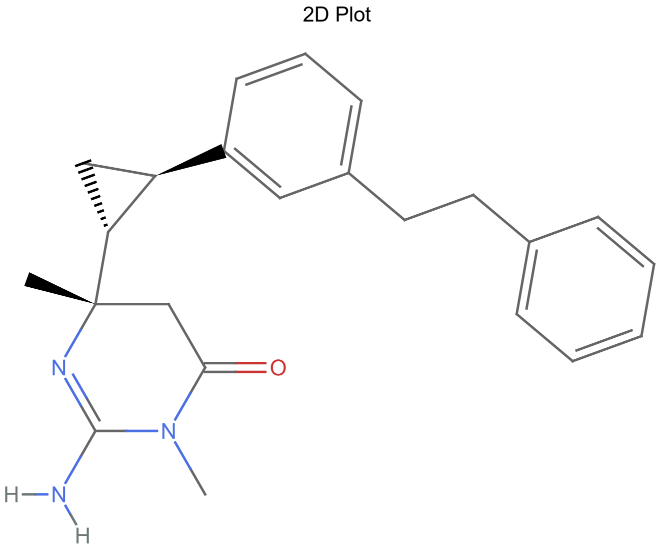

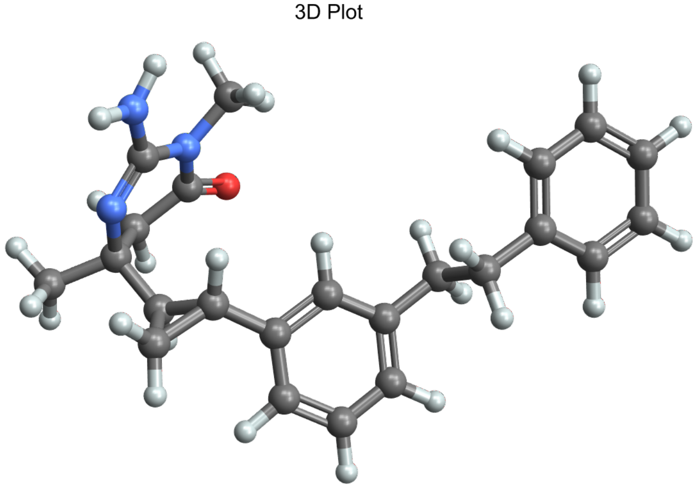

2D Plot

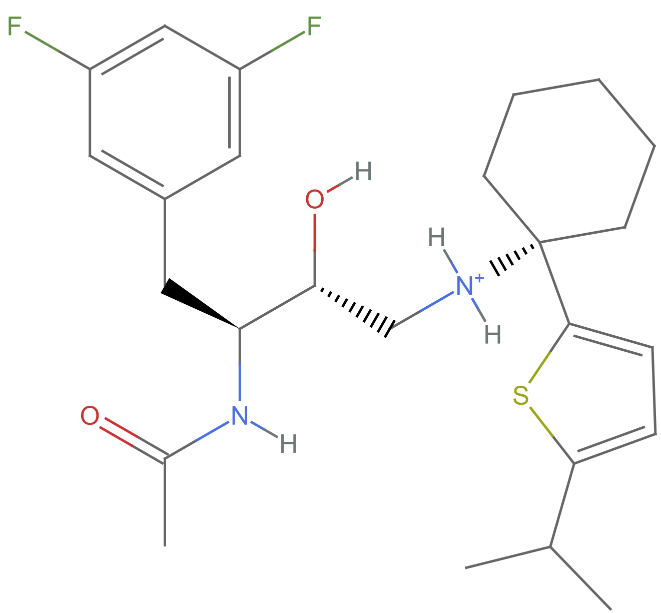

2D Autocorrelation

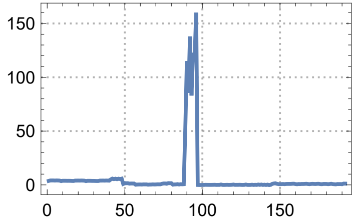

Dataset Index= 1284

Molecule Formula= $C_{25}H_{35}F_2N_2O_2S^+$

3D Plot

3D Autocorrelation

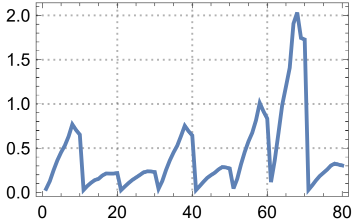

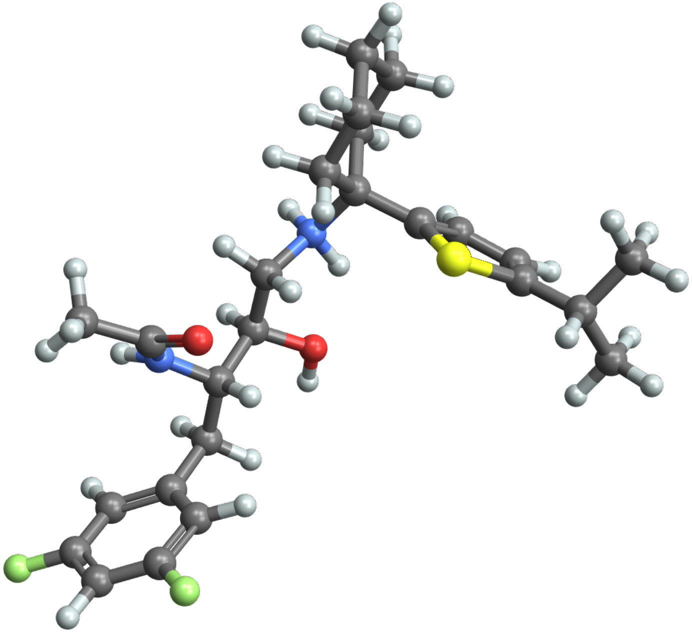

2D Plot

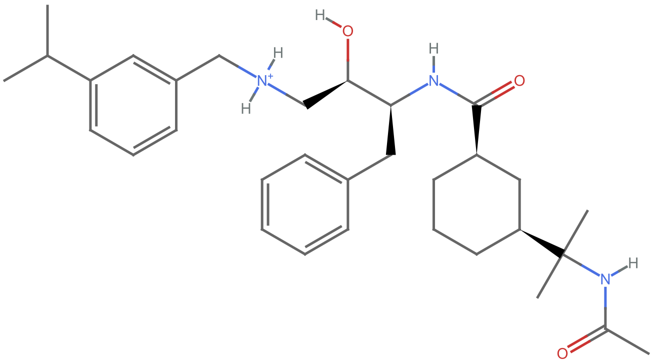

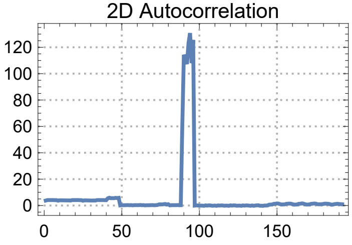

3D Plot

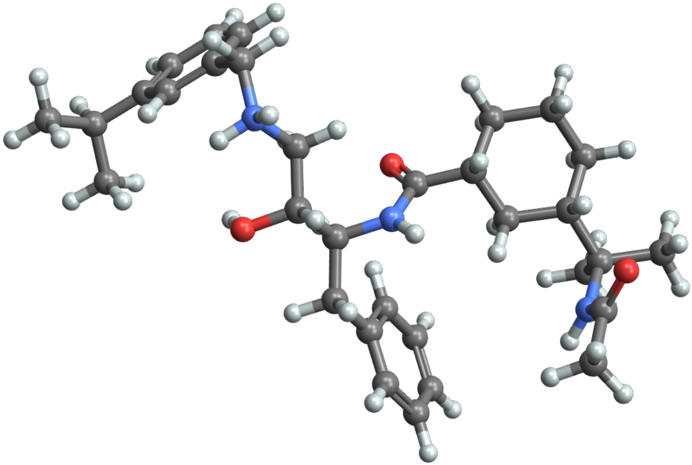

Dataset Index= 1285

Molecule Formula= $C_{32}H_{48}N_3O_3^+$

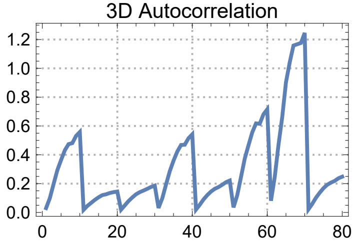

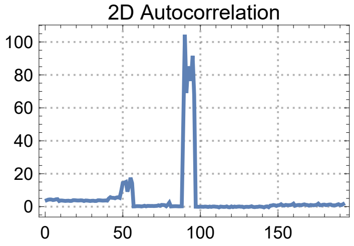

Dataset Index= 1286

Molecule Formula= $C_{19}H_{21}BrCl_2NO_4S^+$

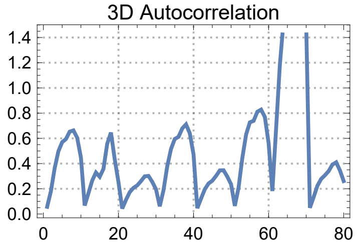

2D Plot

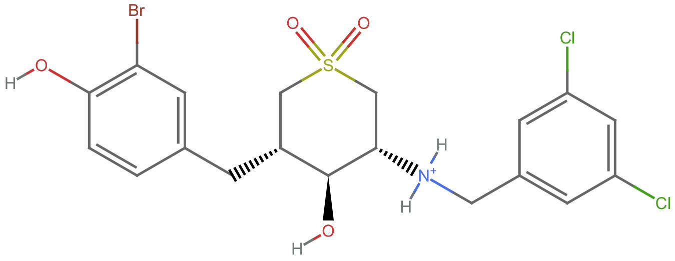

3D Plot

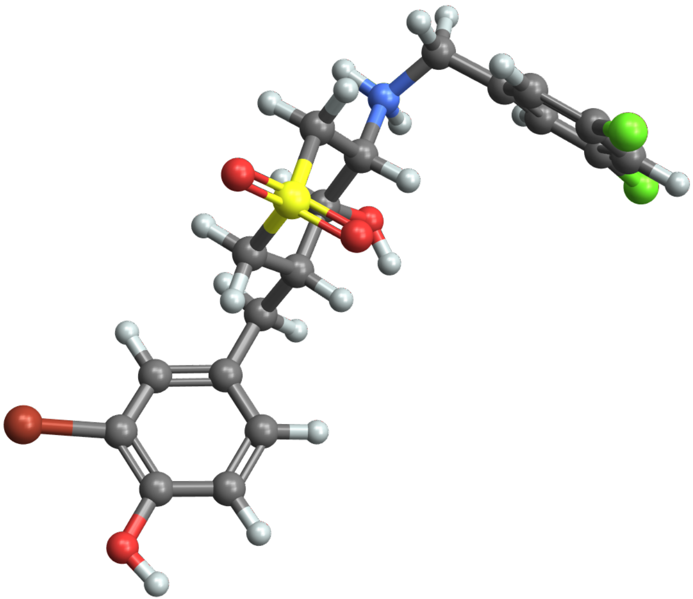

2D Plot

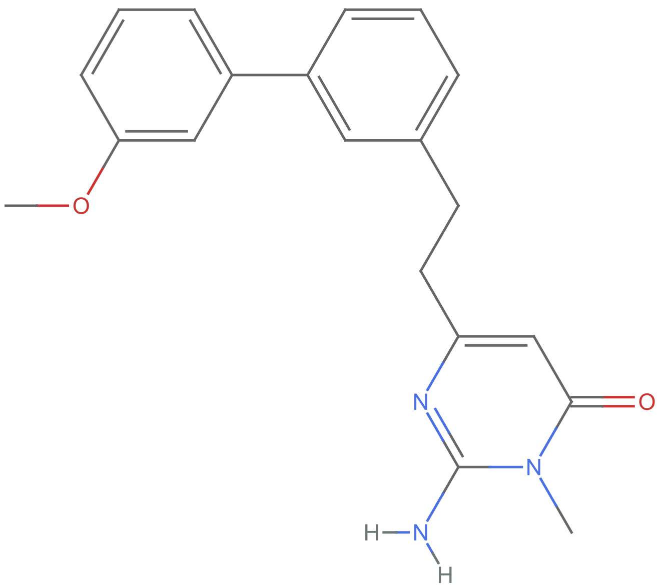

2D Autocorrelation

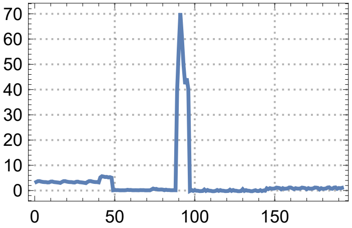

Dataset Index= 1287

Molecule Formula= $C_{20}H_{21}N_3O_2$

3D Autocorrelation

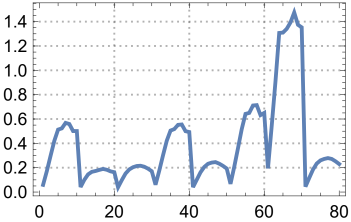

3D Plot

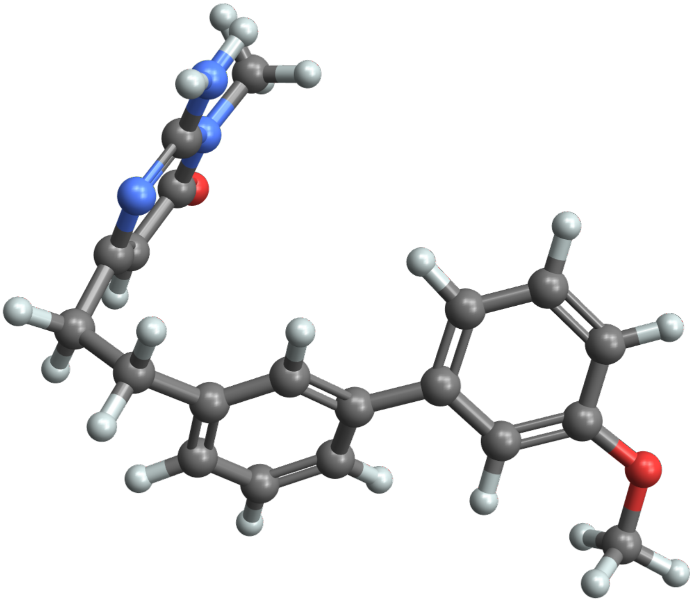

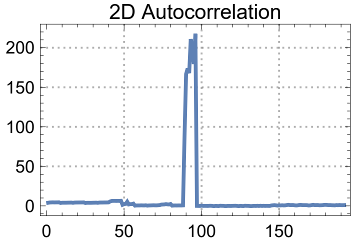

Dataset Index= 1288

Molecule Formula= $C_{30}H_{40}Cl_2N_4O_6$

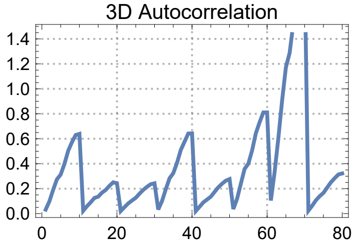

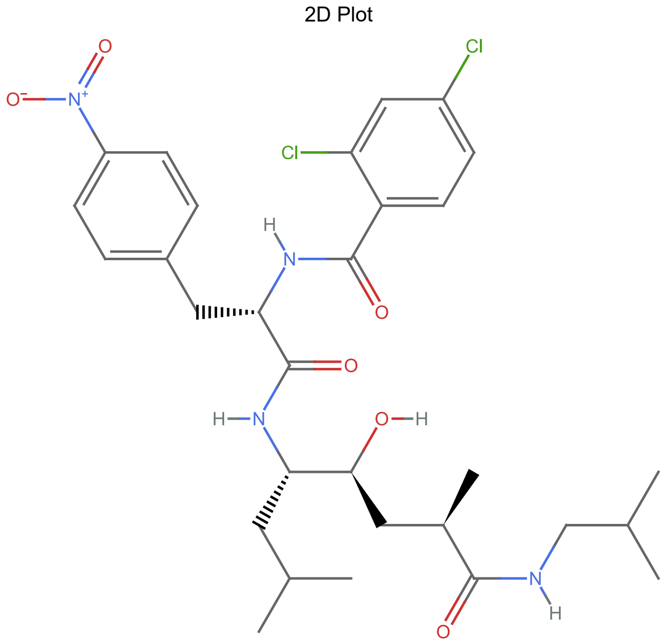

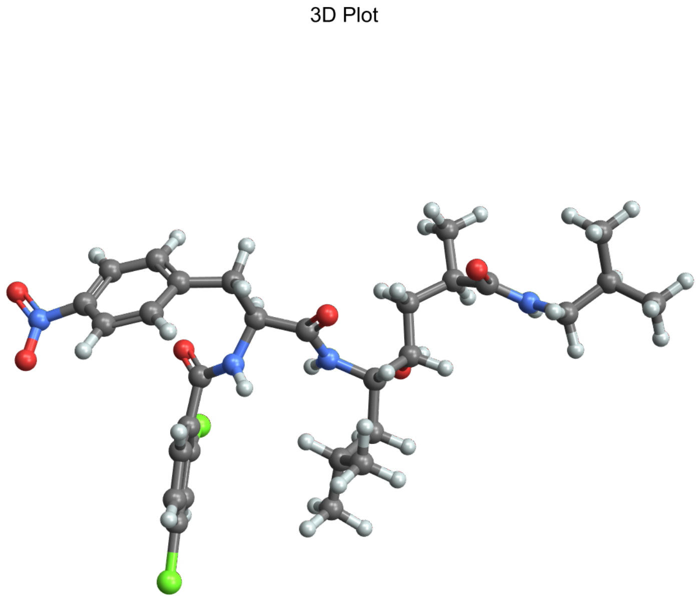

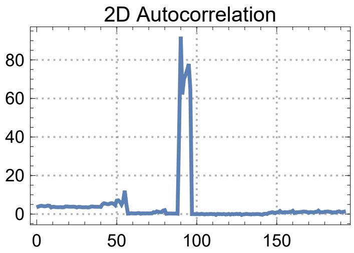

Dataset Index= 1289

Molecule Formula= $C_{21}H_{28}BrN_2O_4S^+$

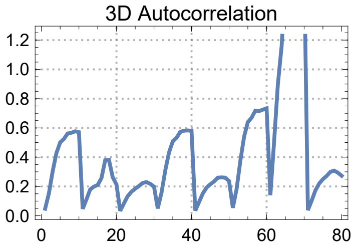

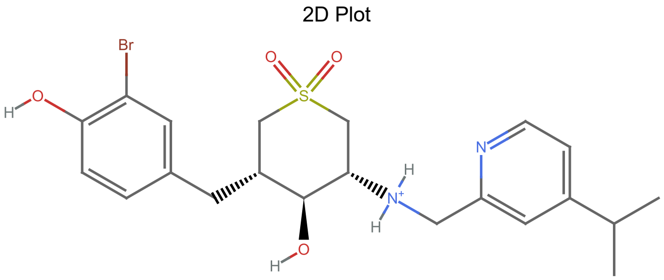

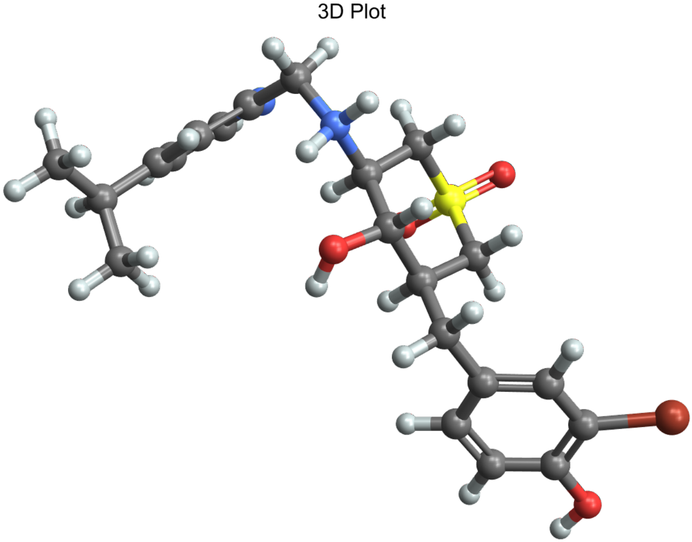

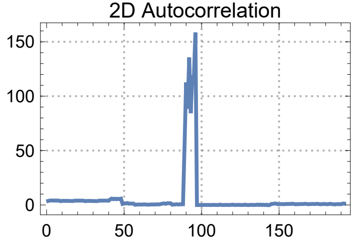

Dataset Index= 1290

Molecule Formula= $C_{24}H_{29}F_2N_2O_2S^+$

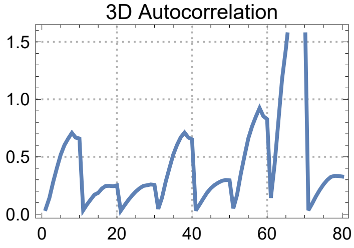

2D Plot

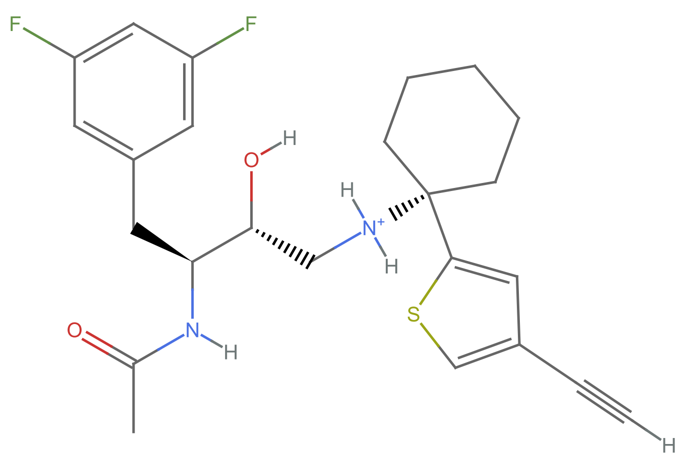

3D Plot

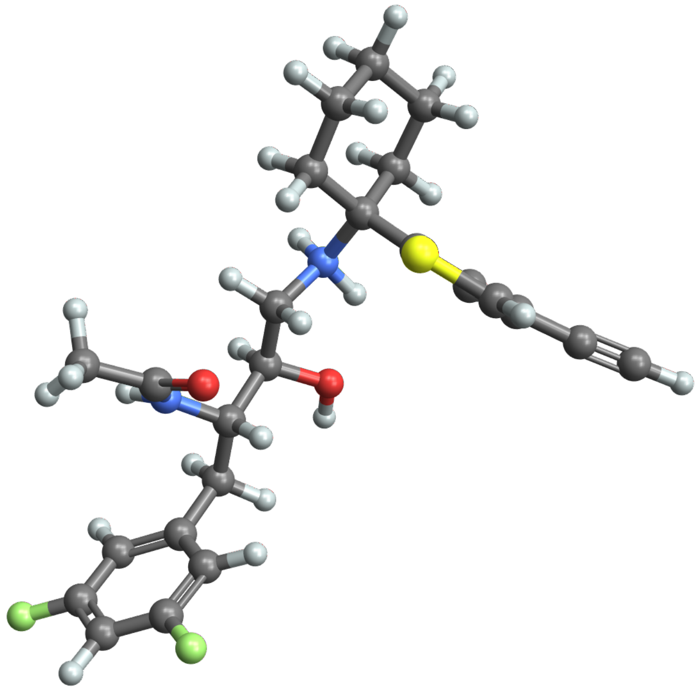

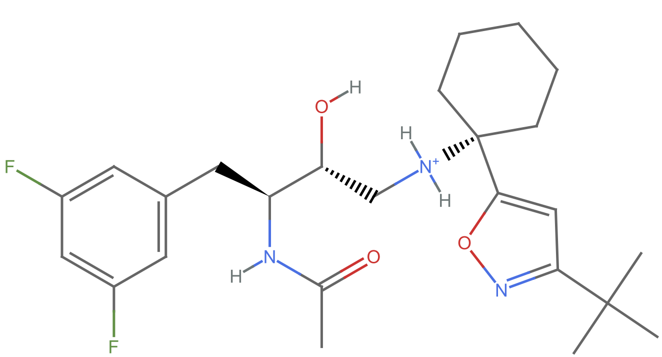

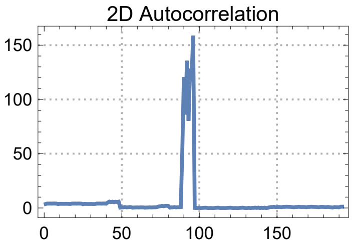

Dataset Index= 1291

Molecule Formula= $C_{25}H_{36}F_2N_3O_3^+$

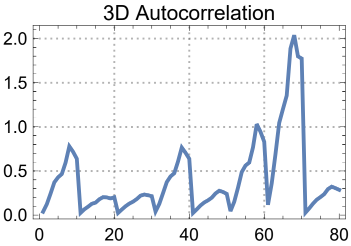

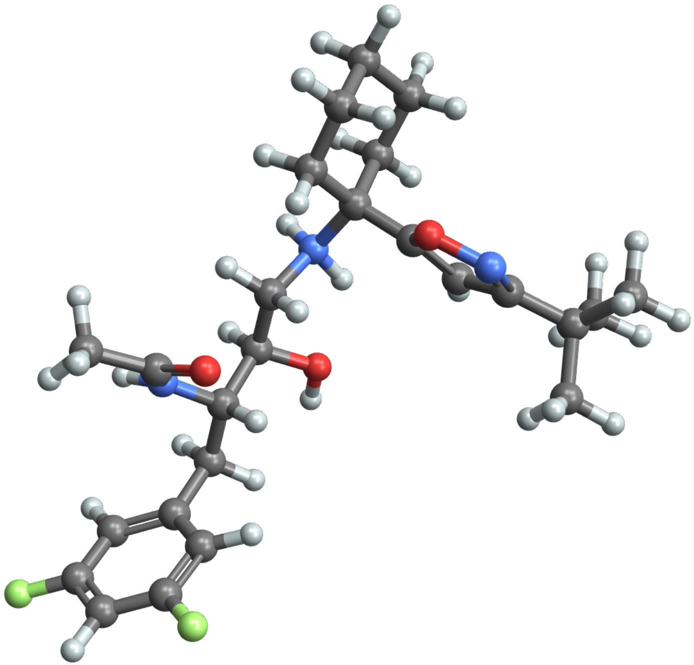

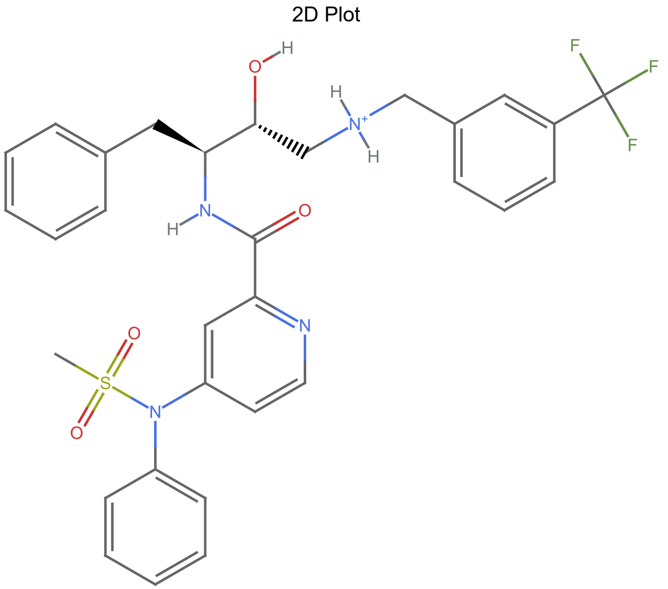

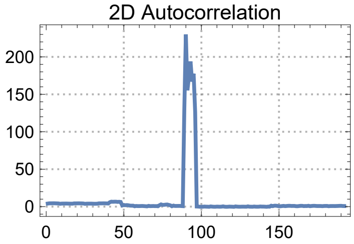

Dataset Index= 1292

Molecule Formula= $C_{31}H_{32}F_3N_4O_4S^+$

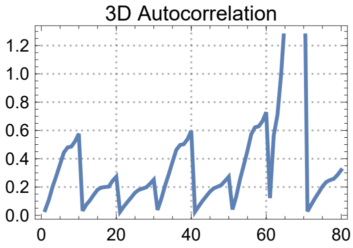

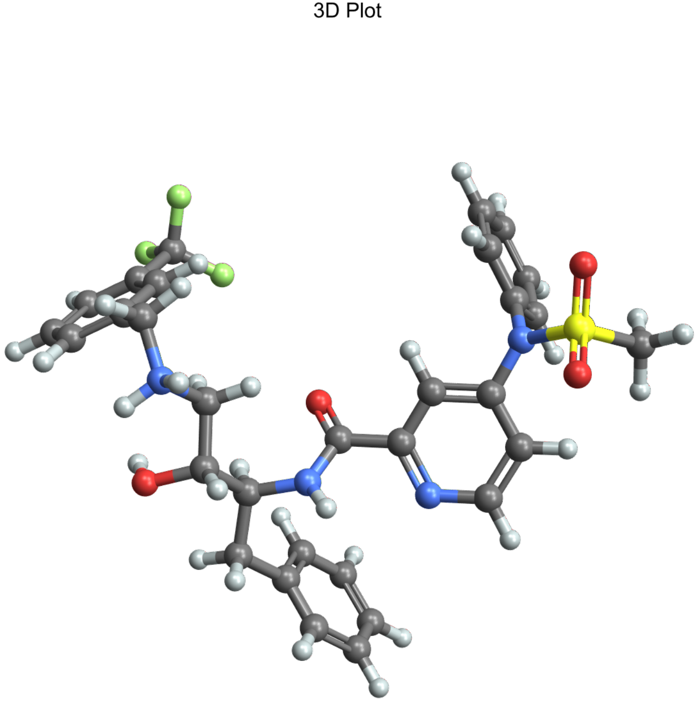

2D Autocorrelation

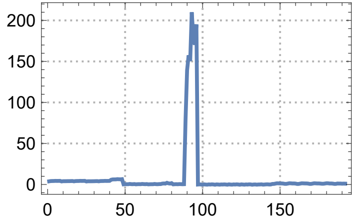

Dataset Index= 1293

Molecule Formula= $C_{34}H_{45}N_3O_4$

3D Autocorrelation

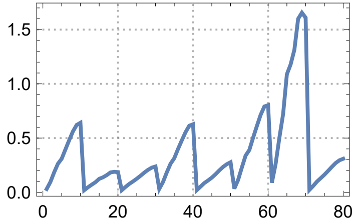

2D Plot

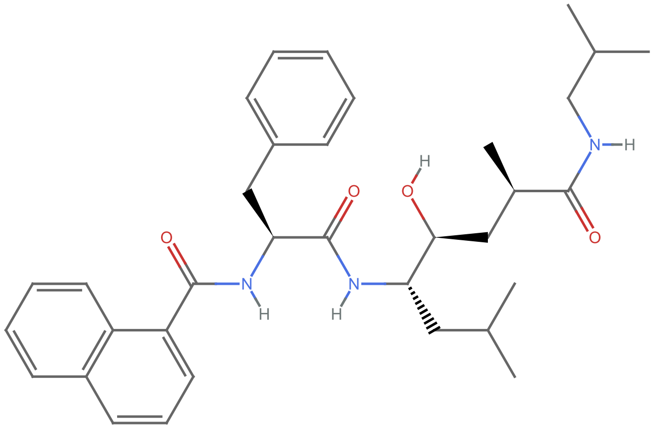

3D Plot

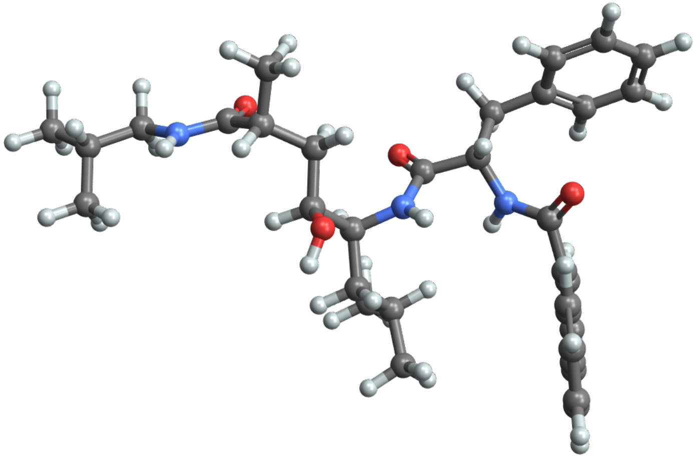

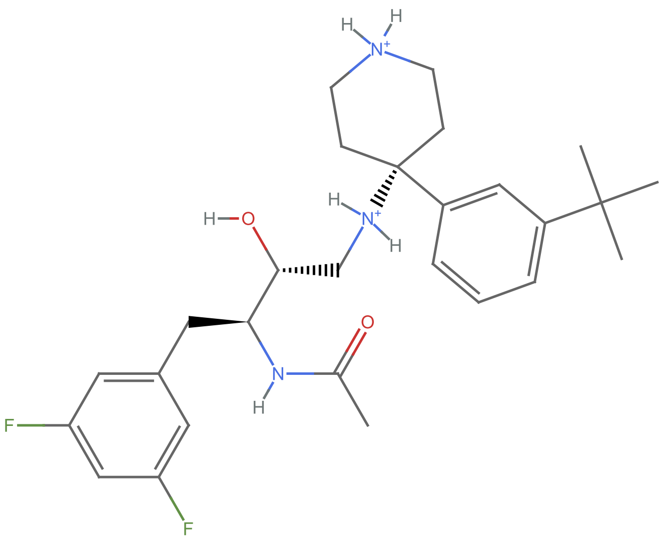

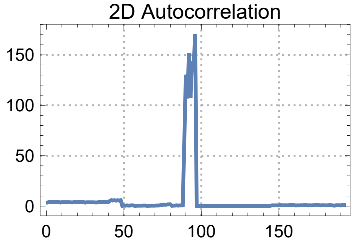

Dataset Index= 1294

Molecule Formula= $C_{27}H_{39}F_2N_3O_2{}^{2+}$

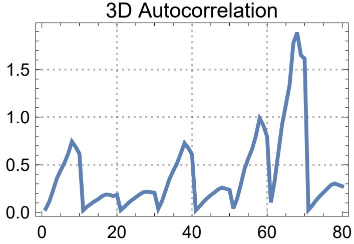

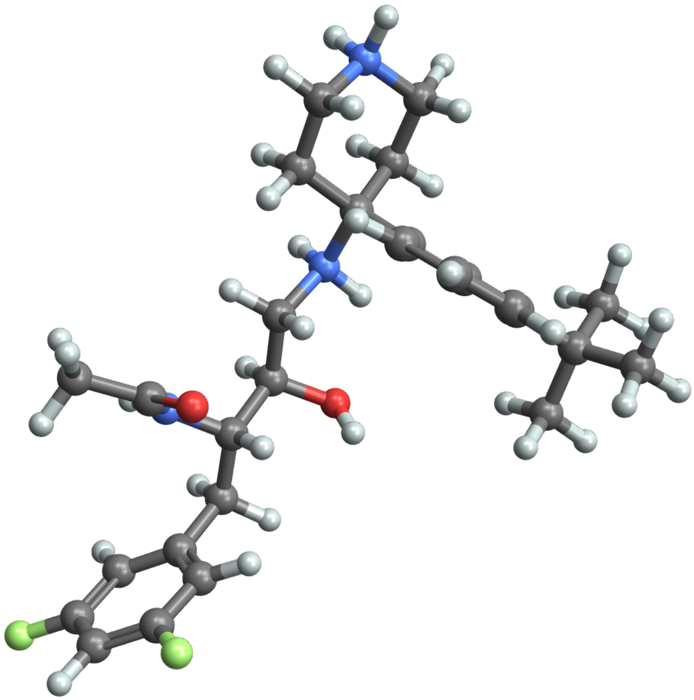

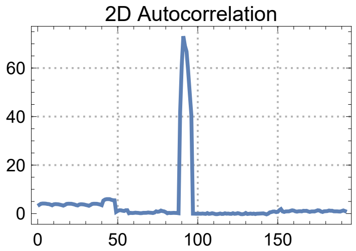

Dataset Index= 1295

Molecule Formula= $C_{22}H_{17}ClN_2O_3$

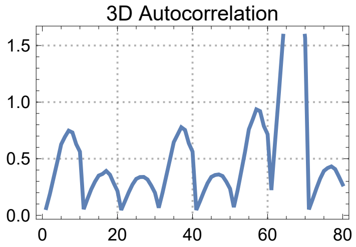

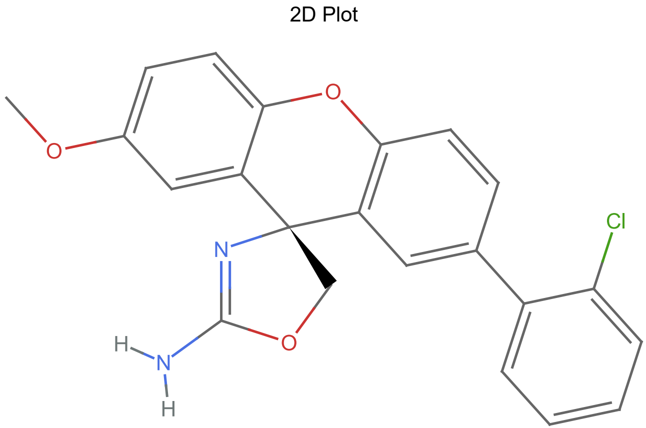

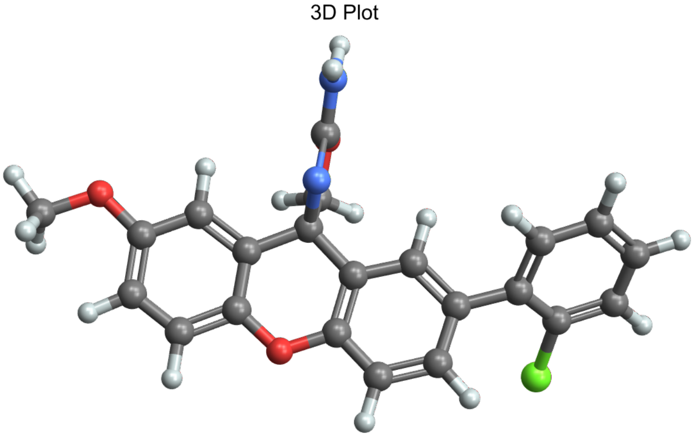

2D Plot

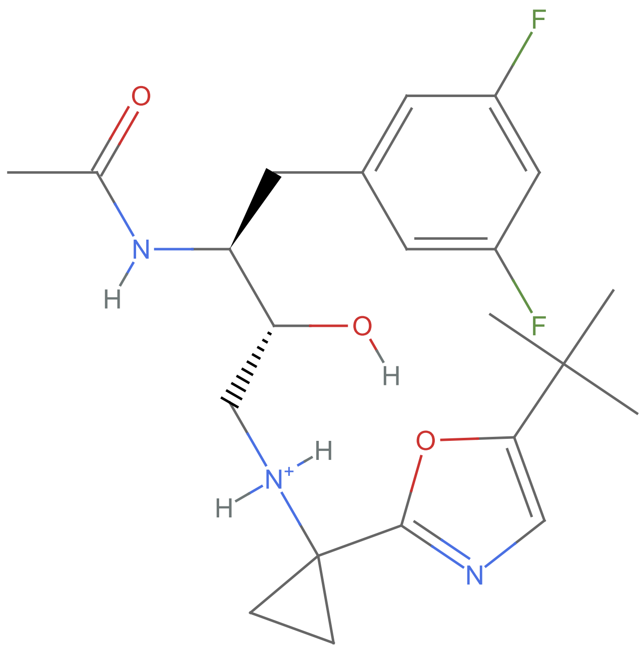

2D Autocorrelation

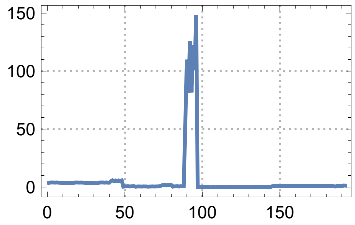

Dataset Index= 1296

Molecule Formula= $C_{22}H_{30}F_2N_3O_3^+$

3D Autocorrelation

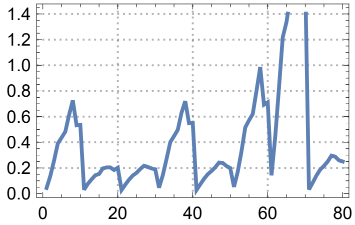

3D Plot

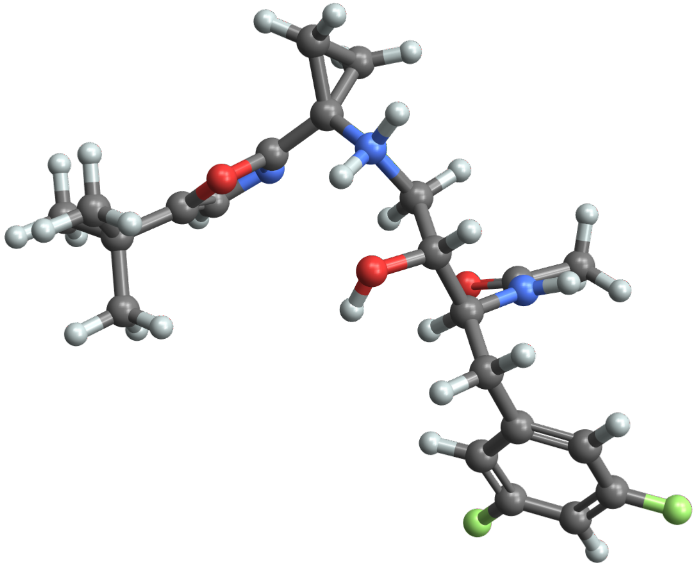

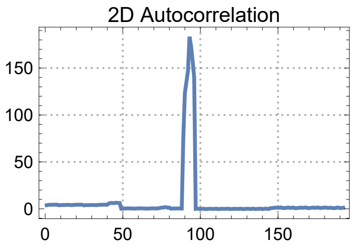

Dataset Index= 1297

Molecule Formula= $C_{34}H_{42}N_3O_4^+$

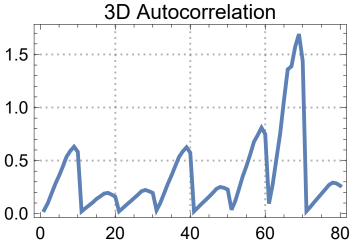

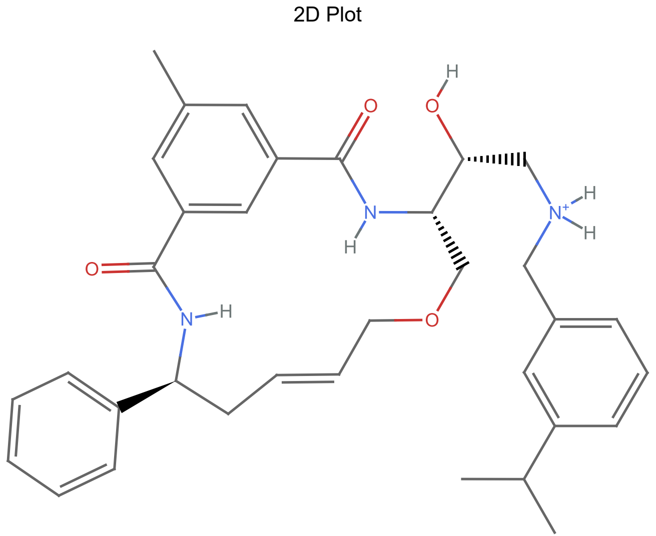

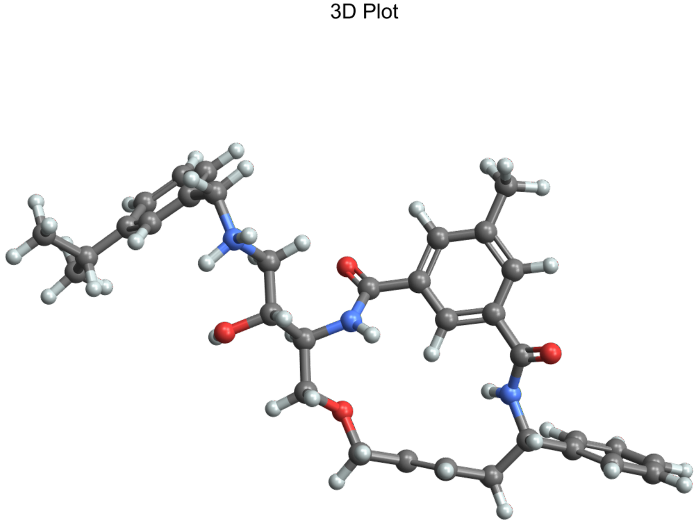

2D Autocorrelation

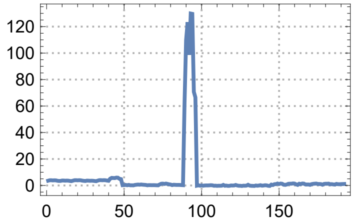

2D Plot

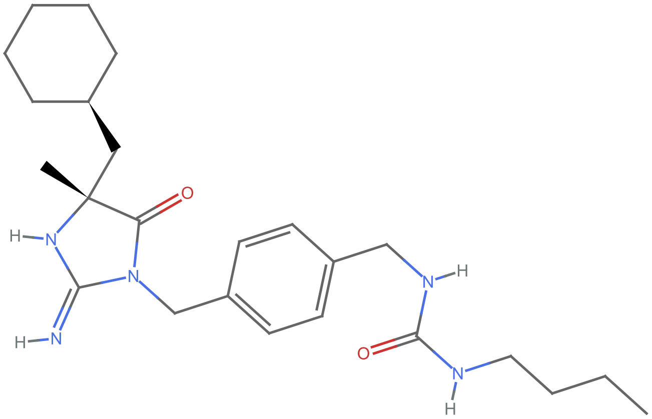

3D Plot

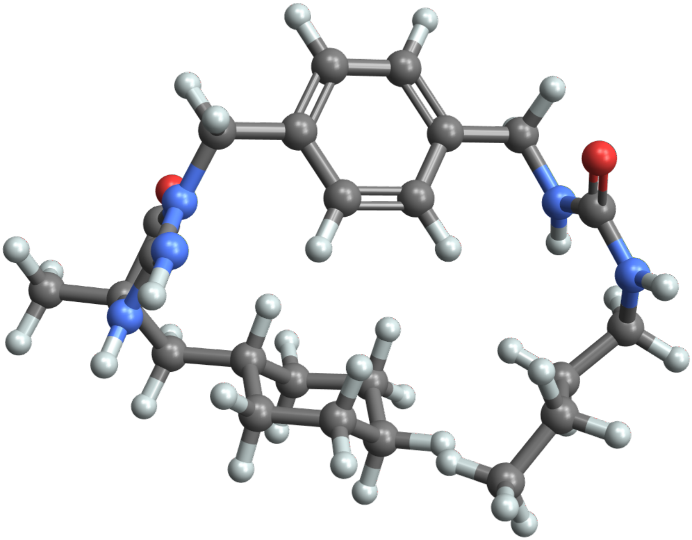

Dataset Index= 1298

Molecule Formula= $C_{24}H_{37}N_5O_2$

3D Autocorrelation

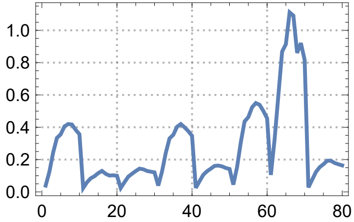

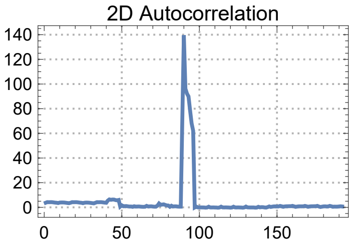

Dataset Index= 1299

Molecule Formula= $C_{23}H_{17}F_3N_2O_4$

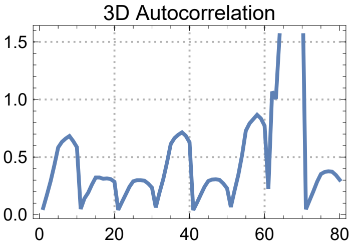

2D Plot

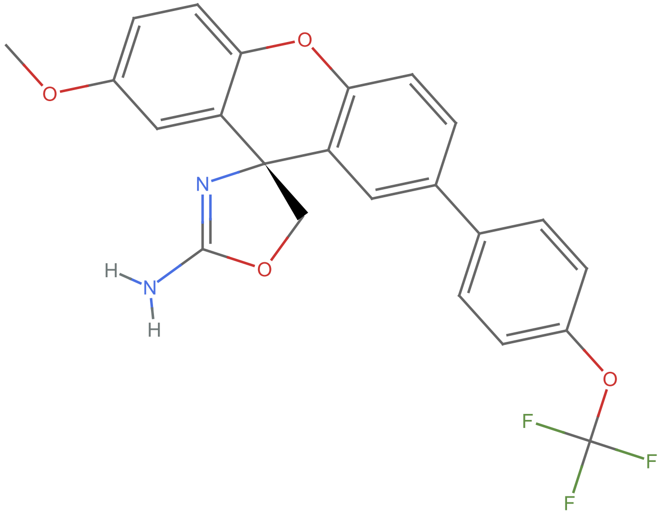

3D Plot

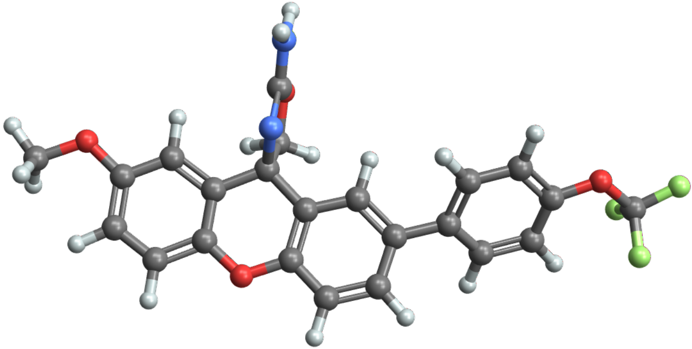

2D Autocorrelation

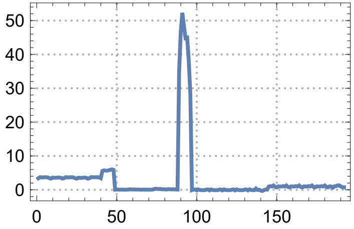

Dataset Index= 1300

Molecule Formula= $C_{22}H_{19}N_3$

3D Autocorrelation

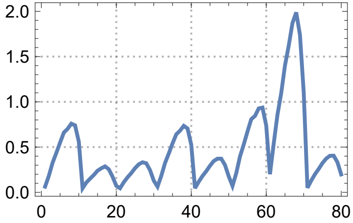

2D Plot

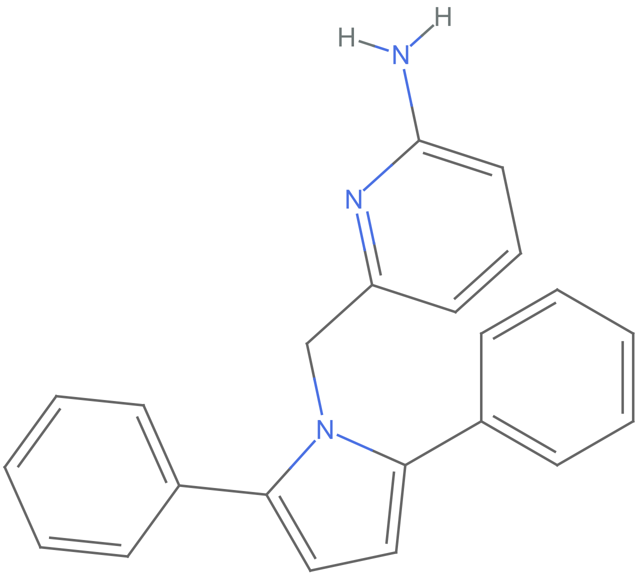

3D Plot

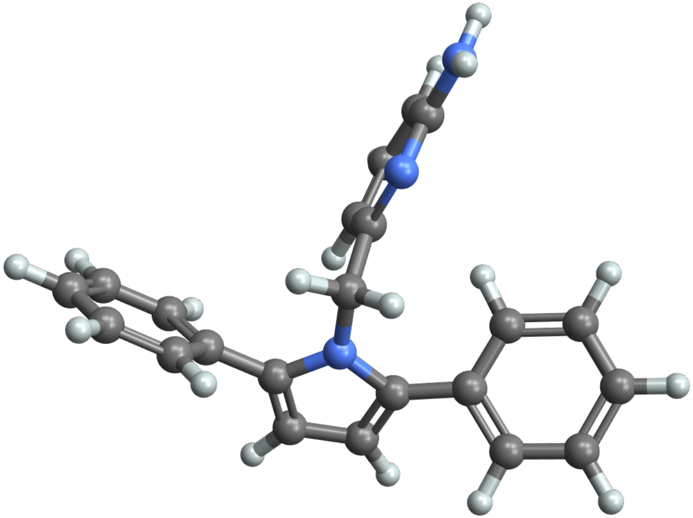

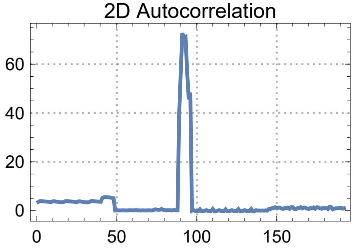

Dataset Index= 1301

Molecule Formula= $C_{26}H_{31}N_3O$

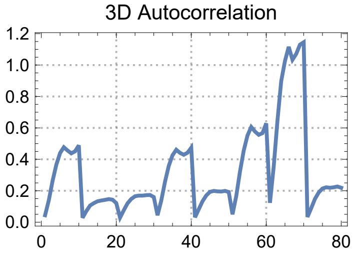

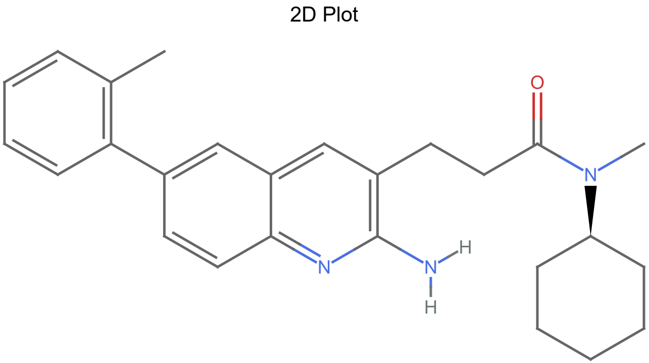

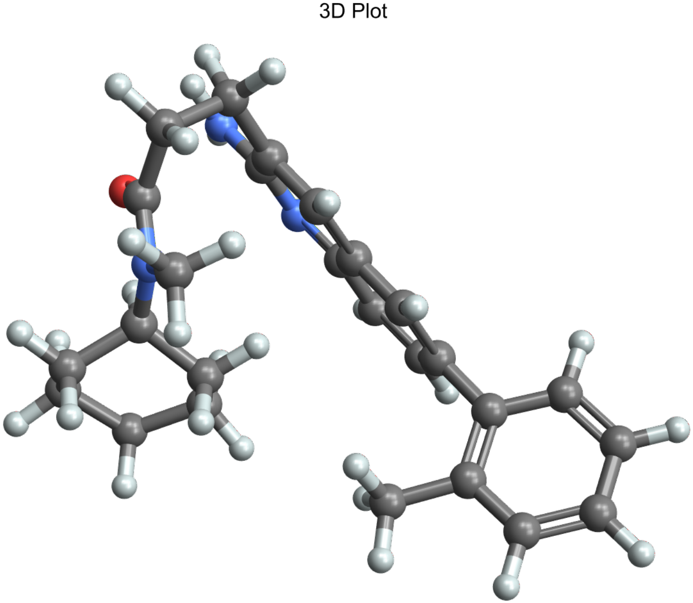

2D Plot

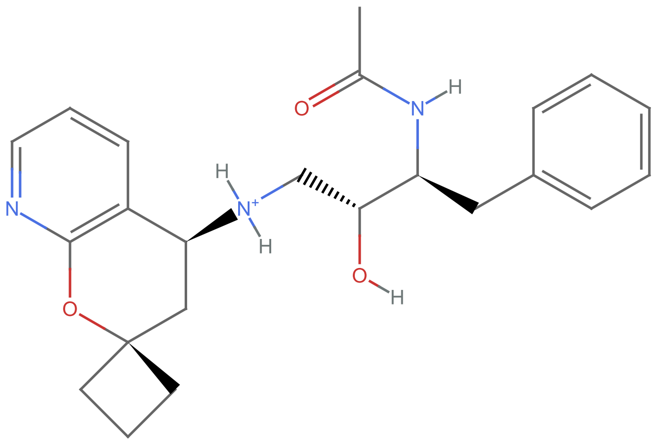

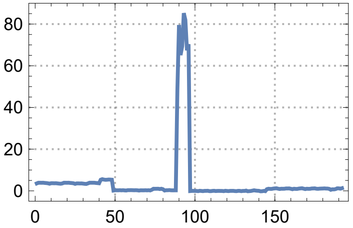

Dataset Index= 1302

Molecule Formula= $C_{23}H_{30}N_3O_3^+$

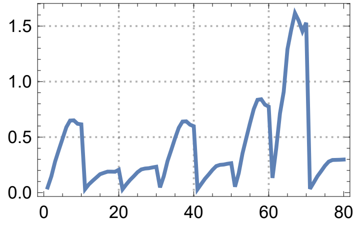

3D Plot

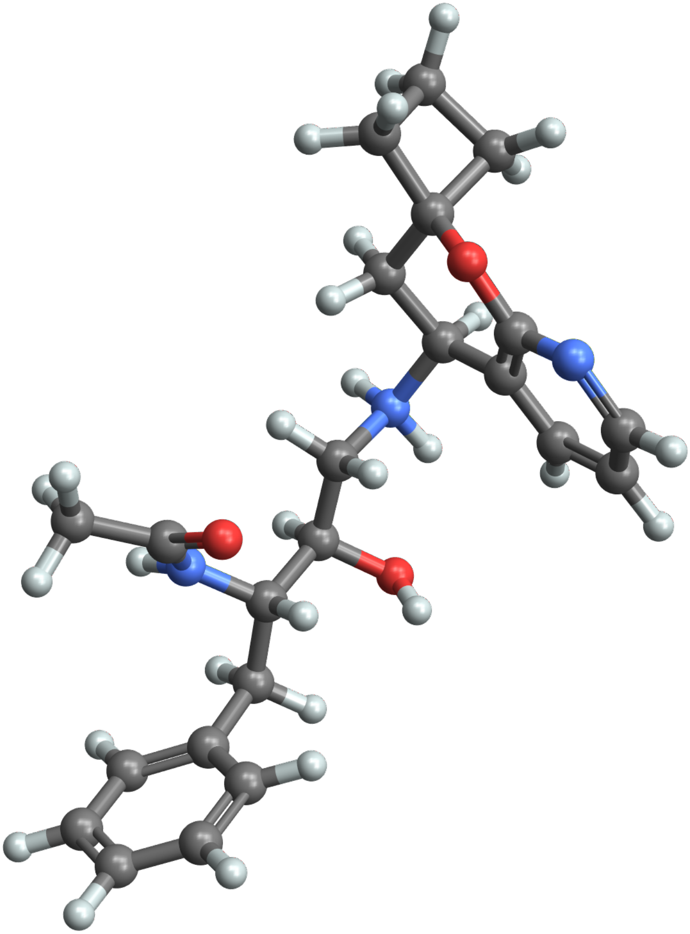

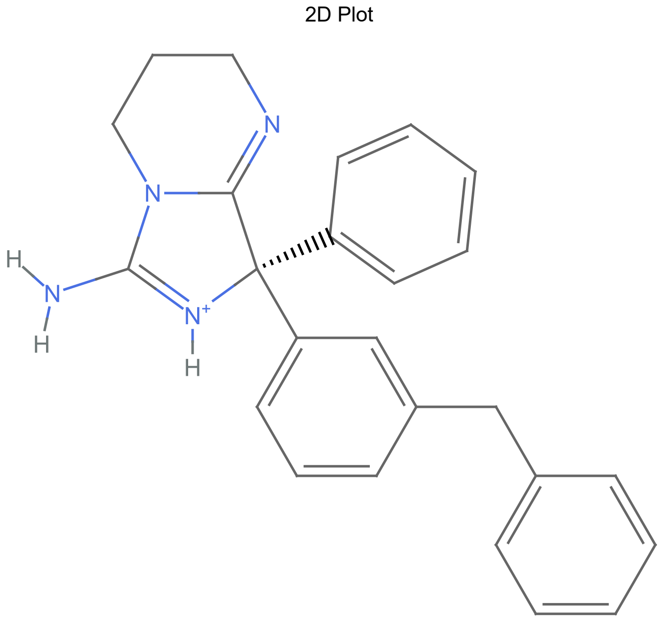

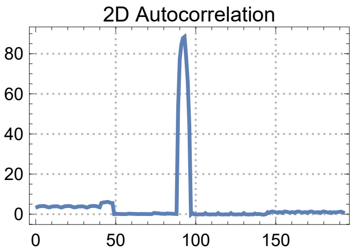

Dataset Index= 1303

Molecule Formula= $C_{25}H_{25}N_4^+$

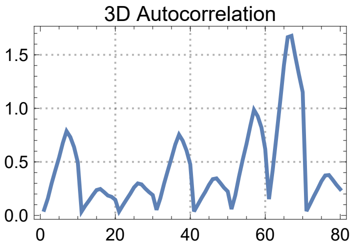

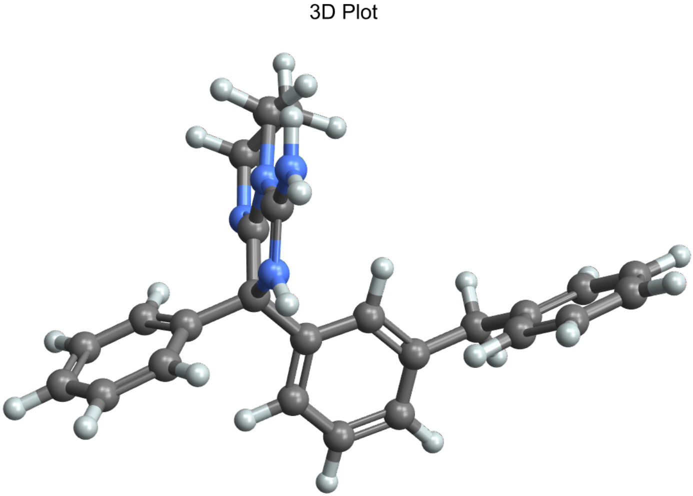

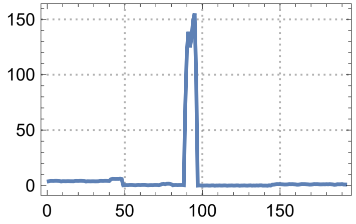

Dataset Index= 1304

Molecule Formula= $C_{30}H_{41}N_4O_4^+$

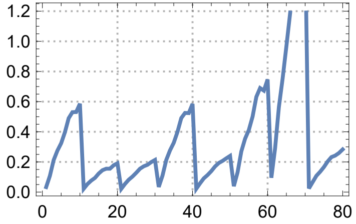

2D Plot

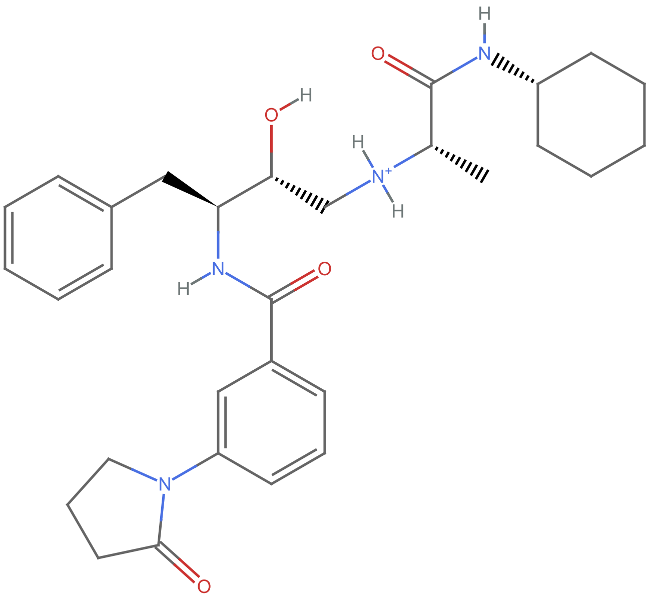

3D Plot

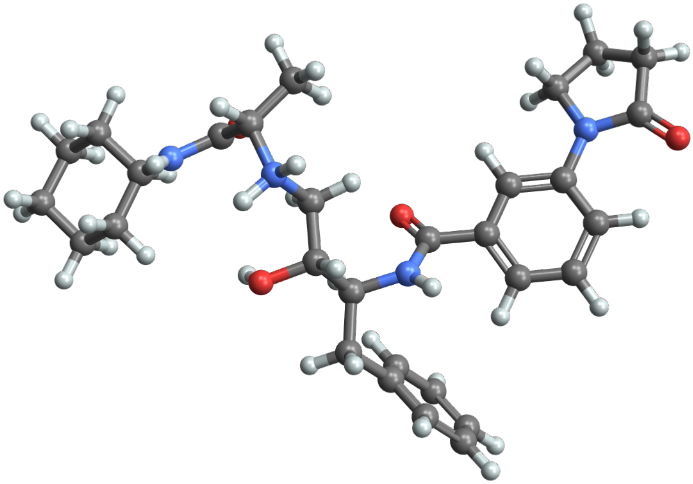

2D Plot

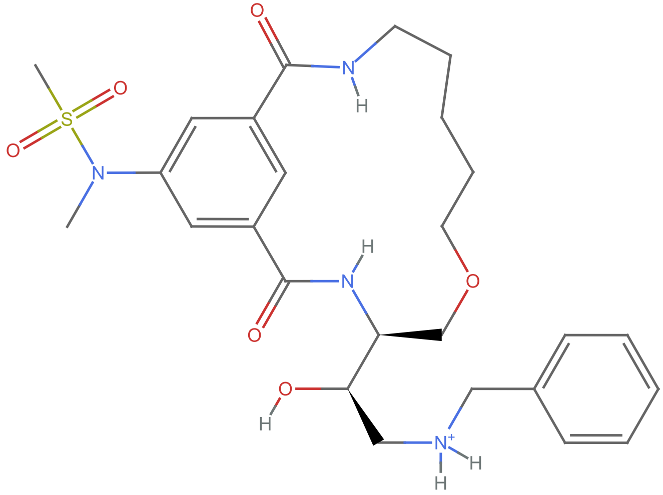

3D Plot

2D Autocorrelation

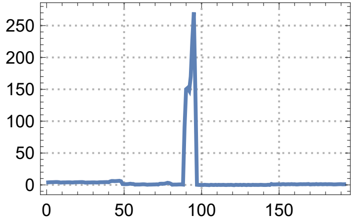

Dataset Index= 1305

Molecule Formula= $C_{26}H_{37}N_4O_6S^+$

3D Autocorrelation

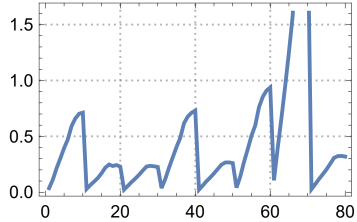

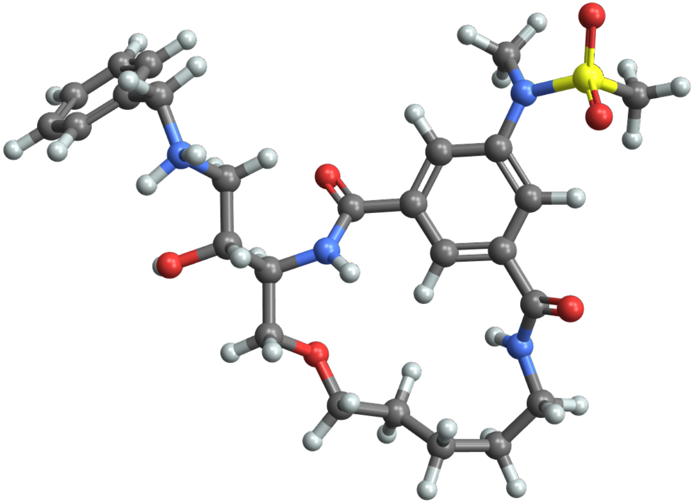

2D Plot

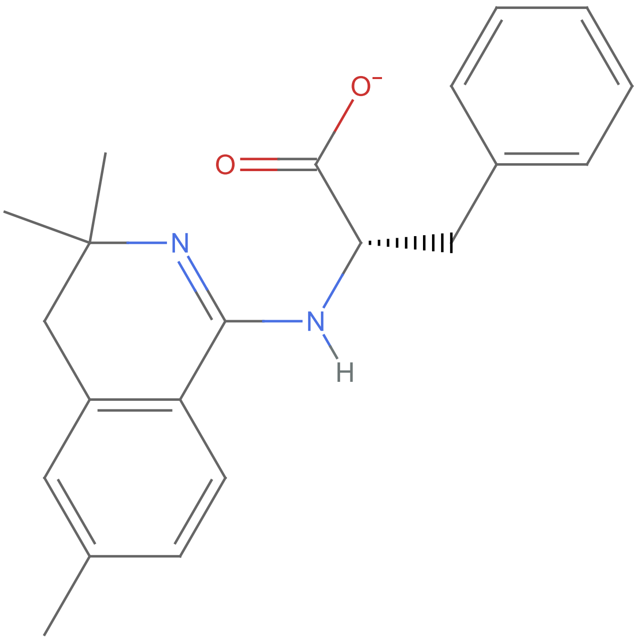

2D Autocorrelation

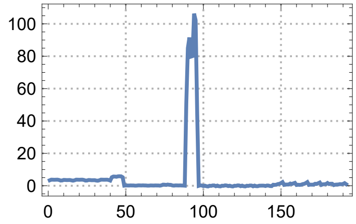

3D Plot

Dataset Index= 1306

Molecule Formula= $C_{21}H_{23}N_2O_2^-$

3D Autocorrelation

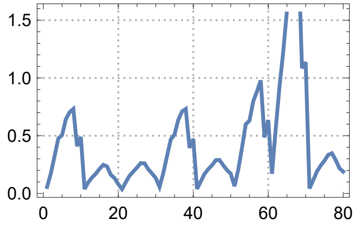

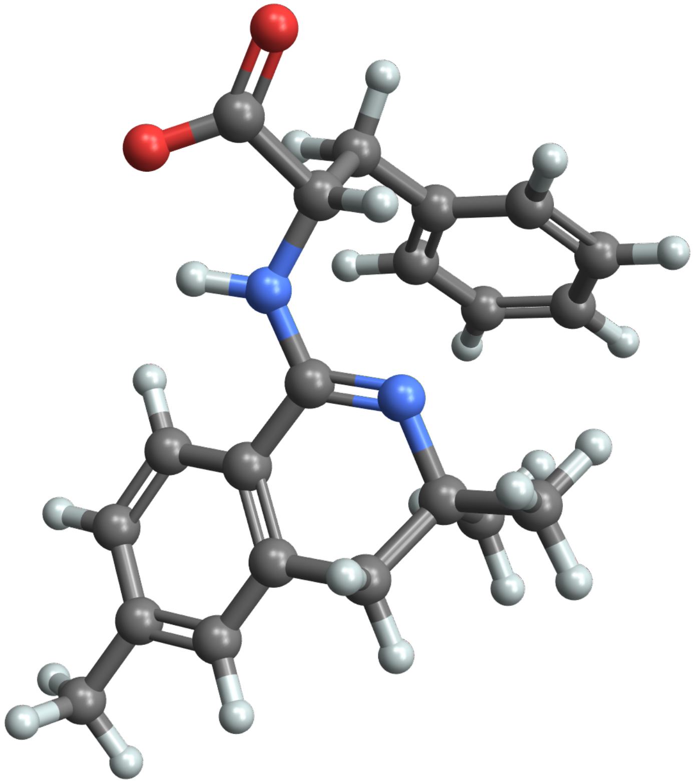

2D Autocorrelation

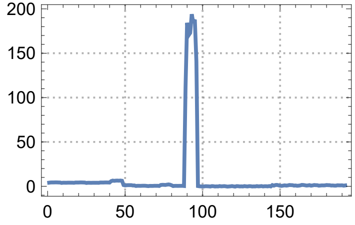

2D Plot

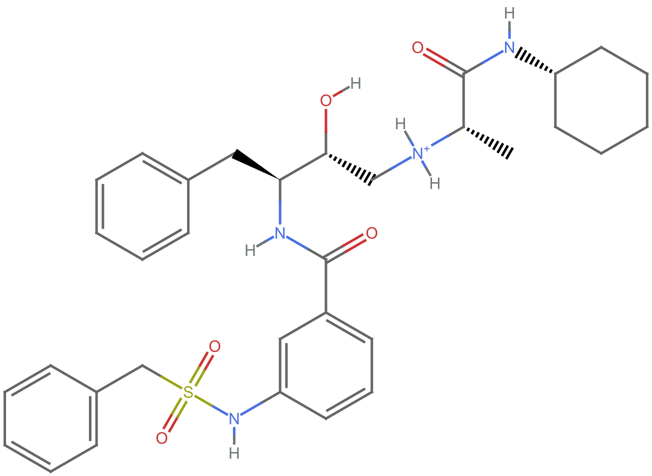

3D Plot

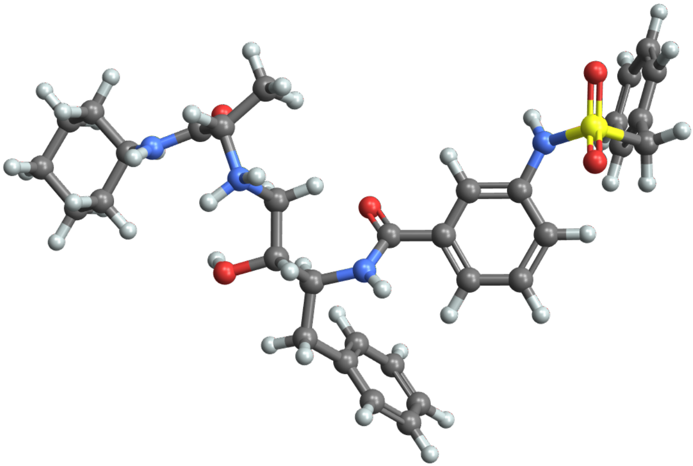

Dataset Index= 1307

Molecule Formula= $C_{33}H_{43}N_{4}O_{5}S^{+}$

3D Autocorrelation

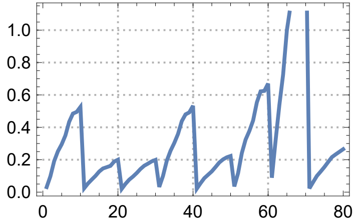

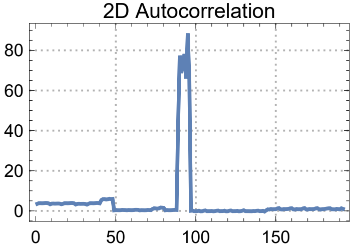

Dataset Index= 1308

Molecule Formula= $C_{19}H_{20}N_4O_4$

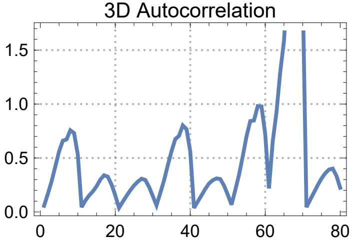

2D Plot

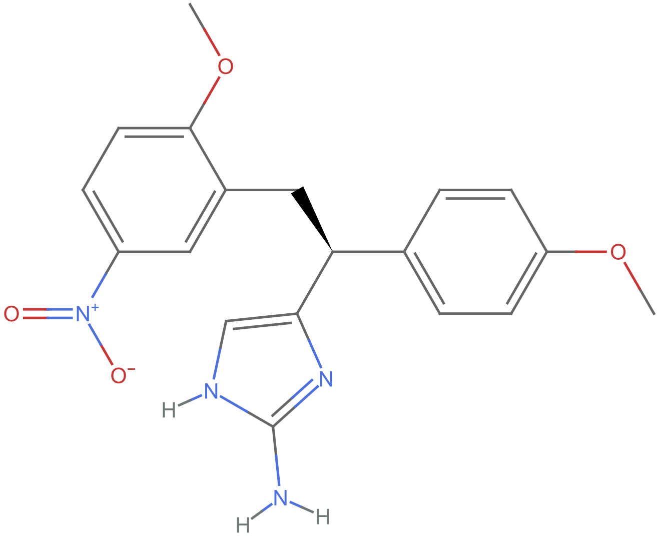

3D Plot

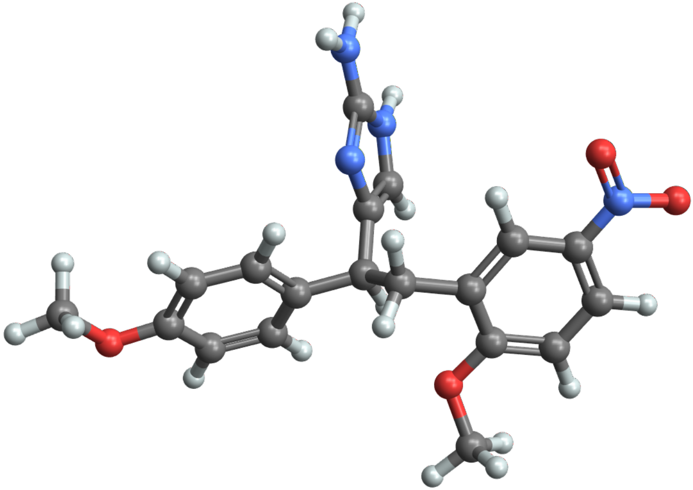

2D Plot

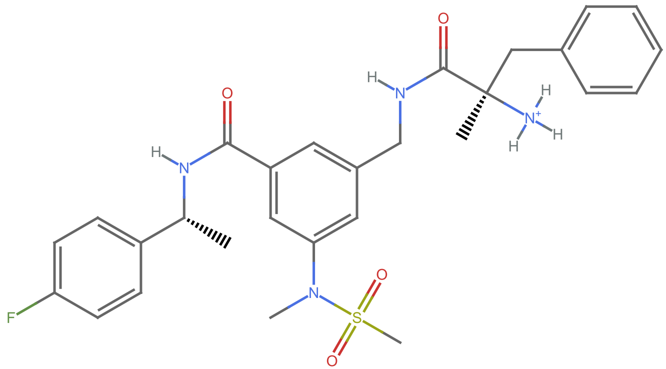

2D Autocorrelation

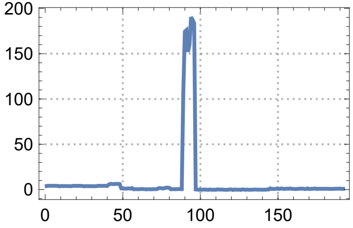

3D Plot

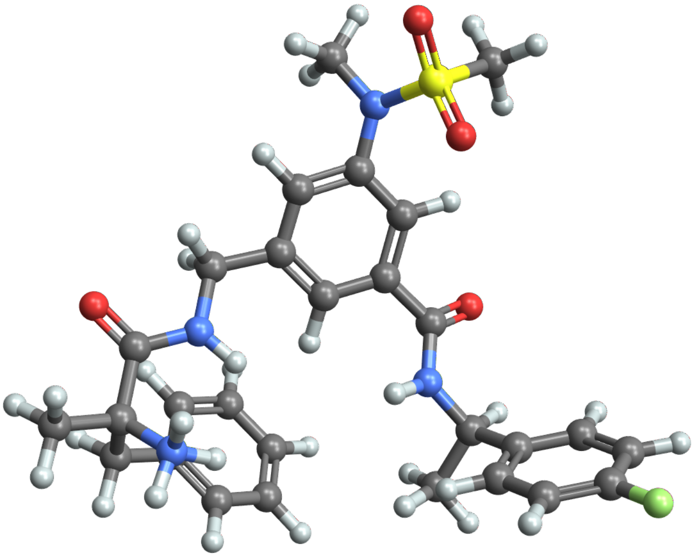

Dataset Index= 1309

Molecule Formula= $C_{28}H_{34}FN_4O_4S^+$

3D Autocorrelation

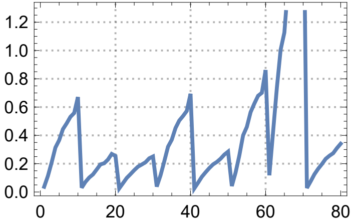

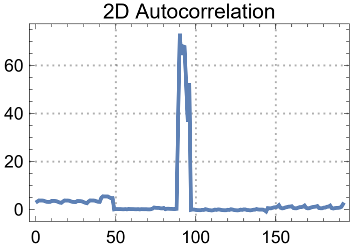

Dataset Index= 1310

Molecule Formula= $C_{19}H_{20}FN_3O$

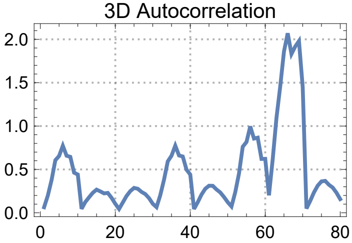

2D Plot

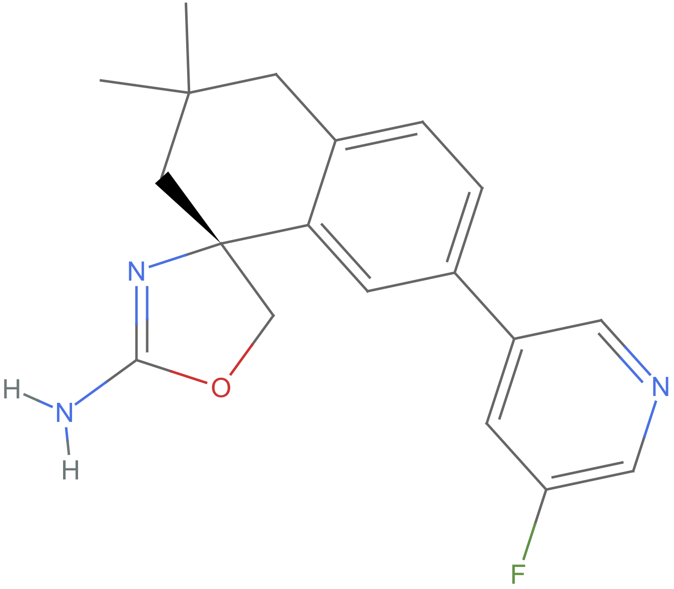

3D Plot

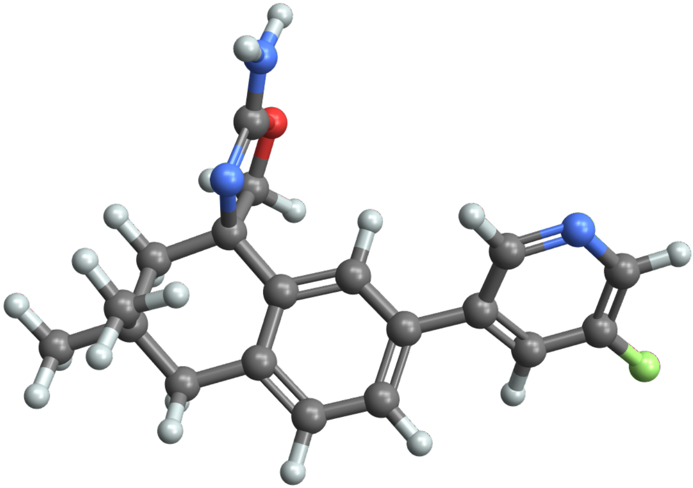

2D Plot

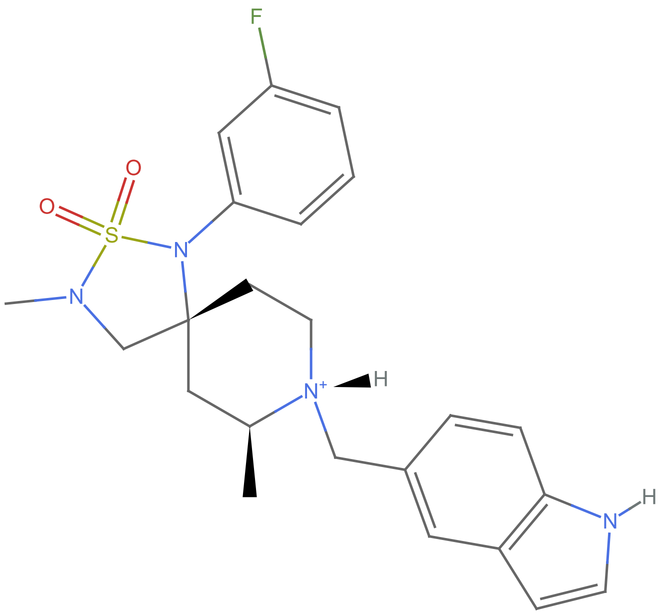

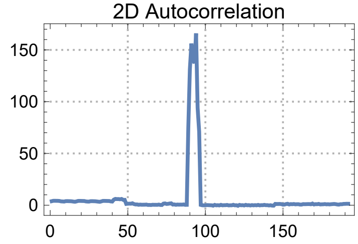

Dataset Index= 1311

Molecule Formula= $C_{23}H_{28}FN_4O_2S^+$

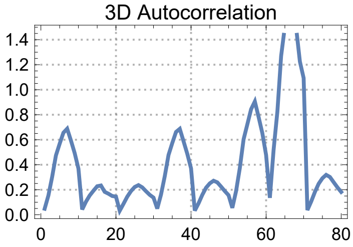

3D Plot

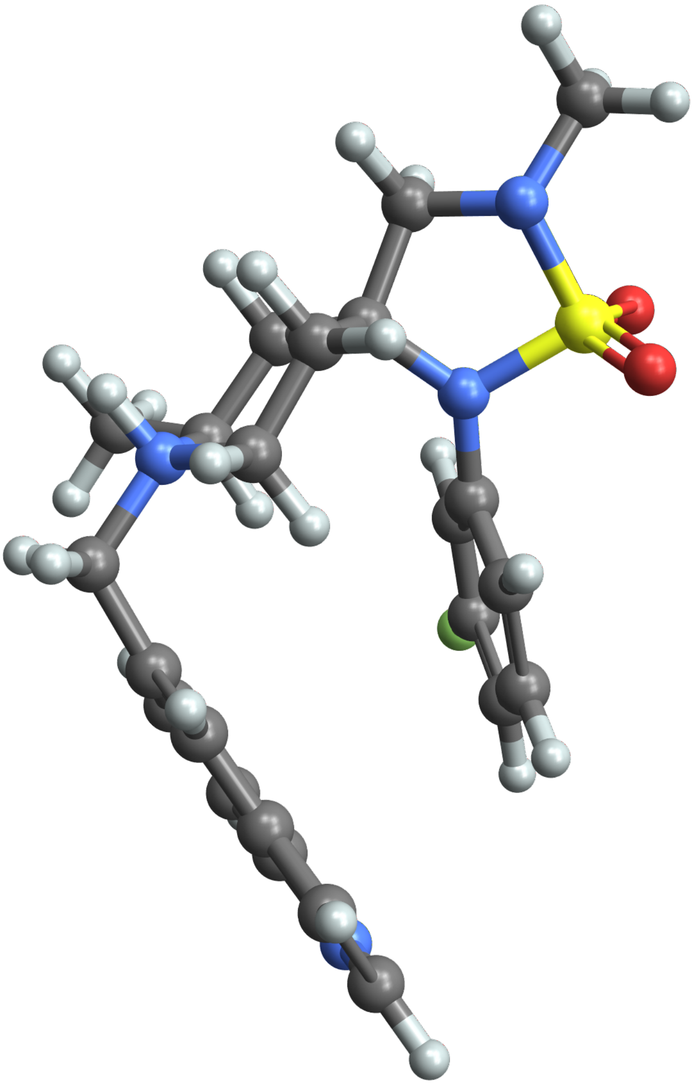

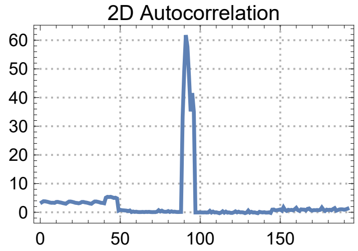

Dataset Index= 1312

Molecule Formula= $C_{19}H_{21}N_3OS$

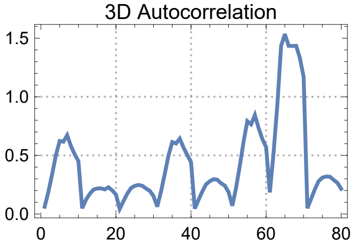

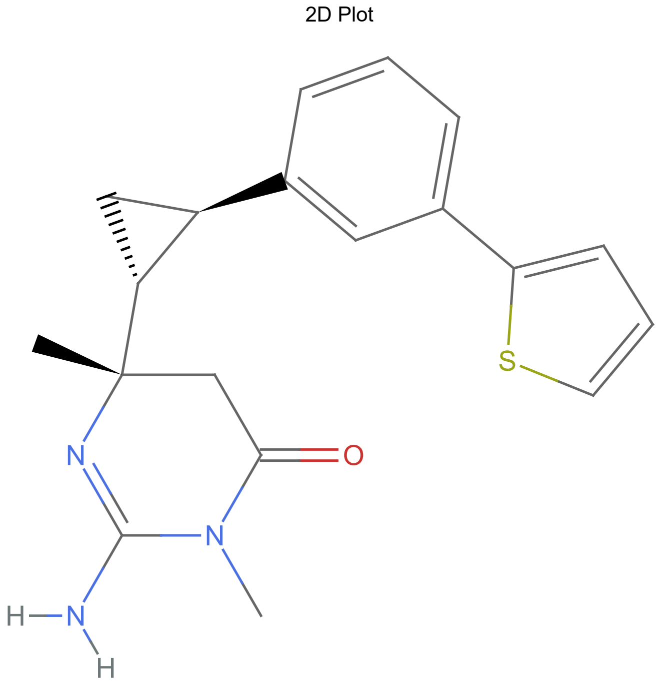

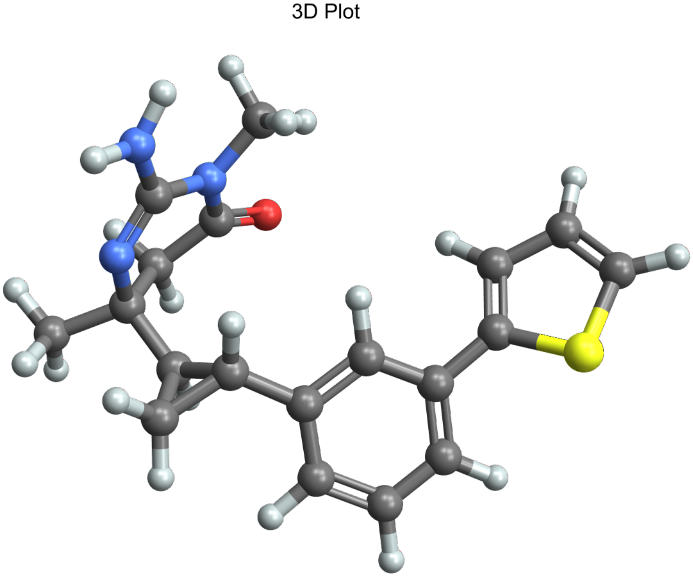

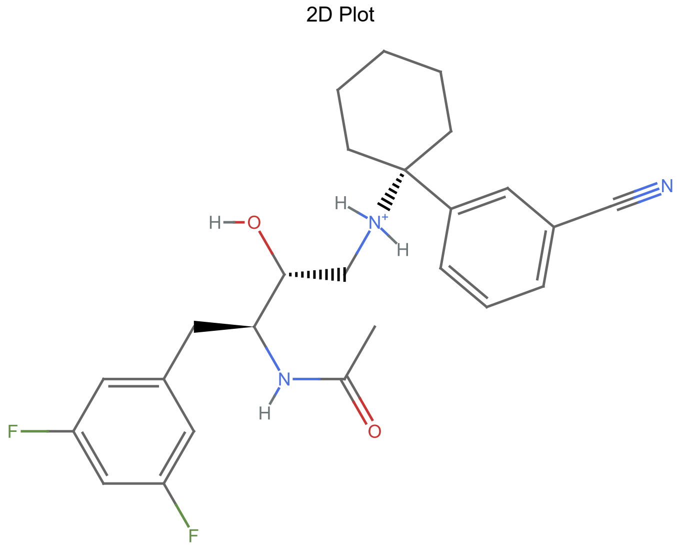

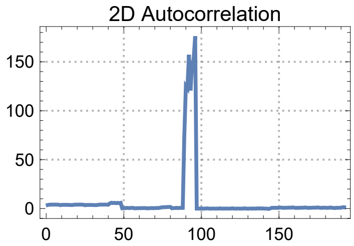

Dataset Index= 1313

Molecule Formula= $C_{25}H_{30}F_2N_3O_2^+$

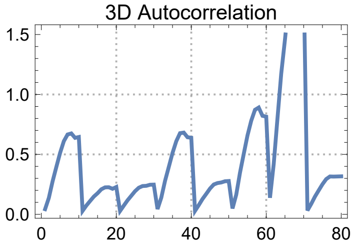

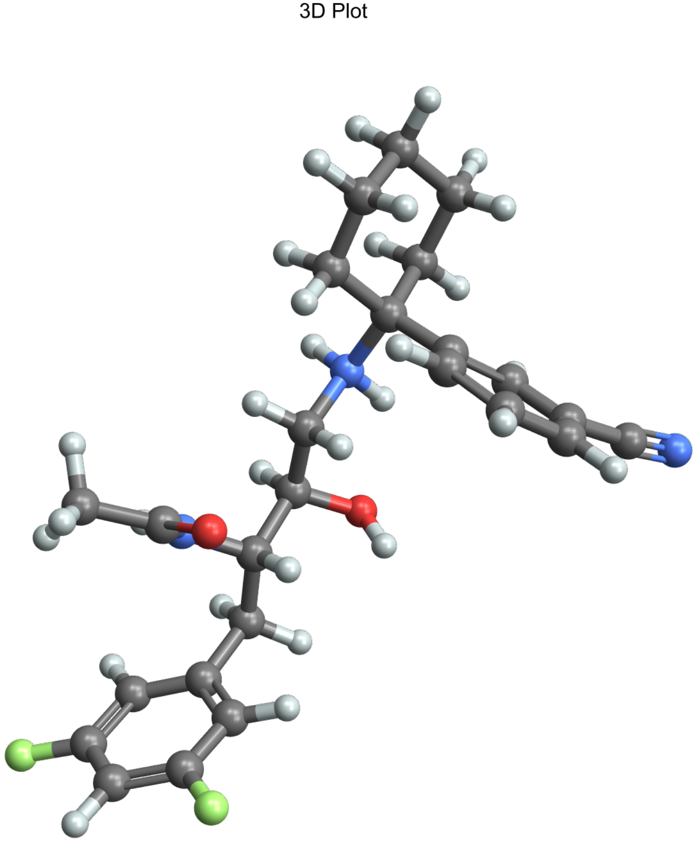

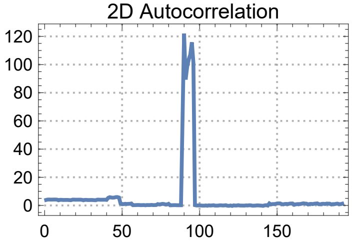

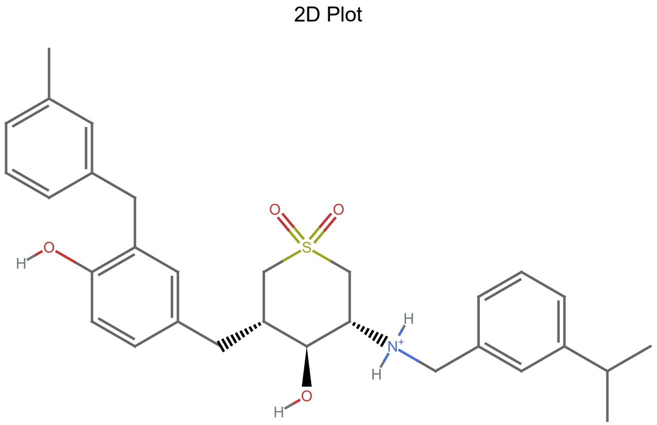

Dataset Index= 1314

Molecule Formula= $C_{30}H_{38}NO_4S^+$

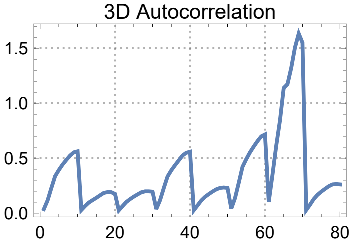

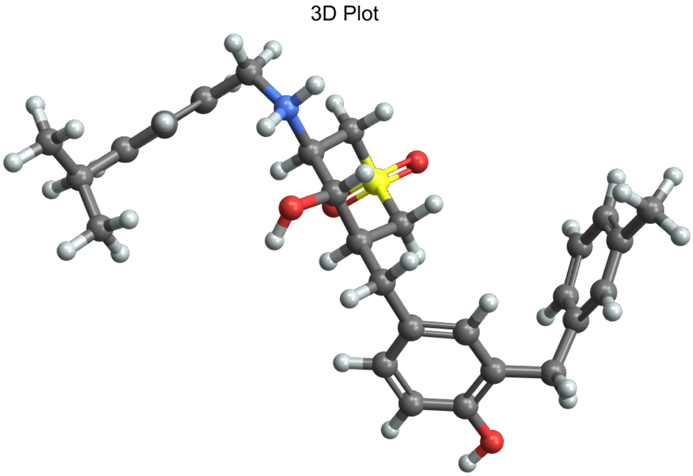

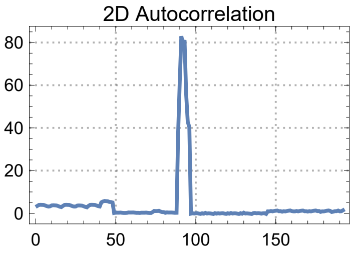

Dataset Index= 1315

Molecule Formula= $C_{19}H_{20}N_4O_2$

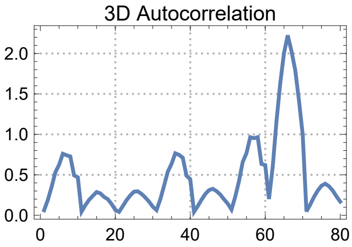

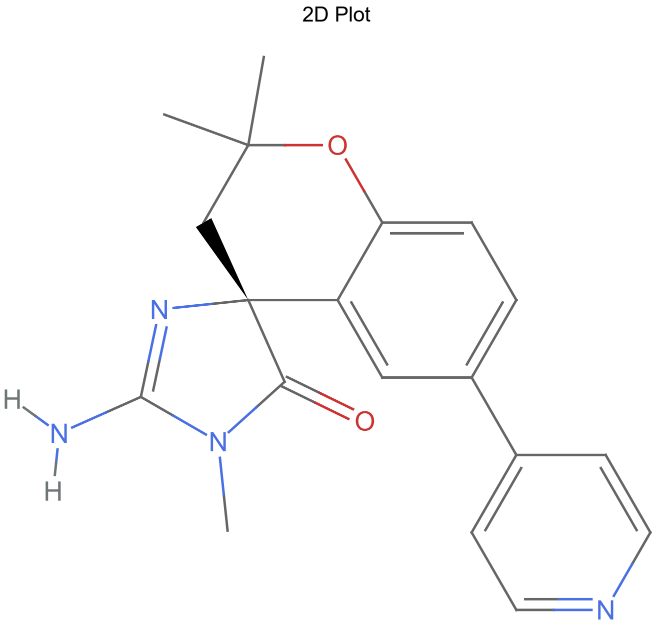

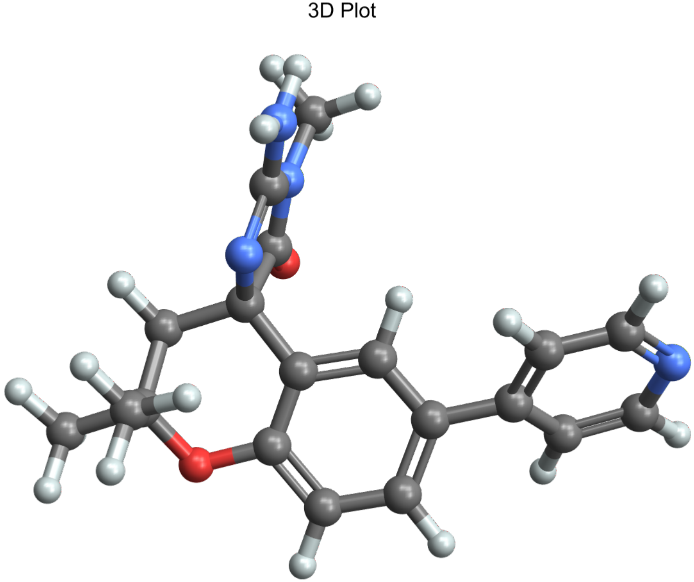

2D Autocorrelation

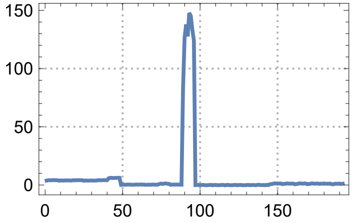

Dataset Index= 1316

Molecule Formula= $C_{32}H_{41}N_4O_3^+$

3D Autocorrelation

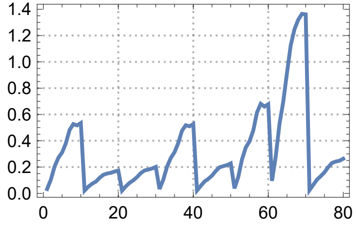

2D Plot

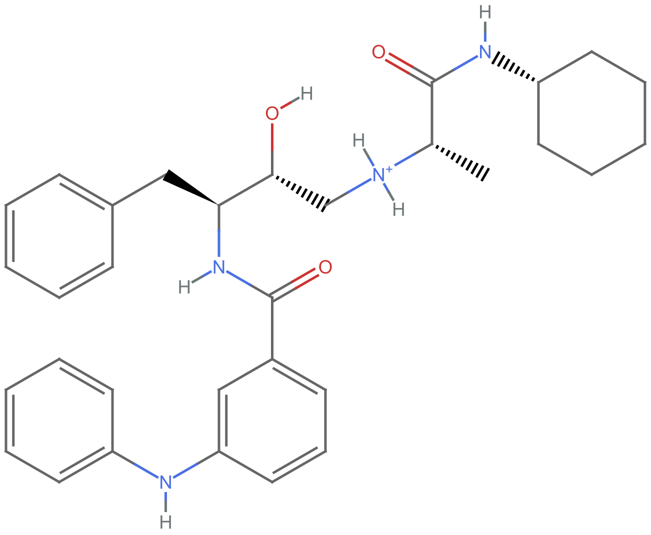

3D Plot

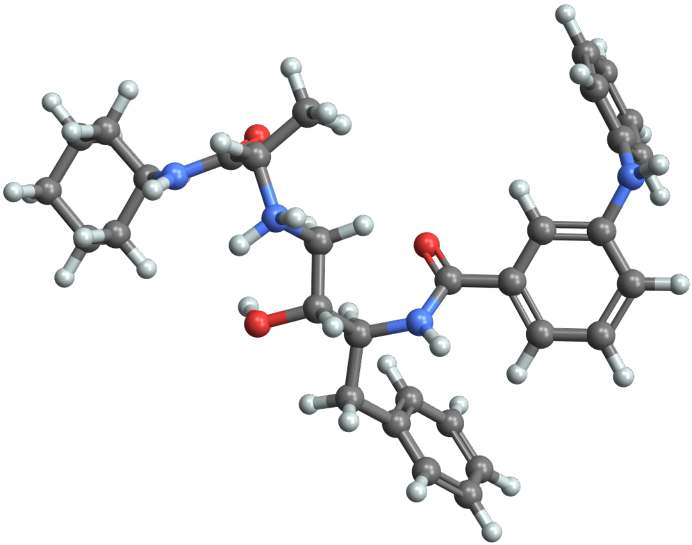

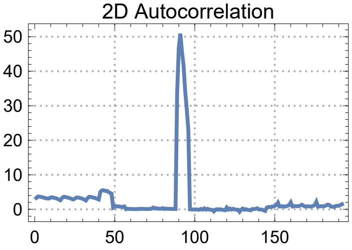

Dataset Index= 1317

Molecule Formula= $C_{17}H_{17}ClN_2O$

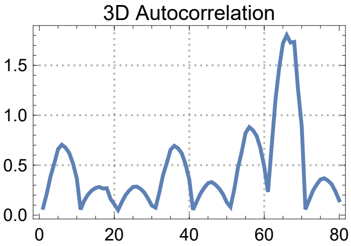

2D Plot

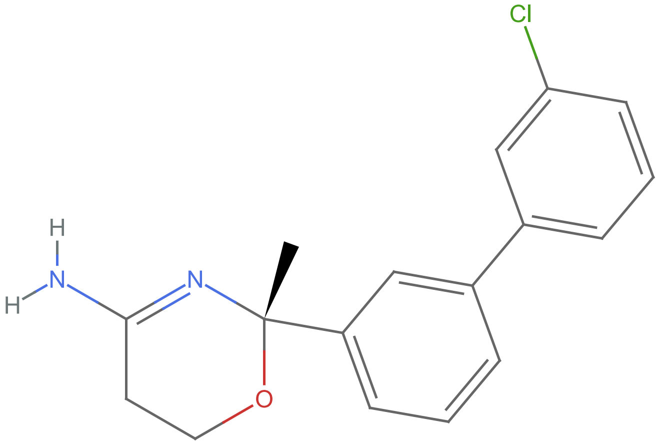

3D Plot

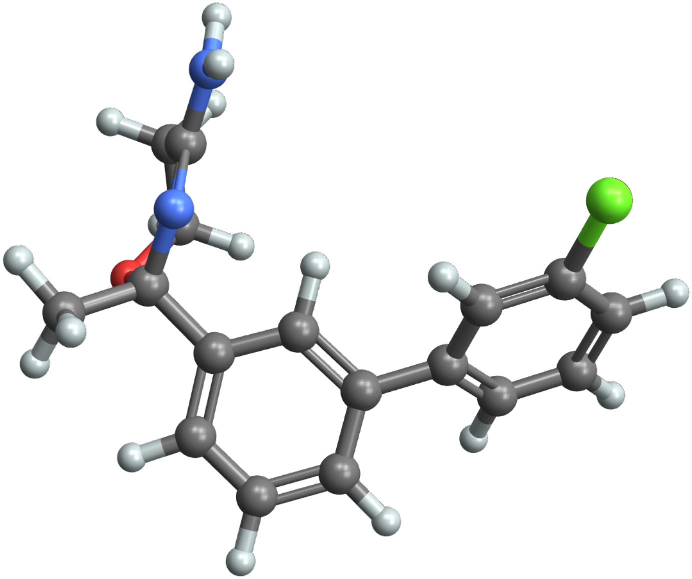

2D Plot

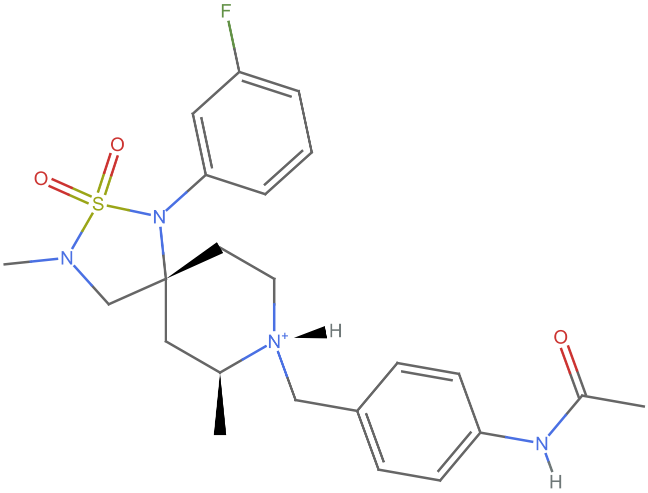

3D Plot

2D Autocorrelation

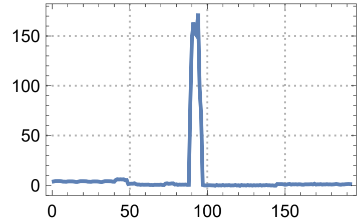

Dataset Index= 1318

Molecule Formula= $C_{23}H_{30}FN_4O_3S^+$

3D Autocorrelation

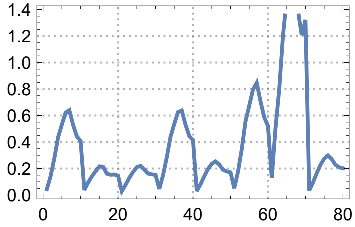

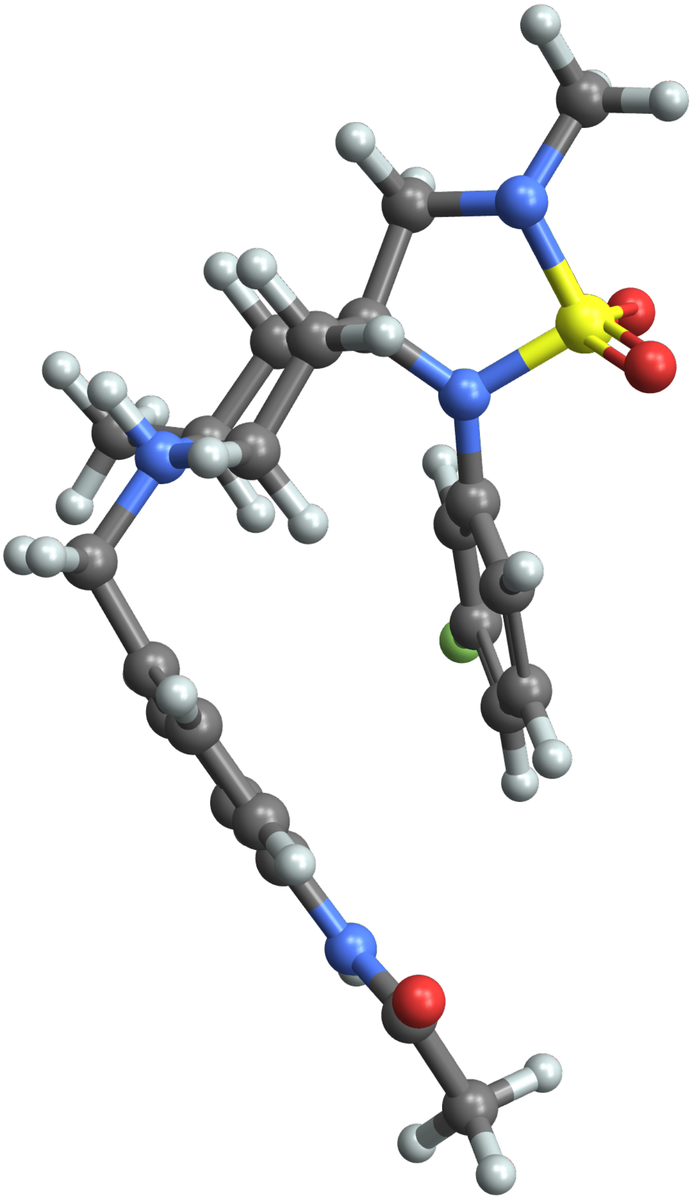

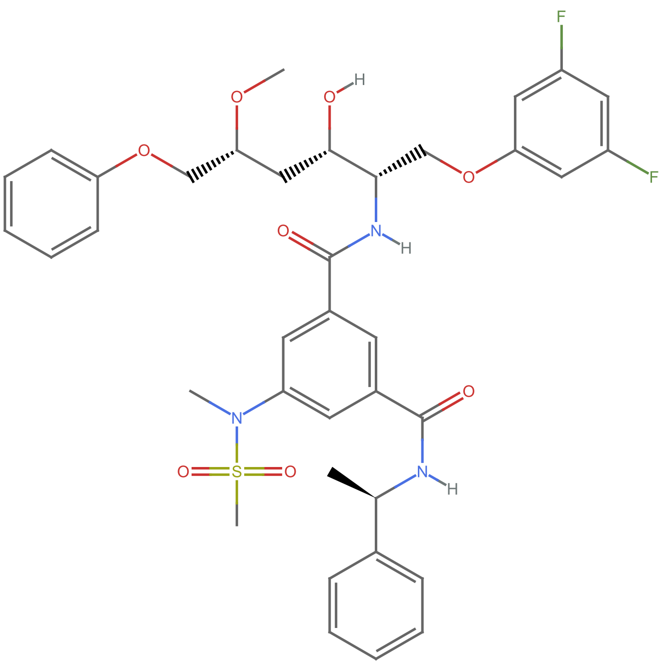

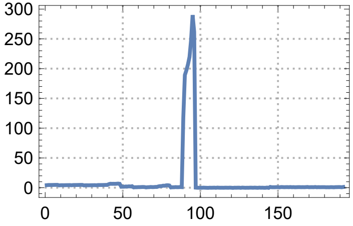

Dataset Index= 1319

Molecule Formula= $C_{37}H_{41}F_2N_3O_8S$

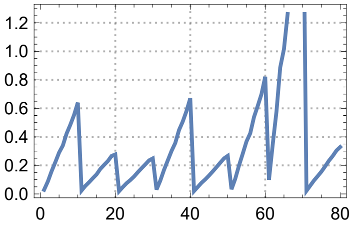

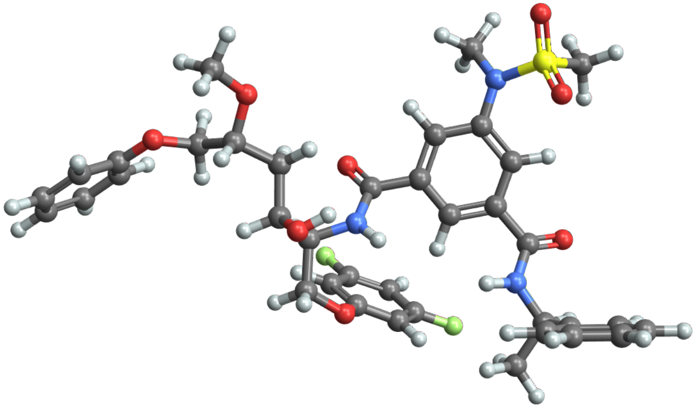

2D Plot

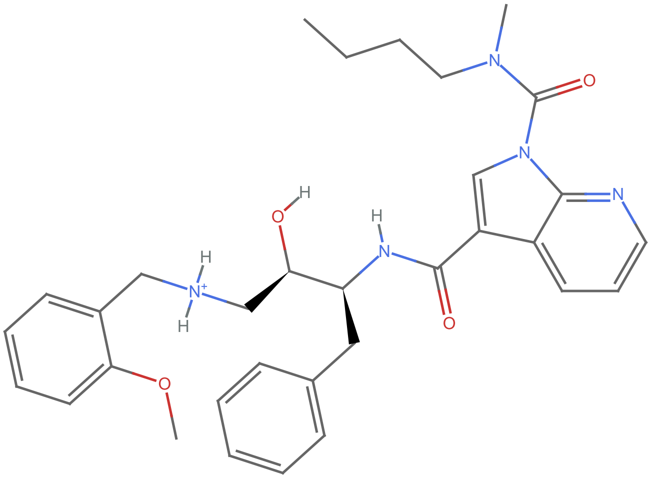

3D Plot

2D Autocorrelation

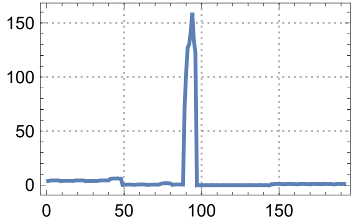

Dataset Index= 1320

Molecule Formula= $C_{32}H_{40}N_5O_4^+$

3D Autocorrelation

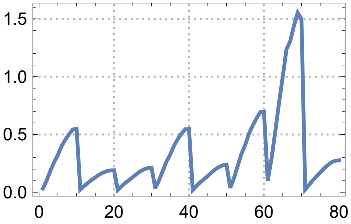

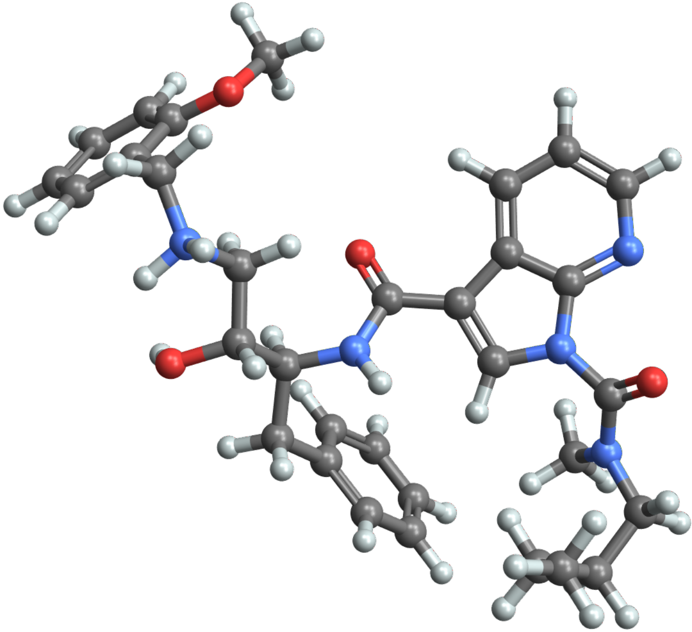

2D Plot

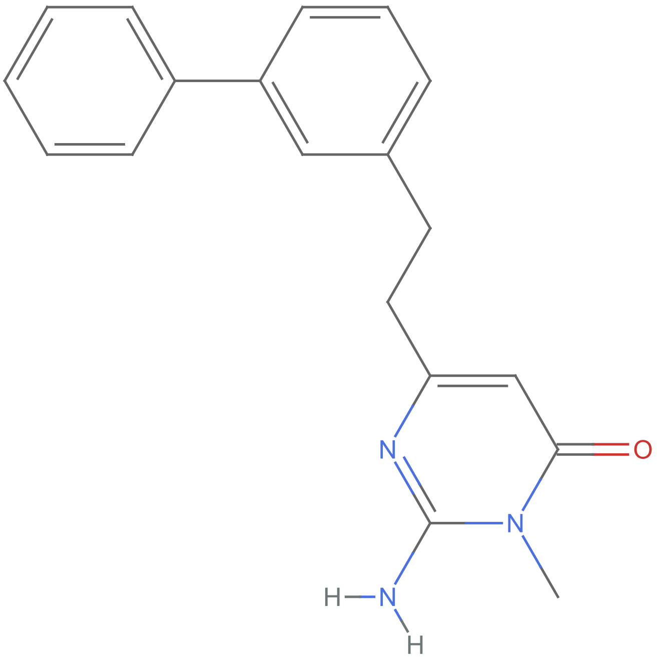

2D Autocorrelation

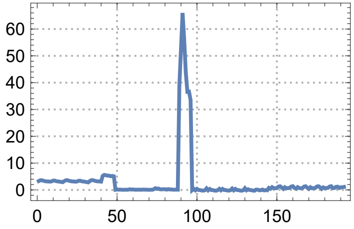

Dataset Index= 1321

Molecule Formula= $C_{19}H_{19}N_3O$

3D Autocorrelation

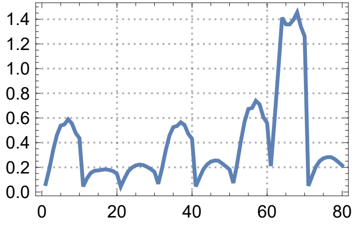

3D Plot

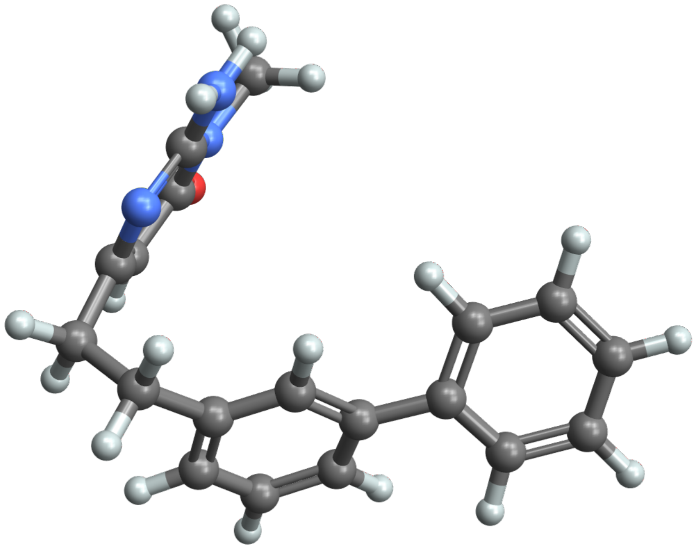

2D Autocorrelation

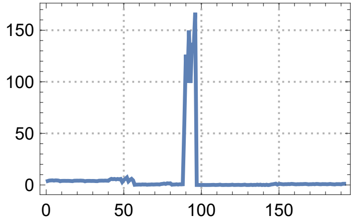

Dataset Index= 1322

Molecule Formula= $C_{28}H_{38}BrF_2N_2O_2^+$

3D Autocorrelation

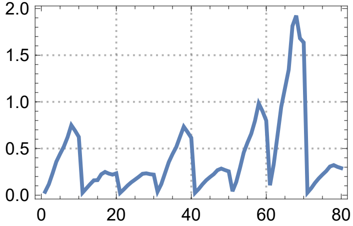

2D Plot

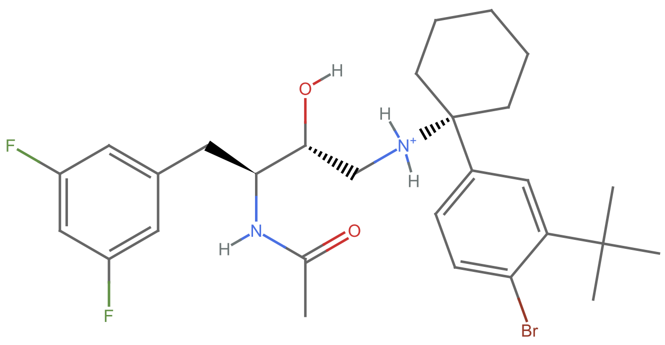

3D Plot

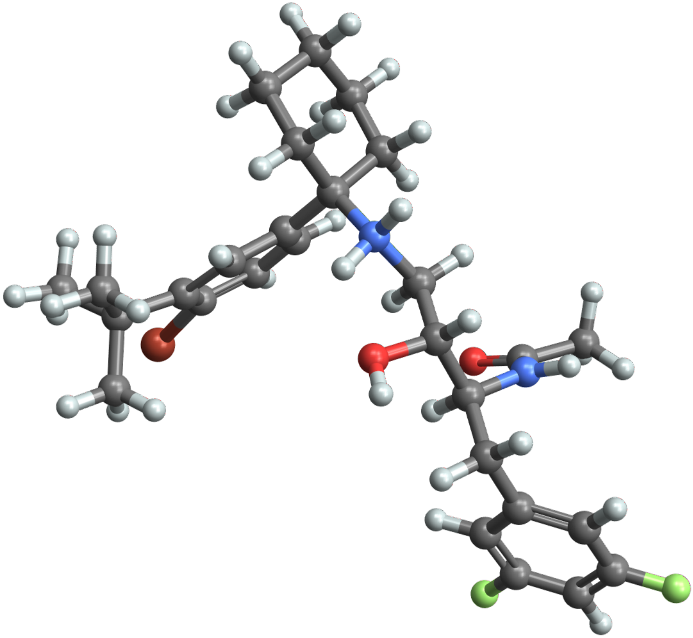

2D Plot

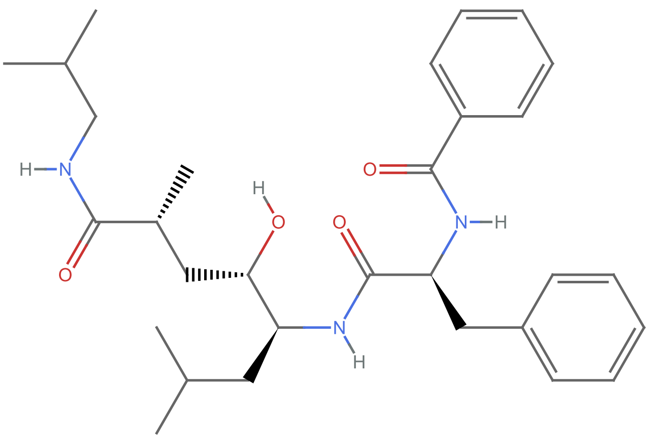

2D Autocorrelation

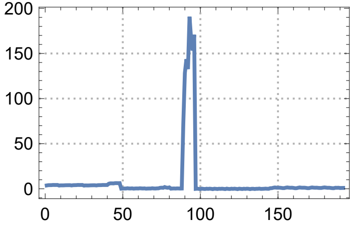

Dataset Index= 1323

Molecule Formula= $C_{30}H_{43}N_3O_4$

3D Plot

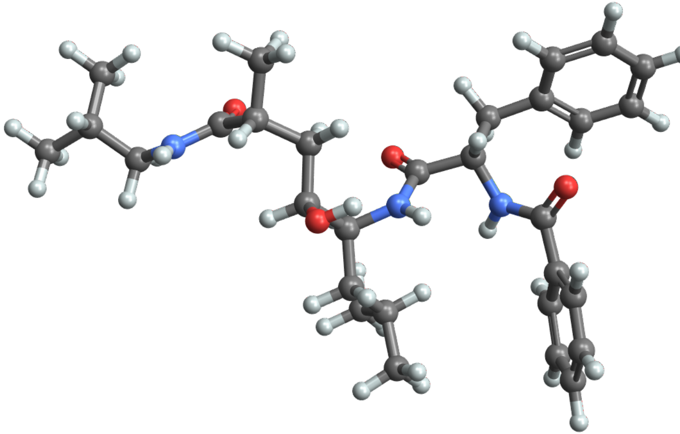

3D Autocorrelation

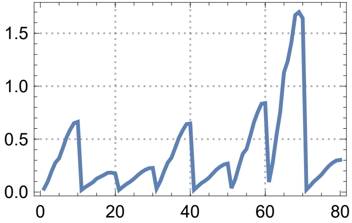

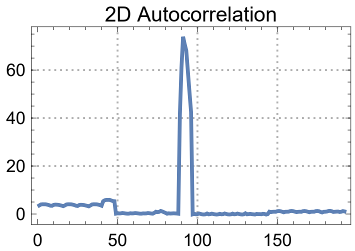

Dataset Index= 1324

Molecule Formula= $C_{23}H_{20}N_2O_3$

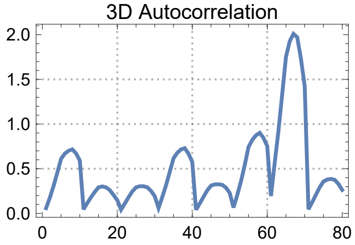

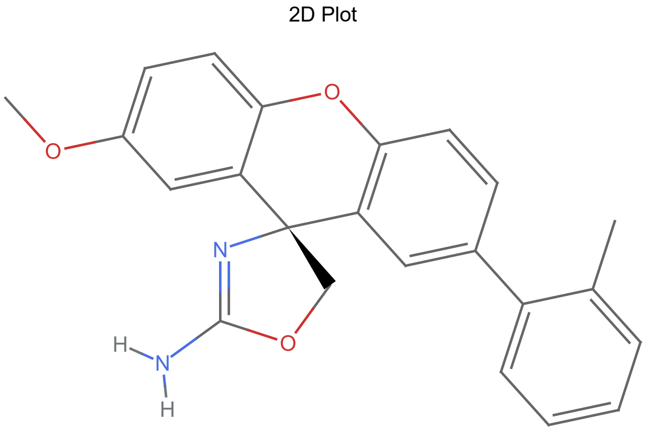

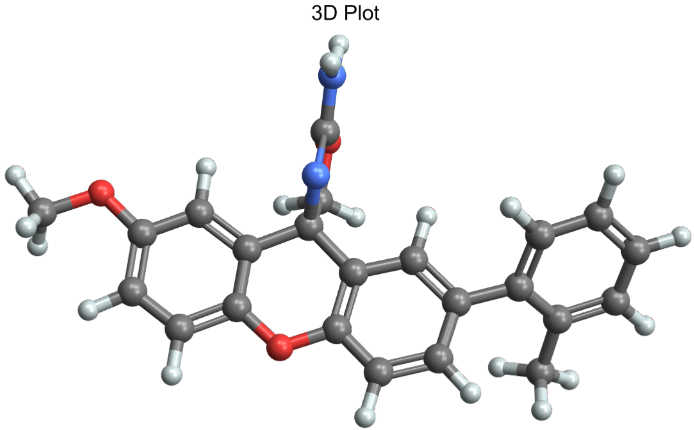

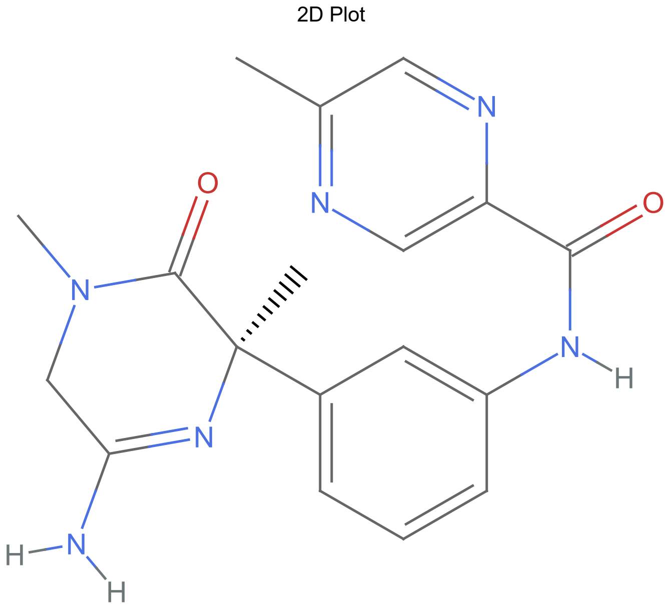

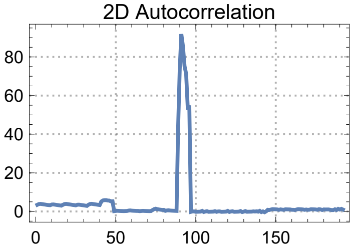

Dataset Index= 1325

Molecule Formula= $C_{18}H_{20}N_6O_2$

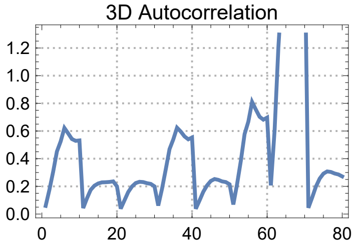

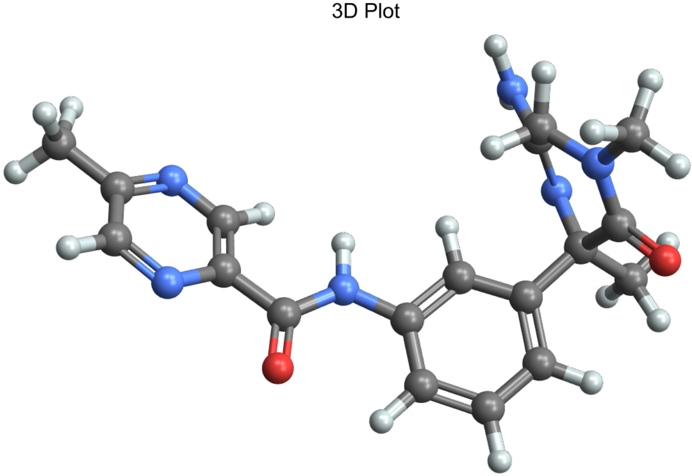

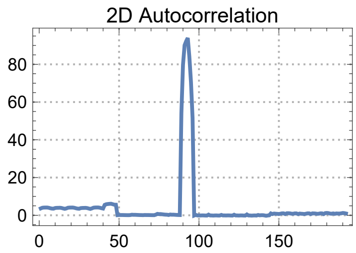

Dataset Index= 1326

Molecule Formula= $C_{26}H_{27}N_4O^+$

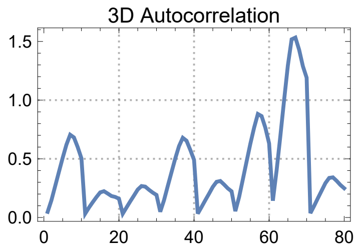

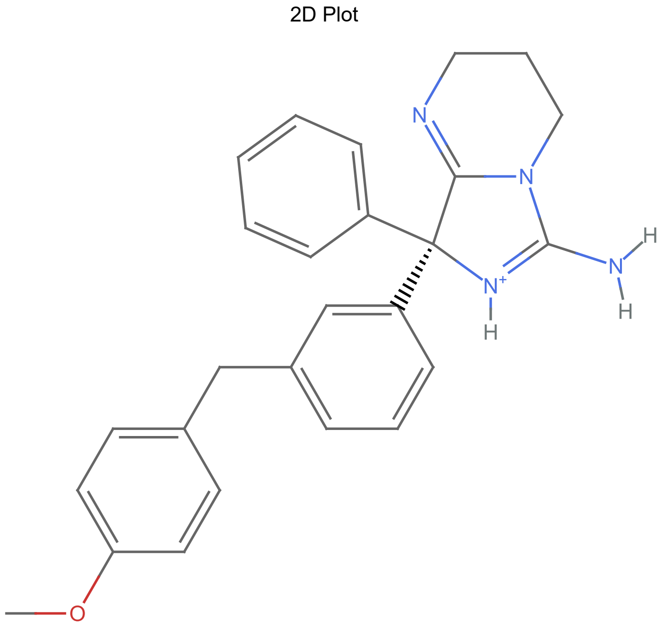

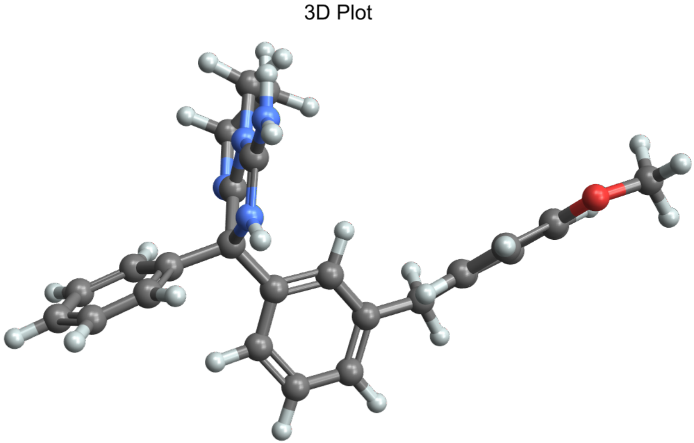

2D Autocorrelation

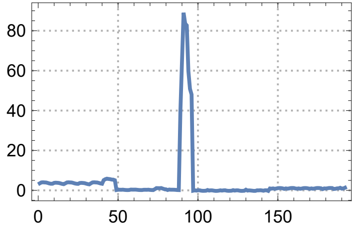

Dataset Index= 1327

Molecule Formula= $C_{21}H_{23}N_3O_3$

3D Autocorrelation

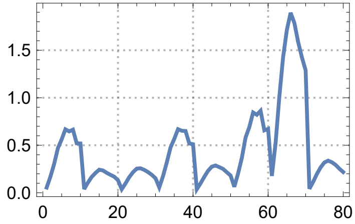

2D Plot

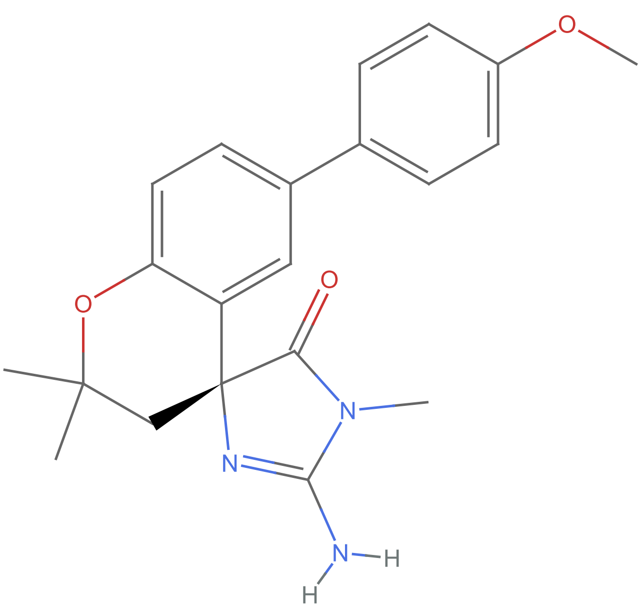

3D Plot

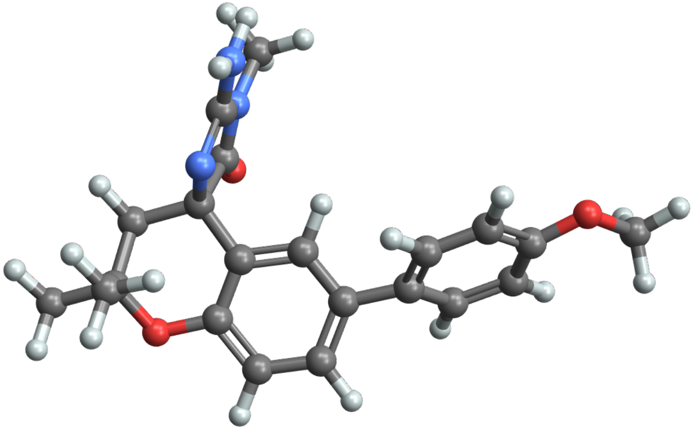

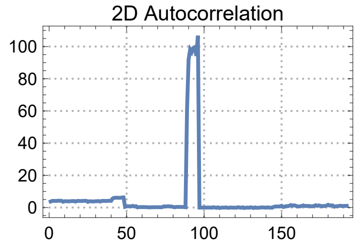

Dataset Index= 1328

Molecule Formula= $C_{27}H_{25}ClN_4O$

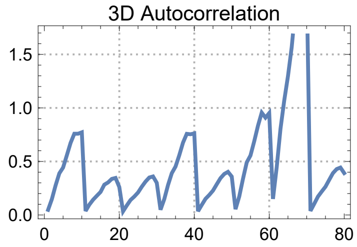

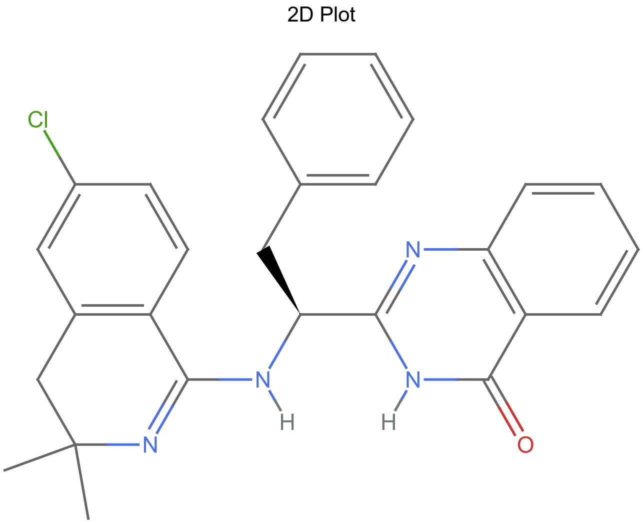

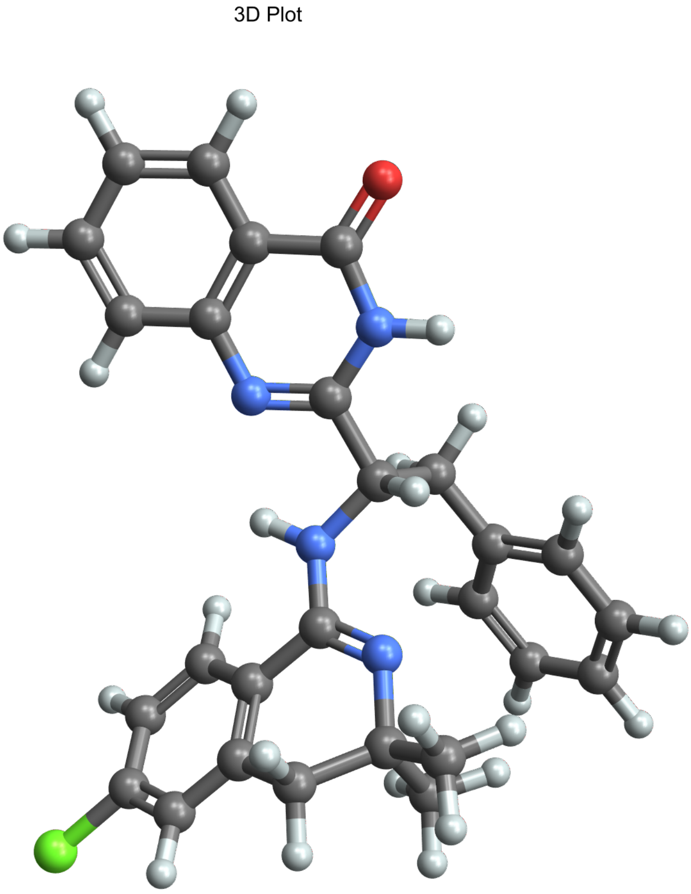

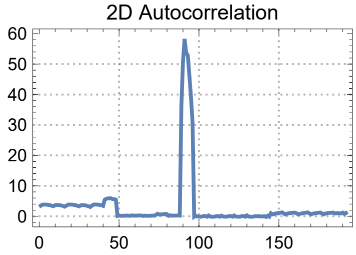

Dataset Index= 1329

Molecule Formula= $C_{21}H_{19}N_3O_2$

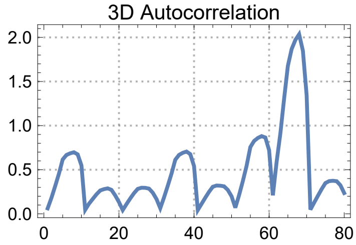

2D Plot

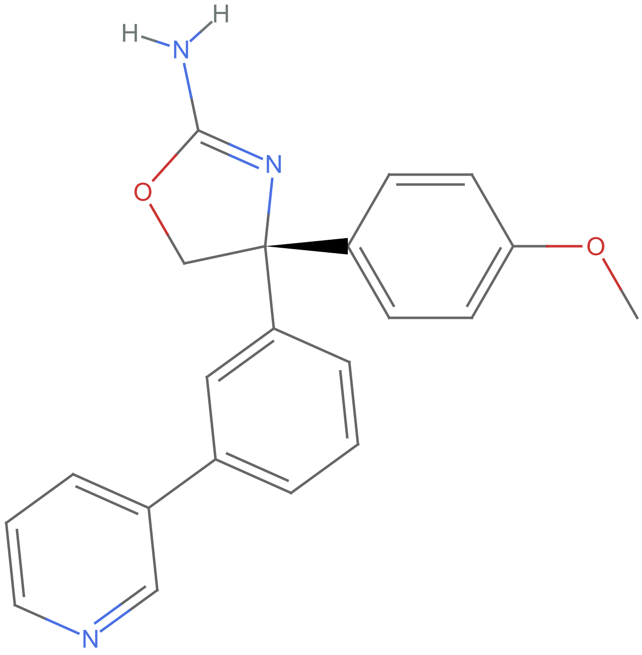

3D Plot

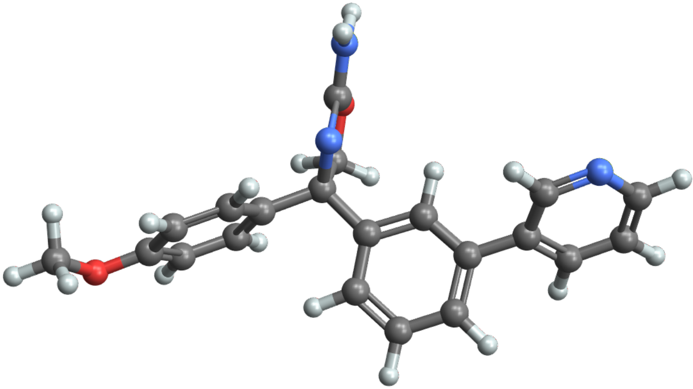

2D Plot

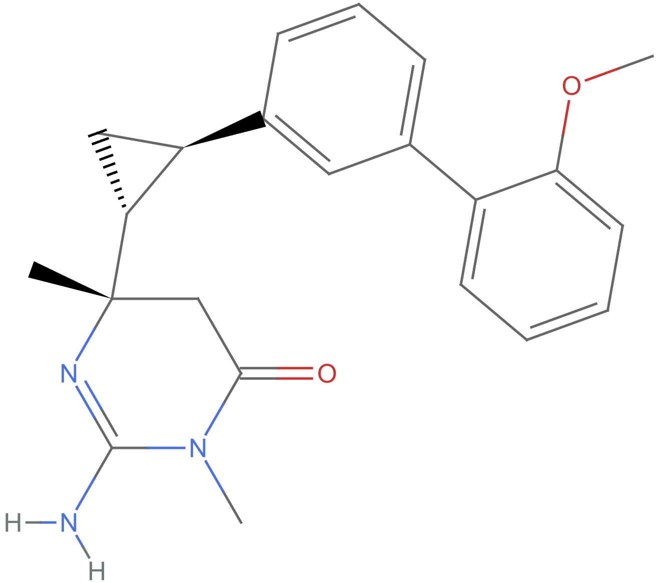

2D Autocorrelation

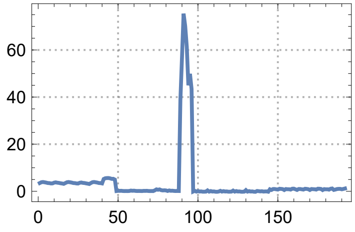

Dataset Index= 1330

Molecule Formula= $C_{22}H_{25}N_3O_2$

3D Autocorrelation

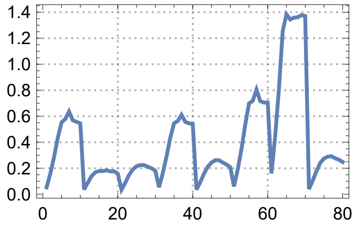

3D Plot

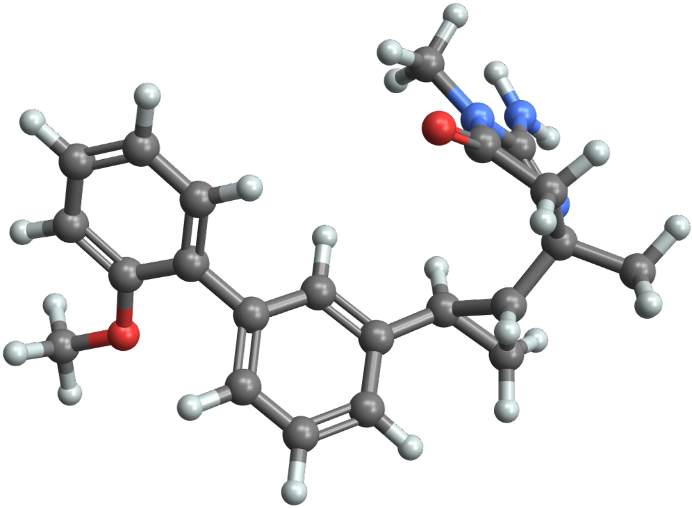

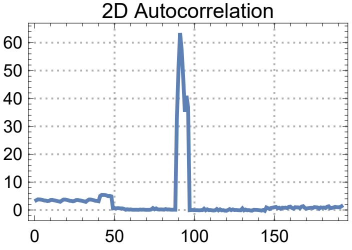

Dataset Index= 1331

Molecule Formula= $C_{19}H_{21}N_3OS$

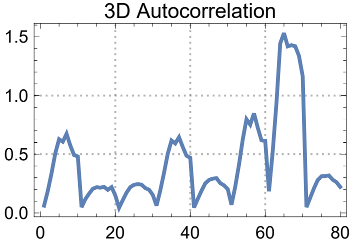

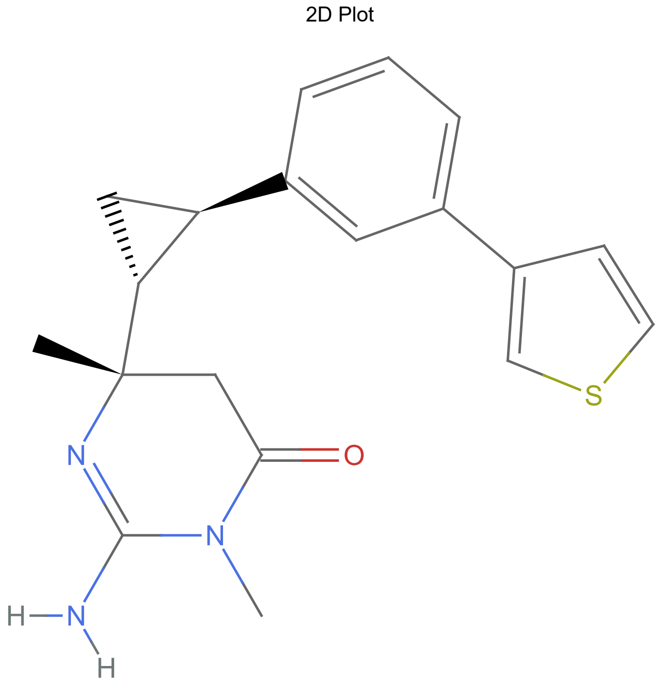

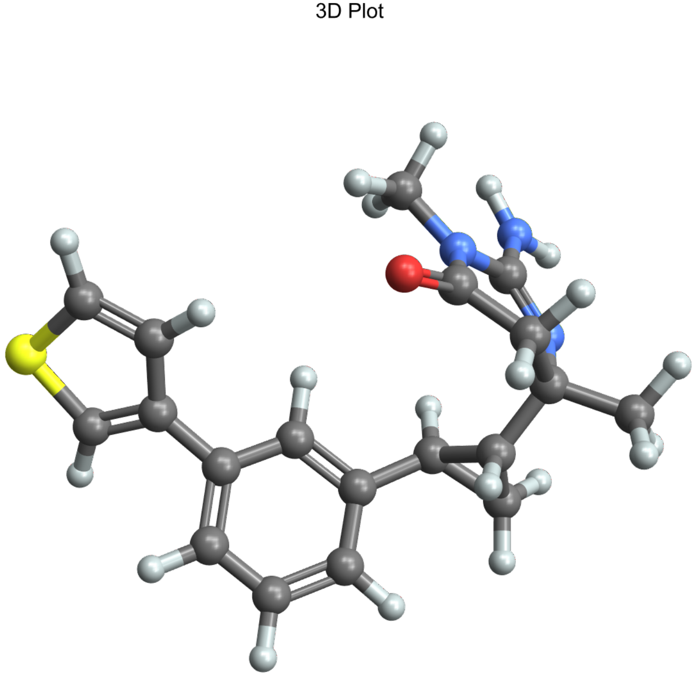

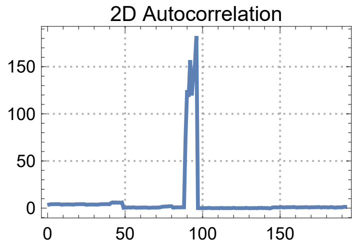

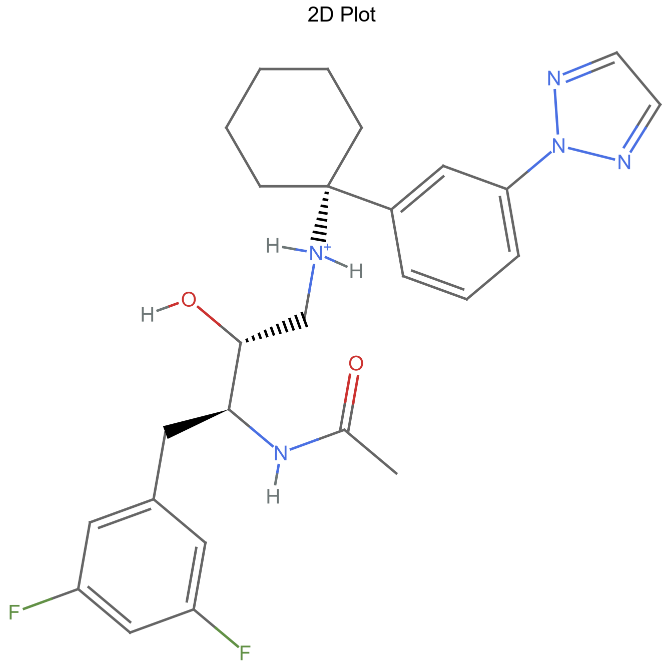

Dataset Index= 1332

Molecule Formula= $C_{26}H_{32}F_2N_5O_2^+$

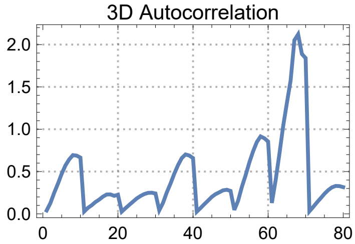

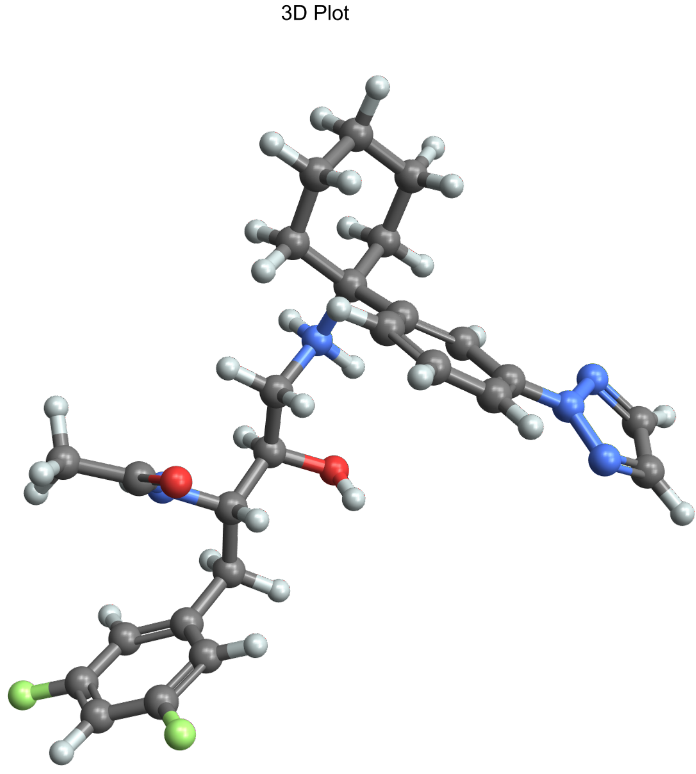

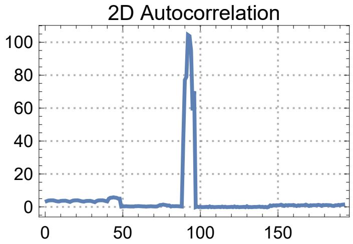

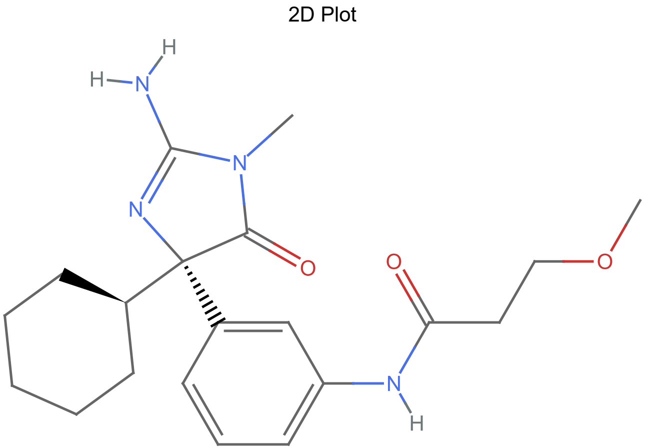

Dataset Index= 1333

Molecule Formula= $C_{20}H_{28}N_4O_3$

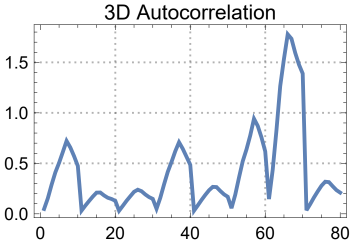

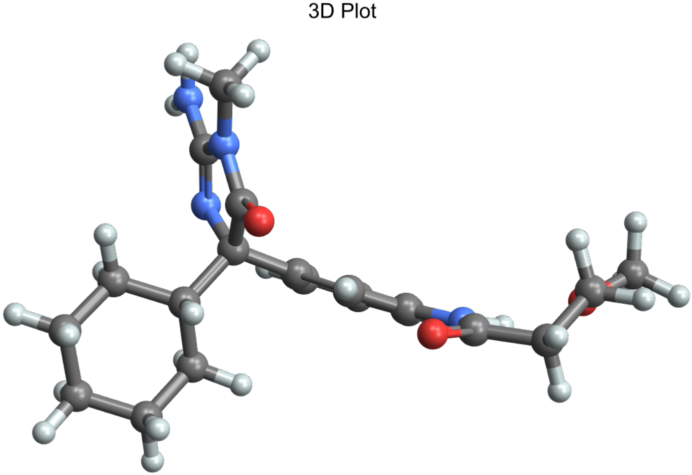

2D Plot

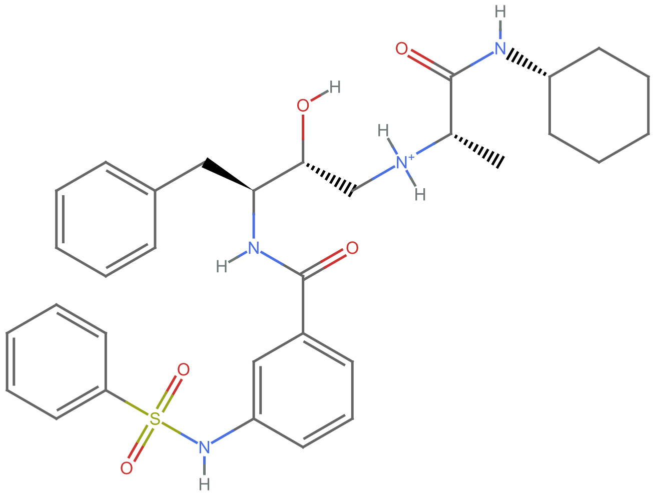

2D Autocorrelation

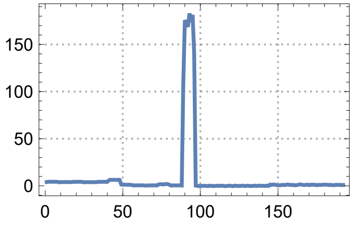

Dataset Index= 1334

Molecule Formula= $C_{32}H_{41}N_4O_5S^+$

3D Plot

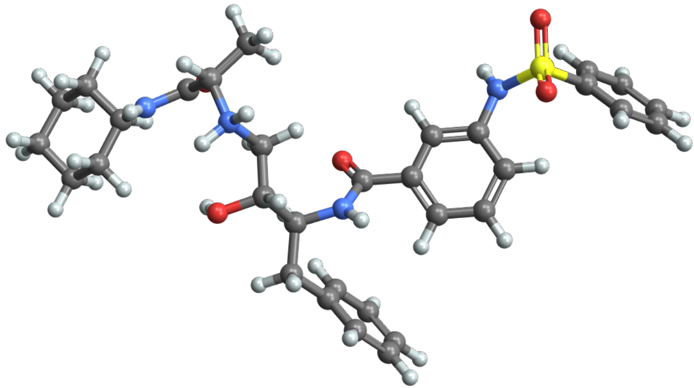

3D Autocorrelation

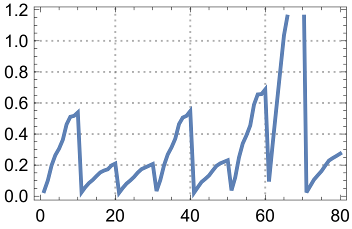

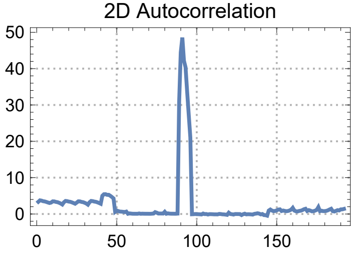

Dataset Index= 1335

Molecule Formula= $C_{17}H_{18}N_2OS$

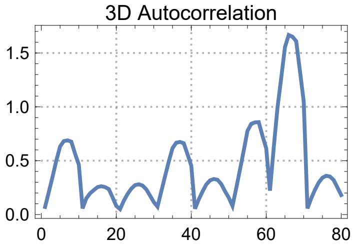

2D Plot

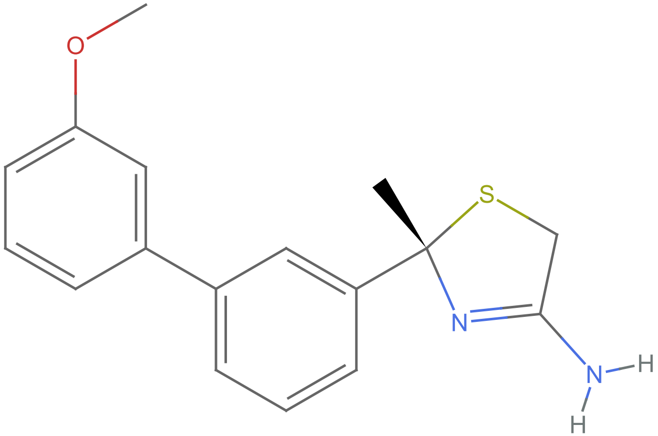

3D Plot

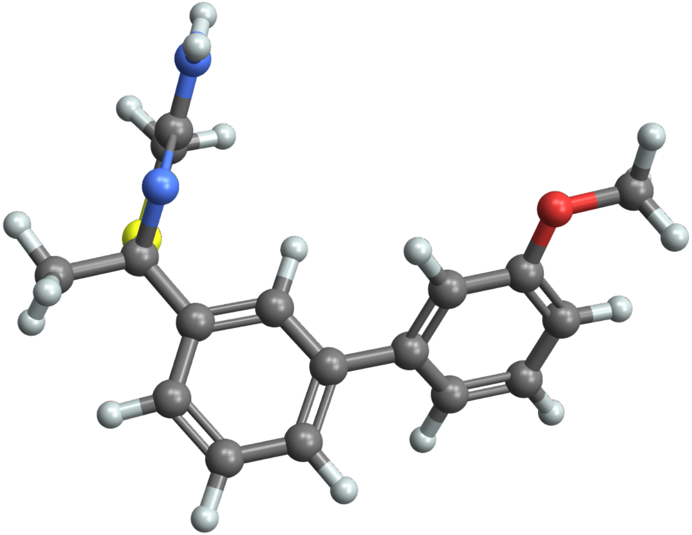

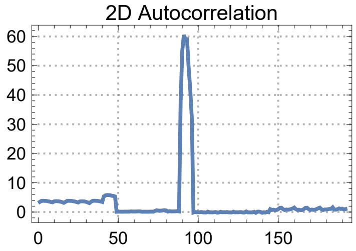

Dataset Index= 1336

Molecule Formula= $C_{21}H_{20}N_4O$

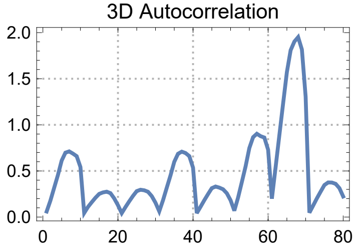

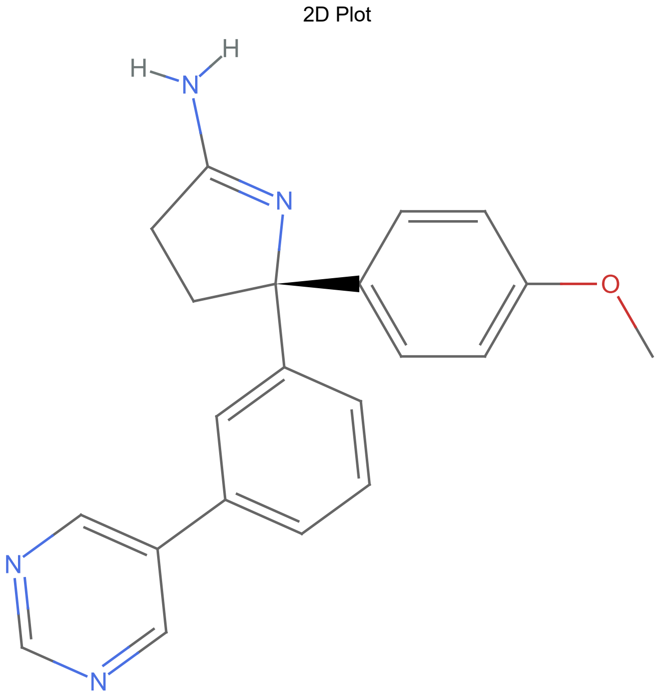

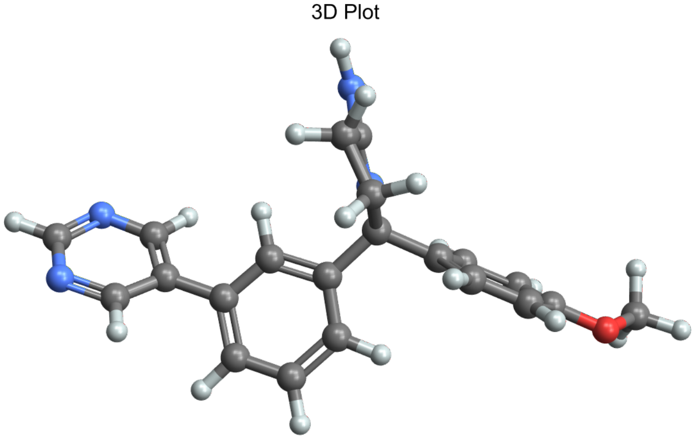

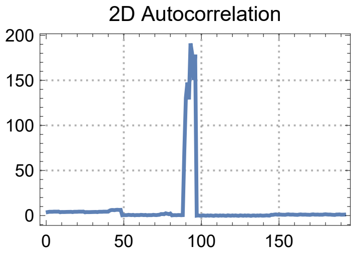

Dataset Index= 1337

Molecule Formula= $C_{31}H_{43}N_3O_6$

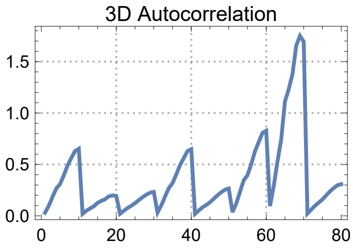

2D Plot

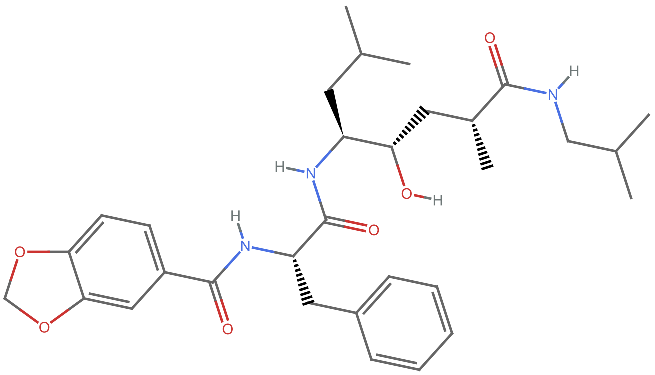

3D Plot

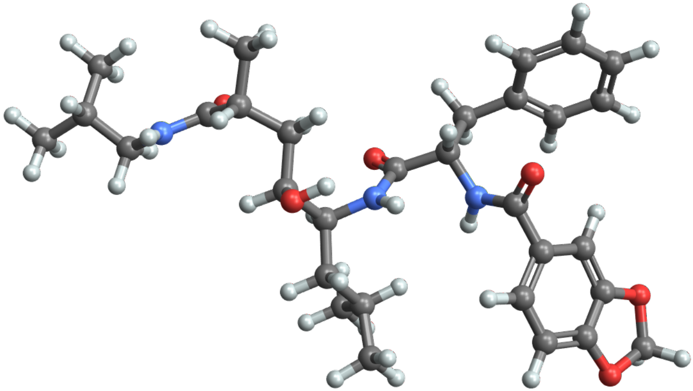

2D Plot

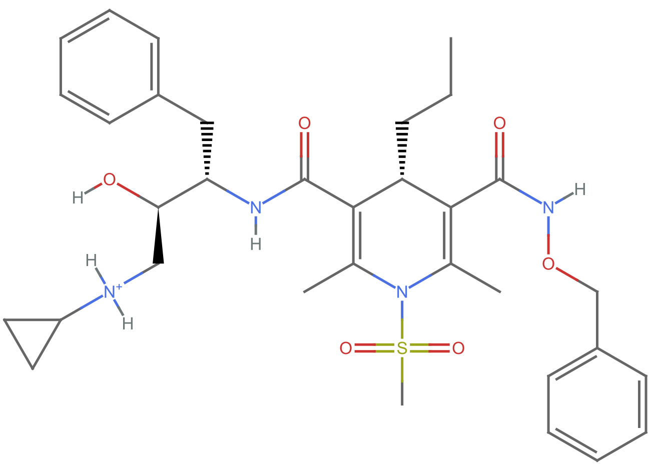

2D Autocorrelation

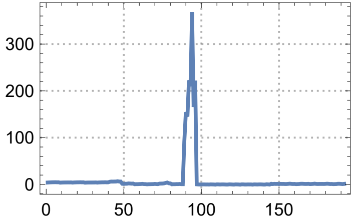

Dataset Index= 1338

Molecule Formula= $C_{33}H_{45}N_4O_6S^+$

3D Plot

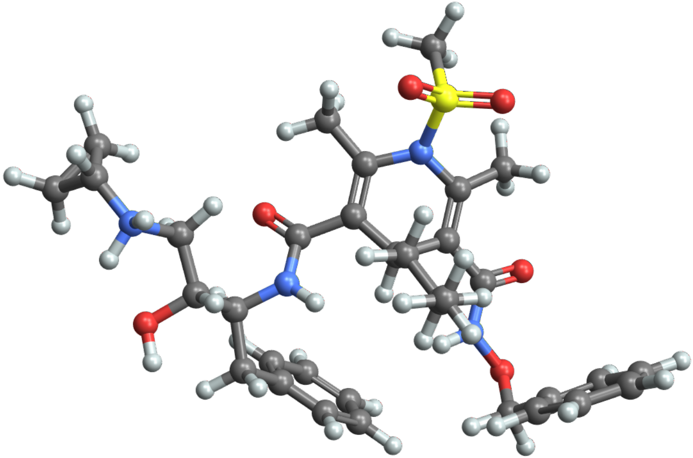

3D Autocorrelation

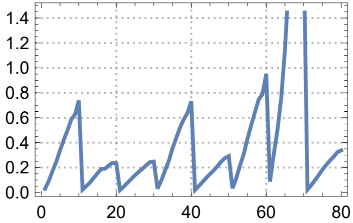

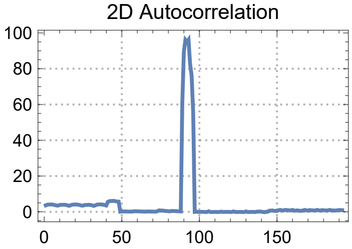

Dataset Index= 1339

Molecule Formula= $C_{25}H_{24}FN_4^+$

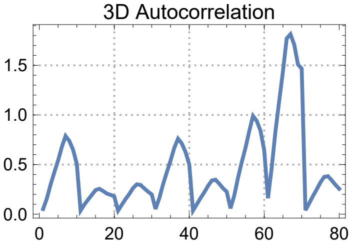

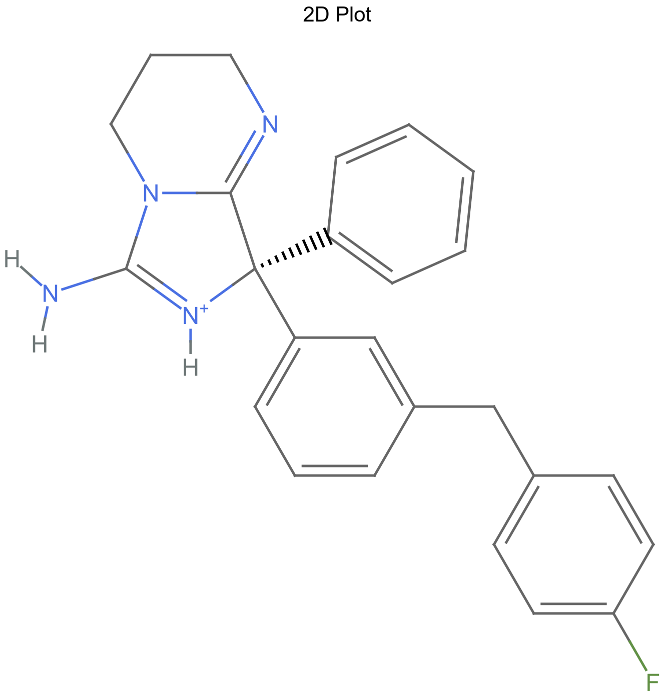

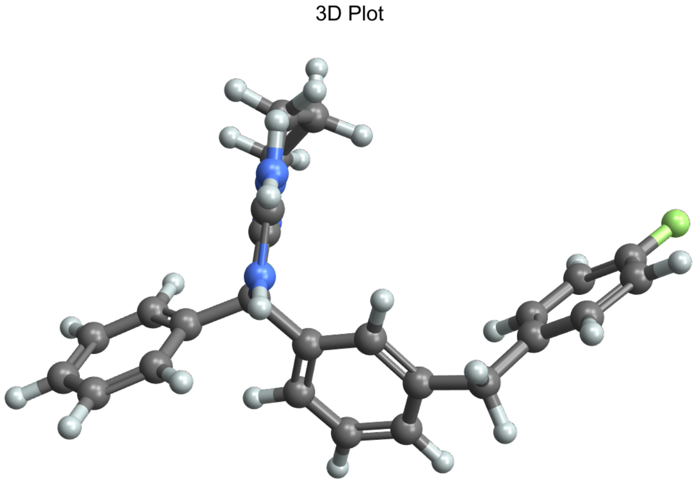

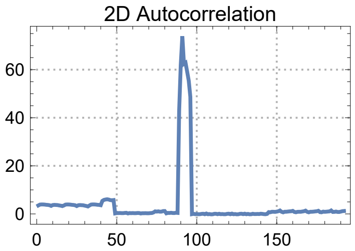

Dataset Index= 1340

Molecule Formula= $C_{21}H_{18}FN_3O_2$

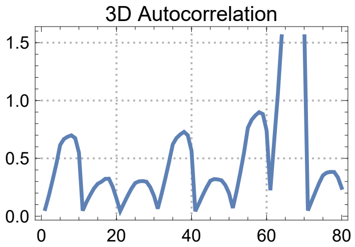

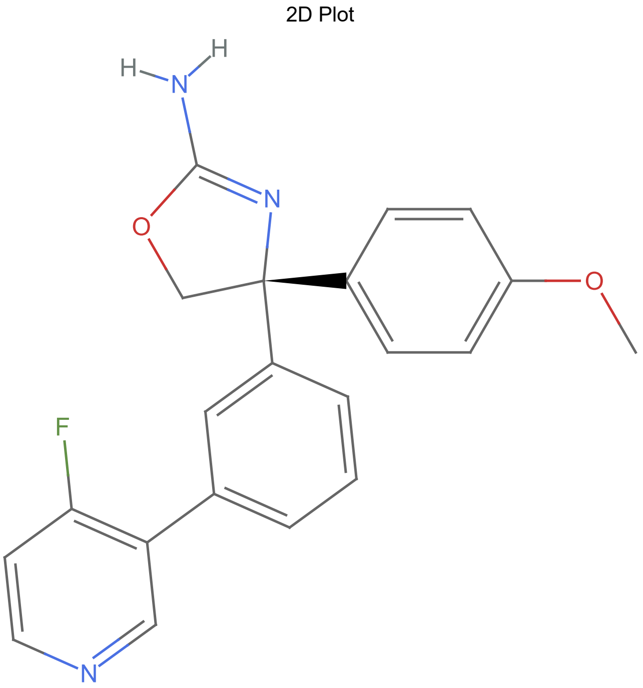

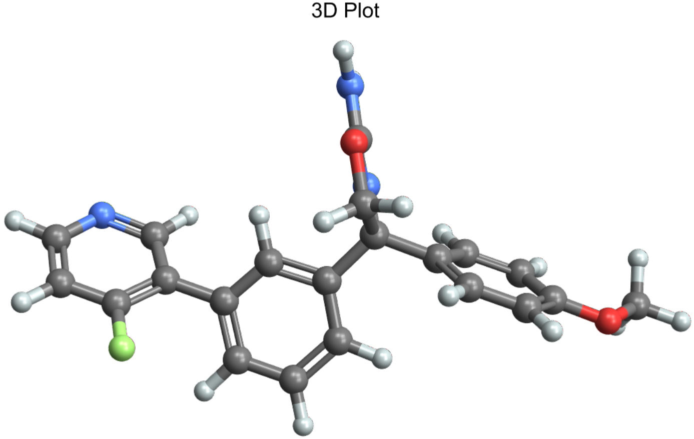

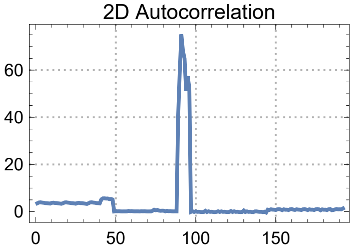

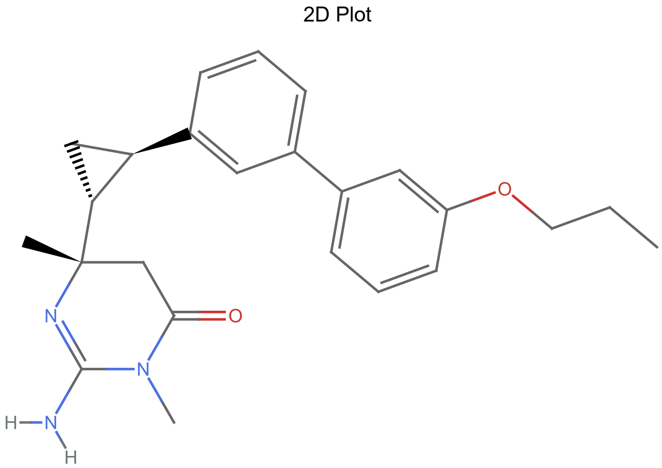

Dataset Index= 1341

Molecule Formula= $C_{24}H_{29}N_3O_2$

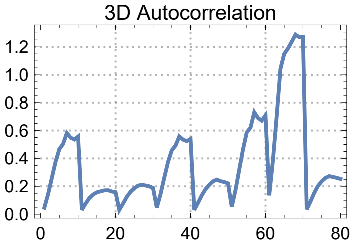

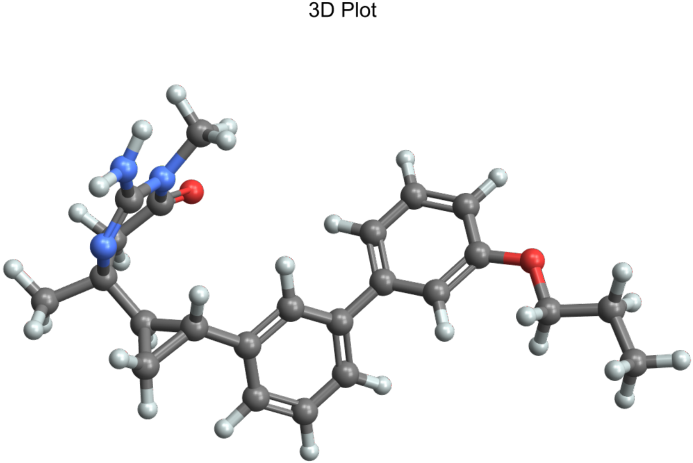

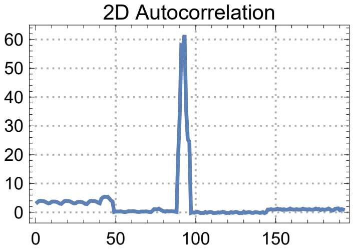

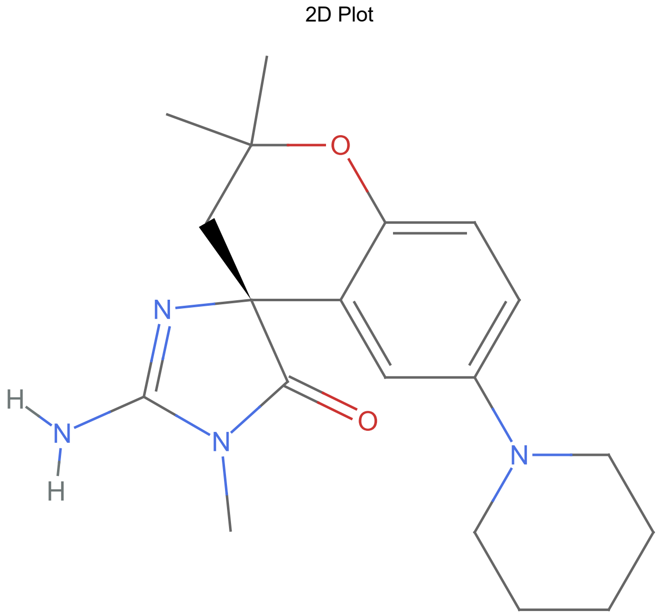

Dataset Index= 1342

Molecule Formula= $C_{19}H_{26}N_4O_2$

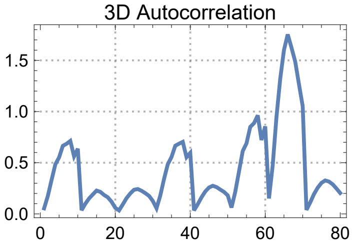

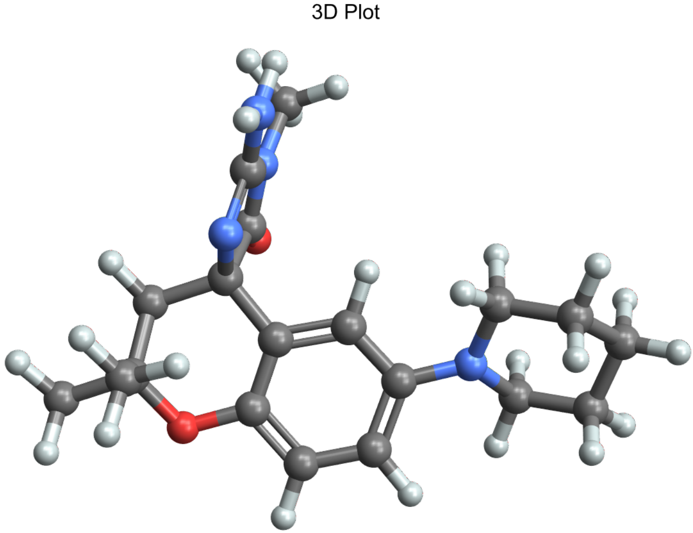

2D Plot

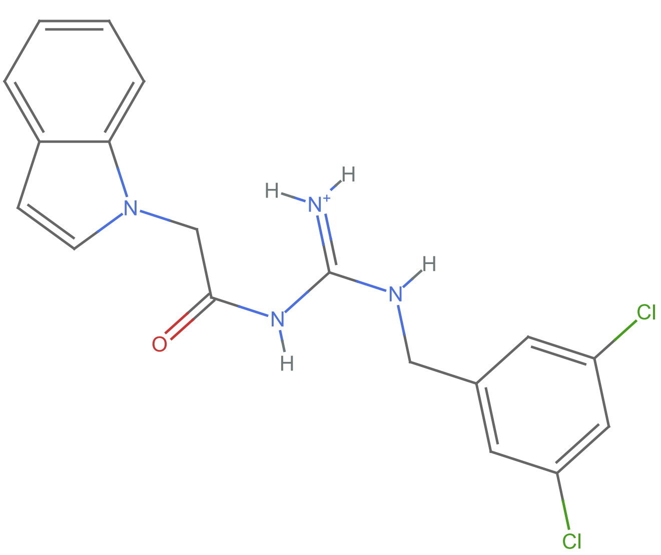

2D Autocorrelation

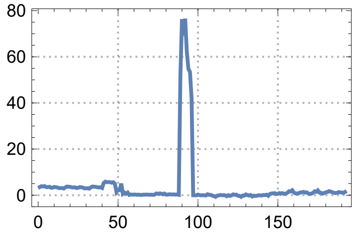

Dataset Index= 1343

Molecule Formula= $C_{18}H_{17}Cl_2N_4O^+$

3D Autocorrelation

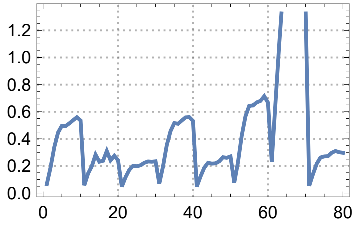

3D Plot

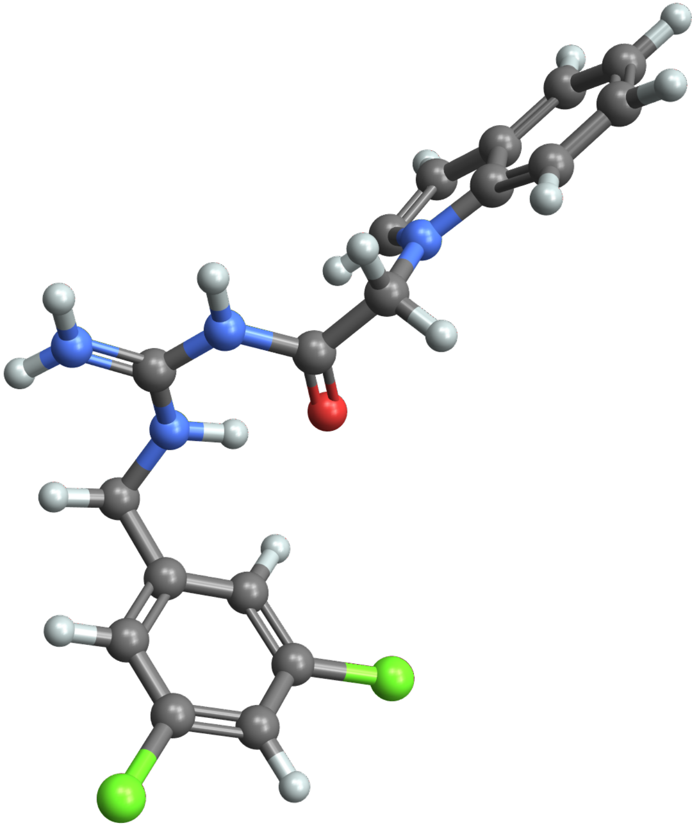

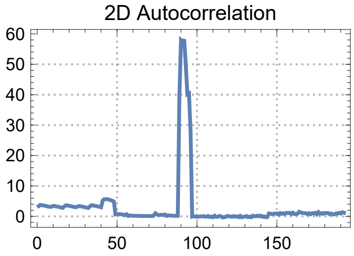

Dataset Index= 1344

Molecule Formula= $C_{16}H_{16}N_4OS$

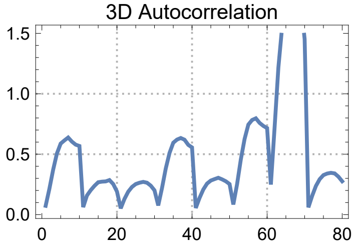

2D Plot

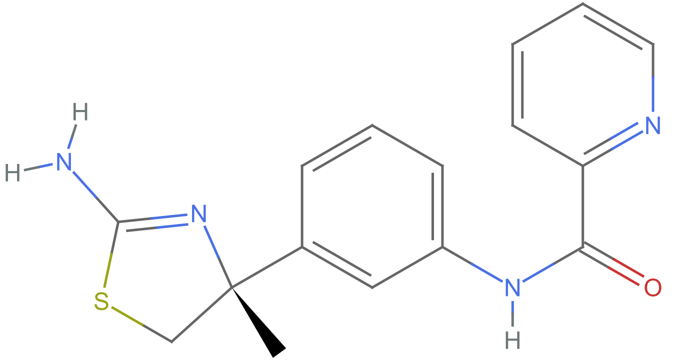

3D Plot

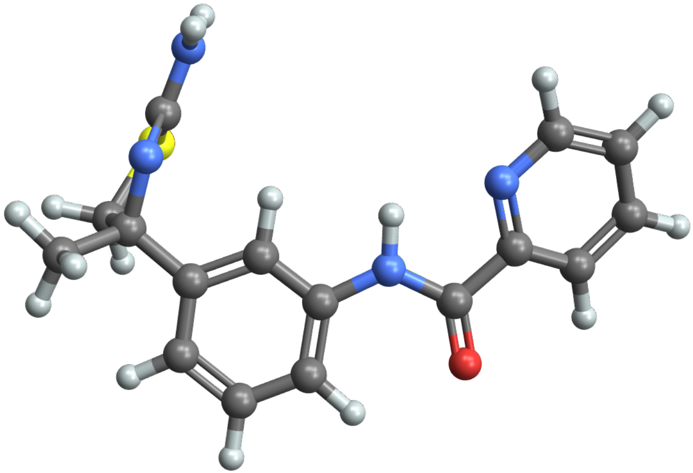

2D Autocorrelation

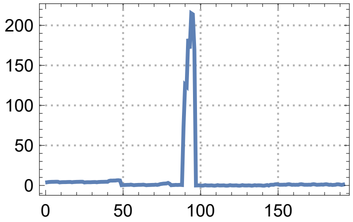

Dataset Index= 1345

Molecule Formula= $C_{35}H_{47}N_4O_6^+$

3D Autocorrelation

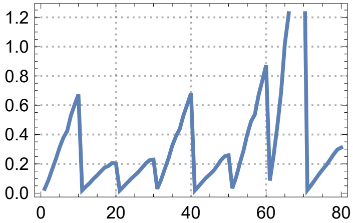

2D Plot

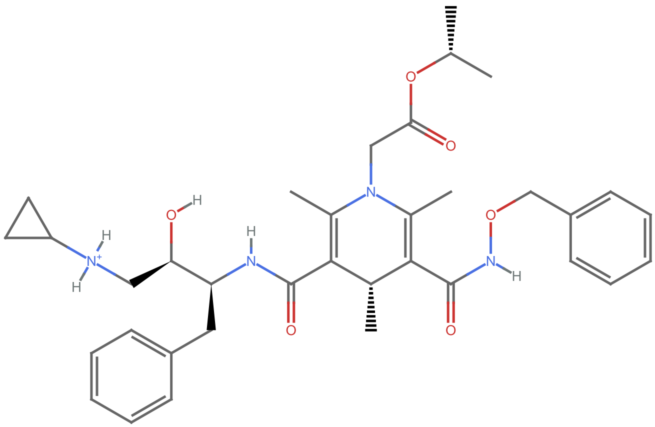

3D Plot

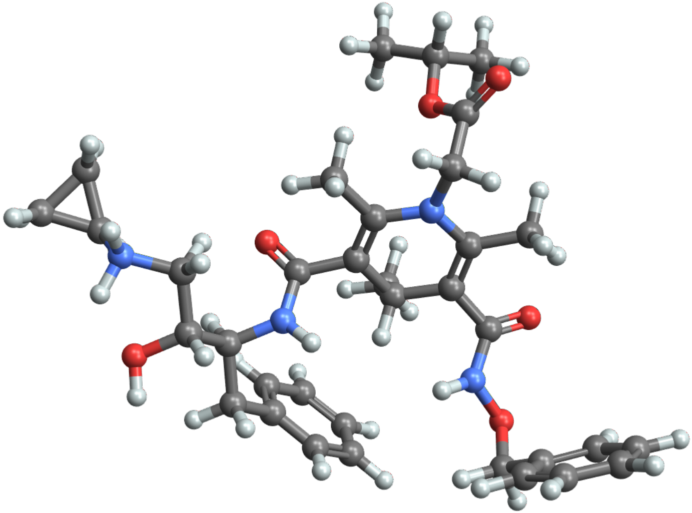

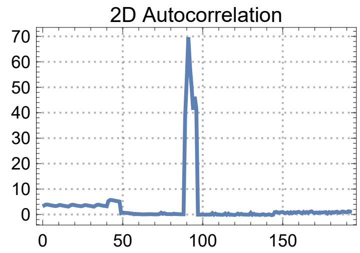

Dataset Index= 1346

Molecule Formula= $C_{22}H_{23}N_3OS$

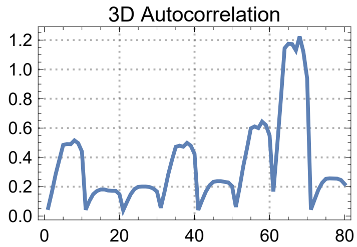

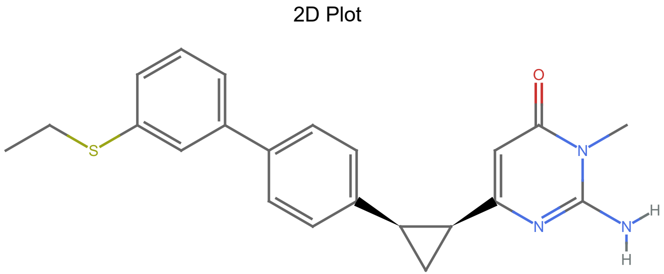

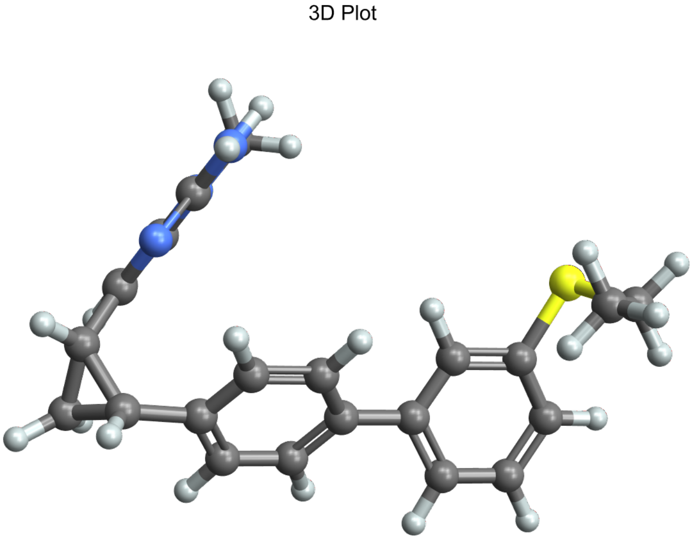

2D Plot

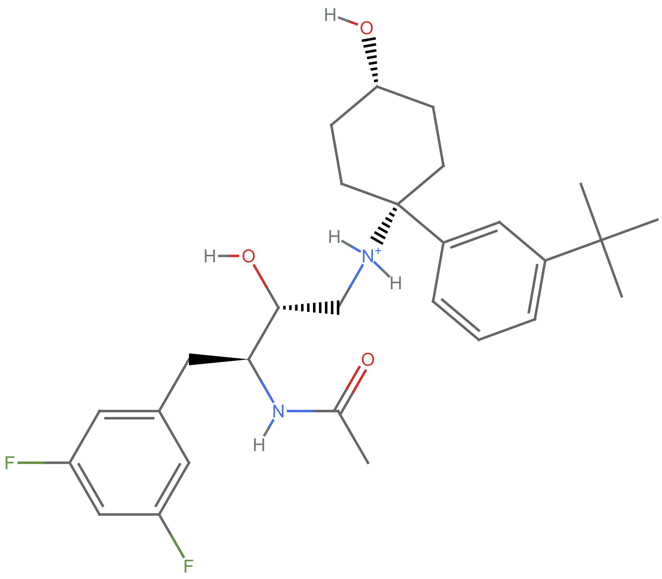

2D Autocorrelation

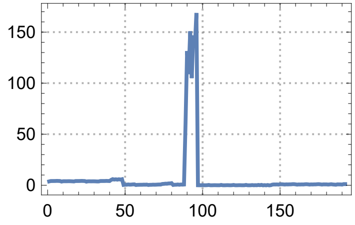

3D Plot

Dataset Index= 1347

Molecule Formula= $C_{28}H_{39}F_2N_2O_3^+$

3D Autocorrelation

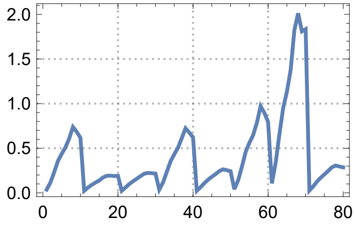

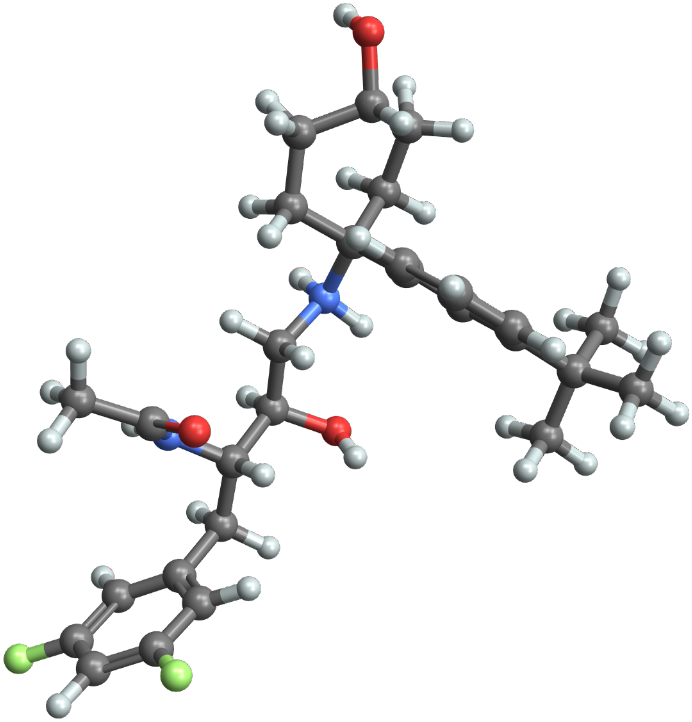

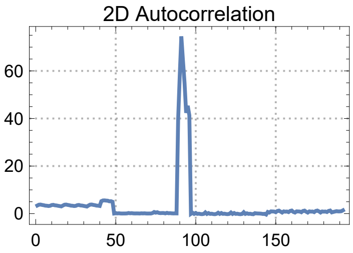

Dataset Index= 1348

Molecule Formula= $C_{21}H_{23}N_3O$

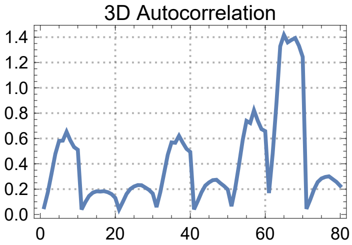

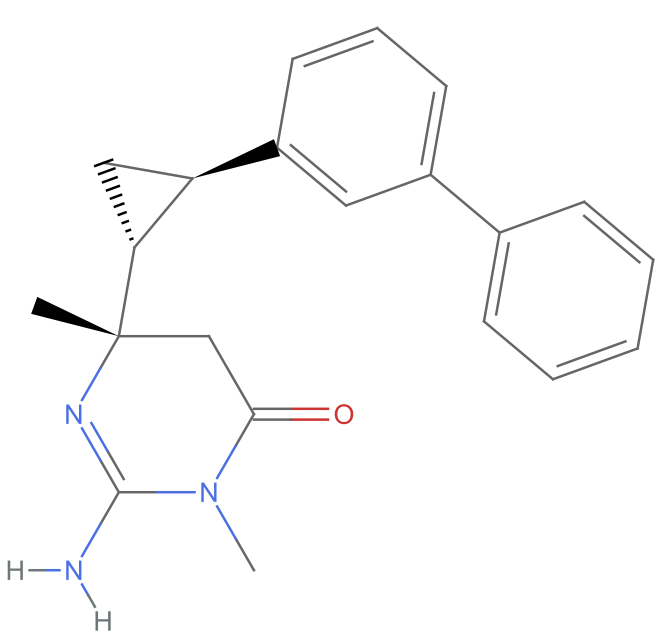

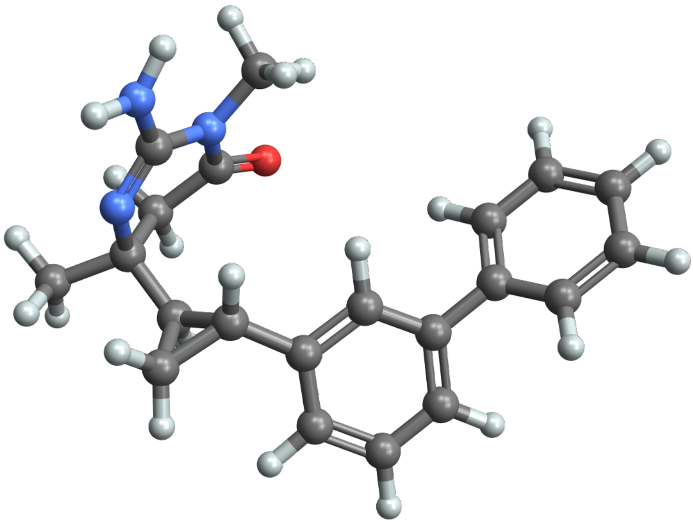

2D Plot

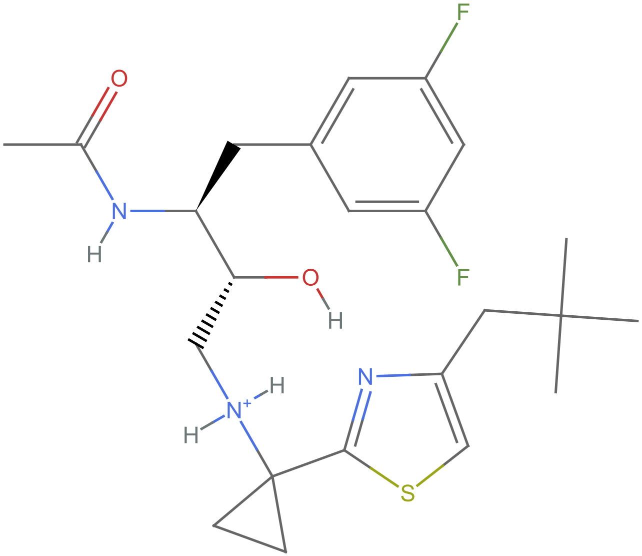

2D Autocorrelation

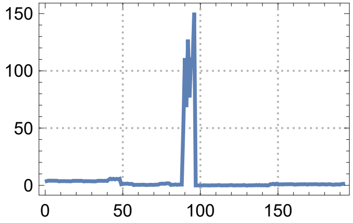

Dataset Index= 1349

Molecule Formula= $C_{23}H_{32}F_2N_3O_2S^+$

3D Autocorrelation

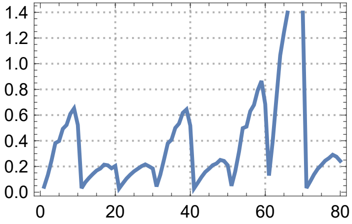

3D Plot

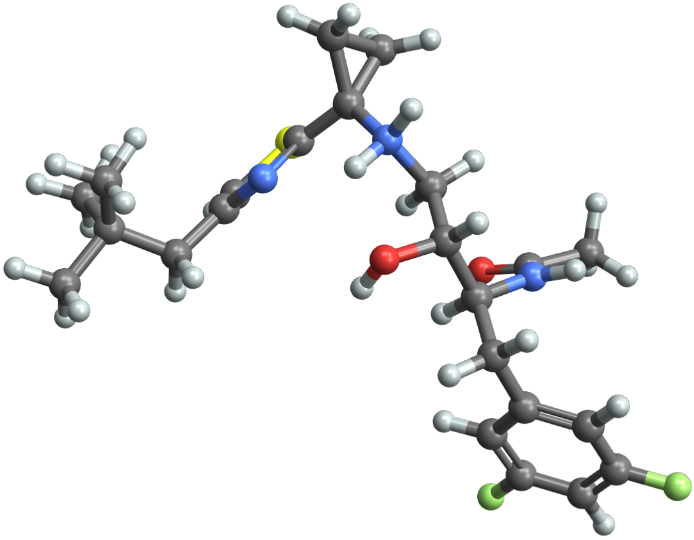

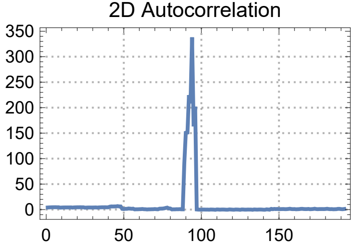

Dataset Index= 1350

Molecule Formula= $C_{32}H_{43}N_4O_6S^+$

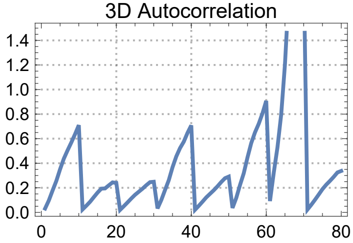

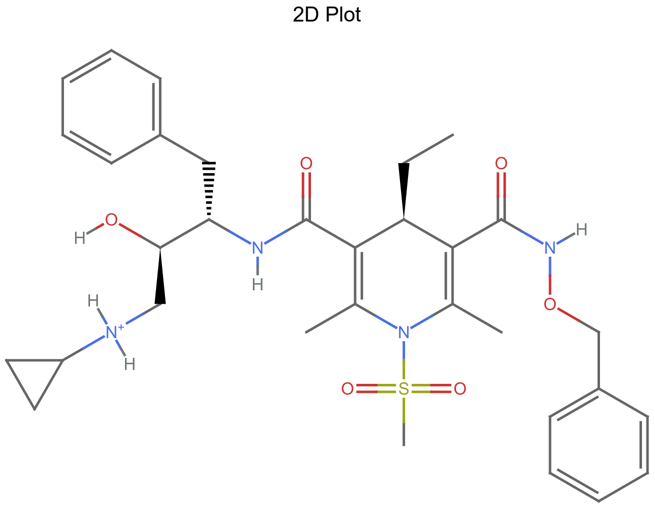

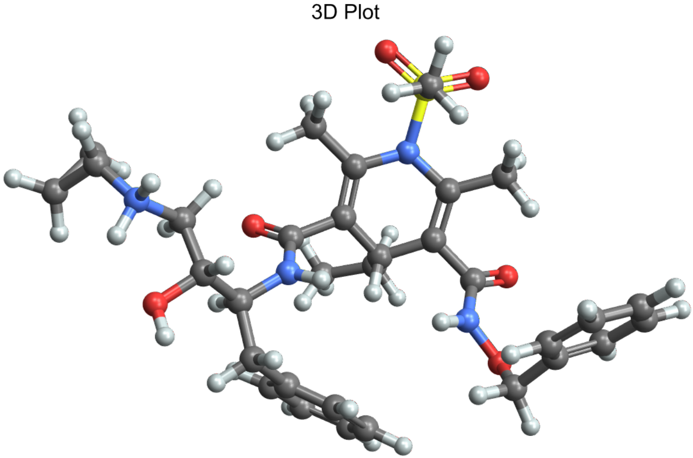

2D Plot

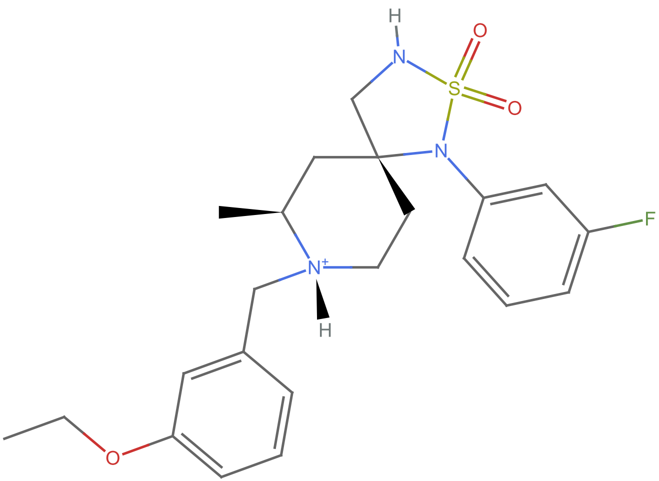

3D Plot

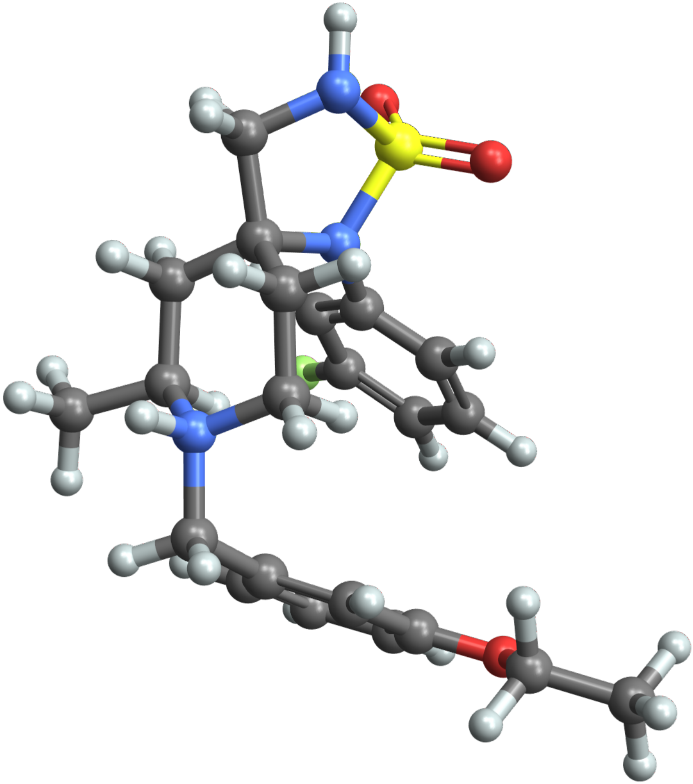

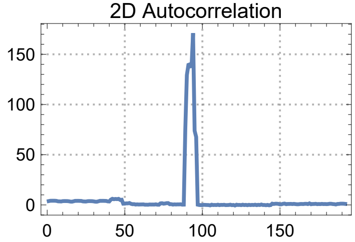

Dataset Index= 1351

Molecule Formula= $C_{22}H_{29}FN_3O_3S^+$

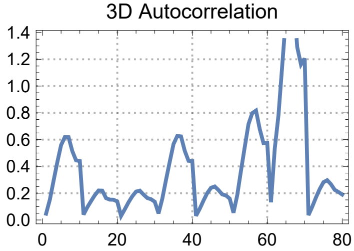

2D Plot

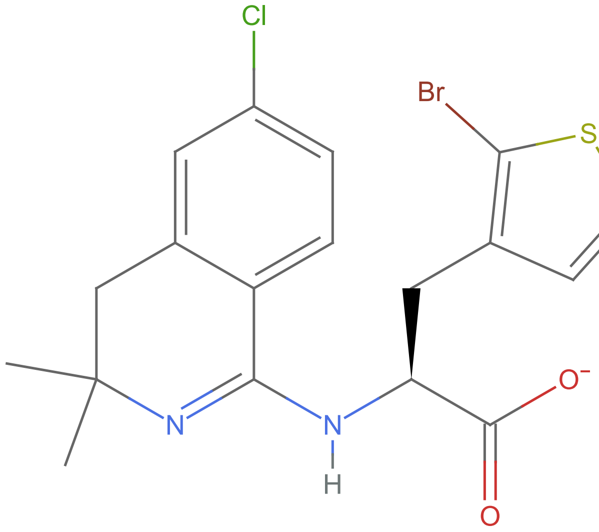

3D Plot

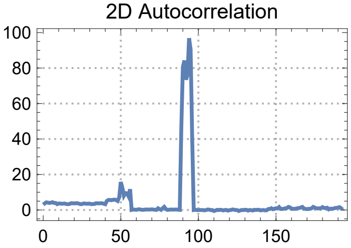

Dataset Index= 1352

Molecule Formula= $C_{18}H_{17}BrClN_2O_2S^-$

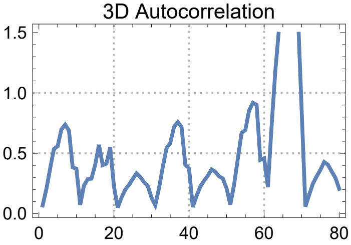

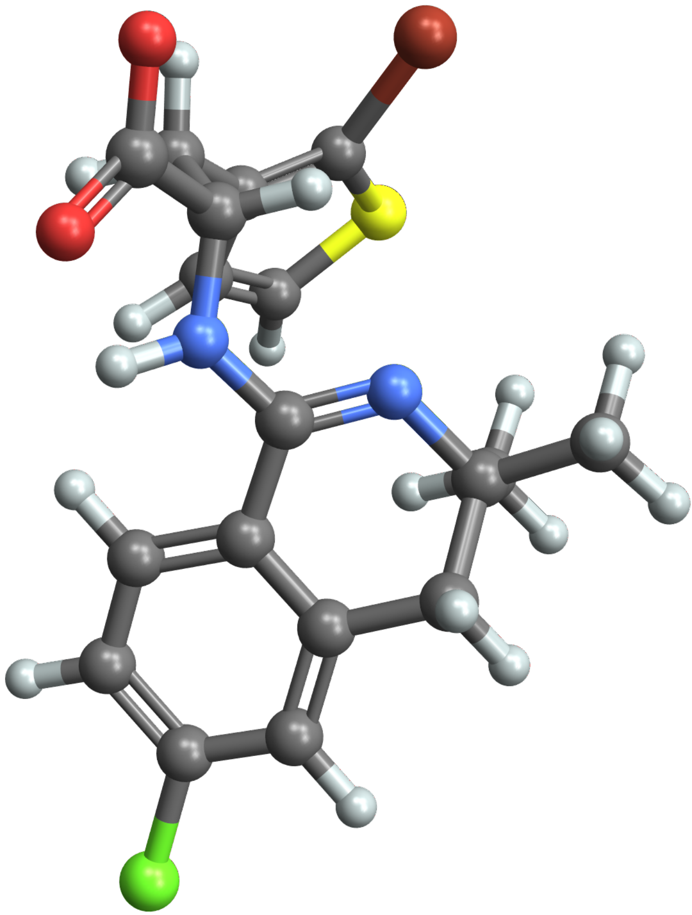

2D Plot

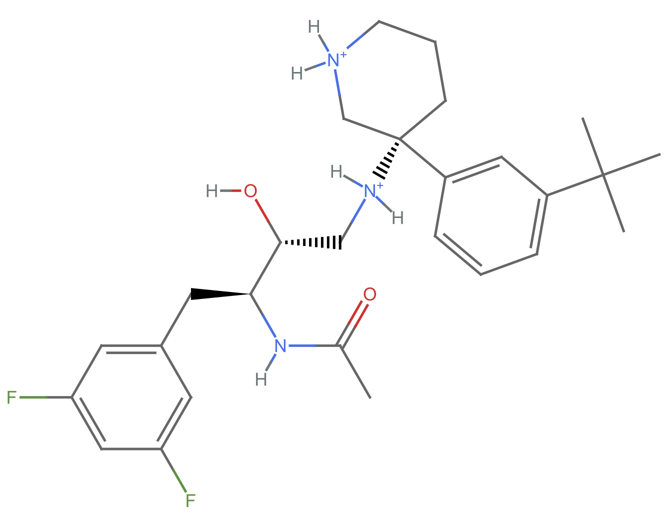

2D Autocorrelation

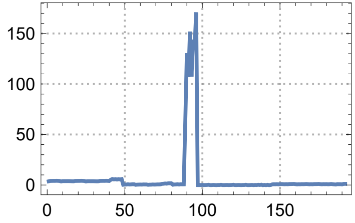

3D Plot

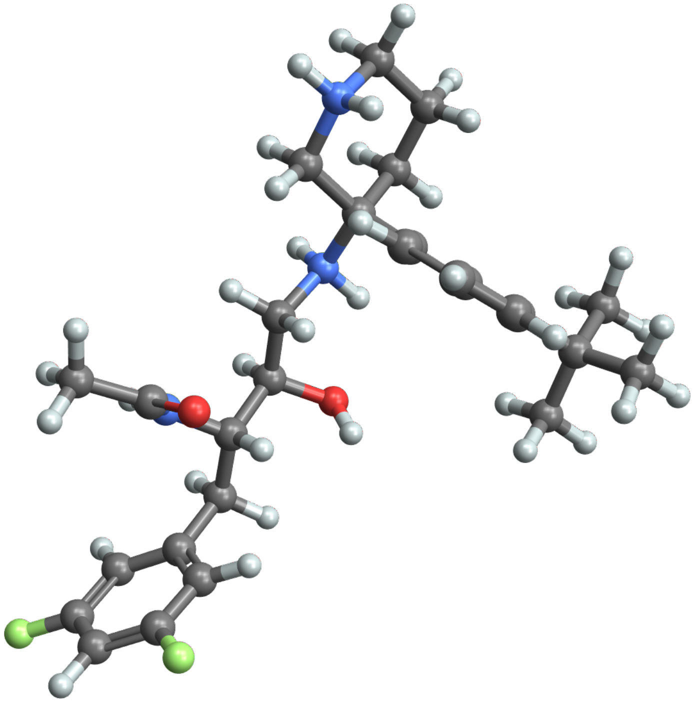

Dataset Index= 1353

Molecule Formula= $C_{27}H_{39}F_2N_3O_2^{2+}$

3D Autocorrelation

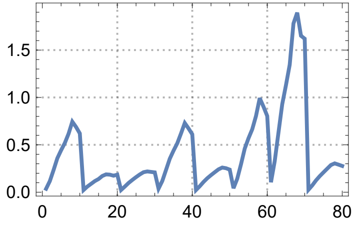

2D Plot

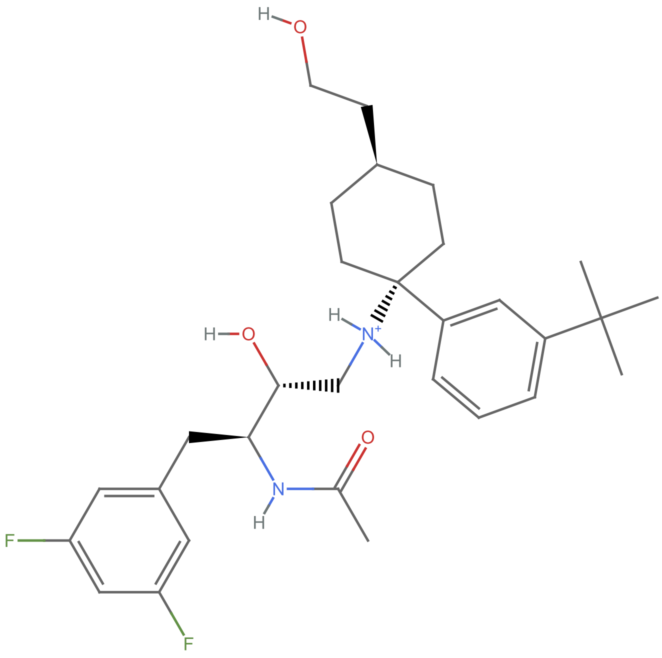

2D Autocorrelation

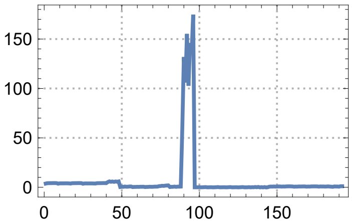

3D Plot

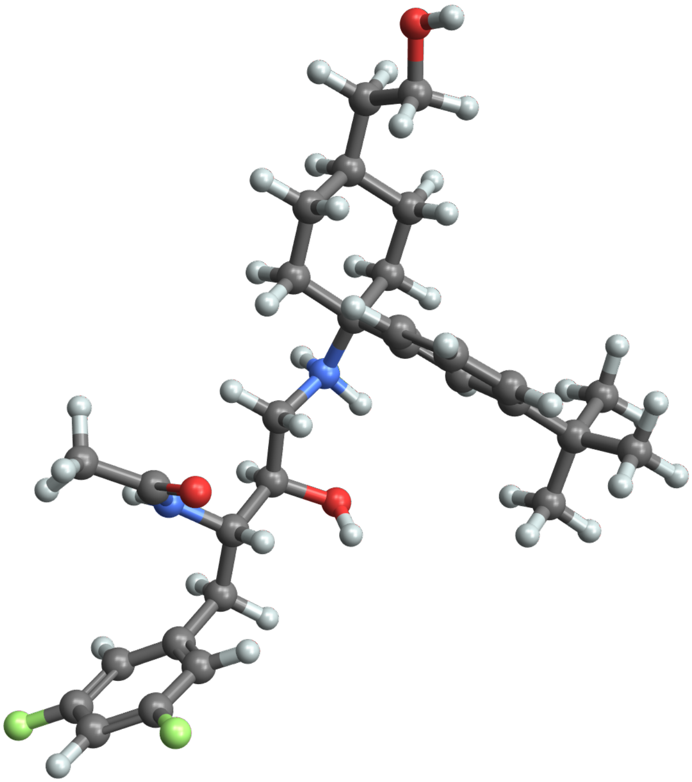

Dataset Index= 1354

Molecule Formula= $C_{30}H_{43}F_2N_2O_3^+$

3D Autocorrelation

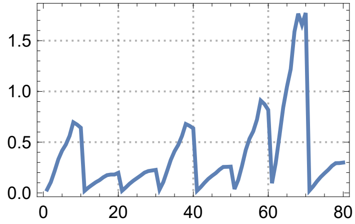

2D Plot

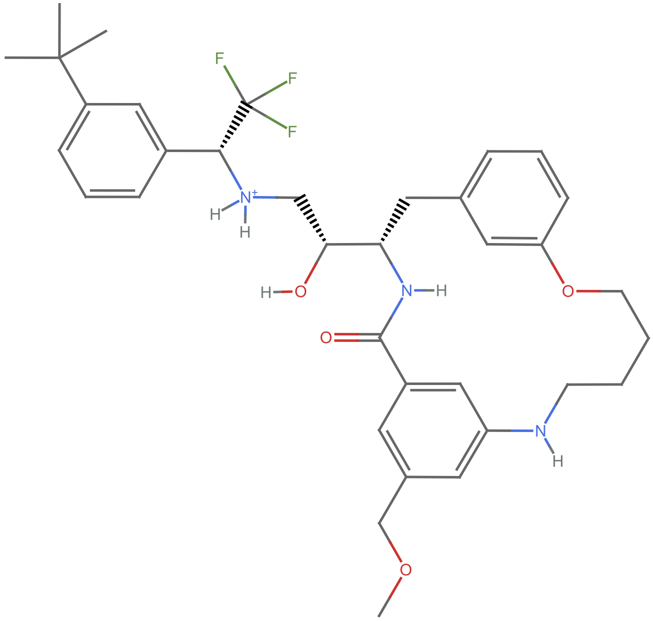

2D Autocorrelation

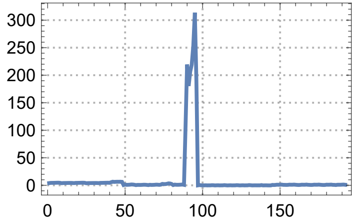

Dataset Index= 1355

Molecule Formula= $C_{35}H_{45}F_{3}N_{3}O_{4}^{+}$

3D Autocorrelation

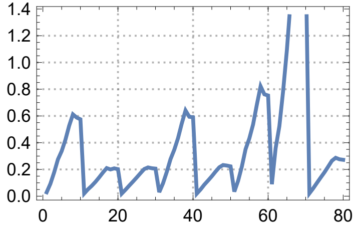

3D Plot

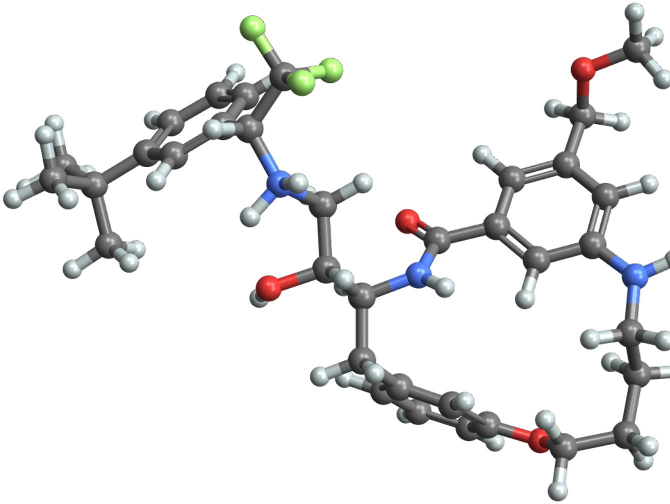

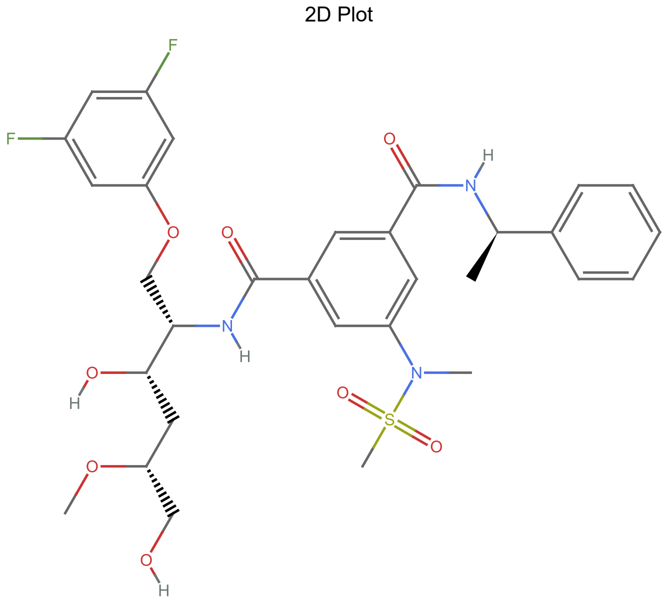

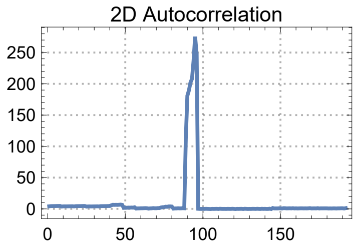

Dataset Index= 1356

Molecule Formula= $C_{31}H_{37}F_2N_3O_8S$

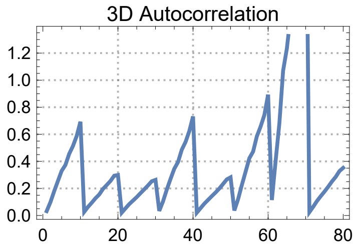

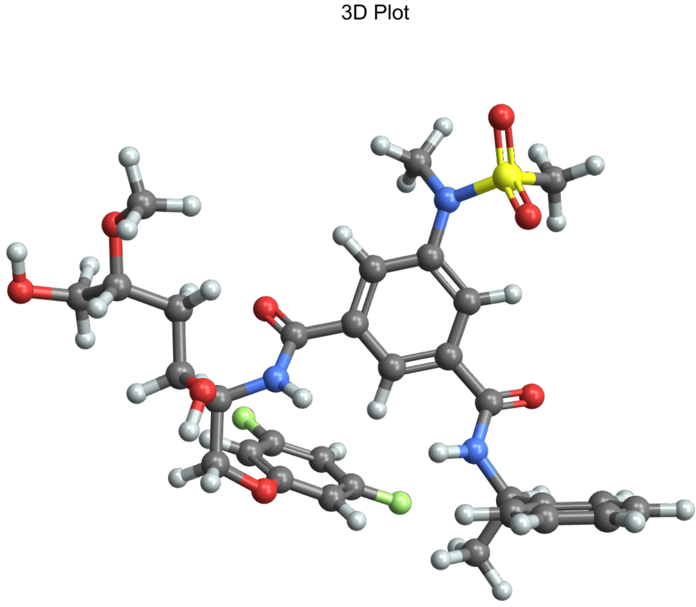

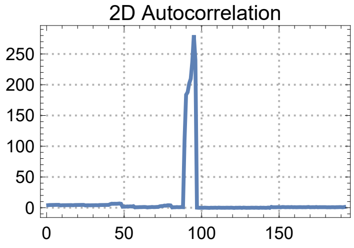

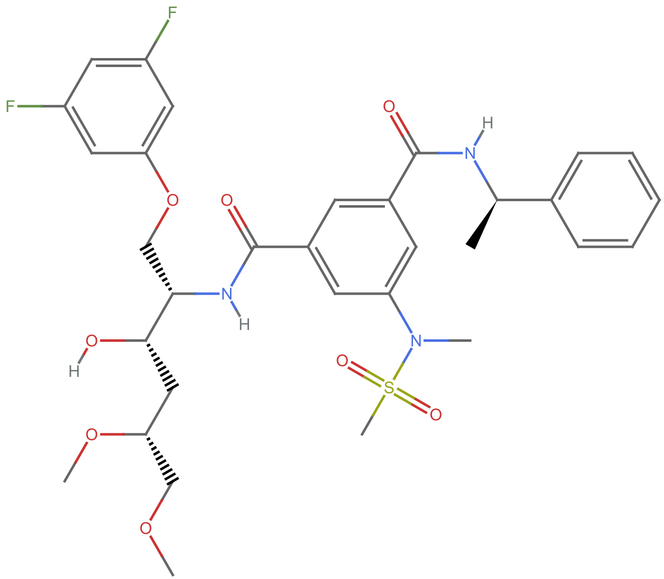

Dataset Index= 1357

Molecule Formula= $C_{32}H_{39}F_2N_3O_8S$

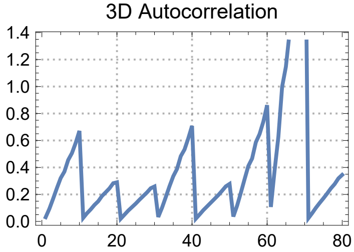

3D Plot

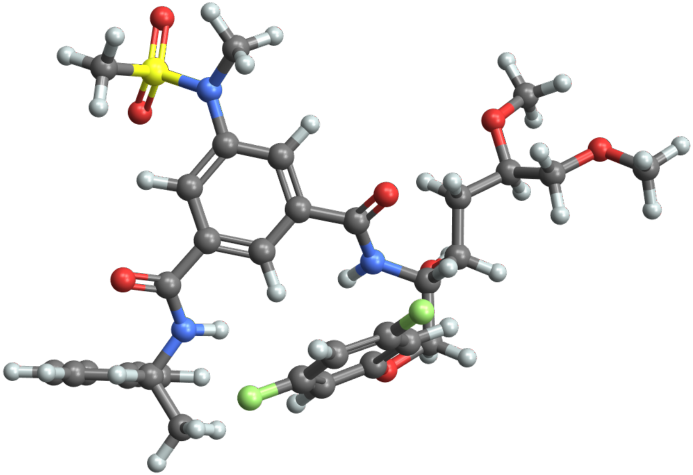

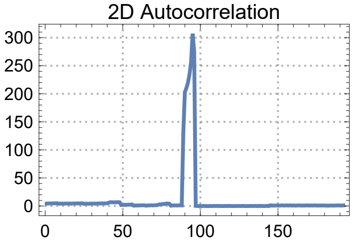

Dataset Index= 1358

Molecule Formula= $C_{38}H_{42}F_3N_3O_8S$

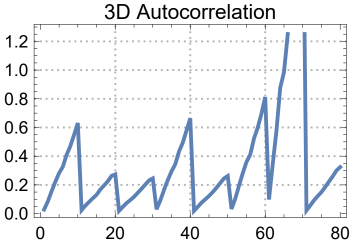

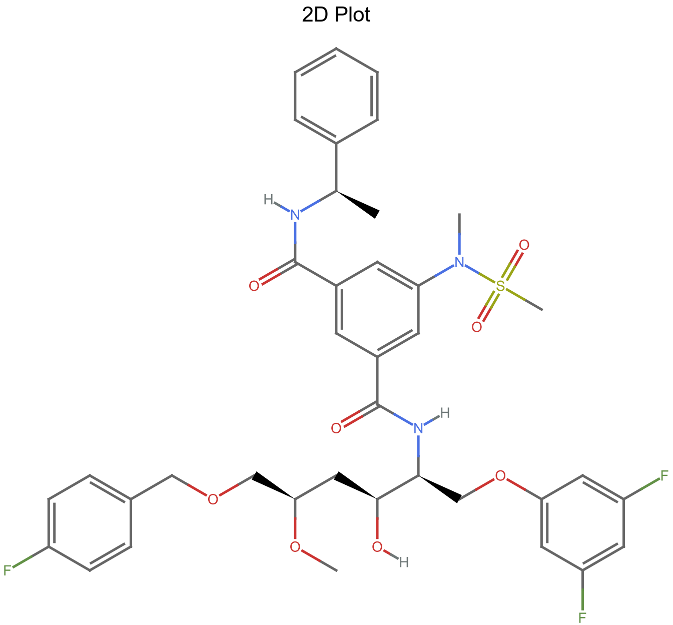

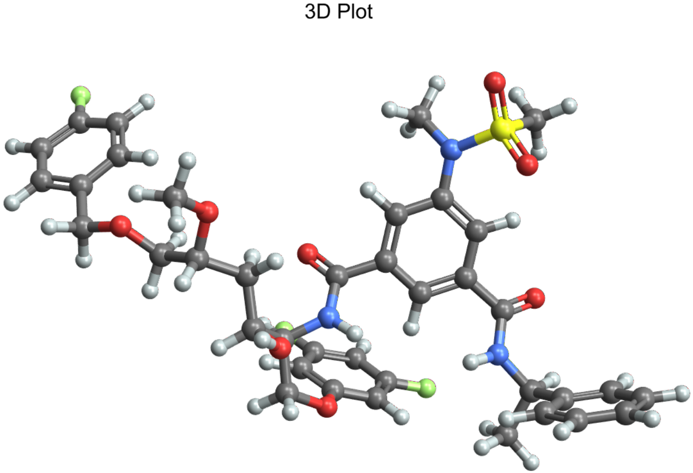

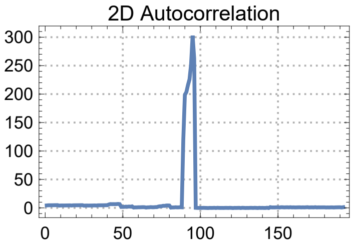

Dataset Index= 1359

Molecule Formula= $C_{39}H_{45}F_2N_3O_9S$

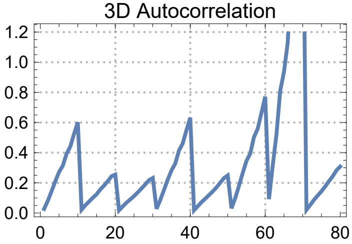

2D Plot

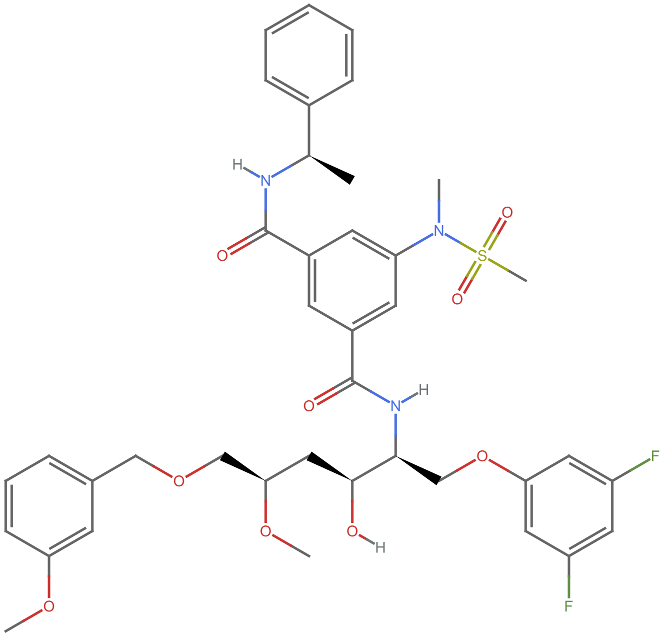

3D Plot

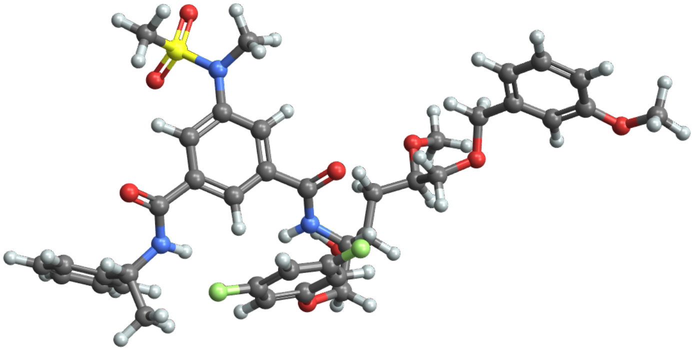

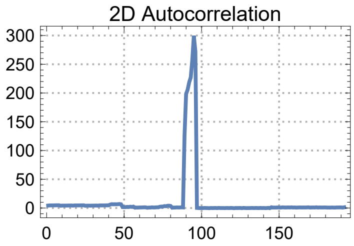

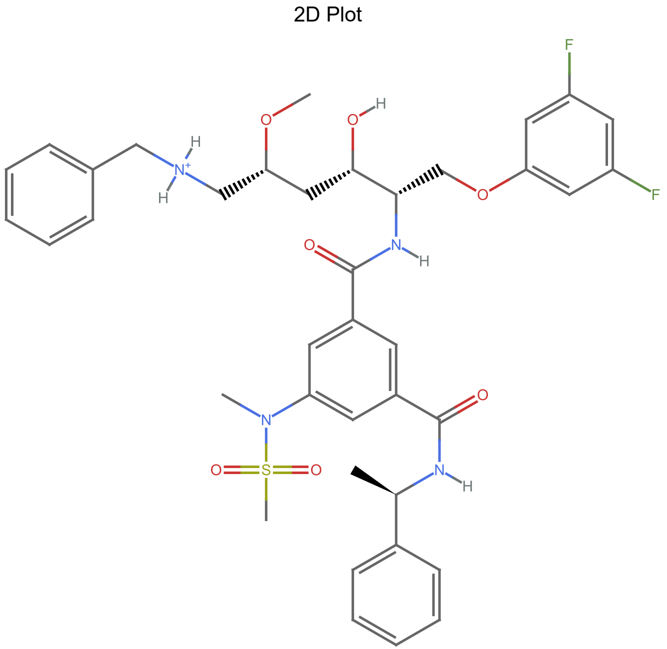

Dataset Index= 1360

Molecule Formula= $C_{38}H_{45}F_2N_4O_7S^+$

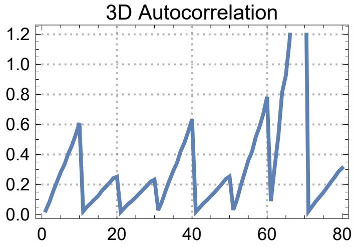

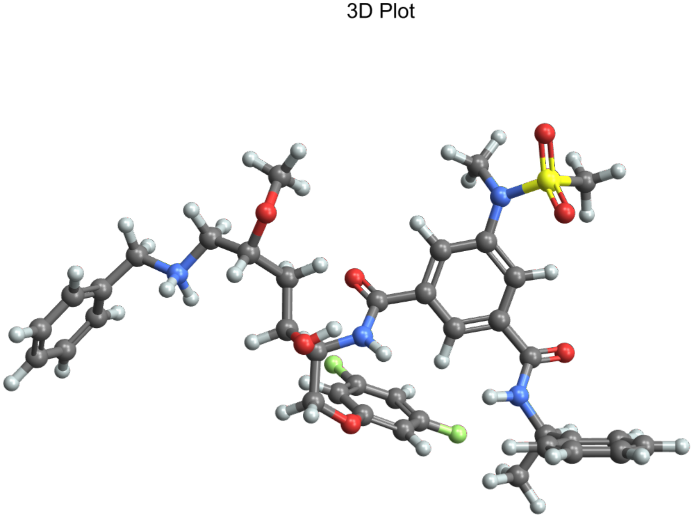

2D Plot

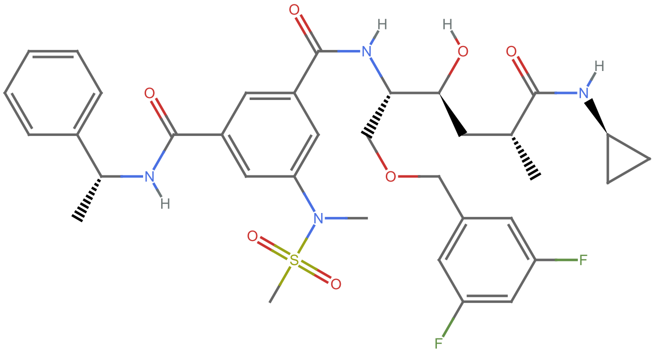

3D Plot

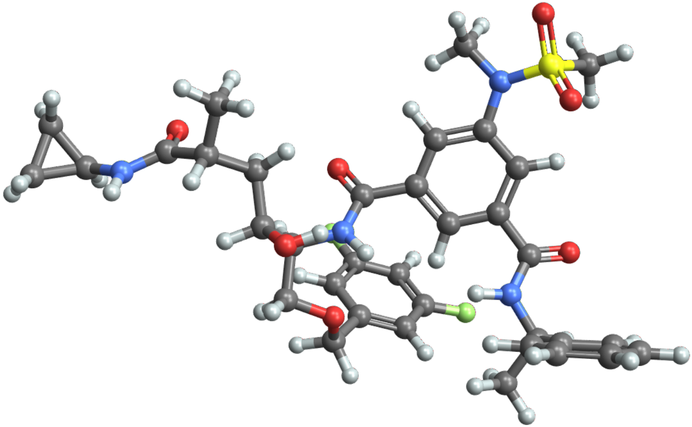

2D Autocorrelation

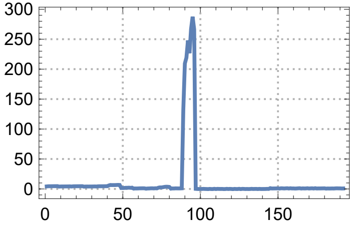

Dataset Index= 1361

Molecule Formula= $C_{35}H_{42}F_2N_4O_7S$

3D Autocorrelation

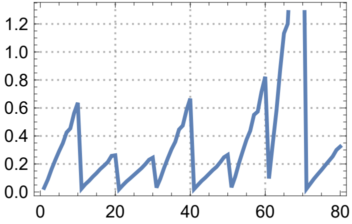

2D Plot

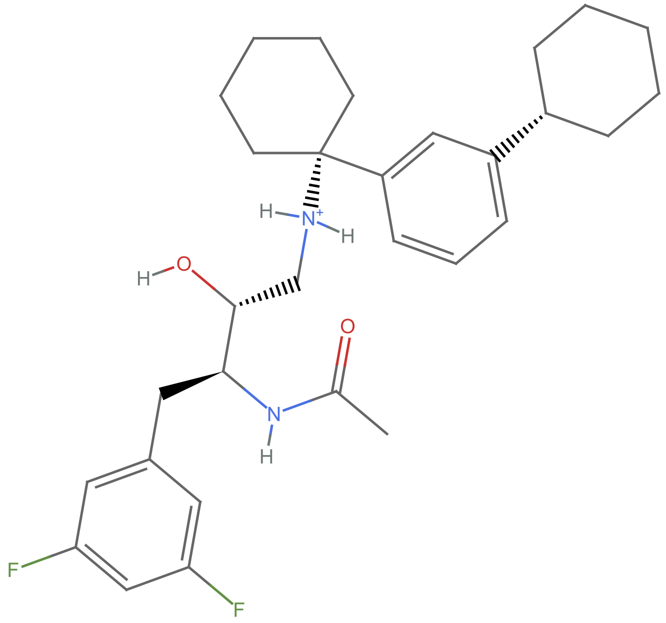

2D Autocorrelation

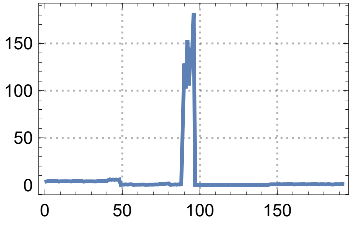

3D Plot

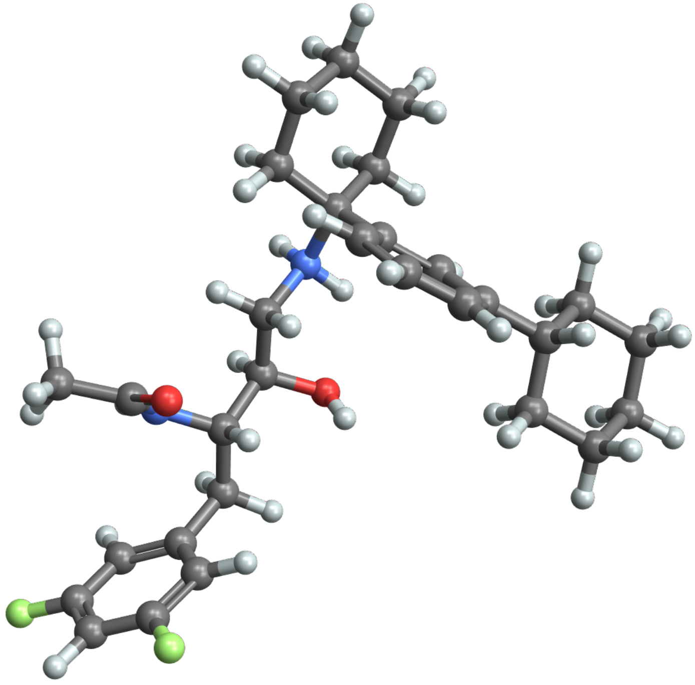

Dataset Index= 1362

Molecule Formula= $C_{30}H_{41}F_2N_2O_2^+$

3D Autocorrelation

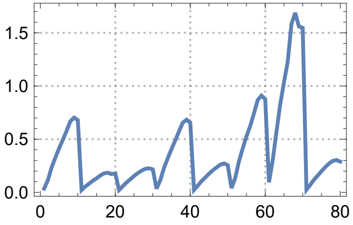

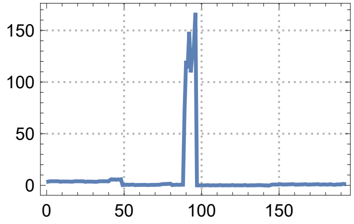

Dataset Index= 1363

Molecule Formula= $C_{24}H_{31}F_2N_2O_2^+$

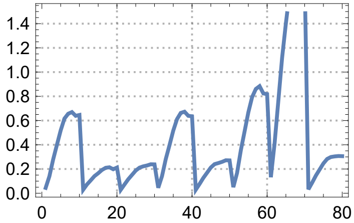

2D Plot

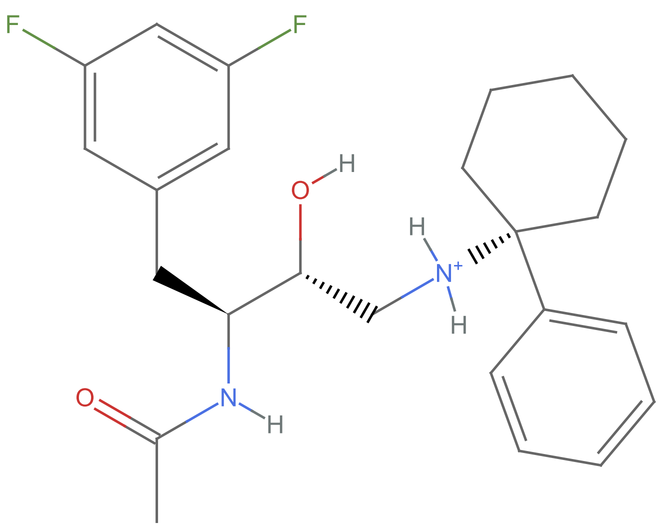

3D Plot

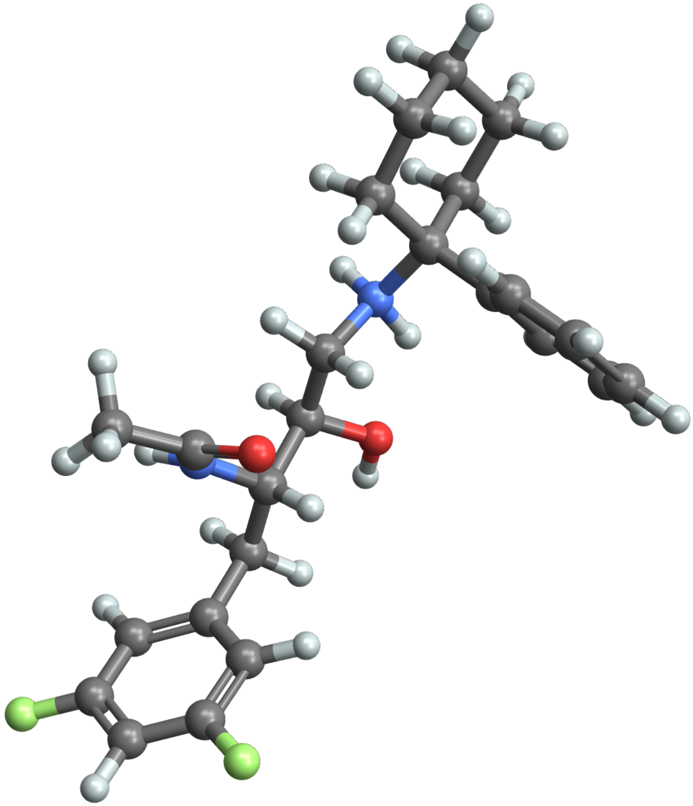

2D Plot

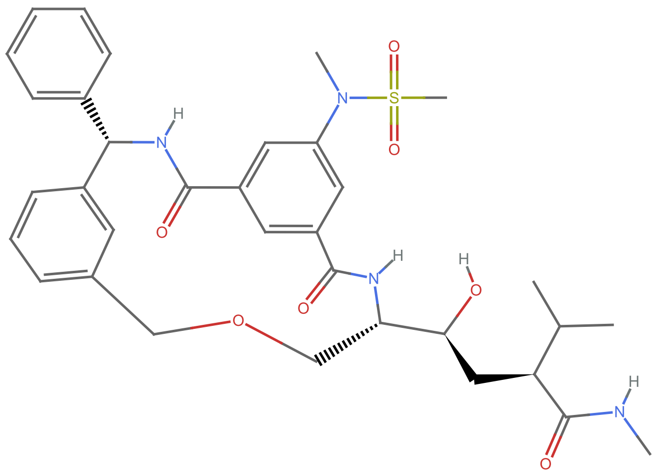

2D Autocorrelation

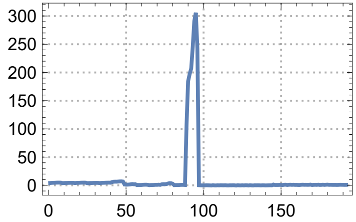

3D Plot

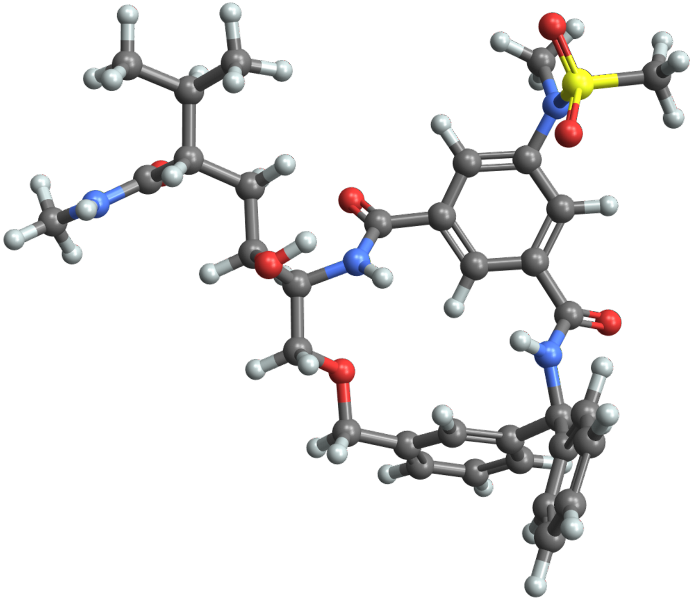

Dataset Index= 1364

Molecule Formula= $C_{34}H_{42}N_4O_7S$

3D Autocorrelation

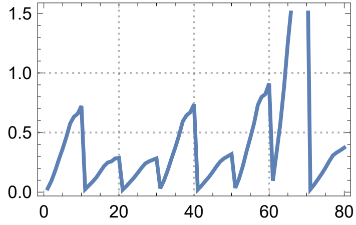

2D Plot

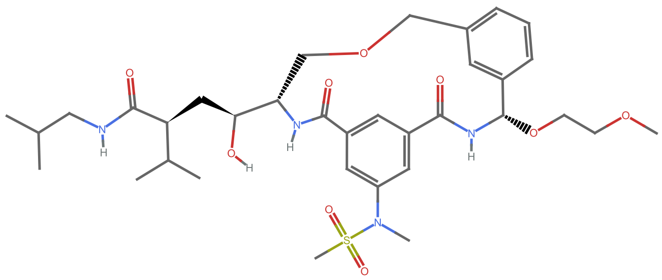

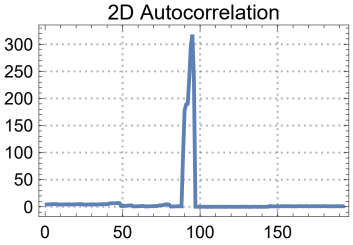

Dataset Index= 1365

Molecule Formula= $C_{34}H_{50}N_4O_9S$

3D Plot

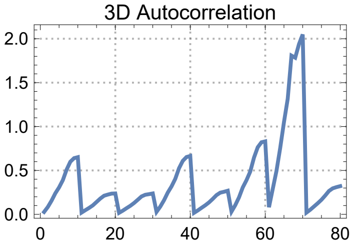

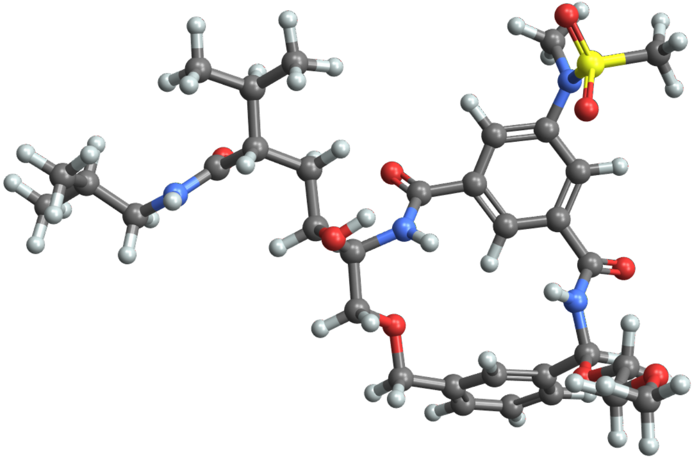

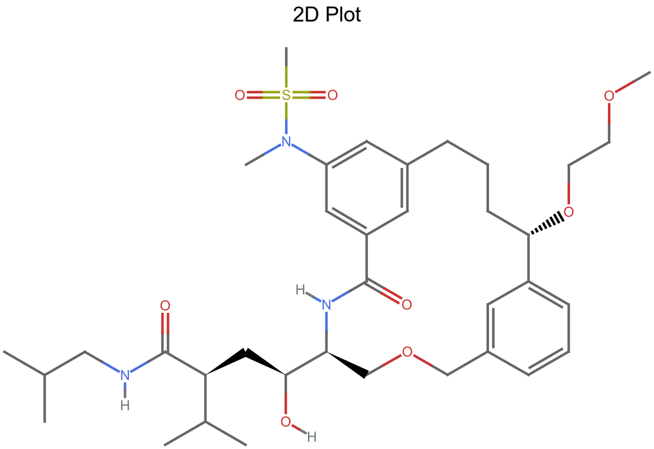

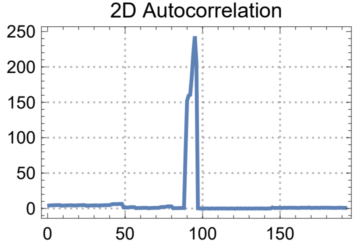

Dataset Index= 1366

Molecule Formula= $C_{36}H_{55}N_3O_8S$

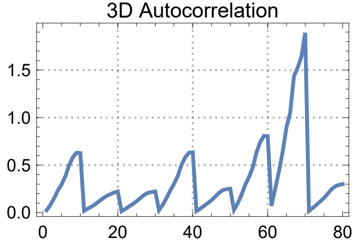

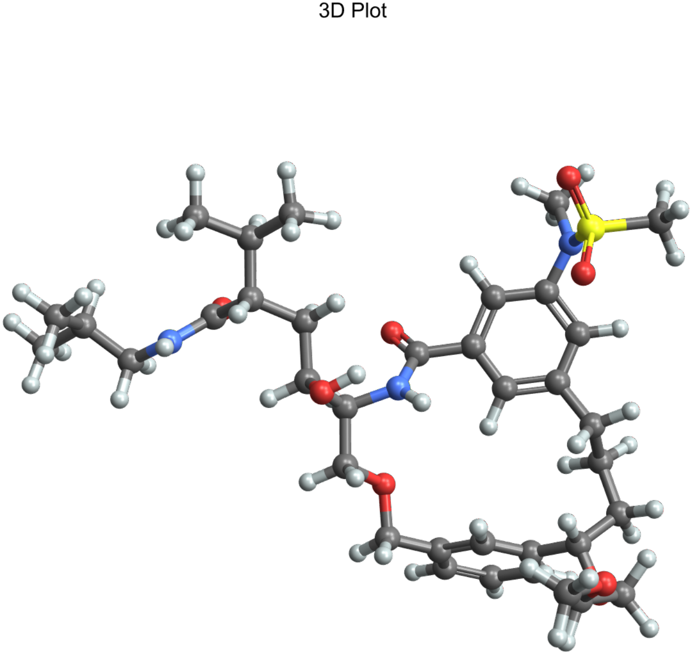

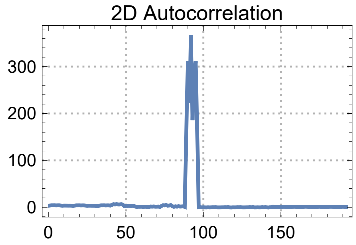

Dataset Index= 1367

Molecule Formula= $C_{15}H_{18}F_7N_2O_4S^+$

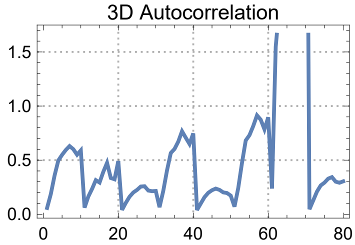

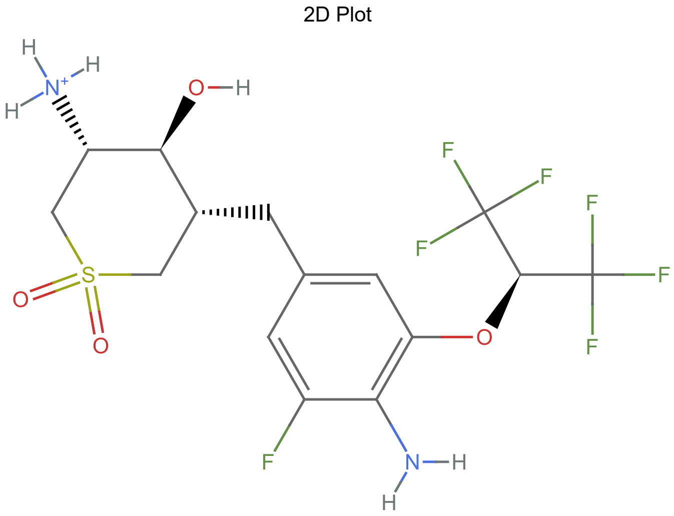

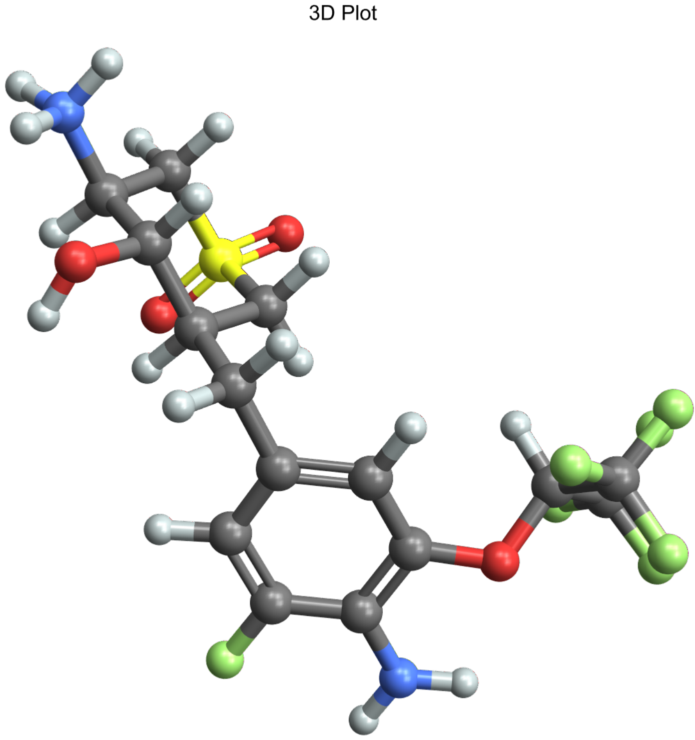

2D Autocorrelation

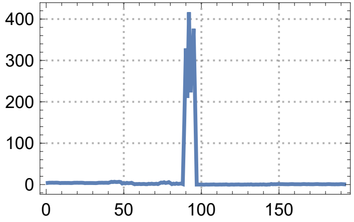

Dataset Index= 1368

Molecule Formula= $C_{23}H_{32}F_7N_2O_5S^+$

3D Autocorrelation

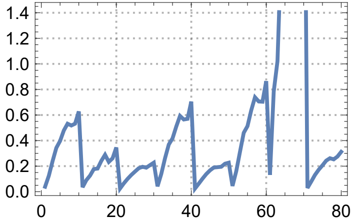

2D Plot

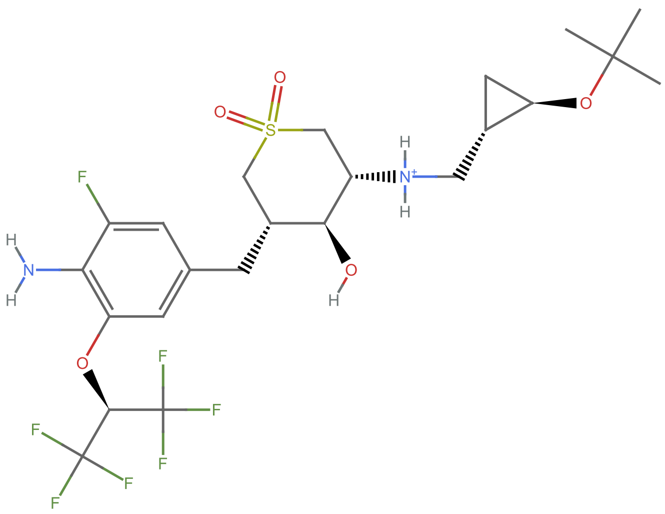

3D Plot

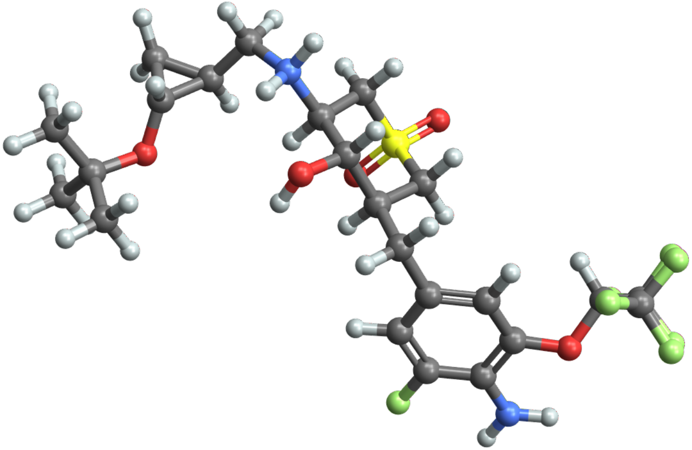

2D Autocorrelation

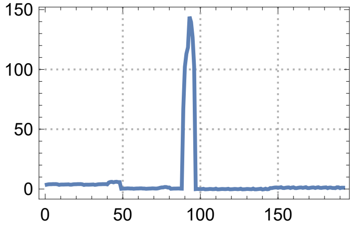

Dataset Index= 1369

Molecule Formula= $C_{25}H_{32}N_3O_4^+$

3D Autocorrelation

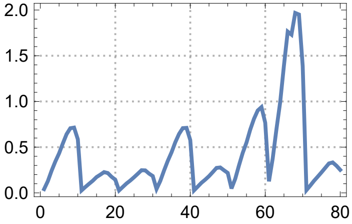

2D Plot

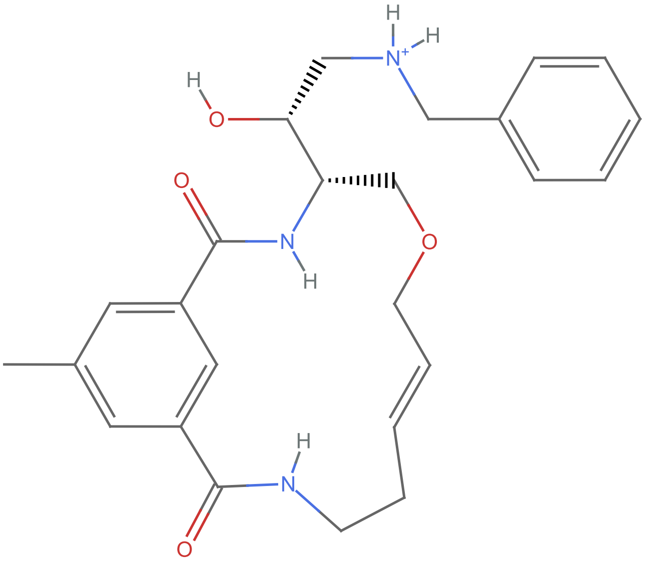

3D Plot

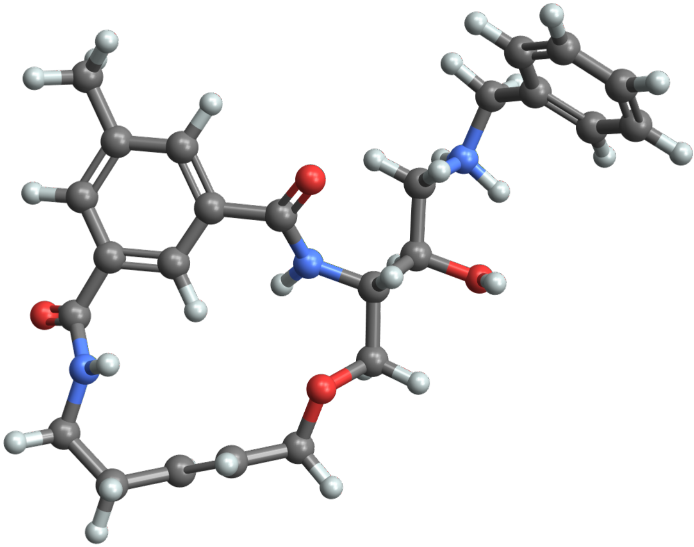

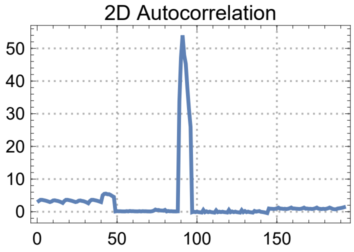

Dataset Index= 1370

Molecule Formula= $C_{18}H_{20}N_2O_2$

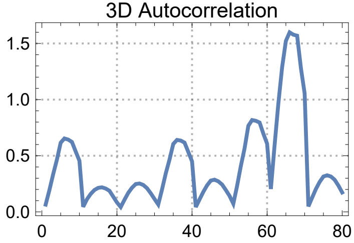

2D Plot

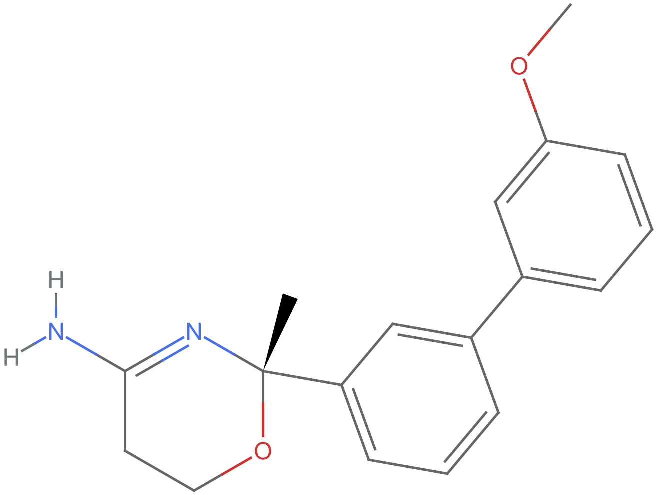

3D Plot

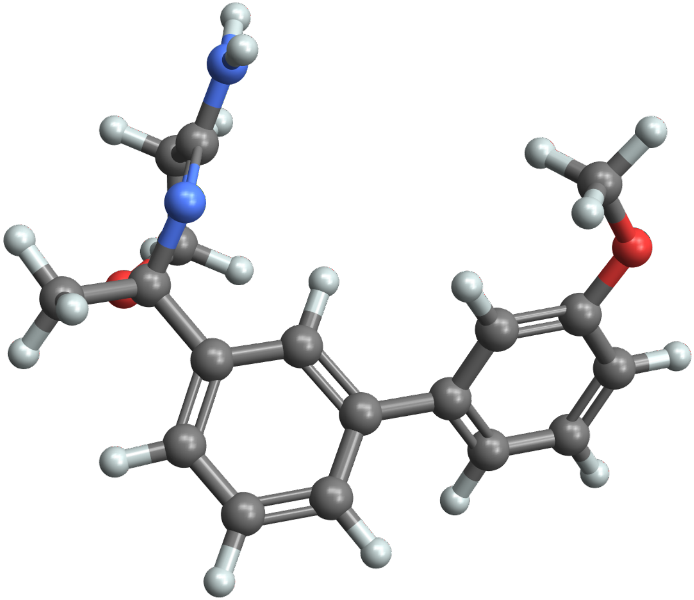

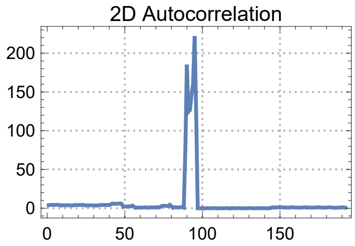

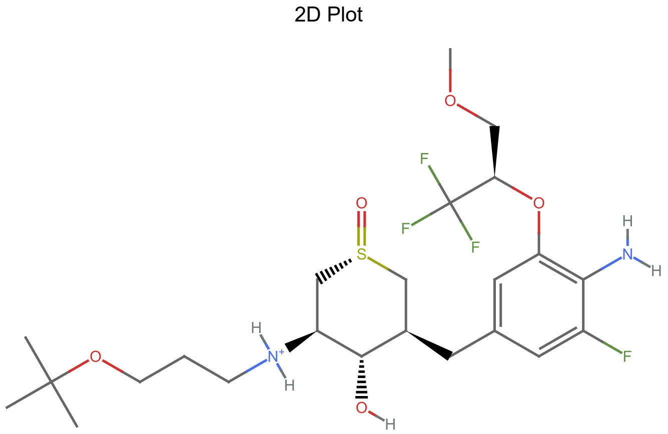

Dataset Index= 1371

Molecule Formula= $C_{23}H_{37}F_4N_2O_5S^+$

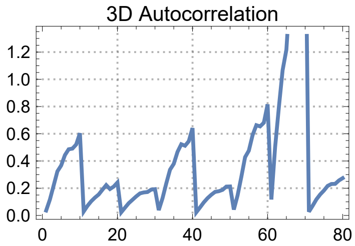

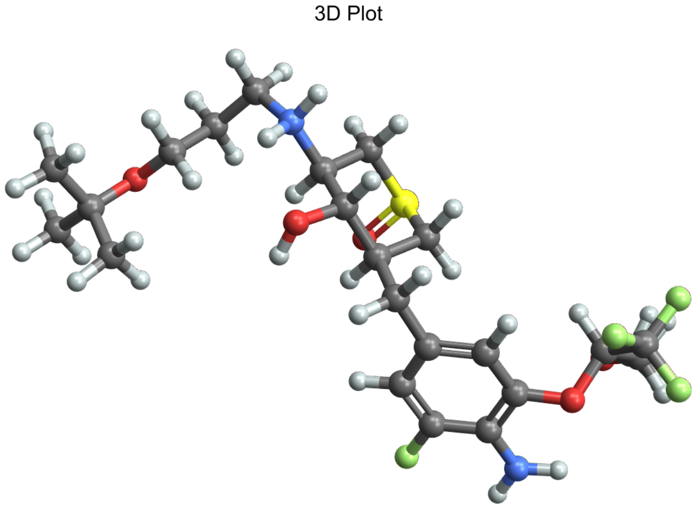

2D Autocorrelation

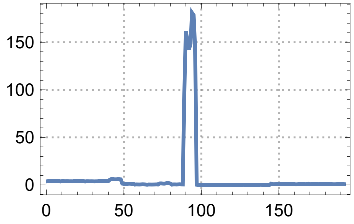

Dataset Index= 1372

Molecule Formula= $C_{28}H_{37}FN_4O_4S^{2+}$

3D Autocorrelation

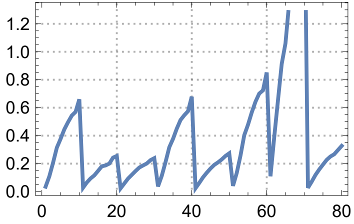

2D Plot

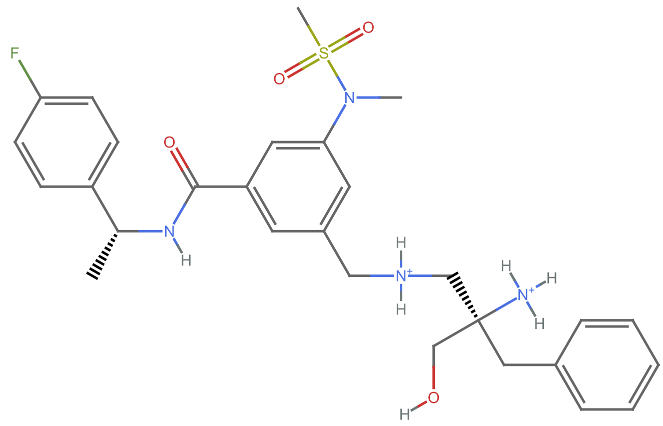

3D Plot

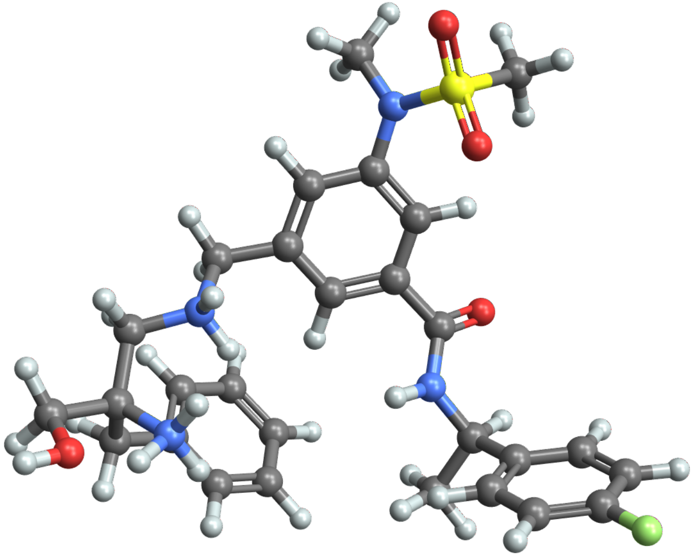

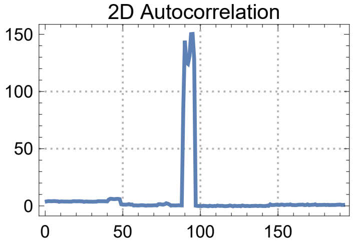

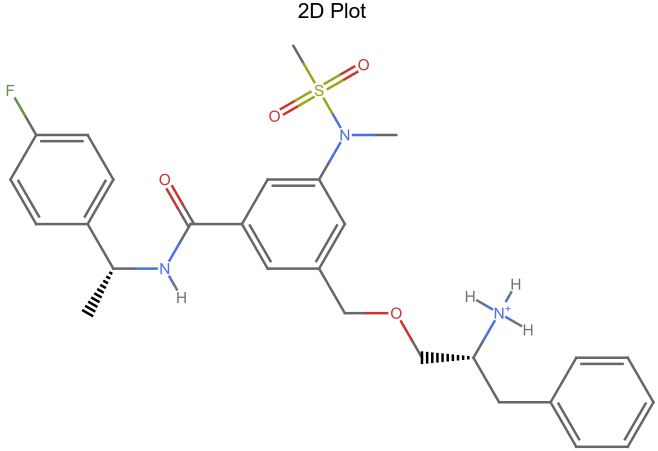

Dataset Index= 1373

Molecule Formula= $C_{27}H_{33}FN_3O_4S^+$

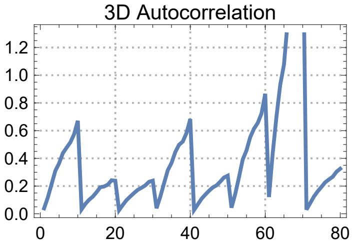

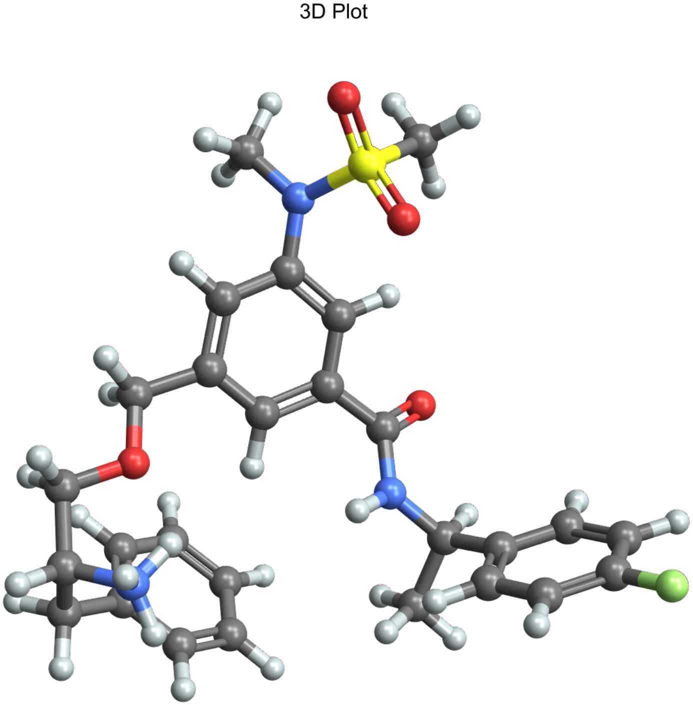

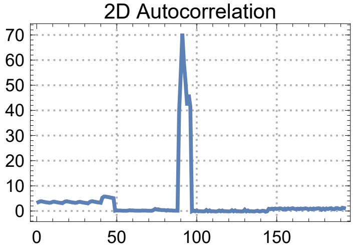

Dataset Index= 1374

Molecule Formula= $C_{22}H_{23}N_3O_2$

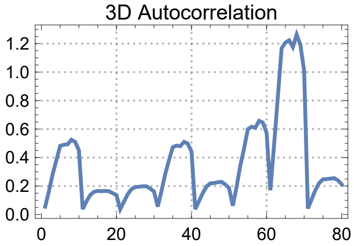

2D Plot

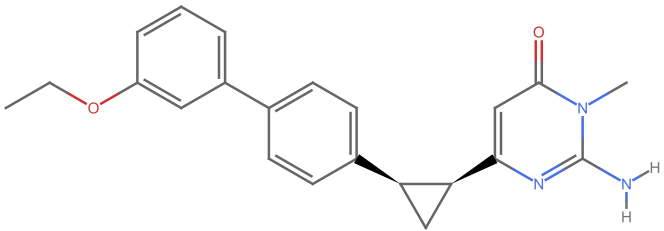

3D Plot

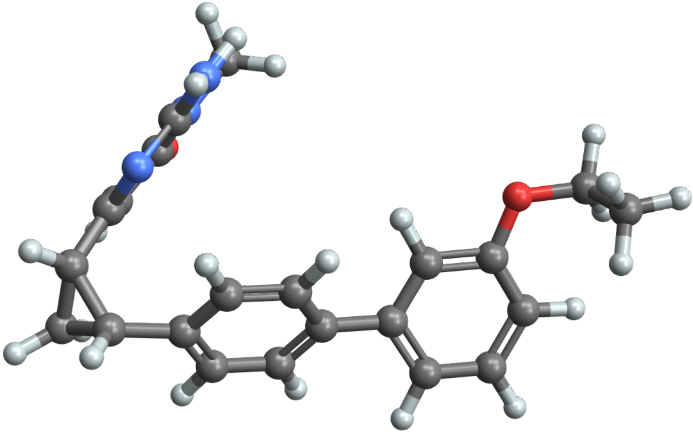

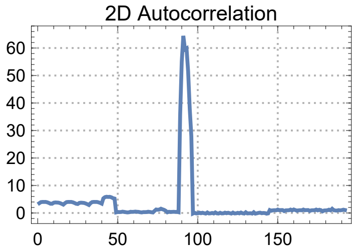

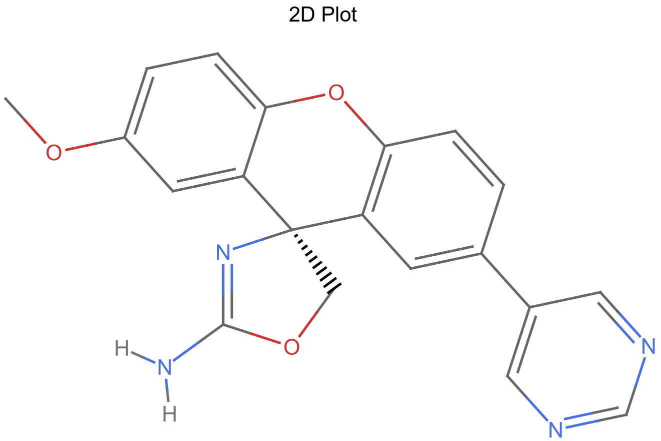

Dataset Index= 1375

Molecule Formula= $C_{20}H_{16}N_4O_3$

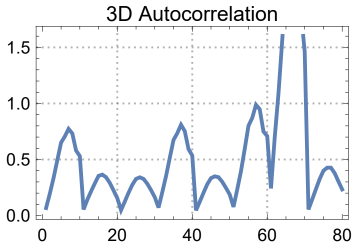

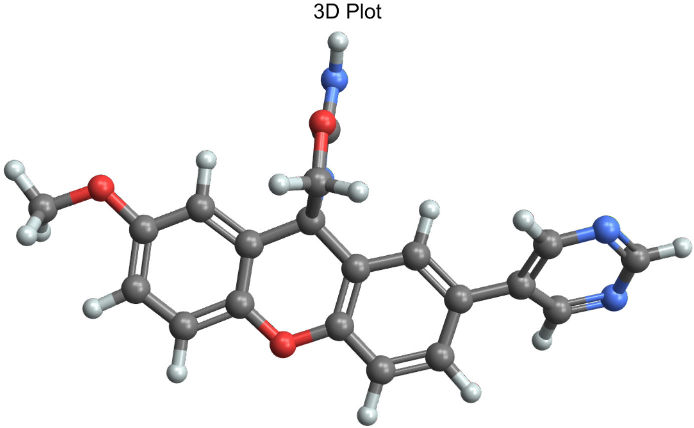

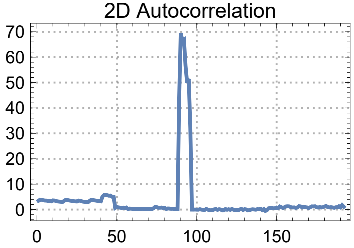

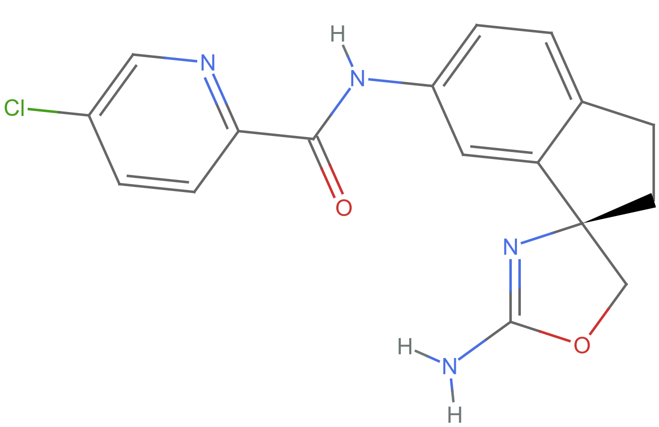

Dataset Index= 1376

Molecule Formula= $C_{17}H_{15}ClN_4O_2$

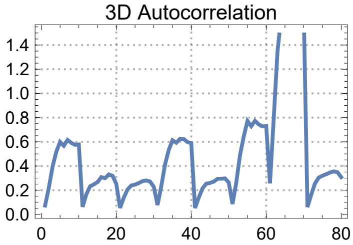

3D Plot

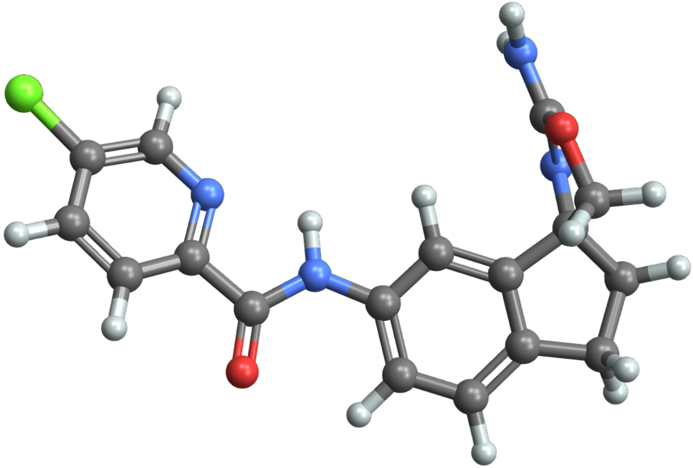

2D Plot

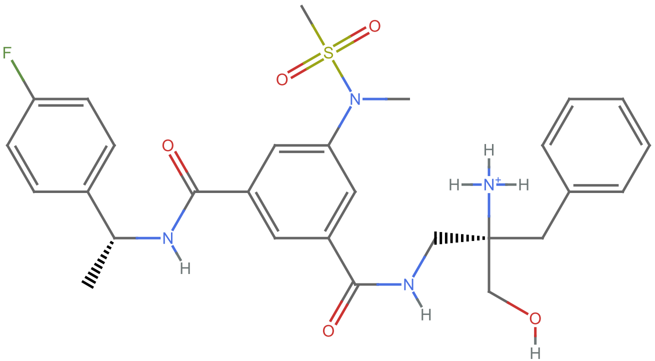

3D Plot

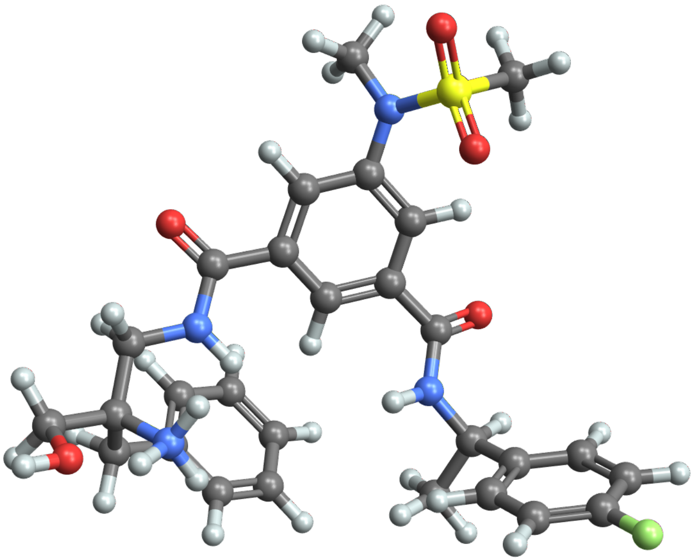

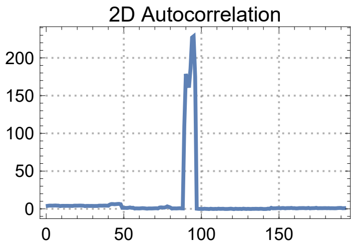

Dataset Index= 1377

Molecule Formula= $C_{28}H_{34}FN_4O_5S^+$

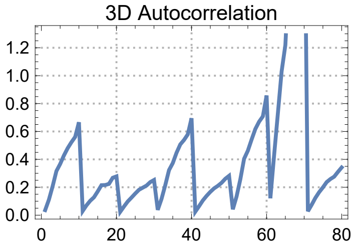

2D Plot

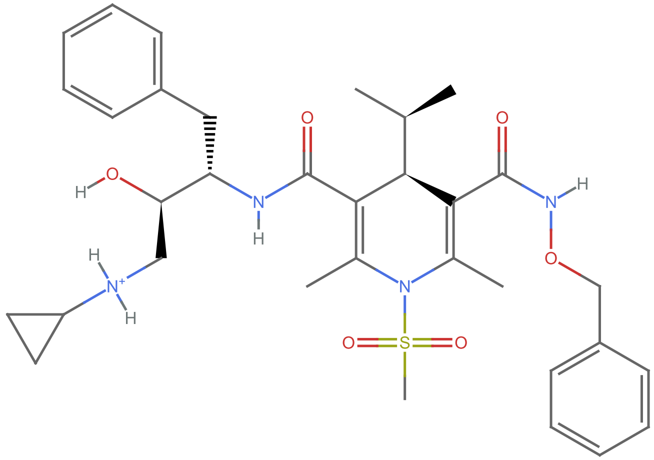

2D Autocorrelation

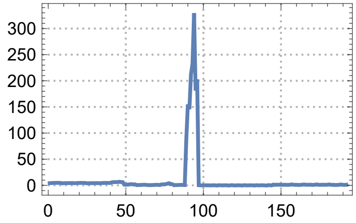

3D Plot

Dataset Index= 1378

Molecule Formula= $C_{33}H_{45}N_4O_6S^+$

3D Autocorrelation

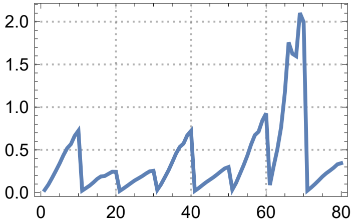

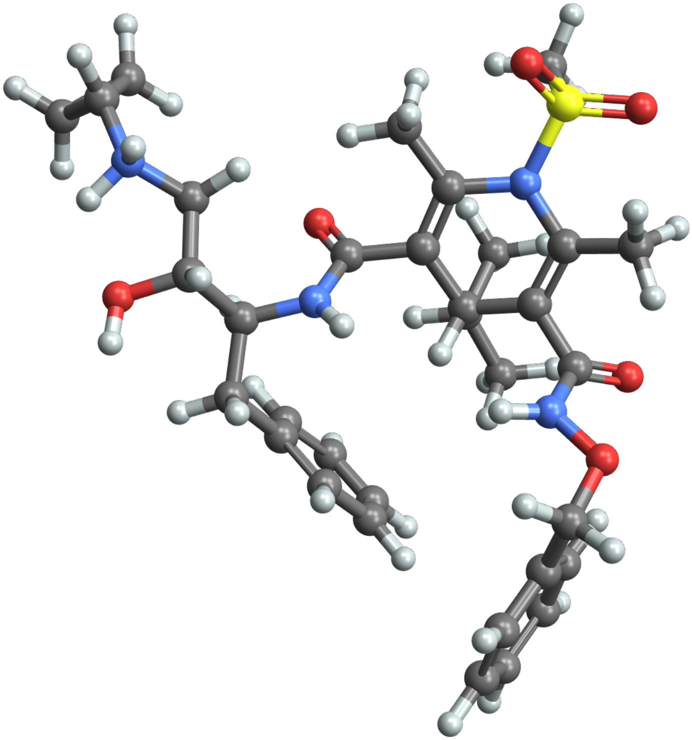

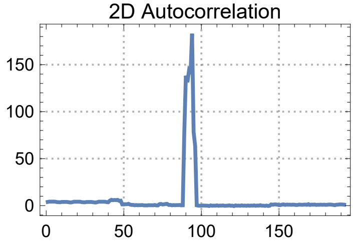

Dataset Index= 1379

Molecule Formula= $C_{25}H_{33}FN_3O_3S^+$

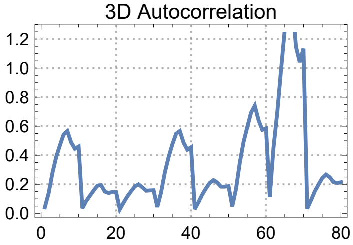

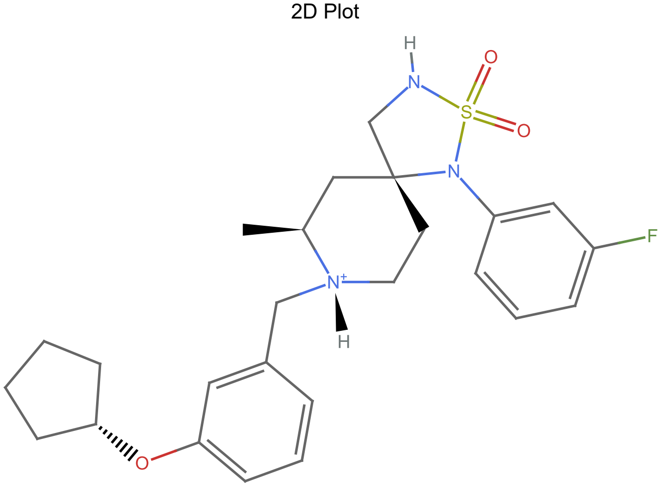

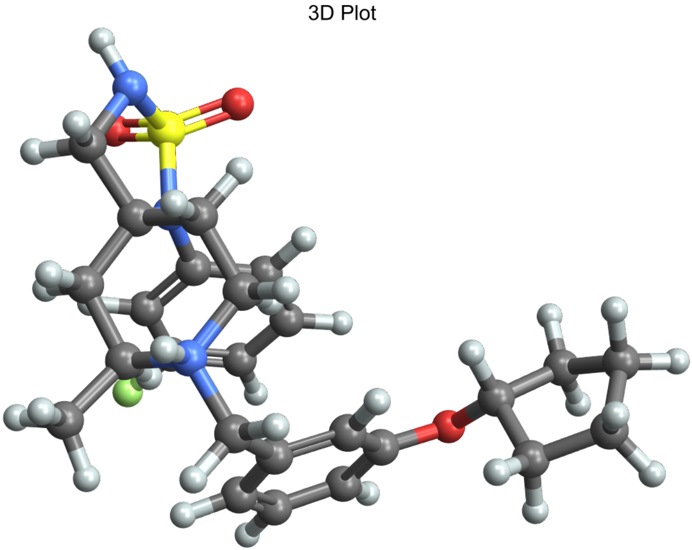

2D Autocorrelation

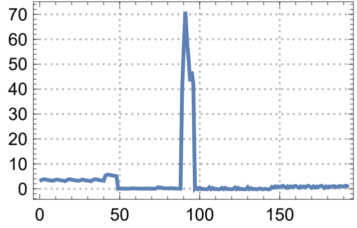

Dataset Index= 1380

Molecule Formula= $C_{23}H_{25}N_3O$

3D Autocorrelation

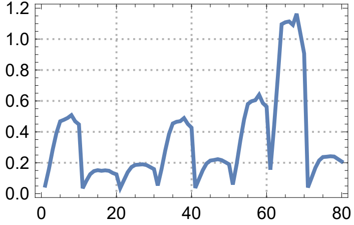

2D Plot

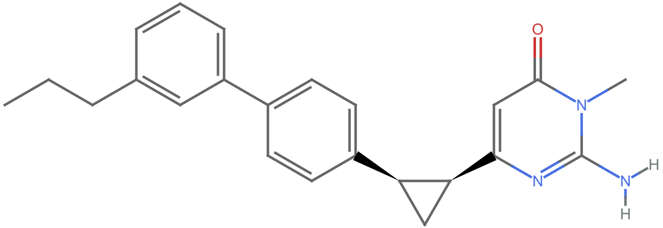

3D Plot

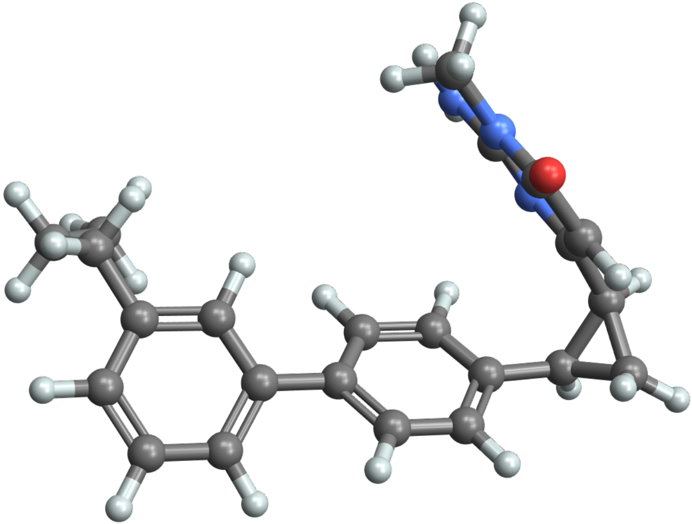

2D Plot

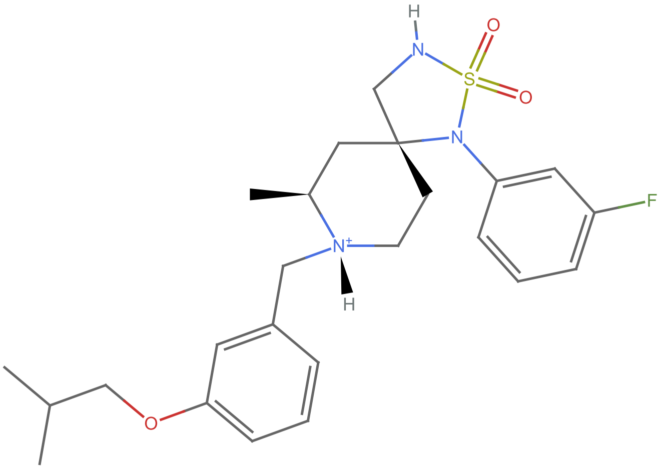

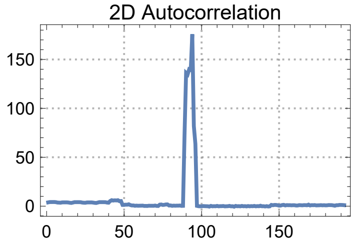

Dataset Index= 1381

Molecule Formula= $C_{24}H_{33}FN_3O_3S^+$

3D Plot

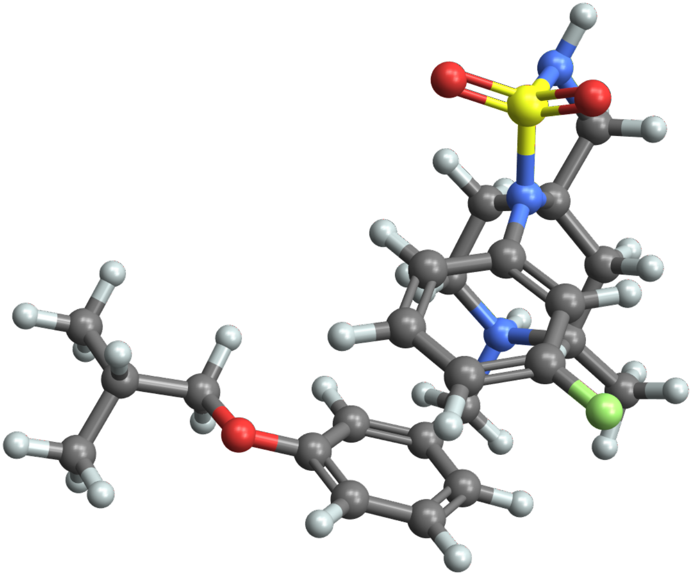

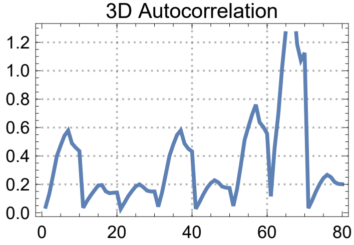

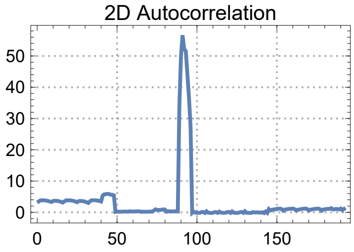

Dataset Index= 1382

Molecule Formula= $C_{20}H_{18}N_4O_2$

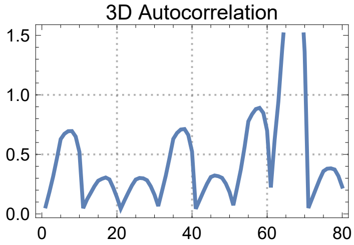

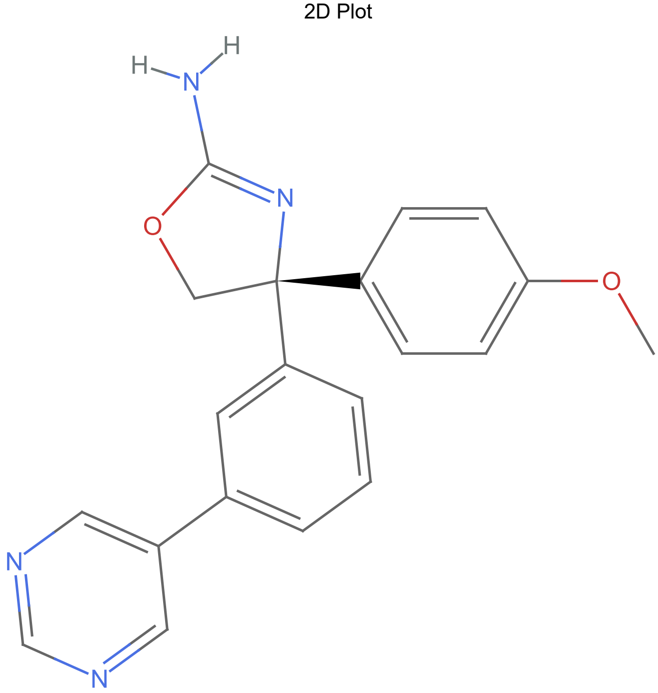

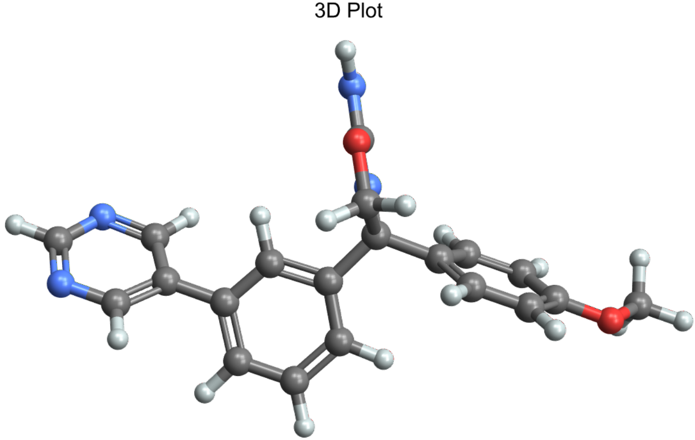

2D Autocorrelation

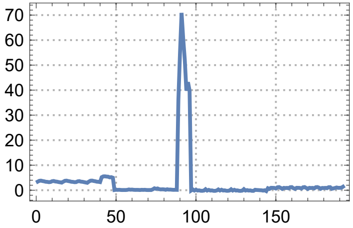

Dataset Index= 1383

Molecule Formula= $C_{20}H_{22}N_4O$

3D Autocorrelation

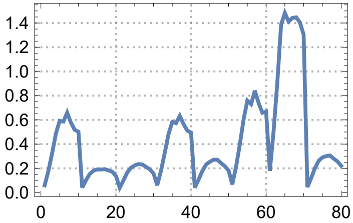

2D Plot

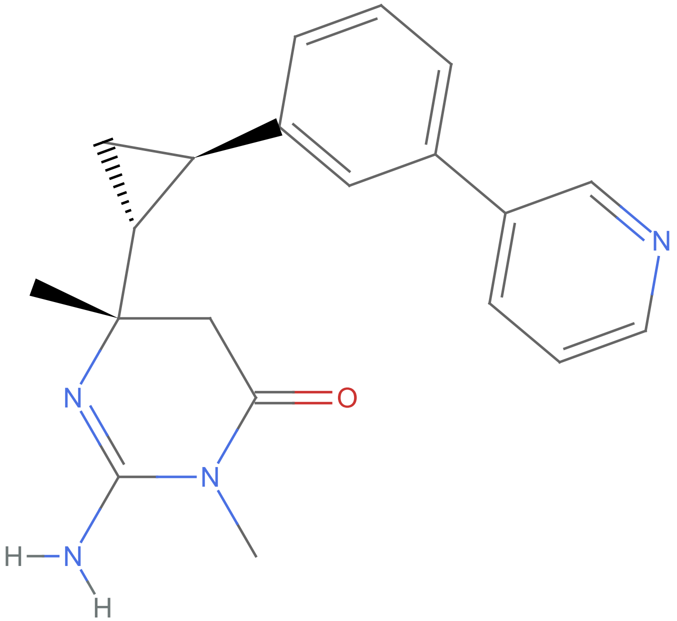

3D Plot

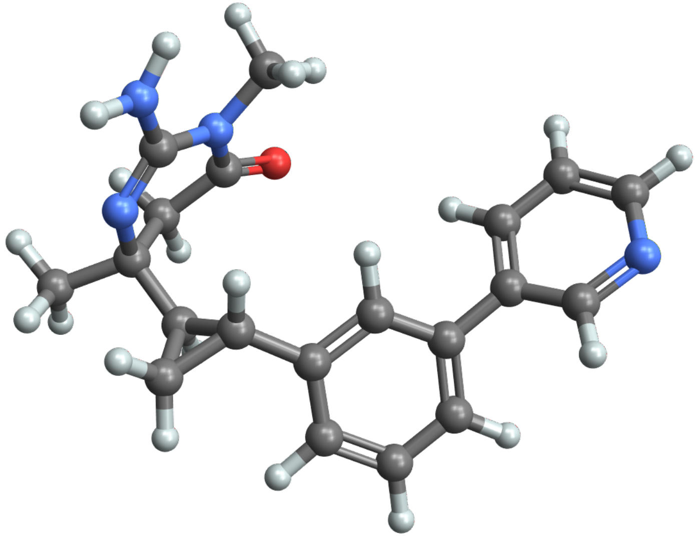

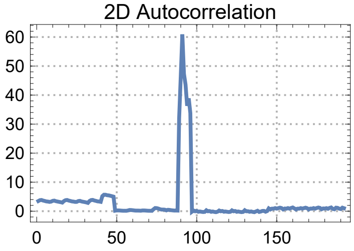

Dataset Index= 1384

Molecule Formula= $C_{19}H_{19}N_5O_2$

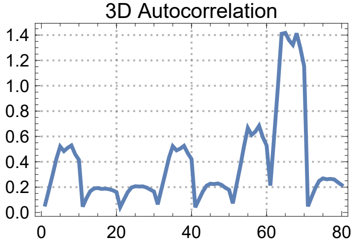

2D Plot

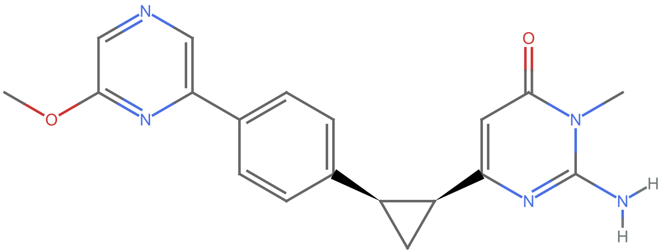

3D Plot

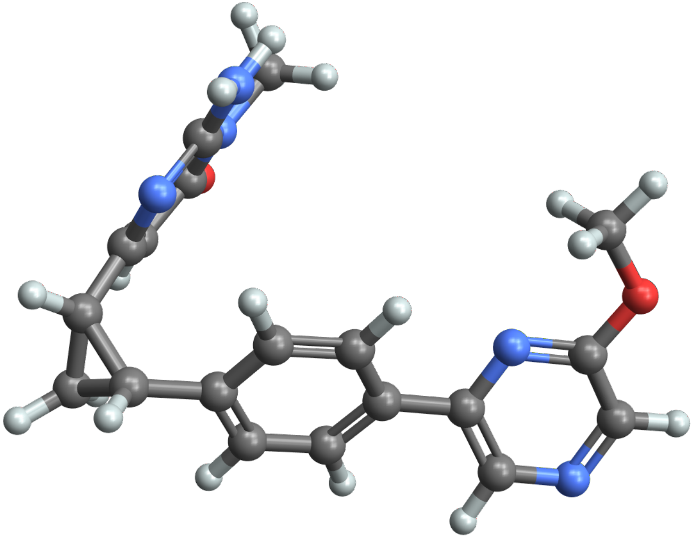

2D Plot

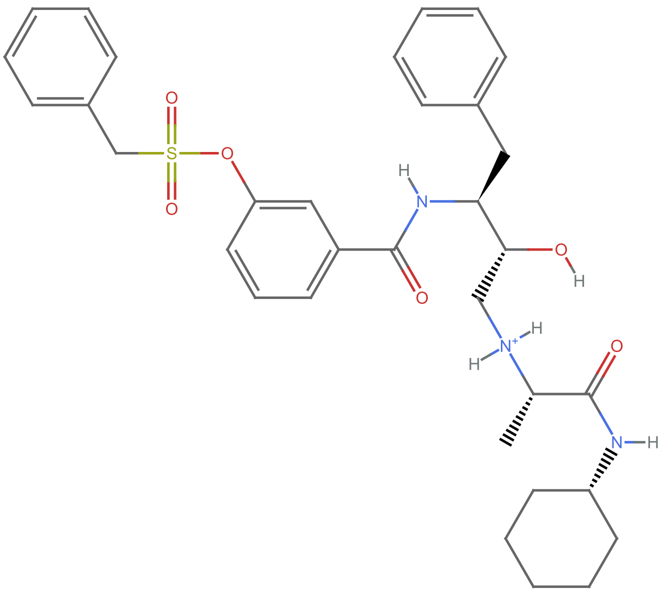

2D Autocorrelation

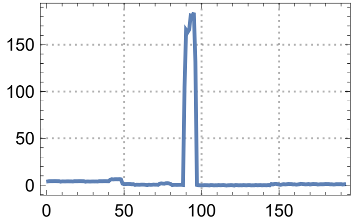

Dataset Index= 1385

Molecule Formula= $C_{33}H_{42}N_3O_6S^+$

3D Autocorrelation

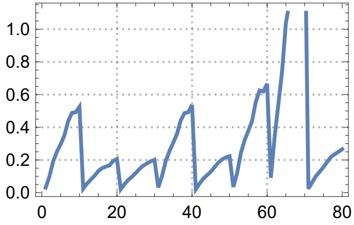

3D Plot

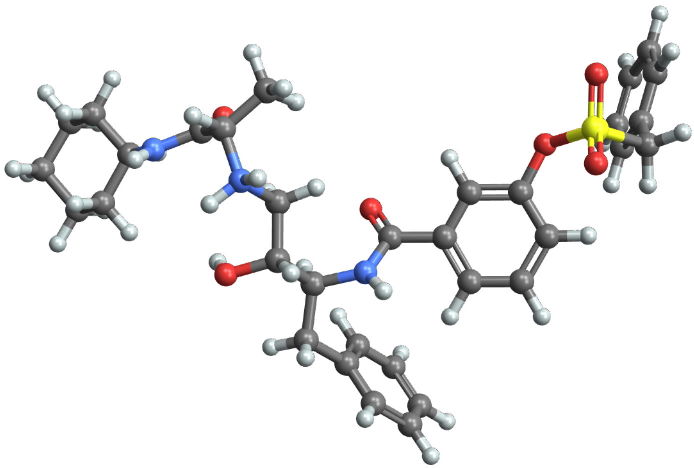

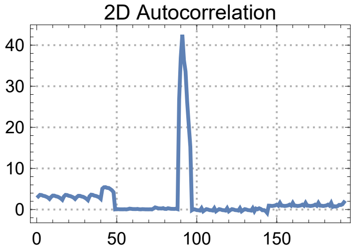

Dataset Index= 1386

Molecule Formula= $C_{16}H_{16}N_4$

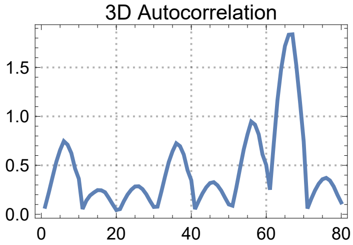

2D Plot

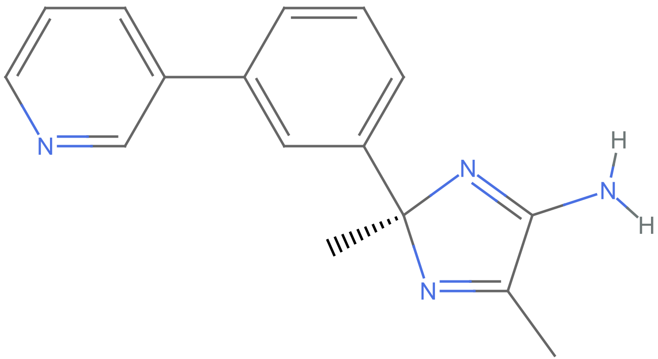

3D Plot

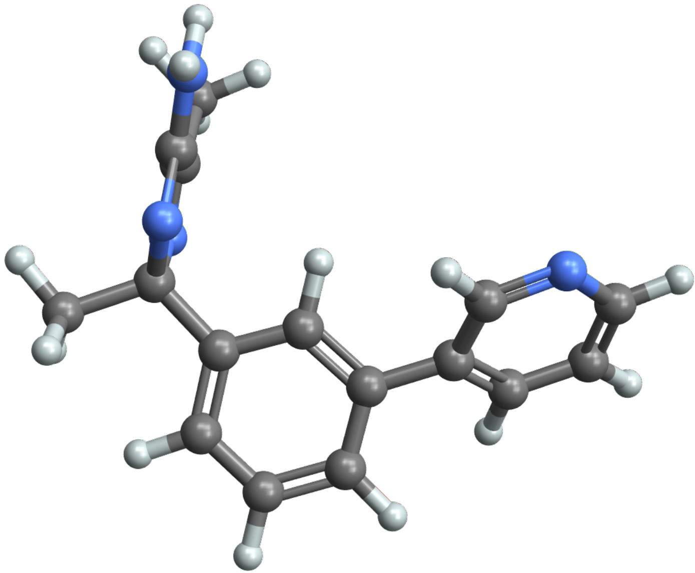

2D Plot

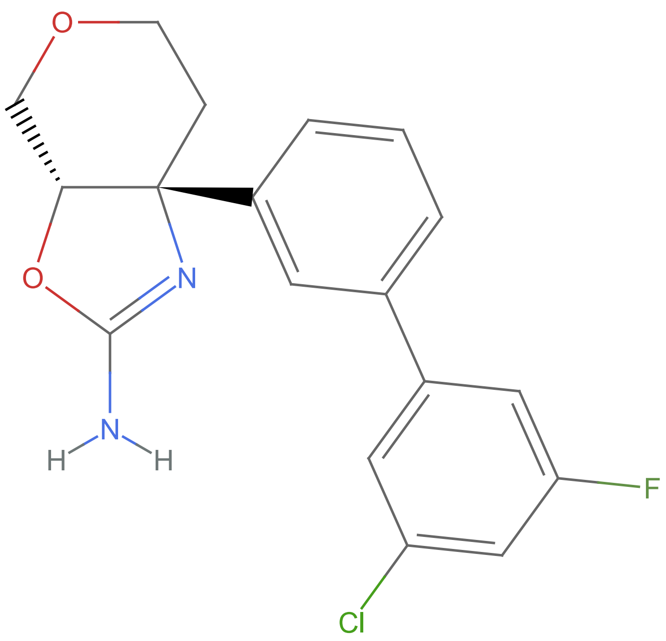

2D Autocorrelation

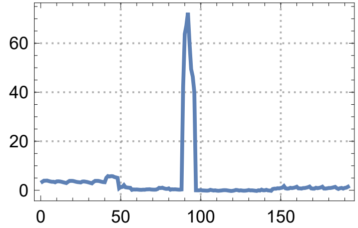

Dataset Index= 1387

Molecule Formula= $C_{18}H_{16}ClFN_2O_2$

3D Autocorrelation

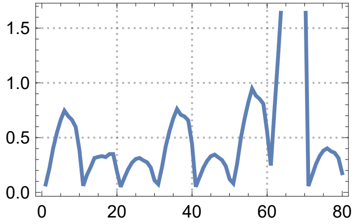

3D Plot

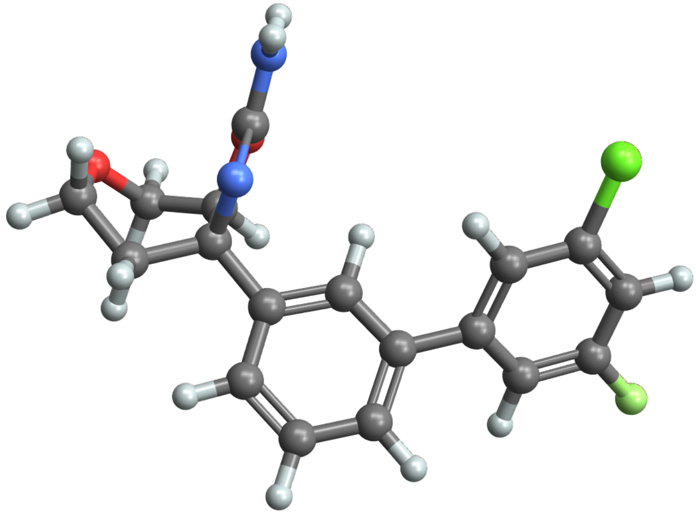

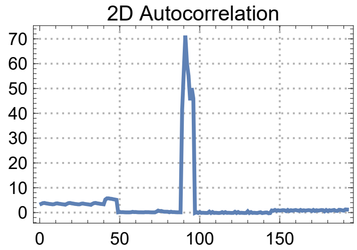

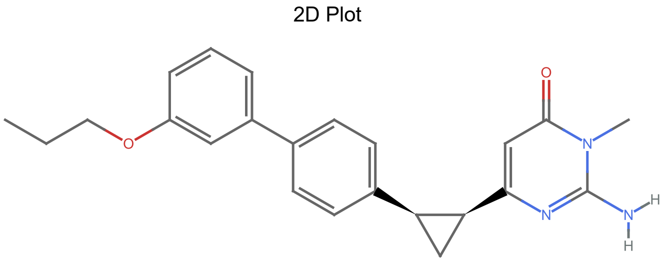

Dataset Index= 1388

Molecule Formula= $C_{23}H_{25}N_3O_2$

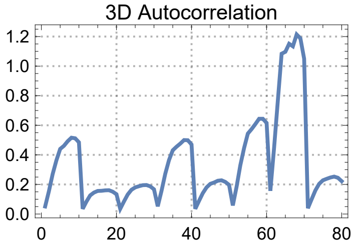

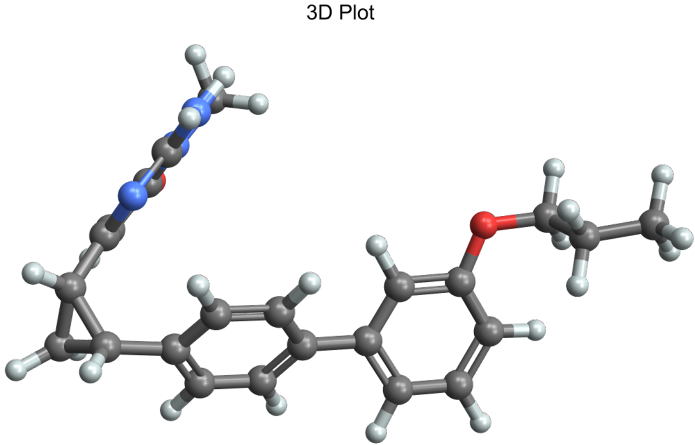

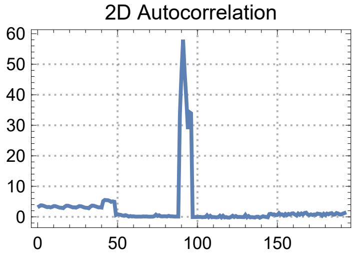

Dataset Index= 1389

Molecule Formula= $C_{18}H_{17}N_3OS$

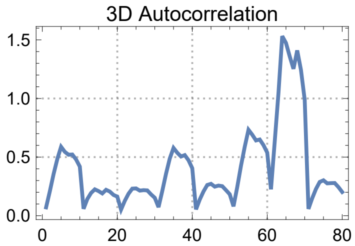

2D Plot

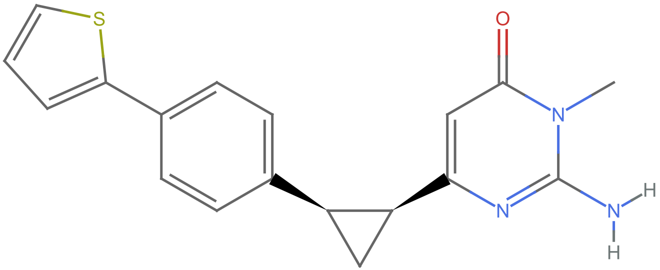

3D Plot

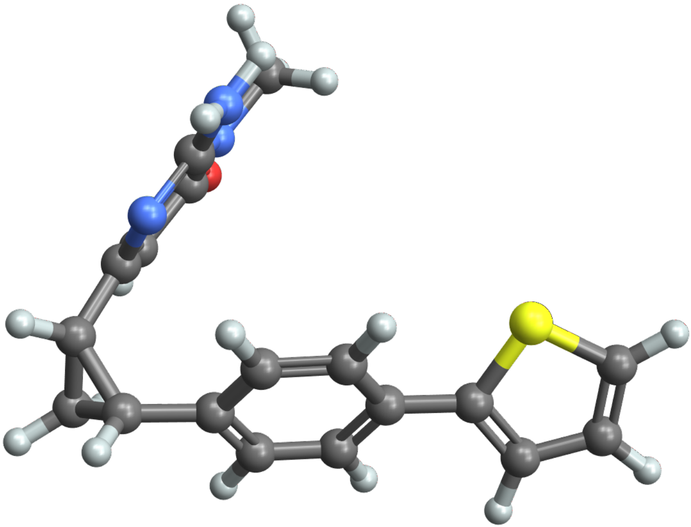

2D Autocorrelation

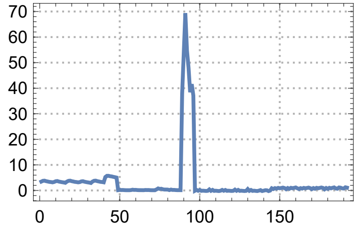

Dataset Index= 1390

Molecule Formula= $C_{21}H_{21}N_3O_2$

3D Autocorrelation

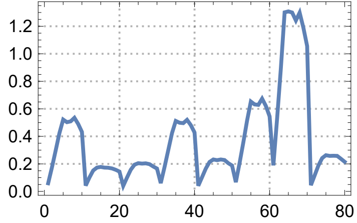

2D Plot

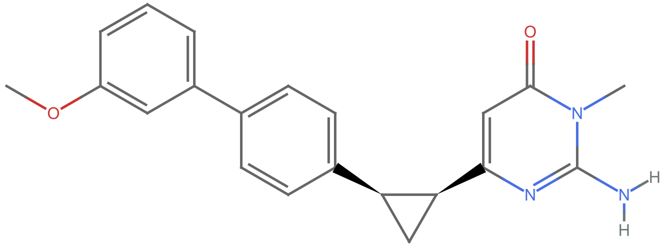

3D Plot

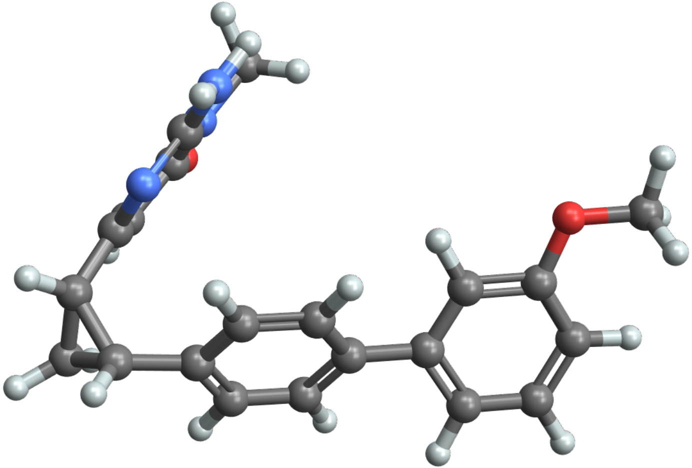

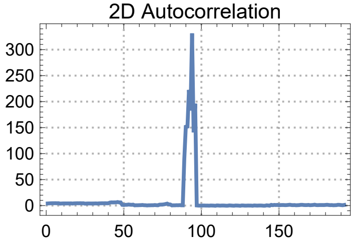

Dataset Index= 1391

Molecule Formula= $C_{31}H_{41}N_4O_6S^+$

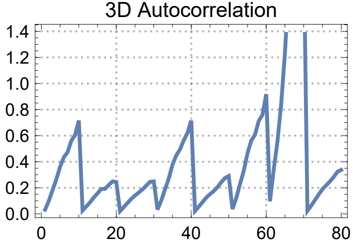

2D Plot

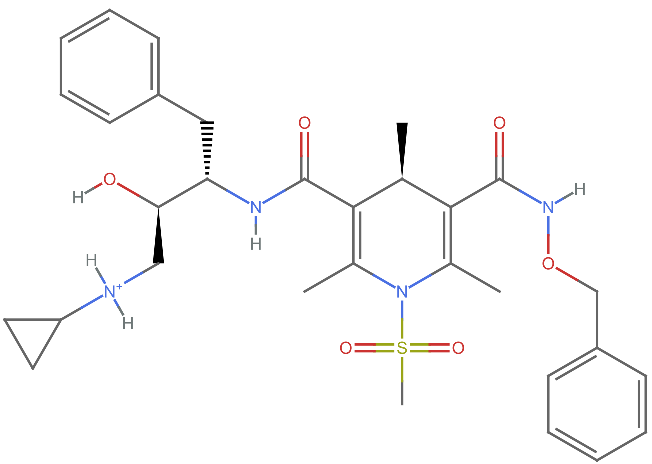

3D Plot

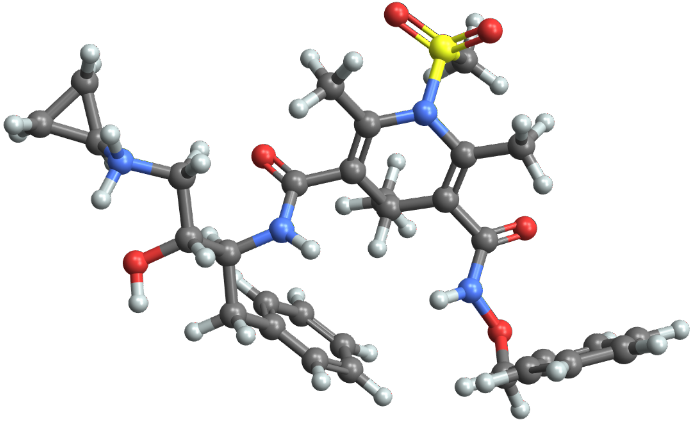

2D Autocorrelation

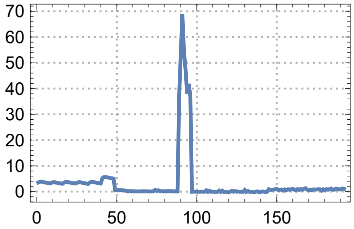

Dataset Index= 1392

Molecule Formula= $C_{21}H_{21}N_3OS$

3D Autocorrelation

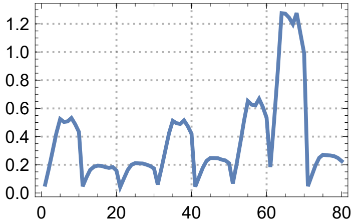

2D Plot

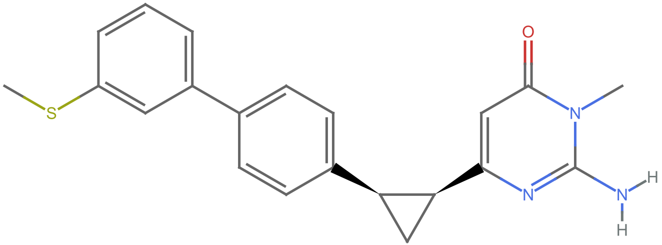

3D Plot

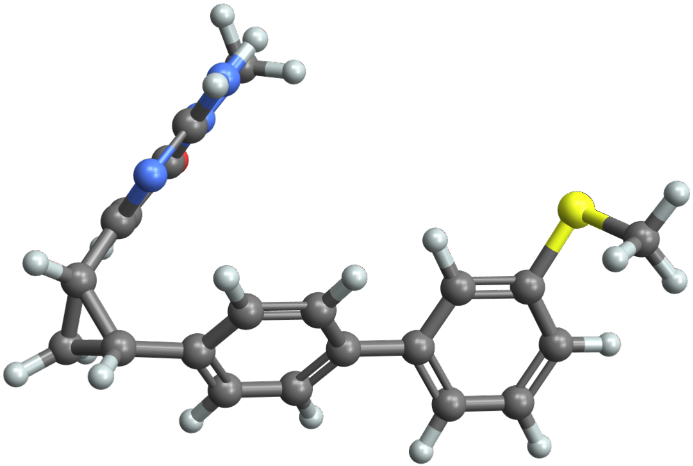

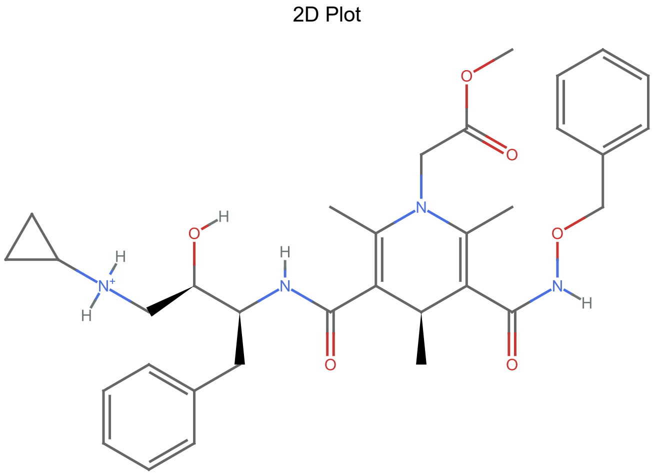

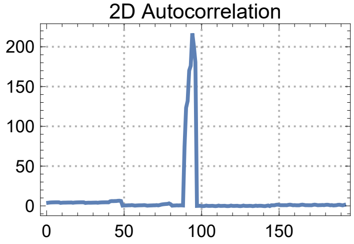

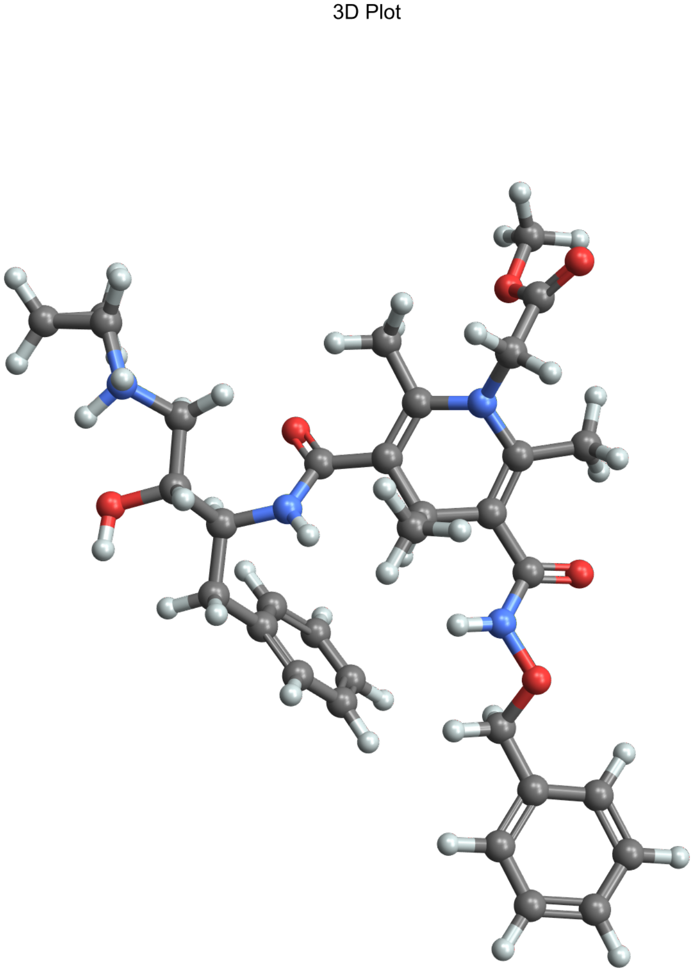

Dataset Index= 1393

Molecule Formula= $C_{33}H_{43}N_4O_6^+$

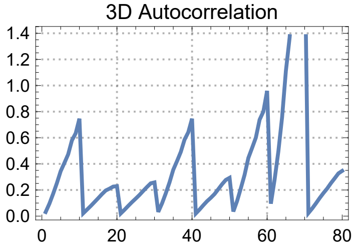

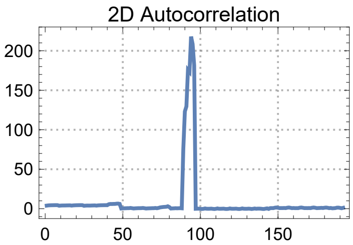

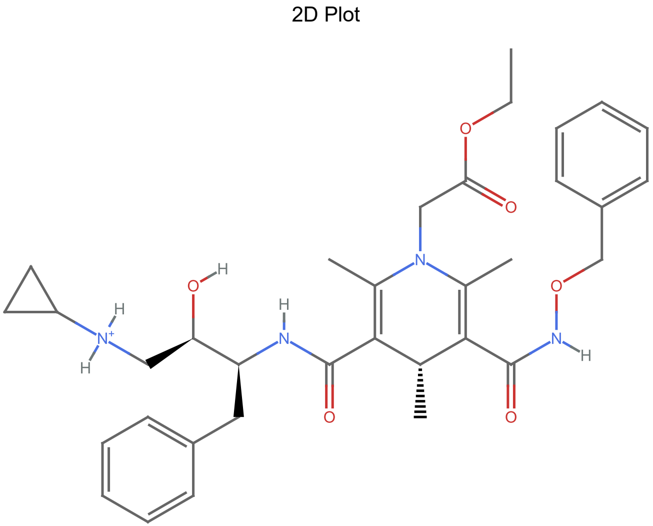

Dataset Index= 1394

Molecule Formula= $C_{34}H_{45}N_4O_6^+$

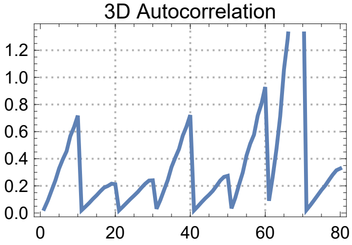

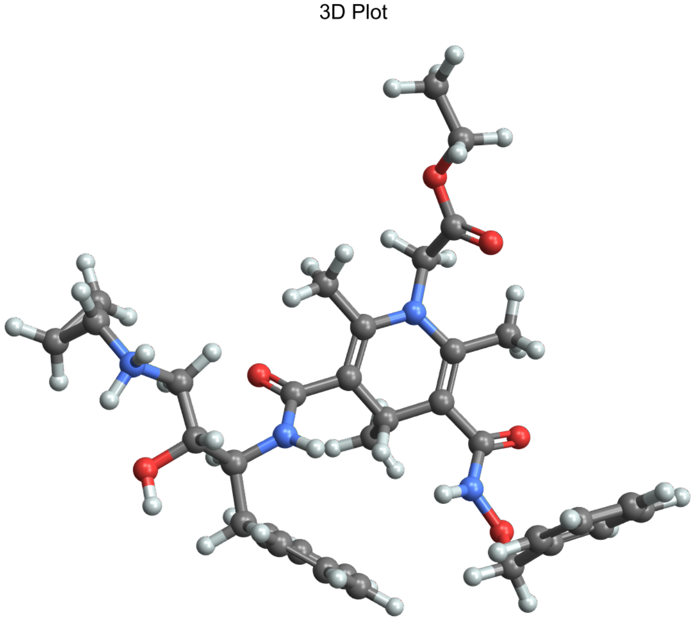

2D Plot

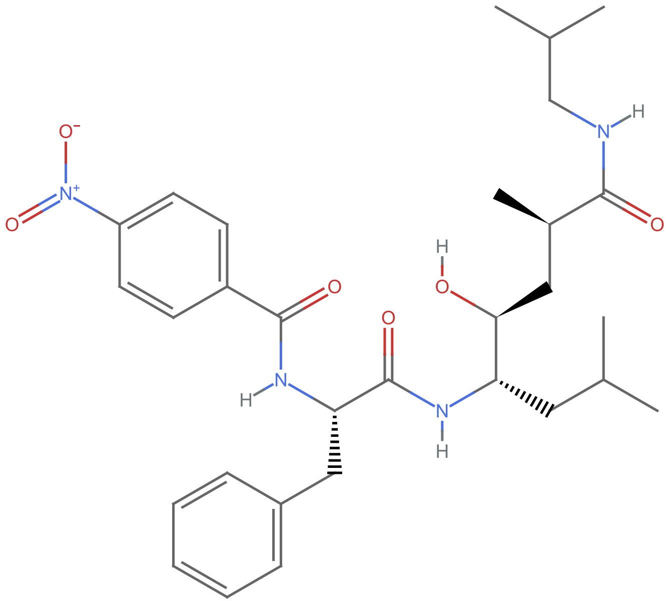

2D Autocorrelation

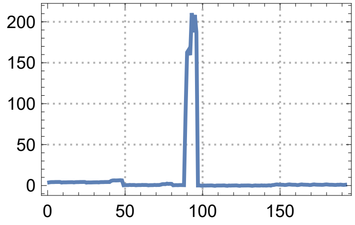

Dataset Index= 1395

Molecule Formula= $C_{30}H_{42}N_4O_6$

3D Plot

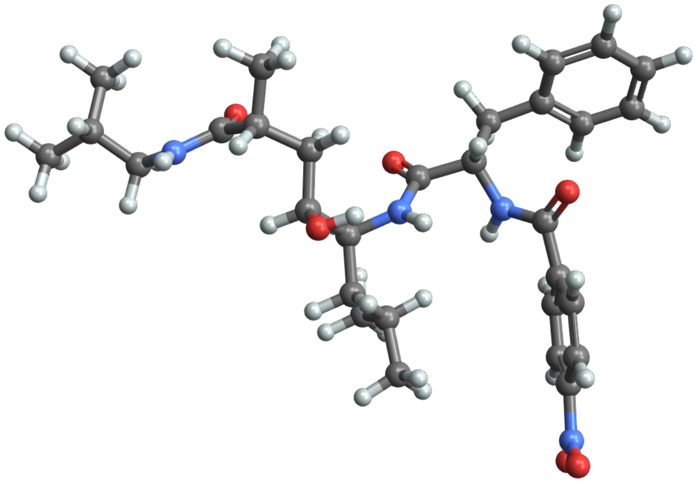

3D Autocorrelation

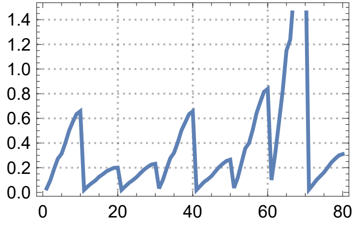

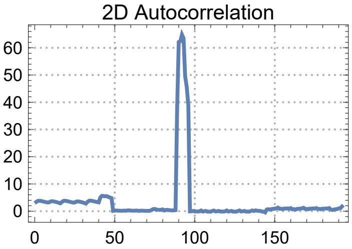

2D Plot

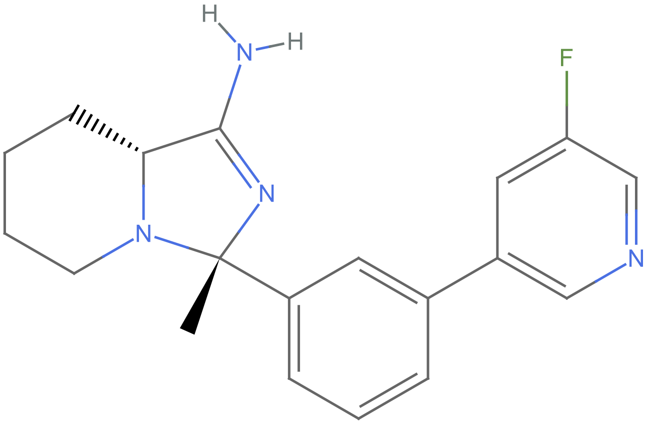

3D Plot

Dataset Index= 1396

Molecule Formula= $C_{19}H_{21}FN_4$

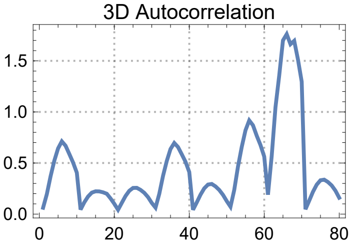

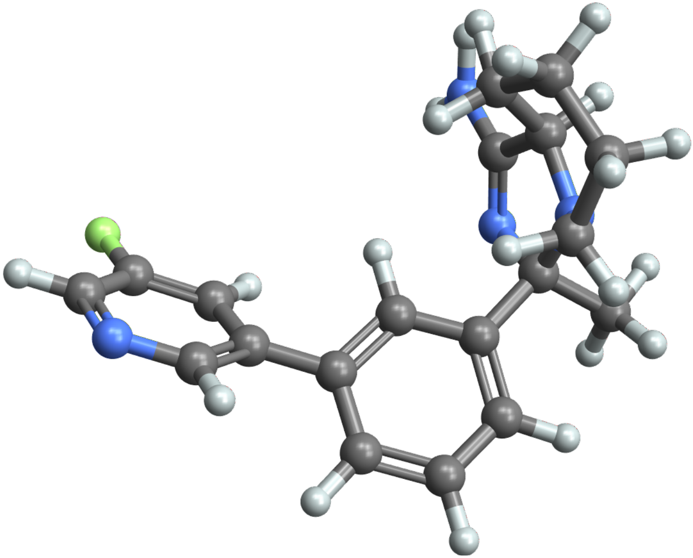

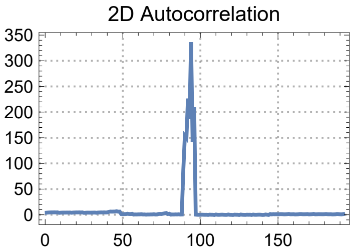

Dataset Index= 1397

Molecule Formula= $C_{32}H_{43}N_4O_5S^+$

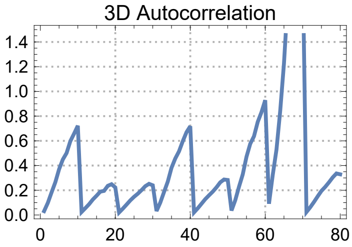

2D Plot

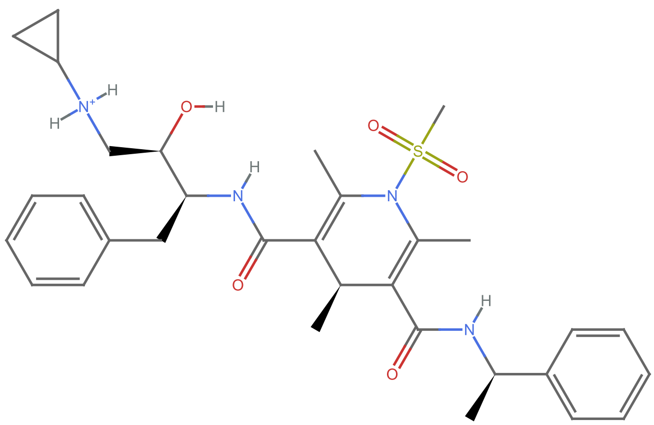

3D Plot

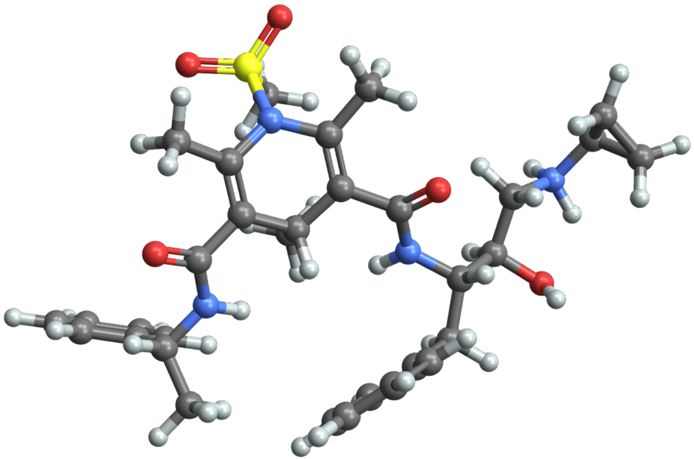

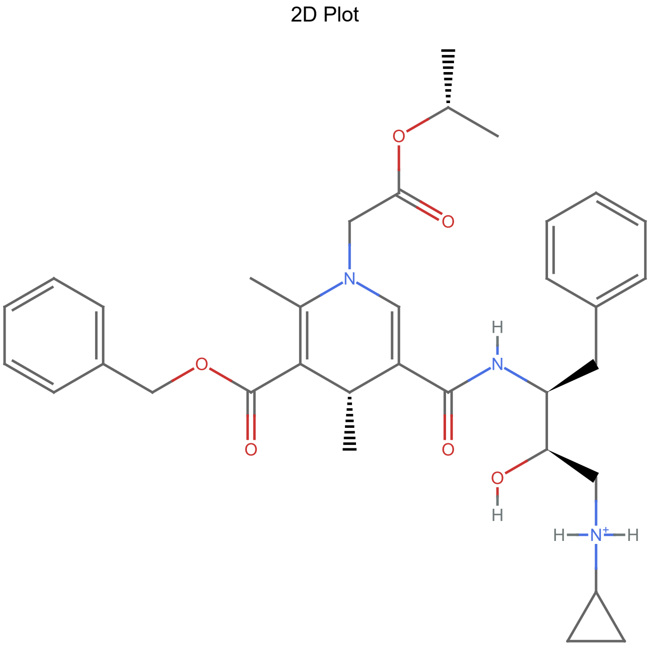

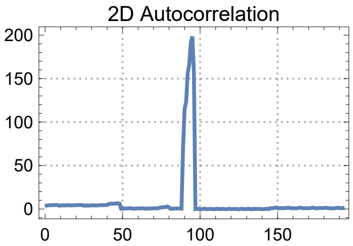

Dataset Index= 1398

Molecule Formula= $C_{34}H_{44}N_3O_6^+$

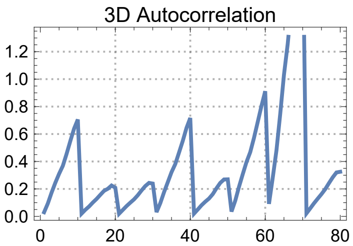

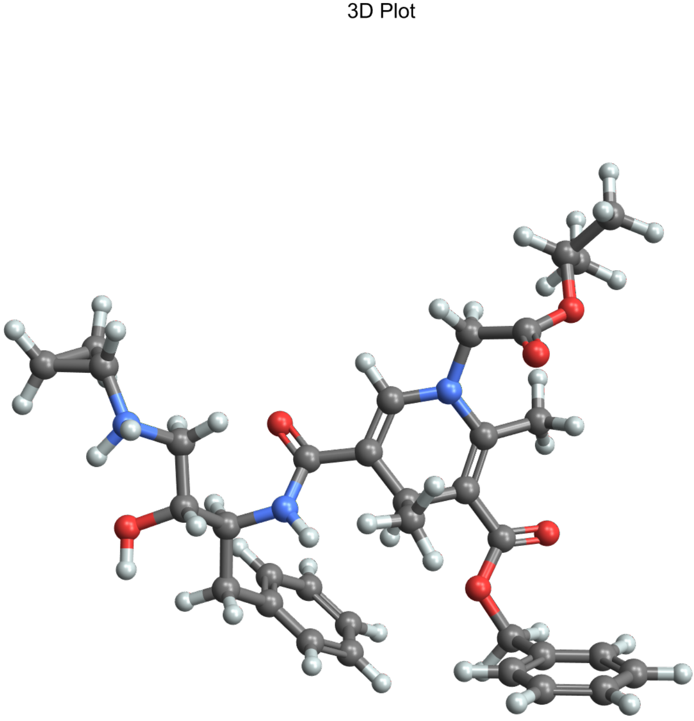

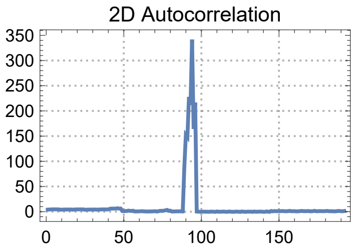

Dataset Index= 1399

Molecule Formula= $C_{33}H_{45}N_4O_5S^+$

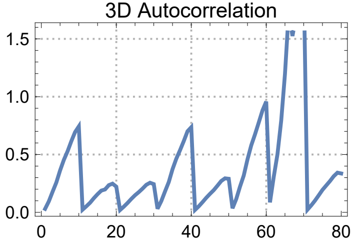

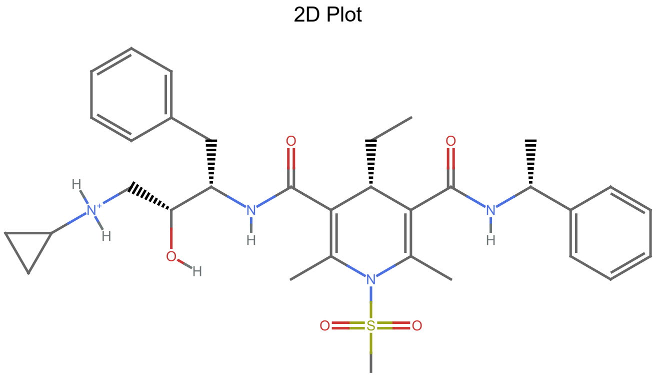

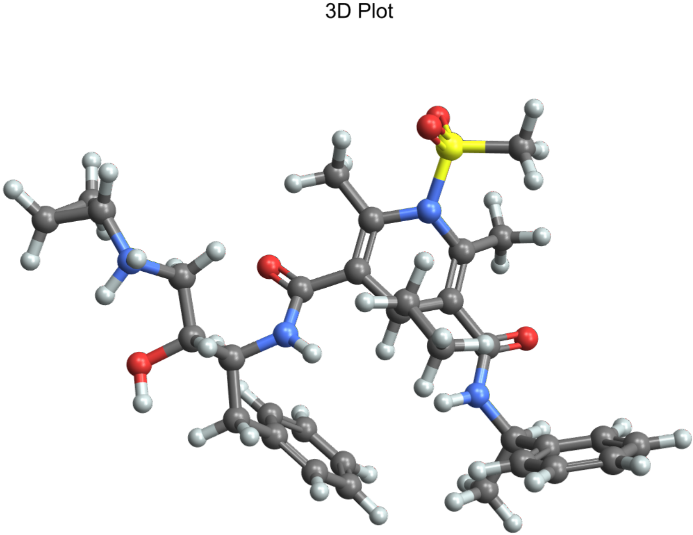

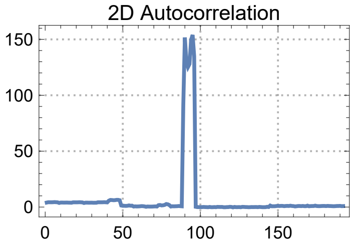

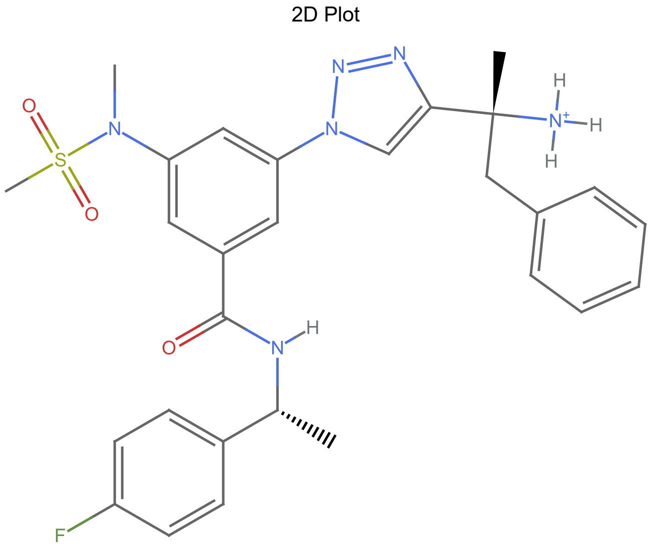

Dataset Index= 1400

Molecule Formula= $C_{28}H_{32}FN_6O_3S^+$

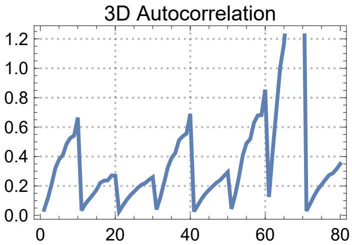

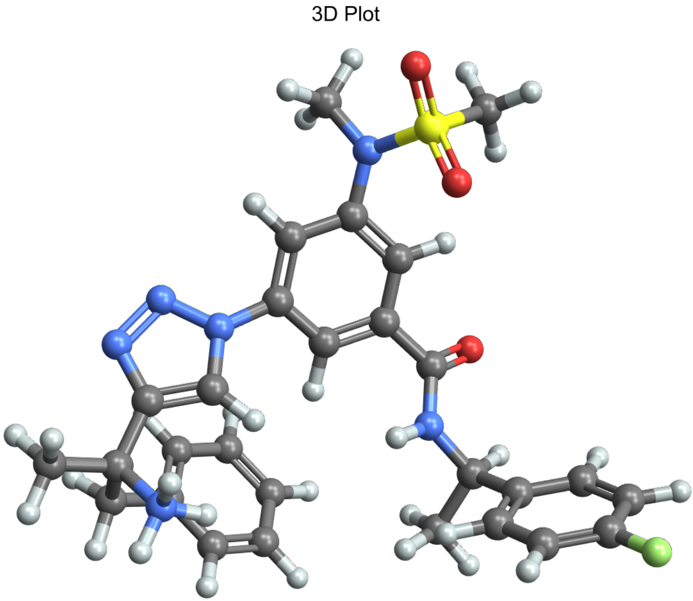

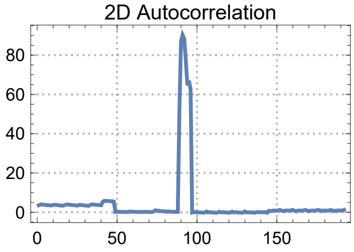

Dataset Index= 1401

Molecule Formula= $C_{23}H_{26}N_4O_2$

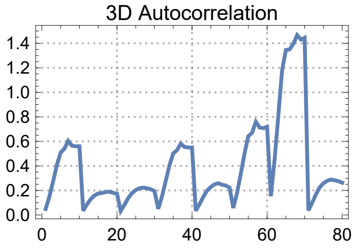

2D Plot

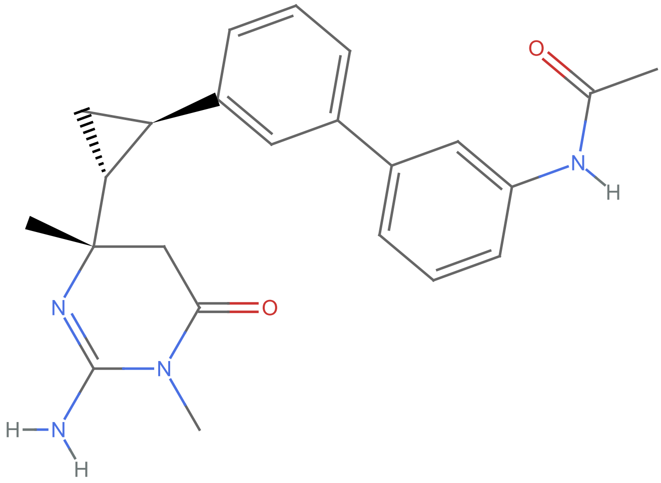

3D Plot

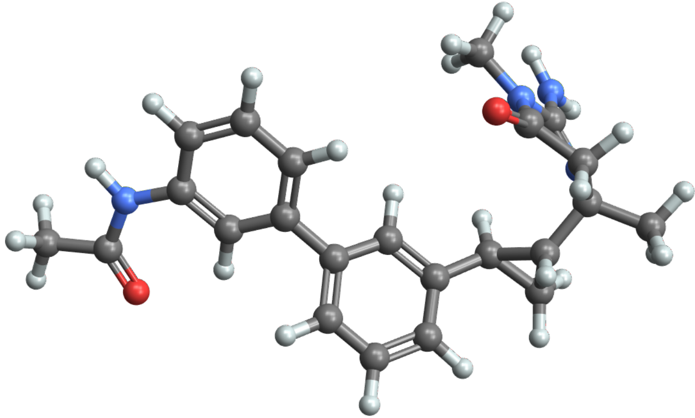

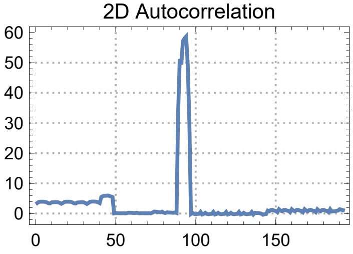

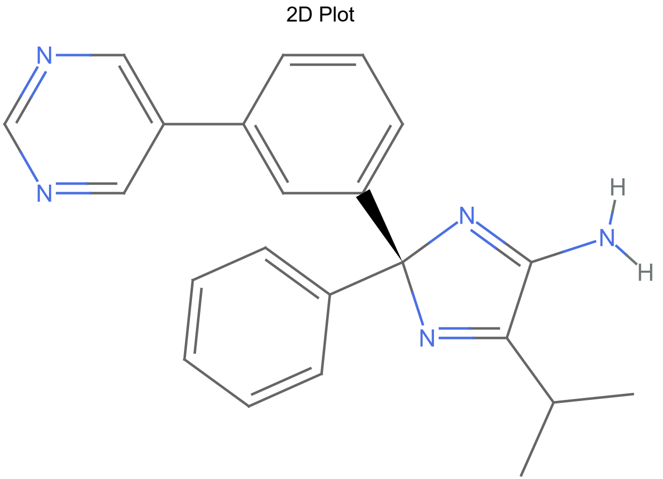

Dataset Index= 1402

Molecule Formula= $C_{22}H_{21}N_5$

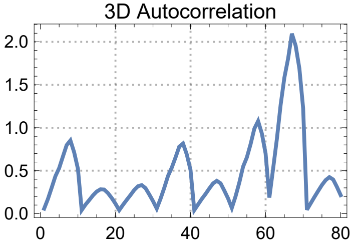

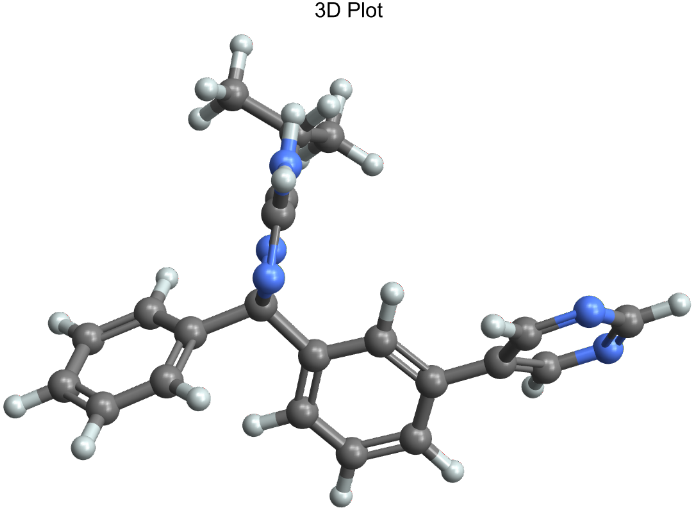

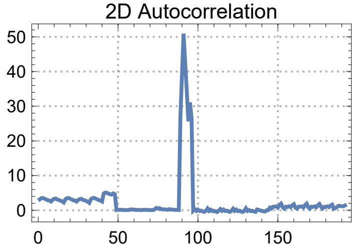

Dataset Index= 1403

Molecule Formula= $C_{15}H_{19}N_3O$

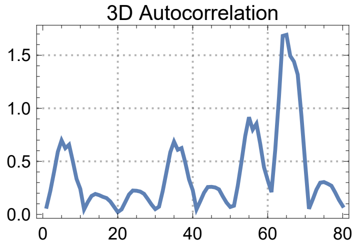

2D Plot

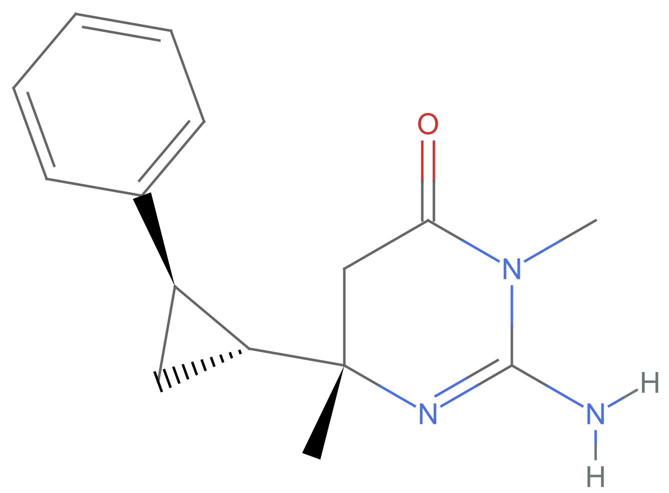

3D Plot

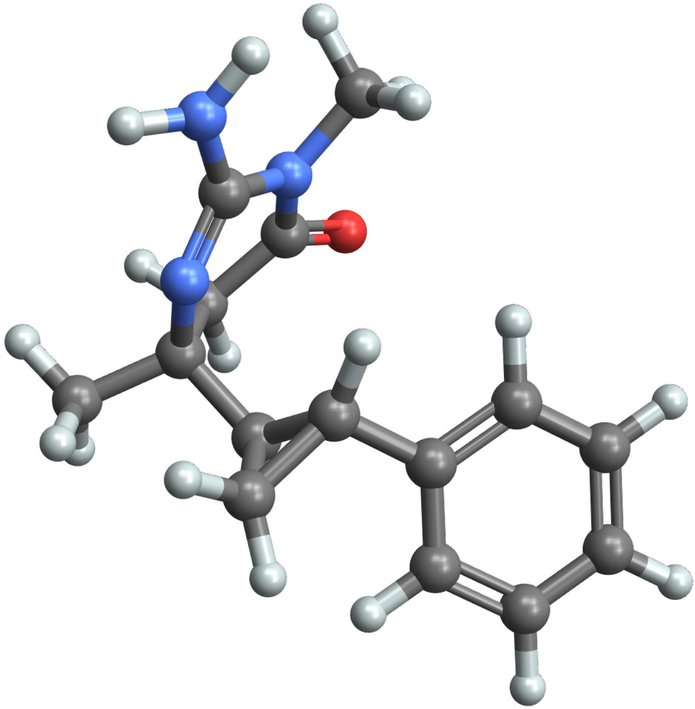

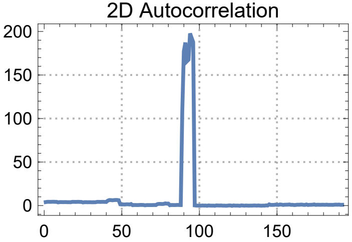

Dataset Index= 1404

Molecule Formula= $C_{28}H_{34}FN_4O_5S^+$

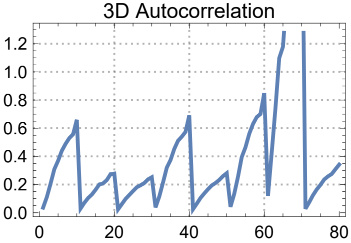

2D Plot

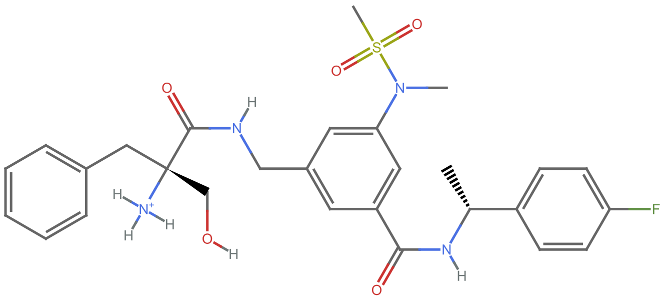

3D Plot

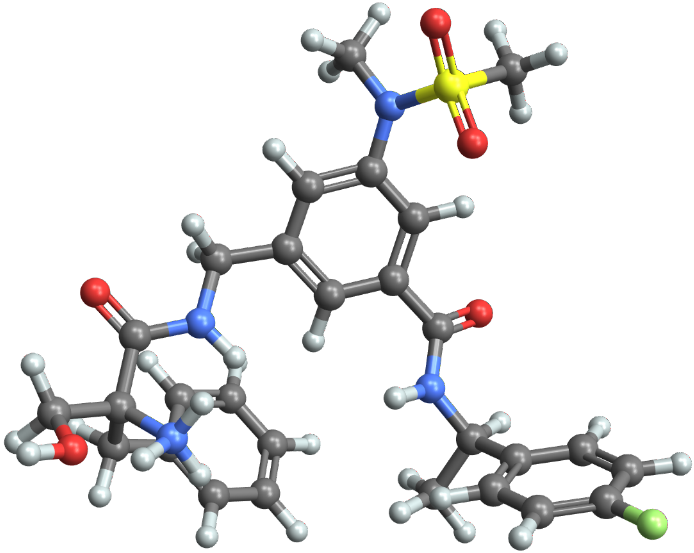

2D Plot

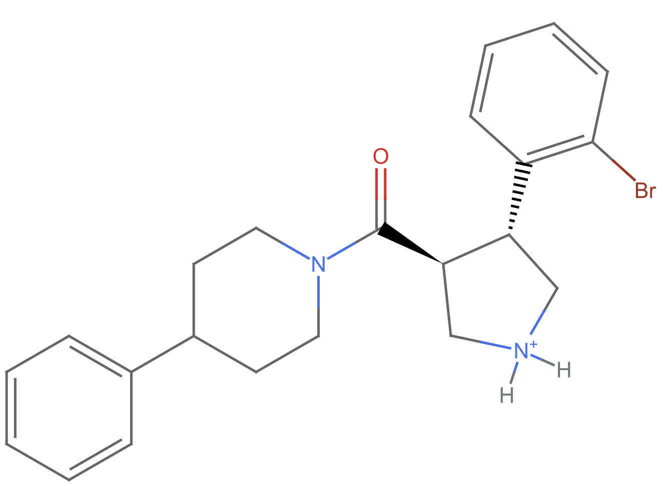

2D Autocorrelation

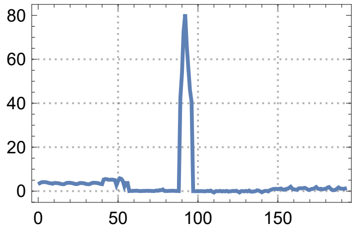

3D Plot

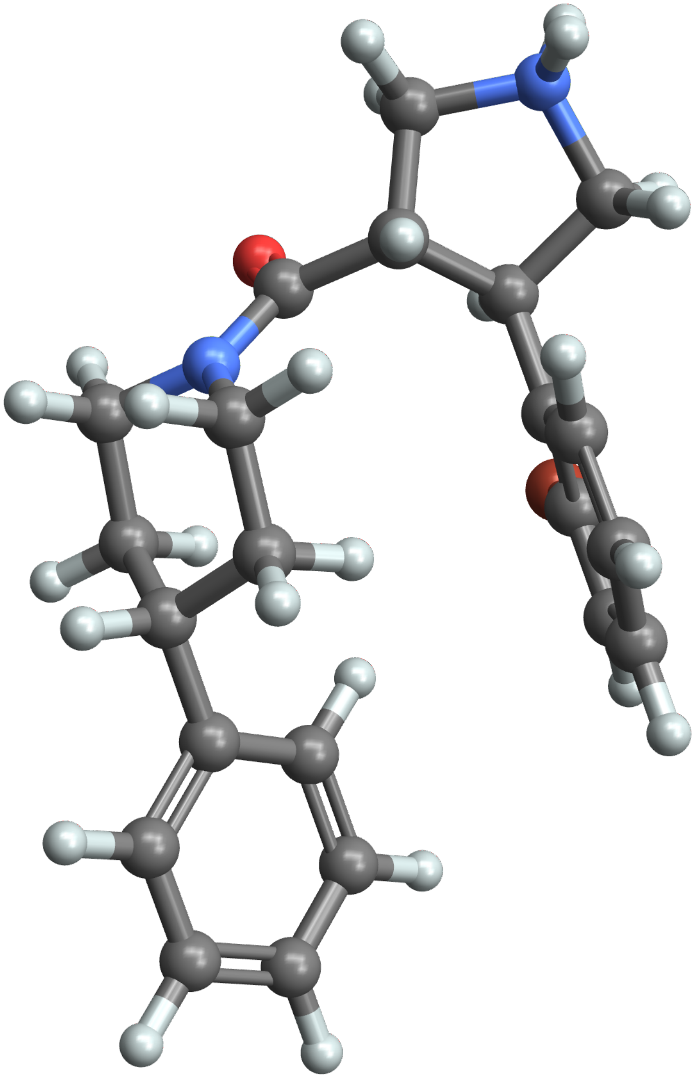

Dataset Index= 1405

Molecule Formula= $C_{22}H_{26}BrN_2O^+$

3D Autocorrelation

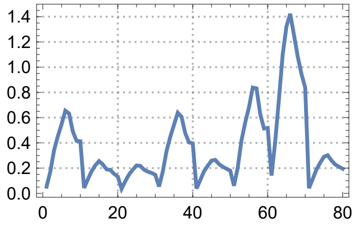

2D Plot

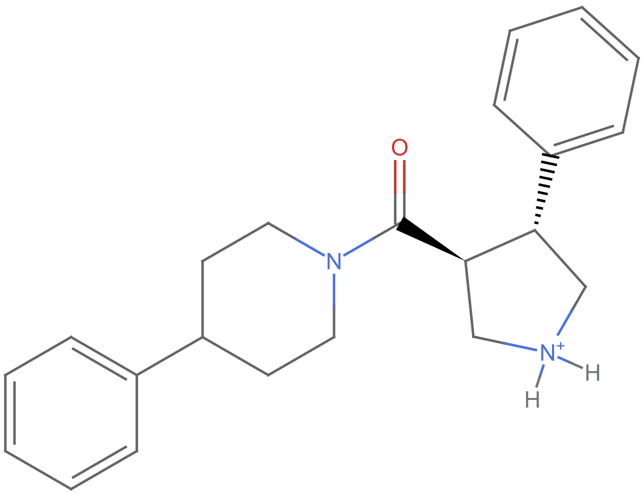

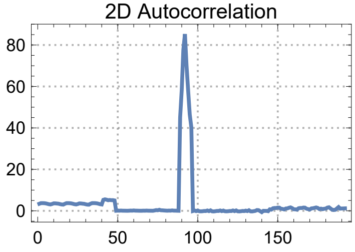

3D Plot

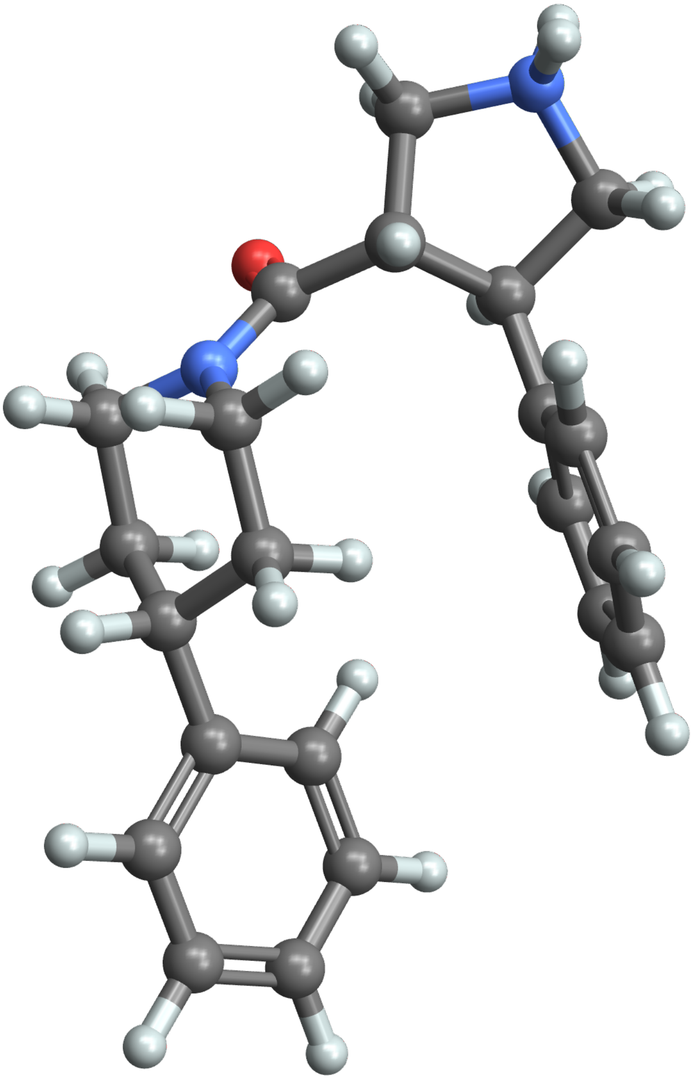

Dataset Index= 1406

Molecule Formula= $C_{22}H_{27}N_2O^+$

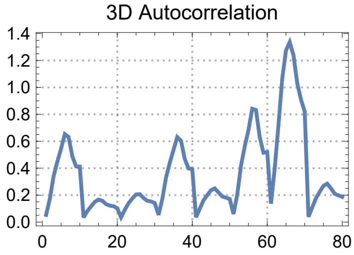

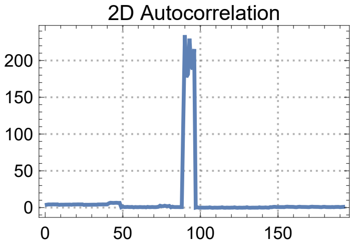

Dataset Index= 1407

Molecule Formula= $C_{33}H_{44}F_3N_3O_4$

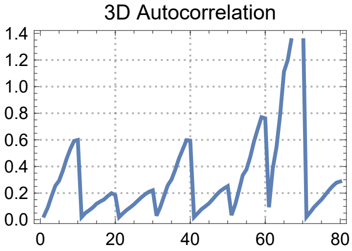

2D Plot

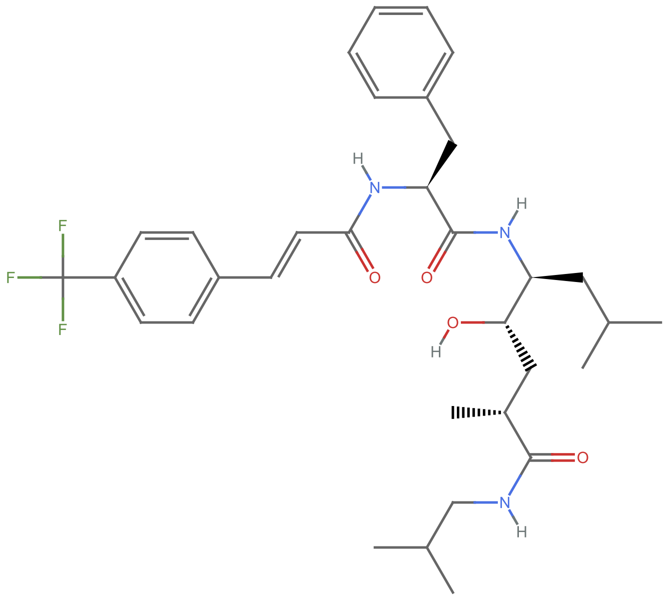

3D Plot

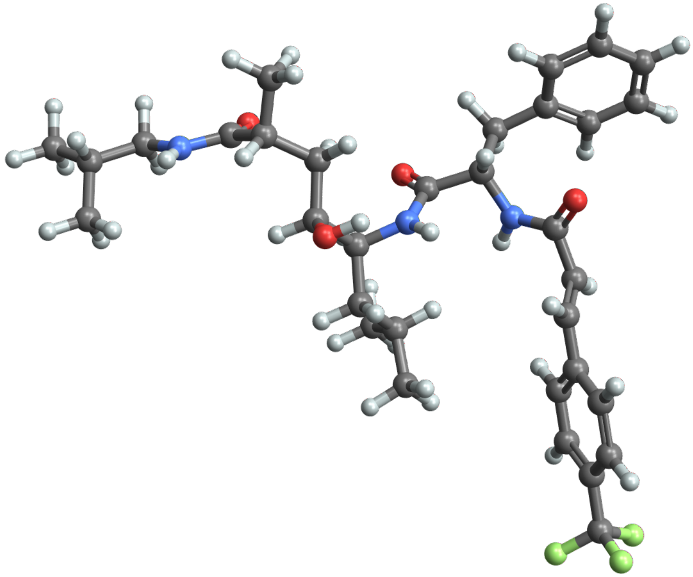

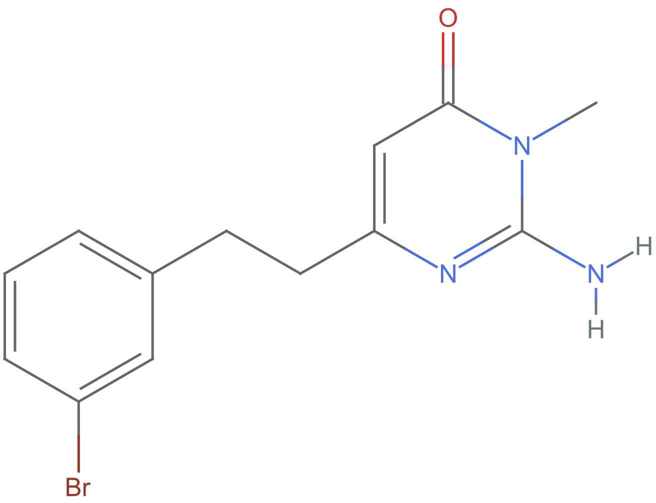

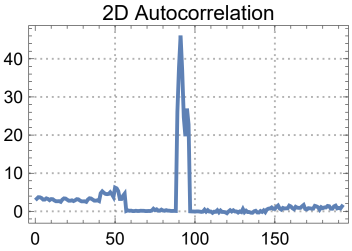

Dataset Index= 1408

Molecule Formula= $C_{13}H_{14}BrN_3O$

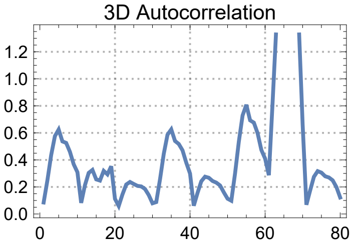

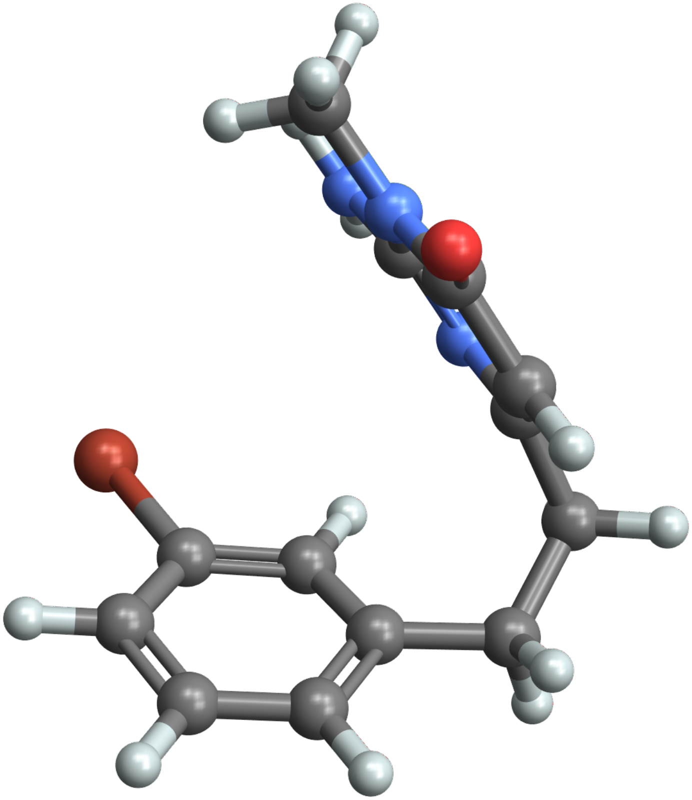

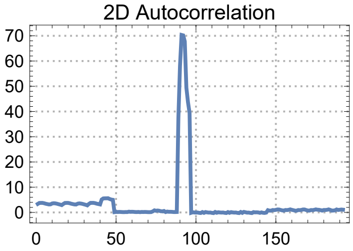

Dataset Index= 1409

Molecule Formula= $C_{19}H_{21}N_3O_2$

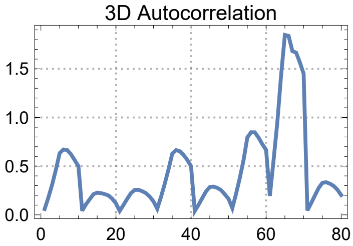

2D Plot

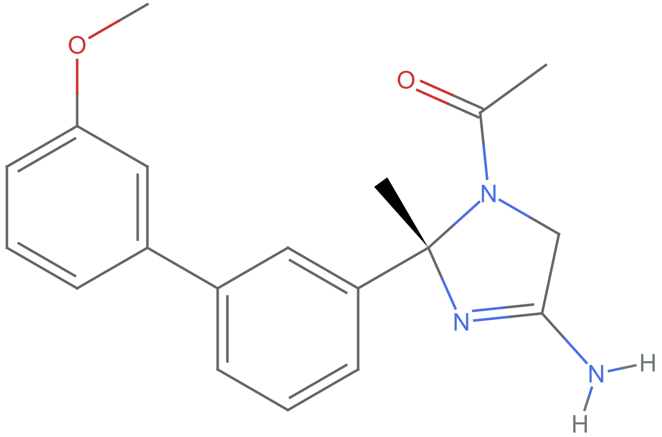

3D Plot

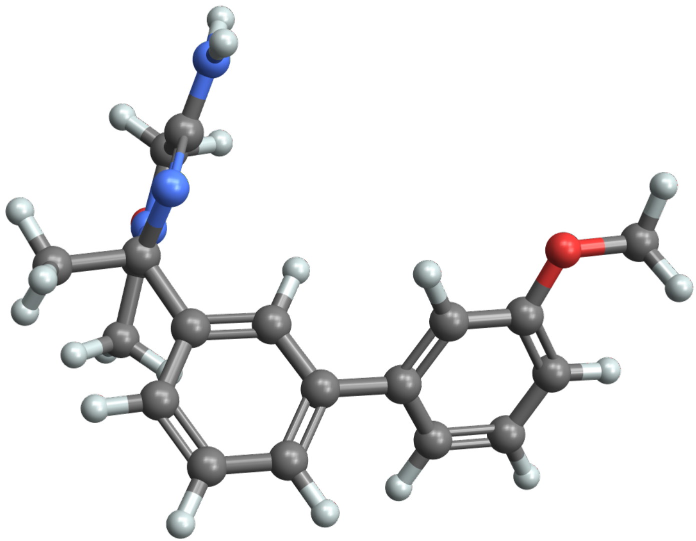

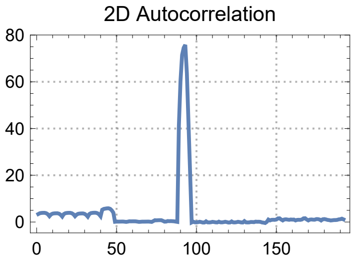

Dataset Index= 1410

Molecule Formula= $C_{19}H_{21}N_4O^+$

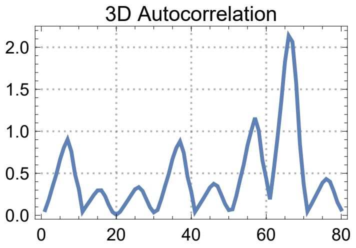

2D Plot

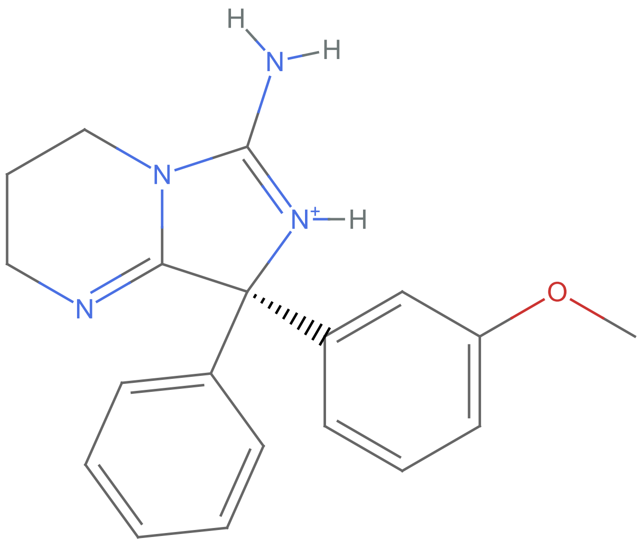

3D Plot

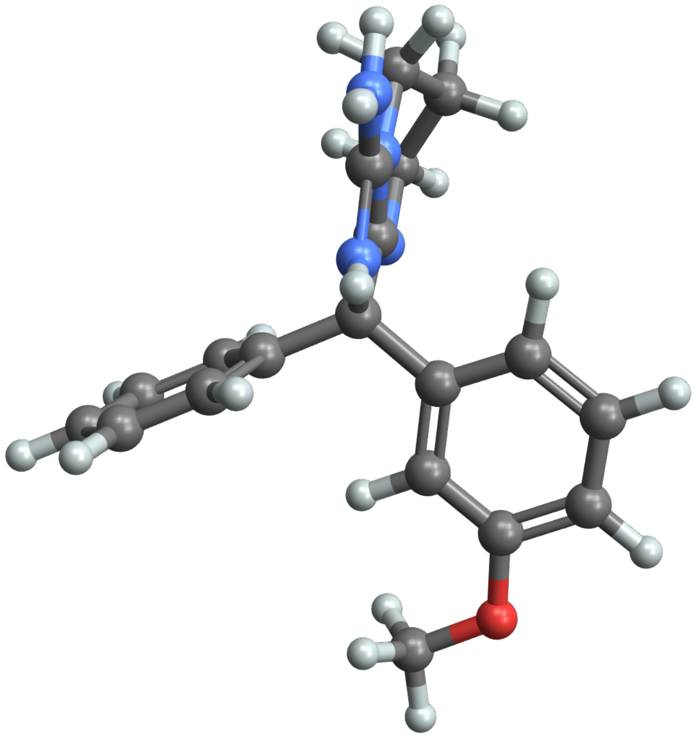

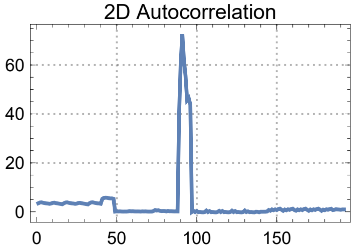

Dataset Index= 1411

Molecule Formula= $C_{22}H_{20}N_4O$

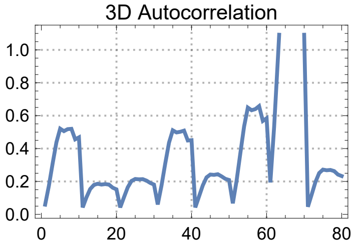

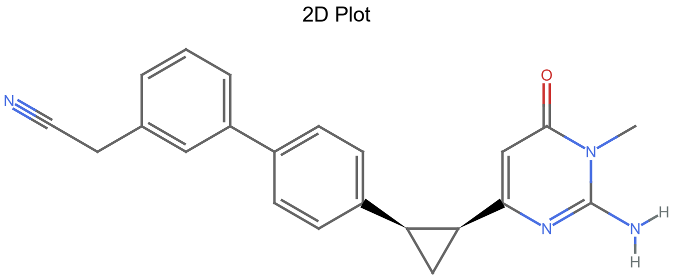

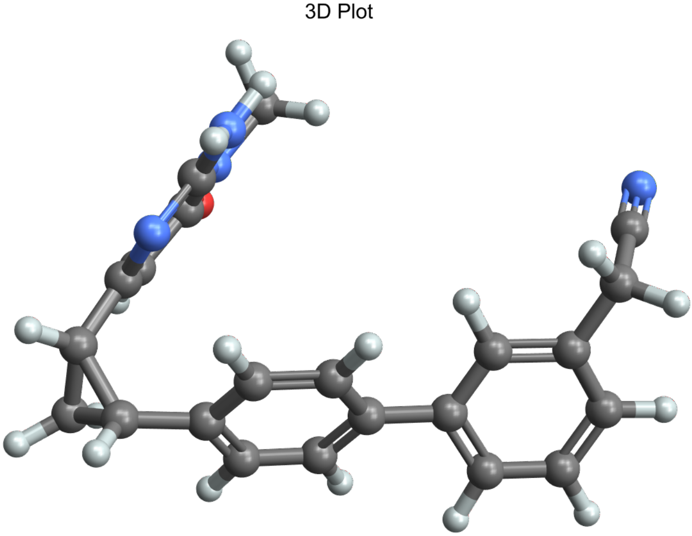

2D Autocorrelation

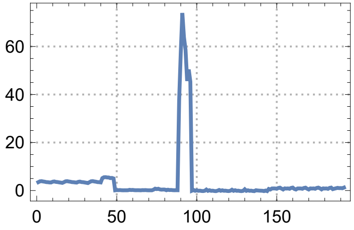

Dataset Index= 1412

Molecule Formula= $C_{22}H_{25}N_3O_2$

3D Autocorrelation

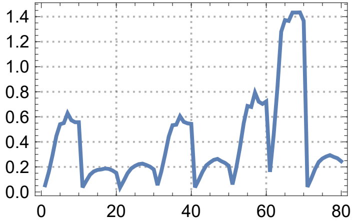

2D Plot

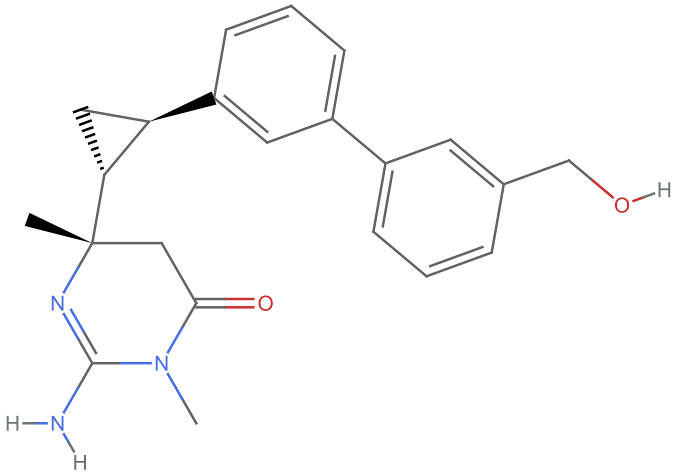

3D Plot

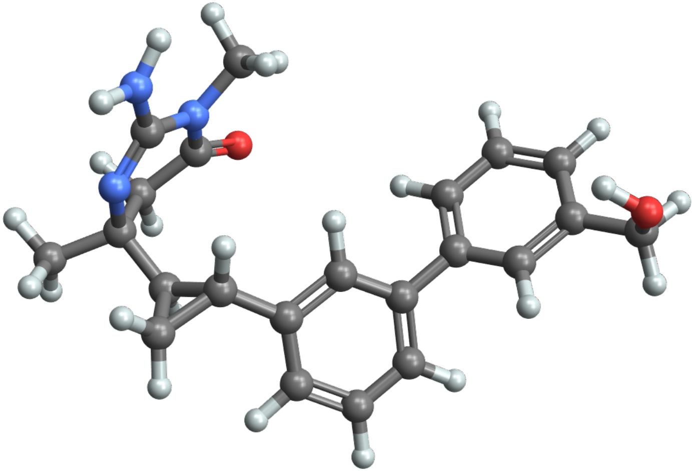

2D Plot

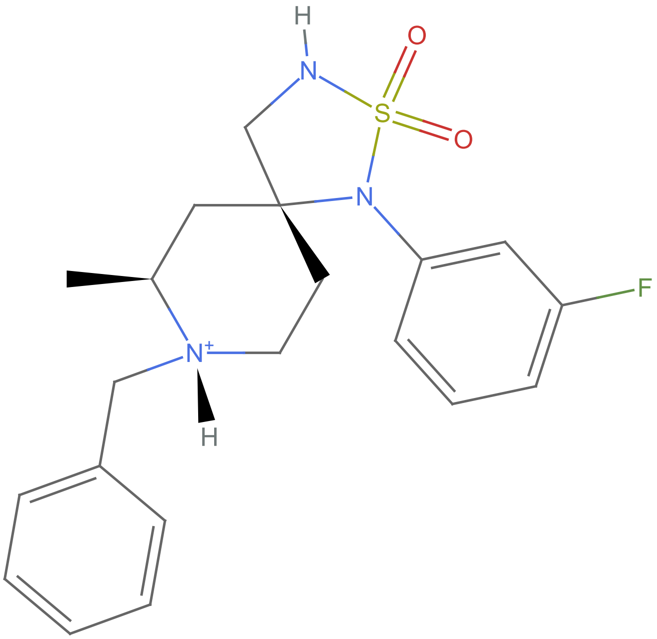

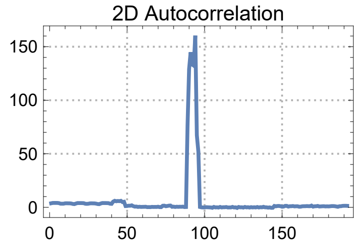

3D Plot

Dataset Index= 1413

Molecule Formula= $C_{20}H_{25}FN_3O_2S^+$

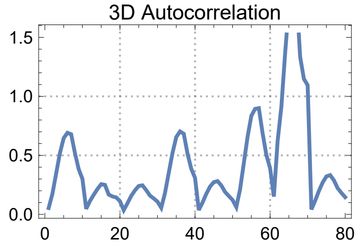

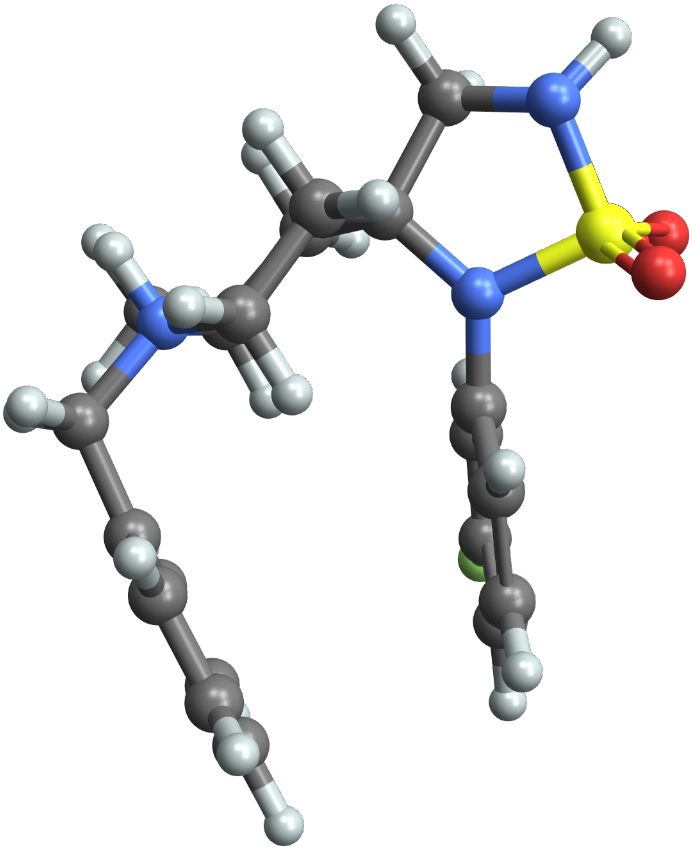

2D Plot

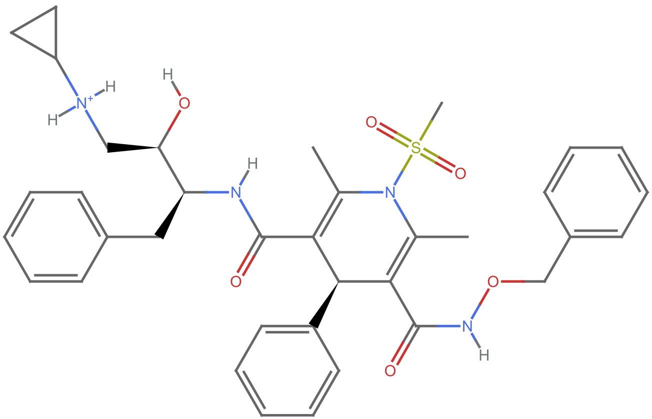

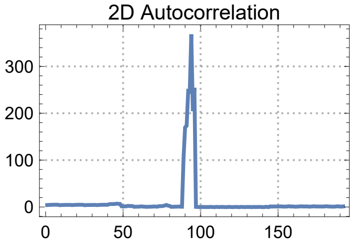

Dataset Index= 1414

Molecule Formula= $C_{36}H_{43}N_4O_6S^+$

3D Plot

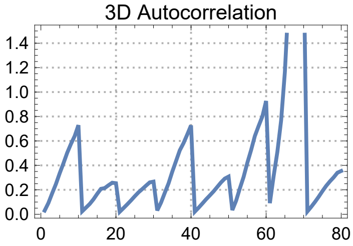

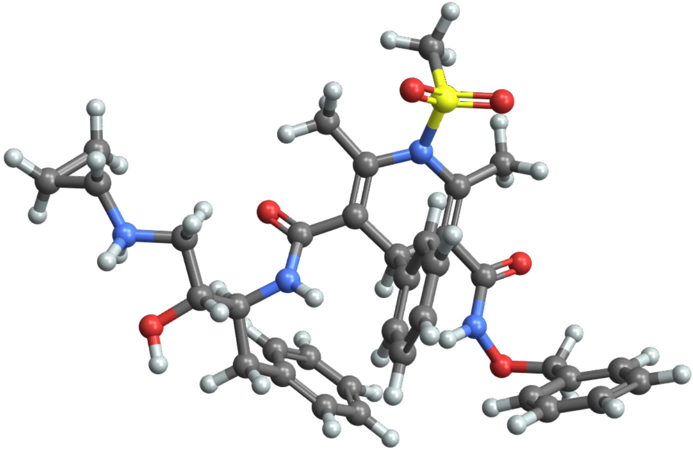

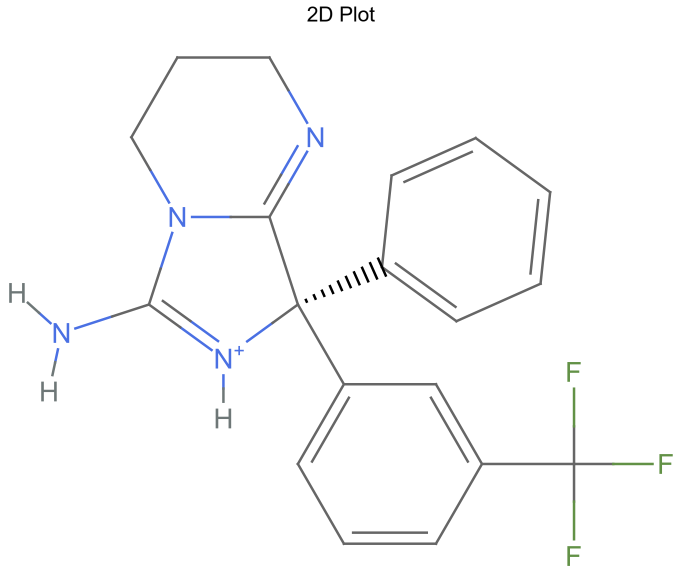

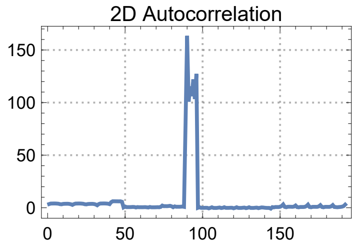

Dataset Index= 1415

Molecule Formula= $C_{19}H_{18}F_3N_4^+$

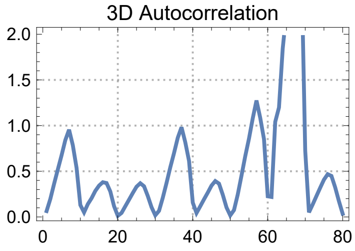

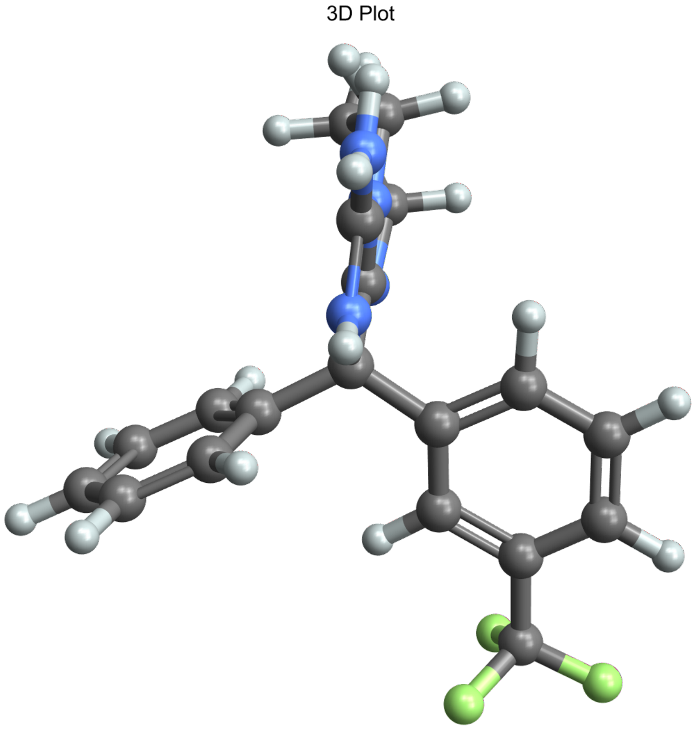

2D Plot

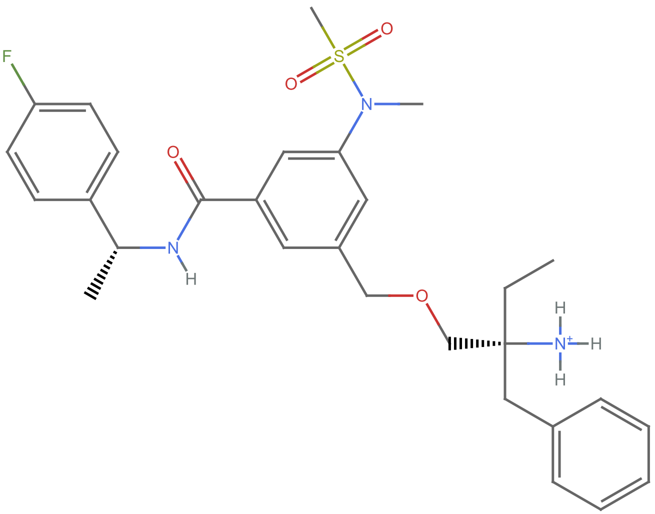

2D Autocorrelation

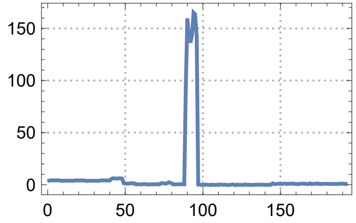

Dataset Index= 1416

Molecule Formula= $C_{29}H_{37}FN_3O_4S^+$

3D Plot

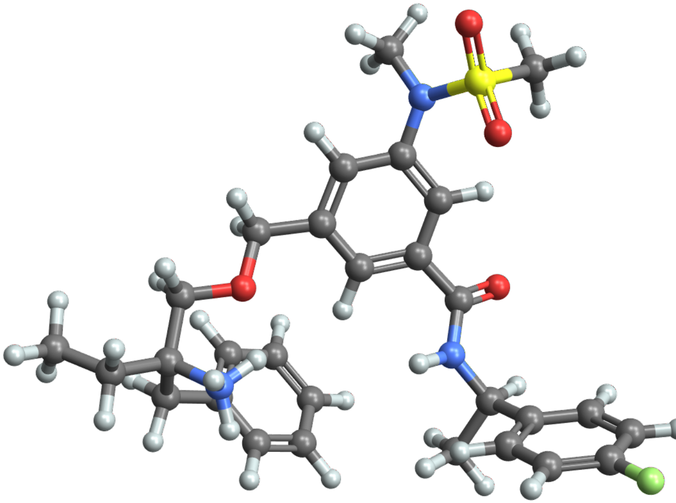

3D Autocorrelation

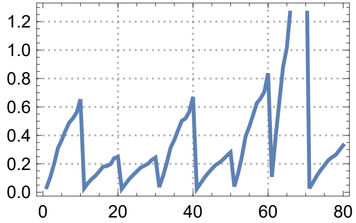

2D Plot

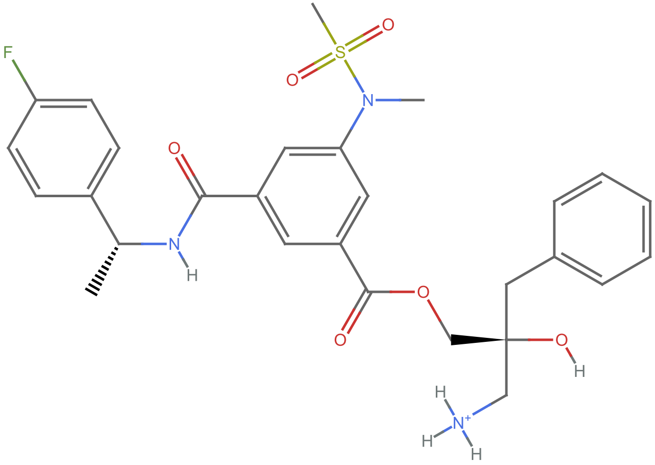

2D Autocorrelation

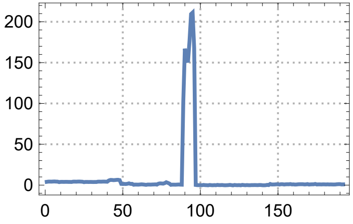

3D Plot

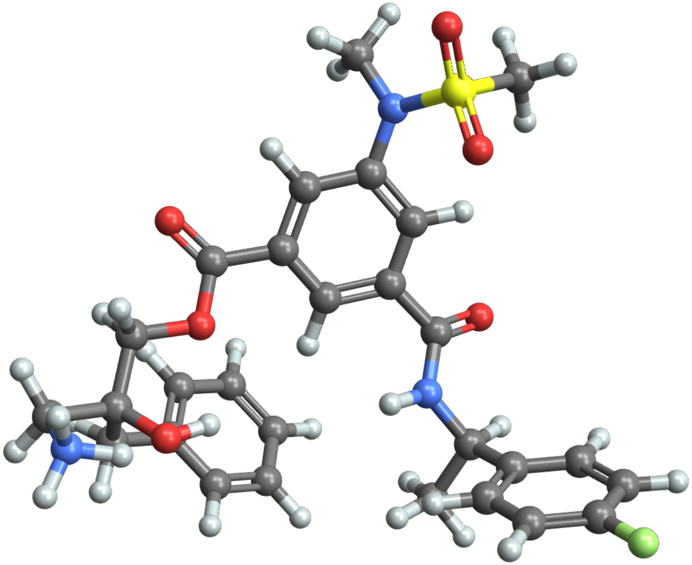

Dataset Index= 1417

Molecule Formula= $C_{28}H_{33}FN_3O_6S^+$

3D Autocorrelation

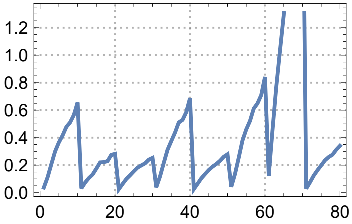

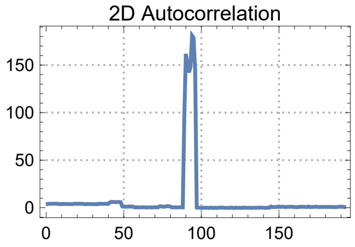

Dataset Index= 1418

Molecule Formula= $C_{29}H_{37}FN_3O_4S^+$

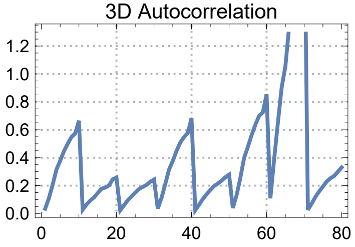

2D Plot

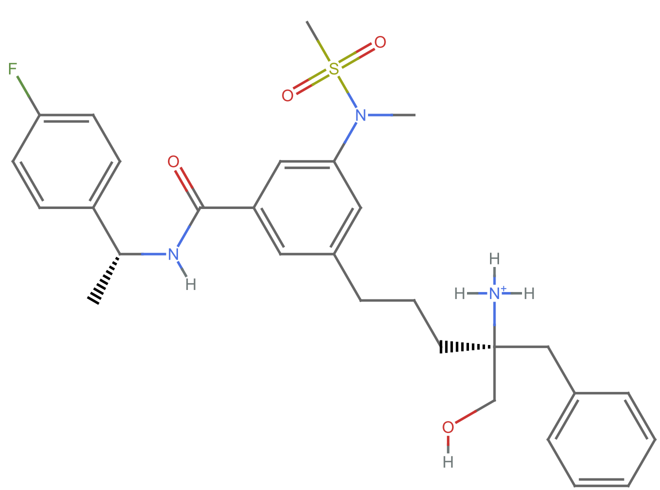

3D Plot

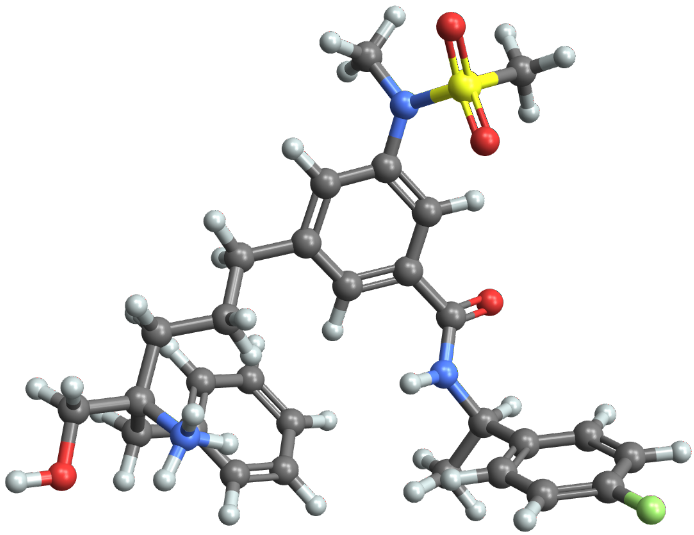

2D Plot

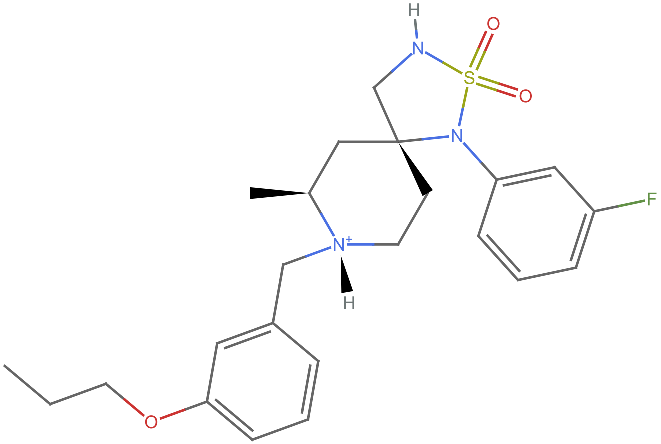

2D Autocorrelation

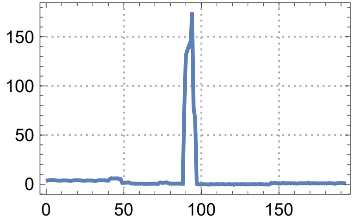

Dataset Index= 1419

Molecule Formula= $C_{23}H_{31}FN_3O_3S^+$

3D Autocorrelation

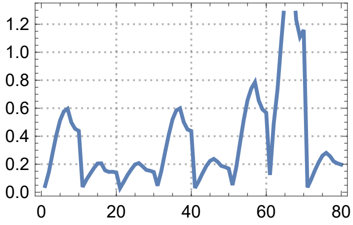

3D Plot

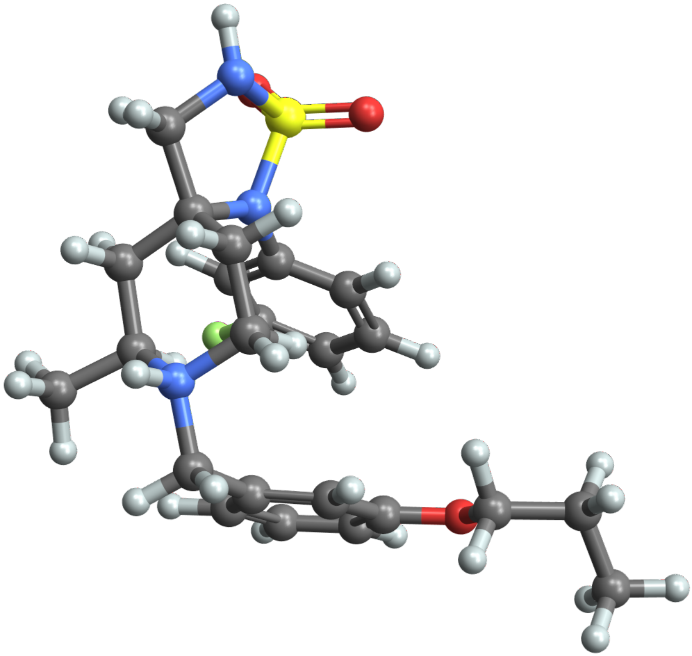

2D Plot

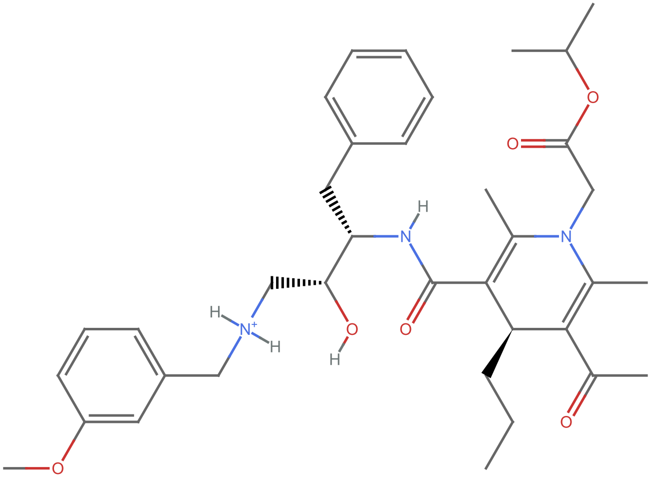

2D Autocorrelation

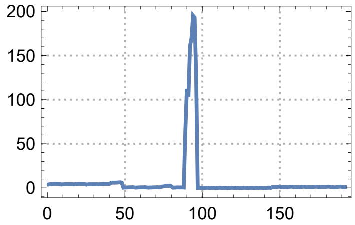

3D Plot

Dataset Index= 1420

Molecule Formula= $C_{36}H_{50}N_{3}O_{6}^{+}$

3D Autocorrelation

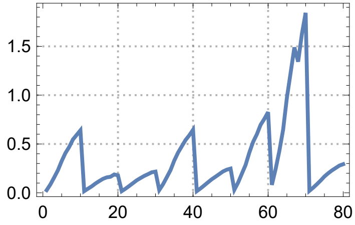

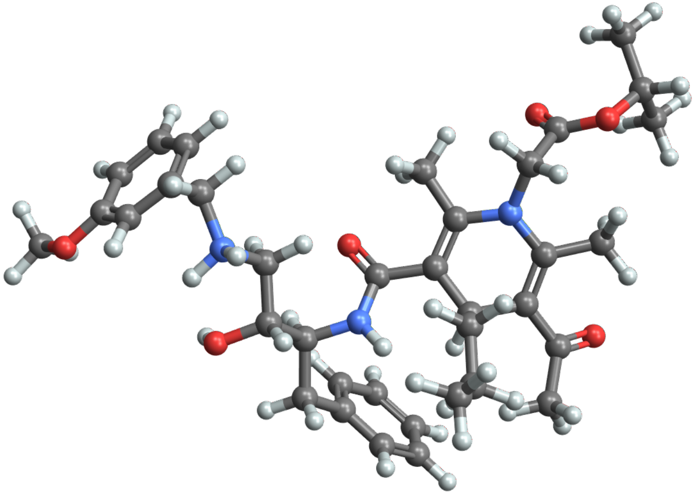

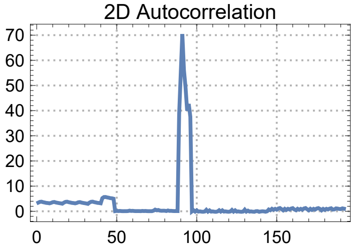

Dataset Index= 1421

Molecule Formula= $C_{22}H_{23}N_3O$

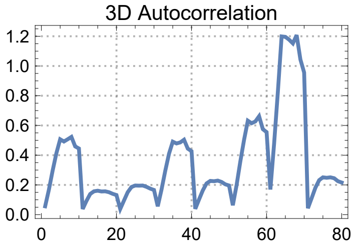

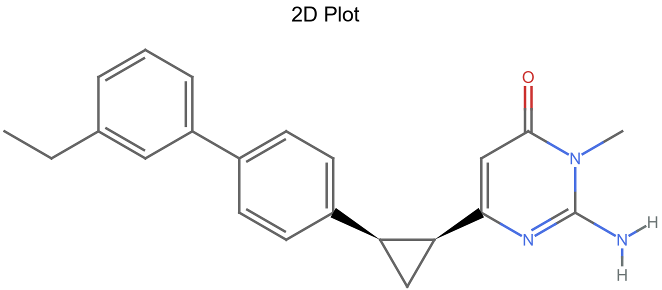

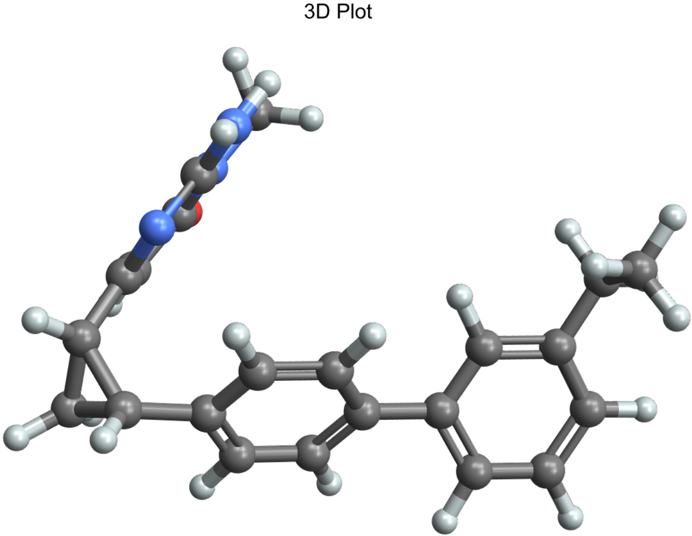

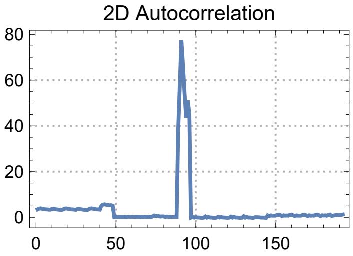

Dataset Index= 1422

Molecule Formula= $C_{22}H_{25}N_3O_2$

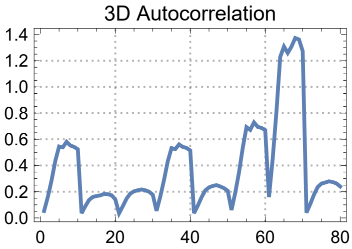

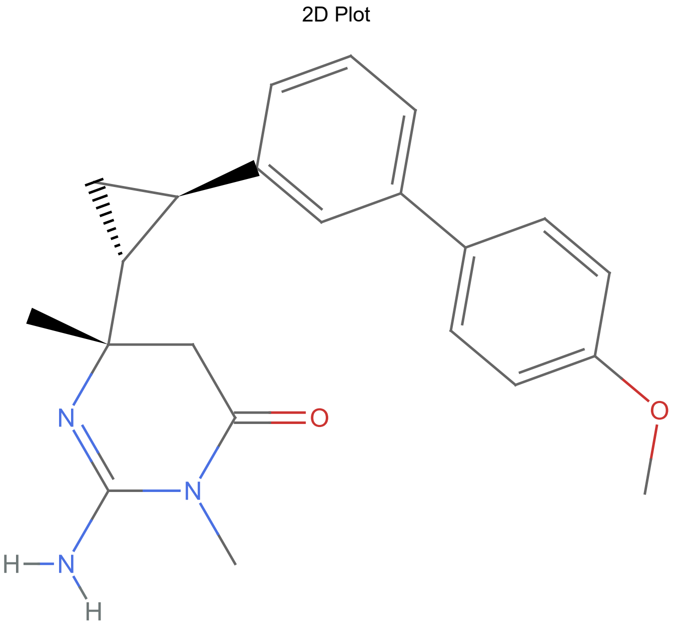

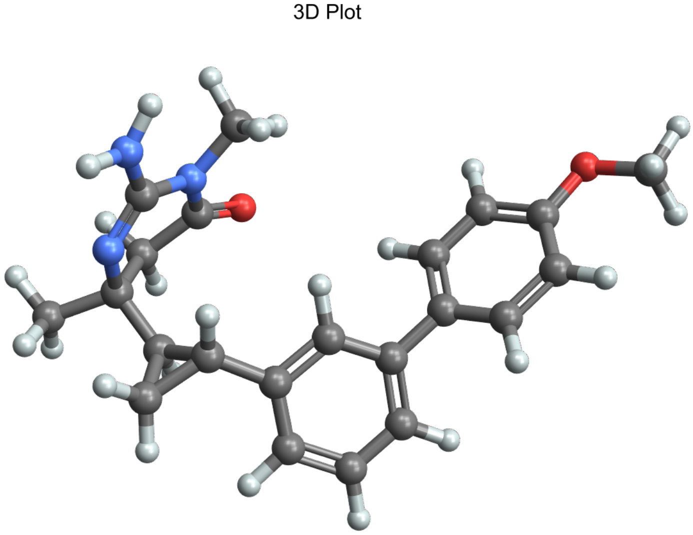

2D Autocorrelation

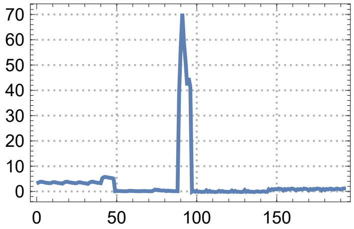

Dataset Index= 1423

Molecule Formula= $C_{22}H_{23}N_3O_2$

3D Autocorrelation

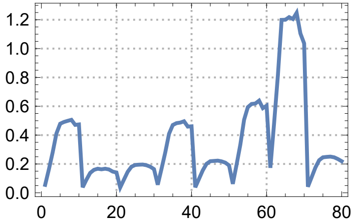

2D Plot

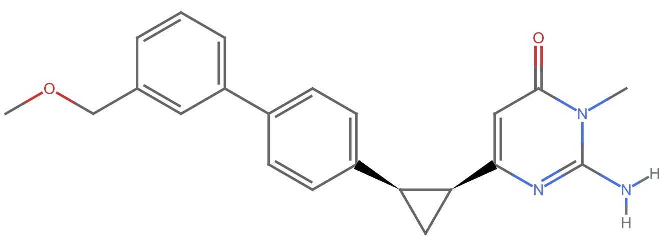

3D Plot

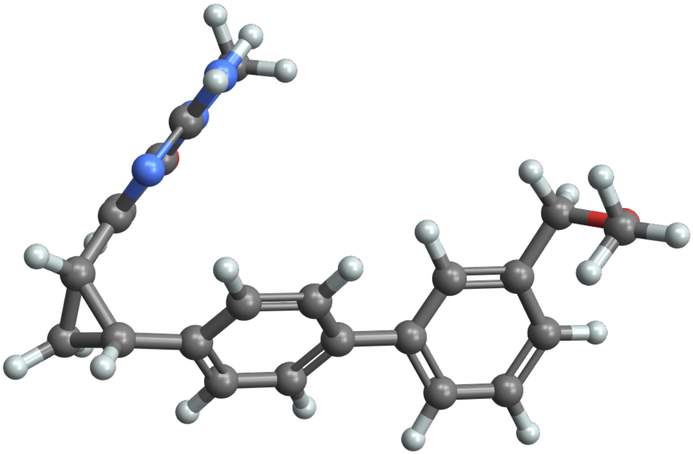

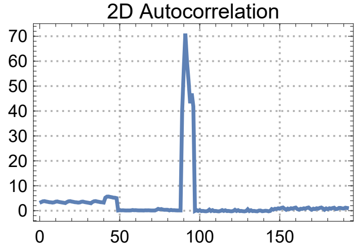

Dataset Index= 1424

Molecule Formula= $C_{22}H_{23}N_3O_2$

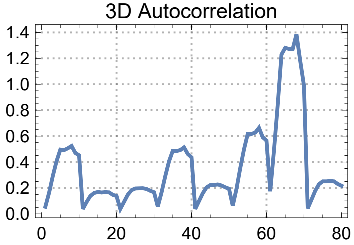

2D Plot

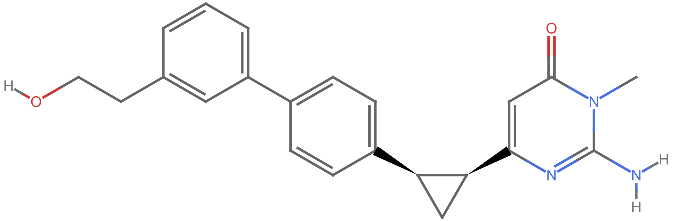

3D Plot

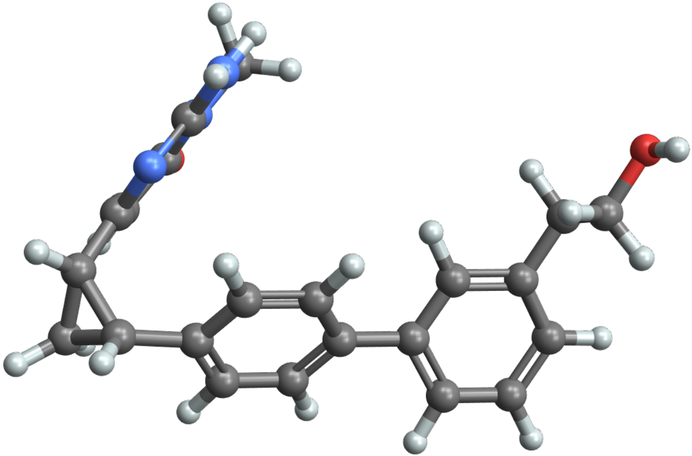

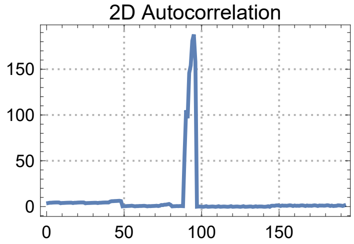

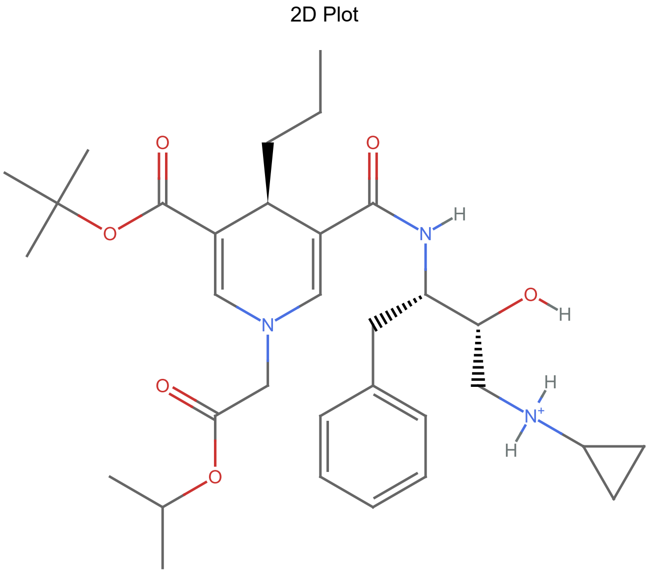

Dataset Index= 1425

Molecule Formula= $C_{32}H_{48}N_3O_6^+$

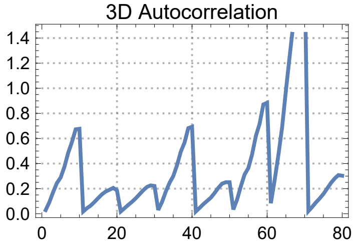

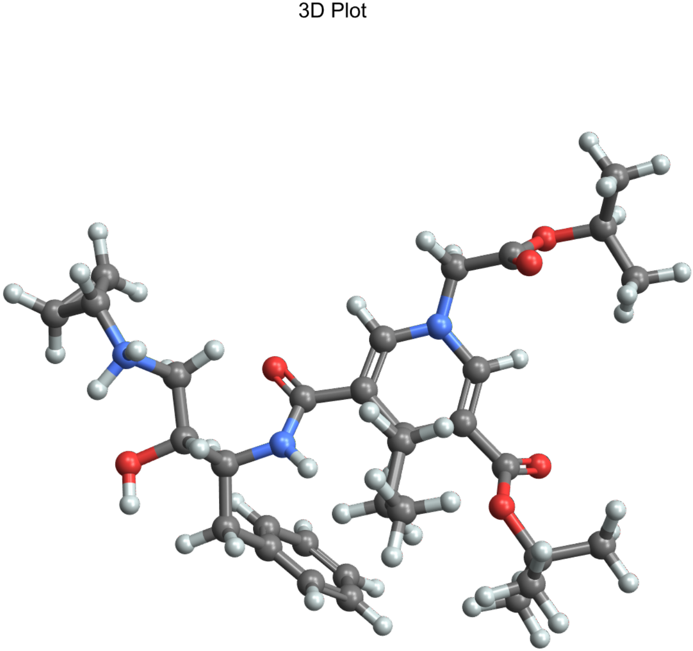

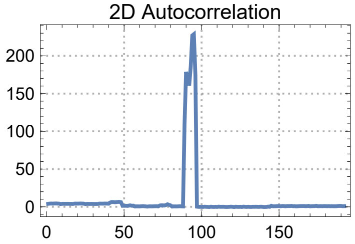

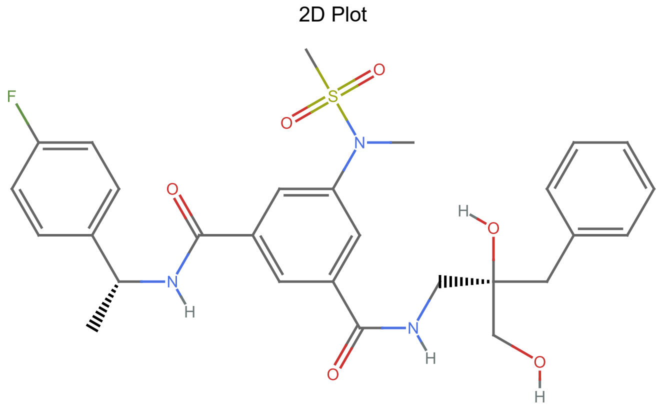

Dataset Index= 1426

Molecule Formula= $C_{28}H_{32}FN_3O_6S$

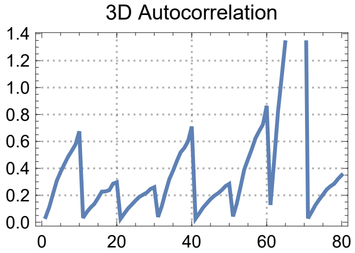

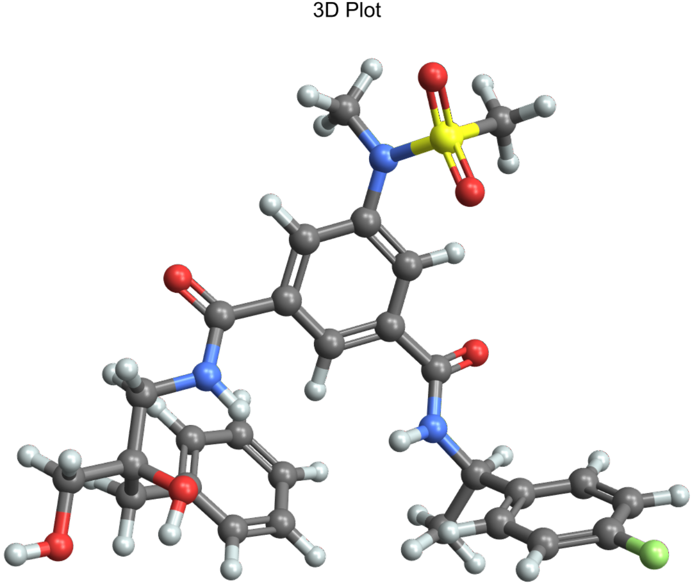

2D Plot

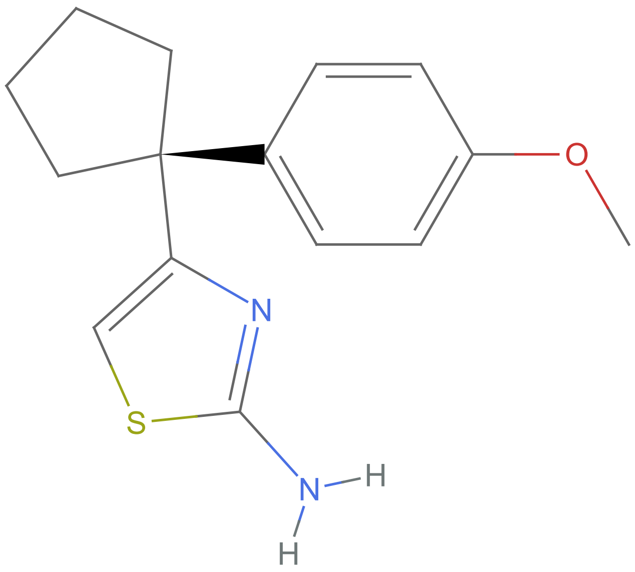

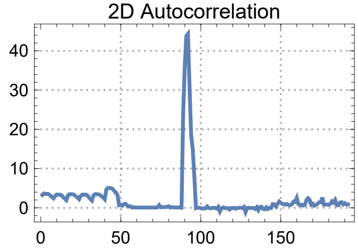

Dataset Index= 1427

Molecule Formula= $C_{15}H_{18}N_2OS$

3D Plot

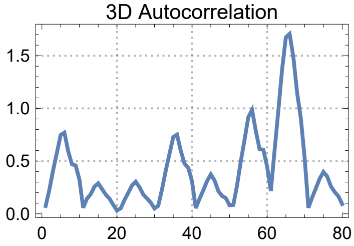

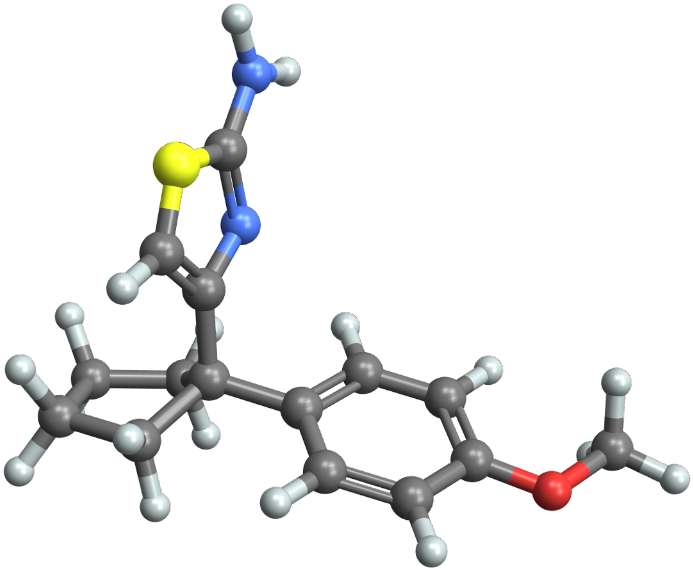

2D Autocorrelation

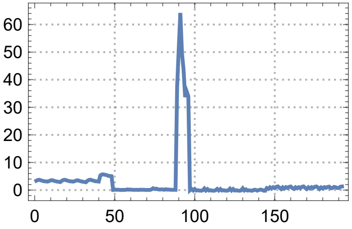

Dataset Index= 1428

Molecule Formula= $C_{20}H_{19}N_3O$

3D Autocorrelation

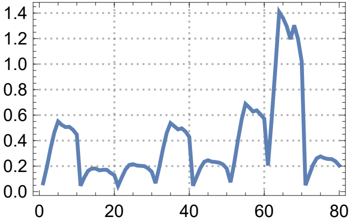

2D Plot

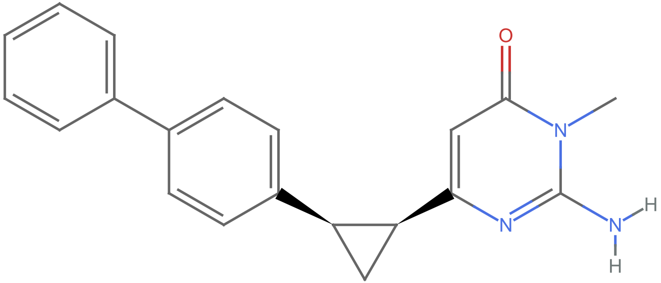

3D Plot

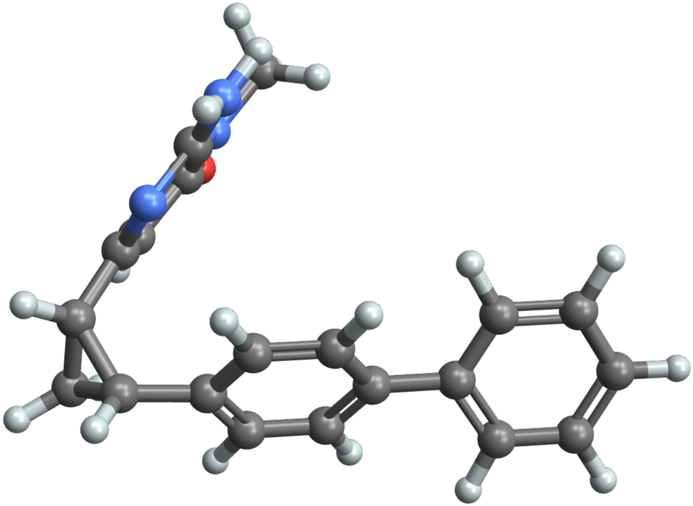

2D Plot

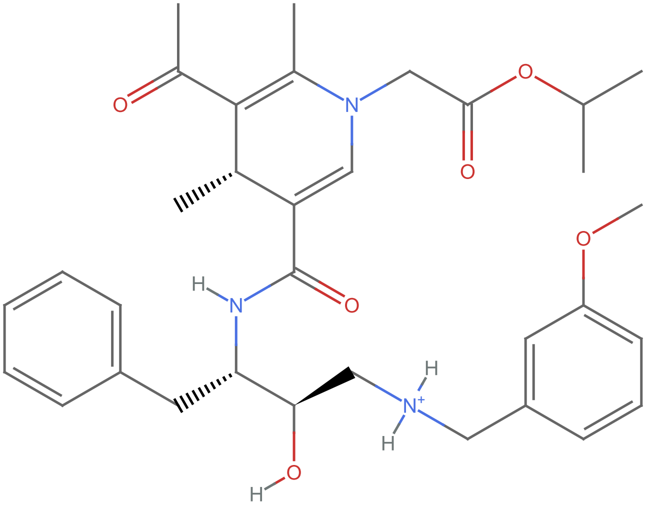

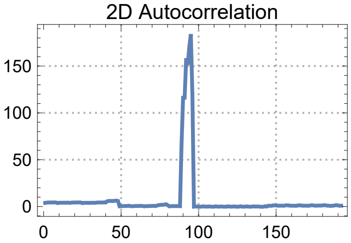

Dataset Index= 1429

Molecule Formula= $C_{33}H_{44}N_{3}O_{6}^{+}$

3D Plot

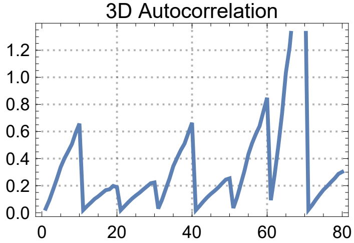

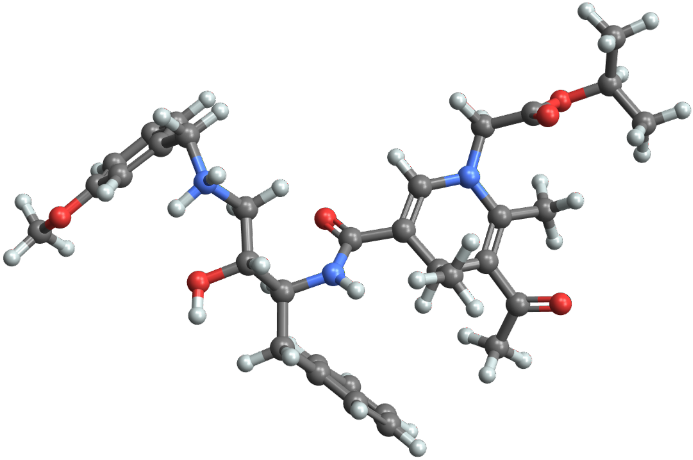

2D Plot

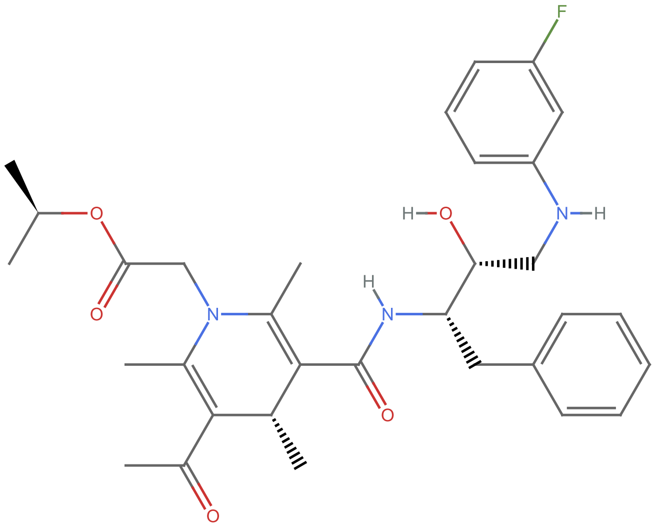

2D Autocorrelation

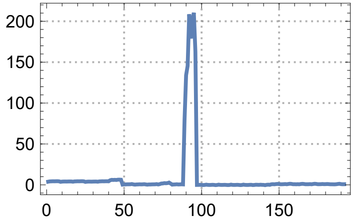

3D Plot

Dataset Index= 1430

Molecule Formula= $C_{32}H_{40}FN_3O_5$

3D Autocorrelation

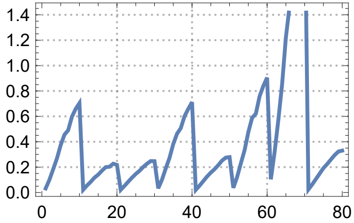

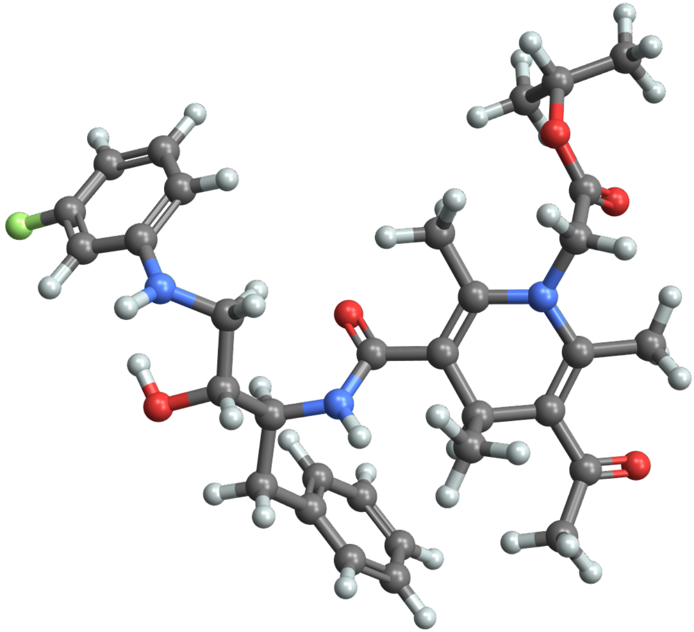

2D Plot

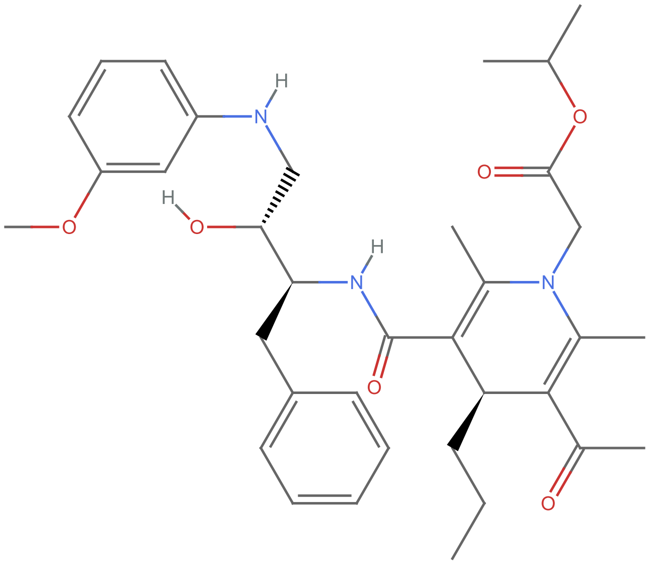

2D Autocorrelation

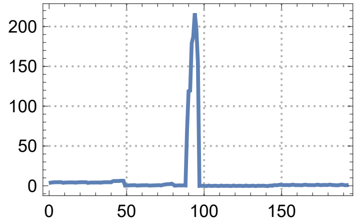

3D Plot

Dataset Index= 1431

Molecule Formula= $C_{35}H_{47}N_3O_6$

3D Autocorrelation

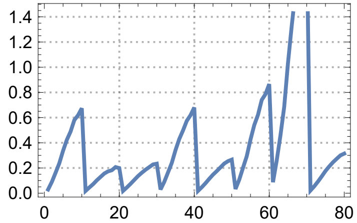

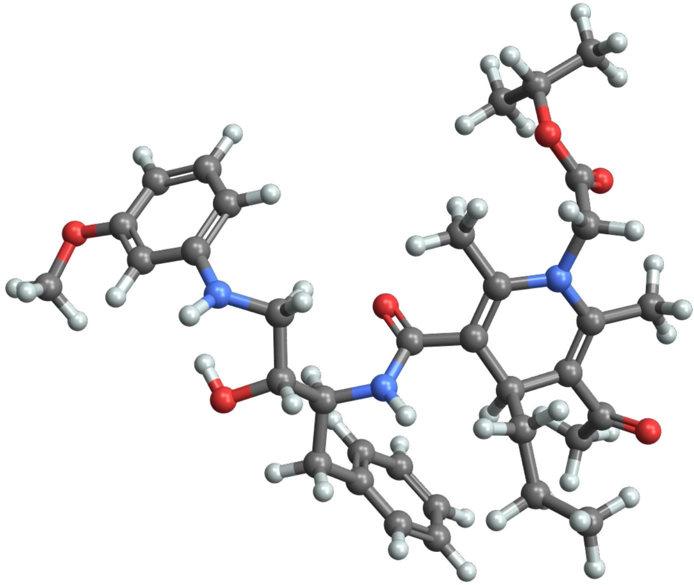

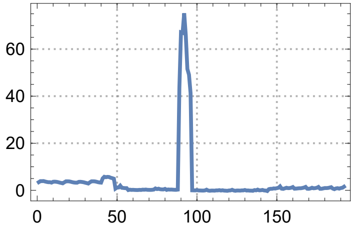

Dataset Index= 1432

Molecule Formula= $C_{19}H_{18}ClFN_2O$

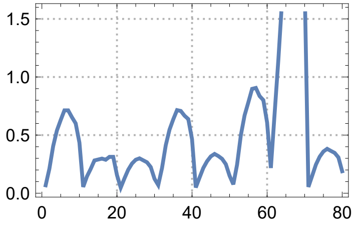

2D Plot

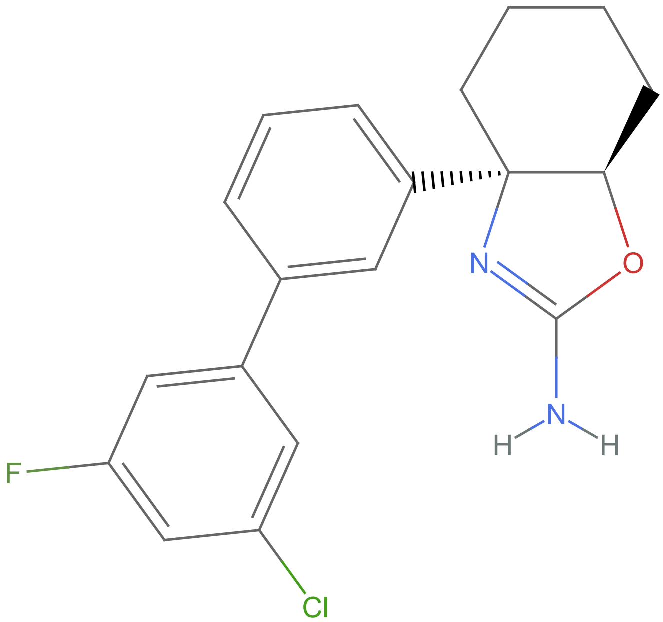

3D Plot

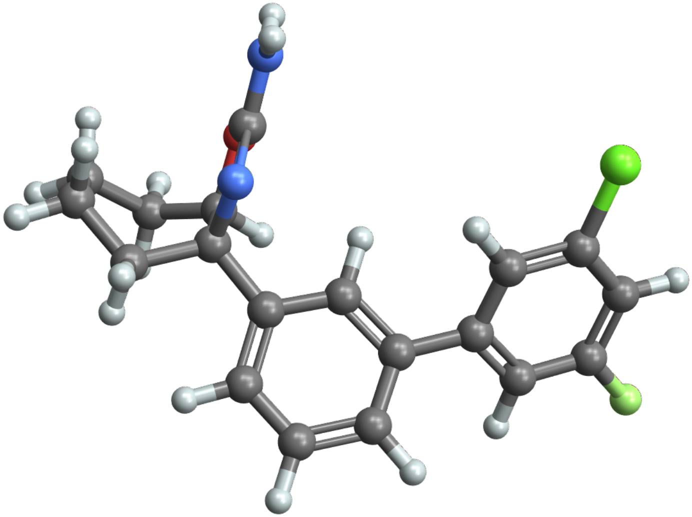

2D Plot

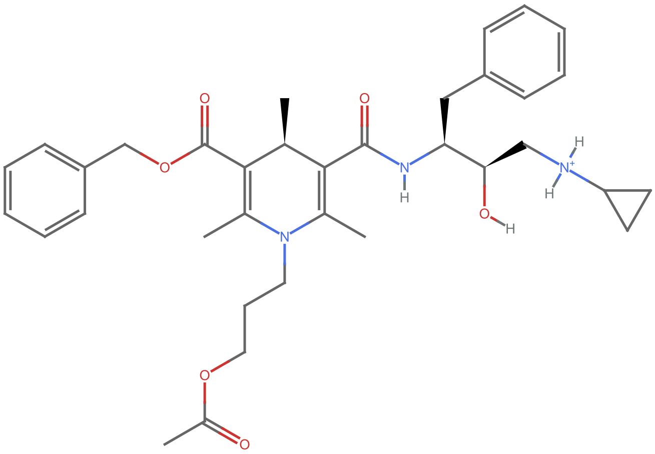

2D Autocorrelation

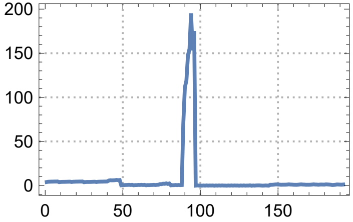

3D Plot

Dataset Index= 1433

Molecule Formula= $C_{35}H_{46}N_3O_6^+$

3D Autocorrelation

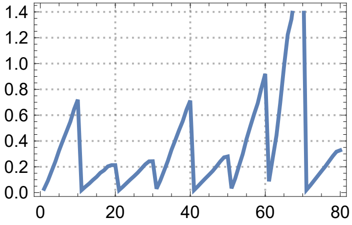

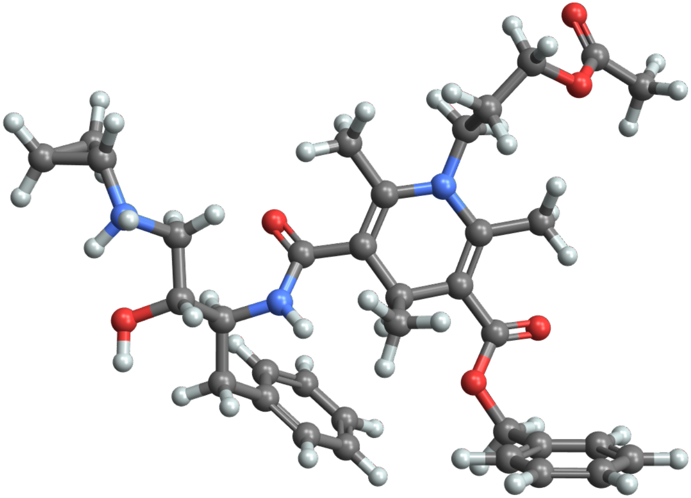

2D Plot

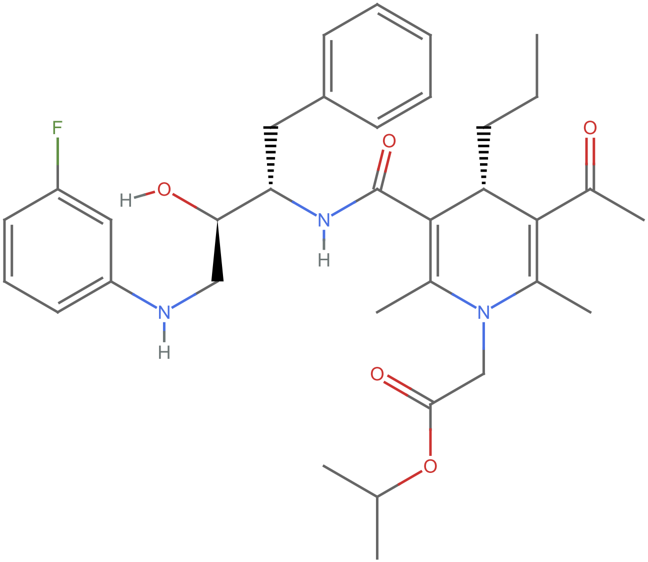

2D Autocorrelation

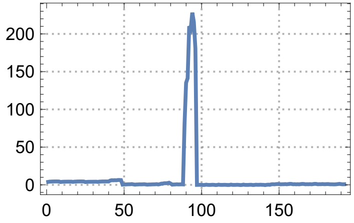

Dataset Index= 1434

Molecule Formula= $C_{34}H_{44}FN_3O_5$

3D Plot

3D Autocorrelation

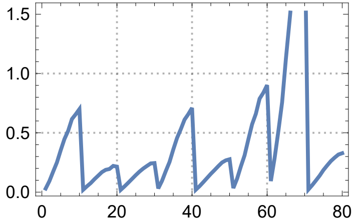

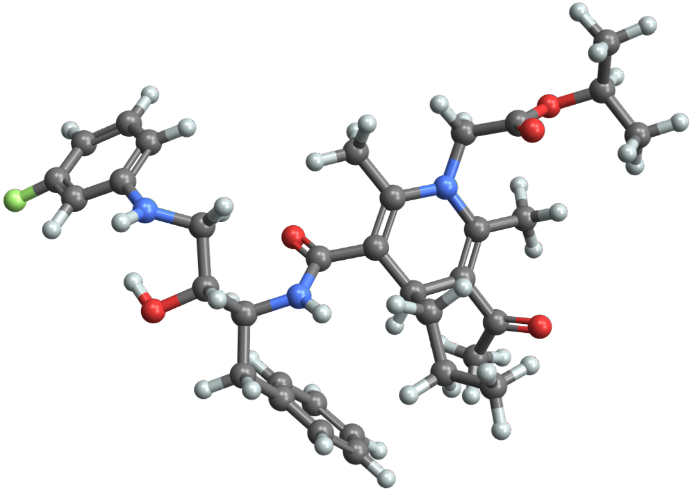

2D Plot

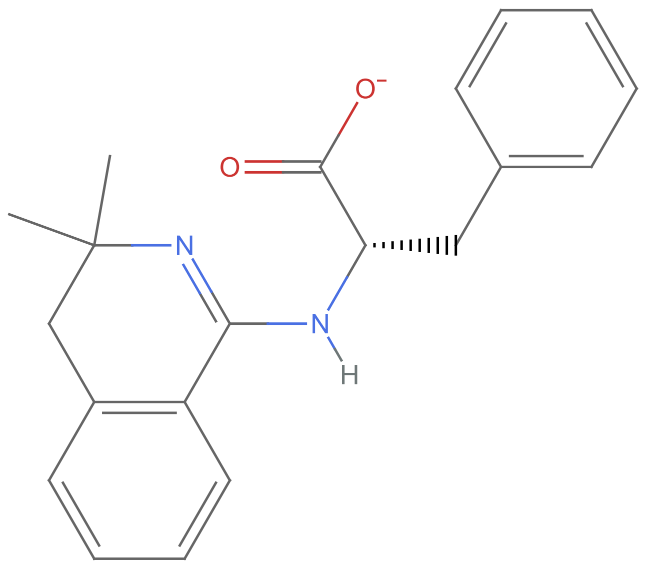

2D Autocorrelation

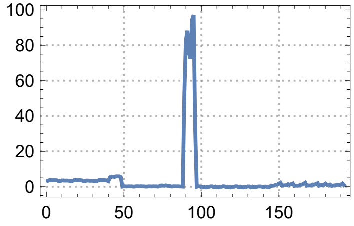

3D Plot

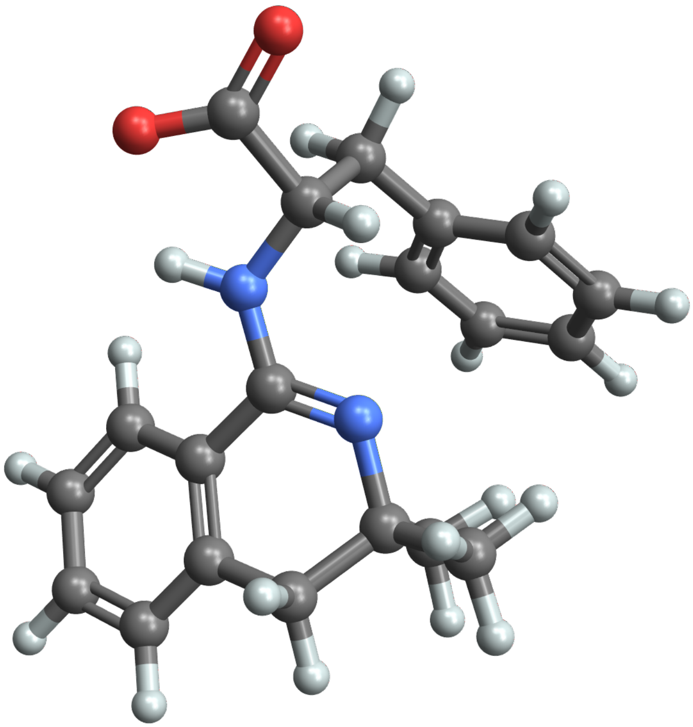

Dataset Index= 1435

Molecule Formula= $C_{20}H_{21}N_2O_2^-$

3D Autocorrelation

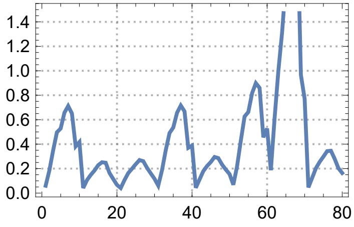

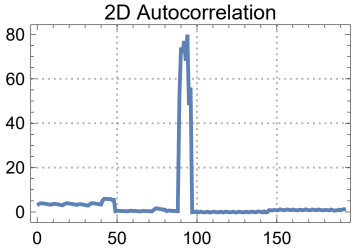

Dataset Index= 1436

Molecule Formula= $C_{18}H_{17}FN_4O_3$

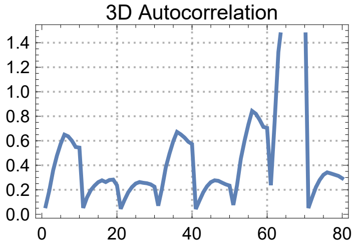

2D Plot

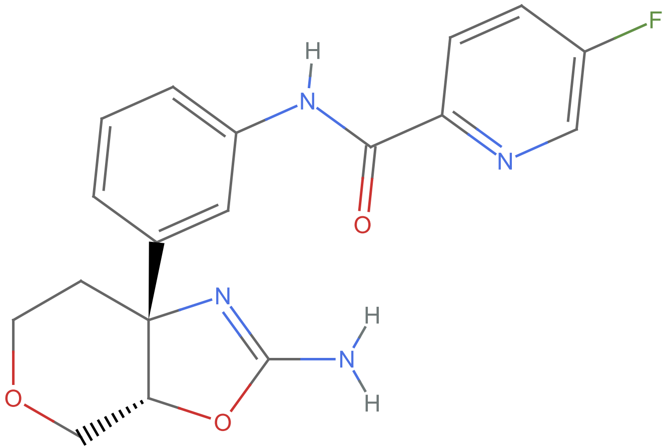

3D Plot

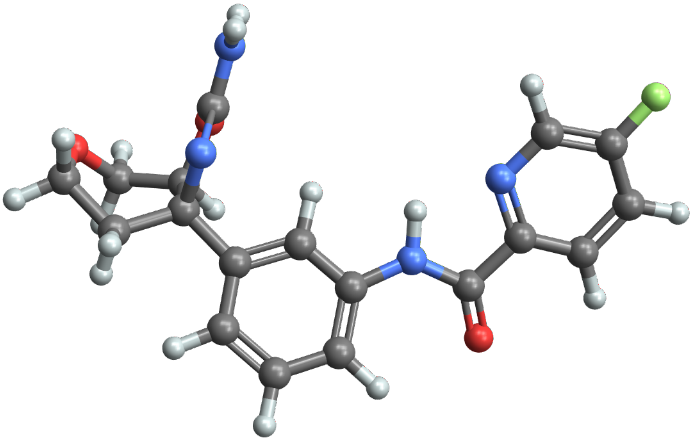

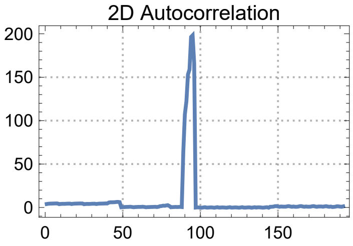

Dataset Index= 1437

Molecule Formula= $C_{36}H_{49}N_4O_5^+$

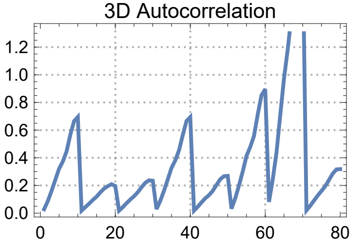

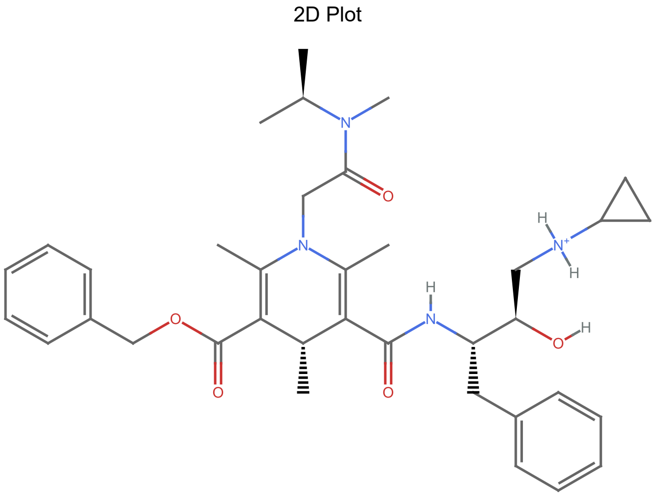

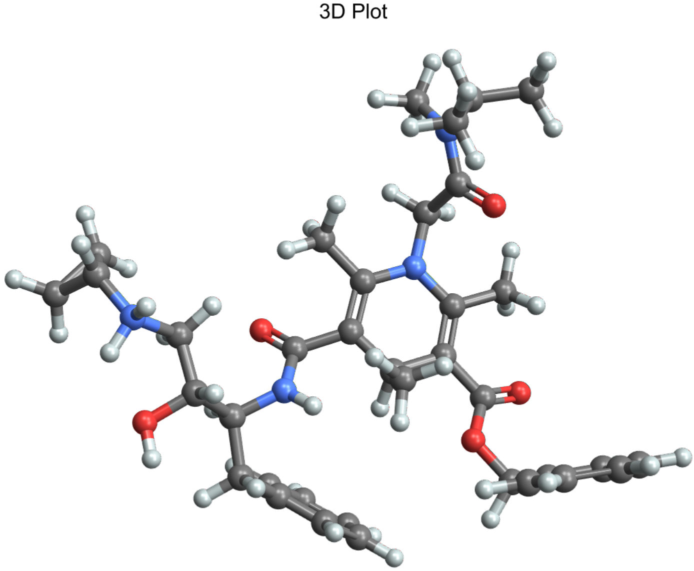

2D Plot

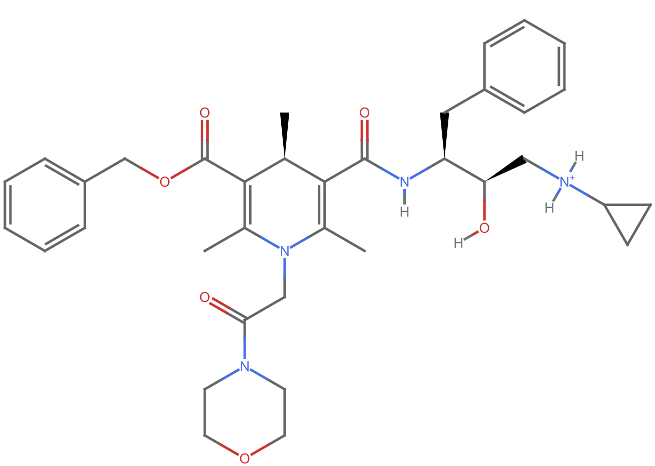

2D Autocorrelation

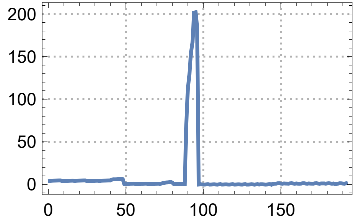

3D Plot

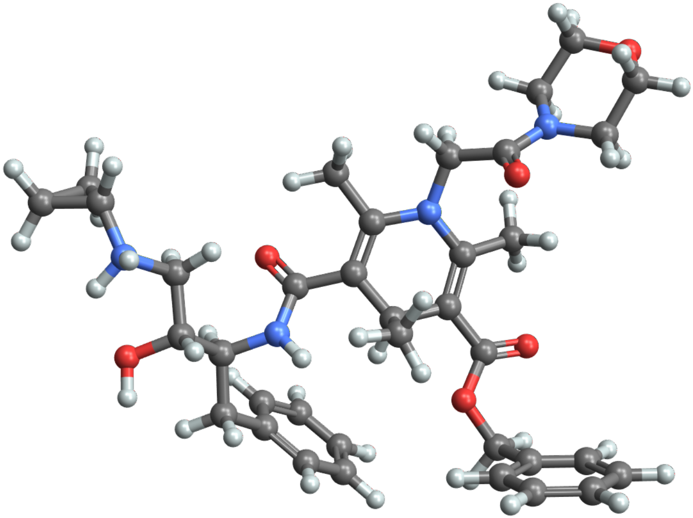

Dataset Index= 1438

Molecule Formula= $C_{36}H_{47}N_4O_6^+$

3D Autocorrelation

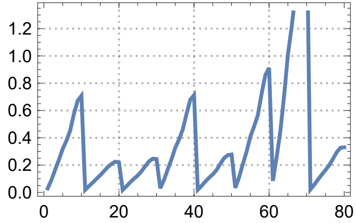

2D Plot

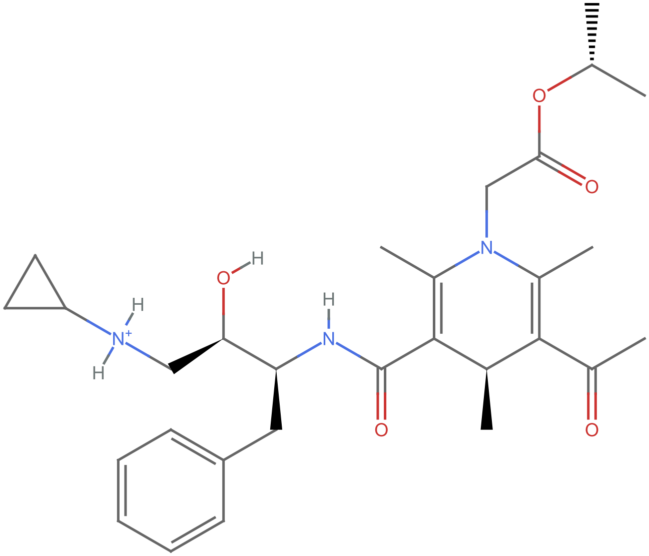

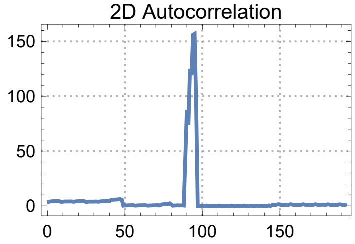

3D Plot

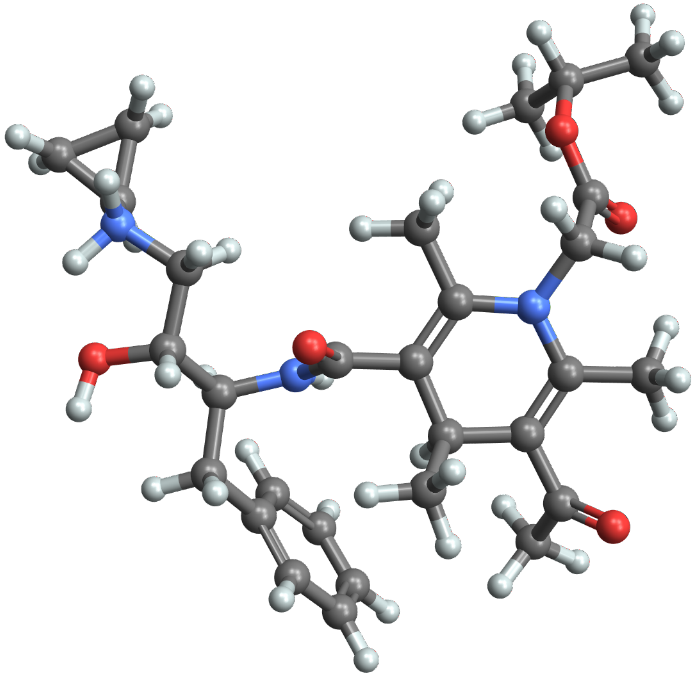

```
Dataset Index=       1439

Molecule Formula=
```
$C_{29}H_{42}N_{3}O_{5}^{+}$

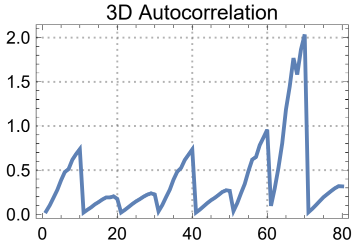

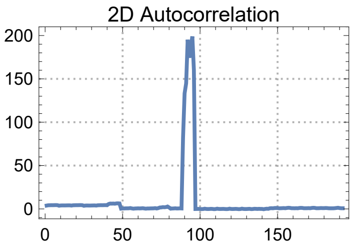

Dataset Index= 1440

Molecule Formula= $C_{31}H_{38}FN_3O_5$

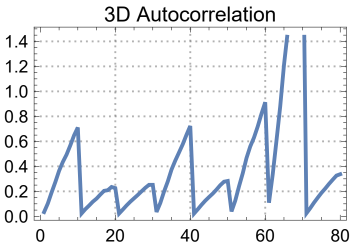

2D Plot

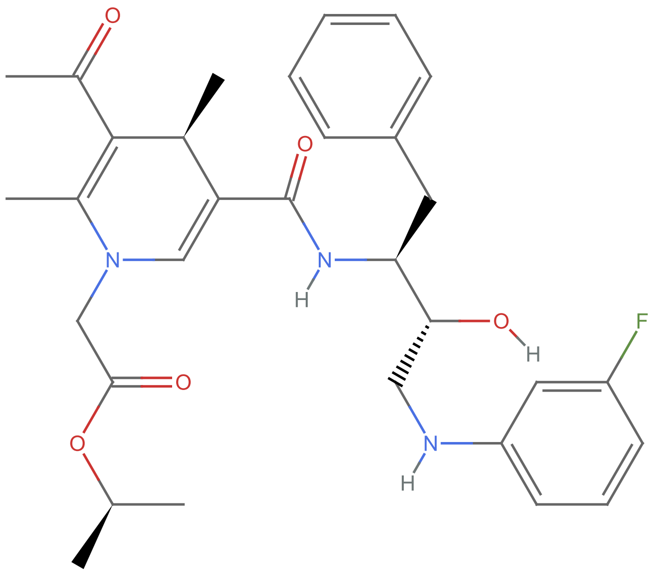

3D Plot

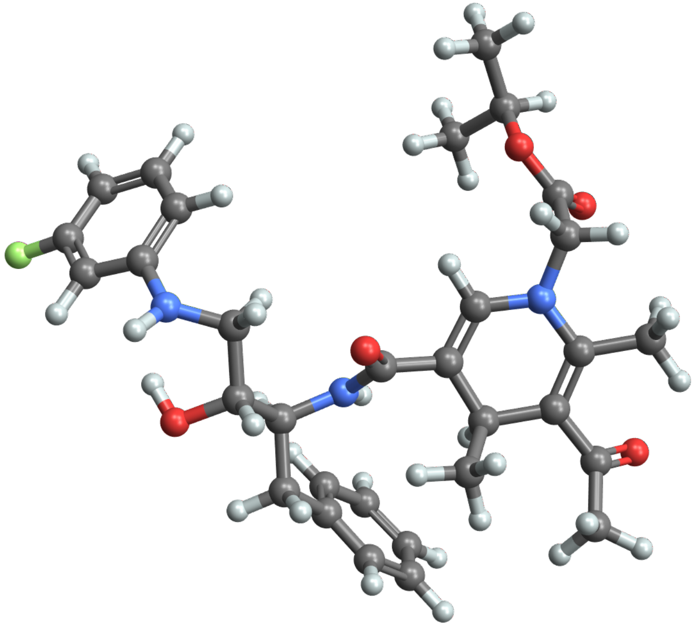

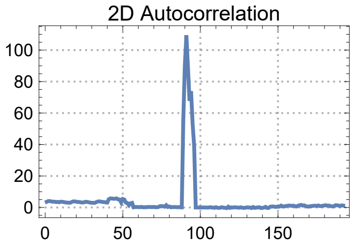

Dataset Index= 1441

Molecule Formula= $C_{19}H_{18}BrN_2O_3^+$

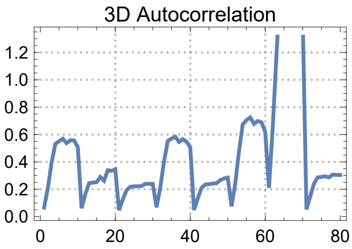

2D Plot

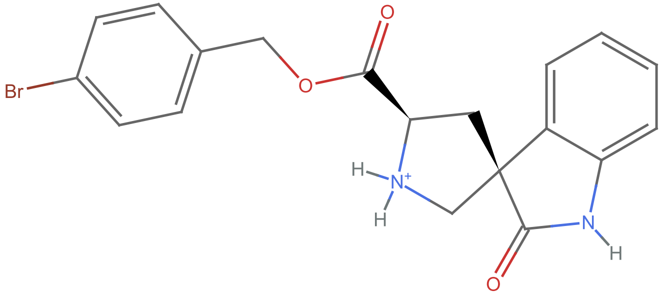

3D Plot

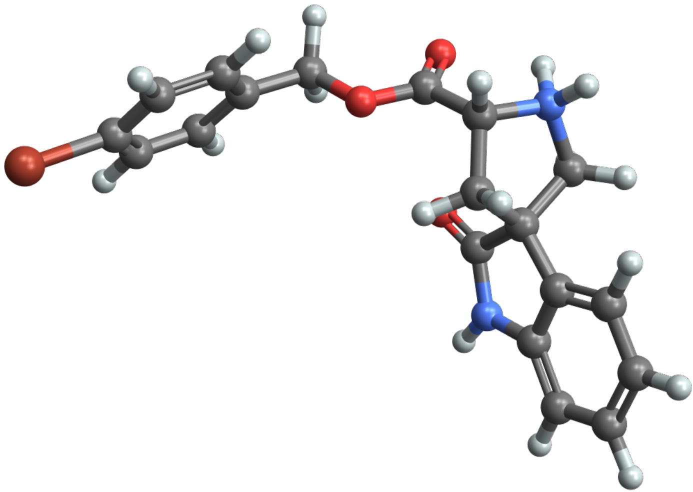

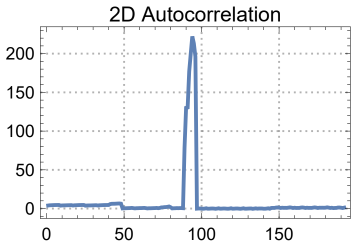

Dataset Index= 1442

Molecule Formula= $C_{36}H_{49}N_4O_5^+$

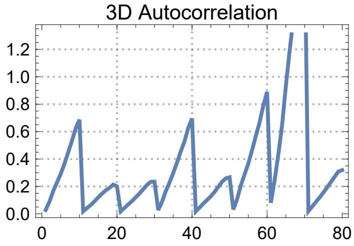

2D Plot

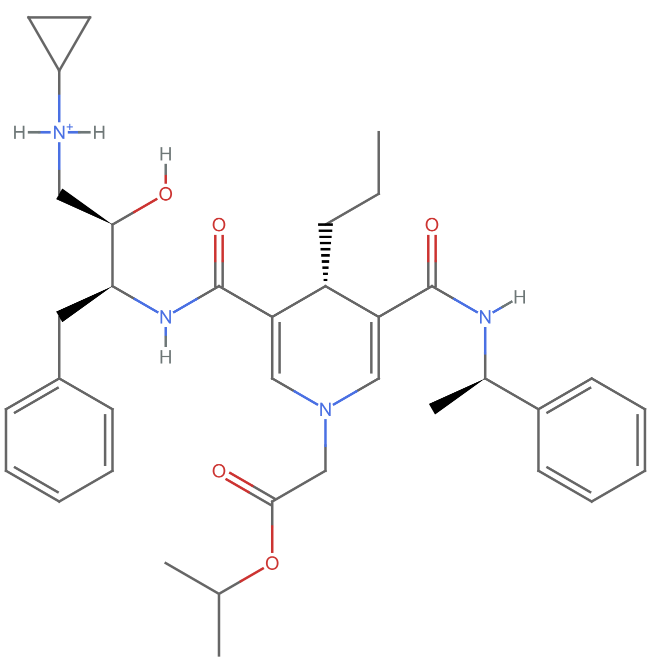

3D Plot

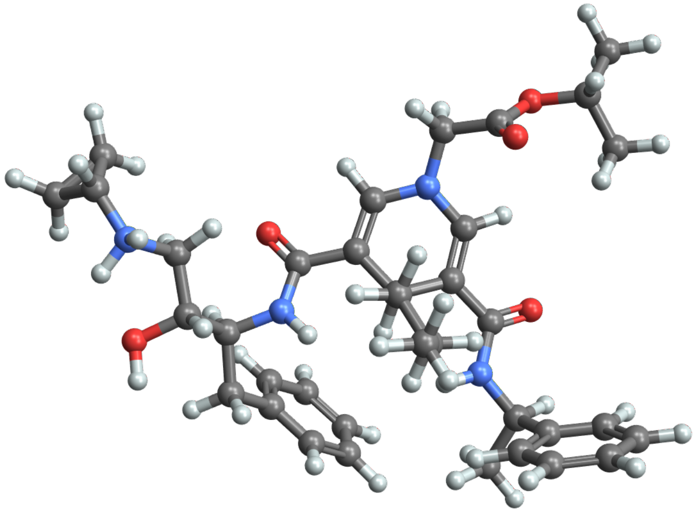

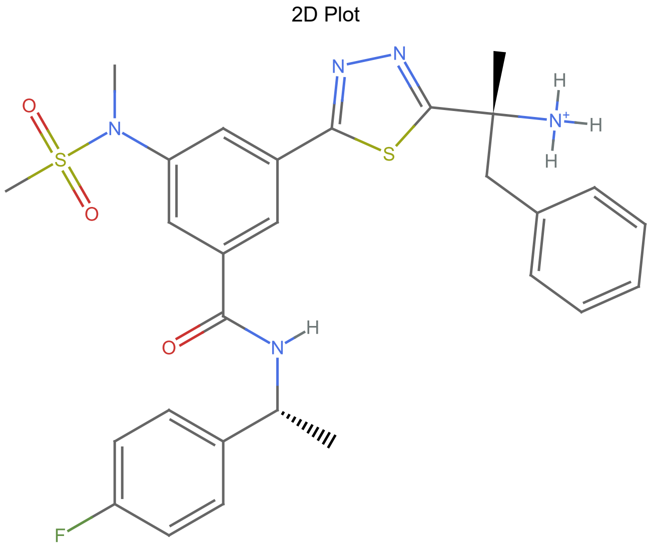

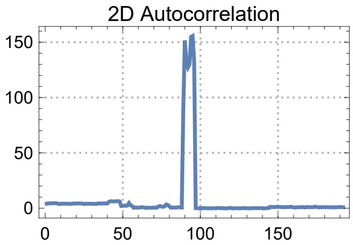

Dataset Index= 1443

Molecule Formula= $C_{28}H_{31}FN_5O_3S_2^+$

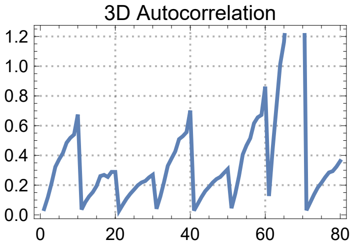

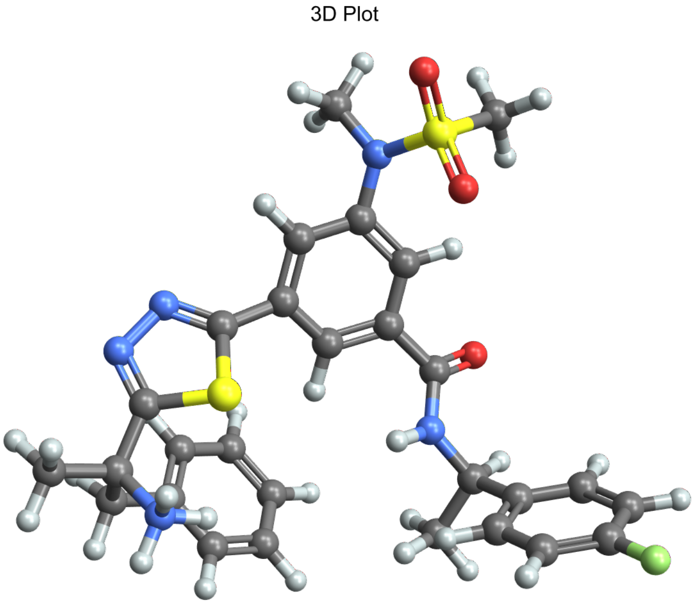

2D Plot

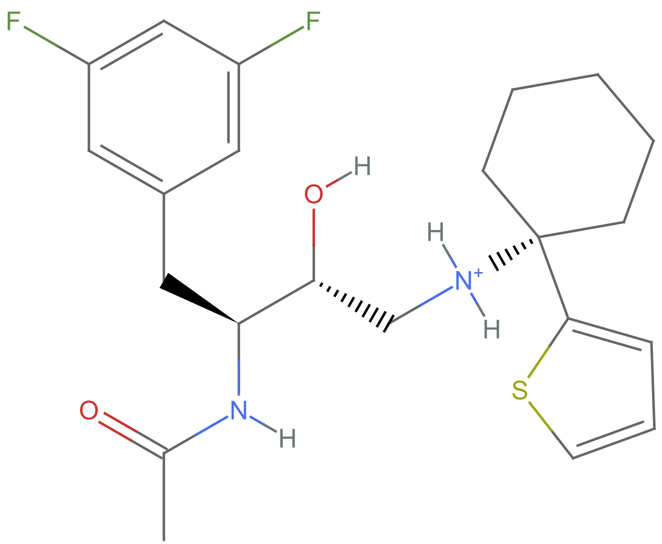

3D Plot

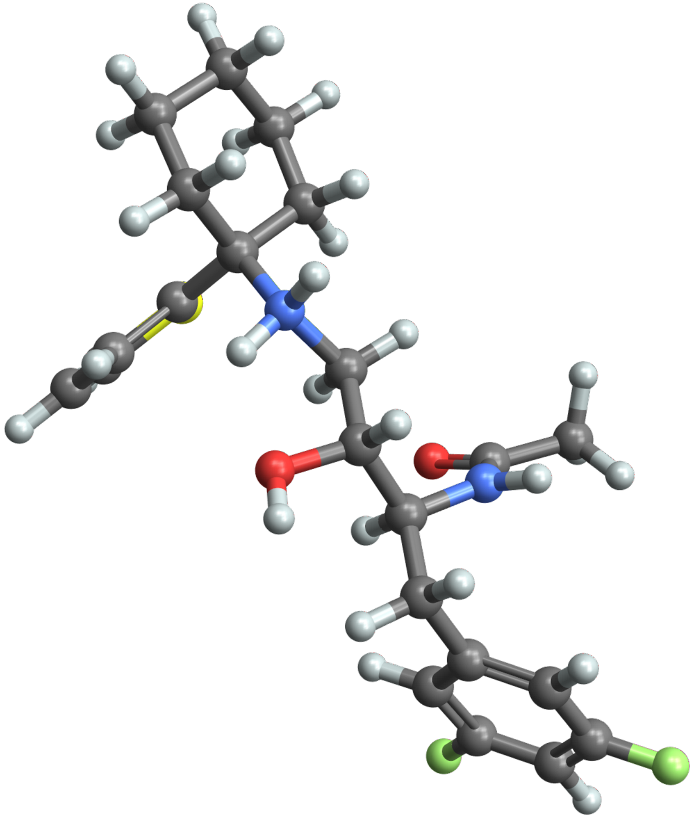

2D Autocorrelation

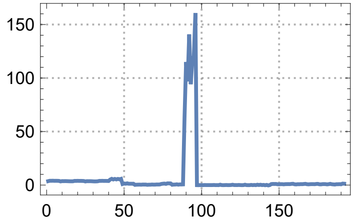

Dataset Index= 1444

Molecule Formula= $C_{22}H_{29}F_2N_2O_2S^+$

3D Autocorrelation

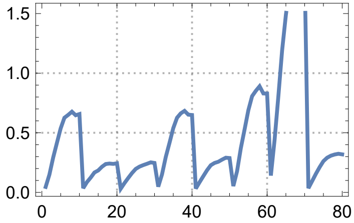

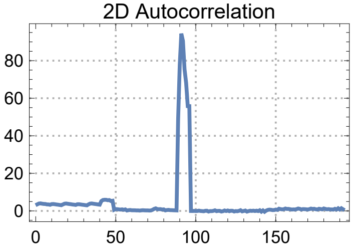

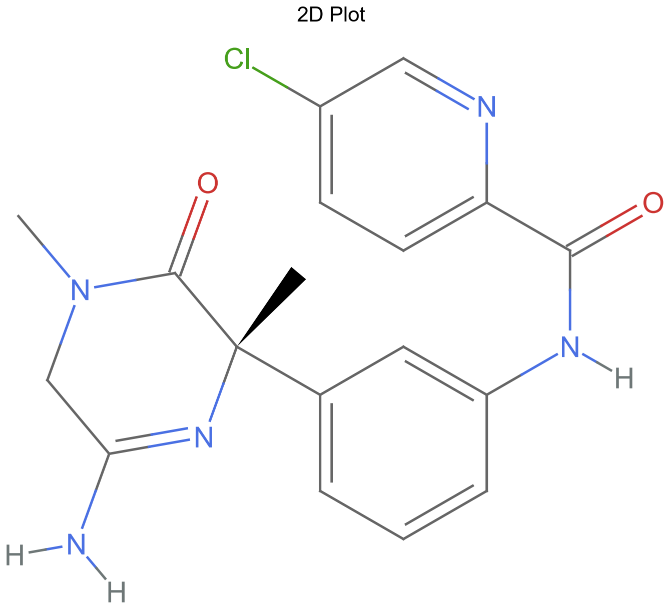

Dataset Index= 1445

Molecule Formula= $C_{18}H_{18}ClN_5O_2$

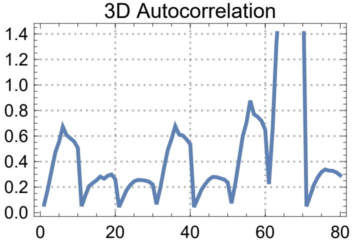

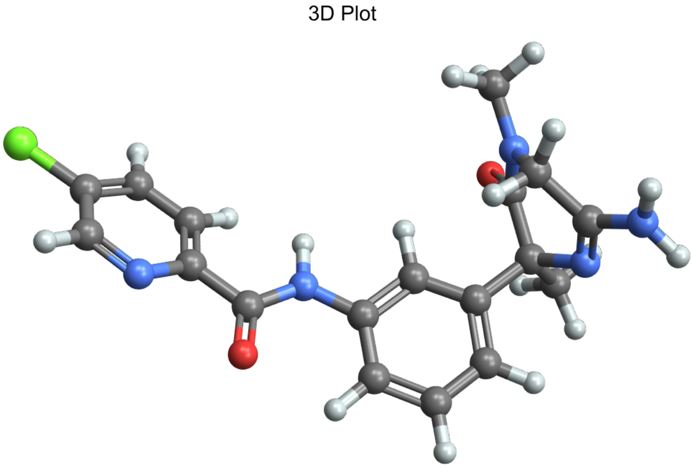

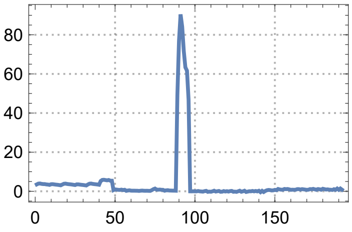

Dataset Index= 1446

Molecule Formula= $C_{18}H_{18}ClN_5O_2$

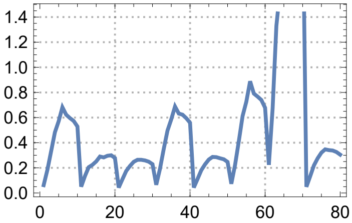

2D Plot

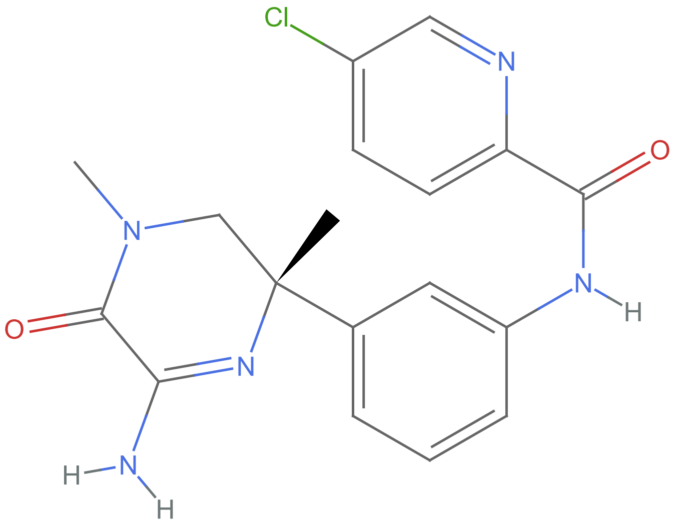

3D Plot

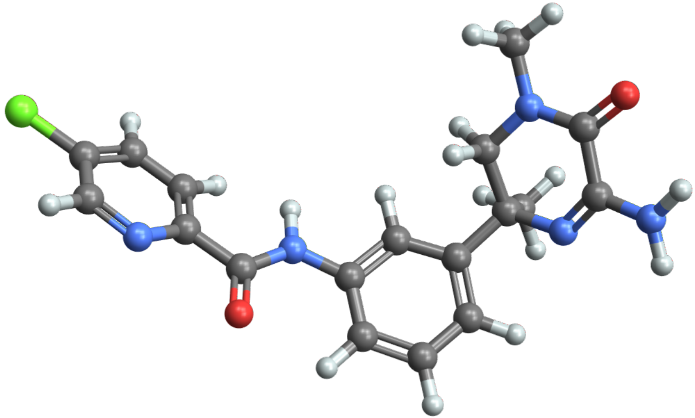

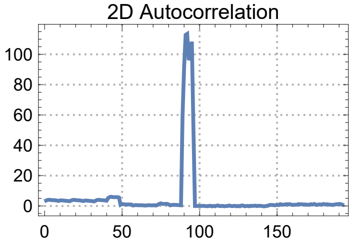

Dataset Index= 1447

Molecule Formula= $C_{18}H_{17}ClFN_5O_2$

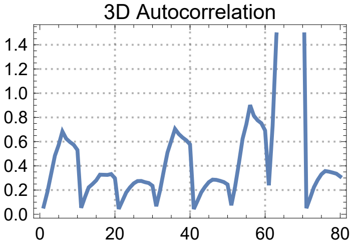

2D Plot

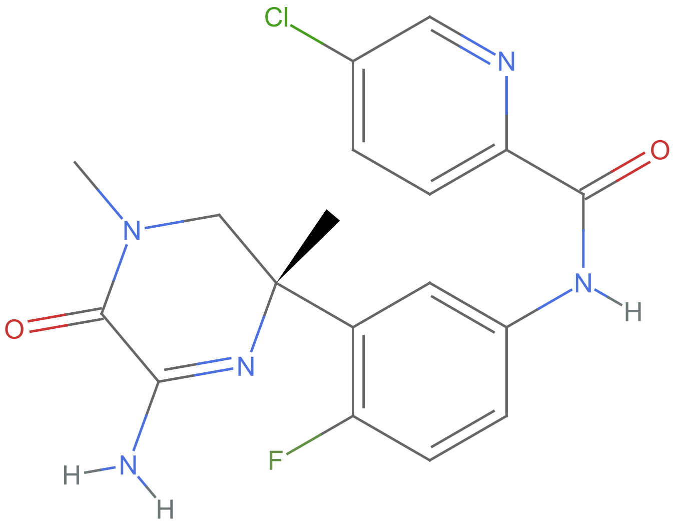

3D Plot

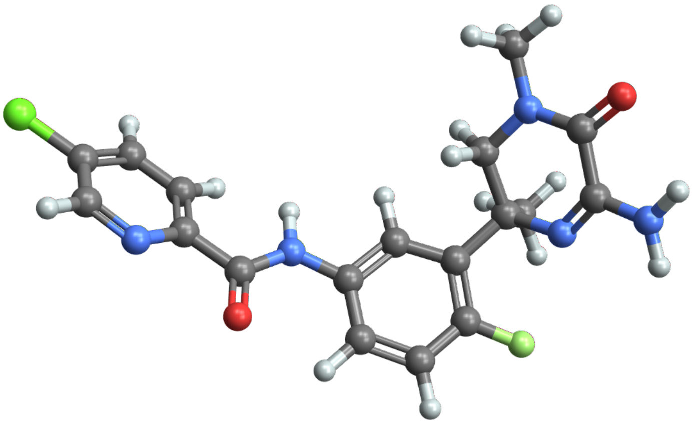

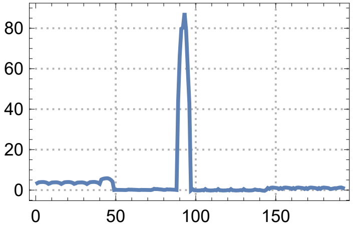

Dataset Index= 1448

Molecule Formula= $C_{22}H_{27}N_4^+$

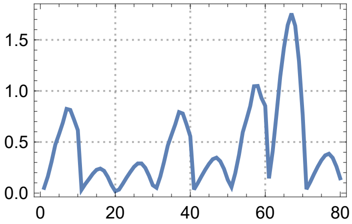

2D Plot

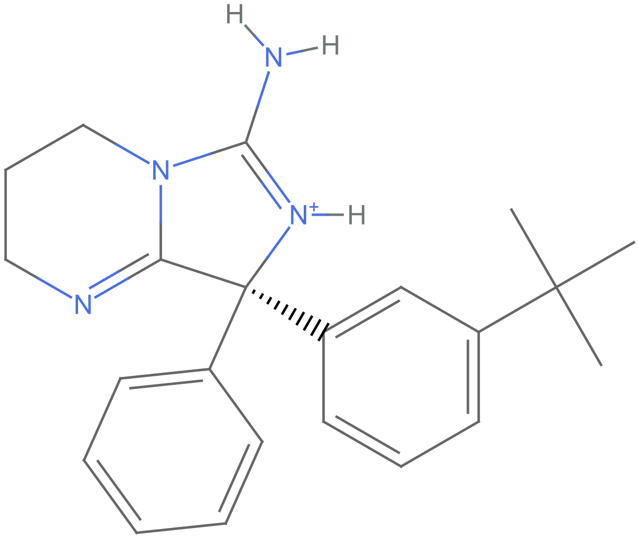

3D Plot

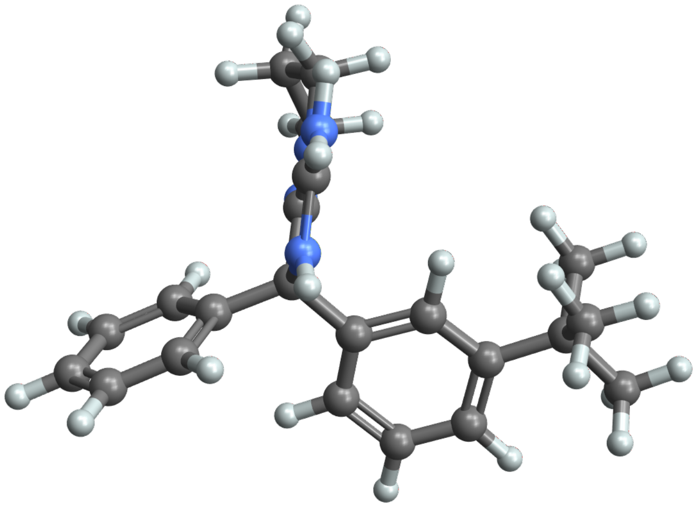

2D Plot

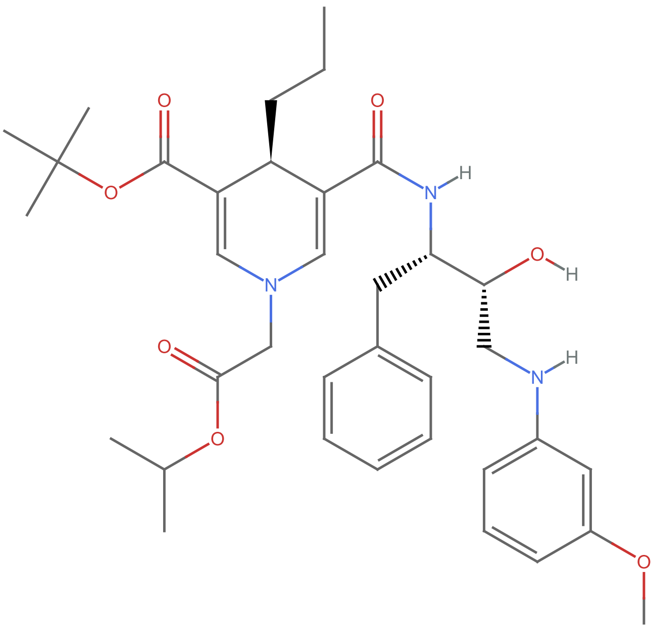

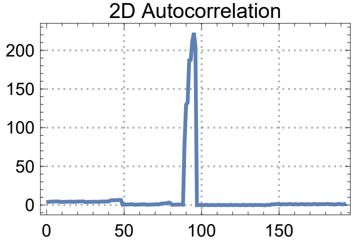

Dataset Index= 1449

Molecule Formula= $C_{36}H_{49}N_3O_7$

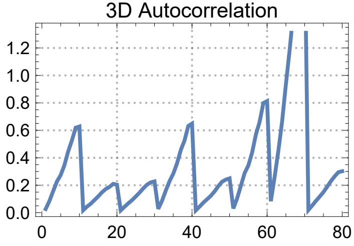

3D Plot

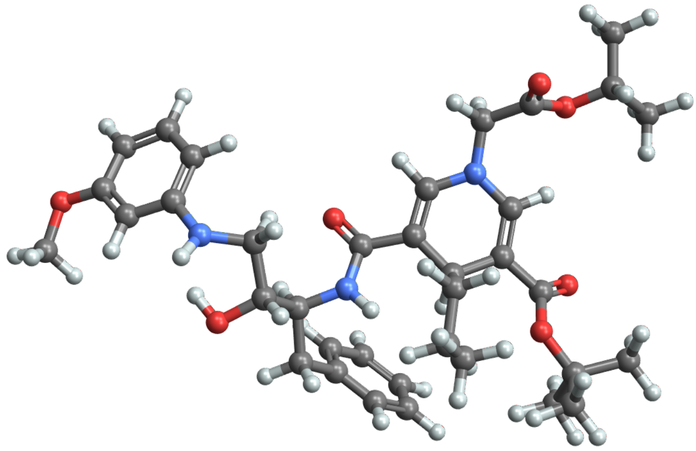

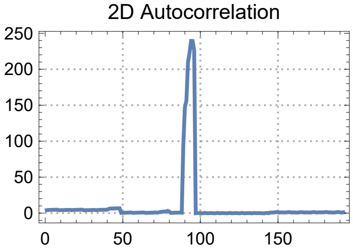

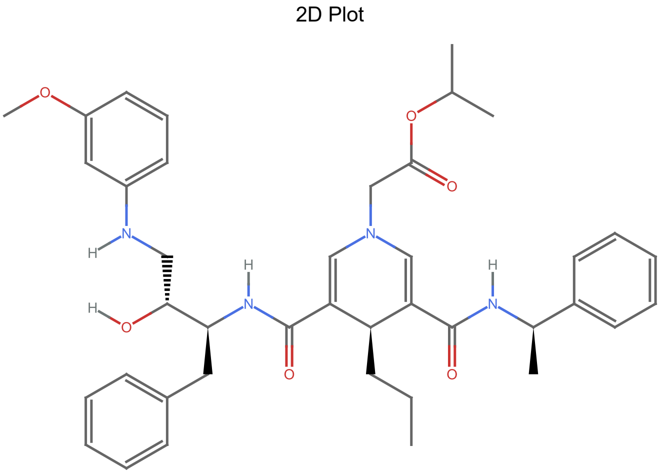

Dataset Index= 1450

Molecule Formula= $C_{40}H_{50}N_4O_6$

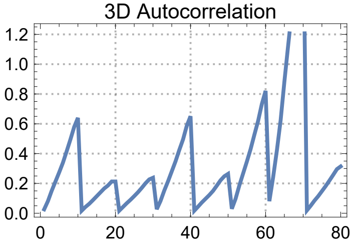

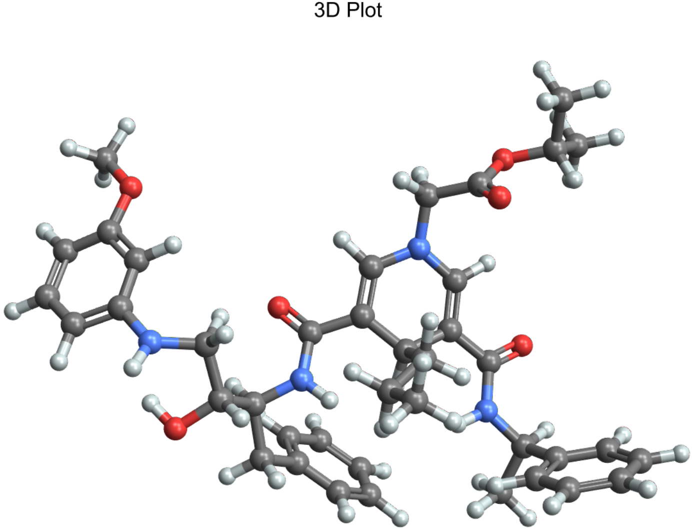

2D Plot

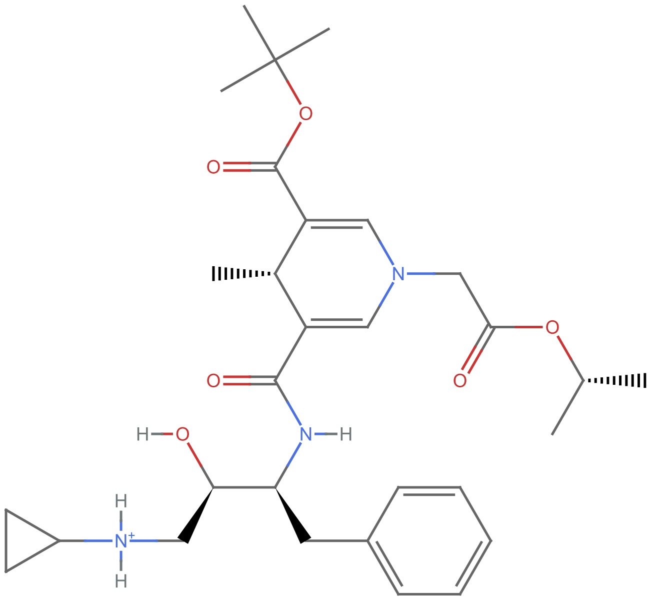

2D Autocorrelation

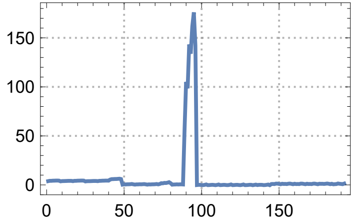

3D Plot

Dataset Index= 1451

Molecule Formula= $C_{30}H_{44}N_3O_6^+$

3D Autocorrelation

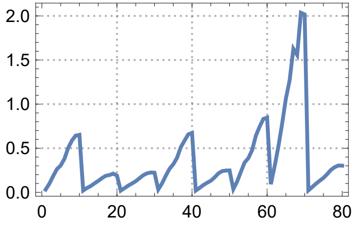

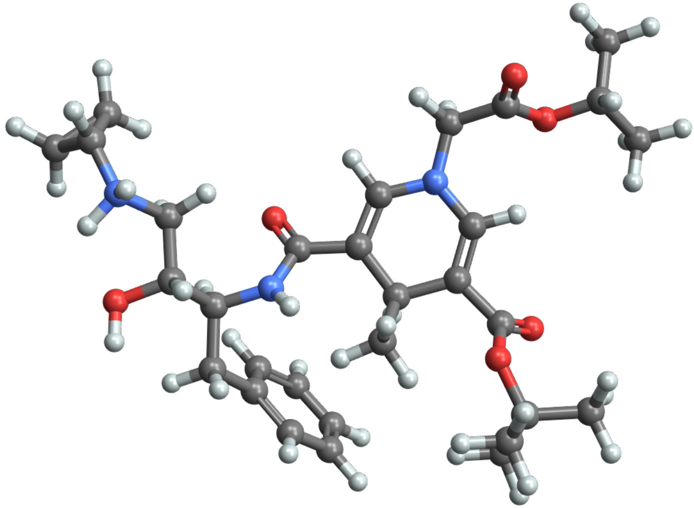

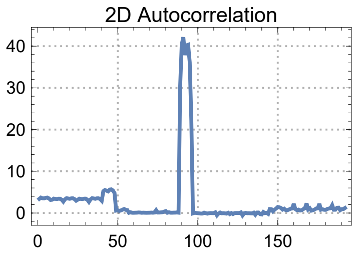

Dataset Index= 1452

Molecule Formula= $C_{18}H_{18}N_2OS$

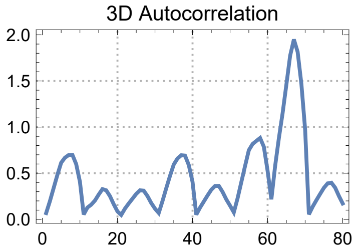

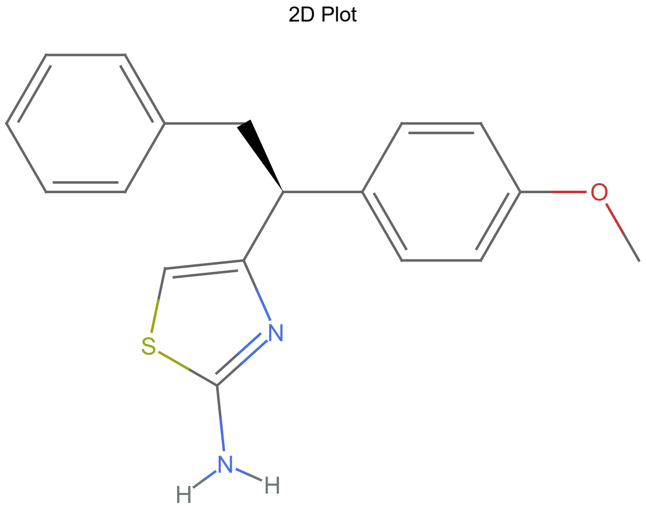

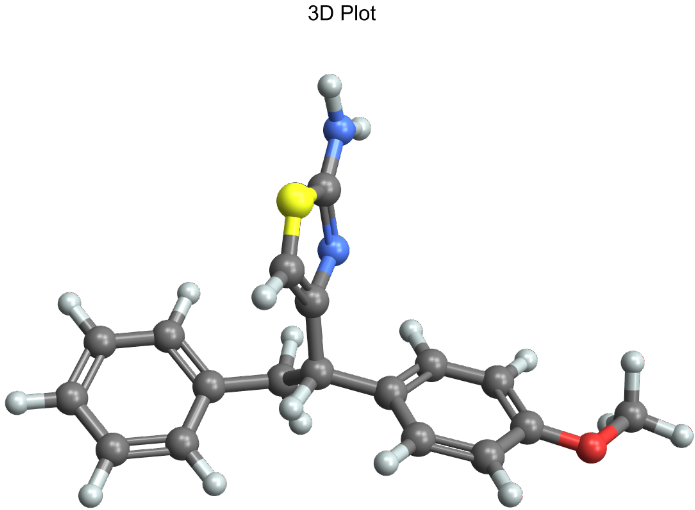

2D Plot

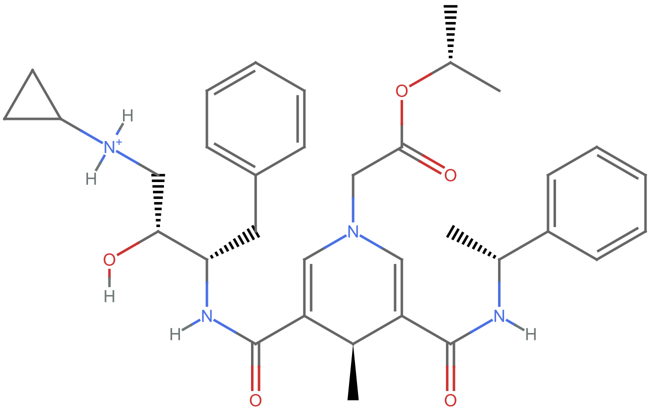

2D Autocorrelation

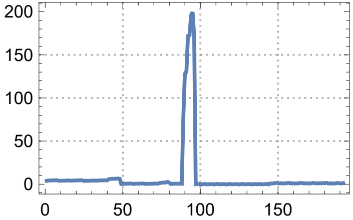

3D Plot

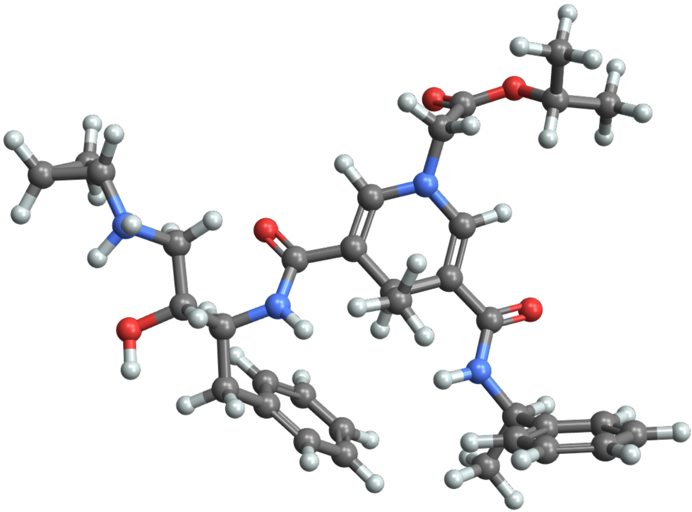

Dataset Index= 1453

Molecule Formula= $C_{34}H_{45}N_4O_5^+$

3D Autocorrelation

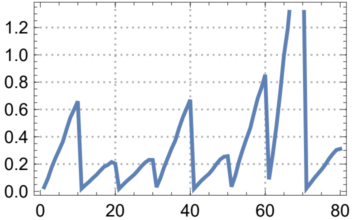

2D Plot

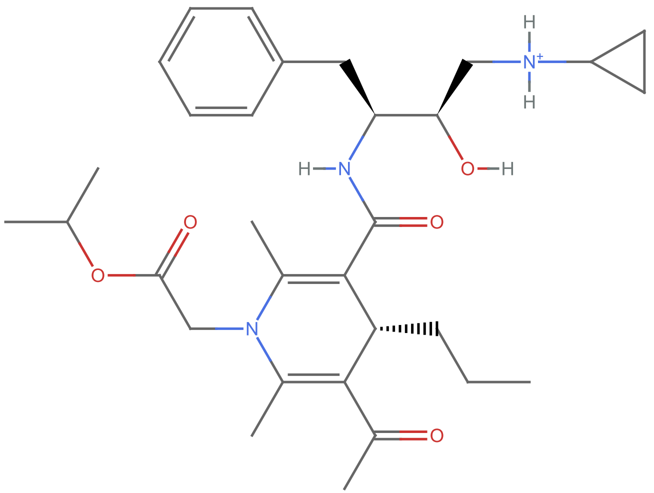

2D Autocorrelation

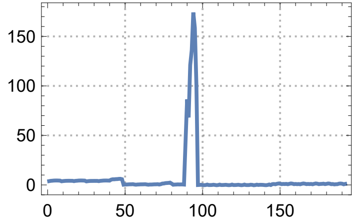

3D Plot

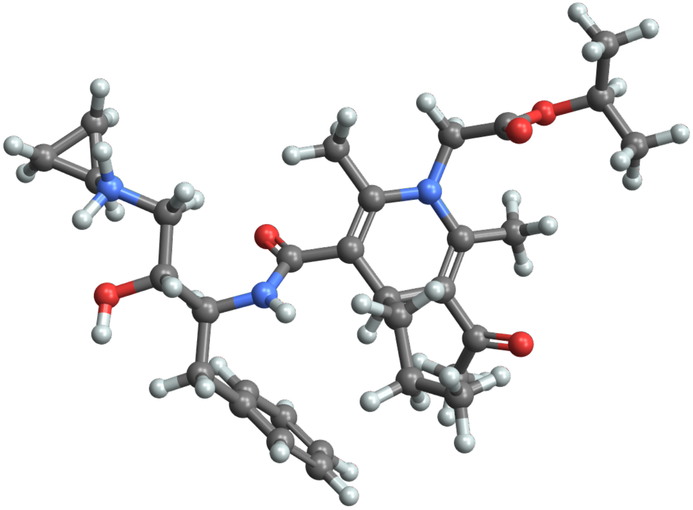

Dataset Index= 1454

Molecule Formula= $C_{31}H_{46}N_3O_5^+$

3D Autocorrelation

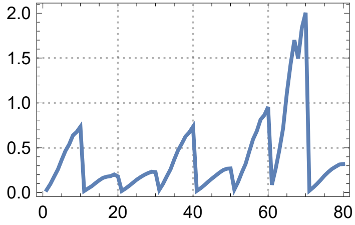

2D Plot

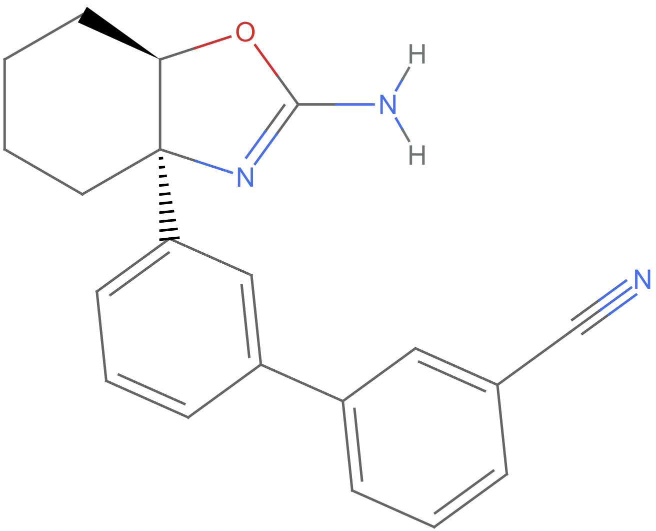

2D Autocorrelation

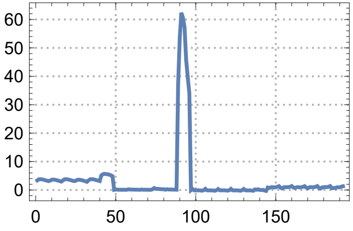

Dataset Index= 1455

Molecule Formula= $C_{20}H_{19}N_3O$

3D Plot

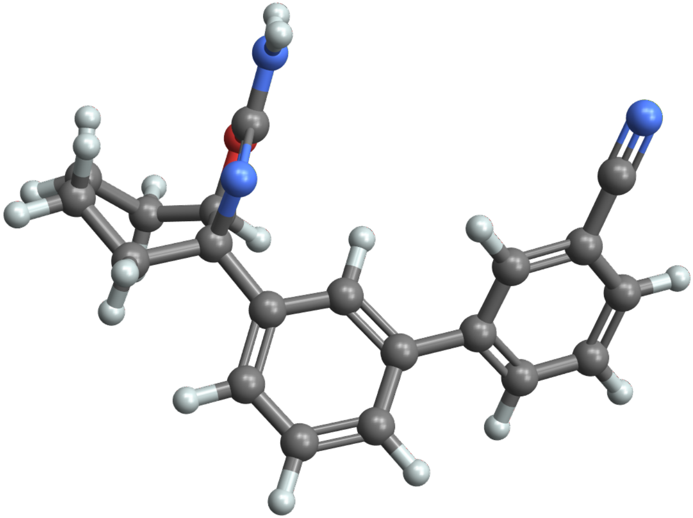

3D Autocorrelation

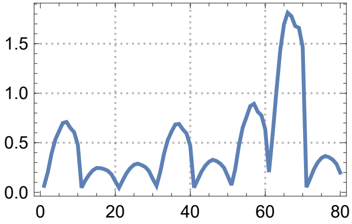

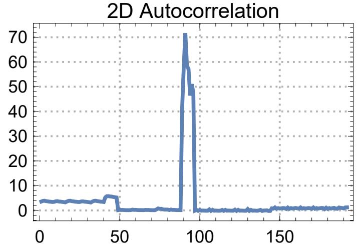

Dataset Index= 1456

Molecule Formula= $C_{24}H_{25}N_3O_2$

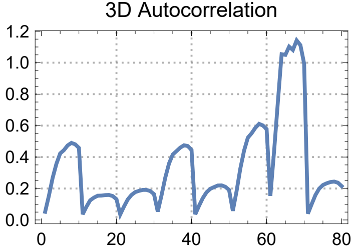

2D Plot

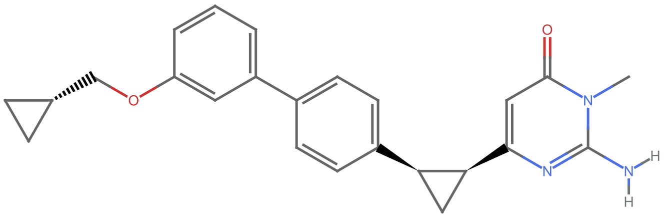

3D Plot

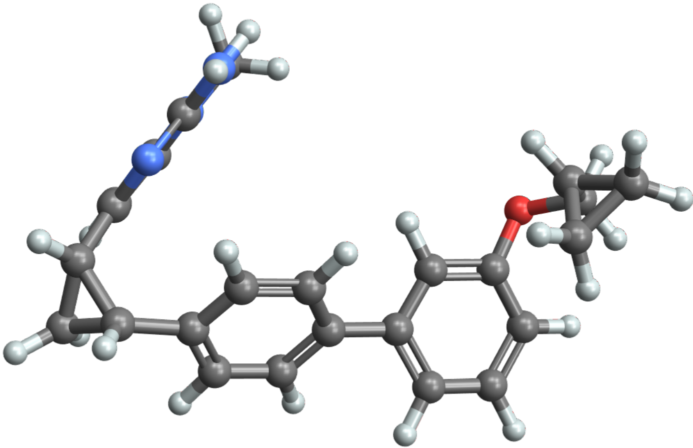

2D Autocorrelation

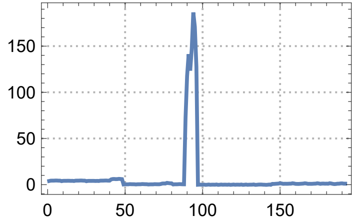

Dataset Index= 1457

Molecule Formula= $C_{35}H_{43}N_4O_4^{+}$

3D Autocorrelation

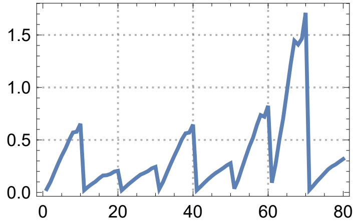

2D Plot

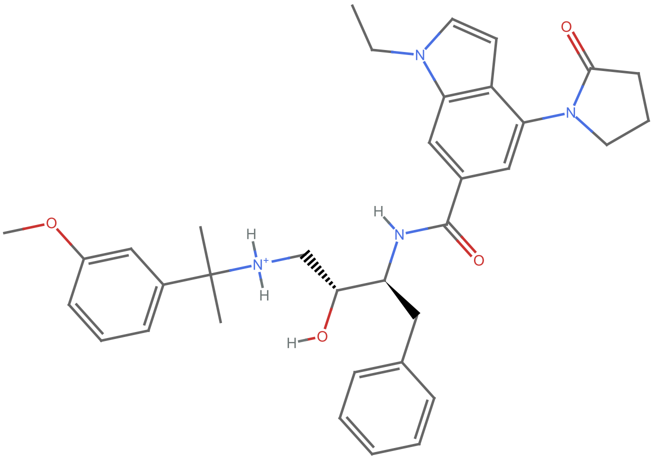

3D Plot

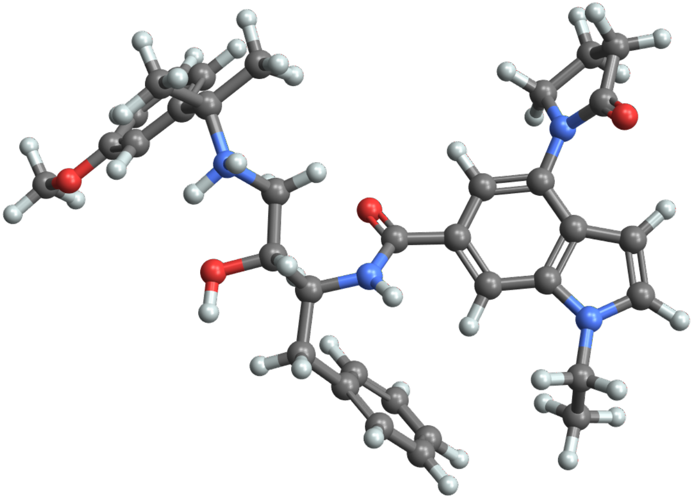

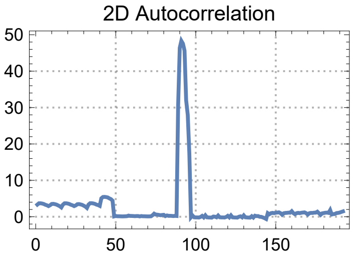

Dataset Index= 1458

Molecule Formula= $C_{17}H_{18}N_4O$

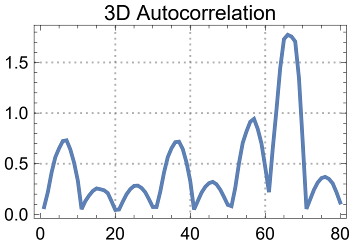

2D Plot

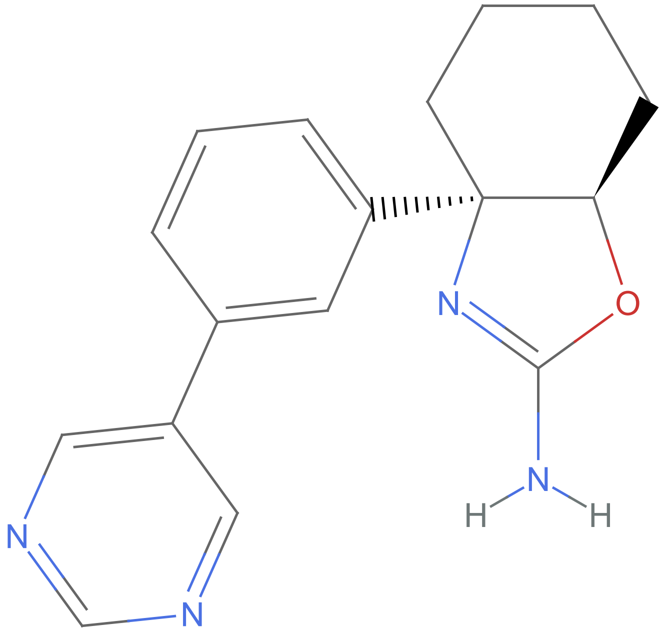

3D Plot

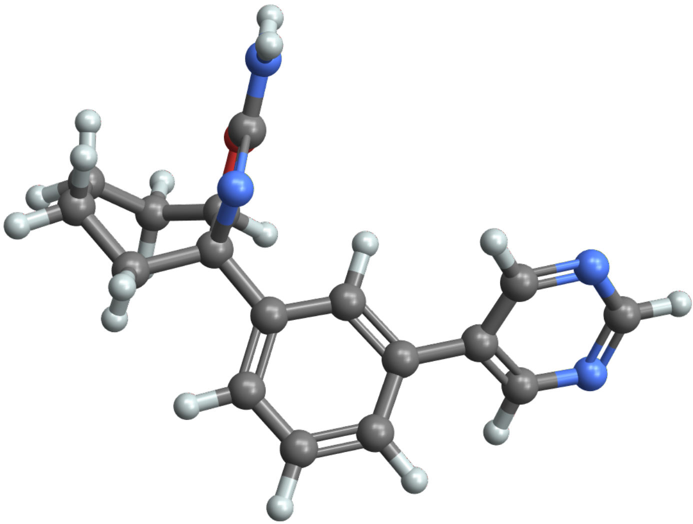

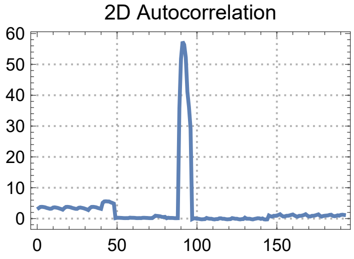

Dataset Index= 1459

Molecule Formula= $C_{19}H_{20}N_2O_3$

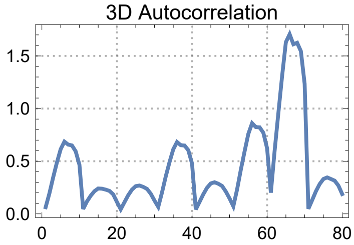

2D Plot

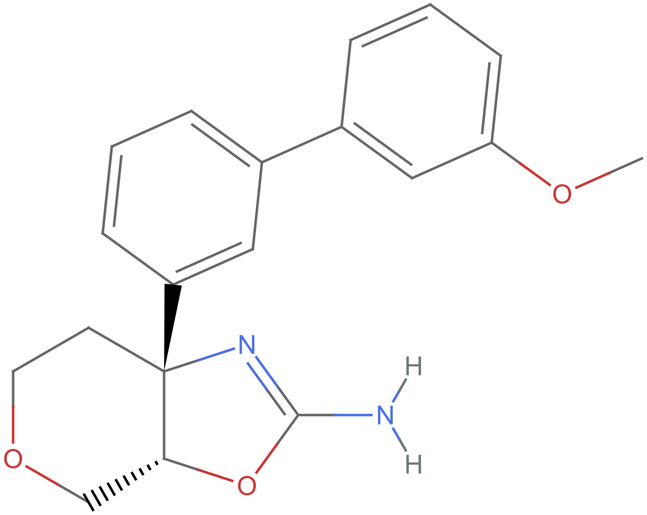

3D Plot

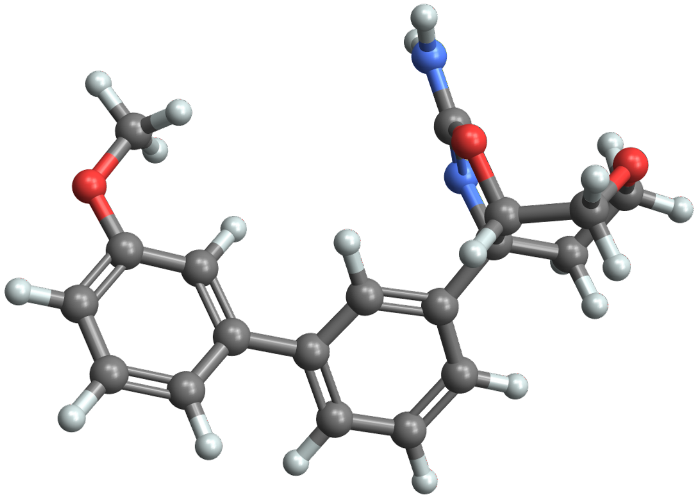

2D Plot

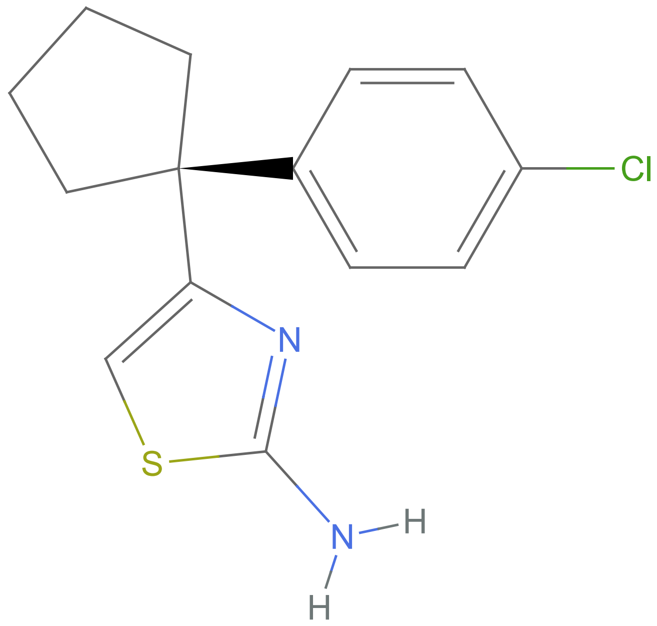

2D Autocorrelation

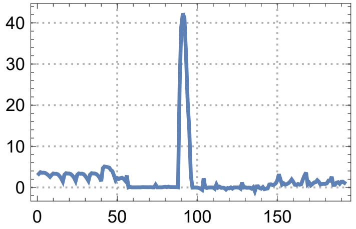

Dataset Index= 1460

Molecule Formula= $C_{14}H_{15}ClN_2S$

3D Plot

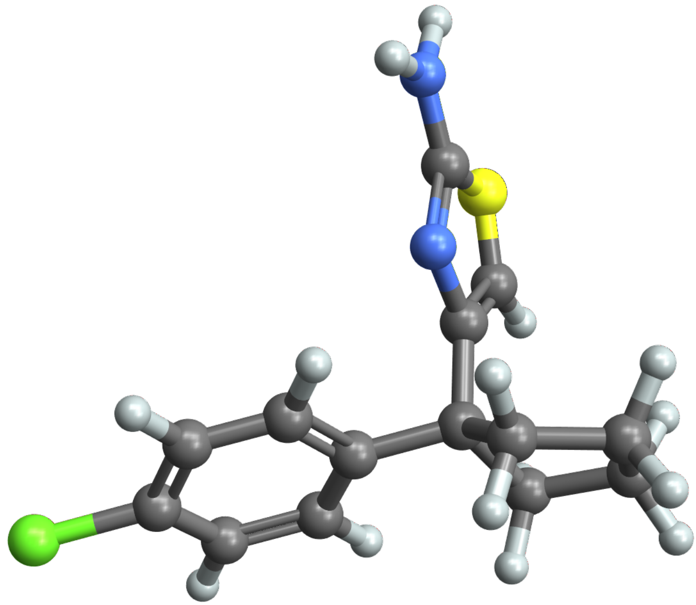

3D Autocorrelation

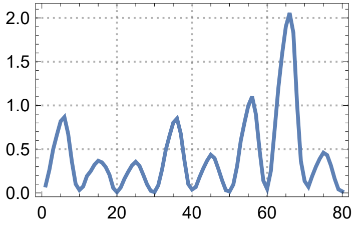

2D Autocorrelation

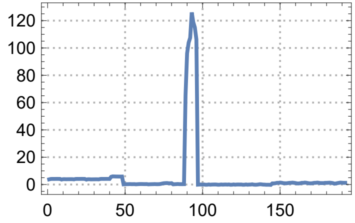

Dataset Index= 1461

Molecule Formula= $C_{31}H_{38}N_3O_3^+$

3D Autocorrelation

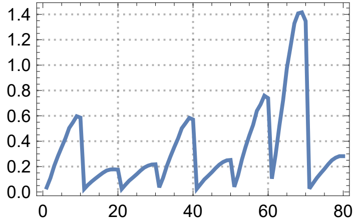

2D Plot

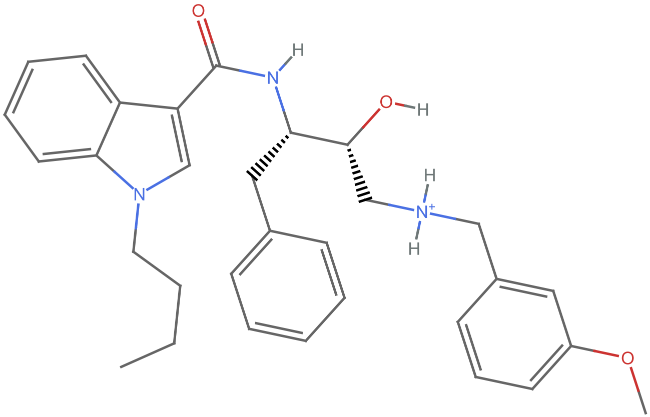

3D Plot

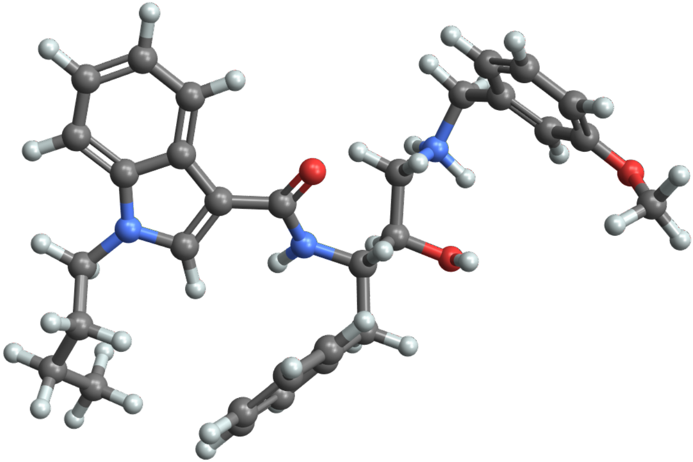

2D Plot

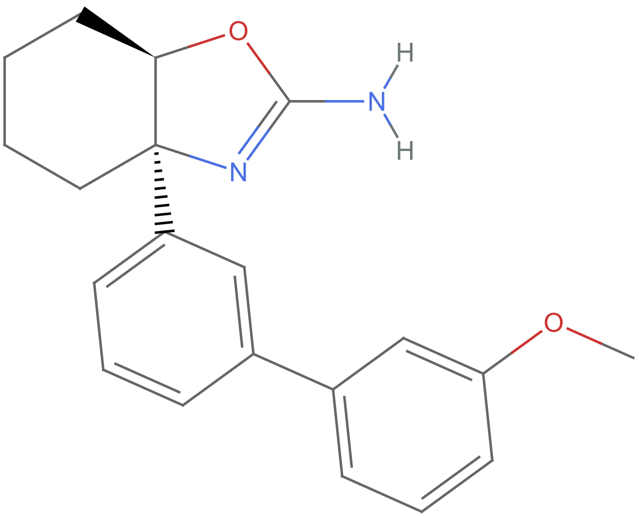

2D Autocorrelation

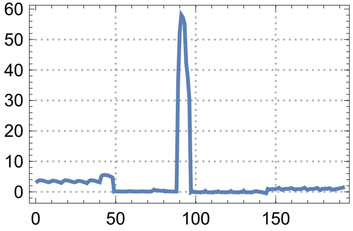

Dataset Index= 1462

Molecule Formula= $C_{20}H_{22}N_2O_2$

3D Plot

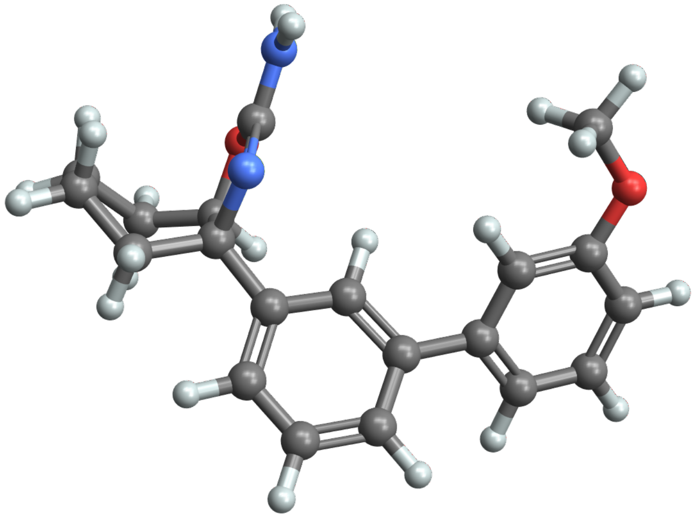

3D Autocorrelation

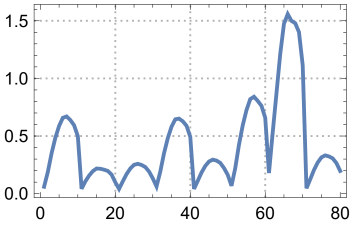

2D Plot

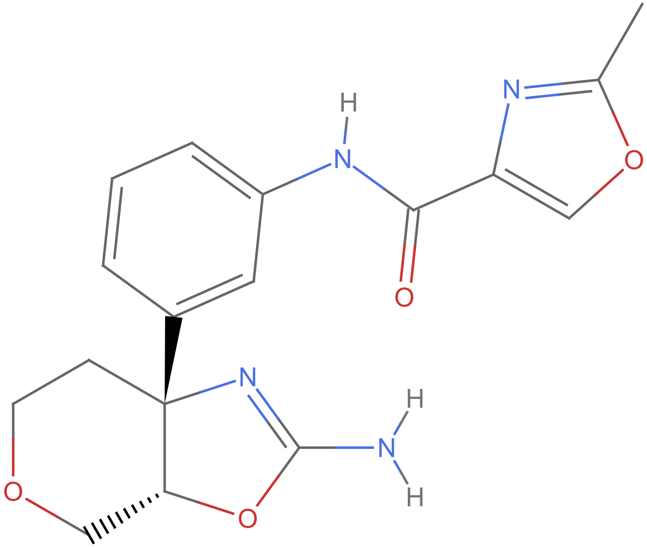

2D Autocorrelation

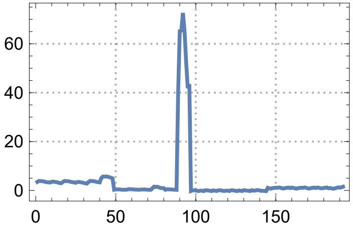

Dataset Index= 1463

Molecule Formula= $C_{17}H_{18}N_4O_4$

3D Autocorrelation

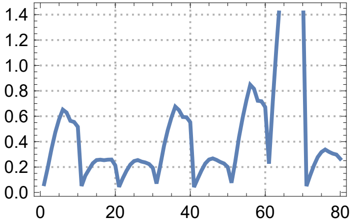

3D Plot

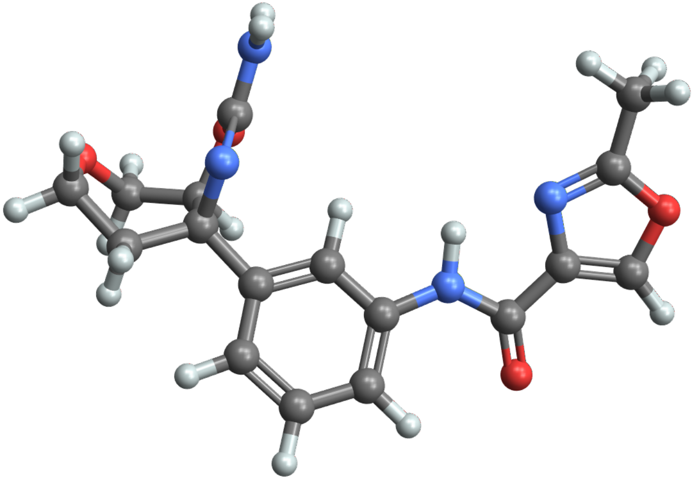

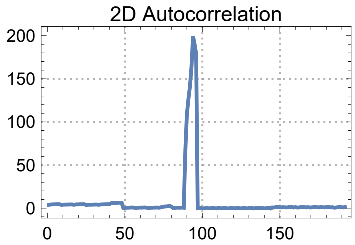

Dataset Index= 1464

Molecule Formula= $C_{34}H_{45}N_4O_5^+$

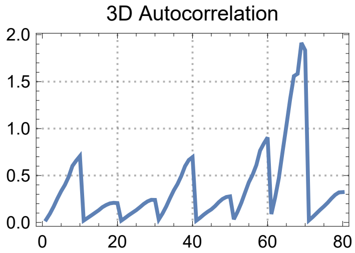

2D Plot

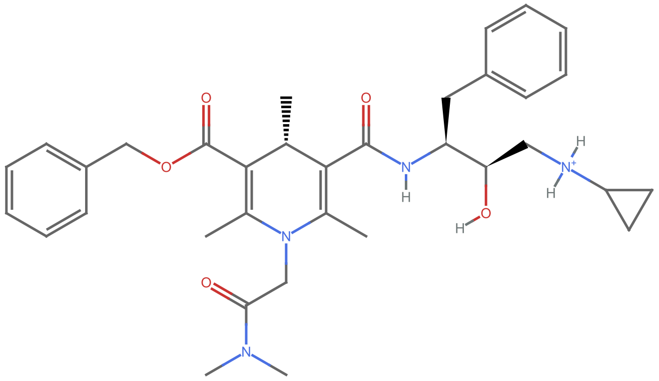

3D Plot

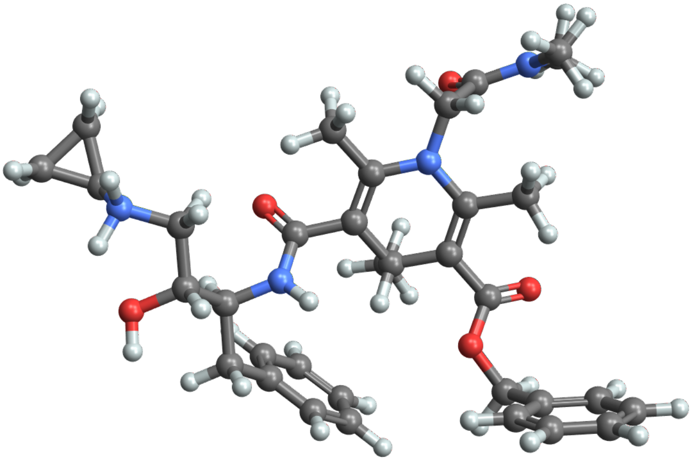

2D Autocorrelation

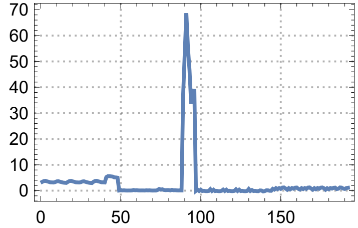

Dataset Index= 1465

Molecule Formula= $C_{21}H_{21}N_3O$

3D Autocorrelation

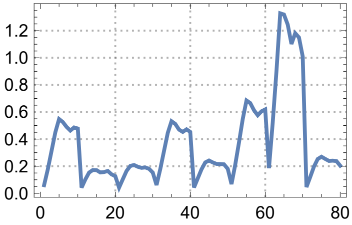

2D Plot

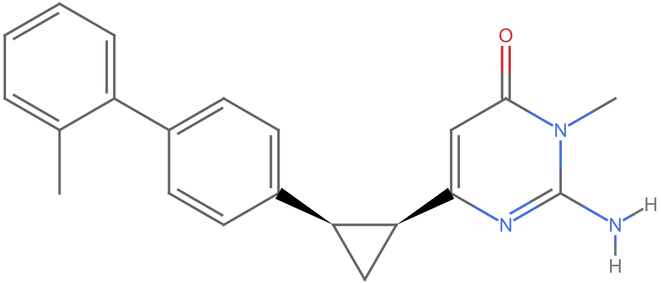

3D Plot

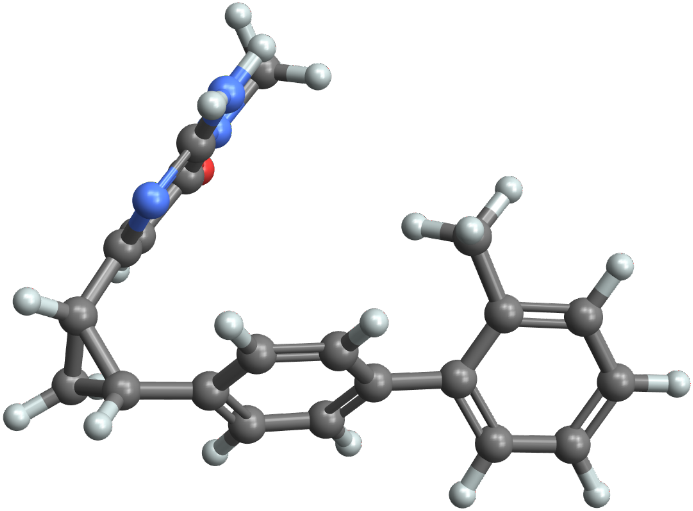

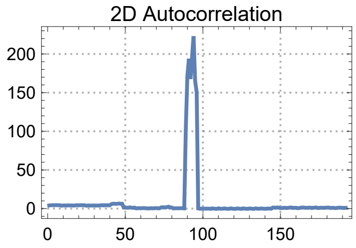

Dataset Index= 1466

Molecule Formula= $C_{34}H_{45}N_4O_5S^+$

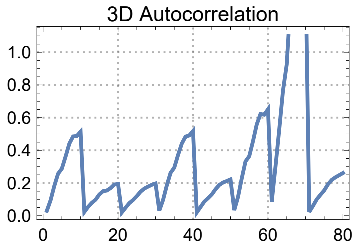

2D Plot

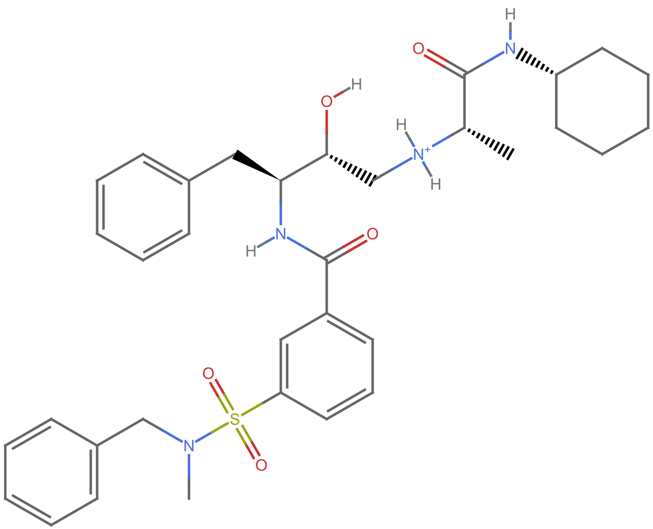

3D Plot

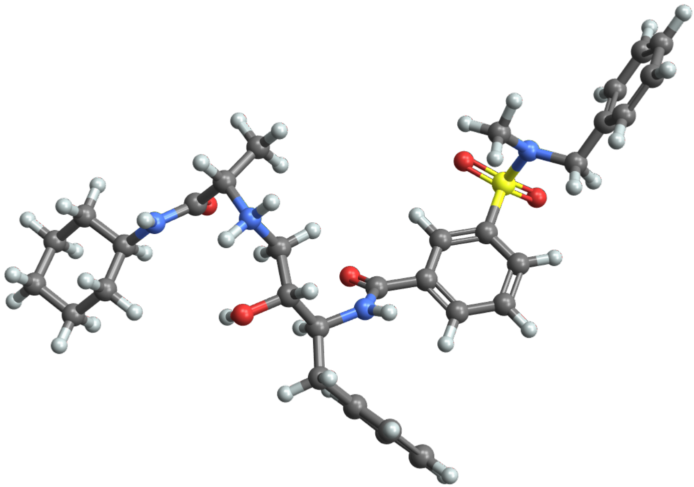

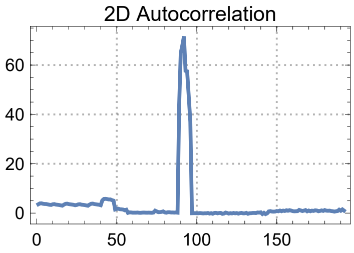

Dataset Index= 1467

Molecule Formula= $C_{18}H_{17}ClN_4OS$

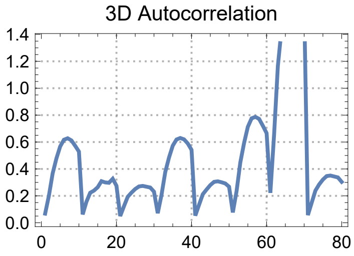

2D Plot

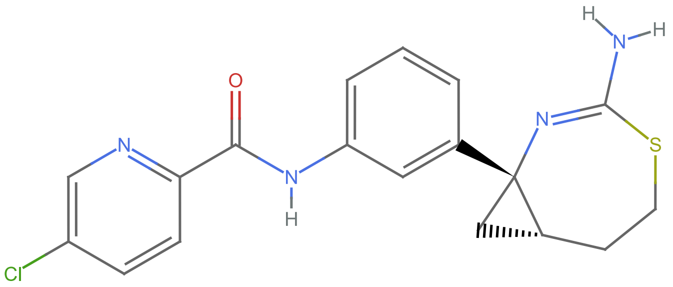

3D Plot

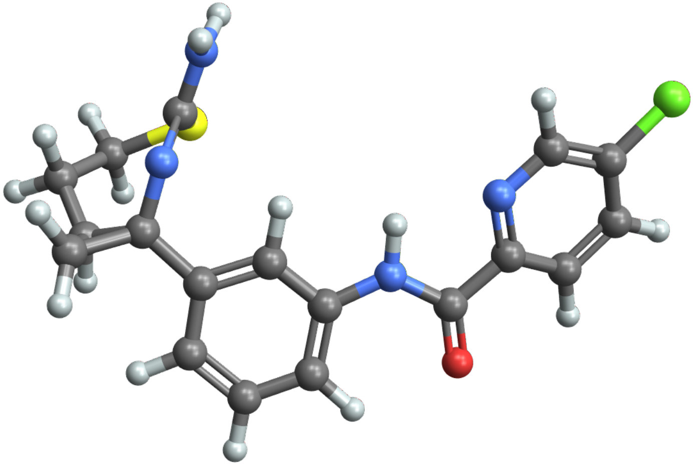

2D Plot

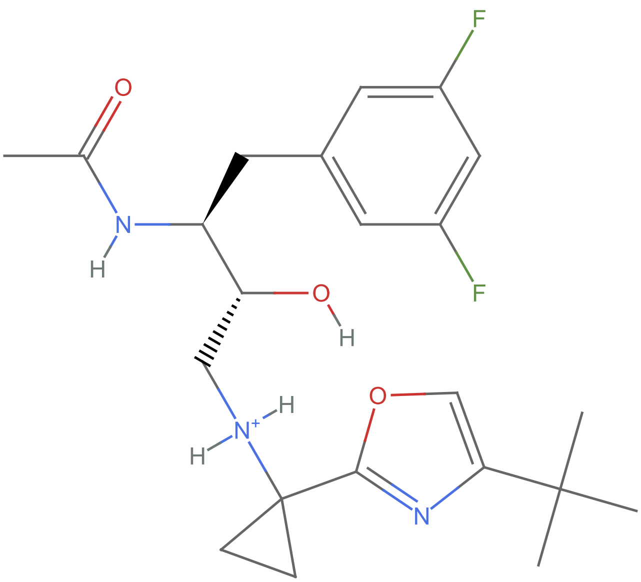

2D Autocorrelation

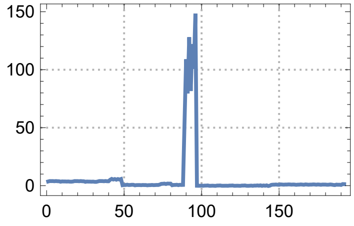

Dataset Index= 1468

Molecule Formula= $C_{22}H_{30}F_2N_3O_3^+$

3D Autocorrelation

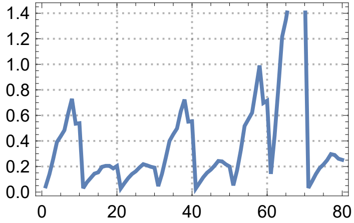

3D Plot

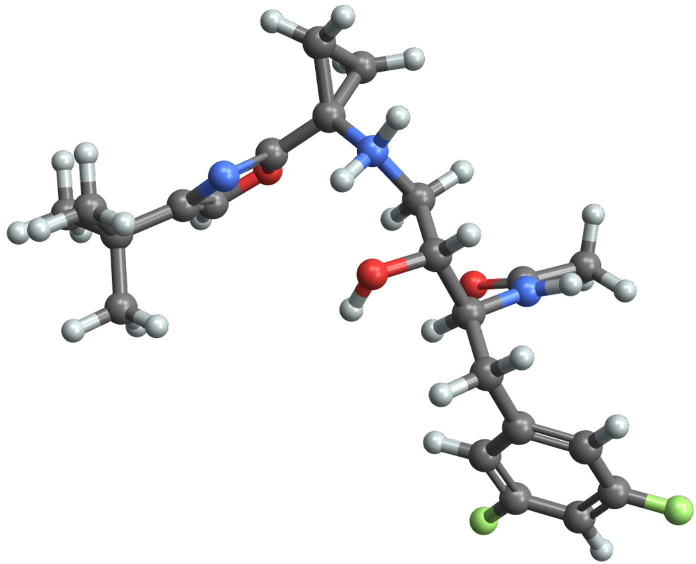

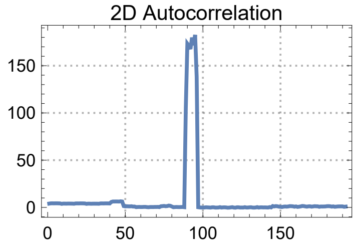

Dataset Index= 1469

Molecule Formula= $C_{33}H_{42}N_3O_5S^+$

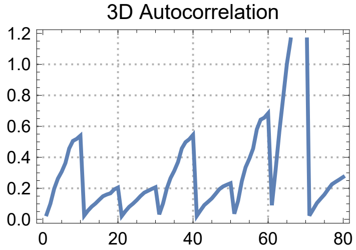

2D Plot

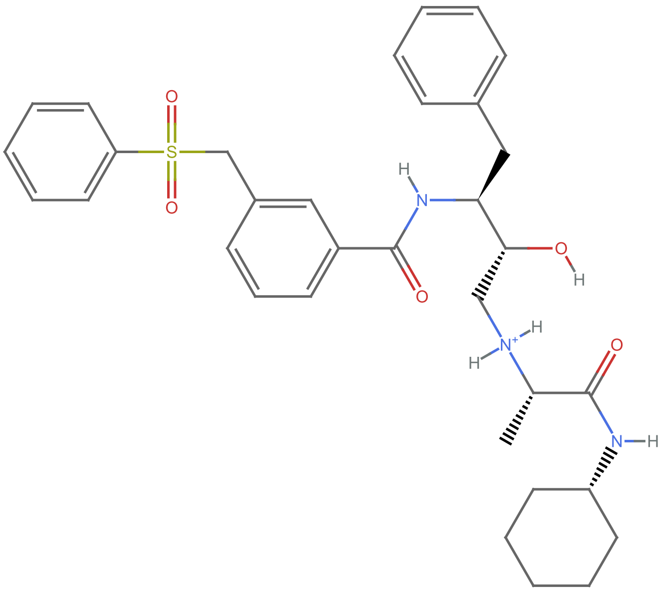

3D Plot

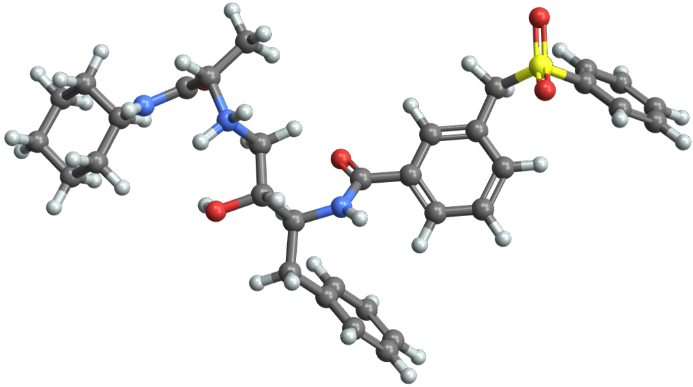

2D Autocorrelation

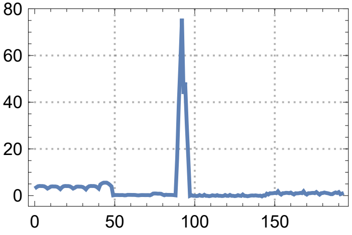

Dataset Index= 1470

Molecule Formula= $C_{22}H_{29}N_3O_2$

3D Autocorrelation

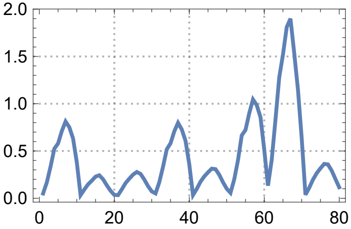

2D Plot

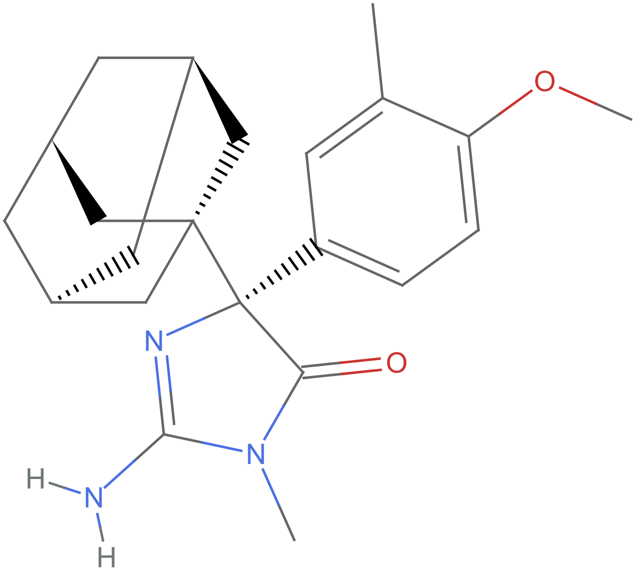

3D Plot

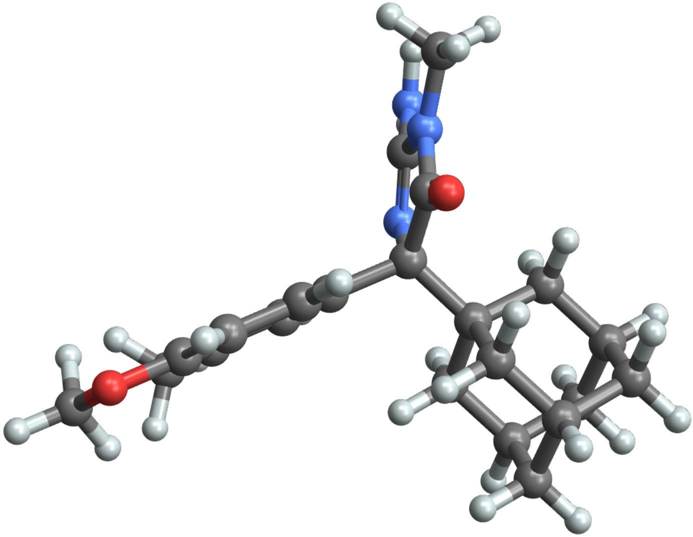

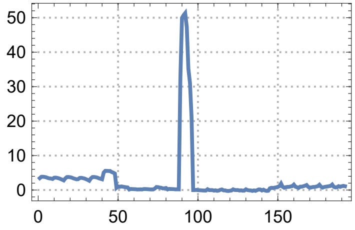

Dataset Index= 1471

Molecule Formula= $C_{17}H_{16}ClN_3O_2$

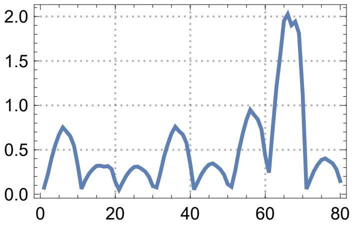

2D Plot

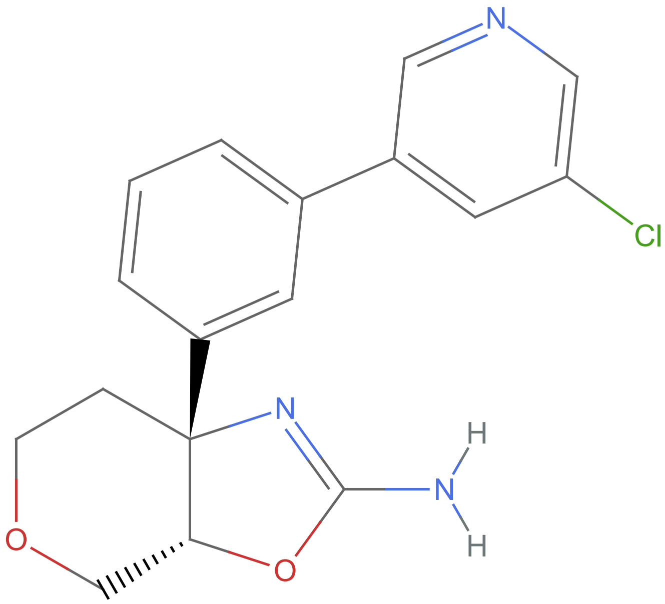

3D Plot

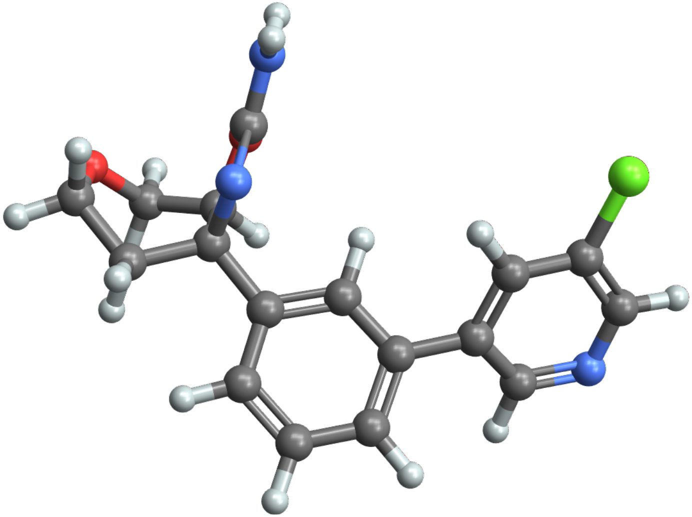

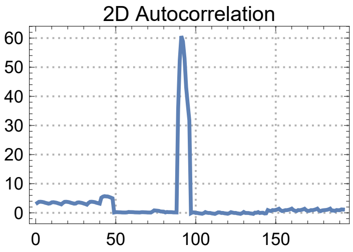

Dataset Index= 1472

Molecule Formula= $C_{19}H_{17}N_3O_2$

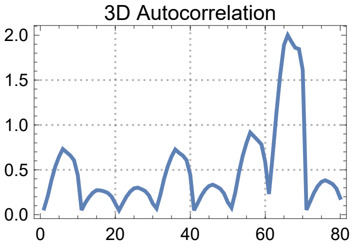

2D Plot

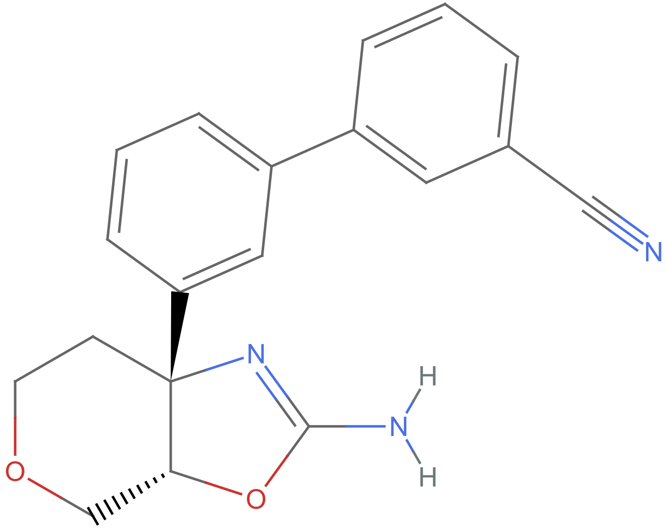

3D Plot

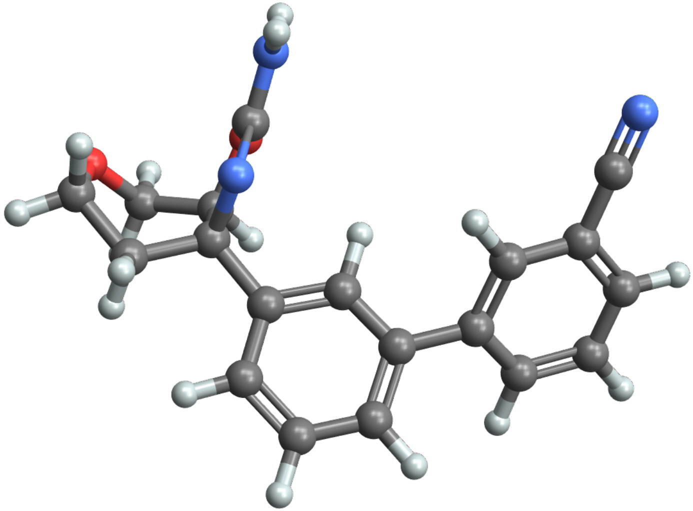

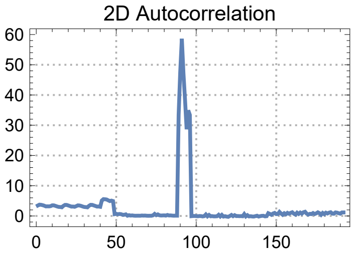

Dataset Index= 1473

Molecule Formula= $C_{18}H_{17}N_3OS$

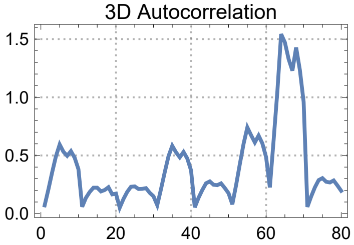

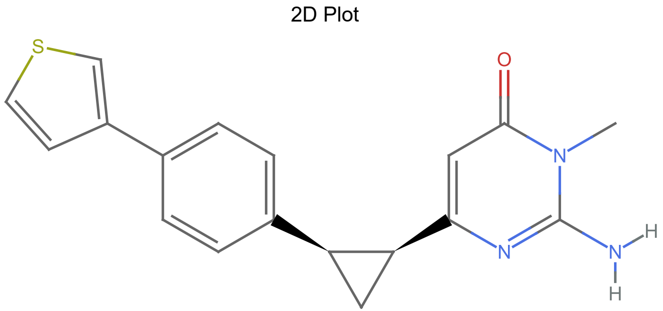

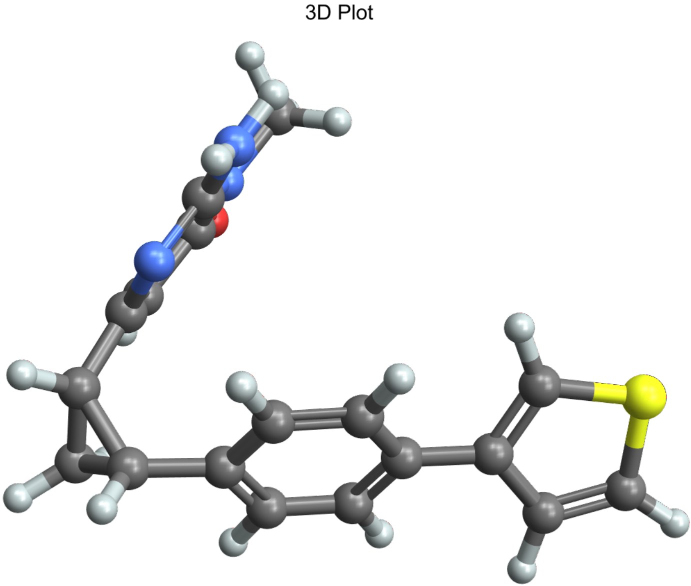

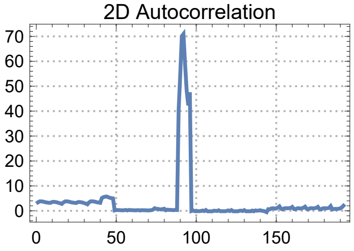

Dataset Index= 1474

Molecule Formula= $C_{18}H_{18}FN_3O$

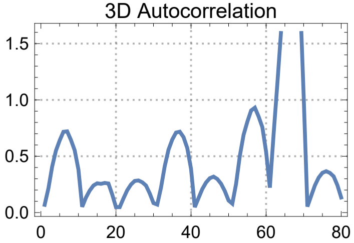

2D Plot

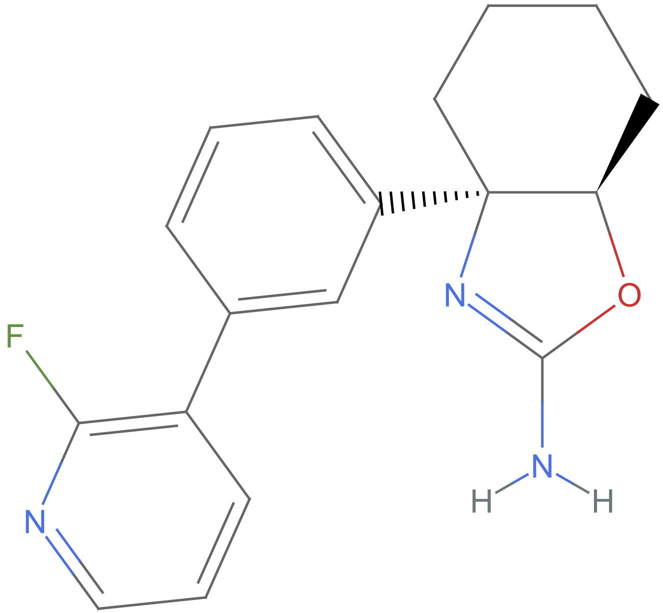

3D Plot

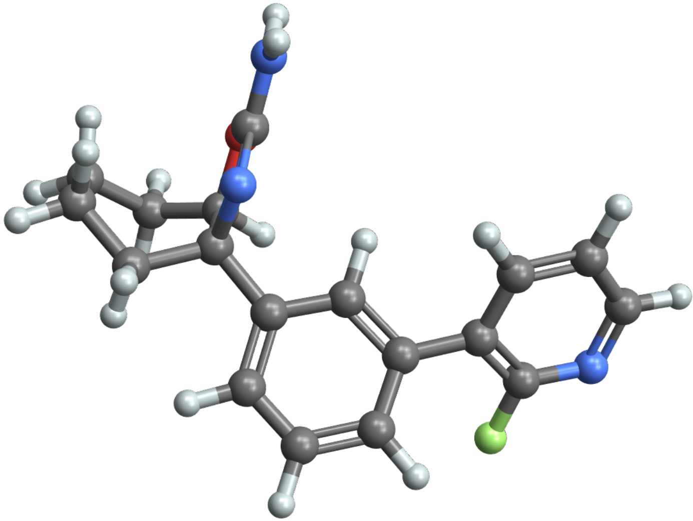

2D Plot

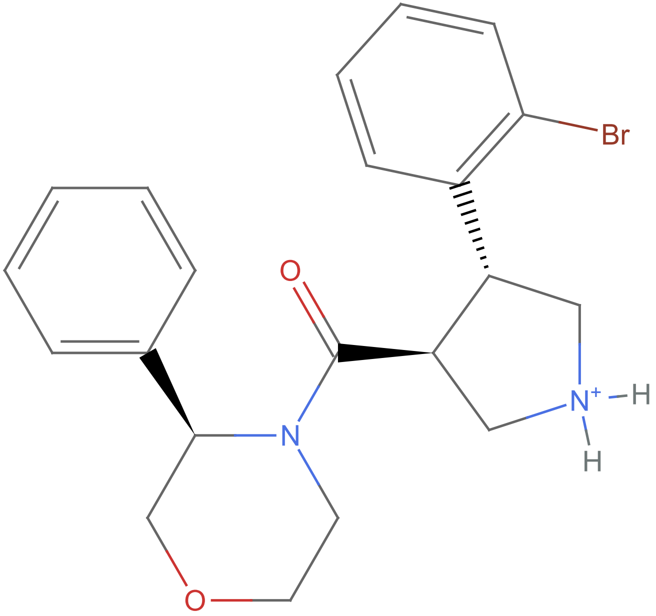

2D Autocorrelation

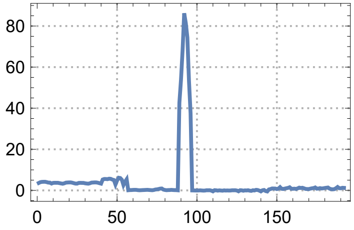

Dataset Index= 1475

Molecule Formula= $C_{21}H_{24}BrN_2O_2^+$

3D Autocorrelation

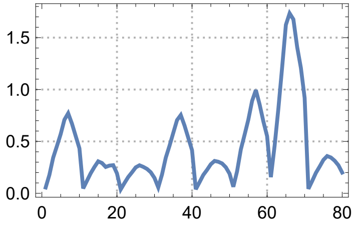

3D Plot

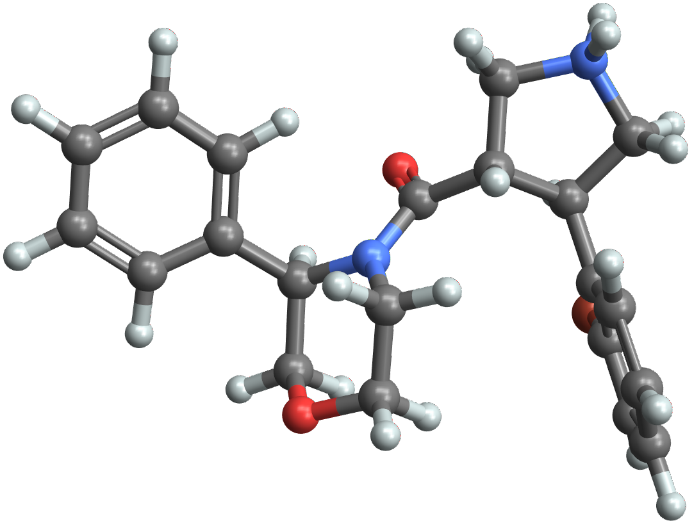

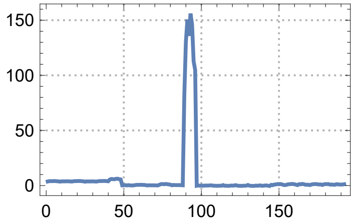

Dataset Index= 1476

Molecule Formula= $C_{28}H_{39}N_5O_2$

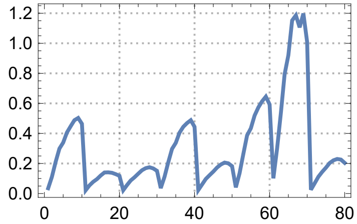

2D Plot

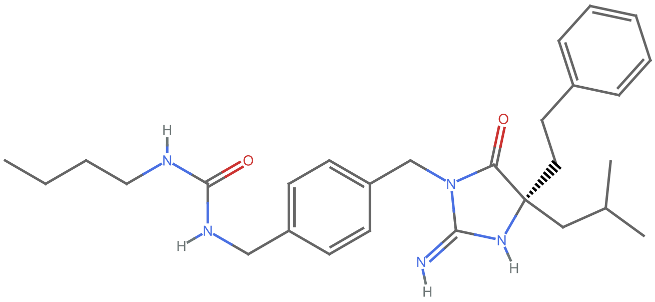

3D Plot

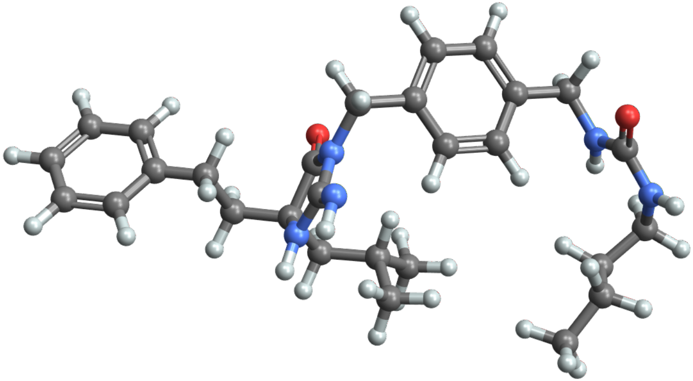

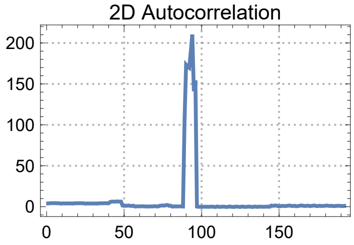

Dataset Index= 1477

Molecule Formula= $C_{33}H_{42}N_3O_5S^+$

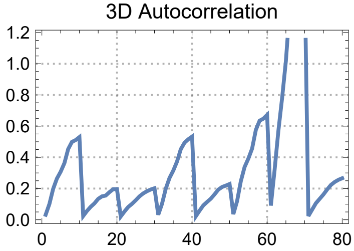

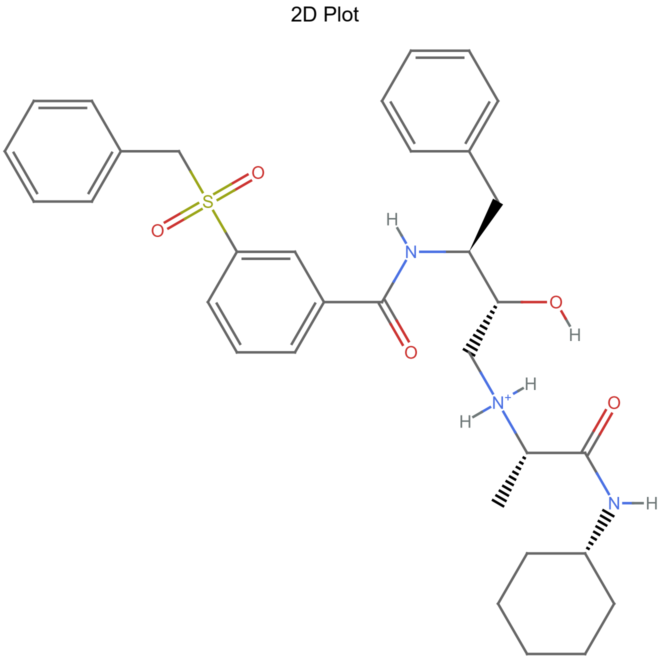

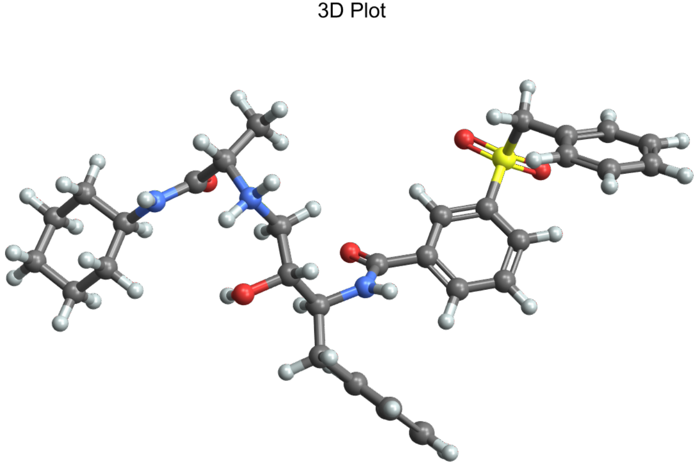

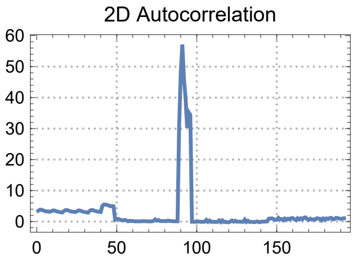

Dataset Index= 1478

Molecule Formula= $C_{19}H_{19}N_3OS$

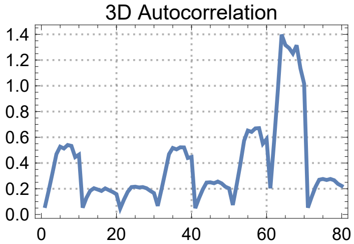

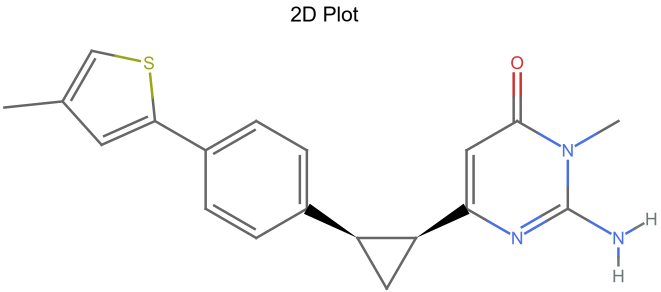

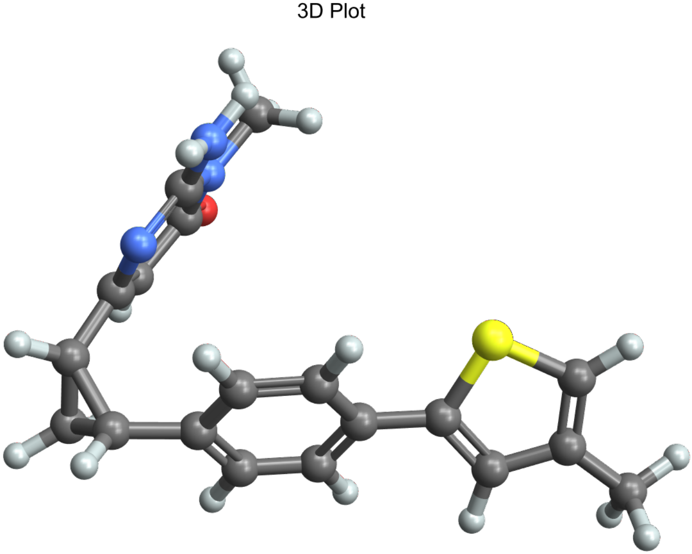

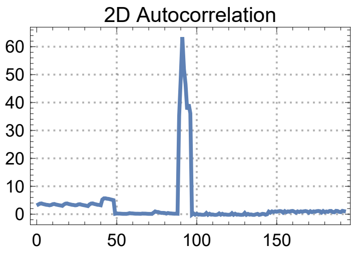

Dataset Index= 1479

Molecule Formula= $C_{20}H_{20}N_4O_2$

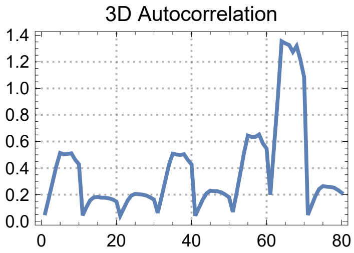

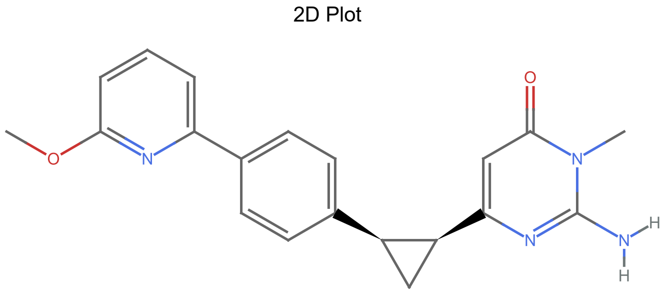

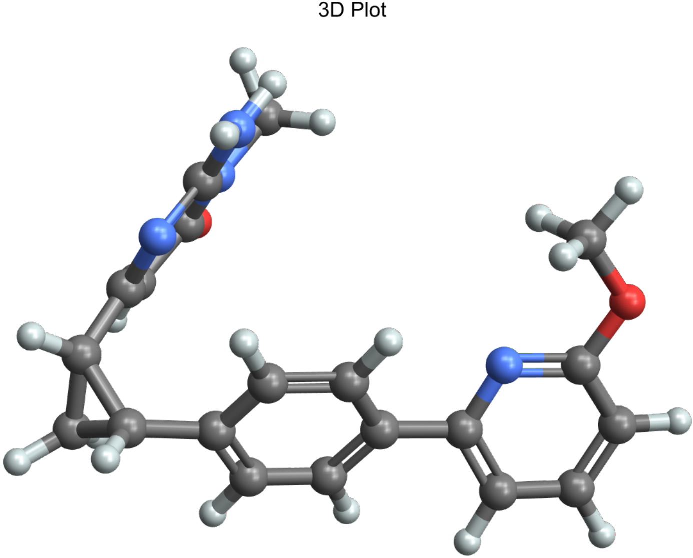

2D Plot

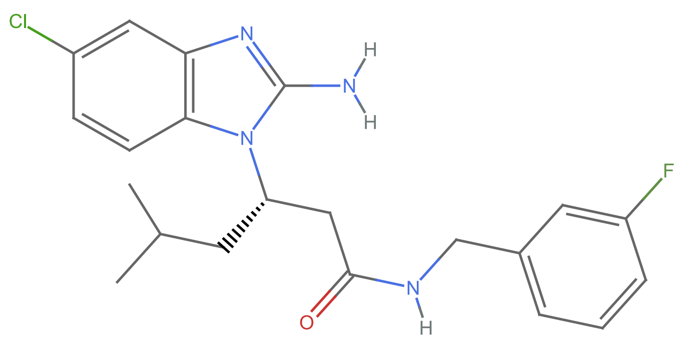

3D Plot

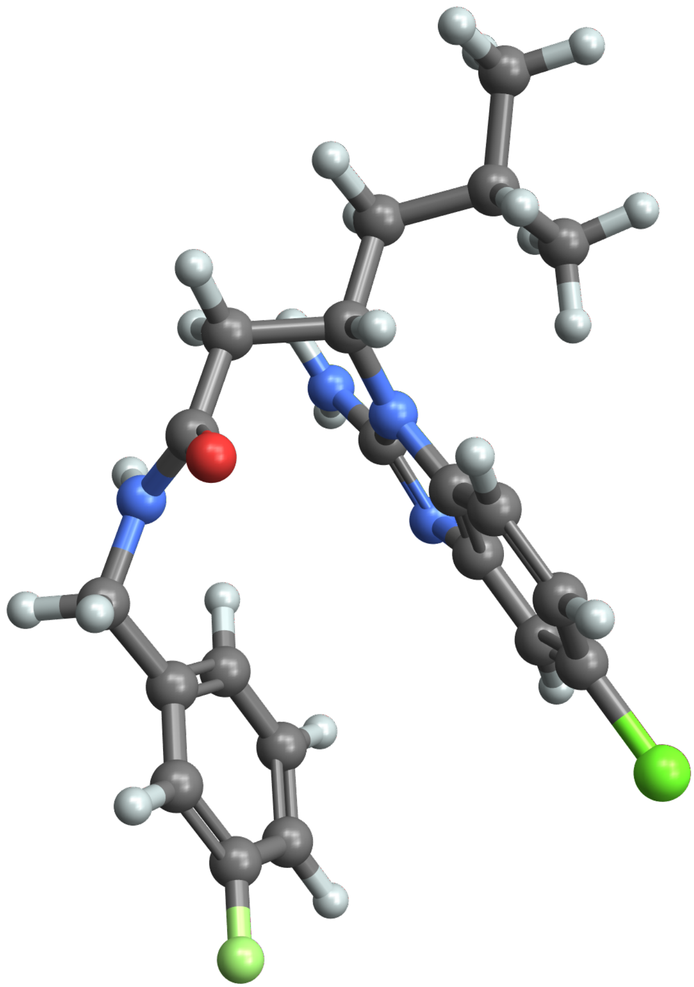

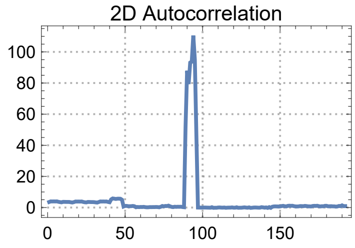

Dataset Index= 1480

Molecule Formula= $C_{21}H_{24}ClFN_4O$

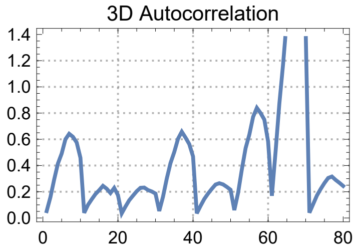

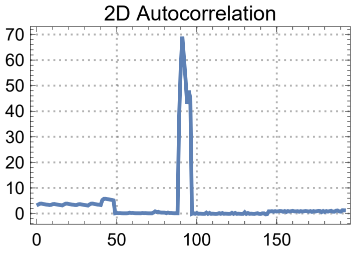

Dataset Index= 1481

Molecule Formula= $C_{23}H_{25}N_3O_2$

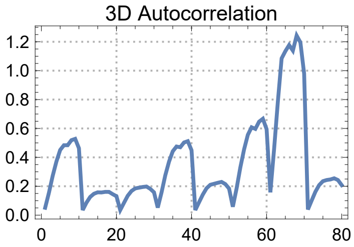

2D Plot

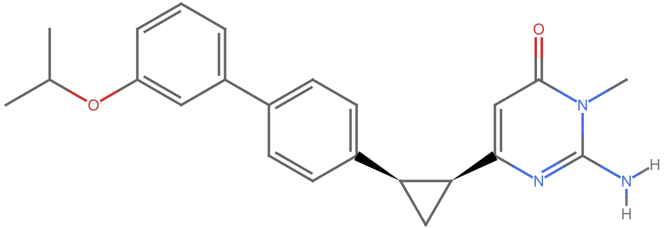

3D Plot

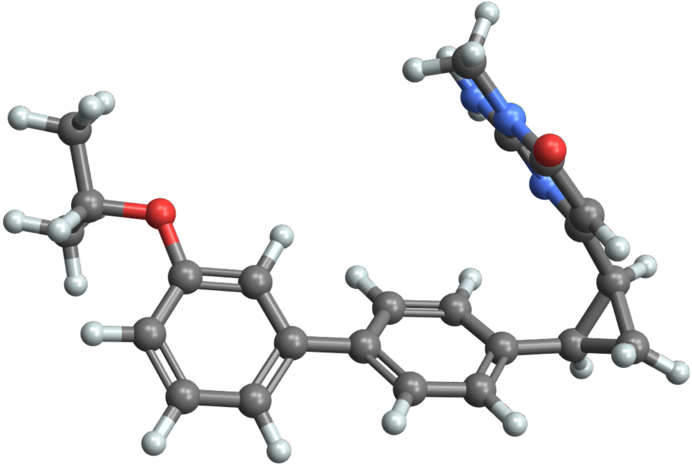

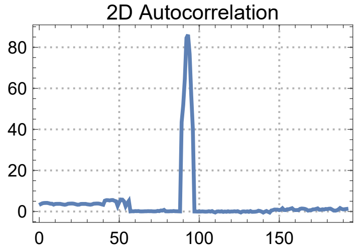

Dataset Index= 1482

Molecule Formula= $C_{22}H_{26}BrN_2O^+$

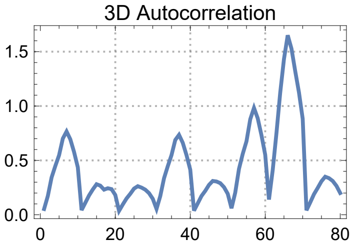

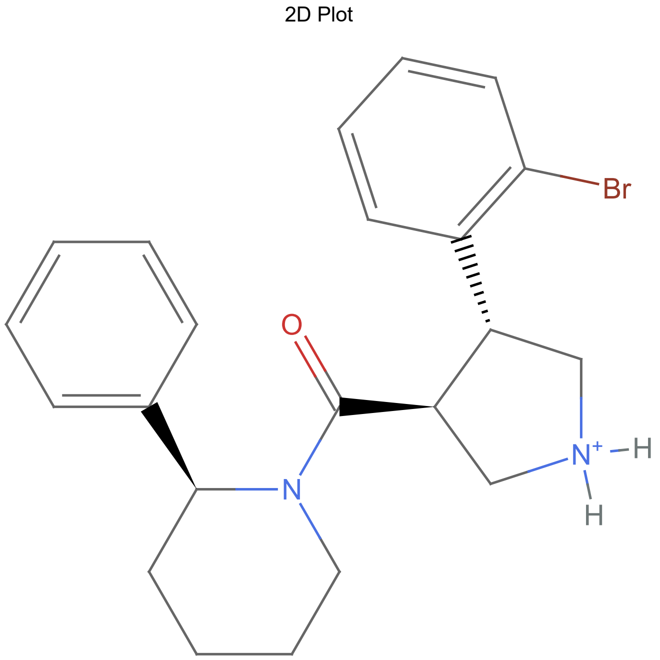

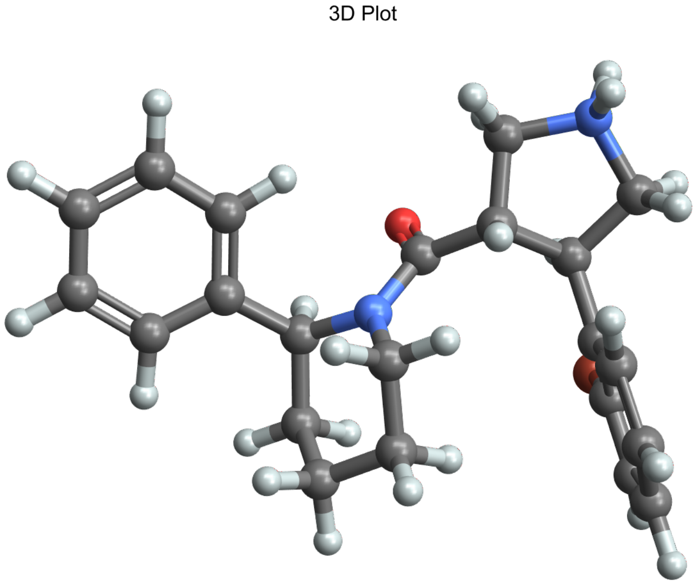

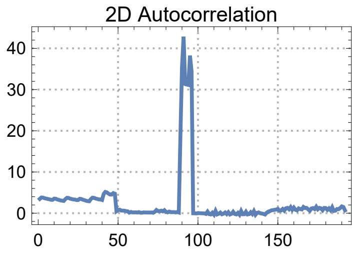

Dataset Index= 1483

Molecule Formula= $C_{19}H_{27}ClN_4O$

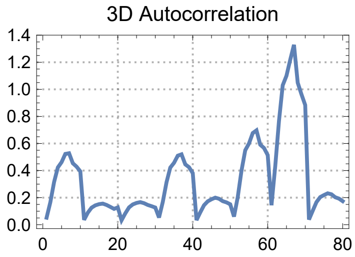

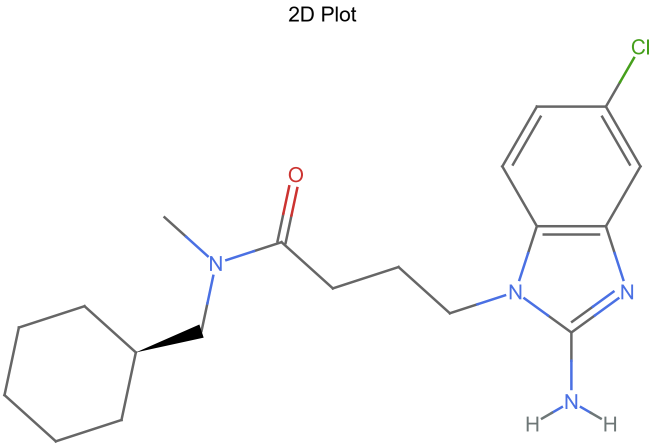

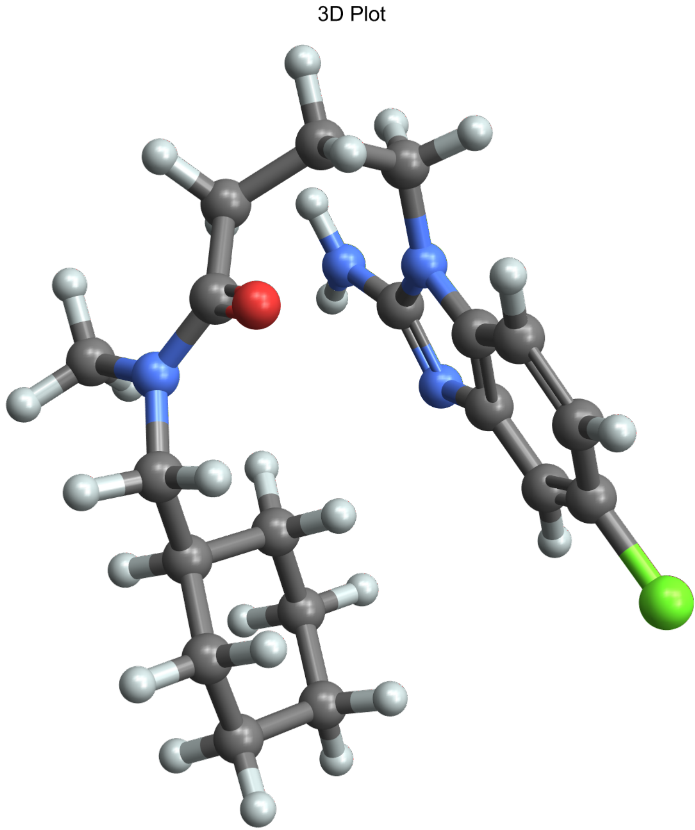

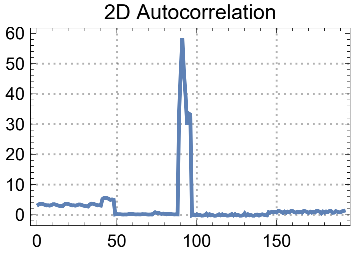

Dataset Index= 1484

Molecule Formula= $C_{18}H_{17}N_3O_2$

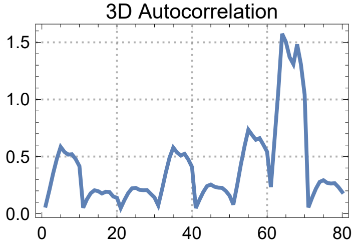

2D Plot

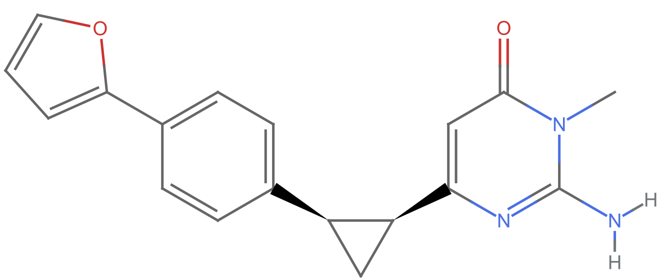

3D Plot

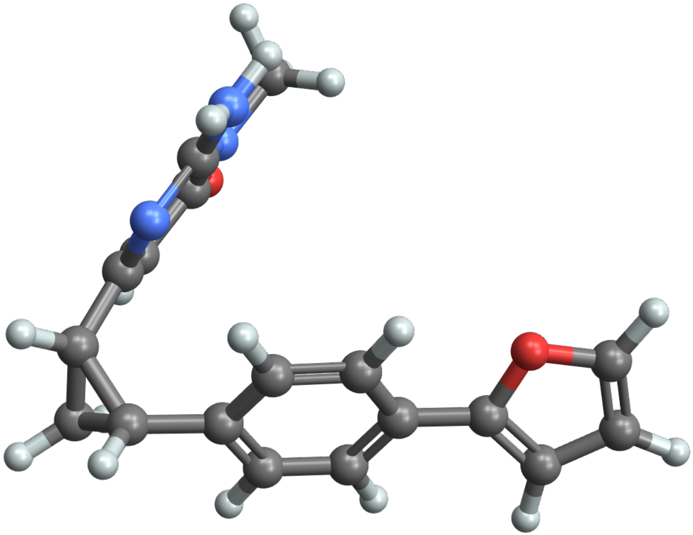

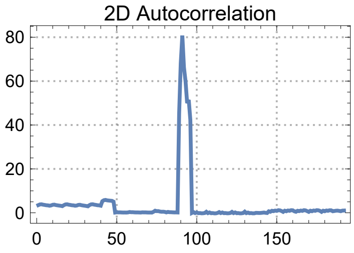


```
Dataset Index=        1485

Molecule Formula=     C21H20N4O2
```

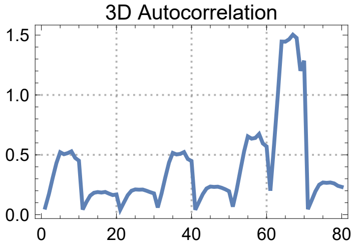

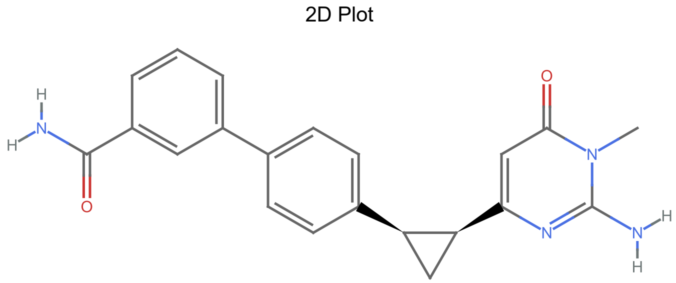

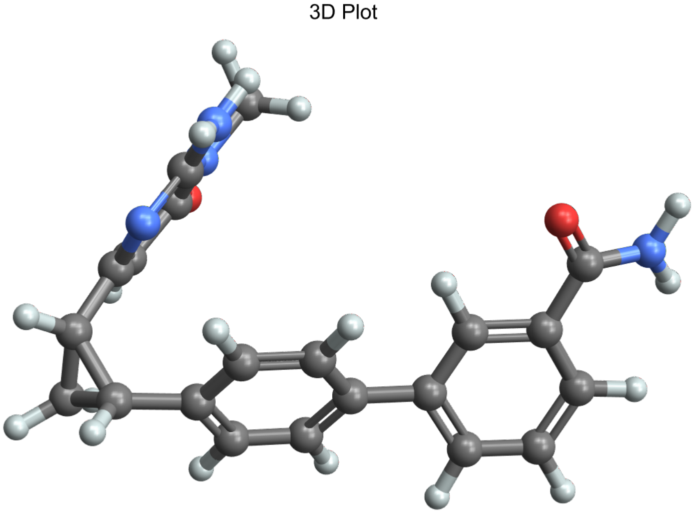

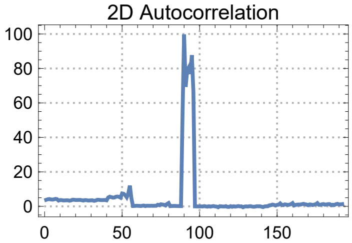

Dataset Index= 1486

Molecule Formula= $C_{19}H_{23}BrNO_4S^+$

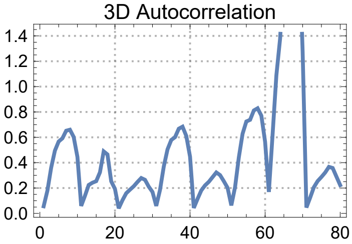

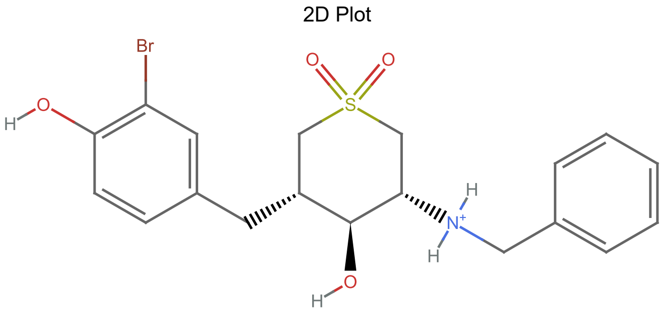

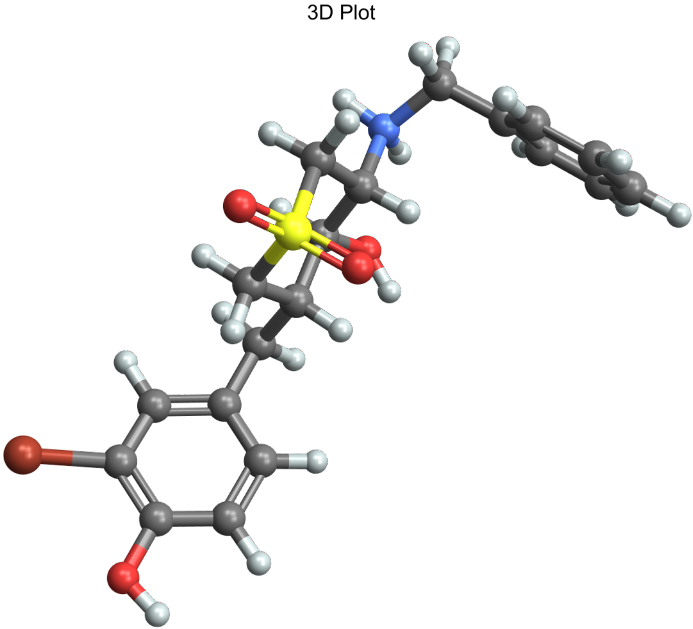

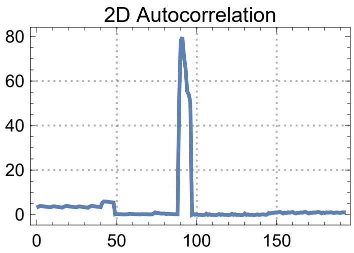

Dataset Index= 1487

Molecule Formula= $C_{22}H_{22}N_4O_2$

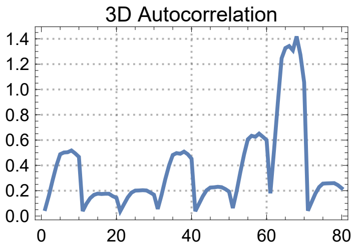

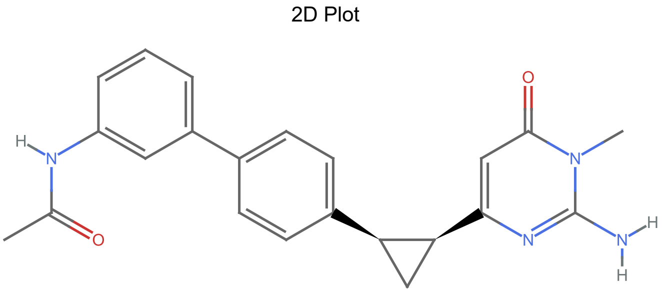

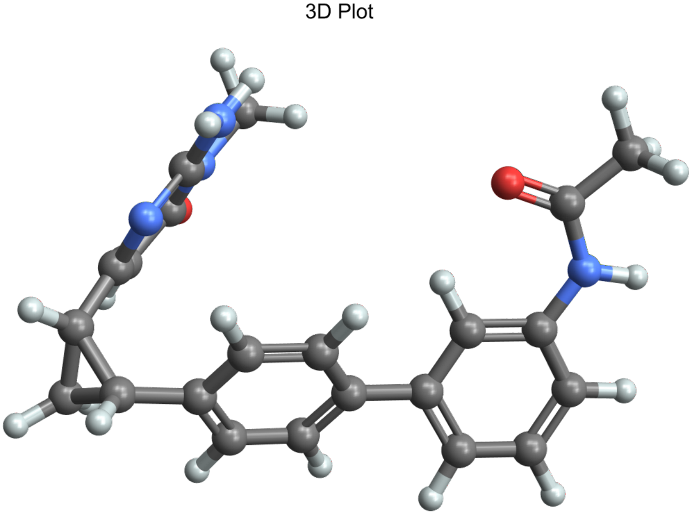

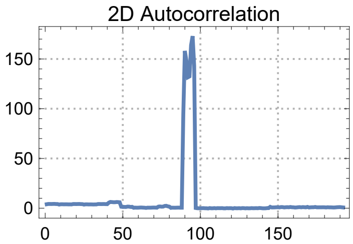

Dataset Index= 1488

Molecule Formula= $C_{28}H_{35}FN_{3}O_{5}S^{+}$

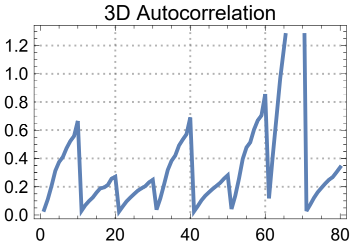

2D Plot

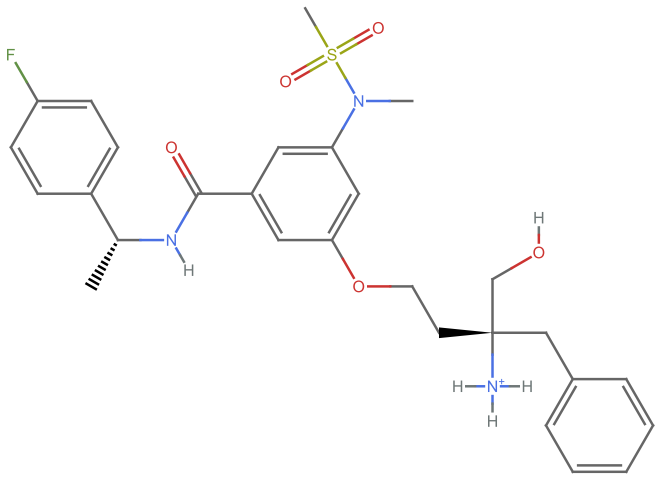

3D Plot

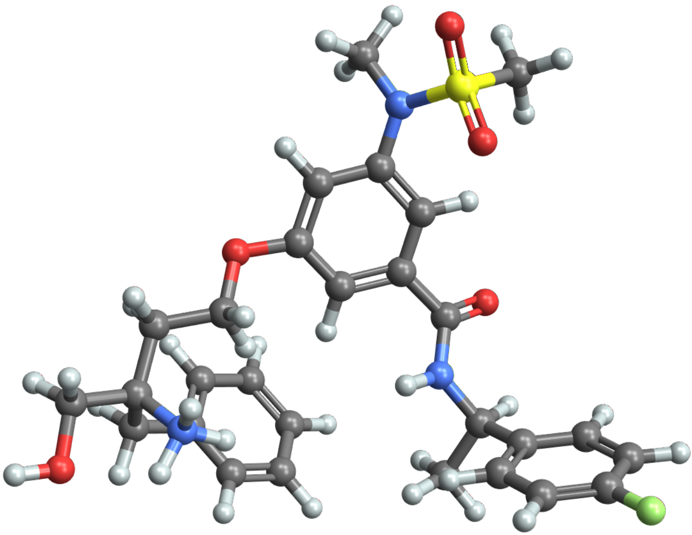

2D Plot

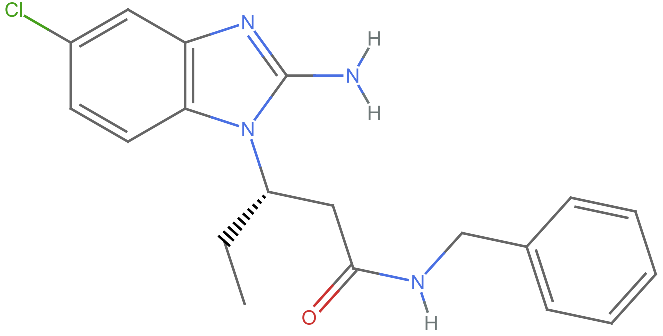

2D Autocorrelation

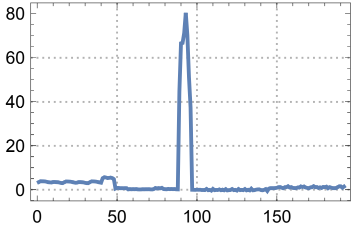


```
Dataset Index=       1489

Molecule Formula=    C19H21ClN4O
```

3D Autocorrelation

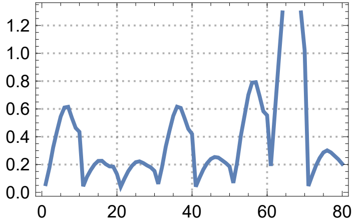

3D Plot

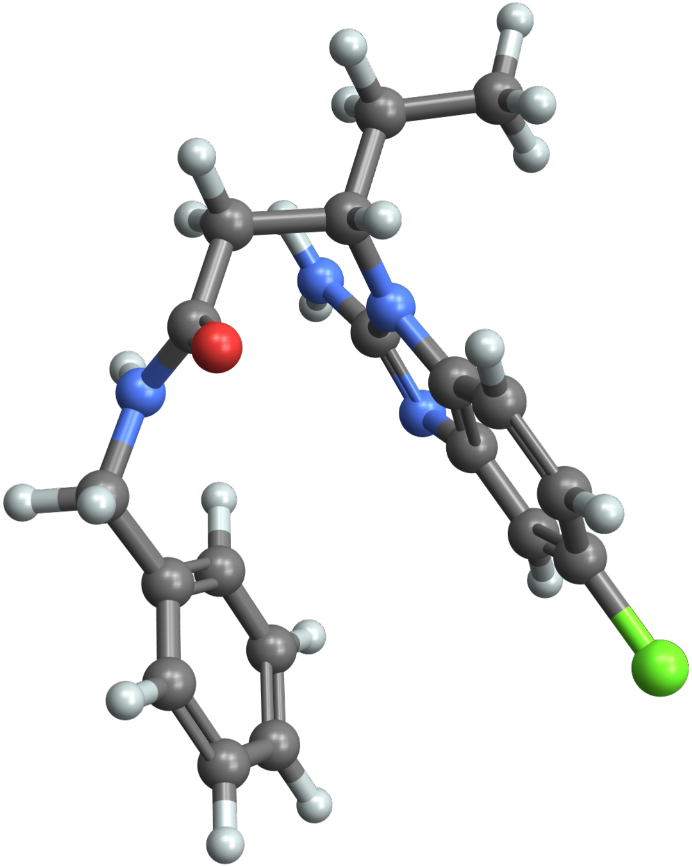

2D Plot

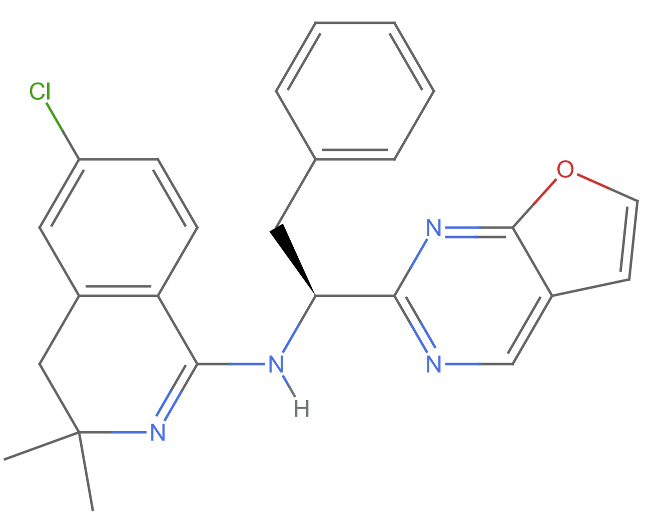

2D Autocorrelation

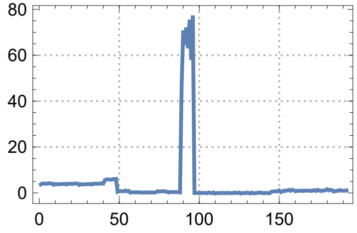

Dataset Index= 1490

Molecule Formula= $C_{25}H_{23}ClN_4O$

3D Autocorrelation

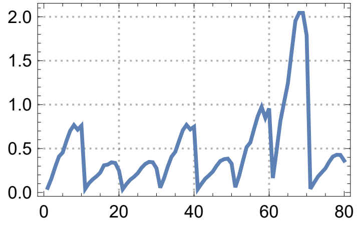

3D Plot

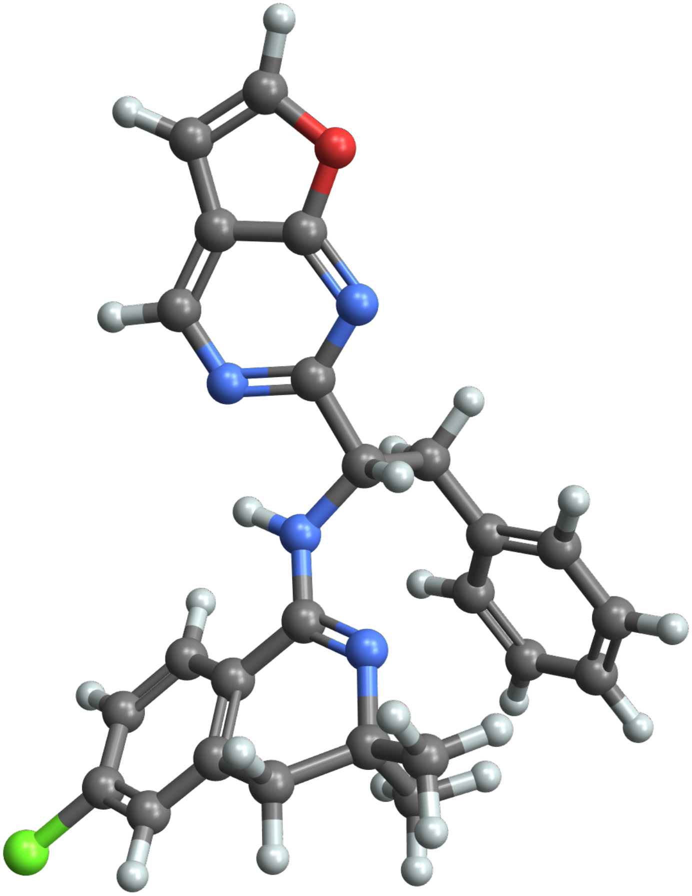

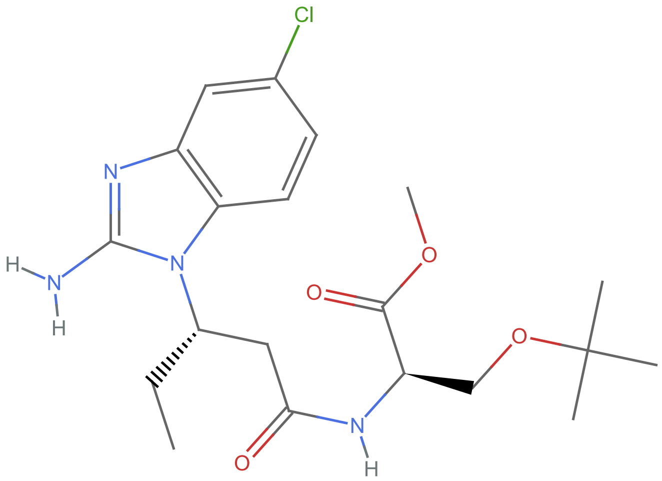

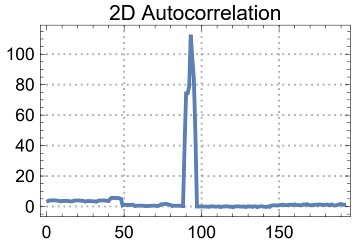

3D Plot

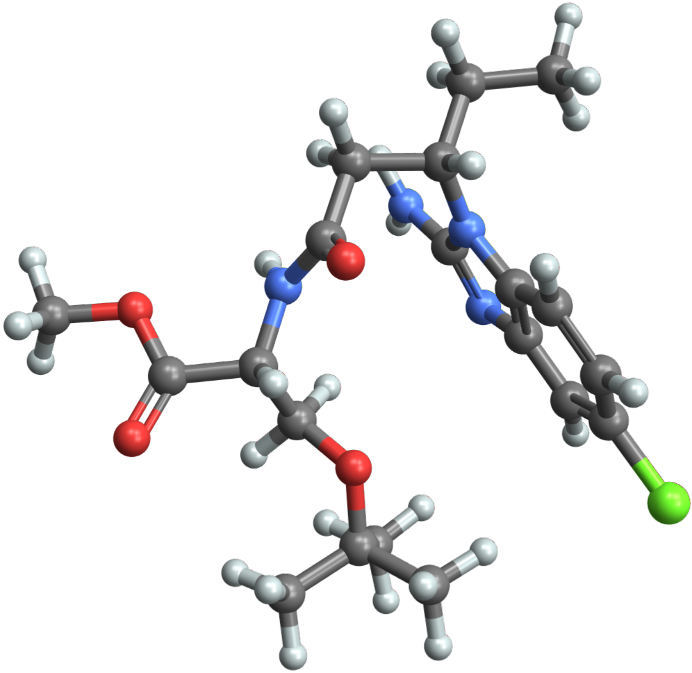

Dataset Index= 1491

Molecule Formula= $C_{20}H_{29}ClN_4O_4$

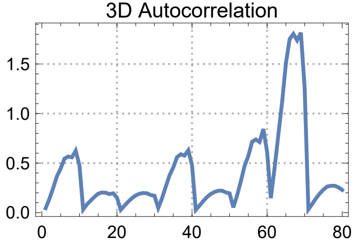

2D Autocorrelation

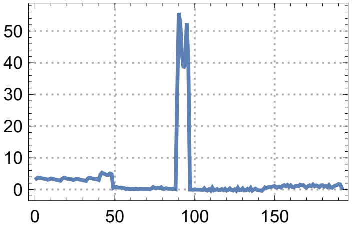

Dataset Index= 1492

Molecule Formula= $C_{18}H_{25}ClN_4O$

3D Autocorrelation

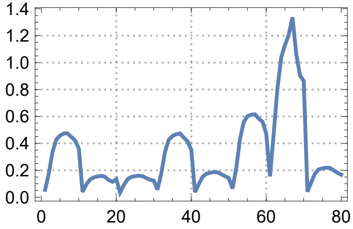

2D Plot

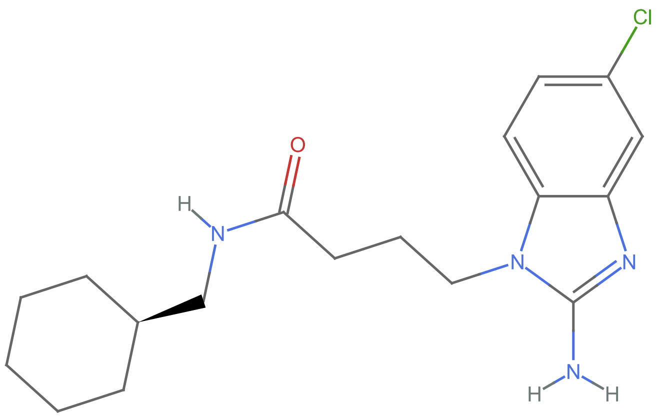

3D Plot

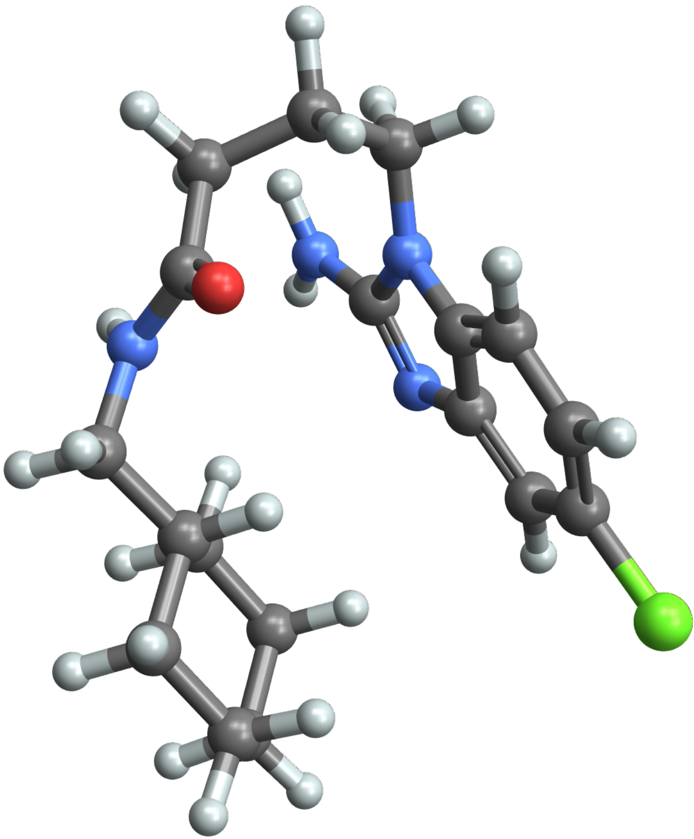

2D Autocorrelation

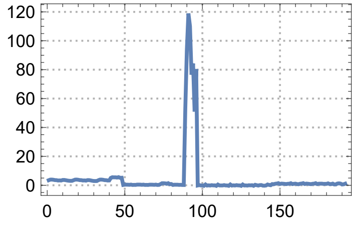

Dataset Index= 1493

Molecule Formula= $C_{19}H_{24}N_3O_4^+$

3D Autocorrelation

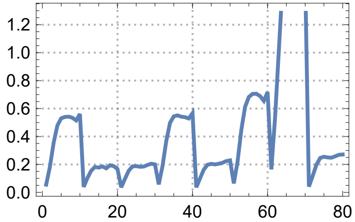

2D Plot

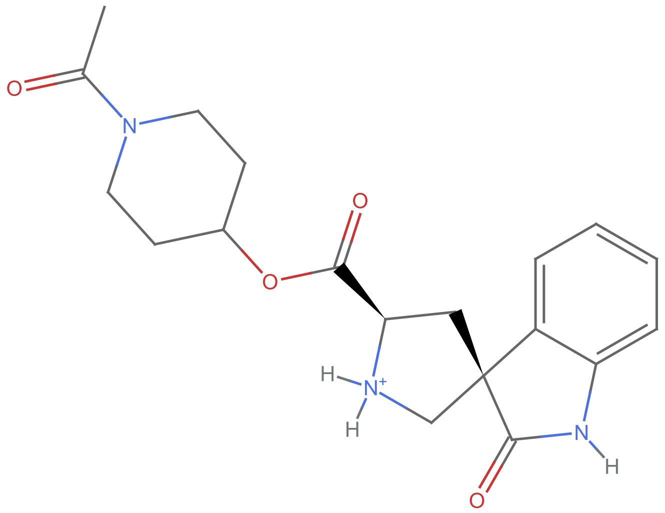

3D Plot

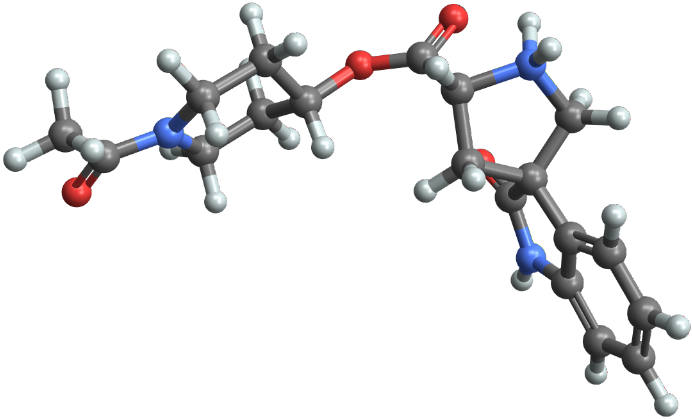

2D Plot

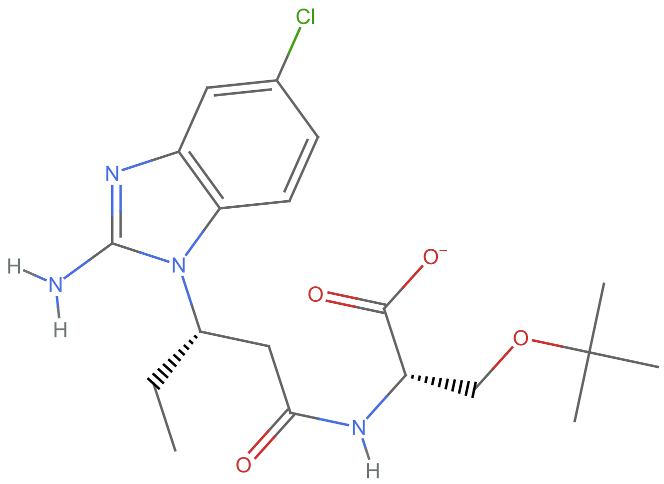

2D Autocorrelation

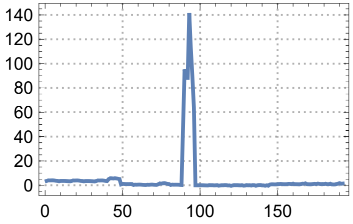

3D Plot

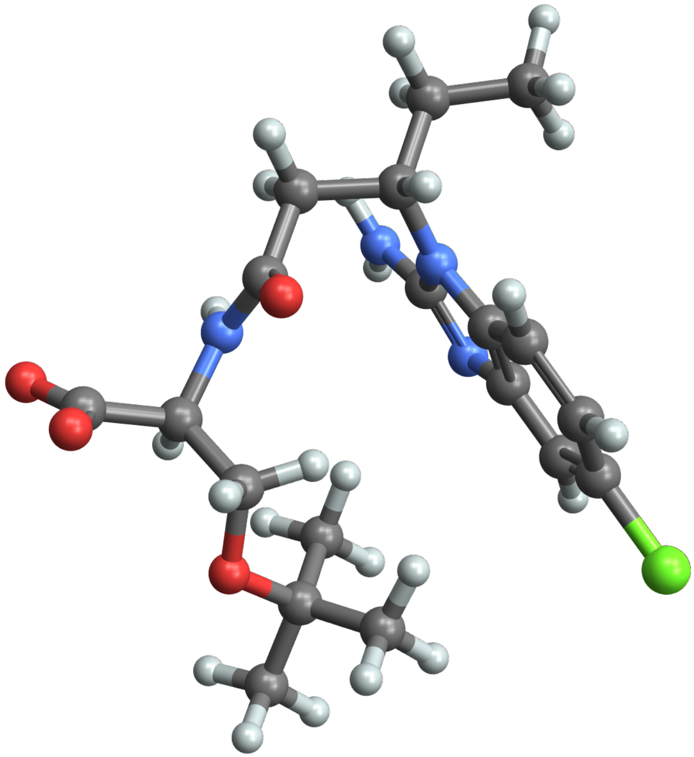

Dataset Index= 1494

Molecule Formula= $C_{19}H_{26}ClN_4O_4^-$

3D Autocorrelation

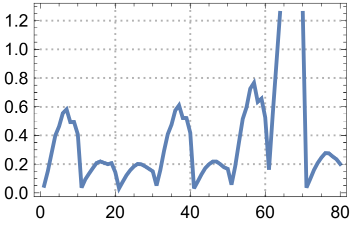

2D Plot

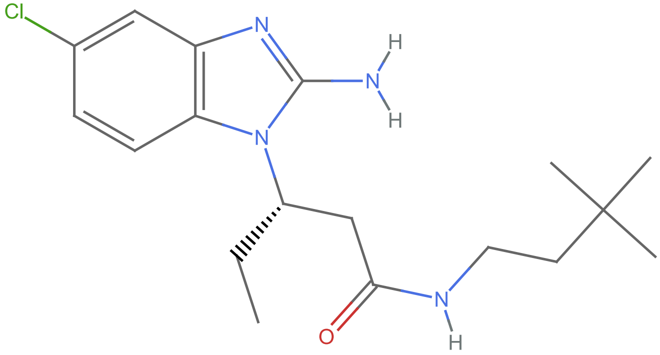

3D Plot

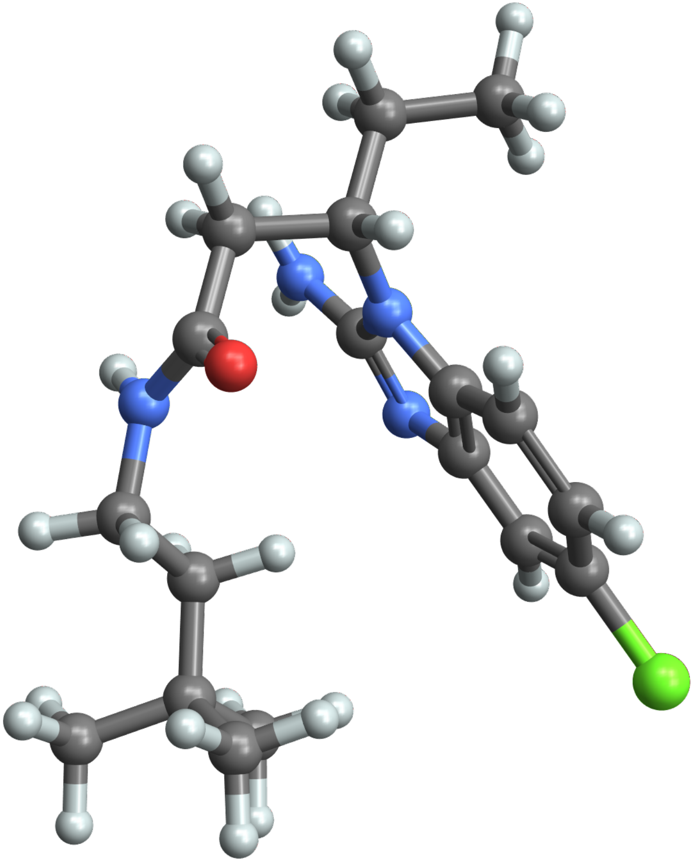

2D Autocorrelation

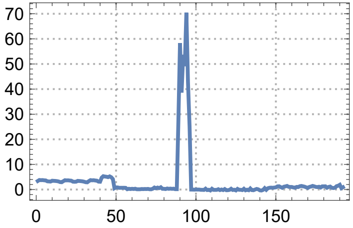

Dataset Index= 1495

Molecule Formula= $C_{18}H_{27}ClN_4O$

3D Autocorrelation

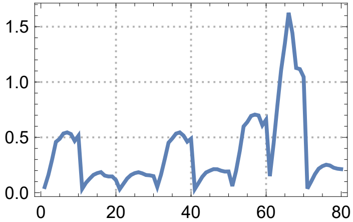

2D Plot

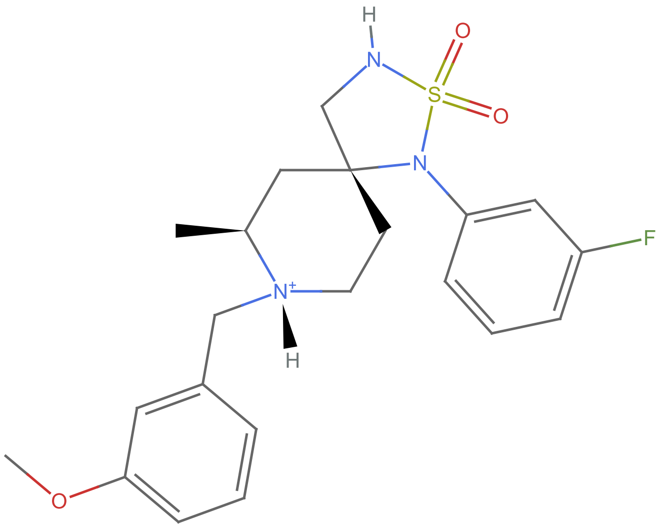

2D Autocorrelation

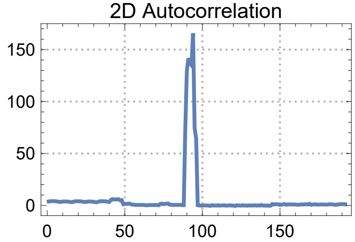

3D Plot

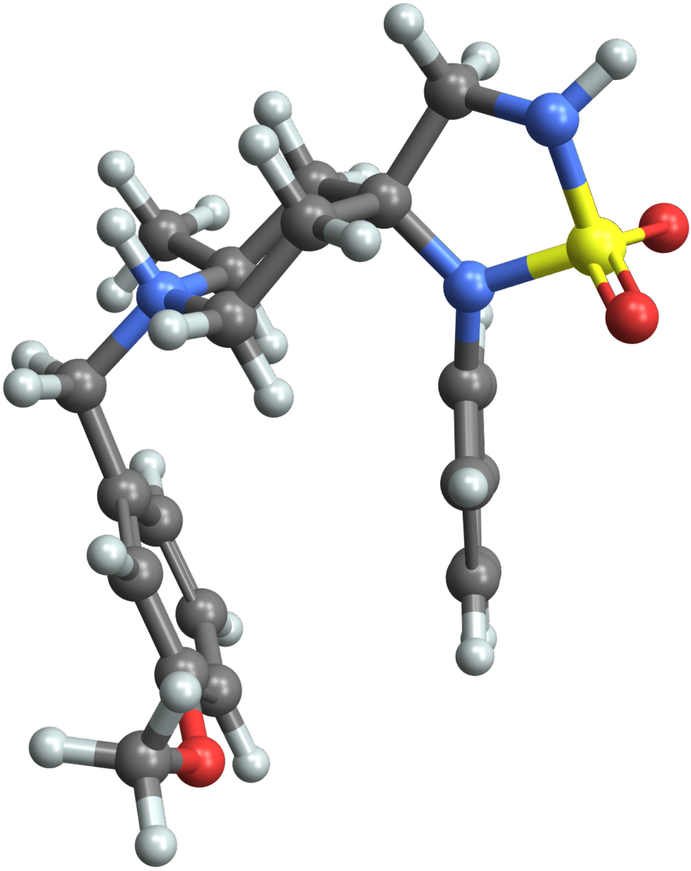

Dataset Index= 1496

Molecule Formula= $C_{21}H_{27}FN_3O_3S^+$

3D Autocorrelation

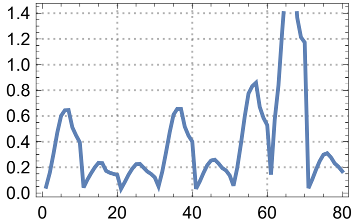

2D Autocorrelation

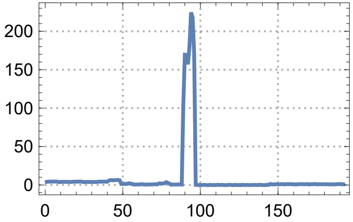

Dataset Index= 1497

Molecule Formula= $C_{29}H_{35}FN_3O_6S^+$

3D Autocorrelation

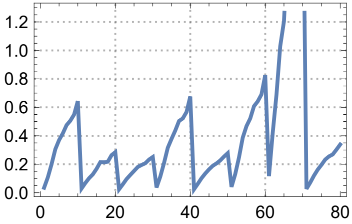

2D Plot

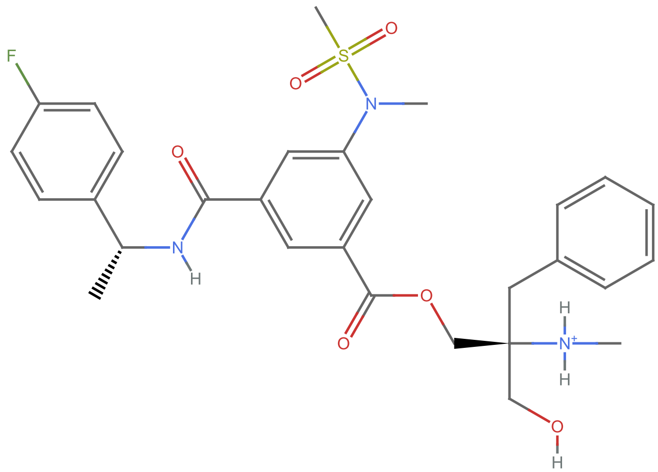

3D Plot

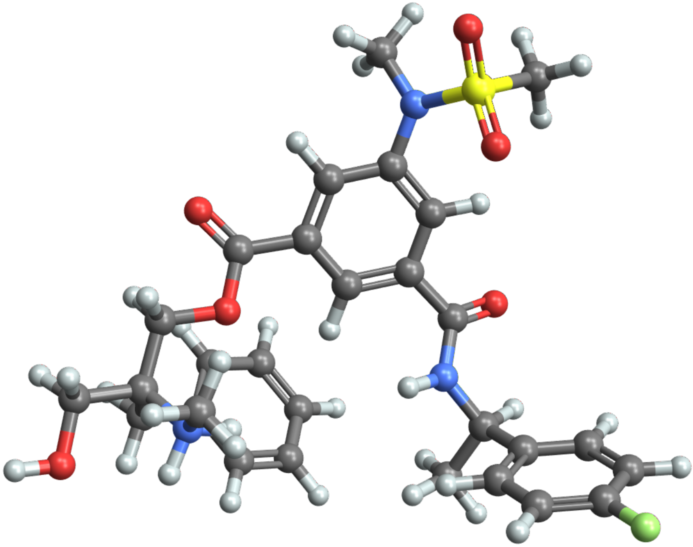

2D Plot

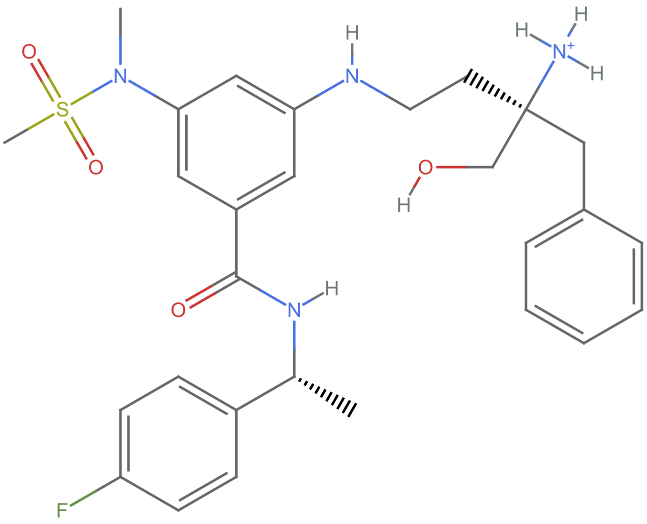

2D Autocorrelation

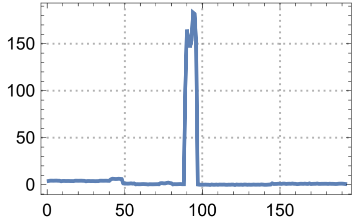

Dataset Index= 1498

Molecule Formula= $C_{28}H_{36}FN_4O_4S^+$

3D Plot

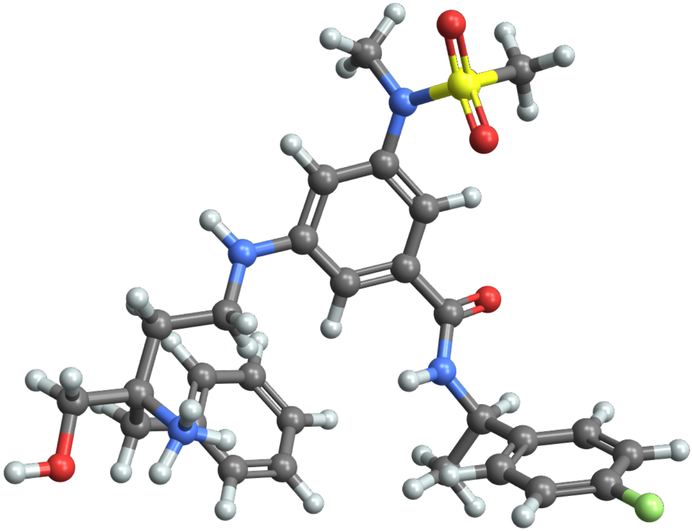

3D Autocorrelation

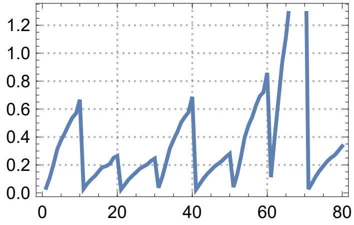

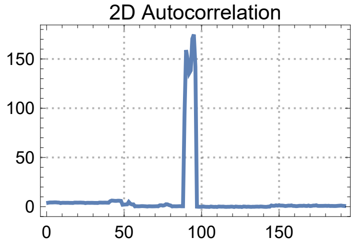

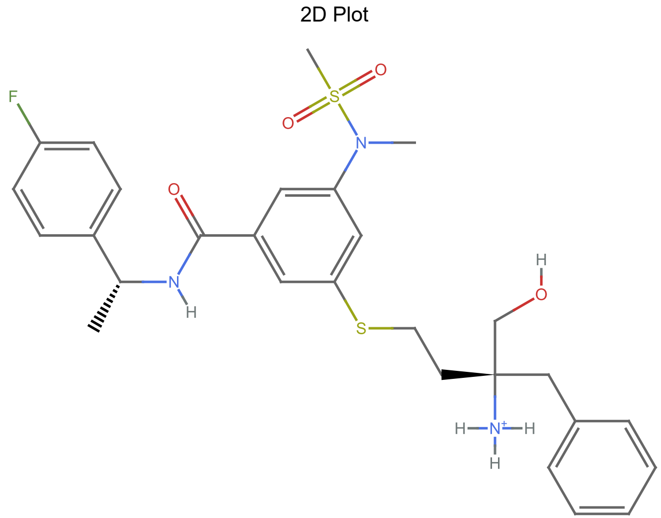

Dataset Index= 1499

Molecule Formula= $C_{28}H_{35}FN_3O_4S_2^+$

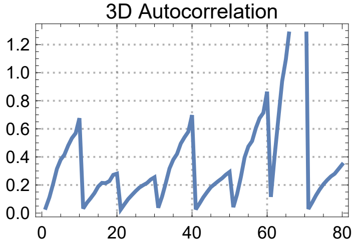

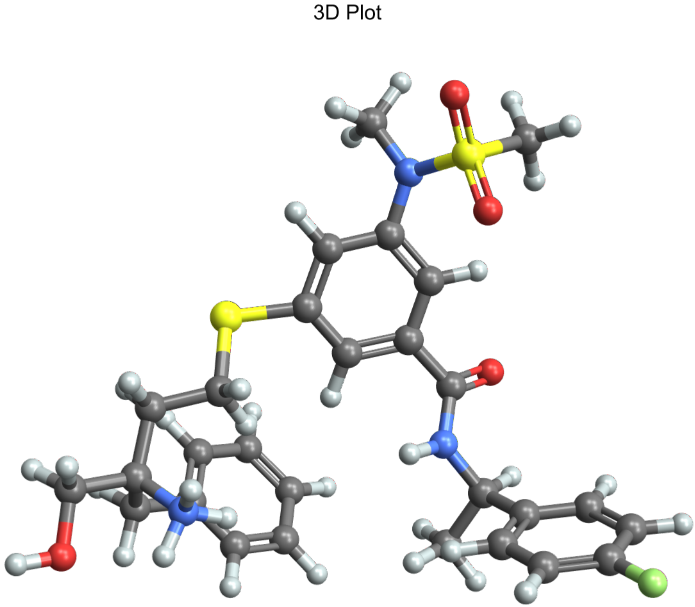

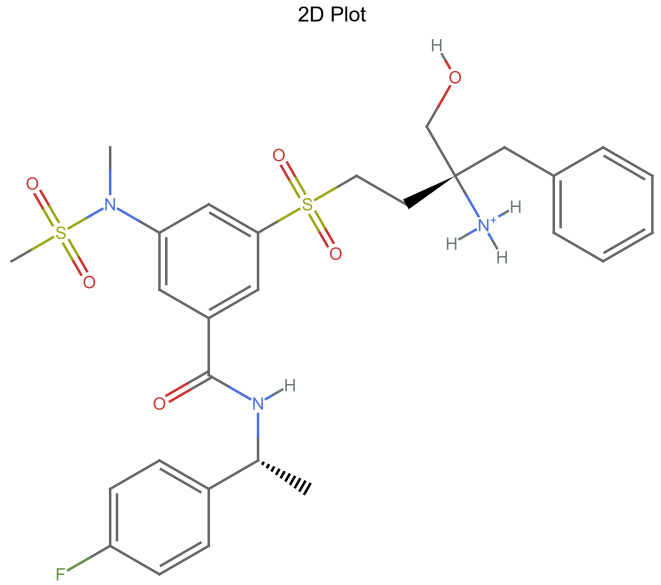

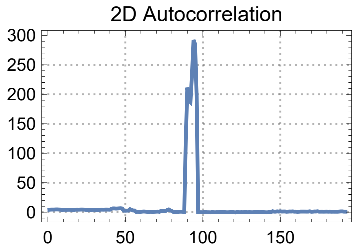

Dataset Index= 1500

Molecule Formula= $C_{28}H_{35}FN_3O_6S_2^+$

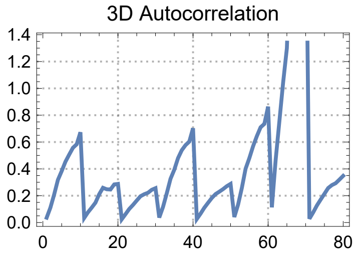

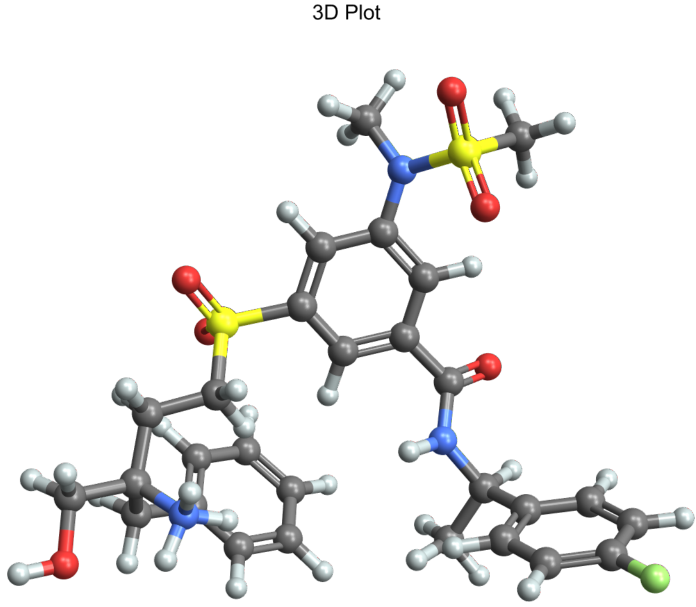

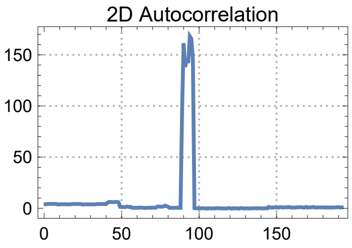

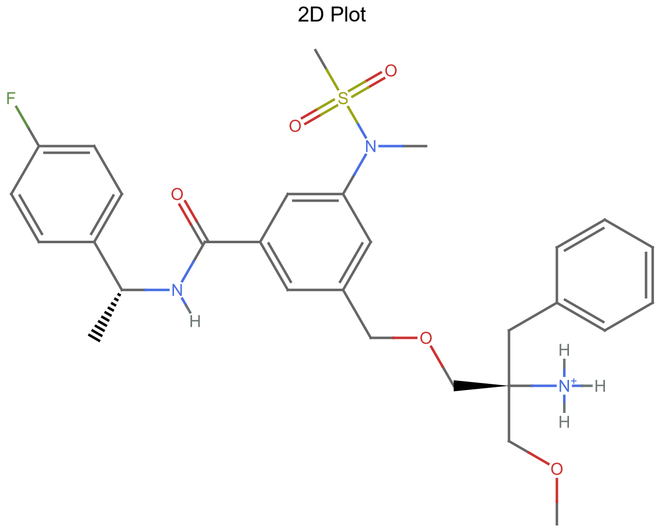

Dataset Index= 1501

Molecule Formula= $C_{29}H_{37}FN_3O_5S^+$

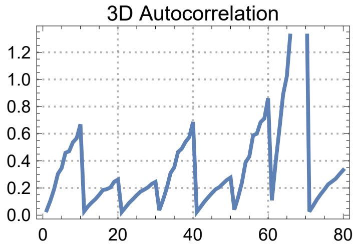

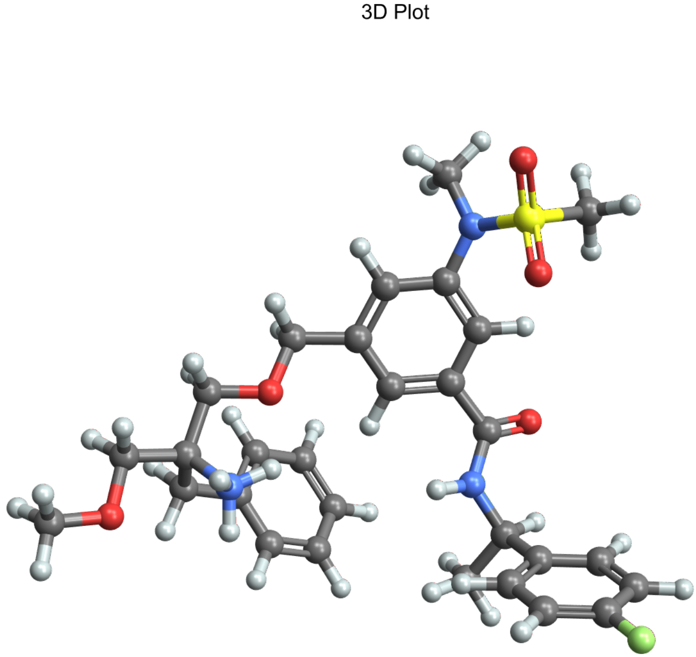

2D Plot

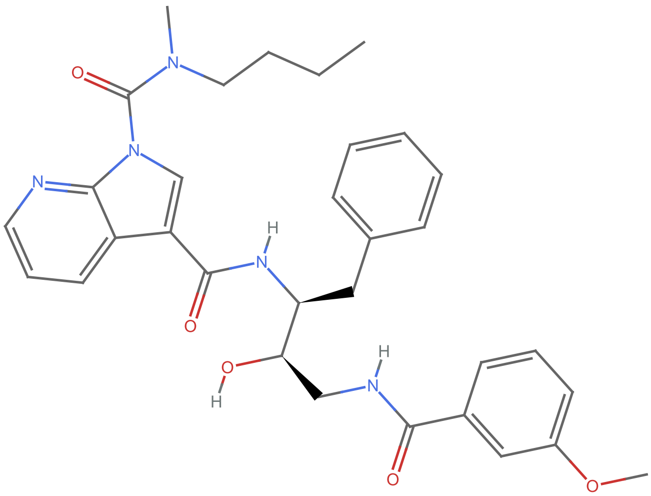

2D Autocorrelation

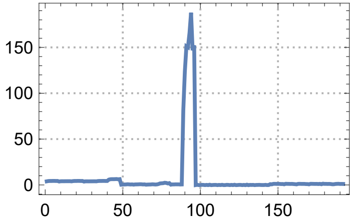

3D Plot

Dataset Index= 1502

Molecule Formula= $C_{32}H_{37}N_5O_5$

3D Autocorrelation

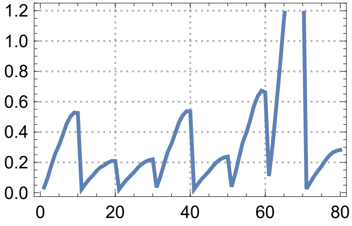

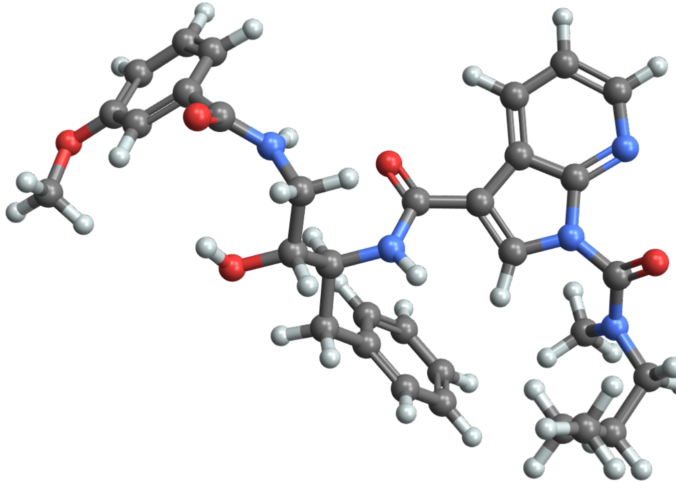

2D Plot

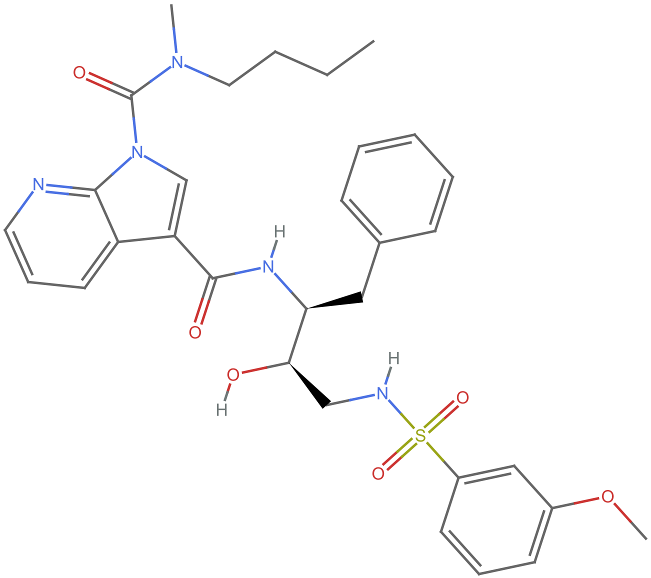

2D Autocorrelation

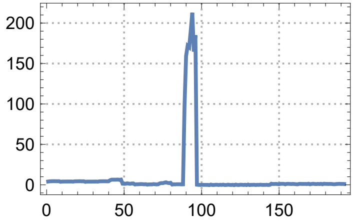

Dataset Index= 1503

Molecule Formula= $C_{31}H_{37}N_5O_6S$

3D Autocorrelation

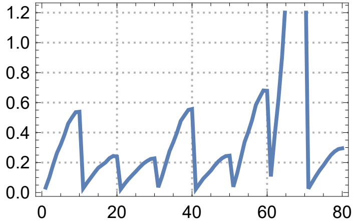

3D Plot

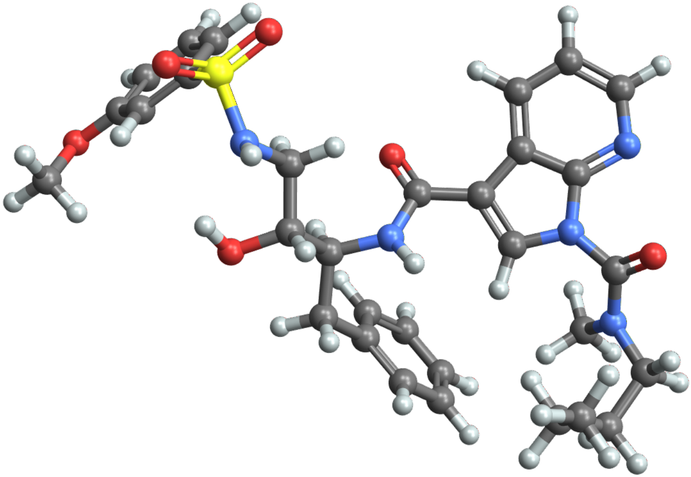

2D Plot

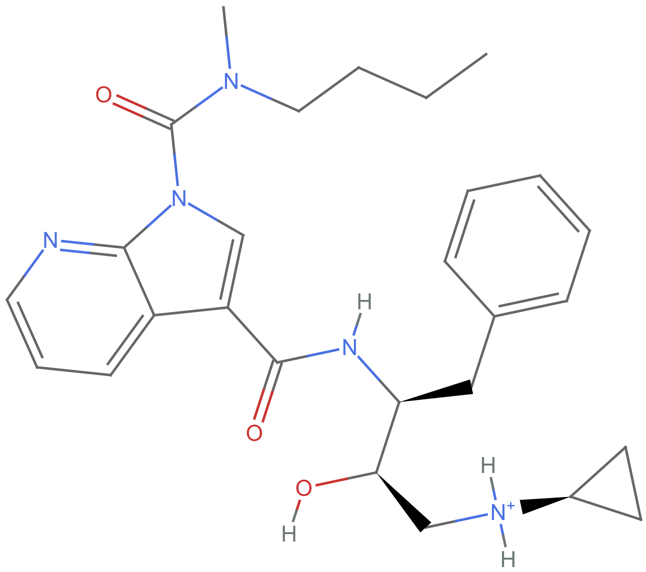

2D Autocorrelation

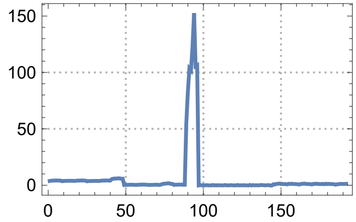

3D Plot

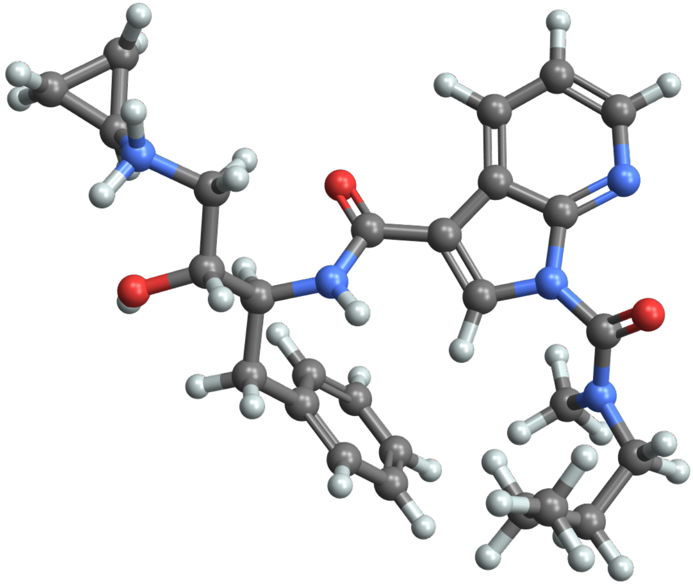

Dataset Index= 1504

Molecule Formula= $C_{27}H_{36}N_5O_3^+$

3D Autocorrelation

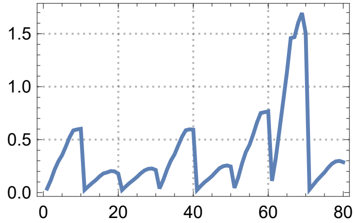

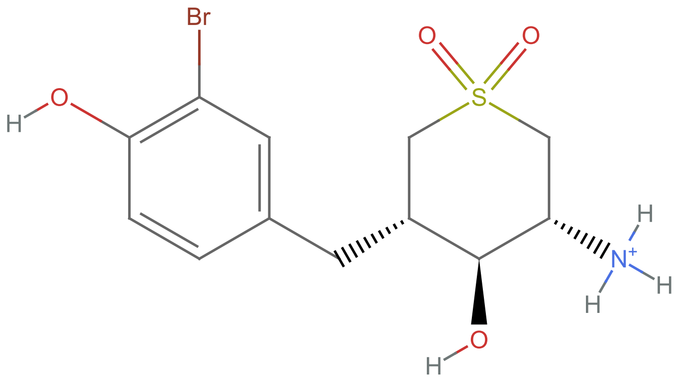

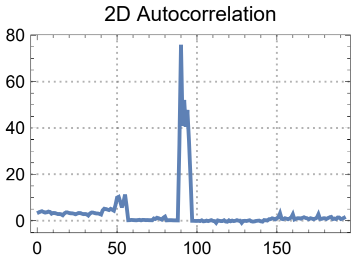

Dataset Index= 1505

Molecule Formula= $C_{12}H_{17}BrNO_4S^+$

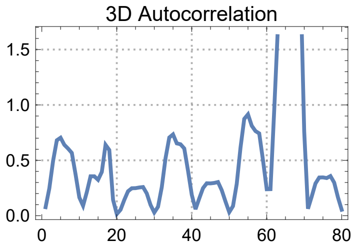

3D Plot

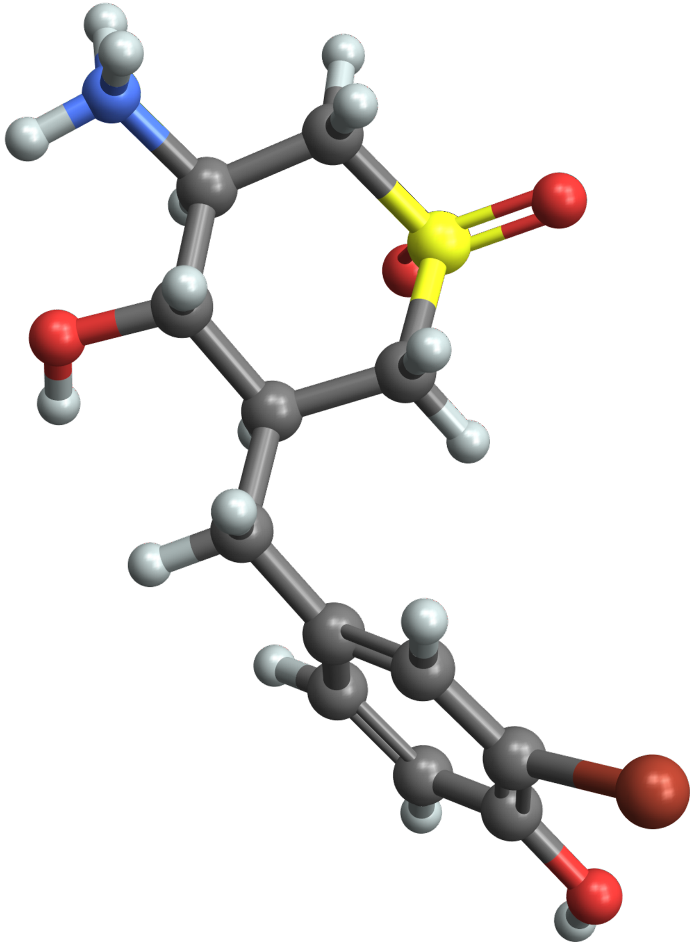

2D Plot

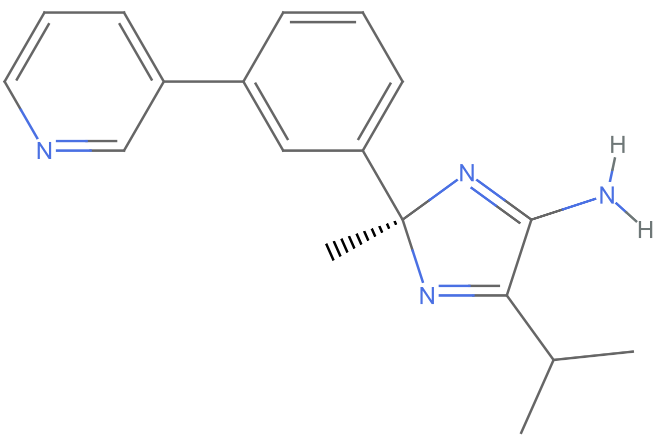

2D Autocorrelation

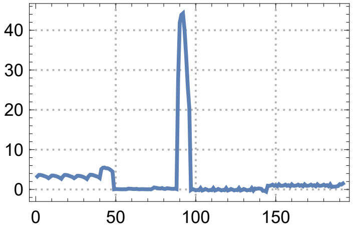

3D Plot

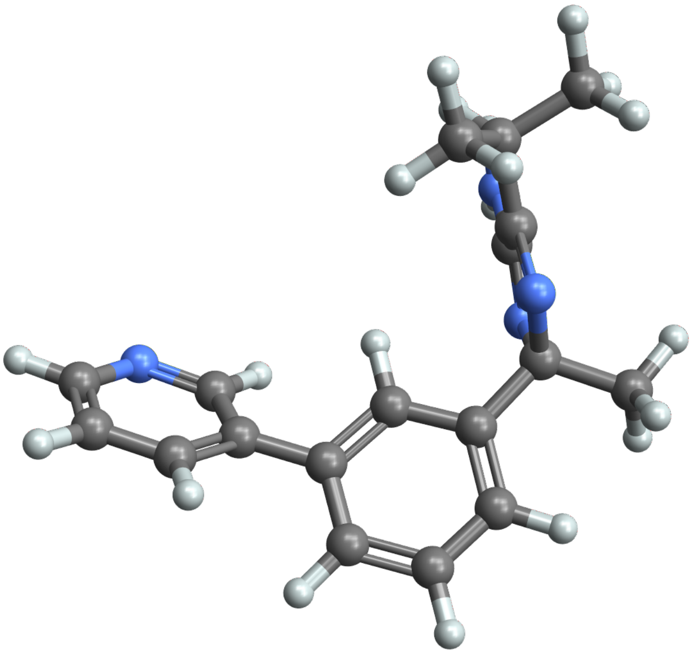

Dataset Index= 1506

Molecule Formula= $C_{18}H_{20}N_4$

3D Autocorrelation

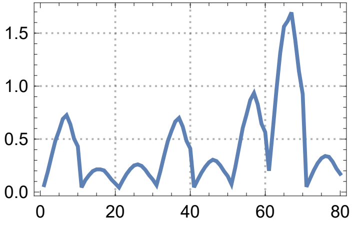

2D Autocorrelation

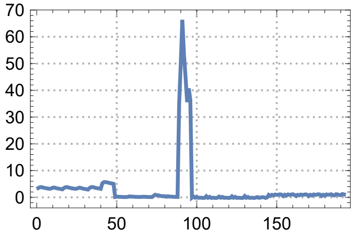

Dataset Index= 1507

Molecule Formula= $C_{20}H_{20}N_4O_2$

3D Autocorrelation

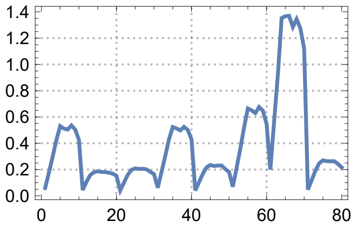

2D Plot

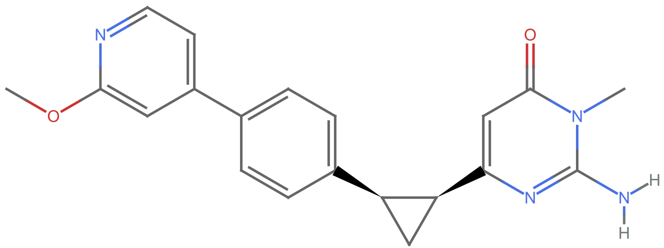

3D Plot

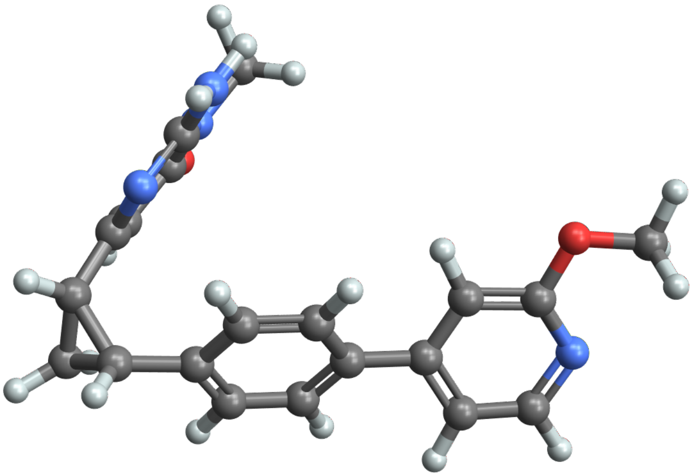

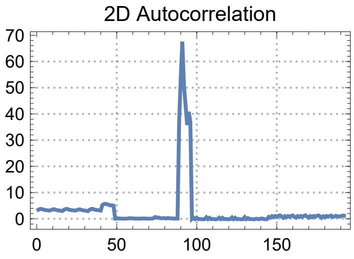

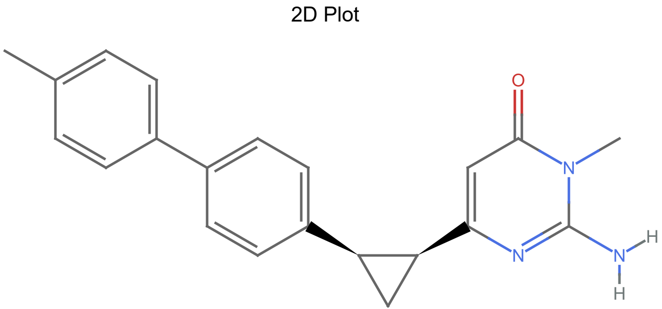

Dataset Index= 1508

Molecule Formula= $C_{21}H_{21}N_3O$

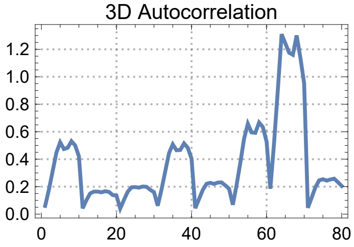

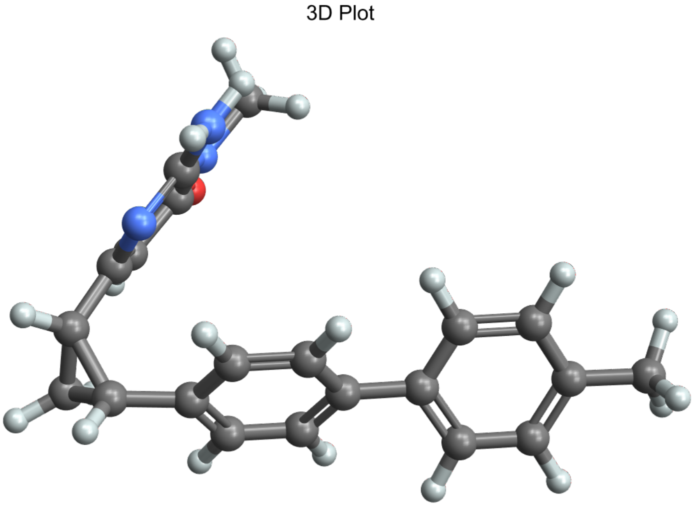

2D Autocorrelation

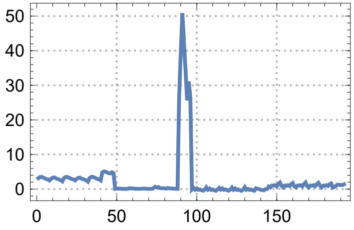

2D Plot

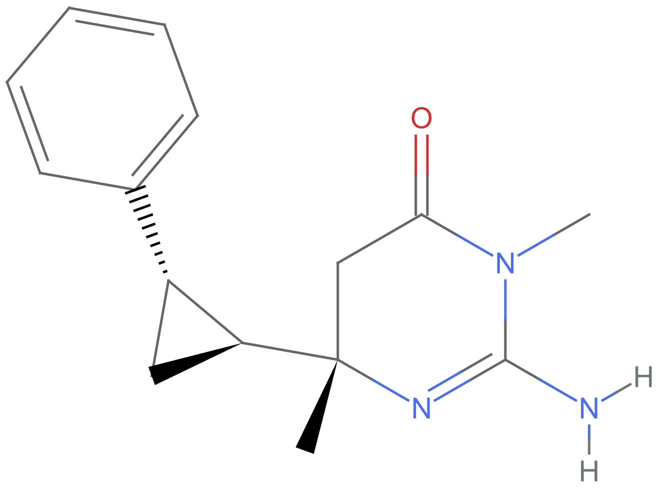

Dataset Index= 1509

Molecule Formula= $C_{15}H_{19}N_3O$

3D Autocorrelation

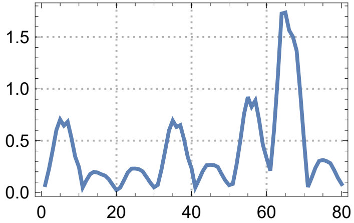

3D Plot

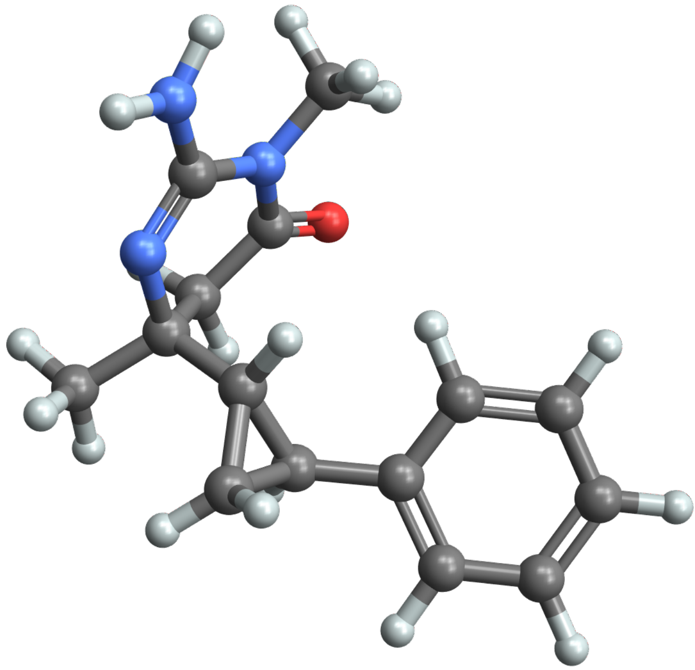

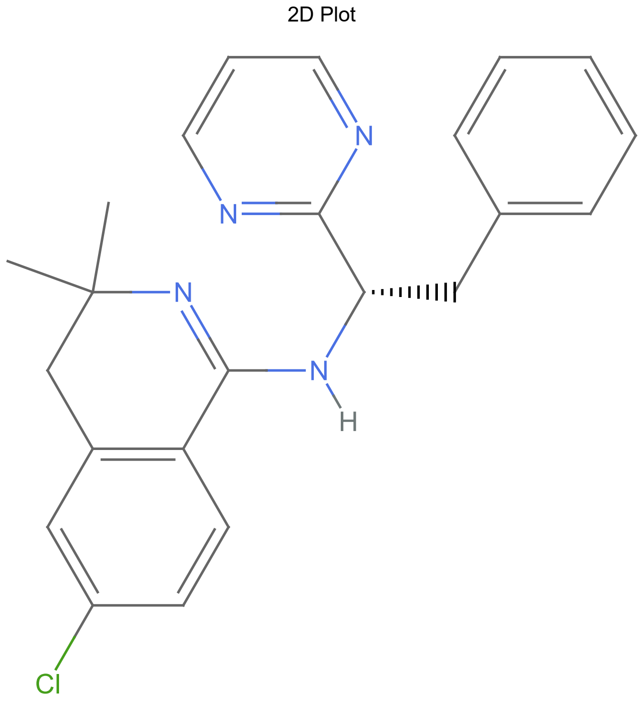

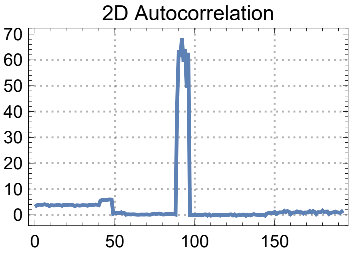

Dataset Index= 1510

Molecule Formula= $C_{23}H_{23}ClN_4$

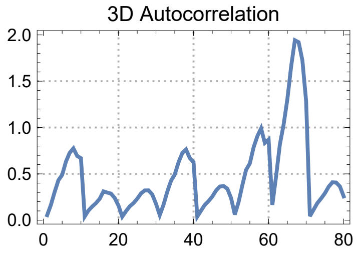

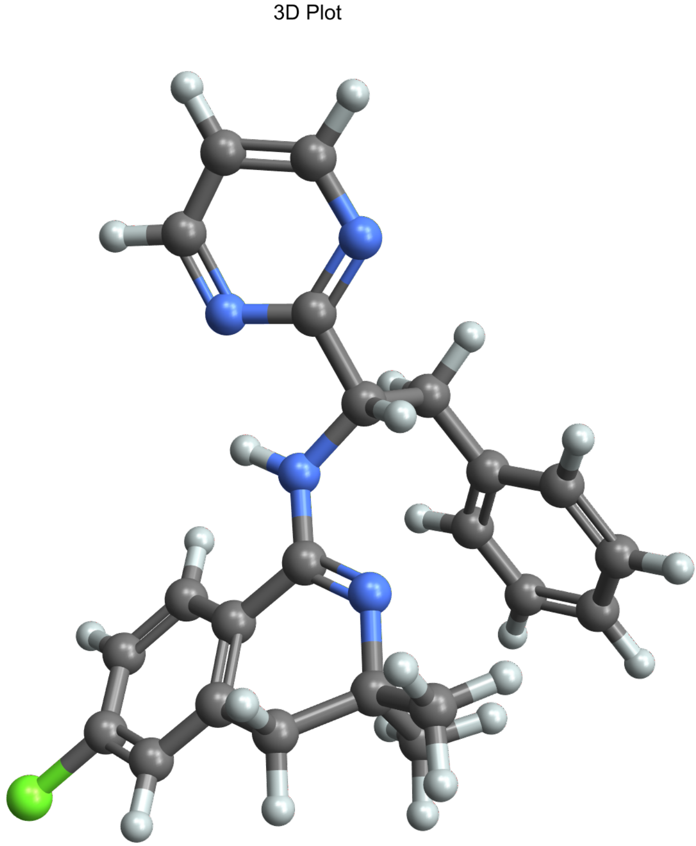

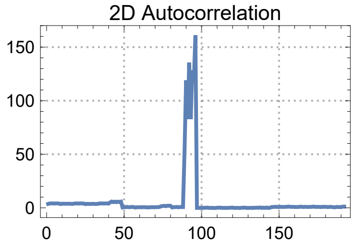

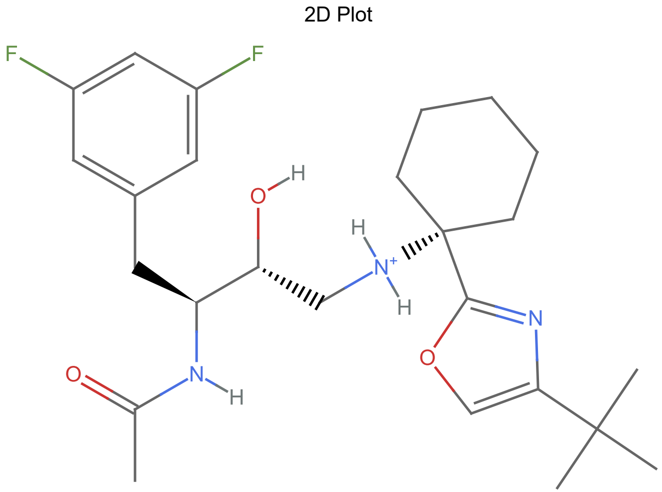

Dataset Index= 1511

Molecule Formula= $C_{25}H_{36}F_2N_3O_3^+$

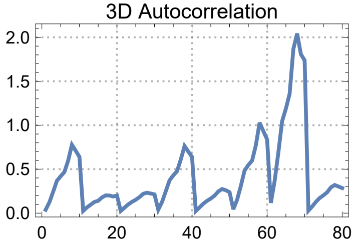

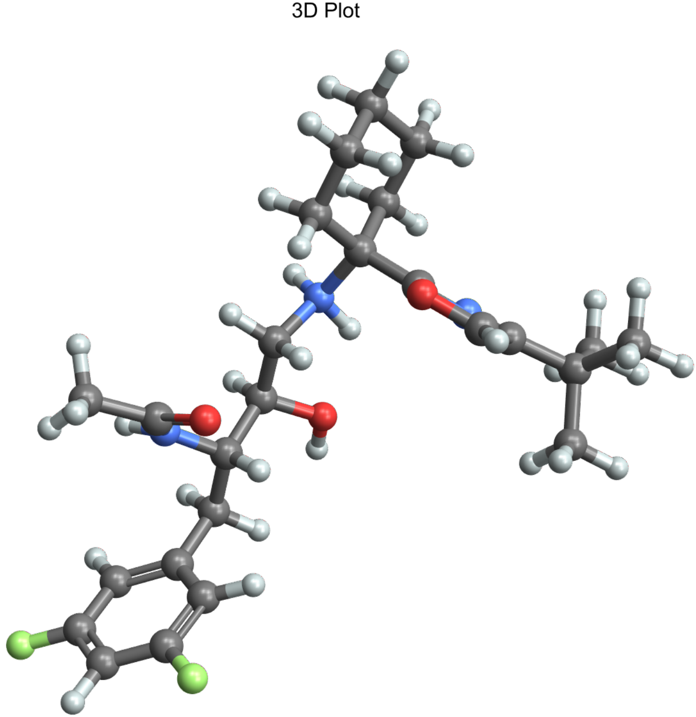

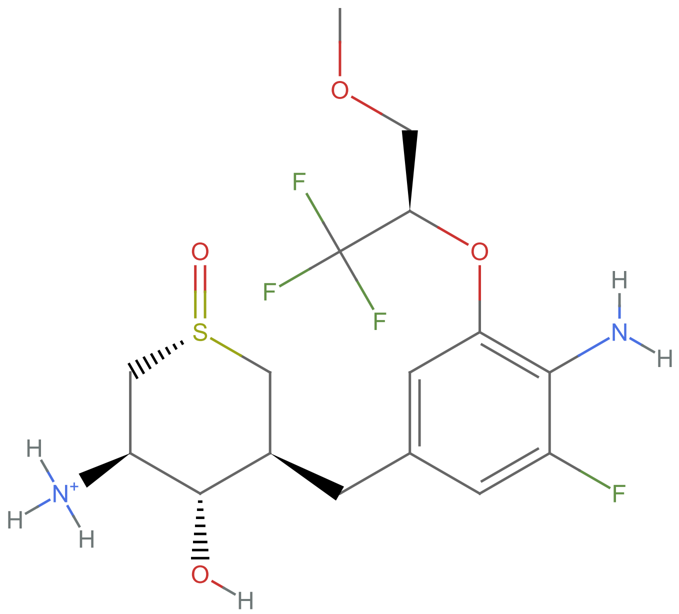

2D Autocorrelation

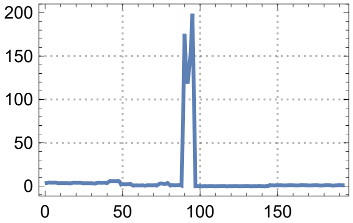

Dataset Index= 1512

Molecule Formula= $C_{16}H_{23}F_4N_2O_4S^+$

3D Autocorrelation

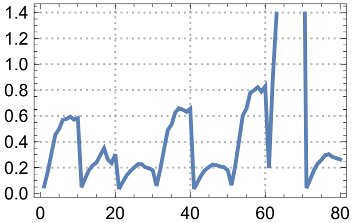

3D Plot

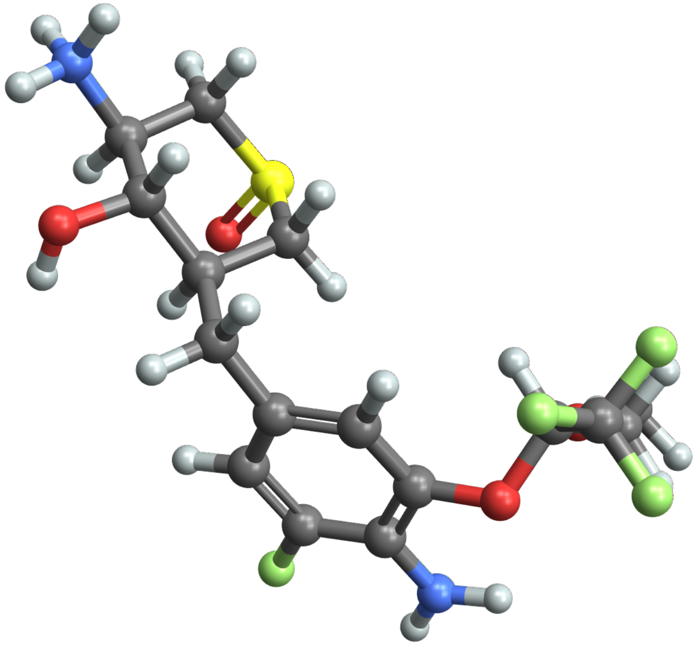

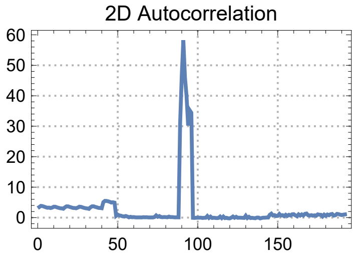

Dataset Index= 1513

Molecule Formula= $C_{19}H_{19}N_3OS$

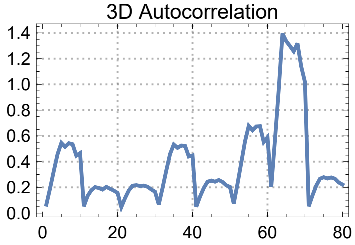

2D Plot

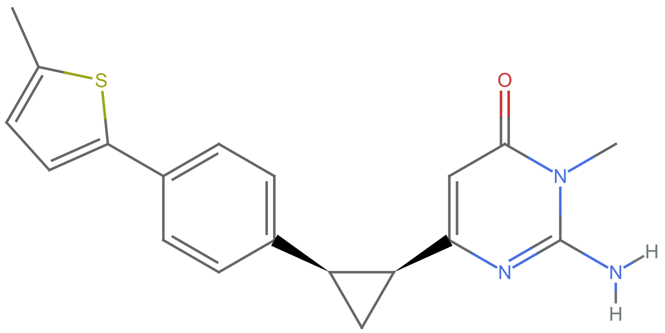

3D Plot

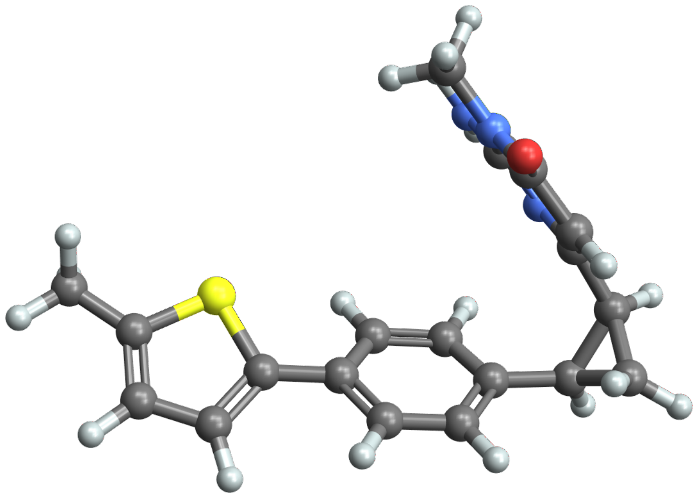

2D Autocorrelation

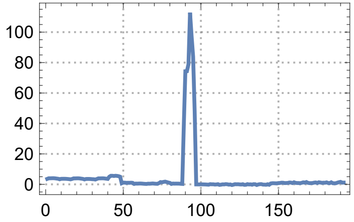

Dataset Index= 1514

Molecule Formula= $C_{20}H_{29}ClN_4O_4$

3D Autocorrelation

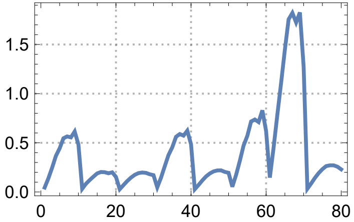

2D Plot

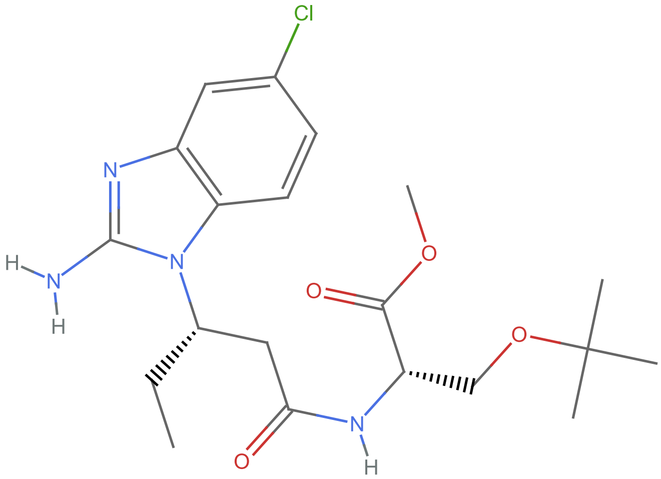

3D Plot

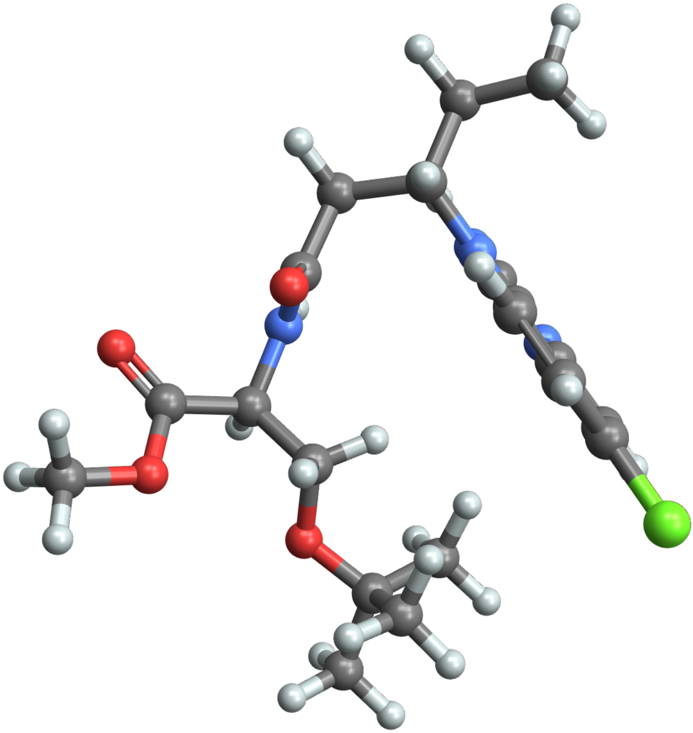

2D Plot

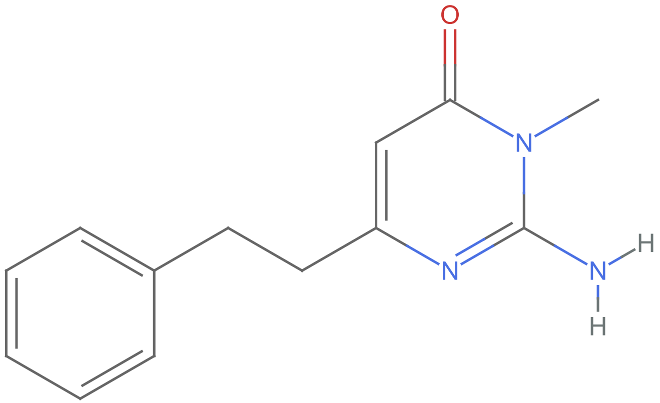

2D Autocorrelation

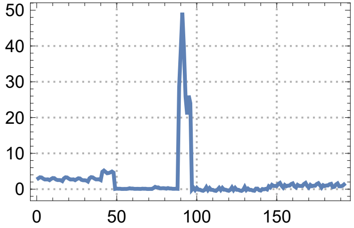

Dataset Index= 1515

Molecule Formula= $C_{13}H_{15}N_3O$

3D Autocorrelation

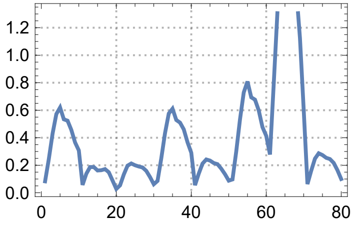

3D Plot

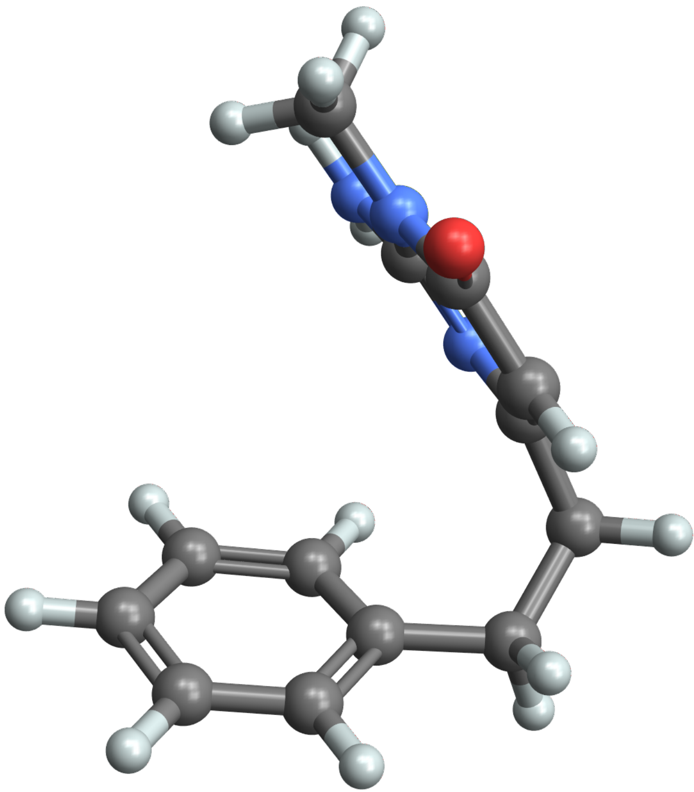

2D Plot

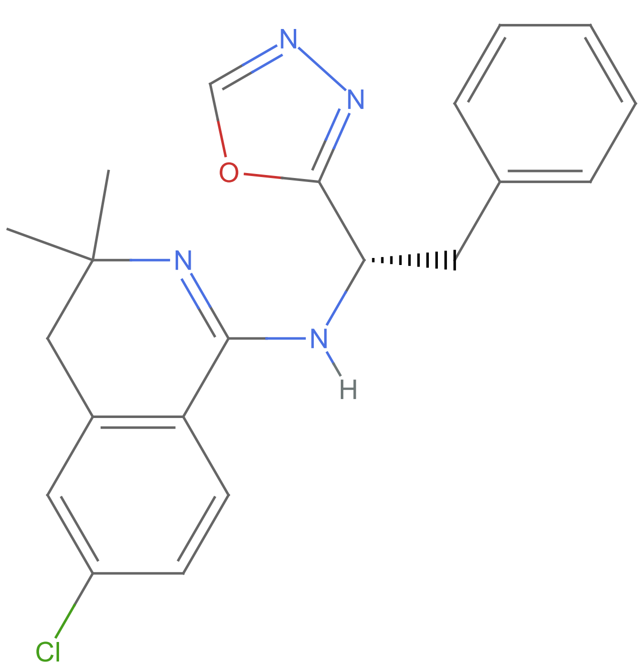

2D Autocorrelation

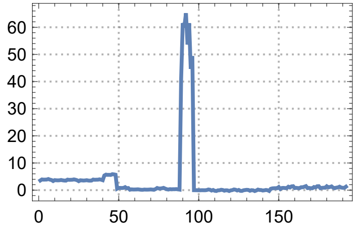

Dataset Index= 1516

Molecule Formula= $C_{21}H_{21}ClN_4O$

3D Autocorrelation

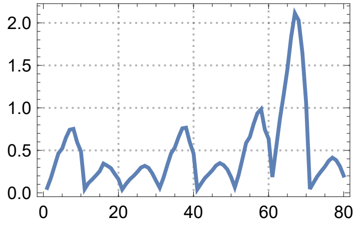

3D Plot

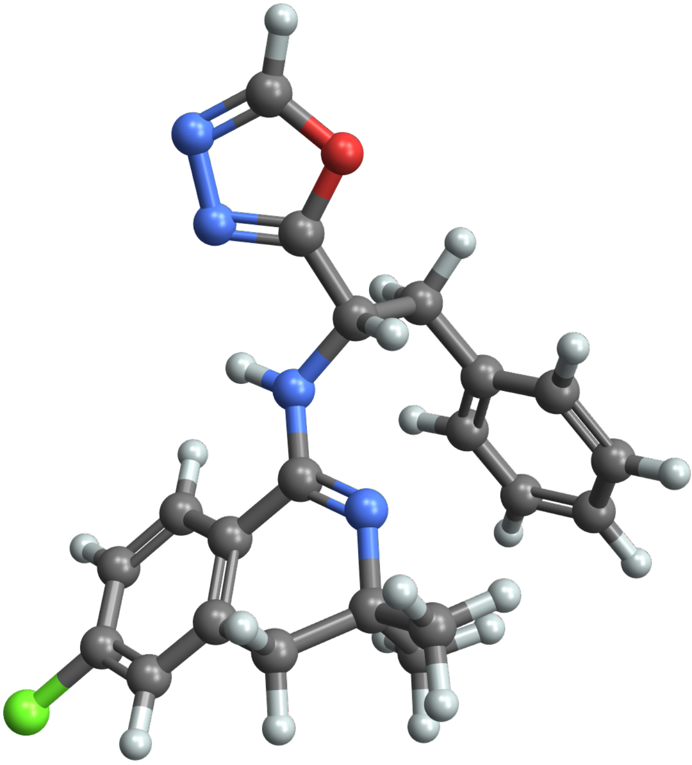

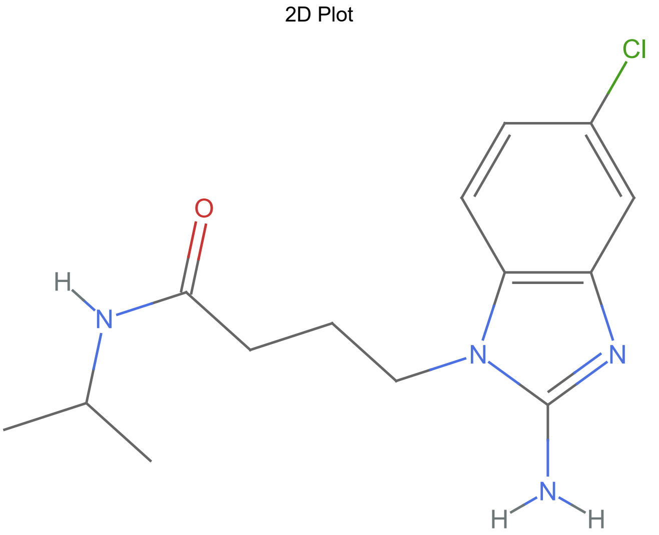

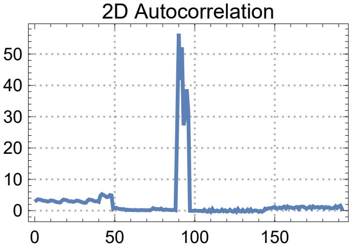

Dataset Index= 1517

Molecule Formula= $C_{14}H_{19}ClN_4O$

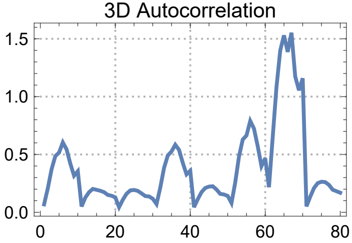

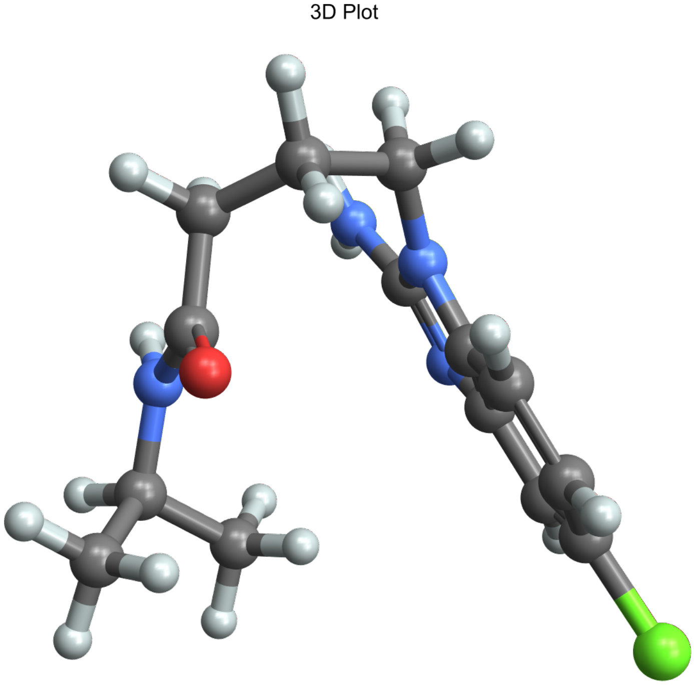

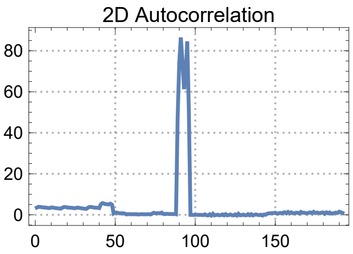

Dataset Index= 1518

Molecule Formula= $C_{19}H_{20}ClFN_4O$

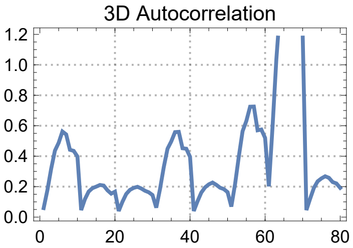

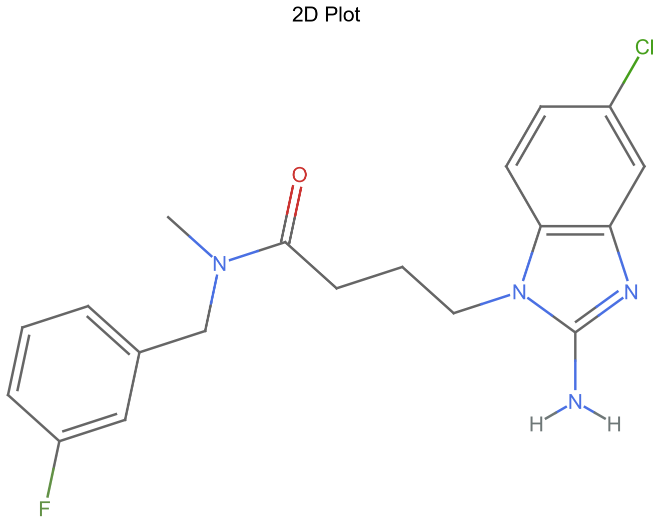

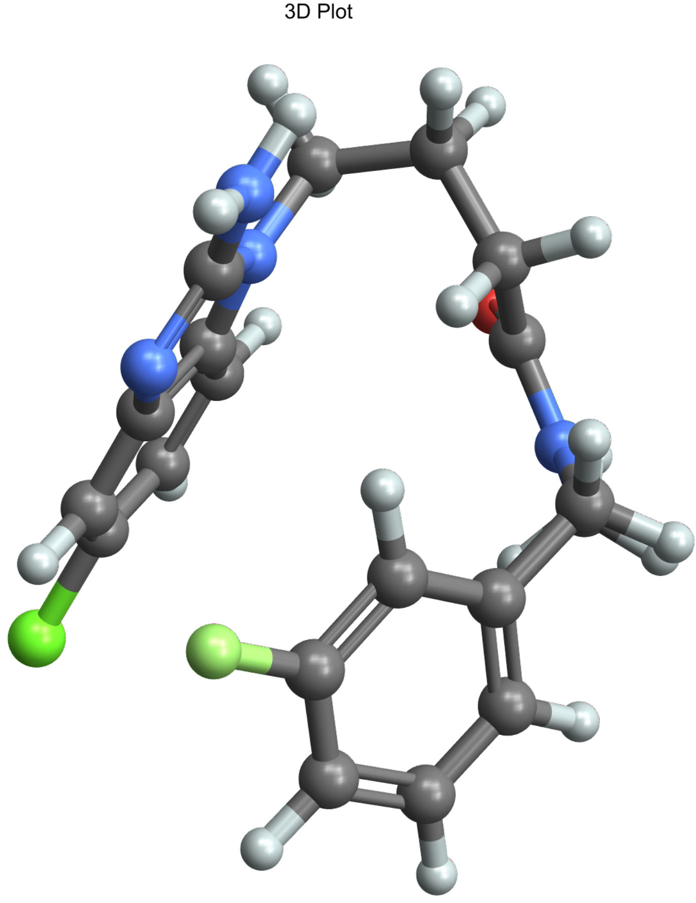

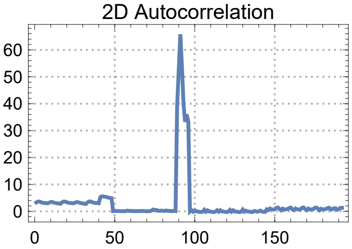

Dataset Index= 1519

Molecule Formula= $C_{19}H_{19}N_3O$

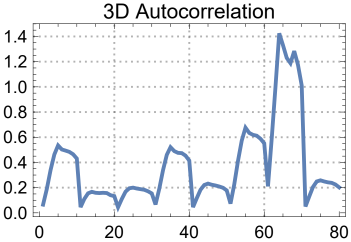

2D Plot

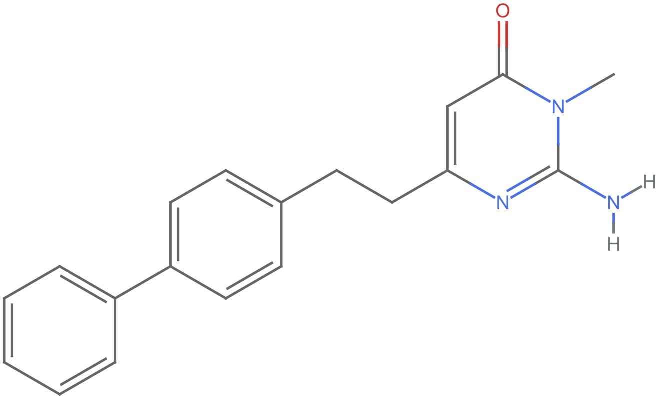

3D Plot

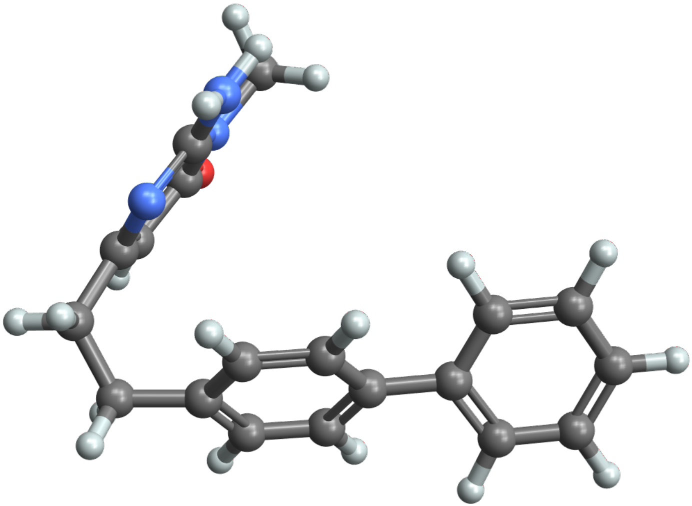

2D Plot

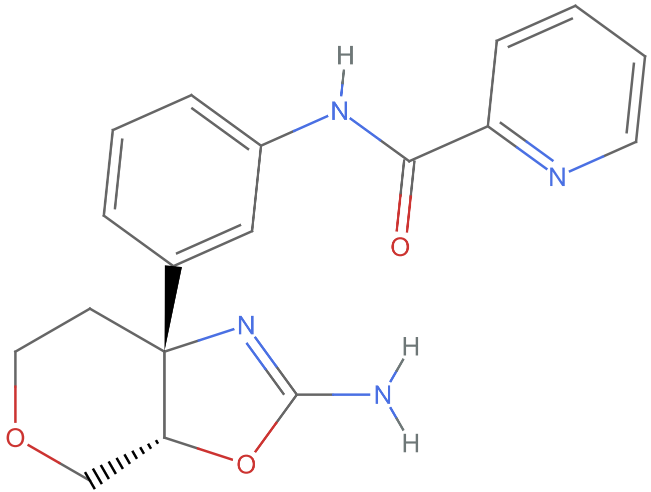

2D Autocorrelation

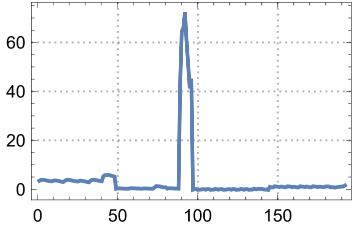

Dataset Index= 1520

Molecule Formula= $C_{18}H_{18}N_4O_3$

3D Plot

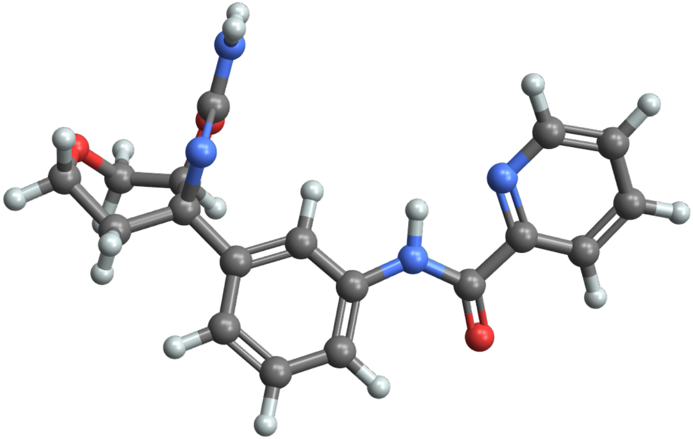

3D Autocorrelation

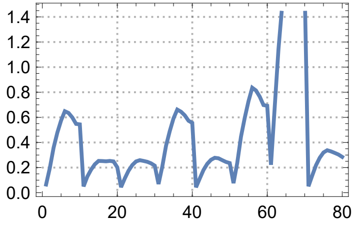

2D Plot

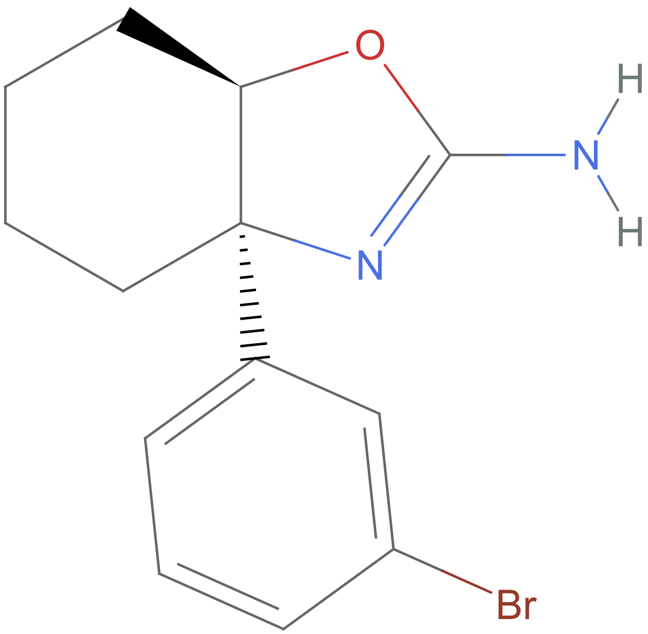

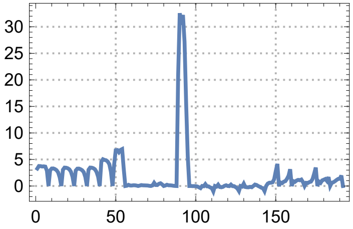

Dataset Index= 1521

Molecule Formula= $C_{13}H_{15}BrN_2O$

3D Plot

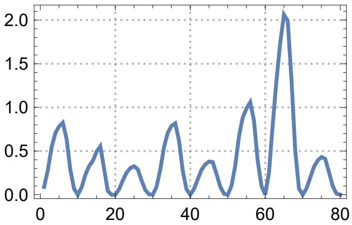

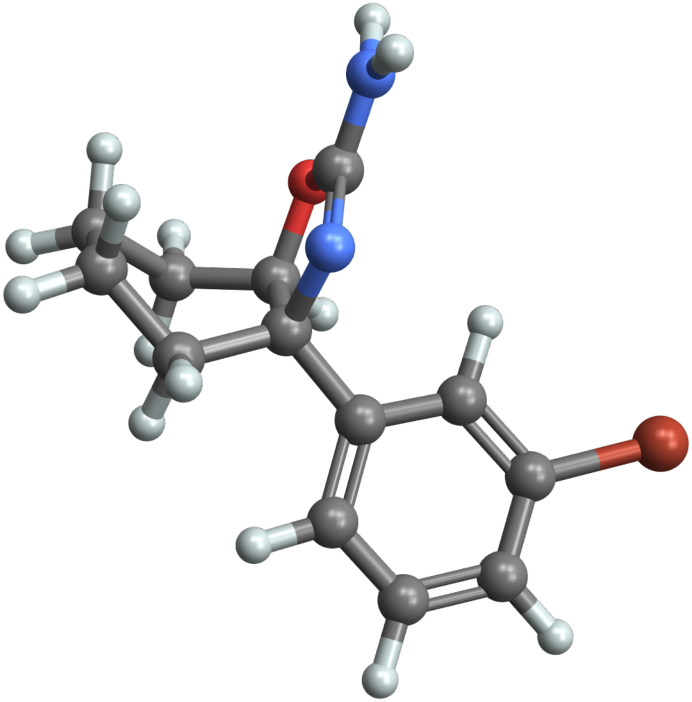

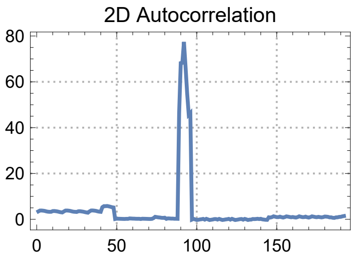

Dataset Index= 1522

Molecule Formula= $C_{19}H_{20}N_4O_2$

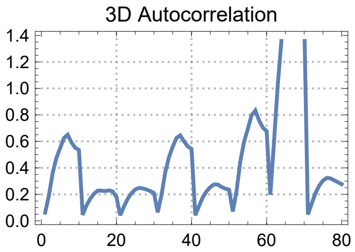

2D Plot

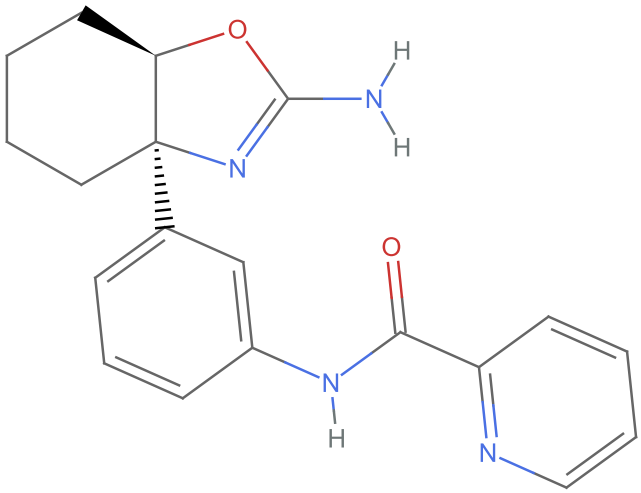

3D Plot

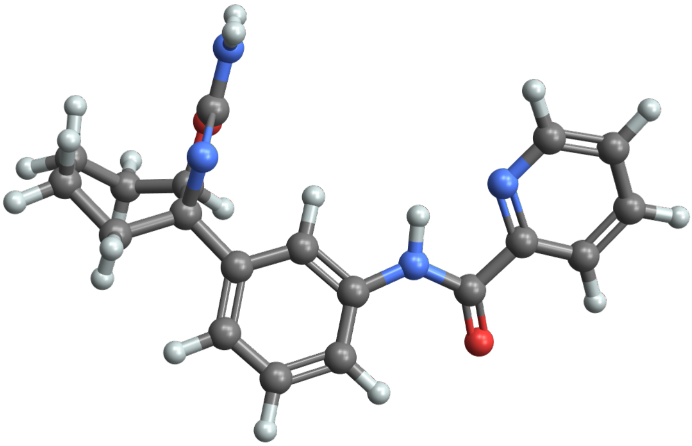

2D Autocorrelation

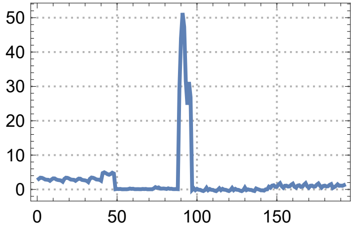

Dataset Index= 1523

Molecule Formula= $C_{14}H_{19}N_3O$

3D Autocorrelation

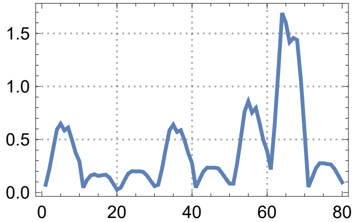

2D Plot

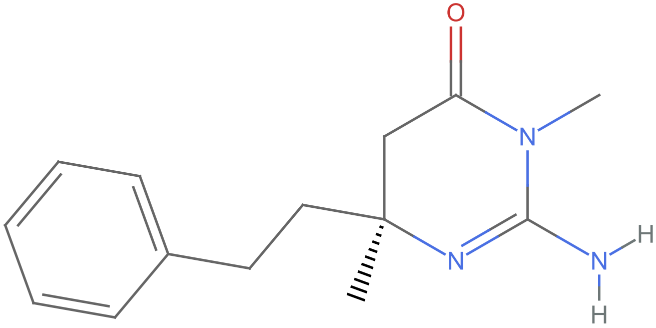

3D Plot

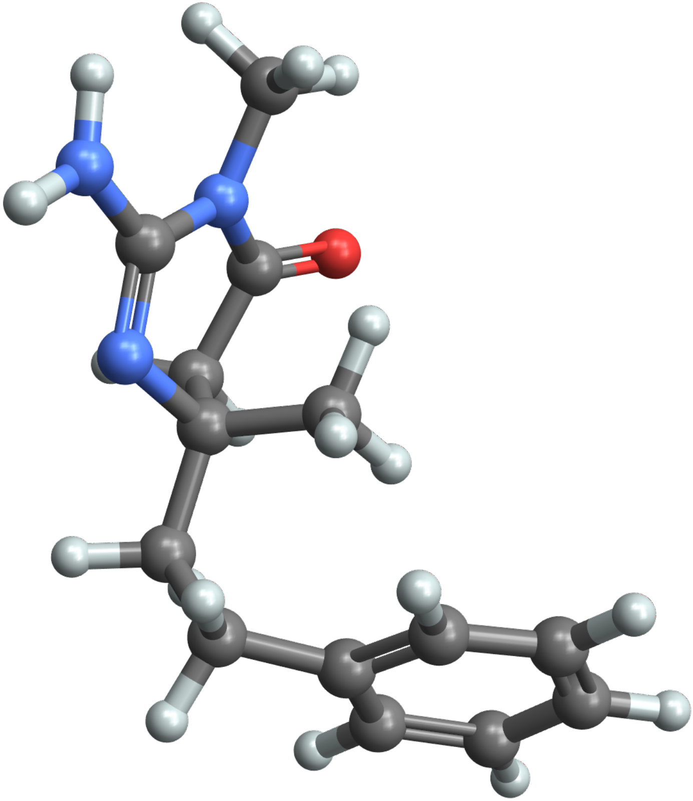

2D Plot

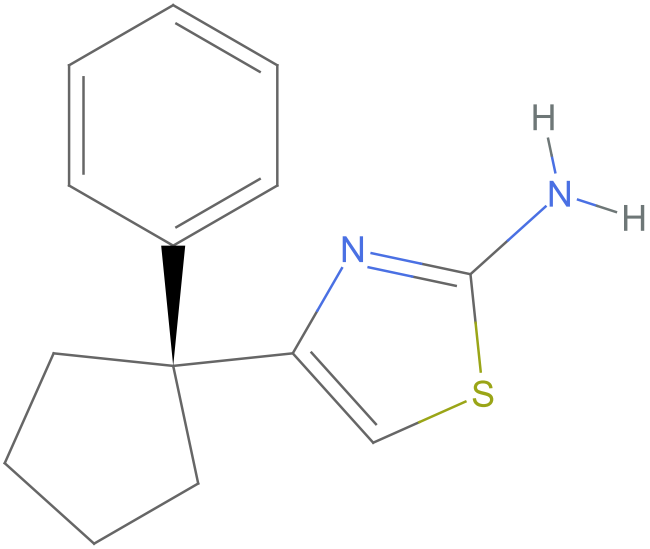

2D Autocorrelation

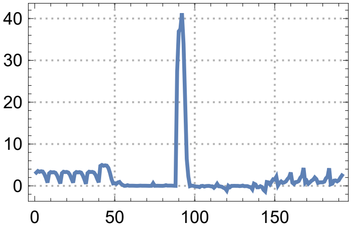

3D Plot

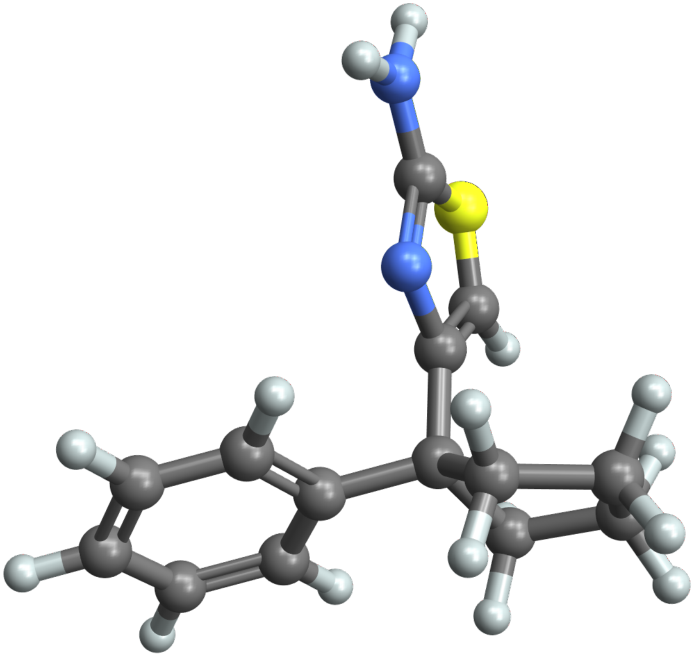

Dataset Index= 1524

Molecule Formula= $C_{14}H_{16}N_2S$

3D Autocorrelation

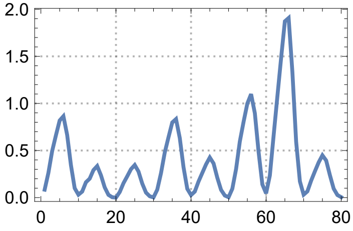

2D Plot

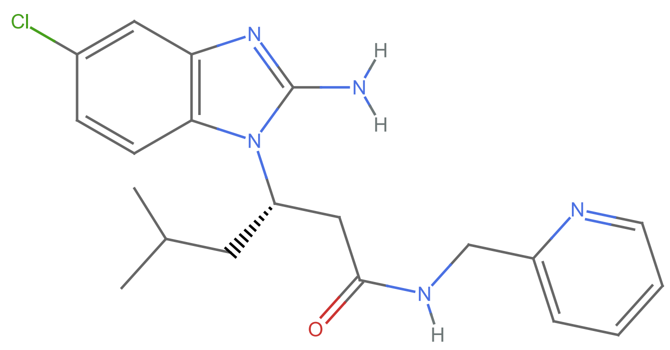

3D Plot

2D Autocorrelation

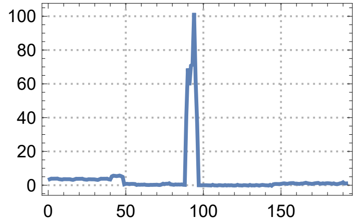

Dataset Index= 1525

Molecule Formula= $C_{20}H_{24}ClN_5O$

3D Autocorrelation

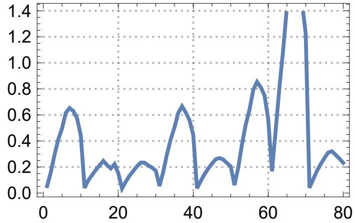

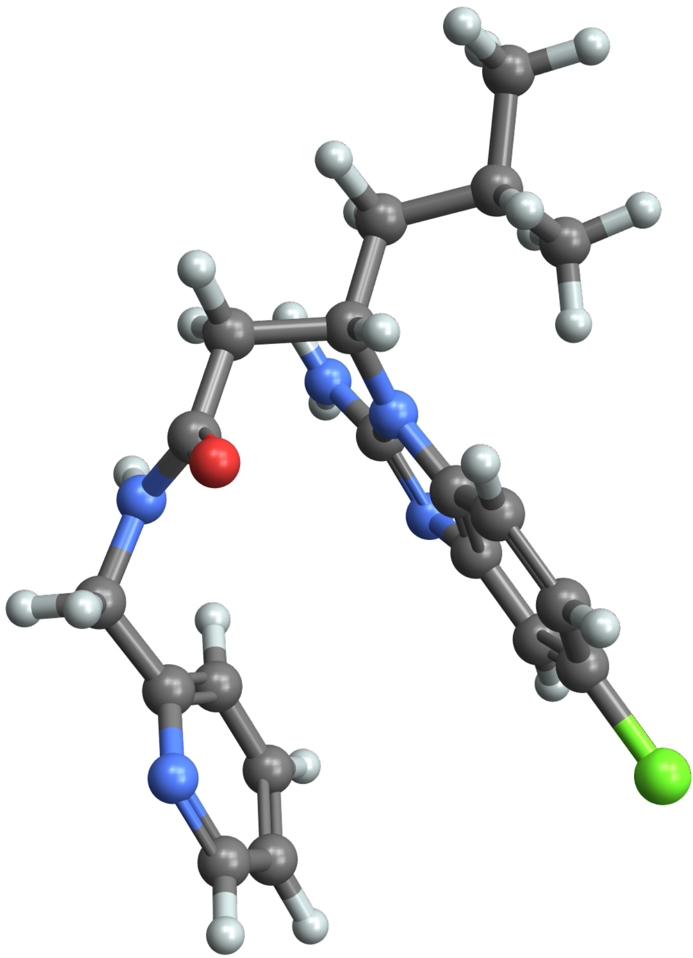

2D Autocorrelation

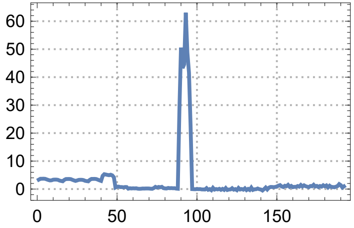

Dataset Index= 1526

Molecule Formula= $C_{16}H_{21}ClN_4O$

3D Autocorrelation

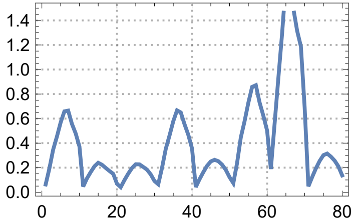

2D Plot

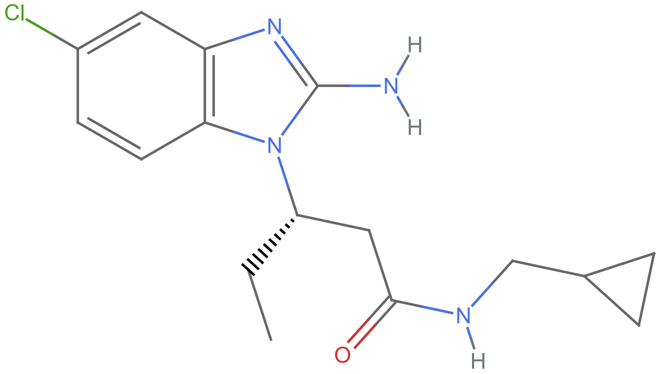

3D Plot

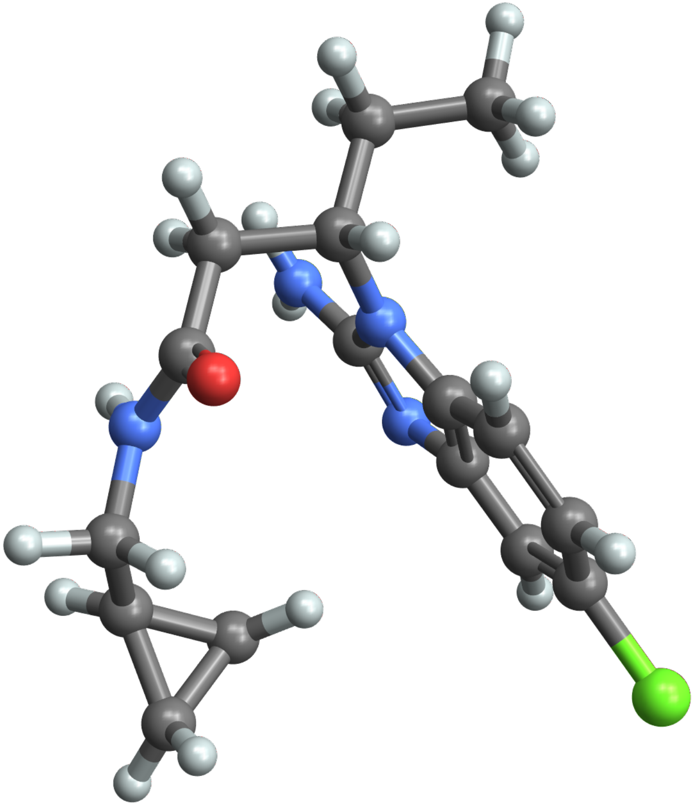

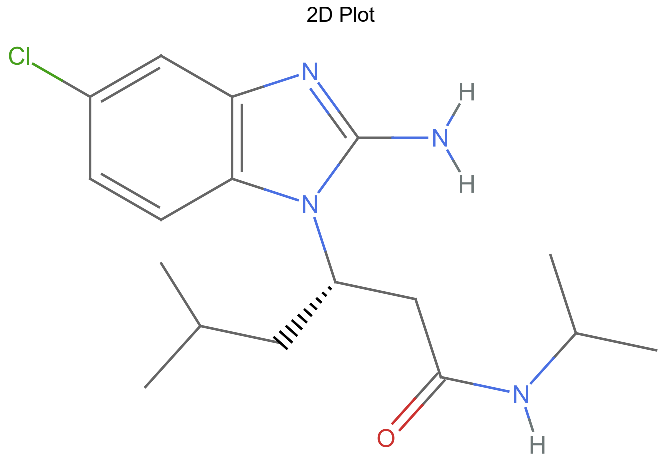

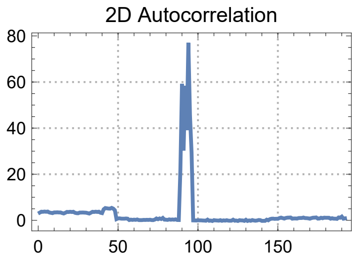

Dataset Index= 1527

Molecule Formula= $C_{17}H_{25}ClN_4O$

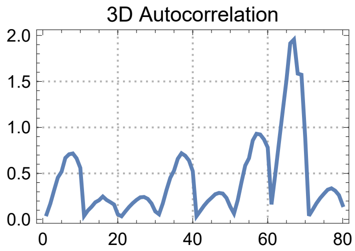

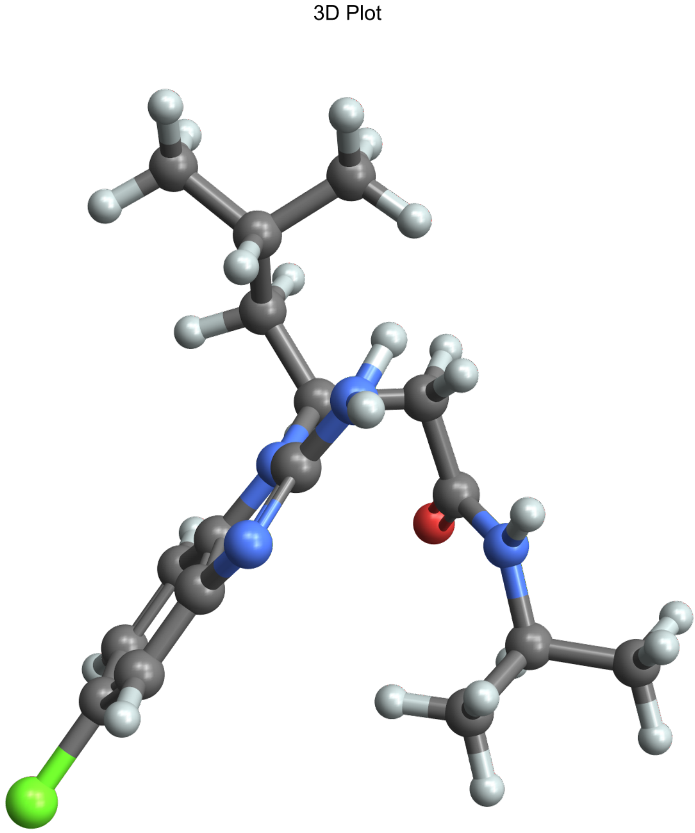

2D Plot

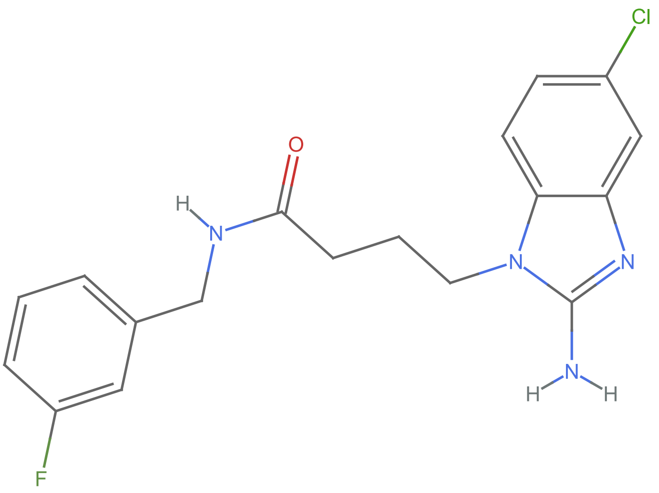

2D Autocorrelation

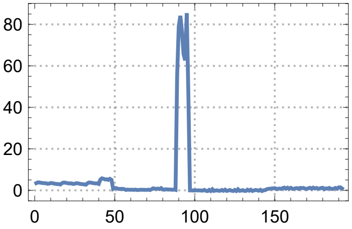

3D Plot

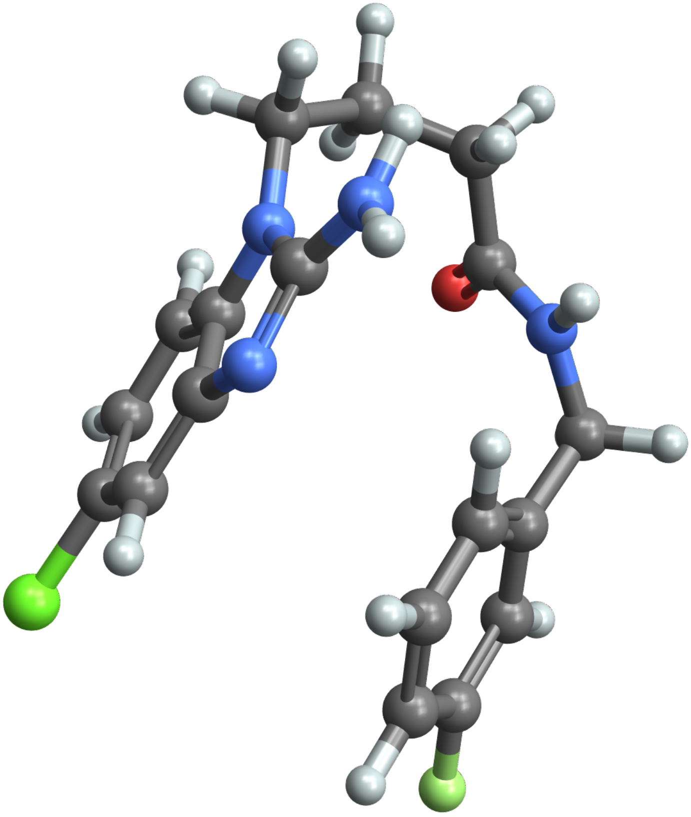

```
Dataset Index=        1528
```

Molecule Formula= $C_{18}H_{18}ClFN_4O$

3D Autocorrelation

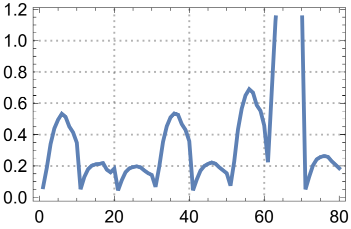

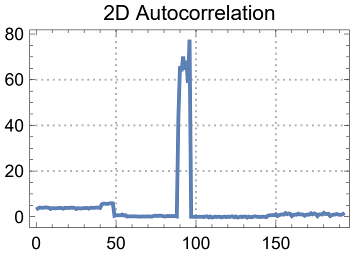

Dataset Index= 1529

Molecule Formula= $C_{24}H_{25}ClN_4$

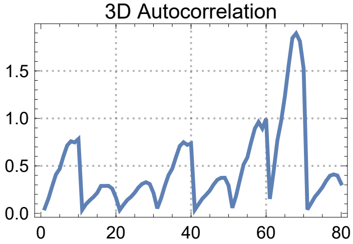

2D Plot

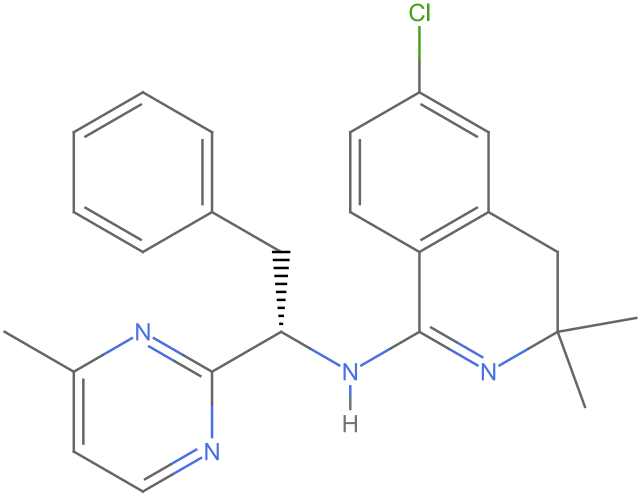

3D Plot

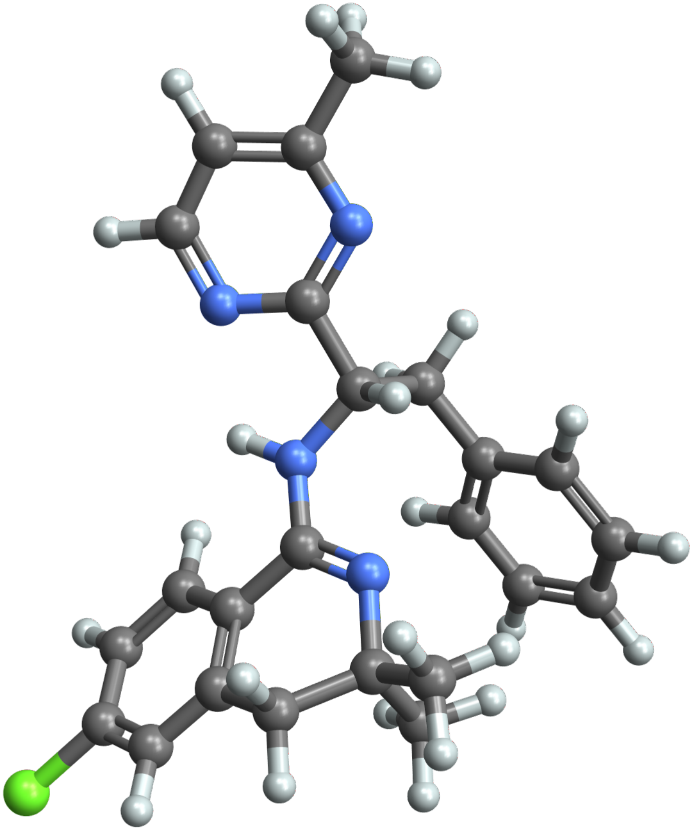

2D Plot

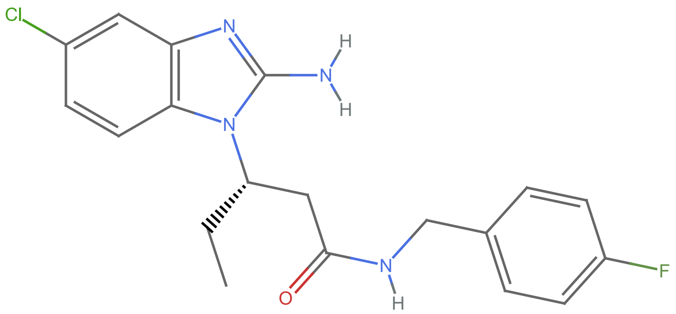

2D Autocorrelation

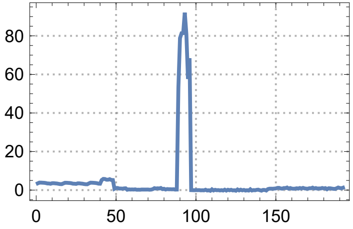

3D Plot

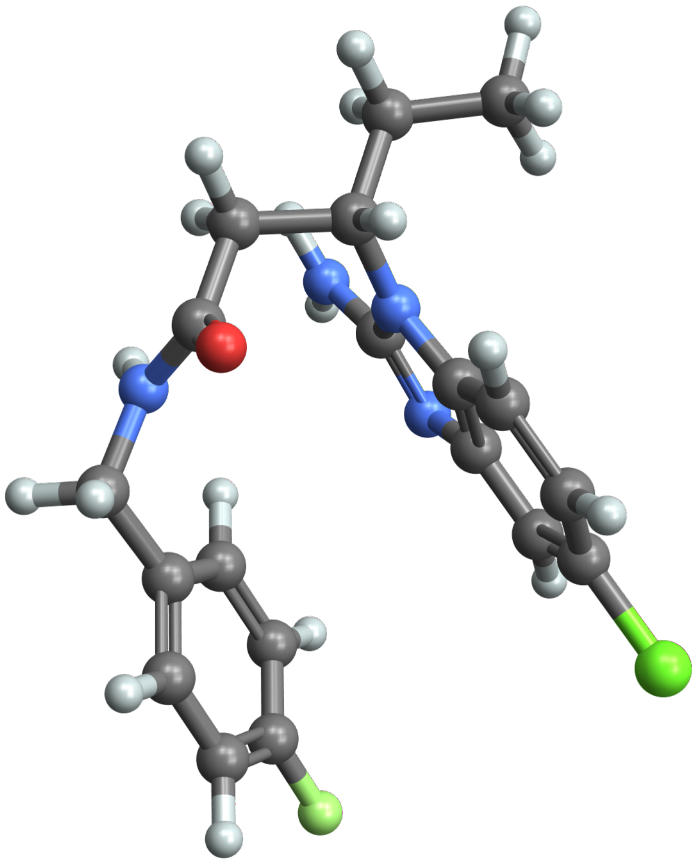

Dataset Index= 1530

Molecule Formula= $C_{19}H_{20}ClFN_4O$

3D Autocorrelation

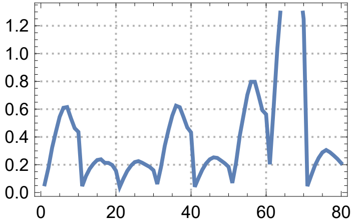

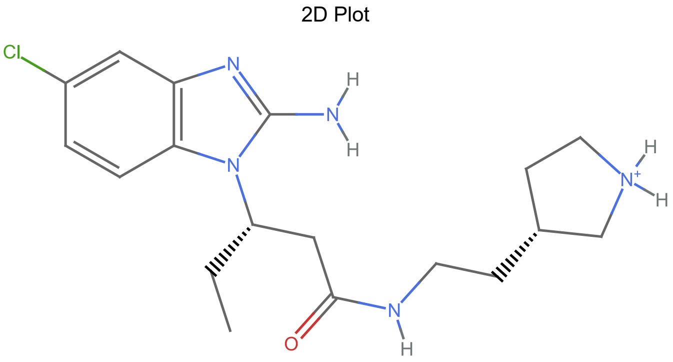

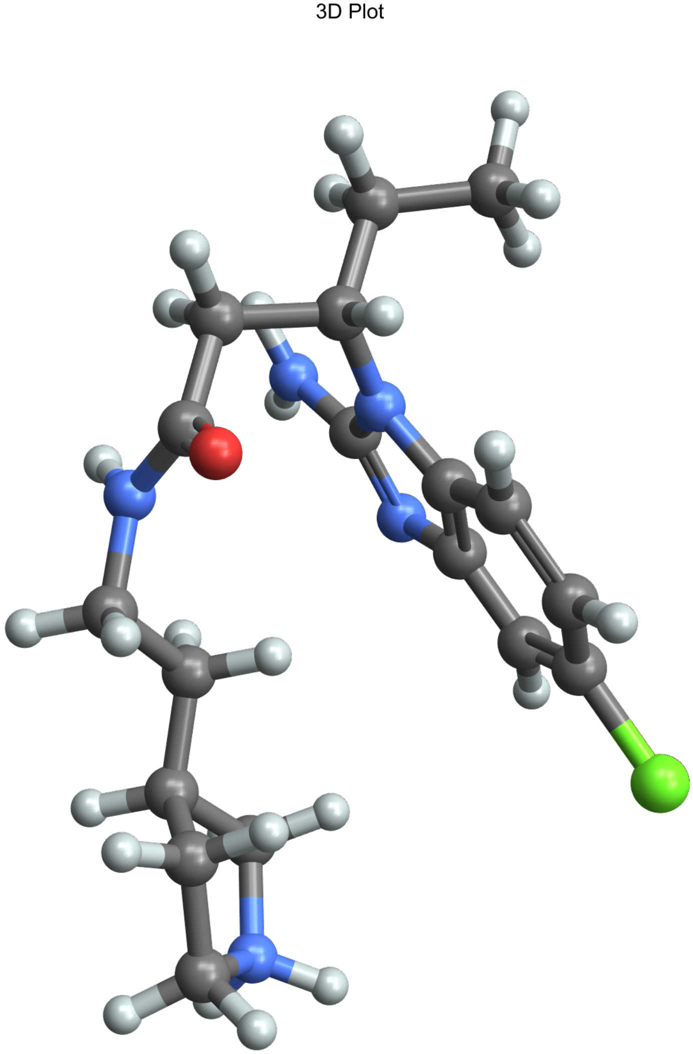

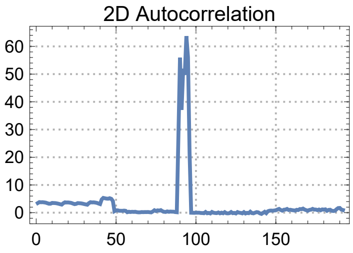

Dataset Index= 1531

Molecule Formula= $C_{18}H_{27}ClN_5O^+$

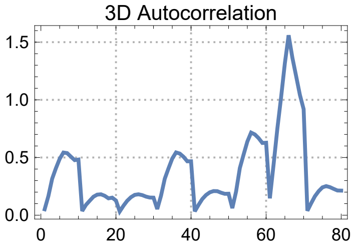

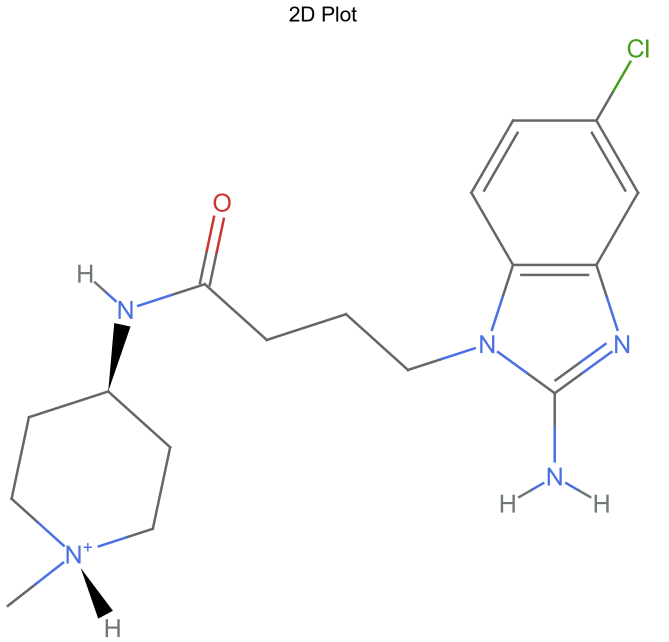

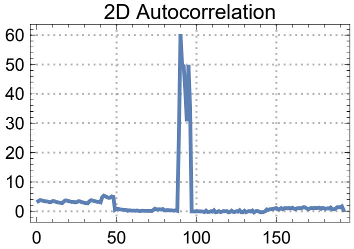

Dataset Index= 1532

Molecule Formula= $C_{17}H_{25}ClN_5O^+$

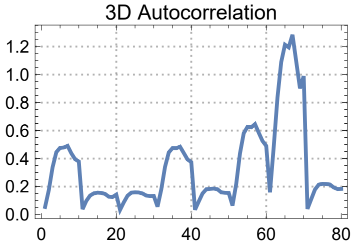

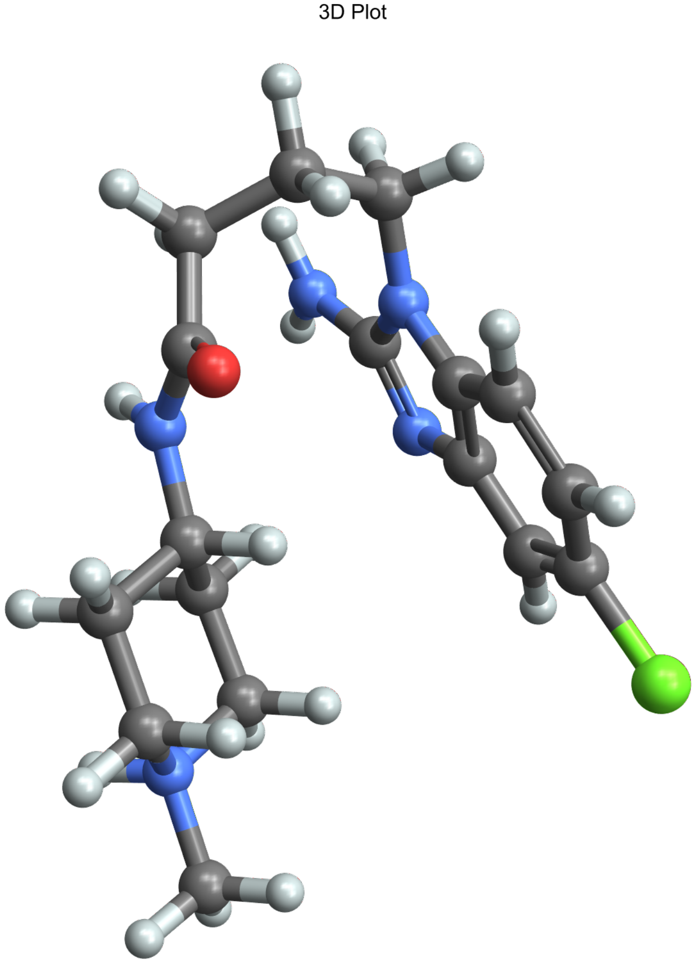

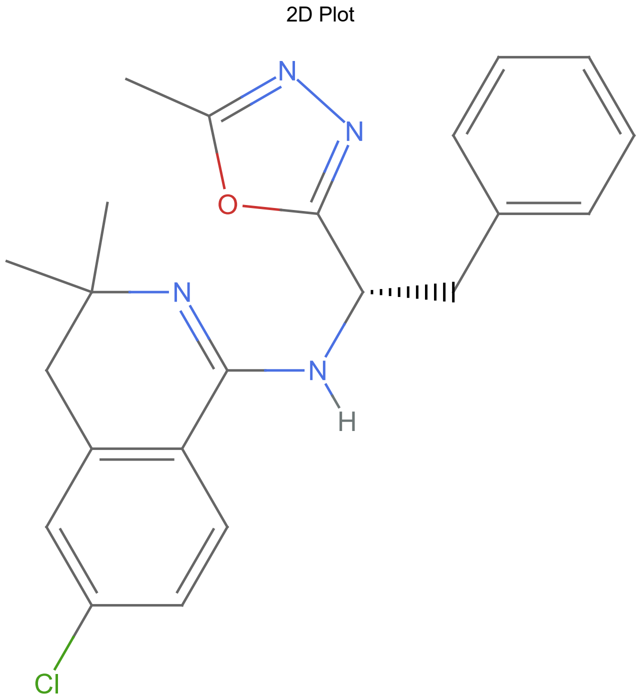

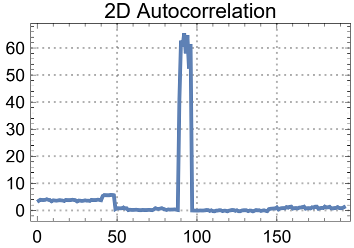

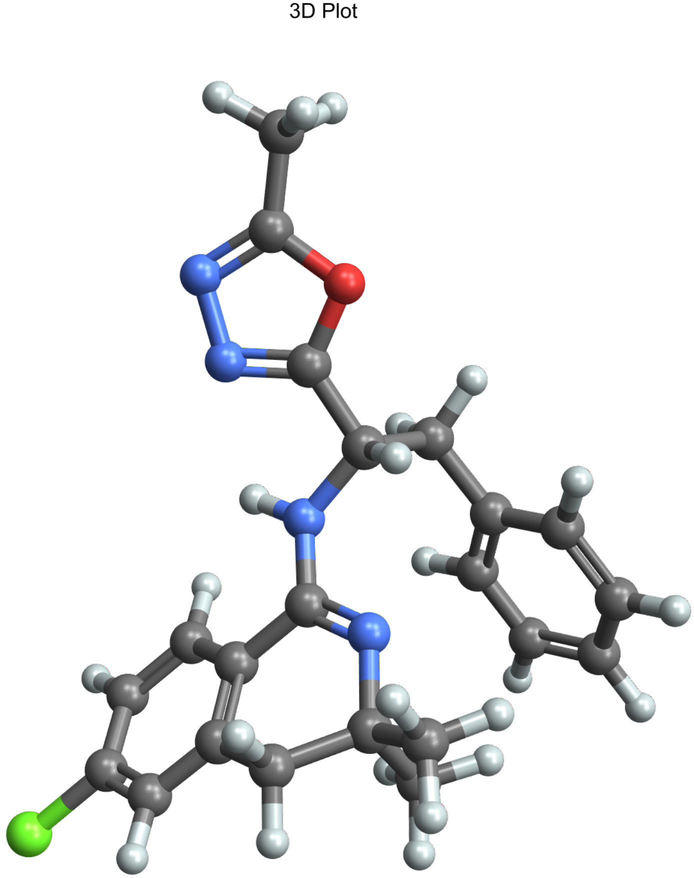

Dataset Index= 1533

Molecule Formula= $C_{22}H_{23}ClN_4O$

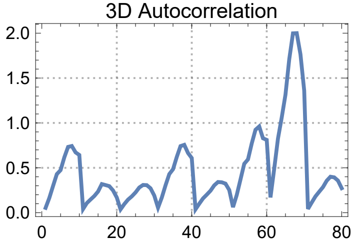

2D Plot

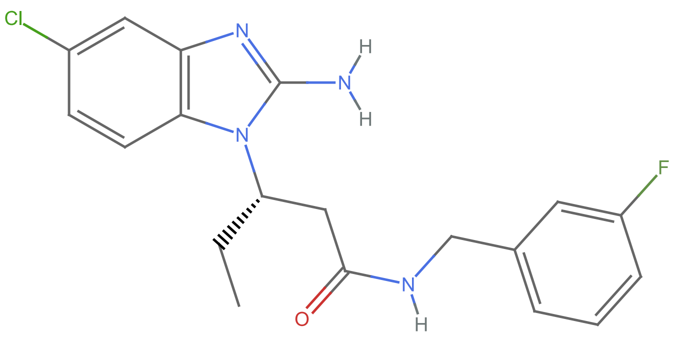

2D Autocorrelation

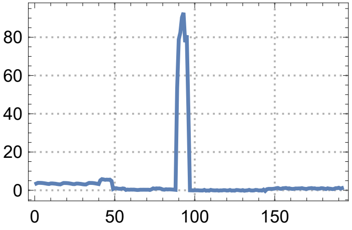

Dataset Index= 1534

Molecule Formula= $C_{19}H_{20}ClFN_4O$

3D Autocorrelation

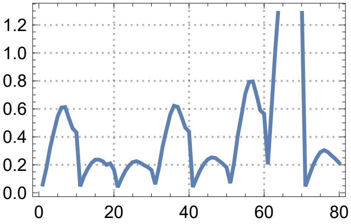

3D Plot

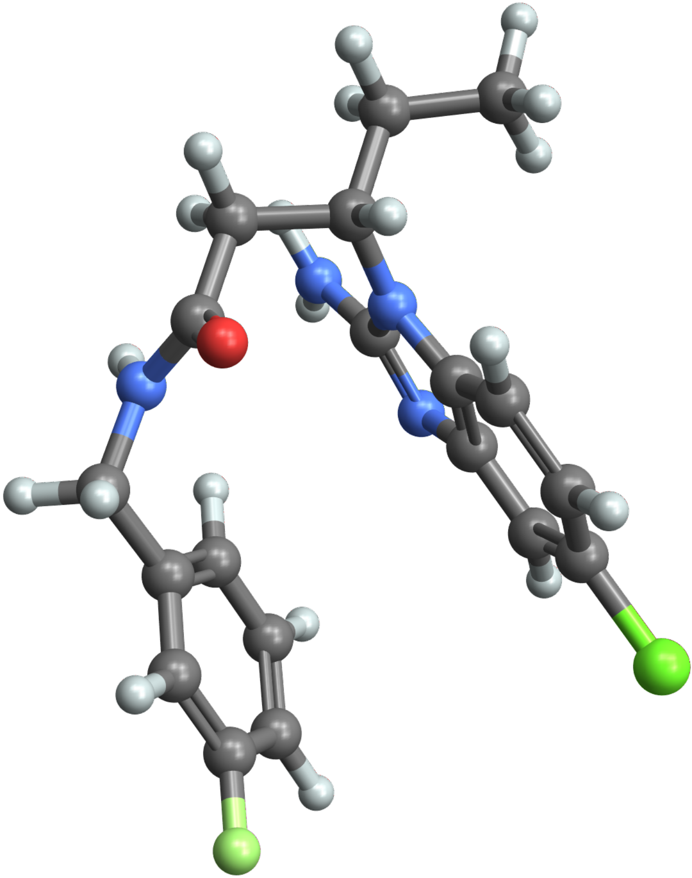

2D Plot

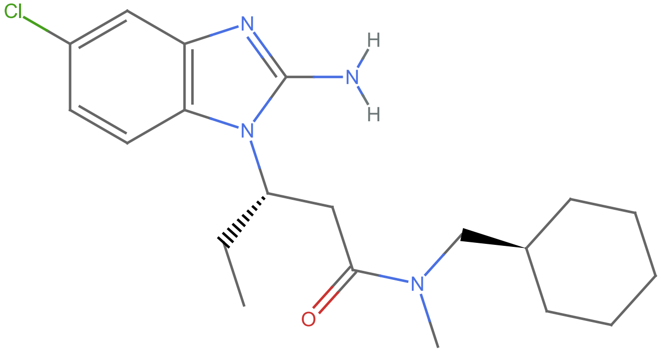

2D Autocorrelation

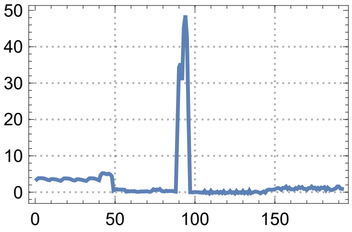

Dataset Index= 1535

Molecule Formula= $C_{20}H_{29}ClN_4O$

3D Autocorrelation

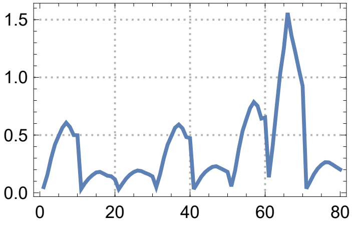

3D Plot

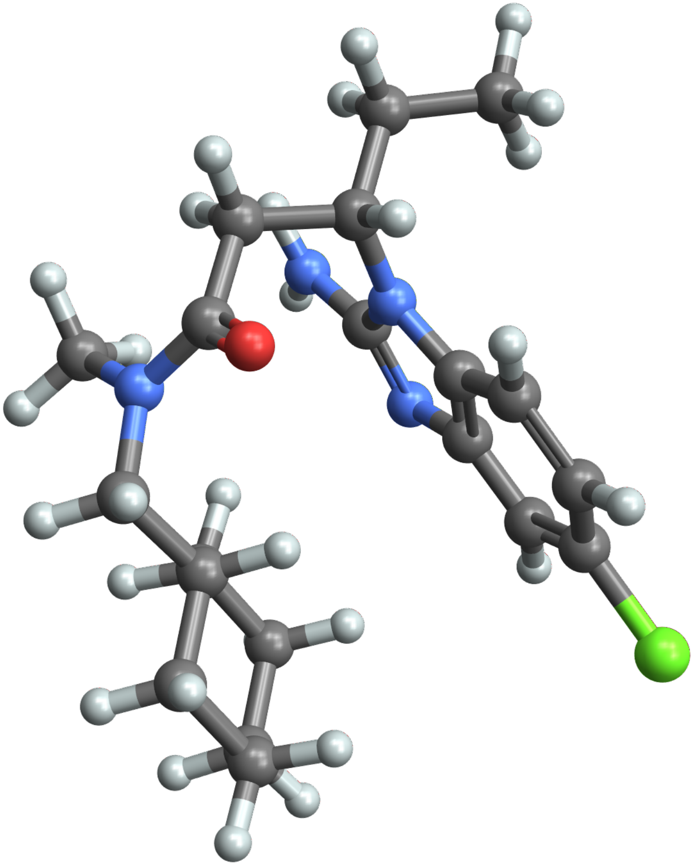

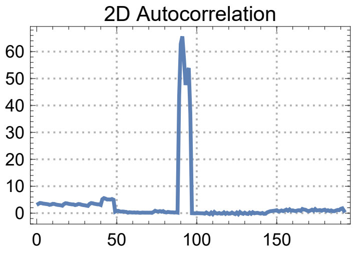

Dataset Index= 1536

Molecule Formula= $C_{17}H_{18}ClN_5O$

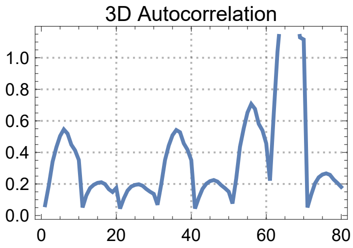

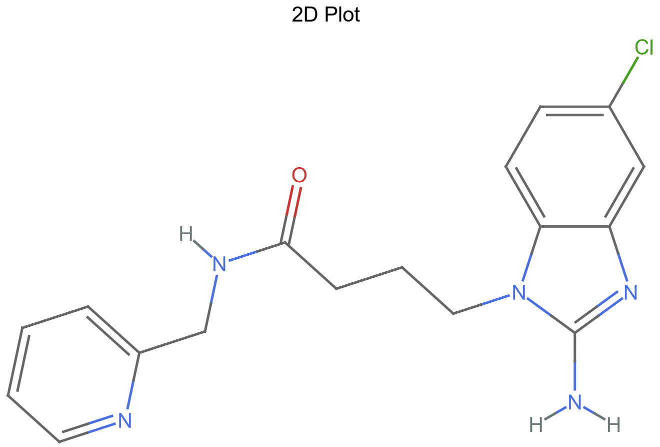

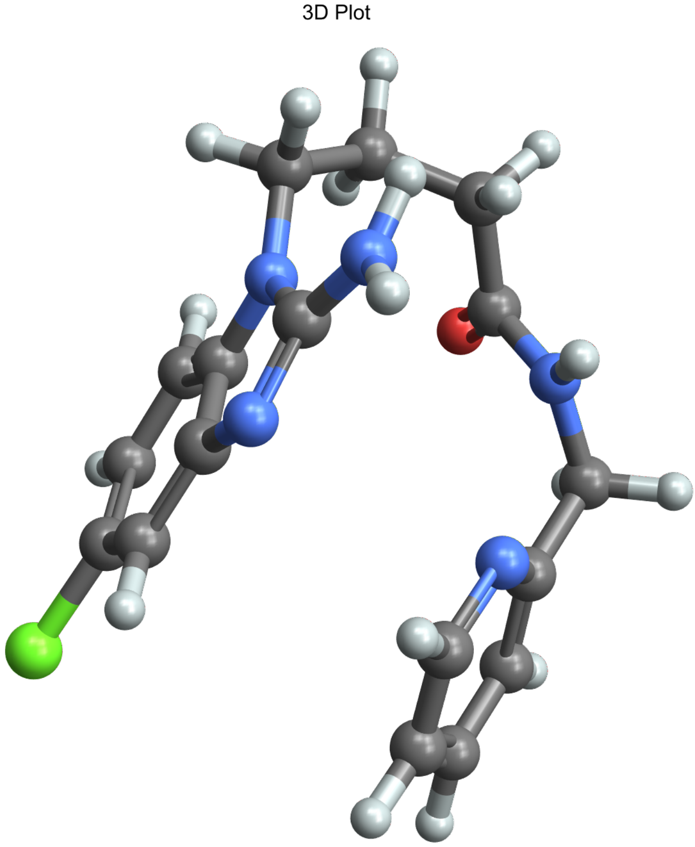

2D Plot

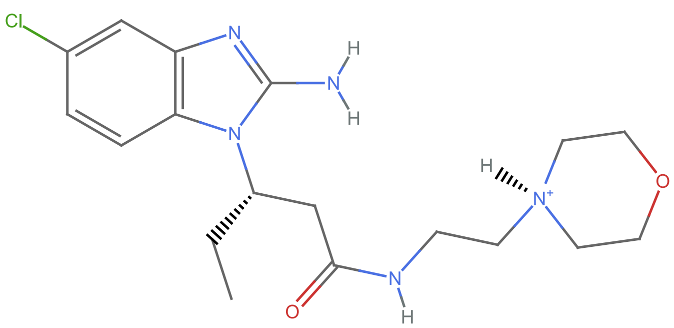

3D Plot

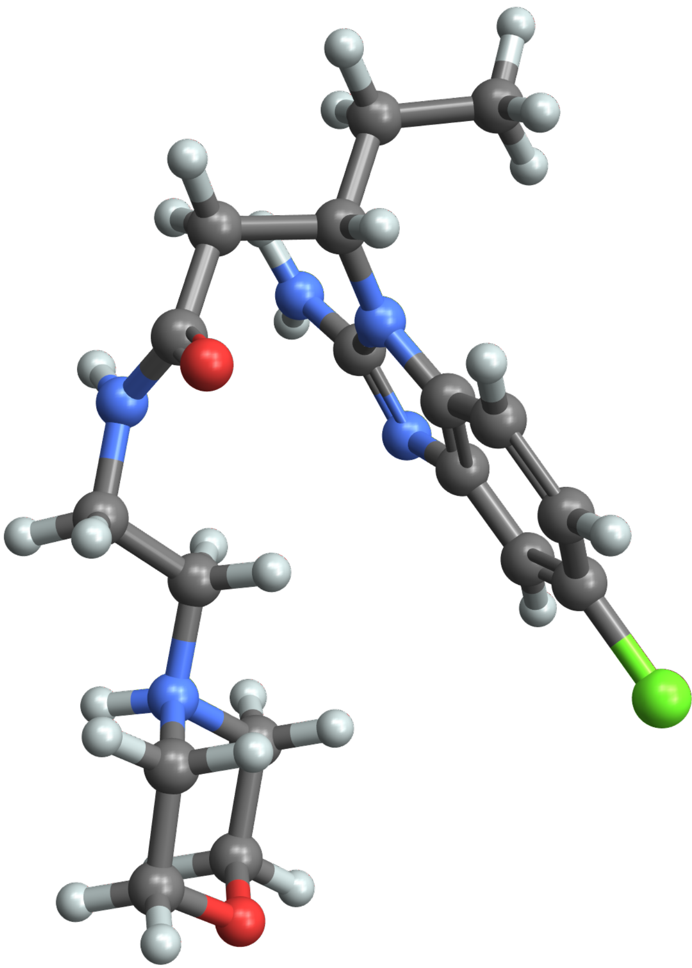

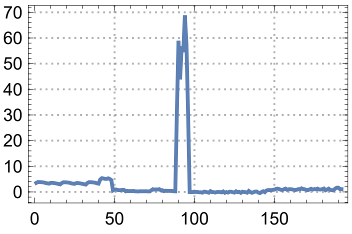

Dataset Index= 1537

Molecule Formula= $C_{18}H_{27}ClN_5O_2^+$

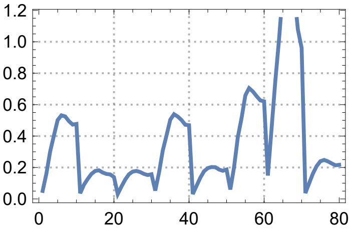

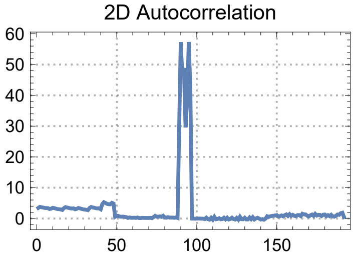

Dataset Index= 1538

Molecule Formula= $C_{17}H_{25}ClN_5O^+$

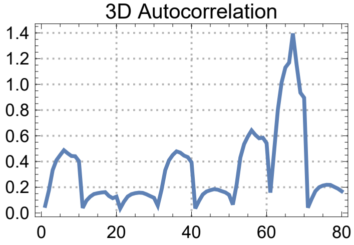

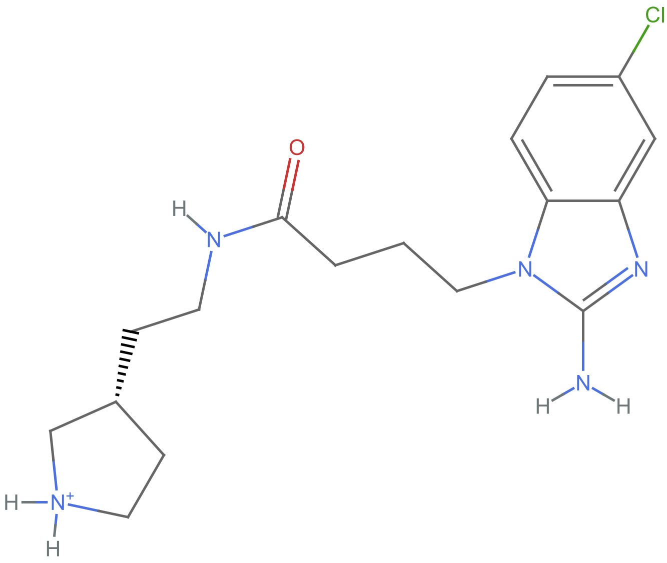

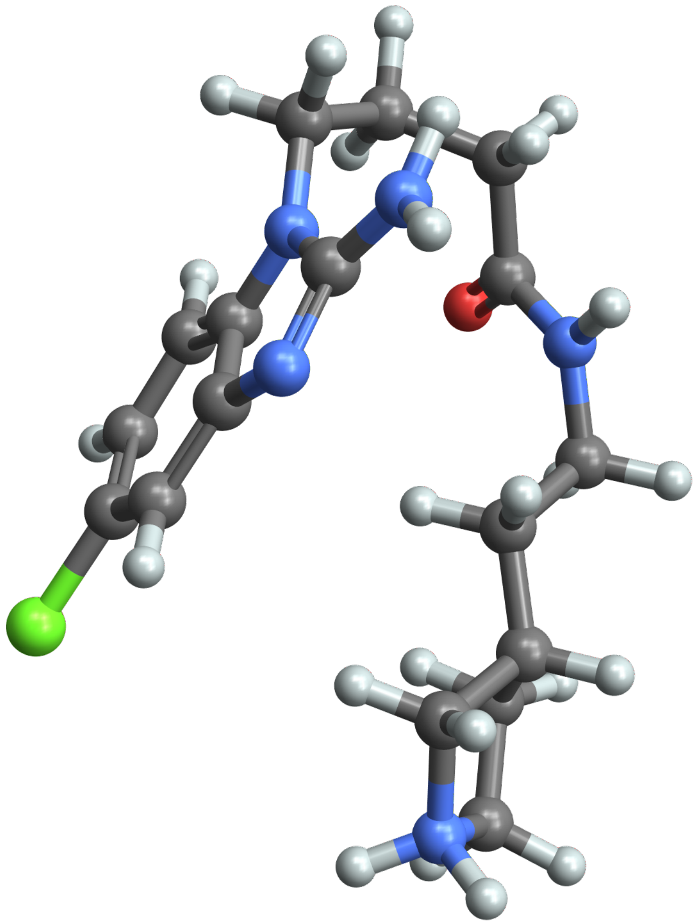

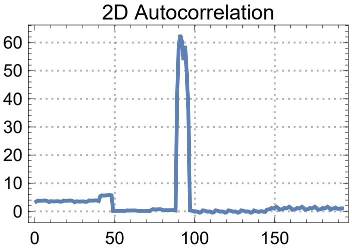

Dataset Index= 1539

Molecule Formula= $C_{20}H_{22}N_6$

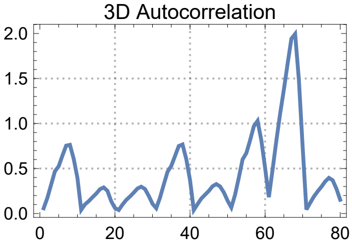

2D Plot

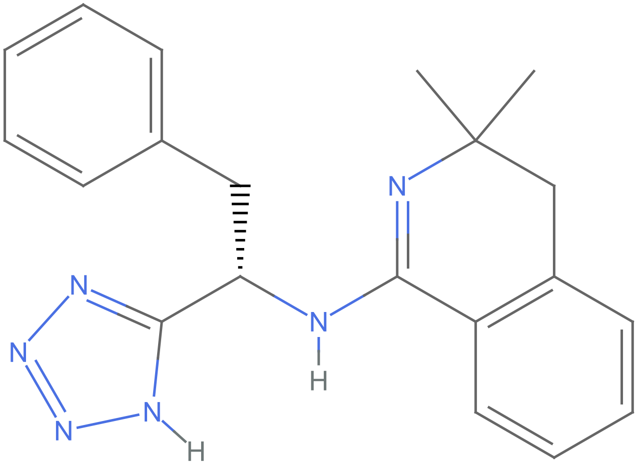

3D Plot

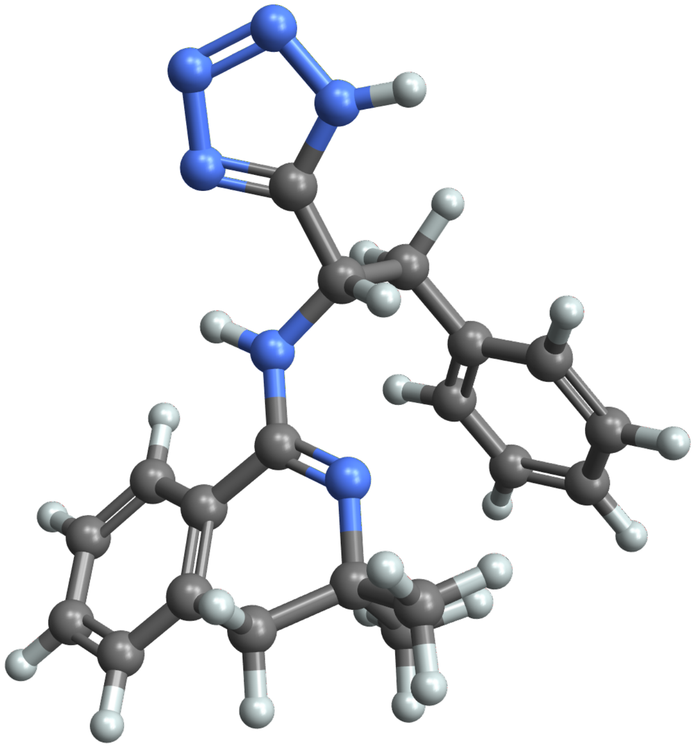

2D Plot

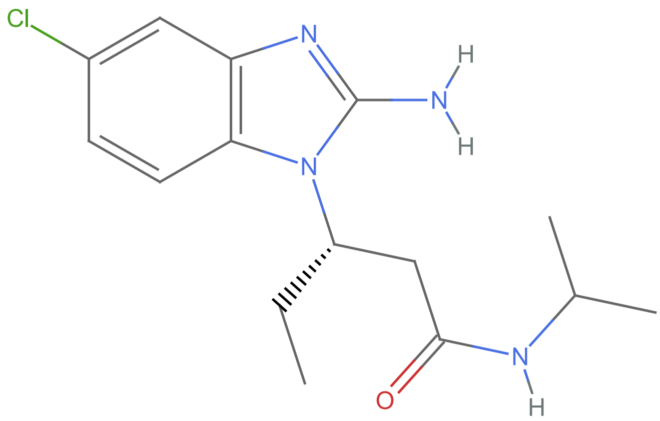

3D Plot

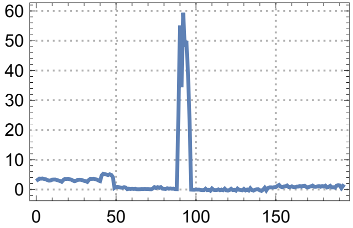

Dataset Index= 1540

Molecule Formula= $C_{15}H_{21}ClN_{4}O$

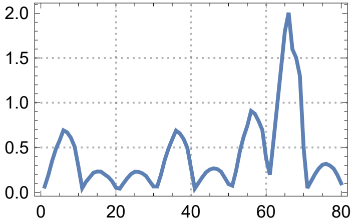

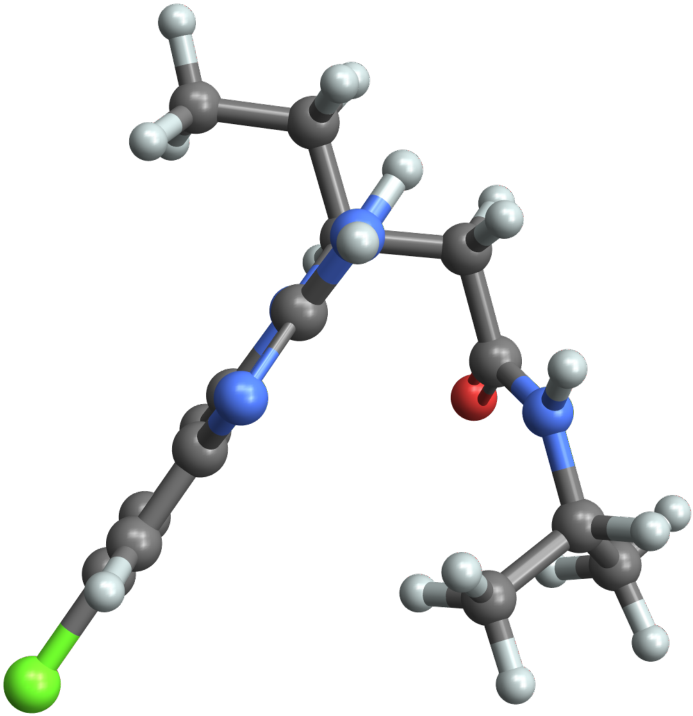

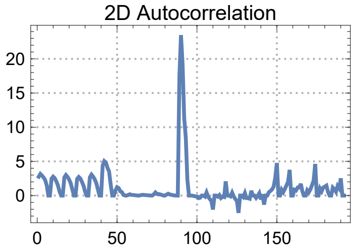

Dataset Index= 1541

Molecule Formula= $C_7H_6ClN_3$

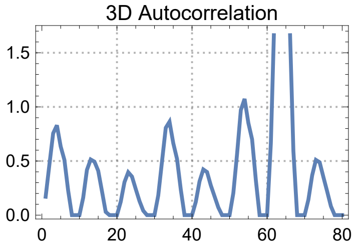

2D Plot

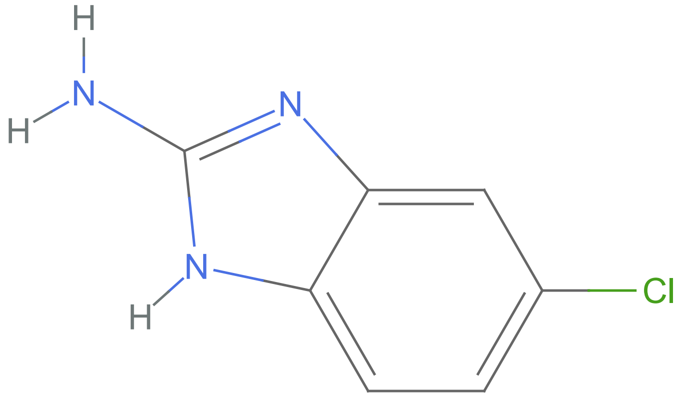

3D Plot

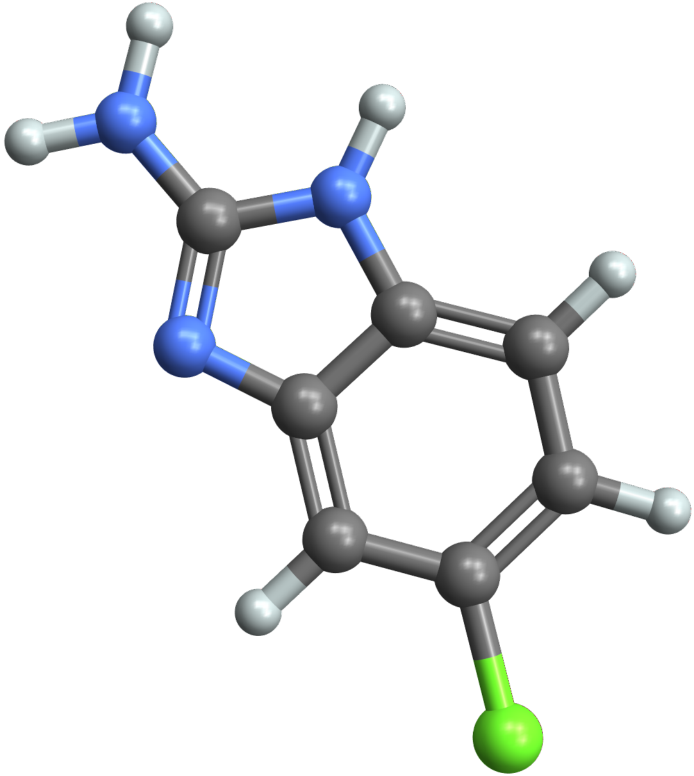

2D Plot

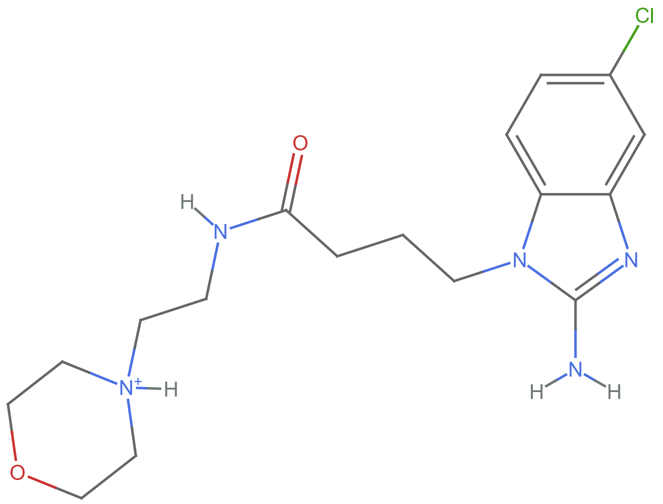

2D Autocorrelation

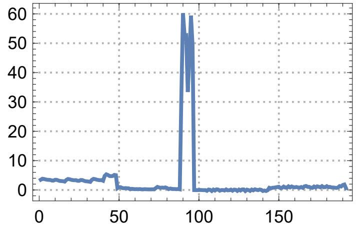

3D Plot

Dataset Index= 1542

Molecule Formula= $C_{17}H_{25}ClN_5O_2^+$

3D Autocorrelation

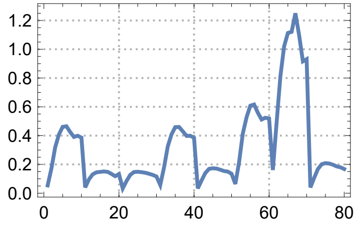

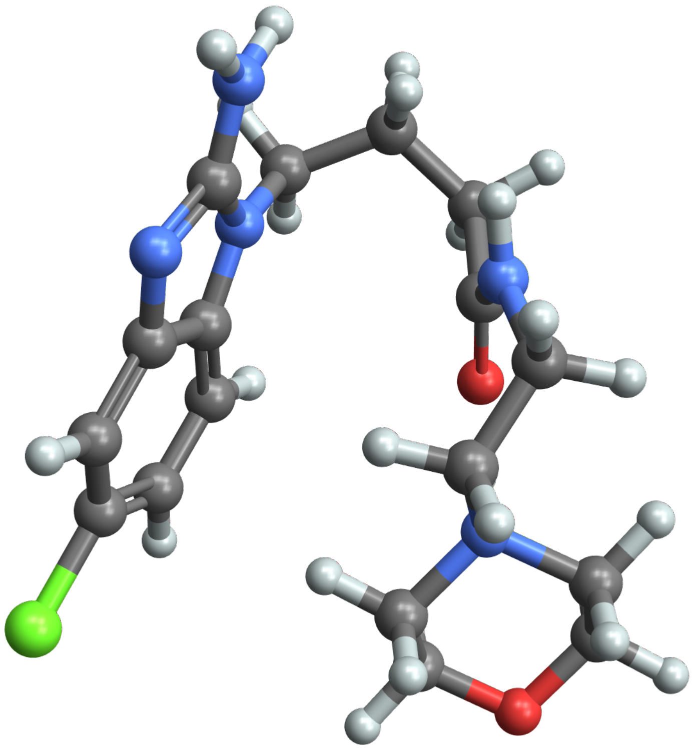

2D Plot

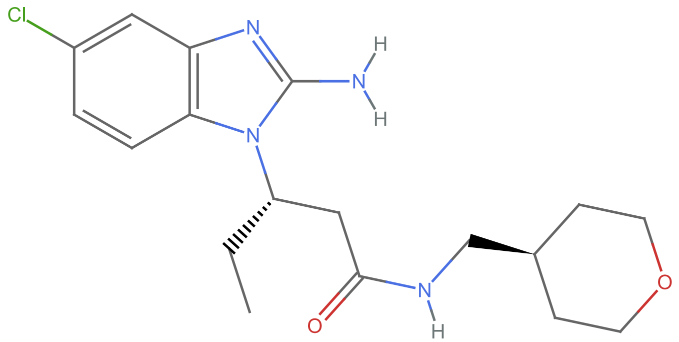

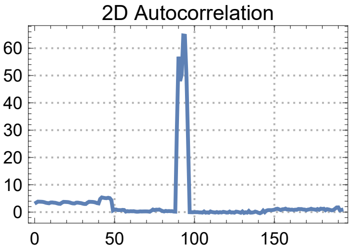

3D Plot

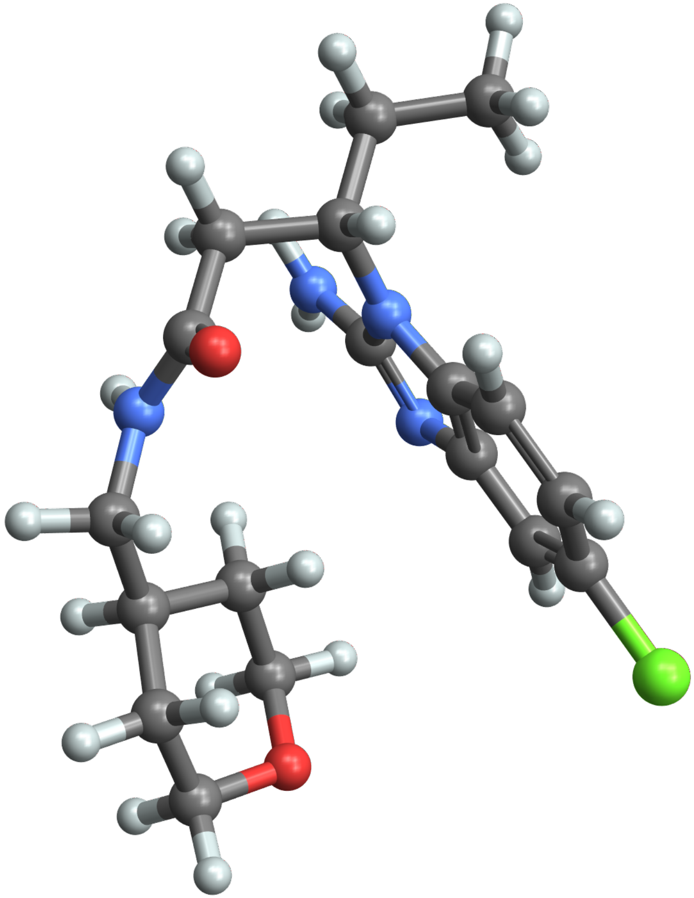

Dataset Index= 1543

Molecule Formula= $C_{18}H_{25}ClN_4O_2$

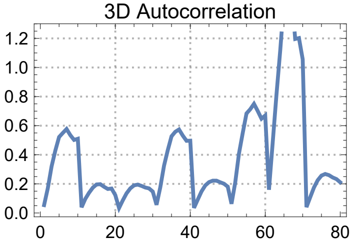

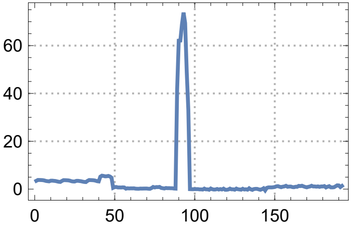

Dataset Index= 1544

Molecule Formula= $C_{18}H_{20}ClN_5O$

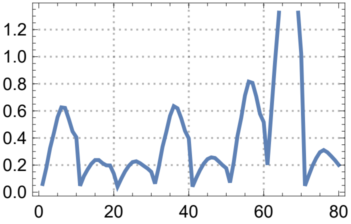

2D Plot

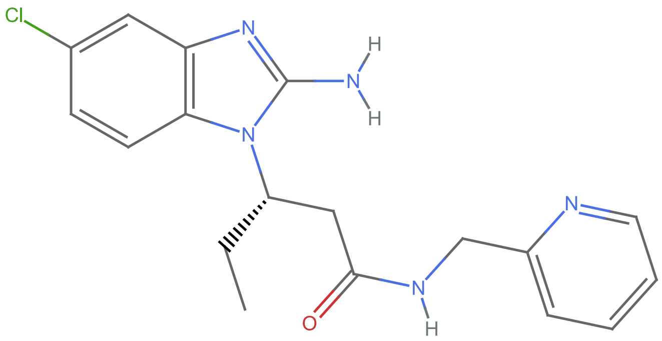

3D Plot

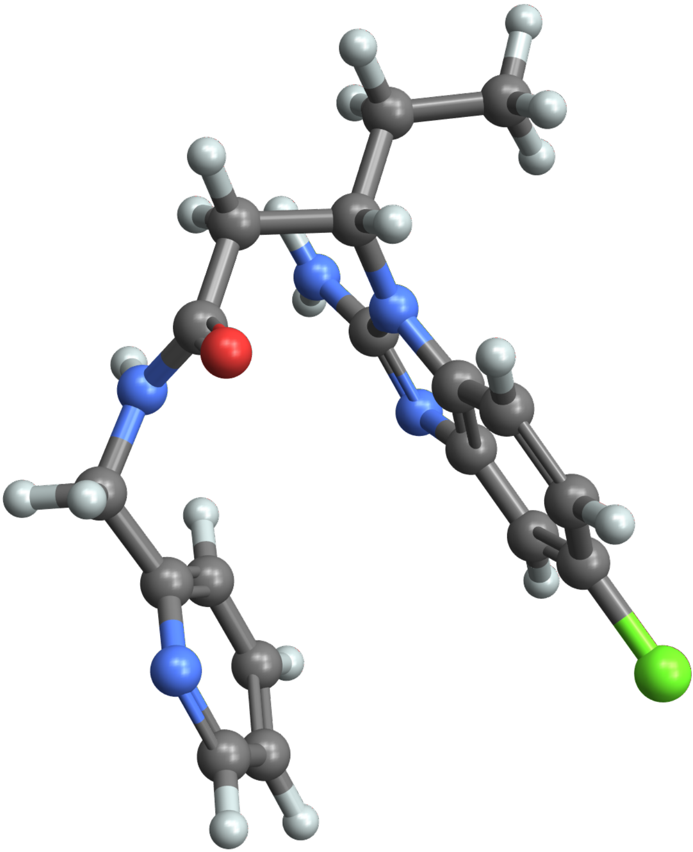

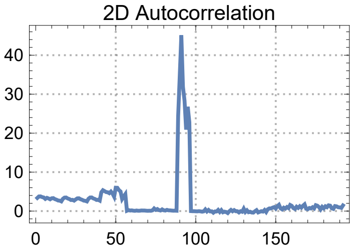

Dataset Index= 1545

Molecule Formula= $C_{14}H_{14}BrN_3O$

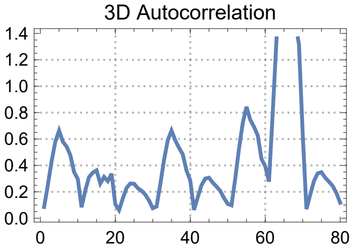

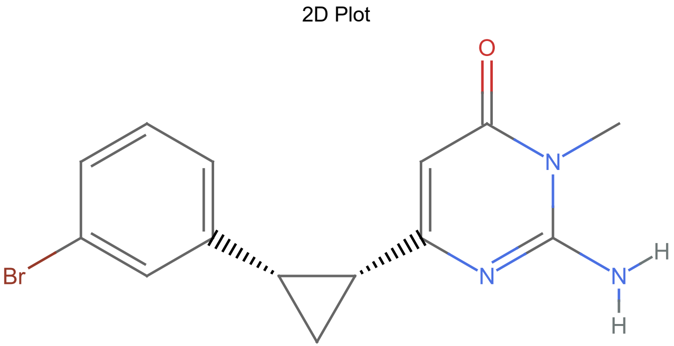

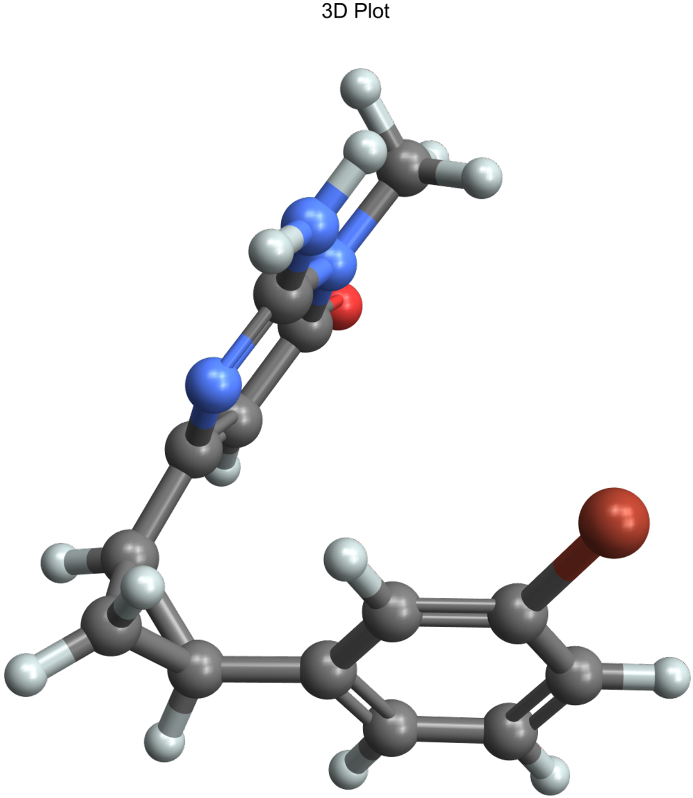

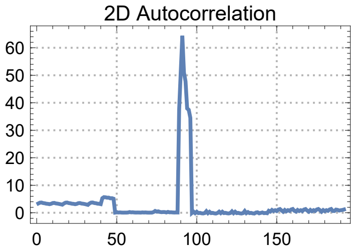

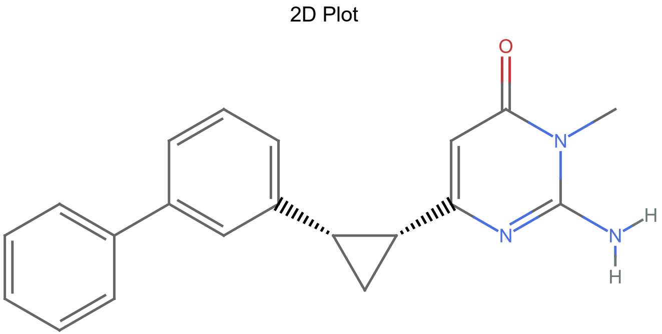

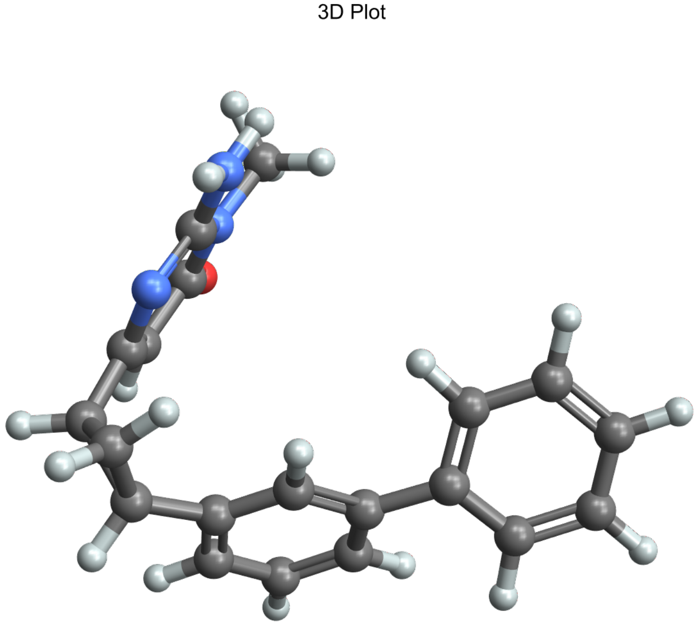

Dataset Index= 1546

Molecule Formula= $C_{20}H_{19}N_3O$

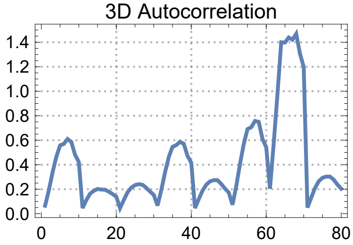

2D Autocorrelation

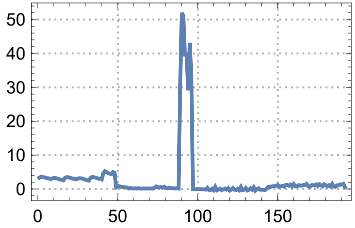

Dataset Index= 1547

Molecule Formula= $C_{15}H_{19}ClN_4O$

3D Autocorrelation

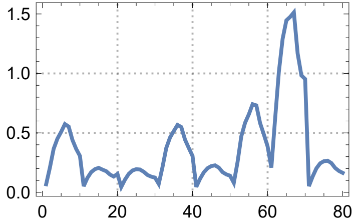

2D Plot

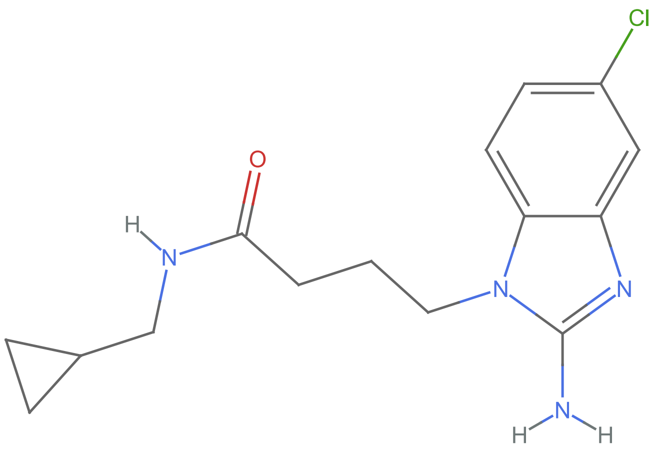

3D Plot

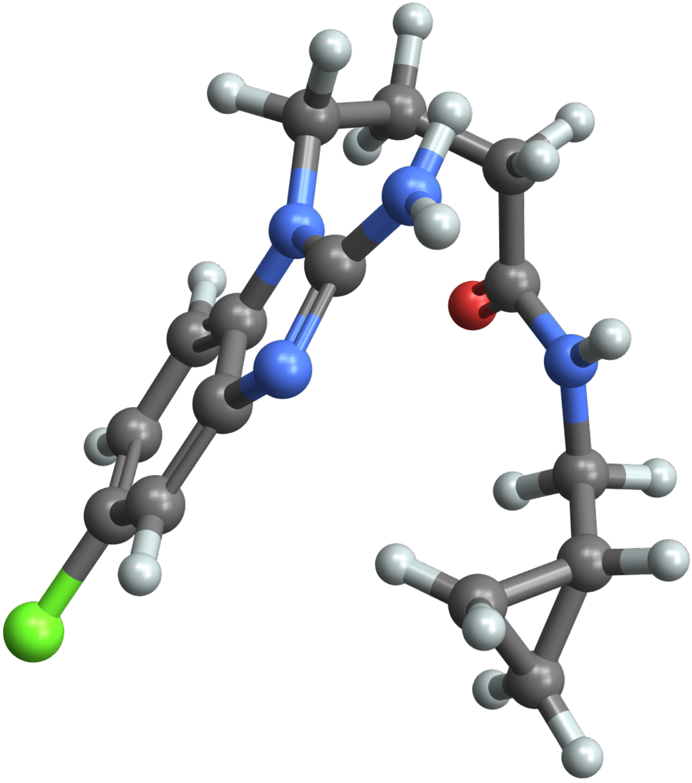

Supplement: Supplementary file 1 [file ijms-25-06890-s001.zip › Plots.pdf]
